# DArTseq-based analysis of genomic relationships among species of tribe Triticeae

**DOI:** 10.1038/s41598-018-34811-y

**Published:** 2018-11-06

**Authors:** Offiong U. Edet, Yasir S. A. Gorafi, Shuhei Nasuda, Hisashi Tsujimoto

**Affiliations:** 1 Arid Land Research Center, Tottori University, Tottori, 680-0001 Japan; 2 United Graduate School of Agricultural Sciences, Tottori University, Tottori, 680-8553 Japan; 3 Agricultural Research Corporation (ARC), P. O. Box 126, Wad Madani, Sudan; 4 Laboratory of Plant Genetics, Graduate School of Agriculture, Kyoto University, Kyoto, 606-8502 Japan

## Abstract

Precise utilization of wild genetic resources to improve the resistance of their cultivated relatives to environmental growth limiting factors, such as salinity stress and diseases, requires a clear understanding of their genomic relationships. Although seriously criticized, analyzing these relationships in tribe Triticeae has largely been based on meiotic chromosome pairing in hybrids of wide crosses, a specialized and labourious strategy. In this study, DArTseq, an efficient genotyping-by-sequencing platform, was applied to analyze the genomes of 34 Triticeae species. We reconstructed the phylogenetic relationships among diploid and polyploid *Aegilops* and *Triticum* species, including hexaploid wheat. Tentatively, we have identified the diploid genomes that are likely to have been involved in the evolution of five polyploid species of *Aegilops*, which have remained unresolved for decades. Explanations which cast light on the progenitor of the A genomes and the complex genomic status of the B/G genomes of polyploid *Triticum* species in the Emmer and Timopheevi lineages of wheat have also been provided. This study has, therefore, demonstrated that DArTseq genotyping can be effectively applied to analyze the genomes of plants, especially where their genome sequence information are not available.

## Introduction

Triticeae is one of the most economically important tribes of the grass family, Poaceae, and includes such globally significant species as bread wheat (*Triticum aestivum* L.), barley (*Hordeum vulgare* L.), and rye (*Secale cereale* L.). Efforts to analyze the genomes of species constituting this tribe to uncover the evolutionary relationships among them have been on for decades^1–5^. These research efforts, beyond attempting to clarify the taxonomy of Triticeae, are largely propelled by the overwhelming role of wild species of this tribe as potential sources of essential alleles for the improvement of their cultivated relatives, especially bread wheat^5–8^.

Analysis of genome differences and phylogenetic relationships in Triticeae has mostly been conducted using cytogenetic approaches that rely on meiotic chromosome pairing in hybrids of wide crosses. However, chromosome pairing is affected by diverse factors, and the reliability of failed chromosome pairing as an indicator of genome dissimilarity has been questioned^9–11^. Molecular cytogenetic methods such as C-banding, fluorescence *in situ* hybridization and genomic *in situ* hybridization have also helped to generate useful information on genome differences and phylogenetic relationships in the Triticeae^1–3,12,13^. Other molecular approaches, including isozyme analysis, variations in low-molecular-weight glutenin subunit and DNA marker systems have provided some explanations on *Aegilops-Triticum* relationships, the origin and differentiation of *Aegilops* species, and intra- and inter-specific variations in the D and U genome clusters of *Aegilops* species^14–17^. Also, a combination of morphology, organelle and nuclear genes reportedly gave insights into the phylogenetic relationships among diploid taxa in Triticeae^18^. Nevertheless, diploid progenitors of *Ae. crassa*, *Ae. vavilovii*, *Ae. juvenalis*, *Ae. columnaris* and *Ae. triaristata*, and the exact progenitor of the B genome of hexaploid wheat and other polyploid *Triticum* species are still in dispute^2,16,19–22^. Also, there are opposing opinions regarding the donors of A genomes of polyploid species in the Emmer (AB group) and Timopheevi (AG group) lineages of wheat^23–29^. In the classification of *Aegilops* species, the justification for including *Ae. speltoides* in section Sitopsis is still under discussion^29–32^. To validate the results so far obtained and fill remaining gaps, more molecular data, especially those with a wide genomic coverage, are needed^31^.

DArTseq is one of the cheap and easy but efficient genotyping-by-sequencing platforms which allow genome-wide marker discovery through restriction enzyme-mediated genome complexity reduction and sequencing of the restriction fragments^33–35^. In this study, we applied DArTseq genotyping to analyze the genomes of 34 species in the tribe Triticeae. DArTseq generates two types of data: SNP and SilicoDArT (http://www.diversityarrays.com/). The former is the nucleotide polymorphisms found in the tag sequences and the latter is the presence/absence variation (PAV) of the tag sequences. The choice of which data to use depends on the research objective. Using both types of data, we clarified the extent of genomic similarity among *Aegilops* species in different sections and clusters and the evolutionary relationships between diploid and polyploid species in *Aegilops* and *Triticum* species. We confirmed the already known genomic constitutions of some polyploid species of *Aegilops* and *Triticum* species, and provided evidence-based explanation for the origin of the unidentified (X) genomes^2,16,19–21^ in *Ae. crassa*, *Ae. columnaris*, *Ae. vavilovii*, *Ae. triaristata*, and *Ae. juvenalis*. The consistency of the outcomes of this study with previous reports and the flexibility of DArTseq genotyping make this marker system suitable for routine applications to analyze plant genomes.

## Results

### Diploid analyzers of polyploid *Aegilops* and *Triticum* species

**Figure 1:**
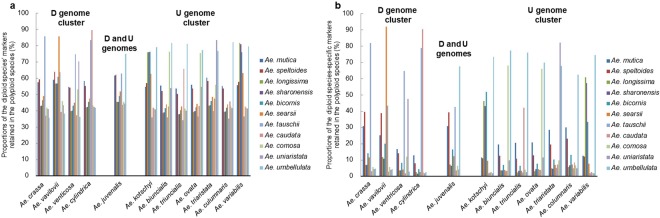
Estimation of the putative diploid progenitors of 12 polyploid *Aegilops* species based on the proportions of diploid species’ SilicoDArT markers retained in the genomes of the polyploid species. (**a**) Analysis based on all the markers of the diploid species. (**b**) Analysis based on the species-specific markers of the diploid species.

To determine the putative progenitors of each of the polyploid species of *Aegilops*, SilicoDArT markers in the diploid genomes of all the *Aegilops* species were used as genome analyzers (Table 1). For each of the diploid species, species-specific markers were selected by filtering markers present in one species but absent in all the others. This made dominant SilicoDArT markers preferred in this analysis, as codominant SNP markers do not give information on PAVs. The progenitors of the polyploid species were estimated based on the proportions of diploid markers that are retained in each polyploid genome (diploid-polyploid monomorphism). Because the number of the species-specific markers is affected by genetic similarity among the diploid species, especially the Sitopsis species, the genomes of the polyploid species of *Aegilops* were first analyzed with all the markers in each diploid genome of *Aegilops* species (Fig. 1a) before being analyzed with diploid species-specific markers (Fig. 1b). This allowed us to determine the suitability of the species-specific markers in estimating the progenitors of the polyploid species. The use of species-specific markers as analyzers reduced the background noise produced by monomorphic markers among the diploid species (Fig. 1b).

**Table 1:** SilicoDArT markers of diploid analyzers of polyploid *Aegilops* species.

Species	Total No. of markers	Species-specific markers (%)
*Ae. mutica*	12238	837 (6.84)
*Ae. speltoides*	9330	699 (7.49)
*Ae. longissima*	18321	761 (4.15)
*Ae. sharonensis*	18205	723 (3.97)
*Ae. bicornis*	16465	598 (3.63)
*Ae. searsii*	15402	1633 (10.60)
*Ae. tauschii*	20288	7420 (36.57)
*Ae. caudata*	19086	6514 (34.13)
*Ae. comosa*	17377	3941 (22.68)
*Ae. uniaristata*	16719	4003 (23.94)
*Ae. umbellulata*	19523	6627 (33.94)

Having confirmed the adequacy of the species-specific markers of the analyzers, polyploid genomes of *Triticum* species were analyzed with only species-specific diploid analyzers (Table 2). Therefore, the conclusions made regarding the progenitors of the polyploid species (*Aegilops* and *Triticum*) are based on the species-specific markers of the analyzers. To select analyzers for the polyploid genomes of *Triticum* species, the genomes of 16 bread wheat-related diploid species were screened on the basis of the proportions of homoeology of their SilicoDArT markers to the total SilicoDArT markers in each of the three genomes of bread wheat. The homoeology of the diploid genomes to each of the genomes of bread wheat was estimated based on the number of markers of the diploid species assigned to each genome of bread wheat (Table 2). This estimation was possible because, in this study, we used DArTseq platform optimized for bread wheat. A diploid species with at least 10% homoeology to any of the three genomes of hexaploid wheat was selected as an analyzer for the corresponding genomes of each of the six polyploid *Triticum* species. With this criterion, a total of 13 analyzers were selected. Species-specific markers of the 13 selected diploid analyzers were then filtered for the analysis of the putative progenitors of the genomes of the polyploid species (Table 2).

**Table 2:** Selection of diploid analyzers of polyploid *Triticum* species with respect to homoeology of their SilicoDArT markers to those of A, B and D genomes of bread wheat.

Species	No. of markers								
	A genome			B genome			D genome		
	Shared	Specific	Total (%)	Shared	Specific	Total (%)	Shared	Specific	Total (%)
Reference genome (bread wheat)	16901	4688	21,589 (100)	13415	7227	20642 (100)	30187	5167	35354 (100)
***T. urartu***	6114	3672	9786 **(45.3)**	1249	95	1344 (6.5)	2763	93	2856 (8.1)
***T. boeoticum***	5492	753	6245 **(28.9)**	1369	71	1436 (7.0)	2870	59	2929 (8.3)
***Ae. mutica***	1824	1348	3172 **(14.7)**	2331	1966	4297 **(20.8)**	4690	2284	6974 **(19.7)**
***Ae. speltoides***	1139	1129	2268 **(10.5)**	2507	4405	6912 **(33.5)**	3335	1883	5218 **(14.8)**
***Ae. longissima***	2301	392	2693 **(12.5)**	3513	755	4268 **(20.7)**	6831	594	7425 **(21.0)**
***Ae. sharonensis***	2294	336	2630 **(12.2)**	3487	769	4256 **(20.6)**	6799	632	7431 **(21.0)**
***Ae. bicornis***	2018	379	2397 **(11.1)**	3026	756	3782 **(18.3)**	6258	638	6896 **(19.5)**
***Ae. searsii***	1915	156	2071 (9.6)	3610	495	3105 **(15.0)**	5558	265	5823 **(16.5)**
***Ae. tauschii***	817	99	916 (4.2)	934	174	1108 (5.4)	8988	7568	16556 **(46.8)**
***Ae. caudata***	1849	1120	2969 **(13.8)**	1955	1013	2968 **(14.4)**	5321	1848	7169 **(20.3)**
***Ae. comosa***	1959	1022	2981 **(13.8)**	2126	1102	3228 **(15.6)**	5545	1904	7449 **(21.1)**
***Ae. uniaristata***	1805	672	2477 **(11.5)**	2049	724	2773 **(13.4)**	5155	1254	6409 **(18.1**)
***Ae. umbellulata***	2014	928	2942 **(13.6)**	2055	1017	3072 **(14.9)**	5174	1653	6827 **(19.3)**
*S. cereale*	699	23	722 (3.3)	959	64	1023 (5.0)	1840	30	1870 (5.3)
*D. villosum*	566	20	586 (2.7)	793	44	837 (4.1)	1538	16	1554 (4.4)
*H. vulgare*	291	8	299 (1.4)	431	19	450 (2.2)	824	9	833 (2.4)

A species whose proportion of markers assigned to any of the bread wheat’s reference genomes (A,B or D) is not less than 10% was selected (emboldened) for the analysis of the corresponding genomes of the polyploid species.

### Genomic differentiation and evolutionary relationships among polyploid and diploid species of *Aegilops*

Before applying the genome analyzers to determine the progenitors of the polyploid species, we used a total of 28,264 polyploid species-specific SilicoDArT markers, ranging from 187 in *Ae. juvenalis* to 4,759 in *Ae. cylindrica* (Table 3), to confirm genomic difference among the 12 polyploid species of *Aegilops*. The polyploid species-species markers were selected in the same manner as the diploid species-specific markers. The relatively low numbers of specific markers in the genomes of *Ae. crassa* and *Ae. juvenalis* is obviously because large proportions of their genomes (D and U genomes) are shared by the other species (Table 3). With the possibility of genomic adjustments during polyploidization^22,36^ and the assumption that the original progenitors of the polyploid species may be different from the accessions of the diploid species used in this study, only diploid analyzers with considerably higher proportions of monomorphism with the polyploid species were taken as the putative progenitors of the polyploid species. Our analysis confirmed the putative diploid progenitors of *Ae. ventricosa* (D^v^N^v^), *Ae. cylindrica* (C^c^D^c^), *Ae. kotschyi* (S^k^U^k^), *Ae. biuncialis* (U^b^M^b^), *Ae. triuncialis* (U^t^C^t^), *Ae. ovata* (U^g^M^g^), and *Ae. variabilis* (S^p^U^p^)^30^ (Fig. 1). Noteworthy is that the proportions of the markers of three Sitopsis species (*Ae. bicornis*, *Ae. longissima*, *and Ae. sharonensis*) retained in the genomes of the two polyploid species with S-related genomes (*Ae. kotschyi* and *Ae. variabilis*) were not reasonably different. This made it difficult to decide which of the Sitopsis species donated the S-related genomes to *Ae. kotschyi* and *Ae. variabilis*, although *Ae. bicornis* and *Ae. longissima*, respectively, seem to be the most likely candidates. This observation confirms the likelihood of a common ancestry of the Sitopsis species^31^. Therefore, the original progenitor of the S-related genomes of the polyploid species may have been an ancient relative of the Sitopsis species, which is probably extinct. Although we adopted the polyploid species-specific markers (Table 3) to differentiate *Ae. kotschyi* (S^k^U^k^) from *Ae. variabilis* (S^p^U^p^) and *Ae. biuncialis* (U^b^M^b^) from *Ae. ovata* (U^g^M^g^), these pairs of species have identical genomic constitutions (same progenitors; Fig. 1), and therefore may be considered to be variants/subspecies of the same species in each case.

**Table 3:** Species-specific SilicoDArT markers of 12 polyploid species of *Aegilops*.

Species	Ploidy	Genome^2,3,16,19–21,31^	No. of markers
*Ae. crassa*	6x	D^cr1^D^cr2^X^cr^	684
*Ae. vavilovii*	6x	D^va^X^va^S^va^	2153
*Ae. ventricosa*	4x	D^v^N^v^	3027
*Ae. cylindrica*	4x	CD	4759
*Ae. juvenalis*	6x	X^j^D^j^U^j^	187
*Ae. kotschyi*	4x	S^k^U^k^	2271
*Ae. biuncialis*	4x	U^b^M^b^	2601
*Ae. triuncialis*	4x	C^t^U^t^	2051
*Ae. ovata* L.	4x	U^g^M^g^	3163
*Ae. triaristata*	6x	U^n^X^n^N^n^	2215
*Ae. columnaris*	4x	U^c^X^c^	2470
*Ae. variabilis*	4x	S^p^U^p^	2683
**Total**	—	—	**28264**

The unidentified diploid progenitors of five polyploid *Aegilops* species (*Ae. triaristata* [U^n^X^n^, U^n^X^n^N^n^], *Ae. crassa* [D^cr^X^cr^, D^cr1^D^cr2^X^cr^], *Ae. juvenalis* [X^j^D^j^U^j^], *Ae. vavilovii* [X^va^D^va^S^va^], and *Ae. columnaris* [U^c^X^c^])^2,16,19–21^ are most likely to be traced to *Ae. speltoides* or *Ae. mutica*. The competing proportions of monomorphic markers between each of the two diploid species (*Ae. speltoides* and *Ae. mutica*) and the genomes of the five polyploid species (Fig. 1) strongly suggest that the unidentified genomes may have been donated by an ancient species closely related to these two diploid species or their direct ancestor(s). Based on these results, we have proposed modifications in the genomic representations of *Ae. crassa*, *Ae. juvenalis*, *Ae. vavilovii*, *Ae. columnaris*, and *Ae. triaristata* (Table 4), in which the genomes of *Ae. speltoides* and *Ae. mutica* are jointly represented as T^s^. This does not suggest that the two diploid species are genomically the same, but shows that we could not, in this study, clearly determine which of the two species may have donated the controversial genome to the five polyploid species. In this proposal, the genome of tetraploid *Ae. crassa* is considered to be constituted of *Ae. tauschii* and T^s^ genomes, while the genome of hexaploid *Ae. crassa* is designated as D^1^D^2^T^s^ (tetraploid *Ae. crassa* x *Ae. tauschii*). *Aegilops vavilovii* is considered to have evolved from the hybridization of tetraploid *Ae. crassa* and *Ae. searsii*, granted that *Ae. crassa* and *Ae. vavilovii* have similar morphological traits and are reported to be sympatric^37^. Furthermore, our analysis shows that *Ae. juvenalis* has T^s^, D and U genomes; hexaploid *Ae. triaristata*, clearly lacks *Ae. comosa* genome but has the genomes of *Ae. umbellulata*, *Ae. uniaristata* and T^s^, and *Ae. columnaris* is composed of *Ae. umbellulata* and T^s^ genomes.

**Table 4:** Proposed modifications in the genomic representations of five polyploid *Aegilop*s species.

Species	Ploidy	Reported genomic formula^2,3,16,19–21^	Proposed genomic formula
*Ae. crassa*	4x, 6x	D^cr^X^cr^, D^cr1^D^cr2^X^cr^	DT^S^, D^1^D^2^T^S^
*Ae. vavilovii*	6x	D^va^X^va^S^va^	DT^S^S^S^
*Ae. juvenalis*	6x	X^j^D^j^U^j^	UDT^S^
*Ae. triaristata*	4x, 6x	U^n^X^n^, U^n^X^n^N^n^	UN, UNT^S^
*Ae. columnaris*	4x	U^c^X^c^	UT^S^

T^S^: joint representation of *Ae. speltoides* and *Ae. mutica*.

### Cluster analysis of diploid and polyploid *Aegilops* species

**Figure 2:**
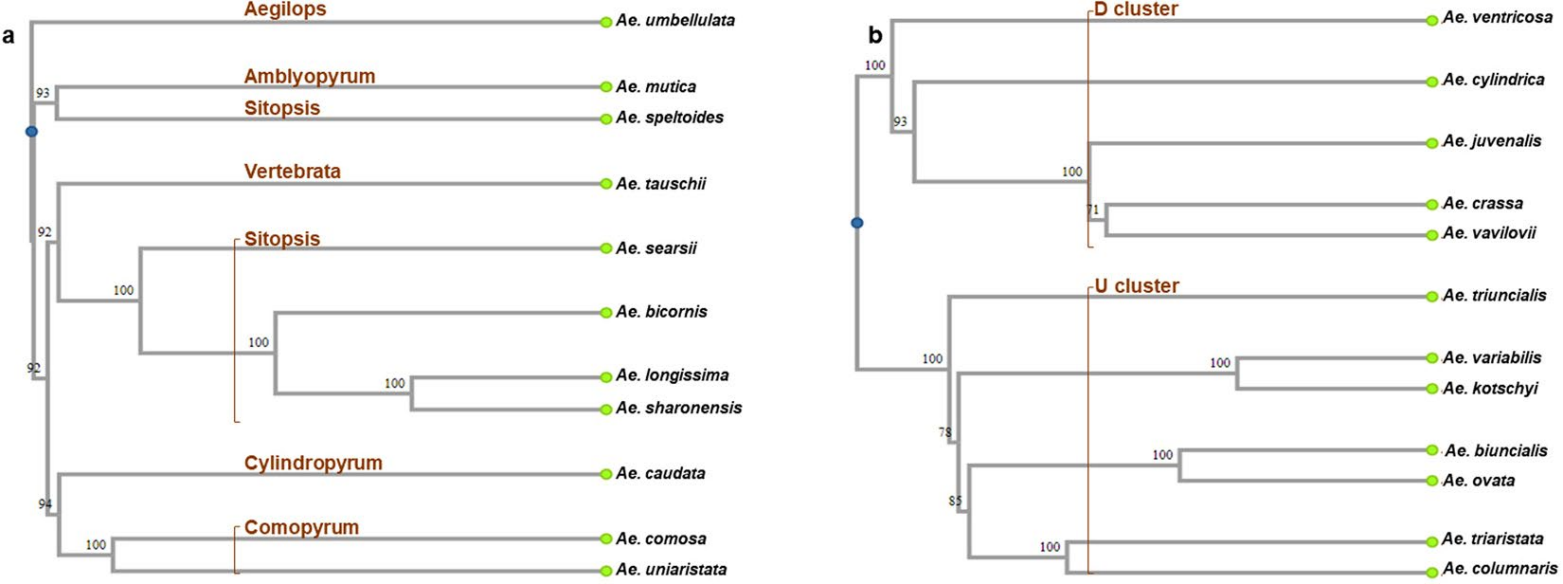
Reconstruction of the evolutionary relationships among *Aegilops* species on the basis of 15,512 SNP markers. (**a**) 11 diploid species. (**b**) 12 polyploid species. The sections of the diploids and two main clusters of the polyploids are labelled in brown^1,16,31^.

A phylogenetic tree (Fig. 2a) constructed with 15,512 frequently called SNP markers separated the diploid *Aegilops* species into the already reported sections^30^, except that *Ae. speltoides* did not cluster with other species in the section Sitopsis, which has been reported by other researchers^18,29,32^ (see online Supplementary Table S1 for information of the markers). *Aegilops umbellulata* (section Aegilops) seemed more distant from the others, whereas *Ae. speltoides* (section Sitopsis) appeared closer to *Ae. mutica* (section Amblyopyrum), and relatively distant from other species of section Sitopsis. Among Sitopsis species, *Ae. longissima* and *Ae. sharonensis* appeared genomically more proximal to each other than to others. The polyploid species of *Aegilops* formed two clusters clearly based on the putative common diploid progenitors, *Ae. tauschii* (D cluster) and *Ae. umbellulata* (U cluster) (Fig. 2b). *Aegilops juvenalis*, bearing both D and U genomes, clustered closely with *Ae. crassa* and *Ae. vavilovii* in the D cluster, indicating a possible evolutionary link between its (*Ae. juvenalis*) genome and the two species in the D cluster. This again suggests the likelihood of the presence of a diploid genome, perhaps T^s^, common to *Ae. juvenalis*, *Ae. crassa* and *Ae. vavilovii* (Fig. 1).

### Genomic and evolutionary relationships in the *Aegilops*-*Triticum* species

**Figure 3:**
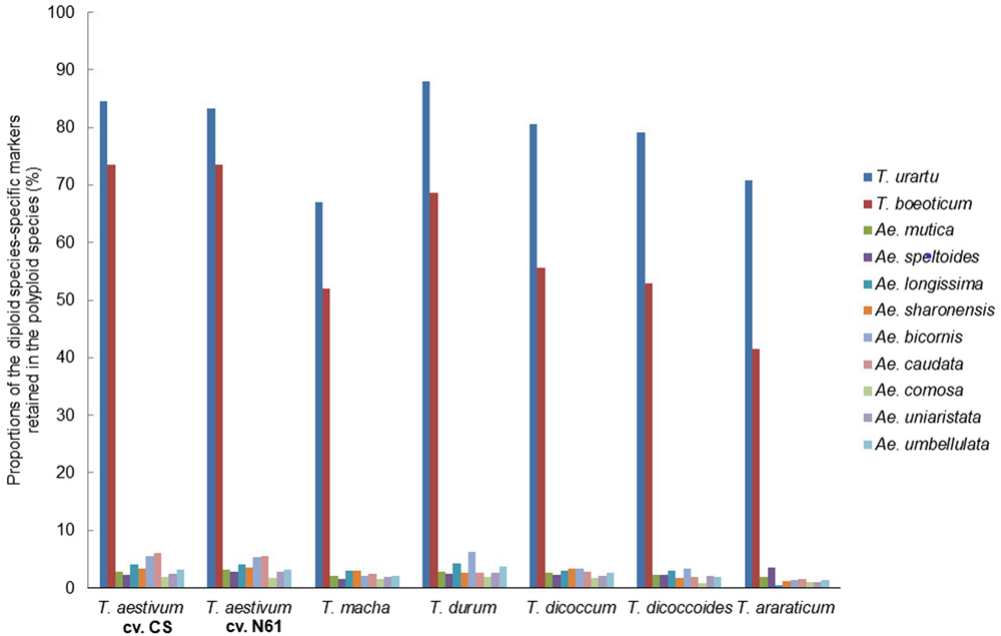
Determination of the donor of the A genomes of six polyploid *Triticum* species using 11 diploid species-specific SilicoDArT markers assigned to the A genome of bread wheat.

**Figure 4:**
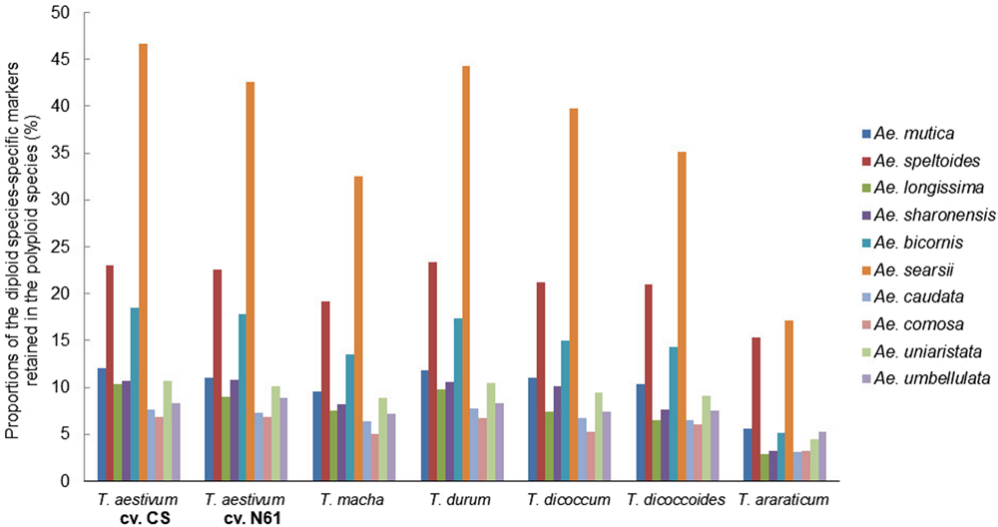
Determination of the donor of the B/G genomes of six polyploid *Triticum* species using 10 diploid species-specific SilicoDArT markers assigned to the B genome of bread wheat.

**Figure 5:**
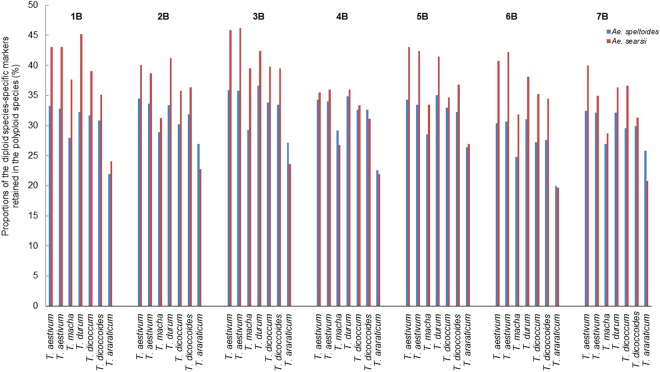
Comparison of the B/G genome chromosomes of polyploid *Triticum* species and the chromosomes of *Ae*. *speltoides* and *Ae. searsii*. The B/G chromosomes are numbered 1B–7B for convenience. This does not change the genomic representation of *T. araraticum*: AAGG.

**Figure 6:**
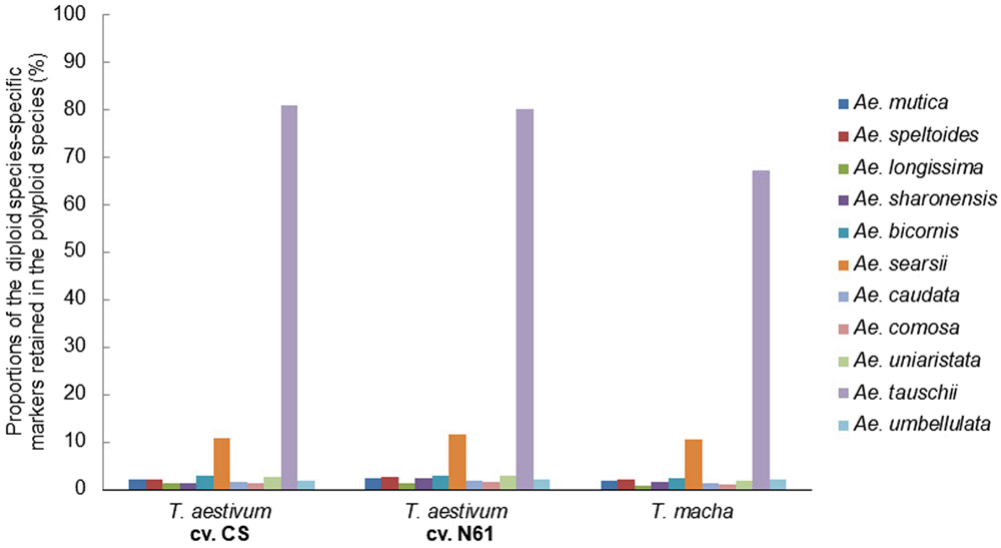
Determination of the donor of the D genomes of two hexaploid *Triticum* species using 11 diploid species-specific SilicoDArT markers assigned to the D genome of bread wheat.

We used species-specific SilicoDArT markers of 13 bread wheat-related diploid species (Table 2) to determine the elementary donors of the A, B and D genomes in six polyploid *Triticum* species. As described for the estimation of the progenitors of the polyploid species of *Aegilops*, the proportions of species-specific markers of the diploid species shared with the genomes of the polyploid species enabled the determination of the progenitors of the genomes of the polyploid *Triticum* species (Figs 3–6).

The genome of *T. urartu* was the closest to the A genomes of all the polyploid species analyzed (Fig. 3), suggesting that *T. urartu* is the most likely donor of the A genome in each of them. The considerable similarity between the A genome of each of the polyploids and *T. boeoticum –*another A genome species *–*suggests a common ancestry of *T. boeoticum* and *T. urartu*. Similarly, *Ae. searsii* seems to be the most closely related to the B/G genomes of the polyploid species (Fig. 4). However, the proportion of *Ae. speltoides* markers assigned to the reference B genome is higher than those of every other diploid species analyzed (Table 2). This strongly suggests an evolutionary link between the genome of *Ae. speltoides* and the B/G genomes of the polyploids. This link is further supported by an almost equal similarity of the genomes of the two diploid species to the G genome of *T. araraticum* (Fig. 4). Using the species-specific markers of *Ae. speltoides* and *Ae. searsii* as analyzers, we found that chromosome 4 S of each of the diploid species are almost equally similar to chromosome 4B/G of each of the polyploids (Fig. 5). But chromosomes 2 S, 3 S and 7 S of *Ae. speltoides* appeared to be more similar to the corresponding chromosomes of *T. araraticum* than those of *Ae. searsii* are. These observations give the impression that the B/G genomes of polyploid *Triticum* species are likely to be recombinant genomes with varying contributions from *Ae. speltoides* and *Ae. searsii*. Analysis of the D genomes of the three hexaploid species unambiguously traced them to *Ae. tauschii* as the sole donor (Fig. 6).

A further analysis using 66, 434 SNP markers consistently called in the six polyploid genomes (see online Supplementary Table S2 for information of the markers) indicated 72% similarity (monomorphism) across their A genomes, B/G genomes and the combined AB/AG genomes. However, higher similarity was observed among the AB genomes: hexaploids, 94%; tetraploids, 90%; hexaploid and tetraploid genomes combined, 84%. The slight differences in the proportions of monomorphic markers in the different groups of the AB genomes suggest that the AB genomes of the hexaploid species originated from the same tetraploid species, whereas those of the tetraploid species may have evolved from different accessions of the elementary A and B genome progenitors (*T. urartu* and *Ae. speltoides/Ae. searsii*, respectively). The lower similarity (84% as compared to 94%) across the hexaploid and tetraploid AB genomes may reflect further modification of AB genomes in hexaploid species resulting from their interaction with the D genome.

## Discussion

The clustering patterns of *Aegilops* species were largely consistent with the established classifications^25,38,39^. Diploid species separated on the basis of their known sections in the genus; polyploid species were delineated following the presence of common diploid progenitor genomes (D and U genomes) among them^1,16,30^. However, as reported previously^18,29,32^, *Ae. speltoides* appeared distant from other species in the section Sitopsis; hence, its inclusion in the section needs to be reconsidered.

Markers specific to each of the 12 polyploid species clearly showed considerable polymorphisms among these genomes, including the genomes of species which arose from the same diploid progenitors. This suggests that genetic modifications, such as chromosomal alterations^2^, may have occurred during independent evolutionary events of those species with identical progenitors. Therefore, without these specific markers, it would be difficult to genomically differentiate *Ae. kotschyi* (S^k^U^k^) from *Ae. variabilis* (S^p^U^p^) and *Ae. biuncialis* (U^b^M^b^) from *Ae. ovata* (U^g^M^g^) because, from the stand point of our result (Fig. 1) and previous studies^30,31^, the species in each pair evolved from the same progenitors. Although each of the species in these two sets are recognized as independent, on the basis of differences in cytoplasm progenitors and/or nuclear genome variation^31,40^, this classification does not seem to be clearly justified. Therefore, in our opinion, each pair should be regarded as variants/subspecies of the same species.

The reported unknown diploid genomes, initially represented as modified M genome and later changed to X, in the genomes of *Ae. triaristata*, *Ae. crassa*, *Ae. juvenalis*, *Ae. vavilovii*, and *Ae. columnaris*^2,16,19–21,30^ is traceable to *Ae. mutica* or *Ae. speltoides*. The small proportions (<10%) of *Ae. comosa*-specific markers shared with the five polyploid species (Fig. 1b) is insufficient to infer the existence of remnants of *Ae. comosa* genome in the polyploid genomes. Assuming *Ae. comosa* was originally involved in the evolution of the polyploids, species-specific elements from other progenitors may have spread and eventually masked *Ae. comosa-*specific elements^41^. Our data suggest that ancient or ancestral forms of *Ae. speltoides* or *Ae. mutica*, which are probably extinct, donated the unidentified genomes to the five polyploid species. From our analysis, it appears that all the polyploid species originally had a genome of such an ancient species (Fig. 1). This observation agrees with the hypothesis that *Ae. mutica* (syn*. Amblyopyrum muticum*) and *Ae. speltoides*, both allogamous species with ancestral traits, diverged earlier than other *Aegilops s*pecies and may therefore be the ancestors of the other *Aegilops* species^22^. Therefore, each diploid *Aegilops* species may have retained a substantial portion of the common ancestral genome (*Ae. speltoides/Ae. mutica* or their ancestor). The difference in the representation of the common progenitor in each of the polyploid species can result from the peculiar evolutionary event(s) of each species. Polyploids that arose from the hybridization of the common diploid ancestor with other diploid species should have larger portions of the genome of the common ancestor than those that did not directly evolve from the common ancestor.

We validated the putative diploid progenitors of the A and D genomes of polyploid *Triticum* species to be *T. urartu* and *Ae. tauschii*, respectively^42–46^. We have also provided information that may help to explain the complex nature of the B/G genomes. The genomes of both *Ae. speltoides* and *Ae. searsii* are similar to the B/G genomes, especially to that of *T. araraticum* (Fig. 4), a relatively less advanced tetraploid genome^47,48^; thus, the B/G genomes of polyploid *Triticum* species may have evolved from an ancestral genome that later differentiated into those of *Ae. speltoides* and *Ae. searsii*. Alternatively, the B/G genome may have arisen from the hybridization of *Ae. speltoides* and *Ae. searsii* before the emergence of the AB/AG genome at different times. The above considerations support earlier postulations that the B genome is the most modified of the three genomes of hexaploid wheat, whereas the A and D genomes are substantially similar to those of *T. urartu* and *Ae. tauschii*, respectively^22^. The previously suggested origin of the A genome of *T. araraticum* from *T. boeoticum*^23,24^ is probably invalid (Fig. 3). Our results agree with the hypothesis that both Emmer and Timopheevi lineages of polyploid wheats have the same sources of elementary A and B/G genomes^25–29^. However, a common ancestry of the A-genome species cannot be ruled out and the A genomes of polyploid *Triticum* species may have evolved from a common ancestor of *T. urartu* and *T. boeoticum* before the differentiation of the two species. Although no karyotypic differences have been detected between these diploid A-genome species^25,49^, low fertility of interspecific F_1_ hybrid plants of these two species has been reported^50^. The latter study, consistent with our result, confirms that the two species are genomically different. As previously documented^47,48^, our study suggests that the A and G genomes in *T. araraticum* are less modified than the A and B genomes in the Emmer lineage.

This study has demonstrated that DArTseq genotyping can be applied to conduct a large scale analysis of plant genomes, mostly because it allocates markers to individual chromosomes, which can be easily extracted and analyzed. This genomic sequence-based platform ensures a wide genomic coverage and is not subject to criticism associated with the factors that affect meiotic chromosome pairing in hybrids of distant crosses, which forms the main anchor of cytogenetic systems of genome analysis^9–11,51–54^. Also, the number of informative markers generated by DArTseq outstrips what is possible with conventional DNA marker procedures^14–17^, making it more robust and reliable. Genotyping of all the available accessions of species in tribe Triticeae using this platform would clarify the genomic relationships between the cultivated and wild species. This information would make the use of the available gene pools for breeding much more precise and would also help to clarify Triticeae taxonomy. As polyploidy and interspecific hybridization are key events in the evolution of higher plants^55^, this genome analysis approach would be useful in other groups of plant species, especially polyploids with unclear phylogeny.

## Methods

### Plant materials

All 23 *Aegilops* species, eight *Triticum* species, and three distant relatives of wheat were analyzed. Except bread wheat, represented by two cultivars ‘Chinese Spring’ (CS) and ‘Norin 61’ (N61), each species was represented by one accession (Table 5). Seedlings were raised in Greenhouses of the Arid Land Research Center, Tottori University and Laboratory of Plant Genetics, Kyoto University, Japan. Depending on growth rate and plant size, fresh leaves were harvested from each 2–4-week-old seedlings and genomic DNA samples were isolated and purified using the cetyl trimethyl ammonium bromide method. Quality check, quantification and concentration adjustment for sequencing and genotyping were accomplished with NanoDrop2000C Spectrophotometer (ThermoScientific). The concentration of each sample was adjusted to 50 ng/μL.

**Table 5:** List of plant materials.

ID	Species	Subspecies/cultivar	Ploidy	Genome	Source
KU–12007	*Ae. mutica* Boiss.	—	2x	T	NBRP
KU–2–5	*Ae. speltoides* Tausch	*typica*	2x	S	NBRP
KU–4–1	*Ae. longissima* Schweinf. et Muschl	*typica*	2x	S^l^	NBRP
KU–5–3	*Ae. sharonensis* Eig	*typica*	2x	S^sh^	NBRP
KU–4–6	*Ae. searsii* Feldman et Kilev ex Hammer	—	2x	S^s^	NBRP
KU–3–1	*Ae. bicornis* (Forssk.) Jaub. et Sp.	*typica*	2x	S^b^	NBRP
KU–2159	*Ae. tauschii* Coss.	*typica*	2x	D	NBRP
KU–5860	*Ae. caudata* L.	*polyathera*	2x	C	NBRP
KU–17–1	*Ae. comosa* Sibth. et Sm.	*comosa*	2x	M	NBRP
KU–19–3	*Ae. uniaristata* Vis.	*typica*	2x	N	NBRP
KU–8–2	*Ae. umbellulata* Zhuk.	*typica*	2x	U	NBRP
KU–7–1	*Ae. cylindrica* Host.	*typica*	4x	CD	NBRP
KU–22–1	*Ae. ventricosa* Tausch	*comosa*	4x	D^v^N^v^	NBRP
KU–9–1	*Ae. ovata* L.	*vulgaris*	4x	U^g^M^g^	NBRP
KU–11–1	*Ae. columnaris* Zhuk.	*typica*	4x	U^c^M^c^	NBRP
KU–13–6	*Ae. kotschyi* Boiss.	*leptostachya*	4x	S^k^U^k^	NBRP
KU–15–1	*Ae. triuncialis* L.	*typica*	4x	C^t^U^t^	NBRP
KU–13–1	*Ae. variabilis* Eig	*intermedia*	4x	S^p^U^p^	NBRP
KU–12–1	*Ae. biuncialis* Vis.	*typica*	4x	U^b^M^b^	NBRP
KU–10–1	*Ae. triaristata* Willd.	*vulgaris*	6x	U^n^M^n^N^n^	NBRP
KU–21–1	*Ae. crassa* Boiss.	*typica*	6x	D^c^M^c^, D^c^D^c^M^c^	NBRP
KU–21–7	*Ae. vavilovii* (Zhuk.) Chennav.	*palaestina*	6x	D^v^M^v^S^v^	NBRP
KU–23–3	*Ae. juvenalis* (Thell.) Eig	*typica*	6x	D^j^M^j^U^j^	NBRP
KU–199–11	*T. urartu* Thum. Ex. Gandil.	*nigrum*	2x	A^u^	NBRP
KT001–001	*T. boeoticum* Boiss.	*boeoticum*	2x	A^b^	NBRP
KT009-17	*T. durum* Desf.	Langdon KU	4x	AB	NBRP
KU–491	*T. dicoccum* (Schrank) Schuebl.	—	4x	AB	NBRP
KU–108–1	*T. dicoccoides* (Koern. Ex Aschers & Graebn.) Schweif	*kotschyanum*	4x	AB	NBRP
KU–196–1	*T. araraticum* Jakubz.	*tumaniani*	4x	AG	NBRP
KT020-003	*T. aestivum* L.	Chinese Spring	6x	ABD	NBRP
KU-260	*T. aestivum* L.	Norin 61	6x	ABD	NBRP
KT018–002	*T. macha* Dekapr. & Menabde	*palaeoimereticum*	6x	ABD	NBRP
TACBOW0071	*S. cereale*	Pektas	2x	R	NBRP
TACBOW0119	*D. villosum*	—	2x	V	NBRP
TACBOW0116	*H. vulgare*	Betzes	2x	H	NBRP

Names and genome symbols of *Aegilops* and *Triticum* species are as in Kilian *et al*.^31^, Bernhardt^58^, and Zhang *et al*.^59^. TACBOW: Tottori Alien Chromosome Bank of Wheat. NBRP: National BioResource Project-Wheat (https://shigen.nig.ac.jp/wheat/komugi/). Additional information on the distant relatives of wheat can be found in Hagras *et al*.^60^.

### Genotyping-by-sequencing and data analysis

Purified DNA samples (1 μg for each sample) were sent to Diversity Arrays Technology Pty Ltd (http://www.diversityarrays.com/) for sequencing and marker identification. Sequences of the genomic representations were aligned to the wheat_ChineseSpring10 reference genome and wheat_ConsensusMap_version_4. This is because our analysis is based on DArTseq platform optimized for hexaploid wheat. DArTseq is a genotyping-by-sequencing system which utilizes Next-Generation-Sequencing platforms (HiSeq. 2500 in our case) to sequence the most informative representations of genomic DNA samples to aid marker discovery. In comparison to the array version of DArT, DArTseq results in higher marker densities^56^. The high marker number generated by this system gives it an edge over previous molecular marker procedures applied for genomic analysis of Triticeae species^14–17^. It, therefore, serves as a cheap alternative to genome analysis by whole genome sequencing, where the sequence information of genomes intended to be analyzed are not available. Two types of data are generated by DArTseq: SNP and SilicoDArT. SNP markers are nucleotide polymorphisms present in the restriction fragments, while SilicoDArT markers represent PAV of the restriction fragments. Therefore, codominant SNP markers are scored “0” (reference allele homozygote), “1” (SNP allele homozygote) and “2” (heterozygote: presence of both reference and SNP alleles), while dominant SilicoDArT markers are scored in a binary fashion, with “1” representing presence of the restriction fragment with the marker sequence and “0” designating its absence.

Frequently called SNP markers (>0.9 call rate) were used for phylogenetic tree constructions and differentiation of the genomes of polyploid species of *Aegilops*, whereas SilicoDArT markers (>0.7 call rate) were used for the determination of putative progenitors of the polyploid *Aegilops* and *Triticum* species. This reduction in call rate was made to accommodate more markers, ensure wider genomic coverage and reduce bias. To estimate the phylogenetic relationships among the 11 diploid and 12 polyploid *Aegilops* species, the raw genotypic data of the two sets (diploid and polyploid) were subjected to cluster analysis. Pearson’s correlation coefficient (**r**) was used as similarity index, and the genetic distances among the species were estimated by transforming the **r** values to distance values, using **d** = **100(1 – r)** (http://genomes.urv.cat/UPGMA/)^57^. Species-specific SilicoDArT markers of the polyploid species of *Aegilops* were used to differentiate their genomes, while species-specific SilicoDArT markers of diploid species of *Aegilops* were used to estimate the diploid-polyploid evolutionary relationships among all the *Aegilops* species. Diploid *Triticum* and *Aegilops* species whose total SilicoDArT markers showed at least 10% homoeology to the total SilicoDArT markers in any of the three genomes of hexaploid wheat were selected as analyzers to determine the putative progenitors of the corresponding genomes of each polyploid *Triticum* species. Species-specific SilicoDArT markers of these selected diploid species were used as analyzers to determine the putative progenitors of each polyploid *Triticum* species. In determining the progenitors of all the polyploid species (*Aegilops* and *Triticum*), the proportions of the species-specific markers of the diploid analyzers retained in the genomes of the polyploid species were used as a basis to draw conclusions on genomic proximity and evolutionary relationships among the species. Species-specific markers of *Ae. speltoides* and *Ae. searsii* were further used to examine the relationship between the seven B/G-genome chromosomes of each of the polyploid *Triticum* species and those of the two diploid species. The two diploid species were chosen based on the close proximity of their genomes to the B/G genomes of the polyploid species.

## Electronic supplementary material

Supplemental information

## Data Availability

The data on which are conclusions are based are included within the article and supplementary files and plant materials can be sourced from KOMUGI database maintained by the National BioResource Project – Wheat, Japan (https://shigen.nig.ac.jp/wheat/komugi/).
