# Supplementary material for: DArTseq-based analysis of genomic relationships among species of tribe Triticeae
Source: Sci Rep. 2018 Nov 6;8:16397. doi: 10.1038/s41598-018-34811-y (PMC6219600; doi:10.1038/s41598-018-34811-y)
Supplement: Supplementary file 1 — Supplemental information [file 41598_2018_34811_MOESM1_ESM.pdf]

DArTseq-based analysis of genomic relationships among species of tribe Triticeae

Offiong U. Edet, Yasir S. A. Gorafi, Shuhei Nasuda and *Hisashi Tsujimoto (tsujim@tottori-u.ac.jp)

Table S1 The 15,512 SNP markers applied to genotype the 23 *Aegilops* species

Allele ID	Trimmed sequence	SNP
1322186|F|0-62:G>T-62:G>T	TGCAGACCAGGATAGCCTCAGATCAAAACCTAATAAATGTACCTGGGGTTGATCATATTTTTGGGGCAA	62:G>T
1342318|F|0-37:C>A-37:C>A	TGCAGACTCTACTGGTGGTATTTCCCGAGGCGAGGGTCAATATGTGCATTCATATCGGTCAGGAAACGA	37:C>A
1322186|F|0-18:C>A-18:C>A	TGCAGACCAGGATAGCCTCAGATCAAAACCTAATAAATGTACCTGGGGTTGATCATATTTTTTGGGCAA	18:C>A
1321642|F|0-67:T>G-67:T>G	TGCAGGACCCAACACAAGTATTCATCCTTTCCAATCTGAAAAAGGTGTTGCCGAAAGCTTACCCTCTTC	67:T>G
1322200|F|0-65:T>G-65:T>G	TGCAGCCACAGCAATCAGAGTACGAACAGAAATTGAAACAACGCAACCGTACTATCTATTTACGATAAT	65:T>G
1322200|F|0-60:T>G-60:T>G	TGCAGCCACAGCAATCAGAGTACGAACAGAAATTGAAACAACGCAACCGTACTATCTATTTACGATAAT	60:T>G
1321642|F|0-38:A>C-38:A>C	TGCAGGACCCAACACAAGTATTCATCCTTTCCAATCTGAAAAAGGTGTTGCCGAAAGCTTACCCTCTTC	38:A>C
1319312|F|0-63:T>G-63:T>G	TGCAGCTGCTGGTACTTTATCTCTGTCGCTTTCAACTCCCACTCCTTCTCCCTTTGGTTTCTATAGGAA	63:T>G
1322190|F|0-62:G>T-62:G>T	TGCAGCACCTACATGGCCACATTTGCACTAACTTTGTTCACTGCTTGGTTTGACTGAGCTGTGAAAGAA	62:G>T
1329644|F|0-29:A>T-29:A>T	TGCAGTAGCTAGAAAAGGTCTCCCCAAAAAAAAATAATAGGAAAGCTCTTATCCTACATCTCCAAGACA	29:A>T
1342327|F|0-63:T>C-63:T>C	TGCAGCTTCGCTGGGCTTTACCCGCGTGGGCGCGGGCTTCTATAAGAGAATGAAAAGCTCTCTTTTTAC	63:T>C
5369712|F|0-16:C>T-16:C>T	TGCAGTTCTTCCTGATCTCGCCCTGCGACCCG	16:C>T
4261115|F|0-24:T>C-24:T>C	TGCAGCCTGCCTCTTCTTTAATTGTAGTATCATGCACTGACCG	24:T>C
4261115|F|0-12:C>T-12:C>T	TGCAGCCTGCCTCTTCTTTAATTGTAGTATCATGCACTGACCG	12:C>T
3947593|F|0-17:G>A-17:G>A	TGCAGGATCTTCAGGAGGGCCATCACCTACCG	17:G>A
3939222|F|0-9:G>A-9:G>A	TGCAGCTCTGGGAGAACAACTTCACCG	9:G>A
6050138|F|0-20:C>T-20:C>T	TGCAGGCCAAGGGGAGCTGCCCCATGTGCCG	20:C>T
1306353|F|0-7:G>C-7:G>C	TGCAGGGGTACATCAAGAGGATGAAGAAGCTGAGCAAGATGTTCGACCG	7:G>C
1016230|F|0-26:G>A-26:G>A	TGCAGGTCTTCATGCGGCTGAAGTACGTAGAGCTCGGCGGCGACGTGCGAAAATACCG	26:G>A
3957382|F|0-12:A>C-12:A>C	TGCAGTGCGAAGAAACAATGTAATCAGCCG	12:A>C
100554760|F|0-17:G>C-17:G>C	TGCAGCTGGGACTATATGCAGGAGGGAGGCCAGGTGATGAAGGACATGGACCG	17:G>C
4261260|F|0-21:T>A-21:T>A	TGCAGGATAAGCAATTATGCGTACGCGTATTCGCCCG	21:T>A
3937560|F|0-8:A>G-8:A>G	TGCAGTGCACGAGAACGGGTTGGCCTGCAAGGACCCG	8:A>G
3950952|F|0-5:G>A-5:G>A	TGCAGGTACGGGAGGCTGTTCCG	5:G>A
4405250|F|0-20:C>T-20:C>T	TGCAGGGCGTGCAGGCCAAGCTGCGCCG	20:C>T
3940229|F|0-25:C>T-25:C>T	TGCAGTCCTTCCTGACCTCCCCCTGCCCGCCG	25:C>T
7353643|F|0-9:A>G-9:A>G	TGCAGGTCAACAGCCTCGCCG	9:A>G
5581467|F|0-20:C>A-20:C>A	TGCAGGAGCGGGCGACGAGGCGGAGCGCGTCGGTGGAGAAGCCG	20:C>A
1013536|F|0-40:C>G-40:C>G	TGCAGAACCATTGTGCGATGCAGCTTTCGTGGAAGGAGTGCAAGCAGCCG	40:C>G
4262112|F|0-19:G>T-19:G>T	TGCAGTGCACGAGAACGGGGTGGCGTGCAAGGACCCG	19:G>T
4261148|F|0-5:C>T-5:C>T	TGCAGCGCCATTGGCTTTTCTTATTTGACCG	5:C>T
3935466|F|0-32:C>G-32:C>G	TGCAGGTACACCTTCTGGCTGACGCTCTGGTACGGCCGCGTCCG	32:C>G
4439683|F|0-13:A>G-13:A>G	TGCAGTGCCAAGAAACAGTATAATCACCCG	13:A>G
4261611|F|0-14:C>A-14:C>A	TGCAGGTACGGCATCTGCGCCACGTCCG	14:C>A
3959878|F|0-15:C>A-15:C>A	TGCAGCAAGCTCCTCCCCGTCGATCCG	15:C>A
3959878|F|0-11:C>T-11:C>T	TGCAGCAAGCTCCTCCCCGTCGATCCG	11:C>T
3944277|F|0-9:C>G-9:C>G	TGCAGTTGACGACGAGGATGTCGATGTCCTCCG	9:C>G
6048558|F|0-15:C>A-15:C>A	TGCAGCCAACGCCATCGCCGAGTGGGGCCGTCCG	15:C>A
1214075|F|0-34:G>A-34:G>A	TGCAGACGAGCTTGCAGCTGGGGCAGTGCACGTAGCAGAGGTCGCGGCAGCCG	34:G>A
3020926|F|0-19:G>A-19:G>A	TGCAGGATTTCTTTGACGGGAAGAAGCTCTGCAAGAAAATTAATCCG	19:G>A
3940722|F|0-15:G>T-15:G>T	TGCAGAGGCTGCCATGGCCG	15:G>T
3941970|F|0-20:G>C-20:G>C	TGCAGCCACGCGACCTGCTCGGCGTACTTCTCCG	20:G>C
3944277|F|0-12:C>T-12:C>T	TGCAGTTGACGACGAGGATGTCGATGTCCTCCG	12:C>T
4990868|F|0-28:A>C-28:A>C	TGCAGGTATGTGTGGTAGGGCAGCAGGGACCG	28:A>C
3020926|F|0-37:A>T-37:A>T	TGCAGGATTTCTTTGACGGGAAGAAGCTCTGCAAGAAAATTAATCCG	37:A>T
4261297|F|0-29:C>T-29:C>T	TGCAGTAAATCTGACACTGACTAGGTTTTCCATTCACCG	29:C>T
4990868|F|0-25:G>A-25:G>A	TGCAGGTATGTGTGGTAGGGCAGCAGGGACCG	25:G>A
32017733|F|0-20:T>C-20:T>C	TGCAGCTTGGGGACGACGGATTTGAACCG	20:T>C
4261638|F|0-8:G>C-8:G>C	TGCAGCTCGTCATCAAGGAGACGCTCCG	8:G>C
3939871|F|0-6:C>A-6:C>A	TGCAGCCACAGTAGATGGCCTGTAATCCAGCACACTGCCG	6:C>A
4005821|F|0-21:C>G-21:C>G	TGCAGCTTGATGATCTTCATGCTGTTGAGCACCTCGGACGTGGACCG	21:C>G
4538356|F|0-8:T>C-8:T>C	TGCAGGTATGTCAGGGTCAGAGCCG	8:T>C
2245281|F|0-5:G>A-5:G>A	TGCAGGCGGCTCTTGAAGCTGGGGTCTGAAATTTGATCCCAGAACTCACGCAGCTCCGCCTTGTCGATG	5:G>A
1153800|F|0-33:C>T-33:C>T	TGCAGCAGAGTCCTACGGGTTCGTCGCTCTCTGCATTTCATGCCATCCG	33:C>T
5582524|F|0-10:G>C-10:G>C	TGCAGCAGGAGGTTGTGGCTACGGAAGTGAACCCCG	10:G>C

1686962|F|0-8:G>A-8:G>A	TGCAGTGAGAAGATGGATCAATTTCTGAAGAGATGCTTCTATCAGTCCG	8:G>A
1052650|F|0-11:C>G-11:C>G	TGCAGCAGCATCACGCCCAGCGAGTAGACGTCCGACTTGACGCCCAGCATCCCCGTCTGCTGGTACTCC	11:C>G
3959878|F|0-9:C>G-9:C>G	TGCAGCAAGCTCCTCCCCGTCGATCCG	9:C>G
3959878|F|0-18:G>A-18:G>A	TGCAGCAAGCTCCTCCCCGTCGATCCG	18:G>A
5002548|F|0-17:A>G-17:A>G	TGCAGCTGCGGGCTGCTAAACAGCTTCATCGGCACCGTGCCGTAGAGTCTTCCG	17:A>G
3026246|F|0-26:T>G-26:T>G	TGCAGCACCACGTCCACCACGGAGCCTAGCGCAAGCCG	26:T>G
3938059|F|0-7:A>G-7:A>G	TGCAGGAAGAGGGAGGGCGCCG	7:A>G
3948597|F|0-10:C>A-10:C>A	TGCAGCTGCTCCGCGGGGTGACCCCCAGGCTCCG	10:C>A
3959093|F|0-19:G>A-19:G>A	TGCAGTCCGCGAGGCCGTCGTCGGGGGAGGCCACGGCGTGGTGCGCCG	19:G>A
4705943|F|0-21:C>T-21:C>T	TGCAGTCCGCGAGGCCGTCGTCGGGGGAGGCCGCGGCGTGGTGCGCCG	21:C>T
5008037|F|0-42:C>G-42:C>G	TGCAGCTGACGTGGAACTACAACTACGGGGCGGCGGGGAAGACCGTCGGGTTCGACGGGCTCAACAACC	42:C>G
3940017|F|0-18:C>T-18:C>T	TGCAGCATCTTGTAGAAGCTGTTGTCCAGCTTGGACGCGCTGGACCG	18:C>T
1052650|F|0-38:G>C-38:G>C	TGCAGCAGCATCACGCCCAGCGAGTAGACGTCCGACTTGACGCCCAGCATCCCCGTCTGCTGGTACTCC	38:G>C
3950735|F|0-19:C>G-19:C>G	TGCAGCCACGCCAGGTGGCCGTCTGGTTCCAGAACCGCCGCGCCCG	19:C>G
7173895|F|0-33:G>A-33:G>A	TGCAGCGAGCGGCTGCTCGTGGACGTGCTCCGCGTAGGCGTCAGGTCCG	33:G>A
5369694|F|0-8:G>C-8:G>C	TGCAGTGCGAAGAAACAGTGTAATCACCCG	8:G>C
32027030|F|0-8:T>C-8:T>C	TGCAGAGCTTGCGGACCTCTCCCTGTGTGCCG	8:T>C
7920170|F|0-5:C>G-5:C>G	TGCAGCTCGCCGAACGAGCCG	5:C>G
5369246|F|0-25:C>G-25:C>G	TGCAGAACCTGGTGTTCCTGGACCTCTACTCGAACAGGTTCACCG	25:C>G
4262003|F|0-6:G>A-6:G>A	TGCAGGGAAGGATGGTGGCAAGAGGATGGGGCTCAAGTTCGACCG	6:G>A
3941580|F|0-26:A>G-26:A>G	TGCAGGCCCTGCGGCGTGACGAAGCGAAAGTCGCCG	26:A>G
3945048|F|0-7:C>A-7:C>A	TGCAGTTCCTGGAGCGCACCG	7:C>A
3949378|F|0-8:G>C-8:G>C	TGCAGGTAGGCCGTGAGCCG	8:G>C
1865587|F|0-53:C>G-53:C>G	TGCAGGTTACCATTGGGGACGTAGTTGTAGAGCAAGAGCTTGACAGACTTGTTCGAGCAGTAACCAAGC	53:C>G
3938526|F|0-30:G>T-30:G>T	TGCAGTCGGTGAGGAGGACGGCGCGGAAGGGGCCCG	30:G>T
4993329|F|0-13:C>T-13:C>T	TGCAGTGGCTCGACGTGTCGACGAACGCGCTCTCCG	13:C>T
1865587|F|0-14:G>A-14:G>A	TGCAGGTTACCATTGGGGACGTAGTTGTAGAGCAAGAGCTTGACAGACTTGTTCGAGCAGTAACCAAGC	14:G>A
7172204|F|0-18:G>T-18:G>T	TGCAGGCGACGGAGCGGCGGAGTGACCG	18:G>T
4262003|F|0-15:T>C-15:T>C	TGCAGGGAAGGATGGTGGCAAGAGGATGGGGCTCAAGTTCGACCG	15:T>C
3954248|F|0-8:G>A-8:G>A	TGCAGCAGGAGAAGCCCTTCCACCG	8:G>A
4262414|F|0-28:C>T-28:C>T	TGCAGCTCCTCCTTGAAGCTCTTCGCCGCAGTGGTCCG	28:C>T
4262003|F|0-39:C>T-39:C>T	TGCAGGGAAGGATGGTGGCAAGAGGATGGGGCTCAAGTTCGACCG	39:C>T
3935071|F|0-11:C>A-11:C>A	TGCAGCAACCCCGTGTCGTGGTCGACGAGCCG	11:C>A
32027657|F|0-23:C>G-23:C>G	TGCAGGAGGACGGTGATGGCGAGCTCGGGGCTGAGCGCGCCGCTGCCCACCATCTCGTTCAGCGTCTCT	23:C>G
980168|F|0-42:C>G-42:C>G	TGCAGGTGAGGTGGACGCAGCGGAAGGGCCCCTGGTGCGCGGCGAGTATGCGGGAGACGGCCGCGGTGA	42:C>G
3033091|F|0-5:C>T-5:C>T	TGCAGCTCCTTGAAGTAGACGCCGTCGAAGGCCGTGCTCGTCCG	5:C>T
5367150|F|0-11:G>A-11:G>A	TGCAGTTCAGGGTGGTGGTGACCG	11:G>A
3939222|F|0-20:T>C-20:T>C	TGCAGCTCTGGGAGAACAACTTCACCG	20:T>C
5369498|F|0-23:G>A-23:G>A	TGCAGGTGGAGGTTGTGGCTACGGAAGTGAACCCCG	23:G>A
3934156|F|0-7:C>T-7:C>T	TGCAGGCCGTGGTGAAGGAGGTGATGCGGCTGCACCCG	7:C>T
4996123|F|0-10:G>C-10:G>C	TGCAGGTGCTGGACCTCAGCCACAACAACCTCTCCG	10:G>C
5366967|F|0-19:T>C-19:T>C	TGCAGCCATTGCGCAGAAATAACCG	19:T>C
4396622|F|0-19:T>C-19:T>C	TGCAGCAAGCTCCTCCCCGTAGATCCG	19:T>C
1131975|F|0-22:G>A-22:G>A	TGCAGGTCTTGGCGCTCTGGCCGTTGTGGGCTCGGAGGGTAAGGTCGAACGCCG	22:G>A
987992|F|0-25:C>G-25:C>G	TGCAGACCATGTATCGGCTCAACGTCGGAGGCCAGGCGATCTCCCCTTCCAAGGACTCCG	25:C>G
7171683|F|0-7:G>A-7:G>A	TGCAGGTGAAGAAGGATGGCCAGTGGATCACCGTCGACCCG	7:G>A
4998550|F|0-17:C>T-17:C>T	TGCAGGAGAGGCTGCGGCGACCTCTGGGTCGGAGCCG	17:C>T
7171683|F|0-22:G>A-22:G>A	TGCAGGTGAAGAAGGATGGCCAGTGGATCACCGTCGACCCG	22:G>A
5369694|F|0-5:T>C-5:T>C	TGCAGTGCGAAGAAACAGTGTAATCACCCG	5:T>C
11313489|F|0-10:G>C-10:G>C	TGCAGGCCGTGTACTCCAGCATGCGCGGCCTCGTCCG	10:G>C
3934168|F|0-25:G>A-25:G>A	TGCAGCTATGTTCAGTGGTGCTCATGTCCG	25:G>A
3942423|F|0-18:G>A-18:G>A	TGCAGGTATTCAAGCCAGGTGGCATAAGTTGACCG	18:G>A
5357322|F|0-26:A>T-26:A>T	TGCAGAGACGCACGTGGCGCGTGACAACCG	26:A>T
1159063|F|0-23:T>A-23:T>A	TGCAGAATCGCCCAGGAATGAGATTCCTCTCCAACATATTCATGCACCG	23:T>A
100520682|F|0-29:C>A-29:C>A	TGCAGCGGTGGTCGTCGTCGATGTGGGGGCTGAGCGGCAGCGACCG	29:C>A
1018927|F|0-23:G>T-23:G>T	TGCAGCTGCTCACCACCATGCTCGGCTTCAACCGTCACCAAGAAAGGATCCACATCCTCAAAGATGTCA	23:G>T
3939599|F|0-5:G>C-5:G>C	TGCAGGAGAAGATCAAGGCCCTGGAGGCAGCCGCCG	5:G>C
3954248|F|0-11:A>G-11:A>G	TGCAGCAGGAGAAGCCCTTCCACCG	11:A>G
3938388|F|0-8:C>A-8:C>A	TGCAGGCTCACCACGCGGAGCGCCG	8:C>A

<table>
<tr><td>3934803|F|0-25:G>A-25:G>A</td><td>TGCAGGATTTCTTTGACGGGAAGAAGCTCTGCAAGAGAATCAATCCG</td><td>25:G>A</td></tr>
<tr><td>32025988|F|0-42:A>G-42:A>G</td><td>TGCAGCCTCTTCAACCCCACGCCCTCGCTCTCCGCCATGGTCATCAACAACTACAAGCTCCG</td><td>42:A>G</td></tr>
<tr><td>4262607|F|0-10:A>G-10:A>G</td><td>TGCAGGCAGTACTCGAGGTTCCACAGCACGTCGCCCATGGACGGCCG</td><td>10:A>G</td></tr>
<tr><td>5001968|F|0-7:T>C-7:T>C</td><td>TGCAGGTTTTGAAGCTCTGGCTGTTGTGGGCTCGGAGGGTAAGGTCGAACGCCG</td><td>7:T>C</td></tr>
<tr><td>7909100|F|0-21:A>G-21:A>G</td><td>TGCAGCTGTGCATCCTCTCGGACGAGCCG</td><td>21:A>G</td></tr>
<tr><td>4262268|F|0-29:G>C-29:G>C</td><td>TGCAGGTTGCCCTTGGGCATGAACTCGTAGACCACGAACCG</td><td>29:G>C</td></tr>
<tr><td>989804|F|0-43:C>A-43:C>A</td><td>TGCAGGGTGCGGAAGTCCTTGAGCGGCCGCGCGCGCGGGTGGGCCACGCTGTTCCG</td><td>43:C>A</td></tr>
<tr><td>3958586|F|0-11:G>A-11:G>A</td><td>TGCAGCACCTTGAACGTGCCGCCCTCCCGCCG</td><td>11:G>A</td></tr>
<tr><td>1017897|F|0-31:G>C-31:G>C</td><td>TGCAGACGTGGAACAACGGGGCCAACAACACGAAGCTGATCGTGCAGGCCG</td><td>31:G>C</td></tr>
<tr><td>3937779|F|0-17:A>G-17:A>G</td><td>TGCAGAACCAGCCGAGTAATGGTTACATCTCACCG</td><td>17:A>G</td></tr>
<tr><td>3028271|F|0-11:A>G-11:A>G</td><td>TGCAGGTGGTCATGGTGGAGGTGCTCACCAGGTTCGCCG</td><td>11:A>G</td></tr>
<tr><td>32025359|F|0-12:T>A-12:T>A</td><td>TGCAGGAAGGAGTAGGCGAACAGGCCG</td><td>12:T>A</td></tr>
<tr><td>3953491|F|0-20:G>A-20:G>A</td><td>TGCAGACCCCCGTCCTGGCAGTAGCCGATGAGGGTCACCACGTTCCG</td><td>20:G>A</td></tr>
<tr><td>4261876|F|0-26:C>T-26:C>T</td><td>TGCAGGTACGGCAGGTTTGGGATGTCCGACTCGTCGGCGAGCCG</td><td>26:C>T</td></tr>
<tr><td>32025403|F|0-16:C>T-16:C>T</td><td>TGCAGTTGAGTTATGGCGGTTTCTGTGATGCTGTCCTGCCTTTCCG</td><td>16:C>T</td></tr>
<tr><td>4993329|F|0-27:C>G-27:C>G</td><td>TGCAGTGGCTCGACGTGTCGACGAACGCGCTCTCCG</td><td>27:C>G</td></tr>
<tr><td>1069659|F|0-11:G>A-11:G>A</td><td>TGCAGGTACCAGTGCAACGAGTGGGGCGTCGGCATGGAGATCAATAACAACGTCCG</td><td>11:G>A</td></tr>
<tr><td>4261876|F|0-18:G>C-18:G>C</td><td>TGCAGGTACGGCAGGTTTGGGATGTCCGACTCGTCGGCGAGCCG</td><td>18:G>C</td></tr>
<tr><td>1148777|F|0-32:A>G-32:A>G</td><td>TGCAGACTCGCCCAGGAATGAGATTCCTCTCCAACATATTCATGCACCG</td><td>32:A>G</td></tr>
<tr><td>3939940|F|0-11:G>C-11:G>C</td><td>TGCAGGAGTTCGTGTCGGAGATCACGAGCATGAGCCG</td><td>11:G>C</td></tr>
<tr><td>5582990|F|0-18:T>C-18:T>C</td><td>TGCAGGATATCAGCTAGGTCACCG</td><td>18:T>C</td></tr>
<tr><td>7173895|F|0-20:G>C-20:G>C</td><td>TGCAGCGAGCGGCTGCTCGTGGACGTGCTCCGCGTAGGCGTCAGGTCCG</td><td>20:G>C</td></tr>
<tr><td>1022649|F|0-11:C>T-11:C>T</td><td>TGCAGATCGTGCCGTGGGGGAACATGGACTGGATGGCGACCAACTTCTACGGGCAGCCG</td><td>11:C>T</td></tr>
<tr><td>100388481|F|0-19:A>G-19:A>G</td><td>TGCAGGGGAAGCCCATCACAGTGTACCG</td><td>19:A>G</td></tr>
<tr><td>100479182|F|0-28:A>G-28:A>G</td><td>TGCAGCATGTGTCGCTCGCCGACAACTCATTCTCCG</td><td>28:A>G</td></tr>
<tr><td>1383897|F|0-37:G>C-37:G>C</td><td>TGCAGGTGATGGAGCGCAAGATGCGGTCCAGCGGCATGGAGCGGCTCATCAAGCGCCG</td><td>37:G>C</td></tr>
<tr><td>3020926|F|0-16:C>A-16:C>A</td><td>TGCAGGATTTCTTTGACGGGAAGAAGCTCTGCAAGAAAATTAATCCG</td><td>16:C>A</td></tr>
<tr><td>987394|F|0-41:G>T-41:G>T</td><td>TGCAGTATCAGTACGCCAACTACTCGGCCAACTACCTCTACGGGGGCAAGGGCGCCATCCG</td><td>41:G>T</td></tr>
<tr><td>3020885|F|0-24:A>G-24:A>G</td><td>TGCAGGAGATCGATACTGTCGTTCAAAGTTCAGAAACCCG</td><td>24:A>G</td></tr>
<tr><td>1147988|F|0-23:C>G-23:C>G</td><td>TGCAGGACGATCATGCCGATCATCACGCCGATCGACATGAACAGCAGTATTCCG</td><td>23:C>G</td></tr>
<tr><td>1055097|F|0-46:C>A-46:C>A</td><td>TGCAGTATCAGTACGCCAACTACTCGGCGAACTACCTCAAAGGTGGCAAGGGCACCATCCG</td><td>46:C>A</td></tr>
<tr><td>100520207|F|0-29:T>C-29:T>C</td><td>TGCAGCATCGGGTTGATGTGGCCCTGGAATGGCAGCGGGAACATCAGTACCCG</td><td>29:T>C</td></tr>
<tr><td>4997209|F|0-7:A>C-7:A>C</td><td>TGCAGCGATGGAAGGAGGAGGAGCAGCGGTCGCAGGACGGCCG</td><td>7:A>C</td></tr>
<tr><td>5367423|F|0-32:A>G-32:A>G</td><td>TGCAGGTACGGCAGGTTTGGGATGTCCGACTCATCGGCGAGCCG</td><td>32:A>G</td></tr>
<tr><td>4262800|F|0-5:G>A-5:G>A</td><td>TGCAGGCCTTGTACATGGCCAAGTGTGCGCCG</td><td>5:G>A</td></tr>
<tr><td>3946889|F|0-19:G>A-19:G>A</td><td>TGCAGTGCATCGTGAGCGAGACGCTGCGGCTGTACCCG</td><td>19:G>A</td></tr>
<tr><td>3946889|F|0-22:G>A-22:G>A</td><td>TGCAGTGCATCGTGAGCGAGACGCTGCGGCTGTACCCG</td><td>22:G>A</td></tr>
<tr><td>1069659|F|0-26:C>T-26:C>T</td><td>TGCAGGTACCAGTGCAACGAGTGGGGCGTCGGCATGGAGATCAATAACAACGTCCG</td><td>26:C>T</td></tr>
<tr><td>5369694|F|0-17:G>A-17:G>A</td><td>TGCAGTGCGAAGAAACAGTGTAATCACCCG</td><td>17:G>A</td></tr>
<tr><td>3935173|F|0-11:T>C-11:T>C</td><td>TGCAGGGGACGTGCACCACCTTATACTGACCG</td><td>11:T>C</td></tr>
<tr><td>1302667|F|0-41:G>A-41:G>A</td><td>TGCAGGTTCTGGTCGCGCTGGAATCCCTTGCCGCAGATCTCGCACACGAACCG</td><td>41:G>A</td></tr>
<tr><td>1044294|F|0-25:C>G-25:C>G</td><td>TGCAGCTGCTGTCGTCGCTGGACACCACCAAGGTCAAGGACAAGAAGGACCG</td><td>25:C>G</td></tr>
<tr><td>3954751|F|0-24:A>G-24:A>G</td><td>TGCAGGCGACGGTGCAGATGATGAACCG</td><td>24:A>G</td></tr>
<tr><td>4261249|F|0-14:G>A-14:G>A</td><td>TGCAGGCCCAGGTAGCAGAGCGAGGAGGCGTCCG</td><td>14:G>A</td></tr>
<tr><td>3953727|F|0-9:T>C-9:T>C</td><td>TGCAGGCCGTGGTCAAGGAGGCGCTCCG</td><td>9:T>C</td></tr>
<tr><td>3949564|F|0-22:C>T-22:C>T</td><td>TGCAGAAGTACCTCGGCAACTACGTCCG</td><td>22:C>T</td></tr>
<tr><td>5363986|F|0-6:C>T-6:C>T</td><td>TGCAGGCTCTTGACCTGTCGGACAACCAGCTCGCCG</td><td>6:C>T</td></tr>
<tr><td>3937925|F|0-7:G>C-7:G>C</td><td>TGCAGCTGGTGTCCAACGACAGGTTCAAGAGCCCG</td><td>7:G>C</td></tr>
<tr><td>22840091|F|0-29:G>C-29:G>C</td><td>TGCAGGCTCACCGCGCTCTCCTTGGACTCGCCG</td><td>29:G>C</td></tr>
<tr><td>6044173|F|0-31:G>C-31:G>C</td><td>TGCAGGTCATGGCCAACATCGCCGACGCGGTGATTGGGGAGACCG</td><td>31:G>C</td></tr>
<tr><td>3947141|F|0-18:T>G-18:T>G</td><td>TGCAGGTACACAAGAACATCTCACCG</td><td>18:T>G</td></tr>
<tr><td>3936328|F|0-8:T>A-8:T>A</td><td>TGCAGGTGTCTTGCGCCGTTTCCG</td><td>8:T>A</td></tr>
<tr><td>3024963|F|0-23:T>G-23:T>G</td><td>TGCAGTTCACGGCGTACATATACTTCGGCGACGCCACCATCAACCG</td><td>23:T>G</td></tr>
<tr><td>7912987|F|0-9:C>G-9:C>G</td><td>TGCAGCCTCCCCATCCCGAGCCTCCG</td><td>9:C>G</td></tr>
<tr><td>5003148|F|0-9:A>T-9:A>T</td><td>TGCAGAATAAATTCTGGCCGAAAGCTTCCG</td><td>9:A>T</td></tr>
<tr><td>1667164|F|0-31:C>A-31:C>A</td><td>TGCAGCCCGTCCATGGCGCGCATGGCGACGTCGAAGAAGACGAGGTTGTGGTTGGCGTACCG</td><td>31:C>A</td></tr>
<tr><td>4439756|F|0-11:G>C-11:G>C</td><td>TGCAGCACGGGGGCGTCACCGCCG</td><td>11:G>C</td></tr>
<tr><td>1062282|F|0-28:A>G-28:A>G</td><td>TGCAGCCACCCGTCCCTCGTCGCCGTGTACAGCGTCCCTCCCGCCGCCGCGTCCACGTACACGTCCTCC</td><td>28:A>G</td></tr>
<tr><td>4004344|F|0-14:T>G-14:T>G</td><td>TGCAGGTTGCAGTGTTGCCTTGTTCACCG</td><td>14:T>G</td></tr>
</table>

<table>
<tr><td>1011276|F|0-29:T>G-29:T>G</td><td>TGCAGCCGCTCGCGCCGAGGGTCGTCGGCTCGGAGGTCACCACGCTGGGCGGGCGCGTCCG</td><td>29:T>G</td></tr>
<tr><td>3033223|F|0-5:C>T-5:C>T</td><td>TGCAGCCTGCCGCTCGCAGCCGACGACTTCTTCTCCG</td><td>5:C>T</td></tr>
<tr><td>3570084|F|0-5:C>A-5:C>A</td><td>TGCAGCTACAAGCGCATCTTCGCGTTCGGTGACTCCCTCATCGACACCG</td><td>5:C>A</td></tr>
<tr><td>7172204|F|0-7:G>C-7:G>C</td><td>TGCAGGCGACGGAGCGGCGGAGTGACCG</td><td>7:G>C</td></tr>
<tr><td>4539647|F|0-7:C>T-7:C>T</td><td>TGCAGGGCGGACGGCGAATGCAGCCG</td><td>7:C>T</td></tr>
<tr><td>3944579|F|0-8:C>A-8:C>A</td><td>TGCAGCAGCGTCGCGATCTGCCG</td><td>8:C>A</td></tr>
<tr><td>32020286|F|0-31:T>C-31:T>C</td><td>TGCAGGATGGGGGCAGCGAGAGGGCCATGGGTTTTCCTCCG</td><td>31:T>C</td></tr>
<tr><td>100494247|F|0-26:C>G-26:C>G</td><td>TGCAGACTATCGGCGCTCATGGCAGACACCG</td><td>26:C>G</td></tr>
<tr><td>5581505|F|0-32:G>C-32:G>C</td><td>TGCAGGCTGAGGTGGTTCAACCAGCTGGACCCGAGGATCAACCG</td><td>32:G>C</td></tr>
<tr><td>1667164|F|0-26:G>C-26:G>C</td><td>TGCAGCCCGTCCATGGCGCGCATGGCGACGTCGAAGAAGACGAGGTTGTGGTTGGCGTACCG</td><td>26:G>C</td></tr>
<tr><td>1092679|F|0-24:T>C-24:T>C</td><td>TGCAGCCACATGTACCACCAGGACTGCATCCTGCCGTGGCTCGCGCTGCGTAACTCGTGTCCTGTGTGC</td><td>24:T>C</td></tr>
<tr><td>32023399|F|0-8:C>G-8:C>G</td><td>TGCAGGTACGGCAGGTTCGGGAAGTCCGTCTCCGACAGGACCCG</td><td>8:C>G</td></tr>
<tr><td>11313330|F|0-25:C>T-25:C>T</td><td>TGCAGACCAATCTGATCAAAGAGAGCATCCG</td><td>25:C>T</td></tr>
<tr><td>3935173|F|0-10:G>A-10:G>A</td><td>TGCAGGGGACGTGCACCACCTTATACTGACCG</td><td>10:G>A</td></tr>
<tr><td>1215300|F|0-17:G>C-17:G>C</td><td>TGCAGTGGCGGTGCAGGGAGGGGTCTGATCACAGGTGAACAGTGGCGCGAACCG</td><td>17:G>C</td></tr>
<tr><td>1059801|F|0-13:G>A-13:G>A</td><td>TGCAGGTCGTCCCGTGGGGCGACATGAGGTGGGCGTCCTTCAGTGCCTTCGGCGCGCCGACGGCCGACA</td><td>13:G>A</td></tr>
<tr><td>32026547|F|0-14:A>C-14:A>C</td><td>TGCAGCAGTGCGTGAAGCGGTGCCAGGAGGACCG</td><td>14:A>C</td></tr>
<tr><td>5361926|F|0-45:C>G-45:C>G</td><td>TGCAGCTTCGGCGTCGACGCGGCCGCGGACGAAGGCTCGCGCTTGCCGTGGTGGCCAGCAGCGGAGGCG</td><td>45:C>G</td></tr>
<tr><td>5010682|F|0-7:T>C-7:T>C</td><td>TGCAGCATGTAGCCACAGCCTCTCCG</td><td>7:T>C</td></tr>
<tr><td>3570862|F|0-23:G>C-23:G>C</td><td>TGCAGAATTCGCTGGAGGTGGACGTCCTCGACCG</td><td>23:G>C</td></tr>
<tr><td>4262160|F|0-16:G>A-16:G>A</td><td>TGCAGAAGGGGCGGCAGCTGGAGACGGTGGAGATGAAGGTCCG</td><td>16:G>A</td></tr>
<tr><td>1011276|F|0-16:G>C-16:G>C</td><td>TGCAGCCGCTCGCGCCGAGGGTCGTCGGCTCGGAGGTCACCACGCTGGGCGGGCGCGTCCG</td><td>16:G>C</td></tr>
<tr><td>1666899|F|0-7:T>C-7:T>C</td><td>TGCAGTATACCCACGCGGTGCTGTTCGAGAACATGCGGCTGTTCCCGCCG</td><td>7:T>C</td></tr>
<tr><td>5364137|F|0-42:T>C-42:T>C</td><td>TGCAGTTGAGTTATGGTGGCTTCTGTGATGCTGTCCTGCCTTTCCG</td><td>42:T>C</td></tr>
<tr><td>6044001|F|0-19:G>C-19:G>C</td><td>TGCAGCTGGCGTCGCCGTCGATGGCCCG</td><td>19:G>C</td></tr>
<tr><td>3027499|F|0-26:T>G-26:T>G</td><td>TGCAGATCTACGCCGCCGCCGAAGACTGTGGCAAGCCG</td><td>26:T>G</td></tr>
<tr><td>4999436|F|0-15:G>C-15:G>C</td><td>TGCAGCGAGCAGCTGGTCGTGGACGTGCTCGGCGTGGGCGTCAGGTCCG</td><td>15:G>C</td></tr>
<tr><td>5007811|F|0-7:C>A-7:C>A</td><td>TGCAGGGCCACGCCGTGAAGCTCGGGTTCCTTGAGCACGACGAGCACGCGCAGAACAGCCTCATCAGCT</td><td>7:C>A</td></tr>
<tr><td>4910284|F|0-10:C>T-10:C>T</td><td>TGCAGCAGGTCGAGCCTGGTCTCCCCG</td><td>10:C>T</td></tr>
<tr><td>3960145|F|0-5:C>G-5:C>G</td><td>TGCAGCAGCGTGTTGATGCCG</td><td>5:C>G</td></tr>
<tr><td>3958329|F|0-32:C>G-32:C>G</td><td>TGCAGCGGGAAGTGCTTCTCGGCGTGCTGCTTCGGGATCACCAGCCG</td><td>32:C>G</td></tr>
<tr><td>1667164|F|0-32:G>A-32:G>A</td><td>TGCAGCCCGTCCATGGCGCGCATGGCGACGTCGAAGAAGACGAGGTTGTGGTTGGCGTACCG</td><td>32:G>A</td></tr>
<tr><td>3958598|F|0-16:C>T-16:C>T</td><td>TGCAGCAAGCTCCTCCCAGTAGATCCG</td><td>16:C>T</td></tr>
<tr><td>3028271|F|0-25:C>G-25:C>G</td><td>TGCAGGTGGTCATGGTGGAGGTGCTCACCAGGTTCGCCG</td><td>25:C>G</td></tr>
<tr><td>1040349|F|0-25:C>T-25:C>T</td><td>TGCAGTGGGTGCGCAACATGGGCTACGTCATCTACGACTACTGCACCGACATGAGCAACCG</td><td>25:C>T</td></tr>
<tr><td>3023394|F|0-41:A>G-41:A>G</td><td>TGCAGAACCTGGTGTTCCTGGACCTGTACTCGAACAGGTTCACCG</td><td>41:A>G</td></tr>
<tr><td>3949863|F|0-40:G>A-40:G>A</td><td>TGCAGGGTTGGCGTCGCTGGGCGGGGGCGCGCTACCAGAAGGTCTTCGGCATACTTGACGGAATTATCG</td><td>40:G>A</td></tr>
<tr><td>2245453|F|0-56:G>A-56:G>A</td><td>TGCAGGATGTCCCTGATCTGGTCGATAGCGTACCCTGCCCTGGGGTCTTCCCTCTCGACGCCCTTGTGG</td><td>56:G>A</td></tr>
<tr><td>1023263|F|0-10:G>C-10:G>C</td><td>TGCAGAGGCGGCTGTGCGAAGCCGAGCGACCTCGCGATCTTGCTCACTGGTGCAGCAGTGTCCG</td><td>10:G>C</td></tr>
<tr><td>1666899|F|0-10:C>T-10:C>T</td><td>TGCAGTATACCCACGCGGTGCTGTTCGAGAACATGCGGCTGTTCCCGCCG</td><td>10:C>T</td></tr>
<tr><td>3022508|F|0-13:C>T-13:C>T</td><td>TGCAGTACCTCACCGAACTGGACCTGAGCAACAACCTGTTCTCCG</td><td>13:C>T</td></tr>
<tr><td>32023406|F|0-13:T>C-13:T>C</td><td>TGCAGGTGATACTTAACGAGATTAGGAACCG</td><td>13:T>C</td></tr>
<tr><td>5371307|F|0-27:A>C-27:A>C</td><td>TGCAGGCGCACAACGACGCGCGCCGCGAGGTCGGCGTGGCACCCCTGACGTGGAACTCGACGCTGGAGC</td><td>27:A>C</td></tr>
<tr><td>3957710|F|0-13:C>G-13:C>G</td><td>TGCAGGCGCTGGTCCGCGGCAACATCGTCCG</td><td>13:C>G</td></tr>
<tr><td>1215401|F|0-33:C>A-33:C>A</td><td>TGCAGTGGGTAACGGCGGAGCTCATGCGGAACCCGAGGGTTCGGCATAAGGCGCAGGAGGAGGTCCGAG</td><td>33:C>A</td></tr>
<tr><td>1215401|F|0-44:C>T-44:C>T</td><td>TGCAGTGGGTAACGGCGGAGCTCATGCGGAACCCGAGGGTTCGGCATAAGGCGCAGGAGGAGGTCCGAG</td><td>44:C>T</td></tr>
<tr><td>5000379|F|0-22:T>C-22:T>C</td><td>TGCAGGTAGGCGAGCCGCGGCATGTCGTCGGCGGTCACCAGCCG</td><td>22:T>C</td></tr>
<tr><td>5578225|F|0-12:G>A-12:G>A</td><td>TGCAGGAGGAAGGGGATGCACCG</td><td>12:G>A</td></tr>
<tr><td>1207029|F|0-5:C>T-5:C>T</td><td>TGCAGCAGCAGCCAGTTCACCTTGGCCAGCACCAGCTGGTCGTCGTTGCAGTAGAAGAGCCG</td><td>5:C>T</td></tr>
<tr><td>32024176|F|0-23:G>C-23:G>C</td><td>TGCAGGATCATCGAGAACGACCTGTTCAAGCACGACTGGAGAAGCCG</td><td>23:G>C</td></tr>
<tr><td>3943230|F|0-15:C>A-15:C>A</td><td>TGCAGAGGCATGACTCCGTGCTCGCCACCCG</td><td>15:C>A</td></tr>
<tr><td>1023263|F|0-28:A>G-28:A>G</td><td>TGCAGAGGCGGCTGTGCGAAGCCGAGCGACCTCGCGATCTTGCTCACTGGTGCAGCAGTGTCCG</td><td>28:A>G</td></tr>
<tr><td>3027758|F|0-10:C>T-10:C>T</td><td>TGCAGCTCGCCATCCTCGCCGCCACCGTCACCTACCAGACCG</td><td>10:C>T</td></tr>
<tr><td>6046651|F|0-37:C>G-37:C>G</td><td>TGCAGCACATCAGCCCATACTTCCTGATCAGTCCATGCGAGTTTCCACAGACCCG</td><td>37:C>G</td></tr>
<tr><td>5582750|F|0-9:C>T-9:C>T</td><td>TGCAGCTGGCGGACGTGCTGCACGCCCG</td><td>9:C>T</td></tr>
<tr><td>3943442|F|0-16:C>T-16:C>T</td><td>TGCAGGAACTGCGTGACGCCCG</td><td>16:C>T</td></tr>
<tr><td>4261134|F|0-20:T>C-20:T>C</td><td>TGCAGGATCATCGAGAACGATCTGTTCAAGCACGACTGGAGGAGCCG</td><td>20:T>C</td></tr>
<tr><td>3022508|F|0-19:G>C-19:G>C</td><td>TGCAGTACCTCACCGAACTGGACCTGAGCAACAACCTGTTCTCCG</td><td>19:G>C</td></tr>
<tr><td>3946001|F|0-25:C>T-25:C>T</td><td>TGCAGCGGCCACGGGAACACGAGAACATGCACCG</td><td>25:C>T</td></tr>
</table>

<table>
<tr><td>3944416|F|0-16:G>A-16:G>A</td><td>TGCAGGCGTCGGCGCTGGCGTTCCG</td><td>16:G>A</td></tr>
<tr><td>1207029|F|0-24:G>C-24:G>C</td><td>TGCAGCAGCAGCCAGTTCACCTTGGCCAGCACCAGCTGGTCGTCGTTGCAGTAGAAGCGCCG</td><td>24:G>C</td></tr>
<tr><td>1862959|F|0-56:C>T-56:C>T</td><td>TGCAGGCGGCTGTCGAAGCTGGTGTCTGAAATTTGATCCCAGAACTCACGCAGCTCCGACTTGTTGATG</td><td>56:C>T</td></tr>
<tr><td>32024078|F|0-7:G>C-7:G>C</td><td>TGCAGATGGACAGGCACCGAGTCGAAGCCGAGCCG</td><td>7:G>C</td></tr>
<tr><td>3960371|F|0-16:G>A-16:G>A</td><td>TGCAGATCCAAGGTGAGCAAGCCAGGTTCGTGAACGTCCTCACCG</td><td>16:G>A</td></tr>
<tr><td>1021367|F|0-7:T>G-7:T>G</td><td>TGCAGACTCTATTGGCGATGAACCAGGTGGCGGAGCACATGGTCCTCGCCG</td><td>7:T>G</td></tr>
<tr><td>4538357|F|0-32:C>A-32:C>A</td><td>TGCAGCTGGAGGTTGTGGCTACGGAAGTGAACCCCG</td><td>32:C>A</td></tr>
<tr><td>3940475|F|0-11:C>T-11:C>T</td><td>TGCAGCGAGTACATGCTGGTGGTGCTGACCG</td><td>11:C>T</td></tr>
<tr><td>1018927|F|0-14:A>T-14:A>T</td><td>TGCAGCTGCTCACCACCATGCTCGGCTTCAACCGTAACCAAGAAAGGATCCACATCCTCAAAGATGTCA</td><td>14:A>T</td></tr>
<tr><td>5363054|F|0-8:T>C-8:T>C</td><td>TGCAGAAGTTGGACCTGAGCTCCAACAACCTCACCG</td><td>8:T>C</td></tr>
<tr><td>5006849|F|0-5:A>C-5:A>C</td><td>TGCAGAAGGCGCAGCAAGACCCCCG</td><td>5:A>C</td></tr>
<tr><td>1023671|F|0-59:T>C-59:T>C</td><td>TGCAGCTCCCGCGCCTGCTCCGCCGCGCGCGCTGCCGCCTCCGCCGCCTGCTCCCCTGCTTTCCTCTCC</td><td>59:T>C</td></tr>
<tr><td>4262404|F|0-5:G>A-5:G>A</td><td>TGCAGGGGTAGCGCCACGGTCGAATGGTCTCCGCTCTACGACCCG</td><td>5:G>A</td></tr>
<tr><td>3954378|F|0-14:C>G-14:C>G</td><td>TGCAGCTGCGGGCACGAGGCGAAGACGCCCTCGTAGGCGGCCG</td><td>14:C>G</td></tr>
<tr><td>3960371|F|0-19:A>G-19:A>G</td><td>TGCAGATCCAAGGTGAGCAAGCCAGGTTCGTGAACGTCCTCACCG</td><td>19:A>G</td></tr>
<tr><td>3935831|F|0-13:G>A-13:G>A</td><td>TGCAGCATGTGTCGCTCGCCGACAACTCGTTCTCCG</td><td>13:G>A</td></tr>
<tr><td>2241528|F|0-28:G>A-28:G>A</td><td>TGCAGAAATTCCTTACATTCACTCTCTCGACTCTGCGCATTTCATCCTCGAGGAGTGTAACTTGTGCTG</td><td>28:G>A</td></tr>
<tr><td>3960371|F|0-30:T>C-30:T>C</td><td>TGCAGATCCAAGGTGAGCAAGCCAGGTTCGTGAACGTCCTCACCG</td><td>30:T>C</td></tr>
<tr><td>1148777|F|0-6:C>A-6:C>A</td><td>TGCAGACTCGCCCAGGAATGAGATTCCTCTCCAACATATTCATGCACCG</td><td>6:C>A</td></tr>
<tr><td>7353208|F|0-11:A>G-11:A>G</td><td>TGCAGTCGGCGAGCAAGCCCCG</td><td>11:A>G</td></tr>
<tr><td>3952428|F|0-13:G>A-13:G>A</td><td>TGCAGAGACGCACGTGGCGCGTGACATCCG</td><td>13:G>A</td></tr>
<tr><td>4262404|F|0-12:G>T-12:G>T</td><td>TGCAGGGGTAGCGCCACGGTCGAATGGTCTCCGCTCTACGACCCG</td><td>12:G>T</td></tr>
<tr><td>4261388|F|0-23:C>G-23:C>G</td><td>TGCAGGATGTCCAGCACCTTGTACGTCGGCCAGTAGAGCCG</td><td>23:C>G</td></tr>
<tr><td>4993334|F|0-6:T>C-6:T>C</td><td>TGCAGGTCGTATCATGTGCTGTCGCCCG</td><td>6:T>C</td></tr>
<tr><td>4995261|F|0-10:T>C-10:T>C</td><td>TGCAGGTGATTCAGGAGGTGTTCAGGGCGGTCCG</td><td>10:T>C</td></tr>
<tr><td>5361936|F|0-28:A>C-28:A>C</td><td>TGCAGCTCGCCCAGCACCCCTACGACGCAGAGGCGATACAGTCGGCGGACGCCGCGTTGGTGGTGGAGC</td><td>28:A>C</td></tr>
<tr><td>5002244|F|0-29:T>C-29:T>C</td><td>TGCAGCTCCTCCTCCTGCGGGTGCTCCCGTGGAACCG</td><td>29:T>C</td></tr>
<tr><td>3942531|F|0-8:G>T-8:G>T</td><td>TGCAGATCGCCTGCGGCAAGTCCATTCTCCG</td><td>8:G>T</td></tr>
<tr><td>4261588|F|0-33:T>C-33:T>C</td><td>TGCAGTCGAGCTGGGCGCGGGAGATCTCGTAGGTTATGACCCG</td><td>33:T>C</td></tr>
<tr><td>1021367|F|0-47:G>A-47:G>A</td><td>TGCAGACTCTATTGGCGATGAACCAGGTGGCGGAGCACATGGTCCTCGCCG</td><td>47:G>A</td></tr>
<tr><td>1004021|F|0-19:G>A-19:G>A</td><td>TGCAGCTCATGCCGAACCTGCACAATCCAGTCCTGAGGTAGTAGGTGCAGTCCG</td><td>19:G>A</td></tr>
<tr><td>3939376|F|0-23:C>G-23:C>G</td><td>TGCAGAAAAGGCGGTCGCAGTTTCTTGGCACTGGAGAAAGCGACCG</td><td>23:C>G</td></tr>
<tr><td>32020395|F|0-26:T>C-26:T>C</td><td>TGCAGTTCTTCGACGCGTCCCAGAACTCGCTCACCG</td><td>26:T>C</td></tr>
<tr><td>2245631|F|0-14:A>C-14:A>C</td><td>TGCAGACGCGTTGAATCGATTCCTTGGTGCGCGGTGATTGCCGAGGGGAGCGAGATGGTAGAGAAGCGC</td><td>14:A>C</td></tr>
<tr><td>1004021|F|0-43:G>A-43:G>A</td><td>TGCAGCTCATGCCGAACCTGCACAATCCAGTCCTGAGGTAGTAGGTGCAGTCCG</td><td>43:G>A</td></tr>
<tr><td>5582091|F|0-22:G>A-22:G>A</td><td>TGCAGTTGGTCTGCTGGTCGGCGAAGAACGGCCAGCTGATCACCG</td><td>22:G>A</td></tr>
<tr><td>4261588|F|0-18:G>A-18:G>A</td><td>TGCAGTCGAGCTGGGCGCGGGAGATCTCGTAGGTTATGACCCG</td><td>18:G>A</td></tr>
<tr><td>2243750|F|0-63:C>G-63:C>G</td><td>TGCAGTGCTCCTGCACCTCCTCGATGTCGTACTGCGTCAGCATCGACGACGCGTTCCCCATCGCCGAGA</td><td>63:C>G</td></tr>
<tr><td>4261388|F|0-26:C>G-26:C>G</td><td>TGCAGGATGTCCAGCACCTTGTACGTCGGCCAGTAGAGCCG</td><td>26:C>G</td></tr>
<tr><td>32019156|F|0-16:T>C-16:T>C</td><td>TGCAGTCGGAGGACACTCTGACGTAGTCCG</td><td>16:T>C</td></tr>
<tr><td>1020654|F|0-19:A>C-19:A>C</td><td>TGCAGCTTGAGGTTCTGAGAAAGATACGCCACCCACACTTGCTGATGCTCCTGGGAGCCTGTCCGAGAT</td><td>19:A>C</td></tr>
<tr><td>4994785|F|0-42:A>G-42:A>G</td><td>TGCAGGAGGCGGCGATGCCGTCGGCAGTGAGGGAGGATCGTCAGATCTGATCACCGATGATCAGCCGAG</td><td>42:A>G</td></tr>
<tr><td>12694081|F|0-17:G>C-17:G>C</td><td>TGCAGCATGAGGACCATGGCCCCG</td><td>17:G>C</td></tr>
<tr><td>1314482|F|0-14:T>C-14:T>C</td><td>TGCAGCTGCAACTCTTGGGCTGCCTCGGCGGGGTGGAGATGGTGCTCAAGGACATCGGGTACCCGAGAT</td><td>14:T>C</td></tr>
<tr><td>3944416|F|0-7:G>A-7:G>A</td><td>TGCAGGCGTCGGCGCTGGCGTTCCG</td><td>7:G>A</td></tr>
<tr><td>4733857|F|0-14:A>G-14:A>G</td><td>TGCAGCCATTGCGCAGAAACAACCG</td><td>14:A>G</td></tr>
<tr><td>1016408|F|0-17:G>A-17:G>A</td><td>TGCAGCCTCTTCAACCCGACGCCGTCGCTCTCCGCCATGGTCGTCAACCACTATAAGCTCCGCGGTAAC</td><td>17:G>A</td></tr>
<tr><td>1016408|F|0-14:C>T-14:C>T</td><td>TGCAGCCTCTTCAACCCGACGCCGTCGCTCTCCGCCATGGTCGTCAACCACTATAAGCTCCGCGGTAAC</td><td>14:C>T</td></tr>
<tr><td>6048317|F|0-23:C>G-23:C>G</td><td>TGCAGCAGCGCCGTCGTGGGGAGCACGAAGGAGACGTTGTTCACGGCCGCCGCGAACCG</td><td>23:C>G</td></tr>
<tr><td>3937257|F|0-10:C>G-10:C>G</td><td>TGCAGTACAACGGGAGCGGACGGCGTTGAAAACGGACCG</td><td>10:C>G</td></tr>
<tr><td>4261200|F|0-13:C>G-13:C>G</td><td>TGCAGGGCATCGTCACGCCG</td><td>13:C>G</td></tr>
<tr><td>1266994|F|0-21:T>C-21:T>C</td><td>TGCAGCAGCAGCCAGTTCACCTTGGCCAGTACGAGCTGGTCGTCGTTGCAGTAGAAGAGCCG</td><td>21:T>C</td></tr>
<tr><td>1863707|F|0-26:G>A-26:G>A</td><td>TGCAGGCGGCTGTCGAAGCTGGTGTCGGAGATCTGGTCCCAGAACTCGAGCAGCTCCGCCTTGCTGATG</td><td>26:G>A</td></tr>
<tr><td>1221582|F|0-26:G>A-26:G>A</td><td>TGCAGCATGGGGCTCCCCAGGAGCACGGAGTGCAAGTGCAGCAGCTTCATGGGGCTCCG</td><td>26:G>A</td></tr>
<tr><td>1016408|F|0-65:T>C-65:T>C</td><td>TGCAGCCTCTTCAACCCGACGCCGTCGCTCTCCGCCATGGTCGTCAACCACTATAAGCTCCGCGGTAAC</td><td>65:T>C</td></tr>
<tr><td>3024045|F|0-25:C>T-25:C>T</td><td>TGCAGGATCCTCGGGCCATTCGTTTCTTTGATCGCCG</td><td>25:C>T</td></tr>
<tr><td>4262756|F|0-17:G>A-17:G>A</td><td>TGCAGCAGCTCCAAGGGGTGCCCG</td><td>17:G>A</td></tr>
<tr><td>4262243|F|0-7:T>C-7:T>C</td><td>TGCAGGTTGCACAGAGCAGCAGGAGCACTAGCCCG</td><td>7:T>C</td></tr>
<tr><td>1016408|F|0-62:C>T-62:C>T</td><td>TGCAGCCTCTTCAACCCGACGCCGTCGCTCTCCGCCATGGTCGTCAACCACTATAAGCTCCGCGGTAAC</td><td>62:C>T</td></tr>
</table>

<table>
<tr><td>1686609|F|0-13:T>C-13:T>C</td><td>TGCAGTTCGTGTTTCCAGGGATGTGGCTCTCGGATTTGACCAAGGGCCG</td><td>13:T>C</td></tr>
<tr><td>3941360|F|0-15:A>G-15:A>G</td><td>TGCAGCCACGTCACCACGATGGGGCTCCG</td><td>15:A>G</td></tr>
<tr><td>1018962|F|0-32:G>C-32:G>C</td><td>TGCAGCCGCTGTGCCATGATCACGTTCCCGCCGACAGTCGCCGTGTTCCG</td><td>32:G>C</td></tr>
<tr><td>1029403|F|0-32:C>T-32:C>T</td><td>TGCAGCAGTGAGGTTGGTATCTGCCCATGGAGCTGGTTGGAGGACAGGTTCAGCCGCTCCAGCTTCACG</td><td>32:C>T</td></tr>
<tr><td>32025816|F|0-14:C>G-14:C>G</td><td>TGCAGCCACGGCCTCGAGGGCAGCATCACCTGCCG</td><td>14:C>G</td></tr>
<tr><td>7917815|F|0-15:G>A-15:G>A</td><td>TGCAGTAGGCAGATCGGCCCG</td><td>15:G>A</td></tr>
<tr><td>1046321|F|0-37:G>A-37:G>A</td><td>TGCAGCGAGACTCGGATTTGGCTGCCCGCGGCACAGAGCATGGAAACGAATCAGGGGAAAGTGAAATCG</td><td>37:G>A</td></tr>
<tr><td>5008450|F|0-12:A>G-12:A>G</td><td>TGCAGAAGAGACAGCGGAGGCGGTTCCG</td><td>12:A>G</td></tr>
<tr><td>100583030|F|0-35:A>G-35:A>G</td><td>TGCAGCGCCCAGGCGGTGTCCGCCATGTACCCTTCACCGCGGATCCTCTCCATCATCTCCTCCACCTTC</td><td>35:A>G</td></tr>
<tr><td>32025073|F|0-22:A>C-22:A>C</td><td>TGCAGCTAGCGTCCGCCACGGCATGCGTCACGCTGTCGGCGGTGCGGCTCGTCAGGCAGGACTTCGGCG</td><td>22:A>C</td></tr>
<tr><td>5006836|F|0-11:T>C-11:T>C</td><td>TGCAGGAGGAGTAGGATGGTGGGCACGCTGTTGCCCCGCGCGTCGTGCGCGCACTGCATCGACTCCGTC</td><td>11:T>C</td></tr>
<tr><td>3572738|F|0-7:G>C-7:G>C</td><td>TGCAGGTGTGCGTCTTCTACCTCGTGCTCCG</td><td>7:G>C</td></tr>
<tr><td>1209156|F|0-5:C>G-5:C>G</td><td>TGCAGCAGCACAACCTCCTCGTGCTCCGCCTCCAGGTCAGGATGCAGCTTGAGCAGCTCCG</td><td>5:C>G</td></tr>
<tr><td>1686609|F|0-31:G>C-31:G>C</td><td>TGCAGTTCGTGTTTCCAGGGATGTGGCTCTCGGATTTGACCAAGGGCCG</td><td>31:G>C</td></tr>
<tr><td>4261595|F|0-23:G>A-23:G>A</td><td>TGCAGGAGTTCGTGTCGGAGATCGTGAGCATGAGCCG</td><td>23:G>A</td></tr>
<tr><td>993875|F|0-40:C>A-40:C>A</td><td>TGCAGAAACTCTTGTATGCCCCAATGGGGGAAGTCCTCATCTTGCAGCCG</td><td>40:C>A</td></tr>
<tr><td>32017691|F|0-29:C>G-29:C>G</td><td>TGCAGCGTCTTCATGTTGAGCGACATCTCCTCCGCCG</td><td>29:C>G</td></tr>
<tr><td>5582080|F|0-11:T>C-11:T>C</td><td>TGCAGTAGACGTTCACGGTGAGCACCGCGCCG</td><td>11:T>C</td></tr>
<tr><td>6048864|F|0-10:G>T-10:G>T</td><td>TGCAGAGTTAGGTGCAGGGCATGGGGTTGCTCGCCG</td><td>10:G>T</td></tr>
<tr><td>4993450|F|0-10:C>T-10:C>T</td><td>TGCAGCCATGCGACCTGCTCGGCGTACTTGTCCG</td><td>10:C>T</td></tr>
<tr><td>3024920|F|0-27:A>T-27:A>T</td><td>TGCAGCTCTTCTCCATGATGCTGCGCCAAGGTGTTCGACCG</td><td>27:A>T</td></tr>
<tr><td>3939112|F|0-13:C>G-13:C>G</td><td>TGCAGCTCAGCGTCAACAAGATGTCCG</td><td>13:C>G</td></tr>
<tr><td>3937257|F|0-33:G>C-33:G>C</td><td>TGCAGTACAACGGGAGCGGACGGCGTTGAAAACGGACCG</td><td>33:G>C</td></tr>
<tr><td>1141725|F|0-5:A>T-5:A>T</td><td>TGCAGAAAACTGCTGCATGTACTGCTGTTCATGAGTTGTTATGTAGCGAACCATCCG</td><td>5:A>T</td></tr>
<tr><td>7173620|F|0-8:T>C-8:T>C</td><td>TGCAGCAGTAGCCAGTTCACCTTGGCCAGTACGAGCTGGTCGTCGTTGCAGTAGAAGAGCCG</td><td>8:T>C</td></tr>
<tr><td>979628|F|0-13:G>A-13:G>A</td><td>TGCAGAACTTCACGGCGAACCACAACAACTTCAGTGGCACGCTGCCGCCGTGCCTCAAGAACTGCACGG</td><td>13:G>A</td></tr>
<tr><td>3940025|F|0-21:T>A-21:T>A</td><td>TGCAGTTGCGGAGGCCCATGCTGCTCCG</td><td>21:T>A</td></tr>
<tr><td>1694890|F|0-65:A>G-65:A>G</td><td>TGCAGGACGTGTTCCGTCGCTTCGCCTTCGACTGCATATGCAAGATCTCCTTCGGCCTCGACCCGAGAT</td><td>65:A>G</td></tr>
<tr><td>1166676|F|0-27:G>A-27:G>A</td><td>TGCAGCCGTTTCGGGTTCTGCGGCAGCGGCTCTGAGTGGTGCGGAGCCG</td><td>27:G>A</td></tr>
<tr><td>2245263|F|0-17:C>A-17:C>A</td><td>TGCAGCAGCAGCGTCTCCAGGTTGGACAGCTCGATGTAGCCGAGGTTGTCGGGGTCGAGCTCCTCCATG</td><td>17:C>A</td></tr>
<tr><td>2245263|F|0-24:G>T-24:G>T</td><td>TGCAGCAGCAGCGTCTCCAGGTTGGACAGCTCGATGTAGCCGAGGTTGTCGGGGTCGAGCTCCTCCATG</td><td>24:G>T</td></tr>
<tr><td>32025844|F|0-22:A>C-22:A>C</td><td>TGCAGCTTCTCCTCGGGGCTTGACGCGAGGAGGTAGGATGTGACGCCG</td><td>22:A>C</td></tr>
<tr><td>3937257|F|0-15:G>A-15:G>A</td><td>TGCAGTACAACGGGAGCGGACGGCGTTGAAAACGGACCG</td><td>15:G>A</td></tr>
<tr><td>1018962|F|0-5:C>T-5:C>T</td><td>TGCAGCCGCTGTGCCATGATCACGTTCCCGCCGACAGTCGCCGTGTTCCG</td><td>5:C>T</td></tr>
<tr><td>1018962|F|0-29:G>T-29:G>T</td><td>TGCAGCCGCTGTGCCATGATCACGTTCCCGCCGACAGTCGCCGTGTTCCG</td><td>29:G>T</td></tr>
<tr><td>3024045|F|0-16:C>T-16:C>T</td><td>TGCAGGATCCTCGGGCCATTCGTTTCTTTGATCGCCG</td><td>16:C>T</td></tr>
<tr><td>5371587|F|0-8:C>T-8:C>T</td><td>TGCAGCAACGCCACCAACGTGCCG</td><td>8:C>T</td></tr>
<tr><td>1062282|F|0-20:C>G-20:C>G</td><td>TGCAGCCACCCGTCCCTCGTCGCCGTGTACAGCGTCCCTCCCGCCGCCGCGTCCACGTACACGTCCTCC</td><td>20:C>G</td></tr>
<tr><td>985682|F|0-33:G>A-33:G>A</td><td>TGCAGGTCCTGAGATGATGATGAAGATGCAGCAGCGCAAGTTCATCCAGGACCG</td><td>33:G>A</td></tr>
<tr><td>1694890|F|0-55:C>T-55:C>T</td><td>TGCAGGACGTGTTCCGTCGCTTCGCCTTCGACTGCATATGCAAGATCTCCTTCGGCCTCGACCCGAGAT</td><td>55:C>T</td></tr>
<tr><td>32021634|F|0-13:A>G-13:A>G</td><td>TGCAGCAGCCGCCACCCTCCCCG</td><td>13:A>G</td></tr>
<tr><td>4989030|F|0-8:T>C-8:T>C</td><td>TGCAGCCGTTTCGGGTTCTGCGGCAGCGGCTCCGACTGGTGCGGAGCCG</td><td>8:T>C</td></tr>
<tr><td>5373111|F|0-11:G>T-11:G>T</td><td>TGCAGCGCTGCGGGAAGAGCTGCCG</td><td>11:G>T</td></tr>
<tr><td>3943684|F|0-5:G>T-5:G>T</td><td>TGCAGGTGTTCTGGTCCGTGGTGCAGCCG</td><td>5:G>T</td></tr>
<tr><td>5358687|F|0-10:G>C-10:G>C</td><td>TGCAGTTGACGACGAGGATGCCGACGTCCTTGGGCTTGACGCCG</td><td>10:G>C</td></tr>
<tr><td>1002448|F|0-54:G>T-54:G>T</td><td>TGCAGGAAGGCCGCCCTGGGCCGCATGGATCTCGCCACCTCGGGGGTGCTGATGGCCG</td><td>54:G>T</td></tr>
<tr><td>5367573|F|0-13:C>G-13:C>G</td><td>TGCAGAAGAAGGACAGCTTGAACTTGGGTAGCCG</td><td>13:C>G</td></tr>
<tr><td>3028258|F|0-14:G>A-14:G>A</td><td>TGCAGTATGCTTGCGCCGACGCCTTTGCATCGTTCGAGGTTGGCCG</td><td>14:G>A</td></tr>
<tr><td>3951089|F|0-10:G>C-10:G>C</td><td>TGCAGGTGCTGTGCTCCCACAAGTCCG</td><td>10:G>C</td></tr>
<tr><td>6045421|F|0-26:G>A-26:G>A</td><td>TGCAGCACCTTCACCACCATGAAGGCGCTGGTGGAATCGAGCCCG</td><td>26:G>A</td></tr>
<tr><td>1020101|F|0-34:C>T-34:C>T</td><td>TGCAGGTGGGGTTCTACTCCAAGTCGTGCCCCAGCGCCGAGTCCACCGTCGCGTCCGTCGTCCG</td><td>34:C>T</td></tr>
<tr><td>5373593|F|0-13:A>C-13:A>C</td><td>TGCAGCTCTAGCGACCTGAGCGCGCCCATCAGCCGCGCCG</td><td>13:A>C</td></tr>
<tr><td>1666899|F|0-40:G>A-40:G>A</td><td>TGCAGTATACCCACGCGGTGCTGTTCGAGAACATGCGGCTGTTCCCGCCG</td><td>40:G>A</td></tr>
<tr><td>5363839|F|0-13:A>G-13:A>G</td><td>TGCAGGCTGGCGCATTTCCCGAGCTCCG</td><td>13:A>G</td></tr>
<tr><td>3020399|F|0-19:T>C-19:T>C</td><td>TGCAGCTGCTCTATCCCCTTGCCGTGCCACAGTATCCG</td><td>19:T>C</td></tr>
<tr><td>4439426|F|0-14:C>G-14:C>G</td><td>TGCAGCCTGCCGTTCCCGCTGAAGATCCG</td><td>14:C>G</td></tr>
<tr><td>3937379|F|0-26:G>C-26:G>C</td><td>TGCAGCGGCGTGCCCATGAAGGTGACGAGCCCG</td><td>26:G>C</td></tr>
<tr><td>32021461|F|0-7:T>C-7:T>C</td><td>TGCAGGCTCCACTCCGACCGAAGGACACGACTCCG</td><td>7:T>C</td></tr>
<tr><td>1041292|F|0-25:G>C-25:G>C</td><td>TGCAGTAATGGGACAGGAACTCCATGGTGAACCGCTTCATCTCCAGGTCGTCCCCGTAGTCCG</td><td>25:G>C</td></tr>
</table>

1020101|F|0-56:G>A-56:G>A	TGCAGGTGGGGTTCTACTCCAAGTCGTGCCCCAGCGCCGAGTCCACCGTCGCGTCCGTCGTCCG	56:G>A
1707771|F|0-27:G>A-27:G>A	TGCAGGTTCGACGCTCGCCTGCTGCTGGAGCGGCTGCGCAACAAGCGGCTGATGTTCGTGGGAGATTCG	27:G>A
3025752|F|0-28:C>G-28:C>G	TGCAGTTGGTGTCCTGGCTGATCAAATTCAGGGTGGTTCCG	28:C>G
3939531|F|0-17:C>G-17:C>G	TGCAGCGGGATCAAGCTCAGGAACATTAGGCTGAGGTACCG	17:C>G
3025752|F|0-32:G>T-32:G>T	TGCAGTTGGTGTCCTGGCTGATCAAATTCAGGGTGGTTCCG	32:G>T
1024013|F|0-14:G>C-14:G>C	TGCAGCTTGTACGTGGACGGCCTCATCACGGCGGCGGCGACGCCGTCCG	14:G>C
4261737|F|0-17:T>C-17:T>C	TGCAGCTGCATGCATGTTGTCTGTTTGCAAATTCCAACCG	17:T>C
3934195|F|0-9:G>C-9:G>C	TGCAGAATTGTGCGCCGTGAGATCTCCG	9:G>C
1038614|F|0-48:A>T-48:A>T	TGCAGCTGATCGGCACGTGCAATGGTCTTCTCTGCCTGTGCGACAACAAGGAGGCACCCG	48:A>T
1010970|F|0-5:G>A-5:G>A	TGCAGGAAGTCGTACAGCTTCACCTGGTTCCACGCCACCTCCATCCCCTGGTACTCGTCGAACGCCCGA	5:G>A
1036613|F|0-5:G>A-5:G>A	TGCAGGTTCTGGTCCCTCTGGAAGCCCTTGCCGCACACCTCGCACACGAACCG	5:G>A
1160898|F|0-52:T>C-52:T>C	TGCAGCTTGTCATAGCCGCGCTTCTTGCGCTGTCCCTGTCCACGACAGCGTCTTCGTCGCCTGCGCCGG	52:T>C
5004792|F|0-27:T>C-27:T>C	TGCAGGGGCAGGGCCAACCCACCCGTGTCAATGGACTTCTTCGGGAACATGGTGCTCTGGGCTTTCCCA	27:T>C
5367197|F|0-11:T>C-11:T>C	TGCAGCTGCTGTGTGTTCGGGTGGAACGCCG	11:T>C
100466643|F|0-44:G>A-44:G>A	TGCAGACAGAAGAGAAGATCAAAAGAGATCGGGCGATGCAGCACGAGATTGGAGGGCAACAACAAGCGC	44:G>A
5363712|F|0-26:A>G-26:A>G	TGCAGGACCGTGACGACGGCGGCGGCAATGTAGTCCGCCTCCG	26:A>G
12697122|F|0-16:C>G-16:C>G	TGCAGGAGACGTACGCCTGCTCGCCG	16:C>G
3024920|F|0-13:C>T-13:C>T	TGCAGCTCTTCTCCATGATGCTGCGCCAAGGTGTTCGACCG	13:C>T
1034740|F|0-15:A>T-15:A>T	TGCAGGAGGAAGAACATGGCCTTCTTTCTTCTTTGCCCACAGAACTGCATAAAGGCCG	15:A>T
1034740|F|0-18:G>A-18:G>A	TGCAGGAGGAAGAACATGGCCTTCTTTCTTCTTTGCCCACAGAACTGCATAAAGGCCG	18:G>A
1010970|F|0-35:C>T-35:C>T	TGCAGGAAGTCGTACAGCTTCACCTGGTTCCACGCCACCTCCATCCCCTGGTACTCGTCGAACGCCCGA	35:C>T
32024837|F|0-23:T>C-23:T>C	TGCAGTTGGGACGCTACCTGGTCTCGCAGCCTCGTCCG	23:T>C
4261574|F|0-5:G>A-5:G>A	TGCAGGTCCTGGCCGCCGCTGAGCCG	5:G>A
1020063|F|0-44:G>T-44:G>T	TGCAGGAGCTCCCTGAGGATCTCCAGGTCGCCGCCCCTCGCCGCGGCGTGCATTGCCCG	44:G>T
3951141|F|0-7:G>C-7:G>C	TGCAGAGGATGATGGCCAGGCTCTTGTTCCG	7:G>C
3947951|F|0-21:A>G-21:A>G	TGCAGGCTCTGGATGGCGCGGACACGCGGCTCCG	21:A>G
1862682|F|0-25:G>A-25:G>A	TGCAGGACGTGTTCCTCAGGCTGACGTTCGACACGACGACCACGCTGGTGTTCGGCGTCGACCCGAGAT	25:G>A
3020710|F|0-30:C>G-30:C>G	TGCAGGTTGTTGTATCTGCTGAAGGTCTTGCAGCACACCG	30:C>G
2245631|F|0-9:G>A-9:G>A	TGCAGACGCGTTGAATCGATTCCTTGGTGCGCGGTGATTGCCGAGGGGAGCGAGATGGTAGAGAAGCGC	9:G>A
32017693|F|0-17:C>G-17:C>G	TGCAGCGTGGAGTCGAACGTGCGCACCTCCG	17:C>G
7915621|F|0-62:A>C-62:A>C	TGCAGGTAGCCGCCACCGAACGTGGGAGGAGAAGGCGTGGGAGACGCGTACAGCGTTGGGGGAGGTGAG	62:A>C
100560467|F|0-7:G>A-7:G>A	TGCAGGTGCGAGTTATCGTACACCCCG	7:G>A
3027505|F|0-11:G>A-11:G>A	TGCAGACATGCGACGGATGGAACCGCCAGTCAGATAGAGCCG	11:G>A
4397689|F|0-20:G>A-20:G>A	TGCAGGTAGACGCCGAGCATGGACACCTGGCCG	20:G>A
1088390|F|0-38:G>A-38:G>A	TGCAGCATCGCCAAGGCGACATTCGTGATCCTGGGCGAGAAGATGGACATCCAGTGCACTGACGAGGAC	38:G>A
32024147|F|0-11:G>A-11:G>A	TGCAGCTATCAGGATCTCGACGCTCCTGGAGATGAAGTGACAGTTTTGGACTACCG	11:G>A
5581545|F|0-19:T>C-19:T>C	TGCAGGTGAAGCTTGTGGGTTACAACCCG	19:T>C
3945781|F|0-16:T>C-16:T>C	TGCAGGTAGGGCAGGTTGGGGAAGTCCGTCTCCGACAGCACCCG	16:T>C
1143285|F|0-20:A>G-20:A>G	TGCAGGACGGTGATGCCACGAGGGTTCCCTGTGCGTTTTCTAGCGAGCGGGACGAGGATCCG	20:A>G
3031511|F|0-8:C>T-8:C>T	TGCAGGAACAGCGTGTCCAGCTTCGCGAGATTCCCGAGCTCCG	8:C>T
4261737|F|0-9:A>G-9:A>G	TGCAGCTGCATGCATGTTGTCTGTTTGCAAATTCCAACCG	9:A>G
4008865|F|0-15:T>C-15:T>C	TGCAGGCCGTAGACGTAGTCGACGATGCACTTCTGCTAGGCGCTGCTGAACACGCCG	15:T>C
3956050|F|0-22:T>G-22:T>G	TGCAGGGCCTTGAGGCGCGACATGATGGCGTGGAGGAGGTCGTCCG	22:T>G
1009308|F|0-37:C>A-37:C>A	TGCAGTTCACTCCGTTGGAGAAGATCATCAGGGACGCCGTGGAGAGCCTCCG	37:C>A
5326403|F|0-23:C>G-23:C>G	TGCAGATGACCCTGTACGAGCTGCTGATAATCTGCGACAAGGAGACGAGTCGGCTGCTGAACCG	23:C>G
3944666|F|0-13:C>T-13:C>T	TGCAGCACCTCCACGTTGGACCG	13:C>T
1041292|F|0-13:C>G-13:C>G	TGCAGTAATGGGACAGGAACTCCATGGTGAACCGCTTCATCTCCAGGTCGTCCCCGTAGTCCG	13:C>G
5371104|F|0-22:G>C-22:G>C	TGCAGGCGGTGATCAAGGAGACGCTCCG	22:G>C
3957199|F|0-15:G>A-15:G>A	TGCAGACGATGAAGCGGTGCGCGCCCCG	15:G>A
6049333|F|0-13:T>C-13:T>C	TGCAGCAGGAGCTTGCCGACACAGTGGGTCTCGACCG	13:T>C
13590222|F|0-5:A>G-5:A>G	TGCAGACGATGAAGCGATGCGCGCCCCG	5:A>G
3031511|F|0-26:G>A-26:G>A	TGCAGGAACAGCGTGTCCAGCTTCGCGAGATTCCCGAGCTCCG	26:G>A
3021021|F|0-5:T>C-5:T>C	TGCAGTGGGCTATTTCACATGCACACTCCAACCGAACCG	5:T>C
7170931|F|0-13:C>T-13:C>T	TGCAGTGCTCCTACCACGGCTGGTCCTTCGACGGCTCCG	13:C>T
986029|F|0-19:C>T-19:C>T	TGCAGCACATCAGCCCATACTTCCTGATCAGTCCATGGGAGTTCCCACAGACCCG	19:C>T
1122854|F|0-49:G>A-49:G>A	TGCAGATTCTTGCTGTGGCATGCGCTTGTATATCTTTGTTCTCTTGTGTGCCG	49:G>A
1052623|F|0-28:T>G-28:T>G	TGCAGCCACGTCCACTGCGGCGTGTACTTGCCGTAGGCAGAGTAGGAGGAGAGCATGATGACGTGCGCC	28:T>G
1041292|F|0-48:C>T-48:C>T	TGCAGTAATGGGACAGGAACTCCATGGTGAACCGCTTCATCTCCAGGTCGTCCCCGTAGTCCG	48:C>T
1041292|F|0-58:G>T-58:G>T	TGCAGTAATGGGACAGGAACTCCATGGTGAACCGCTTCATCTCCAGGTCGTCCCCGTAGTCCG	58:G>T

4262243|F|0-30:G>A-30:G>A	TGCAGGTTGCACAGAGCAGCAGGAGCACTAGCCCG	30:G>A
4008040|F|0-7:C>T-7:C>T	TGCAGGCCGTCTACTCCAGCATGCGCGGCCTCGTCCG	7:C>T
10984460|F|0-9:C>T-9:C>T	TGCAGGAGGCCCTGGAGAAGCTCCG	9:C>T
1017643|F|0-33:T>C-33:T>C	TGCAGGTGGGCAAGGCGCTGGCCAAGGAGGGCGTCTTCGTGGAGTGCCACGTGTTCAACCCGCCGTCCG	33:T>C
5363839|F|0-22:G>A-22:G>A	TGCAGGCTGGCGCATTTCCCGAGCTCCG	22:G>A
3940903|F|0-14:G>A-14:G>A	TGCAGCATGCCCTCGCGCTCCTCCAGGAACTGCTCCG	14:G>A
2245453|F|0-44:G>A-44:G>A	TGCAGGATGTCCCTGATCTGGTCGATAGCGTACCCTGCCCTGGGGTCTTCCCTCTCGACGCCCTTGTGG	44:G>A
32020106|F|0-10:G>C-10:G>C	TGCAGACGCTGTCGCTCGAGTCCAACAACTTCTCCG	10:G>C
5580186|F|0-11:C>G-11:C>G	TGCAGCAGCTTCCTTCCCTCCTCCCCGACGAACCG	11:C>G
1034740|F|0-54:G>A-54:G>A	TGCAGGAGGAAGAACATGGCCTTCTTTCTTCTTTGCCCACAGAACTGCATAAAGGCCG	54:G>A
1014615|F|0-43:G>T-43:G>T	TGCAGGCAGTGCTGCGGCAGCGCCGAGAAGCCCTCCACGCCGAGGATGGCCATGTTGCGGTAGATGAGC	43:G>T
1041292|F|0-31:C>G-31:C>G	TGCAGTAATGGGACAGGAACTCCATGGTGAACCGCTTCATCTCCAGGTCGTCCCCGTAGTCCG	31:C>G
12469790|F|0-20:C>T-20:C>T	TGCAGCTGGTTCTTGTACATCGATCCG	20:C>T
3955058|F|0-10:G>C-10:G>C	TGCAGGCCCTGCTCGCGCACCTGTGGCGTGCGGTGTGCCG	10:G>C
1061265|F|0-11:G>A-11:G>A	TGCAGCTTGCTGAAAGGCAGGGGGATCGGCACGGGGCTGGCATCAGTGAACCACCTCCG	11:G>A
32019176|F|0-5:C>T-5:C>T	TGCAGCTGCTGGCCATGGGCACCAGGCCCG	5:C>T
12696273|F|0-8:T>C-8:T>C	TGCAGAGCTTCTGGGAGCCG	8:T>C
100440224|F|0-14:C>T-14:C>T	TGCAGCCTCTGCTGCTGCTCCCTATCGTGGTCGAACCGCCG	14:C>T
13880648|F|0-5:G>C-5:G>C	TGCAGGTTGGCGTTGCCCGACCG	5:G>C
4993450|F|0-8:T>C-8:T>C	TGCAGCCATGCGACCTGCTCGGCGTACTTGTCCG	8:T>C
3020934|F|0-32:G>A-32:G>A	TGCAGCAACACGGTCTTGAGCAGGAAGTTCTTGACAATCTCCG	32:G>A
991492|F|0-12:G>C-12:G>C	TGCAGGTGCTGAGCAACAAGAAGTTCAAGAGCGCCACGCACAGGGTGGTGAGGAGGCCG	12:G>C
1021258|F|0-8:G>C-8:G>C	TGCAGCATGGAGATGCCGCTCCTCACCCTGCCCCCGATCTCCGCCAGATCGCTCCG	8:G>C
1038581|F|0-10:C>T-10:C>T	TGCAGCTGGGCGCCTGGCCGCTCTACTGGGTCGTGCAGGGCTGCGTCATGACCG	10:C>T
1026860|F|0-13:A>G-13:A>G	TGCAGGGCCCATCAGAAGTGTGGCTGACACTGATGCTGTTGTTGGGCTGGTTCTGGTCCG	13:A>G
3020972|F|0-10:G>A-10:G>A	TGCAGAACGAGTACCCATCGGACCTACACCTTCTTGTGACCG	10:G>A
3024805|F|0-30:C>A-30:C>A	TGCAGAGCCCAAGCCCCACGACCAGATTCCCAGGCCCACCCG	30:C>A
1038581|F|0-7:G>T-7:G>T	TGCAGCTGGGCGCCTGGCCGCTCTACTGGGTCGTGCAGGGCTGCGTCATGACCG	7:G>T
1009308|F|0-14:T>C-14:T>C	TGCAGTTCACTCCGTTGGAGAAGATCATCAGGGACGCCGTGGAGAGCCTCCG	14:T>C
1713915|F|0-7:T>G-7:T>G	TGCAGGTTGTCGGCCAGCTCCGCGATGTCGACGGCGATGCTCTCCTCCGTCCGCGCCG	7:T>G
1050374|F|0-22:C>G-22:C>G	TGCAGAGCTCGGACACGTTCCTCACCATGCTGCTGCTGCTGTCCAAGTACCG	22:C>G
7170761|F|0-20:T>G-20:T>G	TGCAGCTTCTCCTCGGGGCTTGCCTGGAGGAGGTAGGATGTGACGCCG	20:T>G
22841518|F|0-34:G>C-34:G>C	TGCAGTTCACGGCCACCCTCACCTTGGTGAACTCGTCCG	34:G>C
5581941|F|0-23:C>G-23:C>G	TGCAGCAGCGGCCCGACCACCAGCACCG	23:C>G
7170931|F|0-19:C>A-19:C>A	TGCAGTGCTCCTACCACGGCTGGTCCTTCGACGGCTCCG	19:C>A
3571381|F|0-26:C>G-26:C>G	TGCAGGTGGGACAAGGCGTACAGCTTCGCCTACCACCCGACGTCCG	26:C>G
32023383|F|0-29:A>G-29:A>G	TGCAGGCTGTGCGCGGCCGCGATCACCGCATGGTCCG	29:A>G
3574722|F|0-9:C>T-9:C>T	TGCAGCATGCTCTCGCGCTGCTCGAGGAACTGCTCCG	9:C>T
1019171|F|0-13:C>T-13:C>T	TGCAGCACGTGTACCTGAACCTGGGCGGGGAGGGCAGCGGCGACATCCTGGGCCACACGCTCCAGCTCT	13:C>T
3943264|F|0-5:A>C-5:A>C	TGCAGATCAGCCCTCCCTCGCCAAAGGCGCCG	5:A>C
7353345|F|0-15:G>A-15:G>A	TGCAGCAGCGGGTCGGCGCGCCG	15:G>A
3945070|F|0-14:G>C-14:G>C	TGCAGCGAGTTCTGGACCCCTCCGACGACGCACCG	14:G>C
4261261|F|0-19:A>G-19:A>G	TGCAGAGGAGAAATCGAAAAGCACGAACCTTGCCG	19:A>G
7170761|F|0-35:G>A-35:G>A	TGCAGCTTCTCCTCGGGGCTTGCCTGGAGGAGGTAGGATGTGACGCCG	35:G>A
6043670|F|0-19:G>C-19:G>C	TGCAGAGGTTCGAGGTGCAGGGCAACGCCTTCACCG	19:G>C
32021310|F|0-8:C>G-8:C>G	TGCAGATTCTAGCGCTAGGTGTTCCG	8:C>G
1069052|F|0-8:C>G-8:C>G	TGCAGCACCGACAGCTGCACGACGATGTCGCGCGCCCACTTGCCGTACATGGCGTCCAGCAGCGGCACG	8:C>G
1017668|F|0-7:T>C-7:T>C	TGCAGCCTGTGCCTCGCGCGCACGTGCCCGTCATCAAGATGAGCTTCCGCGGCGTGCAGGTGGACCTCC	7:T>C
4998348|F|0-23:G>A-23:G>A	TGCAGGGAGTTTCTATATGAAACGCAAACAGAACCG	23:G>A
3024805|F|0-20:A>C-20:A>C	TGCAGAGCCCAAGCCCCACGACCAGATTCCCAGGCCCACCCG	20:A>C
1004340|F|0-10:T>C-10:T>C	TGCAGTATCCTAGCCTGTTGCTGGAGTTCATGGGGAGCTATCTCACCGAGCTGAGCCTTTTGGATTATG	10:T>C
5360556|F|0-11:G>T-11:G>T	TGCAGACCTTCGGCGAGCCACTGGTGGAGGGTGGCGCGGTCGAGGCGGATCTCGACGGCGGCGCGGTCC	11:G>T
983746|F|0-24:G>A-24:G>A	TGCAGCTTCTGCTTCAGGGAGATCGCCGAGTTTGCGTTCAAGATGAAGGCGTACAGCCACGCGGACCGA	24:G>A
3028678|F|0-30:T>C-30:T>C	TGCAGCGTCTTCATGTTGAGCGACATCTCGTCCGCCG	30:T>C
4440295|F|0-13:C>A-13:C>A	TGCAGGTCCAGGGCCACGCCG	13:C>A
1026860|F|0-10:A>G-10:A>G	TGCAGGGCCCATCAGAAGTGTGGCTGACACTGATGCTGTTGTTGGGCTGATTCTGGTCCG	10:A>G
5363676|F|0-7:G>C-7:G>C	TGCAGCTGCTGCTCGCCGTCGGTCACCAAGCAAAGCCGCCCATCCG	7:G>C
4261204|F|0-9:A>G-9:A>G	TGCAGAGGAACCTCCCTCTTACGCTTCAGATGGGCTGATCCG	9:A>G
5582047|F|0-6:C>T-6:C>T	TGCAGGCGGCCAGGTACCTGCTCGGCTCCCCG	6:C>T

2245768|F|0-44:G>A-44:G>A	TGCAGCGAGGAGTGTAGGAGGAAGCAAATCGAGACGGAGAAGGCGAGACACCG	44:G>A
3959679|F|0-9:A>G-9:A>G	TGCAGACGCACCACTTCGCCTACGAGGAGCTGGAGGAGGCCACCG	9:A>G
1031053|F|0-8:A>G-8:A>G	TGCAGACGATCCGCAAGGCTGCCGTTCTCCATGTACTCGTACACCAAGCAGCCCTTGTCCG	8:A>G
7907133|F|0-8:A>G-8:A>G	TGCAGGGCAGCTACGAGCCG	8:A>G
4003819|F|0-6:A>T-6:A>T	TGCAGCAGGAGCATCAGCAGAGCACATGCTTGGAATGGAAGGCCG	6:A>T
997667|F|0-25:T>C-25:T>C	TGCAGGTACTCCAAGCAGGGCAGGTTCTGAAACTCCATCGGAATTGCGCCG	25:T>C
3947875|F|0-6:T>C-6:T>C	TGCAGCTTGAGGGAGAAGGTCCG	6:T>C
32020228|F|0-6:A>G-6:A>G	TGCAGCATAAAGCAACGGCGAGTGGGAGGAGCCCG	6:A>G
3025434|F|0-7:A>G-7:A>G	TGCAGGGATTGATGATCTTCTGGCCCCGAGCTCTGCCTCGCCG	7:A>G
100488262|F|0-68:A>G-68:A>G	TGCAGGAGCGCGACGGGGTTCTGGGAGGGGCTGTAGCTGGGGCGCCACTCGACGTCGATCCCGACGACA	68:A>G
5003216|F|0-14:T>C-14:T>C	TGCAGCGACTCGTCTGAGTAGGACCCCTTCCGCATGAACCG	14:T>C
4998077|F|0-65:A>C-65:A>C	TGCAGCCGCTCCTCGGCGGCTCTACCGTTGATGAGCAGGCGCGTGGGAATGGAGCGGCCAAGGAGAGAG	65:A>C
100481434|F|0-16:T>C-16:T>C	TGCAGGTGCTCGACCTTAGTTACAATAACCTCTCCG	16:T>C
3020710|F|0-17:G>A-17:G>A	TGCAGGTTGTTGTATCTGCTGAAGGTCTTGCAGCACACCG	17:G>A
100387642|F|0-15:A>G-15:A>G	TGCAGGAATCCATATACAACCTTCCG	15:A>G
4008040|F|0-25:C>T-25:C>T	TGCAGGCCGTCTACTCCAGCATGCGCGGCCTCGTCCG	25:C>T
3946333|F|0-12:G>A-12:G>A	TGCAGGCCACCCGCGCCGACGCCCG	12:G>A
1053523|F|0-40:C>T-40:C>T	TGCAGCTGGATCAGCGATGAGTAGGCGCTGAGAGGGTTGGCCATGAGCTGCTCGTGCGTGCCG	40:C>T
3949598|F|0-5:A>C-5:A>C	TGCAGATCCTGTCCAACGACCG	5:A>C
4261261|F|0-9:G>A-9:G>A	TGCAGAGGAGAAATCGAAAAGCACGAACCTTGCCG	9:G>A
4261261|F|0-29:T>A-29:T>A	TGCAGAGGAGAAATCGAAAAGCACGAACCTTGCCG	29:T>A
1019150|F|0-13:C>G-13:C>G	TGCAGTCGTGGTGCAGGTAGCAGAGCCCGCGCGCCGCCTCCAGCGCGATCCG	13:C>G
1058456|F|0-39:A>T-39:A>T	TGCAGTTCGCGGTGCCCATGGTCCTCACCAACATGTCCTACTACGGCATCCCGCTGGTGTCCGTCATGT	39:A>T
1026249|F|0-41:A>C-41:A>C	TGCAGATGCATCTCGTCGCGCTCATGCAGCAAAGGACCCAGATGCCTCCG	41:A>C
13879537|F|0-7:A>G-7:A>G	TGCAGCAAGATGACCAAGTCGACCTCGCCG	7:A>G
5363676|F|0-28:A>T-28:A>T	TGCAGCTGCTGCTCGCCGTCGGTCACCAAGCAAAGCCGCCCATCCG	28:A>T
1041520|F|0-6:A>C-6:A>C	TGCAGGAGACGACGAGGGAGGAGGAGGAGCTCTTGGGGTGGAAGACGGGGAAGGAGCCG	6:A>C
5365128|F|0-8:C>G-8:C>G	TGCAGATCCAAGGCGCGATGAGGGTGGTCGAGGCCACCAACGACGACAGGGAGCTGAGGATATACATGT	8:C>G
3934238|F|0-20:C>T-20:C>T	TGCAGCGCGCGCGCGTCGGCCAGGAAATGGCCCG	20:C>T
991492|F|0-37:G>A-37:G>A	TGCAGGTGCTGAGCAACAAGAAGTTCAAGAGCGCCACGCACAGGGTGGTGAGGAGGCCG	37:G>A
1021132|F|0-34:C>T-34:C>T	TGCAGGCGTACACCAGGGCACGCTACGGCGAGGACGCGATGATCGTGTACCG	34:C>T
1018396|F|0-22:G>A-22:G>A	TGCAGCTAACCTCGGTTGGACTGTCAAAATCAAGTGTTCATATCTGACGTGATTTGGACCTAGCTAGAA	22:G>A
3935248|F|0-40:G>A-40:G>A	TGCAGGTCATGGCCAACATCGCCGACGCGGTCATCGGGGAGACCG	40:G>A
1021258|F|0-35:G>T-35:G>T	TGCAGCATGGAGATGCCGCTCCTCACCCTGCCCCCGATCTCCGCCAGATCGCTCCG	35:G>T
3940025|F|0-10:G>A-10:G>A	TGCAGTTGCGGAGGCCCATGCTGCTCCG	10:G>A
1043700|F|0-50:C>T-50:C>T	TGCAGTCGGAGCGCCTCCTTATGCAGCAAGCTATTGTTGTGCTCCTCTAGCGCACTCATCTGCATGTTC	50:C>T
5360556|F|0-29:G>A-29:G>A	TGCAGACCTTCGGCGAGCCACTGGTGGAGGGTGGCGCGGTCGAGGCGGATCTCGACGGCGGCGCGGTCC	29:G>A
1091000|F|0-48:C>A-48:C>A	TGCAGGTGGGCAACGACCTCAACCACCGCTTCCTAGCGCGGCTGTCGGCGCGGTACGGCCCCGTGTTCC	48:C>A
1043700|F|0-20:A>G-20:A>G	TGCAGTCGGAGCGCCTCCTTATGCAGCAAGCTATTGTTGTGCTCCTCTAGCGCACTCATCTGCATGTTC	20:A>G
7170761|F|0-12:T>G-12:T>G	TGCAGCTTCTCCTCGGGGCTTGCCTGGAGGAGGTAGGATGTGACGCCG	12:T>G
4261446|F|0-19:C>G-19:C>G	TGCAGGCGCCGATGATGACCCTTCCG	19:C>G
1014615|F|0-35:C>G-35:C>G	TGCAGGCAGTGCTGCGGCAGCGCCGAGAAGCCCTCCACGCCGAGGATGGCCATGTTGCGGTAGATGAGC	35:C>G
4261851|F|0-13:C>G-13:C>G	TGCAGCTGTTGGTCAAGGACGGCGAGAGCCGCGTCACCATCGCCG	13:C>G
1071245|F|0-7:C>T-7:C>T	TGCAGGCCATGAGTGCCAACCAGAGCAGCATGCTGATCCTTCAGGAAACATGCACTGATGCATCCGAGA	7:C>T
3571381|F|0-17:G>C-17:G>C	TGCAGGTGGGACAAGGCGTACAGCTTCGCCTACCACCCGACGTCCG	17:G>C
4261851|F|0-7:G>A-7:G>A	TGCAGCTGTTGGTCAAGGACGGCGAGAGCCGCGTCACCATCGCCG	7:G>A
3956914|F|0-15:A>G-15:A>G	TGCAGGCGTGGGAGCATACATTCGGCGACAAGGTTCGGGAGTTCCG	15:A>G
4262704|F|0-31:C>G-31:C>G	TGCAGGCGCAGGCGGACGACAAGTTCTTCGCCGAGTTCTACCG	31:C>G
3934492|F|0-5:G>A-5:G>A	TGCAGGGCCAGCACGGTGAGGCGGTGCTCCG	5:G>A
3942787|F|0-9:C>T-9:C>T	TGCAGCTGGCGGAGCTCCGTCTCGTACCCCTCCG	9:C>T
7908938|F|0-10:A>C-10:A>C	TGCAGCTTGGATAGCCGCGAGATCAGGCCCG	10:A>C
1018396|F|0-18:G>C-18:G>C	TGCAGCTAACCTCGGTTGGACTGTCAAAATCAAGTGTTCATATCTGACGTGATTTGGACCTAGCTAGAA	18:G>C
1666712|F|0-45:T>C-45:T>C	TGCAGGACGTGTTCATGCGCCTGACGTTCGACCTCACGGCGATGTTCATATTCGGTACGGACCCCGAGA	45:T>C
1004340|F|0-22:G>A-22:G>A	TGCAGTATCCTAGCCTGTTGCTGGAGTTCATGGGGAGCTATCTCACCGAGCTGAGCCTTTTGGATTATG	22:G>A
1262989|F|0-29:G>C-29:G>C	TGCAGGGTGCGGAAGTCCTTGAGCGGCTTGGCCCGCGGGTGGTCGATGTTGTTCCG	29:G>C
3020326|F|0-11:A>G-11:A>G	TGCAGTCAAAGATGTGTAGTGTAGAGAAAACAAGAAACTCACCG	11:A>G
1032506|F|0-39:A>G-39:A>G	TGCAGAAGCCGCAGAGATGACAGCAGAAGATGAGCCACTAAACCCTTGAAACACACCAGCAGCCATTGA	39:A>G
1132668|F|0-35:G>A-35:G>A	TGCAGATGCTGACCTACCTCGCCATGAAGCACGACGCGCGCGTGCAGCGGGTCGACACGGTCCG	35:G>A
1666899|F|0-19:G>T-19:G>T	TGCAGTATACCCACGCGGTGCTGTTCGAGAACATGCGGCTGTTCCCGCCG	19:G>T

3936319|F|0-21:T>A-21:T>A	TGCAGCATTTCCGCTCCTACTTCCAAAGGGTAAACAGGAGTGCACCG	21:T>A
3032674|F|0-34:G>A-34:G>A	TGCAGGTGGAGGAGATGAAGGGCTTGGGCGCGAGGATCCG	34:G>A
4262536|F|0-20:C>G-20:C>G	TGCAGCTCGTCCGCCGCCAGCTCCGCGTCGCCGTCCG	20:C>G
4008040|F|0-16:C>T-16:C>T	TGCAGGCCGTCTACTCCAGCATGCGCGGCCTCGTCCG	16:C>T
1019241|F|0-8:C>T-8:C>T	TGCAGCTTCTTGGTGTCCGTGGTGCTCCAGGAGATGAGGTGGAACAGCCG	8:C>T
1014615|F|0-12:T>C-12:T>C	TGCAGGCAGTGCTGCGGCAGCGCCGAGAAGCCCTCCACGCCGAGGATGGCCATGTTGCGGTAGATGAGC	12:T>C
3944666|F|0-7:C>A-7:C>A	TGCAGCACCTCCACGTTGGACCG	7:C>A
3956664|F|0-25:G>A-25:G>A	TGCAGCTGTAGAAGGTCGACAGGTCGGCGCTCGACCG	25:G>A
1695387|F|0-26:T>C-26:T>C	TGCAGAACACCAACGCCGAGATCTCCTTCTCGTCGGGGCACCGTCCGAAGCCTGATGCGTTCGAGCTCT	26:T>C
7169369|F|0-9:A>G-9:A>G	TGCAGCATGAAGCGCATTCTGTCCATCTGGCCCG	9:A>G
1051309|F|0-39:G>C-39:G>C	TGCAGCGTGCTGCTCGCCCTCATCCTCGTCCTCGCCATCGTCTTCGTCGCGCTCTACTTCACCGTGTTC	39:G>C
4262876|F|0-12:G>C-12:G>C	TGCAGAAGCTGCGCCTGCCTGAGTGCGCCG	12:G>C
1083636|F|0-15:C>T-15:C>T	TGCAGCGACATGTCCCTGGACGAGATGGACGCCGCGGTGAGGAGGATGGAGTTCGTCGTCTTCGACCGA	15:C>T
3934914|F|0-10:T>C-10:T>C	TGCAGGAACATCCACACCGACCG	10:T>C
32022470|F|0-31:G>C-31:G>C	TGCAGCTGTAGAAGGTCGACAGGTCGGCGCTGGACCG	31:G>C
4993981|F|0-55:G>C-55:G>C	TGCAGGACGTGGAGGCTTCGCTGAGGCAGTCGAGGGCGCCGTGGAAGATCGTCGTGGGGCACCACCCCA	55:G>C
32025523|F|0-50:T>C-50:T>C	TGCAGCCCAACATGAGGGAGATTGAGTCCGCCCAGGACCTCGTCGACTCGTTGGCTAACGCCG	50:T>C
5358282|F|0-20:G>C-20:G>C	TGCAGGCTCACCACGCGGAGGGCCG	20:G>C
7155201|F|0-16:C>T-16:C>T	TGCAGCTGGATGTTTACCAAGAACCACCG	16:C>T
4261356|F|0-6:T>C-6:T>C	TGCAGGTGCACTAGTCGATCCG	6:T>C
1044294|F|0-16:G>C-16:G>C	TGCAGCTGCTGTCGTCGCTGGACACCACCAAGGTCAAGGACAAGAAGGACCG	16:G>C
3025086|F|0-26:A>C-26:A>C	TGCAGCAAGTGCCGCACGCCCATCCGAGGCAAGAACCCG	26:A>C
1051488|F|0-22:C>A-22:C>A	TGCAGGGGTGGGACAATGCCACCATCGCTGGTAAGTAGCCCAGATTTGTTTTACCACCTTGTTAGATCA	22:C>A
1695415|F|0-28:G>C-28:G>C	TGCAGATCTCGTGGAACTACAACTACGGGGCGGCAGGGCAGAGCCTCGGCTTCGACGGGCTCAACGACC	28:G>C
3935617|F|0-13:C>G-13:C>G	TGCAGAGCCCAAGCCCCACGACCAGCTTCCCAGGCCCACCCG	13:C>G
32027922|F|0-8:G>A-8:G>A	TGCAGTGGGCCATGGCGGAGCTGATGCGGAGCCCG	8:G>A
4262794|F|0-9:G>C-9:G>C	TGCAGCCATGGCCGCGGTAGTCACCGTAGATGTCCG	9:G>C
1079000|F|0-5:G>T-5:G>T	TGCAGGGCCAGAGTCACCTGGGAGACAACGCCGAGGACCCCAAGGGAGACCTTGGCGGCGTCCAGGTCC	5:G>T
5363838|F|0-11:G>A-11:G>A	TGCAGGCTGGCGCACTTCCCGAGCTCCG	11:G>A
3935016|F|0-5:C>G-5:C>G	TGCAGCTTGAGGGGGAAGGTCCG	5:C>G
1061949|F|0-10:T>C-10:T>C	TGCAGGCATCTGTAGTCCACCAGCGATGTTTCGGCGAGCTGATGCGCCAACCTCTGAACCTCCAGCTCG	10:T>C
1063727|F|0-43:C>T-43:C>T	TGCAGGGTTGGAGCTACGTTGGTGACAGGCTATTGTCGGCAGTCGTGCCGTATGAAGAAACCG	43:C>T
1041520|F|0-28:G>C-28:G>C	TGCAGGAGACGACGAGGGAGGAGGAGGAGCTCTTGGGGTGGAAGACGGGGAAGGAGCCG	28:G>C
1043700|F|0-6:C>T-6:C>T	TGCAGTCGGAGCGCCTCCTTATGCAGCAAGCTATTGTTGTGCTCCTCCAGCGCACTCATCTGCATGTTC	6:C>T
5358200|F|0-29:G>A-29:G>A	TGCAGGCACCATGACATGAGCGGGAAGGCGGACCCG	29:G>A
1075816|F|0-13:C>T-13:C>T	TGCAGACGACAGGCGGCACCATCCTACTGCTCAGCACTGTCCTTGGAATTAAAATCTCCG	13:C>T
3946372|F|0-5:C>T-5:C>T	TGCAGCCTCCCGAGCTCGGGCGGTATCGGCCCG	5:C>T
7173881|F|0-25:G>A-25:G>A	TGCAGAAGAAGTCGACGATGAGGACGGCCG	25:G>A
1079000|F|0-8:C>G-8:C>G	TGCAGGGCCAGAGTCACCTGGGAGACAACGCCGAGGACCCCAAGGGAGACCTTGGCGGCGTCCAGGTCC	8:C>G
3025416|F|0-5:C>G-5:C>G	TGCAGCAGCCGCTTCTCCTGGAGCTCCTGGGACAGGACCACCG	5:C>G
1019161|F|0-5:G>A-5:G>A	TGCAGGAACACGCGGAAGCTGCGCAGCTCGTCGTGGATGACCGACTTGCTCCGCGCGTAGGCCG	5:G>A
3936028|F|0-26:C>G-26:C>G	TGCAGTTGGTGTCCTGGGCGACGAGGCTGAGCCG	26:C>G
1019150|F|0-25:C>G-25:C>G	TGCAGTCGTGGTGCAGGTAGCAGAGCCCGCGCGCCGCCTCCAGCGCGATCCG	25:C>G
3027505|F|0-20:A>T-20:A>T	TGCAGACATGCGACGGATGGAACCGCCAGTCAGATAGAGCCG	20:A>T
7164296|F|0-17:A>G-17:A>G	TGCAGGTCCTTCTCGGGAGTCGACGTCTGCGACGACGCCCACCCCAGTGCCCCAAGGGCTGCTCGGCC	17:A>G
3955155|F|0-7:C>T-7:C>T	TGCAGGACACGTACCTCCCTCGCAATCCG	7:C>T
4993340|F|0-5:C>A-5:C>A	TGCAGCACGGGGAGCAGGAACGGCTTGGACGCGTTGGTGAACTCGCCG	5:C>A
1035262|F|0-5:A>G-5:A>G	TGCAGAATCTCCACCAGATAATTTAAACCAATTCAAAATCCCTAAATTACCCG	5:A>G
1035262|F|0-21:T>C-21:T>C	TGCAGAATCTCCACCAGATAATTTAAACCAATTCAAAATCCCTAAATTACCCG	21:T>C
1035262|F|0-24:A>C-24:A>C	TGCAGAATCTCCACCAGATAATTTAAACCAATTCAAAATCCCTAAATTACCCG	24:A>C
3028857|F|0-30:C>G-30:C>G	TGCAGCTGCCATGATCGGCCGATCTATCCTCGTCTCCCTCCTCCG	30:C>G
5411979|F|0-9:C>T-9:C>T	TGCAGGTGGCCACATCAACCCG	9:C>T
3022428|F|0-33:G>T-33:G>T	TGCAGCAGGTTCTTCCACGGGGATCTGAAGTCTGGTCCG	33:G>T
1047199|F|0-48:T>C-48:T>C	TGCAGCTTCTGCGTCGTCGCCTGCTCCGCCGAGAGATGATGGTGGTGGTCCACGACCG	48:T>C
1083636|F|0-14:C>G-14:C>G	TGCAGCGACATGTCCCTGGACGAGATGGACGCCGCGGTGAGGAGGATGGAGTTCGTCGTCTTCGACCGA	14:C>G
4262423|F|0-10:G>C-10:G>C	TGCAGGCGGCGACGACGACCTGGCAGAGGCTCTTGAGCGGCGAGCCG	10:G>C
100497199|F|0-36:A>G-36:A>G	TGCAGTTAGCTCCCCGACCGTCACCGTCTTGAGCTCACCGTCGGCCG	36:A>G
1019161|F|0-50:C>T-50:C>T	TGCAGGAACACGCGGAAGCTGCGCAGCTCGTCGTGGATGACCGACTTGCTCCGCGCGTAGGCCG	50:C>T
3934835|F|0-44:C>G-44:C>G	TGCAGGCACCGCGCCAGCACCCGCTCCGCCTCCTCCTGCCTGTGCTCCG	44:C>G

4006219|F|0-12:T>C-12:T>C TGCAGTAATTTATCATTTTAATCCGTGGTTGACAAATGGGCCG 12:T>C
32017739|F|0-9:G>T-9:G>T TGCAGGAAGGCACATACAGTTGAAGCATTGAGGCAGCAGTCCG 9:G>T
4261356|F|0-13:G>C-13:G>C TGCAGGTGCACTAGTCGATCCG 13:G>C
4261356|F|0-9:A>G-9:A>G TGCAGGTGCACTAGTCGATCCG 9:A>G
1035262|F|0-16:G>A-16:G>A TGCAGAATCTCCACCAGATAATTTAAACCAATTCAAAATCCCTAAATTACCCG 16:G>A
4538893|F|0-18:G>A-18:G>A TGCAGACGGGAGGAAGAAGACGAATCCG 18:G>A
1044828|F|0-25:G>T-25:G>T TGCAGGGAGAACTGGACGGAGATGTGCGCGGTGCAGCGCGGGCAATTGGCGTCGGCGTGGATCCGAGAT 25:G>T
3934836|F|0-7:C>G-7:C>G TGCAGCTCTACACCAACTTCCTCACCG 7:C>G
1020967|F|0-26:C>G-26:C>G TGCAGATCTGCTCACCAGGGACTACACCTACGACCAGAAGCTGTCCGTCTCCACCGTCAGCTCCTCCGA 26:C>G
3952211|F|0-13:G>T-13:G>T TGCAGAGCCTGCTGCACAGGCTGGGGTCCGACCCG 13:G>T
3032674|F|0-22:C>G-22:C>G TGCAGGTGGAGGAGATGAAGGGCTTGGGCGCGAGGATCCG 22:C>G
7173646|F|0-21:C>G-21:C>G TGCAGATTAGCCGCTCGTTGCCTCCCCG 21:C>G
3020885|F|0-36:C>G-36:C>G TGCAGGAGATCGATACTGTCGTTCAAAGTTCAGAAACCCG 36:C>G
4261851|F|0-10:G>T-10:G>T TGCAGCTGTTGGTCAAGGACGGCGAGAGCCGCGTCACCATCGCCG 10:G>T
4006126|F|0-19:G>A-19:G>A TGCAGCCGCGGATGGTGATGCCGTGGATGATCACGTTGCTCCG 19:G>A
5360641|F|0-40:C>G-40:C>G TGCAGAGGTTCTACCGCCCAGACAGCGGGGAGATACTGCTCGACGGCGACGACATCGGCTCGCTCAACG 40:C>G
32025868|F|0-8:C>G-8:C>G TGCAGGTCCGTGACGAGCGCCACGTACTTGTCCG 8:C>G
1740802|F|0-7:G>C-7:G>C TGCAGACGGTGGCGATGGCGCGGGACGTGCACCAGCAGCGGTACCGCATGGGGGCCG 7:G>C
4994653|F|0-20:T>G-20:T>G TGCAGCAGCGTGCTGATGGTTGCGTTCACCAGCGCGCTGAGCGGCGACGGCCTGAAGCCG 20:T>G
5002107|F|0-31:G>A-31:G>A TGCAGGTCCCGCGCTTTGCCTTCCTCCGTCAGAATCTCCATCAGGCAGCTCTCAAAACTCCG 31:G>A
5365709|F|0-11:A>C-11:A>C TGCAGGCCCAGAGCCGACTCCTCGGCCGCCTCCTTCTCCATCGCCAGCCGCGCCGCGGCCGCCTCCAGC 11:A>C
100560368|F|0-11:G>C-11:G>C TGCAGCTGCATGCCGCCGTCCGTCGCTGCCCAGCCGTGCTCACCACCCG 11:G>C
4997132|F|0-11:A>G-11:A>G TGCAGGCACGAATTGTCGTACACCCCG 11:A>G
5003350|F|0-44:T>C-44:T>C TGCAGCACTTCGAGGTTCGGCAGGTCGCCGATAAATTCGGGAATTTCGCCG 44:T>C
1079000|F|0-35:G>T-35:G>T TGCAGGGCCAGAGTCACCTGGGAGACAACGCCGAGGACCCCAAGGGAGACCTTGGCGGCGTCCAGGTCC 35:G>T
3026919|F|0-16:G>C-16:G>C TGCAGCAGCTGTTCGTGGACATGTTCCAGGCCGACCTCGTCGCCG 16:G>C
3952211|F|0-16:C>T-16:C>T TGCAGAGCCTGCTGCACAGGCTGGGGTCCGACCCG 16:C>T
3954092|F|0-9:T>A-9:T>A TGCAGGGGGTGCATGCCAGGCTGCGCCG 9:T>A
7155201|F|0-8:G>A-8:G>A TGCAGCTGGATGTTTACCAAGAACCACCG 8:G>A
1047199|F|0-38:A>G-38:A>G TGCAGCTTCTGCGTCGTCGCCTGCTCCGCCGAGAGATGATGGTGGTGGTCCACGACCG 38:A>G
3950334|F|0-15:C>T-15:C>T TGCAGGTAAAATAGCCGACGGCAGCAACCCG 15:C>T
3940059|F|0-28:G>A-28:G>A TGCAGGGCCTGTTCCTTGCGGACAATCAGCTGTCCG 28:G>A
1053523|F|0-38:G>A-38:G>A TGCAGCTGGATCAGCGACGAGTAGGCGCTGAGAGGGTTGGCCATGAGCTGCTCGTGCGTGCCG 38:G>A
1053523|F|0-8:G>T-8:G>T TGCAGCTGGATCAGCGACGAGTAGGCGCTGAGAGGGTTGGCCATGAGCTGCTCGTGCGTGCCG 8:G>T
4261415|F|0-11:A>C-11:A>C TGCAGCTTGCGAGGTGAGCCG 11:A>C
3952211|F|0-5:A>G-5:A>G TGCAGAGCCTGCTGCACAGGCTGGGGTCCGACCCG 5:A>G
4261086|F|0-24:G>C-24:G>C TGCAGAGCCTCGCCGACCTGGCGTGGCGCATGGGGCCG 24:G>C
4262100|F|0-16:G>C-16:G>C TGCAGCAGAGTGCCTGGACGATGCGCGTGCGGTGCTCCG 16:G>C
3023022|F|0-31:G>A-31:G>A TGCAGCTGCTCGATGCCGTCGCCGTGCCACAGTATCCG 31:G>A
6045752|F|0-20:G>A-20:G>A TGCAGGGCGAGCAGTGGCTCGTCTTCTCCG 20:G>A
4262100|F|0-19:G>A-19:G>A TGCAGCAGAGTGCCTGGACGATGCGCGTGCGGTGCTCCG 19:G>A
1014038|F|0-8:C>G-8:C>G TGCAGCTACCTAGAGTCAACTCTTAATTTGTACTTAGCATAGGATCAAGTACGTTTCCG 8:C>G
3020583|F|0-5:T>C-5:T>C TGCAGTGCACCTGTCTAATTTGACTAATTGCCACCAGCATGGACCG 5:T>C
7167968|F|0-8:G>C-8:G>C TGCAGCTGGCTCGCTTTTGCTTGCGCCG 8:G>C
1026148|F|0-31:G>C-31:G>C TGCAGAACAAGGTGACCCTGGAGTGGCTTCTGGCCCTGGGTGACAAGCTCGGCATGGTCCGTGTGCCGA 31:G>C
1019150|F|0-28:G>C-28:G>C TGCAGTCGTGGTGCAGGTAGCAGAGCCCGCGCGCCGCCTCCAGCGCGATCCG 28:G>C
1019161|F|0-44:C>T-44:C>T TGCAGGAACACGCGGAAGCTGCGCAGCTCGTCGTGGATGACCGACTTGCTCCGCGCGTAGGCCG 44:C>T
3934294|F|0-20:T>A-20:T>A TGCAGCTTAGGTGCTCGAATTACCCG 20:T>A
4262391|F|0-13:A>T-13:A>T TGCAGCCTGTAGGACCTGCCG 13:A>T
1055737|F|0-42:C>T-42:C>T TGCAGCTTCTGCCCCTTCTCCTCCGCAGCCTCCTTCGCTTTGCTAAGCTCACTACGTAGTTTGTCCGAG 42:C>T
1055737|F|0-29:C>T-29:C>T TGCAGCTTCTGCCCCTTCTCCTCCGCAGCCTCCTTCGCTTTGCTAAGCTCACTACGTAGTTTGTCCGAG 29:C>T
3024533|F|0-13:T>C-13:T>C TGCAGGTGCACATTGTGTACATGGGGGAGCTGCACCCG 13:T>C
1075505|F|0-44:A>C-44:A>C TGCAGTCGGTCTGGAACGGCGCCGCGTACCACGGCGTGCACCACATGCCGCGCGGCGTCCG 44:A>C
5371852|F|0-10:G>A-10:G>A TGCAGGTAGAGGCGGGAGATGCCCG 10:G>A
1014880|F|0-19:G>C-19:G>C TGCAGATCGCCTCCGAGGCGCGCCTCTCCGTGAAGCAGCTCTGCAAGGCGCTGATCCAGCAGGTGGAGG 19:G>C
13589865|F|0-14:G>C-14:G>C TGCAGGTGGCCGCGGGTCAGGGGAGCCG 14:G>C
1020392|F|0-11:C>T-11:C>T TGCAGCTCCAGCTCCGCCTTGAGCCACTTGCAGAACCTGCCGAGGTTGCCGTTGTTGTGGTAGCCAATG 11:C>T
1079188|F|0-68:C>T-68:C>T TGCAGCTGGTGAACTCTCGGCTCCAAGTAACCCAGCACCCGCAGCTTCGGCGCGTCAGCAATGCTGACC 68:C>T
1024820|F|0-51:A>G-51:A>G TGCAGCTGGGGAGGCAGCGGGCCAAGACCAAGGTGATCAGGAAGTCGGCCAACCCCGTGTGGGACGAGG 51:A>G

<table>
<tr><td>5369353|F|0-37:C>T-37:C>T</td><td>TGCAGCAGCCGCTTCTCCTGGAGCTCCTGGGGCAGGACGGCCG</td><td>37:C>T</td></tr>
<tr><td>4411296|F|0-22:G>T-22:G>T</td><td>TGCAGGCACCGCGCCAACACCCGCTTGGCCTCCTCCTGGCGGTGCTCCG</td><td>22:G>T</td></tr>
<tr><td>3027174|F|0-9:T>G-9:T>G</td><td>TGCAGCGCCTTACGCCCGTCCCCGTGCAACGCCAAGCCG</td><td>9:T>G</td></tr>
<tr><td>1059315|F|0-49:C>T-49:C>T</td><td>TGCAGGTGGCGGAGCAGCGAGTCCTCGTCCCAGGAGAAGGTCCAGGGCTCCTCCG</td><td>49:C>T</td></tr>
<tr><td>2249018|F|0-5:C>G-5:C>G</td><td>TGCAGCAGCTTGGTGGTGTCGATGGCGCCCAGCTTCTCGATGTAGATTGGCCG</td><td>5:C>G</td></tr>
<tr><td>5582634|F|0-22:G>C-22:G>C</td><td>TGCAGAAGAAGTCGACGACGAGGACAGCCG</td><td>22:G>C</td></tr>
<tr><td>990605|F|0-53:G>C-53:G>C</td><td>TGCAGCAGGGAGACGGTGTTGGGGAGCTGGAACGACACGTTGTTCATGCTCGCGCCGAACCG</td><td>53:G>C</td></tr>
<tr><td>4262347|F|0-40:C>G-40:C>G</td><td>TGCAGCTCTCGCTGGACCTGATCGAGGAGTGGAGCAAGGCCCACCCG</td><td>40:C>G</td></tr>
<tr><td>4910031|F|0-7:T>C-7:T>C</td><td>TGCAGGGTGGCCGAGTCGCTCCG</td><td>7:T>C</td></tr>
<tr><td>1018265|F|0-14:G>C-14:G>C</td><td>TGCAGGTTCTGGTCGCGCTGGAAGCCCTTGTGGCAGATCTCGCACACGAACCG</td><td>14:G>C</td></tr>
<tr><td>1075505|F|0-5:T>G-5:T>G</td><td>TGCAGTCGGTCTGGAACGGCGCCGCGTACCACGGCGTGCACCACATGCCGCGCGGCGTCCG</td><td>5:T>G</td></tr>
<tr><td>1003806|F|0-35:G>A-35:G>A</td><td>TGCAGCCACATGTACCACCAGGACTGCATCCTGCCGTGGCTCGCGCTGCGCAACTCGTGCCCCGTGTGC</td><td>35:G>A</td></tr>
<tr><td>1018334|F|0-28:C>G-28:C>G</td><td>TGCAGCAGTTCTCGGCGATGAACAAGTTCAAGAAGAAGGCGCTGGGCGTGGTGGCGCGGAACATGCCGA</td><td>28:C>G</td></tr>
<tr><td>3955155|F|0-10:G>T-10:G>T</td><td>TGCAGGACACGTACCTCCCTCGCAATCCG</td><td>10:G>T</td></tr>
<tr><td>4540079|F|0-11:G>C-11:G>C</td><td>TGCAGCTGGCCGTGCTTGAGAAGGCGGCGAGCCG</td><td>11:G>C</td></tr>
<tr><td>3956664|F|0-13:G>T-13:G>T</td><td>TGCAGCTGTAGAAGGTCGACAGGTCGGCGCTCGACCG</td><td>13:G>T</td></tr>
<tr><td>4261836|F|0-22:C>T-22:C>T</td><td>TGCAGCGCATCAGCAGCGTCGACAGCCCG</td><td>22:C>T</td></tr>
<tr><td>4261415|F|0-14:T>G-14:T>G</td><td>TGCAGCTTGCGAGGTGAGCCG</td><td>14:T>G</td></tr>
<tr><td>1019161|F|0-22:G>T-22:G>T</td><td>TGCAGGAACACGCGGAAGCTGCGCAGCTCGTCGTGGATGACCGACTTGCTCCGCGCGTAGGCCG</td><td>22:G>T</td></tr>
<tr><td>2246456|F|0-33:G>A-33:G>A</td><td>TGCAGCTGCCCGTCCACCAGCTTGAACCTGCCTGCCATCCCGTCGAACGTCGTGTTGAGAATCGCCCTA</td><td>33:G>A</td></tr>
<tr><td>3026521|F|0-31:T>C-31:T>C</td><td>TGCAGCTAGCGAGCACATGCATGCGCCGAACTAACAACAGCCG</td><td>31:T>C</td></tr>
<tr><td>6049624|F|0-13:C>G-13:C>G</td><td>TGCAGTAGTTGCCCAGGCTAGCTGCATATGATTAGTTCGGCCG</td><td>13:C>G</td></tr>
<tr><td>3953019|F|0-8:C>T-8:C>T</td><td>TGCAGCACCCCAATGAGTACATCCG</td><td>8:C>T</td></tr>
<tr><td>1079188|F|0-7:G>C-7:G>C</td><td>TGCAGCTGGTGAACTCTCGGCTCCAAGTAACCCAGCACCCGCAGCTTCGGCGCGTCAGCAATGCTGACC</td><td>7:G>C</td></tr>
<tr><td>1083636|F|0-5:C>T-5:C>T</td><td>TGCAGCGACATGTCCCTGGACGAGATGGACGCCGCGGTGAGGAGGATGGAGTTCGTCGTCTTCGACCGA</td><td>5:C>T</td></tr>
<tr><td>3955925|F|0-41:G>C-41:G>C</td><td>TGCAGCTCCTCCACCTCGGCGCAGATGGCGTTCTTCTTGAAGACCCG</td><td>41:G>C</td></tr>
<tr><td>1024820|F|0-7:G>C-7:G>C</td><td>TGCAGCTGGGGAGGCAGCGGGCCAAGACCAAGGTGATCAGGAAGTCGGCCAACCCCGTGTGGGACGAGG</td><td>7:G>C</td></tr>
<tr><td>4394772|F|0-20:G>C-20:G>C</td><td>TGCAGGAGGCAGAGCCTGTAGACCCG</td><td>20:G>C</td></tr>
<tr><td>999436|F|0-33:G>A-33:G>A</td><td>TGCAGCCGTTCCTCAAGTTCCTGTCGGCGACGAGGGCCCCGTTCCTCATCAACTGCTACCCGTTCTTCG</td><td>33:G>A</td></tr>
<tr><td>4261131|F|0-19:C>T-19:C>T</td><td>TGCAGAGAAACAGAGAATTCAGCATGAGACCG</td><td>19:C>T</td></tr>
<tr><td>1016849|F|0-29:T>C-29:T>C</td><td>TGCAGTACCAGATACGGCTTCCCTTCAGTTGTTCGGCATATGTGAATTTGGTGGAATGGGATGCCAAGA</td><td>29:T>C</td></tr>
<tr><td>4397115|F|0-9:T>G-9:T>G</td><td>TGCAGCTCGTAGGAGGTGCTGGTGATGAGCGCGTCGGCACCG</td><td>9:T>G</td></tr>
<tr><td>1017445|F|0-31:C>T-31:C>T</td><td>TGCAGCACATATACCGCGAGGGCAACCAGGTCGCCCACGTCCTGTGCAAAGAAGCATACCAGCGACCGA</td><td>31:C>T</td></tr>
<tr><td>4408932|F|0-12:G>C-12:G>C</td><td>TGCAGAAGGCGTGGAGGCGCCGCCG</td><td>12:G>C</td></tr>
<tr><td>1022281|F|0-16:C>G-16:C>G</td><td>TGCAGTCGCAGAGCCTCATCATGCCGCTGCTCCTGTCGTCCGTGGCCACGCTGGCGCTCCACGTCCCCC</td><td>16:C>G</td></tr>
<tr><td>1023336|F|0-26:C>T-26:C>T</td><td>TGCAGCTGCGGGCTGCTGAACAGCTTCATCGGCACCGTGCCGTAGAGTCTTCCG</td><td>26:C>T</td></tr>
<tr><td>4997432|F|0-23:G>C-23:G>C</td><td>TGCAGCACGCTCATGTTGTGGTGGTGGTGATCATCCGTCGTCCCCTCCG</td><td>23:G>C</td></tr>
<tr><td>32025870|F|0-41:C>G-41:C>G</td><td>TGCAGGTGCCGCAGCGGCGGCGCGCTGGCCTTGCCGAGGTGCCCCAGCTCCG</td><td>41:C>G</td></tr>
<tr><td>100504565|F|0-16:G>A-16:G>A</td><td>TGCAGTAGAGGTAGATGGAGAGATACTTGCCATTGTTGGCTTTCACCCACCCACGCGGGTAGCTATGTA</td><td>16:G>A</td></tr>
<tr><td>5008993|F|0-18:T>C-18:T>C</td><td>TGCAGGCTTATTCGGCCGTTGCGCCCTACTACGCCCAGGGCTACCAGGCCGTGCAAGGTACGTGAGAAA</td><td>18:T>C</td></tr>
<tr><td>100560856|F|0-8:C>A-8:C>A</td><td>TGCAGTCTCTCGGGACCTGGAACGGACCGTCGAAACCG</td><td>8:C>A</td></tr>
<tr><td>5361531|F|0-40:T>C-40:T>C</td><td>TGCAGGGATTCAGGAAGATAGTGGCCGACAGATGGGAGTTTGCCAATGAGTTCTTCAGGAAGGGAGCAA</td><td>40:T>C</td></tr>
<tr><td>7353624|F|0-5:C>T-5:C>T</td><td>TGCAGCTGCGCGATGGAGCCG</td><td>5:C>T</td></tr>
<tr><td>4396872|F|0-13:C>G-13:C>G</td><td>TGCAGAGGCTCATCTGGGTGAAGAGGCCG</td><td>13:C>G</td></tr>
<tr><td>3934914|F|0-17:C>G-17:C>G</td><td>TGCAGGAACATCCACACCGACCG</td><td>17:C>G</td></tr>
<tr><td>3957157|F|0-29:G>C-29:G>C</td><td>TGCAGAATTCGCTGGAGGTGGACCTCCTCGACCG</td><td>29:G>C</td></tr>
<tr><td>3936328|F|0-10:T>G-10:T>G</td><td>TGCAGGTGTCTTGCGCCGTTTCCG</td><td>10:T>G</td></tr>
<tr><td>2246456|F|0-20:C>T-20:C>T</td><td>TGCAGCTGCCCGTCCACCAGCTTGAACCTGCCTGCCATCCCGTCGAACGTCGTGTTGAGAATCGCCCTA</td><td>20:C>T</td></tr>
<tr><td>1018962|F|0-11:T>C-11:T>C</td><td>TGCAGCCGCTGTGCCATGATCACGTTCCCGCCGACAGTCGCCGTGTTCCG</td><td>11:T>C</td></tr>
<tr><td>5580340|F|0-10:C>G-10:C>G</td><td>TGCAGCTTCTCGCAAGAACCGAGCTCCG</td><td>10:C>G</td></tr>
<tr><td>3946372|F|0-11:G>C-11:G>C</td><td>TGCAGCCTCCCGAGCTCGGGCGGTATCGGCCCG</td><td>11:G>C</td></tr>
<tr><td>5580153|F|0-21:C>G-21:C>G</td><td>TGCAGCACCGTCTTCAGCCCCCCGCAGCAGTCCG</td><td>21:C>G</td></tr>
<tr><td>1059094|F|0-46:G>C-46:G>C</td><td>TGCAGTCCAAAAGGAGAGCGATGTTGTATACAAGAGCAGTGGTCAAGATCCG</td><td>46:G>C</td></tr>
<tr><td>1014880|F|0-13:C>G-13:C>G</td><td>TGCAGATCGCCTCCGAGGCGCGCCTCTCCGTGAAGCAGCTCTGCAAGGCGCTGATCCAGCAGGTGGAGG</td><td>13:C>G</td></tr>
<tr><td>3955925|F|0-29:G>A-29:G>A</td><td>TGCAGCTCCTCCACCTCGGCGCAGATGGCGTTCTTCTTGAAGACCCG</td><td>29:G>A</td></tr>
<tr><td>3938070|F|0-28:C>A-28:C>A</td><td>TGCAGGCGCCCATATCGATCCGCCTCACCTCCG</td><td>28:C>A</td></tr>
<tr><td>3934101|F|0-25:T>A-25:T>A</td><td>TGCAGGCCACCCAAATCCGCACCAATTTGGGCGGAAAATTGCACCG</td><td>25:T>A</td></tr>
<tr><td>3957157|F|0-19:G>C-19:G>C</td><td>TGCAGAATTCGCTGGAGGTGGACCTCCTCGACCG</td><td>19:G>C</td></tr>
<tr><td>1019241|F|0-20:G>T-20:G>T</td><td>TGCAGCTTCTTGGTGTCCGTGGTGCTCCAGGAGATGAGGTGGAACAGCCG</td><td>20:G>T</td></tr>
</table>

3942260|F|0-17:G>A-17:G>A	TGCAGCCCGATCTTCGCGGAGATCGTGCCG	17:G>A
1079000|F|0-30:C>G-30:C>G	TGCAGGGCCAGAGTCACCTGGGAGACAACGCCGAGGACCCCAAGGGAGACCTTGGCGGCGTCCAGGTCC	30:C>G
1065045|F|0-53:G>T-53:G>T	TGCAGGTGATTGAGCATGTGGAGGAGGACCAAGCAAGTTTATTCGGCAACGTTGTATTGAGCTCATTCT	53:G>T
1667357|F|0-16:A>T-16:A>T	TGCAGCAACAATGCTAACCATCGAACTACACTCCTCTGCTACATGCTACTGGTTCATTCCG	16:A>T
3030485|F|0-8:C>T-8:C>T	TGCAGCTCCGACTTGCTCCGCGTGGCGCGCACGAACCCCG	8:C>T
32026225|F|0-19:A>C-19:A>C	TGCAGCTTGGCCTTCATGGACAGTAGGTGGAGGGCCTTCCG	19:A>C
3941931|F|0-33:T>C-33:T>C	TGCAGGCAACGCTGGCCAGTGGCTACGTTTAATTTGTACACCG	33:T>C
2245281|F|0-37:C>T-37:C>T	TGCAGGCGGCTCTTGAAGCTGGGGTCTGAAATTTGATCCCAGAACTCACGCAGCTCCGCCTTGTCGATG	37:C>T
1862465|F|0-24:T>C-24:T>C	TGCAGAAGCTCCTCCTGGAAGCCGTCGGCTGGGAAGTCGACGATGGACCAGGTGCCTTCGTCGGCGTTG	24:T>C
1044828|F|0-53:G>C-53:G>C	TGCAGGGAGAACTGGACGGAGATGTGCGCGGTGCAGCGCGGGCAATTGGCGTCGGCGTGGATCCGAGAT	53:G>C
2249018|F|0-29:C>A-29:C>A	TGCAGCAGCTTGGTGGTGTCGATGGCGCCCAGCTTCTCGATGTAGATTGGCCG	29:C>A
100461329|F|0-14:A>G-14:A>G	TGCAGCCTGTCGATATTGTACGCGTTTGGTAGCTTGGTAGCCG	14:A>G
1043228|F|0-43:A>G-43:A>G	TGCAGTAATTGACAAGAACAAGAACCGTAGCAAAGAATGGGGAAGAAAGATCTCACTCACAGGCGACGT	43:A>G
1059094|F|0-40:G>A-40:G>A	TGCAGTCCAAAAGGAGAGCGACGTTGTATACAAGAGCAGTGGTCAAGATCCG	40:G>A
4733745|F|0-6:T>C-6:T>C	TGCAGCTCGTTCTGGGGAGGTTGCCG	6:T>C
5332206|F|0-17:A>G-17:A>G	TGCAGGACGGACGTCTGACCGAACCG	17:A>G
1020364|F|0-45:G>A-45:G>A	TGCAGTGAGCTCAGCGGCGGTGAAGGTGTAGGGCAGGTTGCCGACGTAGATTCGCCCG	45:G>A
3957157|F|0-20:G>T-20:G>T	TGCAGAATTCGCTGGAGGTGGACCTCCTCGACCG	20:G>T
1132668|F|0-58:G>C-58:G>C	TGCAGATGCTGACCTACCTCGCCATGAAGCACGACGCGCGCGTGCAGCGGGTCGACACGGTCCG	58:G>C
1019150|F|0-19:G>A-19:G>A	TGCAGTCGTGGTGCAGGTAGCAGAGCCCGCGCGCCGCCTCCAGCGCGATCCG	19:G>A
1006604|F|0-43:A>G-43:A>G	TGCAGTTCGTGTTTCCAGGGATGTGGCTCTCGGATTTGACCAAAGGCCG	43:A>G
4261502|F|0-5:C>T-5:C>T	TGCAGCTTCGTGGACAGCATGACCATCACCATGATCGAGGGCCG	5:C>T
1016849|F|0-18:T>C-18:T>C	TGCAGTACCAGATACGGCTTCCCTTCAGTTGTTCGGCATATGTGAATTTGGTGGAATGGGATGCCAAGA	18:T>C
4261680|F|0-6:T>C-6:T>C	TGCAGGTTGCCCTAACAGCGACCCG	6:T>C
32017711|F|0-23:G>C-23:G>C	TGCAGCTCGTAGTAGTCGCGCACGGTCCG	23:G>C
3950034|F|0-17:G>C-17:G>C	TGCAGGAGGCAGCCGACGACCAGAGGCCCG	17:G>C
5581548|F|0-7:G>T-7:G>T	TGCAGGTGCCACATAGTTTAGAGCAGCCG	7:G>T
1020364|F|0-15:G>T-15:G>T	TGCAGTGAGCTCAGCGGCGGTGAAGGTGTAGGGCAGGTTGCCGACGTAGATTCGCCCG	15:G>T
1022092|F|0-40:C>G-40:C>G	TGCAGCCACGCCGTGATCCAGCCGAGGTAGATCAGCTCCTCCATGTCCGCCGCCCACCG	40:C>G
1038822|F|0-14:T>A-14:T>A	TGCAGCTCAGAACGTGGCACCCCAGCCACACCCTGCACCTTGAGGCAGAGCCTGAGCAGGAAGGCCTCC	14:T>A
1040984|F|0-32:T>G-32:T>G	TGCAGCCACAACGACGTGCTAACCCACACGCATCACCAAAGCGCCTATATAAACATTAGCTCCG	32:T>G
997479|F|0-41:C>T-41:C>T	TGCAGCTTCTCCCGCCGCTTGCGCTCGGCGATGACGTGCTCCTGCGCGTTCGCCG	41:C>T
4262347|F|0-7:C>T-7:C>T	TGCAGCTCTCGCTGGACCTGATCGAGGAGTGGAGCAAGGCCCACCCG	7:C>T
3938743|F|0-37:C>T-37:C>T	TGCAGCACATATACCGCGAGGGAAACCAGGTCGCCCACGTCCTGTGCAAAGAAGCATACCAGCGACCGA	37:C>T
1079188|F|0-35:C>T-35:C>T	TGCAGCTGGTGAACTCTCGGCTCCAAGTAACCCAGCACCCGCAGCTTCGGCGCGTCAGCAATGCTGACC	35:C>T
5580153|F|0-14:C>G-14:C>G	TGCAGCACCGTCTTCAGCCCCCCGCAGCAGTCCG	14:C>G
4261551|F|0-10:G>C-10:G>C	TGCAGCCCATGCCGCCGAGGTTGAAGCTCCG	10:G>C
3023755|F|0-10:C>A-10:C>A	TGCAGGGGCCCTTCTCCATGCGTATCACCAGCGACTCCG	10:C>A
5368388|F|0-8:C>G-8:C>G	TGCAGCTCCGCCTCGTCGGTCTTGTCGGACTCGATGGACAGCAGCACGTTCTTGCGGCGGACGAACTGG	8:C>G
1018625|F|0-40:A>G-40:A>G	TGCAGCCCAAGGAAGGGCTTGCCATGGTGAACGGCACAGCAGTGGGCTCCG	40:A>G
6042977|F|0-44:C>G-44:C>G	TGCAGCCGCTGCACAGCGCAATCTCGGCGGCGAGGCTAACGTCACGGCTTGGGGCAGAGGTGGCCCGAG	44:C>G
4262614|F|0-17:T>G-17:T>G	TGCAGTCGCCGTTCACGTTCCGCAAGGACGGGCAGGCGAGCCG	17:T>G
1038080|F|0-17:A>G-17:A>G	TGCAGGTCAGGTAATGCAACGACAAGAGCTAACCGCTCGCCAAAGTACAGCCG	17:A>G
986029|F|0-10:C>G-10:C>G	TGCAGCACATCAGCCCATACTTCCTGATCAGTCCATGGGAGTTCCCACAGACCCG	10:C>G
3939310|F|0-12:C>T-12:C>T	TGCAGATCGGTACGCACTAGACCG	12:C>T
3024696|F|0-8:C>T-8:C>T	TGCAGCAGCTGCTCCAGGCCATCGGCTCTAACAACAGCACCACCG	8:C>T
3936994|F|0-10:C>A-10:C>A	TGCAGCGCAGCCATGCATGAATGGACCG	10:C>A
1038822|F|0-17:C>T-17:C>T	TGCAGCTCAGAACGTGGCACCCCAGCCACACCCTGCACCTTGAGGCAGAGCCTGAGCAGGAAGGCCTCC	17:C>T
5580387|F|0-10:C>T-10:C>T	TGCAGGAGTTCGACACGATCCACGGCCCG	10:C>T
986016|F|0-39:A>T-39:A>T	TGCAGCAGCTCTAGTCCCGTCGCCACGTTCCCGAGCGGCAACGACGTCATCCCGCTCGCCGCCGTCGGG	39:A>T
3935542|F|0-7:G>A-7:G>A	TGCAGCTGGCCGTGCCGTTGATGGTCCACCCG	7:G>A
6030873|F|0-15:G>T-15:G>T	TGCAGGTGAGCGTCTGTGCATATTGGGATCCG	15:G>T
1014880|F|0-28:C>G-28:C>G	TGCAGATCGCCTCCGAGGCGCGCCTCTCCGTGAAGCAGCTCTGCAAGGCGCTGATCCAGCAGGTGGAGG	28:C>G
7161674|F|0-13:A>G-13:A>G	TGCAGCTTCCTCAAGCAGCCCACCACGCAGCCG	13:A>G
5364114|F|0-16:G>C-16:G>C	TGCAGTGTGACCCTTAGCACCG	16:G>C
4997078|F|0-13:C>G-13:C>G	TGCAGGCGGTGCGCAGGTCGCTCGACGACATGCCG	13:C>G
1024820|F|0-57:T>C-57:T>C	TGCAGCTGGGGAGGCAGCGGGCCAAGACCAAGGTGATCAGGAAGTCGGCCAACCCCGTGTGGGACGAGG	57:T>C
4002809|F|0-24:A>G-24:A>G	TGCAGCGTACGTACCGCGGTCTTGACGAGCTCATCGTCGTAGGCGCCG	24:A>G
1014880|F|0-67:G>A-67:G>A	TGCAGATCGCCTCCGAGGCGCGCCTCTCCGTGAAGCAGCTCTGCAAGGCGCTGATCCAGCAGGTGGAGG	67:G>A

1062469|F|0-29:C>T-29:C>T	TGCAGTTCTTGCAGCACACCCAGCCCATCCTCCACGAGGTGCAGGACCTGGCGGCGATGAAGCCCATCC	29:C>T
3938799|F|0-17:G>C-17:G>C	TGCAGCGGGACACGAAGGCTCGGAACCGAGGCCCG	17:G>C
1026038|F|0-52:C>T-52:C>T	TGCAGCTCGCGCGCCGCGCCAAGATGATCGGGGACCTGCTGCACCAGCTCCACGCCGCGCAGCTCATGC	52:C>T
4262307|F|0-7:G>A-7:G>A	TGCAGCAGCCACTGGTGATCCACGTCGATGAGGAAGTCCTTGTGGTAGTGCTCCCTCACCTTCTCCCTC	7:G>A
5411947|F|0-5:T>C-5:T>C	TGCAGTTCGATTTATCGTTTTGGTTGTCCG	5:T>C
4262207|F|0-5:T>C-5:T>C	TGCAGTTGTGGGAGAACAACTTCACCG	5:T>C
3951245|F|0-12:A>G-12:A>G	TGCAGTCGGAGCATCCCGAGAGCTTACCG	12:A>G
5367273|F|0-14:G>A-14:G>A	TGCAGGATCGTGTAGTCCTCCCCCTGCGCCG	14:G>A
5580153|F|0-8:C>T-8:C>T	TGCAGCACCGTCTTCAGCCCCCCGCAGCAGTCCG	8:C>T
1051309|F|0-14:C>G-14:C>G	TGCAGCGTGCTGCTCGCCCTCATCCTCGTCCTCGCCATCGTCTTCGTCGCGCTCTACTTCACCGTGTTC	14:C>G
3957157|F|0-28:C>G-28:C>G	TGCAGAATTCGCTGGAGGTGGACCTCCTCGACCG	28:C>G
32020285|F|0-23:G>C-23:G>C	TGCAGGATCTCACGCTCCGTCTCGGCGCGGCTCAGCTTCCG	23:G>C
3026191|F|0-19:G>C-19:G>C	TGCAGCACCACCACCTCCTGGTGCTCCGCGTCCAGGTCCG	19:G>C
1038080|F|0-5:G>A-5:G>A	TGCAGGTCAGGTAATGCAACGACAAGAGCTAACCGCTCGCCAAAGTACAGCCG	5:G>A
3939712|F|0-10:C>G-10:C>G	TGCAGCCGCACTCGTAGCTGCCCCAGGTGTTCTTGCAGTGGCAGTCCG	10:C>G
3935387|F|0-14:C>T-14:C>T	TGCAGATATTGCCACCGAATGCCTGTCTTCCCCTTTCGAGAAAGGAGGAGCAAATCTCCTTGGATGTCC	14:C>T
1023712|F|0-9:C>T-9:C>T	TGCAGACGGCGCCTGCTCGGACGCGGTCCAAGAGGGACGTGAGGTGGTGGAGGACCAGGAGCAGGAGAT	9:C>T
4261315|F|0-28:G>T-28:G>T	TGCAGCACATGCAGGGGGAGGAGGAGACGGCGATGCACCG	28:G>T
3955925|F|0-17:G>A-17:G>A	TGCAGCTCCTCCACCTCGGCGCAGATGGCGTTCTTCTTGAAGACCCG	17:G>A
3942527|F|0-30:A>G-30:A>G	TGCAGTAGTTGCCGAAGCTAGCTGCATGTGATTAGTTAGGCCG	30:A>G
12419533|F|0-31:C>G-31:C>G	TGCAGACGTGGAACAACGGGGCGGAGAACACCAAGCGGATCGTGGCCGCCG	31:C>G
5580368|F|0-7:G>T-7:G>T	TGCAGGAGATTCTGGTCCAGAACGACCACCG	7:G>T
3949378|F|0-7:A>T-7:A>T	TGCAGGTAGGCCGTGAGCCG	7:A>T
1696212|F|0-58:C>T-58:C>T	TGCAGTTCCTCACCAACTCCTGCGTCGTGCTCTTCATGATCCAGAGCGTCGACCGCCTCGTCCTCTGCC	58:C>T
5002652|F|0-39:G>T-39:G>T	TGCAGACCATGGCGCTCGACAGGGTGCTCGAGGAGTGCAGGAGCACCG	39:G>T
4003062|F|0-14:G>A-14:G>A	TGCAGCGAGCGGCGGCACGCGCGTGGTCGCCG	14:G>A
981522|F|0-6:C>T-6:C>T	TGCAGCCCGCTCCGTCTTTGCTGTGATAACGAGGTAAAGTGGCATTTCTTTGTTACATGAGTCCGAGAT	6:C>T
7907133|F|0-6:G>A-6:G>A	TGCAGGGCAGCTACGAGCCG	6:G>A
1040984|F|0-21:A>G-21:A>G	TGCAGCCACAACGACGTGCTAACCCACACGCATCACCAAAGCGCCTATATAAACATTAGCTCCG	21:A>G
6042977|F|0-26:G>A-26:G>A	TGCAGCCGCTGCACAGCGCAATCTCGGCGGCGAGGCTAACGTCACGGCTTGGGGCAGAGGTGGCCCGAG	26:G>A
7928931|F|0-5:C>G-5:C>G	TGCAGCAGCTTGACGACCGCTTCCG	5:C>G
3021984|F|0-20:T>C-20:T>C	TGCAGCAGCAGTTCGTGTCCTTGGCCACCATCGTCCCCG	20:T>C
4396433|F|0-12:G>T-12:G>T	TGCAGCTGCTGCGTGTTCGGGTGGAACGCCG	12:G>T
5004423|F|0-28:G>C-28:G>C	TGCAGCACCAGCTGCGGGGCACGGTGCGGCTCGACCCCTCCG	28:G>C
4538390|F|0-6:T>A-6:T>A	TGCAGCTGGTCAAAGGTTCCG	6:T>A
5363839|F|0-14:T>C-14:T>C	TGCAGGCTGGCGCATTTCCCGAGCTCCG	14:T>C
4261086|F|0-12:C>A-12:C>A	TGCAGAGCCTCGCCGACCTGGCGTGGCGCCTGGGGCCG	12:C>A
1286660|F|0-5:T>C-5:T>C	TGCAGTTTCTCGAGATGAAGCTCGGCTAGCAAGGCACCCTGTGGGATCGCCTGGAGAACCG	5:T>C
7354285|F|0-16:G>T-16:G>T	TGCAGTGCGTGAAGGCGCCTCGGATGGGGAAGGCAAGAAGGTGGATCCCCG	16:G>T
1040349|F|0-14:A>G-14:A>G	TGCAGTGGGTGCGCAACATGGGCTACGTCATCTACGACTACTGCACCGACATGAGCAACCG	14:A>G
1007779|F|0-9:T>C-9:T>C	TGCAGAAGGTTGGCTTGTGCATTCCTCACGACTTGGGGAAACTCTGCTACGCGCACTGGTTGGATCTGG	9:T>C
4261355|F|0-7:C>T-7:C>T	TGCAGAGCACATACCCTGTAAGTGCAATATTATTACCG	7:C>T
1022232|F|0-46:G>A-46:G>A	TGCAGATGACCCTGTACGAGCTGGTGATGATCTGCCACGAGGAGACGGGTCGGCTGCTGAACCG	46:G>A
1059094|F|0-33:G>T-33:G>T	TGCAGTCCAAAAGGAGAGCGACGTTGTATACAAGAGCAGTGGTCAAGATCCG	33:G>T
6042717|F|0-10:A>G-10:A>G	TGCAGCCCATATTCGGCCTGATAAGCGTTGCCACCGACATCCTCGCCTTGGCCGAGTTGGTCACCACCC	10:A>G
1057966|F|0-22:A>T-22:A>T	TGCAGCCCATATTTGGCCTGATAAGCGTTGCCACCGACATCCTCGCCTTGGCCGAGTTGGTCACCACCC	22:A>T
1019940|F|0-10:G>C-10:G>C	TGCAGGTGCTGAGCGCGCTGGACGCGGCCAAGACGCAGTGGTACCACTTCACGGCCATCGTCGTCGCCG	10:G>C
3950362|F|0-5:G>C-5:G>C	TGCAGGGCGCCCTTCAGCCACTCGCCG	5:G>C
1022660|F|0-64:G>A-64:G>A	TGCAGACCATCAACGGCCCCAACTCCGACTTCCCCGTCTACTCGTCGGAGCTCAAGACGGTCAAGGGCA	64:G>A
985416|F|0-8:A>G-8:A>G	TGCAGTGGATCATGGCGAACTTGGTGAAGCATCTGGACGTGCAGGAGGCCGTCCG	8:A>G
3959873|F|0-19:C>G-19:C>G	TGCAGGGCGACAAGACAAGCAAGAACATCCGCGCCG	19:C>G
1059315|F|0-16:G>A-16:G>A	TGCAGGTGGCGGAGCAGCGAGTCCTCGTCCCAGGAGAAGGTCCACGGCTCCTCCG	16:G>A
4412235|F|0-27:A>C-27:A>C	TGCAGGAGCGGGTGCTGAGTTGCCATGAAGCGATGATCCAGGCCACCG	27:A>C
3950362|F|0-22:C>T-22:C>T	TGCAGGGCGCCCTTCAGCCACTCGCCG	22:C>T
1141147|F|0-28:C>A-28:C>A	TGCAGGCGACGATCGCCGCCCGCAACCCCAACTCCCGCGTCGGCATCTACTTCGACCG	28:C>A
3936994|F|0-21:T>G-21:T>G	TGCAGCGCAGCCATGCATGAATGGACCG	21:T>G
3032129|F|0-7:G>A-7:G>A	TGCAGACGCACGCTGGAGAGGCTCAGCTCTGTCAACCGCCG	7:G>A
5582909|F|0-9:C>G-9:C>G	TGCAGTAGTCCATCTGCTTGCCG	9:C>G
1020364|F|0-54:C>G-54:C>G	TGCAGTGAGCTCAGCGGCGGTGAAGGTGTAGGGCAGGTTGCCGACGTAGATTCGCCCG	54:C>G

<table>
<tr><td>6047679|F|0-39:C>G-39:C>G</td><td>TGCAGGGCCGCGCGCTCACCAACGCCGTCGGCAACCTCGCCATCACCG</td><td>39:C>G</td></tr>
<tr><td>4705943|F|0-37:G>T-37:G>T</td><td>TGCAGTCCGCGAGGCCGTCGTCGGGGGAGGCCGCGGCGTGGTGCGCCG</td><td>37:G>T</td></tr>
<tr><td>6030873|F|0-13:C>T-13:C>T</td><td>TGCAGGTGAGCGTCTGTGCATATTGGGATCCG</td><td>13:C>T</td></tr>
<tr><td>5367542|F|0-37:C>A-37:C>A</td><td>TGCAGTGGCTCGACGACGAGCTCACCTCGCTCGCCCTCCCCGAACCCG</td><td>37:C>A</td></tr>
<tr><td>3952467|F|0-7:A>T-7:A>T</td><td>TGCAGGAACTCCACGCGGATGCCG</td><td>7:A>T</td></tr>
<tr><td>5971588|F|0-9:G>A-9:G>A</td><td>TGCAGCACGGTGGTGGCCACGATGCGCAGCCG</td><td>9:G>A</td></tr>
<tr><td>1016260|F|0-26:G>C-26:G>C</td><td>TGCAGCATCCAGTTATGGTACTGCGAGAGGCCGTTGAGCCGCCGCAGGCATCCCGTCCG</td><td>26:G>C</td></tr>
<tr><td>7354276|F|0-52:G>C-52:G>C</td><td>TGCAGTGCACCTCCAAGTGCGTCGCCACCTGCATCCGCGGCGGCGAGGGGCCGCTCAACGTCCG</td><td>52:G>C</td></tr>
<tr><td>22839023|F|0-11:G>C-11:G>C</td><td>TGCAGGATGGCGAGCTTCTGGAGCTTGGAGAAGCCCG</td><td>11:G>C</td></tr>
<tr><td>13881616|F|0-22:C>G-22:C>G</td><td>TGCAGGTGGCCGCGGAGCTGCTCAACGACCG</td><td>22:C>G</td></tr>
<tr><td>100532126|F|0-21:A>G-21:A>G</td><td>TGCAGGTCGGCCGCCGCGAGCACGGGGAAGAAGTCCGAGACGTTCGGGCTGCCG</td><td>21:A>G</td></tr>
<tr><td>100579925|F|0-32:C>A-32:C>A</td><td>TGCAGGAGGCTCGTGAGGCACAAGAGCACGACCAGGTAAGGCGCGGAAGAAGCCATCTCGATTAGCTTA</td><td>32:C>A</td></tr>
<tr><td>11314369|F|0-40:G>C-40:G>C</td><td>TGCAGACGGTGCTCCTGTGGCAGAACCAGCTCGTCGGCACGATACCTCCG</td><td>40:G>C</td></tr>
<tr><td>1126306|F|0-42:A>C-42:A>C</td><td>TGCAGCAGCGCGAGCAGCGCGCGGGCGCCCTCGTCGCGATCGACGTCGCCG</td><td>42:A>C</td></tr>
<tr><td>100463319|F|0-44:C>G-44:C>G</td><td>TGCAGGTGGAAGCTGCGGCGGCCGCAGCGGATGCACAGCGTGTGCGTCTTGTTCCG</td><td>44:C>G</td></tr>
<tr><td>1019171|F|0-43:C>T-43:C>T</td><td>TGCAGCACGTGTACCTGAACCTGGGCGGGGAGGGCAGCGGCGACATCCTGGGCCACACGCTCCAGCTCT</td><td>43:C>T</td></tr>
<tr><td>1038399|F|0-11:C>T-11:C>T</td><td>TGCAGCCTGATCTCCCTGTTGAACCTGGTGATGAAGTCCTCCACCCTCCG</td><td>11:C>T</td></tr>
<tr><td>7171389|F|0-9:C>A-9:C>A</td><td>TGCAGACCACACCTGTCGGCCGCTTCGACGATTCCG</td><td>9:C>A</td></tr>
<tr><td>5370960|F|0-27:A>G-27:A>G</td><td>TGCAGTCGCGCGCCCTCCGCGGCGGGAACGACGCGAAATGGCGACCCG</td><td>27:A>G</td></tr>
<tr><td>32024162|F|0-28:G>C-28:G>C</td><td>TGCAGCTGGCCGTGCTGGAGAAGGCGGCGAGCCG</td><td>28:G>C</td></tr>
<tr><td>5369680|F|0-6:C>T-6:C>T</td><td>TGCAGTCGTAGAGGATGACGGAGCGGAAGCGGCCCG</td><td>6:C>T</td></tr>
<tr><td>1069052|F|0-53:G>A-53:G>A</td><td>TGCAGCACCGACAGCTGCACGACGATGTCGCGCGCCCACTTGCCGTACATGGCGTCCAGCAGCGGCACG</td><td>53:G>A</td></tr>
<tr><td>985416|F|0-40:G>C-40:G>C</td><td>TGCAGTGGATCATGGCGAACTTGGTGAAGCATCTGGACGTGCAGGAGGCCGTCCG</td><td>40:G>C</td></tr>
<tr><td>1130405|F|0-26:C>T-26:C>T</td><td>TGCAGCGACGACACCAAGTACTCCAACGGCCGCGGCCACAGGGACACCG</td><td>26:C>T</td></tr>
<tr><td>5366996|F|0-12:G>A-12:G>A</td><td>TGCAGCCCCTTGGCCTTGATCCG</td><td>12:G>A</td></tr>
<tr><td>1077420|F|0-48:G>A-48:G>A</td><td>TGCAGAGTGCAGGGACTACTTCAGCAAGTCCCTGTTTGTGGTGGGAGAGTTTGGGGGGAACGACTACAA</td><td>48:G>A</td></tr>
<tr><td>1014038|F|0-53:T>C-53:T>C</td><td>TGCAGCTACCTAGAGTCAACTCTTAATTTGTACTTAGCATAGGATCAAGTACGTTTCCG</td><td>53:T>C</td></tr>
<tr><td>6047970|F|0-16:A>G-16:A>G</td><td>TGCAGGACTGGCAGAGACGGTACTGCGAGATGCTGAAGGACAGCGGCAAGGACGTGAGGGTGTTGGAGT</td><td>16:A>G</td></tr>
<tr><td>4261207|F|0-26:G>T-26:G>T</td><td>TGCAGGAAGAGCAGAGGCTTGCCAATGACATCTCTCGTGTTGCCG</td><td>26:G>T</td></tr>
<tr><td>3022355|F|0-14:G>A-14:G>A</td><td>TGCAGCACGTACTGGTGGAACAGGCTCTCCTGGATCCG</td><td>14:G>A</td></tr>
<tr><td>1020008|F|0-35:C>T-35:C>T</td><td>TGCAGCTCCACCTGCACCAGCAACTCCTCAAAAATCTCAACAGAGCCATCCACCACCCTCTTCACAAGC</td><td>35:C>T</td></tr>
<tr><td>4262946|F|0-14:G>C-14:G>C</td><td>TGCAGCCAGCGGTGGAGGGAGCTGCCCCGCACCGCCGCCCG</td><td>14:G>C</td></tr>
<tr><td>1020653|F|0-5:C>A-5:C>A</td><td>TGCAGCTTGGCCATTGCCCGTGGGTTCCCGACCAGCTCCGCCATGGCCCATTCCAGCGTGACGGACGAT</td><td>5:C>A</td></tr>
<tr><td>1026038|F|0-64:C>G-64:C>G</td><td>TGCAGCTCGCGCGCCGCGCCAAGATGATCGGGGACCTGCTGCACCAGCTCCACGCCGCGCAGCTCATGC</td><td>64:C>G</td></tr>
<tr><td>1032506|F|0-65:T>C-65:T>C</td><td>TGCAGAAGCCGCAGAGATGACAGCAGAAGATGAGCCACTAAACCCTTGAAACACACCAGCAGCCATTGA</td><td>65:T>C</td></tr>
<tr><td>5361271|F|0-37:A>G-37:A>G</td><td>TGCAGCTGGTGTGCTTCGGCTGCACCACGCTCCCGACATCCCGCTACGGCCCCTACTGGCGCAACCTCC</td><td>37:A>G</td></tr>
<tr><td>3935387|F|0-49:G>T-49:G>T</td><td>TGCAGATATTGCCACCGAATGCCTGTCTTCCCCTTTCGAGAAAGGAGGAGCAAATCTCCTTGGATGTCC</td><td>49:G>T</td></tr>
<tr><td>1038080|F|0-26:A>T-26:A>T</td><td>TGCAGGTCAGGTAATGCAACGACAAGAGCTAACCGCTCGCCAAAGTACAGCCG</td><td>26:A>T</td></tr>
<tr><td>1031914|F|0-14:C>G-14:C>G</td><td>TGCAGCCTCTTCGCCCCGACGCCGTCTTTCGCCGACATGGTCGTCAGCCG</td><td>14:C>G</td></tr>
<tr><td>1667947|F|0-37:C>T-37:C>T</td><td>TGCAGTGGTTCAGCAAGAGGAAGAAGAAGGCGCTGTTCGACGCCCTGGAGAAGGTTAAATCCG</td><td>37:C>T</td></tr>
<tr><td>3959878|F|0-17:C>T-17:C>T</td><td>TGCAGCAAGCTCCTCCCCGTCGATCCG</td><td>17:C>T</td></tr>
<tr><td>3027282|F|0-20:A>G-20:A>G</td><td>TGCAGCCGCGCCAGGTGGCCATCTGGTTCCAGAACCGCCGCGCCCG</td><td>20:A>G</td></tr>
<tr><td>2246456|F|0-13:C>T-13:C>T</td><td>TGCAGCTGCCCGTCCACCAGCTTGAACCTGCCTGCCATCCCGTCGAACGTCGTGTTGAGAATCGCCCTA</td><td>13:C>T</td></tr>
<tr><td>1038399|F|0-24:C>T-24:C>T</td><td>TGCAGCCTGATCTCCCTGTTGAACCTGGTGATGAAGTCCTCCACCCTCCG</td><td>24:C>T</td></tr>
<tr><td>1003509|F|0-56:C>T-56:C>T</td><td>TGCAGGAGGAAGGTGATGAAGGCCATGAACCTCAAGGGCGTGCTGAGGACGGAGATCCACCCGTCGCTC</td><td>56:C>T</td></tr>
<tr><td>100505022|F|0-10:C>G-10:C>G</td><td>TGCAGTGGTTCAGAAAGAGGAAGAAGAAGGCGCTGTTCGACGCCCTGGAGAAGGTTAAATCCG</td><td>10:C>G</td></tr>
<tr><td>4003335|F|0-29:C>T-29:C>T</td><td>TGCAGGTACCAGTGCAACGAGTGGGGCGTCGGCATGGAGATCGACAGCAACGTCCG</td><td>29:C>T</td></tr>
<tr><td>4262604|F|0-6:C>T-6:C>T</td><td>TGCAGTCCGCCACTGCTCCG</td><td>6:C>T</td></tr>
<tr><td>3949931|F|0-5:C>A-5:C>A</td><td>TGCAGCGTACGGCCATCATATGGAACGTCGTCCG</td><td>5:C>A</td></tr>
<tr><td>3934452|F|0-16:G>C-16:G>C</td><td>TGCAGGGGGAGAAGGCGGCGGTGCGGATGGAGGCCGACCAGTTCCG</td><td>16:G>C</td></tr>
<tr><td>1019940|F|0-52:G>C-52:G>C</td><td>TGCAGGTGCTGAGCGCGCTGGACGCGGCCAAGACGCAGTGGTACCACTTCACGGCCATCGTCGTCGCCG</td><td>52:G>C</td></tr>
<tr><td>3940200|F|0-10:G>T-10:G>T</td><td>TGCAGGAGACGTGCAGCTACATCCG</td><td>10:G>T</td></tr>
<tr><td>4261883|F|0-13:A>C-13:A>C</td><td>TGCAGCAAATCTGAATGTTCTGCACCG</td><td>13:A>C</td></tr>
<tr><td>3027258|F|0-11:C>A-11:C>A</td><td>TGCAGCTGGGACGCTACCTGGTCCCGCAGCCTCGTCCG</td><td>11:C>A</td></tr>
<tr><td>3939712|F|0-38:T>C-38:T>C</td><td>TGCAGCCGCACTCGTAGCTGCCCCAGGTGTTCTTGCAGTGGCAGTCCG</td><td>38:T>C</td></tr>
<tr><td>32027120|F|0-6:A>G-6:A>G</td><td>TGCAGCACGTGGACGACACGCTGGGCGTGTTCCACACCCACGCCGTGGCGGGGTTCCTCGGCGGCGTGA</td><td>6:A>G</td></tr>
<tr><td>32026419|F|0-14:T>C-14:T>C</td><td>TGCAGGCGGACTCGTTGGAGCGGCAGGCCTACGGCTACACGAACGACGGCGTCACCTTCACCG</td><td>14:T>C</td></tr>
<tr><td>1030892|F|0-29:C>T-29:C>T</td><td>TGCAGATTTCGGTGTGGAAGAAATTCAACCTGATCTTCCTCTTCTTCCTACTCCG</td><td>29:C>T</td></tr>
<tr><td>1062469|F|0-40:G>T-40:G>T</td><td>TGCAGTTCTTGCAGCACACCCAGCCCATCCTCCACGAGGTGCAGGACCTGGCGGCGATGAAGCCCATCC</td><td>40:G>T</td></tr>
</table>

4262946|F|0-26:C>T-26:C>T	TGCAGCCAGCGGTGGAGGGAGCTGCCCCGCACCGCGGCCCG	26:C>T
1071743|F|0-7:C>T-7:C>T	TGCAGGGCATCGCCGATACTGCGCAGCAGCTCGGGCAGCTTCTCCTTCTTCCCCACCTCCG	7:C>T
3945602|F|0-16:C>A-16:C>A	TGCAGTCCGTGGAGCCCGCGTCTTATCCG	16:C>A
7344176|F|0-8:C>T-8:C>T	TGCAGTGGCGGTTCGGCGCGCCG	8:C>T
3028034|F|0-7:C>T-7:C>T	TGCAGCACGCCGCCGCCTTCCTCCCGCGCGTCCGCATCGTCTCCG	7:C>T
3021641|F|0-10:C>T-10:C>T	TGCAGGCCCACATGTTCCTGTATGTGCAAGAGAGATGTGCTACCG	10:C>T
2249018|F|0-20:G>A-20:G>A	TGCAGCAGCTTGGTGGTGTCGATGGCGCCCAGCTTCTCGATGTAGATCGGCCG	20:G>A
1021485|F|0-64:A>T-64:A>T	TGCAGGGATTCAGGAAGATAGTGGCCGACAGATGGGAGTTCGCCAACGAGTTCTTCAGGAAGGGAGCAA	64:A>T
5371104|F|0-20:A>T-20:A>T	TGCAGGCGGTGATCAAGGAGACGCTCCG	20:A>T
1020364|F|0-9:C>G-9:C>G	TGCAGTGAGCTCAGCGGCGGTGAAGGTGTAGGGCAGGTTGCCGACGTAGATTCGCCCG	9:C>G
7171635|F|0-27:C>T-27:C>T	TGCAGGCCGTCGCCGAGCCGCTGCGGCCTCCTTATGTCCG	27:C>T
4261201|F|0-17:G>A-17:G>A	TGCAGCATGGAGATCGCGCCCATGCAGCCGTACACCG	17:G>A
3941654|F|0-11:T>A-11:T>A	TGCAGCCTCTTTGGGTCGAGCAGCCG	11:T>A
4262403|F|0-30:C>G-30:C>G	TGCAGGTCTGTCGCCAATGCTGGGGAAGCGCCCG	30:C>G
5367542|F|0-24:C>A-24:C>A	TGCAGTGGCTCGACGACGAGCTCACCTCGCTCGCCCTCCCCGAACCCG	24:C>A
1019056|F|0-6:A>G-6:A>G	TGCAGCAGCACCTGCGCAGGGTCACGGTGCCGCTGCTGGTGCTGCACGGCGCCGACGACATGGTGACCG	6:A>G
4542355|F|0-18:G>C-18:G>C	TGCAGCGACGGTGAGCGAGGGCGCGCGCCG	18:G>C
3024533|F|0-10:C>A-10:C>A	TGCAGGTGCACATTGTGTACATGGGGGAGCTGCACCCG	10:C>A
5008766|F|0-17:G>T-17:G>T	TGCAGCAGAAGCAGCCAGGCGTCGCCG	17:G>T
3956790|F|0-44:T>C-44:T>C	TGCAGCGCGTGCCCACACGGGCCTTGAGGTCGGCGACGAACTCGTCCG	44:T>C
32021463|F|0-7:C>T-7:C>T	TGCAGGCCGTTGATTATTCAATCGCCG	7:C>T
995078|F|0-29:C>G-29:C>G	TGCAGCGCCTTCACCAGCCGCTCGCCCTTCTCGGCCGAGAAGGCCTGCCG	29:C>G
1145549|F|0-39:G>A-39:G>A	TGCAGTTCATGCGGGAGTGGGGGCCGACGGTGATCTACAGGTCGAGGACGGGGACGGAGCGGATGGTCA	39:G>A
1129167|F|0-49:C>A-49:C>A	TGCAGGACACGGACGCCACCAGCGTCGTGCACCGCATCATCTTCAAGGCCTTCGACGAGGCCCG	49:C>A
4261555|F|0-5:G>A-5:G>A	TGCAGGTATTGGTTCCAAACACCG	5:G>A
1046301|F|0-11:A>G-11:A>G	TGCAGCTGAAAAATCCTTCTCCCTTCCAGGTAACTGCTGCCGAACGCCGTCTGCGAGATGACATCTCCG	11:A>G
3949931|F|0-8:A>G-8:A>G	TGCAGCGTACGGCCATCATATGGAACGTCGTCCG	8:A>G
1070948|F|0-7:C>G-7:C>G	TGCAGGTCCAGGTGAACGGGGCGACGAGGCGTGTGGGCGGGGTCTTCGGGACGCCG	7:C>G
1145549|F|0-40:G>A-40:G>A	TGCAGTTCATGCGGGAGTGGGGGCCGACGGTGATCTACAGGTCGAGGACGGGGACGGAGCGGATGGTCA	40:G>A
5369354|F|0-21:T>C-21:T>C	TGCAGGAGCCTGGCGCACGCGTGCGAGTCGACCCCG	21:T>C
5367362|F|0-19:C>T-19:C>T	TGCAGGGGCTGCGGCTGTACGATGCGGACAAATCCATTCTCCG	19:C>T
4262946|F|0-33:G>A-33:G>A	TGCAGCCAGCGGTGGAGGGAGCTGCCCCGCACCGCCGCCCG	33:G>A
7170580|F|0-6:G>A-6:G>A	TGCAGCGGTAGTTCGAATGCAAAGTGCACCG	6:G>A
7922473|F|0-17:T>C-17:T>C	TGCAGCACCACCCCGCATCCG	17:T>C
7333899|F|0-19:C>G-19:C>G	TGCAGGGCACGACCTCGAACCCCACGACCATCCAGCCG	19:C>G
5009326|F|0-23:A>G-23:A>G	TGCAGGAGCGTCTTGACCTGGCCACGCAGCAGCGCCTCGCCAGAGCCG	23:A>G
7911745|F|0-35:C>G-35:C>G	TGCAGGCTGGCGTCGAGCAGCGCGGCCTGCTTGCCCACGTCGGCGTCGGCCACGAAGCCGCCGAACCGA	35:C>G
4993411|F|0-17:C>G-17:C>G	TGCAGCTCGTCGATGATCGCGCGGCCGCCG	17:C>G
1128975|F|0-46:C>T-46:C>T	TGCAGCCGCCGTCCAAGCAGCCCCGCCTCTCCTCCGCCGACTCGAACGCCG	46:C>T
9766732|F|0-14:T>C-14:T>C	TGCAGCAGCAGCAGTATCAGTCCG	14:T>C
1032068|F|0-58:T>C-58:T>C	TGCAGCACATCGGCACGTCCACGTGGCCTGGTTAAGCACCTTCCCTATGGTACATTTTTGCCCCGAGAT	58:T>C
5577813|F|0-19:A>G-19:A>G	TGCAGAAGATCTTGCTGACAACCG	19:A>G
1036296|F|0-10:G>C-10:G>C	TGCAGCATGAGAGGAAATGCCTGGCATAATCAAATGTGTGGAACCCAATCAAGTCGGCATTCAGCAGCG	10:G>C
4262532|F|0-20:C>G-20:C>G	TGCAGCCGCTGCGCAGATTCCTCACCTCCCTTGAACAGGGCCG	20:C>G
3937001|F|0-16:T>A-16:T>A	TGCAGCGGGAGAAGGCTGAGGCCATGATGGAGGCGCGCCAGTTCCG	16:T>A
3946276|F|0-29:G>A-29:G>A	TGCAGCCGTACCTGTCGCGCGGGAAGGACGTCCG	29:G>A
1667327|F|0-8:C>A-8:C>A	TGCAGCCTCCCGCCGTCGAGTCCCAGCCCCTTGGACAGCACCTTTGATACCCTCCG	8:C>A
2241536|F|0-53:G>C-53:G>C	TGCAGAGTCAAAGTCAAGTCTACTGAAGATGAGTAGGTGACCAGAAAAAATATGCTCGATTTGTATCCT	53:G>C
3949931|F|0-6:G>C-6:G>C	TGCAGCGTACGGCCATCATATGGAACGTCGTCCG	6:G>C
1014038|F|0-29:G>C-29:G>C	TGCAGCTACCTAGAGTCAACTCTTAATTTGTACTTAGCATAGGATCAAGTACGTTTCCG	29:G>C
3956790|F|0-30:C>T-30:C>T	TGCAGCGCGTGCCCACACGGGCCTTGAGGTCGGCGACGAACTCGTCCG	30:C>T
3031436|F|0-5:G>A-5:G>A	TGCAGGATGGCCAGCTTCTGGAGCTTGGAGAAGCCCG	5:G>A
12010150|F|0-10:C>T-10:C>T	TGCAGCGGTTCTCGTCGGGAGCAGAGTCCG	10:C>T
32022432|F|0-17:A>G-17:A>G	TGCAGCCTGTCGATGTTATACGCGTTTGGTAGCTTGGTAGCCG	17:A>G
1025266|F|0-16:G>A-16:G>A	TGCAGGACCTGAGGGAGGCGATCGTCAAGGGGCTGGGGCTCCAGTCGGAGGAGCTCAAGGTGTCCGAGA	16:G>A
3030485|F|0-31:C>T-31:C>T	TGCAGCTCCGACTTGCTCCGCGTGGCGCGCACGAACCCCG	31:C>T
1102424|F|0-35:T>G-35:T>G	TGCAGGTCCCAGCGCAGCGTCCACGTGCGCGCCCATCCATGTCCGTCGCCG	35:T>G
1030427|F|0-25:T>C-25:T>C	TGCAGGACCAGATCACGTACAAGGTTGGCAAGCACTACGCCGACTGCCCCAGCTTCCG	25:T>C
4262843|F|0-6:C>T-6:C>T	TGCAGACTATCGGCGCTCATGGCAGAGACCG	6:C>T

<table>
<tr><td>1667327|F|0-42:T>C-42:T>C</td><td>TGCAGCCTCCCGCCGTCGAGTCCCAGCCCCTTGGACAGCACCTTTGATACCCTCCG</td><td>42:T>C</td></tr>
<tr><td>3937716|F|0-5:G>A-5:G>A</td><td>TGCAGGGGCACCCCGAGGGCCG</td><td>5:G>A</td></tr>
<tr><td>4261249|F|0-25:A>C-25:A>C</td><td>TGCAGGCCCAGGTAGCAGAGCGAGGAGGCGTCCG</td><td>25:A>C</td></tr>
<tr><td>3937331|F|0-14:C>A-14:C>A</td><td>TGCAGGCTCCGTATCGCGGTTATCCCCGCAGGGATCTCGCCG</td><td>14:C>A</td></tr>
<tr><td>7350670|F|0-11:G>A-11:G>A</td><td>TGCAGCTGCACGCCCAGGCGCCG</td><td>11:G>A</td></tr>
<tr><td>1024202|F|0-53:C>G-53:C>G</td><td>TGCAGCTCGTAGAGCACGGCGCCGTTGGCTGGCTCGGCCGAATTGTACACGTACCCTCCG</td><td>53:C>G</td></tr>
<tr><td>3023776|F|0-31:C>A-31:C>A</td><td>TGCAGGCCACCCAAATCCGCACCAATTTGGGCGAAAAATTGCACCG</td><td>31:C>A</td></tr>
<tr><td>1025266|F|0-31:G>A-31:G>A</td><td>TGCAGGACCTGAGGGAGGCGATCGTCAAGGGGCTGGGGCTCCAGTCGGAGGAGCTCAAGGTGTCCGAGA</td><td>31:G>A</td></tr>
<tr><td>1024410|F|0-20:C>T-20:C>T</td><td>TGCAGCGCCTTCACCAGCCGCTCGCCCTTCTCGGCCGTGAACGCCTTCCG</td><td>20:C>T</td></tr>
<tr><td>1232096|F|0-32:C>T-32:C>T</td><td>TGCAGGTTCACCAGCTTGCTTATCGTGCTCGGCAGCCTCTCCAGCTGCCCGCACTGGAGGCCCAGGTAC</td><td>32:C>T</td></tr>
<tr><td>3955493|F|0-22:C>A-22:C>A</td><td>TGCAGAACCTCACATCGGGCGGCGACAGCTTCCG</td><td>22:C>A</td></tr>
<tr><td>1024202|F|0-13:G>A-13:G>A</td><td>TGCAGCTCGTAGAGCACGGCGCCGTTGGCTGGCTCGGCCGAATTGTACACGTACCCTCCG</td><td>13:G>A</td></tr>
<tr><td>1128975|F|0-17:C>G-17:C>G</td><td>TGCAGCCGCCGTCCAAGCAGCCCCGCCTCTCCTCCGCCGACTCGAACGCCG</td><td>17:C>G</td></tr>
<tr><td>1021754|F|0-7:G>C-7:G>C</td><td>TGCAGCCGAGCATGAACACCAGCACCAGGCACATACTCAAGTACCTCACCG</td><td>7:G>C</td></tr>
<tr><td>5370994|F|0-24:G>C-24:G>C</td><td>TGCAGAGGAGGAGGCGGCCGTTGCGGAAGTCCCG</td><td>24:G>C</td></tr>
<tr><td>1120978|F|0-30:G>C-30:G>C</td><td>TGCAGCGGCGACCTGCCGTTCGCGCTCGTCGGCAACTACTCTGGCCTCGCCTGCCAGCCCGTCTGGAAC</td><td>30:G>C</td></tr>
<tr><td>5008766|F|0-6:A>G-6:A>G</td><td>TGCAGCAGAAGCAGCCAGGCGTCGCCG</td><td>6:A>G</td></tr>
<tr><td>2245367|F|0-42:G>T-42:G>T</td><td>TGCAGTAAGGGCACTGCAAAGGCACCGCTCAGATTTCGACGAGAGAAACGGTGGTGATATGTGACCGAG</td><td>42:G>T</td></tr>
<tr><td>1128975|F|0-41:T>G-41:T>G</td><td>TGCAGCCGCCGTCCAAGCAGCCCCGCCTCTCCTCCGCCGACTCGAACGCCG</td><td>41:T>G</td></tr>
<tr><td>1667327|F|0-44:T>C-44:T>C</td><td>TGCAGCCTCCCGCCGTCGAGTCCCAGCCCCTTGGACAGCACCTTTGATACCCTCCG</td><td>44:T>C</td></tr>
<tr><td>5324450|F|0-5:C>G-5:C>G</td><td>TGCAGCTCCTTGCCCATGGGCGCGGCACCG</td><td>5:C>G</td></tr>
<tr><td>6037648|F|0-12:C>T-12:C>T</td><td>TGCAGCAGCTTGCTTCCCTCCCCGACAAACCG</td><td>12:C>T</td></tr>
<tr><td>1038399|F|0-26:G>T-26:G>T</td><td>TGCAGCCTGATCTCCCTGTTGAACCTGGTGATGAAGTCCTCCACCCTCCG</td><td>26:G>T</td></tr>
<tr><td>1141147|F|0-7:G>A-7:G>A</td><td>TGCAGGCGACGATCGCCGCCCGCAACCCCAACTCCCGCGTCGGCATCTACTTCGACCG</td><td>7:G>A</td></tr>
<tr><td>1020000|F|0-24:A>G-24:A>G</td><td>TGCAGAGGCCAATGTTGTGTTCTCATCTCTTATGGGTGCTCGGGTAAGCAACCG</td><td>24:A>G</td></tr>
<tr><td>6045527|F|0-5:G>C-5:G>C</td><td>TGCAGGGAGTCGGACCGCAGGTTGTAGCCGTTGAGGTCGACGGACGCGACGGTCCG</td><td>5:G>C</td></tr>
<tr><td>987177|F|0-14:C>T-14:C>T</td><td>TGCAGCTTGTCCCTCAGGTCGTCCAGCTCCTTCCTCACCTCCACCAGCCG</td><td>14:C>T</td></tr>
<tr><td>4993350|F|0-15:C>A-15:C>A</td><td>TGCAGGGGGTGCAGGCCAAGCTGCGCCG</td><td>15:C>A</td></tr>
<tr><td>3028857|F|0-33:C>T-33:C>T</td><td>TGCAGCTGCCATGATCGGCCGATCTATCCTCGTCTCCCTCCTCCG</td><td>33:C>T</td></tr>
<tr><td>1043833|F|0-29:C>A-29:C>A</td><td>TGCAGCAGCAGGTTGTGGTGCATCCCTAGCTTGTTGATATCTTCCAGGCAACCG</td><td>29:C>A</td></tr>
<tr><td>4002656|F|0-19:C>T-19:C>T</td><td>TGCAGCTCGCCATCCTCGCCGCCACCGTCACGTACCAGACCG</td><td>19:C>T</td></tr>
<tr><td>2299065|F|0-47:C>G-47:C>G</td><td>TGCAGCGTCATCACCTCCTCGATCCGCCGCCGCAGCGTGTAGAAGCTCGCGTTCGAGAAGGCCCGAGAT</td><td>47:C>G</td></tr>
<tr><td>32020124|F|0-9:A>G-9:A>G</td><td>TGCAGAGGAAGTCGTACAACCCCCG</td><td>9:A>G</td></tr>
<tr><td>32017617|F|0-17:G>C-17:G>C</td><td>TGCAGCAGCTATGCACCGTAGACGGATAAATCCAGCAGCAACTAGCACCG</td><td>17:G>C</td></tr>
<tr><td>4261129|F|0-19:C>G-19:C>G</td><td>TGCAGGCGGCGTGGCGGATCTCCG</td><td>19:C>G</td></tr>
<tr><td>1215664|F|0-28:T>C-28:T>C</td><td>TGCAGTGGCGGTGCAGGGAGGGGTCTGATCACAGGTGAACAGTGGAGCAAACCG</td><td>28:T>C</td></tr>
<tr><td>1021421|F|0-14:T>C-14:T>C</td><td>TGCAGCTGGGACTATATCCAGGAGGGCAGCCAGGTGGCCAAGGACATGGACCG</td><td>14:T>C</td></tr>
<tr><td>994295|F|0-22:A>G-22:A>G</td><td>TGCAGTGGAACCACCAAGAACAAGCCTGTCCAAGAGGCCATCGCTTGGAGCTTCTGCAAAGGTCCAGTC</td><td>22:A>G</td></tr>
<tr><td>1030892|F|0-10:G>A-10:G>A</td><td>TGCAGATTTCGGTGTGGAAGAAATTCAACCTGATCTTCCTCTTCTTCCTGCTCCG</td><td>10:G>A</td></tr>
<tr><td>3950863|F|0-11:C>T-11:C>T</td><td>TGCAGGTAGAGCATGCCGCCCG</td><td>11:C>T</td></tr>
<tr><td>5006268|F|0-7:G>C-7:G>C</td><td>TGCAGCAGAAGCAGCCAGACGTCGCCG</td><td>7:G>C</td></tr>
<tr><td>3956810|F|0-7:G>A-7:G>A</td><td>TGCAGGAGGGCAGAGAAGCCG</td><td>7:G>A</td></tr>
<tr><td>987437|F|0-10:G>C-10:G>C</td><td>TGCAGGTCCAGGGCCCTGGTTCTACTTATCGTCGTCGCCACCAACACAGCCACCTTCCTCCTCTTCTCC</td><td>10:G>C</td></tr>
<tr><td>3021119|F|0-8:C>G-8:C>G</td><td>TGCAGGCCCACATGTTTCTGTATGTGTAAGACAGATGGGCTACCG</td><td>8:C>G</td></tr>
<tr><td>2299065|F|0-5:C>T-5:C>T</td><td>TGCAGCGTCATCACCTCCTCGATCCGCCGCCGCAGCGTGTAGAAGCTCGCGTTCGAGAAGGCCCGAGAT</td><td>5:C>T</td></tr>
<tr><td>32023415|F|0-27:A>G-27:A>G</td><td>TGCAGTACCTCTCGCTCGCGGCGAACCATCTCACCG</td><td>27:A>G</td></tr>
<tr><td>3029038|F|0-8:G>C-8:G>C</td><td>TGCAGGTCGGCCTCCGTCACGGCCGCCTTCCCCTTGAACGCCTCCCG</td><td>8:G>C</td></tr>
<tr><td>12694981|F|0-26:C>G-26:C>G</td><td>TGCAGGCCCAGGTAGCAGAGCGAGGACGCGTCCG</td><td>26:C>G</td></tr>
<tr><td>1026423|F|0-28:G>A-28:G>A</td><td>TGCAGAAGGCTACCAACAACTTCACCACGATTCTGGGGCAAGGGTCGTTTGGTCCTGTGTACAAGGCTG</td><td>28:G>A</td></tr>
<tr><td>997436|F|0-39:C>T-39:C>T</td><td>TGCAGCACCAATGTTCTGGCCAACCTTCATCGTTGCCATCCTTGCTGCCATCATAGCAAGCCAAGCTAT</td><td>39:C>T</td></tr>
<tr><td>1083842|F|0-14:G>C-14:G>C</td><td>TGCAGCTTCCTCCAGAGGCAGATCATGTGCTGCGGCGACTGGTAGTGCGCGGTGTAGTAGCCGTCGAAG</td><td>14:G>C</td></tr>
<tr><td>3021511|F|0-16:T>C-16:T>C</td><td>TGCAGATGTACAGGACTGTGAAGAATACCGAAAGGCCG</td><td>16:T>C</td></tr>
<tr><td>3946276|F|0-28:C>T-28:C>T</td><td>TGCAGCCGTACCTGTCGCGCGGGAAGGACGTCCG</td><td>28:C>T</td></tr>
<tr><td>991226|F|0-16:G>T-16:G>T</td><td>TGCAGACCGTTGATCAGTCTGAACTGGAGAAATGGTGGAAGAAAAACAAACGCATGTGTCCAGTTACCG</td><td>16:G>T</td></tr>
<tr><td>1026038|F|0-31:A>G-31:A>G</td><td>TGCAGCTCGCGCGCCGCGCCAAGATGATCGGAGACCTGCTGCACCAGCTCCACGCCGCGCAGCTCATGC</td><td>31:A>G</td></tr>
<tr><td>6046794|F|0-27:A>G-27:A>G</td><td>TGCAGGAGAGGCTGCGGTGACCTCTGGATCGGAGCCG</td><td>27:A>G</td></tr>
<tr><td>1038665|F|0-17:G>C-17:G>C</td><td>TGCAGGACAGGAGAGGGGCCCGCGCGCGGCATGCACGTGAAAGCGAGTACGATGCACAATTATGATGTT</td><td>17:G>C</td></tr>
<tr><td>5363515|F|0-13:C>T-13:C>T</td><td>TGCAGCGGATCTGCGATGGAGTGTACCTGAACCG</td><td>13:C>T</td></tr>
<tr><td>5580352|F|0-11:C>G-11:C>G</td><td>TGCAGGAAAAGCAGAGGCTTGGCAAGCACATCCCTGGTGTCACCG</td><td>11:C>G</td></tr>
</table>

6048900|F|0-13:G>A-13:G>A	TGCAGCCTTCCTGGAACATGGTGACCGCCTTTTCTCCGTCAGATTCCCCGCCG	13:G>A
1012031|F|0-62:G>C-62:G>C	TGCAGGTTGTCCAGCGCGTCGCGGAGCAGGTCGGCGGCGCCGCCGTACTGCTCCAGGCAGTGGTCGAAC	62:G>C
12765776|F|0-7:T>C-7:T>C	TGCAGACTATCGGCGCTCATGGCAGAGGCCG	7:T>C
4909330|F|0-29:G>C-29:G>C	TGCAGGTTGGGGTCCTTCTTGTACTTGCAGAGGCAGCTCGCCG	29:G>C
4262532|F|0-27:C>T-27:C>T	TGCAGCCGCTGCGCAGATTCCTCACCTCCCTTGAACAGGGCCG	27:C>T
2299065|F|0-35:C>A-35:C>A	TGCAGCGTCATCACCTCCTCGATCCGCCGCCGCAGCGTGTAGAAGCTCGCGTTCGAGAAGGCCCGAGAT	35:C>A
5369498|F|0-5:G>C-5:G>C	TGCAGGTGGAGGTTGTGGCTACGGAAGTGAACCCCG	5:G>C
3952237|F|0-9:G>T-9:G>T	TGCAGTGAAGCTCCCGATTCGCCG	9:G>T
3952121|F|0-20:G>A-20:G>A	TGCAGGTCCCCCTCGAGGTTGCCCG	20:G>A
1079893|F|0-34:C>T-34:C>T	TGCAGCATCCGCCACCATCGTCCATGTTCCGCTTCTCGGCCACGCTCTGTGTTGCCACCATCGGAAACT	34:C>T
32016474|F|0-17:A>G-17:A>G	TGCAGCGGCACGGGCGGATTCATCCG	17:A>G
32025331|F|0-22:A>T-22:A>T	TGCAGCATGATACACCACCACCAGGTCCTGCACCCG	22:A>T
4995782|F|0-20:T>C-20:T>C	TGCAGCATGCGCTTGCACCGTGCGCACCCGCCG	20:T>C
7165476|F|0-34:A>C-34:A>C	TGCAGCTCATGTACCACTTCGACTGGAGGCTGCCAGCCG	34:A>C
100540426|F|0-13:A>G-13:A>G	TGCAGCTGCACCCACTTATTCTTGCCGATCAACCTACTAGACGCTGCCG	13:A>G
5364476|F|0-8:A>C-8:A>C	TGCAGGGCATCGTACTTGGAGGCCACCTCCCTGAGCATCTCGCCCAGCCGCCG	8:A>C
7164702|F|0-7:T>C-7:T>C	TGCAGAATCTGTTCGTCGTGTCGGACAACCG	7:T>C
32023415|F|0-22:C>G-22:C>G	TGCAGTACCTCTCGCTCGCGGCCAACCATCTCACCG	22:C>G
5361232|F|0-23:T>G-23:T>G	TGCAGCTGCACGGCGGGATGAAGTCCGTGAACCACATATGGCAGGTCGGCACCTCCGTCATCGCCGAGA	23:T>G
5366354|F|0-53:T>C-53:T>C	TGCAGGACGACGTGGGGAGGCTTATCGTTGCTCAGTTCCGCTGGCTTGATTTCTTTGTCGATGCCGACA	53:T>C
989804|F|0-41:G>C-41:G>C	TGCAGGGTGCGGAAGTCCTTGAGCGGCCGCGCGCGCGGGTGGGCCACGCTGTTCCG	41:G>C
32027339|F|0-16:C>G-16:C>G	TGCAGGCCACCCGCGCCGATGCCCG	16:C>G
1012031|F|0-34:C>A-34:C>A	TGCAGGTTGTCCAGCGCGTCGCGGAGCAGGTCGGCCGCGCCGCCGTACTGCTCCAGGCAGTGGTCGAAC	34:C>A
3953801|F|0-14:G>A-14:G>A	TGCAGGAGGGACACGTACACCTCCG	14:G>A
7171389|F|0-5:A>G-5:A>G	TGCAGACCACACCTGTCGGCCGCTTCGACGATTCCG	5:A>G
1232096|F|0-41:C>T-41:C>T	TGCAGGTTCACCAGCTTGCTTATCGTGCTCGGCAGCCTCTCCAGCTGCCCGCACTGGAGGCCCAGGTAC	41:C>T
1038614|F|0-16:G>A-16:G>A	TGCAGCTGATCGGCACGTGCAATGGTCTTCTCTGCCTGTGCGACAACAAGGAGGCACCCG	16:G>A
5581268|F|0-7:C>T-7:C>T	TGCAGAACCTCACGACGGGCGGCGACAGCTTCCG	7:C>T
4262689|F|0-31:G>T-31:G>T	TGCAGTATCTCGGACTCGATGGTGGTGACCCGCTCCAGGACCTCCG	31:G>T
5577872|F|0-25:C>A-25:C>A	TGCAGAGCAGTGGTGTGGAGTTTCGCCCCG	25:C>A
1056040|F|0-48:T>C-48:T>C	TGCAGGTTTGAAATCTGAATACTCTCGTCATGTTGTTTGGTGCAGGTATGAGCCG	48:T>C
3960832|F|0-7:C>G-7:C>G	TGCAGCTCAAGCTGGTGGAGGCCATCAAAGAAGCCG	7:C>G
3952055|F|0-14:G>C-14:G>C	TGCAGCTGCTCGCTGGACAGGGTGAACGTGGCGAGGAGCTGGTCCG	14:G>C
5363219|F|0-26:C>T-26:C>T	TGCAGCAACCAGACCTCGTGCTCTGCCTACGCCG	26:C>T
4261618|F|0-8:C>A-8:C>A	TGCAGGCACGACAGGCGCCGCTGCCTCGCCATCCTCTTCTTCCG	8:C>A
3020934|F|0-8:C>A-8:C>A	TGCAGCAACACGGTCTTGAGCAGGAAGTTCTTGACAATCTCCG	8:C>A
4006298|F|0-15:G>C-15:G>C	TGCAGCACCGACGGCGCCATCAACACCTTAACCTCCG	15:G>C
1038822|F|0-29:A>G-29:A>G	TGCAGCTCAGAACGTGGCACCCCAGCCACACCCTGCACCTTGAGGCAGAGCCTGAGCAGGAAGGCCTCC	29:A>G
1038822|F|0-8:A>G-8:A>G	TGCAGCTCAGAACGTGGCACCCCAGCCACACCCTGCACCTTGAGGCAGAGCCTGAGCAGGAAGGCCTCC	8:A>G
3937142|F|0-29:C>T-29:C>T	TGCAGAAGCTTCTGTTGGCCGACAACAAGCTGTCCG	29:C>T
1026982|F|0-25:C>T-25:C>T	TGCAGGAGACGCTGGACTACGCGTGCGGGCAGGGCGCGGACTGCGCGCCG	25:C>T
3942666|F|0-23:C>A-23:C>A	TGCAGCGAGAAGACCATCCTCACCGTGAACGGCGAGTTCCCCG	23:C>A
32021351|F|0-22:G>A-22:G>A	TGCAGCCTCCTCGCGTCCACCAGCACCG	22:G>A
1029152|F|0-11:C>T-11:C>T	TGCAGGATTTCCTCAACATGTTCTTCAGGGAGCAGTACAAGCCGATCCCGCTGGTCTACAACCTTGTGC	11:C>T
1027276|F|0-37:G>C-37:G>C	TGCAGACGTGGAACAACGGGGCCGAGAACACGAAGCGGATCGTGGCCGCCG	37:G>C
3954921|F|0-7:G>C-7:G>C	TGCAGGGGAAGCCCATCACGGTGTACCG	7:G>C
5582091|F|0-28:C>T-28:C>T	TGCAGTTGGTCTGCTGGTCGGCGAAGAACGGCCAGCTGATCACCG	28:C>T
3034149|F|0-5:C>A-5:C>A	TGCAGCGAGAGCATCACGCCTATCATGCTCTTCGACTCCG	5:C>A
5580134|F|0-17:G>A-17:G>A	TGCAGCAACCAGACCTCGTGCTGTGCCTGCGCCG	17:G>A
1034182|F|0-44:G>A-44:G>A	TGCAGGCCCCCGACCTGGTCCTGGCAGAGGATGGTGAGGAAGTCGCTGTCCGTGTGCGGCACCAGCCCG	44:G>A
6047702|F|0-13:T>C-13:T>C	TGCAGGTTCTGAATCGACTTGGGCAGTGGCCCG	13:T>C
3533170|F|0-16:C>T-16:C>T	TGCAGGCCCTGAAATCCTCGGGTGACGTCTATCTGCTACCG	16:C>T
4002388|F|0-34:C>A-34:C>A	TGCAGGTACGTCGGCTTGCCCATGTCCAGCGACACGCACCG	34:C>A
3951142|F|0-15:A>G-15:A>G	TGCAGAGGTGGGATCACGTCGAGAGGCACCG	15:A>G
5332148|F|0-8:A>G-8:A>G	TGCAGCTTACCCGCAAGCCTTTTCCG	8:A>G
1020648|F|0-35:G>C-35:G>C	TGCAGCTCCCGCACGATGAGCACCATGGAGGAGAAGGCGCGGCCCACCGACTCGCCG	35:G>C
4261140|F|0-17:C>G-17:C>G	TGCAGGTCCTTCTCCTCCAGGAAGAGCGAGAAGCCG	17:C>G
3947831|F|0-8:G>A-8:G>A	TGCAGCGTGGAGTCGAAGGTGCGCACCTCCG	8:G>A
3946135|F|0-21:T>C-21:T>C	TGCAGCGCGTACGTTGACTAATGGAGCCG	21:T>C

994295|F|0-54:T>C-54:T>C	TGCAGTGGAACCACCAAGAACAAGCCTGTCCAAGAGGCCATCGCTTGGAGCTTCTGCAAAGGTCCAGTC	54:T>C
4261822|F|0-23:G>A-23:G>A	TGCAGGCCGTAGGAGAACCTGGCGGCCGCGTAGAGCGCCG	23:G>A
1234784|F|0-59:G>A-59:G>A	TGCAGCTTGGCCATTGCCTGTGGGTTCCCGACCAGCTCCGCCATGGCCCATTCCAGCGTGAGGGACGAT	59:G>A
4262770|F|0-27:G>C-27:G>C	TGCAGGTTGGTGAGCTGTGCCAGCTCTGGCGGGATCGCGCCG	27:G>C
988349|F|0-35:C>A-35:C>A	TGCAGGTACGTGTCGGCCACGATAGCGTCTGGCCTCCGCCCCAGCTCCAGCGCCAGCCG	35:C>A
1232096|F|0-9:A>T-9:A>T	TGCAGGTTCACCAGCTTGCTTATCGTGCTCGGCAGCCTCTCCAGCTGCCCGCACTGGAGGCCCAGGTAC	9:A>T
5411955|F|0-24:C>A-24:C>A	TGCAGCGTGTCGGCGGCCACGAGGCGGCCGTTCATGGCGTAGGGATCGTTGGTGGAGACCATGTCGAAG	24:C>A
1149760|F|0-32:A>G-32:A>G	TGCAGCACGCTCATGTTGTGGTGCTGGTGATCATCCGTCGTCCCCTCCG	32:A>G
1026959|F|0-46:A>G-46:A>G	TGCAGAGGCTCATCTGGGTGGTGAGGCCAGATCTCTTCCAGCTGCCACCG	46:A>G
7173446|F|0-57:A>G-57:A>G	TGCAGAAGCGTTGAATCGATTCCTTGGTGCGCGGCGATTGCCGAGGGGAGCGAGATGATACAGAAGCGC	57:A>G
16795583|F|0-13:A>G-13:A>G	TGCAGGCCCTCCAAATAAGCTTGACCCG	13:A>G
997436|F|0-54:A>T-54:A>T	TGCAGCACCAATGTTCTGGCCAACCTTCATCGTTGCCATCCTTGCTGCCATCATAGCAAGCCAAGCTAT	54:A>T
3934144|F|0-11:G>T-11:G>T	TGCAGCAGTGCGTGCAGCGGTGCCAGCAGGACCG	11:G>T
1707968|F|0-25:C>T-25:C>T	TGCAGCTAGCGTCCGCCACGGCCTGCGTCACGCTGTCGGCGGTGCGGCTCGTCAGGCAGGACTTCGGCG	25:C>T
1136165|F|0-41:G>C-41:G>C	TGCAGGGTGTGCACGTCGGCGACGTGGGAGAGCGCCGTGCGGGAGGCCG	41:G>C
1022735|F|0-8:G>C-8:G>C	TGCAGCTTGGGGAGCAGGTAGTACCTGATCAGCTCGTCGTCGCTCGGCCTGAACCG	8:G>C
3936669|F|0-26:C>T-26:C>T	TGCAGCTGCTGCGGCGTCGGCTGCTGCGAGAAGAAGTGGGGCACGCCG	26:C>T
3952055|F|0-31:C>T-31:C>T	TGCAGCTGCTCGCTGGACAGGGTGAACGTGGCGAGGAGCTGGTCCG	31:C>T
1038665|F|0-56:C>T-56:C>T	TGCAGGACAGGAGAGGGGCCCGCGCGCGGCATGCACGTGAAAGCGAGTACGATGCACAATTATGATGTT	56:C>T
4261207|F|0-10:G>T-10:G>T	TGCAGGAAGAGCAGAGGCTTGCCAATGACATCTCTCGTGTTGCCG	10:G>T
1067877|F|0-33:G>T-33:G>T	TGCAGCTTGGACAGCCACGACGGTATCTGGCCCGTCAGACCGCACTCCTCCATGACGATCAGCCGAGAT	33:G>T
1067877|F|0-5:C>T-5:C>T	TGCAGCTTGGACAGCCACGACGGTATCTGGCCCGTCAGACCGCACTCCTCCATGACGATCAGCCGAGAT	5:C>T
3385533|F|0-13:G>C-13:G>C	TGCAGGAGAGCGTGAGGCGCACGCGTGTGAAGTACCCG	13:G>C
1024410|F|0-32:G>C-32:G>C	TGCAGCGCCTTCACCAGCCGCTCGCCCTTCTCGGCCGTGAAGGCCTTCCG	32:G>C
4262532|F|0-38:G>A-38:G>A	TGCAGCCGCTGCGCAGATTCCTCACCTCCCTTGAACAGGGCCG	38:G>A
4262532|F|0-35:C>G-35:C>G	TGCAGCCGCTGCGCAGATTCCTCACCTCCCTTGAACAGGGCCG	35:C>G
3947529|F|0-16:C>T-16:C>T	TGCAGCTTGGGGACGACGGACTTGAACCG	16:C>T
3947529|F|0-11:G>C-11:G>C	TGCAGCTTGGGGACGACGGACTTGAACCG	11:G>C
5324461|F|0-13:C>T-13:C>T	TGCAGGAGATGAGCACCCGCGTAGCGGACCG	13:C>T
5361353|F|0-47:T>C-47:T>C	TGCAGGACGTCCCTGATCTGGTCGATCGCGTACCCTCCGCTGGGGTCTTCCCTCTCGACGCCATTGTGG	47:T>C
1020648|F|0-8:C>G-8:C>G	TGCAGCTCCCGCACGATGAGCACCATGGAGGAGAAGGCGCGGCCCACCGACTCGCCG	8:C>G
4733803|F|0-16:C>G-16:C>G	TGCAGATGCCGCTGTGCCAGGCACACCG	16:C>G
7173327|F|0-16:G>A-16:G>A	TGCAGTCCGCGAGGCCGCCGTCGGGGGAGGCCGCGGCGTGGTGCGCCG	16:G>A
4262532|F|0-5:C>T-5:C>T	TGCAGCCGCTGCGCAGATTCCTCACCTCCCTTGAACAGGGCCG	5:C>T
32017982|F|0-16:T>G-16:T>G	TGCAGCACCCTTCACGTCCG	16:T>G
22839083|F|0-18:A>G-18:A>G	TGCAGTGAATGAAGCAGGAGGCTGGAAGGACCG	18:A>G
2294672|F|0-13:A>C-13:A>C	TGCAGTGCGCGCCATGCCTCGACTGCTTCCACCAGAGCGGCCCCGTCTTCGACCCG	13:A>C
3957041|F|0-24:A>G-24:A>G	TGCAGGCAGTGCAGCGCCAGCGTCACCGCGCCGCCCG	24:A>G
5008303|F|0-17:T>G-17:T>G	TGCAGGCCAGGATGCAGTGCAGCAACCG	17:T>G
1037648|F|0-34:C>G-34:C>G	TGCAGACCATCAACGGCCCCAACTCCGACTTCCCCGTCTACTCGTCGCAGCTCAAGGAGCTCAAGGGCA	34:C>G
3021100|F|0-13:G>A-13:G>A	TGCAGAGATGAGCGTCTGCACTACATTCTTCATACCG	13:G>A
1141725|F|0-31:T>A-31:T>A	TGCAGAAAACTGCTGCATGTACTGCTGTTCATGAGTTGTTATGTAGCGAACCATCCG	31:T>A
4539437|F|0-7:C>A-7:C>A	TGCAGCACTTTGGCGGGTCGACGGGGTACGGCCACGACGACGCCG	7:C>A
1157517|F|0-9:G>A-9:G>A	TGCAGCTGCGCCCACGCCTCCTCCGTGTCGATCAGGCGGCGCAGCCACGCCG	9:G>A
1046301|F|0-41:G>C-41:G>C	TGCAGCTGAAAAATCCTTCTCCCTTCCAGGTAACTGCTGCCGAACGCCGTCTGCGAGATGACATCTCCG	41:G>C
1024063|F|0-10:G>A-10:G>A	TGCAGCAGCGGTTCCCCGTGGGGGACGGGACGATGGACCTGGTGCGGGCGGGGCACGCGGTGAACCGAG	10:G>A
7917985|F|0-22:A>G-22:A>G	TGCAGCAGGGAAGGAGGGGGGAAGGAGCTCGAGGAGACGGAGGGGTGGTTCCCGTGGAAGCACGACGAC	22:A>G
3935731|F|0-5:G>A-5:G>A	TGCAGGATGTCCCTGATCTGGTCGATCGAGTACCCTCCGCTGGGGTCTTCCCTCTCGACGCCATTGTGG	5:G>A
32027109|F|0-26:G>C-26:G>C	TGCAGCCCCTCGTAGTGCACCAGCGAGCCGCCGTCGTCGGAGAAGTAGGTGGCGTACTTCTCGACCGAG	26:G>C
3941397|F|0-26:G>T-26:G>T	TGCAGTTCCTGTCGGAGGGATCATGGGCGAACCATGACGAGATCGACCTGGAGTTCCTGGGCAACTCCA	26:G>T
4396766|F|0-6:A>G-6:A>G	TGCAGGAAGTTTCTATATGAAACGCGAACAGAACCG	6:A>G
1863668|F|0-25:C>G-25:C>G	TGCAGTACCTGGCCATGGACATCTCCACCGACTACATCGACCACTTCAAGGCCCG	25:C>G
7172934|F|0-11:C>A-11:C>A	TGCAGGAGATGCGCGGGCGCGTGTCGAACCG	11:C>A
4262345|F|0-37:G>A-37:G>A	TGCAGGTGCCCACCCTCAGGAGCTGCGACTTCACGCGGCTCTACTCCG	37:G>A
3935751|F|0-26:G>A-26:G>A	TGCAGCCAGAATGTGCCGTTGGGGTCGCCG	26:G>A
5581347|F|0-16:G>A-16:G>A	TGCAGCATTGCGTCCAGGGCCCTAGCCG	16:G>A
1089132|F|0-17:C>T-17:C>T	TGCAGGTTCGGCCACGACGGATGCCAGGCCGTTCACGTCAACGCCGACGTGGTTGCTGCTGATGTCCCG	17:C>T
7169758|F|0-35:G>C-35:G>C	TGCAGGTAGGGCAGGTTGGGGAAGTCCGTCTCCGAGAGGACCCG	35:G>C
1157517|F|0-35:G>T-35:G>T	TGCAGCTGCGCCCACGCCTCCTCCGTGTCGATCAGGCGGCGCAGCCACGCCG	35:G>T

3956426|F|0-42:A>G-42:A>G	TGCAGCTGCGCCCACGCCTCCTCCGTGTCGATCAGGCGGCGCAACCACGCCG	42:A>G
3937159|F|0-35:A>C-35:A>C	TGCAGTCCAACATCTTCATCGAGGGCGTCTCCCACAAGATCCGCCG	35:A>C
1034182|F|0-26:G>C-26:G>C	TGCAGGCCCCCGACCTGGTCCTGGCAGAGGATGGTGAGGAAGTCGCTGTCCGTGTGCGGCACCAGCCCG	26:G>C
2243289|F|0-8:C>G-8:C>G	TGCAGCTGCGAGCTCAGGTCCTCGATCTTGGCCTCCCCGAAGATCACGTACGTCTCCGACGTCGGGCTC	8:C>G
3940807|F|0-23:G>A-23:G>A	TGCAGCGCGTACGTTGACTAATCGACCCG	23:G>A
1056040|F|0-20:A>G-20:A>G	TGCAGGTTTGAAATCTGAATACTCTCGTCATGTTGTTTGGTGCAGGTATGAGCCG	20:A>G
4262692|F|0-13:C>G-13:C>G	TGCAGGTGTTGTCCACGAGCCG	13:C>G
100513007|F|0-20:G>C-20:G>C	TGCAGCAGCTTCTCCACGATGATGATGATGTTGGCGTAGTGTAATGCGAGCGCCGAGCCGCCGATGGTC	20:G>C
32025865|F|0-7:T>G-7:T>G	TGCAGGGTTTCCAGTCACGGGGCGTGTCGAGGGAGCAGGGCGAGGCGCTGCTCCG	7:T>G
4262345|F|0-13:C>G-13:C>G	TGCAGGTGCCCACCCTCAGGAGCTGCGACTTCACGCGGCTCTACTCCG	13:C>G
4261876|F|0-38:G>C-38:G>C	TGCAGGTACGGCAGGTTTGGGATGTCCGACTCGTCGGCGAGCCG	38:G>C
7353655|F|0-7:C>G-7:C>G	TGCAGCACGCGGCCGACCCG	7:C>G
1666937|F|0-17:C>T-17:C>T	TGCAGCGAGAGCGGCCGCGCGGTGGTGTCGCACCACTCGGTCCTCATCTTCGAGGCCTTCTCCGCGACG	17:C>T
1013852|F|0-33:T>G-33:T>G	TGCAGGGCAGTGGCACTGGCATCTAGCCAGCTATGGCAATTATATATCGCCG	33:T>G
1067877|F|0-39:C>T-39:C>T	TGCAGCTTGGACAGCCACGACGGTATCTGGCCCGTCAGACCGCACTCCTCCATGACGATCAGCCGAGAT	39:C>T
7172508|F|0-16:C>A-16:C>A	TGCAGAGCATCAAAGACGCGGTCGCCG	16:C>A
1157517|F|0-23:C>T-23:C>T	TGCAGCTGCGCCCACGCCTCCTCCGTGTCGATCAGGCGGCGCAGCCACGCCG	23:C>T
1022735|F|0-26:G>C-26:G>C	TGCAGCTTGGGGAGCAGGTAGTACCTGATCAGCTCGTCGTCGCTCGGCCTGAACCG	26:G>C
3028857|F|0-32:T>C-32:T>C	TGCAGCTGCCATGATCGGCCGATCTATCCTCGTCTCCCTCCTCCG	32:T>C
3020583|F|0-15:T>G-15:T>G	TGCAGTGCACCTGTCTAATTTGACTAATTGCCACCAGCATGGACCG	15:T>G
3934181|F|0-20:C>G-20:C>G	TGCAGAAACCACCAAAACGCCCAGCCGAGGAACGTGGCCCG	20:C>G
2245367|F|0-60:G>A-60:G>A	TGCAGTAAGGGCACTGCAAAGGCACCGCTCAGATTTCGACGAGAGAAACGGTGGTGATATGTGACCGAG	60:G>A
3947115|F|0-5:A>G-5:A>G	TGCAGAGCATCAAAGAAGCGGTCGCCG	5:A>G
1864490|F|0-19:G>T-19:G>T	TGCAGCACAGGAATCGGGGGAGAAAGAGGAAAGGGAAAGGAGGGATCGATCAAAAGGAGAGAAAGGCCC	19:G>T
1040306|F|0-14:A>G-14:A>G	TGCAGCATGATTCAATTGAAGCTGTTGTAATTGGAGTTGTAATTTTGAGCCCTGAAAAAGGGGATTTTG	14:A>G
1264077|F|0-41:C>A-41:C>A	TGCAGACGAAGCAGCAGTTCATCTACGGCAGCGTCTCCACCCGCATCAAGCTCGTCCCG	41:C>A
5580134|F|0-19:G>A-19:G>A	TGCAGCAACCAGACCTCGTGCTGTGCCTGCGCCG	19:G>A
1028890|F|0-62:C>T-62:C>T	TGCAGGTGGAGGTGGTGCTCCTGCTGCTGCCTCCTGCTCCCCGCCGCCAACTCCTCCATGGCCGCGGCG	62:C>T
3021021|F|0-32:C>A-32:C>A	TGCAGTGGGCTATTTCACATGCACACTCCAACCGAACCG	32:C>A
1026249|F|0-22:C>A-22:C>A	TGCAGATGCATCTCGTCGCGCTCATGCAGCAAAGGACCCAGATGCCTCCG	22:C>A
5009032|F|0-7:G>A-7:G>A	TGCAGACGTGGCAGACGAACTGCACCGCGCCGATGAGTATCTGCGCCG	7:G>A
1021567|F|0-65:A>C-65:A>C	TGCAGACCTTCTGCACCGTCTACGACTGCAAGCGCTGGTTCGGCGGCATACTGGAACTGCCGCAGATAA	65:A>C
4262345|F|0-18:G>A-18:G>A	TGCAGGTGCCCACCCTCAGGAGCTGCGACTTCACGCGGCTCTACTCCG	18:G>A
7922042|F|0-20:T>C-20:T>C	TGCAGGTGCTCGACCTCAGCTACAACAACCTCTCCG	20:T>C
4262170|F|0-9:A>G-9:A>G	TGCAGGTCGATGCGCGCGTAGAGGTACGTGTCCCTGGACCG	9:A>G
5582908|F|0-16:C>G-16:C>G	TGCAGTAGTCCAACTGCCTGCCG	16:C>G
1038665|F|0-6:A>T-6:A>T	TGCAGGACAGGAGAGGGGCCCGCGCGCGGCATGCACGTGAAAGCGAGTACGATGCACAATTATGATGTT	6:A>T
1034182|F|0-68:G>A-68:G>A	TGCAGGCCCCCGACCTGGTCCTGACAGAGGATGGTGAGGAAGTCGCTGTCCGTGTGCGGCACCAGCCCG	68:G>A
7168223|F|0-17:T>A-17:T>A	TGCAGGTATAAATACACTAGCGCGGATCACCTACCGTTGATTTGCCG	17:T>A
3028775|F|0-17:C>A-17:C>A	TGCAGAAACCACCAAAACGCCCAGCCGAGAAACGCGGCCCG	17:C>A
12771904|F|0-46:C>G-46:C>G	TGCAGAAGAGCTTGGCGGGGTCGTACTCGTAGCCGATGTTCTTGGACGCGTTGCGGCGGTCGAGCCGCG	46:C>G
3947512|F|0-16:G>A-16:G>A	TGCAGGTGGCCAACAAGCTGAACAAGACCCCG	16:G>A
3033212|F|0-26:T>C-26:T>C	TGCAGCCTGTCGATGTTGTACGCGTTTGGTAGCTTGGTAGCCG	26:T>C
4261523|F|0-10:C>G-10:C>G	TGCAGTGGGCCGTGCGCAACTTCATCCG	10:C>G
1707934|F|0-11:G>A-11:G>A	TGCAGCACCTCGAGGCTTGGCAGGTCGCCGACGAAGTCGGGGATGTCGCCGCGCAGCTTGTTCCGAGAT	11:G>A
4404603|F|0-11:C>T-11:C>T	TGCAGCCACTGCGCAGAGACAACCG	11:C>T
32025439|F|0-8:A>G-8:A>G	TGCAGCAGAGGACCAAACACCACCGCCG	8:A>G
4002646|F|0-17:A>G-17:A>G	TGCAGGGACACGCCGTGAAGCTCGGGTTCCTCGAGCACGACGAGCACGCGCAGAACAGCCTCATCAGCT	17:A>G
3955493|F|0-6:A>G-6:A>G	TGCAGAACCTCACATCGGGCGGCGACAGCTTCCG	6:A>G
1019998|F|0-12:C>A-12:C>A	TGCAGGATGAAGCGGGGGAGAAGGCTATGGCTAGCTGCCTTGGGACTAGCAACCG	12:C>A
4002083|F|0-14:G>C-14:G>C	TGCAGGTGAGGAGGGGCGGCAGGTGGTTCCCGCTGGACCCGCTGCCG	14:G>C
4262832|F|0-19:G>C-19:G>C	TGCAGGGCCTCCTCAAGGTGATCGCCG	19:G>C
1242815|F|0-24:A>G-24:A>G	TGCAGACGGGCAGGACCATGACCTACGACATTCATGGCGACGGCGGCCAGCTGACATTCGAGCCGAGAT	24:A>G
1023842|F|0-14:G>C-14:G>C	TGCAGCTCGTGCACGGCCGTGTTCATGTCTGCCACGGCGAAGTCCAGCGCCG	14:G>C
3026056|F|0-28:G>T-28:G>T	TGCAGGAGACGCCGACCCAAGAGCAGAAGGATGTGCCG	28:G>T
1059801|F|0-26:A>G-26:A>G	TGCAGGTCGTCCCGTGGGGCGACATGAGGTGGGCGTCCTTCAGTGCCTTCGGCGCGCCGACGGCCGACA	26:A>G
3939367|F|0-25:A>G-25:A>G	TGCAGAGCAGCAGCGCCAAGGGCGAACTGAGCCG	25:A>G
1061532|F|0-40:G>T-40:G>T	TGCAGAGGAGGTACTGCCTGGTGGCGTGCAAGCAGATGGTGGGTCTCTTCGCAACGGTGTGGGCGCGGC	40:G>T
3953788|F|0-17:C>T-17:C>T	TGCAGTCAGCTCCCCGACAGTCACCGTCTTGAGCTCGCCGTCGGCCG	17:C>T

5371744|F|0-26:G>A-26:G>A	TGCAGTTCAGCCTGACCTCCGTCTGCGTCCCG	26:G>A
987456|F|0-59:G>C-59:G>C	TGCAGGGATAGAGTCATTTTCCAAGCTTGGGGACTCCATCTACTTTGAGGAGAAAGGCGGCAAACCGAG	59:G>C
1019998|F|0-18:A>C-18:A>C	TGCAGGATGAAGCGGGGGAGAAGGCTATGGCTAGCTGCCTTGGGACTAGCAACCG	18:A>C
1012031|F|0-35:G>C-35:G>C	TGCAGGTTGTCCAGCGCGTCGCGGAGCAGGTCGGCGGCGCCGCCGTACTGCTCCAGGCAGTGGTCGAAC	35:G>C
1091381|F|0-60:C>T-60:C>T	TGCAGCACAGCATCGAGTACTGGCTCACGCTGGACCTCCTCGCCTCCGAGCAGGGCGCGCCCACGCCGT	60:C>T
32019004|F|0-26:C>G-26:C>G	TGCAGCTCCACCAGGTGCAGCAGGTACACGGCCACCG	26:C>G
4261907|F|0-16:C>G-16:C>G	TGCAGGAGACGTGCACCGACGCGTCCG	16:C>G
100536452|F|0-25:A>G-25:A>G	TGCAGGCAGCAGCGCGCACGCCATGACTGGACTCTGCCTCCCAAGGGTTTGCGCCAAGAGCGCCGAGAT	25:A>G
1022595|F|0-48:A>G-48:A>G	TGCAGGCAACAAATTCCTGTGTGGCAAGCCAACGGACGTGGTCTGCGAATCCGCGTCGTCGCCCGCCGA	48:A>G
1020648|F|0-29:G>C-29:G>C	TGCAGCTCCCGCACGATGAGCACCATGGAGGAGAAGGCGCGGCCCACCGACTCGCCG	29:G>C
1149760|F|0-5:C>T-5:C>T	TGCAGCACGCTCATGTTGTGGTGCTGGTGATCATCCGTCGTCCCCTCCG	5:C>T
4261736|F|0-20:C>G-20:C>G	TGCAGGCTGATGATGGTGGACGAGGCGGCCAGGACCG	20:C>G
1041280|F|0-34:C>A-34:C>A	TGCAGGCCCAGCACCGCCAACTGCTCCGCCACCGCCAAGAACGTCTGCCCGCCCTACCTCGTCGTCTAC	34:C>A
1015989|F|0-59:C>A-59:C>A	TGCAGACAGAAGAGAAGATCAAAAGAGATCGGGCGATGCAGCACAAGATTGGAGGGCAACAACAAGCGC	59:C>A
32026271|F|0-40:C>G-40:C>G	TGCAGAACGACTTCTACTTCGTGCCGGCGCTGTACGACATCGCCCAGTTCGAGGGCGAGCTCCG	40:C>G
986255|F|0-44:C>T-44:C>T	TGCAGGCGATGCTTGCTCATTGCCCATCCCTGGAGTGCGCAAAGCTCAAGAACATCACCG	44:C>T
4261876|F|0-15:T>C-15:T>C	TGCAGGTACGGCAGGTTTGGGATGTCCGACTCGTCGGCGAGCCG	15:T>C
4262375|F|0-6:G>C-6:G>C	TGCAGCGGATGATGAGCTGATATACCCAGATGTGATGGCACCG	6:G>C
7908954|F|0-17:G>C-17:G>C	TGCAGCCGCTGGAGTGGGGTCCG	17:G>C
1061532|F|0-63:C>T-63:C>T	TGCAGAGGAGGTACTGCCTGGTGGCGTGCAAGCAGATGGTGGGTCTCTTCGCAACGGTGTGGGCGCGGC	63:C>T
1041523|F|0-28:G>T-28:G>T	TGCAGCTCTTCTGCGGCGACACCCTCCTGCTCAAGGGCAAGAAGCGGAAAGACACGGTCTGCATTGCGC	28:G>T
5582355|F|0-22:G>A-22:G>A	TGCAGCGCGCGCGCGTCGGCCAGAAAATGGCCCG	22:G>A
3029961|F|0-5:C>A-5:C>A	TGCAGCAGCTCGGCATCAGTGGGAACCTCGATCGCCG	5:C>A
1253255|F|0-58:G>A-58:G>A	TGCAGAAGAGCTTGGCGGGGTCGTACTCGTAGCCGATGTTCTTGGACGCGTTGCGGCGGTCCAGCCGCG	58:G>A
1046261|F|0-14:A>C-14:A>C	TGCAGGCCATCACGAGAAAGTCCCTATCAGCTGCCCAGGTGAACCAAACGGCCG	14:A>C
1013852|F|0-47:C>G-47:C>G	TGCAGGGCAGTGGCACTGGCATCTAGCCAGCTATGGCAATTATATATCGCCG	47:C>G
1055502|F|0-14:T>G-14:T>G	TGCAGGCCGCCGACTTGGTCCTGGCAGAGCACGGTGAGGAAGTCGCTGTCCGTGTGCGGCACCAGGCCG	14:T>G
991920|F|0-22:A>G-22:A>G	TGCAGGTTGGTTTCAACAATGGAGTCCCCATCACCGAGGAGTTCTACAGCGCCAACATCAGCGGGTGGC	22:A>G
5358459|F|0-31:G>T-31:G>T	TGCAGGTGCTGTTCCTCTGGGGCAATGCGCTGTCCG	31:G>T
1004991|F|0-52:C>T-52:C>T	TGCAGGCTTGTCAGCAGTGCGGATTTAAAGTAGCATGGAGACGGCGACATGGCGTGCCACTGTGAACGG	52:C>T
992043|F|0-10:C>A-10:C>A	TGCAGTCGCACTCACGCTTATCCCATGGGAGACATAATGTCTTGCTTGCACGGCAGGAACAATTCGGGC	10:C>A
3936370|F|0-17:G>A-17:G>A	TGCAGCCAAGCAGCAGAGGAAGAACCG	17:G>A
32024816|F|0-21:A>G-21:A>G	TGCAGCGGAGCTGGGGACGGCAGCGTGATCCG	21:A>G
4261612|F|0-32:C>T-32:C>T	TGCAGCTGGGCACGGAGTGCACCGAGCGGCGGCCCGACCG	32:C>T
1242815|F|0-28:C>G-28:C>G	TGCAGACGGGCAGGACCATGACCTACGACATTCATGGCGACGGCGGCCAGCTGACATTCGAGCCGAGAT	28:C>G
985496|F|0-5:G>C-5:G>C	TGCAGGTCGGCGCGCCCGTGCACGGCACGGCGTTCCAGCGCGTCGCGTCGTGCTTCGTGCAGGGCCTGG	5:G>C
1056056|F|0-20:C>T-20:C>T	TGCAGGTGGTGGCTCCTCATCTTGTCGCCGACGCCGAGGGCGTCAAGCGCGCGGAAGGCGTTGGTCCAT	20:C>T
1043022|F|0-40:C>A-40:C>A	TGCAGGCGTTTCAATGAACCACACGCAAGGCATCAGTGATCACCAGCTCACTGCCATACTGAAACCGAG	40:C>A
5582021|F|0-14:A>G-14:A>G	TGCAGGACGTGCTGAGCCACCCG	14:A>G
3943589|F|0-22:G>C-22:G>C	TGCAGAGCAGCAGCGCCAAGGGGGAACTGAGCCG	22:G>C
1025833|F|0-53:G>T-53:G>T	TGCAGCCTCGCCGCGTGAGCGGCCGCGAGGGCGACGCGCAAGGTGTAGGTGCCGCCG	53:G>T
32027612|F|0-10:G>C-10:G>C	TGCAGTGGCTGGAAGGCAGGGAGCCCG	10:G>C
3959679|F|0-32:G>A-32:G>A	TGCAGACCCACCACTTCGCCTACGAGGAGCTGGAGGAGGCCACCG	32:G>A
4262689|F|0-10:C>T-10:C>T	TGCAGTATCTCGGACTCGATGGTGGTGACCCGCTCCAGGACCTCCG	10:C>T
1110864|F|0-42:A>T-42:A>T	TGCAGCAGGCGATTGAGCAGAGAAGCGCTGCGAGGAAGAAGAAGGACTTTGCGGAGTCGGACCG	42:A>T
3934851|F|0-34:G>A-34:G>A	TGCAGCTTATGATCATCGTGGACGACGCTGGATCGTTTCTTCCG	34:G>A
1218104|F|0-8:A>G-8:A>G	TGCAGCCAACACCAAGCAGAGACCGATCAGGAGGAGCCTGAGCAAGGAGCTAGCCG	8:A>G
991920|F|0-6:T>C-6:T>C	TGCAGGTTGGTTTCAACAATGGAGTCCCCATCACCGAGGAGTTCTACAGCGCCAACATCAGCGGGTGGC	6:T>C
2241644|F|0-18:T>C-18:T>C	TGCAGGTTCATGGAGACGTAACGACTCCAGAATGTCGACAGATGAGAACGTGAACCG	18:T>C
3947951|F|0-17:G>A-17:G>A	TGCAGGCTCTGGATGGCGCGGACACGCGGCTCCG	17:G>A
1036788|F|0-5:G>A-5:G>A	TGCAGGGCGAGGAAGCCCTTGTAGTAGAGGAAGCAGTGGACCATCTTGGCGCAGGCCG	5:G>A
5582021|F|0-5:G>A-5:G>A	TGCAGGACGTGCTGAGCCACCCG	5:G>A
5009032|F|0-28:G>A-28:G>A	TGCAGACGTGGCAGACGAACTGCACCGCGCCGATGAGTATCTGCGCCG	28:G>A
4262813|F|0-25:C>T-25:C>T	TGCAGATGTTGTCGCGCTTCACCGTCAGGATCATGATGGCGTCCG	25:C>T
4439426|F|0-23:G>A-23:G>A	TGCAGCCTGCCGTTCCCGCTGAAGATCCG	23:G>A
994295|F|0-9:A>G-9:A>G	TGCAGTGGAACCACCAAGAACAAGCCTGTCCAAGAGGCCATCGCTTGGAGCTTCTGCAAAGGTCCAGTC	9:A>G
7172638|F|0-17:G>C-17:G>C	TGCAGGCCCAGGTAGCAGAGCGACGACGCCTCCG	17:G>C
12469808|F|0-24:G>A-24:G>A	TGCAGGTGGTGGACTGGGACAACAGCATCATCCG	24:G>A
1149760|F|0-20:G>A-20:G>A	TGCAGCACGCTCATGTTGTGGTGCTGGTGATCATCCGTCGTCCCCTCCG	20:G>A

<table>
<tr><td>4440092|F|0-12:T>A-12:T>A</td><td>TGCAGAACTGCTTCTTTCCAAAACCCG</td><td>12:T>A</td></tr>
<tr><td>4541251|F|0-15:C>G-15:C>G</td><td>TGCAGCTGCTCGAGTCCTCCACCG</td><td>15:C>G</td></tr>
<tr><td>4992336|F|0-19:T>C-19:T>C</td><td>TGCAGGTGTCGTGCGCCGTTTCCG</td><td>19:T>C</td></tr>
<tr><td>1022972|F|0-33:G>A-33:G>A</td><td>TGCAGTGCGGCGCCGACTCGCTCTACGGCGACAGGCTGGGCGAGTTCAGACTGTCCGTGAAAGGCCACG</td><td>33:G>A</td></tr>
<tr><td>6048900|F|0-37:G>A-37:G>A</td><td>TGCAGCCTTCCTGGAACATGGTGACCGCCTTTTCTCCGTCAGATTCCCCGCCG</td><td>37:G>A</td></tr>
<tr><td>1688504|F|0-22:T>C-22:T>C</td><td>TGCAGCCCAACATGAGGGAGATTGAGTCCGCTCAGGACCTCGTCGACTCGCTGGCCAACGCCG</td><td>22:T>C</td></tr>
<tr><td>6044191|F|0-16:C>T-16:C>T</td><td>TGCAGGTGAAGAAGGACGGCCAGTGGATCAGCGTTGACCCG</td><td>16:C>T</td></tr>
<tr><td>32025635|F|0-33:C>G-33:C>G</td><td>TGCAGGGGATTTGGACACCGCCCACAGTCTGCTCGACGAGATGCCCGTCAAGGATGCCGTGGCTTTCAA</td><td>33:C>G</td></tr>
<tr><td>15328426|F|0-13:C>G-13:C>G</td><td>TGCAGCTCGGCGGCGTGAGGACATGGGCCGTGCCG</td><td>13:C>G</td></tr>
<tr><td>3027484|F|0-8:G>C-8:G>C</td><td>TGCAGCCCGAGCTTGCGGGCCAGCTCCGTCTTCCTCTCCG</td><td>8:G>C</td></tr>
<tr><td>1392022|F|0-16:A>G-16:A>G</td><td>TGCAGATACTGTGGGGACAGACGCAGGTGCTCAACGACGGCAACGGCGACCAGACCATCGCGCTGATGC</td><td>16:A>G</td></tr>
<tr><td>100574132|F|0-41:A>G-41:A>G</td><td>TGCAGGATCATGGCGAGCCCCATTTTTGCCTCGAGCAGCGCAAAGTTCTGGCCGATACAGATGCGCGGG</td><td>41:A>G</td></tr>
<tr><td>5357203|F|0-10:A>G-10:A>G</td><td>TGCAGAAACCAAGCCTGTCCTCCTCCG</td><td>10:A>G</td></tr>
<tr><td>4998838|F|0-27:G>C-27:G>C</td><td>TGCAGCGAGCGGCGGCACGCGCGTGGTGGCCG</td><td>27:G>C</td></tr>
<tr><td>4996444|F|0-26:A>T-26:A>T</td><td>TGCAGTGGGATTCCGTGACAAGCAGCAACATGACATTTGGGGCCCG</td><td>26:A>T</td></tr>
<tr><td>1022281|F|0-63:T>C-63:T>C</td><td>TGCAGTCGCAGAGCCTCATCATGCCGCTGCTCCTGTCGTCCGTGGTCACGCTGGCGCTCCACGTCCCCC</td><td>63:T>C</td></tr>
<tr><td>1043022|F|0-23:C>T-23:C>T</td><td>TGCAGGCGTTTCAATGAACCACACGCAAGGCATCAGTGATCACCAGCTCACTGCCATACTGAAACCGAG</td><td>23:C>T</td></tr>
<tr><td>1242815|F|0-5:A>G-5:A>G</td><td>TGCAGACGGGCAGGACCATGACCTACGACATTCATGGCGACGGCGGCCAGCTGACATTCGAGCCGAGAT</td><td>5:A>G</td></tr>
<tr><td>985496|F|0-46:G>A-46:G>A</td><td>TGCAGGTCGGCGCGCCCGTGCACGGCACGGCGTTCCAGCGCGTCGCGTCGTGCTTCGTGCAGGGCCTGG</td><td>46:G>A</td></tr>
<tr><td>3948437|F|0-10:C>G-10:C>G</td><td>TGCAGATTTGCACATGCGCATCCG</td><td>10:C>G</td></tr>
<tr><td>1034182|F|0-65:C>G-65:C>G</td><td>TGCAGGCCCCCGACCTGGTCCTGGCAGAGGATGGTGAGGAAGTCGCTGTCCGTGTGCGGCACCAGCCCG</td><td>65:C>G</td></tr>
<tr><td>5010988|F|0-22:C>T-22:C>T</td><td>TGCAGAACTCCCTCAGCTTCCTCTGCTGCCACACCG</td><td>22:C>T</td></tr>
<tr><td>3936902|F|0-17:G>C-17:G>C</td><td>TGCAGGAGTTCTTCTACGACCAGGTGCCG</td><td>17:G>C</td></tr>
<tr><td>4002821|F|0-24:C>G-24:C>G</td><td>TGCAGCTGCAAGTCAAGGTGCTGGCAACGGACGAGTTCTGGCCG</td><td>24:C>G</td></tr>
<tr><td>3948144|F|0-5:G>C-5:G>C</td><td>TGCAGGTCGATCTTGCGCGCCGACTCGGCCCTCCG</td><td>5:G>C</td></tr>
<tr><td>1052520|F|0-25:G>C-25:G>C</td><td>TGCAGGACAAGAAGCACATCGTGGGGATGACGGGCGACGGCGTGAACGACGCGCCG</td><td>25:G>C</td></tr>
<tr><td>1022595|F|0-16:C>T-16:C>T</td><td>TGCAGGCAACAAATTCCTGTGTGGCAAGCCAACGGACGTGGTCTGCGAATCCGCGTCGTCGCCCGCCGA</td><td>16:C>T</td></tr>
<tr><td>1025257|F|0-29:G>A-29:G>A</td><td>TGCAGGAGCCTCGTGATCATGGCGGCGTCGGTGACGCGGTTACGGCTCAGGCGCACGGTCCG</td><td>29:G>A</td></tr>
<tr><td>1083636|F|0-38:G>C-38:G>C</td><td>TGCAGCGACATGTCCCTGGACGAGATGGACGCCGCGGTGAGGAGGATGGAGTTCGTCGTCTTCGACCGA</td><td>38:G>C</td></tr>
<tr><td>1056584|F|0-37:C>G-37:C>G</td><td>TGCAGTACGACGTGGACTCCCTGGACCAGAACCTGGCCCAGAACCTGGCGGGCGAGATCATCGGCACCC</td><td>37:C>G</td></tr>
<tr><td>5369274|F|0-10:A>G-10:A>G</td><td>TGCAGACGGGACGAAGAAGACGAATCCG</td><td>10:A>G</td></tr>
<tr><td>1022972|F|0-35:C>T-35:C>T</td><td>TGCAGTGCGGCGCCGACTCGCTCTACGGCGACAGGCTGGGCGAGTTCAGACTGTCCGTGAAAGGCCACG</td><td>35:C>T</td></tr>
<tr><td>3030373|F|0-17:C>T-17:C>T</td><td>TGCAGGCCGAGGTCGCGCGCCAGCCGCGCCTTGCGCTCCG</td><td>17:C>T</td></tr>
<tr><td>4404145|F|0-7:G>A-7:G>A</td><td>TGCAGACGACCTGGTGCGTGGCGTTCCG</td><td>7:G>A</td></tr>
<tr><td>1021437|F|0-35:C>G-35:C>G</td><td>TGCAGCCAGTTGAGCAGCCTGCCGATCTTCTTGGACACGAAGACCCAGCCG</td><td>35:C>G</td></tr>
<tr><td>1021132|F|0-28:C>T-28:C>T</td><td>TGCAGGCGTACACCAGGGCACGCTACGGCGAGGATGCGATGATCGTGTACCG</td><td>28:C>T</td></tr>
<tr><td>3948146|F|0-19:G>C-19:G>C</td><td>TGCAGCTCTTCTACAACGGGCTCTCCG</td><td>19:G>C</td></tr>
<tr><td>1004053|F|0-6:G>A-6:G>A</td><td>TGCAGAGAGGGCACATCTCGCTCCCTTTGAGGGTGTCCGCCAGGCATGCGCCATGGAAGACGTGGGGCA</td><td>6:G>A</td></tr>
<tr><td>1063727|F|0-49:G>A-49:G>A</td><td>TGCAGGGTTGGAGCTACGTTGGTGACAGGCTATTGTCGGCAGTCGTGCCGTATGAAGAAACCG</td><td>49:G>A</td></tr>
<tr><td>1036788|F|0-53:G>T-53:G>T</td><td>TGCAGGGCGAGGAAGCCCTTGTAGTAGAGGAAGCAGTGGACCATCTTGGCGCAGGCCG</td><td>53:G>T</td></tr>
<tr><td>4261907|F|0-22:G>T-22:G>T</td><td>TGCAGGAGACGTGCACCGACGCGTCCG</td><td>22:G>T</td></tr>
<tr><td>4261934|F|0-35:G>C-35:G>C</td><td>TGCAGGGGCCTCGAGAAGAAGGAGAAGTTCATGACGCCCTCGCCG</td><td>35:G>C</td></tr>
<tr><td>3952350|F|0-22:G>C-22:G>C</td><td>TGCAGTGGATCATGGCGGAGCTGGTCAAGAACCCG</td><td>22:G>C</td></tr>
<tr><td>4261652|F|0-15:A>G-15:A>G</td><td>TGCAGCCGCGCGTGTAAGGGTTGGCCACCGCAGAAGGCCG</td><td>15:A>G</td></tr>
<tr><td>7173621|F|0-26:T>C-26:T>C</td><td>TGCAGCAGCAGCCAGTTCACCTTGGCTAGCACCAGCTGGTCGTCGTTGCAGTAGAAGAGCCG</td><td>26:T>C</td></tr>
<tr><td>1234784|F|0-39:G>A-39:G>A</td><td>TGCAGCTTGGCCATTGCCTGTGGGTTCCCGACCAGCTCCGCCATGGCCCATTCCAGCGTGAGGGACGAC</td><td>39:G>A</td></tr>
<tr><td>3939103|F|0-36:T>C-36:T>C</td><td>TGCAGGAGCGGGTGCTGAGTTGCCATGCAGCGATGATCCAGGCCACCG</td><td>36:T>C</td></tr>
<tr><td>1056056|F|0-35:G>C-35:G>C</td><td>TGCAGGTGGTGGCTCCTCATCTTGTCGCCGACGCCGAGGGCGTCAAGCGCGCGGAAGGCGTTGGTCCAT</td><td>35:G>C</td></tr>
<tr><td>32023394|F|0-16:C>G-16:C>G</td><td>TGCAGGGGCTCCAGGGCTACGTGCAGAACGTCAGGTTCCTGGCCG</td><td>16:C>G</td></tr>
<tr><td>4262843|F|0-10:C>T-10:C>T</td><td>TGCAGACTATCGGCGCTCATGGCAGAGACCG</td><td>10:C>T</td></tr>
<tr><td>3533831|F|0-29:C>G-29:C>G</td><td>TGCAGCTCCTTGAAGTAGACGCCGTCGAACGCCGTGCTCGTCCG</td><td>29:C>G</td></tr>
<tr><td>2244421|F|0-8:C>T-8:C>T</td><td>TGCAGAGGCACCCCATCGAGCGCGTCGTCGTCGAAGTGCTCGGGTAGCTTGTCGAGGAGGAGCCCCAGG</td><td>8:C>T</td></tr>
<tr><td>1036788|F|0-38:G>C-38:G>C</td><td>TGCAGGGCGAGGAAGCCCTTGTAGTAGAGGAAGCAGTGGACCATCTTGGCGCAGGCCG</td><td>38:G>C</td></tr>
<tr><td>1394604|F|0-31:G>A-31:G>A</td><td>TGCAGGTGATCACCGCGCGGCCGCCCATGGGGCTGACGCATCACGTCGAGAAGGCCATCGAGTCCGAGA</td><td>31:G>A</td></tr>
<tr><td>4405197|F|0-8:G>C-8:G>C</td><td>TGCAGGTGGCGCAACCACAGCCG</td><td>8:G>C</td></tr>
<tr><td>4261129|F|0-16:G>T-16:G>T</td><td>TGCAGGCGGCGTGGCGGATCTCCG</td><td>16:G>T</td></tr>
<tr><td>7916077|F|0-63:C>G-63:C>G</td><td>TGCAGACCTGGGCACGAACAGAGGGCTGTCGTACATCGACATGAGCGACAACCTTCTTGAAGGCAGGAT</td><td>63:C>G</td></tr>
<tr><td>3934308|F|0-47:C>T-47:C>T</td><td>TGCAGGTAGTCCAGCTTCGCCTTCTCCAGATCCGCCATCAAGCGATCCGCCTTGTATATCCG</td><td>47:C>T</td></tr>
<tr><td>7171094|F|0-23:A>G-23:A>G</td><td>TGCAGCCCGTCCATGGCGCGCATAGCGACATCGAAGAAGACGAGGTTGTGGTTGGCGTACCG</td><td>23:A>G</td></tr>
</table>

3934017|F|0-19:A>G-19:A>G	TGCAGCTGCTTGCTGTGCAACCGCCG	19:A>G
1006991|F|0-29:G>C-29:G>C	TGCAGGAGGCCGATCTTGTCCGAGACCTCGGCGTCGGCCGCCGCGTCCTCCG	29:G>C
1004991|F|0-14:C>G-14:C>G	TGCAGGCTTGTCAGCAGTGCGGATTTAAAGTAGCATGGCGACGGCGACATGGCGTGCCACTGTGAACGG	14:C>G
1028890|F|0-20:C>T-20:C>T	TGCAGGTGGAGGTGGTGCTCCTGCTGCTGCCTCCTGCTCCCCGCCGCCAACTCCTCCATGGCCGCCGCG	20:C>T
1056584|F|0-7:C>T-7:C>T	TGCAGTACGACGTGGACTCCCTGGACCAGAACCTGGCCCAGAACCTGGCGGGCGAGATCATCGGCACCC	7:C>T
2244421|F|0-7:G>A-7:G>A	TGCAGAGGCACCCCATCGAGCGCGTCGTCGTCGAAGTGCTCGGGTAGCTTGTCGAGGAGGAGCCCCAGG	7:G>A
3934308|F|0-34:C>T-34:C>T	TGCAGGTAGTCCAGCTTCGCCTTCTCCAGATCCGCCATCAAGCGATCCGCCTTGTATATCCG	34:C>T
32026070|F|0-44:G>A-44:G>A	TGCAGGTAGTCCAGCTTCGCCTTCTCCAGATCCGCCATCACGCGGTCCGCCTTGTATATCCG	44:G>A
4009208|F|0-19:G>C-19:G>C	TGCAGCGCGTCGGCAGCGGGGCCAGCGCGGTCATGGACGCGCCG	19:G>C
7478669|F|0-15:A>G-15:A>G	TGCAGTTCCACCACAAGTCCG	15:A>G
4261131|F|0-16:A>G-16:A>G	TGCAGAGAAACAGAGAATTCAGCATGAGACCG	16:A>G
7353187|F|0-11:C>G-11:C>G	TGCAGGGCATCCCCAAGGCCG	11:C>G
7353655|F|0-8:G>A-8:G>A	TGCAGCACGCGGCCGACCCG	8:G>A
7173599|F|0-12:C>G-12:C>G	TGCAGGTCCACGCGCTGGAAGCCGTGCCCGTTCTTCAGCATGTAGTAGTGCGTGGGCCG	12:C>G
7170779|F|0-45:T>C-45:T>C	TGCAGGAGCAGTGGCGCTGCCCACTGTCCATCCTGCTGCTTACTATTTGTGCGCTTACCCG	45:T>C
6041850|F|0-5:G>C-5:G>C	TGCAGGTCCACGCCGACGGCCAGATCTCCCGCCCG	5:G>C
4262483|F|0-11:C>T-11:C>T	TGCAGCATCGCCTGAATCTCCTCTATCGTCCGCGGCCG	11:C>T
4396785|F|0-20:G>A-20:G>A	TGCAGACGACCTGGTGCATGGCGTTCCG	20:G>A
1090065|F|0-41:G>C-41:G>C	TGCAGGCCCCGCACGAACGCCACGGCGTACTTGCTCGCCGTGGTGGGGTCCTCGCCG	41:G>C
1696698|F|0-42:G>A-42:G>A	TGCAGGTCCTGCTCGCTGTTCTTGTCGATGTTGATGGACAGCGCCAGGTTCCG	42:G>A
7478638|F|0-23:G>A-23:G>A	TGCAGGCTGGCCCTCGCGAGCACGCACCCG	23:G>A
1051733|F|0-41:G>A-41:G>A	TGCAGGAGGGAGAAGCTTACTCCTGGGAGTTTGATGAAGGCGGTTATCCG	41:G>A
3571299|F|0-35:G>T-35:G>T	TGCAGATGCCGCCGATGGCCATGCACGGTCACGGTGACGTGTGCCCG	35:G>T
32024857|F|0-7:T>G-7:T>G	TGCAGGATGGTGGGCCGCCACGTGGAGGAGAATGATATGGGAGGCGCCG	7:T>G
3942396|F|0-19:G>A-19:G>A	TGCAGAACATCCAGAACGCGCTCAACGACGCCG	19:G>A
7170830|F|0-6:C>T-6:C>T	TGCAGGCTGTTAAACTACGTGGAAGTTGCACCG	6:C>T
1107007|F|0-20:C>G-20:C>G	TGCAGCATGGGGCTCCTGAACCGCCTCCCTCCTCGGAACCACGCGCGCTCCGACCCCTGCGCCG	20:C>G
3950610|F|0-19:G>A-19:G>A	TGCAGCTGCATCTGCATCAGGAGAGGTCCATCCG	19:G>A
4261677|F|0-19:G>A-19:G>A	TGCAGATAGGAAGAACCGAGCTCCG	19:G>A
7173582|F|0-10:C>A-10:C>A	TGCAGATCGCCAGGACCCTGGGCAGCCTCGTGGCGCCG	10:C>A
5581268|F|0-10:C>G-10:C>G	TGCAGAACCTCACGACGGGCGGCGACAGCTTCCG	10:C>G
3030373|F|0-25:G>T-25:G>T	TGCAGGCCGAGGTCGCGCGCCAGCCGCGCCTTGCGCTCCG	25:G>T
7172281|F|0-13:C>T-13:C>T	TGCAGTTGTCCTGCTCGGCCGCCGCCAGATCGGCCTCCACCTCCTTCCG	13:C>T
12469752|F|0-19:A>C-19:A>C	TGCAGAGCATAATCCTGCTATGCACCG	19:A>C
32021516|F|0-23:A>G-23:A>G	TGCAGTATCCTGTGGCAGTGCCTATCGGACAGGCCG	23:A>G
1021070|F|0-22:G>C-22:G>C	TGCAGCGCGAGCTCGGTGAGGCGGAAGCCCTTGCGGTGCAGGTACTGCTGCACCTTCCTCTCCATCTCC	22:G>C
3025379|F|0-5:C>G-5:C>G	TGCAGCCCCAGTTCACGCGCCAGCTCCGCCTTCTCCCG	5:C>G
6036196|F|0-29:A>G-29:A>G	TGCAGGCGGAAGTAGAGCAACCCCTCGACAGCGTCGGTGCGGAGCGTGGCGATGCCGCGCGCCATGAAG	29:A>G
100497038|F|0-40:G>C-40:G>C	TGCAGGTGAGCATCACCAGCCAGGCCCTGATATTCGTGACGCGGAGCCG	40:G>C
1149760|F|0-38:C>A-38:C>A	TGCAGCACGCTCATGTTGTGGTGCTGGTGATCATCCGTCGTCCCCTCCG	38:C>A
1001583|F|0-36:T>G-36:T>G	TGCAGCCACCAGGTCGCTCACTTCCCTGCCACCGCCTCCCCCATTGGCTTGTAGCGCTTTGCAATTGCA	36:T>G
4005727|F|0-23:G>A-23:G>A	TGCAGGCTCAGCTCCGTGACGCCGCCGTCGGCATTGCACGACACGCCG	23:G>A
1150315|F|0-22:G>T-22:G>T	TGCAGCCTCACCAGCGGCTCTCGCACGGCGGCGGCGAACCCAAGCTCCG	22:G>T
3957524|F|0-6:C>G-6:C>G	TGCAGGCAGCGACCTAACTCTCGCAGCGCCTGACAAGTTAACCG	6:C>G
7902441|F|0-39:G>C-39:G>C	TGCAGTCGGCGGCGCTGTCCTTCTTCAGCTTCACCAACAGCAAGCCG	39:G>C
3385463|F|0-34:G>C-34:G>C	TGCAGCGGGATCAAGCTCAGGAACATCAGGCTGAGGTACCG	34:G>C
3957680|F|0-9:T>C-9:T>C	TGCAGCCGCTTCACGATCTCGTACTTGTGCTCCG	9:T>C
4261931|F|0-22:C>T-22:C>T	TGCAGCTGCACCGCCGCGGCCACAACCTGCCGTGGAAGCTCCG	22:C>T
2243154|F|0-30:C>G-30:C>G	TGCAGAACACGTATCCACAAGTAGTCGACCCTGTCTGTCGGGTCGCCATCCATCCTTCCACATTTGAGC	30:C>G
1019452|F|0-61:C>T-61:C>T	TGCAGGTGAGCATCATCAGCCAGGCGCTCATCTTCGTGACGCGGTCGCGGAGCTGGTCGTTCGTGGAGC	61:C>T
5578229|F|0-10:G>C-10:G>C	TGCAGGAGGTGAAGGAGAACTCGGTCCG	10:G>C
32024193|F|0-14:A>G-14:A>G	TGCAGGGATGGCCAACGGGAGGCGGGTGGGAAGAGGAAAGAGAAGCCG	14:A>G
2247474|F|0-49:G>A-49:G>A	TGCAGCTCCTCCATCGTGCTCTCCTCCGACGACGTCGACACCTTCACCAGCGACCCGCCCACCGACAGG	49:G>A
1079000|F|0-53:G>T-53:G>T	TGCAGGGCCAGAGTCACCTGGGAGACAACGCCGAGGACCCCAAGGGAGACCTTGGCGGCGTCCAGGTCC	53:G>T
1023110|F|0-11:A>T-11:A>T	TGCAGAACAACACGCTGCGCGGCGGCCTCCCCGCCAGCGTCTCGGCCTGCTCGGAACTCACCCACCTCT	11:A>T
3946756|F|0-10:G>C-10:G>C	TGCAGCGGCTGCTCCTTGCAAACAATGGGTTCTCCG	10:G>C
1036567|F|0-34:G>C-34:G>C	TGCAGTAGAACCAGAGGGCGAGCTTGACGGCGGTGGCCGAGAGCATGATGGAGTAGAGCCATATCAGCT	34:G>C
3027653|F|0-25:G>T-25:G>T	TGCAGCTCTCCAACCTCTCCTCGGGGCCCCTGTTCCTCTCCG	25:G>T
1024352|F|0-53:G>C-53:G>C	TGCAGGCCCCTCACGAAGGCGACGGCGTACTTGCTCGCCGTGATCGGGTCCTCGCCG	53:G>C

4261842|F|0-8:C>G-8:C>G	TGCAGGATCTCAACGTGTCCAACAATCTCCTCTCGGGGAGGATTCCG	8:C>G
991920|F|0-46:C>T-46:C>T	TGCAGGTTGGTTTCAACAATGGAGTCCCCATCACCGAGGAGTTCTACAGCGCCAACATCAGCGGGTGGC	46:C>T
1022186|F|0-7:G>T-7:G>T	TGCAGGAGGCGGCGATGCCGTCGGCAGTGAGGGAGGATCGTCGGATCTGATCACCGATGATCAGCCGAG	7:G>T
4910031|F|0-18:C>G-18:C>G	TGCAGGGTGGCCGAGTCGCTCCG	18:C>G
3949806|F|0-12:T>C-12:T>C	TGCAGGCACCCATATCGATCCGTCTCACCTCCG	12:T>C
3942162|F|0-28:G>A-28:G>A	TGCAGCTGACGTGGAACTACAACTACGGGGCGGCGGGGAAGAGCGTGGGATTCGACGGGCTGAACAACC	28:G>A
4539309|F|0-5:C>T-5:C>T	TGCAGCAATCCCGCGCCGTGGTCGACGAGCCG	5:C>T
5370977|F|0-11:C>T-11:C>T	TGCAGCGCACTCCCTCTGCCGCGAGATGGGCCG	11:C>T
5004301|F|0-13:C>T-13:C>T	TGCAGGTGCTCAACCTGTCGTCGAACCG	13:C>T
4261612|F|0-7:G>C-7:G>C	TGCAGCTGGGCACGGAGTGCACCGAGCGGCGGCCCGACCG	7:G>C
2241644|F|0-21:C>G-21:C>G	TGCAGGTTCATGGAGACGTAACGACTCCAGAATGTCGACAGATGAGAACGTGAACCG	21:C>G
4261677|F|0-6:T>C-6:T>C	TGCAGATAGGAAGAACCGAGCTCCG	6:T>C
984930|F|0-28:G>T-28:G>T	TGCAGCACCTGACGGACAAGAGCGACGTGTACAGCTTCGGCGTGGTGCTGCTGGAGCTCATGTCCGAGA	28:G>T
5580512|F|0-26:G>A-26:G>A	TGCAGGTCGATCTTGCGCGCCGACTCGGCCCGCCG	26:G>A
1263626|F|0-44:G>C-44:G>C	TGCAGCAAGCTAAGCATGCACGAGCACGAGTCGTCCGTCCTCGCGACCAAGGTCATCACCATCTCCGAG	44:G>C
5582389|F|0-8:C>T-8:C>T	TGCAGGTACGGCATCTGGGCCACGTCCG	8:C>T
3945741|F|0-27:C>T-27:C>T	TGCAGATGGGCGTGATGCTCACGACGGCGACCGTGGTGGTCACCGCTGGCATCGTCGTAGTGAGCTTGT	27:C>T
1019021|F|0-7:G>C-7:G>C	TGCAGAAGCTGTGCTTGTAGGAGCTCCTGGCGTGCAGCGTGACGTTGAAAGCCG	7:G>C
1056584|F|0-52:C>T-52:C>T	TGCAGTACGACGTGGACTCCCTGGACCAGAACCTGGCCCAGAACCTGGCGGGCGAGATCATCGGCACCC	52:C>T
5580517|F|0-6:T>A-6:T>A	TGCAGGTGACCTGGGATGAACCG	6:T>A
3025328|F|0-16:C>G-16:C>G	TGCAGGGAGCCCTGCCCCACCGTGCTCGTCACCAGGTCCG	16:C>G
6041850|F|0-30:G>T-30:G>T	TGCAGGTCCACGCCGACGGCCAGATCTCCCGCCCG	30:G>T
1037839|F|0-9:T>C-9:T>C	TGCAGTGCGTGAAGGCTCCTCGGACGGGGAAGGCAAGAAGGTGGATCCCCG	9:T>C
1071878|F|0-67:A>G-67:A>G	TGCAGCCCAGGCGGTGTACGGCGACTGCGAGTGGTACTTCTTCAGCCCGCGGGACCGCAAGTACCCCAA	67:A>G
6047611|F|0-16:A>G-16:A>G	TGCAGGCTGTTAAACTACGTGGAAATTGCGCCG	16:A>G
32021886|F|0-29:A>G-29:A>G	TGCAGCGCGGCGATGGAGTCCCTCCACCCATGGAGGACGCAGGACTCGAAGAAGGTGTCGAGCTTGGAG	29:A>G
100526587|F|0-44:T>C-44:T>C	TGCAGGAGTGTCGGCTGGCCGTTTCATGAATAACCTTTGCTTGTTGGATAGGGAGGGAATAAAGAGAGA	44:T>C
12765966|F|0-17:C>G-17:C>G	TGCAGCGACAGCGACTCCAGCATCCG	17:C>G
4999155|F|0-28:C>T-28:C>T	TGCAGCTCCCTCGTTAACAGGTCGCTCGCCTTCTCCACGGGCCCCG	28:C>T
100557696|F|0-14:C>T-14:C>T	TGCAGCTTATCCAACCCAATACCAGAATCTGCCATCGCCCTCTTGCACGGCTCAACGACTCTCTCCAGC	14:C>T
5004436|F|0-58:G>C-58:G>C	TGCAGACACGGTGTTCTTCCTGGCGTCCAAGCTCCTCAAGACGGTGCCCCAGGTCAGTGTCACTCTCAC	58:G>C
5357870|F|0-29:T>C-29:T>C	TGCAGCGGAGTCGGCCCAAACATGTTGCGTGGCCCG	29:T>C
5007719|F|0-10:G>T-10:G>T	TGCAGACGGCGAGCAGCTTCTGCATGAGGTAGTTCCCGAATGGGTTCACCATGAGGTCGACGGCGTGAC	10:G>T
5361115|F|0-55:T>G-55:T>G	TGCAGCGGTTGGCCGCCGCCTTCAGGCCGCGAATCGACGCGGCGCTGAAGTGGATTACTCGCTCACGGA	55:T>G
5369545|F|0-26:A>G-26:A>G	TGCAGGATCCCCCCGACGAGCGCGTGAAACAGCCGACGCGGCTCGCCG	26:A>G
3934340|F|0-6:C>A-6:C>A	TGCAGTCAGCTCCCCGACCGTCACCGTCTTGAGCTCGCCGTCGGCCG	6:C>A
3937272|F|0-6:A>T-6:A>T	TGCAGTAGACGCTCACCGTGAGCACGGCGCCG	6:A>T
3947066|F|0-26:G>A-26:G>A	TGCAGGTTCCTCTCGAACTCCCTCAGGTAGTCCAGGAGCAAAACGATCACGAAGAGGATGAACCCACCG	26:G>A
3947066|F|0-65:A>G-65:A>G	TGCAGGTTCCTCTCGAACTCCCTCAGGTAGTCCAGGAGCAAAACGATCACGAAGAGGATGAACCCACCG	65:A>G
2243154|F|0-28:C>G-28:C>G	TGCAGAACACGTATCCACAAGTAGTCGACCCTGTCTGTCGGGTCGCCATCCATCCTTCCACATTTGAGC	28:C>G
5358448|F|0-17:A>T-17:A>T	TGCAGGTGACCTCGGACAGACCG	17:A>T
3028878|F|0-20:G>C-20:G>C	TGCAGCGCCTTGTTGTACATGAGCGCGGCGTAGTTCCG	20:G>C
2244196|F|0-32:C>T-32:C>T	TGCAGAAATGGGTTCCAGAAGCTCGACAAGATCAAGGATGCCAACCGCCG	32:C>T
1022972|F|0-27:G>A-27:G>A	TGCAGTGCGGCGCCGACTCGCTCTACGGCGACAGGCTGGGCGAGTTCAGACTGTCCGTGAAAGGCCACG	27:G>A
7169423|F|0-18:T>C-18:T>C	TGCAGCCGCGGCGGCAGGTGGTTCCG	18:T>C
3934144|F|0-7:G>A-7:G>A	TGCAGCAGTGCGTGCAGCGGTGCCAGCAGGACCG	7:G>A
3028678|F|0-31:C>T-31:C>T	TGCAGCGTCTTCATGTTGAGCGACATCTCGTCCGCCG	31:C>T
4993221|F|0-15:A>G-15:A>G	TGCAGACGAGCGGAAACTCATCCG	15:A>G
983897|F|0-43:T>G-43:T>G	TGCAGCTAAACACGCCTCGTGCGGGCAGGGCCTCGCGTAGAACTTCGCGATGTTATCCCCG	43:T>G
1069533|F|0-10:G>A-10:G>A	TGCAGCAGTAGCGCGCGCTGCCGTAGGCGTTGCAGGCGCTCTTGCACGCCACCACCTCACCGCTTGAGC	10:G>A
1242815|F|0-30:T>C-30:T>C	TGCAGACGGGCAGGACCATGACCTACGACATTCATGGCGACGGCGGCCAGCTGACATTCGAGCCGAGAT	30:T>C
5004301|F|0-7:G>C-7:G>C	TGCAGGTGCTCAACCTGTCGTCGAACCG	7:G>C
4261100|F|0-11:G>C-11:G>C	TGCAGGGACGTGTCCGCCGCGGTGCCGTGGCCCG	11:G>C
4262346|F|0-22:G>C-22:G>C	TGCAGGCAGAAGGTGGCCCTCTGGAACAGCCGCATGATGTTGCCG	22:G>C
3949579|F|0-5:A>G-5:A>G	TGCAGAAAGCGGCAGCACATGCGTGGTCACCG	5:A>G
6043774|F|0-14:G>C-14:G>C	TGCAGCATCTCCTGGACCTGCGTGTCCG	14:G>C
1018265|F|0-23:G>C-23:G>C	TGCAGGTTCTGGTCCCGCTGGAAGCCCTTGTGGCAGATCTCGCACACGAACCG	23:G>C
32021417|F|0-31:G>C-31:G>C	TGCAGCTCGCCGTGCTCTACGTCTCGCTGCTGTGCACGTCCCTCGGCTCCG	31:G>C
985496|F|0-13:G>T-13:G>T	TGCAGGTCGGCGCGCCCGTGCACGGCACGGCGTTCCAGCGCGTCGCGTCGTGCTTCGTGCAGGGCCTGG	13:G>T

<table>
<tr><td>1018957|F|0-11:T>C-11:T>C</td><td>TGCAGGTATTCTTGCACGCCGTGCTGGCGAGGAAGCGGAAGATGCAGCTGCGGTTCCG</td><td>11:T>C</td></tr>
<tr><td>4261250|F|0-28:G>C-28:G>C</td><td>TGCAGTGCGAGCCGCCGATGGTGTGCGCGCCG</td><td>28:G>C</td></tr>
<tr><td>4262430|F|0-37:C>T-37:C>T</td><td>TGCAGCACCAGCTGCGGGGCACGGTGCGCCTCGACCCCTCCG</td><td>37:C>T</td></tr>
<tr><td>1055097|F|0-28:G>C-28:G>C</td><td>TGCAGTATCAGTACGCCAACTACTCGGCGAACTACCTCAAAGGTGGCAAGGGCAGCATCCG</td><td>28:G>C</td></tr>
<tr><td>1022186|F|0-27:T>A-27:T>A</td><td>TGCAGGAGGCGGCGATGCCGTCGGCAGTGAGGGAGGATCGTCGGATCTGATCACCGATGATCAGCCGAG</td><td>27:T>A</td></tr>
<tr><td>1040926|F|0-34:C>A-34:C>A</td><td>TGCAGGTGCTCGGCCGCGATGGCCGCTGGGCCGACGTCCAGCCCGTGGAGAACGCCATCGTCATCAACA</td><td>34:C>A</td></tr>
<tr><td>1079008|F|0-17:G>A-17:G>A</td><td>TGCAGGGAGTTGAACGCGAACGTCAGAAGTGGGTGTGCGGCGAGGGCGGACGCCG</td><td>17:G>A</td></tr>
<tr><td>1107007|F|0-26:C>T-26:C>T</td><td>TGCAGCATGGGGCTCCTGAACCGCCTCCCTCCTCGGAACCACGCGCGCTCCGACCCCTGCGCCG</td><td>26:C>T</td></tr>
<tr><td>1136196|F|0-31:C>A-31:C>A</td><td>TGCAGACGCGCCGTTCAGGGACAGCCTGCCGCGGCACGTGCGCGCGCCG</td><td>31:C>A</td></tr>
<tr><td>3934648|F|0-15:C>G-15:C>G</td><td>TGCAGCTCTCCCCTCCTCGCCCATGCACAGACGCCG</td><td>15:C>G</td></tr>
<tr><td>3934279|F|0-25:T>C-25:T>C</td><td>TGCAGGTGCTCGACCTCAGCTACAATAACCTCTCCG</td><td>25:T>C</td></tr>
<tr><td>1020984|F|0-50:G>A-50:G>A</td><td>TGCAGCCTCGGCGGCGCAGAAGGAGCCTCCGCGCAGAGCATCTCGGACTGGAGCACGGCGCAGTAGTGG</td><td>50:G>A</td></tr>
<tr><td>7351934|F|0-5:C>T-5:C>T</td><td>TGCAGCTCAGGGCGGTGGTGACCG</td><td>5:C>T</td></tr>
<tr><td>3940964|F|0-17:C>A-17:C>A</td><td>TGCAGCTTGGCGAGCCGCGAGATCAGGCCCG</td><td>17:C>A</td></tr>
<tr><td>4006298|F|0-11:C>T-11:C>T</td><td>TGCAGCACCGACAGCGCCATCAACACCTTAACCTCCG</td><td>11:C>T</td></tr>
<tr><td>1696481|F|0-50:G>A-50:G>A</td><td>TGCAGGTACTCTGGCTCGAGCGCGAACAGCACGGACACGCCGTCGTCCTTGTCGGGCGCCTGCGTCACG</td><td>50:G>A</td></tr>
<tr><td>1070948|F|0-43:C>T-43:C>T</td><td>TGCAGGTCCAGGTGAACGGGGCGACGAGGCGTGTGGGCGGGGTCTTCGGGACGCCG</td><td>43:C>T</td></tr>
<tr><td>7170823|F|0-53:G>C-53:G>C</td><td>TGCAGGCGCTTCTTCAGGCGCTCGGTGGAGACCGCCGCGGCGGCCGCGGTGTCGCCCG</td><td>53:G>C</td></tr>
<tr><td>7161156|F|0-10:T>C-10:T>C</td><td>TGCAGCCTTCTTGGAACATCGTGACCGCCCTTTCCCCGTCGGATTCCCCGCCG</td><td>10:T>C</td></tr>
<tr><td>32016532|F|0-17:C>G-17:C>G</td><td>TGCAGGATGAGGAAGACCAGCTCCCG</td><td>17:C>G</td></tr>
<tr><td>5370957|F|0-10:C>G-10:C>G</td><td>TGCAGTCCCTCGTCCTCGTCGACAACAACCTCACCG</td><td>10:C>G</td></tr>
<tr><td>4538596|F|0-10:G>A-10:G>A</td><td>TGCAGTGGGTGGACGTCTCGTCGAACGCCTTGACCG</td><td>10:G>A</td></tr>
<tr><td>1019241|F|0-9:T>C-9:T>C</td><td>TGCAGCTTCTTGGTGTCCGTGGTGCTCCAGGAGATGAGGTGGAACAGCCG</td><td>9:T>C</td></tr>
<tr><td>3948297|F|0-17:A>C-17:A>C</td><td>TGCAGCTCAAGCATTCCAAGAGCCCTGACCCG</td><td>17:A>C</td></tr>
<tr><td>12854624|F|0-27:G>A-27:G>A</td><td>TGCAGCTCATCAGGAGGCGCTCAAGGAGGAGATCGAGAGGCTGCGGCAGGTGTACCAGCAGCAGAACCT</td><td>27:G>A</td></tr>
<tr><td>6043621|F|0-28:G>T-28:G>T</td><td>TGCAGACCAAGCTGGCCGCTTACCAGAAGCAACCG</td><td>28:G>T</td></tr>
<tr><td>3947302|F|0-40:G>T-40:G>T</td><td>TGCAGGAGCGGTTGCTGAGTTGCCATGAAGCGATGCTCCAGGCCACCG</td><td>40:G>T</td></tr>
<tr><td>1077816|F|0-11:A>G-11:A>G</td><td>TGCAGAAAAGGAGGTAGGGTGGAGGGGAAGCAGGATGTGATGTGAGAGGGATGATGGGCTAACCGAGAT</td><td>11:A>G</td></tr>
<tr><td>1147583|F|0-38:G>A-38:G>A</td><td>TGCAGGTCCTTGGGGTCCTTCCCGTCGACGGTGCAGCCGACGCTGACGCAGGTGCCGAGGATCTCCTTG</td><td>38:G>A</td></tr>
<tr><td>5582365|F|0-16:C>G-16:C>G</td><td>TGCAGCTCGGCCTCACCCTCTCCG</td><td>16:C>G</td></tr>
<tr><td>32021311|F|0-22:T>G-22:T>G</td><td>TGCAGATTGTGGCGCTAGGTGTTCCG</td><td>22:T>G</td></tr>
<tr><td>5581397|F|0-7:C>A-7:C>A</td><td>TGCAGCGCGTCGTCCTCCTCGGGGCTCCACGGGCCCTTGATCCG</td><td>7:C>A</td></tr>
<tr><td>4398235|F|0-30:C>G-30:C>G</td><td>TGCAGGCTGGTTAGTTGATCGGGCGATCGGCCGACGTACGTGGTCCG</td><td>30:C>G</td></tr>
<tr><td>1055207|F|0-9:T>C-9:T>C</td><td>TGCAGGTTATTGACTCTGGTCAGAGTTATAGTGGTTATTCTATTCTTCACATGGCGCATGAGGCACCGA</td><td>9:T>C</td></tr>
<tr><td>3947359|F|0-26:T>C-26:T>C</td><td>TGCAGAGTTAGGTGCAGGGCATGGGGTTGCTCACCG</td><td>26:T>C</td></tr>
<tr><td>4262789|F|0-7:T>C-7:T>C</td><td>TGCAGTTTGGTAAGGTTACCAAGGACATGTTCACCATGGACTACCG</td><td>7:T>C</td></tr>
<tr><td>3944314|F|0-11:C>T-11:C>T</td><td>TGCAGCAAGCTCACCAGGCTCTCCACGTCCG</td><td>11:C>T</td></tr>
<tr><td>3574874|F|0-14:G>A-14:G>A</td><td>TGCAGCCACTTGACGTCCTTCCAGCTCAGGGTGCGGTCGATTTGGCCG</td><td>14:G>A</td></tr>
<tr><td>1036567|F|0-16:G>A-16:G>A</td><td>TGCAGTAGAACCAGAGGGCGAGCTTGACGGCGGTGGCCGAGAGCATGATGGAGTAGAGCCATGTCAGCT</td><td>16:G>A</td></tr>
<tr><td>1707968|F|0-40:G>C-40:G>C</td><td>TGCAGCTAGCGTCCGCCACGGCCTGCGTCACGCTGTCGGCGGTGCGGCTCGTCAGGCAGGACTTCGGCG</td><td>40:G>C</td></tr>
<tr><td>1061928|F|0-25:G>A-25:G>A</td><td>TGCAGGACGTGGAGGAACTCCTACAGTGCTGGGAGCGGCTCAAGTCGCCG</td><td>25:G>A</td></tr>
<tr><td>5358049|F|0-8:C>G-8:C>G</td><td>TGCAGCTGCCGCTTGCTGATCCG</td><td>8:C>G</td></tr>
<tr><td>5581100|F|0-52:C>T-52:C>T</td><td>TGCAGTGCACGACGCACATGTGCCCCGTGCGCGTCCACTGGCACGTCAAGCTCAACTACCACGACTACT</td><td>52:C>T</td></tr>
<tr><td>3026657|F|0-8:G>A-8:G>A</td><td>TGCAGCAGGATGAGGAATGGCTCCATCAGGAACACCGCCG</td><td>8:G>A</td></tr>
<tr><td>5371587|F|0-6:A>G-6:A>G</td><td>TGCAGCAACGCCACTAACGTGCCG</td><td>6:A>G</td></tr>
<tr><td>32026570|F|0-15:A>G-15:A>G</td><td>TGCAGGGATCATCAGACGCGTGCCTGGGGACGCCACGGGGGAGGAGGTGAACCCG</td><td>15:A>G</td></tr>
<tr><td>100508156|F|0-26:G>A-26:G>A</td><td>TGCAGCACCTCATTGATACCATACATGCCTTCACCG</td><td>26:G>A</td></tr>
<tr><td>3027951|F|0-16:C>G-16:C>G</td><td>TGCAGTCTATCGGGACCTGGAACGGACCGTCCAAGCCG</td><td>16:C>G</td></tr>
<tr><td>1695299|F|0-14:G>A-14:G>A</td><td>TGCAGCCACTGCTCGATGGACGCGCGCTCCAGCGCCCCCGTGCCGATCAGGTCCG</td><td>14:G>A</td></tr>
<tr><td>4261104|F|0-7:A>C-7:A>C</td><td>TGCAGCGACGAATCCCGCAGGCACCG</td><td>7:A>C</td></tr>
<tr><td>1030960|F|0-12:T>C-12:T>C</td><td>TGCAGATGCTAATTAACTTGCTTGTATTCGTGATTGCAGAAACCAAGCGATGATTTGGTCAAGGGTTCT</td><td>12:T>C</td></tr>
<tr><td>4002018|F|0-30:C>T-30:C>T</td><td>TGCAGGCGCTGCACCTCCGCGAGCATGCGGCTCTCGTCGACCAGGGGCGGGCCG</td><td>30:C>T</td></tr>
<tr><td>1230357|F|0-13:C>T-13:C>T</td><td>TGCAGCGTCTCGGCGGCCAGCCGCAGCTGCTGCCCGACGGCGCCCATGCGGAACACGCCG</td><td>13:C>T</td></tr>
<tr><td>4262839|F|0-19:C>A-19:C>A</td><td>TGCAGAGGCGGCGGCTGGGCAGCGCGCCATTTCCTCCG</td><td>19:C>A</td></tr>
<tr><td>3937564|F|0-24:C>T-24:C>T</td><td>TGCAGGCCCTCCAGATAAGCTTGACCCG</td><td>24:C>T</td></tr>
<tr><td>6043621|F|0-19:T>C-19:T>C</td><td>TGCAGACCAAGCTGGCCGCTTACCAGAAGCAACCG</td><td>19:T>C</td></tr>
<tr><td>1696624|F|0-59:G>C-59:G>C</td><td>TGCAGGCACGACGCCGCCGAGTGCGCCAGGCTCCTCCTCGACGGGGAGTACGGGATCACGCCG</td><td>59:G>C</td></tr>
<tr><td>1019021|F|0-21:A>T-21:A>T</td><td>TGCAGAAGCTGTGCTTGTAGGAGCTCCTGGCGTGCAGCGTGACGTTGAAAGCCG</td><td>21:A>T</td></tr>
<tr><td>2373883|F|0-9:T>C-9:T>C</td><td>TGCAGCTCATCAAGAGGCGCTGAAGGAGGAGATCGAGAGGCTGCGGCAGGTGTACCAGCAGCAGAACCT</td><td>9:T>C</td></tr>
</table>

<table>
<tr><td>4262839|F|0-13:G>A-13:G>A</td><td>TGCAGAGGCGGCGGCTGGGCAGCGCGCCATTTCCTCCG</td><td>13:G>A</td></tr>
<tr><td>3946714|F|0-18:C>T-18:C>T</td><td>TGCAGGTAAAGAAGGCGACGACGACGGACCCG</td><td>18:C>T</td></tr>
<tr><td>16709321|F|0-26:A>G-26:A>G</td><td>TGCAGGTTCCCAGGCCACGATTTCTGACCGTCAATTTTTGACCG</td><td>26:A>G</td></tr>
<tr><td>1019596|F|0-9:T>C-9:T>C</td><td>TGCAGGCGATGTGATAAAGACGCTCAAGGACAACGGGAACGAGTACACCTGGGGGCCG</td><td>9:T>C</td></tr>
<tr><td>3026888|F|0-18:G>T-18:G>T</td><td>TGCAGTAACCGCATTAATGGGGAAAATTATTGCAGTCGACTGCGCCG</td><td>18:G>T</td></tr>
<tr><td>7171580|F|0-21:C>T-21:C>T</td><td>TGCAGCTCGCTGCGGATGAAGCCCACCTCCCCCCG</td><td>21:C>T</td></tr>
<tr><td>1023518|F|0-44:C>A-44:C>A</td><td>TGCAGCTGGTGTCCGTGATGTTCGTGGGCCACCTCGGCGAGCTTCCCCTCGCCG</td><td>44:C>A</td></tr>
<tr><td>5363824|F|0-21:G>C-21:G>C</td><td>TGCAGGCTACGGCTCGTGTGTGTTCACCG</td><td>21:G>C</td></tr>
<tr><td>983392|F|0-8:T>C-8:T>C</td><td>TGCAGCTTTTGCTTTCGGTACATAAATGATTTTGTCTTCAAAATGAAAGCCTACAGCCATGTGGACCGA</td><td>8:T>C</td></tr>
<tr><td>5331680|F|0-6:A>T-6:A>T</td><td>TGCAGCAAATCGCCACTGCGCCCG</td><td>6:A>T</td></tr>
<tr><td>3946553|F|0-5:T>C-5:T>C</td><td>TGCAGTCCGCTCGCTACAGCTCCACCG</td><td>5:T>C</td></tr>
<tr><td>3574722|F|0-6:A>G-6:A>G</td><td>TGCAGCATGCTCTCGCGCTGCTCGAGGAACTGCTCCG</td><td>6:A>G</td></tr>
<tr><td>1020063|F|0-19:T>C-19:T>C</td><td>TGCAGGAGCTCCCTGAGGATCTCCAGGTCGCCGCCCCTCGCCGCGGCGTGCATTGCCCG</td><td>19:T>C</td></tr>
<tr><td>4002821|F|0-16:G>A-16:G>A</td><td>TGCAGCTGCAAGTCAAGGTGCTGGCGACGGACGAGTTCTGGCCG</td><td>16:G>A</td></tr>
<tr><td>5363824|F|0-18:T>C-18:T>C</td><td>TGCAGGCTACGGCTCGTGTGTGTTCACCG</td><td>18:T>C</td></tr>
<tr><td>4262614|F|0-37:C>G-37:C>G</td><td>TGCAGTCGCCGTTCACGTTCCGCAAGGACGGGCAGGCCAGCCG</td><td>37:C>G</td></tr>
<tr><td>12855025|F|0-41:C>G-41:C>G</td><td>TGCAGCTCGCCGCCGCGCTCGTACATGCCGTGGCAGCGCACCACGGCCG</td><td>41:C>G</td></tr>
<tr><td>4542665|F|0-8:T>C-8:T>C</td><td>TGCAGCCATGGGTTCTCATGGTACATCCG</td><td>8:T>C</td></tr>
<tr><td>5358113|F|0-14:C>G-14:C>G</td><td>TGCAGGAAGTTGCGCGCCAGCCCCACGGAGACGAGCCCG</td><td>14:C>G</td></tr>
<tr><td>6030746|F|0-31:C>G-31:C>G</td><td>TGCAGGCAGGAGACCGCATGCTCAAACTTTCCGCCG</td><td>31:C>G</td></tr>
<tr><td>3959524|F|0-11:G>A-11:G>A</td><td>TGCAGGTTCGAGGGGTACCAGGACACCCTGTACGTGCACGCCAACCG</td><td>11:G>A</td></tr>
<tr><td>3947125|F|0-10:G>A-10:G>A</td><td>TGCAGAAGGCGGACCTGAGCTACAACCG</td><td>10:G>A</td></tr>
<tr><td>1214870|F|0-51:C>A-51:C>A</td><td>TGCAGAGTGGAACTGAATGACCATGACAGTATCTGGTGCAGCTCGGCGGCCCAGCCAGTGGCCGCCGAG</td><td>51:C>A</td></tr>
<tr><td>2242338|F|0-39:G>C-39:G>C</td><td>TGCAGCAGTCGTGGTGTCTGCATTAGCACCATTCTCGTCGATCCTCATCTGGGTCTTGGCTGGCACGAA</td><td>39:G>C</td></tr>
<tr><td>1041280|F|0-40:A>C-40:A>C</td><td>TGCAGGCCCAGCACCGCCAACTGCTCCGCCACCGCCAAGAACGTCTGCCCGCCCTACCTCGTCGTCTAC</td><td>40:A>C</td></tr>
<tr><td>3937855|F|0-26:G>T-26:G>T</td><td>TGCAGGCTGACCCTGGAGTCGCCTCTGGTGTGCAGCAGCTCCCTGTCCG</td><td>26:G>T</td></tr>
<tr><td>1026148|F|0-49:C>G-49:C>G</td><td>TGCAGAACAAGGTGACCCTGGAGTGGCTTCTGGCCCTGGGTGACAAGCTCGGCATGGTCCGTGTGCCGA</td><td>49:C>G</td></tr>
<tr><td>3959027|F|0-5:G>A-5:G>A</td><td>TGCAGGCCAGATACCCTGTGGAAGGGGAGCTCGCCG</td><td>5:G>A</td></tr>
<tr><td>3960371|F|0-10:A>T-10:A>T</td><td>TGCAGATCCAAGGTGAGGAAGCCAGGTTCGTGAACGTCCTCACCG</td><td>10:A>T</td></tr>
<tr><td>5369353|F|0-31:G>A-31:G>A</td><td>TGCAGCAGCCGCTTCTCCTGGAGCTCCTGGGGCAGGACGGCCG</td><td>31:G>A</td></tr>
<tr><td>2256296|F|0-19:G>A-19:G>A</td><td>TGCAGGCCACCTCGGCGGCGTCCAGGGCGCTCGACCGCTTCGACGTCCTCGACTGCCG</td><td>19:G>A</td></tr>
<tr><td>1215407|F|0-47:G>A-47:G>A</td><td>TGCAGGCGGACTCGCTGGAGCGGCAGGCCTACGGCTTCAAGAACGACGGCCTCAACTTCACCG</td><td>47:G>A</td></tr>
<tr><td>5373676|F|0-6:G>C-6:G>C</td><td>TGCAGGGAGATGATGACGTAGATCTTCCG</td><td>6:G>C</td></tr>
<tr><td>1071878|F|0-30:G>A-30:G>A</td><td>TGCAGCCCAGGCGGTGTACGGCGACTGCGAGTGGTACTTCTTCAGCCCGCGGGACCGCAAGTACCCCAA</td><td>30:G>A</td></tr>
<tr><td>1397609|F|0-8:G>C-8:G>C</td><td>TGCAGGAAGGACTCCTTGGTGCCTTCGTCCTCGCCCAGAATGCCCACGGGCACGGCGCCCACGGACTTG</td><td>8:G>C</td></tr>
<tr><td>4262200|F|0-13:C>T-13:C>T</td><td>TGCAGAAGATGTCCTACTTCAACTGGTGGAACTGCGCGCTCTGCTCCG</td><td>13:C>T</td></tr>
<tr><td>5581339|F|0-16:C>T-16:C>T</td><td>TGCAGCAGTTGGCCTCCTCTCATCCG</td><td>16:C>T</td></tr>
<tr><td>5582004|F|0-8:G>C-8:G>C</td><td>TGCAGCTGGTGCGGCACGGCGTCCG</td><td>8:G>C</td></tr>
<tr><td>2241644|F|0-31:A>T-31:A>T</td><td>TGCAGGTTCATGGAGACGTAACGACTCCAGAATGTCGACAGATGAGAACGTGAACCG</td><td>31:A>T</td></tr>
<tr><td>3938382|F|0-16:C>G-16:C>G</td><td>TGCAGCGAAGCGAGATCGTGAGGCCCG</td><td>16:C>G</td></tr>
<tr><td>7171570|F|0-8:C>G-8:C>G</td><td>TGCAGCTCCCCGCCGCGCTCGTACATGCCGTGGCAGCGGACGACGGCCG</td><td>8:C>G</td></tr>
<tr><td>13120439|F|0-11:G>T-11:G>T</td><td>TGCAGGTGGGCGAGAGGTCGCCG</td><td>11:G>T</td></tr>
<tr><td>32027225|F|0-6:C>T-6:C>T</td><td>TGCAGTCAATCACGGCGAAGGTCTCCG</td><td>6:C>T</td></tr>
<tr><td>32027443|F|0-21:G>C-21:G>C</td><td>TGCAGGTCGGGGCGCATCGGTGGCGGAGGCCGAGAAGACCG</td><td>21:G>C</td></tr>
<tr><td>32022510|F|0-23:C>G-23:C>G</td><td>TGCAGGCTCACCGCGCTCTCCTTCGACTCACCG</td><td>23:C>G</td></tr>
<tr><td>3935882|F|0-19:C>G-19:C>G</td><td>TGCAGACGAGTTGCATATGCCCACTACGTGCGTTCGGCGTGAACGACCG</td><td>19:C>G</td></tr>
<tr><td>5358460|F|0-7:C>G-7:C>G</td><td>TGCAGGTCGACGAGCTGGAGGCGGAGGCGGCAGCGCTGCGCGCCCG</td><td>7:C>G</td></tr>
<tr><td>5364495|F|0-10:T>C-10:T>C</td><td>TGCAGGGGCGTCGCAGCGTACACATGACCG</td><td>10:T>C</td></tr>
<tr><td>7913126|F|0-5:G>T-5:G>T</td><td>TGCAGGCAAGCCGCCAACGACGCCG</td><td>5:G>T</td></tr>
<tr><td>3027991|F|0-10:G>A-10:G>A</td><td>TGCAGCTGCTGGGCCCCGACGAGGAGAAGCCCTCGCCG</td><td>10:G>A</td></tr>
<tr><td>5367804|F|0-7:T>C-7:T>C</td><td>TGCAGTCTCTCAACGTGTCCG</td><td>7:T>C</td></tr>
<tr><td>5009226|F|0-63:A>G-63:A>G</td><td>TGCAGCTCGGCTCTAGTCTACTCCACAGGCTCTGCTCATCTTGAGGAGCGCCGAGAGCACGCCATTCAT</td><td>63:A>G</td></tr>
<tr><td>1021879|F|0-35:C>T-35:C>T</td><td>TGCAGCAGCCGCTCCACCACGTGGCTGAGGCAGCACCCGCGCCTCCCCACCACCAGCACCG</td><td>35:C>T</td></tr>
<tr><td>1865011|F|0-28:C>G-28:C>G</td><td>TGCAGGAAGATCGGCTCGACGTCGGTGGCGACGAACTCGTCCAACGTTGGCGCAGGAGCCG</td><td>28:C>G</td></tr>
<tr><td>3027455|F|0-11:C>A-11:C>A</td><td>TGCAGGCACGGCGCCAGCCCCACCAGCTGCTCCGCGCACTCGCTCCG</td><td>11:C>A</td></tr>
<tr><td>4993350|F|0-7:G>C-7:G>C</td><td>TGCAGGGGGTGCAGGCCAGGCTGCGCCG</td><td>7:G>C</td></tr>
<tr><td>1021567|F|0-43:C>A-43:C>A</td><td>TGCAGACCTTCTGCACCGTCTACGACTGCAAGCGCTGGTTCGGCGGCATACTGGAACTGCCGCAGATAA</td><td>43:C>A</td></tr>
<tr><td>3954296|F|0-7:C>T-7:C>T</td><td>TGCAGCTCCAAGACAACGAGCTTTCCG</td><td>7:C>T</td></tr>
<tr><td>5357415|F|0-23:G>A-23:G>A</td><td>TGCAGATCGGCAGGCACCGAGTCGAAGCCGAGCCG</td><td>23:G>A</td></tr>
</table>

2241644|F|0-9:A>C-9:A>C	TGCAGGTTCATGGAGACGTAACGACTCCAGAATGTCGACAGATGAGAACGTGAACCG	9:A>C
5367336|F|0-7:G>T-7:G>T	TGCAGGCGCGGCGGAGCTGTGGAACCCG	7:G>T
1096523|F|0-52:G>T-52:G>T	TGCAGGTGATGGCGGACGTGATCCACTGGTGCACCCACAAGGACGGCGGGGCGCGGCCGTCGATGAAGC	52:G>T
992105|F|0-66:G>T-66:G>T	TGCAGGTGCAGGCTGGACCTAGTATGCACGATGATCCCAAACACCTCGCCCATGTCCAGCGTCTCCGAG	66:G>T
4261818|F|0-9:G>A-9:G>A	TGCAGCGGGGACCCGACCATGTTTGAGGCCTTCTGGAGGGAGACCG	9:G>A
5372577|F|0-8:C>A-8:C>A	TGCAGCAGCGGCTCTTCTACCCG	8:C>A
991415|F|0-14:T>C-14:T>C	TGCAGTTGCTCCTCTATGGCTCTTCCGCCCACGAGCAGGGCGGTGGGGACGGAGCGGAAAAGGAACAAC	14:T>C
1030325|F|0-27:G>A-27:G>A	TGCAGGCGAGCAGGTACTCCAGGTCGCGCTCCTGCATTATGGTGTGGCAGATGCCGAGCTCCTGGAGGC	27:G>A
32022069|F|0-58:A>C-58:A>C	TGCAGGTGGATTTGGTGACGGAGACGGACAAGGCCTGCGAGGATCTCATCTTCAACCAACTCCG	58:A>C
1025833|F|0-50:G>C-50:G>C	TGCAGCCTCGCCGCGTGAGCGGCCGCGAGGGCGACGCGCAAGGTGTAGGTGCCGCCG	50:G>C
1093237|F|0-38:T>A-38:T>A	TGCAGGCAGCAGCGCGCACGCCATGGCTGGACTCTGCCTCCCAAGGGTTTGCGCCAAGAGCGCCGAGAT	38:T>A
32027469|F|0-14:G>C-14:G>C	TGCAGCAGGAGGATGCCGTCGACGGAGTCGAGGATGCAATGGTTGCTGAAAAGCGGGAGCCG	14:G>C
3947066|F|0-35:G>A-35:G>A	TGCAGGTTCCTCTCGAACTCCCTCAGGTAGTCCAGGAGCAAAACGATCACGAAGAGGATGAACCCACCG	35:G>A
1023497|F|0-10:G>A-10:G>A	TGCAGGCCGCGGGCAACGGGTCCTCGCAGAGCGCCGCGGCGAGGGAGCGCCG	10:G>A
1038581|F|0-22:C>G-22:C>G	TGCAGCTGGGCGCCTGGCCGCTCTACTGGGTCGTGCAGGGCTGCGTCATGACCG	22:C>G
4022587|F|0-32:G>A-32:G>A	TGCAGGGAGCAGGTGATCCTGGCGCCAGCGTCGACGCCGAACGCCCG	32:G>A
1021400|F|0-24:T>C-24:T>C	TGCAGGTACGGCAGCTTGCTGATGTTGGACTCGGTGATGGGCTCGTCGTCGCCGAGCACGTCCCTGATC	24:T>C
3936395|F|0-21:A>G-21:A>G	TGCAGTCCTGCTTCCTCTTGGACACGTTCCTCCTCTTGTCCG	21:A>G
1032900|F|0-9:A>T-9:A>T	TGCAGGATTAGCCGAGAGTAAGAGTGGTCGTGCCTGGACTTGGCTGAGGACGGGAAATGTCTCCATGGA	9:A>T
3960371|F|0-6:T>C-6:T>C	TGCAGATCCAAGGTGAGCAAGCCAGGTTCGTGAACGTCATCACCG	6:T>C
1018433|F|0-6:C>G-6:C>G	TGCAGTCAGCATACCAGGGGCATCGGCGTCAGGGTTGCGGCGGGCTACAACATACTCGCCG	6:C>G
1082372|F|0-15:C>T-15:C>T	TGCAGCTCATGTCCGCCGAATTTCAGATGCCATCGCCGCTGGTGCCGACGCGGGACACCCAGTTCCTGC	15:C>T
7349773|F|0-7:T>C-7:T>C	TGCAGGATCTCCAGCTCCCCG	7:T>C
1023205|F|0-61:C>T-61:C>T	TGCAGCTGCAACTTCTGGGTTGCCTCAGCGGGGTGGAGATGGTGCTCAAGGACATCGGGTACCCGAGAT	61:C>T
1397609|F|0-59:C>G-59:C>G	TGCAGGAAGGACTCCTTGGTGCCTTCGTCCTCGCCCAGAATGCCCACGGGCACGGCGCCCACGGACTTG	59:C>G
3946553|F|0-19:C>A-19:C>A	TGCAGTCCGCTCGCTACAGCTCCACCG	19:C>A
5366989|F|0-11:C>T-11:C>T	TGCAGCCCATCCGCGAGATCTCCG	11:C>T
1018433|F|0-24:G>A-24:G>A	TGCAGTCAGCATACCAGGGGCATCGGCGTCAGGGTTGCGGCGGGCTACAACATACTCGCCG	24:G>A
1036388|F|0-40:T>C-40:T>C	TGCAGATCATACATACACGTAATATAATGGCGTAAAGTTGTTGCAAACCCAACGAGCTCCCCTGGAATG	40:T>C
1210801|F|0-25:C>G-25:C>G	TGCAGGAGGTGCAGAAGGACTGTGTCTGCGAGGCGCTCCTGCGGCTGCCGCCGTTCCTCGTGAAGCCAC	25:C>G
987470|F|0-30:A>T-30:A>T	TGCAGGCCTTCTGGACGGAGGTGCGCACAGAGGGACACGGAGACAAGAAGGACGCGCCGTGGTGGCCTA	30:A>T
981445|F|0-5:C>T-5:C>T	TGCAGCACGCAGCAGAGCTTCCCTACTGCGCCCACGCGGGCCATCTCCCGCACCACCTCCG	5:C>T
2244421|F|0-20:C>T-20:C>T	TGCAGAGGCACCCCATCGAGCGCGTCGTCGTCGAAGTGCTCGGGCAGCTTGTCGAGGAGGAGCCCCAGG	20:C>T
3957007|F|0-8:A>G-8:A>G	TGCAGGGTATGGATCTTGCTGCCGAAGAAGGCGTGGCACACCTCGCCGAGGGAGCCCAGGATGCTGCCC	8:A>G
1075540|F|0-62:G>T-62:G>T	TGCAGCATCTTCTCGTCCGCGCCCTCCCGCGCCACGCAGTAGGTCTGGTTCGTCGTGTCGTTGGCCAGC	62:G>T
2242338|F|0-30:A>G-30:A>G	TGCAGCAGTCGTGGTGTCTGCATTAGCACCATTCTCGTCGATCCTCATCTGGGTCTTGGCTGGCACGAA	30:A>G
1077077|F|0-49:G>T-49:G>T	TGCAGCACGGAGATGGGGCTCGTGTAGTGCATCTTGGGCAACAGCGGTCGCCCGTACTGTGCCAGCATG	49:G>T
1023177|F|0-16:C>G-16:C>G	TGCAGGCGCTGGTGCGCGGTCAGCTGGTGAGGAAGCAGGCCAAGGCCACGCTCCG	16:C>G
1263707|F|0-14:C>G-14:C>G	TGCAGGTCCACGCACCCGTGCCCCAGCGGCGGCTGCTGCCCCCTCAGGCTCTTCTGCATCGGCAGGCAC	14:C>G
1019241|F|0-14:G>C-14:G>C	TGCAGCTTCTTGGTGTCCGTGGTCCTCCAGGAGATGAGGTGGAACAGCCG	14:G>C
3947477|F|0-10:G>C-10:G>C	TGCAGCACGTGGCGCTCAACTTCATCCTCGCCG	10:G>C
4262573|F|0-10:G>A-10:G>A	TGCAGGACGAGCTGGAGGACCCGAACCCGCCG	10:G>A
3938736|F|0-21:T>C-21:T>C	TGCAGCATGAAGAGCATTCTGTCCATCTGGCCCG	21:T>C
4261945|F|0-23:C>G-23:C>G	TGCAGGTAGGCTAGGTCCTTGCCCAGGCACACCCTCGGCCCG	23:C>G
3570079|F|0-8:G>A-8:G>A	TGCAGTGCGTCGTCCACATGGTCTGCGTCGCCGTCGAGTACCG	8:G>A
3948285|F|0-24:C>T-24:C>T	TGCAGCGTGCAGCTTGTTCCCGATCCCCG	24:C>T
4262894|F|0-6:C>T-6:C>T	TGCAGCCTCAAGCTCACCAGCCGCTTGCAGCTGCCAATGTCACCG	6:C>T
3021874|F|0-8:C>T-8:C>T	TGCAGCATCGCACACAAACTGGAAGCCAGCGTCAAGTACTTCCG	8:C>T
999511|F|0-7:A>C-7:A>C	TGCAGCAACTCACCCTCTGACAGCCCGAAGCTCTGGTAAAATGCCATTTTCCGCAGCCATGAGTCCCTC	7:A>C
3940238|F|0-27:G>A-27:G>A	TGCAGGGTGAGGAGCGTCAGGCAGAGCGCTACCTTACGCCG	27:G>A
2241677|F|0-20:A>G-20:A>G	TGCAGGAACCAAATTGCAGTATCAAACTCTCACCTGCATTCGAGATCATTACATGAGAACCTGGCTCCG	20:A>G
3574874|F|0-26:C>G-26:C>G	TGCAGCCACTTGACGTCCTTCCAGCTCAGGGTGCGGTCGATTTGGCCG	26:C>G
3028775|F|0-14:A>G-14:A>G	TGCAGAAACCACCAAAACGCCCAGCCGAGAAACGCGGCCCG	14:A>G
7171736|F|0-19:G>C-19:G>C	TGCAGTGGGTGCGCGACAAGGGCTACGTCATCTACGACTACTGCCTCGACAGGAGCAACCG	19:G>C
3946714|F|0-17:A>G-17:A>G	TGCAGGTAAAGAAGGCGACGACGACGGACCCG	17:A>G
32024120|F|0-18:C>T-18:C>T	TGCAGCCCGAATAGCTCCCCCGGGATCGGCCCG	18:C>T
1027072|F|0-43:G>C-43:G>C	TGCAGGTCGCGCTTAGCGGCAGAGAGAAGGCGGCGACGGCGGCGGAGGCGAGGAAGAGGAGGAAGGAGC	43:G>C
4262348|F|0-14:T>C-14:T>C	TGCAGGTCGAAGCGTGACAGGGCCG	14:T>C
985768|F|0-31:C>G-31:C>G	TGCAGTTGAAGAACACCGACATGTCCCCCTGCACGTGCAGCGCCACCGCCTGGTGCCCGTCCG	31:C>G

7351393|F|0-6:T>C-6:T>C	TGCAGCTTGCCGTAGAGCCG	6:T>C
1865041|F|0-53:G>T-53:G>T	TGCAGGATTATGTTCCCCTGCTTGTCGACGCAGTGGAACAGCCCCAGGAAGTAGCGGCCGTCGTTCACC	53:G>T
6030406|F|0-11:C>G-11:C>G	TGCAGCCAGTCCTCGCGGCTGCCGATGTCCG	11:C>G
4733897|F|0-19:A>C-19:A>C	TGCAGCGTCTTCATCTGCCAGAACTCTCGCTGGATCGCGCCG	19:A>C
1865011|F|0-5:G>C-5:G>C	TGCAGGAAGATCGGCTCGACGTCGGTGGCGACGAACTCGTCCAACGTTGGCGCAGGAGCCG	5:G>C
985897|F|0-46:C>A-46:C>A	TGCAGGCCGCCGCGCTGCTCTGCTTCGTCGCGCTCTCCCTCGTCTCCGCCTTCCG	46:C>A
991415|F|0-47:G>C-47:G>C	TGCAGTTGCTCCTCTATGGCTCTTCCGCCCACGAGCAGGGCGGTGGGGACGGAGCGGAAAAGGAACAAC	47:G>C
3936290|F|0-5:G>C-5:G>C	TGCAGGCCGACGAACGCGAACACGCCG	5:G>C
2246387|F|0-46:C>T-46:C>T	TGCAGGAGTATTACATCAGGCACCTCGACGACGGCGCCGCCGCCTCCGACCAAGACCG	46:C>T
7908723|F|0-13:G>C-13:G>C	TGCAGAGGAGGTCGTACAACCCCCG	13:G>C
1250878|F|0-22:G>T-22:G>T	TGCAGAAGGCGCACAGGCGGCTGTCGCAGGGCAGCGTGGACAGCGCCATGACCATGGAGATCCAGAAGT	22:G>T
1020726|F|0-19:C>T-19:C>T	TGCAGGCCAAGGACCAGATCGGCACCTACTTCTACTTCCCCTCCCTCGCCTTCCACAAGGCCGCCGAGA	19:C>T
1019321|F|0-16:C>G-16:C>G	TGCAGTGGGCCATGGCCGAGCTGATGAGGAACCCGAGGGTGATGCGCAAGGCGCAGGAGGAGGTCCGAG	16:C>G
5582021|F|0-9:T>C-9:T>C	TGCAGGACGTGCTGAGCCACCCG	9:T>C
1135062|F|0-28:C>T-28:C>T	TGCAGCTCAAGGACGCCGTCTACGAGGCCGACGAGGTCGTCGACGAGTTCGAATACCG	28:C>T
1229113|F|0-45:G>C-45:G>C	TGCAGCAGCGCCGTGTTGTAGAGCAGCTCCATCCCGTGGAGGCGGGAGTAGTCCACCTTGTTCTTGAGG	45:G>C
3937735|F|0-20:G>A-20:G>A	TGCAGGCTGTGCGCGGCCGCGATCACCGCGTGGTCCG	20:G>A
2253831|F|0-27:G>A-27:G>A	TGCAGGTCCTCGACGCCGCAGGCCGCGGCGTAGTCGACGCAGCGCTTGAGGTCGGCCTGCGCGAGCTGG	27:G>A
1021778|F|0-37:T>C-37:T>C	TGCAGATGCCTAATGGCGGCGGTCAGATGGCGCACGATGTGTTTGGCTCCTTCCG	37:T>C
4261761|F|0-18:G>A-18:G>A	TGCAGCCGAAGTGGATGGGCGGGCCGCGCACGTCGTAGGTGCCCCG	18:G>A
1047568|F|0-7:T>G-7:T>G	TGCAGCCTCTGCATCCTGCCCCGCTTGGCCTCCCTCAGCCTCTCCGCCTCCGATTCCAGCCTCGCGATC	7:T>G
4262307|F|0-14:G>C-14:G>C	TGCAGCAGCCACTGGTGATCCACGTCGATGAGGAAGTCCTTGTGGTAGTGCTCCCTCACCTTCTCCCTC	14:G>C
1036567|F|0-27:C>T-27:C>T	TGCAGTAGAACCAGAGGGCGAGCTTGACGGCGGTGGCCGAGAGCATGATGGAGTAGAGCCATATCAGCT	27:C>T
32022441|F|0-14:A>G-14:A>G	TGCAGCGCAGCAAGAACTTCACCTACAGGATCAAGGCCCG	14:A>G
7337049|F|0-11:T>C-11:T>C	TGCAGGTACCTTTTGGTATTGGACCG	11:T>C
1865041|F|0-56:G>C-56:G>C	TGCAGGATTATGTTCCCCTGCTTGTCGACGCAGTGGAACAGCCCCAGGAAGTAGCGGCCGTCGTTCACC	56:G>C
4398235|F|0-8:G>C-8:G>C	TGCAGGCTGGTTAGTTGATCGGGCGATCGGCCGACGTACGTGGTCCG	8:G>C
2246387|F|0-50:C>G-50:C>G	TGCAGGAGTATTACATCAGGCACCTCGACGACGGCGCCGCCGCCTCCGACCAAGACCG	50:C>G
2256566|F|0-50:G>A-50:G>A	TGCAGCTCTGGAGGTAGGAGAAGAGGCCGAAGCCGCAGTAGTCGAGGCACGCCTTGTCGAGGTGCGGGT	50:G>A
4262502|F|0-10:G>A-10:G>A	TGCAGATGAAGGACGTGGCCGTCCG	10:G>A
2256566|F|0-46:G>A-46:G>A	TGCAGCTCTGGAGGTAGGAGAAGAGGCCGAAGCCGCAGTAGTCGAGGCACGCCTTGTCGAGGTGCGGGT	46:G>A
1019320|F|0-10:G>A-10:G>A	TGCAGTGGGCGATGGCGGAGCTGATACGGAACCCGCGGGTTATGCGGAAGGCGCAAGACGAGGTCCGAG	10:G>A
3026943|F|0-29:A>G-29:A>G	TGCAGACTCCCCTAATAGACCTCTCCGCCATATTCACGCGCCG	29:A>G
4261364|F|0-20:G>T-20:G>T	TGCAGGCCGATGCACTTGACGCCG	20:G>T
3936573|F|0-22:A>G-22:A>G	TGCAGGTTAATCGTACGAGTAGAAGGACCG	22:A>G
3026943|F|0-9:C>G-9:C>G	TGCAGACTCCCCTAATAGACCTCTCCGCCATATTCACGCGCCG	9:C>G
5581383|F|0-32:G>A-32:G>A	TGCAGCGAGTCGGCTTCTTTCTCGTCGGCCATGAGAAGATTCCG	32:G>A
1036567|F|0-64:C>G-64:C>G	TGCAGTAGAACCAGAGGGCGAGCTTGACGGCGGTGGCCGAGAGCATGATGGAGTAGAGCCATATCAGCT	64:C>G
5361474|F|0-16:G>A-16:G>A	TGCAGGCGGAAGTAGAGCAACCCCTCGACGGCGTCGGTGCGGAGCGTGGCGACGCCGCGCGCCATGAAG	16:G>A
1027981|F|0-30:A>G-30:A>G	TGCAGTGATCGGCATGGAGGGGCTCGCGAGAGGCTTCGCCAAGCCGCCGTCGGACTGGAGCGGCGATCC	30:A>G
1018308|F|0-49:C>T-49:C>T	TGCAGGAAATTCAGAAGTACTTTGTCGATTACATGATAAATGACTCGCTCGGTGCAATCTCAACTGCTC	49:C>T
1023497|F|0-8:G>A-8:G>A	TGCAGGCCGCGGGCAACGGGTCCTCGCAGAGCGCCGCGGCGAGGGAGCGCCG	8:G>A
32024188|F|0-23:G>C-23:G>C	TGCAGGCGGATGCGGAGCGTGTCGAGCGGGTGGCCCGCCAGCACGCCG	23:G>C
1129293|F|0-17:G>A-17:G>A	TGCAGCTGCTGGGGCGCGGTGTACAGCAACGGGACGGGCTACTGCTACCTCATGGACTACCCG	17:G>A
1211371|F|0-36:A>G-36:A>G	TGCAGTACTTCTACGAGCTGGACGTGAGCAACAACAAGCTCGCTCGCTGCGCCTTCCCGACGGACGTGC	36:A>G
1129293|F|0-56:C>A-56:C>A	TGCAGCTGCTGGGGCGCGGTGTACAGCAACGGGACGGGCTACTGCTACCTCATGGACTACCCG	56:C>A
1696628|F|0-44:T>C-44:T>C	TGCAGCTGCGTCTTGACAACGTCGAGTGGTGTGGTCAACGCGGCTGCTAGAGCACCG	44:T>C
986776|F|0-34:G>A-34:G>A	TGCAGCCGAACTGGAATTCCTTGACCGTGTCCGAGCCCAGCGCCAGCGTGTCGGAGCCGTACGTGCCGA	34:G>A
1035361|F|0-18:G>A-18:G>A	TGCAGCCCACACCTTGGGGCCCTCCATGGCGAAGGCGAAAGGGAGGAGGATCACCAGGGACAGCATGGA	18:G>A
1865360|F|0-58:C>A-58:C>A	TGCAGCGTCACCCTCCTGTTCTTCGACAGCTGCAAACGCAAACAAATCGCACCGTAAACCCTAGCCCCG	58:C>A
1035361|F|0-33:G>A-33:G>A	TGCAGCCCACACCTTGGGGCCCTCCATGGCGAAGGCGAAAGGGAGGAGGATCACCAGGGACAGCATGGA	33:G>A
22839965|F|0-13:C>G-13:C>G	TGCAGATGTCCTCCGACTCGGTGGACGCCG	13:C>G
7167221|F|0-19:C>G-19:C>G	TGCAGGTTGCACTTAGCGGCAGAGAGAAGGCGGCGACGGCGGCGGAGGCGAGGAAGAGGAGGAAGGAGC	19:C>G
1047568|F|0-46:C>T-46:C>T	TGCAGCCTCTGCATCCTGCCCCGCTTGGCCTCCCTCAGCCTCTCCGCCTCCGATTCCAGCCTCGCGATC	46:C>T
1017783|F|0-28:G>C-28:G>C	TGCAGTTGGTCTGCTGCTCCGCGAAGAAGGGCCAGCTGAGCATCGGCACGCCGTTGGAGATGCTCTCCA	28:G>C
3934562|F|0-14:G>A-14:G>A	TGCAGAAAGCGGCAGCGCGTGCGTGGTCACCG	14:G>A
5562969|F|0-49:C>T-49:C>T	TGCAGAGCGGCAGGTCGGCGGGGAGCGCCTCCAGGCTGAGGTTGGCGTCCCTCAGCGCGAACACCACCT	49:C>T
3955006|F|0-19:G>A-19:G>A	TGCAGAGGAAGGGGCCGCTGAACCG	19:G>A
5325120|F|0-6:G>A-6:G>A	TGCAGAGGGTCTCGGCGGGAGCAGAGTCCG	6:G>A

1214879|F|0-9:A>G-9:A>G	TGCAGTGGCAGCGCATGGTTGACGACATGCGCGCCCTCGGCAAGCTCAACAGCTGCGTGGCCGTGTGCG	9:A>G
1696628|F|0-41:G>A-41:G>A	TGCAGCTGCGTCTTGACAACGTCGAGCGGTGTGGTCAACGCGGCTGCTAGAGCACCG	41:G>A
4261734|F|0-13:C>T-13:C>T	TGCAGCATCCCCGCGGACACCGACAGCAAGGCGCCCAAGAAGCCG	13:C>T
6041837|F|0-8:G>A-8:G>A	TGCAGGTAGAATAGCCGACGGCAGCAATCCG	8:G>A
4261206|F|0-16:T>C-16:T>C	TGCAGCTCTCGCATGTTGCCGATCTCCG	16:T>C
3029607|F|0-30:A>C-30:A>C	TGCAGAACGCCGTCTGGACCTCACTCTACAAGTGCGTGGAGCCG	30:A>C
1157770|F|0-8:C>T-8:C>T	TGCAGGTCCTGCACCAGACCATGCCCTGCACGCCGATGCTGAAGTTCACCG	8:C>T
2249233|F|0-25:G>C-25:G>C	TGCAGGTGTTCGACGCGATGATCAGGGAGGGACACCAGCCCGACGAGGCCGTGTACGTCGGCGTGCTCA	25:G>C
1016792|F|0-11:G>C-11:G>C	TGCAGCTCCGTGGCCTTCTGCTTGGCGGTGAGCGCCTCCTTGCACGCCGTGCTGTACATGTCCATGGTC	11:G>C
989582|F|0-17:C>T-17:C>T	TGCAGGAAGCGGGTGAGCGGGAGCAGCATGGCCATGGCGAAGTGCTGAGGGATGAGCCACACGCTCATC	17:C>T
3952350|F|0-16:G>C-16:G>C	TGCAGTGGATCATGGCGGAGCTGGTCAAGAACCCG	16:G>C
3960832|F|0-16:G>C-16:G>C	TGCAGCTCAAGCTGGTGGAGGCCATCAAAGAAGCCG	16:G>C
5358063|F|0-5:G>C-5:G>C	TGCAGGTGTCCACATTATCCATGGCCCG	5:G>C
100322982|F|0-61:C>A-61:C>A	TGCAGCGTTTTCTCTCTGTTGCTATTCTCAGCACTTTTGAAAGTATCTATGCAAAGGAATTCGTATTAA	61:C>A
5361677|F|0-46:G>C-46:G>C	TGCAGGTTGGGTCGCCGTGGTTCACGATCCTGTTCGCCCGCAAGACGGAGGAGAAGATGGAGGCGGCAG	46:G>C
12695437|F|0-14:T>C-14:T>C	TGCAGCCAGGAGTGTAGGGAGCATCAGATCACGAAGGACGAGCTCATGGAACATAACTGCACCATCACA	14:T>C
6045482|F|0-17:A>G-17:A>G	TGCAGCCCAGCCTTGGAACTCCTTGACGTCGCCTTCTTGGCCGCGCCG	17:A>G
100302122|F|0-17:G>A-17:G>A	TGCAGCCTCCGTTGCTTGGAGCTCCG	17:G>A
100528469|F|0-21:G>A-21:G>A	TGCAGTTCATGGACCTCTCCAGCAACTCCTTCTCGGGCCAAATCCCCGCTGCCAAGGCAAGTCACAACT	21:G>A
3024864|F|0-19:T>C-19:T>C	TGCAGCACCTCGCGGTGTCTTCCTCCGCTCCTGGGCCCG	19:T>C
100486745|F|0-22:T>C-22:T>C	TGCAGCGCCAGTGCGAGTACCATGCCATCCTCTGCTCCAACCCGCACCCCAACCACGGCGAACGCCACG	22:T>C
4988312|F|0-53:G>C-53:G>C	TGCAGCGACACCTGCCGCTCCATCTGGATGTGCTTGTAGAAGTTGGCGATGAAGAGCTCCGCGCGCCGA	53:G>C
1030427|F|0-22:G>A-22:G>A	TGCAGGACCAGATCACGTACAAGGTTGGCAAGCACTACGCCGACTGCCCCAGCTTCCG	22:G>A
1020726|F|0-61:C>T-61:C>T	TGCAGGCCAAGGACCAGATCGGCACCTACTTCTACTTCCCCTCACTCGCCTTCCACAAGGCCGCCGAGA	61:C>T
2242432|F|0-12:G>A-12:G>A	TGCAGGTGCCAAGGTGAGGCTGGAGTGCAAGCGCTTCGGCACTGAGAAAGTTGAGCGCTCGATCGACGG	12:G>A
1027981|F|0-21:G>A-21:G>A	TGCAGTGATCGGCATGGAGGGGCTCGCGAGAGGCTTCGCCAACCCGCCGTCGGACTGGAGCGGCGATCC	21:G>A
4994507|F|0-35:C>G-35:C>G	TGCAGGGTGAGGTACGAGATCAGGTTCGACCCGAACCCCACCGTCGCCAGCCG	35:C>G
1046898|F|0-15:G>A-15:G>A	TGCAGCTCTCTCCACGGTGGTATTACAAATCTCATGTATTTTAGCTCAGCGCTGGAAAACGAAACAATA	15:G>A
1215407|F|0-7:G>C-7:G>C	TGCAGGCGGACTCGCTGGAGCGGCAGGCCTACGGCTTCAAGAACGACGGCCTCAACTTCACCG	7:G>C
1020726|F|0-6:C>T-6:C>T	TGCAGGCCAAGGACCAGATCGGCACCTACTTCTACTTCCCCTCCCTCGCCTTCCACAAGGCCGCCGAGA	6:C>T
3949342|F|0-15:C>G-15:C>G	TGCAGCCACCAGGGGCGCATGATCCG	15:C>G
4440233|F|0-7:A>C-7:A>C	TGCAGGCAGCAATTTCATGGGCCG	7:A>C
1016289|F|0-5:G>A-5:G>A	TGCAGGATGCAGGCAATGCCCAAGAGCAGGACAAGGCAAGGCATGGAAGAAGTCATATTGATTGATTAA	5:G>A
1018009|F|0-25:C>T-25:C>T	TGCAGTACTGGATGGACGAGAACTACGTCTACAATTGCTTCGCGAGCACCG	25:C>T
995959|F|0-44:C>G-44:C>G	TGCAGCAGGTTCCAGTGCCTGCTCGCCCACGTCTGGAAGAAAATCACGGCGGCGCGGGACCTGAAGCCG	44:C>G
32017761|F|0-25:T>C-25:T>C	TGCAGGCCGCGCTCGACTGGGCGTGTGGCCCG	25:T>C
3029342|F|0-25:C>A-25:C>A	TGCAGGTAGTCGCCGATGTTCACCACCAGTGCCCCCG	25:C>A
4262260|F|0-7:G>C-7:G>C	TGCAGAGGCCGAGCATGAGGCAGTTGTAGGTGAAGGTGTCCG	7:G>C
1081945|F|0-63:A>G-63:A>G	TGCAGGAGGCTGGTGAGGCACAAGAGCACGACAAGGTAAGGCGTGGAAGAAGCCATCTTGATTACTGTA	63:A>G
7171241|F|0-67:T>G-67:T>G	TGCAGGAGGCTCGTGAGGCACAAGAGCACGACAAGGTATGGCGTGCGAGAAGCCATCTTGATTACTGTA	67:T>G
1387672|F|0-7:T>C-7:T>C	TGCAGCTTGGAGATGAGTATCACCTCGTTCTTGAACTCCTCGCACCCCTGCCCG	7:T>C
4734153|F|0-14:C>G-14:C>G	TGCAGGTGGGTGCGCCGCGACGAGCCG	14:C>G
3029038|F|0-24:G>A-24:G>A	TGCAGGTCGGCCTCCGTCACGGCCGCCTTCCCCTTGAACGCCTCCCG	24:G>A
1020967|F|0-42:C>G-42:C>G	TGCAGATCTGCTCACCAGGGACTACACCTACGACCAGAAGCTCTCCGTCTCCACCGTCAGCTCCTCCGA	42:C>G
988075|F|0-38:G>C-38:G>C	TGCAGCAGCGTGCTGATGGTGGCGTTCACCAGCGAGCTGAGCGGCGACGGCCTGAAGCCG	38:G>C
4993716|F|0-10:G>C-10:G>C	TGCAGCGCGTGTTCTACGGCGACAACGCCG	10:G>C
989582|F|0-20:G>C-20:G>C	TGCAGGAAGCGGGTGAGCGGGAGCAGCATGGCCATGGCGAAGTGCTGCGGGATGAGCCACACGCTCATC	20:G>C
1030325|F|0-32:T>A-32:T>A	TGCAGGCGAGCAGGTACTCCAGGTCGCGCTCCTGCATTATGGTGTGGCAGATGCCGAGCTCCTGGAGGC	32:T>A
13879512|F|0-23:T>C-23:T>C	TGCAGATCCCGTCCAGAGTCGAGTGCACAGCCCG	23:T>C
1000560|F|0-44:G>A-44:G>A	TGCAGCATCTGGCGAGCACCCCAACGTTCATCAAGGGACGGCGGGACAAGCCGCCGATATGGCGCGGGT	44:G>A
3938500|F|0-11:G>C-11:G>C	TGCAGCCGCTGGCGCCACCGCAGGACCGACCCG	11:G>C
4537808|F|0-10:C>G-10:C>G	TGCAGGTGGCCCGCGCGCTCGAGTACCTCCACGACCG	10:C>G
3024102|F|0-38:G>T-38:G>T	TGCAGTTCCCATCTGACATCGCCACCGTTCTTCTCGCCGCCG	38:G>T
4995596|F|0-5:A>G-5:A>G	TGCAGATACTCGGGCTCGAGCGCGAACAGCACGGCGATGCCGTCGTCCCTGTCGGCGCCGCGCTGCGTC	5:A>G
5582517|F|0-17:G>T-17:G>T	TGCAGCGGCGTCGGCGTGTGGCCG	17:G>T
3025328|F|0-23:G>A-23:G>A	TGCAGGGAGCCCTGCCCCACCGTGCTCGTCACCAGGTCCG	23:G>A
3948919|F|0-11:G>A-11:G>A	TGCAGCCATGGGTTCTCGTGGTACATCCG	11:G>A
991905|F|0-23:G>T-23:G>T	TGCAGACCGTGGCCATGGCGCGGGAGGTGCACCAGCAGCGGTACCGCATGGGAGCCG	23:G>T
999511|F|0-32:C>T-32:C>T	TGCAGCAACTCACCCTCTGACAGCCCGAAGCTCTGGTAAAATGCCATTTTCCGCAGCCATGAGTCCCTC	32:C>T

993067|F|0-22:C>T-22:C>T	TGCAGCACGCGCACAGGATGCACTCGTACAGCCCGTCCAGCTTCCGCCGCTCGCCCG	22:C>T
1683032|F|0-25:G>C-25:G>C	TGCAGCTGCTGGGGCGCGGTGTACAGCAACATGACGGGCTACTGCTACCTCATGGACTACCCG	25:G>C
1075540|F|0-41:G>T-41:G>T	TGCAGCATCTTCTCGTCCGCGCCCTCCCGCGCCACGCAGTAGGTCTGGTTCGTCGTGTCGTTGGCCAGC	41:G>T
3028112|F|0-23:C>G-23:C>G	TGCAGGTACGCCAGGTCCTTGCCCAGGCATATGCGCGGCCCG	23:C>G
1028626|F|0-22:G>T-22:G>T	TGCAGTAGTAGAGGAAGGCGGAGGCGTTGCCAAGGCAGTTGGCGGTGGAGCCGTCCG	22:G>T
4261166|F|0-20:G>A-20:G>A	TGCAGCATCGCCGCGTCGGTGCGGAGGAAGAAGAAGTTTGTGAGCCCG	20:G>A
1075540|F|0-17:C>T-17:C>T	TGCAGCATCTTCTCGTCCGCGCCCTCCCGCGCCACGCAGTAGGTCTGGTTCGTCGTGTCGTTGGCCAGC	17:C>T
2242931|F|0-44:G>A-44:G>A	TGCAGCGTCACCTTCTGGAGGATCATCAAGAGGAAGTCGACGGAGGACTTCTCCG	44:G>A
7353436|F|0-11:A>G-11:A>G	TGCAGCGCACCATCGTCGCCG	11:A>G
1100627|F|0-58:G>A-58:G>A	TGCAGCCTCTGCATTCTGCCACGCTTAGCCTCCCTCAGCCTCTCCGCCTCCGATTCCAGCCTCGCGATC	58:G>A
983392|F|0-50:C>T-50:C>T	TGCAGCTTTTGCTTTCGGTACATAAATGATTTTGTCTTCAAAATGAAAGCCTACAGCCATGTGGACCGA	50:C>T
4404304|F|0-16:G>A-16:G>A	TGCAGGCGAAGTGGATGGGCGCGCTGCGCACGTCGTAGGAGCCCCG	16:G>A
983392|F|0-53:C>T-53:C>T	TGCAGCTTTTGCTTTCGGTACATAAATGATTTTGTCTTCAAAATGAAAGCCTACAGCCATGTGGACCGA	53:C>T
1055097|F|0-13:C>T-13:C>T	TGCAGTATCAGTACGCCAACTACTCGGCGAACTACCTCAAAGGTGGCAAGGGCAGCATCCG	13:C>T
5363986|F|0-22:C>T-22:C>T	TGCAGGTTCTTGACCTGTCGGACAACCAGCTCGCCG	22:C>T
7339594|F|0-26:G>C-26:G>C	TGCAGCGAGTCGGCTTCTTTCTCGTCGGCCATGAGGAGTTTCCG	26:G>C
3940300|F|0-23:A>G-23:A>G	TGCAGGACGCGGCGGAGGGGGCGAACCG	23:A>G
32022367|F|0-5:G>A-5:G>A	TGCAGGCGGGCGGAAACTCATCCG	5:G>A
32023331|F|0-19:C>G-19:C>G	TGCAGCGGTGGGAGCTGGTCATGACGGCGGCGAGGCGAGGCGACCTCCG	19:C>G
32025362|F|0-14:G>C-14:G>C	TGCAGGAAGTACGGGGTGCGGTGCCCGTGCCCCGACAGGTTCTTCGCCG	14:G>C
5367490|F|0-25:G>A-25:G>A	TGCAGTCGGAGCATCCCGAGAGCTTGCCG	25:G>A
3026968|F|0-28:C>A-28:C>A	TGCAGTACACGGCATTGAGCAAGCGAGCCAGAAGCGCACCG	28:C>A
1166299|F|0-28:C>T-28:C>T	TGCAGTTCATGGACCTCTCCAACAACTCCTTCTCGGGCCAAATCCCCGCTGCCAAGGCAAGTCACAACT	28:C>T
3955006|F|0-16:G>A-16:G>A	TGCAGAGGAAGGGGCCGCTGAACCG	16:G>A
5364137|F|0-22:C>G-22:C>G	TGCAGTTGAGTTATGGTGGCTTCTGTGATGCTGTCCTGCCTTTCCG	22:C>G
12773442|F|0-16:G>A-16:G>A	TGCAGCGCGAGGCAGCGGCCGATCTCCG	16:G>A
5580347|F|0-17:G>A-17:G>A	TGCAGCTTGGTGACGGAGACGATCCAGAAGGTGTCGCCG	17:G>A
3023370|F|0-31:A>G-31:A>G	TGCAGAGAGCATCACCGCCGAGAAATCAACAAGCCACCG	31:A>G
4006219|F|0-8:T>A-8:T>A	TGCAGTAATTTATCATTTTAATCCGTGGTTGACAAATGGGCCG	8:T>A
3022428|F|0-26:G>C-26:G>C	TGCAGCAGGTTCTTCCACGGGGATCTGAAGTCTGGTCCG	26:G>C
5357999|F|0-17:G>A-17:G>A	TGCAGCTCGCGCTCCCAGTCCTCCG	17:G>A
1056670|F|0-22:C>G-22:C>G	TGCAGGCCATGGCGAAGCTGGTCGCCCTCTCCCCCTGCCTCGCGGAAGCCGCCATCTCCG	22:C>G
4261325|F|0-12:C>T-12:C>T	TGCAGGTGCTCGCGCATGCGGAGCCAGTCGTGGACGAAGTCCG	12:C>T
4262208|F|0-8:C>T-8:C>T	TGCAGCGCCTCCACGGCAAGCGTGTCGGAGAGGACGAAGACCG	8:C>T
1087713|F|0-13:T>C-13:T>C	TGCAGCACATGCGTGGCCATCGCACAGCAGCGATTCGTTGGCAAGGCGTCCG	13:T>C
3940186|F|0-25:T>C-25:T>C	TGCAGAATTCCTGGAACCAGGATCCTGCTAACCG	25:T>C
3020630|F|0-16:A>G-16:A>G	TGCAGGTATTTGCAGCACAGTCAAACTTGTGGCACACCG	16:A>G
3026445|F|0-17:G>C-17:G>C	TGCAGGGACAGCTCGTCGGAGTGCAGCGATGACGGTGCCTCCG	17:G>C
7164704|F|0-25:G>A-25:G>A	TGCAGAACGAGTACCCATCTGACCTGCACCTTCTTGTGACCG	25:G>A
1019857|F|0-65:C>A-65:C>A	TGCAGCACGCTGAGGTTGTTGTTGGCCTTCACCGCGTACCCCACGATTGAGCGGAGCCCCTGGAGCGCC	65:C>A
1759701|F|0-25:G>A-25:G>A	TGCAGGTGTACCACGACACATACGCGCTGGTTGTCAGCACGGAGAATATCACCCTAAACGGGTACTTGG	25:G>A
32017960|F|0-14:A>G-14:A>G	TGCAGCTTCGACATATCTCCG	14:A>G
1036567|F|0-5:T>A-5:T>A	TGCAGTAGAACCAGAGGGCGAGCTTGACGGCGGTGGCCGAGAGCATGATGGAGTAGAGCCATGTCAGCT	5:T>A
1863576|F|0-38:G>A-38:G>A	TGCAGCAGGATGGCGAGGATCTCGGAGGCGTACTGCTTGTTGGCCTCGAAGTCGCGGGCCTTGACGCGG	38:G>A
5580150|F|0-16:C>A-16:C>A	TGCAGCACCAGCTCCACGTCGGTGGGGTAGAACCG	16:C>A
1024202|F|0-41:A>G-41:A>G	TGCAGCTCGTAGAGCACGGCGCCGTTGGCTGGCTCGGCCGAATTGTACACGTACCCTCCG	41:A>G
1082071|F|0-36:G>A-36:G>A	TGCAGACGATGCAGAAGAAGAGGTGAACGACCACCCGATCGAGGAGGTCCG	36:G>A
2242065|F|0-50:C>A-50:C>A	TGCAGATAGTGTTCGAGATGATATCGATCGGAACCCTGCTGGCCTTCTACCTGGTGGCCAACGCGCTCA	50:C>A
1019857|F|0-47:T>G-47:T>G	TGCAGCACGCTGAGGTTGTTGTTGGCCTTCACCGCGTACCCCACGATTGAGCGGAGCCCCTGGAGCGCC	47:T>G
5580340|F|0-5:C>T-5:C>T	TGCAGCTTCTCGCAAGAACCGAGCTCCG	5:C>T
3023022|F|0-11:G>T-11:G>T	TGCAGCTGCTCGATGCCTTCGCCGTGCCACAGTATCCG	11:G>T
4406738|F|0-5:G>A-5:G>A	TGCAGGTCATGGCGAGCCGCCGCGTCGTGGCACCCG	5:G>A
5346749|F|0-6:T>C-6:T>C	TGCAGGTGTTCCTCCTCTTCACCG	6:T>C
3934569|F|0-5:C>T-5:C>T	TGCAGCCTCCGCGAAGAAGTCCG	5:C>T
3938500|F|0-5:C>G-5:C>G	TGCAGCCGCTGGCGCCACCGCAGGACCGACCCG	5:C>G
3935849|F|0-5:C>G-5:C>G	TGCAGCATGAGCATCAGCACAGCACATGCTTGGAGTGGAAGGCCG	5:C>G
1028626|F|0-33:G>T-33:G>T	TGCAGTAGTAGAGGAAGGCGGAGGCGTTGCCGAGGCAGTTGGCGGTGGAGCCGTCCG	33:G>T
3942506|F|0-14:G>C-14:G>C	TGCAGGTGGTGGCTGCCAAACTCCG	14:G>C
3936290|F|0-20:C>A-20:C>A	TGCAGGCCGACGAACGCGAACACGCCG	20:C>A

6045431|F|0-7:C>G-7:C>G	TGCAGCACAAGTTTCTCGATCTCGCCG	7:C>G
1036388|F|0-41:T>C-41:T>C	TGCAGATCATACATACACGTAATATAATGGCGTAAAGTTGTTGCAAACCCAACGAGCTCCCCTGGAATG	41:T>C
1029718|F|0-44:G>A-44:G>A	TGCAGCTCGGCCGCGGAGATGAACCCGTCGCCGTTCTCGTCGAAGACCCG	44:G>A
3945159|F|0-15:G>C-15:G>C	TGCAGCTCGGAGCAAGGTGCAGGGCTTCCTGGAGGCCGCGTGCTCCG	15:G>C
5011091|F|0-29:G>T-29:G>T	TGCAGCTTGTCGATGGCCTGGAGCAGGAAGGGCCG	29:G>T
4261945|F|0-11:T>C-11:T>C	TGCAGGTAGGCTAGGTCCTTGCCCAGGCACACCCTCGGCCCG	11:T>C
3936039|F|0-11:G>A-11:G>A	TGCAGGCTGAGGTGGATCAACTACCTGAGGCCG	11:G>A
3024125|F|0-7:C>T-7:C>T	TGCAGTTCCCATCCGACATCGCCACCGTTCTTCTCGCTGCCG	7:C>T
3938500|F|0-9:T>G-9:T>G	TGCAGCCGCTGGCGCCACCGCAGGACCGACCCG	9:T>G
3945927|F|0-31:G>A-31:G>A	TGCAGACGAGTTGCATATGGTCATTACGTGCGTTCGGCGTCAACGACCG	31:G>A
4261573|F|0-19:G>C-19:G>C	TGCAGTGGTAGCACCAGTAGGAGACCG	19:G>C
3949503|F|0-13:A>T-13:A>T	TGCAGCGGCGACGACGACCG	13:A>T
3957524|F|0-37:T>G-37:T>G	TGCAGGCAGCGACCTAACTCTCGCAGCGCCTGACAAGTTAACCG	37:T>G
1053202|F|0-53:G>A-53:G>A	TGCAGTCGTTGTGCCATGATCACGTTCCCGCCGACGGTCGCCGTGTTTCGGACGAACGGCGAGGCCACC	53:G>A
4394631|F|0-11:T>A-11:T>A	TGCAGAATATATTCTGGCCGAAAGCTTCCG	11:T>A
4988139|F|0-15:C>A-15:C>A	TGCAGCTGCACGTCACGCAGCCCGCCG	15:C>A
7169643|F|0-42:C>G-42:C>G	TGCAGGAGCGCCCGAGGTTCAACTCGATCCACATGGGCGTTTCCCGAATCCG	42:C>G
1018677|F|0-41:G>A-41:G>A	TGCAGCATTATCGGTGAGCGCGGATTAGGGCGCCAGCTTATGCTGGAGACCACGGGGAGGTGCGATGCA	41:G>A
4439707|F|0-7:G>C-7:G>C	TGCAGAGGAAGGGTCCGCTGAACCG	7:G>C
3941390|F|0-20:C>T-20:C>T	TGCAGGAAGGGCTTGAGGAACGACCAGCCG	20:C>T
4261408|F|0-22:G>C-22:G>C	TGCAGAACTTGACGAAGCTGCTGCTGCTGAGCAACGAGCTCTCCG	22:G>C
3945561|F|0-17:C>G-17:C>G	TGCAGGCGAAGGATCATCGTCATGGCCTCGCTCGCGGCGCCG	17:C>G
985897|F|0-16:G>C-16:G>C	TGCAGGCCGCCGCGCTGCTCTGCTTCGTCGCGCTCTCCCTCGTCTCCGCCTTCCG	16:G>C
1202383|F|0-13:G>C-13:G>C	TGCAGGACAATGCGGACCACGGCGACAAGTCCGAGGAGACCACGCCGCGGCCGAAGCTGAAGCCCCTGC	13:G>C
996110|F|0-33:A>T-33:A>T	TGCAGACGGCGCGCTGGACGTCGCGGAGGATGGACTCCTTGAGGCTCTCCG	33:A>T
3026943|F|0-7:T>C-7:T>C	TGCAGACTCCCCTAATAGACCTCTCCGCCATATTCACGCGCCG	7:T>C
1056670|F|0-9:T>G-9:T>G	TGCAGGCCATGGCGAAGCTGGTCGCCCTCTCCCCCTGCCTCGCGGAAGCCGCCATCTCCG	9:T>G
996110|F|0-7:G>T-7:G>T	TGCAGACGGCGCGCTGGACGTCGCGGAGGATGGACTCCTTGAGGCTCTCCG	7:G>T
1322114|F|0-17:C>A-17:C>A	TGCAGCCTTTAAGTATCCAAAGTTCTCGAACTTAGATGATGCCCTTAAATGGTATAACGAACACCAGAC	17:C>A
1018677|F|0-47:G>A-47:G>A	TGCAGCATTATCGGTGAGCGCGGATTAGGGCGCCAGCTTATGCTGGAGACCACGGGGAGGTGCGATGCA	47:G>A
1018677|F|0-39:A>T-39:A>T	TGCAGCATTATCGGTGAGCGCGGATTAGGGCGCCAGCTTATGCTGGAGACCACGGGGAGGTGCGATGCA	39:A>T
1023297|F|0-28:C>T-28:C>T	TGCAGTACCTGTCCAAGGAGGAGGCCGCCGTCCACGCGCGGATGCGCCGCCG	28:C>T
2242432|F|0-45:G>C-45:G>C	TGCAGGTGCCAAGGTGAGGCTGGAGTGCAAGCGCTTCGGCACTGAGAAAGTTGAGCGCTCGATCGACGG	45:G>C
4261824|F|0-26:C>T-26:C>T	TGCAGCTGCCGCTCCATCGTCAGCGCCGACATGGTCCG	26:C>T
991905|F|0-13:C>G-13:C>G	TGCAGACCGTGGCCATGGCGCGGGAGGTGCACCAGCAGCGGTACCGCATGGGGGCCG	13:C>G
4262604|F|0-10:C>T-10:C>T	TGCAGTCCGCCACTGCTCCG	10:C>T
4993747|F|0-13:C>G-13:C>G	TGCAGAGGCCGCTCAACAGCTCGGGCAACCG	13:C>G
1012307|F|0-11:C>T-11:C>T	TGCAGGAATCTCCAGATGGAGTACGTGGACCTGTACCTCGTCCACTGGCCG	11:C>T
1087023|F|0-41:G>T-41:G>T	TGCAGGGACACCGTCATGACCATCTGGCATTACCACGGCGGGTGCCAGGTCGGCAGAGTCGTCGACCGC	41:G>T
3952078|F|0-11:G>C-11:G>C	TGCAGCGAATTGAACGCCAACCG	11:G>C
1035361|F|0-57:G>C-57:G>C	TGCAGCCCACACCTTGGGGCCCTCCATGGCGAAGGCGAAAGGGAGGAGGATCACCAGGGACAGCATGGA	57:G>C
3025083|F|0-5:C>G-5:C>G	TGCAGCCCCCTGGGGATGGCAGTCTGGCAGCACCCTATCCCG	5:C>G
3935308|F|0-29:G>A-29:G>A	TGCAGGTGCTCGCGCATGCGGAGCCAATCGTGGACGAAGTCCG	29:G>A
7492208|F|0-16:C>G-16:C>G	TGCAGGTGTGGAGCGCCGCCG	16:C>G
4022553|F|0-14:A>T-14:A>T	TGCAGTGCGCGCTCAGCCTCATGCCGTCGAACGCCGCTGCGCCGCAGCTCGACTGCCTCAACATGCTCA	14:A>T
5000091|F|0-45:G>T-45:G>T	TGCAGGCGGGCGAAGGCGATGTCGATGGGGAGGCTGCGGATCTCCGCCG	45:G>T
3940596|F|0-14:C>T-14:C>T	TGCAGGTGACGTGGCTGCCCGCGTCCG	14:C>T
7153469|F|0-20:T>G-20:T>G	TGCAGAGCCCGAGAGCTGATTGGAAGCGTGGATCCAGTGGACCGCCG	20:T>G
1087023|F|0-14:C>G-14:C>G	TGCAGGGACACCGTCATGACCATCTGGCATTACCACGGCGGGTGCCAGGTCGGCAGAGTCGTCGACCGC	14:C>G
4262859|F|0-22:G>A-22:G>A	TGCAGCCTTTGGCGGTGGCGCAGACGAACTCGTCGCCG	22:G>A
1022281|F|0-40:C>T-40:C>T	TGCAGTCGCAGAGCCTCATCATGCCGCTGCTCCTGTCGTCCGTGGTCACGCTGGCGCTCCACGTCCCCC	40:C>T
1019670|F|0-36:A>G-36:A>G	TGCAGCAAGGACATGCTCACCCTTTTCTGATATGGAATACTCAACAAGTGGGTCTGCCCG	36:A>G
5583061|F|0-10:C>T-10:C>T	TGCAGGCGCTCGCCAGGATGCCG	10:C>T
5325226|F|0-13:G>A-13:G>A	TGCAGACCTGCGTGCTCGACACCCG	13:G>A
3020650|F|0-5:A>G-5:A>G	TGCAGAGATGACACGTGGGAAAGATGCAACCTTTTGAGCCG	5:A>G
3952138|F|0-15:G>T-15:G>T	TGCAGGCGCGGTTCTGGGCCGCGTCCGCCG	15:G>T
3947365|F|0-7:G>A-7:G>A	TGCAGGGGCTCCAGGGGTACGTGCAGAACGTCAGGTTCCTGGCCG	7:G>A
5562969|F|0-52:C>G-52:C>G	TGCAGAGCGGCAGGTCGGCGGGGAGCGCCTCCAGGCTGAGGTTGGCGTCCCTCAGCGCGAACACCACCT	52:C>G
3026968|F|0-7:C>G-7:C>G	TGCAGTACACGGCATTGAGCAAGCGAGCCAGAAGCGCACCG	7:C>G

<table>
<tr><td>3027093|F|0-8:G>T-8:G>T</td><td>TGCAGCGCGAGCTTGAGCGTGTCCATGAGCTCGTCCCCCTCCG</td><td>8:G>T</td></tr>
<tr><td>6037450|F|0-18:A>G-18:A>G</td><td>TGCAGATGCGGAGAAGGGATTGGGTACTGCAAAGTACCCG</td><td>18:A>G</td></tr>
<tr><td>3940515|F|0-29:T>G-29:T>G</td><td>TGCAGCCACTGTCGACAACGACGGTGGCTTCCG</td><td>29:T>G</td></tr>
<tr><td>6047584|F|0-12:G>A-12:G>A</td><td>TGCAGCTCCAAGGACTCCAGCAAGTACATGTTTCCACCTGACACCTTTCTTGGCCG</td><td>12:G>A</td></tr>
<tr><td>6048922|F|0-25:C>A-25:C>A</td><td>TGCAGGCACCCATATCGTTCCGCCTCACCTCCG</td><td>25:C>A</td></tr>
<tr><td>100505260|F|0-46:A>C-46:A>C</td><td>TGCAGTTTTGCTAGACTTAATATGTTGCTAGAAAGTTGCGGTTAACAGTAAGTAGAAGTCAAGGCAGCT</td><td>46:A>C</td></tr>
<tr><td>100557051|F|0-39:A>T-39:A>T</td><td>TGCAGCCTGGGGTTGACGATGGCGATCGAGCCCCACGAGAGGCGGTCCGTGGCGGGCCATGCCGCCGCC</td><td>39:A>T</td></tr>
<tr><td>2276703|F|0-41:G>A-41:G>A</td><td>TGCAGAGAGGCAAGCGAATCGACAGCGCCCTTTGCGAACCAGCTGATTCCAACCTTCACCCG</td><td>41:G>A</td></tr>
<tr><td>5007341|F|0-57:A>T-57:A>T</td><td>TGCAGCTTGTGCAAGAATATTGTACCGAGAAGTGGTTGTTGCAGGGGTGCTAGATGCAAAGCCGTCTGG</td><td>57:A>T</td></tr>
<tr><td>1092464|F|0-5:A>C-5:A>C</td><td>TGCAGAAGCCGCGCCCACTGCTCCTCGTTGTCGCTCCCGAAGTTCTTCTTCGTCTCCTCCACCGCCAGC</td><td>5:A>C</td></tr>
<tr><td>6048922|F|0-7:A>G-7:A>G</td><td>TGCAGGCACCCATATCGTTCCGCCTCACCTCCG</td><td>7:A>G</td></tr>
<tr><td>4993734|F|0-20:G>T-20:G>T</td><td>TGCAGCGACTCCACCGCGCGGGCCACCTCCG</td><td>20:G>T</td></tr>
<tr><td>32021311|F|0-19:T>G-19:T>G</td><td>TGCAGATTGTGGCGCTAGGTGTTCCG</td><td>19:T>G</td></tr>
<tr><td>4733618|F|0-9:A>C-9:A>C</td><td>TGCAGGCTGAGGTGGATAAACTACCTGAGGCCG</td><td>9:A>C</td></tr>
<tr><td>5361483|F|0-48:G>C-48:G>C</td><td>TGCAGGCGGTTGGCCTGCGCGGTGATGGCGTCGATGATGTCGGGGTGGGTGTGGCCGCAGCACACGGTG</td><td>48:G>C</td></tr>
<tr><td>3024682|F|0-15:C>T-15:C>T</td><td>TGCAGAACAAGAACACCCAGATGATCAACCTGCGGATCAACGCGCCG</td><td>15:C>T</td></tr>
<tr><td>2247862|F|0-43:C>T-43:C>T</td><td>TGCAGTGCGCGCCCTGCCTCGACTGCTTCGAGCAGAGCGGGCCCATCTTCGACCCG</td><td>43:C>T</td></tr>
<tr><td>3936395|F|0-25:G>C-25:G>C</td><td>TGCAGTCCTGCTTCCTCTTGGACACGTTCCTCCTCTTGTCCG</td><td>25:G>C</td></tr>
<tr><td>985564|F|0-43:T>C-43:T>C</td><td>TGCAGGACCTGGAGAAGCAGCTGCTCGGCGACGACGACGAGGCTGAGCCG</td><td>43:T>C</td></tr>
<tr><td>1017157|F|0-13:G>A-13:G>A</td><td>TGCAGACGAAGAAGCAGTTCATCTACGGCAGCGTCTCCACCCTCATCAAGCTCGTTCCG</td><td>13:G>A</td></tr>
<tr><td>1088578|F|0-29:C>T-29:C>T</td><td>TGCAGGCTCTGCCACTTGAGCACCCACAGCGGGATGTGCTGCATCAGAGGGTGCACGCTGATGATCACG</td><td>29:C>T</td></tr>
<tr><td>3937614|F|0-13:G>C-13:G>C</td><td>TGCAGCATGTGCTGTCCTTCTTGCCG</td><td>13:G>C</td></tr>
<tr><td>6041941|F|0-10:G>C-10:G>C</td><td>TGCAGTCCACGGTCTACATCAACTCCG</td><td>10:G>C</td></tr>
<tr><td>1085168|F|0-34:C>A-34:C>A</td><td>TGCAGTACGTGTACTGGTCCAACGGGCGGGAGACCGAGAGCCCCAGCGTGGACAACAAGCAGTGCCCCG</td><td>34:C>A</td></tr>
<tr><td>1036567|F|0-37:C>G-37:C>G</td><td>TGCAGTAGAACCAGAGGGCGAGCTTGACGGCGGTGGCCGAGAGCATGATGGAGTAGAGCCATATCAGCT</td><td>37:C>G</td></tr>
<tr><td>1023297|F|0-6:A>G-6:A>G</td><td>TGCAGTACCTGTCCAAGGAGGAGGCCGCCGTCCACGCGCGGATGCGCCGCCG</td><td>6:A>G</td></tr>
<tr><td>5580387|F|0-20:C>T-20:C>T</td><td>TGCAGGAGTTCGACACGATCCACGGCCCG</td><td>20:C>T</td></tr>
<tr><td>3934569|F|0-6:C>A-6:C>A</td><td>TGCAGCCTCCGCGAAGAAGTCCG</td><td>6:C>A</td></tr>
<tr><td>5411820|F|0-5:C>G-5:C>G</td><td>TGCAGCAGCAGGATCCCGTCGACGGAGTCGAGGATGCAGTGGCTGCTGAAAAGCGGGAGCCG</td><td>5:C>G</td></tr>
<tr><td>3938500|F|0-17:C>G-17:C>G</td><td>TGCAGCCGCTGGCGCCACCGCAGGACCGACCCG</td><td>17:C>G</td></tr>
<tr><td>3026888|F|0-22:A>G-22:A>G</td><td>TGCAGTAACCGCATTAATGGGGAAAATTATTGCAGTCGACTGCGCCG</td><td>22:A>G</td></tr>
<tr><td>7940828|F|0-11:C>G-11:C>G</td><td>TGCAGCAGGTGCGCGCGCGCCCG</td><td>11:C>G</td></tr>
<tr><td>3030373|F|0-8:G>A-8:G>A</td><td>TGCAGGCCGAGGTCGCGCGCCAGCCGCGCCTTGCGCTCCG</td><td>8:G>A</td></tr>
<tr><td>1219338|F|0-25:G>C-25:G>C</td><td>TGCAGTACTTCGGCGAGTACCAGGGGAAGCTGCGCGCGCTGGTGGGCGCGTCCCAGGCGACGCAGATCG</td><td>25:G>C</td></tr>
<tr><td>1048758|F|0-25:C>G-25:C>G</td><td>TGCAGTGGCCCATCATCATCATCGGCCTCGCCGTCATGGTCGTCTCCCTCATGGGCTTCGCCG</td><td>25:C>G</td></tr>
<tr><td>4261618|F|0-13:G>A-13:G>A</td><td>TGCAGGCACGACAGGCGCCGCTGCCTCGCCATCCGCTTCTTCCG</td><td>13:G>A</td></tr>
<tr><td>7908954|F|0-10:G>A-10:G>A</td><td>TGCAGCCGCTGGAGTGGGGTCCG</td><td>10:G>A</td></tr>
<tr><td>7916389|F|0-10:G>C-10:G>C</td><td>TGCAGTACCTGAAACTGGTGATCAAGGAGGCGCTCCG</td><td>10:G>C</td></tr>
<tr><td>1123934|F|0-26:C>T-26:C>T</td><td>TGCAGGAGGTCGAGCACCTTCACCCGCTCCTCCATGGGGAGGCTCATGATCCGCCTCGCGCAGTCACGC</td><td>26:C>T</td></tr>
<tr><td>2247426|F|0-22:C>T-22:C>T</td><td>TGCAGGTGTCGTTCGACGAGTACCTCCAGGACCGCCCCCGCGTGTTCCGCGCCATGTTCCCCGACGAGA</td><td>22:C>T</td></tr>
<tr><td>1128525|F|0-50:C>T-50:C>T</td><td>TGCAGGCGGTCCATCTGTAGGGGATCCTGGTGGTGTTGGCGTGCATGGCCCTCTGCACGTCGAGCCGAG</td><td>50:C>T</td></tr>
<tr><td>7167968|F|0-24:G>A-24:G>A</td><td>TGCAGCTGGCTCGCTTTTGCTTGCGCCG</td><td>24:G>A</td></tr>
<tr><td>4993747|F|0-18:G>A-18:G>A</td><td>TGCAGAGGCCGCTCAACAGCTCGGGCAACCG</td><td>18:G>A</td></tr>
<tr><td>3951139|F|0-26:C>G-26:C>G</td><td>TGCAGCTCCTCCTTGTCCCGCTCCTTCTCCCAGCTCGCGAACCG</td><td>26:C>G</td></tr>
<tr><td>3942534|F|0-18:T>C-18:T>C</td><td>TGCAGCATAAACTCCTCCTCGTCGGCGCCTCCG</td><td>18:T>C</td></tr>
<tr><td>1088798|F|0-34:C>G-34:C>G</td><td>TGCAGCACAAGATGAGCGCCAAGGAGTCCGACACCTGGTCCAAGGTGCTCCG</td><td>34:C>G</td></tr>
<tr><td>2247426|F|0-37:C>G-37:C>G</td><td>TGCAGGTGTCGTTCGACGAGTACCTCCAGGACCGCCCCCGCGTGTTCCGCGCCATGTTCCCCGACGAGA</td><td>37:C>G</td></tr>
<tr><td>6038176|F|0-15:G>C-15:G>C</td><td>TGCAGGTCCGATCGTGGCGGTTATGCCATCGGCAGCGCTGGCTGCCACGCCG</td><td>15:G>C</td></tr>
<tr><td>7170747|F|0-22:G>C-22:G>C</td><td>TGCAGCTGTCCATCTGCGCCGTGGCCGCCG</td><td>22:G>C</td></tr>
<tr><td>6046159|F|0-36:G>A-36:G>A</td><td>TGCAGAAGGCGGCCCAGATGGGCGCGACGACGTCGAGTGGCTACGGCGTGAGCTTCACCCCTGGCCACC</td><td>36:G>A</td></tr>
<tr><td>5372976|F|0-38:T>C-38:T>C</td><td>TGCAGCGCGTCGAGGCCCGCCCGCATCACGGCGAACTCTTCGGCGGCGGCCCG</td><td>38:T>C</td></tr>
<tr><td>32022467|F|0-31:C>G-31:C>G</td><td>TGCAGCTGGAGGCGGCGGGGCGGAAGCGCCACGGGCCG</td><td>31:C>G</td></tr>
<tr><td>2247634|F|0-13:T>G-13:T>G</td><td>TGCAGGAACAGCATGGGCTCCGCCGACGGGCTCACCGCCTGCCGCAGCATCGACCCCATGATCAGCGAC</td><td>13:T>G</td></tr>
<tr><td>3028376|F|0-19:G>A-19:G>A</td><td>TGCAGATGAACTCGTACAGGTCCTTGACGGCCGAGATGAGCGCCG</td><td>19:G>A</td></tr>
<tr><td>1863696|F|0-12:C>T-12:C>T</td><td>TGCAGGGGGCTACGGAACACTCGAGGAGCTTCTGGAAGTAATCACGTGGGCCCAGCTTGGCATCCACGA</td><td>12:C>T</td></tr>
<tr><td>3029371|F|0-20:G>A-20:G>A</td><td>TGCAGGCCGTGACCCTCCAGGAACACCCCGTCCGCCG</td><td>20:G>A</td></tr>
<tr><td>6046283|F|0-28:T>C-28:T>C</td><td>TGCAGCGGGTCGTCAACTCCCTGGCTGTTGAGATGTTCGCTGGACTCGCGTCCGAGGACGCCAGCAAGG</td><td>28:T>C</td></tr>
<tr><td>1024899|F|0-7:G>A-7:G>A</td><td>TGCAGACGGTGCTCCTGTGGCAGAACCAGCTCGTCGGCACCATACCCCCG</td><td>7:G>A</td></tr>
<tr><td>1088798|F|0-21:A>G-21:A>G</td><td>TGCAGCACAAGATGAGCGCCAAGGAGTCCGACACCTGGTCCAAGGTGCTCCG</td><td>21:A>G</td></tr>
</table>

3940554|F|0-7:C>A-7:C>A	TGCAGCTCCTCGACCTCACCAACAACACGCTCACCG	7:C>A
1211371|F|0-19:G>C-19:G>C	TGCAGTACTTCTACGAGCTGGACGTGAGCAACAACAAGCTCGCTCGCTGCGCCTTCCCGACGGACGTGC	19:G>C
1132341|F|0-21:T>C-21:T>C	TGCAGCTGCCTCGAACGACTCTCCACGGCCAGGAGGCAAAGCCGTCCGTCCTCCG	21:T>C
4395906|F|0-8:G>A-8:G>A	TGCAGGCTGACGTGTGGAGCTTCGACCG	8:G>A
32020320|F|0-27:A>G-27:A>G	TGCAGGGGCGGCCTCAAGAAAGCAGGGATCAACGGCACCG	27:A>G
986776|F|0-61:C>G-61:C>G	TGCAGCCGAACTGGAATTCCTTGACCGTGTCCGAGCCCAGCGCCAGCGTGTCGGAGCCGTACGTGCCGA	61:C>G
1696619|F|0-26:C>G-26:C>G	TGCAGGTCCTGCGGGGTGTACATGGCCGCCATCTCGACTCGTTGCAATGCCGCCGCCGCTGCGAGGGAG	26:C>G
5580340|F|0-17:A>T-17:A>T	TGCAGCTTCTCGCAAGAACCGAGCTCCG	17:A>T
1088578|F|0-14:C>T-14:C>T	TGCAGGCTCTGCCACTTGAGCACCCACAGCGGGATGTGCTGCATCAGAGGGTGCACGCTGATGATCACG	14:C>T
997852|F|0-44:C>G-44:C>G	TGCAGCAGCAGCACGCCCAGAGAATAGATGTCCGACTTGACGCCCAGCTTGCCCGTTTGCTGGTACTCC	44:C>G
3027466|F|0-8:A>G-8:A>G	TGCAGTTGATCCTGCTCCTTGGGTGGTTCGGGAAGCTCGCCG	8:A>G
4439426|F|0-6:C>T-6:C>T	TGCAGCCTGCCGTTCCCGCTGAAGATCCG	6:C>T
3945031|F|0-22:T>C-22:T>C	TGCAGGAGGCAGAGCCTGTAGATCCG	22:T>C
1108835|F|0-58:G>C-58:G>C	TGCAGCAGCACCTAGGGCAGCCCGATATCAAGGTGAAAGAATCTCTCATCTGCCTCACGTTTTCCCTTT	58:G>C
988391|F|0-37:C>G-37:C>G	TGCAGGCCGCCGCGTCGGCGTGGTCCGTGCGCAGCGGCCTCGCCGTGCCCATGGCCAACAGCACCAAGC	37:C>G
1061569|F|0-46:G>C-46:G>C	TGCAGACGGCGCTGCGGGCCAGCGCCGCCAACCTCAAGCAGGACCTGTACGCCATCATCCAGGCGAGGC	46:G>C
1080037|F|0-13:C>T-13:C>T	TGCAGCACCCCAACGAGTACATCCGCGGCGTCACGCTGCGGTTCCTCTGCCGCCTCTCCGAGCCGAGAT	13:C>T
993664|F|0-8:G>C-8:G>C	TGCAGCCTGTCCAGCACGTTCTCCACGCCCACGTCCTCCCTCAGGCTCTCCTCGCAGGCCTTCTTCAGG	8:G>C
1012307|F|0-29:C>T-29:C>T	TGCAGGAATCTCCAGATGGAGTACGTGGACCTGTACCTTGTCCACTGGCCG	29:C>T
6049052|F|0-13:C>A-13:C>A	TGCAGCCACCCGTCCCTCGTCGCCGTGTACAGCGTCCCGCCCGCCGCAGCGTCGATGTAAACGTCCTCC	13:C>A
5366786|F|0-17:G>C-17:G>C	TGCAGACGGCCTACCGCGTCAACGTCGGCGGCCCG	17:G>C
1231121|F|0-10:T>C-10:T>C	TGCAGGTGGCTTCGAGGAACATTGGCGGCTGCTCGCCCAATTCCGACGGCCGCGCCGTGGACGCCGAGA	10:T>C
1021257|F|0-56:G>T-56:G>T	TGCAGCCTCTTCAACCCGACGCCCTCGCTCTCCGCCATGGTCGTCAACAAGTACAAGCTCCG	56:G>T
4733790|F|0-8:C>A-8:C>A	TGCAGGATCTCGACGACGAGGCCG	8:C>A
3022240|F|0-23:C>G-23:C>G	TGCAGTGGTTTCCCTGTTTGGCTCAAGTATGTCCCCG	23:C>G
3936528|F|0-23:A>T-23:A>T	TGCAGCTCCCTCCACATTTTCGCATCCG	23:A>T
999511|F|0-59:T>C-59:T>C	TGCAGCAACTCACCCTCTGACAGCCCGAAGCTCTGGTAAAATGCCATTTTCCGCAGCCATGAGTCCCTC	59:T>C
1090998|F|0-18:A>G-18:A>G	TGCAGAGAGGACTGAGGTAGTGTCTGCCTTTCGGAGCGACGGGAAGTGGTCCCCCCACACCACGAGGTC	18:A>G
3946784|F|0-12:C>T-12:C>T	TGCAGGAGTACGCGCAGAAAGCCG	12:C>T
1088113|F|0-68:C>T-68:C>T	TGCAGCGGCGTCGGCATCCTCGGGATCGCCTGGGCCTTCGGCGGCATGATCTTCGTCCTCGTCTACTGC	68:C>T
6044084|F|0-14:G>C-14:G>C	TGCAGGCAGCACGGGATATCGAGATGGCCATTCCG	14:G>C
1303748|F|0-15:C>G-15:C>G	TGCAGGTCGCGCACGCGGACGCGCAGCGCGTCGCCTCCGCCGTCACCCGCATCCTCGACGCTCACCCGA	15:C>G
1108835|F|0-48:T>G-48:T>G	TGCAGCAGCACCTAGGGCAGCCCGATATCAAGGTGAAAGAATCTCTCATCTGCCTCACGTTTTCCCTTT	48:T>G
1696973|F|0-31:G>A-31:G>A	TGCAGATGTTCTCGCCGCCGCTGATGATCACGTCCTTTGACCTGTCCTTTACCTCGATGTACCCATCCG	31:G>A
1025266|F|0-25:C>G-25:C>G	TGCAGGACCTGAGGGAGGCGATCGTCAAGGGGCTGGGGCTCCAGTCGGAGGAGCTCAAGGTGTCCGAGA	25:C>G
3022831|F|0-5:G>A-5:G>A	TGCAGGGGCTTCCATGTCACCTTCGTCAACTCCGAGTTCAACCACCG	5:G>A
1047731|F|0-39:G>A-39:G>A	TGCAGTGGGATCGGAGCAGGAGAAGAAGGCCGCCGCCACGGTGGAAGTGGAAGGCATCCCGTTCCCGCG	39:G>A
1698307|F|0-68:G>T-68:G>T	TGCAGCAGCTGCTGGTGCGACCTCTTGCAGTGGGCAATGGCGCCCTGGATGGCCCCCTCCTCCGACCCG	68:G>T
7167304|F|0-31:A>G-31:A>G	TGCAGTGGTTCAGCAAGAGGAAGAAGAAGGCACTGTTCGACGCCCTGGAGAAGGTTAAATCCG	31:A>G
4261408|F|0-8:T>C-8:T>C	TGCAGAACTTGACGAAGCTGCTGCTGCTGAGCAACGAGCTCTCCG	8:T>C
993344|F|0-14:T>C-14:T>C	TGCAGCTTGGCCATTACCCGCGGGTTTCCGATGAGCTCCGCCATGACCCATTCCAGCGTGATAGATGAC	14:T>C
4261350|F|0-18:G>A-18:G>A	TGCAGGAGTACTTCGACAGGGTGTTCGAGGCGTCCGCCG	18:G>A
1047731|F|0-18:G>C-18:G>C	TGCAGTGGGATCGGAGCAGGAGAAGAAGGCCGCCGCCACGGTGGAAGTGGAAGGCATCCCGTTCCCGCG	18:G>C
6048495|F|0-22:G>A-22:G>A	TGCAGTTCTTGCAGCACACCCAGCCCATCCTCCACGAGGTTCAGGACCTGGCAGCGATGAAGCCCATCC	22:G>A
2252437|F|0-35:C>G-35:C>G	TGCAGCAGGAAGGGCGCCCAGTAGAACTCCACCGTCGCGTTGTACTCCCTGGCGGTGAACACGGTGTGC	35:C>G
1019045|F|0-35:G>A-35:G>A	TGCAGCAGCAGGTTGTGGTGCATCCCTAGCTTGTTGTAATCTTCCAGGCAGCCG	35:G>A
4003872|F|0-28:G>C-28:G>C	TGCAGTCGGAGGTGTTCTCGATGCAGACGGTGCAGCCG	28:G>C
1862702|F|0-62:G>A-62:G>A	TGCAGATATTGCCACCGAATGCCTGTCTCTCCCTTTCGAGAAAGGAGGAGCAAATCTCCTTGGATGTCC	62:G>A
1230214|F|0-8:C>T-8:C>T	TGCAGGCCCTGCACGCTGCCGTTGCCGTGGCAGGAGACGCCGTACCAGGAGGAGTGGTTGCCGTTGCAC	8:C>T
1036388|F|0-49:C>A-49:C>A	TGCAGATCATACATACACGTAATATAATGGCGTAAAGTTGTTGCAAACCCAACGAGCTCCCCTGGAATG	49:C>A
3957169|F|0-7:C>G-7:C>G	TGCAGCGCAGGTCGTGCAGCACGGCGAACACCTCCGAGAGGAGCCCCG	7:C>G
1033214|F|0-59:G>C-59:G>C	TGCAGCCGCTTCACCAAGCGGCTGTACAACTACAGCAGCACCGTGAAGCTCGACCCGTCGTTCAACCCG	59:G>C
6046824|F|0-7:T>G-7:T>G	TGCAGGCTCTGGTGCGCGGCCACCTCGTCCG	7:T>G
1061650|F|0-5:G>C-5:G>C	TGCAGGGTGAGCACGTCGGTCTTCATGTTGGGCTCGTTCTCCGCCACGCCCG	5:G>C
3029384|F|0-20:G>A-20:G>A	TGCAGGATGAGCCAGAGCGCGTAGAAGAAGCAGCTGCCG	20:G>A
1030325|F|0-52:G>A-52:G>A	TGCAGGCGAGCAGGTACTCCAGGTCGCGCTCCTGCATTATGGTGTGGCAGATGCCGAGCTCCTGGAGGC	52:G>A
1696619|F|0-30:A>C-30:A>C	TGCAGGTCCTGCGGGGTGTACATGGCCGCCATCTCGACTCGTTGCAATGCCGCCGCCGCTGCGAGGGAA	30:A>C
1696619|F|0-61:C>T-61:C>T	TGCAGGTCCTGCGGGGTGTACATGGCCGCCATCTCGACTCGTTGCAATGCCGCCGCCGCTGCGAGGGAG	61:C>T
4734209|F|0-17:C>A-17:C>A	TGCAGCCAGTTGCCGAACACCG	17:C>A

5366989|F|0-7:C>G-7:C>G	TGCAGCCCATCCGCGAGATCTCCG	7:C>G
4261197|F|0-16:G>C-16:G>C	TGCAGCAGGAGTACGAGCTGCTCTCCG	16:G>C
5411936|F|0-9:C>T-9:C>T	TGCAGAGGCCGCTCAATAGTTCGGGTAACCG	9:C>T
3934101|F|0-33:G>A-33:G>A	TGCAGGCCACCCAAATCCGCACCAATTTGGGCGGAAAATTGCACCG	33:G>A
3024682|F|0-24:T>G-24:T>G	TGCAGAACAAGAACACCCAGATGATCAACCTGCGGATCAACGCGCCG	24:T>G
6049064|F|0-23:A>G-23:A>G	TGCAGGAGTTCTTCGCCATGATCACCAAGGACTCGGCGCGGGCTTGCTACGGGCCCAAGCACGTCGAGG	23:A>G
3946784|F|0-13:G>A-13:G>A	TGCAGGAGTACGCGCAGAAAGCCG	13:G>A
2248694|F|0-6:C>T-6:C>T	TGCAGCCTCATCGAGATCGACGGCGTGGTGCACGAGTTCCAAGCAGTTCCG	6:C>T
3941841|F|0-21:G>A-21:G>A	TGCAGCACGGGCGTGGCGCCCGCGTCGTCCG	21:G>A
4440011|F|0-29:T>C-29:T>C	TGCAGCATGGGAGACGGCGGGGCATCGTCTACTACCG	29:T>C
986901|F|0-25:C>A-25:C>A	TGCAGAAGACGCCGTCCTGGTTCTGCGCCACGATGAACTCCGACCTCCCTCTCCTCCTCCG	25:C>A
7153469|F|0-28:T>C-28:T>C	TGCAGAGCCCGAGAGCTGATTGGAAGGGTGGATCCAGTGGACCGCCG	28:T>C
1029079|F|0-37:G>C-37:G>C	TGCAGGCCGTCCTGGCGCGCGCCCACGGCGAGATGCAGGACTCGTTCGTCTGGCTCTTCCAGCACATCT	37:G>C
1696619|F|0-64:G>C-64:G>C	TGCAGGTCCTGCGGGGTGTACATGGCCGCCATCTCGACTCGTTGCAATGCCGCCGCCGCTGCGAGGGAG	64:G>C
991905|F|0-42:A>T-42:A>T	TGCAGACCGTGGCCATGGCGCGGGAGGTGCACCAGCAGCGGTACCGCATGGGAGCCG	42:A>T
1017783|F|0-49:G>A-49:G>A	TGCAGTTGGTCTGCTGCTCCGCGAAGAAGGGCCAGCTGAGCATCGGCACGCCGTTAGAGATGCTCTCCA	49:G>A
4261218|F|0-20:C>T-20:C>T	TGCAGCTCGCGGTACGAGAACTGCACCG	20:C>T
32026035|F|0-34:A>C-34:A>C	TGCAGGAACGTGTTCCAGTCCCTGCGCTTCTGCGACTCGTACCTGCTCAGCTGCGCAGGCGGCTTAGGC	34:A>C
32026435|F|0-14:G>A-14:G>A	TGCAGGTAGCCGCCGCCGAACGTGGGAGGAGATGGCGTGGGAGAAGCATACAGCGTTGGGGGCGGTGAG	14:G>A
32024150|F|0-5:T>C-5:T>C	TGCAGTTCCAGCAGGTTGAGCCGCGGGAGGTAGAGCAAGCCG	5:T>C
2243499|F|0-67:G>T-67:G>T	TGCAGCTGCCCGAGTACCCCAACAAGGAGGAGCTCGACGCGGTCCTCAAGACGATCGAGACCTTCCCGC	67:G>T
5577089|F|0-10:G>C-10:G>C	TGCAGGAGGCGATGGAGTTCCTGAGGAGGGTGGAGACGCAGCTCAACTCCCTCTCCATCTCCG	10:G>C
5000611|F|0-14:A>G-14:A>G	TGCAGCAGCGCGCAAAAGACGCTCCCCCAGGCCCCCGCGCCCACGCCG	14:A>G
1090065|F|0-8:C>G-8:C>G	TGCAGGCCCCGCACGAACGCCACGGCGTACTTGCTCGCCGTGGTGGGGTCCTCGCCG	8:C>G
3025772|F|0-16:C>A-16:C>A	TGCAGCTGCACACCACCAGGAGCCCCGACTTCCTGGGCATTGGCCCG	16:C>A
4993794|F|0-29:G>T-29:G>T	TGCAGCTGGTAGCTGAGGTGCAGGTCCTCGCCG	29:G>T
4009311|F|0-11:T>A-11:T>A	TGCAGCAGCCGTAGTGGAGGAAGACGCCGAGCGGCGGCAGGATGATGGCGAGCAGGATCTCGAGGAAGG	11:T>A
6048512|F|0-19:G>A-19:G>A	TGCAGAGAGAGATGCGTCAGATAGATTGAATTGCTGACGAGGAACAGCGCTGCACGGGGCCCGCACCGC	19:G>A
100554023|F|0-15:T>C-15:T>C	TGCAGAACAACAACATCACAGGGCGGCTGCCGCCG	15:T>C
3939446|F|0-51:C>T-51:C>T	TGCAGCTTCTCGACCTCAAGAGGGGCCACCACCATCTCGGCTCCCCCGTCGCCCCGCCGTTGGCTCTCC	51:C>T
4004083|F|0-14:G>C-14:G>C	TGCAGCTGGTAGCTGAGGTGGAGGTCCTCGCCG	14:G>C
3955240|F|0-5:C>G-5:C>G	TGCAGCGCCTGCTTGGCGCCGTCGAGCCCG	5:C>G
986901|F|0-31:G>A-31:G>A	TGCAGAAGACGCCGTCCTGGTTCTGCGCCACGATGAACTCCGACCTCCCTCTCCTCCTCCG	31:G>A
12470620|F|0-7:T>A-7:T>A	TGCAGGCTGAGGCACCCCCG	7:T>A
1694876|F|0-16:G>A-16:G>A	TGCAGCGGCAGTCGCAGGACGTCGACCTGGGCATGGTGGATGTCAAGATTCAGAAGCTCATGCCCGAGC	16:G>A
986787|F|0-62:G>A-62:G>A	TGCAGCCACACCGAGGCGAGGTTCCACAGCGCAAGCGCCACCGCGGAGGCCTGCGTGGAAACGAAGCTG	62:G>A
5582053|F|0-6:G>A-6:G>A	TGCAGGGATACGACGCGGTGGCCCTGTCCG	6:G>A
2247175|F|0-41:C>A-41:C>A	TGCAGCATCATCTCGATCTCCTTGACGACGTCGCTCATCGACGGTCGGGCGGCAGCGCCCTCCTCCACG	41:C>A
999436|F|0-46:C>G-46:C>G	TGCAGCCGTTCCTCAAGTTCCTGTCGGCGACGAGGGCCCCGTTCCTCATCAACTGCTACCCGTTCTTCG	46:C>G
4261408|F|0-28:G>A-28:G>A	TGCAGAACTTGACGAAGCTGCTGCTGCTGAGCAACGAGCTCTCCG	28:G>A
6050035|F|0-23:G>C-23:G>C	TGCAGCTCGTCGATGACGGCGCGGCCGCCG	23:G>C
4261325|F|0-28:C>T-28:C>T	TGCAGGTGCTCGCGCATGCGGAGCCAGTCGTGGACGAAGTCCG	28:C>T
3951895|F|0-18:G>C-18:G>C	TGCAGATGCCGCCCATGGGCATGCACGGCCACGGCGACGTGTACCCG	18:G>C
1864156|F|0-34:C>T-34:C>T	TGCAGAGCAACGCGCTCGCCGTCCTCGAGGCCGTCGACATGCCCGCCGCGGACCAGATCATGGACGCCG	34:C>T
1045227|F|0-54:C>G-54:C>G	TGCAGATCACAAGGCGCGGTGTTGTAACTGGTGCGACCAGGAAATGAGCTTGCACAGCTGTCCG	54:C>G
3937200|F|0-29:C>T-29:C>T	TGCAGCCCGAATAGCTCCTTCGGGATCGGCCCG	29:C>T
7173428|F|0-32:C>T-32:C>T	TGCAGGCGGCGACGACGACCTGGCAGAGGCTCCTGAGCGGCGAGCCG	32:C>T
5373676|F|0-13:T>C-13:T>C	TGCAGGGAGATGATGACGTAGATCTTCCG	13:T>C
1738362|F|0-64:G>C-64:G>C	TGCAGTTCGCGGTGCCGATGGTCCTCACCAACATGTCCTACTACGGCATCCCGCTGGTGTCCGTGATGT	64:G>C
986901|F|0-10:G>A-10:G>A	TGCAGAAGACGCCGTCCTGGTTCTGCGCCACGATGAACTCCGACCTCCCTCTCCTCCTCCG	10:G>A
1019320|F|0-64:C>G-64:C>G	TGCAGTGGGCGATGGCGGAGCTGATACGGAACCCGCGGGTTATGCGGAAGGCGCAAGACGAGGTCCGAG	64:C>G
1019320|F|0-27:G>A-27:G>A	TGCAGTGGGCGATGGCGGAGCTGATACGGAACCCGCGGGTTATGCGGAAGGCGCAAGACGAGGTCCGAG	27:G>A
5006275|F|0-14:A>G-14:A>G	TGCAGGTGATACTCAACGAGATTAGGAACCG	14:A>G
1085272|F|0-58:G>C-58:G>C	TGCAGCTGGAGCGCGTCAACGTCTACTACAACGAGGCCTCGTGCGGCCGCTTCGTGCCGCGCGCCGTGC	58:G>C
1864156|F|0-46:C>G-46:C>G	TGCAGAGCAACGCGCTCGCCGTCCTCGAGGCCGTCGACATGCCCGCCGCCGACCAGATCATGGACGCCG	46:C>G
3024682|F|0-23:A>C-23:A>C	TGCAGAACAAGAACACCCAGATGATCAACCTGCGGATCAACGCACCG	23:A>C
3026621|F|0-35:G>C-35:G>C	TGCAGCCCGTTGCTGGAGCTCAGCTGCCTCAGGAAGTGCTCCCCG	35:G>C
1047731|F|0-60:G>A-60:G>A	TGCAGTGGGATCGGAGCAGGAGAAGAAGGCCGCCGCCACGGTGGAAGTGGAAGGCATCCCGTTCCCGCG	60:G>A
2249233|F|0-5:G>C-5:G>C	TGCAGGTGTTCGACGCGATGATCAGGGAGGGACACCAGCCCGACGAGGCCGTGTACGTCGGCGTGCTCA	5:G>C

1020726|F|0-28:C>T-28:C>T	TGCAGGCCAAGGACCAGATCGGCACCTACTTCTACTTCCCCTCCCTCGCCTTCCACAAGGCCGCCGAGA	28:C>T
100415942|F|0-21:C>T-21:C>T	TGCAGCGAGGATGTTAGGGCGCCTCGCAGGCCCTCCG	21:C>T
1004053|F|0-68:A>G-68:A>G	TGCAGAGAGGGCACATCTCGCTCCCTTTGAGGGTGTCCGCCAGGCACGCGCCATGGAAGACGTGGGGCA	68:A>G
1089514|F|0-20:G>T-20:G>T	TGCAGGGGACCAGGTGGGCCGCCGCCGTGTCCGACGGCCGCAAGGCCGACCCG	20:G>T
5578244|F|0-8:C>T-8:C>T	TGCAGGTACCTCAGGAAATCCCCG	8:C>T
3934500|F|0-24:C>T-24:C>T	TGCAGCTGCTCATCCGCCACTTCTCCCG	24:C>T
1003778|F|0-43:G>C-43:G>C	TGCAGCCCCGCGCAGCGCGGCCGCCAGCGGCGCCAGCTCCAGCGCCACCCG	43:G>C
5361542|F|0-68:T>G-68:T>G	TGCAGGGCCTACTCCGCCGCGAACGTCAGCGGCGGGATCAACCGCGGGTGCGTCATCTGGGGCGTCGAT	68:T>G
1695387|F|0-67:C>T-67:C>T	TGCAGAACACCAACGCCGAGATCTCCTTCTCGTCGGGGCACCGTCCAAAGCCTGATGCGTTCGAGCTCT	67:C>T
2250319|F|0-27:T>C-27:T>C	TGCAGACGTCGATGGCGCACTCGATCTTGAGGCGGTCACCGAGGACGTACCGCGAGCCG	27:T>C
3029446|F|0-32:G>T-32:G>T	TGCAGCGTCTTCATCTGACCGACCTCTCGCGGGATCGCGCCG	32:G>T
4539688|F|0-14:G>A-14:G>A	TGCAGTTCCACCACGAGTCCG	14:G>A
4261697|F|0-14:G>C-14:G>C	TGCAGCGCGTACACGCGCGTGCCGCCGTCGAACCGCCGCGTCCG	14:G>C
2251731|F|0-39:G>T-39:G>T	TGCAGGAGGAGCAGGATGGTGGGCACGCTGTTCCCCCGCGCGTCGTGCGCGCACTGCATCGACTCCGTC	39:G>T
2251379|F|0-28:C>A-28:C>A	TGCAGGATGTCCCTGATCTGGTCGATCGCGTACCCTGCGCTGGGGTCTTCCCTCTCGACGCCATTGTGG	28:C>A
3025772|F|0-19:G>A-19:G>A	TGCAGCTGCACACCACCAGGAGCCCCGACTTCCTGGGCATCGGCCCG	19:G>A
1128424|F|0-5:C>T-5:C>T	TGCAGCGCGTGGGGCAGGAGTTCTTCGCACTGCCGCAGGAGGAGAAGCAGCGGTACGCGATGGACCCGA	5:C>T
1021513|F|0-51:G>C-51:G>C	TGCAGGACGCACGTTCTTGTACTGGTTCGGAGCACGGCCGACCCTGTGCGTGGCCGACGTGAGCATGGT	51:G>C
5562969|F|0-31:C>G-31:C>G	TGCAGAGCGGCAGGTCGGCGGGGAGCGCCTCCAGGCTGAGGTTGGCGTCCCTCAGCGCGAACACCACCT	31:C>G
100561590|F|0-46:T>G-46:T>G	TGCAGAGATCAACCAAGGGGTTCAAGGAGAAGCTGGGCGCGGGCGGTTTCGGCGCCGTGTACCGCGGCG	46:T>G
4002601|F|0-6:T>C-6:T>C	TGCAGGTGCTGCTTGCCATCGCGCTGTCGGAGAAGCCG	6:T>C
13877253|F|0-25:C>A-25:C>A	TGCAGGGGGCTCGCCGACATCAAGGCCATGGCGCCGACCGCGGTCCG	25:C>A
1047731|F|0-9:A>G-9:A>G	TGCAGTGGGATCGGAGCAGGAGAAGAAGGCCGCCGCCACGGTGGAAGTGGAAGGCATCCCGTTCCCGCG	9:A>G
1045227|F|0-49:T>C-49:T>C	TGCAGATCACAAGGCGCGGTGTTGTAACTGGTGCGACCAGGAAATGAGCTTGCACAGCTGTCCG	49:T>C
6048864|F|0-20:A>C-20:A>C	TGCAGAGTTAGGTGCAGGGCATGGGGTTGCTCGCCG	20:A>C
993664|F|0-23:C>A-23:C>A	TGCAGCCTGTCCAGCACGTTCTCCACGCCCACGTCCTCTCTCAGGCTCTCCTCGCAGGCCTTCTTCAGG	23:C>A
3939963|F|0-6:G>A-6:G>A	TGCAGCGCCCTCGCCGAAAGCGTGAAGCGCTGGGCGCCG	6:G>A
1206254|F|0-47:C>T-47:C>T	TGCAGCCGCTTCGGCTGGTGCGGCTCCACCTCGGACTACTGCGGCGACGGATGCCAGAGCCAGTGCTCC	47:C>T
994321|F|0-16:C>G-16:C>G	TGCAGCTCGCCGTGCTCTACGTCTCGCTGCTCTTCACGTCCCTCGGCTCCG	16:C>G
2251731|F|0-19:T>A-19:T>A	TGCAGGAGGAGCAGGATGGTGGGCACGCTGTTCCCCCGCGCGTCGTGCGCGCACTGCATCGACTCCGTC	19:T>A
3940554|F|0-21:A>C-21:A>C	TGCAGCTCCTCGACCTCACCAACAACACGCTCACCG	21:A>C
32021380|F|0-17:A>G-17:A>G	TGCAGCGAGAAGAGCTCAAGCGGCCCG	17:A>G
1023546|F|0-11:G>T-11:G>T	TGCAGCAGGGCGTTGTGCACCCAGGAGAGCGTGTTAAGGTCCTTGATGCAGCCGCTGCGCGGGCCGTAC	11:G>T
1089514|F|0-19:C>T-19:C>T	TGCAGGGGACCAGGTGGGCCGCCGCCGTGTCCGACGGCCGCAAGGCCGACCCG	19:C>T
4261077|F|0-20:G>A-20:G>A	TGCAGGTTGATGAAGTACTGGTTGTCGAAGGCGTGCGGCGTCACCG	20:G>A
4261077|F|0-35:C>T-35:C>T	TGCAGGTTGATGAAGTACTGGTTGTCGAAGGCGTGCGGCGTCACCG	35:C>T
1045227|F|0-21:T>C-21:T>C	TGCAGATCACAAGGCGCGGTGTTGTAACTGGTGCGACCAGGAAATGAGCTTGCACAGCTGTCCG	21:T>C
1204519|F|0-58:G>A-58:G>A	TGCAGCAGCTCTCCAGGGTTCTCGCCTCCCTGGGGTACAACGAGTTGGCGTCGGAGGCGCCGCTCCTCG	58:G>A
1037648|F|0-25:C>G-25:C>G	TGCAGACCATCAACGGCCCCAACTCCGACTTCCCCGTCTACTCGTCGCAGCTCAAGGAGCTCAAGGGCA	25:C>G
7911386|F|0-10:T>G-10:T>G	TGCAGCGGCATGGAGCTATCCG	10:T>G
4261582|F|0-10:C>T-10:C>T	TGCAGCGCGGCGTAGTGGTACCG	10:C>T
1863576|F|0-14:G>A-14:G>A	TGCAGCAGGATGGCGAGGATCTCGGAGGCGTACTGCTTGTTGGCCTCGAAGTCGCGGGCCTTGACGCGG	14:G>A
1020792|F|0-34:G>T-34:G>T	TGCAGGTCCCGTGGTACTTCCGTTGCCCAATCTCGCTGGAGCTCATGCAGGACCCCGTCACCGTGGCAA	34:G>T
7333805|F|0-10:T>C-10:T>C	TGCAGGAGGTTGCAGAGCGCCG	10:T>C
6048922|F|0-10:C>T-10:C>T	TGCAGGCACCCATATCGATCCGCCTCACCTCCG	10:C>T
3941518|F|0-12:C>G-12:C>G	TGCAGAGCGCGGCGTCCGTGCACCG	12:C>G
5371565|F|0-19:G>T-19:G>T	TGCAGACGGAGCTGAAGCCGACGCGGATGCCG	19:G>T
1694706|F|0-16:C>T-16:C>T	TGCAGGTCTGCTCCGCCGCGGACGACGGCCAGCTCGAGTGGAACGGGAACGACGACGGCGACTGCTGCT	16:C>T
991153|F|0-13:G>T-13:G>T	TGCAGCGCCAGATGCGTCTTGTCGGACCCCTTCTCCACCGTGAAGCTCACCG	13:G>T
5578244|F|0-17:A>G-17:A>G	TGCAGGTACCTCAGGAAATCCCCG	17:A>G
5347964|F|0-45:G>A-45:G>A	TGCAGGCGGGTTCGGGTCGACCTTGCTGGGTCTCAACAAGAAGACGTACGAGCTTGCCG	45:G>A
12470620|F|0-11:G>C-11:G>C	TGCAGGCTGAGGCACCCCCG	11:G>C
1084317|F|0-33:G>C-33:G>C	TGCAGGCGTGCGCCGTGTACCTGTTGCAGGTGAGCGTCACCTCGAACAGGCCG	33:G>C
1067770|F|0-7:G>C-7:G>C	TGCAGGCGAACATCAACTACGCGTGCTCCAACGTGAGCTGCGCCGTGATCCAGCCG	7:G>C
992169|F|0-32:G>A-32:G>A	TGCAGGCGTATGCGCCGCTAGGCAGGATGAAAGTCGTGGCGGATAACCCG	32:G>A
3951619|F|0-22:G>A-22:G>A	TGCAGATCCTGAACGTGCGGGTGGTGGGCACCG	22:G>A
1263707|F|0-36:T>C-36:T>C	TGCAGGTCCACGCACCCGTGCCCCAGCGGCGGCTGCTGCCCCCTCAGGCTCTTCTGCATCGGCAGGCAC	36:T>C
1095280|F|0-8:C>G-8:C>G	TGCAGGCGCCGCCGCGGCAGGACACGTGGGGGTTCTCGCCGCTGGATGCGCAGGCCGCGTACATGCCGA	8:C>G
3944859|F|0-26:C>T-26:C>T	TGCAGAACAACAACATCACGGGGCGGCTGCCGCCG	26:C>T

1128525|F|0-7:G>A-7:G>A	TGCAGGCGGTCCATCTGTAGGGGATCCTGGTGGTGTTGGCGTGCATGGCCCTCTGCACGTCGAGCCGAG	7:G>A
4003872|F|0-7:G>C-7:G>C	TGCAGTCGGAGGTGTTCTCGATGCAGACGGTGCAGCCG	7:G>C
4262498|F|0-52:C>G-52:C>G	TGCAGTTCCTGCTCAACCCCAAGTTCGTGGAGGCGGAGTGGTTCCTCCACGCCGCGCTGGGCCGCGGCA	52:C>G
1001439|F|0-35:G>A-35:G>A	TGCAGCTCACCGACCTCCAGCGGCAGGGCCTCGGCGGCGAGGAGAGCGGCGGCACGGGGTTCTCGCCGC	35:G>A
988391|F|0-5:G>A-5:G>A	TGCAGGCCGCCGCGTCGGCGTGGTCCGTGCGCAGCGGCCTCGCCGTGCCCATGGCCAACAGCACCAAGC	5:G>A
3947762|F|0-5:G>A-5:G>A	TGCAGGTACCTCTTGGTATTGGACCG	5:G>A
2276508|F|0-13:G>A-13:G>A	TGCAGGAGCGCCCGAGGTTCAACTCGATCCACATGGGCGTTTGCCAAGTCCG	13:G>A
1231121|F|0-24:G>A-24:G>A	TGCAGGTGGCTTCTAGGAACATTGGCGGCTGCTCGCCCAATTCCGACGGCCGCGCCGTGGACGCCGAGA	24:G>A
5582547|F|0-18:G>C-18:G>C	TGCAGGGGTTCACGTGGAGCATGCCGCCCG	18:G>C
4394806|F|0-20:T>C-20:T>C	TGCAGACGAGCGGAAGCTCATCCG	20:T>C
1219338|F|0-34:C>T-34:C>T	TGCAGTACTTCGGCGAGTACCAGGGGAAGCTGCGCGCGCTGGTGGGCGCGTCCCAGGCGACGCAGATCG	34:C>T
4262697|F|0-28:T>C-28:T>C	TGCAGCAGGATCTCGTTCCACGTGTCGATGGGGCCCG	28:T>C
1207119|F|0-16:C>G-16:C>G	TGCAGAAGGTTGTGCTCAAGCTGGACGTGCACGACGACAGGCACAAGGCCAAGGCGCTCAAGGCCGTCT	16:C>G
1234784|F|0-17:C>T-17:C>T	TGCAGCTTGGCCATTGCCTGTGGGTTCCCGACCAGCTCCGCCATGGCCCATTCCAGCGTGAGGGACGAT	17:C>T
4262113|F|0-8:A>G-8:A>G	TGCAGCTGATCCATGGGCGGATGGTGGACCATCTCTTCCG	8:A>G
3023419|F|0-32:A>C-32:A>C	TGCAGAGGTGCATGGACTCCAGCTCGCTCGTCAACACCTACCCG	32:A>C
32025637|F|0-7:A>G-7:A>G	TGCAGGGAGCTCCGCGCCGCGGCGGAGGGGCGCGACCAGCAGCAAGAGGGGGGCGACCACGCGGCGGGA	7:A>G
3953808|F|0-31:A>G-31:A>G	TGCAGCATGATACACCACCACCTGGTCCTGCACCCG	31:A>G
1021225|F|0-41:C>G-41:C>G	TGCAGGTCCTGCAAGTAGAAGGAAGGCTTGGTGGGGCGGAGCGCAAGGTAGACGACGAGCGCGATGAAT	41:C>G
1066408|F|0-37:C>G-37:C>G	TGCAGGACACCACGCTCGGCTCCCTGCTCTCCTCGCTCATGCAGCACTGCGACCCGCCGCAGCGCCGAG	37:C>G
3027857|F|0-35:A>G-35:A>G	TGCAGCTTCTTGACGATCTCGTACTTGTGCTCGGGAAAGACACCG	35:A>G
3027857|F|0-32:G>T-32:G>T	TGCAGCTTCTTGACGATCTCGTACTTGTGCTCGGGAAAGACACCG	32:G>T
3934648|F|0-23:T>C-23:T>C	TGCAGCTCTCCCCTCCTCGCCCATGCACAGACGCCG	23:T>C
986416|F|0-47:G>T-47:G>T	TGCAGCAGCTGCTGGTGAGACCGCTTGCAGTGGGCAATGGCGCCCTGGATGGCCCCCTCCTCCGACCCG	47:G>T
3026910|F|0-19:C>G-19:C>G	TGCAGCTCAAGGAATAGGTCGAGTGGCACTCGCACTAGCTTGCCG	19:C>G
1046269|F|0-46:C>G-46:C>G	TGCAGGACGTCTACGGCGAGGTGTTCCGCGTGCTCAAGCCAGGGGGCCTCTACGTCTCCTACGAGTGGG	46:C>G
1035766|F|0-22:G>C-22:G>C	TGCAGGAGTTCGTCAACCACGGGGGCGTCATCTTCAAGGTTTACGTCGTCGGCGGCCACGTCACCTGCG	22:G>C
1022719|F|0-13:G>C-13:G>C	TGCAGGGCACCGTGACGCCGTCGCGGGAGCGCGAGCTCGCCGCGTCCAAGCCG	13:G>C
1695138|F|0-27:A>G-27:A>G	TGCAGAAGATGTTCGAGGACCAGCTCAAGGCCAGCGGCAACACCGCGCCG	27:A>G
994321|F|0-7:C>T-7:C>T	TGCAGCTCGCCGTGCTCTACGTCTCGCTGCTCTTCACGTCCCTCGGCTCCG	7:C>T
1046269|F|0-28:C>T-28:C>T	TGCAGGACGTCTACGGCGAGGTGTTCCGCGTGCTCAAGCCAGGGGGCCTCTACGTCTCCTACGAGTGGG	28:C>T
2265833|F|0-47:G>T-47:G>T	TGCAGAGGCTGAACTTCGCCGTCGACAGGAGCCTTGCGGCTGACCACGCCGTCGGTCTCTGCGGGATCA	47:G>T
1075616|F|0-7:G>C-7:G>C	TGCAGTAGAGGTAGATAGAGAGATACTTGCCATTGTTGGCCTTCACCCACCCACGCGGGTAGCAATGTA	7:G>C
1055207|F|0-29:A>T-29:A>T	TGCAGGTTATTGACTCTGGTCAGAGTTATAGTGGTTATTCTATTCTTCACATGGCGCATGAGGCACCGA	29:A>T
3022831|F|0-11:C>T-11:C>T	TGCAGGGGCTTCCATGTCACCTTCGTCAACTCCGAGTTCAACCACCG	11:C>T
7164908|F|0-19:C>T-19:C>T	TGCAGATTCTGCTCTGCGTCCTGCCCCCG	19:C>T
32021463|F|0-18:C>G-18:C>G	TGCAGGCCGTTGATTATTCAATCGCCG	18:C>G
4261352|F|0-17:C>G-17:C>G	TGCAGGATGATGACGGCCCTGAGCCG	17:C>G
32017985|F|0-15:A>T-15:A>T	TGCAGCTCGCGGTCGAACCG	15:A>T
32017583|F|0-20:A>G-20:A>G	TGCAGAGCGTCGTAGGACACAACCG	20:A>G
7173791|F|0-19:A>G-19:A>G	TGCAGCTCGAAGAAGCCATATTGGATGCCG	19:A>G
7911378|F|0-15:G>A-15:G>A	TGCAGCCGCCGCGCAGGCCCG	15:G>A
32026546|F|0-5:T>C-5:T>C	TGCAGTAATCTATCATTTTAATCCGTGGTTGACAAATGGGCCG	5:T>C
2242355|F|0-65:A>G-65:A>G	TGCAGCTGCACATTACGGTCCTTGAACGCTGAATCATGGCCCAAGAAGTCGAAATCCTGACATGAAGCA	65:A>G
4993914|F|0-18:C>T-18:C>T	TGCAGGTTGTATCATGCACCGTCGCCCG	18:C>T
1034514|F|0-32:T>G-32:T>G	TGCAGCCAGGCGGGGGTGGTGAGGTTGTTGAATTGGTCGGCGATGCTGATGTAGGTGTCTCCG	32:T>G
2256826|F|0-21:C>T-21:C>T	TGCAGCTGAAACGCTCGGCTTCGAGGGCTACGCGCCGCCGTACCTGAGCCCGCTGGCGTCGGGGAAGAA	21:C>T
1023798|F|0-17:C>T-17:C>T	TGCAGGGGAGGTACGAGCTGGGGCGCGTGCTGGGGCACGGCAACTTCGGCCG	17:C>T
5562969|F|0-37:G>C-37:G>C	TGCAGAGCGGCAGGTCGGCGGGGAGCGCCTCCAGGCTGAGGTTGGCGTCCCTCAGCGCGAACACCACCT	37:G>C
1045189|F|0-22:G>C-22:G>C	TGCAGCAGCTCTCCGACGAGCTGCGCTCCGACGTCATCTTCCACGTCTCCAAGACCG	22:G>C
1046269|F|0-55:C>T-55:C>T	TGCAGGACGTCTACGGCGAGGTGTTCCGCGTGCTCAAGCCAGGGGGCCTCTACGTCTCCTACGAGTGGG	55:C>T
1021513|F|0-12:G>A-12:G>A	TGCAGGACGCACGTTCTTGTACTGGTTCGGAGCACGGCCGACCCTGTGCGTGGCCGACGTGAGCATGGT	12:G>A
3943304|F|0-9:G>A-9:G>A	TGCAGATGGGGACCAAGTTCAGGGTGCTCGAGGCGGACTTGGCGCGGTCGCGGTGGGCGAGGCTGAGGA	9:G>A
3943304|F|0-19:C>T-19:C>T	TGCAGATGGGGACCAAGTTCAGGGTGCTCGAGGCGGACTTGGCGCGGTCGCGGTGGGCGAGGCTGAGGA	19:C>T
5358448|F|0-15:A>C-15:A>C	TGCAGGTGACCTCGGACAGACCG	15:A>C
1049979|F|0-44:C>T-44:C>T	TGCAGGGCTTACTGCTACCAGCGAGGCTACGACCTGTTGAAAAGCTATTGCAAGCCG	44:C>T
3020650|F|0-11:C>G-11:C>G	TGCAGAGATGACACGTGGGAAAGATGCAACCTTTTGAGCCG	11:C>G
16662544|F|0-5:A>C-5:A>C	TGCAGACGCGACTTCACCACGTCCAGCGGGTAGCAGCAGACCCAGCTGGCGACTCCCGCGAGGCCGCCG	5:A>C
10984362|F|0-14:T>G-14:T>G	TGCAGCAGCGTGTTTATGCCG	14:T>G

1667327|F|0-37:G>A-37:G>A	TGCAGCCTCCCGCCGTCGAGTCCCAGCCCCTTGGACAGCACCTTTGATACCCTCCG	37:G>A
977494|F|0-61:G>A-61:G>A	TGCAGCTACGGGGACGAAGAGGGGAAGCTCAATCTTGGGTACGATGCCATGGCGATGGCGAGCACCGAG	61:G>A
3028561|F|0-13:C>A-13:C>A	TGCAGATGTACTCCGAGTGCGGGGCGCCGCGGGACGCCAACCG	13:C>A
1694959|F|0-8:G>A-8:G>A	TGCAGGAGGATTAGCCACAGGACGCACCACCAGAACCACGGCCACGTCGAGAATGACGAGTCCTGGCAT	8:G>A
4261841|F|0-8:G>A-8:G>A	TGCAGAGCGTCGTGACGGACGCGAGCCG	8:G>A
1039845|F|0-56:T>C-56:T>C	TGCAGATGGTAAACCAAAAAGGATGGATGGTGGTGATTGAGAAGGATATATACCCTTTTGTACTTGGAT	56:T>C
7169820|F|0-19:A>G-19:A>G	TGCAGTCCGAGCAAGCTAAACTCTTTGTTGGTGCAGTCCAGAGGATTATTATTCCG	19:A>G
3027393|F|0-23:C>G-23:C>G	TGCAGCACCACGTCCACCACGGACCCGAACGCCAGCCG	23:C>G
1091381|F|0-13:C>A-13:C>A	TGCAGCACAGCATCGAGTACTGGCTCACGCTGGACCTCCTCGCCTCCGAGCAGGGCGCGCCCACGCCGT	13:C>A
5363444|F|0-5:T>C-5:T>C	TGCAGTCTTGGCAGGGACGCGAAGATATCCG	5:T>C
1394376|F|0-22:C>A-22:C>A	TGCAGCTCGAGCGCGTCAACGTCTACTACAACGAGGCCTCCTGCGGCCGCTTCGTGCCGCGCGCCGTGC	22:C>A
2244710|F|0-19:C>G-19:C>G	TGCAGTGAGGCATCCAGCTCGACTTCGCCATGCGGGTCGCGCTCCACGTTCGTCCCATCGCCG	19:C>G
3032865|F|0-22:C>T-22:C>T	TGCAGGCCTACCTTGGGAACTCCCCGTCGCGGCGCATCCG	22:C>T
4993003|F|0-11:A>G-11:A>G	TGCAGCAGAGTAGTAGGAGCCATGTCCG	11:A>G
1090275|F|0-22:C>A-22:C>A	TGCAGTTCCCGCCGCTTCATCGCCGCGAAATCCATATTGGCCGCGCGCACGAATCCAAACCCCACCCCG	22:C>A
4002965|F|0-5:C>G-5:C>G	TGCAGCGCAGAGCTACCGTCGAGGCCCTTGGCCCTGTTGTGCCG	5:C>G
1045189|F|0-13:C>T-13:C>T	TGCAGCAGCTCTCCGACGAGCTGCGCTCCGACGTCATCTTCCACGTCTCCAAGACCG	13:C>T
1049979|F|0-37:T>C-37:T>C	TGCAGGGCTTACTGCTACCAGCGAGGCTACGACCTGTTGAAAAGCTATTGCAAGCCG	37:T>C
3026693|F|0-14:G>T-14:G>T	TGCAGCCCCAGCGCGCGCGCCAGCTGCATCTTGCGCTCCG	14:G>T
1695387|F|0-46:A>G-46:A>G	TGCAGAACACCAACGCCGAGATCTCCTTCTCGTCGGGGCACCGTCCAAAGCCTGATGCGTTCGAGCTCT	46:A>G
4006126|F|0-10:G>T-10:G>T	TGCAGCCGCGGATGGTGATGCCGTGGATGATCACGTTGCTCCG	10:G>T
1041672|F|0-20:G>A-20:G>A	TGCAGTCCGACATCAGGGAGGTCGCAAGCAAGTGCGCCGACGCCGCCAAGATGGCCG	20:G>A
3942452|F|0-16:G>T-16:G>T	TGCAGGATCTGCATTGGCACGTACAACGCCGCCG	16:G>T
6043924|F|0-25:C>G-25:C>G	TGCAGCGGGAGGCGGCCATGGCGGCCCGCGAGCGGCGCCGCATCCAGGCGCGGGTCAACCG	25:C>G
5577846|F|0-22:G>A-22:G>A	TGCAGACGACGGCGAAGGAGGAGCCCCG	22:G>A
6048006|F|0-13:G>A-13:G>A	TGCAGGGGATGCTGGAGAGGCTGCGGCTGGAGAAGGAGAAGGCGGAGGAGATGGTGCGGGAGCGGGACG	13:G>A
7167193|F|0-23:G>A-23:G>A	TGCAGGTGCAGGCTGGACCTAGTGTGCACGATGATCCCAAACACCTCGCCCATGTCCAGCGCCTCCGAG	23:G>A
4262697|F|0-5:C>T-5:C>T	TGCAGCAGGATCTCGTTCCACGTGTCGATGGGGCCCG	5:C>T
4408081|F|0-6:T>C-6:T>C	TGCAGCTCTTGGCGGTCGCCG	6:T>C
7354026|F|0-20:G>T-20:G>T	TGCAGGTTCGCGCCCTTCTGGGCCCTCACGGCGGCCAGCGTCCG	20:G>T
6045495|F|0-11:G>A-11:G>A	TGCAGCCGCGCGTCGACCACCTCTGCCG	11:G>A
7168845|F|0-54:G>A-54:G>A	TGCAGCTTGCGGCGGCGCTTGGCGGAGGCCTCGTCGTCGACGACGGCGGAGGAGGCGGAGGAGGAGGAC	54:G>A
3936669|F|0-14:C>T-14:C>T	TGCAGCTGCTGCGGCGTCGGCTGCTGCGAGAAGAAGTGGGGCACGCCG	14:C>T
1049351|F|0-6:C>G-6:C>G	TGCAGGCCATGAGCCCGTACAGCGAGTTGTGGGCATAGTTGATGAGGATCCG	6:C>G
7168845|F|0-29:C>G-29:C>G	TGCAGCTTGCGGCGGCGCTTGGCGGAGGCCTCGTCGTCGACGACGGCGGAGGAGGCGGAGGAGGAGGAC	29:C>G
5367409|F|0-17:C>A-17:C>A	TGCAGGGTGTTGTTTAGCGGTGACAGGTGGGCCCG	17:C>A
3947365|F|0-40:G>C-40:G>C	TGCAGGGGCTCCAGGGGTACGTGCAGAACGTCAGGTTCCTGGCCG	40:G>C
4262656|F|0-23:C>G-23:C>G	TGCAGCTTCCCAGCGTCGCCTATCGACGACGGTATCGCGCCG	23:C>G
4262125|F|0-28:G>A-28:G>A	TGCAGAAGTACGACCACCTCCTGGCCGCGCGGTCCATCACCGACGCCG	28:G>A
3951141|F|0-23:T>C-23:T>C	TGCAGAGGATGATGGCCAGGCTCTTGTTCCG	23:T>C
3939963|F|0-35:G>A-35:G>A	TGCAGCGCCCTCGCCGAAAGCGTGAAGCGCTGGGCGCCG	35:G>A
3943304|F|0-29:G>C-29:G>C	TGCAGATGGGGACCAAGTTCAGGGTGCTCGAGGCGGACTTGGCGCGGTCGCGGTGGGCGAGGCTGAGGA	29:G>C
1694706|F|0-55:C>T-55:C>T	TGCAGGTCTGCTCCGCCGCGGACGACGGCCAGCTCGAGTGGAACGGGAACGACGACGGCGACTGCTGCT	55:C>T
2241765|F|0-17:C>T-17:C>T	TGCAGTTGGAATATCCGCCTTCCTTCGAGATAACTGCTACCGAAAGCTGTGCGGGAAATGACATCCCCG	17:C>T
2241677|F|0-25:G>A-25:G>A	TGCAGGAACCAAATTGCAGTATCAAGCTCTCACCTGCATTCGAGATCATTACATGAGAACCTGGCTCCG	25:G>A
3031436|F|0-14:C>G-14:C>G	TGCAGGATGGCCAGCTTCTGGAGCTTGGAGAAGCCCG	14:C>G
7909099|F|0-16:G>C-16:G>C	TGCAGTAGACGCTCACGGTGAGCAAGGCGCCG	16:G>C
4006245|F|0-44:G>C-44:G>C	TGCAGCCAGACAAGCAGCGTGCTCGACAGGAGGGAGCTGAGGTTGATCATGAAGTAGTACCAGTTGAAG	44:G>C
6041761|F|0-28:T>C-28:T>C	TGCAGGCTCCGTATCGCGGTTATCCCCGTAGAGATCTCGCCG	28:T>C
6046738|F|0-13:A>G-13:A>G	TGCAGCGGGCGATATGGGTGACGCAGAAAGGGCCCG	13:A>G
7353345|F|0-18:C>T-18:C>T	TGCAGCAGCGGGTCGGCGCGCCG	18:C>T
5363832|F|0-12:C>G-12:C>G	TGCAGGCTCGGTCTCCGCGGCGGGCTCCG	12:C>G
3026990|F|0-23:G>C-23:G>C	TGCAGCTTCACCGACTTGCCATCGAGCTCAACGGTCCG	23:G>C
996110|F|0-22:G>A-22:G>A	TGCAGACGGCGCGCTGGACGTCGCGGAGGATGGACTCCTTGAGGCTCTCCG	22:G>A
32017700|F|0-5:T>C-5:T>C	TGCAGTTCAAGTGGGCGCATCCG	5:T>C
7478638|F|0-14:C>G-14:C>G	TGCAGGCTGGCCCTCGCGAGCACGCACCCG	14:C>G
991152|F|0-67:G>T-67:G>T	TGCAGCGCCGAGTTCTGCACGAAGAGGACGATGCCGCACTTGGTGGCGCGGAAGTCGTCCCACAGCGGG	67:G>T
1022756|F|0-7:C>T-7:C>T	TGCAGCTCAGCGCGGCCGAGAGGAGGCTTGTGCAGACCTACAACCAGAAGCCG	7:C>T
6047715|F|0-11:G>T-11:G>T	TGCAGTCACTGGTCCTCGTCGACAACAACCTCACCG	11:G>T

7342807|F|0-7:G>T-7:G>T	TGCAGGAGGCCCTCAAGAAGCTCCG	7:G>T
1099286|F|0-7:G>T-7:G>T	TGCAGCCGCACCTCCAGACCATCACGGACACCCTCCAGGTAGGTACCCG	7:G>T
7170554|F|0-25:G>C-25:G>C	TGCAGCACTCGCTCTGCGGCGTCGGGGTGTCCG	25:G>C
3026592|F|0-20:G>C-20:G>C	TGCAGGCACCGCGTGGTGACGGGGATCTCGAAGTCCACCCG	20:G>C
3023062|F|0-21:C>T-21:C>T	TGCAGTATGATCTTGTGCTTCCTCACCACCCTCTGCCCCGCCG	21:C>T
1005165|F|0-7:G>A-7:G>A	TGCAGAGGCGCGGCGCGGCCGTGGGCGGGATCGGGGTGCAGGGCCACGTGACGTACCCG	7:G>A
3946843|F|0-27:G>C-27:G>C	TGCAGGTTGTGGTAGTCGCCGACGTTGGTGGTGTCCG	27:G>C
4993716|F|0-7:C>T-7:C>T	TGCAGCGCGTGTTCTACGGCGACAACGCCG	7:C>T
1696212|F|0-17:T>G-17:T>G	TGCAGTTCCTCACCAACTCCTGCGTCGTGCTCTTCATGATCCAGAGCGTCGACCGCCTCGTCCTCTGCC	17:T>G
3024886|F|0-8:A>C-8:A>C	TGCAGGACAACAAGGCCACCGCCGCCATGGAGCTCCCCG	8:A>C
3025739|F|0-16:C>T-16:C>T	TGCAGCAGGTCCTTGGCGAGGTCGATGGACAGCAGCCCG	16:C>T
1049979|F|0-7:C>T-7:C>T	TGCAGGGCTTACTGCTACCAGCGAGGCTACGACCTGTTGAAAAGCTATTGCAAGCCG	7:C>T
3942452|F|0-10:G>T-10:G>T	TGCAGGATCTGCATTGGCACGTACAACGCCGCCG	10:G>T
5412038|F|0-7:C>G-7:C>G	TGCAGGTCCTGTCCACCGACTTCATCGACGGCGACTTCGTCGACGCCTGCGCCG	7:C>G
1046301|F|0-47:C>G-47:C>G	TGCAGCTGAAAAATCCTTCTCCCTTCCAGGTAACTGCTGCCGAACGCCGTCTGCGAGATGACATCTCCG	47:C>G
2242355|F|0-50:G>A-50:G>A	TGCAGCTGCACATTACGGTCCTTGAACGCTGAATCATGGCCCAAGAAGTCGAAATCCTGACATGAAGCA	50:G>A
1095280|F|0-7:G>A-7:G>A	TGCAGGCGCCGCCGCGGCAGGACACGTGGGGGTTCTCGCCGCTGGATGCGCAGGCCGCGTACATGCCGA	7:G>A
6043924|F|0-13:G>T-13:G>T	TGCAGCGGGAGGCGGCCATGGCGGCCCGCGAGCGGCGCCGCATCCAGGCGCGGGTCAACCG	13:G>T
3943668|F|0-40:C>G-40:C>G	TGCAGAAGAAGGCGTCCGAGCTCGCCACGCTCTGCGCGGTCCCG	40:C>G
4397570|F|0-8:C>T-8:C>T	TGCAGGGACAGCGGCGCGATCCTCGACTCGGACAGCGCCCG	8:C>T
1025289|F|0-33:A>G-33:A>G	TGCAGTGTCTGAGCAAAGGCCTGGTATTTGCGAATATCAGCATCACTAACACTCCG	33:A>G
12470363|F|0-5:G>A-5:G>A	TGCAGGAGCGCAGAGAAGCCG	5:G>A
12470363|F|0-14:G>T-14:G>T	TGCAGGAGCGCAGAGAAGCCG	14:G>T
1027276|F|0-46:C>G-46:C>G	TGCAGACGTGGAACAACGGGGCCGAGAACACGAAGCGGATCGTGGCCGCCG	46:C>G
1053022|F|0-23:G>A-23:G>A	TGCAGCTCCAAGAACTCCAGCTAGTACATGTTTCCACCTGACGCCTTTCTTGCCCG	23:G>A
1038471|F|0-14:C>A-14:C>A	TGCAGGAACAGCGCCCGCCGCCCCACCTTGTCCGCGGTCACCACGGCCACGAAGGTGGAGAACACGTTC	14:C>A
3946718|F|0-19:C>T-19:C>T	TGCAGCAGGAGCTCGCCGACGTTGTGGGGCTCGACCG	19:C>T
5577850|F|0-31:C>A-31:C>A	TGCAGACGCTTCTCCTCAGCGGCAACCAGCTCGCCG	31:C>A
5010632|F|0-12:T>C-12:T>C	TGCAGGACGAAGTGCAGCCG	12:T>C
3940440|F|0-11:A>T-11:A>T	TGCAGGTGTCGACGTGCGCGGTGGCCCG	11:A>T
5368539|F|0-9:G>A-9:G>A	TGCAGGCGCGTCAATGGCGTCCTTTACCGCCATCAATGGCGTCCTTTACTGTCACATCGAAACGCAGAC	9:G>A
3936531|F|0-22:G>C-22:G>C	TGCAGATCGCCTCCCAGTACAGGTCCCTCGTCGCGATCTGCTCGCCG	22:G>C
32025076|F|0-42:A>G-42:A>G	TGCAGCTCCCGCGCCTGCTCCGCCGCGCGCGCCGCCGCCTCCACCGTCTGCTCCCCTGCCTTCCTCTCC	42:A>G
5010438|F|0-35:A>G-35:A>G	TGCAGGGCGTCGAACTTGTATTCCTCGCTGATGCTACATGGGACGTGCACCACCTTGTACTGCCCGAGA	35:A>G
100418164|F|0-5:C>G-5:C>G	TGCAGCTGGGCATTGGTGAAGGACCG	5:C>G
7354204|F|0-9:A>G-9:A>G	TGCAGGGGCACCACAGCGTACACATGACCG	9:A>G
100585612|F|0-17:C>T-17:C>T	TGCAGCCGTCGACGGTGCCGACGTCTTTTTCACCG	17:C>T
100519689|F|0-5:T>A-5:T>A	TGCAGTGCCCACGCCGCCGCGAGCCACAGCGCCGCCAGAGGAGAGCGCCG	5:T>A
100575407|F|0-13:C>G-13:C>G	TGCAGGAGATCGCCCAGAACAGGACCACCACGCCG	13:C>G
100520563|F|0-26:C>G-26:C>G	TGCAGCGCGCCGAGGCGGTCGGCGATCGGCGCGAAGTAGGGCAGCAGGGTGCTCCG	26:C>G
5371241|F|0-26:C>G-26:C>G	TGCAGCGGGTTCGGCCCTGCCCCGTCCCTCGCCGCCATCATCGCCAACCGCTACCGCATGCCCACCGAC	26:C>G
5000424|F|0-11:G>C-11:G>C	TGCAGCAACGTGCTCCTCGACCACAACTACCGCGCCCGCGTCTCCGACTTCGGCACCGCCAAGGTCGGC	11:G>C
3026621|F|0-28:T>G-28:T>G	TGCAGCCCGTTGCTGGAGCTCAGCTGCCTCAGGAAGTGCTCCCCG	28:T>G
5577850|F|0-22:C>T-22:C>T	TGCAGACGCTTCTCCTCAGCGGCAACCAGCTCGCCG	22:C>T
4262125|F|0-19:C>A-19:C>A	TGCAGAAGTACGACCACCTCCTGGCCGCGCGGTCCATCACCGACGCCG	19:C>A
4006298|F|0-23:C>T-23:C>T	TGCAGCACCGACAGCGCCATCAACACCTTAACCTCCG	23:C>T
1020561|F|0-27:G>C-27:G>C	TGCAGTGGAGAGCACGAGGAGCTGGAGGGTGTGCCACATGGAACGGGGTTGAATTGGCTCTCGCGAATT	27:G>C
5348065|F|0-6:G>A-6:G>A	TGCAGGGTGAACTTGGCGCCGTTGGCGCACCG	6:G>A
1009527|F|0-29:G>C-29:G>C	TGCAGGCGTGGTGGAGCGAGGTGCGAAACGTGGGCCACGGCGACCTGCGCGACGCGCCGTGGTGGCCGA	29:G>C
4262275|F|0-28:G>C-28:G>C	TGCAGGACTACGAGCTGCTCGACCAGGCGGGCTCGGGCGGCCCG	28:G>C
1130103|F|0-17:C>G-17:C>G	TGCAGCTTCTTCATCACCCGCGGGGTGCGCATGATCTCGGACATCGCCCACTCCGTCGACGAGCCGAGA	17:C>G
5361542|F|0-14:C>G-14:C>G	TGCAGGGCCTACTCCGCCGCGAACGTCAGCGGCGGGATCAACCGCGGGTGCGTCATCTGGGGCGTCGAT	14:C>G
1253974|F|0-12:C>G-12:C>G	TGCAGGGGAAGGCAGCGGCGGCATGCGCGCGCGGCGCAGAGAGCGGCAGCTCCATGGCGAGAGCTAGCC	12:C>G
978564|F|0-46:G>C-46:G>C	TGCAGGTGGGTGGGTGGACGATCACCTACGAGGGCCTCACGTTCGTGACGATCCGCGGAGCCG	46:G>C
1100300|F|0-8:T>C-8:T>C	TGCAGAGATGAGACTGAGAGAAGTTACATTGAGGAAGCAGAGGAGGGAGCCCGCCAGCGATCCG	8:T>C
5582039|F|0-34:C>T-34:C>T	TGCAGGCCACCTCCGACGACATGGCGGCGGACGGCCTCACCGCCCG	34:C>T
7170554|F|0-22:C>T-22:C>T	TGCAGCACTCGCTCTGCGGCGTCGGGGTGTCCG	22:C>T
5010632|F|0-7:C>T-7:C>T	TGCAGGACGAAGTGCAGCCG	7:C>T
3940734|F|0-18:G>C-18:G>C	TGCAGCAGCAGAGAAAATGTCTCCTCCATGTCCG	18:G>C

<table>
<tr><td>1051500|F|0-68:T>C-68:T>C</td><td>TGCAGGGGGCTCAACTTCTTGGCGATCATCGGGCTGATCCTCTGCACGGCGTCCTTCGGCATCAACCTT</td><td>68:T>C</td></tr>
<tr><td>1000580|F|0-27:G>C-27:G>C</td><td>TGCAGCAGGTTCCTCGCGAGCCCCACGGCGATGAGCCCCGCGCTGCACCCCATGCCG</td><td>27:G>C</td></tr>
<tr><td>1020725|F|0-67:G>T-67:G>T</td><td>TGCAGGCCGACGAGCCACTGGTTGGCGGCTAGACGTGCGGAGGGATGCGAGGCGTGATTAAGTGCGTGC</td><td>67:G>T</td></tr>
<tr><td>3936028|F|0-21:C>T-21:C>T</td><td>TGCAGTTGGTGTCCTGGGCGACGAGGCTGAGCCG</td><td>21:C>T</td></tr>
<tr><td>7167539|F|0-13:A>G-13:A>G</td><td>TGCAGATGCCGCTATGCCAGGCACACCG</td><td>13:A>G</td></tr>
<tr><td>3960245|F|0-46:C>T-46:C>T</td><td>TGCAGGACGTGTTCCGCCGCTTCGCTTTCGACTGCATATGCAAGATCTCCTTCGGCCTCGACCCCGAGA</td><td>46:C>T</td></tr>
<tr><td>1106820|F|0-19:C>T-19:C>T</td><td>TGCAGATCGCGAACGACAGCGCCAGCGCCGCGCCGCCGTGCAGCACGGACATGCCGCCCCG</td><td>19:C>T</td></tr>
<tr><td>6048164|F|0-6:T>G-6:T>G</td><td>TGCAGCTCGGAACACACTGCTTGAAGGTGACGGGATGTCCG</td><td>6:T>G</td></tr>
<tr><td>1081341|F|0-29:G>T-29:G>T</td><td>TGCAGTCTGCCCCTGCTGCCACTGCCACCGCGGCGCCTCCTCCTCGTCGTCCTCGTCGCCATGCTCACC</td><td>29:G>T</td></tr>
<tr><td>5367126|F|0-7:C>T-7:C>T</td><td>TGCAGTGCCTCTGCCTCCTCGCCG</td><td>7:C>T</td></tr>
<tr><td>3944527|F|0-29:C>T-29:C>T</td><td>TGCAGCATCCTGTACAACCGCGGGGTGTACCCG</td><td>29:C>T</td></tr>
<tr><td>2245453|F|0-26:A>C-26:A>C</td><td>TGCAGGATGTCCCTGATCTGGTCGATAGCGTACCCTGCCCTGGGGTCTTCCCTCTCGACGCCCTTGTGG</td><td>26:A>C</td></tr>
<tr><td>3024886|F|0-20:G>A-20:G>A</td><td>TGCAGGACAACAAGGCCACCGCCGCCATGGAGCTCCCCG</td><td>20:G>A</td></tr>
<tr><td>1045054|F|0-21:C>A-21:C>A</td><td>TGCAGTCCACATCCGACAGCACAGCACCGACACAAATCTCCTCTCACGCACTCACACACACCG</td><td>21:C>A</td></tr>
<tr><td>1134693|F|0-14:T>C-14:T>C</td><td>TGCAGCCACTCCCCTCCGCTTGCGCTCTGCACCACCATGACGAGCAGCGTTCCCAGGTAGTTCCCGAGC</td><td>14:T>C</td></tr>
<tr><td>3941966|F|0-57:G>A-57:G>A</td><td>TGCAGCCACTTCGGGTCCAACGTCTCGGCATCCTCCAGGTTCGGGACCTCGAAGACCGCCTGCACGTCC</td><td>57:G>A</td></tr>
<tr><td>4002562|F|0-34:G>A-34:G>A</td><td>TGCAGCGCCAGCTCGTCTCCGCCGCCTGCGCCGAGGACAAGTCCG</td><td>34:G>A</td></tr>
<tr><td>1025479|F|0-28:C>G-28:C>G</td><td>TGCAGGCGTCGGTGATCCGCGACCTGGTCCTCCTCTCCTGCGTCGGCCTGCGCCCCGTGCTCGTGCACG</td><td>28:C>G</td></tr>
<tr><td>3026320|F|0-12:G>A-12:G>A</td><td>TGCAGTGATCAAGCTGTGGTCGGGCGGTAGCAGTCCTGCCG</td><td>12:G>A</td></tr>
<tr><td>32025399|F|0-10:G>T-10:G>T</td><td>TGCAGTGCGGGGCAGTGGCCGAGCTGCTGGAGCCG</td><td>10:G>T</td></tr>
<tr><td>1713599|F|0-18:A>C-18:A>C</td><td>TGCAGGCGGAAGTAGTAGAGGCCCTCGATGGCGTCAGTGCGGATGGTTGTGACGCCGCGCGCCATGAAG</td><td>18:A>C</td></tr>
<tr><td>1669863|F|0-23:G>C-23:G>C</td><td>TGCAGGTGGTACAGGATGTGGTCGAACATGATGTTCCTCTGGTCCATCTACTCCGTCTTCTACACCCCC</td><td>23:G>C</td></tr>
<tr><td>1862865|F|0-8:T>C-8:T>C</td><td>TGCAGGTCTTCTCCACCGACTTCATCGACGGCGACTTCGTCGACGCCTGCGCCG</td><td>8:T>C</td></tr>
<tr><td>1021070|F|0-44:C>T-44:C>T</td><td>TGCAGCGCGAGCTCGGTGAGGCGGAAGCCCTTGCGGTGCAGGTACTGCTGCACCTTCCTCTCCATCTCC</td><td>44:C>T</td></tr>
<tr><td>2245281|F|0-63:T>C-63:T>C</td><td>TGCAGGCGGCTCTTGAAGCTGGGGTCTGAAATTTGATCCCAGAACTCACGCAGCTCCGCCTTGTCGATG</td><td>63:T>C</td></tr>
<tr><td>6045780|F|0-10:G>C-10:G>C</td><td>TGCAGGTCGTGGCGGAGCAACGTGTGCTCCAAGTACCG</td><td>10:G>C</td></tr>
<tr><td>1695126|F|0-59:G>A-59:G>A</td><td>TGCAGCAGCATGTACTCCAGCAGGGGCATCATCGCCAACTCCTTCAGCGAGCTCGAGTCGGTCTACATC</td><td>59:G>A</td></tr>
<tr><td>1016778|F|0-13:G>C-13:G>C</td><td>TGCAGTCGTCGCGGAACCCCACGCCCAGGGGCTCCAGCTTCAACCACACCGACTTCTACTCCATGGTCG</td><td>13:G>C</td></tr>
<tr><td>6043924|F|0-22:G>C-22:G>C</td><td>TGCAGCGGGAGGCGGCCATGGCGGCCCGCGAGCGGCGCCGCATCCAGGCGCGGGTCAACCG</td><td>22:G>C</td></tr>
<tr><td>984861|F|0-23:C>T-23:C>T</td><td>TGCAGCTTAGGAATCAGTTCCTCCTGCTCCAGGACGCAGGGCAGCACGGTCCG</td><td>23:C>T</td></tr>
<tr><td>2242931|F|0-8:C>G-8:C>G</td><td>TGCAGCGTCACCTTCTGGAGGATCATCAAGAGGAAGTCGACGGAGGACTTCTCCG</td><td>8:C>G</td></tr>
<tr><td>3027720|F|0-28:T>C-28:T>C</td><td>TGCAGGTTCCCGAGCTCCTTTGGGATGGTGCCGTCCAGGTGGTTCCG</td><td>28:T>C</td></tr>
<tr><td>4261787|F|0-19:C>G-19:C>G</td><td>TGCAGAAGCCCGTGCCAGCCGTGACGTACAGCCTCAGCCG</td><td>19:C>G</td></tr>
<tr><td>3027991|F|0-32:T>G-32:T>G</td><td>TGCAGCTGCTAGGCCCCGACGAGGAGAAGCCCTCGCCG</td><td>32:T>G</td></tr>
<tr><td>1124420|F|0-41:T>G-41:T>G</td><td>TGCAGAGCGGCGCACACCTGAGCCGCCGCTTCTTCCTTGTCTCCGTCCAGCCTTCCCCTCAGGACCCGA</td><td>41:T>G</td></tr>
<tr><td>1130186|F|0-31:G>A-31:G>A</td><td>TGCAGGCGCAGACGCAGACGCACCCGCCGTCGCCGAGGTACCGCGGCGTCGTCGACTGCTTCCG</td><td>31:G>A</td></tr>
<tr><td>986230|F|0-17:A>C-17:A>C</td><td>TGCAGGTACGCGTACGCAGCGTGTTTTACCAGCGGGTGGCGTATCATGTGTATGTCCCCACAGCTGCCG</td><td>17:A>C</td></tr>
<tr><td>4999261|F|0-28:C>A-28:C>A</td><td>TGCAGTTCATCGATGGTATGTTCGTCTGCATCACCG</td><td>28:C>A</td></tr>
<tr><td>1045054|F|0-44:C>T-44:C>T</td><td>TGCAGTCCACATCCGACAGCACAGCACCGACACAAATCTCCTCTCACGCACTCACACACACCG</td><td>44:C>T</td></tr>
<tr><td>5578314|F|0-14:G>A-14:G>A</td><td>TGCAGGGACGGCTCGGGCTCCTTCCCCAGGAGCCG</td><td>14:G>A</td></tr>
<tr><td>1863114|F|0-38:C>T-38:C>T</td><td>TGCAGAGACGATGTCAAGAACAAGGCGCACGCCATCATCGACAAGTACGCCGAGCGCGGCCTCCGAGAT</td><td>38:C>T</td></tr>
<tr><td>4261499|F|0-5:G>A-5:G>A</td><td>TGCAGGAACATGAGCTTGTCGCGGAGCCTCCG</td><td>5:G>A</td></tr>
<tr><td>1204519|F|0-6:A>T-6:A>T</td><td>TGCAGCAGCTCTCCAGGGTTCTCGCCTCCCTGGGGTACAACGAGTTGGCGTCGGAGGCGCCGCTCCTCG</td><td>6:A>T</td></tr>
<tr><td>1018051|F|0-26:C>T-26:C>T</td><td>TGCAGGATGATCTCCCCGTGGTGCGGCACGGACAGGTAGCTCTTCTCCGCCCTGCGGATGACCTCGGCG</td><td>26:C>T</td></tr>
<tr><td>4009512|F|0-14:T>C-14:T>C</td><td>TGCAGCAAGATATCTGACACCACCGCGCCGCTGCCCG</td><td>14:T>C</td></tr>
<tr><td>4261948|F|0-7:G>T-7:G>T</td><td>TGCAGTCGTGGTGCGGGTTCTACCAGAGCCTCCG</td><td>7:G>T</td></tr>
<tr><td>1130186|F|0-26:C>T-26:C>T</td><td>TGCAGGCGCAGACGCAGACGCACCCGCCGTCGCCGAGGTACCGCGGCGTCGTCGACTGCTTCCG</td><td>26:C>T</td></tr>
<tr><td>2249018|F|0-47:T>C-47:T>C</td><td>TGCAGCAGCTTGGTGGTGTCGATGGCGCCCAGCTTCTCGATGTAGATTGGCCG</td><td>47:T>C</td></tr>
<tr><td>5348065|F|0-23:G>C-23:G>C</td><td>TGCAGGGTGAACTTGGCGCCGTTGGCGCACCG</td><td>23:G>C</td></tr>
<tr><td>3026990|F|0-20:A>G-20:A>G</td><td>TGCAGCTTCACCGACTTGCCATCGAGCTCGACGGTCCG</td><td>20:A>G</td></tr>
<tr><td>32027057|F|0-8:C>T-8:C>T</td><td>TGCAGGTACACAAGAACATCTCGCCG</td><td>8:C>T</td></tr>
<tr><td>4261239|F|0-35:C>G-35:C>G</td><td>TGCAGCTTATCCATCTCGCCGATGGTCGCCGGGATCGCGCCG</td><td>35:C>G</td></tr>
<tr><td>5363832|F|0-16:G>T-16:G>T</td><td>TGCAGGCTCGGTCTCCGCGGCGGGCTCCG</td><td>16:G>T</td></tr>
<tr><td>1093145|F|0-27:A>G-27:A>G</td><td>TGCAGCGCGCCCTCCTTCTCGGCGACGACGTCGTTGAGCCGCGCGTTCTCGACCCG</td><td>27:A>G</td></tr>
<tr><td>1021829|F|0-14:T>G-14:T>G</td><td>TGCAGGGGGAACTCTTTGTCGTCCTCGTCGGTGCGCCCCTACGTCCGCTCCAAGAACCCGCGCCTCCGC</td><td>14:T>G</td></tr>
<tr><td>1020730|F|0-24:G>T-24:G>T</td><td>TGCAGCTTGCTGAGGACGGCGAGGGCGCTTGCGGCGGAAAGATCCATGGACGCGGGGGAGGCCTCGCCG</td><td>24:G>T</td></tr>
<tr><td>3027991|F|0-8:C>G-8:C>G</td><td>TGCAGCTGCTAGGCCCCGACGAGGAGAAGCCCTCGCCG</td><td>8:C>G</td></tr>
<tr><td>3940266|F|0-22:C>A-22:C>A</td><td>TGCAGGAGAAGGGCGGGAAGGGCGATGCCGTCCGCCG</td><td>22:C>A</td></tr>
<tr><td>1095280|F|0-58:G>A-58:G>A</td><td>TGCAGGCGCCGCCGCGGCAGGACACGTGGGGGTTCTCGCCGCTGGATGCGCAGGCCGCGTACATGCCGA</td><td>58:G>A</td></tr>
</table>

3570214|F|0-19:G>A-19:G>A	TGCAGTACAGCAGATGAGCGCCATGAACTCCG	19:G>A
1230460|F|0-8:A>T-8:A>T	TGCAGCTGACGTGGAACTACAACTACGGGGCGGCGGGGCAGAAGCTCGGCTTCAACGGGCTCGACAACC	8:A>T
5006275|F|0-22:T>G-22:T>G	TGCAGGTGATACTCAACGAGATTAGGAACCG	22:T>G
1320600|F|0-68:C>A-68:C>A	TGCAGTCGGCACACGCACGCGCCCGCCATCATGCCTCCTTGGTTCGGGACGAAGCCAAGCGAGACATAC	68:C>A
32020118|F|0-7:T>C-7:T>C	TGCAGAGTCCTGTTGTCGCCGTGCTGCCAACGGACATGGCCG	7:T>C
100486511|F|0-12:T>G-12:T>G	TGCAGCCTGAGCTCACCTGGAAGTCGCCGCAGCAGATGAGGAGGTCGATCTTGATGCCCTCGGCCGCCT	12:T>G
32020576|F|0-25:G>C-25:G>C	TGCAGAGGCAGCTGAAGAGGCCGCAGGCCAGGCACTTGAACGGGCAGCACAGGCACGACGCCGCGCGGC	25:G>C
1190972|F|0-48:C>A-48:C>A	TGCAGGCTGCCTGACGTTCTTCTGCCCGTGCGTCGTCTTCGGGAGGATCGCCGAGATCGTCGACATGGG	48:C>A
6044258|F|0-5:T>C-5:T>C	TGCAGTCTTGGCGAGCCGCGGGCTCCG	5:T>C
3934500|F|0-7:G>C-7:G>C	TGCAGCTGCTCATCCGCCACTTCTCCCG	7:G>C
1695269|F|0-63:C>T-63:C>T	TGCAGGTCACCAGCATGGACCGCTACGTCAGGTACCACGTGAGGGAGTTCGAGAGGGCCTTCGCCCTCA	63:C>T
3953861|F|0-20:C>A-20:C>A	TGCAGTTGGTTAAGCGTCAGCCTTTCACCG	20:C>A
5411987|F|0-24:G>A-24:G>A	TGCAGCCCTCCCACGCCTCCCGCGGAATCACCG	24:G>A
3959309|F|0-23:G>T-23:G>T	TGCAGGATCACCTGGAAGAGGTTGCCCACCACCG	23:G>T
1045227|F|0-57:C>T-57:C>T	TGCAGATCACAAGGCGCGGTGTTGTAACTGGTGCGACCAGGAAATGAGCTTGCACAGCTGTCCG	57:C>T
1022595|F|0-37:G>A-37:G>A	TGCAGGCAACAAATTCCTGTGTGGCAAGCCAACGGACGTGGTCTGCGAATCCGCGTCGTCGGCCGCCGA	37:G>A
1088578|F|0-20:C>T-20:C>T	TGCAGGCTCTGCCACTTGAGCACCCACAGCGGGATGTGCTGCATCAGAGGGTGCACGCTGATGATCACG	20:C>T
1067023|F|0-56:G>A-56:G>A	TGCAGCAGCACCACCCCGAAGCTGTACACGTCGGACTTCATGCTGACCTGGCCGCCGCGGTCGTACTCC	56:G>A
22840626|F|0-14:C>G-14:C>G	TGCAGGTCCATCATCGCGTTCACCAGGCTCGCCG	14:C>G
7333812|F|0-14:C>T-14:C>T	TGCAGGATGATGGACGATCCG	14:C>T
4999261|F|0-26:T>G-26:T>G	TGCAGTTCATCGATGGTATGTTCGTCTGCATCACCG	26:T>G
5367722|F|0-5:G>C-5:G>C	TGCAGGAGCTCGCCGCAGCGCCCG	5:G>C
1695363|F|0-7:G>T-7:G>T	TGCAGCTGCTCTTCTTGGAGGGCAACCTCTTCACCAAAGAGCTGCCGAACGTCGACATGCCATCCCTCT	7:G>T
4002413|F|0-20:G>T-20:G>T	TGCAGTGGAGCAGTCAAGAAGTATCCCAAGAACAGAATGCTCGGCCG	20:G>T
2242751|F|0-20:C>T-20:C>T	TGCAGGACGTGTTCATGCGGCTGACGTTCGACCTCACGGCGATGTTCGTGTTCGGTGTCGACCCCGAGA	20:C>T
5579693|F|0-10:G>C-10:G>C	TGCAGGCGCGGTTCGAGGAGCTGGAGCTGCCGCTGCTGGCCGTGCACGGCGGCGACGACACGGTGTGCG	10:G>C
1016778|F|0-7:G>C-7:G>C	TGCAGTCGTCGCGGAACCCCACGCCCAGGGGCTCCAGCTTCAACCACACCGACTTCTACTCCATGGTCG	7:G>C
7344176|F|0-17:G>A-17:G>A	TGCAGTGGCGGTTCGGCGCGCCG	17:G>A
1230844|F|0-42:T>G-42:T>G	TGCAGGGGAGGCTGCGGAGCACGCAGCCGCTGACCAAGGGCGTGGTGGCGGAGTGCGTGCTGGGCGCGA	42:T>G
1021070|F|0-26:G>T-26:G>T	TGCAGCGCGAGCTCGGTGAGGCGGAAGCCCTTGCGGTGCAGGTACTGCTGCACCTTCCTCTCCATCTCC	26:G>T
7353729|F|0-8:C>T-8:C>T	TGCAGCGGCGCCTACGCGACGCCG	8:C>T
5363289|F|0-21:T>C-21:T>C	TGCAGCAGCGGGCACGGCGGGTAGTGATTCACCCG	21:T>C
1047001|F|0-45:G>C-45:G>C	TGCAGACCGTCGCCACCGACTTCCAGCGGGTCTTCAAGATCCACGGCAAGCTCTACATCGGCCTCTCCG	45:G>C
7918507|F|0-8:T>C-8:T>C	TGCAGAAGTTATCCCTTGATGCCAAGACTGAAGCCATCGACGCGCCG	8:T>C
32027629|F|0-9:T>A-9:T>A	TGCAGGTGCTCGCGGCGCGGACGAGGTGGGCAGGCGAAGGGAGGCGCAGGAACACCAGCTCGAGAAGCT	9:T>A
4261239|F|0-6:T>G-6:T>G	TGCAGCTTATCCATCTCGCCGATGGTCGCCGGGATCGCGCCG	6:T>G
3023203|F|0-31:A>G-31:A>G	TGCAGGGGATATACGTGCTGAGGTTGGACTTATTACCTGGTGTACCG	31:A>G
1054604|F|0-10:C>T-10:C>T	TGCAGCCGCACAGCGCCACGTCGGTGTTGAGAAGCGCCACGACGGAGATGGCGAGGATGACAGTGGACA	10:C>T
1039845|F|0-16:A>G-16:A>G	TGCAGATGGTAAACCAAAAAGGATGGATGGTGGTGATTGAGAAGGATATATACCCTTTTGTACTTGGAT	16:A>G
3942452|F|0-23:C>T-23:C>T	TGCAGGATCTGCATTGGCACGTACAACGCCGCCG	23:C>T
1028737|F|0-31:G>T-31:G>T	TGCAGTCGGCGATGTACTACGAGCGCGGTGCGGTGATCGGGTGCCACCACTGCCCG	31:G>T
3936463|F|0-8:G>C-8:G>C	TGCAGGACGTGCATTGCCTGGGCGACAGACGGCCGCTCGCTCCGTTCCG	8:G>C
991152|F|0-5:C>T-5:C>T	TGCAGCGCCGAGTTCTGCACGAAGAGGACGAGGCCGCACTTGGTGGCGCGGAAGTCGTCCCACAGCGGG	5:C>T
985964|F|0-7:T>C-7:T>C	TGCAGCGTTACCAGCCGTTCGGGTCGCCGCCTGGCGAGTACCACCATTTCGGCCCG	7:T>C
6044258|F|0-17:G>T-17:G>T	TGCAGTCTTGGCGAGCCGCGGGCTCCG	17:G>T
1696089|F|0-9:C>T-9:C>T	TGCAGGTCACCGTGCCGCTGAAGAAGGTCGCCAAGACGGAAGCGCTGGCCATCGGCGACCCTACCCGTC	9:C>T
5582490|F|0-12:C>T-12:C>T	TGCAGAAAGCGGCAGCACGTGCGTGGTTGCCG	12:C>T
5363717|F|0-34:C>G-34:C>G	TGCAGGACGTGTACGGGGAGGTGTACCGCGTGCTCAAGCCG	34:C>G
986255|F|0-25:A>G-25:A>G	TGCAGGCGATGCTTGCTCATTGCCCATCCCTGGAGTGCGCAAAGCTCAAGAACATCACCG	25:A>G
1015949|F|0-31:G>C-31:G>C	TGCAGGTGCACCCCAACTCGTACGCGCTGGTGATCAGCATGGAGAACATCACCCTCAACTGGTACTTCG	31:G>C
3942016|F|0-23:T>A-23:T>A	TGCAGATCCCAGTGGTCGACATGTCCTTGCCG	23:T>A
5332136|F|0-21:G>A-21:G>A	TGCAGCTGGTGCGGTAGTGGTGGCGGCCGTCACCG	21:G>A
3022242|F|0-26:A>C-26:A>C	TGCAGGATCCTGTTCCTGCTCACCCTATCCCTCCCCCG	26:A>C
4002189|F|0-25:C>T-25:C>T	TGCAGCTGGGCGGCGACGTGGGCGGCGGCCGCCTCAGCACCGACCCG	25:C>T
4002189|F|0-43:C>T-43:C>T	TGCAGCTGGGCGGCGACGTGGGCGGCGGCCGCCTCAGCACCGACCCG	43:C>T
988349|F|0-6:T>C-6:T>C	TGCAGGTACGTGTCGGCCACGATAGCGTCTGGCCTCCGCCCCAGCTCCAGCGCCAGCCG	6:T>C
3023262|F|0-18:A>G-18:A>G	TGCAGGAAGTTGGTCAGCATTAGCTGCTTCAGGCTTCTGGCCG	18:A>G
1099286|F|0-10:C>T-10:C>T	TGCAGCCGCACCTCCAGACCATCACGGACACCCTCCAGGTAGGTACCCG	10:C>T
1038979|F|0-41:A>G-41:A>G	TGCAGCAAACCATCTCAGCGGATGACGCTGCACTTCTCGGCACCCGTGTCTCCG	41:A>G

988075|F|0-34:A>C-34:A>C	TGCAGCAGCGTGCTGATGGTGGCGTTCACCAGCGAGCTCAGCGGCGACGGCCTGAAGCCG	34:A>C
3960467|F|0-17:C>T-17:C>T	TGCAGCCTTGGCAGGGACGCGAAGAAATCCG	17:C>T
4995194|F|0-31:C>T-31:C>T	TGCAGGGCTTCCTGGTCTTCAACGCCGTCGGCGGTGGAACCG	31:C>T
5325236|F|0-17:T>A-17:T>A	TGCAGGAGGAGAAGGCCTTCAACCG	17:T>A
4261761|F|0-7:G>C-7:G>C	TGCAGCCGAAGTGGATGGGCGGGCCGCGCACGTCGTAGGTGCCCCG	7:G>C
4262807|F|0-26:G>A-26:G>A	TGCAGCGGAGCTTCGCCGAGGTGCAGGCGATCCTGGAGAAGAACCG	26:G>A
4006132|F|0-20:G>A-20:G>A	TGCAGAACGAGGGCAACGTCGTCACCATCAAGACGGGCGCCTCCG	20:G>A
1230597|F|0-41:G>A-41:G>A	TGCAGCCTCGTGAGCATCGGCTCGGTGACGTGGAAGGAGTAGCCCGCCATGTCGACGCCG	41:G>A
1230597|F|0-5:C>T-5:C>T	TGCAGCCTCGTGAGCATCGGCTCGGTGACGTGGAAGGAGTAGCCCGCCATGTCGACGCCG	5:C>T
32023345|F|0-9:C>T-9:C>T	TGCAGCTGACCATTCTGAAGCGGTACTCTATGCCG	9:C>T
1047731|F|0-26:A>T-26:A>T	TGCAGTGGGATCGGAGCAGGAGAAGAAGGCCGCCGCCACGGTGGAAGTGGAAGGCATCCCGTTCCCGCG	26:A>T
3954112|F|0-8:C>A-8:C>A	TGCAGGGGCGATTGGATCCG	8:C>A
3029371|F|0-21:A>T-21:A>T	TGCAGGCCGTGACCCTCCAGGAACACCCCGTCCGCCG	21:A>T
2361445|F|0-31:T>C-31:T>C	TGCAGGCTGGCGTCGAGCAGCGCGGCCTGCTTGCCGACGTCGGCGTCGGCCACGAAGCCGCCGAACCGA	31:T>C
1696795|F|0-67:G>A-67:G>A	TGCAGCTCCTCAAGGCCGTCTTGCTGGCCTTCGCGTGGGCCTTCGACGCGCAGCAGGTGTTCATCTCGG	67:G>A
1696089|F|0-62:A>G-62:A>G	TGCAGGTCACCGTGCCGCTGAAGAAGGTCGCCAAGACGGAAGCGCTGGCCATCGGCGACCCTACCCGTC	62:A>G
3943304|F|0-46:G>C-46:G>C	TGCAGATGGGGACCAAGTTCAGGGTGCTCGAGGCGGACTTGGCGCGGTCGCGGTGGGCGAGGCTGAGGA	46:G>C
1013635|F|0-25:G>A-25:G>A	TGCAGTTCACGGTGAAGGACTCTGTGCGGGAGAGCGTGTCCAGCGCGTCCGAGAACCTCTCCTCGTCAT	25:G>A
3940132|F|0-14:G>T-14:G>T	TGCAGCAGATATGTGAATTCAAGATGGTGGCACCG	14:G>T
3022242|F|0-30:C>T-30:C>T	TGCAGGATCCTGTTCCTGCTCACCCTATCCCTCCCCCG	30:C>T
4541529|F|0-20:C>G-20:C>G	TGCAGGATCTGGACCACCTCCGCCATGGACGGCCG	20:C>G
3025862|F|0-22:G>A-22:G>A	TGCAGTAGGAGTAGAGCTTGTCGTCCAGGACGCACTCCCG	22:G>A
4398235|F|0-12:A>G-12:A>G	TGCAGGCTGGTTAGTTGATCGGGCGATCGGCCGACGTACGTGGTCCG	12:A>G
991152|F|0-26:G>A-26:G>A	TGCAGCGCCGAGTTCTGCACGAAGAGGACGATGCCGCACTTGGTGGCGCGGAAGTCGTCCCACAGCGGG	26:G>A
1099286|F|0-44:A>G-44:A>G	TGCAGCCGCACCTCCAGACCATCACGGACACCCTCCAGGTAGGTACCCG	44:A>G
17568760|F|0-10:G>A-10:G>A	TGCAGCACCAGCTCCACCGTCGGCACGGCCG	10:G>A
2361445|F|0-29:C>T-29:C>T	TGCAGGCTGGCGTCGAGCAGCGCGGCCTGCTTGCCGACGTCGGCGTCGGCCACGAAGCCGCCGAACCGA	29:C>T
5580502|F|0-35:G>A-35:G>A	TGCAGGTACGGCAGGCTTGGGATGTCCGACTCGTCGGCCAGCCG	35:G>A
3945401|F|0-22:C>T-22:C>T	TGCAGCCAACGAAGTGGTGCGCCGTCTTCGATGTCCAGAGCTCTTCCCCCAGCCTGCTCCCGCCACTCT	22:C>T
1070864|F|0-14:G>C-14:G>C	TGCAGCTTGGCGATGAGCTGGATCTCGTTCTTGAACTCCACAAGCCCCTGCCCG	14:G>C
4992336|F|0-14:G>A-14:G>A	TGCAGGTGTCGTGCGCCGTTTCCG	14:G>A
1020207|F|0-23:A>G-23:A>G	TGCAGAACTGTGAGAATTGTCCAAAGTCATGACTTACAGGCCACCGACGATTGCGGAGAAGATGGCCGT	23:A>G
1038161|F|0-11:G>A-11:G>A	TGCAGGTAGCCGCCACCGAACGTGGGAGGAGATGGCGTGGGAGACGCGTACAGCGTTGGGGGCGGCGAG	11:G>A
3940008|F|0-5:C>T-5:C>T	TGCAGCGGCATGGAACTATCCG	5:C>T
3024696|F|0-11:C>T-11:C>T	TGCAGCAGCTGCTCCAGGCCATCGGCTCTAACAACAGCACCACCG	11:C>T
1221266|F|0-14:A>G-14:A>G	TGCAGAGCAGCAGCAGGATCCATCTCGTACGGCTGGCTGTTGCCGTCACCG	14:A>G
1719278|F|0-28:G>C-28:G>C	TGCAGAACGTGAGCTGCGCCAAGGCGGGGAGGATCCAGGGCAAGGACCTCAACTACCCGTCGATCATGG	28:G>C
7173895|F|0-10:G>A-10:G>A	TGCAGCGAGCGGCTGCTCGTGGACGTGCTCCGCGTAGGCGTCAGGTCCG	10:G>A
1038161|F|0-26:A>G-26:A>G	TGCAGGTAGCCGCCACCGAACGTGGGAGGAGATGGCGTGGGAGACGCGTACAGCGTTGGGGGCGGTGAG	26:A>G
5411854|F|0-19:C>G-19:C>G	TGCAGAGCCTGAGCTCCAGCGGCGGATCCCGTCCG	19:C>G
3945217|F|0-19:G>T-19:G>T	TGCAGCTGTGCATCCTCTCGGGCGAGCCG	19:G>T
3940642|F|0-7:C>T-7:C>T	TGCAGTGCGCGAACCCCACGGTGTGGGCGCCG	7:C>T
4261618|F|0-34:T>G-34:T>G	TGCAGGCACGACAGGCGCCGCTGCCTCGCCATCCTCTTCTTCCG	34:T>G
3959309|F|0-8:C>G-8:C>G	TGCAGGATCACCTGGAAGAGGTTGCCCACCACCG	8:C>G
1019894|F|0-19:A>T-19:A>T	TGCAGCTCGGAGAGAGGGGAAACGGGGAGGAGGAGGCGGACGAGACGGATGGGGAATTAAGGAGAGAAT	19:A>T
1024820|F|0-58:G>C-58:G>C	TGCAGCTGGGGAGGCAGCGGGCCAAGACCAAGGTGATCAGGAAGTCGGCCAACCCCGTGTGGGACGAGG	58:G>C
32016321|F|0-29:G>C-29:G>C	TGCAGAAAGAGCATGCGGTTCAGCATGTTGTCCG	29:G>C
6046145|F|0-35:C>G-35:C>G	TGCAGAACAGCCACATCACCGAGGCCATGCTGCACCGCAAGATGAGCTACGGGAGGGCCTCCGAGGACG	35:C>G
4994752|F|0-36:T>G-36:T>G	TGCAGGGGGGAGAAACGAACACCAATAAGCAACACGTCCGTCTGAACCCTTTCTAGAAGCTTCCGAGAA	36:T>G
2371769|F|0-13:C>T-13:C>T	TGCAGAAGGAGCACGAGCAGTCGCCGCCGCGGCCCTCGAACCGCAAGTCGCAGTTCGAGGAGATGACGA	13:C>T
5005875|F|0-54:C>G-54:C>G	TGCAGCTCGTCCAGCACGCCTCGCCCCCCTGCGTGGATGCAGAAGTGCTCCAGCCCAAGAGTGAAGTCG	54:C>G
32018922|F|0-33:T>C-33:T>C	TGCAGCAGCGTGATCCCCCTCGGGTTCTTGGTGTGCCTCCG	33:T>C
100442175|F|0-16:T>A-16:T>A	TGCAGGTACAGTTGCATTTCCAGGCCATGCCTAGCCG	16:T>A
7907482|F|0-64:T>C-64:T>C	TGCAGCTGACCAGCAGCATCGGGTCCATCATCGGGGGCTTCTCCGCCCTGCTGCTGGCGCCGACTACCA	64:T>C
100548194|F|0-9:A>T-9:A>T	TGCAGGACCACGGCGATGGCGCGGTCCACGCGATCGGCGTCGTCGCGGAGCGCCG	9:A>T
3945070|F|0-9:T>C-9:T>C	TGCAGCGAGTTCTGGACCCCTCCGACGACGCACCG	9:T>C
989265|F|0-35:C>T-35:C>T	TGCAGGTCCACTCGGCCCCGCCGCGCCGCCCGATCCCGATCCGACGGCCCG	35:C>T
7172841|F|0-9:C>T-9:C>T	TGCAGCAGCCTGCCGTCAGCAACGACGGATACGCCG	9:C>T
17568760|F|0-11:C>T-11:C>T	TGCAGCACCAGCTCCACCGTCGGCACGGCCG	11:C>T

4261881|F|0-31:T>C-31:T>C	TGCAGCTCCGAGGCGCGCGCCGCCGTGCTCGTGCCACGGGCCG	31:T>C
1013852|F|0-5:G>T-5:G>T	TGCAGGGCAGTGGCACTGGCATCTAGCCAGCTATGGCAATTATATATCGCCG	5:G>T
7173103|F|0-19:T>C-19:T>C	TGCAGCAGCTTCCTCCCCTTCTCCCCGACGAACCG	19:T>C
1862657|F|0-61:G>A-61:G>A	TGCAGCACGGGAAGACGGTGTTCATGGACATGCTCGTGGAGCAGACCCACGAGGTGGACACGTTCGACT	61:G>A
1038161|F|0-57:G>C-57:G>C	TGCAGGTAGCCGCCACCGAACGTGGGAGGAGATGGCGTGGGAGACGCGTACAGCGTTGGGGGCGGCGAG	57:G>C
4261841|F|0-13:G>C-13:G>C	TGCAGAGCGTCGTGACGGACGCGAGCCG	13:G>C
5346555|F|0-14:T>C-14:T>C	TGCAGGCTGGCTGGTGGACGCCG	14:T>C
3020650|F|0-20:A>G-20:A>G	TGCAGAGATGACACGTGGGAAAGATGCAACCTTTTGAGCCG	20:A>G
4261239|F|0-23:G>C-23:G>C	TGCAGCTTATCCATCTCGCCGATGGTCGCCGGGATCGCGCCG	23:G>C
1021210|F|0-8:C>T-8:C>T	TGCAGTGCCTTGGTCCTCTCCTCCTCGGAGCTGGGCTGCGCCGCCACGGCGGACATGCCGTCCACCCTC	8:C>T
3960644|F|0-29:C>G-29:C>G	TGCAGTATCCTGTGGCAGTGCCTGTCGGACAGCCCG	29:C>G
988488|F|0-29:T>G-29:T>G	TGCAGCACAGGTTGTGCAGTCCCGCACTCTCACCAGCAACACTCATGCACGACCTTGCGGGGCCTCCGC	29:T>G
977215|F|0-8:G>C-8:G>C	TGCAGGGTGAGGAAGGTTCGGCCCTTGAGGAGGGCCCTCAAGGGCCTCAAGGCCTGGATCACCG	8:G>C
1003778|F|0-37:T>C-37:T>C	TGCAGCCCCGCGCAGCGCGGCCGCCAGCGGCGCCAGCTCCAGCGCCACCCG	37:T>C
3938349|F|0-10:T>C-10:T>C	TGCAGGTGGGTGTGGACCTGCTGCGGTCCG	10:T>C
3027570|F|0-29:G>A-29:G>A	TGCAGCGCAACGGGTGTCAGTCCTTCTGGGCACCATCCG	29:G>A
1253974|F|0-8:A>G-8:A>G	TGCAGGGGAAGGCAGCGGCGGCATGCGCGCGCGGCGCAGAGAGCGGCAGCTCCATGGCGAGAGCTAGCC	8:A>G
5366854|F|0-17:T>C-17:T>C	TGCAGATTCAGAAGCGATTCGCCG	17:T>C
1099286|F|0-25:G>C-25:G>C	TGCAGCCGCACCTCCAGACCATCACGGACACCCTCCAGGTAGGTACCCG	25:G>C
7352152|F|0-29:C>T-29:C>T	TGCAGTTGATCGCCGATGTTGATGACCAGCGCCCCG	29:C>T
7173349|F|0-8:G>C-8:G>C	TGCAGGACGCGCAGCGACACCCCGATCTCCGCCGCCGTCTGCTGCGGCGTCGAGTTGGACGTCGCGTAG	8:G>C
3027570|F|0-21:C>A-21:C>A	TGCAGCGCAACGGGTGTCAGTCCTTCTGGGCACCATCCG	21:C>A
1865011|F|0-29:G>C-29:G>C	TGCAGGAAGATCGGCTCGACGTCGGTGGCGACGAACTCGTCCAACGTTGGCGCAGGAGCCG	29:G>C
3935369|F|0-5:G>C-5:G>C	TGCAGGTGCACGACTCGCCG	5:G>C
991226|F|0-10:T>A-10:T>A	TGCAGACCGTTGATCAGTCTGAACTGGAGAAATGGTGGAAGAAAAACAAACGCATGTGTCCAGTTACCG	10:T>A
4261787|F|0-28:C>T-28:C>T	TGCAGAAGCCCGTGCCAGCCGTGACGTACAGCCTCAGCCG	28:C>T
1089360|F|0-67:C>T-67:C>T	TGCAGAACGCCAACATGCTGGCGCCCAACAACAGCGAGACCCACCCGTGGCACCTCCACGGCCACGACT	67:C>T
2246068|F|0-49:C>G-49:C>G	TGCAGGCATGGTCGTGTCGAGCAACGCATGCATGGCGCCTCCTGGGGTTCGAGGTGTCTGCCTCCTGCA	49:C>G
1070984|F|0-6:C>A-6:C>A	TGCAGCCTGCTCGTCCAGCAACGCCGCGTGGGCGTCGTAGTACATGTGGTGGCCACCG	6:C>A
1042706|F|0-51:T>G-51:T>G	TGCAGAAACGATAATTACCTAACCCGACAAGCACTGAGGCACCGACGAGAGTGCACGGTGATTCAACCA	51:T>G
5582399|F|0-10:G>A-10:G>A	TGCAGTTCAGGAGGGTGCGCTGCAAGTACGCGCCCG	10:G>A
1694930|F|0-17:A>G-17:A>G	TGCAGCAGCCTTCGAAGAGCATTCTCCAAGGTCTGGTTCTCCTCTAGAACCAGCACCGTTTGCTTCTGC	17:A>G
1088701|F|0-48:T>C-48:T>C	TGCAGCGCAGGAATTCGGGCTGCCCCTGCTGCCGCCGTCCAAGGCCAATCGCTCCGACTTGAGTCACGG	48:T>C
1013635|F|0-55:C>T-55:C>T	TGCAGTTCACGGTGAAGGACTCTGTGCGGGAGAGCGTGTCCAGCGCGTCCGAGAACCTCTCCTCGTCAT	55:C>T
1013635|F|0-31:G>T-31:G>T	TGCAGTTCACGGTGAAGGACTCTGTGCGGGAGAGCGTGTCCAGCGCGTCCGAGAACCTCTCCTCGTCAT	31:G>T
981336|F|0-12:G>C-12:G>C	TGCAGCATGACGGTGACGAACGTGAAGAAGAAGCAGGCGGCCAGGGCTTCCACGGCCG	12:G>C
1049979|F|0-22:G>T-22:G>T	TGCAGGGCTTACTGCTACCAGCGAGGCTACGACCTGTTGAAAAGCTATTGCAAGCCG	22:G>T
989564|F|0-15:C>T-15:C>T	TGCAGAGCGAGCCGACGGACGCAGAGGTCGCCGAGGCCATGAACATGCCG	15:C>T
3027570|F|0-31:A>G-31:A>G	TGCAGCGCAACGGGTGTCAGTCCTTCTGGGCACCATCCG	31:A>G
3025434|F|0-18:T>C-18:T>C	TGCAGGGGCTGATGATCTTCTGGCCCCGAGCTCTGCCTCGCCG	18:T>C
4262125|F|0-6:A>G-6:A>G	TGCAGAAGTACGACCACCTCCTGGCCGCGCGGTCCATCACCGACGCCG	6:A>G
32020117|F|0-12:T>C-12:T>C	TGCAGAGCCCACTTGATGAGCACCTGCCCCG	12:T>C
988865|F|0-13:T>C-13:T>C	TGCAGGAGAAGCATGCCGCTGCCATTGATAACATCCTGAGGGACCTGCACTGGTCTAGCCG	13:T>C
4261628|F|0-9:C>T-9:C>T	TGCAGGTGCCTGGAGCAGTCCG	9:C>T
1018388|F|0-13:T>C-13:T>C	TGCAGAGTCTGCATTGCATCGCTTGTTGCAGCAGCTTGAAGAACAGAGCTTTCGCTAAGGGCATCAGCC	13:T>C
7171988|F|0-59:A>G-59:A>G	TGCAGTTGGAATATCCGCCTTCCTTCGAGATAACTGCTACCGAAAGCTGTGCGAGAAATAACATCCCCG	59:A>G
6048345|F|0-20:G>C-20:G>C	TGCAGCAGCATCACGCCGAGGGAGTACACGTCCGACTTGACCCCCAGCATCCCCGTCTGCTGGTACTCC	20:G>C
7349741|F|0-5:C>G-5:C>G	TGCAGCTACCTGGGCAGCCCCG	5:C>G
4002569|F|0-25:G>C-25:G>C	TGCAGTCCAAGAGCGTGTGCGTGGTGGGGGGCGGCGTGGCCG	25:G>C
1695363|F|0-14:T>C-14:T>C	TGCAGCTGCTCTTCTTGGAGGGCAACCTCTTCACCAAAGAGCTGCCGAACGTCGACATGCCATCCCTCT	14:T>C
1695126|F|0-60:G>C-60:G>C	TGCAGCAGCATGTACTCCAGCAGGGGCATCATCGCCAACTCCTTCAGCGAGCTCGAGTCGGTCTACATT	60:G>C
1695363|F|0-28:C>T-28:C>T	TGCAGCTGCTCTTCTTGGAGGGCAACCTCTTCACCAAAGAGCTGCCGAACGTCGACATGCCATCCCTCT	28:C>T
1114550|F|0-50:G>A-50:G>A	TGCAGCCACCGTAGGCTCCCACAGCGCTGCCATAGCCACCCGAGTCCGTGGTCGCCG	50:G>A
990057|F|0-41:C>T-41:C>T	TGCAGCAGCGGCGGCAGGCCCAGCCACGAGGTGGCCAACTACATCCAGAGGCTCATCGCTGCCACGCTC	41:C>T
1020494|F|0-6:G>A-6:G>A	TGCAGTGCCTGGATGCAGCCGTCTTACTGTTACAGCACGGCGGAGGCGCCG	6:G>A
32024215|F|0-13:T>C-13:T>C	TGCAGGTGACCGTTGGCGGCCTTGACCCCG	13:T>C
32021973|F|0-55:A>C-55:A>C	TGCAGGAGAGCATCTCGACGGTGCAGTCGTCGCGGGTCGGGGTGACGCGGAGGTCAAGGACCG	55:A>C
32022450|F|0-34:A>G-34:A>G	TGCAGCGGAGGCTGATCGAGTTCCTCGCCGCGCGACCAGAGTGGCGGAGGTCCG	34:A>G
5363606|F|0-36:G>C-36:G>C	TGCAGCTCCGAGGCGCGCGCCGCCGTGCTCGTGCCAGGGGCCG	36:G>C

<table>
<tr><td>2250377|F|0-15:C>T-15:C>T</td><td>TGCAGGTGTTGTCGACGAGGCGGAGCAGCGTCTCGTAGCCCTTGGTGTTGATTGGGGTGGAGCTGAGCT</td><td>15:C>T</td></tr>
<tr><td>1394604|F|0-7:G>T-7:G>T</td><td>TGCAGGTGATCACCGCGCGGCCGCCCATGGGGCTGACGCATCACGTCGAGAAGGCCATCGAGTCCGAGA</td><td>7:G>T</td></tr>
<tr><td>1124420|F|0-26:C>T-26:C>T</td><td>TGCAGAGCGGCGCACACCTGAGCCGCCGCTTCTTCCTTGTCTCCGTCCAGCCTTCCCCTCAGGACCCGA</td><td>26:C>T</td></tr>
<tr><td>4005867|F|0-32:C>G-32:C>G</td><td>TGCAGCAGCGTCGCCTCCGCCGACTTCGAGGGCCTCTTCTCCCCCTCCG</td><td>32:C>G</td></tr>
<tr><td>4993562|F|0-6:G>A-6:G>A</td><td>TGCAGCGGGCGGCGACGGCGAGGCAGGGCTGACCCG</td><td>6:G>A</td></tr>
<tr><td>7342808|F|0-5:A>T-5:A>T</td><td>TGCAGAAGGGCCCGATCTCGTTCCCG</td><td>5:A>T</td></tr>
<tr><td>1027333|F|0-20:T>C-20:T>C</td><td>TGCAGTGAAGTGTGATGAACTGAGGTGGCTAGGAGCGCTCGCGTATATGTACTGGTCCGTCGAGCACCG</td><td>20:T>C</td></tr>
<tr><td>5367154|F|0-7:C>G-7:C>G</td><td>TGCAGCTCCCACGGCTGGGCCGAGTCGCCG</td><td>7:C>G</td></tr>
<tr><td>1696089|F|0-39:A>G-39:A>G</td><td>TGCAGGTCACCGTGCCGCTGAAGAAGGTCGCCAAGACGGAAGCGCTGGCCATCGGCGACCCTACCCGTC</td><td>39:A>G</td></tr>
<tr><td>1080966|F|0-33:C>G-33:C>G</td><td>TGCAGATGATGCAGTCCTTCCTGCGGGTGCAGTCCTCCGCCACGCGCTGGGGCGCCG</td><td>33:C>G</td></tr>
<tr><td>1070984|F|0-37:T>A-37:T>A</td><td>TGCAGCCTGCTCGTCCAGCAACGCCGCGTGGGCGTCGTAGTACATGTGGTGGCCACCG</td><td>37:T>A</td></tr>
<tr><td>1685839|F|0-16:G>A-16:G>A</td><td>TGCAGGGCCACGCCACGCTTGAGGCCGAGGTGGCGCGGCTGAGGGGCCTCCTCTTCGACGTCCG</td><td>16:G>A</td></tr>
<tr><td>3222464|F|0-5:C>T-5:C>T</td><td>TGCAGCCGACGAACGAGAACCCCGTGTCCTCCAGCATGCTCTGCCG</td><td>5:C>T</td></tr>
<tr><td>1091459|F|0-19:G>A-19:G>A</td><td>TGCAGGTGGGTGGGTGGACGATCACCTACGAGGGCTTGACGTTCGTGACGATTCGCGGAGCCG</td><td>19:G>A</td></tr>
<tr><td>3027570|F|0-9:A>G-9:A>G</td><td>TGCAGCGCAACGGGTGTCAGTCCTTCTGGGCACCATCCG</td><td>9:A>G</td></tr>
<tr><td>1242527|F|0-37:G>C-37:G>C</td><td>TGCAGGTGATCCTGCACCAGCAGCTGCAAGTGCTGGTGGAGAGGTACCG</td><td>37:G>C</td></tr>
<tr><td>3957222|F|0-6:G>C-6:G>C</td><td>TGCAGGGCAGACCGCACAAGGGACCTCGCATACGCCTCTGCGATCAGGCCGTTGTGCTTGACAAGCGCC</td><td>6:G>C</td></tr>
<tr><td>1022998|F|0-34:G>C-34:G>C</td><td>TGCAGGTGCTGAGCGCGCTGGACGGCGCCAAGACGCAGTTGTACCACTTCAGGGCGATCGTTGTCGCCG</td><td>34:G>C</td></tr>
<tr><td>4262795|F|0-31:C>T-31:C>T</td><td>TGCAGGACTTGACGGGGTATATGAGGATGGACGCCACCGTCGCCGCCG</td><td>31:C>T</td></tr>
<tr><td>1034492|F|0-25:C>G-25:C>G</td><td>TGCAGCCCAAGGTCCCGTTCTTCACCGTCATCACCGACCTCAACACCTGCCACCCGACATGGTAACCAA</td><td>25:C>G</td></tr>
<tr><td>6044014|F|0-8:T>C-8:T>C</td><td>TGCAGCTTTTCCCGCCGCTTGCGCTCGGCGATGACGTGGTCCTGCGCGTTCGCCG</td><td>8:T>C</td></tr>
<tr><td>5582399|F|0-19:C>G-19:C>G</td><td>TGCAGTTCAGGAGGGTGCGCTGCAAGTACGCGCCCG</td><td>19:C>G</td></tr>
<tr><td>4261150|F|0-19:A>G-19:A>G</td><td>TGCAGGTCCCGTCGAGGCAAGCCCTGGCGGAGCGGCGCCG</td><td>19:A>G</td></tr>
<tr><td>3023262|F|0-23:C>G-23:C>G</td><td>TGCAGGAAGTTGGTCAGCATTAGCTGCTTCAGGCTTCTGGCCG</td><td>23:C>G</td></tr>
<tr><td>2255238|F|0-28:A>G-28:A>G</td><td>TGCAGACCGCCCCGTCATCGAACAGCACATCCACGTCGATGGACACCTCGACAACCCG</td><td>28:A>G</td></tr>
<tr><td>10984254|F|0-19:G>T-19:G>T</td><td>TGCAGTTGGCGTGGGAGGAGGCGAATCCG</td><td>19:G>T</td></tr>
<tr><td>1020725|F|0-7:C>T-7:C>T</td><td>TGCAGGCCGAGGAGCCACTGGTTGGCGGCTAGACGTGCGGAGGGATGCGAGGCGTGATTAAGTGCGTGC</td><td>7:C>T</td></tr>
<tr><td>1167323|F|0-7:T>C-7:T>C</td><td>TGCAGCGTCTGTCCATGTCGCCCGAGATTGCCCGCGCAATCGTCGGCCACAGCGGCGTCCGTCCACTCA</td><td>7:T>C</td></tr>
<tr><td>7344279|F|0-13:G>A-13:G>A</td><td>TGCAGAACAACCAGCTTTCCG</td><td>13:G>A</td></tr>
<tr><td>3942640|F|0-7:C>G-7:C>G</td><td>TGCAGTCCCTGGTCCTCGTCGACAACAACCTCACCG</td><td>7:C>G</td></tr>
<tr><td>4910031|F|0-5:G>T-5:G>T</td><td>TGCAGGGTGGCCGAGTAGCTCCG</td><td>5:G>T</td></tr>
<tr><td>3934836|F|0-23:A>G-23:A>G</td><td>TGCAGCTCTACACCAACTTCCTCACCG</td><td>23:A>G</td></tr>
<tr><td>3026500|F|0-26:G>C-26:G>C</td><td>TGCAGTCAGGGCAGTGCTCCTTGGGAGCCACATGCCCTACCG</td><td>26:G>C</td></tr>
<tr><td>3934511|F|0-20:A>G-20:A>G</td><td>TGCAGCTTCGAGAGGTTGGCAAGAGAGGCCG</td><td>20:A>G</td></tr>
<tr><td>4990816|F|0-13:G>A-13:G>A</td><td>TGCAGCAGGCGGCGGCGCGCGCGTGGTCGCCG</td><td>13:G>A</td></tr>
<tr><td>1032068|F|0-60:C>G-60:C>G</td><td>TGCAGCACATCGGCACGTCCACGTGGCCTGGTTAAGCACCTTCCCTATGGTACATTTTTGCCCCGAGAT</td><td>60:C>G</td></tr>
<tr><td>1076445|F|0-28:C>T-28:C>T</td><td>TGCAGGTGTATCACCCGAACATCAACTCCAACGGCAGCATCTGCCTGGACATCCTCAAGGAGCAGTGGA</td><td>28:C>T</td></tr>
<tr><td>3027442|F|0-20:G>T-20:G>T</td><td>TGCAGGTCGAGGAAGGCGTGGCCCATCTTGTCGTCGTACTTGAACCG</td><td>20:G>T</td></tr>
<tr><td>3064908|F|0-32:G>A-32:G>A</td><td>TGCAGCAGCGTCGGCGCGAACGAACGAGGAAAGTCCG</td><td>32:G>A</td></tr>
<tr><td>1037736|F|0-22:A>T-22:A>T</td><td>TGCAGATTTGTGGTATTGCTTCAAAGCTTGCAGGGTCTTCAAGAACCTTGCAAGACAAGGTATCTCTCC</td><td>22:A>T</td></tr>
<tr><td>1021070|F|0-40:G>A-40:G>A</td><td>TGCAGCGCGAGCTCGGTGAGGCGGAAGCCCTTGCGGTGCAGGTACTGCTGCACCTTCCTCTCCATCTCC</td><td>40:G>A</td></tr>
<tr><td>12470452|F|0-23:A>G-23:A>G</td><td>TGCAGAGCCACAGGCGACCACACACCG</td><td>23:A>G</td></tr>
<tr><td>1024669|F|0-57:T>C-57:T>C</td><td>TGCAGATGGGGCAGGTCGGCCTCGTCGTCGTCTCGTCCCGCGAGGCGGCCAGGGAGGTCATGAAGGTGC</td><td>57:T>C</td></tr>
<tr><td>1232096|F|0-20:T>G-20:T>G</td><td>TGCAGGTTCACCAGCTTGCTTATCGTGCTCGGCAGCCTCTCCAGCTGCCCGCACTGGAGGCCCAGGTAC</td><td>20:T>G</td></tr>
<tr><td>3944157|F|0-7:G>C-7:G>C</td><td>TGCAGGTGTCGCACCCGACCAAGGAGGCGCCG</td><td>7:G>C</td></tr>
<tr><td>985898|F|0-43:A>G-43:A>G</td><td>TGCAGGCCCTAGAGAGCTCCAGCACCGTGGTGTCAGTGGCGACAGCATGCTCCCCG</td><td>43:A>G</td></tr>
<tr><td>32025563|F|0-45:A>G-45:A>G</td><td>TGCAGCTCCGCCATGCCGCTCTGCTGCCACGCGACCGCCTCACGCAGCGCGGCCGCCTCGTCGCCGAGA</td><td>45:A>G</td></tr>
<tr><td>4261171|F|0-36:A>G-36:A>G</td><td>TGCAGATGCTTAACTGGGTCCGCCTTGTGGCGAAGAATCCG</td><td>36:A>G</td></tr>
<tr><td>7172986|F|0-12:T>G-12:T>G</td><td>TGCAGCGGACTCTCCGTGGCCAGCTCCG</td><td>12:T>G</td></tr>
<tr><td>7172177|F|0-19:T>C-19:T>C</td><td>TGCAGGAGAAGATCAAGGCTCTCGAGGCAGCCACCG</td><td>19:T>C</td></tr>
<tr><td>1862625|F|0-17:G>A-17:G>A</td><td>TGCAGAGTGAAGTTGACGTGGTCCTGCACGCCCTCGAGGTGCTTCTGGGCGGCGACGTTCACCTCCTCC</td><td>17:G>A</td></tr>
<tr><td>4261171|F|0-34:G>A-34:G>A</td><td>TGCAGATGCTTAACTGGGTCCGCCTTGTGGCGAAGAATCCG</td><td>34:G>A</td></tr>
<tr><td>1667027|F|0-24:C>T-24:C>T</td><td>TGCAGCTGCCCAAGGGCATGGAGGCCGTGAACCACATCTGGCAGGTAGGGTCGACGGTGGCCAATGGGG</td><td>24:C>T</td></tr>
<tr><td>2250377|F|0-37:G>C-37:G>C</td><td>TGCAGGTGTTGTCGACGAGGCGGAGCAGCGTCTCGTAGCCCTTGGTGTTGATTGGGGTGGAGCTGAGCT</td><td>37:G>C</td></tr>
<tr><td>977215|F|0-49:A>G-49:A>G</td><td>TGCAGGGTGAGGAAGGTTCGGCCCTTGAGGAGGGCCCTCAAGGGCCTCAAGGCCTGGATCACCG</td><td>49:A>G</td></tr>
<tr><td>1254491|F|0-28:C>T-28:C>T</td><td>TGCAGGGCAAGCTGGACCCCCGCCACCCCAGTTTTGTCAAGCCCATGACCCAGTCTCATGTCACCGAGA</td><td>28:C>T</td></tr>
<tr><td>4262277|F|0-7:C>G-7:C>G</td><td>TGCAGGTCGGGTACTACGACACGCTGTGCCCG</td><td>7:C>G</td></tr>
<tr><td>1041969|F|0-59:C>T-59:C>T</td><td>TGCAGCCTCATCTCCTCCCTGAACTTCCTGATGAACATCTCCGCCCTCACGTCCACCTCCCCCATCCCA</td><td>59:C>T</td></tr>
<tr><td>4262278|F|0-18:C>T-18:C>T</td><td>TGCAGTATTATTAAAAAACGACAGCACAGGCAGCATGCAGAGACCG</td><td>18:C>T</td></tr>
</table>

1013635|F|0-58:C>T-58:C>T	TGCAGTTCACGGTGAAGGACTCTGTGCGGGAGAGCGTGTCCAGCGCGTCCGAGAACCTCTCGTCGTCAT	58:C>T
1024456|F|0-63:C>G-63:C>G	TGCAGAGGACGCTGGACTACGCGTGCGGCAGCGCCGCCGACTGCAAGCCCATCCAGCCCAACGCCACCT	63:C>G
5581498|F|0-8:C>A-8:C>A	TGCAGGCGCTTGATGGCCGCCGTGGTGCCG	8:C>A
977055|F|0-18:G>C-18:G>C	TGCAGGTTCGGCCACGACGGATGCCAGGCCGTTCACGTCCACGCCGACGTGGTTACTGCTGATGTCCCG	18:G>C
1221524|F|0-16:G>T-16:G>T	TGCAGGAGGTGAAGCTGGCCATGTTCCACCTCTACCGCCGCTACGTGTTCCGCCG	16:G>T
2321212|F|0-5:C>T-5:C>T	TGCAGCTGGCCGCGCACGACTGCTCGCACTCCGCCGCCGTCTTGTACGTCTCGAACGGCAGCTGCTCCT	5:C>T
5411830|F|0-7:C>G-7:C>G	TGCAGCGCAATGCGGAGGAGCAGGAGATCATCTCCAGGGACCG	7:C>G
1041369|F|0-37:G>C-37:G>C	TGCAGGAGCACTCCAGCTTCATGGCGCCGCCCCCCTCGGACAGCTCCACCATGCAGATCCG	37:G>C
1089360|F|0-23:C>G-23:C>G	TGCAGAACGCCAACATGCTGGCGCCCAACAACAGCGAGACCCACCCGTGGCACCTCCACGGCCACGACT	23:C>G
4261060|F|0-17:T>C-17:T>C	TGCAGATAGCAACTTCGTGGGGTTCAGTTACGTGCGGCCG	17:T>C
4408436|F|0-5:G>T-5:G>T	TGCAGGTAGGGGAGCTTGTGCGTGTCCG	5:G>T
4408436|F|0-23:G>C-23:G>C	TGCAGGTAGGGGAGCTTGTGCGTGTCCG	23:G>C
3939963|F|0-11:C>T-11:C>T	TGCAGCGCCCTCGCCGAAAGCGTGAAGCGCTGGGCGCCG	11:C>T
2321212|F|0-57:G>A-57:G>A	TGCAGCTGGCCGCGCACGACTGCTCGCACTCCGCCGCCGTCTTGTACGTCTCGAACGGCAGCTGCTCCT	57:G>A
3945135|F|0-29:G>A-29:G>A	TGCAGGAAGGAGATGGCCCCCACGCAGCCGTACACCG	29:G>A
1029852|F|0-43:C>T-43:C>T	TGCAGAAGGTGGCCAAGATGGTGGACGAAGTGGCGGCGGAGATCGCGACGGACGAGTCCCTACCGATAT	43:C>T
6044226|F|0-6:A>C-6:A>C	TGCAGTACGGCGTTTCGGCGTCCG	6:A>C
4261787|F|0-8:C>G-8:C>G	TGCAGAAGCCCGTGCCAGCCGTGACGTACAGCCTCAGCCG	8:C>G
3943030|F|0-7:C>T-7:C>T	TGCAGTACGCCATCGACGCCGTCCATTCGTTCACCG	7:C>T
1025257|F|0-53:C>T-53:C>T	TGCAGGAGCCTCGTGATCATGGCGGCGTCGGTGACGCGGTTACGGCTCAGGCGCACGGTCCG	53:C>T
3029607|F|0-9:C>T-9:C>T	TGCAGAACGCCGTCTGGACCTCACTCTACAAGTGCGTGGAGCCG	9:C>T
1067023|F|0-11:C>G-11:C>G	TGCAGCAGCACCACCCCGAAGCTGTACACGTCGGACTTCATGCTGACCTGGCCGCCGCGGTCGTACTCC	11:C>G
4261628|F|0-15:C>G-15:C>G	TGCAGGTGCCTGGAGCAGTCCG	15:C>G
1024456|F|0-6:G>A-6:G>A	TGCAGAGGACGCTGGACTACGCGTGCGGCAGCGCCGCCGACTGCAAGCCCATCCAGCCCAACGCCACCT	6:G>A
1070952|F|0-47:G>A-47:G>A	TGCAGCGTGATCAAGACTGCTGCTCCCAAGACCAAGGCCACAAGGAAGACCCG	47:G>A
985898|F|0-21:G>A-21:G>A	TGCAGGCCCTAGAGAGCTCCAGCACCGTGGTGTCAGTGGCGACAGCATGCTCCCCG	21:G>A
4261139|F|0-12:T>C-12:T>C	TGCAGCTCACCGTTCGTTCTTCTCGCTCCG	12:T>C
1111757|F|0-41:G>A-41:G>A	TGCAGCCACTGTAGGCTCCCACAGCGCTGCCATAGCCACCTGAGCCCGTGGTCGCCG	41:G>A
7917298|F|0-41:G>T-41:G>T	TGCAGGTAGAGGAAGGGGTCGGCGACGAAGTTGCTGGGGTAGCCG	41:G>T
3024793|F|0-5:G>C-5:G>C	TGCAGGTTCCCAGGCCACGATTTCTGGCCGTCAATTCTTGACCG	5:G>C
1694794|F|0-29:G>C-29:G>C	TGCAGGCCATCGACGTCGTTGGCACGGAGGACGCTGAGGATCTGTGGGTCGGAGTGCTCGCCAAACCCG	29:G>C
1017611|F|0-17:G>A-17:G>A	TGCAGTACGTGTCCAACGTGATCATCCACAACCTGCACGTGCACGACTGCGTGCCG	17:G>A
3937294|F|0-10:C>G-10:C>G	TGCAGATCGCCGCGGCCGCCGCCTGCTGCGCGCCG	10:C>G
7172983|F|0-5:C>T-5:C>T	TGCAGCCCCGTGGAGGTGGTGTCGGTGCCG	5:C>T
3943030|F|0-28:G>C-28:G>C	TGCAGTACGCCATCGACGCCGTCCATTCGTTCACCG	28:G>C
1054728|F|0-61:C>T-61:C>T	TGCAGAGGCACCCCAACACCGAGGACGAGATGGTGCAGCTGCTCCAAGTCGCCATGGCGTGCGTCGCCG	61:C>T
3020630|F|0-22:A>G-22:A>G	TGCAGGTATTTGCAGCACAGTCAAACTTGTGGCACACCG	22:A>G
1061423|F|0-41:G>C-41:G>C	TGCAGCAGCGCCGTGGTCGGGAGCACGAAGGAGACGTTGTTGATGGACGCCGCGAACCG	41:G>C
6038176|F|0-17:C>A-17:C>A	TGCAGGTCCGATCGTGGCGGTTATGCCATCGGCAGCGCTGGCTGCCACGCCG	17:C>A
3943030|F|0-9:C>T-9:C>T	TGCAGTACGCCATCGACGCCGTCCATTCGTTCACCG	9:C>T
1024350|F|0-5:G>T-5:G>T	TGCAGGCGCAGTTCGGCAACAAGTGGGCGAGGATCGCCACCTACCTGCCG	5:G>T
1088113|F|0-59:C>T-59:C>T	TGCAGCGGCGTCGGCATCCTCGGGATCGCCTGGGCCTTCGGCGGCATGATCTTCGTCCTCGTCTACTGC	59:C>T
3029515|F|0-29:C>G-29:C>G	TGCAGCACGGTGTCCGCCGCCTCGGCGCTCGGCGACCACCGCCG	29:C>G
1203622|F|0-34:G>T-34:G>T	TGCAGATGAAAGGCATCGGCTACGAGTACATGGAGCAGGACCTGTTCACCAAGGGCGAGCTCCTCCGCA	34:G>T
3385510|F|0-10:G>C-10:G>C	TGCAGCTCTCGCGCTGCGCCTCGCTCGCCGCCCTCAACCTCAGCTCCG	10:G>C
1065940|F|0-43:C>T-43:C>T	TGCAGGGCAGCATGTCCAAGCTCAAATCGCAACTGAAACCTTTCTGGCACAAGTACCCGTATGAGGAGG	43:C>T
3937159|F|0-31:C>G-31:C>G	TGCAGTCCAACATCTTCATCGAGGGCGTCTCCCACAAGATCCGCCG	31:C>G
5325034|F|0-7:G>A-7:G>A	TGCAGGAGAACCAGAAGCTGAAGAAGCAGGTGGAGAAGATGAGGAAGAAGCACGAGGTGGAGATCGAGA	7:G>A
1078294|F|0-46:G>A-46:G>A	TGCAGAGCGTGACGCTGATCCCGTCGGTTTGATTTTGCTGGCCCTCGTTCAGATTGTAAGAGCCGAGAT	46:G>A
1862531|F|0-34:C>T-34:C>T	TGCAGTTGCGCGCGCACAGCGCCTCGCAGTCTCCCGCGGACGGCGCGTGCTCGAACCCCAGCAGCTCCT	34:C>T
1023527|F|0-7:C>G-7:C>G	TGCAGCTCACGGCGCGGCCCTCGGCGGCGAGGAGGAGGCGGTTGAAGGCCG	7:C>G
3959413|F|0-5:G>T-5:G>T	TGCAGGGCGCCGACGGCGGGCAGAACGGGGAGTTCTCGCCG	5:G>T
1864536|F|0-7:C>T-7:C>T	TGCAGAGCAGCAGCAGGATCCATCTCGTATGGCTGGCTGTTGCCATCACCG	7:C>T
1024669|F|0-34:G>A-34:G>A	TGCAGATGGGGCAGGTCGGCCTCGTCGTCGTCTCGTCCCGCGAGGCGGCCAGGGAGGTCATGAAGGTGC	34:G>A
3939204|F|0-14:T>G-14:T>G	TGCAGGCGCTGTGGTCGCAGCCG	14:T>G
23919947|F|0-6:C>T-6:C>T	TGCAGCCCCTCCACGTATCGCCGCCG	6:C>T
1051500|F|0-9:C>T-9:C>T	TGCAGGGGGCTCAACTTCTTGGCGATCATCGGGCTGATCCTCTGCACGGCGTCGTTCGGCATCAACCTT	9:C>T
4262292|F|0-31:T>C-31:T>C	TGCAGGAAGTCGCTGGCGCCGATGCTGAAGATGTAGAGGGCGCCG	31:T>C
2242932|F|0-5:C>G-5:C>G	TGCAGCCGCACGATCTCCGTGATCTTGTTCGACTTCTTGCCCCCGAGCTGCTCCATCTCCG	5:C>G

5358348|F|0-22:T>C-22:T>C	TGCAGGGCTATTACATGTGGGATTCCTTTGCCGCCG	22:T>C
4261150|F|0-34:G>A-34:G>A	TGCAGGTCCCGTCGAGGCAAGCCCTGGCGGAGCGGCGCCG	34:G>A
1064241|F|0-41:T>C-41:T>C	TGCAGGTGATTGAGCATGTGGAGGAGGACCAAGCAAGTTTATTCGGCAACGTTGCATTGAGTTCATTCT	41:T>C
2361445|F|0-26:C>T-26:C>T	TGCAGGCTGGCGTCGAGCAGCGCGGCCTGCTTGCCGACGTCGGCGTCGGCCACGAAGCCGCCGAACCGA	26:C>T
1055233|F|0-10:T>C-10:T>C	TGCAGAGCATTCCAGAGTCAATGATCGCGCGGCTGCTGAACGTGGTGGTGCTGGACGTGCGCTCCAACC	10:T>C
5010988|F|0-19:C>T-19:C>T	TGCAGAACTCCCTCAGCTTCCTCTGCTGCCACACCG	19:C>T
4009326|F|0-41:G>A-41:G>A	TGCAGGACTTGATGGGGTATATGAGGATGGACGCCACCGTCGCCGCCG	41:G>A
12695035|F|0-13:C>T-13:C>T	TGCAGTTCCTGTGCGCGGTGCCG	13:C>T
1038836|F|0-14:C>T-14:C>T	TGCAGCCCAAGGAGCTGTACAAGATCGCCAAGGACATCGCCCCCGCGCCG	14:C>T
1150940|F|0-29:A>G-29:A>G	TGCAGAGGACGCTGGACTACGCGTGCGGCAGCGCCGCCGACTGCAAGCCCATCCAGCGCAACGCCGCCT	29:A>G
3934476|F|0-11:A>G-11:A>G	TGCAGGATCTCACGCTCCGTCTCCGCGCGGCTCAGCTTCCG	11:A>G
6043983|F|0-20:A>C-20:A>C	TGCAGCTGCATCCCGCCGTCAGCCGCTGCCCAGCCGTGCTCACCACCCG	20:A>C
1034492|F|0-68:A>C-68:A>C	TGCAGCCCAAGGTCCCGTTCTTCACCGTCATCACCGACCTCAACACCTGCCACCCGACATGGTAACCAA	68:A>C
1025155|F|0-27:T>C-27:T>C	TGCAGCAATGTGCATCTTCTCCAGATTTACAAGCGACGGTGCTGAAACCGAGGTGTGCATGCCGCCGCC	27:T>C
1038979|F|0-38:G>C-38:G>C	TGCAGCAAACCATCTCAGCGGATGACGCTGCACTTCTCGGCACCCGTGTCTCCG	38:G>C
1205880|F|0-59:C>G-59:C>G	TGCAGCGAGCTGCCGTTGCGCACGCTCAGCTCCGCCGTGGGCGCGCCCGTGGCGTCCGTCCCGAACCGA	59:C>G
3023650|F|0-11:C>T-11:C>T	TGCAGGATGAGCCAGAGCGCATAAAAGAAGCAGCTGCCG	11:C>T
7353729|F|0-10:C>A-10:C>A	TGCAGCGGCGCCTACGCGACGCCG	10:C>A
5363732|F|0-25:C>A-25:C>A	TGCAGGAGGCAGTTCGAGACGAAGGCTGTCGGGACGAACCCG	25:C>A
3024360|F|0-27:C>A-27:C>A	TGCAGCGCTCGCCAAGGACTTCCTCACCAACTTCGCCG	27:C>A
3024793|F|0-35:T>C-35:T>C	TGCAGGTTCCCAGGCCACGATTTCTGGCCGTCAATTCTTGACCG	35:T>C
4999261|F|0-21:T>C-21:T>C	TGCAGTTCATCGATGGTATGTTCGTCTGCATCACCG	21:T>C
5324478|F|0-13:C>A-13:C>A	TGCAGGCGGGGGGCGACGCGGCGGCCAAGCCG	13:C>A
1020207|F|0-31:A>G-31:A>G	TGCAGAACTGTGAGAATTGTCCAAAGTCATGACTTACAGGCCACCGACGATGGCGGAGAAGATGGCCGT	31:A>G
1020730|F|0-38:A>T-38:A>T	TGCAGCTTGCTGAGGACGGCGAGGGCGCTTGCGGCGGAAAGATCCATGGACGCGGGGGAGGCCTCGCCG	38:A>T
5373735|F|0-5:C>A-5:C>A	TGCAGCACCTCCTGAGCGTACTCCG	5:C>A
986239|F|0-40:C>T-40:C>T	TGCAGGGCCAGGTCCTGGTGCTCCTTCTTGCACACCTGGTCGGCGCCGATGTTGCCGTACCG	40:C>T
3935245|F|0-10:T>C-10:T>C	TGCAGTTTCTTGTTCCCATTTTAATCTTGGGGCTTGCATTTGGTGTCCG	10:T>C
1695009|F|0-65:C>T-65:C>T	TGCAGGCGTCGTCGGCGTAGCTGAACACGGCCTCGCGGTCGATGGCGGTGATGAGGCTCTTCTCGCTGA	65:C>T
1695009|F|0-64:G>A-64:G>A	TGCAGGCGTCGTCGGCGTAGCTGAACACGGCCTCGCGGTCGATGGCGGTGATGAGGCTCTTCTCGCTGA	64:G>A
3222153|F|0-27:T>C-27:T>C	TGCAGCTGGGAAATACTGTAACGACATTGGTGTAGGACTTGCACCG	27:T>C
1144861|F|0-5:C>G-5:C>G	TGCAGCGAGCAGGCGGAGATGATCCTGGTGTACGAGCACATGGAGAAAGGCACGCTGCGGAGCCACCTG	5:C>G
1718514|F|0-30:G>A-30:G>A	TGCAGGGACCTGGGCGATGAAGACTCCGACGAGCAGCGCCAGTGCAAGCGCCG	30:G>A
5358456|F|0-20:C>G-20:C>G	TGCAGGTGCGAGGGCATGTCCACCTATCCCCG	20:C>G
5000494|F|0-23:G>T-23:G>T	TGCAGGTACGTCATGGCGAGGCTGGCGTGCATCGCCGCGCCGTACGGGAGCGCGCCTTCGTCGACGAGG	23:G>T
3024360|F|0-6:G>C-6:G>C	TGCAGCGCTCGCCAAGGACTTCCTCACCAACTTCGCCG	6:G>C
3946410|F|0-24:C>T-24:C>T	TGCAGCACCTTCCTCTGCCTCTCGCTGTAGCCG	24:C>T
2247983|F|0-41:C>G-41:C>G	TGCAGCAGCAGCTTCGAGGCCAGGGTCTTCGAGCTCGCGCGCGCCTCCAACCCG	41:C>G
1069445|F|0-20:G>A-20:G>A	TGCAGCCACCCGAAGGCCGTGCCGCGGTGGTCGTCGTCGGTGGAGTCGGCCACCAGTGACTGGACCGCC	20:G>A
3027323|F|0-7:T>C-7:T>C	TGCAGTATCTGCGCCACGTTGCGGTCATCCTGGACGAAGCGCTTCCG	7:T>C
6041850|F|0-9:A>T-9:A>T	TGCAGGTCCACGCCGACGGCCAGATCTCCCGCCCG	9:A>T
1073290|F|0-7:G>C-7:G>C	TGCAGTCGGTGCGGCTCAAGTACGTCAAGCTGGGGTACCACTACCTCATCACCCACGGCATGTACCTCC	7:G>C
2246054|F|0-17:C>T-17:C>T	TGCAGCTACCTCCTCCACGTCGACGCCGACGACGAGGCGCTGATCCAAATCCCCG	17:C>T
4262244|F|0-19:T>G-19:T>G	TGCAGCTGCCTCGAACGACTCCCCACGGCCAGGAGGCAGAGCCG	19:T>G
1023049|F|0-5:A>G-5:A>G	TGCAGATTCAGATCCAACGGTAGAATCAGGTGATATTGACGGTACCACCG	5:A>G
3943060|F|0-11:G>T-11:G>T	TGCAGCCAGAAGGTGCCGTTGGGGTCGCCG	11:G>T
7902441|F|0-7:G>C-7:G>C	TGCAGTCGGCGGCGCTGTCCTTCTTCAGCTTCACCAACAGCAAGCCG	7:G>C
1019516|F|0-13:C>G-13:C>G	TGCAGGCAGCGTTCGGCGGGGAGGGCGGCGAGGGGGCATGCATGAGGGTTAACTTCTACCCGAGGTGCC	13:C>G
1123751|F|0-16:G>C-16:G>C	TGCAGAGGAACGTCGCGCTATACCGGAAGATCGTGCCGCCCGTGGCGGAGCACTCCCCG	16:G>C
1064430|F|0-40:T>A-40:T>A	TGCAGCTCTGGCCACGGTGGTATTACAAATCTCATGTATTTTAGCCCAGCGCTGGAAAACGAAACAATA	40:T>A
4261571|F|0-7:G>T-7:G>T	TGCAGTTGCGCCTGATCTCGCCG	7:G>T
7172960|F|0-25:G>T-25:G>T	TGCAGGTGATCCTGCACCAGCAGCTGCAAGTGCTGGTCGAGAGGCACCG	25:G>T
4262278|F|0-36:G>A-36:G>A	TGCAGTATTATTAAAAAACGACAGCACAGGCAGCATGCAGAGACCG	36:G>A
9766478|F|0-18:C>G-18:C>G	TGCAGCATACTAGGGATGCTATTTCTTTTCTCCG	18:C>G
998072|F|0-39:C>T-39:C>T	TGCAGTGGGTGAGCCTGGTGATCATCATCGGGGCACTGGCGTGCAGCCTCGCGATAAGGCCGCTGTCAC	39:C>T
7913650|F|0-10:T>C-10:T>C	TGCAGGCAGGTAGGGGGCTCCG	10:T>C
6044272|F|0-11:C>G-11:C>G	TGCAGTGGACGCTCCTCCACTTCTTCTCCCCG	11:C>G
1029079|F|0-6:C>G-6:C>G	TGCAGGCCGTCCTGGCGCGCGCCCACGGCGAGATGCAGGACTCGTTCGTCTGGCTCTTCCAGCACATCT	6:C>G
1087713|F|0-47:G>A-47:G>A	TGCAGCACATGCGTGGCCATCGCACAGCAGCGATTCGTTGGCAAGGCGTCCG	47:G>A

32017963|F|0-16:A>G-16:A>G	TGCAGGGCCACGTGCAAAACCCG	16:A>G
32020257|F|0-17:G>C-17:G>C	TGCAGCTTCCGACTGGCGCCCATGGCATCCG	17:G>C
32026628|F|0-13:T>C-13:T>C	TGCAGGCGGAGAGTACGCACGAGAGGAACAGGTACGCGGGAGCGGCGTACCCGTACGAGCTGCCATGGC	13:T>C
6045740|F|0-6:A>G-6:A>G	TGCAGGAGTGGCCGAGGCGGACGCTCTCCG	6:A>G
16709379|F|0-13:T>C-13:T>C	TGCAGGTGTATGCTACGATCAGGACGGGGTCCATGGCGATCGCGGCGTCCACGCTCGACTCGCTGCTGG	13:T>C
1069445|F|0-37:C>T-37:C>T	TGCAGCCACCCGAAGGCCGTGCCGCGGTGGTCGTCGTCGGTGGAGTCGGCCACCAGTGACTGGACCGCC	37:C>T
4999624|F|0-12:G>T-12:G>T	TGCAGCCTGCGGGACACTTGCCG	12:G>T
100579388|F|0-6:G>C-6:G>C	TGCAGTGCGTGTGCCGACGCCGAGCGCCCGCCGCCACGGCGGAGCACGAGCGGCAACGTGAATGACGGA	6:G>C
10984064|F|0-7:C>A-7:C>A	TGCAGGGCGACGCTGTCCACCCAGATGTCCACCG	7:C>A
5355611|F|0-34:T>C-34:T>C	TGCAGTTTGTTGTCTCGGAGAAGAGGAGAAATTGTTGGTCCTGTGATATCTGCACGCCGTTTGGGAAGA	34:T>C
1006991|F|0-9:C>G-9:C>G	TGCAGGAGGCCGATCTTGTCCGAGACCTCGGCGTCGGCCGCCGCGTCCTCCG	9:C>G
1020984|F|0-22:G>C-22:G>C	TGCAGCCTCGGCGGCGCAGAAGGAGCCTCCGCACAGAGCATCTCGGACTGGAGCACGGCGCAGTAGTGG	22:G>C
1094102|F|0-9:G>C-9:G>C	TGCAGGTCGGCGCGGAGCACGCTGGCGGCGGCGTCGTGGGACTGCGCGACGTCGCGCCACATCTGCGCC	9:G>C
4262072|F|0-15:G>A-15:G>A	TGCAGATCTCGCGCTGATGAGCGGCGCGCAGCTCTTCATCCG	15:G>A
2242928|F|0-16:G>A-16:G>A	TGCAGGTTGGGCGGCAGGTTGTAGAATGGCATCGACGACGGGTCTGGCAGTCCG	16:G>A
5581942|F|0-22:G>A-22:G>A	TGCAGCAGCGGGCCCAGCACCAGCACCG	22:G>A
992762|F|0-28:A>G-28:A>G	TGCAGGGGCCTGGGCGATGAAGACTCCGACGAGCAACGCCAGTGCAAGCACCG	28:A>G
4993329|F|0-22:G>A-22:G>A	TGCAGTGGCTCGACGTGTCGACGAACGCGCTCTCCG	22:G>A
4262292|F|0-28:A>T-28:A>T	TGCAGGAAGTCGCTGGCGCCGATGCTGAAGATGTAGAGGGCGCCG	28:A>T
1862895|F|0-32:C>A-32:C>A	TGCAGCTCGCTGGTGTTTGTGCTCGAGTCTGGCTCGGAGGGCTCCGTCTCCG	32:C>A
1053022|F|0-37:C>T-37:C>T	TGCAGCTCCAAGAACTCCAGCTAGTACATGTTTCCACCTGACACCTTTCTTGCCCG	37:C>T
1103635|F|0-54:C>T-54:C>T	TGCAGCAGCAGCACGTTGTGCATCTGTGCGAAGAGGTTGAGCCCGATGAGGCAGCCGTTGCCGTCGTAC	54:C>T
1085343|F|0-44:G>A-44:G>A	TGCAGCATGTACCGCCCGTTGTTCATCAGGAAGATGCTGCTCAGGGCCG	44:G>A
5567404|F|0-22:G>A-22:G>A	TGCAGGTCTCCGCCGTGATGTCGGCCG	22:G>A
998072|F|0-26:A>G-26:A>G	TGCAGTGGGTGAGCCTGGTGATCATCATCGGGGCACTGGCGTGCAGCCTCGCGATAAGGCCGCTGTCAC	26:A>G
1104443|F|0-14:T>C-14:T>C	TGCAGGATCATCTGTTCCAGGGTGACCGCGGCCGCCGCATGCGGGCCTCCACCG	14:T>C
1105435|F|0-9:C>T-9:C>T	TGCAGCCTTCCTGGAACATGGTGACCGCCTTTTCTCCATCTGACTCCCCGCCG	9:C>T
1020137|F|0-31:C>T-31:C>T	TGCAGAGCATATACGAGACGGTGAAGAGCCTCCAGGAGAAAGGGATGCTGCCG	31:C>T
4395246|F|0-5:G>A-5:G>A	TGCAGGCTGGTGCAGGGCATCAGAGACGCCG	5:G>A
7161514|F|0-41:C>T-41:C>T	TGCAGCTCCGCGGGGCACAGCGCGTTCACGTCGCCCACGCACCCG	41:C>T
1024214|F|0-29:G>C-29:G>C	TGCAGCGGCCTCTCCGCCGAGGAGGTGGAGTCTCCCTACGTCAACTTCGGCCGCCG	29:G>C
1037736|F|0-29:G>C-29:G>C	TGCAGATTTGTGGTATTGCTTCAAAGCTTGCAGGGTCTTCAAGAACCTTGCAAGACAAGGTATCTCTCC	29:G>C
1125483|F|0-52:C>T-52:C>T	TGCAGCGCACAAGACAGAAAAGCATGCACAGCCAACAAGCACAAGCACACCGCCATGCTCGTCTTGGCA	52:C>T
3385510|F|0-27:C>T-27:C>T	TGCAGCTCTCGCGCTGCGCCTCGCTCGCCTCCCTCAACCTCAGCTCCG	27:C>T
1036296|F|0-28:A>G-28:A>G	TGCAGCATGAGAGGAAATGCCTGGCATAATCAAATGTGTGGAACCCAATCAAGTCGGCATTCAGCAGCG	28:A>G
3027088|F|0-23:C>G-23:C>G	TGCAGCGACCCGTTGGGCATGAGCTCGTACACGAGCATCATCCG	23:C>G
1738574|F|0-32:C>T-32:C>T	TGCAGGAAGTACGGCGTGCGGTGCCCGTGCCCCGACAGGTTCTTCGCCG	32:C>T
5370409|F|0-53:T>A-53:T>A	TGCAGTTCATGCGGGAGTGGGGGCCGACGGTGATCTACAGGTCGAGGACGGGGTCGGAGCGGATGGTCA	53:T>A
1069445|F|0-29:G>A-29:G>A	TGCAGCCACCCGAAGGCCGTGCCGCGGTGGTCGTCGTCGGTGGAGTCGGCCACCAGTGACTGGACCGCC	29:G>A
1061423|F|0-14:G>C-14:G>C	TGCAGCAGCGCCGTGGTCGGGAGCACGAAGGAGACGTTGTTGATGGACGCCGCGAACCG	14:G>C
3026592|F|0-8:C>T-8:C>T	TGCAGGCACCGCGTGGTGACGGGGATCTCGAAGTCCACCCG	8:C>T
1016339|F|0-19:C>T-19:C>T	TGCAGCACACCATGTTCCGCGACACGCCCGCCCCCTTCCACTGCCAGTCCTGCGGCGACGCCGCCGTCA	19:C>T
3944031|F|0-18:A>G-18:A>G	TGCAGCATCTGCCTGGAGACGGCGTCGGAGCCG	18:A>G
5346555|F|0-5:G>C-5:G>C	TGCAGGCTGGCGGGTGGACGCCG	5:G>C
1027421|F|0-53:C>T-53:C>T	TGCAGGCAAGCGTCCAGCCAGGTCGGCGGTGGCTCCTGTGGACCGCCGTTGACCCTGGGCTGCGCCGAG	53:C>T
12341528|F|0-7:G>A-7:G>A	TGCAGCTGAGGACGTCGATCGCGGGGTGCCTGCTGGTGTCGGACAACCTGGAAGGGTCGTCCAGGCCGC	7:G>A
11313554|F|0-15:A>G-15:A>G	TGCAGTGGTTAACCTATGGGCTGGCCG	15:A>G
1033214|F|0-50:G>C-50:G>C	TGCAGCCGCTTCACCAAGCGGCTGTACAACTACAGCAGCACCGTGAAGCTGGACCCGTCGTTCAACCCG	50:G>C
6046855|F|0-19:C>T-19:C>T	TGCAGGGCGCGACCCTGGTCGTCATCAGGCCGTCCG	19:C>T
1262642|F|0-14:G>A-14:G>A	TGCAGCAGAGGCCCGAACACCAGCGGCGGGCACACGGTCACCACGTCCAGCCCG	14:G>A
1034492|F|0-29:A>G-29:A>G	TGCAGCCCAAGGTCCCGTTCTTCACCGTCATCACCGACCTCAACACCTGCCACCCGACATGGTAACCAA	29:A>G
3947817|F|0-9:T>C-9:T>C	TGCAGGATTTCAGGTCTGCCACTCGCTGGGAGGAGGCACTGGCTCCG	9:T>C
4396686|F|0-11:C>T-11:C>T	TGCAGCTGGCGCTGCTGCTGTTTGCAATGGCCCG	11:C>T
3957784|F|0-25:C>T-25:C>T	TGCAGAGCACGTCCATGCCGGCCATCTCCTCGATGGCCGTCATGCGCTTGGTGATGGCGCCCTGCTTCG	25:C>T
989829|F|0-23:C>T-23:C>T	TGCAGGCTGCGCCACACCTTCTGCGTGGCCGTGATACCGACGCCGATGCTGATGCCG	23:C>T
4010179|F|0-9:A>G-9:A>G	TGCAGGGGAAGCGGTTCTTCGCCG	9:A>G
7923660|F|0-9:G>C-9:G>C	TGCAGCTGCGGCATCCGCCCG	9:G>C
3941936|F|0-25:G>C-25:G>C	TGCAGGTGGCGGACATGCACTACGCGGACGGGCGGAGCACCG	25:G>C
7164783|F|0-14:A>G-14:A>G	TGCAGACGGCTCGGAGTCTCGACATCCACCG	14:A>G

<table>
<tr><td>1069445|F|0-38:G>C-38:G>C</td><td>TGCAGCCACCCGAAGGCCGTGCCGCGGTGGTCGTCGTCGGTGGAGTCGGCCACCAGTGACTGGACCGCC</td><td>38:G>C</td></tr>
<tr><td>1144861|F|0-23:C>A-23:C>A</td><td>TGCAGCGAGCAGGCGGAGATGATCCTGGTGTACGAGCACATGGAGAAAGGCACGCTGCGGAGCCACCTG</td><td>23:C>A</td></tr>
<tr><td>998527|F|0-32:C>A-32:C>A</td><td>TGCAGCGACGCGCTCTGCTTCTGCGCCGCGTCCAGCTTCGCCGCCGCGCCG</td><td>32:C>A</td></tr>
<tr><td>3026479|F|0-15:C>G-15:C>G</td><td>TGCAGATCAGTTGTACTTGAACAGACAGGGACGGAGCAGGCCTTCCG</td><td>15:C>G</td></tr>
<tr><td>3029032|F|0-17:G>A-17:G>A</td><td>TGCAGGTTCTTCTCGGGGGCCGTGGTCTGCGATGACGCCG</td><td>17:G>A</td></tr>
<tr><td>1102865|F|0-34:C>G-34:C>G</td><td>TGCAGCAGATGCTCGGCGTCTACGACGAGATCCTCGCCAAGAACCAGTACCTCGCCG</td><td>34:C>G</td></tr>
<tr><td>4261586|F|0-19:T>A-19:T>A</td><td>TGCAGGACTATGGACTTGGTGCTGAGCCCCG</td><td>19:T>A</td></tr>
<tr><td>1019516|F|0-27:G>C-27:G>C</td><td>TGCAGGCAGCGTTCGGCGGGGAGGGCGGCGAGGGGGCATGCATGAGGGTTAACTTCTACCCGAGGTGCC</td><td>27:G>C</td></tr>
<tr><td>1067877|F|0-20:C>T-20:C>T</td><td>TGCAGCTTGGACAGCCACGACGGTATCTGGCCCGTCAGACCGCACTCCTCCATGACGATCAGCCGAGAT</td><td>20:C>T</td></tr>
<tr><td>998072|F|0-37:G>C-37:G>C</td><td>TGCAGTGGGTGAGCCTGGTGATCATCATCGGGGCACTGGCGTGCAGCCTCGCGATAAGGCCGCTGTCAC</td><td>37:G>C</td></tr>
<tr><td>1077471|F|0-7:A>G-7:A>G</td><td>TGCAGTAATTTAGTATGTTGTTTTTACCTATAGTGCCACCAAGTTCAATGACACAAACATCAGCTGGCT</td><td>7:A>G</td></tr>
<tr><td>1013083|F|0-14:G>A-14:G>A</td><td>TGCAGGGCGTTCTTGTCACCAGCTTGGATGATAACAGCAGCCGTATGGTGGCAGTGAAGGCCCTGTTCA</td><td>14:G>A</td></tr>
<tr><td>2241765|F|0-13:T>C-13:T>C</td><td>TGCAGTTGGAATATCCGCCTTCCTTCGAGATAACTGCTACCGAAAGCTGTGCGGGAAATGACATCCCCG</td><td>13:T>C</td></tr>
<tr><td>6046667|F|0-32:G>C-32:G>C</td><td>TGCAGCAGCTTCGCCCCGCTCGGCGCGCCCCCGACCATCGTCTCGCCG</td><td>32:G>C</td></tr>
<tr><td>4261660|F|0-16:G>T-16:G>T</td><td>TGCAGCAGTAGCATGAGCCAGTAGCGGCACGTAGCGTCCG</td><td>16:G>T</td></tr>
<tr><td>4262795|F|0-16:G>A-16:G>A</td><td>TGCAGGACTTGACGGGGTATATGAGGATGGACGCCACCGTCGCCGCCG</td><td>16:G>A</td></tr>
<tr><td>4995431|F|0-12:T>C-12:T>C</td><td>TGCAGGGCGCTCTTCCCGTGCTTGACCTCTCCG</td><td>12:T>C</td></tr>
<tr><td>1023226|F|0-13:G>A-13:G>A</td><td>TGCAGCGGATCGGGGTCGGGATGCTGCTCCAGGTGGTGACCATGGTGATCACGGCAGGGATAGAGAACC</td><td>13:G>A</td></tr>
<tr><td>3948732|F|0-9:G>A-9:G>A</td><td>TGCAGGCGAGTCTGGGGGCACCG</td><td>9:G>A</td></tr>
<tr><td>1037736|F|0-45:C>G-45:C>G</td><td>TGCAGATTTGTGGTATTGCTTCAAAGCTTGCAGGGTCTTCAAGAACCTTGCAAGACAAGGTATCTCTCC</td><td>45:C>G</td></tr>
<tr><td>4396629|F|0-32:G>C-32:G>C</td><td>TGCAGCAGGATCTCGTTCCACGTGTCGATGGGGCCG</td><td>32:G>C</td></tr>
<tr><td>1079032|F|0-10:G>C-10:G>C</td><td>TGCAGGCGGTGGAGGGCAACATCCACTTCCGCGACGTGCGGTTCGCGTACCCGTCCCGCCCG</td><td>10:G>C</td></tr>
<tr><td>4397794|F|0-19:G>C-19:G>C</td><td>TGCAGTTGGTCTGCTGGTCGGCGAAGAAAGGCCAGCTGATCACCG</td><td>19:G>C</td></tr>
<tr><td>4406927|F|0-8:A>G-8:A>G</td><td>TGCAGAGCATCAAAGAAGCCGTCGCCG</td><td>8:A>G</td></tr>
<tr><td>7172849|F|0-5:A>G-5:A>G</td><td>TGCAGACTCTGGATGGCGCGGACACACGGCTCCG</td><td>5:A>G</td></tr>
<tr><td>1037736|F|0-16:T>G-16:T>G</td><td>TGCAGATTTGTGGTATTGCTTCAAAGCTTGCAGGGTCTTCAAGAACCTTGCAAGACAAGGTATCTCTCC</td><td>16:T>G</td></tr>
<tr><td>3022223|F|0-17:C>G-17:C>G</td><td>TGCAGAGGCAACATCCACTATCATGAGAAGGTTTCCG</td><td>17:C>G</td></tr>
<tr><td>978197|F|0-16:T>G-16:T>G</td><td>TGCAGTTGTCGTGGTCTTGCGCGAGCCTCTTGATGATGAGGAGGCCGAGTAGGTTGCACTCCTCGTCCG</td><td>16:T>G</td></tr>
<tr><td>5365818|F|0-15:G>C-15:G>C</td><td>TGCAGGGCTGGTCCTGGCTTCGCTCGCCGTGGTGCTGGCAACCACGCTGTGGACGGCGGCGGTGCACCT</td><td>15:G>C</td></tr>
<tr><td>2253851|F|0-29:G>C-29:G>C</td><td>TGCAGCTCCTCGAGGTACACGCTGCTGGTGCCATCGAGGAAGCTGTCGGTGAGGCGCGAGAGCGGCACG</td><td>29:G>C</td></tr>
<tr><td>5350155|F|0-5:C>G-5:C>G</td><td>TGCAGCTGCTCGTCTCCGCGGGGCCGCCG</td><td>5:C>G</td></tr>
<tr><td>2253851|F|0-11:G>C-11:G>C</td><td>TGCAGCTCCTCGAGGTACACGCTGCTGGTGCCATCGAGGAAGCTGTCGGTGAGGCGCGAGAGCGGCACG</td><td>11:G>C</td></tr>
<tr><td>4002569|F|0-15:T>C-15:T>C</td><td>TGCAGTCCAAGAGCGTGTGCGTGGTGGGGGGCGGCATGGCCG</td><td>15:T>C</td></tr>
<tr><td>1031593|F|0-8:A>G-8:A>G</td><td>TGCAGGGCAAAGACACACCGCACAAGGATGTGCTGAATTGACTCCTCAACTCGGTCACACAAGGGGAAA</td><td>8:A>G</td></tr>
<tr><td>5580368|F|0-20:A>C-20:A>C</td><td>TGCAGGAGATTCTGGTCCAGAACGACCACCG</td><td>20:A>C</td></tr>
<tr><td>3937961|F|0-18:G>A-18:G>A</td><td>TGCAGAAAGCGGCAGCACGTGCGTGGTCACCG</td><td>18:G>A</td></tr>
<tr><td>3959942|F|0-8:T>G-8:T>G</td><td>TGCAGGCATTACTATGTCAGCATGAGCATTGGGGATCCG</td><td>8:T>G</td></tr>
<tr><td>992371|F|0-35:C>T-35:C>T</td><td>TGCAGCATCGGGTTGATGTGGCCCTGGAACGGCAGCGGGAACACTAGCACCCG</td><td>35:C>T</td></tr>
<tr><td>4261550|F|0-20:C>G-20:C>G</td><td>TGCAGCTGCGGGCCGACGTGCTCAAGCAGCTGGACGGCGCGCCG</td><td>20:C>G</td></tr>
<tr><td>1016339|F|0-56:G>A-56:G>A</td><td>TGCAGCACACCATGTTCCGCGACACGCCCGCCCCCTTCCACTGCCAGTCCTGCGGCGACGCCGCCGTCA</td><td>56:G>A</td></tr>
<tr><td>5372180|F|0-10:G>C-10:G>C</td><td>TGCAGCAGGTGCGTCACCGCCACCG</td><td>10:G>C</td></tr>
<tr><td>1024350|F|0-43:C>T-43:C>T</td><td>TGCAGGCGCAGTTCGGCAACAAGTGGGCGAGGATCGCCACCTACCTGCCG</td><td>43:C>T</td></tr>
<tr><td>3021842|F|0-16:A>G-16:A>G</td><td>TGCAGTCAGTGGAGCAATCCTTCGATACCGTACATACCTCGTCCG</td><td>16:A>G</td></tr>
<tr><td>1016715|F|0-5:T>G-5:T>G</td><td>TGCAGTCGGTTGGTGCGGCATCCTCTGTCCCTGACCACCACAACATGCTCATCGGCACACTGTTCTTAG</td><td>5:T>G</td></tr>
<tr><td>3947004|F|0-6:A>G-6:A>G</td><td>TGCAGGACGGCGGCGTCGACCG</td><td>6:A>G</td></tr>
<tr><td>2250762|F|0-8:C>A-8:C>A</td><td>TGCAGCTGCTGGACTTCGTGCTCTTCGACTGGGACCGCGTGCTCCGCCCCG</td><td>8:C>A</td></tr>
<tr><td>3033998|F|0-6:A>G-6:A>G</td><td>TGCAGCACCCGTCGATTTTAAACTGCCGTAGCTAAGCCG</td><td>6:A>G</td></tr>
<tr><td>1221211|F|0-16:C>A-16:C>A</td><td>TGCAGCAAGTCGGGAGCCATCATCCCCTGGAACACGTCCTCCTCCAAGAAGCTCAGGAAGCCCCCGAGA</td><td>16:C>A</td></tr>
<tr><td>3022332|F|0-5:G>A-5:G>A</td><td>TGCAGGGTAACCATCTCGAAGAAGGAGTCCTCTGGCCCG</td><td>5:G>A</td></tr>
<tr><td>5363711|F|0-10:G>A-10:G>A</td><td>TGCAGGACAAGGTGGTGTACGGCACGGCGGACCG</td><td>10:G>A</td></tr>
<tr><td>1020908|F|0-35:G>C-35:G>C</td><td>TGCAGCTCAGAGCAGTATGAGGCCATGGCGATCTCGGCGCCCTTGAAACCATAGTCCAAGCTCGGGTTG</td><td>35:G>C</td></tr>
<tr><td>4539232|F|0-22:T>A-22:T>A</td><td>TGCAGTAAGAATGTGTTAGTACTGCACCCG</td><td>22:T>A</td></tr>
<tr><td>1018026|F|0-6:T>C-6:T>C</td><td>TGCAGGTGGGTTCGGGTCAACCTTGCTGGGCCTGAACAAGAAGACGTACGAGCTGGCCG</td><td>6:T>C</td></tr>
<tr><td>2242932|F|0-57:T>G-57:T>G</td><td>TGCAGCCGCACGATCTCCGTGATCTTGTTCGACTTCTTGCCCCCGAGCTGCTCCATCTCCG</td><td>57:T>G</td></tr>
<tr><td>3947568|F|0-19:G>C-19:G>C</td><td>TGCAGCCGCCTGGCGACACGTACACGCGGCGGTCGGCGTTCGACGCCATCCTTGCCG</td><td>19:G>C</td></tr>
<tr><td>1066921|F|0-20:A>T-20:A>T</td><td>TGCAGGAAGAGGTAAGGAGGAATGTACCCAACGGGCAAGAGATGGCCGCCGAAGACGATCTCCCCAACA</td><td>20:A>T</td></tr>
<tr><td>2242932|F|0-41:C>G-41:C>G</td><td>TGCAGCCGCACGATCTCCGTGATCTTGTTCGACTTCTTGCCCCCGAGCTGCTCCATCTCCG</td><td>41:C>G</td></tr>
<tr><td>3944157|F|0-16:G>C-16:G>C</td><td>TGCAGGTGTCGCACCCGACCAAGGAGGCGCCG</td><td>16:G>C</td></tr>
<tr><td>1019953|F|0-44:G>A-44:G>A</td><td>TGCAGGAAGATCTTGAGGATGTTCTTGGAGAAGCCGCTCACCATGGCCACCCCG</td><td>44:G>A</td></tr>
</table>

5009032|F|0-25:C>G-25:C>G	TGCAGACGTGGCAGACGAACTGCACCGCGCCGATGAGTATCTGCGCCG	25:C>G
3935245|F|0-20:T>C-20:T>C	TGCAGTTTCTTGTTCCCATTTTAATCTTGGGGCTTGCATTTGGTGTCCG	20:T>C
1065875|F|0-58:C>T-58:C>T	TGCAGGTGGCCCCCAAGCCCAACACGTTCATGCCGTTCGGGAACGGGACCCACTCGTGCCCCG	58:C>T
1022735|F|0-29:C>G-29:C>G	TGCAGCTTGGGGAGCAGGTAGTACCTGATCAGCTCGTCGTCGCTCGGCCTGAACCG	29:C>G
1766375|F|0-56:C>G-56:C>G	TGCAGGCTCTCGTGGAGGCCCTGCCAGGGCCAGGCCTTCTGGGCGGCGTCCTCGGCCGCCGCCTTCTTG	56:C>G
3937608|F|0-23:A>G-23:A>G	TGCAGGCCCAGCGTAAGCGACACATCGCCG	23:A>G
993630|F|0-31:C>G-31:C>G	TGCAGCGGTTCCCGCGCGGCCAGGCGGCGTCCTCGTCCGCCGACGCGCACGAGGTCCG	31:C>G
1037736|F|0-37:T>C-37:T>C	TGCAGATTTGTGGTATTGCTTCAAAGCTTGCAGGGTCTTCAAGAACCTTGCAAGACAAGGTATCTCTCC	37:T>C
1201716|F|0-7:T>C-7:T>C	TGCAGAATTCGTCGAGGAGCTCCTGGGCCGCGCGCGTGTACTTGGAGTTGCGCAGCACGCCG	7:T>C
32027923|F|0-8:C>G-8:C>G	TGCAGCAGCAGCAGCAGAGGCTCCG	8:C>G
100571995|F|0-12:T>G-12:T>G	TGCAGCAGCGTTTTCGACGGGTCGTCCAAGTCTGTGCGCCCCG	12:T>G
7170923|F|0-21:A>T-21:A>T	TGCAGTGCAAGCTCGTCTTCCACGCGCAGCCACCGCTCCAGTCCCCCG	21:A>T
3023764|F|0-22:A>G-22:A>G	TGCAGTGCCCGATCACACTGGAACTCATGACTGACCCG	22:A>G
7352307|F|0-9:G>A-9:G>A	TGCAGGCGGGCGAGCGACCCG	9:G>A
4002189|F|0-23:G>A-23:G>A	TGCAGCTGGGCGGCGACGTGGGCGGCGGCCGCCTCAGCACCGACCCG	23:G>A
1130803|F|0-8:G>C-8:G>C	TGCAGCTTGGCGTTGAAGGTGAGGGAGTCGTTGTTGAGCCGCTCCACGCAGCCGCCCCCGCCGCCGCCG	8:G>C
3938577|F|0-5:G>C-5:G>C	TGCAGGTGCTGAGGGTGGCCCTGAACCCG	5:G>C
1018548|F|0-50:G>T-50:G>T	TGCAGGTCATCATGTCGTTCCACGCGTGCGGCGGCAACGTCGGCGACGCCGTGAACATCCCGATACCGA	50:G>T
1240822|F|0-43:C>T-43:C>T	TGCAGCGCGAAGCCCCGCAGCTTCCGCAGCGACGACTTCATCTCGCGCCCGCCG	43:C>T
4262208|F|0-11:C>G-11:C>G	TGCAGCGCCTCCACGGCAAGCGTGTCGGAGAGGACGAAGACCG	11:C>G
1069445|F|0-30:T>A-30:T>A	TGCAGCCACCCGAAGGCCGTGCCGCGGTGGTCGTCGTCGGTGGAGTCGGCCACCAGTGACTGGACCGCC	30:T>A
2266494|F|0-34:G>T-34:G>T	TGCAGCGCCTCCTGCTCCCGCAGCCGCTTCACGAGGTGTGTCGACGGCGAGCGCGCCAGCTTCTTGAGG	34:G>T
4993471|F|0-10:C>T-10:C>T	TGCAGCTGCACCGCCTCCTCGCGGCCGACGACACCG	10:C>T
4411656|F|0-14:C>G-14:C>G	TGCAGCACCTGCTGCGCGTACTCCG	14:C>G
5357262|F|0-24:T>C-24:T>C	TGCAGATTTTCCTGATCTCCCCGTTGTCGCCG	24:T>C
1120742|F|0-28:A>G-28:A>G	TGCAGCACGCACGCATCCATGATGCAACACACATCAGCCAGAAACCATGCAAGACCAACACCATTTCCA	28:A>G
998527|F|0-27:G>A-27:G>A	TGCAGCGACGCGCTCTGCTTCTGCGCCGCGTCCAGCTTCGCCGCCGCGCCG	27:G>A
1148898|F|0-10:G>C-10:G>C	TGCAGAACCTGAAGACGCTGCTCCTCTACTCCAACGCCCTCGTCGGCACCATCCCTCCG	10:G>C
2243185|F|0-28:C>A-28:C>A	TGCAGGTTCATGTACTTGCTCCGCGCCGCCAGCGCCTTCACCTCCTCGATGGCCTTGTTGTCCACCCGC	28:C>A
986686|F|0-16:C>T-16:C>T	TGCAGGCCACTCCAAACAGGCTTCGCATGCTGAGCCTATACTCTGCCATCACCG	16:C>T
5325386|F|0-12:T>C-12:T>C	TGCAGGTGGACGTCGTCGAGAGCATGGTGAGGCGGGTGTTCGAGGAGATGGGCGACGCTGAAGCCGAGA	12:T>C
1006991|F|0-35:G>T-35:G>T	TGCAGGAGGCCGATCTTGTCCGAGACCTCGGCGTCGGCCGCCGCGTCCTCCG	35:G>T
4022600|F|0-5:C>T-5:C>T	TGCAGCCTCTTGGGGTCCAGAACATGGTTGCCGAGGGAGGGGCTCCG	5:C>T
5567404|F|0-9:C>G-9:C>G	TGCAGGTCTCCGCCGTGATGTCGGCCG	9:C>G
986089|F|0-8:A>G-8:A>G	TGCAGGCAAGGGGATAAGATGCTCGCCATCCCAAGAAGCCAGCTGCTCAAGGTCGGAATGTCTTACGAG	8:A>G
1862895|F|0-8:G>A-8:G>A	TGCAGCTCGCTGGTGTTTGTGCTCGAGTCTGGCTCGGAGGGCTCCGTCTCCG	8:G>A
5366787|F|0-10:C>A-10:C>A	TGCAGACGGCCTACCGCCTCAACGTGGGCGGCCCG	10:C>A
3027570|F|0-19:G>C-19:G>C	TGCAGCGCAACGGGTGTCAGTCCTTCTGGGCACCATCCG	19:G>C
3943030|F|0-25:T>C-25:T>C	TGCAGTACGCCATCGACGCCGTCCATTCGTTCACCG	25:T>C
4398370|F|0-14:C>G-14:C>G	TGCAGGCTGGCCAGCTCGTCCCTCTTCTCCG	14:C>G
1023369|F|0-52:G>C-52:G>C	TGCAGCCGCGGATCATCGCGTGCGGGAACAAGGTGGCGACGTACGCCATGGCGGTGCGGTTCCTCGCCG	52:G>C
3936171|F|0-11:G>A-11:G>A	TGCAGCTGCACGTCAAGCAGCCCGCCG	11:G>A
7169527|F|0-7:A>G-7:A>G	TGCAGCTAACACATGCATGGTACTGGCACCG	7:A>G
32027259|F|0-18:C>A-18:C>A	TGCAGACGGCAGCTTATTCAGGGGCTAGTTTAAAGCGAGGACGGCAATGCGCTGGGTCCGCGAAGGCCG	18:C>A
1206626|F|0-10:G>A-10:G>A	TGCAGCAGGTGGACACAAGGTTCGTGTACGACCTCAAAGACGCCAAGCTGTCCTTTGCGTCGGAGCCGT	10:G>A
100589405|F|0-33:G>A-33:G>A	TGCAGCGACCTCATCCACAAGATGCTCGCCGAGGCGCCG	33:G>A
3959219|F|0-11:G>A-11:G>A	TGCAGCTTGCCGATCTCCCTGGGTATCTCGCCG	11:G>A
7173299|F|0-10:T>C-10:T>C	TGCAGCGGCGTGCGCATGAAGGTGATGAGCCCG	10:T>C
1028132|F|0-32:C>G-32:C>G	TGCAGCGACAACGTCACGGTGCGGGCCCTCGGCGGCGTCTCTGGCTTCGCCG	32:C>G
3940807|F|0-22:C>G-22:C>G	TGCAGCGCGTACGTTGACTAATCGACCCG	22:C>G
2279513|F|0-64:G>A-64:G>A	TGCAGCTCCGCCCCGTCGACCACAAGGCCGTCGCGGAGGAGTGCTCCCTCGCGCTCCAGGCCGAGCCGC	64:G>A
985897|F|0-7:C>T-7:C>T	TGCAGGCCGCCGCGCTGCTCTGCTTCGTCGCGCTCTCCCTCGTCTCCGCCTTCCG	7:C>T
1035705|F|0-60:C>G-60:C>G	TGCAGCGGTTCCTATCTTGTGGAAGGCATGCCTGGAGCAGAAGGCGAGCTGGGTGTAGGACGGGGAAGA	60:C>G
4993471|F|0-13:C>T-13:C>T	TGCAGCTGCACCGCCTCCTCGCGGCCGACGACACCG	13:C>T
5010763|F|0-7:A>G-7:A>G	TGCAGGCAGGAGGCTCGGTCACGGTCAGACAGCTCGAGTCGGCCGACGACGACTCGTGCGGCGGCTCCG	7:A>G
996602|F|0-31:G>T-31:G>T	TGCAGCTCGCCCAGCACCCCTACGACGCCGAGGCGATACAGTCGGCGGACGCCGCGTTGGTCGTGGAGC	31:G>T
4412211|F|0-7:A>C-7:A>C	TGCAGTGAAGAACCGTGACGCGGCGCGGTTCCCTTGATGTGTCCG	7:A>C
1007409|F|0-25:G>A-25:G>A	TGCAGCAGCGGGTCGGCGTGCGACAGCAGCGCCACGAGCGGCCGCACCGCCCCG	25:G>A
4005798|F|0-12:G>A-12:G>A	TGCAGGTACACGGCGCCGCGGTAGGGATGAGCGGCCG	12:G>A

2245264|F|0-19:C>T-19:C>T	TGCAGATGAAGTAGTAGGTCTTGGTCTCGTTGAGCTCGAAGGCGAAGCTGCGGCCCTTGCTGTAGTTGT	19:C>T
987965|F|0-17:G>A-17:G>A	TGCAGCTGGGCGGCCTGGACCACCTCTCTGTTGGACAGGGTGTACTCGCCCGTCTCGCTGGCCTTCCTC	17:G>A
3953435|F|0-26:C>A-26:C>A	TGCAGGAAGGCGTTCCCCTCGGCGCTCTGCCTCGGCGGTTCGGCCG	26:C>A
4411706|F|0-8:G>A-8:G>A	TGCAGGCGGGAGGAGTTGAGGAGGAACCTGGATGACGTCGCCG	8:G>A
1020494|F|0-44:G>A-44:G>A	TGCAGTGCCTGGATGCAGCCGTCTTACTGTTACAGCACGGCGGAGGCGCCG	44:G>A
2245264|F|0-52:G>A-52:G>A	TGCAGATGAAGTAGTAGGTCTTGGTCTCGTTGAGCTCGAAGGCGAAGCTGCGGCCCTTGCTGTAGTTGT	52:G>A
4262805|F|0-19:G>A-19:G>A	TGCAGCTGCGGGTGAACGGGGCGCCG	19:G>A
3936422|F|0-16:C>T-16:C>T	TGCAGCACTCCATGCCCACCTCCGTGCTCTCCG	16:C>T
1397912|F|0-29:C>T-29:C>T	TGCAGCACGCACCCATCCATGACGCAACACACATCAGCCAGAAACCATGCAAAACCAACACCATTTCGA	29:C>T
4394733|F|0-17:G>C-17:G>C	TGCAGCGCGACGCAGCGGCCGATCTCCG	17:G>C
1084756|F|0-28:C>T-28:C>T	TGCAGCGAGGAGGAGGGGACGCCCATGGCGTCCTTGAGGAAGAAGTTGACGCCCAGCCAGGGGAAGAGC	28:C>T
1385721|F|0-6:C>A-6:C>A	TGCAGCCTCGGGCTCTTCGACTGGAAGTACGACCACATCCTCTCGGGCTCCG	6:C>A
4261849|F|0-7:C>G-7:C>G	TGCAGCTCCCTGAGCACGGCGTCGCGGCTCTTGAGGAACCCG	7:C>G
6041561|F|0-14:A>T-14:A>T	TGCAGCTCTCGCCGACTTTCGATCCACCG	14:A>T
1036446|F|0-36:T>A-36:T>A	TGCAGTGGAGGATCTTGGCCAGCTTCAGCATCGGCGTGACGTGCCCCTGCGCCG	36:T>A
5367448|F|0-23:A>G-23:A>G	TGCAGGTGCTGAAGGTGTACCTGAACCCG	23:A>G
2250762|F|0-40:G>A-40:G>A	TGCAGCTGCTGGACTTCGTGCTCTTCGACTGGGACCGCGTGCTCCGCCCCG	40:G>A
1058456|F|0-16:C>G-16:C>G	TGCAGTTCGCGGTGCCCATGGTCCTCACCAACATGTCCTACTACGGCATCCCGCTGGTGTCCGTCATGT	16:C>G
1073290|F|0-31:G>T-31:G>T	TGCAGTCGGTGCGGCTCAAGTACGTCAAGCTGGGGTACCACTACCTCATCACCCACGGCATGTACCTCC	31:G>T
3941407|F|0-11:A>G-11:A>G	TGCAGCAGGGGACCGAACACCACCGCCG	11:A>G
1129938|F|0-6:G>T-6:G>T	TGCAGCGCATCGCCTGGGTCGGCGACGACCACGAGCGCTTCCTCTCCAAGTTCAAGGACCGCGTCGACC	6:G>T
4262500|F|0-27:C>T-27:C>T	TGCAGAAGAACGCGGCCAAGAAGGGAGCCCTGGCCGTGTCGCCG	27:C>T
6049824|F|0-20:A>G-20:A>G	TGCAGCCCCACGTCACTGAAAGCCACGGTGAGGAACACCAGGGCCGTGAAGACGGCGCGCATGAAGTCC	20:A>G
1024214|F|0-35:C>G-35:C>G	TGCAGCGGCCTCTCCGCCGAGGAGGTGGAGTCTCCCTACGTCAACTTCGGCCGCCG	35:C>G
996034|F|0-28:C>T-28:C>T	TGCAGATGGCGTCCTCTGCTGGTAATGGCAAGGAGATGGCCGCGCTCGAGTCCG	28:C>T
3935119|F|0-21:G>A-21:G>A	TGCAGAGTGAAGTAAAGACGAGGCCGAAGACGAATCGGGCAGGATGTTTCACCTCGCGACTCCG	21:G>A
5360098|F|0-6:A>T-6:A>T	TGCAGCAGGAGCCTGTGGCCACGGAAATGAACCCCG	6:A>T
6049531|F|0-13:A>G-13:A>G	TGCAGATGTTCTCACCGCCGCTGATGATCACGTCCTTTGACCTGTCTTTTATCTCGATGTACCCGTCCG	13:A>G
1042706|F|0-14:T>A-14:T>A	TGCAGAAACGATAATTACCTAACCCGACAAGCACTGAGGCACCGACGAGAGTGCACGGTGATTCAACCA	14:T>A
992762|F|0-20:A>G-20:A>G	TGCAGGGGCCTGGGCGATGAAGACTCCGACGAGCAACGCCAGTGCAAGCACCG	20:A>G
7169452|F|0-12:G>C-12:G>C	TGCAGTGGACGAGAGCAAGACTGAACCG	12:G>C
3026500|F|0-10:G>A-10:G>A	TGCAGTCAGGGCAGTGCTCCTTGGGAGCCACATGCCCTACCG	10:G>A
1211131|F|0-14:G>C-14:G>C	TGCAGCAGCACCTGGCCGATGCTCCTGAGCTCCTCCTGCTCCTCCCTCTCCTCCG	14:G>C
1694779|F|0-16:G>A-16:G>A	TGCAGTCGGACGAGCCGTCGACGGACGCAAGCACCACCACGACGTCCACGACCACGATGAGCATGACGG	16:G>A
5360641|F|0-34:A>C-34:A>C	TGCAGAGGTTCTACCGCCCAGACAGCGGGGAGATACTGCTCGACGGCGACGACATCGGCTCGCTCAACG	34:A>C
1095335|F|0-25:C>G-25:C>G	TGCAGCCGTTCATGGCGGACGGCACCACGTACACGCACGACCAGTTCATCCGCCTCCTCGTCGCCACTC	25:C>G
1091000|F|0-19:C>G-19:C>G	TGCAGGTGGGCAACGACCTCAACCACCGCTTCCTAGCGCGGCTGTCGGCGCGGTACGGCCCCGTGTTCC	19:C>G
6046855|F|0-27:G>A-27:G>A	TGCAGGGCGCGACCCTGGTCGTCATCAGGCCGTCCG	27:G>A
3940182|F|0-11:C>T-11:C>T	TGCAGGAATTCCTTCATGTTGCTGGCGGCGATCAGCCCGCGGCCGCCG	11:C>T
4733964|F|0-25:C>G-25:C>G	TGCAGCGGTCCACCCACACGTGCCTCGACCCG	25:C>G
1107629|F|0-6:A>G-6:A>G	TGCAGGAAGACAAGCGTGCCGCGGATGAGGAGAGCCAGCCTCACTTCCTGATCGTGACGTACCCGAGAT	6:A>G
1696619|F|0-22:T>C-22:T>C	TGCAGGTCCTGCGGGGTGTACATGGCCGCCATCTCGACTCGTTGCAATGCCGCCGCCGCTGCGAGGGAG	22:T>C
1073290|F|0-13:G>C-13:G>C	TGCAGTCGGTGCGGCTCAAGTACGTCAAGCTGGGGTACCACTACCTCATCACCCACGGCATGTACCTCC	13:G>C
5368388|F|0-16:C>T-16:C>T	TGCAGCTCCGCCTCGTCGGTCTTGTCGGACTCGATGGACAGCAGCACGTTCTTGCGGCGGACGAACTGG	16:C>T
5357754|F|0-9:C>T-9:C>T	TGCAGCCTCCTCCACCCGCCGTACTCCG	9:C>T
2266494|F|0-31:C>G-31:C>G	TGCAGCGCCTCCTGCTCCCGCAGCCGCTTCACGAGGTGTGTCGACGGCGAGCGCGCCAGCTTCTTGAGG	31:C>G
3953953|F|0-8:T>C-8:T>C	TGCAGCTCTGAGTAGCTGTATGCCCG	8:T>C
1005371|F|0-40:C>T-40:C>T	TGCAGTTGATGTCCGCGGGCCCGCTGGTGGACGTGGGCCTCGCGGCGCTGCGGTCTCCG	40:C>T
986239|F|0-5:G>T-5:G>T	TGCAGGGCCAGGTCCTGGTGCTCCTTCTTGCACACCTGGTCGGCGCCGATGTTGCCGTACCG	5:G>T
4262500|F|0-32:G>A-32:G>A	TGCAGAAGAACGCGGCCAAGAAGGGAGCCCTGGCCGTGTCGCCG	32:G>A
5363167|F|0-13:C>G-13:C>G	TGCAGATTCTCTCCGTGTCGCGCAACCTGCTCTCCG	13:C>G
1005371|F|0-46:G>A-46:G>A	TGCAGTTGATGTCCGCGGGCCCGCTGGTGGACGTGGGCCTCGCGGCGCTGCGGTCTCCG	46:G>A
10984460|F|0-18:A>C-18:A>C	TGCAGGAGGCCCTGGAGGAGCTCCG	18:A>C
1069454|F|0-33:G>T-33:G>T	TGCAGTGCGGCCACGAGTTCCACCTCGGTAAGGGGCTATGGTCCGATCTGTTGATGCGTTATTGCACTG	33:G>T
2260336|F|0-11:C>A-11:C>A	TGCAGCGGCACCACGTCCTCGGGCCACGTCTTGGTCACGTCCAGCGGGTCGAAGTCGAACCGCTCCTCG	11:C>A
998527|F|0-41:C>A-41:C>A	TGCAGCGACGCGCTCTGCTTCTGCGCCGCGTCCAGCTTCGCCGCCGCGCCG	41:C>A
3937911|F|0-10:G>T-10:G>T	TGCAGGGAGCGGTTGGAGATGGTGGCGCCCG	10:G>T
13880622|F|0-8:C>G-8:C>G	TGCAGCGCCGCGGTTAGCATGATCGGGTCGACCAGCGTCCG	8:C>G
1018027|F|0-36:C>G-36:C>G	TGCAGGTGCAAATGGCGAACGTGACGAGGCTGTGCGCCAGCAAGAGCATCGTGACGGTGAACGGGGAGT	36:C>G

32026453|F|0-35:A>C-35:A>C	TGCAGTCGCTCTCCCGCCAGCGCAGCTGCACCGAGATCCCTCGCTTCGCGGAGGCCGACGCCGACGCCG	35:A>C
100467626|F|0-13:T>G-13:T>G	TGCAGATGGAGCCTCCGCGGCCGCCGCCGCTCAAGTCCGTGTCGACGAGCTGCGACCTGCACCCGAGAT	13:T>G
100483905|F|0-5:G>A-5:G>A	TGCAGGACACGGTCCTCGCGATCATCGACCACACCTACGCCGCGGCGCTCCAGATCGTCGGGGCGATGC	5:G>A
5355619|F|0-22:C>T-22:C>T	TGCAGTTTTGCTAGACTTAATACGTTGCTCGAAAGTTGCGGTTAACCGTAAGTAGAAGTCAAGGCAGCT	22:C>T
32026568|F|0-14:G>C-14:G>C	TGCAGGCTCAGCTCGGTGACGCCGCCGTCGGCATTGCACGCCACGCCG	14:G>C
100580404|F|0-38:A>G-38:A>G	TGCAGTCTGCCGTGGCCGCGGCCGTGGCGGTCGTCCTCACCCCCGCCG	38:A>G
100558520|F|0-65:A>G-65:A>G	TGCAGGTTCTGCGTGGGGAGGTAGGCGGAGAAGACGAAGAGGAAGGCGGCCGACAGGAGGTGAACATCG	65:A>G
5006767|F|0-20:C>G-20:C>G	TGCAGGATCTTCGTTGCTACCTGGAATGTGGGCGGCCGATCCCCTCCCG	20:C>G
1094457|F|0-67:G>A-67:G>A	TGCAGGGACGGCAGATCCCCATTGGCAGGGTCACCTCAGGGGCCGAGTAGATGTACTTGGTCACTTGGT	67:G>A
5365660|F|0-35:G>C-35:G>C	TGCAGGAGGACGGTGATGGCGAGGTCGGGGCTGAGGACGCCGCTGCTCACCATCTCGTCCAGCGTCTCC	35:G>C
981263|F|0-13:C>T-13:C>T	TGCAGAACGTGGCCATCATGCTCAAGGCGCTCAATGTGACCGCAGACGAGGTGATCGGAGCACTCGTGC	13:C>T
4994840|F|0-22:T>G-22:T>G	TGCAGCTCAACGCCGTTACGGCTCTGCTCAACCTCTCCATTCTCGATGCCAACAAGAAGCGCATCATGC	22:T>G
6030374|F|0-9:A>G-9:A>G	TGCAGCAGTAACTCATCATGCGTGCAGCAGGCCG	9:A>G
3935519|F|0-25:C>T-25:C>T	TGCAGTGAGTTGATGTTGCCCCATGCCCCG	25:C>T
5364124|F|0-16:T>G-16:T>G	TGCAGTTCATGTTCACTTACCTCTTCTTCCCCG	16:T>G
1230750|F|0-52:C>A-52:C>A	TGCAGCACGCCGCCGCCGACGGCATGAGCCCCGAACAGAGGCTCGTCGTGGACGCCATCGCCCG	52:C>A
1017713|F|0-50:C>T-50:C>T	TGCAGAGAGATCAGGGGATGCAGGCATCTGGAGGAGATGGTCACCAACTGCGTGCAGAGGGTGGAGGCT	50:C>T
990539|F|0-14:C>T-14:C>T	TGCAGCGGCCACCCCTCCCTCGTTGCTCTGCGTGGCGTCGTGCGCACACCCG	14:C>T
1016715|F|0-37:C>T-37:C>T	TGCAGTCGGTTGGTGCGGCATCCTCTGTCCCTGACCACCACAACATGCTCATCGGCACACTGTTCTTAG	37:C>T
3024418|F|0-32:C>G-32:C>G	TGCAGCTGCATCACGCCGTTCTTGAACACCCACACGCCG	32:C>G
2266494|F|0-6:G>T-6:G>T	TGCAGCGCCTCCTGCTCCCGCAGCCGCTTCACGAGGTGTGTCGACGGCGAGCGCGCCAGCTTCTTGAGG	6:G>T
7169350|F|0-22:C>T-22:C>T	TGCAGCAGGAGCGGGAGCACTGCGTCACGGAGGTACCG	22:C>T
4001967|F|0-30:A>T-30:A>T	TGCAGCCAGGCGGGCACCGATTTCCTGGCAATAGTTTACTCCG	30:A>T
1077471|F|0-15:T>A-15:T>A	TGCAGTAATTTAGTATGTTGTTTTTACCTATAGTGCCACCAAGTTCAATGACACAAACATCAGCTGGCT	15:T>A
3937748|F|0-15:C>T-15:C>T	TGCAGTTGCAGCTAGCGCGGCCG	15:C>T
1074139|F|0-62:G>A-62:G>A	TGCAGCCCCACGTCGCTAAACACCACCGTGAGGAAAACCACGGCGGCGAAGAAGGAGTGCACGAAGTCC	62:G>A
4262086|F|0-23:C>A-23:C>A	TGCAGCTTGTACCTCCCCTCGGGCGTCTTGAAGTAGGTCTTGAGCCCG	23:C>A
1051713|F|0-44:C>G-44:C>G	TGCAGCTACCATATGCCCCACAGAGTGCATACACCTCACACTGCCTCCGCGGCTGTGACCAGAACAAGA	44:C>G
1018027|F|0-37:C>A-37:C>A	TGCAGGTGCAAATGGCGAACGTGACGAGGCTGTGCGCCAGCAAGAGCATCGTGACGGTGAACGGGGAGT	37:C>A
1215048|F|0-38:A>C-38:A>C	TGCAGACGGAGGCGGATGCGGCGGATCTCGTCGCGCGGAGGGTTGACGACGCCG	38:A>C
4005798|F|0-17:G>A-17:G>A	TGCAGGTACACGGCGCCGCGGTAGGGATGAGCGGCCG	17:G>A
1017267|F|0-53:C>T-53:C>T	TGCAGGTTGAACGTGCCCGTGACCACGGCCGTCTCCGCGACCACGCTCCGCATCTCCTCGGTGTCGTTG	53:C>T
987148|F|0-36:G>A-36:G>A	TGCAGCGCACACGTCCGTCCACTCATCATCACACCCGAACGGATTAGACAAACGGCTCAAAAGGCTTTC	36:G>A
3022182|F|0-18:T>C-18:T>C	TGCAGGAATCCATCCAATTTGCGCCCTCTCCTTTCCGCCCG	18:T>C
4993728|F|0-10:G>T-10:G>T	TGCAGGTGACGGACATCGTGCCG	10:G>T
3946119|F|0-20:G>T-20:G>T	TGCAGCGAGGGGCGCCCTTCGCCG	20:G>T
7165452|F|0-14:C>T-14:C>T	TGCAGCTACGACGACGTGATCAGCCCTGTCTCCG	14:C>T
4262292|F|0-35:G>A-35:G>A	TGCAGGAAGTCGCTGGCGCCGATGCTGAAGATGTAGAGGGCGCCG	35:G>A
6030374|F|0-13:C>A-13:C>A	TGCAGCAGTAACTCATCATGCGTGCAGCAGGCCG	13:C>A
985354|F|0-46:G>T-46:G>T	TGCAGACGTTCAAGACCTGGCGGAGCTGGGCCAACGGGCCCTTCGTGTTCAACACCCG	46:G>T
1244246|F|0-13:G>C-13:G>C	TGCAGCTCGAGGTGACCAGGCTGAGCACGCCGTGGGGCTACCTGAGCGCCGTGGCCTTGGCCGTGACGA	13:G>C
5373733|F|0-14:G>A-14:G>A	TGCAGGACCTCGCGGGCGGCCTCCG	14:G>A
996602|F|0-55:G>A-55:G>A	TGCAGCTCGCCCAGCACCCCTACGACGCCGAGGCGATACAGTCGGCGGACGCCGCGTTGGTCGTGGAGC	55:G>A
1018977|F|0-46:C>T-46:C>T	TGCAGAGGCTTCTGATTCCACACCTGACCAATGCATCATGATGTTGCTTAATCATTTTATCTCCACGAT	46:C>T
2279513|F|0-61:C>T-61:C>T	TGCAGCTCCGCCCCGTCGACCACAAGGCCGTCGCGGAGGAGTGCTCCCTCGCGCTCCAGGCCGAGCCGC	61:C>T
7173995|F|0-5:A>C-5:A>C	TGCAGAGGCACGACGCCGCGTTCGCCACCCG	5:A>C
1213799|F|0-8:C>G-8:C>G	TGCAGGTTCACGTCGGAGCTCGGGGGCCAGTTTGGCGATTTCTCGGCGTCCCTCGGAGGAGCGGCTGCC	8:C>G
996034|F|0-21:G>C-21:G>C	TGCAGATGGCGTCCTCTGCTGGTAATGGCAAGGAGATGGCCGCGCTCGAGTCCG	21:G>C
3027991|F|0-22:G>A-22:G>A	TGCAGCTGCTAGGCCCCGACGAGGAGAAACCCTCGCCG	22:G>A
1213799|F|0-65:T>A-65:T>A	TGCAGGTTCACGTCGGAGCTCGGGGGCCAGTTTGGCGATTTCTCGGCGTCCCTCGGAGGAGCGGCTGCC	65:T>A
6048317|F|0-17:G>C-17:G>C	TGCAGCAGCGCCGTCGTGGGGAGCACGAAGGAGACGTTGTTCACGGCCGCCGCGAACCG	17:G>C
1016715|F|0-16:G>A-16:G>A	TGCAGTCGGTTGGTGCGGCATCCTCTGTCCCTGACCACCACAACATGCTCATCGGCACACTGTTCTTAG	16:G>A
2247862|F|0-40:G>C-40:G>C	TGCAGTGCGCGCCCTGCCTCGACTGCTTCGAGCAGAGCGGGCCCATCTTCGACCCG	40:G>C
5363131|F|0-19:C>G-19:C>G	TGCAGAGCGTGCGGAGCAGCGCCG	19:C>G
4002481|F|0-24:C>G-24:C>G	TGCAGGACACGTCCGCGCAGTGCACCCACCGCCCGTGCTCGCCG	24:C>G
5006251|F|0-15:C>G-15:C>G	TGCAGGCTACTACGACGACGACACCG	15:C>G
1399012|F|0-9:A>T-9:A>T	TGCAGCAGAAACTTGTGGGTGAAGAACTTCTTTCCACAGCCCTCAACCG	9:A>T
983722|F|0-17:C>A-17:C>A	TGCAGCTGCAACAACCCCTGACTCTTGTCCTTTCCATGCTCACACTGGGCCATCGCGGCCGCTTATGTG	17:C>A
1019879|F|0-23:C>T-23:C>T	TGCAGGGACAAGGACGAAGGCGACGCGATCAAGGGACTCCTCGCAACGGCGTCGGCGGCCACGTACGAC	23:C>T

<table>
<tr><td>3949517|F|0-32:G>A-32:G>A</td><td>TGCAGGCTGCGCTGGTTGAGGCGCTTGACGGCGAGGACGACGCGGCCGCCG</td><td>32:G>A</td></tr>
<tr><td>993908|F|0-14:C>A-14:C>A</td><td>TGCAGTTCCAGAGGCCAAGAAAGGCACAAAGGTTACCAACCAAGCTCCCTCCAACAAAACCCTAACCAC</td><td>14:C>A</td></tr>
<tr><td>3945442|F|0-16:C>T-16:C>T</td><td>TGCAGCCGCGCGTCGACCACCTCCGCCG</td><td>16:C>T</td></tr>
<tr><td>5324461|F|0-14:A>G-14:A>G</td><td>TGCAGGAGATGAGCACCCGCGTAGCGGACCG</td><td>14:A>G</td></tr>
<tr><td>22840966|F|0-15:A>G-15:A>G</td><td>TGCAGCAGCGACGCGACGCCGCCG</td><td>15:A>G</td></tr>
<tr><td>3949035|F|0-16:G>C-16:G>C</td><td>TGCAGCGCAGGAGTACGTCGAAAAACGACGGCCG</td><td>16:G>C</td></tr>
<tr><td>3937735|F|0-14:G>A-14:G>A</td><td>TGCAGGCTGTGCGCGGCCGCGATCACCGCGTGGTCCG</td><td>14:G>A</td></tr>
<tr><td>2243185|F|0-32:C>G-32:C>G</td><td>TGCAGGTTCATGTACTTGCTCCGCGCCGCCAGCGCCTTCACCTCCTCGATGGCCTTGTTGTCCACCCGC</td><td>32:C>G</td></tr>
<tr><td>984930|F|0-49:C>G-49:C>G</td><td>TGCAGCACCTGACGGACAAGAGCGACGTGTACAGCTTCGGCGTGGTGCTCCTGGAGCTCATGTCCGAGA</td><td>49:C>G</td></tr>
<tr><td>1051713|F|0-5:C>G-5:C>G</td><td>TGCAGCTACCATATGCCCCACAGAGTGCATACACCTCACACTGCCTCCGCGGCTGTGACCAGAACAAGA</td><td>5:C>G</td></tr>
<tr><td>22839965|F|0-16:C>T-16:C>T</td><td>TGCAGATGTCCTCCGACTCGGTGGACGCCG</td><td>16:C>T</td></tr>
<tr><td>3947374|F|0-8:G>T-8:G>T</td><td>TGCAGCGGGACGGCGTCGATGGGTTCCGTGGCCATTGCTTCCG</td><td>8:G>T</td></tr>
<tr><td>5325034|F|0-28:G>A-28:G>A</td><td>TGCAGGAGAACCAGAAGCTGAAGAAGCAGGTGGAGAAGATGAGGAAGAAGCACGAGGTGGAGATCGAGA</td><td>28:G>A</td></tr>
<tr><td>993630|F|0-27:C>T-27:C>T</td><td>TGCAGCGGTTCCCGCGCGGCCAGGCGGCGTCCTCGTCCGCCGACGCGCACGAGGTCCG</td><td>27:C>T</td></tr>
<tr><td>4262391|F|0-5:C>T-5:C>T</td><td>TGCAGCCTGTAGGACCTGCCG</td><td>5:C>T</td></tr>
<tr><td>5346922|F|0-20:T>A-20:T>A</td><td>TGCAGTGCATCAACGTCAGCTCCG</td><td>20:T>A</td></tr>
<tr><td>4993728|F|0-16:C>T-16:C>T</td><td>TGCAGGTGACGGACATCGTGCCG</td><td>16:C>T</td></tr>
<tr><td>1285274|F|0-25:C>T-25:C>T</td><td>TGCAGGCGCCCGCCAGGGACGTCGTCGCCGTGCTCTCGGCCTACGTGCGCAAGCTCGAGGAGGAGTCCG</td><td>25:C>T</td></tr>
<tr><td>6049347|F|0-11:C>G-11:C>G</td><td>TGCAGCGGGACCACGACACCGCCCTGCGCCTCCTCCACCCCTTCACCG</td><td>11:C>G</td></tr>
<tr><td>1029006|F|0-6:C>A-6:C>A</td><td>TGCAGGCGGCGTCCAGGGGCCGTCCACGCGGAGGCTGACGAGCAGCGACGAGAGGCTGGAGGCGGCGGC</td><td>6:C>A</td></tr>
<tr><td>5332423|F|0-6:G>C-6:G>C</td><td>TGCAGGGCCATCAAGTGAGTGCCG</td><td>6:G>C</td></tr>
<tr><td>4002046|F|0-13:C>T-13:C>T</td><td>TGCAGGCGCACGCCGCCGCGGACGACCTCTGGATCTCCATCTCCG</td><td>13:C>T</td></tr>
<tr><td>3027571|F|0-38:C>T-38:C>T</td><td>TGCAGCGACAGGTGCAGCAGCTGGTTGAGGTGGCCCTGCGCCG</td><td>38:C>T</td></tr>
<tr><td>1019104|F|0-55:G>A-55:G>A</td><td>TGCAGACCTCGGAGATGGCCAGGATGGCCAAGACGTACGGCAAGTTCTGGTGCACGTGGCAGGTGGACC</td><td>55:G>A</td></tr>
<tr><td>5357481|F|0-26:C>T-26:C>T</td><td>TGCAGCAAGCCGAGCGAGAAGAACCCCTCCG</td><td>26:C>T</td></tr>
<tr><td>1029381|F|0-9:T>C-9:T>C</td><td>TGCAGCTTGTGGTGTCGGAGCTGTGCCGCGTCCAGGAGCTGGTGCGGCAGCTGGAGCTGCACCTACACG</td><td>9:T>C</td></tr>
<tr><td>1667127|F|0-28:C>T-28:C>T</td><td>TGCAGGGCGGGATCCCGTTCGAGAAGTCCAAGGGCCAGCACATCCTGCGGAACCCG</td><td>28:C>T</td></tr>
<tr><td>1005020|F|0-53:G>C-53:G>C</td><td>TGCAGCACGTCGCGGGCGCACGCGGCGGACGAGGCCACGACGGCGGTGGTGGCGCCGAGCTTGATGGAT</td><td>53:G>C</td></tr>
<tr><td>2244426|F|0-29:G>A-29:G>A</td><td>TGCAGTCTGTCGTCAGTTCATCTGCATGCGCCGCAAGGATCAGCACAGCGCCACCGCTCGATCCGCGGA</td><td>29:G>A</td></tr>
<tr><td>4003300|F|0-27:G>T-27:G>T</td><td>TGCAGCTGCTGGGGCGCGGTGTACAACGGCGGGAGCGGCTACTGCTACCTCATCGACTTTCCCGTGGAG</td><td>27:G>T</td></tr>
<tr><td>1756996|F|0-14:C>G-14:C>G</td><td>TGCAGCGAGGCGCTCTTCAAGTTCGCGGTGTTCGTGCTGGTGCAGGCGCTGGTGTACCTCATTCTGTCC</td><td>14:C>G</td></tr>
<tr><td>1767452|F|0-29:C>T-29:C>T</td><td>TGCAGGTCGATGGACTGAGGCACGGAGGTCTCCTCCATCTTGGCGGCAAGAGACAGGCACGCCACCGAT</td><td>29:C>T</td></tr>
<tr><td>32026388|F|0-46:A>G-46:A>G</td><td>TGCAGGACTGGCAGCGGCGGTACGCCGACGTGCTGCGGCGGAAGGGAGTAAAGGTTCAGGTGGCGGAGT</td><td>46:A>G</td></tr>
<tr><td>32027390|F|0-14:G>C-14:G>C</td><td>TGCAGGCCCTACCTGGCCAAGTCCCTCTTCCTCCTCGGCTCCATCGGCGGCAACGATTACAACGCGATG</td><td>14:G>C</td></tr>
<tr><td>32024421|F|0-19:G>C-19:G>C</td><td>TGCAGCGAGGAGTGCAGGAGGGAGCAGATGGAGGCGGACGAGGAGATGGAGAGGAAGGAGAAGAGCGCC</td><td>19:G>C</td></tr>
<tr><td>32023602|F|0-10:A>T-10:A>T</td><td>TGCAGCACGCACTCGGACTCGGCGTCCAGCGCGCTCGTCGCCTCGTCCAGGAGCAGGATGACCG</td><td>10:A>T</td></tr>
<tr><td>22839978|F|0-14:G>C-14:G>C</td><td>TGCAGCAGCGGGCAGGGCGGGTAGTGGTTCACCCG</td><td>14:G>C</td></tr>
<tr><td>32025330|F|0-46:G>C-46:G>C</td><td>TGCAGCATCGCCGCGTCCCTGGGCGCCACGGGCTGCGCCGCCAGCTGCCCG</td><td>46:G>C</td></tr>
<tr><td>5363694|F|0-23:G>C-23:G>C</td><td>TGCAGCTTGTACTGCTGGAAGACGCCCG</td><td>23:G>C</td></tr>
<tr><td>3950847|F|0-28:C>A-28:C>A</td><td>TGCAGGACATGCTGCCGTCCTTCATCCACGGCGCCG</td><td>28:C>A</td></tr>
<tr><td>1072043|F|0-16:C>T-16:C>T</td><td>TGCAGCAGCACCTGGCCGACCCATGGCACAACACCATCATCCAGACCATCTCCG</td><td>16:C>T</td></tr>
<tr><td>7165452|F|0-17:G>A-17:G>A</td><td>TGCAGCTACGACGACGTGATCAGCCCTGTCTCCG</td><td>17:G>A</td></tr>
<tr><td>4261849|F|0-10:T>C-10:T>C</td><td>TGCAGCTCCCTGAGCACGGCGTCGCGGCTCTTGAGGAACCCG</td><td>10:T>C</td></tr>
<tr><td>1385721|F|0-44:G>A-44:G>A</td><td>TGCAGCCTCGGGCTCTTCGACTGGAAGTACGACCACATCCTCTCGGGCTCCG</td><td>44:G>A</td></tr>
<tr><td>1021118|F|0-14:C>T-14:C>T</td><td>TGCAGGTTCACAAACTGCTGCTTCTTGGTCTTGTGGTTGGAGAAGGACACCTTGTTGGCCCG</td><td>14:C>T</td></tr>
<tr><td>3946119|F|0-16:C>G-16:C>G</td><td>TGCAGCGAGGGGCGCCCTTCGCCG</td><td>16:C>G</td></tr>
<tr><td>1052623|F|0-38:A>G-38:A>G</td><td>TGCAGCCACGTCCACTGCGGCGTGTACTTGCCGTAGGCAGAGTAGGAGGAGAGCATGATGACGTGCGCC</td><td>38:A>G</td></tr>
<tr><td>1022972|F|0-48:G>A-48:G>A</td><td>TGCAGTGCGGCGCCGACTCGCTCTACGGCGACAGGCTGGGCGAGTTCAGACTGTCCGTGAAAGGCCACG</td><td>48:G>A</td></tr>
<tr><td>7487734|F|0-11:C>T-11:C>T</td><td>TGCAGCTCCTGCCTCGACGAGCCGACGCCG</td><td>11:C>T</td></tr>
<tr><td>2247862|F|0-19:C>T-19:C>T</td><td>TGCAGTGCGCGCCCTGCCTCGACTGCTTCGAGCAGAGCGGGCCCATCTTCGACCCG</td><td>19:C>T</td></tr>
<tr><td>7170134|F|0-32:C>G-32:C>G</td><td>TGCAGCGCGCACGCGTGCGCGTCGTCGTTGGCCGCGATGTTGGAGCGCACCAGGCAACGGAGCTCCATC</td><td>32:C>G</td></tr>
<tr><td>6045374|F|0-20:A>C-20:A>C</td><td>TGCAGATCGTGCCGTACGGCAGGCTGGAGGAGATCGCCCG</td><td>20:A>C</td></tr>
<tr><td>1038464|F|0-25:C>A-25:C>A</td><td>TGCAGTTCAGCTGGTACCTGACGGGCACCATGGTGTTCGTCGTCGTGCTCTACCTCCAGGTCAGCAGGC</td><td>25:C>A</td></tr>
<tr><td>987965|F|0-32:G>C-32:G>C</td><td>TGCAGCTGGGCGGCCTGGACCACCTCTCTGTTGGACAGGGTGTACTCGCCCGTCTCGCTGGCCTTCCTC</td><td>32:G>C</td></tr>
<tr><td>1038464|F|0-29:A>C-29:A>C</td><td>TGCAGTTCAGCTGGTACCTGACGGGCACCATGGTGTTCGTCGTCGTGCTCTACCTCCAGGTCAGCAGGC</td><td>29:A>C</td></tr>
<tr><td>1028890|F|0-65:C>G-65:C>G</td><td>TGCAGGTGGAGGTGGTGCTCCTGCTGCTGCCTCCTGCTCCCCGCCGCCAACTCCTCCATGGCCGCCGCG</td><td>65:C>G</td></tr>
<tr><td>3026592|F|0-32:G>A-32:G>A</td><td>TGCAGGCACCGCGTGGTGACGGGGATCTCGAAGTCCACCCG</td><td>32:G>A</td></tr>
<tr><td>3028103|F|0-31:G>A-31:G>A</td><td>TGCAGGTGTACGTGTTCGCGCTGTTCAACGAGGACCAGAAGCCCG</td><td>31:G>A</td></tr>
<tr><td>4262731|F|0-23:T>C-23:T>C</td><td>TGCAGGCTCGCCTCCCTGAACCGTGAGCTCCCCG</td><td>23:T>C</td></tr>
</table>

1098241|F|0-13:C>G-13:C>G	TGCAGTAGCAATTCGTCGAACAGGTGGTGTGCGTCTTCAGGGCTGAGCGTCCCG	13:C>G
3027653|F|0-31:G>C-31:G>C	TGCAGCTCTCCAACCTCTCCTCGGGGCCCCTGTTCCTCTCCG	31:G>C
985692|F|0-16:T>C-16:T>C	TGCAGAGCCAACACGGTGAGCCTGTTCGGTAGCCCCAGCTGCGTGGGGATGCTCCCG	16:T>C
1018026|F|0-12:C>T-12:C>T	TGCAGGTGGGTTCGGGTCAACCTTGCTGGGCCTGAACAAGAAGACGTACGAGCTGGCCG	12:C>T
2244920|F|0-46:C>T-46:C>T	TGCAGAACGTCGTGCAGATGATCTCGGTCATGTTCGTCGGCCATCTCGGTGAGCTCGCTCTCTCGAGTG	46:C>T
3949483|F|0-13:T>C-13:T>C	TGCAGAGCCGAGATGCTCGGTCCG	13:T>C
5567404|F|0-11:G>C-11:G>C	TGCAGGTCTCCGCCGTGATGTCGGCCG	11:G>C
3951478|F|0-19:G>A-19:G>A	TGCAGGGTCTCTTCGCGCGGCTCACGGCGTCCG	19:G>A
6037613|F|0-8:T>C-8:T>C	TGCAGCACTTCAAGGTTCGGCAGGTCGCCGATAAACTCGGGAATCTCGCCG	8:T>C
4398416|F|0-11:G>T-11:G>T	TGCAGCAAGCCGAGCAAGAAGAAGCCCTCCG	11:G>T
987148|F|0-20:A>G-20:A>G	TGCAGCGCACACGTCCGTCCACTCATCATCGCACCCGAACGGATTAGACAAACGGCTCAAAAGGCTTTC	20:A>G
4733924|F|0-8:G>C-8:G>C	TGCAGCGCGGACTGGCACGTGGGGCCCG	8:G>C
32020352|F|0-19:A>G-19:A>G	TGCAGGTGGGGGTGAAGGCACCAGGGACGCCGAAGAGGATGACCTTCTTGCCG	19:A>G
5582514|F|0-14:G>A-14:G>A	TGCAGCGCGGACACGGCGGACGACTGGAACCG	14:G>A
1244246|F|0-10:G>A-10:G>A	TGCAGCTCGAGGTGACCAGGCTGAGCACGCCGTGGGGCTACCTGAGCGCCGTGGCCTTGGCCGTCACGA	10:G>A
2243824|F|0-37:C>T-37:C>T	TGCAGCGGCGTCTGCAACCAAATACAACCAACCCCATCAACACCCTGCATGTCGACCACATAGATCGCG	37:C>T
5580325|F|0-11:C>T-11:C>T	TGCAGCTGCGTCTTCAGCAGCGGCGGGAACGACGCCG	11:C>T
3942803|F|0-37:C>A-37:C>A	TGCAGTTCTCGAACGCTTGCGGCGCCATCTGCACCACCAAGAAGGGAGCCATCCCG	37:C>A
996034|F|0-41:G>A-41:G>A	TGCAGATGGCGTCCTCTGCTGGTAATGGCAAGGAGATGGCCGCGCTCGAGTCCG	41:G>A
7172127|F|0-19:T>G-19:T>G	TGCAGCGCCCTCGCCGAAATCGTGAAGCGCTGCGCGCCG	19:T>G
1008117|F|0-5:G>C-5:G>C	TGCAGGTCGGCCAGCGCCTTGGGGGAGAGGTTGAAGTGCGTGGACTCGGCGGGCTCGGTGCCGCACCAC	5:G>C
1025427|F|0-37:C>A-37:C>A	TGCAGTCGCTGCTGCTCTGGCAGAACCAGCTCGTCGGCGCGATCCCGCCG	37:C>A
1025764|F|0-22:G>C-22:G>C	TGCAGGACAAGTACAAGCAGGCGAGGCTGGCCCTCGGCGACGTAACTCCG	22:G>C
100515999|F|0-32:A>T-32:A>T	TGCAGGGCGCGAGCGGGCGGCCGCAGAGGCACATGTTGGGCGCGAAGTGCTTGGGGCCGAACCTGTGCG	32:A>T
7168021|F|0-7:A>G-7:A>G	TGCAGGAATCCGCGGGAAGCAACAGCCTCTCCG	7:A>G
3948183|F|0-8:G>A-8:G>A	TGCAGCAAGTGGTTCTGCATGATGTCCCG	8:G>A
1038464|F|0-53:C>A-53:C>A	TGCAGTTCAGCTGGTACCTGACGGGCACCATGGTGTTCGTCGTCGTGCTCTACCTCCAGGTCAGCAGGC	53:C>A
7930549|F|0-11:C>G-11:C>G	TGCAGGCCCTTCTGCACGCCGTCGGATGCCG	11:C>G
992772|F|0-8:G>A-8:G>A	TGCAGGGCGGGCACGCCGAAGCCACAGGCGTACCGAGGAGGCAGCAAGACGGTCGGCCG	8:G>A
4993539|F|0-20:C>A-20:C>A	TGCAGCCTGCGGGCTCGCGGCGGGCAGCCG	20:C>A
1040262|F|0-14:G>A-14:G>A	TGCAGCCAGGATGCGCCCCACTTTGGAGACGCAATTATTGAGGAAGGAATTCATGGCGCCGAGGTGGAG	14:G>A
993630|F|0-6:G>A-6:G>A	TGCAGCGGTTCCCGCGCGGCCAGGCGGCGTCCTCGTCCGCCGACGCGCACGAGGTCCG	6:G>A
1087697|F|0-23:C>G-23:C>G	TGCAGCGCCACGTTGTGCACCCACGACAGCGTGTTGAGGTCCTTGATGCACCCGCTCTTGCCGCCGTAC	23:C>G
3949503|F|0-7:G>A-7:G>A	TGCAGCGGCGACGACGACCG	7:G>A
3955724|F|0-34:C>G-34:C>G	TGCAGACGGCGGAGCAGAGCACGTTGTCGAGGGGCCCG	34:C>G
3957141|F|0-23:G>A-23:G>A	TGCAGACGCAGAGCGCCATCGACGCGCTCGGCGCCCG	23:G>A
1115540|F|0-59:G>A-59:G>A	TGCAGCACGAAGGCGACCAGCGTGGGCGCCCTCATCACCACCTCGTTCACCTCGCGCCAGTCGCTCATC	59:G>A
6043043|F|0-43:C>T-43:C>T	TGCAGCTACCATATGCCCCACAGAGTGCATACACCTCACACTGCGTTCGCGGCTGTGACCAGAACAAGA	43:C>T
1278460|F|0-48:T>G-48:T>G	TGCAGCCTGTGGCTCCAGCGGCGGCGACAGCACGCCCACCACCACATTTGCGGGAGGAGGGGGGATCGG	48:T>G
3028103|F|0-20:C>T-20:C>T	TGCAGGTGTACGTGTTCGCGCTCTTCAACGAGGACCAGAAGCCCG	20:C>T
3953435|F|0-23:G>T-23:G>T	TGCAGGAAGGCGTTCCCCTCGGCGCTCTGCCTCGGCGGTTCGGCCG	23:G>T
3574707|F|0-22:C>T-22:C>T	TGCAGGTCCTCGACGCCGCAGGCCGCGGCGTAGTCGACGCAGCGCTTGAGGTCGGCCTGCGACAGCTGG	22:C>T
6048473|F|0-25:C>T-25:C>T	TGCAGGGCGTCGAACTTGTATTCCTCGCTGATGCTGCATGGGATGTGCACCACCTTGTACTGCCCGAGA	25:C>T
5364124|F|0-22:C>T-22:C>T	TGCAGTTCATGTTCACTTACCTCTTCTTCCCCG	22:C>T
1038464|F|0-28:C>G-28:C>G	TGCAGTTCAGCTGGTACCTGACGGGCACCATGGTGTTCGTCGTCGTGCTCTACCTCCAGGTCAGCAGGC	28:C>G
3027859|F|0-20:C>T-20:C>T	TGCAGCTTCAGCTGCTCGATCTCCAACCGAACCTCTCGGCCG	20:C>T
1667127|F|0-25:G>A-25:G>A	TGCAGGGCGGGATCCCGTTCGAGAAGTCCAAGGGCCAGCACATCCTGCGGAACCCG	25:G>A
6045430|F|0-10:C>G-10:C>G	TGCAGCACGGCCGTCGCGCCCGCGACGTCCG	10:C>G
3936827|F|0-6:T>C-6:T>C	TGCAGCTTGAGTAGGACGCCGAGCAGGTCCTCATCTCTCTCTGGCTCCG	6:T>C
988647|F|0-25:G>C-25:G>C	TGCAGAAGAACCTGGAGCTGTCGCCGCGGCACGTGGAGGCGTCGCGCATGACGCTGCACCG	25:G>C
3958751|F|0-17:G>A-17:G>A	TGCAGTACAGCGTCAAGGTCGGGCGGCCATACACCCG	17:G>A
1021204|F|0-46:G>C-46:G>C	TGCAGTTGAGGGCGAGGAGGGCGCGGATGCGGTGGAAGAGGTTCTTGAGGTAGACCTTGTCCCTGACGA	46:G>C
1053007|F|0-43:G>A-43:G>A	TGCAGCTGGGCTACAACCAGCTCTCAGGGAGCATCCCAACTCAGCTGGGTGAGCTCAAGAAGCTCACTG	43:G>A
1053007|F|0-64:C>T-64:C>T	TGCAGCTGGGCTACAACCAGCTCTCAGGGAGCATCCCAACTCAGCTGGGTGAGCTCAAGAAGCTCACTG	64:C>T
2243824|F|0-17:C>T-17:C>T	TGCAGCGGCGTCTGCAACCAAATACAACCAACCCCATCAACACCCTGCATGTCGACCACATAGATCGCG	17:C>T
1068619|F|0-22:C>T-22:C>T	TGCAGGTGCTCACGCTTTGCCTCCAGGCCACGAAGAACGTTAACCTCATGATGCTCCG	22:C>T
6048906|F|0-5:C>T-5:C>T	TGCAGCGGCATCAGCCAGAGGTCGTGCAGCCCATGCATGGCGCCG	5:C>T
985665|F|0-50:C>T-50:C>T	TGCAGCACCAGGTGCACCAGCAGGTCCAGGCTCTCCAGCGCCGTCACCAGCGCGCCG	50:C>T
5358014|F|0-7:C>G-7:C>G	TGCAGCTCTGCTGCGGGCGCCG	7:C>G

1211131|F|0-35:C>T-35:C>T	TGCAGCAGCACCTGGCCGATGCTCCTGAGCTCCTCCTGCTCCTCCCTCTCCTCCG	35:C>T
1047019|F|0-46:C>A-46:C>A	TGCAGAGATTCCATCTGATCCGATCAGGTTCTCTGCATGCTCGGCGCAAAAGTCTTCTTCTGATGCAGC	46:C>A
3938954|F|0-17:G>C-17:G>C	TGCAGGTTTTTGTAGTAGGAGTTGTCGAATCCCAACGGCGTCGGGTCGAGGAACGCGGAGCTCTGGCTG	17:G>C
2266494|F|0-14:C>T-14:C>T	TGCAGCGCCTCCTGCTCCCGCAGCCGCTTCACGAGGTGTGTCGACGGCGAGCGCGCCAGCTTCTTGAGG	14:C>T
1088913|F|0-45:G>A-45:G>A	TGCAGTGGAGATGGGAGCGTGTGCGACGAAGCCTGCTGATCTCAAGGTAGAAGGTGAGGCGCCG	45:G>A
1077420|F|0-11:A>G-11:A>G	TGCAGAGTGCAAGGACTACTTCAGCAAGTCCCTGTTTGTGGTGGGAGAGTTTGGGGGGAACGACTACAA	11:A>G
1696589|F|0-65:C>T-65:C>T	TGCAGGTACTACTCGACCTGGGGACACGTCGCGACGCTGGCGGAGGAGATGAAGAAGGGCGCCGACTCC	65:C>T
1047086|F|0-62:C>T-62:C>T	TGCAGACCGCTCAGCCAAACCATTCAAGACAGTGGCAAAGGTGAACTCATCAGGCCGAACCCCGCAGCC	62:C>T
1083206|F|0-43:G>T-43:G>T	TGCAGCTGACGGAGTGGCACATGGAGCCGTCGCGGATGACGCTGCACCG	43:G>T
1318538|F|0-14:C>G-14:C>G	TGCAGGGTGGAGGACGAGAGGTTGGCGGAAGGGAGGACGAGGGCGGTGACGGCGGCCG	14:C>G
1213704|F|0-5:G>C-5:G>C	TGCAGGAAGTTAACGAGCGCAGACTGGTGAAACACGATCTTGGCAGCTGGAGCGCAGGACCACGCTCGG	5:G>C
11314822|F|0-13:A>G-13:A>G	TGCAGCCACTTGTACTGCGCCGTGCCG	13:A>G
3022350|F|0-12:T>C-12:T>C	TGCAGAGGCAAATCTGCTCTTCTTGGAGCAGCCTGCCG	12:T>C
1087697|F|0-50:C>G-50:C>G	TGCAGCGCCACGTTGTGCACCCACGACAGCGTGTTGAGGTCCTTGATGCACCCGCTCTTGCCGCCGTAC	50:C>G
1069017|F|0-56:C>G-56:C>G	TGCAGCTCCTTCTCCAACCGCTTCTCCGAGGACTCCGACTTCGTCCGAAGCCTGGCCTCCAACTGCTCC	56:C>G
6048922|F|0-16:G>A-16:G>A	TGCAGGCACCCATATCGTTCCGCCTCACCTCCG	16:G>A
1225163|F|0-23:G>A-23:G>A	TGCAGCCGCTGCCTGACAAGTTCGTGAGGGAGCTCAACGGCCGTGCGCTCCG	23:G>A
1017298|F|0-28:G>A-28:G>A	TGCAGAAAAATGTGCATTTCCTCACAAAGGTTGCTGGGCTGGAGATATCATACATTGCTCAAAGGCCGA	28:G>A
4993794|F|0-20:C>G-20:C>G	TGCAGCTGGTAGCTGAGGTGCAGGTCCTCGCCG	20:C>G
3029332|F|0-25:G>A-25:G>A	TGCAGGTGCACAACCAGATGCGGCAGCTTGGTTGTGATCCG	25:G>A
3955724|F|0-33:G>C-33:G>C	TGCAGACGGCGGAGCAGAGCACGTTGTCGAGGGGGCCG	33:G>C
1033462|F|0-11:G>A-11:G>A	TGCAGCCATGCGACCTGCTCGGCGTACTTGTCGGGGGTCGCGTTGGGGTCGTTGCCGCACTCCACGTTG	11:G>A
6030878|F|0-23:G>A-23:G>A	TGCAGGTGCGTCTTGACGACGACGGCCG	23:G>A
1224551|F|0-31:C>T-31:C>T	TGCAGGTCCTCCTCCCGCAGCTCGCCGTCGACGGCGGTGGAGACCTCCTCCCTTAGCCTGTCCTGCATC	31:C>T
1863078|F|0-21:C>T-21:C>T	TGCAGCGGCGATCCTGTGGAGCACGAGTAGGGGCAGCTGGTTCTCGACCATGAGCATGTCGCGCTGGAC	21:C>T
1033462|F|0-36:G>T-36:G>T	TGCAGCCATGCGACCTGCTCGGCGTACTTGTCGGGGGTCGCGTTGGGGTCGTTGCCGCACTCCACGTTG	36:G>T
1059110|F|0-37:G>C-37:G>C	TGCAGGGGTGGGATAATGCGACCATTGCAGGTAATTCGTTCGTTCGTGCTTCCG	37:G>C
1338735|F|0-18:T>C-18:T>C	TGCAGCAGCCCACCTTCCTCCGTGAACCCCAGGCGTCCCGCGAGCGGGTAGTACTGCACCAGGGCCTGC	18:T>C
6048549|F|0-26:T>G-26:T>G	TGCAGCAGATATGTGAGGTCAAGATGTTGGCACCG	26:T>G
1024066|F|0-34:C>T-34:C>T	TGCAGCACTGCGACTGCACCGCGCGCATCGTCGCCTTGAACTCCTCGCTGCGGTACACGTTGGTGGTCG	34:C>T
7351790|F|0-15:C>T-15:C>T	TGCAGCAGCCCCAGCCGATCGCCG	15:C>T
4395665|F|0-8:T>G-8:T>G	TGCAGTACTCGAGCGAGCAGACCTTCAAGGAGGCCG	8:T>G
3951478|F|0-13:C>T-13:C>T	TGCAGGGTCTCTTCGCGCGGCTCACGGCGTCCG	13:C>T
5578229|F|0-23:G>A-23:G>A	TGCAGGAGGTGAAGGAGAACTCGGTCCG	23:G>A
989354|F|0-31:A>C-31:A>C	TGCAGCTTCAGATCCGTAACGACCAGATCGCACGTTGATGGACGTGGGAAGTCCAACTGCGCCG	31:A>C
12469755|F|0-16:G>A-16:G>A	TGCAGAGGTGCAAGTCGGAGCCG	16:G>A
1242326|F|0-61:C>G-61:C>G	TGCAGGTGCAGGAGGAGCTGGTGGGCGACATGGAGAAGATCGTGGAGGTGCTGAGGGGGCTCGACAAGG	61:C>G
1008117|F|0-41:G>C-41:G>C	TGCAGGTCGGCCAGCGCCTTGGGGGAGAGGTTGAAGTGCGTGGACTCGGCGGGCTCGGTGCCGCACCAC	41:G>C
6042763|F|0-9:G>A-9:G>A	TGCAGCTTGGACAGCCCACCGAAGCTCCTCGGGATGGGGCCCGTGAGCATGTTGAGCGACAGGTCAATG	9:G>A
2256046|F|0-16:C>A-16:C>A	TGCAGCTGCCCGTCCACAAGCTTGAACCTGCCGGCCATCCCGTCGAACGTTGTGTTGAGCACCGCTCTG	16:C>A
4406089|F|0-10:C>T-10:C>T	TGCAGCTTCGCGTCGCGGAAGCAATTGCCCGCGTCGAGCACCGTGCGCGCCGACGAGTTGGACCGCATG	10:C>T
2283143|F|0-51:G>C-51:G>C	TGCAGCGCTGCCAGCCCCTCCTCCCCGTCGCCGAGGAGCTCCTCATCGTCAGCCGCTGCGTCGAGTCGC	51:G>C
1224551|F|0-32:G>A-32:G>A	TGCAGGTCCTCCTCCCGCAGCTCGCCGTCGACGGCGGTGGAGACCTCCTCCCTTAGCCTGTCCTGCATC	32:G>A
1013635|F|0-49:C>G-49:C>G	TGCAGTTCACGGTGAAGGACTCTGTGCGGGAGAGCGTGTCCAGCGCGTCCGAGAACCTCTCCTCGTCAT	49:C>G
4262557|F|0-10:C>A-10:C>A	TGCAGGCCCTCGGGATCGCGCTGCTCTGCGCCAGCACGCGCCCG	10:C>A
986912|F|0-55:G>T-55:G>T	TGCAGAAACTCACCTTGGGTGATCTTGTTGAGGCCACCAATGGCTTCCACAATGAGAGCTTGATTGGGT	55:G>T
1046261|F|0-44:C>T-44:C>T	TGCAGGCCATCACGAGAAAGTCCCTATCAGCTGCCCAGGTGAACCAAACGGCCG	44:C>T
987148|F|0-13:T>C-13:T>C	TGCAGCGCACACGTCCGTCCACTCATCATCACACCCGAACGGATTAGACAAACGGCTCAAAAGGCTTTC	13:T>C
4002481|F|0-40:G>C-40:G>C	TGCAGGACACGTCCGCGCAGTGCACCCACCGCCCGTGCTCGCCG	40:G>C
7351477|F|0-13:C>T-13:C>T	TGCAGTGCCCCGACGGGCCG	13:C>T
1019413|F|0-37:T>C-37:T>C	TGCAGGGCGATGGCACGGAGGCGCAGGTGACGTGGGATGACCAGCAGAACATCAACCG	37:T>C
1264332|F|0-43:C>T-43:C>T	TGCAGCAAACCATCTGCAACATGTCGCACCTGAGCATCCTGACCTTCAAATACAACCTAGCGAAGCTCC	43:C>T
5011056|F|0-15:T>C-15:T>C	TGCAGTTGCGCCTGATCTGGGTGACGGCTCCG	15:T>C
7169350|F|0-15:A>G-15:A>G	TGCAGCAGGAGCGGGAGCACTGCGTCACGGAGGTACCG	15:A>G
4002481|F|0-5:G>A-5:G>A	TGCAGGACACGGCCGCGCAGTGCACCCACCGCCCGTGCTCGCCG	5:G>A
1230460|F|0-38:C>A-38:C>A	TGCAGCTGACGTGGAACTACAACTACGGGGCGGCGGGGCAGAAGCTCGGCTTCAACGGGCTCGACAACC	38:C>A
4734153|F|0-15:C>A-15:C>A	TGCAGGTGGGTGCGCCGCGACGAGCCG	15:C>A
3022350|F|0-30:G>A-30:G>A	TGCAGAGGCAAATCTGCTCTTCTTGGAGCAGCCTGCCG	30:G>A
1068553|F|0-17:G>A-17:G>A	TGCAGGGAGAGCGCCCTGGTGATGTTGCGCAGCACGGCCTGCTCGTGCTGCTGCTCCTGCACGCTCGGG	17:G>A

<table>
<tr><td>5325034|F|0-64:C>A-64:C>A</td><td>TGCAGGAGAACCAGAAGCTGAAGAAGCAGGTGGAGAAGATGAGGAAGAAGCACGAGGTGGAGATCGAGA</td><td>64:C>A</td></tr>
<tr><td>3029817|F|0-17:G>A-17:G>A</td><td>TGCAGGAGGTGGTCGTGGTGGTGGTGATGCTGCACGCCG</td><td>17:G>A</td></tr>
<tr><td>4262476|F|0-23:G>C-23:G>C</td><td>TGCAGGTGGCAGGGCAGCCAGTCGGCCG</td><td>23:G>C</td></tr>
<tr><td>3943188|F|0-30:T>C-30:T>C</td><td>TGCAGAACTTGCGGTCCGATGCTTCCTCCGTCCG</td><td>30:T>C</td></tr>
<tr><td>1214876|F|0-28:C>T-28:C>T</td><td>TGCAGAGCTAGGTGGATAGAGTAGCTGGCGGCTAGGCGCTAGTTGCAGGAAGGAAAGGTTGGATGGAGC</td><td>28:C>T</td></tr>
<tr><td>4404352|F|0-10:G>T-10:G>T</td><td>TGCAGGACGGGACCGTGGCGTGGGGCAAGGCCGCCACCTTCCGCGCGTCCCCCTACCCG</td><td>10:G>T</td></tr>
<tr><td>2246955|F|0-30:A>G-30:A>G</td><td>TGCAGGTGGTGCCGTGGGGCCACATGGAGCACTCGGCCATGATATTCTACGGCGTGCAGGCCAAGGAGA</td><td>30:A>G</td></tr>
<tr><td>2283143|F|0-44:A>G-44:A>G</td><td>TGCAGCGCTGCCAGCCCCTCCTCCCCGTCGCCGAGGAGCTCCTCATCGTCAGCCGCTGCGTCGAGTCGC</td><td>44:A>G</td></tr>
<tr><td>4262765|F|0-11:G>C-11:G>C</td><td>TGCAGGAAGGTGACCACCGCGTGGATGGACTCCG</td><td>11:G>C</td></tr>
<tr><td>32023330|F|0-15:A>T-15:A>T</td><td>TGCAGCGGTGGGAGCAGGTGATGACGGCGGCGAGGCGAGGCGACCTCCG</td><td>15:A>T</td></tr>
<tr><td>1021673|F|0-15:A>G-15:A>G</td><td>TGCAGACCACGACGCAGTTAACCGCCCCAGCAAAGGAGGTTCTCTTCTCCGCCG</td><td>15:A>G</td></tr>
<tr><td>5006251|F|0-21:C>T-21:C>T</td><td>TGCAGGCTACTACGACGACGACACCG</td><td>21:C>T</td></tr>
<tr><td>3953435|F|0-35:C>T-35:C>T</td><td>TGCAGGAAGGCGTTCCCCTCGGCGCTCTGCCTCGGCGGTTCGGCCG</td><td>35:C>T</td></tr>
<tr><td>1029006|F|0-51:G>A-51:G>A</td><td>TGCAGGCGGCGTCCAGGGGCCGTCCACGCGGAGGCTGACGAGCAGCGACGAGAGGCTGGAGGCGGCGGC</td><td>51:G>A</td></tr>
<tr><td>4261894|F|0-32:C>T-32:C>T</td><td>TGCAGCTGCCGCTTCACCCACTTCACGATGTCCTCGTCCTCGCCG</td><td>32:C>T</td></tr>
<tr><td>4009317|F|0-25:G>A-25:G>A</td><td>TGCAGAGGTAGAGCGCGATGGCCCTGCCGAGCTTGGAGGTGGCGCCG</td><td>25:G>A</td></tr>
<tr><td>7170771|F|0-19:G>C-19:G>C</td><td>TGCAGGACACCAGCATCGTGACGCCG</td><td>19:G>C</td></tr>
<tr><td>1024410|F|0-41:G>C-41:G>C</td><td>TGCAGCGCCTTCACCAGCCGCTCGCCCTTCTCGGCCGTGAAGGCCTTCCG</td><td>41:G>C</td></tr>
<tr><td>1031760|F|0-34:G>T-34:G>T</td><td>TGCAGCTTGCGCCGCAGGAACCCCCTGGAGAGCAGCACCTTGGTGCCGACCCTCTTGTCGTCCCAGCTG</td><td>34:G>T</td></tr>
<tr><td>3028103|F|0-13:G>C-13:G>C</td><td>TGCAGGTGTACGTGTTCGCGCTCTTCAACGAGGACCAGAAGCCCG</td><td>13:G>C</td></tr>
<tr><td>1020552|F|0-61:G>A-61:G>A</td><td>TGCAGAGGAAGAAGCGGCTGGTGGCCTACAAGGTGTACGACGTGGAGGGCAAGGTGAAGGGGTCGGTGC</td><td>61:G>A</td></tr>
<tr><td>2248179|F|0-15:T>G-15:T>G</td><td>TGCAGGGGAGGCGATTAAGCAGTCGCCGTCGATGCCGACGACCCCGACCACGCCG</td><td>15:T>G</td></tr>
<tr><td>985692|F|0-50:G>C-50:G>C</td><td>TGCAGAGCCAACACGGTGAGCCTGTTCGGTAGCCCCAGCTGCGTGGGGATGCTCCCG</td><td>50:G>C</td></tr>
<tr><td>1151484|F|0-29:C>A-29:C>A</td><td>TGCAGCTTGAGGAGGACGCCGAGCAGGTCCTCATCTCTCTCTGGCTCCG</td><td>29:C>A</td></tr>
<tr><td>1031695|F|0-25:C>T-25:C>T</td><td>TGCAGGCCTACTTCAACGCCTACTACGCGTCCATCGGGGTGGCCTTCACGGTCGCCGTCACCG</td><td>25:C>T</td></tr>
<tr><td>5373570|F|0-9:G>C-9:G>C</td><td>TGCAGGTGCGCCACGGCGCCCG</td><td>9:G>C</td></tr>
<tr><td>5373570|F|0-11:C>G-11:C>G</td><td>TGCAGGTGCGGCACGGCGCCCG</td><td>11:C>G</td></tr>
<tr><td>1667729|F|0-7:T>C-7:T>C</td><td>TGCAGGCTGACCAGGGGTGGTCGAGGCGGGTGACGTTGGCGTGCATCGCCTTCTGCACGTCCG</td><td>7:T>C</td></tr>
<tr><td>1089043|F|0-32:G>A-32:G>A</td><td>TGCAGGACGTGCGCGCCCAGGCCGACTACGACGGCTGGTACCACAACCAGTTCCTCGTCGTCAACGACT</td><td>32:G>A</td></tr>
<tr><td>2242439|F|0-64:C>A-64:C>A</td><td>TGCAGGCCATCATCGAGGGCTCCAGGGAGACCTGGACCTACGCCATCTTCTGGCAGTCCTCCACCGACG</td><td>64:C>A</td></tr>
<tr><td>1087697|F|0-11:G>A-11:G>A</td><td>TGCAGCGCCACGTTGTGCACCCACGACAGCGTGTTGAGGTCCTTGATGCACCCGCTCTTGCCGCCGTAC</td><td>11:G>A</td></tr>
<tr><td>1740524|F|0-32:A>C-32:A>C</td><td>TGCAGAAATCTGTGGGGAATACGTGGTGGAGGAGGCGGTGGTGGTGGAGGCGTCGCCG</td><td>32:A>C</td></tr>
<tr><td>32025541|F|0-24:C>T-24:C>T</td><td>TGCAGCGCCTGCACGGCTTCTCGCCGTCCCCGCAGCACGCGCAGGTCCATAAGCTCTCCTGGGAGCAGG</td><td>24:C>T</td></tr>
<tr><td>3024772|F|0-16:C>T-16:C>T</td><td>TGCAGGACTATGACGCCGAGACGTGGGCATTTCGGTACCG</td><td>16:C>T</td></tr>
<tr><td>32026817|F|0-11:G>C-11:G>C</td><td>TGCAGCAGCAGGTCCGCCGCCGCCAGCTCCG</td><td>11:G>C</td></tr>
<tr><td>3020538|F|0-19:A>G-19:A>G</td><td>TGCAGCTTATTCGGGATTCAAAATCACCATCATCTTTAATCCG</td><td>19:A>G</td></tr>
<tr><td>32026371|F|0-17:G>C-17:G>C</td><td>TGCAGCTGGTGGCACAGGGTGTTGGTCTCCAGGAAGAACTCGAGGAACACCTTGCTCTCGGAGTCGCCG</td><td>17:G>C</td></tr>
<tr><td>5355098|F|0-67:C>G-67:C>G</td><td>TGCAGGAGTCTTCGCCTGGGGCGACCGTAGAAGCGGGGGAGCTGGTGGAGGGGATGATGGCCAAGCTCT</td><td>67:C>G</td></tr>
<tr><td>5363639|F|0-22:G>C-22:G>C</td><td>TGCAGCTGAGCCCGTGCCCCATGGCCTCCG</td><td>22:G>C</td></tr>
<tr><td>5364263|F|0-16:G>C-16:G>C</td><td>TGCAGCAGGGGAGGGCGGCGCCGAACCCGCCGCACGCCGAGCACGGGTCCG</td><td>16:G>C</td></tr>
<tr><td>100557699|F|0-59:C>T-59:C>T</td><td>TGCAGCTTATCCAATCCAATACCTGAATCTGCCATCGCCCTCTTGCACGGCTCAACGACCCTCTCCAGC</td><td>59:C>T</td></tr>
<tr><td>100524149|F|0-46:A>G-46:A>G</td><td>TGCAGCACATGAACAAGGGGATCTTCTCCTGGGCCACACTCATCTCACGCATACAGAGCGAATCGCCGA</td><td>46:A>G</td></tr>
<tr><td>22831862|F|0-5:G>C-5:G>C</td><td>TGCAGGACCTCCAGGCCGACGCCG</td><td>5:G>C</td></tr>
<tr><td>4994297|F|0-31:A>C-31:A>C</td><td>TGCAGCAGATCGGCGTGCTCGGGGAGGACGAAGGCGGCGCGGCCG</td><td>31:A>C</td></tr>
<tr><td>3935824|F|0-10:C>G-10:C>G</td><td>TGCAGAACGCCATCATCATGCCGCCG</td><td>10:C>G</td></tr>
<tr><td>1124420|F|0-23:C>G-23:C>G</td><td>TGCAGAGCGGCGCACACCTGAGCCGCCGCTTCTTCCTTGTCTCCGTCCAGCCTTCCCCTCAGGACCCGA</td><td>23:C>G</td></tr>
<tr><td>1010739|F|0-48:G>A-48:G>A</td><td>TGCAGCTGTGCGGCCCGTGGCATGTAGGGAATGGTCAAATGGAGGAGGGGGGTATGTCAGAGCAAGAGA</td><td>48:G>A</td></tr>
<tr><td>986230|F|0-32:C>A-32:C>A</td><td>TGCAGGTACGCGTACGCCGCGTGTTTTACCAGCGGGTGGCGTATCATGTGTATGTCCCCACAGCTGCCG</td><td>32:C>A</td></tr>
<tr><td>3942478|F|0-5:C>G-5:C>G</td><td>TGCAGCCACTGCCACTGCAAGCCCCG</td><td>5:C>G</td></tr>
<tr><td>12770201|F|0-23:C>T-23:C>T</td><td>TGCAGAACAGAATCACAAACCAACCCACCG</td><td>23:C>T</td></tr>
<tr><td>5357999|F|0-11:C>T-11:C>T</td><td>TGCAGCTCGCGCTCCCAGTCCTCCG</td><td>11:C>T</td></tr>
<tr><td>7165106|F|0-23:C>T-23:C>T</td><td>TGCAGCGAAGGGGAAGAAGGGCGCGGCCG</td><td>23:C>T</td></tr>
<tr><td>1012307|F|0-21:T>C-21:T>C</td><td>TGCAGGAATCTCCAGATGGAGTACGTGGACCTGTACCTTGTCCACTGGCCG</td><td>21:T>C</td></tr>
<tr><td>3026721|F|0-7:C>T-7:C>T</td><td>TGCAGTTCTTGATCAGCTGGATGTCGCGGTTCCCGCAGTACCG</td><td>7:C>T</td></tr>
<tr><td>4002481|F|0-13:C>G-13:C>G</td><td>TGCAGGACACGTCCGCGCAGTGCACCCACCGCCCGTGCTCGCCG</td><td>13:C>G</td></tr>
<tr><td>1694774|F|0-43:G>A-43:G>A</td><td>TGCAGCATGGGCGACAGGTCGGAGAAGGACTTGTAGGCCGCCAGCTTGAGGGCGACGTCGAGCGGTGAG</td><td>43:G>A</td></tr>
<tr><td>3942104|F|0-25:C>T-25:C>T</td><td>TGCAGATGAGCCCCACGGCGAAGCTCGGGGAGGTGATGTCGCGCCCG</td><td>25:C>T</td></tr>
<tr><td>3945252|F|0-19:A>C-19:A>C</td><td>TGCAGGGATCATCGGAACCATGGTCACCG</td><td>19:A>C</td></tr>
<tr><td>6046819|F|0-18:C>G-18:C>G</td><td>TGCAGGCGATGGCGCAACCTAATCGCGGGCGACGGCGGCGGCTTCCTCCGCCG</td><td>18:C>G</td></tr>
</table>

<table>
<tr><td>4261322|F|0-19:T>C-19:T>C</td><td>TGCAGACGCCGAGCGAGGTTGATCTGCTGGACGACGGCTACCG</td><td>19:T>C</td></tr>
<tr><td>1221211|F|0-49:A>G-49:A>G</td><td>TGCAGCAAGTCGGGAGCCATCATCCCCTGGAACACGTCCTCCTCCAAGAAGCTCAGGAAGCCCCCGAGA</td><td>49:A>G</td></tr>
<tr><td>3026293|F|0-35:C>T-35:C>T</td><td>TGCAGCGACATCGCCGAGGGGGTTGCCTACCTGCACCACCACTCACCG</td><td>35:C>T</td></tr>
<tr><td>1077816|F|0-19:C>T-19:C>T</td><td>TGCAGAAAAGGAGGTAGGGCGGAGGGGAAGCAGGATGTGATGTGAGAGGGATGATGGGCTAACCGAGAT</td><td>19:C>T</td></tr>
<tr><td>998552|F|0-11:A>T-11:A>T</td><td>TGCAGCCGCGGAGCTGGCGGCGGCGCTGGGAGTGTCCTCGGATCGTACGCTGTTTCTGTGCAGGTAAGC</td><td>11:A>T</td></tr>
<tr><td>7907133|F|0-16:G>C-16:G>C</td><td>TGCAGGGCAGCTACGAGCCG</td><td>16:G>C</td></tr>
<tr><td>2283143|F|0-28:C>T-28:C>T</td><td>TGCAGCGCTGCCAGCCCCTCCTCCCCGTCGCCGAGGAGCTCCTCATCGTCAGCCGCTGCGTCGAGTCGC</td><td>28:C>T</td></tr>
<tr><td>32027084|F|0-13:G>T-13:G>T</td><td>TGCAGCCAGCCAAGGCAAGCCG</td><td>13:G>T</td></tr>
<tr><td>7169350|F|0-34:A>G-34:A>G</td><td>TGCAGCAGGAGCGGGAGCACTGCGTCACGGAGGTACCG</td><td>34:A>G</td></tr>
<tr><td>3022182|F|0-10:C>A-10:C>A</td><td>TGCAGGAATCCATCCAATTTGCGCCCTCTCCTTTCCGCCCG</td><td>10:C>A</td></tr>
<tr><td>1099230|F|0-10:C>T-10:C>T</td><td>TGCAGGTGTTCGTCGCCGCCTTCACCAAGAGGAAGCAAATCTGCCCTACCAATACTGGCAACGCCGAGA</td><td>10:C>T</td></tr>
<tr><td>1058180|F|0-9:C>T-9:C>T</td><td>TGCAGCAAGCTTCCAGTTTCATTCCTTGGACCACACCATAGATTCAAATAACAAATAGAGTTACTTTCT</td><td>9:C>T</td></tr>
<tr><td>3064378|F|0-26:C>A-26:C>A</td><td>TGCAGTAATAGCTTCAAAGTCGTCAGCGAAAAGCATTCTGGTAGATATGATTAGTGTGATGCGTCCATC</td><td>26:C>A</td></tr>
<tr><td>3028381|F|0-23:T>A-23:T>A</td><td>TGCAGAGCGAGGTGGTTCCAGGTTGCCCATCATGGATTGCCG</td><td>23:T>A</td></tr>
<tr><td>3937317|F|0-6:G>C-6:G>C</td><td>TGCAGGGCGCCGATGGCGGGCAGAACGGGGAGTTCTCGCCG</td><td>6:G>C</td></tr>
<tr><td>7171179|F|0-7:C>G-7:C>G</td><td>TGCAGCTCCTGGTGGTAGCTGCACGACTCGTTGTGGTTGGGCGTCTTGCACGCAACGTGCGGGAAACGG</td><td>7:C>G</td></tr>
<tr><td>7155281|F|0-10:C>T-10:C>T</td><td>TGCAGCTTGTCGGCCTCCTTCTTGCGGCCG</td><td>10:C>T</td></tr>
<tr><td>3958507|F|0-9:C>T-9:C>T</td><td>TGCAGCTCGCGCTCCTCCTCCGCCGTCAGCGCCGCCG</td><td>9:C>T</td></tr>
<tr><td>4406428|F|0-25:G>A-25:G>A</td><td>TGCAGGCGCTGGGAGAGGACAGGCGGCCG</td><td>25:G>A</td></tr>
<tr><td>4406935|F|0-8:T>C-8:T>C</td><td>TGCAGCGATCTCATCCACGAGATGCTCGCCGACGCGCCG</td><td>8:T>C</td></tr>
<tr><td>2250796|F|0-41:A>C-41:A>C</td><td>TGCAGTACGTGATCACCGTCGGCATCGGCTCCCCTGTCGTGACCCAGACCATGATGATCGACACCAGCG</td><td>41:A>C</td></tr>
<tr><td>1020552|F|0-33:T>C-33:T>C</td><td>TGCAGAGGAAGAAGCGGCTGGTGGCCTACAAGGTGTACGACGTGGAGGGCAAGGTGAAGGGGTCGGTGC</td><td>33:T>C</td></tr>
<tr><td>986029|F|0-43:C>T-43:C>T</td><td>TGCAGCACATCAGCCCATACTTCCTGATCAGTCCATGGGAGTTCCCACAGACCCG</td><td>43:C>T</td></tr>
<tr><td>3948919|F|0-17:G>A-17:G>A</td><td>TGCAGCCATGGGTTCTCGTGGTACATCCG</td><td>17:G>A</td></tr>
<tr><td>1018977|F|0-17:C>T-17:C>T</td><td>TGCAGAGGCTTCTGATTCCACACCTGACCAATGCATCATGATGTTGCTTAATCATTTTATCTCCACGAT</td><td>17:C>T</td></tr>
<tr><td>5346239|F|0-19:A>C-19:A>C</td><td>TGCAGCTGGTTGTGTCGGGACAGCAGGGTAACCCG</td><td>19:A>C</td></tr>
<tr><td>3951245|F|0-8:G>A-8:G>A</td><td>TGCAGTCGGAGCATCCCGAGAGCTTACCG</td><td>8:G>A</td></tr>
<tr><td>987148|F|0-68:C>T-68:C>T</td><td>TGCAGCGCACACGTCCGTCCACTCATCATCACACCCGAACGGATTAGACAAACGGCTCAAAAGGCTTTC</td><td>68:C>T</td></tr>
<tr><td>1070952|F|0-39:A>G-39:A>G</td><td>TGCAGCGTGATCAAGACTGCTGCTCCCAAGACCAAGGCCACAAGGAAGACCCG</td><td>39:A>G</td></tr>
<tr><td>5363149|F|0-16:G>A-16:G>A</td><td>TGCAGAGGATGCTTGTGTGCCCGCCG</td><td>16:G>A</td></tr>
<tr><td>5359391|F|0-26:T>C-26:T>C</td><td>TGCAGGTGAGGCTGACGAAGGTGAAGTTCCCCG</td><td>26:T>C</td></tr>
<tr><td>32019278|F|0-6:A>T-6:A>T</td><td>TGCAGAAGCCTACGCGCGTCCG</td><td>6:A>T</td></tr>
<tr><td>4394733|F|0-10:C>G-10:C>G</td><td>TGCAGCGCGACGCAGCGGCCGATCTCCG</td><td>10:C>G</td></tr>
<tr><td>985665|F|0-41:C>G-41:C>G</td><td>TGCAGCACCAGGTGCACCAGCAGGTCCAGGCTCTCCAGCGCCGTCACCAGCGCGCCG</td><td>41:C>G</td></tr>
<tr><td>4261923|F|0-10:C>G-10:C>G</td><td>TGCAGATTCTCAGCCTGCTGCCGCTCAAGTGCGCCATCCGCACCG</td><td>10:C>G</td></tr>
<tr><td>3936592|F|0-5:G>C-5:G>C</td><td>TGCAGGGGATCCTGGGCTTCCTCAGCCGCACCG</td><td>5:G>C</td></tr>
<tr><td>1229132|F|0-10:C>T-10:C>T</td><td>TGCAGGTGGACGGGCAGCTCCTCACCGACATCCCGGCGGGGACCTTCGACGTCCG</td><td>10:C>T</td></tr>
<tr><td>3023203|F|0-16:G>A-16:G>A</td><td>TGCAGGGGATATACGTGCTGAGGTTGGACTTATTACCTGGTGTACCG</td><td>16:G>A</td></tr>
<tr><td>990763|F|0-14:G>C-14:G>C</td><td>TGCAGAAGCAGGTGGACGGCCTGGAGAAGAAGAAGGAGGAGCTGACGCGGGCCAGCTGCAAGCCCGAGA</td><td>14:G>C</td></tr>
<tr><td>6044650|F|0-31:G>C-31:G>C</td><td>TGCAGGACACCACGCTCGGATCCCTGCTCTCGTCGCTCATGCAGCACTGCGACCCGCCGCAGCGCCGAG</td><td>31:G>C</td></tr>
<tr><td>981702|F|0-5:T>C-5:T>C</td><td>TGCAGTAATTTAGTGTGTTGTTTTTACCTATAGTGCCACCAAGTTCTATGACACAAACATCAGCTGGCT</td><td>5:T>C</td></tr>
<tr><td>1286660|F|0-8:C>T-8:C>T</td><td>TGCAGTTTCTCGAGATGAAGCTCGGCTAGCAAGGCACCCTGTGGGATCGCCTGGAGAACCG</td><td>8:C>T</td></tr>
<tr><td>3941804|F|0-12:G>A-12:G>A</td><td>TGCAGTGGCGGCGCATAAGAGGCAACTTGTTGAGCCG</td><td>12:G>A</td></tr>
<tr><td>1119657|F|0-6:A>G-6:A>G</td><td>TGCAGGAGGGTGGGCCGCCACGTGGAGGAGAATGATATGGGAGGCGCCG</td><td>6:A>G</td></tr>
<tr><td>5581526|F|0-5:G>T-5:G>T</td><td>TGCAGGGTCCCGTGCAACTGGCCG</td><td>5:G>T</td></tr>
<tr><td>4261923|F|0-7:T>C-7:T>C</td><td>TGCAGATTCTCAGCCTGCTGCCGCTCAAGTGCGCCATCCGCACCG</td><td>7:T>C</td></tr>
<tr><td>4910031|F|0-9:G>T-9:G>T</td><td>TGCAGGGTGGCCGAGTCGCTCCG</td><td>9:G>T</td></tr>
<tr><td>2276508|F|0-43:C>A-43:C>A</td><td>TGCAGGAGCGCCCGAGGTTCAACTCGATCCACATGGGCGTTTGCCAAGTCCG</td><td>43:C>A</td></tr>
<tr><td>7173751|F|0-35:C>T-35:C>T</td><td>TGCAGCATCTTGTAGAAGCTGTTGTCTAGTTTGGACGCGCTGGACCG</td><td>35:C>T</td></tr>
<tr><td>1696801|F|0-26:C>T-26:C>T</td><td>TGCAGCGACAGGTCGAAGTCGGAGAGCATGATGTGGCCGTCGTCGCGCACCAGCACGTTCTCCG</td><td>26:C>T</td></tr>
<tr><td>3947063|F|0-14:G>C-14:G>C</td><td>TGCAGCAGCAGCTTGACGCGCTCCACCG</td><td>14:G>C</td></tr>
<tr><td>987148|F|0-61:A>T-61:A>T</td><td>TGCAGCGCACACGTCCGTCCACTCATCATCGCACCCGAACGGATTAGACAAACGGCTCAAAAGGCTTTC</td><td>61:A>T</td></tr>
<tr><td>1278436|F|0-5:C>G-5:C>G</td><td>TGCAGCTCGACGCAGCCTCGGCGCTGGTTGGCCTGCGCGCGCTTGAGCGCGGCGACGAGCGCGTTGGAG</td><td>5:C>G</td></tr>
<tr><td>5357203|F|0-16:G>C-16:G>C</td><td>TGCAGAAACCGAGCCTGTCCTCCTCCG</td><td>16:G>C</td></tr>
<tr><td>7170386|F|0-61:C>G-61:C>G</td><td>TGCAGTGAAGTGTGATGAACCGAGGTGGCCAGGAGCGCTCGCGTATATGTACTGGTCCGTCCAGCACCG</td><td>61:C>G</td></tr>
<tr><td>3025891|F|0-14:C>A-14:C>A</td><td>TGCAGCGTCCTGAGCACCACGTCCATCGTGTTGCCATGCGCCG</td><td>14:C>A</td></tr>
<tr><td>977536|F|0-67:T>C-67:T>C</td><td>TGCAGCGAGGTGGCGGACATCATGCGGTGCGTCAAGGTGGCGCTGCTTTGCGTGCAGGACAACGCCATG</td><td>67:T>C</td></tr>
<tr><td>1089221|F|0-17:G>T-17:G>T</td><td>TGCAGGCGTCGTGGCGAGAGCGCGCCGCCACCACCTGTACGTGTACCTCCGCCCGCTCTGAACGCCGAG</td><td>17:G>T</td></tr>
<tr><td>1205880|F|0-56:C>G-56:C>G</td><td>TGCAGCGAGCTGCCGTTGCGCACGCTCAGCTCCGCCGTGGGCGCGCCCGTGGCGTCCGTCCCGAACCGA</td><td>56:C>G</td></tr>
</table>

5325262|F|0-7:A>G-7:A>G	TGCAGCAATTGCAGAGAAACTGACCTGGATCTCGCGCTTGATGTGTTCGAAATCGTCGAATCGGCGGCC	7:A>G
5000238|F|0-31:G>A-31:G>A	TGCAGCGCGCGAGCCTTGGGCGCCTGGCGCCGAACGCCG	31:G>A
3953325|F|0-19:G>A-19:G>A	TGCAGATGCCGACGTACGCGAACCCGATGCCG	19:G>A
1010818|F|0-51:G>A-51:G>A	TGCAGGGATGGGCGGCCTGGGCGTGGAGGAGACGGCGCTGGTGTCGGCGCTGGGGCGGTGGAGGAAGCA	51:G>A
980124|F|0-6:T>A-6:T>A	TGCAGGTGGCGCATGGCCAGGCAGCGCACGATCTCCAGGCCCCGCAGCCTCTCGCCG	6:T>A
1694779|F|0-7:G>C-7:G>C	TGCAGTCGGACGAGCCGTCGACGGACGCAAGCACCACCACGACGTCCACGACCACGATGAGCATGACGG	7:G>C
3941804|F|0-5:T>C-5:T>C	TGCAGTGGCGGCGCATAAGAGGCAACTTGTTGAGCCG	5:T>C
1068553|F|0-65:C>G-65:C>G	TGCAGGGAGAGCGCCCTGGTGATGTTGCGCAGCACGGCCTGCTCGTGCTGCTGCTCCTGCACGCTCGGG	65:C>G
3941023|F|0-11:T>G-11:T>G	TGCAGCATCGGTAGGACACACGCTGGGCGCCG	11:T>G
5323637|F|0-8:C>T-8:C>T	TGCAGGCGCCGTGGGCCTCCACGGGCAGCCG	8:C>T
3947941|F|0-24:T>G-24:T>G	TGCAGCTCATGTCGGAGCTCACCCTCGGGCCG	24:T>G
4261923|F|0-31:C>G-31:C>G	TGCAGATTCTCAGCCTGCTGCCGCTCAAGTGCGCCATCCGCACCG	31:C>G
1077594|F|0-49:C>G-49:C>G	TGCAGACGGACCTGGACTACGCGTGCTCCCAGGTGGGCGTGGACTGCGGCGCCATCCAGCCCG	49:C>G
1093145|F|0-28:C>T-28:C>T	TGCAGCGCGCTCTCCTTCTCGGCGACGACGTCGTTGAGCCGCGCGTTCTCGACCCG	28:C>T
5371733|F|0-10:G>C-10:G>C	TGCAGTAGTCGAACAGCTTGCCG	10:G>C
998477|F|0-41:G>A-41:G>A	TGCAGCGTGGTGTCGCCGTTGCGGAGGAAGAAGAGCACGCGGTTGAGGAAGACGGCGCCGATGAGCCGA	41:G>A
984092|F|0-11:G>A-11:G>A	TGCAGCATGTCGCAGTCGAAGCGCCTCATGACATACTGGTTCTCGTCCCCG	11:G>A
2249622|F|0-8:G>A-8:G>A	TGCAGCGCGTCATCGTCTTCGGCGACATGGGGAAGGCGGAGAGGGACGGGTCGAACGAGTACGCCAACT	8:G>A
1037736|F|0-52:A>G-52:A>G	TGCAGATTTGTGGTATTGCTTCAAAGCTTGCAGGGTCTTCAAGAACCTTGCAAGACAAGGTATCTCTCC	52:A>G
1038725|F|0-9:G>T-9:G>T	TGCAGCCTTGGAGCTCTTTCCTGAGAGCAAGTGCGCAGGATATAATGCTGGCCG	9:G>T
3023134|F|0-34:C>T-34:C>T	TGCAGAAGCTGACCATGCTGGACCTGAGCAACAACAGCTTCTCCG	34:C>T
1021179|F|0-7:A>G-7:A>G	TGCAGAGAGGCTACGACCAGGTGGTGCACGACGTGGACCTCCAGAAGCTCCCG	7:A>G
3938266|F|0-9:C>G-9:C>G	TGCAGCGTGCTCTTGGTCCAGCCG	9:C>G
1100382|F|0-8:G>C-8:G>C	TGCAGCGAGGAGGAAGGGACGCCCATGGCGTCCTTGAGGAAGAAGTTGACGCCGAGCCAGGGGAAGAGC	8:G>C
4993892|F|0-21:C>T-21:C>T	TGCAGGCGATGAGCGGGGGCGCGTACCG	21:C>T
989354|F|0-32:C>T-32:C>T	TGCAGCTTCAGATCCGTAACGACCAGATCGCACGTTGATGGACGTGGGAAGTCCAACTGCGCCG	32:C>T
5350074|F|0-6:A>G-6:A>G	TGCAGCAGCGTACCCTCCGCCG	6:A>G
1020792|F|0-67:A>G-67:A>G	TGCAGGTCCCGTGGTACTTCCGTTGCCCAATCTCGCTGGAGCTCATGCAGGACCCCGTCACCGTGGCAA	67:A>G
995676|F|0-10:G>T-10:G>T	TGCAGGGCGTGCACATGGTGAACTGCTCCTACAACAGCCTGTGGATGGGCCAGTTCGTACGCCCGAGAT	10:G>T
4005390|F|0-15:C>G-15:C>G	TGCAGGATTCCAGGTCTGCCACTCGCTGGGAGGAGGCACTGGCTCCG	15:C>G
3935637|F|0-29:G>C-29:G>C	TGCAGCCGCGAGTTGGGGACGGTGACCATGACCTCCGCTCCG	29:G>C
1023927|F|0-11:G>T-11:G>T	TGCAGGGTGTAGGTGCCCTCCGCCTCCACCAGCAGCAGCTTGTGGCCCTGGAGCCG	11:G>T
1025077|F|0-14:G>A-14:G>A	TGCAGCATGCGGATGGCCGTGTTGTAGGCGCGGACAATGCCGTAGCATCGCGCGGCGAGGTAGGCCGAG	14:G>A
3022831|F|0-8:C>G-8:C>G	TGCAGGGGCTTCCATGTCACCTTCGTCAACTCCGAGTTCAACCACCG	8:C>G
6038140|F|0-12:T>G-12:T>G	TGCAGGGGTACGTCAGGGACGCCCG	12:T>G
2262365|F|0-56:G>C-56:G>C	TGCAGCTCGGCGACGGTCTCGAAGAGCTGCGTCTGGAGGGAGGTGAGCATCGACATGGCCTCCTTGGTG	56:G>C
4538433|F|0-14:G>T-14:G>T	TGCAGGTGACCGTCGGCGGCCTTGGCCCCG	14:G>T
6043875|F|0-19:C>G-19:C>G	TGCAGCGACCTCACAACTTCATACATGTCCG	19:C>G
1163252|F|0-10:C>T-10:C>T	TGCAGGTGGGCTACTACAAGAAGACGTGCCCTCGCGTGGAGGCCATCGTGAGGGATGAGGTGAAGCGCT	10:C>T
1051566|F|0-53:G>C-53:G>C	TGCAGGGAGATGTACAAGTGCATCCAGGACGAGCTGCTGCCGCTGCTCCTCGAGCCCATGACCATCGCC	53:G>C
100302250|F|0-6:G>A-6:G>A	TGCAGCGATGTAGCCGCTGCCTCCG	6:G>A
3028310|F|0-39:T>C-39:T>C	TGCAGCTCCTGGGAGCCCCCAGCCTGCTCCGCCGCCGCGTCCG	39:T>C
989611|F|0-28:T>C-28:T>C	TGCAGAAGGTCCTGCGCCACCACCACTCTCACTCTGCTTCTCTCGGGGAGGACCGCAAGGGGGAGAGCA	28:T>C
1058456|F|0-12:T>G-12:T>G	TGCAGTTCGCGGTGCCGATGGTCCTCACCAACATGTCCTACTACGGCATCCCGCTGGTGTCCGTCATGT	12:T>G
3960287|F|0-63:A>C-63:A>C	TGCAGATGGTACAGGATGTGGTCGAACATGATGTTCCTGTGGTCCATCTACTCCGTCTTCTACACCCCC	63:A>C
1031592|F|0-46:A>T-46:A>T	TGCAGGTCCACTGATTTGAATCTTGACGACGAAACCGTCAATCTGAACAAGCTCAAGGCTTCCACCTCC	46:A>T
998527|F|0-43:C>T-43:C>T	TGCAGCGACGCGCTCTGCTTCTGCGCCGCGTCCAGCTTCGCCGCCGCGCCG	43:C>T
1024345|F|0-68:G>A-68:G>A	TGCAGGCTCCAGTCGGCCCCCGCCGCCATGAGCATCTCAGCTGCCGCGGCGTCGCCGAAGCGGACGGCG	68:G>A
5358302|F|0-26:G>C-26:G>C	TGCAGGCTGGGGAAGTACGGCGGGCTGGACCG	26:G>C
990910|F|0-20:C>G-20:C>G	TGCAGCTTAGCCGCCGACGACGGAAGGCTGCCTCTGAACTTGTTATTCATGATGTAGAGCCTCGTCATG	20:C>G
5324000|F|0-11:G>A-11:G>A	TGCAGATCGTCGTGGGAAAGAAGCAGCAGCGTGCCG	11:G>A
1405545|F|0-35:T>C-35:T>C	TGCAGGTAGAAACCATGGATCGTATGTAGGAGCACTCGCCTAAGTGACCAGGAATACTGGTGGGTCACC	35:T>C
2244426|F|0-6:C>A-6:C>A	TGCAGTCTGTCGTCAGTTCATCTGCATGCGCCGCAAGGATCAGCACAGCGCCACCGCTCGATCCGCGGA	6:C>A
32025653|F|0-67:C>G-67:C>G	TGCAGGTGCTCCGCATGGTCGGCTTCGCGAGGGAGTTCGGCAGGTTCGCCGACGTCGCCGTCGTGCTCG	67:C>G
7172903|F|0-23:C>G-23:C>G	TGCAGCGCGCTCTCCTTCTCGGCCACGACGTCGTTGAGCCGCGCGTTCTCGACCCG	23:C>G
4261853|F|0-26:C>A-26:C>A	TGCAGGTATTCCTGTGCGGCGGACGGCTTATACTCGGGCCG	26:C>A
4261236|F|0-28:G>T-28:G>T	TGCAGGTACAGTTGCAATTCCAGGCCATGCCTAGCCG	28:G>T
3944926|F|0-19:C>T-19:C>T	TGCAGTTCATGCGCGGCTACGCGGCGGCGCGGGCGTGGGACCG	19:C>T
16794981|F|0-12:G>A-12:G>A	TGCAGGGGTTCAGCGAGCAGCGGGTCCG	12:G>A

<table>
<tr><td>6043605|F|0-7:G>A-7:G>A</td><td>TGCAGAAGTTCGCCGAGTCGGTGTGCCCG</td><td>7:G>A</td></tr>
<tr><td>3954823|F|0-7:G>C-7:G>C</td><td>TGCAGGAGATGGCGCACGCCG</td><td>7:G>C</td></tr>
<tr><td>1318538|F|0-11:G>C-11:G>C</td><td>TGCAGGGTGGAGGACGAGAGGTTGGCGGAAGGGAGGACGAGGGCGGTGACGGCGGCCG</td><td>11:G>C</td></tr>
<tr><td>1091061|F|0-47:C>T-47:C>T</td><td>TGCAGAGAGCACGTCCATGGTGATGAAGAACGCGCCGCCTGCGTCCACGGCGGTGCCCAGCACGGAGGA</td><td>47:C>T</td></tr>
<tr><td>3955529|F|0-16:C>T-16:C>T</td><td>TGCAGGTGAACGGGCTCACCG</td><td>16:C>T</td></tr>
<tr><td>1080576|F|0-28:C>T-28:C>T</td><td>TGCAGCTGCTCGGCTTCGCGGACGCCGTCGCCATGTCTGCGCGCGCCACCGAGAAGCTCTACCGCACGC</td><td>28:C>T</td></tr>
<tr><td>1035804|F|0-65:C>T-65:C>T</td><td>TGCAGCCGCGGTTAACGCTGGCAACGAGGCATCCCTCGGTGGTGGCCATGGGAACATGGTAGTCGCGCC</td><td>65:C>T</td></tr>
<tr><td>1031592|F|0-6:T>C-6:T>C</td><td>TGCAGGTCCACTGATTTGAATCTTGACGACGAAACCGTCAATCTGAACAAGCTCAAGGCTTCCACCTCC</td><td>6:T>C</td></tr>
<tr><td>3028381|F|0-20:G>A-20:G>A</td><td>TGCAGAGCGAGGTGGTTCCAGGTTGCCCATCATGGATTGCCG</td><td>20:G>A</td></tr>
<tr><td>3959736|F|0-26:G>C-26:G>C</td><td>TGCAGCGCGGCGATGGAGTCCCTCCAGCCGTGGAGGACGCAGGACTCGAAGAAGGTGTCGAGCTTGGAG</td><td>26:G>C</td></tr>
<tr><td>6044000|F|0-28:C>G-28:C>G</td><td>TGCAGCTGGCGGCGGCGGGGCTCGCCGTCGGCTTCTTCGTGTACCG</td><td>28:C>G</td></tr>
<tr><td>7170807|F|0-26:A>T-26:A>T</td><td>TGCAGGCCATCAAGTCGGCCCTCGGCACCGCGCCG</td><td>26:A>T</td></tr>
<tr><td>3573393|F|0-23:C>G-23:C>G</td><td>TGCAGGTACGCCGACGGCGTCGCCTTCGACCCCGAGCCG</td><td>23:C>G</td></tr>
<tr><td>32021507|F|0-10:T>C-10:T>C</td><td>TGCAGGTGGCTATGGCGTGCGTGGCCACCGTGCCG</td><td>10:T>C</td></tr>
<tr><td>3957940|F|0-23:C>T-23:C>T</td><td>TGCAGAGCCCACCTGATGAGCACCTGCCCCG</td><td>23:C>T</td></tr>
<tr><td>1696470|F|0-23:G>C-23:G>C</td><td>TGCAGGTCGAGCGTGACCTCGACGAGCTCCTCGCCGTCGTCGGAGCTGCGGTCGGAGCCGCGCGGCGAG</td><td>23:G>C</td></tr>
<tr><td>2256296|F|0-10:C>G-10:C>G</td><td>TGCAGGCCACCTCGGCGGCGTCCAGGGCGCTCGACCGCTTCGACGTCCTCGACTGCCG</td><td>10:C>G</td></tr>
<tr><td>3022350|F|0-33:T>A-33:T>A</td><td>TGCAGAGGCAAATCTGCTCTTCTTGGAGCAGCCTGCCG</td><td>33:T>A</td></tr>
<tr><td>3064906|F|0-5:C>G-5:C>G</td><td>TGCAGCCGCGTCCAGCGGCCGTACTCGCGCGGAATCTCGCCG</td><td>5:C>G</td></tr>
<tr><td>1057485|F|0-19:G>C-19:G>C</td><td>TGCAGGCGCAGAGCAAGGTGATGGTGCTGGCCTGGATCAGCCTCGCCGCGCTCGCCGCGCACGTCGCCA</td><td>19:G>C</td></tr>
<tr><td>4261314|F|0-28:C>A-28:C>A</td><td>TGCAGGCCGCGCCGCCCTCGCCGCCGCGCGAGATCCCCG</td><td>28:C>A</td></tr>
<tr><td>2242803|F|0-49:C>A-49:C>A</td><td>TGCAGGGCCTCGTGCGCGAGCTCGAGGACGGTGGCAATGCATCTGCGAGCTTCGTGTTCGCTGACGCCG</td><td>49:C>A</td></tr>
<tr><td>1061236|F|0-9:A>T-9:A>T</td><td>TGCAGGAGGAGGCATGCCACGGTGGCGCCGAGCAGCTGGGCGACCCAGTAGAAGAGCCCG</td><td>9:A>T</td></tr>
<tr><td>6041514|F|0-9:T>C-9:T>C</td><td>TGCAGACAATCCATCGGTGACCCCG</td><td>9:T>C</td></tr>
<tr><td>1016654|F|0-65:G>C-65:G>C</td><td>TGCAGCCACGTCCACTGCGGCGTGTACTTGCCGTAGGCGGAGTAGGAGGAGAGCATGATGACGTGGGCG</td><td>65:G>C</td></tr>
<tr><td>1007223|F|0-59:C>T-59:C>T</td><td>TGCAGCGCGCGCCTCACGTCGGCGAAGAGGTCCGCGCAGCCGTCGCCGCAGCTGAGCGTCGCCTTGACG</td><td>59:C>T</td></tr>
<tr><td>2246569|F|0-25:T>C-25:T>C</td><td>TGCAGCGGGTCGTCAACTCCCTGGCTGTCGAGATGTTCGCTGGACTCGCGTCTGAGGACGCCAGCAAGG</td><td>25:T>C</td></tr>
<tr><td>993908|F|0-22:G>A-22:G>A</td><td>TGCAGTTCCAGAGGCCAAGAAAGGCACAAAAGTTACCAACCAAGCTCCCTCCAACAAAACCCTAACCAC</td><td>22:G>A</td></tr>
<tr><td>7170937|F|0-22:T>G-22:T>G</td><td>TGCAGTGGATCATGGCGAACCTTGTCCG</td><td>22:T>G</td></tr>
<tr><td>5000238|F|0-10:G>A-10:G>A</td><td>TGCAGCGCGCGAGCCTTGGGCGCCTGGCGCCGAACGCCG</td><td>10:G>A</td></tr>
<tr><td>3021702|F|0-32:C>T-32:C>T</td><td>TGCAGAAGAGAACAAGCACAAGAGCTCCAACTCGACCTCCG</td><td>32:C>T</td></tr>
<tr><td>4406428|F|0-8:C>T-8:C>T</td><td>TGCAGGCGCTGGGAGAGGACAGGCGGCCG</td><td>8:C>T</td></tr>
<tr><td>982614|F|0-17:A>G-17:A>G</td><td>TGCAGTGCTTCATGGGCATCAGCATGATCACCTGCGTCGCGCTGTACGACCGCGTCCTGGTGCCCGTGG</td><td>17:A>G</td></tr>
<tr><td>3955296|F|0-14:T>C-14:T>C</td><td>TGCAGTTCCTCAGCTTGTCCAACAACAGCTTCACCG</td><td>14:T>C</td></tr>
<tr><td>3949931|F|0-18:T>C-18:T>C</td><td>TGCAGCGTACGGCCATCATATGGAACGTCGTCCG</td><td>18:T>C</td></tr>
<tr><td>1101684|F|0-29:C>T-29:C>T</td><td>TGCAGCACCGTGGCGTTGCGAGGGAGGAACACCATGTTGGCGAGCCCAGCACCATGCACGCCCACCATC</td><td>29:C>T</td></tr>
<tr><td>1106013|F|0-9:C>T-9:C>T</td><td>TGCAGAAGACGTTGTCCTGGGTCCTCAGCACCGCCTCGGCGAGCGAGGCCGAGGAGACCACGATGGCCC</td><td>9:C>T</td></tr>
<tr><td>1106013|F|0-18:G>T-18:G>T</td><td>TGCAGAAGACGTTGTCCTGGGTCCTCAGCACCGCCTCGGCGAGCGAGGCCGAGGAGACCACGATGGCCC</td><td>18:G>T</td></tr>
<tr><td>3949606|F|0-17:A>G-17:A>G</td><td>TGCAGCTCCAAGACAACAAGCTTTCCG</td><td>17:A>G</td></tr>
<tr><td>1338735|F|0-35:T>G-35:T>G</td><td>TGCAGCAGCCCACCTTCCTCCGTGAACCCCAGGCGTCCCGCGAGCGGGTAGTACTGCACCAGGGCCTGC</td><td>35:T>G</td></tr>
<tr><td>7337045|F|0-29:A>G-29:A>G</td><td>TGCAGGGTGGAGGACGAGAGGTTGGCCGAAGGGAGGACGAGGGCGGTGACGGCGGCCG</td><td>29:A>G</td></tr>
<tr><td>7487734|F|0-14:C>G-14:C>G</td><td>TGCAGCTCCTGCCTCGACGAGCCGACGCCG</td><td>14:C>G</td></tr>
<tr><td>3945565|F|0-11:G>T-11:G>T</td><td>TGCAGCGGCGAGTGGTTTTAGGGTTTGAGAGACGAAGCCG</td><td>11:G>T</td></tr>
<tr><td>6044843|F|0-44:G>A-44:G>A</td><td>TGCAGCATGCCCTCCTTGGCCGCCTCGGCGATGAACTGCTTCTCGAAGTCGGCCCCCTTGGCCAGGGTG</td><td>44:G>A</td></tr>
<tr><td>1031695|F|0-7:C>T-7:C>T</td><td>TGCAGGCCTACTTCAACGCCTACTACGCGTCCATCGGGGTGGCCTTCACGGTCGCCGTCACCG</td><td>7:C>T</td></tr>
<tr><td>1095280|F|0-43:G>C-43:G>C</td><td>TGCAGGCGCCGCCGCGGCAGGACACGTGGGGGTTCTCGCCGCTGGATGCGCAGGCCGCGTACATGCCGA</td><td>43:G>C</td></tr>
<tr><td>5001575|F|0-5:G>A-5:G>A</td><td>TGCAGGCGGTAGATCACGTCCG</td><td>5:G>A</td></tr>
<tr><td>980124|F|0-16:C>T-16:C>T</td><td>TGCAGGTGGCGCATGGCCAGGCAGCGCACGATCTCCAGGCCCCGCAGCCTCTCGCCG</td><td>16:C>T</td></tr>
<tr><td>4394624|F|0-5:G>A-5:G>A</td><td>TGCAGGCCCGCCATCAGCAGGCAGCCG</td><td>5:G>A</td></tr>
<tr><td>2246692|F|0-30:A>G-30:A>G</td><td>TGCAGCCACACCTGTCGATGCCCTTGCCAAACTCCTCACTGGAGCTAGCAGTCGGGGGAATCGGTGGCA</td><td>30:A>G</td></tr>
<tr><td>1009692|F|0-44:A>G-44:A>G</td><td>TGCAGATCAAGGGCAGAGCACACAACCCCATAGCTCCCCTTGCCAATGACCTCCTGGATCTTGTACCGA</td><td>44:A>G</td></tr>
<tr><td>5580218|F|0-17:G>C-17:G>C</td><td>TGCAGCCCGATGCACTTGACGCCG</td><td>17:G>C</td></tr>
<tr><td>2243102|F|0-32:C>T-32:C>T</td><td>TGCAGCCTGTTCTGCCCCACGCCGTCCCTGGTCGACATGATCGTGAACAAGTACAAGCTGCGGAGCGAC</td><td>32:C>T</td></tr>
<tr><td>32024776|F|0-14:C>A-14:C>A</td><td>TGCAGAACATCCAGCGCGCCCTCGACGAGGCCG</td><td>14:C>A</td></tr>
<tr><td>32026723|F|0-27:A>G-27:A>G</td><td>TGCAGGGCGAACTCGTTGTGGTATACGACAACGAGCACGGCGGCGAGGAGCGCGAGGGGAAGCAGGAGG</td><td>27:A>G</td></tr>
<tr><td>32018917|F|0-13:C>T-13:C>T</td><td>TGCAGCAGCCGCGCGGCGAGCACGCGCGCCCG</td><td>13:C>T</td></tr>
<tr><td>32020863|F|0-16:C>G-16:C>G</td><td>TGCAGGGCCAGGGCTCCCTCACGCTGCCCCGCACCCTCAGCGCCAAGACGGTCGACGAGGTGTGGCGCA</td><td>16:C>G</td></tr>
<tr><td>4262354|F|0-27:T>C-27:T>C</td><td>TGCAGGCCAAGCAAAATCGAAACAGAGTGGGGTGCCGAGAGGCCG</td><td>27:T>C</td></tr>
<tr><td>16794819|F|0-15:A>G-15:A>G</td><td>TGCAGCTGCCTCGCCATGGCAGCGCTGGCGGCCACAGTCGCCGCCGCTCTCCTGCTCTCGTTCGCCGAG</td><td>15:A>G</td></tr>
</table>

<table>
<tr><td>1025550|F|0-23:G>A-23:G>A</td><td>TGCAGCGCGTAGGCGACGTCGGCGACGGGGAAGAACTGCTGCATGTGGATGACGGGCGCCCAGTGGTGG</td><td>23:G>A</td></tr>
<tr><td>32023398|F|0-11:C>G-11:C>G</td><td>TGCAGGTAGCTCAGGAAGGTGTGGAACGGGCCG</td><td>11:C>G</td></tr>
<tr><td>7169561|F|0-29:A>G-29:A>G</td><td>TGCAGCTCGTCCGCCGCCAGCTCCGCGTCACCGTCCG</td><td>29:A>G</td></tr>
<tr><td>3027176|F|0-17:G>T-17:G>T</td><td>TGCAGCGAGCTGCGCGTGCCCCAGATGCACAGCCTGCGCCG</td><td>17:G>T</td></tr>
<tr><td>7865070|F|0-31:C>T-31:C>T</td><td>TGCAGACCCAGATGCTCATGGGAGACGGCGGCGCGTCGGCCG</td><td>31:C>T</td></tr>
<tr><td>6041176|F|0-10:G>C-10:G>C</td><td>TGCAGACGCTGACCGTGCCCAAGGTGCCGCCG</td><td>10:G>C</td></tr>
<tr><td>3936827|F|0-8:G>A-8:G>A</td><td>TGCAGCTTGAGTAGGACGCCGAGCAGGTCCTCATCTCTCTCTGGCTCCG</td><td>8:G>A</td></tr>
<tr><td>1055207|F|0-20:C>G-20:C>G</td><td>TGCAGGTTATTGACTCTGGTCAGAGTGATAGTGGTTATTCTATTCTTCACATGGCGCATGAGGCACCGA</td><td>20:C>G</td></tr>
<tr><td>2244426|F|0-13:C>G-13:C>G</td><td>TGCAGTCTGTCGTCAGTTCATCTGCATGCGCCGCAAGGATCAGCACAGCGCCACCGCTCGATCCGCGGA</td><td>13:C>G</td></tr>
<tr><td>4993206|F|0-10:C>T-10:C>T</td><td>TGCAGAACTGCTACTTCCCACAACCCG</td><td>10:C>T</td></tr>
<tr><td>5357203|F|0-15:T>C-15:T>C</td><td>TGCAGAAACCGAGCCTGTCCTCCTCCG</td><td>15:T>C</td></tr>
<tr><td>5358113|F|0-17:C>G-17:C>G</td><td>TGCAGGAAGTTGCGCGCCAGCCCCACGGAGACGAGCCCG</td><td>17:C>G</td></tr>
<tr><td>1023927|F|0-32:C>G-32:C>G</td><td>TGCAGGGTGTAGGTGCCCTCCGCCTCCACCAGCAGCAGCTTGTGGCCCTGGAGCCG</td><td>32:C>G</td></tr>
<tr><td>1667027|F|0-18:T>C-18:T>C</td><td>TGCAGCTGCCCAAGGGCATGGAGGCCGTGAACCACATCTGGCAGGTAGGGTCGACGGTGGCCAATGGGG</td><td>18:T>C</td></tr>
<tr><td>4010179|F|0-13:G>A-13:G>A</td><td>TGCAGGGGAAGCGGTTCTTCGCCG</td><td>13:G>A</td></tr>
<tr><td>7352102|F|0-13:C>G-13:C>G</td><td>TGCAGTACAGCGGCTCGTGCCG</td><td>13:C>G</td></tr>
<tr><td>3943230|F|0-6:G>A-6:G>A</td><td>TGCAGAGGCATGACTCCGTGCTCGCCACCCG</td><td>6:G>A</td></tr>
<tr><td>3948743|F|0-9:T>G-9:T>G</td><td>TGCAGAAGATGTCGATGTCAGGCTTGCCCG</td><td>9:T>G</td></tr>
<tr><td>3940182|F|0-8:T>C-8:T>C</td><td>TGCAGGAATTCCTTCATGTTGCTGGCGGCGATCAGCCCGCGGCCGCCG</td><td>8:T>C</td></tr>
<tr><td>1041714|F|0-6:C>T-6:C>T</td><td>TGCAGGCAAAGCTAGCATGGGGGAGAAAGAGTGGTACTTCTTCAGCATGCGCGACCG</td><td>6:C>T</td></tr>
<tr><td>6046909|F|0-16:C>T-16:C>T</td><td>TGCAGTCCATCTACCACATGAGGCCCG</td><td>16:C>T</td></tr>
<tr><td>3940392|F|0-21:C>T-21:C>T</td><td>TGCAGGTCAAGGGGAGCTGCCCCATGTGCCG</td><td>21:C>T</td></tr>
<tr><td>1018717|F|0-28:T>G-28:T>G</td><td>TGCAGGCCATGCAATTTCCTGAGCTTTATTGTCATTAGAGACTCCGAATGTATGGTGAAATTGTCCAGG</td><td>28:T>G</td></tr>
<tr><td>1020888|F|0-59:C>A-59:C>A</td><td>TGCAGGCAGAAGCGCGCCCCTTGGAAGACGCGCATGAAGGTGCTGGGCACGAGGCAGTTCTTCTCGCTC</td><td>59:C>A</td></tr>
<tr><td>4993576|F|0-24:C>T-24:C>T</td><td>TGCAGGAGCTCTTCCCCTCCTGCGCCGTCCTGCCCG</td><td>24:C>T</td></tr>
<tr><td>7917108|F|0-16:C>T-16:C>T</td><td>TGCAGCGGATCTGCGACGGGGTGTACCTGAACCG</td><td>16:C>T</td></tr>
<tr><td>5363113|F|0-20:A>C-20:A>C</td><td>TGCAGAGACAGACATCCCTGACCCG</td><td>20:A>C</td></tr>
<tr><td>1074139|F|0-8:C>T-8:C>T</td><td>TGCAGCCCCACGTCGCTAAACACCACCGTGAGGAAAACCACGGCGGCGAAGAAGGAGTGCACGAAGTCC</td><td>8:C>T</td></tr>
<tr><td>5367166|F|0-6:T>C-6:T>C</td><td>TGCAGCTCGCCATGGCCACCCG</td><td>6:T>C</td></tr>
<tr><td>1278436|F|0-32:C>T-32:C>T</td><td>TGCAGCTCGACGCAGCCTCGGCGCTGGTTGGCCTGCGCGCGCTTGAGCGCGGCGACGAGCGCGTTGGAG</td><td>32:C>T</td></tr>
<tr><td>4396424|F|0-8:G>A-8:G>A</td><td>TGCAGCGGGTCCAGCCCGCCGACGGCCACCATCGCCG</td><td>8:G>A</td></tr>
<tr><td>1119657|F|0-15:C>T-15:C>T</td><td>TGCAGGAGGGTGGGCCGCCACGTGGAGGAGAATGATATGGGAGGCGCCG</td><td>15:C>T</td></tr>
<tr><td>5373735|F|0-11:C>T-11:C>T</td><td>TGCAGCACCTCCTGAGCGTACTCCG</td><td>11:C>T</td></tr>
<tr><td>3941084|F|0-8:T>C-8:T>C</td><td>TGCAGCCTTCCTTTTCCTCCCTCCG</td><td>8:T>C</td></tr>
<tr><td>3937256|F|0-17:C>A-17:C>A</td><td>TGCAGGAGGGTCGACACCACCGTCTACATCGTCTCCCTCGCCG</td><td>17:C>A</td></tr>
<tr><td>1863286|F|0-22:C>G-22:C>G</td><td>TGCAGGCGGCGTCGGACGCGGCCAAGAAGCGGCACTCGGACCTCAAGCTCGTCGTCTTCGACATCTACA</td><td>22:C>G</td></tr>
<tr><td>1251556|F|0-46:G>C-46:G>C</td><td>TGCAGCTGTCCCCGAGCCTGCGCAGGACGTCCATCACCTCCTCCGCGGTGATCCTGCCGTCCCTGTCCC</td><td>46:G>C</td></tr>
<tr><td>989944|F|0-24:G>A-24:G>A</td><td>TGCAGCTCCCGCGCCTGCTCCGCCGCGCGGGCCGCCGCCTCCGCCGCCTGCTCCCCTGCCTTCCTCTCC</td><td>24:G>A</td></tr>
<tr><td>4261153|F|0-37:A>T-37:A>T</td><td>TGCAGGGGCTGATGATCTTCTGGCCCCGAGCTCTGCCACACCG</td><td>37:A>T</td></tr>
<tr><td>3955860|F|0-26:C>G-26:C>G</td><td>TGCAGCATGCGCTTGCACCGCGCGCACCCGCCG</td><td>26:C>G</td></tr>
<tr><td>7907382|F|0-21:G>T-21:G>T</td><td>TGCAGCCGCGCCTCGAGCGCCGCCTTGGGCGTGCCCCGCACGGGCTCGCACTCGAACTCCCACCGCAGC</td><td>21:G>T</td></tr>
<tr><td>4536993|F|0-20:A>G-20:A>G</td><td>TGCAGTGCCTGACAAGTGACAAGCCACCG</td><td>20:A>G</td></tr>
<tr><td>32020828|F|0-59:T>G-59:T>G</td><td>TGCAGGCCGCAGATCCCCATCATCTGCTGCTCCGTCAGCGGATCCAGCTGCCCGATCAGTATCTGCATG</td><td>59:T>G</td></tr>
<tr><td>6041176|F|0-11:A>G-11:A>G</td><td>TGCAGACGCTGACCGTGCCCAAGGTGCCGCCG</td><td>11:A>G</td></tr>
<tr><td>1080576|F|0-24:C>T-24:C>T</td><td>TGCAGCTGCTCGGCTTCGCGGACGCCGTCGCCATGTCTGCGCGCGCCACCGAGAAGCTCTACCGCACGC</td><td>24:C>T</td></tr>
<tr><td>1865011|F|0-52:C>T-52:C>T</td><td>TGCAGGAAGATCGGCTCGACGTCGGTGGCGACGAACTCGTCCAACGTTGGCGCAGGAGCCG</td><td>52:C>T</td></tr>
<tr><td>990910|F|0-14:C>T-14:C>T</td><td>TGCAGCTTAGCCGCCGACGACGGAAGGCTGCCTCTGAACTTGTTATTCATGATGTAGAGCCTCGTCATG</td><td>14:C>T</td></tr>
<tr><td>996524|F|0-36:C>T-36:C>T</td><td>TGCAGGAGCAGTGGCGCTGCCCACTGTCCATCCTGCCGCTTACTACTTGTGCGCTTACCCG</td><td>36:C>T</td></tr>
<tr><td>5350206|F|0-7:C>T-7:C>T</td><td>TGCAGGGCGCGGCGGAGTTCCG</td><td>7:C>T</td></tr>
<tr><td>1278436|F|0-29:G>T-29:G>T</td><td>TGCAGCTCGACGCAGCCTCGGCGCTGGTTGGCCTGCGCGCGCTTGAGCGCGGCGACGAGCGCGTTGGAG</td><td>29:G>T</td></tr>
<tr><td>6043666|F|0-23:G>A-23:G>A</td><td>TGCAGAGGCGTAGCAGCCACTTCGACGCGCGCGAGGTGTTCCCCGAGCCG</td><td>23:G>A</td></tr>
<tr><td>5338375|F|0-38:G>C-38:G>C</td><td>TGCAGCTGCTGGGGCGCGGTGTACAACGGCGGGAGCGGGTACTGCTACCTCATCGACTTTCCCGTGGAG</td><td>38:G>C</td></tr>
<tr><td>3021702|F|0-14:A>C-14:A>C</td><td>TGCAGAAGAGAACAAGCACAAGAGCTCTAACTCGACCTCCG</td><td>14:A>C</td></tr>
<tr><td>1033069|F|0-46:C>T-46:C>T</td><td>TGCAGTACGAGAAGACCCACTCCCCCTCCGTGCTGCACTACAACCACAGCTCTGGTGGCAAGACTGTCT</td><td>46:C>T</td></tr>
<tr><td>5580227|F|0-7:G>T-7:G>T</td><td>TGCAGCCGCACGGGCACCGTGATGTCCACGTCGAACGGCACCGACCG</td><td>7:G>T</td></tr>
<tr><td>12695246|F|0-6:G>A-6:G>A</td><td>TGCAGCGTCTCCTGCCGCTCCG</td><td>6:G>A</td></tr>
<tr><td>3934689|F|0-22:G>C-22:G>C</td><td>TGCAGCACTCCATGCCCACCTCGGTGCTCTCCG</td><td>22:G>C</td></tr>
<tr><td>1296761|F|0-41:A>C-41:A>C</td><td>TGCAGGTAATTACTCATCGCATGTTCATGCACGAAGTAGCTAGCTTGATGCTTAATTACTTGTGTTATG</td><td>41:A>C</td></tr>
<tr><td>4262391|F|0-9:T>C-9:T>C</td><td>TGCAGCCTGTAGGACCTGCCG</td><td>9:T>C</td></tr>
</table>

1033462|F|0-8:T>C-8:T>C	TGCAGCCATGCGACCTGCTCGGCGTACTTGTCGGGGGTCGCGTTGGGGTCGTTGCCGCACTCCACGTTG	8:T>C
1069017|F|0-37:A>C-37:A>C	TGCAGCTCCTTCTCCAACCGCTTCTCCGAGGACTCCGACTTCGTCCGAAGCCTCGCCTCCAACTGCTCC	37:A>C
32027259|F|0-37:A>G-37:A>G	TGCAGACGGCAGCTTATTAAGGGGCTAGTTTAAAGCGAGGACGGCAATGCGCTGGGTCCGCGAAGGCCG	37:A>G
1022874|F|0-10:T>C-10:T>C	TGCAGGGGATTTGGACACCGCCCATAGTCTGCTGGACGAGATGCCCGTCAAGGATGCTGTGGCTGTCAA	10:T>C
985692|F|0-14:G>A-14:G>A	TGCAGAGCCAACACGGTGAGCCTGTTCAGTAGCCCCAGCTGCGTGGGGATGCTCCCG	14:G>A
989015|F|0-16:G>A-16:G>A	TGCAGCAGAGGCTGTTGCTTAAACTACCAGCACATACACCGCGTAGATGATCCCCG	16:G>A
4261067|F|0-16:T>C-16:T>C	TGCAGCTTCTTGAGCCTCCCGATGCTCGCCG	16:T>C
13880404|F|0-12:G>T-12:G>T	TGCAGTGCAAGGGGGAGCCCG	12:G>T
3953293|F|0-16:G>C-16:G>C	TGCAGCTCCTCCACGGGGAACCCG	16:G>C
1865360|F|0-36:C>G-36:C>G	TGCAGCGTCACCCTCCTGTTCTTCGACAGCTGCAAACGCAAACAAATCGCACCGTAAACCCTAGCCCCG	36:C>G
7155245|F|0-14:G>A-14:G>A	TGCAGCTTCATCTCGGAGACCAACCCCG	14:G>A
5582052|F|0-10:C>T-10:C>T	TGCAGGGGCACTGCCCCACTGCCG	10:C>T
2262365|F|0-50:C>G-50:C>G	TGCAGCTCGGCGACGGTCTCGAAGAGCTGCGTCTGGAGGGAGGTGAGCATCGACATGGCCTCCTTGGTG	50:C>G
1107522|F|0-7:G>C-7:G>C	TGCAGAAGGCGTTGTCCTGGGTCCTGAGCACCGCCTCGGCGAGCGAGGCCGAGGAGACCACGATGGCCC	7:G>C
1027781|F|0-10:G>A-10:G>A	TGCAGCTGCCGCGCCAGGTAGGCGGCGTACCTCGCGACGTCCTCGCGGCCG	10:G>A
12469814|F|0-18:G>A-18:G>A	TGCAGTTTTCTCCGCGGTGTCCG	18:G>A
1025550|F|0-8:G>A-8:G>A	TGCAGCGCGTAGGCGACGTCGGCGACGGGGAAGAACTGCTGCATGTGGATGACGGGCGCCCAGTGGTGG	8:G>A
7909289|F|0-32:G>T-32:G>T	TGCAGTTCATGCGCGGCTACGCGGCCGCGCGGGCGTGGGACCG	32:G>T
1064678|F|0-26:G>C-26:G>C	TGCAGCAGCTTGGACCGCTTGTGCACGACCATCATGGTGAACCACGGAATGCTGCCG	26:G>C
988647|F|0-23:C>G-23:C>G	TGCAGAAGAACCTGGAGCTGTCGCCGCGGCACGTGGAGGCGTCGCGCATGACGCTGCACCG	23:C>G
12695248|F|0-13:C>G-13:C>G	TGCAGGTGCACACCGAGGGCCG	13:C>G
1117334|F|0-60:A>G-60:A>G	TGCAGCGATATCAATTCAGCGGAACAATGTGAGAGAAGCGAGCACTGTGCGGAGGGAGAAAAGCTGAGG	60:A>G
2242803|F|0-32:G>C-32:G>C	TGCAGGGCCTCGTGCGCGAGCTCGAGGACGGTGGCAATGCATCTGCGAGCTTCGTGTTCGCTGACGCCG	32:G>C
998498|F|0-29:C>G-29:C>G	TGCAGCGGCAGCACCGCCGTCACCGTCCTCAAGCTCGTACGTGGCACTGGCGCCG	29:C>G
2241885|F|0-32:G>A-32:G>A	TGCAGATGGTGCCGTTCCACGCTGGGAGCAACGAGGGCCTACTCGAGCAATTCTCATGGGACGATCCCT	32:G>A
3027174|F|0-11:G>A-11:G>A	TGCAGCGCCTTGCGCCCGTCCCCGTGCAACGCCAAGCCG	11:G>A
4262749|F|0-27:G>A-27:G>A	TGCAGCCGCCGCCCGCCCTCGTCCTCCGCGGCGATCAGCCG	27:G>A
1033069|F|0-22:C>T-22:C>T	TGCAGTACGAGAAGACCCACTCCCCCTCCGTGCTGCACTACAACCACAGCTCTGGTGGCAAGACTGTCT	22:C>T
3949260|F|0-34:T>G-34:T>G	TGCAGCGCGTACAGCCCGCCGAGGCTGTGCGCCATGAGGATCGCCG	34:T>G
4993665|F|0-11:G>A-11:G>A	TGCAGAAGCATGGCCATTGCCGTTCTGCTCTCCG	11:G>A
986886|F|0-42:A>G-42:A>G	TGCAGAATGGCCCAGCACCGATGCCAATGGTTGGGATTTTCAATGCTGATGTTGCAGCTGCTGCCACCG	42:A>G
5580225|F|0-19:T>C-19:T>C	TGCAGCCGCAACTGCCTGATGGTCCACTCGCCG	19:T>C
1088798|F|0-7:C>G-7:C>G	TGCAGCACAAGATGAGCGCCAAGGAGTCCGACACCTGGTCCAAGGTGCTCCG	7:C>G
3020654|F|0-5:T>C-5:T>C	TGCAGTTCCTCCTAATATTGCAAATGATGGTGCACCG	5:T>C
1089043|F|0-25:C>G-25:C>G	TGCAGGACGTGCGCGCCCAGGCCGACTACGACGGCTGGTACCACAACCAGTTCCTCGTCGTCAACGACT	25:C>G
3940890|F|0-15:G>T-15:G>T	TGCAGGCCGCGCGGCGTGATCCG	15:G>T
1211582|F|0-20:G>C-20:G>C	TGCAGCGCGCACGCGTGCGCGTCGTCGTTGGCGGCGATGTTGGAGCGCACCAGGCAGCGGAGCTCCATC	20:G>C
5332172|F|0-11:C>T-11:C>T	TGCAGCTTGTACGGTGCGGCGGCGGCCG	11:C>T
5367362|F|0-25:G>A-25:G>A	TGCAGGGGCTGCGGCTGTACGATGCGGACAAATCCATTCTCCG	25:G>A
4995596|F|0-17:G>C-17:G>C	TGCAGGTACTCGGGCTCGAGCGCGAACAGCACGGCGATGCCGTCGTCCCTGTCGGCGCCGCGCTGCGTC	17:G>C
6044300|F|0-19:C>T-19:C>T	TGCAGTTGCTGGCCATGGACACCAGGCCCG	19:C>T
7166490|F|0-45:C>A-45:C>A	TGCAGGAGGAAGGTGATGAAGGCCATGAACCTCAAGGGCGTGCTGCGGACGGAGATCCACCCGTCGCTC	45:C>A
5363474|F|0-7:A>C-7:A>C	TGCAGCGAACCGCGGAGGAGATCGGGACCGCCGCCG	7:A>C
100515410|F|0-44:G>A-44:G>A	TGCAGGATGTCGCGGGCGCCCGACGCGGAGGAGGCGACGATGGCGGTGGTCGTGCCGAGCTTCAAGGAC	44:G>A
5361289|F|0-10:G>C-10:G>C	TGCAGCTTCTGAGCGACGGTACAGTCGTGCGCTCTGCGGCCTTGCCCTTCCCCGTCGACGGCAACGTCG	10:G>C
5341766|F|0-61:C>G-61:C>G	TGCAGGGCATGTGGTTCTACCAGATGGCGTTCACGCTCTATGGGCCGATGCTCCCCGCGGGCTGCCACC	61:C>G
1051566|F|0-33:T>C-33:T>C	TGCAGGGAGATGTACAAGTGCATCCAGGACGAGTTGCTGCCGCTGCTCCTGGAGCCCATGACCATCGCC	33:T>C
100581206|F|0-44:G>C-44:G>C	TGCAGGAAGCCCACCATGAGCGCCGTCCACGACACCACGTTCCTGTCGGGCATTCCGTCGAACACCTGG	44:G>C
1863509|F|0-31:C>T-31:C>T	TGCAGGAGTTCTATGCCATGATCGCCAAGGACTCGGCGAGGGCTTGCTACGGGCCCAAGCACGTCGAGG	31:C>T
22840848|F|0-20:C>G-20:C>G	TGCAGCCTGCGGCGCACCACCACGCCG	20:C>G
100535641|F|0-37:C>G-37:C>G	TGCAGCTGATGCGGACGCGGACCGACAGGGAGCTGGGCCGCCTCCTCGCGTCGGCGTCGGCGCTCGCGG	37:C>G
3944531|F|0-20:G>T-20:G>T	TGCAGGCGCAGAAGCTGGTGGTGCCG	20:G>T
6030670|F|0-26:C>G-26:C>G	TGCAGCTTCTACGCCTCCACGTCCCTCCCG	26:C>G
4005907|F|0-11:G>A-11:G>A	TGCAGCAGCCTGATGTGGCTGGACCTCAACAGCAACAGGCTCACCG	11:G>A
2283143|F|0-16:C>G-16:C>G	TGCAGCGCTGCCAGCCCCTCCTCCCCGTCGCCGAGGAGCTCCTCATCGTCAGCCGCTGCGTCGAGTCGC	16:C>G
5332191|F|0-20:G>A-20:G>A	TGCAGGAACGGCGGGCCGCCGTGCGACCCG	20:G>A
7491865|F|0-17:G>C-17:G>C	TGCAGCGGCCTCGCCGTGGCCAGCTCCG	17:G>C
3949139|F|0-6:G>A-6:G>A	TGCAGCGCCTGCTTGGCGCCGTCCAGCCCG	6:G>A
1089043|F|0-40:C>T-40:C>T	TGCAGGACGTGCGCGCCCAGGCCGACTACGACGGCTGGTACCACAACCAGTTCCTCGTCGTCAACGACT	40:C>T

2246955|F|0-61:C>G-61:C>G	TGCAGGTGGTGCCGTGGGGCCACATGGAGCACTCGGCCATGATATTCTACGGCGTGCAGGCCAAGGAGA	61:C>G
1338735|F|0-62:G>A-62:G>A	TGCAGCAGCCCACCTTCCTCCGTGAACCCCAGGCGTCCCGCGAGCGGGTAGTACTGCACCAGGGCCTGC	62:G>A
6047916|F|0-11:G>A-11:G>A	TGCAGCATCTCGTGAGCAACCTTAAGGCCTTCCTTGTACCTCCCTTCTCTAAAGAACCCGCGCATAAGA	11:G>A
3031222|F|0-31:A>G-31:A>G	TGCAGGTGCACCCGAACATCAAGGAGGCGGAAAGGTACCG	31:A>G
5581397|F|0-29:C>G-29:C>G	TGCAGCGCGTCGTCCTCCTCGGGGCTCCACGGGCCCTTGATCCG	29:C>G
6046095|F|0-17:T>C-17:T>C	TGCAGGACAGCTGCATTTTCAAAAGCAAAAGAATCTGAATTCCATCAATATTTATTCTTGTCTCCCGAG	17:T>C
4005725|F|0-26:C>G-26:C>G	TGCAGGAAGTCGAAGTGGTGCAGCGTCACGAGCGGCACCACCG	26:C>G
1105546|F|0-64:A>G-64:A>G	TGCAGCGACCTGCCCATCACGCAGTACTTCGACCTCGCCATGCAGGCCAGGCTGCTCAAGCGCCACCGC	64:A>G
1338735|F|0-7:G>A-7:G>A	TGCAGCAGCCCACCTTCCTCCGTGAACCCCAGGCGTCCCGCGAGCGGGTAGTACTGCACCAGGGCCTGC	7:G>A
5000238|F|0-27:C>G-27:C>G	TGCAGCGCGCGAGCCTTGGGCGCCTGGCGCCGAACGCCG	27:C>G
7172618|F|0-10:C>T-10:C>T	TGCAGGATGACGACGGCCCTGAGCCG	10:C>T
3024574|F|0-28:C>G-28:C>G	TGCAGCAGCTCAGCATCCGCTCCAACCCCTCGCTGTCCG	28:C>G
5582028|F|0-25:T>C-25:T>C	TGCAGGAGGTTGACGCTGCCATTGCTAAGCTGGAGGGTGAGACCG	25:T>C
1031695|F|0-52:C>T-52:C>T	TGCAGGCCTACTTCAACGCCTACTACGCGTCCATCGGGGTGGCCTTCACGGTCGCCGTCACCG	52:C>T
1090765|F|0-53:G>C-53:G>C	TGCAGCCTGCGCGACTTCGCCGACGGCCTGCTGGACGTGACCTGCGGCAGCACGGACGTGACCGTCTCC	53:G>C
4733634|F|0-13:G>T-13:G>T	TGCAGCATCCATCGCTTGAGCTGGCCG	13:G>T
2243220|F|0-7:G>C-7:G>C	TGCAGGGGCCCTACGTGTACTACCTCTACAGCCAGTACGGGTTCGACAAGGGCGACATCGGCCGCCTCT	7:G>C
1216830|F|0-43:G>T-43:G>T	TGCAGCTGGTGTCGGTGATGTTCGTGGGCCACCTCGGCGAGCTGCCCCTCGCCG	43:G>T
6038140|F|0-15:A>G-15:A>G	TGCAGGGGTACGTCAAGGACGCCCG	15:A>G
4261623|F|0-8:G>A-8:G>A	TGCAGTGCGGATCCAGCAGCCGCGGGGGCAATGCCGCCGCCG	8:G>A
1078289|F|0-28:C>A-28:C>A	TGCAGTCCTGCGCGCCCCTGGCGGCGACCCCGAACCCGAGCGGCACGTCGGCCATGGACATGACGACGA	28:C>A
3956159|F|0-28:C>T-28:C>T	TGCAGGCCATCAAGTCGGCCCTCGGCTCCGCGCCG	28:C>T
3956159|F|0-16:G>C-16:G>C	TGCAGGCCATCAAGTCGGCCCTCGGCTCCGCGCCG	16:G>C
1024315|F|0-43:C>T-43:C>T	TGCAGTACCTGACGAGCGGCGGCGGGTCCAAGGCGTGGGGCGGCGTCTACGCGCCG	43:C>T
3948943|F|0-16:G>T-16:G>T	TGCAGATGACCCATCCGTCCTCCTGCGCCGACGACTGGTCCCCG	16:G>T
1020924|F|0-20:C>G-20:C>G	TGCAGATTGATGTCCATCGCCGAGGAGACACTAACGGAGTTTCTGTCCAAGGCCACCG	20:C>G
32027761|F|0-15:G>A-15:G>A	TGCAGAGTAGAAGGCGTGAAGCGTACCATGGAGCCCCGCGTTGGGAAGGTGGAGCTCGGTAACATGTCC	15:G>A
3024418|F|0-26:C>G-26:C>G	TGCAGCTGCATCACGCCGTTCTTGAACACCCACACGCCG	26:C>G
1018677|F|0-17:G>T-17:G>T	TGCAGCATTATCGGTGAGCGCGGATTAGGGCGCCAGCTTATGCTGGAGACCACGGGGAGGTGCGATGCA	17:G>T
995663|F|0-5:G>A-5:G>A	TGCAGGGCCTCGTACTTGGAGGCCACCTCCCTGAGCATCTCGCCCAGCCGCCG	5:G>A
1075847|F|0-14:G>C-14:G>C	TGCAGGTAGTACTGGCGCAGCCCCTCGCCTCCGCCACGCGCGCCCTTGGCCGACTCGTCGCCG	14:G>C
6046603|F|0-7:G>A-7:G>A	TGCAGAGGTGGAGCTCCAGAACCCG	7:G>A
3950780|F|0-17:A>C-17:A>C	TGCAGCTCGCAAGCTGCATCGGGCTCATCGCCG	17:A>C
3938322|F|0-15:A>C-15:A>C	TGCAGCGCCAAACCAAAGCAAGCCGTCGTCAACCG	15:A>C
1039693|F|0-34:C>T-34:C>T	TGCAGATGTACGTGGAGAGCTGCCTGGCGCACCACGACGAGGTGCTGGCCATCAAGGACGCCGTCATCA	34:C>T
4993562|F|0-18:C>T-18:C>T	TGCAGCGGGCGGCGACGGCGAGGCAGGGCTGACCCG	18:C>T
22839067|F|0-27:G>C-27:G>C	TGCAGGTGCTCGACCTCTCCTTCAACGGCTTCAACGGGACCCTGCCCG	27:G>C
3941390|F|0-26:G>C-26:G>C	TGCAGGAAGGGCTTGAGGAACGACCAGCCG	26:G>C
4262464|F|0-20:C>A-20:C>A	TGCAGCACACCTTCCCCATGCTAGCCTGAGCAGAGAGGTTGCTTCCCG	20:C>A
983041|F|0-20:G>C-20:G>C	TGCAGGTACGTGAGCATGTCGCCGAACGCGGCGGCGTCGCGGGCGAAGGCGCCCG	20:G>C
987004|F|0-22:C>G-22:C>G	TGCAGGTCCTGGAGGACTTGCTCGCCCTCGGCGGCAAGGATTTCTTCGCCG	22:C>G
992922|F|0-25:C>T-25:C>T	TGCAGCTCCCACTGGCATCTAAATGCGGCTACATAAGGAAGCTGGTGTCGGGGGCCAACGGGTCCAGGG	25:C>T
5363156|F|0-10:C>T-10:C>T	TGCAGAGGGCCAGCTCCGCGGCAGCCG	10:C>T
5364124|F|0-19:C>T-19:C>T	TGCAGTTCATGTTCACTTACCTCTTCTTCCCCG	19:C>T
1035804|F|0-61:G>T-61:G>T	TGCAGCCGCGGTTAACGCTGGCAACGAGGCATCCCTCAGTGGTGGCCATGGGAACATGGTAGTCGCGCC	61:G>T
1018717|F|0-44:C>T-44:C>T	TGCAGGCCACGCAATTTCCTGAGCTTTATTGTCATTAGAGACTCCGAATGTATGGTGAAATTGTCCAGG	44:C>T
1695126|F|0-53:C>G-53:C>G	TGCAGCAGCATGTACTCCAGCAGGGGCATCATCGCCAACTCCTTCAGCGAGCTCGAGTCGGTCTACATT	53:C>G
32024136|F|0-26:G>A-26:G>A	TGCAGCGCGCTCCGGAGCAGCACCCCGATGTGCTCCTTCTCCCG	26:G>A
32027762|F|0-64:C>T-64:C>T	TGCAGAGTAGAAGGCATGAAGCGTACCATGGAGCCGCGCGTTGGGAAGGTGGAGCTCGGTAACACGTCC	64:C>T
3024574|F|0-17:C>A-17:C>A	TGCAGCAGCTCAGCATCCGCTCCAACCCCTCGCTGTCCG	17:C>A
7155245|F|0-19:C>A-19:C>A	TGCAGCTTCATCTCGGAGACCAACCCCG	19:C>A
1095248|F|0-31:G>C-31:G>C	TGCAGCGCAACGCCACGCGGTACGGCGCGGCGGCCACGGCCGTGATCCG	31:G>C
985564|F|0-19:G>A-19:G>A	TGCAGGACCTGGAGAAGCAGCTGCTCGGCGACGACGACGAGGCTGAGCCG	19:G>A
1087736|F|0-41:C>A-41:C>A	TGCAGCGTCTCCCGCAACGTGGGGAACGTGACCCTGTACGCCCTCCGCCG	41:C>A
3025523|F|0-28:T>C-28:T>C	TGCAGCCTCCCCCAGTGTATCTCCCACATGTCGGTGCTCAGCCCG	28:T>C
6044249|F|0-16:G>A-16:G>A	TGCAGTCGCCTCTGAGGGGGGAGTCTGCACCG	16:G>A
1095390|F|0-14:G>A-14:G>A	TGCAGAGCGCAGGAGTGCGGTATGGAAGGCTACGAGATGGACCTGGAACGGACACTGCGGATGCCGTCC	14:G>A
5367178|F|0-6:T>G-6:T>G	TGCAGCTGATGCTTTCCCGTCTTCTCCG	6:T>G
6048591|F|0-14:G>T-14:G>T	TGCAGCTGCGTGAAGCCCCTGTCGTGGCCG	14:G>T

988364|F|0-23:C>G-23:C>G	TGCAGGGCGCGGCCCATGGGCTCCGCGAAGGAGCCCTCCTGCGCGTAGAAGTCGTTGGAGGAGTAGCCG	23:C>G
6044111|F|0-20:T>G-20:T>G	TGCAGGCGATGGGCATCAACTCCAGCGGCACCG	20:T>G
6047432|F|0-31:C>G-31:C>G	TGCAGAAGCTGGACCTCTCGCGGAACGACCTCTCCG	31:C>G
1214919|F|0-49:T>C-49:T>C	TGCAGCTCTGGAAGTGGGAGCTCCTCGCGCTCGCGCTCGTCTGCGGCCGTCTCGTCTCCG	49:T>C
1047086|F|0-7:C>T-7:C>T	TGCAGACCGCTCAGCCAAACCATTCAAGACAGTGGCAAAGGTGAACTCATCAGGCCGAACCCCGCAGCC	7:C>T
1023927|F|0-31:G>C-31:G>C	TGCAGGGTGTAGGTGCCCTCCGCCTCCACCAGCAGCAGCTTGTGGCCCTGGAGCCG	31:G>C
1206894|F|0-32:G>C-32:G>C	TGCAGCTCGTAGTCGTCCTGCACGGCCTCGTCGGACGGGTAGTAGGCCGCCACGTAGGCCTCGCACCAC	32:G>C
7352275|F|0-7:C>A-7:C>A	TGCAGCCCGTGGCTCCAGCCG	7:C>A
1084212|F|0-35:G>C-35:G>C	TGCAGACGACTGTGGCAGCAAAGGCCCTCATGCTTGAAGCCCATCCACTGTAGCATCACATGTATGGCA	35:G>C
1694707|F|0-5:G>A-5:G>A	TGCAGGAAGTCGATGTAGTGCAGCGCGCCATGGATGAACTGCGTGCCGCCGCCG	5:G>A
4537825|F|0-32:C>G-32:C>G	TGCAGTTCGCCATGGCGTGATGCGTCCGTACGCGATGCGCGGATACACGCGGATTCGGCTGCCG	32:C>G
2248101|F|0-49:A>G-49:A>G	TGCAGATCCTTGGCCCCCTCTGCTTCGACATAGGCCACACTGCGCTGACAATGGTGGCGTCGCACGTTG	49:A>G
7172638|F|0-23:C>G-23:C>G	TGCAGGCCCAGGTAGCAGAGCGACGACGCCTCCG	23:C>G
5324000|F|0-23:C>A-23:C>A	TGCAGATCGTCGTGGGAAAGAGGCAGCAGCGTGCCG	23:C>A
3020882|F|0-26:G>C-26:G>C	TGCAGGGTTCCTTCACCTAATGATCAGATGATCATAGCCCG	26:G>C
990539|F|0-47:A>G-47:A>G	TGCAGCGGCCACCCCTCCCTCGTTGCTCTGCGTGGCGTCGTGCGCACACCCG	47:A>G
5411980|F|0-17:C>G-17:C>G	TGCAGGTCGTCCATGCTCAGGCCG	17:C>G
1037736|F|0-20:T>C-20:T>C	TGCAGATTTGTGGTATTGCTTCAAAGCTTGCAGGGTCTTCAAGAACCTTGCAAGACAAGGTATCTCTCC	20:T>C
5357203|F|0-14:C>A-14:C>A	TGCAGAAACCGAGCCTGTCCTCCTCCG	14:C>A
7910049|F|0-19:G>C-19:G>C	TGCAGCTCTCGCCGTCTTTGGCTCCACCG	19:G>C
32019039|F|0-11:A>G-11:A>G	TGCAGGATGCGACGGCAGCATCGCCG	11:A>G
32023592|F|0-26:A>G-26:A>G	TGCAGCACAGACTCGGGTGCATCCCAAGGGTGGTGTATCCATTCCGTTGGTAGCCTCGCCAGCTCCGGC	26:A>G
32020862|F|0-5:C>G-5:C>G	TGCAGCGCCAGAGCTCGCTCACCCTGCCCCGCACCCTCAGCGCCAAGACGGTCGACGAGGTGTGGCGCA	5:C>G
3955546|F|0-15:T>C-15:T>C	TGCAGAAGCTGCGGGTGGAGCTGGATGCCGTGGTGGGCAGGTCCCG	15:T>C
100502329|F|0-13:T>C-13:T>C	TGCAGCTCGAGGCTGTCCGAGCAGGTGCCATGAGTCAGCTGCTCATGATGGTGCAAGGCGGCTGCTCGG	13:T>C
7174087|F|0-16:C>T-16:C>T	TGCAGTTGAGGCGGATCTCCCCCTGCGTGCCG	16:C>T
3953791|F|0-11:A>C-11:A>C	TGCAGCCGTAGAGCATACAGCTGGTTGTTCTCGACGCCGAGGACTGTCAGGTACCGCCG	11:A>C
32021749|F|0-21:A>G-21:A>G	TGCAGATCTGAAGGAGATCGCATCCGTGGTCGAGGCGGGGTCGCTGACCAAGGAGGTCCGCCG	21:A>G
32023851|F|0-25:T>G-25:T>G	TGCAGTACCACCCCGACGTGTGCCCTCCGTCGCGCAGGGCCGAGTCCACGGCGCTCTTCGTCGAGCTCC	25:T>G
7170857|F|0-16:G>C-16:G>C	TGCAGGTGAGCATCACGAGCCAGGCCCTGATATTCGTGACCCGCAGCCG	16:G>C
32027116|F|0-38:A>G-38:A>G	TGCAGCGAGTTGTTCGATATGTCGAGGTAGAAGAGGCGATCGAGCTCGCCGAGCAGCGGCGGGATCGGG	38:A>G
1024976|F|0-47:C>T-47:C>T	TGCAGGTGCAGGTACGCCGCCTGGAACGACGACGTCGTCGCGAGTGCCGCCGCCACGGCGGCCTCCTTT	47:C>T
1056934|F|0-10:C>T-10:C>T	TGCAGACCGTCGCCACCGACTTCCAGAGGGTGTTCAAGATCCACGGCAAGCTGTACATCGGCCTCTCCG	10:C>T
3948206|F|0-10:C>T-10:C>T	TGCAGAACCTCGACCTCTCCAAGAACTCGCTCGCCG	10:C>T
5357203|F|0-7:A>T-7:A>T	TGCAGAAACCGAGCCTGTCCTCCTCCG	7:A>T
3944131|F|0-26:G>C-26:G>C	TGCAGCATGCGCTTGCACCGCGCGCAGCCGCCCG	26:G>C
6030670|F|0-14:C>T-14:C>T	TGCAGCTTCTACGCCTCCACGTCCCTCCCG	14:C>T
100439845|F|0-5:C>T-5:C>T	TGCAGCATGCGCTTGCACCACGCGCAGCCGCCCG	5:C>T
2248101|F|0-13:C>G-13:C>G	TGCAGATCCTTGGCCCCCTCTGCTTCGACATAGGCCACACTGCGCTGACAATGGTGGCGTCGCACGTTG	13:C>G
7172638|F|0-8:C>G-8:C>G	TGCAGGCCCAGGTAGCAGAGCGACGACGCCTCCG	8:C>G
1697137|F|0-38:G>A-38:G>A	TGCAGGCCCCTCCAGCTCTCCTTGTAGTTCCAGTACGTGTTGAGGCAGTGCCCGAGCGCGTCGATCATC	38:G>A
3935636|F|0-11:C>A-11:C>A	TGCAGCAGCTGCGCCGCCGACGGCCGCTTCGCCG	11:C>A
1043324|F|0-19:C>A-19:C>A	TGCAGCTCCTCCTCCACGGCCTTCATCTTGGCCTCCCACTCGGCGAGCCG	19:C>A
6046929|F|0-11:G>A-11:G>A	TGCAGTTGATCGCCGATGTTGATGACTAGCGCGCCG	11:G>A
985215|F|0-23:C>T-23:C>T	TGCAGCTTCAAGGGACACATCCGCGTCTCTGCCACGGAGGCGCTGGAGTGCTATTTCAAACTCCTTTTT	23:C>T
3937231|F|0-26:G>T-26:G>T	TGCAGGAACTTGGCGGTGGGGAAGTTGACGGCGAAGGAGAGCGCGCCG	26:G>T
1080470|F|0-19:G>C-19:G>C	TGCAGGAGAGCGTGCGCGGGAGCGACGTGTTCCTGGTGCAGCCCACCTGCCCGCCG	19:G>C
2247689|F|0-29:C>T-29:C>T	TGCAGCCTCACGCTGAGCGACGTCGAGGGCCAGCTGCACGGCGTCAACCTCGACAACCTCCTCCGCACG	29:C>T
5354592|F|0-45:G>T-45:G>T	TGCAGCATCTCCTGCACCTGCGCCTCCTGCTTCCCGCGCAGCCGCGGGTCGAGGAGCTCCGCCACCCCC	45:G>T
1087736|F|0-29:G>A-29:G>A	TGCAGCGTCTCCCGCAACGTGGGGAACGTGACCCTGTACGCCCTCCGCCG	29:G>A
2283143|F|0-67:G>A-67:G>A	TGCAGCGCTGCCAGCCCCTCCTCCCCGTCGCCGAGGAGCTCCTCATCGTCAGCCGCTGCGTCGAGTCGC	67:G>A
5581906|F|0-5:A>T-5:A>T	TGCAGAAGTGGTGGTGCTTGCCG	5:A>T
1739894|F|0-66:G>C-66:G>C	TGCAGTCGCTGGTGACCGAGCACGGGCTCCCCGCTGCCGAGGCCTCTCCCCCTTCCCCTCCTGTTGGCA	66:G>C
1041714|F|0-27:A>G-27:A>G	TGCAGGCAAAGCTAGCATGGGGGAGAAAGAGTGGTACTTCTTCAGCATGCGCGACCG	27:A>G
3949155|F|0-16:C>T-16:C>T	TGCAGCGGCACGACTCCGCGTTCGCCGCCCG	16:C>T
3026721|F|0-15:G>A-15:G>A	TGCAGTTCTTGATCAGCTGGATGTCGCGGTTCCCGCAGTACCG	15:G>A
2251044|F|0-57:A>C-57:A>C	TGCAGGTACCCAGCGTACGACAGGCTTCTGCCCTGCCCGTCGAAGGACGACCCTCCGAGGATCGAGCAC	57:A>C
4002580|F|0-19:C>T-19:C>T	TGCAGGAGTGTCGGCTGGCCGTTTCATGAATAACCTTTGCTTGTCGGATAGGGAGGGAATAAAGAGAGA	19:C>T
1047019|F|0-41:C>G-41:C>G	TGCAGAGATTCCATCTGATCCGATCAGGTTCTCTGCATGCTCGGCGCAAAAGTCTTCTTCTGATGCAGC	41:C>G

1064678|F|0-9:T>C-9:T>C	TGCAGCAGCTTGGACCGCTTGTGCACGACCATCATGGTGAACCACGGGATGCTGCCG	9:T>C
1020842|F|0-5:A>C-5:A>C	TGCAGAGGTTCTTCCTCGGAGGGAGACTGATATTCGGACCCGATGCCAAGTCCCTGCTCGTCTCGGTTG	5:A>C
1146832|F|0-67:C>T-67:C>T	TGCAGATGCATTAATTGGCGTTATTATGGCAGGCATGAGTACACACGTAGAGTGTATTTGTTGCCGCCT	67:C>T
4262979|F|0-17:G>C-17:G>C	TGCAGCAAGCAGAAGGTGAAGCGGGGGCTATGGTCCCCG	17:G>C
1076645|F|0-58:G>A-58:G>A	TGCAGCGTGACCTCCTCGCCAACTTCCTCAGCTTCCAGGTCTTCTCCTTCCTCCCGCCGCTCACCAAGC	58:G>A
1152398|F|0-40:G>C-40:G>C	TGCAGCGACGACACCAAGTTCTCCGACGGCACGGGCCACCGTGACACCG	40:G>C
1667190|F|0-22:G>A-22:G>A	TGCAGATCGGATTCGCCCTCAAGAAGGTGTTCGACGAGGGCATAACCAGCCG	22:G>A
998477|F|0-14:G>A-14:G>A	TGCAGCGTGGTGTCGCCGTTGCGGAGGAAGAAGAGCACGCGGTTGAGGAAGACGGCGCCGATGAGCCGA	14:G>A
5411898|F|0-11:A>T-11:A>T	TGCAGCCGCAGATACTCAGCTTCCCG	11:A>T
4006159|F|0-12:C>T-12:C>T	TGCAGGAAGGCGCTCGTCATGAGCCCGCGCGAGAACGCGCCG	12:C>T
4262733|F|0-14:C>G-14:C>G	TGCAGCGCGAGCACCAGCTTCCG	14:C>G
4002386|F|0-32:C>G-32:C>G	TGCAGGGCCTTGAGGCGCGACAGGATGGCGTGCAGGAGTTCGTCCG	32:C>G
3940568|F|0-19:A>G-19:A>G	TGCAGGAGGGAAGACAAAGATAACCCCGCCG	19:A>G
1167502|F|0-29:C>G-29:C>G	TGCAGCACCCCCTCGAACGGGTACTTGACCAGCGACAGGTAGTGGAACCACAGCCAGTAGGCCG	29:C>G
100508155|F|0-25:T>C-25:T>C	TGCAGTACCTCATTGACTACATACATACCTTCACCG	25:T>C
5364084|F|0-13:T>G-13:T>G	TGCAGTGCCTGCGTGCCTGCCCG	13:T>G
4261708|F|0-5:C>G-5:C>G	TGCAGCGCGGGCTTGACCAGGCCCTCGTCGGGCCAGCCCG	5:C>G
1056569|F|0-50:T>C-50:T>C	TGCAGTAGTTGGCCAGATTCAGTGTAAGCACCTGTTGATATAAGTTAATCTGCATCCCCG	50:T>C
4262426|F|0-37:G>T-37:G>T	TGCAGGCGTCCGTGAACGCGGACGGGGCCTTCACCAAGGTTTTCGCCG	37:G>T
1022281|F|0-56:C>T-56:C>T	TGCAGTCGCAGAGCCTCATCATGCCGCTGCTCCTGTCGTCCGTGGCCACGCTGGCGCTCCACGTCCCCC	56:C>T
4262464|F|0-31:A>G-31:A>G	TGCAGCACACCTTCCCCATGCTAGCCTGAGCAGAGAGGTTGCTTCCCG	31:A>G
985696|F|0-67:G>C-67:G>C	TGCAGAGCAACCTGGCCGTGGCGGAGATCTCCAAGATCGCGTCCTCGCTCCTGCCGTTCGGGGAGGAGG	67:G>C
1047019|F|0-45:G>A-45:G>A	TGCAGAGATTCCATCTGATCCGATCAGGTTCTCTGCATGCTCGGCGCAAAAGTCTTCTTCTGATGCAGC	45:G>A
984746|F|0-22:G>C-22:G>C	TGCAGTGCACCTCCAAGTGCGTGGCCACCTGCATCCGCGGCGGCGAGGGGCCCATCAACGTCCG	22:G>C
10984309|F|0-17:C>A-17:C>A	TGCAGGGGTGGGAGAAGCGGATCGACCG	17:C>A
32018965|F|0-8:T>C-8:T>C	TGCAGCGATCACCTCGATCAGCCCG	8:T>C
7173317|F|0-12:G>A-12:G>A	TGCAGGAGGGGAGCCGCATGCTCCG	12:G>A
1022874|F|0-12:G>A-12:G>A	TGCAGGGGATTTGGACACCGCCCGCAGTCTGCTGGACGAGATGCCCGTCAAGGATGCTGTGGCTGTCAA	12:G>A
3955135|F|0-14:A>G-14:A>G	TGCAGGGTTTGGTGAGGAGGAGGTGGACGGGTCGTTAGATTAGCCG	14:A>G
4910325|F|0-29:G>A-29:G>A	TGCAGGCCGCCATGCAGGCTGACGTGGATGCCG	29:G>A
984837|F|0-37:C>T-37:C>T	TGCAGGAGATGAAGCACATCTGCGGGATGACGGGCGACGGCGTGAACGACGCGCCG	37:C>T
3024946|F|0-5:C>T-5:C>T	TGCAGCTGTCTTTCCAGGACTCGTCGGCAACCACGGTGTCGCCG	5:C>T
3949299|F|0-5:C>G-5:C>G	TGCAGCGATTGCCTTCACCAGGAACCG	5:C>G
5331503|F|0-9:T>G-9:T>G	TGCAGACCTTGATCAGGCCCG	9:T>G
1024352|F|0-22:C>T-22:C>T	TGCAGGCCCCTCACGAAGGCGACGGCGTACTTGCTCACCGTGATCGGGTCCTCGCCG	22:C>T
1156192|F|0-50:C>G-50:C>G	TGCAGGCACACCACCGTGGCGGCGCCGCCCATGAACCCGACGATGGCGGCCTGCGACAAAAAGTCCACC	50:C>G
4396759|F|0-10:G>A-10:G>A	TGCAGCAGCAGCGGACGGCCGCACGCCG	10:G>A
1212241|F|0-25:C>T-25:C>T	TGCAGCACATCAAGTCCATCGCCGACGAGATGGAGTTGCGCCGCCGCGAGCTGCGGCTCTACGCCAACA	25:C>T
5006251|F|0-13:G>A-13:G>A	TGCAGGCTACTACGACGACGACACCG	13:G>A
32023768|F|0-11:A>G-11:A>G	TGCAGGACTTCATCGCCAGCCACAGCCCCGACGCGGACGCCGCAAAGGCCGCCGCCGACGCCCGTAAGG	11:A>G
3957321|F|0-13:C>T-13:C>T	TGCAGGAGGCGCGCGCGCTCCTCGAGTACAACCCCCG	13:C>T
2245281|F|0-21:G>C-21:G>C	TGCAGGCGGCTCTTGAAGCTGGGGTCTGAAATTTGATCCCAGAACTCACGCAGCTCCGCCTTGTCGATG	21:G>C
3935858|F|0-9:C>G-9:C>G	TGCAGGTCCCCCGCCGCGCCCGTCGTCACCTCCG	9:C>G
1087736|F|0-6:G>C-6:G>C	TGCAGCGTCTCCCGCAACGTGGGGAACGTGACCCTGTACGCCCTCCGCCG	6:G>C
5367166|F|0-7:C>A-7:C>A	TGCAGCTCGCCATGGCCACCCG	7:C>A
1093465|F|0-38:G>T-38:G>T	TGCAGTGCTTCCTTGCCTCCATCATGGTGGCCATAGCAGACAATGATGGCATTCCAGGATACAGAGTTC	38:G>T
1021637|F|0-32:C>T-32:C>T	TGCAGCCTCCTCACGTTCTTGGTGCTGTTGTCCCGCTTGTCCAGCTGGTCCGCTCCGAACGCCAGCGCG	32:C>T
32021289|F|0-17:A>G-17:A>G	TGCAGAGGGCCAGCTCCACGGCAGCCG	17:A>G
3936848|F|0-23:C>A-23:C>A	TGCAGCAGCTGGAGTCGTCGACGCGGCAGAGCAAGCTGCGGCCG	23:C>A
1022874|F|0-57:T>G-57:T>G	TGCAGGGGATTTGGACACCGCCCATAGTCTGCTGGACGAGATGCCCGTCAAGGATGCTGTGGCTGTCAA	57:T>G
981258|F|0-16:T>C-16:T>C	TGCAGCCCAGAAGGATTTATCTTGTGATGAAAGCAGAGTGCGAGGAGCAGAAGAGAAAATATTTTCAGG	16:T>C
1095248|F|0-17:C>A-17:C>A	TGCAGCGCAACGCCACGCGGTACGGCGCGGCGGCCACGGCCGTGATCCG	17:C>A
1068553|F|0-18:G>A-18:G>A	TGCAGGGAGAGCGCCCTGGTGATGTTGCGCAGCACGGCCTGCTCGTGCTGCTGCTCCTGCACGCTCGGG	18:G>A
1092720|F|0-44:A>G-44:A>G	TGCAGTCCTGCGCGCCCCTGGCGGCGACCCCAAACCCGAGGGGCACGTCGGCCATGGACATGACGACGA	44:A>G
3939963|F|0-9:C>A-9:C>A	TGCAGCGCCCTCGCCGAAAGCGTGAAGCGCTGGGCGCCG	9:C>A
1242527|F|0-16:C>A-16:C>A	TGCAGGTGATCCTGCACCAGCAGCTGCAAGTGCTGGTGGAGAGGTACCG	16:C>A
985294|F|0-9:G>A-9:G>A	TGCAGCAGCGTGTCAATGGGCCATGGACAAGTGTTGCAGAACCTCGCCATCTCCG	9:G>A
1233475|F|0-5:G>A-5:G>A	TGCAGGCCGCGGGGCACGACGTAGAGCTCGTTCTTCTTCACGACCTTGGAGTATAGCCG	5:G>A
1126981|F|0-38:T>A-38:T>A	TGCAGCATCACCACACCGAAGCAGTACACGTCGCGGCCTGTGGCCGCCTCCGTCGTTGCCGCTTCTTCC	38:T>A

<table>
<tr><td>986352|F|0-20:C>A-20:C>A</td><td>TGCAGCGGCTGCGCCATGATCTCGCTCATCACGCTCTCGTTGTACCCGAGCCCGCACACGTAGGGGATG</td><td>20:C>A</td></tr>
<tr><td>982151|F|0-49:C>G-49:C>G</td><td>TGCAGAAGCTCCTCGTCCTCGCCTCGCTCGCCGTGTGGTCCCGCCTCCCCGTCCCCTTCGCCG</td><td>49:C>G</td></tr>
<tr><td>982151|F|0-40:C>G-40:C>G</td><td>TGCAGAAGCTCCTCGTCCTCGCCTCGCTCGCCGTGTGGTCCCGCCTCCCCGTCCCCTTCGCCG</td><td>40:C>G</td></tr>
<tr><td>985696|F|0-16:C>T-16:C>T</td><td>TGCAGAGCAACCTGGCCGTGGCGGAGATCTCCAAGATCGCGTCCTCGCTCCTGCCGTTCGGGGAGGAGG</td><td>16:C>T</td></tr>
<tr><td>7168665|F|0-11:C>T-11:C>T</td><td>TGCAGCATCACCACACCGAAGCAGTACACGTCGCGGCCTGTGGCCGCCTCCGTCGTTGTGGCTTCTTCC</td><td>11:C>T</td></tr>
<tr><td>3028027|F|0-22:G>A-22:G>A</td><td>TGCAGCCACCCGTAGAAGAAGAGCGCCGGGATCATCCACCG</td><td>22:G>A</td></tr>
<tr><td>5358459|F|0-8:T>C-8:T>C</td><td>TGCAGGTGTTGTTCCTCTGGGGCAATGCGCTGTCCG</td><td>8:T>C</td></tr>
<tr><td>3029456|F|0-10:A>G-10:A>G</td><td>TGCAGCGGCAAGTTCGGGCCCGACCACCCGTACATCAACCCG</td><td>10:A>G</td></tr>
<tr><td>1065215|F|0-20:G>A-20:G>A</td><td>TGCAGGAACTTCTGGATGGGGAAGTTGGCCGCGTACGCGAAGATCTGCGGGATGAGGCCGTAGGCGAAC</td><td>20:G>A</td></tr>
<tr><td>1029349|F|0-64:G>T-64:G>T</td><td>TGCAGGCCCTGCACGCTGCCGTTGCCGTGGCAGGAGACGCCGTACCAGGAGGAGTGGTTGCCGTGGCAC</td><td>64:G>T</td></tr>
<tr><td>6039808|F|0-14:A>G-14:A>G</td><td>TGCAGCCCTACCGTATCCAGCCCCAGCGCCAGCGGCACGCTCCCTGGCATTGGCGAAACTCTACTGCTC</td><td>14:A>G</td></tr>
<tr><td>4396875|F|0-5:C>G-5:C>G</td><td>TGCAGCGAGTCCACGGCCAGCGGCATGTTCTCCTGGTCCG</td><td>5:C>G</td></tr>
<tr><td>1075847|F|0-32:G>A-32:G>A</td><td>TGCAGGTAGTACTGGCGCAGCCCCTCGCCTCCGCCACGCGCGCCCTTGGCCGACTCGTCGCCG</td><td>32:G>A</td></tr>
<tr><td>3023143|F|0-17:C>T-17:C>T</td><td>TGCAGCAGCATGTTGTACACGTCCCTGTGCGCCTTCTCGACCTCGTCGAACAGCACCACCGCGTACGGC</td><td>17:C>T</td></tr>
<tr><td>4262304|F|0-35:T>A-35:T>A</td><td>TGCAGACGCAGATGCTCATGGGAGACGGCGGCGCGTCAGCCG</td><td>35:T>A</td></tr>
<tr><td>1076645|F|0-18:C>G-18:C>G</td><td>TGCAGCGTGACCTCCTCGCCAACTTCCTCAGCTTCCAGGTCTTCTCCTTCCTCCCGCCGCTCACCAAGC</td><td>18:C>G</td></tr>
<tr><td>1022332|F|0-17:C>T-17:C>T</td><td>TGCAGCTCCAGGTCGCACTCGCCGTTGGCGCTCCGCGTGGCGAGCTCCGCCG</td><td>17:C>T</td></tr>
<tr><td>4734178|F|0-8:C>T-8:C>T</td><td>TGCAGGCACTGCGCTCGAGGCGCTGAGGTCCATTGGTGCCAAGTTTGACGGCACCG</td><td>8:C>T</td></tr>
<tr><td>7169618|F|0-20:G>T-20:G>T</td><td>TGCAGGAACTCGTCGTCGCAGAGGAGGTGGCCCATGGTGTCCG</td><td>20:G>T</td></tr>
<tr><td>1163710|F|0-31:G>T-31:G>T</td><td>TGCAGTGCTGTCATGCTGCGCACATCTCGGGGCGCTAGAGAAGGGACGGGAGGTCCATGAGTATGTGAT</td><td>31:G>T</td></tr>
<tr><td>5411812|F|0-5:T>C-5:T>C</td><td>TGCAGTCGTACGGCTACTGCCG</td><td>5:T>C</td></tr>
<tr><td>32027359|F|0-52:G>C-52:G>C</td><td>TGCAGGAGAAGTCCATGGGCAGGGTGAAGCCGTCGACGACCGATATGCCGTAGCTGCCCG</td><td>52:G>C</td></tr>
<tr><td>1347849|F|0-23:G>T-23:G>T</td><td>TGCAGCGGAGAGGAGGGAGGCGAGAGCGTGAAACGCGGGGAAGCAGTGTCGGAAGCCCTTCTGGCGGCC</td><td>23:G>T</td></tr>
<tr><td>1116406|F|0-55:T>C-55:T>C</td><td>TGCAGCGCCGTGGCCAACGCCATGGCTGTGCAGTACAACAAGGCGGCCGAGGGCGTCCTCAGCGGCATG</td><td>55:T>C</td></tr>
<tr><td>3944126|F|0-7:C>G-7:C>G</td><td>TGCAGGTCGGCGTCGACCTGCTGCGCGCCG</td><td>7:C>G</td></tr>
<tr><td>1017611|F|0-11:T>G-11:T>G</td><td>TGCAGTACGTGTCCAACGTGATCATCCACAACCTGCACGTGCACGACTGCGTGCCG</td><td>11:T>G</td></tr>
<tr><td>3949729|F|0-23:A>T-23:A>T</td><td>TGCAGGTGGACGGGCAGCTCCTCACCGACACCCCG</td><td>23:A>T</td></tr>
<tr><td>1252331|F|0-13:G>T-13:G>T</td><td>TGCAGATGATCGTGGAGAAGGAGATCGTGGGCGGCGCCGACCTGCGGGAGCTCCTCCACCG</td><td>13:G>T</td></tr>
<tr><td>1697137|F|0-14:G>A-14:G>A</td><td>TGCAGGCCCCTCCAGCTCTCCTTGTAGTTCCAGTACGTGTTGAGGCAGTGCCCGAGCGCGTCGATCATC</td><td>14:G>A</td></tr>
<tr><td>1156192|F|0-40:C>T-40:C>T</td><td>TGCAGGCACACCACCGTGGCGGCGCCGCCCATGAACCCGACGATGGCGGCCTGCGACAGAAAGTCCACC</td><td>40:C>T</td></tr>
<tr><td>1338735|F|0-11:A>G-11:A>G</td><td>TGCAGCAGCCCACCTTCCTCCGTGAACCCCAGGCGTCCCGCGAGCGGGTAGTACTGCACCAGGGCCTGC</td><td>11:A>G</td></tr>
<tr><td>5359303|F|0-6:C>T-6:C>T</td><td>TGCAGGCGGTTCAGCACCACCG</td><td>6:C>T</td></tr>
<tr><td>5370854|F|0-8:G>A-8:G>A</td><td>TGCAGCTCGGCGGGCTGAGGACGTGGGCCGTGCCG</td><td>8:G>A</td></tr>
<tr><td>982151|F|0-23:T>G-23:T>G</td><td>TGCAGAAGCTCCTCGTCCTCGCCTCGCTCGCCGTGTGGTCCCGCCTCCCCGTCCCCTTCGCCG</td><td>23:T>G</td></tr>
<tr><td>2244920|F|0-49:T>C-49:T>C</td><td>TGCAGAACGTCGTGCAGATGATCTCGGTCATGTTCGTCGGCCATCTCGGTGAGCTCGCTCTCTCGAGTG</td><td>49:T>C</td></tr>
<tr><td>1024349|F|0-10:G>C-10:G>C</td><td>TGCAGGCGCAGTTCGGGAACAAGTGGGCGAGGATCGCGACGTACCTGCCCG</td><td>10:G>C</td></tr>
<tr><td>32023351|F|0-11:G>C-11:G>C</td><td>TGCAGCTTCGAGAGGTTGGCGAGAGAGGCCG</td><td>11:G>C</td></tr>
<tr><td>3939049|F|0-6:G>A-6:G>A</td><td>TGCAGAGGACCGCCTTCGGGTCGGTGTCGAGCAGCACCG</td><td>6:G>A</td></tr>
<tr><td>1138469|F|0-52:T>C-52:T>C</td><td>TGCAGGATGAGAGGAGCAAAGGCTGGCGAGTGCAGGCCGTCTCTGTTGCACCTGACAGATTCGAGAGCC</td><td>52:T>C</td></tr>
<tr><td>1121130|F|0-38:C>G-38:C>G</td><td>TGCAGGGCATCTACGTCAAGGTACTCTTCCTGTGGATCCCCGTCGCCGAGGTCGACCGCGCCG</td><td>38:C>G</td></tr>
<tr><td>7160835|F|0-17:G>A-17:G>A</td><td>TGCAGCAGCGGCGCCGCGCTCTGCCG</td><td>17:G>A</td></tr>
<tr><td>4261708|F|0-31:G>T-31:G>T</td><td>TGCAGCGCGGGCTTGACCAGGCCCTCGTCGGGCCAGCCCG</td><td>31:G>T</td></tr>
<tr><td>1003268|F|0-62:G>C-62:G>C</td><td>TGCAGGTAGAAGCGGGGCTTGTGGGGGCGGAGCACCAGGTAGACGATGAGGATGATGAAGAGGGCGAGG</td><td>62:G>C</td></tr>
<tr><td>3941517|F|0-17:C>G-17:C>G</td><td>TGCAGGCTGGACGCCAGCGCCG</td><td>17:C>G</td></tr>
<tr><td>2247666|F|0-21:C>G-21:C>G</td><td>TGCAGACGCCTCCCTTCCTTGCCGTCCATGACCCTCTCGATGCAGTCCTTCACCTCGTCTCTGCTCATG</td><td>21:C>G</td></tr>
<tr><td>4004161|F|0-34:C>A-34:C>A</td><td>TGCAGGGCCAGAGCGTGGAGAACAAGTCCAGCGGCGTCTGGAGCGTCGACGCTGAGCTCTGGAACGGCC</td><td>34:C>A</td></tr>
<tr><td>2243702|F|0-13:T>A-13:T>A</td><td>TGCAGGACGGCGGTGTCGAGGTTCTGCTCCCGCTTGAGCTTGTCCTTGAGGAACCAGTCGCACACCCGC</td><td>13:T>A</td></tr>
<tr><td>3025523|F|0-14:G>A-14:G>A</td><td>TGCAGCCTCCCCCAGTGTATCTCCCACATGTCGGTGCTCAGCCCG</td><td>14:G>A</td></tr>
<tr><td>2241885|F|0-41:C>T-41:C>T</td><td>TGCAGATGGTGCCGTTCCACGCTGGGAGCAACGAGGGCCTACTCGAGCAATTCTCATGGGACGATCCCT</td><td>41:C>T</td></tr>
<tr><td>3949313|F|0-31:C>G-31:C>G</td><td>TGCAGCGGCTGGCCAAGTCGACGCGGCGGACCATCATCACCACCATCCACCAGCCGTCCAGCCG</td><td>31:C>G</td></tr>
<tr><td>988647|F|0-27:G>A-27:G>A</td><td>TGCAGAAGAACCTGGAGCTGTCGCCGCGGCACGTGGAGGCGTCGCGGATGACGCTGCACCG</td><td>27:G>A</td></tr>
<tr><td>1056012|F|0-23:C>A-23:C>A</td><td>TGCAGTACTTCTTCCCCATCTTCCACTGGGGCTCCAACTACAGCCTCCGCCTCCTCCGCTCCGACGCCG</td><td>23:C>A</td></tr>
<tr><td>3954911|F|0-9:G>A-9:G>A</td><td>TGCAGCGTCGTCAACAACCG</td><td>9:G>A</td></tr>
<tr><td>3949686|F|0-11:G>T-11:G>T</td><td>TGCAGCGTCGGGGACACCTCGCCG</td><td>11:G>T</td></tr>
<tr><td>7337147|F|0-7:C>T-7:C>T</td><td>TGCAGCCCGCGGCGCTCTCGCCG</td><td>7:C>T</td></tr>
<tr><td>3953345|F|0-20:C>G-20:C>G</td><td>TGCAGCAGGCGCTGGACACGCTGCTGGGGCGGCGGAGCCTCAAGATGAGCGACTTCGTGGTGGGCGAGC</td><td>20:C>G</td></tr>
<tr><td>989354|F|0-46:G>C-46:G>C</td><td>TGCAGCTTCAGATCCGTAACGACCAGATCGCACGTTGATGGACGTGGGAGGTCCAACTGCGCCG</td><td>46:G>C</td></tr>
<tr><td>1025510|F|0-56:C>G-56:C>G</td><td>TGCAGCTTGTTGACGATCGGGATCACCGAGGAGCCCACCACCGCCGCGGCCTCGGCCGCCGCCGCCGCC</td><td>56:C>G</td></tr>
<tr><td>1200708|F|0-62:G>C-62:G>C</td><td>TGCAGGAAGCGCAGCTCGTCGGACTCGGACCAGATGCGCTGGAACTTGAAGGGCGGGTTCTGGTTCTGG</td><td>62:G>C</td></tr>
</table>

1080037|F|0-25:C>T-25:C>T	TGCAGCACCCCAACGAGTACATCCGCGGCGTCACGCTGCGGTTCCTCTGCCGCCTCTCCGAGCCGAGAT	25:C>T
4733962|F|0-22:C>G-22:C>G	TGCAGATCCTCCTCCTCTCGCTCGCCG	22:C>G
996524|F|0-23:C>G-23:C>G	TGCAGGAGCAGTGGCGCTGCCCACTGTCCATCCTGCTGCTTACTACTTGTGCGCTTACCCG	23:C>G
12694099|F|0-16:G>T-16:G>T	TGCAGCCTGTACGATCGCCCG	16:G>T
1021430|F|0-57:G>A-57:G>A	TGCAGCCTCTTCGGCAAGCAGGCGCCCCAGCCGCAGCACCGCGCGGGCGGCGGCAGCGCCAAGGGGAAG	57:G>A
3941516|F|0-17:G>C-17:G>C	TGCAGGTCGAGCGCCTCGCCGCGGAGCTTGTCGGGGAGCGCGTCGACGAGCGCGATCTCGTCGGCCAGG	17:G>C
1075519|F|0-43:G>T-43:G>T	TGCAGCCACCTTATCCAACTATTGTGCGTATCTGGTCAGGAAGGCACTGGTGCCG	43:G>T
1052066|F|0-24:T>G-24:T>G	TGCAGGCTTTGTATCCAACTTCAGTGTGGATCCACCATTACTACCTGCTCAATGCTTTCAACCAATATC	24:T>G
2247504|F|0-34:G>A-34:G>A	TGCAGGAGGAGGTGCTCGAGGTACCACATCAGGGGCGACAAGGCAGGCACGGTGGAGAGCTTCATCGAC	34:G>A
3029114|F|0-11:C>G-11:C>G	TGCAGCTCCGTCTCGAACTCCATGTCCATCCGCGGCGTGCACTCCTCCG	11:C>G
1230628|F|0-25:G>A-25:G>A	TGCAGTCGTCGCTCATCATGTCGGCGGTGGTGTCTATGGTCATCGCCGTCCTGTGGCTGTTCACGGAGC	25:G>A
4261226|F|0-8:C>G-8:C>G	TGCAGGGACACGATGACGGCGGCGCCGCCCATGAACCCG	8:C>G
1022533|F|0-64:A>G-64:A>G	TGCAGAAAAATGTGCATTTCCTCACAAAGGTTGCTGGGCTGGAGATATCTTACATTGCTCAAAGACCGA	64:A>G
998178|F|0-49:G>C-49:G>C	TGCAGGTTGGGAAGCAGCGGTTCAAGACCAAGGTGGTCAAGATGAACCTGAACCCG	49:G>C
1063684|F|0-37:A>C-37:A>C	TGCAGCGTAGTTTAGGTGTACATGAGCTATGCTTTTCATCGTATTTGCTGCACTGCCG	37:A>C
1164753|F|0-6:T>G-6:T>G	TGCAGTTACCTGGGCCATGCGGCGAACCCACGCCAGCTTGTTGTGATTCTCGTAGCCGATGACGGTGGC	6:T>G
1116406|F|0-6:G>A-6:G>A	TGCAGCGCCGTGGCCAACGCCATGGCTGTGCAGTACAACAAGGCGGCCGAGGGCGTCCTCAGCGGCATG	6:G>A
1021430|F|0-54:A>G-54:A>G	TGCAGCCTCTTCGGCAAGCAGGCGCCCCAGCCGCAGCACCGCGCGGGCGGCGGCAGCGCCAAGGGGAAG	54:A>G
3951327|F|0-6:C>T-6:C>T	TGCAGTCGCTGATGAGCTCCTGCGTCCCGCCGCCG	6:C>T
987470|F|0-28:A>C-28:A>C	TGCAGGCCTTCTGGACGGAGGTGCGCACAGAGGGACACGGAGACAAGAAGGACGCGCCGTGGTGGCCTA	28:A>C
1101657|F|0-20:G>A-20:G>A	TGCAGAAGCGTCCACCGCGAGGTCTCGGGGACTCCCCAGATCACCGCCCG	20:G>A
1032034|F|0-39:A>G-39:A>G	TGCAGCAGAGGGCCGAACACCAGCGAGGGACACACGGTCACCACGCTTAGCCCG	39:A>G
1278088|F|0-36:G>A-36:G>A	TGCAGCCCCAACAGTACCCTCAACCTCGTGCTCCCCGCACTAAGAGATGTCCACGGCTTGCCCCCGAGA	36:G>A
1031592|F|0-18:A>T-18:A>T	TGCAGGTCCACTGATTTGAATCTTGACGACGAAACCGTCAATCTGAACAAGCTCAAGGCTTCCACCTCC	18:A>T
32022437|F|0-21:C>G-21:C>G	TGCAGCGACGCCGCGTACGCGCCCG	21:C>G
4537825|F|0-50:G>A-50:G>A	TGCAGTTCGCCATGGCGTGATGCGTCCGTACGCGATGCGCGGATACACGCGGATTCGGCTGCCG	50:G>A
1021565|F|0-63:T>C-63:T>C	TGCAGACGCTCATGGACAAGGGCTGCGACGTCAACGCGAGGGCGGAGGCTGGCGATGTCGCCGTCATCC	63:T>C
3935103|F|0-17:G>C-17:G>C	TGCAGGTTCTCGAACGGGCCCTTGCCG	17:G>C
1064471|F|0-39:C>T-39:C>T	TGCAGCCTCATCTTTGTCACAGGTCAACCTCGGAATCTCCCTCGCCCTACCCTACCAACAGATCGTCGG	39:C>T
4262823|F|0-34:T>C-34:T>C	TGCAGCAGCTTGCCGATCTTGTCCTTCACCTCGCTCAGCTCCCAGCCG	34:T>C
7908723|F|0-21:C>A-21:C>A	TGCAGAGGAGGTCGTACAACCCCCG	21:C>A
4398173|F|0-8:C>G-8:C>G	TGCAGCGACGCCGCGTAGGCGCCCG	8:C>G
5348311|F|0-8:G>A-8:G>A	TGCAGTTCGTCGGCGAAGGCTTCCGCGCCG	8:G>A
1251687|F|0-25:T>C-25:T>C	TGCAGAAGGTTGTGCTCAAGCTGGATGTGCACGACGACAGGCACAAGGCCAAGGCGCTAAAGGCCGTCT	25:T>C
1041714|F|0-21:G>T-21:G>T	TGCAGGCAAAGCTAGCATGGGGGAGAAAGAGTGGTACTTCTTCAGCATGCGCGACCG	21:G>T
4262746|F|0-11:G>A-11:G>A	TGCAGCCTTCGGCAGCGGCGGTGGCATCGGCAGCACGGTGCCCG	11:G>A
1695050|F|0-21:T>C-21:T>C	TGCAGCAACGGTTGTCGCCGCTGTCGACGTTCTTGCTGTCCAGGTACCAGCCG	21:T>C
1020654|F|0-21:A>G-21:A>G	TGCAGCTTGAGGTTCTGAGCAAGATACGCCACCCACACTTGCTGATGCTCCTGGGAGCCTGTCCGAGAT	21:A>G
1167502|F|0-8:C>G-8:C>G	TGCAGCACCCCCTCGAACGGGTACTTGACCAGCGACAGGTAGTGGAACCACAGCCAGTAGGCCG	8:C>G
5411812|F|0-7:G>A-7:G>A	TGCAGTCGTACGGGTACTGCCG	7:G>A
1862758|F|0-28:C>T-28:C>T	TGCAGATCCTGGGCCTGTTCCAGGTGCTCCCCCGCCTCGTCGTCTTCGACCTCGACTACACCCTGTGGC	28:C>T
3945777|F|0-15:T>C-15:T>C	TGCAGCGCCTGCTGGTCGCCGCGGACCG	15:T>C
5369649|F|0-17:C>A-17:C>A	TGCAGGTCGGCGTGGACCTGCTCCG	17:C>A
5332191|F|0-17:G>T-17:G>T	TGCAGGAACGGCGGGCCGCCGTGCGACCCG	17:G>T
5578233|F|0-15:T>C-15:T>C	TGCAGGATGCCCCACTGCGTGAAGACGTCCCG	15:T>C
1090765|F|0-5:C>T-5:C>T	TGCAGCCTGCGCGACTTCGCCGACGGCCTGCTGGACGTGACCTGCGGCAGCACGGACGTGACCGTCTCC	5:C>T
1065215|F|0-8:C>T-8:C>T	TGCAGGAACTTCTGGATGGGGAAGTTGGCCGCGTACGCGAAGATCTGCGGGATGAGGCCGTAGGCGAAC	8:C>T
984837|F|0-40:C>T-40:C>T	TGCAGGAGATGAAGCACATCTGCGGGATGACGGGCGACGGCGTGAACGACGCGCCG	40:C>T
1696543|F|0-47:C>G-47:C>G	TGCAGGAGAACATCGACTTCGTGTGCGGAGGGGGCATGGACTGTGGTCCGATACGGCCG	47:C>G
3945817|F|0-8:C>G-8:C>G	TGCAGTTGCTGAGGCAGACCTTGAAGTCCG	8:C>G
4995765|F|0-8:G>A-8:G>A	TGCAGCTTGAACTCGCCCTTCTCCCCGTTCCATCCG	8:G>A
1211582|F|0-23:G>A-23:G>A	TGCAGCGCGCACGCGTGCGCGTCGTCGTTGGCGGCGATGTTGGAGCGCACCAGGCAGCGGAGCTCCATC	23:G>A
4987034|F|0-11:G>C-11:G>C	TGCAGGCTCTCGTGGAGGCCCTGCCATGGCCAGGCCTTGTGGGCGGCGTCCTCGGCCGCCGCCTTCTTG	11:G>C
1862758|F|0-10:G>C-10:G>C	TGCAGATCCTGGGCCTGTTCCAGGTGCTCCCCCGCCTCGTCGTCTTCGACCTCGACTACACCCTGTGGC	10:G>C
2252024|F|0-25:C>G-25:C>G	TGCAGCGTCCGCAAGAACATGGTCCCCGTCCTCGACTACCTCGGCAAGATCGGCGTCCGCCGCGACGAG	25:C>G
2249364|F|0-14:G>C-14:G>C	TGCAGTGGCTGCTCGTCACCATGTCCGACAAGTCCATCCTTGTCATCGAGGACATCGACTGCTGCTGCG	14:G>C
1264981|F|0-14:C>G-14:C>G	TGCAGCGACTTGGACGTGAACTTGAGGTCGAAGATCTGCGCCATCAGCTTCTCCG	14:C>G
3935858|F|0-11:C>T-11:C>T	TGCAGGTCCCCCGCCGCGCCCGTCGTCACCTCCG	11:C>T
982151|F|0-22:C>T-22:C>T	TGCAGAAGCTCCTCGTCCTCGCCTCGCTCGCCGTGTGGTCCCGCCTCCCCGTCCCCTTCGCCG	22:C>T

982130|F|0-56:A>G-56:A>G	TGCAGCTCCTTGACGCTGGCCTCTGTTTCGTAGCCGCCGATGCTGGCCTCCTTGGCAACTTCGCGATGC	56:A>G
4262464|F|0-9:C>T-9:C>T	TGCAGCACACCTTCCCCATGCTAGCCTGAGCAGAGAGGTTGCTTCCCG	9:C>T
1069212|F|0-58:C>A-58:C>A	TGCAGCGCGTCGCCTACATCAACATCACCACCTACCCCTTCACCGCCATCTTCCTCATCTTCTACACCA	58:C>A
6048232|F|0-10:G>C-10:G>C	TGCAGGGCCTGCTCAAGGTGATAGCCG	10:G>C
3934409|F|0-7:C>G-7:C>G	TGCAGGGCGAGAAGGCGGCGGTGCGGATGGAGGCCGACCAGTTCCG	7:C>G
1252331|F|0-34:C>T-34:C>T	TGCAGATGATCGTGGAGAAGGAGATCGTGGGCGGCGCCGACCTGCGGGAGCTCCTCCACCG	34:C>T
1252331|F|0-22:G>A-22:G>A	TGCAGATGATCGTGGAGAAGGAGATCGTGGGCGGCGCCGACCTGCGGGAGCTCCTCCACCG	22:G>A
3957579|F|0-5:T>C-5:T>C	TGCAGTGCCTTGGTCCTCTCCTCCTCGAAGCTGGGCTGCGCTGCCACGGCGGACATGCCGTCCACCCTC	5:T>C
5411787|F|0-23:G>C-23:G>C	TGCAGCAAGGAGGTGGAGGAGGCGCTGTTCCG	23:G>C
1387546|F|0-12:A>G-12:A>G	TGCAGTGGAGGCAACTGTCGAGTTCCTGAACAAGGCGGTGAAGCCAGTGCTTGTCGCCG	12:A>G
4262426|F|0-16:C>T-16:C>T	TGCAGGCGTCCGTGAACGCGGACGGGGCCTTCACCAAGGTTTTCGCCG	16:C>T
1061244|F|0-7:C>T-7:C>T	TGCAGGACGAGTCCTGGAACGTCACCCGCTTCCTGCTGCTCTCCAAGCCGCCGTCGCCG	7:C>T
3951850|F|0-18:G>T-18:G>T	TGCAGCCAGATCATCATCGTGCCG	18:G>T
7491660|F|0-12:A>C-12:A>C	TGCAGCGGTTCAATGCCACCG	12:A>C
7349785|F|0-13:A>G-13:A>G	TGCAGGCGCTGGAAGCGGACCG	13:A>G
1021556|F|0-13:A>G-13:A>G	TGCAGATCGTGGGACGGCACGCGTTGCTCTTTGACGACGACGCGGCGGCGGAGGTCGTAAACTCCGAGA	13:A>G
3960893|F|0-6:T>C-6:T>C	TGCAGATGAGTACTACTTCAAGGCGCTGCCG	6:T>C
1075847|F|0-31:C>T-31:C>T	TGCAGGTAGTACTGGCGCAGCCCCTCGCCTCCGCCACGCGCGCCCTTGGCCGACTCGTCGCCG	31:C>T
3533508|F|0-7:C>A-7:C>A	TGCAGACCAAGATCGTTGAGGCGCTGCACCGCGAGGTGTCCG	7:C>A
1203512|F|0-28:G>C-28:G>C	TGCAGCTGGGCAAGATGAGCATCGCGTCGTGGGCCAGCAAGGAGGATCGCATCCGCGCCTCGCCGAGAT	28:G>C
7351471|F|0-5:C>T-5:C>T	TGCAGCCGCTTGTTGTGCCG	5:C>T
13880404|F|0-17:C>T-17:C>T	TGCAGTGCAAGGGGGAGCCCG	17:C>T
6030640|F|0-24:A>G-24:A>G	TGCAGCTGCTCCTCCTGCTCGGGCACCTTCCTCTCCACCGCCG	24:A>G
4260678|F|0-24:C>T-24:C>T	TGCAGGTGGTGAACGTGACGTACGCCCTGTGGACGTGGCACCG	24:C>T
998178|F|0-7:T>C-7:T>C	TGCAGGTTGGGAAGCAGCGGTTCAAGACCAAGGTGGTCAAGATGAACCTGAACCCG	7:T>C
4009837|F|0-10:C>T-10:C>T	TGCAGGGCCGCTGGTGCTATGGAACCACCG	10:C>T
1019952|F|0-64:T>C-64:T>C	TGCAGGAGCTGTCGTACCAGGAGAGCTTCCACGGGGCCTTCTCCGCCGCCAACGCCGCGGTGCGTTTGA	64:T>C
1031592|F|0-36:G>A-36:G>A	TGCAGGTCCACTGATTTGAATCTTGACGACGAAACCGTCAATCTGAACAAGCTCAAGGCTTCCACCTCC	36:G>A
4262170|F|0-33:C>T-33:C>T	TGCAGGTCGATGCGCGCGTAGAGGTACGTGTCCCTGGACCG	33:C>T
6043090|F|0-7:C>G-7:C>G	TGCAGCTCGAGGAGCACGACGCCCACCAGCGCTGGGACCTCGGGCTGAGCGACGCCGACGAGTGGGAGC	7:C>G
4984932|F|0-22:G>C-22:G>C	TGCAGATCACGCCGAACACCGGGAACATGAGCCACCG	22:G>C
1121130|F|0-10:C>T-10:C>T	TGCAGGGCATCTACGTCAAGGTACTCTTCCTGTGGATCCCCGTCGCCGAGGTCGACCGCGCCG	10:C>T
32023736|F|0-19:G>A-19:G>A	TGCAGCTGCTGGTCCACGTGGTAGGAATCGACGTCGAAGTTGGTGACCGCGGCGAGGCCCCG	19:G>A
22840069|F|0-8:C>G-8:C>G	TGCAGGAACGTGTTCCAATCGCGGCGCTTCTGCGACTCGTACCG	8:C>G
7174087|F|0-19:C>T-19:C>T	TGCAGTTGAGGCGGATCTCCCCCTGCGTGCCG	19:C>T
5358409|F|0-11:T>C-11:T>C	TGCAGGTAGTCTTGGTCGGGCCG	11:T>C
6046485|F|0-44:G>T-44:G>T	TGCAGTTTCTGTGGAGTTTGCCCCTTGCTTGTCTGGATATCTTTGCTCTCAGGAATGAAAAGGATCTTC	44:G>T
32016449|F|0-15:A>C-15:A>C	TGCAGCCGCTTCGTGAGGGTCTCTCCG	15:A>C
7911392|F|0-5:C>G-5:C>G	TGCAGCCCGAACCCGTACTCGCCG	5:C>G
6044699|F|0-22:C>T-22:C>T	TGCAGAAAGAGAGAGAGGAATACGAGGTTGTGATTGAGGATGGCAAGTTCATGTACAAGAACAGCAGGC	22:C>T
5360702|F|0-26:C>T-26:C>T	TGCAGATTAGTAATAGTTCGGTTAGCCTTTTCCTTCCACACCAACTCGTCACGTTCTCCCCAATAATTG	26:C>T
100535970|F|0-7:G>T-7:G>T	TGCAGCTGGTGAAGAACAGGAGATACGCAGCTAGGTAGGACTGTAGCGGAAAACGAATCTCACCCCCAA	7:G>T
100577772|F|0-8:T>C-8:T>C	TGCAGCAGTGCGATGGGGACGGGATCGTGTTCGCGTCGTCGGTGGTGACCG	8:T>C
1146832|F|0-20:T>C-20:T>C	TGCAGATGCATTAATTGGCGTTATTATGGCAGGCATGAGTACACACGTAGAGTGTATTTGTTGCCGCCT	20:T>C
32025334|F|0-8:G>A-8:G>A	TGCAGCCCGTCCTTGGAGCTCCTCGACGTTGCCTTCTTGGCTGCGCCG	8:G>A
14394184|F|0-15:G>A-15:G>A	TGCAGACGGAACCATGGCCG	15:G>A
1266085|F|0-56:G>C-56:G>C	TGCAGCCGCGGCGGCAGGTGGAGCGGCAGGAGCTTGCCCCTGTAGAACAGCTCGTCGGCCG	56:G>C
5372007|F|0-45:A>G-45:A>G	TGCAGGGCGACGAAGCAGCCGTCGAGCGTGACGAAGACGGCCGCCACGGGCCAAAACTTGCCG	45:A>G
100349983|F|0-10:C>T-10:C>T	TGCAGATCCGCGTCGACGAAGGCACCG	10:C>T
100525814|F|0-41:A>G-41:A>G	TGCAGCTCGACGCTGAAGGCAGCAACAGCAACAACTACAGGACCATGCAGCAGATCACCTGGGCTGCCG	41:A>G
100540624|F|0-8:G>C-8:G>C	TGCAGGAGGTGGAGGACGAGCAGGCCAAGGGGTACGCGCCGTCGCTGCGGCACGCCG	8:G>C
5365892|F|0-31:G>C-31:G>C	TGCAGGTGGCGCTCAACCACAGCAAACTCGTGGACCGCCAGCTCAGGTCGCTCTACGCCCCGCCACCGA	31:G>C
13881102|F|0-31:G>C-31:G>C	TGCAGCAGGTCGTCGCAGAGCGCGGCGTCCAGGTCGCCGCATGCCCCGCCACCGCCGCCG	31:G>C
5008935|F|0-15:G>A-15:G>A	TGCAGCTTCCGCCTAGCACCATCCCTCCCTCCCTGGCGCACCCTCTCGCCAGCAGCTTACTAATGGCGG	15:G>A
1065215|F|0-14:G>C-14:G>C	TGCAGGAACTTCTGGATGGGGAAGTTGGCCGCGTACGCGAAGATCTGCGGGATGAGGCCGTAGGCGAAC	14:G>C
7915260|F|0-23:C>G-23:C>G	TGCAGCGCGTACTCGAGGTTCCACAGCACGTCGCCCATGGAGAGGCGGTCGCTGCCGAACTCGCAGAGG	23:C>G
4009329|F|0-38:T>G-38:T>G	TGCAGGCCGTGCGCCAGGACGTCGAGGCCGTAGAGGTCTGCGGCGCGCGGGCTGGCGATGGCGGCCGTG	38:T>G
4544061|F|0-5:C>T-5:C>T	TGCAGCAGGGTGTTGCGGGTGAAGACGTCGGGTGCGGCGCCG	5:C>T
1076645|F|0-19:C>G-19:C>G	TGCAGCGTGACCTCCTCGCCAACTTCCTCAGCTTCCAGGTCTTCTCCTTCCTCCCGCCGCTCACCAAGC	19:C>G

<table>
<tr><td>4262498|F|0-61:C>G-61:C>G</td><td>TGCAGTTCCTGCTCAACCCCAAGTTCGTGGAGGCGGAGTGGTTCCTCCACGCCGCGCTGGGCCGCGGCA</td><td>61:C>G</td></tr>
<tr><td>3028163|F|0-10:C>G-10:C>G</td><td>TGCAGCGGGACAAGTCCGAGGCCATGATGGAGGCGCGCCAGTTCCG</td><td>10:C>G</td></tr>
<tr><td>4397991|F|0-5:C>G-5:C>G</td><td>TGCAGCACGATAGACTTGGTGCTGAGGCCCG</td><td>5:C>G</td></tr>
<tr><td>1089820|F|0-20:G>A-20:G>A</td><td>TGCAGTGTTGTTATTGCCAGGCTCTCCTTGCCATGTGATCGTCCACCCCCCACATTGGTTGCCCAGGTT</td><td>20:G>A</td></tr>
<tr><td>1021430|F|0-29:G>A-29:G>A</td><td>TGCAGCCTCTTCGGCAAGCAGGCGCCCCAGCCGCAGCACCGCGCGGGCGGCGGCAGCGCCAAGGGGAAG</td><td>29:G>A</td></tr>
<tr><td>999506|F|0-24:A>G-24:A>G</td><td>TGCAGCGCGGCCTCGGGCTGTCGGACAAGCAGGTGGAGCCGTCGCGCATGGCGCTGCACCG</td><td>24:A>G</td></tr>
<tr><td>1104443|F|0-26:C>G-26:C>G</td><td>TGCAGGATCATCTGTTCCAGGGTGACCGCGGCCGCCGCATGCGGGCCTCCACCG</td><td>26:C>G</td></tr>
<tr><td>12420121|F|0-10:C>T-10:C>T</td><td>TGCAGCAGTCCCGTGGTTTCGGCAGTGAGTTCCG</td><td>10:C>T</td></tr>
<tr><td>1863003|F|0-16:G>C-16:G>C</td><td>TGCAGACGGGCTCGGCGGTGTCGACGGGCCTGGCTCTCTCTCTGGAGGACCG</td><td>16:G>C</td></tr>
<tr><td>5358176|F|0-22:T>C-22:T>C</td><td>TGCAGGATCCTGGTGGACGGCGTGCCGATCCG</td><td>22:T>C</td></tr>
<tr><td>1050727|F|0-26:C>T-26:C>T</td><td>TGCAGGAACGTCATCTTCTCCAGGAACCCGAGCTCCGCCGTGGCCAGCGTGAGCACGTAGACGAAGAGC</td><td>26:C>T</td></tr>
<tr><td>3023973|F|0-16:G>A-16:G>A</td><td>TGCAGAAGCTGTATCTGTTCAACAACATGCTCCACGGCCCG</td><td>16:G>A</td></tr>
<tr><td>4394806|F|0-19:A>G-19:A>G</td><td>TGCAGACGAGCGGAAGCTCATCCG</td><td>19:A>G</td></tr>
<tr><td>1051316|F|0-27:C>T-27:C>T</td><td>TGCAGCGCCATCATCATGGGCACCTGCCTGGCCCAGTTCCAGACCTTCTCCATCCAGCAGGGCTCCACC</td><td>27:C>T</td></tr>
<tr><td>7172589|F|0-12:G>C-12:G>C</td><td>TGCAGCGTCGTCGCCGACGTCTCGCTGCCG</td><td>12:G>C</td></tr>
<tr><td>5366914|F|0-42:T>C-42:T>C</td><td>TGCAGCAGCTTCGCCCCGCTCGGCGCGCCCCCCACCATCGCCTCGCCG</td><td>42:T>C</td></tr>
<tr><td>4262238|F|0-24:G>A-24:G>A</td><td>TGCAGACGTTCGTACGTACCGTCGGTGCATGCCTGTGACCG</td><td>24:G>A</td></tr>
<tr><td>4982009|F|0-6:G>A-6:G>A</td><td>TGCAGCGACGCCACCAAGGAGTTCGCCG</td><td>6:G>A</td></tr>
<tr><td>1345106|F|0-60:T>G-60:T>G</td><td>TGCAGAAAACTATTAAGTCATCCAATTCATGCCGCATTGCAAGATTACTCTACCACTCTATAAATGGTA</td><td>60:T>G</td></tr>
<tr><td>5369628|F|0-32:C>A-32:C>A</td><td>TGCAGGTCATGGCGTGCCGCCGCGTCGTGGCACCCG</td><td>32:C>A</td></tr>
<tr><td>6041547|F|0-15:G>C-15:G>C</td><td>TGCAGCTCCTGGGAGGTCCCAGCCTGCTCCGCCGCCGCGTCCG</td><td>15:G>C</td></tr>
<tr><td>3959524|F|0-26:C>G-26:C>G</td><td>TGCAGGTTCGAGGGGTACCAGGACACCCTGTACGTGCACGCCAACCG</td><td>26:C>G</td></tr>
<tr><td>1021956|F|0-47:G>T-47:G>T</td><td>TGCAGCTGCTCTTCCAACTGCCGCTTGTTCCCGCGGCGCCGTGACCAGAGGGTGCTCCCCTCGGGCTTC</td><td>47:G>T</td></tr>
<tr><td>1061595|F|0-14:T>G-14:T>G</td><td>TGCAGGTAGGGTGCTCTTAGGCTGAAGACTTGTCGCCGCCAAAGGCATTCTCCAGAACACCG</td><td>14:T>G</td></tr>
<tr><td>1129678|F|0-68:C>T-68:C>T</td><td>TGCAGCACCCTGGCACGCAGCACACGGTACCTCCTCTCTGACACGCCCCGCAGGATGGCCTTCAGCTCC</td><td>68:C>T</td></tr>
<tr><td>7172180|F|0-22:G>A-22:G>A</td><td>TGCAGGAGATGAGCACCCGCGTGGCCGACCG</td><td>22:G>A</td></tr>
<tr><td>3026721|F|0-25:G>A-25:G>A</td><td>TGCAGTTCTTGATCAGCTGGATGTCGCGGTTCCCGCAGTACCG</td><td>25:G>A</td></tr>
<tr><td>1050158|F|0-60:A>T-60:A>T</td><td>TGCAGAGGCTCTCAGGACGAGCAAGCGAGAGATGTCAGTCGCCACAAGAGGTGAGAGCAAAATCTCCTC</td><td>60:A>T</td></tr>
<tr><td>3951139|F|0-5:T>C-5:T>C</td><td>TGCAGTTCCTCCTTGTCCCGCTCCTTCTCCCAGCTCGCGAACCG</td><td>5:T>C</td></tr>
<tr><td>3940847|F|0-23:C>G-23:C>G</td><td>TGCAGGTAGTCGCCGACGTTGACCACCAGCGCCCCCG</td><td>23:C>G</td></tr>
<tr><td>1127120|F|0-43:T>G-43:T>G</td><td>TGCAGGTGCTTCCCTGGTTGGTGGCAGTGCTGCTGTGCCCCTGTTCGTCCACGACGGCGCTAGCCGAGA</td><td>43:T>G</td></tr>
<tr><td>995875|F|0-43:C>G-43:C>G</td><td>TGCAGCGCTCCTGGATCGTGCTCTTCCTCTTCGCGCTGGCGCTCACGCCCACCTACGTCTTCACCGAGC</td><td>43:C>G</td></tr>
<tr><td>3946376|F|0-14:G>C-14:G>C</td><td>TGCAGCGTGACGCTGCCCTGGCACGGCCCG</td><td>14:G>C</td></tr>
<tr><td>7349785|F|0-15:C>A-15:C>A</td><td>TGCAGGCGCTGGAAGCGGACCG</td><td>15:C>A</td></tr>
<tr><td>993664|F|0-10:C>T-10:C>T</td><td>TGCAGCCTGTCCAGCACGTTCTCCACGCCCACGTCCTCCCTCAGGCTCTCCTCGCAGGCCTTCTTCAGG</td><td>10:C>T</td></tr>
<tr><td>1024669|F|0-28:C>G-28:C>G</td><td>TGCAGATGGGGCAGGTCGGCCTCGTCGTCGTCTCGTCCCGCGAGGCGGCCAGGGAGGTCATGAAGGTGC</td><td>28:C>G</td></tr>
<tr><td>982130|F|0-23:T>C-23:T>C</td><td>TGCAGCTCCTTGACGCTGGCCTCTGTTTCGTAGCCGCCGATGCTGGCCTCCTTGGCAACTTCGCGATGC</td><td>23:T>C</td></tr>
<tr><td>1050727|F|0-29:G>C-29:G>C</td><td>TGCAGGAACGTCATCTTCTCCAGGAACCCGAGCTCCGCCGTGGCCAGCGTGAGCACGTAGACGAAGAGC</td><td>29:G>C</td></tr>
<tr><td>3945135|F|0-8:G>A-8:G>A</td><td>TGCAGGAAGGAGATGGCCCCCACGCAGCCGTACACCG</td><td>8:G>A</td></tr>
<tr><td>3957784|F|0-31:G>A-31:G>A</td><td>TGCAGAGCACGTCCATGCCGGCCATCTCCTCGATGGCCGTCATGCGCTTGGTGATGGCGCCCTGCTTCG</td><td>31:G>A</td></tr>
<tr><td>7353729|F|0-11:C>G-11:C>G</td><td>TGCAGCGGCGCCTACGCGACGCCG</td><td>11:C>G</td></tr>
<tr><td>3947352|F|0-22:C>T-22:C>T</td><td>TGCAGTGATGCGCATGCGTTGGCCGCCG</td><td>22:C>T</td></tr>
<tr><td>1075269|F|0-40:C>T-40:C>T</td><td>TGCAGGAGCACACCGCCGTCTTCTTCAGGGTGTTCAGGCACCATGACAAGTACAGAGTGCTCATGTGCA</td><td>40:C>T</td></tr>
<tr><td>6041176|F|0-9:T>C-9:T>C</td><td>TGCAGACGCTGACCGTGCCCAAGGTGCCGCCG</td><td>9:T>C</td></tr>
<tr><td>1696543|F|0-55:G>A-55:G>A</td><td>TGCAGGAGAACATCGACTTCGTGTGCGGAGGGGGCATGGACTGTGGTCCGATACGGCCG</td><td>55:G>A</td></tr>
<tr><td>3027466|F|0-5:C>T-5:C>T</td><td>TGCAGCTGATCCTGCTCCTTGGGTGGTTCGGGAAGCTCGCCG</td><td>5:C>T</td></tr>
<tr><td>4262646|F|0-64:C>G-64:C>G</td><td>TGCAGACGGCGGCGGCCTGGCGCTCGGAGTAGCTCCCCTTGGCGGTGATGCGGTCGAAGAGCTCCCCGC</td><td>64:C>G</td></tr>
<tr><td>3941706|F|0-12:G>C-12:G>C</td><td>TGCAGCGCCCAGGCGTACTGGATGCCGCCG</td><td>12:G>C</td></tr>
<tr><td>12470493|F|0-37:G>T-37:G>T</td><td>TGCAGGTCGGCGCGCGCCTTGGGGGAGAGGTTGAAGTGCGGGGACCCG</td><td>37:G>T</td></tr>
<tr><td>6040177|F|0-17:G>C-17:G>C</td><td>TGCAGCCCGATGAACGCGAGAAACAGCCCGATCCCCGCGGACGACGCGATCCGCACGGGCTTGGGGATC</td><td>17:G>C</td></tr>
<tr><td>1003268|F|0-68:G>C-68:G>C</td><td>TGCAGGTAGAAGCGGGGCTTGTGGGGGCGGAGCACCAGGTAGACGATGAGGATGATGAAGAGGGCGAGG</td><td>68:G>C</td></tr>
<tr><td>999506|F|0-25:C>G-25:C>G</td><td>TGCAGCGCGGCCTCGGGCTGTCGGACAAGCAGGTGGAGCCGTCGCGCATGGCGCTGCACCG</td><td>25:C>G</td></tr>
<tr><td>6043666|F|0-10:T>C-10:T>C</td><td>TGCAGAGGCGTAGCAGCCACTTCGACGCGCGCGAGGTGTTCCCCGAGCCG</td><td>10:T>C</td></tr>
<tr><td>1067333|F|0-18:G>A-18:G>A</td><td>TGCAGAGAGGGAAAAGCAGGGGAGCAGGTCGCTGGGCTGCCACGCGTCGTCCACCG</td><td>18:G>A</td></tr>
<tr><td>1121130|F|0-52:C>T-52:C>T</td><td>TGCAGGGCATCTACGTCAAGGTACTCTTCCTGTGGATCCCCGTCGCCGAGGTCGACCGCGCCG</td><td>52:C>T</td></tr>
<tr><td>5971365|F|0-11:G>A-11:G>A</td><td>TGCAGGGAAGCGCCAACCAGGACGAGCAGCGGCAGTACTCCG</td><td>11:G>A</td></tr>
<tr><td>4262721|F|0-36:T>C-36:T>C</td><td>TGCAGGTATAAATACACAAGCGCGCATCACCTACCGTTGATTTGCCG</td><td>36:T>C</td></tr>
<tr><td>995666|F|0-31:A>G-31:A>G</td><td>TGCAGGGGTCGTGCGGGCTGAGCAAGGAGCGAGCACGACGCAGAGACGGCCTTCCG</td><td>31:A>G</td></tr>
<tr><td>3026657|F|0-5:T>C-5:T>C</td><td>TGCAGTAGGATGAGGAATGGCTCCATCAGGAACACCGCCG</td><td>5:T>C</td></tr>
</table>

<table>
<tr><td>4396798|F|0-23:C>T-23:C>T</td><td>TGCAGCGCCCCTGCGCCCAGCGCCGCCGCTCCG</td><td>23:C>T</td></tr>
<tr><td>1069052|F|0-11:C>G-11:C>G</td><td>TGCAGCACCGACAGCTGCACGACGATGTCGCGCGCCCACTTGCCGTACATGGCGTCCAGCAGCGGCACG</td><td>11:C>G</td></tr>
<tr><td>1091043|F|0-16:C>T-16:C>T</td><td>TGCAGACGGAGCACCACGCGGCGGCCATGCACAACGCCTACGCCGCAGCGGACCCCTACTGGCACCTCA</td><td>16:C>T</td></tr>
<tr><td>1862758|F|0-43:C>A-43:C>A</td><td>TGCAGATCCTGGGCCTGTTCCAGGTGCTCCCCCGCCTCGTCGTCTTCGACCTCGACTACACCCTGTGGC</td><td>43:C>A</td></tr>
<tr><td>1090179|F|0-63:G>C-63:G>C</td><td>TGCAGAGAAGACTGAGGTAGTGTCTACCTTTCGGAGCGACGGGAGGTGGTCCCCCCACACCACGAGGTC</td><td>63:G>C</td></tr>
<tr><td>2248101|F|0-58:G>C-58:G>C</td><td>TGCAGATCCTTGGCCCCCTCTGCTTCGACATAGGCCACACTGCGCTGACAATGGTGGCGTCGCACGTTG</td><td>58:G>C</td></tr>
<tr><td>1072922|F|0-43:C>A-43:C>A</td><td>TGCAGGACGAGGAACGCGACCACCGCGGCCACGAGCGCGAAGACATAGATTGAGGTGCGCAGCAGCCGA</td><td>43:C>A</td></tr>
<tr><td>3942640|F|0-17:G>A-17:G>A</td><td>TGCAGTCCCTGGTCCTCGTCGACAACAACCTCACCG</td><td>17:G>A</td></tr>
<tr><td>3954902|F|0-8:G>C-8:G>C</td><td>TGCAGGTAGGACATGAGCTCGTGCGGCTCGATCGCCG</td><td>8:G>C</td></tr>
<tr><td>1039732|F|0-55:C>T-55:C>T</td><td>TGCAGGTGTTTGTGAGAGATTTCGTGCCCAGCGCCATGGCGTTTCTGATGAATGGCGAGCCG</td><td>55:C>T</td></tr>
<tr><td>7166718|F|0-50:A>G-50:A>G</td><td>TGCAGCAGCTCGACGGCGAGCACGTCGGACGGCGCGGTGGCCATGGAGATAACGTAGGCCCCGAAGCTG</td><td>50:A>G</td></tr>
<tr><td>1090847|F|0-21:C>G-21:C>G</td><td>TGCAGCACGTCCTCCAGACGGCGCTCGTGCTCTGGCTCGTCCTCAACGCCACCG</td><td>21:C>G</td></tr>
<tr><td>7166490|F|0-38:C>T-38:C>T</td><td>TGCAGGAGGAAGGTGATGAAGGCCATGAACCTCAAGGGCGTGCTGCGGACGGAGATCCACCCGTCGCTC</td><td>38:C>T</td></tr>
<tr><td>1694734|F|0-52:C>T-52:C>T</td><td>TGCAGTGCGTGGTGGCGCACCTGTGGCGGACCATGACGATGGCGCGCGGGCTCGACGGCGGGGAGAGCA</td><td>52:C>T</td></tr>
<tr><td>3944978|F|0-17:G>C-17:G>C</td><td>TGCAGCGTCAGGAACTCGGCCG</td><td>17:G>C</td></tr>
<tr><td>1024408|F|0-15:T>C-15:T>C</td><td>TGCAGCGCGAGGCGGTGGAAGGCCTTCTTGACCTCGCCCCTGGAGGCGCCCG</td><td>15:T>C</td></tr>
<tr><td>7172280|F|0-9:T>C-9:T>C</td><td>TGCAGTTGGTCCTGATGGTGCCCTGGGTGCCG</td><td>9:T>C</td></tr>
<tr><td>3944031|F|0-24:T>A-24:T>A</td><td>TGCAGCATCTGCCTGGAGACGGCGTCGGAGCCG</td><td>24:T>A</td></tr>
<tr><td>1066648|F|0-29:A>G-29:A>G</td><td>TGCAGACGTTCATCTGCCTGACCATCATCACCTTCGTCCCTGTGTACGACCG</td><td>29:A>G</td></tr>
<tr><td>1078999|F|0-33:C>A-33:C>A</td><td>TGCAGGGCCTGGGCGCGCCAGCCGACGCCTGCGCCTTGAGCCTGGAGATGAACTCCCG</td><td>33:C>A</td></tr>
<tr><td>5411992|F|0-8:G>T-8:G>T</td><td>TGCAGGTGGCGTGCGCCGTCGCCCG</td><td>8:G>T</td></tr>
<tr><td>7167041|F|0-10:C>T-10:C>T</td><td>TGCAGGAGGCCGTCCCCTCTGGCGAGGAGGACGCCGAGCACGACGAGCTGGAAGATGCTCTGGCCGAGA</td><td>10:C>T</td></tr>
<tr><td>1089186|F|0-26:C>G-26:C>G</td><td>TGCAGGAACGCGAACTCGCGCTCCACCTCGTCGGGCAGCGACGCGTGCGCGGCCCACCCG</td><td>26:C>G</td></tr>
<tr><td>1069212|F|0-13:C>T-13:C>T</td><td>TGCAGCGCGTCGCCTACATCAACATCACCACCTACCCGTTCACCGCCATCTTCCTCATCTTCTACACCA</td><td>13:C>T</td></tr>
<tr><td>1342351|F|0-49:A>G-49:A>G</td><td>TGCAGGCGCGGCAGGCGGCGTGCGCGTCGTCCCCGCAGGCCGCGCAGGGAGGATGGGCCCCCG</td><td>49:A>G</td></tr>
<tr><td>4544061|F|0-29:G>T-29:G>T</td><td>TGCAGCAGGGTGTTGCGGGTGAAGACGTCGGGTGCGGCGCCG</td><td>29:G>T</td></tr>
<tr><td>5331938|F|0-6:G>A-6:G>A</td><td>TGCAGCGACACGAAGACGAGCATCCG</td><td>6:G>A</td></tr>
<tr><td>6045191|F|0-31:G>C-31:G>C</td><td>TGCAGTTCCTCCTGATCTCGCCGTTGGCAGCGGTCTTGAGCTCGATGGCAGCCATCTTGACCATCGCCT</td><td>31:G>C</td></tr>
<tr><td>4008942|F|0-19:G>A-19:G>A</td><td>TGCAGGCGTCCTTCCTGGAGGTGTTCGTCCGCGGCCG</td><td>19:G>A</td></tr>
<tr><td>1048955|F|0-40:C>A-40:C>A</td><td>TGCAGCCTCTTCAGCTGGGCCTTCAGGGCCAACCACGGGCCTCCCTACCTCATGCACTCCCAGCCACTC</td><td>40:C>A</td></tr>
<tr><td>1229132|F|0-49:C>A-49:C>A</td><td>TGCAGGTGGACGGGCAGCTCCTCACCGACATCCCGGCGGGGACTTTCGACGTCCG</td><td>49:C>A</td></tr>
<tr><td>100481144|F|0-19:C>T-19:C>T</td><td>TGCAGGGGCTCCAGGGGTACGTGCAGAACGTCAGGTTCCTCGCCG</td><td>19:C>T</td></tr>
<tr><td>2247634|F|0-14:G>C-14:G>C</td><td>TGCAGGAACAGCATGGGCTCCGCCGACGGGCTCACCGCCTGCCGCAGCATCGACCCCATGATCAGCGAC</td><td>14:G>C</td></tr>
<tr><td>1068553|F|0-61:C>A-61:C>A</td><td>TGCAGGGAGAGCGCCCTGGTGATGTTGCGCAGCACGGCCTGCTCGTGCTGCTGCTCCTGCACGCTCGGG</td><td>61:C>A</td></tr>
<tr><td>1053137|F|0-8:T>C-8:T>C</td><td>TGCAGATCTCCGACCATCTTCATCAGGATCTCCCCCACATCAGACTGCTTCGCCGTGAGGGACTCGTGG</td><td>8:T>C</td></tr>
<tr><td>978496|F|0-58:T>G-58:T>G</td><td>TGCAGCACACCGAGTCGGGGGAGGTGACGGTGGAGATGGAATGGATGGCTCTAAACATTTAGAGAATCC</td><td>58:T>G</td></tr>
<tr><td>3954596|F|0-28:G>A-28:G>A</td><td>TGCAGCTCGGAACACGCTGCTCGAAGGTGACGGGATGTCCG</td><td>28:G>A</td></tr>
<tr><td>978496|F|0-5:C>A-5:C>A</td><td>TGCAGCACACCGAGTCGGGGGAGGTGACGGTGGAGATGGAATGGATGGCTCTAAACATTTAGAGAATCC</td><td>5:C>A</td></tr>
<tr><td>4262823|F|0-38:C>T-38:C>T</td><td>TGCAGCAGCTTGCCGATCTTGTCCTTCACCTCGCTCAGCTCCCAGCCG</td><td>38:C>T</td></tr>
<tr><td>5365560|F|0-28:C>T-28:C>T</td><td>TGCAGCTGCTCGACCTGTCGTCCAACTCCCTCACGGGCGCCATCCCGCCTGAGCTCGGCAAGCTCCAGG</td><td>28:C>T</td></tr>
<tr><td>1089106|F|0-47:C>A-47:C>A</td><td>TGCAGGAAGTGCGCGGCCATCTGCCACGCGTCCCCCTGCGCCGCCCGCACCAGGGCCAGGTAGAAGTAG</td><td>47:C>A</td></tr>
<tr><td>1021661|F|0-10:G>A-10:G>A</td><td>TGCAGATCCTGCGCATGACGGGCTTCGCGCGGGACTTCGGCCAGTGCGCCGACGTGGCCGCCGTGCTGG</td><td>10:G>A</td></tr>
<tr><td>3941582|F|0-12:G>T-12:G>T</td><td>TGCAGGAAATCCGCCATCGCTCGCCCG</td><td>12:G>T</td></tr>
<tr><td>1055737|F|0-50:A>C-50:A>C</td><td>TGCAGCTTCTGCCCCTTCTCCTCCGCAGCCTCCTTCGCTTTGCTAAGCTCACTACGTAGTTTGTCCGAG</td><td>50:A>C</td></tr>
<tr><td>6037485|F|0-5:G>A-5:G>A</td><td>TGCAGGGAGGGCTCTGTCGACCCG</td><td>5:G>A</td></tr>
<tr><td>3951936|F|0-18:G>A-18:G>A</td><td>TGCAGGATAGTGGTTAACGTACCG</td><td>18:G>A</td></tr>
<tr><td>3951601|F|0-20:C>T-20:C>T</td><td>TGCAGGAACGCCCTCAGGGCCTCCG</td><td>20:C>T</td></tr>
<tr><td>1220141|F|0-46:C>T-46:C>T</td><td>TGCAGCGGCAGGAAGGGCCGTTGCTCATCCTGGGGTTTCTCATGGGCATCAACGCCG</td><td>46:C>T</td></tr>
<tr><td>993594|F|0-5:C>G-5:C>G</td><td>TGCAGCTCGGCTCTACTCTCCTCCACAGGCTCTGCTCATCTTGAGGAGCGCCGAGAGCACGCCGTTCAT</td><td>5:C>G</td></tr>
<tr><td>1055670|F|0-47:C>T-47:C>T</td><td>TGCAGGAACTTCTGGATGGGGAAGTTGAGCGCGTAGGCGAAGAGCTGCGGGATCATGTAGAGCGAGAAC</td><td>47:C>T</td></tr>
<tr><td>1061244|F|0-19:C>T-19:C>T</td><td>TGCAGGACGAGTCCTGGAACGTCACCCGCTTCCTGCTGCTCTCCAAGCCGCCGTCGCCG</td><td>19:C>T</td></tr>
<tr><td>2244447|F|0-32:G>C-32:G>C</td><td>TGCAGGATCATGGCGAGCCCCATTTTAGCCTCGAGCAGCGCGAAGTTCTGGCCGACACAGATCCGCGGG</td><td>32:G>C</td></tr>
<tr><td>1080223|F|0-39:A>G-39:A>G</td><td>TGCAGATCGCGCGCGGCATGGAGCACCTCCACTCCAGGAAGATCTACCACGGCGAGCTCAACCCGTCCA</td><td>39:A>G</td></tr>
<tr><td>4262749|F|0-29:G>C-29:G>C</td><td>TGCAGCCGCCGCCCGCCCTCGTCCTCCGCGGCGATCAGCCG</td><td>29:G>C</td></tr>
<tr><td>4993593|F|0-24:C>G-24:C>G</td><td>TGCAGGCTCTGGATGGCGCGGACACGCGCCTCCG</td><td>24:C>G</td></tr>
<tr><td>1023858|F|0-16:G>C-16:G>C</td><td>TGCAGCGGAGAGGAGGGAGGCGAGAGCGTGGAACGCAGGGAAGCAGTGTCGGAAGCCCTTCTGGCGGCC</td><td>16:G>C</td></tr>
<tr><td>7353650|F|0-5:G>C-5:G>C</td><td>TGCAGGCCGTCGCGTTCGTCCG</td><td>5:G>C</td></tr>
<tr><td>1089867|F|0-44:G>A-44:G>A</td><td>TGCAGCCCGATGCCGCCCACGGGGGCACCCTTCTCCATGAGCGCGTTGACCTGCGCGATGTACTTCTCC</td><td>44:G>A</td></tr>
<tr><td>1048955|F|0-35:C>T-35:C>T</td><td>TGCAGCCTCTTCAGCTGGGCCTTCAGGGCCAACCACGGGCCTCCCTACCTCATGCACTCCCAGCCACTC</td><td>35:C>T</td></tr>
</table>

3385251|F|0-14:C>T-14:C>T	TGCAGCAGCTTCGCCCCGCTCGGCGCGCCCCCCACCATCGTCTCGCCG	14:C>T
1037142|F|0-49:A>G-49:A>G	TGCAGCCTCTTCGCGCCCATCCCGTCCTTCACCGACATGATCATCCGCAAGTACAAGATGCGCAGCGAC	49:A>G
1264981|F|0-38:C>T-38:C>T	TGCAGCGACTTGGACGTGAACTTGAGGTCGAAGATCTGCGCCATCAGCTTCTCCG	38:C>T
1694966|F|0-5:C>A-5:C>A	TGCAGCGGGTCGTCGAAGACGCCGACGGTGAGCACGGTGCACGGGTCGTACACGGGCCACGTGTACTGC	5:C>A
1053742|F|0-61:C>T-61:C>T	TGCAGAACAGCTCTTCAAACCCATCAGATGTTTTCTGGCAGAGCTTTGACTACCCCACGGACACATTTT	61:C>T
4262857|F|0-9:G>A-9:G>A	TGCAGATTCGCCGTCGCCATCGCGGACGCACCAACTGCAACCG	9:G>A
1003268|F|0-12:C>G-12:C>G	TGCAGGTAGAAGCGGGGCTTGTGGGGGCGGAGCACCAGGTAGACGATGAGGATGATGAAGAGGGCGAGG	12:C>G
3023097|F|0-17:C>G-17:C>G	TGCAGCTCCACGCGCTTCAGCCTCCACGATGCCTCTTTCCG	17:C>G
7342807|F|0-13:C>G-13:C>G	TGCAGGAGGCCCTCAAGAAGCTCCG	13:C>G
7923024|F|0-5:C>A-5:C>A	TGCAGCGGCACGTCGCCTTCTTCGACCG	5:C>A
3947352|F|0-8:T>C-8:T>C	TGCAGTGATGCGCATGCGTTGGCCGCCG	8:T>C
4262857|F|0-35:T>A-35:T>A	TGCAGATTCGCCGTCACCATCGCGGACGCACCAACTGCAACCG	35:T>A
1130186|F|0-28:G>C-28:G>C	TGCAGGCGCAGACGCAGACGCACCCGCCGTCGCTGAGGTACCGCGGCGTCGTCGACTGCTTCCG	28:G>C
6045666|F|0-35:C>G-35:C>G	TGCAGGAGGTGGAGGTCGGGGGACTTGGCGCGGAGCGCGCCG	35:C>G
1044289|F|0-14:G>A-14:G>A	TGCAGCTTCGGGTCGGCCCAGGAGACCCCGCAGCTCAACCTGGCGGGCCACTGCGACCCCTACTCCAAC	14:G>A
985043|F|0-41:T>A-41:T>A	TGCAGTTTGTTGTCTCGGAGAAGAGGAGAAATTGCTGGTCCTGTGATATCTGCACGCCGTTTGGGAAGA	41:T>A
999506|F|0-13:C>T-13:C>T	TGCAGCGCGGCCTCGGGCTGTCGGACAAGCAGGTGGAGCCGTCGCGCATGGCGCTGCACCG	13:C>T
1051808|F|0-14:C>A-14:C>A	TGCAGGAACGGCACCATCCTGCCCAAGACCATGGACGAGGCGCAGTCCAAGTCGGCGTTCCAGATGGAG	14:C>A
32024203|F|0-23:C>G-23:C>G	TGCAGGGCGATGGGCGACCAGCTCGAGGGCGACGAGGGGCAGCACATGAAGTACCG	23:C>G
1041369|F|0-40:C>G-40:C>G	TGCAGGAGCACTCCAGCTTCATGGCGCCGCCCCCCTCGGACAGCTCCACCATGCAGATCCG	40:C>G
3025654|F|0-29:C>T-29:C>T	TGCAGCGACTTCATCCGCTCCGCGATCCTCTCCCGCCG	29:C>T
3026225|F|0-20:A>C-20:A>C	TGCAGGATGGGGGCAGCGAGAGGGCCATGGGCTTTCCTCCG	20:A>C
1037648|F|0-47:C>G-47:C>G	TGCAGACCATCAACGGCCCCAACTCCGACTTCCCCGTCTACTCGTCGCAGCTCAAGGAGCTCAAGGGCA	47:C>G
3945784|F|0-18:C>T-18:C>T	TGCAGTGACGACAACGAGCGCCTTGAATGCCGCCG	18:C>T
6048211|F|0-28:A>G-28:A>G	TGCAGGCAGGCTACAGGCTGGTCATGGCAACCCAGTTCAGGAGGTTCACGTACCG	28:A>G
1061496|F|0-22:T>C-22:T>C	TGCAGATGAAGGAGTTTTATTCTGGTGAAAAGATACTGCCCAAGAAACCCGTGTACTACAAAACAGATG	22:T>C
5324578|F|0-40:C>A-40:C>A	TGCAGCAGCATCCGTGAGTTGGTGATGTATCGCCCCTCAGCCTCCTCGACGACGGTGTGGACTCTGAGG	40:C>A
1666353|F|0-16:C>G-16:C>G	TGCAGGTGAGGTAAGTCTCCGAGGGCAGGTAAGGGTGGAAGTTCGAGGCTGTCCATTCACCG	16:C>G
7173535|F|0-15:G>T-15:G>T	TGCAGTGAAGCGGAGGCTCAGCCG	15:G>T
7156710|F|0-8:C>A-8:C>A	TGCAGTGGCTCATCGGCTTCGCCGTCTTCTGGATGCCCG	8:C>A
3934562|F|0-22:G>A-22:G>A	TGCAGAAAGCGGCAGCGCGTGCGTGGTCACCG	22:G>A
1230151|F|0-11:T>C-11:T>C	TGCAGCCGCGGTCGGCGAGCGGCGGGAGCGGGGCGGTGACGCGGCGCGACTCTCGTTTGTTCCG	11:T>C
985215|F|0-9:A>C-9:A>C	TGCAGCTTCAAGGGACACATCCGCGTCTCTGCCACGGAGGCGCTGGAGTGCTATTTCAAACTCCTTTTG	9:A>C
4262072|F|0-12:G>C-12:G>C	TGCAGATCTTGCGCTGATGAGCGGCGCGCAGCTCTTCATCCG	12:G>C
1001684|F|0-17:C>A-17:C>A	TGCAGCACCGAGAGCTGCACGATGATGTCGCGCGCCCACTTGCCGTACATGGCGTCCAGCAGCGGCACG	17:C>A
7158524|F|0-26:T>C-26:T>C	TGCAGCCTCTTCGGCAAGCAGGCGCCTCAGCCACAGCACCGCGCGGGCGGCGGCAGCGCCAAGGGGAAG	26:T>C
1044629|F|0-8:A>G-8:A>G	TGCAGCACATTATAAATACCTGGCCAGACACACCAGCTGAATGCATCAGTTCTCCATCTTCCTCTTCCA	8:A>G
2246968|F|0-21:C>G-21:C>G	TGCAGCGAGGAGTGCAGGCATCAGCAGATCGAGGTGGACAGGGCGAGGCACCG	21:C>G
5358509|F|0-25:C>T-25:C>T	TGCAGTAGAGGCGGCAAAGGGTGGACGGATCCG	25:C>T
1017881|F|0-10:C>G-10:C>G	TGCAGCCGCTCACCACCAGCCTTTGGCTCGCCAGCCTCGCCTTCTTCTGCTTCACCG	10:C>G
6045666|F|0-29:G>A-29:G>A	TGCAGGAGGTGGAGGTCGGGGGACTTGGCGCGGAGCGCGCCG	29:G>A
5323499|F|0-12:C>T-12:C>T	TGCAGACGACGACGCAGACCTCCG	12:C>T
978496|F|0-38:G>C-38:G>C	TGCAGCACACCGAGTCGGGGGAGGTGACGGTGGAGATGGAATGGATGGCTCTAAACATTTAGAGAATCC	38:G>C
1863557|F|0-16:G>A-16:G>A	TGCAGCGATGAAGACGGCGACAGCGGCGATCGAGCAGCTGGTTTCAGCACGGAAGAGGTCGGGGCTTTC	16:G>A
4262746|F|0-33:C>T-33:C>T	TGCAGCCTTCGGCAGCGGCGGTGGCATCGGCAGCACGGTGCCCG	33:C>T
6037784|F|0-13:G>A-13:G>A	TGCAGCGCCAAGGGCACAATCAGGAACCGCAAGCCG	13:G>A
1050727|F|0-8:C>T-8:C>T	TGCAGGAACGTCATCTTCTCCAGGAACCCGAGCTCCGCCGTTGCCAGCGTGAGCACGTAGACGAAGAGC	8:C>T
3948659|F|0-16:C>T-16:C>T	TGCAGCTGCCAGAGCTCGCGCCG	16:C>T
3946426|F|0-27:G>A-27:G>A	TGCAGCCGCCCTCCGTCGGAGATCTGGGCTCATCCG	27:G>A
3028072|F|0-7:C>T-7:C>T	TGCAGTTCATGTCAACAAGGGTGAACTACCCTGGCAGCGCCG	7:C>T
1021890|F|0-55:C>T-55:C>T	TGCAGAGATGGGGCGGCATCAAGAACATCACCGTGGATGAGGCCAAGGCCAGGATCGCCGACATCCTGG	55:C>T
5331938|F|0-17:G>C-17:G>C	TGCAGCGACACGAAGACGAGCACCCG	17:G>C
7352966|F|0-9:C>A-9:C>A	TGCAGGAACCTCCACACCGACCG	9:C>A
1061244|F|0-35:C>T-35:C>T	TGCAGGACGAGTCCTGGAACGTCACCCGCTTCCTGCTGCTCTCCAAGCCGCCGTCGCCG	35:C>T
3064839|F|0-6:C>T-6:C>T	TGCAGGCCCTGCTCGCGCACCTCTGGCGTGCCGTGTGCCGAGCCCG	6:C>T
2247755|F|0-19:A>G-19:A>G	TGCAGTACTCCATGACGAGACAGTGGAACTTGTCGGTCTCGAAGTGGGTGTAGAGCGTGGGGAGGAAGG	19:A>G
1696722|F|0-58:C>T-58:C>T	TGCAGCGCCTTGCCGAGGAGGAGCTCGTTGGTGTGCGGGCCGTAGATGTCGGAGGTGTCGAGGAGGGTG	58:C>T
7170747|F|0-20:G>A-20:G>A	TGCAGCTGTCCATCTGCGCCGTGGCCGCCG	20:G>A
5581091|F|0-24:G>A-24:G>A	TGCAGGTTACCTGAACGACCCAGAGTCCACCAAAAACACCATCGACAAGGATGGATGGCTGCACACCGA	24:G>A

<table>
<tr><td>1696446|F|0-25:C>T-25:C>T</td><td>TGCAGTTTGTGACATACTTCTGCTGCTGATTGACTGGCCTGAAGTCTGAACCTCATGGAGACCGTTTCG</td><td>25:C>T</td></tr>
<tr><td>5578236|F|0-20:C>A-20:C>A</td><td>TGCAGGATGGACAGCTCGCTCCCAGCCGCGGACGGAGCCG</td><td>20:C>A</td></tr>
<tr><td>3946426|F|0-9:C>T-9:C>T</td><td>TGCAGCCGCCCTCCGTCGGAGATCTGGGCTCATCCG</td><td>9:C>T</td></tr>
<tr><td>1020233|F|0-53:A>G-53:A>G</td><td>TGCAGCTGGAGATGGCGGCGGCGTAGGTGGTTGTGAGGAGTGGGGAGGGGGAGAGCCG</td><td>53:A>G</td></tr>
<tr><td>3942729|F|0-20:C>T-20:C>T</td><td>TGCAGGAGCTCGTGCAGCCTCCGTTCCAGCACACGGGCCG</td><td>20:C>T</td></tr>
<tr><td>4261226|F|0-35:C>G-35:C>G</td><td>TGCAGGGACACGATGACGGCGGCGCCGCCCATGAACCCG</td><td>35:C>G</td></tr>
<tr><td>993664|F|0-40:T>G-40:T>G</td><td>TGCAGCCTGTCCAGCACGTTCTCCACGCCCACGTCCTCCCTCAGGCTCTCCTCGCAGGCCTTCTTCAGG</td><td>40:T>G</td></tr>
<tr><td>1090765|F|0-32:G>C-32:G>C</td><td>TGCAGCCTGCGCGACTTCGCCGACGGCCTGCTGGACGTGACCTGCGGCAGCACGGACGTGACCGTCTCC</td><td>32:G>C</td></tr>
<tr><td>985536|F|0-51:C>A-51:C>A</td><td>TGCAGGCGGAGAGCACGCACGAGAGGAACAGGTACGCGGGAGCGGCGTACCCGTACGACCTGCCCTGGC</td><td>51:C>A</td></tr>
<tr><td>1089186|F|0-29:G>C-29:G>C</td><td>TGCAGGAACGCGAACTCGCGCTCCACCTCGTCGGGCAGCGACGCGTGCGCGGCCCACCCG</td><td>29:G>C</td></tr>
<tr><td>999506|F|0-53:C>A-53:C>A</td><td>TGCAGCGCGGCCTCGGGCTGTCGGACAAGCAGGTGGAGGCGTCGCGCATGGCGCTGCACCG</td><td>53:C>A</td></tr>
<tr><td>7353643|F|0-11:A>G-11:A>G</td><td>TGCAGGTCAACAGCCTCGCCG</td><td>11:A>G</td></tr>
<tr><td>5578225|F|0-16:T>G-16:T>G</td><td>TGCAGGAGGAAGAGGATGCACCG</td><td>16:T>G</td></tr>
<tr><td>985043|F|0-32:T>C-32:T>C</td><td>TGCAGTTTGTTGTCTCGGAGAAGAGGAGAAATTGCTGGTCCTGTGATATCTGCACGCCGTTTGGGAAGA</td><td>32:T>C</td></tr>
<tr><td>1044289|F|0-16:C>G-16:C>G</td><td>TGCAGCTTCGGGTCGGCCCAGGAGACCCCGCAGCTCAACCTGGCGGGCCACTGCGACCCCTACTCCAAC</td><td>16:C>G</td></tr>
<tr><td>1034916|F|0-39:T>C-39:T>C</td><td>TGCAGTGGGTCGGCCTGGTGCTCATCATCGGGGCATTGGTGTGCAGCCTCACGATAAAGCCCTTGTCCC</td><td>39:T>C</td></tr>
<tr><td>1093143|F|0-38:G>A-38:G>A</td><td>TGCAGCGGGAGCGAGGCGCGGAGGTAGGCGCGCATGACGGTGCTGACGACGAACGGGTCCGCGTCGGCC</td><td>38:G>A</td></tr>
<tr><td>3385533|F|0-20:A>G-20:A>G</td><td>TGCAGGAGAGCGTGAGGCGCACGCGTGTGAAGTACCCG</td><td>20:A>G</td></tr>
<tr><td>5363717|F|0-16:G>C-16:G>C</td><td>TGCAGGACGTGTACGGGGAGGTGTACCGCGTGCTCAAGCCG</td><td>16:G>C</td></tr>
<tr><td>5578188|F|0-6:T>C-6:T>C</td><td>TGCAGCTTCCGCGGCTTCACCGTCACATGCACGCCG</td><td>6:T>C</td></tr>
<tr><td>1264847|F|0-11:G>C-11:G>C</td><td>TGCAGGGTCTTGAGGAAGTTGTTGTCGTCCGACTCTGTGTCGTGTGTGTGCCACACTGAGTTGCTCCCG</td><td>11:G>C</td></tr>
<tr><td>1089106|F|0-5:G>A-5:G>A</td><td>TGCAGGAAGTGCGCGGCCATCTGCCACGCGTCCCCCTGCGCCGCCCGCACCAGGGCCAGGTAGAAGTAG</td><td>5:G>A</td></tr>
<tr><td>3950735|F|0-7:A>G-7:A>G</td><td>TGCAGCCACGCCAGGTGGCCGTCTGGTTCCAGAACCGCCGCGCCCG</td><td>7:A>G</td></tr>
<tr><td>1136347|F|0-39:C>T-39:C>T</td><td>TGCAGTGTACCGCAGGCTTTGTGGCAAGGATGTTGTGTACGAGTACCCG</td><td>39:C>T</td></tr>
<tr><td>1201605|F|0-11:C>A-11:C>A</td><td>TGCAGCTCTGACAGCTGCCCGAGCTCCATGGGGATCGGCCCCTCCAGCGAGTTCTCCGACAGGCGCAGC</td><td>11:C>A</td></tr>
<tr><td>1057036|F|0-27:T>C-27:T>C</td><td>TGCAGTTCAGACATTCATTCTGGCGTATATTACCATGCGATGTGATTGGGACGAAGAGGTCATTTCTTT</td><td>27:T>C</td></tr>
<tr><td>4261626|F|0-5:T>C-5:T>C</td><td>TGCAGTACCTCATTGACTACGTACATACCTTCACCG</td><td>5:T>C</td></tr>
<tr><td>4022563|F|0-19:C>G-19:C>G</td><td>TGCAGCGCCTCACCGCGCGCTCCAGCCGCTACCTCTGGATCCCCG</td><td>19:C>G</td></tr>
<tr><td>2252543|F|0-22:G>A-22:G>A</td><td>TGCAGATGATGAGCCTCGACGCGCGCAAGTCCGTCGAGCGCTTCATCGAGACGCACGGGCCCG</td><td>22:G>A</td></tr>
<tr><td>3946565|F|0-5:C>T-5:C>T</td><td>TGCAGCGTCATCCTGGGGACGTCCTCCTGCGCCG</td><td>5:C>T</td></tr>
<tr><td>1694734|F|0-27:G>A-27:G>A</td><td>TGCAGTGCGTGGTGGCGCACCTGTGGCGGACCATGACGATGGCGCGCGGGCTCGACGGCGGGGAGAGCA</td><td>27:G>A</td></tr>
<tr><td>977215|F|0-23:C>G-23:C>G</td><td>TGCAGGGTGAGGAAGGTTCGGCCCTTGAGGAGGGCCCTCAAGGGCCTCAAGGCCTGGATCACCG</td><td>23:C>G</td></tr>
<tr><td>5365507|F|0-54:G>A-54:G>A</td><td>TGCAGCTCCCCGTGCAGCCTCCACGCCGCCGCCAGGTCGCGGTCCATGGCCCGCGCCGTGGACCGAGAT</td><td>54:G>A</td></tr>
<tr><td>3960529|F|0-10:T>C-10:T>C</td><td>TGCAGCCCCTTGGGGAAGATGGCCG</td><td>10:T>C</td></tr>
<tr><td>4537904|F|0-9:G>A-9:G>A</td><td>TGCAGGCGAGCTTGATGGCCTCGTCCCGCGTGGACGTGCCG</td><td>9:G>A</td></tr>
<tr><td>32021972|F|0-30:G>C-30:G>C</td><td>TGCAGGAGAGCATCTCGACGGTGCAGTCGTGGCGGGTCGGGGTGACGCGGAGGTCCAGGACCG</td><td>30:G>C</td></tr>
<tr><td>7925934|F|0-49:C>G-49:C>G</td><td>TGCAGCACGGACCCGTCGACGTCGCCGTCACCGCCGTCCCCTCGCCGCCCCCAGAGCCG</td><td>49:C>G</td></tr>
<tr><td>13880969|F|0-5:G>A-5:G>A</td><td>TGCAGGCCTTGTGGGGACCG</td><td>5:G>A</td></tr>
<tr><td>5002936|F|0-14:A>G-14:A>G</td><td>TGCAGCACGGAGGCAGCCCCG</td><td>14:A>G</td></tr>
<tr><td>9764450|F|0-14:C>G-14:C>G</td><td>TGCAGCAGCGACGCCACGCCGCCG</td><td>14:C>G</td></tr>
<tr><td>4262594|F|0-33:C>T-33:C>T</td><td>TGCAGAAGCTGCAAGAGAAATGGAAGTCCACGGCGGCGCCGTACCCG</td><td>33:C>T</td></tr>
<tr><td>1019894|F|0-49:T>A-49:T>A</td><td>TGCAGCTCGGAGAGAGGGGAAACGGGGAGGAGGAGGCGGACGAGACGGATGGGGAATTAAGGAGAGAAT</td><td>49:T>A</td></tr>
<tr><td>5580487|F|0-23:A>G-23:A>G</td><td>TGCAGGGCTGTAAGGACTGCGTCACCGTCGTCCCCG</td><td>23:A>G</td></tr>
<tr><td>2260280|F|0-64:C>T-64:C>T</td><td>TGCAGGATCGCCTTGGCTACGACCAGCCCAGCAAGGCCGTCGAGTGGCTCATCAAGGCTGCCGCCGCCG</td><td>64:C>T</td></tr>
<tr><td>981258|F|0-24:T>C-24:T>C</td><td>TGCAGCCCAGAAGGATTTATCTTGTGATGAAAGCGGAGTGCGAGGAGCAGAAGAGAAAATATTTTCAGG</td><td>24:T>C</td></tr>
<tr><td>1666656|F|0-5:G>A-5:G>A</td><td>TGCAGGTGCGTGCCTGCGCCGTCGTCGCCCTTCTCGAGCAGCCTGGCGAAGCAGAGGAGCGCGCGGGAC</td><td>5:G>A</td></tr>
<tr><td>1113218|F|0-9:G>C-9:G>C</td><td>TGCAGCAGCGCGAGCAGCGCGCGGGCGCCCTCGTCGCGATCGCCCTCGCCG</td><td>9:G>C</td></tr>
<tr><td>4396615|F|0-16:G>C-16:G>C</td><td>TGCAGAGCTCGGGCGAGGCGCGGATCCG</td><td>16:G>C</td></tr>
<tr><td>1080223|F|0-34:C>T-34:C>T</td><td>TGCAGATCGCGCGCGGCATGGAGCACCTCCACTCCAAGAAGATCTACCACGGCGAGCTCAACCCGTCCA</td><td>34:C>T</td></tr>
<tr><td>5580487|F|0-31:C>G-31:C>G</td><td>TGCAGGGCTGTAAGGACTGCGTCACCGTCGTCCCCG</td><td>31:C>G</td></tr>
<tr><td>4261821|F|0-16:C>G-16:C>G</td><td>TGCAGTACCTCTCGCTCGCGGCGAACCGTCTCACCG</td><td>16:C>G</td></tr>
<tr><td>1020233|F|0-13:G>A-13:G>A</td><td>TGCAGCTGGAGATGGCGGCGGCGTAGGTGGTTGTGAGGAGTGGGGAGGGGGAGAGCCG</td><td>13:G>A</td></tr>
<tr><td>1023798|F|0-25:C>G-25:C>G</td><td>TGCAGGGGAGGTACGAGCTGGGGCGCGTGCTGGGGCACGGCAACTTCGGCCG</td><td>25:C>G</td></tr>
<tr><td>5578282|F|0-12:C>G-12:C>G</td><td>TGCAGGCGGCGGCGCAGCGGACGAGGTCCCCG</td><td>12:C>G</td></tr>
<tr><td>1695399|F|0-22:C>T-22:C>T</td><td>TGCAGCAGCTCGACCTCTCCTCCAACGCTTTCAACGGCTCCATCCCGCCG</td><td>22:C>T</td></tr>
<tr><td>5358509|F|0-23:G>A-23:G>A</td><td>TGCAGTAGAGGCGGCAAAGGGTGGACGGATCCG</td><td>23:G>A</td></tr>
<tr><td>2299974|F|0-35:T>C-35:T>C</td><td>TGCAGATGCTCGACCTGCTCCGCCTCACCGTGCCGTCCCTGCTCGCCTTCCTCCGCTCCCTCCCCTCCG</td><td>35:T>C</td></tr>
<tr><td>1047412|F|0-22:T>C-22:T>C</td><td>TGCAGTGCATGCTGCCAGAGTTTTCGGCGTGCTGCGAAGAGTTGGTGGGCAGATGGACGGAGTCCATCG</td><td>22:T>C</td></tr>
<tr><td>4262594|F|0-38:C>G-38:C>G</td><td>TGCAGAAGCTGCAAGAGAAATGGAAGTCCACGGCGGCGCCGTACCCG</td><td>38:C>G</td></tr>
</table>

<table>
<tr><td>3951601|F|0-11:C>G-11:C>G</td><td>TGCAGGAACGCCCTCAGGGCCTCCG</td><td>11:C>G</td></tr>
<tr><td>1695415|F|0-34:G>A-34:G>A</td><td>TGCAGATCTCGTGGAACTACAACTACGGGGCGGCGGGGCAGAGCCTCGGCTTCGACGGGCTCAACGACC</td><td>34:G>A</td></tr>
<tr><td>984784|F|0-7:G>C-7:G>C</td><td>TGCAGGTGGACGGCTGCTACGTGCGGTACGACGCCAGCGACTTCGTCGGCCG</td><td>7:G>C</td></tr>
<tr><td>985964|F|0-37:G>C-37:G>C</td><td>TGCAGCGTTACCAGCCGTTCGGGTCGCCGCCTGGCGAGTACCACCATTTCGGCCCG</td><td>37:G>C</td></tr>
<tr><td>1070948|F|0-5:G>T-5:G>T</td><td>TGCAGGTCCAGGTGAACGGGGCGACGAGGCGTGTGGGCGGGGTCTTCGGGACGCCG</td><td>5:G>T</td></tr>
<tr><td>5367368|F|0-6:G>T-6:G>T</td><td>TGCAGGGAGGGCCCCGCGCGGGCCG</td><td>6:G>T</td></tr>
<tr><td>5357979|F|0-5:C>T-5:C>T</td><td>TGCAGCTCCTCCCGCTCGTCCCTCAGCCG</td><td>5:C>T</td></tr>
<tr><td>2254577|F|0-28:C>T-28:C>T</td><td>TGCAGCACCTCAGCGGCCAGCCCAACATCGCCGAGTTCAGGGGCGCCTTCGAGGACGCCGAGAGCGTGC</td><td>28:C>T</td></tr>
<tr><td>7352283|F|0-16:G>A-16:G>A</td><td>TGCAGCGGCGCTCCTGGCCG</td><td>16:G>A</td></tr>
<tr><td>1026798|F|0-8:C>G-8:C>G</td><td>TGCAGGTTCACCAGCGCCTTGGCGCGGTGGAGCGCCTCGTCGGTGCGGCACCG</td><td>8:C>G</td></tr>
<tr><td>7169859|F|0-13:T>G-13:T>G</td><td>TGCAGTGGTTCGGTGACAGCAGCGGCCAGGCGGCGCCG</td><td>13:T>G</td></tr>
<tr><td>1023496|F|0-61:G>C-61:G>C</td><td>TGCAGGCGGAGGTGTTCTTCCTTGGGCAACTGAGGCACAGGAACCTGGTGAAGCTGGTCGGGTACTGCT</td><td>61:G>C</td></tr>
<tr><td>3022485|F|0-8:G>C-8:G>C</td><td>TGCAGGAAGCCTTCCACCATGGCCTCGTGGCTCACCG</td><td>8:G>C</td></tr>
<tr><td>4262746|F|0-21:T>C-21:T>C</td><td>TGCAGCCTTCGGCAGCGGCGGTGGCATCGGCAGCACGCTGCCCG</td><td>21:T>C</td></tr>
<tr><td>2275535|F|0-37:C>T-37:C>T</td><td>TGCAGGAGCAGGCCGTCGACATGGTGCTCACTGACTACTGCATGCCTGAGATGACCG</td><td>37:C>T</td></tr>
<tr><td>1048955|F|0-25:G>C-25:G>C</td><td>TGCAGCCTCTTCAGCTGGGCCTTCAGGGCCAACCACGGGCCTCCCTACCTCATGCACTCCCAGCCACTC</td><td>25:G>C</td></tr>
<tr><td>1040482|F|0-65:A>G-65:A>G</td><td>TGCAGATCTTTTCCCAGTTTTATGTGAAACCGTGTTCCTCGCGATCTGCCCGATTGCCGTTAAGGAGAT</td><td>65:A>G</td></tr>
<tr><td>1020082|F|0-8:T>C-8:T>C</td><td>TGCAGATCTCCAAGATGCCTGAGCATAGGGAACTCAAGAGGACGGCCCTCACCGACCTCTCTGGCGGCG</td><td>8:T>C</td></tr>
<tr><td>5577912|F|0-18:C>G-18:C>G</td><td>TGCAGATGACGCGCACCCCCGTGGCGCCG</td><td>18:C>G</td></tr>
<tr><td>7339075|F|0-13:A>G-13:A>G</td><td>TGCAGTCGATCCAAGAAATCATTGGCCGCTCGAGTGGCGGCAGATCCATCACGGACCTCTGGGTCCGAG</td><td>13:A>G</td></tr>
<tr><td>1694865|F|0-6:C>A-6:C>A</td><td>TGCAGGCGTTCGACGACACCAAGGCGGGCGTCAAGGGCCTCGTCGACGCCG</td><td>6:C>A</td></tr>
<tr><td>1079174|F|0-45:G>C-45:G>C</td><td>TGCAGCTTCGGCGGCGACACCTTCAAAGGCTTTTGCGGGGGCGGGGGCGACAGCTTTGAATGCTTCTGC</td><td>45:G>C</td></tr>
<tr><td>3957502|F|0-39:T>C-39:T>C</td><td>TGCAGTTGACGATGAGTATGTCGATGGCCTCGGGGGCGATGCCG</td><td>39:T>C</td></tr>
<tr><td>3573560|F|0-13:G>T-13:G>T</td><td>TGCAGCTCGAGGAGGGCCTGCGTCGCGGCGAAGGAGTCCG</td><td>13:G>T</td></tr>
<tr><td>995663|F|0-47:C>G-47:C>G</td><td>TGCAGGGCCTCGTACTTGGAGGCCACCTCCCTGAGCATCTCGCCCAGCCGCCG</td><td>47:C>G</td></tr>
<tr><td>7342844|F|0-8:G>C-8:G>C</td><td>TGCAGCACGGTGTGGCTGCCGATGTACCCCGCGCCGCCG</td><td>8:G>C</td></tr>
<tr><td>1030286|F|0-38:G>A-38:G>A</td><td>TGCAGGAGTGGGAGCTCCTCCTCGCCTCCCCCACCGCCGCCGCGGCGCCCG</td><td>38:G>A</td></tr>
<tr><td>993527|F|0-21:C>A-21:C>A</td><td>TGCAGGACGTGACGGTGGCCGCGTACGCCCTGGAGGCCTGGGTCGCGGCGAACCG</td><td>21:C>A</td></tr>
<tr><td>3064764|F|0-31:G>A-31:G>A</td><td>TGCAGCAGCGCCGTGGCGGACATGCTCGCCAGGCCGCCGTTGCCCG</td><td>31:G>A</td></tr>
<tr><td>1696490|F|0-8:T>C-8:T>C</td><td>TGCAGTTCTTGTCGGAGGGATCATGGGCGAACCACGACGAGATCGACCTGGAGTTCCTGGGCAACTCCA</td><td>8:T>C</td></tr>
<tr><td>32027038|F|0-42:T>C-42:T>C</td><td>TGCAGATCGTGCCGTGGACGGCGCGGCAGTGGTAGCACACGATGTGCCCG</td><td>42:T>C</td></tr>
<tr><td>4008942|F|0-16:G>A-16:G>A</td><td>TGCAGGCGTCCTTCCTGGAGGTGTTCGTCCGCGGCCG</td><td>16:G>A</td></tr>
<tr><td>1008777|F|0-14:G>A-14:G>A</td><td>TGCAGGTACATCTGGAGCGTGAAGCTGAAGACGCACGCGAACATGACGGGGATGTACCAGAGCGAGATG</td><td>14:G>A</td></tr>
<tr><td>1061236|F|0-8:G>C-8:G>C</td><td>TGCAGGAGGAGGCATGCCACGGTGGCGCCGAGCAGCTGGGCGACCCAGTAGAAGAGCCCG</td><td>8:G>C</td></tr>
<tr><td>5370897|F|0-10:C>G-10:C>G</td><td>TGCAGGCCCTCGACGGCGCGGAGGCCCG</td><td>10:C>G</td></tr>
<tr><td>2260280|F|0-61:C>T-61:C>T</td><td>TGCAGGATCGCCTTGGCTACGACCAGCCCAGCAAGGCCGTCGAGTGGCTCATCAAGGCTGCCGCCGCCG</td><td>61:C>T</td></tr>
<tr><td>990451|F|0-13:C>T-13:C>T</td><td>TGCAGGAGGTGCGCGCTTTTGCTTTTGCGTCGCTCGCCTTTCCTCTCTCGCCG</td><td>13:C>T</td></tr>
<tr><td>991324|F|0-55:C>T-55:C>T</td><td>TGCAGAGCGCCATCCAGGCCGCCATCGCGCACTGCAAGAACTCCATCGCCGTGGCCAAGCAGTAGTACG</td><td>55:C>T</td></tr>
<tr><td>7172507|F|0-7:T>G-7:T>G</td><td>TGCAGACTTCGATCCAGCCTTGGTCGGGGTCCCAGTCGGGAAACGGCAGGCCG</td><td>7:T>G</td></tr>
<tr><td>5002926|F|0-12:G>C-12:G>C</td><td>TGCAGCCAGAGCGAGCACGAGGCCGAGGCCG</td><td>12:G>C</td></tr>
<tr><td>1667190|F|0-10:A>G-10:A>G</td><td>TGCAGATCGGATTCGCCCTCAAGAAGGTGTTCGACGAGGGCATAACCAGCCG</td><td>10:A>G</td></tr>
<tr><td>2254889|F|0-32:A>G-32:A>G</td><td>TGCAGGTCCTGGCGGCGGAGCTGCCGACGGGCACCATCCGCTACTCCTCCAAGATCGTCTCCATCGACG</td><td>32:A>G</td></tr>
<tr><td>3574545|F|0-16:G>C-16:G>C</td><td>TGCAGCTGGCGCCCAAGAAGGTCACCCCCAGGGTCACCG</td><td>16:G>C</td></tr>
<tr><td>3946690|F|0-8:T>C-8:T>C</td><td>TGCAGGTCTTCAGGGTTTACAATGCCG</td><td>8:T>C</td></tr>
<tr><td>6045617|F|0-10:G>C-10:G>C</td><td>TGCAGTTGGAGAGGTTCTACCAGCCAAAGAAGAGCCG</td><td>10:G>C</td></tr>
<tr><td>1020691|F|0-10:T>C-10:T>C</td><td>TGCAGTTGTCTTTCCGTAGCACCCATAGGAACGGCCTGTTGGACTGGCTCATGGCGTCCGCTATCTCGG</td><td>10:T>C</td></tr>
<tr><td>1218327|F|0-59:G>A-59:G>A</td><td>TGCAGCATCTCGTGAGCAACCTTAAGGCCTTCCTTGTACCTGCCCTCTCTGAAGAACCCGCGCATAAGA</td><td>59:G>A</td></tr>
<tr><td>6046744|F|0-11:C>T-11:C>T</td><td>TGCAGCGTTAACGAATCGTGCGCCAAAATCTGAATTATCAACCG</td><td>11:C>T</td></tr>
<tr><td>1214870|F|0-5:A>C-5:A>C</td><td>TGCAGAGTGGAACTGAATGACCATGACAGTATCTGGTGCAGCTCGGCGGCCCAGCCAGTCGCCGCCGAG</td><td>5:A>C</td></tr>
<tr><td>1052648|F|0-47:A>G-47:A>G</td><td>TGCAGCAAGCTCACGAGCTCCTCCTCCGACTGTGACGAAACCATACAAAGGTACAGTTTGCAACAGAAG</td><td>47:A>G</td></tr>
<tr><td>989113|F|0-28:G>C-28:G>C</td><td>TGCAGAAGGCCATCGGCGAGCACGACGCGGCCGCCGTGACGCGGAGGCTGGACGAGTACGAGGCGGCGC</td><td>28:G>C</td></tr>
<tr><td>4262786|F|0-26:T>C-26:T>C</td><td>TGCAGCGGCGGCGCTTGGTCGCCATCTCCTACCTCCG</td><td>26:T>C</td></tr>
<tr><td>5578447|F|0-19:G>T-19:G>T</td><td>TGCAGTGCATGGGCATGGGGATCACGTCGCCG</td><td>19:G>T</td></tr>
<tr><td>2361499|F|0-52:C>T-52:C>T</td><td>TGCAGGCGGCGACACGCCACAGCCCAGCGCGCACCTCGTCGCGCAGCTGCTCCCGCTCCCACCCGCAGA</td><td>52:C>T</td></tr>
<tr><td>6048017|F|0-34:C>A-34:C>A</td><td>TGCAGGTGGATTTGGTGACGGAGACGGACAAGGCCTGTGAGGATCTCATCTTCAACCACCTCCG</td><td>34:C>A</td></tr>
<tr><td>6048017|F|0-11:T>C-11:T>C</td><td>TGCAGGTGGATTTGGTGACGGAGACGGACAAGGCCTGTGAGGATCTCATCTTCAACCACCTCCG</td><td>11:T>C</td></tr>
<tr><td>5369294|F|0-7:G>C-7:G>C</td><td>TGCAGAGGAGTGGAGGTAGGGGTGGCGCCGCCATGGCCGCGGGCCG</td><td>7:G>C</td></tr>
<tr><td>1079587|F|0-13:C>T-13:C>T</td><td>TGCAGATGACCTGCAACCTCGGGTCCATCGTGGGGGGCTCCTTCGGCGTGCTCCTCGCGCCG</td><td>13:C>T</td></tr>
<tr><td>12856456|F|0-14:G>A-14:G>A</td><td>TGCAGGTCTTCAGGGTTTATAATGCCG</td><td>14:G>A</td></tr>
</table>

<table>
<tr><td>1272975|F|0-48:G>A-48:G>A</td><td>TGCAGAATCCATTTGCAAGAATCTAATCTACGCTCAGATTGTCTTGGCGCCG</td><td>48:G>A</td></tr>
<tr><td>7168097|F|0-23:A>G-23:A>G</td><td>TGCAGGCATAACGGAAATGGGCAACTGCGCAACGGCTGTCCCTGTCCG</td><td>23:A>G</td></tr>
<tr><td>982130|F|0-18:G>A-18:G>A</td><td>TGCAGCTCCTTGACGCTGGCCTCTGTTTCGTAGCCGCCGATGCTGGCCTCCTTGGCAACTTCGCGATGC</td><td>18:G>A</td></tr>
<tr><td>4262907|F|0-13:G>A-13:G>A</td><td>TGCAGGAGTTCGAGTGGCGCGCGCACCCGTCGCAGCCG</td><td>13:G>A</td></tr>
<tr><td>984784|F|0-16:C>T-16:C>T</td><td>TGCAGGTGGACGGCTGCTACGTGCGGTACGACGCCAGCGACTTCGTCGGCCG</td><td>16:C>T</td></tr>
<tr><td>988587|F|0-5:A>G-5:A>G</td><td>TGCAGAGGGTAGCTGCGCAGGAACACCCGCCTGGTGCTGTAGTCCTCGTACCG</td><td>5:A>G</td></tr>
<tr><td>4262244|F|0-14:A>G-14:A>G</td><td>TGCAGCTGCCTCGAACGACTCCCCACGGCCAGGAGGCAGAGCCG</td><td>14:A>G</td></tr>
<tr><td>4004154|F|0-22:C>G-22:C>G</td><td>TGCAGTCCAACCCCGTCTGCCTCTGCCAGCTGCTCGCCG</td><td>22:C>G</td></tr>
<tr><td>6043883|F|0-11:C>T-11:C>T</td><td>TGCAGCGAGCACTCGAAGTCGGCCGCGGACAGCACCCCCTCCG</td><td>11:C>T</td></tr>
<tr><td>1220141|F|0-9:A>G-9:A>G</td><td>TGCAGCGGCAGGAAGGGCCGTTGCTCATCCTGGGGTTTCTGATGGGCATCAACGCCG</td><td>9:A>G</td></tr>
<tr><td>3952878|F|0-19:C>G-19:C>G</td><td>TGCAGGACCGCAAGAGGACCAAGGTGCCCGCCG</td><td>19:C>G</td></tr>
<tr><td>1216830|F|0-46:C>G-46:C>G</td><td>TGCAGCTGGTGTCCGTGATGTTCGTGGGCCACCTCGGCGAGCTGCCCCTCGCCG</td><td>46:C>G</td></tr>
<tr><td>6044168|F|0-11:G>A-11:G>A</td><td>TGCAGGTAGAAGGTGTATGCTTCGGGGGTGGTGGGGAGCCG</td><td>11:G>A</td></tr>
<tr><td>3940596|F|0-23:T>G-23:T>G</td><td>TGCAGGTGACGTGGCTGCCCGCGTCCG</td><td>23:T>G</td></tr>
<tr><td>12694184|F|0-21:A>G-21:A>G</td><td>TGCAGGTGGAGGAGATGAAGGACTTGGGCGCGAGGATCCG</td><td>21:A>G</td></tr>
<tr><td>1020842|F|0-7:G>A-7:G>A</td><td>TGCAGAGGTTCTTCCTCGGAGGGAGACTGATATTCGGACCCGATGCCAAGTCCCTGCTCGTCTCGGTTG</td><td>7:G>A</td></tr>
<tr><td>5370780|F|0-5:C>A-5:C>A</td><td>TGCAGCTTGAACGACATCTCCG</td><td>5:C>A</td></tr>
<tr><td>4394186|F|0-7:C>G-7:C>G</td><td>TGCAGCTCAGCAATGCCGCGGAGGCGCAGGCCGAGTGCCTGCTCCG</td><td>7:C>G</td></tr>
<tr><td>7164760|F|0-7:C>T-7:C>T</td><td>TGCAGACCGTGCCAGGGTACGACATGGCCG</td><td>7:C>T</td></tr>
<tr><td>1076602|F|0-26:C>G-26:C>G</td><td>TGCAGCTCCAGGCCCAGCTCCTCCTCCACGTCGTCGGACGCCGAGATCAGCAGCAGCTCGCACCCCTCG</td><td>26:C>G</td></tr>
<tr><td>2249981|F|0-61:A>G-61:A>G</td><td>TGCAGCACATGGAGCTGCTCGAGGAGGGTGGCGACGGCGACGAGCGGTTCGAACTCTTCGAAACGGAAG</td><td>61:A>G</td></tr>
<tr><td>3957901|F|0-18:T>A-18:T>A</td><td>TGCAGAACTGGTCGGCGATGGTGGGCCACGTCAGAGCCG</td><td>18:T>A</td></tr>
<tr><td>4261076|F|0-13:C>G-13:C>G</td><td>TGCAGCTCCAGGTCCAGATGATGTCGACGATGGGGACGGCCG</td><td>13:C>G</td></tr>
<tr><td>2249981|F|0-25:G>A-25:G>A</td><td>TGCAGCACATGGAGCTGCTCGAGGAGGGTGGCGACGGCGACGAGCGGTTCGAACTCTTCGAAACGGAAG</td><td>25:G>A</td></tr>
<tr><td>1141256|F|0-15:C>T-15:C>T</td><td>TGCAGGAGATCGCCGCGTCCAGGGGCAAGAGCGTGGCGCAGGTGTGCCTGAGGTGGGTGTACGAGCAGG</td><td>15:C>T</td></tr>
<tr><td>987633|F|0-13:G>A-13:G>A</td><td>TGCAGCACGCGGAGGACATGCACGAGTGGATCGTCGGCAACCTGCGCCGCACCG</td><td>13:G>A</td></tr>
<tr><td>3023337|F|0-5:A>G-5:A>G</td><td>TGCAGAGCCTCATGGACTTCTACTACGCCAGCGTGCCG</td><td>5:A>G</td></tr>
<tr><td>5567165|F|0-20:G>A-20:G>A</td><td>TGCAGCATCTCGCGGCGGCAGCGCTTGATCCG</td><td>20:G>A</td></tr>
<tr><td>6049034|F|0-12:T>C-12:T>C</td><td>TGCAGAGAGGACTGAGGTAGTGTCTACGTTTCGGAGCGACGGGAGGTGGTCCCCTCACACCACGAGGTC</td><td>12:T>C</td></tr>
<tr><td>992207|F|0-19:G>C-19:G>C</td><td>TGCAGTTCTGCGACGAGGTGGGCGTCAAGCGCCACGTCCTCAAGGTGTGGATGCACAACAACAAGCACA</td><td>19:G>C</td></tr>
<tr><td>1066921|F|0-26:C>A-26:C>A</td><td>TGCAGGAAGAGGTAAGGAGGAATGTACCCAACGGGCAAGAGATGGCCGCCGAAGACGATCTCCCCAACA</td><td>26:C>A</td></tr>
<tr><td>7167958|F|0-26:G>C-26:G>C</td><td>TGCAGCTGCTGGAGCGCCACGACGGCGACGGCGACCG</td><td>26:G>C</td></tr>
<tr><td>1108292|F|0-47:C>A-47:C>A</td><td>TGCAGCATGAGGCCGCACCTCCTCCAGGAGAGCACCTGGGTTAGCCCCAGAGAGAGGGACTTGTCCGAG</td><td>47:C>A</td></tr>
<tr><td>5971434|F|0-17:C>T-17:C>T</td><td>TGCAGCGGCGACATGGGCGGCGGCGGCGCAGCCG</td><td>17:C>T</td></tr>
<tr><td>4262196|F|0-12:C>T-12:C>T</td><td>TGCAGCCCACCACGCACTGCCACCCCG</td><td>12:C>T</td></tr>
<tr><td>5360829|F|0-22:T>G-22:T>G</td><td>TGCAGCAGCTGCTGGTGGGACCTCTTGCAGTGGGCAATGGCGCCCTGGATGGCCCCCTCCTCCGACCCG</td><td>22:T>G</td></tr>
<tr><td>1014668|F|0-8:G>T-8:G>T</td><td>TGCAGCTTGAGCGGGCATTTGGCCTGGAACTTGCACTTGGTGAAGTGCGCCTGGTACCTGACCTCGCCC</td><td>8:G>T</td></tr>
<tr><td>1863204|F|0-19:C>T-19:C>T</td><td>TGCAGCCGCTGCGCCTCGCCTTCGAGACCAAGCACATCAAGCTCGTCGAGCCG</td><td>19:C>T</td></tr>
<tr><td>4005725|F|0-29:G>C-29:G>C</td><td>TGCAGGAAGTCGAAGTGGTGCAGCGTCACGAGCGGCACCACCG</td><td>29:G>C</td></tr>
<tr><td>1051607|F|0-8:A>C-8:A>C</td><td>TGCAGGTTATGTCTCTATCATGCCAGGAAGAGCGCGAGCATGTTCTGGACCCG</td><td>8:A>C</td></tr>
<tr><td>4396479|F|0-7:G>T-7:G>T</td><td>TGCAGCAGCGCTGGAACCAAGTTCTTCCG</td><td>7:G>T</td></tr>
<tr><td>3951601|F|0-6:A>G-6:A>G</td><td>TGCAGGAACGCCCTCAGGGCCTCCG</td><td>6:A>G</td></tr>
<tr><td>12419580|F|0-16:G>C-16:G>C</td><td>TGCAGTGCGCGAACCCGACGGTGTGGGCGCCG</td><td>16:G>C</td></tr>
<tr><td>2247679|F|0-5:G>A-5:G>A</td><td>TGCAGGAGCACCGCCTGCCGCTCTCCAACCTCGACCTCATCCTGCCCCCCATCGACGTCAGCGTCTTCT</td><td>5:G>A</td></tr>
<tr><td>7909099|F|0-24:A>C-24:A>C</td><td>TGCAGTAGACGCTCACGGTGAGCAAGGCGCCG</td><td>24:A>C</td></tr>
<tr><td>5582365|F|0-19:C>G-19:C>G</td><td>TGCAGCTCGGCCTCACCCTCTCCG</td><td>19:C>G</td></tr>
<tr><td>7907162|F|0-7:G>C-7:G>C</td><td>TGCAGCTGGTGCAGAACGCCG</td><td>7:G>C</td></tr>
<tr><td>5581409|F|0-11:G>C-11:G>C</td><td>TGCAGCGTCACGGTGAGGTAGGACGCCG</td><td>11:G>C</td></tr>
<tr><td>1075269|F|0-9:A>G-9:A>G</td><td>TGCAGGAGCACACCGCCGTCTTCTTCAGGGTGTTCAGGCACCATGACAAGTACAGAGTGCTCATGTGCA</td><td>9:A>G</td></tr>
<tr><td>1093145|F|0-26:G>C-26:G>C</td><td>TGCAGCGCGCTCTCCTTCTCGGCGACGACGTCGTTGAGCCGCGCGTTCTCGACCCG</td><td>26:G>C</td></tr>
<tr><td>5581591|F|0-8:C>A-8:C>A</td><td>TGCAGTGGCACATCGTCCTCCTCCTCGGCGCCG</td><td>8:C>A</td></tr>
<tr><td>981489|F|0-66:C>T-66:C>T</td><td>TGCAGCACATGAACAAGGGGATCTTCTCCTGGGCCACCCTCATCTCGCGCATTCAGAGCGAGTCCCCGA</td><td>66:C>T</td></tr>
<tr><td>2242683|F|0-8:C>T-8:C>T</td><td>TGCAGCTGCTGGATGTACTCGATCGCGTCCTTGATGATCGAGGCCTTGTCCATCTGCAACGACGAGGGA</td><td>8:C>T</td></tr>
<tr><td>5365560|F|0-61:G>A-61:G>A</td><td>TGCAGCTGCTCGACCTGTCGTCCAACTCCCTCACGGGCGCCATCCCGCCTGAGCTCGGCAAGCTCCAGG</td><td>61:G>A</td></tr>
<tr><td>3951620|F|0-13:C>T-13:C>T</td><td>TGCAGCAGCTGTTCCACCTGCACGAGGACGGCGCCG</td><td>13:C>T</td></tr>
<tr><td>1055670|F|0-17:G>T-17:G>T</td><td>TGCAGGAACTTCTGGATGGGGAAGTTGAGCGCGTAGGCGAAGAGCTGCGGGATCATGTAGAGCGAGAAC</td><td>17:G>T</td></tr>
<tr><td>3949462|F|0-5:A>C-5:A>C</td><td>TGCAGATGGCGGGGTGCGCGGTGCCGCTGTTCCG</td><td>5:A>C</td></tr>
<tr><td>4262167|F|0-28:C>A-28:C>A</td><td>TGCAGGGGCCCTTCTCGCTGCGCGTCACCGACGAGTCCG</td><td>28:C>A</td></tr>
<tr><td>987633|F|0-8:G>A-8:G>A</td><td>TGCAGCACGCGGAGGACATGCACGAGTGGATCGTCGGCAACCTGCGCCGCACCG</td><td>8:G>A</td></tr>
</table>

5582934|F|0-13:G>C-13:G>C	TGCAGGCCGTCGTGAAGGAGTCGCTCCG	13:G>C
1697137|F|0-50:C>T-50:C>T	TGCAGGCCCCTCCAGCTCTCCTTGTAGTTCCAGTACGTGTTGAGGCAGTGCCCGAGCGCGTCGATCATC	50:C>T
32020219|F|0-8:A>G-8:A>G	TGCAGCGCAACGCCTACATGAGCTACAGCCG	8:A>G
22838434|F|0-12:A>G-12:A>G	TGCAGCGCGACGACTACGCAGACGATCTTCAAGGCAATGCCTCGATAGCGCGGTACCTCGTGGACTCCA	12:A>G
5361773|F|0-35:G>A-35:G>A	TGCAGTCTGCACGAAGTGAGTGAGTTGAGTTACTCGCATGGTCGAGGCTCCCGTACCCTGCGACGACGT	35:G>A
1865366|F|0-10:A>C-10:A>C	TGCAGCCCTGAGACGGCGTCGAACGCGCACGGGCCAGCCGCCAGCCCGTGCCCG	10:A>C
5004798|F|0-42:A>G-42:A>G	TGCAGCGGAGAGGAGGGAGGCGAGAGCGTGGAACGCAGGGAAACAGTGTCGGAAGCCCTTCTGGCGGCC	42:A>G
7917698|F|0-41:T>C-41:T>C	TGCAGCTGGAAGTGCAGGATGAGGGTGATGCTTTCGCCATCTGATGCCGACCACATGGTACCGTTCATC	41:T>C
7917544|F|0-51:T>C-51:T>C	TGCAGACGGCAGCTTATTAAGGGGCTAGTTTAAAGCGGGGGCGGCAATGCGTTGGGTCCGCGAAGGCCG	51:T>C
5363256|F|0-38:T>G-38:T>G	TGCAGCACTACGCCGCGGCGTTCGACGCGGTGGGCGCGTCCG	38:T>G
4997965|F|0-26:T>C-26:T>C	TGCAGCAGGATGCTGGGAATGAAATATGAGTCGCAGAGGGGATACATTGGACTGGAGTACTATGGACGG	26:T>C
3954911|F|0-5:C>G-5:C>G	TGCAGCGTCGTCAACAACCG	5:C>G
2247504|F|0-44:A>G-44:A>G	TGCAGGAGGAGGTGCTCGAGGTACCACATCAGGGGCGACAAGGCAGGCACGGTGGAGAGCTTCATCGAC	44:A>G
4993471|F|0-12:G>A-12:G>A	TGCAGCTGCACCGCCTCCTCGCGTCCGACGACACCG	12:G>A
1040482|F|0-49:C>G-49:C>G	TGCAGATCTTTTCCCAGTTTTATGTGAAACCGTGTTCCTCGCGATCTGCCCGATTGCCGTTAAGGAGAT	49:C>G
6045706|F|0-11:A>C-11:A>C	TGCAGGCCGTGACCCTCGAGGAACACCCCGTCTGCCG	11:A>C
4261647|F|0-7:C>T-7:C>T	TGCAGGCCCTCCACCTCGGCCGCGTTCCACGGCCTCAGGGCGCCG	7:C>T
2247679|F|0-25:C>T-25:C>T	TGCAGGAGCACCGCCTGCCGCTCTCCAACCTCGACCTCATCCTGCCCCCCATCGACGTCAGCGTCTTCT	25:C>T
3952878|F|0-14:A>G-14:A>G	TGCAGGACCGCAAGAGGACCAAGGTGCCCGCCG	14:A>G
1141256|F|0-40:G>A-40:G>A	TGCAGGAGATCGCCGCGTCCAGGGGCAAGAGCGTGGCGCAGGTGTGCCTGAGGTGGGTGTACGAGCAGG	40:G>A
1079587|F|0-22:G>C-22:G>C	TGCAGATGACCTGCAACCTCGGGTCCATCGTGGGGGGCTCCTTCGGCGTGCTCCTCGCGCCG	22:G>C
2268315|F|0-7:G>C-7:G>C	TGCAGGAGCGCGGCGGGGTTCTGCACGCGGCTGTAGCTGGGACGCCACTCGACGTCGAGCCCGACGAGG	7:G>C
3570049|F|0-62:G>T-62:G>T	TGCAGCCACCTCCTCAAGGACATCGAGCAGATCCGCCTCCGCTACCGCCCGCTCAAGGCCGCGCTCCGC	62:G>T
2289249|F|0-36:A>G-36:A>G	TGCAGGCTGGTGAGATGGGTGAGGTGAACCACATCTACGGCCTCCTCCTTGTCCTCGATTGATTTCTCA	36:A>G
1062844|F|0-19:G>C-19:G>C	TGCAGAACGGGCAGCAGCAGCACCTGACCTTCACCCGCTGCTCCAACGCCAGGGCCAGCTTCCTCAGGG	19:G>C
6040333|F|0-53:C>A-53:C>A	TGCAGCTGCTGGTGGTAGCTGCACGACTCGTTGTGGTTGGGCGTCTTGCACGCCACGTGCGGGAAACGG	53:C>A
5350156|F|0-14:C>T-14:C>T	TGCAGCTGGCGGCGCGGCAGAGACCTCGCTTCCG	14:C>T
4262684|F|0-5:T>C-5:T>C	TGCAGTTGGTCCTGATGGTGCCTTGGTTGCCG	5:T>C
4022563|F|0-34:C>G-34:C>G	TGCAGCGCCTCACCGCGCGCTCCAGCCGCTACCTCTGGATCCCCG	34:C>G
4008942|F|0-22:G>A-22:G>A	TGCAGGCGTCCTTCCTGGAGGTGTTCGTCCGCGGCCG	22:G>A
1072118|F|0-50:C>G-50:C>G	TGCAGCACGATAGAGTAGATCACGTCGGCGTACTTGACGGCGAGGTTGAGCGACATGCACCG	50:C>G
3937499|F|0-8:C>T-8:C>T	TGCAGCCTCCGTTGCTTAGAGCTCCG	8:C>T
4262788|F|0-10:C>G-10:C>G	TGCAGCTCTCCCTCGCCATCCGCATCACCTCCGAGGCCTTCCTCGCCG	10:C>G
2242683|F|0-65:G>C-65:G>C	TGCAGCTGCTGGATGTACTCGATCGCGTCCTTGATGATCGAGGCCTTGTCCATCTGCAACGACGAGGGA	65:G>C
3028072|F|0-31:T>C-31:T>C	TGCAGTTCATGTCAACAAGGGTGAACTACCCTGGCAGCGCCG	31:T>C
1152164|F|0-17:C>T-17:C>T	TGCAGGGCTGGTCCTGGCTTCGCTCGCCGTGGTGCTGGCAACCTCGCTGAGGACGGCGGCGGTGCACCT	17:C>T
5324496|F|0-19:G>A-19:G>A	TGCAGTGACGTTTGAGTTCGACGGCGAGAGTTCCG	19:G>A
1093200|F|0-7:C>T-7:C>T	TGCAGACCCCGCCCAGACCGCTGCATCACGCCAAAACTCTAGCCCTAGAGGCCTCCTCTGCCGCCGCCA	7:C>T
1075269|F|0-19:C>A-19:C>A	TGCAGGAGCACACCGCCGTCTTCTTCAGGGTGTTCAGGCACCATGACAAGTACAGAGTCCTCATGTGCA	19:C>A
7353540|F|0-5:C>T-5:C>T	TGCAGCGCGCCGTCCACCGCCG	5:C>T
3959679|F|0-7:G>A-7:G>A	TGCAGACGCACCACTTCGCCTACGAGGAGCTGGAGGAGGCCACCG	7:G>A
1321129|F|0-19:T>C-19:T>C	TGCAGCATGCACATTGCTATCTCTTTCTGTGCCCATTACAGCAACTTTTTCTGGTGTATTATTGCCGAG	19:T>C
1025759|F|0-32:A>T-32:A>T	TGCAGGAGGCCCTCCGCCGCGACCATGCCGCCAGCACGCGGCGGCGCTTGTTGAAGGGGTTCTCCGAGA	32:A>T
5350155|F|0-10:C>G-10:C>G	TGCAGCTGCTCGTCTCCGCGGGGCCGCCG	10:C>G
4261647|F|0-32:C>A-32:C>A	TGCAGGCCCTCCACCTCGGCCGCGTTCCACGGCCTCAGGGCGCCG	32:C>A
3943205|F|0-10:G>A-10:G>A	TGCAGTCGGAGGACACCCTGACGTAGTCCG	10:G>A
1093486|F|0-31:G>A-31:G>A	TGCAGGCGGACTGCGACCGAGACGTGAAGGAGGCGGCGAGGGCCGTGCTGAGGGTGCACTCCG	31:G>A
4262072|F|0-9:C>T-9:C>T	TGCAGATCTCGCGCTGATGAGCGGCGCGCAGCTCTTCATCCG	9:C>T
2245927|F|0-47:C>T-47:C>T	TGCAGGCGCGCCTCGCCGCCGTCAAATGGATCCTTGAAGTGAGCACTCACTCAGCTCCTCGCTCTCCCG	47:C>T
4396798|F|0-24:G>A-24:G>A	TGCAGCGCCCCTGCGCCCAGCGCCGCCGCTCCG	24:G>A
5358176|F|0-20:C>A-20:C>A	TGCAGGATCCTGGTGGACGGCGTGCCGATCCG	20:C>A
5370854|F|0-9:G>T-9:G>T	TGCAGCTCGGCGGGGTGAGGACGTGGGCCGTGCCG	9:G>T
4538909|F|0-7:C>G-7:C>G	TGCAGCTCAGGCGCCCGCGGTCGCTCCG	7:C>G
4409602|F|0-10:G>A-10:G>A	TGCAGGGGGAGGGGATGATTGCCTGGCCCG	10:G>A
12470406|F|0-7:G>C-7:G>C	TGCAGGTGGTGAAGTATCTGATTATGGCCTTCGATATCGTCAACTCTGTAGACGACCAAGGGAATACAG	7:G>C
1146832|F|0-54:T>C-54:T>C	TGCAGATGCATTAATTGGCGTTATTATGGCAGGCATGAGTACACACGTAGAGTGTATTTGTTGCCGCCT	54:T>C
2242803|F|0-28:C>T-28:C>T	TGCAGGGCCTCGTGCGCGAGCTCGAGGACGGTGGCAATGCATCTGCGAGCTTCGTGTTCGCTGACGCCG	28:C>T
4262170|F|0-5:G>A-5:G>A	TGCAGGTCGATGCGCGCGTAGAGGTACGTGTCCTTGGACCG	5:G>A
100445751|F|0-7:G>C-7:G>C	TGCAGGCGCTTGGCGTCGCCCG	7:G>C

4261647|F|0-35:C>G-35:C>G	TGCAGGCCCTCCACCTCGGCCGCGTTCCACGGCCTCAGGGCGCCG	35:C>G
6045689|F|0-17:A>G-17:A>G	TGCAGGCATCGGAGCGCAACCCGCTCCCGCGTCTCCTCCG	17:A>G
988587|F|0-28:T>G-28:T>G	TGCAGAGGGTAGCTGCGCAGGAACACCCTCCTGGTGCTGTAGTCCTCGTACCG	28:T>G
1079174|F|0-37:G>A-37:G>A	TGCAGCTTCGGCGGCGACACCTTCAAAGGCTTTTGCGGGGGCGGGGGCGACAGCTTTGAATGCTTCTGC	37:G>A
1026243|F|0-21:A>G-21:A>G	TGCAGGGGTCGGTGAGGGACGAGGCGGCGGAGCCGAGGTTGGTGAGCACGCGGCGGAAGTCGGCGGCGA	21:A>G
5365560|F|0-16:G>A-16:G>A	TGCAGCTGCTCGACCTGTCGTCCAACTCCCTCACGGGCGCCATCCCGCCTGAGCTCGGCAAGCTCCAGG	16:G>A
4262890|F|0-5:G>T-5:G>T	TGCAGGATGTCCCCCACGCGGTACCG	5:G>T
3027720|F|0-6:T>C-6:T>C	TGCAGGTTCCCGAGCTCCTTTGGGATGGCGCCGTCCAGGTGGTTCCG	6:T>C
977583|F|0-41:C>G-41:C>G	TGCAGCCTCTTGTCCATGTACTCCCTCACCGCTCCGACGCCCTTGGCCACCACCTCCAGGGGCGGCACG	41:C>G
1253942|F|0-11:C>A-11:C>A	TGCAGATGCGCCACCACCCACGCGGCGAACACCAGCTTCCAGCCCAGCGGGCCCCACGACTCGTACCCG	11:C>A
4262746|F|0-19:G>A-19:G>A	TGCAGCCTTCGGCAGCGGCGGTGGCATCGGCAGCACGCTGCCCG	19:G>A
3957544|F|0-8:C>G-8:C>G	TGCAGCTGCCGCTCCACGGCCTCCGCCCG	8:C>G
2243729|F|0-10:G>A-10:G>A	TGCAGTTCTCGATGTGCAGGTCCTCGAGGACGGGGCACTGGTCGCCGAGATCCCGCCCGAAACTAGGGC	10:G>A
1044289|F|0-44:G>A-44:G>A	TGCAGCTTCGGGTCGGCCCAGGAGACCCCGCAGCTCAACCTGGCGGGCCACTGCGACCCCTACTCCAAC	44:G>A
5578133|F|0-17:C>T-17:C>T	TGCAGCTCCGAGAACCCCCGCTCGTTGCCG	17:C>T
5579624|F|0-44:C>G-44:C>G	TGCAGCGGTTGGCCGCCGCCTTCAGGCCGCGAATCGACGCGGCGCTGAAGTGGATGACTCGCTCGCGGA	44:C>G
5411967|F|0-32:C>A-32:C>A	TGCAGCGCGGAGACCATCGCGGCGGCGGCCCCCGAGGTGACGGCCTCCG	32:C>A
7173863|F|0-29:C>T-29:C>T	TGCAGGGTCGTACACAGCCAGCGTCCGCTCGGAGCCTGCGTAGCCCG	29:C>T
6043682|F|0-5:A>G-5:A>G	TGCAGATCCTTCAACTAATTCTCTCCG	5:A>G
3027900|F|0-32:C>G-32:C>G	TGCAGCAGCATCGCGTAGAGGATGTGGTTCCACGCGTCCG	32:C>G
3957568|F|0-8:C>G-8:C>G	TGCAGCACCCACGCCGTGCTCAGCCCGCCCGCCGTCGAGAGGCTCTCCG	8:C>G
986253|F|0-31:G>A-31:G>A	TGCAGGCGCAGGGGTAGCGCAGCGGGTGCTCGTCGTCGCCGCGGTTGCGGCAGATCCG	31:G>A
3935430|F|0-7:C>G-7:C>G	TGCAGGACAAGCTTGGTGGCGAGCCG	7:C>G
994127|F|0-34:G>A-34:G>A	TGCAGGTCATGTATGGCCGTAATGCAGTCCTTCAGCTCGGATGCCCTGTACCCG	34:G>A
3024070|F|0-17:C>T-17:C>T	TGCAGCTACCTCCTCAGCCTCCTCCGCCCCGCCCTCATCCG	17:C>T
1090096|F|0-61:C>A-61:C>A	TGCAGGAAATCCAGGCTGGGCCTGCCGTGCTTCATGCAGTTCTGGTAGTCCTGGATGAAGGCGCACGTC	61:C>A
3574101|F|0-11:C>G-11:C>G	TGCAGCAACGCCAGCAGCGACGACTGGAACGGCTCAGCCG	11:C>G
7906813|F|0-17:T>A-17:T>A	TGCAGTGCAGCCCAGGCTCCG	17:T>A
1004996|F|0-16:A>G-16:A>G	TGCAGCAGCTCAAGAGAGTGGACGACTGGCTCAACCATGTCGTGCGGCAGGAGAGGATGCTTCCGAGAT	16:A>G
1088261|F|0-7:C>T-7:C>T	TGCAGGTCGCGGAGAAGCCCAGCATCGCGCTCTCCATCGCCTCCGCCCTCTCCG	7:C>T
1003887|F|0-27:C>T-27:C>T	TGCAGCACCTTCACGGCAACCATGGTGCCGTCCCGCAGCGTGCCGCGGTACACGCGGCTGTAGCTGCCT	27:C>T
1102865|F|0-16:C>A-16:C>A	TGCAGCAGATGCTCGGCGTCTACGACGAGATCCTCGCCAAGAACCAGTACCTCGCCG	16:C>A
6049162|F|0-8:A>C-8:A>C	TGCAGGATATTCAGGAGGACCATTACCTACCG	8:A>C
6048914|F|0-19:T>G-19:T>G	TGCAGCTGCTCCGTGGGGTTACCCCCAGGCTCCG	19:T>G
4005999|F|0-14:T>C-14:T>C	TGCAGCGCGGCGGTTAGCATGATCGGGTCCACCAGCGTCCG	14:T>C
1133314|F|0-11:A>G-11:A>G	TGCAGGAAGTCATACATCTTCTGCGGGACGAACCGCTCCCGCGCGGCCGTCACCTGTTCGGGGAGGTAC	11:A>G
986769|F|0-14:G>A-14:G>A	TGCAGCGAGAAGGAGGCGCTGGTGTCCTTGGGGTGCTTGGAGACGAGGATCCCGACGCCCTGGCCCCGC	14:G>A
1018552|F|0-18:A>G-18:A>G	TGCAGGAGGGCGGGCGGAAGCTCAAGGTGGACGCCTGGTTCGGCACCCGCAAGACCATGGCCGCCATCC	18:A>G
3949770|F|0-17:C>A-17:C>A	TGCAGCCGCTTCACCGCCGCCGTGGCGCCG	17:C>A
1012889|F|0-31:C>T-31:C>T	TGCAGTACTCGCTCTACTTCGTCTACCTCGGCCTCGTCGTCTGCGCATCCTCGTACCTGGGTGAGTTCA	31:C>T
1005371|F|0-5:T>C-5:T>C	TGCAGTTGATGTCCGCGGGCCCGCTGGTGGACGTGGGCCTCGCGGCGCTGCGGTCTCCG	5:T>C
12341528|F|0-13:G>C-13:G>C	TGCAGCTGAGGACGTCGATCGCGGGGTGCCTGCTGGTGTCGGACAACCTGGAAGGGTCGTCCAGGCCGC	13:G>C
1319093|F|0-49:C>T-49:C>T	TGCAGAAGCTGACGCCATGGGCGGACGGGCCGAGCATGGTGACGCAGTGCCCGATCCAGCCCAACGGCT	49:C>T
3946718|F|0-15:C>T-15:C>T	TGCAGCAGGAGCTCGCCGACGTTGTGGGGCTCGACCG	15:C>T
4262721|F|0-14:C>T-14:C>T	TGCAGGTATAAATACACAAGCGCGCATCACCTACCGTTGATTTGCCG	14:C>T
3952878|F|0-26:C>G-26:C>G	TGCAGGACCGCAAGAGGACCAAGGTGCCCGCCG	26:C>G
2243729|F|0-48:G>C-48:G>C	TGCAGTTCTCGATGTGCAGGTCCTCGAGGACGGGGCACTGGTCGCCGAGATCCCGCCCGAAACTAGGGC	48:G>C
984784|F|0-34:C>G-34:C>G	TGCAGGTGGACGGCTGCTACGTGCGGTACGACGCCAGCGACTTCGTCGGCCG	34:C>G
1020233|F|0-23:T>G-23:T>G	TGCAGCTGGAGATGGCGGCGGCGTAGGTGGTTGTGAGGAGTGGGGAGGGGGAGAGCCG	23:T>G
1022708|F|0-58:G>C-58:G>C	TGCAGGTTCTGCGTGGGGAGGTAGGCGGAGAAGACGAAGAGGAAGGCGGCCGACAGGAGGTGAACGTCG	58:G>C
1026246|F|0-24:C>T-24:C>T	TGCAGGGCTGGTCCTGGCTTCGCTCGCCGTGGTGCTGGCAACCACGCTGTGGAAGGCTGCGGTGCACCT	24:C>T
4009326|F|0-19:T>A-19:T>A	TGCAGGACTTGATGGGGTATACGAGGATGGACGCCACCGTCGCCGCCG	19:T>A
13871883|F|0-13:C>T-13:C>T	TGCAGGTCATGTCCAGGACCG	13:C>T
1059098|F|0-43:A>G-43:A>G	TGCAGGTAAGTGTAGGTAACTTCAGTGTACTGTAGCAAATAACATTTTGTCAGGAGATCACGCTCAAGA	43:A>G
5363999|F|0-28:G>C-28:G>C	TGCAGTACATGGACCTCGCCAACAACTCGCTCGCCG	28:G>C
1023867|F|0-9:T>C-9:T>C	TGCAGCCCATGGGCAGCTGCTACAACCCCAACACGATCCAGGCGCACGCCTCCTACGCCTTCAACGCCT	9:T>C
32027438|F|0-27:T>G-27:T>G	TGCAGCCCATGCCCGAGAGGTTGAAGATCTTAATGTCCGCGCGCATCCG	27:T>G
7353596|F|0-8:G>A-8:G>A	TGCAGCTCGAAGAAGCCGTGTTGGATACCG	8:G>A
1023099|F|0-33:A>G-33:A>G	TGCAGAGCGACGTGTGTATTTGTGGCGTGGGGGACGCAGCATTGGATCGCCCTCATTCCG	33:A>G

989113|F|0-52:C>G-52:C>G	TGCAGAAGGCCATCGGCGAGCACGACGCGGCCGCCGTGACGCGGAGGCTGGACGAGTACGAGGCGGCGC	52:C>G
4262196|F|0-7:C>G-7:C>G	TGCAGCCCACCACGCACTGCCACCCCG	7:C>G
4262907|F|0-22:G>T-22:G>T	TGCAGGAGTTCGAGTGGCGCGCGCACCCGTCGCAGCCG	22:G>T
1090275|F|0-26:G>A-26:G>A	TGCAGTTCCCGCCGCTTCATCGCCGCGAAATCCATATTGGCCGCGCGCACGAATCCAAACCCCACCCCG	26:G>A
4993562|F|0-17:G>A-17:G>A	TGCAGCGGGCGGCGACGGCGAGGCAGGGCTGACCCG	17:G>A
1081828|F|0-55:C>A-55:C>A	TGCAGTTCGACAGGTACAGATACGTCAGGTTCTTGAGGTTCCCAATGCTCGGAGGCATCTCCCCTTGAT	55:C>A
4005179|F|0-35:A>G-35:A>G	TGCAGCAGATACTTGTGCGTGAAGAACTTCTTCCCACAGCCCTCGACCG	35:A>G
3961213|F|0-29:C>T-29:C>T	TGCAGACGTGGCACGTCCCCACCGCCAGCCCGAGCATGTCGCGGCAGGGCGAGCAGATCAGGTGCCCAG	29:C>T
4397492|F|0-5:C>G-5:C>G	TGCAGCAGCCTGGCGCACGCGTGCGCGTCGACCCCG	5:C>G
990515|F|0-22:C>T-22:C>T	TGCAGCTCATATCATTCGGCGACGCCATTTCTTCCTCCAGCCGCTCGCCG	22:C>T
2251741|F|0-55:C>T-55:C>T	TGCAGCGAGGACTGGAAATTGGAGAGCAGGCGCGTGAGGTCCCGTGGCCGTATGGCGAGGCGGCTGGGG	55:C>T
3028072|F|0-37:C>T-37:C>T	TGCAGTTCATGTCAACAAGGGTGAACTACCCTGGCAGCGCCG	37:C>T
7170733|F|0-13:T>G-13:T>G	TGCAGCTGCACGATGCATCGGTGAACGATCTCCTGATACCCGCCAAGCCTCCG	13:T>G
32025371|F|0-19:G>T-19:G>T	TGCAGGAGCGGCGCGACGCGCGCCATCTCCTCCTTGGCCGCTGCGTACCG	19:G>T
3949686|F|0-6:G>T-6:G>T	TGCAGCGTCGGGGACACCTCGCCG	6:G>T
4005941|F|0-7:C>T-7:C>T	TGCAGTGCACGTCCTCGGACAGGGACAGGTTCAGGGGGAAGGCCG	7:C>T
32026236|F|0-10:G>C-10:G>C	TGCAGGGTTCGACGGCGCGTCCTTGCCG	10:G>C
5361701|F|0-43:A>T-43:A>T	TGCAGTACAGGAAGGAGTATATGGCGGCGCAGACCGTGAACGGAATCGCGATGGCCGTGTACAGCGCCT	43:A>T
32024545|F|0-55:C>A-55:C>A	TGCAGGCCAGGAAGCTCTCACTCGGAGGAGGCATCGGCGGCAAGCTCAAGGCTGTCGGGGAGATGGGGG	55:C>A
5354789|F|0-14:T>C-14:T>C	TGCAGCGCGTCCTGTGCCACCGACGTCGACGCCGCCATCGACACGGCGAGCAGCACCGCCGCCAGCTGA	14:T>C
3026535|F|0-6:A>T-6:A>T	TGCAGCAGCTGCTCCAGCTCCTTGACGAAGTTGATGGCCCCG	6:A>T
4993540|F|0-26:C>T-26:C>T	TGCAGACGGCGGCGGCGCGGACGAGGCTCCTGGGGTCGTCCGCCG	26:C>T
1025335|F|0-51:G>C-51:G>C	TGCAGCAGCAGCAGCCAATGGAGAGCTTCTTCGCGTGCAGCGATGGCGGCGGCATTGGGTCGTTGGGGA	51:G>C
3031666|F|0-14:C>T-14:C>T	TGCAGCGTCCGCGGCATCGGCGGCATGTCGACGTGCACGACGCCCTCCAGCGCCTGCACGGCCTGCGCC	14:C>T
5581409|F|0-23:C>G-23:C>G	TGCAGCGTCACGGTGAGGTAGGACGCCG	23:C>G
5971256|F|0-26:G>C-26:G>C	TGCAGGCTGGTGAGGCCGACGGAGCGGAGCCCGTCCAGGATCTCCG	26:G>C
1095087|F|0-14:C>G-14:C>G	TGCAGCATCGGGTTCAGGTGGCCCTGGAACGGCAGCGGGAACAGCAGCACCCGCCG	14:C>G
6046782|F|0-34:T>C-34:T>C	TGCAGGACACGTACAGGGGGAGGGTGTGCGAGTGTCCG	34:T>C
1017783|F|0-55:A>G-55:A>G	TGCAGTTGGTCTGCTGCTCCGCGAAGAAGGGCCAGCTGAGCATCGGCACGCCGTTAGAGATGCTCTCCA	55:A>G
983735|F|0-8:G>C-8:G>C	TGCAGCTTGGAGCAGCACCTGCCGATAGCCTTGAGGCCTTCATTGGCAACACCAGAGCATGCTTCAATG	8:G>C
3029515|F|0-14:C>T-14:C>T	TGCAGCACGGGGTCCGCCGCCTCGGCGCTCGGCGACCACCGCCG	14:C>T
1064061|F|0-45:G>A-45:G>A	TGCAGGAACGCCATCCTCCTCATCCGCCGCAGCTGCTCCGTGGTGGTGGCGTCGTGCGCCTCGCTCTGC	45:G>A
3028714|F|0-13:T>C-13:T>C	TGCAGCATGTCGATGTACCCGTCGAGGGCCCTGGCGTGCAGCCCG	13:T>C
6049153|F|0-7:C>G-7:C>G	TGCAGCGCGTCGGCAGTGGGGCCAGCGCGGTCATGGACGCGCCG	7:C>G
4005941|F|0-33:G>A-33:G>A	TGCAGTGCACGTCCTCGGACAGGGACAGGTTCAGGGGGAAGGCCG	33:G>A
1085343|F|0-28:G>A-28:G>A	TGCAGCATGTACCGCCCGTTGTTCATCAGGAAGATGCTGCTCAGGGCCG	28:G>A
3022332|F|0-26:G>A-26:G>A	TGCAGGGTAACCATCTCGAAGAAGGAGTCCTCTGGCCCG	26:G>A
6046172|F|0-58:C>G-58:C>G	TGCAGACGCCGACGTACGTGGTGCGCGCGCGCCTGGGCACCCCGCCGCAGCAGCTCCTCCTCGCCGTCG	58:C>G
1025047|F|0-16:C>G-16:C>G	TGCAGCGGGGTGAGGACCCTGAATGGCAAGGCGTCCACCTCGGCCGACCG	16:C>G
6043924|F|0-34:G>C-34:G>C	TGCAGCGGGAGGCGGCCATGGCGGCCCGCGAGCGGCGCCGCATCCAGGCGCGGGTCAACCG	34:G>C
3935723|F|0-58:G>A-58:G>A	TGCAGATCGGCCTCCCCGTGGTGCCCTTCGCGCGCGCCATGGACGACGTGCTCGAGACGCTCTTCCTCC	58:G>A
1021077|F|0-22:G>T-22:G>T	TGCAGCAGGAGCAGCCGCAGCTGGTGCCGCACTTGCACTGGGTGCAGCCGCAGCCGCCATCCTCCGCCG	22:G>T
5332172|F|0-14:T>C-14:T>C	TGCAGCTTGTACGGTGCGGCGGCGGCCG	14:T>C
1067301|F|0-43:G>A-43:G>A	TGCAGAGCGTGTACGTGGAGCACGAGGTGGAGGCGCTGATCCAGGTGGCGCTGCTGTGCACGCAGGGGT	43:G>A
32024106|F|0-28:G>C-28:G>C	TGCAGCAGGCCTTCGGCTACCAGCACGGGCCG	28:G>C
3939518|F|0-17:G>C-17:G>C	TGCAGGTGGCCGCGCGTGAGGGGGGCCG	17:G>C
1034916|F|0-10:C>A-10:C>A	TGCAGTGGGTCGGCCTGGTGCTCATCATCGGGGCATTGGTGTGCAGCCTCACGATAAAGCCCTTGTCCC	10:C>A
4008657|F|0-17:G>C-17:G>C	TGCAGGTAGCACCAGTCGAGCCTGTGCTCCTCCTTGCGCTGCCCG	17:G>C
3946690|F|0-17:T>C-17:T>C	TGCAGGTCTTCAGGGTTTACAATGCCG	17:T>C
1077776|F|0-40:A>G-40:A>G	TGCAGCTTTTCTCCGTGGTTACTGCCCTGCTACTCCACGGACTTTATTTCTCACCG	40:A>G
1207964|F|0-34:A>C-34:A>C	TGCAGAGGCGAGCGGGTGGTTGTGCTGGGCTACAAACTCAGTGACACGGAAGGCATTGGCCGAAGGGAG	34:A>C
1223619|F|0-20:C>G-20:C>G	TGCAGGTCGAGCGTGACCTCCACCAGCTCTTCCCCGTCCTCGGAGGCCG	20:C>G
1051808|F|0-11:C>T-11:C>T	TGCAGGAACGGCACCATCCTGCCCAAGACCATGGACGAGGCGCAGTCCAGGTCGGCGTTCCAGATGGAG	11:C>T
1092174|F|0-16:A>G-16:A>G	TGCAGTGAATTAATGGATGCAACGAGATCAGAAACCGTTGCCAGACCACGACGCCGACACCGACACCGA	16:A>G
7167193|F|0-59:C>T-59:C>T	TGCAGGTGCAGGCTGGACCTAGTGTGCACGATGATCCCAAACACCTCGCCCATGTCCAGCGCCTCCGAG	59:C>T
1069859|F|0-63:C>G-63:C>G	TGCAGCTCCGCTGAGGCCTCAGAATGAGCTCAAGCACGCAAGACCCTGCGCGACCACGGACAACGACCG	63:C>G
1058292|F|0-22:T>C-22:T>C	TGCAGATGCTGCTCCTGGACAGTTGCTTCGTCCTCGTCGTGAGCATGATGCTCAGCAAGACCG	22:T>C
1148893|F|0-44:C>T-44:C>T	TGCAGAACGTCCTCCCCACGCAGCTCCTCTCCCTCCTCCACTCGCTCTGGCAGTCGCTCCAGGACTCGC	44:C>T
1051808|F|0-59:C>T-59:C>T	TGCAGGAACGGCACCATCCTGCCCAAGACCATGGACGAGGCGCAGTCCAAGTCGGCGTTCCAGATGGAG	59:C>T

<table>
<tr><td>3934915|F|0-11:G>T-11:G>T</td><td>TGCAGGCTCTCGGGGTTGAGCTTCTTGACGGCGACGGGCATGCCG</td><td>11:G>T</td></tr>
<tr><td>988829|F|0-19:G>C-19:G>C</td><td>TGCAGGCGCCCAGCAGCGCGCCCCAGACCACCGAGTGCTCCTGGCAGTCCG</td><td>19:G>C</td></tr>
<tr><td>1719641|F|0-34:T>C-34:T>C</td><td>TGCAGCTCTACAACAACAGCCTGAGCGGAGAGGTTCCTGCCGGGATCACTCAGTCGAGGAAGCTCAGAG</td><td>34:T>C</td></tr>
<tr><td>1279123|F|0-40:G>C-40:G>C</td><td>TGCAGCGGATGGGCGTGGGGAGGGTCCTGGTGGTTCTGGCGATGGCGACGGCGGCGCTGGTGGAGAGCC</td><td>40:G>C</td></tr>
<tr><td>3028714|F|0-41:C>T-41:C>T</td><td>TGCAGCATGTCGATGTACCCGTCGAGGGCCCTGGCGTGCAGCCCG</td><td>41:C>T</td></tr>
<tr><td>1025759|F|0-52:G>C-52:G>C</td><td>TGCAGGAGGCCCTCCGCCGCGACCATGCCGCCAGCACGCGGCGGCGCTTGTTGAAGGGGTTCTCCGAGA</td><td>52:G>C</td></tr>
<tr><td>1025479|F|0-10:G>C-10:G>C</td><td>TGCAGGCGTCGGTGATCCGCGACCTGGTCCTCCTCTCCTGCGTCGGCCTGCGCCCCGTGCTCGTGCACG</td><td>10:G>C</td></tr>
<tr><td>7161502|F|0-20:C>G-20:C>G</td><td>TGCAGCTCCAGGTACGTGATCGACGTGCCG</td><td>20:C>G</td></tr>
<tr><td>1092512|F|0-32:G>A-32:G>A</td><td>TGCAGAGGACGCTGGACTACGCGTGTGGCCACGGCGCCGACTGCGCCCCGCTGCTCCCCG</td><td>32:G>A</td></tr>
<tr><td>4398173|F|0-17:G>C-17:G>C</td><td>TGCAGCGACGCCGCGTAGGCGCCCG</td><td>17:G>C</td></tr>
<tr><td>5369360|F|0-11:G>T-11:G>T</td><td>TGCAGCAGCTCGTGCTGAACTACGTGCCCG</td><td>11:G>T</td></tr>
<tr><td>4262636|F|0-23:G>A-23:G>A</td><td>TGCAGGACTGGAGATCAGCACAGGCCGTCTTTTGCTCCACAACGCCG</td><td>23:G>A</td></tr>
<tr><td>7350881|F|0-6:G>C-6:G>C</td><td>TGCAGGGTTCAGGTGGGACCG</td><td>6:G>C</td></tr>
<tr><td>1052648|F|0-7:G>A-7:G>A</td><td>TGCAGCAGGCTCACGAGCTCCTCCTCCGACTGTGACGAAACCATACAAAGGTACAGTTTGCAACAGAAG</td><td>7:G>A</td></tr>
<tr><td>5324392|F|0-9:A>G-9:A>G</td><td>TGCAGGTCCATCACGACAAGAAGCTGACGGCGCTCCG</td><td>9:A>G</td></tr>
<tr><td>5332338|F|0-24:G>A-24:G>A</td><td>TGCAGGCGCTCCCCGACGCGCGACGCGCCG</td><td>24:G>A</td></tr>
<tr><td>3534305|F|0-8:C>G-8:C>G</td><td>TGCAGGATCATGGCGAGCCCCATTTTAGCCTCGAGCAGTGCGAAGCTCTGGCCGACGCAGATCCGCGGG</td><td>8:C>G</td></tr>
<tr><td>5366398|F|0-5:G>A-5:G>A</td><td>TGCAGGCGGCGGAGGCCAAGCTGGACATGGCGCGGGCGGAGCTCGAGTCCGCCAAGGGAGAGAGCCTCC</td><td>5:G>A</td></tr>
<tr><td>1005372|F|0-67:G>C-67:G>C</td><td>TGCAGTTGAGGATCCGCGGGTCGCGGGCGACGACGTGGGCGATGTCGGCCTTGGAGAGGCGGAGGGTGG</td><td>67:G>C</td></tr>
<tr><td>4396183|F|0-11:C>G-11:C>G</td><td>TGCAGCAGCGGCATCAGCCG</td><td>11:C>G</td></tr>
<tr><td>3951850|F|0-7:A>G-7:A>G</td><td>TGCAGCCAGATCATCATCGTGCCG</td><td>7:A>G</td></tr>
<tr><td>3955448|F|0-21:G>A-21:G>A</td><td>TGCAGCTGAATCTCATGTACAGGTTCCTCCCG</td><td>21:G>A</td></tr>
<tr><td>4261550|F|0-6:T>C-6:T>C</td><td>TGCAGCTGCGGGCCGACGTGCTCAAGCAGCTGGACGGCGCGCCG</td><td>6:T>C</td></tr>
<tr><td>2277895|F|0-14:C>G-14:C>G</td><td>TGCAGAGCGGGCAGCTGGTGTGGGAGGCAAGCCACATGTCGACGCACTCGGTGTGGAAGCCGTGGCCGC</td><td>14:C>G</td></tr>
<tr><td>1005586|F|0-15:T>C-15:T>C</td><td>TGCAGGCAAGCCTTTTCCGTCGCTCTTGATGGATAGCAGCAGGAGGAGAGCCTTCTTCAATCTCGCAGG</td><td>15:T>C</td></tr>
<tr><td>1067301|F|0-35:C>T-35:C>T</td><td>TGCAGAGCGTGTACGTGGAGCACGAGGTGGAGGCGCTGATCCAGGTGGCGCTGCTGTGCACGCAGGGGT</td><td>35:C>T</td></tr>
<tr><td>32027588|F|0-32:T>G-32:T>G</td><td>TGCAGCATGGCGTTGCTCCACGGGAAGCCGCCTTTTGCGGAGTATGCCCCG</td><td>32:T>G</td></tr>
<tr><td>7172958|F|0-15:G>A-15:G>A</td><td>TGCAGGTCGGGGCGCGTCGGTCGCCGACGCCGAGAAGACCG</td><td>15:G>A</td></tr>
<tr><td>100582063|F|0-12:A>T-12:A>T</td><td>TGCAGGCGAAGCAGACGCCAACCGAACCCCCATGAAAAAGAAGCTTAAAAATGCGTTCGTGCTCGTGAA</td><td>12:A>T</td></tr>
<tr><td>6046415|F|0-59:C>G-59:C>G</td><td>TGCAGGTAGCGCGCGGCGCAGCGGAGCATGGCGACGTTGGCAACCGTGATGTCGACGTGCACGCCGTAG</td><td>59:C>G</td></tr>
<tr><td>991324|F|0-16:G>A-16:G>A</td><td>TGCAGAGCGCCATCCAGGCCGCCATCGCGCACTGCAAGAACTCCATCGCCGTGGCCAAGCAGTAGTACG</td><td>16:G>A</td></tr>
<tr><td>4008942|F|0-10:C>G-10:C>G</td><td>TGCAGGCGTCCTTCCTGGAGGTGTTCGTCCGCGGCCG</td><td>10:C>G</td></tr>
<tr><td>4262167|F|0-17:C>T-17:C>T</td><td>TGCAGGGGCCCTTCTCGCTGCGCGTCACCGACGAGTCCG</td><td>17:C>T</td></tr>
<tr><td>3938519|F|0-14:G>T-14:G>T</td><td>TGCAGATTTCAGTCGGGTCCG</td><td>14:G>T</td></tr>
<tr><td>1250878|F|0-10:G>A-10:G>A</td><td>TGCAGAAGGCGCACAGGCGGCTGTCGCAGGGCAGCGTGGACAGCGCCATGACCATGGAGATCCAGAAGT</td><td>10:G>A</td></tr>
<tr><td>5580226|F|0-6:C>T-6:C>T</td><td>TGCAGCCGCACCGCGCACGGCAGCGCCG</td><td>6:C>T</td></tr>
<tr><td>7172612|F|0-20:G>A-20:G>A</td><td>TGCAGCTTGGTGAGCCCCGCGAGCGACTCCG</td><td>20:G>A</td></tr>
<tr><td>4262636|F|0-31:T>C-31:T>C</td><td>TGCAGGACTGGAGATCAGCACAGGCCGTCTTTTGCTCCACAACGCCG</td><td>31:T>C</td></tr>
<tr><td>3957568|F|0-38:G>C-38:G>C</td><td>TGCAGCACCCACGCCGTGCTCAGCCCGCCCGCCGTCGAGAGGCTCTCCG</td><td>38:G>C</td></tr>
<tr><td>1059056|F|0-5:G>T-5:G>T</td><td>TGCAGGTGTATGCCACGATCAGGACGGGGTCCATGGCGATCGCGGCGTCCACGCTGGACTCGCTGCTGG</td><td>5:G>T</td></tr>
<tr><td>1203264|F|0-50:G>C-50:G>C</td><td>TGCAGCGCCATCCAGCCAGGCAAGCCCTGCTACGAGCCCAACACCAAGCTGGCGCACGCGTCGTACGCC</td><td>50:G>C</td></tr>
<tr><td>1023454|F|0-11:G>A-11:G>A</td><td>TGCAGTCCGTCGTCCTCGGGTCGCAGCTGAAGCACCTGCTGGGGAGCCTCGGCTCCTCCG</td><td>11:G>A</td></tr>
<tr><td>1129938|F|0-40:C>T-40:C>T</td><td>TGCAGCGCATCGCCTGGGTCGGCGACGACCACGAGCGCTTCCTCTCCAAGTTCAAGGACCGCGTCGACC</td><td>40:C>T</td></tr>
<tr><td>4261607|F|0-7:C>T-7:C>T</td><td>TGCAGCTCCAGCATGGCGTTGTACTTGTACAGCCG</td><td>7:C>T</td></tr>
<tr><td>3947004|F|0-14:G>A-14:G>A</td><td>TGCAGGACGGCGGCGTCGACCG</td><td>14:G>A</td></tr>
<tr><td>984784|F|0-25:G>C-25:G>C</td><td>TGCAGGTGGACGGCTGCTACGTGCGGTACGACGCCAGCGACTTCGTCGGCCG</td><td>25:G>C</td></tr>
<tr><td>2245927|F|0-54:C>T-54:C>T</td><td>TGCAGGCGCGCCTCGCCGCCGTCAAATGGATCCTTGAAGTGAGCACTCACTCAGCTCCTCGCTCTCCCG</td><td>54:C>T</td></tr>
<tr><td>1022708|F|0-5:G>A-5:G>A</td><td>TGCAGGTTCTGCGTGGGGAGGTAGGCGGAGAAGACGAAGAGGAAGGCGGCCGACAGGAGGTGAACGTCG</td><td>5:G>A</td></tr>
<tr><td>1053162|F|0-66:G>A-66:G>A</td><td>TGCAGAGGCGAGCGGGTGGTTGTGCTCGGCGACAAATTCAGTGACACGGAAGGCATTGGCCGAAGGGAG</td><td>66:G>A</td></tr>
<tr><td>1021437|F|0-41:C>G-41:C>G</td><td>TGCAGCCAGTTGAGCAGCCTGCCGATCTTCTTGGACACGAACACCCAGCCG</td><td>41:C>G</td></tr>
<tr><td>1151000|F|0-38:G>A-38:G>A</td><td>TGCAGGTACAGTCCTAGCATGCGGTATAGTTGGGCGCCGCAGGCCTGCCCGCATAGCGTCTCCAGGGCG</td><td>38:G>A</td></tr>
<tr><td>1028020|F|0-9:A>G-9:A>G</td><td>TGCAGGTCGATGGACTGAGGCACGGAAGTTTCGTCCATCTTGGCAGCAAGAGACAGGCACGCCACCGAT</td><td>9:A>G</td></tr>
<tr><td>992207|F|0-46:G>C-46:G>C</td><td>TGCAGTTCTGCGACGAGGTGGGCGTCAAGCGCCACGTCCTCAAGGTGTGGATGCACAACAACAAGCACA</td><td>46:G>C</td></tr>
<tr><td>1382098|F|0-26:C>G-26:C>G</td><td>TGCAGCCTGAGCCGACCGCCCTCGCGCGACGCCCGCAGGAGCTCCTTCCCGGGGATGACAACCTCCACC</td><td>26:C>G</td></tr>
<tr><td>3946714|F|0-8:A>C-8:A>C</td><td>TGCAGGTAAAGAAGGCGACGACGACGGACCCG</td><td>8:A>C</td></tr>
<tr><td>3946717|F|0-19:C>T-19:C>T</td><td>TGCAGCGCGAGTTCCGCGGCGCCGTCAGGTACGCCATGGAACGCCG</td><td>19:C>T</td></tr>
<tr><td>4261396|F|0-17:C>T-17:C>T</td><td>TGCAGCGCCTTGGCGTCCCTCTCTCGTCGCTGCTCCG</td><td>17:C>T</td></tr>
<tr><td>3951817|F|0-8:C>T-8:C>T</td><td>TGCAGCAACGGCACGTCGACCGATGGGTTCCTTCCG</td><td>8:C>T</td></tr>
<tr><td>3948024|F|0-8:C>T-8:C>T</td><td>TGCAGGTACGAGTTGTCGTACACCCCG</td><td>8:C>T</td></tr>
</table>

<table>
<tr><td>1092625|F|0-14:G>T-14:G>T</td><td>TGCAGGCGAGGATTGTGCTCGGGGAGGACCCAGGCGACGCGCCGCTCGCCGTCGCGGTGCACCCCACCG</td><td>14:G>T</td></tr>
<tr><td>3937409|F|0-31:G>C-31:G>C</td><td>TGCAGACGTGGCACGTCCCCGCCGCCACCCCGAGCATGTCGCGGCAGGGCGAGCAGATCAGGTGCCCAG</td><td>31:G>C</td></tr>
<tr><td>1033462|F|0-19:C>T-19:C>T</td><td>TGCAGCCATGCGACCTGCTCGGCGTACTTGTCGGGGGTCGCGTTGGGGTCGTTGCCGCACTCCACGTTG</td><td>19:C>T</td></tr>
<tr><td>2247075|F|0-49:C>A-49:C>A</td><td>TGCAGGAGCACCGCCTGCCGCTGTCTAACCTGGACCTCCTCCTGCCGCCCATCGACGTCGGCGTCTTCT</td><td>49:C>A</td></tr>
<tr><td>1759191|F|0-33:G>C-33:G>C</td><td>TGCAGCAGCGACGACGACGCCATCGTCGGCATGGTGACCTGCGTCTGGAGGAACGGCTGCTGGAGCTGG</td><td>33:G>C</td></tr>
<tr><td>6047853|F|0-14:G>A-14:G>A</td><td>TGCAGGCGGCGACAGAGACAGGCGACCGTCGGCCG</td><td>14:G>A</td></tr>
<tr><td>1020517|F|0-33:G>T-33:G>T</td><td>TGCAGGCCAGCATCATCTCGCCCTAGCTCCATGGCTAGCGGGCATTTGACATGGATTCCG</td><td>33:G>T</td></tr>
<tr><td>6040570|F|0-35:A>G-35:A>G</td><td>TGCAGGTCCTGCAAGTAGAAGGAAGGCTTGGTGGGACGGAGCGCAAGGTAGACAACGAGCACGATGAAT</td><td>35:A>G</td></tr>
<tr><td>1044692|F|0-34:A>G-34:A>G</td><td>TGCAGCTCATGAGGCCATTAAAAGATGCAGCCTGACAGAGCTCTGGACGGCCG</td><td>34:A>G</td></tr>
<tr><td>7173323|F|0-17:C>G-17:C>G</td><td>TGCAGGTAGGGGAGCCTCTGCGTGTCCG</td><td>17:C>G</td></tr>
<tr><td>22838970|F|0-34:C>G-34:C>G</td><td>TGCAGCTCCCCTACGGCATCGACTTCCCGCAGTCCAGGCCCACCG</td><td>34:C>G</td></tr>
<tr><td>1083022|F|0-7:C>G-7:C>G</td><td>TGCAGCTCCTCTCGGTGGCCTGCCTCTCGCTGGCGGCCAAGGTGGAGGAGCACCG</td><td>7:C>G</td></tr>
<tr><td>1023449|F|0-15:C>T-15:C>T</td><td>TGCAGTGGAGGCGCACGGTGGACGACCTGCTGGCGCTGGGCAAGCTCAACAACTGCCTCGCCGTGTGCG</td><td>15:C>T</td></tr>
<tr><td>3026535|F|0-20:C>T-20:C>T</td><td>TGCAGCAGCTGCTCCAGCTCCTTGACGAAGTTGATCGCCCCG</td><td>20:C>T</td></tr>
<tr><td>1020842|F|0-16:C>G-16:C>G</td><td>TGCAGAGGTTCTTCCTCGGAGGGAGACTGATATTCGGACCCGATGCCAAGTCCCTGCTCGTCTCGGTTG</td><td>16:C>G</td></tr>
<tr><td>1057485|F|0-28:G>C-28:G>C</td><td>TGCAGGCGCAGAGCAAGGTGATGGTGCTGGCCTGGATCAGCCTCGCCGCGCTCGCCGCGCACGTCGCCA</td><td>28:G>C</td></tr>
<tr><td>1087845|F|0-13:T>C-13:T>C</td><td>TGCAGCAGAGTTATGATCATGAGAAATCACATTTCAGATACTAGACGATATCGCCG</td><td>13:T>C</td></tr>
<tr><td>2254577|F|0-56:G>A-56:G>A</td><td>TGCAGCACCTCAGCGGCCAGCCCAACATCGCCGAGTTCAGGGGCGCCTTCGAGGACGCCGAGAACGTGC</td><td>56:G>A</td></tr>
<tr><td>3946714|F|0-16:G>C-16:G>C</td><td>TGCAGGTAAAGAAGGCGACGACGACGGACCCG</td><td>16:G>C</td></tr>
<tr><td>1042149|F|0-40:A>G-40:A>G</td><td>TGCAGGAGGCCATGGAGTTCCTGAGGAGGGTGGAGACGCAACTCAACTCCCTCTCCATCTCCG</td><td>40:A>G</td></tr>
<tr><td>16796264|F|0-31:G>C-31:G>C</td><td>TGCAGCAGGCGGCGGCGCGCGCGTGGTCGCAGGAAGGGTCGTCCCGCCG</td><td>31:G>C</td></tr>
<tr><td>1697137|F|0-5:G>A-5:G>A</td><td>TGCAGGCCCCTCCAGCTCTCCTTGTAGTTCCAGTACGTGTTGAGGCAGTGCCCGAGCGCGTCGATCATC</td><td>5:G>A</td></tr>
<tr><td>1667190|F|0-47:A>G-47:A>G</td><td>TGCAGATCGGATTCGCCCTCAAGAAGGTGTTCGACGAGGGCATAACCAGCCG</td><td>47:A>G</td></tr>
<tr><td>6038041|F|0-10:A>C-10:A>C</td><td>TGCAGGCCACAAACACCTACCCG</td><td>10:A>C</td></tr>
<tr><td>4262109|F|0-14:C>G-14:C>G</td><td>TGCAGGTGAGTGCGCGTGTGGCCG</td><td>14:C>G</td></tr>
<tr><td>3952140|F|0-22:G>C-22:G>C</td><td>TGCAGGCCCCATTCCGACCGAAGGACACGACTCCG</td><td>22:G>C</td></tr>
<tr><td>5358402|F|0-18:G>C-18:G>C</td><td>TGCAGGTACCCTTCTTCGGCCTCGCCGTCTTGCACAGCCG</td><td>18:G>C</td></tr>
<tr><td>3028155|F|0-7:C>G-7:C>G</td><td>TGCAGCTCATGCTCAAGGGGTGCGAGCCGCTGCCGCGCCG</td><td>7:C>G</td></tr>
<tr><td>3934544|F|0-16:A>G-16:A>G</td><td>TGCAGTCAAGCCGCCAACGACGCCG</td><td>16:A>G</td></tr>
<tr><td>5581941|F|0-9:G>A-9:G>A</td><td>TGCAGCAGCGGCCCGACCACCAGCACCG</td><td>9:G>A</td></tr>
<tr><td>1004893|F|0-34:G>C-34:G>C</td><td>TGCAGTACAGCCACGCCGATCCCAACCGCACGGTGGTGAACCCGTACGTGCACTTCGGGCAGCCGTCGG</td><td>34:G>C</td></tr>
<tr><td>986352|F|0-5:C>T-5:C>T</td><td>TGCAGCGGCTGCGCCATGATCTCGCTCATCACGCTCTCGTTGTACCCGAGGCCGCACACGTAGGGGATG</td><td>5:C>T</td></tr>
<tr><td>1021567|F|0-7:C>A-7:C>A</td><td>TGCAGACCTTCTGCACCGTCTACGACTGCAAGCGCTGGTTCGGCGGCATACTGGAACTGCCGCAGATAA</td><td>7:C>A</td></tr>
<tr><td>1064108|F|0-45:C>G-45:C>G</td><td>TGCAGTGCTTGGCGTAGATGAGGAAGAGGAAGAGCCCCAGGAGGACGCCCGTGAGCACGCCCACGATGA</td><td>45:C>G</td></tr>
<tr><td>1003268|F|0-14:G>C-14:G>C</td><td>TGCAGGTAGAAGCGGGGCTTGTGGGGGCGGAGCACCAGGTAGACGATGAGGATGACGAAGAGGGCGAGG</td><td>14:G>C</td></tr>
<tr><td>1099015|F|0-37:G>A-37:G>A</td><td>TGCAGCGGTGGGAGCAGGTGATGACGGCGGCGAGGCGGGGCGACCTCCG</td><td>37:G>A</td></tr>
<tr><td>3028486|F|0-19:C>T-19:C>T</td><td>TGCAGCTCATGTACCACTTCGACTGGACGCTGCCCGCCG</td><td>19:C>T</td></tr>
<tr><td>5372200|F|0-5:C>T-5:C>T</td><td>TGCAGCTCCGAGATGCCTTTCTCCCTCCCG</td><td>5:C>T</td></tr>
<tr><td>1109610|F|0-33:G>T-33:G>T</td><td>TGCAGCGAGGCCCGCAGCGCGCTCACCCGCTGCGCCAGCGCGTCCATCGTCTCCG</td><td>33:G>T</td></tr>
<tr><td>3028721|F|0-35:G>C-35:G>C</td><td>TGCAGCACGGGGAACATCACCTGGGCGACGATCACGCCG</td><td>35:G>C</td></tr>
<tr><td>1126318|F|0-9:T>G-9:T>G</td><td>TGCAGTGGCTCCCCCTGAACAGACGGAAGAGGGGGAGAAGGAGGCCCCG</td><td>9:T>G</td></tr>
<tr><td>3029786|F|0-19:G>C-19:G>C</td><td>TGCAGGCGCAGCTCGCCTCGCTCAAGGCGCACGCCCCGCCG</td><td>19:G>C</td></tr>
<tr><td>7344173|F|0-12:T>C-12:T>C</td><td>TGCAGCAGCATCTCCATTGCCG</td><td>12:T>C</td></tr>
<tr><td>1092174|F|0-23:G>T-23:G>T</td><td>TGCAGTGAATTAATGGATGCAACGAGATCAGAAACCGTTGCCAGACCACGACGCCGACACCGACACCGA</td><td>23:G>T</td></tr>
<tr><td>32022402|F|0-9:T>A-9:T>A</td><td>TGCAGCACTTCCAGCTCAACGTTGGCACCG</td><td>9:T>A</td></tr>
<tr><td>4262733|F|0-8:G>C-8:G>C</td><td>TGCAGCGCGAGCACCAGCTTCCG</td><td>8:G>C</td></tr>
<tr><td>1022518|F|0-10:C>G-10:C>G</td><td>TGCAGAGCCTCCCCTCGCAGATGCAGGCCATCAAGGTGATGGCGACGCTGGACATCCTCTCCTCGCACT</td><td>10:C>G</td></tr>
<tr><td>3952024|F|0-32:C>T-32:C>T</td><td>TGCAGGTCGAGCGTGACCTCCACCAGCTCCTCCCCGTCCTCGGAGGCCG</td><td>32:C>T</td></tr>
<tr><td>989034|F|0-23:G>A-23:G>A</td><td>TGCAGATGTCGTCCTCACCGCGTGGCGGGGGGATCAAGCGCAGGTTCGGCACCG</td><td>23:G>A</td></tr>
<tr><td>7160619|F|0-13:G>C-13:G>C</td><td>TGCAGATGGGGCTGCACGTCGGGGGCCG</td><td>13:G>C</td></tr>
<tr><td>32025472|F|0-39:C>A-39:C>A</td><td>TGCAGATCCTTGGCCCCCTCTGCTTCGACATAGGCCACACTGCGTTGACAATGGTGGGGTCGCACGTTG</td><td>39:C>A</td></tr>
<tr><td>2247075|F|0-67:C>G-67:C>G</td><td>TGCAGGAGCACCGCCTGCCGCTGTCTAACCTGGACCTCCTCCTGCCGCCCATCGACGTCGGCGTCTTCT</td><td>67:C>G</td></tr>
<tr><td>7166937|F|0-58:T>G-58:T>G</td><td>TGCAGCTGCTGCGGCGGGCGGCGAGGCGGAGCCGCCACCGCATGTCCAGGCTCGTCGCTCGGACCACCG</td><td>58:T>G</td></tr>
<tr><td>1023867|F|0-50:T>G-50:T>G</td><td>TGCAGCCCATGGGCAGCTGCTACAACCCCAACACGATCCAGGCGCACGCCTCCTACGCCTTCAACGCCT</td><td>50:T>G</td></tr>
<tr><td>3939333|F|0-14:G>C-14:G>C</td><td>TGCAGCGCGTTGCAGGTCGCCGCCTCCGCGCCCGCCG</td><td>14:G>C</td></tr>
<tr><td>6043352|F|0-25:T>G-25:T>G</td><td>TGCAGTGCATGCTGCCAGAGTTTTCTGCGTGCTGTGAAGAGTTGGTGGGCCGATGGACGGAGTCCGTCG</td><td>25:T>G</td></tr>
<tr><td>1020022|F|0-61:C>T-61:C>T</td><td>TGCAGCACCTCCCGTCTCGTATCGGCCACCTCCGCCTCCAGCTTCTTGATGGAGCTCACGACCGCGAGC</td><td>61:C>T</td></tr>
<tr><td>1078294|F|0-13:G>A-13:G>A</td><td>TGCAGAGCGTGACGCTGATCCCGTCGGTTTGATTTTGCTGGCCCTCGTTCAGATTGTAAGAGCCGAGAT</td><td>13:G>A</td></tr>
<tr><td>1079174|F|0-11:C>T-11:C>T</td><td>TGCAGCTTCGGCGGCGACACCTTCAAAGGCTTTTGCGGGGGCGGGGGCGACAGCTTTGAATGCTTCTGC</td><td>11:C>T</td></tr>
</table>

1043324|F|0-38:C>T-38:C>T	TGCAGCTCCTCCTCCACGGCCTTCATCTTGGCCTCCCACTCGGCGAGCCG	38:C>T
4261401|F|0-23:G>A-23:G>A	TGCAGCCCCCGAACGTGGTCGTCGACGAGCAGCATGGTCATGCCGCCG	23:G>A
5411797|F|0-13:C>G-13:C>G	TGCAGGAGGTCTTCGTCCTGCTCCCG	13:C>G
1162679|F|0-36:T>C-36:T>C	TGCAGGAAGTCGCCGACGTTCACCACCAGCGACGCCTCCCCCATCGCCG	36:T>C
2249233|F|0-67:C>G-67:C>G	TGCAGGTGTTCGACGCGATGATCAGGGAGGGACACCAGCCCGACGAGGCCGTGTACGTCGGCGTGCTCA	67:C>G
5364114|F|0-8:G>T-8:G>T	TGCAGTGTGACCCTTACCACCG	8:G>T
990424|F|0-23:G>A-23:G>A	TGCAGGCCGCCGACGTTGTCCTGGTGGAGGATGGTCAGCGACGTCGGGTCGCAATGCGGGCCCGTGCCC	23:G>A
3956856|F|0-32:C>T-32:C>T	TGCAGCTCAGAGAAGTGCAGGATGCAGACGTACCCG	32:C>T
4262979|F|0-23:G>A-23:G>A	TGCAGCAAGCAGAAGGTGAAGCGGGGGCTATGGTCCCCG	23:G>A
992610|F|0-13:G>C-13:G>C	TGCAGTTCAGTGGGCAGTCTCAGCCGTGCGGGCTGCTGCTCTCCAGCACCG	13:G>C
7353569|F|0-11:G>A-11:G>A	TGCAGCTCATCGATGACCGCGCGGCCGCCG	11:G>A
13590277|F|0-17:C>A-17:C>A	TGCAGCGTCACTTTCCTCGCCG	17:C>A
3025561|F|0-7:G>A-7:G>A	TGCAGGCGCCCTTCTCGGTCCGCCTCACCTCCAGCTCCG	7:G>A
4262109|F|0-5:G>C-5:G>C	TGCAGGTGAGTGCGCGTGTGGCCG	5:G>C
3948259|F|0-14:G>A-14:G>A	TGCAGAGGAACAGCGCTCACCG	14:G>A
32025652|F|0-64:G>A-64:G>A	TGCAGGTGCTGCGCATGGTGGGCTTCGCGCGCGAGTTCGGCAGGTTCGCCGACGTCGCCGTCGTGCTGG	64:G>A
3939369|F|0-28:C>T-28:C>T	TGCAGGGCATCGTGTCGGAGGCCAGGGCCGTGTTCCG	28:C>T
7349815|F|0-14:C>A-14:C>A	TGCAGGTGCACGCCCAGCCG	14:C>A
1169049|F|0-13:A>G-13:A>G	TGCAGCCTTCCAAAAATACTGCCCAACTGCCTGTGATGGATCACCAGCTGTACGGGGACCCGTCCGCGA	13:A>G
1044692|F|0-39:G>A-39:G>A	TGCAGCTCATGAGGCCATTAAAAGATGCAGCCTGACAGAGCTCTGGACGGCCG	39:G>A
3941326|F|0-8:C>G-8:C>G	TGCAGCCGCACCGTGCCGCTGGAGGGCAACTCGCCG	8:C>G
1306480|F|0-17:A>G-17:A>G	TGCAGGTATCGATTATGAGTACAAGGCGGTGAACCTCCTCAAGGGCGAGCAGTCTGATCCCG	17:A>G
2244584|F|0-21:G>C-21:G>C	TGCAGATTGCTCGAAGCAGCAGACCTCGACGCCCGCGTTGCCCTTGTGCTCCCCTTCCG	21:G>C
2246457|F|0-12:C>T-12:C>T	TGCAGCTCGTGCCCCCGAGCCGCGCATAAGACGGATCAAGAGCATGAACGCGTTCGACATCATCGCCTC	12:C>T
4261821|F|0-28:T>C-28:T>C	TGCAGTACCTCTCGCTCGCGGCGAACCGTCTCACCG	28:T>C
5411655|F|0-28:T>C-28:T>C	TGCAGATGGTTTGGTGCACCCACTGGACTGCCCCCG	28:T>C
1087736|F|0-34:T>A-34:T>A	TGCAGCGTCTCCCGCAACGTGGGGAACGTGACCCTGTACGCCCTCCGCCG	34:T>A
6043217|F|0-6:T>C-6:T>C	TGCAGGTGGTGGTAGAAGGTGAGGTTGCCGTCGTAGACGTGGAGCATCAGCGTCGTCGCCTGCCTCTCC	6:T>C
1012889|F|0-52:G>A-52:G>A	TGCAGTACTCGCTCTACTTCGTCTACCTCGGCCTCGTCGTCTGCGCATCCTCGTACCTGGGTGAGTTCA	52:G>A
3945266|F|0-43:C>T-43:C>T	TGCAGGTGAAGCAGCTCGAGGATGCGGTGCAGGAGCTGGTGGCCTCGTACGAGGACTGCGCGTACCTCG	43:C>T
7911484|F|0-7:A>G-7:A>G	TGCAGCCATGCCCATCACCG	7:A>G
1277774|F|0-24:G>C-24:G>C	TGCAGAAGATGGAGGGCGCGTTCGGCGAGTGCTCCAAGGGCAAGGCCTTCTTCGGCGGCGACGGCCCGA	24:G>C
1000441|F|0-28:C>T-28:C>T	TGCAGCGGATTGGCTTCGCCTCGTCGCTCTCATTCACCATCTTCATCGCGCTCATGGGGCTGGTGGCAC	28:C>T
2241884|F|0-11:G>A-11:G>A	TGCAGGACCTTGTAGTTCTGGATCATGTCGTACTCGTTCTTGGCGTCGAAGTTGACCTTGTGCATGGGC	11:G>A
1064108|F|0-18:T>C-18:T>C	TGCAGTGCTTGGCGTAGATGAGGAAGAGGAAGAGCCCCAGGAGGACGCCCGTGAGCACGCCCACGATGA	18:T>C
7164908|F|0-20:C>T-20:C>T	TGCAGATTCTGCTCTGCGTCCTGCCCCCG	20:C>T
1026243|F|0-64:G>C-64:G>C	TGCAGGCGTCGGTGAGGGACGAGGCGGCGGAGCCGAGGTTGGTGAGCACGCGGCGGAAGTCGGCGGCGA	64:G>C
3942534|F|0-24:G>C-24:G>C	TGCAGCATAAACTCCTCCTCGTCGGCGCCTCCG	24:G>C
7345550|F|0-14:C>T-14:C>T	TGCAGATTGTGGTGCACCGACCG	14:C>T
1003085|F|0-43:G>A-43:G>A	TGCAGAAAAGGGAGAGCAAGACCAATATCATCTCCAGGACTAAGCAAAGACCCACTCCATCTCCGAAAC	43:G>A
2257770|F|0-61:C>T-61:C>T	TGCAGCAGCCGCCGTCGAAGCACGGGTACGGCGCCGTCAGGAACTGCGCCACGATCTCCGACTCCTCCT	61:C>T
985898|F|0-10:A>T-10:A>T	TGCAGGCCCTAGAGAGCTCCAGCACCGTGGTGTCAGTGGCGACAGCATGCTCCCCG	10:A>T
4262684|F|0-7:G>T-7:G>T	TGCAGTTGGTCCTGATGGTGCCTTGGTTGCCG	7:G>T
100445823|F|0-5:T>A-5:T>A	TGCAGTCCCTTAACGTGTCCG	5:T>A
2246968|F|0-20:T>C-20:T>C	TGCAGCGAGGAGTGCAGGCATCAGCAGATCGAGGTGGACAGGGCGAGGCACCG	20:T>C
978496|F|0-55:C>T-55:C>T	TGCAGCACACCGAGTCGGGGGAGGTGACGGTGGAGATGGAATGGATGGCTCTAAACATTTAGAGAATCC	55:C>T
5411797|F|0-19:G>C-19:G>C	TGCAGGAGGTCTTCGTCCTGCTCCCG	19:G>C
1136441|F|0-35:G>A-35:G>A	TGCAGCGCGCCCCCCTCCTTGGTCCTCCGATGGGAGGTGGCGCCGAAGCCG	35:G>A
6048864|F|0-31:C>T-31:C>T	TGCAGAGTTAGGTGCAGGGCATGGGGTTGCTCGCCG	31:C>T
1020137|F|0-7:C>T-7:C>T	TGCAGAGCATATACGAGACGGTGAAGAGCCTCCAGGAGAAAGGGATGCTGCCG	7:C>T
1024971|F|0-50:C>G-50:C>G	TGCAGGTTGGCGTACGCGTACGCCTCGCACGAGCAGTTGGCGGCGCACCTCGCCGCGCACTCCTCCGCG	50:C>G
1060399|F|0-22:G>T-22:G>T	TGCAGATTACGTGCGTCGTCACGCTCTACATTTGGTGCGCCGCCACCAACCCCG	22:G>T
12695246|F|0-13:G>C-13:G>C	TGCAGCGTCTCCTGCCGCACCG	13:G>C
7161886|F|0-8:C>A-8:C>A	TGCAGGCACGGGAGACATGATCACCTGCTCCCCG	8:C>A
5411123|F|0-47:C>A-47:C>A	TGCAGGATGCGGATGCGCTCGCTGATGCGCTCCCGCCGCATCCTCGCCGCCACGCTCTGCGGGTCCGTC	47:C>A
6046250|F|0-25:C>T-25:C>T	TGCAGCCCTAAACCAAGCACAACACCAACAGAACCCAACGCCTCTCCTCCTAGGGCTCCACCAGCCGCC	25:C>T
1057847|F|0-39:G>T-39:G>T	TGCAGGATGCATCCCAGCATCCGCGTAGCCACCAATGAGGGCAGACCACGAGACAACATTCCGCTCCGA	39:G>T
4406018|F|0-13:G>A-13:G>A	TGCAGTGCTGGGAGACCGACGCCGTCGTGCCCGTCGGCGGCCG	13:G>A
7350355|F|0-17:T>C-17:T>C	TGCAGAATGTCTCAGGCTACCG	17:T>C

<table>
<tr><td>1306480|F|0-6:T>C-6:T>C</td><td>TGCAGGTATCGATTATGAGTACAAGGCGGTGAACCTCCTCAAGGGCGAGCAGTCTGATCCCG</td><td>6:T>C</td></tr>
<tr><td>1003085|F|0-57:C>T-57:C>T</td><td>TGCAGAAAAGGGAGAGCAAGACCAATATCATCTCCAGGACTAAGCAAAGACCCACTCCATCTCCGAAAC</td><td>57:C>T</td></tr>
<tr><td>2257770|F|0-28:C>G-28:C>G</td><td>TGCAGCAGCCGCCGTCGAAGCACGGGTACGGCGCCGTCAGGAACTGCGCCACGATCTCCGACTCCTCCT</td><td>28:C>G</td></tr>
<tr><td>991319|F|0-36:T>C-36:T>C</td><td>TGCAGAGGCGGTCGGTGGCGAGCCTGCCCTTGTGGGTGCTGCTCTTGTACTCCTTCCAGGTGAACTCCC</td><td>36:T>C</td></tr>
<tr><td>1048955|F|0-62:G>A-62:G>A</td><td>TGCAGCCTCTTCAGCTGGGCCTTCAGGGCCAACCACGGGCCTCCCTACCTCATGCACTCCCAGCCACTC</td><td>62:G>A</td></tr>
<tr><td>3943902|F|0-5:T>C-5:T>C</td><td>TGCAGTCGATGAACCCGCACCG</td><td>5:T>C</td></tr>
<tr><td>2254643|F|0-47:T>C-47:T>C</td><td>TGCAGCGTGAGCACGGGCCCTCGATGGGCCGCCGCCGCCATCTACATTGTTGGCTTCTGGTTCCTCGAC</td><td>47:T>C</td></tr>
<tr><td>991324|F|0-65:T>C-65:T>C</td><td>TGCAGAGCGCCATCCAGGCCGCCATCGCGCACTGCAAGAACTCCATCGCCGTGGCCAAGCAGTAGTACG</td><td>65:T>C</td></tr>
<tr><td>5578133|F|0-8:C>T-8:C>T</td><td>TGCAGCTCCGAGAACCCCCGCTCGTTGCCG</td><td>8:C>T</td></tr>
<tr><td>3945162|F|0-5:C>T-5:C>T</td><td>TGCAGCCGCCACCTCCCGCGCTCCTCCG</td><td>5:C>T</td></tr>
<tr><td>2257770|F|0-10:G>A-10:G>A</td><td>TGCAGCAGCCGCCGTCGAAGTACGGGTACGGCGCCGTCAGGAACTGCGCCACGATCTCCGACTCCTCCT</td><td>10:G>A</td></tr>
<tr><td>988829|F|0-34:G>A-34:G>A</td><td>TGCAGGCGCCCAGCAGCGCGCCCCAGACCACCGAGTGCTCCTGGCAGTCCG</td><td>34:G>A</td></tr>
<tr><td>4009560|F|0-17:G>A-17:G>A</td><td>TGCAGCTCCCCCTCGTCGATGCTGCCGCTGCCGTCGCGGTCGACGGCCCG</td><td>17:G>A</td></tr>
<tr><td>1079587|F|0-49:G>C-49:G>C</td><td>TGCAGATGACCTGCAACCTCGGGTCCATCGTGGGGGGCTCCTTCGGCGTGCTCCTCGCGCCG</td><td>49:G>C</td></tr>
<tr><td>4261106|F|0-5:C>T-5:C>T</td><td>TGCAGCTCGTTCTCGGGAGGTTGCCG</td><td>5:C>T</td></tr>
<tr><td>3022223|F|0-14:C>T-14:C>T</td><td>TGCAGAGGCAACATCCAGTATCATGAGAAGGTTTCCG</td><td>14:C>T</td></tr>
<tr><td>1018682|F|0-43:A>G-43:A>G</td><td>TGCAGATCGGTCGTAGCTTTCGTGTGAGAAAGGATGACATGCCACTATCATCACTAAACGGGTGTCGTT</td><td>43:A>G</td></tr>
<tr><td>4009024|F|0-19:T>A-19:T>A</td><td>TGCAGCAGCGGCATCAAACTCGTCGGCCCTCAACGTCGTGCACCG</td><td>19:T>A</td></tr>
<tr><td>5370820|F|0-5:G>C-5:G>C</td><td>TGCAGGGCCGACACGGCGGAGCTCTGGAACCG</td><td>5:G>C</td></tr>
<tr><td>1018617|F|0-41:G>A-41:G>A</td><td>TGCAGGATGCTCATGGTGATGGCTCCCCCCAGGCACAGAAGGGTCCCCACTATCTTGGCTCTTGTGTAC</td><td>41:G>A</td></tr>
<tr><td>6044001|F|0-7:C>G-7:C>G</td><td>TGCAGCTCGCGTCGCCGTCCATGGCCCG</td><td>7:C>G</td></tr>
<tr><td>3027026|F|0-22:G>A-22:G>A</td><td>TGCAGATGCTCCCCGTCATGCCGCTCGACGCCGACGTGTTCCG</td><td>22:G>A</td></tr>
<tr><td>1024561|F|0-6:C>T-6:C>T</td><td>TGCAGGCGTTCGCCCTCGTCCTGAACAAGCGCCTCGCCGACGACGACGCCG</td><td>6:C>T</td></tr>
<tr><td>1050629|F|0-19:C>T-19:C>T</td><td>TGCAGAACTCCAACATGCTCAACAACAAGAGCGAGACGCACCCATGGCATCTCCATGGGCACGACTTCT</td><td>19:C>T</td></tr>
<tr><td>1023335|F|0-33:C>T-33:C>T</td><td>TGCAGCTGCGTCTTGGCCATGATCTCCGTCCTCCTCAGCTCCATCTCGCCCTTCTTCACCTCGGCCTCG</td><td>33:C>T</td></tr>
<tr><td>1038471|F|0-27:T>C-27:T>C</td><td>TGCAGGAACAGCGCCCGCCGCCCCACCTTGTCCGCGGTCACCACGGCCACGAAGGTGGAGAACACGTTC</td><td>27:T>C</td></tr>
<tr><td>3934650|F|0-10:G>C-10:G>C</td><td>TGCAGTGGGGGAGGAGCTACACGCTCAAGTCCTTCCG</td><td>10:G>C</td></tr>
<tr><td>1125483|F|0-23:A>C-23:A>C</td><td>TGCAGCGCACAAGACAGAAAAGCATGCACAGCCAACAAGCACAAGCACACCGCCATGCTCGTCTTGGCA</td><td>23:A>C</td></tr>
<tr><td>1228527|F|0-36:C>T-36:C>T</td><td>TGCAGAGGTACCGCGAGGGCGAGGAGCTCGCGGCGGCGCTCAACAACCACAGCAGGTCACCG</td><td>36:C>T</td></tr>
<tr><td>1028439|F|0-23:G>C-23:G>C</td><td>TGCAGCGCGCCGAGGCGGTCGGCGATGGGCGCGAAGTAGGGCAGCAGGGTGCTCCG</td><td>23:G>C</td></tr>
<tr><td>2279291|F|0-29:G>A-29:G>A</td><td>TGCAGGCTGGTGACGCGCGGCGGCTCGGCGAACTCGTAGTCGTACCTGAAGTAGTCGAGCACCATGTCC</td><td>29:G>A</td></tr>
<tr><td>2263602|F|0-43:G>A-43:G>A</td><td>TGCAGGCCGCCGACATGTTCGAGCTGGCCCGCCTCGCCGACGAGTACGGCTCCG</td><td>43:G>A</td></tr>
<tr><td>5581387|F|0-7:C>G-7:C>G</td><td>TGCAGCGCCGCACCGAGCCCAAGCTGCCG</td><td>7:C>G</td></tr>
<tr><td>1048239|F|0-17:T>C-17:T>C</td><td>TGCAGCCCTAAACCAAGTACATCGCCAACAGAACCCAACGCCTCTCCTCCTAGGGCTCCACCAGCCGCC</td><td>17:T>C</td></tr>
<tr><td>1040262|F|0-41:G>A-41:G>A</td><td>TGCAGCCAGGATGCGCCCCACTTTGGAGACGCAATTATTGAGGAAGGAATTCATGGCGCCGAGGTGGAG</td><td>41:G>A</td></tr>
<tr><td>5369577|F|0-5:G>A-5:G>A</td><td>TGCAGGCGGTCGCCTATCTCCG</td><td>5:G>A</td></tr>
<tr><td>1112647|F|0-64:G>A-64:G>A</td><td>TGCAGCTTCTTCATGCTGTGCTGCTGCTTCTTCTGCCATGAGGCCTGCGAGCACTGCCGCAAGCGCTTC</td><td>64:G>A</td></tr>
<tr><td>1092174|F|0-65:C>T-65:C>T</td><td>TGCAGTGAATTAATGGATGCAACGAGATCAGAAACCGTTGCCAGACCACGACGCCGACACCGACACCGA</td><td>65:C>T</td></tr>
<tr><td>991319|F|0-33:G>T-33:G>T</td><td>TGCAGAGGCGGTCGGTGGCGAGCCTGCCCTTGTGGGTGCTGCTCTTGTACTCCTTCCAGGTGAACTCCC</td><td>33:G>T</td></tr>
<tr><td>1064108|F|0-13:G>A-13:G>A</td><td>TGCAGTGCTTGGCGTAGATGAGGAAGAGGAAGAGCCCCAGGAGGACGCCCGTGAGCACGCCCACGATGA</td><td>13:G>A</td></tr>
<tr><td>5581481|F|0-17:A>C-17:A>C</td><td>TGCAGGCCAAGATCGTCAAGATAGCGCTGCCG</td><td>17:A>C</td></tr>
<tr><td>1021754|F|0-27:G>A-27:G>A</td><td>TGCAGCCGAGCATGAACACCAGCACCAGGCACATACTCAAGTACCTCACCG</td><td>27:G>A</td></tr>
<tr><td>4006298|F|0-30:A>G-30:A>G</td><td>TGCAGCACCGACGGCGCCATCAACACCTTAACCTCCG</td><td>30:A>G</td></tr>
<tr><td>1863451|F|0-28:C>T-28:C>T</td><td>TGCAGGTGGTGAAGTATCTGATTGTGGCCTTCGATATCGTCAACTCTGTAGATGACCAAGGGAATACAG</td><td>28:C>T</td></tr>
<tr><td>32026958|F|0-28:T>C-28:T>C</td><td>TGCAGGCGCAGCTCGGCTACCGCTCGGTTGACTTCCTGCCCTACGCGTCCCACGTCCGCAAGACCGCGG</td><td>28:T>C</td></tr>
<tr><td>1028439|F|0-38:G>T-38:G>T</td><td>TGCAGCGCGCCGAGGCGGTCGGCGATGGGCGCGAAGTAGGGCAGCAGGGTGCTCCG</td><td>38:G>T</td></tr>
<tr><td>3948890|F|0-8:T>C-8:T>C</td><td>TGCAGGCATTGGCATTGGCGGACAACCG</td><td>8:T>C</td></tr>
<tr><td>13881091|F|0-24:T>C-24:T>C</td><td>TGCAGCAATGTCTTCGGCCACCCCTCCTCCGCCG</td><td>24:T>C</td></tr>
<tr><td>4262684|F|0-15:T>C-15:T>C</td><td>TGCAGTTGGTCCTGATGGTGCCTTGGTTGCCG</td><td>15:T>C</td></tr>
<tr><td>11914563|F|0-22:T>C-22:T>C</td><td>TGCAGACGCTGCTGGTCAGCAATAACCGTCTCGCCG</td><td>22:T>C</td></tr>
<tr><td>10984430|F|0-7:C>A-7:C>A</td><td>TGCAGGGCTCCTACAACGAGCACATGTCCG</td><td>7:C>A</td></tr>
<tr><td>6045638|F|0-38:A>C-38:A>C</td><td>TGCAGCTTGGCGAGGTTCCAGATCGACGGTGGAATGTTACCG</td><td>38:A>C</td></tr>
<tr><td>32025370|F|0-10:C>G-10:C>G</td><td>TGCAGGAGCGCCGCCACGCGCGCCATCTCCTCCTTGGCCGCCGCGTACCG</td><td>10:C>G</td></tr>
<tr><td>32026808|F|0-23:A>G-23:A>G</td><td>TGCAGATTGGCTACTCGGCACCAACTGAGTTCGAGATCAGCCG</td><td>23:A>G</td></tr>
<tr><td>6038028|F|0-23:A>G-23:A>G</td><td>TGCAGGCAGCCCACGAGGCACGTAAGCGCCGTGGCCCAGTACCG</td><td>23:A>G</td></tr>
<tr><td>5008744|F|0-23:A>G-23:A>G</td><td>TGCAGGCCTTCGACGACACCAAGACGGGCGTCAAGGGCCTCGTCGACGCCG</td><td>23:A>G</td></tr>
<tr><td>100386879|F|0-14:G>C-14:G>C</td><td>TGCAGCGGCCTCGTGAGCTCCTCCCG</td><td>14:G>C</td></tr>
<tr><td>5358266|F|0-30:G>C-30:G>C</td><td>TGCAGGCGGCGTCGTCGAAGGTGCTGCCGCGGCCG</td><td>30:G>C</td></tr>
<tr><td>5341360|F|0-17:A>G-17:A>G</td><td>TGCAGAAGCTCCTGCACAGCGCCTACAAGCGCGGGGACACGTCCGTGGAGGTGCGCGTGCGCCGCCCCG</td><td>17:A>G</td></tr>
</table>

100549876|F|0-14:C>T-14:C>T	TGCAGAGTCGGCCACATTATTCTCATGGTACTGATAATGCGGGCGAGGGGCAGGGCGGTGATGGTGTCT	14:C>T
100353238|F|0-16:A>G-16:A>G	TGCAGTCGCTGCGCCCACCCCCG	16:A>G
100530080|F|0-41:A>C-41:A>C	TGCAGTACATGGAGACAAACCCACTGCTGACATGGAAATCGATACCGCTGCACATCCCGCCCTTGATGG	41:A>C
7913348|F|0-25:A>C-25:A>C	TGCAGCCCAGGACGCGCCGCCGCGAAAGGGCCG	25:A>C
32023693|F|0-34:C>T-34:C>T	TGCAGCGGATGGGCGTGGGGAGGGTCCTGGTGGTCCTGGCCATGGCGACGGCGGCGCTGGTGGAGAGCC	34:C>T
7168999|F|0-59:A>G-59:A>G	TGCAGGTCGCGCCCGTACGGCGGGAAGTTGCTCTTCAGCACCGTGCCGATCACGTTGTTATTCCCCGTG	59:A>G
100479288|F|0-31:A>G-31:A>G	TGCAGCCCCAGGGGGACTCGTACACGCGGCGATCGGCGTTCGACGCCGTGCTCGCCG	31:A>G
5355313|F|0-53:T>G-53:T>G	TGCAGGTCCAGCATCTCGCCGCGGACCCTGTCGGCCTCGGCGTCCACGAGCGCTATCTCGTCGGCGAGG	53:T>G
1023911|F|0-16:G>C-16:G>C	TGCAGAAGGCGAGGCGGGCGGTGGAGAGGTAGAGCGTGCCGATGACGGGGCCG	16:G>C
5578165|F|0-13:C>T-13:C>T	TGCAGCTGCGCCTCCTTCAGCTCGTGTGGTCCG	13:C>T
3023337|F|0-28:C>T-28:C>T	TGCAGAGCCTCATGGACTTCTACTACGCCAGCGTGCCG	28:C>T
1064061|F|0-44:G>C-44:G>C	TGCAGGAACGCCATCCTCCTCATCCGCCGCAGCTGCTCCGTGGTGGTGGCGTCGTGCGCCTCGCTCTGC	44:G>C
1264847|F|0-14:G>T-14:G>T	TGCAGGGTCTTGAGGAAGTTGTTGTCGTCCGACTCTGTGTCGTGTGTGTGCCACACTGAGTTGCTCCCG	14:G>T
2279291|F|0-56:G>C-56:G>C	TGCAGGCTGGTGACGCGCGGCGGCTCGGCGAACTCGTAGTCGTACCTGAAGTAGTCGAGCACCATGTCC	56:G>C
32027924|F|0-21:C>T-21:C>T	TGCAGCGGGACGAGGACACCGCCGTCCGCCTCCTCCACCCCTTCACCG	21:C>T
4262313|F|0-13:C>T-13:C>T	TGCAGGGCCAGTTCACCGACATGGTCACGGCCG	13:C>T
3027026|F|0-10:C>T-10:C>T	TGCAGATGCTCCCCGTCATGCCGCTCGACGCCGACGTGTTCCG	10:C>T
3944810|F|0-10:A>G-10:A>G	TGCAGAAGGAAGAGGACCGAAAGCCG	10:A>G
13590251|F|0-20:T>C-20:T>C	TGCAGCAGCCAGGCAGAAGCTGCCGATCAGGCAGCCG	20:T>C
4262737|F|0-31:G>A-31:G>A	TGCAGCAGCCGCAGATCATCCACCGCGACCTGAAGCCG	31:G>A
3029515|F|0-23:G>C-23:G>C	TGCAGCACGGTGTCCGCCGCCTCGGCGCTCGGCGACCACCGCCG	23:G>C
1003085|F|0-36:G>A-36:G>A	TGCAGAAAAGGGAGAGCAAGACCAATATCATCTCCAGGACTAAGCAAAGACCCACTCCATCTCCGAAAC	36:G>A
3943268|F|0-17:C>T-17:C>T	TGCAGGCAGTGCAGCGCCAGCGTCGCCGCGCCGCCCG	17:C>T
2247548|F|0-37:A>G-37:A>G	TGCAGATGTAGAAGTCGTCGCCGTAGAGAGCCCTGACAGCATCGACGGGCTTCACCGCCG	37:A>G
1109610|F|0-38:C>T-38:C>T	TGCAGCGAGGCCCGCAGCGCGCTCACCCGCTGCGCCAGCGCGTCCATCGTCTCCG	38:C>T
1083022|F|0-19:C>G-19:C>G	TGCAGCTCCTCTCGGTGGCCTGCCTCTCGCTGGCGGCCAAGGTGGAGGAGCACCG	19:C>G
980135|F|0-18:C>G-18:C>G	TGCAGGTGGCCGTCTGGGCGTGCATGGTCATGTCGGTGATGCTGGTGGTGGAGGCCCTGTACAACTGCG	18:C>G
5369531|F|0-16:C>T-16:C>T	TGCAGGAGAACATCGCCGACCG	16:C>T
1071080|F|0-9:A>G-9:A>G	TGCAGGGCGATGGGCGACCAGCTGGAGGGCGACGAGGGGCAGCACATCAAGTACCG	9:A>G
3938270|F|0-16:C>T-16:C>T	TGCAGCAGGCCTTCGGCTACCAGCACGGCCCG	16:C>T
6041655|F|0-15:T>C-15:T>C	TGCAGGAGCTCGACGTCTCGCACAACCAGCTCACCG	15:T>C
1089106|F|0-14:G>A-14:G>A	TGCAGGAAGTGCGCGGCCATCTGCCAAGCGTCCCCCTGCGCCGCCCGCACCAGGGCCAGGTAGAAGTAG	14:G>A
3948890|F|0-7:A>G-7:A>G	TGCAGGCATTGGCATTGGCGGACAACCG	7:A>G
1306480|F|0-15:T>C-15:T>C	TGCAGGTATCGATTATGAGTACAAGGCGGTGAACCTCCTCAAGGGCGAGCAGTCTGATCCCG	15:T>C
3029170|F|0-25:G>A-25:G>A	TGCAGCAGCGCGTAGTAGCTCAGCGGGACGCGCCCGAAGCCG	25:G>A
1088578|F|0-44:C>G-44:C>G	TGCAGGCTCTGCCACTTGAGCACCCACAGCGGGATGTGCTGCATCAGAGGGTGCACGCTGATGATCACG	44:C>G
4006245|F|0-8:G>A-8:G>A	TGCAGCCAGACAAGCACCGTGCTCGACAGGAGGGAGCTGAGGTTGATCATGAAGTAGTACCAGTTGAAG	8:G>A
6041707|F|0-25:C>G-25:C>G	TGCAGGCCAGACACCCTGTGGAAGGCGAGCTCGCCG	25:C>G
5358402|F|0-23:G>A-23:G>A	TGCAGGTACCCTTCTTCGGCCTCGCCGTCTTGCACAGCCG	23:G>A
1090096|F|0-9:T>C-9:T>C	TGCAGGAAATCCAGGCTGGGCCTGCCGTGCTTCATGCAGTTCTGGTAGTCCTGGATGAAGGCGCACGTC	9:T>C
4405451|F|0-27:G>C-27:G>C	TGCAGCTTTATTGAGGCCATGCCCTACGGCCG	27:G>C
3027422|F|0-10:G>A-10:G>A	TGCAGTTCCCGTCGGACATAGCCACCGTTCTTCTCGCCGCCG	10:G>A
22839067|F|0-13:C>T-13:C>T	TGCAGGTGCTCGACCTCTCCTTCAACGCCTTCAACGGCACCCTGCCCG	13:C>T
1229529|F|0-64:G>C-64:G>C	TGCAGGACATGCGGGAGGTGACGAGGCCGTAGAGCTTCATGACGTTGGGGTGGTCGAGGCGGCGGAGGA	64:G>C
992480|F|0-16:G>A-16:G>A	TGCAGAGGATCTGGCCGTTGCGGAGGCCGAGGCGGAACTCCTCCTCCGACGGCTCCTCCG	16:G>A
1018617|F|0-35:C>T-35:C>T	TGCAGGATGCTCATGGTGATGGCTCCCCCCAGGCACAGAAGGGTCCCCACTATCTTGGCTCTTGTGTAC	35:C>T
3021318|F|0-39:T>A-39:T>A	TGCAGATGGTATCGTTCCACGCTGGGAACAACGAGGGCCTACTCGAGCAATTCTCATGGGACGACCCCT	39:T>A
1056795|F|0-20:G>A-20:G>A	TGCAGCCCCACCCCGAGCCCGCGCTCCCTCCTCACCTGCTCCACGCCCACCAGCGCCG	20:G>A
5578282|F|0-6:C>T-6:C>T	TGCAGGCGGCGGCGCAGCGGACGAGGTCCCCG	6:C>T
7155514|F|0-7:T>C-7:T>C	TGCAGGATAGGCTTAGCAAGGCCACCG	7:T>C
3942522|F|0-31:C>T-31:C>T	TGCAGTACATGGACCTCGCCAACAACTCCCTCGCCG	31:C>T
3032895|F|0-9:T>C-9:T>C	TGCAGGCACTCCACCACGTCGTCCGCCGAGAACCCCG	9:T>C
3937435|F|0-34:C>T-34:C>T	TGCAGGTGCTCGACCTCTCCTTCAACGCCTTCAACGGGACCCTGCCCG	34:C>T
3948067|F|0-5:C>A-5:C>A	TGCAGCAGGCGCTGGCGGCGCTGGAGGCGGCGAGCGCCG	5:C>A
3026693|F|0-17:G>C-17:G>C	TGCAGCCCCAGCGCGCGGGCCAGCTGCATCTTGCGCTCCG	17:G>C
1060399|F|0-10:G>C-10:G>C	TGCAGATTACGTGCGTCGTCACGCTCTACATTTGGTGCGCCGCCACCAACCCCG	10:G>C
987594|F|0-50:C>G-50:C>G	TGCAGCCCCACCAGCGCCGCCGCCGCCAGCTGCCACGCGTTGTCGCCCTTCTCCAGCCACTCCG	50:C>G
1321129|F|0-28:G>A-28:G>A	TGCAGCATGCACATTGCTATCTCTTTCTGTGCCCATTACAGCAACTTTTTCTGGTGTATTATTGCCGAG	28:G>A
4993513|F|0-6:C>T-6:C>T	TGCAGGCCGCGAGATCAGACCG	6:C>T

5324000|F|0-21:A>G-21:A>G	TGCAGATCGTCGTGGGAAAGAAGCAGCAGCGTGCCG	21:A>G
6047870|F|0-61:T>C-61:T>C	TGCAGGGACACGCCGTGAAGCTCGGGTTCCTCGAGCACGACGAGCACGCGCAGAACAGCCTTGTCAGCT	61:T>C
7169068|F|0-39:T>C-39:T>C	TGCAGTCTGCCCCTGCTGCCACTGCCACCGCGGCGCCTCTTCCTCGTCGTCCTCGTCGCCATGCTGACC	39:T>C
7161886|F|0-16:A>G-16:A>G	TGCAGGCACGGGAGACACGATCACCTGCTCCCCG	16:A>G
4405669|F|0-18:T>C-18:T>C	TGCAGGGTTCTTCGAGTCTCGGCGTGCGTGCTGCGGGACGGGGACGATCGAGACGTCGGTGCTGTGCCA	18:T>C
5580253|F|0-23:C>T-23:C>T	TGCAGCGGCGGCGGGTGGCTCCTCCACCG	23:C>T
1043238|F|0-41:G>A-41:G>A	TGCAGGTGGCTCTTGACGTGCGCCAGCGTCAGATCCTTGACGTTCATCAGCTCCAGCACCGACTTCGGC	41:G>A
1109610|F|0-18:G>A-18:G>A	TGCAGCGAGGCCCGCAGCGCGCTCACCCGCTGCGCCAGCGCGTCCATCGTCTCCG	18:G>A
2245527|F|0-17:G>A-17:G>A	TGCAGCGTGTACTCGATGTAGTACAGGCCGTTTGATGCCTTGGAGTCGATGAGCTTCGCTGCGAGCCCC	17:G>A
1000447|F|0-23:C>G-23:C>G	TGCAGCGCCGTCCCGCACAGCATCCCCGCCCAGATGCCCTGGGGGCCGAGCCG	23:C>G
2241884|F|0-29:G>A-29:G>A	TGCAGGACCTTGTAGTTCTGGATCATGTCGTACTCGTTCTTGGCGTCGAAGTTGACCTTGTGCATGGGC	29:G>A
3021692|F|0-34:T>C-34:T>C	TGCAGGTAACCCAATCCCCTCTACTACGTCGTTTTGACCG	34:T>C
1140846|F|0-28:G>A-28:G>A	TGCAGGCGTCCATATGGAGCAAGGACGTGGAGTTCTCCTTCGGCGAGATCCTGGCGGCGACGGAGCACT	28:G>A
1146832|F|0-64:C>G-64:C>G	TGCAGATGCATTAATTGGCGTTATTATGGCAGGCATGAGTACACACGTAGAGTGTATTTGTTGCCGCCT	64:C>G
2245527|F|0-41:G>A-41:G>A	TGCAGCGTGTACTCGATGTAGTACAGGCCGTTTGATGCCTTGGAGTCGATGAGCTTCGCGGCGAGCCCC	41:G>A
2245527|F|0-14:G>A-14:G>A	TGCAGCGTGTACTCGATGTAGTACAGGCCGTTTGATGCCTTGGAGTCGATGAGCTTCGCTGCGAGCCCC	14:G>A
1020188|F|0-23:C>A-23:C>A	TGCAGCTTGAGGGAGATGCCGCGCACGGGGCCGCGCTGGATGCGGCGCATCAGATGGGTGGTGAAGCCG	23:C>A
1006037|F|0-9:G>A-9:G>A	TGCAGCGGCGGGATCGCGCGCTCAAGATCTTCATGAACGAGGGTCGGTCGTGGCACGGCATCAACCACC	9:G>A
1150140|F|0-33:G>C-33:G>C	TGCAGTGGATCATCTGGTGAACACGGTGAGCCTGGGCCACGACTTCCTCTTCGAGGAGGCCG	33:G>C
5366805|F|0-8:G>A-8:G>A	TGCAGAGCGCGAGGCGAATCCG	8:G>A
3027442|F|0-10:T>G-10:T>G	TGCAGGTCGATGAAGGCGTGGCCCATCTTGTCGTCGTACTTGAACCG	10:T>G
989265|F|0-24:G>A-24:G>A	TGCAGGTCCACTCGGCCCCGCCGCGCCGCCCGATCCCGATCCGACGGCCCG	24:G>A
4262633|F|0-6:A>T-6:A>T	TGCAGCAGTTTGCGCGCGCGCGACCGCTGCGCGGATAGCCG	6:A>T
989503|F|0-27:T>C-27:T>C	TGCAGCACAACAACCTGAGTGGCGAGATCCCAGCGGTGCTCGCGGCGCTGCCGTCGGTCACCGAGGTGG	27:T>C
5581409|F|0-20:G>C-20:G>C	TGCAGCGTCACGGTGAGGTAGGACGCCG	20:G>C
1023867|F|0-49:C>T-49:C>T	TGCAGCCCATGGGCAGCTGCTACAACCCCAACACGATCCAGGCGCACGCCTCCTACGCCTTCAACTCCT	49:C>T
4262238|F|0-34:G>A-34:G>A	TGCAGACGTTCGTACGTACCGTCGGTGCATGCCTGTGACCG	34:G>A
1667480|F|0-16:A>G-16:A>G	TGCAGGGGTTCCAGTCACGGGGCGTGTCGAGGGAGCAGGGCGAGGCGCTGCTCCG	16:A>G
5332344|F|0-10:C>T-10:C>T	TGCAGGCGGACGGCGATGCTGTCCCCG	10:C>T
977583|F|0-11:G>T-11:G>T	TGCAGCCTCTTGTCCATGTACTCCCTCACCGCTCCGACGCCCTTGGCCACCACCTCCAGGGGCGGCACG	11:G>T
1089536|F|0-68:C>T-68:C>T	TGCAGGGTGTTCCAGAAGCAGAAAGTGGACGGCGAGCAAGACAACGCCCGCTCCTCTTCGCCGACCTTC	68:C>T
1087708|F|0-47:T>G-47:T>G	TGCAGACGCAAACCCAAACCAAGCGAAAAATATCCACTGAATCTCTCTCGCTGCTCGCGGCCGCGGCGA	47:T>G
1117708|F|0-58:G>A-58:G>A	TGCAGCTGCGCTCCAGGCCCTGCTGGAACACGATCTTGCCCGAGGAGTTCATGCGCGGGGAGTCCACGG	58:G>A
7162190|F|0-16:C>A-16:C>A	TGCAGGGGGCGGCCTCCGAATACCTAGCGAGACCG	16:C>A
3940266|F|0-25:T>C-25:T>C	TGCAGGAGAAGGGCGGGAAGGGCGATGCCGTCCGCCG	25:T>C
1087708|F|0-48:C>T-48:C>T	TGCAGACGCAAACCCAAACCAAGCGAAAAATATCCACTGAATCTCTCTCGCTGCTCGCGGCCGCGGCGA	48:C>T
4397587|F|0-20:G>C-20:G>C	TGCAGGTACAGCGCCGCCATGAGCTGCCG	20:G>C
989034|F|0-13:C>G-13:C>G	TGCAGATGTCGTCCTCACCGCGTGGCGGGGGGATCAAGCGCAGGTTCGGCACCG	13:C>G
6048155|F|0-23:C>G-23:C>G	TGCAGCGACTGCGGGCAGAGCTTCCCAATCTCCG	23:C>G
1024561|F|0-40:C>T-40:C>T	TGCAGGCGTTCGCCCTCGGCCTGAACAAGCGCCTCGCCGACGACGACGCCG	40:C>T
1141218|F|0-14:G>A-14:G>A	TGCAGGCACTGCGCGCATCCCCCCGCCCCGACCGTCTCCACACACTGCGCCATCGCGTACACGCCGAGA	14:G>A
989944|F|0-62:C>T-62:C>T	TGCAGCTCCCGCGCCTGCTCCGCCGCGCGCGCCGCCGCCTCCGCCGCCTGCTCCCCTGCCTTCCTCTCC	62:C>T
1017643|F|0-49:C>T-49:C>T	TGCAGGTGGGCAAGGCGCTGGCCAAGGAGCGCGTCTTCGTGGAGTGCCACGTGTTCAACCCGCCGTCCG	49:C>T
1014875|F|0-6:G>T-6:G>T	TGCAGCGGTAGAAGGCCGACAGGTCGGCGTTCACCCGCAGCGCCACCGCCTGGTGCTTGGCCG	6:G>T
7165106|F|0-6:G>C-6:G>C	TGCAGCGAAGGGGAAGAAGGGCGCGGCCG	6:G>C
5580607|F|0-13:A>G-13:A>G	TGCAGTTCATTTCAAGAAGCAGCTTCCCCG	13:A>G
5578236|F|0-14:C>T-14:C>T	TGCAGGATGGACAGCTCGCTCCCAGCCGCGGACGGAGCCG	14:C>T
1094245|F|0-46:G>A-46:G>A	TGCAGAGGATCAGGGACTGCGTCCAGAGGTGGTACCACGACGAGGGGTTCGTGTGCGCTCAGGTGATCA	46:G>A
3029114|F|0-31:G>A-31:G>A	TGCAGCTCCGTCTCGAACTCCATGTCCATCCGCGGCGTGCACTCCTCCG	31:G>A
991599|F|0-52:C>G-52:C>G	TGCAGGCCTTGCAGAGCGTGCCGAGGGAGTTGGCGAGGGAGGGCGCCTCCTCCGCTCCG	52:C>G
1004575|F|0-22:G>A-22:G>A	TGCAGGCGGCGCGCTGCGCCGTGGCGATGGCGAGGGGAGGCGGCGCGCTGGTCATGAGCAGCAGGGCGC	22:G>A
1004575|F|0-49:G>A-49:G>A	TGCAGGCGGCGCGCTGCGCCGTGGCGATGGCGAGGGGAGGCGGCGCGCTGGTCATGAGCAGCAGGGCGC	49:G>A
1091441|F|0-11:C>T-11:C>T	TGCAGGAACTTCTGCGTCGGGAAGGTGAGCGCCAGCGAGAACATCTGCGGGAGGATGCGCAGCGTGAAG	11:C>T
1134607|F|0-32:G>A-32:G>A	TGCAGGACCTCGGCGATGGCGCGGTCCACGCGGTCGGCGTCCTCGCGGAGCGCCG	32:G>A
3948820|F|0-5:G>C-5:G>C	TGCAGGCGCCGCAGGGTTCTCCG	5:G>C
4404372|F|0-10:C>G-10:C>G	TGCAGCTGCTCGCCCGCTACCCG	10:C>G
1695356|F|0-66:G>A-66:G>A	TGCAGGCTCCAGATATGGAAGCTCGCCACCCGCCTCTGGAACGCCGTCGTCGACCGCGCCAACTCCGCC	66:G>A
4006159|F|0-23:C>A-23:C>A	TGCAGGAAGGCGCTCGTCATGAGCTCGCGCGAGAACGCGCCG	23:C>A
1395408|F|0-55:G>T-55:G>T	TGCAGGCCGTTCATGGCGTACAGGGACGCCGAGATCCGCCGCTCCACCTTCATCCGCTCGAACTCCACC	55:G>T

22839078|F|0-6:T>C-6:T>C	TGCAGTTAAGTTAAATGTTGGGGGGCCTTGTAGCCG	6:T>C
996241|F|0-42:T>C-42:T>C	TGCAGTTACCCAGGCACAGCTGCCTGCACTCGGCCAGGGTCATGTCGGGGCGCACCGTCGCTTCCGTCG	42:T>C
1022518|F|0-13:C>G-13:C>G	TGCAGAGCCTCCCCTCGCAGATGCAAGCCATCAAGGTGATGGCGACGCTGGACATCCTCTCCTCACACT	13:C>G
4262976|F|0-17:C>G-17:C>G	TGCAGCAGGTTCTTGTACACCCCG	17:C>G
982614|F|0-37:C>A-37:C>A	TGCAGTGCTTCATGGGCATCAGCATGATCACCTGCGTCGCGCTGTATGACCGCGTCCTGGTGCCCGTGG	37:C>A
5581409|F|0-8:C>G-8:C>G	TGCAGCGTCACGGTGAGGTAGGACGCCG	8:C>G
3020870|F|0-25:G>A-25:G>A	TGCAGTGGATATTTGGGTTCGTGACGTTCTTCTTCCCCG	25:G>A
1020022|F|0-11:C>G-11:C>G	TGCAGCACCTCCCGTCTCGTATCGGCCACCTCCGCCTCCAGCTTCTTGATGGAGCTCACGACCGCGAGC	11:C>G
4733518|F|0-55:G>A-55:G>A	TGCAGGCCCAGGCACGGAGCAGCGGGCGCGGCACGGCTGCGCCGTCGGTGTTCGAGGGCGTGGGGAGGA	55:G>A
13590277|F|0-10:C>T-10:C>T	TGCAGCGTCACTTTCCTCGCCG	10:C>T
1049199|F|0-62:T>A-62:T>A	TGCAGGTTGCTGAGGTGGAGATCCGAAAAGGACGGACCGAATTGCGCATGCCTGGCTCCCTGTATCCCC	62:T>A
991153|F|0-6:G>A-6:G>A	TGCAGCGCCAGATGCGTCTTGTCGGACCCCTTCTCCACCGTGAAGCTCACCG	6:G>A
4006123|F|0-5:G>A-5:G>A	TGCAGGAGAGATTTGGAGCCGCGTTGCTAGGTAAGGACTCCG	5:G>A
1102016|F|0-31:C>G-31:C>G	TGCAGGTGACCCACTTCAAGTGCGGAGGCGTCGCCCTCGGCATAGGCATGCAGCACCACGTCGCCGACG	31:C>G
5582090|F|0-5:T>C-5:T>C	TGCAGTTGGCCGAGCTCGGCGATCTCCG	5:T>C
32025638|F|0-31:G>A-31:G>A	TGCAGGGGGCTCCGCGCCGCGGCGGAGGGGCGCGACCAGCAGCAAGAGGGGGGCGACGACGCGGCGGGA	31:G>A
7170849|F|0-12:C>T-12:C>T	TGCAGGGGCGTACGACGAGGAGGAGGCCGCAGCCCG	12:C>T
32025609|F|0-47:C>G-47:C>G	TGCAGGCGGATCGTCCACAACACGGTGGGCAGCGACAAGGCGGTGGCCAAGCTCTTCAGCGGGATGTCG	47:C>G
1141256|F|0-22:G>A-22:G>A	TGCAGGAGATCGCCGCGTCCAGGGGCAAGAGTGTGGCGCAGGTGTGCCTGAGGTGGGTGTACGAGCAGG	22:G>A
986636|F|0-23:G>A-23:G>A	TGCAGGTGGGCCCAGATAGCCAAGCACCTGCCTGGCAGAACGGACAATGAGGTGAAGAACTTTTGGAAC	23:G>A
12770736|F|0-20:T>C-20:T>C	TGCAGGTCCCCGAGCGTGTCTGGAACGGCGCCG	20:T>C
978197|F|0-19:C>T-19:C>T	TGCAGTTGTCGTGGTCTTGCGCGAGCCTCTTGATGATGAGGAGGCCGAGTAGGTTGCACTCCTCGTCCG	19:C>T
7166096|F|0-9:T>C-9:T>C	TGCAGTCCATGTCCGTGGGAGTCCCG	9:T>C
4009024|F|0-8:C>T-8:C>T	TGCAGCAGCGGCATCAAACTCGTCGGCCCTCAACGTCGTGCACCG	8:C>T
1153489|F|0-28:C>G-28:C>G	TGCAGTTCCCCGTCGGCCGCATCGGCCGCTTCCTCAAGAAGGGCCGCTACGCGCAACGCGTCGGCATGG	28:C>G
2369270|F|0-16:C>G-16:C>G	TGCAGGACCGCCTCGGCTTCGACCAGCCCAGCAAGGCCGTCGAGTGGCTCATCAACGCCGCCTCCGCCG	16:C>G
1020517|F|0-27:T>A-27:T>A	TGCAGGCCAGCATCATCTCGCCCTAGCTCCATGGTTAGCGGGCATTTGACATGGATTCCG	27:T>A
1092278|F|0-53:G>A-53:G>A	TGCAGCGACTCCAGCGTGTCCGACTTGTCGCCGTCCGTGGCCGCCGTGGCCGCGCGCACCACGAGGCTC	53:G>A
1695269|F|0-28:G>C-28:G>C	TGCAGGTCACCAGCATGGACCGCTACGTGAGGTACCACGTGAGGGAGTTCGAGAGGGCCTTCGCCCTCA	28:G>C
3025796|F|0-25:G>T-25:G>T	TGCAGAAGCTATCCCTTGATGCCAAGACTGAAGCCATCGACGCGCCG	25:G>T
1004996|F|0-40:C>G-40:C>G	TGCAGCAGCTCAAGAGAGTGGACGACTGGCTCAACCATGTCGTGCGGCAGGAGAGGATGCTTCCGAGAT	40:C>G
12771904|F|0-61:G>C-61:G>C	TGCAGAAGAGCTTGGCGGGGTCGTACTCGTAGCCGATGTTCTTGGACGCGTTGCGGCGGTCGAGCCGCG	61:G>C
7168556|F|0-5:G>A-5:G>A	TGCAGGGCTGCGAGCAGCGGTTCATCTGGGTGCTGCGCGACGCCGACCGCGCCGACATCTTCGCGGAGG	5:G>A
1666709|F|0-25:C>T-25:C>T	TGCAGGGAGCGGTGACGCACGAGGACTTCGAGGGCCACCGTGGCACGATCAAGGCCG	25:C>T
1147132|F|0-16:C>T-16:C>T	TGCAGCTGGAGGAGCACGACGCGCACCAGCGCTGGGACCTCGCGCTGAGCGACGCCGACGAGTGGGAGC	16:C>T
5332338|F|0-26:G>A-26:G>A	TGCAGGCGCTCCCCGACGCGCGACGCGCCG	26:G>A
2252070|F|0-33:A>C-33:A>C	TGCAGCTCGTCCTGCACCCCCGCGAACAGACAAACCAGATCAGGCAACCCTGGACCGACGGGACGGGAT	33:A>C
6045579|F|0-31:C>G-31:C>G	TGCAGCTCCCCCTCGTCGATGCTGCCGCTGCCGTCGCGGTCGGCGGCCCG	31:C>G
3023151|F|0-35:T>C-35:T>C	TGCAGTGGGATTCCGTGACAAGCAGCTACATGACATTTGGGGCCCG	35:T>C
1141256|F|0-49:G>C-49:G>C	TGCAGGAGATCGCCGCGTCCAGGGGCAAGAGCGTGGCGCAGGTGTGCCTGAGGTGGGTGTACGAGCAGG	49:G>C
1124105|F|0-34:A>G-34:A>G	TGCAGCGCCTCCTTGTTCAGCGCCTTCCCCTCCAACGCCTGTCACTTTGCCTCGTCCGTCAGAACCCAC	34:A>G
1169049|F|0-66:C>A-66:C>A	TGCAGCCTTCCAAAAATACTGCCCAACTGCCTGTGATGGATCACCAGCTGTACGGGGACCCGTCCACGA	66:C>A
1092578|F|0-38:C>A-38:C>A	TGCAGATGGCCCACCACCACCGTGTACGTCGTTTCCCTCGTCGGCGCCGTGCTCCTCTACATTGTGCTG	38:C>A
1020026|F|0-10:T>C-10:T>C	TGCAGATTTCTGCACCTTTATCCCACTCTGTGATGAACGTTCCCAAAACCTTGGCTCAAGAACCGAGAT	10:T>C
1063795|F|0-49:C>A-49:C>A	TGCAGGGGCAGGTGAACGTCACCACGGCGCACTCCCTGGACATCATGGGCAGCTCCTACCCTCCGTCCG	49:C>A
3942749|F|0-12:G>A-12:G>A	TGCAGGGAGGTCGGCCAGCAGGACCG	12:G>A
1083484|F|0-49:C>A-49:C>A	TGCAGACCTCGACCTCCTCGGCGTCGGAGGACTCGTCCATGTCGGAGGCCGTGGAGGCGCTCCCGTCGT	49:C>A
1034916|F|0-22:C>T-22:C>T	TGCAGTGGGTCGGCCTGGTGCTCATCATCGGGGCATTGGTGTGCAGCCTCACGATAAAGCCCTTGTCCC	22:C>T
6044000|F|0-22:C>T-22:C>T	TGCAGCTGGCGGCGGCGGGGCTCGCCGTCGGCTTCTTCGTCTACCG	22:C>T
5582381|F|0-10:A>G-10:A>G	TGCAGGCGACAGAGCGGCGGCCTGACCG	10:A>G
1265035|F|0-31:G>T-31:G>T	TGCAGCACCTTCCTCTCCGCGCTCTCCAGGAGCTCCTCCCACCAGGAGCTCCTCCAAGCCTTCTCGTCG	31:G>T
1020682|F|0-22:C>G-22:C>G	TGCAGACGAGCGGGACGGAGAGCGACTTGCAGACCCTGGGGAGGGAGATGGCGAGGGTGCGGTTGATGG	22:C>G
6030532|F|0-9:A>C-9:A>C	TGCAGCGGCAGCTGCGGCAACGGCCG	9:A>C
3937435|F|0-26:G>A-26:G>A	TGCAGGTGCTCGACCTCTCCTTCAACGCCTTCAACGGGACCCTGCCCG	26:G>A
1044692|F|0-40:C>G-40:C>G	TGCAGCTCATGAGGCCATTAAAAGATGCAGCCTGACAGAGCTCTGGACGGCCG	40:C>G
2248262|F|0-22:T>G-22:T>G	TGCAGCGAGAGGAGCACCTTCTTGAGCCGCTCCCTGGCTTGGGCGTCGATGAGGTTCCCCTCGCCGTCG	22:T>G
4993554|F|0-16:T>G-16:T>G	TGCAGGTATCTAGAGTTACCTTCCG	16:T>G
1109610|F|0-31:G>C-31:G>C	TGCAGCGAGGCCCGCAGCGCGCTCACCCGCTGCGCCAGCGCGTCCATCGTCTCCG	31:G>C
4002876|F|0-44:G>C-44:G>C	TGCAGGAACAGCGTGGGCGCCTGGTGGTACTTCACCAGGGACTTGCCG	44:G>C

3026116|F|0-22:T>G-22:T>G	TGCAGCTCGGAGCAAGGTGCAGTCTTTCCTGGAGGCCGCGTGCTCCG	22:T>G
7343602|F|0-20:C>T-20:C>T	TGCAGCTCCGAGATACCCTTCTCCCTCCCG	20:C>T
986769|F|0-41:G>C-41:G>C	TGCAGCGAGAAGGAGGCGCTGGTGTCCTTGGGGTGCTTGGAGACGAGGATCCCGACGCCCTGGCCCCTC	41:G>C
7352259|F|0-9:G>C-9:G>C	TGCAGCAATGCCGTCGTGCCG	9:G>C
1021637|F|0-11:C>G-11:C>G	TGCAGCCTCCTCACGTTCTTGGTGCTGTTGTCCCGCTTGTCCAGCTGGTCCGCTCCGAACGCCAGCGCG	11:C>G
7917497|F|0-10:G>C-10:G>C	TGCAGGCGGCGGCGGCGCCCG	10:G>C
4002269|F|0-8:T>C-8:T>C	TGCAGCTGTTTCTCGCCGTCGGTCACCAAGCAAAGCCGCCCGTCCG	8:T>C
2253331|F|0-22:G>C-22:G>C	TGCAGGAGCGCGAGTCCCGCCTGCGCACCGAGCTGCTCGAGCACAAGATCCTCAAGGAGACCGTCGTCA	22:G>C
6041821|F|0-35:C>G-35:C>G	TGCAGGGGGCTCGCCGACATCAGGGACATGGCGCCCACCTTCGACCG	35:C>G
1119431|F|0-19:G>A-19:G>A	TGCAGCGCCTCATCGAGGCGCGCGCCACCGTGCCCGTCGCCGCCCAGCGCCTCTCCCTCGACCCGAGAT	19:G>A
5367370|F|0-10:G>C-10:G>C	TGCAGGGAGGGGGCTGCTTCGCCCCG	10:G>C
4396666|F|0-16:G>C-16:G>C	TGCAGCGGTACAGCGCGAGCACCG	16:G>C
4397615|F|0-6:C>G-6:C>G	TGCAGGCGGTGGGGTCGCCTCAGCCCCGCCCCGATCCG	6:C>G
1000343|F|0-26:G>A-26:G>A	TGCAGCTGCTGCGCGCAGGCCTCGCAGTGCATGTTGACGAGCAGCTCCACGGTGCGGGCCTCGCTGACG	26:G>A
1230357|F|0-56:C>G-56:C>G	TGCAGCGTCTCGGCGGCCAGCCGCAGCTGCTGCCCGACGGCGCCCATGCGGAACACCCCG	56:C>G
5579624|F|0-31:A>G-31:A>G	TGCAGCGGTTGGCCGCCGCCTTCAGGCCGCGAATCGACGCGGCGCTGAAGTGGATGACTCGCTCGCGGA	31:A>G
1199683|F|0-53:A>G-53:A>G	TGCAGGCGGACGCGGCAGAGCTTGCAGCGGAGCAGCTCGTGCGGCAGGCCGCGATCGCCGCACATGCAG	53:A>G
5579643|F|0-62:C>G-62:C>G	TGCAGCTCCTTGTCGTCGATGGTCCCGCTGCCGTCGCGGTCGGCGGCCTGGAAGCAGGCCACCACGTTG	62:C>G
3031528|F|0-25:G>A-25:G>A	TGCAGCTTGGCGTACGGGTCGCCCAGCCCCTGCGCCTCCGCCGCCG	25:G>A
1100936|F|0-14:G>A-14:G>A	TGCAGGAAATTACGGATTAAGAGGTCAATTAAAGTGCCCGCCATTAGCAACAAAATATCTGATATACCT	14:G>A
1130803|F|0-56:C>G-56:C>G	TGCAGCTTGGCGTTGAAGGTGAGGGAGTCGTTGTTGAGCCGCTCCACGCAGCCGCCCCCGCCGCCGCCG	56:C>G
5324703|F|0-13:C>T-13:C>T	TGCAGCTCGCGTACGCCGTCGGCGGCGGCTGCCCG	13:C>T
988617|F|0-19:G>C-19:G>C	TGCAGACTATAACTGAAGCGGACATTCGGAAGAGGCCGATGTGGCACACGTACCCG	19:G>C
2369270|F|0-65:G>T-65:G>T	TGCAGGACCGCCTCGGCTTCGACCAGCCCAGCAAGGCCGTCGAGTGGCTCATCAACGCCGCCTCCGCCG	65:G>T
6047398|F|0-8:G>C-8:G>C	TGCAGAGAGGATGTCAAGAACAAGGCGCACGCCATCATCGACAAGTACGCCGAGCGCGGCCTCCGAGAT	8:G>C
7172672|F|0-18:T>G-18:T>G	TGCAGTAGCACGTGAGCATGGTGTTCCACGACGCGGGGGTCCTGTCCG	18:T>G
5411830|F|0-31:C>A-31:C>A	TGCAGCGCAATGCGGAGGAGCAGGAGATCATCTCCAGGGACCG	31:C>A
3574722|F|0-10:T>C-10:T>C	TGCAGCATGCTCTCGCGCTGCTCGAGGAACTGCTCCG	10:T>C
1025753|F|0-28:G>T-28:G>T	TGCAGGACGTGAGCCAGAGCGAGGAGGTGTTCCAGGCCGAGCTGAGCGCGATCGGCAGGATCTACCACA	28:G>T
5581409|F|0-14:G>A-14:G>A	TGCAGCGTCACGGTGAGGTAGGACGCCG	14:G>A
2251962|F|0-14:G>A-14:G>A	TGCAGGCAGCTCCAGGTCGAAGTGGAACAGCAGCGCGGCGAGCGCGAGCTCGATGTGCGCCAGCCCAAA	14:G>A
5970951|F|0-17:G>A-17:G>A	TGCAGGACCGTCGGGCAGGATGGCGAAGCCTGATGGCAGGAGCGCGACGTAGTCTGGGTCGCCGCCGTT	17:G>A
3944480|F|0-20:G>C-20:G>C	TGCAGCTTCAAGTACGCCACGTTCGCCTACTACGCCG	20:G>C
3950315|F|0-7:G>C-7:G>C	TGCAGCTGATCATTCTGAAGTGGTACTCTATGCCG	7:G>C
4539232|F|0-11:T>C-11:T>C	TGCAGTAAGAATGTGTTAGTACTGCACCCG	11:T>C
2375135|F|0-34:C>G-34:C>G	TGCAGATCCTGCTCGAGTACATGGACGGCGGCTCCCTCGACGGCCGCCGCATCGCCGACGAGCCCTTCC	34:C>G
5324703|F|0-19:C>G-19:C>G	TGCAGCTCGCGTACGCCGTCGGCGGCGGCTGCCCG	19:C>G
1001684|F|0-38:C>T-38:C>T	TGCAGCACCGAGAGCTGCACGATGATGTCGCGCGCCCACTTGCCGTACATGGCGTCCAGCAGCGGCACG	38:C>T
5992804|F|0-17:A>G-17:A>G	TGCAGGCGAAGGTGCGCACCGCGGAGCCG	17:A>G
5332795|F|0-7:G>A-7:G>A	TGCAGTTGCGAATCAGAGAAGTCACCG	7:G>A
1087708|F|0-46:C>T-46:C>T	TGCAGACGCAAACCCAAACCAAGCGAAAAATATCCACTGAATCTCTCTCGCTGCTCGCGGCCGCGGCGA	46:C>T
1862657|F|0-19:G>A-19:G>A	TGCAGCACGGGAAGACGGTGTTCATGGACATGCTCGTGGAGCAGACCCACGAGGTGGACACGTTCGACT	19:G>A
1072043|F|0-30:A>C-30:A>C	TGCAGCAGCACCTGGCCGACCCATGGCACAACACCATCATCCAGACCATCTCCG	30:A>C
1024096|F|0-64:C>G-64:C>G	TGCAGAAGCGCACCGAGGTGAAGCTCCAGGACCTGCCCGTGGTGCTCGGCGCCTGCTGCGTGCTCCACA	64:C>G
3959004|F|0-10:C>T-10:C>T	TGCAGATCAACTACACCTTCGACATACGCCG	10:C>T
1003085|F|0-40:T>C-40:T>C	TGCAGAAAAGGGAGAGCAAGACCAATATCATCTCCAGGACTAAGCAAAGACCCACTCCATCTCCGAAAC	40:T>C
5366915|F|0-5:C>A-5:C>A	TGCAGCAGCTTGCCATCGGCAACGACGGATACGCCG	5:C>A
3959219|F|0-23:T>A-23:T>A	TGCAGCTTGCCGATCTCCCTGGGTATCTCGCCG	23:T>A
4439587|F|0-8:C>A-8:C>A	TGCAGTTGCGTTATGGTGGCTTCTGTGACGCTGTCCTGCCTTTCCG	8:C>A
5360515|F|0-31:G>C-31:G>C	TGCAGAAGGTCGGGGTGTGGCGGGATAAGGGGAAGAGCAAGGTGACGGAGCTTGAGGAGAGGGTGGAGG	31:G>C
1024841|F|0-60:G>A-60:G>A	TGCAGCGCAGCGTTTGCTGTGCATGCACCGCGCATGTGCCTGGTCTCTATCTGTTCGCCTGTGCCCTCC	60:G>A
100450291|F|0-20:A>G-20:A>G	TGCAGCTCCTTGAGGGACAGATTCTTCATGTGGATGGGGTAGTTGACGGTGTCCAGCAGCGGCGTCGGC	20:A>G
1863316|F|0-29:G>C-29:G>C	TGCAGCTTTGTGTAGTTGCCGTGCGCGTCGACGTGCGTCAGGGCCACGCGCAGGCCCTCCAGGCGGCCC	29:G>C
3957231|F|0-30:T>C-30:T>C	TGCAGGCGCTGGTGAGCGTGGACATGCTGGTGCTGTTCGTGGCGACCATCTGCGGCGTCGGCGGCACGC	30:T>C
1025267|F|0-44:G>A-44:G>A	TGCAGGACCTCGGCGATGGCGCGGTCCACGTGGTCGGCGTCGTCGCGGAGCGCCG	44:G>A
2248262|F|0-56:C>T-56:C>T	TGCAGCGAGAGGAGCACCTTCTTGAGCCGCTCCCTGGCTTGGGCGTCGATGAGGTTCCCCTCGCCGTCG	56:C>T
3024222|F|0-22:G>A-22:G>A	TGCAGAGAGCATCACCGCCGAGGAATCAACAAGCCACCG	22:G>A
1087708|F|0-56:G>A-56:G>A	TGCAGACGCAAACCCAAACCAAGCGAAAAATATCCACTGAATCTCTCTCGCTGCTCGCGGCCGCGGCGA	56:G>A
989503|F|0-46:G>T-46:G>T	TGCAGCACAACAACCTGAGTGGCGAGATCCCAGCGGTGCTCGCGGCGCTGCCGTCGGTCACCGAGGTGG	46:G>T

5324118|F|0-23:C>G-23:C>G	TGCAGGCCGCGCTGTCGGTTGCGCGCCCG	23:C>G
986239|F|0-26:C>G-26:C>G	TGCAGGGCCAGGTCCTGGTGCTCCTTCTTGCACACCTGGTCGGCGCCGATGTTGCCGTACCG	26:C>G
1061244|F|0-40:C>G-40:C>G	TGCAGGACGAGTCCTGGAACGTCACCCGCTTCCTGCTGCTCTCCAAGCCGCCGTCGCCG	40:C>G
3942104|F|0-22:G>A-22:G>A	TGCAGATGAGCCCCACGGCGAAGCTCGGGGAGGTGATGTCGCGCCCG	22:G>A
1090062|F|0-25:C>T-25:C>T	TGCAGGTAGAAGATGACGTGCCGCCCGTTGGTGCACACCCTCAGCACGGCGGTGTCCATGTCGTGCCGA	25:C>T
2244447|F|0-17:C>T-17:C>T	TGCAGGATCATGGCGAGCCCCATTTTAGCCTCGAGCAGCGCGAAGCTCTGGCCGACACAGATCCGCGGG	17:C>T
2375135|F|0-28:C>G-28:C>G	TGCAGATCCTGCTCGAGTACATGGACGGCGGCTCCCTCGACGGCCGCCGCATCGCCGACGAGCCCTTCC	28:C>G
4262161|F|0-31:C>G-31:C>G	TGCAGAACTGGTCGGCGAAGTTGGGCCACGTCAGCGCCG	31:C>G
1043238|F|0-49:G>A-49:G>A	TGCAGGTGGCTCTTGACGTGCGCCAGCGTCAGATCCTTGACGTTCATCAGCTCCAGCACCGACTTCGGC	49:G>A
3938270|F|0-24:A>G-24:A>G	TGCAGCAGGCCTTCGGCTACCAGCACGGCCCG	24:A>G
5367216|F|0-11:G>C-11:G>C	TGCAGCTTCGCGGCTCGCGGGCTCCG	11:G>C
2265717|F|0-26:G>A-26:G>A	TGCAGCAGCGGAAGGAGGCCGCGCACGTCGTCGAGCTCGAGAGGCGCTTCAAGGTGCTCGACGGCCTCA	26:G>A
1169049|F|0-21:C>T-21:C>T	TGCAGCCTTCCAAAAATACTGCCCAACTGCCTGTGATGGATCACCAGCTGTACGGGGACCCGTCCGCGA	21:C>T
988002|F|0-39:A>G-39:A>G	TGCAGCGGCTTCACCCCGACGCCGAGCTTCACCGACATCATCGTGAACAAGTACAAGCTCCGAGGAGAC	39:A>G
6045193|F|0-22:A>G-22:A>G	TGCAGTTGACGACGAGCAAGTCAATGGCGTCGGGGCGGATGCCCGTCTTGGCGAGCAGGTCGTCGATGG	22:A>G
3024886|F|0-17:A>G-17:A>G	TGCAGGACAACAAGGCCACCGCCGCCATGGAGCTCCCCG	17:A>G
7352622|F|0-9:C>T-9:C>T	TGCAGCTCGCGTACGCCGTCGGAGGCGGCTGCCCG	9:C>T
3028155|F|0-31:G>A-31:G>A	TGCAGCTCATGCTCAAGGGGTGCGAGCCGCTGCCACGCCG	31:G>A
1020517|F|0-34:C>T-34:C>T	TGCAGGCCAGCATCATCTCGCCCTAGCTCCATGGCTAGCGGGCATTTGACATGGATTCCG	34:C>T
1018656|F|0-14:A>G-14:A>G	TGCAGCGAAAGAGGAAGAGAAATCGCGTGAAAGTACGTCGCCGAGAAGCAGGTGGCAGAAATGGAGGCG	14:A>G
4002585|F|0-16:G>A-16:G>A	TGCAGCTGCCAACCGCGCACGACGGCGGCTTCACGGCGTCTGGGCCG	16:G>A
1040251|F|0-49:C>T-49:C>T	TGCAGCCCAGAGAACTTGGACTGGTTGTACAGCATGTGCGGCGGCATCGCGAAGGCCG	49:C>T
3934321|F|0-15:C>G-15:C>G	TGCAGAGCATCATCACCTCCG	15:C>G
1863287|F|0-56:C>A-56:C>A	TGCAGGCGGCGGAGCTTGAAGGAGGAGAGCTTCTGCGGGCCGTCCTTGACGGAGGGCTCCTCCTCGAAG	56:C>A
7346500|F|0-8:C>T-8:C>T	TGCAGGGACGGCATCTCCCCG	8:C>T
7164716|F|0-37:G>A-37:G>A	TGCAGAAGCCGCAGGCGGCGACGCGCGGGGAGGGTGGGTCGAGCGCCG	37:G>A
1695019|F|0-34:C>T-34:C>T	TGCAGGACCTCCAGCAGCTGCACCTCGGGACCGCCGCTCACAACTATTTCCAGGCATCGTCGAGCTCAA	34:C>T
2243250|F|0-29:G>A-29:G>A	TGCAGTATGGTGAGAGCGTCCAGCTTCCCGCGGTTGAAGTCGTCGGGGATGTCGTCGTCGATGAACGGG	29:G>A
4261492|F|0-31:G>C-31:G>C	TGCAGCTGCGCAAGAATGCCTTCTCTGGCGAGCTCCG	31:G>C
3031666|F|0-17:C>T-17:C>T	TGCAGCGTCCGCGGCATCGGCGGCATGTCGACGTGCACGACGCCCTCCAGCGCCTGCACGGCCTGCGCC	17:C>T
1024768|F|0-48:C>T-48:C>T	TGCAGGTGAGGCTGACGAAGGGGAATGGCCCAGGATGTGCCTTGAGGGCGGCGGAGACGGCGTCGGTGA	48:C>T
1024841|F|0-12:T>C-12:T>C	TGCAGCGCAGCGTTTGCTGTGCATGCACCGCGCATGTGCCTGGTCTCTATCTGTTCGCCTGTGCCCTCC	12:T>C
3960408|F|0-6:C>G-6:C>G	TGCAGCCTCCACAAGGAGCGCAAGCAGGAGGCCCCG	6:C>G
1865366|F|0-16:C>A-16:C>A	TGCAGCCCTGCGACGGCGTCGAACGCGCACGGGCCAGCCGCCAGCCCGTGCCCG	16:C>A
1865366|F|0-50:C>G-50:C>G	TGCAGCCCTGCGACGGCGTCGAACGCGCACGGGCCAGCCGCCTGCCCGTGCCCG	50:C>G
1051488|F|0-54:C>T-54:C>T	TGCAGGGGTGGGACAATGCCACCATCGCTGGTAAGTAGCCCAGATTTGTTTTACCACCTTGTTAGATCA	54:C>T
1045535|F|0-7:T>A-7:T>A	TGCAGACTGAATTTAAGATCTATGCAGTAACTTTTAATCAGAACACCTTGCCCG	7:T>A
4262563|F|0-10:G>A-10:G>A	TGCAGATGACGCCGTCGGCGAGGGAGGACCCAACCCGCCG	10:G>A
1036711|F|0-15:T>G-15:T>G	TGCAGGTCAGTGACCTTGGTGTGGCGGCGCTATGTGGGCTAGAGGTCCTGTACCTTGCCAAGGCGCCGA	15:T>G
1029718|F|0-14:C>G-14:C>G	TGCAGCTCGGCCGCCGAGATGAACCCGTCGCCGTTCTCGTCGAAGACCCG	14:C>G
2241884|F|0-59:G>A-59:G>A	TGCAGGACCTTGTAGTTCTGGATCATGTCGTACTCGTTCTTGGCGTCGAAGTTGACCTTGTGCATGGGC	59:G>A
1049199|F|0-26:A>G-26:A>G	TGCAGGTTGCTGAGGTGGAGATCCGAAAAGGACGGACCGAATTGCGCATGCCTGGCTCCCTGTATCCCT	26:A>G
3026756|F|0-7:T>C-7:T>C	TGCAGCGTGCTAACCGAAAGCGCGAAGCACTTGGCGCCG	7:T>C
32022494|F|0-24:C>T-24:C>T	TGCAGGAGTGGCAGACGGGGCAGGCCGCCG	24:C>T
2277895|F|0-65:C>T-65:C>T	TGCAGAGCGGGCAGCTGGTGTGGGAGGCAAGCCACATGTCGACGCACTCGGTGTGGAAGCCGTGGCCGC	65:C>T
1219873|F|0-52:C>T-52:C>T	TGCAGGGGAGAGAAACGAACACCAATAAGCAACACGGCCGTCTGAACTCATTCTAGAAGCTTCCGAGAA	52:C>T
5324703|F|0-16:C>T-16:C>T	TGCAGCTCGCGTACGCCGTCGGCGGCGGCTGCCCG	16:C>T
1136150|F|0-5:G>A-5:G>A	TGCAGGCACTGCGCGCAGCCCCCCGCCCCGACCGTCTCCACGCACTGCGCCATCGCGTACACGCCGAGA	5:G>A
3946240|F|0-11:G>A-11:G>A	TGCAGGTAGTCGCCCACGTTCACCACCAGCGCCCCCGCCACTGCCG	11:G>A
1154987|F|0-18:G>T-18:G>T	TGCAGCGCCGTGAGCGACGGGGGCGCGGCGGTGGTGTGGTTCAAGCATGACCTCCGCCTCGACGACCAC	18:G>T
990929|F|0-38:G>A-38:G>A	TGCAGGTGCTGGACCTGTTCACCGCGAGCATCGTCTCCGTCGCGGACGACTACGTGGCGCCG	38:G>A
2248262|F|0-44:G>A-44:G>A	TGCAGCGAGAGGAGCACCTTCTTGAGCCGCTCCCTGGCTTGGGCGTCGATGAGGTTCCCCTCGCCGTCG	44:G>A
3029450|F|0-5:C>T-5:C>T	TGCAGCGGGTCATAGCCGCCGATGACGAGCAGCACCG	5:C>T
1089106|F|0-53:G>C-53:G>C	TGCAGGAAGTGCGCGGCCATCTGCCACGCGTCCCCCTGCGCCGCCCGCACCAGGGCCAGGTAGAAGTAG	53:G>C
993067|F|0-13:C>G-13:C>G	TGCAGCACGCGCACAGGATGCACTCGTACAGCCCGTCCAGCTTCCGCCGCTCGCCCG	13:C>G
2243878|F|0-13:G>A-13:G>A	TGCAGCTTGGGCCGCCTCGTCTGTCATACTCGAAGCCGAACGCCATCCTCAATGGCGTCGAGATCATGA	13:G>A
3027062|F|0-10:T>C-10:T>C	TGCAGGGGATTGTTTTCCTATGGCTGAGCGCCATGATACCCG	10:T>C
3948826|F|0-8:C>A-8:C>A	TGCAGCTGCCCGTCTACCAGCTTGAACCTGCCG	8:C>A
4261492|F|0-16:T>C-16:T>C	TGCAGCTGCGCAAGAATGCCTTCTCTGGCGAGCTCCG	16:T>C

100572255|F|0-27:G>T-27:G>T	TGCAGGCAGTGCAGCGCCAGCGTCGCCGCACCGCCCG	27:G>T
3938507|F|0-7:T>C-7:T>C	TGCAGTCTGCATCAACCGCCG	7:T>C
989922|F|0-14:A>T-14:A>T	TGCAGCTGCTGATCAAGCCAGTTGCTGGTCTCCTTGAGCTGCGACAGCCG	14:A>T
3944557|F|0-46:G>A-46:G>A	TGCAGACGCCGACGTACGTGGTGCGCGCGCCTCGGCACCCCGCCGCAGCAGCTCCTGCTCGCCGTCG	46:G>A
1094223|F|0-38:G>A-38:G>A	TGCAGGGCGCGAGTGGGCGGCCGCAGAGGCACTTGTTGGGCGCGAAGTGCTTGGGGCCGAACCTGTGCG	38:G>A
1020517|F|0-17:T>C-17:T>C	TGCAGGCCAGCATCATCTCGCCCTAGCTCCATGGCTAGCGGGCATTTGACATGGATTCCG	17:T>C
32025343|F|0-5:G>C-5:G>C	TGCAGGGATAGCGGCGCGATCCTCGACTCGGACAGCGCCCG	5:G>C
2244584|F|0-8:G>C-8:G>C	TGCAGATTGCTCGAAGCAGCAGACCTCGACGCCCGCGTTGCCCTTGTGCTCCCCTTCCG	8:G>C
3028721|F|0-23:G>C-23:G>C	TGCAGCACGGGGAACATCACCTGGGCGACGATCACGCCG	23:G>C
1071245|F|0-49:A>G-49:A>G	TGCAGGCCATGAGTGCCAACCAGAGCAGCATGCTGATCCTTCAGGAAACATGCACTGATGCATCCGAGA	49:A>G
1150140|F|0-36:C>G-36:C>G	TGCAGTGGATCATCTGGTGAACACGGTGAGCCTGGGCCACGACTTCCTCTTCGAGGAGGCCG	36:C>G
1318131|F|0-11:C>G-11:C>G	TGCAGGAGCGACGCGTTCTTGGGGAACTGGAACGAGTTCCTGTTCATGAACGCCACCAGCGCGAACCCC	11:C>G
7922145|F|0-10:C>A-10:C>A	TGCAGCCTCGCGGACCGCCG	10:C>A
25956594|F|0-16:A>G-16:A>G	TGCAGCACCGCCGTCGACTGGTCCGACCG	16:A>G
100564604|F|0-11:T>C-11:T>C	TGCAGAGAGTGTGCGACGACGGCGGCGGAGCGGTTGGCTTCCACGAGCGGCTGCACGCCG	11:T>C
980448|F|0-35:C>G-35:C>G	TGCAGGAACTTCTGCGTGGGGAAGGTGAGCGCGAGCGAGAACATCTGCGGGAGGATGCGGAGCGTGAAG	35:C>G
1059062|F|0-10:G>C-10:G>C	TGCAGTCGCTGCCACCAAACCCCGACGAGGACGCGCTCTTCGCGGAGTACTGCCGATTGGCGAGCCTCT	10:G>C
3020870|F|0-16:G>A-16:G>A	TGCAGTGGATATTTGGGTTCGTGACGTTCTTCTTTCCCG	16:G>A
3024881|F|0-28:T>C-28:T>C	TGCAGGCCGATTCCCGCGCCTTCAAGAGTGCCTCCTACCG	28:T>C
999233|F|0-13:G>A-13:G>A	TGCAGGGCTCGGCGGCGCTCGGGGTCGGGATCCTGAACGAGGCCGCCCG	13:G>A
2361851|F|0-37:G>A-37:G>A	TGCAGGACCTCCTCGGCTTCGACAAGGCCAGCAAGACGGTGGAGTGGCTCCTCACGCAGTCCAAGCCGA	37:G>A
1165550|F|0-8:G>C-8:G>C	TGCAGCATGCTGCCGAGCTTCCTCACCGACCCGTTCCCCGCGGACCCCCTGGTGGAGGACTGCGACGGC	8:G>C
1062651|F|0-10:G>A-10:G>A	TGCAGGTTTGGAATCTGAATACTCTCGTCGTGTTGTTTGGTGCAGGTATGAGCCG	10:G>A
1029559|F|0-34:C>T-34:C>T	TGCAGATCATGGCCCTGCACTCCTCCAGCCCCATCTTCTCGGACCCCAGCAGCTTGCCCAGCATCCGCC	34:C>T
988800|F|0-47:G>C-47:G>C	TGCAGGGCGCGGCCATCAAGCCGCTGCTGGCCTCCATTGCCACGGGGGTCATCATCTGGTTCATCCCTG	47:G>C
4397615|F|0-7:G>A-7:G>A	TGCAGGCGGTGGGGTCGCCTCAGCCCCGCCCCGATCCG	7:G>A
989353|F|0-6:T>A-6:T>A	TGCAGCTTCAGCCGAGGGGGTGCCGCGTGACGTGCTTGGCCGACGCGGGGGGAGCCG	6:T>A
2249981|F|0-64:G>C-64:G>C	TGCAGCACATGGAGCTGCTCGAGGAGGGTGGCGACGGCGACGAGCGGTTCGAACTCTTCGAAACGGAAG	64:G>C
5580116|F|0-14:G>C-14:G>C	TGCAGATACGACGCGCTTCAAGTCTCCCCG	14:G>C
1029735|F|0-39:A>T-39:A>T	TGCAGCGTGGCGGTGGAGGAGCCGATGGAGTGCCTGGGCACGGGGCTGGTGAGCCCG	39:A>T
989922|F|0-41:C>T-41:C>T	TGCAGCTGCTGATCAAGCCAGTTGCTGGTCTCCTTGAGCTGCGACAGCCG	41:C>T
7174043|F|0-19:G>A-19:G>A	TGCAGAAGGTGCCCTCGCAGCGGCACAGGAACCCCAGCAGCCCCACCTTCTTGCGGCACGCCGCGCAGC	19:G>A
1008796|F|0-65:T>C-65:T>C	TGCAGCACCGTTTTCGTCACCTGCTCCTGGCCCATCGGCTGCGCCGTGGAGTTGAGCCCAGCCAATGGG	65:T>C
1039417|F|0-14:G>A-14:G>A	TGCAGTTCTCATCGGCGATTGACACGCTTCGTGCTTGTTTCAGGTGTATCAGGAGGAGTCAGACAAGCC	14:G>A
1024096|F|0-13:C>G-13:C>G	TGCAGAAGCGCACCGAGGTGAAGCTCCAGGACCTGCCCGTGGTGCTCGGCGCCTGCTGCGTGCTCCACA	13:C>G
2271963|F|0-13:C>T-13:C>T	TGCAGCTGCTGGACCACCCCTTCCTCCCCACCCTCTACGCCCACTTCGAGACCGACAAGTTCGCCTGCC	13:C>T
5581387|F|0-23:C>T-23:C>T	TGCAGCGCCGCACCGAGCCCAAGCTGCCG	23:C>T
3028898|F|0-20:C>T-20:C>T	TGCAGCCCCTACCGCGCCGACCTGTCCGACCGCCCCTGCGCCGCCG	20:C>T
4396458|F|0-25:C>T-25:C>T	TGCAGGCGAACTTGATGGCCTCGTCCCGCGTGGAGGTGCCG	25:C>T
1024576|F|0-25:C>T-25:C>T	TGCAGGAGGGCGACCTCGCCATCTGCCCCGAGGGGACGACGTCCCGAGAGCCGTTCCTGCTCCG	25:C>T
7166296|F|0-16:G>T-16:G>T	TGCAGTTTTTTTAGGCGGTGCGGGGAGCCG	16:G>T
981402|F|0-13:C>T-13:C>T	TGCAGCAGCAGCACGATAACCACCACCGACGCCACGAAGGAGGTGGAGTTGCAGTAGAAGAAGGCCCGA	13:C>T
5582655|F|0-17:C>G-17:C>G	TGCAGCGAGCTCTGGACCCCGCCGACGGCGCACCG	17:C>G
1023480|F|0-17:G>A-17:G>A	TGCAGGTCGGCCGCCGCGAGCGCGGGGAAGAAGTCGGACACGTTCGGGCTGCCG	17:G>A
5363868|F|0-23:C>T-23:C>T	TGCAGGGCGACGACGTGCCCGTGCTGCCG	23:C>T
5580341|F|0-8:G>C-8:G>C	TGCAGCTTGAGGAGGTGCCTCCG	8:G>C
2244584|F|0-39:G>T-39:G>T	TGCAGATTGCTCGAAGCAGCAGACCTCGACGCCCGCGTTGCCCTTGTGCTCCCCTTCCG	39:G>T
7940674|F|0-15:C>T-15:C>T	TGCAGAGGGCGTGCGCGGGCGACGTGCCG	15:C>T
1863287|F|0-57:T>G-57:T>G	TGCAGGCGGCGGAGCTTGAAGGAGGAGAGCTTCTGCGGGCCGTCCTTGACGGAGGGCTCCTCCTCGAAG	57:T>G
4734171|F|0-26:G>C-26:G>C	TGCAGCGGCTTGGTGCAGAGCGCGCAGGCGTCCAGCTCCAGCTCCG	26:G>C
7167958|F|0-25:A>C-25:A>C	TGCAGCTGCTGGAGCGCCACGACGGAGACGGCGACCG	25:A>C
1026265|F|0-7:C>G-7:C>G	TGCAGGCCGCCATGAAGCAGGCCTCCCGAAGGAGCTCGCCGACCGATTCTTGGGTGGGCAAGCTGCGGA	7:C>G
1217107|F|0-28:C>G-28:C>G	TGCAGCTGGTCCCGCCGCTCAACTTCGCCATGGTGGACCACGGCGTGTACCGCTCCG	28:C>G
1084864|F|0-5:G>A-5:G>A	TGCAGGTACGTCTCGACGAACTTGCGCTTGAACCACCTCAGCCTCATCCAGTTCATGATAATCGGCTGC	5:G>A
5363712|F|0-14:G>C-14:G>C	TGCAGGACCGTGACGACGGCGGCGGCGATGTAGTCCGCCTCCG	14:G>C
991324|F|0-66:A>G-66:A>G	TGCAGAGCGCCATCCAGGCCGCCATCGCGCACTGCAAGAACTCCATCGCCGTGGCCAAGCAGTAGTACG	66:A>G
1024345|F|0-47:G>C-47:G>C	TGCAGGCTCCAGTCGGCCCCCGCCGCCATGAGCATCTCAGCTGCCGCGGCGTCGCCGAAGCGGACGGCA	47:G>C
6040210|F|0-47:A>C-47:A>C	TGCAGCCTCTCCTTCTTCACCTCCCTCAGGCGCTCTTCCTCCTGTGCAGGCCTCATCCTTTTGCCGCCG	47:A>C
3949109|F|0-22:G>A-22:G>A	TGCAGCAGTCGGCGCTGGGGTCGGCGGCGCCG	22:G>A

<table>
<tr><td>2252070|F|0-30:C>T-30:C>T</td><td>TGCAGCTCGTCCTGCACCCCCGCGAACAGACAAACCAGATCAGGCAACCCTGGACCGACGGGACGGGAT</td><td>30:C>T</td></tr>
<tr><td>7161755|F|0-22:A>C-22:A>C</td><td>TGCAGGACGACGTCGACAGCGAAGTCGACCCG</td><td>22:A>C</td></tr>
<tr><td>7923298|F|0-6:G>T-6:G>T</td><td>TGCAGCGGTGCCCTTGTCGCCGCCG</td><td>6:G>T</td></tr>
<tr><td>2258071|F|0-35:C>T-35:C>T</td><td>TGCAGGAAGGTGAGCTTCTCGAGGAAGCCGAGCTGCGCGTTGCCCACGGCGAGCGCGAAGACGAGGAGC</td><td>35:C>T</td></tr>
<tr><td>998441|F|0-37:C>A-37:C>A</td><td>TGCAGCTCCTGGACCTCAGCAACAACTCCCTCAGCGGCGGCATCCCGAGCAGCCTCTGCAACTGCTCGG</td><td>37:C>A</td></tr>
<tr><td>988800|F|0-50:A>G-50:A>G</td><td>TGCAGGGCGCGGCCATCAAGCCGCTGCTGGCCTCCATTGCCACGGGGGTCATCATCTGGTTCATCCCTG</td><td>50:A>G</td></tr>
<tr><td>12772357|F|0-52:C>T-52:C>T</td><td>TGCAGGACCTCCTCGGCTTCGACAAGGCCAGCAAGACGGTGGAGTGGCTCCTCACGCAGTCCAAGCCGG</td><td>52:C>T</td></tr>
<tr><td>2258071|F|0-47:G>A-47:G>A</td><td>TGCAGGAAGGTGAGCTTCTCGAGGAAGCCGAGCTGCGCGTTGCCCACGGCGAGCGCGAAGACGAGGAGC</td><td>47:G>A</td></tr>
<tr><td>2257770|F|0-67:C>G-67:C>G</td><td>TGCAGCAGCCGCCGTCGAAGCACGGGTACGGCGCCGTCAGGAACTGCGCCACGATCTCCGACTCCTCCT</td><td>67:C>G</td></tr>
<tr><td>1143356|F|0-41:C>G-41:C>G</td><td>TGCAGCTGGAAGTCGAGGTTGAGGTGCTTGAAGTTGCGGGTCAGCGTCCCCCGCGGCCCCGTCACCGAG</td><td>41:C>G</td></tr>
<tr><td>4262945|F|0-16:G>C-16:G>C</td><td>TGCAGGACGTGTTCACGCGCTGGTCCTTCGACACGACCTGCAACCTCGTCTTCGGCGTCGACCCCGAGA</td><td>16:G>C</td></tr>
<tr><td>4261716|F|0-32:C>T-32:C>T</td><td>TGCAGGAGGTTGCCGAGCACGGGAAGCTGTTTCGGCGGCCCG</td><td>32:C>T</td></tr>
<tr><td>5325226|F|0-7:C>T-7:C>T</td><td>TGCAGACCTGCGTGCTGGACACCCG</td><td>7:C>T</td></tr>
<tr><td>4993003|F|0-8:A>G-8:A>G</td><td>TGCAGCAGAGTGGTAGGAGCCATGTCCG</td><td>8:A>G</td></tr>
<tr><td>1219453|F|0-22:C>T-22:C>T</td><td>TGCAGAGCGTCATCCAGATGATCTCCGTCATGTTCGTCGGGCACCTGGGCGAGCTCGCGCTGGCCAGCG</td><td>22:C>T</td></tr>
<tr><td>2246457|F|0-5:C>T-5:C>T</td><td>TGCAGCTCGTGCCCCCGAGCCGCGCATAAGACGGATCAAGAGCATGAACGCGTTCGACATCATCGCCTC</td><td>5:C>T</td></tr>
<tr><td>1306202|F|0-37:C>T-37:C>T</td><td>TGCAGACAAAGGAACTGATAGCAGAGATTGAATTGCTCGAAGAGGAAATCGCGAACCGCGAGCAGCACG</td><td>37:C>T</td></tr>
<tr><td>1093363|F|0-32:G>C-32:G>C</td><td>TGCAGCGCGACGGGGCTGAGCCCGAGCCCGCGGGTGAGGTGGAAGTTGAGCGCCAGCCACGGGAAGCAC</td><td>32:G>C</td></tr>
<tr><td>1040251|F|0-17:G>T-17:G>T</td><td>TGCAGCCCAGAGAACTTGGACTGGTTGTACAGCATGTGCGGCGGCATCGCGAAGGCCG</td><td>17:G>T</td></tr>
<tr><td>12766093|F|0-29:G>C-29:G>C</td><td>TGCAGCTTCTTTATTGTGTCGATCTGCTTGGCCG</td><td>29:G>C</td></tr>
<tr><td>1025984|F|0-15:T>C-15:T>C</td><td>TGCAGCTTGGATGAATGGGTTAGAGATCTGTTCAATGGAGGTTGACACCG</td><td>15:T>C</td></tr>
<tr><td>1061245|F|0-18:A>C-18:A>C</td><td>TGCAGGACGAAACCGTTGAGCTCCCACTGTCTCCCCACGACCTGCGCTTCGCTCTGGTCTACCG</td><td>18:A>C</td></tr>
<tr><td>4262109|F|0-16:T>G-16:T>G</td><td>TGCAGGTGAGTGCGCGTGTGGCCG</td><td>16:T>G</td></tr>
<tr><td>3030984|F|0-30:G>A-30:G>A</td><td>TGCAGTGCCCTACGGGGTCACGGCCAAGGCGTCGGCGCCG</td><td>30:G>A</td></tr>
<tr><td>988800|F|0-31:C>T-31:C>T</td><td>TGCAGGGCGCGGCCATCAAGCCGCTGCTGGCCTCCATTGCCACGGGGGTCATCATCTGGTTCATCCCTG</td><td>31:C>T</td></tr>
<tr><td>981402|F|0-20:C>G-20:C>G</td><td>TGCAGCAGCAGCACGATGACCACCACCGACGCCACGAAGGAGGTGGAGTTGCAGTAGAAGAAGGCCCGA</td><td>20:C>G</td></tr>
<tr><td>1063764|F|0-22:C>T-22:C>T</td><td>TGCAGCATGTGCAGGCAGCTGACCAAACTCTTTCCTCTCCACCTGGTCATCGTCCTTCTCCTCGGCGTC</td><td>22:C>T</td></tr>
<tr><td>2252222|F|0-43:G>C-43:G>C</td><td>TGCAGGCGCCGTCGAGGCCAACGTTCTGCGTCGACGACATGGAGGGTTTCTCGGGCATGTACTCGGGTG</td><td>43:G>C</td></tr>
<tr><td>3028721|F|0-17:C>T-17:C>T</td><td>TGCAGCACGGGGAACATCACCTGGGCGACGATCACGCCG</td><td>17:C>T</td></tr>
<tr><td>4261492|F|0-13:G>C-13:G>C</td><td>TGCAGCTGCGCAAGAATGCCTTCTCTGGCGAGCTCCG</td><td>13:G>C</td></tr>
<tr><td>1029504|F|0-28:T>A-28:T>A</td><td>TGCAGATGAGATCGCCAAGCTGTTCCCCTGCCTGTACGGCCAGCCGTCGGCCGCCGTGGTGCAGTCCGC</td><td>28:T>A</td></tr>
<tr><td>2251962|F|0-16:T>C-16:T>C</td><td>TGCAGGCAGCTCCAGGTCGAAGTGGAACAGCAGCGCGGCGAGCGCGAGCTCGATGTGCGCCAGCCCAAA</td><td>16:T>C</td></tr>
<tr><td>5372181|F|0-15:A>G-15:A>G</td><td>TGCAGCTGCTGCTTCACCGCCACCG</td><td>15:A>G</td></tr>
<tr><td>4002481|F|0-11:T>G-11:T>G</td><td>TGCAGGACACGTCCGCGCAGTGCACCCACCGCCCGTGCTCGCCG</td><td>11:T>G</td></tr>
<tr><td>1039167|F|0-49:G>C-49:G>C</td><td>TGCAGAGCTCGCGGTCGCAGTTGCCGTTCTGCCACAGCGCGCACATCTTGGTCTTGTACGCCG</td><td>49:G>C</td></tr>
<tr><td>2248694|F|0-11:C>T-11:C>T</td><td>TGCAGCCTCATCGAGATCGACGGCGTGGTGCACGAGTTCCAAGCAGTTCCG</td><td>11:C>T</td></tr>
<tr><td>7167447|F|0-14:C>T-14:C>T</td><td>TGCAGACGCGGAGCCACCGAGAGCCG</td><td>14:C>T</td></tr>
<tr><td>1694774|F|0-14:C>G-14:C>G</td><td>TGCAGCATGGGCGACAGGTCGGAGAAGGACTTGTAGGCCGCCAGCTTGAGGGCGACGTCGAGCGGTGAG</td><td>14:C>G</td></tr>
<tr><td>999806|F|0-16:G>A-16:G>A</td><td>TGCAGACCCCACGGCCGCAGTACGAGTTCACGGACTTCCCGTGGCCGCCG</td><td>16:G>A</td></tr>
<tr><td>1026265|F|0-52:G>A-52:G>A</td><td>TGCAGGCCGCCATGAAGCAGGCCTCCCGAAGGAGCTCGCCGACCGATTCTTGGGTGGGCAAGCTGCGGA</td><td>52:G>A</td></tr>
<tr><td>1046618|F|0-68:C>A-68:C>A</td><td>TGCAGGAACTCGGACGACGGCCCGTTGCGCACCGTGGCGTTGAACCCGCAGAGGTGCTGTTGGAGCTCC</td><td>68:C>A</td></tr>
<tr><td>1863316|F|0-40:G>A-40:G>A</td><td>TGCAGCTTCGTGTAGTTGCCGTGCGCGTCGACGTGCGTCAGGGCCACGCGCAGGCCCTCCAGGCGGCCC</td><td>40:G>A</td></tr>
<tr><td>1028777|F|0-27:G>A-27:G>A</td><td>TGCAGCGCCGTCTCCGCGTGGCGGGCAGCGTCGGCGTGGTGGAGTTGCCACCTGTCCCACCACCACGGC</td><td>27:G>A</td></tr>
<tr><td>2242683|F|0-59:C>A-59:C>A</td><td>TGCAGCTGCTGGATGTACTCGATCGCGTCCTTGATGATCGAGGCCTTGTCCATCTGCAACGACGAGGCA</td><td>59:C>A</td></tr>
<tr><td>1103078|F|0-13:G>T-13:G>T</td><td>TGCAGAGCACCGAGGAGAAGTGCAGCACTTGGGAGAAGCGCTCGCGGTTCTTCATGGACGAGTATATCG</td><td>13:G>T</td></tr>
<tr><td>3937863|F|0-13:C>T-13:C>T</td><td>TGCAGCAGGCGATCGAGCTCCAGAGCCG</td><td>13:C>T</td></tr>
<tr><td>2247075|F|0-19:G>A-19:G>A</td><td>TGCAGGAGCACCGCCTGCCGCTGTCTAACCTGGACCTCCTCCTGCCGCCCATCGACGTCGGCGTCTTCT</td><td>19:G>A</td></tr>
<tr><td>1093481|F|0-22:C>T-22:C>T</td><td>TGCAGGTTGGCGGTGACCGCCGCGCGACGGCGCGCCTTGAAGATGGACACGCACTCCTTGCCGTACCCG</td><td>22:C>T</td></tr>
<tr><td>32023369|F|0-12:A>G-12:A>G</td><td>TGCAGGATCTGCACGGCGCTCCAGCTGGCCCCG</td><td>12:A>G</td></tr>
<tr><td>1098064|F|0-26:T>G-26:T>G</td><td>TGCAGCAGGCGGCGGCGCGCGCGTGGTCGCCCGAGGGGTCGTCGCGCCG</td><td>26:T>G</td></tr>
<tr><td>1341019|F|0-54:T>G-54:T>G</td><td>TGCAGATGAACGGGAACCTGACGATTGGCAAGGCCATGAGGTGGTGGGAGAAGGTGACGCACCCCAACA</td><td>54:T>G</td></tr>
<tr><td>4538778|F|0-11:C>T-11:C>T</td><td>TGCAGCGATCCCTCGATGAGGCCGCCG</td><td>11:C>T</td></tr>
<tr><td>1129293|F|0-53:G>C-53:G>C</td><td>TGCAGCTGCTGGGGCGCGGTGTACAGCAACGGGACGGGCTACTGCTACCTCATGGACTACCCG</td><td>53:G>C</td></tr>
<tr><td>1741300|F|0-47:C>G-47:C>G</td><td>TGCAGCAGCTCCCGTGCAGCCTCCCAGCCCGTGCCGTCTCCGTCGTGCCTGCTGGGGAAGCCCG</td><td>47:C>G</td></tr>
<tr><td>6046289|F|0-48:A>G-48:A>G</td><td>TGCAGCGTCACCACGCGCCATGCGCCGACGACCGCGCTGGCCGACGCGACATGCTCGTCTTTGAGGATG</td><td>48:A>G</td></tr>
<tr><td>996789|F|0-52:C>T-52:C>T</td><td>TGCAGCACGCCCTCCAGACGGCGCTCGTGCTCTGGCTCATCCTCAACGCCATCGGCGCCAACACCAGCT</td><td>52:C>T</td></tr>
<tr><td>6045785|F|0-17:C>G-17:C>G</td><td>TGCAGGTCGTCGGCGGGCGGGTCGTAGGGGACCG</td><td>17:C>G</td></tr>
<tr><td>5357363|F|0-10:G>A-10:G>A</td><td>TGCAGAGCTCGTCGGGGATGCCGCCG</td><td>10:G>A</td></tr>
<tr><td>2271963|F|0-55:C>G-55:C>G</td><td>TGCAGCTGCTGGACCACCCCTTCCTCCCCACCCTCTACGCCCACTTCGAGACCGACAAGTTCGCCTGCC</td><td>55:C>G</td></tr>
</table>

<table>
<tr><td>12469795|F|0-26:C>T-26:C>T</td><td>TGCAGGAGGTACGTGCGGTCCAGCGCCGTCGCGCCG</td><td>26:C>T</td></tr>
<tr><td>5411723|F|0-9:A>G-9:A>G</td><td>TGCAGCGAGAGCGGCACCACGCCG</td><td>9:A>G</td></tr>
<tr><td>1026265|F|0-42:C>T-42:C>T</td><td>TGCAGGCCGCCATGAAGCAGGCCTCCCGAAGGAGCTCGCCGACCGATTCTTGGGTGGGCAAGCTGCGGA</td><td>42:C>T</td></tr>
<tr><td>6043611|F|0-6:G>A-6:G>A</td><td>TGCAGAGTTCGCTCGAGGTGGACCTCCTCGACCG</td><td>6:G>A</td></tr>
<tr><td>5332545|F|0-8:C>G-8:C>G</td><td>TGCAGGTGCAGTCGGTCCTCCG</td><td>8:C>G</td></tr>
<tr><td>1025077|F|0-43:A>G-43:A>G</td><td>TGCAGCATGCGGATGGCCGTGTTGTAGGCGCGGACAATGCCGTAGCATCGCGCGGCGAGGTAGGCCGAG</td><td>43:A>G</td></tr>
<tr><td>3956818|F|0-8:T>G-8:T>G</td><td>TGCAGACGTAGAGCAGAGCCGCCGCGCGCAGTGGCCG</td><td>8:T>G</td></tr>
<tr><td>989371|F|0-30:G>A-30:G>A</td><td>TGCAGCTGCCAAGTTTGGCGTCCCACCGCCGCCGCCGTTCCTCTCCCTGTATATGACCGACGACGAGGT</td><td>30:G>A</td></tr>
<tr><td>1049199|F|0-29:G>A-29:G>A</td><td>TGCAGGTTGCTGAGGTGGAGATCCGAAAAGGACGGACCGAATTGCGCATGCCTGGCTCCCTGTATCCCT</td><td>29:G>A</td></tr>
<tr><td>3936050|F|0-31:G>T-31:G>T</td><td>TGCAGCGCGCGCGCGAACCGCGCCTCCCATCGCCCG</td><td>31:G>T</td></tr>
<tr><td>1022580|F|0-38:T>G-38:T>G</td><td>TGCAGGGCCTCCGCGACGGCCACGTAGTTGACGGCTCCTCCGATGTGCCG</td><td>38:T>G</td></tr>
<tr><td>981402|F|0-62:G>C-62:G>C</td><td>TGCAGCAGCAGCACGATGACCACCACCGACGCCACGAAGGAGGTGGAGTTGCAGTAGAAGAAGGCCCGA</td><td>62:G>C</td></tr>
<tr><td>1058180|F|0-21:T>G-21:T>G</td><td>TGCAGCAAGCTTCCAGTTTCATTCCTTGGACCACACCATAGATTCAAATAACAAATAGAGTTACTTTCT</td><td>21:T>G</td></tr>
<tr><td>5581561|F|0-25:T>C-25:T>C</td><td>TGCAGGTGGTTGTCCTCGAACGGGATCTTGCCG</td><td>25:T>C</td></tr>
<tr><td>1046618|F|0-47:G>A-47:G>A</td><td>TGCAGGAACTCGGACGACGGCCCGTTGCGCACCGTGGCGTTGAACCCGCAGAGGTGCTGTTGGAGCTCC</td><td>47:G>A</td></tr>
<tr><td>1093481|F|0-59:G>C-59:G>C</td><td>TGCAGGTTGGCGGTGACCGCCGCGCGACGGCGCGCCTTGAAGATGGACACGCACTCCTTGCCGTACCCG</td><td>59:G>C</td></tr>
<tr><td>5580095|F|0-19:C>G-19:C>G</td><td>TGCAGACGTGGAACAACGGCGCGGAGAACACGAAGCGGATCGTGGCCGCCG</td><td>19:C>G</td></tr>
<tr><td>4396458|F|0-37:G>C-37:G>C</td><td>TGCAGGCGAACTTGATGGCCTCGTCCCGCGTGGAGGTGCCG</td><td>37:G>C</td></tr>
<tr><td>1022063|F|0-20:G>A-20:G>A</td><td>TGCAGGAATGCTGCACGGACGCGACGGGGTCGTATGTGGTGTACGCGCCG</td><td>20:G>A</td></tr>
<tr><td>6045433|F|0-14:C>A-14:C>A</td><td>TGCAGCAGCAACGCCGTCCAGCCGCCG</td><td>14:C>A</td></tr>
<tr><td>3946426|F|0-5:C>T-5:C>T</td><td>TGCAGCCGCCCTCCGTCGGAGATCTGGGCTCATCCG</td><td>5:C>T</td></tr>
<tr><td>5579499|F|0-13:G>T-13:G>T</td><td>TGCAGACGCGGGAGGCGCGCGGGAGGGAGGACGGCTGCGGGGGTAGGCGGAGGAGGATCTCGGAGACCA</td><td>13:G>T</td></tr>
<tr><td>2246569|F|0-6:G>A-6:G>A</td><td>TGCAGCGGGTCGTCAACTCCCTGGCTGTCGAGATGTTCGCTGGACTCGCGTCTGAGGACGCCAGCAAGG</td><td>6:G>A</td></tr>
<tr><td>1149940|F|0-19:C>A-19:C>A</td><td>TGCAGACGGCCTTCTTCTACGTCTCGCTCTACCTGGTGGCCATTGCGCAGAGCGGCCACAAGCCCTGCG</td><td>19:C>A</td></tr>
<tr><td>2253331|F|0-10:C>T-10:C>T</td><td>TGCAGGAGCGCGAGTCCCGCCTGCGCACCGAGCTGCTCGAGCACAAGATCCTCAAGGAGACCGTCGTCA</td><td>10:C>T</td></tr>
<tr><td>2253331|F|0-7:G>C-7:G>C</td><td>TGCAGGAGCGCGAGTCCCGCCTGCGCACCGAGCTGCTCGAGCACAAGATCCTCAAGGAGACCGTCGTCA</td><td>7:G>C</td></tr>
<tr><td>992371|F|0-41:C>G-41:C>G</td><td>TGCAGCATCGGGTTGATGTGGCCCTGGAACGGCAGCGGGAACACTAGCACCCG</td><td>41:C>G</td></tr>
<tr><td>6046275|F|0-27:T>C-27:T>C</td><td>TGCAGCGCGGGTCGGCGCAGGCGGAGGTAGCGCAGCCGCGGAGGAGCAGCTTCAGCGTGGTCCG</td><td>27:T>C</td></tr>
<tr><td>1669837|F|0-57:G>A-57:G>A</td><td>TGCAGGACCGTCGGGCAGGATGGCGAAGCCTGATGGCAGGAGAGCGACGTAGTCTGGGTCGCCGCCGTT</td><td>57:G>A</td></tr>
<tr><td>3025019|F|0-38:C>T-38:C>T</td><td>TGCAGAAGCTCACCACCCTCGACCTGGCGCACAACTTCCTGTCCG</td><td>38:C>T</td></tr>
<tr><td>1072922|F|0-14:C>G-14:C>G</td><td>TGCAGGACGAGGAACGCGACCACCGCGGCCACGAGCGCGAAGACATAGATTGAGGTGCGCAGCAGCCGA</td><td>14:C>G</td></tr>
<tr><td>2242168|F|0-7:C>G-7:C>G</td><td>TGCAGTCCACCCAGGAGGAGGAGCAGATCGACACCCAGCTCAAGGGCAAGGCCTCATCCG</td><td>7:C>G</td></tr>
<tr><td>989371|F|0-24:A>G-24:A>G</td><td>TGCAGCTGCCAAGTTTGGCGTCCCACCGCCGCCGCCGTTCCTCTCCCTGTATATGACCGACGACGAGGT</td><td>24:A>G</td></tr>
<tr><td>5372200|F|0-24:C>T-24:C>T</td><td>TGCAGCTCCGAGATGCCTTTCTCCCTCCCG</td><td>24:C>T</td></tr>
<tr><td>3941813|F|0-11:G>A-11:G>A</td><td>TGCAGCTCGGAGAAATGCATGATTGCGATGTACCCCG</td><td>11:G>A</td></tr>
<tr><td>1064161|F|0-54:A>G-54:A>G</td><td>TGCAGCTGCTTCAACCCCACGCCGTCCTTCACCGACATGATCGTAAACAAGTACAAGCTGCGAGGCGAC</td><td>54:A>G</td></tr>
<tr><td>1154987|F|0-29:G>A-29:G>A</td><td>TGCAGCGCCGTGAGCGACGGGGGCGCGGCGGTGGTGTGGTTCAAGCATGACCTCCGCCTCGACGACCAC</td><td>29:G>A</td></tr>
<tr><td>1023501|F|0-55:C>T-55:C>T</td><td>TGCAGGAGGTGGACAACATGACGCTGCGGAGGTTCCTGCGCGCGCGCGACCACGACGTGGGCAAGGCCT</td><td>55:C>T</td></tr>
<tr><td>2246457|F|0-27:A>T-27:A>T</td><td>TGCAGCTCGTGCCCCCGAGCCGCGCATAAGACGGATCAAGAGCATGAACGCGTTCGACATCATCGCCTC</td><td>27:A>T</td></tr>
<tr><td>3030819|F|0-43:C>T-43:C>T</td><td>TGCAGAACAAGCCCGTCCCTATCTGCTACGACGGGTTCGAGCCCTCCG</td><td>43:C>T</td></tr>
<tr><td>1108636|F|0-46:C>T-46:C>T</td><td>TGCAGCCGTTCGCGCTCCGCCTGAACAAGCGCCTCGCCGACGACCACGCCG</td><td>46:C>T</td></tr>
<tr><td>16794825|F|0-29:G>A-29:G>A</td><td>TGCAGTGACATAAGCCTGACAGGCATTGCGTGACGACGATCGGCTTGCCTGGCATCCAGGCTTTGCGGT</td><td>29:G>A</td></tr>
<tr><td>32021432|F|0-9:C>G-9:C>G</td><td>TGCAGCTTCCAGAAGGTGAGGGAGGCGCTCCG</td><td>9:C>G</td></tr>
<tr><td>32021918|F|0-68:G>T-68:G>T</td><td>TGCAGCTCCTTGGTGGAGATCATGCGGACGTCGGCGGCGTCCTTGCTGGCGGCCGGCATTGCGTCGGCG</td><td>68:G>T</td></tr>
<tr><td>7340667|F|0-46:T>C-46:T>C</td><td>TGCAGCACACCACCATGTACGGCTCCCGCACCATCATGTTCCTCACTGACGACGGCACGCTGCGCCTCC</td><td>46:T>C</td></tr>
<tr><td>12772357|F|0-43:G>C-43:G>C</td><td>TGCAGGACCTCCTCGGCTTCGACAAGGCCAGCAAGACGGTGGAGTGGCTCCTCACGCAGTCCAAGCCGG</td><td>43:G>C</td></tr>
<tr><td>100515910|F|0-13:C>T-13:C>T</td><td>TGCAGGGAGTGTACACATTTGATGAGAAGGCGGGCGCAATCAAGGTGGAGACATTCTGTGTCCATGGTT</td><td>13:C>T</td></tr>
<tr><td>1067755|F|0-52:T>G-52:T>G</td><td>TGCAGGCTGCCTGTCTTTTGTGGAGAGAGATGTGGGAGATGGCGTGTCATGTTTGGTTTGGTGCTCCCT</td><td>52:T>G</td></tr>
<tr><td>32026940|F|0-55:T>C-55:T>C</td><td>TGCAGCTTATCCAATCCAATACCTGAATCTGCCATCGCCCTCTTGCACGGCTCAATGACTCTGTCCAGC</td><td>55:T>C</td></tr>
<tr><td>7351961|F|0-18:A>C-18:A>C</td><td>TGCAGCTGGTCGTTGGAGAGCAGCTGCTCCTCCG</td><td>18:A>C</td></tr>
<tr><td>100488852|F|0-49:C>T-49:C>T</td><td>TGCAGGCGCTCAAGGCCAAGGAGGCGCAGAAGAAGTCCGCGCAGGCCGACGAGGAAGCTGGCTCCCTGT</td><td>49:C>T</td></tr>
<tr><td>100539276|F|0-27:A>T-27:A>T</td><td>TGCAGGGCTTCGTGATCTCAGATTGGAAGGGCATTGACAAGATCACGACCCCTGAAGGGTCGGACTACC</td><td>27:A>T</td></tr>
<tr><td>100543593|F|0-5:T>C-5:T>C</td><td>TGCAGTGAGGACGCGTGGAATACATGGAGCGTGCTCGGGTGCCGCCAGGGGCGCGTCCTCATCATGAAC</td><td>5:T>C</td></tr>
<tr><td>4984932|F|0-20:A>G-20:A>G</td><td>TGCAGATCACGCCGAACACCAGGAACATGAGCCACCG</td><td>20:A>G</td></tr>
<tr><td>100550826|F|0-7:G>C-7:G>C</td><td>TGCAGCGGCTGGCGTACCTCAACACGACGGTGTACCCGTTCACCTCCATCTTCCTCCTAGTCTACTGCC</td><td>7:G>C</td></tr>
<tr><td>4261731|F|0-16:G>C-16:G>C</td><td>TGCAGGGGCTTAAAGGGGAACCG</td><td>16:G>C</td></tr>
<tr><td>4262825|F|0-23:G>A-23:G>A</td><td>TGCAGCTTGTCGAGGTCCTTGGCGAGGTCCACGGGGGCCG</td><td>23:G>A</td></tr>
<tr><td>2251962|F|0-56:G>A-56:G>A</td><td>TGCAGGCAGCTCCAGGTCGAAGTGGAACAGCAGCGCGGCGAGCGCGAGCTCGATGTGCGCCAGCCCGAA</td><td>56:G>A</td></tr>
<tr><td>6045100|F|0-23:G>A-23:G>A</td><td>TGCAGGTGGTGAAGTATCTGATTGTGGCTTTCGATATCGTCAACTCTGTAGACGACCAAGGGAATACAG</td><td>23:G>A</td></tr>
</table>

5368641|F|0-24:T>C-24:T>C	TGCAGGTCGGCGACGGCGAACTTGTCAGGCGGCCCGTGCCCGCGATCGGCTTGGGTGAGGAGGCCCATG	24:T>C
3025103|F|0-10:C>G-10:C>G	TGCAGGGCACCCGTGACAGGATCTCCATGAGAGCGTCCTCCG	10:C>G
1052066|F|0-51:A>G-51:A>G	TGCAGGCTTTGTATCCAACTTCAGGGTGGATCCACCATTACTACCTGCTCAATGCTTTCAACCAATATC	51:A>G
1017442|F|0-23:C>T-23:C>T	TGCAGCATCTTGCGGCGAGCCGCCGCCTTCAGCACGGAGGAAACGGTGAAGGTGAGCGGCTTGATCCCC	23:C>T
5581832|F|0-17:G>A-17:G>A	TGCAGGTCGAGGACGCCGTAGGTGGTGGAGGTGAGGGAGACGACGTGGTCGACGCGGCCGCGCACGGTG	17:G>A
1267399|F|0-14:G>T-14:G>T	TGCAGGGGCGACAGGGCTGCGTAGTTGATGGCAGCATGCATGGTGTGGTGGCTCACGGCTCCCGCCGAG	14:G>T
3944993|F|0-20:G>C-20:G>C	TGCAGCGGCCACGGGAACACGAGAACATGCGCCG	20:G>C
1031533|F|0-20:C>G-20:C>G	TGCAGGTTCTCGTTGCCCACCGAGATGATGAACTCCACGCCCGTGTTGGCGAACGCCGTCAGCACCTTG	20:C>G
5411898|F|0-18:G>A-18:G>A	TGCAGCCGCAGATACTCAGCTTCCCG	18:G>A
2256219|F|0-27:A>G-27:A>G	TGCAGCAGCAGCCACCCCGCGAACCCCACCACGTTCGCCAGCATCGACGCCGACCCCAGCACCCACCGC	27:A>G
1023480|F|0-12:G>A-12:G>A	TGCAGGTCGGCCGCCGCGAGCGCGGGGAAGAAGTCCGACACGTTCGGGCTGCCG	12:G>A
12735261|F|0-9:A>G-9:A>G	TGCAGCGTCAGCTCCAGCCG	9:A>G
1098289|F|0-16:C>T-16:C>T	TGCAGGTCGACTGCGGCGGCGAGGGCAGCGGCGTCTGGTTCACGGAGGCCGCGGCCG	16:C>T
7163395|F|0-18:A>G-18:A>G	TGCAGAGCGAATTACAAGAAGGAAAGCCCACCTGTACGGGTACAGGATGATATTCTTGACTCTCCTGTT	18:A>G
1069490|F|0-54:C>T-54:C>T	TGCAGCATGTACACCAGCGCCGACTGGTCGTCCGACTCGTTGAACACCTTGTCCCTGAACGTGGACGTG	54:C>T
991905|F|0-52:A>G-52:A>G	TGCAGACCGTGGCCATGGCGCGGGAGGTGCACCAGCAGCGGTACCGCATGGGAGCCG	52:A>G
13142884|F|0-17:G>A-17:G>A	TGCAGCAGGCCGTTGAAGCCG	17:G>A
993067|F|0-28:C>T-28:C>T	TGCAGCACGCGCACAGGATGCACTCGTACAGCCCGTCCAGCTTCCGCCGCTCGCCCG	28:C>T
2265607|F|0-22:G>C-22:G>C	TGCAGCGGCTGTCGATGTCCTCGACGATGTCGGTGCCGTGGTCCTCCCTGTACCGCCG	22:G>C
6045653|F|0-10:C>T-10:C>T	TGCAGGACGACGTCGACAGCGAAATCGACCCG	10:C>T
1020458|F|0-22:T>C-22:T>C	TGCAGCTGTTCGTCTGGTTTGGTTTTCTGGTGATCTCATGGCTTCCTCTCATTTACAGAGGTGACCCGA	22:T>C
1253585|F|0-40:G>C-40:G>C	TGCAGCGTCTGGTTCGGCGGCAGGTCCCGCTCGCTGTTGGGTCCCGTCAGAAGGACGGACGCGTCGCAG	40:G>C
988449|F|0-22:C>G-22:C>G	TGCAGCTGGCCGTGGACGCCGCCGCCGCCAAGTTCCTCGCCGCCGAGAAGCTGGAGGTGGTGGACTACA	22:C>G
1056824|F|0-17:G>C-17:G>C	TGCAGCATCACCACCATGGCCGACCGCGCGTCGCCTTCCTCGTACACGCTGGTGCAGTGGTTGTGCAGC	17:G>C
3024537|F|0-38:G>A-38:G>A	TGCAGGCAGTCCATGCTGTAGCTCTTCCTCATGGAGGCGCCG	38:G>A
4439652|F|0-6:T>G-6:T>G	TGCAGCTGTAGCGATCGGCCG	6:T>G
5324118|F|0-25:C>T-25:C>T	TGCAGGCCGCGCTGTCGGTTGCGCGCCCG	25:C>T
1023680|F|0-8:T>A-8:T>A	TGCAGCGCTGACCTCGGGAAGGGAGCTGAGAAGGCCGCGGGAGGAGGCCG	8:T>A
1165550|F|0-23:C>A-23:C>A	TGCAGCATGCTGCCGAGCTTCCTCACCGACCCGTTCCCCGCGGACCCCCTGGTGGAGGACTGCGACGGC	23:C>A
1045535|F|0-6:C>T-6:C>T	TGCAGACTGAATTTAAGATCTATGCAGTAACTTTTAATCAGAACACCTTGCCCG	6:C>T
4261744|F|0-7:C>T-7:C>T	TGCAGACCGACGGCGAAACGACGACCCATCGCTCTCCG	7:C>T
3028898|F|0-17:C>T-17:C>T	TGCAGCCCCTACCGCGCCGACCTGTCCGACCGCCCCTGCGCCGCCG	17:C>T
3031666|F|0-50:C>T-50:C>T	TGCAGCGTCCGCGGCATCGGCGGCATGTCGACGTGCACGACGCCCTCCAGCGCCTGCACGGCCTGCGCC	50:C>T
1083484|F|0-64:G>T-64:G>T	TGCAGACCTCGACCTCCTCGGCGTCGGAGGACTCGTCCATGTCGGAGGCCGTGGAGGCGCTCCCGTCGT	64:G>T
4262343|F|0-7:G>C-7:G>C	TGCAGACGCTCATCCTCGACGACAACATGATCGGCGGCGAGGTGCCG	7:G>C
7923298|F|0-9:G>T-9:G>T	TGCAGCGGTGCCCTTGTCGCCGCCG	9:G>T
996241|F|0-47:G>A-47:G>A	TGCAGTTACCCAGGCACAGCTGCCTGCACTCGGCCAGAGTCATGTCGGGGCGCACCGTCGCTTCCGTCG	47:G>A
32023251|F|0-13:G>C-13:G>C	TGCAGACGCAGACGTTCCTCGCCCTGCTGTGCCG	13:G>C
3023563|F|0-22:C>G-22:C>G	TGCAGGAGGTTTCTGCGTATGTCGCCGTGCATCCCACCG	22:C>G
5367126|F|0-8:C>T-8:C>T	TGCAGTGCCTCTGCATCCTCGCCG	8:C>T
4009024|F|0-33:C>T-33:C>T	TGCAGCAGCGGCATCAAACTCGTCGGCCCTCAACGTCGTGCACCG	33:C>T
2242463|F|0-30:G>A-30:G>A	TGCAGATACCCTTGATTTTTAAAATGCTTCGACTGCACGTCGAAGGAATCGCGAAAAACCAAGTGAGTC	30:G>A
1032467|F|0-59:C>G-59:C>G	TGCAGAATGCACTTGTAGGTGTAGCCATTAAGCCTCAACCCCCACTTCATGGCCAGCTCCAGGCACTTG	59:C>G
1008796|F|0-12:T>C-12:T>C	TGCAGCACCGTTTTCGTCACCTGCTCCTGGCCCATCGGCTGCGCCGTGGAGTTGAGCCCAGTCAATGGG	12:T>C
3954530|F|0-13:C>A-13:C>A	TGCAGGCGCTCGCCATGGACCGCGCCGTCGGCCGCTTCACCGTGCCG	13:C>A
1014663|F|0-49:G>C-49:G>C	TGCAGGCAGGCTACAGGCTGGTCAGGGCGACCCAGTTCAGGAGGTTCACGTACCG	49:G>C
4262636|F|0-19:A>G-19:A>G	TGCAGGACTGGAGATCAGCACAGGCCGTCTTTTGCTCCACAACGCCG	19:A>G
997853|F|0-47:G>A-47:G>A	TGCAGAGCGCAGGAGTGCGGTATGGAAGGCTACGAGATGGACCTGGAGCGGACACTGCGGATGCCGTCC	47:G>A
1107737|F|0-15:G>A-15:G>A	TGCAGACAGGGCAAAGACCATCCTTGCTCTGGCCTCCCCTGACTCCAAGCCCAGTGATCTCAGAAGGCC	15:G>A
1036908|F|0-11:G>A-11:G>A	TGCAGCTTGTCGGCCAAGCTCTTGGCCCCCTTGAGCGAGAGCACGAGCCAGACGAAGGAGAGGAACTCC	11:G>A
1214019|F|0-59:C>G-59:C>G	TGCAGGTTCTTCTCGGCAAGGAAGCACGGCTTGGTGGATGCTGTAGTGCGCAACAGCTCCGCTGATAAA	59:C>G
1148893|F|0-55:G>C-55:G>C	TGCAGAACGTCCTCCCCACGCAGCTCCTCTCCCTCCTGCACTCGCTCTGGCAGTCGCTCCAGGACTCGC	55:G>C
1092449|F|0-33:C>T-33:C>T	TGCAGGCTGGGCAACCTCATCCTTGGATGGCGCCTCAGAAGAATCGGCCTCGCCATCCGTAGTGACACC	33:C>T
5370975|F|0-10:G>C-10:G>C	TGCAGTGGGCGATGGCGGAGCTGGTGAAGAACCCG	10:G>C
1071379|F|0-68:C>T-68:C>T	TGCAGGAGCTGCTCTGCGCCATGGTCCTCGCCGAGTGCGTCTACAAGGTTCCCTAACCTCCCACCTTCC	68:C>T
2275535|F|0-46:T>C-46:T>C	TGCAGGAGCAGGCCGTCGACATGGTGCTCACTGACTACTGCATGCCTGAGATGACCG	46:T>C
5331503|F|0-6:C>T-6:C>T	TGCAGACCTTGATCAGGCCCG	6:C>T
7907164|F|0-13:C>T-13:C>T	TGCAGGAGCTCGACCTCTCCG	13:C>T
997000|F|0-10:C>A-10:C>A	TGCAGAACACCACCCAGTTCGGCCTCATGGCCGTCAGCCTCGGCTTCTACGACACCCTCATGTCCTGCT	10:C>A

5368108|F|0-22:G>C-22:G>C	TGCAGATCTCGATGAGCTCGTCGAGGCCCTCGGACTCGGCGGCGACCTTGGACGCCGCCGACGACGACA	22:G>C
32027215|F|0-5:C>G-5:C>G	TGCAGCCGCTGCACGTCGGCGAGCATGCGGGTCTCGTCGACCAGCGGCGGGCCG	5:C>G
1058248|F|0-13:T>C-13:T>C	TGCAGATTAGTAATAGTTCGGTTAGCTTTTTCCTTCCACACCAACTCGTCACGTTCTCCCCAATAATTC	13:T>C
3937911|F|0-19:T>C-19:T>C	TGCAGGGAGCGGTTGGAGATGGTGGCGCCCG	19:T>C
1029559|F|0-29:C>T-29:C>T	TGCAGATCATGGCCCTGCACTCCTCCAGCCCCATCTTCTCGGACCCCAGCAGCTTGCCCAGCATCCGCC	29:C>T
7339075|F|0-29:T>G-29:T>G	TGCAGTCGATCCAAGAAATCATTGGCCGCTCGAGTGGCGGCAGATCCATCACGGACCTCTGGGTCCGAG	29:T>G
1695019|F|0-17:C>T-17:C>T	TGCAGGACCTCCAGCAGCTGCACCTCGGGACCGCCGCTCACAACTATTTCCAGGCATCGTCGAGCTCAA	17:C>T
987534|F|0-30:C>T-30:C>T	TGCAGCTGCACCACCTCCTCGCCGTCTCCACCTCGCCGCCGCGGCTGCCTCTGCTCCCGCGTGGGCCGC	30:C>T
5331503|F|0-13:C>G-13:C>G	TGCAGACCTTGATCAGGCCCG	13:C>G
1103078|F|0-59:G>A-59:G>A	TGCAGAGCACCGAGGAGAAGTGCAGCACTTGGGAGAAGCGCTCGCGGTTCTTCATGGACGAGTATATCG	59:G>A
5367503|F|0-25:C>T-25:C>T	TGCAGTCGAACGGGCGGGCAGCCGCCGCGGCGCACTGTGCCG	25:C>T
2242889|F|0-13:G>A-13:G>A	TGCAGGTGCTTGCGGAGATCGACGATAGGTTCTTGAAGGCGTCAGAGAGCGCCG	13:G>A
2257132|F|0-44:C>G-44:C>G	TGCAGCCGCAGCCTCCTCGACAACTTCCGCTTCTGCTCCCTCGGCTGCAAGGTCGCCG	44:C>G
7923420|F|0-15:C>T-15:C>T	TGCAGGCCTTGTACACGGCCACGTGTGCGCCG	15:C>T
2253187|F|0-32:A>G-32:A>G	TGCAGGTAGCGCGCGGCGCAGCGGAGCATGGCAACGTTGGCAACCGTGATGTCGACGTGGACGCCGTAG	32:A>G
1008777|F|0-32:G>A-32:G>A	TGCAGGTACATCTGGAGCGTGAAGCTGAAGACGCACGCGAACATGACGGGGATGTACCAGAGCGAGATG	32:G>A
1001628|F|0-24:G>T-24:G>T	TGCAGCAGCGCAGCCTCTTGCGGCGCCGTGGCGTCGTTGAGGAGGAGCTGCTGGTTGCGGGCCTTGAGG	24:G>T
1092956|F|0-17:C>G-17:C>G	TGCAGGAGGGGGTTCCCCACGGCGCAGGCGCTCGGGGGGCACATGAACGTGCACCG	17:C>G
1126318|F|0-12:C>T-12:C>T	TGCAGTGGCTCCCCCTGAACAGACGGAAGAGGGGGAGAAGGAGGCCCCG	12:C>T
4261716|F|0-11:G>A-11:G>A	TGCAGGAGGTTGCCGAGCACGGGAAGCTGTTTCGGCGGCCCG	11:G>A
1021733|F|0-47:G>A-47:G>A	TGCAGCTGGTGGACTACGGCGTCAACAAGGTCTGGGAGATGAACAACGTCGGGGTAATATTCTCTCTCC	47:G>A
3027714|F|0-33:C>G-33:C>G	TGCAGTCGCAGAGCATCGTCCTGCCCGTGACGGCCAGCTCCG	33:C>G
999233|F|0-31:C>A-31:C>A	TGCAGGGCTCGGCGGCGCTCGGGGTCGGGATCCTGAACGAGGCCGCCCG	31:C>A
1018968|F|0-55:G>A-55:G>A	TGCAGCTGGAGGCCGTCCGAGCGGGTGCCATGAGTCAGCTGCTCATGATGGTGCAGGGCGGCTGCTCGG	55:G>A
5363755|F|0-13:G>C-13:G>C	TGCAGGCGGCGGTGGGGGTGTCGGCGCCG	13:G>C
6047702|F|0-25:A>G-25:A>G	TGCAGGTTCTGAATCGACTTGGGCAATGGCCCG	25:A>G
3942164|F|0-11:C>T-11:C>T	TGCAGGCTGCACGCTGCCACTGTTCCTTGCATCAGCCTCGAACTCGGCGCCACCTCTGGTGCTCGATCA	11:C>T
3957169|F|0-43:C>A-43:C>A	TGCAGCGCAGGTCGTGCAGCACGGCGAACACCTCCGAGAGGAGCCCCG	43:C>A
5363055|F|0-10:T>C-10:T>C	TGCAGAAGGCTTTCCGCGTCCG	10:T>C
3935509|F|0-17:C>T-17:C>T	TGCAGCGCGATCCTCTCCTCGTCCTCCATTGCCTCCG	17:C>T
3024290|F|0-34:C>T-34:C>T	TGCAGGTTCTGATCCTCACCGTGAATAGGCTCAACGGCACAATCCCG	34:C>T
4262667|F|0-28:C>T-28:C>T	TGCAGACGTTCAACGGCACGTACCTGAACACGACGTTCCCG	28:C>T
1094957|F|0-45:C>T-45:C>T	TGCAGGCGGTGAGGCTGCGGTGGTTCTGCGAGCAGAGCGGCGTGGCGCTCTTCACGGCGAGGAACCGAG	45:C>T
7160619|F|0-15:A>G-15:A>G	TGCAGATGGGGCTGCACGTCGGGGGCCG	15:A>G
1113744|F|0-68:G>C-68:G>C	TGCAGCCCCCCCTGCGCGACGCGGAAGTTGGCGTCCCGCGTCAGATCCACGCAGGTGTCCACCAGAGAG	68:G>C
5369579|F|0-13:C>G-13:C>G	TGCAGGCGTCCGTCAATGCCGACGGGACCTTCGGCGAGGTCTTCGCCG	13:C>G
1087731|F|0-46:C>A-46:C>A	TGCAGCAGACGTTCGTCGTCTCCGACGCCACCCGCCCCGACATCCCCATCATCTACGCCAGCGCCGAGA	46:C>A
984092|F|0-18:T>A-18:T>A	TGCAGCATGTCGCAGTCGTAGCGCCTCATGACATACTGGTTCTCGTCCCCG	18:T>A
1093363|F|0-68:C>G-68:C>G	TGCAGCGCGACGGGGCTGAGCCCGAGCCCGCGGGTGAGGTGGAAGTTGAGCGCCAGCCACGGGAAGCAC	68:C>G
1863287|F|0-20:G>A-20:G>A	TGCAGGCGGCGGAGCTTGAAGGAGGAGAGCTTCTGCGGGCCGTCCTTGACGGAGGGCTCCTCCTCGAAG	20:G>A
2248694|F|0-26:G>A-26:G>A	TGCAGCCTCATCGAGATCGACGGCGTGGTGCACGAGTTCCAAGCAGTTCCG	26:G>A
1008796|F|0-43:C>A-43:C>A	TGCAGCACCGTTTTCGTCACCTGCTCCTGGCCCATCGGCTGCGCCGTGGAGTTGAGCCCAGTCAATGGG	43:C>A
1242896|F|0-20:G>A-20:G>A	TGCAGCACCGAGAGCTGCACGATGACGTCGCGCGCCCACTTGCCGTACATGGCATCCAGCAGCGACACG	20:G>A
1020022|F|0-56:C>G-56:C>G	TGCAGCACCTCCCGTCTCGTATCGGCCACCTCCGCCTCCAGCTTCTTGATGGAGCTCACGACCGCGAGC	56:C>G
4262834|F|0-22:G>A-22:G>A	TGCAGCTCGTCGATCTGCGCCAGCTGGGCCG	22:G>A
5411723|F|0-18:A>T-18:A>T	TGCAGCGAGAGCGGCACCACGCCG	18:A>T
1165550|F|0-32:G>C-32:G>C	TGCAGCATGCTGCCGAGCTTCCTCACCGACCCGTTCCCCGCGGACCCCCTGGTGGAGGACTGCGACGGC	32:G>C
7916190|F|0-5:C>G-5:C>G	TGCAGCCTTCTCAGCCCCGCGAGGCCG	5:C>G
4261716|F|0-38:C>G-38:C>G	TGCAGGAGGTTGCCGAGCACGGGAAGCTGTTTCGGGGGCCCG	38:C>G
6047498|F|0-5:C>T-5:C>T	TGCAGCGCTCGGAGGCGCGACCACGGCGGCGGTATTGCGCCG	5:C>T
2361851|F|0-33:A>G-33:A>G	TGCAGGACCTCCTCGGCTTCGACAAGGCCAGCAAGACGGTGGAGTGGCTCCTCACGCAGTCCAAGCCGA	33:A>G
992826|F|0-59:G>A-59:G>A	TGCAGGATGAAGAGCGAGGCCCAGGCGAGGGTGGCGATGATGATGAAGAGGGAGCCCAGGAACCACTCC	59:G>A
3023973|F|0-26:A>T-26:A>T	TGCAGAAGCTGTATCTGTTCAACAACATGCTCCACGGCCCG	26:A>T
4262945|F|0-28:C>T-28:C>T	TGCAGGACGTGTTCACGCGCTGGTCCTTCGACACGACCTGCAACCTCGTCTTCGGCGTCGACCCCGAGA	28:C>T
1021673|F|0-29:A>G-29:A>G	TGCAGACCACGACGCAGTTAACCGCCCCAACAAAGGAGGTTCTCTTCTCCGCCG	29:A>G
3064906|F|0-23:C>G-23:C>G	TGCAGCCGCGTCCAGCGGCCGTACTCGGGCGGAATCTCGCCG	23:C>G
1100956|F|0-16:T>C-16:T>C	TGCAGCGACGCCGAGTTGACCCTCAGGTCGCCTTGGTCAAACAAGAAGCCGCCGAGCCG	16:T>C
2243310|F|0-24:G>C-24:G>C	TGCAGCAGCCCGTTGTTCCCCGTGGTGTTCAGCCCCAGCCTCGATATGATCGACTCGTTGTGCACCTTG	24:G>C
3029232|F|0-18:C>T-18:C>T	TGCAGAAGGTCGGGAATGCCCTCAAGCGGTCCGCGGGCTCCG	18:C>T

5339963|F|0-17:G>C-17:G>C	TGCAGCACGGCGCGGTAGGCCTTGGTGGGCCGTGCCACCTCGAACTGGGACCTGAAGTCGACGTCCACC	17:G>C
1233858|F|0-22:G>A-22:G>A	TGCAGCACCTCGCCAAGCTGAAGAAGCTCTGGAAGCTCAGCGTCAACGTCACCACCGACGCCAACGTGG	22:G>A
1047152|F|0-8:G>A-8:G>A	TGCAGAAGGCGCCCAGCCAATCCCTGAAGCAGCTCATCACTCTCACCGCCAAGGAGGATTCTGCGGTCG	8:G>A
18732705|F|0-7:G>T-7:G>T	TGCAGTCGCCGCTGCACGTGGCCGCCTACTACGGCTCGACGGATGCCATGGTGGAGCTGCTCAAGCAGT	7:G>T
988829|F|0-43:G>A-43:G>A	TGCAGGCGCCCAGCAGCGCGCCCCAGACCACCGAGTGCTCCTGGCAGTCCG	43:G>A
6038109|F|0-17:G>C-17:G>C	TGCAGGGATCATCGGAAGCATGGTCACCG	17:G>C
1125919|F|0-9:C>T-9:C>T	TGCAGATCGCGCACACGGAGAACTCGCGGGATAAGTGGGCCAATGGCGAGCCGCCG	9:C>T
4733924|F|0-20:G>C-20:G>C	TGCAGCGCGGACTGGCACGTGGGGCCCG	20:G>C
5579546|F|0-17:C>G-17:C>G	TGCAGCACCTTGCGGCCCGTCATGCCCAGCAGGAACGGCTCCGTGGAGGCGGCCAGGTAGCGGCCGTAC	17:C>G
7343602|F|0-11:G>A-11:G>A	TGCAGCTCCGAGATACCCTTCTCCCTCCCG	11:G>A
2256549|F|0-59:T>C-59:T>C	TGCAGGCTGCGCGCTGCCACTGTTCCTTGCATCAGCCTCGAACTCGGCGCCACCTCTCGTGCTCGATCA	59:T>C
5581381|F|0-32:G>A-32:G>A	TGCAGCGAGAGGTGTTCACGAAGAGAGGCGGCGGCCG	32:G>A
1150140|F|0-46:C>T-46:C>T	TGCAGTGGATCATCTGGTGAACACGGTGAGCCTGGGCCACGACTTCCTCTTCGAGGAGGCCG	46:C>T
1051134|F|0-32:G>A-32:G>A	TGCAGTCTCCTCTTACGCTTCTTCTCCTCCTTGGCCCGCCTCACGATCGCGCACAGCGCCTCGTCGCCG	32:G>A
3935011|F|0-11:C>T-11:C>T	TGCAGCGCCTCCCCGATGTCGTGCGCCTGGCTGAGCGGCATGTCGCCG	11:C>T
2279291|F|0-60:A>G-60:A>G	TGCAGGCTGGTGACGCGCGGCGGCTCGGCGAACTCGTAGTCGTACCTGAAGTAGTCGAGCACCATGTCC	60:A>G
5367441|F|0-13:G>C-13:G>C	TGCAGGTGAGGCGGGAGCAGCCCG	13:G>C
32020241|F|0-8:C>G-8:C>G	TGCAGCTCCACGGTGGTGAGCGCCG	8:C>G
7167716|F|0-17:C>G-17:C>G	TGCAGCCCGTCGCGGCCCTCCGCGAGCTCCGAGCGCCG	17:C>G
5355279|F|0-23:A>G-23:A>G	TGCAGGGGGCTCCGCGCCGCGGCAGAGGAGCACGACCAGCAGCAAGAGGGGGGCGACGACGCGGCGGGA	23:A>G
32025092|F|0-14:A>G-14:A>G	TGCAGCTGGAGCGCAAACTCGAGGTTCCAGAGGACGTCGCCCATGGACGGCCTGTCGACGCTGTGATCG	14:A>G
32026451|F|0-10:C>G-10:C>G	TGCAGTCCTCCAGGCGGTCGAGGAGGTTATCGGCAACCGCGTCTACTAGTGAATCAGTTTCCCGCATCG	10:C>G
32017776|F|0-16:T>G-16:T>G	TGCAGGCTGTGGCTGTTGCTCCCGATGAGAGGAAGCCCG	16:T>G
6044974|F|0-38:A>G-38:A>G	TGCAGCTGCTGCGCGCAGGCCTCGCAGTGCATGTTGACAAGCAGCTCCACGGTGCGTGCCTCGGTGACC	38:A>G
1865366|F|0-20:G>A-20:G>A	TGCAGCCCTGCGACGGCGTCGAACGCGCACGGGCCAGCCGCCAGCCCGTGCCCG	20:G>A
3941326|F|0-11:C>T-11:C>T	TGCAGCCGCACCGTGCCGCTGGAGGGCAACTCGCCG	11:C>T
1397912|F|0-12:C>G-12:C>G	TGCAGCACGCACCCATCCATGACGCAACACACATCAGCCAGAAACCATGCAAAACCAACACCATTTCGA	12:C>G
5324478|F|0-19:C>G-19:C>G	TGCAGGCGGGGGGCGACGCCGCGGCCAAGCCG	19:C>G
1034899|F|0-34:G>A-34:G>A	TGCAGACGCACTACAAGCGCAAGCACGGCCTCAAGCCCTTCCTCTGCCGCCG	34:G>A
4004843|F|0-8:G>A-8:G>A	TGCAGTAGGCACCATGACATAGATCTCCAGCGCATTGAGCAATGGCGGCAGCGTCGAGTTGGCGGTCGG	8:G>A
1022580|F|0-35:T>C-35:T>C	TGCAGGGCCTCCGCGACGGCCACGTAGTTGACGGCTCCTCCGATGTGCCG	35:T>C
12778601|F|0-41:C>T-41:C>T	TGCAGGTGGCCTTCCCGCTGGATCCTCAGGAGCACGTCGAGCAAGTCCTCCTTGTCGTCATCGGCTGCT	41:C>T
1032467|F|0-44:C>T-44:C>T	TGCAGAATGCACTTGTAGGTGTAGCCATTAAGCCTCAACCCCCACTTCATGGCCAGCTCCAGGCACTTG	44:C>T
1865366|F|0-9:G>C-9:G>C	TGCAGCCCTGCGACGGCGTCGAACGCGCACGGGCCAGCCGCCAGCCCGTGCCCG	9:G>C
7349724|F|0-8:G>C-8:G>C	TGCAGCGCGTACGCCATCCG	8:G>C
3023764|F|0-31:C>G-31:C>G	TGCAGTGCCCGATCACACTGGAACTCATGACCGACCCG	31:C>G
1017748|F|0-7:C>T-7:C>T	TGCAGCTCCTGGACCTCGGCAACAACTCCCTCAGCGGCGGCATCCCGAGCCGCCTCTGCAACTGCTCGG	7:C>T
1214816|F|0-35:G>A-35:G>A	TGCAGCGGTTCTCTTTTGAGCTGTCGCCGACGTACGTGCATGCGCCCTATGCCGTCCTTACACTGCACC	35:G>A
3024189|F|0-12:C>A-12:C>A	TGCAGCGCCACCCACTTCCAGGAGGACCTCTTCACTTTGCTCTGCCG	12:C>A
7349677|F|0-11:G>T-11:G>T	TGCAGCAGGAAGTGACGCCCG	11:G>T
4910380|F|0-16:G>C-16:G>C	TGCAGCTCGCCGTCTCGATTTGGTTCGAAGGGAGCTCGCGCGGCCTAGTCGGCAACGGCGGGAGCCCCG	16:G>C
1141294|F|0-46:G>A-46:G>A	TGCAGCTGTTCGACAACCAGATCCATGGTGCCATCCCTCCTTTGCTGGGGGCGAGGAGCACACTGTCGG	46:G>A
983722|F|0-39:T>G-39:T>G	TGCAGCTGCAACAACCCCTGACTCTTGTCCTTTCCATGCTCACACTGGGCCATCGCGGCCGCTTATGTG	39:T>G
32026876|F|0-54:A>G-54:A>G	TGCAGAGGCTGATCGTGGAGGCGAGCCAGGGCGGCGCCGACGAGGTGCGCGTCGACGGCCTGCCCGAGA	54:A>G
3029342|F|0-11:G>A-11:G>A	TGCAGGTAGTCGCCGATGTTCACCACCAGTGCCCCCG	11:G>A
1021673|F|0-17:T>C-17:T>C	TGCAGACCACGACGCAGTTAACCGCCCCAACAAAGGAGGTTCTCTTCTCCGCCG	17:T>C
6030762|F|0-6:C>G-6:C>G	TGCAGGCCTTCGACTGGGAGCTGCCG	6:C>G
7154646|F|0-12:G>C-12:G>C	TGCAGCGCACACGATCGATCGACCACCG	12:G>C
7154646|F|0-21:A>G-21:A>G	TGCAGCGCACACGATCGATCGACCACCG	21:A>G
1023501|F|0-25:G>T-25:G>T	TGCAGGAGGTGGACAACATGACGCTGCGGAGGTTCCTGCGCGCGCGCGACCACGACGTGGGCAAGGCCT	25:G>T
12470885|F|0-13:G>T-13:G>T	TGCAGCGGAGGCTGGAGCGCCG	13:G>T
1074522|F|0-36:T>G-36:T>G	TGCAGATCCAAAAGAAAGCCATGCTGAAAACCCCCATCTTTCCTCCCCCG	36:T>G
979973|F|0-52:C>G-52:C>G	TGCAGTCGGAGTAGGCGACGGCGTGGCGCTTGAACACGCCGAACACCGCCACCCGCCCG	52:C>G
4540866|F|0-20:C>A-20:C>A	TGCAGCAATGTCTTCGGCCACCCCCCCTCCGCCG	20:C>A
7916190|F|0-6:C>T-6:C>T	TGCAGCCTTCTCAGCCCCGCGAGGCCG	6:C>T
3941804|F|0-14:A>G-14:A>G	TGCAGTGGCGGCGCATAAGAGGCAACCTGTTGAGCCG	14:A>G
3939543|F|0-10:C>G-10:C>G	TGCAGGTGGCCGCGGAGCTGCTGAACGACCG	10:C>G
1165550|F|0-14:G>C-14:G>C	TGCAGCATGCTGCCGAGCTTCCTCACCGACCCGTTCCCCGCGGACCCCCTGGTGGAGGACTGCGACGGC	14:G>C
5323637|F|0-22:G>T-22:G>T	TGCAGGCGCCGTGGGCCTCCACGGGCAGCCG	22:G>T

<table>
<tr><td>988735|F|0-11:G>A-11:G>A</td><td>TGCAGTATCGCGCCGTGGCCGCCCCCACCCATCTCGTGTTTGACGTTGCCGTCCGCGTCCAGGAAGAGG</td><td>11:G>A</td></tr>
<tr><td>4262825|F|0-35:G>T-35:G>T</td><td>TGCAGCTTGTCGAGGTCCTTGGCGAGGTCCACGGGGGCCG</td><td>35:G>T</td></tr>
<tr><td>992922|F|0-53:G>A-53:G>A</td><td>TGCAGCTCCCACTGGCATCTAAATGCGGCTACATAAGGAAGCTGGTGTCGGGGGCCAACGGGTCCAGGG</td><td>53:G>A</td></tr>
<tr><td>1863122|F|0-59:G>A-59:G>A</td><td>TGCAGTTCCACGGGTTCCACGGCGTGAAGCAGGAGGAGAAGAAGCTGGGCCAGAAGTTCGTGGTCGACG</td><td>59:G>A</td></tr>
<tr><td>2257132|F|0-17:C>G-17:C>G</td><td>TGCAGCCGCAGCCTCCTCGACAACTTCCGCTTCTGCTCCCTCGGCTGCAAGGTCGCCG</td><td>17:C>G</td></tr>
<tr><td>7343602|F|0-7:C>T-7:C>T</td><td>TGCAGCTCCGAGATACCCTTCTCCCTCCCG</td><td>7:C>T</td></tr>
<tr><td>5346922|F|0-15:T>C-15:T>C</td><td>TGCAGTGCATCAACGTCAGCTCCG</td><td>15:T>C</td></tr>
<tr><td>1080966|F|0-27:T>C-27:T>C</td><td>TGCAGATGATGCAGTCCTTCCTGCGGGTGCAGTCCTCCGCCACGCGCTGGGGCGCCG</td><td>27:T>C</td></tr>
<tr><td>4005735|F|0-7:T>C-7:T>C</td><td>TGCAGCGTGCGCCTGCGGCAGCTCGTGATCTCAGTGTGCGGCCCCG</td><td>7:T>C</td></tr>
<tr><td>3027872|F|0-17:C>G-17:C>G</td><td>TGCAGCTCCACGCCGTCCACGCCCAGCAGCTTCACGGCGCGCATCCCG</td><td>17:C>G</td></tr>
<tr><td>1087015|F|0-29:G>A-29:G>A</td><td>TGCAGGGCGAGAAGGAACTGGAGGCTCTTGCCGTTGCAGAGGTGCCTCGGGAACGGCCCGACGAATCCG</td><td>29:G>A</td></tr>
<tr><td>990983|F|0-10:G>C-10:G>C</td><td>TGCAGGGCGGGCACAGGGTGGGCAACGTGAAGGTGATGAAGAACGTGGCCAACACCAGCGTGCTCTGGT</td><td>10:G>C</td></tr>
<tr><td>1229816|F|0-58:C>T-58:C>T</td><td>TGCAGACGATGCGGCACCACCAGCTGGAGGCGGCGGAGGCGCTGCGGTCCTTCTCCGACGCCAACGCGG</td><td>58:C>T</td></tr>
<tr><td>981918|F|0-19:G>A-19:G>A</td><td>TGCAGGCCGCGGAGGCCGAGCTGTCCATGGCGAAGAACGAGCTGGAGAGCATCAAGGCGGGGGCGTTCC</td><td>19:G>A</td></tr>
<tr><td>6046432|F|0-10:A>G-10:A>G</td><td>TGCAGTACCCACAAGGGCACTGCCTGTCTGCGGGGGAGCTCGCTGCCATCCTGGGCGCGCAGTACATGC</td><td>10:A>G</td></tr>
<tr><td>3029232|F|0-22:C>G-22:C>G</td><td>TGCAGAAGGTCGGGAATGCCCTCAAGCGGTCCGCGGGCTCCG</td><td>22:C>G</td></tr>
<tr><td>1029018|F|0-10:A>G-10:A>G</td><td>TGCAGGCAGCACTGCACCACCATCACTGGCTGTGCCCCTCGCACAGGCCATGGCGGTGGAGTGCAGGAG</td><td>10:A>G</td></tr>
<tr><td>5357435|F|0-17:C>A-17:C>A</td><td>TGCAGATGCTCGTCGTTCGATATGGCCG</td><td>17:C>A</td></tr>
<tr><td>3022240|F|0-5:T>C-5:T>C</td><td>TGCAGTGGTTTCCCTGTTTGGCTCAAGTATGTCCCCG</td><td>5:T>C</td></tr>
<tr><td>5971515|F|0-45:G>C-45:G>C</td><td>TGCAGCGTCTGTCCATGTCGCCCGAGATTGCCCGCGCAATCGTCGGCCACAGCGGCGTCCGCCCACTCA</td><td>45:G>C</td></tr>
<tr><td>13880634|F|0-12:C>T-12:C>T</td><td>TGCAGCTCCTCACCCGCGCCGTCGGCCG</td><td>12:C>T</td></tr>
<tr><td>1066703|F|0-55:C>G-55:C>G</td><td>TGCAGTAGCTGTCCACCACCCGCTTGTAGGTCAGCTCCATGGGCCTCAGCTTGCGCTGGATCATGTACC</td><td>55:C>G</td></tr>
<tr><td>3025772|F|0-32:C>T-32:C>T</td><td>TGCAGCTGCACACCACCAGGAGCCCCGACTTCCTGGGCATTGGCCCG</td><td>32:C>T</td></tr>
<tr><td>32024839|F|0-17:A>C-17:A>C</td><td>TGCAGCTGTCCAACAACAAGCTCACGGCAACCGACGCCG</td><td>17:A>C</td></tr>
<tr><td>1022580|F|0-11:C>T-11:C>T</td><td>TGCAGGGCCTCCGCGACGGCCACGTAGTTGACGGCTCCTCCGATGTGCCG</td><td>11:C>T</td></tr>
<tr><td>32022971|F|0-21:T>G-21:T>G</td><td>TGCAGGCTGCCTGTCTTTTGTTGAGAGAGATGTGGGAGATGGCGTGTCATGTGTGGTCTGGTGCTCCCT</td><td>21:T>G</td></tr>
<tr><td>1246961|F|0-13:A>G-13:A>G</td><td>TGCAGCTTCACCAACCCCTTGAGCGCGCGCAGAGCCTTCTTGGCCTGCGATAATTCAACACGCATTGAT</td><td>13:A>G</td></tr>
<tr><td>32023694|F|0-68:T>C-68:T>C</td><td>TGCAGCGGCATGGTCGACCTGCTCGCCAAGAGCGGCCGCCTCGGGGACGCGCTCAGGGCGTTCGCGGAT</td><td>68:T>C</td></tr>
<tr><td>1018305|F|0-30:T>C-30:T>C</td><td>TGCAGGTTGGTTGCTTAGCGCCTACCTCCTTGGCGGATTTGGTGATGATGCCAAAGGAAGCAATGGTGT</td><td>30:T>C</td></tr>
<tr><td>1008629|F|0-16:C>T-16:C>T</td><td>TGCAGCCGTACTTCGGCGGCGAGGAGCGCACGGAGGCGGAGCTGAGGCTGGAGGGCGTGGCGCCGAGAT</td><td>16:C>T</td></tr>
<tr><td>1864138|F|0-33:G>C-33:G>C</td><td>TGCAGATGAGATCGCCAAGCTGTTCCCCTGCCTGTACGGCCAGCCGTCGGCCGCCGTGGTGCCGTCCGC</td><td>33:G>C</td></tr>
<tr><td>1077792|F|0-50:A>G-50:A>G</td><td>TGCAGCTTGAACAGGGAGAAGGACAGGCAGAGATCCTTGGTGTGCGGATAAGCCGCGAGGAGCGAGTCT</td><td>50:A>G</td></tr>
<tr><td>1253585|F|0-59:C>G-59:C>G</td><td>TGCAGCGTCTGGTTCGGCGGCAGGTCCCGCTCGCTGTTGGGTCCCGTCAGAAGGACGGACGCGTCGCAG</td><td>59:C>G</td></tr>
<tr><td>12419879|F|0-64:T>C-64:T>C</td><td>TGCAGGGGGCGCTCGCGGAACGGCGGGCGCGTGGTGAGCTCGGGCCTGTCGATGGAGCGCCTGCTGGAG</td><td>64:T>C</td></tr>
<tr><td>3024290|F|0-39:C>G-39:C>G</td><td>TGCAGGTTCTGATCCTCACCGTGAATAGGCTCAACGGCACAATCCCG</td><td>39:C>G</td></tr>
<tr><td>987534|F|0-16:C>A-16:C>A</td><td>TGCAGCTGCACCACCTCCTCGCCGTCTCCACCTCGCCGCCGCGGCTGCCTCTGCTCCCGCGTGGGCCGC</td><td>16:C>A</td></tr>
<tr><td>1027829|F|0-11:C>G-11:C>G</td><td>TGCAGCGACGGCGACAGGACCACCATCAGCATGGCGGCGGAGAAGAAGCCG</td><td>11:C>G</td></tr>
<tr><td>1097490|F|0-68:C>A-68:C>A</td><td>TGCAGCAGGCAGTGGTTGGAGAAGCCCCGCTTCAGCGTGCCGTACGTGAACACCAGCGTCCTCTTCGCC</td><td>68:C>A</td></tr>
<tr><td>5581381|F|0-31:C>T-31:C>T</td><td>TGCAGCGAGAGGTGTTCACGAAGAGAGGCGGCGGCCG</td><td>31:C>T</td></tr>
<tr><td>1029559|F|0-40:G>C-40:G>C</td><td>TGCAGATCATGGCCCTGCACTCCTCCAGCCCCATCTTCTCGGACCCCAGCAGCTTGCCCAGCATCCGCC</td><td>40:G>C</td></tr>
<tr><td>6047648|F|0-21:G>C-21:G>C</td><td>TGCAGGCGGCGGCCACGTCGGGGGCGAGCGCGGCCG</td><td>21:G>C</td></tr>
<tr><td>1060558|F|0-53:C>T-53:C>T</td><td>TGCAGGAGCAGTCGTCGGAGTTGACAGAGCTGATGGACGGGATGCTCAACAAGCTGTCCAGCGCTCTGT</td><td>53:C>T</td></tr>
<tr><td>3023592|F|0-17:T>G-17:T>G</td><td>TGCAGGTGCCCCCGCACTATGAACAGCATCCTCTGCACCG</td><td>17:T>G</td></tr>
<tr><td>1064678|F|0-47:G>A-47:G>A</td><td>TGCAGCAGCTTGGACCGCTTGTGCACGACCATCATGGTGAACCACGGGATGCTGCCG</td><td>47:G>A</td></tr>
<tr><td>2247862|F|0-44:A>G-44:A>G</td><td>TGCAGTGCGCGCCCTGCCTCGACTGCTTCGAGCAGAGCGGGCCCATCTTCGACCCG</td><td>44:A>G</td></tr>
<tr><td>3943330|F|0-27:T>A-27:T>A</td><td>TGCAGCCCGAGCGCTGCCTCCTCCCCCTCGCCG</td><td>27:T>A</td></tr>
<tr><td>7349819|F|0-10:C>A-10:C>A</td><td>TGCAGGTGCTCAAGGAAGGCCG</td><td>10:C>A</td></tr>
<tr><td>4261736|F|0-29:G>C-29:G>C</td><td>TGCAGGCTGATGATGGTGGACGAGGCGGCGAGGACCG</td><td>29:G>C</td></tr>
<tr><td>3947054|F|0-23:G>C-23:G>C</td><td>TGCAGCTTGAACTCGCCCTTCTCGCCGTTCCATCCG</td><td>23:G>C</td></tr>
<tr><td>5577906|F|0-10:G>C-10:G>C</td><td>TGCAGATCCTGGACCTCGACTCCG</td><td>10:G>C</td></tr>
<tr><td>3949826|F|0-15:C>A-15:C>A</td><td>TGCAGATACGACGTGCTTCAAGTTTCCCCG</td><td>15:C>A</td></tr>
<tr><td>1043522|F|0-41:G>A-41:G>A</td><td>TGCAGCATCCTGTACAGGAGCAGAGCGGACATGCGGGCCTGGTCGCGCACCGCCTCCAGGTCGCGGTGC</td><td>41:G>A</td></tr>
<tr><td>7354190|F|0-23:G>C-23:G>C</td><td>TGCAGCGAGTTGGTGTGGAGCGCGAGCGACCG</td><td>23:G>C</td></tr>
<tr><td>3934979|F|0-17:T>G-17:T>G</td><td>TGCAGGTCCACGTTGTCTATCCCGACGCCG</td><td>17:T>G</td></tr>
<tr><td>999492|F|0-17:C>T-17:C>T</td><td>TGCAGCGGCGTGACGTGCCCGAGGCGCGACGGGTGGAGGGTCTCGGAGGTGATGTTGCACCG</td><td>17:C>T</td></tr>
<tr><td>4410247|F|0-20:G>A-20:G>A</td><td>TGCAGCATAAACTCCTCCTCGTCGCCGCCTCCG</td><td>20:G>A</td></tr>
<tr><td>7163806|F|0-19:C>G-19:C>G</td><td>TGCAGCGGATGGGCGTGGGCAGGGTCCTGGTGGTTCTGGCGATGGCGACGGCGGCGCTGGTGGAGAGCC</td><td>19:C>G</td></tr>
<tr><td>2253851|F|0-32:A>G-32:A>G</td><td>TGCAGCTCCTCGAGGTACACGCTGCTGGTGCCATCGAGGAAGCTGTCGGTGAGGCGCGAGAGCGGCACG</td><td>32:A>G</td></tr>
<tr><td>1034899|F|0-25:C>T-25:C>T</td><td>TGCAGACGCACTACAAGCGCAAGCACGGCCTCAAGCCCTTCCTCTGCCGCCG</td><td>25:C>T</td></tr>
</table>

3021702|F|0-27:C>T-27:C>T	TGCAGAAGAGAACAAGCACAAGAGCTCCAACTCGACCTCCG	27:C>T
5581381|F|0-28:C>T-28:C>T	TGCAGCGAGAGGTGTTCACGAAGAGAGGCGGCGGCCG	28:C>T
1049393|F|0-28:C>G-28:C>G	TGCAGGATGATCACCTCGCTCAGCGGCACCCTCTTCCGCACCGCGCCCTCCTCCG	28:C>G
3942350|F|0-19:A>G-19:A>G	TGCAGCAGCACCTGGGTTCACTGGCCAGCTCCG	19:A>G
1113245|F|0-11:C>G-11:C>G	TGCAGCAATCACGCGCGTGCCGAAGAACGTCGTCGTCCATCCCTCACTTCAATGGCCTGAGGATCCTGC	11:C>G
1713534|F|0-29:G>A-29:G>A	TGCAGCGCGAGGTACTGATCGTAGAGCTGGAGGACGACGTCGAGGTTGGCGGCGGAGTTCCGATTCCGA	29:G>A
5582028|F|0-22:T>C-22:T>C	TGCAGGAGGTTGACGCTGCCATTGCTAAGCTGGAGGGTGAGACCG	22:T>C
1025762|F|0-13:G>C-13:G>C	TGCAGGACGGGACGGTGGCGTGGGGCAAGGCCGCCACCTTCCGCGCGTCCCCCTACCCG	13:G>C
6048017|F|0-37:T>C-37:T>C	TGCAGGTGGATTTGGTGACGGAGACGGACAAGGCCTGTGAGGATCTCATCTTCAACCACCTCCG	37:T>C
1297527|F|0-7:T>C-7:T>C	TGCAGTTTGATGAGGCAGAGAAGCTGTGCCGCAAAGCCCTTGAGATCCACCG	7:T>C
2247887|F|0-17:C>G-17:C>G	TGCAGCTCGGTGTCCCTCGCCGCCAGCATCCGCTTCAGAACGTTGTTCTCGTCCTCGATCGCGCGCAGC	17:C>G
988829|F|0-40:C>A-40:C>A	TGCAGGCGCCCAGCAGCGCGCCCCAGACCACCGAGTGCTCCTGGCAGTCCG	40:C>A
988344|F|0-37:C>G-37:C>G	TGCAGGTCCACGACGTGGCCTCCGACGTCCCCGCCGCCCACCGCCGCGCGCTCTACTCCTCCG	37:C>G
2258075|F|0-8:G>C-8:G>C	TGCAGCTTGGCGTCGCGGAAGCAATTGCCCGCGTCGAGGACCGTGCGCGCCGACGAGCTGGACCGCATG	8:G>C
2247548|F|0-39:C>T-39:C>T	TGCAGATGTAAAAGTCGTCGCCGTAGAGAGCCCTGACAGCATCGACGGGCTTCACCGCCG	39:C>T
5358221|F|0-6:C>T-6:C>T	TGCAGGCCCACGCAAGGTATGTCCCG	6:C>T
992863|F|0-46:G>C-46:G>C	TGCAGGACACCAGCATCCAGGGCGCCGAGAGCCACCCGCTGCACCTGCACGGCTACGACTTCCACGTCG	46:G>C
3021717|F|0-24:T>A-24:T>A	TGCAGCTTCACCATATCCAACTACTGCACCCACACCATCTGGCCG	24:T>A
1127066|F|0-34:G>C-34:G>C	TGCAGATCATCACGGCGCGGCCGCCCATGGGGCTGACCCACCACGTGGCGCGCGCGCTGGACCACGGCA	34:G>C
1093065|F|0-13:G>C-13:G>C	TGCAGCGCAGCCTGACGCTGTCGGACGAGCAGGTGGAGGCGTCGCGGATGACGCTGCACCG	13:G>C
1018507|F|0-21:G>A-21:G>A	TGCAGACCCAGACCTGAAGGCGTCGGAAGTGACGGTGAAGGGCGTGTTTGAGGAGGTCAAGCTGGCCGA	21:G>A
3026703|F|0-37:C>T-37:C>T	TGCAGATGTGAGTTGGGGATGGTGACCATGACCTCTGCGCCG	37:C>T
1018472|F|0-52:G>T-52:G>T	TGCAGCCCGATCATGGCGAACACGTCCGACAGCCCGAACAGCACGTACTGCGGCACCATCCACCACAGG	52:G>T
1250602|F|0-17:T>G-17:T>G	TGCAGTGTCTTGGAAGCTCTTGATGAGATCTTGAGCGGCGCGCAGGTTCTCGCCGTCCCGCTCCGCTCG	17:T>G
5325247|F|0-7:G>A-7:G>A	TGCAGGCGGGAGAGCGCGGCGGGGGCGACGGCCG	7:G>A
3024189|F|0-22:A>T-22:A>T	TGCAGCGCCACCCACTTCCAGGAGGACCTCTTCACTTTGCTCTGCCG	22:A>T
3944009|F|0-12:C>T-12:C>T	TGCAGCTTTGGGCACGAGGTGAAGACAGCCTCGTAGGCGGCCG	12:C>T
1023297|F|0-31:C>G-31:C>G	TGCAGTACCTGTCCAAGGAGGAGGCCGCCGTCCACGCGCGGATGCGCCGCCG	31:C>G
1033651|F|0-7:C>T-7:C>T	TGCAGCTCCCCATGGAATCATGTTGCTGATCTACCTTGTCTCAATAACAGCGCTAGTCAGATACACCAG	7:C>T
3946894|F|0-10:C>G-10:C>G	TGCAGGGCAGCGGCACCCAGAGCTCCTGGCCGACGGCGACCCG	10:C>G
3024189|F|0-35:T>C-35:T>C	TGCAGCGCCACCCACTTCCAGGAGGACCTCTTCACTTTGCTCTGCCG	35:T>C
7902469|F|0-24:G>T-24:G>T	TGCAGTTTCTCCTGATCTCTCCCTGGCCGCCG	24:G>T
2250331|F|0-29:G>C-29:G>C	TGCAGGTTGTCGGCGAGCTCCTCGATGTCGAGGGCGATGCTCTTCTCAGACGGCGCCG	29:G>C
3938410|F|0-10:G>T-10:G>T	TGCAGACACAGGGGCATGCCG	10:G>T
1229132|F|0-23:A>G-23:A>G	TGCAGGTGGACGGGCAGCTCCTCACCGACATCCCGGCGGGGACCTTCGACGTCCG	23:A>G
7172805|F|0-47:G>C-47:G>C	TGCAGGGACGAGATGCGCAGGGTGCTCGGCGTCGCGCTGCTCTGCGCGTCCAGCCTCCCGATCAACCGA	47:G>C
1021420|F|0-28:G>A-28:G>A	TGCAGCTGGTCTATCTGGCGGCGGCCACGGTGTTCTACTACGAAGCCATGGAGAGCAAGGAGGTGGTGC	28:G>A
1024456|F|0-57:C>G-57:C>G	TGCAGAGGACGCTGGACTACGCGTGCGGCAGCGCCGCCGACTGCAAGCCCATCCAGCCCAACGCCACCT	57:C>G
1063871|F|0-28:G>A-28:G>A	TGCAGATCATTGCCGTGCACTCGTCCACGCCCAGTTCGTGAGAGCCCAGCCTGCTCAGCGTCCGCCTCA	28:G>A
2276045|F|0-53:G>A-53:G>A	TGCAGGCGCGTCAGTTCCGCCTCGAGCTCCGCTTCCATCTGATCCATCGCAGCGACAGCATCTCCAACG	53:G>A
7907111|F|0-10:G>C-10:G>C	TGCAGCAGCAGCCTCGAGCCG	10:G>C
6040548|F|0-13:A>G-13:A>G	TGCAGGGGGAGGGACGGTACGTCGGGTCGCCGTCACGCCGCTGTAGCCACAACTGTAGTCTACTGGCGC	13:A>G
3954530|F|0-23:G>A-23:G>A	TGCAGGCGCTCGCCATGGACCGCGCCGTCGGCCGCTTCACCGTGCCG	23:G>A
3949987|F|0-11:A>G-11:A>G	TGCAGGGGACGAGGGGAAAGTGGGGCTGCTGCGGCGGCTCGTCCCCG	11:A>G
1046695|F|0-52:G>C-52:G>C	TGCAGATTCGTCTAGAAAATCTAACTCGTGAAAAGCAGCTCGCAAATTCCCTGGAGTTAGGCAGGCCCA	52:G>C
1040382|F|0-30:C>G-30:C>G	TGCAGGGTGTTGTGGTAGTGCTGAACACCCCTGGACCATGGAGATGGAGAGGACATTGCCAATTGGAAC	30:C>G
1094583|F|0-16:G>C-16:G>C	TGCAGCACGAAGGCGAGCAGCGTTGGCGCCCTCATCACCACCTCCTTCACCTCGCGCCAGTCGCTCGTC	16:G>C
1027589|F|0-7:C>T-7:C>T	TGCAGAGCCCACGCCGCCGCGAGCCACAGCGCCGCCAGAGGAGAGCGCCG	7:C>T
1041707|F|0-20:T>G-20:T>G	TGCAGGGCATCTGCTTCGCCTTGGGGCTCCTCGTCTCGCAGGTCCTCAACGAGCTCATCAAGCACTCCG	20:T>G
1002591|F|0-44:G>A-44:G>A	TGCAGCGGGAGGTGCACGTTGAGGTCGCAGGCGGCGGCGAGCGGGCAGTCGGCGCCG	44:G>A
5581942|F|0-14:C>G-14:C>G	TGCAGCAGCGGGCCCAGCACCAGCACCG	14:C>G
1040382|F|0-52:A>G-52:A>G	TGCAGGGTGTTGTGGTAGTGCTGAACACCCCTGGACCATGGAGATGGAGAGGACATTGCCAATTGGAAC	52:A>G
1068761|F|0-28:T>C-28:T>C	TGCAGGCAAGCTCCCACTGATCCTGTTGTTGTTCAGGTTGATCCTGTACAGAGACTGGCATCTGGCAAT	28:T>C
4262472|F|0-8:G>C-8:G>C	TGCAGCTGGACGCCCAAGCTGGTGTTGAGAGGGAACTTGTTGCCG	8:G>C
5581377|F|0-10:T>G-10:T>G	TGCAGCGACCTCTTCACTGCTTTAGCGCGCGATTGGGTGATGACCGTGGATGGTGCGCCG	10:T>G
3934608|F|0-20:C>T-20:C>T	TGCAGGCCCCGACGGCCGAGCCCCACGGCGCCG	20:C>T
5360252|F|0-18:T>G-18:T>G	TGCAGTTCGTCGACCTGCTGGGCGGCGTGCCG	18:T>G
3030025|F|0-43:C>G-43:C>G	TGCAGAGGAGCGAGCAGCTGCGGGAGCTCTACGAGTCCCTCCTCTCCG	43:C>G
5332157|F|0-12:G>A-12:G>A	TGCAGCTTCGTGGCCCTGTACGACCG	12:G>A

4910359|F|0-6:A>G-6:A>G	TGCAGAAGCACGACGCCGTCTTCGCCACCCG	6:A>G
7167027|F|0-32:C>G-32:C>G	TGCAGGAGCGCGTCGACGCGGAACGGCGAGCCCAGGAACCACGCCGACGAGGCGTCCGTCTCCACCACG	32:C>G
5370896|F|0-5:G>T-5:G>T	TGCAGGCCCTGGACGGCGCGGAGGCCCG	5:G>T
1241882|F|0-23:G>T-23:G>T	TGCAGCTGAAGAAGAACAAGCTGGCGGGGACCTGCAAGCTCAAGCTGAAGCTCGACTCGCCG	23:G>T
992808|F|0-15:G>A-15:G>A	TGCAGGCCCCGCCCAGACCGCTGCATCACGCCAAAACTCTAGCCCTAGCGTCCTCCTCTGCCGCCGCCA	15:G>A
4262240|F|0-16:G>C-16:G>C	TGCAGACGCAGAACGTGGTGGTGCCCGTGATGCTCAGCTCCG	16:G>C
3956789|F|0-5:C>G-5:C>G	TGCAGCTCCGCAAAAAGCCG	5:C>G
6035470|F|0-49:T>G-49:T>G	TGCAGGCGAAGAGGAGCACCTTCTGGTCCCTGTCCTCCTTGGGATACAATATGTTGTTGCTGCGCCCCC	49:T>G
5579549|F|0-10:C>A-10:C>A	TGCAGCACGGCGCGGTAGGCCTTGGTGGGCCGTGCGACCTCGAACTGGGACCTGAAGTCGACGTCCACG	10:C>A
1106971|F|0-5:A>C-5:A>C	TGCAGAGGCGTCTCGTCCTCGCCGCCCTGGCTCAGCGTCTCCATCAGGTTCCCCCACTCCGTCCGCATC	5:A>C
994161|F|0-39:G>A-39:G>A	TGCAGGGCCCACGCGCCCGTGGCTCCCAAGGTGATGTTCGCGGATTGGCTCAACATGGACTACATCGGT	39:G>A
5581047|F|0-8:G>A-8:G>A	TGCAGGAGGAGGACGAACTTGAACATGGTGTCACCCCTCGGGATGAACCCCAGCCTGTCCGCCAGGGCC	8:G>A
3952299|F|0-5:G>C-5:G>C	TGCAGGCAGTGCAGCGCCAGCGTTGCCGCGCCGCCCG	5:G>C
1023936|F|0-14:G>A-14:G>A	TGCAGTGCCAGCGCGCCCTGGACCTTTTGGTCCTGCTCTTCCTTGGCGCGCCG	14:G>A
1060558|F|0-16:G>T-16:G>T	TGCAGGAGCAGTCGTCGGAGTTGACAGAGCTGATGGACGGGATGCTCAACAAGCTGTCCAGCGCTCTGT	16:G>T
6049316|F|0-49:G>C-49:G>C	TGCAGACGGTGGTGGTGACCACCAAGTGGGAGACGTTCGACAGCAGCCCGCCG	49:G>C
1024822|F|0-59:G>C-59:G>C	TGCAGCTGCTCTTCCTCCACCGTCTTGACCACGGCGTGGGTCACGGCGACGCTGCCGCAGCCG	59:G>C
986230|F|0-56:C>T-56:C>T	TGCAGGTACGCGTACGCCGCGTGTTTTACCAGCGGGTGGCGTATCATGTGTATGTCCCCACAGCTGCCG	56:C>T
4262472|F|0-39:T>C-39:T>C	TGCAGCTGGACGCCCAAGCTGGTGTTGAGAGGGAACTTGTTGCCG	39:T>C
5357621|F|0-26:C>G-26:C>G	TGCAGCATGTTCATGGGGTTGAGGTACGGCAGCCG	26:C>G
1057496|F|0-20:A>G-20:A>G	TGCAGGCGATCCAGCAGTTCACCAGGCTGACGTACCTGGACCTGGAGGACACCG	20:A>G
5369301|F|0-6:T>A-6:T>A	TGCAGATCAAACCATGCAGCCG	6:T>A
3943891|F|0-8:C>G-8:C>G	TGCAGCAGCCGCGTGGCGAGCACGCGCGCCCG	8:C>G
1049393|F|0-41:C>G-41:C>G	TGCAGGATGATCACCTCGCTCAGCGGCACCCTCTTCCGCACCGCGCCCTCCTCCG	41:C>G
1026636|F|0-5:C>G-5:C>G	TGCAGCCGCCTCTCCCACTGGCCCTTGGAGGAGGAGGCGGGCCTCTGCGCCGCCG	5:C>G
2250331|F|0-47:A>G-47:A>G	TGCAGGTTGTCGGCGAGCTCCTCGATGTCGAGGGCGATGCTCTTCTCAGACGGCGCCG	47:A>G
2276045|F|0-5:G>A-5:G>A	TGCAGGCGCGTCAGTTCCGCCTCGAGCTCCGCTTCCATCTGATCCATCGCAGCGACAGCATCTCCAACG	5:G>A
5372877|F|0-16:G>T-16:G>T	TGCAGCAGCTGGTCCAGGGTGGCGCGGAGGCCG	16:G>T
6047856|F|0-33:C>G-33:C>G	TGCAGGTGGTGGCGCACGGGTCGGTCTCGGCTCCGTGTTGAACGGACCG	33:C>G
7166937|F|0-37:C>T-37:C>T	TGCAGCTGCTGCGGCGGGCGGCGAGGCGGAGCCGCCACCGCATGTCCAGGCTCGTCGCGCGGACCACCG	37:C>T
7166937|F|0-10:G>T-10:G>T	TGCAGCTGCTGCGGCGGGCGGCGAGGCGGAGCCGCCACCGCATGTCCAGGCTCGTCGCGCGGACCACCG	10:G>T
32022373|F|0-25:G>C-25:G>C	TGCAGACGCAGAGCGCCATCGACGCGCTCGTCGCCCG	25:G>C
996429|F|0-22:G>T-22:G>T	TGCAGAGCCGCATCCCCCGAGAGCTGTCCGTGATGCTGGGACGCGAGCGAGGGGCGTGGCGGCTGATCC	22:G>T
3945516|F|0-18:T>C-18:T>C	TGCAGCGCGACGCCTACATGAGCTACAGCCG	18:T>C
1028793|F|0-29:G>C-29:G>C	TGCAGCCGCTGGACGCGGGTGTCGGCGGCGCGTCGGCGGCGCCGCCAATCCG	29:G>C
1094084|F|0-23:C>G-23:C>G	TGCAGCGGGCTGGGCATGTTCCTCAACAGCACGGCGGCGGTGGACGACCGCTCGCTGCGGTGCCTGTGC	23:C>G
1094084|F|0-47:C>T-47:C>T	TGCAGCGGGCTGGGCATGTTCCTCAACAGCACGGCGGCGGTGGACGACCGCTCGCTGCGGTGCCTGTGC	47:C>T
981900|F|0-59:C>T-59:C>T	TGCAGGCGGATCGTCCACAACACGGTGGGCAGCGACAAGGCGGTGGCGAAGCTCTTCAGCGGGATGTCG	59:C>T
1051522|F|0-52:C>T-52:C>T	TGCAGGGCCAGCTCAAGGACCTCAAGGCGGAGCTCTCCCTCCTCCGCGTCGCCAAGGTCACCG	52:C>T
5411865|F|0-8:G>C-8:G>C	TGCAGGAGGAGGTTGAGGAAGAGGGCGAGCCG	8:G>C
7168837|F|0-68:C>T-68:C>T	TGCAGCTTCGTGTAGTTGCCATGCGCGTCGACGTGCGTCAGGGCCACGCGCAGGCCCTCCAGGCGGCCC	68:C>T
4397739|F|0-5:C>A-5:C>A	TGCAGCGGAGGGTGAAGATGCCG	5:C>A
4405358|F|0-20:A>G-20:A>G	TGCAGGTACCTGCGCATGGCACCG	20:A>G
990204|F|0-16:C>A-16:C>A	TGCAGAACACGCAAAGCCTCCTCTTCTGTGGTCTTCCTCCTCCTCCGAGACCAATAGAGCGCCCGAGAT	16:C>A
1397091|F|0-42:A>G-42:A>G	TGCAGACGGTGGTGGTGACCACCAAGTGGGAGACGTTCGACAACAGCCCCCCG	42:A>G
1087015|F|0-34:T>C-34:T>C	TGCAGGGCGAGAAGGAACTGGAGGCTCTTGCCGTTGCAGAGGTGCCTCGGGAACGGCCCGACGAATCCG	34:T>C
1028698|F|0-8:C>G-8:C>G	TGCAGGCCCTACCTCGCCAGGTCCCTCTTCCTCCTCGGCTCCATCGGCGGCAACGACTACAACGCCATG	8:C>G
3028359|F|0-34:C>T-34:C>T	TGCAGCAGACGAGGGTGCCCGTCGTCATCGATTACTACTCCCG	34:C>T
988439|F|0-40:C>G-40:C>G	TGCAGCTTGAGGACGGTGGTCTTCTGCGTGTCGGTGAGCACGCTTCCGCCCCCG	40:C>G
3951404|F|0-20:C>T-20:C>T	TGCAGCTCTGAGTAGCTGTACGCCCG	20:C>T
992871|F|0-41:G>A-41:G>A	TGCAGGAAGCCCAGCTCCGCCTGGTCCGCCCCCATGTACAGGCCCTGGGCGCGGCCGACGGCCGCCGAG	41:G>A
1024768|F|0-25:T>G-25:T>G	TGCAGGTGAGGCTGACGAAGGGGAATGGCCCAGGATGTGCCTTGAGGGCGGCGGAGACGGCGTCGGTGA	25:T>G
1863122|F|0-32:G>A-32:G>A	TGCAGTTCCACGGGTTCCACGGCGTGAAGCAGGAGGAGAAGAAGCTGGGCCAGAAGTTCGTGGTCGACG	32:G>A
5361126|F|0-31:G>A-31:G>A	TGCAGCGTGCCATGGCGTTCCTCGAGGACGAGTTCCACGCGCTGCTCGAGCCCAAGACGGAGACCTGCA	31:G>A
1032467|F|0-25:C>T-25:C>T	TGCAGAATGCACTTGTAGGTGTAGCCATTAAGCCTCAACCCCCACTTCATGGCCAGCTCCAGGCACTTG	25:C>T
1088814|F|0-51:C>T-51:C>T	TGCAGAAATGGCGCAAGAGAGTTGGAAAGAGGCAGAGGATACGGGTGTCCACGCGCCCGAGGCTCCGAT	51:C>T
2252222|F|0-49:C>G-49:C>G	TGCAGGCGCCGTCGAGGCCAACGTTCTGCGTCGACGACATGGAGGGTTTCTCGGGCATGTACTCGGGTG	49:C>G
3939236|F|0-27:G>T-27:G>T	TGCAGGCGAACTTGATGGCCTCGTCCCGCGTGGCCGTGCCG	27:G>T
1862984|F|0-26:G>A-26:G>A	TGCAGCCTGGACGGGAAGTAGACGAAGTGCCAGGGGGACGGGTGGGCGACGTCGAGGCGGCCGCCGAAG	26:G>A

1002591|F|0-35:G>A-35:G>A	TGCAGCGGGAGGTGCACGTTGAGGTCGCAGGCGGCGGCGAGCGGGCAGTCGGCGCCG	35:G>A
3030387|F|0-14:T>C-14:T>C	TGCAGGATGCCCCATTGCGTGAACACGTCCCGCGTCTGGAAGCACCG	14:T>C
2253440|F|0-19:C>T-19:C>T	TGCAGGCCGCTCGGCGAGACCGTCGCCGTCAAGGTGCTCGACTTCGAGCGCACCAACAGCAACCTGGTA	19:C>T
988344|F|0-35:G>A-35:G>A	TGCAGGTCCACGACGTGGCCTCCGACGTCCCCGCCGCCCACCGCCGCGCGCTCTACTCCTCCG	35:G>A
7338616|F|0-5:C>G-5:C>G	TGCAGCAGGAGCGGTATGCCG	5:C>G
1865080|F|0-22:C>G-22:C>G	TGCAGCTCATCCACGAGAACCTCGCCAACTTCGCCGCCGAGCTCGGGGTGCCCTTCCAGTTCGTCGTGT	22:C>G
1075589|F|0-35:C>T-35:C>T	TGCAGCACGAAGGTGAAGAAGCCCGAGTCCGTGTGCGCGATCAGGCCCAGCGCCCGCTTCGGGTCCGAG	35:C>T
2257165|F|0-37:C>T-37:C>T	TGCAGAGCACGTCCATGCCGGCCATCTCCTCGATGGCCGTCATGCGCTTGGTGATGGCGCCCTGCTGCG	37:C>T
7172910|F|0-5:C>T-5:C>T	TGCAGCGGGTCGAACCCGCCGATCACCACCATCGCCG	5:C>T
4397718|F|0-23:G>C-23:G>C	TGCAGCGTCTGGGTGCACACGCGGTGCGCGCGCTACGTCCG	23:G>C
1023881|F|0-23:G>A-23:G>A	TGCAGCACGTCGCTGTCGGCATTGGCTAAATAGCGCAGGGAGGAGGAGGGGCCGCGGCTTGCGGGGGAA	23:G>A
1036908|F|0-53:G>A-53:G>A	TGCAGCTTGTCGGCCAAGCTCTTGGCCCCGTTGAGCGAGAGCACGAGCCAGACGAAGGAGAGGAACTCC	53:G>A
5000416|F|0-7:G>A-7:G>A	TGCAGGCGCAGCTCGGCTACCGCTCGGTCGACTTCCTGCCCTACGCGTCCCACGTCCGCAAGACGGCGG	7:G>A
1106312|F|0-58:C>G-58:C>G	TGCAGAAGGTGCCTTCGCAGCGGCACAGGAATCCCAGCAGCCCCACCTTCTTGCGGCACGCCGCGCACC	58:C>G
1049393|F|0-42:G>A-42:G>A	TGCAGGATGATCACCTCGCTCAGCGGCACCCTCTTCCGCACCGCGCCCTCCTCCG	42:G>A
5580511|F|0-16:A>T-16:A>T	TGCAGGTCCTGGACGAAGCTGCCG	16:A>T
1044757|F|0-41:C>G-41:C>G	TGCAGTGGAACAAGGTCTGCAACATCTTCACCCGCTTCTGCCAGCAGGTCGCCG	41:C>G
3025772|F|0-40:T>C-40:T>C	TGCAGCTGCACACCACCAGGAGCCCCGACTTCCTGGGCATTGGCCCG	40:T>C
12735261|F|0-5:C>G-5:C>G	TGCAGCGTCAGCTCGAGCCG	5:C>G
3020975|F|0-13:G>A-13:G>A	TGCAGCAATGCTCGATCTTCCCCATCTGGGTGTCATTGTACCG	13:G>A
1059056|F|0-55:G>C-55:G>C	TGCAGGTGTATGCCACGATCAGGACGGGGTCCATGGCGATCGCGGCGTCCACGCTGGACTCGCTGCTGG	55:G>C
978246|F|0-8:C>G-8:C>G	TGCAGTTCCGTCCGTCTCCTGCCGCACCAAATCCGCCCACCGTGGTTGCTACATCGGAGATTCAAGAAG	8:C>G
1043522|F|0-44:G>C-44:G>C	TGCAGCATCCTGTACAGGAGCAGAGCGGACATGCGGGCCTGGTCGCGCACCGCCTCCAGGTCGCGGTGC	44:G>C
1230783|F|0-20:C>T-20:C>T	TGCAGGGGGTGGAGGTGGAGCGGCCTTTGGCTTTGGCTGGATCCCAGCGGCAGCCTCAACGTCAGCCGC	20:C>T
3946705|F|0-11:C>T-11:C>T	TGCAGGGTCTTCATCCTGCTGCTGCTCCG	11:C>T
1862758|F|0-31:C>G-31:C>G	TGCAGATCCTGGGCCTGTTCCAGGTGCTCCCCCGCCTCGTCGTCTTCGACCTCGACTACACCCTGTGGC	31:C>G
1028367|F|0-56:G>A-56:G>A	TGCAGGCCGTCGCGCCAGTGGCCGTCGTAGACGTCGCCGTTGGCGTAGGCCTGCGTGCCGCGGCCGTGC	56:G>A
1026502|F|0-19:C>T-19:C>T	TGCAGTAGTCGCAGGCTAGCGCGTCGGCAGAGAGCTCCTTCTCCATGGCCG	19:C>T
1093465|F|0-27:T>C-27:T>C	TGCAGTGCTTCCTTGCCTCCATCATGGTGGCCATAGCAGACAATGATGGCATTCCAGGATACAGAGTTC	27:T>C
5358220|F|0-9:A>T-9:A>T	TGCAGGCCCACCAGATAAGCTTGACCCG	9:A>T
5580176|F|0-20:G>C-20:G>C	TGCAGCAGGCGTATCACGCTGCGCCGCGCGTCGTACCG	20:G>C
3946842|F|0-9:A>G-9:A>G	TGCAGTTACAATGGTATTGGTACCTGTGAGATCATTCCCGCAGCACCG	9:A>G
3029515|F|0-10:T>C-10:T>C	TGCAGCACGGTGTCCGCCGCCTCGGCGCTCGGCGACCACCGCCG	10:T>C
7909245|F|0-10:G>T-10:G>T	TGCAGTCCACGCTGTGGCCGCTGGGCCACCCG	10:G>T
7166937|F|0-34:C>T-34:C>T	TGCAGCTGCTGCGGCGGGCGGCGAGGCGGAGCCGCCACCGCATGTCCAGGCTCGTCGCGCGGACCACCG	34:C>T
5368108|F|0-37:G>C-37:G>C	TGCAGATCTCGATGAGCTCGTCGAGGCCCTCGGACTCGGCGGCGACCTTGGACGCCGCCGACGACGACA	37:G>C
996571|F|0-7:G>T-7:G>T	TGCAGCTGGTGCCGACGCAGAAGATCGGCGCCATCCCGCAGCCGCTGGAGGAGCTCCTCCTCCACTCCT	7:G>T
996571|F|0-13:G>C-13:G>C	TGCAGCTGGTGCCGACGCAGAAGATCGGCGCCATCCCGCAGCCGCTGGAGGAGCTCCTCCTCCACTCCT	13:G>C
1106971|F|0-14:G>A-14:G>A	TGCAGAGGCGTCTCGTCCTCGCCGCCCTGGCTCAGCGTCTCCATCAGGTTCCCCCACTCCGTCCGCATC	14:G>A
2256566|F|0-49:C>G-49:C>G	TGCAGCTCTGGAGGTAGGAGAAGAGGCCGAAGCCGCAGTAGTCGAGGCACGCCTTGTCGAGGTGCGGGT	49:C>G
3021288|F|0-25:T>C-25:T>C	TGCAGCCCTATATCTGCCTGGAAATTCACACCTGTACAACCG	25:T>C
5373111|F|0-5:C>A-5:C>A	TGCAGCGCTGCGGGAAGAGCTGCCG	5:C>A
1145809|F|0-47:T>C-47:T>C	TGCAGGTTGGAGAACAGAGGCTGGGCAATGGGGATGTGTTCAGTCTGTTGGACCATCTGGCCAACATCG	47:T>C
4394667|F|0-11:G>A-11:G>A	TGCAGCCTGCGGTACATGTGCCG	11:G>A
11315169|F|0-9:C>T-9:C>T	TGCAGAAGGCGCGGAGGCGCCGCCG	9:C>T
2251724|F|0-36:C>A-36:C>A	TGCAGGGTGGTGGGCGACATCGACGCGCTGGTGGCACTGGACCTGGACTCGGCGAGCGTGCGCGGGCTG	36:C>A
5579705|F|0-7:C>G-7:C>G	TGCAGGGCGCGGAGGTGTCGGTGGAGAACGTCCGCAGCAGCTTCCCTCTGGGCGCCGCCATGAGCCGCG	7:C>G
12773585|F|0-20:G>A-20:G>A	TGCAGGCACGCGCACGCCTTGAGCAGGAAGGGGAAGGTGAGGTGGTCCG	20:G>A
2252593|F|0-14:G>A-14:G>A	TGCAGCCGTAGCGCGTACAGCTGGTTGTTCTCGACGCCGAGGACTGTCAGGTACCGCCG	14:G>A
1666914|F|0-46:C>G-46:C>G	TGCAGGCGTGACATCCGCGACGTTGGAGGGGGACAAGATCATCATCCTCGGCGACGGCGTCGACCCGAT	46:C>G
5357481|F|0-15:G>A-15:G>A	TGCAGCAAGCCGAGCGAGAAGAACCCCTCCG	15:G>A
979973|F|0-16:G>C-16:G>C	TGCAGTCGGAGTAGGCGACGGCGTGGCGCTTGAACACGCCGAACACCGCCACCCGCCCG	16:G>C
6047437|F|0-5:A>G-5:A>G	TGCAGACCAAGATGGTCAAGATAGCGGTGCCG	5:A>G
4262323|F|0-15:C>T-15:C>T	TGCAGTGCGCGGCCGCTCATTCCGCCGACCGCCGCCGTGCCG	15:C>T
2362620|F|0-11:G>A-11:G>A	TGCAGCCCCAGGGGGACTCGTACACGCGGCGGTCGGCGTTCGACGCCGTGCTCGCCG	11:G>A
32023350|F|0-5:A>C-5:A>C	TGCAGATGTTCCAGGAGTACAAGGCCAGGGTGGGGGGCACCATCCCG	5:A>C
7168187|F|0-20:T>C-20:T>C	TGCAGGGGCGTCAAGTACCATGAGCCG	20:T>C
6040344|F|0-17:A>G-17:A>G	TGCAGCTGGCCGTGGACAACGCCGCCGCCAAGTTCCTCGCGGCGGAGAAGCTGGAGGTGGTGGACTACA	17:A>G
32026724|F|0-9:C>G-9:C>G	TGCAGGGCGCCGTCGCCGAGCGGGCAGGCGGCCTCGAAGGTGGCCCCCTCCTCGAGCGCGAAGTCCTCC	9:C>G

32022455|F|0-7:C>T-7:C>T	TGCAGCGCCGTCTGGCGCGCGCTCCGCGACTCCG	7:C>T
100397547|F|0-28:T>C-28:T>C	TGCAGCAGAGCACCATCAAGAAGTGGGCTGGCAAGAAGGAGAACGTCGCCGACGCGCAGGCCACCTTCC	28:T>C
100450297|F|0-45:G>C-45:G>C	TGCAGCTCCTTGGTGGAGATCATGCGCACGTCGGCGGCGTCCTTGGTGGCGGCCGGCATTGCGTCGGCG	45:G>C
100494093|F|0-38:G>A-38:G>A	TGCAGAAGCTGCGGGCGGAGCTGGACGCCGTGGTGGGCGCGTCCCG	38:G>A
7164268|F|0-54:C>T-54:C>T	TGCAGGGTCTCGAGATGCATCTTCCTCCTCATGCTGGCGATGAACTCTTCGAATCTCTTGTTCATCTCG	54:C>T
32022657|F|0-7:C>G-7:C>G	TGCAGCTCATGAAGGGCTCCCG	7:C>G
100531441|F|0-20:G>C-20:G>C	TGCAGCTGCTCGAGCGCCGTGACGACCTGCGCCATGCCG	20:G>C
100553971|F|0-37:C>T-37:C>T	TGCAGGTTTGGAAGGGGATCACCGATGGGGAGCTCGACGTGCTGGACGTATTTGAGGATGAGGAGTATG	37:C>T
4997306|F|0-33:C>G-33:C>G	TGCAGGTCGGGGCGCATCGGTCGCCGAGGCCGACAAGACCG	33:C>G
3934839|F|0-11:G>C-11:G>C	TGCAGCCCCATGAGGCGCCG	11:G>C
100416518|F|0-10:T>C-10:T>C	TGCAGCTCGCTGTGCAGGACTTCCAGTCGTGCGACCG	10:T>C
1341985|F|0-13:C>T-13:C>T	TGCAGCTGGTGTACGAGCTGCTGCTCAAGTTCATCGGGTCCAACTCCCTGGATGCCAAGATAGGGAAGA	13:C>T
100532256|F|0-8:C>G-8:C>G	TGCAGGTTCTTCTCCAGCTCGTCCAGCACGGCCCGCCCGCCG	8:C>G
5005882|F|0-23:C>T-23:C>T	TGCAGGCCCAGGTACGCGTCCTCCCGCGCCGCGCCG	23:C>T
32027814|F|0-60:T>C-60:T>C	TGCAGGAGAAGGCCACGCTCCTGGCGTTGAAACGCTCGCTCACCTTGCTGTCGCCCTCGGTTCTGGCGG	60:T>C
100540585|F|0-25:G>C-25:G>C	TGCAGGACCCCCGCATCGCGGCGGCGGCCCCCGCCCTCATACGCGCCGCCACAGCCTCCG	25:G>C
5372257|F|0-22:G>A-22:G>A	TGCAGGCTCTCCATCTGCGGCAGGGCGGCGGCCG	22:G>A
1152835|F|0-12:T>C-12:T>C	TGCAGCTGCTCTTCCTCCACCGTCTTGACCACGGCGTGGGTCACGGCGACACTGCCGCAGCCG	12:T>C
1207911|F|0-28:G>A-28:G>A	TGCAGCACCTGACGGACAAGAGCGACGTGTACAGCTTCGGGGTGCTGCTGCTGGAGCTGCTGTCCGAGA	28:G>A
3949212|F|0-23:G>A-23:G>A	TGCAGCTCACGCGCCCGACGTCCGCGTCGCCCG	23:G>A
5411802|F|0-6:C>T-6:C>T	TGCAGGCGCGCGCGGCCACGCGGTGGCGACCG	6:C>T
7173978|F|0-17:C>G-17:C>G	TGCAGCAGCTGGTCCAGCGTCGCGCGGAGGCCG	17:C>G
999492|F|0-11:G>C-11:G>C	TGCAGCGGCGTGACGTGCCCGAGGCGCGACGGGTGGAGGGTCTCGGAGGTGATGTTGCACCG	11:G>C
4262544|F|0-19:G>T-19:G>T	TGCAGGCGCCGTCGCGCGCGGCCCAGCCGTCGAGCGCGTCCCG	19:G>T
1052066|F|0-15:C>G-15:C>G	TGCAGGCTTTGTATCCAACTTCAGTGTGGATCCACCATTACTACCTGCTCAATGCTTTCAACCAATATC	15:C>G
1213983|F|0-47:C>T-47:C>T	TGCAGCTGCTCCATGTCGCTCGGGTACACCGCGATGGCGCCCCGCCGCGGCAGGCTCGGCCG	47:C>T
995752|F|0-68:G>C-68:G>C	TGCAGGAGGACGGTGATGGCGAGGTCGGGGCTGAGCACGCCGCTGCTCACCATCTCGTCCAGCGTCTCG	68:G>C
3025393|F|0-22:T>C-22:T>C	TGCAGTCTCCAGCCCCATACCCTTGTCACCGCCGATCTTTGACCG	22:T>C
7340639|F|0-30:G>A-30:G>A	TGCAGGTGGGCAAGGCGCTGGCCAAGGAGCGCGTCTTCGTGGAGTGCCACGTGTTTAACCCGCCGTCCG	30:G>A
1006331|F|0-40:C>T-40:C>T	TGCAGCCTAGCAAGTACAAGGACATGGCTCAGGTGGACGCCTGCAATGGCCTCCTTCTCTACCTAAGCT	40:C>T
989810|F|0-28:G>T-28:G>T	TGCAGGGGCTCGGGGTCAACCCCGCCGTGCACGAGGTCGCCGACGAGGCCGCGCTCGCCG	28:G>T
4262153|F|0-5:A>G-5:A>G	TGCAGATGACCTCCCACGCGCGCTCGAAGCCGCCGCGGTACCCG	5:A>G
986316|F|0-17:G>T-17:G>T	TGCAGCTGGCGGGACGCGGACCAGGTGCGGGACACGCTCCAGGACAGCGACCTGAAGTGGGAGCCGCCG	17:G>T
3935520|F|0-23:G>A-23:G>A	TGCAGCCACCTGTGCTGCGCCGTGCCG	23:G>A
1165688|F|0-64:G>C-64:G>C	TGCAGTGCTTCTTCGTCGCCACCAAGTGCGTCTGGCTGCTCCACCTGCTCGCCTTCTCCTTCGCGCCGC	64:G>C
986001|F|0-60:C>T-60:C>T	TGCAGTTTCCACTGCTGCAATAACTATTTATGCGAAAAAGGCGCTCCAAAAATTGCAAGCCGAGGATGA	60:C>T
4002415|F|0-9:T>C-9:T>C	TGCAGGTCGTCGCGGAGCAACGTGTGCTCCAAGTACCG	9:T>C
1019869|F|0-37:G>A-37:G>A	TGCAGTACTCCTCGTCCGACCAGCAGTGTTCGTCCATGGTGGGGCCCTCGGGGGGGTTGGGGTTGATGA	37:G>A
1068761|F|0-54:C>T-54:C>T	TGCAGGCAAGCTCCCACTGATCCTGTTGTTGTTCAGGTTGATCCTGTACAGAGACTGGCATCTGGCAAT	54:C>T
5582607|F|0-30:C>G-30:C>G	TGCAGCATGAACGGCAATGGCACCCAATCCCAGTGCGGGTATCGAGTCGAGTACGGAGGAGGGTCAAAG	30:C>G
4396339|F|0-18:G>A-18:G>A	TGCAGCCGCGGTCGAGCCGCCCG	18:G>A
7173513|F|0-14:G>A-14:G>A	TGCAGCTCGGAGAAGTGCATGACGATGAAATATCCCG	14:G>A
977536|F|0-47:T>C-47:T>C	TGCAGCGAGGTGGCGGACATCATGCGGTGCGTCAAGGTGGCGCTGCTTTGCGTGCAGGACAACGCCATG	47:T>C
7169811|F|0-31:G>A-31:G>A	TGCAGTCAGGGCAGTGCTCCTTGGGAGCCACGTGCCCTACCG	31:G>A
2252593|F|0-35:G>A-35:G>A	TGCAGCCGTAGCGCGTACAGCTGGTTGTTCTCGACGCCGAGGACTGTCAGGTACCGCCG	35:G>A
1696589|F|0-23:A>G-23:A>G	TGCAGGTACTACTCGACCTGGGGACACGTCGCGACGCTGGCGGAGGAGATAAAGAAGGGCGCCGACTCC	23:A>G
988344|F|0-28:C>G-28:C>G	TGCAGGTCCACGACGTGGCCTCCGACGTCCCCGCCGCCCACCGCCGCGCGCTCTACTCCTCCG	28:C>G
1074522|F|0-15:A>C-15:A>C	TGCAGATCCAAAAGAAAGCCATGCTGAAAACCCCCATGTTTCCTCCCCCG	15:A>C
1021854|F|0-10:C>G-10:C>G	TGCAGCTGTCCGAGTCGGAGTCCGACGTCCAGGGCAGGCCCATCTCCTCCG	10:C>G
2242889|F|0-25:T>C-25:T>C	TGCAGGTGCTTGCGGAGATCGACGATAGGTTCTTGAAGGCGTCAGAGAGCGCCG	25:T>C
1105209|F|0-38:C>T-38:C>T	TGCAGAGCACCGAGGAGAAGAGCAGCACTTGGGAGAAGCGCTCGCGGTTCCTCATGGACGAGTATATCG	38:C>T
6042714|F|0-13:G>C-13:G>C	TGCAGCCCAAGGTGCCGTTCTTCACCGTCATCACCGACCTCAACACCTGCCACCCGACATGGTAACCGA	13:G>C
987565|F|0-13:C>G-13:C>G	TGCAGCGCTTCTCCCGCCACGTGATGCACGAGCTCGTCCGCCTCGTCTTCGCCCGCCTCCCGCTGATAG	13:C>G
5005882|F|0-10:G>A-10:G>A	TGCAGGCCCAGGTACGCGTCCTCCCGCGCCGCGCCG	10:G>A
1022580|F|0-17:G>T-17:G>T	TGCAGGGCCTCCGCGACGGCCACGTAGTTGACGGCTCCTCCGATGTGCCG	17:G>T
986877|F|0-12:C>G-12:C>G	TGCAGACCAGCCCCAAGTGCCTGTGCGTGCTCGTCAAGGACCGCGACGATCCCG	12:C>G
1073887|F|0-59:C>G-59:C>G	TGCAGGAACTTGGTGATGGGCTGGATCACCGCGTTGGCGAAGAGCCCCGGGATGAGCCACACGATGTAC	59:C>G
3950343|F|0-26:A>G-26:A>G	TGCAGGAGATCGCGCAGAACAGGGCCACGACGCCG	26:A>G
1074522|F|0-9:A>G-9:A>G	TGCAGATCCAAAAGAAAGCCATGCTGAAAACCCCCATCTTTCCTCCCCCG	9:A>G

3939901|F|0-10:C>T-10:C>T	TGCAGCCGAGCTCCCAGACGTACTGCCG	10:C>T
1031760|F|0-32:C>G-32:C>G	TGCAGCTTGCGCCGCAGGAACCCCCTGGAGAGCAGCACCTTGGTGCCGACCCTCTTGTCGTCCCAGCTG	32:C>G
3956442|F|0-20:C>A-20:C>A	TGCAGGCCCAGCGCGCCGAGCGCCTGGTACACGGACTTCATCGCCG	20:C>A
991450|F|0-30:G>A-30:G>A	TGCAGTGGCGCCGCATGGTAGACGACCTGCGCTCCAAGGGGGCGCTGCGCAACTGCATCTCCGTCTGCG	30:G>A
1075589|F|0-57:T>C-57:T>C	TGCAGCACGAAGGTGAAGAAGCCCGAGTCCGTGTGCGCGATCAGGCCCAGCGCCCGCTTCGGGTCCGAG	57:T>C
3956546|F|0-15:A>G-15:A>G	TGCAGGCCAACATCAAGAAGTCCGTCAACCTCGAGGCCCTCGCCGCCG	15:A>G
988735|F|0-22:C>T-22:C>T	TGCAGTATCGCGCCGTGGCCGCCCCCACCCATCTCGTGCTTGACGTTGCCGTCCGCGTCCAGGAAGAGG	22:C>T
1063972|F|0-12:G>C-12:G>C	TGCAGGAGAACAGATGCATCTCACCAAGGCAAGAACTGGCCGTGCAAACTCAGGCCAAGAGACACAAGG	12:G>C
983722|F|0-58:C>G-58:C>G	TGCAGCTGCAACAACCCCTGACTCTTGTCCTTTCCATGCGCACACTGGGCCATCGCGGCCGCTTATGTG	58:C>G
13142884|F|0-5:C>T-5:C>T	TGCAGCAGGCCGTTGAAGCCG	5:C>T
3027178|F|0-23:G>C-23:G>C	TGCAGCGACGTGCTCGTCGACGGGCTCTTCATCAGGTCCCCG	23:G>C
5363323|F|0-11:G>C-11:G>C	TGCAGCATCCTGGTGAGCGGGCTGCCCCCG	11:G>C
4005686|F|0-35:C>T-35:C>T	TGCAGGCACGCCCTGGCCACGCCCATCACGGTGGCCACGCTCCCCG	35:C>T
1089536|F|0-59:G>A-59:G>A	TGCAGGGTGTTCCAGAAGCAGAAAGTGGACGGCGAGCAAGACAACGCCCGCTCCTCTTCGCCGACCTTC	59:G>A
1068761|F|0-68:T>A-68:T>A	TGCAGGCAAGCTCCCACTGATCCTGTTGTTGTTCAGGTTGATCCTGTACAGAGACTGGCATCTGGCAAT	68:T>A
7161358|F|0-25:C>A-25:C>A	TGCAGCGGGATCCCGCGAGGCGGCTCGGGTACGCCG	25:C>A
7907111|F|0-17:G>A-17:G>A	TGCAGCAGCAGCCTCGAGCCG	17:G>A
7170520|F|0-5:C>G-5:C>G	TGCAGCAAACTTTGATCGCGACCG	5:C>G
2320865|F|0-45:A>G-45:A>G	TGCAGGGCCAGGGCTCGCTCACGCTGCCCCGCACCCTCAGCGCCAAGACGGTCGACGAGGTGTGGCGCA	45:A>G
1011593|F|0-41:C>A-41:C>A	TGCAGAGGGACGCGATGCCGTCGCACCTGGCGCTGACGGTGCGGGGCTCGCTGTACCCGCAGCCGAGAT	41:C>A
1053162|F|0-26:C>G-26:C>G	TGCAGAGGCGAGCGGGTGGTTGTGCTCGGCGACAAATTCAGTGACACGGAAGGCATTGGCCGAAGGGAG	26:C>G
1022649|F|0-37:G>C-37:G>C	TGCAGATCGTGCCGTGGGGGAACATGGACTGGATGGCGACCAACTTCTACGGGCAGCCG	37:G>C
5000416|F|0-25:G>C-25:G>C	TGCAGGCGCAGCTCGGCTACCGCTCGGTCGACTTCCTGCCCTACGCGTCCCACGTCCGCAAGACGGCGG	25:G>C
999492|F|0-44:G>A-44:G>A	TGCAGCGGCGTGACGTGCCCGAGGCGCGACGGGTGGAGGGTCTCGGAGGTGATGTTGCACCG	44:G>A
990763|F|0-43:G>C-43:G>C	TGCAGAAGCAGGTGGACGGCCTGGAGAAGAAGAAGGAGGAGCTGACGCGGGCCAGCTGCAAGCCCGAGA	43:G>C
1091204|F|0-32:C>T-32:C>T	TGCAGTTGGACGGAGCGCACAACACCTCATCTCGTTCATCATCTTCGCTGGCTTGACAGCCG	32:C>T
4003312|F|0-27:A>G-27:A>G	TGCAGGAACTTGGTGATGGGCTGGATCACGGCGTTGGCGAAGAGCCCCG	27:A>G
2268151|F|0-29:C>T-29:C>T	TGCAGGGCGCTGAGCCGCTTCACGGCCGCCACGATGCCGTCCTCGAGGACCGCCTTGTACGTGCTCCCG	29:C>T
1253152|F|0-14:G>A-14:G>A	TGCAGCTCGGCACCGTGCGGACGGTGGTGGTCTCGTCGGCTGAGGCGGCGCGCGAGGTGATGAAGATGC	14:G>A
992480|F|0-22:G>C-22:G>C	TGCAGAGGATCTGGCCGTTGCGGAGGCCGAGGCGGAACTCCTCCTCCGACGGCTCCTCCG	22:G>C
991902|F|0-48:A>G-48:A>G	TGCAGACGACGAAGCCGCACCTCCTCCGCGAGCACCAGGTGCATCCGCAGCCG	48:A>G
5367503|F|0-18:C>T-18:C>T	TGCAGTCGAACGGGCGGGCAGCCGCCGCGGCGCACTGTGCCG	18:C>T
1006331|F|0-30:A>T-30:A>T	TGCAGCCTAGCAAGTACAAGGACATGGCTCAGGTGGACGCCTGCAATGGCCTCCTTCTCTACCTAAGCT	30:A>T
1007011|F|0-32:T>C-32:T>C	TGCAGGACTGCGCCGTCCGCCGAGCGCTCCGCTGCGGACAGGAGCTCCACGCGCGGCTGCTCCGCTCCG	32:T>C
1079033|F|0-14:C>G-14:C>G	TGCAGGCGGCGGGACGGCGCGCGCTTGCCGAGGAGCTTCTTCTTGAGCTTGGTGTTCCAGTAGTTCTTG	14:C>G
7168594|F|0-34:G>A-34:G>A	TGCAGATGTCCTCGTGGACGAACACCTCGAGGCAGATCGCGCACACCAGCTTCTCCGACGCCCTCCTGC	34:G>A
990204|F|0-59:C>T-59:C>T	TGCAGAACACGCAAAGCCTCCTCTTCTGTGGTCTTCCTCCTCCTCCGAGACCAATAGAGCGCCCGAGAT	59:C>T
7340714|F|0-50:C>T-50:C>T	TGCAGCGACCTTGCCCCCTCGTCGTCTTCGGCGCCATCGTCCCCTCCTCCCACGCCGCCGCTGCCTTCG	50:C>T
3943516|F|0-7:C>T-7:C>T	TGCAGGGCATCCTGCACACGGTGGCCG	7:C>T
998634|F|0-31:G>A-31:G>A	TGCAGCAGGAGGCGGCGGAGGAGGAGGCCAGGAGGAAAGCCGAGGAGGAGGAGGCGGCGGCCAAGAAGA	31:G>A
6044128|F|0-14:A>G-14:A>G	TGCAGGCTCCCCAGATCGCCTAGCTCCG	14:A>G
7172018|F|0-12:C>G-12:C>G	TGCAGAACTTGCCGTTCGATGCTTCCTCTGTCCG	12:C>G
2268462|F|0-30:T>C-30:T>C	TGCAGCGTGCCGTAGAGCCGCACCTTCCCGTCGGCGCCCTCGTCGGCGGCGAGCTCGGTGGCCTCGAAC	30:T>C
5411889|F|0-14:G>C-14:G>C	TGCAGAACCTCGACGTGTCCAGGAACTCGCTCTCCG	14:G>C
1113765|F|0-9:C>T-9:C>T	TGCAGCCACCGTAGGCTCACACAGCGCTGCCATAGCCACCTGAGCCCGTGGTCGCCG	9:C>T
2336803|F|0-20:G>C-20:G>C	TGCAGGGCGCCCTGGCCGTCGAGGCCCGAGCAGAGCACCGCGACGGGCGCGTCGGCCATCTCCGCGAGC	20:G>C
7167781|F|0-13:C>G-13:C>G	TGCAGCGCAGAAGCCGTTCTGCTCCAACCCG	13:C>G
6037758|F|0-5:C>G-5:C>G	TGCAGCGAATAAGCGGTTGGCCG	5:C>G
1262989|F|0-35:C>T-35:C>T	TGCAGGGTGCGGAAGTCCTTGAGCGGCTTGGCCCGCGGGTGGTCGATGTTGTTCCG	35:C>T
7165606|F|0-8:G>A-8:G>A	TGCAGCTTGGTGATGATGTCCACCTCCCG	8:G>A
995885|F|0-10:C>T-10:C>T	TGCAGCGCGACGGCTACGCAGACGATCTTCAAGGCAATGCCAAGATAGCGCGGTACCTCGTGGACTCCA	10:C>T
1341985|F|0-42:A>G-42:A>G	TGCAGCTGGTGTACGAGCTGCTGCTCAAGTTCATCGGGTCCAACTCCCTGGATGCCAAGATAGGGAAGA	42:A>G
4261625|F|0-12:C>G-12:C>G	TGCAGGGGCGGCCTCAAGAAAGCAGGGGTCAACGGCACCG	12:C>G
7170555|F|0-21:A>G-21:A>G	TGCAGCACTGCCACGAGCGCAACATCCTGCACCG	21:A>G
1093813|F|0-14:G>T-14:G>T	TGCAGGGCGGCGTCGCGGCGGACGGGGAGATCCAGCACGGGGTCGTAGAGGACGGAGACGACGGACAGG	14:G>T
7907111|F|0-16:A>G-16:A>G	TGCAGCAGCAGCCTCGAGCCG	16:A>G
1219453|F|0-20:A>T-20:A>T	TGCAGAGCGTCATCCAGATGATCTCCGTCATGTTCGTCGGGCACCTGGGCGAGCTCGCGCTGGCCAGCG	20:A>T
6047267|F|0-8:C>G-8:C>G	TGCAGGCACCAGCGCGCACGCCATGGTTGGACTCAGCCTCCCAAGGGTTTGCGCCAAGAGCGCCGAGAT	8:C>G
981918|F|0-58:G>A-58:G>A	TGCAGGCCGCGGAGGCCGAGCTGTCCATGGCGAAGAACGAGCTGGAGAGCATCAAGGCGGGGGCGTTCC	58:G>A

1040610|F|0-47:C>G-47:C>G	TGCAGCGGGTTCGGCCCTGCCCCGTCGCTCACCGCCATCATCGCCAACCGCTACCGCATGCCCAGCGAC	47:C>G
1088740|F|0-35:G>C-35:G>C	TGCAGCATCCTGGCCCGCTCCTCGCACATGCTGACGAGCCGCTCCTCGGCGCGCTCCATGGTCTCCCGA	35:G>C
7343602|F|0-26:C>G-26:C>G	TGCAGCTCCGAGATACCCTTCTCCCTCCCG	26:C>G
1077177|F|0-13:T>G-13:T>G	TGCAGCAACGCACTCACGTCTTTGTTGTCTTCGCTGCTCGGCGCCGCTCCATACTCTGTAACGTCATAA	13:T>G
1147720|F|0-36:C>T-36:C>T	TGCAGGGATGCGACGCGTCGGTGCTGATCACGAGCGCGGGCAACGCCGCGGAGGTGAACAACAACAAGC	36:C>T
2251238|F|0-23:C>T-23:C>T	TGCAGGATCTCCACCTGCGGGTACTTCTCCCTGAAGCCTTCGATGAGCTCCTTGCTGAGCGGCGCGCCG	23:C>T
3957306|F|0-14:C>G-14:C>G	TGCAGCTTCCTCCCCACCACCGCCG	14:C>G
1021154|F|0-38:C>G-38:C>G	TGCAGCTCGGCATTGGGCGGCAGGATGACGGGCTTCTGCACGACGATGCCGCCGCCGCCGTCGGCGGGG	38:C>G
1002654|F|0-61:C>A-61:C>A	TGCAGCCTCGGTGCCATGGAGACGGAGCCTCCTCCTGCTCCCGACCCTTGCGCTCCTGTGGCAGCTCTG	61:C>A
5363533|F|0-17:A>C-17:A>C	TGCAGCGGCGGTCCACGAGGCCACTGGACGCCG	17:A>C
1020026|F|0-43:T>C-43:T>C	TGCAGATTTCTGCACCTTTATCCCACTCTGTGATGAACGTTCCTAAAACCTTGGCTCAAGAACCGAGAT	43:T>C
3032956|F|0-16:C>G-16:C>G	TGCAGGACCGTGACCACTGCGGCGGCGATGTAGTCCGCCTCCG	16:C>G
990582|F|0-47:G>C-47:G>C	TGCAGCCAGCCGACGCTGGTCGGCTACTCCCTCTGCGAGCACCACCTGGGCAAGGGCCG	47:G>C
2247444|F|0-25:A>G-25:A>G	TGCAGGGAACCCATCCTCGAGTCCAAGATCGCCAGCTGGGAGACGACCCAGTTGGGGTTGTTCCTCCCC	25:A>G
5581279|F|0-6:A>C-6:A>C	TGCAGAAGCGGCGGCGAGGAATGGCTTCGTCGGAGAAGCGCCG	6:A>C
1147720|F|0-40:C>T-40:C>T	TGCAGGGATGCGACGCGTCGGTGCTGATCACGAGCGCGGGCAACGCCGCGGAGGTGAACAACAACAAGC	40:C>T
1730522|F|0-31:A>T-31:A>T	TGCAGCTTCTCGTATTACGCCATGCCAGGCTACATCTTCCAGAGTCTGACGTCCATCGCATGGGTGTGC	31:A>T
1143827|F|0-26:G>A-26:G>A	TGCAGAGGGAACTCGAGGGGCACGCCGAAGGCGGCCATGAGGAGGATGAGGCCGCGGGCGTCCCTGGCG	26:G>A
1694835|F|0-10:G>T-10:G>T	TGCAGTCGGGGCTCCCCCTCGACGACCGCCCTGAGGGCGCCCGCTCGCCGTCCCCTGAGCCAATATACG	10:G>T
993114|F|0-21:G>A-21:G>A	TGCAGATGGCGGTGGAGAACGGGTGGGGCGGCCGCGACTCCCGCGCCAAGGCCGACCG	21:G>A
1044757|F|0-31:C>G-31:C>G	TGCAGTGGAACAAGGTCTGCAACATCTTCACCCGCTTCTGCCAGCAGGTCGCCG	31:C>G
2246664|F|0-37:C>T-37:C>T	TGCAGAGCTCCATGACGAGGTAGAACTTGTCGGCGTCCTCGAAGACGGCCTTGAGCGTGACGACGCCGA	37:C>T
1229816|F|0-13:G>C-13:G>C	TGCAGACGATGCGGCACCACCAGCTGGAGGCGGCGGAGGCGCTGCGGTCCTTCTCCGACGCCAACGCGG	13:G>C
1011068|F|0-18:G>A-18:G>A	TGCAGCTGATGCGGACGCGGACCGACAGGGAGCTGGGGCGCCTCCTCGCGTCGGCGTCGGCGCTCGCGG	18:G>A
3935771|F|0-10:A>G-10:A>G	TGCAGTCGATAAACCCGCACCG	10:A>G
1165688|F|0-16:C>G-16:C>G	TGCAGTGCTTCTTCGTCGCCACCAAGTGCGTCTGGCTGCTCCACCTGCTCGCCTTCTCCTTCGCGCCGC	16:C>G
1025722|F|0-8:G>C-8:G>C	TGCAGGGTGATGAGCAGGCCGCTGGCGAGGTCGGCGGGGAAGCTCGCCG	8:G>C
1074522|F|0-30:C>A-30:C>A	TGCAGATCCAAAAGAAAGCCATGCTGAAAACCCCCATCTTTCCTCCCCCG	30:C>A
4734163|F|0-7:G>C-7:G>C	TGCAGGCGTCCTTCGACGGCGTCCCG	7:G>C
3024290|F|0-25:T>C-25:T>C	TGCAGGTTCTGATCCTCACCGTGAATAGGCTCAACGGCACAATCCCG	25:T>C
1245138|F|0-39:C>A-39:C>A	TGCAGATGCGCCATTATTCTGCGGGGACCAGGGTGTCAGCTCGAGGCGCCG	39:C>A
1095755|F|0-17:G>T-17:G>T	TGCAGCCTGAGGGTGGAGGCGACGACGATGGCGAGGATGGCGAAGCAGGCCGGGATGAGCCCG	17:G>T
1026636|F|0-35:G>C-35:G>C	TGCAGCCGCCTCTCCCACTGGCCCTTGGAGGAGGAGGCGGGCCTCTGGGCCGCCG	35:G>C
3026681|F|0-19:G>A-19:G>A	TGCAGCTGATCCAGATACAGAAGCAGCACAAGCTCATCGCCCCG	19:G>A
6046574|F|0-10:C>G-10:C>G	TGCAGAAGGCCACGTTCATGTTGGTGAGGTAGAGCGTGCCG	10:C>G
1272296|F|0-18:C>T-18:C>T	TGCAGCAGCATGGACATCCTCGCTGCCTGCTGGGCCTGGTAATGGTGGAAAGGCAGGGGGGTGTTGTAG	18:C>T
32025839|F|0-28:G>C-28:G>C	TGCAGCTGCTCGAGCGCGGTCACCACCTGCGCCATGCCG	28:G>C
32024330|F|0-28:G>C-28:G>C	TGCAGAGCTACTGCTTCGCCAAGATGGCGGAGGTGGGGTCCCGCCCCTGCATCATCGACGGGCAGACGG	28:G>C
32025471|F|0-31:A>G-31:A>G	TGCAGATCCCGTCACTTCCTTCCTCTACCGCACGCTCCACGTCAGGCGAGTACGTAGGTCCCTGTCAGG	31:A>G
998634|F|0-18:A>C-18:A>C	TGCAGCAGGAGGCGGCGGAGGAGGAGGCCAGGAGGAAGGCCGAGGAGGAGGAGGCGGCGGCCAAGAAGA	18:A>C
4262855|F|0-9:G>A-9:G>A	TGCAGAGGGGAACATCGGGGAGGGCGTCGCGGCGGAGGGGCCG	9:G>A
1017267|F|0-22:C>G-22:C>G	TGCAGGTTGAACGTGCCCGTGACCACGGCCGTCTCCGCGACCACGCTCCGCATCTCCTCGGTGTCGTTG	22:C>G
3026518|F|0-31:G>T-31:G>T	TGCAGCTCCTCGAGAAGAAGGAGGAGCATCCGTGCTCCATCTCCCCG	31:G>T
5361455|F|0-8:A>C-8:A>C	TGCAGGCGAGGTTCGAGGAGGTGGAGCTGCCGCTGCTGGCGGTTCACGGCGGCGACGACACGGTGTGCG	8:A>C
1069212|F|0-37:C>G-37:C>G	TGCAGCGCGTCGCCTACATCAACATCACCACCTACCCCTTCACCGCCATCTTCCTCATCTTCTACACCA	37:C>G
1063972|F|0-39:C>T-39:C>T	TGCAGGAGAACAGATGCATCTCACCAAGGCAAGAACTGGCCGTGCAAACTCAGGCCAAGAGACACAAGG	39:C>T
5363515|F|0-19:A>G-19:A>G	TGCAGCGGATCTGCGATGGAGTGTACCTGAACCG	19:A>G
1264847|F|0-29:C>A-29:C>A	TGCAGGGTCTTGAGGAAGTTGTTGTCGTCCGACTCCGTGTCGTGTGTGTGCCACACTGAGTTGCTCCCG	29:C>A
1207439|F|0-25:T>C-25:T>C	TGCAGCAGTTTCAGAACCTGGTTCGTTTCCTGCTGCGAAGCAACGGCGCCGCCCCCGAGCTGGCCAAGA	25:T>C
3948073|F|0-8:A>T-8:A>T	TGCAGTCAAAGTACCAGATTCCG	8:A>T
32026948|F|0-64:G>A-64:G>A	TGCAGCTTGTGGTGTCGGAGCTGTGCCGCGTCCAGGAGCTGGTGCGGCAGCTGGAGCTGCACCTGCACG	64:G>A
1062548|F|0-48:G>A-48:G>A	TGCAGATCAGCAAGGTCACCTACCAGTCAGTACCAGGCAATCGCGAGCGAAAGCGGCGAATTCCGAGAT	48:G>A
4262153|F|0-12:C>G-12:C>G	TGCAGATGACCTCCCACGCGCGCTCGAAGCCGCCGCGGTACCCG	12:C>G
1007011|F|0-6:A>G-6:A>G	TGCAGGACTGCGCCGTCCGCCGAGCGCTCCGCTGCGGACAGGAGCTCCACGCGCGGCTGCTCCGCTCCG	6:A>G
1207439|F|0-62:G>C-62:G>C	TGCAGCAGTTTCAGAACCTGGTTCGTTTCCTGCTGCGAAGCAACGGCGCCGCCCCCGAGCTGGCCAAGA	62:G>C
1075589|F|0-53:C>T-53:C>T	TGCAGCACGAAGGTGAAGAAGCCCGAGTCCGTGTGCGCGATCAGGCCCAGCGCCCGCTTCGGGTCCGAG	53:C>T
5372907|F|0-8:C>T-8:C>T	TGCAGCTCCGCCACGGCCTCAGCCG	8:C>T
6048529|F|0-17:C>G-17:C>G	TGCAGACGTACACCGTCCGCTCCGTGCGGCCG	17:C>G
12469796|F|0-11:G>T-11:G>T	TGCAGGATGTAGGTGAAGTTGGCGCCCG	11:G>T

<table>
<tr><td>4996960|F|0-19:C>T-19:C>T</td><td>TGCAGGCGTGGTGGCACGACGTCCGCACCGTCGGCCACGGCGACCG</td><td>19:C>T</td></tr>
<tr><td>1071379|F|0-62:A>C-62:A>C</td><td>TGCAGGAGCTGCTCTGCGCCATGGTCCTCGCCGAGTGCGTCTACAAGGTTCCCTAACCTCCCACCTTCC</td><td>62:A>C</td></tr>
<tr><td>7913266|F|0-32:G>A-32:G>A</td><td>TGCAGATGCAGGTGCAGATGATGTGGATGGGCGGCGGCATGGCGCCGCCG</td><td>32:G>A</td></tr>
<tr><td>32024801|F|0-15:C>T-15:C>T</td><td>TGCAGCAGCTGGAGTCGTCGACGAGGCAGAGCAAGCTGCGGCCG</td><td>15:C>T</td></tr>
<tr><td>4262667|F|0-34:G>A-34:G>A</td><td>TGCAGACGTTCAACGGCACGTACCTGAACACGACGTTCCCG</td><td>34:G>A</td></tr>
<tr><td>5582494|F|0-5:A>G-5:A>G</td><td>TGCAGACAACGACTCCAAACCG</td><td>5:A>G</td></tr>
<tr><td>4262834|F|0-26:G>C-26:G>C</td><td>TGCAGCTCGTCGATCTGCGCCAGCTGGGCCG</td><td>26:G>C</td></tr>
<tr><td>32026448|F|0-46:C>G-46:C>G</td><td>TGCAGTACTTCTACGAGCTGGACCTCAGCAACAACAAGCTCGCGCCCGCTCCGTTCCCGACGGACGTGC</td><td>46:C>G</td></tr>
<tr><td>991326|F|0-26:G>A-26:G>A</td><td>TGCAGAGCCATGCATGCACACCGACAGCATTGCACGATCGGCAGCTACGCGACGCACGCCGTGGCGATG</td><td>26:G>A</td></tr>
<tr><td>1089789|F|0-52:C>G-52:C>G</td><td>TGCAGGGCGCGTGCGTGATGAACACGCTGGACTCCGTGGTGGACGCGCTGGCCAGGGACCCCG</td><td>52:C>G</td></tr>
<tr><td>5357754|F|0-14:C>G-14:C>G</td><td>TGCAGCCTCCTCCACCCGCCGTACTCCG</td><td>14:C>G</td></tr>
<tr><td>7920116|F|0-12:G>A-12:G>A</td><td>TGCAGACACAGCGGCATGCCG</td><td>12:G>A</td></tr>
<tr><td>3956546|F|0-31:C>G-31:C>G</td><td>TGCAGGCCAACATCAAGAAGTCCGTCAACCTCGAGGCCCTCGCCGCCG</td><td>31:C>G</td></tr>
<tr><td>3938821|F|0-18:G>C-18:G>C</td><td>TGCAGCTGGAGGCGGCGGGGCGGAAGCGCCAGGGGCCG</td><td>18:G>C</td></tr>
<tr><td>1278807|F|0-11:C>T-11:C>T</td><td>TGCAGGAGGCGCTGGAGCGGCTGATGAAGGGGCGGACGACGGTGCTGGTGGCGCACCG</td><td>11:C>T</td></tr>
<tr><td>1087976|F|0-10:C>G-10:C>G</td><td>TGCAGGGCGTCTACTGCCCCACCAGCGTCCTCTTCTACGGCCCCTCCGCCG</td><td>10:C>G</td></tr>
<tr><td>2248959|F|0-14:C>G-14:C>G</td><td>TGCAGCGAGGGCAGCGGAGCTGGCCGTTGGGATTATTCGAATGGCCTCCTTCAGCACCACGTCCGAGAT</td><td>14:C>G</td></tr>
<tr><td>4261740|F|0-17:T>G-17:T>G</td><td>TGCAGGTCGGCCCCGCCTCCG</td><td>17:T>G</td></tr>
<tr><td>4398145|F|0-18:A>C-18:A>C</td><td>TGCAGCGCGCCTTCCTGGAGATGACCGCCGCCG</td><td>18:A>C</td></tr>
<tr><td>3935035|F|0-10:C>A-10:C>A</td><td>TGCAGCGCATCGGGATCGGGTTCCTCTCGGCCACCGTGGCCACCG</td><td>10:C>A</td></tr>
<tr><td>1087976|F|0-34:C>T-34:C>T</td><td>TGCAGGGCGTCTACTGCCCCACCAGCGTCCTCTTCTACGGCCCCTCCGCCG</td><td>34:C>T</td></tr>
<tr><td>1228561|F|0-28:C>T-28:C>T</td><td>TGCAGGCGGCAACGGGGCTTTGCAGGAGCTTCCACGGGTGACGGCGGCGACTACGCCCGCTCTAGGCCC</td><td>28:C>T</td></tr>
<tr><td>1021890|F|0-37:T>G-37:T>G</td><td>TGCAGAGGTGGGGCGGCATCAAGAACATCACCGTGGATGAGGCCAAGGCCAGGATCGCCGACATCCTGG</td><td>37:T>G</td></tr>
<tr><td>3026681|F|0-38:G>A-38:G>A</td><td>TGCAGCTGATCCAGATACAGAAGCAGCACAAGCTCATCGCCCCG</td><td>38:G>A</td></tr>
<tr><td>1750586|F|0-17:C>A-17:C>A</td><td>TGCAGAAGATACACCCGCACGGGACACGTAGTCGACTATCCTGATAGCATCAGAAACGCGCAGAGCCGC</td><td>17:C>A</td></tr>
<tr><td>2361445|F|0-11:G>C-11:G>C</td><td>TGCAGGCTGGCGTCGAGCAGCGCGGCCTGCTTGCCGACGTCGGCGTCGGCCACGAAGCCGCCGAACCGA</td><td>11:G>C</td></tr>
<tr><td>1088814|F|0-58:G>A-58:G>A</td><td>TGCAGAAATGGCGCAAGAGAGTTGGAAAGAGGCAGAGGATACGGGTGTCCACGCGCCCGAGGCTCCGAT</td><td>58:G>A</td></tr>
<tr><td>1000567|F|0-7:T>C-7:T>C</td><td>TGCAGCATATGGAAGTGGACCGTGCCACTATCCACACACCAGCGACGGCTGGCCG</td><td>7:T>C</td></tr>
<tr><td>1007221|F|0-28:G>C-28:G>C</td><td>TGCAGCGCGTGGAGGCGCTGGTGGACGTGGCCAACGTGGCGTCGCAGCGGGTGCTGGAGAAGGCCGAGA</td><td>28:G>C</td></tr>
<tr><td>5324990|F|0-13:C>T-13:C>T</td><td>TGCAGCTGCCCGTCCACAAGCTTGAACCTGCCG</td><td>13:C>T</td></tr>
<tr><td>1863313|F|0-54:T>A-54:T>A</td><td>TGCAGGACACGAGCATCCAGGGCGCCGAGAGCCACCCGCTGCACCTGCACGGGTTCGACTTCTTTGTCG</td><td>54:T>A</td></tr>
<tr><td>3574957|F|0-8:G>A-8:G>A</td><td>TGCAGGACGTGCATGGCCTGCGCCATGGATGGCCGCTCGCTCCG</td><td>8:G>A</td></tr>
<tr><td>4910745|F|0-6:C>G-6:C>G</td><td>TGCAGTCGAGCAGAGGAGGAACCCG</td><td>6:C>G</td></tr>
<tr><td>2246664|F|0-46:G>A-46:G>A</td><td>TGCAGAGCTCCATGACGAGGTAGAACTTGTCGGCGTCCTCGAAGACGGCCTTGAGCGTGACGACGCCGA</td><td>46:G>A</td></tr>
<tr><td>4733890|F|0-5:G>A-5:G>A</td><td>TGCAGGGCGCCTCTGCTCCG</td><td>5:G>A</td></tr>
<tr><td>3948676|F|0-13:A>G-13:A>G</td><td>TGCAGCGGGATCAAGCTCAAGAACATCAGGCTGAGGTACCG</td><td>13:A>G</td></tr>
<tr><td>4262274|F|0-53:C>G-53:C>G</td><td>TGCAGAGAGCCGCGCAGATCGGCTCCGTCACGTCCTCCAACACCTCGATGCCGCTCGCGGGGGCCTTCG</td><td>53:C>G</td></tr>
<tr><td>4393753|F|0-22:C>G-22:C>G</td><td>TGCAGGAGACCATCGCGTCCGTCTGCAAGTCCATCTTCAGCCGCCCCG</td><td>22:C>G</td></tr>
<tr><td>3025685|F|0-22:T>C-22:T>C</td><td>TGCAGGAGGATCGACACCACCGTGTACGTCGTCTGCCTCGCCG</td><td>22:T>C</td></tr>
<tr><td>996579|F|0-59:G>C-59:G>C</td><td>TGCAGCTGCTCGTCGCTCACCACCCACGCGCCCTGCGTCCGCTGGATCTCGTCCATGGCGGCGTTGAAC</td><td>59:G>C</td></tr>
<tr><td>2243334|F|0-49:C>A-49:C>A</td><td>TGCAGCTGGTGGCACAGCGTGTTGGTCTCCAGGAAGAACTCGAGGAACACCTTGCTCTCGGAGTCGCCC</td><td>49:C>A</td></tr>
<tr><td>4663946|F|0-6:C>T-6:C>T</td><td>TGCAGCCTCCCACAATGCATCTACATCCACCCG</td><td>6:C>T</td></tr>
<tr><td>3946544|F|0-19:C>T-19:C>T</td><td>TGCAGCAACCCGAACCCATCGGACCG</td><td>19:C>T</td></tr>
<tr><td>3953025|F|0-19:G>A-19:G>A</td><td>TGCAGCAGAGCCGCGGCAAGGAGCAGGCCGCCG</td><td>19:G>A</td></tr>
<tr><td>986801|F|0-18:A>C-18:A>C</td><td>TGCAGCAGGATGCTGGGAATGAAATACGAGTCGCAGAGGGGATACATTGGACTGGAGTATTATGGACGG</td><td>18:A>C</td></tr>
<tr><td>1025047|F|0-29:G>A-29:G>A</td><td>TGCAGCGGGGTGAGGACCCTGAACGGCAAGGCGTCCACCTCGGCCGACCG</td><td>29:G>A</td></tr>
<tr><td>3029043|F|0-38:C>A-38:C>A</td><td>TGCAGGTAGTCGCCGACGTTCACCACCAGCGCCCCCGCCTCCGCCG</td><td>38:C>A</td></tr>
<tr><td>12010231|F|0-7:C>G-7:C>G</td><td>TGCAGCTCGGTGGTCTGCCG</td><td>7:C>G</td></tr>
<tr><td>4261450|F|0-5:C>G-5:C>G</td><td>TGCAGCCTCGGGAGGTCGCAGAGCGCCATCGGGAGCTTGCCG</td><td>5:C>G</td></tr>
<tr><td>4262544|F|0-5:G>A-5:G>A</td><td>TGCAGGCGCCGTCGCGCGCGGCCCAGCCGTCGAGCGCGTCCCG</td><td>5:G>A</td></tr>
<tr><td>5580606|F|0-9:C>G-9:C>G</td><td>TGCAGTTCACCCTGACCTGCCCGCTGGATCCG</td><td>9:C>G</td></tr>
<tr><td>5358639|F|0-22:C>G-22:C>G</td><td>TGCAGTGTGCCTCGACGGGAGCCCGCCG</td><td>22:C>G</td></tr>
<tr><td>7924900|F|0-18:G>C-18:G>C</td><td>TGCAGCAGAAGCAGCGCAGCCG</td><td>18:G>C</td></tr>
<tr><td>5332344|F|0-16:T>C-16:T>C</td><td>TGCAGGCGGACGGCGATGCTGTCCCCG</td><td>16:T>C</td></tr>
<tr><td>1023496|F|0-55:C>G-55:C>G</td><td>TGCAGGCGGAGGTGTTCTTCCTTGGGCAACTGAGGCACAGGAACCTGGTGAAGCTCGTCGGGTACTGCT</td><td>55:C>G</td></tr>
<tr><td>4261513|F|0-5:G>C-5:G>C</td><td>TGCAGGCCGTGCAGGGTGAACCCGTTCGCCGACGACTCCTTCTCCG</td><td>5:G>C</td></tr>
<tr><td>1077594|F|0-28:C>G-28:C>G</td><td>TGCAGACGGACCTGGACTACGCGTGCTCCCAGGTGGGCGTGGACTGCGGCGCCATCCAGCCCG</td><td>28:C>G</td></tr>
<tr><td>7153661|F|0-8:G>T-8:G>T</td><td>TGCAGATCGTGTCCAAGAAGGCGCCGCCGCCG</td><td>8:G>T</td></tr>
<tr><td>5363584|F|0-17:G>A-17:G>A</td><td>TGCAGCTACCTCGTGCTGCTGTCTTACCG</td><td>17:G>A</td></tr>
</table>

3945756|F|0-16:C>T-16:C>T	TGCAGGACAAGCTTGGCGGCGAGCCG	16:C>T
3947468|F|0-13:A>T-13:A>T	TGCAGGTACGCCGACGGCGTCGTCTTCGATCCCAAGCCG	13:A>T
1863313|F|0-46:G>C-46:G>C	TGCAGGACACGAGCATCCAGGGCGCCGAGAGCCACCCGCTGCACCTGCACGGGTTCGACTTCTTTGTCG	46:G>C
977314|F|0-38:C>G-38:C>G	TGCAGGAGGAGGACGAACTTGAACATGGTGTCGCCCCTCGGGATGAACCCCAGCCTGTCCGCCAGGGCG	38:C>G
1028037|F|0-15:G>A-15:G>A	TGCAGGGCAGCGTCAGGCTCCAGCTCATCTGCCTCACCGCCGTCCTCCTCGGCGCCG	15:G>A
981028|F|0-40:G>C-40:G>C	TGCAGATCATGCCGCAGGCCATCTGCACGCGCTACGGGCTGAGCATGGGGGCCAAGGCGGCGCCCATCG	40:G>C
3953851|F|0-17:C>T-17:C>T	TGCAGCCCCGCCGTTCTCGGGCTCACCG	17:C>T
3028898|F|0-26:C>G-26:C>G	TGCAGCCCCTACCGCGCCGACCTCTCCGACCGCCCCTGCGCCGCCG	26:C>G
1023936|F|0-15:C>T-15:C>T	TGCAGTGCCAGCGCGCCCTGGACCTTTTGGTCCTGCTCTTCCTTGGCGCGCCG	15:C>T
3951435|F|0-24:C>T-24:C>T	TGCAGGCACACCCATCAAGCTCCTCGGCCGCCCG	24:C>T
5358034|F|0-6:T>A-6:T>A	TGCAGCTGCGGCAGCGGCGCCGCGGCCG	6:T>A
1025722|F|0-44:C>G-44:C>G	TGCAGGGTGATGAGCAGGCCGCTGGCGAGGTCGGCGGGGAAGCTCGCCG	44:C>G
1688504|F|0-31:T>C-31:T>C	TGCAGCCCAACATGAGGGAGATTGAGTCCGCTCAGGACCTCGTCGACTCGCTGGCCAACGCCG	31:T>C
3030892|F|0-9:G>T-9:G>T	TGCAGCTCGGTGTCGACCAGCGACTCGTCGTACGACCCCG	9:G>T
986801|F|0-14:G>T-14:G>T	TGCAGCAGGATGCTGGGAATGAAATACGAGTCGCAGAGGGGATACATTGGACTGGAGTATTATGGACGG	14:G>T
1750586|F|0-12:A>G-12:A>G	TGCAGAAGATACACCCGCACGGGACACGTAGTCGACTATCCTGATAGCATCAGAAACGCGCAGAGCCGC	12:A>G
3957434|F|0-5:G>A-5:G>A	TGCAGGGCATCCACAGAGCCG	5:G>A
5324392|F|0-5:G>A-5:G>A	TGCAGGTCCATCACGACAAGAAGCTGACGGCGCTCCG	5:G>A
2243102|F|0-8:G>C-8:G>C	TGCAGCCTGTTCTGCCCCACGCCGTCCCTGGTCGACATGATCGTGAACAAGTACAAGCTGCGGAGCGAC	8:G>C
1286530|F|0-30:A>T-30:A>T	TGCAGCTCCCGCCACGACCTGGCGTCGATGAACTGCTTGACGCGCACCAGGTTCCTCGCGTGCGTCCTC	30:A>T
1862625|F|0-41:C>T-41:C>T	TGCAGAGTGAAGTTGACATGGTCCTGCACGCCCTCGAGGTGCTTCTGGGCGGCGACGTTCACCTCCTCC	41:C>T
2280796|F|0-30:C>T-30:C>T	TGCAGCACGGACCCGTCGACGTCGCCGTCACCGCCCTCCCCTCGCCGCCGCCAGAGCCG	30:C>T
4537861|F|0-15:T>C-15:T>C	TGCAGGAGCAGCAGGTGAATGTGCCG	15:T>C
5578237|F|0-11:G>A-11:G>A	TGCAGGATGGGGTCTCGCTCTGCGCCGCCG	11:G>A
1114839|F|0-43:G>A-43:G>A	TGCAGAGCGCGTGCCAGACGGCGGCCGTGCGGGCGCTGTGGGCGGGCGCGGTGCGGGACCCG	43:G>A
5411705|F|0-43:C>G-43:C>G	TGCAGGCGGGCTCGGCTGAGCTGGAGAGAGCCGCGAAGGGATCCTGCTGCTGCGGCTGGAGGTGGTGGC	43:C>G
7162821|F|0-25:G>A-25:G>A	TGCAGTTGGCCCTGATCTTGCCGCTGCTGCCG	25:G>A
991902|F|0-26:C>T-26:C>T	TGCAGACGACGAAGCCGCACCTCCTCCGCGAGCACCAGGTGCATCCGCAGCCG	26:C>T
1021567|F|0-19:C>A-19:C>A	TGCAGACCTTCTGCACCGTCTACGACTGCAAGCGCTGGTTCGGCGGCATACTGGAACTGCCGCAGATAA	19:C>A
32027567|F|0-56:A>G-56:A>G	TGCAGGTACCGCTACGCGTACGGCGACGGCGCCTACTCGGCTGGCGGCCTGGGAACAGAGACGTTCACG	56:A>G
3574957|F|0-13:T>C-13:T>C	TGCAGGACGTGCATGGCCTGCGCCATGGATGGCCGCTCGCTCCG	13:T>C
6041260|F|0-22:G>A-22:G>A	TGCAGATTATTGGCTCCATATTGGGGGTGTCTGCCG	22:G>A
1695880|F|0-17:G>C-17:G>C	TGCAGCTCGATGGCGGTGGCGGTCAGCGGGAGCGCCGAGTAGTTGGAGTTCACCATGGTCCCCG	17:G>C
3941566|F|0-11:A>T-11:A>T	TGCAGTCTTCCATCTCCAGGTCGTCTTCCTCCCG	11:A>T
6043664|F|0-13:G>T-13:G>T	TGCAGAGGAAGGGGCCGCTAAACCG	13:G>T
3024772|F|0-20:A>G-20:A>G	TGCAGGACTATGACGCCGAGACGTGGGCATTTCGATACCG	20:A>G
4910583|F|0-5:C>G-5:C>G	TGCAGCCGCACGACGTGGAGGGTCTCCG	5:C>G
1028020|F|0-10:T>C-10:T>C	TGCAGGTCGATGGACTGAGGCACGGAAGTTTCGTCCATCTTGGCAGCAAGAGACAGGCACGCCACCGAT	10:T>C
995885|F|0-33:G>A-33:G>A	TGCAGCGCGACGGCTACGCAGACGATCTTCAAGGCAATGCCAAGATAGCGCGGTACCTCGTGGACTCCA	33:G>A
5580517|F|0-10:C>T-10:C>T	TGCAGGTGACCTGGGATGAACCG	10:C>T
991014|F|0-8:C>G-8:C>G	TGCAGGCCCCCCTGCGCGACGCGGAAGTTGGCGTCCCGCGTCAGATCCACGCAGGTGTCCACCAGCGAG	8:C>G
1390823|F|0-15:G>C-15:G>C	TGCAGGAGTACAAGAGCCACGCCGACAGCTTCATCTGCTCCATGGTGCCCG	15:G>C
6045120|F|0-30:A>G-30:A>G	TGCAGGTTGGGCGCCTCGGGGGTGGAGGCCACGCGGAAGAGGAACGGCAGCAGCTCGGCCCAAGTGTTC	30:A>G
2243741|F|0-8:C>G-8:C>G	TGCAGCATCCGCTCCACGGCGTCGATGCCGTACACCTCCAGGTAGCTGAGGATGCAGTAGAACCCGAGA	8:C>G
1088506|F|0-30:T>G-30:T>G	TGCAGCGGGAACGAGGGCATCCCCACTGTGTGGCACCAGTCGCCGCCGTAGGCAAAAGGCCACCCGTGC	30:T>G
3024772|F|0-10:T>C-10:T>C	TGCAGGACTATGACGCCGAGACGTGGGCATTTCGATACCG	10:T>C
5332362|F|0-10:G>A-10:G>A	TGCAGGCGGGGGACGCGCTGCCGCTCCCGATCCG	10:G>A
1054075|F|0-16:C>T-16:C>T	TGCAGCGGGGCAACGCCGTGGTGGTCATGTCGGGGTTCGCCATGGACTCCGTGATGAGGGCCGTGCAGC	16:C>T
2269540|F|0-13:G>A-13:G>A	TGCAGGCGGCGAGGGAGTCCCCCACGCTGGCGCTGCTGGACTGGTTGGACGGCTTCGAGGATGCCGCCG	13:G>A
5411644|F|0-33:G>C-33:G>C	TGCAGGTCTGCTGGTGAGGCTGGACAGCGGCAGGGGTCGCCTCAAGGAGACGGTAGAGGCGCTGAGGGT	33:G>C
3960705|F|0-17:G>A-17:G>A	TGCAGGCGCTTCGGGGCGCCCACGGTGCTGCCG	17:G>A
1064161|F|0-30:A>G-30:A>G	TGCAGCTGCTTCAACCCCACGCCGTCCTTCACCGACATGATCGTAAACAAGTACAAGCTGCGAGGCGAC	30:A>G
3023670|F|0-22:C>T-22:C>T	TGCAGCCCTCACAGGCCTGGTCCCTCTTCAAGCTCCG	22:C>T
6048346|F|0-8:G>T-8:G>T	TGCAGCAGGACCTTGAGAATCCTATCCGCCAGCCTCAATTCCTCACGGGGCCTCCGCCTGGAACCGAGA	8:G>T
3939822|F|0-8:G>C-8:G>C	TGCAGGAAGACGGTGGCCCACATGGTGATGTGGCCG	8:G>C
1100936|F|0-26:A>G-26:A>G	TGCAGGAAATTACGGATTAAGAGGTCAATTAAAGTGCCCGCCATTAGCAACAAAATATCTGATATACCT	26:A>G
1001684|F|0-5:C>G-5:C>G	TGCAGCACCGAGAGCTGCACGATGATGTCGCGCGCCCACTTGCCGTACATGGCGTCCAGCAGCGGCACG	5:C>G
1216236|F|0-23:C>T-23:C>T	TGCAGGCAGCTGTGGAGGAGCGCCGCCAAAGGCCGCAACAGCGAAGCGGTGCAGCTCGTCGGCACGTGC	23:C>T
7940658|F|0-16:C>G-16:C>G	TGCAGGCGCCCGTGCTCAAGAAGTACCCCGCGAAGCTGTCCGTCACCTCCTACACCTGCTTCTTCGGCG	16:C>G

1085226|F|0-49:C>A-49:C>A	TGCAGGCGCCCGTGCTCAAGAAGTACCCCGCGAGGCTGTCCGTCACCTCCTACACCTGCTTCTTCGGCG	49:C>A
1221328|F|0-10:C>A-10:C>A	TGCAGGCGTACACCGCCCTGGCCCCCTCCCTGTGCAGCAGCTCGGCGCCCTTGTGCATCGTCCCGAGAT	10:C>A
1090394|F|0-37:C>A-37:C>A	TGCAGGAGAGCATCTCGACGGTGCAGTCGTCGCGGGTCGGGGTGACGCGGAGGTCCAGGACCG	37:C>A
4993251|F|0-15:T>A-15:T>A	TGCAGGGCGCGGAGGTTCCGCTGGTAACAAGTTCCG	15:T>A
992506|F|0-40:T>C-40:T>C	TGCAGACGTTCGCCGCCGCGGACAACGAGCTGATCGGCGGTGTGCCTGATGAGCTCGGCGATTGCCGAG	40:T>C
5332362|F|0-7:G>T-7:G>T	TGCAGGCGGGGGACGCGCTGCCGCTCCCGATCCG	7:G>T
3939901|F|0-16:G>A-16:G>A	TGCAGCCGAGCTCCCAGACGTACTGCCG	16:G>A
5372257|F|0-8:C>T-8:C>T	TGCAGGCTCTCCATCTGCGGCAAGGCGGCGGCCG	8:C>T
7918807|F|0-22:G>C-22:G>C	TGCAGGGGTACGACGCGGTGGCGCTGTCCG	22:G>C
32025862|F|0-28:A>C-28:A>C	TGCAGGGACAGGAGGAGGAAGGCGATGAACCACCG	28:A>C
32025791|F|0-10:T>C-10:T>C	TGCAGACCTTTATCAGCGTCACCATCGTCGCCTTCATCCCG	10:T>C
32027257|F|0-36:T>C-36:T>C	TGCAGACCAAGATTTCACAGAGCTCGTGCAGAAAGTTCCCGATGGCTGCCTGTTCACCATAGTCTCCGA	36:T>C
32022093|F|0-27:C>G-27:C>G	TGCAGTATCCAGTGGCAGTGCCTGTCGCGCAACCTGCCGCGGGCGGCGACCTGCCACGACGACGAGAGC	27:C>G
7917766|F|0-55:C>T-55:C>T	TGCAGGTGCAGATGCAGCGGAGCACGGCTGGGAAAGGCGCTGAAAAGATGAGCGTCGATGATGGCTAGC	55:C>T
1027722|F|0-53:G>T-53:G>T	TGCAGCGCGTACTGGACGCCGTCCACGTCGCCTGCCGCCGCGAGCCTCCTGACGGTGGCCTCATGGGCC	53:G>T
32025367|F|0-25:G>C-25:G>C	TGCAGGAGATGGCCTTCTCCTGCGGGGACGACGTCAGAGGAGGCTATCCG	25:G>C
32027329|F|0-12:C>T-12:C>T	TGCAGCAGCACGCATGAGCAAGCCG	12:C>T
13880485|F|0-14:G>A-14:G>A	TGCAGAAGAAGGCCGTCGCCG	14:G>A
4262323|F|0-7:C>T-7:C>T	TGCAGTGCGCGGCCGCTCATTCCGCCGACCGCCGCCGTGCCG	7:C>T
977990|F|0-28:C>G-28:C>G	TGCAGCTCACGCCCTCCAACTCCAACCTCACCCTCCTCTACAACTGCAAGGCCG	28:C>G
1050193|F|0-42:A>G-42:A>G	TGCAGAAGGTCACCAACCCCAAGACGATCCAGAAGATCGCCAAGTCCAAGAACCGAGGTCAGCTCAGGG	42:A>G
1041707|F|0-41:G>C-41:G>C	TGCAGGGCATCTGCTTCGCCTTGGGGCTCCTCGTCTCGCAGGTCCTCAACGAGCTCATCAAGCACTCCG	41:G>C
1229816|F|0-11:C>T-11:C>T	TGCAGACGATGCGGCACCACCAGCTGGAGGCGGCGGAGGCGCTGCGGTCCTTCTCCGATGCCAACGCGG	11:C>T
998634|F|0-8:G>C-8:G>C	TGCAGCAGGAGGCGGCGGAGGAGGAGGCCAGGAGGAAGGCCGAGGAGGAGGAGGCGGCGGCCAAGAAGA	8:G>C
1667127|F|0-7:C>T-7:C>T	TGCAGGGCGGGATCCCGTTCGAGAAGTCCAAGGGCCAGCACATCCTGCGGAACCCG	7:C>T
3941815|F|0-16:C>T-16:C>T	TGCAGAGGAACAGCGCCCACCG	16:C>T
3956241|F|0-11:C>T-11:C>T	TGCAGGCGCCGCCCCCGCCGCCG	11:C>T
987565|F|0-61:G>C-61:G>C	TGCAGCGCTTCTCCCGCCACGTGATGCACGAGCTCGTCCGCCTCGTCTTCGCCCGCCTCCCGCTGATAG	61:G>C
1040382|F|0-50:G>A-50:G>A	TGCAGGGTGTTGTGGTAGTGCTGAACACCCCTGGACCATGGAGATGGAGAGGACATTGCCAATTGGAAC	50:G>A
1025764|F|0-28:G>C-28:G>C	TGCAGGACAAGTACAAGCAGGCGAGGCTGGCCCTCGGCGACGTAACTCCG	28:G>C
7487642|F|0-19:C>T-19:C>T	TGCAGTACGCGGGGTACCACGAGCGGCTGCTGCGCCTGGTGCGCGTCGGCGCCGCCATCGCCTACGACA	19:C>T
1002654|F|0-37:C>T-37:C>T	TGCAGCCTCGGTGCCATGGAGACGGAGCCTCCTCCTGCTCCCGACCCTTGCGCTCCTGTGGCAGCTCTG	37:C>T
6047257|F|0-37:G>A-37:G>A	TGCAGGACTGGCAGCGGCGGTACGCCGACGTGCTGCGGCGGCAGGGGATAAAGGTCCAGGTGGCGGAGT	37:G>A
990929|F|0-22:C>G-22:C>G	TGCAGGTGCTGGACCTGTTCACCGCGAGCATCGTCTCCGTCGCGGACGACTACGTCGCGCCG	22:C>G
1001639|F|0-49:G>T-49:G>T	TGCAGCAGCGGGCCGAGGACCAAGGGCGGGCACACGGTGACCACGTCCAGGCCG	49:G>T
1028698|F|0-65:C>G-65:C>G	TGCAGGCCCTACCTCGCCAGGTCCCTCTTCCTCCTCGGCTCCATCGGCGGCAACGACTACAACGCCATG	65:C>G
7172920|F|0-22:C>G-22:C>G	TGCAGCTCGTTCTTGGGAGGTTCCCG	22:C>G
1021154|F|0-20:C>G-20:C>G	TGCAGCTCGGCATTGGGCGGCAGGATGACGGGCTTCTGCACGACGATGCCGCCGCCGCCGTCGGCGGGG	20:C>G
5582462|F|0-10:C>A-10:C>A	TGCAGGGCGACAACGTCGGCGACCGCGTCTTCAAGATACCAAACCCCATGTACTTCGTCCCGTGAAAAC	10:C>A
4262684|F|0-26:T>C-26:T>C	TGCAGTTGGTCCTGATGGTGCCTTGGTTGCCG	26:T>C
1060660|F|0-20:G>C-20:G>C	TGCAGCGACCTCTTGAGCTCGGTGAAAAGGTCACCCCAGCCATCGGTGCCCGTGTCGCTGACCACGGTC	20:G>C
7168053|F|0-9:A>C-9:A>C	TGCAGGAGGAGTTCGGCGGGGCGGAGGGCGCCG	9:A>C
1096095|F|0-8:G>C-8:G>C	TGCAGGGCGAGGACTCGCAGATTGCAGAACCCCCCTGCAATGACGTCCAGATCGTCGTCGATAACACCG	8:G>C
4394186|F|0-16:C>G-16:C>G	TGCAGCTCAGCAATGCCGCGGAGGCGCAGGCCGAGTGCCTGCTCCG	16:C>G
2251238|F|0-26:C>G-26:C>G	TGCAGGATCTCCACCTGCGGGTACTTCTCCCTGAAGCCTTCGATGAGCTCCTTGCTGAGCGGCGCGCCG	26:C>G
4393753|F|0-16:G>C-16:G>C	TGCAGGAGACCATCGCGTCCGTCTGCAAGTCCATCTTCAGCCGCCCCG	16:G>C
4262909|F|0-8:G>A-8:G>A	TGCAGGATGGGCGCGACGGCCG	8:G>A
988449|F|0-33:T>G-33:T>G	TGCAGCTGGCCGTGGACGCCGCCGCCGCCAAGTTCCTCGCCGCCGAGAAGCTGGAGGTGGTGGACTACA	33:T>G
6045813|F|0-10:T>C-10:T>C	TGCAGTACTTTCTCACCACGGGCCCCGTCGCCG	10:T>C
998634|F|0-16:G>A-16:G>A	TGCAGCAGGAGGCGGCGGAGGAGGAGGCCAGGAGGAAGGCCGAGGAGGAGGAGGCGGCGGCCAAGAAGA	16:G>A
1026363|F|0-53:C>G-53:C>G	TGCAGCATCTCCTGCACCTGCGCCTCCTGCTTCCCGCGCAGCCGCGGGTCCAGCAGCTCCGCCACGCCC	53:C>G
3947654|F|0-24:G>C-24:G>C	TGCAGCGGCGGAGCCCAAACCGCCGTACACGCCG	24:G>C
983897|F|0-21:C>T-21:C>T	TGCAGGTAAACACGCCTCGTGCGGGCAGGGCCTCGCGTAGAACTTCGCGATGTTATCCCCG	21:C>T
1037142|F|0-32:C>T-32:C>T	TGCAGCCTCTTCGCGCCCATCCCGTCCTTCACCGACATGATCATCCGCAAGTACAAGATGCGCAGCGAC	32:C>T
1750586|F|0-38:T>C-38:T>C	TGCAGAAGATACACCCGCACGGGACACGTAGTCGACTATCCTGATAGCATCAGAAACGCGCAGAGCCGC	38:T>C
1026222|F|0-13:C>A-13:C>A	TGCAGGTGCTGGCCATCCTGCCGTCGTACGCGGGCAAGACGGTGCTGGAGCTCGGCGCCG	13:C>A
1694835|F|0-61:A>G-61:A>G	TGCAGTCGGGGCTCCCCCTCGACGACCGCCCTGAGGGCGCCCGCTCGCCGTCCCCTGAGCCAATATACG	61:A>G
7352307|F|0-17:C>T-17:C>T	TGCAGGCGGGCGAGCGACCCG	17:C>T
3029043|F|0-7:A>G-7:A>G	TGCAGGTAGTCGCCGACGTTCACCACCAGCGCCCCCGCCTCCGCCG	7:A>G

<table>
<tr><td>1025502|F|0-24:A>G-24:A>G</td><td>TGCAGGACCTCCCGTGGCGTGCGGATGCTGGCGGACCAGTTCACCGCCGAGCCGCCGCCGACGGTGGTG</td><td>24:A>G</td></tr>
<tr><td>2247873|F|0-44:A>G-44:A>G</td><td>TGCAGCACGATTGCAGCAGCGGTGGTAGCGTTGTCGACGAGGGAAATCTCGGAGACGTCCCCAGCGTTG</td><td>44:A>G</td></tr>
<tr><td>1040206|F|0-25:G>C-25:G>C</td><td>TGCAGCCGATGACGTAGACGACGAGGAGCGCGACGGTGGCGGCGACGAGCGCCACGTTGGCCCG</td><td>25:G>C</td></tr>
<tr><td>4537861|F|0-13:G>A-13:G>A</td><td>TGCAGGAGCAGCAGGTGAATGTGCCG</td><td>13:G>A</td></tr>
<tr><td>5579756|F|0-25:C>G-25:C>G</td><td>TGCAGTGGGTGAGGGAGAACATGGACTTCAACACCACCAGGGTCTTCTTCACCAGCATGTCGCCCACCC</td><td>25:C>G</td></tr>
<tr><td>4996938|F|0-10:G>A-10:G>A</td><td>TGCAGAACCCGCCGACGTACGCCG</td><td>10:G>A</td></tr>
<tr><td>3026721|F|0-8:T>C-8:T>C</td><td>TGCAGTTCTTGATCAGCTGGATGTCGCGGTTCCCGCAGTACCG</td><td>8:T>C</td></tr>
<tr><td>3959462|F|0-26:G>C-26:G>C</td><td>TGCAGCGCCGCCCGCAGCTCGTACATGAGCCG</td><td>26:G>C</td></tr>
<tr><td>12777657|F|0-30:G>C-30:G>C</td><td>TGCAGGAAGCACGGCGTCCCGAGGGAGTTCGCCGCGCGCCCAGCCG</td><td>30:G>C</td></tr>
<tr><td>1025619|F|0-12:C>T-12:C>T</td><td>TGCAGAGGTCCACGGTGGAGGTGGTCGGGCACAAGGCGCACGGAGCCGTGCCTCAGCCG</td><td>12:C>T</td></tr>
<tr><td>5370807|F|0-25:C>A-25:C>A</td><td>TGCAGCCTGCCGCTCGCAGTCGACGCCTTCTTCTCCG</td><td>25:C>A</td></tr>
<tr><td>5367703|F|0-10:C>G-10:C>G</td><td>TGCAGCTCGCCTACGCCGTCGGCGGCGGCTGCCCG</td><td>10:C>G</td></tr>
<tr><td>4544220|F|0-17:T>C-17:T>C</td><td>TGCAGGCTCGAGGGACCTGCGGCGTGGGACGTGCTCGCCAACTTCGAGCACCG</td><td>17:T>C</td></tr>
<tr><td>5366905|F|0-20:G>T-20:G>T</td><td>TGCAGCAGCCTCGTGGTCTGGACTGCGCCG</td><td>20:G>T</td></tr>
<tr><td>32024782|F|0-16:A>C-16:A>C</td><td>TGCAGACAGACCCGCGATCGGCGCCG</td><td>16:A>C</td></tr>
<tr><td>1096712|F|0-20:C>T-20:C>T</td><td>TGCAGGAACTTGGTGATGGGCTGGATCACCGCGTTGGCGAAGAGCCCCG</td><td>20:C>T</td></tr>
<tr><td>7353683|F|0-13:G>C-13:G>C</td><td>TGCAGATGTTCTCGCCGCCG</td><td>13:G>C</td></tr>
<tr><td>3940678|F|0-11:G>C-11:G>C</td><td>TGCAGCCCGCCGACGCGGTCGTCCTGGAAGAGCAGGATGAGGCCGCCG</td><td>11:G>C</td></tr>
<tr><td>985224|F|0-49:C>T-49:C>T</td><td>TGCAGCTGCCCATCTGGTGCGCCGTGCAGCACAGGCTCCAGTTCTCGCTCTTGGACTGCGGCCCGCGCA</td><td>49:C>T</td></tr>
<tr><td>1044289|F|0-66:A>G-66:A>G</td><td>TGCAGCTTCGGGTCGGCCCAGGAGACCCCGCAGCTCAACCTGGCGGGCCACTGCGACCCCTACTCCAAC</td><td>66:A>G</td></tr>
<tr><td>10984282|F|0-11:C>A-11:C>A</td><td>TGCAGGTCCGCCGCGCCGATGAACCCGTCCGCGTCGCCGTCGAACACGGCGAACGCGCGCCGCACCTCT</td><td>11:C>A</td></tr>
<tr><td>1088080|F|0-22:G>T-22:G>T</td><td>TGCAGGCGCTCCAGGCCGAGGCGGCCACCGCCTCCTCCATGCTCTCCTCCG</td><td>22:G>T</td></tr>
<tr><td>1143427|F|0-7:G>T-7:G>T</td><td>TGCAGCGGGCCGCCGAGTCCGCCATCCGCTCCATCGGCCTCGGCTACGACGTCGCCGCCTACGTCCGAG</td><td>7:G>T</td></tr>
<tr><td>5363584|F|0-20:G>A-20:G>A</td><td>TGCAGCTACCTCGTGCTGCTGTCTTACCG</td><td>20:G>A</td></tr>
<tr><td>5411931|F|0-12:G>C-12:G>C</td><td>TGCAGTCGGTGGGCTTCAGCGCGGCGCCG</td><td>12:G>C</td></tr>
<tr><td>7906790|F|0-17:C>T-17:C>T</td><td>TGCAGCGGGGACGGCGGCATGCCG</td><td>17:C>T</td></tr>
<tr><td>7351634|F|0-8:T>C-8:T>C</td><td>TGCAGGTGTGGTGGCGAGTGGCAGGTGTGGCTGCCTTGGTCCGATGCTGCCGCCACCACCACCGTGGCG</td><td>8:T>C</td></tr>
<tr><td>1167323|F|0-61:T>C-61:T>C</td><td>TGCAGCGTCTGTCCATGTCGCCCGAGATTGCCCGCGCAATCGTCGGCCACAGCGGCGTCCGTCCACTCA</td><td>61:T>C</td></tr>
<tr><td>1864692|F|0-31:C>T-31:C>T</td><td>TGCAGGACATGTACAACAGCCTGACGGCGTGCAAGGAGCTCGTCAGAGTCCTCGGCAACATCTGGGGGC</td><td>31:C>T</td></tr>
<tr><td>3948533|F|0-14:A>G-14:A>G</td><td>TGCAGAGGCGAGGCACTGAAACGAACTAGGGCCG</td><td>14:A>G</td></tr>
<tr><td>3951327|F|0-10:G>C-10:G>C</td><td>TGCAGTCGCTGATGAGCTCCTGCGTCCCGCCGCCG</td><td>10:G>C</td></tr>
<tr><td>3020834|F|0-30:A>G-30:A>G</td><td>TGCAGAGCTAGAAGGCCTCCCTGTTGAGCTACTCCACCG</td><td>30:A>G</td></tr>
<tr><td>989664|F|0-19:C>A-19:C>A</td><td>TGCAGTTGGCCAGGCACAGCTGTCTGCACTCGTCCAGCGTCATGTCGGCGTGCACCGTCGCGTTAGTCG</td><td>19:C>A</td></tr>
<tr><td>12470497|F|0-15:C>G-15:C>G</td><td>TGCAGTGGGGGAGGACCTACACGCTCAAGTCCTTCCG</td><td>15:C>G</td></tr>
<tr><td>7170749|F|0-10:C>T-10:C>T</td><td>TGCAGCTGTCCTTCCAGGACTCGTCGGCAACCACGGTGTCGCCG</td><td>10:C>T</td></tr>
<tr><td>6047648|F|0-19:G>A-19:G>A</td><td>TGCAGGCGGCGGCCACGTCGGGGGCGAGCACGGCCG</td><td>19:G>A</td></tr>
<tr><td>2263602|F|0-10:C>G-10:C>G</td><td>TGCAGGCCGCCGACATGTTCGAGCTGGCCCGCCTCGCCGACGAGTACGGCTCCG</td><td>10:C>G</td></tr>
<tr><td>1020713|F|0-28:C>G-28:C>G</td><td>TGCAGGTGAGCTCCGACGGCAGCTTGCTCAGGAACGTGACGGTGTACACCG</td><td>28:C>G</td></tr>
<tr><td>5372336|F|0-8:G>C-8:G>C</td><td>TGCAGGTCGACGACCGCGCCGCCG</td><td>8:G>C</td></tr>
<tr><td>6038028|F|0-7:A>T-7:A>T</td><td>TGCAGGCAGCCCACGAGGCACGTAAGCGCCGTGGCCCAGTACCG</td><td>7:A>T</td></tr>
<tr><td>1012318|F|0-51:A>C-51:A>C</td><td>TGCAGGAAATGTGGGAAATGACAAGATTGTGCAACAGAAGAAAGCTAACCCAGTTGCTCTGTTTCTCTC</td><td>51:A>C</td></tr>
<tr><td>1012318|F|0-12:G>T-12:G>T</td><td>TGCAGGAAATGTGGGAAATGACAAGATTGTGCAACAGAAGAAAGCTAACCCAGTTGCTCTGTTTCTCTC</td><td>12:G>T</td></tr>
<tr><td>1026246|F|0-6:G>T-6:G>T</td><td>TGCAGGGCTGGTCCTGGCTTCGCTCGCCGTGGTGCTGGCAACCACGCTGTGGAAGGCTGCGGTGCACCT</td><td>6:G>T</td></tr>
<tr><td>7168831|F|0-17:T>C-17:T>C</td><td>TGCAGCTGTCCCCGAGCTTGCGCAGGACGTCCATCACCTCCTCGGCGGTGATCCTGCCGTCCCTGTCCC</td><td>17:T>C</td></tr>
<tr><td>32022438|F|0-5:A>C-5:A>C</td><td>TGCAGAGAGTAGATCTCCACGCCG</td><td>5:A>C</td></tr>
<tr><td>982890|F|0-17:A>G-17:A>G</td><td>TGCAGTTTGTTGGGAGGATCAAGTTCTCCATGGAACCCGTCGCCTACACCG</td><td>17:A>G</td></tr>
<tr><td>16795578|F|0-16:C>G-16:C>G</td><td>TGCAGCTGGGGTTCCTCTGCGTGCTCCTGCCGCTCGTCTTCCACGCCGCGCCG</td><td>16:C>G</td></tr>
<tr><td>1042793|F|0-7:A>T-7:A>T</td><td>TGCAGGTAGAGCTAATGCAGCATTGCATTTGCAGCTCGCACAAATTTGATGGTGATCCTGCCCTGGCAT</td><td>7:A>T</td></tr>
<tr><td>4262323|F|0-18:A>G-18:A>G</td><td>TGCAGTGCGCGGCCGCTCATTCCGCCGACCGCCGCCGTGCCG</td><td>18:A>G</td></tr>
<tr><td>1318131|F|0-18:T>C-18:T>C</td><td>TGCAGGAGCGACGCGTTCTTGGGGAACTGGAACGAGTTCCTGTTCATGAACGCCACCAGCGCGAACCCC</td><td>18:T>C</td></tr>
<tr><td>1060998|F|0-29:A>G-29:A>G</td><td>TGCAGCTTCTCGTTGAAGAGCACGAGGGGATCCTTGTCCCTGAGCACGCCGTCGTCGTCGTACGCGTCC</td><td>29:A>G</td></tr>
<tr><td>5360515|F|0-43:G>T-43:G>T</td><td>TGCAGAAGGTCGGGGTGTGGCGGGATAAGGGGAAGAGCAAGGTGACGGAGCTTGAGGAGAGGGTGGAGG</td><td>43:G>T</td></tr>
<tr><td>2254316|F|0-14:A>G-14:A>G</td><td>TGCAGGACGAGAGGAAGAATCTGCCCGTGTACAAGTTCAAGGACGACCTGCTCAAGGCCATCGAAAAGT</td><td>14:A>G</td></tr>
<tr><td>3948376|F|0-7:G>C-7:G>C</td><td>TGCAGCTGGCCGACGTGCTGCACGCCCG</td><td>7:G>C</td></tr>
<tr><td>1026364|F|0-47:C>G-47:C>G</td><td>TGCAGCATCCGCACCACGGTGCTGGGCGGCAGCTGGTGCGACGCCATCGTGCCCCAGTTCACCCCGAGC</td><td>47:C>G</td></tr>
<tr><td>7353268|F|0-7:A>T-7:A>T</td><td>TGCAGATATGCTAACCTCCATGGCCG</td><td>7:A>T</td></tr>
<tr><td>3942413|F|0-10:G>A-10:G>A</td><td>TGCAGGAGGCGGACCACGGCGGCCGTCTCGAGAGGGACGACATATGGAACATGATCCAGTCGCAGAAGC</td><td>10:G>A</td></tr>
<tr><td>1328631|F|0-26:A>G-26:A>G</td><td>TGCAGGCAGAGCTAATGCAGCATTGCATTTGCAGCTCGCACAAATTTGATGGTGATCTTGCCCTAGCAT</td><td>26:A>G</td></tr>
<tr><td>5359918|F|0-5:G>A-5:G>A</td><td>TGCAGGCTGAGGTGGTTAAACTACCTCCG</td><td>5:G>A</td></tr>
</table>

5580152|F|0-25:C>T-25:C>T	TGCAGCACCGCCAGTTGCTCCGCGCCGTCCG	25:C>T
4261945|F|0-29:T>C-29:T>C	TGCAGGTAGGCTAGGTCCTTGCCCAGGCATACCCTCGGCCCG	29:T>C
3957939|F|0-16:C>T-16:C>T	TGCAGAAGCCCCGCGACCTGCGAATCGCACGTCGTCGTGTACCCG	16:C>T
1066609|F|0-15:G>T-15:G>T	TGCAGTGCGATAACAGGTGCAGGTACGCGCTCGTGGTGTCCACGGAGATTCTCACCTCGGATTACTACT	15:G>T
7172017|F|0-13:C>G-13:C>G	TGCAGAACTTGCGCTCCGATGCTTCCTCTGTCCG	13:C>G
1212403|F|0-16:C>T-16:C>T	TGCAGTGTTTGTGGAGCTCCCGCTGATGGATGCGATGGATTCGGTCGCCGTGATGGAGGTGGCGCCGAG	16:C>T
4002390|F|0-9:T>C-9:T>C	TGCAGCGGGTGCACCACTCCGCCGACGCCACCGACCGCGCCG	9:T>C
1213658|F|0-25:G>A-25:G>A	TGCAGCGCATGGGCGTCGGGCTCGTGATCGGCATGGGGGCGATGGTGGTGGCAGGCGTCGTGGAGGTGG	25:G>A
1753649|F|0-44:A>G-44:A>G	TGCAGCTCCACCTGCACCAGCAACTCCTCAAAAATCTCAACAGAACCATCCACCACCCTCTTCACAAGC	44:A>G
1062548|F|0-15:T>C-15:T>C	TGCAGATCAGCAAGGTCACCTACCAGTCAGTACCAGGCAATCGCGAGCGAAAGCGGCGAATTCCGAGAT	15:T>C
3948748|F|0-5:C>T-5:C>T	TGCAGCGCCCAGCCGAACTGCACGCCG	5:C>T
3021180|F|0-18:C>T-18:C>T	TGCAGCTTCGCCTACTACCTGATCCCCAGCTACCTGTTCCCG	18:C>T
1144182|F|0-50:C>T-50:C>T	TGCAGTCTGCACGAAGTGAGTGAGTTGAGTTACTCACATGGTCGAGGCTCCCGTACCCTGCGACGACGT	50:C>T
2246123|F|0-18:A>G-18:A>G	TGCAGCTTGAGCGGGCAGACGGCCTCGATCTTGCACTTGGTGTAGCGCGCCGTGTAGCTCACCTTGCGG	18:A>G
2243741|F|0-38:C>G-38:C>G	TGCAGCATCCGCTCCACGGCGTCGATGCCGTACACCTCCAGGTAGCTGAGGATGCAGTAGAACCCGAGA	38:C>G
7172588|F|0-14:C>G-14:C>G	TGCAGCGTCGACGCCGAGGTCTCGCTCCCG	14:C>G
3956088|F|0-14:G>A-14:G>A	TGCAGCACCGCGCAGTTGCCGTAGTACCCG	14:G>A
992863|F|0-20:G>A-20:G>A	TGCAGGACACCAGCATCCAGGGCGCCGAGAGCCACCCGCTGCACCTGCACGGCTACGACTTCCACGTCG	20:G>A
1214876|F|0-65:G>A-65:G>A	TGCAGAGCTAGGTGGATAGAGTAGCTGGCGGCTAGGCGCTAGTTGCAGGAAGGAAAGGTTGGATGGAGC	65:G>A
1011593|F|0-47:T>A-47:T>A	TGCAGAGGGACGCGATGCCGTCGCACCTGGCGCTGACGGTGCGGGGCTCGCTGTACCCGCAGCCGAGAT	47:T>A
2256332|F|0-56:C>T-56:C>T	TGCAGCCGTTGACGACACATGCGTTCCGTGCCCATCGATGTCCCTCGGACTCGAATCGGTGAGGCCACC	56:C>T
1089789|F|0-59:C>G-59:C>G	TGCAGGGCGCGTGCGTGATGAACACGCTGGACTCCGTGGTGGACGCGCTGGCCAGGGACCCCG	59:C>G
2299974|F|0-38:C>A-38:C>A	TGCAGATGCTCGACCTGCTCCGCCTCACCGTGCCGTCCCTGCTCGCCTTCCTCCGCTCCCTCCCCTCCG	38:C>A
6046104|F|0-7:G>C-7:G>C	TGCAGGCGGACTGCGACCGAGACGTGAAGGAGACGGCGAGGGCCGTGCTGAGCATGCACTCCG	7:G>C
3952290|F|0-12:C>T-12:C>T	TGCAGGGACATGCTCGTCTCCGCGTCCG	12:C>T
1254491|F|0-13:G>A-13:G>A	TGCAGGGCAAGCTGGACCCCCGCCACCCCAGTTTTGTCAAGCCCATGACCCAGTCTCATGTCACCGAGA	13:G>A
3935392|F|0-10:C>T-10:C>T	TGCAGCTGAACTCAACCTCGCGGGGCCG	10:C>T
4398369|F|0-13:T>C-13:T>C	TGCAGGAGCTTCATCGCCGCCG	13:T>C
1159349|F|0-30:C>T-30:C>T	TGCAGCGCGTGCCCACACGGGCCTTGAGGTCGGCGACGAACTCGTCCGGGAAGTGCACGGCCAGGTTCT	30:C>T
7940722|F|0-8:C>T-8:C>T	TGCAGGCGCCGCCGCCGCCGCCG	8:C>T
2362796|F|0-61:C>G-61:C>G	TGCAGCAGTACTGCGCCGTCCCGAACGCCTCGCACGCGCTCTTGCACGCCACGCCGCCCGCCCCCGCCG	61:C>G
3948659|F|0-13:G>A-13:G>A	TGCAGCTGCCAGAGCTCGCGCCG	13:G>A
4261474|F|0-17:C>G-17:C>G	TGCAGCTTCTTCACCTCCTCGATGGTGAAGTCCG	17:C>G
4261664|F|0-33:G>A-33:G>A	TGCAGCAGCCTGAGCTCCACCGTCGAGTCCTTCGGCGGCCCGCGGGCGCGGCCG	33:G>A
7906784|F|0-9:C>A-9:C>A	TGCAGCCTGCGGACCTACCCCG	9:C>A
3946931|F|0-10:C>T-10:C>T	TGCAGCCGCTCCGCCGCCTCCG	10:C>T
986352|F|0-24:C>T-24:C>T	TGCAGCGGCTGCGCCATGATCTCGCTCATCACGCTCTCGTTGTACCCGAGGCCGCACACGTAGGGGATG	24:C>T
3957169|F|0-22:G>A-22:G>A	TGCAGCGCAGGTCGTGCAGCACGGCGAACACCTCCGAGAGGAGCCCCG	22:G>A
1027358|F|0-8:G>A-8:G>A	TGCAGGTTGGGCGCCTCGGGGGTGGAGGCCGCGCGGAAGAGGAACGGCAGCAGCTCGGCCCAGGTGTTC	8:G>A
4734116|F|0-34:C>A-34:C>A	TGCAGTCCAACGCCACCTCCCAGGTCGCGCTCGTCGGCACCGACTTCTGCCCCATCGAGAGCCTCGACT	34:C>A
4983122|F|0-22:G>T-22:G>T	TGCAGCGGCGAGCACGGGCCGTGCCG	22:G>T
2251556|F|0-64:C>T-64:C>T	TGCAGACCTCGCGCAAGAGGTACTTCGTGCTCGAGGACTCCGCGCTCCGCTGCTTCAAGGCCGCCCCGA	64:C>T
1158392|F|0-14:C>A-14:C>A	TGCAGGCTCGAGGGCCCCGCGGCGTGGGACGTGCTCGCCAACTTCGAGCACCG	14:C>A
1207965|F|0-8:C>T-8:C>T	TGCAGAGGCAGCTAAGAAGAAATTTGACATGGAAATCAAGCTGCCCCTGTCCG	8:C>T
1862702|F|0-28:C>T-28:C>T	TGCAGATATTGCCACCGAATGCCTGTCTCTCCCTTTCGAGAAAGGAGGAGCAAATCTCCTTGGATGTCC	28:C>T
32020826|F|0-44:A>C-44:A>C	TGCAGGCCCTGCTGCTGGTGCTGCTGGCTCTGGCGGCGGTGGGGAGGGTAGTAAAAGCGGTCGCCTTTG	44:A>C
7153491|F|0-22:G>C-22:G>C	TGCAGAGCGTGCCCAAGGCGCGGCCCCG	22:G>C
22838405|F|0-11:C>A-11:C>A	TGCAGCAGCAGCGATGCGCCG	11:C>A
32024420|F|0-50:G>C-50:G>C	TGCAGCGAGGACTGCCGCTACGAGCAGATGCACTTCGACGCCGCCATGGCGAGGCAGCAGGCCAGCGCC	50:G>C
6043889|F|0-5:A>C-5:A>C	TGCAGAGCCAGCCCCACGCCCGCCTCCATTGCTCGCCG	5:A>C
1024312|F|0-49:C>G-49:C>G	TGCAGTACGCGGGGTACCACGAGCGGCTGCTGCGCCTGGTGCGCGTCGGCGGCGCCATCGCCTACGACA	49:C>G
32025509|F|0-56:G>C-56:G>C	TGCAGCATCCGCGCGTAGCATCCGTCGGGGTGATGGTTGAGCAGGTGCGAGTGCGAGCCGTGCTCCACC	56:G>C
7172476|F|0-41:T>C-41:T>C	TGCAGCGCCGCGCTCGACGTCGGCGCCGAGCTCGCCGTCCCTACGTACTTCTTCCTCACCACCTGCATC	41:T>C
7907587|F|0-45:C>G-45:C>G	TGCAGGTTGTTTGAGGAGGCGGAGAACGGCGTCGGGCAATGGCGACTGCGGCAGCACGGCGCGCCAGAA	45:C>G
13880983|F|0-15:G>C-15:G>C	TGCAGGTCGTCCATAGTCAGGCCG	15:G>C
5368583|F|0-5:T>G-5:T>G	TGCAGTGAGAATCAAAGCCAAGATAGATGTTAGTGCCATACGTTCATGACAAATTAATCGATCTAGCTA	5:T>G
7173276|F|0-6:A>G-6:A>G	TGCAGCACGCCGAGGCGGTCGGCGATGGGCGCGAAGTATGGCAGCAGGGTGCTCCG	6:A>G
5367455|F|0-56:A>C-56:A>C	TGCAGGTGGCGCTGGACTACGCGTGCGGGCAGGGCGCGGACTGCTCGCCGATCCAGACCG	56:A>C
5369599|F|0-7:G>C-7:G>C	TGCAGGGGCTGCTCAAGGTGGCCGCCG	7:G>C

3959624|F|0-19:C>G-19:C>G	TGCAGATGGGCCAGGCCCCCTTGTCGCTGTCGCTCCACGGGCCGCCG	19:C>G
1112225|F|0-12:G>A-12:G>A	TGCAGGAGTTCCGTCTTGTCGTGCCTGTAGAGGAGGAAGTTGATGAGGGCGCTGAGGACCTCGACGACC	12:G>A
1026364|F|0-8:C>G-8:C>G	TGCAGCATCCGCACCACGGTGCTGGGCGGCAGCTGGTGCGACGCCATCGTGCCCCAGTTCACCCCGAGC	8:C>G
3959908|F|0-11:G>A-11:G>A	TGCAGGGGCTCGGCTACGGGCTCTACAGCTCCG	11:G>A
1025293|F|0-46:C>G-46:C>G	TGCAGCGTCGCCGTGCGGCGTTTCCTGTACATGATGTCGGGCACGGCCCG	46:C>G
1088080|F|0-10:C>T-10:C>T	TGCAGGCGCTCCAGGCCGAGGCGGCCACCGCCTCCTCCATGCTCTCCTCCG	10:C>T
4261814|F|0-31:C>T-31:C>T	TGCAGCGGTTCACGGCGGCCACCCTGGAGCACGGCATGCACCCGCCG	31:C>T
1001639|F|0-39:A>G-39:A>G	TGCAGCAGCGGGCCGAGGACCAAGGGCGGGCACACGGTGACCACGTCCAGGCCG	39:A>G
2284516|F|0-15:C>T-15:C>T	TGCAGCGGGTACCAGCGCGTCCCGAGGGAGCGGTTGTCGGCGTCGAGCACGGCGGAGAGGGGCACCTTG	15:C>T
1088080|F|0-31:C>G-31:C>G	TGCAGGCGCTCCAGGCCGAGGCGGCCACCGCCTCCTCCATGCTCTCCTCCG	31:C>G
5332578|F|0-24:C>T-24:C>T	TGCAGGTTTGGTACTCCTCGCCGCCGTCGCCCCG	24:C>T
5581942|F|0-11:G>C-11:G>C	TGCAGCAGCGGGCCCAGCACCAGCACCG	11:G>C
5580214|F|0-9:C>T-9:C>T	TGCAGCCGACGGCGGCCAGCGGCGACGAGCGGCCGAGGTGGTTCTTGCACCCG	9:C>T
1666656|F|0-38:C>G-38:C>G	TGCAGGTGCGTGCCTGCGCCGTCGTCGCCCTTCTCGAGCAGCCTGGCGAAGCAGAGGAGCGCGCGGGAC	38:C>G
4002610|F|0-12:G>A-12:G>A	TGCAGCTCCCCCGTCGCCGACCCATTCGTCCCGAGCCCAGCAGCCG	12:G>A
3222153|F|0-6:T>G-6:T>G	TGCAGCTGGGAAATACTGTAACGACATTGGTGTAGGACTTGCACCG	6:T>G
1766597|F|0-35:C>T-35:C>T	TGCAGTGAACGGGGAAATGAAAAGAGCATAACACGCGTGACTGACCAAGAGCGGTACCTGCAAAGCGTC	35:C>T
996571|F|0-19:G>A-19:G>A	TGCAGCTGGTGCCGACGCAGAAGATCGGCGCCATCCCGCAGCCGCTGGAGGAGCTCCTCCTCCACTCCT	19:G>A
995885|F|0-40:C>G-40:C>G	TGCAGCGCGACGGCTACGCAGACGATCTTCAAGGCAATGCCAAGATAGCGCGGTACCTCGTGGACTCCA	40:C>G
4261474|F|0-23:G>C-23:G>C	TGCAGCTTCTTCACCTCCTCGATGGTGAAGTCCG	23:G>C
1033651|F|0-68:G>C-68:G>C	TGCAGCTCCCCATGGAATCATGTTGCTGATCTACCTTGTCTCAATAACAGCGCTAGTCAGATACACCAG	68:G>C
995799|F|0-15:A>G-15:A>G	TGCAGCTGTAGCGGCACCAAGACGGAGGCAGAACGTGGGGACACGAGGCTCGTGGTGATCGTGGGGGCA	15:A>G
1215372|F|0-43:A>G-43:A>G	TGCAGGCGTGCATGCACAGAGTGGCCACCGAGCCAGCGGCGCGAGGCTCGCGGTGGCCG	43:A>G
987565|F|0-21:T>G-21:T>G	TGCAGCGCTTCTCCCGCCACGTGATGCACGAGCTCGTCCGCCTCGTCTTCGCCCGCCTCCCGCTGATAG	21:T>G
5363657|F|0-25:T>G-25:T>G	TGCAGCTGGCCGCCGAGGAGGAACCTGCGCCG	25:T>G
3943207|F|0-26:A>G-26:A>G	TGCAGATCAAACGCGCGGATGATTCAACGAGCGACCG	26:A>G
1143356|F|0-67:A>T-67:A>T	TGCAGCTGGAAGTCGAGGTTGAGGTGCTTGAAGTTGCGGGTCAGCGTGCCCCGCGGCCCCGTCACCGAG	67:A>T
1007634|F|0-22:G>C-22:G>C	TGCAGAGCAGCCAGGTGCTGCGGAACGGCGGGACGAGCTGCTGCATGCTGGGGCCG	22:G>C
990929|F|0-25:G>C-25:G>C	TGCAGGTGCTGGACCTGTTCACCGCGAGCATCGTCTCCGTCGCGGACGACTACGTGGCGCCG	25:G>C
5579682|F|0-37:C>T-37:C>T	TGCAGGCCAGGAAGCTCTCACTCGGAGGAGGCATCGGCGGCAAGCTCAAGGCTGTAGGGGAGATGGGGG	37:C>T
4262544|F|0-28:G>T-28:G>T	TGCAGGCGCCGTCGCGCGCGGCCCAGCCGTCGAGCGCGTCCCG	28:G>T
3943475|F|0-23:C>T-23:C>T	TGCAGCCACACCTGTCGATGCCCCTGCCGAACTCGTCACTGGAGCTAGCAGTCGGGGGAATCGGTGGCA	23:C>T
1051134|F|0-14:A>C-14:A>C	TGCAGTCTCCTCTTACGCTTCTTCTCCTCCTTGGCCCGCCTCACGATCGCGCACAGCGCCTCGTCGCCG	14:A>C
5368424|F|0-20:C>G-20:C>G	TGCAGCTGCTGCGCCACCTCCGCCACGGCGGCCACGAACTCCCGCAGCGGCCGCCCCGAGCGCTTCACC	20:C>G
989503|F|0-67:G>C-67:G>C	TGCAGCACAACAACCTGAGTGGCGAGATCCCAGCGGTGCTCGCGGCGCTGCCGTCGGTCACCGAGGTGG	67:G>C
4396447|F|0-22:C>G-22:C>G	TGCAGAGCAAGGTCGGCCTGCTCGTCGACTTCGCCG	22:C>G
1862625|F|0-26:C>G-26:C>G	TGCAGAGTGAAGTTGACATGGTCCTGCACGCCCTCGAGGTGCTTCTGGGCGGCGACGTTCACCTCCTCC	26:C>G
1216431|F|0-48:C>T-48:C>T	TGCAGCAGACCAGCGGTCCAAGGCCAAGGCCAGCGTCTTCTCCCGCATCAGCTTCCCCG	48:C>T
1216431|F|0-24:C>G-24:C>G	TGCAGCAGACCAGCGGTCCAAGGCCAAGGCCAGCGTCTTCTCCCGCATCAGCTTCCCCG	24:C>G
4396851|F|0-9:C>A-9:C>A	TGCAGGATACATGTCGTCGCCG	9:C>A
2248596|F|0-53:G>A-53:G>A	TGCAGCTTCTCAGCGACGGTACAGTCGTGCGCTCTGCGGCCTTGCCCTTCCCTGTCGACGGCAACGTCG	53:G>A
1002654|F|0-22:C>T-22:C>T	TGCAGCCTCGGTGCCATGGAGACGGAGCCTCCTCCTGCTCCCGACCCTTGCGCTCCTGTGGCAGCTCTG	22:C>T
1027592|F|0-30:A>G-30:A>G	TGCAGTGGATCATGGCCGAACTGGTCAAGAACCCAGCAATCCAGGAGAAGCTCTACGAGGAGATCAAAG	30:A>G
1864938|F|0-12:G>C-12:G>C	TGCAGGACAACGGCGTCTTCTTCGACCACTTCAAGAACTCCATCAAGCGCATGGGCCAGATCGGCGTCC	12:G>C
1004893|F|0-19:T>C-19:T>C	TGCAGTACAGCCACGCCGATCCCAACCGCACGGTCGTGAACCCGTACGTGCACTTCGGGCAGCCGTCGG	19:T>C
3026700|F|0-31:T>C-31:T>C	TGCAGCACGCTCTGCTCCGTCCTCCTCGCCGTCATCCTCGTGGCCG	31:T>C
1106804|F|0-16:C>G-16:C>G	TGCAGCGCGGCATCGTCGCCACCAACCAGATCGCGCACAACGTGACGTCCACGCTGCACATCATCTCGG	16:C>G
5363219|F|0-28:A>G-28:A>G	TGCAGCAACCAGACCTCGTGCTCTGCCTACGCCG	28:A>G
2284516|F|0-6:G>T-6:G>T	TGCAGCGGGTACCAGCGCGTCCCGAGGGAGCGGTTGTCGGCGTCGAGCACGGCGGAGAGGGGCACCTTG	6:G>T
992458|F|0-9:C>A-9:C>A	TGCAGATCCCGTCACTTCCTTCCTCTACCGCGCGCTCCACGTCAGGCAAGCACGTAGGTCCCTGTCAGG	9:C>A
2250351|F|0-44:C>G-44:C>G	TGCAGCTCCTTGTCGTCGATCATCCCGCTGCCGTCGCGGTCCGCCGCCTGGAAGCACGCCACCACGTTC	44:C>G
6048615|F|0-16:C>G-16:C>G	TGCAGGCGCTGGGAGACGAGAGGCGGCCG	16:C>G
1082688|F|0-34:C>T-34:C>T	TGCAGCCGCCGCACCACCGACTGCCCGTCCGCGCCACGCGCAGCCGCCG	34:C>T
1099675|F|0-10:G>C-10:G>C	TGCAGGGGCGGTACGAGATGGGCCGTGTGCTCGGCCACGGCAACTTCGGGCGGGTGCACATCGCGCGGG	10:G>C
5331964|F|0-5:C>T-5:C>T	TGCAGCGCCTCCGCGTGCTCGCCG	5:C>T
991874|F|0-7:G>A-7:G>A	TGCAGAGGGCGTGCGAGCGGCACGCGTGCCTCGTGACGCCGCCGCACAGGGACATCGCCG	7:G>A
1694868|F|0-19:C>G-19:C>G	TGCAGGAGAAGTCCATGGGCAGGGTGAAGCGGTCGACGACCGATATGCCGTACCTGCCCG	19:C>G
3027787|F|0-30:C>T-30:C>T	TGCAGCAGTACGCCTCCGTCCCGAACGCCTCGCACGCGCTCCG	30:C>T
4003327|F|0-13:C>T-13:C>T	TGCAGCGCGTGCCCACGCGGGCCTTGAGGTCGGCGATGAACTCATCCG	13:C>T

2281902|F|0-34:G>C-34:G>C	TGCAGGTCTCGGCGACGCGGGGGTCGCGGTCGCCGAGCGCGGCCATCTGGATCTCGAAGCCCTTGCCGT	34:G>C
1060558|F|0-8:C>A-8:C>A	TGCAGGAGCAGTCGTCGGAGTTGACAGAGCTGATGGACGGGATGCTCAACAAGCTGTCCAGCGCTCTGT	8:C>A
7165379|F|0-17:T>G-17:T>G	TGCAGCGGCAGGCGTCGTCGGGGTCCCTGACGCAGGGCCCGTCGGGGGAGCTCCG	17:T>G
1096650|F|0-12:G>C-12:G>C	TGCAGCCGACGGGCCACCGACAGGGTACCAACTCCTCAAAAGTAGCAGCAAATCGGCCCCCGACGCCGA	12:G>C
7165379|F|0-12:C>T-12:C>T	TGCAGCGGCAGGCGTCGTCGGGGTCCCTGACGCAGGGCCCGTCGGGGGAGCTCCG	12:C>T
1021179|F|0-22:G>C-22:G>C	TGCAGAGAGGCTACGACCAGGTGGTGCACGACGTGGACCTCCAGAAGCTCCCG	22:G>C
4261552|F|0-13:G>C-13:G>C	TGCAGTCGAAGGCGGCGTCCAGGGCGCTCGGAGCCATCAGGCCG	13:G>C
1007634|F|0-45:T>C-45:T>C	TGCAGAGCAGCCAGGTGCTGCGGAACGGCGGGACGAGCTGCTGCATGCTGGGGCCG	45:T>C
2251741|F|0-23:G>A-23:G>A	TGCAGCGAGGACTGGAAATTGGAGAGCAGGCGCGTGAGGTCCCGTGGCCGTATGGCGAGGCGGCTGGGG	23:G>A
2251741|F|0-11:C>T-11:C>T	TGCAGCGAGGACTGGAAATTGGAGAGCAGGCGCGTGAGGTCCCGTGGCCGTATGGCGAGGCGGCTGGGG	11:C>T
6046747|F|0-7:G>C-7:G>C	TGCAGCTGAAGGACGCCGTCTACGAGGCCGACGAGGTCGTCGACGAGTTCGAATACCG	7:G>C
4003312|F|0-9:T>C-9:T>C	TGCAGGAACTTGGTGATGGGCTGGATCACGGCGTTGGCGAAGAGCCCCG	9:T>C
6048536|F|0-16:A>C-16:A>C	TGCAGAGATCACACTCATGAACGTGAAGTGCGTCAATAAGAAGACCG	16:A>C
7913266|F|0-37:C>T-37:C>T	TGCAGATGCAGGTGCAGATGATGTGGATGGGCGGCGGCATGGCGCCGCCG	37:C>T
1024214|F|0-14:C>G-14:C>G	TGCAGCGGCCTCTCCGCCGAGGAGGTGGAGTCTCCCTACGTCAACTTCGGCCGCCG	14:C>G
1067812|F|0-32:G>C-32:G>C	TGCAGGATGAGGAAGGCGACCACCAGGGCCACGAGCGCGAAGACGTAGATGGACGTGCGTAGTTTCCGA	32:G>C
7349664|F|0-11:A>C-11:A>C	TGCAGCACCGAATGCTCCCCG	11:A>C
2246126|F|0-16:T>C-16:T>C	TGCAGACCGCACCAGATGTCGTTCTGGTTGGACTCGGTGATGCCACGGTCGATCACAGCACTGGCCTCC	16:T>C
1015270|F|0-16:C>A-16:C>A	TGCAGACCGTCCAGACCGCACGGGCTGACGCCGACGCGCTCGCGTGGACGCCG	16:C>A
3941185|F|0-22:G>A-22:G>A	TGCAGGTGGCGTTGTCGTAGCCGCTCCCG	22:G>A
3026623|F|0-18:T>A-18:T>A	TGCAGCCACCAGGAAACGTGCTCCACGGGACGCTGCCG	18:T>A
3940266|F|0-13:C>A-13:C>A	TGCAGGAGAAGGGCGGGAAGGGCGATGCCGTCCGCCG	13:C>A
1079033|F|0-17:C>T-17:C>T	TGCAGGCGGCGGGACGGCGCGCGCTTGCCGAGGAGCTTCTTCTTGAGCTTGGTGTTCCAGTAGTTCTTG	17:C>T
5324886|F|0-20:G>A-20:G>A	TGCAGGTCCAGCCCCAGCGCGTCCTCGGGCGCGGCGGCCATCGGCTCCG	20:G>A
986877|F|0-7:C>T-7:C>T	TGCAGACCAGCCCCAAGTGCCTGTGCGTGCTCGTCAAGGACCGCGACGATCCCG	7:C>T
5582379|F|0-17:C>G-17:C>G	TGCAGGCCGAGGAGGAGCGGCGAGACGTCGACGGAGGCGCCCG	17:C>G
32026809|F|0-31:T>C-31:T>C	TGCAGATTGGCTACTCGGCACCAGCTGAGGCTGGGATCAGCCG	31:T>C
990204|F|0-23:T>C-23:T>C	TGCAGAACACGCAAAGCCTCCTCTTCTGTGGTCTTCCTCCTCCTCCGAGACCAATAGAGCGCCCGAGAT	23:T>C
4734064|F|0-7:A>G-7:A>G	TGCAGCAAAGCTAGCTCCTCCG	7:A>G
1024352|F|0-24:G>C-24:G>C	TGCAGGCCCCTCACGAAGGCGACGGCGTACTTGCTCACCGTGATGGGGTCCTCGCCG	24:G>C
3956546|F|0-34:G>C-34:G>C	TGCAGGCCAACATCAAGAAGTCCGTCAACCTCGAGGCCCTCGCAGCCG	34:G>C
7350701|F|0-24:A>G-24:A>G	TGCAGCTGACACATGCATGGTACTAACACCG	24:A>G
1040382|F|0-17:G>C-17:G>C	TGCAGGGTGTTGTGGTAGTGCTGAACACCCCTGGACCATGGAGATGGAGAGGACATTGCCAATTGGAAC	17:G>C
1090899|F|0-45:C>A-45:C>A	TGCAGAGCGCGTAGAGCGCCGTGGCGGCGTCCTTCTTCCCGCGCGCGCCGCCG	45:C>A
1150103|F|0-6:C>G-6:C>G	TGCAGACACACGGGCTCCCGCGCGGGCTGCTCCCCGCGGGGATCGCGGCGTTCTCGCACGACCCGAGAT	6:C>G
980448|F|0-68:G>C-68:G>C	TGCAGGAACTTCTGCGTGGGGAAGGTGAGCGCGAGCGAGAACATCTGCGGGAGGATGCGGAGCGTGAAG	68:G>C
6047648|F|0-30:C>T-30:C>T	TGCAGGCGGCGGCCACGTCGGGGGCGAGCGCGGCCG	30:C>T
1051808|F|0-53:G>A-53:G>A	TGCAGGAACGGCACCATCCTGCCCAAGACCATGGACGAGGCGCAGTCCAAGTCGGCGTTCCAGATGGAG	53:G>A
1863204|F|0-28:C>A-28:C>A	TGCAGCCGCTGCGCCTCGCCTTCGAGACCAAGCACATCAAGCTCGTCGAGCCG	28:C>A
4398415|F|0-12:C>A-12:C>A	TGCAGACAAGTGCAACCACCG	12:C>A
1019953|F|0-17:G>C-17:G>C	TGCAGGAAGATCTTGAGGATGTTCTTGGAGAAGCCGCTCACCATGGCCACCCCG	17:G>C
1011077|F|0-46:G>C-46:G>C	TGCAGCTCTGGGCGCTCCGCATCCCCCGCGAGCTCGCCTCCGCCGTGACCCGCCTGTTTCGCTCCGAGA	46:G>C
4734116|F|0-7:C>T-7:C>T	TGCAGTCCAACGCCACCTCCCAGGTCGCGCTCGTCGGCACCGACTTCTGCCCCATCGAGAGCCTCGACT	7:C>T
1075099|F|0-12:C>T-12:C>T	TGCAGGTCCATTCCAGTTCGTCGCAGAGCCCTACAACGGCACCATACCGCGCCTGCAAATCAACCCCTA	12:C>T
5580543|F|0-22:G>A-22:G>A	TGCAGTACGTGCAGGGGCAGGCGCGGTCGCCG	22:G>A
1023911|F|0-19:G>C-19:G>C	TGCAGAAGGCGAGGCGGGCGGTGGAGAGGTAGAGCGTGCCGATGACGGGGCCG	19:G>C
1050193|F|0-64:C>T-64:C>T	TGCAGAAGGTCACCAACCCCAAGACGATCCAGAAGATCGCCAAGTCCAAGAACCGAGGTCAGCTCAGGG	64:C>T
17568760|F|0-14:C>G-14:C>G	TGCAGCACCAGCTCCACCGTCGGCACGGCCG	14:C>G
32020155|F|0-13:C>T-13:C>T	TGCAGCACTTGGTCGCCGACGCCAGCACCG	13:C>T
16794995|F|0-19:G>C-19:G>C	TGCAGTTCATCGGGCAGGGGTTCCGCTCCG	19:G>C
1863941|F|0-38:T>C-38:T>C	TGCAGGGTGCTCACCTCGATGCCGCTCCTCCTCCACCCTTCCTCTCCTCCG	38:T>C
7488203|F|0-13:C>T-13:C>T	TGCAGTACCTCGACTCCACCG	13:C>T
7165959|F|0-14:G>A-14:G>A	TGCAGGTCGCGCTCGAGGTCCTCAAAGGTGGGGCCCGCCG	14:G>A
3029977|F|0-19:C>T-19:C>T	TGCAGCAATGGACATCGTGCGTCTTCAGCACCTCCTTGGCGGCCTCCG	19:C>T
6044636|F|0-44:G>A-44:G>A	TGCAGCCACCCGAACGCGGCGCCCCTGGTGTGGTCGTCCGTGTAGTCGGCGACCAGCGAGTTGATGGCC	44:G>A
4537703|F|0-16:C>T-16:C>T	TGCAGGCTGCGGTGGACAAACTACCTCAGACCCG	16:C>T
1075430|F|0-37:C>G-37:C>G	TGCAGCGGCAGTGGACCTTCTGGTACGACATCCAGACCAAGCCCAAGCCCG	37:C>G
1053988|F|0-20:G>C-20:G>C	TGCAGCTTCCTGACGCTGCTGATGACCGCCTCCTGCCTCGGCCTCTTCCTCGCCACCACGCGCCGAGAT	20:G>C
1092246|F|0-11:G>C-11:G>C	TGCAGCGACCTGCCCTGGGCGCGCTGGATCCGCGGATGGACGGCCGAGGAGAATCGCCACGGTGATCTC	11:G>C

2336803|F|0-50:G>C-50:G>C	TGCAGGGCGCCCTGGCCGTCGAGGCCCGAGCAGAGCACCGCGACGGGCGCGTCGGCCATCTCCGCGAGC	50:G>C
1863941|F|0-7:T>C-7:T>C	TGCAGGGTGCTCACCTCGATGCCGCTCCTCCTCCACCCTTCCTCTCCTCCG	7:T>C
7911253|F|0-12:T>C-12:T>C	TGCAGCGTCACCTTCGCGCCG	12:T>C
4261738|F|0-35:G>A-35:G>A	TGCAGCAGCTGTTCCTCGACGACAACAGCTTCGTCGGGGAGCTGCCG	35:G>A
3943964|F|0-14:C>G-14:C>G	TGCAGCACCTGGGCCGCGGCCGCGCCGCCGCCG	14:C>G
1021154|F|0-51:C>G-51:C>G	TGCAGCTCGGCATTGGGCGGCAGGATGACGGGCTTCTGCACGACGATGCCGCCGCCGCCGTCGGAGGGG	51:C>G
1297790|F|0-14:G>C-14:G>C	TGCAGGAGCAAGCTGAGGCACTTGCTCGCCGCGGAGGGGGAGCCAGGGAGGGAGCCG	14:G>C
1026222|F|0-10:G>A-10:G>A	TGCAGGTGCTGGCCATCCTGCCGTCGTACGCGGGCAAGACGGTGCTGGAGCTCGGCGCCG	10:G>A
1042793|F|0-65:G>A-65:G>A	TGCAGGTAGAGCTAATGCAGCATTGCATTTGCAGCTCGCACAAATTTGATGGTGATCCTGCCCTGGCAT	65:G>A
1149419|F|0-8:C>A-8:C>A	TGCAGGAGCGAAAACACGAGAACATTGAGATTAAGAGAGATCTAGCGGGAAACGGAACAAATCAAGCGC	8:C>A
1225917|F|0-36:G>A-36:G>A	TGCAGCGGCTGGTGGAGGCGGAGGGCCGCCCCGTCGGCGGCACCGACGCCTCCG	36:G>A
2256906|F|0-40:C>G-40:C>G	TGCAGTCGGCGGCGCGCACCTGGAGCTTCATCTGCGCGGGCAAGTACCCCATCGACCACCCGTACGCGA	40:C>G
2246126|F|0-38:G>A-38:G>A	TGCAGACCGCACCAGATGTCGTTCTGGTTGGACTCGGTGATGCCACGGTCGATCACAGCACTGGCCTCC	38:G>A
7167999|F|0-26:G>C-26:G>C	TGCAGCTTGGGCGACTTGGGCGTCGGGGGCGGCGAGGACGCGCCG	26:G>C
1081642|F|0-11:C>G-11:C>G	TGCAGGATGCGCAGCGAGGAGTTCCTCTCGCCGCCGAACGCCGCGGCCGGGATGGCGCCG	11:C>G
4542748|F|0-8:C>G-8:C>G	TGCAGCAGCGAGACGAGGCCG	8:C>G
5370763|F|0-6:G>A-6:G>A	TGCAGCGGAGCGAGATCGTCAGGCCCG	6:G>A
1231257|F|0-41:G>A-41:G>A	TGCAGCTGCCGCCGAGGCGTCGTGGGGCGCCCCGACGGCGAGGTCGCGTCCATCCGCGGAAGGGCCGCG	41:G>A
998786|F|0-19:G>C-19:G>C	TGCAGAGGGTGGAGAGGTCGTGGGGCTTCGTGTGCGCGGGGAGGTACGGACCGACCACCCGTTCATCA	19:G>C
4002610|F|0-17:C>G-17:C>G	TGCAGCTCCCCCGTCGCCGACCCATTCGTCCCGAGCCCAGCAGCCG	17:C>G
6044050|F|0-18:C>G-18:C>G	TGCAGGACGATGGACTTGCTGCTGAGCCCCG	18:C>G
1064623|F|0-17:T>C-17:T>C	TGCAGCCAAACATGAAATCATCAATGCTGAAGTTGTTTCCTAATGTGAGCTTGTCCTTGACCAAGCTTC	17:T>C
7354190|F|0-12:G>C-12:G>C	TGCAGCGAGTTGGTGTGGAGCGCGAGCGACCG	12:G>C
1064623|F|0-24:T>C-24:T>C	TGCAGCCAAACATGAAATCATCAATGCTGAAGTTGTTTCCTAATGTGAGCTTGTCCTTGACCAAGCTTC	24:T>C
2243334|F|0-67:C>T-67:C>T	TGCAGCTGGTGGCACAGCGTGTTGGTCTCCAGGAAGAACTCGAGGAACACCTTGCTCTCGGAGTCGCCC	67:C>T
1113245|F|0-10:A>G-10:A>G	TGCAGCAATCACGCGCGTGCCGAAGAACGTCGTCGTCCATCCCTCACTTCAATGGCCTGAGGATCCTGC	10:A>G
100530568|F|0-29:G>A-29:G>A	TGCAGCACCTGCGCCGCCGCCGCGCCGCCGCCG	29:G>A
3027023|F|0-24:C>A-24:C>A	TGCAGCAACAACGGCCACAACGCGCGTACCTGCCCCGCCCGCTCCG	24:C>A
2253831|F|0-61:T>A-61:T>A	TGCAGGTCCTCGACGCCGCAGGCCGCGGCGTAGTCGACGCAGCGCTTGAGGTCGGCCTGCGTGAGCTGG	61:T>A
1719278|F|0-30:G>A-30:G>A	TGCAGAACGTGAGCTGCGCCAAGGCGGGGAGGATCCAGGGCAAGGACCTCAACTACCCGTCGATCATGG	30:G>A
5582325|F|0-6:T>C-6:T>C	TGCAGATATGGCAACCTCCATGGCCG	6:T>C
4002610|F|0-13:T>C-13:T>C	TGCAGCTCCCCCGTCGCCGACCCATTCGTCCCGAGCCCAGCAGCCG	13:T>C
1398794|F|0-17:T>C-17:T>C	TGCAGCTCGATCTCTGCTTTCGGCTGCACCATGCAGACCTGCTTGGTGATGTCGTCGCTCCTCTCCTCC	17:T>C
1087023|F|0-56:A>G-56:A>G	TGCAGGGACACCGTCATGACCATCTGGCATTACCACGGCGGGTGCCAGGTCGGCAGAGTCGTCGACCGC	56:A>G
5411723|F|0-6:G>A-6:G>A	TGCAGCGAGAGCGGCACCACGCCG	6:G>A
4910730|F|0-42:C>G-42:C>G	TGCAGGTGGTAGCTCCATCGGCATGTCCAACTTTCGGGTGGTCGCCGTGACCTACGAAAGCGATGTTTT	42:C>G
6048757|F|0-25:G>A-25:G>A	TGCAGCAGCACCGCGTTGGTGCGCAGGAAGAAGAAGTTTGTGAGCGCGGCCCCGTGCACGCCCATGAGC	25:G>A
4543858|F|0-10:G>C-10:G>C	TGCAGAACCTGCTCGTCGCCG	10:G>C
23919648|F|0-20:A>G-20:A>G	TGCAGGACGACGTCGACAGCAAAATCGACCCG	20:A>G
13880606|F|0-27:T>G-27:T>G	TGCAGCCTGCGGCGGACCACCACGCCCTCCGTCGCCGACGCCG	27:T>G
6040726|F|0-5:C>T-5:C>T	TGCAGCTTCACATACGGGTCGCTGAACCCGTTGAAATCCATGGCGCGCAGGTTGCGGGCTCCGATCACA	5:C>T
2263181|F|0-57:A>C-57:A>C	TGCAGGAACGCGTCGTACACCACGGCGCGCACGGGCCGGCCCTTCTCCGCCTCGGACAGCAGCAGCTCG	57:A>C
6048185|F|0-13:C>G-13:C>G	TGCAGCTGCTGGACCGCCACGACGCCCACAGCGACCG	13:C>G
4002390|F|0-19:C>A-19:C>A	TGCAGCGGGTGCACCACTCCGCCGACGCCACCGACCGCGCCG	19:C>A
2243334|F|0-41:G>C-41:G>C	TGCAGCTGGTGGCACAGCGTGTTGGTCTCCAGGAAGAACTCGAGGAACAACTTGCTCTCCGAGTCGCCC	41:G>C
1217790|F|0-37:C>G-37:C>G	TGCAGATCCTGATCAACGTGCCGTTCTCCTACTACGCCTTCACCACCGTCTTCTTCCCGTTCCTGACGC	37:C>G
992863|F|0-40:G>T-40:G>T	TGCAGGACACCAGCATCCAGGGCGCCGAGAGCCACCCGCTGCACCTGCACGGCTACGACTTCCACGTAG	40:G>T
4002610|F|0-36:C>T-36:C>T	TGCAGCTCCCCCGTCGCCGACCCATTCGTCCCGAGCCCAGCATCCG	36:C>T
1231257|F|0-8:C>T-8:C>T	TGCAGCTGCCGCCGAGGCGTCGTGGGGCGCCCCGACGGCGAGGTCGCGTCCATCCGCGGAAGGGCCGCG	8:C>T
2251099|F|0-20:C>G-20:C>G	TGCAGGCCCTTGAAGTACTCCACGTCGAAGGCGTTGCTGGTGGCGTCGAGCTTGGCCGTGCCGCCGTCG	20:C>G
1090832|F|0-5:T>C-5:T>C	TGCAGTTCCATGATCGCGGCGGCGAGCGCCTCGTCCAGCACCAGCCTCTCCAGCACCGTCATGCCGCTC	5:T>C
982890|F|0-22:G>A-22:G>A	TGCAGTTTGTTGGGAGGATCAAGTTCTCCATGGAACCCGTCGCCTACACCG	22:G>A
1094102|F|0-21:C>T-21:C>T	TGCAGGTCGGCGCGGAGCACGCTGGCGGCGGCGTCGTGGGACTGCGCGACGTCGCGCCACATCTGCGCC	21:C>T
5582379|F|0-20:C>A-20:C>A	TGCAGGCCGAGGAGGAGCGGCGAGACGTCGACGGAGGCGCCCG	20:C>A
1069859|F|0-11:T>A-11:T>A	TGCAGCTCCGCTGAGGCCTCAGAATGAGCTCAAGCACGCAAGACCCTGCGCGACCACGGACAACGACCG	11:T>A
5578096|F|0-17:G>A-17:G>A	TGCAGCGGGGAGGGGGTGCTCTTCGTCGAGGCCGACGCGCCCG	17:G>A
7352327|F|0-5:T>G-5:T>G	TGCAGTGCCAAGAACGGCCG	5:T>G
1089113|F|0-36:T>C-36:T>C	TGCAGAACTGGTCGGCGATGGTGGGCCACGTCAGAGTCGGCACGCCGTAGGAGACGGCCTCCAGCGTCG	36:T>C
3944888|F|0-13:G>A-13:G>A	TGCAGAACCCGCCGACGTAGGCCG	13:G>A

1380699|F|0-19:A>C-19:A>C	TGCAGAAAGAGAGAGAGGAATATGAGGTTGTGATTGAGGATGGCAAGTTTATGTACAAGAACAGCAGGC	19:A>C
3949616|F|0-13:G>T-13:G>T	TGCAGCTTGTACCGCAGGGCGACGGCCG	13:G>T
1766794|F|0-35:G>A-35:G>A	TGCAGCCGCTGGATCCAGCTCGGTGCATTCTACCCGTTCGCGAGGGCGCACATGGAGAGAACCACCGAA	35:G>A
2250961|F|0-34:C>T-34:C>T	TGCAGGGCCACCCCAACGTGATCGGCCTCCATGGCCTCATCACCTCCCGCTCCTCCGCCTGCATCTACC	34:C>T
1771526|F|0-58:C>G-58:C>G	TGCAGCTTGCGGACTTCATAGGGTTAGATGTTTGCCTCTCAGTACTTAGGGTGCTCCACAACGGGCTAG	58:C>G
6048580|F|0-12:A>G-12:A>G	TGCAGCGTCTTCATCTGACCGACCTCTCGCGGGATCTCGCCG	12:A>G
3946705|F|0-17:G>C-17:G>C	TGCAGGGTCTTCATCCTGCTGCTGCTCCG	17:G>C
1042145|F|0-60:G>T-60:G>T	TGCAGAGCTCGCCTACTTGAGGGTGACGACCAAGTGCGACGTGTACAGCTTCGGCGTGGTGGCCATGGA	60:G>T
1389546|F|0-43:C>T-43:C>T	TGCAGCTGGAGCGTGTCAACGTCTACTACAATGAGGCCTCCTGCGGTCGTTTTGTGCCCCGTGCTGTCC	43:C>T
4005629|F|0-20:G>C-20:G>C	TGCAGGTTCCGCCGCTTGTAGCCGACGAGGCGCCGCGACGGCCG	20:G>C
3949453|F|0-7:C>T-7:C>T	TGCAGAGCCACAGGCGACCTCACACCG	7:C>T
3937238|F|0-7:C>T-7:C>T	TGCAGCACTACCAGCTCAACGTTGGTACCG	7:C>T
1213658|F|0-16:C>T-16:C>T	TGCAGCGCATGGGCGTCGGGCTCGTGATCGGCATGGGGGCGATGGTGGTGGCAGGCGTCGTGGAGGTGG	16:C>T
1214567|F|0-25:G>A-25:G>A	TGCAGGAGAGCTGCACGGACACGTCGGGGTGCTCCATGGTGGTGCACTCGCCCATCGACATCCCGAGAT	25:G>A
1714098|F|0-52:C>T-52:C>T	TGCAGCCGCCTGGCGACACGTACACGCGGCGGTCGGCGTTCGACGCCATCCTCGCCG	52:C>T
992992|F|0-58:C>T-58:C>T	TGCAGCCCTCAGCGCCGAGGACCGCGTCGGCCTCGTCAATGCCCTGAAGGTACGGTTACTCGCTGTTCG	58:C>T
3942059|F|0-58:C>G-58:C>G	TGCAGAAGGAGTCGTCGGACGCCTCGGACGCGCACGACACGTTGAGCGACTGCATGTTCCCGTTCTTCC	58:C>G
1053607|F|0-62:G>C-62:G>C	TGCAGCAGCTCCCGCAGCTCCGCGCCGCCCGTGATCCCGTTCTCCACCACCATCTCCGCCATGGACCGC	62:G>C
1073887|F|0-41:G>C-41:G>C	TGCAGGAACTTGGTGATGGGCTGGATCACCGCGTTGGCGAAGAGCCCAGGGATGAGCCACACGATGTAC	41:G>C
4395607|F|0-9:G>T-9:G>T	TGCAGATCCGTCAACTAATTCTCTCCG	9:G>T
13881125|F|0-19:A>G-19:A>G	TGCAGCGACGCCGTCCACAAGATGCTGAGGGAGACGCCG	19:A>G
5358200|F|0-11:T>C-11:T>C	TGCAGGCACCATGACATGAGCGGGAAGGCGGACCCG	11:T>C
1007221|F|0-31:C>G-31:C>G	TGCAGCGCGTGGAGGCGCTGGTGGACGTGGCCAACGTGGCGTCGCAGCGGGTGCTGGAGAAGGCCGAGA	31:C>G
1081642|F|0-17:G>C-17:G>C	TGCAGGATGCGCAGCGAGGAGTTCCTCTCGCCGCCGAACGCCGCGGCCGGGATGGCGCCG	17:G>C
1695188|F|0-27:T>C-27:T>C	TGCAGGCTCTTGAGCTCTGAATCAAAATCTTTCTGCATGGACACGGCGCGGCGGCTGAACTGCTCGTCG	27:T>C
5577817|F|0-13:T>C-13:T>C	TGCAGAAGCGCCGTCGGGACGATCCCGTCGCCTCCG	13:T>C
12469813|F|0-7:G>C-7:G>C	TGCAGTTGAGAACCACCCTCACACCATCCG	7:G>C
1046617|F|0-8:G>T-8:G>T	TGCAGGAAGGGCCTCAGGAAGGACCAGCCCGTGCCGACCAGCACGATGACCGCGAAGAGGATGACGCCC	8:G>T
7168053|F|0-25:G>A-25:G>A	TGCAGGAGGAGTTCGGCGGGGCGGAGGGCGCCG	25:G>A
2336803|F|0-38:C>G-38:C>G	TGCAGGGCGCCCTGGCCGTCGAGGCCCGAGCAGAGCACCGCGACGGGCGCGTCGGCCATCTCCGCGAGC	38:C>G
4261578|F|0-19:G>T-19:G>T	TGCAGATGCAGGCCCCGTCGCTGACGCGCGCGCCG	19:G>T
7172660|F|0-24:T>G-24:T>G	TGCAGGTGACCGTCGGCGGCCTTGTCCCCG	24:T>G
1028020|F|0-50:A>G-50:A>G	TGCAGGTCGATGGACTGAGGCACGGAAGTTTCGTCCATCTTGGCAGCAAGAGACAGGCACGCCACCGAT	50:A>G
1204046|F|0-61:G>T-61:G>T	TGCAGGGGCTCGGCCTCGTGCACCCGCAGGCCGTGCACCGCTTCTACGACGAGCTCCACGCGTACCTCG	61:G>T
3022763|F|0-28:G>C-28:G>C	TGCAGCTAAGCCAACACACATGCTGACAGATGTCTTTCAGCCCCCG	28:G>C
1024054|F|0-55:G>A-55:G>A	TGCAGCCAGCCGAAGGCGGCGCCCCTGGTGTGGTCGTCCGTGTAGTCGGCGACCAGCGAGTTGATGGCC	55:G>A
1024054|F|0-41:G>A-41:G>A	TGCAGCCAGCCGAAGGCGGCGCCCCTGGTGTGGTCGTCCGTGTAGTCGGCGACCAGCGAGTTGATGGCC	41:G>A
5331680|F|0-16:T>A-16:T>A	TGCAGCAAATCGCCACTGCGCCCG	16:T>A
1007221|F|0-13:G>A-13:G>A	TGCAGCGCGTGGAGGCGCTGGTGGACGTGGCCAACGTGGCGTCGCAGCGGGTGCTGGAGAAGGCCGAGA	13:G>A
1002433|F|0-58:C>G-58:C>G	TGCAGGAGACGATGTGGCGGAGGAGGTGGCGGCCGACGCCGCCGCAGCCGAGGAGCACCACCG	58:C>G
1070262|F|0-45:C>T-45:C>T	TGCAGAAGGGCACCCAGGCCCTGGCAAAGGCGTAGGCCTTCCTGGCCGCGAGCAGGGTCCGCGGCGACC	45:C>T
4261567|F|0-5:G>C-5:G>C	TGCAGGCCGCGGCCGATGCCGATGATGATCTGGTGCCG	5:G>C
1075430|F|0-35:A>T-35:A>T	TGCAGCGGCAGTGGACCTTCTGGTACGACATCCAGACCAAGCCCAAGCCCG	35:A>T
1075430|F|0-7:G>A-7:G>A	TGCAGCGGCAGTGGACCTTCTGGTACGACATCCAGACCAAGCCCAAGCCCG	7:G>A
1124006|F|0-25:G>A-25:G>A	TGCAGGTGGGCTCCATCCGACGGCCGCAGTTGACCTTCCAGGCCCCGCCTCCGCGGCCCGCCG	25:G>A
2265552|F|0-49:G>C-49:G>C	TGCAGCTGGAGGTGGAGCCGTCGACGCGCGACCCGCGCGACGAGGGCACGGCGGACGCCTGGATCGACC	49:G>C
6048569|F|0-7:G>A-7:G>A	TGCAGCGGCGGTGAGTCCGAGCACGCGCCG	7:G>A
22838457|F|0-29:A>G-29:A>G	TGCAGTGGCCACTCCCTGCTGAGGGATCCACGGCACAACAAGGGGATGTCGTTCACGGAGAAGGAGCGG	29:A>G
32023664|F|0-22:A>G-22:A>G	TGCAGCCTGAGCGCACCTGGAAATCGCCGCAGCAGATGAGGAGGTCAATCTTGATGCCCTCGGCCGCCT	22:A>G
32025595|F|0-47:T>C-47:T>C	TGCAGGACAGTGTTGCAAGCGAAGACCGTGACTCCAGTGAGCAGGTATACACAGCCAAGGACCCAACCT	47:T>C
7168823|F|0-56:T>G-56:T>G	TGCAGCTGCTGCGCGCAGGCCTCGCAGTGCATGTTGACGAGCAGCTCCACGGTGCGTGCCTCGCTGACG	56:T>G
1162736|F|0-34:C>T-34:C>T	TGCAGCACGCGCTTGGCGCCCGTGACCTCCAGGACCCTCCTCGCCCTCATCTGGCTGCCGCCGTCGCCG	34:C>T
6042825|F|0-5:G>A-5:G>A	TGCAGGCGGCAGCTTATTAAGGGGCTAGTTTAAAGCGGGGGCGGCAATGCGCTGGGTCCGCGAAGGCCG	5:G>A
1766608|F|0-40:G>A-40:G>A	TGCAGTCCTCGAGGCGGTCGAGGAGGTTATCGGCGACCGCGTCTACCAGTGGGTCAGTTTCCCGCATCG	40:G>A
6041845|F|0-5:G>C-5:G>C	TGCAGGTATCTAGAAATGCATTCCG	5:G>C
1039421|F|0-48:G>A-48:G>A	TGCAGTTCCTCGGCCATCCTGCCCATGTTCCCATGATCGGAAGCAGCAGGGGTAGCCAGAGCTGGTGCA	48:G>A
1083022|F|0-25:C>G-25:C>G	TGCAGCTCCTCTCGGTGGCCTGCCTCTCGCTGGCGGCCAAGGTGGAGGAGCACCG	25:C>G
100446086|F|0-5:C>G-5:C>G	TGCAGCTCGGCCACGGCCCCG	5:C>G
1072118|F|0-44:G>A-44:G>A	TGCAGCACGATAGAGTAGATCACGTCGGCGTACTTGACGGCGAGGTTGAGCGACATGCACCG	44:G>A

1046610|F|0-17:A>C-17:A>C	TGCAGCGCATGTCGTACATCAACGTCGGCATCTACCCGTTCACGTCCCTCTTCCTCATCATGTACTGCC	17:A>C
995078|F|0-5:C>G-5:C>G	TGCAGCGCCTTCACCAGCCGCTCGCCCTTCTCGGCCGAGAAGGCCTGCCG	5:C>G
7170299|F|0-32:G>C-32:G>C	TGCAGGCTCTCCCGGCCCTCGGGCTCCTCGAAGGAGCCCTGGTCGGCGTACCACGAGTTGGAGGAGTAG	32:G>C
4002529|F|0-8:G>C-8:G>C	TGCAGCCCGTCGCGGCCGTCCGCCAGCTCCGAGCGCCG	8:G>C
7165379|F|0-28:G>C-28:G>C	TGCAGCGGCAGGCGTCGTCGGGGTCCCTGACGCAGGGCCCGTCGGGGGAGCTCCG	28:G>C
32018940|F|0-14:A>G-14:A>G	TGCAGCCCAGCCTCAGCACCGACACGCCG	14:A>G
986316|F|0-44:C>G-44:C>G	TGCAGCTGGCGGGACGCGGACCAGGTGCGGGACACGCTCCAGGACAGCGACCTGAAGTGGGAGCCGCCG	44:C>G
7350840|F|0-10:G>C-10:G>C	TGCAGGCGTCGAGCGGCCAGCCG	10:G>C
3953275|F|0-11:G>A-11:G>A	TGCAGCAGCGTGTGTGAGCAAGCCG	11:G>A
5971400|F|0-41:A>G-41:A>G	TGCAGGCCCGCTGCTGAACGGGAACTTCGAGCAGGCGCCGAACAGGTCGCAGATGAACGGCTCGAGGGT	41:A>G
1102385|F|0-43:C>G-43:C>G	TGCAGGCCTTCGTCCCCGATCTCGAGGTCGCCGTCGCCGACCTCCTCGCCTTCTTCCG	43:C>G
2253714|F|0-12:G>A-12:G>A	TGCAGCGTTCACGTACTCGAGGGAGCTGCACGACGTGGTGGAGGTGGGCAAGGCCG	12:G>A
1026087|F|0-31:G>C-31:G>C	TGCAGCACCTCGGGTCCAGGGTCTACGAGCGGCTGCCGTCCATGTCGTCCG	31:G>C
1076373|F|0-47:G>A-47:G>A	TGCAGGGCCGCCTCCTGACGGGGTTCGGCGAGCACACGGACCCGCAGGTCATCTCCGTGCTCCGCTCCA	47:G>A
4002344|F|0-7:G>T-7:G>T	TGCAGCCGTCGTCCGAGCAAACCTTGTAGATGGCGAGCCTGGCGCCCG	7:G>T
3941346|F|0-10:G>T-10:G>T	TGCAGGATCTGCATTGGCACGTACAACGCCGCCGGCATAGAGCCGCCG	10:G>T
7172024|F|0-13:A>G-13:A>G	TGCAGAAGGGCCAAGAGCCCATCTTCGGCGCCG	13:A>G
993148|F|0-12:A>G-12:A>G	TGCAGAGTACACAGCGGCAGTGGCACCCACACGGCCATTAGCCGAGACCCTCTGCTTGAGCTGCCTGTG	12:A>G
1302338|F|0-68:G>T-68:G>T	TGCAGTGTCTTGGAAGCTCTTGATGAGATCTTGAGCGGCGCACAGGTGCTCGCCGTCCCGCTCCGCTCG	68:G>T
3935737|F|0-16:C>G-16:C>G	TGCAGGGGTTCGCCGTCGACCG	16:C>G
1204046|F|0-7:G>C-7:G>C	TGCAGGGGCTCGGCCTCGTGCACCCGCAGGCCGTGCACCGCTTCTACGACGAGCTCCACGCGTACCTCG	7:G>C
3934563|F|0-7:G>C-7:G>C	TGCAGCTGGAGCGGATCAACGTCTACTACAACGAGGCGAGCGGGGGCCG	7:G>C
7168136|F|0-20:T>C-20:T>C	TGCAGGCGTCGCGGGCCTTCTTCCG	20:T>C
2253714|F|0-19:A>C-19:A>C	TGCAGCGTTCACGTACTCGAGGGAGCTGCACGACGTGGTGGAGGTGGGCAAGGCCG	19:A>C
1053607|F|0-32:G>C-32:G>C	TGCAGCAGCTCCCGCAGCTCCGCGCCGCCCGTGATCCCGTTCTCCACCACCATCTCCGCCATGGACCGC	32:G>C
4261664|F|0-31:T>A-31:T>A	TGCAGCAGCCTGAGCTCCACCGTCGAGTCCTTCGGCGGCCCGCGGGCGCGGCCG	31:T>A
4540164|F|0-23:G>C-23:G>C	TGCAGCCAGTTGCCGACGATGGGGGCGCCG	23:G>C
3953982|F|0-31:G>A-31:G>A	TGCAGAGCACGGACGACGCAGAGGTCCACATGTCGTCGGCCTCGACGGCGACGACGTCAGGGCGCGGCT	31:G>A
1027592|F|0-43:G>A-43:G>A	TGCAGTGGATCATGGCCGAACTGGTCAAGAACCCAGAAATCCAGGAGAAGCTCTACGAGGAGATCAAAG	43:G>A
13880978|F|0-10:C>G-10:C>G	TGCAGCGGGGCACCTTCCACCG	10:C>G
2365472|F|0-52:T>G-52:T>G	TGCAGCTGGAGCGCGAACTCGAGGTTCCAGAGGACGTCGCCCATGGACGGCCTGTCGACGCTGCGGTCG	52:T>G
3023313|F|0-21:A>G-21:A>G	TGCAGAAAACAATTCAACTAGACCAGCTCCTTCCGCCG	21:A>G
992922|F|0-64:C>G-64:C>G	TGCAGCTCCCACTGGCATCTAAATGCGGCTACATAAGGAAGCTGGTGTCGGGGGCCAACGGGTCCAGGG	64:C>G
1695198|F|0-58:C>G-58:C>G	TGCAGGACAATGGCATCAAGAAGGTGAAGCTGTTCGACGCGGACGCGGAGCCGCTGGGCGCGCTCGCCG	58:C>G
7350492|F|0-14:G>A-14:G>A	TGCAGCAGCAGCGGGCGGCCGCACGCCG	14:G>A
1865360|F|0-18:T>C-18:T>C	TGCAGCGTCACCCTCCTGTTCTTCGACAGCTGCAAACGTAAACAAATCGCACCGTAAACCCTAGCCCCG	18:T>C
3953851|F|0-14:T>C-14:T>C	TGCAGCCCCGCCGTTCTCGGGCTCACCG	14:T>C
5324497|F|0-13:G>A-13:G>A	TGCAGTGCGGGGGGTTCGGCCG	13:G>A
985479|F|0-43:C>G-43:C>G	TGCAGGTGTTGTAGGACTGGCAATGGCAACACTTTCTCTGTCCCGCCATGCCCG	43:C>G
1863330|F|0-18:T>C-18:T>C	TGCAGCTGCTGGTCCACGTAGTAGGAATCGACGTCGAAGTTGGTGACCGCGGCGAGGCCCCG	18:T>C
5363584|F|0-23:T>C-23:T>C	TGCAGCTACCTCGTGCTGCTGTCTTACCG	23:T>C
4005686|F|0-14:G>C-14:G>C	TGCAGGCACGCCCTGGCCACGCCCATCACGGTGGCCACGCTCCCCG	14:G>C
5580214|F|0-25:C>T-25:C>T	TGCAGCCGACGGCGGCCAGCGGCGACGAGCGGCCGAGGTGGTTCTTGCACCCG	25:C>T
5581322|F|0-22:G>A-22:G>A	TGCAGCAGAGGCTGTTGCTTAAGCCACCAGCACGTACACCGCGTAGATGATCCCCG	22:G>A
1091482|F|0-56:G>A-56:G>A	TGCAGGGAGTCGTCGGAGTAGGTCCCCTTGCGCATGAAGCGCGACAGGAGGTCGTCGTGCGGCGTGGAC	56:G>A
7172944|F|0-14:T>A-14:T>A	TGCAGGTGTCGACGTGCGCGGTTGCCCG	14:T>A
2281902|F|0-22:G>A-22:G>A	TGCAGGTCTCGGCGACGCGGGGGTCGCGGTCGCCGAGCGCGGCCATCTGGATCTCGAAGCCCTTGCCGT	22:G>A
5363323|F|0-26:C>G-26:C>G	TGCAGCATCCTGGTGAGCGGGCTGCCCCCG	26:C>G
1007634|F|0-13:G>A-13:G>A	TGCAGAGCAGCCAGGTGCTGCGGAACGGCGGGACGAGCTGCTGCATGCTGGGGCCG	13:G>A
4262823|F|0-44:G>C-44:G>C	TGCAGCAGCTTGCCGATCTTGTCCTTCACCTCGCTCAGCTCCCAGCCG	44:G>C
3944050|F|0-23:C>T-23:C>T	TGCAGATCCATCACGACAAGAAGCTGACGGCGCTCCG	23:C>T
2256569|F|0-20:C>G-20:C>G	TGCAGCTCCGCGTCGCCGTCCACCTCCTTGTCGCTGTGGTAGAAGTGGGCGCAGTAGCTGCGCACCCAC	20:C>G
4999155|F|0-30:T>G-30:T>G	TGCAGCTCCCTCGTTAACAGGTCGCTCGTCTTCTCCACGGGCCCCG	30:T>G
5412030|F|0-47:G>A-47:G>A	TGCAGTTCGCGGTGCCGATGGTCCTCACCAACATGTCCTACTACGGCGTCCCGCTGGTGTCCGTCATGT	47:G>A
1013053|F|0-55:G>T-55:G>T	TGCAGAACGAGCATGGCTGGAGCGCGCTCCAGGAGGCTATCTGCGCGCGTGAGGAGGCGCTCGCACGCG	55:G>T
1021042|F|0-61:G>C-61:G>C	TGCAGGTTCCAGAGCACGTCCTCCACTGAGGGCCTCTGCGTTGGCTCCTTGGCCAGGCAGCGGAGGCAG	61:G>C
1000839|F|0-13:G>A-13:G>A	TGCAGAAGGAGTCGTCGGACGCCTCCGAGGCGCACGACACGTTGAGCGACTGCATGTTCCCGTTCTTCC	13:G>A
2242135|F|0-16:C>A-16:C>A	TGCAGACAAAACATTCCCGAACCATATCACCGTGTTCAGATCTTCAGTTGACCACTCAACATCGACAGC	16:C>A
977117|F|0-32:C>T-32:C>T	TGCAGGTGAGCGCGCCACGCCTGCGCGCGCTCCGCGTCACCGAGTGCTTCAGGCTGGAGTCCAGCGCAG	32:C>T

<table>
<tr><td>6048403|F|0-22:G>T-22:G>T</td><td>TGCAGCAGCTTCTCCACGATCAGGATGACGTTGGCGTAGTGTGATGCCAGCGCCGAGCCGCCCACGGTC</td><td>22:G>T</td></tr>
<tr><td>3935636|F|0-17:C>G-17:C>G</td><td>TGCAGCAGCTGCGCCGCCGACGGCCGCTTCGCCG</td><td>17:C>G</td></tr>
<tr><td>1277774|F|0-44:G>C-44:G>C</td><td>TGCAGAAGATGGAGGGCGCGTTCGGCGAGTGCTCCAAGGGCAAGGCCTTCTTCGGCGGCGACGGCCCGA</td><td>44:G>C</td></tr>
<tr><td>1070154|F|0-33:C>G-33:C>G</td><td>TGCAGAGGTGGAGCTGATGAAGGAGAGGTTCTCCAAGCTGCTGCTGGGCGAGGACATGTCCG</td><td>33:C>G</td></tr>
<tr><td>4261689|F|0-20:C>G-20:C>G</td><td>TGCAGCAGCACGGCGCGGCTCGGGAGGAACACCATGTTGGTCAGCCCG</td><td>20:C>G</td></tr>
<tr><td>1020713|F|0-40:G>A-40:G>A</td><td>TGCAGGTGAGCTCCGACGGCAGCTTGCTCAGGAACGTGACGGTGTACACCG</td><td>40:G>A</td></tr>
<tr><td>1023426|F|0-35:T>G-35:T>G</td><td>TGCAGAACCTGAAGACGCTGCTCCTCTACTCCAACTCCCTCGTCGGCACCATCCCTCCG</td><td>35:T>G</td></tr>
<tr><td>2242200|F|0-6:G>C-6:G>C</td><td>TGCAGGGCCGAGGGCTTCGACAACGGCCATTGTGACAACCTCTTTACATGCATCTGCTACAGAAACTGC</td><td>6:G>C</td></tr>
<tr><td>992992|F|0-67:C>T-67:C>T</td><td>TGCAGCCCTCAGCGCCGAGGACCGCGTCGGCCTCGTCAATGCCCTGAAGGTACGGTTACTCGTTGTTCG</td><td>67:C>T</td></tr>
<tr><td>3952616|F|0-5:G>C-5:G>C</td><td>TGCAGGAGCCTCTTCTGGCCG</td><td>5:G>C</td></tr>
<tr><td>3030529|F|0-40:G>A-40:G>A</td><td>TGCAGCGCGAGTTCCGCGGCGCCGTCAGGTACGCCATGGAGCGCCG</td><td>40:G>A</td></tr>
<tr><td>3022242|F|0-7:T>C-7:T>C</td><td>TGCAGGATCCTGTTCCTGCTCACCCTATCCCTCCCCCG</td><td>7:T>C</td></tr>
<tr><td>1093774|F|0-58:G>A-58:G>A</td><td>TGCAGCAGGTCGGGATACGCGCGCGGTCCGCGCTCGTCGCCGTCGTGTACGAGAAGGGGCTGGCCCTCT</td><td>58:G>A</td></tr>
<tr><td>1021523|F|0-5:C>T-5:C>T</td><td>TGCAGCTGATGTTCGTGGACCAGAGAAAGGAGAGAACAGAGCTGCTGCGAGAACTACTCAGGAGCGGAG</td><td>5:C>T</td></tr>
<tr><td>1696973|F|0-64:A>G-64:A>G</td><td>TGCAGATGTTCTCGCCGCCGCTGATGATCACGTCCTTTGACCTGTCCTTTACCTCGATGTACCCATCCG</td><td>64:A>G</td></tr>
<tr><td>1114845|F|0-15:C>T-15:C>T</td><td>TGCAGCTTCTTCATGCTGTGCTGCTGCTTCTTCTGCCATGAGGCGTGCGAGCACTGCCTCAAGCGCTTC</td><td>15:C>T</td></tr>
<tr><td>3025764|F|0-6:C>T-6:C>T</td><td>TGCAGGCCGCCAACAGCAACCTCCCCGCGCCCACCTCCG</td><td>6:C>T</td></tr>
<tr><td>3938821|F|0-21:G>A-21:G>A</td><td>TGCAGCTGGAGGCGGCGGGGCGGAAGCGCCAGGGGCCG</td><td>21:G>A</td></tr>
<tr><td>32025654|F|0-46:A>G-46:A>G</td><td>TGCAGGTGGCGCTCAACCACAGCAAACTCGTCGACCGCCAGCTCAGATCGCTCTACGCCCCGCCACCGA</td><td>46:A>G</td></tr>
<tr><td>1707920|F|0-25:G>A-25:G>A</td><td>TGCAGGCAGAGGAGCGGCAGATGGAGGCGGAGGTGTCCGCGCTCGAGTCCGCCACCG</td><td>25:G>A</td></tr>
<tr><td>5367722|F|0-13:C>G-13:C>G</td><td>TGCAGGAGCTCGCCGCAGCGCCCG</td><td>13:C>G</td></tr>
<tr><td>32027441|F|0-8:T>C-8:T>C</td><td>TGCAGCTCTCCCCTCGTCGCCCATGCACAGACGCCG</td><td>8:T>C</td></tr>
<tr><td>1153776|F|0-56:G>C-56:G>C</td><td>TGCAGGCCGCTCAAGGGGAGCTTCTACGTCGGCGTCACCTTCCTGCGCCTGCTGCCGCACGCGTATGAA</td><td>56:G>C</td></tr>
<tr><td>3942374|F|0-25:C>G-25:C>G</td><td>TGCAGCACTTGGTTGCCGACGCCAGCACCG</td><td>25:C>G</td></tr>
<tr><td>977990|F|0-21:C>G-21:C>G</td><td>TGCAGCTCACGCCCTCCAACTCCAACCTCACCCTCCTCTACAACTGCAAGGCCG</td><td>21:C>G</td></tr>
<tr><td>1053988|F|0-50:C>T-50:C>T</td><td>TGCAGCTTCCTGACGCTGCTGATGACCGCCTCCTGCCTCGGCCTCTTCCTCGCCACCACGCGCCGAGAT</td><td>50:C>T</td></tr>
<tr><td>15306770|F|0-10:C>A-10:C>A</td><td>TGCAGGAGAGCGGCCTTGCCG</td><td>10:C>A</td></tr>
<tr><td>1696722|F|0-11:G>C-11:G>C</td><td>TGCAGCGCCTTGCCGAGGAGGAGCTCGTTGGTGTGCGGGCCGTAGATGTCGGAGGTGTCGAGGAGGGTG</td><td>11:G>C</td></tr>
<tr><td>1020888|F|0-20:T>C-20:T>C</td><td>TGCAGGCAGAAGCGCGCCCCTTGGAAGACGCGCATGAAGGTGCTGGGCACGAGGCAGTTCTTCTCGCTC</td><td>20:T>C</td></tr>
<tr><td>7167407|F|0-21:T>G-21:T>G</td><td>TGCAGAAGCGGCGCGGAGGCGTTGCTGTCGCCG</td><td>21:T>G</td></tr>
<tr><td>4993581|F|0-5:A>C-5:A>C</td><td>TGCAGAGGCCTCCTGGACACGCCG</td><td>5:A>C</td></tr>
<tr><td>1276799|F|0-29:G>A-29:G>A</td><td>TGCAGCGAGATGGCCACCGACAGGCTGCCGTCGCGGCTGGCGCTGGGGAGCACGAACGCGAGGCCCTCG</td><td>29:G>A</td></tr>
<tr><td>5367206|F|0-8:G>T-8:G>T</td><td>TGCAGCTTGCCGCCGCGCGGCTCGCCGCGGGCCG</td><td>8:G>T</td></tr>
<tr><td>7170457|F|0-8:T>C-8:T>C</td><td>TGCAGACCTTGTACTTCGCCGACCG</td><td>8:T>C</td></tr>
<tr><td>1153776|F|0-39:T>C-39:T>C</td><td>TGCAGGCCGCTCAAGGGGAGCTTCTACGTCGGCGTCACCTTCCTGCGCCTGCTGCCGCACGCGTATGAA</td><td>39:T>C</td></tr>
<tr><td>997849|F|0-62:G>A-62:G>A</td><td>TGCAGAGCTGCGAGCAGCGGTTCATCTGGGTGCTGCGCGACGCCGACCGCGCCGACATCTTCGCGGAGG</td><td>62:G>A</td></tr>
<tr><td>1102396|F|0-53:A>G-53:A>G</td><td>TGCAGGAGGCGGAGGCCGACCCCGCCAAGCGTCCCCGCCGACCCCTGCGGGCTATTCATCCACAGTCCG</td><td>53:A>G</td></tr>
<tr><td>4261738|F|0-22:C>T-22:C>T</td><td>TGCAGCAGCTGTTCCTCGACGACAACAGCTTCGTCGGGGAGCTGCCG</td><td>22:C>T</td></tr>
<tr><td>1696722|F|0-55:T>C-55:T>C</td><td>TGCAGCGCCTTGCCGAGGAGGAGCTCGTTGGTGTGCGGGCCGTAGATGTCGGAGGTGTCGAGGAGGGTG</td><td>55:T>C</td></tr>
<tr><td>1002433|F|0-46:G>A-46:G>A</td><td>TGCAGGAGACGATGTGGCGGAGGAGGTGGCGGCCGACGCCGCCGCAGCCGAGGAGCACCACCG</td><td>46:G>A</td></tr>
<tr><td>2248596|F|0-49:C>T-49:C>T</td><td>TGCAGCTTCTCAGCGACGGTACAGTCGTGCGCTCTGCGGCCTTGCCCTTCCCTGTCGACGGCAACGTCG</td><td>49:C>T</td></tr>
<tr><td>4986796|F|0-7:C>G-7:C>G</td><td>TGCAGCTCGCCTTCACCGCCACCTTCTTCGCCG</td><td>7:C>G</td></tr>
<tr><td>1070921|F|0-67:C>T-67:C>T</td><td>TGCAGCTCCGCTCCGTCAGCCTCGGGGACCCGTCCCTGTCCGCCACCGCGCAGGTCACGCTCGCCTCCC</td><td>67:C>T</td></tr>
<tr><td>4407572|F|0-14:C>G-14:C>G</td><td>TGCAGGTCGAGCGTCACCTCCACCAGCTCCTCCCCGTCCTCTGAGGCCG</td><td>14:C>G</td></tr>
<tr><td>3032678|F|0-20:G>A-20:G>A</td><td>TGCAGGTGCGAGACGATGCTGCCGTCCTCCGCCCGCCACACGCCG</td><td>20:G>A</td></tr>
<tr><td>5367153|F|0-8:C>G-8:C>G</td><td>TGCAGCTCCAGGATGCCCCCGCCG</td><td>8:C>G</td></tr>
<tr><td>1216931|F|0-62:A>T-62:A>T</td><td>TGCAGCATGAGCTCGTAGTTCTTATGCCCCTTGGAGATGGTCTGCCCAGGTCGCTTCCCCTCAGGCGTG</td><td>62:A>T</td></tr>
<tr><td>1102396|F|0-20:C>G-20:C>G</td><td>TGCAGGAGGCGGAGGCCGACCCCGCCAAGCGTCCCCGCCGACCCCTGCGGGCTATTCATCCACAGTCCG</td><td>20:C>G</td></tr>
<tr><td>6046289|F|0-20:T>C-20:T>C</td><td>TGCAGCGTCACCACGCGCCATGCGCCGACGACCGCGCTGGCCGACGCGACATGCTCGTCTTTGAGGATG</td><td>20:T>C</td></tr>
<tr><td>987565|F|0-52:C>T-52:C>T</td><td>TGCAGCGCTTCTCCCGCCACGTGATGCACGAGCTCGTCCGCCTCGTCTTCGCCCGCCTCCCGCAGATAG</td><td>52:C>T</td></tr>
<tr><td>1075122|F|0-41:A>G-41:A>G</td><td>TGCAGGTAGGAGTGTGACCCATTGCCCGCCTCGTCCCCGAAATCCTGCCCCG</td><td>41:A>G</td></tr>
<tr><td>3934013|F|0-18:T>A-18:T>A</td><td>TGCAGAGTCTCCTTCTTCTCCG</td><td>18:T>A</td></tr>
<tr><td>3942684|F|0-25:C>T-25:C>T</td><td>TGCAGGCGGCGTCCGCGGCCGGGATCGTCCG</td><td>25:C>T</td></tr>
<tr><td>3025764|F|0-28:G>C-28:G>C</td><td>TGCAGGCCGCCAACAGCAACCTCCCCGCGCCCACCTCCG</td><td>28:G>C</td></tr>
<tr><td>1864917|F|0-45:C>A-45:C>A</td><td>TGCAGATGCTCCCCGACATGCCGCTCGACGCCGACGTGTTCCGGCCGCCGCCTGGCGACAATGCGCCGA</td><td>45:C>A</td></tr>
<tr><td>1039421|F|0-38:G>A-38:G>A</td><td>TGCAGTTCCTCGGCCATCCTGCCCATGTTCCCATGATCGGAAGCAGCAGGGGTAGCCAGAGCTGGTGCA</td><td>38:G>A</td></tr>
<tr><td>992992|F|0-65:T>C-65:T>C</td><td>TGCAGCCCTCAGCGCCGAGGACCGCGTCGGCCTCGTCAATGCCCTGAAGGTACGGTTACTCGTTGTTCG</td><td>65:T>C</td></tr>
<tr><td>4262772|F|0-8:G>C-8:G>C</td><td>TGCAGGTCGCCGATGGCGGGGGGCTGGTAGGCGCCGCCGCCG</td><td>8:G>C</td></tr>
<tr><td>5582181|F|0-13:A>G-13:A>G</td><td>TGCAGATGGCGGTACAGATCCG</td><td>13:A>G</td></tr>
</table>

<table>
<tr><td>1707920|F|0-17:C>A-17:C>A</td><td>TGCAGGCAGAGGAGCGGCAGATGGAGGCGGAGGTGTCCGCGCTCCAGTCCGCCACCG</td><td>17:C>A</td></tr>
<tr><td>3026234|F|0-23:G>C-23:G>C</td><td>TGCAGCGCCTCCACCTTCCGCCCGAGGATGCACGCCCG</td><td>23:G>C</td></tr>
<tr><td>3946186|F|0-17:C>G-17:C>G</td><td>TGCAGCCGCGCCGACGGCGACAGGGTGCAGATTCTGTCGAAGGCCGTGGCCGCCGCGAGCTCCAGCTCC</td><td>17:C>G</td></tr>
<tr><td>32021406|F|0-10:C>T-10:C>T</td><td>TGCAGCGGGACGGCTGCCTGCTCCG</td><td>10:C>T</td></tr>
<tr><td>1202124|F|0-53:C>A-53:C>A</td><td>TGCAGCTCGTTGTACGCCTCATAGAGAAGCCCCCGCGCGCTCTCCTCCTCATCCTCACCCG</td><td>53:C>A</td></tr>
<tr><td>2242135|F|0-23:A>G-23:A>G</td><td>TGCAGACAAAACATTCCCGAACCATATCACCGTGTTCAGATCTTCAGTTGACCACTCAACATCGACAGC</td><td>23:A>G</td></tr>
<tr><td>1205783|F|0-15:G>A-15:G>A</td><td>TGCAGGCATGTGTCGGCCACGATAGCTTCTGGCCTCCGCCCCAGCTCCAGCACCAGCCG</td><td>15:G>A</td></tr>
<tr><td>1214806|F|0-11:A>G-11:A>G</td><td>TGCAGTGGCGGATGGCGGACACGCGAGGCGAAACGTGGGGGAGCAGGATGCGCCGCGGCGCCGCGCAGC</td><td>11:A>G</td></tr>
<tr><td>1153776|F|0-14:G>C-14:G>C</td><td>TGCAGGCCGCTCAAGGGGAGCTTCTACGTCGGCGTCACCTTCGTGCGCCTGCTGCCGCACGCGTATGAA</td><td>14:G>C</td></tr>
<tr><td>1050193|F|0-67:G>T-67:G>T</td><td>TGCAGAAGGTCACCAACCCCAAGACGATCCAGAAGATCGCCAAGTCCAAGAACCGAGGTCAGCTCAGGG</td><td>67:G>T</td></tr>
<tr><td>1050193|F|0-38:G>T-38:G>T</td><td>TGCAGAAGGTCACCAACCCCAAGACGATCCAGAAGATCGCCAAGTCCAAGAACCGAGGTCAGCTCAGGG</td><td>38:G>T</td></tr>
<tr><td>1049504|F|0-35:C>T-35:C>T</td><td>TGCAGGAATGGCACCATCCTGCCCAAGTCCATGGACGCGGCTCAGTCAAAATCGGCGTTCCAAATGCAG</td><td>35:C>T</td></tr>
<tr><td>1133865|F|0-12:A>G-12:A>G</td><td>TGCAGTTCCACCACGCGTCGGGCGACATGTACATCGCCGACGCGTACCTGGGGCTGATGAGGGTGGGGC</td><td>12:A>G</td></tr>
<tr><td>3935850|F|0-25:C>T-25:C>T</td><td>TGCAGAGCGACTGCCTCCACTTCTGCCTCCCCG</td><td>25:C>T</td></tr>
<tr><td>2281902|F|0-37:C>G-37:C>G</td><td>TGCAGGTCTCGGCGACGCGGGGGTCGCGGTCGCCGAGCGCGGCCATCTGGATCTCGAAGCCCTTGCCGT</td><td>37:C>G</td></tr>
<tr><td>1202124|F|0-28:G>T-28:G>T</td><td>TGCAGCTCGTTGTACGCCTCATAGAGAAGCCCCCGCGCGCTCTCCTCCTCATCCTCACCCG</td><td>28:G>T</td></tr>
<tr><td>1025259|F|0-34:C>T-34:C>T</td><td>TGCAGGAGATGGCCTTCTCCTGCGGCGACGACGTCAGAGGAGGCTACCCG</td><td>34:C>T</td></tr>
<tr><td>5332251|F|0-10:G>A-10:G>A</td><td>TGCAGGATCCGACGGGGAGGGCGAGCTTCTGCCG</td><td>10:G>A</td></tr>
<tr><td>989564|F|0-22:A>G-22:A>G</td><td>TGCAGAGCGAGCCGACGGACGCAGAGGTCGCCGAGGCCATGAACATGCCG</td><td>22:A>G</td></tr>
<tr><td>4001967|F|0-32:T>A-32:T>A</td><td>TGCAGCCAGGCGGGCACCGATTTCCTGGCAATTGTTTACTCCG</td><td>32:T>A</td></tr>
<tr><td>1013053|F|0-22:C>T-22:C>T</td><td>TGCAGAACGAGCATGGCTGGAGCGCGCTCCAGGAGGCTATCTGCGCGCGTGAGGAGGCGGTCGCACGCG</td><td>22:C>T</td></tr>
<tr><td>5332330|F|0-7:G>A-7:G>A</td><td>TGCAGGCGATCGCCTCCAGGTTGTACCG</td><td>7:G>A</td></tr>
<tr><td>3942803|F|0-10:G>A-10:G>A</td><td>TGCAGTTCTCGAACGCTTGCGGCGCCATCTGCACCACCAAGAAGGGAGCCATCCCG</td><td>10:G>A</td></tr>
<tr><td>5332578|F|0-6:T>C-6:T>C</td><td>TGCAGGTTTGGTCCTCCTCGCCGCCGTCGCCCCG</td><td>6:T>C</td></tr>
<tr><td>1022722|F|0-41:G>C-41:G>C</td><td>TGCAGGGACTGCTTGTAGTCGCTGGCGGCGGTGATGGTGACGACGAGGGAGATGGTGAGCATGATGCCG</td><td>41:G>C</td></tr>
<tr><td>5364104|F|0-16:A>C-16:A>C</td><td>TGCAGTGGCTCGACCTATCAGGCAACCAGCTCACCG</td><td>16:A>C</td></tr>
<tr><td>5580143|F|0-5:C>A-5:C>A</td><td>TGCAGCAATGCGCCACACACCG</td><td>5:C>A</td></tr>
<tr><td>1212149|F|0-38:G>A-38:G>A</td><td>TGCAGCGCGCGCAGCCGCACCGCGGCGCGCCTGGCCTCGCGCAGCACGGCGAGGAGCTCCCCG</td><td>38:G>A</td></tr>
<tr><td>5332031|F|0-15:G>C-15:G>C</td><td>TGCAGCGTGGCCAGGGCGCGCCGCCG</td><td>15:G>C</td></tr>
<tr><td>3935392|F|0-22:G>C-22:G>C</td><td>TGCAGCTGAACTCAACCTCGCGGGGCCG</td><td>22:G>C</td></tr>
<tr><td>7171576|F|0-11:C>G-11:C>G</td><td>TGCAGCTCCTCCCGCCGCTGCTTCAGCTTCCG</td><td>11:C>G</td></tr>
<tr><td>3954453|F|0-10:G>C-10:G>C</td><td>TGCAGAACGGGAGCGGGGGCAGCGTCACCCCGACCGTCCAGTGGCCG</td><td>10:G>C</td></tr>
<tr><td>7168113|F|0-13:C>T-13:C>T</td><td>TGCAGGCCGTCTTCGCCCGCCTGCCG</td><td>13:C>T</td></tr>
<tr><td>986780|F|0-26:C>T-26:C>T</td><td>TGCAGCCCCGACGAGGTGATCTTCGGCGCCGACTTCGCGCAGTCGCTCTACTGCCACCTCCTCTGCGGC</td><td>26:C>T</td></tr>
<tr><td>1023455|F|0-14:C>T-14:C>T</td><td>TGCAGTCCCACTGGCGCGCAGACCTGCGAGACGGTCCGTGGCTCCAACCTGAGCCTGCTCCTCCTGGGC</td><td>14:C>T</td></tr>
<tr><td>986780|F|0-65:C>T-65:C>T</td><td>TGCAGCCCCGACGAGGTGATCTTCGGCGCCGACTTCGCGCAGTCGCTCTACTGCCACCTCCTCTGCGGC</td><td>65:C>T</td></tr>
<tr><td>3946939|F|0-7:C>T-7:C>T</td><td>TGCAGTTCTTGGGCAACCCG</td><td>7:C>T</td></tr>
<tr><td>3946939|F|0-5:T>C-5:T>C</td><td>TGCAGTTCTTGGGCAACCCG</td><td>5:T>C</td></tr>
<tr><td>6041847|F|0-7:G>C-7:G>C</td><td>TGCAGGTGACCACGCAGGCGCCGCCGCCGCCCATGTCGGTGGCCTCCG</td><td>7:G>C</td></tr>
<tr><td>1218327|F|0-35:G>A-35:G>A</td><td>TGCAGCATCTCGTGAGCAACCTTAAGGCCTTCCTTGTACCTGCCCTCTCTGAAGAACCCGCGCATAAGA</td><td>35:G>A</td></tr>
<tr><td>5347251|F|0-25:C>T-25:C>T</td><td>TGCAGATCGCGCACGGGGTCCCGTCCCCG</td><td>25:C>T</td></tr>
<tr><td>5358121|F|0-19:A>C-19:A>C</td><td>TGCAGGACCTCACCCTGCGACACCTCCCG</td><td>19:A>C</td></tr>
<tr><td>4261686|F|0-36:A>T-36:A>T</td><td>TGCAGGAATTATTCTTGGCCCAGCATGGAAGTGGGCAACACAGCCCCG</td><td>36:A>T</td></tr>
<tr><td>3029119|F|0-7:A>G-7:A>G</td><td>TGCAGCTAGAGCAGCTCACCGCGCTCCCTGACCACGCCATGCCG</td><td>7:A>G</td></tr>
<tr><td>32024130|F|0-9:G>A-9:G>A</td><td>TGCAGCGACGCAGATTAGCGATTTGACCG</td><td>9:G>A</td></tr>
<tr><td>989201|F|0-27:G>T-27:G>T</td><td>TGCAGTGCACCGTTGCTCTGCCTCCGTGGACTTCATGGAGGAAACGATGAGGCCGTACCCG</td><td>27:G>T</td></tr>
<tr><td>2250961|F|0-59:T>A-59:T>A</td><td>TGCAGGGCCACCCCAACGTGATCGGCCTCCATGGCCTCATCACCTCCCGCTCCTCCGCCTGCATCTACC</td><td>59:T>A</td></tr>
<tr><td>3574545|F|0-20:G>A-20:G>A</td><td>TGCAGCTGGCGCCCAAGAAGGTCACCCCCAGGGTCACCG</td><td>20:G>A</td></tr>
<tr><td>4262734|F|0-7:G>A-7:G>A</td><td>TGCAGAGGTACCCTGACATGAGCAAGGCTCTGATGCAGGCCG</td><td>7:G>A</td></tr>
<tr><td>4910945|F|0-13:C>T-13:C>T</td><td>TGCAGCGCGTCCCCGTATCTGCCCTCCTCGTCGACAAGGCAATGGCAATGGCAATGCCAACGGTGGCAA</td><td>13:C>T</td></tr>
<tr><td>4262512|F|0-11:A>G-11:A>G</td><td>TGCAGTCGTCGATGCACAGCTGCCG</td><td>11:A>G</td></tr>
<tr><td>1319093|F|0-16:A>G-16:A>G</td><td>TGCAGAAGCTGACGCCATGGGCGGACGGGCCGAGCATGGTGACGCAGTGCCCGATCCAGCCCAACGGCT</td><td>16:A>G</td></tr>
<tr><td>3026915|F|0-5:C>G-5:C>G</td><td>TGCAGCGAGGGGAAGGCGGCACCAGCACTGCTGCCACCG</td><td>5:C>G</td></tr>
<tr><td>988005|F|0-11:C>G-11:C>G</td><td>TGCAGCGGCATCAGCCACAGGTTGTGGAGCTCGTGCGTGACATTGGACACCTCCTCGGCATCCACCTCC</td><td>11:C>G</td></tr>
<tr><td>5360684|F|0-26:A>C-26:A>C</td><td>TGCAGATGAAGTAGTTCGTCCCGGGGACGAGGGTCACGCGGTCGCTCCCCGAGGTGTAGGTGTGCGTCG</td><td>26:A>C</td></tr>
<tr><td>1699288|F|0-45:G>A-45:G>A</td><td>TGCAGGTAAGCTCCCACTGATCCTGTTGTTGTTCAGGTTGATCCTGTACAGAGACTGGCATCTAGCAAT</td><td>45:G>A</td></tr>
<tr><td>4002607|F|0-22:G>A-22:G>A</td><td>TGCAGAGATTGCAAAGGAGCTGGACGTGCAGGGGCGTGGGATCCCG</td><td>22:G>A</td></tr>
<tr><td>986306|F|0-17:G>A-17:G>A</td><td>TGCAGCTTGGACAGCCCGCCGAAGCTCCTCGGGATGGGGCCCGTGAGCATGTTGAGCGAGAGGTCAATG</td><td>17:G>A</td></tr>
<tr><td>1034613|F|0-51:C>A-51:C>A</td><td>TGCAGCTGGCAGTGCTCACCTATCATAGATAAATAAAAATCACAGCTAGGACAAGCGAATAATACACGG</td><td>51:C>A</td></tr>
</table>

3960705|F|0-8:C>A-8:C>A	TGCAGGCGCTTCGGGGCGCCCACGGTGCTGCCG	8:C>A
1027737|F|0-10:G>C-10:G>C	TGCAGGCGGGGCTGAGCTGGGCGTGCGGGCAGGGCCAGGCCGACTGCTCCGCGATCCAGCCCG	10:G>C
1071006|F|0-5:C>G-5:C>G	TGCAGCCGCGGCACCGCCCAAGGGCGACAAGTCGGCGGCCAAGAAGAAGGGCGGATGGCTCAAGAACAT	5:C>G
1018977|F|0-62:C>G-62:C>G	TGCAGAGGCTTCTGATTCCACACCTGACCAATGCATCATGATGTTGCTTAATCATTTTATCTCCACGAT	62:C>G
1217790|F|0-28:C>G-28:C>G	TGCAGATCCTGATCAACGTGCCGTTCTCCTACTACGCCTTCACCACCGTCTTCTTCCCGTTCCTGACGC	28:C>G
1209817|F|0-25:G>C-25:G>C	TGCAGTTGGTGGACGGCACGGCGGAGAGCTCCAGCCTCCCCGTCCACTCCTCCCCG	25:G>C
1153577|F|0-29:C>T-29:C>T	TGCAGCTGAGACTGCACAAGGTAGGCACCCAGTTTCTTCGCTGATGGATCATTCGGCTTGCTGCCGGCC	29:C>T
4733881|F|0-19:C>G-19:C>G	TGCAGCTGCACACCACGCGCTCGTGGGACTTCCTCGACACGCAGTCCG	19:C>G
1766794|F|0-26:A>G-26:A>G	TGCAGCCGCTGGATCCAGCTCGGTGCATTCTACCCGTTCGCGAGGGCGCACACGGAGAGAACCACCGAA	26:A>G
1025259|F|0-35:A>C-35:A>C	TGCAGGAGATGGCCTTCTCCTGCGGCGACGACGTCAGAGGAGGCTACCCG	35:A>C
5000424|F|0-42:T>G-42:T>G	TGCAGCAACGTCCTCCTCGACCACAACTACCGCGCCCGCGTCTCCGACTTCGGCACCGCCAAGGTCGGC	42:T>G
4909682|F|0-17:G>A-17:G>A	TGCAGAGCCTCGCGGCTGCTGTGGTCCG	17:G>A
5580275|F|0-26:G>A-26:G>A	TGCAGCGGTCGACGGTGCTCCTCATGGCGACCG	26:G>A
3941346|F|0-23:C>T-23:C>T	TGCAGGATCTGCATTGGCACGTACAACGCCGCCGGCATAGAGCCGCCG	23:C>T
3956491|F|0-40:G>A-40:G>A	TGCAGGGCGTGGTGCTCCGCCCGCCCTCGCACCTCCCGCAGCCG	40:G>A
3029316|F|0-14:A>G-14:A>G	TGCAGTAACGCAGTAAATCCAGCGCGCGCAGCAGTGGCGCCG	14:A>G
7173892|F|0-8:C>G-8:C>G	TGCAGCAGCAGTTCGTGTCCTTGGCCACCATCGTGCCCG	8:C>G
988457|F|0-57:T>C-57:T>C	TGCAGCTGCCGATGACGTCGTTGAAGCTGGGGGACTGCATCATCTCCAGCACGGTGTTGCGGCCGAGAT	57:T>C
1004688|F|0-35:C>A-35:C>A	TGCAGCTTGTGCGGCCCCTTCGCCTCGGCCGCACCCGCGTCGCCCTCGGCGGCGACCTCCG	35:C>A
1034613|F|0-15:T>A-15:T>A	TGCAGCTGGCAGTGCTCACCTATCATAGATAAATAAAAATCACAGCTAGGACAAGCGAATAATACACGG	15:T>A
988005|F|0-13:G>A-13:G>A	TGCAGCGGCATCAGCCACAGGTTGTGGAGCTCGTGCGTGACATTGGACACCTCCTCGGCATCCACCTCC	13:G>A
1214806|F|0-27:G>T-27:G>T	TGCAGTGGCGGATGGCGGACACGCGAGGCGAAACGTGGGGGAGCAGGATGCGCCGCGGCGCCGCGCAGC	27:G>T
985521|F|0-29:T>C-29:T>C	TGCAGGGCCTGTGGTTCTACCAGACGGCGTTCACGCTCTACGGGCCGATGCTCCCCGAGGGGTGCCACC	29:T>C
6040521|F|0-25:T>G-25:T>G	TGCAGGGAGGAGGCGGAGGCGGGGCTGGGCATAAGAGGAGCGGCTCCATGGATGGAGCCAGCTCGCCGT	25:T>G
4262167|F|0-34:G>A-34:G>A	TGCAGGGGCCCTTCTCGCTGCGCGTCACCGACGAGTCCG	34:G>A
2242200|F|0-65:C>T-65:C>T	TGCAGGGCCGAGGGCTTCGACAACGGCCATTGTGACAACCTCTTTACATGCATCTGCTACAGAAACTGC	65:C>T
997705|F|0-22:C>G-22:C>G	TGCAGCAGGCGGCGGCGATGCACGGGCTCATGCTGCCTTGCGATGCGCAGAGCAGGGCGGTCGGCTCCG	22:C>G
985659|F|0-24:G>A-24:G>A	TGCAGCAGCATGGACATCTGCGCTGCCTGCTGGGCCTGGTAATGGTGGACAAGCAGGGGGGTGTTGTAG	24:G>A
1017505|F|0-16:T>C-16:T>C	TGCAGTTCTTGAAGGCTCACGGGAGGGAGCTGTTTCTGAGCTGCGGCTCCAACGCCACCGCCGATGCCG	16:T>C
1019946|F|0-13:G>C-13:G>C	TGCAGGCGCAGGAGGACGTCGTGGGCGAGATGAAGGAGAACGCGTCCAAGACGCTCGTCCGCGTCACCA	13:G>C
1707920|F|0-7:A>G-7:A>G	TGCAGGCAGAGGAGCGGCAGATGGAGGCGGAGGTGTCCGCGCTCGAGTCCGCCACCG	7:A>G
1023925|F|0-64:A>C-64:A>C	TGCAGTTCACGCAGATCTGCAACCTCTGCTACCCGTGGCCGTGCAGCGACCCGAGCCACTGGGGAGGCG	64:A>C
3956646|F|0-8:G>C-8:G>C	TGCAGCAGGCGGACGGCGACGGCGAGCGGTGCAGCACCG	8:G>C
1217790|F|0-42:C>G-42:C>G	TGCAGATCCTGATCAACGTGCCGTTCTCCTACTACGCCTTCACCACCGTCTTCTTCCCGTTCCTGACGC	42:C>G
3959212|F|0-5:G>A-5:G>A	TGCAGGAAGGTGTTCCAGTCGCGGCGCTTCTGCGACTCGTACCG	5:G>A
3946186|F|0-7:G>A-7:G>A	TGCAGCCGCGCCGACGGCGACAGGGTGCAGATTCTGTCGAAGGCCGTGGCCGCCGCGAGCTCCAGCTCC	7:G>A
1136150|F|0-17:G>T-17:G>T	TGCAGGCACTGCGCGCAGCCCCCCGCCCCGACCGTCTCCACACACTGCGCCATCGCGTACACGCCGAGA	17:G>T
1059183|F|0-53:C>A-53:C>A	TGCAGTACGAGGCGCGCATGCTCCAGAAGGAGCTGGAGGTGAGGAGCCAGGAGCGGGAGTACGACCTCA	53:C>A
2257077|F|0-17:G>A-17:G>A	TGCAGGTTGGCGACCTCGGGGTCGAAGTAGGCGGACGTCAGGATCTCGTGCGGCAGCAGCGCGCCCGCC	17:G>A
7914816|F|0-8:G>T-8:G>T	TGCAGGTTGGTCAGGCTCCG	8:G>T
2257077|F|0-67:C>A-67:C>A	TGCAGGTTGGCGACCTCGGGGTCGAAGTAGGCGGACGTCAGGATCTCGTGCGGCAGCAGCGCGCCCGCC	67:C>A
12769221|F|0-43:G>T-43:G>T	TGCAGTCGAGGACGCGGCAGCGGAGATGGCGGCGAGGAGGCGGGAGGTGGGAACGGGCAGGGGCATGGC	43:G>T
1046321|F|0-21:C>T-21:C>T	TGCAGCGAGACTCGGATTTGGCTGCCCGCGGCACAGAACATGGAAACGAATCAGGGGAAAGTGAAATCG	21:C>T
981417|F|0-64:T>C-64:T>C	TGCAGTTATTTTGTCACTCAACACAAGGATCAGGATCTAAAGATGCTTTGATGTTGTGCTGCGCTGTGC	64:T>C
5332131|F|0-29:G>C-29:G>C	TGCAGCTGGGCGTGGAGAAGGACTGGCGGGCGTGGTCGCCG	29:G>C
1035638|F|0-32:T>C-32:T>C	TGCAGCTGAGACTGCACAAGGTAGGCACCCAGTTTCTTCGCTGATGGATCATTCGGCTTGCTGCCGAGA	32:T>C
1075099|F|0-21:C>G-21:C>G	TGCAGGTCCATTCCAGTTCGTCGCAGAGCCCTACAACGGCACCATACCGCGCCTGCAAATCAACCCCTA	21:C>G
2250546|F|0-6:A>G-6:A>G	TGCAGCACCTGAGCGCCCATGTCGATCGCCTGCGAGGAGACCGTGGTCGGCCTGCCCG	6:A>G
1071006|F|0-36:G>A-36:G>A	TGCAGCCGCGGCACCGCCCAAGGGCGACAAGTCGGCGGCCAAGAAGAAGGGCGGATGGCTCAAGAACAT	36:G>A
1081186|F|0-20:A>G-20:A>G	TGCAGAATCCATTTGCAAGAATCTAATCTACGCTCAGATAGTCCTGGCACCG	20:A>G
4262581|F|0-26:A>G-26:A>G	TGCAGCTTGTCGAGGTACTGAGCCCCATGGCCCTTCTGGATGGTGCCG	26:A>G
1694707|F|0-29:G>A-29:G>A	TGCAGGAAGTCGATGTAGTGCAGCGCGCCGTGGATGAACTGCGTGCCGCCGCCG	29:G>A
4261845|F|0-14:G>T-14:G>T	TGCAGCGGCACCGCGCACGCCAGAGCCGCGCCGTCGCCCG	14:G>T
1023925|F|0-25:C>A-25:C>A	TGCAGTTCACGCAGATCTGCAACCTCTGCTACCCGTGGCCGTGCAGCGACCCGAGCCACTGGGGAGGCG	25:C>A
1156567|F|0-45:G>T-45:G>T	TGCAGGTGTGGTGGTGAGTGGCAAGTGTGGCTGCCTTGGTCCGATGCTGCCGCCGCCACCACCGTGGCG	45:G>T
1060814|F|0-38:C>G-38:C>G	TGCAGAGCACCAGTTCCAACCCTTGTCCTTGACCTGTGCATTGAAGGAAAGCTTCAGCCTCAGCTTCAA	38:C>G
988005|F|0-28:G>C-28:G>C	TGCAGCGGCATCAGCCACAGGTTGTGGAGCTCGTGCGTGACATTGGACACCTCCTCGGCATCCACCTCC	28:G>C
2252204|F|0-37:C>T-37:C>T	TGCAGACCTTCTTCGACATGGTGGACAAGGACGCCGACGGCAGGATCACCGAGGCGGAGGTCGGGCAGA	37:C>T
1130281|F|0-7:C>T-7:C>T	TGCAGGCCCTGCATCCTGCACATGCTGGAGCACGTGAGCGCCTCGAGCGCCG	7:C>T

<table>
<tr><td>13142884|F|0-7:G>A-7:G>A</td><td>TGCAGCAGGCCGTTAAAGCCG</td><td>7:G>A</td></tr>
<tr><td>4407908|F|0-17:G>A-17:G>A</td><td>TGCAGGGCGTTCACCTCGTCGTAGTCGTAGTAGACGGTGGCGCCG</td><td>17:G>A</td></tr>
<tr><td>32026363|F|0-23:C>G-23:C>G</td><td>TGCAGCTCCTGCTGCGCGGCAGCCAGCTCTTTCCTCAGAGACGCATGGCTCGCGGCGAGCCGTTCGTTC</td><td>23:C>G</td></tr>
<tr><td>1020677|F|0-67:G>C-67:G>C</td><td>TGCAGATTGGTAGCACCTGCGTGAACGGGACGAGCTTCTGGTGCTCCTGCAACAGCTCCGTCAAGTAGT</td><td>67:G>C</td></tr>
<tr><td>1023947|F|0-7:C>G-7:C>G</td><td>TGCAGTCCGCCGAGAACGAGGTGCTGGGGCTGGGGCAGACGCAGAAGGGCGGGCCCGCGGCCGTCATGC</td><td>7:C>G</td></tr>
<tr><td>5324497|F|0-18:C>G-18:C>G</td><td>TGCAGTGCGGGGGGTTCGCCCG</td><td>18:C>G</td></tr>
<tr><td>32026372|F|0-12:A>G-12:A>G</td><td>TGCAGCTGTTGCAGTATTCCTCCAGCTTGTACCAACAGCCGATGCTGCCGCAGCAGCTCCAGCAGCAGC</td><td>12:A>G</td></tr>
<tr><td>7169242|F|0-7:A>C-7:A>C</td><td>TGCAGATAGCGCACGCGGTCCCGTCCCCG</td><td>7:A>C</td></tr>
<tr><td>32019041|F|0-6:T>A-6:T>A</td><td>TGCAGGTCATACCAATTTGGCCG</td><td>6:T>A</td></tr>
<tr><td>32026210|F|0-11:C>G-11:C>G</td><td>TGCAGCGGAGCCAGATAGCCATGCTCTCGCCCTCCGCGTCGTCTTTGCCCCCCG</td><td>11:C>G</td></tr>
<tr><td>32027236|F|0-15:C>T-15:C>T</td><td>TGCAGTTCGCCCTGACCTCGGCGTGGCGGCCG</td><td>15:C>T</td></tr>
<tr><td>32024280|F|0-15:A>G-15:A>G</td><td>TGCAGCATTCTCCGAAGCCG</td><td>15:A>G</td></tr>
<tr><td>32025931|F|0-32:A>G-32:A>G</td><td>TGCAGAAGACGAAGGTGATCGAGACCTTCCAGAGCCAGGCCAACAAGACCGACGAGGGCATCACCATCT</td><td>32:A>G</td></tr>
<tr><td>1092037|F|0-62:G>C-62:G>C</td><td>TGCAGCGGGCGGAGGAAGTCGGCGAGCAGCATGTAGAGGCCGTAGAGCACGAGCAGCGCCGTGGCGAGC</td><td>62:G>C</td></tr>
<tr><td>7171186|F|0-26:G>C-26:G>C</td><td>TGCAGCTCGTCCAGCACACCTCGCCCGCCAGCGTGGACGCAGAAGTGCTCCAGCGCCAGAGTGAAGTCG</td><td>26:G>C</td></tr>
<tr><td>5357223|F|0-12:T>C-12:T>C</td><td>TGCAGAACGCGATCAAAATCGAAACCGTCAGAGAAGATAAAACCG</td><td>12:T>C</td></tr>
<tr><td>5369298|F|0-46:A>G-46:A>G</td><td>TGCAGAGGGATGGGAGCAGCAGGTGGCGACGGCAAAGGTGTGCGGGAATGGCCG</td><td>46:A>G</td></tr>
<tr><td>7909597|F|0-29:T>C-29:T>C</td><td>TGCAGCGACTGCGGGCAGAGCTTGCCGATTTCCG</td><td>29:T>C</td></tr>
<tr><td>100581292|F|0-41:C>T-41:C>T</td><td>TGCAGGCCGTGAGCACGCCCGTGAACGTCACCGCATCTGGCCTGAACCCCCTCCTCCGCATTGCCTCGA</td><td>41:C>T</td></tr>
<tr><td>100485124|F|0-26:G>A-26:G>A</td><td>TGCAGCAAGCGCGGCCGCGGGCCCGTGATGGCCAGGACTGTCGCGGCCACCATGTTCGTCGTGCTCGGC</td><td>26:G>A</td></tr>
<tr><td>5373336|F|0-16:T>C-16:T>C</td><td>TGCAGGCCCTTCTGCATGCCGTCAGATGCCG</td><td>16:T>C</td></tr>
<tr><td>4985870|F|0-8:C>A-8:C>A</td><td>TGCAGCGGCAGCTGCCTCCTCGCGAACTTGCACTCCAAGGGCGGCAGGACCG</td><td>8:C>A</td></tr>
<tr><td>7354061|F|0-41:T>G-41:T>G</td><td>TGCAGGCGGATCGTCCACAACACAGTGGGCAGCGACAAGGCTGTGGCGAAGCTCTTCAGCGGGATGTCG</td><td>41:T>G</td></tr>
<tr><td>1679384|F|0-38:A>C-38:A>C</td><td>TGCAGCACGGCTACACACTCGTGCTCGTTCCCTGGCTGAACCTCCCATATCCACACAGCCTTGTCCCGG</td><td>38:A>C</td></tr>
<tr><td>6048250|F|0-11:A>C-11:A>C</td><td>TGCAGGTGCCGAAGCGGCGGCGCGCTGGCCTTGCCGAGGTGGCCCAGCTCCG</td><td>11:A>C</td></tr>
<tr><td>32021913|F|0-11:T>C-11:T>C</td><td>TGCAGCTCCCTTCCCGACTCGATGAGCGGCAAGGTGGTGCTCTGCGAGAGCCGCGGCTTCAAGGAGCAC</td><td>11:T>C</td></tr>
<tr><td>7169366|F|0-14:A>G-14:A>G</td><td>TGCAGCATCTTCAGACCACCCAACGCCGCCG</td><td>14:A>G</td></tr>
<tr><td>100503342|F|0-30:C>G-30:C>G</td><td>TGCAGGCCGAGGGCAAGATGCCGCTGCCGACCAGCACTGACGTTGCCTCGCGGATGCTCGATGGCGCAG</td><td>30:C>G</td></tr>
<tr><td>32027258|F|0-56:G>A-56:G>A</td><td>TGCAGACCATCAACGGCCCCAACTCCGACTTCCCCGTCTACTCGTCGGAGCTCAAGGTGGTCAAGGGCA</td><td>56:G>A</td></tr>
<tr><td>5363800|F|0-31:T>C-31:T>C</td><td>TGCAGGCGCTCGGCGGCGTCCTGGAGATCTTTCAGGTGGCCG</td><td>31:T>C</td></tr>
<tr><td>1072921|F|0-38:G>A-38:G>A</td><td>TGCAGGACGCAGCCCCTGGAGACGTCGGCGAAGCACTCGTGGCCGAGCTCCTCGTCGGACGCGAAGACC</td><td>38:G>A</td></tr>
<tr><td>100516268|F|0-20:A>C-20:A>C</td><td>TGCAGGTCGCGCCCGTACGGAGGGAAGTTGCTCTTCAGCACCGTGCCGATCACGTTGTTGTTGCCCGTG</td><td>20:A>C</td></tr>
<tr><td>2269789|F|0-20:G>C-20:G>C</td><td>TGCAGCTGCCCGCGGCATAGGCCCCCATGAGACCGAACTGGTCGAGCAGCATGGCGGCCTGGAGCGCGC</td><td>20:G>C</td></tr>
<tr><td>4262512|F|0-5:T>A-5:T>A</td><td>TGCAGTCGTCGATGCACAGCTGCCG</td><td>5:T>A</td></tr>
<tr><td>1018154|F|0-20:C>T-20:C>T</td><td>TGCAGGTACTACACCTCGTTCCAGCTCAGCGAGAAGAGCGACGTGTACAGCTTCGGGATCGTGCTGCTG</td><td>20:C>T</td></tr>
<tr><td>1062010|F|0-11:G>A-11:G>A</td><td>TGCAGCTTCTCGTATTACGCCCTGCCAGGCTTCATCTTCCCGAGCCTGACGTCCATCGCATGGGTGTGC</td><td>11:G>A</td></tr>
<tr><td>5580214|F|0-18:G>A-18:G>A</td><td>TGCAGCCGACGGCGGCCAGCGGCGACGAGCGGCCGAGGTGGTTCTTGCACCCG</td><td>18:G>A</td></tr>
<tr><td>7917211|F|0-5:G>T-5:G>T</td><td>TGCAGGAAGTCGCCCACGTTCACCACCAGCGCCCCG</td><td>5:G>T</td></tr>
<tr><td>5358121|F|0-25:C>G-25:C>G</td><td>TGCAGGACCTCACCCTGCGCCACCTCCCG</td><td>25:C>G</td></tr>
<tr><td>4407908|F|0-26:G>A-26:G>A</td><td>TGCAGGGCGTTCACCTCGTCGTAGTCGTAGTAGACGGTGGCGCCG</td><td>26:G>A</td></tr>
<tr><td>1060660|F|0-32:A>G-32:A>G</td><td>TGCAGCGACCTCTTGAGCTCGGTGAAAAGGTCACCCCAGCCATCGGTGCCCGTGTCGCTGACCACGGTC</td><td>32:A>G</td></tr>
<tr><td>1100382|F|0-53:G>C-53:G>C</td><td>TGCAGCGAGGAGGAAGGGACGCCCATGGCGTCCTTGAGGAAGAAGTTGACGCCGAGCCAGGGGAAGAGC</td><td>53:G>C</td></tr>
<tr><td>1068752|F|0-32:C>G-32:C>G</td><td>TGCAGGATCCCCGTCGCCACAGGCATCATCATCACCGTGCACGCCGTGTTGTGGATCCACATGCTGACG</td><td>32:C>G</td></tr>
<tr><td>4261450|F|0-7:T>G-7:T>G</td><td>TGCAGCCTCGGGAGGTCGCAGAGCGCCATGGGGAGCTTGCCG</td><td>7:T>G</td></tr>
<tr><td>1162736|F|0-24:A>G-24:A>G</td><td>TGCAGCACGCGCTTGGCGCCCGTGACCTCCAGGACCCTCCTCGCCCTCATCTGGCTGCCGCCGTCGCCG</td><td>24:A>G</td></tr>
<tr><td>1162736|F|0-36:C>T-36:C>T</td><td>TGCAGCACGCGCTTGGCGCCCGTGACCTCCAGGACCCTCCTCGCCCTCATCTGGCTGCCGCCGTCGCCG</td><td>36:C>T</td></tr>
<tr><td>5367153|F|0-7:C>T-7:C>T</td><td>TGCAGCTCCAGGATGCCCCCGCCG</td><td>7:C>T</td></tr>
<tr><td>994225|F|0-62:C>G-62:C>G</td><td>TGCAGGATGGTCGCCGCCCATGTCCACTGCGACACGATCACGCTGGCGATCAGCCCCGTCTTCACGCTC</td><td>62:C>G</td></tr>
<tr><td>5581554|F|0-10:T>C-10:T>C</td><td>TGCAGGTGCTTGACGTCGTGGTCTCCG</td><td>10:T>C</td></tr>
<tr><td>2250546|F|0-5:C>T-5:C>T</td><td>TGCAGCACCTGAGCGCCCATGTCGATCGCCTGCGAGGAGACCGTGGTCGGCCTGCCCG</td><td>5:C>T</td></tr>
<tr><td>3953613|F|0-31:G>T-31:G>T</td><td>TGCAGCTAGGCGGGCCGAGCTGGGCGGTGCCGCTGGGCCG</td><td>31:G>T</td></tr>
<tr><td>988449|F|0-40:C>G-40:C>G</td><td>TGCAGCTGGCCGTGGACGCCGCCGCCGCCAAGTTCCTCGCCGCCGAGAAGCTGGAGGTGGTGGACTACA</td><td>40:C>G</td></tr>
<tr><td>1035804|F|0-37:A>G-37:A>G</td><td>TGCAGCCGCGGTTAACGCTGGCAACGAGGCATCCCTCAGTGGTGGCCATGGGAACATGGTAGTCGCGCC</td><td>37:A>G</td></tr>
<tr><td>2249021|F|0-64:C>G-64:C>G</td><td>TGCAGAACAGCGCCAAGGGGAGGGAGGTCGTCGAGCGGATCTGCCGCTACCTCGAGGCCGAGGCCGCCG</td><td>64:C>G</td></tr>
<tr><td>3026535|F|0-5:C>T-5:C>T</td><td>TGCAGCAGCTGCTCCAGCTCCTTGACGAAGTTGATGGCCCCG</td><td>5:C>T</td></tr>
<tr><td>3957231|F|0-49:C>A-49:C>A</td><td>TGCAGGCGCTGGTGAGCGTGGACATGCTGGTGCTGTTCGTGGCGACCATCTGCGGCGTCGGCGGCACGC</td><td>49:C>A</td></tr>
<tr><td>1029272|F|0-18:C>G-18:C>G</td><td>TGCAGTCGCCGTCCGCGGCCAGGCGGCCGACCATGGAGCACGCCGTGACGGTGCTCTCGGCGGCCATCA</td><td>18:C>G</td></tr>
<tr><td>1026114|F|0-52:C>A-52:C>A</td><td>TGCAGCCAGAGGACGGTCGGCCATGGCTTGGTGGGCGTGGACACCCTCTGGGCCTGGGGGCTCTTGTAG</td><td>52:C>A</td></tr>
<tr><td>985521|F|0-46:G>C-46:G>C</td><td>TGCAGGGCCTGTGGTTCTACCAGACGGCGTTCACGCTCTACGGGCCGATGCTCCCCGAGGGGTGCCACC</td><td>46:G>C</td></tr>
</table>

5359391|F|0-28:C>T-28:C>T	TGCAGGTGAGGCTGACGAAGGTGAAGTTCCCCG	28:C>T
1408775|F|0-34:C>T-34:C>T	TGCAGGTCGGGTTCCCCCCTAAGCCGCTCGACGACGCGACCAAGACCATTGAGGAAGCCG	34:C>T
1149940|F|0-37:G>A-37:G>A	TGCAGACGGCCTTCTTCTACGTCTCGCTCTACCTGGTGGCCATCGCGCAGAGCGGCCACAAGCCCTGCG	37:G>A
5001575|F|0-13:T>C-13:T>C	TGCAGGCGGTAGATCACGTCCG	13:T>C
982151|F|0-34:G>C-34:G>C	TGCAGAAGCTCCTCGTCCTCGCCTCGCTCGCCGTGTGGTCCCGCCTCCCCGTCCCCTTCGCCG	34:G>C
3941039|F|0-19:C>G-19:C>G	TGCAGGTGGTGCCGTTCGGCGGGCTGGAGTGGCTGTCCCG	19:C>G
1072921|F|0-14:C>T-14:C>T	TGCAGGACGCAGCCCCTGGAGACGTCGGCGAAGCACTCGTGGCCGAGCTCCTCGTCGGACGCGAAGACC	14:C>T
1864269|F|0-41:G>A-41:G>A	TGCAGGTTGTGCGCCAGGGCGTCGAACGCAATGCAGAGCAGGAGCTCCAGCTGCTGCTGCGGCTGCGGC	41:G>A
1708221|F|0-12:G>A-12:G>A	TGCAGCTGCACAGCGGGATGAAGGCGGTGAACCACATATGGCAGGTCGGCACCTCCGTCACCGCCGAGA	12:G>A
1000580|F|0-13:T>C-13:T>C	TGCAGCAGGTTCCTCGCGAGCCCCACGGCGATGAGCCCCGCGCTGCACCCCATGCCG	13:T>C
1164941|F|0-44:G>A-44:G>A	TGCAGCTCGACGCCATCATCGGCCACCACCAGAGCACCAGCCTAGCCCTGCCG	44:G>A
1133865|F|0-16:G>A-16:G>A	TGCAGTTCCACCACGCGTCGGGCGACATGTACATCGCCGACGCGTACCTGGGGCTGATGAGGGTGGGGC	16:G>A
1000731|F|0-22:G>A-22:G>A	TGCAGATACGCAGCCACGCCCAGAAGTACTTCCTCAAGGTCCAGAGGCTGGGCCTCGCCGCCG	22:G>A
985906|F|0-15:G>T-15:G>T	TGCAGGCACCAAGGAGAATCTGAGGGAGATGTTCGGCTCGGTGGGGAAGAGGTTCAGCGAGGCCGCGCG	15:G>T
1685839|F|0-33:C>T-33:C>T	TGCAGGGCCACGCCACGCTCGAGGCCGAGGTGGCGCGGCTGAGGGGCCTCCTCTTCGACGTCCG	33:C>T
1090065|F|0-10:G>T-10:G>T	TGCAGGCCCCGCACGAACGCCACGGCGTACTTGCTCGCCGTGGTGGGGTCCTCGCCG	10:G>T
1019099|F|0-57:T>G-57:T>G	TGCAGCAACTTCTCCAGAGCGGAGACCACAGCAGCATGGCTCTGGTCGCCCACAATCTCCG	57:T>G
1209668|F|0-15:A>T-15:A>T	TGCAGCACATCCTCCAGGGCTGCTCCAAGCTGGAGAAGCTGGAGATAAGGGACTGCCCGTTCGGCGACG	15:A>T
3020531|F|0-18:A>G-18:A>G	TGCAGTTGGAAGAGAAGTAGTGGACCTTTGCCTTGACCG	18:A>G
2245520|F|0-20:G>A-20:G>A	TGCAGCAGAGCCACCACGGCGAACTCCTTCTCCCTCGCAGGGCCGTCCTCGATGGTCTCAACCAGTGCG	20:G>A
988005|F|0-38:G>T-38:G>T	TGCAGCGGCATCAGCCACAGGTTGTGGAGCTCGTGCGTGACATTGGACACCTCCTCGGCATCCACCTCC	38:G>T
996421|F|0-67:C>G-67:C>G	TGCAGGGGCTGTCCAAGTACGACCTCGTGGACATGGACGGCGACGCGCAGGTGCGGTGCTTCCCGCGCG	67:C>G
988647|F|0-46:C>G-46:C>G	TGCAGAAGAACCTGGAGCTGTCGCCGCGGCACGTGGAGGCGTCGCGCATGACGCTGCACCG	46:C>G
5332746|F|0-15:A>G-15:A>G	TGCAGTGGGGACGCCAGCATACATTGCACCG	15:A>G
1004688|F|0-43:C>T-43:C>T	TGCAGCTTGTGCGGCCCCTTCGCCTCGGCCGCACCCGCGTCGCCCTCGGCCGCGACCTCCG	43:C>T
2278631|F|0-50:A>C-50:A>C	TGCAGCTCCCCTCCGACTGCAACGTCGACGGCATCCGCGCCAAGTTCGAGAACGAGGCGCTCACCATCA	50:A>C
4262298|F|0-35:G>T-35:G>T	TGCAGCGCCTCGATGCCCTCCTCGACGGAGGCGGCGGCCCCGCCG	35:G>T
1695026|F|0-44:G>C-44:G>C	TGCAGCTCTCCTACTCCATCCTCGACCAGGAAGGCCATGGCGAGGCCCCAGGTGATGTGCACATCCAGA	44:G>C
5324854|F|0-26:G>A-26:G>A	TGCAGCCTCAGCGCCGCCGTCGGCATGAGCGGCCCG	26:G>A
4993523|F|0-15:C>T-15:C>T	TGCAGTTGAGCCAGACGAGGTCGCTGCCG	15:C>T
4262820|F|0-35:T>A-35:T>A	TGCAGGAGGTCGCCGAGCTGGCGTCCCCGTTCGGGTCGTCCCCG	35:T>A
3957000|F|0-6:T>C-6:T>C	TGCAGGTGCTGGCGCCCTTCCCCG	6:T>C
5581293|F|0-7:G>A-7:G>A	TGCAGAGGGGCGGAGAGCGGCCG	7:G>A
1201646|F|0-32:G>C-32:G>C	TGCAGCCCATGATCAATGCGGCAGCATCCTTTGGAGATCCAGAGCTGCTCGGCGACATCCAGATCGGCC	32:G>C
1089503|F|0-15:A>G-15:A>G	TGCAGTACCGTTGGCAGCCACGAGCAGGTCCTGGAGCATGCTGCTGCTGCTCCG	15:A>G
1023947|F|0-31:G>C-31:G>C	TGCAGTCCGCCGAGAACGAGGTGCTGGGGCTGGGGCAGACGCAGAAGGGCGGGCCCGCGGCCGTCATGC	31:G>C
5580395|F|0-5:G>A-5:G>A	TGCAGGCACATACGACGGTCGCGTTCATGGCCG	5:G>A
1184439|F|0-63:G>A-63:G>A	TGCAGACTCGAGCTTCGTGCCGCCGCTGGTGTACGCGATGATGGGGAGCTCCAAGGACCTGGCGGTGGG	63:G>A
987258|F|0-6:G>A-6:G>A	TGCAGAGCGGGCAAGTGCTGCTGGTGCGCAGCCACGTCCTGAGGCAGCGCCTGTGGAACATGTGGCCGC	6:G>A
5332656|F|0-11:C>T-11:C>T	TGCAGTCGAACCGCACCGCCACCG	11:C>T
1864269|F|0-5:G>A-5:G>A	TGCAGGTTGTGCGCCAGGGCGTCGAACGCAATGCAGAGCAGGAGCTCCAGCTGCTGCTGCGGCTGCGGC	5:G>A
3028420|F|0-10:C>G-10:C>G	TGCAGTACTTCTACGAGCTGGACCTGAGCAACAACAAGCTGGCGCCG	10:C>G
1021700|F|0-50:G>C-50:G>C	TGCAGTCTCTCCAGCAGCTCCCCAAGGCTTGTCCCGTTGAACAGGAACGAGTGCCCCTCATCTCCCTCA	50:G>C
7352997|F|0-7:A>G-7:A>G	TGCAGGTAGTGGCTGCCAAACTCCG	7:A>G
997705|F|0-61:C>G-61:C>G	TGCAGCAGGCGGCGGCGATGCACGGGCTCATGCTGCCTTGCGATGCGCAGAGCAGGGCGGTCGGCTCCG	61:C>G
985906|F|0-55:A>G-55:A>G	TGCAGGCACCAAGGAGAATCTGAGGGAGATGTTCGGCTCGGTGGGGAAGAGGTTCAGCGAGGCCGCGCG	55:A>G
1023097|F|0-5:G>A-5:G>A	TGCAGGGCGCCGCCCTCCTTGGTCCTCCGATGGGAGGTGGCGCCGAAGCCG	5:G>A
7165592|F|0-20:A>G-20:A>G	TGCAGCTTCGAGAAGGTGAGAGAGATGCTCCG	20:A>G
1022998|F|0-32:A>G-32:A>G	TGCAGGTGCTGAGCGCGCTGGACGGCGCCAAGACGCAGTTGTACCACTTCAGGGCGATCGTTGTCGCCG	32:A>G
1862625|F|0-44:C>G-44:C>G	TGCAGAGTGAAGTTGACATGGTCCTGCACGCCCTCGAGGTGCTTCTGGGCGGCGACGTTCACCTCCTCC	44:C>G
1241882|F|0-28:G>T-28:G>T	TGCAGCTGAAGAAGAACAAGCTGGCGGGGACCTGCAAGCTCAAGCTGAAGCTCGACTCGCCG	28:G>T
4396424|F|0-23:G>C-23:G>C	TGCAGCGGGTCCAGCCCGCCGACGGCCACCATCGCCG	23:G>C
988005|F|0-47:C>G-47:C>G	TGCAGCGGCATCAGCCACAGGTTGTGGAGCTCGTGCGTGACATTGGACACCTCCTCGGCATCCACCTCC	47:C>G
5332131|F|0-18:A>C-18:A>C	TGCAGCTGGGCGTGGAGAAGGACTGGCGGGCGTGGTCGCCG	18:A>C
5578047|F|0-5:T>C-5:T>C	TGCAGTGATTGTGTCTGACCGCCG	5:T>C
1694868|F|0-55:G>A-55:G>A	TGCAGGAGAAGTCCATGGGCAGGGTGAAGCCGTCGACGACCGATATGCCGTACCTGCCCG	55:G>A
1021523|F|0-7:G>C-7:G>C	TGCAGCTGATGTTCGTGGACCAGAGAAAGGAGAGAACGGAGCTGCTGCGAGAACTACTCAGGAGCGGAG	7:G>C
2338810|F|0-14:C>T-14:C>T	TGCAGCGCCACCAGCTGCTGCTCCAGCTGCTTCACGTAGTCGATGGCTCCCCCCACCACCGTCGCTTGG	14:C>T
4663806|F|0-24:T>G-24:T>G	TGCAGCCAGAGCAGCAGCGGCGTGTCGGCCG	24:T>G

6040203|F|0-43:G>C-43:G>C	TGCAGCCGTTGACGACACATGCGTTCCGTGCCCATCGATGTCCGTCGGACTCGAATCGGCGAGGCCACC	43:G>C
1069006|F|0-5:C>G-5:C>G	TGCAGCTCTGCTGCATCTCCCTCGGCCTTCTCCTGATGGCCGCCGTCTGCCCGCACGTCCCGCTGCTCA	5:C>G
10592804|F|0-7:G>C-7:G>C	TGCAGGCGTTCGGCGTGGCGCCG	7:G>C
7172983|F|0-26:G>C-26:G>C	TGCAGCCCCGTGGAGGTGGTGTCGGTGCCG	26:G>C
1023501|F|0-58:G>A-58:G>A	TGCAGGAGGTGGACAACATGACGCTGCGGAGGTTCCTGCGCGCGCGCGACCACGACGTGGGCAAGGCCT	58:G>A
3027739|F|0-40:G>C-40:G>C	TGCAGGCACCACCGAGTCGACAACCACTCACGAGAACAACGGCGCCG	40:G>C
977460|F|0-22:G>A-22:G>A	TGCAGGCGCCGCACTTGGGCTCGTGGACCGAGTGGTACGACACCTTCATGTGCTCCACAAGGTGCTCCA	22:G>A
4262953|F|0-22:C>T-22:C>T	TGCAGAAGGTCGACGAGGAGGGCGCTGCCGCCGCCGTCATCAGGCCG	22:C>T
5363149|F|0-5:A>C-5:A>C	TGCAGAGGATGCTTGTGTGCCCGCCG	5:A>C
1221328|F|0-37:C>T-37:C>T	TGCAGGCGTACACCGCCCTGGCCCCCTCCCTGTGCAGCAGCTCGGCGCCCTTGTGCATCGTCCCGAGAT	37:C>T
2242683|F|0-67:G>C-67:G>C	TGCAGCTGCTGGATGTACTCGATCGCGTCCTTGATGATCGAGGCCTTGTCCATCTGCAACGACGAGGGA	67:G>C
6049469|F|0-28:A>G-28:A>G	TGCAGCAGCTGTTCGTGGACATGTTCCAAGCTGACCTCGTCACCG	28:A>G
2257077|F|0-11:G>A-11:G>A	TGCAGGTTGGCGACCTCGGGGTCGAAGTAGGCGGACGTCAGGATCTCGTGCGGCAGCAGCGCGCCCGCC	11:G>A
1695363|F|0-58:G>A-58:G>A	TGCAGCTGCTCTTCTTGGAGGGCAACCTCTTCACCAAAGAGCTGCCGAACGTCGACATGCCATCCCTCT	58:G>A
977479|F|0-34:G>C-34:G>C	TGCAGCTCAAGGGGCACAGGTCGGTCGGCGGCGTGCGCGCCTCCATCTACAACGCCATGCCGCTCTCCG	34:G>C
1207965|F|0-18:G>C-18:G>C	TGCAGAGGCAGCTAAGAAGAAATTTGACATGGAAATCAAGCTGCCCCTGTCCG	18:G>C
1408775|F|0-37:G>T-37:G>T	TGCAGGTCGGGTTCCCCCCTAAGCCGCTCGACGACGCGACCAAGACCATTGAGGAAGCCG	37:G>T
6041551|F|0-11:C>G-11:C>G	TGCAGCTCCAGCCCGAGGAGGCCGCCCCACGTCGCCG	11:C>G
6048618|F|0-8:G>C-8:G>C	TGCAGGCTGGAGGGACCCGCGGCGTGGGACGTGCTCGCCAACTTCGAGCACCG	8:G>C
1001628|F|0-47:C>T-47:C>T	TGCAGCAGCGCAGCCTCTTGCGGCGCCGTGGCGTCGTTGAGGAGGAGCTGCTGGTTGCGGGCCTTGAGG	47:C>T
1130281|F|0-37:G>T-37:G>T	TGCAGGCCCTGCATCCTGCACATGCTGGAGCACGTGAGCGCCTCGAGCGCCG	37:G>T
1167275|F|0-13:G>T-13:G>T	TGCAGGAACCTGGGGTCCGCCCCCTCCGACGGCACCAGATCCTTGCCGCGGAAGTACGGGTGCAGGAGC	13:G>T
1667521|F|0-41:C>G-41:C>G	TGCAGCTTCTCCCCCATCATGCCCTGGCTCACGTCCAGCACCGTCACCAGGTCGATGGGCGCGCGCCGC	41:C>G
3027422|F|0-34:C>T-34:C>T	TGCAGTTCCCGTCGGACATAGCCACCGTTCTTCTCGCCGCCG	34:C>T
4261623|F|0-5:T>A-5:T>A	TGCAGTGCGGATCCAGCAGCCGCGGGGGCAATGCCGCCGCCG	5:T>A
1067333|F|0-48:G>C-48:G>C	TGCAGAGAGGGAAAAGCAGGGGAGCAGGTCGCTGGGCTGCCACGCGTCGTCCACCG	48:G>C
994225|F|0-11:C>T-11:C>T	TGCAGGATGGTCGCCGCCCACGTCCACTGCGACACGATCACGCTGGCGATCAGCCCCGTCTTCACGCTC	11:C>T
3028486|F|0-27:G>C-27:G>C	TGCAGCTCATGTACCACTTCGACTGGAGGCTGCCCGCCG	27:G>C
1209668|F|0-64:C>T-64:C>T	TGCAGCACATCCTCCAGGGCTGCTCCAAGCTGGAGAAGCTGGAGATAAGGGACTGCCCGTTCGGCGACG	64:C>T
1059051|F|0-7:G>C-7:G>C	TGCAGTCGTCCAGGAACCCCACGCCGCGGGGCTCCAGCTTCAACCACGCCGAGTTCTTCAACATCGTCG	7:G>C
3938457|F|0-24:A>G-24:A>G	TGCAGCCCAGGACGCGCCGCCGCGACAGGGCCG	24:A>G
2242135|F|0-46:G>A-46:G>A	TGCAGACAAAACATTCCCGAACCATATCACCGTGTTCAGATCTTCAGTTGACCACTCAACATCGACAGC	46:G>A
1020717|F|0-29:T>A-29:T>A	TGCAGGTAGTCCAGCTTCGCCTTCTCCAGTTCCGCCATCACGCGATCCGCCTTGTATATCCG	29:T>A
7353503|F|0-6:T>C-6:T>C	TGCAGCTTGACGCCCTCCCCG	6:T>C
32026223|F|0-11:G>T-11:G>T	TGCAGCTTGAGGAGGACGCCGAGCAGGTCCTCGTCTCTCTCTGGCTCCG	11:G>T
1285274|F|0-64:G>A-64:G>A	TGCAGGCGCCCGCCAGGGACGTCGTCGCCGTGCTCTCGGCCTACGTGCGCAAGCTCGAGGAGGAGTCCG	64:G>A
3027714|F|0-19:C>G-19:C>G	TGCAGTCGCAGAGCATCGTCCTGCCCGTGACGGGCAGCTCCG	19:C>G
3025379|F|0-13:C>G-13:C>G	TGCAGCCCCAGCTCGCGCGCCAGCTCCGCCTTCTCCCG	13:C>G
5358045|F|0-22:T>G-22:T>G	TGCAGCTGCTTGCTGGCCCCTCTCCG	22:T>G
1092037|F|0-26:C>T-26:C>T	TGCAGCGGGCGGAGGAAGTCGGCGAGCAGCATGTAGAGGCCGTAGAGCACGAGCAGCGCCGTGGCGAGC	26:C>T
1070154|F|0-25:A>C-25:A>C	TGCAGAGGTGGAGCTGATGAAGGAGAGGTTCTCCAAGCTGCTGCTGGGCGAGGACATGTCCG	25:A>C
1236784|F|0-23:T>C-23:T>C	TGCAGCTTGCTGACTTCATAGGGTTAGATGTTTGCCTTTCAGTACTTAGGGTGCTCCACAACGGGCTAG	23:T>C
4262823|F|0-11:G>C-11:G>C	TGCAGCAGCTTGCCGATCTTGTCCTTCACCTCGCTCAGCTCCCAGCCG	11:G>C
6046087|F|0-7:C>T-7:C>T	TGCAGCGCCCCATGGGGTTCACTCTCGGCCACGGCATCCACGAGCGGCACGCCCTCCTGCTCGAGGTCG	7:C>T
6030890|F|0-11:G>A-11:G>A	TGCAGGTGGGCGCTGGTGAAGGACCG	11:G>A
5582046|F|0-7:G>A-7:G>A	TGCAGGCGCACTACCGCCGCATCCCCG	7:G>A
32027466|F|0-9:T>C-9:T>C	TGCAGCACCTCCACCATTGCAGGTTCGACCATGAAATCCACTACCAACACTGGTTTTATCACATCCGGA	9:T>C
7171889|F|0-14:G>T-14:G>T	TGCAGCTTGGAGATGATGGTGGGGCGGCGGAAGCGGGCGGGGTCGACCAGGACGAGGTGGGCGATCTGG	14:G>T
3028027|F|0-8:C>G-8:C>G	TGCAGCCACCCGTAGAAGAAGAGCGCCGGGATCATCCACCG	8:C>G
3025335|F|0-14:C>G-14:C>G	TGCAGCGCCAGCACCCCCATGAACAGCTTCACCTTCTCCCG	14:C>G
7169164|F|0-5:A>G-5:A>G	TGCAGAAGGAAGAGGGCCGAAAGCCG	5:A>G
981402|F|0-17:G>A-17:G>A	TGCAGCAGCAGCACGATGACCACCACCGACGCCACGAAGGAGGTGGAGTTGCAGTAGAAGAAGGCCCGA	17:G>A
1021968|F|0-34:G>A-34:G>A	TGCAGCCGACCAACGAGGTCCTGGAGGGCCTCCTGTCGGACCCCAAGGCCG	34:G>A
4989011|F|0-61:C>G-61:C>G	TGCAGTGGCTGTCGGCGCGGCCGCAGTGGGCGGCCTTCGGCGGCCGCGACCACTTCATGGTCGTCGGCA	61:C>G
3027988|F|0-31:C>G-31:C>G	TGCAGCTGTCCAACAACCAGCTCACGGCGACCGACGCCG	31:C>G
1221328|F|0-13:C>G-13:C>G	TGCAGGCGTACACCGCCCTGGCCCCCTCCCTGTGCAGCAGCTCGGCGCCCTTGTGCATCGTCCCGAGAT	13:C>G
2253805|F|0-9:T>A-9:T>A	TGCAGAGTTTGCGGCATGCTCGAACTGCGACCTGATCTCCCTTGCCACCTCAATATCATCGTTGTAGGA	9:T>A
5582539|F|0-17:G>A-17:G>A	TGCAGGTAGCAGAACCCGTCGCCG	17:G>A
6044786|F|0-5:G>A-5:G>A	TGCAGGTGGTACAGGATGTGGTCGAACATGATGTTCCTCTGGTCCATCTACTCTGTTTTCTACACCCCC	5:G>A

100367445|F|0-52:C>G-52:C>G	TGCAGGAGAAGGAGATGATCGACAACCTCTCCAACAAAAACACGCTCATGTCCGTTCGGGAGCTCGCCA	52:C>G
1020677|F|0-12:G>A-12:G>A	TGCAGATTGGTAGCACCTGCGTGAACGGGACGAGCTTCTGGTGCTCCTGCAACAGCTCCGTCAAGTAGT	12:G>A
1229332|F|0-15:T>G-15:T>G	TGCAGCCAGGTCATCTGCTGGCAGTCGGCGCTCGTCATGCCGAGCTCCG	15:T>G
1091295|F|0-19:A>G-19:A>G	TGCAGTAGCGCATCCACGCACCCCTCCAGGATTGCGACCGCTCATCGCACCTGAGGCAACCACTCCCAA	19:A>G
1056012|F|0-68:G>A-68:G>A	TGCAGTACTTCTTCCCCATCTTCCACTGGGGCTCCAACTACAGCCTCCGCCTCCTCCGCTCCGACGCCG	68:G>A
2284516|F|0-20:C>G-20:C>G	TGCAGCGGGTACCAGCGCGTCCCGAGGGAGCGGTTGTCGGCGTCGAGCACGGCGGAGAGGGGCACCTTG	20:C>G
1766608|F|0-46:C>A-46:C>A	TGCAGTCCTCGAGGCGGTCGAGGAGGTTATCGGCGACCGCGTCTACCAGTGGGTCAGTTTCCCGCATCG	46:C>A
1019805|F|0-22:T>C-22:T>C	TGCAGCCTGCTATTCTTCCCACTGAAGATCTGGTTAAGGATCACCGCCAGCCTCACGCCCCCCTGAGCG	22:T>C
1024561|F|0-5:G>C-5:G>C	TGCAGGCGTTCGCCCTCGGCCTGAACAAGCGCCTCGCCGACGACGACGCCG	5:G>C
1092037|F|0-31:T>G-31:T>G	TGCAGCGGGCGGAGGAAGTCGGCGAGCAGCATGTAGAGGCCGTAGAGCACGAGCAGCGCCGTGGCGAGC	31:T>G
2249108|F|0-31:G>C-31:G>C	TGCAGCAAATCTGGTGGTTCGAGGTGGTCGGGGAGATCGACTTCCGCTTCCCG	31:G>C
1059051|F|0-31:C>A-31:C>A	TGCAGTCGTCCAGGAACCCCACGCCGCGGGGCTCCAGCTTCAACCACGCCGAGTTCTTCAACATCGTCG	31:C>A
1241882|F|0-37:G>A-37:G>A	TGCAGCTGAAGAAGAACAAGCTGGCGGGGACCTGCAAGCTCAAGCTGAAGCTCGACTCGCCG	37:G>A
985768|F|0-25:G>C-25:G>C	TGCAGTTGAAGAACACCGACATGTCGCCCTGCACGTGCAGCGCCACCGCCTGGTGCCCGTCCG	25:G>C
4003062|F|0-10:G>T-10:G>T	TGCAGCGAGCGGCGGCACGCGCGTGGTCGCCG	10:G>T
1079174|F|0-43:G>C-43:G>C	TGCAGCTTCGGCGGCGACACCTTCAAAGGCTTTTGCGGGGGCGGGGGCGACAGCTTTGAATGCTTCTGC	43:G>C
1066550|F|0-31:G>C-31:G>C	TGCAGCTGCACCGTCGCGGCCACAACCTGCCGTGGAAGCTGAAGCAGAAGAACCCCAAGGAGACGCGGC	31:G>C
1059051|F|0-19:C>G-19:C>G	TGCAGTCGTCCAGGAACCCCACGCCGCGGGGCTCCAGCTTCAACCACGCCGAGTTCTTCAACATCGTCG	19:C>G
5580275|F|0-28:G>C-28:G>C	TGCAGCGCTCGACGGTGCTCCTCATGGCGACCG	28:G>C
989720|F|0-27:T>C-27:T>C	TGCAGTCGTCGCTCATCATGTCGGCGGTGGTGTCCGTGGCCATCGCCGTCATCTGGCTGTTCACGGAGC	27:T>C
7168615|F|0-59:G>C-59:G>C	TGCAGCAACGTCCTCCTCGACCACAACTACCGCGCCCGCGTCTCCGACTTCGGCACCGCGAAGGTCGGC	59:G>C
4395722|F|0-5:G>C-5:G>C	TGCAGGAGCAGAACGCTCGTGCCG	5:G>C
32019169|F|0-25:G>C-25:G>C	TGCAGTTCTTCGCCCCCAACCCGACGTGCGGCCTCTCCCCCTTCGTCCG	25:G>C
1049393|F|0-38:C>T-38:C>T	TGCAGGATGATCACCTCGCTCAGCGGCACCCTCTTCCGCACCGCGCCCTCCTCCG	38:C>T
1864269|F|0-16:G>A-16:G>A	TGCAGGTTGTGCGCCAGGGCGTCGAACGCAATGCAGAGCAGGAGCTCCAGCTGCTGCTGCGGCAGCGGC	16:G>A
4002344|F|0-30:T>C-30:T>C	TGCAGCCGTCGTCCGAGCAAACCTTGTAGATGGCGAGCCTGGCGCCCG	30:T>C
4989011|F|0-20:C>A-20:C>A	TGCAGTGGCTGTCGGCGCGGCCGCAGTGGGCGGCCTTCGGCGGCCGCGACCACTTCATGGTCGTCGGCA	20:C>A
991902|F|0-11:A>G-11:A>G	TGCAGACGACGAAGCCGCACCTCCTCCGCGAGCACCAGGTGCATACGCAGCCG	11:A>G
996112|F|0-40:C>G-40:C>G	TGCAGACGCCGCGCACGCTCTACGAGTTCTCCGACTTCCCCTGGCCGCCG	40:C>G
3027265|F|0-11:C>G-11:C>G	TGCAGCGTCGGCCAGCAGTTCGTCTACTCCAGCTTCGCCG	11:C>G
4261325|F|0-26:G>A-26:G>A	TGCAGGTGCTCGCGCATGCGGAGCCAGTCGTGGACGAAGTCCG	26:G>A
4405074|F|0-20:C>T-20:C>T	TGCAGGTTCGCGAACGACCGCGACTTGCCG	20:C>T
1004688|F|0-21:G>A-21:G>A	TGCAGCTTGTGCGGCCCCTTCGCCTCGGCCGCACCCGCGTCGCCCTCGGCCGCGACCTCCG	21:G>A
985906|F|0-39:G>C-39:G>C	TGCAGGCACCAAGGAGAATCTGAGGGAGATGTTCGGCTCGGTGGGGAAGAGGTTCAGCGAGGCCGCGCG	39:G>C
32026311|F|0-11:C>A-11:C>A	TGCAGCAGTGCCACCTCTTGCGGCGCCGTGGCGTCGTTGAGGAGGAGCTGCTGGTTGCGGGCCTTGAGG	11:C>A
4910641|F|0-13:G>C-13:G>C	TGCAGCTCACGCGGCCGACGTCGGCGTCGCCCG	13:G>C
4733518|F|0-37:T>G-37:T>G	TGCAGGCCCAGGCACGGAGCAGCGGGCGCGGCACGGCTGCGCCGTCGGTGTTCGAAGGCGTGGGGAGGA	37:T>G
3031363|F|0-6:C>T-6:C>T	TGCAGGCTGGGTAGGTCCAGCACACCCTGGTTCGCCGCCG	6:C>T
2249021|F|0-8:A>T-8:A>T	TGCAGAACAGCGCCAAGGGGAGGGAGGTCGTCGAGCGGATCTGCCGCTACCTCGAGGCCGAGGCCGCCG	8:A>T
5581037|F|0-7:G>T-7:G>T	TGCAGCTGGTGTTGATCCGCTTGTGGCTCGCGCGGTGGCCGCCCAGCGCCTGGTAGGAGCTGAACACCC	7:G>T
2251962|F|0-27:C>G-27:C>G	TGCAGGCAGCTCCAGGTCGAAGTGGAACAGCAGCGCGGCGAGCGCGAGCTCGATGTGCGCCAGTCCAAA	27:C>G
3021874|F|0-10:T>C-10:T>C	TGCAGCATCGTACACAAACTGGAAGCCAGCGTCAAGTACTTCCG	10:T>C
3064797|F|0-31:G>T-31:G>T	TGCAGTTCCACGGCTTCCACGGCGTGAAGCAGGAGGAGAAGAAGCTCGGGCAGAAGTTCGTGGTCGACG	31:G>T
1695183|F|0-46:C>T-46:C>T	TGCAGGGCGACGAAGCAGCCGTCGAGCGTGAGGAAGACGGCCGCCGCGGCCCAGAACTTGCCG	46:C>T
7172024|F|0-28:C>A-28:C>A	TGCAGAAGGGCCAAGAGCCCATCTTCGGCGCCG	28:C>A
1127561|F|0-11:C>T-11:C>T	TGCAGCGCCTCCGCGAACAGCCGCTTCCCCTCCGCCGACGCGAACTCCACCGCCG	11:C>T
5370854|F|0-14:G>C-14:G>C	TGCAGCTCGGCGGGGTGAGGACGTGGGCCGTGCCG	14:G>C
3023670|F|0-5:C>G-5:C>G	TGCAGCCCTCACAGGCCTGGTCCCTCTTCAAGCTCCG	5:C>G
6045563|F|0-18:A>G-18:A>G	TGCAGCGTCATCCTGGGGACGTCCTTCTGCGCCG	18:A>G
11314453|F|0-7:C>G-7:C>G	TGCAGCTCAGGCCGTGCCCCATCGCCTCCG	7:C>G
32022452|F|0-23:A>G-23:A>G	TGCAGCGGCCCACAGGCGGTGGTATACTGCCG	23:A>G
4262551|F|0-35:C>G-35:C>G	TGCAGTCTGCCGTGGCCGCGGCCGTGGCGGTCGTCCTCGCCCCCGCCG	35:C>G
1024595|F|0-46:T>C-46:T>C	TGCAGCTGGTGTGCTTCGGCTGCACCACGCTCCCGACGTCCCGCTATGGCCCCTACTGGCGCAACCTCC	46:T>C
1251556|F|0-43:C>G-43:C>G	TGCAGCTGTCCCCGAGCCTGCGCAGGACGTCCATCACCTCCTCCGCGGTGATCCTGCCGTCCCTGTCCC	43:C>G
2252204|F|0-63:G>C-63:G>C	TGCAGACCTTCTTCGACATGGTGGACAAGGACGCCGACGGCAGGATCACCGAGGCGGAGGTCGGGCAGA	63:G>C
1046321|F|0-29:G>A-29:G>A	TGCAGCGAGACTCGGATTTGGCTGCCCGCGGCACAGAACATGGAAACGAATCAGGGGAAAGTGAAATCG	29:G>A
1125324|F|0-64:C>G-64:C>G	TGCAGGGCCGCGTGAACGTGACCACCGCGCACTCGCTGGACATCATGGGCGTCTCCTACCCGCCCTCCG	64:C>G
5325248|F|0-11:G>A-11:G>A	TGCAGGCGGGAGAGCGTGGCGGGGGCGACCGCCG	11:G>A
1096095|F|0-13:C>T-13:C>T	TGCAGGGCGAGGACTCGCAGATTGCAGAACCCCCCTGCAATGACGTCCAGATCGTCGTCGATAACACCG	13:C>T

1181960|F|0-11:C>A-11:C>A TGCAGTGCCAGCGGGTGAGCGACTTCCTCATCGCCGCCTCCTACCTCTCCATCCCGCTGGAGCTGCTCT 11:C>A

3940115|F|0-26:G>T-26:G>T TGCAGGACGGGTACGCCGTGGTGGCCGCCGACGGGCCCG 26:G>T

7171009|F|0-6:G>A-6:G>A TGCAGAGCGGCAACGGCTACATCAGCTACGGCGCGCTGCGGCGGGACAACGTGCCCTGCTCCGTGCGCG 6:G>A

7345550|F|0-7:T>C-7:T>C TGCAGATTGTGGTGCACCGACCG 7:T>C

993221|F|0-28:C>G-28:C>G TGCAGAACGTGGTCCAGATGATCTCCGTCATGTTCGTGGGCCACCTCGGCGAGCTCGCGCTCTCCGCCG 28:C>G

1166279|F|0-16:C>G-16:C>G TGCAGTTGGAGATGAGCACCAGCCTCATGACGCCCTCCCTCTCCGCCAGCAGCCCCGTCATCACGGCGT 16:C>G

1212149|F|0-43:G>T-43:G>T TGCAGCGCGCGCAGCCGCACCGCGGCGCGCCTGGCCTCGCGCAGCACGGCGAGGAGCTCCCCG 43:G>T

1020254|F|0-36:C>G-36:C>G TGCAGTTGGAGCTGCTATAGGTGTTGTGGGGGTAGCCGCCTACAGGGTTTATGCGGCTAGGAGGAACTC 36:C>G

1167275|F|0-26:C>T-26:C>T TGCAGGAACCTGGGGTCCGCGCCCTCCGACGGCACCAGATCCTTGCCGCGGAAGTACGGGTGCAGGAGC 26:C>T

4002529|F|0-11:G>T-11:G>T TGCAGCCCGTCGCGGCCGTCCGCCAGCTCCGAGCGCCG 11:G>T

1064449|F|0-17:G>A-17:G>A TGCAGCCTGCACGCGTCGCGCCGCCGCTTGCCGACGCCGAACACGAAGCCCTTGAACACGCGCAGCTCC 17:G>A

3027265|F|0-8:C>T-8:C>T TGCAGCGTCGGCCAGCAGTTCGTCTACTCCAGCTTCGCCG 8:C>T

3028037|F|0-22:G>C-22:G>C TGCAGCAGCACCACGACTACCTGCCCGCCTTCGACGCCGCCACCG 22:G>C

1212122|F|0-9:G>A-9:G>A TGCAGCTACGCCCTCAGCTACCGCGACGGGTCCTACTCCCGCGGCGTGCTGGCGCACGACAAGCTGAGC 9:G>A

1091531|F|0-10:C>T-10:C>T TGCAGTGGTTCGCCGACAAGGGCCTCACCGCCTACAGCATCTCGTGCGGCACCTCCCTGCTGTACAACA 10:C>T

32017660|F|0-28:G>C-28:G>C TGCAGCCTGAACACGTCGCGGTTCCACCGCCG 28:G>C

15306867|F|0-49:T>C-49:T>C TGCAGCGACAGGTCGAAGTCGGAGAGCATGATGTGGCCGTCGTCGCGCATCAGCACGTTCTCCG 49:T>C

4733860|F|0-30:A>T-30:A>T TGCAGCACTACCGCTCCCGCTCCGCCTCCTACGCGCGCTCCCCG 30:A>T

4005778|F|0-22:G>C-22:G>C TGCAGCAGAGCAGCTGGAGCCCGAGCTTGAGCGGCGCCG 22:G>C

1217150|F|0-67:C>T-67:C>T TGCAGCGACCTGAGCAGGAGCAGCAGCAGCTTCCTCCTCCGCTTCCCCTTGTTCTTGCCGCCCGCCTCC 67:C>T

1695363|F|0-46:G>C-46:G>C TGCAGCTGCTCTTCTTGGAGGGCAACCTCTTCACCAAAGAGCTGCCGAACGTCGACATGCCATCCCTCT 46:G>C

1035705|F|0-24:A>G-24:A>G TGCAGCGGTTCCTATCTTGTGGAAAGCATGCCTGGAGCAGAAGGCGAGCTGGGTGTAGGACGGGGAAGA 24:A>G

998928|F|0-13:G>A-13:G>A TGCAGCCGATGAGGTAGAGGAAGACGAGCGAGACGGTGGCGACGACGAGCGCGATGTTGGCCTTGTGCC 13:G>A

4261067|F|0-17:C>G-17:C>G TGCAGCTTCTTGAGCCTCCCGATGCTCGCCG 17:C>G

7168265|F|0-17:G>T-17:G>T TGCAGGTGGTTGTCCTCGAACGGCACCTTGCCG 17:G>T

1038525|F|0-47:G>T-47:G>T TGCAGCTTGAGCTTGAGGTCCCTGATGATGTGGAAGAGGCGCTTGTAGCCG 47:G>T

1026052|F|0-31:C>A-31:C>A TGCAGCGCGAAGTTGTGCGGAAGAGAAGGCACCTGCATGGAGAAGCCCTGCATCTGCGCCGCCGCCGAG 31:C>A

4394508|F|0-15:A>G-15:A>G TGCAGTGCCTGACAAACGACAAGCCACCG 15:A>G

4262734|F|0-28:T>C-28:T>C TGCAGAGGTACCCTGACATGAGCAAGGCTCTGATGCAGGCCG 28:T>C

1063492|F|0-49:C>A-49:C>A TGCAGGTGAAGGTGCTGGTGCTGTCGTCGTAGGCGTAGCTGTAGGCGCGCGGGCACGCCATCTTGAAGA 49:C>A

987468|F|0-20:G>A-20:G>A TGCAGGCGGTGGTAGTAGGTGAGGTTGCCGTCGTAGACGTGGAGCATCAGCGTCGTCGCCTGCCGCTCC 20:G>A

6049356|F|0-14:G>A-14:G>A TGCAGCTCGGTGAAGTGCAGGATGGCTATGTACCCCG 14:G>A

1238129|F|0-19:G>C-19:G>C TGCAGCGACGACATCATCGGGATGGTGTTGCCCAGGCCTGCCCTCATCGCCTCGTCGAGCCGCATCTCC 19:G>C

1022238|F|0-15:T>C-15:T>C TGCAGATACGCTCTCTCCGTTAGCCGAGTGGTGGTCAGCTAGCGAGCTGAGGCCACTCGTACGCAGTGG 15:T>C

3027337|F|0-27:A>G-27:A>G TGCAGGGCCGAGAAGATGAACTCCACCACCACCATGGCCGCCGCCG 27:A>G

100571945|F|0-27:A>G-27:A>G TGCAGCAGTCCGCGTCGTGCACCTTTAAAGCCTCCCGCGCGGCCTCCG 27:A>G

2243475|F|0-57:G>A-57:G>A TGCAGGGTCAGTCTCGATAACTTTGAACTCCACGCTTGTCATTCCGCCTCTCACAAGGAAAAGGTCCCC 57:G>A

7900173|F|0-7:G>C-7:G>C TGCAGCTGACGGCCGTCGACGGCCGCCGCGCCTCCGTGGTCGTGCCCAACGACCCCGACCTCATCGACA 7:G>C

1021253|F|0-13:G>A-13:G>A TGCAGCGCCTGGCGTACCTCAACACGACGGTGTACCCGTTCACCTCCATCTTTCTCCTGCTCTACTGCC 13:G>A

7917211|F|0-29:C>G-29:C>G TGCAGGAAGTCGCCCACGTTCACCACCAGCGCCCCG 29:C>G

1026265|F|0-37:G>A-37:G>A TGCAGGCCGCCATGAAGCAGGCCTCCCGAAGGAGCTCGCCGACCGATTCTTGGGTGGGCAAGCTGCGGA 37:G>A

1013738|F|0-28:C>G-28:C>G TGCAGTCGCTGCTCAGCTGGCGCGACCCCAGGGCGTCCTGCCTCTTCGTCTTCTTCTGCCTCATCGCCG 28:C>G

4261309|F|0-16:C>T-16:C>T TGCAGGAGGCTGCCGCCGCCCG 16:C>T

9721886|F|0-29:G>A-29:G>A TGCAGACGTAGAGCAGAGCCGCCGCGCGCGGTGGCCG 29:G>A

5971458|F|0-61:G>C-61:G>C TGCAGCAGCCGTAGCGGAGGAAGACGCCGAGCGGCGGCAGGATGACGGCGAGCAGGATCTCGACGAAGG 61:G>C

5332517|F|0-20:C>G-20:C>G TGCAGGTCCACGACGGGCTGCTCTACACCG 20:C>G

1026114|F|0-26:C>T-26:C>T TGCAGCCAGAGGACGGTCGGCCATGGCTTGGTGGGCGTGGACACCCTCTGGGCCTGGGGGCTCTTGTAG 26:C>T

1056125|F|0-7:G>T-7:G>T TGCAGCCGCTCCCCCTGCGTGGCCATGTCGCCGACGACGGTCTGTATCCTGCCG 7:G>T

989201|F|0-50:G>A-50:G>A TGCAGTGCACCGCTGCTCTGCCTCCGTGGACTTCATGGAGGAAACGATGAGGCCGTACCCG 50:G>A

3029607|F|0-13:C>G-13:C>G TGCAGAACGCCGTCTGGACCTCACTCTACAAGTGCGTGGAGCCG 13:C>G

7170775|F|0-19:C>T-19:C>T TGCAGGAGAAGCACGCCGCCGCCATTGATAACATCCTGAGGGACCTGCACTGGTCCAGCCG 19:C>T

6047336|F|0-6:C>T-6:C>T TGCAGTCCCACTGACGCGCAGACCTGCGAGACGGTCCGTGGCTCCAACCTGAGTCTGCTCCTCCTGGGC 6:C>T

5581387|F|0-13:C>G-13:C>G TGCAGCGCCGCACCGAGCCCAAGCTGCCG 13:C>G

1020945|F|0-67:C>G-67:C>G TGCAGTGCACGCTCCAGCAATCCGAACCCACCCAACCACCGCACGCTCCGCGGATCCTCCTCGTGCTCC 67:C>G

1060936|F|0-41:C>T-41:C>T TGCAGTTGGAAGACGTAGCGCACCCCGTCTGGAACTTCCACCCTCCGACGGAAGTCGTGGGCGAGGAAG 41:C>T

1004575|F|0-16:C>T-16:C>T TGCAGGCGGCGCGCTGCGCCGTGGCGATGGCGAGGGGAGGCGGCGCGCTGGTCATGAGCAGCAGGGCGC 16:C>T

7911393|F|0-13:C>T-13:C>T TGCAGCGCGCCACCTCGCCG 13:C>T

1028719|F|0-65:G>A-65:G>A TGCAGGACGGCACGAATACCCGCGGCATCTTCGGGCGCGAGACGGCGACGGTGGCGGTGTCGGGCGGGA 65:G>A

7335922|F|0-14:G>A-14:G>A TGCAGCAGCGGGTCGGGCCG 14:G>A

987258|F|0-22:G>C-22:G>C	TGCAGAGCGGGCAAGTGCTGCTGGTGCGCAGCCACGTCCTGAGGCAGCGCCTGTGGAACATGTGGCCGC	22:G>C
5358156|F|0-5:G>C-5:G>C	TGCAGGAGGCTCGCGCCCATCTCCG	5:G>C
1021317|F|0-16:C>G-16:C>G	TGCAGGCCATCAAGCACGACCTCTCCGACCCCTACGGCTTCCTCCGCTCCTGGAACGACACCG	16:C>G
1089129|F|0-30:T>C-30:T>C	TGCAGCACATTCAAAGGTTGAGCGGCAAAGTTTGGCTGAAAGAATTGGTATACATATCCGAATATCGCA	30:T>C
1081642|F|0-18:G>A-18:G>A	TGCAGGATGCGCAGCGAGGAGTTCCTCTCGCCGCCGAACGCCGCGGCCGGGATGGCGCCG	18:G>A
7168869|F|0-26:G>T-26:G>T	TGCAGGAGCGCGACGGGGTTCTGCGCGCGGCTGTAGCTGGGGCGCCACTCGACGTCCAGCCCCACGATG	26:G>T
4394561|F|0-8:G>A-8:G>A	TGCAGGACGAGGAGGGAGCGCAGCGTGTCCCG	8:G>A
992302|F|0-38:C>G-38:C>G	TGCAGCGAGTCCATCGCCTGGAAGGCCTGCGCCAACCCCAGGGGCGTCCGCTGGGCCTACACCG	38:C>G
993148|F|0-15:G>A-15:G>A	TGCAGAGTACACAGCGGCAGTGGCACCCACACGGCCATTAGCCGAGACCCTCTGCTTGAGCTGCCTGTG	15:G>A
1021317|F|0-23:T>G-23:T>G	TGCAGGCCATCAAGCACGACCTCTCCGACCCCTACGGCTTCCTCCGCTCCTGGAACGACACCG	23:T>G
3950056|F|0-22:A>G-22:A>G	TGCAGCCCGAGGTGCTGTGCCGACACCTCAACCG	22:A>G
2338810|F|0-53:C>T-53:C>T	TGCAGCGCCACCAGCTGCTGCTCCAGCTGCTTCACGTAGTCGATGGCTCCCCCACCACCGTCGCTTGG	53:C>T
3022287|F|0-9:T>G-9:T>G	TGCAGACTCTCGCAAGATTCCTCTCCGACATGTTCATGGATCCG	9:T>G
4407215|F|0-23:G>A-23:G>A	TGCAGGCGGATCGTCCACAACACGGTGGGCAGCGACAAGGCGGTGGCGAAGCTCTTCAACGGGATGTCG	23:G>A
1865066|F|0-7:G>A-7:G>A	TGCAGTCGCACGCCAGGAAGCTCAAGGAGGACGTGGCGACGCTCGAGGCGCGGCCG	7:G>A
5580192|F|0-5:C>G-5:C>G	TGCAGCAGGTGGCGGCCCCGCCGCGAGCGCCG	5:C>G
1051692|F|0-19:G>C-19:G>C	TGCAGCTTCTACTGGGCGAGGCGATCACGTCCATGCTCCGATCCGCACATCACGCCTCGGCCTGCCGCG	19:G>C
1708221|F|0-16:G>C-16:G>C	TGCAGCTGCACAGCGGGATGAAGGCGGTGAACCACATATGGCAGGTCGGCACCTCCGTCACCGCCGAGA	16:G>C
4991910|F|0-20:G>A-20:G>A	TGCAGCGGGATCAAGCTCAGGAACATCAGGCTGAGCTACCG	20:G>A
3947815|F|0-13:C>T-13:C>T	TGCAGTGCCAACGCGCACACCG	13:C>T
3025739|F|0-25:T>C-25:T>C	TGCAGCAGGTCCTTGGCGAGGTCGATGGACAGCAGCCCG	25:T>C
5324678|F|0-10:G>C-10:G>C	TGCAGCAGCTGGTGCTGAACAACGTGACCG	10:G>C
3025561|F|0-22:C>A-22:C>A	TGCAGGCGCCCTTCTCGGTCCGCCTCACCTCCAGCTCCG	22:C>A
990479|F|0-30:C>T-30:C>T	TGCAGCTTCTGCTGGACCATGGCGTTGTGGCGCTGCGCGAAGTCGTTGTACGTCTTCAGGCACCCGACG	30:C>T
22838457|F|0-45:A>C-45:A>C	TGCAGTGGCCACTCCCTGCTGAGGGATCCGCGGCACAACAAGGGGATGTCGTTCACGGAGAAGGAGCGG	45:A>C
32024419|F|0-41:T>C-41:T>C	TGCAGCGAGGACTGCCGCTACGAGCAGATGCACTTCGACGCTGCCATGACCAGGCAGCAGGCCAGCGCC	41:T>C
1001758|F|0-26:T>C-26:T>C	TGCAGATGAGCAGGGCGAGGGAGCTCTTGGAGGAGATGACGACGGACGCCG	26:T>C
1139120|F|0-26:C>T-26:C>T	TGCAGCACGCCGAGGCACACCGAGCACGTCGCCGCCTCCTCCCCACCGCCCGTCACGTTGTGCTTCACG	26:C>T
2256569|F|0-59:G>C-59:G>C	TGCAGCTCCGCGTCGCCGTCCACCTCCTTGTCGCTGTGGTAGAAGTGGGCGCAGTAGCTGCGCACCCAC	59:G>C
1066912|F|0-32:G>A-32:G>A	TGCAGCATGGCATTGGAGCCGAGGCGGAGCGCGTACTTGAGCGTGAGGTGGGTGGCGAAGGGCGTGAGG	32:G>A
1180126|F|0-31:C>A-31:C>A	TGCAGCAGACGCGGTTCCACTCGCAGCCGATCCTCCACGCGTCCCAGATCAACGACGGGACGCGGAGGG	31:C>A
1106728|F|0-18:G>T-18:G>T	TGCAGCGCCGACATCTCCGCCATTTCTAGCGCCTGGGCCAGGTTCGGCCTCTCCTCCAGGCGCATGATC	18:G>T
992371|F|0-20:A>G-20:A>G	TGCAGCATCGGGTTGATGTGACCCTGGAACGGCAGCGGGAACACTAGCACCCG	20:A>G
6045340|F|0-11:G>A-11:G>A	TGCAGCGCGGAGAGGTTGGCCAGCGCGGCGGGCAGCGGCCCG	11:G>A
6045340|F|0-5:C>A-5:C>A	TGCAGCGCGGAGAGGTTGGCCAGCGCGGCGGGCAGCGGCCCG	5:C>A
3028565|F|0-21:C>G-21:C>G	TGCAGATAAGCGCCACTGCGACCCTCCTCGCAGCCGCCAGCCG	21:C>G
1218775|F|0-67:C>G-67:C>G	TGCAGTTCCTCCGAACCTCACCCTGCGCACCCGTGAGCGGCTTGATGTTCCCCATGTTGATCATGGCCG	67:C>G
5325267|F|0-5:C>A-5:C>A	TGCAGCGAGTTGTTCGATATGTCGAGGTAGAAGAGGCGGTCGAGCTCGCCGAGCAGCGGCGGGATCGGG	5:C>A
2307492|F|0-14:G>A-14:G>A	TGCAGCTCCGCCTTGCGGCAGGTCTCCCCCAGCGCCTCGAGGAGGCGCGAGCATGGCCAGGAGAAGGCG	14:G>A
2256569|F|0-26:C>G-26:C>G	TGCAGCTCCGCGTCGCCGTCCACCTCCTTGTCGCTGTGGTAGAAGTGGGCGCAGTAGCTGCGCACCCAC	26:C>G
3023313|F|0-9:C>T-9:C>T	TGCAGAAAACAATTCAACTAGACCAGCTCCTTCCGCCG	9:C>T
5580176|F|0-8:G>C-8:G>C	TGCAGCAGGCGTATCACGCTGCGCCGCGCGTCGTACCG	8:G>C
100516738|F|0-35:T>A-35:T>A	TGCAGTAGCAGGCCGGACGGTGTTCTGCAAGTGGCTACGGCTTATGCGTATAACCTGGGTTAGGGTTCT	35:T>A
1034117|F|0-31:T>C-31:T>C	TGCAGGGGATAAAGATCAAGCAGCTAGCCTGTGGGGATAGCCACTGTCTGGCCGTCACCATGGCCGAGA	31:T>C
5366905|F|0-5:C>G-5:C>G	TGCAGCAGCCTCGTGGTCTGGACTGCGCCG	5:C>G
2257077|F|0-14:C>G-14:C>G	TGCAGGTTGGCGACCTCGGGGTCGAAGTAGGCGGACGTCAGGATCTCGTGCGGCAGCAGCGCGCCCGCC	14:C>G
2276045|F|0-7:G>A-7:G>A	TGCAGGCGCGTCAGTTCCGCCTCGAGCTCCGCTTCCATCTGATCCATCGCAGCGACAGCATCTCCAACG	7:G>A
1028245|F|0-50:G>C-50:G>C	TGCAGCACCAGGATGAGCGGCTGCGGCAGGATGACGCAGTAGCACACTGAGGGGAACCCCATGGCCGAG	50:G>C
1100680|F|0-63:A>G-63:A>G	TGCAGAGCACCGAGGAGAAGAGCAGCACTTGGGAGAAGCGCTCTCGGTTCTTCATGGACGAGTATATCG	63:A>G
2273793|F|0-21:G>A-21:G>A	TGCAGGGCGTCGAACTTGTATGCCTTGTTGATGCTGCATGGGACGTGCACCACCTTGTACTGCCCGAGA	21:G>A
1053988|F|0-13:C>T-13:C>T	TGCAGCTTCCTGACGCTGCTGATGACCGCCTCCTGCCTCGGCCTCTTCCTCGCCACCACGCGCCGAGAT	13:C>T
1247354|F|0-47:C>G-47:C>G	TGCAGGCTCCTCGCCAAGGCCGCCTCGTCCACCTTCCCGTCCTACTCCTACAGCGGCGGCGCCCAGCTC	47:C>G
7171943|F|0-23:C>G-23:C>G	TGCAGGTCGGCCAGCGCCTTGGGCGAGAGGTTGAAGTGCGTGGACTCGGCGGGCTCGGTTCCGCACCAC	23:C>G
4002650|F|0-29:A>G-29:A>G	TGCAGGAGGAGCAGGAAGAGACGGCAGCGACTGCCGCGCCG	29:A>G
32022991|F|0-19:G>C-19:G>C	TGCAGGTGAAGGGCTTCGTGGGGGACGACCAGCTCGGGGAGGCGCTGGAGGGCGCCGACCTCGTCATCA	19:G>C
5580123|F|0-28:A>G-28:A>G	TGCAGATGAACTTTGTCGCGGAACACCCACGACCG	28:A>G
100531402|F|0-15:T>C-15:T>C	TGCAGCTGCCATCCGTTCCAGTACTGCGCACCTGTGCCGCAGTTGGCATCTGCCGCCG	15:T>C
7168819|F|0-49:G>A-49:G>A	TGCAGCTGGGGTGGAGGAGGGGTCGACGGCGGGGCCGCAGGCGGGGGGCGCCGAGATCGCCTTCTTCGA	49:G>A
12469813|F|0-25:A>G-25:A>G	TGCAGTTGAGAACCACCCTCACACCATCCG	25:A>G

1067736|F|0-19:T>C-19:T>C	TGCAGGGAGTGTATACATTTGATGAGAAGGCGGGCGCAATCAAGGTGGAGACATTCTGTGTCCATGGTT	19:T>C
1023386|F|0-11:C>A-11:C>A	TGCAGCAGCACCACCTCCTGGTGCTCCGCCTCCAGGTCAGGATGCAGCTTCAGAAGCTCCG	11:C>A
9721886|F|0-20:G>A-20:G>A	TGCAGACGTAGAGCAGAGCCGCCGCGCGCGGTGGCCG	20:G>A
1048912|F|0-65:C>T-65:C>T	TGCAGCTTCTTCATAAGGTGTGGCTTCCGTATGAGCTCCGCCATGGCAAACTCCAGCGTCATATACGAG	65:C>T
5580214|F|0-7:G>C-7:G>C	TGCAGCCGACGGCGGCCAGCGGCGACGAGCGGCCGAGGTGGTTCTTGCACCCG	7:G>C
4733772|F|0-8:T>G-8:T>G	TGCAGCTCTGCCACGTCCCCG	8:T>G
2365472|F|0-29:G>C-29:G>C	TGCAGCTGGAGCGCGAACTCGAGGTTCCAGAGGACGTCGCCCATGGACGGCCTGTCGACGCTGCGGTCG	29:G>C
1045600|F|0-21:A>C-21:A>C	TGCAGTGCGAGCTGCCGCCGAAACATACTGCCAGCTCCTCTCATCGCAGAGTGACAACAATGTCAGGCT	21:A>C
1021897|F|0-31:C>A-31:C>A	TGCAGACGTGGAAGCAGCAGGCCATCTTCTCCGACCCCAAGAACCTCACCG	31:C>A
1092174|F|0-41:C>G-41:C>G	TGCAGTGAATTAATGGATGCAACGAGATCAGAAACCGTTGCCAGACCACGACGCCGACACCGACACCGA	41:C>G
2254082|F|0-34:G>C-34:G>C	TGCAGAACTCCATGAGGAGGCAGAAGAACTTGTCGGTCTCGAAGTGGGAGTAGAGCGTGGGGAGGAAGG	34:G>C
997705|F|0-10:G>A-10:G>A	TGCAGCAGGCGGCGGCGATGCACGGGCTCATGCTGCCTTGCGATGCGCAGAGCAGGGCGGTCGGCTCCG	10:G>A
1071014|F|0-44:T>A-44:T>A	TGCAGGTACAGCAGCACAGGCGGCGGCAGGGGCAGCGCGACCGCTCCGTTATTATGCGCGCGGCCGAGA	44:T>A
2242135|F|0-60:A>G-60:A>G	TGCAGACAAAACATTCCCGAACCATATCACCGTGTTCAGATCTTCAGTTGACCACTCAACATCGACAGC	60:A>G
1057821|F|0-49:A>G-49:A>G	TGCAGGCGCATGCGGATGCGGGGGATCTCGTCGCGCGTCGGGTTGATCAAGCCG	49:A>G
7169399|F|0-11:C>T-11:C>T	TGCAGCCCGAACAGCTCCTTCGGGATCGGCCCG	11:C>T
4261273|F|0-16:G>A-16:G>A	TGCAGTCCTTCGCCAGGTCCATCGTCGTCCCGTCGCCG	16:G>A
1004730|F|0-44:C>A-44:C>A	TGCAGCTGCCCCGTCGCGCCGTCCAGGATCAGCCGCGACAGCGCCGACGGGTCGCTGACGTAGTAGGCG	44:C>A
2250961|F|0-19:G>C-19:G>C	TGCAGGGCCACCCCAACGTGATCGGCCTCCATGGCCTCATCACCTCCCGCTCCTCCGCCTGCATCTACC	19:G>C
1865066|F|0-37:G>C-37:G>C	TGCAGTCGCACGCCAGGAAGCTCAAGGAGGACGTGGCGACGCTCGAGGCGCGGCCG	37:G>C
3032678|F|0-29:C>T-29:C>T	TGCAGGTGCGAGACGATGCTGCCGTCCTCCGCCCGCCACACGCCG	29:C>T
1230783|F|0-6:G>A-6:G>A	TGCAGGGGGTGGAGGTGGAGCGGCCTTTGGCTTTGGCTGGATCCCAGCGGCAGCCTCAACGTCAGCCGC	6:G>A
1153776|F|0-61:C>T-61:C>T	TGCAGGCCGCTCAAGGGGAGCTTCTACGTCGGCGTCACCTTCCTGCGCCTGCTGCCGCACGCGTATGAA	61:C>T
4002513|F|0-15:T>A-15:T>A	TGCAGACATATTCACTGTGGCTGATCCACTGGAAGGGAGCACCG	15:T>A
1228668|F|0-13:G>A-13:G>A	TGCAGGCCAGGCTGGGCGTCCTGGGCTCCGACCAGGCGCTGTTCCTGGACGCGCGGACCAGGCCGCTCG	13:G>A
1026114|F|0-8:G>T-8:G>T	TGCAGCCAGAGGACGGTCGGCCATGGCTTGGTGGGCGTGGACACCCTCTGGGCCTGGGGGCTCTTGTAG	8:G>T
1001408|F|0-38:C>T-38:C>T	TGCAGCTGCCCCGTCGCGCCGTCCAGGATCAGCCGCGACAGAGCCGACGGGTCGCTGACGTAGTAGGCA	38:C>T
3941813|F|0-10:A>C-10:A>C	TGCAGCTCGGAGAAATGCATGATTGCGATGTACCCCG	10:A>C
3941811|F|0-25:G>C-25:G>C	TGCAGGCCCCGCTGTTGGTGCAGACGAAGATGACGTCGATAGTCGCGGGGCCCG	25:G>C
5366355|F|0-59:G>A-59:G>A	TGCAGGACGACGTGGGGAGGCTTATCGTTGCTCAGTTCCGCTGGCTTGATTTCCTTGTCGATGCCGACA	59:G>A
1864017|F|0-68:C>T-68:C>T	TGCAGCTTCTCGAACACCCGCGCGCCGCCCTCGTCGCCGATGCCGAAGCTGGCCCTGGCGATGATCTCC	68:C>T
1096626|F|0-30:C>G-30:C>G	TGCAGGCGCGCGATGACGGCGTGCAGCAGGCCGATGGTCTGCTCCTGCCTCATCGCGGTGGCCTCCATC	30:C>G
4987790|F|0-34:G>C-34:G>C	TGCAGCGCCGCCAGGCGTTCGACGCGTACGCCGAGTACCAGCCCG	34:G>C
3942624|F|0-24:T>C-24:T>C	TGCAGCCTCGCCACCGCCCCCATGTCGCCCG	24:T>C
7169164|F|0-15:G>A-15:G>A	TGCAGAAGGAAGAGGGCCGAAAGCCG	15:G>A
1060936|F|0-53:G>A-53:G>A	TGCAGTTGGAAGACGTAGCGCACCCCGTCTGGAACTTCCACCCTCCGACGGAAGTCGTCGGCGAGGAAG	53:G>A
3959405|F|0-13:C>G-13:C>G	TGCAGAGCCTCTGCGAGCCG	13:C>G
3947012|F|0-12:A>G-12:A>G	TGCAGCTCCCTGAGAACCCTGCCGAGTTATACGAGATCAGCAGAAGCAAAGCTTCTCCTGAGGAGGAGT	12:A>G
1020082|F|0-47:C>G-47:C>G	TGCAGATCTCCAAGATGCCTGAGCACAGGGAACTCAAGAGGACGGCCCTCACCGACCTCTCTGGCGGCG	47:C>G
6047868|F|0-13:T>C-13:T>C	TGCAGGGACACGCTGTGAAGCTGGGGTTCCTCGAGCACGACGAGCACGCGCAGAACAGCCTCATCAGCT	13:T>C
989931|F|0-57:T>A-57:T>A	TGCAGCTGACCCGCGCCGCCTCGTAGAGCTCGTCGGAGATGAGCCCCATGCCGTGCATGAAAGGCACCT	57:T>A
1228668|F|0-31:C>T-31:C>T	TGCAGGCCAGGCTGGGCGTCCTGGGCTCCGACCAGGCGCTGTTCCTGGACGCGCGGACCAGGCCGCTCG	31:C>T
7902460|F|0-13:C>G-13:C>G	TGCAGTTCATCGGCCAGGGCTTCCGCTCCG	13:C>G
5331711|F|0-29:G>A-29:G>A	TGCAGCACCGCCGCGACCATATGCATGGAGCCG	29:G>A
986886|F|0-25:G>A-25:G>A	TGCAGAATGGCCCAGCACCGATGCCGATGGTTGGGATTTTCAATGCTGATGTTGCAGCTGCTGCCACCG	25:G>A
1095280|F|0-46:T>G-46:T>G	TGCAGGCGCCGCCGCGGCAGGACACGTGGGGGTTCTCGCCGCTGGATGCGCAGGCCGCGTACATGCCGA	46:T>G
992506|F|0-13:C>T-13:C>T	TGCAGACGTTCGCCGCCGCGGACAACGAGCTGATCGGGGGTGTGCCTGATGAGCTCGGCGATTGCCGAG	13:C>T
1018717|F|0-9:C>T-9:C>T	TGCAGGCCACGCAATTTCCTGAGCTTTATTGTCATTAGAGACTCCGAATGTATGGTGAAATTGTCCAGG	9:C>T
4733860|F|0-23:G>A-23:G>A	TGCAGCACTACCGCTCCCGCTCCGCCTCCTACGCGCGCTCACCG	23:G>A
1020908|F|0-17:T>C-17:T>C	TGCAGCTCAGAGCAGTATGAGGCCATGGCGATCTCCGCGCCCTTGAAACCATAGTCCAAGCTCGGGTTG	17:T>C
7353362|F|0-16:C>T-16:C>T	TGCAGGTTGTGGACGCCGCCG	16:C>T
1694710|F|0-47:G>A-47:G>A	TGCAGGTTTGGAAGGGGATCACTGACAGGGAGCTCGATGTGCTGGACGTCTTTGAGGATGAGGAGTATG	47:G>A
1020656|F|0-58:C>G-58:C>G	TGCAGCTGTGGAAGACGGCCTTCTACGACCAGCAGGCGATGCAGGCCACCGAGTTCCTCCTCAGCGACG	58:C>G
6047303|F|0-28:G>A-28:G>A	TGCAGGTACTCGGGCTCCAGCGCGAACAGCACGGCGATGCCGTCGTCCCTGTCCGCGCCGCGCTGCGTC	28:G>A
1030488|F|0-13:G>A-13:G>A	TGCAGCTGGTTGTGACGCGAGATCTGCTCTTATTTTAGGTTGCTGGGAGGAGCGACGCTGCCAGCGCGC	13:G>A
1021663|F|0-10:G>A-10:G>A	TGCAGAGCTGGTGGTGACACTGGAGGGCCTCTGCAAAGACCCGAGCAGGTTCCTGACCTACGACGAGGA	10:G>A
985798|F|0-10:C>G-10:C>G	TGCAGTGCACCACCCACATGTGCCCCGTGCGCGTCCACTGGCACGTCAAGCTCAACTACCACGACTACT	10:C>G
2316759|F|0-32:G>T-32:G>T	TGCAGCTCGGCCACGCCTACCGCCTCATCGCCGTCGACGAGGTCACCAGGGCCGTGCAGGCCAGGAAGG	32:G>T
4395066|F|0-8:G>C-8:G>C	TGCAGAAAGAAGCGGGCGCGGCCG	8:G>C

1027875|F|0-56:G>C-56:G>C	TGCAGCAGCTTCTCCACGATCATGATGATGTTGGCGTAGTGTGATGCCAGCGCCGAGCCGCCCACGGTC	56:G>C
1865066|F|0-34:G>C-34:G>C	TGCAGTCGCACGCCAGGAAGCTCAAGGAGGACGTGGCGACGCTCGAGGCGCGGCCG	34:G>C
3953445|F|0-15:C>G-15:C>G	TGCAGCGCGGAGGCGCGCGGGCAGGTGGCCCCGTCGCACGCCG	15:C>G
2280698|F|0-47:C>T-47:C>T	TGCAGCTTCACCTTGATGGTGTCGAACGGGTGGCCGACGACCAGCTGCGCCGCGCCGCCCACCGTGCCG	47:C>T
992506|F|0-16:C>G-16:C>G	TGCAGACGTTCGCCGCCGCGGACAACGAGCTGATCGGGGGTGTGCCTGATGAGCTCGGCGATTGCCGAG	16:C>G
4910545|F|0-8:C>G-8:C>G	TGCAGCGACGAGGCGTACCAGTCGACGTGCGCCGCGAACTCGCTGGGCATGGCCGCGTGCGAGCAGTGC	8:C>G
1007221|F|0-36:T>C-36:T>C	TGCAGCGCGTGGAGGCGCTGGTGGACGTGGCCAACGTGGCGTCGCAGCGGGTGCTGGAGAAGGCCGAGA	36:T>C
1341019|F|0-67:C>T-67:C>T	TGCAGATGAACGGGAACCTGACGATTGGCAAGGCCATGAGGTGGTGGGAGAAGGTGACGCACCCCAACA	67:C>T
1063492|F|0-59:A>C-59:A>C	TGCAGGTGAAGGTGCTGGTGCTGTCGTCGTAGGCGTAGCTGTAGGCGCGCGGGCACGCCATCTTGAAGA	59:A>C
7342613|F|0-8:T>A-8:T>A	TGCAGGTGTCGTGCGCCGCCG	8:T>A
1120667|F|0-33:T>C-33:T>C	TGCAGCTTCTGCTGGACCATGGCGTTGTGGCGCTGCGCGAAGTCGTTGTACGTTTTCAGGCAGCCGACG	33:T>C
5363343|F|0-45:T>C-45:T>C	TGCAGCCAACGGCGGACGGCGCCTCCGCGATCTCTGCCGCGGCGGTGGCCG	45:T>C
1060399|F|0-46:G>C-46:G>C	TGCAGATTACGTGCGTCGTCACGCTCTACATTTGGTGCGCCGCCACGAACCCCG	46:G>C
1863289|F|0-56:G>A-56:G>A	TGCAGGCGCTCCGTCTCGGTGAAGCGGGAGGCGTAGGTCTCGGCGACGCCGTTGCGGATCCGCAGGGCG	56:G>A
1094719|F|0-31:C>T-31:C>T	TGCAGCAGGAGCAGCCGCACGCGGTGCCGCACTTGCAGGTGGTGCAGTCGCAGCCGCCG	31:C>T
3021288|F|0-27:A>G-27:A>G	TGCAGCCCTATATCTGCCTGGAAATTCACACCTGTACAACCG	27:A>G
1047152|F|0-7:G>A-7:G>A	TGCAGAAGGCGCCCAGCCAATCCCTGAAGCAGCTCATCACTCTCACCGCCAAGGAGGATTCTGCGGTCG	7:G>A
4989011|F|0-8:C>A-8:C>A	TGCAGTGGCTGTCGGCGCGGCCGCAGTGGGCGGCCTTCGGCGGCCGCGACCACTTCATGGTCGTCGGCA	8:C>A
1039421|F|0-66:G>A-66:G>A	TGCAGTTCCTCGGCCATCCTGCCCATGTTCCCATGATCGGAAGCAGCAGGGGTAGCCAGAGCTGGTGCA	66:G>A
5579604|F|0-20:G>T-20:G>T	TGCAGCGAGGTGGTGGAGAAGTTCGTGGAGCCGACGAGAGGCGTGGGGATGGAGCTGCTGCGGCTGCTG	20:G>T
3024280|F|0-17:C>G-17:C>G	TGCAGCGACGCCGTGCCCTGCACCAACATCACCCTCTCCG	17:C>G
1228668|F|0-28:C>A-28:C>A	TGCAGGCCAGGCTGGGCGTCCTGGGCTCCGACCAGGCGCTGTTCCTGGACGCGCGCACCAGGCCGCTCG	28:C>A
1045011|F|0-60:T>A-60:T>A	TGCAGCAATGGCAACAGAATAGTACAGATCGGGTAGGTGCCCTGCCTGCCCGATGCCTTCTCCGACACG	60:T>A
1270721|F|0-26:C>T-26:C>T	TGCAGGTGTTTGGACGCGGTCCTGCACGCGGAGCACAAGGAGACGCTCGAGCAGATGCGCGCCTACTAC	26:C>T
989600|F|0-66:C>G-66:C>G	TGCAGACACCATGGACTCGGTGAAGGACGTGCTGGGGAAGATGGGCAAGAGGTTCGGCGAGGCCGCCCG	66:C>G
1069988|F|0-54:C>T-54:C>T	TGCAGCCAACCATTTCGCCGAGCGGATGGCCGTTCGCAAGTCATGGATGATTGCCACAAGGAGATCGTC	54:C>T
1864956|F|0-61:C>T-61:C>T	TGCAGTACCACCCCGACGTGTGCCCGCCGTCGCGCAGGGCCGAGTCCACGGAGCTCTTCGTCGAGCTCC	61:C>T
978496|F|0-64:A>G-64:A>G	TGCAGCACACCGAGTCGGGGGAGGTGACGGTGGAGATGGAATGGATGGCTCTAAACATTTAGAGAATCC	64:A>G
995872|F|0-52:G>C-52:G>C	TGCAGCGTAGGGGCTCTGGTGGGTGTAGCCGTACGTTGGCGTTATTGCCTCTGCCTCTGGCTCCGAGAT	52:G>C
3948194|F|0-8:A>C-8:A>C	TGCAGCTGAAGCCTCTGGAGTTGCGCATCGACCG	8:A>C
1023254|F|0-64:C>A-64:C>A	TGCAGATGCTGCGGCCGCACCGCGAATCCATAAACGTCCCTGCGCCAAACCTCAACAACATCAGCCACA	64:C>A
1027830|F|0-33:G>T-33:G>T	TGCAGCGACGAGGCGTACCAGTCGCCGTGCGCCGCGAACTCGCTGGGCATGGCGGCGTGCGAGCAGTGG	33:G>T
32024185|F|0-22:C>G-22:C>G	TGCAGGCGCAGAGGAGCAGTATCGTCCTGGACAGCACCG	22:C>G
2258675|F|0-8:G>A-8:G>A	TGCAGGCAGCCGCTGAGCATGGACGTCCACGACACCACGTTACGGCCAAGGATCCTGTCGAACACCTTG	8:G>A
3941292|F|0-10:G>A-10:G>A	TGCAGGGACAGGAGGAGGAAGGCGATGACCCACCG	10:G>A
1062809|F|0-38:C>T-38:C>T	TGCAGGAGCTCCCGCAGCGTGAGCCCGCGCTTGTTCACCGCCATGCCCGCCCTCTCCGACGCCATGGTC	38:C>T
1133018|F|0-16:G>T-16:G>T	TGCAGGTCAGGCTCGTGGGGATGGTGAGCGGCAAGGCGGAGCGGAACACGGACGTCTCCTTCACGCTCG	16:G>T
7169366|F|0-11:C>T-11:C>T	TGCAGCATCTTCAGGCCACCCAACGCCGCCG	11:C>T
989931|F|0-16:C>T-16:C>T	TGCAGCTGACCTGCGCCGCCTCGTAGAGCTCGTCGGAGATGAGCCCCATGCCGTGCATGAAAGGCACCT	16:C>T
980448|F|0-17:G>C-17:G>C	TGCAGGAACTTCTGCGTGGGGAAGGTGAGCGCGAGCGAGAACATCTGCGGGAGGATGCGGAGCGTGAAG	17:G>C
3938219|F|0-22:A>G-22:A>G	TGCAGGTGTACTACAATCTTGGAGAACTGAAGCCG	22:A>G
1667137|F|0-13:C>G-13:C>G	TGCAGGCCGAGTCCGACTTCGTCTACGTCCTCGACGACGACATGATCCCCG	13:C>G
3938378|F|0-22:C>T-22:C>T	TGCAGCCAGTATGTATTTGGTTCACCG	22:C>T
4006159|F|0-35:C>G-35:C>G	TGCAGGAAGGCGCTCGTCATGAGCCCGCGCGAGAACGCGCCG	35:C>G
1218554|F|0-41:G>C-41:G>C	TGCAGGAAGAGGTCCACTTGCATCCTGCTCCTGATCGTCTCGCTGAACCGCTGGCCCACGGCGAGACCG	41:G>C
5357845|F|0-21:C>T-21:C>T	TGCAGCGCGGAACTTTCGGTGCCTGATCCG	21:C>T
1862417|F|0-30:T>A-30:T>A	TGCAGCTCGGCGGCGTCCATGCTCTGGATCTGCGTCGACGACGGCGGCCAGGAGGCGGAGGACGACGAG	30:T>A
1066912|F|0-62:C>G-62:C>G	TGCAGCATGGCATTGGAGCCGAGGCGGAGCGCGTACTTGAGCGTGAGGTGGGTGGCGAAGGGCGTGAGG	62:C>G
5341583|F|0-61:G>A-61:G>A	TGCAGCGGCAGGGCACGAAGAACTTCATCGGCCGCGGCCTGCTCATGGCCAACGGCGCCAGGTGGTCGC	61:G>A
4985470|F|0-23:G>A-23:G>A	TGCAGGCGCTCCTCCGCGGCGTCGACATTGCCG	23:G>A
1206525|F|0-7:C>T-7:C>T	TGCAGGACCTGGTGGTGGCCATGCTGGAGCTGTGGAGCCTGATGGACACGCCGCCG	7:C>T
5580325|F|0-16:G>A-16:G>A	TGCAGCTGCGTCTTCAGCAGCGGCGGGAACGACGCCG	16:G>A
1068773|F|0-26:C>A-26:C>A	TGCAGGAGGTGCTGACGGTGGTGCCGCGGGTGTGCAAGTCCTGGGGGCGCGTCGTGGCCG	26:C>A
1068773|F|0-25:G>T-25:G>T	TGCAGGAGGTGCTGACGGTGGTGCCGCGGGTGTGCAAGTCCTGGGGGCGCGTCGTGGCCG	25:G>T
2250829|F|0-67:G>A-67:G>A	TGCAGAAGGGGTACTCGTTCATCATGTCGGCCGTGATCGACGAGGAGGCCGCCGCTAGGCAGAGGGCGA	67:G>A
7161829|F|0-16:C>T-16:C>T	TGCAGGAGTACGACAGCGGCGGCGGCGTCCG	16:C>T
32026686|F|0-6:T>C-6:T>C	TGCAGCTGACGAAGAATCCATGCCTGCGGTGGACGGTGGTGATCAAAGTGTGGGTTTCTTGCGGCTTGG	6:T>C
7172661|F|0-16:C>T-16:C>T	TGCAGGTGACCGTCGGCGGCCTTAGCCCCG	16:C>T
1022920|F|0-40:G>C-40:G>C	TGCAGCAGGCGCTGGACACGCTGCTGGGGCGGCGGAGCCTGAAGATGAGCGACTTCGTGGTGGGCGAGC	40:G>C

1864138|F|0-62:C>A-62:C>A	TGCAGATGAGATCGCCAAGCTGTTCCCCTGCCTGTACGGCCAGCCGTCGGCCGCCGTGGTGCCGTCCGC	62:C>A
1394364|F|0-14:C>G-14:C>G	TGCAGCTGGTCGAGCGCCTTGAGGAGGAACGGCTTGAATCTGGTGACATTCACCATGACATCCTGCTTC	14:C>G
3937802|F|0-34:T>C-34:T>C	TGCAGCAGCGCCGTGGCGGACATGCTCGCCAGGCTGCCGTTGCCCG	34:T>C
7168072|F|0-28:G>T-28:G>T	TGCAGGATGTGGTGAGGGCGCTGGCGATGGGGTCATGCCTGCCG	28:G>T
5579604|F|0-5:C>G-5:C>G	TGCAGCGAGGTGGTGGAGAAGTTCGTGGAGCCGACGAGAGGCGTGGGGATGGAGCTGCTGCGGCTGCTG	5:C>G
1075099|F|0-22:G>C-22:G>C	TGCAGGTCCATTCCAGTTCGTCGCAGAGCCCTACAACGGCACCATACCGCGCCTGCAAATCAACCCCTA	22:G>C
4993342|F|0-13:C>G-13:C>G	TGCAGCGGCGGCTCACGCGGCTGTACCCG	13:C>G
5358121|F|0-13:C>G-13:C>G	TGCAGGACCTCACCCTGCGCCACCTCCCG	13:C>G
3959812|F|0-6:C>T-6:C>T	TGCAGCCTCGCCTGCATGGCCTTGCCG	6:C>T
7351890|F|0-14:G>A-14:G>A	TGCAGCGCCGCCAGGCGTTCGACGCGTACGCCGAGTATCAGCCCG	14:G>A
1117708|F|0-16:G>C-16:G>C	TGCAGCTGCGCTCCAGGCCCTGCTGGAACACGATCTTGCCCGAGGAGTTCATACGCGGGGAGTCCACGG	16:G>C
986435|F|0-20:G>A-20:G>A	TGCAGCAAGAGATCCATGGCGGCGCCCCGCCGCCGTTGCACGTCCAGCACCG	20:G>A
5357765|F|0-11:C>T-11:C>T	TGCAGCCTGAGCGAGAGCCGCCG	11:C>T
2245372|F|0-49:C>T-49:C>T	TGCAGACACGCACTGATCACGATGTTCCAGGAGACGGCGTCGACCTCGGCCATGGCTCTGAATGCCCGC	49:C>T
1080223|F|0-36:A>G-36:A>G	TGCAGATCGCGCGCGGCATGGAGCACCTCCACTCCAAGAAGATCTACCACGGCGAGCTCAACCCGTCCA	36:A>G
991319|F|0-15:T>C-15:T>C	TGCAGAGGCGGTCGGTGGCGAGCCTGCCCTTGTGGGTGCTGCTCTTGTACTCCTTCCAGGTGAACTCCC	15:T>C
6040211|F|0-66:T>C-66:T>C	TGCAGCCTGAGCTCTTCTTCAAGATGTCCCACGACATCTACAACTACGGCGAAGGGTCAGTACTCCTAC	66:T>C
1696537|F|0-58:C>T-58:C>T	TGCAGGTGCACGCGGCACGGATGAGGTGGGCAGGCGAGGGGAGGCGCAGGAACACCAGCTCGAGAAGCT	58:C>T
1229270|F|0-8:C>T-8:C>T	TGCAGCGCCGCCGCCGTCGCGGCCAGCATCGCGTACGCCAGCAGCTGGTCGCCG	8:C>T
1021154|F|0-64:C>A-64:C>A	TGCAGCTCGGCATTGGGCGGCAGGATGACGGGCTTCTGCACGACGATGCCGCCGCCGCCGTCGGCGGGG	64:C>A
1096626|F|0-53:C>G-53:C>G	TGCAGGCGCGCGATGACGGCGTGCAGCAGGCCGATGGTCTGCTCCTGCCTCATCGCGGTGGCCTCCATC	53:C>G
999482|F|0-60:T>C-60:T>C	TGCAGCGTCACCTCCCCAGGCCCAGGCGTCTCCTTCTCCACAATCTCCACCTTCCTCCCTTCGTCCTCC	60:T>C
1094719|F|0-9:A>C-9:A>C	TGCAGCAGGAGCAGCCGCACGCGGTGCCGCACTTGCAGGTGGTGCAGTCGCAGCCGCCG	9:A>C
1096523|F|0-61:C>G-61:C>G	TGCAGGTGATGGCGGACGTGATCCACTGGTGCACCCACAAGGACGGCGGGGCGCGGCCGTCCATGAAGC	61:C>G
5580451|F|0-7:G>C-7:G>C	TGCAGGCGGGCTCGGAGCGGGCGGCTCCGCTCCCG	7:G>C
1093292|F|0-36:C>A-36:C>A	TGCAGGCGCTCTCGTCCGAAACCCCCTCCGCCTCCGCCGCGGCGTCGCTCCTCGCCGCCGCCGCGCGCG	36:C>A
5338383|F|0-27:C>T-27:C>T	TGCAGAAAGCGGCAGCAGGTGCGTGGTCACCG	27:C>T
1341985|F|0-37:G>A-37:G>A	TGCAGCTCGTGTACGAGCTGCTGCTCAAGTTCATCGGGTCCAACTCCCTGGATGCCAAGATAGGGAAGA	37:G>A
983652|F|0-62:T>C-62:T>C	TGCAGGAGGGTGTTGATGCGGACGAGCATAGCCGCGCGTGTCGCCTCGGCGGGCAGAACGTGTCCGTCC	62:T>C
1695017|F|0-29:T>G-29:T>G	TGCAGGATCTTGGGCTCGAAGCCGTGGCCTTCGGGGTACCTGGCGTATCCCCGCGTGGCCGTGCGCCCG	29:T>G
2262079|F|0-10:G>A-10:G>A	TGCAGCAACTGCGTCACCCAGGCCGTGCAGCGCGTCGCCGTCGAGTGCGGCGGCGCCCCCTCCG	10:G>A
4262298|F|0-23:G>T-23:G>T	TGCAGCGCCTCGATGCCCTCCTCGACGGAGGCGGCGGCCCCGCCG	23:G>T
5581339|F|0-19:C>T-19:C>T	TGCAGCAGTTGGCCTCCTCCCATCCG	19:C>T
1150528|F|0-46:T>C-46:T>C	TGCAGGCTCGGTTTTTACTGGCAAAGCTAAATCCATCCGTGACATATAACTCTGATAACCCTGCTCCCG	46:T>C
1008440|F|0-23:C>G-23:C>G	TGCAGCTCGTGCTTCTCCGTCTCCGTCTCGTGGACGAGCTGCATGGCCACCCGCGCCGCCTCCCGAGAT	23:C>G
984675|F|0-28:A>G-28:A>G	TGCAGGTTTTCACCACGCTGATATACGGAGCTGGGATTGTGAGGGACGCGGCCCGTGCACGTCGGCTGT	28:A>G
1020973|F|0-54:C>T-54:C>T	TGCAGGAGATGGTCACTGTAATAACCCACCGTTGCTGTAGTATCACCGCCTATCCTTCTCTCCATTTCT	54:C>T
2258302|F|0-10:C>G-10:C>G	TGCAGCGCCCCATGGGGTTCACGCTCGGCCACGGCATCCACGAGCGGCACGCCCTGCTGCTCGAGGTCG	10:C>G
992114|F|0-29:G>A-29:G>A	TGCAGGTCGCAGACCATCTTGAAGCGGACGTCGGCCTGCATGAGCGCGCGCGCGATCTCGTTGAGGCAC	29:G>A
1021663|F|0-25:G>A-25:G>A	TGCAGAGCTGGTGGTGACACTGGAGGGCCTCTGCAAAGACCCGAGCAGGTTCCTGACCTACGACGAGGA	25:G>A
1025726|F|0-13:G>A-13:G>A	TGCAGGGGGCTCCGCGCCGCGGCGGAGGGGCACGACCAGCAGCAAGAGGGGGGCGACGACGCGGCGGGA	13:G>A
4261481|F|0-8:T>C-8:T>C	TGCAGTTGTCCATTGCCGAACCCCG	8:T>C
1094719|F|0-8:G>T-8:G>T	TGCAGCAGGAGCAGCCGCACGCGGTGCCGCACTTGCAGGTGGTGCAGTCGCAGCCGCCG	8:G>T
5359298|F|0-11:C>A-11:C>A	TGCAGGCGGGGCGGACCTACTTCGTGCTCCCCGCGGCCCG	11:C>A
5324496|F|0-30:T>A-30:T>A	TGCAGTGACGTTTGAGTTCGACGGCGAGAGTTCCG	30:T>A
7491661|F|0-5:C>A-5:C>A	TGCAGCGATTCAATGTCACCG	5:C>A
5331746|F|0-28:T>C-28:T>C	TGCAGCAGCAGCGGAGCCAGCAGCTGAATCTGAGCCAGATCCAAACCG	28:T>C
32021403|F|0-10:T>C-10:T>C	TGCAGCGGCTTGTTGCCTTCTCGTCGCGCATCGTCGCCG	10:T>C
5002768|F|0-19:A>G-19:A>G	TGCAGGAACTGCACTGTGCAGGAATAATGGGCTCTTTTAATGAGGACGGCGACGTTCACCCGTGTAATC	19:A>G
12778063|F|0-9:C>A-9:C>A	TGCAGGTCCCGGGCCCTGGTTCTACTTATCGTCGTCGCCACCAACACCGCCATCTTCCTCCTCTTCTCC	9:C>A
100349618|F|0-11:C>T-11:C>T	TGCAGACAGATCTGGCCACACCG	11:C>T
5341819|F|0-10:G>C-10:G>C	TGCAGGTGCGGCGCGCCATGCTGGTGTGCCGCGTCGTGGCGGGACGCGTCGTTCGCGGCGGCGATGGCG	10:G>C
100547606|F|0-5:C>A-5:C>A	TGCAGCTCGATGGCACGATCGTAGCGCCGAGCGAGCCCACGACGTGGCCG	5:C>A
5364126|F|0-6:C>T-6:C>T	TGCAGTCCGTCCTGATCTCGGCGTGGCGGCCG	6:C>T
100387809|F|0-14:G>C-14:G>C	TGCAGGAGCTCTACGAGCTGTTGCTTGCCG	14:G>C
1032233|F|0-39:C>G-39:C>G	TGCAGAGTGTTTTAATGGCCGCACAGTGAAACTGCGTCCCTTTCCCTATCATCGCCTTCAATGAGCCAG	39:C>G
100498706|F|0-51:C>T-51:C>T	TGCAGTTGCCTCTGGTTTTATCTGTGGAGATGGTATATGGATGTTCCCTTCCTCCTTGCTTTCTCTTGC	51:C>T
100502361|F|0-65:G>C-65:G>C	TGCAGCTGGCGATTGAGGAGGAGGACGACGCCACCAGCAAGCTGGCGCAGCCGATGCAGCCCTCCGAGA	65:G>C
5369553|F|0-8:T>C-8:T>C	TGCAGGCATGGCGAGGCAAACACACGTGAGCTACCGCCCGCTGAGCGCCCACGGCGCCG	8:T>C

990385|F|0-49:G>A-49:G>A	TGCAGGGGTGGGCCAAGAGGAAGCGATCGCGCCGCCAGCGCTCCGAGGAGGAGAACCTCGCGCTCTGCC	49:G>A
2260353|F|0-26:G>C-26:G>C	TGCAGCGATGAGCGGGGGTCGACGGAGAGGGTGAAGCTGAGCCGCGGGCAGGTCGTGTTCCAGGCGTCC	26:G>C
1166279|F|0-12:T>C-12:T>C	TGCAGTTGGAGATGAGCACCAGCCTCAAGACGCCCTCCCTCTCCGCCAGCAGCCCCGTCATCACGGCGT	12:T>C
1215939|F|0-43:G>C-43:G>C	TGCAGAGCGACGTGTACGCGTTCGGCGTCGTCCTCCTGGAGCTGCTCACCG	43:G>C
2258923|F|0-43:C>T-43:C>T	TGCAGCGCGTGCAGTCGGAGGCGCTGCGCGCGCTGGGGCCGCGCGACTTCGACCCCAAGCTCTACGTCG	43:C>T
1025267|F|0-41:G>C-41:G>C	TGCAGGACCTCGGCGATGGCGCGGTCCACGCGGTCGGCGTCGTCGCGGAGCGCCG	41:G>C
3023313|F|0-11:A>G-11:A>G	TGCAGAAAACAATTCAACTAGACCAGCTCCTTCCGCCG	11:A>G
6037519|F|0-21:T>C-21:T>C	TGCAGAGGTGGTGGTGGAGCCTGTGGCGGCCCG	21:T>C
1359255|F|0-49:C>A-49:C>A	TGCAGGAGTTCAAGACGGACTACAACCAGACCCACTTCATCCGCAACCCCGAGTTCAAGGAGGCCGCCG	49:C>A
3953445|F|0-29:G>C-29:G>C	TGCAGCGCGGAGGCGCGCGGGCAGGCGGCGCCGTCGCACGCCG	29:G>C
5368032|F|0-66:C>T-66:C>T	TGCAGACACGCGCTGATCACGATGTTCCAGGAGACGGCGTCGACCTCGGCCATTGCTCTGAATGCCCGC	66:C>T
1089655|F|0-37:G>T-37:G>T	TGCAGAACGACACGCACTACACACTGTCAGCGTGGTTGCAGGTGAGCCATGGTGTCGCCGCCGACGTGT	37:G>T
4982323|F|0-23:G>T-23:G>T	TGCAGGGCCTCCTGGGGAGCCTCGGCCTGCCTCCCG	23:G>T
3032678|F|0-32:C>G-32:C>G	TGCAGGTGCGAGACGATGCTGCCGTCCTCCGCCCGCCACACGCCG	32:C>G
1865066|F|0-5:T>A-5:T>A	TGCAGTCACACGCCAGGAAGCTCAAGGAGGACGTGGCGACGCTCGAGGCGCGGCCG	5:T>A
3942805|F|0-7:C>T-7:C>T	TGCAGCTCGCCGTCGGCCATGGCACATCCACGCCG	7:C>T
988457|F|0-56:T>C-56:T>C	TGCAGCTGCCGATGACGTCGTTGAAGCTGGGGGACTGCATCATCTCCAGCACGGTGTTGCGGCCGAGAT	56:T>C
5582514|F|0-8:G>C-8:G>C	TGCAGCGCGGACACGGCGGACGACTGGAACCG	8:G>C
5346073|F|0-5:C>T-5:C>T	TGCAGCGGCTCGTTGCCGTCGACGCTATCCG	5:C>T
6040063|F|0-6:A>T-6:A>T	TGCAGCAAACCTGCGTCCATTCTGTACGCGTAGCTCGAAACGGTCATGTCCTCTGCCGCGGCGGGTGAA	6:A>T
992804|F|0-7:C>G-7:C>G	TGCAGGCCGCGCTGCTCAAGGAGTACCCCAACAAGCTGCTCGTCACGGCGACGCAGTGCGTCTTCAGCG	7:C>G
4408483|F|0-7:C>T-7:C>T	TGCAGGCCGAGTCCGACTTCGTCTACGTCCTCGACGACGACATGATACCCG	7:C>T
1068434|F|0-10:C>T-10:C>T	TGCAGGTAGACGCCGAGCATGGGGAGCTTCTTGGCGCCGTAGGCCTGCCCGCACAGCGTCTCCAGCGCG	10:C>T
1075544|F|0-40:C>G-40:C>G	TGCAGCATCCGCCTACCTACACCCTCGGCAAGCGGCGCACCGACCACAACCTGCTCGTGCCG	40:C>G
3025428|F|0-20:C>T-20:C>T	TGCAGGTTGTAGGCAAGGAGCTTCTGGCCGAGCCCAATGCCCCG	20:C>T
1864269|F|0-66:G>C-66:G>C	TGCAGGTTGTGCGCCAGGGCGTCGAACGCAATGCAGAGCAGGAGCTCCAGCTGCTGCTGCGGCTGCGGC	66:G>C
985906|F|0-11:A>C-11:A>C	TGCAGGCACCAAGGAGAATCTGAGGGAGATGTTCGGCTCGGTGGGGAAGAGGTTCAGCGAGGCCGCGCG	11:A>C
1023880|F|0-43:T>C-43:T>C	TGCAGCAGCCAGTGGAACTGCCCCTCCACGCCCATGTGGTCCGTGGAGTGGAGGTGGAAGACGAGCAGC	43:T>C
1027515|F|0-38:G>T-38:G>T	TGCAGCCGCTGGTCCGACGGCGACGTCGGCTGCGGGACGTGCTCTGGGTCCG	38:G>T
1862722|F|0-36:G>C-36:G>C	TGCAGGGGCTTCCTCGGCGGCCGAGGCCGAGCAGTCGTCCTGCTCGACGGAGCTTCCGTCCG	36:G>C
4261326|F|0-23:A>G-23:A>G	TGCAGTGGTTCGGGGACAGCAGCAGCCAGGCGGCGCCG	23:A>G
1030422|F|0-23:G>A-23:G>A	TGCAGATCCCCGCCGACCAGGCCGCGCTCGCGCTCGGGACGCTCGCCGCGGTCCTCCCCG	23:G>A
1023455|F|0-22:C>T-22:C>T	TGCAGTCCCACTGGCGCGCAGACCTGCGAGACGGTCCGTGGCTCCAACCTGAGCCTGCTCCTCCTGGGC	22:C>T
2256549|F|0-10:G>A-10:G>A	TGCAGGCTGCGCGCTGCCACTGTTCCTTGCATCAGCCTCGAACTCGGCGCCACCTCCCGTGCTCGATCA	10:G>A
1101954|F|0-17:A>G-17:A>G	TGCAGCGTCACCATGAAATCCGCCATGAAGAAGATCCACGGCGCCACGGCCGCCTTGAAGGTCCACGCC	17:A>G
1101954|F|0-33:A>C-33:A>C	TGCAGCGTCACCATGAAATCCGCCATGAAGAAGATCCACGGCGCCACGGCCGCCTTGAAGGTCCACGCC	33:A>C
4990825|F|0-21:T>C-21:T>C	TGCAGGTCGACCACGTGTAGTTCATGGCGCCG	21:T>C
1089540|F|0-37:C>G-37:C>G	TGCAGAGGCGGAGCAGGTCGCAGGAGGAGAGCACGAGCGAGACCATCGCGAGGCCGTTGGTGATCCGCA	37:C>G
3940105|F|0-20:G>T-20:G>T	TGCAGCAGCGTCGTCGACGGGTCGTCCAGGTCCGTGCGCCCCG	20:G>T
7492655|F|0-5:C>T-5:C>T	TGCAGCGGCGCGCAGGGCGCCG	5:C>T
1167323|F|0-19:A>G-19:A>G	TGCAGCGTCTGTCCATGTCACCCGAGATTGCCCGCGCAATCGTCGGCCACAGCGGCGTCCGTCCACTCA	19:A>G
1062010|F|0-40:A>C-40:A>C	TGCAGCTTCTCGTATTACGCCCTGCCAGGCTTCATCTTCCAGAGCCTGACGTCCATCGCATGGGTGTGC	40:A>C
1110940|F|0-59:G>A-59:G>A	TGCAGGTACGCGTACGCCGCGTGCTTCACGAGCGGGTGGCGTATCATGTGCACGTCCCCGCCGCCTCCG	59:G>A
16795833|F|0-13:A>G-13:A>G	TGCAGATTCAGAAACGATTGCCCG	13:A>G
1220649|F|0-46:C>A-46:C>A	TGCAGACACGGTGTTCTTCCTGGCGTCCAAGCTCCTCAAGACGGTCCCCCAGGTCAGTCACTCTCACTT	46:C>A
1070921|F|0-45:C>A-45:C>A	TGCAGCTCCGCTCCGTCAGCCTCGGGGACCCGTCCCTGTCCGCCACCGCGCAGGTCACGCTCGCCTCCC	45:C>A
4395284|F|0-26:G>C-26:G>C	TGCAGTATCCTGCTCAGCGCCATCTTGGCCTCGATGAGCGCGAAGTTCTGGCCGACGCAGACCCGTGGG	26:G>C
9721822|F|0-8:T>G-8:T>G	TGCAGAGCTCGGCGTCCGTGCACCG	8:T>G
992992|F|0-36:C>G-36:C>G	TGCAGCCCTCAGCGCCGAGGACCGCGTCGGCCTCGTCAATGCCCTGAAGGTACGGTTACTCGCTGTTCG	36:C>G
2248798|F|0-54:G>C-54:G>C	TGCAGCCGCACACCGTCAACTTCCTCAACGTGGTCATCGACAACACCCGCGCGGGCCTGATGCCGCAGA	54:G>C
1059725|F|0-22:C>G-22:C>G	TGCAGTAGTAGATGACGGCCTGCGGGTCCTGGTAGATCATGGCGCGGCAGTAGGGGCACCGCCCGAACC	22:C>G
985659|F|0-59:G>T-59:G>T	TGCAGCAGCATGGACATCTGCGCTGCCTGCTGGGCCTGGTAATGGTGGACAAGCAGGGGGGTGTTGTAG	59:G>T
1021154|F|0-47:G>A-47:G>A	TGCAGCTCGGCATTGGGCGGCAGGATGACGGGCTTCTGCACGACGATGCCGGCGCCGCCGTCGGCGGGG	47:G>A
3946078|F|0-9:T>G-9:T>G	TGCAGGGCCTCCTTGCACTGCCGCTTGCCG	9:T>G
3027882|F|0-11:G>A-11:G>A	TGCAGCGACGTGTGGTTCAAGTACTCGCCGCCTCCGCGCAAGCCG	11:G>A
32026046|F|0-14:G>A-14:G>A	TGCAGGACGGCACGGTGACCCGCGGCATCTTCGGGCGCGAGACGGCGACGGTGGCGGTGTCGGGCGGGC	14:G>A
5369546|F|0-7:T>C-7:T>C	TGCAGGATCCCCCCGACGAGCGCCTGGAACAGCCGACGCGGCTGGCCG	7:T>C
4993892|F|0-16:G>C-16:G>C	TGCAGGCGATGAGCGGGGGCGCGTACCG	16:G>C
5324113|F|0-21:C>T-21:C>T	TGCAGGCAGGCTAATCTTTCGCGGCCG	21:C>T

4993510|F|0-7:C>G-7:C>G	TGCAGGGCGACAACATGAGCAAGAACATCCGCGCCG	7:C>G
2245372|F|0-47:G>A-47:G>A	TGCAGACACGCACTGATCACGATGTTCCAGGAGACGGCGTCGACCTCGGCCATGGCTCTGAATGCCCGC	47:G>A
1096095|F|0-37:C>A-37:C>A	TGCAGGGCGAGGACTCGCAGATTGCAGAACCCCCCTGCAATGACGTCCAGATCGTCGTCGATAACACCG	37:C>A
1064339|F|0-13:G>C-13:G>C	TGCAGGATTTCCTGAACATGTTCTTCAAGGAGCAGTACAAGCCGATCCCGCTGGTCTACAACCTTGTGC	13:G>C
4002427|F|0-16:C>G-16:C>G	TGCAGGAGGCGATCGGCCTTGACATGGACGCGATGACGTCCG	16:C>G
2244489|F|0-14:G>A-14:G>A	TGCAGGACGACAGCGAGTCCGAGTCAGATTCAGAGTCTATCGAGATCGCGGACCTCAAGAAGCGCATGT	14:G>A
1083484|F|0-28:G>A-28:G>A	TGCAGACCTCGACCTCCTCGGCGTCGGAGGACTCGTCCATGTCGGAGGCCGTGGAGGCGCTCCCGTTGT	28:G>A
3942386|F|0-7:C>G-7:C>G	TGCAGCTCATCGTGTACGGGCCCG	7:C>G
7169452|F|0-5:C>T-5:C>T	TGCAGCGGACGAGAGCAAGACTGAACCG	5:C>T
1068773|F|0-11:C>T-11:C>T	TGCAGGAGGTGCTGACGGTGGTGCCGCGGGTGTGCAAGTCCTGGGGGCGCGTCGTGGCCG	11:C>T
1139882|F|0-7:A>G-7:A>G	TGCAGCCACCGCCGCCGCCGCATCCAAGATGATCTCAATCCTCCCTCCAAGCCGCGCGCGTGGTCCATG	7:A>G
3956890|F|0-7:T>C-7:T>C	TGCAGGCTGTGCCGACGACGAGGATCGCGGTGGAGATGACCG	7:T>C
3952827|F|0-16:G>C-16:G>C	TGCAGCTGGCCTTCACGTCCACCTTCTTCGCCG	16:G>C
3026320|F|0-33:T>C-33:T>C	TGCAGTGATCAAGCTGTGGTCGGGTGGTAGCAGTCCTGCCG	33:T>C
1139882|F|0-62:G>C-62:G>C	TGCAGCCACCGCCGCCGCCGCATCCAAGATGATCTCAATCCTCCCTCCAAGCCGCGCGCGTGGTCCATG	62:G>C
1696537|F|0-66:G>C-66:G>C	TGCAGGTGCACGCGGCACGGATGAGGTGGGCAGGCGAGGGGAGGCGCAGGAACACCAGCTCGAGAAGCT	66:G>C
4261481|F|0-18:A>T-18:A>T	TGCAGTTGTCCATTGCCGAACCCCG	18:A>T
32021829|F|0-63:C>T-63:C>T	TGCAGCATGTACCGCCCGTTGTTCATCAGGAAGATGCTGCTCAGCGCCAGGTCCTTGTACAGCCGAGAC	63:C>T
5357559|F|0-22:C>T-22:C>T	TGCAGCAGCAGGAGCCAGCGGCCGCCG	22:C>T
1071840|F|0-19:C>T-19:C>T	TGCAGGAGTTCAAGAACGCCGTGCGCAAGATGGGCCGCGTCGGCGTGCTCACCG	19:C>T
3952787|F|0-11:G>C-11:G>C	TGCAGTGGATCGAAACTCTTGTCCG	11:G>C
1696537|F|0-27:G>C-27:G>C	TGCAGGTGCACGCGGCACGGATGAGGTGGGCAGGCGAGGGGAGGCGCAGGAACACCAGCTCGAGAAGCT	27:G>C
1006032|F|0-44:C>T-44:C>T	TGCAGCGGGAGCGCGTCCATGTCGGCGCGCAGCGCGACGAAGGGCGGGCGCCCG	44:C>T
4989393|F|0-25:C>T-25:C>T	TGCAGCGGCGGCTCACGCGCCTGTACCCG	25:C>T
5371853|F|0-7:C>T-7:C>T	TGCAGCACATGGCCGAGTTCCG	7:C>T
978702|F|0-16:G>C-16:G>C	TGCAGGCGTTCGGCGAGCACGTACACGTGTCCGTGCACAGCTGGGCGACGGACGTGAAGAAGAAGGCGA	16:G>C
5580239|F|0-9:G>C-9:G>C	TGCAGCCTCGGCCAGGCTCGTGAAGGATTTGGACTTGCCG	9:G>C
2255361|F|0-50:A>G-50:A>G	TGCAGTGGAAGCGCAAGGGCCTCATCGAGAAGATCATGGACCCCAACCTCAACGGCAAGGTCAACCCGA	50:A>G
1084864|F|0-41:C>T-41:C>T	TGCAGGTACGTCTCGACGAACTTGCGCTTGAACCACCTCAGCCTCATCCAGTTCATGATAATCGGCTGC	41:C>T
2247195|F|0-46:T>C-46:T>C	TGCAGGACGACGTGGGGAGGCTTATCGTTGCTCAGTTCCGCTGGCTTGATTTCCTTGTCGACGCCGACA	46:T>C
3028081|F|0-7:C>T-7:C>T	TGCAGTGCCACGGGGCCAACGACGCGTCGAGCGACGCGCTCTCCG	7:C>T
1014386|F|0-24:C>A-24:C>A	TGCAGAAAAGCAAGCAAAGCCAGGCCTGACACGCGACTAGACTATCTTGTCTTACTTCGGAGGGCCACC	24:C>A
1696816|F|0-8:A>G-8:A>G	TGCAGATTACCAAGGTCGAAGAGGCTGCTGGGTATGCTCCCCTCAAGGCCATTGGAGTGGAGGTTGAGC	8:A>G
1130281|F|0-16:T>C-16:T>C	TGCAGGCCCTGCATCCTGCACATGCTGGAGCACGTGAGCGCCTCGAGCGCCG	16:T>C
1066793|F|0-38:G>A-38:G>A	TGCAGCGACTTCATCTTGAAGTTGGCCGCGAAGGAGAGGAGGTAGAGGTTCCAGAGCAGGTTGCCGAGA	38:G>A
1228668|F|0-19:C>G-19:C>G	TGCAGGCCAGGCTGGGCGTCCTGGGCTCCGACCAGGCGCTGTTCCTGGACGCGCGCACCAGGCCGCTCG	19:C>G
1746362|F|0-67:A>T-67:A>T	TGCAGGTTGGGTCGCCGTGGTTCACGATCCTGTTCGCCCGCAAGACCGAGGAGAAGATGGAGGCGGCAG	67:A>T
1092909|F|0-41:C>G-41:C>G	TGCAGCGTGTCGGCGGCGGCGAAGCGCAGGTCCATGGCGTACAGGTTGCTGCCG	41:C>G
1110940|F|0-47:G>A-47:G>A	TGCAGGTACGCGTACGCCGCGTGCTTCACGAGCGGGTGGCGTATCATGTGCACGTCCCCGCCGCCTCCG	47:G>A
7168021|F|0-16:A>G-16:A>G	TGCAGGAGTCCGCGGGAAGCAACAGCCTCTCCG	16:A>G
32023644|F|0-23:T>G-23:T>G	TGCAGCCGAGACCGCACCGCCTCTCCCAGCGACACGTTCGGCGCCGTGTCCGCGTTCTTCAGCCACGGG	23:T>G
12778601|F|0-15:C>T-15:C>T	TGCAGGTGGCCTTCCCGCTGGATCCTCAGGAGCACGTCGAGCAAGTCCTCCTTGTCGTCATCGGCTGCT	15:C>T
7171491|F|0-38:C>T-38:C>T	TGCAGCATCGCCGCGTTGGTGCGGAGGAAGAAGAAGTTCGTGAGCCCG	38:C>T
1714496|F|0-8:T>C-8:T>C	TGCAGGTATCAGTGCAATGAGTGGGGCGTCGGCATGGAGATCGATAGCAACGTCCG	8:T>C
3026225|F|0-23:A>G-23:A>G	TGCAGGATGGGGGCAGCGAGAGGACCATGGGCTTTCCTCCG	23:A>G
1036082|F|0-6:G>A-6:G>A	TGCAGAGTTGCTCTTGGTGATGGAGGAGACGACCGAGAGCGGCACCTGCTTGGCCCACCGCTTGATGTC	6:G>A
5582368|F|0-12:C>A-12:C>A	TGCAGCTCGTCCCCGCCGTCGCCG	12:C>A
12772079|F|0-16:C>T-16:C>T	TGCAGCAGAACAGCTACGCCAAGCAGCAGCACGAGCCCGCCGACGACGACAACAACGGCAACGGCAACG	16:C>T
4396805|F|0-10:C>T-10:C>T	TGCAGCCGTTCTCCAAGGAGGAGTGGAGCTCCCACCG	10:C>T
32025632|F|0-11:T>G-11:T>G	TGCAGGGATGCTCGTGCCCTCCTTCCAGGCTCCGCCCTGGCTTCTATTGGTACGATAAGGTATCCGAGA	11:T>G
1063795|F|0-64:G>C-64:G>C	TGCAGGGGCAGGTGAACGTCACCACGGCGCACTCCCTGGACATCATGGGCAGCTCCTACCCTCCGTCCG	64:G>C
4734196|F|0-5:G>C-5:G>C	TGCAGGCGCCGCAGCTGCCG	5:G>C
6041361|F|0-11:C>G-11:C>G	TGCAGCCACGCCCACAACATGGCCCGCAACAACCCG	11:C>G
3027337|F|0-35:G>C-35:G>C	TGCAGGGCCGAGAAGATGAACTCCACCACCACCATGGCCGCCGCCG	35:G>C
7172228|F|0-27:G>A-27:G>A	TGCAGGGGCCTGACGCGGCGCCGCCTGGCTACCTGACGCTGTCACTCACGCCCGCCG	27:G>A
2255388|F|0-14:A>G-14:A>G	TGCAGGCCCAGGGCACGGCGGAGCCCGCCTGCGATCTCGTTGACGAAATCAGTGGGCGGCATGACGGCG	14:A>G
4990825|F|0-28:G>A-28:G>A	TGCAGGTCGACCACGTGTAGTTCATGGCGCCG	28:G>A
1110940|F|0-17:C>A-17:C>A	TGCAGGTACGCGTACGCCGCGTGCTTCACGAGCGGGTGGCGTATCATGTGCACGTCCCCGCCGCCTCCG	17:C>A
3949826|F|0-13:T>C-13:T>C	TGCAGATACGACGTGCTTCAAGTTTCCCCG	13:T>C

7349677|F|0-15:C>A-15:C>A	TGCAGCAGGAAGTGACGCCCG	15:C>A
1021118|F|0-8:C>G-8:C>G	TGCAGGTTCACAAACTGCTGCTTCTTGGTCTTGTGGTTGGAGAAGGACACCTTGTTGGCCCG	8:C>G
1087499|F|0-20:A>G-20:A>G	TGCAGCTGCCTCGCCGTCGCAGCGCTGGCGGCCACAGTCGCCGCCGCTCTCCTGCTCTCGTTCGCCGAG	20:A>G
5341583|F|0-49:C>T-49:C>T	TGCAGCGGCAGGGCACGAAGAACTTCATCGGCCGCGGCCTGCTCATGGCCAACGGCGCCAGGTGGTCGC	49:C>T
2316759|F|0-48:G>A-48:G>A	TGCAGCTCGGCCACGCCTACCGCCTCATCGCCGTCGACGAGGTCACCAGGGCCGTGCAGGCCAGGAAGG	48:G>A
1096626|F|0-31:C>T-31:C>T	TGCAGGCGCGCGATGACGGCGTGCAGCAGGCCGATGGTCTGCTCCTGCCTCATCGCGGTGGCCTCCATC	31:C>T
1048912|F|0-55:G>A-55:G>A	TGCAGCTTCTTCATAAGGTGTGGCTTCCGTATGAGCTCCGCCATGGCAAACTCCAGCGTCATATACGAG	55:G>A
16662633|F|0-14:C>A-14:C>A	TGCAGCACGCGCAGCGACACCCCGATCTCCCCCGCCGTCTGCTGCGGCGTCACGTTCGACGTCGCGTAG	14:C>A
5580268|F|0-20:G>C-20:G>C	TGCAGCGCGCCCTGCCAGGGGCCGAGCTCCG	20:G>C
32021449|F|0-24:T>C-24:T>C	TGCAGGAGTGCCAGGGCTTGGTGGTGGCCATGGAGCAGGCCG	24:T>C
1694710|F|0-34:C>G-34:C>G	TGCAGGTTTGGCAGGGGATCACTGACAGGGAGCTCGATGTGCTGGACATCTTTGAGGATGAGGAGTATG	34:C>G
7172020|F|0-36:G>A-36:G>A	TGCAGAAGAAGCGCCCCCGCGTCGGCGCCATGGATCGGGAGGCGCCTCCG	36:G>A
1000247|F|0-10:C>G-10:C>G	TGCAGGATGCCGAGGAGCGCGAGGTCGCCCAGCGGCTCATGGCGCTCGCTGGGATCACTGATTGTGAGT	10:C>G
5359167|F|0-8:C>G-8:C>G	TGCAGCTTCACGCTAAAGCCG	8:C>G
7164282|F|0-26:G>C-26:G>C	TGCAGGTACTACACCTCGTTCCAGCTGAGCGAGAAGAGCGACGTGTACAGCTTCGGGATCGTGCTGCTG	26:G>C
1021123|F|0-30:G>C-30:G>C	TGCAGGTCAGGGTGAGCATGCGGAAGGTGCGGTACCTGCGGGAGAGCTCCGTCATGTACTCCG	30:G>C
3027265|F|0-29:C>A-29:C>A	TGCAGCGTCGGCCAGCAGTTCGTCTACTCCAGCTTCGCCG	29:C>A
4663844|F|0-11:G>A-11:G>A	TGCAGCACGACGGTCCTGGCCTGCCG	11:G>A
6041361|F|0-8:C>G-8:C>G	TGCAGCCACGCCCACAACATGGCCCGCAACAACCCG	8:C>G
1004730|F|0-23:C>T-23:C>T	TGCAGCTGCCCCGTCGCGCCGTCCAGGATCAGCCGCGACAGCGCCGACGGGTCGCTGACGTAGTAGGCG	23:C>T
3941543|F|0-10:G>A-10:G>A	TGCAGGGGTGGCCGAGGCGGACGCTCTCCG	10:G>A
3023313|F|0-28:C>A-28:C>A	TGCAGAAAACAATTCAACTAGACCAGCTCCTGCCGCCG	28:C>A
3026640|F|0-20:C>T-20:C>T	TGCAGACTTTGACGTATTGCCCATCGGCCAAATATCTTAGGCGACCG	20:C>T
1075047|F|0-50:G>C-50:G>C	TGCAGGTTGGACAGCGCGTCCTTGAGCTCCTGCGACACCCGCGGCAGCGCGTCGGACGACCCGCGCCCC	50:G>C
4395196|F|0-10:C>T-10:C>T	TGCAGTTGGCCGCCAGCCTCCG	10:C>T
1253942|F|0-44:C>T-44:C>T	TGCAGATGCGCCACCACCCACGCGGCGAACACCAGCTTCCAGCCCAGCGGGCCCCACGACTCGTACCCG	44:C>T
1076347|F|0-22:T>C-22:T>C	TGCAGGGTGATGTATTTACAGTTACTGTACAGGGTTTTTGGCTGGCCTCCCGATCAAACGACTTTGCTG	22:T>C
5324914|F|0-40:C>G-40:C>G	TGCAGATCTGGTGCATCGACCGCGGCGGCCCGCTCTTCACCGCCGTGTTCCAGCCCGTGCAGACCGTCG	40:C>G
1239225|F|0-41:C>A-41:C>A	TGCAGCTCAAGGAGTTCGCGCCGCCGTTCCTGGACAGAAGCCTGTCCGACAAGGACATCGTGACGGGGG	41:C>A
991623|F|0-5:G>C-5:G>C	TGCAGGAGGTTATTGGCGAGGCCCACGGAGATGAGCCCCGCGCTGCACCCCATGCCG	5:G>C
1229270|F|0-30:G>A-30:G>A	TGCAGCGCCGCCGCCGTCGCGGCCAGCATCGCGTACGCCAGCAGCTGGTCGCCG	30:G>A
996761|F|0-31:C>T-31:C>T	TGCAGCAGCCGCCGCCGCTGCACCTGCTCCCCGCGGCCGCCACGTGCTACGGGAAACCCATGGCCGAGA	31:C>T
3956937|F|0-13:G>C-13:G>C	TGCAGCTGGCGGCGGAGTTCGCCGCGTCCTGGGTCCCCTTCTGCCG	13:G>C
992506|F|0-65:C>A-65:C>A	TGCAGACGTTCGCCGCCGCGGACAACGAGCTGATCGGGGGTGTGCCTGATGAGCTCGGCGATTGCCGAG	65:C>A
4262335|F|0-8:C>G-8:C>G	TGCAGCATCGAGATCGCGCCCATGCAGCCGTACACCG	8:C>G
4262229|F|0-11:G>A-11:G>A	TGCAGCCTCCCGGCCTGGCTGGTGCTCGACAACAAGCCACCG	11:G>A
4398082|F|0-22:C>G-22:C>G	TGCAGGCCCCGAGGCCCTGCTCCCCG	22:C>G
4006086|F|0-7:G>A-7:G>A	TGCAGCAGCCAAAGCCCACCGACCACGCCCAACCCGACGGCCAGCCCG	7:G>A
1022722|F|0-23:G>A-23:G>A	TGCAGGGACTGCTTGTAGTCGCTGGCGGCGGTGATCGTGACGACGAGGGAGATGGTGAGCATGATGCCG	23:G>A
990367|F|0-41:G>C-41:G>C	TGCAGGTCGTTGCGGTGCTCGTGGAGGATCCTGGCGGCGCCGAGCAGCATCGCCGTGTGCGCGTCGTGG	41:G>C
1694710|F|0-17:A>G-17:A>G	TGCAGGTTTGGAAGGGGATCACTGACAGGGAGCTCGATGTGCTGGACGTCTTTGAGGATGAGGAGTATG	17:A>G
1051596|F|0-55:C>G-55:C>G	TGCAGTACCAGGACTTCGCCAACCGCTACCAGCTCTGCCTCTCCCACCTCGCCGACGCCGCGCGCGAGG	55:C>G
1237017|F|0-29:G>C-29:G>C	TGCAGCAGGGAAGGAGGGGGGAGGGAGCTGGAGGAGACGGAGGGGTGGTTCCCGTGGAAGCACGACGAC	29:G>C
32023252|F|0-35:C>A-35:C>A	TGCAGACGGAGGCGGATGCGGCGGATCTCGTCGCGCGGCGGGTTGACGACGCCG	35:C>A
1006032|F|0-25:C>A-25:C>A	TGCAGCGGGAGCGCGTCCATGTCGGCGCGCAGCGCGACGAAGGGCGGGCGCCCG	25:C>A
1025753|F|0-34:G>A-34:G>A	TGCAGGACGTGAGCCAGAGCGAGGAGGTGTTCCAGGCCGAGCTGAGCGCGATCGGCAGGATCTACCACA	34:G>A
32017813|F|0-23:A>T-23:A>T	TGCAGTACGTGCAGCAGGGCGGGACGCCG	23:A>T
4994757|F|0-37:C>T-37:C>T	TGCAGCGGAAGAAGTACCAGCCGTTCCCGTTCTTCCACGTCGACAGGGAGAAGAAGTGCCG	37:C>T
1093226|F|0-9:A>C-9:A>C	TGCAGATCCACGCCGACCAGGCCGCGCTGGCGCTCGGGGCGCTCGCCGCCGTGCTCCCCG	9:A>C
5363645|F|0-28:G>C-28:G>C	TGCAGCTGCCAACCGCGCACGACGGCGGGTTCACGGCGTCGGGGCCG	28:G>C
32027391|F|0-40:G>C-40:G>C	TGCAGGCCCTCCATCCGCGCCGCCATGGCGTCGCCGACGAGCCTGACCTCCCTCTCGATGCTCGCCGCC	40:G>C
1230187|F|0-17:C>G-17:C>G	TGCAGCGCGGAGACGAACGCGGAGGACGGCGGCGAGGGCGAGCCGAGGTAGGGCGAGACGGGGCTCGGC	17:C>G
16796556|F|0-37:T>C-37:T>C	TGCAGCTCGTAGGAGGTGCTGGTGATGAGCGCGTCGGTGCCG	37:T>C
5578366|F|0-23:G>A-23:G>A	TGCAGGTAGGGCAGCAGCCCCTTGGCCG	23:G>A
7341568|F|0-7:G>C-7:G>C	TGCAGGCGGAAGGCGACGGCCCG	7:G>C
1092909|F|0-26:C>G-26:C>G	TGCAGCGTGTCGGCGGCGGCGAAGCGCAGGTCCATGGCGTACAGGTTGCTGCCG	26:C>G
1696722|F|0-29:G>T-29:G>T	TGCAGCGCCTTGCCGAGGAGGAGCTCGTTGGTGTGCGGGCCGTAGATGTCGGAGGCGTCGAGGAGGGTG	29:G>T
1128170|F|0-21:A>T-21:A>T	TGCAGGTACCATTGATCACACATGTTCTAACGACACTTCAGCCCCAACACCG	21:A>T
4002650|F|0-23:G>A-23:G>A	TGCAGGAGGAGCAGGAAGAGACGGCCGCGGCTGCCGCGCCG	23:G>A

6041361|F|0-23:C>A-23:C>A	TGCAGCCACGCCCACAACATGGCCCGCAACAACCCG	23:C>A
5346073|F|0-9:T>G-9:T>G	TGCAGCGGCTCGTTGCCGTCGACGCTATCCG	9:T>G
7492291|F|0-35:G>C-35:G>C	TGCAGGCGTCCGTCAACGCCGACGGGACCTTCGGCGAGGTTTTCGCCG	35:G>C
1027219|F|0-50:G>A-50:G>A	TGCAGCACCTGCGCGCTGACGGAGTTGAAGAGGATCTCCACGAAGCCGTTGATGACGGCGGGCTTGGCC	50:G>A
1121829|F|0-16:A>C-16:A>C	TGCAGTTCCCCGTGGGAAGGATCGCCAGGTTCCTCAAGGCCGGCAAGTACGCCGAGCGCGTCGGAGCCG	16:A>C
1298072|F|0-13:C>A-13:C>A	TGCAGAACCTCATCCAGATGATCTCCGTCATGTACGTCGGCCACCTCGGCGAGCTCGCCCTCGCCGAGA	13:C>A
4993768|F|0-13:G>T-13:G>T	TGCAGGGGCTGGGGCTCGGGGAGCCG	13:G>T
986348|F|0-8:C>T-8:C>T	TGCAGCGTCTTGACGTACTTGATCGCGTCGCCCAGCACCGACGCCTTGTCCATCTTCTTGAGGCCCGAG	8:C>T
1021879|F|0-7:G>A-7:G>A	TGCAGCAGCCGCTCCACCACGTGGCTGAGGCAGCACCCGCGCCTCCCCACCACCAGCACCG	7:G>A
3030984|F|0-27:G>T-27:G>T	TGCAGTGCCCTACGGGGTCACGGCCAAGGCGTCGGCGCCG	27:G>T
2251559|F|0-35:G>A-35:G>A	TGCAGAAGGGGATCAAGGGAGCCATCGAGTTCAGGGACGTGCACTTCAGCTACCCGACGCGGCCGAGAT	35:G>A
11309801|F|0-17:G>C-17:G>C	TGCAGGTGGAACTTCACGATGCCG	17:G>C
5357979|F|0-25:G>T-25:G>T	TGCAGCTCCGCCCGCTCGTCCCTCAGCCG	25:G>T
3026623|F|0-23:C>G-23:C>G	TGCAGCCACCAGGAAACGTGCTCCACGGGACGCTGCCG	23:C>G
3950456|F|0-17:G>A-17:G>A	TGCAGCCAGCTTCTGCCGTCCTCCCCG	17:G>A
1021492|F|0-51:T>G-51:T>G	TGCAGGTCCCCATCGCCATCGGAGGTCCAGGGCTTGCGCCAGGGGCTACATTCCG	51:T>G
978702|F|0-10:C>T-10:C>T	TGCAGGCGTTCGGCGAGCACGTACACGTGTCCGTGCACAGCTGGGCGACGGACGTGAAGAAGAAGGCGA	10:C>T
1089540|F|0-42:C>T-42:C>T	TGCAGAGGCGGAGCAGGTCGCAGGAGGAGAGCACGAGCGAGACCATCGCGAGGCCGTTGGTGATCCGCA	42:C>T
3029171|F|0-5:C>A-5:C>A	TGCAGCAGCGCGCCGAGCGCGCCCAGCCCGAGCTCGCCG	5:C>A
1070921|F|0-31:G>C-31:G>C	TGCAGCTCCGCTCCGTCAGCCTCGGGGACCCGTCCCTGTCCGCCACCGCGCAGGTCACGCTCGCCTCCC	31:G>C
1002042|F|0-50:A>G-50:A>G	TGCAGGGTGCCACGACGGAGGAGATGCATGGAGAAGAGGAAGAGGAAAGGAGACAGCTCCTCGTGCCTC	50:A>G
5580382|F|0-25:G>A-25:G>A	TGCAGGAGGGCGGGCTGCTGGGAGCGGAGACCCG	25:G>A
1153924|F|0-40:G>C-40:G>C	TGCAGACCAACGTGTACGTCCGCGGCAGCGGCGACGGGCGGATCATCGGGCGGGAGATGCGGTTCCACC	40:G>C
1038161|F|0-65:T>C-65:T>C	TGCAGGTAGCCGCCACCGAACGTGGGAGGAGATGGCGTGGGAGACGCGTACAGCGTTGGGGGCGGTGAG	65:T>C
1055251|F|0-26:C>G-26:C>G	TGCAGCCTCTTCTCCCCGACGCCCTCCCTCTCCGCCATGATCATCAACAAGTACAAGCTCCGCAGCAAC	26:C>G
988366|F|0-56:C>T-56:C>T	TGCAGGGCCTTGGTCATGTTGAGGGTGACGCCGTAGTCCTGGCCGCCCTGGAAGGCCTGGCAGAGGCAG	56:C>T
1285274|F|0-10:C>A-10:C>A	TGCAGGCGCCCGCCAGGGACGTCGTCGCCGTGCTCTCGGCCTACGTGCGCAAGCTCGAGGAGGAGTCCG	10:C>A
32025584|F|0-60:G>A-60:G>A	TGCAGCTTGGAGATGATGGTGGGGCGGCGGAAGCGGGCGGGATCAACCAGGACGAGGCGGGCGATCTGG	60:G>A
985174|F|0-37:G>C-37:G>C	TGCAGGCGCACCTGGCCGACAGCAACCTGGGCGCCGTGGTGGAGGAGTGCATGCAGCACTACGACGAGC	37:G>C
1862754|F|0-14:C>T-14:C>T	TGCAGCACGCGGTCCCGCGGCCACTGCTGGGTGAGGGGCAGCTCGAGCAGCTGCCTCCAGAACTCGGAG	14:C>T
4993790|F|0-21:G>C-21:G>C	TGCAGAGCTTGACGATGTCGTGGACGACGCCG	21:G>C
1091531|F|0-25:C>G-25:C>G	TGCAGTGGTTCGCCGACAAGGGCCTCACCGCCTACAGCATCTCGTGCGGCACCTCCCTGCTGTACAACA	25:C>G
1314388|F|0-48:T>C-48:T>C	TGCAGGAGCACGCCGCGCACGACGGCGGGGAGGGCGACGAGGTTCTTGTCCTCCTCCTCGGCCCGCGTG	48:T>C
4408937|F|0-5:C>A-5:C>A	TGCAGCCTGGGCAGCGCAATGCCGTCGACCCCGCCG	5:C>A
1214567|F|0-43:G>T-43:G>T	TGCAGGAGAGCTGCACGGACACGTCGGGGTGCTCCATGGTGGTGCACTCGCCCATCGACATCCCGAGAT	43:G>T
1000247|F|0-19:C>G-19:C>G	TGCAGGATGCCGAGGAGCGCGAGGTCGCCCAGCGGCTCATGGCGCTCGCTGGGATCACTGATTGTGAGT	19:C>G
3029171|F|0-14:G>C-14:G>C	TGCAGCAGCGCGCCGAGCGCGCCCAGCCCGAGCTCGCCG	14:G>C
2252110|F|0-40:G>A-40:G>A	TGCAGGTCAGCAGCGCCACGTCCGCCGCCGTGTCGATCATGTTGTCCATGAAGAAGACGTAGGCGGTCA	40:G>A
6045080|F|0-43:G>T-43:G>T	TGCAGGGCGACATCGCCGTGGGGCTCGGGTTCGCGGCGTTCGCGTTCCTTGCGCTGGCGGCGCTCGCGA	43:G>T
2244217|F|0-20:G>C-20:G>C	TGCAGCTCGCCCTTCATCATGCCGCCGAACTCCAGCACGCTCCACGCCAGCATCGTCGAGCTGAACGCC	20:G>C
5577937|F|0-7:C>G-7:C>G	TGCAGCACGACGGTCCTGGCTTGCCG	7:C>G
5581911|F|0-6:C>T-6:C>T	TGCAGACGTACGCCACCTTGCAGCCG	6:C>T
5582333|F|0-5:C>A-5:C>A	TGCAGCACGGCGTGGCTGCCGATGTAGCCCGCCCCGCCG	5:C>A
1216931|F|0-23:A>G-23:A>G	TGCAGCATGAGCTCGTAGTTCTTATGCCCCTTGGAGATGGTCTGCCCAGGTCGCTTCCCCTCAGGCGTG	23:A>G
5364471|F|0-8:G>C-8:G>C	TGCAGGGAGAGGAGGAGGAAGGCGATAACCCACCG	8:G>C
7167525|F|0-19:C>G-19:C>G	TGCAGATCAAGCTGGGTGTCCCGAGGCCG	19:C>G
32027806|F|0-20:C>G-20:C>G	TGCAGGTAGTCGCCGACGTTCACCACCAGCGCCCCCG	20:C>G
3935275|F|0-8:C>T-8:C>T	TGCAGTCCCTGGACGCCAGCAACAACCGCCTCACCG	8:C>T
3948194|F|0-24:G>A-24:G>A	TGCAGCTGAAGCCTCTGGAGTTGCGCATCGACCG	24:G>A
5346073|F|0-17:G>A-17:G>A	TGCAGCGGCTCGTTGCCGTCGACGCTATCCG	17:G>A
5363660|F|0-8:G>A-8:G>A	TGCAGCTGGCGGCGGAGTTCGCCGCGTCGTGGGTGCCGTTCTGCCG	8:G>A
3025057|F|0-5:C>T-5:C>T	TGCAGCGGTCAAGGCATCAACCTCGGACTGCGCGAGCCG	5:C>T
1253942|F|0-21:G>A-21:G>A	TGCAGATGCGCCACCACCCACGCGGCGAACACCAGCTTCCAGCCCAGCGGGCCCCACGACTCGTACCCG	21:G>A
2244489|F|0-10:C>G-10:C>G	TGCAGGACGACAGCGAGTCCGAGTCAGGTTCAGAGTCTATCGAGATCGCGGACCTCAAGAAGCGCATGT	10:C>G
985174|F|0-19:C>T-19:C>T	TGCAGGCGCACCTGGCCGACAGCAACCTGGGCGCCGTGGTGGAGGAGTGCATGCAGCACTACGACGAGC	19:C>T
4262551|F|0-32:G>T-32:G>T	TGCAGTCTGCCGTGGCCGCGGCCGTGGCGGTCGTCCTCGCCCCCGCCG	32:G>T
1036082|F|0-27:G>A-27:G>A	TGCAGAGTTGCTCTTGGTGATGGAGGAGACGACTGAGAGCGGCACCTGCTTGGCCCACCGCTTGATGTC	27:G>A
3960077|F|0-5:C>G-5:C>G	TGCAGCACGCGCAGCGACACCCCGATCTCCGCCGCCGTCTGCTGCGGCGTCACGTTCGACGTCGCGTAG	5:C>G
1140846|F|0-37:C>G-37:C>G	TGCAGGCGTCCATATGGAGCAAGGACGTGGAGTTCTCCTTCGGCGAGATCCTGGCGGCGACGGAGCACT	37:C>G

<table>
<tr><td>2241770|F|0-56:A>G-56:A>G</td><td>TGCAGCTTCTTGATCTCCTTCTTGAGGTCCGCCTCGAACTTCTCCTTCTGGTTCGCATTCTCGGTGTCG</td><td>56:A>G</td></tr>
<tr><td>5363660|F|0-22:C>A-22:C>A</td><td>TGCAGCTGGCGGCGGAGTTCGCCGCGTCGTGGGTGCCGTTCTGCCG</td><td>22:C>A</td></tr>
<tr><td>5338374|F|0-15:C>A-15:C>A</td><td>TGCAGACGGCGGAGGCGCGCGGGAGGGAGGACGGGAGCGGCGGCAGGCGGAGGAGGATCTCGGAGAGCA</td><td>15:C>A</td></tr>
<tr><td>1180126|F|0-28:G>C-28:G>C</td><td>TGCAGCAGACGCGGTTCCACTCGCAGCCGATCCTCCACGCGTCCCAGATCAACGACGGGACGCGGAGGG</td><td>28:G>C</td></tr>
<tr><td>1206525|F|0-19:C>G-19:C>G</td><td>TGCAGGACCTGGTGGTGGCCATGCTGGAGCTGTGGAGCCTGATGGACACGCCGCCG</td><td>19:C>G</td></tr>
<tr><td>1862865|F|0-49:C>T-49:C>T</td><td>TGCAGGTCTTCTCCACCGACTTCATCGACGGCGACTTCGTGGACGCCTGCGCCG</td><td>49:C>T</td></tr>
<tr><td>4262354|F|0-13:A>G-13:A>G</td><td>TGCAGGCCAAGCAAAATCGAAACAGAGTGGGGTGCCGAGAGGCCG</td><td>13:A>G</td></tr>
<tr><td>1205521|F|0-29:C>T-29:C>T</td><td>TGCAGCGCCCAGTGCTTCTGGATGAAGGACGACTCCCAGCTCCGCTCCTTGTCCGCCAGCGACGACGGC</td><td>29:C>T</td></tr>
<tr><td>1060660|F|0-65:G>C-65:G>C</td><td>TGCAGCGACCTCTTGAGCTCGGTGAAAAGGTCACCCCAGCCATCGGTGCCCGTGTCGCTGACCACGGTC</td><td>65:G>C</td></tr>
<tr><td>1000247|F|0-7:T>C-7:T>C</td><td>TGCAGGATGCCGAGGAGCGCGAGGTCGCCCAGCGGCTCATGGCGCTCGCTGGGATCACTGATTGTGAGT</td><td>7:T>C</td></tr>
<tr><td>3955149|F|0-11:T>A-11:T>A</td><td>TGCAGGGACTCTGGCAGGGCACCCGAGAGCCCGTTCCGCGACACGTCGATCGCCTCCAGGAACGGCAGC</td><td>11:T>A</td></tr>
<tr><td>3945784|F|0-24:G>C-24:G>C</td><td>TGCAGTGACGACAACGAGCGCCTTGAATGCCGCCG</td><td>24:G>C</td></tr>
<tr><td>993046|F|0-59:A>G-59:A>G</td><td>TGCAGCAGCTCGGGGCTGATGCTCAGGTGCACGGCGCCGCTGCCTTTCTTGGACCTCCCACGCCTCCGA</td><td>59:A>G</td></tr>
<tr><td>4262703|F|0-23:G>T-23:G>T</td><td>TGCAGCACGCGGCTGATACCGCCGCCCCCGCCCG</td><td>23:G>T</td></tr>
<tr><td>1070154|F|0-9:G>C-9:G>C</td><td>TGCAGAGGTGGAGCTGATGAAGGAGCGGTTCTCCAAGCTGCTGCTGGGCGAGGACATGTCCG</td><td>9:G>C</td></tr>
<tr><td>1000264|F|0-30:A>C-30:A>C</td><td>TGCAGGTGGGTAAGGTGGAGATCCGATAAAAATGGACCAAATTGCGAATGCCTGGCTCCCTGTATGCCT</td><td>30:A>C</td></tr>
<tr><td>1215372|F|0-32:G>C-32:G>C</td><td>TGCAGGCGTGCATGCACAGAGTGGCCACCGAGGCAGCGGCGCGAGGCTCGCGGTGGCCG</td><td>32:G>C</td></tr>
<tr><td>3032678|F|0-41:G>A-41:G>A</td><td>TGCAGGTGCGAGACGATGCTGCCGTCCTCCGCCCGCCACACGCCG</td><td>41:G>A</td></tr>
<tr><td>12776281|F|0-14:T>A-14:T>A</td><td>TGCAGGTCGGCCACTCCGCCGTCGTCCCCATCTTCGCCGAGGGACTCGATGATCTGATGCGGGAGCAGC</td><td>14:T>A</td></tr>
<tr><td>1106007|F|0-43:G>C-43:G>C</td><td>TGCAGGATCTCGGCCTCCAGTTCACCCCTGTCAACGACTCTCTGTACGAGACGGTGAAGAGCCTCCAGG</td><td>43:G>C</td></tr>
<tr><td>989503|F|0-17:A>C-17:A>C</td><td>TGCAGCACAACAACCTGAGTGGCGAGATCCCAGCGGTGCTCGCGGCGCTGCCGTCGGTCACCG</td><td>17:A>C</td></tr>
<tr><td>5368791|F|0-13:G>A-13:G>A</td><td>TGCAGTTGACGATGAGCACGTCGATGTCGGCTGGCTTGATGACGGCCGTCTTGGCGAACACGTCGTCGA</td><td>13:G>A</td></tr>
<tr><td>3027787|F|0-19:C>A-19:C>A</td><td>TGCAGCAGTACGCCTCCGTCCCGAACGCCTCGCACGCGCTCCG</td><td>19:C>A</td></tr>
<tr><td>12420016|F|0-11:T>A-11:T>A</td><td>TGCAGAACAACTACCTCACCG</td><td>11:T>A</td></tr>
<tr><td>3025523|F|0-38:C>G-38:C>G</td><td>TGCAGCCTCCCCCAGTGTATCTCCCACATGTCGGTGCTCAGCCCG</td><td>38:C>G</td></tr>
<tr><td>1211528|F|0-64:G>A-64:G>A</td><td>TGCAGGCGCTGGTGCGCGGCCGCCGCGTGCGCAAGCAGCTGGCCGTCACGGTCAAGTGCATGCAGGCGC</td><td>64:G>A</td></tr>
<tr><td>32022578|F|0-16:C>A-16:C>A</td><td>TGCAGTTCCCCGTGGGCAGGATCGCCAGGTTCCTCAAGGCCG</td><td>16:C>A</td></tr>
<tr><td>6044066|F|0-7:A>G-7:A>G</td><td>TGCAGGAAGTGCGGCTCGCCCTGCCG</td><td>7:A>G</td></tr>
<tr><td>1865063|F|0-47:C>A-47:C>A</td><td>TGCAGTGACAAGAAAACGCTGAAGAAGTGGTTCTTCATCGACAAGACCGTGGGCTAAGAAAATCCAGCT</td><td>47:C>A</td></tr>
<tr><td>32026395|F|0-17:C>G-17:C>G</td><td>TGCAGGATGGAGAAGACCGCCGCGACGGTGCCGCGGAGGTAGTTGGCGTCCAGCGTCATGGCGATGTGC</td><td>17:C>G</td></tr>
<tr><td>100576224|F|0-5:G>C-5:G>C</td><td>TGCAGGAGCAGCTTGCCTTGGACACGAGCGCTAGTGGCAGTGGACGTGGTCTGCACGTCGGAGCCGAGA</td><td>5:G>C</td></tr>
<tr><td>5358034|F|0-19:G>C-19:G>C</td><td>TGCAGCTGCGGCAGCGGCGGCGCGGCCG</td><td>19:G>C</td></tr>
<tr><td>7167631|F|0-26:C>G-26:C>G</td><td>TGCAGCAGGATCGACCTCTTCAAGAACTCCG</td><td>26:C>G</td></tr>
<tr><td>6048008|F|0-22:C>T-22:C>T</td><td>TGCAGGGGCGGATCGCCAAGATCGCGGAGCAGGAGAGGGGCCAGGCTGACCCGCGGTATGGCGGCGAGT</td><td>22:C>T</td></tr>
<tr><td>4262855|F|0-35:A>G-35:A>G</td><td>TGCAGAGGGGAACATCGGGGAGGGCGTCGCGGCGGAGGGGCCG</td><td>35:A>G</td></tr>
<tr><td>977165|F|0-6:G>C-6:G>C</td><td>TGCAGGGCATCGCGCAGAGCGGCAGCGTCGTGATCATGTCCATCCACCAGCCGAGCTACCGCATCCTCG</td><td>6:G>C</td></tr>
<tr><td>5581581|F|0-14:G>C-14:G>C</td><td>TGCAGTCGCCCGCCGTCGTGCCCAGCTCCG</td><td>14:G>C</td></tr>
<tr><td>2259806|F|0-11:T>G-11:T>G</td><td>TGCAGGAAGTTTGTGGCTGAGTTCCAGAGCGTGGGGAGGGCGCGGCTGCCGACGAAGGCGTCGACCTCG</td><td>11:T>G</td></tr>
<tr><td>4006284|F|0-31:C>T-31:C>T</td><td>TGCAGTGCTGGTGCCTCAACTTCCGCGGCCGCGTGACCATCGCCTCGGTGAAGAACTTCCAGCTGACCG</td><td>31:C>T</td></tr>
<tr><td>5579587|F|0-31:G>T-31:G>T</td><td>TGCAGCCGCACACCGTCAACTTCCTCAACGTGGTCATCGACAACACCCGCGCCAGCCTGATGCCGCAGA</td><td>31:G>T</td></tr>
<tr><td>5580382|F|0-17:C>T-17:C>T</td><td>TGCAGGAGGGCGGGCTGCTGGGAGCGGAGACCCG</td><td>17:C>T</td></tr>
<tr><td>1025726|F|0-54:G>A-54:G>A</td><td>TGCAGGGGGCTCCGCGCCGCGGCGGAGGGGCACGACCAGCAGCAAGAGGGGGGCGACGACGCGGCGGGA</td><td>54:G>A</td></tr>
<tr><td>1110940|F|0-49:G>A-49:G>A</td><td>TGCAGGTACGCGTACGCCGCGTGCTTCACGAGCGGGTGGCGTATCATGTGCACGTCCCCGCCGCCTCCG</td><td>49:G>A</td></tr>
<tr><td>1059849|F|0-14:C>G-14:C>G</td><td>TGCAGCTGATAAAGCAAGTGCTCACAGACAGGACGGGCCTGTATCAGAAGGACTTCATGATCCCCGTGC</td><td>14:C>G</td></tr>
<tr><td>1098311|F|0-24:G>A-24:G>A</td><td>TGCAGCGACCGCTTCACTGCTTTAGCGCGCGAATGGGTGATGACCGTGGATGGTGCGCCG</td><td>24:G>A</td></tr>
<tr><td>1002042|F|0-23:A>G-23:A>G</td><td>TGCAGGGTGCCACGACGGAGGAGATGCATGGAGAAGAGGAAGAGGAAAGGAGACAGCTCCTCGTGCCTC</td><td>23:A>G</td></tr>
<tr><td>1094719|F|0-19:C>G-19:C>G</td><td>TGCAGCAGGAGCAGCCGCACGCGGTGCCGCACTTGCAGGTGGTGCAGTCGCAGCCGCCG</td><td>19:C>G</td></tr>
<tr><td>2251559|F|0-8:G>C-8:G>C</td><td>TGCAGAAGGGGATCAAGGGAGCCATCGAGTTCAGGGACGTGCACTTCAGCTACCCGACGCGGCCGAGAT</td><td>8:G>C</td></tr>
<tr><td>5992708|F|0-11:C>G-11:C>G</td><td>TGCAGCTGCGACGCGCCGTGCGGGTGGCCG</td><td>11:C>G</td></tr>
<tr><td>986316|F|0-47:C>G-47:C>G</td><td>TGCAGCTGGCGGGACGCGGACCAGGTGCGGGACACGCTCCAGGACAGCGACCTGAAGTGGGAGCCGCCG</td><td>47:C>G</td></tr>
<tr><td>1075047|F|0-44:C>A-44:C>A</td><td>TGCAGGTTGGACAGCGCGTCCTTGAGCTCCTGCGACACCCGCGGCAGCGCGTCGGACGACCCGCGCCCC</td><td>44:C>A</td></tr>
<tr><td>4261309|F|0-17:G>A-17:G>A</td><td>TGCAGGAGGCTGCCGCCGCCCG</td><td>17:G>A</td></tr>
<tr><td>3941460|F|0-11:G>T-11:G>T</td><td>TGCAGCATCGCGCTCGACGTCGCCGCCGAGCTTGGCGTCCCCG</td><td>11:G>T</td></tr>
<tr><td>6045622|F|0-5:C>T-5:C>T</td><td>TGCAGCTGGGAAGCTACTTGGTCCCACAGCCTCGTCCG</td><td>5:C>T</td></tr>
<tr><td>1039946|F|0-48:T>C-48:T>C</td><td>TGCAGGAAAAATGAGAAACTACAGTCATCATCTAAAATTTGTCCTTTGTCTAGTCCTCGGAAGGCGGCG</td><td>48:T>C</td></tr>
<tr><td>5010854|F|0-66:G>C-66:G>C</td><td>TGCAGCGAGTTGTTCGATATGTCGAGGTAGAAGAGGCGGTCGAGCTCGCCGAGCAGTGGCGGGATCGGG</td><td>66:G>C</td></tr>
<tr><td>1167494|F|0-37:G>A-37:G>A</td><td>TGCAGCAGAAGTACACGCAGAGCGGAGGGGTGCGCCCGTTCGGGCTCTCCACGCTTATAGTTGGCTTCG</td><td>37:G>A</td></tr>
<tr><td>7351326|F|0-16:T>C-16:T>C</td><td>TGCAGACCTTGCTGCATCCG</td><td>16:T>C</td></tr>
<tr><td>6047394|F|0-10:G>C-10:G>C</td><td>TGCAGAATGGGCCGCGGAGGGGCAGCGACTACGACAGCGGGCTGGCACCGCAGTAGGTTCATTTTAGTA</td><td>10:G>C</td></tr>
</table>

<table>
<tr><td>2308539|F|0-21:A>T-21:A>T</td><td>TGCAGCCGCGCGGCGACTCATACATGCGCCGCTCCTCCGTCGACGCCATCATGGCCG</td><td>21:A>T</td></tr>
<tr><td>5011470|F|0-19:A>G-19:A>G</td><td>TGCAGGCTGCCGACAGAGAAGAGACGGCGGCCG</td><td>19:A>G</td></tr>
<tr><td>32023390|F|0-20:G>C-20:G>C</td><td>TGCAGGGCCAGGCGCGGCGCGGATACCG</td><td>20:G>C</td></tr>
<tr><td>1667137|F|0-28:C>G-28:C>G</td><td>TGCAGGCCGAGTCCGACTTCGTCTACGTCCTCGACGACGACATGATCCCCG</td><td>28:C>G</td></tr>
<tr><td>5367470|F|0-16:C>G-16:C>G</td><td>TGCAGTACACGGTGGGCGCGGCGGGGTACTACGGGATCAGCGTGGCCG</td><td>16:C>G</td></tr>
<tr><td>7337147|F|0-5:C>G-5:C>G</td><td>TGCAGCCCGCGGCGCTCTCGCCG</td><td>5:C>G</td></tr>
<tr><td>7337147|F|0-10:G>C-10:G>C</td><td>TGCAGCCCGCGGCGCTCTCGCCG</td><td>10:G>C</td></tr>
<tr><td>1249368|F|0-13:A>G-13:A>G</td><td>TGCAGCCTCAGGGAGATTACATAGAGCTTCACCAGAAGCGCCATGGCCG</td><td>13:A>G</td></tr>
<tr><td>5361549|F|0-6:G>A-6:G>A</td><td>TGCAGGGCGGGCTCGGCGAGCGGGCAGGCGGCCTCGAAGGTGGCCCCCTCCTCGAGCGCGAAGTCCTCC</td><td>6:G>A</td></tr>
<tr><td>1164636|F|0-35:C>T-35:C>T</td><td>TGCAGCACGGTCGTCTCCTTGGGCAGCGCCGTCGGCCCCACCATCACCTTGGGCCCCAGGAGCACGTCC</td><td>35:C>T</td></tr>
<tr><td>7913398|F|0-19:C>G-19:C>G</td><td>TGCAGCGCAACGCCACGCGCTACGGCGCGGCGGCCACGGCCGTGATCCG</td><td>19:C>G</td></tr>
<tr><td>32025957|F|0-10:A>C-10:A>C</td><td>TGCAGATCAGAGGCGAGCGGAACAAGGAGCAGGAGGAGAAGACCGACACCTGGCACCGCGTGGAGCGCA</td><td>10:A>C</td></tr>
<tr><td>22836228|F|0-13:T>C-13:T>C</td><td>TGCAGTTCTTCCGTGCGGACAAGGCGGCCG</td><td>13:T>C</td></tr>
<tr><td>2260066|F|0-7:C>G-7:C>G</td><td>TGCAGCGCCTTGAGTCCGCGGCCGCCCTCGGCAACGGTGGCGGCCTCGAGACAGAGCACTACAGCCCAT</td><td>7:C>G</td></tr>
<tr><td>1118231|F|0-62:C>T-62:C>T</td><td>TGCAGTCAGTGCAACATCGTCTGGCCCCAATCCCTCGCCCCTCATCCTGTCAAACAGCGCCACCGCCTC</td><td>62:C>T</td></tr>
<tr><td>6044111|F|0-19:C>T-19:C>T</td><td>TGCAGGCGATGGGCATCAACTCCAGCGGCACCG</td><td>19:C>T</td></tr>
<tr><td>1209465|F|0-64:G>T-64:G>T</td><td>TGCAGTAGGCGGGCGCGGACAGGGACACGGCGAGCGCCATCCCCAGAACCGCGCGGCGGCCGAGGCCGA</td><td>64:G>T</td></tr>
<tr><td>1040251|F|0-5:C>G-5:C>G</td><td>TGCAGCCCAGAGAACTTGGACTGGTTGTACAGCATGTGCGGCGGCATCGCGAAGGCCG</td><td>5:C>G</td></tr>
<tr><td>5001534|F|0-10:C>T-10:C>T</td><td>TGCAGATGGGCGTGATGCTCACGACGGCGACCGTGGTGGTCACCGCGGGCATCGTCGTCGTGAGCTTGT</td><td>10:C>T</td></tr>
<tr><td>5581757|F|0-23:A>G-23:A>G</td><td>TGCAGAAGCCGCGCGTACGGCGTATCCTCCAGCGCCACGTGGCGGACGGCAGCGCCGCTGCCGTCGCCC</td><td>23:A>G</td></tr>
<tr><td>3030819|F|0-13:C>A-13:C>A</td><td>TGCAGAACAAGCCCGTCCCTATCTGCTACGACGGGTTCGAGCCCTCCG</td><td>13:C>A</td></tr>
<tr><td>4988565|F|0-23:C>G-23:C>G</td><td>TGCAGCAGCGCGCAGAAGACGCTCCCCCAGGCCCCCGCGCCCACGCCG</td><td>23:C>G</td></tr>
<tr><td>1090646|F|0-61:G>C-61:G>C</td><td>TGCAGCGCGTGCAGGCGCACCTCCCGAACGCGACGCAGGTGGTGGGCCTGCTGACCCTGCTGCTCGCCG</td><td>61:G>C</td></tr>
<tr><td>1202680|F|0-27:G>A-27:G>A</td><td>TGCAGCTGGTGTCCAACGACAGGTTCAGGAGCGTCGCGCACCGCGTGGTGGCCAACAGCGTGGGGCCCC</td><td>27:G>A</td></tr>
<tr><td>2265607|F|0-31:G>C-31:G>C</td><td>TGCAGCGGCTGTCGATGTCCTCGACGATGTCGGTGCCGTGGTCCTCCCTGTACCGCCG</td><td>31:G>C</td></tr>
<tr><td>1695198|F|0-40:G>C-40:G>C</td><td>TGCAGGACAATGGCATCAAGAAGGTGAAGCTGTTCGACGCGGACGCGGAGCCGCTGGGCGCGCTCGCCG</td><td>40:G>C</td></tr>
<tr><td>3533625|F|0-14:C>T-14:C>T</td><td>TGCAGGTACATTTGCCGAAGAAGCTGCACCGCCACGCCGTCGCCG</td><td>14:C>T</td></tr>
<tr><td>4993593|F|0-28:C>G-28:C>G</td><td>TGCAGGCTCTGGATGGCGCGGACACGCGCCTCCG</td><td>28:C>G</td></tr>
<tr><td>1215939|F|0-13:G>A-13:G>A</td><td>TGCAGAGCGACGTGTACGCGTTCGGCGTCGTCCTCCTGGAGCTGCTCACCG</td><td>13:G>A</td></tr>
<tr><td>1862986|F|0-31:G>A-31:G>A</td><td>TGCAGCCGCGCGGGGACAGCTACACGCGGCGGTCCCTGTTCGACTGCATGGTGGCCG</td><td>31:G>A</td></tr>
<tr><td>6043007|F|0-26:C>G-26:C>G</td><td>TGCAGCGAGAGCGGCAGCTGCACCAACATCCACGTCAACTCCCTGGCGATCACAGCTGCGTACGGCGGA</td><td>26:C>G</td></tr>
<tr><td>3950480|F|0-8:A>G-8:A>G</td><td>TGCAGGTCACCGCGTTCTCGCCCCTCATCGACGTCATCAACCGCCGCGACGGCACCGTCAACCGCGGCC</td><td>8:A>G</td></tr>
<tr><td>2254577|F|0-63:G>A-63:G>A</td><td>TGCAGCACCTCAGCGGCCAGCCCAACATCGCCGAGTTCAGGGGCGCCTTCGAGGACGCCGAGAGCGTGC</td><td>63:G>A</td></tr>
<tr><td>7172832|F|0-10:T>G-10:T>G</td><td>TGCAGAAGTCTGTCAGCAAGGGGTGGTGCTGCGGGCGCACGTAGGTGCGGAACTCGGCGGGGTCGCGGT</td><td>10:T>G</td></tr>
<tr><td>7350798|F|0-7:A>G-7:A>G</td><td>TGCAGGCAGAGGGCGGGATCCCG</td><td>7:A>G</td></tr>
<tr><td>1862754|F|0-44:G>C-44:G>C</td><td>TGCAGCACGCGGTCCCGCGGCCACTGCTGGGTGAGGGGCAGCTCGAGCAGCTGCGTCCAGAACTCGGAG</td><td>44:G>C</td></tr>
<tr><td>4733973|F|0-22:G>C-22:G>C</td><td>TGCAGCTCCACTGGAAGGAGCTGGCCCGCGACATCGAGACCG</td><td>22:G>C</td></tr>
<tr><td>7339075|F|0-31:G>C-31:G>C</td><td>TGCAGTCGATCCAAGAAATCATTGGCCGCTCGAGTGGCGGCAGATCCATCACGGACCTCTGGGTCCGAG</td><td>31:G>C</td></tr>
<tr><td>1124662|F|0-12:G>A-12:G>A</td><td>TGCAGCGTCGACGACACCCTCAAGGCCCGCGTCGCCATCTTACGCGGCTATGGCCTCTCCGAAGACCAC</td><td>12:G>A</td></tr>
<tr><td>5580150|F|0-28:A>G-28:A>G</td><td>TGCAGCACCAGCTCCACGTCGGTGGGGTAGAACCG</td><td>28:A>G</td></tr>
<tr><td>3027502|F|0-19:G>A-19:G>A</td><td>TGCAGAGGACCGCCTTCGGGTCGGTGTCCAGCAGCACCG</td><td>19:G>A</td></tr>
<tr><td>5372257|F|0-10:C>T-10:C>T</td><td>TGCAGGCTCTCCATCTGCGGCAAGGCGGCGGCCG</td><td>10:C>T</td></tr>
<tr><td>1318673|F|0-7:C>T-7:C>T</td><td>TGCAGAACATCTACTTCTCCTACGGGATGGCGTACGGCGGCGGCGGCTTCGCCATCAGCAGGCCGCTGG</td><td>7:C>T</td></tr>
<tr><td>1156192|F|0-58:A>G-58:A>G</td><td>TGCAGGCACACCACCGTGGCGGCGCCGCCCATGAACCCGACGATGGCGGCCTGCGACAAAAAGTCCACC</td><td>58:A>G</td></tr>
<tr><td>6030645|F|0-19:C>T-19:C>T</td><td>TGCAGCTGGAGCGCCTCGGCCTGGCCG</td><td>19:C>T</td></tr>
<tr><td>985914|F|0-32:G>A-32:G>A</td><td>TGCAGCTGGCCATACAATGCCTCCAATATCTTGTCCCCCTTGGGCAGCTTCTCTGCCAGGTTGAATGCC</td><td>32:G>A</td></tr>
<tr><td>996362|F|0-18:C>A-18:C>A</td><td>TGCAGGTTCACGAGCGCCCTGGCGCGGTGGAGGCTGGCCTTGCTGTGGCAGCGCACCGTGTCGCGGCCG</td><td>18:C>A</td></tr>
<tr><td>1739440|F|0-24:A>G-24:A>G</td><td>TGCAGGACGTGGAGGTGCACGTCAAGGGGGATATTGGGTATGTCACCTGCCTGGAGATGGTGAAGACGA</td><td>24:A>G</td></tr>
<tr><td>2244008|F|0-7:G>A-7:G>A</td><td>TGCAGGCGGCCAGAAGAAAAAAGTCACTGGTCTTTCCATAGATTAGGACAGCTACACAAAGCAGCACAG</td><td>7:G>A</td></tr>
<tr><td>1091531|F|0-43:G>C-43:G>C</td><td>TGCAGTGGTTCGCCGACAAGGGCCTCACCGCCTACAGCATCTCGTGCGGCACCTCCCTGCTGTACAACA</td><td>43:G>C</td></tr>
<tr><td>992506|F|0-19:G>A-19:G>A</td><td>TGCAGACGTTCGCCGCCGCGGACAACGAGCTGATCGGCGGTGTGCCTGATGAGCTCGGCGATTGCCGAG</td><td>19:G>A</td></tr>
<tr><td>1100173|F|0-37:C>G-37:C>G</td><td>TGCAGATCATGCCGCAGGCCATCTGCACGCGCTACGGCCTCAGCATGGGGGCCAAGGCAGCGCCCATCG</td><td>37:C>G</td></tr>
<tr><td>1153924|F|0-49:G>A-49:G>A</td><td>TGCAGACCAACGTGTACGTCCGCGGCAGCGGCGACGGGCGGATCATCGGGCGGGAGATGCGGTTCCACC</td><td>49:G>A</td></tr>
<tr><td>7167662|F|0-6:G>A-6:G>A</td><td>TGCAGCGTGATACACCACCAGCTGGTCCTGCACCCG</td><td>6:G>A</td></tr>
<tr><td>4733973|F|0-7:C>G-7:C>G</td><td>TGCAGCTCCACTGGAAGGAGCTGGCCCGCGACATCGAGACCG</td><td>7:C>G</td></tr>
<tr><td>4262411|F|0-29:C>T-29:C>T</td><td>TGCAGCTTGGACATCGAGTATGATCAGGGCGAGGCCG</td><td>29:C>T</td></tr>
<tr><td>4005999|F|0-35:C>T-35:C>T</td><td>TGCAGCGCGGCGGTTAGCATGATCGGGTCCACCAGCGTCCG</td><td>35:C>T</td></tr>
<tr><td>2243403|F|0-37:G>A-37:G>A</td><td>TGCAGGAGAAGGAGATGATCGACAACCTCTCCAACAAGAACACGCTCATGTCCGTGCGGGAGCTCGCCA</td><td>37:G>A</td></tr>
<tr><td>1314388|F|0-47:G>C-47:G>C</td><td>TGCAGGAGCACGCCGCGCACGACGGCGGGGAGGGCGACGAGGTTCTTGTCCTCCTCCTCGGCCCGCGTG</td><td>47:G>C</td></tr>
</table>

2248971|F|0-62:C>G-62:C>G TGCAGGTACTCCGCGGGCTTCCCGAGGCCGAAGGGGTCGAAGCCGTAGTCGCCGACGAGCGTCCCGTCG 62:C>G

1202084|F|0-50:A>G-50:A>G TGCAGGAGCAAGCTCAGGCACTTGCTCGCCGCGGAGGGGGAGCCAGGGAGAGAGCCG 50:A>G

3950480|F|0-20:C>G-20:C>G TGCAGGTCACCGCGTTCTCGCCCCTCATCGACGTCATCAACCGCCGCGACGGCACCGTCAACCGCGGCC 20:C>G

7352786|F|0-8:G>C-8:G>C TGCAGGGCGTGTGCGGCCCG 8:G>C

1090096|F|0-11:G>C-11:G>C TGCAGGAAATCGAGGCTGGGCCTGCCGTGCTTCATGCAGTTCTGGTAGTCCTGGATGAAGGCGCACGTC 11:G>C

3936463|F|0-23:G>A-23:G>A TGCAGGACGTGCATTGCCTGGGCGACAGACGGCCGCTCGCTCCGTTCCG 23:G>A

1075544|F|0-7:T>C-7:T>C TGCAGCATCCGCCTACCTACACCCTCGGCAAGCGGCGCACCGACCACAACCTGCTCGTGCCG 7:T>C

4398082|F|0-16:C>G-16:C>G TGCAGGCCCCGAGGCCCTGCTCCCCG 16:C>G

1002042|F|0-11:A>G-11:A>G TGCAGGGTGCCACGACGGAGGAGATGCATGGAGAAGAGGAAGAGGAAAGGAGACAGCTCCTCGTGCCTC 11:A>G

5357363|F|0-21:C>T-21:C>T TGCAGAGCTCGTCGGGGATGCCGCCG 21:C>T

1046837|F|0-55:G>C-55:G>C TGCAGGTGTGCAAGGTGTATGTGTCCGTGTTCGGGGACGAGAGGGGGAAGAAGGTGGCCATGGCCGAGA 55:G>C

985174|F|0-28:G>A-28:G>A TGCAGGCGCACCTGGCCGACAGCAACCTGGGCGCCGTGGTGGAGGAGTGCATGCAGCACTACGACGAGC 28:G>A

1126648|F|0-22:C>A-22:C>A TGCAGCGCCTGCCGCGGATCGTCGGCTACGGGAACGCCGCTGACCTCGCCCTCACCG 22:C>A

7339615|F|0-16:C>T-16:C>T TGCAGCGGCCCGTAGACGAACAGTGCCG 16:C>T

4538265|F|0-36:A>T-36:A>T TGCAGCCTTCATTCCAATATCGAACACGTCCCCCAAACTCTCCTGCAACGCGCCACTCTGAGCATCAGA 36:A>T

6041795|F|0-11:G>C-11:G>C TGCAGGGCCTCGTCGGCCTCGACATGTCGCCGCCG 11:G>C

7171969|F|0-48:C>G-48:C>G TGCAGTGCTCCGAGTCCTTCAAGAGGGTCGCGCCGTCGCCGTTGCCTTCCTCCAAGCATCTCGACGCCG 48:C>G

1093137|F|0-51:T>G-51:T>G TGCAGGTCCCCATCGCCATCGGAGGTCCAGGGCTTGCGCCAGGGGCTACATTCCGGGAGGACCTTCCGA 51:T>G

2299791|F|0-46:C>G-46:C>G TGCAGCGCGTCGGCGTCGAGCCGCAGTTCCGCAACGGCCTCCGCGTCACCGACGCGCTCACCATGGAGG 46:C>G

1075878|F|0-10:G>C-10:G>C TGCAGAAGCCGCCGAACACGCCGCACGCCTCGCTCCACGTCACCTCCTTGTCGCCGTTGTACGCCAGGT 10:G>C

1026382|F|0-53:C>G-53:C>G TGCAGCACGTGCAGGTACTCCAGCCCCGACGCGATGCCCACCGCCACGCGGAGCCGCGCCG 53:C>G

7353674|F|0-11:A>G-11:A>G TGCAGGACCTCAACCTCTCCG 11:A>G

7173892|F|0-19:C>G-19:C>G TGCAGCAGCAGTTCGTGTCCTTGGCCACCATCGTGCCCG 19:C>G

1000446|F|0-68:C>T-68:C>T TGCAGCGCCTCCCTCATGCTGCTCCGCGGCTGCGGCAATGGCTGCTCCGACATTGCTCCCGCCCTCCTC 68:C>T

5363309|F|0-17:G>A-17:G>A TGCAGCAGGTCGGGCTCGTCGAGGAGGCCG 17:G>A

4002486|F|0-31:G>C-31:G>C TGCAGCAGAGCGCGCTGACGACGTCGCGCGCGCGCCG 31:G>C

4261441|F|0-13:C>T-13:C>T TGCAGCTGAAGTCCCGCGTCGGGCCCAAGAAGCGGGGCAGCGCCG 13:C>T

5360214|F|0-7:C>A-7:C>A TGCAGCTCCACGCGCACCTCCTCACCGCCG 7:C>A

4987790|F|0-30:C>G-30:C>G TGCAGCGCCGCCAGGCGTTCGACGCGTACGCCGAGTACCAGCCCG 30:C>G

3950480|F|0-40:C>T-40:C>T TGCAGGTCACCGCGTTCTCGCCCCTCATCGACGTCATCAACCGCCGCGACGGCACCGTCAACCGCGGCC 40:C>T

5579583|F|0-43:G>A-43:G>A TGCAGCCCTCGTCGCTGCCCGCGCCGTCGTCCTCCTCCGACTGGATGCCGCCCGCCACGGGGCCCATCA 43:G>A

3028172|F|0-26:C>G-26:C>G TGCAGCGAGAAGACCATCCTCACCGTCAATGGCGAGTTCCCCG 26:C>G

32017955|F|0-10:T>C-10:T>C TGCAGCGCACTGAGAGCGTCCG 10:T>C

993046|F|0-20:G>A-20:G>A TGCAGCAGCTCGGGGCTGATGCTCAGGTGCACGGCGCCGCTGCCTTTCTTGGACCTCCCACGCCTCCGA 20:G>A

4262411|F|0-25:A>T-25:A>T TGCAGCTTGGACATCGAGTATGATCAGGGCGAGGCCG 25:A>T

3026234|F|0-11:C>G-11:C>G TGCAGCGCCTCCACCTTCCGCCCGAGGATGCACGCCCG 11:C>G

3950637|F|0-13:C>T-13:C>T TGCAGCTCCAGCACGGCGTTGTACTTGTACAGCCG 13:C>T

1060936|F|0-58:C>G-58:C>G TGCAGTTGGAAGACGTAGCGCACCCCGTCTGGAACTTCCACCCTCCGACGGAAGTCGTCGGCGAGGAAG 58:C>G

5357760|F|0-6:C>T-6:C>T TGCAGCCTCGCGAGCCGCGAGATCAGGCCCG 6:C>T

6045025|F|0-35:C>A-35:C>A TGCAGGAGCGGGCGCGGGAGGTAGAGGACGGCGAGCAGGGAGGCGGCCGCGGCTGCGGCGAGGAGGGGG 35:C>A

1096987|F|0-14:G>C-14:G>C TGCAGCAGACACGGGGTGGGAGTTGATGCCAAGGCCAAGGGAAGGGCGGCGGCCG 14:G>C

3028565|F|0-25:C>T-25:C>T TGCAGATAAGCGCCACTGCGACCCTCCTCGCAGCCGCCAGCCG 25:C>T

1694795|F|0-34:C>G-34:C>G TGCAGGACCTCGGCTTCACCTTCTCCGAGACCGACGTCGACGCCGAGCTCGCGTCCATCGTGCCGAGAT 34:C>G

1002042|F|0-29:G>T-29:G>T TGCAGGGTGCCACGACGGAGGAGATGCATGGAGAAGAGGAAGAGGAAAGGAGACAGCTCCTCGTGCCTC 29:G>T

5373571|F|0-7:G>C-7:G>C TGCAGCAGCGCCACGGGGCCCG 7:G>C

1026941|F|0-27:C>A-27:C>A TGCAGATGAAGTAGTTGGTGCCGCGGGCGAGCGTGACGCGGTCGCTGCCG 27:C>A

1153924|F|0-52:G>C-52:G>C TGCAGACCAACGTGTACGTCCGCGGCAGCGGCGACGGGCGGATCATCGGGCGGGAGATGCGGTTCCACC 52:G>C

6044943|F|0-25:C>A-25:C>A TGCAGCTCCATCGGGATCGACTTCCCTGGACCAGAGGTAGATGCTGGATTCCTGCACGCCTGCAAATCC 25:C>A

989353|F|0-16:G>A-16:G>A TGCAGCTTCAGCCGAGGGGGTGCCGCGTGACGTGCTTGGCCGACGCGGGGGGAGCCG 16:G>A

4909682|F|0-11:G>A-11:G>A TGCAGAGCCTCGCGGCTGCTGTGGTCCG 11:G>A

982259|F|0-65:C>A-65:C>A TGCAGCCTAGAGCACAGCGCGGCGCGCAATAATTTAGATGGAGGCGTCGTGCAAGTGCCCGATCTCTCG 65:C>A

1095562|F|0-30:C>T-30:C>T TGCAGAGAGTGCGCGACGACGGCGGCGGAGCGGTTGGCTTCCATTAGAGGCTGCACGCCG 30:C>T

5004837|F|0-11:C>G-11:C>G TGCAGGCTCACCGCGCTCTCCTTGGACTCCCCG 11:C>G

3951277|F|0-8:G>C-8:G>C TGCAGGAAGGGGTGGGGATTGGGGTCAGGGTAGTCCG 8:G>C

4262338|F|0-27:G>A-27:G>A TGCAGGTACCAGCTGCCCACGGACGAGGCCACGGAGCCG 27:G>A

1066550|F|0-67:G>C-67:G>C TGCAGCTGCACCGTCGCGGCCACAACCTGCCCTGGAAGCTGAAGCAGAAGAACCCCAAGGAGACGCGGC 67:G>C

1027926|F|0-22:T>G-22:T>G TGCAGGATCTGAGGAAGATGCTTGACGAAGGGAAGGAGCTCGGGATGCCG 22:T>G

985642|F|0-45:G>A-45:G>A TGCAGCCAGGAGGAGGACGAGGCGGGGTTCAACCTGCTGCCGCTGGTGATGGCCGCCTTCGTCATCATG 45:G>A

1025759|F|0-12:T>G-12:T>G TGCAGGAGGCCCTCCGCCGCGACCATGCCGCCAGCACGCGGCGGCGCTTGTTGAAGGGGTTCTCCGAGA 12:T>G

4262338|F|0-35:G>A-35:G>A	TGCAGGTACCAGCTGCCCACGGACGAGGCCACGGAGCCG	35:G>A
1238553|F|0-52:G>C-52:G>C	TGCAGTCGTACCAGCAGTCCCTCCACGCGCTCCAGTCCATACCGCTCTGCGTGCTCGTGCCGCACCTCT	52:G>C
2243952|F|0-53:C>T-53:C>T	TGCAGGTTGAGCATGAGCTCGTAGTTCTTGTGCCCCTTGGCAATGGTCTCCCCCTGCCGCTTCGACACC	53:C>T
1014386|F|0-33:C>A-33:C>A	TGCAGAAAAGCAAGCAAAGCCAGGCCTGACACGCGACTAGACTATCTTGTCTTACTTCGGAGGGCCACC	33:C>A
2316759|F|0-7:C>G-7:C>G	TGCAGCTCGGCCACGCCTACCGCCTCATCGCCGTCGACGAGGTCACCAGGGCCGTGCAGGCCAGGAAGG	7:C>G
6046579|F|0-39:G>T-39:G>T	TGCAGAATGGAGGAGGCCGCGGCGGGAAGCAGCAGCACGGCTTCCG	39:G>T
3938865|F|0-11:C>G-11:C>G	TGCAGGATCTGCGCGGCGCTCCAGCTGGCCCCG	11:C>G
1061439|F|0-25:C>T-25:C>T	TGCAGAGGGTCATCAAGCAGGAGTTCTCAGGGTGCACGGTGATCACCATAGCGCACAGGGTTCCCACCG	25:C>T
5357979|F|0-9:T>G-9:T>G	TGCAGCTCCTCCCGCTCGTCCCTCAGCCG	9:T>G
7167629|F|0-19:G>C-19:G>C	TGCAGCAGGAGCCCGAGCTGGCCGTCTTGGGGCGGGCGCCG	19:G>C
5581332|F|0-17:G>C-17:G>C	TGCAGCAGGATCTCGTTGAACGTGTCGATCGGGCCG	17:G>C
6043738|F|0-8:A>G-8:A>G	TGCAGCACAGATGGAGGCGCCATTGCGCGCGTGCCCTTAGTACCG	8:A>G
7353046|F|0-6:C>T-6:C>T	TGCAGCCACCAGCGGCGGGCCG	6:C>T
7909226|F|0-22:G>A-22:G>A	TGCAGGTTGAGCATGAGGCCGTGGCTCCG	22:G>A
7902195|F|0-12:G>A-12:G>A	TGCAGAGGAGAAGTCGAAAAGCACGAACCTTGCCG	12:G>A
3030819|F|0-40:G>A-40:G>A	TGCAGAACAAGCCCGTCCCTATCTGCTACGACGGGTTCGAGCCCTCCG	40:G>A
4398082|F|0-7:C>T-7:C>T	TGCAGGCCCCGAGGCCCTGCTCCCCG	7:C>T
1022995|F|0-36:C>T-36:C>T	TGCAGGTTGGACGCGCCGTGCATCGCGAAGTACTCGCGCACCATCTCCCG	36:C>T
1052066|F|0-45:T>C-45:T>C	TGCAGGCTTTGTATCCAACTTCAGTGTGGATCCACCATTACTACCTGCTCAATGCTTTCAACCAATATC	45:T>C
1092416|F|0-26:C>T-26:C>T	TGCAGGTCCTCCTCCGTCAACAGCGTCTTCTTCCCGCCGCTCCCAGCCTTGTCGTATGGCTGCGCCATC	26:C>T
1155372|F|0-41:C>T-41:C>T	TGCAGCGAGTAGATCTCGGCGTTGGTGAGGTTGCTGGGGCGCGGCGTGGTGCTGGAGAACCCCATGGAG	41:C>T
3961317|F|0-8:G>C-8:G>C	TGCAGCTCGAGCTTGGCCTGGTCGGCGCCG	8:G>C
7169473|F|0-8:C>G-8:C>G	TGCAGCGCCGCGGCCGACCTCTTGCCCTTCCG	8:C>G
1062809|F|0-49:C>A-49:C>A	TGCAGGAGCTCCCGCAGCGTGAGCCCGCGCTTGTTCACCGCCATGCCCGCCCTCTCCGACGCCATGGTC	49:C>A
6042966|F|0-6:G>C-6:G>C	TGCAGCGCGACGTAGGCGTCGCGGAGGCGCGGGTTGGCGAAGGCGTAGGACGGGTCGACGAAGACGTCG	6:G>C
2243952|F|0-68:C>A-68:C>A	TGCAGGTTGAGCATGAGCTCGTAGTTCTTGTGCCCCTTGGCAATGGTCTCCCCCTGCCGCTTCGACACC	68:C>A
988777|F|0-21:G>C-21:G>C	TGCAGGTAGTCGGCGAGCCAGGAGGCCTGCTGCTCCTGCTGCTGCATCCCCG	21:G>C
5577898|F|0-22:C>G-22:C>G	TGCAGATACACCAGATGGTGGGCGACCG	22:C>G
1021492|F|0-49:A>T-49:A>T	TGCAGGTCCCCATCGCCATCGGAGGTCCAGGGCTTGCGCCAGGGGCTACATTCCG	49:A>T
990124|F|0-42:A>G-42:A>G	TGCAGATACTGTGGGGGCAGACGAAGGTGCTCAACGACGGCAACGGCGACCAGACCATCGCGCTGATGC	42:A>G
4262491|F|0-19:C>G-19:C>G	TGCAGACTGGGCAGCTGGCCACGGTGCCCCCGAAGCAGCGCACGCCG	19:C>G
7352786|F|0-7:C>G-7:C>G	TGCAGGGCGTGTGCGGCCCG	7:C>G
1080483|F|0-26:G>A-26:G>A	TGCAGGACCTCGCCGACGGCCTCGGCGCCGTCTTCCAGGACTACGTCTCCTTCCTCGCCTCCTGCGGCA	26:G>A
993046|F|0-14:G>A-14:G>A	TGCAGCAGCTCGGGGCTGATGCTCAGGTGCACGGCGCCGCTGCCTTTCTTGGACCTCCCACGCCTCCGA	14:G>A
3029171|F|0-7:G>C-7:G>C	TGCAGCAGCGCGCCGAGCGCGCCCAGGCCGAGCTCGCCG	7:G>C
1096988|F|0-36:G>T-36:G>T	TGCAGCAGAAGCACGCACCTGTGACGATGGCTGTGAGATGGCTGGCGTCGATGCCG	36:G>T
4261557|F|0-12:G>T-12:G>T	TGCAGGTGGTGGGGGTTGACGCCGAGGAAGTGCGCGTACTGCTCCG	12:G>T
1127365|F|0-43:G>C-43:G>C	TGCAGTGGCGCCGCATGGTGGACGACCTCCGCGCCAAGGGCTCGCTGCGCAACTGCATCTCCATCTGCG	43:G>C
7351468|F|0-16:G>C-16:G>C	TGCAGAAGACGGTGGCGCCG	16:G>C
1084317|F|0-49:G>C-49:G>C	TGCAGGCGTGCGCCGTGTACCTGTTGCAGGTGAGCGTCACCTCGAACAGGCCG	49:G>C
3949987|F|0-14:G>C-14:G>C	TGCAGGGGACGAGGGGAAAGTGGGGCTGCTGCGGCGGCTCGTCCCCG	14:G>C
5578004|F|0-13:G>C-13:G>C	TGCAGCCGATGAAGAGACGGTCGCCG	13:G>C
3939691|F|0-14:C>G-14:C>G	TGCAGGGAGAAGAGCCGTGCCG	14:C>G
32024937|F|0-15:T>C-15:T>C	TGCAGACAATGGAATTCCCCG	15:T>C
6041795|F|0-5:G>T-5:G>T	TGCAGGGCCTCGTCGGCCTCGACATGTCGCCGCCG	5:G>T
4910345|F|0-65:G>T-65:G>T	TGCAGCTCGTCGATCACCGCTCGCCCGCCCGCGTGGATGCAGAAGTGCTCGAATGCCGTGCGGAAGTCC	65:G>T
1238553|F|0-40:A>C-40:A>C	TGCAGTCGTACCAGCAGTCCCTCCACGCGCTCCAGTCCATACCGCTCTGCGTGCTCGTGCCGCACCTCT	40:A>C
7341314|F|0-5:G>C-5:G>C	TGCAGGGTGGCCCGCGTCTTCTCCAGGCCG	5:G>C
1024266|F|0-28:C>G-28:C>G	TGCAGAGGTCGGAGAGCTCCTTGCGGCCCTCCACAATGTCGTCCAGGAAGTCGTCTAGCTGGCACGCCA	28:C>G
12470887|F|0-7:C>G-7:C>G	TGCAGCGCAAGATCGCCGAGCTCCG	7:C>G
1029743|F|0-9:C>G-9:C>G	TGCAGCGACCGCCGAGATGTACGACTCAGAGCTCAAGTTCTTGGCCGCCG	9:C>G
2253720|F|0-14:G>A-14:G>A	TGCAGCCGCTTGAAGTCCCGCATCAGGCGCTTTCTCGACGGCGTCGACATCTCCGCCGCCG	14:G>A
1050802|F|0-8:G>C-8:G>C	TGCAGGGGGTGGAGGTGGAGCGGCCTTTGGCTTTGGCTGGATGCCAACGACTGCCTCAACGTCAGCCGC	8:G>C
4262855|F|0-15:C>T-15:C>T	TGCAGAGGGGAACATCGGGGAGGGCGTCGCGGCGGAGGGGCCG	15:C>T
7170730|F|0-25:G>C-25:G>C	TGCAGCTGACCCAGAGCAACGGGGCGGACCTCTGGGCGCCG	25:G>C
1062809|F|0-26:G>A-26:G>A	TGCAGGAGCTCCCGCAGCGTGAGCCCGCGCTTGTTCACCGCCATGCCCGCCCTCTCCGACGCCATGGTC	26:G>A
1019982|F|0-6:G>A-6:G>A	TGCAGTGTGATATTCTGGAGGTATCTTGCTGACGGATTTCCTATGCGATGCTCAATGTGCAGGTGAAAA	6:G>A
985914|F|0-29:C>T-29:C>T	TGCAGCTGGCCATACAATGCCTCCAATATCTTGTCCCCCTTGGGCAGCTTCTCTGCCAGGTTGAATGCC	29:C>T
4262647|F|0-10:C>A-10:C>A	TGCAGTTCATCACCATGGACCCCG	10:C>A

5372236|F|0-6:G>A-6:G>A	TGCAGGGCACGATAGCGAACCCATGGCCCG	6:G>A
4733969|F|0-12:C>G-12:C>G	TGCAGAGCAGCACCAGGATCCATCGGGAATGGCTGGTTGTTGCCGTCACCG	12:C>G
1041802|F|0-20:C>T-20:C>T	TGCAGCAGGCGCGAAATCTCCAGGAGGCGCGAGTTGAGGTTGTGCACCTCGGGATCCATCTCGGCGGCG	20:C>T
3951277|F|0-23:G>A-23:G>A	TGCAGGAAGGGGTGGGGATTGGGGTCAGGGTAGTCCG	23:G>A
1076165|F|0-8:G>A-8:G>A	TGCAGTCAGTGCAACATCGTCTGGTCCCAATCCCTCACCTCTCATCCTGTCAAACAGCGCCACCGCCTC	8:G>A
1110311|F|0-36:G>T-36:G>T	TGCAGCGCCACCCGCGCCCGTTCACGCGGCTGCACCGCGACCCCTCCATCAGCACCGCCG	36:G>T
2278806|F|0-25:C>G-25:C>G	TGCAGCTCCTCTTCGACGCCTGCGTCGAGGCCTGCATCCGCTTCCTCGAGGCCGTGCCCTGGTCCGAGG	25:C>G
3028384|F|0-37:G>A-37:G>A	TGCAGAGACTGTAGCTGCGCCTTGCCGCTGAGGCCGCGCTGCCG	37:G>A
994237|F|0-55:G>A-55:G>A	TGCAGGAGGCAGGCGGCGCGGTTGCTGTGGAAGACGGCGCGGTCGGGATGGCCACGCGGGGCAAGGCGG	55:G>A
990511|F|0-49:C>T-49:C>T	TGCAGCTCCTCCGCGACGCGCATGGCACGCCTTTCATCCGCGACGTCGCCGCCG	49:C>T
4262953|F|0-34:C>A-34:C>A	TGCAGAAGGTCGACGAGGAGGGCGCTGCCGCCGCCGTCATCAGGCCG	34:C>A
1021420|F|0-63:T>C-63:T>C	TGCAGCTGGTCTATCTGGCGGCGGCCACGGTGTTCTACTACGAAGCCATGGAGAGCAAGGAGGTGGTGC	63:T>C
1089221|F|0-8:T>C-8:T>C	TGCAGGCGTCGTGGCGAGAGCGCGCCGCCACCACCTGTACGTGTACCTCCGCCCGCTCTGAACGCCGAG	8:T>C
1027224|F|0-7:C>G-7:C>G	TGCAGCACAAGGGCAGCCCGTGGCGGGCCAAGGTGTTCCTGGAGAACGACTACTACTTCCAGGGCTACT	7:C>G
1064011|F|0-25:T>C-25:T>C	TGCAGTTTTGCTGTGCTCATAAGATTTGATATACAGAATGCTGAATCTGAATCTCTTTTCTTGGAGAAG	25:T>C
4394353|F|0-10:G>A-10:G>A	TGCAGCGACAGCGACTCGAGCATCCG	10:G>A
4987790|F|0-16:G>C-16:G>C	TGCAGCGCCGCCAGGCGTTCGACGCGTACGCCGAGTACCAGCCCG	16:G>C
2243952|F|0-62:C>A-62:C>A	TGCAGGTTGAGCATGAGCTCGTAGTTCTTGTGCCCCTTGGCAATGGTCTCCCCCTGCCGCTTCGACACC	62:C>A
983777|F|0-20:C>T-20:C>T	TGCAGCTGGCCATACAATGCCTCCAATATCTTGTCCCCCTTGGGCAGCTTCTCTGCCAGGTTGAAGGCC	20:C>T
7340523|F|0-11:G>T-11:G>T	TGCAGCACGGCGGCGTGCGCGCCG	11:G>T
7343466|F|0-7:C>A-7:C>A	TGCAGCACCACCCCGCACCCG	7:C>A
1068826|F|0-26:G>A-26:G>A	TGCAGGAACGAGGAGTAGATGGCGCAGAAGGGCTTGAGCCCCTCGCAGGCCAGGCCG	26:G>A
3945488|F|0-19:G>T-19:G>T	TGCAGCTGCCCGTCTACCAGCTTGAACTTGCCG	19:G>T
1218104|F|0-51:A>C-51:A>C	TGCAGCCAACACCAAGCAGAGACCGATCAGGAGGAGCCTGAGCAAGGAGCTAGCCG	51:A>C
3949301|F|0-7:T>G-7:T>G	TGCAGACTGGGCTCAACTACGCGTGCGGCCCCG	7:T>G
991037|F|0-19:T>G-19:T>G	TGCAGGAGCTTGCGGTTCTTGGCCATGGCCTCCATGGTGGCGATGCCCATGGCCGCGTCCATGGCGCGC	19:T>G
5370710|F|0-10:A>G-10:A>G	TGCAGAGCCAACACCGCGCCGCCCTCCG	10:A>G
980135|F|0-28:G>C-28:G>C	TGCAGGTGGCCGTCTGGGCGTGCATGGTGATGTCGGTGATGCTGGTGGTGGAGGCCCTGTACAACTGCG	28:G>C
1019869|F|0-52:C>G-52:C>G	TGCAGTACTCCTCGTCCGACCAGCAGTGTTCGTCCATGGTGGGGCCCTCGGGCGGGTTGGGGTTGATGA	52:C>G
6043795|F|0-5:G>C-5:G>C	TGCAGGCGTCAGGAAACGTGCTCCACGGGACGCTACCG	5:G>C
5582088|F|0-19:C>G-19:C>G	TGCAGTGGGCGCTCCTCCTCGCGTCGCTGGCCGCGGCGGGGCACCG	19:C>G
6044008|F|0-37:G>C-37:G>C	TGCAGCTGTCGAGGGCCGTGACGGTGGACGAGTCGGCGGACGCGTTCCCGAACCCG	37:G>C
22841032|F|0-7:G>C-7:G>C	TGCAGACGTTCATCAGCGTCAGCATCGTGGTCTTCATCCCG	7:G>C
1244951|F|0-29:C>T-29:C>T	TGCAGGATGTTGCGCCACGGGTGCTGCACCAGCTTCGACTCCTCGCTCGCCACCACCAGGTCCGCGTAC	29:C>T
5327412|F|0-12:A>G-12:A>G	TGCAGCTTGCAAATAGTGACAACATACGTGTATACGGCCTTGCCCTGAGTATCAGATGTATATTTGCTG	12:A>G
3941850|F|0-23:A>G-23:A>G	TGCAGCAGCACCACTCCTCCCGAAGCGACCTGTTCGAGCCGAGCTCCG	23:A>G
5368608|F|0-40:T>G-40:T>G	TGCAGGGGCGCTTGGCCAGCGCGGGTGCAGCCATGGCCGCTGAGGCGGGAGTCGCTACTAGTAGCGGCC	40:T>G
100542237|F|0-35:C>T-35:C>T	TGCAGACAACTACCTCATCAACTGCGGCTCCACGGCCGACGCTAATCTCCACGATGGGAGGGTCTTCAA	35:C>T
5365171|F|0-34:T>C-34:T>C	TGCAGCAACGGCATGAAGCTCGTGGTCAACGTCGTCGCCGCCGCCGCCGCCTCGCCGCCCAAGGCGTCC	34:T>C
3028230|F|0-11:C>T-11:C>T	TGCAGAAAATCCCGGTGAAGATGTGCCCCAGGAGGCCG	11:C>T
32021484|F|0-26:T>G-26:T>G	TGCAGGGCGTCCGCCGCGGCGCGCGCTGCGCCCCG	26:T>G
1089507|F|0-52:A>G-52:A>G	TGCAGACGGACATCCACACTGCGCGGCAGGCCCTGCGCGACGCGCTCTCTCTAGAGCCCTCACAGCCCG	52:A>G
7167631|F|0-19:T>G-19:T>G	TGCAGCAGGATCGACCTCTTCAAGAACTCCG	19:T>G
5581550|F|0-24:C>T-24:C>T	TGCAGGTGCCTATGTAACGCAGGGCCCG	24:C>T
4002655|F|0-25:C>T-25:C>T	TGCAGCGCCTCCGCGAGCGCCGCGGCGCCCACGCGCCCG	25:C>T
3954247|F|0-9:C>G-9:C>G	TGCAGCAGTCGCCGACGGCTTGGCGAAGGGAGGACCG	9:C>G
1150469|F|0-6:C>T-6:C>T	TGCAGTCGCTCTGGAACGGCGCCGCGTACCACGGCGTGCACCACCTGCCGTGCGGCGTCAGGTACAACT	6:C>T
1046837|F|0-19:T>C-19:T>C	TGCAGGTGTGCAAGGTGTATGTGTCCGTGTTCGGGGACGAGAGGGGGAAGAAGGTGGCCATGGCCGAGA	19:T>C
4261788|F|0-11:C>T-11:C>T	TGCAGGCCGCGCCGCCCTCGCCTCGGCGCGAGATCCCCG	11:C>T
1096988|F|0-31:T>C-31:T>C	TGCAGCAGAAGCACGCACCTGTGACGATGGCTGTGAGATGGCTGGCGTCGATGCCG	31:T>C
1090139|F|0-34:C>G-34:C>G	TGCAGATGAAGTAGTTCGTCCCAGGGACGAGCGTCACGCGGTCGCTCCCCGAGGTGTAGGTGTGCGTCG	34:C>G
1041202|F|0-5:G>A-5:G>A	TGCAGGGACGCGGCCATGTCCGCCTACGTCTTCGTTCTCGTCGCCTTGGTTGGCTGCTACGTCTCCGCC	5:G>A
1164510|F|0-19:C>T-19:C>T	TGCAGGCCTACTACTTCAGCAAGGACGAGCGGCTCATGGTGTACGAGTTCGTCGCCACGGGCAGCCTCT	19:C>T
1089507|F|0-58:C>G-58:C>G	TGCAGACGGACATCCACACTGCGCGGCAGGCCCTGCGCGACGCGCTCTCTCTAGAGCCCTCACAGCCCG	58:C>G
2260066|F|0-40:C>A-40:C>A	TGCAGCGCCTTGAGTCCGCGGCCGCCCTCGGCAACGGTGGCGGCCTCGAGACAGAGCACTACAGCCCAT	40:C>A
988498|F|0-41:G>A-41:G>A	TGCAGCGACGTGTGGTTCAGGTACTCGCCGCCTCCGCGTAAGCCCACGCCCAAGAAGACGCGGAGGCCG	41:G>A
1398552|F|0-19:C>T-19:C>T	TGCAGGGCGTCGCCGACGGCACCGTGGTGAAGAAGCTGGTGCCCAACCG	19:C>T
1029183|F|0-13:C>G-13:C>G	TGCAGATCGGGCCCCGCCGCGCCGCTCGCCGTGTCGTCCGCCGCCGCGCCG	13:C>G
1696660|F|0-25:G>C-25:G>C	TGCAGGACACCACGCTGGGCTCGCTGCTCTCGGCGCTCATGCAGCACTGCGATCCCCCGCAGCGAAGGT	25:G>C

4262890|F|0-11:C>G-11:C>G	TGCAGGATGTCCCCCACGCGGTACCG	11:C>G
1135515|F|0-9:A>G-9:A>G	TGCAGCTCCATCTCCCCGCGGCCACGGATGTCCCGCACCGACACGTACCTGTGCACGCTCGGCGCGTCA	9:A>G
988366|F|0-25:T>C-25:T>C	TGCAGGGCCTTGGTCATGTTGAGGGTGACGCCGTAGTCCTGGCCGCCCTGGAAGGCCTGGCAGAGGCAG	25:T>C
100416934|F|0-29:G>A-29:G>A	TGCAGGAAGGGGGCTGTGAAACGACGTGCGCGGTGCCGTTCTGTCGCCG	29:G>A
1062809|F|0-16:G>A-16:G>A	TGCAGGAGCTCCCGCAGCGTGAGCCCGCGCTTGTTCACCGCCATGCCCGCCCTCTCCGACGCCATGGTC	16:G>A
5346073|F|0-20:G>C-20:G>C	TGCAGCGGCTCGTTGCCGTCGACGCTATCCG	20:G>C
4909682|F|0-5:A>G-5:A>G	TGCAGAGCCTCGCGGCTGCTGTGGTCCG	5:A>G
1863625|F|0-54:C>A-54:C>A	TGCAGAAGGCGATCGACTACGCGTGCGGGCAGGGCGCGGACTGCACGGCCATCGCGCAGAGCGGGGCGT	54:C>A
991279|F|0-24:G>T-24:G>T	TGCAGATGCCCTGGGTCTCCACCCGCACCACGCCCTTCCTGAACGTGAAGAGCTCCG	24:G>T
993076|F|0-19:C>T-19:C>T	TGCAGCACAACTACTTCACCAACCCGTGGGCGATCGTCTCCGTCGTCGCCG	19:C>T
1029962|F|0-19:C>G-19:C>G	TGCAGTCCCTGCCCAGTGGCAACCTCTCCCTGCTCTGCGATGTGCTGGTCCG	19:C>G
999482|F|0-14:C>G-14:C>G	TGCAGCGTCACCTCCCCAGGCCCAGGCGTCTCCTTCTCCACAATCTCCACCTTCCTCCCCTCGTCCTCC	14:C>G
7170993|F|0-28:A>G-28:A>G	TGCAGAAGGTGGCCAAGATGGTGGACGAAGTAGCGGCGGAGATCGCGACGGACGAGTCCCTACCGATCT	28:A>G
6036220|F|0-11:G>A-11:G>A	TGCAGGCTGGTGAGATGCGTAAGGTGAACCACGTCTACTTCTTTCTCCTTGCCCTTGAGTGAGTTTTCA	11:G>A
989931|F|0-8:A>G-8:A>G	TGCAGCTGACCTGCGCCGCCTCGTAGAGCTCGTCGGAGATGAGCCCCATGCCGTGCATGAAAGGCACCT	8:A>G
5360744|F|0-26:C>T-26:C>T	TGCAGCACAATGGCGTTGGGGGAGGACGCCGACGAATCGACGGAGAGGAGCCCCGAGCGAAGGAGCAGC	26:C>T
6043838|F|0-17:G>C-17:G>C	TGCAGCCTGCGGCGCACGACGACGCCG	17:G>C
1094267|F|0-54:T>G-54:T>G	TGCAGCTTCCTCTGGACGGCCGCCACGTCGCCAGCGCGCACGAGGCCGTGGATGTTCTCGGACTTGGGC	54:T>G
1096975|F|0-11:G>A-11:G>A	TGCAGCGAGCGGCGGAGCACGAGGAGCGGGCGGCGGCCGTACACTCTGGCGATGCCG	11:G>A
5579606|F|0-66:T>C-66:T>C	TGCAGCGATCGCGCTCCTGGTGAAAGGGTTCCCTCCCGTAGTCCCCTTGCCCGCGGCGGGGCAGGTTAT	66:T>C
3943702|F|0-27:G>T-27:G>T	TGCAGGCGCCCACACGATCGGGCAATCGCAGTGCCG	27:G>T
3029763|F|0-40:G>T-40:G>T	TGCAGGGCGGAGAGGTTGCAGAAGATGGCCGCCGGGATGGGCCCG	40:G>T
4540440|F|0-22:G>C-22:G>C	TGCAGGGGAGGTACAGCGTGGTGGGGGCGCAGCCG	22:G>C
1714231|F|0-24:T>C-24:T>C	TGCAGATCTCGACGCCATGAAGGCTCTGCTCGACAAGCTCGTGGTGCTCAAGCTCAACGGAGGCCTCGG	24:T>C
7920121|F|0-6:C>A-6:C>A	TGCAGCCAGTCCACGGCGGCCG	6:C>A
5582396|F|0-15:C>T-15:C>T	TGCAGTGCGTCGAGGCCTCGCCGTCGGCCCG	15:C>T
5578464|F|0-5:T>C-5:T>C	TGCAGTGGGGGCGGTTTTCGGGCCG	5:T>C
3960231|F|0-15:G>A-15:G>A	TGCAGGAGCGGGACGGCGTGCAGCTCCCCG	15:G>A
3943156|F|0-32:C>G-32:C>G	TGCAGCCGTCAGGAAACGTGCTCCACGGGACGCTCCCG	32:C>G
1254052|F|0-36:G>T-36:G>T	TGCAGCGGCCATGGGAGTCCCGCAGCGCAGGCAGCAGGTGCGACGACGGCCAGCCATCTGGATGCTGTC	36:G>T
5369294|F|0-16:T>G-16:T>G	TGCAGAGGAGTGGAGGTAGGGGTGGCGCCGCCATGGCCGCGGGCCG	16:T>G
1121829|F|0-58:C>T-58:C>T	TGCAGTTCCCCGTGGGAAGGATCGCCAGGTTCCTCAAGGCCGGCAAGTACGCCGAGCGCGTCGGAGCCG	58:C>T
1123953|F|0-61:C>A-61:C>A	TGCAGCTGCACCTGCCGCTGGTCGAGGCCTCCAACGTGCTCACGAACTACCACGGCACCACCAACCTCA	61:C>A
1024832|F|0-37:A>G-37:A>G	TGCAGCTAATGGAGAAGAAGGTGGTGGCGGTGGTGGGACCGCAGTCGTCGGGGATCGCCCACGTGGTGT	37:A>G
7353196|F|0-19:C>G-19:C>G	TGCAGGTTGTAGAGCGCGCCCCG	19:C>G
1022995|F|0-13:C>T-13:C>T	TGCAGGTTGGACGCGCCATGCATCGCGAAGTACTCGCGCACCATCTCCCG	13:C>T
2308539|F|0-19:A>C-19:A>C	TGCAGCCGCGCGGCGACTCATACATGCGCCGCTCCTCCGTCGACGCCATCATGGCCG	19:A>C
1095765|F|0-34:C>T-34:C>T	TGCAGATTGCCTGCGCAGCCCGTGGGCGGGACGACGGCGGGGAGGTGGTGGAACCG	34:C>T
1135515|F|0-44:G>A-44:G>A	TGCAGCTCCATCTCCCCGCGGCCACGGATGTCCCGCACCGACACGTACCTGTGCACGCTCGGCGCGTCA	44:G>A
1051522|F|0-34:C>G-34:C>G	TGCAGGGCCAGCTCAAGGACCTCAAGGCGGAGCTCTCCCTCCTCCGCGTCGCCAAGGTCACCG	34:C>G
5577181|F|0-63:G>A-63:G>A	TGCAGTGCAGGACACGGCTCGTTTGGTCGATGGTCACGGTCAGGGGTTACCCGTCGTGACACCGATCTG	63:G>A
1247354|F|0-38:G>C-38:G>C	TGCAGGCTCCTCGCCAAGGCCGCCTCGTCCACCTTCCCGTCCTACTCCTACAGCGGCGGCGCCCAGCTC	38:G>C
1063492|F|0-16:G>A-16:G>A	TGCAGGTGAAGGTGCTGGTGCTGTCGTCGTAGGCGTAGCTGTAGGCGCGCGGGCACGCCATCTTGAAGA	16:G>A
6045022|F|0-20:A>G-20:A>G	TGCAGGAGAGCTGCACGGACACGTCGGGGTGCTCCATGGTGGTGTACTCGCCCATTGACATCCCGAGAT	20:A>G
3950480|F|0-11:G>C-11:G>C	TGCAGGTCGCCGCGTTCTCGCCCCTCATCGACGTCATCAACCGCCGCGACGGCACCGTCAACCGCGGCC	11:G>C
2244008|F|0-8:G>C-8:G>C	TGCAGGCAGCCAGAAGAAAAAAGTCACTGGTCTTTCCATAGATTAGGACAGCTACACAAAGCAGCACAG	8:G>C
5360929|F|0-18:G>C-18:G>C	TGCAGCCCCAACAGTACCGTCAACCTCGTGCTCCCCGCACTAAGGGATGTCCACGGCTTGCCCCCGAGA	18:G>C
4404381|F|0-14:C>G-14:C>G	TGCAGGACGAGGAGCGAGCGCAGCGTGTCCCG	14:C>G
1022410|F|0-13:G>C-13:G>C	TGCAGTCCAACGCGGACGACGCCGTGGTGCACAAGATCTCCGACCGCATGGTGCGCAACGGCTCCATCG	13:G>C
1118231|F|0-37:C>G-37:C>G	TGCAGTCAGTGCAACATCGTCTGGCCCCAATCCCTCGCCCCTCATCCTGTCAAACAGCGCCACCGCCTC	37:C>G
7169410|F|0-16:G>T-16:G>T	TGCAGCCCTTGCAGGAGGAGCGGCCG	16:G>T
1017442|F|0-35:G>T-35:G>T	TGCAGCATCTTGCGGCGAGCCGCCGCCTTCAGCACGGAGGAAACGGTGAAGGTGAGCGGCTTGATCCCC	35:G>T
986636|F|0-62:C>T-62:C>T	TGCAGGTGGGCCCAGATAGCCAAGCACCTGCCTGGCAGAACGGACAATGAGGTGAAGAACTTCTGGAAC	62:C>T
7340523|F|0-13:C>T-13:C>T	TGCAGCACGGCGGCGTGCGCGCCG	13:C>T
3938219|F|0-7:G>C-7:G>C	TGCAGGTGTACTACAATCTTGGAGAACTGAAGCCG	7:G>C
1041802|F|0-8:G>C-8:G>C	TGCAGCAGGCGCGAAATCTCCAGGAGGCGCGAGTTGAGGTTGTGCACCTCGGGATCCATCTCGGCGGCG	8:G>C
1072027|F|0-63:T>C-63:T>C	TGCAGCCTGAGCTCTTCTTCAAGATGTCCCACGACATCTACAACTACGGCGAAGGGTCAGTACTCCCCA	63:T>C
1063608|F|0-9:A>G-9:A>G	TGCAGGACAAGAGAGGGGCCCGCGCGCGGCATGCACGTGAAACCGAGTACGATGCACAATTATGATGTT	9:A>G
7350668|F|0-7:A>G-7:A>G	TGCAGGCAACCCATCGGCGACCCCG	7:A>G

1021501|F|0-5:G>C-5:G>C	TGCAGGGGAGGGGAGTGCAATGGACTTGGCTGGGATCTAGGTGAGCTCTCCG	5:G>C
1309881|F|0-22:C>T-22:C>T	TGCAGGAGCTGGTGCCCAACGGCAGCAAGGTGGACATGGTCACCATGCTCGACAAGGCCATCACTTACG	22:C>T
3954530|F|0-10:C>T-10:C>T	TGCAGGCGCTCGCCATGGACCGCGCCGTCGGCCGCTTCACCGTGCCG	10:C>T
13879655|F|0-10:A>C-10:A>C	TGCAGTCAAGACACCAACGACGCCG	10:A>C
3029171|F|0-9:G>T-9:G>T	TGCAGCAGCGCGCCGAGCGCGCCCAGCCCGAGCTCGCCG	9:G>T
4262274|F|0-22:C>T-22:C>T	TGCAGAGAGCCGCGCAGATCGGCTCCGTCACGTCCTCCAACACCTCGATGCCGCTCGCGGGGGCCTTTG	22:C>T
2248902|F|0-59:C>G-59:C>G	TGCAGGTACTGCACGCTCACGGTGCAGAGCACGGCGTCGAAGGAGGCGCTCTCGAGCTCCAGCCGCTGC	59:C>G
1028379|F|0-47:C>T-47:C>T	TGCAGGAGCAGCTGCTGCCGTTCGCGTCCACGGCGGCGGGCAAGCCGCTCCTCGCCGCGCTCCAGGCCG	47:C>T
4005802|F|0-5:G>A-5:G>A	TGCAGGAGCAGCAAGCAGAGTGGCAGACAGGCGTAGCCATCACCG	5:G>A
1150469|F|0-50:C>T-50:C>T	TGCAGTCGCTCTGGAACGGCGCCGCGTACCACGGCGTGCACCACCTGCCGCGCGGCGTCAGGTACAACT	50:C>T
1696589|F|0-41:G>T-41:G>T	TGCAGGTACTACTCGACCTGGGGACACGTCGCGACGCTGGCGGAGGAGATAAAGAAGGGCGCCGACTCC	41:G>T
1066193|F|0-41:G>A-41:G>A	TGCAGCCACCGCGACATGGTCTTCTCGGTGGGGTTGAGGGAGCTCGCCGCATCAGCACACTGCATGTCG	41:G>A
1000589|F|0-17:C>A-17:C>A	TGCAGCAGGATGGTGAGCGCCGACGGGTCGCAGTGCGGCCCCGTGCCCAGCGTCCG	17:C>A
1092922|F|0-10:C>T-10:C>T	TGCAGATGGGCGTGCTCTACGTGTGCCTCTACCTCATCGCGCTCGGCACCG	10:C>T
990124|F|0-36:A>G-36:A>G	TGCAGATACTGTGGGGGCAGACGAAGGTGCTCAACGACGGCAACGGCGACCAGACCATCGCGCTGATGC	36:A>G
6046273|F|0-58:C>G-58:C>G	TGCAGCGCATGGGCGTCGGGCTCGTGATCGGCATGGGGGCGATGGTGGTGGCGGGCGTCGTGGAGGTGG	58:C>G
7170822|F|0-13:C>T-13:C>T	TGCAGGCGCTGCTCGCCGTCGCGCTGTCGGAGAAGCCG	13:C>T
4395748|F|0-8:C>A-8:C>A	TGCAGCTGCGGGCCGAGACCTTCAGCGTCGCCCG	8:C>A
1314388|F|0-17:C>T-17:C>T	TGCAGGAGCACGCCGCGCACGACGGCGGGGAGGGCGACGAGGTTCTTGTCCTCCTCCTCGGCCCGCGTG	17:C>T
1106467|F|0-23:A>G-23:A>G	TGCAGCCACTTCTTGTAGTAGACACAAGTGACTGGCGAGATGACCCCATCCG	23:A>G
4006021|F|0-14:A>G-14:A>G	TGCAGCCCGACGTCAGAGAGGGCGAGGTTGTCGAGCACGGCGCCGACGGGGGCCAGCTTGCGGAACCCG	14:A>G
1044204|F|0-10:C>T-10:C>T	TGCAGTCGCTCGTGAATCTGTTAAAGATGATCTCGGAGCAGAAGTCTCCTATAACCAGGAGGAAAAGCC	10:C>T
5369589|F|0-17:G>A-17:G>A	TGCAGGCAAGTACAGGGGCCG	17:G>A
2248034|F|0-53:C>G-53:C>G	TGCAGCTTCCAGTGGAGGTCCTGGTTGATGAAGCTGAGCGACTCGATGGACGACGCGATGGCGCTGCTC	53:C>G
3024946|F|0-7:G>A-7:G>A	TGCAGCTGTCTTTCCAGGACTCGTCGGCAACCACGGTGTCGCCG	7:G>A
1200118|F|0-23:T>A-23:T>A	TGCAGAGGATGCACCCCAACGGGTCGTGGGAGCAGTGGCGGTTCGTCGGCGGCACGGGCCTGCTCGGCA	23:T>A
1028775|F|0-31:G>C-31:G>C	TGCAGCGCGGCTTCGGCTCCCTCGTCCGCGAGCCCTGCGGCCTCAGCCCG	31:G>C
7492652|F|0-6:T>C-6:T>C	TGCAGGTGCCTCGCCGCCGCCG	6:T>C
993195|F|0-22:C>T-22:C>T	TGCAGAATGTGCACGGCCTCCGCCGCCGCGAGTTCAGGGCCACGCTCGCGCACCTGCACTCTATGGCCA	22:C>T
981730|F|0-23:C>T-23:C>T	TGCAGATCCTCGTCCAGCACCACCCGCAACCGTGTCATGTCGTCCTCAAGCAGATGGTCAGCTCGCCGC	23:C>T
1113245|F|0-15:C>T-15:C>T	TGCAGCAATCACGCGCGTGCCGAAGAACGTCGTCGTCCATCCCTCACTTCAATGGCCTGAGGATCCTGC	15:C>T
7155644|F|0-21:G>A-21:G>A	TGCAGGCCAGCTGGGCTGGGCGCCG	21:G>A
5357559|F|0-14:C>T-14:C>T	TGCAGCAGCAGGAGCCAGCGGCTGCCG	14:C>T
1864599|F|0-41:T>C-41:T>C	TGCAGTGCTCCGAGTCCTTCAAGAGGGTCGCGCCGTCGCCGTTGCCCTGCTCCAAGCATCTCGACGCCG	41:T>C
1096346|F|0-12:A>G-12:A>G	TGCAGTTGAGAGATAAATGACGCACCTGCTCGCTGTGGCCCTGTGGAAAGTAGACGACAAGGCTGCCCA	12:A>G
1079151|F|0-45:C>G-45:C>G	TGCAGGAGAAGACCGCAAAGAAGGCGGCGCAGGCCACGCAGGGGGCCGACGACGCCAAGAAGAAGAACG	45:C>G
5357837|F|0-28:G>A-28:G>A	TGCAGCGCGAAGGAGGACAGGGACGAGCGGCAGGCCG	28:G>A
1027808|F|0-5:C>A-5:C>A	TGCAGCGGCGGCACCGTCGCCGCGGGCTCCTCCTCTTTCACCTCCTGCTGGGCTCGCACCG	5:C>A
7492260|F|0-41:T>C-41:T>C	TGCAGAAGCCCCGCGACCTGCGAATCGCACGTCGTCGTGTATCCG	41:T>C
4396300|F|0-32:G>A-32:G>A	TGCAGGTTCTTCTCCAGCTCGTCGATCACCGCGCGCCCGCCG	32:G>A
1044204|F|0-54:C>A-54:C>A	TGCAGTCGCTCGTGAATCTGTTAAAGATGATCTCGGAGCAGAAGTCTCCTATAACCAGGAGGAAAAGCC	54:C>A
3944995|F|0-16:G>C-16:G>C	TGCAGATTGGCTACTCGGCACCAGCTGAGGCCAGGATCAGCCG	16:G>C
5341583|F|0-16:G>C-16:G>C	TGCAGCGGCAGGGCACGAAGAACTTCATCGGCCGCGGCCTGCTCATGGCCAACGGCGCCAGGTGGTCGC	16:G>C
7353650|F|0-6:C>G-6:C>G	TGCAGGCCGTCGCGCTCGTCCG	6:C>G
3956723|F|0-25:C>T-25:C>T	TGCAGCACTACCAGCTCAACGTTGGCACCG	25:C>T
2260345|F|0-24:G>A-24:G>A	TGCAGCGCGTCCTGCGCCACCGACGTCGACGCCGCCATCGACACGGCGAGCAGCACCGCCGCCAGCTGA	24:G>A
5000416|F|0-16:C>G-16:C>G	TGCAGGCGCAGCTCGGCTACCGCTCGGTCGACTTCCTGCCCTACGCGTCCCACGTCCGCAAGACGGCGG	16:C>G
1027349|F|0-44:G>C-44:G>C	TGCAGCAGGATGGTGAGCGCCGTGGGGTCGCAGTGCGGGCCCGTGCCCAGCGTGCGCTCCG	44:G>C
3960893|F|0-17:T>C-17:T>C	TGCAGATGAGTACTACTTCAAGGCGCTGCCG	17:T>C
32021637|F|0-6:C>T-6:C>T	TGCAGCCACGGTCGAGCCGCCCG	6:C>T
1129334|F|0-10:G>C-10:G>C	TGCAGAGCCCGAGGGATGCCTGCGCCGCGCCGCTGAGGTCGCCGTCGCCGAGGGTCGTCGCCG	10:G>C
994237|F|0-53:A>G-53:A>G	TGCAGGAGGCAGGCGGCGCGGTTGCTGTGGAAGACGGCGCGGTCGGGATGGCCACGCGGGGCAAGGCGG	53:A>G
1006951|F|0-28:C>T-28:C>T	TGCAGGCCATGCCGCTGGTGCCGCTCAACGACTGCCCGCGCCACGCGCGGTACATGGACGGCGTCTTCG	28:C>T
5364986|F|0-19:C>T-19:C>T	TGCAGAACGAGTTCGCGCGCGGCGGCGAGTGCTTCGTGCGCGGCGCGCAGATCTTCGACAACTCGCCGC	19:C>T
991480|F|0-5:T>A-5:T>A	TGCAGTCCCTGGAGGACGCCGTGGGGCGGGGCAACGTCACCATCTTCGCGCCCCG	5:T>A
13026185|F|0-14:A>G-14:A>G	TGCAGGACAACGCCATCTCCG	14:A>G
981720|F|0-31:G>C-31:G>C	TGCAGATCGTGGTGATGAACATGCTCATGGGGGCCTCCACCGTCAGCAGCATCCTCTCCACCTACCTCT	31:G>C
1147918|F|0-22:A>G-22:A>G	TGCAGGATCCGAACCCGAGCGGAGGAGTTGGGGAAGGCGGCGTAGAAGTCCTCGAGGGCCATGGGGATG	22:A>G
1022410|F|0-22:C>G-22:C>G	TGCAGTCCAACGCGGACGACGCCGTGGTGCACAAGATCTCCGACCGCATGGTGCGCAACGGCTCCATCT	22:C>G

<table>
<tr><td>2247936|F|0-29:G>T-29:G>T</td><td>TGCAGGAACGTCTCGCTCTCCCCCGACGAGATGAGCGCATCGATCGTGCTCTCCTCCGCCGTCTCCCCC</td><td>29:G>T</td></tr>
<tr><td>3028947|F|0-34:C>T-34:C>T</td><td>TGCAGAGGAAGACGTGCGGGACGTCCTCCGTGTCCGCGACCCCG</td><td>34:C>T</td></tr>
<tr><td>1063156|F|0-49:G>A-49:G>A</td><td>TGCAGACCTTCTCGGTGCAGCAGGGCAGCGCCATGGACACCGTCCTCGGGGGCGCCTTCCGCGTCCCGC</td><td>49:G>A</td></tr>
<tr><td>1094041|F|0-33:C>A-33:C>A</td><td>TGCAGAAATCGGCGGCGCTCTCCTGTTCGCCCTCGCCGCCGCCGCGGCCCTCTTCGCCGCCGTCTCCAC</td><td>33:C>A</td></tr>
<tr><td>32027320|F|0-37:G>C-37:G>C</td><td>TGCAGCCGTTCTTCGGCGGCGAGGCGGCGACCGCGTCGGAGCAGCGGCTCCGCCACGCGCCGTTCGGGG</td><td>37:G>C</td></tr>
<tr><td>2256575|F|0-26:C>G-26:C>G</td><td>TGCAGCGGGTTCACGATCGACTCCAGCGACCCCAGCGGGTAGTAGGAGTGGAGGACGTGGAAGCGGAGC</td><td>26:C>G</td></tr>
<tr><td>1728583|F|0-9:C>T-9:C>T</td><td>TGCAGCAGACGCCCATGGGCACGGCGATCTCCGCGAAGACCGCCTTCGCCAGCGCGGCGGCGTTCTCCC</td><td>9:C>T</td></tr>
<tr><td>6043992|F|0-26:C>T-26:C>T</td><td>TGCAGCTGCGTCTTGACAACGTCGAGCGGTGTAGTCAACGCGGCTGCGAGAGCACCG</td><td>26:C>T</td></tr>
<tr><td>32021379|F|0-9:G>C-9:G>C</td><td>TGCAGCGACGATATTCGGGCAGCTGTGGAGGCTGGAGCCGCTGCTGCCG</td><td>9:G>C</td></tr>
<tr><td>3026623|F|0-17:G>T-17:G>T</td><td>TGCAGCCACCAGGAAACGTGCTCCACGGGACGCTGCCG</td><td>17:G>T</td></tr>
<tr><td>989471|F|0-25:C>T-25:C>T</td><td>TGCAGCAGTCCTCGGGCCGCCCGAACGCCTCGCACGCGCTCTTGCAGGCGATGCCG</td><td>25:C>T</td></tr>
<tr><td>1076165|F|0-5:T>C-5:T>C</td><td>TGCAGTCAGTGCAACATCGTCTGGTCCCAATCCCTCACCTCTCATCCTGTCAAACAGCGCCACCGCCTC</td><td>5:T>C</td></tr>
<tr><td>7170100|F|0-20:A>G-20:A>G</td><td>TGCAGCCGCTGCTTGACGGTATCCATCGGGGTGAAGACCGCGTCGCTCGCGATGGTGGCGAGCACCCCC</td><td>20:A>G</td></tr>
<tr><td>6040300|F|0-29:C>G-29:C>G</td><td>TGCAGCTCCCTCCTCTCCTCCTCGCCGCCCCCGAACTCGCAGAGGGAGTAGGCCACCATGTCCTCCTCG</td><td>29:C>G</td></tr>
<tr><td>1023680|F|0-9:G>T-9:G>T</td><td>TGCAGCGCTGACCTCGGGAAGGGAGCTGAGAAGGCCGCGGGAGGAGGCCG</td><td>9:G>T</td></tr>
<tr><td>1096346|F|0-58:A>G-58:A>G</td><td>TGCAGTTGAGAGATAAATGACGCACCTGCTCGCTGTGGCCCTGTGGAAAGTAGACGACAAGGCTGCCCA</td><td>58:A>G</td></tr>
<tr><td>1126085|F|0-14:C>T-14:C>T</td><td>TGCAGCGCCTTGTCCCTGGCCATCCCGCCCTCCACGGCGACGAACAGCAGGATCCCCAGCGTGGCGGCG</td><td>14:C>T</td></tr>
<tr><td>4984856|F|0-5:C>T-5:C>T</td><td>TGCAGCGCCGCGGGCGGTATCTGGAACCCCGACGACCCGACCCTCCG</td><td>5:C>T</td></tr>
<tr><td>5577181|F|0-19:C>G-19:C>G</td><td>TGCAGTGCAGGACACGGCTCGTTTGGTCGATGGTCACGGTCAGGGGTTACCCGTCGTGACACCGATCTG</td><td>19:C>G</td></tr>
<tr><td>2250320|F|0-55:C>T-55:C>T</td><td>TGCAGACGGAGGCGCAGAGCTTCGACGTGCCCAGCGCCGACATGATCTACAGCCTCGCCTACCTGCCGC</td><td>55:C>T</td></tr>
<tr><td>1020254|F|0-39:G>C-39:G>C</td><td>TGCAGTTGGAGCTGCTATAGGTGTTGTGGGGGTAGCCGCGTACAGGGTTTATGCGGCTAGGAGGAACTC</td><td>39:G>C</td></tr>
<tr><td>1093168|F|0-5:C>A-5:C>A</td><td>TGCAGCGACCGCTTCACTGCTTTAGCGCGCGATTGGGTGATAACCGTGGATGGTGCGCCG</td><td>5:C>A</td></tr>
<tr><td>7350668|F|0-12:A>G-12:A>G</td><td>TGCAGGCAACCCATCGGCGACCCCG</td><td>12:A>G</td></tr>
<tr><td>7168960|F|0-20:C>T-20:C>T</td><td>TGCAGGGATGTGAGCTGGTGCGGGAGGAATTTCTCTGGCGCTGGAAAGCTCATTCCTGAGCCG</td><td>20:C>T</td></tr>
<tr><td>32020151|F|0-13:G>C-13:G>C</td><td>TGCAGCACCTCGGGTCCAGGGTCTACGAGCGCCTGCCGTCCATGTCGTCCG</td><td>13:G>C</td></tr>
<tr><td>1000446|F|0-26:C>A-26:C>A</td><td>TGCAGCGCCTCCCTCATGCTGCTCCGCGGCTGCGGCAATGGCTGCTCCGACATTGCTCCCGCCCTCCTC</td><td>26:C>A</td></tr>
<tr><td>3025428|F|0-32:C>G-32:C>G</td><td>TGCAGGTTGTAGGCAAGGAGCTTCTGGCCGAGCCCAATGCCCCG</td><td>32:C>G</td></tr>
<tr><td>1047412|F|0-65:A>G-65:A>G</td><td>TGCAGTGCATGCTGCCAGAGTTTTCGGCGTGCTGCGAAGAGTTGGTGGGCAGATGGACGGAGTCCATCG</td><td>65:A>G</td></tr>
<tr><td>5368791|F|0-30:C>T-30:C>T</td><td>TGCAGTTGACGATGAGCACGTCGATGTCGGCTGGCTTGATGACGGCCGTCTTGGCGAACACGTCGTCGA</td><td>30:C>T</td></tr>
<tr><td>1123953|F|0-58:C>A-58:C>A</td><td>TGCAGCTGCACCTGCCGCTGGTCGAGGCCTCCAACGTGCTCACGAACTACCACGGCACCACCAACCTCA</td><td>58:C>A</td></tr>
<tr><td>1063156|F|0-53:G>A-53:G>A</td><td>TGCAGACCTTCTCGGTGCAGCAGGGCAGCGCCATGGACACCGTCCTCGGGGGCGCCTTCCGCGTCCCGC</td><td>53:G>A</td></tr>
<tr><td>993046|F|0-7:G>T-7:G>T</td><td>TGCAGCAGCTCGGGGCTGATGCTCAGGTGCACGGCGCCGCTGCCTTTCTTGGACCTCCCACGCCTCCGA</td><td>7:G>T</td></tr>
<tr><td>4394737|F|0-19:G>A-19:G>A</td><td>TGCAGGAGGAAGAGGATGTGCCG</td><td>19:G>A</td></tr>
<tr><td>1179008|F|0-40:G>C-40:G>C</td><td>TGCAGCCGAGGGCGAGCGGGTAGGCGGCGAGGTCGTCGGGGCCGAGGCCGAGGGAGCGGAGGAACTCGA</td><td>40:G>C</td></tr>
<tr><td>1026498|F|0-10:C>G-10:C>G</td><td>TGCAGTCGCTCTTCGGGACGCGGCTGCCCACGGTCATAGGCGGCTCCTACGCCTTCGTCATCCCGAGAT</td><td>10:C>G</td></tr>
<tr><td>1099370|F|0-58:C>T-58:C>T</td><td>TGCAGGTCTATGGACCAGAGGCCACAATGACTGGAATCTGCAAGCAAGCCATTGATTGCGTCATGGCCG</td><td>58:C>T</td></tr>
<tr><td>3959148|F|0-15:T>A-15:T>A</td><td>TGCAGCACCACCTCCTGCTGCTGCACCGCGCCG</td><td>15:T>A</td></tr>
<tr><td>5582181|F|0-11:G>A-11:G>A</td><td>TGCAGATGGCGGTACAGATCCG</td><td>11:G>A</td></tr>
<tr><td>3951864|F|0-19:A>G-19:A>G</td><td>TGCAGCGCAGCAACCCGCGAAAGCGGCCACCACCG</td><td>19:A>G</td></tr>
<tr><td>5323908|F|0-44:A>T-44:A>T</td><td>TGCAGGGTTCAGGTGGGATCGGCAGTGGCTGGTGGTGAACGCCAAGGGCAGGGCGCTCACGCAGAGGGT</td><td>44:A>T</td></tr>
<tr><td>1220624|F|0-11:C>T-11:C>T</td><td>TGCAGATGCTACGCGCGCCTGCACCCAAGGGCTGTCAACTGCCGCAAGAAGAAGTGTGGCCACAGCAAC</td><td>11:C>T</td></tr>
<tr><td>5368502|F|0-19:G>C-19:G>C</td><td>TGCAGGATGAAATGTCGTCGTTCCAAGCGCCGTCGAGCCTACTGACTGATCAGGCCAGCCCCAACGTGA</td><td>19:G>C</td></tr>
<tr><td>1099776|F|0-7:A>G-7:A>G</td><td>TGCAGCAACTATGCACCCTAGACGGACAAATCCAGCAGCAACTAGCACCG</td><td>7:A>G</td></tr>
<tr><td>987498|F|0-8:C>G-8:C>G</td><td>TGCAGCTTCACGTGGTTGTAGTAGCCCACGCCCAGGAAGGCGATCCCGTAGCCGAAGAGGTTGATCGGG</td><td>8:C>G</td></tr>
<tr><td>3961212|F|0-6:G>C-6:G>C</td><td>TGCAGCGACCATATTCGGGCAGCTGTGGAGGCTGGAGCCGCTGCTGCCG</td><td>6:G>C</td></tr>
<tr><td>1214965|F|0-40:C>T-40:C>T</td><td>TGCAGTGTCAAGCTTACGTAACTGCTTTGTGTCGGGGAAGCGGCGGGACTCGGATCATTTGCGGCCGCG</td><td>40:C>T</td></tr>
<tr><td>978877|F|0-49:A>T-49:A>T</td><td>TGCAGCTTGATGCATGGTATATTTTGTTGCTCTGGAAAGAAGAAAGAAAATTTTACAGGTACGTGTATC</td><td>49:A>T</td></tr>
<tr><td>3956776|F|0-49:A>T-49:A>T</td><td>TGCAGCTTGGACAGCAGCTCGGAGATCTGCTCCTCCGAGATCGACGGCGAGCTCCCG</td><td>49:A>T</td></tr>
<tr><td>1032233|F|0-18:C>A-18:C>A</td><td>TGCAGAGTGTTTTAATGGCCGCACAGTGAAACTGCGTCCCTTTCCCTATCATCGCCTTCAATGAGCCAG</td><td>18:C>A</td></tr>
<tr><td>1666709|F|0-7:A>G-7:A>G</td><td>TGCAGGGAGCGGTGACGCACGAGGACTTCGAGGGCCACCGTGGCACGATCAAGGCCG</td><td>7:A>G</td></tr>
<tr><td>4262304|F|0-37:A>G-37:A>G</td><td>TGCAGACGCAGATGCTCATGGGAGACGGCGGCGCGTCAGCCG</td><td>37:A>G</td></tr>
<tr><td>1126085|F|0-53:C>G-53:C>G</td><td>TGCAGCGCCTTGTCCCTGGCCATCCCGCCCTCCACGGCGACGAACAGCAGGATCCCCAGCGTGGCGGCG</td><td>53:C>G</td></tr>
<tr><td>1089507|F|0-31:C>T-31:C>T</td><td>TGCAGACGGACATCCACACTGCGCGGCAGGCCCTGCGCGACGCGCTCTCTCTAGAGCCCTCACAGCCCG</td><td>31:C>T</td></tr>
<tr><td>991219|F|0-5:C>A-5:C>A</td><td>TGCAGCAGCCCATGGCGCGAGGGCGATGTGACTCTTGAATACCTCTCTGAACCACGGAACCTTCTTATC</td><td>5:C>A</td></tr>
<tr><td>2254908|F|0-64:G>A-64:G>A</td><td>TGCAGCTGGAGAACTACGTCTACTCCTTCGAGTTCCCCGTGGACCAGGGAAGCTGGAGGGCGACGGCGC</td><td>64:G>A</td></tr>
<tr><td>2262079|F|0-14:C>A-14:C>A</td><td>TGCAGCAACTGCGTCACCCAGGCCGTGCAGCGCGTCGCCGTCGAGTGCGGCGGCGCCCCCTCCG</td><td>14:C>A</td></tr>
<tr><td>2242042|F|0-33:T>G-33:T>G</td><td>TGCAGGCAGGCCACATCCGATCGATCGTATCGATCACATAAAGCATCACATGTTGTATCGTCATCATTG</td><td>33:T>G</td></tr>
<tr><td>1183371|F|0-41:C>T-41:C>T</td><td>TGCAGCCTGCCGCCCCTCTCGCCGCTGTCGCCGTACCCGCACTTCGCCTTCCCGCAGCTCTCGGTCGTC</td><td>41:C>T</td></tr>
<tr><td>6048382|F|0-5:A>G-5:A>G</td><td>TGCAGAGCCTACTCCGCCGCGAACGTGAGCGGCGGGACCAATCGCGGGTGCGTCATTTGGGGCTTCGAT</td><td>5:A>G</td></tr>
</table>

1215068|F|0-41:G>T-41:G>T	TGCAGGAGGAGGACGCGGCGGGTGAGCAGCGCGTAGAGGAAGGCGGACGCGATGGAGAGCACGCGGTCG	41:G>T
5971636|F|0-6:C>T-6:C>T	TGCAGCCGTCGTTGGTGAGACCG	6:C>T
1219453|F|0-37:C>G-37:C>G	TGCAGAGCGTCATCCAGATGATCTCCGTCATGTTCGTCGGGCACCTGGGCGAGCTCGCGCTGGCCAGCG	37:C>G
3020932|F|0-19:G>A-19:G>A	TGCAGTGGGCTCACTTTCCGCATATCCACTTTGGCCTCATGCCG	19:G>A
1082679|F|0-35:C>G-35:C>G	TGCAGCGACGCGGGCGCCTTCTTCTGGAGCCTGCTCGGCGGGCGGTTCTTGCAGCCTAGCACCTCGCCG	35:C>G
1013644|F|0-31:C>T-31:C>T	TGCAGCTGCTGAGGATCGAGGAGGAGCTCGGCGACGCGGCGGTGTACGCCG	31:C>T
3949909|F|0-17:G>C-17:G>C	TGCAGCCACTGCGGCGTGCAGAAGACCCCGCAGTGGCGCGCGGGGCCG	17:G>C
7353650|F|0-14:C>G-14:C>G	TGCAGGCCGTCGCGCTCGTCCG	14:C>G
2243310|F|0-7:G>C-7:G>C	TGCAGCAGCCCGTTGTTCCCCGTGGTGTTCAGCCCCAGCCTCGATATGATCGACTCGTTGTGCACCTTG	7:G>C
3023963|F|0-13:C>G-13:C>G	TGCAGCGCCACTTCGAGTCCTCGGTGATCCTCTTCCG	13:C>G
3954247|F|0-5:C>T-5:C>T	TGCAGCAGTCGCCGACGGCTTGGCGAAGGGAGGACCG	5:C>T
1126085|F|0-47:C>T-47:C>T	TGCAGCGCCTTGTCCCTGGCCATCCCGCCCTCCACGGCGACGAACAGCAGGATCCCCAGCGTGGCGGCG	47:C>T
1066912|F|0-50:G>C-50:G>C	TGCAGCATGGCATTGGAGCCGAGGCGGAGCGCGTACTTGAGCGTGAGGTGGGTGGCGAAGGGCGTGAGG	50:G>C
7168102|F|0-6:C>A-6:C>A	TGCAGGCCATCTACGAGGCCGAGTCCCCG	6:C>A
1283131|F|0-47:C>G-47:C>G	TGCAGCTCGACATCGTCAGCCCACCACTGCTCCAGCCCGTGCGCGGCCAGGAACTTGCTCGGCCCCTTG	47:C>G
1001643|F|0-65:C>T-65:C>T	TGCAGCAGCCTGGCGCTCACCCGTCCCTGCTTCCTCACCATCTCCACCACCTCTCCGCGCACGGGCTCC	65:C>T
993679|F|0-5:C>G-5:C>G	TGCAGCCGCTGGCGCCGCCTCCTCTCCGACCCCGCCTTCCGCCGCCGCTTCCGCATTCACCACCGCCGC	5:C>G
1863313|F|0-52:G>C-52:G>C	TGCAGGACACGAGCATCCAGGGCGCCGAGAGCCACCCGCTGCACCTGCACGGGTTCGACTTCTTTGTCG	52:G>C
3957502|F|0-22:G>A-22:G>A	TGCAGTTGACGATGAGTATGTCGATGGCCTCGGGGGCGACGCCG	22:G>A
5578080|F|0-19:G>C-19:G>C	TGCAGCGGATGGGCTTGTCGGTGCCG	19:G>C
3953613|F|0-7:A>C-7:A>C	TGCAGCTAGGCGGGCCGAGCTGGGCGGTGCCGCTGGGCCG	7:A>C
6046579|F|0-35:C>T-35:C>T	TGCAGAATGGAGGAGGCCGCGGCGGGAAGCAGCAGCACGGCTTCCG	35:C>T
1094041|F|0-40:G>A-40:G>A	TGCAGAAATCGGCGGCGCTCTCCTGTTCGCCCTCGCCGCCGCCGCGGCCCTCTTCGCCGCCGTCTCCAC	40:G>A
3029089|F|0-32:G>C-32:G>C	TGCAGGAACAGCGTGGGCGCCTGGTGGTACTTGACCAGGGACTTGCCG	32:G>C
1143535|F|0-58:T>G-58:T>G	TGCAGCCCCACCATGGTGAACACGTCCGCCGCGCCGAACAGCACGTACTGCGGCACCATCCACCACAGG	58:T>G
5361130|F|0-9:C>A-9:C>A	TGCAGCGTGCTGTAGAGCCGCACCTTCCCATCGGCGCCCTCGTCGGCGGCGAGCTCGGTGGCCTCGAAC	9:C>A
5346156|F|0-23:G>T-23:G>T	TGCAGCTCCCGCGCCTCCGCCTCGCCG	23:G>T
1091228|F|0-49:G>A-49:G>A	TGCAGCGCCGCTCTTCTGGACGGTGAAGGAGACGGGTGCTGGCGGCGACGTGCTCGGGTCGGACCCGTT	49:G>A
1129334|F|0-46:G>T-46:G>T	TGCAGAGCCCGAGGGATGCCTGCGCCGCGCCGCTGAGGTCGCCGTCGCCGAGGGTCGTCGCCG	46:G>T
1095544|F|0-29:C>G-29:C>G	TGCAGCATCCTGGCGAAAGGCACGTTGAGCACGCCGCAGATGGCCACGGGGGCCAGCGCGGGGAGCGCG	29:C>G
1000127|F|0-22:G>C-22:G>C	TGCAGGTGAGCGACGCCATCTCGTCCTTCACCGCCGAGGCGAAGGTGCCCACGCCGCCG	22:G>C
1000127|F|0-46:G>C-46:G>C	TGCAGGTGAGCGACGCCATCTCGTCCTTCACCGCCGAGGCGAAGGTGCCCACGCCGCCG	46:G>C
1062105|F|0-66:T>C-66:T>C	TGCAGCTCTGCAAGGAGGAGTAGCTCTTGTGTCGTAGCTTATGAGTGAGCAGTGAACCTCCATGTTTCA	66:T>C
1135062|F|0-52:G>A-52:G>A	TGCAGCTCAAGGACGCCGTCTACGAGGCCGACGAGGTCGTCGACGAGTTCGAGTACCG	52:G>A
1164636|F|0-59:G>C-59:G>C	TGCAGCACGGTCGTCTCCTTGGGCAGCGCCGTCGGCCCCACCATCACCTTGGGCCCCAGGAGCACGTCC	59:G>C
3027026|F|0-15:A>T-15:A>T	TGCAGATGCTCCCCGACATGCCGCTCGACGCCGACGTGTTCCG	15:A>T
1123953|F|0-43:G>A-43:G>A	TGCAGCTGCACCTGCCGCTGGTCGAGGCCTCCAACGTGCTCACGAACTACCACGGCACCACCAACCTCA	43:G>A
997377|F|0-41:C>A-41:C>A	TGCAGGCCGACCATGGTGAACACGTCCGCCGCGCCGAACAGCACGTACTGCGGCACCATCCACCACAGG	41:C>A
7161541|F|0-8:G>A-8:G>A	TGCAGCTGGTGCTGGAGATGAGCGCCCG	8:G>A
32021559|F|0-32:T>G-32:T>G	TGCAGGAGGTGTTCCGCCTGCTCCCGCCCGCCTCCG	32:T>G
32025604|F|0-16:C>G-16:C>G	TGCAGGAGATGCGGCTCCGCGGGGTGGCGTGCCCCGAGGGAGCCCTCGTCGCCGCCATCTGCGCCTTCG	16:C>G
7921859|F|0-14:T>C-14:T>C	TGCAGAGCGGGCGGTGCAGTGGCAGTGCCG	14:T>C
1089507|F|0-59:T>A-59:T>A	TGCAGACGGACATCCACACTGCGCGGCAGGCCCTGCGCGACGCGCTCTCTCTAGAGCCCTCACAGCCCG	59:T>A
3960633|F|0-24:T>A-24:T>A	TGCAGCACCACCGCCTGCGTCGGCTGGATGATCTGGTTCGTCAGCCCG	24:T>A
3029763|F|0-10:A>G-10:A>G	TGCAGGGCGGAGAGGTTGCAGAAGATGGCCGCCGGGATGGGCCCG	10:A>G
999236|F|0-65:G>A-65:G>A	TGCAGGGCCCGCGCCCCCCGCGGCTGTCCGTCAGCAAGGACTCCCACAAGATCAGGAAGCCGCCCGTCG	65:G>A
1126085|F|0-5:C>T-5:C>T	TGCAGCGCCTTGTCCCTGGCCATCCCGCCCTCCACGGCGACGAACAGCAGGATCCCCAGCGTGGCGGCC	5:C>T
1013644|F|0-10:G>A-10:G>A	TGCAGCTGCTGAGGATCGAGGAGGAGCTCGGCGACGCGGCGGTGTACGCCG	10:G>A
1057391|F|0-28:T>G-28:T>G	TGCAGCATTAACTTCAACATCACCACCTTGATCCAGACATCGTGCAGATTGGGGCAAGCACAAACTCCG	28:T>G
1091962|F|0-25:G>C-25:G>C	TGCAGCCGCAGCAGCAGGCCATCCTGCGCCAGGGCTCCATGACGCTGCCCCGCACGCTCAGCCAGATGA	25:G>C
997947|F|0-11:T>C-11:T>C	TGCAGAACTCCTTGACCATCAGGGCCTCCTGCGGCAGGTCTGTCGTGACGACGTGCTTGCTCGGCTGCG	11:T>C
2262079|F|0-36:G>C-36:G>C	TGCAGCAACTGCGTCACCCAGGCCGTGCAGCGCGTCGCCGTCGAGTGCGGCGGCGCCCCCTCCG	36:G>C
7906789|F|0-5:C>T-5:C>T	TGCAGCGGCACATATGCCGCCG	5:C>T
6046823|F|0-12:C>T-12:C>T	TGCAGGCGCTGGCCATGGACCGCGCCGTGGGCCGCTTCACGGTGCCG	12:C>T
1024832|F|0-58:C>A-58:C>A	TGCAGCTAATGGAGAAGAAGGTGGTGGCGGTGGTGGGACCGCAGTCGTCGGGGATCGCCCACGTGGTGT	58:C>A
3935905|F|0-10:C>A-10:C>A	TGCAGAAGGCCATTCACCCG	10:C>A
3024682|F|0-43:A>G-43:A>G	TGCAGAACAAGAACACCCAGATGATCAACCTGCGGATCAACGCACCG	43:A>G
1027556|F|0-32:G>C-32:G>C	TGCAGCACCGAGATGTTGTACGGCTCCTCGTGGATGGGCTTGACGAAGAGCACGCTGGTGGCGCGCGCG	32:G>C
1023636|F|0-10:C>T-10:C>T	TGCAGGCCGTCGCCACGCCGCGCCTGGAGAATGAGGAGCTCTACAAGAGGGTCCCG	10:C>T

1098241|F|0-41:G>C-41:G>C	TGCAGTAGCAATTCGTCGAACAGGTGGTGTGCGTCTTCAGGGCTGAGCGTCCCG	41:G>C
3030416|F|0-25:T>C-25:T>C	TGCAGGACAATCTGGAGTGGGAGGATGTGCAGTGGTCGCAGACCG	25:T>C
3935538|F|0-26:G>C-26:G>C	TGCAGCTCCTCGAGGTACACGCTGCTGGTGCCGTCGAGGAAGCTGTCGGTGAGCCTCGAGAGCGGGACG	26:G>C
14394363|F|0-18:A>G-18:A>G	TGCAGCAGGCGGCGGCGCACGCGTGGTCGCCCGAGGGGTCGTCGCGCCG	18:A>G
988123|F|0-40:G>A-40:G>A	TGCAGATCTCCACGGCGGAGTGCGTCGGCAAGCTGACGGCGATCTCGCTGTGGGGATTGGCCG	40:G>A
22841466|F|0-17:G>A-17:G>A	TGCAGGCGGTAGGGCAAGAACCCG	17:G>A
6044005|F|0-56:T>C-56:T>C	TGCAGCTGGGCTGCGAACCGCGGGCTCAGCGTCGGGTCGGCTGTCGGCCGCAGCCGTCCG	56:T>C
3956646|F|0-31:C>T-31:C>T	TGCAGCAGGCGGACGGCGACGGCGAGCGGTGCAGCACCG	31:C>T
1009707|F|0-23:C>G-23:C>G	TGCAGCTGTTCACGGAACACCCTCAGTATGTCGGCGAACGTGACGATCCCG	23:C>G
5579606|F|0-46:T>G-46:T>G	TGCAGCGATCGCGCTCCTGGTGAAAGGGTTCCCTCCCGTAGTCCCCTTGCCCGCGGCGGGGCAGGTTAT	46:T>G
10592804|F|0-8:T>G-8:T>G	TGCAGGCGTTCGGCGTGGCGCCG	8:T>G
7172589|F|0-10:T>A-10:T>A	TGCAGCGTCGTCGCCGACGTCTCGCTGCCG	10:T>A
1030286|F|0-40:G>C-40:G>C	TGCAGGAGTGGGAGCTCCTCCTCGCCTCCCCCACCGCCGCGGCGGCGCCCG	40:G>C
4396380|F|0-50:C>G-50:C>G	TGCAGGCGGCGCGGCAGCAGGCACACGCCGCAGTGGTTGTCGATGGCCTTCACGGTGCAGAGGGTGAGG	50:C>G
2257132|F|0-14:C>G-14:C>G	TGCAGCCGCAGCCTCCTCGACAACTTCCGCTTCTGCTCCCTCGGCTGCAAGGTCGCCG	14:C>G
1091228|F|0-48:C>T-48:C>T	TGCAGCGCCGCTCTTCTGGACGGTGAAGGAGACGGGTGCTGGCGGCGACGTGCTCGGGTCGGACCCGTT	48:C>T
2249828|F|0-16:G>C-16:G>C	TGCAGTTGACGACGAGGAAGTCAATGGCGTCGGGGCTGATGCCCGTCTTGGCGAGCAGGTCGTCGATGG	16:G>C
4261863|F|0-19:A>G-19:A>G	TGCAGGACACGTACAGGGGAAGGGTGTGCGAGTGCCCG	19:A>G
6047598|F|0-44:T>C-44:T>C	TGCAGCTGCACCCGCTTGTTCTTGCCGATCACCCTGCTAGACGCTGCCG	44:T>C
6047598|F|0-29:C>T-29:C>T	TGCAGCTGCACCCGCTTGTTCTTGCCGATCACCCTGCTAGACGCTGCCG	29:C>T
977990|F|0-32:C>T-32:C>T	TGCAGCTCACGCCCTCCAACTCCAACCTCACCCTCCTCTACAACTGCAAGGCCG	32:C>T
991219|F|0-8:C>A-8:C>A	TGCAGCAGCCCATGGCGCGAGGGCGATGTGACTCTTGAATACCTCTCTGAACCACGGAACCTTCTTATC	8:C>A
3950480|F|0-19:G>T-19:G>T	TGCAGGTCACCGCGTTCTCGCCCCTCATCGACGTCATCAACCGCCGCGACGGCACCGTCAACCGCGGCC	19:G>T
4002390|F|0-29:A>G-29:A>G	TGCAGCGGGTGCACCACTCCGCCGACGCCACCGACCGCGCCG	29:A>G
5367317|F|0-5:G>A-5:G>A	TGCAGGCCGTGCGCCTGCGGCAGGCTCGGCTCCG	5:G>A
7161541|F|0-7:C>G-7:C>G	TGCAGCTCGTGCTGGAGATGAGCGCCCG	7:C>G
1073903|F|0-67:T>G-67:T>G	TGCAGGTGAGCATCATCAGCCAGGCGCTCATCTTCGTGACGCGGTCGCGGGGGTGGTCGTTCCG	67:T>G
7171826|F|0-8:G>T-8:G>T	TGCAGCAGGCCGTCCTCGTTGACGAAGCCCCACAGGAGGCCGAGCATGCCGCCGATCACCGTACCGAGA	8:G>T
3947359|F|0-7:T>C-7:T>C	TGCAGAGTTAGGTGCAGGGCATGGGGTTGCTCACCG	7:T>C
5582039|F|0-40:C>T-40:C>T	TGCAGGCCACCTCCGACGACATGGCGGCGGACGGCCTCACCGCCCG	40:C>T
7902388|F|0-19:G>C-19:G>C	TGCAGGGAGTGCAGTGCGCGATGGCCG	19:G>C
1082679|F|0-54:C>G-54:C>G	TGCAGCGACGCGGGCGCCTTCTTCTGGAGCCTGCTCGGCGGGCGGTTCTTGCAGCCTAGCACCTCGCCG	54:C>G
2251743|F|0-36:C>T-36:C>T	TGCAGCAGCTCCGACTGGGACTCCGACTCCTCCTCCCCCGTCCTCGACCGCGCGCCGCACCGCAACACC	36:C>T
4440386|F|0-9:A>G-9:A>G	TGCAGCAGGATGTTGCGGGTGAAGACGTCGGGGGCGGCGCCG	9:A>G
32018451|F|0-31:C>G-31:C>G	TGCAGGTGCTGGGCCTGTCGGGGTGCAACCTCACGGGGCCGATCCCGCGGGGCGCGTTCGCGGCGCTGG	31:C>G
6046670|F|0-34:T>C-34:T>C	TGCAGCAGGCGCTGGCGGCGCTGGAGGCGGCGAGTGCCG	34:T>C
32023774|F|0-28:A>G-28:A>G	TGCAGGAGGCCGAGGCCGCCGCGCACCAAGCCGTCACCACCCTCGCCAAGCGCGGGGAGGAGGCCATCG	28:A>G
1864017|F|0-10:C>G-10:C>G	TGCAGCTTCTCGAACACCCGCGCGCCGCCCTCGTCGCCGATGCCGAAGCTGGCCCTGGCGATGATCTCC	10:C>G
1018520|F|0-67:C>A-67:C>A	TGCAGGTGTCGGACGCGATGGACGTGGAGATGCGCGCGGGGCTCGCCTCCGAGGACGGGAGCAAGCTCA	67:C>A
1080483|F|0-29:G>A-29:G>A	TGCAGGACCTCGCCGACGGCCTCGGCGCCGTCTTCCAGGACTACGTCTCCTTCCTCGCCTCCTGCGGCA	29:G>A
4909962|F|0-11:G>C-11:G>C	TGCAGGCCACCGTGATGCGCCGTGGCGTCGCCATCGGCATGGCCGTGCACCACACCGCCTGCGACGGGG	11:G>C
7168102|F|0-10:C>G-10:C>G	TGCAGGCCATCTACGAGGCCGAGTCCCCG	10:C>G
1028038|F|0-45:A>C-45:A>C	TGCAGGGACGGCAGATCCCCATTGGCAGGGTCACGTCGGGGGCTGAGTAGATGTACTTGGTTACTTGGT	45:A>C
3948661|F|0-16:G>C-16:G>C	TGCAGCGCAGCAACCCGCGAAAGCGGCCACCGCCG	16:G>C
5367368|F|0-7:A>C-7:A>C	TGCAGGGAGGGCCCCGCGCGGGCCG	7:A>C
1001643|F|0-30:T>C-30:T>C	TGCAGCAGCCTGGCGCTCACCCGTCCCTGCTTCCTCACCATCTCCACCACCTCTCCGCGCACGGGCTCC	30:T>C
994831|F|0-61:G>C-61:G>C	TGCAGGTGGGGGCGGCGCTGGAGACGTCCATCAACGAAACGCTGGCGGAGCTCAGCGCCCCGCTGCTGG	61:G>C
1006951|F|0-49:G>C-49:G>C	TGCAGGCCATGCCGCTGGTGCCGCTCAACGACTGCCCGCGCCACGCGCGGTACATGGACGGCGTCTTCG	49:G>C
1093985|F|0-56:C>T-56:C>T	TGCAGGTGCCGAGCAGGTAGGGTGCCGCCGCCTTGACGGCCTCGGACACGGCGTCGCTGTCCCCGAGAT	56:C>T
985521|F|0-10:G>C-10:G>C	TGCAGGGCCTGTGGTTCTACCAGACGGCGTTCACGCTCTACGGGCCGATGCTCCCCGAGGGGTGCCACC	10:G>C
1038385|F|0-33:C>T-33:C>T	TGCAGCTGACGAAGAATCCATGCCTGCGGTGGACGGTGGTGATCAAAGTGTGAGTTTCTTGCAGCTTGG	33:C>T
5567238|F|0-34:G>A-34:G>A	TGCAGCGGCTCCCGCCGAGCAGCCGCCGCAGGGCGCGCCTCGTCTGCCG	34:G>A
981730|F|0-34:T>C-34:T>C	TGCAGATCCTCGTCCAGCACCACCCGCAACCGTGTCATGTCGTCCTCAAGCAGATGGTCAGCTCGCCGC	34:T>C
2267392|F|0-58:C>T-58:C>T	TGCAGCGGCATGGTCGACCTGCTCGCCAAGAGCGGCCGCCTCGGGGACGCGCTCAGGGCGTTCGCGGAC	58:C>T
5332362|F|0-25:C>G-25:C>G	TGCAGGCGGGGGACGCGCTGCCGCTCCCGATCCG	25:C>G
3948768|F|0-24:A>G-24:A>G	TGCAGCGCGGACCAAAAGGATTGCATCCCG	24:A>G
3938865|F|0-14:G>T-14:G>T	TGCAGGATCTGCGCGGCGCTCCAGCTGGCCCCG	14:G>T
1094041|F|0-61:G>T-61:G>T	TGCAGAAATCGGCGGCGCTCTCCTGTTCGCCCTCGCCGCCGCCGCGGCCCTCTTCGCCGCCGTCTCCAC	61:G>T
1695399|F|0-43:C>A-43:C>A	TGCAGCAGCTCGACCTCTCCTCCAACGCTTTCAACGGCTCCATCCCGCCG	43:C>A

<table>
<tr><td>1013425|F|0-17:G>T-17:G>T</td><td>TGCAGGGGCGTCCACACGCCCGAGCTACGGCCCGCGTCGCCGTCCTCGGCTGGCCTGCCCTGCGCCATG</td><td>17:G>T</td></tr>
<tr><td>3023785|F|0-31:C>T-31:C>T</td><td>TGCAGAACAAGGTGTGCCTGGACCACTTCATCGGCCTCAGCTCCG</td><td>31:C>T</td></tr>
<tr><td>1001920|F|0-35:G>A-35:G>A</td><td>TGCAGAACGGCGTCGTCTGGTCCGTCTCCAAGGACGTCACCAGGCACGCGTCCCTCCTCGCCGCCGAGA</td><td>35:G>A</td></tr>
<tr><td>3947834|F|0-22:C>G-22:C>G</td><td>TGCAGGCGGTGCGGAGGTCGCTCGACGACATGCCG</td><td>22:C>G</td></tr>
<tr><td>986045|F|0-10:G>C-10:G>C</td><td>TGCAGATTCAGATCCAACGGTAGAATCGGGTGATATTAACGGTACCACCG</td><td>10:G>C</td></tr>
<tr><td>1026052|F|0-65:C>G-65:C>G</td><td>TGCAGCGCGAAGTTGTGCGGGAGAGAAGGCACCTGCATGGAGAAGCCCTGCATCTGCGCCGCCGCCGAG</td><td>65:C>G</td></tr>
<tr><td>4410385|F|0-6:G>C-6:G>C</td><td>TGCAGCGATGGAAATCGGCCAAACCG</td><td>6:G>C</td></tr>
<tr><td>4262855|F|0-21:G>A-21:G>A</td><td>TGCAGAGGGGAACATCGGGGAGGGCGTCGCGGCGGAGGGGCCG</td><td>21:G>A</td></tr>
<tr><td>1385984|F|0-31:T>C-31:T>C</td><td>TGCAGCCCAAGGAAGGCCTTGCCATGGTGAATGGCACGGCTGTGGGCTCTGGTCTTGCATCGACCGTGC</td><td>31:T>C</td></tr>
<tr><td>4262338|F|0-23:C>T-23:C>T</td><td>TGCAGGTACCAGCTGCCCACGGACGAGGCCACGGAGCCG</td><td>23:C>T</td></tr>
<tr><td>1027224|F|0-55:C>T-55:C>T</td><td>TGCAGCACAAGGGCAGCCCGTGGCGGGCCAAGGTGTTCCTGGAGAACGACTACTACTTCCAGGGCTACT</td><td>55:C>T</td></tr>
<tr><td>1690848|F|0-64:C>G-64:C>G</td><td>TGCAGGACTGGCAGCGGCGGTACGGCGCCATGCTGCGGCGGAAGGGGAAGGCGGTCAAGGTGCTCGAGT</td><td>64:C>G</td></tr>
<tr><td>1024832|F|0-49:G>C-49:G>C</td><td>TGCAGCTAATGGAGAAGAAGGTGGTGGCGGTGGTGGGACCGCAGTCGTCGGGGATCGCCCACGTGGTGT</td><td>49:G>C</td></tr>
<tr><td>988123|F|0-47:C>G-47:C>G</td><td>TGCAGATCTCCACGGCGGAGTGCGTCGGCAAGCTGACGGCGATCTCGCTGTGGGGATTGGCCG</td><td>47:C>G</td></tr>
<tr><td>2278806|F|0-46:C>G-46:C>G</td><td>TGCAGCTCCTCTTCGACGCCTGCGTCGAGGCCTGCATCCGCTTCCTCGAGGCCGTGCCCTGGTCCGAGG</td><td>46:C>G</td></tr>
<tr><td>1213836|F|0-13:C>T-13:C>T</td><td>TGCAGCGCATGGGCGTCGGGCTCGTGATCGGCATGGGGGCGATGGTGGTGGCGGGCGTGGTGGAGGTGG</td><td>13:C>T</td></tr>
<tr><td>1106335|F|0-41:G>A-41:G>A</td><td>TGCAGAAGTGAAGCAGGGGCATGATTTGCAGCCCCCGATCCGTCGTCGTCGCGGCCCATCCAGCGGTGC</td><td>41:G>A</td></tr>
<tr><td>1024657|F|0-23:C>G-23:C>G</td><td>TGCAGCACCTGCCTCCTCTCCTCCTCCGCGCCAGGGGGAGCGCGCGCGCGCGCCAGCCAAGGTGAGAGC</td><td>23:C>G</td></tr>
<tr><td>1063492|F|0-18:T>A-18:T>A</td><td>TGCAGGTGAAGGTGCTGGTGCTGTCGTCGTAGGCGTAGCTGTAGGCGCGCGGGCACGCCATCTTGAAGA</td><td>18:T>A</td></tr>
<tr><td>4405729|F|0-26:T>C-26:T>C</td><td>TGCAGGATCGTGTAGTCCTCCCCCCTTGCCG</td><td>26:T>C</td></tr>
<tr><td>4398245|F|0-5:C>T-5:C>T</td><td>TGCAGCGACCACCTCGATCAGCCCG</td><td>5:C>T</td></tr>
<tr><td>3944888|F|0-19:G>C-19:G>C</td><td>TGCAGAACCCGCCGACGTAGGCCG</td><td>19:G>C</td></tr>
<tr><td>6042708|F|0-16:C>G-16:C>G</td><td>TGCAGCAGATCGCCTCCCTCAACGGCAACAGCATCACGTCCAGCTTCTCCATGCTGCACGCCTGCACCA</td><td>16:C>G</td></tr>
<tr><td>5970733|F|0-6:G>A-6:G>A</td><td>TGCAGCGCGATGCGCGCCAGGAAGAAGATGTCGAACAGGGTGCGGATCACGGCGGTGGAGATGGCGAGG</td><td>6:G>A</td></tr>
<tr><td>7168693|F|0-37:G>C-37:G>C</td><td>TGCAGCCGAAGACGACGCCGTGGAACGTGTCGTCGCCGATGGTCAGCTTGTCGACGGCCAGCGTGCCCC</td><td>37:G>C</td></tr>
<tr><td>3028081|F|0-28:G>C-28:G>C</td><td>TGCAGTGCCACGGGGCCAACGACGCGTCGAGCGACGCGCTCTCCG</td><td>28:G>C</td></tr>
<tr><td>1000275|F|0-53:G>T-53:G>T</td><td>TGCAGGACTTCCGTCGCTGGCTCTCTTCCCAGGGAGCGGAGACGGGCTCGGTGGCTCCG</td><td>53:G>T</td></tr>
<tr><td>999128|F|0-7:C>T-7:C>T</td><td>TGCAGTACCTCCGCCGCATGGCCCTGGACCGCCCGCTGCCCGCCGCCGTCATCCCCGTCGTCCACGCCG</td><td>7:C>T</td></tr>
<tr><td>7168693|F|0-67:C>T-67:C>T</td><td>TGCAGCCGAAGACGACGCCGTGGAACGTGTCGTCGCCGATGGTCAGCTTGTCGACGGCCAGCGTGCCCC</td><td>67:C>T</td></tr>
<tr><td>988123|F|0-27:G>A-27:G>A</td><td>TGCAGATCTCCACGGCGGAGTGCGTCGGCAAGCTGACGGCGATCTCGCTGTGGGGATTGGCCG</td><td>27:G>A</td></tr>
<tr><td>4003062|F|0-6:G>A-6:G>A</td><td>TGCAGCGAGCGGCGGCACGCGCGTGGTCGCCG</td><td>6:G>A</td></tr>
<tr><td>1069104|F|0-18:G>C-18:G>C</td><td>TGCAGCTACTCTTCAGCGGGGAGGAAAGGGAGCATGTGTTCCTCGCCTCCTCTGGCTCGGAGGCGTCAA</td><td>18:G>C</td></tr>
<tr><td>1044204|F|0-20:T>C-20:T>C</td><td>TGCAGTCGCTCGTGAATCTGTTAAAGATGATCTCGGAGCAGAAGTCTCCTATAACCAGGAGGAAAAGCC</td><td>20:T>C</td></tr>
<tr><td>1212605|F|0-26:C>T-26:C>T</td><td>TGCAGCTTGCCCATCACCCTCGGGCTCCGCATCAGCTCCGCCATGCCCCAGTCCAGCGACGCCG</td><td>26:C>T</td></tr>
<tr><td>1865366|F|0-42:A>T-42:A>T</td><td>TGCAGCCCTGCGACGGCGTCGAACGCGCACGGGCCAGCCGCCAGCCCGTGCCCG</td><td>42:A>T</td></tr>
<tr><td>1263850|F|0-55:A>C-55:A>C</td><td>TGCAGAATGCAATGGCATGCCTCTCTTCCTCGCCACCGTCGGCCGTGCCATGTCCAACAAACGTACAGC</td><td>55:A>C</td></tr>
<tr><td>4993665|F|0-5:A>G-5:A>G</td><td>TGCAGAAGCATGGCCATTGCCGTTCTGCTCTCCG</td><td>5:A>G</td></tr>
<tr><td>4990224|F|0-11:C>G-11:C>G</td><td>TGCAGCCGCGTCTTATGGCGGTAGAGCTCGATGAGTAGCGCCGTCAGGTCGCCCGCCGCCTCGGCGTCC</td><td>11:C>G</td></tr>
<tr><td>1027556|F|0-57:G>A-57:G>A</td><td>TGCAGCACCGAGATGTTGTACGGCTCCTCGTGGATGGGCTTGACGAAGAGCACGCTGGTGGCGCGCGCG</td><td>57:G>A</td></tr>
<tr><td>4409602|F|0-22:C>T-22:C>T</td><td>TGCAGGGGGAGGGGATGATTGCCTGGCCCG</td><td>22:C>T</td></tr>
<tr><td>5971049|F|0-19:G>C-19:G>C</td><td>TGCAGAAGCGGGGGTGGCGGACGAGCGTGGCCTCCAACCCG</td><td>19:G>C</td></tr>
<tr><td>2266482|F|0-38:G>A-38:G>A</td><td>TGCAGCGGAAGCTGCCTCCTCGCGAACTTGCACTCGAAGGGCGGCAGGACCG</td><td>38:G>A</td></tr>
<tr><td>4329543|F|0-11:G>A-11:G>A</td><td>TGCAGAGCGCCGTGGAAATTGACCTGTCAGAGAACAAGCTGACAGGAGCCATCCCGAGCGAGCTCGGCA</td><td>11:G>A</td></tr>
<tr><td>5368502|F|0-34:G>C-34:G>C</td><td>TGCAGGATGAAATGTCGTCGTTCCAAGCGCCGTCGAGCCTACTGACTGATCAGGCCAGCCCCAACGTGA</td><td>34:G>C</td></tr>
<tr><td>4394353|F|0-8:C>G-8:C>G</td><td>TGCAGCGACAGCGACTCGAGCATCCG</td><td>8:C>G</td></tr>
<tr><td>12770671|F|0-17:A>C-17:A>C</td><td>TGCAGGGACAGCTGGTTAAGCCG</td><td>17:A>C</td></tr>
<tr><td>3027416|F|0-6:A>T-6:A>T</td><td>TGCAGAACGCGACCAAAATCGAAACCATCAGAGAAGATAAAACCG</td><td>6:A>T</td></tr>
<tr><td>3028565|F|0-6:T>C-6:T>C</td><td>TGCAGATAAGCGCCACTGCGACCCTCCTCGCAGCCGCCAGCCG</td><td>6:T>C</td></tr>
<tr><td>1023925|F|0-19:C>T-19:C>T</td><td>TGCAGTTCACGCAGATCTGCAACCTCTGCTACCCGTGGCCGTGCAGCGACCCGAGCCACTGGGGAGGCG</td><td>19:C>T</td></tr>
<tr><td>4993725|F|0-18:C>T-18:C>T</td><td>TGCAGGGGCCTGACGCGGCGCCGCCG</td><td>18:C>T</td></tr>
<tr><td>4537469|F|0-8:T>A-8:T>A</td><td>TGCAGCGGTGGGTGAAGATGCCG</td><td>8:T>A</td></tr>
<tr><td>1865063|F|0-5:T>C-5:T>C</td><td>TGCAGTGACAAGAAAACGCTGAAGAAGTGGTTCTTCATCGACAAGACAGTGGGCTAAGAAAATCCTGCT</td><td>5:T>C</td></tr>
<tr><td>988831|F|0-67:C>T-67:C>T</td><td>TGCAGGCGCAGAGCAAGAACATGATCATCACCTACCTCGCCTTCGTCAACCTCGGCCTCCACCTGTTCC</td><td>67:C>T</td></tr>
<tr><td>7168594|F|0-28:G>C-28:G>C</td><td>TGCAGATGTCCTCGTGGACGAACACCTCGAGGCAGATCGCGCACACCAGCTTCTCCGACGCCCTCCTGC</td><td>28:G>C</td></tr>
<tr><td>3949260|F|0-11:C>G-11:C>G</td><td>TGCAGCGCGTACAGCCCGCCGAGGCTGTGCGCCATGAGGATCGCCG</td><td>11:C>G</td></tr>
<tr><td>1116567|F|0-54:G>A-54:G>A</td><td>TGCAGCGAGGCCGCGTGGAACACCTGGTGCGTGCTCGGGTGCCGCCAGGGGCGCGTCCTCATCATGAAC</td><td>54:G>A</td></tr>
<tr><td>32025166|F|0-52:A>G-52:A>G</td><td>TGCAGGTTGGTGTAGTAGCTGCCGTCGAACTTGAACGGCGTGGCGCCGTCCAAGTCCACCGTGGACGTG</td><td>52:A>G</td></tr>
<tr><td>5581983|F|0-8:G>C-8:G>C</td><td>TGCAGCGCGCCCCAGTGCAGCGCGAGCGCGCCGCCG</td><td>8:G>C</td></tr>
<tr><td>1743483|F|0-21:G>T-21:G>T</td><td>TGCAGACGGGGCAGGAGCTGTGCGCCTCCAGCCACGGCAGGAGGCACGCGTCGTGGAAGCGGTGCTTGC</td><td>21:G>T</td></tr>
</table>

13117196|F|0-16:G>A-16:G>A	TGCAGGCCCCGCCCAGGCCGCTGCATCACGCCAAAACTCTAACCCTAGCGGCCTCCTCTGCCGCTGCCG	16:G>A
7170057|F|0-37:T>C-37:T>C	TGCAGCAGGTCGGGATACGCGCGCGGTCCGCGCTCGTTGCCGTCGTGTACGAGAAGGGGCTGGCCCTCT	37:T>C
5361672|F|0-59:A>G-59:A>G	TGCAGGTTGATCTCCTGCACGGTCTCCACCTCCAGGTCGCTGAGGGGCAGCTTGACGATACCCAGCCTC	59:A>G
32022468|F|0-17:A>G-17:A>G	TGCAGTTGGCCGCCGACACGACGACAACGTTCCGCATGGCCGTGAACTGCCG	17:A>G
32027394|F|0-46:T>G-46:T>G	TGCAGGCGGCGGCGGACATGACCACGCTGCTCAAGGCCCTCGTCAGTGAGGTGGCGACCTTGCGGAGCA	46:T>G
1026498|F|0-59:A>G-59:A>G	TGCAGTCGCTCTTCGGGACGCGGCTGCCCACGGTCATAGGCGGCTCCTACGCCTTCGTCATCCCGAGAT	59:A>G
1023307|F|0-44:G>A-44:G>A	TGCAGGTAGGGGATGCCGTTGGGGTGGCCCGTGATGCCGCGGGCGAAGGGCACGAAGAGGCGCTCGTAG	44:G>A
1029774|F|0-16:C>T-16:C>T	TGCAGCCACCCAAGGACGGCGAGTGGTACGAGCGGCTCGCCTACGGCCTGCCCAAGGCCAGGAAGAGCG	16:C>T
4261360|F|0-7:G>C-7:G>C	TGCAGGGGTGGCCTTTCCACGCCATGCCGCCCAGCTTCGACGCCG	7:G>C
3952896|F|0-5:G>A-5:G>A	TGCAGGTCCTGGAGCTGGCCG	5:G>A
4993523|F|0-9:G>A-9:G>A	TGCAGTTGAGCCAGACGAGATCGCTGCCG	9:G>A
2245675|F|0-34:G>A-34:G>A	TGCAGCCATTGGCGATGTAAGGATTGCCCTGGTAGTGCTCGGAGCACTTGCACACGTAGCTGAGTGCGT	34:G>A
5357283|F|0-17:C>T-17:C>T	TGCAGACGACTTGCCAGCAAGGAGGCCG	17:C>T
3953411|F|0-10:C>G-10:C>G	TGCAGGACCTCATGCTCAGCGACAACAACCTCACCG	10:C>G
1002591|F|0-52:C>A-52:C>A	TGCAGCGGGAGGTGCACGTTGAGGTCGCAGGCGGCGGCGAGCGGGCAGTCGGCGCCG	52:C>A
999252|F|0-37:G>T-37:G>T	TGCAGGCTGCTCAGCATCCGCGCCTCCCGCACCTCCCGCCGCCGCACGCGCACCGCCACCGCCG	37:G>T
3943724|F|0-26:C>T-26:C>T	TGCAGCGACCTCCTCACTTTCGACGACCCGCCACCGTCCTCCG	26:C>T
4262300|F|0-28:G>A-28:G>A	TGCAGTCGATCTCGACCCTGCCGTCCTCGTCCCGCGCGAGGCGGCCG	28:G>A
4262798|F|0-7:G>C-7:G>C	TGCAGACGTTCGCGCAGGAGCTGGGGAAGCTGGAACCG	7:G>C
1077776|F|0-25:C>A-25:C>A	TGCAGCTTTTCTCCGTGGTTACTGCCCTGCTACTCCACGGACTTTATTTCTCACCG	25:C>A
1026406|F|0-33:T>C-33:T>C	TGCAGCTGCCACTCGGCGGCCGTGGACGGGAGGTTGGTGCCGTCGCCGTCGTTATTGCCCG	33:T>C
1689110|F|0-16:G>A-16:G>A	TGCAGTCCACGGAGCGGCAGTGCGCTCTCCCGGCGTCCTCGCACTCCTCCAACAACACATCCCTGAAGT	16:G>A
5580121|F|0-19:G>C-19:G>C	TGCAGATCGTGCCGTACGGGCGGCTGGAGGAGATCGCCCG	19:G>C
3938193|F|0-7:G>T-7:G>T	TGCAGCAGCGCGTGTGAGCAAGCCG	7:G>T
1075930|F|0-38:T>C-38:T>C	TGCAGAAACAGGCGCTGGAGCGGGTGCAGGCGGAGGCGTAACATTGGAAGGCTTCGTGGCACCAGAACT	38:T>C
4261199|F|0-22:G>A-22:G>A	TGCAGGAGTGCCAGGGCTTGGTGGCGGCCATGGAGCAGGCCG	22:G>A
4396300|F|0-29:C>G-29:C>G	TGCAGGTTCTTCTCCAGCTCGTCGATCACCGCGCGCCCGCCG	29:C>G
1026226|F|0-19:C>G-19:C>G	TGCAGGTCGCGGGCCTCTCCCTCTTCCTCTACGGCTTCTTCCCCGTCAAGCCCACGCTCCCG	19:C>G
1212605|F|0-6:T>C-6:T>C	TGCAGCTTGCCCATCACCCTCGGGCTCCGCATCAGCTCCGCCATGCCCCAGTCCAGCGACGCCG	6:T>C
2260280|F|0-19:C>T-19:C>T	TGCAGGATCGCCTTGGCTACGACCAGCCCAGCAAGGCCGTCGAGTGGCTCATCAAGGCTGCCGCCGCCG	19:C>T
7172979|F|0-10:C>T-10:C>T	TGCAGTTCGACGAGGCAGAGAAACTGTGCCGCAAAGCCCTTGAGATCCACCG	10:C>T
5358263|F|0-19:G>C-19:G>C	TGCAGGCGCTGCCCCGCGCGCTCGTCGCCG	19:G>C
1151440|F|0-14:G>C-14:G>C	TGCAGCGCGTACACGGTCCGCAGCAGGTGCCGCCGCTCGTACTTGCCCG	14:G>C
5581358|F|0-6:G>C-6:G>C	TGCAGCGGAGGCGGCAAGAGGCGGGCGGATCCG	6:G>C
1094041|F|0-53:T>C-53:T>C	TGCAGAAATCGGCGGCGCTCTCCTGTTCGCCCTCGCCGCCGCCGCGGCCCTCTTCGCCGCCGTCTCCAC	53:T>C
29430036|F|0-13:G>C-13:G>C	TGCAGCCGAAGACGACGCCGTGGAACGAGTCGTCGCCGATGGTCAGCGTGTCCACGGCCAGCGTGCCCC	13:G>C
3021065|F|0-31:T>A-31:T>A	TGCAGCTTCAGAGTGACCTCCTGCTTCACCTTGCCTCCG	31:T>A
3025517|F|0-5:A>C-5:A>C	TGCAGATCCGCCTTGTTCTTCCCCCTCAGCTCGTGCACCTTGATCCG	5:A>C
5582011|F|0-8:C>A-8:C>A	TGCAGCTTCGGCCGCCTCATCGGCACCACCG	8:C>A
1088814|F|0-33:A>G-33:A>G	TGCAGAAATGGCGCAAGAGAGTTGGAAAGAGGCAGAGGATACGGGTGTCCACGCGCCCGAGGCTCCGAT	33:A>G
4262491|F|0-28:C>A-28:C>A	TGCAGACTGGGCAGCTGGCCACGGTGCCCCCGAAGCAGCGCACGCCG	28:C>A
32020958|F|0-10:A>C-10:A>C	TGCAGTGCCCAGACGACCTGGAGCGGCTCTACTGCGAGATCCACCTCCTCAAGACGCTCAAGCACAAGA	10:A>C
4005735|F|0-13:T>C-13:T>C	TGCAGCGTGCGCCTGCGGCAGCTCGTGATCTCAGTGTGCGGCCCCG	13:T>C
6046172|F|0-11:A>T-11:A>T	TGCAGACGCCGACGTACGTGGTGCGCGCGCGCCTGGGCACCCCGCCGCAGCAGCTCCTCCTCGCCGTCG	11:A>T
1000127|F|0-13:C>T-13:C>T	TGCAGGTGAGCGACGTCATCTCGTCCTTCACCGCCGAGGCGAAGGTGCCCACGCCGCCG	13:C>T
2267244|F|0-31:C>T-31:C>T	TGCAGGACGGGCTCTTCGACCTCACCGAGGACGGCGGCGGCGGCGTGGTGATGGACACCG	31:C>T
7170058|F|0-28:T>C-28:T>C	TGCAGCAGGTCGGGATACGCGCGCGGTCTGCGCTCGTCGCCGTCGTGTACGAGAAGGGGCTGGCCCTCT	28:T>C
1074290|F|0-46:G>C-46:G>C	TGCAGCCGACGGTGAACGCGAGCACGGAGCACGTGATGAAGTACCTGACGGGGTCGGCCAAGACGTACG	46:G>C
3937540|F|0-14:G>A-14:G>A	TGCAGGACGCGAGGGTCGAGGAGGCGTGGGCGCTGGAGGAGGCTATGCGGCTGCCG	14:G>A
3949518|F|0-23:A>G-23:A>G	TGCAGGGGGAAGTTGGGGCGGCGACCTCCACCG	23:A>G
7172305|F|0-8:G>A-8:G>A	TGCAGCACGGCAGCGCAGAGCTGCCG	8:G>A
4262475|F|0-22:T>G-22:T>G	TGCAGCGACGCCTTCCTCGCGATCGGCATGTCGACGAACGCCG	22:T>G
1008647|F|0-11:G>A-11:G>A	TGCAGCCCGCGGACCAGCCGATGCTGTTCCCGCCTGCGCCGCCGTCGGCGTCACCG	11:G>A
997727|F|0-50:C>G-50:C>G	TGCAGCACGCTCTCCGACTCGGCGTCCAGCGCGCTCGTCGCCTCGTCCAGCAGCAGGATGGCCG	50:C>G
3944091|F|0-20:C>G-20:C>G	TGCAGCGGCGGCCGCTTGCTCGACGAGATGGCCAGGGACGGCACCTTCTCCGAGCAGCGAGCCGCCCTT	20:C>G
1000439|F|0-45:A>G-45:A>G	TGCAGCTTTAGCTAGGTCAGTTGACAAGCAAGGCGTGTACACGGCATGCACACGCTTGCCTTCATCTCA	45:A>G
1080483|F|0-31:C>T-31:C>T	TGCAGGACCTCGCCGACGGCCTCGGCGCCGTCTTCCAGGACTACGTCTCCTTCCTCGCCTCCTGCGGCA	31:C>T
3222409|F|0-36:T>C-36:T>C	TGCAGCGCGGCCAGGCCGCCGAGCCACGCGGGCACGTCGCCG	36:T>C
3944211|F|0-41:C>T-41:C>T	TGCAGGTGGCAGAGCACGCGGTCGGCGGCGCCGTCGATCGCCTGCACCG	41:C>T

<table>
<tr><td>993522|F|0-12:A>C-12:A>C</td><td>TGCAGGAGCGACATGCTGTACGTGTCCGACCCGTGCGAGCACGCGGTGCCCG</td><td>12:A>C</td></tr>
<tr><td>4002585|F|0-23:G>A-23:G>A</td><td>TGCAGCTGCCAACCGCGCACGACGGCGGCTTCACGGCGTCGGGGCCG</td><td>23:G>A</td></tr>
<tr><td>3938586|F|0-11:G>A-11:G>A</td><td>TGCAGCATGGGGGACGGCGGGGCATAGTCTACTACCG</td><td>11:G>A</td></tr>
<tr><td>2247504|F|0-23:C>T-23:C>T</td><td>TGCAGGAGGAGGTGCTCGAGGTACCACATCAGGGGCGACAAGGCAGGCACGGTGGGGAGCTTCATCGAC</td><td>23:C>T</td></tr>
<tr><td>1010561|F|0-14:C>G-14:C>G</td><td>TGCAGGTACTTGACCGCCTTGGTCCTGGTGATGTACTTGGCGGCGTTCCCCTCCTTCTTCTTGCCCTGG</td><td>14:C>G</td></tr>
<tr><td>1694844|F|0-46:G>C-46:G>C</td><td>TGCAGCTCTGCGCGGGCCACCGCTGCCTCGTCTTCCACCTCGCCCAGGTCGACGCCGAAGCCGTCCCCG</td><td>46:G>C</td></tr>
<tr><td>4396380|F|0-68:G>A-68:G>A</td><td>TGCAGGCGGCGCGGCAGCAGGCACACGCCGCAGTGGTTGTCGATGGCCTTCACGGTGCAGAGGGTGAGG</td><td>68:G>A</td></tr>
<tr><td>3024064|F|0-11:T>A-11:T>A</td><td>TGCAGGTGACTTGGTGCTCAATGCGTGGTTCGGCATCCCG</td><td>11:T>A</td></tr>
<tr><td>1026430|F|0-20:C>G-20:C>G</td><td>TGCAGACGACGACCTGCTCGCTGGTTTCGGAACCAAGTCAGAGGCTAGGGAGGTGCCG</td><td>20:C>G</td></tr>
<tr><td>3945057|F|0-17:C>G-17:C>G</td><td>TGCAGTTATACTTGTAACAGAGGGATTCCCG</td><td>17:C>G</td></tr>
<tr><td>4398245|F|0-17:T>C-17:T>C</td><td>TGCAGCGACCACCTCGATCAGCCCG</td><td>17:T>C</td></tr>
<tr><td>984858|F|0-5:C>T-5:C>T</td><td>TGCAGCTTGCCGCCGAGGCCCCTGGCGAAGGCCTGGGCGATGAAGAAGCGCGCGAAGCGGTGGATGCGC</td><td>5:C>T</td></tr>
<tr><td>999128|F|0-40:C>T-40:C>T</td><td>TGCAGTACCTCCGCCGCATGGCCCTGGACCGCCCGCTGCCCGCCGCCGTCATCCCCGTCGTCCACGCCG</td><td>40:C>T</td></tr>
<tr><td>6043658|F|0-31:T>C-31:T>C</td><td>TGCAGAGCCGCCAGAACCCGACCTTCTACTATGTGCCGTTCACCG</td><td>31:T>C</td></tr>
<tr><td>3029763|F|0-20:G>A-20:G>A</td><td>TGCAGGGCGGAGAGGTTGCAGAATATGGCCGCCGGGATGGGCCCG</td><td>20:G>A</td></tr>
<tr><td>1027289|F|0-10:G>A-10:G>A</td><td>TGCAGAAGCCGAAGACGAAGCAGTAGATGATGGAGCAGATGCCAGCGAAGACACCGACCGCGCCGACGA</td><td>10:G>A</td></tr>
<tr><td>5580382|F|0-29:A>C-29:A>C</td><td>TGCAGGAGGGCGGGCTGCTGGGAGCGGAGACCCG</td><td>29:A>C</td></tr>
<tr><td>5579606|F|0-29:T>A-29:T>A</td><td>TGCAGCGATCGCGCTCCTGGTGAAAGGGTTCCCTCCCGTAGTCCCCTTGCCCGCGGCGGGGCAGGTTAT</td><td>29:T>A</td></tr>
<tr><td>996500|F|0-24:A>G-24:A>G</td><td>TGCAGGATGGAGGCGGCGGAGGAGACGAGGACGGCGAGGTGGAGCAGGAGGCGCGCGGGGGCGGAGGAT</td><td>24:A>G</td></tr>
<tr><td>1745270|F|0-14:C>T-14:C>T</td><td>TGCAGCCTGGCGGACGCGATCGGGGAGGACTCCGTGGCCGTGGTGAGGATGTCGACGGTGGCGTGCGAC</td><td>14:C>T</td></tr>
<tr><td>3944091|F|0-62:C>T-62:C>T</td><td>TGCAGCGGCGGCCGCTTGCTCGACGAGATGGCCAGGGACGGCACCTTCTCCGAGCAGCGGGCCGCCCTT</td><td>62:C>T</td></tr>
<tr><td>3947094|F|0-22:G>A-22:G>A</td><td>TGCAGCCCTTCTCCAAGGAGGAGTGGAGCTCCCACCG</td><td>22:G>A</td></tr>
<tr><td>5579606|F|0-23:A>C-23:A>C</td><td>TGCAGCGATCGCGCTCCTGGTGAAAGGGTTCCCTCCCGTAGTCCCCTTGCCCGCGGCGGGGCAGGTTAT</td><td>23:A>C</td></tr>
<tr><td>997947|F|0-12:T>G-12:T>G</td><td>TGCAGAACTCCTTGACCATCAGGGCCTCCTGCGGCAGGTCTGTCGTGACGACGTGCTTGCTCGGCTGCG</td><td>12:T>G</td></tr>
<tr><td>986325|F|0-37:G>A-37:G>A</td><td>TGCAGCTGCACCGCCGCGGCCACAACCTGCCGTGGAAGCTGAAGCAGAAGAACCCGAACCAGGTGCAGC</td><td>37:G>A</td></tr>
<tr><td>1126085|F|0-26:G>T-26:G>T</td><td>TGCAGCGCCTTGTCCCTGGCCATCCCGCCCTCCACGGCGACGAACAGCAGGATCCCCAGCGTGGCGGCG</td><td>26:G>T</td></tr>
<tr><td>1053893|F|0-40:T>G-40:T>G</td><td>TGCAGGTTCTGCCACACCACCGTCCACAGCACCGCGATGATGCTGAGCACCACCCCG</td><td>40:T>G</td></tr>
<tr><td>4396190|F|0-23:G>A-23:G>A</td><td>TGCAGCTTCCGCGCGCTGTTGGAGGACGCATCCG</td><td>23:G>A</td></tr>
<tr><td>4262407|F|0-28:C>T-28:C>T</td><td>TGCAGTCCGTCGAGCACGACGGAGCCGACGAACAGCCCG</td><td>28:C>T</td></tr>
<tr><td>6047341|F|0-23:C>A-23:C>A</td><td>TGCAGTCCTTCCTGATCTCGAAGCGGGTGCCCTCGGCGGGGATCTCGCTGAGCTTGGCCATGGCCTTGC</td><td>23:C>A</td></tr>
<tr><td>2251559|F|0-19:A>G-19:A>G</td><td>TGCAGAAGGGGATCAAGGGAGCCATCGAGTTCAGGGACGTGCACTTCAGCTACCCGACGCGGCCGAGAT</td><td>19:A>G</td></tr>
<tr><td>7352259|F|0-11:C>G-11:C>G</td><td>TGCAGCAATGCCGTCGTGCCG</td><td>11:C>G</td></tr>
<tr><td>979989|F|0-51:A>G-51:A>G</td><td>TGCAGTCCACGGAGCGGCAGTGCGCTCTCCCGGCGTCCTCGCACTCCTCCAACAGCACATCTCTGAAGT</td><td>51:A>G</td></tr>
<tr><td>3020490|F|0-26:C>T-26:C>T</td><td>TGCAGCTTCTGTTCGAGAAGTGGCACCTGCTACTTGCCG</td><td>26:C>T</td></tr>
<tr><td>3958517|F|0-12:G>T-12:G>T</td><td>TGCAGCTCCATCGGGATCGACTTCCATGGACCAGAGGTAAATGCTGGATTCCTGCACGCCTGCAAATCC</td><td>12:G>T</td></tr>
<tr><td>5358623|F|0-18:C>G-18:C>G</td><td>TGCAGTGGCTGGCCCTGGCGGCGGCCTGCGCCG</td><td>18:C>G</td></tr>
<tr><td>1027581|F|0-57:G>C-57:G>C</td><td>TGCAGTGGATCATGGCGAACCTGGTGAAGCGCCCACACATGCAAGAGGCTGTCCGTAGGGAGATCAACG</td><td>57:G>C</td></tr>
<tr><td>1263734|F|0-56:G>T-56:G>T</td><td>TGCAGGATGTACGTCGTGCCCATCGGCGTGTACAGCACCTTCTGGCTGTTGAGGCAGGGGTGCGTGCGC</td><td>56:G>T</td></tr>
<tr><td>1027100|F|0-22:C>A-22:C>A</td><td>TGCAGGCCTGCACGACAGTGGCCCGCACGGTGGTGGCACCCTGATCGGCGCCG</td><td>22:C>A</td></tr>
<tr><td>5580573|F|0-16:T>C-16:T>C</td><td>TGCAGTCGTTCGACGCTAGCAGTAATCTCCTCACCG</td><td>16:T>C</td></tr>
<tr><td>1211113|F|0-11:A>G-11:A>G</td><td>TGCAGCAGGTCAGGAGCCATCATCCCCTGGAACACGTCCTCCTCCAAGAAGCTCAGGAAGCCCCCGAGA</td><td>11:A>G</td></tr>
<tr><td>984858|F|0-40:T>C-40:T>C</td><td>TGCAGCTTGCCGCCGAGGCCCCTGGCGAAGGCCTGGGCGATGAAGAAGCGCGCGAAGCGGTGGATGCGC</td><td>40:T>C</td></tr>
<tr><td>2242643|F|0-43:A>G-43:A>G</td><td>TGCAGCTGGAGTGACGCCCTTCGTCGTTGCAGGGGTCGAGTTCAGCAAAAGAATTGTGAGTTCCGAGAT</td><td>43:A>G</td></tr>
<tr><td>1139882|F|0-34:T>C-34:T>C</td><td>TGCAGCCACCGCCGCCGCCGCATCCAAGATGATCTCAATCCTCCCTCCAAGCCGCGCGCGTGGTCCATG</td><td>34:T>C</td></tr>
<tr><td>984858|F|0-63:A>G-63:A>G</td><td>TGCAGCTTGCCGCCGAGGCCCCTGGCGAAGGCCTGGGCGATGAAGAAGCGCGCGAAGCGGTGGATGCGC</td><td>63:A>G</td></tr>
<tr><td>12775414|F|0-20:T>C-20:T>C</td><td>TGCAGCGTCAGGCAGTACCCTGTGGCGTGCCG</td><td>20:T>C</td></tr>
<tr><td>2266482|F|0-20:C>T-20:C>T</td><td>TGCAGCGGAAGCTGCCTCCTCGCGAACTTGCACTCGAAGGGCGGCAGGACCG</td><td>20:C>T</td></tr>
<tr><td>1017794|F|0-53:G>A-53:G>A</td><td>TGCAGGTAGGTGATCAGGACCAGCCACGCGTTCACGATCAGCAGCGGCACGCCGTAGACCCGCACCACC</td><td>53:G>A</td></tr>
<tr><td>7353542|F|0-8:C>A-8:C>A</td><td>TGCAGCTGCCCGCTGAGGCCG</td><td>8:C>A</td></tr>
<tr><td>1224593|F|0-6:A>G-6:A>G</td><td>TGCAGCAGGCCTGCGGCGACGCGCCGTGCATGGTGGTGGCGTACGCTCCGATTGACGCGGGAGAGCTCA</td><td>6:A>G</td></tr>
<tr><td>29430036|F|0-39:T>C-39:T>C</td><td>TGCAGCCGAAGACGACGCCGTGGAACGAGTCGTCGCCGATGGTCAGCGTGTCCACGGCCAGCGTGCCCC</td><td>39:T>C</td></tr>
<tr><td>1012318|F|0-48:C>T-48:C>T</td><td>TGCAGGAAATGTGGGAAATGACAAGATTGTGCAACAGAAGAAAGCTAACCCAGTTGCCCTGTTTCTCTC</td><td>48:C>T</td></tr>
<tr><td>1256487|F|0-22:C>T-22:C>T</td><td>TGCAGAGACGGCCCCGCCCTCGCCCTCTCCCTCTTCAACCGTGTCTGCCGAGAAGAAGCCGGCATGCGG</td><td>22:C>T</td></tr>
<tr><td>4261716|F|0-35:G>C-35:G>C</td><td>TGCAGGAGGTTGCCGAGCACGGGAAGCTGTTTCGGGGGCCCG</td><td>35:G>C</td></tr>
<tr><td>984858|F|0-50:C>T-50:C>T</td><td>TGCAGCTTGCCGCCGAGGCCCCTGGCGAAGGCCTGGGCGATGAAGAAGCGCGCGAAGCGGTGGATGCGC</td><td>50:C>T</td></tr>
<tr><td>1027581|F|0-39:T>G-39:T>G</td><td>TGCAGTGGATCATGGCGAACCTGGTGAAGCGCCCACACATGCAAGAGGCTGTCCGTAGGGAGATCAACG</td><td>39:T>G</td></tr>
<tr><td>3939026|F|0-25:G>T-25:G>T</td><td>TGCAGGAGGTGTTCCGCCTGCTCCCGCCCGCCGCCG</td><td>25:G>T</td></tr>
<tr><td>5363464|F|0-14:G>A-14:G>A</td><td>TGCAGCGAGGCCGAGGACGATCCG</td><td>14:G>A</td></tr>
<tr><td>1001758|F|0-46:C>G-46:C>G</td><td>TGCAGATGAGCAGGGCGAGGGAGCTCTTGGAGGAGATGACGACGGACGCCG</td><td>46:C>G</td></tr>
</table>

996500|F|0-59:G>T-59:G>T	TGCAGGATGGAGGCGGCGGAGGAGACGAGGACGGCGAGGTGGAGCAGGAGGCGCGCGGGGGCGGAGGAT	59:G>T
3023785|F|0-29:A>C-29:A>C	TGCAGAACAAGGTGTGCCTGGACCACTTCATCGGCCTCAGCTCCG	29:A>C
1092464|F|0-42:T>C-42:T>C	TGCAGAAGCCGCGCCCACTGCTCCTCGTTGTCGCTCCCGAAGTTCTTCTTCGTCTCCTCCACCGCCAGC	42:T>C
1029735|F|0-26:G>A-26:G>A	TGCAGCGTGGCGGTGGAGGAGCCGATGGAGTGCCTGGGCACGGGGCTGGTGAGCCCG	26:G>A
5365411|F|0-17:C>G-17:C>G	TGCAGCGCCGACACGAACGCGGAGGACGGCGGCGACGGCGAGTAGAGGTGCGGGGTGAAGGAGATGGCC	17:C>G
1026364|F|0-50:G>T-50:G>T	TGCAGCATCCGCACCACGGTGCTGGGCGGCAGCTGGTGCGACGCCATCGTGCCCCAGTTCACCCCGAGC	50:G>T
996500|F|0-35:G>T-35:G>T	TGCAGGATGGAGGCGGCGGAGGAGACGAGGACGGCGAGGTGGAGCAGGAGGCGCGCGGGGGCGGAGGAT	35:G>T
991147|F|0-20:C>T-20:C>T	TGCAGCGGCGCCGCCACGGACGACACGCCGCTGGACGACAACCCCAACTTCAACCCG	20:C>T
2246596|F|0-55:G>C-55:G>C	TGCAGTTCGGCAAGGTGTCCAAGGACATGTTCACCATGGACTACCGCTACCCGCTGTCGGCGTTCCAGG	55:G>C
1023936|F|0-23:C>T-23:C>T	TGCAGTGCCAGCGCGCCCTGGACCTTTTGGTCCTGCTCTTCCTTGGCGCGCCG	23:C>T
2253366|F|0-29:C>T-29:C>T	TGCAGAACGACTTCTACTTCGTGCCGGCGCTGTACGACATGGCCGAGTTCGAAGGCGAGCTCCG	29:C>T
6047053|F|0-7:C>T-7:C>T	TGCAGGACGTGAGCCAGAGCGAGGAGGTGTTCCAGGCCGAGCTGGGCGCGATCGGCAGGATCTACCACA	7:C>T
5369291|F|0-7:C>G-7:C>G	TGCAGAGCTTGACGATGTCGTGGACGGCACCG	7:C>G
1014875|F|0-25:C>G-25:C>G	TGCAGCGGTAGAAGGCCGACAGGTCCGCGTTCACCCGCAGCGCCACCGCCTGGTGCTTGGCCG	25:C>G
977314|F|0-52:G>A-52:G>A	TGCAGGAGGAGGACGAACTTGAACATGGTGTCGCCCCTCGGGATGAACCCCAGCCTGTCCGCCAGGGCG	52:G>A
32021634|F|0-16:C>A-16:C>A	TGCAGCAGCCGCCGCCCTCCCCG	16:C>A
6046854|F|0-7:G>C-7:G>C	TGCAGGGGGCTGACGGGGGCCAGAACGGGGAGTTCTCGCCG	7:G>C
22840147|F|0-37:C>T-37:C>T	TGCAGTGGGATTCCGTGACAAGCAGCTACATGACATTCGGGGCCCG	37:C>T
12683704|F|0-8:G>A-8:G>A	TGCAGGCCGTGCGCGAACCG	8:G>A
3025841|F|0-13:G>C-13:G>C	TGCAGCACATCGCGGCCGCCGACTACGACATGCTCCTCCTCCCCG	13:G>C
1070126|F|0-37:G>A-37:G>A	TGCAGATATTCAACAAGAGGTGGAAGGAGGCGGTGGCGGAGGACATGTGCTGGACGGACCCCTACCCGC	37:G>A
2266482|F|0-14:C>G-14:C>G	TGCAGCGGAAGCTGCCTCCTCGCGAACTTGCACTCGAAGGGCGGCAGGACCG	14:C>G
1027289|F|0-15:C>T-15:C>T	TGCAGAAGCCGAAGACGAAGCAGTAGATGATGGAGCAGATGCCAGCGAAGACACCGACCGCGCCGACGA	15:C>T
1076308|F|0-5:G>C-5:G>C	TGCAGGTGACCCACCCGTACGTCTTGGCGTCCACCAGGAGCTCCAAGACGCGCCG	5:G>C
1004818|F|0-19:C>G-19:C>G	TGCAGCGGTTCCTGTCGCTCAACGCGGCGCGCCACCACCACCTCATCCTCCGCGCCTTCGCGGACGTCT	19:C>G
1051765|F|0-8:G>A-8:G>A	TGCAGGACGTTGCTGATCTGCGGGCCATGCTTGAACGCCTGGCCGCTGATGCCG	8:G>A
5355233|F|0-52:T>C-52:T>C	TGCAGGGAACAGACGGTGGTGAGCAGGAGATCAAGGACGTGGCGGCTGGAGGTGGGGAGCAGAGCAATA	52:T>C
5373576|F|0-11:T>A-11:T>A	TGCAGCCATCCTCGTGTTGATGCTCTCGCTCCCG	11:T>A
7167027|F|0-52:C>T-52:C>T	TGCAGGAGCGCGTCGACGCGGAACGGCGAGCCTAGGAACCACGCCGACGAGGCGTCCGTCTCCACCACG	52:C>T
1076801|F|0-8:C>T-8:C>T	TGCAGCAGCAGCAGCCACGAGATCCAGGGCGCGAACAGGTAGTTCATGCCGACGTGGTGCCG	8:C>T
13879505|F|0-20:C>A-20:C>A	TGCAGAGCGGTGACGTGGACCTGCGGGCTGTTGCAGCCG	20:C>A
1237462|F|0-17:C>T-17:C>T	TGCAGCCGCACGATCCCCCGCAGCGCCCTCAGCGCCCTCCTGGCCTGCACGAGCACCCAAAAGAAGCGC	17:C>T
3958517|F|0-6:T>A-6:T>A	TGCAGCTCCATCGGGATCGACTTCCATGGACCAGAGGTAAATGCTGGATTCCTGCACGCCTGCAAATCC	6:T>A
3957047|F|0-22:C>T-22:C>T	TGCAGCGCCTCTCCGCGTCCCCCGACGTCGCGCGCGCCATCGCCG	22:C>T
4734172|F|0-16:G>A-16:G>A	TGCAGCCTCCGCGGCAGGAAGGAGCCCGCGCTGGCCTCCCCG	16:G>A
4262153|F|0-7:G>C-7:G>C	TGCAGATGACCTCCCACGCGCGCTCGAAGCCGCCGCGGTACCCG	7:G>C
1001059|F|0-11:G>A-11:G>A	TGCAGTATCACGGGGGAGGCCAGGTGGAAGACGCCGTGGACGCCCTCGACCGCCG	11:G>A
994225|F|0-20:T>C-20:T>C	TGCAGGATGGTCGCCGCCCATGTCCACTGCGACACGATCACGCTGGCGATCAGCCCCGTCTTCACGCTC	20:T>C
7352297|F|0-16:G>C-16:G>C	TGCAGGAGCTGGACGGGCCG	16:G>C
2260116|F|0-26:T>C-26:T>C	TGCAGCAAGCACACTGAGGGCAAGCGTCCGCTCTACGCCATCAGCTTCAACTTCATCGACGCCCGCTAC	26:T>C
3937575|F|0-20:G>T-20:G>T	TGCAGCGTGGCCGTCTGCCTGGAAGCCG	20:G>T
4005469|F|0-15:C>T-15:C>T	TGCAGAGGGCGGTGACCCAGTGGACGCTGGACCCG	15:C>T
1025842|F|0-22:G>A-22:G>A	TGCAGCCCTGCAAGCCGCCGCCGCACGAGACGGACCACCTCGGCGACGCCGTGTTCACCG	22:G>A
1027556|F|0-62:G>C-62:G>C	TGCAGCACCGAGATGTTGTACGGCTCCTCGTGGATGGGCTTGACGAAGAGCACGCTGGTGGCGCGCGCG	62:G>C
1076308|F|0-45:A>G-45:A>G	TGCAGGTGACCCACCCGTACGTCTTGGCGTCCACCAGGAGCTCCAAGACGCGCCG	45:A>G
1217398|F|0-23:A>G-23:A>G	TGCAGGAGCTGCTGTTTGCCCGTACCATCCTCGCAGACAAGTTCGGCGGCGACTTTGCTGTCGCGGCCA	23:A>G
1019445|F|0-16:T>G-16:T>G	TGCAGTCCCTCCTGGCTCTGTCCGACAGGGAGAGCGTCGTGTACAGCTACGGCATGGTGCTGCTGGAGA	16:T>G
5581345|F|0-16:C>G-16:C>G	TGCAGCATGGCGCTGGCGTCGTCCCCG	16:C>G
1207029|F|0-57:C>A-57:C>A	TGCAGCAGCAGCCAGTTCACCTTGGCCAGCACCAGCTGGTCGTCGTTGCAGTAGAAGCGCCG	57:C>A
1039946|F|0-29:A>G-29:A>G	TGCAGGAAAAATGAGAAACTACAGTCATCATCTAAAATTTGTCCTTTGTCTAGTCCTCGGAAGGCGGCG	29:A>G
1214806|F|0-31:A>G-31:A>G	TGCAGTGGCGGATGGCGGACACGCGAGGCGAAACGTGGGGGAGCAGGATGCGCCGCGGCGCCGCGCAGC	31:A>G
3021717|F|0-11:C>G-11:C>G	TGCAGCTTCACCATATCCAACTACTGCACCCACACCATCTGGCCG	11:C>G
4261550|F|0-24:A>G-24:A>G	TGCAGCTGCGGGCCGACGTGCTCAAGCAGCTGGACGGCGCGCCG	24:A>G
12770991|F|0-5:G>A-5:G>A	TGCAGGAGGTGTTCCGCCTGCTCCCTCCCGCCGCCG	5:G>A
1024166|F|0-5:G>A-5:G>A	TGCAGGTACACCGTGGCCGTGGCCGACAGCTGCACCAGCGTGAAGAGCTCCGCCATGAGCAGCAGCCAC	5:G>A
3022763|F|0-15:A>G-15:A>G	TGCAGCTAAGCCAACACACATGCTGACAGATGTCTTTCAGCCCCCG	15:A>G
1210661|F|0-46:C>T-46:C>T	TGCAGCACTGCTGTCTCTCCGAATCCGAGGACGATGCCGCCAGCTGCGGCAGCCAGCAGCAGCTCGCGG	46:C>T
992330|F|0-38:G>C-38:G>C	TGCAGCCGCGCCGTCACCTGCTGGATCTGCTCGGCGGTGGGGGTGCGCAGCCCCTGCCG	38:G>C
4993731|F|0-23:A>G-23:A>G	TGCAGGAGCTCTTCCCCTCCTGCACCGTCATGCCCG	23:A>G

1027807|F|0-56:C>G-56:C>G	TGCAGCGGCGGCACGGTCGCCGCGGGCTCCTCCTCTTTCACCTCCTGCTGGGCTCGCACCG	56:C>G
32025007|F|0-25:A>C-25:A>C	TGCAGCACGGCGATGTCCCAGGGCGATTCCTTGACCGACTTCACGTAGAACACGGCGGCGGTCCCGTTC	25:A>C
988831|F|0-40:G>C-40:G>C	TGCAGGCGCAGAGCAAGAACATGATCATCACCTACCTCGCGTTCGTCAACCTCGGCCTCCACCTGTTCC	40:G>C
32022446|F|0-17:A>C-17:A>C	TGCAGCGCTGGTCGCAGACCCTCCCCG	17:A>C
5358058|F|0-16:G>A-16:G>A	TGCAGCTGGTGGCGCAGCGCGCGATCCG	16:G>A
4395837|F|0-12:C>T-12:C>T	TGCAGCATTCTCCGAGGCCG	12:C>T
5373460|F|0-7:A>G-7:A>G	TGCAGACACAGAGCATCGTCTTGCCG	7:A>G
100513828|F|0-11:G>A-11:G>A	TGCAGCGACCCGTCTACCCACCTCTTCTGGGACGGCATCCATTACACCGAGGCCGCCAGCAAGTACATC	11:G>A
7918346|F|0-22:T>C-22:T>C	TGCAGGTTCTTTCACCTCTTGTTCCTATTAGGCAGGTGGCCTTTCTCAGGTTCTGCAAACAGTTGGGTG	22:T>C
100501064|F|0-7:A>G-7:A>G	TGCAGCCACCCGTCTGGGTCGCGTTCCTGCTGTTCGGCCTCCTCCTGGGCGTGCCGTACGGGGTGTTCT	7:A>G
6044630|F|0-16:G>A-16:G>A	TGCAGCACGATAGAGTGGATCACGTCGGCGTATTTGACGGCGAGGTTGAGCGACATGCACCG	16:G>A
5361213|F|0-32:A>C-32:A>C	TGCAGCTCGTTCGCCTTCTGCTTGGCGTTCAGAGCCTCCTTGCAGGCAGTGCTGTACATGTCCATGGTC	32:A>C
100543764|F|0-25:G>A-25:G>A	TGCAGCGGCACAACACCCTGAAAGGGGGCTCGGTTCGCAAGGACAGCAAAGAGCCGAGCTTCAGGGTGG	25:G>A
5363609|F|0-12:A>G-12:A>G	TGCAGCTCCGCGACCGCGATCGGCGGCCCG	12:A>G
6041317|F|0-20:T>G-20:T>G	TGCAGCAGCACGCGCGCCGCTCCCTCGCTGCTGGTCCCGCCG	20:T>G
5372975|F|0-11:C>G-11:C>G	TGCAGCGCGTCCAGATTCGCCCGCATCACGGCGAACTCTTCAGCGGCTGCCCG	11:C>G
5358346|F|0-22:C>G-22:C>G	TGCAGGGCGTCCGCCGCGGCGCCCGCGGCGCCCCG	22:C>G
4986415|F|0-7:G>C-7:G>C	TGCAGGGGCTGCTCAGGAAGCTCGGCGCCG	7:G>C
3959326|F|0-17:C>A-17:C>A	TGCAGTACAGGCACGAGCGGGGTCTCTTCGAGCAGAAAGCCAGGCGCTGGACCCGCAGATACGCCTCGG	17:C>A
1078072|F|0-20:G>A-20:G>A	TGCAGCAGGTGAAGCTCGCCGTGGTGCACCTGTACCGACGGTACGTCTTCCGCCACTCGCCG	20:G>A
5372180|F|0-17:C>G-17:C>G	TGCAGCAGGTGCGTCACCGCCACCG	17:C>G
3943724|F|0-34:C>A-34:C>A	TGCAGCGACCTCCTCACTTTCGACGACCCGCCACCGTCCTCCG	34:C>A
988774|F|0-11:T>C-11:T>C	TGCAGGTGCACTTCGGCACGCGGCTCCCCGCCGTCATGGGCGGCTCCTTCACCTACATCTACGCCGCCG	11:T>C
994831|F|0-43:G>C-43:G>C	TGCAGGTGGGGGCGGCGCTGGAGACGTCCATCAACGAAACGCTGGCGGAGCTCAGCGCCCCGCTGCTGG	43:G>C
1092416|F|0-14:C>T-14:C>T	TGCAGGTCCTCCTCCGTCAACAGCGTCTTCTTCCCGCCGCTCCCAGCCTTGTCGTATGGCTGCGCCATC	14:C>T
1150469|F|0-8:C>G-8:C>G	TGCAGTCGCTCTGGAACGGCGCCGCGTACCACGGCGTGCACCACCTGCCGCGCGGCGTCAGGTACAACT	8:C>G
1080483|F|0-25:C>T-25:C>T	TGCAGGACCTCGCCGACGGCCTCGGCGCCGTCTTCCAGGACTACGTCTCCTTCCTCGCCTCCTGCGGCA	25:C>T
5579663|F|0-52:C>G-52:C>G	TGCAGCTTGTGGTGTCGGAACTGTGCCGCGTCCAGGAGCTGGTGCGGCAGCTCGAGCTGCACCTGCACG	52:C>G
10993237|F|0-7:C>T-7:C>T	TGCAGCACGTACTGTCCTTCTTGCCG	7:C>T
3958517|F|0-38:A>G-38:A>G	TGCAGCTCCATCGGGATCGACTTCCATGGACCAGAGGTAAATGCTGGATTCCTGCACGCCTGCAAATCC	38:A>G
1024657|F|0-29:G>A-29:G>A	TGCAGCACCTGCCTCCTCTCCTCCTCCGCGCCAGGGGGAGCGCGCGCGCGCCAGCCAAGGTGAGAGC	29:G>A
1021497|F|0-24:T>C-24:T>C	TGCAGGTACATCTGGAGCGTGAAGTTGAAGACGCTGGCGAACATGACGGGGATGTACCATATGGAGATG	24:T>C
2247100|F|0-5:C>A-5:C>A	TGCAGCCCTTGGTGGTTTCGACGGTGCACAGGGTGTCGAGGGCATCGTCGCTCCG	5:C>A
5324019|F|0-28:G>A-28:G>A	TGCAGCACGACGGCCACCACCACCACCCGTCCCCG	28:G>A
1862907|F|0-6:C>G-6:C>G	TGCAGCCTCCCTGCACACAACCTGTTCGACGAAACGCCTCCGAGCTCTGACCCCATGGCGGAGGCGTCG	6:C>G
999236|F|0-13:C>G-13:C>G	TGCAGGGCCCGCGCCCCCCGCGGCTGTCCGTCAGCAAGGACTCCCACAAGGTCAGGAAGCCGCCCGTCG	13:C>G
986643|F|0-20:G>C-20:G>C	TGCAGGTACGACGACGAATGGGCGGCGCCGTACAGCTGCTCCGCCCTCGTCGTCGCCATGCCCAGGCCG	20:G>C
2289816|F|0-35:G>A-35:G>A	TGCAGCTCCTTGAGGCGCTTGAGCCTCATGCGGTCGCGCCACATGCGGCGCTCCAGCTCCTCGATGCCG	35:G>A
5578466|F|0-14:G>C-14:G>C	TGCAGTGTATCAGCGCCGCCG	14:G>C
7172983|F|0-6:C>G-6:C>G	TGCAGCCCCGTGGAGGTGGTGTCGGTGCCG	6:C>G
1206575|F|0-29:G>C-29:G>C	TGCAGCTCCACGAAGGAGAGACACAGCGCGACAGAGATGCTCATGGCCTGCCCG	29:G>C
3935840|F|0-14:G>T-14:G>T	TGCAGCTGATGTGGGCTATGGGCGGCCGCATGGCGCTGGCGCCG	14:G>T
12774825|F|0-52:C>A-52:C>A	TGCAGTACAGGGACCAGCGGGGTCTCTTCGAGCAGAAGGCCAGGCGCTGGACCCGCAGATACGCCTCGG	52:C>A
5581983|F|0-31:C>T-31:C>T	TGCAGGGCGCCCCAGTGCAGCGCGAGCGCGCCGCCG	31:C>T
7350579|F|0-9:T>C-9:T>C	TGCAGCCTCTTGAGCCCCGCGAGGCCG	9:T>C
5367368|F|0-15:G>A-15:G>A	TGCAGGGAGGGCCCCGCGCGGGCCG	15:G>A
5372257|F|0-14:C>G-14:C>G	TGCAGGCTCTCCATCTGCGGCAAGGCGGCGGCCG	14:C>G
4982813|F|0-68:A>C-68:A>C	TGCAGCCAAGGCGGGTTCATGGAGGAAGCCTGGATATACTTCAGAGCCATGACAGACAAGTTCCGCATA	68:A>C
4262475|F|0-29:G>C-29:G>C	TGCAGCGACGCCTTCCTCGCGATCGGCATGTCGACGAACGCCG	29:G>C
2248164|F|0-31:C>G-31:C>G	TGCAGCATGGGGATCTTGACGTCCAGCAGCTCGGATATGGTCTCCTCGATGGCCGACTTCTCCATGAGC	31:C>G
1224593|F|0-40:G>A-40:G>A	TGCAGCAGGCCTGCGGCGACGCGCCGTGCATGGTGGTGGCGTACGCTCCGATTGACGCGGGAGAGCTCA	40:G>A
4261072|F|0-38:C>A-38:C>A	TGCAGGTGGCTGGAGACGATGAAGCCGCCCTTCTTGCCCCCG	38:C>A
7918664|F|0-5:G>C-5:G>C	TGCAGGGGACGAGGGGAAAGCGGGGCTGCTGCGGCGGCTCGTCCCCG	5:G>C
1024166|F|0-8:C>G-8:C>G	TGCAGGTACACGGTGGCCGTGGCCGACAGCTGCACCAGCGTGAAGAGCTCCGCCATGAGCAGCAGCCAC	8:C>G
5354977|F|0-63:T>C-63:T>C	TGCAGCTGGGGCATGGTGCAGAACGACCTGGTGCACGCGTGGGGCCTCGACTACAAGCTCGGCTACTGC	63:T>C
6044636|F|0-8:C>G-8:C>G	TGCAGCCACCCGAACGCGGCGCCCCTGGTGTGGTCGTCCGTGTAGTCGGCGACCAGCGAGTTGATGGCC	8:C>G
32025564|F|0-7:T>C-7:T>C	TGCAGCTTGACGCTGAAGGCAGCAACAGCAACAACTACAGGGCCATGCAGCAGATCACCTGGGCTGCCG	7:T>C
1127561|F|0-20:C>G-20:C>G	TGCAGCGCCTCCGCGAACAGCCGCTTCCCCTCCGCCGACGCGAACTCCACCGCCG	20:C>G
5331473|F|0-6:A>T-6:A>T	TGCAGAAGGCCGTGGCCAACATCGCCG	6:A>T

1023254|F|0-26:T>G-26:T>G	TGCAGATGCTGCGGCCGCACCGCGAATCCATAAACGTCCCTGCGCCAAACCTCAACAACATCAGCCACA	26:T>G
2373210|F|0-5:G>A-5:G>A	TGCAGGCCGTGGCGCGCACCGTCATGGGAGCCAACCTTCAGAAGCCACAGAGGGTGAGGATGGGCCCGT	5:G>A
3951277|F|0-17:A>G-17:A>G	TGCAGGAAGGGGTGGGGATTGGGGTCAGGGTAGTCCG	17:A>G
7906789|F|0-17:C>A-17:C>A	TGCAGCGGCACATATGCCGCCG	17:C>A
1106335|F|0-33:C>T-33:C>T	TGCAGAAGTGAAGCAGGGGCATGATTTGCAGCCCCCGATCCGTCGTCGTCGCGGCCCATCCAGCGGTGC	33:C>T
985354|F|0-40:A>C-40:A>C	TGCAGACGTTCAAGACCTGGCGGAGCTGGGCCAACGGGCCATTCGTGTTCAACACCCG	40:A>C
7171541|F|0-20:G>A-20:G>A	TGCAGCGACGGGAAGGCGGCGCCAGCACTGCTTCCACCG	20:G>A
7172131|F|0-20:G>C-20:G>C	TGCAGCGCCGCGACCCAGATGCCGCCG	20:G>C
32018964|F|0-41:G>C-41:G>C	TGCAGCGACAGCGTCGTCAGGCTCGTCAGCCGCCCGAACGTGTCCG	41:G>C
5363439|F|0-11:G>A-11:G>A	TGCAGCCTGCCGTGCCCGAAGCTCGCTTCCCG	11:G>A
5410401|F|0-32:A>G-32:A>G	TGCAGCATCCCGCCTCCGTCGACGATCCTCCCAAGGCGGAGCGTCTCCAGCGACGCCATGTCGACGCTC	32:A>G
1055414|F|0-7:C>A-7:C>A	TGCAGTCCATCACCACGATGCCGATCCTGGTGAAAGGAGTGATCACCGCAGAGGACAGCAGGCTCGCCG	7:C>A
3937180|F|0-19:C>A-19:C>A	TGCAGGAGCTCCACCACCTCAACCTCGGCGCCG	19:C>A
1020121|F|0-47:C>G-47:C>G	TGCAGCCCGCGGTCCATCGGCAGCAGCGAGTTCCTCAACCCCATCGCCCACGAGGTGGTGCTCAGCACC	47:C>G
7172601|F|0-8:G>C-8:G>C	TGCAGCTCGAGCAGGTTGAGCCGCGGGAGGTAGAGGAAGCCG	8:G>C
1695198|F|0-61:G>C-61:G>C	TGCAGGACAATGGCATCAAGAAGGTGAAGCTGTTCGACGCGGACGCGGAGCCGCTGGGCGCGCTCGCCG	61:G>C
1081642|F|0-39:G>A-39:G>A	TGCAGGATGCGCAGCGAGGAGTTCCTCTCGCCGCCGAACGCCGCGGCCGGGATGGCGCCG	39:G>A
1201696|F|0-44:C>T-44:C>T	TGCAGAGTGAAGTAAAGACGAAGCCGAAGACGAATCGGGCAGCACGTTTCACCTCGCGACTCCG	44:C>T
5009958|F|0-28:G>C-28:G>C	TGCAGCAGGACCCCGAGCTCGCCGTCTTGGGGCGGGCGCCG	28:G>C
6045025|F|0-11:G>C-11:G>C	TGCAGGAGCGGGCGCGGGAGGTAGAGGACGGCGAGCAGGGAGGCGGCCGCGGCTGCGGCGAGGAGGGGG	11:G>C
1864140|F|0-19:G>A-19:G>A	TGCAGATCTGAAGGAGATCGCGTCCGTGGTCGAGGCGGGGTCGCTGACCAAGGAGGTCCGCCG	19:G>A
2243499|F|0-43:G>C-43:G>C	TGCAGCTGCCCGAGTACCCCAACAAGGAGGAGCTCGACGCGGTGCTCAAGACGATCGAGACCTTCCCGC	43:G>C
1148571|F|0-33:G>C-33:G>C	TGCAGATTCTGGTTGTGCTGACCTCGCCCGATCGTCCGTCTCCATCCACAGCCG	33:G>C
1020797|F|0-18:C>G-18:C>G	TGCAGGGAGGCTGTTCGTCTACTGGTACGGGAGCGAGCCACGGGTGTGCGTGACGGACGCCG	18:C>G
2260280|F|0-58:T>C-58:T>C	TGCAGGATCGCCTTGGCTACGACCAGCCCAGCAAGGCCGTCGAGTGGCTCATCAAGGCTGCCGCCGCCG	58:T>C
3934321|F|0-14:A>G-14:A>G	TGCAGAGCATCATCACCTCCG	14:A>G
2289816|F|0-23:C>T-23:C>T	TGCAGCTCCTTGAGGCGCTTGAGCCTCATGCGGTCGCGCCACATGCGGCGCTCCAGCTCCTCGATGCCG	23:C>T
2258501|F|0-11:C>G-11:C>G	TGCAGCTCCTCCGACATTGCCCAGAAGCACGACTCGAGGCTGGAACCCAAGGGGATGCGGTGCGACTGC	11:C>G
1024458|F|0-8:C>T-8:C>T	TGCAGAGACCATGCCTGTGATCACTGCGTTCCAGGAAAAGTTGTTCCTTTCCG	8:C>T
984393|F|0-38:C>G-38:C>G	TGCAGAGCTCCTGGGACGCAGGCGTCATAGACGGCGCCCTCCACCTCACCACCGACGACGTCTACCAGC	38:C>G
2243430|F|0-67:C>T-67:C>T	TGCAGTACTACGTCCCCCTCTTCGGCTCCTTCGAGAACCTCCTCCAGGCGCTCAAGTACAACTCCTACC	67:C>T
5580068|F|0-13:C>T-13:C>T	TGCAGAAGAACAGCGCGATAGCAGGGGCGATCGCCG	13:C>T
4262407|F|0-29:G>A-29:G>A	TGCAGTCCGTCGAGCACGACGGAGCCGACGAACAGCCCG	29:G>A
5582496|F|0-21:T>G-21:T>G	TGCAGACACGCCCTCCCCGTCTTCCCCG	21:T>G
5583162|F|0-16:A>C-16:A>C	TGCAGGTCGACGACCGAGGCGCCG	16:A>C
7351424|F|0-5:T>C-5:T>C	TGCAGTCCGCGCCGCTGGCCG	5:T>C
3941590|F|0-11:G>C-11:G>C	TGCAGCTGGATGGGGCCCCG	11:G>C
13877676|F|0-15:A>G-15:A>G	TGCAGTTGCTCGGTCACCGCCG	15:A>G
32025999|F|0-47:C>T-47:C>T	TGCAGCGTCTCATATTGGGAAGTGATCACACACTAAAGTGATTTGCCCATCGAGGAGAAAAAGGCTAAG	47:C>T
1862907|F|0-43:G>A-43:G>A	TGCAGCCTCCCTGCACACAACCTGTTCGACGAAACGCCTCCGAGCTCTGACCCCATGGCGGAGGCGTCG	43:G>A
5369436|F|0-14:T>G-14:T>G	TGCAGCGCCTGCTTTGCGCTCTCGAGCCCG	14:T>G
1318141|F|0-12:T>C-12:T>C	TGCAGAACCCGATGGCGTTCCCGTCGGAGAGCCACCCGATCCACCTCCACGGCTTCAACTTCTTCGTGC	12:T>C
6037684|F|0-18:G>A-18:G>A	TGCAGCCACGTCGGCCGCGATGGAAGCGGCCG	18:G>A
7156721|F|0-13:G>T-13:G>T	TGCAGTGGGAGCCGCCCGAGTTCGCGCGCTCGCCCG	13:G>T
4002464|F|0-22:G>C-22:G>C	TGCAGGCGGTGCTCAAGGAGACGCTGCGGCTGCGCCCGCCG	22:G>C
1333911|F|0-58:G>T-58:G>T	TGCAGGCCGCGTCGGTGGTGTTGACGGTGGGGTCGGACTGGTGCTTGTACCCGATGACGGTGGCCACGG	58:G>T
7156721|F|0-19:G>A-19:G>A	TGCAGTGGGAGCCGCCCGAGTTCGCGCGCTCGCCCG	19:G>A
989600|F|0-45:C>G-45:C>G	TGCAGACACCATGGACTCGGTGAAGGACGTGCTGGGGAAGATGGGCAAGAGGTTCGGCGAGGCCGCCCG	45:C>G
1088829|F|0-44:C>G-44:C>G	TGCAGCACGGAGTTCATGAAGCAGGTGTTGCCGAGGTTGTTGAGCCCGCGCAGGCCCGCCG	44:C>G
1092791|F|0-31:G>A-31:G>A	TGCAGAAGAAGCGCCCCCGCGTCGGCGCCATGGATCAGGAGGCGCTGCCG	31:G>A
5358263|F|0-10:G>C-10:G>C	TGCAGGCGCTGCCCCGCGCCCTCGTCGCCG	10:G>C
2247504|F|0-18:A>C-18:A>C	TGCAGGAGGAGGTGCTCGAGGTACCACATCAGGGGCGACAAGGCAGGCACGGTGGGGAGCTTCATCGAC	18:A>C
1020797|F|0-57:C>T-57:C>T	TGCAGGGAGGCTGTTCGTCTACTGGTACGGGAGCGAGCCACGGGTGTGCGTGACGGACGCCG	57:C>T
2244237|F|0-21:G>A-21:G>A	TGCAGGAAGTATGCATCGACTGAATCTAGTGGACAGCGAAATGCAGCTCTACATAGTACAGCTCACGAG	21:G>A
991134|F|0-35:C>G-35:C>G	TGCAGCTACGACTACAAGTCGGCGGCCAGGGACGCCATCGCCAGGGAGAACCCCGTCGTGCGCGCCGCC	35:C>G
988777|F|0-23:G>C-23:G>C	TGCAGGTAGTCGGCGAGCCAGGAGGCCTGCTGCTCCTGCTGCTGCATCCCCG	23:G>C
1713501|F|0-25:C>G-25:C>G	TGCAGGACGTGCTCCTCCGCCTCACCTTCGACAACGTGTGCATGATCGCCTTCGGCGCCGACCCCGAGA	25:C>G
5354734|F|0-5:C>G-5:C>G	TGCAGCCTGGCGGACGCGATCGGGGACGACTCCGTGGCCGTGGTGAGGATGTCGACCGTGGCGTGCGAC	5:C>G
7902447|F|0-29:T>G-29:T>G	TGCAGTGGGCGGCGGACAACCTGCTCCGCTCCG	29:T>G

2242108|F|0-8:T>A-8:T>A	TGCAGAATTCAGCTGCTAATTCCACAATACAGTCGACACGAAATAGAAGCTAGAGCAGCGAGATCGAGA	8:T>A
1044204|F|0-46:T>C-46:T>C	TGCAGTCGCTCGTGAATCTGTTAAAGATGATCTCGGAGCAGAAGTCTCCTATAACCAGGAGGAAAAGCC	46:T>C
6045605|F|0-10:G>C-10:G>C	TGCAGCTGCCGTTCAAGGCCCTCGCCG	10:G>C
1013349|F|0-62:C>G-62:C>G	TGCAGGTGGCGGCGGAGCACGACGCCATCGGTGGCGGCCCTGGGGTCGGCGCGGACGTTGTCCACCACG	62:C>G
1087882|F|0-19:T>C-19:T>C	TGCAGCCCGATCACATGCATGGCTGGCCTGATCGTCCCAAAAGGTCTCGGCGTTACGTGTCGCCGCGCC	19:T>C
994366|F|0-15:C>G-15:C>G	TGCAGCGCCACGGTGCCGCCCATGGAGAAGCCGTAGAGGAACCTCTTCTTCGTCTTGTTCTCCG	15:C>G
1213983|F|0-34:T>C-34:T>C	TGCAGCTGCTCCATGTCGCTCGGGTACACCGCGATGGCGCCCCGCCGCGGCAGGCTCGGCCG	34:T>C
5358423|F|0-30:G>A-30:G>A	TGCAGGTCCTGGAGCGTGCCGTCCAGCGCCGCCGGCACGTCCTCCG	30:G>A
3950445|F|0-11:G>A-11:G>A	TGCAGGTCGCCGCCGCGGGGCCG	11:G>A
4262407|F|0-5:T>G-5:T>G	TGCAGTCCGTCGAGCACGACGGAGCCGACGAACAGCCCG	5:T>G
1089751|F|0-25:C>T-25:C>T	TGCAGCGCTCCTGGATCATCCTCGCCGTCGCCGCGCTCCTCATGGTGCCCTTCTACGTCTTCGCCGAGC	25:C>T
7353542|F|0-14:G>C-14:G>C	TGCAGCTGCCCGCTGAGGCCG	14:G>C
3941804|F|0-26:T>C-26:T>C	TGCAGTGGCGGCGCATAAGAGGCAACTTGTTGAGCCG	26:T>C
1002591|F|0-50:G>A-50:G>A	TGCAGCGGGAGGTGCACGTTGAGGTCGCAGGCGGCGGCGAGCGGGCAGTCGGCGCCG	50:G>A
1090859|F|0-10:C>G-10:C>G	TGCAGTGCGTCGCCATCTCGTACAACATCGGGTGGCTGGACGCCGAGCACCCGCTCCTGCGCGACAACG	10:C>G
1046610|F|0-25:C>G-25:C>G	TGCAGCGCATGTCGTACATCAACGTCGGCATCTACCCGTTCACGTCCCTCTTCCTCATCATGTACTGCC	25:C>G
32027959|F|0-10:T>C-10:T>C	TGCAGCACGGTGCTGGCGACAATGCGCAGCCG	10:T>C
1055414|F|0-25:C>T-25:C>T	TGCAGTCCATCACCACGATGCCGATCCTGGTGAAAGGAGTGATCACCGCAGAGGACAGCAGGCTCGCCG	25:C>T
4262407|F|0-20:G>T-20:G>T	TGCAGTCCGTCGAGCACGACGGAGCCGACGAACAGCCCG	20:G>T
4261273|F|0-10:C>T-10:C>T	TGCAGTCCTTCGCCAGGTCCATCGTCGTCCCGTCGCCG	10:C>T
1024557|F|0-23:G>A-23:G>A	TGCAGGGACCTGTCTGCCAGGTCGTGCATGTTGAAGAGGACGTGCTTGTTCAGAGGGTGGCCG	23:G>A
1118336|F|0-10:G>A-10:G>A	TGCAGGCGACGGTAAGCAAAGCTTCGTTGTGGTGTCAACCAGGTCATTTCAGACACAAGATGTCTGCTC	10:G>A
3026518|F|0-13:G>A-13:G>A	TGCAGCTCCTCGAGAAGAAGGAGGAGCATCCGTGCTCCATCTCCCCG	13:G>A
2251743|F|0-49:G>A-49:G>A	TGCAGCAGCTCCGACTGGGACTCCGACTCCTCCTCCCCCGTCCTCGACCGCGCGCCGCACCGCAACACC	49:G>A
1149940|F|0-41:A>G-41:A>G	TGCAGACGGCCTTCTTCTACGTCTCGCTCTACCTGGTGGCCATTGCGCAGAGCGGCCACAAGCCCTGCG	41:A>G
4397949|F|0-16:T>C-16:T>C	TGCAGGAGCTGCTGCGTCGGCAGCAGGAACCG	16:T>C
1092791|F|0-20:G>A-20:G>A	TGCAGAAGAAGCGCCCCCGCGTCGGCGCCATGGATCAGGAGGCGCCGCCG	20:G>A
3948676|F|0-19:A>G-19:A>G	TGCAGCGGGATCAAGCTCAAGAACATCAGGCTGAGGTACCG	19:A>G
3029707|F|0-34:T>C-34:T>C	TGCAGTACGCGCGCCCCGAAGGGATCGACGTGCTTCTCCG	34:T>C
1018785|F|0-50:C>G-50:C>G	TGCAGGCACATGGCCGCCTCCAGAGTGTTGCGGATGCAGGTGAGGTACAGCCGCAGCGTGTTGGCCTGC	50:C>G
5359215|F|0-10:G>C-10:G>C	TGCAGGACGCGCTGAACAAGCTACTCCG	10:G>C
984858|F|0-35:G>T-35:G>T	TGCAGCTTGCCGCCGAGGCCCCTGGCGAAGGCCTGGGCGATGAAGAAGCGCGCGAAGCGGTGGATGCGC	35:G>T
3934563|F|0-37:G>C-37:G>C	TGCAGCTGGAGCGGATCAACGTCTACTACAACGAGGCGAGCGGGGGCCG	37:G>C
3949974|F|0-11:C>T-11:C>T	TGCAGCAGCGACGCGGCGCCGCCG	11:C>T
6041335|F|0-26:G>A-26:G>A	TGCAGCAGTCTTCTGAAAACCTTCTCGCTTCCG	26:G>A
1215457|F|0-49:G>A-49:G>A	TGCAGGGGCGGTGGAGGCTGGTCTACAGCAGCGCCTTCTCGTCGCGGACGCTCGGCGGCAGCCGCCCCG	49:G>A
984858|F|0-17:G>C-17:G>C	TGCAGCTTGCCGCCGAGGCCCCTGGCGAAGGCCTGGGCGATGAAGAAGCGCGCGAAGCGGTGGATGCGC	17:G>C
1154341|F|0-7:C>T-7:C>T	TGCAGGACCCCCGCATCGCGGCGGCCGCCCCCGCCCTCATACGCGCCGCCACAGCCTCCG	7:C>T
1082028|F|0-43:G>C-43:G>C	TGCAGCGCGCCCTGGTCGGCGGCGGCATCGCCAAGTCCTCGGCGGCTCTGTTCCTCGCCTTCTTCAGGG	43:G>C
1045011|F|0-50:T>C-50:T>C	TGCAGCAATGGCAACAGAATAGTACAGATCGGGTAGGTGCCCTGCCTGCCTGATGCCTTCTCCGACACG	50:T>C
1067063|F|0-10:C>T-10:C>T	TGCAGCCCCGCCGACTCCTCCGTCTCCTTCACGCTCTCCGAGATCACCGCCAACCTCAGCAACCACGCG	10:C>T
1106335|F|0-54:C>T-54:C>T	TGCAGAAGTGAAGCAGGGGCATGATTTGCAGCCCCCGATCCGTCGTCGTCGCGGCCCATCCAGCGGTGC	54:C>T
6038092|F|0-13:T>A-13:T>A	TGCAGGCTCGCCGTGCGGCTGTCCTTGCGGCCG	13:T>A
4993879|F|0-11:A>G-11:A>G	TGCAGGTTGTCACGAAGACTGCACCGCTGCCG	11:A>G
1747435|F|0-50:T>C-50:T>C	TGCAGCGTCTCATATTGGGAAGTGATCACACACTAAAGTGATTTGCCTATTGAGGAGAAAAAGGCTAAG	50:T>C
5326242|F|0-7:C>T-7:C>T	TGCAGAGCGAAGGCTTGAGCTTGTCGAACCAGTCGAGCTGGACGCTGAGCGACAGGTTGGTCCAGAGCT	7:C>T
32027381|F|0-53:C>A-53:C>A	TGCAGTTCACGGTGTAGCAGCTGTCGGGCCGGGAGAGCGCGTCCGAGAACCGCCCCGCGGCCTGCTGAT	53:C>A
7169387|F|0-10:G>A-10:G>A	TGCAGCCAGTGTGTATTTGGTTCACCG	10:G>A
32021524|F|0-30:C>A-30:C>A	TGCAGTGCGGCGGCTGCGACGAAATGCTCGCGGTGAAGCTGCCG	30:C>A
7921408|F|0-9:C>T-9:C>T	TGCAGCTGCCCTTCCTCCACCGTCTTGACCACGGCGTGGGTCACGGCGACGCTGCCGCAGCCG	9:C>T
1333911|F|0-40:G>A-40:G>A	TGCAGGCCGCGTCGGTGGTGTTGACGGTGGGGTCGGACTGGTGCTTGTACCCGATGACGGTGGCCACGG	40:G>A
7492555|F|0-5:G>C-5:G>C	TGCAGGAGGGCGGCTGGCCCG	5:G>C
1095217|F|0-25:G>T-25:G>T	TGCAGCGCCGCGTCGGGGAGGCGCTGGCCGTGAACGCGGTGAACCGCATGCACCGCGTCCCCG	25:G>T
5006063|F|0-5:G>C-5:G>C	TGCAGGGGGATGACCACCATGAGCACCTTCTTCTCACGGTCCGCCAGGTAGGGCTTGAGGAAGGACCAG	5:G>C
5367125|F|0-15:A>T-15:A>T	TGCAGCGTACGGCCAACATATGGAACATCGTCCG	15:A>T
3949563|F|0-6:G>A-6:G>A	TGCAGGGCGGCCGCGTGTTCGAGAAGGCCGCCGTGAACGTGTCCGTCGTCTACGGAGTCATGCCCCCAG	6:G>A
1089751|F|0-38:C>T-38:C>T	TGCAGCGCTCCTGGATCATCCTCGCCGTCGCCGCGCTCCTCATGGTGCCCTTCTACGTCTTCGCCGAGC	38:C>T
5577051|F|0-41:C>A-41:C>A	TGCAGCTCCTGCCGCTCCGCCGTCGACATCTCGGGCGCTGGCGCGCCGCCCTTGGCCCCGCTGGCGCCT	41:C>A
1055414|F|0-5:T>A-5:T>A	TGCAGTCCATCACCACGATGCCGATCCTGGTGAAAGGAGTGATCACCGCAGAGGACAGCAGGCTCGCCG	5:T>A

1019099|F|0-7:A>G-7:A>G	TGCAGCAACTTCTCCAGAGCGGAGACCACAGCAGCATGGCTCTGGTCGCCCACAATCTCCG	7:A>G
1063793|F|0-26:C>T-26:C>T	TGCAGCAGCACCCCGACCTCCGTCAGCGACAGCACGTCCTCCAGTATGACCTTGGCGAATATCCGAGAT	26:C>T
1132535|F|0-11:C>G-11:C>G	TGCAGCCTCCCCTGCGTGCAGGCCCACAAGCGCCGCACCGCCTGCTCCG	11:C>G
2242108|F|0-16:T>C-16:T>C	TGCAGAATTCAGCTGCTAATTCCACAATACAGTCGACGCGAAATAGAAGCTAGAGCAGCGAGATCGAGA	16:T>C
3940456|F|0-27:T>A-27:T>A	TGCAGCTGGTGATGTTGCTGGGCCGCGTGCTCGGCTGCCG	27:T>A
1168067|F|0-6:G>A-6:G>A	TGCAGCGGCGGGTCGTGCCTGAGCTGCCCGAAGCCGTCGTGCCCCGAGTGCTCCTGCGGGTGCGTGTGG	6:G>A
7916085|F|0-19:G>A-19:G>A	TGCAGAGCAGAGGGATCACGTCGTTCGTGCCG	19:G>A
1298868|F|0-6:G>A-6:G>A	TGCAGTGGTCGCAGGCTACCGCGTCGGCAGAGAGCTCCTTCTCCATGTCCG	6:G>A
1214625|F|0-35:C>G-35:C>G	TGCAGCCGCAGGATCATGGCCAGCGCCTCGTCGGCCCCCGACGACGCCGCCTGCCTCTCCTCCTCCAGC	35:C>G
1029980|F|0-58:G>C-58:G>C	TGCAGCTGGTTTATCTCGGCACCCGCATCATAAACAAGAACTGGATCAATCCCTTCGGGAGGCACTGCT	58:G>C
1215457|F|0-40:G>C-40:G>C	TGCAGGGGCGGTGGAGGCTGGTCTACAGCAGCGCCTTCTCGTCGCGGACGCTCGGCGGCAGCCGCCCCG	40:G>C
3027225|F|0-14:G>C-14:G>C	TGCAGTATCTCCGTGGCCCTCCTGTTGGCGTCGGTCTCGCCGTCGCCG	14:G>C
986769|F|0-67:G>T-67:G>T	TGCAGCGAGAAGGAGGCGCTGGTGTCCTTGGGGTGCTTGGAGACGAGGATCCCGACGCCCTGGCCCCGC	67:G>T
4988931|F|0-33:C>A-33:C>A	TGCAGCTCAAGCTCCACGCCTTCCTCCTCTGGTCCTCCGTCGGCTTCCTCATGCCGATCGGCGTGCTGC	33:C>A
4262475|F|0-30:T>G-30:T>G	TGCAGCGACGCCTTCCTCGCGATCGGCATGTTGACGAACGCCG	30:T>G
1007185|F|0-16:G>A-16:G>A	TGCAGCGGTGCGTGGAGACGATCATGGGCGACGGGGAGGCCGCGTTGGGGATCCG	16:G>A
3950548|F|0-24:G>A-24:G>A	TGCAGGCCCTTTGGCGCACCCGCCGCACCG	24:G>A
3949826|F|0-23:T>C-23:T>C	TGCAGATACGACGTGCTTCAAGTTTCCCCG	23:T>C
2245644|F|0-30:G>A-30:G>A	TGCAGGCTTCGATGATTAGTTCATTGATTGGTTGGCCTCGCACGGGCGCGGGATAATTGTTTCTCCGAG	30:G>A
1044033|F|0-9:A>C-9:A>C	TGCAGCTCCAACTTCGCTGTCACAGAGAGGACACAGGACGCAGGGCGCACGGTTTCCGTGGGCTACAAC	9:A>C
3951138|F|0-12:G>C-12:G>C	TGCAGCGGCGGAGTCTCTCCG	12:G>C
988364|F|0-8:G>C-8:G>C	TGCAGGGCGCGGCCCATGGGCTCCGCGAAGGAGCCCTCCTGCGCGTAGAAGTCGTTGGAGGAGTAGCCG	8:G>C
1068761|F|0-66:A>T-66:A>T	TGCAGGCAAGCTCCCACTGATCCTGTTGTTGTTCAGGTTGATCCTGTACAGAGACTGGCATCTGGCAAT	66:A>T
4991038|F|0-29:G>T-29:G>T	TGCAGGTCGAGGAGATCGGGCAGTCGGCCGCCG	29:G>T
1022238|F|0-18:G>C-18:G>C	TGCAGATACGCTCTCCCCGTTAGCCGAGTGGTGGTCAGCTAGCGAGCTGAGGCCACTCGTACGCAGTGG	18:G>C
12419870|F|0-66:T>A-66:T>A	TGCAGCTGCTGGATGTACTCGACGGCGTCCCTGAGGATGGACACCTTATCCATCTGCCAGGAAGAATGG	66:T>A
5367153|F|0-13:T>C-13:T>C	TGCAGCTCCAGGATGCCCCCGCCG	13:T>C
2267244|F|0-25:C>G-25:C>G	TGCAGGACGGGCTCTTCGACCTCACCGAGGACGGCGGCGGCGGCGTGGTGATGGACACCG	25:C>G
1135493|F|0-66:C>A-66:C>A	TGCAGCTGCGCGCTTGCGACGGCATCGGAGATGGCCCCTCCTCGGCTGCCTTCTCAGGATGCCTAGCCG	66:C>A
2244900|F|0-53:C>T-53:C>T	TGCAGGTTCAGCTTCCCCATCTTATGGTCCAGCTTCGAAGCAGCTGATGAGCCCACATCAAACCGTGGC	53:C>T
1132187|F|0-19:G>A-19:G>A	TGCAGCTGGCGGAGATCAAGCACGCCCGCCTCGCCATGGTCGCCTTCCTCGGCTTCGCCGTGCAGGCCG	19:G>A
1203472|F|0-29:T>C-29:T>C	TGCAGGCACAGGTTCCACTCCCAGTGCATTGTGCCGTGGCTGGACATGCACAGCTCCTGCCCG	29:T>C
5371616|F|0-13:T>C-13:T>C	TGCAGCCGTCCGATGCGAGGCTCCG	13:T>C
996500|F|0-11:G>T-11:G>T	TGCAGGATGGAGGCGGCGGAGGAGACGAGGACGGCGAGGTGGAGCAGGAGGCGCGCGGGGGCGGAGGAT	11:G>T
1022746|F|0-35:C>T-35:C>T	TGCAGCTTGCCGTTCCTGGAGTTGATGAGGTGGTACTTCTCGTTGATTCGCCCGTTGTTCACCACGTCC	35:C>T
986325|F|0-64:G>T-64:G>T	TGCAGCTGCACCGCCGCGGCCACAACCTGCCGTGGAAGCTGAAGCAGAAGAACCCGAACCAGGTGCAGC	64:G>T
2247100|F|0-11:G>A-11:G>A	TGCAGCCCTTGGTGGTTTCGACGGTGCACAGGGTGTCGAGGGCATCGTCGCTCCG	11:G>A
1022998|F|0-61:T>C-61:T>C	TGCAGGTGCTGAGCGCGCTGGACGGCGCCAAGACGCAGTTGTACCACTTCAGGGCGATCGTTGTCGCCG	61:T>C
1667521|F|0-68:C>A-68:C>A	TGCAGCTTCTCCCCCATCATGCCCTGGCTCACGTCCAGCACCGTCACCAGGTCGATGGGCGCGCGCCGC	68:C>A
1078072|F|0-29:C>T-29:C>T	TGCAGCAGGTGAAGCTCGCCGTGGTGCACCTGTACCGACGGTACGTCTTCCGCCACTCGCCG	29:C>T
1024816|F|0-10:T>G-10:T>G	TGCAGCTTCCTCCCGCAGAACACCTCCACCTCCGCCGCGTACGTCGCCGTCACCG	10:T>G
5580519|F|0-13:C>G-13:C>G	TGCAGGTGATTCTCGAGTACGGCCGCGCCG	13:C>G
1038958|F|0-24:G>C-24:G>C	TGCAGCCCGCTCCTCTGGCTCTACGTGATCGCCATGTTCCTGCTCGTCATCGGCATGTTCTTCGTCACC	24:G>C
990006|F|0-55:C>T-55:C>T	TGCAGCCGCCGATGCGGCAGCTCTCTGCGGCGTCATCCACCAACCTCGCGCCCGACTACGCCG	55:C>T
1129587|F|0-22:C>T-22:C>T	TGCAGGAGTTCCCCAGCGAGTCCCTGGAGGTGCTGCGGGCGGCGGTGCGGAGCATGGTCGACGCTCCGA	22:C>T
1015621|F|0-30:T>G-30:T>G	TGCAGCGGCGGGATCGCGCGCTCAAGATCTTCATGAACGAGGGTCGCTCATGGCACGGCATCAACCACC	30:T>G
5361457|F|0-37:C>T-37:C>T	TGCAGGCGCACCGCGACGACAGCATCATGACGGCGATCGTGCAGCACGGCGTGGAGGGCCTCGAGGTGC	37:C>T
1022238|F|0-8:C>A-8:C>A	TGCAGATACGCTCTCCCCGTTAGTCGAGTGGTGGTCAGCTAGCGAGCTGAGGCCACTCGTACGCAGTGG	8:C>A
1017145|F|0-31:G>A-31:G>A	TGCAGCCCGACATCCAGACGAACCGCATGTTGTAGAAATGGTGCATGCCG	31:G>A
988457|F|0-31:G>C-31:G>C	TGCAGCTGCCGATGACGTCGTTGAAGCTGGGGGACTGCATCATCTCCAGCACGGTGTTGCGGCCGAGAT	31:G>C
986643|F|0-52:T>C-52:T>C	TGCAGGTACGAGGACGAATGGGCGGCGCCGTACAGCTGCTCCGCCCTCGTCGTCGCCATGCCCAGGCCG	52:T>C
7154544|F|0-38:G>A-38:G>A	TGCAGCCTGGGCGCGTGGCAGGTGGCCTCGATGGAGTAGGCGCCGTCGAAGGACGCGTCCG	38:G>A
985874|F|0-28:G>A-28:G>A	TGCAGGGGAGCAGCGGGAGGGAGCTCGTGGCGGCGCTCATGGGGAACCCCG	28:G>A
1278033|F|0-5:C>A-5:C>A	TGCAGCTCGGCGATGGTGTCGACGCGAACGGCGCGGCAGAGCTCGCGAAGCTGGATGCGGCGGCGCTCA	5:C>A
5357788|F|0-11:G>C-11:G>C	TGCAGCGACGTGGGCGGCGGGCCG	11:G>C
1766529|F|0-47:T>C-47:T>C	TGCAGAGCCTCATCGAGCTCCTGCTTGTCATCGGCAGTGATATCCTTTGCATACAACTGCCTCAGGCAA	47:T>C
23919872|F|0-47:G>C-47:G>C	TGCAGCCTGGAGATGGCCTTGGCGGCGAGCTTGTACGACTTGAGCGCGCCGGCAGCGGTGACGGGCACG	47:G>C
988774|F|0-25:C>G-25:C>G	TGCAGGTGCACTTCGGCACGCGGCTCCCGGCCGTCATGGGCGGCTCCTTCACCTACATCTACGCCGCCG	25:C>G
2245675|F|0-46:C>T-46:C>T	TGCAGCCATTGGCGATGTAAGGATTGCCCTGGTAGTGCTCGGAGCACTTGCACACGTAGCTGAGTGCGT	46:C>T

4261633|F|0-27:C>T-27:C>T	TGCAGCAGGCGGTGGTGGCGAACAATCCGCTCGCCCCG	27:C>T
7166904|F|0-44:C>G-44:C>G	TGCAGCTCCAGGAGCCCACCCTCCACCTTCTCAGCCTCGGAGGACGACAGCATGGCCGCCCGCATCGCC	44:C>G
5367344|F|0-11:T>G-11:T>G	TGCAGGCGGGCTCGGAGCGTGCGGCGCCGCTCCCG	11:T>G
3934655|F|0-5:G>C-5:G>C	TGCAGGGCTTCGTGGACGGCCTGCCG	5:G>C
1114839|F|0-44:A>G-44:A>G	TGCAGAGCGCGTGCCAGACGGCGGCCGTGCGGGCGCTGTGGGCGAGCGCGGTGCGGGACCCG	44:A>G
977117|F|0-9:G>A-9:G>A	TGCAGGTGAGCGCGCCACGCCTGCGCGCGCTCCGCGTCACCGAGTGCTTCAGGCTGGAGTCCAGCGCAG	9:G>A
7352275|F|0-5:C>G-5:C>G	TGCAGCCCGTGGCTCCAGCCG	5:C>G
987847|F|0-43:A>C-43:A>C	TGCAGGTTGGACACCAGGCCGCCGACGTACGTGTCCCGCGGCGACACCACCAGGTTGTGCGTCTCCTTG	43:A>C
987847|F|0-11:C>T-11:C>T	TGCAGGTTGGACACCAGGCCGCCGACGTACGTGTCCCGCGGCGACACCACCAGGTTGTGCGTCTCCTTG	11:C>T
1862570|F|0-41:G>C-41:G>C	TGCAGCTTCCTCGGATGGAGAATGGGGAGATCGAGGCCGCGGAGGATGGGCTGCCG	41:G>C
1005067|F|0-62:G>C-62:G>C	TGCAGGAAGTCCCAGGACCTGGTGGTGTGGAGCTGGTACACGGGGTCGGCGAACACGGAGACGACGCCG	62:G>C
996971|F|0-17:T>C-17:T>C	TGCAGAATGGCCCATCCTGTGGGTGCAAAGGCAAGGAAGCTGGCGAATATGTCACCAACTGTAAGGTGC	17:T>C
2245675|F|0-10:G>A-10:G>A	TGCAGCCATTGGCGATGTAAGGATTGCCCTGGTAGTGCTCGGAGCACTTGCACACGTAGCTGAGTGCGT	10:G>A
1019160|F|0-45:A>C-45:A>C	TGCAGGACCTTGCCGCACGAGTCGCAGGAGACGAACTCGCCCTCCATCCTGACGCGGCGGAGGCCGCCG	45:A>C
1026222|F|0-31:G>C-31:G>C	TGCAGGTGCTGGCCATCCTGCCGTCGTACGCGGGCAAGACGGTGCTGGAGCTCGGCGCCG	31:G>C
1132535|F|0-32:C>T-32:C>T	TGCAGCCTCCCCTGCGTGCAGGCCCACAAGCGCCGCACCGCCTGCTCCG	32:C>T
5368321|F|0-58:G>C-58:G>C	TGCAGCGCATCGGCGTCGGGATGGTGCTCTCGCTCGTGTCGATGGTGGTCGCGGCGCTGGTGGAGACGC	58:G>C
1070126|F|0-26:G>C-26:G>C	TGCAGATATTCAACAAGAGGTGGAAGGAGGCGGTGGCGGAGGACATGTGCTGGACGGACCCCTACCCGC	26:G>C
6045063|F|0-9:G>T-9:G>T	TGCAGGCGGGAGGCTCGGTCACGGTCAGACAGCTCGAGTCGGCCGACGACGACTCGTGCGGGGGCTCCG	9:G>T
7165493|F|0-26:G>A-26:G>A	TGCAGCTCGCCGCCCTGCGTGTCCAGGATGCACCG	26:G>A
1023335|F|0-51:C>T-51:C>T	TGCAGCTGCGTCTTGGCCATGATCTCCGTCCTCCTCAGCTCCATCTCGCCCCTCTTCACCTCCGCCTCG	51:C>T
6045706|F|0-17:G>C-17:G>C	TGCAGGCCGTGACCCTCGAGGAACACCCCGTCTGCCG	17:G>C
1001480|F|0-5:C>T-5:C>T	TGCAGCGGCCGCTTGCAAGGGAGCAGTCGGGAGGGGTTGGAAGAGCTGCCGTAGTGGGGCGGAGCAGGA	5:C>T
7167521|F|0-7:G>A-7:G>A	TGCAGATGCGTCGGCGCAGCTCCCG	7:G>A
12683534|F|0-15:T>C-15:T>C	TGCAGCCGAACACCATGCCG	15:T>C
1203637|F|0-7:C>T-7:C>T	TGCAGAGCGCCGTGGAAATTGACCTGTCGGAGAACAAGCTGACAGGAGTCATCCCCAGCGAGCTCGGCA	7:C>T
2368253|F|0-29:C>G-29:C>G	TGCAGGTGCTCCAGCACGTCGCGGCTGGCCGCGTGCATGCAGAAGTGCTCGAACGCGCGCTTGAAGTCC	29:C>G
4544238|F|0-38:G>C-38:G>C	TGCAGCGGTGGTCGTCGTCGATGTGGGGGATGAGCGGCGGCGACCG	38:G>C
1215457|F|0-43:G>T-43:G>T	TGCAGGGGCGGTGGAGGCTGGTCTACAGCAGCGCCTTCTCGTCGCGGACGCTCGGCGGCAGCCGCCCCG	43:G>T
7902426|F|0-21:C>T-21:C>T	TGCAGTAAGATTTAACTTCTTCCCGTCGACCG	21:C>T
4004143|F|0-17:C>G-17:C>G	TGCAGCGCCACCCACCTCGTCGTGTACCTCGGTATCCCGCGCTGCCG	17:C>G
4004143|F|0-27:C>T-27:C>T	TGCAGCGCCACCCACCTCGTCGTGTACCTCGGTATCCCGCGCTGCCG	27:C>T
1294997|F|0-49:G>A-49:G>A	TGCAGCTGGGGTTCCTGTGCGTGCTCCTGCCGCTCGTCTTCCACGCCGCGCCG	49:G>A
1027371|F|0-14:C>T-14:C>T	TGCAGGTGCCGCAGCGGCGGCGCGCTGGCCTTGCCGAGGTGGCCCAGCTCCG	14:C>T
1007185|F|0-28:C>G-28:C>G	TGCAGCGGTGCGTGGAGACGATCATGGGCGACGGGGAGGCCGCGTTGGGGATCCG	28:C>G
7165493|F|0-8:G>A-8:G>A	TGCAGCTCGCCGCCCTGCGTGTCCAGGATGCACCG	8:G>A
1278033|F|0-47:A>G-47:A>G	TGCAGCTCGGCGATGGTGTCGACGCGAACGGCGCGGCAGAGCTCGCGAAGCTGGATGCGGCGGCGCTCA	47:A>G
991285|F|0-32:G>T-32:G>T	TGCAGATCGCGCGCGGGATGGAGTACCTGCACGCCAAGGGAATATACCACGGCGAGCTGAACCCGTCCA	32:G>T
3028376|F|0-24:T>G-24:T>G	TGCAGATGAACTCGTACAGGTCCTTGACGGCCGAGATGAGCGCCG	24:T>G
1241869|F|0-46:C>G-46:C>G	TGCAGTCGACGTTGGAGAGGTCGCCCTCGCACTGCGCCAGCGCGTACACCTGCTGGAAGCTGGTGGCGA	46:C>G
4395722|F|0-17:C>G-17:C>G	TGCAGGAGCAGAACGCTCGTGCCG	17:C>G
4536708|F|0-11:C>G-11:C>G	TGCAGGAAGTCCCCCACGTTCACCACCAGCGCCCCCGCGACCG	11:C>G
1092386|F|0-9:G>T-9:G>T	TGCAGCGCGGCTGAAGCCACCGCCTCGGCCGATGAGGCGCCTGCTCCCCCG	9:G>T
1238727|F|0-53:G>T-53:G>T	TGCAGCAGCGCCTTGAGCTGGTCCTGCGCCAGGGCCTTGGTCGGGAACACGTAGAGCGCGCAGGCCACC	53:G>T
1087882|F|0-38:A>G-38:A>G	TGCAGCCCGATCACATGCATGGCTGGCCTGATCGTCCCAAAAGGTCTCGGCGTTACGTGTCGCCGCGCC	38:A>G
3936131|F|0-13:G>C-13:G>C	TGCAGTACCTGCGGAACCACAAGCCGCCGCTGACGCTGGCGCGGTGCAGCGGCGCGCACGTGATCGAGT	13:G>C
4538986|F|0-9:C>T-9:C>T	TGCAGCTGGCGCAGGTCGCCGATCCG	9:C>T
1018520|F|0-10:G>A-10:G>A	TGCAGGTGTCGGACGCGATGGACGTGGAGATGCGCGCGGGGCTCGCCTCCGAGGACGGGAGCAAGCTCA	10:G>A
4022593|F|0-17:G>A-17:G>A	TGCAGAAGCTCAGAGAGGTCGTCCTCAATGACGACGAAGCCTCCGCCG	17:G>A
1230090|F|0-58:C>G-58:C>G	TGCAGGGGGTGTGGGTGTGGCGGCCCTCGTAGGTGGTGATCACCATGCGGCAGTCCTCCGACAGCCGCT	58:C>G
6044975|F|0-68:C>G-68:C>G	TGCAGCTGCTGCGCGCAGGCCTCGCAGTGCATGTTGACGAGCAGCTCCACGGTGCGGGCCTCGCTGACC	68:C>G
1016997|F|0-68:T>C-68:T>C	TGCAGGCCGCAGATCCCCATCATCTGCTGCTCCGTCAGCGGATCCAGCTGCCCGATCAGGATCTGCATT	68:T>C
1696921|F|0-18:C>A-18:C>A	TGCAGAAGCTGTCGAGGGCGGGGTGGGAGCGGAGGTCCTTGTCCATGGCCTTGTAGGCGCGCGCGAAGG	18:C>A
1060520|F|0-34:C>T-34:C>T	TGCAGGCCGTGGTGGAGCTCAAGGACTTCTTGGACCACACCGCCATGCTCGCCATGCCCAGCCAGAGGA	34:C>T
2256681|F|0-47:C>T-47:C>T	TGCAGGATCCTGGGAGACGTCTTCATGGGCCCCTACCACACCGTCTTCGACTACGGCAAGCTGCGGGTC	47:C>T
2373210|F|0-7:C>T-7:C>T	TGCAGGCCGTGGCGCGCACCGTCATGGGAGCCAACCTTCAGAAGCCACAGAGGGTGAGGATGGGCCCGT	7:C>T
3028790|F|0-33:C>A-33:C>A	TGCAGTGTACCTGAACGGGAACAAGTTTGCTGGCGCGGTGCCG	33:C>A
1023927|F|0-21:G>A-21:G>A	TGCAGGGTGTAGGTGCCCTCCGCCTCCACCAGCAGCAGCTTGTGGCCCTGGAGCCG	21:G>A
7350802|F|0-5:G>T-5:G>T	TGCAGGCAGTGCTCAACCTCCCG	5:G>T

4262164|F|0-23:G>A-23:G>A	TGCAGCCCGCGGTCCAGGTTAAGGTGGAGGAGTCGGCGCCG	23:G>A
1164636|F|0-11:C>G-11:C>G	TGCAGCACGGTCGTCTCCTTGGGCAGCGCCGTCGGCCCCACCATCACCTTGGGCCCCAGGAGCACGTCC	11:C>G
992102|F|0-28:G>C-28:G>C	TGCAGGTGCTTCACGCGCTGGACGTGGCGAGGACGCAGAGGTACCACGCGTGGGCGGTGGTGATCGCCG	28:G>C
1217621|F|0-6:G>A-6:G>A	TGCAGAGTTGCGCCTGAGCCGAAGCCGTCCCCGTTGATGAGCAGGAAGCTCGTCTGCCTAACCATAAAC	6:G>A
1128587|F|0-35:G>C-35:G>C	TGCAGCCACTTCTTGTAGTAGACGCAAGTGGTCGGGGAGATGACCCCATCCG	35:G>C
1078072|F|0-46:C>G-46:C>G	TGCAGCAGGTGAAGCTCGCCGTGGTGCACCTGTACCGACGGTACGTCTTCCGCCACTCGCCG	46:C>G
1029980|F|0-67:C>A-67:C>A	TGCAGCTGGTTTATCTCGGCACCCGCATCATAAACAAGAACTGGATCAATCCCTTCGGGAGGCACTGCT	67:C>A
3934655|F|0-22:G>C-22:G>C	TGCAGGGCTTCGTGGACGGCCTGCCG	22:G>C
5582062|F|0-11:C>G-11:C>G	TGCAGGTAGGCCAGCCGCGGCACGTCGTCGGCGGTGACCAGCCG	11:C>G
7170898|F|0-13:C>G-13:C>G	TGCAGTACATCGTCAGGGGCGGGCGGAACCTCTTCCCGCTGCTCCCG	13:C>G
7165493|F|0-11:G>A-11:G>A	TGCAGCTCGCCGCCCTGCGTGTCCAGGATGCACCG	11:G>A
15306324|F|0-7:T>C-7:T>C	TGCAGCTTGTCTCCGACGCCGAGATCCCG	7:T>C
1026048|F|0-16:G>A-16:G>A	TGCAGCGGCATTGCTCGTCGCCACGTCGCTGCTGGTCGCCACCCTCCTCACCTCGCCGCTGCCGTTGCT	16:G>A
3028694|F|0-10:T>A-10:T>A	TGCAGCGACGTGCTGGGATCCAGGCCGTAGGAAAAGCGCCCCG	10:T>A
5357321|F|0-25:G>A-25:G>A	TGCAGAGACCGCTACGCGCCACCTCGGCGGCGCCG	25:G>A
4535744|F|0-10:C>A-10:C>A	TGCAGCGGCGCGCACGGGACGCCG	10:C>A
3027770|F|0-28:G>T-28:G>T	TGCAGCGCCACGTACGCCTTCTCCAGCCGCGAGTTGTTCGTCGCCG	28:G>T
7170519|F|0-13:G>T-13:G>T	TGCAGGAAACTTGGATCGCGACCG	13:G>T
3945659|F|0-25:C>T-25:C>T	TGCAGAACATCCAGAAGGCCCTTGACGAGGCCG	25:C>T
1026052|F|0-20:A>G-20:A>G	TGCAGCGCGAAGTTGTGCGGAAGAGAAGGCACCTGCATGGAGAAGCCCTGCATCTGCGCCGCCGCCGAG	20:A>G
7900077|F|0-50:C>G-50:C>G	TGCAGATCGCGCAGCATCTGCATGAAGTCGGGCTGGTCGAGGTAGGCGCGCGTGGTGGGGTCGGCGGCG	50:C>G
1021420|F|0-22:G>A-22:G>A	TGCAGCTGGTCTATCTGGCGGCGGCCACGGTGTTCTACTACGAAGCCATGGAGAGCAAGGAGGTGGTGC	22:G>A
6040063|F|0-14:G>C-14:G>C	TGCAGCAAACCTGCGTCCATTCTGTACGCGTAGCTCGAAACGGTCATGTCCTCTGCCGCGGCGGGTGAA	14:G>C
1017145|F|0-46:G>A-46:G>A	TGCAGCCCGACATCCAGACGAACCGCATGTTGTAGAAATGGTGCATGCCG	46:G>A
3026761|F|0-23:G>C-23:G>C	TGCAGCGCCTCCATCTGCATGGCGGCCTTCTCCTCCTGCAGCCG	23:G>C
1009565|F|0-14:C>G-14:C>G	TGCAGGCCCGCCTTCACCGACCGCGACACCGACTTGCTCCCCTTCGGCTTGCCCCTCCCGCCGCCGAGA	14:C>G
1002433|F|0-31:G>A-31:G>A	TGCAGGAGACGATGTGGCGGAGGAGGTGGCGGCCGACGCCGCCGCAGCCGAGGAGCACCACCG	31:G>A
1001498|F|0-10:C>G-10:C>G	TGCAGCGCGCCGCCGCGCTCTACAACCCGCAGCTGGTGCAGATGACCAAGGACCTCAACGCTCAGTTCG	10:C>G
1203916|F|0-40:G>A-40:G>A	TGCAGAGGACTTCTCATGCCGACGCATGCTACTCTTCACAGCCGTTGCTGGTGCTAGTTCAGAATCCTA	40:G>A
987847|F|0-8:G>T-8:G>T	TGCAGGTTGGACACCAGGCCGCCGACGTACGTGTCCCGCGGCGACACCACCAGGTTGTGCGTCTCCTTG	8:G>T
5370780|F|0-11:C>T-11:C>T	TGCAGCTTGGACGACATCTCCG	11:C>T
3574957|F|0-23:C>A-23:C>A	TGCAGGACGTGCATGGCCTGCGCCATGGATGGCCGCTCGCTCCG	23:C>A
985932|F|0-66:T>A-66:T>A	TGCAGCTTGTGCAAGAATATTGTACCGAGAAGTGGTTGTTGCAGGGGTGCTAGATGCTAAGCCGTCTGG	66:T>A
3024148|F|0-9:C>T-9:C>T	TGCAGCAATCCTTGAGTACCTTACTGCTGAGGTCCTTGAGCTGGCCG	9:C>T
1696921|F|0-16:G>C-16:G>C	TGCAGAAGCTGTCGAGGGCGGGGTGGGAGCGGAGGTCCTTGTCCATGGCCTTGTAGGCGCGCGCGAAGG	16:G>C
3942162|F|0-49:A>G-49:A>G	TGCAGCTGACGTGGAACTACAACTACGGGGCGGCGGGGAAGAGCGTGGGATTCGACGGGCTGAACAACC	49:A>G
2248164|F|0-14:C>G-14:C>G	TGCAGCATGGGGATCTTGACGTCCAGCAGCTCGGATATGGTCTCCTCGATGGCCGACTTCTCCATGAGC	14:C>G
1050629|F|0-22:C>T-22:C>T	TGCAGAACTCCAACATGCTCAACAACAAGAGCGAGACGCACCCGTGGCATCTCCATGGTCACGACTTCT	22:C>T
7169690|F|0-16:G>A-16:G>A	TGCAGGCGCCGCCCTCGTGGGTGCCCG	16:G>A
3954402|F|0-7:A>G-7:A>G	TGCAGTCATGGTACACGGAGGCCGTGCAGGTGGGGCACCCG	7:A>G
3943426|F|0-31:G>C-31:G>C	TGCAGCTGCGGCTCAACGACGGGCTGGTCACGTCGTACCG	31:G>C
993977|F|0-36:C>A-36:C>A	TGCAGTTCGGACGGCGCCTAGCATGACGGAGGCGGCCGTGGGGAGGATCGCGCAGGGGACCAAGGTGCT	36:C>A
4544261|F|0-11:C>T-11:C>T	TGCAGGGCGTACGCGGCGGCGGACGTCAGCGGGGGCATCAACCGCGGGTGCGTCGTCTGGGCCGTGGAT	11:C>T
2253524|F|0-62:A>C-62:A>C	TGCAGCAGCCACTCGATGGTCTCGCCGTCGGACTTGTGGCCGAGCTCCCGCGTGAGCTGGAAAACCCGC	62:A>C
2244267|F|0-7:C>G-7:C>G	TGCAGCTCCTCCACCTCGTCGAGCGTTTCCCGTCCGAGCCGTGCGCCGAAGCACATGTACACGAGCAGC	7:C>G
4261691|F|0-7:A>G-7:A>G	TGCAGGCAGTGCTCAAGGAGACCTTGCGGCTGCGGCCGCCG	7:A>G
1183371|F|0-50:C>G-50:C>G	TGCAGCCTGCCGCCCCTCTCGCCGCTGTCGCCGTACCCGCACTTCGCCTTCCCGCAGCTCTCGGTCGTC	50:C>G
985874|F|0-7:G>C-7:G>C	TGCAGGGGAGCAGCGGGAGGGAGCTCGTGGCGGCGCTCATGGGGAACCCCG	7:G>C
3026761|F|0-38:C>G-38:C>G	TGCAGCGCCTCCATCTGCATGGCGGCCTTCTCTTCCTGCAGCCG	38:C>G
5358629|F|0-5:T>C-5:T>C	TGCAGTGGGTACACGATCCCG	5:T>C
2243334|F|0-68:C>G-68:C>G	TGCAGCTGGTGGCACAGCGTGTTGGTCTCCAGGAAGAACTCGAGGAACACCTTGCTCTCGGAGTCGCCC	68:C>G
4396300|F|0-20:G>A-20:G>A	TGCAGGTTCTTCTCCAGCTCGTCGATCACCGCGCGCCCGCCG	20:G>A
999236|F|0-43:C>A-43:C>A	TGCAGGGCCCGCGCCCCCCGCGGCTGTCCGTCAGCAAGGACTCCCACAAGGTCAGGAAGCCGCCCGTCG	43:C>A
1147720|F|0-34:C>T-34:C>T	TGCAGGGATGCGACGCGTCGGTGCTGATCACGAGCGCGGGCAACGCCGCGGAGGTGAACAACAACAAGC	34:C>T
2253117|F|0-43:C>G-43:C>G	TGCAGTCCTCGTCGAAGGGCACCTCCTTGTGGAAGATGCAGCACTTTTTGGAGCTCTTGCGGCCGAGGG	43:C>G
1001496|F|0-13:T>C-13:T>C	TGCAGCGCGCGCGTGCTGACGGGTCGCTCGCGGCGCGGGGTGTTTCTGCTGCCGCCGCGGCCCAGAAGA	13:T>C
2248204|F|0-62:C>G-62:C>G	TGCAGCGGATACCTGGCGCCTGAATACGTGGTGCACGGGCAGCTGACCAAGAAGGCCGACGTCTACAGC	62:C>G
2245675|F|0-13:G>A-13:G>A	TGCAGCCATTGGCGATGTAAGGATTGCCCTGGTAGTGCTCGGAGCACTTGCACACGTAGCTGAGTGCGT	13:G>A
5357837|F|0-8:G>C-8:G>C	TGCAGCGCGAAGGAGGACAGGGACGAGCGGCAGGCCG	8:G>C

5324496|F|0-9:G>T-9:G>T	TGCAGTGACGTTTGAGTTCGACGGCGAGAGTTCCG	9:G>T
1072859|F|0-27:G>C-27:G>C	TGCAGGCATGCCTAGGTGCACCTCGTCGGTCAACTACCAAACGGAGACAGCGACCATGTCGGAGCTCAT	27:G>C
4404463|F|0-8:T>C-8:T>C	TGCAGCCCTGCAATCTGGGCGAGCTCCG	8:T>C
3942133|F|0-14:C>A-14:C>A	TGCAGCTCATGCGGCGGTACCCCG	14:C>A
7173613|F|0-13:T>C-13:T>C	TGCAGCTGCATGGTAGCGTCGCCTCCCTGCGAGATGGGCGCCG	13:T>C
1063793|F|0-11:C>G-11:C>G	TGCAGCAGCACCCCGACCTCCGTCAGCGACAGCACGTCCTCCAGTATGACCTTGGCGAATATCCGAGAT	11:C>G
3940456|F|0-25:C>A-25:C>A	TGCAGCTGGTGATGTTGCTGGGCCGCGTGCTCGGCTGCCG	25:C>A
1002222|F|0-34:C>T-34:C>T	TGCAGGTGCGGAAGGCGGCGCCGCCTAAGCGGACCACGAGCAAGGACCG	34:C>T
987258|F|0-36:T>C-36:T>C	TGCAGAGCGGGCAAGTGCTGCTGGTGCGCAGCCACGTCCTGAGGCAGCGCCTGTGGAACATGTGGCCGC	36:T>C
1018520|F|0-32:C>T-32:C>T	TGCAGGTGTCGGACGCGATGGACGTTGAGATGCGCGCGGGGCTCGCCTCCGAGGACGGGAGCAAGCTCA	32:C>T
1059990|F|0-6:G>C-6:G>C	TGCAGCGCTTGCCTAAACATCCCAGAGCCGCGGGGCACGCCTAGAGCTTCGGCGCATGCCACCATCTCC	6:G>C
1055548|F|0-59:G>C-59:G>C	TGCAGGAGCTTGATGATGTTGCTGGCGCCGAAGACGCGGTGCGCGGTGGCGAACTTGTGGGGGTCCGTG	59:G>C
1094957|F|0-65:C>A-65:C>A	TGCAGGCGGTGAGGCTGCGGTGGTTCTGCGAGCAGAGCGGCGTGGCGCTCTTCACGGCGAGGAACCGAG	65:C>A
3953982|F|0-44:A>G-44:A>G	TGCAGAGCACGGACGACGCAGAGGTCCACATGTCGTCGGCCTCGACGGCGACGACGTCAGGGCGCGGCT	44:A>G
1115062|F|0-55:A>G-55:A>G	TGCAGAGCATCGGGAGGAACGACTGCGTCTGCACGAACGTCAGGCCGAGGCCGATAGACACGTTCATGG	55:A>G
1111422|F|0-50:A>G-50:A>G	TGCAGATTGAGGAGCACGGCGATCATGCTCTTCTTGTCGCCTTCGGTGCCAGCGTCGTCGAGCCTCCGA	50:A>G
6040114|F|0-64:T>G-64:T>G	TGCAGCAGCAGAAAGGCGGCAGTTGCAGCCAGGAGAGCGACTGAATGAATCTTGTAAACACTCATTGGT	64:T>G
2257103|F|0-13:C>T-13:C>T	TGCAGGAAGGTGGCCGGCCAGCCGCAGCCCGTGCCCATCATCGCCGAGGTCGATCTCTACAAGTTCAAC	13:C>T
6044731|F|0-57:A>C-57:A>C	TGCAGAGCAGCACGTAGTCGGCGGCCGCCGAACTCGCCGCGCCCGCGTCCTTGGCTGAACGCCCTCGCT	57:A>C
985642|F|0-17:C>G-17:C>G	TGCAGCCAGGAGGAGGACGAGGCGGGGTTCAACCTGCTGCCGCTGGTGATGGCCGCCTTCGTCATCATG	17:C>G
1090093|F|0-13:G>A-13:G>A	TGCAGGGGTACGGGCTGACGGAGAGCACGGCCATCGGCGCGTCCACGGACTCGGCCGAGGAGAGCCGCC	13:G>A
1094202|F|0-7:G>A-7:G>A	TGCAGCGGGAGCGAGGCGCGGAGGTAGGCTCGCATGACGGTGCTGACGACGAACGCGTCCGCGTCGGCC	7:G>A
994119|F|0-32:G>A-32:G>A	TGCAGGTGCACCCCAGCTCGTACGCGCTGGTGGTCAGCATGGAGAACATCACCCTCAACTGGTACTTCG	32:G>A
4398159|F|0-13:G>T-13:G>T	TGCAGGCGCCCGCGCTGCTGCCG	13:G>T
1102684|F|0-23:T>C-23:T>C	TGCAGCAGGTCGTCGCCCTTGGGTTCAACCTTCTGGCGCATCTCCACCACCTTCTTGTGGGAGTTGGAA	23:T>C
2258676|F|0-22:C>A-22:C>A	TGCAGGCACCACAGGTCGCGCACGCCCACGATCGCCAGCTCCTCGAGGTCGCCGACGCAGAGGTCCAGC	22:C>A
1863122|F|0-46:G>C-46:G>C	TGCAGTTCCACGGCTTCCACGGCGTGAAGCAGGAGGAGAAGAAGCTGGGCCAGAAGTTCGTGGTCGACG	46:G>C
5361457|F|0-32:G>A-32:G>A	TGCAGGCGCACCGCGACGACAGCATCATGACGGCGATCGTGCAGCACGGCGTGGAGGGCCTCGAGGTGC	32:G>A
1125324|F|0-16:C>T-16:C>T	TGCAGGGCCGCGTGAACGTGACCACCGCGCACTCGCTGGACATCATGGGCGTCTCCTACCCGCCCTCCG	16:C>T
5367344|F|0-8:G>A-8:G>A	TGCAGGCGGGCTCGGAGCGTGCGGCGCCGCTCCCG	8:G>A
980685|F|0-59:G>A-59:G>A	TGCAGCCGCTTGTCGTCCGTGGTCGCCGTCTTGTGCACCGCCTTCTTCTTCCTGCACCAGACCCCAGCC	59:G>A
3026761|F|0-27:T>C-27:T>C	TGCAGCGCCTCCATCTGCATGGCGGCCTTCTCCTCCTGCAGCCG	27:T>C
990006|F|0-22:C>T-22:C>T	TGCAGCCGCCGATGCGGCAGCTCTCTGCGGCGTCATCCACCAACCTCGCGCCCGACTACGCCG	22:C>T
3028790|F|0-38:T>C-38:T>C	TGCAGTGTACCTGAACGGGAACAAGTTTGCTGGCGCGGTGCCG	38:T>C
3937769|F|0-16:G>T-16:G>T	TGCAGGCCTTCAGGGAGACCCACTTCCCGGCCCTCGTGACGCGCCTCCG	16:G>T
991014|F|0-5:G>C-5:G>C	TGCAGGCCCCCCTGCGCGACGCGGAAGTTGGCGTCCCGCGTCAGATCCACGCAGGTGTCCACCAGCGAG	5:G>C
1051566|F|0-50:G>C-50:G>C	TGCAGGGAGATGTACAAGTGCATCCAGGACGAGCTGCTGCCGCTGCTCCTGGAGCCCATGACCATCGCC	50:G>C
7171152|F|0-9:G>T-9:G>T	TGCAGCGGGGGAGTGGTGGGAGGTAGCGAAGGCGACCATCGCGGCGTACACAGGACTGCCGCCG	9:G>T
1026902|F|0-12:T>C-12:T>C	TGCAGCATGGCGTTGCTCCACGGGAAGCCGCCGCTGGCGGAGTATGGCCCG	12:T>C
3938322|F|0-11:A>T-11:A>T	TGCAGCGCCAAACCAAAGCAAGCCGTCGTCAACCG	11:A>T
4993540|F|0-17:C>A-17:C>A	TGCAGACGGCGGCGGCGCGGACGAGGCTCCTCGGGTCGTCCGCCG	17:C>A
5363541|F|0-7:G>C-7:G>C	TGCAGCGGCCCCTCGGCGGCGGTGCCGAGGCGGTGGCCG	7:G>C
1862739|F|0-41:C>G-41:C>G	TGCAGCTCCGCCTCGTCCGTCTTGTCCGAGTCGATGCTGAGCAGCACGTTCTTGCGCCGCACGAACCTG	41:C>G
2242218|F|0-65:C>G-65:C>G	TGCAGTGGAAGCATCCATATGTCATGAAAGTACCATGCATAGTCCCCCACCCGCTGCACATCAACCGCC	65:C>G
3952851|F|0-17:C>T-17:C>T	TGCAGGAGCCGCAGGAACGCGGTGGCGCGCCGCCG	17:C>T
1027722|F|0-32:T>C-32:T>C	TGCAGCGCGTACTGGACGCCGTCCACGTCGCCTGCCGCCGCGAGCCTCCTGACGGTGGCCTCATGGGCC	32:T>C
2252070|F|0-45:A>G-45:A>G	TGCAGCTCGTCCTGCACCCCCGCGAACAGACAAACCAGATCAGGCAACCCTGGACCGACGGGACGGGAT	45:A>G
1091041|F|0-41:C>G-41:C>G	TGCAGCAGGGCGGTGACGAAGGTGAAGAAGAAGCAGGTGACCAGGGCTTCCACGGCCG	41:C>G
5357524|F|0-19:T>G-19:T>G	TGCAGCACGTTGGCCAGGGTCGGCCG	19:T>G
1001059|F|0-10:C>T-10:C>T	TGCAGTATCACGGGGGAGGCCAGGTGGAAGACGCCGTGGACGCCCTCGACCGCCG	10:C>T
1024793|F|0-26:C>G-26:C>G	TGCAGGCACAGCTCGCCGTCGCCGTTCGCCGCCATGGCCGAGAACATCTGCCG	26:C>G
987045|F|0-7:G>C-7:G>C	TGCAGGCGGAGGCCGAGGTCGGGGAGGACGGCAGGATGCGGGTCACCGTGCGCAAGTCCACCAGCTCGC	7:G>C
1064534|F|0-44:C>T-44:C>T	TGCAGCATGATCTTGGCCGTGCCCTTGTCTTTGAAGGTGATGGCCCGCTGCGCCGCGCCGATGGTGTCC	44:C>T
1078072|F|0-25:G>A-25:G>A	TGCAGCAGGTGAAGCTCGCCGTGGTGCACCTGTACCGACGGTACGTCTTCCGCCACTCGCCG	25:G>A
1243336|F|0-10:G>C-10:G>C	TGCAGCGCGCGTCCGAGGCCTACAACCCGCAGATGGAGGCCATGCTGAACGAGCTCAACGCCG	10:G>C
993195|F|0-61:C>T-61:C>T	TGCAGAATGTGCACGGCCTCCGCCGCCGCGAGTTCAGGGCCACGCTCGCGCACCTGCACTCCATGGCCA	61:C>T
987045|F|0-31:C>A-31:C>A	TGCAGGCGGAGGCCGAGGTCGGGGAGGACGGCAGGATGCGGGTCACCGTGCGCAAGTCCACCAGCTCGC	31:C>A
1092909|F|0-8:G>A-8:G>A	TGCAGCGTGTCGGCGGCGGCGAAGCGCAGGTCCATGGCGTACAGGTTGCTGCCG	8:G>A
3938912|F|0-19:G>A-19:G>A	TGCAGAACCTGACGCTGGCGTGATGCGAGGCGTGGGTTCCACTCCG	19:G>A

<table>
<tr><td>6049430|F|0-31:G>C-31:G>C</td><td>TGCAGCCGTACTTCGGCGGCGAGGAGCGCACGGAGGCGGAGGTGAGGCTGGAGGGCGTGGCGCCGAGAT</td><td>31:G>C</td></tr>
<tr><td>7154544|F|0-41:G>A-41:G>A</td><td>TGCAGCCTGGGCGCGTGGCAGGTGGCCTCGATGGAGTAGGCGCCGTCGAAGGACGCGTCCG</td><td>41:G>A</td></tr>
<tr><td>1214625|F|0-5:C>T-5:C>T</td><td>TGCAGCCGCAGGATCATGGCCAGCGCCTCGTCGGCCCCCGACGACGCCGCCTGCCTCTCCTCCTCCAGC</td><td>5:C>T</td></tr>
<tr><td>1215639|F|0-47:G>A-47:G>A</td><td>TGCAGCTCCTTGGTGGAGATCATGCGGACGTCGGCGGCGTCCTTGCTGGCGGCCGGCATTGCGTCGGCT</td><td>47:G>A</td></tr>
<tr><td>999236|F|0-16:C>T-16:C>T</td><td>TGCAGGGCCCGCGCCCCCCGCGGCTGTCCGTCAGCAAGGACTCCCACAAGGTCAGGAAGCCGCCCGTCG</td><td>16:C>T</td></tr>
<tr><td>1069006|F|0-25:C>T-25:C>T</td><td>TGCAGCTCTGCTGCATCTCCCTCGGCCTTCTCCTGATGGCCGCCGTCTGCCCGCACGTCCCGCTGCTCA</td><td>25:C>T</td></tr>
<tr><td>7169155|F|0-16:T>C-16:T>C</td><td>TGCAGAACGGCGGCGGTGGCAACCTGTCCG</td><td>16:T>C</td></tr>
<tr><td>1203916|F|0-6:G>A-6:G>A</td><td>TGCAGAGGACTTCTCATGCCGACGCATGCTACTCTTCACAGCCGTTGCTGGTGCTAGTTCAGAATCCTA</td><td>6:G>A</td></tr>
<tr><td>5578361|F|0-14:G>C-14:G>C</td><td>TGCAGGTACTTTTGGTGGCTGGACTGCGCTCCG</td><td>14:G>C</td></tr>
<tr><td>1107104|F|0-5:G>A-5:G>A</td><td>TGCAGGTGGCCGCCCTTCTTGCCGTGGAGCACCTCGCCGAGGCTGCCGTTGGGCATGTACTCGTACACC</td><td>5:G>A</td></tr>
<tr><td>993977|F|0-12:G>A-12:G>A</td><td>TGCAGTTCGGACGGCGCCTAGCATGACGGAGGCGGCCGTGGGGAGGATCGCGCAGGGGACCAAGGTGCT</td><td>12:G>A</td></tr>
<tr><td>3956442|F|0-17:G>C-17:G>C</td><td>TGCAGGCCCAGCGCGCCGAGCGCCTGGTACACGGACTTCATCGCCG</td><td>17:G>C</td></tr>
<tr><td>5582347|F|0-17:C>T-17:C>T</td><td>TGCAGCGCCAGCGTCTTCGGGGTGTCGCCG</td><td>17:C>T</td></tr>
<tr><td>7918764|F|0-23:C>G-23:C>G</td><td>TGCAGGCAGAGGCGGCCGCCGCTCACGGTGCGCGGGCGAGCGGCGAGGAAGCAAGGCCG</td><td>23:C>G</td></tr>
<tr><td>3936493|F|0-20:C>T-20:C>T</td><td>TGCAGCCACAGGCTTCTTGGCTTTGCGCTTAGGCGCTGCCTGGCCG</td><td>20:C>T</td></tr>
<tr><td>1318213|F|0-46:C>G-46:C>G</td><td>TGCAGATGCGGCAGGCGCTCTTCTACGAGGACATGCAGAGCGTGCTCGCGCGCGTGCACGCCG</td><td>46:C>G</td></tr>
<tr><td>1129014|F|0-55:C>A-55:C>A</td><td>TGCAGGTCGGCGGTCGCCGAGTCCCGCACCTCGCGCCAGCCCTTGGAGATGAAGGCGAACGCGCCGTCG</td><td>55:C>A</td></tr>
<tr><td>1028838|F|0-6:A>G-6:A>G</td><td>TGCAGCACCTTCACGGCAACCATGGTGCCGTCCCGCAGGGTGCCACGGTACACGCGGCCGTAGCTGCCC</td><td>6:A>G</td></tr>
<tr><td>1213506|F|0-16:T>G-16:T>G</td><td>TGCAGCGCGGGTCGGCTCAGGCGGAGGCGGCGCAGCCGCGGCGGAGCAGCTTCAGCGTGGTCCG</td><td>16:T>G</td></tr>
<tr><td>7923298|F|0-21:G>A-21:G>A</td><td>TGCAGCGGTGCCCTTGTCGCCGCCG</td><td>21:G>A</td></tr>
<tr><td>1072859|F|0-45:G>A-45:G>A</td><td>TGCAGGCATGCCTAGGTGCACCTCGTCGGTCAACTACCAAACGGAGACAGCGACCATGTCGGAGCTCAT</td><td>45:G>A</td></tr>
<tr><td>1007185|F|0-45:T>C-45:T>C</td><td>TGCAGCGGTGCGTGGAGACGATCATGGGCGACGGGGAGGCCGCGTTGGGGATCCG</td><td>45:T>C</td></tr>
<tr><td>1863335|F|0-17:C>T-17:C>T</td><td>TGCAGCTCGCTGGTCACCACATACCTGCCGCCACTCCTCTCCCTCGACCACACTGTGGTGGAGGGGGAC</td><td>17:C>T</td></tr>
<tr><td>1183371|F|0-53:G>T-53:G>T</td><td>TGCAGCCTGCCGCCCCTCTCGCCGCTGTCGCCGTACCCGCACTTCGCCTTCCCGCAGCTCTCGGTCGTC</td><td>53:G>T</td></tr>
<tr><td>1001498|F|0-63:A>G-63:A>G</td><td>TGCAGCGCGCCGCCGCGCTCTACAACCCGCAGCTGGTGCAGATGACCAAGGACCTCAACGCTCAGTTCG</td><td>63:A>G</td></tr>
<tr><td>982259|F|0-38:T>C-38:T>C</td><td>TGCAGCCTAGAGCACAGCGCGGCGCGCAATAATTTAGATGGAGGCGTCGTGCAAGTGCCCGATCTCTCG</td><td>38:T>C</td></tr>
<tr><td>1217398|F|0-63:C>T-63:C>T</td><td>TGCAGGAGCTGCTGTTTGCCCGTACCATCCTCGCAGACAAGTTCGGCGGCGACTTTGCTGTCGCGGCCA</td><td>63:C>T</td></tr>
<tr><td>1695356|F|0-47:C>G-47:C>G</td><td>TGCAGGCTCCAGATATGGAAGCTCGCCACCCGCCTCTGGAACGCCGTCGTCGACCGCGCCAACTCCGCC</td><td>47:C>G</td></tr>
<tr><td>990006|F|0-25:T>C-25:T>C</td><td>TGCAGCCGCCGATGCGGCAGCTCTCTGCGGCGTCATCCACCAACCTCGCGCCCGACTACGCCG</td><td>25:T>C</td></tr>
<tr><td>1001498|F|0-7:C>G-7:C>G</td><td>TGCAGCGCGCCGCCGCGCTCTACAACCCGCAGCTGGTGCAGATGACCAAGGACCTCAACGCGCAGTTCG</td><td>7:C>G</td></tr>
<tr><td>5005239|F|0-11:C>T-11:C>T</td><td>TGCAGCTCCCGCTCCGACGCGGACGGCGAGGCCG</td><td>11:C>T</td></tr>
<tr><td>5582090|F|0-11:A>G-11:A>G</td><td>TGCAGTTGGCCAAGCTCGGCGATCTCCG</td><td>11:A>G</td></tr>
<tr><td>2260353|F|0-20:G>A-20:G>A</td><td>TGCAGCGATGAGCGGGGGTCGACGGAGAGGGTGAAGCTGAGCCGCGGGCAGGTCGTGTTCCAGGCGTCC</td><td>20:G>A</td></tr>
<tr><td>996571|F|0-55:C>G-55:C>G</td><td>TGCAGCTGGTGCCGACGCAGAAGATCGGCGCCATCCCGCAGCCGCTGGAGGAGCTCCTCCTCCACTCCT</td><td>55:C>G</td></tr>
<tr><td>1070886|F|0-47:A>G-47:A>G</td><td>TGCAGCTGTGCAAATATGTCATCCAATTTGCCGACGAAGCCGTTGCCACGGAGATCCAAGTACTTCAGT</td><td>47:A>G</td></tr>
<tr><td>1027297|F|0-23:T>G-23:T>G</td><td>TGCAGTTTTTGCTGCCCCAGTCGTGTCCGACCTGGCACTTCCCTTTCCACCTTGATGGCACCG</td><td>23:T>G</td></tr>
<tr><td>3030480|F|0-23:G>A-23:G>A</td><td>TGCAGCTCGCCTCGGTCCACCGCGCCCTCGCCGTCCGCCCG</td><td>23:G>A</td></tr>
<tr><td>5577028|F|0-42:G>C-42:G>C</td><td>TGCAGCGGCGACGGCATGGGCGACCTCTGGCCCGTCGGCTTCGGCGGCGGCGTCGCCATGGGCTCCTCC</td><td>42:G>C</td></tr>
<tr><td>981702|F|0-14:G>A-14:G>A</td><td>TGCAGTAATTTAGTGTGTTGTTTTTACCTATAGTGCCACCAAGTTCTATGACACAAACATCAGCTGGCT</td><td>14:G>A</td></tr>
<tr><td>1072263|F|0-11:C>G-11:C>G</td><td>TGCAGATTGACCAGGCAGAGCGCGCGCTCCAGGCTCTCGCGGCAGACACCG</td><td>11:C>G</td></tr>
<tr><td>6043876|F|0-16:G>T-16:G>T</td><td>TGCAGCGACGCCTTCCGCGCGATCGGCATGTCCACGAACGCCG</td><td>16:G>T</td></tr>
<tr><td>1072859|F|0-20:C>A-20:C>A</td><td>TGCAGGCATGCCTAGGTGCACCTCGTCGGTCAACTACCAAACGGAGACAGCGACCATGTCGGAGCTCAT</td><td>20:C>A</td></tr>
<tr><td>995197|F|0-29:G>A-29:G>A</td><td>TGCAGCAGCAGAGAGAAGGCCTCCTCCATGTTGAGCTTGTCGGGCGTCTGGCCCGCCG</td><td>29:G>A</td></tr>
<tr><td>3934979|F|0-8:C>G-8:C>G</td><td>TGCAGGTCCACGTTGTCTATCCCGACGCCG</td><td>8:C>G</td></tr>
<tr><td>5581798|F|0-8:C>T-8:C>T</td><td>TGCAGCTCCCCGCACTGCGGCGCCGAGCACGGCACGGCGGCGTACGTGCTGGACGCGCGCGGGTTGTAG</td><td>8:C>T</td></tr>
<tr><td>5567238|F|0-22:C>T-22:C>T</td><td>TGCAGCGGCTCCCGCCGAGCAGCCGCCGCAGGGCGCGCCTCGTCTGCCG</td><td>22:C>T</td></tr>
<tr><td>4009326|F|0-21:C>T-21:C>T</td><td>TGCAGGACTTGATGGGGTATACGAGGATGGACGCCACCGTCGCCGCCG</td><td>21:C>T</td></tr>
<tr><td>4538937|F|0-12:T>C-12:T>C</td><td>TGCAGAAGCCTGTGTGTAGCTGTGCCG</td><td>12:T>C</td></tr>
<tr><td>1027289|F|0-16:G>A-16:G>A</td><td>TGCAGAAGCCGAAGACGAAGCAGTAGATGATGGAGCAGATGCCAGCGAAGACACCGACCGCGCCGACGA</td><td>16:G>A</td></tr>
<tr><td>977460|F|0-34:G>A-34:G>A</td><td>TGCAGGCGCCGCACTTGGGCTCGTGGACCGAGTGGTACGACACCTTCATGTGCTCCACAAGGTGCTCCA</td><td>34:G>A</td></tr>
<tr><td>996709|F|0-10:G>A-10:G>A</td><td>TGCAGCCCGCGAACCGCGCGATGCGCGCCATGTACGCGCGCACGGAGATGCCG</td><td>10:G>A</td></tr>
<tr><td>1122632|F|0-7:C>G-7:C>G</td><td>TGCAGAACAGCGACAGGTACGCCAGCGAGATGTCCTCCCACAGGTCCCACACCAGCCCGCCCGACCACT</td><td>7:C>G</td></tr>
<tr><td>988439|F|0-44:T>C-44:T>C</td><td>TGCAGCTTGAGGACGGTGGTCTTCTGCGTGTCGGTGAGCACGCTTCCGCCCCCG</td><td>44:T>C</td></tr>
<tr><td>1020022|F|0-59:G>A-59:G>A</td><td>TGCAGCACCTCCCGTCTCGTATCGGCCACCTCCGCCTCCAGCTTCTTGATGGAGCTCACGACCGCGAGC</td><td>59:G>A</td></tr>
<tr><td>3947895|F|0-23:G>C-23:G>C</td><td>TGCAGCTCCCGCACGAGGTGCACGAGCCGCCGCGCCG</td><td>23:G>C</td></tr>
<tr><td>3959631|F|0-6:C>T-6:C>T</td><td>TGCAGGCCGTAGACGCAGTCGACGATGCGCTTCTGCTCGGCGGTGCTGAACACGCCGGCGTGGAGCTCC</td><td>6:C>T</td></tr>
<tr><td>2253366|F|0-26:G>T-26:G>T</td><td>TGCAGAACGACTTCTACTTCGTGCCGGCGCTGTACGACATGGCCGAGTTCGAAGGCGAGCTCCG</td><td>26:G>T</td></tr>
<tr><td>1207849|F|0-51:G>T-51:G>T</td><td>TGCAGCCGCTCCGTGGGCGTGGCGAAGCTCTCCTCGACGTCACGCAGCACCGCCATGGCCCG</td><td>51:G>T</td></tr>
<tr><td>2244267|F|0-19:C>G-19:C>G</td><td>TGCAGCTCCTCCACCTCGTCGAGCGTTTCCCGTCCGAGCCGTGCGCCGAAGCACATGTACACGAGCAGC</td><td>19:C>G</td></tr>
</table>

<table>
<tr><td>1166916|F|0-22:C>A-22:C>A</td><td>TGCAGAACGTGGCTATCATGCTCAAGGCGCTCAATGTGACCGCGGACGAGGTGATCGGAGCACTCATGC</td><td>22:C>A</td></tr>
<tr><td>1318673|F|0-49:C>T-49:C>T</td><td>TGCAGAACATCTACTTCTCCTACGGGATGGCGTACGGCGGCGGCGGCTTCGCCATCAGCAGGCCGCTGG</td><td>49:C>T</td></tr>
<tr><td>7170982|F|0-6:G>A-6:G>A</td><td>TGCAGCGACGCGACGTGGCGCGCGCCG</td><td>6:G>A</td></tr>
<tr><td>7902166|F|0-13:G>A-13:G>A</td><td>TGCAGAAGGAGCGGCCCACGGTGTGGGCGCCG</td><td>13:G>A</td></tr>
<tr><td>3944019|F|0-32:G>C-32:G>C</td><td>TGCAGCAGCGGCGAGGCATCGGCATCGTCGTCGTCCG</td><td>32:G>C</td></tr>
<tr><td>1023254|F|0-33:A>G-33:A>G</td><td>TGCAGATGCTGCGGCCGCACCGCGAATCCATAAACGTCCCTGCGCCAAACCTCAACAACATCAGCCACA</td><td>33:A>G</td></tr>
<tr><td>3948841|F|0-8:C>A-8:C>A</td><td>TGCAGGCGCTCTACACCGCCG</td><td>8:C>A</td></tr>
<tr><td>3028230|F|0-28:C>G-28:C>G</td><td>TGCAGAAAATCCCGGTGAAGATGTGCCCCAGGAGGCCG</td><td>28:C>G</td></tr>
<tr><td>1387967|F|0-32:C>T-32:C>T</td><td>TGCAGGTAGGCGAGGTCCTTGCCGAGGCACGTCCTCGGCCCGCCGTTGAACGCCACGAAGCGGTACGCG</td><td>32:C>T</td></tr>
<tr><td>1225729|F|0-20:C>T-20:C>T</td><td>TGCAGGCGGCAACGGGACTGCGCAGGAGCTTCCACGGGTGACGGCGGCGACTACGCCCGCTCTAGGCCC</td><td>20:C>T</td></tr>
<tr><td>991285|F|0-56:C>T-56:C>T</td><td>TGCAGATCGCGCGCGGGATGGAGTACCTGCACGCCAAGGGAATATACCACGGCGAGCTGAACCCGTCCA</td><td>56:C>T</td></tr>
<tr><td>4407817|F|0-7:G>C-7:G>C</td><td>TGCAGGTGCAGGACGCCGCCTTCTCCAACCG</td><td>7:G>C</td></tr>
<tr><td>23105021|F|0-28:G>A-28:G>A</td><td>TGCAGCACTACGACAAGAGGGACCTCAAGTCCCCGACCTTCAGGACGTGGAACGACGACTCCGTCAGGA</td><td>28:G>A</td></tr>
<tr><td>5582379|F|0-14:G>C-14:G>C</td><td>TGCAGGCCGAGGAGGAGCGGCGAGACGTCGACGGAGGCGCCCG</td><td>14:G>C</td></tr>
<tr><td>1092791|F|0-42:C>T-42:C>T</td><td>TGCAGAAGAAGCGCCCCCGCGTCGGCGCCATGGATCAGGAGGCGCCGCCG</td><td>42:C>T</td></tr>
<tr><td>1863335|F|0-15:A>T-15:A>T</td><td>TGCAGCTCGCTGGTCACCACGTACCTGCCGCCACTCCTCTCCCTCGACCACACTGTGGTGGAGGGGGAC</td><td>15:A>T</td></tr>
<tr><td>3935691|F|0-29:A>G-29:A>G</td><td>TGCAGCCTCTCCTCGGATATGGTCAGGATACCCG</td><td>29:A>G</td></tr>
<tr><td>990178|F|0-12:C>T-12:C>T</td><td>TGCAGACATCGGCGTGATGAGCGGAGCGTCGCTGTACATCCAGAAGGACCTGAAGATCAACGACACCCA</td><td>12:C>T</td></tr>
<tr><td>1701694|F|0-11:T>C-11:T>C</td><td>TGCAGCTGGAGTGGCGACAGAAGGGACACGGGCTTGCCGAGGAACTTGGTGGCGAGGGTGCAGACACGG</td><td>11:T>C</td></tr>
<tr><td>4006245|F|0-16:G>C-16:G>C</td><td>TGCAGCCAGACAAGCAGCGTGCTCGACAGGAGGGAGCTGAGGTTGATCATGAAGTAGTACCAGTTGAAG</td><td>16:G>C</td></tr>
<tr><td>7919750|F|0-13:A>C-13:A>C</td><td>TGCAGGAGGTCGTATCCCTCGCCGCCCCG</td><td>13:A>C</td></tr>
<tr><td>6048197|F|0-11:C>G-11:C>G</td><td>TGCAGGAAGTCCCCCACGTTCACCACCAGCGACGCCTCCCCCATCGCCG</td><td>11:C>G</td></tr>
<tr><td>5000494|F|0-62:C>G-62:C>G</td><td>TGCAGGTACGTCATGGCGAGGCTGGCGTGCATCGCCGCGCCGTACGGGAGCGCGCCTTCGTCCACGAGG</td><td>62:C>G</td></tr>
<tr><td>3940504|F|0-10:C>G-10:C>G</td><td>TGCAGGGTGTCCAAGGGCGTGCGGGAGCTGCCG</td><td>10:C>G</td></tr>
<tr><td>32027560|F|0-66:T>G-66:T>G</td><td>TGCAGGAACGTGTTCCAGTCCCGCCGCTTCTGCGACTCGTACCTGCTCAGCTGCGGCGGCGGCGGCTGC</td><td>66:T>G</td></tr>
<tr><td>32018985|F|0-23:T>G-23:T>G</td><td>TGCAGCGGTGGACGTGGGTGTGGTCGGCCG</td><td>23:T>G</td></tr>
<tr><td>32025953|F|0-30:T>C-30:T>C</td><td>TGCAGAGGAGGATCGGCGGCGAGCTGGAGGTGGTGGGCATGGCGAGTCCTGCGGCTCATCGGAGTCAGA</td><td>30:T>C</td></tr>
<tr><td>7170811|F|0-34:T>C-34:T>C</td><td>TGCAGGCCGCCACGCTCAACCCGTCAGCGTGCTCTGCCTCCG</td><td>34:T>C</td></tr>
<tr><td>5354840|F|0-25:T>C-25:T>C</td><td>TGCAGCGGTACGTGTGGGCCGACGGTGCGTGGAACAACTTCTGGTACCACCCGACCGACCCGTGCGACA</td><td>25:T>C</td></tr>
<tr><td>13880613|F|0-25:G>C-25:G>C</td><td>TGCAGCGCCACGCCGCGCGGGAGAAGACCG</td><td>25:G>C</td></tr>
<tr><td>32027500|F|0-14:A>G-14:A>G</td><td>TGCAGGCAGTCGGCAGCGTGCTCCTTGGACCCGAGCACGGTGCCGTAGTCCAGCAGGCTGACCATGACG</td><td>14:A>G</td></tr>
<tr><td>32024329|F|0-13:T>C-13:T>C</td><td>TGCAGAGCTACTGTGACGTGTCGGTGGTGAGCGGCTGGTCCACCGTGGGGTACGTCGGCCACGGCCTCG</td><td>13:T>C</td></tr>
<tr><td>7170283|F|0-32:C>G-32:C>G</td><td>TGCAGGCGAGCATGTAGTCGAGGCCACGGTCGCTGATGGTGATCAGGAACATGTAGAGCTCCCG</td><td>32:C>G</td></tr>
<tr><td>7940662|F|0-44:T>G-44:T>G</td><td>TGCAGTATCCAGTGGCAGTGCCTGTCGGGCAACCTGCCGCGGGCTGCGACCTGCCACGACGACGAGAGC</td><td>44:T>G</td></tr>
<tr><td>5006461|F|0-12:T>C-12:T>C</td><td>TGCAGTGGCGTATGTGCTTACCTTCCAGAGGCCGACCTCGAGGCCGTACTTCTCGGTGATCTCCTCGGC</td><td>12:T>C</td></tr>
<tr><td>5355064|F|0-64:T>C-64:T>C</td><td>TGCAGGAGAGCAAGCTGCGGGAGGCGCTGGAGGAGGCCTCCGAGGACGGGTGCCTCACCAAGTCTCGCG</td><td>64:T>C</td></tr>
<tr><td>5000413|F|0-60:C>A-60:C>A</td><td>TGCAGAAGGCCTTTGAGGTGTGGTGGAGGAGGACGTTTGTGCGGCCTTACGTCGAACAGCCGGGGAGCC</td><td>60:C>A</td></tr>
<tr><td>5360942|F|0-5:T>C-5:T>C</td><td>TGCAGTCCGTCGACCTGCTGCTTCGGCTGCTGCCTCTGCGGCGGGAACGGCGCCCACGCCGAGTCCGTC</td><td>5:T>C</td></tr>
<tr><td>5370455|F|0-36:G>C-36:G>C</td><td>TGCAGAGGAGGAGGCCGCCGCAGCACTGCTGGATCTGGACGCGCTCGTAGCGGCCTCGCAGGAACGGGA</td><td>36:G>C</td></tr>
<tr><td>5003212|F|0-27:A>G-27:A>G</td><td>TGCAGGGGTGCTGCGACGAGCTGGACCACGCCTTCGCGGACCCCG</td><td>27:A>G</td></tr>
<tr><td>5340625|F|0-64:T>C-64:T>C</td><td>TGCAGGAGCTCGACACGGTGTTCCTTTACAAGAACAACATCGGCGGCAAGATACCCAAGGAGCTTGGCC</td><td>64:T>C</td></tr>
<tr><td>22840107|F|0-36:G>A-36:G>A</td><td>TGCAGGGTGGAGGACGAGAGGTTGGCCGAAGGGAGGGCGAGGGCGGTGACGGCGGCCG</td><td>36:G>A</td></tr>
<tr><td>100567663|F|0-17:C>T-17:C>T</td><td>TGCAGGTCTTCAGGGTTCACAATGCCGGCCAGAATTTTCAGCCTCTCCGTGTAGGGCACGTTGCTGTCC</td><td>17:C>T</td></tr>
<tr><td>1028366|F|0-40:G>T-40:G>T</td><td>TGCAGGCGCTCGTGTCGTGGCGCGACCCGCGCGCCACAGGGATGTTCGTGGTGCTCTGCGTCGTGGTGG</td><td>40:G>T</td></tr>
<tr><td>100550264|F|0-31:A>G-31:A>G</td><td>TGCAGCACGTGGTGTTCGGCATCGCGGCGTCAGCGCGCATGTGGGAGAAGCGGAAGGAGTACATTAAAA</td><td>31:A>G</td></tr>
<tr><td>1214625|F|0-41:C>G-41:C>G</td><td>TGCAGCCGCAGGATCATGGCCAGCGCCTCGTCGGCCCCCGACGACGCCGCCTGCCTCTCCTCCTCCAGC</td><td>41:C>G</td></tr>
<tr><td>2256019|F|0-31:A>G-31:A>G</td><td>TGCAGGTGTCGGCGCTGATCGACCACGTCGCACGCGTCGACTGGTCCCTCCTCGACCGCGTCCCCGAGA</td><td>31:A>G</td></tr>
<tr><td>2246877|F|0-47:G>A-47:G>A</td><td>TGCAGGGCGCGGATCTTGTGCACGTAGGGCAGGAACAGGTCGTGGAAGTCGAGCATGAAGAGCCTCTTG</td><td>47:G>A</td></tr>
<tr><td>1694913|F|0-35:C>G-35:C>G</td><td>TGCAGGTCCTGGTCGAACGAGCGCGCCCAGTAGGCCACATCATGGGTGCTCACATACTTGTAATGCTTC</td><td>35:C>G</td></tr>
<tr><td>5581500|F|0-16:G>C-16:G>C</td><td>TGCAGGCGGCCGTGGGGGTGTCCGCGCCG</td><td>16:G>C</td></tr>
<tr><td>5011165|F|0-28:G>T-28:G>T</td><td>TGCAGATACTGCTGCGGTGCGAGCAGGCGCACCG</td><td>28:G>T</td></tr>
<tr><td>7169155|F|0-10:C>T-10:C>T</td><td>TGCAGAACGGCGGCGGTGGCAACCTGTCCG</td><td>10:C>T</td></tr>
<tr><td>1060520|F|0-65:A>C-65:A>C</td><td>TGCAGGCCGTGGTGGAGCTCAAGGACTTCTTGGACCACACCGCCATGCTCGCCATGCCCAGCCAGAGGA</td><td>65:A>C</td></tr>
<tr><td>7165965|F|0-14:C>T-14:C>T</td><td>TGCAGGTCGGGGCGCTCCTGCGTCCGTGCTCCG</td><td>14:C>T</td></tr>
<tr><td>1021064|F|0-19:C>T-19:C>T</td><td>TGCAGCTCCTCCTCCATGCCCCTCCGCTCGTCCCGCAGCCTCTGCACCGCCTGGATCGTTCCCCGCACC</td><td>19:C>T</td></tr>
<tr><td>3956535|F|0-34:G>C-34:G>C</td><td>TGCAGCTCACCGAGCGCGGGCGGGGGCTCCTGGCGTCCCG</td><td>34:G>C</td></tr>
<tr><td>7921933|F|0-49:C>T-49:C>T</td><td>TGCAGCGCCAGCGCGAAGTAGTCGAACTCGCGCTGCTCCTTCCCGACGGCGGCCG</td><td>49:C>T</td></tr>
<tr><td>5368583|F|0-8:G>A-8:G>A</td><td>TGCAGGGAGAATCAAAGCCAAGATAGATGTTAGTGCCATACGTTCATGACAAATTAATCGATCTAGCTA</td><td>8:G>A</td></tr>
<tr><td>1072263|F|0-43:A>G-43:A>G</td><td>TGCAGATTGACCAGGCAGAGCGCGCGCTCCAGGCTCTCGCGGCAGACACCG</td><td>43:A>G</td></tr>
</table>

2258676|F|0-35:C>A-35:C>A	TGCAGGCACCACAGGTCGCGCACGCCCACGATCGCCAGCTCCTCGAGGTCGCCGACGCAGAGGTCCAGC	35:C>A
7167814|F|0-23:G>C-23:G>C	TGCAGCGCGAGGCGGACGCGGCGGGGCCAGCCG	23:G>C
3027494|F|0-10:C>A-10:C>A	TGCAGCACATCGCCGCCGCCGACTACGACATGCTGCTCCTCCCCG	10:C>A
5324914|F|0-46:G>C-46:G>C	TGCAGATCTGGTGCATCGACCGCGGCGGCCCGCTCTTCACCGCCGTGTTCCAGCCCGTGCAGACCGTCG	46:G>C
2250844|F|0-43:C>T-43:C>T	TGCAGGCGCCGAGCCAGTCGATGGGGATCGGGACGACCAACAGCCGCAACCTGAGCCAGATGCTGAGCC	43:C>T
1164941|F|0-20:G>A-20:G>A	TGCAGCTCGACGCCATCATCGGCCACCACCAGAGCACCAGCCTAGCCCTGCCG	20:G>A
1218327|F|0-41:G>T-41:G>T	TGCAGCATCTCGTGAGCAACCTTAAGGCCTTCCTTGTACCTGCCCTCTCTGAAGAACCCGCGCATAAGA	41:G>T
5001087|F|0-43:T>C-43:T>C	TGCAGGCCGAGGGCAAGATGCCGCTGCCGAGCAGCACTGACGTTGCCTCGCGGATGCTCGATGGCGCGG	43:T>C
3029171|F|0-26:C>G-26:C>G	TGCAGCAGCGCGCCGAGCGCGCCCAGCCCGAGCTCGCCG	26:C>G
2247422|F|0-63:C>A-63:C>A	TGCAGTGATCCATGCCGACGACATCGAGATATCGCTGTGCGACGGCAACTCGGAGGACGAGCGCCGCCG	63:C>A
1067955|F|0-37:C>G-37:C>G	TGCAGCGTGTCCGCGGCCAGGAAGCGGAGGTTCATCTCGTAGGGGTCGTTGGTGGAGATGAAGTGGAAG	37:C>G
4440010|F|0-13:T>C-13:T>C	TGCAGATTCCTGTTTTCTTGCAGCGGTTGGGGTCTCGGGTGGGCCG	13:T>C
7161321|F|0-41:T>C-41:T>C	TGCAGCGGCATGCGGAACTCGCCAAGCGCGAATCCCGCGGCTGCGTACTCCG	41:T>C
6045121|F|0-25:T>C-25:T>C	TGCAGGTTGGTTGCTTAGCGCCTACTTCCTTGGTGGATTTGGTGATGATGCCAAAGGAAGCAATGGTGT	25:T>C
3028878|F|0-26:C>G-26:C>G	TGCAGCGCCTTGTTGTACATGAGCGCCGCGTAGTTCCG	26:C>G
994189|F|0-13:C>T-13:C>T	TGCAGGCGGCCGCCGCTCTCGGTCAGGTTGGTGAACATCACCGCGTGGAACTGCTCCG	13:C>T
3937092|F|0-6:G>A-6:G>A	TGCAGAGGAGCACGTCGTGGCCG	6:G>A
3942865|F|0-18:A>T-18:A>T	TGCAGAAGCTGCGGTGGTACTGCCAGGTGTGCGAGAAGCAGTGCCGCGACGAGAACGGCTTCGAGTGCC	18:A>T
4262191|F|0-13:G>A-13:G>A	TGCAGGGCAAGAAGGCGACGACGTGCGCATCCATGGCCG	13:G>A
995015|F|0-55:G>C-55:G>C	TGCAGCTGCTGCTCAACGAGAAGAACGAGGCGCGGATCGCCGCCTTCCTCGTGCTGCTCCG	55:G>C
4262191|F|0-27:C>G-27:C>G	TGCAGGGCAAGAAGGCGACGACGTGCGCATCCATGGCCG	27:C>G
3945320|F|0-5:G>C-5:G>C	TGCAGGAGCAACACCGTCAACGCGGCTGCCG	5:G>C
3957231|F|0-58:C>G-58:C>G	TGCAGGCGCTGGTGAGCGTGGACATGCTGGTGCTGTTCGTGGCGACCATCTGCGGCGTCGGCGGCACGC	58:C>G
980135|F|0-10:C>G-10:C>G	TGCAGGTGGCCGTCTGGGCGTGCATGGTGATGTCGGTGATGCTGGTGGTGGAGGCCCTGTACAACTGCG	10:C>G
4544261|F|0-60:G>A-60:G>A	TGCAGGGCGTACGCGGCGGCGGACGTCAGCGGGGGCATCAACCGCGGGTGCGTCATCTGGGCCGTGGAT	60:G>A
1695118|F|0-29:G>A-29:G>A	TGCAGCGCGCGGTCACAGTCCTTGACGACGTGCTTGTGGAGCTCGAGCTGGAAGTAGCAGAAGGCCCGA	29:G>A
3935997|F|0-26:C>T-26:C>T	TGCAGCCACTCGATGATGGCGTCGGGCGACACGCCGCCG	26:C>T
1022558|F|0-52:G>A-52:G>A	TGCAGTACGGCGCCCAGCCCCTAATCTCCAAGCGCTGCGTCGGGTACGCCGCGCACCTTCGTATCACCT	52:G>A
12764166|F|0-53:A>G-53:A>G	TGCAGTGGCGGGACGGCTGCCCCGTGGAGCAGCCCGTTCTCGATCAGCGGAGCAGCGACAATGACGACG	53:A>G
1694944|F|0-16:G>A-16:G>A	TGCAGAACCTGCGCTCGCTGTCGCTCGACTCGTGCAAGGTGACGGCCTGCGAGATCAAGAAGCTTCAGC	16:G>A
1102451|F|0-46:C>G-46:C>G	TGCAGCCGTGGCAACAGGGGTGTCCAGCGGTCTGGTCATGCTTCTGCGCAAGATGATGCGTGCAGACTG	46:C>G
1059990|F|0-5:C>T-5:C>T	TGCAGCGCTTGCCTAAACATCCCAGAGCCGCGGGGCACGCCTAGAGCTTCGGCGCATGCCACCATCTCC	5:C>T
7169055|F|0-40:G>A-40:G>A	TGCAGTCGACCACGGCGTCCTTCTCCCACCCCATGTCGCGGCCCTTCGTGCTCCTCAGGGTCCAGGTGG	40:G>A
3948533|F|0-18:A>C-18:A>C	TGCAGAGGCGAGGCACTGAAACGAACTAGGGCCG	18:A>C
1080536|F|0-16:T>C-16:T>C	TGCAGCTTGCTTTCGGTGAGAAAACTCACCCAAATTCGTGAATTGCAGGAGCCTTGGACAGTCGTTCTC	16:T>C
1087690|F|0-31:T>C-31:T>C	TGCAGCAGTACTCGCAGTCCCCTGTCCATTCTTCGCCTCATCACCACCACCTCAATTCCATGAGGTACT	31:T>C
1133246|F|0-23:C>T-23:C>T	TGCAGGAGGGCGTGATCCCGGCGCTGGTGTCGCTGACGGCGAACGGCACGGGCAAGGCCAAGGACAAGG	23:C>T
1026333|F|0-46:T>C-46:T>C	TGCAGCGCGTTGTGGCTGAGGTCCAGGATGCTGAGCTGCTGGAACCTGAGGTTCGGCACCTGGCCGAGA	46:T>C
4262191|F|0-6:G>A-6:G>A	TGCAGGGCAAGAAGGCGACGACGTGCGCATCCATGGCCG	6:G>A
100583542|F|0-17:C>T-17:C>T	TGCAGCTTGGACAGCAGCTCGGAGATCTGGTCCTCCGAGATCGACGGCGAGCTCCCGGCGCGCCGCGTG	17:C>T
1089628|F|0-29:T>C-29:T>C	TGCAGCAATGGCAACAGAATAGTACAGATTGGGTAGGTGCCCTGCCTGCCCGATGCCGTCTCCGACACG	29:T>C
4396487|F|0-8:G>A-8:G>A	TGCAGCGAGTGCGGGCTGGCGGTGCCG	8:G>A
990479|F|0-43:C>G-43:C>G	TGCAGCTTCTGCTGGACCATGGCGTTGTGGCGCTGCGCGAAGTCGTTGTACGTCTTCAGGCACCCGACG	43:C>G
2252327|F|0-68:G>A-68:G>A	TGCAGCTCAGGGAGGTGCCCTCTGACGACGCCACCTGTGACGTCGCCCTTGACTGCACGCGGCCATGGG	68:G>A
2247422|F|0-66:C>A-66:C>A	TGCAGTGATCCATGCCGACGACATCGAGATATCGCTGTGCGACGGCAACTCGGAGGACGAGCGCCGCCG	66:C>A
7351372|F|0-12:G>C-12:G>C	TGCAGCGGCCCTGAAACCCG	12:G>C
1067955|F|0-31:T>C-31:T>C	TGCAGCGTGTCCGCGGCCAGGAAGCGGAGGTTCATCTCGTAGGGGTCGTTGGTGGAGATGAAGTGGAAG	31:T>C
7172655|F|0-20:A>G-20:A>G	TGCAGGTCGACTGCGGCGGCAACGGCAGCGGCGTCTGGTTCACCGAGGCCGCCGCCG	20:A>G
7171129|F|0-40:A>G-40:A>G	TGCAGCGCCTGCACGGCTTCTCGCTGTCCCCGCAGCACGCACAGGTCCATAAGCTCTCCTGGGAGCAGG	40:A>G
32023285|F|0-13:C>T-13:C>T	TGCAGCAGCGGACCGATCTCCTGGCCCCG	13:C>T
3030480|F|0-9:C>T-9:C>T	TGCAGCTCGCCTCGGTCCACCGCGCCCTCGCCGTCCGCCCG	9:C>T
2253450|F|0-10:A>C-10:A>C	TGCAGCGCGGAGTTCGAGGCGCAGGTGGCCACGCTCGCCGACGTCCCTTCCTCCATCCCTCTCGACTCC	10:A>C
1212169|F|0-65:G>C-65:G>C	TGCAGCCGCGGGTTCGGCTCCACCCGCAGCGAGCCGCCGAGCTGGTTCCCCGACACGTCCACGGCGCGG	65:G>C
100569606|F|0-15:C>A-15:C>A	TGCAGAATGTCCGCACAAAACTTAACCAAGTTGCTTCTGTAAGTCCAAAAGCTTTCTTTTCGTGCTCTT	15:C>A
4396523|F|0-8:C>T-8:C>T	TGCAGCGGCTCCATGCGCCTGCCG	8:C>T
5357587|F|0-5:C>A-5:C>A	TGCAGCAGGCAGCAGGTGACCTCCTCCCCG	5:C>A
1153753|F|0-46:G>A-46:G>A	TGCAGGGGCTGGGGAGGTACTGGGGCGTGGGCGGCAGGAGGTGCGGGTCGTGCCG	46:G>A
2246609|F|0-40:C>A-40:C>A	TGCAGCCCCACCGCGCCCTCTCAGACAAGGTGGAGCTGATCGAGATCGACCTCTCCGAGGAATCCGCCT	40:C>A
5578451|F|0-19:C>G-19:C>G	TGCAGTGCGAGCACCGCCTCGTCATCCCCG	19:C>G

13879505|F|0-35:G>T-35:G>T	TGCAGAGCGGTGACGTGGACCTGCGGGCTGTTGCAGCCG	35:G>T
1666874|F|0-62:A>G-62:A>G	TGCAGACGGACATCAACATGGCGCGCCGCGCGCTGCGCGAGGCGCTCACCACGCTCGACGACATCAAGC	62:A>G
1087692|F|0-17:G>C-17:G>C	TGCAGCGCGGAGAAGACGACGTAGGTGGACTGCTGCACCACGGCCACGTAGTAGATGATCCCG	17:G>C
5581757|F|0-11:C>T-11:C>T	TGCAGAAGCCGCGCGTACGGCGTATCCTCCAGCGCCACGTGGCGGACGGCAGCGCCGCTGCCGTCGCCC	11:C>T
1023916|F|0-25:G>C-25:G>C	TGCAGAACACGGCCATCCTGGGGTCGGAGAACCACCCGCTGCACCTGCACGGCTTCAACTTCTACGTGC	25:G>C
2246609|F|0-52:C>G-52:C>G	TGCAGCCCCACCGCGCCCTCTCAGACAAGGTGGAGCTGATCGAGATCGACCTCTCCGAGGAATCCGCCT	52:C>G
1097886|F|0-16:C>T-16:C>T	TGCAGCTGGCACGGACCGCCGCGAGGCCAGCGAGGCGCGCGCTGGTGGTGGAGGCCCGCGGGGGCAGGG	16:C>T
5581509|F|0-14:G>C-14:G>C	TGCAGGGACATGCTGGTCTCCGCGTCCG	14:G>C
5331711|F|0-10:C>A-10:C>A	TGCAGCACCGCCGCGACCATATGCATGGAGCCG	10:C>A
4538027|F|0-8:G>A-8:G>A	TGCAGGTAGCCGAGGAAGGTGTGGAACGGGCCG	8:G>A
1028366|F|0-34:C>T-34:C>T	TGCAGGCGCTCGTGTCGTGGCGCGACCCGCGCGCCACAGGGATGTTCGTGGTGCTCTGCGTCGTGGTGG	34:C>T
1096762|F|0-34:C>T-34:C>T	TGCAGTACGGCGCCCAGCCCCTAATCTCCAAGCGCTGCGTCGGGTACGCCGCGCACCTTCATATCATCT	34:C>T
3955049|F|0-31:T>C-31:T>C	TGCAGAGCATATACTGTCATTTCGTACCTGATGCCG	31:T>C
7921933|F|0-5:C>T-5:C>T	TGCAGCGCCAGCGCGAAGTAGTCGAACTCGCGCTGCTCCTTCCCGACGGCGGCCG	5:C>T
996709|F|0-14:C>T-14:C>T	TGCAGCCCGCGAACCGCGCGATGCGCGCCATGTACGCGCGCACGGAGATGCCG	14:C>T
3939216|F|0-7:G>A-7:G>A	TGCAGCAGCTGGATATTGGCGGTGAAATCTCCAACCAGGAGCCGCTTCCG	7:G>A
1695186|F|0-13:T>A-13:T>A	TGCAGGGAGCAGTTCGCCGAGCTCGACTACTGCATCACCAGCCCGCCGCCATGGAGTGCGTGCCCTCGC	13:T>A
4982323|F|0-32:C>T-32:C>T	TGCAGGGCCTCCTGGGGAGCCTCGGCCTGCCTCCCG	32:C>T
1088823|F|0-33:G>A-33:G>A	TGCAGTGACTGCACCGCCTCTCTACCCTAATGGGCACCACGGGCTAGGGCTAGGGCTCTTCCTTGACGT	33:G>A
1095342|F|0-49:C>T-49:C>T	TGCAGCGCGTCGGCGTCGGCCTCGTGCTCTCCGTCGTCTCCATGTGCATCGCCGCCGTCGTGGAGATGC	49:C>T
1023426|F|0-22:G>C-22:G>C	TGCAGAACCTGAAGACGCTGCTGCTCTACTCCAACTCCCTCGTCGGCACCATCCCTCCG	22:G>C
980685|F|0-55:A>G-55:A>G	TGCAGCCGCTTGTCGTCCGTGGTCGCCGTCTTGTGCACCGCCTTCTTCTTCCTGCACCAGACCCCAGCC	55:A>G
1067955|F|0-50:G>A-50:G>A	TGCAGCGTGTCCGCGGCCAGGAAGCGGAGGTTCATCTCGTAGGGGTCGTTGGTGGAGATGAAGTGGAAG	50:G>A
5582067|F|0-11:T>C-11:T>C	TGCAGGTGGGCTTCGATGCGGCACGGCGTGGCCG	11:T>C
1002619|F|0-59:G>T-59:G>T	TGCAGCGCCACGGACACGGTGGCCCTGCCGCAGAACCGACCGAAGTAGGAGCACACCTCGCCCCACGAC	59:G>T
1696921|F|0-21:G>A-21:G>A	TGCAGAAGCTGTCGAGGGCGGGGTGGAGCGGAGGTCCTTGTCCATGGCCTTGTAGGCGCGCGCGAAGG	21:G>A
993195|F|0-7:T>C-7:T>C	TGCAGAATGTGCACGGCCTCCGCCGCCGCGAGTTCAGGGCCACGCTCGCGCACCTGCACTCCATGGCCA	7:T>C
1061023|F|0-52:C>G-52:C>G	TGCAGTCCGTGGTGAAGGAGACGCTCCGCCTCCGCATGGCCATCCCGCTCCTCGTGCCCCACATGAACC	52:C>G
4910150|F|0-19:C>G-19:C>G	TGCAGTCGGTGCTCCACGCCGCGGTGCCG	19:C>G
5578225|F|0-18:C>T-18:C>T	TGCAGGAGGAAGAGGATGCACCG	18:C>T
7342607|F|0-14:G>C-14:G>C	TGCAGCGCGGCGACGGCCTCCG	14:G>C
5373570|F|0-10:G>A-10:G>A	TGCAGGTGCGGCACGGCGCCCG	10:G>A
1359255|F|0-61:G>A-61:G>A	TGCAGGAGTTCAAGACGGACTACAACCAGACCCACTTCATCCGCAACCCCGAGTTCAAGGAGGCCGCCG	61:G>A
16794962|F|0-13:A>G-13:A>G	TGCAGCGCATCGGAATAGGGTTCCTCTCGGCCACCGTGGCCACCG	13:A>G
5361383|F|0-22:A>T-22:A>T	TGCAGGAGGTTGGGCGGGATAGAGGTGGGGCGGCCGCCGCGGCTGCCGCCACGGAGGCGCAGCACCAGG	22:A>T
1251387|F|0-59:G>A-59:G>A	TGCAGCACGGAGTTGAGCGCGCCGCGGCTCTCCTTCACCACGCAGTGCAGCTCCAGGACGCTGTTGATG	59:G>A
1019236|F|0-25:G>C-25:G>C	TGCAGGCGAACGGCGGCAGCAAGGTGGCGAAGAAGATCAGCCCGAAGGACAAGTACTGGGTGGCCGCCG	25:G>C
1067955|F|0-28:G>A-28:G>A	TGCAGCGTGTCCGCGGCCAGGAAGCGGAGGTTCATCTCGTAGGGGTCGTTGGTGGAGATGAAGTGGAAG	28:G>A
12470689|F|0-5:C>T-5:C>T	TGCAGCGCTTGCCTGAACATCCCG	5:C>T
3934505|F|0-21:G>A-21:G>A	TGCAGCGGATGTTGCTTGTGCGAAGACCG	21:G>A
1023916|F|0-14:A>G-14:A>G	TGCAGAACACGGCCATCCTGGGGTCGGAGAACCACCCGCTGCACCTGCACGGCTTCAACTTCTACGTGC	14:A>G
3028020|F|0-23:G>T-23:G>T	TGCAGCCCAGCCTTGGAGCTCCTGGACGTCGCCTTCTTGGCCGCGCCG	23:G>T
1096562|F|0-38:G>T-38:G>T	TGCAGCAGCGCCCCAGGCCAAGAGGCAGCAGGACGGCGGGCCTACCGCCG	38:G>T
1090419|F|0-40:C>T-40:C>T	TGCAGCAGAACGTGTCCTGGGCGCTCGGCTTCGGCGTCTCCGCGCTCTGCCTGCTCCTGGCGGCCGCGG	40:C>T
7169799|F|0-8:C>G-8:C>G	TGCAGTACCTCATCGACGCCGTCCATTCGTTCTCCG	8:C>G
4394612|F|0-28:C>G-28:C>G	TGCAGGAGATCGCGCAGAACAGGGCCACCACGCCG	28:C>G
1097886|F|0-61:G>A-61:G>A	TGCAGCTGGCACGGACCGCCGCGAGGCCAGCGAGGCGCGCGCTGGTGGTGGAGGCCCGCGGGGGCAGGG	61:G>A
3944480|F|0-23:C>T-23:C>T	TGCAGCTTCAAGTACGCCACGTTCGCCTACTACGCCG	23:C>T
980685|F|0-5:C>T-5:C>T	TGCAGCCGCTTGTCGTCCGTGGTCGCCGTCTTGTGCACCGCCTTCTTCTTCCTGCACCAGACCCCAGCC	5:C>T
1001496|F|0-52:C>T-52:C>T	TGCAGCGCGCGCGTGCCGACGGGTCGCTCGCGGCGCGGGGTGTTTCTGCTGCCGCCGCGGCCCAGAAGA	52:C>T
1128688|F|0-34:C>G-34:C>G	TGCAGCGCATCGGGGTCGGCCTCGGCACCGTCGCCTTCTCCATGGTCGCCGCCGCCACCGTCGAGCGCC	34:C>G
5411951|F|0-12:C>A-12:C>A	TGCAGGCAACCGCCGCCGCCGTCGCCG	12:C>A
978081|F|0-13:C>T-13:C>T	TGCAGAAGCCCAACGCGGTGGTGGCCGCGGACGGGAGCGGCAACTTCAAGACCATCACGGAGGCCCTCA	13:C>T
1240847|F|0-41:C>T-41:C>T	TGCAGCAGCGCAGTCGCGGACATCTGCGGGAGCCCGCCGCCCGCCTCAGACGAGCTGTAGAGCGAAGGG	41:C>T
2256421|F|0-13:C>T-13:C>T	TGCAGGGGATGGTCGAGGCAGCAGTCCATGCCGCTGACAAGGAGGTGGCTAAGGCTGCCAGGAGGATAA	13:C>T
4408582|F|0-9:T>C-9:T>C	TGCAGTGTGTGGCGTGGGGCCG	9:T>C
1004365|F|0-64:A>G-64:A>G	TGCAGTATCCAGTGGCAGTGCCTGTCGGAGAGCCTGGCTCGGTTGTCGCGGAGCGGCCGCGGCGACGAG	64:A>G
1235036|F|0-40:G>C-40:G>C	TGCAGTCCATCAAGTGGGGCACCGACTACTTCATCAAGGCGCACACCGCGCCCAACGAGCTCTGGACCC	40:G>C
5367367|F|0-22:C>A-22:C>A	TGCAGGGAGGGCCAGCGGGAGGCGGGTGGGAAGAGGAAAGAGAAGCCG	22:C>A

<table>
<tr><td>7342214|F|0-17:T>C-17:T>C</td><td>TGCAGGAACCCGAGGCATGGAGACAGGTCAGAGAGCATGCCG</td><td>17:T>C</td></tr>
<tr><td>5363956|F|0-18:A>C-18:A>C</td><td>TGCAGGCGCCCACACGATAGGGCAATCTCAGTGCCG</td><td>18:A>C</td></tr>
<tr><td>1218352|F|0-9:C>T-9:C>T</td><td>TGCAGCGCGCTGATGACGGTGCGGAGCGCGCGGCTCGGGGACCCGCGGTTGGCGAGCATGATCTCGCCG</td><td>9:C>T</td></tr>
<tr><td>6045546|F|0-12:T>C-12:T>C</td><td>TGCAGCGCTCGATGGTGCTCCTCGTGGCCACCG</td><td>12:T>C</td></tr>
<tr><td>3029897|F|0-29:C>G-29:C>G</td><td>TGCAGCGACTTCTTGAGCGCGAACCACACCGTCGGCGCCG</td><td>29:C>G</td></tr>
<tr><td>1017775|F|0-34:G>A-34:G>A</td><td>TGCAGGCCGAGCTGGTGAGGTACCTGATGAGGCAGGAGGAGTGGAGGAGGTGGGGCGGCAAGGACCACC</td><td>34:G>A</td></tr>
<tr><td>32025906|F|0-8:G>A-8:G>A</td><td>TGCAGCCAGGCGCCCCGCCG</td><td>8:G>A</td></tr>
<tr><td>1080536|F|0-39:G>T-39:G>T</td><td>TGCAGCTTGCTTTCGGTGAGAAAACTCACCCAAATTCGTGAATTGCAGGAGCCTTGGACAGTCGTTCTC</td><td>39:G>T</td></tr>
<tr><td>2251187|F|0-22:G>T-22:G>T</td><td>TGCAGGCGTCGCCCAGGTGGTCGTCGGCCACCCTTTCGACACCGTGAAGGTAATTCTCTTTCCTCTCTT</td><td>22:G>T</td></tr>
<tr><td>1178910|F|0-20:C>T-20:C>T</td><td>TGCAGGACGTGGGACTCGTCCTCGTCGGCGTACATGTACTTGTTTTGGTGGCTCCGCAGCCGCACCGTC</td><td>20:C>T</td></tr>
<tr><td>4009326|F|0-12:T>C-12:T>C</td><td>TGCAGGACTTGATGGGGTATACGAGGATGGACGCCACCGTCGCCGCCG</td><td>12:T>C</td></tr>
<tr><td>5367216|F|0-16:A>G-16:A>G</td><td>TGCAGCTTCGCGGCTCACGGGCTCCG</td><td>16:A>G</td></tr>
<tr><td>3028898|F|0-23:C>G-23:C>G</td><td>TGCAGCCCCTACCGCGCCGACCTCTCCGACCGCCCCTGCGCCGCCG</td><td>23:C>G</td></tr>
<tr><td>3028116|F|0-28:C>T-28:C>T</td><td>TGCAGGGGTGCTGCGACGAGCTGGACCGCTCCTTCGCGGACCCCG</td><td>28:C>T</td></tr>
<tr><td>2259538|F|0-5:G>C-5:G>C</td><td>TGCAGGGCCTCGAGGAACTTGTCGTGCTCGGGCTCGGTCCAGCTCTCGCGGGACTTGGTGATGGTGTAG</td><td>5:G>C</td></tr>
<tr><td>3934505|F|0-7:G>A-7:G>A</td><td>TGCAGCGGATGTTGCTTGTGCGAAGACCG</td><td>7:G>A</td></tr>
<tr><td>4262703|F|0-26:C>T-26:C>T</td><td>TGCAGCACGCGGCTGATACCGCCGCCCCCGCCCG</td><td>26:C>T</td></tr>
<tr><td>3938865|F|0-26:G>C-26:G>C</td><td>TGCAGGATCTGCGCGGCGCTCCAGCTGGCCCCG</td><td>26:G>C</td></tr>
<tr><td>5580310|F|0-17:C>T-17:C>T</td><td>TGCAGCTCCGTCAGACCCTGCGGGTTCCCG</td><td>17:C>T</td></tr>
<tr><td>2247690|F|0-27:A>T-27:A>T</td><td>TGCAGCCATGTCGGTGCAGGAGTACCTAGAGAAGCACCTGCTCCCGCGCAAGATCGAGGAGGCCGTGAA</td><td>27:A>T</td></tr>
<tr><td>4409632|F|0-10:G>A-10:G>A</td><td>TGCAGCCGACGATCTTCCAGTGCCG</td><td>10:G>A</td></tr>
<tr><td>1055548|F|0-20:G>A-20:G>A</td><td>TGCAGGAGCTTGATGATGTTGCTGGCGCCGAAGACGCGGTGCGCGGTGGCGAACTTGTGGGGGTCCGTG</td><td>20:G>A</td></tr>
<tr><td>1695029|F|0-22:G>C-22:G>C</td><td>TGCAGCTCCTGCTTGGTGAAGAGCGTGAAGGACATTCCCTGCTTGTTGGACTTCTTCATCTCCTCGAAC</td><td>22:G>C</td></tr>
<tr><td>1023062|F|0-28:A>C-28:A>C</td><td>TGCAGCCCTTCTCCTTCCCCGAGTGGGAAGAGCACCGTCGTCTGTCGGCCG</td><td>28:A>C</td></tr>
<tr><td>1204782|F|0-16:C>G-16:C>G</td><td>TGCAGGACAAGATGACCGCCAGGGAGATCGTCACATGGCTCGCCGTGCTCAAGGCGGAGGAGGAGCTCA</td><td>16:C>G</td></tr>
<tr><td>985956|F|0-10:G>A-10:G>A</td><td>TGCAGCTCCAGGAGCCCACCCTCCACCTTCTCAGCCTCGGAGGAGGACAGCATGGCCGCCCGCATCGCC</td><td>10:G>A</td></tr>
<tr><td>1141772|F|0-13:C>G-13:C>G</td><td>TGCAGTTCTTCGCCCCCAACCCGACCTGCGGCCTCTCCCCCTTCGTCCG</td><td>13:C>G</td></tr>
<tr><td>5411951|F|0-6:C>T-6:C>T</td><td>TGCAGGCAACCGCCGCCGCCGTCGCCG</td><td>6:C>T</td></tr>
<tr><td>3026597|F|0-26:C>G-26:C>G</td><td>TGCAGCTTGCCGCCACCCCCTCCCACCGAGAGGTCCCCG</td><td>26:C>G</td></tr>
<tr><td>7166938|F|0-52:G>A-52:G>A</td><td>TGCAGCTGCTGTGGCTCGTGGCTTGACGGTGGTTGGCTTTGGCGTAGTTGTTGTGGCTGATGTCGCTGC</td><td>52:G>A</td></tr>
<tr><td>1063793|F|0-53:G>T-53:G>T</td><td>TGCAGCAGCACCCCGACCTCCGTCAGCGACAGCACGTCCTCCAGTATGACCTTGGCGAATATCCGAGAT</td><td>53:G>T</td></tr>
<tr><td>985521|F|0-40:C>T-40:C>T</td><td>TGCAGGGCCTGTGGTTCTACCAGACGGCGTTCACGCTCTACGGGCCGATGCTCCCCGAGGGGTGCCACC</td><td>40:C>T</td></tr>
<tr><td>3952851|F|0-26:G>A-26:G>A</td><td>TGCAGGAGCCGCAGGAACGCGGTGGCGCGCCGCCG</td><td>26:G>A</td></tr>
<tr><td>993977|F|0-24:G>C-24:G>C</td><td>TGCAGTTCGGACGGCGCCTAGCATGACGGAGGCGGCCGTGGGGAGGATCGCGCAGGGGACCAAGGTGCT</td><td>24:G>C</td></tr>
<tr><td>2247548|F|0-10:G>A-10:G>A</td><td>TGCAGATGTAGAAGTCGTCGCCGTAGAGAGCCCTGACAGCATCGACGGGCTTCACCGCCG</td><td>10:G>A</td></tr>
<tr><td>3024189|F|0-20:C>G-20:C>G</td><td>TGCAGCGCCACCCACTTCCACGAGGACCTCTTCACTTTGCTCTGCCG</td><td>20:C>G</td></tr>
<tr><td>1020656|F|0-67:C>T-67:C>T</td><td>TGCAGCTGTGGAAGACGGCCTTCTACGACCAGCAGGCGATGCAGGCCACCGAGTTCCTCCTCAGCGACG</td><td>67:C>T</td></tr>
<tr><td>5369393|F|0-5:C>G-5:C>G</td><td>TGCAGCCGCCGCACGATCTCGTACTTGTGCTCCG</td><td>5:C>G</td></tr>
<tr><td>5578076|F|0-7:G>A-7:G>A</td><td>TGCAGCGGAAGGATGAGGTGGAGATGCACCCCGCCG</td><td>7:G>A</td></tr>
<tr><td>5366355|F|0-61:T>C-61:T>C</td><td>TGCAGGACGACGTGGGGAGGCTTATCGTTGCTCAGTTCCGCTGGCTTGATTTCCTTGTCGATGCCGACA</td><td>61:T>C</td></tr>
<tr><td>7920472|F|0-17:T>G-17:T>G</td><td>TGCAGCGCCGCGACCCATATCCCGCCG</td><td>17:T>G</td></tr>
<tr><td>1863497|F|0-58:C>T-58:C>T</td><td>TGCAGGCCGAGGGCAAGATGCCGCTGCCGAGCAGCACTGACGTTGCCTCGCGGATGCTCGATGGAGCAG</td><td>58:C>T</td></tr>
<tr><td>5582039|F|0-16:G>C-16:G>C</td><td>TGCAGGCCACCTCCGAGGACATGGCGGCGGACGGCCTCACCGCCCG</td><td>16:G>C</td></tr>
<tr><td>1092737|F|0-38:G>C-38:G>C</td><td>TGCAGCCGCTCCTCGGCGGCTCTACCATTGACGAGCAGGCGCGTGGGAATGGAGCGGCCAAGGAGCGAG</td><td>38:G>C</td></tr>
<tr><td>5581383|F|0-35:A>G-35:A>G</td><td>TGCAGCGAGTCGGCTTCTTTCTCGTCGGCCATGAGAAGATTCCG</td><td>35:A>G</td></tr>
<tr><td>3957000|F|0-16:C>A-16:C>A</td><td>TGCAGGTGCTGGCGCCCTTCCCCG</td><td>16:C>A</td></tr>
<tr><td>12777317|F|0-68:C>T-68:C>T</td><td>TGCAGGCGGGCGGACGTCGAGAGCTTCGGCGAGGTCCGCCTGGCGCGCTGCAAGGGGGTGCGCGCCGAC</td><td>68:C>T</td></tr>
<tr><td>4911118|F|0-37:T>C-37:T>C</td><td>TGCAGAATGTCCGCACAAAACTTAACCAAGTTGCTTCTGTAAGTCCACAAGCTTTGATTTCGTGCTCTT</td><td>37:T>C</td></tr>
<tr><td>1103684|F|0-50:G>A-50:G>A</td><td>TGCAGATGCCCGCGGCGTGCAGGGTCCAGGCGCCGCCCGCCAGCCAGTGCGACTGTAAGTTCACATTGC</td><td>50:G>A</td></tr>
<tr><td>23889496|F|0-7:G>C-7:G>C</td><td>TGCAGCTGGCCCTCAACGTGCCCACCG</td><td>7:G>C</td></tr>
<tr><td>2247690|F|0-21:G>A-21:G>A</td><td>TGCAGCCATGTCGGTGCAGGAGTACCTAGAGAAGCACCTGCTCCCGCGCAAGATCGAGGAGGCCGTGAA</td><td>21:G>A</td></tr>
<tr><td>1090093|F|0-55:C>G-55:C>G</td><td>TGCAGGGGTACGGGCTGACGGAGAGCACGGCCATCGGCGCGTCCACGGACTCGGCCGAGGAGAGCCGCC</td><td>55:C>G</td></tr>
<tr><td>3939216|F|0-23:G>A-23:G>A</td><td>TGCAGCAGCTGGATATTGGCGGTGAAATCTCCAACCAGGAGCCGCTTCCG</td><td>23:G>A</td></tr>
<tr><td>1049522|F|0-55:C>T-55:C>T</td><td>TGCAGTGCTTCCTGATTGAAGTTGTGTGCGCGTGCATCTCAGGCTCGTTGTTTCCCCCG</td><td>55:C>T</td></tr>
<tr><td>3957572|F|0-17:A>G-17:A>G</td><td>TGCAGCAAGGATCCACAAGTGATGCCGCCG</td><td>17:A>G</td></tr>
<tr><td>1264244|F|0-58:G>C-58:G>C</td><td>TGCAGCTGGCGTTCCAGAGCATCGGCGTGGTGTACGGCGACGTGGGGACGTCGCCGCTGTACGTGTACT</td><td>58:G>C</td></tr>
<tr><td>980685|F|0-66:G>A-66:G>A</td><td>TGCAGCCGCTTGTCGTCCGTGGTCGCCGTCTTGTGCACCGCCTTCTTCTTCCTGCACCAGACCCCAGCC</td><td>66:G>A</td></tr>
<tr><td>2241770|F|0-29:C>G-29:C>G</td><td>TGCAGCTTCTTGATCTCCTTCTTGAGGTCCGCCTCGAACTTCTCCTTCTGGTTCGCATTCTCGGTGTCG</td><td>29:C>G</td></tr>
<tr><td>7902161|F|0-22:T>C-22:T>C</td><td>TGCAGAACCTTGGGCGGGCGCTTATCTTTGCGGCCG</td><td>22:T>C</td></tr>
</table>

2252327|F|0-16:G>C-16:G>C	TGCAGCTCAGGGAGGTGCCCTCTGACGACGCCACCTGTGACGTCGCCCTTGACTGCACGCGGCCATGGG	16:G>C
1128688|F|0-61:C>A-61:C>A	TGCAGCGCATCGGGGTCGGCCTCGGCACCGTCGCCTTCTCCATGGTCGCCGCCGCCACCGTCGAGCGCC	61:C>A
3959378|F|0-20:C>G-20:C>G	TGCAGCGAGCACTCGAAGTCCGCCGAAGACAGCACCCCCTCCG	20:C>G
1019803|F|0-18:G>A-18:G>A	TGCAGGTTGAACGTGCCCGTGAGCTCGGCCGTCTCGGCGACCACGCTCCGCATCTCCTCGGTGTCGTTG	18:G>A
4733780|F|0-26:C>A-26:C>A	TGCAGCAGCGGCGACACCAGCCACCCCGCCGCCGCGCCG	26:C>A
1136655|F|0-24:A>G-24:A>G	TGCAGCGGGGTGAGGACCCTGAACAGCAAGGCGTCCACGGCGGCCGACCG	24:A>G
1062823|F|0-28:G>A-28:G>A	TGCAGAAGAGCCTGCGGAACTGCGGGCTGGCGAGGAGGTACCTCCACTCCGAGGTCTTGACCGCGGCAG	28:G>A
1146686|F|0-31:G>C-31:G>C	TGCAGGGCGCGGAGGTGTCGGTGGAGAACGTGCGCAGCAGCTTCCCTCTGGGCGCGGCCATGAGCCGCG	31:G>C
994650|F|0-62:T>C-62:T>C	TGCAGAAACGAGAACACCAAAAAGACCTACCAGGTTGTGGTAGTTGGCGAGCTTGGCCATGGTGGTGCT	62:T>C
988966|F|0-32:C>T-32:C>T	TGCAGCCCGAGAGGAAGCCATGATGGTCGCGGCGACCGCGGTTCAAGAGCAAGCTATGTCGGTCGCAGC	32:C>T
1103684|F|0-52:C>T-52:C>T	TGCAGATGCCCGCGGCGTGCAGGGTCCAGGCGCCGCCCGCCAGCCAGTGCGACTGTAAGTTCACATTGC	52:C>T
2251743|F|0-42:C>G-42:C>G	TGCAGCAGCTCCGACTGGGACTCCGACTCCTCCTCCCCGTCCTCGACCGCGCGCCGCACGGCAACACC	42:C>G
5341289|F|0-46:A>G-46:A>G	TGCAGTTGGGCGCCACCGCCTAGCATCTGCGTCGCCGCTTGATTTAACTGCATCGCCGCCGTGCCGTCG	46:A>G
6041888|F|0-17:C>T-17:C>T	TGCAGGTGTGGCCGCGGCCCTACCGTCTCCTCACCG	17:C>T
1864269|F|0-63:T>A-63:T>A	TGCAGGTTGTGCGCCAGGGCGTCGAACGCAATGCAGAGCAGGAGCTCCAGCTGCTGCTGCGGCTGCGGC	63:T>A
3027453|F|0-19:A>C-19:A>C	TGCAGGCCGATTCCCGCGCATTCAAGAGCGCCTCCTACCG	19:A>C
2265324|F|0-28:A>T-28:A>T	TGCAGGTTGCCGGCGCTGGCGTCGAGGGACTGGTCGTCGAGGCCGACCAGCGTGGCCG	28:A>T
1050629|F|0-43:G>A-43:G>A	TGCAGAACTCCAACATGCTCAACAACAAGAGCGAGACGCACCCGTGGCATCTCCATGGTCACGACTTCT	43:G>A
1025085|F|0-23:A>G-23:A>G	TGCAGCAGCGACACCAGCACGGTAGCCTCGCCCAGGTGGACAATCTGCTGGATGCACCTGACGACCTGG	23:A>G
7171043|F|0-19:G>A-19:G>A	TGCAGCACCGTGGACGCCAGGGCGTGGACGCACGGCGGCCGCGTCGCCGCCCTGGTGTTCGTCCGCGAC	19:G>A
3028420|F|0-25:G>C-25:G>C	TGCAGTACTTCTACGAGCTGGACCTGAGCAACAACAAGCTGGCGCCG	25:G>C
4002413|F|0-8:G>A-8:G>A	TGCAGTGGGGCAGTCAAGAAGTATCCCAAGAACAGAATGCTCGGCCG	8:G>A
2245236|F|0-54:C>A-54:C>A	TGCAGGCCGAGGGACCCGAGCTTCGAGCTCATCTCCATGAACCTGTCCACCTTCCACTTCCG	54:C>A
1195887|F|0-31:G>A-31:G>A	TGCAGAAGGTCATGCAGTACTCGCAGAAGGCGAGGCACTTGTTGGGGCAGGTCTTGGGGCAGGAGACGA	31:G>A
12469748|F|0-6:G>C-6:G>C	TGCAGGGCATGAAAAGCCCG	6:G>C
1019556|F|0-44:G>A-44:G>A	TGCAGGTAGTGCAGGTTCGTCAGGCCATCCTCCGTCACCTTGTCGTGGCCG	44:G>A
1022518|F|0-64:A>G-64:A>G	TGCAGAGCCTCCCCTCGCAGATGCAGGCCATCAAGGTGATGGCGACGCTGGACATCCTCTCCTCACACT	64:A>G
990516|F|0-16:G>C-16:G>C	TGCAGCTCAAGTTCGGGGTGGATGAATCGTACAAGCTGACCGTGCCTGCAACCG	16:G>C
1001554|F|0-6:C>A-6:C>A	TGCAGCCCTTCCTCGGCGGCGACACGGCCTACCTCCTGCGCTTCTCGTCGCAGGTCGGGGAGTGGGTCA	6:C>A
980639|F|0-13:T>G-13:T>G	TGCAGCGACGGCCTCCAGTGCCCCCTCGTCTCGTGATCGTCGTGGTAGCACACCGTGCCG	13:T>G
1265236|F|0-13:G>C-13:G>C	TGCAGGACGGCGGGGGGCTGCTCGCCTCCGACCAGCTCCTCTACAACGACAACCGCACGCGGCCCCTCG	13:G>C
5363799|F|0-7:G>A-7:G>A	TGCAGGCGCTCCTCCACGCGCCGCCGCTCCG	7:G>A
5567238|F|0-14:C>A-14:C>A	TGCAGCGGCTCCCGCCGAGCAGCCGCCGCAGGGCGCGCCTCGTCTGCCG	14:C>A
7167914|F|0-10:C>T-10:C>T	TGCAGCTCCTCGCCCTCGTCTCCGATCTCCG	10:C>T
1062823|F|0-49:C>G-49:C>G	TGCAGAAGAGCCTGCGGAACTGCGGGCTGGCGAGGAGGTACCTCCACTCCGAGGTCTTAACCGCGGCAG	49:C>G
985588|F|0-38:T>G-38:T>G	TGCAGCTGCTGGAGCAGGGAGAGGACAATGGCAAGTCATCGGCCGCTCCTCAGGTGTCATCGCTCAAGT	38:T>G
2242955|F|0-61:A>C-61:A>C	TGCAGCTTTGTACGAGCCGAGCGCTCTGGCCGTCGATCAAACGGATTCATCCTTTATCAGGAGCAGCAA	61:A>C
5324394|F|0-7:G>C-7:G>C	TGCAGCTGGTCGTCCCTGACCG	7:G>C
1026369|F|0-22:G>C-22:G>C	TGCAGCAGCTGGGCGGCGACGCGGTGACGAGGCAGATACTCAAGTGCACGCGGTGGCAGCTGGAGGAGA	22:G>C
996221|F|0-68:G>C-68:G>C	TGCAGTTGCTCCTCTATGGCTCTTCCGCCCACGAGCAGGGCGGTGGGGACGGAGCGGAAAAGGAACAAG	68:G>C
1865142|F|0-44:G>C-44:G>C	TGCAGCTCGAGCGGGTGGTGCGTCGACGGCAGCACCAATGCGGTGACCTTGAGCGCGGCGCCG	44:G>C
1107797|F|0-8:C>G-8:C>G	TGCAGCCACCTTATCCAACTATTGTACGTATCTGGTCAGCAAGGATCTGGTGCCG	8:C>G
1690848|F|0-61:G>A-61:G>A	TGCAGGACTGGCAGCGGCGGTACGGCGCCATGCTGCGGCGGAAGGGGAAGGCGGTCAAGGTGCTCGAGT	61:G>A
3028224|F|0-7:G>A-7:G>A	TGCAGAAGGGGAACGTGAAGAAGGAGTACGTGAACCGCGCCCG	7:G>A
1695118|F|0-26:G>A-26:G>A	TGCAGCGCGCGGTCACAGTCCTTGACGACGTGCTTGTGGAGCTCGAGCTGGAAGTAGCAGAAGGCCCGA	26:G>A
2252327|F|0-25:C>T-25:C>T	TGCAGCTCAGGGAGGTGCCCTCTGACGACGCCACCTGTGACGTCGCCCTTGACTGCACGCGGCCATGGG	25:C>T
1028471|F|0-8:G>C-8:G>C	TGCAGCATGGACGGCACGACGAAAATTGCGGCCGCCGCGAGAAGCGAGACGGCCACCCACGGCGACGGG	8:G>C
4262191|F|0-28:A>G-28:A>G	TGCAGGGCAAGAAGGCGACGACGTGCGCATCCATGGCCG	28:A>G
5373818|F|0-7:C>A-7:C>A	TGCAGGGCCACGTCAGCAACCCG	7:C>A
1007364|F|0-19:A>G-19:A>G	TGCAGGTCGTGCAAGAGACATGGACTAGGACGGGGGGCCAGGTGACGGACTACACGCTCTACACGGGCG	19:A>G
1019236|F|0-7:G>A-7:G>A	TGCAGGCGAACGGCGGCAGCAAGGTGGCGAAGAAGATCAGCCCGAAGGACAAGTACTGGGTGGCCGCCG	7:G>A
4544261|F|0-54:A>G-54:A>G	TGCAGGGCGTACGCGGCGGCGGACGTCAGCGGGGGCATCAACCGCGGGTGCGTCATCTGGGCCGTGGAT	54:A>G
1090720|F|0-20:C>G-20:C>G	TGCAGGATTATTGTGCCCGACAGCACGAGCACCGCACCCAGGAGGCACAGGCAGCCGACGACCCAGGCT	20:C>G
4005778|F|0-19:C>A-19:C>A	TGCAGCAGAGCAGCTGGAGCCCGAGCTTGAGCGGCGCCG	19:C>A
990138|F|0-35:T>C-35:T>C	TGCAGAGGGGATGGCTTCTTAGCCTTTCTTTGTCATGAACATGTTAGATTTGCTGGATTAGCATGCCTG	35:T>C
997633|F|0-5:C>G-5:C>G	TGCAGCCGCGCCGTGACGTCCTGCATGACGCCGTTCCACGTGTCGGTCCTCCCCGTGGCGGCCACGAAG	5:C>G
1067955|F|0-14:G>C-14:G>C	TGCAGCGTGTCCGCGGCCAGGAAGCGGAGGTTCATCTCGTAGGGGTCGTTGGTGGAGATGAAGTGGAAG	14:G>C
986473|F|0-11:C>A-11:C>A	TGCAGACGTCGCGGGAGCCCACGCCGAGGCAGTCCAGCTTCAACCAGTCCGACTTCTACTCCATGTTCA	11:C>A
4539051|F|0-7:C>G-7:C>G	TGCAGCTCGGCCTCCTCTCCG	7:C>G

<table>
<tr><td>1002300|F|0-22:C>T-22:C>T</td><td>TGCAGGGCGTCGGCGACGTTGACGAAGAAGGAGTCGGGGTCGGGCGGCACGGCGGCCCAGGAGCCATCG</td><td>22:C>T</td></tr>
<tr><td>6047341|F|0-34:G>A-34:G>A</td><td>TGCAGTCCTTCCTGATCTCGAAGCGGGTGCCCTCGGCGGGGATCTCGCTGAGCTTGGCCATGGCCTTGC</td><td>34:G>A</td></tr>
<tr><td>7492139|F|0-16:A>C-16:A>C</td><td>TGCAGTCGGAGCATCCAGAGAGCTTACCG</td><td>16:A>C</td></tr>
<tr><td>1041202|F|0-10:C>T-10:C>T</td><td>TGCAGGGACGCGGCCATGTCCGCCTACGTCTTCGTTCTCGTCGCCTTGGTTGGCTGCTACGTCTCCGCC</td><td>10:C>T</td></tr>
<tr><td>1102099|F|0-20:C>T-20:C>T</td><td>TGCAGGAGGTTTCTCACGACCTCTAGGGAGATCACCCCCGCGCTGCACCCCATCCCG</td><td>20:C>T</td></tr>
<tr><td>1203637|F|0-10:C>G-10:C>G</td><td>TGCAGAGCGCCGTGGAAATTGACCTGTCGGAGAACAAGCTGACAGGAGTCATCCCCAGCGAGCTCGGCA</td><td>10:C>G</td></tr>
<tr><td>5580523|F|0-24:G>C-24:G>C</td><td>TGCAGGTGGCGGACATGCACTACGGGGACGGGCGGAGCACCG</td><td>24:G>C</td></tr>
<tr><td>3023420|F|0-10:A>G-10:A>G</td><td>TGCAGAGGAGAAGGGCTGAGAGCAAGAAACAGAAGCACCG</td><td>10:A>G</td></tr>
<tr><td>1025720|F|0-22:G>A-22:G>A</td><td>TGCAGGTGCTGTCGCCGCTGGTGCCGATCAGGGAGGTGACGTTCCTGCGCTTCTGCAAGCAGCACGCCG</td><td>22:G>A</td></tr>
<tr><td>1747435|F|0-31:A>G-31:A>G</td><td>TGCAGCGTCTCATATTGGGAAGTGATCACACACTAAAGTGATTTGCCTATCGAGGAGAAAAAGGCTAAG</td><td>31:A>G</td></tr>
<tr><td>6035785|F|0-53:G>C-53:G>C</td><td>TGCAGCAGGCCCGTGAAGCCCTGCGCGAGGCGGTCGGCGATGTCCTGGCCGTGGCGCTGCACCCCCTCC</td><td>53:G>C</td></tr>
<tr><td>1019236|F|0-35:A>G-35:A>G</td><td>TGCAGGCGAACGGCGGCAGCAAGGTGGCGAAGAAGATCAGCCCGAAGGACAAGTACTGGGTGGCCGCCG</td><td>35:A>G</td></tr>
<tr><td>1864269|F|0-57:T>G-57:T>G</td><td>TGCAGGTTGTGCGCCAGGGCGTCGAACGCAATGCAGAGCAGGAGCTCCAGCTGCTGCTGCGGCTGCGGC</td><td>57:T>G</td></tr>
<tr><td>7161899|F|0-5:G>A-5:G>A</td><td>TGCAGGCAGCACTTCTTGCTGCTGTATCCCG</td><td>5:G>A</td></tr>
<tr><td>2252238|F|0-23:C>A-23:C>A</td><td>TGCAGCCCGAACTCGCCGCTCGACTTGCTCATCAGGACGAACTCGTCCATCCGCTTGCCGCCG</td><td>23:C>A</td></tr>
<tr><td>4004832|F|0-7:G>C-7:G>C</td><td>TGCAGTCGGCGGCGGCCTCAACGAACCAAGCGCCGTCGCCG</td><td>7:G>C</td></tr>
<tr><td>5582352|F|0-7:C>G-7:C>G</td><td>TGCAGCGCCTCAGCGCCGAGGTGCTCACCCG</td><td>7:C>G</td></tr>
<tr><td>4397193|F|0-15:G>C-15:G>C</td><td>TGCAGCGCCTGGTCGGCAACAGCGGCAACGCGGCCG</td><td>15:G>C</td></tr>
<tr><td>3034574|F|0-34:G>C-34:G>C</td><td>TGCAGGCATTTCCGCAGCCCGCCGCGGCAACGTAGGCATCGCAAATCGACGCCGCCTGACACCGTGAGT</td><td>34:G>C</td></tr>
<tr><td>5411755|F|0-8:C>T-8:C>T</td><td>TGCAGGTTCCCCGTCCGACGTCCCG</td><td>8:C>T</td></tr>
<tr><td>1178910|F|0-38:C>T-38:C>T</td><td>TGCAGGACGTGGGACTCGTCCTCGTCGGCGTACATGTACTTGTTTTGGTGGCTCCGCAGCCGCACCGTC</td><td>38:C>T</td></tr>
<tr><td>5577051|F|0-56:C>T-56:C>T</td><td>TGCAGCTCCTGCCGCTCCGCCGTCGACATCTCGGGCGCTGGCGCGCCGCCCTTGGCCCCGCTGGCGCCT</td><td>56:C>T</td></tr>
<tr><td>1063721|F|0-31:C>T-31:C>T</td><td>TGCAGCCCGCTCCTCTGGCTCTACGTGATCGCCATGTTCCTGCTCGTCGTCGGCATGTTCTTCCTCACC</td><td>31:C>T</td></tr>
<tr><td>1021222|F|0-31:C>G-31:C>G</td><td>TGCAGGTGACGAAGCGCGACGGCGACCCGCCCAAGAGCGAGTGGCAGAAGTGGAACTGGATATCCGACG</td><td>31:C>G</td></tr>
<tr><td>996156|F|0-52:G>T-52:G>T</td><td>TGCAGAACGCGCTGCTGGAGTTCGTGCGGGTCGTGGAGGCCAAGGAGCAGACGGTGGCCG</td><td>52:G>T</td></tr>
<tr><td>32027961|F|0-8:C>G-8:C>G</td><td>TGCAGCATCTCCTTGCTCCACGGGAGCCCGTCGGCATCGGCGCCG</td><td>8:C>G</td></tr>
<tr><td>1028131|F|0-62:G>T-62:G>T</td><td>TGCAGCGACGTGGGGTTGGCGGCCAGCGCCTTGGAGGCGAGGGGCCACATGTTGAGCGGCGAGCCGCCG</td><td>62:G>T</td></tr>
<tr><td>4006086|F|0-31:A>G-31:A>G</td><td>TGCAGCAGCCAAAGCCCGCCGACCACGCCCAACCCGACGGCCAGCCCG</td><td>31:A>G</td></tr>
<tr><td>1053258|F|0-67:G>A-67:G>A</td><td>TGCAGTTGTCCAACTCCTTTACAAAAACTTCTACAGGCTCTACTACTCAGCCTATAAGCTGCCTCCTGG</td><td>67:G>A</td></tr>
<tr><td>5577174|F|0-52:G>C-52:G>C</td><td>TGCAGTCGATCTCGACCCTGCCGTCCTCGTCGCGCGCGAGGCGGCCGGCCATGGGGTAGAAGGGCACGA</td><td>52:G>C</td></tr>
<tr><td>4396050|F|0-8:G>C-8:G>C</td><td>TGCAGCTGGACGACATCCCG</td><td>8:G>C</td></tr>
<tr><td>1105546|F|0-29:T>C-29:T>C</td><td>TGCAGCGACCTGCCCATCACGCAGTACTTTGACCTCGCCATGCAGGCCAGGCTGCTCAAGCGCCACCGC</td><td>29:T>C</td></tr>
<tr><td>5357737|F|0-13:G>C-13:G>C</td><td>TGCAGCCGTTCTCGAGGGAGGAGTGGAGCTCCCACCG</td><td>13:G>C</td></tr>
<tr><td>1059990|F|0-51:G>A-51:G>A</td><td>TGCAGCGCTTGCCTAAACATCCCAGAGCCGCGGGGCACGCCTAGAGCTTCGGCGCATGCCACCATCTCC</td><td>51:G>A</td></tr>
<tr><td>6047307|F|0-52:C>G-52:C>G</td><td>TGCAGGTCATCATGTCGTTCCACGCGTGCGGCGGCAACGTCGGCGACGCCGTCAACATCCCGATACCGA</td><td>52:C>G</td></tr>
<tr><td>32020864|F|0-46:A>G-46:A>G</td><td>TGCAGGGCCTGCGCGCCGTTGACGGCGAGGACCCGCCCAGGATACTACACCTCAACCCTAGGCTGAGGG</td><td>46:A>G</td></tr>
<tr><td>32016349|F|0-12:A>C-12:A>C</td><td>TGCAGACGGTCCATACATACGCGTGCGGCCG</td><td>12:A>C</td></tr>
<tr><td>5365277|F|0-55:C>G-55:C>G</td><td>TGCAGCATCGTGCGCGCGGGATTGCGCGGATTGGTGCTGGGGCACCGTGCGCGCGCGCGTGTGGCTCGC</td><td>55:C>G</td></tr>
<tr><td>3960718|F|0-28:G>A-28:G>A</td><td>TGCAGGTGATGAGCACTTCAGGGAAGTCGTCGTGGCCG</td><td>28:G>A</td></tr>
<tr><td>32023687|F|0-10:A>G-10:A>G</td><td>TGCAGCGCGAAACGTTCCCTGATGTGATGAGGCTCATGGACAAGCACGAGCAGATCGACCGCATCCTTT</td><td>10:A>G</td></tr>
<tr><td>5358346|F|0-25:C>T-25:C>T</td><td>TGCAGGGCGTCCGCCGCGGCGCGCGCGGCGCCCCG</td><td>25:C>T</td></tr>
<tr><td>1081003|F|0-26:G>A-26:G>A</td><td>TGCAGTTGAGGATCCGCGGGTCGCGGGCGACGATGTGGGCGATGTCGGCCTTGGAGAAGCGGAGGGTGG</td><td>26:G>A</td></tr>
<tr><td>2251783|F|0-28:G>T-28:G>T</td><td>TGCAGACGCGCACGGAGACGACGCCGCCGATCTGGGCGAAGACGTCGAAGAGCTGCGCGTCCTGCACGC</td><td>28:G>T</td></tr>
<tr><td>2246123|F|0-17:G>C-17:G>C</td><td>TGCAGCTTGAGCGGGCAGACGGCCTCGATCTTGCACTTGGTGTAGCGCGCCGTGTAGCTCACCTTGCGG</td><td>17:G>C</td></tr>
<tr><td>6040123|F|0-19:A>G-19:A>G</td><td>TGCAGCAGCTGTCGCGCGGAGCGCGGAGTTCGAGCGCGGATAGGCGCGTGGAAGCGGCACTGGAGGCGG</td><td>19:A>G</td></tr>
<tr><td>1128688|F|0-31:C>G-31:C>G</td><td>TGCAGCGCATCGGGGTCGGCCTCGGCACCGTCGCCTTCTCCATGGTCGCCGCCGCCACCGTCGAGCGCC</td><td>31:C>G</td></tr>
<tr><td>5332167|F|0-32:C>T-32:C>T</td><td>TGCAGCTTGCGCAGCTGGCCGAGCTGCGGCGGCACCG</td><td>32:C>T</td></tr>
<tr><td>989478|F|0-32:G>C-32:G>C</td><td>TGCAGCAGCCCCACGGCCACCACCACGTCCATGCCCGCCTTCACCAGCGCCAGCAGCCG</td><td>32:G>C</td></tr>
<tr><td>4733935|F|0-50:T>C-50:T>C</td><td>TGCAGGCTGGCCTCGAGCAGCGCGGCCTGCTTGCCGACGTCGGCGTCGGCTACGAAGCCGCCGAACCGA</td><td>50:T>C</td></tr>
<tr><td>2251206|F|0-61:T>C-61:T>C</td><td>TGCAGCAGCCTGCTCACCGTCGACATGAGGGGCCTCCTCTTCCCGACCACGACCACCACTGTCGCCGCC</td><td>61:T>C</td></tr>
<tr><td>1120791|F|0-30:A>G-30:A>G</td><td>TGCAGACAGGGCAAAGACCATCCTTGCTCTAGCCTCCCCTGACTCCAAGCCCAGTGACCTCAGAAGGCC</td><td>30:A>G</td></tr>
<tr><td>1027759|F|0-13:A>C-13:A>C</td><td>TGCAGGAGCCATGACGGAAGTCGTGGTGCGGGGTGATCCTGAGGCGGCCGCCG</td><td>13:A>C</td></tr>
<tr><td>1228175|F|0-41:C>A-41:C>A</td><td>TGCAGACGAAGCAGCAGTTCATCTACGGCAGCGTCTCCACCCGCATCAAGCTCGTCCCAGGGAACTCCG</td><td>41:C>A</td></tr>
<tr><td>7170555|F|0-19:T>C-19:T>C</td><td>TGCAGCACTGCCACGAGCGTAACATCCTGCACCG</td><td>19:T>C</td></tr>
<tr><td>989876|F|0-11:C>T-11:C>T</td><td>TGCAGGAGGGACTCGTCGGCGAGGAGGAAGAAGCCGATCTGCTTGGGGTCCGCCCGCGCGAAGGAGGAG</td><td>11:C>T</td></tr>
<tr><td>1021037|F|0-66:G>A-66:G>A</td><td>TGCAGGTTGTACCCTCTTCTTAGGCTCCCGTGGCTTTGACTCAGCCTTCTTGATGCCACCCTTGGCGGC</td><td>66:G>A</td></tr>
<tr><td>1182699|F|0-49:G>A-49:G>A</td><td>TGCAGCTCGACGCTGAAGGCAGCAACAGCAACAACTACAGGGCCATGCAGCAGATCACCTGGGCTGCCG</td><td>49:G>A</td></tr>
<tr><td>1106728|F|0-53:C>T-53:C>T</td><td>TGCAGCGCCGACATCTCCGCCATTTCTAGCGCCTGGGCCAGGTTCGGCCTCTCCTCCAGGCGCATGATC</td><td>53:C>T</td></tr>
<tr><td>990479|F|0-41:G>C-41:G>C</td><td>TGCAGCTTCTGCTGGACCATGGCGTTGTGGCGCTGCGCGAAGTCGTTGTACGTCCTCAGGCACCCGACG</td><td>41:G>C</td></tr>
</table>

12775319|F|0-7:G>A-7:G>A	TGCAGCAGCTCGCCCAGCGTCACCACCG	7:G>A
5369354|F|0-32:C>G-32:C>G	TGCAGGAGCCTGGCGCACGCGTGCGAGTCGACCCCG	32:C>G
990516|F|0-8:A>C-8:A>C	TGCAGCTCAAGTTCGGGGTGGATGAATCGTACAAGCTGACCGTGCCTGCAACCG	8:A>C
2247355|F|0-41:C>T-41:C>T	TGCAGCATCCTCATCTGCATCTGCTTCGGCGCCAAGATCCCCGACGAGCTCATCGTGGAGATCGAGGAG	41:C>T
3942413|F|0-28:C>G-28:C>G	TGCAGGAGGCGGACCACGGCGGCCGTCTCGAGAGGGACGACATATGGAACATGATCCAGTCGCAGAAGC	28:C>G
6041888|F|0-32:A>G-32:A>G	TGCAGGTGTGGCCGCGGCCCTACCGTCTCCTCACCG	32:A>G
5332443|F|0-19:G>A-19:G>A	TGCAGGGGAACACCATCGCGGTGCTCGACACCG	19:G>A
2306194|F|0-37:C>T-37:C>T	TGCAGCGCAACGACGTCGCCGCTGCCATCGCGCGCACCGACGTCTTCGACTTCCTCGTCGACATTGTGC	37:C>T
989600|F|0-51:G>A-51:G>A	TGCAGACACCATGGACTCGGTGAAGGACGTGCTGGGGAAGATGGGCAAGAGGTTCGGCGAGGCCGCCCG	51:G>A
5581952|F|0-11:G>C-11:G>C	TGCAGCCAGAGGAGCAACGGCGTGTCGACCG	11:G>C
7491847|F|0-13:C>T-13:C>T	TGCAGCAGAGTGGCAGGAGCCATGTCCG	13:C>T
3026293|F|0-11:C>T-11:C>T	TGCAGCGACATCGCCGAGGGGGTTGCCTACCTGCACCACCACTCGCCG	11:C>T
1059183|F|0-13:G>A-13:G>A	TGCAGTACGAGGCGCGCATGCTCCAGAAGGAGCTGGAGGTGAGGAGCCAGGAGCGGGAGTACGACCTCA	13:G>A
2247936|F|0-28:A>C-28:A>C	TGCAGGAACGTCTCGCTCTCCCCCGACGAGATGAGCGCATCGATCGTGCTCTCCTCCGCCGTCTCCCCC	28:A>C
5371632|F|0-14:G>C-14:G>C	TGCAGCGCGAGCGTGTTCGGGGTGTCGCCG	14:G>C
4910730|F|0-49:A>G-49:A>G	TGCAGGTGGTAGCTCCATCGGCATGTCCAACTTTCGGGTGGTCGCCGTGACCTACGAAAGCGATGTTTT	49:A>G
1022238|F|0-23:C>T-23:C>T	TGCAGATACGCTCTCCCCGTTAGCCGAGTGGTGGTCAGCTAGCGAGCTGAGGCCACTCGTACGCAGTGG	23:C>T
2251206|F|0-23:C>T-23:C>T	TGCAGCAGCCTGCTCACCGTCGACATGAGGGGCCTCCTCTTCCCGACCACGACCACCACTGTCGCCGCC	23:C>T
2247355|F|0-50:C>A-50:C>A	TGCAGCATCCTCATCTGCATCTGCTTCGGCGCCAAGATCCCCGACGAGCTCATCGTGGAGATCGAGGAG	50:C>A
32022895|F|0-19:A>C-19:A>C	TGCAGCTGACCAGCAGCATAGGGTCCATCATCGGGGGCTTCTCCGCCCTGCTGCTGGCGCCGACCACCA	19:A>C
1357131|F|0-68:A>G-68:A>G	TGCAGGGCACAGACGGCGGTGAACAGGAGATCAAGGACGTGGCGGCTGGAGGTGGGGAGCAGAGCAATA	68:A>G
1063972|F|0-9:A>G-9:A>G	TGCAGGAGAACAGATGCATCTCACCAAGGCAAGAACTGGCCGTGCAAACTCAGGCCAAGAGACACAAGG	9:A>G
3934408|F|0-16:G>C-16:G>C	TGCAGTTCTTCCGCGCGGACAAGGCGGCCG	16:G>C
32025864|F|0-17:G>A-17:G>A	TGCAGGGCGTCGCCGACGGTACCGTGGTGAAGAAGCTGGTGCCCAACCG	17:G>A
32019378|F|0-11:A>C-11:A>C	TGCAGAGGAGGAGGCGGAGCCCCAGAGGCCTGCGGCTGAGGCGCGTGCTGGCCGTTCATTGGCGGCTGG	11:A>C
7351962|F|0-22:G>C-22:G>C	TGCAGCTGGTGATGTTGCTGGGGCGCGTGCTCGGCTGCCG	22:G>C
980226|F|0-62:A>G-62:A>G	TGCAGGGTGTGGAGGAGGAAATTGCGGACGATCTCGGTCTCCCCGCGCATGAGGAAGGCAAGAGCGGAG	62:A>G
1202124|F|0-23:G>C-23:G>C	TGCAGCTCGTTGTACGCCTCATAGAGAAGCCCCCGCGCGCTCTCCTCCTCATCCTCACCCG	23:G>C
1863335|F|0-38:C>T-38:C>T	TGCAGCTCGCTGGTCACCACATACCTGCCGCCACTCCTCTCCCTCGACCACACTGTGGTGGAGGGGGAC	38:C>T
100503874|F|0-7:C>T-7:C>T	TGCAGGGCTGGGGAAGGTGGAGGGCGAGAAGGAGGAGGAGCGGGAGGCGTACCTGCGGCTCGCCGTGAC	7:C>T
2258576|F|0-17:C>T-17:C>T	TGCAGAGCACCGTCGCGCTGCTGCCGTGGTCCGAGGACCTCAGGCTCGTCGCGCGCTGCGTCGACTCCA	17:C>T
1025619|F|0-43:A>G-43:A>G	TGCAGAGGTCCACGGTGGAGGTGGTCGGGCACAAGGCGCACGGAGCCGTGCCGCAGCCG	43:A>G
1021897|F|0-7:A>G-7:A>G	TGCAGACATGGAAGCAGCAGGCCATCTTCTCCGACCCCAAGAACCTCACCG	7:A>G
998431|F|0-31:C>T-31:C>T	TGCAGCTCTTCACGTCGTCGCTCTACCTGGCCGCGCTCGTCACCAGCCTAGGGGCGTCCATGGCGTGCA	31:C>T
3953445|F|0-24:G>C-24:G>C	TGCAGCGCGGAGGCGCGCGGGCAGGCGGCGCCGTCGCACGCCG	24:G>C
1863316|F|0-8:C>G-8:C>G	TGCAGCTTCGTGTAGTTGCCGTGCGCGTCGACGTGCGTCAGGGCCACGCGCAGGCCCTCCAGGCGGCCC	8:C>G
1205521|F|0-53:C>G-53:C>G	TGCAGCGCCCAGTGCTTCTGGATGAAGGACGACTCCCAGCTCCGCTCCTTGTCCGCCAGCGACGACGGC	53:C>G
985942|F|0-5:C>T-5:C>T	TGCAGCTGTTGATGCAGCTCATCTCCACCGTCATGGTGGGGCACCTCGGCGAGGTCGCGCTCGCTGGCG	5:C>T
6046154|F|0-48:A>G-48:A>G	TGCAGAAGATACACCCGCACGGGACACGTAGTCGATTATCCTGATAGCATCAGAAACGCGCAGAGCCGC	48:A>G
4261689|F|0-23:G>C-23:G>C	TGCAGCAGCACGGCGCGGCTCGGGAGGAACACCATGTTGGTCAGCCCG	23:G>C
6049258|F|0-13:A>G-13:A>G	TGCAGCATGAACGACAATGGCACCCAGTCCCAGTGCGGGTATCGAGTCGAGTACGGAGGAGGGTCAAAG	13:A>G
5374266|F|0-13:A>G-13:A>G	TGCAGGTGGCGCAACCATAGCCG	13:A>G
1092000|F|0-17:G>C-17:G>C	TGCAGCGCGTACGCCAGGCGCACGATGTCCCGCCGCTCGCGCCGCTTGGGCGTGGTCAGCACCCCGACG	17:G>C
4262191|F|0-9:A>G-9:A>G	TGCAGGGCAAGAAGGCGACGACGTGCGCATCCATGGCCG	9:A>G
1053258|F|0-36:G>A-36:G>A	TGCAGTTGTCCAACTCCTTTACAAAAACTTCTACAGGCTCTACTACTCAGCCTATAAGCTGCCTCCTGG	36:G>A
1064334|F|0-14:G>A-14:G>A	TGCAGGCACCAGGCGGACACAGCGCCGCGACGGCCGACGCCCCAACCACGCCGTAACAGCGAGATGCCA	14:G>A
1081125|F|0-11:G>A-11:G>A	TGCAGACCGTCGTCGCCGTCACGCTGGCCACCACGCTGCTGGGCATTCCGTACCCGCGGAGCTTTGTGG	11:G>A
3030819|F|0-5:A>C-5:A>C	TGCAGAACAAGCCCGTCCCTATCTGCTACGACGGGTTCGAGCCCTCCG	5:A>C
1023925|F|0-16:C>G-16:C>G	TGCAGTTCACGCAGATCTGCAACCTCTGCTACCCGTGGCCGTGCAGCGACCCGAGCCACTGGGGAGGCG	16:C>G
2259109|F|0-64:A>C-64:A>C	TGCAGCTGACGTGGAACTACAACTACGGGGCGGCGGGGAAGAGCGTGGGGTTCGACGGGCTCAAAAACC	64:A>C
1264244|F|0-55:G>A-55:G>A	TGCAGCTGGCGTTCCAGAGCATCGGCGTGGTGTACGGCGACGTGGGGACGTCGCCGCTCTACGTGTACT	55:G>A
985942|F|0-49:C>T-49:C>T	TGCAGCTGTTGATGCAGCTCATCTCCACCGTCATGGTGGGGCACCTCGGCGAGGTCGCGCTCGCTGGTG	49:C>T
1357114|F|0-27:G>A-27:G>A	TGCAGGGCTGGGGAAGGTGGAGGGCGGGAAGGAGGAGGAGCGGGAGGCGTACCTGCGGCTCGCCGTGAC	27:G>A
4006165|F|0-8:G>C-8:G>C	TGCAGCGGGACGGTCTGGTTGAAGGCGTTGGACGCGAGGCTGAGGCCG	8:G>C
1083263|F|0-65:C>A-65:C>A	TGCAGCACAGACTCGGGTGCATCCCAGGGGTGGTGTATCCATTCCGTTGGTAGCCTCGCCAGCTCCGAG	65:C>A
980226|F|0-41:C>A-41:C>A	TGCAGGGTGTGGAGGAGGAAATTGCGGACGATCTCGGTCTCCCCGCGCATGAGGAAGGCAAGAGCGGAG	41:C>A
1112909|F|0-26:G>A-26:G>A	TGCAGGAGTACAAGGCCCACGCCGACGGCTTCATCTGCTCCATGGTGCCCG	26:G>A
1071080|F|0-47:C>G-47:C>G	TGCAGGGCGATGGGCGACCAGCTGGAGGGCGACGAGGGGCAGCACATCAAGTACCG	47:C>G
7902442|F|0-16:C>G-16:C>G	TGCAGTCGTGGTGCGGCTTCTACCAGAGCCTCCG	16:C>G

3961280|F|0-9:G>C-9:G>C	TGCAGCCGCGTGCGGCCGTCGGCCCG	9:G>C
1234850|F|0-20:T>G-20:T>G	TGCAGCCCGACGTCGGAGAGTGCGAGGTTGTCCAGCACGGCGCCGACGGGGGCCAGCTTGCGGAACCCG	20:T>G
5582436|F|0-14:C>T-14:C>T	TGCAGCCTCAGCGCCGCCGTCGGCATATGCGGCCCG	14:C>T
5347188|F|0-20:C>T-20:C>T	TGCAGAGCCGAGATGCTCGGCCCG	20:C>T
1020980|F|0-29:C>A-29:C>A	TGCAGCTTGATATGGTTGTAGTAGCCAACCCCTAGGAAGGCGATGCCGTAGCCGAAGAGGTTGATGGGG	29:C>A
1209486|F|0-20:G>A-20:G>A	TGCAGGAGCTCCCGCCTCCCGCCAGCCACCGCCTTCTCGTTCGCCTTCACGAACCCG	20:G>A
6042727|F|0-65:G>A-65:G>A	TGCAGCTGCCTCAACGTGGAGAAAATAGCGGGCTGCTCCAGAGAAAAACCTCCGCCATCGTCATCGCGT	65:G>A
2260066|F|0-10:T>C-10:T>C	TGCAGCGCCTTGAGTCCGCGGCCGCCCTCGGCAACGGTGGCGGCCTCGAGACAGAGCACTACAGCCCAT	10:T>C
1215684|F|0-62:C>G-62:C>G	TGCAGCGAGATGGCCACCGACAGGCTGCCGTCGCGGCTGGCGCTGGGGAGCACGAACGCGAGCCCCTCG	62:C>G
1022191|F|0-44:C>T-44:C>T	TGCAGGAACAAGGTGGTGGCCTGCGCAAAGCACTTCGTCGGCGACGGCGGCACGGTGAACGGCATCAAC	44:C>T
5577028|F|0-11:C>T-11:C>T	TGCAGCGGCGACGGCATGGGCGACCTCTGGCCCGTCGGCTTCGGCGGCGGCGTCGCCATGGGCTCCTCC	11:C>T
1096095|F|0-54:T>C-54:T>C	TGCAGGGCGAGGACTCGCAGATTGCAGAACCCCCCTGCAATGACGTCCAGATCGTCGTCGATAACACCG	54:T>C
4005798|F|0-29:A>G-29:A>G	TGCAGGTACACGGCGCCGCGGTAGGGATGAGCGGCCG	29:A>G
2261024|F|0-44:C>T-44:C>T	TGCAGCACGAGCCCGACAGCCACGGCGCCGAGGCTCACCAGCGCCACCTTGAGCGTCGACAGCGCGAGC	44:C>T
3025819|F|0-9:C>T-9:C>T	TGCAGGAGGCTCCTGCGTATCTCGCCATGCATCCCGCCG	9:C>T
1027759|F|0-25:G>A-25:G>A	TGCAGGAGCCATGACGGAAGTCGTGGTGCGGGGTGATCCTGAGGCGGCCGCCG	25:G>A
3941453|F|0-10:T>G-10:T>G	TGCAGTACCTTGACCTGTCCG	10:T>G
1049669|F|0-13:T>C-13:T>C	TGCAGCTTCTGCGTGGTCTGGTCCAGCTCCTGCTTCAGAGAAGAAACCAAGGTGGCGTCCGAGGACACC	13:T>C
988691|F|0-6:G>A-6:G>A	TGCAGTGTGTTGCTTGCATCCCCTGGGACCAAAATGCTGCGAAGTTTTGTGGACAGTCTTCCG	6:G>A
1209817|F|0-10:C>G-10:C>G	TGCAGTTGGTCGACGGCACGGCGGAGAGCTCCAGCCTCCCCGTCCACTCCTCCCCG	10:C>G
1002827|F|0-32:C>G-32:C>G	TGCAGCAACGGCATGAAGCTCGTGGTCAACGTCGCCGCCGCCACCGCCGCCTCGCCGCCCAAGGCGTCC	32:C>G
5578330|F|0-12:G>A-12:G>A	TGCAGGGCGCGGGGGTTCCACGTGAAGCCG	12:G>A
4734029|F|0-19:G>T-19:G>T	TGCAGGAGGAGGTGTACGGGACGGGGTCGAGCCTGCCG	19:G>T
1025480|F|0-49:G>A-49:G>A	TGCAGGCGTCCTTCACGGGCGCGCTCCACTCCCGCGTGGACGGCGGCGGGAGCGTGCTGGTGGCACTGT	49:G>A
3024183|F|0-34:T>C-34:T>C	TGCAGGGGCCATTCGCCATCCGCATCACCAGCGATACCG	34:T>C
3941646|F|0-8:G>C-8:G>C	TGCAGCGGGGGAGCCCAAACCGCCGTACACGCCG	8:G>C
6040123|F|0-8:C>T-8:C>T	TGCAGCAGCTGTCGCGCGGAGCGCGGAGTTCGAGCGCGGATAGGCGCGTGGAAGCGGCACTGGAGGCGG	8:C>T
6045443|F|0-19:T>C-19:T>C	TGCAGCAGCGAGGCGAGAGTTTGGCCTCCACGCCG	19:T>C
1049482|F|0-50:C>T-50:C>T	TGCAGGACGACCTGCACCACGGAGCCGAACCTGAGCTTGTACAGCTTGGTCGCCG	50:C>T
1741166|F|0-18:G>T-18:G>T	TGCAGGCCCTCCATCCGCGCCGCCATGGCGTCGCCCACGGCCCTGACCTCCCGCTCGATCCCCGCCGCC	18:G>T
5578332|F|0-9:T>C-9:T>C	TGCAGGGCGTGGTGGTCGGGGCGGTCACCG	9:T>C
999296|F|0-11:C>T-11:C>T	TGCAGGCATCGCTGACCGCGGCGGCCCCGCCGCCGCCGCCATCGTGGGAGCTGCCG	11:C>T
3939216|F|0-20:G>A-20:G>A	TGCAGCAGCTGGATATTGGCGGTGAAATCTCCAACCAGGAGCCGCTTCCG	20:G>A
986348|F|0-62:G>C-62:G>C	TGCAGCGTCTTGACGTACTTGATCGCGTCGCCCAGCACCGACGCCTTGTCCATCTTCTTGAGGCCCGAG	62:G>C
5581798|F|0-29:C>T-29:C>T	TGCAGCTCCCCGCACTGCGGCGCCGAGCACGGCACGGCGGCGTACGTGCTGGACGCGCGCGGGTTGTAG	29:C>T
1237244|F|0-16:A>G-16:A>G	TGCAGCCCATCCCGGCAAGGTGCACGCTGCGCACGTCCTCGCGGAGCTTGTACCG	16:A>G
1028471|F|0-14:C>A-14:C>A	TGCAGCATGGACGGCACGACGAAAATTGCGGCCGCCGCGAGAAGCGAGACGGCCACCCACGGCGACGGG	14:C>A
5577874|F|0-16:G>A-16:G>A	TGCAGAGCATACAGGCGGCGCAGATGCTGCCCCG	16:G>A
7902416|F|0-8:T>C-8:T>C	TGCAGGTGTGCGCTGCCATGTCCG	8:T>C
1338752|F|0-44:T>C-44:T>C	TGCAGCGGCCAAGCAGAAGGTGAAGAAAAATGCCAAGAAGGAGGTGGTGGCGGCCGCGGTAGATGATGG	44:T>C
1090899|F|0-46:G>C-46:G>C	TGCAGAGCGCGTAGAGCGCCGTGGCGGCGTCCTTCTTCCCGCGCGCGCCGCCG	46:G>C
5363987|F|0-11:C>T-11:C>T	TGCAGGTTGGACAAGCTCGCGACCTGCGCCG	11:C>T
1023921|F|0-16:C>T-16:C>T	TGCAGCTCGCCCTGCCCCTCCTGCTGGTGGCCATGGCCTTCGCGCTTCAGAACTTCGCCAAAACCAAAA	16:C>T
2247351|F|0-10:C>G-10:C>G	TGCAGCGTGCCATGGCGTTCCTCGAGGACGAGTTCCACGCGCTGCTCGAGCCCAAGTCGGACACCTGCA	10:C>G
1104410|F|0-25:C>T-25:C>T	TGCAGGGGCGCTACGAGATGGGCCGCGTGCTCGGCCACGGCAACTTCGGGCGGGTGCACATCGCGCGGG	25:C>T
4394517|F|0-21:C>A-21:C>A	TGCAGCTGGATGCAGATTGCACGGCCG	21:C>A
1025479|F|0-31:G>C-31:G>C	TGCAGGCGTCGGTGATCCGCGACCTGGTCCTGCTCTCCTGCGTCGGCCTGCGCCCCGTGCTCGTGCACG	31:G>C
4261480|F|0-16:T>A-16:T>A	TGCAGAAGCGCGGGTATCGGGCGAGCTGGGCGGCGATGCCG	16:T>A
2249417|F|0-37:G>A-37:G>A	TGCAGATGTCCAGCTTCAAGGACGACTCGGGCCACCTGGCCTGGGACTGCTCCGCGTGGGTGCGCACCT	37:G>A
1019805|F|0-11:A>G-11:A>G	TGCAGCCTGCTATTCTTCCCACCGAAGATCTGGTTAAGGATCACCGCCAGCCTCACGCCCCCCTGAGCG	11:A>G
5364336|F|0-29:G>T-29:G>T	TGCAGCGCCACGCTCACCGCGAGCGCCTCGCCG	29:G>T
4262146|F|0-23:G>C-23:G>C	TGCAGCGGGACGGCGGCGAAGGAGTCGGAGCTCTCGAAGCCG	23:G>C
1698163|F|0-53:G>C-53:G>C	TGCAGCTCCCTCCTCTCCTCCTCGCCGCCGCCGAACTCGCAGAGGGAGTAGGCGACCATGTCCTCCTCG	53:G>C
7161321|F|0-32:T>C-32:T>C	TGCAGCGGCATGCGGAACTCGCCAAGCGCGAATCCCGCGGCCGCGTACTCCG	32:T>C
6044914|F|0-22:G>A-22:G>A	TGCAGCGCCTGCACGGCTTCTCGCTGTCCCCGCAGCACGCGCAGGTCCATAAGCTCTCGTGGGAGCAGG	22:G>A
994831|F|0-7:G>C-7:G>C	TGCAGGTGGGGGCGGCGCTGGAGACGTCCATCAACGAAACGCTGGCAGAGCTCAGCGCCCCGCTGCTGG	7:G>C
7162797|F|0-11:C>G-11:C>G	TGCAGTTCTTCCAGCAGGGGTCGCTCCCG	11:C>G
2244667|F|0-23:G>C-23:G>C	TGCAGGAACTTCGTCGAGCTCGCGCGCCGCAAATACTACGACAACGTCGTCTTCCACCGCATCATCAAG	23:G>C
3029508|F|0-7:G>C-7:G>C	TGCAGCAGGCTCTGCTCCCTCCTCCTCGCCGTCATCCTCGTGGCCG	7:G>C

<table>
<tr><td>7169940|F|0-45:T>A-45:T>A</td><td>TGCAGAACACCACCCAGTTCGGCCTCATGGCCGTCAGCCTCGGCTTCTACGACACGCTCATGTCCTGCT</td><td>45:T>A</td></tr>
<tr><td>5364126|F|0-9:T>C-9:T>C</td><td>TGCAGTTCGTCCTGATCTCGGCGTGGCGGCCG</td><td>9:T>C</td></tr>
<tr><td>1111609|F|0-19:C>T-19:C>T</td><td>TGCAGGCCACCGTCCGCCGCGTCACCAAATCGTACGCGGTCTGCAAGTACATCCCG</td><td>19:C>T</td></tr>
<tr><td>32023343|F|0-12:T>C-12:T>C</td><td>TGCAGCTCGCAGTGCAGAAGGCAGCCCTTCTCCAGCAGCGACCTCCCCG</td><td>12:T>C</td></tr>
<tr><td>7168053|F|0-21:T>C-21:T>C</td><td>TGCAGGAGGAGTTCGGCGGGGTGGAGGGCGCCG</td><td>21:T>C</td></tr>
<tr><td>32026228|F|0-20:G>C-20:G>C</td><td>TGCAGGAGCTGGAGGACGAGGCGGCCAAGGGGTACGCGCCGTCGCTGCGCCACGCCG</td><td>20:G>C</td></tr>
<tr><td>32024487|F|0-66:A>G-66:A>G</td><td>TGCAGCTGCAAACGAGCTGTACAAGGTAGCCAAATCGCTGGGCCTCGCCGACCAGGACTTCTCCGCAGT</td><td>66:A>G</td></tr>
<tr><td>1111624|F|0-9:T>G-9:T>G</td><td>TGCAGGAGCTGGAGGACGAGCAGGCCAAGGGGTACGCGCCGTCGCTGCGCCACGCCG</td><td>9:T>G</td></tr>
<tr><td>6045573|F|0-33:C>G-33:C>G</td><td>TGCAGCTACTGTGCGTAGCTCTCGTGTAGGCAACTTCCTAGGCTGGTCCTCCCG</td><td>33:C>G</td></tr>
<tr><td>4987663|F|0-52:G>C-52:G>C</td><td>TGCAGCGCGCCGCCGCGCTCTACAACCCGCAGCTGGTGCAGATGACCAAGGAGCTCAACGCCCAGTTCG</td><td>52:G>C</td></tr>
<tr><td>1029980|F|0-17:G>A-17:G>A</td><td>TGCAGCTGGTTTATCTCGGCACCCGCATCATAAACAAGAACTGGATCAATCCCTTCGGGAGGCACTGCT</td><td>17:G>A</td></tr>
<tr><td>3028382|F|0-25:A>G-25:A>G</td><td>TGCAGAGCCGCGAGACGGACTCTGAAATCTCCACCCACGACTCCG</td><td>25:A>G</td></tr>
<tr><td>1097886|F|0-30:C>T-30:C>T</td><td>TGCAGCTGGCACGGACCGCCGCGAGGCCAGCGAGGCGCGCGCTGGTGGTGGAGGCCCGCGGGGGCAGGG</td><td>30:C>T</td></tr>
<tr><td>1696848|F|0-32:C>G-32:C>G</td><td>TGCAGGTCCGAGTCCCCCGCGTAGCGCCCCGTCCCGTCGATCCCGTGCTCGTCGCAGATCACCTCCCAG</td><td>32:C>G</td></tr>
<tr><td>1696572|F|0-27:C>T-27:C>T</td><td>TGCAGAGGTTCGAGAAGGCGGACGCCGCGGGCGAGGCGCGGCGGATGGTCTGCGCCGCCGACAGCAACA</td><td>27:C>T</td></tr>
<tr><td>4262854|F|0-13:C>T-13:C>T</td><td>TGCAGAAGCGAGGCGCTCGTGAAGGCGACGTGACATCCG</td><td>13:C>T</td></tr>
<tr><td>1000330|F|0-11:A>G-11:A>G</td><td>TGCAGCTGGTCACCCAGGGAAGACTGCGATTGGATCGTCTTCTCCTCCTCCG</td><td>11:A>G</td></tr>
<tr><td>1065387|F|0-62:C>G-62:C>G</td><td>TGCAGCATCTTGATCACGTCCACCGTGTCGTCCAGGTAGTACTTGGCCATGCTCGGCTTCTTCCCCACG</td><td>62:C>G</td></tr>
<tr><td>1062823|F|0-46:C>A-46:C>A</td><td>TGCAGAAGAGCCTGCGGAACTGCGGGCTGGCGAGGAGGTACCTCCACTCCGAGGTCTTAACCGCGGCAG</td><td>46:C>A</td></tr>
<tr><td>1178910|F|0-11:G>C-11:G>C</td><td>TGCAGGACGTGGGACTCGTCCTCGTCGGCGTACATGTACTTGTTTTGGTGGCTCCGCAGCCGCACCGTC</td><td>11:G>C</td></tr>
<tr><td>1178910|F|0-53:C>T-53:C>T</td><td>TGCAGGACGTGGGACTCGTCCTCGTCGGCGTACATGTACTTGTTTTGGTGGCTCCGCAGCCGCACCGTC</td><td>53:C>T</td></tr>
<tr><td>2244667|F|0-5:G>A-5:G>A</td><td>TGCAGGAACTTCGTCGAGCTCGCGCGCCGCAAATACTACGACAACGTCGTCTTCCACCGCATCATCAAG</td><td>5:G>A</td></tr>
<tr><td>1065076|F|0-60:G>A-60:G>A</td><td>TGCAGAGAGCCAAAGCCAAGAAACCAAGAACCAAGCAGATGATGCCAGCAAGCCTAACGAGGCCGCCGA</td><td>60:G>A</td></tr>
<tr><td>3031628|F|0-32:G>A-32:G>A</td><td>TGCAGCTCCACCGCCGAGGCGCGCTGCTGCGCGTGCGAGCTCATGCCG</td><td>32:G>A</td></tr>
<tr><td>3027219|F|0-16:G>T-16:G>T</td><td>TGCAGTTCAATGCCAAGGAGGCGGACTGCGTCCAGGTCCAGCTCGCCAAGTTCCTGGTGGAGAACGCCA</td><td>16:G>T</td></tr>
<tr><td>1019946|F|0-17:A>G-17:A>G</td><td>TGCAGGCGCAGGAGGACATCGTGGGCGAGATGAAGGAGAACGCGTCCAAGACGCTCGTCCGCGTCACCA</td><td>17:A>G</td></tr>
<tr><td>994427|F|0-32:C>G-32:C>G</td><td>TGCAGCATCCGCGCCACCATCAGCGCGATGTGCGTCGTCCCCATGGCCAAGCCCG</td><td>32:C>G</td></tr>
<tr><td>1000346|F|0-23:C>G-23:C>G</td><td>TGCAGCTGCGCGTCGCGTATGGCCACGGCCTTGGCCGCCTCCACCTCCGCCACCCTCGCCTGCTGCTCC</td><td>23:C>G</td></tr>
<tr><td>1021386|F|0-21:C>A-21:C>A</td><td>TGCAGTCCTGCTCCTCGCTGGCGTCTTCATCCTCTCGCGCCGCCTCTCTCGCGACCCCGCAGGTTCGTG</td><td>21:C>A</td></tr>
<tr><td>1065387|F|0-53:C>G-53:C>G</td><td>TGCAGCATCTTGATCACGTCCACCGTGTCGTCCAGGTAGTACTTGGCCATGCTCGGCTTCTTCCCCACG</td><td>53:C>G</td></tr>
<tr><td>1001476|F|0-5:C>T-5:C>T</td><td>TGCAGCGGCGTCGTGGTCTCGCAGGGAGGGCCGAAGTAGTTGCCGTTGAGCGCGCCG</td><td>5:C>T</td></tr>
<tr><td>7168871|F|0-7:G>A-7:G>A</td><td>TGCAGGAGCTCGACACGGTGTTCCTTTACAAGAACAACATCGGCGGCAAGATACCCAAGGAGCTCGGCA</td><td>7:G>A</td></tr>
<tr><td>5363350|F|0-21:G>A-21:G>A</td><td>TGCAGCCACCGCGGATGGCGCGCGGCCG</td><td>21:G>A</td></tr>
<tr><td>3954688|F|0-5:C>T-5:C>T</td><td>TGCAGCTTCGTGTGGGCCTTCGTGCCG</td><td>5:C>T</td></tr>
<tr><td>7173795|F|0-8:C>G-8:C>G</td><td>TGCAGCTGCCGTCGCGGCGGAGGCCTCACTTCCG</td><td>8:C>G</td></tr>
<tr><td>1075616|F|0-40:C>T-40:C>T</td><td>TGCAGTAGAGGTAGATAGAGAGATACTTGCCATTGTTGGCCTTCACCCACCCACGCGGGTAGCAATGTA</td><td>40:C>T</td></tr>
<tr><td>3939068|F|0-5:C>G-5:C>G</td><td>TGCAGCATGTCCCCCACGTTCACCACCACCGCGCCCTCCACCG</td><td>5:C>G</td></tr>
<tr><td>4910997|F|0-26:G>A-26:G>A</td><td>TGCAGCGACAGCGGTGGACGTGGCTGGAATTTCTCGGGGGTGTTAAAACTAGGCGGGCGGGGATTGGTG</td><td>26:G>A</td></tr>
<tr><td>5356108|F|0-56:C>T-56:C>T</td><td>TGCAGCTTGGCCAACGGAGCGTTGCAGAGCACGGTGATGACGACGACGACCAGGGACGAGACCATGGCG</td><td>56:C>T</td></tr>
<tr><td>3954340|F|0-11:G>C-11:G>C</td><td>TGCAGCACGCTGACGGTCTCGCCG</td><td>11:G>C</td></tr>
<tr><td>3027858|F|0-20:A>G-20:A>G</td><td>TGCAGCTTCGACGTGCACCCATGCTGCGCGGCCATGACGCGCCG</td><td>20:A>G</td></tr>
<tr><td>1001059|F|0-6:A>G-6:A>G</td><td>TGCAGTATCACGGGGGAGGCCAGGTGGAAGACGCCGTGGACGCCCTCGACCGCCG</td><td>6:A>G</td></tr>
<tr><td>6043994|F|0-17:A>C-17:A>C</td><td>TGCAGCTGGAGAGGGCGAAGGTCGGGGCTGGACCG</td><td>17:A>C</td></tr>
<tr><td>993522|F|0-7:G>C-7:G>C</td><td>TGCAGGAGCGACATGCTGTACGTGTCCGACCCGTGCGAGCACGCGGTGCCCG</td><td>7:G>C</td></tr>
<tr><td>1248702|F|0-19:C>G-19:C>G</td><td>TGCAGAAGCTGCACCCGATCAGCCGCCGCTTCTCGTAGAAGAGCGAGGGGCGGGAGAACCAGTTGGCCG</td><td>19:C>G</td></tr>
<tr><td>1024970|F|0-52:G>A-52:G>A</td><td>TGCAGCTGGAAGTGCAGGATGAGGGTGATGCTTTCGCCATCCGATGCCGACCGCATGGTACCGTTCATC</td><td>52:G>A</td></tr>
<tr><td>6050236|F|0-7:A>G-7:A>G</td><td>TGCAGCAACGGCCGCGCCGCGACGTCGCGGCTGGTGATGACCG</td><td>7:A>G</td></tr>
<tr><td>7168265|F|0-23:C>G-23:C>G</td><td>TGCAGGTGGTTGTCCTCGAACGGCACCTTGCCG</td><td>23:C>G</td></tr>
<tr><td>988194|F|0-46:C>A-46:C>A</td><td>TGCAGAAGGAGCTGGCGGAGCTGCGCGCGCTCAAGACGGTGCACCCCTTCTACATGCACCTCCCGAGAT</td><td>46:C>A</td></tr>
<tr><td>3026049|F|0-8:C>G-8:C>G</td><td>TGCAGGGCCCCTTCTCTCTCCGCATCCGCAGCGAATCCG</td><td>8:C>G</td></tr>
<tr><td>1696774|F|0-25:G>C-25:G>C</td><td>TGCAGGGGAGCTGGTGGAGGTAGGGGGTGTTGGCGCCATGCTTGGTGGCGCCGAAGAAGGCGGTGGCGT</td><td>25:G>C</td></tr>
<tr><td>2251944|F|0-64:G>C-64:G>C</td><td>TGCAGAACTCGTAGAGCTTGCCGCGGTTGGAGAAGATGATGAGCGCGACCTCGGCATCGCAGAGGACGG</td><td>64:G>C</td></tr>
<tr><td>3027455|F|0-16:A>G-16:A>G</td><td>TGCAGGCACGGCGCCAACCCCACCAGCTGCTCCGCGCACTCGCTCCG</td><td>16:A>G</td></tr>
<tr><td>7172025|F|0-13:G>C-13:G>C</td><td>TGCAGGAGTTCCTGGCGCAGCCGCTCCCG</td><td>13:G>C</td></tr>
<tr><td>3944480|F|0-14:C>T-14:C>T</td><td>TGCAGCTTCAAGTACGCCACGTTCGCCTACTACGCCG</td><td>14:C>T</td></tr>
<tr><td>1006032|F|0-50:C>G-50:C>G</td><td>TGCAGCGGGAGCGCGTCCATGTCGGCGCGCAGCGCGACGAAGGGCGGGCGCCCG</td><td>50:C>G</td></tr>
<tr><td>6044914|F|0-53:C>T-53:C>T</td><td>TGCAGCGCCTGCACGGCTTCTCGCTGTCCCCGCAGCACGCGCAGGTCCATAAGCTCTCGTGGGAGCAGG</td><td>53:C>T</td></tr>
<tr><td>2264326|F|0-26:G>A-26:G>A</td><td>TGCAGCATCTCCCCCTGGCCGGTGCCGACGGCGTGCCTCGTCGACTCCACCACCGACTTCCTCCGCAGC</td><td>26:G>A</td></tr>
<tr><td>2249062|F|0-8:G>A-8:G>A</td><td>TGCAGGTCGAGGCCGTCGAAGCGCTTGCCGCGCATTCGGAGGTTCTCCACCTCCACCACCG</td><td>8:G>A</td></tr>
</table>

1227022|F|0-49:C>T-49:C>T	TGCAGCGCACCACCATGTACGGCTCCCGCACCATCATGTTCCTCACCGACGACGGCACGCTGCGCCTCC	49:C>T
2254889|F|0-16:A>G-16:A>G	TGCAGGTCCTGGCGGCAGAGCTGCCGACGGGCACCATCCGCTACTCCTCCAAGATCGTCTCCATCGACG	16:A>G
12735303|F|0-5:G>C-5:G>C	TGCAGGCGCTTGATGGCGGCCG	5:G>C
1201557|F|0-40:C>T-40:C>T	TGCAGGCGGCGGGGAGGCCGAGCGCCCTCGTGACGTTGAGCTTCAAGTCCAGGCCG	40:C>T
1144317|F|0-46:C>T-46:C>T	TGCAGCTGGGCAAGATGAGCATCGCGTCCTGGGCCAGCAAGGAGGACCGCATCCGCACCTCGCCGAGAT	46:C>T
12777317|F|0-11:G>C-11:G>C	TGCAGGCGGGCGGACGTCGAGAGCTTCGGCGAGGTCCGCCTGGCGCGCTGCAAGGGGGTGCGCGCCGAC	11:G>C
12470469|F|0-15:G>A-15:G>A	TGCAGCGCGCGCAGAGGAAGCTGCGGGACAAGGAGCACGAGGTGTCCCG	15:G>A
1027810|F|0-41:G>T-41:G>T	TGCAGCGGCGTCGGGAAGAAGGCGTGGCCCTTGAGCTGGCTGGTGGCGTTGCTCAGCTGGAGCAGCCCG	41:G>T
1338752|F|0-66:T>G-66:T>G	TGCAGCGGCCAAGCAGAAGGTGAAGAAAAATGCCAAGAAGGAGGTGGTGGCGGCCGCGGTAGATGATGG	66:T>G
7162123|F|0-17:A>T-17:A>T	TGCAGGGCCACCACCAGAGCTACCCCG	17:A>T
1044824|F|0-28:G>A-28:G>A	TGCAGGGCGGGACGCAGATGATCATCTCGCAGATCCTGGTGGGCACCTTCATCGGGCTCCAGTTCGGGC	28:G>A
5367554|F|0-6:T>C-6:T>C	TGCAGTTCTCGCGGCGTCTCCG	6:T>C
6041704|F|0-14:G>C-14:G>C	TGCAGGCATGTGCAGAACAACAGACTAACCG	14:G>C
12772640|F|0-7:G>C-7:G>C	TGCAGTTGACGATGAGCACGTCGATGTCGGCTGGCTTGATCGCGGCCGCCTTGGCGAACACGTCGTCGA	7:G>C
1044032|F|0-53:G>A-53:G>A	TGCAGTGCCAGTTTCTGGGCCTTCTCGTCTTTGGAGAGCCCCTTGCCGACCTTGGCAATCCTCTTCCCA	53:G>A
4537569|F|0-11:G>T-11:G>T	TGCAGCTCGACGGTGGTGAGCGCCG	11:G>T
3028532|F|0-23:C>G-23:C>G	TGCAGCCACTTGCGGGTCACATCCGACAGGCCCTCCGCCG	23:C>G
996459|F|0-55:G>C-55:G>C	TGCAGGCGGCGGCGGACATGACCACGCTGCTCAAGGCCCTCGTCAAGGAGCTGACGACCTTGCGGAGCA	55:G>C
990385|F|0-7:G>C-7:G>C	TGCAGGGGTGGGCCAAGAGGAAGCGATCGCGCCGCCAGCGCTCCGAGGAGGAGAACCTCGCGCTCTGCC	7:G>C
7172093|F|0-43:G>A-43:G>A	TGCAGCATGGCGTTGCTCCACGGGAAGCCGCCGCTGGCGGAGTGTGCCCCG	43:G>A
4006083|F|0-35:G>T-35:G>T	TGCAGGCTTTTATAAGGGCGTGGATGAAGGCGTGCGCGTCGCCG	35:G>T
7169580|F|0-8:C>T-8:C>T	TGCAGCTGCCCCCACGATGACGCCGCACCG	8:C>T
5359797|F|0-10:G>C-10:G>C	TGCAGAAGCTGAGGCTGGGGCTGCCG	10:G>C
1025238|F|0-5:G>T-5:G>T	TGCAGGGCAGGGCCAACGACGCGACGGAGGGCGTGGTGGACGTCGTTCCCG	5:G>T
5358245|F|0-25:C>T-25:C>T	TGCAGACGAGGACGGAGGGGGGCGACCCTGTCACCG	25:C>T
7917793|F|0-8:G>T-8:G>T	TGCAGAGCGTGCGGCGGTGCCG	8:G>T
1088713|F|0-17:G>A-17:G>A	TGCAGATCATGGACGCCGTCATCCAGGAGCACCAGGAGAGGAAAGCCGCCG	17:G>A
2259105|F|0-33:G>A-33:G>A	TGCAGCTGGCGCGCGCTCCCGCGGGACGAGGATGTCGTGGTCGATTTCGACCCCTGGCGGTTCGAAGGA	33:G>A
2250536|F|0-22:C>T-22:C>T	TGCAGGACCAGTCGAAGGACGCCACGCACACGTTCCCGGCCCGCTTCTTCCACTCGCAGTCTGCAAAAC	22:C>T
1027829|F|0-14:C>G-14:C>G	TGCAGCGACGGCGACAGCACCACCATCAGCATGGCGGCGGAGAAGAAGCCG	14:C>G
2247131|F|0-68:G>A-68:G>A	TGCAGGAGCTTCGGGTGGTCAGGGTACTGGTAGAAGAACTTATCGACGATGTCCTCCTCTGTGAGCACG	68:G>A
1021890|F|0-7:A>G-7:A>G	TGCAGAGATGGGGCGGCATCAAGAACATCACCGTGGATGAGGCCAAGGCCAGGATCGCCGACATCCTGG	7:A>G
10984571|F|0-7:T>C-7:T>C	TGCAGCCTAATTCGAGCGCCG	7:T>C
1696572|F|0-61:C>T-61:C>T	TGCAGAGGTTCGAGAAGGCGGACGCCGCGGGCGAGGCGCGGCGCATGGTCTGCGCCGCCGACAGCAACA	61:C>T
1674720|F|0-17:T>C-17:T>C	TGCAGCCGCTCGGACGATCCCACGGGGACCTCCCAGGCGGCCTCAGCCATGTCGCGGCTGGTCCCGAGA	17:T>C
1027759|F|0-28:C>T-28:C>T	TGCAGGAGCCATGACGGAAGTCGTGGTGCGGGGTGATCCTGAGGCGGCCGCCG	28:C>T
993575|F|0-25:C>T-25:C>T	TGCAGCTGGCCGTGTACCTCTGCGCCGCCATGTCCCTCATGCTCTTCGCCGAGCGCCTCTACATGGGGC	25:C>T
5363608|F|0-26:G>C-26:G>C	TGCAGCTCCGCGGCCGCGATCGGCGGGCCG	26:G>C
1025326|F|0-35:C>G-35:C>G	TGCAGCATGGTGAGGTCCGCCGCGAAGCCCGCCGTCAGGTTGGTGTGCCG	35:C>G
1096346|F|0-46:A>G-46:A>G	TGCAGTTGAGAGATAAATGACGCACCTGCTCGCTGTGGCCCTGTGGAAAGTAGACGACAAGGCTGCCCA	46:A>G
12763892|F|0-21:A>G-21:A>G	TGCAGCATGACGAGGTTGGAGACAGGCTCGCCGATGCAGCTGTAGCGCGAGTAGCAGGCGCTCAGCTCC	21:A>G
1044824|F|0-52:C>T-52:C>T	TGCAGGGCGGGACGCAGATGATCATCTCGCAGATCCTGGTGGGCACCTTCATCGGGCTCCAGTTCGGGC	52:C>T
7922140|F|0-10:C>G-10:C>G	TGCAGGCGCTCGCCAAGGCCG	10:C>G
1014668|F|0-17:T>C-17:T>C	TGCAGCTTGAGCGGGCATTTGGCCTGGAACTTGCACTTGGTGAAGTGCGCCTGGTACCTGACCTCGCCC	17:T>C
1022225|F|0-23:G>A-23:G>A	TGCAGCAATGGCCGCCGCGGTTGGAGCCCGAGCCAGCCCCGATGATGTCGGCCG	23:G>A
1070629|F|0-14:A>G-14:A>G	TGCAGGTTACACTCACTTACTGGTTCAGACAGAGTCCAAACGCTTTCATGTCAAATTCAGCAGTATAAT	14:A>G
3024946|F|0-22:A>G-22:A>G	TGCAGCTGTCTTTCCAGGACTCATCGGCAACCACGGTGTCGCCG	22:A>G
3957256|F|0-13:C>G-13:C>G	TGCAGGAATTGCTCGCGTTGGGTGACCG	13:C>G
1004574|F|0-8:G>C-8:G>C	TGCAGGCGGCGCGGCGAGGCTACCGTTCCGTTGTGGTCACGGGGCTCCTGGCCG	8:G>C
5971089|F|0-20:C>G-20:C>G	TGCAGCTCGGTCAGCCCCTGCGGGTTCCCG	20:C>G
5363931|F|0-11:T>C-11:T>C	TGCAGGTCCAGTGGCGCCAGGTTGTTGTCGCCG	11:T>C
5346012|F|0-8:A>G-8:A>G	TGCAGCGGAAGTGGCTCATGGCGTTCGTCTCCG	8:A>G
991877|F|0-39:T>G-39:T>G	TGCAGAGGGACCCTCCCACGGAAGCCGAATGTACGGAATTGTCCACTCTATCCCCTTGGCCAGCTCGTA	39:T>G
1027417|F|0-10:A>C-10:A>C	TGCAGGCAGGATACAGGCTGGTCATGGCGACCCAGTTCAGGAGGTTCACGTACCG	10:A>C
12683592|F|0-8:C>T-8:C>T	TGCAGCGCCGCAAGCCACCG	8:C>T
985942|F|0-8:T>A-8:T>A	TGCAGCTGTTGATGCAGCTCATCTCCACCGTCATGGTGGGGCACCTCGGCGAGGTCGCGCTCGCTGGCG	8:T>A
1865063|F|0-59:A>G-59:A>G	TGCAGTGACAAGAAAACGCTGAAGAAGTGGTTCTTCATCGACAAGACAGTGGGCTAAGAAAATCCTGCT	59:A>G
1090093|F|0-19:G>C-19:G>C	TGCAGGGGTACGGGCTGACGGAGAGCACGGCCATCGGCGCGTCCACGGACTCGGCCGAGGAGAGCCGCC	19:G>C
981720|F|0-34:C>G-34:C>G	TGCAGATCGTGGTGATGAACATGCTCATGGGGGCCTCCACCGTCAGCAGCATCCTCTCCACCTACCTCT	34:C>G

1669231|F|0-31:T>C-31:T>C	TGCAGGCGGCCACGGGCCTCGCCACGCTCTTTGACGTGGTCACCCAGGGGTGGCTGGACGCCGACATGC	31:T>C
2250536|F|0-67:A>G-67:A>G	TGCAGGACCAGTCGAAGGACGCCACGCACACGTTCCCGGCCCGCTTCTTCCACTCGCAGTCTGCAAAAC	67:A>G
1215801|F|0-22:C>T-22:C>T	TGCAGCTCTACAAGGCTCACTCCGACAGCTACATCTGCTCGCTCGTCCCCG	22:C>T
1027759|F|0-46:G>A-46:G>A	TGCAGGAGCCATGACGGAAGTCGTGGTGCGGGGTGATCCTGAGGCGGCCGCCG	46:G>A
5580081|F|0-13:G>C-13:G>C	TGCAGACCATGGCGACCAGCCCCG	13:G>C
2245504|F|0-44:A>G-44:A>G	TGCAGGACGTCGAGGAAGCGGTCGAGGAGGAGCGCGTCGTACTCAATGAGCTTGTCGTCCTCGGACACC	44:A>G
1093353|F|0-18:T>C-18:T>C	TGCAGGGCGAACTCGTTGTGGTATACGGCGACGAGCACGGCGGCGAGGAGCGCGAGGGGAAGCAGGAGG	18:T>C
1106723|F|0-44:C>G-44:C>G	TGCAGGATGCCGAGGAAAAGGATGAAGATGCCGAGGCCACGGTGCGCTGGGATGTCGGCCTGGATCTTG	44:C>G
990314|F|0-61:G>A-61:G>A	TGCAGTAGGTCTTGCAGCCTGGGCACATGACGATGCACTCGTTGGGGCAGCGGACCTTGTCGCAGGTGG	61:G>A
5356108|F|0-18:G>C-18:G>C	TGCAGCTTGGCCAACGGAGCGTTGCAGAGCACGGTGATGACGACGACGACCAGGGACGAGACCATGGCG	18:G>C
1085853|F|0-9:A>G-9:A>G	TGCAGCCCCACCAACTCCCTGAAAATTATCACGGCTCTCTGAAACAAGCCTGCGTGAGCGAACCCAGAG	9:A>G
2268541|F|0-8:G>A-8:G>A	TGCAGCCTGAGCGACAGCCGCGACGAGGCGGAGCAGGTCATCTTTGCAGCCATCGACGACCTGCTCGCC	8:G>A
7352152|F|0-32:C>G-32:C>G	TGCAGTTGATCGCCGATGTTGATGACCAGCGCCCCG	32:C>G
1044824|F|0-10:G>A-10:G>A	TGCAGGGCGGGACGCAGATGATCATCTCGCAGATCCTGGTGGGCACCTTCATCGGGCTCCAGTTCGGGC	10:G>A
981730|F|0-68:C>A-68:C>A	TGCAGATCCTCGTCCAGCACCACCCGCAACCGTGTCATGTCGTCCTCAAGCAGATGGTCAGCTCGCCGC	68:C>A
1049669|F|0-10:G>T-10:G>T	TGCAGCTTCTGCGTGGTCTGGTCCAGCTCCTGCTTCAGAGAAGAAACCAAGGTGGCGTCCGAGGACACC	10:G>T
3945144|F|0-16:G>A-16:G>A	TGCAGCTCGCCGTGCAGGACTTCCAGTCGTGCGACCG	16:G>A
32027502|F|0-19:A>G-19:A>G	TGCAGGCGACGGTAAGCAAAGCTTCGTTGTGGTGTCAACCAGCTCATTTCAGACGCAAGATGTCTGCTC	19:A>G
4999891|F|0-20:C>G-20:C>G	TGCAGCGGGAAGTGCTTCTCCGCGTGCTGCTTCGGTATCACCAGCCG	20:C>G
4262191|F|0-7:C>T-7:C>T	TGCAGGGCAAGAAGGCGACGACGTGCGCATCCATGGCCG	7:C>T
991564|F|0-19:G>C-19:G>C	TGCAGGGGTACCGCGCGCTGAAGAAGGCGTTCGCGGCGCAGATGAAGCTGCCG	19:G>C
12763892|F|0-23:A>G-23:A>G	TGCAGCATGACGAGGTTGGAGACAGGCTCGCCGATGCAGCTGTAGCGCGAGTAGCAGGCGCTCAGCTCC	23:A>G
1863083|F|0-13:G>T-13:G>T	TGCAGCGCGAGACGTTCCCTGATGTGATGAGGCTCATGGACAAGCACGAGCAGATCGACCGCATCCTTT	13:G>T
1226743|F|0-49:G>A-49:G>A	TGCAGGCGCTCGCCATGGACCGCGCCGTCGGCCGCTTCACCGTGCCGGCGGGCTCCATGTTCGTCGCCA	49:G>A
3021068|F|0-13:G>A-13:G>A	TGCAGTTCTTGGTGCAGCTCCACTGCTTTGGCCCCATCCCCG	13:G>A
7346442|F|0-7:G>A-7:G>A	TGCAGGCGCTCCGCGAGTCCACGGCGGCCG	7:G>A
3028382|F|0-37:C>G-37:C>G	TGCAGAGCCGCGAGACGGACTCTGAAATCTCCACCCACGACTCCG	37:C>G
1218775|F|0-19:A>C-19:A>C	TGCAGTTCCTCCGAACCTCACCCTGCGCACCCGTGAGCGGCTTGATGTTCCCCATGTTGATCATGGCCG	19:A>C
5356108|F|0-44:G>A-44:G>A	TGCAGCTTGGCCAACGGAGCGTTGCAGAGCACGGTGATGACGACGACGACCAGGGACGAGACCATGGCG	44:G>A
5580329|F|0-21:C>A-21:C>A	TGCAGCTGGCGCCACCTCAACCACCCG	21:C>A
7169631|F|0-16:C>G-16:C>G	TGCAGGACCTCACCCTCCGCCACCTCCCG	16:C>G
6041723|F|0-31:G>C-31:G>C	TGCAGGCCGCCAACAGCAACCTCCCCGCGCCGACTTCCG	31:G>C
1018656|F|0-15:A>C-15:A>C	TGCAGCGAAAGAGGAAGAGAAATCGCGTGAAAGTACGTCGCCGAGAAGCAGGTGGCAGAAATGGAGGCG	15:A>C
988366|F|0-32:G>A-32:G>A	TGCAGGGCCTTGGTCATGTTGAGGGTGACGCCGTAGTCCTGGCCGCCCTGGAAGGCCTGGCAGAGGCAG	32:G>A
988366|F|0-44:G>A-44:G>A	TGCAGGGCCTTGGTCATGTTGAGGGTGACGCCGTAGTCCTGGCCGCCCTGGAAGGCCTGGCAGAGGCAG	44:G>A
1082056|F|0-43:C>T-43:C>T	TGCAGATCTCCACCATGTTCACGCACCACCAGAAGACCGTCATCGTGGACCACGACATGCCCCAGAGCG	43:C>T
7339758|F|0-37:C>G-37:C>G	TGCAGGGCCAGAAGCGGCAGCATCCCGCTCGACGGGCCGCTGCCCCAGCCGACGCCG	37:C>G
3952704|F|0-65:T>C-65:T>C	TGCAGCGTGGGTACAACATCGAGTCGTCGACCTCGGGAAGCTGCTCCCCTCTCGCGCCCTCATGATCAT	65:T>C
7164771|F|0-8:A>G-8:A>G	TGCAGACGAGTTATGATGCGCCGCCG	8:A>G
1088789|F|0-53:C>T-53:C>T	TGCAGGCTGAGGTGGAGCAGCGCCGAGCAGTTGGCGAGCGCCGACGGGATGGTCCCCTCAAGAAGGTTG	53:C>T
1006951|F|0-61:C>T-61:C>T	TGCAGGCCATGCCGCTGGTGCCGCTCAACGACTGCCCGCGCCACGCGCGGTACATGGACGGCGTCTTCG	61:C>T
1065426|F|0-14:C>T-14:C>T	TGCAGCACCTTCCGCACCCCCGCCTCGCCGTCCACCGCCAGCGAGTACAGCACCG	14:C>T
4910307|F|0-35:C>T-35:C>T	TGCAGCCGCTCCAGGACAGGACCAGCGACGCCTACCGCTGGATGGTGCACCACGGCGAGAGGTACCGCG	35:C>T
5579546|F|0-41:C>G-41:C>G	TGCAGCACCTTGCGGCCCGTCATGCCCAGCAGGAACGGCTCCGTGGAGGCGGCCAGGTAGCGGCCGTAC	41:C>G
1027759|F|0-21:C>G-21:C>G	TGCAGGAGCCATGACGGAAGTCGTGGTGCGGGGTGATCCTGAGGCGGCCGCCG	21:C>G
3958346|F|0-62:G>A-62:G>A	TGCAGCGCGGCGCGGCGCGCGCCCGAGGAGCGGATGCGGTGCGCCGTCTTGTGGAGCTCGAAGTTGTAG	62:G>A
1004847|F|0-26:G>C-26:G>C	TGCAGCGCCGTCATGAGCAGCAGCACGACGGCGGCGAGCAGCGTGAGCAGCCTCCACGACCTGAACACG	26:G>C
1026620|F|0-42:C>T-42:C>T	TGCAGCGGCTGGCCTACGTGAACTGCGTCGCCTACCCGCTCACCTCCATCCCGCTCACCGTCTACTGCG	42:C>T
4396248|F|0-37:A>G-37:A>G	TGCAGGGCGAGACCCAGGCACATCAGGGCCCGCCGCCATACCG	37:A>G
1231004|F|0-5:A>C-5:A>C	TGCAGAGCGTGCGCGATGAGGTGGTGATACGGCTGCTCCTCCACCTGCTCGTGCTTGGGTGCTGACGGC	5:A>C
3025425|F|0-13:G>T-13:G>T	TGCAGTTCCCCGTGGGAAGGATCGCCAGGTTCCTCAAGGCCG	13:G>T
1864394|F|0-10:C>T-10:C>T	TGCAGCTCGGCAAGGTCAAAAGGAACATCGAAAGCCGGCTTCCGGAAACCGCAGTCCTCGCTGTCCCCA	10:C>T
1039352|F|0-55:A>G-55:A>G	TGCAGGAAGTTGGCTCTTGCCTGACACTGGACAAGTTTGAGAAGATGGTGCGGCCATTCGCCATGCCCC	55:A>G
2249062|F|0-5:G>C-5:G>C	TGCAGGTCGAGGCCGTCGAAGCGCTTGCCGCGCATTCGGAGGTTCTCCACCTCCACCACCG	5:G>C
1088357|F|0-39:C>T-39:C>T	TGCAGCACCATCGTGGGAGGCTAGGGTTTGCTCGGGCACCGCGCTCTCTGGTCTCCG	39:C>T
5580535|F|0-5:G>C-5:G>C	TGCAGGTCGCGCCGCCGCCGTCGCCG	5:G>C
7166904|F|0-8:C>A-8:C>A	TGCAGCTCCAGGAGCCCACCCTCCACCTTCTCAGCCTCGGAGGACGACAGCATGGCCGCCCGCATCGCC	8:C>A
981900|F|0-58:G>A-58:G>A	TGCAGGCGGATCGTCCACAACACGGTGGGCAGCGACAAGGCGGTGGCGAAGCTCTTCAGCGGGATGTCG	58:G>A
3958346|F|0-17:C>G-17:C>G	TGCAGCGCGGCGCGGCGCGCGCCCGAGGAGCGGATGCGGTGCGCCGTCTTGTGGAGCTCGAAGTTGTAG	17:C>G

986790|F|0-25:G>T-25:G>T	TGCAGCCAACTCGGGTCCTGGCGAGGATGGGAGTTGAAGGCGAGGAAGGGAGGAGGAGGTGGACGCGAC	25:G>T
1214365|F|0-29:G>A-29:G>A	TGCAGCTGCGCAAGAATGCCTTCTCTGGCGAGCTCCGGCCGGCGGGGCGGTTGCCTTCGGGCGCCACGG	29:G>A
7172199|F|0-11:A>G-11:A>G	TGCAGGCCGTCAGCGAGCTGGCGGGCGGCATGGAGGCGCCAAGCGTGCGGCCG	11:A>G
1061961|F|0-29:G>C-29:G>C	TGCAGGAAGTAGAGGTGCTCGTCCTCGGCGAGGCAGTAGAAGAGCGCGAGCGAGACGCGGCGGAGCGAC	29:G>C
1864394|F|0-50:G>A-50:G>A	TGCAGCTCGGCAAGGTCAAAAGGAACATCGAAAGCCGGCTTCCGGAAACCGCAGTCCTCGCTGTCCCCA	50:G>A
998377|F|0-30:C>T-30:C>T	TGCAGGAACGACGGCGGCGAGCGTGAGGGGCGAGGTGGACAAGAGCAGTGCTCTCCTGGTTGTGGCCGA	30:C>T
2249398|F|0-48:A>G-48:A>G	TGCAGGGCCACATCAACTCGATGCTTCATTTCGCCGCGGGGCTCCTCGACGCCG	48:A>G
32022392|F|0-16:A>C-16:A>C	TGCAGATGAACTTTGTAGCCGAACACCCGCAACCG	16:A>C
32027434|F|0-25:G>T-25:G>T	TGCAGCAGTCCGCGTCGTGCGTCTTGAGAGCCTCCCGTGCGACCTCCG	25:G>T
32026049|F|0-38:G>A-38:G>A	TGCAGGATGCCCTTGGTCGTGAAGTCGAACGCCATGGCGGGGCCACCGACCGCCTTCACCCAGTTCACC	38:G>A
32026645|F|0-38:C>A-38:C>A	TGCAGCACGTTGTGTTCGGCATCGCGGCGTCGGCGCGCCTGTGGGAGAAGCGGAAGGAGTACATTAAGA	38:C>A
100352968|F|0-6:C>T-6:C>T	TGCAGGCTGCAAATATGACCG	6:C>T
1093935|F|0-21:C>T-21:C>T	TGCAGTCTCCACCACACGGACCTGCCACCATGACGGCGGCTCCTCTGTTATCGTTGACGGATGCGGCGA	21:C>T
5363855|F|0-11:A>C-11:A>C	TGCAGGGCCAGAGCCGTGTAAGGCTTCGCCGCGCCG	11:A>C
32022542|F|0-7:T>C-7:T>C	TGCAGGTTTTCGCGGTGGCCATGCTGTGCATCAGCCACCGCGCCGACGACCG	7:T>C
3028420|F|0-40:G>C-40:G>C	TGCAGTACTTCTACGAGCTGGACCTGAGCAACAACAAGCTGGCGCCG	40:G>C
1050679|F|0-49:C>T-49:C>T	TGCAGTGGTGGTGGCGATGTGTAGTGAGCATGGAGAGGCGTATCTGGTCCCTCATATGCTCTAACCAGC	49:C>T
5581911|F|0-22:C>G-22:C>G	TGCAGACGTACGCCACCTTGCACCCG	22:C>G
1696590|F|0-17:T>G-17:T>G	TGCAGGGTGAGGTACGATATCAGGTTCGAGCCGAACCCCACCGTCGCCAGCCG	17:T>G
6042708|F|0-13:C>T-13:C>T	TGCAGCAGATCGCCTCCCTCAACGGCAACAGCATCACGTCCAGCTTCTCCATGCTGCACGCCTGCACCA	13:C>T
1013425|F|0-5:G>A-5:G>A	TGCAGGGGCGTCCACACGCCCGAGCCACGGCCCGCGTCGCCGTCCTCGGCTGGCCTGCCCTGCGCCATG	5:G>A
1065076|F|0-47:G>A-47:G>A	TGCAGAGAGCCAAAGCCAAGAAACCAAGAACCAAGCAGATGATGCCAGCAAGCCTAACGAGGCCGCCGA	47:G>A
990138|F|0-13:G>A-13:G>A	TGCAGAGGGGATGGCTTCTTAGCCTTTCTTTGTCATGAACATGTTAGATTTGCTGGATTAGCATGCCTG	13:G>A
5580275|F|0-7:G>C-7:G>C	TGCAGCGGTCGACGGTGCTCCTCATGGCGACCG	7:G>C
1143830|F|0-41:G>C-41:G>C	TGCAGCCGCGACGGCGACGAGCACGACTTCTTCCGCGCCGCGCAGGCCG	41:G>C
6030176|F|0-20:G>A-20:G>A	TGCAGAAGCTGGCCATGGACGTCCTCACCG	20:G>A
4990903|F|0-7:G>C-7:G>C	TGCAGCAGCAGCTCCACGTCGGTGGGGTGGAACCG	7:G>C
1317625|F|0-32:A>G-32:A>G	TGCAGCGGGTCGGCCTGGGCCTCGTCTTCTCCATCCTCTCCGTGGCCTGGGCCG	32:A>G
12764043|F|0-56:C>G-56:C>G	TGCAGGATCCGCGACGACGGCTGGTGGATGGTCAGCACCACGGTGCTCCCTGCGCACGCGATGTCGTGC	56:C>G
1008802|F|0-41:G>C-41:G>C	TGCAGCACCATCTGCTTCGCGGAGGCGGCGGCGCCGCCAGGGCCGCCGCTAATCTGGTGCTGCCGAGAT	41:G>C
3064706|F|0-14:C>G-14:C>G	TGCAGGAAGTCGCCCACGTTCACCACCAGCGACGCCTCCCCGTCGCCG	14:C>G
21743558|F|0-14:G>T-14:G>T	TGCAGACTTTATTGGGCGCCG	14:G>T
6042858|F|0-28:A>G-28:A>G	TGCAGATCGGGTTCAGATTTAGGCCGAAATAGCACTCGCTCGAGGCGTACATAGTGCACGCCAGAGGCA	28:A>G
1237382|F|0-25:C>T-25:C>T	TGCAGCAGCCACCGCCAGGCACACTCACCCTGCCCATGGCATAACTGATGCACGGGGCCAGGTAGCTCA	25:C>T
4005941|F|0-13:C>G-13:C>G	TGCAGTGCACGTCCTCGGACAGGGACAGGTTCAGGGGGAAGGCCG	13:C>G
1093935|F|0-28:C>A-28:C>A	TGCAGTCTCCACCACACGGACTTGCCACCATGACGGCGGCTCCTCTGTTATCGTTGACGGATGCGGCGA	28:C>A
1030576|F|0-7:C>G-7:C>G	TGCAGCTCATGAAGGACTCCCGCTGGGTCGCCGTGAAACCCCGCCCCGACGCGCTCATCGTCAACATCG	7:C>G
1862754|F|0-54:G>C-54:G>C	TGCAGCACGCGGTCCCGCGGCCACTGCTGGGTGAGGGGCAGCTCGAGCAGCTGCGTCCAGAACTCGGAG	54:G>C
5348291|F|0-5:T>C-5:T>C	TGCAGTGTCAGCGGTCTCAGCGGCGTGCCG	5:T>C
1061961|F|0-11:G>A-11:G>A	TGCAGGAAGTAGAGGTGCTCGTCCTCGGCGAGGCAGTAGAAGAGCGCGAGCGAGACGCGGCGGAGCGAC	11:G>A
1025238|F|0-35:G>C-35:G>C	TGCAGGGCAGGGCCAACGACGCGACGGAGGGCGTGGTGGACGTCGTTCCCG	35:G>C
2265996|F|0-7:C>T-7:C>T	TGCAGTTCCTACCGTCCTGCTCCATCGATGCGGAGAGTCTGCAAGAGCTCCGCGAGTTCGAGTTGGCCG	7:C>T
3031628|F|0-20:G>T-20:G>T	TGCAGCTCCACCGCCGAGGCGCGCTGCTGCGAGTGCGAGCTCATGCCG	20:G>T
1696572|F|0-19:G>C-19:G>C	TGCAGAGGTTCGAGAAGGCGGACGCCGCGGGCGAGGCGCGGCGGATGGTCTGCGCCGCCGACAGCAACA	19:G>C
1182699|F|0-10:C>T-10:C>T	TGCAGCTCGACGCTGAAGGCAGCAACAGCAACAACTACAGGGCCATGCAGCAAATCACCTGGGCTGCCG	10:C>T
1081945|F|0-11:G>C-11:G>C	TGCAGGAGGCTGGTGAGGCACAAGAGCACGACAAGGTAAGGCGTGGAAGAAGCCATCTTGATTACTGTA	11:G>C
4439793|F|0-13:C>T-13:C>T	TGCAGGCGCTCGGCGGCGTCCTGGAGATCTTCCATGTGGCCG	13:C>T
1026371|F|0-68:G>C-68:G>C	TGCAGCAGCGCCGTGTTGCTGCGCCGCAGCCGCTCGTTCTCGTCCATCAGCACCGCCGCGCTGGACGCG	68:G>C
1093342|F|0-30:C>T-30:C>T	TGCAGATCCCCGCCGACCAGGCCGCGCTCGCGCTCGCGACGCTCGCCGCGGTGCTCCCCG	30:C>T
1000839|F|0-19:C>G-19:C>G	TGCAGAAGGAGTCGTCGGACGCCTCCGAGGCGCACGACACGTTGAGCGACTGCATGTTCCCGTTCTTCC	19:C>G
3936873|F|0-20:G>C-20:G>C	TGCAGGGTCTGCTGCCTCAGGAGGTCGCCCTGCCG	20:G>C
6030689|F|0-23:C>G-23:C>G	TGCAGGAACCTGGAGACGTCCGCCTACGTGATCGCGGTCGTGGCCG	23:C>G
6044098|F|0-24:C>T-24:C>T	TGCAGGCCGCCTCCGAGTCGCGGCCGCCG	24:C>T
1008802|F|0-22:A>T-22:A>T	TGCAGCACCATCTGCTTCGCGGAGGCGGCGGCGCCGCCAGGGCCGCCGCTAATCTGGTGCTGCCGAGAT	22:A>T
5357844|F|0-11:A>C-11:A>C	TGCAGCGCGCGAGCCGCCTGGCGCCG	11:A>C
5368321|F|0-31:G>C-31:G>C	TGCAGCGCATCGGCGTCGGGATGGTGCTCTCGCTCGTGTCGATGGTGGTCGCGGCGCTGGTGGAGACGC	31:G>C
990314|F|0-54:C>G-54:C>G	TGCAGTAGGTCTTGCAGCCTGGGCACATGACGATGCACTCGTTGGGGCAGCGGACCTTGTCGCAGGTGG	54:C>G
1138584|F|0-28:T>G-28:T>G	TGCAGGCGGTCCATCTGTAGGGGATCCTTGTAATGTTTGCGTGCATGGCCCTCTGCACGTCGAGCCGAG	28:T>G
1087023|F|0-53:T>C-53:T>C	TGCAGGGACACCGTCATGACCATCTGGCATTACCACGGCGGGTGCCAGGTCGGTAGAGTCGTCGACCGC	53:T>C

6048928|F|0-6:C>T-6:C>T	TGCAGGCGTCAACGTGCGCGGTGGCCCG	6:C>T
1864394|F|0-43:G>C-43:G>C	TGCAGCTCGGCAAGGTCAAAAGGAACATCGAAAGCCGGCTTCCGGAAACCGCAGTCCTCGCTGTCCCCA	43:G>C
5371573|F|0-16:A>C-16:A>C	TGCAGATCAGGAGGAAAGGCGGCTGGGTCCCG	16:A>C
1134776|F|0-46:G>T-46:G>T	TGCAGAGGCACTCCTCCCGGCCGGTGCCGCTGCACGTGGAGCAGCGGGGCCGCGCGTCCCACCTGGACG	46:G>T
1240822|F|0-11:G>C-11:G>C	TGCAGCGCGAAGCCCCGCAGCTTCCGCAGCGACGACTTCATCTCGCGCCCGCCG	11:G>C
1063871|F|0-34:T>C-34:T>C	TGCAGATCATTGCCGTGCACTCGTCCACGCCCAGTTCGTGAGAGCCCAGCCTGCTCAGCGTCCGCCTCA	34:T>C
2253524|F|0-53:G>A-53:G>A	TGCAGCAGCCACTCGATGGTCTCGCCGTCGGACTTGTGGCCGAGCTCCCGCGTGAGCTGGAAAACCCGC	53:G>A
1096951|F|0-22:G>A-22:G>A	TGCAGGACAGCATCCCCAAGGTGTACCTCGCCGCCGCGGCCGCCGCGCTGTACGCGCGCGCGTCGTCGT	22:G>A
1010561|F|0-68:G>A-68:G>A	TGCAGGTACTTGACCGCCTTGGTCCTGGTGATGTACTTGGCGGCGTTCCCCTCCTTCTTCTTGCCCTGG	68:G>A
32025930|F|0-59:A>G-59:A>G	TGCAGAAGACGAAGGTGATCGAGACCTTCCAGGCCCAGGCCAACAAGACCGACGAGGGCATCACCATCT	59:A>G
1017794|F|0-23:C>A-23:C>A	TGCAGGTAGGTGATCAGGACCAGCCACGCGTTCACGATCAGCAGCGGCACGCCGTAGACCCGCACCACC	23:C>A
1000127|F|0-15:C>T-15:C>T	TGCAGGTGAGCGACGCCATCTCGTCCTTCACCGCCGAGGCGAAGGTGCCCACGCCGCCG	15:C>T
1022573|F|0-14:C>G-14:C>G	TGCAGGTACTTCCTCGTCGTCTCGTCGTTCATCACGTGGGCGACGCCGTTGGCGTGGATCTTCTCCCGA	14:C>G
6046359|F|0-25:A>G-25:A>G	TGCAGGATGAAATGTCGTCGTTCCAAGCGCCGTCCAGCCTGCTGACTGATCAGGCCAGCCCCAACGTGA	25:A>G
1005586|F|0-48:G>A-48:G>A	TGCAGGCAAGCCTTTTCCGTCGCTCTTGATGGATAGCAGCAGGAGGAGGGCCTTCTTCAACCTCGCAGG	48:G>A
6041704|F|0-23:A>G-23:A>G	TGCAGGCATGTGCAGAACAACAGACTAACCG	23:A>G
2247246|F|0-56:C>A-56:C>A	TGCAGGAGAGGGAGTCTCTCGGCCGCCTCGAAGAGCATAAGCACGTCCGACGCAGACGTGTAGCACAGC	56:C>A
1006951|F|0-40:C>G-40:C>G	TGCAGGCCATGCCGCTGGTGCCGCTCAACGACTGCCCGCGCCACGCGCGGTACATGGACGGCGTCTTCG	40:C>G
3027571|F|0-5:C>A-5:C>A	TGCAGCGACAGGTGCAGCAGCTGGTTGAGGTGCCCCTGCGCCG	5:C>A
1207726|F|0-20:C>G-20:C>G	TGCAGGCACAGGTTCCACTCCCAGTGCATCGTGCCGTGGCTGGAGATGCACAGCTCCTGCCCG	20:C>G
32027347|F|0-9:T>G-9:T>G	TGCAGCTGCTGCTTCACCGACGCCG	9:T>G
1096951|F|0-44:G>A-44:G>A	TGCAGGACAGCATCCCCAAGGTGTGCCTCGCCGCCGCGGCCGCCGCGCTGTACGCGCGCGCGTCGTCGT	44:G>A
2247246|F|0-68:C>T-68:C>T	TGCAGGAGAGGGAGTCTCTCGGCCGCCTCGAAGAGCATAAGCACGTCCGACGCAGACGTGTAGCACAGC	68:C>T
3952729|F|0-13:C>G-13:C>G	TGCAGACGCCGTCCCCGACGGCGCAGGACCCGACCATGGACCCCG	13:C>G
3959151|F|0-8:A>C-8:A>C	TGCAGAACAATCCCATGTCGTCGATGCCGACGCCGACGCCG	8:A>C
32025546|F|0-9:G>A-9:G>A	TGCAGCGGCGCAGCCACTGCCTGATGACGATGACGATCTGATCGAGTAGAGAGAGGATCATGAGTCTGT	9:G>A
1096951|F|0-38:G>A-38:G>A	TGCAGGACAGCATCCCCAAGGTGTGCCTCGCCGCCGCGGCCGCCGCGCTGTACGCGCGCGCGTCGTCGT	38:G>A
3952704|F|0-62:T>G-62:T>G	TGCAGCGTGGGTACAACATCGAGTCGTCGACCTCGGGAAGCTGCTCCCCTCTCGCGCCCTCATGATCAT	62:T>G
32025987|F|0-24:A>G-24:A>G	TGCAGCCGCGTCGAGGAGGGGTTCACCGACCAGACCGACGGGCTCATGGGCCTCGGCGGCGACGCGCAG	24:A>G
1066043|F|0-43:G>A-43:G>A	TGCAGGTGACGGAGAAGTTCAAGGAGCGGGTCGTGGACGAAGGGTATAACCCTAGCTACGGAGCGCGGC	43:G>A
5362833|F|0-9:T>C-9:T>C	TGCAGGCCTTGACGCGGGGGCGCGCCTCGTCGGCGAGCTGCACGGTGACGTTCGAACCATCCTTGGCCA	9:T>C
7338596|F|0-9:T>C-9:T>C	TGCAGACCTTGTGGGGACCG	9:T>C
1019996|F|0-40:G>C-40:G>C	TGCAGGCCGTGATCAAGGAGACGCTGCGGCTGCACTCCCCGATCCCGCTCCTCGTCCCCCACATGAACC	40:G>C
1748935|F|0-11:T>C-11:T>C	TGCAGGACTGATGAGTCTCCATAGAGCTCGATGGCGGACAAGTAAGGCACATAGTACTGCACGACCCCG	11:T>C
1088789|F|0-26:G>A-26:G>A	TGCAGGCTGAGGTGGAGCAGCGCCGAGCAGTTGGCGAGCGCCGACGGGATGGTCCCCTCAAGAAGGTTG	26:G>A
1017210|F|0-37:A>G-37:A>G	TGCAGGAGCGAAAACACGAGAACATTGAGATTAAGAGAGATCTAGCGGGACACGGAACAAATCAAGCGC	37:A>G
4262728|F|0-31:C>T-31:C>T	TGCAGACGCTGGCGCGGGAGCTCCTGGACCGCCACGGCCG	31:C>T
1028367|F|0-68:G>C-68:G>C	TGCAGGCCGTCGCGCCAGTGGCCGTCGTAGACGTCGCCGTTGGCGTAGGCCTGCGTGCCGCGGCCGTGG	68:G>C
984423|F|0-15:A>G-15:A>G	TGCAGCACTTTCTAGAAGGGTGTTGATGACGGGGGTTCTGTGGAGGGAGGGGGTGGAGGCACGTCGCCT	15:A>G
4910775|F|0-17:C>T-17:C>T	TGCAGCTTATTCGGGATCCGAAATCACCATAATCTTTAATCCG	17:C>T
1061961|F|0-56:G>C-56:G>C	TGCAGGAAGTAGAGGTGCTCGTCCTCGGCGAGGCAGTAGAAGAGCGCGAGCGAGACGCGGCGGAGCGAC	56:G>C
7161955|F|0-9:T>C-9:T>C	TGCAGGCCGTGCGGACGAGCGCGCTGTCCCG	9:T>C
4538874|F|0-6:C>A-6:C>A	TGCAGCCTTCCGAGCTCGGGCGGTATCGGCCCG	6:C>A
4398160|F|0-16:T>C-16:T>C	TGCAGAGCGCGCGCCGTGCGCCCG	16:T>C
1324604|F|0-47:C>T-47:C>T	TGCAGGGGAGGGCAGTGGAACTCAGGAGGGAGGTGCCACAGGGAGACCGAGCCGATATTCAACCAGACG	47:C>T
12010054|F|0-23:G>C-23:G>C	TGCAGGGCCGAGAAAGGTGCTCCGATCCCCGCCG	23:G>C
3954786|F|0-40:T>G-40:T>G	TGCAGCTTATCCAATCCAATACCTGAATCTGCCATCGCCCTCTTGCACGGCTCAACAACTCTCTCCAGC	40:T>G
1024563|F|0-24:G>T-24:G>T	TGCAGGCGGCGCGTGCGGGTACGGGAACCTGTACAGCACGGGGTACGGCACGAACACGGCGGCGCTGAG	24:G>T
1103598|F|0-61:C>T-61:C>T	TGCAGTACACGCACGCGGTGCTGTACGAGAACATGCGCCTGTTCCCGCCCGTGCAGTTCGACTCCAAGT	61:C>T
6040123|F|0-42:G>A-42:G>A	TGCAGCAGCTGTCGCGCGGAGCGCGGAGTTCGAGCGCGGATAGGCGCGTGGAAGCGGCACTGGAGGCGG	42:G>A
1318135|F|0-8:T>A-8:T>A	TGCAGGGGTTGGGACCCTCTGTGGTTCTACCTTCATGGCGGCATTCAGTTCGTCGGCTTCATCCTCGGT	8:T>A
1025326|F|0-17:C>T-17:C>T	TGCAGCATGGTGAGGTCCGCCGCGAAGCCCGCCGTCAGGTTCGTGTGCCG	17:C>T
2262698|F|0-31:C>A-31:C>A	TGCAGGCGCCCGACGTCGACGCCTTGCGCGCCCCCGTCGACGAGTTCGGCCCCGTCGACACCG	31:C>A
5581461|F|0-9:G>A-9:G>A	TGCAGGAGAGGATCGAGAAGCTGCCG	9:G>A
6050212|F|0-10:G>C-10:G>C	TGCAGAGCTCGACGATGCCGCGGACGACGCCG	10:G>C
1091647|F|0-39:C>T-39:C>T	TGCAGCAGGCCCTTGGAGAAGGCCAGCGACCGCAGGCGCCTCCGCTCCTCCGCCTCCG	39:C>T
2313198|F|0-8:T>C-8:T>C	TGCAGTGGTGTCTCCTCCTCCACGGCCGACGCGACGTCTGGCTGCGCCTCGACGACCCTCCCAGGAGGC	8:T>C
7168655|F|0-56:A>G-56:A>G	TGCAGCAGGAAGCAGCCGTCGAGGAACATCATCGGCTTGAAGTCGTCGTCGCCGAGATCCCCCAGCCTG	56:A>G
1696797|F|0-11:C>G-11:C>G	TGCAGCTCCGACGCCGAGATGAAGCCGTCGCCGTCCACGTCGAACACCTTGAAGGCCTCCCGCATCTCC	11:C>G

987969|F|0-6:T>C-6:T>C	TGCAGCTGCGGTGGGGCTCTCCTTCTTGCAGTTCACCAACATGAACTCCATGCGCAACCTGTTCATCGT	6:T>C
1124006|F|0-17:C>A-17:C>A	TGCAGGTGGGCTCCATCCGACGGCCGCAGTTGACCTTCCAGGCCCCGCCTCCGCGGCCCGCCG	17:C>A
1152074|F|0-58:C>G-58:C>G	TGCAGCACCACCAGGACGCGCTGGTGCTGGCGTGGCCGCGGCTGCACGAGGCCGTGCTCGACCCCGCCG	58:C>G
23919919|F|0-10:C>G-10:C>G	TGCAGATAGTCTTCGAGATGATATCGATCGGAACCCTGCTGGCCTTCTACCTGGTGGCTAACGCGCTCA	10:C>G
2247957|F|0-20:T>C-20:T>C	TGCAGGGCGCTGAGCCGCTTTACGGCCGCCACGATGCCATCCTCGAGGACCGCCTTGTACGTGCTACCG	20:T>C
1378274|F|0-27:C>T-27:C>T	TGCAGCGGCACAGCCACTGCCTGATGACGATGACGATCTGATCGAGTAGAGAGAGGATCATGAGTCTGT	27:C>T
1030576|F|0-58:C>T-58:C>T	TGCAGCTCATGAAGGACTCCCGCTGGGTCGCCGTGAAACCCCGCCCCGACGCGCTCATCGTCAACATCG	58:C>T
12778480|F|0-44:G>A-44:G>A	TGCAGGTCCCCTAACCAGAGTGTCCTGACCTCGTTGGGCCCAGCGGGCGCCTGCGTCGGGGCAGCCGAG	44:G>A
12683257|F|0-8:C>T-8:C>T	TGCAGGTGCTTGGACGCGGTCCTGCACGCGGAGCACAAGGAGACGCTCGAGCAGATGCGCGCCTACTAC	8:C>T
6050212|F|0-13:G>A-13:G>A	TGCAGAGCTCGACGATGCCGCGGACGACGCCG	13:G>A
1237382|F|0-44:C>T-44:C>T	TGCAGCAGCCACCGCCAGGCACACTCACCCTGCCCATGGCATAACTGATGCACGGGGCCAGGTAGCTCA	44:C>T
4261756|F|0-34:C>T-34:C>T	TGCAGCGTCACCACGCACCTCGCCTCCGCCCCGTCCCCGCTCCCCG	34:C>T
5371744|F|0-9:G>A-9:G>A	TGCAGTTCAGCCTGACCTCCGTCTGCGTCCCG	9:G>A
2252238|F|0-64:G>C-64:G>C	TGCAGCCCGAACTCGCCGCTCGACTTGCTCATCAGGACGAACTCGTCCATCCGCTTGCCGCCG	64:G>C
3385534|F|0-15:A>G-15:A>G	TGCAGCTCCCTCCACATTTTCACATCCGGGCTTGGGACCG	15:A>G
5363749|F|0-6:C>T-6:C>T	TGCAGGCACGAGTTGTCGTAGACCCCG	6:C>T
32024591|F|0-55:G>A-55:G>A	TGCAGGTCGCGCGCCATGTACACGTTGCTGTACGTACCCTGCCCGATCTTGTCCAGCCGCTCGAAGGTG	55:G>A
1076331|F|0-8:G>A-8:G>A	TGCAGGTAGGACCTCTTGATGAACTCCAAGCAGTCAGCAATGGCCTGGTCGGCATGGAAGGAAGCCGAG	8:G>A
1003846|F|0-50:A>T-50:A>T	TGCAGCAGGTCGTCGCAGAGCGCGGCGTCCACGTCGCCGCATGCCCCGCCACCGCCGCCG	50:A>T
999296|F|0-17:G>T-17:G>T	TGCAGGCATCGCTGACCGCGGCGGCCCCGCCGCCGCCGCCATCGTGGGAGCTGCCG	17:G>T
1220240|F|0-44:G>A-44:G>A	TGCAGCGGCACCTCCAGCTCGTGCGGCGCCACGGCCCACGGGTAGAGCTGCACCGTGTAGCCCCACGCG	44:G>A
7170987|F|0-14:G>T-14:G>T	TGCAGCGAGGTGGTGGAGAAGTTCGTGGAGCCGACGAGAGGCGTGGGGATGGAGCTTTTGAGGCTGCTG	14:G>T
1004688|F|0-50:C>G-50:C>G	TGCAGCTTGTGCGGCCCCTTCGCCTCGGCCGCACCCGCGTCGCCCTCGGCCGCGACCTCCG	50:C>G
2264154|F|0-26:C>G-26:C>G	TGCAGGAGCGTCTTCGCCCTCGACTCCCCGCCGCTCTGGGGGCTCGAGTCCGTCTGCGGCCGCCGCCCG	26:C>G
7331306|F|0-13:G>A-13:G>A	TGCAGGAGCTCGAGCTCGCCG	13:G>A
1000839|F|0-25:C>G-25:C>G	TGCAGAAGGAGTCGTCGGACGCCTCCGAGGCGCACGACACGTTGAGCGACTGCATGTTCCCGTTCTTCC	25:C>G
1017213|F|0-38:G>A-38:G>A	TGCAGCGTGTCGGCGGCGATGAAGCGCAGGTTCATGGTGTAGGGGTTGTTGGTGGAGATGAAGTGGAAG	38:G>A
3937692|F|0-35:C>T-35:C>T	TGCAGCCACGCGCGCAGCCTCCGCACTGACACGCTCCCG	35:C>T
2245504|F|0-62:G>C-62:G>C	TGCAGGACGTCGAGGAAGCGGTCGAGGAGGAGCGCGTCGTACTCAATGAGCTTGTCGTCCTCGGACACC	62:G>C
1178910|F|0-44:T>C-44:T>C	TGCAGGACGTGGGACTCGTCCTCGTCGGCGTACATGTACTTGTTTTGGTGGCTCCGCAGCCGCACCGTC	44:T>C
12763892|F|0-17:G>T-17:G>T	TGCAGCATGACGAGGTTGGAGACAGGCTCGCCGATGCAGCTGTAGCGCGAGTAGCAGGCGCTCAGCTCC	17:G>T
3939450|F|0-12:G>A-12:G>A	TGCAGCTCTTCCGCCTCCACGCCTCCGTCTGCGACCG	12:G>A
7171459|F|0-8:G>T-8:G>T	TGCAGCACGAGCAGTGTGGCCGTGAGCAGCCAGCCGTTCTCCCCGCCG	8:G>T
3935840|F|0-5:C>G-5:C>G	TGCAGCTGATGTGGGCTATGGGCGGCCGCATGGCGCTGGCGCCG	5:C>G
1019445|F|0-37:T>C-37:T>C	TGCAGTCCCTCCTGGCTCTGTCCGACAGGGAGAGCGTTGTGTACAGCTACGGCATGGTGCTGCTGGAGA	37:T>C
5970733|F|0-24:A>T-24:A>T	TGCAGCGCGATGCGCGCCAGGAAGAAGATGTCGAACAGGGTGCGGATCACGGCGGTGGAGATGGCGAGG	24:A>T
2246111|F|0-65:T>G-65:T>G	TGCAGATTACCGAGCCAGTGCCACTGGAACTGCGAGAAACTATCCACGATGGTGCGGCAATCGGTTTCG	65:T>G
1002791|F|0-14:C>T-14:C>T	TGCAGCACCCGCAGCTCCAGCGGGCCGATGGCGCGGGACCGCCGCGACCCGACGTAGCCG	14:C>T
1076346|F|0-32:C>G-32:C>G	TGCAGCTTCCTTAGCCTCTCATCATCAGTAAACCCGAACCTCTGCGCGGCCATTGCCCACAGCTCTTTG	32:C>G
1167345|F|0-10:T>C-10:T>C	TGCAGCGCCGTCATCAGCGGGGTCTCCAGCCCGAACATCCTGCCCTCCATTCTCGCTCGCTGCTGATCT	10:T>C
3951544|F|0-16:G>A-16:G>A	TGCAGGCGCTCTGCATGGACCGCGCCGACTTCGAGGCCAGGTACCCGCCCCGCGGCGCGCCCG	16:G>A
1025238|F|0-22:G>C-22:G>C	TGCAGGGCAGGGCCAACGACGCGACGGAGGGCGTGGTGGACGTCGTCCCCG	22:G>C
1021353|F|0-34:T>C-34:T>C	TGCAGCACCTGGTGGAGGTGTCGGTGGCCAAGCGTGGGCTGGCGGCGAGGACGTGGTGGAACGAGCAGA	34:T>C
2244688|F|0-42:G>A-42:G>A	TGCAGTAGCCAAGTCTCCCGCCATGTCCAGCAGGTTGCAACTGCTCTCGAGGGACGCCGTCGCCGTCTG	42:G>A
2251783|F|0-16:G>C-16:G>C	TGCAGACGCGCACGGAGACGACGCCGCCGATCTGGGCGAAGACGTCGAAGAGCTGCGCGTCCTGCACGC	16:G>C
1089751|F|0-34:G>A-34:G>A	TGCAGCGCTCCTGGATCATCCTCGCCGTCGCCGCGCTCCTCATGGTGCCCTTCTACGTCTTCGCCGAGC	34:G>A
1696797|F|0-8:C>G-8:C>G	TGCAGCTCCGACGCCGAGATGAAGCCGTCGCCGTCGACGTCGAACACCTTGAAGGCCTCCCGCATCTCC	8:C>G
1206279|F|0-12:T>A-12:T>A	TGCAGCACGTCGTGTCCGTCGCCGACGTGATGGAGCTGTGCCGCCGCGAGCTGCGGCTCTACGCCAACA	12:T>A
2283560|F|0-38:C>A-38:C>A	TGCAGCAGCCCCGTCGAGCCTTCCACGACGAGCCGCGTCAGCAGCGTCGGGGAGCTGATGTAGTAGGTG	38:C>A
1026620|F|0-31:C>T-31:C>T	TGCAGCGGCTGGCCTACGTGAACTGCGTCGCCTACCCGCTCACCTCCATCCCGCTCACCGTCTACTGCG	31:C>T
988652|F|0-23:G>C-23:G>C	TGCAGAACGCCGTGAAAGGTAAGGCACGCTCATCACCTTTCGCTTTCATGAATGCTGCCATGCTGGTGC	23:G>C
1092026|F|0-20:C>T-20:C>T	TGCAGAACTGGTCGGCGAAGCAGGGCCATGTCAGCGTCGGCACGCCGTGGGAGATGGCCTCCAGCGTGG	20:C>T
1056121|F|0-22:T>C-22:T>C	TGCAGGCCCATCAGCTCCGCGTTCAGCGTCCACCGCCACTCCTTCCTCCCCGACACGCCGCCGTTCCTC	22:T>C
12778480|F|0-11:T>G-11:T>G	TGCAGGTCCCCTAACCAGAGTGTCCTGACCTCGTTGGGCCCAGCGGGCGCCTGCGTCGGGGCAGCCGAG	11:T>G
5581832|F|0-50:G>C-50:G>C	TGCAGGTCGAGGACGCCGTAGGTGGTGGAGGTGAGGGAGACGACGTGGTCGACGCGGCCGCGCACGGTG	50:G>C
32022764|F|0-11:A>G-11:A>G	TGCAGATTGATATCAGATAATCATTAGGTTCAGTTCAAAACACCCCCAAATCCACCAATCACATATATG	11:A>G
5004255|F|0-22:A>G-22:A>G	TGCAGTCGTAGAACGGCGGCGCATGGAAGCACGGCTCCATGGACACGTCCCG	22:A>G
6040325|F|0-28:A>G-28:A>G	TGCAGCTGCCTCCTGGGAAATGCACATCACCGAAGCAGAACCAGGTTCAGTGTGGCTCCTGCTCAGAGG	28:A>G
12470363|F|0-8:C>T-8:C>T	TGCAGGAGCGCAGAGAAACCG	8:C>T

5363939|F|0-5:G>A-5:G>A	TGCAGGTCGCCGACGGTGGCGGCGGCCGCGTCCG	5:G>A
1200695|F|0-17:G>C-17:G>C	TGCAGGCACATGAGCGCGAGCTGCGCGCAGAGCGTGGCCTGGCCCCTGTCGTAGGCGCCCTCCAGCCGC	17:G>C
1075340|F|0-46:C>A-46:C>A	TGCAGTGGGTGGTGACCGCCTTCTCCGTGGCGTCGCTGCCCAACACCATCATCATGGGCGTGCCGCTCC	46:C>A
1093935|F|0-8:C>G-8:C>G	TGCAGTCTCCACCACACGGACTTGCCACCATGACGGCGGCTCCTCTGTTATCGTTGACGGATGCGGCGA	8:C>G
1092880|F|0-15:C>A-15:C>A	TGCAGGCGCTGGCGCCTCCTCGTCTCCGACCCAGGATTCTCCCGCCGCTTCCGCATCCACCACCGCCGC	15:C>A
1006037|F|0-46:G>C-46:G>C	TGCAGCGGCGGGATCGCGCGCTCAAGATCTTCATGAACGAGGGTCGGTCGTGGCACGGCATCAACCACC	46:G>C
1110141|F|0-10:G>C-10:G>C	TGCAGAGCATGGGGAGGAACGACTGCGTCTGCACGAACGTCAGGCCGAGGCCGATGGACACGTTCATGG	10:G>C
4404476|F|0-19:T>C-19:T>C	TGCAGTTCGCCAGCGCGGATGACCTGCCGCTGCCG	19:T>C
1018682|F|0-31:A>G-31:A>G	TGCAGATCGGTCGTAGCTTTCGTGTGAGAAAAGATGACATGCCACTATCATCACTAAACGGGTGTCGTT	31:A>G
6048201|F|0-16:T>G-16:T>G	TGCAGGACGTGACGGTTGCCGCGTACGCCCTGGAGGCATGGGTCGCGGCGAACCG	16:T>G
7162797|F|0-19:G>T-19:G>T	TGCAGTTCTTCCAGCAGGGGTCGCTCCCG	19:G>T
7492652|F|0-8:C>T-8:C>T	TGCAGGTGCCTCGCCGCCGCCG	8:C>T
13879560|F|0-13:C>T-13:C>T	TGCAGCCTCCGCCCCTCCTCCTCATCGCCG	13:C>T
32018344|F|0-26:C>G-26:C>G	TGCAGGAGCAGCGCCTTGACGCTGGCCGTGACCTGCTGCACGCACTTGCTCCAGTGGCGCTCCAGGTTG	26:C>G
995193|F|0-14:C>G-14:C>G	TGCAGCAGCCACTGCACGGTCTCGCCGTCCGACTTGTGGCCGAGCTCGCGCGTGAGCTGGAAGATCCGC	14:C>G
1000264|F|0-18:A>T-18:A>T	TGCAGGTGGGTAAGGTGGAGATCCGATAAAAATGGACCAAATTGCGAATGCCTGGCTCCCTGTATGCCT	18:A>T
1008647|F|0-35:C>G-35:C>G	TGCAGCCCGCGGACCAGCCGATGCTGTTCCCGCCTCGCCGCCGTCGGCGTCACCG	35:C>G
1010442|F|0-21:G>T-21:G>T	TGCAGTGCCTCTACTACCTCGGGCTCGGCCTCTTCATGGCGCTCCTCGTCGGCACCCGCGTCCCGCGCC	21:G>T
986237|F|0-7:G>A-7:G>A	TGCAGGGGCAGGCCGACCTTTGGCGCCACAGCCTCTACTACATGACCTCCATGGCATTCCAGTGCGCCG	7:G>A
1008802|F|0-20:G>C-20:G>C	TGCAGCACCATCTGCTTCGCGGAGGCGGCGGCGCCGCCAGGGCCGCCGCTAATCTGGTGCTGCCGAGAT	20:G>C
1056121|F|0-17:C>T-17:C>T	TGCAGGCCCATCAGCTCCGCGTTCAGCGTCCACCGCCACTCCTTCCTCCCCGACACGCCGCCGTTCCTC	17:C>T
3026049|F|0-6:G>C-6:G>C	TGCAGGGCCCCTTCTCTCTCCGCATCCGCAGCGAATCCG	6:G>C
3029716|F|0-35:C>G-35:C>G	TGCAGGTTCGACGTGTCTGACGCCATCTGCATCAGCGCCG	35:C>G
7338838|F|0-16:C>T-16:C>T	TGCAGACCCAGCAGAGCAACATCCTCGACTCCGCGGCCGTCTCTCCGCAGCGCCTCTTCGCCGACGCCT	16:C>T
1074182|F|0-10:G>C-10:G>C	TGCAGCTCTCGGTCATCGGCTTCGTGCTCCACTTCATCTTCACCCAGAGCAGCCCGCTCTGGATCCTGC	10:G>C
1081801|F|0-32:C>G-32:C>G	TGCAGCGCCTTCATCTTCTCGTTGATCTTGCACCGCCGCCTCTTCTCGGAGAGGTTGTGCACCTCGGCG	32:C>G
1001480|F|0-17:A>G-17:A>G	TGCAGCGGCCGCTTGCAAGGGAGCAGTCGGGAGGGGTTGGAAGAGCTGCCGTAGTGGGGCGGAGCAGGA	17:A>G
2252132|F|0-17:G>C-17:G>C	TGCAGCGACGAGATCGAGAGCCTCGAGCGGGAGATCGGCGACCGAGCGTCCGAGAGATGGACTGCCGCC	17:G>C
12768489|F|0-19:C>G-19:C>G	TGCAGAAGCTCATCGTGCTCGCGCTCCTCACCCTCTGGAGCCACCTCTCCCGCAACGGCTCCCTCGAGT	19:C>G
991877|F|0-18:C>T-18:C>T	TGCAGAGGGACCCTCCCACGGAAGCCGAATGTACGGAATTGTCCACTCTATCCCCTTGGCCAGCTCGTA	18:C>T
1038385|F|0-35:G>A-35:G>A	TGCAGCTGACGAAGAATCCATGCCTGCGGTGGACGGTGGTGATCAAAGTGTGAGTTTCTTGCGGCTTGG	35:G>A
4261756|F|0-38:G>C-38:G>C	TGCAGCGTCACCACGCACCTCGCCTCCGCCCCGTCCCCGCTCCCCG	38:G>C
1093342|F|0-22:C>A-22:C>A	TGCAGATCCCCGCCGACCAGGCCGCGCTCGCGCTCGCGACGCTCGCCGCGGTGCTCCCCG	22:C>A
1021353|F|0-13:G>C-13:G>C	TGCAGCACCTGGTGGAGGTGTCGGTGGCCAAGCGTGGGCTGGCGGCGAGGACGTGGTGGAACGAGCAGA	13:G>C
1120263|F|0-26:G>A-26:G>A	TGCAGGTATCCGAGCGACGTGGACAAGCACATCCTGGCTCGCCAGACGGGCCTCTCACGGAGCCAGGTA	26:G>A
1093097|F|0-53:T>G-53:T>G	TGCAGCCGCGCAGGCGCGAACGGCCTCCCCGAGCGCCATGGCGCGCCGCCTCATCGCGAGCCCTTCCTC	53:T>G
1096975|F|0-47:G>A-47:G>A	TGCAGCGAGCGGCGGAGCACGAGGAGCGGGCGGCGGCCGTACACTCTGGCGATGCCG	47:G>A
7169061|F|0-31:G>C-31:G>C	TGCAGTCGGCCAGGCACGCCTGCCGGCACTGGTCCAGCGTCATGTCGGCGTGCACCGTCGCGTTGGTCG	31:G>C
3939140|F|0-18:C>T-18:C>T	TGCAGACATGAGGGCGTTCGACGAGATGCGGAAGCTGGAGATGTTCTTCAGGGAGGAGACCCGCCGAGA	18:C>T
1078576|F|0-17:G>C-17:G>C	TGCAGGTTCTCGGCCCAGGGCGAGAGGCCCTCGCAGCTCTTGAGGACGACGATGGAGTCGCGCCAGGAG	17:G>C
1018520|F|0-25:G>T-25:G>T	TGCAGGTGTCGGACGCGATGGACGTGGAGATGCGCGCGGGGCTCGCCTCCGAGGACGGGAGCAAGCTCA	25:G>T
7169617|F|0-27:A>G-27:A>G	TGCAGGAATTCCTTCATGTTGCTGGCGACGATCAGCCCGCGGCCGCCG	27:A>G
1220240|F|0-34:C>G-34:C>G	TGCAGCGGCACCTCCAGCTCGTGCGGCGCCACGGCCCACGGGTAGAGCTGCACCGTGTAGCCCCACGCG	34:C>G
6038049|F|0-17:T>G-17:T>G	TGCAGGCCAGCTACGCGTCTCAGGCCG	17:T>G
1000437|F|0-25:G>C-25:G>C	TGCAGCGGCAGGAAGGGCCGTTGCTGATCCTTGGGTTCCTCATGGGCATCAACGCCG	25:G>C
1008440|F|0-25:T>C-25:T>C	TGCAGCTCGTGCTTCTCCGTCTCCGTCTCGTGGACGAGCTGCATGGCCACCCGCGCCGCCTCCCGAGAT	25:T>C
1076331|F|0-11:C>T-11:C>T	TGCAGGTAGGACCTCTTGATGAACTCCAAGCAGTCAGCAATGGCCTGGTCGGCATGGAAGGAAGCCGAG	11:C>T
4539611|F|0-6:C>T-6:C>T	TGCAGGCGCTGGCGTCTCCTCATCTCCGACCCCG	6:C>T
3031226|F|0-32:A>G-32:A>G	TGCAGGTGATGTGGGCTATGGGCGGCCGCATGACGCTGGCGCCG	32:A>G
12764043|F|0-14:C>T-14:C>T	TGCAGGATCCGCGACGACGGCTGGTGGATGGTCAGCACCACGGTGCTCCCTGCGCACGCGATGTCGTGC	14:C>T
3026535|F|0-35:G>C-35:G>C	TGCAGCAGCTGCTCCAGCTCCTTGACGAAGTTGATGGCCCCG	35:G>C
1078576|F|0-11:G>C-11:G>C	TGCAGGTTCTCGGCCCAGGGCGAGAGGCCCTCGCAGCTCTTGAGGACGACGATGGAGTCGCGCCAGGAG	11:G>C
990251|F|0-39:G>A-39:G>A	TGCAGCAGCAGATCCTTGCCTACATGTGCCTCAAGTTCAGGGCGGAGAGCCTGAACCAGCAGCAGCTCA	39:G>A
990251|F|0-14:C>T-14:C>T	TGCAGCAGCAGATCCTTGCCTACATGTGCCTCAAGTTCAGGGCGGAGAGCCTGAACCAGCAGCAGCTCA	14:C>T
1248967|F|0-47:C>T-47:C>T	TGCAGGCACGTGTTGTTCTGCTAGCTGAAGCAAAGCAGCAGCACTCACGGCAATGGTGGGAAGCAAAAG	47:C>T
7171580|F|0-31:C>T-31:C>T	TGCAGCTCGCTGCGGATGAAGCCCACCTCCCCCCG	31:C>T
3952704|F|0-6:G>T-6:G>T	TGCAGCGTGGGTACAACATCGAGTCGTCGACCTCGGGAAGCTGCTCCCCTCTCGCGCCCTCATGATCAT	6:G>T
5580395|F|0-22:G>C-22:G>C	TGCAGGCACATACGACGGTCGCGTTCATGGCCG	22:G>C
12419562|F|0-22:C>T-22:C>T	TGCAGGAGAAGATCATCGTCTGCGGGGCCG	22:C>T

32022977|F|0-7:G>C-7:G>C	TGCAGGGGGTGCAGAGCATGTACGCCGTGGGGAAGGTGAAGATGTGCGCGTCCGAGTTCCACCTCGGCG	7:G>C
1026371|F|0-8:C>G-8:C>G	TGCAGCAGCGCCGTGTTGCTGCGCCGCAGCCGCTCGTTCTCGTCCATCAGCACCGCCGCGCTGGACGCG	8:C>G
5331574|F|0-16:A>C-16:A>C	TGCAGAGCTTGCTGGGAGCCTGCCG	16:A>C
6044920|F|0-57:C>G-57:C>G	TGCAGCGGATCGGGGCCGGGATGCTGCTCCAGGTGGTGACCATGGTGATCACGGCAGCGATAGAGAACC	57:C>G
2249398|F|0-28:T>C-28:T>C	TGCAGGGCCACATCAACTCGATGCTTCATTTCGCCGCGGGGCTCCTCGACGCCG	28:T>C
7350802|F|0-6:C>A-6:C>A	TGCAGGCAGTGCTCAACCTCCCG	6:C>A
1079022|F|0-31:A>T-31:A>T	TGCAGGCTGACATCTGCAAGAAAGACTCTGAAATCGGTTGGCTTCGGCCCGAGCTGGACGAGCTGGAAA	31:A>T
6040493|F|0-49:G>C-49:G>C	TGCAGGCGGGCTCGGCTGAGCTGGAGAGAGCCGCGAAGGGATCCTGCTGGTGGGGCTGGAGGTGGTGGC	49:G>C
987433|F|0-22:C>T-22:C>T	TGCAGGTGACTCAGAAGGAGGTCCCCACGCACAAGGCGATGGGGGTGCCGCTAAACGCGTACATGCTCC	22:C>T
5346063|F|0-11:G>C-11:G>C	TGCAGCGGCGGGAGGCAGGCCCG	11:G>C
2252023|F|0-61:A>G-61:A>G	TGCAGCTCCCGCCGAGGCACCGCCTGTAAGGAGAGGAACCTGTCGATGACGTACATTGTCAAGTACATG	61:A>G
1044032|F|0-65:C>G-65:C>G	TGCAGTGCCAGTTTCTGGGCCTTCTCGTCTTTGGAGAGCCCCTTGCCGACCTTGGCAATCCTCTTCCCA	65:C>G
2244688|F|0-66:C>G-66:C>G	TGCAGTAGCCAAGTCTCCCGCCATGTCCAGCAGGTTGCAACTGCTCTCGAGGGACGCCGTCGTCGTCTG	66:C>G
1027779|F|0-26:G>C-26:G>C	TGCAGCTGCGCGTAGGCCGCCTTGACGGCCGAGACGCCCGCGAACAGATTGGCGAGGAGGGAGTCCGCG	26:G>C
1021051|F|0-29:G>A-29:G>A	TGCAGGATGTACTCTACCGAGTCCTTGGAGCGCGCGCGGGCCAGCAGCGGGAGGTGCTCGTGCCCCCAG	29:G>A
1147808|F|0-28:G>A-28:G>A	TGCAGGCGGACCTGGGCGAGACGGTCGGGGCGTTCAAGGAGGCCGTGGCCGCCAGCTGCGACGTGCCGA	28:G>A
1215118|F|0-58:G>C-58:G>C	TGCAGAGCTACTGCGACGTGTCGGTGGTGAGCGGCTGGTCCACCGTGGGGTACGTCGGGCACGGCCTCG	58:G>C
2245750|F|0-5:T>C-5:T>C	TGCAGTACTCATCAAGCTGCTGCTCGAGAAAGGCTACGCGGTCAAGACGACCGTCAGGGACCCCGAGAT	5:T>C
2255492|F|0-26:G>A-26:G>A	TGCAGCGCGAGGCCGTCGAAGAAGGCGGCGTCGAGCGAGAGGCTCCTCATATGCGCGCCCCCGCCACCG	26:G>A
1076331|F|0-56:G>A-56:G>A	TGCAGGTAGGACCTCTTGATGAACTCCAAGCAGTCAGCAATGGCCTGGTCGGCATGGAAGGAAGCCGAG	56:G>A
991480|F|0-7:C>G-7:C>G	TGCAGTCCCTGGAGGACGCCGTGGGGCGGGGCAACGTCACCATCTTCGCGCCCCG	7:C>G
1089373|F|0-27:C>G-27:C>G	TGCAGCAAGCAGGAGCAGTCCTCAGCGCCCCCGCCACACACGCGGCACGCGGCGCCCGACGCCGCCGAC	27:C>G
6045109|F|0-32:T>C-32:T>C	TGCAGGTGCCCAGCAGGTAGGGCGCCGCCGCCTTGACGGCCTCGGACACGGCGTCGCTGTCCCCGAGAT	32:T>C
1266085|F|0-11:C>G-11:C>G	TGCAGCCGCGGCGGGAGGTGGAGCGGCAGGAGCTTGCCCCTGTAGAACAGCTCGTCCGCCG	11:C>G
12768897|F|0-14:A>G-14:A>G	TGCAGGAACTGTGCACATGGATCGAAGGACGCAACCTGAAGCGGGCGGCGGTGACGAACGCGCCGAGGG	14:A>G
7168871|F|0-5:G>C-5:G>C	TGCAGGAGCTCGACACGGTGTTCCTTTACAAGAACAACATCGGCGGCAAGATACCCAAGGAGCTCGGCA	5:G>C
1125324|F|0-19:G>C-19:G>C	TGCAGGGCCGCGTGAACGTGACCACCGCGCACTCGCTGGACATCATGGGCGTCTCCTACCCGCCCTCCG	19:G>C
1001492|F|0-5:C>A-5:C>A	TGCAGCGCGTGGTACCCTCCGTCAGCGAACGCCCAGGTGCGGCACACGTTGAGCCCCGCCCCG	5:C>A
1102385|F|0-22:T>C-22:T>C	TGCAGGCCTTCGTCCCCGATCTTGAGGTCGCCGTCGCCGACCTCCTCGCCTTCTTCCG	22:T>C
5332129|F|0-20:C>A-20:C>A	TGCAGCTGGCTCAGCTGGTTCAGCTCTTCCG	20:C>A
5970911|F|0-9:C>T-9:C>T	TGCAGTTCACGACGCCAGCCACGCCG	9:C>T
6045118|F|0-35:C>G-35:C>G	TGCAGGTTCTGCGTGGGGAGGTAGGCGGAGAAGACCAAGAGGAAGGCGGCGGACAGGAGGTGAACGTCG	35:C>G
5579734|F|0-19:A>G-19:A>G	TGCAGGTTCGAGAGGCACAAGCTGCGGATGGCGGAGGCGAGGCGGCGGGCGGTGTACGAGGCGGAGGGG	19:A>G
1863625|F|0-28:G>A-28:G>A	TGCAGAAGGCGATCGACTACGCGTGCGGGCAGGGCGCGGACTGCACGGCCATCGCGCAGAGCGGGGCGT	28:G>A
5971256|F|0-32:G>C-32:G>C	TGCAGGCTGGTGAGGCCGACGGAGCGGAGCCCGTCCAGGATCTCCG	32:G>C
100563778|F|0-68:A>C-68:A>C	TGCAGTTCGGCACGGCGTTCGAACAGCTTGTCAATGCTCACCAGGACTCAAAAACAACACTGGACTCCA	68:A>C
2252271|F|0-8:T>C-8:T>C	TGCAGGGATGGCTCGATGATGCCTCCGCGGAGCCTGAGGACGAGATGGAGGGTCGACTCCTTCTGGATG	8:T>C
1250733|F|0-59:C>A-59:C>A	TGCAGCATGTACCGCCCGTTGTTCATCAGGAAGATGCTGCTCAGCGCCAGGTCCTTGTACAGCTTCGAC	59:C>A
3027282|F|0-10:C>G-10:C>G	TGCAGCCGCGCCAGGTGGCCATCTGGTTCCAGAACCGCCGCGCCCG	10:C>G
7913978|F|0-16:C>G-16:C>G	TGCAGTTCTTCGACGCCTCCCAGAACCAGCTCACCG	16:C>G
1010442|F|0-25:C>T-25:C>T	TGCAGTGCCTCTACTACCTCGGGCTCGGCCTCTTCATGGCGCTCCTCGTCGGCACCCGCGTCCCGCGCC	25:C>T
1279676|F|0-31:T>A-31:T>A	TGCAGCAGATGCCCCCTGTTTTGCCTGAAAATTTTCTTCTTCGGTACTTTTGCCCTCTGCTGCTTCAGC	31:T>A
5579734|F|0-31:C>T-31:C>T	TGCAGGTTCGAGAGGCACAAGCTGCGGATGGCGGAGGCGAGGCGGCGGGCGGTGTACGAGGCGGAGGGG	31:C>T
2256685|F|0-61:G>A-61:G>A	TGCAGGAACTGTGCACATGGATCGAAGGCCGCAACCTGAAGCGGGCGGCGGTGACGAACGCGCCGAGGG	61:G>A
1089638|F|0-34:A>G-34:A>G	TGCAGATGCAGGAGGCGAGCCTTCGAACGAACTTAGTTCAGGCGAAGCAGAAGCGTGATCACGACATGG	34:A>G
2246609|F|0-22:A>G-22:A>G	TGCAGCCCCACCGCGCCCTCTCAGACAAGGTGGAGCTGATCGAGATCGACCTCTCCGAGGAATCCGCCT	22:A>G
982108|F|0-26:A>T-26:A>T	TGCAGCTGAAATATCTATCCTACAAGACACCTAAGTGGGATCGGCCAAACCTTAGTTCTGCTGCAAAAT	26:A>T
7921933|F|0-50:G>C-50:G>C	TGCAGCGCCAGCGCGAAGTAGTCGAACTCGCGCTGCTCCTTCCCGACGGCGGCCG	50:G>C
1002791|F|0-34:G>A-34:G>A	TGCAGCACCCGCAGCTCCAGCGGGCCGATGGCGCGGGACCGCCGCGACCCGACGTAGCCG	34:G>A
1026226|F|0-34:C>G-34:C>G	TGCAGGTCGCGGGCCTCTCCCTCTTCCTCTACGGCTTCTTCCCCGTCAAGCCCACGCTCCCG	34:C>G
1240720|F|0-25:G>C-25:G>C	TGCAGAGCCTCGTGTCCCGGGAGGCGGACCCGCTGGACTCGCAGGTGGTGTCCGTCGCCATGGTCAACG	25:G>C
4396050|F|0-16:C>A-16:C>A	TGCAGCTGGACGACATCCCG	16:C>A
7353363|F|0-5:C>A-5:C>A	TGCAGCCCGCGACAGCGCCG	5:C>A
3021836|F|0-21:G>C-21:G>C	TGCAGACCTTCATCAGCGTCAGCATCGTGGCCTTCATCCCG	21:G>C
2250710|F|0-59:C>A-59:C>A	TGCAGGTGCATCGGGTGGTCCCAGTCGAACTTGTTCATGGCCAGGTCCCGCTGGATCTGCTCCTCCGTC	59:C>A
1057036|F|0-13:T>A-13:T>A	TGCAGTTCAGACATTCATTCTGGCGTATATTACCATGCGATGTGATTGGGACGAAGAGGTCATTTCTTT	13:T>A
5355071|F|0-5:G>C-5:G>C	TGCAGGAGCGCGACGGGGTTCTGGGAGGGGCCGTAGCTGGGGCGCCACTCGACGTCGATCCCGACGACG	5:G>C
2261504|F|0-37:G>C-37:G>C	TGCAGTACCTGCGCAACCACAAGCCGCCGCTGACACTGGCGCGGTGCAGCGGCGCGCACGTCATCGAGT	37:G>C
7940722|F|0-13:G>C-13:G>C	TGCAGGCGCCGCCGCCGCCGCCG	13:G>C

<table>
<tr><td>3937246|F|0-14:C>T-14:C>T</td><td>TGCAGGTCCTGGAGCGTGCCGTCCAGCGCCACCG</td><td>14:C>T</td></tr>
<tr><td>2283560|F|0-56:G>T-56:G>T</td><td>TGCAGCAGCCCCGTCGAGCCTTCCACGACGAGCCGCGTCAGCAGCGTCGGGGAGCTGATGTAGTAGGTG</td><td>56:G>T</td></tr>
<tr><td>1093097|F|0-57:G>T-57:G>T</td><td>TGCAGCCGCGCAGGCGCGAACGGCCTCCCCGAGCGCCATGGCGCGCCGCCTCATCGCGAGCCCTTCCTC</td><td>57:G>T</td></tr>
<tr><td>1025720|F|0-17:C>T-17:C>T</td><td>TGCAGGTGCTGTCGCCGCTGGTGCCGATCAGGGAGGTGACGTTCCTGCGCTTCTGCAAGCAGCACGCCG</td><td>17:C>T</td></tr>
<tr><td>5368518|F|0-21:C>G-21:C>G</td><td>TGCAGGCCCGCTGCTGAACGGCAACTTCGAGCAGGCGCCGAACAGGTCCCAGATGAACGGCTCGAGGGT</td><td>21:C>G</td></tr>
<tr><td>1695396|F|0-25:C>T-25:C>T</td><td>TGCAGGACGTCCACCCCGACGTCGGCGACGCGCTCCTGGGCTTCGTCTACGACCCGCTCGACCCCGCCG</td><td>25:C>T</td></tr>
<tr><td>6046371|F|0-49:G>C-49:G>C</td><td>TGCAGGCCTTCATCGCGCCGTTCGGGTCGGCGTCCGTGTCCTGGAACACGTCCCGCCAGACGTGCTCCT</td><td>49:G>C</td></tr>
<tr><td>5582001|F|0-12:A>T-12:A>T</td><td>TGCAGCTCTCGCACACGAAGTCCCAGTGCGGGCCG</td><td>12:A>T</td></tr>
<tr><td>4002018|F|0-17:C>T-17:C>T</td><td>TGCAGGCGCTGCACCTCCGCGAGCATGCGGTTCTCGTCGACCAAGGGCGGGCCG</td><td>17:C>T</td></tr>
<tr><td>1165473|F|0-64:C>T-64:C>T</td><td>TGCAGCGGTACGTGTGGGCCGACGGCGCGTGGAACAACTTCTGGTACCACCCGACCGACCCGTGCGACA</td><td>64:C>T</td></tr>
<tr><td>2245539|F|0-56:G>A-56:G>A</td><td>TGCAGGCCTGCTCGTCGACCAGTGCATAGAGGTCCCTGAAGATCTCGTTGATCTCGGTGATCACCTGCT</td><td>56:G>A</td></tr>
<tr><td>6047341|F|0-10:C>T-10:C>T</td><td>TGCAGTCCTTCCTGATCTCGAAGCGGGTGCCCTCGGCGGGGATCTCGCTGAGCTTGGCCATGGCCTTGC</td><td>10:C>T</td></tr>
<tr><td>1228454|F|0-13:C>G-13:C>G</td><td>TGCAGAAGCAGCTCAGGCCCTACATGGAGTCCATCGTGCCGCTCCCCGGCATCTATGACCCG</td><td>13:C>G</td></tr>
<tr><td>1156230|F|0-31:C>T-31:C>T</td><td>TGCAGCTGCAAGTCAAGGTGCTGGCGACGGACGAGTTCTGGCCGGCGCAAGGGGGAACGGCGCCGAGAT</td><td>31:C>T</td></tr>
<tr><td>5581360|F|0-7:T>G-7:T>G</td><td>TGCAGCCTCATGAGCGCCTGCAGCCG</td><td>7:T>G</td></tr>
<tr><td>994364|F|0-29:G>A-29:G>A</td><td>TGCAGCGCGAAGATGTGGGCGACGCAGCCGTAGACGGGGTCGCGCAGCCG</td><td>29:G>A</td></tr>
<tr><td>4009329|F|0-44:G>A-44:G>A</td><td>TGCAGGCCGTGCGCGAGGACGTCGAGGCCGTAGAGGTCGGCGGCGCGCGGGCTGGCGATGGCGGCCGTG</td><td>44:G>A</td></tr>
<tr><td>1090310|F|0-8:G>C-8:G>C</td><td>TGCAGAGTGCAGAAGCGCACCTGGTGGACGAGGTGCACGGTGGGCGCGAGGAAGAAGACGATCCGAGAT</td><td>8:G>C</td></tr>
<tr><td>2283560|F|0-65:G>T-65:G>T</td><td>TGCAGCAGCCCCGTCGAGCCTTCCACGACGAGCCGCGTCAGCAGCGTCGGGGAGCTGATGTAGTAGGTG</td><td>65:G>T</td></tr>
<tr><td>1129334|F|0-24:C>G-24:C>G</td><td>TGCAGAGCCCGAGGGATGCCTGCGCCGCGCCGCTGAGGTCGCCGTCGCCGAGGGTCGTCGCCG</td><td>24:C>G</td></tr>
<tr><td>12776600|F|0-7:G>C-7:G>C</td><td>TGCAGACGGACCCGCGCGCGCCGCCG</td><td>7:G>C</td></tr>
<tr><td>23919587|F|0-24:C>T-24:C>T</td><td>TGCAGGCCTGCTCGTCGACCAGCGCGTAGAGGTCCCTGAAGATCTCGTTGATCTCGGTGATCACCTGCT</td><td>24:C>T</td></tr>
<tr><td>1865125|F|0-18:C>T-18:C>T</td><td>TGCAGGTGTACTGGTGGGCGCTGCGCGCGTTGCGGTCGAGGAAGCCGAGCGCGTGGCCG</td><td>18:C>T</td></tr>
<tr><td>3958379|F|0-19:C>G-19:C>G</td><td>TGCAGGCTGGCCAGGCGGCCGAGGGACGCCGGGATGGCGCCG</td><td>19:C>G</td></tr>
<tr><td>1091125|F|0-30:C>T-30:C>T</td><td>TGCAGCGCTCCTGGCTGCTGCTCACCGCCACGGCCGTCTGCATGCTCCCGCTCTACCTCTTCGCCACCC</td><td>30:C>T</td></tr>
<tr><td>4982261|F|0-10:G>T-10:G>T</td><td>TGCAGTTCCTGGCCATGCTCCCCGACCTCCGCCCCAAAGTCAGGGTGATCGCGGAATGCGGCAACTGGT</td><td>10:G>T</td></tr>
<tr><td>1095573|F|0-38:A>G-38:A>G</td><td>TGCAGGGGGAGATGTGCCGCCTGGCGCTGCGCGAGCCCACCAAGGACGGGGCCGAGCACCTCCGCGCCG</td><td>38:A>G</td></tr>
<tr><td>2281139|F|0-33:C>T-33:C>T</td><td>TGCAGGATCCTCGACCTCCTCTCCTTGGGGTGCCGAGAGAACTGGTCCGCGCCGAAGGCCATGGAGCAC</td><td>33:C>T</td></tr>
<tr><td>1862570|F|0-11:G>C-11:G>C</td><td>TGCAGCTTCCTGGGATGGAGAATGGGGAGATCGAGGCCGCGGAGGATGGGCTGCCG</td><td>11:G>C</td></tr>
<tr><td>1240245|F|0-27:T>C-27:T>C</td><td>TGCAGGAGCGCGACGGGGTTCTGCGCGTGGCTGTAGCTGGGGCGCCACTCCACGTCCAGCCCCACGATG</td><td>27:T>C</td></tr>
<tr><td>3952787|F|0-16:T>C-16:T>C</td><td>TGCAGTGGATCGAAACTCTTGTCCG</td><td>16:T>C</td></tr>
<tr><td>4404476|F|0-15:C>T-15:C>T</td><td>TGCAGTTCGCCAGCGCGGATGACCTGCCGCTGCCG</td><td>15:C>T</td></tr>
<tr><td>32024349|F|0-23:T>C-23:T>C</td><td>TGCAGATTTAAGCCGTATCCACTTAGGATGGCCGTGCTCACGACGATCTATGGGTTTGGACTTTACAAG</td><td>23:T>C</td></tr>
<tr><td>7169505|F|0-12:G>A-12:G>A</td><td>TGCAGCGGCTGGGGAGTCGGTGGCCG</td><td>12:G>A</td></tr>
<tr><td>6035845|F|0-25:C>G-25:C>G</td><td>TGCAGCCCTGCGAGGTGTGGTCGGGCACGGCGGGCGTCATGCTCTTGCCCTTGGTGGTGTCGATCCTCT</td><td>25:C>G</td></tr>
<tr><td>5354953|F|0-8:A>C-8:A>C</td><td>TGCAGCTGACGCTGCTGCTGCTGGAGCAGGAGCACGGTGTGGTTGAAGGCGCCGTCGACGGCGTGGAGC</td><td>8:A>C</td></tr>
<tr><td>32021335|F|0-12:C>G-12:C>G</td><td>TGCAGCAGCAAGCAGCTACCAGCGCCG</td><td>12:C>G</td></tr>
<tr><td>7173141|F|0-29:C>G-29:C>G</td><td>TGCAGCTTCCCGAAGGTGCTGATCGACGGCCCGTACGGCGCGCCG</td><td>29:C>G</td></tr>
<tr><td>24505073|F|0-17:T>C-17:T>C</td><td>TGCAGCTCGATCTCGCCTGCGGCATGGCTCCCCGCGCCTCAGCCG</td><td>17:T>C</td></tr>
<tr><td>5361174|F|0-42:T>C-42:T>C</td><td>TGCAGCTCCTCAGCCCGCTCGACGACGCCATCGCCATTGTTGTTCCCTCGATGCTGGCTGCTCCTTGCT</td><td>42:T>C</td></tr>
<tr><td>100560039|F|0-10:T>C-10:T>C</td><td>TGCAGCAGCATTACTCCGCCCGAGGCGACCTCTTCGAGCCGAGCTCCG</td><td>10:T>C</td></tr>
<tr><td>100573669|F|0-9:G>A-9:G>A</td><td>TGCAGCGGCGCCGCCTTGGGGAGCGTCAGCCCACCTCCTTCACCCATGTCAACCTCAGCATCACCAACC</td><td>9:G>A</td></tr>
<tr><td>5370858|F|0-17:T>C-17:T>C</td><td>TGCAGCTGCGCCCACGCTTCCTCTGTGTCGATCAGGCGGTGCAACCACGCCG</td><td>17:T>C</td></tr>
<tr><td>1026259|F|0-43:G>T-43:G>T</td><td>TGCAGGCGGCGGTGGAGCTCCAGGAGGCGCTCGCGCTGCGCCCGCACGTTCTTGGCGGCCATCATGGCC</td><td>43:G>T</td></tr>
<tr><td>100440256|F|0-16:T>C-16:T>C</td><td>TGCAGCCTGGGCTGGATCGAGGCTCCCG</td><td>16:T>C</td></tr>
<tr><td>14385088|F|0-22:T>C-22:T>C</td><td>TGCAGAGGCGGCGGCTGGGCAGTGCGCCATTTCCTCCG</td><td>22:T>C</td></tr>
<tr><td>5360609|F|0-26:A>G-26:A>G</td><td>TGCAGAGCGCCCTGAATCTCCGCCATATACTTGTTGTCTTCGTCTAACCATCGGGCGTACTCCATGTCG</td><td>26:A>G</td></tr>
<tr><td>7170760|F|0-8:G>C-8:G>C</td><td>TGCAGCTTGGAGAGGTTGGCAAGAGAGGCCG</td><td>8:G>C</td></tr>
<tr><td>2266783|F|0-59:G>A-59:G>A</td><td>TGCAGACCGTGAGCGCGATCGCCCTCGGGAGCGAGACAGAGAGGGCCCCGTGGAAGGCGGTGTGAAGGA</td><td>59:G>A</td></tr>
<tr><td>5355023|F|0-38:T>C-38:T>C</td><td>TGCAGGAACAAGGTGGTGGCCTGCGCAAAGCACTTCGTTGGCGACGGCGGCACGGTGAACGGCATCAAC</td><td>38:T>C</td></tr>
<tr><td>4910125|F|0-26:C>G-26:C>G</td><td>TGCAGGTTCCGCCGCTTGTAGCCGACCAGGCGCCGCGACGGCCG</td><td>26:C>G</td></tr>
<tr><td>100586364|F|0-40:T>C-40:T>C</td><td>TGCAGGCCGCCGCTGCCATGGCGCGCATCGTCGGCGACGGTGACGCCGAGTGCTGCTTCCGTGCGCGGT</td><td>40:T>C</td></tr>
<tr><td>1698163|F|0-20:C>G-20:C>G</td><td>TGCAGCTCCCTCCTCTCCTCCTCGCCGCCGCCGAACTCGCAGAGGGAGTAGGCCACCATGTCCTCCTCG</td><td>20:C>G</td></tr>
<tr><td>1263957|F|0-49:G>A-49:G>A</td><td>TGCAGGCCATCGTGCGCGGCCGGCAGGTGCGGAAGCAGGCGGCGGTGACGCTCCG</td><td>49:G>A</td></tr>
<tr><td>1089751|F|0-26:G>A-26:G>A</td><td>TGCAGCGCTCCTGGATCATCCTCGCCGTCGCCGCGCTCCTCATGGTGCCCTTCTACGTCTTCGCCGAGC</td><td>26:G>A</td></tr>
<tr><td>7353362|F|0-5:G>C-5:G>C</td><td>TGCAGGTTGTGGACGCCGCCG</td><td>5:G>C</td></tr>
<tr><td>3944521|F|0-6:G>A-6:G>A</td><td>TGCAGGGCCTTTTCTGGCGGTGGCGCCG</td><td>6:G>A</td></tr>
<tr><td>7164282|F|0-14:C>T-14:C>T</td><td>TGCAGGTACTACACCTCGTTCCAGCTGAGCGAGAAGAGCGACGTGTACAGCTTCGGGATCGTGCTGCTG</td><td>14:C>T</td></tr>
<tr><td>4734172|F|0-23:G>C-23:G>C</td><td>TGCAGCCTCCGCGGCAGGAAGGAGCCCGCGCTGGCCTCCCCG</td><td>23:G>C</td></tr>
</table>

1865063|F|0-65:A>T-65:A>T	TGCAGTGACAAGAAAACGCTGAAGAAGTGGTTCTTCATCGACAAGACAGTGGGCTAAGAAAATCCAGCT	65:A>T
1074182|F|0-53:G>C-53:G>C	TGCAGCTCTCGGTCATCGGCTTCGTGCTCCACTTCATCTTCACCCAGAGCAGCGCGCTCTGGATCCTGC	53:G>C
3947093|F|0-26:C>G-26:C>G	TGCAGGTAAGCCACTCTCGAGCACTACAAGCTCCTGAAACACGATTGATACCG	26:C>G
1250733|F|0-50:G>A-50:G>A	TGCAGCATGTACCGCCCGTTGTTCATCAGGAAGATGCTGCTCAGCGCCAGGTCCTTGTACAGCTTCGAC	50:G>A
1089105|F|0-25:C>G-25:C>G	TGCAGGAGTCGGCGCTCAACACCCTCGCCAGGCTCGCCTCCCACCTCGCCGCCG	25:C>G
1082688|F|0-29:C>T-29:C>T	TGCAGCCGCCGCACCACCGACTGCCCGTCCGCGCCACGCGCAGCCGCCG	29:C>T
1082688|F|0-14:C>G-14:C>G	TGCAGCCGCCGCACCACCGACTGCCCGTCCGCGCCACGCGCAGCCGCCG	14:C>G
1318673|F|0-46:C>G-46:C>G	TGCAGAACATCTACTTCTCCTACGGGATGGCGTACGGCGGCGGCGGCTTCGCCATCAGCAGGCCGCTGG	46:C>G
1718142|F|0-20:C>G-20:C>G	TGCAGGTAGTCGCCGACGTTCACCACCAGCGCCCCGCCTCCGCCGGCACGTCCACCCACGCCG	20:C>G
1089638|F|0-15:C>T-15:C>T	TGCAGATGCAGGAGGCGAGCCTTCGGACGAACTTAGTTCAGGCGAAGCAGAAGCGTGATCACGACATGG	15:C>T
3022980|F|0-9:C>G-9:C>G	TGCAGCATCCCACACAAACTGGAAGCCAGCGTCAAGTACTTCCG	9:C>G
1200695|F|0-54:G>C-54:G>C	TGCAGGCACATGAGCGCGAGCTGCGCGCAGAGCGTGGCCTGGCCCCTGTCGTAGGCGCCCTCCAGCCGC	54:G>C
2256549|F|0-56:C>T-56:C>T	TGCAGGCTGCGCGCTGCCACTGTTCCTTGCATCAGCCTCGAACTCGGCGCCACCTCCCGTGCTCGATCA	56:C>T
7167621|F|0-14:C>G-14:C>G	TGCAGCAGCTGGTCCGCCAGCGCGTAGTCCTCCTTCCCCG	14:C>G
2250322|F|0-16:G>C-16:G>C	TGCAGAAGAACCTCGGGCTGTCCGACGAGCAGGTGGAGGCGTCGCGGATGACGCTGCACAAGTTCGGGA	16:G>C
1322188|F|0-47:A>G-47:A>G	TGCAGTGGAGGAGCTTGCTCAAGAACCGAGAGCCGGCCAGGGACTGGACGGCCCTCGTTCGGGAAGGTC	47:A>G
23919587|F|0-10:C>T-10:C>T	TGCAGGCCTGCTCGTCGACCAGCGCGTAGAGGTCCCTGAAGATCTCGTTGATCTCGGTGATCACCTGCT	10:C>T
1009326|F|0-23:C>A-23:C>A	TGCAGTAGTGGCGGCGCCTACCCCCGCGCGACTCCTCCTCGTCTCCGCCCGCCGCGCTGCTCCGCGATT	23:C>A
1024854|F|0-62:G>C-62:G>C	TGCAGCCACCCGACGCCCGCCGAGCGGCCCAAGGCGCAGGCCATCTCGCAGATCCTGCTGCGGTCCATG	62:G>C
5324019|F|0-13:C>T-13:C>T	TGCAGCATGACGGCCACCACCACCACCCGTCCCCG	13:C>T
2280310|F|0-35:C>G-35:C>G	TGCAGCCTGGCGGACGCCTCGTCGAACTCCACCGTCGCGCGGCCGTCCTCCCCCG	35:C>G
3937246|F|0-30:A>G-30:A>G	TGCAGGTCCTGGAGCGTGCCGTCCAGCGCCACCG	30:A>G
3033840|F|0-59:A>T-59:A>T	TGCAGGTGCACTGCACTGGAAGGTCGTATCAGTGTACATCAACCGAATGGGCCTGGTAAATGCTCATGT	59:A>T
2261925|F|0-7:G>T-7:G>T	TGCAGGCGCTCTGCATGGATCGCGCCGACTTCGAGGCCAGGTACCCGCCCCGCGACGCGCCCG	7:G>T
1213103|F|0-25:T>C-25:T>C	TGCAGGCGCTGGTGCGCGGCCGCCGTGTGCGCAAGCAGCTGGCCGTCACGGTCAAGTGCATGCAAGCGC	25:T>C
2245073|F|0-49:G>C-49:G>C	TGCAGTGCGCGCTCAGCCTCATGCCGTCGAACGCCGCCGCGCCGCAGCTGGACTGCCTCAACATGCTCA	49:G>C
2279245|F|0-65:C>T-65:C>T	TGCAGGGGCTCTCCCAGATGGTTCTGCACCTCGTCGAGGGTGAGGCTGTACAGAGACCCCTGCCTCGCC	65:C>T
3028663|F|0-14:C>A-14:C>A	TGCAGCTTCGCCGCCGCCGACGACGCCGACGTGCCCTCGCCCGCCG	14:C>A
4993438|F|0-22:T>C-22:T>C	TGCAGCGCGTCTTCTACGGCGATAACGCCG	22:T>C
25258780|F|0-44:G>A-44:G>A	TGCAGACCGTGAGCGCGATCGCCCTCGGGAGTGCGACGGAGAGGGCCCCGTGGAAGGCGGTGTGAAGGA	44:G>A
4993427|F|0-14:C>G-14:C>G	TGCAGACGTAGGCGCTGAAGCCG	14:C>G
6044098|F|0-17:T>C-17:T>C	TGCAGGCCGCCTCCGAGTCGCGGCCGCCG	17:T>C
6049722|F|0-11:A>T-11:A>T	TGCAGGCGCCCATATCGATCCGCCTTACCTTTAGCTCCG	11:A>T
1863625|F|0-25:C>T-25:C>T	TGCAGAAGGCGATCGACTACGCGTGCGGGCAGGGCGCGGACTGCACGGCCATCGCGCAGAGCGGGGCGT	25:C>T
2250322|F|0-9:A>G-9:A>G	TGCAGAAGAACCTCGGGCTGTCCGACGAGCAGGTGGAGGCGTCGCGGATGACGCTGCACAAGTTCGGGA	9:A>G
1228454|F|0-16:G>A-16:G>A	TGCAGAAGCAGCTCAGGCCCTACATGGAGTCCATCGTGCCGCTCCCCGGCATCTATGACCCG	16:G>A
3022522|F|0-21:T>C-21:T>C	TGCAGCCACACTCTTTGAGCTTGGGGCTCAGGTGCATGCCCG	21:T>C
6038048|F|0-7:C>T-7:C>T	TGCAGGCCAGCTACGCGGCTGAGGCCG	7:C>T
2268151|F|0-32:G>A-32:G>A	TGCAGGGCGCTGAGCCGCTTCACGGCCGCCACGATGCCGTCCTCGAGGACCGCCTTGTACGTGCTCCCG	32:G>A
989467|F|0-44:T>C-44:T>C	TGCAGCATCACCTCCGACACCCACACCGCATACGCCCTCTTCTCTCTGCCCTCGCGCCCCG	44:T>C
1195849|F|0-58:G>A-58:G>A	TGCAGCAGCGGGAACGGCACCGAAGGCGACGAGCTCTCCATGCTCCTCCGCCGCCGCTGCTGCGGTGCG	58:G>A
4261742|F|0-8:G>A-8:G>A	TGCAGGAAGAGCAGAGGCTTGGCAACCACATCCCTGGTGTCACCG	8:G>A
1032056|F|0-5:C>A-5:C>A	TGCAGCACATTATGCATCTTCTCTTCAGGCAAGCTCTCCAGCCCCGCCCTAAGCATGTTCTTCTCCTCC	5:C>A
5580536|F|0-7:T>C-7:T>C	TGCAGGTTGGGATCCGTTGTGCTCAGCGACCCG	7:T>C
3570121|F|0-47:G>A-47:G>A	TGCAGCAGGCCCGTGAAGCCCTGCGCGAGGCGGTCGGCGATGTCCTGGCCGTGCCGCTGCACCCCCTCC	47:G>A
7158947|F|0-15:C>T-15:C>T	TGCAGCTTGTACGGCCCGACGGCCCGCGGCGCGCGGAGCTCGAGGAGCGCGACGGGGAGCGGCGCGAGC	15:C>T
1200695|F|0-53:G>C-53:G>C	TGCAGGCACATGAGCGCGAGCTGCGCGCAGAGCGTGGCCTGGCCCCTGTCGTAGGCGCCCTCCAGCCGC	53:G>C
1027592|F|0-36:A>C-36:A>C	TGCAGTGGATCATGGCCGAACTGGTCAAGAACCCAGAAATCCAGGAGAAGCTCTACGAGGAGATCAAAG	36:A>C
5363939|F|0-14:G>C-14:G>C	TGCAGGTCGCCGACGGTGGCGGCGGCCGCGTCCG	14:G>C
2252132|F|0-38:C>G-38:C>G	TGCAGCGACGAGATCGAGAGCCTCGAGCGGGAGATCGGCGACCGAGCGTCCGAGAGATGGACTGCCGCC	38:C>G
6038048|F|0-19:T>C-19:T>C	TGCAGGCCAGCTACGCGGCTGAGGCCG	19:T>C
993268|F|0-11:T>C-11:T>C	TGCAGGTATGGTTCTGCAAGAACAAGCTGCCCATGTGAAAAGAGACAACCTTTGCGATCAAGCCAACCC	11:T>C
993268|F|0-58:C>T-58:C>T	TGCAGGTATGGTTCTGCAAGAACAAGCTGCCCATGTGAAAAGAGACAACCTTTGCGATCAAGCCAACCC	58:C>T
1244635|F|0-16:A>C-16:A>C	TGCAGTTCTTCAACGTAAGCTACAACAGGCTCTGCGGCCCTGTGCCGACCG	16:A>C
6042917|F|0-29:C>G-29:C>G	TGCAGGAGCGCCGTGGTGGGGAGCACGAGCGAGACGTTGTTTACGGCCGCCGCGAACTGCGTGCCGTTG	29:C>G
2249108|F|0-49:C>A-49:C>A	TGCAGCAAATCTGGTGGTTCGAGGTGGTCGGGGAGATCGACTTCCGCTTCCCG	49:C>A
2250322|F|0-13:C>G-13:C>G	TGCAGAAGAACCTCGGGCTGTCCGACGAGCAGGTGGAGGCGTCGCGGATGACGCTGCACAAGTTCGGGA	13:C>G
1120263|F|0-56:A>T-56:A>T	TGCAGGTATCCGAGCGACGTGGACAAGCACATCCTGGCTCGCCAGACGGGCCTCTCACGGAGCCAGGTA	56:A>T
1214563|F|0-28:A>C-28:A>C	TGCAGGTACCGCATGAACGGGAGCTTGGAGTCGTCGGGGAAGGGGAGACCCCG	28:A>C

1044032|F|0-26:G>A-26:G>A	TGCAGTGCCAGTTTCTGGGCCTTCTCGTCTTTGGAGAGCCCCTTGCCGACCTTGGCAATCCTCTTCCCA	26:G>A
5580329|F|0-5:C>T-5:C>T	TGCAGCTGGCGCCACCTCAACAACCCG	5:C>T
990006|F|0-34:A>C-34:A>C	TGCAGCCGCCGATGCGGCAGCTCTCTGCGGCGTCATCCACCAACCTCGCGCCCGACTACGCCG	34:A>C
7164893|F|0-46:G>C-46:G>C	TGCAGATGCTGCGGGTGCAACCGAGGTGGGCGGATCAGGCGGACGCGGAGGCGGCCG	46:G>C
1143410|F|0-60:T>G-60:T>G	TGCAGCGTCGATCTGCGCGGACGCAGTGGAGAAAGCGGCTTCGTCCCCCGAGGATGCCGCTGAGATCAT	60:T>G
1143410|F|0-36:G>A-36:G>A	TGCAGCGTCGATCTGCGCGGACGCAGTGGAGAAAGCGGCTTCGTCCCCCGAGGACGCCGCTGAGATCAT	36:G>A
1050486|F|0-46:C>T-46:C>T	TGCAGCATGTGCAGGCAGCTGACCAAACTCTTTCCTCTCCACCTGACCATCGTCCTTCTCCTCGGCGTC	46:C>T
5358266|F|0-25:G>T-25:G>T	TGCAGGCGGCGTCGTCGAAGGTGCTGCCGCCGCCG	25:G>T
3027502|F|0-28:C>G-28:C>G	TGCAGAGGACCGCCTTCGGGTCGGTGTCCAGCAGCACCG	28:C>G
4910284|F|0-17:G>C-17:G>C	TGCAGCAGGTTGAGCCTGGTCTCCCCG	17:G>C
1126888|F|0-44:G>A-44:G>A	TGCAGCTCCTGGAGAGAGAACCACTCCTTCCCGTCCTTCCCTGCGTCCGCCG	44:G>A
1085630|F|0-11:A>C-11:A>C	TGCAGGCCTTCATCGCGCCCTTCGGGTCGGCGTCCGTGTCCTGGAACGCGTCCCGGCAGACGTGCTCCT	11:A>C
3027770|F|0-38:C>G-38:C>G	TGCAGCGCCACGTACGCCTTCTCCAGCCGCGAGTTGTTCGTCGCCG	38:C>G
1060520|F|0-29:T>C-29:T>C	TGCAGGCCGTGGTGGAGCTCAAGGACTTCTTGGACCACACCGCCATGCTCGCCATGCCCAGCCAGAGGA	29:T>C
988498|F|0-44:C>G-44:C>G	TGCAGCGACGTGTGGTTCAGGTACTCGCCGCCTCCGCGTAAGCCCACGCCCAAGAAGACGCGGAGGCCG	44:C>G
6035958|F|0-28:T>C-28:T>C	TGCAGCGTTCTCTGAGGTACTCCTCGAGTGGGCTGAGGTTGGCGTGCTCGGTGCTAGAGATGGACTCGG	28:T>C
1257776|F|0-63:A>C-63:A>C	TGCAGGCCCTGGACCGCGGCGAGCAGGAGCTGGCCTTCTACACGGCCTTCTCCGCCCACCCCGACGTGC	63:A>C
7350668|F|0-17:C>T-17:C>T	TGCAGGCAACCCATCGGCGACCCCG	17:C>T
1126888|F|0-29:C>G-29:C>G	TGCAGCTCCTGGAGAGAGAACCACTCCTTCCCGTCCTTCCCTGCGTCCGCCG	29:C>G
6039964|F|0-28:G>A-28:G>A	TGCAGAGCGTTTTAATGGCCGCACAGTGGAACTGCGTCCCTTTCCCTATCATCGCCTTCAATGAGCCAG	28:G>A
5348124|F|0-19:C>T-19:C>T	TGCAGGTGTACACGATCGTCGCGGCCG	19:C>T
2250347|F|0-12:C>T-12:C>T	TGCAGCTTCTGGCGGGGCTCCAGCTTGGTCTGCGTGGCGAACATGGACTCGAGCGACTTGATCTGCTCC	12:C>T
2249077|F|0-38:G>A-38:G>A	TGCAGACGTGGGCATACTACGACCAGGGGCAGCTGGACGAGATCATCGACAGCTCCCTGGGCGACGACC	38:G>A
985853|F|0-47:G>A-47:G>A	TGCAGGTGGCGAGGGGTGATACGCTTCACCTTGAGATCCTTGCTGGCGTTGCCG	47:G>A
1006595|F|0-57:C>T-57:C>T	TGCAGTTGAGGACGCGGCAGCGGAGATGGCGGCGAGGAGGCGGGAGGTGGGAACGGGCAGGGGCATGGC	57:C>T
1178910|F|0-26:G>C-26:G>C	TGCAGGACGTGGGACTCGTCCTCGTCGGCGTACATGTACTTGTTTTGGTGGCTCCGCAGCCGCACCGTC	26:G>C
3021028|F|0-23:A>G-23:A>G	TGCAGGCTCACCCAAGATTCTCCACCATATTCACTTCTCCCG	23:A>G
1019650|F|0-27:G>C-27:G>C	TGCAGGCAGCCTGGTCTTCCTCTTCTTGACGGACGCCACCACCACCCTGAGGATGTCCCTGAGCGGGCT	27:G>C
1003777|F|0-28:C>T-28:C>T	TGCAGCCCGAACACGGCGGCGATGGCGACGACGGTGAGGTACGCGACGCCCTCGGCGGCGCCGAGGAGG	28:C>T
986252|F|0-28:C>G-28:C>G	TGCAGGCGCTGCTGCTCGCCTTCCTCTCCCTCTGCCTGCTGCTCCTCTCCGCGCTCCTCTCGCTCCACC	28:C>G
1088789|F|0-20:C>G-20:C>G	TGCAGGCTGAGGTGGAGCAGCGCCGAGCAGTTGGCGAGCGCCGACGGGATGGTCCCCTCAAGAAGGTTG	20:C>G
1028336|F|0-12:T>C-12:T>C	TGCAGGTGCAGATGCAGCGGAGCGCGGCTGGGAAAGGCGCTGAAAAGATGAGCGTTGATGATGGCTAGC	12:T>C
3943634|F|0-8:G>A-8:G>A	TGCAGCGTGGGGATGAGGATGGCCATGGCTAGCTGTGGCCG	8:G>A
1024458|F|0-15:T>A-15:T>A	TGCAGAGACCATGCCTGTGATCACTGCGTTCCAGGAAAAGTTGTTCCTTTCCG	15:T>A
32023395|F|0-24:G>C-24:G>C	TGCAGGGGGTGGTCGGCCCCGACGGCGCCTTCACCGACGTCTGCATCGGCTGGCCG	24:G>C
1864394|F|0-60:C>T-60:C>T	TGCAGCTCGGCAAGGTCAAAAGGAACATCGAAAGCCGGCTTCCGGAAACCGCAGTCCTCGCTGTCCCCA	60:C>T
1127636|F|0-5:C>A-5:C>A	TGCAGCACTACCTTCCAGCAGTCAGCCGCGGCCTCGGCCACCTCGCCCTCCCCCG	5:C>A
2249077|F|0-58:G>A-58:G>A	TGCAGACGTGGGCATACTACGACCAGGGGCAGCTGGACGAGATCATCGACAGCTCCCTGGGCGACGACC	58:G>A
1070571|F|0-22:C>G-22:C>G	TGCAGTACGCGTGCAGGAAAACCTTGGCGGACAGCAGGCCGCGCGTCCGCGGGAGGTTCGCCAAGAACG	22:C>G
7169487|F|0-18:G>A-18:G>A	TGCAGCGCTCCGAGACGGGGAAGATGATGGCCG	18:G>A
3028382|F|0-31:C>T-31:C>T	TGCAGAGCCGCGAGACGGACTCTGAAATCTCCACCCACGACTCCG	31:C>T
4003106|F|0-50:A>G-50:A>G	TGCAGCGGCACCACGTCCTCGGGCCACGTCTTGGTCACGTCCAGCGGGTCAAAGTCGAAGCGGTCCTCG	50:A>G
1674720|F|0-47:C>T-47:C>T	TGCAGCCGCTCGGACGACCCCACGGGGACCTCCCACGCGGCCTCAGCCATGTCGCGGCTGGTCCCGAGA	47:C>T
5582651|F|0-5:G>T-5:G>T	TGCAGGCTGCTCCGAAGTCCCG	5:G>T
995643|F|0-52:C>G-52:C>G	TGCAGGTCCGCGACGACGTGCCGCTCATCGGGTTCATCGGGCGGCTGGACCACCAGAAGGGCGTGGACA	52:C>G
7170982|F|0-8:C>G-8:C>G	TGCAGCGACGCGACGTGGCGCGCGCCG	8:C>G
1076346|F|0-44:C>T-44:C>T	TGCAGCTTCCTTAGCCTCTCATCATCAGTAAACCCGAACCTCTGCGCGGCCATTGCCCACAGCTCTTTG	44:C>T
1201557|F|0-52:G>A-52:G>A	TGCAGGCGGCGGGGAGGCCGAGCGCCCTCGTGACGTTGAGCTTCAAGTCCAGGCCG	52:G>A
1200695|F|0-11:G>C-11:G>C	TGCAGGCACATGAGCGCGAGCTGCGCGCAGAGCGTGGCCTGGCCCCTGTCGTAGGCGCCCTCCAGCCGC	11:G>C
7173292|F|0-20:T>C-20:T>C	TGCAGCGCCCCTCGAGCGACTCCAGGAAGAGCGGGACCGCGAGCAGGCCG	20:T>C
7351860|F|0-14:A>G-14:A>G	TGCAGCGTGCTCTGACACAGGAAGCACTGGCCG	14:A>G
1008561|F|0-18:C>T-18:C>T	TGCAGCGCCAACGTGTTCCTGGACAACGCCGAGTACCGCCAGTTCCTCCGCAAGCAGTTCGGGTGCACC	18:C>T
32027204|F|0-21:T>C-21:T>C	TGCAGCCCTGCAAGCCGCCGCTGCACGAGACGGACCACCTCGGCGGCGCCGTGTTCACCG	21:T>C
1027581|F|0-43:A>G-43:A>G	TGCAGTGGATCATGGCGAACCTGGTGAAGCGCCCACACATGCAAGAGGCTGTCCGTAGGGAGATCAACG	43:A>G
993268|F|0-19:G>C-19:G>C	TGCAGGTATGGTTCTGCAAGAACAAGCTGCCCATGTGAAAAGAGACAACCTTTGCGATCAAGCCAACCC	19:G>C
6037424|F|0-11:C>T-11:C>T	TGCAGAAGAGACGGCGGAGGCGGTTCCG	11:C>T
1165473|F|0-32:A>G-32:A>G	TGCAGCGGTACGTGTGGGCCGACGGCGCGTGGAACAACTTCTGGTACCACCCGACCGACCCGTGCGACA	32:A>G
1263957|F|0-46:G>C-46:G>C	TGCAGGCCATCGTGCGCGGCCGGCAGGTGCGGAAGCAGGCGGCGGTGACGCTCCG	46:G>C
23919587|F|0-13:G>C-13:G>C	TGCAGGCCTGCTCGTCGACCAGCGCGTAGAGGTCCCTGAAGATCTCGTTGATCTCGGTGATCACCTGCT	13:G>C

5581400|F|0-6:G>A-6:G>A	TGCAGCGGCCCGTAGACGAACAATGCCG	6:G>A
1129014|F|0-53:G>A-53:G>A	TGCAGGTCGGCGGTCGCCGAGTCCCGCACCTCGCGCCAGCCCTTGGAGATGAAGGCGAACGCGCCGTCG	53:G>A
1129014|F|0-8:G>A-8:G>A	TGCAGGTCGGCGGTCGCCGAGTCCCGCACCTCGCGCCAGCCCTTGGAGATGAAGGCGAACGCGCCGTCG	8:G>A
1029081|F|0-10:G>A-10:G>A	TGCAGCGCGTGGACGACACGCTGGGCGTGTTCCACACCCACGCCGTGGCGGGGTTCCTCGGCGGCGTGA	10:G>A
1864191|F|0-8:G>A-8:G>A	TGCAGAACGTGGCCCTCGCGATCATCGACCACACCTACGCCGCGGCGCTCCAGATCGTCGGGGGGACGC	8:G>A
1130006|F|0-34:A>T-34:A>T	TGCAGGGGGCACTGCTGCCGTCCGTCCAGAGCGCAGGAGCCCGCGCGGCCG	34:A>T
1128282|F|0-10:C>T-10:C>T	TGCAGAGCAACCAGGGGTTCGGCCTCGTCGGGTTCAGCACGTCCACGCTGGTCACGCTCGGCGCGTGCC	10:C>T
1125977|F|0-56:C>G-56:C>G	TGCAGCGCGTGCAAGGTGGAGATGCAGCCGTGGCACTTCTGGCGCAAGTACGGGGCCAAGCAGTTCCAG	56:C>G
3939812|F|0-19:C>G-19:C>G	TGCAGCTGCTCGCCGCGGGCTTCCGCGCCGTCGTCGTCGACAGCCTCGAGAACTCCTCCGAGCTCGCCG	19:C>G
1093097|F|0-33:C>T-33:C>T	TGCAGCCGCGCAGGCGCGAACGGCCTCCCCGAGCGCCATGGCGCGCCGCCTCATCGCGAGCCCTTCCTC	33:C>T
2253450|F|0-14:C>T-14:C>T	TGCAGCGCGGAGTTCGAGGCGCAGGTGGCCACGCTCGCCGACGTCCCTTCCTCCATCCCTCTCGACTCC	14:C>T
1093821|F|0-37:G>C-37:G>C	TGCAGCGCCTGCCGCGGATCGTCGGCTACGGGAACGCGGCGGACCTCGCGCTCACCG	37:G>C
3953373|F|0-19:C>T-19:C>T	TGCAGGTCGGCGGTGAGGACGGAGGAGGATGTGGCGGCGCCG	19:C>T
990516|F|0-40:C>T-40:C>T	TGCAGCTCAAGTTCGGGGTGGATGAATCGTACAAGCTGACCGTGCCTGCAACCG	40:C>T
32027999|F|0-18:C>T-18:C>T	TGCAGCCCGACGCCATCTCCACCATGCCG	18:C>T
1019160|F|0-56:G>T-56:G>T	TGCAGGACCTTGCCGCACGAGTCGCAGGAGACGAACTCGCCCTCCATCCTGACGCGGCGGAGGCCGCCG	56:G>T
7170575|F|0-15:T>A-15:T>A	TGCAGCAGGCCAGCCTCTGTCAACAAATTGCCGCCG	15:T>A
1026226|F|0-27:T>A-27:T>A	TGCAGGTCGCGGGCCTCTCCCTCTTCCTCTACGGCTTCTTCCCCGTCAAGCCCACGCTCCCG	27:T>A
1213121|F|0-12:G>C-12:G>C	TGCAGGCAGATGGGCTGAGGCAGCAGACCCTGGACAAGCTGCACGGCATGCTGACGGTCCGACAGGCCG	12:G>C
995761|F|0-12:G>C-12:G>C	TGCAGGACTGCGGCGTGTTTCCCGACGGCGAGTACTTCGCTTTGCACCGCCG	12:G>C
1084594|F|0-13:C>T-13:C>T	TGCAGGCCGTCGGCGGCACGGGCGTCAACGTGGTCGTCGGGGCGCCTAACGACGTGCTCTCCAACCTCG	13:C>T
2261623|F|0-41:C>G-41:C>G	TGCAGCTCGCCGCCCTGCGTGTCCAGGATGCACCGGGAATCCCCGACCGACGCCACGGTGACCGTGAAC	41:C>G
7169737|F|0-41:T>C-41:T>C	TGCAGGGGCAGTTCGAGATCCTGTCCCTCACGGGCACGGTGTTGCCGCCGCCG	41:T>C
3026293|F|0-44:A>G-44:A>G	TGCAGCGACATCGCCGAGGGGGTTGCCTACCTGCACCACCACTCACCG	44:A>G
11913165|F|0-15:G>A-15:G>A	TGCAGGCATGTCGCCGCCGCCG	15:G>A
1003777|F|0-53:G>A-53:G>A	TGCAGCCCGAACACGGCGGCGATGGCGACGACGGTGAGGTACGCGACGCCCTCGGCGGCGCCGAGGAGG	53:G>A
7172927|F|0-29:A>G-29:A>G	TGCAGCTGCTCGGCGCGGGAGGCGGCAAGACCG	29:A>G
7172055|F|0-10:A>G-10:A>G	TGCAGAGGGCACGATCCCCACGGCGCCG	10:A>G
3940409|F|0-24:T>C-24:T>C	TGCAGCACGACGCCGTCGACTTAGTCCGCTTCCACGCCGTCTGCAAGCCATGGCGGGACTCCCG	24:T>C
4540866|F|0-22:C>T-22:C>T	TGCAGCAATGTCTTCGGCCACCCCCCCTCCGCCG	22:C>T
4540866|F|0-23:C>T-23:C>T	TGCAGCAATGTCTTCGGCCACCCCCCCTCCGCCG	23:C>T
4398259|F|0-12:C>A-12:C>A	TGCAGAATCCCCCGTCTTGGTGCCG	12:C>A
1074097|F|0-29:A>G-29:A>G	TGCAGGACGAGATTCCCAGTGTTCAGGAGAACAGCAATGGTGTCGGTGGTTGTGACTCTTGCTTGAGAA	29:A>G
1863576|F|0-65:A>G-65:A>G	TGCAGCAGGATGGCGAGGATCTCGGAGGCGTACTGCTTGTTGGCCTCGAAGTCGCGGGCCTTGACACGG	65:A>G
32021389|F|0-11:G>C-11:G>C	TGCAGCGCCTCGGCCTCGTCCGCCGCCG	11:G>C
5582396|F|0-7:C>T-7:C>T	TGCAGTGCGTCGAGGCCTCGCCGTCGGCCCG	7:C>T
2250347|F|0-11:G>C-11:G>C	TGCAGCTTCTGGCGGGGCTCCAGCTTGGTCTGCGTGGCGAACATGGACTCGAGCGACTTGATCTGCTCC	11:G>C
5331507|F|0-17:T>C-17:T>C	TGCAGACGAAGAGGGACTTGAGGCCG	17:T>C
1210801|F|0-64:G>A-64:G>A	TGCAGGAGGTGCAGAAGGACTGTGTGTGCGAGGCGCTCCTGCGGCTGCCGCCGTTCCTCGTGAAGCCAC	64:G>A
4544201|F|0-17:G>C-17:G>C	TGCAGGGCCAAGAAGAAGGGGGACCTCGGCGCCGTTCTCCG	17:G>C
32026310|F|0-14:C>T-14:C>T	TGCAGCAGCCGTAGCGGAGGAAGACGCCGAGCGGCGGCAGGATGATGGCGAGCAGGATCTCGAGGAAGG	14:C>T
3026986|F|0-35:T>G-35:T>G	TGCAGGAAGTACTCCATCCCCGACGGCTTCACCCCTCTCCTCGCCG	35:T>G
5324019|F|0-7:C>T-7:C>T	TGCAGCACGACGGCCACCACCACCACCCGTCCCCG	7:C>T
4262581|F|0-23:C>G-23:C>G	TGCAGCTTGTCGAGGTACTGAGCCCCATGGCCCTTCTGGATGGTGCCG	23:C>G
16786217|F|0-6:G>A-6:G>A	TGCAGCGCGCAGGCGTATGAGCCG	6:G>A
1231549|F|0-22:G>A-22:G>A	TGCAGCGCGCGCAGAGGAAGCTGCGGGACAAGGAGCGCGAGGTGTCCCG	22:G>A
1028059|F|0-61:A>G-61:A>G	TGCAGGCCCACGCCGCCGTCGGGACGGCGCTGGTGGAGATGTACGCCATGTGCGGCGACGTAGCCCGCG	61:A>G
3030782|F|0-5:A>G-5:A>G	TGCAGACACGGCGCCAGCCCCACCAGCTGCTCTGCGCACTCGCTCCG	5:A>G
984905|F|0-27:C>T-27:C>T	TGCAGCCACATTGCTGTTTCCTGGCGCCGCCCCACCGCTGCACTTCGTCGCCG	27:C>T
1202071|F|0-46:C>T-46:C>T	TGCAGGCAGATGGGCTGAGGCAGCAGACCCTGCACAAGCTGCACGGCATGCTGACGGTCCGACAGGCCG	46:C>T
7153206|F|0-34:G>T-34:G>T	TGCAGAAGGAGCTCGCCGAGTGGCAGGTCGGCCCGCCCGCCG	34:G>T
1124105|F|0-55:C>T-55:C>T	TGCAGCGCCTCCTTGTTCAGCGCCTTCCCCTCCAACGCCTGTCACTTTGCCTCGTCCGTCAGAACCCAC	55:C>T
1036584|F|0-28:C>G-28:C>G	TGCAGTTCGGGTGGTACCTGTCCTTCATCACCGTGTTCGTCCTCACGGTCTACCTCTACGTGTGCAGCA	28:C>G
1026636|F|0-38:G>A-38:G>A	TGCAGCCGCCTCTCCCACTGGCCCTTGGAGGAGGAGGCGGGCCTCTGCGCCGCCG	38:G>A
3947152|F|0-18:C>G-18:C>G	TGCAGATTGGACACGAGCCCCGAGGGGAGGGGATTGGGCCTCCG	18:C>G
4261881|F|0-20:T>C-20:T>C	TGCAGCTCCGAGGCGCGCGCTGCCGTGCTCGTGCCACGGGCCG	20:T>C
992442|F|0-59:C>A-59:C>A	TGCAGATGGCGGAGGTGTGGCGCGAGGAGAGGGTCCAGATGAAGCTCTCCGACGCCAAGCTCGCGCTGG	59:C>A
1019796|F|0-28:G>T-28:G>T	TGCAGTCGCTCTGTCTCAAGGTCATCACGGCAAGCGTCACCACCGTGGCCG	28:G>T
1212200|F|0-25:G>C-25:G>C	TGCAGCTGGGCCTGGGCCTCGGCGCGGCGGAGGCTGTCGCGGAAGCACTTGGAGCAGAGGCCGCCGAGA	25:G>C

<table>
<tr><td>5580440|F|0-19:C>T-19:C>T</td><td>TGCAGGCGCCGCAGGGTTCCCCG</td><td>19:C>T</td></tr>
<tr><td>2277326|F|0-65:G>A-65:G>A</td><td>TGCAGGATAGGGTTAGGGCGGTCGCAGCAGTACTGCTCGACGCCGAGCACGAGATCCCTGGCTCCGAGA</td><td>65:G>A</td></tr>
<tr><td>3222400|F|0-11:C>G-11:C>G</td><td>TGCAGCGCGTACACCGTCCGCAGCAGGTGCCGCCGCTCGTACTTGCCCG</td><td>11:C>G</td></tr>
<tr><td>1023407|F|0-39:G>C-39:G>C</td><td>TGCAGAGACCTCTGCGCGGAGTGCGGCGGAGTAAGGGTCGGAGAACACAGGGTCAAGGTCCG</td><td>39:G>C</td></tr>
<tr><td>3027337|F|0-42:G>A-42:G>A</td><td>TGCAGGGCCGAGAAGATGAACTCCACCACCACCATGGCCGCCGCCG</td><td>42:G>A</td></tr>
<tr><td>3958109|F|0-22:T>G-22:T>G</td><td>TGCAGCCGCAAGTGGGAGGAAATGCCG</td><td>22:T>G</td></tr>
<tr><td>1001740|F|0-17:T>A-17:T>A</td><td>TGCAGGAGCCACCAGAATAGGGTGAGAAAGCCTTTCCACCCATTCAAGGCGAACCCCTTCCAGCCCAGG</td><td>17:T>A</td></tr>
<tr><td>2280310|F|0-34:T>C-34:T>C</td><td>TGCAGCCTGGCGGACGCCTCGTCGAACTCCACCGTCGCGCGGCCGTCCTCCCCCG</td><td>34:T>C</td></tr>
<tr><td>1001740|F|0-10:A>G-10:A>G</td><td>TGCAGGAGCCACCAGAATAGGGTGAGAAAGCCTTTCCACCCATTCAAGGCGAACCCCTTCCAGCCCAGG</td><td>10:A>G</td></tr>
<tr><td>3937346|F|0-10:T>A-10:T>A</td><td>TGCAGCTCGCTCCAATGAGCTTTCCCAGCCCG</td><td>10:T>A</td></tr>
<tr><td>996917|F|0-19:C>G-19:C>G</td><td>TGCAGAGCTTCCTCGGCCTCCGCCTCCTCCTCGTGGCCGCCCTCGCCGCCTTCCTCCTCCTCTTCTCCG</td><td>19:C>G</td></tr>
<tr><td>5368032|F|0-57:C>T-57:C>T</td><td>TGCAGACACGCGCTGATCACGATGTTCCAGGAGACGGCGTCGACCTCGGCCATTGCTCTGAATGCCCGC</td><td>57:C>T</td></tr>
<tr><td>1018388|F|0-58:G>T-58:G>T</td><td>TGCAGAGTCTGCATTGCATCGCTTGTTGCAGCAGCTTGAAGAACAGAGCTTTCGCTAAGGGCATCAGCC</td><td>58:G>T</td></tr>
<tr><td>1018924|F|0-10:C>T-10:C>T</td><td>TGCAGGCCAGCAGGGCTGAGCATCTCGCGGACGCAGACGCGGCGGAGCAGACGCCAGGTGGGACCGACG</td><td>10:C>T</td></tr>
<tr><td>1082056|F|0-37:C>G-37:C>G</td><td>TGCAGATCTCCACCATGTTCACGCACCACCAGAAGACCGTCATCGTGGACCACGACATGCCCCAGAGCG</td><td>37:C>G</td></tr>
<tr><td>1863028|F|0-47:G>A-47:G>A</td><td>TGCAGGTGGCGCGGGTGGTCCCAGTCGAACTTGTTCATGGCGAGGTCGCGCCTCACCTGCTCCTCCGTC</td><td>47:G>A</td></tr>
<tr><td>1239225|F|0-59:G>A-59:G>A</td><td>TGCAGCTCAAGGAGTTCGCGCCGCCGTTCCTGGACAGAAGCCTGTCCGACAAGGACATCGTGACGGGGG</td><td>59:G>A</td></tr>
<tr><td>5971403|F|0-61:C>G-61:C>G</td><td>TGCAGGGCCCGCGCCCGCCGCGGCTCAGCGTGCGCCCCGAGTCGCACGCCATCAAGAAGCCCTCCGAGA</td><td>61:C>G</td></tr>
<tr><td>994995|F|0-38:C>G-38:C>G</td><td>TGCAGGAAGATCTTGAGCACGTTCTTGGAGAAGCCGCTCACCATGGCGGCGCCG</td><td>38:C>G</td></tr>
<tr><td>985853|F|0-44:G>A-44:G>A</td><td>TGCAGGTGGCGAGGGGTGATACGCTTCACCTTGAGATCCTTGCTGGCGTTGCCG</td><td>44:G>A</td></tr>
<tr><td>32020478|F|0-8:G>C-8:G>C</td><td>TGCAGGGAGTCTGGTCTCCCG</td><td>8:G>C</td></tr>
<tr><td>1209199|F|0-16:C>G-16:C>G</td><td>TGCAGAGCGGCAACGGCTACATCAGCTACGGCGCGCTGCGGAGGGACAACGTGCCCTGCTCCGTGCGCG</td><td>16:C>G</td></tr>
<tr><td>11913165|F|0-14:C>G-14:C>G</td><td>TGCAGGCATGTCGCCGCCGCCG</td><td>14:C>G</td></tr>
<tr><td>3934298|F|0-5:G>C-5:G>C</td><td>TGCAGGCGCGGGAGGTCGCAGAGCGAGGCCGGGAAGGCGCCG</td><td>5:G>C</td></tr>
<tr><td>1014251|F|0-36:G>A-36:G>A</td><td>TGCAGAACCGCCGCCGCCGTCAGCAGGAGCCCCGCGGCTGCGGCGAGGCTCATCCACGGCGGCGCCGAG</td><td>36:G>A</td></tr>
<tr><td>2252950|F|0-20:C>G-20:C>G</td><td>TGCAGGTACTTGATGTCGAACGTCGGCACGACGATGTTGGTGACGGTGTCGGCGACGGTCACGTCGTTG</td><td>20:C>G</td></tr>
<tr><td>2340595|F|0-7:G>T-7:G>T</td><td>TGCAGCGGTCGAAGCACGACGCGGAGCTGCGCATGCTGGCGTGCGTGCACACCACCCGCAACGTGCCGT</td><td>7:G>T</td></tr>
<tr><td>2321663|F|0-20:C>T-20:C>T</td><td>TGCAGCAGCATCTTCTCCTCCAGCGGCAGCTCGAAGAACTTCTTGCTCTCGGCGAACAACGCCTCCAGC</td><td>20:C>T</td></tr>
<tr><td>986694|F|0-11:C>T-11:C>T</td><td>TGCAGCAATTCCACGATTGGTTATCCTGCAAAGCAAGGAAGAAGCCACGGAGCCCATCGTGGTCGTCTC</td><td>11:C>T</td></tr>
<tr><td>1004000|F|0-8:A>C-8:A>C</td><td>TGCAGATAATGATGGCGGCCACGGCGCTCTACGCCTTCCCGTCGCTGCGGCACCTCCCG</td><td>8:A>C</td></tr>
<tr><td>1179478|F|0-9:A>T-9:A>T</td><td>TGCAGAACCAGCATCTTTGAAGACATCAGGTCACCGACAGTTGCAATCTCCTTCTGAATGCCCACGGCC</td><td>9:A>T</td></tr>
<tr><td>991658|F|0-10:G>T-10:G>T</td><td>TGCAGCTGGCGATTGAGGAGGAGGACGACGCCACCAGCAAGCTGGCGCAGCCGATGCAGCCCTCCCGAG</td><td>10:G>T</td></tr>
<tr><td>5367222|F|0-35:G>A-35:G>A</td><td>TGCAGCTTTGACAGGACGGCGGCGGCCCGATCACCGCCGAGCAGGAAATAGGGGTGCCG</td><td>35:G>A</td></tr>
<tr><td>5339963|F|0-35:C>G-35:C>G</td><td>TGCAGCACGGCGCGGTAGGCCTTGGTGGGCCGTGCCACCTCGAACTGGGACCTGAAGTCGACGTCCACC</td><td>35:C>G</td></tr>
<tr><td>1244764|F|0-55:G>A-55:G>A</td><td>TGCAGAGCTTGCGCCAGCGCCGCAGCGCCTCCTCGGACGAGTTCTTGCCCTTGACGCCCCCGAAGTTCT</td><td>55:G>A</td></tr>
<tr><td>5578004|F|0-16:A>T-16:A>T</td><td>TGCAGCCGATGAAGAGACGGTCGCCG</td><td>16:A>T</td></tr>
<tr><td>1103059|F|0-49:C>T-49:C>T</td><td>TGCAGGAGGGCGACCTGGGCACCGTCTCCATGGGGGTCCTCTACACCTCCTTCACGCTCTTCGCGGCCG</td><td>49:C>T</td></tr>
<tr><td>6044664|F|0-30:C>G-30:C>G</td><td>TGCAGGTATCGATTATGAGTACAAGGCGGTCAACCTCCTCAAGGGCGAGCAGTCTGATCCCG</td><td>30:C>G</td></tr>
<tr><td>7168792|F|0-61:G>C-61:G>C</td><td>TGCAGCTCAACGCCGTGACGGCGCTGCTCAACCTCTCCATTCTTGACGCCAACAAGAAGCGGATCATGC</td><td>61:G>C</td></tr>
<tr><td>32026152|F|0-65:G>A-65:G>A</td><td>TGCAGCGCGGCCTCCCACTCCTCGTCGCCCTCCTCCTGCTCCTTCGCCTCCACTGCGGCGGGAGGGGGC</td><td>65:G>A</td></tr>
<tr><td>1068218|F|0-24:C>T-24:C>T</td><td>TGCAGACTATGCAAGGAGTAAAAGCGAAAGCCATGGCACCAGCCGCTTGCCACCGCCAACCCAAGACAG</td><td>24:C>T</td></tr>
<tr><td>100581396|F|0-32:A>C-32:A>C</td><td>TGCAGGGTGGAGACGAGCGCGCCGACCAGCGCAGCGCCATCCCTGTCGACGGCGCGCGCCAGGCACCGC</td><td>32:A>C</td></tr>
<tr><td>5359918|F|0-15:T>C-15:T>C</td><td>TGCAGGCTGAGGTGGTTAAACTACCTCCG</td><td>15:T>C</td></tr>
<tr><td>2279245|F|0-8:C>T-8:C>T</td><td>TGCAGGGGCTCTCCCAGATGGTTCTGCACCTCGTCGAGGGTGAGGCTGTACAGAGACCCCTGCCTCGCC</td><td>8:C>T</td></tr>
<tr><td>988186|F|0-17:A>G-17:A>G</td><td>TGCAGAATCCCCCATACAGATACCTCCTTGGGGTCCTCTACCTGGAGCCG</td><td>17:A>G</td></tr>
<tr><td>3934004|F|0-8:G>T-8:G>T</td><td>TGCAGGCGGAGGCTGCGCTTCTGGAGGTCGAGGAGGGAGGGCCG</td><td>8:G>T</td></tr>
<tr><td>7344288|F|0-10:C>T-10:C>T</td><td>TGCAGTTCCCCGTCGGCCGCATCGGGCGCTTCCTCAAGAAGGGCCGCTACGCGCCGCGCGTCGGCATGG</td><td>10:C>T</td></tr>
<tr><td>3028714|F|0-40:G>A-40:G>A</td><td>TGCAGCATGTCGATGTACCCGTCGAGGGCCCTGGCGTGCAGCCCG</td><td>40:G>A</td></tr>
<tr><td>1026259|F|0-38:C>G-38:C>G</td><td>TGCAGGCGGCGGTGGAGCTCCAGGAGGCGGTCGCGCTGCGCCCGCACGTTCTTGGCGGCCATCATGGCC</td><td>38:C>G</td></tr>
<tr><td>7171395|F|0-5:A>G-5:A>G</td><td>TGCAGACGCATGAGCGGCGCGTCGAGCCG</td><td>5:A>G</td></tr>
<tr><td>7158947|F|0-41:G>A-41:G>A</td><td>TGCAGCTTGTACGGCCCGACGGCCCGCGGCGCGCGGAGCTCGAGGAGCGCGACGGGGAGCGGCGCGAGC</td><td>41:G>A</td></tr>
<tr><td>1037781|F|0-44:G>A-44:G>A</td><td>TGCAGTGTTCTTCCTGTAGTCCACCACAGTGCCACTACTCTGTGGCTCTGCTCTCTTTAAAGAAACAGC</td><td>44:G>A</td></tr>
<tr><td>1202577|F|0-42:C>G-42:C>G</td><td>TGCAGTGACGCCTAAGCCGTATTGTGGCAGTCCCAGCTTCGTCTTCGTGAAGTCGTCATACGCCTCTTC</td><td>42:C>G</td></tr>
<tr><td>12776698|F|0-11:A>G-11:A>G</td><td>TGCAGCGCCGCAACCCAGATCCCGCCG</td><td>11:A>G</td></tr>
<tr><td>1064534|F|0-36:G>A-36:G>A</td><td>TGCAGCATGATCTTGGCCGTGCCCTTGTCTTTGAAGGTGATGGCCCGCTGCGCCGCGCCGATGGTGTCC</td><td>36:G>A</td></tr>
<tr><td>1009966|F|0-5:C>G-5:C>G</td><td>TGCAGCCCGTGCACGATCCCCGTGGCGAACGTCGCGAAGCTGATCTCCTTCTTCTTGCCGCCG</td><td>5:C>G</td></tr>
<tr><td>5363342|F|0-7:T>A-7:T>A</td><td>TGCAGCCTCAGGAATTCGCGGCCG</td><td>7:T>A</td></tr>
<tr><td>2280310|F|0-9:G>T-9:G>T</td><td>TGCAGCCTGGCGGACGCCTCGTCGAACTCCACCGTCGCGCGGCCGTCCTCCCCCG</td><td>9:G>T</td></tr>
<tr><td>1088294|F|0-5:C>G-5:C>G</td><td>TGCAGCATCCTCTTGCTCAGGGTGGTGTTCCAGTAGTTCTTGATCTCATTGTCTGTTCGCCCCG</td><td>5:C>G</td></tr>
</table>

4410898|F|0-29:C>G-29:C>G TGCAGCAGAGGAACCGCTGCATGATCATGCCCCCCGTGCCGGCCGAGCGGCGGTTGGAGCCG 29:C>G
1156758|F|0-44:C>G-44:C>G TGCAGCTCCTTGAGGGACAGGTTCTTCATGTGGATGGGGTAGTTCACGGTGTCCAGCAGCGGCGTCGGC 44:C>G
1168249|F|0-11:C>T-11:C>T TGCAGCAAGGGCTGCACGGGTACCACTTCGCCGACGGCGACATCGGCTCACAGCTGGCCGACGGGTTCC 11:C>T
1708237|F|0-59:G>A-59:G>A TGCAGATCCCGCCCGACCAGGCGGCGCTCGCGATCGGCATGCTCGCGGCGGTCCTCCCCGCGGAGGACC 59:G>A
1342642|F|0-29:C>T-29:C>T TGCAGGCCCTGCTGCTGGTGCTGCTGGCTCTGGCGGCGGTGGGGCGGGTAGTAAAAGCGGTCGCCTTTG 29:C>T
985878|F|0-50:C>T-50:C>T TGCAGGGCGCGGGAGCTGTTCTACTACCTCAAGGGGGGACAGGTGGACTACGGCGTGGAGCACAGCCAG 50:C>T
1089971|F|0-24:G>A-24:G>A TGCAGGCGCGAATCTGATGGCCGCGCTACAAACGCCATCCCCATTCGCCG 24:G>A
32022878|F|0-14:G>C-14:G>C TGCAGCTCCAGCGGGACGCTGGAGCTCGTGGCGGCGTTCACGGCGGTGTGCCTCGCACTCTACGGGGTG 14:G>C
1125214|F|0-19:C>G-19:C>G TGCAGTGCATGCAGCAGGCCGTCGCGCGGAGGAGGTGCGGCCGTACAGACGGCAAGAAGTGGCGCTGCG 19:C>G
1042601|F|0-17:C>T-17:C>T TGCAGCTTGCGCACCAACTATCAGAGAAACATATTCTCAGAACACAGACGTTACTGTGCGACATGCTAC 17:C>T
7161928|F|0-5:G>C-5:G>C TGCAGGCCATGGCCGACTTGAGGTTCCCG 5:G>C
994859|F|0-7:C>G-7:C>G TGCAGGTCCTTGCCGCCGCGGACGCGCACCTCCACGGAGGTGTCGCCGCGCTTGAACGCCTTGTGCAGC 7:C>G
1201557|F|0-28:C>G-28:C>G TGCAGGCGGCGGGGAGGCCGAGCGCCCTCGTGACGTTGAGCTTCAAGTCCAGGCCG 28:C>G
992968|F|0-14:G>T-14:G>T TGCAGCCTCTGGTGGATGACGTGCACGTTGTGCTGCGCGTTGAGGTTGAGGAGGCCGCAGGCGGGCACG 14:G>T
2351339|F|0-28:C>G-28:C>G TGCAGATCCTGCTCGAGTACATGGACGGCGGGTCCCTGGACGGCCGCCGCATCGCGTCCGAGTCGTTCC 28:C>G
7343170|F|0-10:G>T-10:G>T TGCAGCAGGTGGCTGGGGTCTACAAGATGCTTCCGCCGCTTCTGTCGACGGTGCACGAGCAGACGGGGA 10:G>T
1117912|F|0-26:C>T-26:C>T TGCAGCTTGCAAGTAGTGACAACATACGTGTATACGGCCTTGCTCTGTGTATCAGATGTATATTTTCTG 26:C>T
13879505|F|0-19:C>T-19:C>T TGCAGAGCGGTGACGTGGACCTGCGGGCTGTTGCAGCCG 19:C>T
1087273|F|0-20:T>C-20:T>C TGCAGATCGAGGCGCTCATATTGCTCTGCTACATCGCGCTGCATGTCCCG 20:T>C
4002650|F|0-10:G>A-10:G>A TGCAGGAGGAGCAGGAAGAGACGGCCGCGGCTGCCGCGCCG 10:G>A
984905|F|0-13:C>A-13:C>A TGCAGCCACATTGCTGTTTCCTGGCGCCGCCCCACCGCTGCACTTCGTCGCCG 13:C>A
2266783|F|0-29:A>G-29:A>G TGCAGACCGTGAGCGCGATCGCCCTCGGGAGCGAGACAGAGAGGGCCCCGAGGAAGGCGGTGTGAAGGA 29:A>G
1398429|F|0-49:C>T-49:C>T TGCAGTACCAGAAGGAGTGCGTGAACCAGGTGCTCAAGGCCGCCATGGCCATCAACGCGCAGGTGCTGG 49:C>T
4733749|F|0-20:G>C-20:G>C TGCAGCAGCGCGTAGTAGCTGAGGGGCACGCGGCCGAAGCCG 20:G>C
1178758|F|0-37:C>G-37:C>G TGCAGCGGTCGCGGCTCGGGTTCAGCTCCTCCCTGCTCTTCCGCCAGACCACCTGCAACAACTTCCGCC 37:C>G
1200695|F|0-24:G>A-24:G>A TGCAGGCACATGAGCGCGAGCTGCGCGCAGAGCGTGGCCTGGCCCCTGTCGTAGGCGCCCTCCAGCCGC 24:G>A
16327952|F|0-25:C>G-25:C>G TGCAGGCCGCGTCGGCGGCGTTGGGCCCG 25:C>G
2363879|F|0-51:G>A-51:G>A TGCAGTGGCGCGGTTGGTGCGCGCGCGGTTGGCGTGCTTGCGGTCGAGGCGGCTGAAGAAGTACCACTG 51:G>A
984905|F|0-36:G>A-36:G>A TGCAGCCACATTGCTGTTTCCTGGCGCCGCCCCACCGCTGCACTTCGTCGCCG 36:G>A
2254586|F|0-10:G>A-10:G>A TGCAGATCCAGCTGAACAGGTACAAGTGCGGTGGTCTCGTGGTCGGGATATGCTCCCACCACCATACCG 10:G>A
1149419|F|0-50:A>C-50:A>C TGCAGGAGCGAAAACACGAGAACATTGAGATTAAGAGAGATCTAGCGGGAAACGGAACAAATCAAGCGC 50:A>C
1150172|F|0-61:T>C-61:T>C TGCAGCGCGTACGCCAGCCGCACGATGTCGCGCCGCTCCCGCCGCTTCGGCATCGTCAGGATGCCGACC 61:T>C
1081143|F|0-7:A>G-7:A>G TGCAGACACTTGATCTGTCGTCCAACAACATCACAGGCAGCATCCCTGTTTCATTGGCTCAGATTCCAA 7:A>G
12419289|F|0-25:G>A-25:G>A TGCAGGAGAAGCTTCTGGAGGCTAGGATGGATGATCGCAAGCCCTCGCCCAGCTCTGCTGCCAAGCACA 25:G>A
4544201|F|0-10:A>G-10:A>G TGCAGGGCCAAGAAGAAGGGGGACCTCGGCGCCGTTCTCCG 10:A>G
7902265|F|0-23:G>A-23:G>A TGCAGCCGTACCTGTCGCGCGGGGAGGACGTCCG 23:G>A
1097298|F|0-14:C>A-14:C>A TGCAGCAATGGCCGCCGCGGTTGGAGCCCGAGCCTGCCCCGATGATGTCGGCCG 14:C>A
12765831|F|0-11:C>T-11:C>T TGCAGATGATTCGCCAGATGCCG 11:C>T
3958379|F|0-5:G>T-5:G>T TGCAGGCTGGCCAGGCGGCGGAGGGACGCCGGGATGGCGCCG 5:G>T
2263307|F|0-29:C>A-29:C>A TGCAGCATGCCCTTGAGCCTCGTCGCCCCCTCGCGCGCCTCGATCAGGTCGTTCACCGCCGCGGCCGCC 29:C>A
4538937|F|0-9:C>T-9:C>T TGCAGAAGCCTGTGTGTAGCTGTGCCG 9:C>T
2245084|F|0-17:G>A-17:G>A TGCAGAGCATCCCTCCAGATATCCCATTGCCTCCTCGATACCTTCGGGATTGTAGGCGTCGCGCAGCAT 17:G>A
986003|F|0-26:T>G-26:T>G TGCAGCCAGCTTGAGCTGTACAAACGTCCTCAGACGCCTTGCCTCCTCTACACCTCTGAATCTGCTGGT 26:T>G
4910307|F|0-44:G>A-44:G>A TGCAGCCGCTCCAGGACAGGACCAGCGACGCCTACCGCTGGATGGTGCACCACGGCGAGAGGTACCGCG 44:G>A
3021028|F|0-5:G>A-5:G>A TGCAGGCTCACCCAAGATTCTCCACCATATTCACTTCTCCCG 5:G>A
3021561|F|0-13:A>T-13:A>T TGCAGCTTCAGGGAGACCTCCTGCTTCACCTTGCCTCCG 13:A>T
1130145|F|0-5:A>G-5:A>G TGCAGACTCTCCCAAGATTCCTCTCCAACATGTTCACGGAGCCGGGACGGCGGATCCG 5:A>G
16530667|F|0-13:C>T-13:C>T TGCAGGAAGGCCCCTGGCCCG 13:C>T
1001090|F|0-31:G>C-31:G>C TGCAGTACAGGAAGGAGTATATGGCGGCGCAGACCGTGAACGGTATCGCGATGGCCGTGTACAGCGCCT 31:G>C
1027779|F|0-8:C>T-8:C>T TGCAGCTGCGCGTAGGCCGCCTTGACGGCCGAGACGCCCGCGAACAGGTTGGCGAGGAGGGAGTCCGCG 8:C>T
1229444|F|0-31:A>G-31:A>G TGCAGCGCGGGTCGGCGCAGGCGGAGGTGGCACAGCCGCGGCGGAGCAGCTTCAGCGTGGTCCG 31:A>G
5367013|F|0-50:G>C-50:G>C TGCAGCCGCCTCTCCCACTGGCCCTTGGAGGAGGAGGCGGGCCTCTGCGCGGCCG 50:G>C
989201|F|0-12:T>C-12:T>C TGCAGTGCACCGTTGCTCTGCCTCCGTGGACTTCATGGAGGAAACGATGAGGCCGTACCCG 12:T>C
1037781|F|0-29:T>C-29:T>C TGCAGTGTTCTTCCTGTAGTCCACCACAGTGCCACTACTCTGTGGCTCTGCTCTCTTTAAAGAAACAGC 29:T>C
1865125|F|0-43:G>C-43:G>C TGCAGGTGTACTGGTGGGCGCTGCGCGCGTTGCGGTCGAGGAAGCCGAGCGCGTGGCCG 43:G>C
100516947|F|0-19:C>G-19:C>G TGCAGTCGCTGGCGACGGCCGCCGACGCGCTCACCGCGGCCCGCATCCTGGTGCAGGCCAGCCAGGGCG 19:C>G
5581983|F|0-5:G>C-5:G>C TGCAGGGCGCCCCAGTGCAGCGCGAGCGCGCCGCCG 5:G>C
3956491|F|0-25:C>G-25:C>G TGCAGGGCGTGGTGCTCCGCCCGCCCTCGCACCTCCCGCAGCCG 25:C>G
2260644|F|0-29:G>C-29:G>C TGCAGCTCCGCCGCGGAGATGTACCCGTCGCCGTTCTCGTCGAACACGGCGAACGCCTCCCTCATCTCG 29:G>C

5579587|F|0-52:C>G-52:C>G	TGCAGCCGCACACCGTCAACTTCCTCAACGTGGTCATCGACAACACCCGCGCCAGCCTGATGCCGCAGA	52:C>G
990541|F|0-31:A>C-31:A>C	TGCAGCGCCAGTGCGAGTACCACGCCATCCTATGCTCCAACCCGCACCCCAACCACGGCGACCGCCACG	31:A>C
2294962|F|0-60:A>G-60:A>G	TGCAGCCGCCGCTGCATCCACGTCATCCCCCTCGCCGATGGGGACGAGGCGGAGGACCTCAGAAATGGA	60:A>G
1009326|F|0-34:C>T-34:C>T	TGCAGTAGTGGCGGCGCCTACCCCCGCGCGACTCCTCCTCGTCTCCGCCCGCCGCGCTGCTCCGCGATT	34:C>T
1136150|F|0-41:G>A-41:G>A	TGCAGGCACTGCGCGCAGCCCCCCGCCCCGACCGTCTCCACGCACTGCGCCATCGCGTACACGCCGAGA	41:G>A
32026661|F|0-8:C>G-8:C>G	TGCAGCCTCCGCAGGAGCTGCTGGTTGGCTGCGCGGAGCTTCTTGACCTCCTCCTCCAGGAACGCGGCG	8:C>G
7351931|F|0-24:T>C-24:T>C	TGCAGCTAGGCGGGCCAAGCTGGGTGGTGCCGCTGGGCCG	24:T>C
32024384|F|0-56:T>G-56:T>G	TGCAGCATCGTGCGCGCGGGATTGCGCGGATTGGTGCTGGGGCACCGTGCGCGCGGTCGTGTGGCTCGC	56:T>G
1112493|F|0-14:G>C-14:G>C	TGCAGGAGCGCCGTGGTGGGGAGCACGAGGGAGACGTTGTTCACGGCCGCCGCGAACTGCGTGCCGTTG	14:G>C
5360744|F|0-32:C>G-32:C>G	TGCAGCACAATGGCGTTGGGGGAGGACGCCGACGAATCGACGGAGAGGAGCCCCGAGCGAAGGAGCAGC	32:C>G
1694876|F|0-10:A>G-10:A>G	TGCAGCGGCAATCGCAGGACGTCGACCTGGGCATGGTGGATGTCAAGATTCAGAAGCTCATGCCCGAGC	10:A>G
1263957|F|0-29:C>A-29:C>A	TGCAGGCCATCGTGCGCGGCCGGCAGGTGCGGAAGCAGGCGGCCGTGACGCTCCG	29:C>A
1245799|F|0-9:G>A-9:G>A	TGCAGGCACGGCGAGGCAAACACACGTGAGCTACCGCCCGCTGAGCGCCTACGGCGCCG	9:G>A
1251387|F|0-5:C>G-5:C>G	TGCAGCACGGAGTTGAGCGCGCCGCGGCTCTCCTTCACCACGCAGTGCAGCTCCAGGACGCTGTTGATG	5:C>G
3947192|F|0-9:A>G-9:A>G	TGCAGCGCCACCCACTTGGCGCCGAACCGCGGGATCTGCCGCTGCCG	9:A>G
7164971|F|0-14:G>C-14:G>C	TGCAGCACCGCCGTGGGCTGCTCCGACCG	14:G>C
3029450|F|0-14:A>G-14:A>G	TGCAGCGGGTCATAACCGCCGATGACGAGCAGCACCG	14:A>G
2243137|F|0-59:A>C-59:A>C	TGCAGAACATAGTCATCCCCACCTTCGACATCAAGCTGCTCCAGCCAACCATCTTCTCCAGATACGACG	59:A>C
4992294|F|0-8:C>G-8:C>G	TGCAGCCTCGGCAGCGCAATGCCGTCGACCCCGCCG	8:C>G
32021422|F|0-7:C>G-7:C>G	TGCAGCTCTTCCGCCTCCACGCCCACGTCTGCGACCG	7:C>G
6039944|F|0-24:G>C-24:G>C	TGCAGACGCAAATGAGCAGCACGGGGTCAGACCTCATGCAGCTCACCTCGGGATACACCGACGAGTGCT	24:G>C
1694954|F|0-15:G>C-15:G>C	TGCAGGGCGTGGCGCGACACCGTCGACGCCCGCCTGCGCGGCCACCTGCTCTCGCGCTCCGTGCGCGGC	15:G>C
2254668|F|0-33:G>A-33:G>A	TGCAGACGGTGCTCCCGCTGGGCAGGGTGAAGAGGATCATGCGGGTCGACAGCGAGATAAAGAAGGTGA	33:G>A
3030599|F|0-20:G>C-20:G>C	TGCAGCATGGCGTTGCTCCAGGGGAACCCGTCGGCGTCGCCGCCG	20:G>C
2252445|F|0-13:G>A-13:G>A	TGCAGAGGCAGAAGAGCGATCGGCACCTGGAAGAGAGCTCCATCTCCATCGACCAGCTCCACAGGCTGA	13:G>A
1043083|F|0-51:C>T-51:C>T	TGCAGGCCGAGGTTGCCTTTCATCGTCTCATAGAAGCCCCAATTCCGAATGCTCAAGCTGCCGTCGTCG	51:C>T
1067775|F|0-39:A>T-39:A>T	TGCAGGCCTCAATGTCCTGTGCGGTGTTGGAATTCTCACAACGTGTTTCGGAATCAAACAAGCAGGATG	39:A>T
1226743|F|0-23:G>A-23:G>A	TGCAGGCGCTCGCCATGGACCGCGCCGTCGGCCGCTTCACCGTGCCGGCGGGCTCCATGTTCGTCGCCA	23:G>A
1056404|F|0-66:T>C-66:T>C	TGCAGAATGTCTCCCCCAAATAGATCGCCCTAGCCACAGACACAGAAGCATCAGCGCAGGCTCTCGTCT	66:T>C
1218474|F|0-7:G>A-7:G>A	TGCAGGGGGGGAGGCGGAGGCGGAGCTGGGCATAAGAGGAGCGGCTCCATGGATGGAGCGAGCTCGCCGT	7:G>A
1095342|F|0-32:G>A-32:G>A	TGCAGCGCGTCGGCGTCGGCCTCGTGCTCTCCGTCGTCTCCATGTGCATCGCCGCCGTCGTGGAGATGC	32:G>A
12772134|F|0-56:C>A-56:C>A	TGCAGCCAGCAGCACTACGACGGTGACGGTGCTCACCGCGAGCTACTCCCGCTCAACCCTCACCTCTCT	56:C>A
1263957|F|0-43:G>C-43:G>C	TGCAGGCCATCGTGCGCGGCCGGCAGGTGCGGAAGCAGGCGGCGGTGACGCTCCG	43:G>C
991018|F|0-32:G>A-32:G>A	TGCAGGCAGCGCCACCGCCCCTTGAGCCTCTGGAAGGCGCCCTGTGCTGCTGCGCGCGCGGCGGCGATG	32:G>A
1224060|F|0-46:G>C-46:G>C	TGCAGCAGCTGGAGTTCACGGTGCACACCTTGAAGGTGAGAGACACGACATCCACAGGAGCTCCGTTGA	46:G>C
1027829|F|0-16:G>A-16:G>A	TGCAGCGACGGCGACAGCACCACCATCAGCATGGCGGCGGAGAAGAAGCCG	16:G>A
5363349|F|0-14:G>A-14:G>A	TGCAGCCAGCGCCCGCCTCCGCCACCG	14:G>A
6047937|F|0-47:G>A-47:G>A	TGCAGCGGGGGGCCGTTCCTCATGGCCTCCGCGGGCGTGCACGTCATGGACTTGGTGCAGGCGGACGCT	47:G>A
4009329|F|0-65:C>G-65:C>G	TGCAGGCCGTGCGCGAGGACGTCGAGGCCGTAGAGGTCGGCGGCGCGCGGGCTGGCGATGGCGGCCGTG	65:C>G
3027739|F|0-31:C>G-31:C>G	TGCAGGCACCACCGAGTCGACAACCACTCACCAGAACAACGGCGCCG	31:C>G
1029018|F|0-45:G>C-45:G>C	TGCAGGCAGCACTGCACCACCATCACTGGCTGTGCCCCTCGCACAGGCCATGGCGGTGGAGTGCAGGAG	45:G>C
2243137|F|0-40:C>A-40:C>A	TGCAGAACATAGTCATCCCCACCTTCGACATCAAGCTGCTCCAGCCCACCATCTTCTCCAGATACGACG	40:C>A
6045409|F|0-21:C>T-21:C>T	TGCAGCAATCCTTGAGTACCTCACTGCCGAGGTCCTTGAGCTGGCCG	21:C>T
995805|F|0-23:T>G-23:T>G	TGCAGCTGGATGAGAAGGAGGGCTTGGGCAGGCGCGCCGACCTCAAGGCCAGTGTTCTCATGAACCATA	23:T>G
2262791|F|0-17:G>T-17:G>T	TGCAGCGCGGCGTCCCCGAGCCCGTTGCCCGCGTAGCCCTCGATCATGAGGTGCCAGGAGGCCATGTCC	17:G>T
2363879|F|0-45:G>A-45:G>A	TGCAGTGGCGCGGTTGGTGCGCGCGCGGTTGGCGTGCTTGCGGTCGAGGCGGCTGAAGAAGTACCACTG	45:G>A
2254908|F|0-25:C>A-25:C>A	TGCAGCTGGAGAACTACGTCTACTCCTTCGAGTTCCCCGTGGACCAGGGAAGCTGGAGGGCGACGGCGC	25:C>A
5363342|F|0-16:C>T-16:C>T	TGCAGCCTCAGGAATTCGCGGCCG	16:C>T
7163719|F|0-60:A>G-60:A>G	TGCAGCCGTGCACGAGCCACCGCATCGCCAGCAGGATGTTGGACTTGGTCGGCGTCCTGTACGGGTCGC	60:A>G
1203916|F|0-22:C>A-22:C>A	TGCAGAGGACTTCTCATGCCGACGCATGCTACTCTTCACAGCCGTTGCTGGTGCTAGTTCAGAATCCTA	22:C>A
12470689|F|0-9:T>C-9:T>C	TGCAGCGCTTGCCTGAACATCCCG	9:T>C
3951864|F|0-31:A>G-31:A>G	TGCAGCGCAGCAACCCGCGAAAGCGGCCACCACCG	31:A>G
1027506|F|0-20:G>C-20:G>C	TGCAGCGACGTGATGGCGCCGACGCAGCCGTAGATGGGGTCGCGCACGCGCGCGTTCGCCTCGTACACC	20:G>C
5580261|F|0-24:G>T-24:G>T	TGCAGCGCCGCGACAGGAAGCTCCGCCCG	24:G>T
1000263|F|0-10:C>G-10:C>G	TGCAGGAGGTCGTGGATTTCCTCAAGAACCCCGACAAGTACACCGCGCTCGGGGCCAAGATCCCCAAGG	10:C>G
1026620|F|0-55:C>G-55:C>G	TGCAGCGGCTGGCCTACGTGAACTGCGTCGCCTACCCGCTCACCTCCATCCCGCTCACCGTCTACTGCG	55:C>G
987730|F|0-37:G>A-37:G>A	TGCAGAAGCTGGGCGTGAAGAAGATCCTGGTGAACCAGCTGCACCCCGTCGGCTGCACGCCGTGGCTGA	37:G>A
12773432|F|0-38:G>A-38:G>A	TGCAGCGCAACGAGGTGTTCAACAGGGACAACATCCCGGCCTGGGTGACCTACGCCG	38:G>A
6049856|F|0-17:A>C-17:A>C	TGCAGGTGGGCGTGGACATGCTCCGATCCG	17:A>C

<table>
<tr><td>2242218|F|0-62:A>G-62:A>G</td><td>TGCAGTGGAAGCATCCATATGTCATGAAAGTACCATGCATAGTCCCCCACCCGCTGCACATCAACCGCC</td><td>62:A>G</td></tr>
<tr><td>1076165|F|0-24:T>C-24:T>C</td><td>TGCAGTCAGTGCAACATCGTCTGGTCCCAATCCCTCACCTCTCATCCTGTCAAACAGCGCCACCGCCTC</td><td>24:T>C</td></tr>
<tr><td>1086644|F|0-44:T>C-44:T>C</td><td>TGCAGCGGAGCGAGATGGCCATGCTCTCGCCCTCCGCGTCGTCTTTGCTCCCCG</td><td>44:T>C</td></tr>
<tr><td>6041655|F|0-19:G>A-19:G>A</td><td>TGCAGGAGCTCGACGTCTCGCACAACCAGCTCACCG</td><td>19:G>A</td></tr>
<tr><td>1204117|F|0-16:C>G-16:C>G</td><td>TGCAGCTCGGTGGCCTCTTCATATGGACGTACACCTACAGTCTGATGCAGAAGTCTGGCAAGCTGTACC</td><td>16:C>G</td></tr>
<tr><td>3934240|F|0-11:C>T-11:C>T</td><td>TGCAGGCACAGCACGGCGGTGAGCAGCACCGCCAGACCG</td><td>11:C>T</td></tr>
<tr><td>1378383|F|0-25:C>G-25:C>G</td><td>TGCAGGCCATGGACTCCTCCCACGTCGCGCTCGTGGCCCTCCTCCTCCGCTCCGAGGGCTTCGAGCACT</td><td>25:C>G</td></tr>
<tr><td>3021561|F|0-11:G>A-11:G>A</td><td>TGCAGCTTCAGGGAGACCTCCTGCTTCACCTTGCCTCCG</td><td>11:G>A</td></tr>
<tr><td>1079552|F|0-51:A>G-51:A>G</td><td>TGCAGATTAGTTCCGCCGTTCTTTTTGTACTCTACAAAGGCGGATTAGGAAATGCTAGCTGTTTGGTTT</td><td>51:A>G</td></tr>
<tr><td>7166938|F|0-32:T>C-32:T>C</td><td>TGCAGCTGCTGTGGCTCGTGGCTTGACGGTGGTTGGCTTTGGCGTAGTTGTTGTGGCTGATGTCGCTGC</td><td>32:T>C</td></tr>
<tr><td>29308187|F|0-50:C>A-50:C>A</td><td>TGCAGCTGGGGCGCCGTCTTCTTCCCCAAGCACTGGCGCGAGTTCTACGCCTACATGGCCGCGCGCTTC</td><td>50:C>A</td></tr>
<tr><td>1088597|F|0-8:T>G-8:T>G</td><td>TGCAGTCCTCCCTCTGCCCCACCGGCATCCTCGCCGACGGCTCCACATTGTACACCGCCG</td><td>8:T>G</td></tr>
<tr><td>1020797|F|0-10:C>T-10:C>T</td><td>TGCAGGGAGGCTGTTCGTCTACTGGTACGGGAGCGAGCCACGGGTGTGCGTGACGGACGCCG</td><td>10:C>T</td></tr>
<tr><td>7168391|F|0-8:G>A-8:G>A</td><td>TGCAGTTAGCGCGGACCTTGCCCG</td><td>8:G>A</td></tr>
<tr><td>4538308|F|0-6:G>A-6:G>A</td><td>TGCAGGGCTTGGGCCTTGCCG</td><td>6:G>A</td></tr>
<tr><td>1385984|F|0-61:C>G-61:C>G</td><td>TGCAGCCCAAGGAAGGCCTTGCCATGGTGAACGGCACGGCTGTGGGCTCTGGTCTTGCATCCACCGTGC</td><td>61:C>G</td></tr>
<tr><td>1027506|F|0-34:T>C-34:T>C</td><td>TGCAGCGACGTGATGGCGCCGACGCAGCCGTAGATGGGGTCGCGCACGCGCGCGTTCGCCTCGTACACC</td><td>34:T>C</td></tr>
<tr><td>5582042|F|0-32:C>G-32:C>G</td><td>TGCAGGCCGTGGCCGCTGTAGTGGAAGAAGAGCGAGTCCCCG</td><td>32:C>G</td></tr>
<tr><td>7337230|F|0-12:C>T-12:C>T</td><td>TGCAGCGGCTCACGCAGCAGCTCCG</td><td>12:C>T</td></tr>
<tr><td>3934371|F|0-19:T>G-19:T>G</td><td>TGCAGCCGCAATGGCATCTTGGCCG</td><td>19:T>G</td></tr>
<tr><td>1013846|F|0-28:G>C-28:G>C</td><td>TGCAGCCGCGGGGGCTGCCCGTGATGCGGCCGCATCAGATGGTGCATGCGCCGCTGCCGCGGCCGAGAT</td><td>28:G>C</td></tr>
<tr><td>1013846|F|0-10:G>A-10:G>A</td><td>TGCAGCCGCGGGGGCTGCCCGTGATGCGGCCGCATCAGATGGTGCATGCGCCGCTGCCGCGGCCGAGAT</td><td>10:G>A</td></tr>
<tr><td>1167275|F|0-20:G>C-20:G>C</td><td>TGCAGGAACCTGGGGTCCGCGCCCTCCGACGGCACCAGATCCTTGCCGCGGAAGTACGGGTGCAGGAGC</td><td>20:G>C</td></tr>
<tr><td>2250322|F|0-19:G>C-19:G>C</td><td>TGCAGAAGAACCTCGGGCTGTCCGACGAGCAGGTGGAGGCGTCGCGGATGACGCTGCACAAGTTCGGCA</td><td>19:G>C</td></tr>
<tr><td>994834|F|0-13:G>C-13:G>C</td><td>TGCAGGTGGCGCTGGACTACGCGTGCGGGCAGGGCGCGGACTGCTCGCCGATCCAGCCCG</td><td>13:G>C</td></tr>
<tr><td>5411903|F|0-8:T>A-8:T>A</td><td>TGCAGCTGTCGGAGTGGCACATGGAGCCGTCGCGGATGACGCTGCACCG</td><td>8:T>A</td></tr>
<tr><td>1028366|F|0-37:A>G-37:A>G</td><td>TGCAGGCGCTCGTGTCGTGGCGCGACCCGCGCGCCACAGGGATGTTCGTGGTGCTCTGCGTCGTGGTGG</td><td>37:A>G</td></tr>
<tr><td>1093935|F|0-32:A>G-32:A>G</td><td>TGCAGTCTCCACCACACGGACTTGCCACCATGACGGCGGCTCCTCTGTTATCGTTGACGGATGCGGCGA</td><td>32:A>G</td></tr>
<tr><td>984905|F|0-45:C>T-45:C>T</td><td>TGCAGCCACATTGCTGTTTCCTGGCGCCGCCCCACCGCTGCACTTCGTCGCCG</td><td>45:C>T</td></tr>
<tr><td>4393846|F|0-26:A>G-26:A>G</td><td>TGCAGCAGTGGTTGTTGGGGCACGTCACCGCGTTCGCCCCG</td><td>26:A>G</td></tr>
<tr><td>3023078|F|0-7:C>T-7:C>T</td><td>TGCAGGCCATCCCCTACGCCATGCTCCTCCTGCTCGTCCCG</td><td>7:C>T</td></tr>
<tr><td>2252271|F|0-26:G>A-26:G>A</td><td>TGCAGGGATGGCTCGATGATGCCTCCGCGGAGCCTGAGGACGAGATGGAGGGTCGACTCCTTCTGGATG</td><td>26:G>A</td></tr>
<tr><td>1090419|F|0-58:G>T-58:G>T</td><td>TGCAGCAGAACGTGTCCTGGGCGCTCGGCTTCGGCGTCTCCGCGCTCTGCCTGCTCCTGGCGGCCGCGG</td><td>58:G>T</td></tr>
<tr><td>1864394|F|0-8:G>C-8:G>C</td><td>TGCAGCTCGGCAAGGTCAAAAGGAACATCGAAAGCCGGCTTCCGGAAACCGCAGTCCTCGTTGTCCCCA</td><td>8:G>C</td></tr>
<tr><td>1062099|F|0-9:C>T-9:C>T</td><td>TGCAGCCTGCTGGAGGAGACCGCCGACTACGTCCGCTGCCTCCGCGCGCAGGTGCAGCTCATGCAGGGC</td><td>9:C>T</td></tr>
<tr><td>1244001|F|0-13:G>A-13:G>A</td><td>TGCAGTGCACGTCGTGCCACGGCTCCCTCGGCCCGCCGTGCCGCAGGGAGGCGTGCCTGAAGTTGTTCT</td><td>13:G>A</td></tr>
<tr><td>3950799|F|0-23:C>G-23:C>G</td><td>TGCAGCAGGTGCGCCGACGAGCACAGCACGTACCG</td><td>23:C>G</td></tr>
<tr><td>24505088|F|0-8:A>G-8:A>G</td><td>TGCAGCAAAATCGGCAACGCCCG</td><td>8:A>G</td></tr>
<tr><td>1032056|F|0-34:T>C-34:T>C</td><td>TGCAGCACATTATGCATCTTCTCTTCAGGTAAGCTCTCCAGCCCCGCCCTAAGCATGTTCTTCTCCTCC</td><td>34:T>C</td></tr>
<tr><td>5581569|F|0-25:A>C-25:A>C</td><td>TGCAGTACTTCCTCACCACGGGCCCAGTCGCCG</td><td>25:A>C</td></tr>
<tr><td>3022447|F|0-20:A>G-20:A>G</td><td>TGCAGGACGAGGTAACACGAATCGCCAACGGCAAGCCAACCATCGAGGAAGACGATCTGAGGATGATGG</td><td>20:A>G</td></tr>
<tr><td>1261735|F|0-46:G>C-46:G>C</td><td>TGCAGCTCGCCAACGTGAAGACGGCGCTGGGGAGGCGGGAGGAGGCGCTCGCCGACCTGCGCGCCAGCG</td><td>46:G>C</td></tr>
<tr><td>3945862|F|0-5:C>A-5:C>A</td><td>TGCAGCACCAGCCGCACCTCGGCGGCGACGCCG</td><td>5:C>A</td></tr>
<tr><td>1001476|F|0-23:C>G-23:C>G</td><td>TGCAGCGGCGTCGTGGTCTCGCACGGAGGGCCGAAGTAGTTGCCGTTGAGCGCGCCG</td><td>23:C>G</td></tr>
<tr><td>1246961|F|0-67:A>G-67:A>G</td><td>TGCAGCTTCACCAGCCCCTTGAGCGCGCGCAGAGCCTTCTTGGCCTGCGATAATTCAACACGCATTGAT</td><td>67:A>G</td></tr>
<tr><td>4262426|F|0-35:A>G-35:A>G</td><td>TGCAGGCGTCCGTGAACGCGGACGGGGCCTTCACCAAGGTTTTCGCCG</td><td>35:A>G</td></tr>
<tr><td>1168249|F|0-37:C>T-37:C>T</td><td>TGCAGCAAGGGCTGCACGGGTACCACTTCGCCGACGGCGACATCGGCTCACAGCTGGCCGACGGGTTCC</td><td>37:C>T</td></tr>
<tr><td>1012259|F|0-32:G>T-32:G>T</td><td>TGCAGGATGTTGTAGAGCACCCATCCGAGGGCGGGCGCCACCACGAAGAGCAGCAGCAGGCCCCGAGAT</td><td>32:G>T</td></tr>
<tr><td>1094201|F|0-22:G>C-22:G>C</td><td>TGCAGCGGGTCCTGCTCGTGCTGGCCCTCGTCGGCACCTGCATGGTCATCGGCGACGGCGTCCTCACGC</td><td>22:G>C</td></tr>
<tr><td>1094106|F|0-14:A>G-14:A>G</td><td>TGCAGGCTCGGGTCACGGATCTCCGCGGCGTACTTGCCCCACGGCCGCTGCCGCACGCCACGGAACCGC</td><td>14:A>G</td></tr>
<tr><td>7353670|F|0-16:G>C-16:G>C</td><td>TGCAGCACAACTTCCTGACCG</td><td>16:G>C</td></tr>
<tr><td>5577051|F|0-47:G>A-47:G>A</td><td>TGCAGCTCCTGCCGCTCCGCCGTCGACATCTCGGGCGCTGGCGCGCCGCCCTTGGCTCCGCTGGCGCCT</td><td>47:G>A</td></tr>
<tr><td>32026706|F|0-6:A>G-6:A>G</td><td>TGCAGGATGCATCCCAGCATCCGCGTAGCCACCAATGAGTGCAGACCACGAGACAACATTCCGCTCCGG</td><td>6:A>G</td></tr>
<tr><td>6042969|F|0-37:A>C-37:A>C</td><td>TGCAGCCCTCGTCGCTGCCCGCGCCGTCGTCCTCCTCAGACTGGATGCCGCCCGCCACGGGGCCCATCA</td><td>37:A>C</td></tr>
<tr><td>1090701|F|0-28:T>C-28:T>C</td><td>TGCAGGTAGAAGATGACGTGCCGCCCGTTGGTGCACACCCTCAGCGCGGCGACGCCCATGTCATGCCGA</td><td>28:T>C</td></tr>
<tr><td>12774225|F|0-7:A>C-7:A>C</td><td>TGCAGCGAGGCGTAGTGGTACCG</td><td>7:A>C</td></tr>
<tr><td>1022063|F|0-34:C>T-34:C>T</td><td>TGCAGGAATGCTGCACGGACGCGACGGGGTCGTACGTGGTGTACGCGCCG</td><td>34:C>T</td></tr>
<tr><td>4394812|F|0-28:C>A-28:C>A</td><td>TGCAGCTCATCGCGTGCGGGGCGGTCACCGCCG</td><td>28:C>A</td></tr>
<tr><td>1263850|F|0-12:T>G-12:T>G</td><td>TGCAGAATGCAATGGCCTGCCTCTCTTCCTCGCCACCGTCGGCCGTGCCATGTCCAACAAACGTACAGC</td><td>12:T>G</td></tr>
</table>

997633|F|0-14:G>C-14:G>C	TGCAGCCGCGCCGTGACGTCCTGCATGACGCCGTTCCACGTGTCGGTCCTCCCCGTGGCGGCCACGAAG	14:G>C
1080669|F|0-16:C>T-16:C>T	TGCAGCGCCAGGCTTGCAGTCAAACGGCTTGGATTATCTTAGTGCTGTCGCTCCAACACAACGGCGACA	16:C>T
1026222|F|0-19:G>C-19:G>C	TGCAGGTGCTGGCCATCCTGCCGTCGTACGCGGGCAAGACGGTGCTGGAGCTCGGCGCCG	19:G>C
1083859|F|0-7:G>C-7:G>C	TGCAGCGGGTCGGGCTGGGGCTCGTCTTCTCCATCCTCTCCGTGGCCTGGGCCG	7:G>C
1025864|F|0-5:G>A-5:G>A	TGCAGGGACATGCCGAGGAAGGCGAGCATGGCGAGGCGGCCGTTCTTGATCTCCTTGAGCTTGAGCTCC	5:G>A
1133759|F|0-13:C>G-13:C>G	TGCAGCTCTCCGCCGCCGCCGTCGGCCTCTCCGTCGTCTCCAACTTCTCCTTCGGCTTCCTCCTCGGCA	13:C>G
1030576|F|0-17:T>G-17:T>G	TGCAGCTCATGAAGGACTCCCGCTGGGTCGCCGTGAAACCCCGCCCCGACGCGCTCATCGTCAACATCG	17:T>G
1228823|F|0-17:G>C-17:G>C	TGCAGCTGCCTCGCCGTGGCAGCGCTGGCGGCCACAGTCGCCGCCGCTCTCCTGCTCTCGTTCGCCGAG	17:G>C
5578035|F|0-8:C>T-8:C>T	TGCAGCGACCTCACGAGCCCCTCCGCCG	8:C>T
12850977|F|0-52:A>G-52:A>G	TGCAGCCGATCACGTCGTCGAAGAGCTGGACGGCGAGCTCGGCCGACCCGCTATCCACGTTGTGGAGTT	52:A>G
1019650|F|0-51:T>G-51:T>G	TGCAGGCAGCCTGGTCTTCCTCTTCTTGACGGACGCCACCACCACCCTGAGTATGTCCCTGAGCGGGCT	51:T>G
1024457|F|0-49:A>G-49:A>G	TGCAGAGACTGGTCCATGAGTTGCTTGAGAAAGAGCAGAATCCGACTGTAAAATCAAGGCTGGCACTTG	49:A>G
989810|F|0-49:C>G-49:C>G	TGCAGGGGCTCGGGGTCAACCCCGCCGTGCACGAGGTCGCCGACGAGGCCGCGCTCGCCG	49:C>G
1696795|F|0-20:C>T-20:C>T	TGCAGCTCCTCAAGGCCGTCCTGCTGGCCTTCGCGTGGGCCTTCGACGCGCAGCAGGTGTTCATCTCGG	20:C>T
1081801|F|0-37:G>T-37:G>T	TGCAGCGCCTTCATCTTCTCGTTGATCTTGCACCGCCGCCTCTTCTCGGAGAGGTTGTGCACCTCGGCG	37:G>T
1025366|F|0-13:A>C-13:A>C	TGCAGAGGATGGGACTTGGCCTGGTCCTCGGCGCAATGGCCATGGCCACCGCCG	13:A>C
985878|F|0-38:A>G-38:A>G	TGCAGGGCGCGGGAGCTGTTCTACTACCTCAAGGGGGGACAGGTGGACTACGGCGTGGAGCACAGCCAG	38:A>G
1863988|F|0-47:G>A-47:G>A	TGCAGGATGCCCTTGGTCGTGAAGTCGAACGCCATGGCAGGGCCGCCGACCGCCTTGGCCCAGTTCACC	47:G>A
4262164|F|0-19:T>G-19:T>G	TGCAGCCCGCGGTCCAGGTTAAGGTGGAGGAGTCGGCGCCG	19:T>G
5580268|F|0-9:C>G-9:C>G	TGCAGCGCGCCCTGCCAGGGGCCGAGCTCCG	9:C>G
1027779|F|0-44:C>G-44:C>G	TGCAGCTGCGCGTAGGCCGCCTTGACGGCCGAGACGCCCGCGAACAGATTGGCGAGGAGGGAGTCCGCG	44:C>G
7351751|F|0-19:T>C-19:T>C	TGCAGATGAAGTAGCGCTTTCCG	19:T>C
2254908|F|0-19:C>G-19:C>G	TGCAGCTGGAGAACTACGTCTACTCCTTCGAGTTCCCCGTGGACCAGGGAAGCTGGAGGGCGACGGCGC	19:C>G
2245958|F|0-8:G>A-8:G>A	TGCAGGACGAGTATGCCATTGGTGGCGTTCCCCAGCTTCATCTTCTCGACGCAGCTCCTGACCACGGCC	8:G>A
4984118|F|0-16:C>T-16:C>T	TGCAGCCGCTCCAGGACAGGACCAGCGACGCCTACCGCTGGATGGTGCACCACGGTGAGAGGTACCGCG	16:C>T
1080945|F|0-9:C>T-9:C>T	TGCAGATGTCGGTGTCATGAGTGGCGCAATCATCTACATCCAGAAAGATCTCCACATCACCGAGTTTCA	9:C>T
5357980|F|0-6:T>G-6:T>G	TGCAGCTCCGCGGTCGGGGGCTCCGTGCCG	6:T>G
6047672|F|0-10:G>A-10:G>A	TGCAGGGGCAGGAATGCCTCGCTGCCG	10:G>A
980661|F|0-8:C>G-8:C>G	TGCAGCCTCTTCAACCCGACGCCGTCGCTGGCGTCCATGATCATCCACCATTACAAGATGCGGGAGGAC	8:C>G
23919584|F|0-24:A>G-24:A>G	TGCAGGAGCTGGTGCCCAACGGCAACAAGGTGGACATGGTCACCATGCTCGACAAGGCCATCACTTACG	24:A>G
1056404|F|0-13:C>G-13:C>G	TGCAGAATGTCTCCCCCAAATAGATCGCCCTAGCCACAGACACAGAAGCATCAGCGCAGGCTCTCGTCT	13:C>G
5411526|F|0-25:C>G-25:C>G	TGCAGGACTGGCAGCGGCGGTACGCCGACGTGCTGCGGCGGAAGGGGAAGTCCGTGGAGGTGGTGGAGT	25:C>G
1022741|F|0-27:T>A-27:T>A	TGCAGGAACCCCGTGTCCTTGGCCGACTCGCCCACGCTGGCGAGCCGCGCGCTGAACGTCGTGCACCGA	27:T>A
1129585|F|0-9:C>A-9:C>A	TGCAGAACGCCAACAGCAGCGTCGCCTCCTGACACTAGGAAAGGCCAGCCG	9:C>A
3949303|F|0-34:T>C-34:T>C	TGCAGCGCCATCTCCAGGAACCTCATCATCTCCGTCTCCTCGTCGCCG	34:T>C
4393846|F|0-16:G>A-16:G>A	TGCAGCAGTGGTTGTTGGGGCACGTCACCGCGTTCGCCCCG	16:G>A
12772134|F|0-16:A>G-16:A>G	TGCAGCCAGCAGCACTACGACGGTGACGGTGCTCACCGCGAGCTACTCCCGCTCAACCCTCACCTCTCT	16:A>G
4910284|F|0-11:G>C-11:G>C	TGCAGCAGGTTGAGCCTGGTCTCCCCG	11:G>C
994427|F|0-20:C>G-20:C>G	TGCAGCATCCGCGCCACCATCAGCGCGATGTGGGTCGTCCCCATGGCCAAGCCCG	20:C>G
1056404|F|0-31:A>G-31:A>G	TGCAGAATGTCTCCCCCAAATAGATCGCCCTAGCCACAGACACAGAAGCATCAGCGCAGGCTCTCGTCT	31:A>G
2256096|F|0-48:C>G-48:C>G	TGCAGCAAAGTCGTCGAGGTCACCAGCTCCGTCATCGTCCAGGGTGATCGACTCCTCCAGCGGGCGGTG	48:C>G
1015137|F|0-5:C>G-5:C>G	TGCAGCAAGACGACCGCGCCGACGTCGGCTTCGCCACGCCGCTGGCCACGGGTGGAGTAATTGGGCCGC	5:C>G
2261623|F|0-26:G>A-26:G>A	TGCAGCTCGCCGCCCTGCGTGTCCAGGATGCACCGAGAATCCCCGACCGACGCCACGGTGACCGTGAAC	26:G>A
1202071|F|0-19:G>A-19:G>A	TGCAGGCAGATGGGCTGAGGCAGCAGACCCTGCACAAGCTGCACGGCATGCTGACGGTCCGACAGACCG	19:G>A
1089511|F|0-43:C>T-43:C>T	TGCAGGTTGTCCTGGAGCAGCAGCCTTGCCGTCTTCGTGAAGACGGTGGCGCCGCCG	43:C>T
1027556|F|0-61:C>G-61:C>G	TGCAGCACCGAGATGTTGTACGGCTCCTCGTGGATGGGCTTGACGAAGAGCACGCTGGTGGCGCGCGCG	61:C>G
986367|F|0-56:G>A-56:G>A	TGCAGCGACGCCGAGTAGCGCACCGCCTCCCGCACCGTCAGCGTCGTCATCAGCACGTCGTCCTGCGTC	56:G>A
1863229|F|0-19:G>A-19:G>A	TGCAGAGCGCGGCGGCGCCGAGCGCGCTGCGGGTGTCGAGACGGCCCAGGATCTGGAGGATCAGGTCGT	19:G>A
2250320|F|0-37:C>T-37:C>T	TGCAGACGGAGGCGCAGAGCTTCGACGTGCCCAGCGCCGACATGATCTACAGCCTCGCCTACCTGCCGC	37:C>T
993268|F|0-22:C>A-22:C>A	TGCAGGTATGGTTCTGCAAGAACAAGCTGCCCATGTGAAAAGAGACAACCTTTGCGATCAAGCCAACCC	22:C>A
2259386|F|0-34:G>A-34:G>A	TGCAGCCCGCCGTACCTGCCCTGGCCGACGCACAGCTCCTCGTTGCCGCGCACGTCGAGCCGCCTGCCG	34:G>A
5005126|F|0-5:G>C-5:G>C	TGCAGGGCAGGACCGATGGTCGTCGCCG	5:G>C
6045685|F|0-38:C>T-38:C>T	TGCAGGCATAACGGAAATGGGCAGCTGCGCAACGGCTGCCCCTGTCCG	38:C>T
993268|F|0-31:C>T-31:C>T	TGCAGGTATGGTTCTGCAAGAACAAGCTGCCCATGTGAAAAGAGACAACCTTTGCGATCAAGCCAACCC	31:C>T
7927919|F|0-38:T>C-38:T>C	TGCAGGCGCTGGCGCCACCACGCATCCGACCGCAAGTTTCTCCGCCGTTTCCGCGAGCTCCAACCGCCC	38:T>C
7916309|F|0-11:G>C-11:G>C	TGCAGGATGCGGAGCGAGGAGTTCCTCTCGCCGCCGAACGCCGCGGCCG	11:G>C
100485639|F|0-10:C>G-10:C>G	TGCAGCAGCTCTGCGCGGGGTCGGCGGGGTCGGCGCTGGCGCACCTGGACCTCACCACGCCG	10:C>G
5008870|F|0-14:T>G-14:T>G	TGCAGCAGCGTGGTTTTCGTGGGCAACATCCCGTACCAGGCGAGCGAGGAGGAGCTCCG	14:T>G
5363129|F|0-7:T>C-7:T>C	TGCAGAGTGGGCGGCGCAGTGGCAGTGCCG	7:T>C

5356348|F|0-34:C>G-34:C>G	TGCAGTCGCCCAAGTTCAAGGAGATGATCGAGCGCACGTCGGCCGCCGAGAGCAGCACCACCACGTCCT	34:C>G
100557251|F|0-34:T>C-34:T>C	TGCAGCGGCACGACGAGGCGGACTCGGCGCTCCGTGGCGCGCCGAGGTTCGGCGTCAACGAGTCCACCA	34:T>C
5365752|F|0-42:A>G-42:A>G	TGCAGGCGGGCCGACGTCGAGAGCTTCGGCGAGGTCCGCCTGACGCGCTGCAAGGGGGTGCGCGCCGAC	42:A>G
1252133|F|0-5:G>A-5:G>A	TGCAGGCTGAGGTGGACCAACTACCTGAGGCCCGACCTCAAGCGGGGGCTCCTCTCCGACGAGGAGCAG	5:G>A
1009951|F|0-26:A>G-26:A>G	TGCAGCCGCGCGCCGCCGCCGCCGCCACCGCTGCCGTGGCGCTTCTTCTGGATCTTGCCGCCG	26:A>G
1089511|F|0-36:G>A-36:G>A	TGCAGGTTGTCCTGGAGCAGCAGCCTTGCCGTCTTCGTGAAGACGGTGGCGCCGCCG	36:G>A
7345916|F|0-5:C>T-5:C>T	TGCAGCGAACCAGCAGATCCG	5:C>T
1026620|F|0-5:C>A-5:C>A	TGCAGCGGCTGGCCTACGTGAACTGCGTCGCCTACCCGCTCACCTCCATCCCGCTGACCGTCTACTGCG	5:C>A
2254908|F|0-46:G>C-46:G>C	TGCAGCTGGAGAACTACGTCTACTCCTTCGAGTTCCCCGTGGACCAGGGAAGCTGGAGGGCGACGGCGC	46:G>C
5971564|F|0-23:G>T-23:G>T	TGCAGCCGCCCGCAGCAGTACACGGCCCGCGGAACGCTCTACGGCTGCCG	23:G>T
1028336|F|0-23:A>G-23:A>G	TGCAGGTGCAGATGCAGCGGAGCACGGCTGGGAAAGGCGCTGAAAAGATGAGCGTTGATGATGGCTAGC	23:A>G
3029909|F|0-18:A>G-18:A>G	TGCAGCCGCGCGTGTAAGAGTTGGCCACCGCGGAAGGCCG	18:A>G
3023078|F|0-13:C>G-13:C>G	TGCAGGCCATCCCCTACGCCATGCTCCTCCTGCTCGTCCCG	13:C>G
12850977|F|0-41:G>A-41:G>A	TGCAGCCGATCACGTCGTCGAAGAGCTGGACGGCGAGCTCGGCCGACCCGCTATCCACGTTGTGGAGTT	41:G>A
3026715|F|0-8:A>C-8:A>C	TGCAGAAGATATCCGTGAGCACCAACATGCTCGACGGGAAGCTGCCG	8:A>C
1105859|F|0-20:A>G-20:A>G	TGCAGGGCGACGTCCCCAAGAACTTCACCAGCGGCATGCCTTTCGTCGCCG	20:A>G
1079552|F|0-32:T>G-32:T>G	TGCAGATTAGTTCCGCCGTTCTTTTTGTACTCTACAAAGGCGGATTAGGAAATGCTAGCTGTTTGGTTT	32:T>G
1024773|F|0-8:C>T-8:C>T	TGCAGGGTCACCTCGGCGGGCCGCGGCGCGAGGAAGCGCACGGCGAGGCCG	8:C>T
7166785|F|0-65:A>T-65:A>T	TGCAGCCGCCTCAGGAGCTGCTGGTTGGCGGCGCGGAGCTTCTTGACCTCCTCCTCGAGGAAGGCAGCG	65:A>T
1090705|F|0-32:G>C-32:G>C	TGCAGGATGATGAGCGGCAGCGCGTAGTCCAGGGGGTTCTCCCCCTGGAACGCGCCGTGCGACGCCACC	32:G>C
1863241|F|0-32:C>T-32:C>T	TGCAGACAACTACCTCATCAACTGCGGCTCCACGGTCGACGCTAATCTCCACGATGGGAGGGTCTTCAA	32:C>T
3031905|F|0-14:C>G-14:C>G	TGCAGCACCGCGAACGCGATGCCGACCGCCGCCGCCACCG	14:C>G
2361057|F|0-9:G>C-9:G>C	TGCAGGTTGGCGGCGCTGGCGTCGAGGGACTGGTCGTTGAGGCCGACCAGTGTGGCCG	9:G>C
1863555|F|0-20:C>T-20:C>T	TGCAGCGCCTGGGTGCGAACCGCGGCGACGAAGGCGAGGTCGCCGTAGGGGGCGAGGCCCTCGAAGACA	20:C>T
1051874|F|0-26:G>A-26:G>A	TGCAGCTTCTCGTCGCCGATGAGCGGGCGGGCATGGACCGCCACCTTGACGCAGCAATCCTCGCCGTGC	26:G>A
985568|F|0-23:A>G-23:A>G	TGCAGGAACGTGTTCCAGTCCCTACGCTTCTGCGCCTCGTACCTGCTCAGCTGCGCAGGCGGCTTCGGC	23:A>G
1088357|F|0-36:C>A-36:C>A	TGCAGCACCATCGTGGGAGGCTAGGGTTTGCTCGGGCACCGCGCTCTCTGGTCTCCG	36:C>A
4005696|F|0-44:G>C-44:G>C	TGCAGCGCCAACTACCCACTGATGCAGAAGCTGCGCGCCGTGCTGGTCGACCACGCCCTGACCGCCGAG	44:G>C
1039058|F|0-26:C>T-26:C>T	TGCAGGTACGAGAAGAGTTATATGCACAGGGACGTTGTCACGCACGTCGCCGTTTCCCCTGCTGACTTC	26:C>T
2252771|F|0-21:C>A-21:C>A	TGCAGCTGCGCCGATCGTCTCCTCGATATTTCAATCATGTCGATGTCGGCGCTCGTATCTGCCCTCCGC	21:C>A
4993586|F|0-13:C>T-13:C>T	TGCAGTGCTGGAACGGAGGCGGTGGCGCCG	13:C>T
3028576|F|0-22:T>C-22:T>C	TGCAGAATGTAGACAGGAGGCCTGACCACAGGCCAGTGCACAGCCG	22:T>C
1080945|F|0-27:A>G-27:A>G	TGCAGATGTCGGTGTCATGAGTGGCGCAATCATCTACATCCAGAAAGATCTCCACATCACCGAGTTTCA	27:A>G
5580593|F|0-7:G>C-7:G>C	TGCAGTGGTCGACGTGGTGGTGCACCTCCCCG	7:G>C
4992169|F|0-17:A>G-17:A>G	TGCAGGAAGGAGAAGGCAAACAGGCCG	17:A>G
3023078|F|0-28:C>G-28:C>G	TGCAGGCCATCCCCTACGCCATGCTCCTCCTGCTCGTCCCG	28:C>G
1042888|F|0-23:T>C-23:T>C	TGCAGATATAAGTTGGATGTTGTTGCTGCTGGGAAGAACTTCACGAAAATAAGGAAAGCAATCACTGCC	23:T>C
2250175|F|0-56:C>G-56:C>G	TGCAGCGTGCTCCTCGACATCAACGACGGCGACCGCCTCGCCTTCTTCCGCCTCACCCCCG	56:C>G
995805|F|0-58:C>G-58:C>G	TGCAGCTGGATGAGAAGGAGGGCTTGGGCAGGCGCGCCGACCTCAAGGCCAGTGTTCTCATGAACCATA	58:C>G
1215993|F|0-7:G>A-7:G>A	TGCAGGCGCCGCTCTCCCTCCGCCTCACGTCCAGCTCTGGCAAGAAGCTCGTCGCCAGCAACGTCATCC	7:G>A
3028774|F|0-16:G>A-16:G>A	TGCAGAAACCACCAAAGCGCCCAGCCGAGGAACGCGGCCCG	16:G>A
1080669|F|0-5:C>T-5:C>T	TGCAGCGCCAGGCTTGCAGTCAAACGGCTTGGATTATCTTAGTGCTGTCGCTCCAACACAACGGCGACA	5:C>T
1089143|F|0-38:G>A-38:G>A	TGCAGCCTTACTTGGTTGTGCTTCCAGCAGAACTGCACGACCTCCACTGGCATGGCAAGGCCGCTCCCT	38:G>A
5324413|F|0-17:C>G-17:C>G	TGCAGCATCCGCATGAGCACGTCCTCCTCGGCGGCGGGCTCGGCGCCG	17:C>G
988333|F|0-63:A>T-63:A>T	TGCAGGTGGCGGACCTGCGCGCCGTCACCACCAAGAACCCCGTCGGCCACCAGTCGGAGCGCTACCGCA	63:A>T
3570100|F|0-31:C>G-31:C>G	TGCAGGGCGACATGCGGGAGCTCCCGTTCACCCTGGCCG	31:C>G
1066043|F|0-46:C>T-46:C>T	TGCAGGTGACGGAGAAGTTCAAGGAGCGGGTCGTGGACGAAGGGTACAACCCTAGCTACGGAGCGCGGC	46:C>T
5581037|F|0-64:C>A-64:C>A	TGCAGCTGGTGTTGATCCGCTTGTGGCTCGCGCGGTGGCCGCCCAGCGCCTGGTAGGAGCTGAACACCC	64:C>A
3022866|F|0-11:C>G-11:C>G	TGCAGGAACTCCTCGTCCTTCTCGAGCCTCTGCATGGGCTCCG	11:C>G
6042996|F|0-67:A>C-67:A>C	TGCAGCCTGGGGTTGACGACGGCGATCGAGCCCCACGAGTGGCGGTCCGTGGCGGGCCATGCCGCCGAC	67:A>C
4261756|F|0-33:T>C-33:T>C	TGCAGCGTCACCACGCACCTCGCCTCCGCCCCGTCCCCGCTCCCCG	33:T>C
3934298|F|0-6:C>T-6:C>T	TGCAGGCGCGGGAGGTCGCAGAGCGAGGCCGGGAAGGCGCCG	6:C>T
4002442|F|0-11:G>C-11:G>C	TGCAGCAGGTAGGGGCTGTGCTCGGCGGCGAGGCGGTTCGGCTTCCG	11:G>C
1261735|F|0-22:G>T-22:G>T	TGCAGCTCGCCAACGTGAAGACGGCGCTGGGGAGGCGGGAGGAGGCGCTCGCCGACCTGCGCGCCAGCG	22:G>T
995676|F|0-58:A>G-58:A>G	TGCAGGGCGTGCACATGGTGAACTGCTCCTACAACAGCCTGTGGATGGGCCAGTTCGTACGCCCGAGAT	58:A>G
32020283|F|0-18:T>C-18:T>C	TGCAGGATCCTCGACCTCTTCTCCTTGGGGTGCCG	18:T>C
5580139|F|0-11:C>T-11:C>T	TGCAGCAAGGACCCACAGGTAATGCCGCCG	11:C>T
1696351|F|0-23:C>T-23:C>T	TGCAGTTTCATGTGCTACTTCCACCTTTTCGGGTTCGTGGAGCAGAGCTGGACCATGGTCGCTGCCTTC	23:C>T
5363892|F|0-7:A>G-7:A>G	TGCAGGGATGGCCGAGGCGGACGCTCTCCG	7:A>G

6045398|F|0-26:A>C-26:A>C	TGCAGCAACAGCAGCAGCCGCGTGAGAAGGCCG	26:A>C
2259595|F|0-34:A>G-34:A>G	TGCAGCGGCGCGTTCGCCAACCCCAACACCTGCCACCCCACCGCCTACTCGCAGGCGTTCAAGCTGGCG	34:A>G
7170909|F|0-10:G>C-10:G>C	TGCAGTCCCTGGACGCTAGCAACAACCGCCTCACCG	10:G>C
2245236|F|0-48:A>T-48:A>T	TGCAGGCCGAGGGACCCGAGCTTCGAGCTCATCTCCATGAACCTGTCCACCTTCAACTTCCG	48:A>T
2252834|F|0-13:A>G-13:A>G	TGCAGCGAGTAGTAGTCGTGTGGCCAGCAGTCATCGAGCTTGACGTGCGGCCTCTCGCCGTCGGCCCAC	13:A>G
3940446|F|0-15:T>C-15:T>C	TGCAGCGCCGCTGCGTGGGACCG	15:T>C
2252771|F|0-51:C>G-51:C>G	TGCAGCTGCGCCGATCGTCTCCTCGATATTTCAATCATGTCGATGTCGGCGCTCGTATCTGCCCTCCGC	51:C>G
3941858|F|0-7:C>T-7:C>T	TGCAGTTCTCCCCGTGCCTGAGCTACGCGCCCCCG	7:C>T
3028172|F|0-29:T>C-29:T>C	TGCAGCGAGAAGACCATCCTCACCGTCAATGGCGAGTTCCCCG	29:T>C
977718|F|0-10:C>G-10:C>G	TGCAGCAGCTCAAGGCGCTGCTGGGCATCGTGCACTTCACCACCCAGATGGGCATCGTCCCCGTCATGG	10:C>G
1228883|F|0-5:T>C-5:T>C	TGCAGTTTCTTGGACGGCGCCGACCGCCGCGTGCTCGCGCGCTGGTTCCAGCTCCTTGCCACCAAGGGC	5:T>C
5366169|F|0-16:C>T-16:C>T	TGCAGAGCATCACCACCATGCCCATCCTCGTCAAGGGAGTCATCACCGCGGAGGACGGTAACCCATCTC	16:C>T
1087690|F|0-32:T>G-32:T>G	TGCAGCAGTACTCGCAGTCCCCTGTCCATTCTTCGCCTCATCACCACCACCTCAACTCCATGAGGTACT	32:T>G
2259409|F|0-10:C>T-10:C>T	TGCAGCACCTCGTCCCTGCTGGTCGCGCTCAGCTCCTTGAGCAGCCGCTCGTACCTTTGCGCCGTCGAC	10:C>T
5581525|F|0-13:C>G-13:C>G	TGCAGGGGGTGGTCGGCCCCGACGCCGCCTTCACCGACGTCTGCATCGGCTGGCCG	13:C>G
1025427|F|0-34:G>C-34:G>C	TGCAGTCGCTGCTGCTCTGGCAGAACCAGCTCGTGGGCGCGATCCCGCCG	34:G>C
4993479|F|0-21:G>A-21:G>A	TGCAGCTCCTCCTTGCCGTGCGGCCG	21:G>A
2249414|F|0-11:C>G-11:C>G	TGCAGCACCACCACGTGGAACAGCTCGCTGTTCATCGACCTCAGCCCGTCCACCACCACGTCCCTCGAG	11:C>G
3028173|F|0-24:C>T-24:C>T	TGCAGCGACGCCGTCCACAGGATGCTGAGGGCGACGCCG	24:C>T
7169823|F|0-7:C>T-7:C>T	TGCAGTCCTCCTTCTCCAGGTCGTCTTCCTCCCG	7:C>T
1106335|F|0-10:A>C-10:A>C	TGCAGAAGTGAAGCAGGGGCATGATTTGCAGCCCCCGATCCGTCGTCGTCGCGGCCCATCCAGCGGTGC	10:A>C
5368351|F|0-5:C>T-5:C>T	TGCAGCGGCTCGGCGGTGCTGCCGCGGAGGAGCTCGGCGCCGCCGAGCCAGACGCCGAAGATCCTGTCG	5:C>T
1708249|F|0-55:A>T-55:A>T	TGCAGAGTCTGCATTGCATCGCTTGTTGCAGCAGCTTGAAGAACAGAGCTTTCGCAAATGGCATCAGCC	55:A>T
3952390|F|0-20:G>T-20:G>T	TGCAGGCCGCGCTCGACGCCGTCGCCG	20:G>T
1130166|F|0-29:C>A-29:C>A	TGCAGTCGCTGGCGACGGCCGCGGACGCGCTCACCGCGGCGCGCATCCTGGTGCAGGCCAGCCAGGGCG	29:C>A
2255577|F|0-13:G>A-13:G>A	TGCAGGCGCTGGAGGGCGCGTGCAGGCAGAAGGGCGAGGAGTTGGGCAGCGCCGCCCTGCAAATCGACC	13:G>A
1863625|F|0-22:G>C-22:G>C	TGCAGAAGGCGATCGACTACGCGTGCGGGCAGGGCGCGGACTGCACGGCCATCGCGCAGAGCGGGGCGT	22:G>C
1002827|F|0-42:A>G-42:A>G	TGCAGCAACGGCATGAAGCTCGTGGTCAACGTCGCCGCCGCCACCGCCGCCTCGCCGCCCAAGGCGTCC	42:A>G
2256621|F|0-30:C>A-30:C>A	TGCAGAGCATCCCTCCAGATATCCCATTGCCTCCTCGATGCCTTCGATATTGTAAGCGTCACGCAGCAT	30:C>A
1204117|F|0-58:C>G-58:C>G	TGCAGCTCGGTGGCCTCTTCATATGGACGTACACCTACAGTCTGATGCAGAAGTCTGGCAAGCTGTACC	58:C>G
1130145|F|0-9:T>G-9:T>G	TGCAGACTCTCCCAAGATTCCTCTCCAACATGTTCACGGAGCCGGGACGGCGGATCCG	9:T>G
998188|F|0-28:G>A-28:G>A	TGCAGGTGACGGAGAAGCTGGTGGTGTCGGGGTACATCAAGGACACCATCGGGGAGGAGAGCGTGTTCC	28:G>A
3948768|F|0-18:G>A-18:G>A	TGCAGCGCGGACCAAAAGGATTGCATCCCG	18:G>A
1695118|F|0-14:A>G-14:A>G	TGCAGCGCGCGGTCACAGTCCTTGACAACGTGCTTGTGGAGCTCGAGCTGGAAGTAGCAGAAGGCCCGA	14:A>G
1021646|F|0-34:T>C-34:T>C	TGCAGCACTTCAAGAAGCCGTTCGCTCATGAGCATGAAGAACTGAAGACCCTGCTGGAGGCCGTCCAGT	34:T>C
1261735|F|0-7:C>T-7:C>T	TGCAGCTCGCCAACGTGAAGACGGCGCTGGGGAGGCGGGAGGAGGCGCTCGCCGACCTGCGCGCCAGCG	7:C>T
1696351|F|0-50:G>C-50:G>C	TGCAGTTTCATGTGCTACTTCCACCTTTTCGGGTTCGTGGAGCAGAGCTGGACCATGGTCGCTGCCTTC	50:G>C
3023078|F|0-29:C>G-29:C>G	TGCAGGCCATCCCCTACGCCATGCTCCTCCTGCTCGTCCCG	29:C>G
2246111|F|0-68:G>A-68:G>A	TGCAGATTACCGAGCCAGTGCCACTGGAACTGCGAGAAACTATCCACGATGGTGCGGCAATCGGTTTCG	68:G>A
1128780|F|0-26:A>G-26:A>G	TGCAGCACGTCCTCGCCTGCCTCCTCATCGCCCTCGCCGCCGCCGCGCTCGCCG	26:A>G
1056404|F|0-54:C>T-54:C>T	TGCAGAATGTCTCCCCCAAATAGATCGCCCTAGCCACAGACACAGAAGCATCAGCGCAGGCTCTCGTCT	54:C>T
1089511|F|0-26:T>C-26:T>C	TGCAGGTTGTCCTGGAGCAGCAGCCTTGCCGTCTTCGTGAAGACGGTGGCGCCGCCG	26:T>C
1000346|F|0-14:G>C-14:G>C	TGCAGCTGCGCGTCGCGTATGGCCACGGCCTTGGCCGCCTCCACCTCCGCCACCCTCGCCTGCTGCTCC	14:G>C
6043749|F|0-22:A>C-22:A>C	TGCAGCAGGTGGGCCAGGGACAACGCCG	22:A>C
7169281|F|0-7:G>A-7:G>A	TGCAGCAGCGGTTGTCGCCGTTGTCGATATTCTTGCTGTCCAGGTACCAGCCG	7:G>A
6042795|F|0-50:T>G-50:T>G	TGCAGAAGCACGTGGCGTTCTTCGACGCCGACAACGACGGCATCGTCTCCTTCTCCGAGACAGAGCAAG	50:T>G
1012783|F|0-8:C>T-8:C>T	TGCAGCAACTCTGAATGTGTATGGCAGCAATCAAAACCACTGATGCACAAGAACGCCGCTCTCCCGAGA	8:C>T
1152074|F|0-40:G>C-40:G>C	TGCAGCACCACCAGGACGCGCTGGTGCTGGCGTGGCCGCGGCTGCACGAGGCCGTGCTGGACCCCGCCG	40:G>C
6049268|F|0-16:T>C-16:T>C	TGCAGCTCCGCCTCCCTAGAAGCCTCTCCTTTCTGTTGCCGTGGCGGGTGATCCAGTACGAGCAGGCCG	16:T>C
2250181|F|0-22:C>A-22:C>A	TGCAGATGGTCAGCCTCACGCTCCGCGTCCTCGCCCGCCCCGATGTGGAACGCCTTCCTGAAATCTTCA	22:C>A
6038167|F|0-22:T>G-22:T>G	TGCAGGTAGTGCAGGTTTGTCATGCCATCCTCCGTCACCTTGTCGTGGCCG	22:T>G
1863555|F|0-35:G>C-35:G>C	TGCAGCGCCTGGGTGCGTACCGCGGCGACGAAGGCGAGGTCGCCGTAGGGGGCGAGGCCCTCGAAGACA	35:G>C
5582014|F|0-22:G>C-22:G>C	TGCAGCTTGTACCGCATGGCGAGGGCCG	22:G>C
5357878|F|0-7:G>C-7:G>C	TGCAGCGGCCGCACGGCGAGTAGGCCG	7:G>C
1001087|F|0-38:T>C-38:T>C	TGCAGTACCAGCAGCCGCTGGTTCTGCGAGAAGCCGTGTGTGGTGAACGGCGGCGCGTACATGGTCACC	38:T>C
2252132|F|0-32:G>C-32:G>C	TGCAGCGACGAGATCGAGAGCCTCGAGCGGGAGATCGGCGACCGAGCGTCCGAGAGATGGACTGCCGCC	32:G>C
1238129|F|0-56:C>G-56:C>G	TGCAGCGACGACATCATCGGGATGGTGTTGCCCAGGCCTGCCCTCATCGCCTCGTCCAGCCGCATCTCC	56:C>G
2268151|F|0-59:C>G-59:C>G	TGCAGGGCGCTGAGCCGCTTCACGGCCGCCACGATGCCGTCCTCGAGGACCGCCTTGTACGTGCTCCCG	59:C>G
1023816|F|0-63:T>A-63:T>A	TGCAGGAGGCCGTCTGGGACGCTGACATTGTCATCAACGGCCTGCCGTCCACGGAGACGAGGGTTGTGT	63:T>A

7165352|F|0-5:T>C-5:T>C	TGCAGTGCGTTCCCTGGGAGTTCGCGGCGCGGCTCGCGGCCG	5:T>C
12763869|F|0-11:T>G-11:T>G	TGCAGCACTTCTTCAAGCGCCACCTCGGCCGCCACGCGCGCCGCCTCGCCGCCCTCGTCGACCCCTACC	11:T>G
1240720|F|0-16:T>C-16:T>C	TGCAGAGCCTCGTGTCTCGGGAGGCGGACCCGCTGGACTCGCAGGTGGTGTCCGTCGCCATGGTCAACG	16:T>C
5581037|F|0-37:G>C-37:G>C	TGCAGCTGGTGTTGATCCGCTTGTGGCTCGCGCGGTGGCCGCCCAGCGCCTGGTAGGAGCTGAACACCC	37:G>C
5359298|F|0-7:G>T-7:G>T	TGCAGGCGGGGCGGACCTACTTCGTGCTCCCCGCGGCCCG	7:G>T
2255577|F|0-52:C>T-52:C>T	TGCAGGCGCTGGAGGGCGCGTGCAGGCAGAAGGGCGAGGAGTTGGGCAGCGCCGCCCTGCAAATCGACC	52:C>T
7172275|F|0-18:C>G-18:C>G	TGCAGTTGCGCCTGATCTCGCCAGCGTTTCCG	18:C>G
2261623|F|0-8:G>A-8:G>A	TGCAGCTCGCCGCCCTGCGTGTCCAGGATGCACCGAGAATCCCCGACCGACGCCACGGTGACCGTGAAC	8:G>A
990314|F|0-42:T>G-42:T>G	TGCAGTAGGTCTTGCAGCCTGGGCACATGACGATGCACTCGTTGGGGCAGCGGACCTTGTCGCAGGTGG	42:T>G
996545|F|0-29:C>G-29:C>G	TGCAGGAACGACGGCGGCGAGCGTGAGGGCTGAGGTGGACAAGAGCAGTGCTCTCCTGGTTGTGGCCGA	29:C>G
2258830|F|0-31:C>G-31:C>G	TGCAGTCGAACTTGACCGCCACGGGCGTCCTCGACGTGCCCTCGATGTCCTCGTACGACACGTCGCTGA	31:C>G
1088400|F|0-6:G>A-6:G>A	TGCAGGGCGGTGTCGTTGCCCTTGAACGCCTCGTTGCCGACGGTGATGCAGGTGATGCGCGCGCCGAGA	6:G>A
1249581|F|0-22:C>G-22:C>G	TGCAGAACAACAACATCAATGGCCGAATCCCGGCAGAGATTGGCAGGCTCACGAAGCTTAGGACGCTTG	22:C>G
5411980|F|0-14:G>A-14:G>A	TGCAGGTCGTCCATGCTCAGGCCG	14:G>A
4004515|F|0-33:G>A-33:G>A	TGCAGGAACATGAGCTTCCGCGACGCCAGGAACGCGTTGTTCCG	33:G>A
2261626|F|0-31:G>A-31:G>A	TGCAGCTCCGTCACCGCCTCCGTCTCCGCCAGCACGCCGCCGAGCACGGCGAAGAAGTCGTGGTCGCGG	31:G>A
7336038|F|0-64:A>C-64:A>C	TGCAGAGGAGGAGGCCGCCGCAGCACTGCTGGATCTCGACGCGCTCGTAGCGCCCTCGCAGGAAAGGGA	64:A>C
6041233|F|0-15:A>G-15:A>G	TGCAGATGATCATCAACCGCCGCCCATCTCGGTGGTTTCCTTCGGCGGCCCGAAGACCG	15:A>G
998509|F|0-13:G>C-13:G>C	TGCAGCGCGCCCAGCTTCTGCACCAGGAAGGACTTGGAGAGGAGGATCCG	13:G>C
1215091|F|0-42:G>C-42:G>C	TGCAGCGGCGGCAGCGTGTCGGCGATGGTGACCTGCACGGTGGCCGTCTGCCG	42:G>C
987472|F|0-23:A>G-23:A>G	TGCAGGCCGTCGGTGAGCTGGCGAGCGGCATGGAGGCGCCAAGCGTGCGGCCG	23:A>G
3944019|F|0-9:G>A-9:G>A	TGCAGCAGCGGCGAGGCATCGGCATCGTCGTCGTCCG	9:G>A
6045525|F|0-32:T>G-32:T>G	TGCAGCGAGCGGCGGAGCACGAGGAGCGGGCGTCGGCCGTACACTCTGGCGATGCCG	32:T>G
2247711|F|0-55:G>C-55:G>C	TGCAGCGGCCGCGCTTTGGTCGGCTCAGCATCTGGAATCCTCACAAAGTGTTCAGGCTCCATTCGACCA	55:G>C
4003272|F|0-16:T>C-16:T>C	TGCAGGCCGCCACGCTTAACCCGTCGGCGTGCTCCGCCTCCG	16:T>C
12768972|F|0-28:C>T-28:C>T	TGCAGGCCAAGATAATCAAGTCGGTCGACGTGTCGTACGGCGGGGAGAGCGGCTTCAACCAGGCCATTG	28:C>T
987045|F|0-67:G>C-67:G>C	TGCAGGCGGAGGCCGAGGTCGGGGAGGACGGCAGGATGCGGGTCACCGTGCGCAAGTCCACCAGCTCGC	67:G>C
2247211|F|0-64:G>T-64:G>T	TGCAGGATCTTGCTGGGCATTCGCCGCCGTGGGTGCAATCGAAGGACTACACAAGATCAAGAGAGGGAC	64:G>T
1095483|F|0-33:G>A-33:G>A	TGCAGCGCAAGCGGCCGCCGAGGCTGACGGTGAGCAAGAATCGTCTGGACAAGGGCGCGGCGGAGCAGG	33:G>A
1018924|F|0-41:G>A-41:G>A	TGCAGGCCAGCAGGGCTGAGCATCTCGCGGACGCAGACGCGGCGGAGCAGACGCCAGGTGGGACCGACG	41:G>A
1024266|F|0-24:G>C-24:G>C	TGCAGAGGTCGGAGAGCTCCTTGCGGCCCTCCACAATGTCGTCCAGGAAGTCGTCCAGCTGGCACGCCA	24:G>C
1252133|F|0-42:C>A-42:C>A	TGCAGGCTGAGGTGGACCAACTACCTGAGGCCCGACCTCAAGCGGGGGCTCCTCTCCGACGAGGAGCAG	42:C>A
1863241|F|0-50:A>G-50:A>G	TGCAGACAACTACCTCATCAACTGCGGCTCCACGGTCGACGCTAATCTCCACGATGGGAGGGTCTTCAA	50:A>G
992552|F|0-52:T>C-52:T>C	TGCAGAACACGCAGCCGCTGCCGACGTTCAGCAACTTCGGCGACGACGTCTATTTCGTGGCGGACCAGC	52:T>C
1200118|F|0-34:G>C-34:G>C	TGCAGAGGATGCACCCCAACGGGTCGTGGGAGCAGTGGCGGTTCGTCGGCGGCACGGGCCTGCTCGGCA	34:G>C
2351339|F|0-58:C>G-58:C>G	TGCAGATCCTGCTCGAGTACATGGACGGCGGGTCCCTGGACGGCCGCCGCATCGCGTCCGAGTCGTTCC	58:C>G
3937786|F|0-60:C>T-60:C>T	TGCAGCGCGAGCTCGAGCTCGCCGAACTGGCCGTGGTGAACCGAGCTCGGATGGACCTCGCTCATGACC	60:C>T
1284172|F|0-53:G>A-53:G>A	TGCAGGAGTCGAATCCATGGACAAAAATGATCTTGTACTTGGCCTTCTCCTTTGGGACGCCAGATTCCA	53:G>A
3951874|F|0-5:G>C-5:G>C	TGCAGGTCCAGCGACTGGAGCGCGCCCATCAGCCGCGCCG	5:G>C
32019301|F|0-8:G>C-8:G>C	TGCAGGAGGTGGACCTGGCCG	8:G>C
32020239|F|0-10:T>C-10:T>C	TGCAGCTCGGTAAGGTCAAAAGGAACATCGAAAACCG	10:T>C
1398010|F|0-10:A>G-10:A>G	TGCAGATAGCAGCTACGGCGGCAGGAGCTTGGGATTTACAGGGACGGGTGGGGAATAGCATGGATTAAT	10:A>G
7352269|F|0-12:C>T-12:C>T	TGCAGCATGTCCCGCGCCTCCG	12:C>T
1023915|F|0-31:G>C-31:G>C	TGCAGAACAGCCACATCACCGAGGCCATGCTGCACGGCAGGATGAGCTACGGGAGGGCCTCCGAGGACG	31:G>C
1341728|F|0-13:C>T-13:C>T	TGCAGTGCCCCGACGACCTGGAGCGCCTCTACTGCGAGATCCACCTCCTCAAGACGCTCAAGCACAAGA	13:C>T
1100113|F|0-52:C>T-52:C>T	TGCAGCAGAAGAAGCCATGGACGAAAATGATCTTGTACTTGGCCTGCTCCTTCGGGACGCCG	52:C>T
998211|F|0-40:C>G-40:C>G	TGCAGGTCCGCGCCGACGTGCCGCTGCTCGGCTTCATCGGCCGCCTGGACGGGCAGAAGGGCGTGGAGA	40:C>G
1025059|F|0-17:G>A-17:G>A	TGCAGCGACGCCCGCATGTACTTGTCGTAGCACTGGAACTCGCCGATGCCCG	17:G>A
1214808|F|0-7:G>A-7:G>A	TGCAGAAGGAGAAATCGTCCTTGGAGATGGAGGTGCGGCAGCAGCGCCGCACCTCTGATGAGCGCTGCG	7:G>A
1059807|F|0-65:A>C-65:A>C	TGCAGGTCCCAGTCGTTGAGGTGCTCCATGCGCAGAAGCAGGCCGCACATGAACGGCGACCAGGGATTT	65:A>C
5578273|F|0-16:C>T-16:C>T	TGCAGGCGCAGCGGCACTTCGTGCTCCACCG	16:C>T
3955944|F|0-20:G>C-20:G>C	TGCAGCGGCGGCATGTGCGGGCACTACACGCAGATCGTGTGGCGCGGCAGCACCCGCGTGGGCTGCGCC	20:G>C
4536355|F|0-11:C>A-11:C>A	TGCAGGAGAAGCTGGAGCAGCTGCCG	11:C>A
1863241|F|0-36:C>T-36:C>T	TGCAGACAACTACCTCATCAACTGCGGCTCCACGGTCGACGCTAATCTCCACGATGGGAGGGTCTTCAA	36:C>T
1316101|F|0-37:G>T-37:G>T	TGCAGCGTTTTCTCTCTGTTGCTATTCTCAGCACTTTGGAAAGTATCTATGCAAAGGAATTAGTATTAA	37:G>T
4982323|F|0-17:A>C-17:A>C	TGCAGGGCCTCCTGGGGAGCCTCGGCCTGCCTCCCG	17:A>C
7168931|F|0-46:C>T-46:C>T	TGCAGGCGGCGGCCGTGGCGGCGGGGCACGAGAACTGGGGGACAAGCGGGCCGCACGACGCCG	46:C>T
12850977|F|0-48:C>T-48:C>T	TGCAGCCGATCACGTCGTCGAAGAGCTGGACGGCGAGCTCGGCCGACCCGCTATCCACGTTGTGGAGTT	48:C>T
7902430|F|0-22:C>G-22:C>G	TGCAGTACTACTGCGTGCTGGGCGCGGTGGCCG	22:C>G

1127579|F|0-63:C>G-63:C>G TGCAGCGAGGCGCTGTTCAAGTTCGCGGTGTTCGTCCTGGTGCAGGCGCTGGTGTACCTCATCCTGTCC 63:C>G
7341769|F|0-8:C>G-8:C>G TGCAGCCCCACGGCCTCCCG 8:C>G
5331760|F|0-12:C>T-12:C>T TGCAGCAGCCGCCGCGGCCCGTGAGCCCCG 12:C>T
1696351|F|0-21:C>G-21:C>G TGCAGTTTCATGTGCTACTTCCACCTTTTCGGGTTCGTGGAGCAGAGCTGGACCATGGTCGCTGCCTTC 21:C>G
3022242|F|0-24:A>C-24:A>C TGCAGGATCCTGTTCCTGCTCACCATATCCCTCCCCCG 24:A>C
7172198|F|0-8:C>G-8:C>G TGCAGGCCCTCGGTGAGCTGGCGGGCGGCATGGAGGCGCCAAGCGTGCGGCCG 8:C>G
1003846|F|0-55:C>T-55:C>T TGCAGCAGGTCGTCGCAGAGCGCGGCGTCCACGTCGCCGCATGCCCCGCCACCGCCGCCG 55:C>T
3026430|F|0-13:C>T-13:C>T TGCAGAAGATATCCGTGAGCACCAACATGCTCGATGGGGAGCTGCCG 13:C>T
1027599|F|0-6:C>G-6:C>G TGCAGACCAGCACGTAGTCGGCGGCCGCCGAACTCGCCGCGCCCGCGTCCTTGGCTGCACGCCCTCGCT 6:C>G
1200870|F|0-28:G>A-28:G>A TGCAGTACATGGAGACAAACCCGCTGCTGACATGGAAATCGCCGTCGCCGCACATCCCGCCCTTGACGG 28:G>A
1024576|F|0-10:C>T-10:C>T TGCAGGAGGGCGACCTCGCCATCTGCCCCGAGGGGACGACGTCCCGAGAGCCGTTCCTGCTCCG 10:C>T
1024557|F|0-19:G>A-19:G>A TGCAGGGACCTGTCTGCCAGGTCGTGCATGTTGAAGAGTACGTGCTTGTTCAGAGGGTGGCCG 19:G>A
989217|F|0-67:G>T-67:G>T TGCAGTTGCATGCATACTTGCATAGCGGTGAAAGACGGTGAGCGAAAGAGGAGGAAAGCTCTCTCTCGG 67:G>T
1152074|F|0-43:G>C-43:G>C TGCAGCACCACCAGGACGCGCTGGTGCTGGCGTGGCCGCGGCTGCACGAGGCCGTGCTCGACCCCGCCG 43:G>C
6045098|F|0-7:G>C-7:G>C TGCAGGTGGGCGTCTACTTCGGCGGTCTGTACATCGTTGCCTTGGGCAACGGCGGTACAAAGCCTAACA 7:G>C
5368351|F|0-11:G>C-11:G>C TGCAGCGGCTCGGCGGTGCTGCCGCGGAGGAGCTCGGCGCCGCCGAGCCAGACGCCGAAGATCCTGTCG 11:G>C
5581470|F|0-7:C>G-7:C>G TGCAGGACGCGCTCGAGGAGAGCGAGAAGGCCG 7:C>G
4910225|F|0-12:C>T-12:C>T TGCAGGATCTTGCTGGGCATTCGCCGCTGTGGGTGCAATCGAAGGACTACACAAGATCAAGAGAGGGTC 12:C>T
3028633|F|0-35:G>T-35:G>T TGCAGGGCGTCGGCGTCCGGGAAGTAGATGCGCATGCCG 35:G>T
2251292|F|0-14:G>C-14:G>C TGCAGCGGGTTGAAGGTGAGCATCCTCTCGATGAGGTCCAGCGCGGCGGGCTGCACCTTGGGGAACTGT 14:G>C
996761|F|0-34:C>G-34:C>G TGCAGCAGCCGCCGCCGCTGCACCTGCTCCCCGCCGCCGCCACGTGCTACGGGAAACCCATGGCCGAGA 34:C>G
1159042|F|0-7:C>G-7:C>G TGCAGCTCGCTGGCGTTGGCCACGCCGAGAACGGCCGACTGAAACGTGCCGAAGTTCCCGACGATCTTC 7:C>G
991279|F|0-43:G>C-43:G>C TGCAGATGCCCTGGGTCTCCACCCGCACCACGCCCTTCCTGAAGGTGAAGAGCTCCG 43:G>C
4544061|F|0-32:T>G-32:T>G TGCAGCAGGGTGTTGCGGGTGAAGACGTCGGGTGCGGCGCCG 32:T>G
1694736|F|0-10:T>C-10:T>C TGCAGGGAGCTCGCGAGGAGATGGAGGACGAGGTCGTCCTCCGCCCTGACTCCCTCCTCCACGGCTTCT 10:T>C
2252284|F|0-17:G>C-17:G>C TGCAGCGACACGATGATGGCCGCGCCCGCCATGAACCCCACCAGCGTCGCCTTGGACAGGAAGTCGATG 17:G>C
1250281|F|0-10:C>T-10:C>T TGCAGAGATTCGTGGCGCTCAAGATCCAGAAGAGCGCGAGGGACTACGCGCACGCCGCGCTCCACGAGA 10:C>T
4396641|F|0-13:C>G-13:C>G TGCAGTACCTCGCCCTCTCCG 13:C>G
1309033|F|0-8:G>A-8:G>A TGCAGCTCGCAGCGCAGAAGGCAGCCCTTCTCCAGCAGCGACCTCCCCG 8:G>A
5363049|F|0-14:G>A-14:G>A TGCAGAACGCCATCGTGATGCCGCCG 14:G>A
5323908|F|0-29:T>C-29:T>C TGCAGGGTTCAGGTGGGATCGGCAGTGGCTGGTGGTGAACGCCAAGGGCAGGGCGCTCACGCAGAGGGT 29:T>C
7929944|F|0-7:A>T-7:A>T TGCAGCCAGACATAACTGCCCCG 7:A>T
7487970|F|0-11:C>A-11:C>A TGCAGCACAACCGCCTCACCG 11:C>A
3029716|F|0-8:C>G-8:C>G TGCAGGTTCGACGTGTCTGACGCCATCTGCATCAGCGCCG 8:C>G
1022310|F|0-5:G>C-5:G>C TGCAGGGACACCTGCCGCTCCATCTGGATGTGCTTGTAGAAGTTGGCGATGAACTGCTCCGCGCGCCGA 5:G>C
6049325|F|0-22:G>C-22:G>C TGCAGATCAGGAGGAAAGGAGGGTGGGTCCCG 22:G>C
2260887|F|0-25:G>A-25:G>A TGCAGGGGCAGTTCGAGATCCTGTCGCTCACGGGCACGGTGCTGCCGCCGCCG 25:G>A
7170445|F|0-13:C>G-13:C>G TGCAGAAGGAGGGCCTGCACGCCAAGCACCCCGTCATCTTCGTGCCG 13:C>G
7338837|F|0-31:A>G-31:A>G TGCAGACCATGTTGCACCAGCCGTTGAGCACAATGTTGAAGCCCTTGGTCTCGAACGGGAACTCCTTCT 31:A>G
1031984|F|0-61:C>G-61:C>G TGCAGCGCGTGGCCTACTTCAACGTGGGCATGTACCCGTTCACCTCCATGTTCCTCATCGTCTACTGCG 61:C>G
998838|F|0-8:T>A-8:T>A TGCAGACGTAGGCGTCGCCCGCGAGCCCGTCCTTCCAGGCGGCGCAGTAGAGCTGCTCCGCGAAGGCGA 8:T>A
991772|F|0-44:C>T-44:C>T TGCAGCATCGTGCGCGCGGGATTGCGCGGATTGGTGCTGGGGCACCGTGCGCGCGGGCGTGTGGCTCGC 44:C>T
3029089|F|0-38:C>G-38:C>G TGCAGGAACAGCGTGGGCGCCTGGTGGTACTTGACCAGCGACTTGCCG 38:C>G
3025796|F|0-43:C>G-43:C>G TGCAGAAGCTATCCCTTGATGCCAAGACTGAAGCCATCGACGCCCCG 43:C>G
1128488|F|0-52:T>C-52:T>C TGCAGCAGGAGCCACCCGTGCAGCGGGATCAGCATGCAGGACGTGAAGTTGATGTACGACAGGCGCGTC 52:T>C
3955613|F|0-7:G>A-7:G>A TGCAGGCGCCAATAAGTTTTCATCGCAGGCTTTTTACCG 7:G>A
1090663|F|0-13:G>C-13:G>C TGCAGGTCTTGCAGACGAACTCCCCCTCCCCCGACGTGGCCACCACTCTGCCCCGCCGCGCCCTCTTCC 13:G>C
5581052|F|0-29:G>C-29:G>C TGCAGGCAGGAGTGCGCCACGGCCGCGGCGCCGCGCGCCGCCCGCGTCGAGTACTGCGACTCCATGCCG 29:G>C
5578457|F|0-12:A>G-12:A>G TGCAGTGCTGTCAGCAGTCTCCG 12:A>G
1005621|F|0-22:G>A-22:G>A TGCAGGTGTACCAGATGGCGAGGTAGATGAGGAAGGTGAGGATGCCGATGGCGAAGGAGAGGCCCATGA 22:G>A
32027973|F|0-10:C>T-10:C>T TGCAGGGACGCCTGGTAGCTGACGAAACCCATGAACCAGAACTCGAAGCCGTCGTCGGTGACCAGCTGG 10:C>T
4394812|F|0-29:G>A-29:G>A TGCAGCTCATCGCGTGCGGGGCGGTCACCGCCG 29:G>A
100594792|F|0-32:G>A-32:G>A TGCAGCTCGTCGATCACGGCGCGCCCGCCCGCGTGGATGCAGAAGTGCTCGAACGCCATGCGGAAGTCG 32:G>A
1007687|F|0-14:T>C-14:T>C TGCAGACCCCACGGTCACAGTACGAGTTCACGGACTTCCCGTGGCCGCCG 14:T>C
3029521|F|0-20:G>T-20:G>T TGCAGCACCATGGGGAGCGAGCCGTGGTGGTTCGCGGCGTTGCCG 20:G>T
1005621|F|0-6:T>C-6:T>C TGCAGGTGTACCAGATGGCGAGGTAGATGAGGAAGGTGAGGATGCCGATGGCGAAGGAGAGGCCCATGA 6:T>C
2242955|F|0-41:C>T-41:C>T TGCAGCTTTGTACGAGCCGAGCGCTCTGGCCGTCGATCAAACGGATTCATCCTTTATCAGGAGCAGCAA 41:C>T
1156567|F|0-54:G>A-54:G>A TGCAGGTGTGGTGGTGAGTGGCAAGTGTGGCTGCCTTGGTCCGATGCTGCCGCCGCCACCACCGTGGCG 54:G>A
4019082|F|0-28:C>G-28:C>G TGCAGCCGACCCTGGCCACCGAGTACGCCAGCCAGGCCG 28:C>G

<table>
<tr><td>6043748|F|0-35:G>A-35:G>A</td><td>TGCAGCAGATCGGCGTGCTCGGGGAGGACGACGGCGGCGCGGCCG</td><td>35:G>A</td></tr>
<tr><td>7167186|F|0-37:G>C-37:G>C</td><td>TGCAGGTCGTGCAAGAGACATGGACTAGGACGGGGGGGCATGTGACGGACTACACGCTCTACACGGGCG</td><td>37:G>C</td></tr>
<tr><td>7492421|F|0-7:A>G-7:A>G</td><td>TGCAGGTAAACGGGCTCACCG</td><td>7:A>G</td></tr>
<tr><td>1070126|F|0-61:C>T-61:C>T</td><td>TGCAGATATTCAACAAGAGGTGGAAGGAGGCGGTGGCGGAGGACATGTGCTGGACGGACCCCTACCCGC</td><td>61:C>T</td></tr>
<tr><td>7161755|F|0-23:G>A-23:G>A</td><td>TGCAGGACGACGTCGACAGCGAAGTCGACCCG</td><td>23:G>A</td></tr>
<tr><td>5577014|F|0-19:T>G-19:T>G</td><td>TGCAGCGAGAGCGGCAGCTTCACGAAGATCCACGTCAATTCCGTGGAGATCACCGCTGCGGACGGCGGA</td><td>19:T>G</td></tr>
<tr><td>4733783|F|0-24:G>A-24:G>A</td><td>TGCAGGGATGGAGAGCGACGACGAGATAGGGACGGTGCCG</td><td>24:G>A</td></tr>
<tr><td>1284172|F|0-19:G>C-19:G>C</td><td>TGCAGGAGTCGAATCCATGGACAAAAATGATCTTGTACTTGGCCTTCTCCTTTGGGACGCCAGATTCCA</td><td>19:G>C</td></tr>
<tr><td>1015137|F|0-25:C>T-25:C>T</td><td>TGCAGCAAGACGACCGCGCCGACGTCGGCTTCGCCACGCCGCTGGCCACGGGTGGAGTAATTGGGCCGC</td><td>25:C>T</td></tr>
<tr><td>1094397|F|0-12:A>C-12:A>C</td><td>TGCAGGGATGGGAGGTCTGGGCGTGGAGGAGACGGCGCTGGTGTCGGCGCTGGGGCGGTGGAGGAAGCA</td><td>12:A>C</td></tr>
<tr><td>1117315|F|0-41:G>A-41:G>A</td><td>TGCAGCGGCGCGGAGGAGAAGGAGGAGGATGTGTGGAACGCGGAGGAGTAGTTCTGCGGCACGGAGCCC</td><td>41:G>A</td></tr>
<tr><td>3959730|F|0-44:G>C-44:G>C</td><td>TGCAGGTGCTGGGCGGCGGAGGCGCGGGGGCGGTGAAGCGGAACGAGGGGCGGGGGAAGCTGCTCCCCA</td><td>44:G>C</td></tr>
<tr><td>3029786|F|0-35:G>C-35:G>C</td><td>TGCAGGCGCAGCTCGCCTCGCTCAAGGCGCACGCCGCGCCG</td><td>35:G>C</td></tr>
<tr><td>5361439|F|0-32:G>C-32:G>C</td><td>TGCAGGCCCCACTCGGCGAGGCTGACCTGCTCGCGCGGCAGCTGCGGGTCGATGGGCGCCCGCGCGCAC</td><td>32:G>C</td></tr>
<tr><td>7155323|F|0-20:C>T-20:C>T</td><td>TGCAGGAACGGCCGGCCATGCGCGTCCG</td><td>20:C>T</td></tr>
<tr><td>1064179|F|0-13:A>G-13:A>G</td><td>TGCAGTACTCCTCATTGGACCAGCAGTGTTCGTCCAGGACGGGGCCCTCGGGGAGCTTGGGGTTGATGA</td><td>13:A>G</td></tr>
<tr><td>1666813|F|0-65:A>C-65:A>C</td><td>TGCAGAAGTCGGTCAGCGCGGGGTGGTGCTGCGGGCGCACGTAGGTGCGGAACTCGGCGGAGTCGAGGT</td><td>65:A>C</td></tr>
<tr><td>1024561|F|0-18:G>T-18:G>T</td><td>TGCAGGCGTTCGCCCTCGGCCTGAACAAGCGCCTCGCCGACGACGACGCCG</td><td>18:G>T</td></tr>
<tr><td>1863053|F|0-49:C>G-49:C>G</td><td>TGCAGGACGACGTGGGCGGGCTGGAGGTCCTCGTCGACGGCGACTGGCGCCCCGTCCGCCCCGTCCCCG</td><td>49:C>G</td></tr>
<tr><td>2298663|F|0-53:C>A-53:C>A</td><td>TGCAGAAGCTCATGATGCTCGCCATGCTCACCGCCTGGAGCCACCTCTCCCGCCGCGGCAGCCTCGAGT</td><td>53:C>A</td></tr>
<tr><td>2258923|F|0-18:A>G-18:A>G</td><td>TGCAGCGCGTGCAGTCGGAGGCGCTGCGCGCGCTGGGGCCGCGCGACTTCGACCCCAAGCTCTACGTCG</td><td>18:A>G</td></tr>
<tr><td>12768972|F|0-25:C>G-25:C>G</td><td>TGCAGGCCAAGATAATCAAGTCGGTCGACGTGTCGTACGGCGGCGAGAGCGGCTTCAACCAGGCCATTG</td><td>25:C>G</td></tr>
<tr><td>2246988|F|0-14:T>C-14:T>C</td><td>TGCAGATCAGCGGGTATGTCACTGCATGTACCTTTTCTGTCGAGGGCGCTCCACTGGAGATTCCACAGC</td><td>14:T>C</td></tr>
<tr><td>1088789|F|0-23:C>G-23:C>G</td><td>TGCAGGCTGAGGTGGAGCAGCGCCGAGCAGTTGGCGAGCGCCGACGGGATGGTCCCCTCAAGAAGGTTG</td><td>23:C>G</td></tr>
<tr><td>1202071|F|0-31:G>T-31:G>T</td><td>TGCAGGCAGATGGGCTGAGGCAGCAGACCCTGCACAAGCTGCACGGCATGCTGACGGTCCGACAGACCG</td><td>31:G>T</td></tr>
<tr><td>3959003|F|0-22:G>A-22:G>A</td><td>TGCAGGGGAATCGCCCATGGAGGGCTCCCG</td><td>22:G>A</td></tr>
<tr><td>2252852|F|0-16:C>G-16:C>G</td><td>TGCAGAGGACGAGATTCAGCTCCCAGAACTTTTTGCTGCGCTCGCGCAGGACGAGTAGGCCGCCGCGGC</td><td>16:C>G</td></tr>
<tr><td>1214808|F|0-51:C>T-51:C>T</td><td>TGCAGAAGGAGAAATCGTCCTTGGAGATGGAGGTGCGGCAGCAGCGCCGCACCTCTGATGAGCGCTGCG</td><td>51:C>T</td></tr>
<tr><td>1068826|F|0-8:A>C-8:A>C</td><td>TGCAGGAAAGAGGAGTAGATGGCGCAGAAGGGCTTGAGCCCCTCGCAGGCCAGGCCG</td><td>8:A>C</td></tr>
<tr><td>988507|F|0-10:C>G-10:C>G</td><td>TGCAGCCGACCAGCCCCACGGCGGTGAAGCAGGTGAGCGTGGTGTGGGACGCGTACGAGGCCGCCAAGG</td><td>10:C>G</td></tr>
<tr><td>1020290|F|0-24:C>A-24:C>A</td><td>TGCAGCATCGCCGAGAGATCGTTGCTGGCTAGCTCGTCCCATGACCAGTTCCAGTAGGCCTGTGGGGGT</td><td>24:C>A</td></tr>
<tr><td>7166994|F|0-40:C>A-40:C>A</td><td>TGCAGGAAGCCCATTGCTGTCTGGTCCGCCCCTATGTAGACGCCCTGGGCCCGTCCCACGGCCGCCGAC</td><td>40:C>A</td></tr>
<tr><td>1864417|F|0-68:G>C-68:G>C</td><td>TGCAGCGTCGGCTTGCAGTTCTCCCTCTTGCGCATTTCGTCGAGCAGCTGGAGGGCGTCCAGAAACCTG</td><td>68:G>C</td></tr>
<tr><td>3029521|F|0-21:C>G-21:C>G</td><td>TGCAGCACCATGGGGAGCGAGCCGTGGTGGTTCGCGGCGTTGCCG</td><td>21:C>G</td></tr>
<tr><td>2251466|F|0-14:C>T-14:C>T</td><td>TGCAGGGCGGAGGACTACTTCAAGCGTGCGGTGGCCATCGAGCCGCCCGACGGCGAGGCGATGCGGAGG</td><td>14:C>T</td></tr>
<tr><td>1249581|F|0-52:G>A-52:G>A</td><td>TGCAGAACAACAACATCAATGGCCGAATCCCGGCAGAGATTGGCAGGCTCACGAAGCTTAGGACGCTTG</td><td>52:G>A</td></tr>
<tr><td>5970348|F|0-13:C>T-13:C>T</td><td>TGCAGGACCAGCTCGGCTTCGACAAGGCCAGCAAGACGGTGGACTGGCTGCTCACCCAGTCCAAGCCAG</td><td>13:C>T</td></tr>
<tr><td>1062296|F|0-7:G>C-7:G>C</td><td>TGCAGAGGAGCGGGTGGGGCGGGTACGAGGTGAGGCACGACGCCGAGGGGCACTGCTTCTTCCTCTGCT</td><td>7:G>C</td></tr>
<tr><td>32024977|F|0-40:G>C-40:G>C</td><td>TGCAGAGTGAAGGCTTGAGCTTCTCGAACCAGTCGAGCTGGACGCTGAGCGACAGGTTGGTCCAGAGCT</td><td>40:G>C</td></tr>
<tr><td>1001498|F|0-61:T>G-61:T>G</td><td>TGCAGCGCGCCGCCGCGCTCTACAACCCGCAGCTGGTGCAGATGACCAAGGACCTCAACGCTCAGTTCG</td><td>61:T>G</td></tr>
<tr><td>2334099|F|0-27:A>T-27:A>T</td><td>TGCAGCCGAAGACGACGCCGTGGAACGAGTCCTCGCCGATGGTCAGCTTGTCGACGGCCAGCGTGCCCC</td><td>27:A>T</td></tr>
<tr><td>1696538|F|0-16:C>A-16:C>A</td><td>TGCAGGTACCAGTGCACTGAGTGGGGCGTCGGCGTGGAGATCGACAGCAACGTCCG</td><td>16:C>A</td></tr>
<tr><td>32026956|F|0-49:G>C-49:G>C</td><td>TGCAGGCCCTCAGCGGCTCCAACATCGGCCTCATCATCGACGTGGCCAAGGAGGACCTCGCCTCCCTCG</td><td>49:G>C</td></tr>
<tr><td>1014911|F|0-13:C>G-13:C>G</td><td>TGCAGCGCCGCACCGTGGCGCAGGAGGACGAGCTGTCGCGGGAGATGGCGCGGGTGCAGGAGGGCCACG</td><td>13:C>G</td></tr>
<tr><td>3024203|F|0-22:A>G-22:A>G</td><td>TGCAGTCGACGCAGGAGGAGGAACAGATCGACACCCAGCTCAAGGGCAAGGCCTCCACCG</td><td>22:A>G</td></tr>
<tr><td>1020554|F|0-8:G>T-8:G>T</td><td>TGCAGAATGTGAAGAGGGTGAGCCCATGGCTGGTTGAGCTGGTCTCAAACATGCCG</td><td>8:G>T</td></tr>
<tr><td>7167668|F|0-17:A>G-17:A>G</td><td>TGCAGCATGTGCAGCGGAGGCAGTATCCTGGGGCCG</td><td>17:A>G</td></tr>
<tr><td>32027291|F|0-32:C>T-32:C>T</td><td>TGCAGCTTGGGGGCGGAAACAATTTTCACCGTCGCTCTGCCGCTCGGCAAATGGATGCCCACCAGTTCT</td><td>32:C>T</td></tr>
<tr><td>1214689|F|0-11:G>A-11:G>A</td><td>TGCAGTGGGCGGCGCCGAGGGAGCCGCTCTGCCAGACGCAGGCGGACTGCGAGGACGGCGCCAACGCCA</td><td>11:G>A</td></tr>
<tr><td>3934162|F|0-32:C>T-32:C>T</td><td>TGCAGCGCCGCGCTGCTGTGCACGCGGTTGTTCGCCG</td><td>32:C>T</td></tr>
<tr><td>9766637|F|0-16:C>G-16:C>G</td><td>TGCAGCTCATGCGGCGCTACCCCG</td><td>16:C>G</td></tr>
<tr><td>1088337|F|0-68:T>C-68:T>C</td><td>TGCAGCAGCAGCACGTTGTGCATCTGCGCGAAGAGGTTGAGCCCGATGAGGCAGCCGTTGCCGTCGTAT</td><td>68:T>C</td></tr>
<tr><td>7165352|F|0-6:G>C-6:G>C</td><td>TGCAGTGCGTTCCCTGGGAGTTCGCGGCGCGGCTCGCGGCCG</td><td>6:G>C</td></tr>
<tr><td>1059807|F|0-59:C>G-59:C>G</td><td>TGCAGGTCCCAGTCGTTGAGGTGCTCCATGCGCAGAAGCAGGCCGCACATGAACGGCGACCAGGGATTT</td><td>59:C>G</td></tr>
<tr><td>1023792|F|0-31:T>C-31:T>C</td><td>TGCAGGTGCTCAAGGACGCCAGCATCAACGGTGGCGCGCTCCAGCTCACGCCG</td><td>31:T>C</td></tr>
<tr><td>24505227|F|0-64:G>C-64:G>C</td><td>TGCAGGCCATGCAGGCGATGCTGTTCCGCTCCAACCGCCTCGTCGGCGTGCCGCTGGCCAGGCTGCTCC</td><td>64:G>C</td></tr>
<tr><td>1091125|F|0-7:C>T-7:C>T</td><td>TGCAGCGCTCCTGGCTGCTGCTCACCGCCACGGCCGTCTGCATGCTCCCGCTCTACCTCTTCGCCACCC</td><td>7:C>T</td></tr>
<tr><td>2252834|F|0-44:G>T-44:G>T</td><td>TGCAGCGAGTAGTAGTCGTGTGGCCAGCAGTCATCGAGCTTGACGTGCGGCCTCTCGCCGTCGGCCCAC</td><td>44:G>T</td></tr>
<tr><td>987584|F|0-22:C>T-22:C>T</td><td>TGCAGCCTCAGGTCGTCCAGGACGGGCGCGGCCGCGGCCACCTTGGAGCACCG</td><td>22:C>T</td></tr>
</table>

1863289|F|0-35:G>C-35:G>C	TGCAGGCGCTCCGTCTCGGTGAAGCGGGAGGCGTAGGTCTCGGCGACGCCGTTGCGGATCCGCAGGGCG	35:G>C
7345916|F|0-8:G>T-8:G>T	TGCAGCGAGCCAGCAGATCCG	8:G>T
1080669|F|0-60:A>G-60:A>G	TGCAGCGCCAGGCTTGCAGTCAAACGGCTTGGATTATCTTAGTGCTGTCGCTCCAACACAACGGCGACA	60:A>G
1272644|F|0-24:G>A-24:G>A	TGCAGAAACGAAAACACCAAAAAGGCCTACCAGGTTGTGGTAGTTGGCGAGCTTGGCCATGGTGGTGCT	24:G>A
1861486|F|0-11:C>T-11:C>T	TGCAGATCCAGCTCAACAGGTACAGGTGCAGTGGTCTCGTGGTCGGCATATGCTCCCACCATCAGGCCG	11:C>T
1864417|F|0-8:C>G-8:C>G	TGCAGCGTCGGCTTGCAGTTCTCCCTCTTGCGCATTTCGTCGAGCAGCTGGAGGGCGTCCAGAAACCTG	8:C>G
3025064|F|0-12:C>T-12:C>T	TGCAGCACCAGGCGCTACTCTCGGTCACCACATTCCCG	12:C>T
1309033|F|0-35:C>T-35:C>T	TGCAGCTCGCAGCGCAGAAGGCAGCCCTTCTCCAGCAGCGACCTCCCCG	35:C>T
1020945|F|0-5:T>C-5:T>C	TGCAGTGCACGCTCCAGCAATCCGAACCCACCCAACCACCGCACGCTCCGCGGATCCTCCTCGTGCTCC	5:T>C
1127579|F|0-35:C>G-35:C>G	TGCAGCGAGGCGCTGTTCAAGTTCGCGGTGTTCGTCCTGGTGCAGGCGCTGGTGTACCTCATCCTGTCC	35:C>G
7351993|F|0-6:G>A-6:G>A	TGCAGGGTGATGCTTTTGCACAATAAACTCACTAACAGCAACCG	6:G>A
6044953|F|0-50:G>C-50:G>C	TGCAGCTCCTTGACGCTGGCCTCTGTTTCGTAGCCGCCGATGCTGGGCTCGTTGGCAACATCGCGATGC	50:G>C
991599|F|0-16:A>C-16:A>C	TGCAGGCCTTGCAGAGAGTGCCGAGGGAGTTGGCGAGGGAGGGCGCCTCCTCCGCTCCG	16:A>C
4909658|F|0-17:C>T-17:C>T	TGCAGCGCCTGCTTTATCTCACAGAGAACTCTTGCCG	17:C>T
12768859|F|0-20:G>A-20:G>A	TGCAGCTGCCGCATGTCGCCGACAGATGCTGCTGGGGCCGGCTTCAGCTCCGTCCTCCCGTCGATCATC	20:G>A
23919706|F|0-34:T>C-34:T>C	TGCAGCGGTTGGCCGCCGCCTTCAGGCCGCGAATTGACGCGGCGCTGAAGTGGATGACTCGCTCACGGA	34:T>C
5358138|F|0-13:G>A-13:G>A	TGCAGGAGAGGCGGCGGGCGGCGCCG	13:G>A
1120263|F|0-41:C>G-41:C>G	TGCAGGTATCCGAGCGACGTGGACAAGCACATCCTGGCTCGCCAGACGGGCCTCTCACGAAGCCAGGTA	41:C>G
1018240|F|0-42:A>G-42:A>G	TGCAGAGTATAGCCGCGTGTGGTGTGGGTTTTATAGGACGGCAGGAGGTGGGGAAATGTGGCGGCACGC	42:A>G
3959730|F|0-8:C>T-8:C>T	TGCAGGTGCTGGGCGGCGGAGGCGCGGGGGCGGTGAAGCGGAACGAGGGGCGGGGGAAGCTGCTCCCCA	8:C>T
7165352|F|0-28:G>A-28:G>A	TGCAGTGCGTTCCCTGGGAGTTCGCGGCGCGGCTCGCGGCCG	28:G>A
7866346|F|0-26:C>A-26:C>A	TGCAGAACAACAACATCACGGGGCGGCTGCCG	26:C>A
1088597|F|0-23:A>G-23:A>G	TGCAGTCCTCCCTCTGCCCCACCAGCATCCTCGCCGACGGCTCCACATTGTACACCGCCG	23:A>G
5361439|F|0-44:C>T-44:C>T	TGCAGGCCCCACTCGGCGAGGCTGACCTGCTCGCGCGGCAGCTGCGGGTCGATGGGCGCCCGCGCGCAC	44:C>T
1301249|F|0-36:C>G-36:C>G	TGCAGGTTACTGGAGAAGGTCTGGCCGAACCTCCAGCTGGGCGGCACCACGTTGGAGAAGACGAGCGTC	36:C>G
1167398|F|0-23:G>T-23:G>T	TGCAGCCGCTGTTCAAGCGGTCGGTGACGTTCGTGACGCGCGACGACTCGGACTTCGCGGACCAGGTGG	23:G>T
2252532|F|0-44:C>T-44:C>T	TGCAGCATGCTCAGCTTCCACCTCCACTCGTCGATGTTCGCCACCGACGACGACGGCTGCGGTTAGCAT	44:C>T
1112493|F|0-53:G>A-53:G>A	TGCAGGAGCGCCGTGGTGGGGAGCACGAGGGAGACGTTGTTCACGGCCGCCGCGAACTGCGTGCCGTTG	53:G>A
5580259|F|0-5:C>T-5:C>T	TGCAGCGCCACCGTCTCGATTCCG	5:C>T
5580259|F|0-13:T>C-13:T>C	TGCAGCGCCACCGTCTCGATTCCG	13:T>C
5363289|F|0-26:A>G-26:A>G	TGCAGCAGCGGGCACGGCGGGTAGTGATTCACCCG	26:A>G
1024689|F|0-5:A>G-5:A>G	TGCAGAGCAATGGAGAGGTCCTTGTAGGTGTTGTACATCTTGAGATCCACCTTGCGCAGGTAGGGCGCG	5:A>G
999128|F|0-66:C>A-66:C>A	TGCAGTACCTCCGCCGCATGGCCCTGGACCGCCCGCTGCCCGCCGCCGTCATCCCCGTCGTCCACGCCG	66:C>A
980765|F|0-65:C>G-65:C>G	TGCAGGAAGGAGCGCGCGATGGCGTAGAGCATCTCCGCCTCCACGCCCAGCCGCTGCGTGAAGCTCAGC	65:C>G
1027554|F|0-32:C>T-32:C>T	TGCAGCACGGCCGCCAGGTGCGCCGACGTGCCCCCGATCACCCAGAACTGCTCGTTGCGCCACCACTCC	32:C>T
3940561|F|0-32:G>C-32:G>C	TGCAGCGAGGCGAGCCAGCGGCGCGCGCCCAGGGCCTGGCCG	32:G>C
3025428|F|0-35:A>G-35:A>G	TGCAGGTTGTAGGCAAGGAGCTTCTGGCCGAGCCCAATGCCCCG	35:A>G
3956879|F|0-34:T>C-34:T>C	TGCAGCCCGCGCCGCACCTCCCCGACATGGCCGATGCCGCGCCG	34:T>C
3385510|F|0-29:G>T-29:G>T	TGCAGCTCTCGCGCTGCGCCTCGCTCGCCGCCCTCAACCTCAGCTCCG	29:G>T
3938168|F|0-16:C>G-16:C>G	TGCAGGCGGCGGCGGTCGCGTTCGGGGAGAAGCTCCCGATTCCG	16:C>G
1117458|F|0-5:G>C-5:G>C	TGCAGGTGTCCACGTCGCTCCGGGAAGCGGACTTCTCGTACAACAACTTCTCCG	5:G>C
3955944|F|0-59:G>C-59:G>C	TGCAGCGGCGGCATGTGCGGGCACTACACGCAGATCGTGTGGCGCGGCAGCACCCGCGTGGGGTGCGCC	59:G>C
4019082|F|0-16:C>G-16:C>G	TGCAGCCGACCCTGGCCACCGAGTACGCCAGCCAGGCCG	16:C>G
997568|F|0-57:G>A-57:G>A	TGCAGCGGGCGGAGCCTGTCCAGGCGCCAGATGGAGCCCCACGACTCCCTCATCGGCGTCCAGTACCCC	57:G>A
3959201|F|0-20:G>T-20:G>T	TGCAGCGCCAGCTCCACGTCGATGCCGCCGCTGTCGCCGCCGCGGCCCG	20:G>T
3027872|F|0-26:G>C-26:G>C	TGCAGCTCCACGCCGTCCACGCCCAGGAGCTTCACGGCGCGCATCCCG	26:G>C
7168169|F|0-11:G>A-11:G>A	TGCAGGGCGTCGGCGTCGGGGAAGTAGATGCGCATGCCG	11:G>A
1045626|F|0-67:C>T-67:C>T	TGCAGTAGCATTAGCTGTTCTTCAGGTTCCCCTCAAAAGCCTCCTCAAAAACACATAAAAAGGATTCCG	67:C>T
998838|F|0-67:G>A-67:G>A	TGCAGACGTAGGCGTCGCCCGCGAGCCCGTCCTTCCAGGCGGCGCAGTAGAGCTGCTCCGCGAAGGCGA	67:G>A
1089511|F|0-29:C>T-29:C>T	TGCAGGTTGTCCTGGAGCAGCAGCCTTGCCGTCTTCGTGAAGACGGTGGCGCCGCCG	29:C>T
3029521|F|0-29:G>A-29:G>A	TGCAGCACCATGGGGAGCGAGCCGTGGTGGTTCGCGGCGTTGCCG	29:G>A
1210668|F|0-20:A>G-20:A>G	TGCAGCACCACGCAAGAACGAAACGCCAGCGTCGGCTACTCGCCCTCCCGTCACCTGAAGAACTACAAC	20:A>G
3021065|F|0-5:C>G-5:C>G	TGCAGCTTCAGGGTGACCTCCTGCTTCACCTTGCCTCCG	5:C>G
7343582|F|0-7:C>T-7:C>T	TGCAGAGCGCGGCGTGGCCGGCGCGGCAGAGGCAGCACACCACGTGCCCG	7:C>T
2250320|F|0-19:C>T-19:C>T	TGCAGACGGAGGCGCAGAGCTTCGACGTGCCCAGCGCCGACATGATCTACAGCCTCGCCTACCTGCCGC	19:C>T
5358058|F|0-19:G>C-19:G>C	TGCAGCTGGTGGCGCAGCGGGCGATCCG	19:G>C
1058145|F|0-38:G>A-38:G>A	TGCAGACGGTACCGAGCGGCCCGACAGAAGAAAGAAAAGACATGGCGTCAGTGGCGATGAACTCTCAGC	38:G>A
5580300|F|0-8:G>A-8:G>A	TGCAGCGTGTCGTTTGAGGAGGCGGCCGCCG	8:G>A
5567231|F|0-20:T>G-20:T>G	TGCAGCGGATGAACGGCAGCTACGGGAGAGGTTTCCAAGGCGGCCG	20:T>G

32026299|F|0-33:G>A-33:G>A	TGCAGATTTTCTCCAGCTCAGAGGGCCCCAACAGTCCACGAACTCTCTGAAGGCCTTGTCACCAACGGC	33:G>A
2252834|F|0-45:T>A-45:T>A	TGCAGCGAGTAGTAGTCGTGTGGCCAGCAGTCATCGAGCTTGACGTGCGGCCTCTCGCCGTCGGCCCAC	45:T>A
6044613|F|0-14:A>G-14:A>G	TGCAGCGCCGACACAAACGCGGAGGACGGCGGCGAGGGCGAGTAGAGGTGCGGGGTGAAGGAGATGGCC	14:A>G
2277878|F|0-65:G>A-65:G>A	TGCAGCGACTCGTCAGGCAGCTTCCCGCTCTCCGTGAACATGCTGCCGATCTGGCATTGCTGGATGATC	65:G>A
3385596|F|0-47:G>A-47:G>A	TGCAGGGGGCACTGCTGCCGCCCGTCCAGAGCGCAGGAGCCCGCGCGGCCG	47:G>A
1165027|F|0-17:G>C-17:G>C	TGCAGCAGACTGTACCCGACGGCAGCGCCGTTGGCGGCCACCAGGAGCACCAGCGCCTTCATGTCGGTG	17:G>C
7171260|F|0-38:T>C-38:T>C	TGCAGGCCCCGCCCAGACCGCTGCATCACGCCAAAACTTTAGCCCTAGCGGCCTCCTCTGCCGCCGCCA	38:T>C
1130145|F|0-15:G>A-15:G>A	TGCAGACTCTCCCAAGATTCCTCTCCAACATGTTCACGGAGCCGGGACGGCGGATCCG	15:G>A
2306579|F|0-31:A>G-31:A>G	TGCAGACGTCGGCGTGCTGCAAGCACGCCACAGCCTACGACCTGGAGGACTGGGGCGGCGTGGCCCGCT	31:A>G
12778480|F|0-36:G>T-36:G>T	TGCAGGTCCCCTAACCAGAGTGTCCTGACCTCGTTGGGCCCAGCGGGCGCCTGCGTCGGGGCAGCCGAG	36:G>T
2251466|F|0-38:C>A-38:C>A	TGCAGGGCGGAGGACTACTTCAAGCGTGCGGTGGCCATCGAGCCGCCCGACGGCGAGGCGATGCGGAGG	38:C>A
5971693|F|0-11:G>C-11:G>C	TGCAGCGGGATGAAGCTCAGGAACATCAGGCTGAGGTACCG	11:G>C
1236203|F|0-8:T>C-8:T>C	TGCAGTTCTTGATCAGCTGGATGTCGCGGTTCCCGCAGTACCGGCACCGGGATTTCTACATCGCCGAGA	8:T>C
4261294|F|0-6:C>G-6:C>G	TGCAGCCGAGAGGTTAATTTGCTGCGCTGCATGTGCCG	6:C>G
7351018|F|0-11:C>T-11:C>T	TGCAGTTTAACCAATAACTGCTCCG	11:C>T
1227022|F|0-35:A>C-35:A>C	TGCAGCGCACCACCATGTACGGCTCCCGCACCATCATGTTCCTCACCGACGACGGCACGCTGCGCCTCC	35:A>C
5368583|F|0-40:C>T-40:C>T	TGCAGGGAGAATCAAAGCCAAGATAGATGTTAGTGCCATACGTTCATGACAAATTAATCGATCTAGCTA	40:C>T
993076|F|0-31:C>G-31:C>G	TGCAGCACAACTACTTCACCAACCCGTGGGCCATCGTCTCCGTCGTCGCCG	31:C>G
4397866|F|0-22:G>C-22:G>C	TGCAGTTCAACCTGACCTCCGTGGGCGTCCCG	22:G>C
3024125|F|0-37:T>C-37:T>C	TGCAGTTCCCATCCGACATCGCCACCGTTCTTCTCGCTGCCG	37:T>C
12683541|F|0-7:G>T-7:G>T	TGCAGCCGCTGGGGGACACCCCG	7:G>T
3948012|F|0-10:G>C-10:G>C	TGCAGGCCGCGCCCGACGGGTACAGGGTGGGCAGCTCCG	10:G>C
4022563|F|0-16:G>A-16:G>A	TGCAGCGCCTCACCGCGCGCTCCAGCCGCTACCTCTGGATCCCCG	16:G>A
5357352|F|0-13:G>C-13:G>C	TGCAGAGCGCGCGGCGCGCGCCCG	13:G>C
5580158|F|0-5:G>C-5:G>C	TGCAGGACGCGCTTCTCGAACTCCTCGTCGGCGGCGGGGGCGGTGGCGACCG	5:G>C
1048569|F|0-11:A>C-11:A>C	TGCAGACGCACAAGCAACAGCATCCCCCTCACTCCACAGCAAGTAGACGGCGCGGGGACGCTCCAAAAG	11:A>C
3948768|F|0-5:C>T-5:C>T	TGCAGCGCGGACCAAAAGGATTGCATCCCG	5:C>T
1207249|F|0-7:C>T-7:C>T	TGCAGGGCTGCGCGCTGCACGCGGTGGCGACGAGCTACGGCGCGATCGCGGCGTCGCAGCCG	7:C>T
1207329|F|0-26:A>C-26:A>C	TGCAGCTGGGGCGCCGTCTTCTTCCCAAAGCACTGGCGCGAGTTCTACGCCTACATGGCGGCACGCTTC	26:A>C
1090123|F|0-29:G>C-29:G>C	TGCAGGCAGTCGGCGGCGTGCTCCTTGGAGCCCAGCACGGTGCCGTAGTCCAGCAGGTTGACCATGACG	29:G>C
1026254|F|0-17:C>T-17:C>T	TGCAGGCTGCCGAGCACCGACATCCCCCGCATGCTCACCATCCCGAGGCAGGCCACGCCG	17:C>T
2252334|F|0-37:C>T-37:C>T	TGCAGCACCGCAACCTGGTGAGGATCCTGGGCTGCTGCATCCATGGCCACGAGAAGATGCTGGTGTACG	37:C>T
5360825|F|0-59:G>C-59:G>C	TGCAGCAGCTCGACGGCGAGCACGTCGGACGGCGCGGTGGCCATGGAGATGACGTAGGCGCCGAAGCTG	59:G>C
4006284|F|0-64:C>G-64:C>G	TGCAGTGCTGGTGCCTCAACTTCCGCGGCCGCGTGACCATCGCCTCGGTGAAGAACTTCCAGCTCACCG	64:C>G
7921933|F|0-44:G>C-44:G>C	TGCAGCGCCAGCGCGAAGTAGTCGAACTCGCGCTGCTCCTTCCCGACGGCGGCCG	44:G>C
5359215|F|0-7:C>T-7:C>T	TGCAGGACGCGCTGAACAAGCTACTCCG	7:C>T
1049482|F|0-5:G>C-5:G>C	TGCAGGACGACCTGCACCACGGAGCCGAACCTGAGCTTGTACAGCTTGGTCGCCG	5:G>C
996638|F|0-8:C>G-8:C>G	TGCAGCGTCGCCTCCGCCTCGTCCTCCGTGAAGTCGGCCCCGCCGATCAGCTGCTCCAGCACCTGGCGC	8:C>G
1035374|F|0-17:G>C-17:G>C	TGCAGGGGCAACCAGGTGTACATCCGCGGGGAGGACGTCTGCGTGGCCAACAGCATGTCCAGGTTCGGC	17:G>C
10992853|F|0-8:G>C-8:G>C	TGCAGGAGGGCGTGATCCCG	8:G>C
2252334|F|0-21:G>A-21:G>A	TGCAGCACCGCAACCTGGTGAGGATCCTGGGCTGCTGCATCCATGGCCACGAGAAGATGCTGGTGTACG	21:G>A
5366831|F|0-11:T>C-11:T>C	TGCAGAGTCTGTGACTGTTCTGCGGCGCCGCCG	11:T>C
1694905|F|0-34:C>T-34:C>T	TGCAGTTCACCGACAAGCGCTTCCAGCCCGTCCACGACCTCACCATCGGCGTCGAGTTCGGCGCCCGAG	34:C>T
989876|F|0-32:G>C-32:G>C	TGCAGGAGGGACTCGTCGGCGAGGAGGAAGAAGCCGATCTGCTTGGGGTCCGCCCGTGCGAAGGAGGAG	32:G>C
1094226|F|0-17:C>G-17:C>G	TGCAGGCCGTCGGCGCACGTCTGCTGGTTGGTCAGGATGGCCGACAGCAGCGTCTGCACGTCCTCGGCC	17:C>G
2266496|F|0-35:C>A-35:C>A	TGCAGCGCCGAGGTCGACCGCCTCACCGACGCCGTCGACGCGCTCGAGCGCCTCGCCG	35:C>A
5580416|F|0-8:A>G-8:A>G	TGCAGGCCATCGGGGGCAACCTGCCG	8:A>G
1088783|F|0-39:G>A-39:G>A	TGCAGAGGATCAGGGACTGCGTCCAGAGGTGGTACCACGGTGAGGGGTTCGTGTGCGCTCAGGTGATCA	39:G>A
3959798|F|0-22:C>G-22:C>G	TGCAGAAGAAGCACGTCCGCGGCGCTGGCGGTCCG	22:C>G
999128|F|0-25:G>C-25:G>C	TGCAGTACCTCCGCCGCATGGCCCTGGACCGCCCGCTGCCCGCCGCCGTCATCCCCGTCGTCCACGCCG	25:G>C
5361672|F|0-21:G>C-21:G>C	TGCAGGTTGATCTCCTGCACGGTCTCCACCTCCAGGTCGCTGAGGGGCAGCTTGACGATGCCCAGCCTC	21:G>C
1022191|F|0-26:A>G-26:A>G	TGCAGGAACAAGGTGGTGGCCTGCGCAAAGCACTTCGTCGGCGACGGCGGCACGGTGAACGGCATCAAC	26:A>G
4995728|F|0-10:A>G-10:A>G	TGCAGAACGTAGGGCTGATGGCGCCGCTGCCG	10:A>G
3026611|F|0-32:C>A-32:C>A	TGCAGCGCGCGCTCCACCTGCGCCATGTCCACCTCCTCCCCG	32:C>A
1007625|F|0-19:C>G-19:C>G	TGCAGAGCCTCGTGTCCCGCGAGGCGGACCCGCTGGACTCACAGGTGGTGTCGGTGGCCATGGTCAACG	19:C>G
996709|F|0-43:C>G-43:C>G	TGCAGCCCGCGAACCGCGCGATGCGCGCCATGTACGCGCGCACCGAGATGCCG	43:C>G
991147|F|0-44:C>A-44:C>A	TGCAGCGGCGCCGCCACGGACGACACGCCGCTGGACGACAACCCCAACTTCAACCCG	44:C>A
32019606|F|0-8:A>G-8:A>G	TGCAGCTCAAGGACCTGGTTCTGGAGCTTCTTGATGTGATCGATGGCGTCCGCCAGGGTGGACTCCTTG	8:A>G
13881146|F|0-6:G>T-6:G>T	TGCAGCGTACCCATCTCGCCGATGGTCGCCG	6:G>T

<table>
<tr><td>6045206|F|0-33:C>A-33:C>A</td><td>TGCAGTTTTGCTAGACTTAATATGTTGCTCGAACGTTGCGGTTAACCGTAAGTAGAAGTCAAGGCAGCT</td><td>33:C>A</td></tr>
<tr><td>1024090|F|0-13:C>T-13:C>T</td><td>TGCAGCAGTTCTACTGCATGACCCTGAGGATGAGCATAGACTGCAACGGGTGCTACCAGAAGATCAGGA</td><td>13:C>T</td></tr>
<tr><td>15306747|F|0-28:T>G-28:T>G</td><td>TGCAGGGGCTGGGGAGGTACTGGGGCGTTGGCGGGCGGAGGTGCGGGTCATGCCG</td><td>28:T>G</td></tr>
<tr><td>7167412|F|0-20:C>G-20:C>G</td><td>TGCAGAAGGCCCCATACGGCCAGCCG</td><td>20:C>G</td></tr>
<tr><td>6046367|F|0-40:G>C-40:G>C</td><td>TGCAGGCCATGGACTCCTCCCACGTCGCCCTCGTCGCCCTGCTCCTCCGCTCCGAGGGCTTCGAGCACT</td><td>40:G>C</td></tr>
<tr><td>4982491|F|0-61:A>G-61:A>G</td><td>TGCAGATGTCGTAGATGGCCTCGTTGTCGAGCAGGATGGAGACATCGGTGTGCTCCAGCAGAGAGTGGG</td><td>61:A>G</td></tr>
<tr><td>7172425|F|0-42:T>A-42:T>A</td><td>TGCAGGGCAATGGCGAGCAGGCCACGGACGCGCGCTACCACGTCGCCGCGTCGGCGGCGCCG</td><td>42:T>A</td></tr>
<tr><td>4262986|F|0-19:A>G-19:A>G</td><td>TGCAGGAGGGCTTCTCGGCAGCGTCCTCGATCACGCCG</td><td>19:A>G</td></tr>
<tr><td>32023742|F|0-19:C>G-19:C>G</td><td>TGCAGCTTCCTCCGTCCAGCCGTCCACCCGATGCCGCTCCTCGCCACCCCACCCTATATAACACCGCGC</td><td>19:C>G</td></tr>
<tr><td>5361299|F|0-50:A>G-50:A>G</td><td>TGCAGCTTGTCGTCGGAATCGTGTTGGCATGTGTGGCAGCGGCATTGATCACATTGGCGCTGCTGGCCT</td><td>50:A>G</td></tr>
<tr><td>5365864|F|0-43:T>C-43:T>C</td><td>TGCAGGTCCGCGGCATGTCGCAGGACCTCAGCTGGGACCACGCTGCCGAGCTCTACGAGAACGTCCTCG</td><td>43:T>C</td></tr>
<tr><td>5359410|F|0-14:T>C-14:T>C</td><td>TGCAGGTTCCAGAGTACGTCGTCGACGGAAGGCCG</td><td>14:T>C</td></tr>
<tr><td>5360769|F|0-48:A>G-48:A>G</td><td>TGCAGCACCTGGTGGAGGTGTCGGTGGCCAAGCGTGGGCTGGCGGCGAAGACGTGGTGGAACGAGCAGA</td><td>48:A>G</td></tr>
<tr><td>5363701|F|0-41:C>G-41:C>G</td><td>TGCAGGAACTTGGTGATGGGCTGGATCACCGCGTTGGCGAACAGCCCCG</td><td>41:C>G</td></tr>
<tr><td>5001448|F|0-23:G>T-23:G>T</td><td>TGCAGCTGCCGTCGGTGATTGCAGTCCACGATGGAGCGGTGACGACAACGGCGGCCACACACCAGGTGT</td><td>23:G>T</td></tr>
<tr><td>2253158|F|0-56:C>G-56:C>G</td><td>TGCAGCAGCAGCCTGTCCTCCGCCGACGACTCGAACCCGTGCAGGAACGACATGTCCGCGCCGCCGCCC</td><td>56:C>G</td></tr>
<tr><td>1865036|F|0-48:G>C-48:G>C</td><td>TGCAGGTGCACCATCTTGGCGTACACGCCGCCTTCGCCGCCGTCCATGGCGACGAGCTCGTCGTGCGTG</td><td>48:G>C</td></tr>
<tr><td>1017027|F|0-31:T>C-31:T>C</td><td>TGCAGCTCGCGGCGGAACATGAAGTGGGAGATGAAGCCGCCGAGGCTGATGAAGACGGGGATTAGGGAG</td><td>31:T>C</td></tr>
<tr><td>3028633|F|0-5:G>C-5:G>C</td><td>TGCAGGGCGTCGGCGTCCGGGAAGTAGATGCGCATGCCG</td><td>5:G>C</td></tr>
<tr><td>1862419|F|0-37:A>C-37:A>C</td><td>TGCAGCTCGATCTTGGCAAATCTCTCAAATGGATGTCAAAAATGCTTTCCTTCATGGTGATCTACATGA</td><td>37:A>C</td></tr>
<tr><td>998838|F|0-51:G>C-51:G>C</td><td>TGCAGACGTAGGCGTCGCCCGCGAGCCCGTCCTTCCAGGCGGCGCAGTAGAGCTGCTCCGCGAAGGCGA</td><td>51:G>C</td></tr>
<tr><td>32025307|F|0-14:C>A-14:C>A</td><td>TGCAGAGACTGTAGCTGTGCCTTGCCGCTGAGGCCGCGCTGCCG</td><td>14:C>A</td></tr>
<tr><td>1010317|F|0-22:C>G-22:C>G</td><td>TGCAGACGGGGCAGGAGCTGTTCGCCTCCAGCCACGGCAGCAGGCACACGTCGTGGAAGCGGTGCTTGC</td><td>22:C>G</td></tr>
<tr><td>1695224|F|0-68:A>G-68:A>G</td><td>TGCAGCCACGCGGGGCGGTTTGACCCTCCGCCGCGGCCGTCGCCCATCGCCGCCGACGGCAGTCGACCA</td><td>68:A>G</td></tr>
<tr><td>15321646|F|0-8:G>C-8:G>C</td><td>TGCAGCTCGGAGAAGTGCAGGATGCAGACGTACCCG</td><td>8:G>C</td></tr>
<tr><td>23919539|F|0-18:C>A-18:C>A</td><td>TGCAGAAACACGCCAATCCACCAGTCGATCAGCGCCAGGAACCTCCACGACAACGAGATCTGGGGAGGG</td><td>18:C>A</td></tr>
<tr><td>2252532|F|0-32:G>T-32:G>T</td><td>TGCAGCATGCTCAGCTTCCACCTCCACTCGTCGATGTTCGCCACCGACGACGACGGCTGCGGTTAGCAT</td><td>32:G>T</td></tr>
<tr><td>1201821|F|0-35:T>C-35:T>C</td><td>TGCAGGAACGTGTTCCAGTCCCTGCGCTTCTGCGCTTCATACCTGCTCAGCTGAGCAGGCGGCTTCGGC</td><td>35:T>C</td></tr>
<tr><td>5324801|F|0-46:T>C-46:T>C</td><td>TGCAGGTGCTGCCGCGCGCGCTTGTGGCAGGCTTCGTCAAGACAGATATTGACTTCCAGCGCAAGGGTG</td><td>46:T>C</td></tr>
<tr><td>5582489|F|0-54:A>T-54:A>T</td><td>TGCAGGAGTCGTAGCCGGCCTTGCTGACCTCGACCACGTCGTGCGCCGTGGGCGAGTACTTGAAGACGA</td><td>54:A>T</td></tr>
<tr><td>3934442|F|0-24:A>T-24:A>T</td><td>TGCAGCGACCGCCCGCTCTCCTTCACCGCGCCGTCGGGCCAGACCGCCTGGAGCTACTACAAGATCATG</td><td>24:A>T</td></tr>
<tr><td>32027790|F|0-44:G>A-44:G>A</td><td>TGCAGGTACTTCCTCGTCGTCTCGTCGTTCATCACGTGGGCGACGCTGTTGGTGTGGATCTTCTCCCGA</td><td>44:G>A</td></tr>
<tr><td>2252453|F|0-40:G>C-40:G>C</td><td>TGCAGTTGACGACGAGGATGTCGATGTCCTTGGGCTTGAGGCCCGTGCGGCGGACGAGGTCGTCGATGG</td><td>40:G>C</td></tr>
<tr><td>5361439|F|0-14:G>A-14:G>A</td><td>TGCAGGCCCCACTCGGCGAGGCTGACCTGCTCGCGCGGCAGCTGCGGGTCGATGGGCGCCCGCGCGCAC</td><td>14:G>A</td></tr>
<tr><td>1035374|F|0-62:G>C-62:G>C</td><td>TGCAGGGGCAACCAGGTGTACATCCGAGGGGAGGACGTCTGCGTGGCCAACAGCATGTCCAGGTTCGGC</td><td>62:G>C</td></tr>
<tr><td>1865036|F|0-35:G>C-35:G>C</td><td>TGCAGGTGCACCATCTTGGCGTACACGCCGCCTTCGCCGCCGTCCATGGCGACGAGCTCGTCGTGCGTG</td><td>35:G>C</td></tr>
<tr><td>1027589|F|0-17:G>C-17:G>C</td><td>TGCAGAGCCCACGCCGCGGCGAGCCACAGCGCCGCCAGAGGAGAGCGCCG</td><td>17:G>C</td></tr>
<tr><td>12768972|F|0-11:A>G-11:A>G</td><td>TGCAGGCCAAGATAATCAAGTCGGTCGACGTGTCGTACGGCGGCGAGAGCGGCTTCAACCAGGCCATTG</td><td>11:A>G</td></tr>
<tr><td>6045447|F|0-14:G>C-14:G>C</td><td>TGCAGCAGGATGGTGAGAGCCGACGGGTCGCAGTGCGGCCCTGTCCCCAGCGTCCG</td><td>14:G>C</td></tr>
<tr><td>1011588|F|0-5:A>C-5:A>C</td><td>TGCAGAGGGCCGCCGAGTCCGCCATCCGCTCCATCGGCCTCGGCTACGACGTCGCCGCCGACGTCCGAG</td><td>5:A>C</td></tr>
<tr><td>1111533|F|0-44:C>G-44:C>G</td><td>TGCAGCAGCGCCGTCGTCGGGAGCACGAAGGAGACGTTGTTCACCGCCGCCGCGAACCG</td><td>44:C>G</td></tr>
<tr><td>2244267|F|0-25:T>C-25:T>C</td><td>TGCAGCTCCTCCACCTCGTCGAGCGTTTCCCGTCCGAGCCGTGCGCCGAAGCACATGTACACGAGCAGC</td><td>25:T>C</td></tr>
<tr><td>1030791|F|0-11:G>A-11:G>A</td><td>TGCAGCTTGCGGAGGTCCTCGGTGGGGAAGTTGTCGTCGCCCATGTAGTTGGACAGCCATTCCAGCTCC</td><td>11:G>A</td></tr>
<tr><td>1024090|F|0-49:G>A-49:G>A</td><td>TGCAGCAGTTCTACTGCATGACCCTGCGGATGAGCATAGACTGCAACGGGTGCTACCAGAAGATCAGGA</td><td>49:G>A</td></tr>
<tr><td>3934298|F|0-23:C>G-23:C>G</td><td>TGCAGGCGCGGGAGGTCGCAGAGCGAGGCCGGGAAGGCGCCG</td><td>23:C>G</td></tr>
<tr><td>1166279|F|0-27:A>T-27:A>T</td><td>TGCAGTTGGAGATGAGCACCAGCCTCAAGACGCCCTCCCTCTCCGCCAGCAGCCCCGTCATCACGGCGT</td><td>27:A>T</td></tr>
<tr><td>5971669|F|0-5:C>G-5:C>G</td><td>TGCAGCCACAACAGGACGCCGCCG</td><td>5:C>G</td></tr>
<tr><td>1151056|F|0-19:G>A-19:G>A</td><td>TGCAGAATTCGTCGTTGCGGCCGAGGCAGGAGAGGATCCCCGAGTCCCAGCTTTCACGCGGCGCGGCGC</td><td>19:G>A</td></tr>
<tr><td>2280707|F|0-20:G>C-20:G>C</td><td>TGCAGCCTGGGCGCGTGGCAGGTGGCCTCGATGGAGTAGGCGCCGTCGAAGGAGGCGTCGTCGAAGGGC</td><td>20:G>C</td></tr>
<tr><td>2260271|F|0-19:C>T-19:C>T</td><td>TGCAGGCCCAGGTCCTCGACGCGAACCTCGGCACCATTGTCGAAGACTGCATGCGGCACCACGACGAGC</td><td>19:C>T</td></tr>
<tr><td>1102684|F|0-11:G>A-11:G>A</td><td>TGCAGCAGGTCGTCGCCCTTGGGTTCAACCTTCTGGCGCATCTCCACCACCTTCTTGTGGGAGTTGGAA</td><td>11:G>A</td></tr>
<tr><td>1069344|F|0-24:G>C-24:G>C</td><td>TGCAGTTTCTGGGGTGCACGCTCGGCTGGAGAAGAAGGACGGGAACCTGGTGGTCACGGACATGGACAG</td><td>24:G>C</td></tr>
<tr><td>6046309|F|0-68:T>C-68:T>C</td><td>TGCAGCTGCCGCATGTCGCCGACAGATGCTGCTGGTGCCGGCTTCAGCTCCGTCCTCCCGTCGATCATT</td><td>68:T>C</td></tr>
<tr><td>6014212|F|0-13:G>C-13:G>C</td><td>TGCAGCAGCGCCCGTGGCCAAGAGGCAGCAGGACGGCGGGTCTGCCGCCG</td><td>13:G>C</td></tr>
<tr><td>1090882|F|0-5:G>C-5:G>C</td><td>TGCAGGAGGTCATCGCCCTTGGGTTCAACCTTCTGGCGCATCTCCACCACCTTCTTGTGGGAGTTGGAA</td><td>5:G>C</td></tr>
<tr><td>997377|F|0-8:G>C-8:G>C</td><td>TGCAGCCCGACCATGGTGAACACGTCCGCCGCGCCGAACAGCACGTACTGCGGCACCATCCACCACAGG</td><td>8:G>C</td></tr>
<tr><td>1164426|F|0-50:G>A-50:G>A</td><td>TGCAGCTGCCCGCTGAGGCCAGTCGCCCACGGCCTGACGGTGACCACCTTGCTGGACCG</td><td>50:G>A</td></tr>
<tr><td>5358183|F|0-20:A>G-20:A>G</td><td>TGCAGGATGCGGCGGGACGGACTGCCG</td><td>20:A>G</td></tr>
<tr><td>992903|F|0-23:C>T-23:C>T</td><td>TGCAGCTGGAAGTTGCGGAACTTCTCGGGGCAGGAGAAGGCGGAGGTGGAGGGCGCGGCGGGGCGCGGG</td><td>23:C>T</td></tr>
</table>

2246640|F|0-65:C>T-65:C>T	TGCAGCATCCTCCTGAGCAGCAAGATCCCTTCCTCGGTCCTCCCCTCCTCGAAGATCCACAGCGCCAGC	65:C>T
1204117|F|0-40:T>C-40:T>C	TGCAGCTCGGTGGCCTCTTCATATGGACGTACACCTACAGTCTGATGCAGAAGTCTGGCAAGCTGTACC	40:T>C
1003723|F|0-20:C>G-20:C>G	TGCAGCGACGGCGCGTAGAACGGCACGTTGTAGGCGTCCACCTTGTCCTGCTCCACCAGCCCCTGCGAC	20:C>G
1094245|F|0-35:T>C-35:T>C	TGCAGAGGATCAGGGACTGCGTCCAGAGGTGGTACTACGACGAGGGGTTCGTGTGCGCTCAGGTGATCA	35:T>C
4910567|F|0-10:A>C-10:A>C	TGCAGCAGCGAGGCGAGAGCTTGTCCTCCACGCCG	10:A>C
1103078|F|0-8:A>G-8:A>G	TGCAGAGCACCGAGGAGAAGTGCAGCACTTGGGAGAAGCGCTCGCGGTTCTTCATGGACGAGTATATCG	8:A>G
1090394|F|0-16:T>G-16:T>G	TGCAGGAGAGCATCTCTACGGTGCAGTCGTCGCGGGTCGGGGTGACGCGGAGGTCCAGGACCG	16:T>G
3023698|F|0-8:G>T-8:G>T	TGCAGATGGTTCGAGAACATGATCTAATGTTTTCCCG	8:G>T
5364598|F|0-16:C>T-16:C>T	TGCAGTTGCTCGGTGGCCGCCG	16:C>T
1318141|F|0-15:C>T-15:C>T	TGCAGAACCCGATGGCCTTCCCGTCGGAGAGCCACCCGATCCACCTCCACGGCTTCAACTTCTTCGTGC	15:C>T
100511221|F|0-11:C>T-11:C>T	TGCAGGGCCCTCCTCCTGGCCG	11:C>T
4397193|F|0-31:G>A-31:G>A	TGCAGCGCCTGGTCGGCAACAGCGGCAACGCGGCCG	31:G>A
1204782|F|0-53:G>A-53:G>A	TGCAGGACAAGATGACCGCCAGGGAGATCGTCACATGGCTCGCCGTGCTCAAGGCGGAGGAGGAGCTCA	53:G>A
1863028|F|0-32:G>T-32:G>T	TGCAGGTGGCGCGGGTGGTCCCAGTCGAACTTGTTCATGGCGAGGTCGCGCCTCACCTGCTCCTCCGTC	32:G>T
1089698|F|0-50:G>A-50:G>A	TGCAGCCAGAATGTGGTTTCATTTCAGACGAGCGCGACGCCTAGCGGGTCGGGAGGCAGCATGCCGAGA	50:G>A
1027501|F|0-41:C>T-41:C>T	TGCAGCGCGGCCTTGTTCATGAGGATGGCGCACATGCACGCCGCCTGCGTGGCCGCCGCCTCCTTGAGC	41:C>T
5582333|F|0-32:C>G-32:C>G	TGCAGCACGGCGTGGCTGCCGATGTAGCCCGCCCCGCCG	32:C>G
999516|F|0-40:G>C-40:G>C	TGCAGCGCAAGGAGTCGGTGGAGTGGGTCAACATGGTGCTGGGCAAGCTCTGGAAGGTGTACCGCCCCG	40:G>C
4397882|F|0-16:G>T-16:G>T	TGCAGGCCTTCAGGGAGACCCACTTCCCG	16:G>T
3029511|F|0-14:G>T-14:G>T	TGCAGCAGCCCGTAGGCGAATATCGCCGGGATCATCCACCG	14:G>T
1092779|F|0-13:G>C-13:G>C	TGCAGCGCCGCCTGTACGAGCAGGGCGGGCTCAAGACGGAGGGCATCTTCCGCGTGGCCGCCGACGGCG	13:G>C
1100449|F|0-25:A>G-25:A>G	TGCAGTACGTGTACTGGTCCAACGGAAGAGAGACCGAGAGCCCCAGCGTGGACAACAAGCAGTGCCCCG	25:A>G
1031984|F|0-7:C>G-7:C>G	TGCAGCGCGTGGCCTACTTCAACGTGGGCATGTACCCGTTCACCTCCATGTTCCTCATCGTCTACTGCG	7:C>G
984247|F|0-17:G>C-17:G>C	TGCAGCATCTCCCACATGACCTGCACGTCCTCGTACGCGCACGTCTGCACGTCGTCGTGGAGCTTCACC	17:G>C
7352269|F|0-11:C>G-11:C>G	TGCAGCATGTCCCGCGCCTCCG	11:C>G
3027662|F|0-28:G>C-28:G>C	TGCAGCGCCGCCCCTTCATGTACGTCGTGCGCGACCTCCG	28:G>C
2247711|F|0-8:C>T-8:C>T	TGCAGCGGCCGCGCTTTGGTCGGCTCAGCATCTGGAATCCTCACAAAGTGTTCAGGCTCCATTCGACCA	8:C>T
1022995|F|0-17:G>A-17:G>A	TGCAGGTTGGACGCGCCGTGCATCGCGAAGTACTCGCGCACCATCTCCCG	17:G>A
1030008|F|0-20:G>A-20:G>A	TGCAGCGTCGTGCGGGCGCAGCGGCGGTGAAACTGGCGATGTGCAGAGGAAGGGGATTTGGGGTGGTGG	20:G>A
5581324|F|0-8:A>G-8:A>G	TGCAGCAGATGGGCCAGGGACACCGCCG	8:A>G
1862771|F|0-32:G>A-32:G>A	TGCAGAACCCTAGGTGCAGCGACCTCGACGGCGTCCCGTCGCTCTCGGCGGCCGCGTACGCGCTCCTGC	32:G>A
2253158|F|0-28:A>T-28:A>T	TGCAGCAGCAGCCTGTCCTCCGCCGACGACTCGAACCCGTGCAGGAACGACATGTCCGCGCCGCCGCCC	28:A>T
1204824|F|0-8:G>T-8:G>T	TGCAGCGCGCCGTGGAGCCTGGCGGAGCCGCGGAGGCTGTTGCACGCCAGCAGGCGCAGCTCGTCCTCC	8:G>T
5364126|F|0-8:G>T-8:G>T	TGCAGTTCGTCCTGATCTCGGCGTGGCGGCCG	8:G>T
12470475|F|0-17:C>G-17:C>G	TGCAGCTGGCGCAGGTCCCCGATCCG	17:C>G
1022602|F|0-66:C>T-66:C>T	TGCAGGACATCATCCACGCCAAGCTCCCCAATGCCGTGCTCGGACAGGTACACATACCTAACCTTTCCT	66:C>T
986473|F|0-10:G>C-10:G>C	TGCAGACGTCGCGGGAGCCCACGCCGCGGCAGTCCAGCTTCAACCAGTCCGACTTCTACTCCATGTTCA	10:G>C
992903|F|0-35:G>T-35:G>T	TGCAGCTGGAAGTTGCGGAACTTCTCGGGGCAGGAGAAGGCGGAGGTGGAGGGCGCGGCGGGGCGCGGG	35:G>T
1020035|F|0-61:C>T-61:C>T	TGCAGAAATCTGTGACCTCGTGCGTCTCAGGGCTCTCCACAAGGTAGCTGTCCACCACATCCTCCTGGG	61:C>T
6045519|F|0-32:A>G-32:A>G	TGCAGCGACGACACCAAGTTCTCCGACGGCACAGGCCACCGCGACACCG	32:A>G
5582001|F|0-10:G>A-10:G>A	TGCAGCTCTCGCACACGAAGTCCCAGTGCGGGCCG	10:G>A
1002604|F|0-35:G>A-35:G>A	TGCAGCGGAGGGTGCTCGTGGGCATCGCCATCGGGGTCGGCGCGGGCGGCGTCGTGGTCGCCG	35:G>A
1080669|F|0-17:A>G-17:A>G	TGCAGCGCCAGGCTTGCAGTCAAACGGCTTGGATTATCTTAGTGCTGTCGCTCCAACACAACGGCGACA	17:A>G
3026430|F|0-16:G>C-16:G>C	TGCAGAAGATATCCGTGAGCACCAACATGCTCGATGGGGAGCTGCCG	16:G>C
1237382|F|0-20:A>G-20:A>G	TGCAGCAGCCACCGCCAGGCACACTCACCCTGCCCATGGCATAACTGATGCACGGGGCCAGGTAGCTCA	20:A>G
2262503|F|0-61:G>A-61:G>A	TGCAGAGGAGGAGGCCCCCGCAGCACTGCTGGATCTCGACGCGCTCGTAGCGCCCTCGTAGGAACGGGA	61:G>A
6050365|F|0-14:G>C-14:G>C	TGCAGGTAGTCGCCGATGTTCACCACCAGCGCCCCCGCCTCCGCCG	14:G>C
1094924|F|0-67:A>C-67:A>C	TGCAGCCGCGTCGAGGAGGGGTTCGGCGACAAGACCGACGGGCTCATGGGGCTCGGCGGCGACGCGCAG	67:A>C
7168368|F|0-10:T>C-10:T>C	TGCAGTGCGGTGGCTGCGACGAAATGCTTGAGGTGACGTTGCCG	10:T>C
2243202|F|0-55:A>G-55:A>G	TGCAGAGGAGGAAGCTGATCATCGAGAAGTACAGCAGCATGGACGAGATACTAAGAGAGTGCAACAGCA	55:A>G
3940936|F|0-17:A>C-17:A>C	TGCAGGCCGTTGTCGTAAGGCTTGTCGCGGTGGCCGATGCCG	17:A>C
1218352|F|0-38:G>T-38:G>T	TGCAGCGCGCTGATGACGGTGCGGAGCGCGCGGCTCGGGGACCCGCGGTTGGCGAGCATGATCTCGCCG	38:G>T
23919991|F|0-7:G>C-7:G>C	TGCAGCAGCGGCTCAAGCCCGCCGCCGTGCTCTGGGGCTGGCCG	7:G>C
4993342|F|0-19:G>C-19:G>C	TGCAGCGGCGGCTCACGCGGCTGTACCCG	19:G>C
5985495|F|0-44:T>C-44:T>C	TGCAGCTCCGCAAAGGCCTCAGAATGAGCTCAAGCACGCAAGACTCTGCGCGACCACGGACAACGACCA	44:T>C
3023805|F|0-7:G>A-7:G>A	TGCAGCTGGAGCATCACGCCGATCATGCTCTTCGACTCCG	7:G>A
2289355|F|0-35:C>T-35:C>T	TGCAGTACTGGCCGTGGAACCGACTCGAATTCGACCCGCAGCCTGTCGATGCTGGCAAACAGATTTTTC	35:C>T
1017794|F|0-13:T>C-13:T>C	TGCAGGTAGGTGATCAGGACCAGCCACGCGTTCACGATCAGCAGCGGCACGCCGTAGACCCGCACCACC	13:T>C
5367545|F|0-10:C>T-10:C>T	TGCAGTGGGACGGCAGCGGGGGCCACGCCG	10:C>T

5579675|F|0-61:C>T-61:C>T	TGCAGGAGGCGGAGCGGCGCATGGACCTGGCCCGCGTCGCCGACCTCAAGTACGGCGCCCTCCAGGAGA	61:C>T
1256514|F|0-19:G>A-19:G>A	TGCAGCTCCCCGCCGACGAGTACATCGTTGGCGCCGCCGACGAGGACGACGACGGCTGCGGCACCGAGA	19:G>A
992877|F|0-11:C>A-11:C>A	TGCAGGAAAGGCGTGAGATGGGATTCCTGGAAGTTGGCGTTGTACACCATGCCGAAGACGAAGCCCAAG	11:C>A
1215238|F|0-14:C>G-14:C>G	TGCAGCGCCTCGCGCGGCACGTTCTCCAGCAGCCACGTCGGCCACCCGTCAACCAGCTGCTCGCGGTCG	14:C>G
3021793|F|0-25:T>C-25:T>C	TGCAGGAGAAGCTGGAGCGCCTGCCTGAGATCGAGCGCGCCTTCGTGCACCTTGATTACGAGTTCACCC	25:T>C
7330975|F|0-7:A>G-7:A>G	TGCAGGCAGGCGAACTGACCG	7:A>G
2245232|F|0-8:C>G-8:C>G	TGCAGCAGCGCGTCGAACTCGTCCTGGTTGAGCTGCTTGTTAATCTCGGCGTTGAACAGCTGCTCCATC	8:C>G
1001208|F|0-43:C>T-43:C>T	TGCAGGGGCCGCAGCTGCCGCCGTTCCTGTCCAAGACGTACGACCTCGTCAGCGAGCCGCAGCTGGACG	43:C>T
1039591|F|0-22:G>A-22:G>A	TGCAGTCCTTCGTAATCCACAGGAAGGATGGAGATCAGAAACTCACAACGCCATGGCCATACATGACGC	22:G>A
3940655|F|0-19:A>G-19:A>G	TGCAGAACCATCCCATGACATCCATGCCGACGCCGACGCCG	19:A>G
3938199|F|0-6:C>G-6:C>G	TGCAGGCCCCGAGCTGCGTCTCCCG	6:C>G
1089698|F|0-36:A>G-36:A>G	TGCAGCCAGAATGTGGTTTCATTTCAGACGAGCGCGACGCCTAGCGGGTCGGGAGGCAGCATGCCGAGA	36:A>G
1865080|F|0-49:G>C-49:G>C	TGCAGCTCATCCACGAGAACCTCGCCAACTTCGCCGCCGAGCTCGGGGTGCCCTTCCAGTTCGTCGTGT	49:G>C
32017989|F|0-11:C>A-11:C>A	TGCAGGTCAGCCAGGTCCCCG	11:C>A
32018162|F|0-55:A>G-55:A>G	TGCAGCCACATTGCGGCGACGAAGACGAGCCATCGGTTGGTGGAGAAGGCCCTGAACCTGTCGGCGAGC	55:A>G
7168532|F|0-22:G>C-22:G>C	TGCAGACGCATGTGAAAAGCCAGCGTGGTCCTCGTGGAACTCCATCTATCCATCTATCCGCACCGAGAT	22:G>C
4909795|F|0-20:G>C-20:G>C	TGCAGGAACGTGTTCCAGTCGCGCCGCTTCTGCGACTCGTACCTGCTCAGCTGCTGCTGCGGCGGCGGC	20:G>C
5363793|F|0-11:A>G-11:A>G	TGCAGGCGCAGACTGTCGGCTCCG	11:A>G
6037812|F|0-20:A>G-20:A>G	TGCAGCGCGGGCTTGAGCCTATCCAGGAGGAGGGCGACGTCCCCG	20:A>G
32024373|F|0-7:A>G-7:A>G	TGCAGCAACCGCATCATCTCCTCCTCCACGCGCGGCTCGCTGCCGATGACGGAGTCGAACACCTCGGAC	7:A>G
4734188|F|0-7:C>G-7:C>G	TGCAGGCCCTGGGCAACGCCGCGCTCGGCGGGGACCG	7:C>G
3222334|F|0-8:C>T-8:C>T	TGCAGCAGCAAGAACACCAGCGACAGCAGCGCCGCGGTTCCG	8:C>T
12470640|F|0-5:A>C-5:A>C	TGCAGATCATGCGCGACCCG	5:A>C
2321922|F|0-12:G>A-12:G>A	TGCAGAGGTGCAGAGCTTGACGCCGCTGGTCACCTGCATACTCCAGAGGCTCGGCACCGACATCGACCG	12:G>A
981567|F|0-31:C>G-31:C>G	TGCAGCAACCTTTTCTCTGGCGTCGGAGACTCTTTCCAGGATCTCTACCTCTGCTCGGGCGGGCCAGAG	31:C>G
4002585|F|0-40:T>G-40:T>G	TGCAGCTGCCAACCGCGCACGACGGCGGCTTCACGGCGTCTGGGCCG	40:T>G
4397905|F|0-7:G>A-7:G>A	TGCAGCAGCAAGGAGCTGCCAGCGCCG	7:G>A
1000447|F|0-44:G>C-44:G>C	TGCAGCGCCGTCCCGCACAGCATCCCCGCCCAGATGCCCTGGGGGCCGAGCCG	44:G>C
12773785|F|0-8:T>C-8:T>C	TGCAGTTGTAGCTAGCGCGGCCG	8:T>C
1200118|F|0-13:C>G-13:C>G	TGCAGAGGATGCACCCCAACGGGTCGTGGGAGCAGTGGCGGTTCGTCGGCGGCACGGGCCTGCTCGGCA	13:C>G
1091254|F|0-14:G>A-14:G>A	TGCAGAAGGCCGCCGTCGGAGAGGTACCCGTCGCCCGCGCCTCCAGCGTCCCCG	14:G>A
5005239|F|0-14:C>G-14:C>G	TGCAGCTCCCGCTCCGACGCGGACGGCGAGGCCG	14:C>G
22839993|F|0-8:C>G-8:C>G	TGCAGCCACGACACGAGCGTCCCG	8:C>G
1246053|F|0-8:G>C-8:G>C	TGCAGGCGGCGCGTGCGGGTACGGTAACCTCTACAGTACGGGGTACGGCACGAACACGGCGGCGCTGAG	8:G>C
7169690|F|0-21:T>C-21:T>C	TGCAGGCGCCGCCCTCGTGGGTGCCCG	21:T>C
1026498|F|0-28:G>C-28:G>C	TGCAGTCGCTCTTCGGGACGCGGCTGCCGACGGTCATAGGCGGCTCCTACGCCTTCGTCATCCCGAGAT	28:G>C
1002604|F|0-23:A>G-23:A>G	TGCAGCGGAGGGTGCTCGTGGGCATCGCCATCGGGGTCGGCGCGGGCGGCGTCGTGGTCGCCG	23:A>G
12763916|F|0-55:C>T-55:C>T	TGCAGCCCTAAATATTACAATTTCCCACAAATTAGTTGCTGCACGAAGAGCGCTACATAGAAAAGCTGA	55:C>T
12470809|F|0-11:C>G-11:C>G	TGCAGCGCGTGCGCGCCCTCGCCG	11:C>G
4002680|F|0-10:T>C-10:T>C	TGCAGGGGGCTAACCAGGCGCTGTTCGACGGGTTCGGGGCCGCCG	10:T>C
1017794|F|0-40:G>A-40:G>A	TGCAGGTAGGTGATCAGGACCAGCCACGCGTTCACGATCAGCAGCGGCACGCCGTAGACCCGCACCACC	40:G>A
1696848|F|0-23:G>C-23:G>C	TGCAGGTCCGAGTCCCCCGCGTAGCGCCCCGTCCCGTCGATCCCGTGCTCGTCGCAGATCACCTCCCAG	23:G>C
1096951|F|0-49:G>A-49:G>A	TGCAGGACAGCATCCCCAAGGTGTGCCTCGCCGCCGCGGCCGCCGCGCTGTACGCGCGCGCGTCGTCGT	49:G>A
1026254|F|0-53:C>T-53:C>T	TGCAGGCTGCCGAGCACCGACATCCCCCGCATGCTCACCATCCCGAGGCAGGCCACGCCG	53:C>T
5357831|F|0-18:C>A-18:C>A	TGCAGCGCCGCCTCGACGCGCGGGCCG	18:C>A
1152355|F|0-7:T>G-7:T>G	TGCAGAATTCGTCGTTGCGGCCGAGGCAGGAGAGGATCCCAGAGTCCCAGCTTTCACGCGGCTCGGCGC	7:T>G
991011|F|0-11:A>C-11:A>C	TGCAGGCCTTCATCGCGCCCTTCGGGTCGGCGTCCGTGTCCTGGAACGCGTCCCG	11:A>C
1002604|F|0-14:C>G-14:C>G	TGCAGCGGAGGGTGCTCGTGGGCATCGCCATCGGGGTCGGCGCGGGCGGCGTCGTGGTCGCCG	14:C>G
1026708|F|0-65:G>A-65:G>A	TGCAGAGACGGCCCCGCCCTCGCCCTCACCCTCTTCAACCGTGTCTGCCGAGAAGAAGCCGGCATGCGG	65:G>A
2281032|F|0-5:G>C-5:G>C	TGCAGGAGCCACTCGATGGTCTCGCCGTCCGTCTTGTGCCCCAGCTCCCGCGTCAGCTGGAACACCCGC	5:G>C
1092142|F|0-43:G>C-43:G>C	TGCAGGTGGCGCCGCGGGGCTCGCACGCGCTGGTGGTGTCCACGGAGACCATCACGCCCAACTACTACG	43:G>C
989471|F|0-46:C>G-46:C>G	TGCAGCAGTCCTCGGGCCGCCCGAACGCCTCGCACGCGCTCTTGCACGCGATGCCG	46:C>G
5971197|F|0-65:C>T-65:C>T	TGCAGTGGTAGGCGTCGGAGGAGGAGACGACCCTGGCCGGCAGCCAGATGAGGAGGCAGCACCTGCCGA	65:C>T
4261531|F|0-8:A>G-8:A>G	TGCAGGCCAGTGACAATCGTGGTCAACGAGCGCACGATCTCCG	8:A>G
32025616|F|0-24:A>G-24:A>G	TGCAGGCAGCAGCAACATCTCCTCAAGTTCAACATGGAGCAGAACATTAACAAAACTGCTTCGATCAAG	24:A>G
3951792|F|0-12:G>T-12:G>T	TGCAGCCAGGTCGCGTCGCCG	12:G>T
7336038|F|0-26:T>G-26:T>G	TGCAGAGGAGGAGGCCGCCGCAGCACTGCTGGATCTCGACGCGCTCGTAGCGCCCTCGCAGGAACGGGA	26:T>G
1029458|F|0-37:C>A-37:C>A	TGCAGCCATCGCAAGGAGATGCTTCAGTGACTGTCGGCGACGGAGGGTGGAAGAAGGGGGTGCCGAGAT	37:C>A
1028699|F|0-13:G>C-13:G>C	TGCAGGCCCCGCTGCTCGCCGTCGGCTTCGTCGCCTTCCTCGTCTCGCTCCCG	13:G>C

<table>
<tr><td>1022410|F|0-40:C>G-40:C>G</td><td>TGCAGTCCAACGCGGACGACGCCGTGGTGCACAAGATCTCCGACCGCATGGTGCGCAACGGCTCCATCG</td><td>40:C>G</td></tr>
<tr><td>2255220|F|0-11:G>C-11:G>C</td><td>TGCAGCCACCTGCTCAAGGACATCGAGCAGATCCGCCTGCGCTACCGCCCGCTCAAGGCCGCGCTCCGC</td><td>11:G>C</td></tr>
<tr><td>1024863|F|0-8:C>G-8:C>G</td><td>TGCAGCAGCTGCTGCTGCTTCAGGAAGTTGTGCAGGGTAATGTGCTTCGTCGGCTCGGTGCTGCTGGCA</td><td>8:C>G</td></tr>
<tr><td>1210386|F|0-27:A>C-27:A>C</td><td>TGCAGGTGAAGAGCTTCATCCCGCGGGAGTTCTGCACCGACTCCTCCTCGTACTCGTAGCCGCCATCGC</td><td>27:A>C</td></tr>
<tr><td>7165711|F|0-13:A>G-13:A>G</td><td>TGCAGGATCTGAGAAAGATGCTGGACGAAGGGAAGGAGCTCGGGATGCCG</td><td>13:A>G</td></tr>
<tr><td>2247497|F|0-41:G>A-41:G>A</td><td>TGCAGATTGAGGGAGGAGACACCTCCGCCGACGGGTGTAGCGAGACCGCCCTTGAGGCAGACCTTGTTG</td><td>41:G>A</td></tr>
<tr><td>991011|F|0-6:C>T-6:C>T</td><td>TGCAGGCCTTCATCGCGCCCTTCGGGTCGGCGTCCGTGTCCTGGAACGCGTCCCG</td><td>6:C>T</td></tr>
<tr><td>1116567|F|0-39:T>G-39:T>G</td><td>TGCAGCGAGGCCGCGTGGAACACCTGGTGCGTGCTCGGGTGCCGCCAGGGGCGCGTCCTCATCATGAAC</td><td>39:T>G</td></tr>
<tr><td>6041995|F|0-25:G>C-25:G>C</td><td>TGCAGTGGCACATCGTCCTGCTCCTGGGCGCCG</td><td>25:G>C</td></tr>
<tr><td>1382098|F|0-50:G>T-50:G>T</td><td>TGCAGCCTGAGCCGACCGCCCTCGCGCGACGCCCGCAGGAGCTCCTTCCCGGGGATGACAACCTCCACC</td><td>50:G>T</td></tr>
<tr><td>1067012|F|0-58:C>T-58:C>T</td><td>TGCAGCAGCAGAAAGGCGGCAGTTGCAGCCAGGAGAGCGACTGAATGAATCTTGTAAACACTCAGCGGT</td><td>58:C>T</td></tr>
<tr><td>1716255|F|0-6:T>C-6:T>C</td><td>TGCAGATGCAGAGCGACCTCGACGCTCTCGCCGGGAAGCTGGACCTCAACCTCCG</td><td>6:T>C</td></tr>
<tr><td>4006089|F|0-56:G>C-56:G>C</td><td>TGCAGGAGCACGCCCTTGACCACGTCCCTCTTGGACGCCATGTTCTTGGTCTCCTCGTCCCTCCTCGAG</td><td>56:G>C</td></tr>
<tr><td>1089801|F|0-16:G>C-16:G>C</td><td>TGCAGATCGCGAACGAGAGCGCCAGCGCCGCGCCGCCGTGCAGCACCGACATGCTGCCCCG</td><td>16:G>C</td></tr>
<tr><td>5358090|F|0-5:C>G-5:C>G</td><td>TGCAGCTTGTTGATGGCCGCGCCCG</td><td>5:C>G</td></tr>
<tr><td>3946379|F|0-66:A>T-66:A>T</td><td>TGCAGCTACGACTTCCTCGGCGAGCCCGACGTGTCGCTCGGGATCAACACGCTCTCCTACTGCGGCAGC</td><td>66:A>T</td></tr>
<tr><td>1862771|F|0-25:C>T-25:C>T</td><td>TGCAGAACCCTAGGTGCAGCGACCTCGACGGCGTCCCGTCCCTCTCGGCGGCCGCGTACGCGCTCCTGC</td><td>25:C>T</td></tr>
<tr><td>1027501|F|0-52:C>T-52:C>T</td><td>TGCAGCGCGGCCTTGTTCATGAGGATGGCGCACATGCACGCCGCCTGCGTGGCCGCCGCCTCCTTGAGC</td><td>52:C>T</td></tr>
<tr><td>3959730|F|0-16:C>T-16:C>T</td><td>TGCAGGTGCTGGGCGGCGGAGGCGCGGGGGCGGTGAAGCGGAACGAGGGGCGGGGGAAGCTGCTCCCCA</td><td>16:C>T</td></tr>
<tr><td>3024864|F|0-25:C>A-25:C>A</td><td>TGCAGCACCTCGCGGTGTCCTCCTCCGCTCCTGGGCCCG</td><td>25:C>A</td></tr>
<tr><td>3949747|F|0-23:G>C-23:G>C</td><td>TGCAGCCAGTTGCCGAAGATGGGGACCG</td><td>23:G>C</td></tr>
<tr><td>1022589|F|0-16:A>C-16:A>C</td><td>TGCAGGCGCTCAAGGCAAAGGAGGCGCAGAAGAAGTCCGCGCAGGCCGATGAGGAGGCTGGCTCCCTGT</td><td>16:A>C</td></tr>
<tr><td>3025223|F|0-38:C>G-38:C>G</td><td>TGCAGGCTGGCTGCATGATCACTGACCACCTGGAACTGCACCG</td><td>38:C>G</td></tr>
<tr><td>3029511|F|0-11:G>A-11:G>A</td><td>TGCAGCAGCCCGTAGGCGAATATCGCCGGGATCATCCACCG</td><td>11:G>A</td></tr>
<tr><td>7165875|F|0-26:G>A-26:G>A</td><td>TGCAGGGCGTTGCCCGTGACATCGGCGAGCGGCCG</td><td>26:G>A</td></tr>
<tr><td>1092142|F|0-19:C>T-19:C>T</td><td>TGCAGGTGGCGCCGCGGGGCTCGCACGCGCTGGTGGTGTCCACGGAGACCATCACGCCCAACTACTACG</td><td>19:C>T</td></tr>
<tr><td>1002316|F|0-11:A>C-11:A>C</td><td>TGCAGGGACCGAGTGGACGTGCGCGGCGCACATCATCACGGCGGTGATCGGGTCCG</td><td>11:A>C</td></tr>
<tr><td>1092773|F|0-65:T>C-65:T>C</td><td>TGCAGGGGTAGGGCCGACCCTCCTGTGCCGATGGACTTCTTCGGGAACATGGTGCTCTGGGCTTTTCCA</td><td>65:T>C</td></tr>
<tr><td>100472532|F|0-50:A>G-50:A>G</td><td>TGCAGGTGCTCGACGTCAGCTCCAACATGCTCAACGGCACGCTCCCGGCGAACTTCGGAGGCGCCGCGC</td><td>50:A>G</td></tr>
<tr><td>1159021|F|0-9:C>G-9:C>G</td><td>TGCAGCGGCCCTCCTCTCGGCCACCTCCACACCGTCTCGCTCCTCTCCTCCTCCGCTGCCTCGCTCTCC</td><td>9:C>G</td></tr>
<tr><td>1045626|F|0-22:C>T-22:C>T</td><td>TGCAGTAGCATTAGCTGTTCTTCAGGTTCCCCTCAAAAGCCTCCTCAAAAACACATAAAAAGGATTCCG</td><td>22:C>T</td></tr>
<tr><td>1397989|F|0-55:C>G-55:C>G</td><td>TGCAGATGGCCTCGTACTCCGCCAGGTAGTCGTCCAGCGTCCTGATCAGCGTCAGCGCGTTCGTCTTGC</td><td>55:C>G</td></tr>
<tr><td>1022043|F|0-14:G>C-14:G>C</td><td>TGCAGGGGGATGACGACCATGAGCACCTTCTTCTCGCGGTCCGCCAGGTAGGGCTTGAGGAAGGACCAG</td><td>14:G>C</td></tr>
<tr><td>1092142|F|0-61:C>T-61:C>T</td><td>TGCAGGTGGCGCCGCGGGGCTCGCACGCGCTGGTGGTGTCCACGGAGACCATCACGCCCAACTACTACG</td><td>61:C>T</td></tr>
<tr><td>1091254|F|0-37:C>T-37:C>T</td><td>TGCAGAAGGCCGCCGTCGGAGAGGTACCCGTCGCCCGCGCCTCCAGCGTCCCCG</td><td>37:C>T</td></tr>
<tr><td>2351339|F|0-43:C>G-43:C>G</td><td>TGCAGATCCTGCTCGAGTACATGGACGGCGGGTCCCTGGACGGCCGCCGCATCGCGTCCGAGTCGTTCC</td><td>43:C>G</td></tr>
<tr><td>4261531|F|0-19:T>C-19:T>C</td><td>TGCAGGCCGGTGACAATCGTGGTCAACGAGCGCACGATCTCCG</td><td>19:T>C</td></tr>
<tr><td>1053944|F|0-12:T>C-12:T>C</td><td>TGCAGCTCTGGGTCCGCGCGGAGGCCAGAGGGAGCGACAATGGGTGGTTTCTGGAGAGGGAGATGCTCA</td><td>12:T>C</td></tr>
<tr><td>32021781|F|0-20:A>G-20:A>G</td><td>TGCAGCAATCGAGGGGATGAAGAGCCAGATGGGCGGCGCCGCCAGCGAGGAGACGGTGAGCGACCACGA</td><td>20:A>G</td></tr>
<tr><td>1126452|F|0-29:C>T-29:C>T</td><td>TGCAGCGCCTGGCCCTGGCCGTGGTTGAACTGGGCGAGCCCGCCCTTGGTGAAGTTGAACGCGTTGAAC</td><td>29:C>T</td></tr>
<tr><td>1318141|F|0-16:G>C-16:G>C</td><td>TGCAGAACCCGATGGCGTTCCCGTCGGAGAGCCACCCGATCCACCTCCACGGCTTCAACTTCTTCGTGC</td><td>16:G>C</td></tr>
<tr><td>1061439|F|0-52:A>G-52:A>G</td><td>TGCAGAGGGTCATCAAGCAGGAGTTCTCAGGGTGCACGGTGATCACCATAGCACACAGGGTTCCCACCG</td><td>52:A>G</td></tr>
<tr><td>2245958|F|0-11:T>G-11:T>G</td><td>TGCAGGACGAGTATGCCATTGGTGGCGTTCCCCAGCTTCATCTTCTCGACGCAGCTCCTGACCACGGCC</td><td>11:T>G</td></tr>
<tr><td>1378383|F|0-37:C>G-37:C>G</td><td>TGCAGGCCATGGACTCCTCCCACGTCGCGCTCGTGGCCCTCCTCCTCCGCTCCGAGGGCTTCGAGCACT</td><td>37:C>G</td></tr>
<tr><td>990556|F|0-59:C>A-59:C>A</td><td>TGCAGCCGCTGCTCCAAGGCCTACGCCGTCCACTCCGACTACAAGGCCCACCTCAAGACCTGCGGCACC</td><td>59:C>A</td></tr>
<tr><td>5357601|F|0-20:G>A-20:G>A</td><td>TGCAGCAGGTCGGCCGCCGTGTGGCGCCG</td><td>20:G>A</td></tr>
<tr><td>5354875|F|0-29:C>G-29:C>G</td><td>TGCAGCTACGTGACCGAGGGCGGCGACGTCGAGGTCGACAACGTCGACGAGCCCCTCATCCTCGACGGC</td><td>29:C>G</td></tr>
<tr><td>1027945|F|0-64:T>A-64:T>A</td><td>TGCAGAACATCTTCTTCTCGTACGGGATGGCGTTCGGCGGCGGCGGCTTCGCCATCAGCCAGCCTCTGG</td><td>64:T>A</td></tr>
<tr><td>7351914|F|0-37:G>C-37:G>C</td><td>TGCAGCGGTGGTCGTCGTCGATGTGGGGGATGAGCGGGCGCGACCG</td><td>37:G>C</td></tr>
<tr><td>1698386|F|0-14:G>C-14:G>C</td><td>TGCAGGATGTTGGCGGATGTCCACGTGGTGGCGTAGAGACGGCCGCGGAAAAGGAGGACGCTCTGGAGC</td><td>14:G>C</td></tr>
<tr><td>2247497|F|0-5:A>G-5:A>G</td><td>TGCAGATTGAGGGAGGAGACACCTCCGCCGACGGGTGTAGCGAGACCGCCCTTGAGGCAGACCTTGTTG</td><td>5:A>G</td></tr>
<tr><td>32016487|F|0-13:C>T-13:C>T</td><td>TGCAGCGTGATGACGGCCTTCTCCCG</td><td>13:C>T</td></tr>
<tr><td>32025020|F|0-35:G>C-35:G>C</td><td>TGCAGCAGCGCCAGCAGCTTCGCCACGCCGCCGCTGGCCACCATGTCGTCCAGCACCCTCTCCGTGGGC</td><td>35:G>C</td></tr>
<tr><td>1024863|F|0-11:C>T-11:C>T</td><td>TGCAGCAGCTGCTGCTGCTTCAGGAAGTTGTGCAGGGTAATGTGCTTCGTCGGCTCGGTGCTGCTGGCA</td><td>11:C>T</td></tr>
<tr><td>11914769|F|0-49:T>C-49:T>C</td><td>TGCAGATGCTGACAGAGGGGCCATGGGAAGAACACGATGAGGTCGACCTTGAATTCCTGGGGAACAGCT</td><td>49:T>C</td></tr>
<tr><td>7172763|F|0-11:C>G-11:C>G</td><td>TGCAGCGGCGTCGGCATCCTGGGCATCGCGTGGGCGTTTGGCGGCATGATCTTCGTGCTCGTCTACTGC</td><td>11:C>G</td></tr>
<tr><td>4019082|F|0-10:C>G-10:C>G</td><td>TGCAGCCGACCCTGGCCACCGAGTACGCCAGCCAGGCCG</td><td>10:C>G</td></tr>
<tr><td>1862771|F|0-55:G>C-55:G>C</td><td>TGCAGAACCCTAGGTGCAGCGACCTCGACGGCGTCCCGTCGCTCTCGGCGGCCGCGTACGCGCTCCTGC</td><td>55:G>C</td></tr>
<tr><td>986686|F|0-40:T>C-40:T>C</td><td>TGCAGGCCACTCCAAACAGGCTTCGCATGCTGAGCCTATATTCTGCCATCACCG</td><td>40:T>C</td></tr>
</table>

<table>
<tr><td>995193|F|0-29:C>G-29:C>G</td><td>TGCAGCAGCCACTGCACGGTCTCGCCGTCCGACTTGTGGCCGAGCTCGCGCGTGAGCTGGAAGATCCGC</td><td>29:C>G</td></tr>
<tr><td>7351634|F|0-14:C>T-14:C>T</td><td>TGCAGGTGTGGTGGCGAGTGGCAGGTGTGGCTGCCTTGGTCCGATGCTGCCGCCACCACCACCGTGGCG</td><td>14:C>T</td></tr>
<tr><td>1022995|F|0-14:G>C-14:G>C</td><td>TGCAGGTTGGACGCGCCGTGCATCGCGAAGTACTCGCGCACCATCTCCCG</td><td>14:G>C</td></tr>
<tr><td>2272026|F|0-36:G>T-36:G>T</td><td>TGCAGCCAGGTGCTCCTCCTTCCTTCTCCTTGGTCGGTCGGTCGGTCGAGGAGTTAAGGAAGAAGAACC</td><td>36:G>T</td></tr>
<tr><td>1001090|F|0-13:G>A-13:G>A</td><td>TGCAGTACAGGAAGGAGTATATGGCGGCGCAGACCGTGAACGGTATCGCGATGGCCGTGTACAGCGCCT</td><td>13:G>A</td></tr>
<tr><td>986473|F|0-26:C>A-26:C>A</td><td>TGCAGACGTCGCGGGAGCCCACGCCGCGGCAGTCCAGCTTCAACCAGTCCGACTTCTACTCCATGTTCA</td><td>26:C>A</td></tr>
<tr><td>1167494|F|0-55:C>T-55:C>T</td><td>TGCAGCAGAAGTACACGCAGAGCGGAGGGGTGCGCCCGTTCGGGCTCTCCACGCTCATAGTTGGCTTCG</td><td>55:C>T</td></tr>
<tr><td>1090478|F|0-10:G>C-10:G>C</td><td>TGCAGGTGACGGCTGTAACCGATCCCACCTCACCGTTTACGCAGTCCCTAGCAGCGACGCTGCCCGAGA</td><td>10:G>C</td></tr>
<tr><td>5371569|F|0-21:A>C-21:A>C</td><td>TGCAGAGGTGGAGCTCCAGGAACCG</td><td>21:A>C</td></tr>
<tr><td>990506|F|0-8:G>A-8:G>A</td><td>TGCAGCTCGTTCGCCTTCTGCTTGGCGTTCAGCGCCTCCTTGCAGGCGGTGCTGTACATGTCCATGGTC</td><td>8:G>A</td></tr>
<tr><td>6040254|F|0-67:C>G-67:C>G</td><td>TGCAGCGCGTCCTGCGCCACCGACGTCGACGCCGCCATCGACACGGCGAGCAGCACCGCCGCCAGCTCA</td><td>67:C>G</td></tr>
<tr><td>4398155|F|0-8:G>C-8:G>C</td><td>TGCAGGTGGCCGCCGAACGCCG</td><td>8:G>C</td></tr>
<tr><td>1111609|F|0-28:A>C-28:A>C</td><td>TGCAGGCCACCGTCCGCCGCGTCACCAAATCGTACGCGGTCTGCAAGTACATCCCG</td><td>28:A>C</td></tr>
<tr><td>1206915|F|0-7:T>G-7:T>G</td><td>TGCAGCTTGAGCGGGCACTTGGCCAGGACCTTGCACTTGGTGAAGTGCGCCTGGTACCTGACCTCGCCC</td><td>7:T>G</td></tr>
<tr><td>992903|F|0-14:G>T-14:G>T</td><td>TGCAGCTGGAAGTTGCGGAACTTCTCGGGGCAGGAGAAGGCGGAGGTGGAGGGCGCGGCGGGGCGCGGG</td><td>14:G>T</td></tr>
<tr><td>1010818|F|0-37:T>C-37:T>C</td><td>TGCAGGGATGGGCGGCCTGGGCGTGGAGGAGACGGCGTTGGTGTCGGCGCTGGGGCGGTGGAGGAAGCA</td><td>37:T>C</td></tr>
<tr><td>1001087|F|0-20:G>A-20:G>A</td><td>TGCAGTACCAGCAGCCGCTGGTTCTGCGAGAAGCCGTGTGTGGTGAACGGCGGCGCGTACATGGTCACC</td><td>20:G>A</td></tr>
<tr><td>2260637|F|0-43:C>T-43:C>T</td><td>TGCAGCTCTACTCCAAGGAGGTCAGCCGCCGCCTCCTCGACTTCGTCAAGTCCCGCTCCGCCGCCGCCA</td><td>43:C>T</td></tr>
<tr><td>1863497|F|0-67:A>G-67:A>G</td><td>TGCAGGCCGAGGGCAAGATGCCGCTGCCGAGCAGCACTGACGTTGCCTCGCGGATGCTCGATGGCGCAG</td><td>67:A>G</td></tr>
<tr><td>1209668|F|0-40:G>C-40:G>C</td><td>TGCAGCACATCCTCCAGGGCTGCTCCAAGCTGGAGAAGCTGGAGATAAGGGACTGCCCGTTCGGCGACG</td><td>40:G>C</td></tr>
<tr><td>1001740|F|0-42:T>C-42:T>C</td><td>TGCAGGAGCCACCAGAATAGGGTGAGAAAGCCTTTCCACCCATTCAAGGCGAACCCCTTCCAGCCCAGG</td><td>42:T>C</td></tr>
<tr><td>5580310|F|0-14:A>C-14:A>C</td><td>TGCAGCTCCGTCAGACCCTGCGGGTTCCCG</td><td>14:A>C</td></tr>
<tr><td>3064632|F|0-17:T>G-17:T>G</td><td>TGCAGCACGTCGAGTCCTTCGCCGACGAGATGGAGTTGCGCCGCTGCGAGCTGCGGCTCTACGCCAACA</td><td>17:T>G</td></tr>
<tr><td>25949778|F|0-17:A>G-17:A>G</td><td>TGCAGGCGATGAGGGACACCTTCGACGGGACGCTGATGGTCGGCGGAGGGTACGGCAGGGAGGAGGGGA</td><td>17:A>G</td></tr>
<tr><td>5361369|F|0-8:C>G-8:C>G</td><td>TGCAGGAGCGCGGCGTGGAGCGCGCGCAGCTCGGTGGCCTTGGCGATGGCCGCCTGGATTTCCTGGCGC</td><td>8:C>G</td></tr>
<tr><td>5579600|F|0-35:A>G-35:A>G</td><td>TGCAGCCTTCCGTTGCCCTGCACCCTAGGGTTCATAAAGTCTTCGGCGAGGAGGTCGACGAGGGCCATG</td><td>35:A>G</td></tr>
<tr><td>5363675|F|0-5:C>T-5:C>T</td><td>TGCAGCTGTTGGACCTCGGCGACAACGCTCTCTCCG</td><td>5:C>T</td></tr>
<tr><td>5363906|F|0-19:T>C-19:T>C</td><td>TGCAGGGTTCCGCCGCGCGTCCTTGCCG</td><td>19:T>C</td></tr>
<tr><td>7353049|F|0-7:C>G-7:C>G</td><td>TGCAGCGCCGTGCGGCAGCCG</td><td>7:C>G</td></tr>
<tr><td>2247497|F|0-35:T>C-35:T>C</td><td>TGCAGATTGAGGGAGGAGACACCTCCGCCGACGGGTGTAGCGAGACCGCCCTTGAGGCAGACCTTGTTG</td><td>35:T>C</td></tr>
<tr><td>6045193|F|0-36:G>T-36:G>T</td><td>TGCAGTTGACGACGAGCAAGTCAATGGCGTCGGGGCGGATGCCCGTCTTGGCGAGCAGGTCGTCGATGG</td><td>36:G>T</td></tr>
<tr><td>7169150|F|0-7:G>C-7:G>C</td><td>TGCAGAAGAGCGAGGGCGACAGTAGGCCCCTGGGGCGCGCCTGGCCG</td><td>7:G>C</td></tr>
<tr><td>7340694|F|0-30:C>T-30:C>T</td><td>TGCAGCATGTCGTTGTGGTGGCCGCGGGAGCTGATGGCCTGGTAGATGACGTCCGACTCCTTGAACGCG</td><td>30:C>T</td></tr>
<tr><td>32023003|F|0-8:T>C-8:T>C</td><td>TGCAGGTTTGACGCCCCCTGCTCCAGTATCCGCATCGCGTCCTCTGGCACACCAGCAGCAAAACAGCGG</td><td>8:T>C</td></tr>
<tr><td>32025950|F|0-14:G>A-14:G>A</td><td>TGCAGAGCGCCCTGGATCTCCGCCATGTACTTGCTGTCTTCGTCTAACCATCGGGCGTACTCCATGTCG</td><td>14:G>A</td></tr>
<tr><td>32023804|F|0-24:A>G-24:A>G</td><td>TGCAGGCGCGCGGCGAGTTCGCGGAGGCGCTGGCGTCCTTCACGGCGGTGGTGAGCGGGTACAAGGACC</td><td>24:A>G</td></tr>
<tr><td>5357832|F|0-13:T>C-13:T>C</td><td>TGCAGCGCCGCTGTGTGGGACCG</td><td>13:T>C</td></tr>
<tr><td>1020327|F|0-19:G>A-19:G>A</td><td>TGCAGGCGGAGACGCCGTCGCTGGGGGACTTCGCGTACTCGGTGGAGGTGGGGACGCGGAAGAAGCCGC</td><td>19:G>A</td></tr>
<tr><td>2266496|F|0-8:C>G-8:C>G</td><td>TGCAGCGCCGAGGTGGACCGCCTCACCGACGCCGTCGACGCGCTCGAGCGCCTCGCCG</td><td>8:C>G</td></tr>
<tr><td>1217332|F|0-32:T>C-32:T>C</td><td>TGCAGCAGCAACATGGAGGAGAACAAGCAGCATAGCTGGAAAAAGATGTCAACTGCAAAGCAGTACAAG</td><td>32:T>C</td></tr>
<tr><td>1023671|F|0-32:T>C-32:T>C</td><td>TGCAGCTCCCGCGCCTGCTCCGCCGCGCGCGCTGCCGCCTCCGCCGCCTGCTCCCCTGCCTTCCTCTCC</td><td>32:T>C</td></tr>
<tr><td>3939881|F|0-14:T>C-14:T>C</td><td>TGCAGGAGCGGGTGTTGAGTTGCCATGAAGCCATGATTCAGGCCACCG</td><td>14:T>C</td></tr>
<tr><td>2272026|F|0-15:C>T-15:C>T</td><td>TGCAGCCAGGTGCTCCTCCTTCCTTCTCCTTGGTCGGTCGGTCGGTCGAGGAGTTAAGGAAGAAGAACC</td><td>15:C>T</td></tr>
<tr><td>1022746|F|0-47:T>C-47:T>C</td><td>TGCAGCTTGCCGTTCCTGGAGTTGATGAGGTGGTACTTCTCGTTGATTCGCCCGTTGTTCACCACGTCC</td><td>47:T>C</td></tr>
<tr><td>7338594|F|0-17:G>A-17:G>A</td><td>TGCAGAATCTTTCGGGCGCCG</td><td>17:G>A</td></tr>
<tr><td>4910606|F|0-24:C>T-24:C>T</td><td>TGCAGTTCCTCCCCCCGCCCTTCTCCAACCTCACGCAGCTCGTCGACAGCTTCAAGGCCAAGAACATGA</td><td>24:C>T</td></tr>
<tr><td>7352972|F|0-15:T>C-15:T>C</td><td>TGCAGGAGATGACTGTACTCCG</td><td>15:T>C</td></tr>
<tr><td>5369428|F|0-28:T>C-28:T>C</td><td>TGCAGCGAGTTCTGGACCCCTCCGACGATGCACCG</td><td>28:T>C</td></tr>
<tr><td>995663|F|0-7:T>C-7:T>C</td><td>TGCAGGGTCTCGTACTTGGAGGCCACCTCCCTGAGCATCTCGCCCAGCCGCCG</td><td>7:T>C</td></tr>
<tr><td>1047138|F|0-57:A>G-57:A>G</td><td>TGCAGGTATGCCACAGACGGAAGAGATGTCCTTTCACAGATTAAGGCTGGAGATAAAATTCGCTCCGCC</td><td>57:A>G</td></tr>
<tr><td>4991983|F|0-29:A>T-29:A>T</td><td>TGCAGCTCCCCGTCGTCCCCCTCTCGCCCACCCTCTCCTTCGCTCGCGATGGGGTCCGCGGCGTCGACC</td><td>29:A>T</td></tr>
<tr><td>1225998|F|0-21:G>A-21:G>A</td><td>TGCAGAGCTCCGACTCGCCCAGCAGCCCGCTCAGGCTGTCCCTCCAGCTCGTCAGCGCCACCGTCGCCG</td><td>21:G>A</td></tr>
<tr><td>6047501|F|0-10:A>G-10:A>G</td><td>TGCAGCAGCAAGAACACCAGCGCCAGCAGTGCCGCGGTTCCG</td><td>10:A>G</td></tr>
<tr><td>2256197|F|0-53:C>G-53:C>G</td><td>TGCAGCGCGAACTCGAGGCACCAGAGCACGTCCCCCATGGTGGGCCGCTCCACCCCGTACTCGGCGAGG</td><td>53:C>G</td></tr>
<tr><td>1202403|F|0-5:C>G-5:C>G</td><td>TGCAGCTGGGGCCAGTGTGTGCTGCTGGTCGGCCATTGCTTGGAAGAACGGGAAGCCTTGCATCTGCTG</td><td>5:C>G</td></tr>
<tr><td>1223679|F|0-20:C>A-20:C>A</td><td>TGCAGGGAGCTTGAATCGCGCGTCGGCACTGGTAACGCACCCAGCTTCCTTCCTCACTCCGCTCGAAAT</td><td>20:C>A</td></tr>
<tr><td>3939302|F|0-14:G>C-14:G>C</td><td>TGCAGGTGATCCTGGAGCAGCAGCTGCAAGTGCTGGTGGAGAGGTACCG</td><td>14:G>C</td></tr>
<tr><td>1250512|F|0-66:G>C-66:G>C</td><td>TGCAGCAGAGATGGAGTGGCTCAACGAGTACGAGAAGCTGGTCATCAGGATGGACACCCCCAAGGTGGT</td><td>66:G>C</td></tr>
<tr><td>6046101|F|0-35:T>C-35:T>C</td><td>TGCAGGATGTTGTAGAGCACCCAGCCGAGGGCGGGTGCCACCACGAAGAGCAGCAGCAGGCCCCGAGAT</td><td>35:T>C</td></tr>
</table>

4909784|F|0-13:A>G-13:A>G	TGCAGGTCGAGGAAATCGGGCAGTCGGCCTCCG	13:A>G
1074053|F|0-43:G>A-43:G>A	TGCAGGCAACACAGCAAAATAGAAGCCCAAGAGATATTTGACTGACGATGGTTGCTTGGAACATATATG	43:G>A
4990067|F|0-15:G>T-15:G>T	TGCAGCGGCCCACAGGCGGTGGTGTACTGCCG	15:G>T
3026761|F|0-32:T>C-32:T>C	TGCAGCGCCTCCATCTGCATGGCGGCCTTCTCTTCCTGCAGCCG	32:T>C
6047259|F|0-13:C>T-13:C>T	TGCAGGAGCTCCGCGCCATCAAGTTCGCCCCGCCGCCACCGCCCAACAGCGCCAGCCAACACCCCGAGA	13:C>T
989664|F|0-6:T>C-6:T>C	TGCAGTTGGCCAGGCACAACTGTCTGCACTCGTCCAGCGTCATGTCGGCGTGCACCGTCGCGTTAGTCG	6:T>C
7351632|F|0-26:C>G-26:C>G	TGCAGGTGGCGGCGGAGCACGACGCCCTCGGTGGCGGCCCTGGGGTCGGCGCGGACGTTGTCGACGACG	26:C>G
5580989|F|0-28:G>C-28:G>C	TGCAGCACCTCGCCAAGCTGAAGAAGCTGTGGAAGCTCAGCGTCAACGTCACCACCGACGCCAACGTCG	28:G>C
32027561|F|0-60:T>G-60:T>G	TGCAGGAACGTGTTCCAGTCCCGCCGCTTCTGCGACTCGTACCTGCTCAGCTGCTGCTGCTGCGGCGGC	60:T>G
5581979|F|0-7:T>A-7:T>A	TGCAGCGTGGCGTCAGGGGTCGCCG	7:T>A
1028245|F|0-5:A>C-5:A>C	TGCAGAACCAGGATGAGCGGCTGCGGCAGGATGACGCAGTAGCACACTGAGGGGAACCCCATGGCCGAG	5:A>C
998431|F|0-61:G>C-61:G>C	TGCAGCTCTTCACGTCGTCGCTCTACCTGGCCGCGCTCGTCACCAGCCTAGGGGCGTCCATGGCGTGCA	61:G>C
1695138|F|0-30:T>C-30:T>C	TGCAGAAGATGTTCGAGGACCAGCTCAAGGTCAGCGGCAACACCGCGCCG	30:T>C
7168094|F|0-14:C>A-14:C>A	TGCAGGCGGTGCGGCGGTCGCTCGACGACATGCCG	14:C>A
983127|F|0-7:G>A-7:G>A	TGCAGGGGAAACGAACCGAGTCAAAGGGGAAGCAACTAGAATCAGATATCACATACCTTATAGCAAAGC	7:G>A
7349800|F|0-10:C>G-10:C>G	TGCAGGGCACCGACATGCCG	10:C>G
1005839|F|0-14:C>T-14:C>T	TGCAGGAGCTCCTGCACGGCGCGCTCGTGGCGCTCCCGCGCGGCGCGGTGGTCGTCGGAGACCTCGTAG	14:C>T
1027942|F|0-46:G>C-46:G>C	TGCAGAAGCTCCTGCACGCCGCCTACAAGCGCGGGGACACGTCCGTGGAGGTGCGCGTGCGGCGCCCCG	46:G>C
984927|F|0-43:C>G-43:C>G	TGCAGCACGACCTCCTTGTGGCGATTATGTCCCGCGTGGGCGCCCCCGATTTACTTTCTGGCGGCGCAC	43:C>G
5581422|F|0-8:G>C-8:G>C	TGCAGCTCGAGGCCGAGGCGGCCGCCCCACGTCGCCG	8:G>C
5357234|F|0-22:G>C-22:G>C	TGCAGAAGCAGGTGACGCAGTCGCCG	22:G>C
2254347|F|0-6:T>C-6:T>C	TGCAGATGTCGGCGTGATGAGCGGCTGCATCATCTTCATCCAGAAGGACCTCCACATCGACGAGGTGCA	6:T>C
1021663|F|0-30:C>G-30:C>G	TGCAGAGCTGGTGGTGACACTGGAGGGCCTCTGCAAAGACCCGAGCAGGTTCCTGACCTACGACGAGGA	30:C>G
1251195|F|0-49:G>C-49:G>C	TGCAGTTCGGGCGCGCGCGGCGGGACTTCGTGCTGCGCTCCCACGGCGGGGAGGGCTTCTCCGTCGCCG	49:G>C
32019408|F|0-61:C>G-61:C>G	TGCAGATGATCGAGCGCGAGAAGGCCAAGGCCAAGGCGGCCATGGAGGCGGCGGAGGCGTCCCAGAGGA	61:C>G
1213790|F|0-22:A>G-22:A>G	TGCAGGCCGTGGCCAAGGAGACACTGCGGCTTCACCCGACGGGCCCGCTGGTGGTGCGGCAGTCCCCGA	22:A>G
1159021|F|0-38:G>A-38:G>A	TGCAGCGGCCCTCCTCTCGGCCACCTCCACACCGTCTCGCTCCTCTCCTCCTCCGCTGCCTCGCTCTCC	38:G>A
1251314|F|0-5:C>T-5:C>T	TGCAGCTGGAGCTCCACCAGCTCCCGGCATTTCCCGATTTCTCGTGGGATCGTTCCG	5:C>T
1024631|F|0-17:G>T-17:G>T	TGCAGCCGACTGACTACGTCATGCCGCTGGTGCTGGTCACCCCGTCGGACGGCGCCGCTATCCAGAAGT	17:G>T
1094223|F|0-13:T>C-13:T>C	TGCAGGGCGCGAGTGGGCGGCCGCAGAGGCACTTGTTGGGCGCGAAGTGCTTGGGGCCGAACCTGTGCG	13:T>C
5580505|F|0-20:G>C-20:G>C	TGCAGGTAGTACTGGCGCAGGCCCTCCCCGCCGCCG	20:G>C
2242889|F|0-22:T>C-22:T>C	TGCAGGTGCTTGCGGAGATCGATGATAGGTTCTTGAAGGCGTCAGAGAGCGCCG	22:T>C
1106971|F|0-47:A>G-47:A>G	TGCAGAGGCGTCTCGTCCTCGCCGCCCTGGCTCAGCGTCTCCATCAGATTCCCCCACTCCGTCCGCATC	47:A>G
1695299|F|0-23:T>G-23:T>G	TGCAGCCACTGCTCGATGGACGCTCGCTCCAGCGCCCCCGTGCCGATCAGGTCCG	23:T>G
2267348|F|0-38:C>T-38:C>T	TGCAGCTCGCGGAAGTCGCGCTCGAAGCCCTCGACCCTCCTCTCGCCGCGCCG	38:C>T
1397989|F|0-46:C>T-46:C>T	TGCAGATGGCCTCGTACTCCGCCAGGTAGTCGTCCAGCGTCCTGATCAGCGTCAGCGCGTTCGTCTTGC	46:C>T
2247465|F|0-19:T>G-19:T>G	TGCAGTGAAATTGCAAGAATCACTTTATACGCTATTTCGAAATCGAATGAATTGAGCAACTGTATTTCC	19:T>G
32024620|F|0-16:G>A-16:G>A	TGCAGTCGTAGACGGTGGCCGAGGCCGAGGCCACTGTCGCGAGGAGGCGGGAGCCGTCGCTGGAGAAGG	16:G>A
12768999|F|0-12:C>T-12:C>T	TGCAGGCGGCGGCGGATGGCCTGCACCTGTGCCTCCATGGCCGTCACCGACGTGTCCACGGTGGTGAGG	12:C>T
1005761|F|0-47:C>G-47:C>G	TGCAGGCGGCGGCGGATGGCCTGCACCTGTGCCTCCATGGCCGTCACCGACGTGTCCACGGCGGTGAGG	47:C>G
1029912|F|0-41:G>C-41:G>C	TGCAGCTCCACGCCAATGCCTCTCAGGAGGCCCAGCTGAGAGAGTTCATCTCGTCCAGGAAGAACAGCG	41:G>C
5363273|F|0-22:G>C-22:G>C	TGCAGCAGCAGAGGGCGCAGCTGCCG	22:G>C
1863780|F|0-21:C>G-21:C>G	TGCAGCGACACCGATGGACGTCGAGGCAGAAGCCCAACAACACCAGCCATGCCG	21:C>G
5578096|F|0-38:C>G-38:C>G	TGCAGCGGGGAGGGGGTGCTCTTCGTCGAGGCCGACGCCCCCG	38:C>G
32025930|F|0-49:C>G-49:C>G	TGCAGAAGACGAAGGTGATCGAGACCTTCCAGGCCCAGGCCAACAAGACCGACGAGGGCATCACCATCT	49:C>G
7170471|F|0-20:G>T-20:G>T	TGCAGAGCGGCACACGGCAGGCCTTGTCCG	20:G>T
4993373|F|0-5:C>T-5:C>T	TGCAGCCCTTTGGTCAGATGCCG	5:C>T
992184|F|0-38:C>G-38:C>G	TGCAGGCACGACCGCATGCCCTTCCTGGCGTCGGCGTCCGCCGCCGAGGCCG	38:C>G
1057947|F|0-48:G>C-48:G>C	TGCAGCCGATCTGCAAGCCACACCACATGAATCAGAATTCAGAAACACGAACAAACAGAACCGCAGATC	48:G>C
2244237|F|0-50:A>C-50:A>C	TGCAGGAAGTATGCATCGACTGAATCTAGTGGACAGCGAAATGCAGCTCTACATAGTACAGCTCACGAG	50:A>C
6039882|F|0-19:T>G-19:T>G	TGCAGAACATCTTCTTCTCTTACGGGATGGCGTTCGGCGGCGGCGGCTTTGCCATCAGCCAGCCTCTGG	19:T>G
1262203|F|0-65:C>A-65:C>A	TGCAGGACAAGCAGTGCGCCGGCAAGGACTTCGTGGTGCTCATCGCGCGCCTCCTCGTCGCCGAGCTCT	65:C>A
1025267|F|0-30:T>C-30:T>C	TGCAGGACCTCGGCGATGGCGCGGTCCACGTGGTCGGCGTCGTCGCGGAGCGCCG	30:T>C
1057947|F|0-13:C>A-13:C>A	TGCAGCCGATCTGCAAGCCACACCACATGAATCAGAATTCAGAAACACGAACAAACAGAACCGCAGATC	13:C>A
12419432|F|0-13:C>T-13:C>T	TGCAGGCGCTCTGCGCCGCCG	13:C>T
1025596|F|0-62:A>G-62:A>G	TGCAGCACAACAGGCAGTACTCCCTGGGGCCGCAGACGTTCTACGAGCCGAGCTGCGAGGCGACGAACG	62:A>G
3025482|F|0-26:A>C-26:A>C	TGCAGCATCTTGTAGAAGCTGTTGTCAAGCTTGGATGCGCTGGACCG	26:A>C
2252962|F|0-17:G>A-17:G>A	TGCAGGAAGCGGAGCGCGCGGGAGAGGTCCTTGCTCTCGACGGCGTCCTCGAACTCGCTCCACTCCCTG	17:G>A
5580213|F|0-11:A>C-11:A>C	TGCAGCCCAAGAGGCAGCTCCCGCCCG	11:A>C

<table>
<tr><td>2252962|F|0-65:C>A-65:C>A</td><td>TGCAGGAAGCGGAGCGCGCGGGAGAGGTCCTTGCTCTCGACGGCGTCCTCGAACTCGCTCCACTCCCTG</td><td>65:C>A</td></tr>
<tr><td>1150481|F|0-9:A>G-9:A>G</td><td>TGCAGCTCCACCATGCCGCTCTGCTGCCACGCGACCGCCTCACGCGGCGCGGCCGCCTCGTCGCCGAGA</td><td>9:A>G</td></tr>
<tr><td>981764|F|0-43:C>G-43:C>G</td><td>TGCAGATCACGGACGTGCAGCTGGAGATCATGATGGGCATCCTCAGCGTGTACGCGCTCATCGGGTCCT</td><td>43:C>G</td></tr>
<tr><td>1863780|F|0-48:A>G-48:A>G</td><td>TGCAGCGACACCGATGGACGTCGAGGCAGAAGCCCAACAACACCAGCCATGCCG</td><td>48:A>G</td></tr>
<tr><td>1117458|F|0-13:G>A-13:G>A</td><td>TGCAGGTGTCCACGTCGCTCCGGGAAGCGGACTTCTCGTACAACAACTTCTCCG</td><td>13:G>A</td></tr>
<tr><td>32020478|F|0-6:G>A-6:G>A</td><td>TGCAGGGAGTCTGGTCTCCCG</td><td>6:G>A</td></tr>
<tr><td>5579734|F|0-50:C>G-50:C>G</td><td>TGCAGGTTCGAGAGGCACAAGCTGCGGATGGCGGAGGCGAGGCGGCGGGCCGTGTACGAGGCGGAGGGG</td><td>50:C>G</td></tr>
<tr><td>6037975|F|0-7:T>C-7:T>C</td><td>TGCAGGATCTGCGTGCGCTCCCCG</td><td>7:T>C</td></tr>
<tr><td>977232|F|0-20:T>C-20:T>C</td><td>TGCAGGCCGTGGATGCAGGCTGCGTGGAAGGTGCATATCTCATGGAAGTTGAACTTCTTCCACGCCTCG</td><td>20:T>C</td></tr>
<tr><td>1227137|F|0-12:T>C-12:T>C</td><td>TGCAGCGACGCCTTGATCAGCCTGGCGGGGTGCCTGAGCTACGTGCAGGAGGGGAGCACGGTGGCGACG</td><td>12:T>C</td></tr>
<tr><td>12766259|F|0-8:T>A-8:T>A</td><td>TGCAGTACTGGTGGCACCTCCCG</td><td>8:T>A</td></tr>
<tr><td>3934237|F|0-16:C>T-16:C>T</td><td>TGCAGAGGCGGCGGCACACGCCGTTGAAGGGGCCGATGGCCG</td><td>16:C>T</td></tr>
<tr><td>992184|F|0-32:G>T-32:G>T</td><td>TGCAGGCACGACCGCATGCCCTTCCTGGCGTCGGCGTCCGCCGCCGAGGCCG</td><td>32:G>T</td></tr>
<tr><td>3940561|F|0-29:C>G-29:C>G</td><td>TGCAGCGAGGCGAGCCAGCGGCGCGCGCCCAGGGCCTGGCCG</td><td>29:C>G</td></tr>
<tr><td>7338616|F|0-17:C>G-17:C>G</td><td>TGCAGCAGGAGCGGTATCCCG</td><td>17:C>G</td></tr>
<tr><td>3951829|F|0-29:T>G-29:T>G</td><td>TGCAGCAGCTTCCCGACGAGCCAGGCGGCTATATAGCCG</td><td>29:T>G</td></tr>
<tr><td>1009565|F|0-38:G>C-38:G>C</td><td>TGCAGGCCCGCCTTCACCGACCGCGACACCGACTTGCTGCCCTTCGGCTTGCCCCTCCCGCCGCCGAGA</td><td>38:G>C</td></tr>
<tr><td>4983663|F|0-22:G>A-22:G>A</td><td>TGCAGCTGGTCGTTGGAGCGCAGCTGCTCCTCCG</td><td>22:G>A</td></tr>
<tr><td>1090901|F|0-49:C>G-49:C>G</td><td>TGCAGAAGGTGATGGCGCATCTCTCGCGACGGCTCAAGGACACGGCCTCCGACTCGTCCGTGCGGGACG</td><td>49:C>G</td></tr>
<tr><td>1212605|F|0-65:G>T-65:G>T</td><td>TGCAGCTTGCCCATCACCCTCGGGCTCCGCATCAGCTCCGCCATGCCCCAGTCCAGCGACGCCG</td><td>65:G>T</td></tr>
<tr><td>2247246|F|0-14:T>C-14:T>C</td><td>TGCAGGAGAGGGAGTCTCTCGGCCGCCTCGAAGAGCATAAGCACGTCCGACGCAGACGTGTAGCACAGC</td><td>14:T>C</td></tr>
<tr><td>2252771|F|0-5:C>T-5:C>T</td><td>TGCAGCTGCGCCGATCGTCTCCTCGATATTTCAATCATGTCGATGTCGGCGCTCGTATCTGCCCTCCGC</td><td>5:C>T</td></tr>
<tr><td>1863340|F|0-32:G>A-32:G>A</td><td>TGCAGCTCAAAGAACCCGTGCTCGATCCCGGCGATCTTGAATGCCTCGGCAGCGGTCACCTTGCTGCCA</td><td>32:G>A</td></tr>
<tr><td>1022558|F|0-66:C>T-66:C>T</td><td>TGCAGTACGGCGCCCAGCCCCTAATCTCCAAGCGCTGCGTCGGGTACGCCGCGCACCTTCATATCACCT</td><td>66:C>T</td></tr>
<tr><td>1027942|F|0-61:G>C-61:G>C</td><td>TGCAGAAGCTGCTGCACGCCGCCTACAAGCGCGGGGACACGTCCGTGGAGGTGCGCGTGCGGCGCCCCG</td><td>61:G>C</td></tr>
<tr><td>5580422|F|0-22:G>A-22:G>A</td><td>TGCAGGCCGCCGTGATCCCACAGTCCCG</td><td>22:G>A</td></tr>
<tr><td>5582001|F|0-19:G>C-19:G>C</td><td>TGCAGCTCTCGCACACGAAGTCCCAGTGCGGGCCG</td><td>19:G>C</td></tr>
<tr><td>1026430|F|0-41:A>T-41:A>T</td><td>TGCAGACGACGACCTGCTCGCTGGTTTCGGAACCAAGTCAGAGGCTAGGGAGGTGCCG</td><td>41:A>T</td></tr>
<tr><td>5359509|F|0-11:C>T-11:C>T</td><td>TGCAGTGGCTCCCGCTGCTCTCCGCGCTGGCGGCCCG</td><td>11:C>T</td></tr>
<tr><td>5581448|F|0-15:T>C-15:T>C</td><td>TGCAGCTTCTTCAACTTCCCGATGCTCGCCG</td><td>15:T>C</td></tr>
<tr><td>1084609|F|0-46:C>G-46:C>G</td><td>TGCAGGAGTTCTTCTACGACCAGGTCCCCGACGCGCTGCGCAGCCTCGGGCTGGCCTTCTTCCTCAGCA</td><td>46:C>G</td></tr>
<tr><td>32023804|F|0-67:C>G-67:C>G</td><td>TGCAGGCGCGCGGCGAGTTCGCGGGGGCGCTGGCGTCCTTCACGGCGGTGGTGAGCGGGTACAAGGACC</td><td>67:C>G</td></tr>
<tr><td>100531075|F|0-11:T>C-11:T>C</td><td>TGCAGCGCTGCTGCCCTATGAAAGGCCG</td><td>11:T>C</td></tr>
<tr><td>100397634|F|0-65:A>G-65:A>G</td><td>TGCAGCAGCAGGCGGAACAGGGAAGAGTAGGGCGCGTGTGAAAGTATGACAACGGACTTCTGCTCACCG</td><td>65:A>G</td></tr>
<tr><td>100417091|F|0-31:C>T-31:C>T</td><td>TGCAGGAGCCTCGTCAGGCTGTCGCAGATGGCGGAGGAGCCCATCCCG</td><td>31:C>T</td></tr>
<tr><td>100366392|F|0-17:T>C-17:T>C</td><td>TGCAGCTCCATCGTCTCTATCCTCCTCGCCTCCGTCCGCCAGTCCGTGCTCGTCATCTCCACCGACCCC</td><td>17:T>C</td></tr>
<tr><td>5361579|F|0-16:A>G-16:A>G</td><td>TGCAGGGGGTGTGGGTATGGCGGCCCTCGTAGGTGGTGATCACCATGCGGCAGTCCTCCGACAGCCGCT</td><td>16:A>G</td></tr>
<tr><td>1097915|F|0-50:G>A-50:G>A</td><td>TGCAGCCACGACACGAGCGTCCCGGCGACGAAGATGCCGAGGTTGATGGCGCCGAAGAACCAGGTGAAG</td><td>50:G>A</td></tr>
<tr><td>1027392|F|0-34:G>C-34:G>C</td><td>TGCAGGGGCTGGGGAGGTACTGGGGCGTGGGCGGGAGGAGGTGCGGGTCGTGCCG</td><td>34:G>C</td></tr>
<tr><td>1092599|F|0-31:T>C-31:T>C</td><td>TGCAGGTCGGCGCGGAGCACGTTGGCGGCGGTGTCGTGGGACTGCGCGACGTCGCGCCACATCTGAGCC</td><td>31:T>C</td></tr>
<tr><td>4986915|F|0-57:C>T-57:C>T</td><td>TGCAGAGCGGGCGCGGGCTGCTGTCCTCCGACGAGGTGCTGCTGACGCACCCCAAGACGCGGCGGTTCG</td><td>57:C>T</td></tr>
<tr><td>7339758|F|0-29:C>G-29:C>G</td><td>TGCAGGGCCAGAAGCGGCAGCATCCCGCTCGACGGGCCGCTGCCCCAGCCGACGCCG</td><td>29:C>G</td></tr>
<tr><td>5371683|F|0-16:G>C-16:G>C</td><td>TGCAGGAGCTGGCGCGGCATCCTCACCGACCCCG</td><td>16:G>C</td></tr>
<tr><td>5331558|F|0-13:G>A-13:G>A</td><td>TGCAGAGCCCGCTGCTGCCGCCGCCG</td><td>13:G>A</td></tr>
<tr><td>983127|F|0-12:G>A-12:G>A</td><td>TGCAGGGGAAACGAACCGAGTCAAAGGGGAAGCAACTAGAATCAGATATCACATACCTTATAGCAAAGC</td><td>12:G>A</td></tr>
<tr><td>1049648|F|0-11:C>T-11:C>T</td><td>TGCAGCTGGGCCCCAAATTCCACCTCATCGGCTTCTCCATGGGCGGCGAAATCATGTGGAGCTGCCTCA</td><td>11:C>T</td></tr>
<tr><td>1022741|F|0-68:A>T-68:A>T</td><td>TGCAGGAACCCCGTGTCCTTGGCCGACTCGCCCACGCTGGCGAGCCGCGCGCTGAACGTCGTGCACCGA</td><td>68:A>T</td></tr>
<tr><td>985914|F|0-65:T>G-65:T>G</td><td>TGCAGCTGGCCATACAATGCCTCCAATATCTTGTCCCCCTTGGGCAGCTTCTCTGCCAGGTTGAATGCC</td><td>65:T>G</td></tr>
<tr><td>2260901|F|0-21:A>G-21:A>G</td><td>TGCAGGCGCTGGCGCAGCCTCATCTCCGACCCAGCCTTCTCCCGCCGCTTCCGCCTCCACCACCGCCGC</td><td>21:A>G</td></tr>
<tr><td>1863340|F|0-14:C>G-14:C>G</td><td>TGCAGCTCAAAGAACCCGTGCTCGATCCCGGCGATCTTGAATGCCTCGGCAGCGGTCACCTTGCTGCCA</td><td>14:C>G</td></tr>
<tr><td>12772592|F|0-13:G>A-13:G>A</td><td>TGCAGTCTGCACGGAGTGAGTGAGTTGAGTTACTCACATGGTCGAGGCTCCCGTACCCTGCGACGACGT</td><td>13:G>A</td></tr>
<tr><td>993575|F|0-34:C>G-34:C>G</td><td>TGCAGCTGGCCGTGTACCTCTGCGCCGCCATGTCCCTCATGCTCTTCGCCGAGCGCCTCTACATGGGGC</td><td>34:C>G</td></tr>
<tr><td>12772134|F|0-23:T>C-23:T>C</td><td>TGCAGCCAGCAGCACTACGACGGTGACGGTGCTCACCGCGAGCTACTCCCGCTCAACCCTCACCTCTCT</td><td>23:T>C</td></tr>
<tr><td>3954303|F|0-11:G>A-11:G>A</td><td>TGCAGCGCCAAGAGGTTCCCGAGCGACGCCGGGATGTGACCG</td><td>11:G>A</td></tr>
<tr><td>3958398|F|0-6:A>G-6:A>G</td><td>TGCAGGATGTCCAAGGGCGTGCGGGAGCTGCCG</td><td>6:A>G</td></tr>
<tr><td>5357787|F|0-11:T>C-11:T>C</td><td>TGCAGCGACGGTATCGACGCGACCGCGGCCG</td><td>11:T>C</td></tr>
<tr><td>1022979|F|0-37:T>A-37:T>A</td><td>TGCAGTCCGTCCTTGTGAATTTCGTCAAGGGTGTTGGTAACCTTAGCCATGCAGAATGGCGGTCATTCT</td><td>37:T>A</td></tr>
<tr><td>1057947|F|0-33:A>G-33:A>G</td><td>TGCAGCCGATCTGCAAGCCACACCACATGAATCAGAATTCAGAAACACGAACAAACAGAACCGCAGATC</td><td>33:A>G</td></tr>
<tr><td>7172182|F|0-39:C>T-39:C>T</td><td>TGCAGGAGCAGCTGGTCACCGACCTGCTCGACATCGGCGCGCTCGCCG</td><td>39:C>T</td></tr>
</table>

2253639|F|0-47:G>C-47:G>C	TGCAGCGCGTACGAGTACATGAGGTTGTCGAAGCCGATGAGGGTGCCGAGCCCGAGGTAGATGGGCACG	47:G>C
6043170|F|0-31:T>G-31:T>G	TGCAGGAGGGCGCTTGGATGATGGGAGGAGTTGTTGGCCATGGCGGGGAAGGGTTGGGGACGCCGAGGC	31:T>G
1173088|F|0-13:A>C-13:A>C	TGCAGCTGCATCGACAACTCTAGGTTCCATAGCACGTCGCCCATGGCCGGCCGCTCCGTGCCTTGGTGC	13:A>C
4910385|F|0-16:C>T-16:C>T	TGCAGCTCAGCGCCGTCGAGAGGCTCAGCCTCCGCGGCGCCAACGTCAGCGGCGCGCTCGCCGCGGCGG	16:C>T
1689110|F|0-61:C>T-61:C>T	TGCAGTCCACGGAGCGGCAGTGCGCTCTCCCGGCGTCCTCGCACTCCTCCAACAGCACATCCCTGAAGT	61:C>T
994451|F|0-28:G>C-28:G>C	TGCAGCAGCAGAAGGGCCTGCTGTCGTCGGACCAGGTGCTCTTCGCCGACCGCCGCTCCCG	28:G>C
7167228|F|0-55:A>G-55:A>G	TGCAGTACACGCACGCGGTGCTGTACGAGAACATGCGCCTGTTCCCGCCCGTGCAATTCGACTCCAAGT	55:A>G
4543725|F|0-7:A>G-7:A>G	TGCAGCCACAGATACTCAGCTTCCCG	7:A>G
5580177|F|0-23:T>C-23:T>C	TGCAGCAGCCTCGGCACGGCGGTTCAGTGGGTGGTGGAGGCCGTCCCCCG	23:T>C
2290993|F|0-65:A>G-65:A>G	TGCAGCGCGGTCACCGTGGTGTCGACGCTGGCCGTGAGGAACTCCGTGCAGAGGCTCACCATCTCAGCC	65:A>G
1078576|F|0-68:G>C-68:G>C	TGCAGGTTCTCGGCCCAGGGCGAGAGGCCCTCGCAGCTCTTGAGGACGACGATGGAGTCGCGCCAGGAG	68:G>C
5367134|F|0-20:G>T-20:G>T	TGCAGCTAGCAACCCACTCTGGCCG	20:G>T
1319024|F|0-65:G>C-65:G>C	TGCAGGGCGGTGTTGACGCAGTGCACGGCGCACAGCTTCCCCTCCTGCACCTCGTGGTACAGCAGGCCC	65:G>C
10992853|F|0-5:G>C-5:G>C	TGCAGGAGGGCGTGATCCCG	5:G>C
1091975|F|0-15:T>C-15:T>C	TGCAGGTTGTATCTGTTATGGACCAAGTTCATACTGGTTTGGGCGGTGTACAGTTCCTTCTTTACGCCC	15:T>C
5345384|F|0-10:A>G-10:A>G	TGCAGAGGCTAAGCTTACAGGACAACAACCTCTCCG	10:A>G
1221427|F|0-30:G>C-30:G>C	TGCAGAGCTCAGTATCTTTCCAGAAGCATCGGTGCCTCCAGCGACATACGCCTTTTCACCGACGCTTGT	30:G>C
1698804|F|0-15:T>C-15:T>C	TGCAGTTGCTCCTGATCTGGCCGTTGCGGCCAGTGATTGGCGAGATGTTGCCCATCTTCACCATGGACT	15:T>C
1177014|F|0-19:G>A-19:G>A	TGCAGAGCGCGTGAACGACGGCATTGTACGAGGAGCACACAGGCACCGTCTCTGCCATGACCCTCCTGG	19:G>A
5579635|F|0-55:G>C-55:G>C	TGCAGCTCAGCGCCGTCGAGAGGCTCAGCCTCCGCGGCGCCAACGTCAGCGGCGCGCTCTCCGCCGCGG	55:G>C
2244237|F|0-25:C>T-25:C>T	TGCAGGAAGTATGCATCGACTGAATCTAGTGGACAGCGAAATGCAGCTCTACATAGTACAGCTCACGAG	25:C>T
1088797|F|0-68:G>A-68:G>A	TGCAGCAGCGCGAAGTGGAAGAACTTGCTGTTCACCGACGAGATGTCCTTCACCACCGTGTTCTCGTTG	68:G>A
1221427|F|0-15:C>A-15:C>A	TGCAGAGCTCAGTATCTTTCCAGAAGCATCGGTGCCTCCAGCGACATACGCCTTTTCACCGACGCTTGT	15:C>A
1252133|F|0-50:C>A-50:C>A	TGCAGGCTGAGGTGGACCAACTACCTGAGGCCCGACCTCAAGCGGGGGCTCCTCTCCGACGAGGAGCAG	50:C>A
994366|F|0-17:G>T-17:G>T	TGCAGCGCCACGGTGCCGCCCATGGAGAAGCCGTAGAGGAACCTCTTCTTCGTCTTGTTCTCCG	17:G>T
32022446|F|0-14:C>T-14:C>T	TGCAGCGCTGGTCGCAGACCCTCCCCG	14:C>T
1154680|F|0-46:C>T-46:C>T	TGCAGCGCGCGCACGGCCGCGTCCCAGCTGCCTAGCACGTCGCGGACGAAGAAGGCCTCGGCGTGGGCG	46:C>T
2253639|F|0-44:G>A-44:G>A	TGCAGCGCGTACGAGTACATGAGGTTGTCGAAGCCGATGAGGGTGCCCAGCCCGAGGTAGATGGGCACC	44:G>A
5369433|F|0-11:G>A-11:G>A	TGCAGCGCCGCGCCGACTGCAAGGACTCGTGGCCCG	11:G>A
5366970|F|0-11:T>A-11:T>A	TGCAGCCAGAATGCGGAGACCG	11:T>A
996579|F|0-26:T>C-26:T>C	TGCAGCTGCTCGTCGCTCACCACCCATGCGCCCTGCGTCCGCTGGATCTCGTCCATGGCGGCGTTGAAC	26:T>C
996535|F|0-14:G>C-14:G>C	TGCAGGACGCGGATGCGCTCGCTGATGCGCTCCCGCCGCAGCCTCGCCGCCACGCTCTGCGGGTCCTTG	14:G>C
3959627|F|0-8:G>C-8:G>C	TGCAGGGCGGAGGTCGTCGGTGGGAGGTTGTTCCCCACCCG	8:G>C
7917413|F|0-12:A>G-12:A>G	TGCAGCCGCCCTACACGCCACCG	12:A>G
4733884|F|0-39:C>T-39:C>T	TGCAGCGAGTCGGCTTCTTTCTCGTCGGCCATGAGGAGACTCCG	39:C>T
1144549|F|0-42:C>T-42:C>T	TGCAGGCGGGCGCCGACATCCTTGAGTTGCTCCTCAAGGTCGCTGTCCGCCCCGTCCGCCGCCATTTGA	42:C>T
1397989|F|0-28:G>C-28:G>C	TGCAGATGGCCTCGTACTCCGCCAGGTAGTCGTCCAGCGTCCTGATCAGCGTCAGCGCGTTCGTCTTGC	28:G>C
1221427|F|0-42:G>A-42:G>A	TGCAGAGCTCAGTATCTTTCCAGAAGCATCGGTGCCTCCAGCGACATACGCCTTTTCACCGACGCTTGT	42:G>A
3934782|F|0-56:T>C-56:T>C	TGCAGCACGTCGAGTCCGTCGCCGACGAGATGGAGCTGCGCCGCCGCGAGCTGCGGTTCTACGCCAACA	56:T>C
1025085|F|0-17:T>C-17:T>C	TGCAGCAGCGACACCAGTACGGTAGCCTCGCCCAGGTGGACAATCTGCTGGATGCACCTGACGACCTGG	17:T>C
5331884|F|0-19:C>T-19:C>T	TGCAGCCGCCGCAGCGACCCGACCACCGCCG	19:C>T
7172304|F|0-17:T>G-17:T>G	TGCAGGGGACTCATGCTTCCGCCGCCG	17:T>G
12696962|F|0-6:A>C-6:A>C	TGCAGCACCCGCCGCTTGCCG	6:A>C
3948468|F|0-16:G>C-16:G>C	TGCAGGGGGAGGACGCGCTCTTCGCGGGGCTGGGCGAGCTCCCG	16:G>C
1211100|F|0-40:C>G-40:C>G	TGCAGCGGCTCGTGGGCCGCCACGGCGCGCGCAACTGGTCCCTCATCAGCAAGTCCATCCCG	40:C>G
1088797|F|0-47:C>G-47:C>G	TGCAGCAGCGCGAAGTGGAAGAACTTGCTGTTCACCGACGAGATGTCCTTCACCACCGTGTTCTCGTTG	47:C>G
32025458|F|0-49:G>C-49:G>C	TGCAGACGGCGGCGGCGACGTCTTCGTTCATGGTGCTGTTCTGCGCGTCGATGTCGATGGTCCAGTTCA	49:G>C
3942865|F|0-26:G>A-26:G>A	TGCAGAAGCTGCGGTGGTACTGCCAGGTGTGCGAGAAGCAGTGCCGCGACGAGAACGGCTTCGAGTGCC	26:G>A
1026848|F|0-36:C>T-36:C>T	TGCAGCTTCCTCCGTCCAGGCGTCCACCCGATGCCGCTCCTCGCCACCCCACCCTATATAACGCCGCGC	36:C>T
1695414|F|0-14:G>C-14:G>C	TGCAGCACGCAGGCGAGGGACGACACGAGCGCCTGCCGCTCGAGCTCCTCCTTGGAGCCGCGGTCCAGG	14:G>C
1088797|F|0-5:C>T-5:C>T	TGCAGCAGCGCGAAGTGGAAGAACTTGCTGTTCACCGACGAGATGTCCTTCACCACCGTGTTCTCGTTG	5:C>T
2252818|F|0-25:A>T-25:A>T	TGCAGGCGAGCATGTAGTCGAGGCCACGGTCGGTGATGGTGATCAGGAACATGTAGAGCTCCCG	25:A>T
2263309|F|0-19:A>G-19:A>G	TGCAGCAGTGCTCGATCTGACCGAGGGACGTGTCGTCGCGCCGCGACGGCGGCAGGTCGTGCACCACCG	19:A>G
988333|F|0-19:C>A-19:C>A	TGCAGGTGGCGGACCTGCGCGCCGTCACCACCAAGAACCCCGTCGGCCACCAGTCGGAGCGCTACCGCA	19:C>A
5578273|F|0-7:G>T-7:G>T	TGCAGGCGCAGCGGCACTTCGTGCTCCACCG	7:G>T
5332366|F|0-7:G>C-7:G>C	TGCAGGCGGTGGGGCGCGGCCTCGACGCCGCCG	7:G>C
5581798|F|0-26:G>A-26:G>A	TGCAGCTCCCCGCACTGCGGCGCCGAGCACGGCACGGCGGCGTACGTGCTGGACGCGCGCGGGTTGTAG	26:G>A
5361397|F|0-11:C>G-11:C>G	TGCAGGATGGACACGACGACGGTGGCGGACATGTGGGCGGTGTAGACGCGGCCCCGCGGCGGGTCGGGC	11:C>G
7167437|F|0-7:A>C-7:A>C	TGCAGACATTCTCGGGGCTGATGGAGCACCTGGCCG	7:A>C

<table>
<tr><td>12769221|F|0-6:C>T-6:C>T</td><td>TGCAGTCGAGGACGCGGCAGCGGAGATGGCGGCGAGGAGGCGGGAGGTGGGAACGGGCAGGGGCATGGC</td><td>6:C>T</td></tr>
<tr><td>3031528|F|0-22:A>C-22:A>C</td><td>TGCAGCTTGGCGTACGGGTCGCACAGCCCCTGCGCCTCCGCCGCCG</td><td>22:A>C</td></tr>
<tr><td>992968|F|0-57:T>C-57:T>C</td><td>TGCAGCCTCTGGTGGATGACGTGCACGTTGTGCTGCGCGTTGAGGTTGAGGAGGCCGTAGGCGGGCACG</td><td>57:T>C</td></tr>
<tr><td>1165027|F|0-38:C>T-38:C>T</td><td>TGCAGCAGACTGTACCCGACGGCAGCGCCGTTGGCGGCCACCAGGAGCACCAGCGCCTTCATGTCGGTG</td><td>38:C>T</td></tr>
<tr><td>2373437|F|0-55:G>C-55:G>C</td><td>TGCAGGAGGCCGAGGCCGCCGCGCACCAGGCCGTCACCACCCTCGCCATGCGCGGGGAGGAGGCCATCG</td><td>55:G>C</td></tr>
<tr><td>1027263|F|0-7:G>C-7:G>C</td><td>TGCAGAGGACTTCTCCAGAGCGGTACGCGCCATCTCGGAGCTCGCCGCCG</td><td>7:G>C</td></tr>
<tr><td>2281920|F|0-65:C>A-65:C>A</td><td>TGCAGGCCCTCAGCGGCTCCAACATCGGCCTCATCATCGACGTGGCCAACGAGGACCTCGCCTCCCTCG</td><td>65:C>A</td></tr>
<tr><td>5346445|F|0-7:C>T-7:C>T</td><td>TGCAGGCCAGGCCGCGGCTGCCG</td><td>7:C>T</td></tr>
<tr><td>6036459|F|0-58:T>G-58:T>G</td><td>TGCAGTGGATCATGGCCGAACTGGTCAAGAACCCAGCAATCCAGGAGAAGCTCTACGATGAGATCAAAG</td><td>58:T>G</td></tr>
<tr><td>1027807|F|0-14:G>C-14:G>C</td><td>TGCAGCGGCGGCACGGTCGCCGCGGGCTCCTCCTCTTTCACCTCCTGCTGGGCTCGCACCG</td><td>14:G>C</td></tr>
<tr><td>7338775|F|0-13:C>G-13:C>G</td><td>TGCAGCTCGTCCTCCCCGCCG</td><td>13:C>G</td></tr>
<tr><td>2265717|F|0-28:T>C-28:T>C</td><td>TGCAGCAGCGGAAGGAGGCCGCGCACGTTGTCGAGCTCGAGAGGCGCTTCAAGGTGCTCGACGGCCTCA</td><td>28:T>C</td></tr>
<tr><td>7491866|F|0-20:T>C-20:T>C</td><td>TGCAGCGGCGACATGGGCGGTGGCGGCGCAGCCG</td><td>20:T>C</td></tr>
<tr><td>1204744|F|0-16:C>T-16:C>T</td><td>TGCAGGCGCTGGGACTCGCCGATGAGCTTGGCGAGGAGGGCGACGAAGGGGTCGCGGTCCAGGACTCCG</td><td>16:C>T</td></tr>
<tr><td>1251963|F|0-49:G>A-49:G>A</td><td>TGCAGATGGCCGCGTTCTACACCTCCCTCTACCTGGTGGCCCTGGCGCAGGGCGGGCACAAGCCGTGCG</td><td>49:G>A</td></tr>
<tr><td>1695187|F|0-36:A>G-36:A>G</td><td>TGCAGGGACATCGATGTCCTGCCCACACATCAGCGGACTGGCCGCGCTGCTCAAGGCGGCGCACCCCAA</td><td>36:A>G</td></tr>
<tr><td>13026144|F|0-17:A>C-17:A>C</td><td>TGCAGCCACCGCAGGTCATTCTCCG</td><td>17:A>C</td></tr>
<tr><td>2245090|F|0-32:T>C-32:T>C</td><td>TGCAGCTTCACCAGCCCCCTGAGCGCGCACAGTGCCTTCCTCGCCTGCGCGTCGATCCCGAGAAACGAA</td><td>32:T>C</td></tr>
<tr><td>5354962|F|0-23:C>T-23:C>T</td><td>TGCAGCTGCTCGAAGAAGAGGACCTGCACCACCACGCGCAGCGGCAGCCTCTCGTTCTGCGCCGCGTGG</td><td>23:C>T</td></tr>
<tr><td>1092026|F|0-28:T>C-28:T>C</td><td>TGCAGAACTGGTCGGCGAAGCAGGGCCATGTCAGCGTCGGCACGCCGTGGGAGATGGCCTCCAGCGTGG</td><td>28:T>C</td></tr>
<tr><td>7172030|F|0-25:A>C-25:A>C</td><td>TGCAGACCAGCACGCCCGCCGCCGCAGCCGTGGTGACGGCGCCG</td><td>25:A>C</td></tr>
<tr><td>2244900|F|0-59:G>A-59:G>A</td><td>TGCAGGTTCAGCTTCCCCATCTTATGGTCCAGCTTCGAAGCAGCTGATGAGCCCACATCGAACCGTGGC</td><td>59:G>A</td></tr>
<tr><td>7925064|F|0-65:A>G-65:A>G</td><td>TGCAGCTGCCGTCGGTGATTGCATTCCAAGATGGAGCGGTGACGACAACGGCGGCCACGCACCAGATGT</td><td>65:A>G</td></tr>
<tr><td>5354562|F|0-53:G>C-53:G>C</td><td>TGCAGCAGGATGGTGGAGCGGTCGACGACGATGGTGCGCACCAGCGTGGCGTCGGTCTCGAGCCGCGAC</td><td>53:G>C</td></tr>
<tr><td>1095052|F|0-10:C>G-10:C>G</td><td>TGCAGATGCCCTGCGACCGCGAGAACTTCTACCCGTTCCCGCCCTTCATGTCCG</td><td>10:C>G</td></tr>
<tr><td>1093739|F|0-50:A>C-50:A>C</td><td>TGCAGCGCCTGCTGCATCAGGAGGGCCTGCTGCTGTTGCTGCTGCTGCTGACGGAGCCTCTGCTGCTGC</td><td>50:A>C</td></tr>
<tr><td>1098189|F|0-21:C>G-21:C>G</td><td>TGCAGCTCGGGTTTGATTGGTCTACCATTGCCGTAAGTGCATCTTCAGAGTCGGGTGTAGGTTGGCCGA</td><td>21:C>G</td></tr>
<tr><td>7171687|F|0-18:G>A-18:G>A</td><td>TGCAGGTGGAGGGGGTGAGCACCAAGGAGGACGTGTCCTACTACGCCG</td><td>18:G>A</td></tr>
<tr><td>1024554|F|0-10:G>C-10:G>C</td><td>TGCAGGGCGGGACCATGGAGGGCTTCTTCAACCTCATCTCCTACTTCCAGACGCAGTCGGAGCCGAGAT</td><td>10:G>C</td></tr>
<tr><td>1106479|F|0-42:A>C-42:A>C</td><td>TGCAGCAGCAGCACGTTGTGCATCTGCGCGAAGAGGTTGAGCACCACGAGGCAGCCGTTGCCGTCGTAC</td><td>42:A>C</td></tr>
<tr><td>12775319|F|0-14:C>G-14:C>G</td><td>TGCAGCAGCTCGCCCAGCGTCACCACCG</td><td>14:C>G</td></tr>
<tr><td>1245487|F|0-51:A>T-51:A>T</td><td>TGCAGTCGGCGGTGCAGGGCGAGCTGTGGAGGATGCGGGACAAGGAGGGCAACGCTCCCGGGATGATCG</td><td>51:A>T</td></tr>
<tr><td>988194|F|0-37:G>A-37:G>A</td><td>TGCAGAAGGAGCTGGCGGAGCTGCGCGCGCTCAAGACGGTGCACCCCTTCTACATGCACCTCCCGAGAT</td><td>37:G>A</td></tr>
<tr><td>1696256|F|0-44:C>G-44:C>G</td><td>TGCAGCCTTCCGTTGCCCTGCACCCTAGGGTTCATGAAGTCTTCCGCGAGGAGGTCGACGAGGGCCATG</td><td>44:C>G</td></tr>
<tr><td>990470|F|0-65:A>G-65:A>G</td><td>TGCAGGACAGTGTTGCAAGCGAAGACCGTGACTCCAGTGAGCAGGTACACACAGCCAAGGACCCAATCT</td><td>65:A>G</td></tr>
<tr><td>1864140|F|0-24:G>C-24:G>C</td><td>TGCAGATCTGAAGGAGATCGCGTCGGTGGTCGAGGCGGGGTCGCTGACCAAGGAGGTCCGCCG</td><td>24:G>C</td></tr>
<tr><td>1146929|F|0-62:C>G-62:C>G</td><td>TGCAGAGATCGTGCCAGCGCCACCTGCCGCCCCAGGGAAGAAGCCCTGTACCACGCAAACCCCACGGTT</td><td>62:C>G</td></tr>
<tr><td>3024455|F|0-9:C>T-9:C>T</td><td>TGCAGAAAGCACGTGATGGGGTTCGATGGAAACGATTGGTTTGACCG</td><td>9:C>T</td></tr>
<tr><td>4994901|F|0-7:C>G-7:C>G</td><td>TGCAGCGCTTCGAGTTCGGGCTGTCGCCGTCCTACAGGCACGCCCCGTTCCCG</td><td>7:C>G</td></tr>
<tr><td>4261727|F|0-9:A>G-9:A>G</td><td>TGCAGATGAAGCCCGCCCCGTACAACATCGTGCTCCG</td><td>9:A>G</td></tr>
<tr><td>3029106|F|0-41:T>C-41:T>C</td><td>TGCAGCTGCCGCACTCAGGCCTAACGTGGTCGCAGCAGCTGTGCCG</td><td>41:T>C</td></tr>
<tr><td>1125398|F|0-61:G>A-61:G>A</td><td>TGCAGCACCTGTCGGCCACCCCGACGTTCATCGCCGCCGTGCAGGCGCAGGCCGACATGGCGCCGAGAT</td><td>61:G>A</td></tr>
<tr><td>3956374|F|0-23:C>A-23:C>A</td><td>TGCAGGACGGGTTCTACGGCACCCGCACCAACTGCACCGTCGACGTCGAGGCCGTCGTGCGGGGCGTCG</td><td>23:C>A</td></tr>
<tr><td>2252962|F|0-49:C>G-49:C>G</td><td>TGCAGGAAGCGGAGCGCGCGGGAGAGGTCCTTGCTCTCGACGGCGTCCTCGAACTCGCTCCACTCCCTG</td><td>49:C>G</td></tr>
<tr><td>988133|F|0-52:C>T-52:C>T</td><td>TGCAGATAAGATTAAAAAGTGTGCCTTCTTGCAGGAGGAGCATGAAGCAATTCTGAAGAAAAGAGAAGA</td><td>52:C>T</td></tr>
<tr><td>1032056|F|0-50:A>G-50:A>G</td><td>TGCAGCACATTATGCATCTTCTCTTCAGGCAAGCCCTCCAGCCCCGCCCTAAGCATGTTCTTCTCCTCC</td><td>50:A>G</td></tr>
<tr><td>3024805|F|0-19:C>G-19:C>G</td><td>TGCAGAGCCCAAGCCCCACCACCAGATTCCCAGGCCCACCCG</td><td>19:C>G</td></tr>
<tr><td>3385526|F|0-5:C>A-5:C>A</td><td>TGCAGCACACGGTGATCCCGGAGCGGCCCATGTTCTACCCG</td><td>5:C>A</td></tr>
<tr><td>1127737|F|0-24:A>G-24:A>G</td><td>TGCAGCGGCGTCGGCGTGTGGCCGATGAGCCGCCTCCAGAGCGGGAGCAGGACCCG</td><td>24:A>G</td></tr>
<tr><td>2257769|F|0-48:G>A-48:G>A</td><td>TGCAGCAGCCTGAAGCCGACGTTGGAGGCGGCGAGCCGCGTGTCGACGGAGGCCTGCTCCAGCCCCTCC</td><td>48:G>A</td></tr>
<tr><td>2338168|F|0-19:T>C-19:T>C</td><td>TGCAGGAGCTCGAGCTCGCTGGGAACGGCCTCGGCGGCAAACTGCCGTCGTTCATCGACGAGCTCTTGC</td><td>19:T>C</td></tr>
<tr><td>1202978|F|0-44:G>C-44:G>C</td><td>TGCAGCGAGCCCTCCGAGATTTTGACCTCCACGAGCGCAAGCTTGTTGAGCAGGGGAAACTGAAGCGTC</td><td>44:G>C</td></tr>
<tr><td>5578260|F|0-8:G>A-8:G>A</td><td>TGCAGGCCGCGCACCCTGTCCTGCCACCG</td><td>8:G>A</td></tr>
<tr><td>1027501|F|0-12:T>C-12:T>C</td><td>TGCAGCGCGGCCTTGTTCATGAGGATGGCGCACATGCACGCCGCCTGCGTGGCCGCCGCCTCCTTGAGC</td><td>12:T>C</td></tr>
<tr><td>6036060|F|0-62:A>C-62:A>C</td><td>TGCAGCTTCTTCTGCTGCGTCTTCACGGTCCTGCTGATGATCTTGTAGTTCCAGCGCTGCTGAACGTAC</td><td>62:A>C</td></tr>
<tr><td>1012054|F|0-35:G>C-35:G>C</td><td>TGCAGTCAGCCTCCAAGGAGAAGGTGCAAACTCCTGAGATAGAGAGGCCG</td><td>35:G>C</td></tr>
<tr><td>32022834|F|0-18:A>G-18:A>G</td><td>TGCAGCCGCCGTGATGGCACCAGTCCCAGCCGCTCCCAAGAGCTCCCTCGAGGTCTTGCTCGAGACCAT</td><td>18:A>G</td></tr>
<tr><td>32023291|F|0-16:T>C-16:T>C</td><td>TGCAGCGCGATCTCGCTCGCGGCATGGCTCCCCGCGCCTCAGCCG</td><td>16:T>C</td></tr>
<tr><td>1022812|F|0-29:G>A-29:G>A</td><td>TGCAGACGCACTACAAGCGCAGGCACTGTGCCAAGCCATTCCTCTGCCGCAAGTGCGGCAAGGCGCTCG</td><td>29:G>A</td></tr>
</table>

992184|F|0-11:C>G-11:C>G	TGCAGGCACGACCGCATGCCCTTCCTGGCGTCGGCGTCCGCCGCCGAGGCCG	11:C>G
1032871|F|0-65:A>G-65:A>G	TGCAGGGGCGCTTGGCCAGCGCGGGTGCAGCCATGGCCGCGGAGGCGAGAGGCGCTACTAGTAGCAGGC	65:A>G
5971550|F|0-41:T>A-41:T>A	TGCAGCGTCGACAACACCCTCAAGGCCCGCGTCGCCACCTTTCGCGGCTATGGCCTCTCCGAGGACCAC	41:T>A
986673|F|0-10:A>C-10:A>C	TGCAGGCTGGATGGCTGGTAGTGACATTGACAAGAACAATATTGTTGCCG	10:A>C
32027660|F|0-29:G>A-29:G>A	TGCAGAGAGCGGCGAGCGCTTCAACAGGGGCAGAAGACGCCGAGGCTCCTCCCTCCG	29:G>A
2254347|F|0-12:C>T-12:C>T	TGCAGATGTCGGCGTGATGAGCGGCTGCATCATCTTCATCCAGAAGGACCTCCACATCGACGAGGTGCA	12:C>T
1219765|F|0-30:G>A-30:G>A	TGCAGCCAGGAGTGCAGGGAGCATCAGATCGCGAAGGACGAGCTCATGGAACAGAACTGCACCATCACA	30:G>A
4733768|F|0-15:T>G-15:T>G	TGCAGTCGAACGGGCTGGCTGCCGCCGCGGCGCACTGTGCCG	15:T>G
3934237|F|0-22:G>A-22:G>A	TGCAGAGGCGGCGGCACACGCCGTTGAAGGGGCCGATGGCCG	22:G>A
1695187|F|0-18:C>T-18:C>T	TGCAGGGACATCGATGTCCTGCCCACACATCAGCGGACTGGCCGCGCTGCTCAAGGCGGCGCACCCCAA	18:C>T
1028775|F|0-45:A>G-45:A>G	TGCAGCGCGGCTTCGGCTCCCTCGTCCGCGAGCCCTGCGGCCTCAACCCG	45:A>G
1696577|F|0-64:C>T-64:C>T	TGCAGTTCACGGTCCCCGCCCTCAAAGGCGGCACGCTCGACTCCCTCCTCGCCCTCAGCGACGACCTCA	64:C>T
996653|F|0-37:A>T-37:A>T	TGCAGAAGGAACTGAACAGATACATCCCTACTGCCGCAGCATTTGGTGGAGTGTGCATCGGCGCATTGA	37:A>T
1095408|F|0-47:A>G-47:A>G	TGCAGGCGCTTCTTCAGGCGCTCGGTGGAGACCGCCGCGGCCGCCGCAGTGTCCCCCG	47:A>G
1023358|F|0-17:C>G-17:C>G	TGCAGCGAGGAGATCCTCAACCCGTCGGTGCCCATGGCGCTCAGGCTCTCCG	17:C>G
995365|F|0-30:C>T-30:C>T	TGCAGACACGGCGCATTTACCTTTGTCCCGCCACGCGGTGAAAGCTAGCGCACGTCTGTCTGGTGGTAA	30:C>T
998540|F|0-8:G>C-8:G>C	TGCAGCCTGGAGTGCAGGCAGCAGCAGATGAACCTGGACGAGCTCAAGGACAAGAAGTGCTTCCCGAGA	8:G>C
4993802|F|0-9:C>T-9:C>T	TGCAGGAAGCTGCCGAGGGCGTCGCCG	9:C>T
3943944|F|0-7:G>T-7:G>T	TGCAGCAGGTGGCTGGTGGCCTTGTCGACCCGCGACGCCGCCG	7:G>T
5578381|F|0-5:G>C-5:G>C	TGCAGGTGGCGGCGCGCGACGCCATCCCG	5:G>C
2351339|F|0-64:G>C-64:G>C	TGCAGATCCTGCTCGAGTACATGGACGGCGGGTCCCTGGACGGCCGCCGCATCGCGTCCGAGTCGTTCC	64:G>C
1025284|F|0-8:C>G-8:C>G	TGCAGCTCCCCGAAGGGCACGTCGGCGAGCACGCGCTCCAGCTTCCTCTCCCGCTCCGCTAACTGGTCG	8:C>G
1023404|F|0-50:A>G-50:A>G	TGCAGAGGGATGGGAGCAGCAGGTGGCGACGGCAAAGGTGTGCGGGGATGACCG	50:A>G
7925088|F|0-8:C>G-8:C>G	TGCAGGAGCAGGCGCGGCGCGGGAAGAAGCGGCCTCTCTCGGTGGCGGAGGAGAGGGGGTATCGCGAGT	8:C>G
1102054|F|0-14:A>G-14:A>G	TGCAGCCTGTCCTCAAGAAAGTGCTTCCGCATGTGGCCGCCGAGCGCCTGCCCG	14:A>G
4002258|F|0-37:C>G-37:C>G	TGCAGACCAGGGGGTGCTCGCGGCTGAGGCGCATACCCATGCGGCCG	37:C>G
2252445|F|0-19:T>G-19:T>G	TGCAGAGGCAGAAGAGCGATCGGCACCTGGAAGAGAGCTCCATCTCCATCGACCAGCTCCACAGGCTGA	19:T>G
5577920|F|0-17:C>T-17:C>T	TGCAGCAAAGAAAGAACCCCCG	17:C>T
1009738|F|0-63:T>G-63:T>G	TGCAGCTGCCCTGCATCATGTGGCTCGCCATCTACAAGCCCAAAAGATTCAGCCTCTCATGGTTCACCA	63:T>G
7940658|F|0-33:A>G-33:A>G	TGCAGGCGCCCGTGCTCAAGAAGTACCCCGCGAAGCTGTCCGTCACCTCCTACACCTGCTTCTTCGGCG	33:A>G
7353569|F|0-8:A>G-8:A>G	TGCAGCTCATCGATGACCGCGCGGCCGCCG	8:A>G
7155323|F|0-14:G>C-14:G>C	TGCAGGAACGGCCGGCCATGCGCGTCCG	14:G>C
1203749|F|0-21:G>C-21:G>C	TGCAGGGGGGAGAAACGAACAGCAATAAGCAACGCGGCCGTCTGAACCCTTTCTAGAAGCTTCCGAGAA	21:G>C
1018656|F|0-23:T>C-23:T>C	TGCAGCGAAAGAGGAAGAGAAATTGCGTGAAAGTACGTCGCCGAGAAGCAGGTGGCAGAAATGGAGGCG	23:T>C
988133|F|0-14:A>G-14:A>G	TGCAGATAAGATTAAAAAGTGTGCCTTCTTGCAGGAGGAGCATGAAGCAATTCTGAAGAAAAGAGAAGA	14:A>G
1021826|F|0-50:C>T-50:C>T	TGCAGGTGCGAGGGCACGTCCACCTATCCGCGGACCTACGATCTCATCCACGCCGACTCGGTGTTCACT	50:C>T
2251892|F|0-30:A>G-30:A>G	TGCAGAATCCACCACCACGAGATGTTTTCGAAACCCCAGAACGTCACAGCGGCGGAAGCCGATAAACCA	30:A>G
6030814|F|0-23:C>A-23:C>A	TGCAGGGCAACCGCCTGAACGGCCCCATCCCG	23:C>A
1001554|F|0-8:T>C-8:T>C	TGCAGCCCTTCCTCGGCGGCGACACGGCCGACCTCCTGCGCTTCTCGTCGCAGGTCGGGGAGTGGGTCA	8:T>C
5578265|F|0-28:G>T-28:G>T	TGCAGGCCGTGCTCGTCTCCCCCGCCGCGCTGGCCG	28:G>T
5411876|F|0-7:G>A-7:G>A	TGCAGTTGGCGCGTATCTCGCCGCTGCTCCCG	7:G>A
4986915|F|0-23:T>G-23:T>G	TGCAGAGCGGGCGCGGGCTGCTGTCCTCCGACGAGGTGCTGCTGACGCACCCCAAGACGCGGCGGTTCG	23:T>G
2252834|F|0-27:C>T-27:C>T	TGCAGCGAGTAGTAGTCGTGTGGCCAGCAGTCATCGAGCTTGACGAGCGGCCTCTCGCCGTCGGCCCAC	27:C>T
1030190|F|0-13:C>A-13:C>A	TGCAGTCCCACGGCGTCGCCTTCCGCGAGCGCGACGTCTCCATGGACCGCGGCTTCCG	13:C>A
2251292|F|0-68:T>G-68:T>G	TGCAGCGGGTTGAAGGTGAGCATCCTCTCGATGAGGTCCAGCGCGGCGGGCTGCACCTTGGGGAACTGT	68:T>G
3029761|F|0-30:C>G-30:C>G	TGCAGGGGCGGAGACACCGCTCCGGGATTTCCGCTCCG	30:C>G
984867|F|0-41:A>G-41:A>G	TGCAGCTGTTGGTAAAGGGGCAAGTCCATGGGGCTAGGCGAATCATAGAAAGGAAACATGCTTTAGTGA	41:A>G
7340601|F|0-5:T>G-5:T>G	TGCAGTACAGCTTCGAGCCG	5:T>G
5581445|F|0-17:C>T-17:C>T	TGCAGCTGTCGCAGGAGCTCATGGCCG	17:C>T
5357985|F|0-14:C>G-14:C>G	TGCAGCTCCTGCCCCACGGGCGCCGTCACCCGCCG	14:C>G
5580381|F|0-27:C>T-27:C>T	TGCAGGAGGCGGTTGAGCGCGGGGTGCCTGGTGATCTGGTGGTGGCCG	27:C>T
2242108|F|0-37:A>G-37:A>G	TGCAGAATTCAGCTGCTAATTCCACAATACAGTCGACACGAAATAGAAGCTAGAGCAGCGAGATCGAGA	37:A>G
1001208|F|0-58:G>A-58:G>A	TGCAGGGGCCGCAGCTGCCGCCGTTCCTGTCCAAGACGTACAACCTCGTCAGCGAGCCGCAGCTGGACG	58:G>A
5367350|F|0-22:G>C-22:G>C	TGCAGGCGTGGTGGCACGACGTGCGCACCGTCGGCCACGGCGACCG	22:G>C
2261024|F|0-50:G>C-50:G>C	TGCAGCACGAGCCCGACAGCCACGGCGCCGAGGCTCACCAGCGCCACCTTGAGCGTCGACAGCGCGAGC	50:G>C
3024408|F|0-14:A>G-14:A>G	TGCAGAAAGGCCAGACCTTTGCTCGTGGAGAGGTACCG	14:A>G
1695414|F|0-41:G>C-41:G>C	TGCAGCACGCAGGCGAGGGACGACACGAGCGCCTGCCGCTCGAGCTCCTCCTTGGAGCCGCGGTCCAGG	41:G>C
1320455|F|0-16:C>T-16:C>T	TGCAGTTTGGGAAGGTCGCGAGGGACATGTTCACCATGGACTACCGCTACCCTCTGTCGGCGTTCCAGG	16:C>T
4260627|F|0-37:G>C-37:G>C	TGCAGGAGGCGTGGCTGGAGGAGTTCGTCGACGCGCGGCTGCGCGGCGACTTCAGCCACCCGCAGGCGG	37:G>C

3030533|F|0-12:G>C-12:G>C	TGCAGCGCCGACGGCGCCCGCGTCGTCGAGCTGCGCCTGCCG	12:G>C
4986834|F|0-22:C>G-22:C>G	TGCAGCTCAGGCGCCCGCGGTCCCTCCG	22:C>G
987160|F|0-27:C>T-27:C>T	TGCAGCCGCGACTTGTGCCATGACAGGCAGGAGCGGCGAAGGTTCTTCTGGACCAAGAAGGCCAAGAGG	27:C>T
12695468|F|0-9:T>C-9:T>C	TGCAGATCATCATGGATACCCTGGTTGCCGCCAGGCAGGTCAACAAGGCCGTCCAACTGCTCCAGAGCG	9:T>C
1023241|F|0-19:C>T-19:C>T	TGCAGAACGGCTGCGAAAACGGCCCGAAGTTTTGATTAGGATCGTCTGCCATCTCTATGCCCAGCAACT	19:C>T
1220382|F|0-29:G>A-29:G>A	TGCAGCTGTTGGTAAAGGGGTAAGTCCATGGGGCTCGGGGAATCATAGAAAGGAAACATGCTTTAGTGA	29:G>A
1007026|F|0-7:A>C-7:A>C	TGCAGGAATGCGCCGTCCGCCGAGCGCTCCGCTGCGGACAGGAGCTCCACGCGCGGCTGCTCCGTTCCG	7:A>C
6047684|F|0-41:C>G-41:C>G	TGCAGGGTCACCTCGGCGGGCCGCGGCGCGAGGAAGCGCACCGCAAGGCCG	41:C>G
4261235|F|0-20:G>C-20:G>C	TGCAGCCGCCGCAGGCTCGCGACGGTGAACACGGCCG	20:G>C
12775376|F|0-23:T>C-23:T>C	TGCAGGGACAGCGGCGCGATCCTTGACTCGGACAGCGCCCG	23:T>C
32027151|F|0-37:C>T-37:C>T	TGCAGGTGCGGATTCGCTCGCTGGTACGCCGCCAGTGCACGCCGCCCATCCGTATGATTCGGTTCCTGT	37:C>T
7169676|F|0-7:C>G-7:C>G	TGCAGGCCGCCGTGGGGGTGTCGGCGCCG	7:C>G
32018090|F|0-25:A>G-25:A>G	TGCAGATGTCGTAGATGGCCTCGTTATCGAGCAGGATGGAGACATCGGTGTGCTCCAGCAGGGAGTGGG	25:A>G
3949377|F|0-11:G>C-11:G>C	TGCAGGTTCCAGAGCACGTCGTCGACGGAAGGCCG	11:G>C
2248029|F|0-54:C>T-54:C>T	TGCAGTGACGCGCCGAAGACGTAGGGACTGTAGTTCTCCCTGTCGTAGTAGTACCGCATGACCACCCGC	54:C>T
994451|F|0-25:G>C-25:G>C	TGCAGCAGCAGAAGGGCCTGCTGTCGTCGGACCAGGTGCTCTTCGCCGACCGCCGCTCCCG	25:G>C
1088797|F|0-40:A>C-40:A>C	TGCAGCAGCGCGAAGTGGAAGAACTTGCTGTTCACCGACGAGATGTCCTTCACCACCGTGTTCTCGTTG	40:A>C
994895|F|0-52:G>C-52:G>C	TGCAGGGAGTGGAACCGCGCACTTGGGGTGGGATAGCATCCATCATGTTCCAGCAGCAGCAGCCGCCGC	52:G>C
1047138|F|0-26:T>C-26:T>C	TGCAGGTATGCCACAGACGGAAGAGATGTCCTTTCACAGATTAAGGCTGGAGATAAAATTCGCTCCGCC	26:T>C
2266783|F|0-50:T>A-50:T>A	TGCAGACCGTGAGCGCGATCGCCCTCGGGAGCGAGACAGAGAGGGCCCCGTGGAAGGCGGTGTGAAGGA	50:T>A
1368203|F|0-37:C>G-37:C>G	TGCAGCCCAGCGACACCTTCCCGCGGAGGCACAACTCCGCCACCCCCGCCGAGCAGGCCGTCATGGCGT	37:C>G
988567|F|0-24:G>A-24:G>A	TGCAGATGCCGAGGAAGATGCTGAGCAAGCTGTGGTCCAGCAAGGCGAGCAGCGGGGAGAACAGCGCCG	24:G>A
2245873|F|0-19:C>T-19:C>T	TGCAGGAGGACTCCCAAGTCGTGGTTGCTGTGCTCGACACTTTGTCAAACCTTAACCTCGATGCGCTGT	19:C>T
7911392|F|0-11:C>G-11:C>G	TGCAGGCCGAACCCGTACTCGCCG	11:C>G
1026037|F|0-48:C>T-48:C>T	TGCAGCTCCGACCCGATCAGCGACTGCATCCGCGCGCGCTCCCTGTCCCGCGGGTACGTGCGGTACAGC	48:C>T
3025064|F|0-31:A>G-31:A>G	TGCAGCACCAGGCGCTACTCTCGGTCACCACATTCCCG	31:A>G
2251292|F|0-50:C>T-50:C>T	TGCAGCGGGTTGAAGGTGAGCATCCTCTCGATGAGGTCCAGCGCGGCGGGCTGCACCTTGGGGAACTGT	50:C>T
4983200|F|0-28:C>G-28:C>G	TGCAGCCGCGGGTGCACGGCTGCGAGGCCTCCGTGGACTTCTTCGAGAACCAGAGCTTCTACCCGCCCA	28:C>G
5354395|F|0-5:A>C-5:A>C	TGCAGATCGACGGCACGATCGTGGCGCCGAGCGAGCCCACCACGTGGCCGGCCAACAGCAAGCGCAACT	5:A>C
100549396|F|0-22:C>T-22:C>T	TGCAGAAGATTGTGATCAGAGCCGATCTCGTCGGCAAGAAGTGCACGAGTGGGATCCTGTCAATCGTTT	22:C>T
7167507|F|0-15:G>C-15:G>C	TGCAGAGGGCGGCGTGGAGGCCGCCGTCGCCG	15:G>C
5578090|F|0-19:A>G-19:A>G	TGCAGCGGCTGGAGGGTCGATGGCCG	19:A>G
1022920|F|0-10:A>G-10:A>G	TGCAGCAGGCACTGGACACGCTGCTGGGGCGGCGGAGCCTGAAGATGAGCGACTTCGTGGTGGGCGAGC	10:A>G
1092636|F|0-11:G>C-11:G>C	TGCAGAAGCATGGAGGTGAGCCCGACGATCTGTGCCCGCACGGCCCGCGGCGCCG	11:G>C
5363381|F|0-17:C>A-17:C>A	TGCAGCCCGCCGCGGGCCAGGATCTCCTTGCCG	17:C>A
1717652|F|0-43:T>G-43:T>G	TGCAGCGTGCTGTAAAGCCCGATCCTCCCCACTAAGAAGAGCCTTTTGCGAGCTCCGGCGACGAGATAC	43:T>G
3029783|F|0-5:G>C-5:G>C	TGCAGGCGCTTGAAGTCGCGCATCAGGCGCTTCCTCGCCG	5:G>C
7906816|F|0-5:T>A-5:T>A	TGCAGTAGTACCCTTGCCCCG	5:T>A
3939903|F|0-24:T>C-24:T>C	TGCAGTCGAAGGCGGCGTCCAGGGTTCTCGGAGCCATCAGGCCG	24:T>C
1003040|F|0-63:G>A-63:G>A	TGCAGAAGCTGCGGTGGTACTGCCAGATGTGCGAGAAGCAGTGCCGCGACGAGAACGGCTTCAGGTGCC	63:G>A
1097915|F|0-26:G>A-26:G>A	TGCAGCCACGACACGAGCGTCCCGGCGACGAAGATGCCGAGGTTGATGGCGCCGAAGAACCAGGTGAAG	26:G>A
1201985|F|0-35:T>C-35:T>C	TGCAGTTGACGACGAGCACGCCGATGTCCTTGGGCTTGACGCCCGTCTTGGCGAACAGCTGGTCCAGCG	35:T>C
5992634|F|0-5:C>T-5:C>T	TGCAGCGCCATATTATATGTCTGCTTCCG	5:C>T
5578265|F|0-13:C>T-13:C>T	TGCAGGCCGTGCTCGTCTCCCCCGCCGCGCTGGCCG	13:C>T
22841466|F|0-19:A>G-19:A>G	TGCAGGCGGTAGGGCAAGAACCCG	19:A>G
1027781|F|0-18:T>G-18:T>G	TGCAGCTGCCGCGCCAGGTAGGCGGCGTACCTCGCGACGTCCTCGCGGCCG	18:T>G
7167385|F|0-21:A>T-21:A>T	TGCAGAAAGCATGTGATGGGGATCGATGGAAACGATTGGTTTGACCG	21:A>T
32025951|F|0-17:G>C-17:G>C	TGCAGAGCGCCCTGAATGTCCGCCATGTACTTGTTGTCTTCGTCTAACCATCGGGCGTACTCCATGTCG	17:G>C
1027806|F|0-24:C>T-24:C>T	TGCAGCGGGTTGGGCCCGCTCACGCGGCAGTGCAGGTGCAGCGTCATCTCCCCGCCGCCCG	24:C>T
1012397|F|0-5:C>T-5:C>T	TGCAGCTGCCGCCGCCGCTCGCGGCCGCGGCCTACGCCGCCCTGCTCTCCCCG	5:C>T
5992634|F|0-7:C>G-7:C>G	TGCAGCGCCATATTATATGTCTGCTTCCG	7:C>G
1342351|F|0-57:C>T-57:C>T	TGCAGGCGCGGCAGGCGGCGTGCGCGTCGTCCCCGCAGGCCGCGCAGGGAGGATGGGCCCCCG	57:C>T
1027421|F|0-47:A>G-47:A>G	TGCAGGCAAGCGTCCAGCCAGGTCGGCGGTGGCTCCTGTGGACCGCCATTGACCCTGGGCTGCGCCGAG	47:A>G
1863340|F|0-26:C>A-26:C>A	TGCAGCTCAAAGAACCCGTGCTCGATCCCGGCGATCTTGAATGCCTCGGCAGCGGTCACCTTGCTGCCA	26:C>A
5370829|F|0-40:T>C-40:T>C	TGCAGCGCGTGCCCACGCGGGCCTTGAGGTCGGCGACGAATTCGTCCG	40:T>C
1240323|F|0-25:C>G-25:C>G	TGCAGCGGGAGGTGCAGGAGCTGCGCGCGCTCAAGCTGCTCGCCCCGCCG	25:C>G
5581411|F|0-8:C>G-8:C>G	TGCAGCGTCGCGTTGGTGAGGCCGAGGAAGCCGCCGTGGCTGCCG	8:C>G
2316497|F|0-15:T>C-15:T>C	TGCAGGCCGTCGCCCTGCTTGACGAAGGACTCGAACTTGAGCGGCAGCCCCTTGCCG	15:T>C
1051874|F|0-32:A>G-32:A>G	TGCAGCTTCTCGTCGCCGATGAGCGGGCGGGCATGGACCGCCACCTTGACGCAGCAATCCTCGCCGTGC	32:A>G

5582347|F|0-8:C>G-8:C>G TGCAGCGCCAGCGTCTTCGGGGTGTCGCCG 8:C>G
7925602|F|0-23:G>C-23:G>C TGCAGGAGAACGGCGGCTCGTCAGTCCAGGGCCCG 23:G>C
995571|F|0-13:T>G-13:T>G TGCAGTATCCAGTTGCAGTGCCTGTCGGGCAACCTGCCGCGGGCGGCGACCTGCCACGACGACGAGAGC 13:T>G
1024243|F|0-57:G>C-57:G>C TGCAGCAGGCTGTTGTGGTACGTGGAGAGGTCGTTGAACTTCTTGAGGCAGCCGAGGGCGTCGTAGTCG 57:G>C
1206575|F|0-27:G>T-27:G>T TGCAGCTCCACGAAGGAGAGACACAGCGCGACAGAGATGCTCATGGCCTGCCCG 27:G>T
6039999|F|0-10:T>C-10:T>C TGCAGAGGTTTGACAAGGCGGACGCCGCGGGCGAGGCGCGGCGGATGGTCTGCGCCGCCGACAGCAACA 10:T>C
7343490|F|0-14:G>C-14:G>C TGCAGCTCCCCCCAGGACCG 14:G>C
1091375|F|0-6:G>A-6:G>A TGCAGCGCTTTCTAGGAGGGTGTTGATGACGGGGGTTCTGTGGAGAGAGGGGGTGGAGGCACGTCGCCT 6:G>A
1029183|F|0-16:A>C-16:A>C TGCAGATCGGGCCCCGACGCGCCGCTCGCCGTGTCGTCCGCCGCCGCGCCG 16:A>C
985222|F|0-9:G>C-9:G>C TGCAGCTGCGTCAACGTGGAGAAAATAGCGGGCTGCTCCAGAGAAAAATCTCCGCCATCGTCATCGCGT 9:G>C
32019101|F|0-21:G>A-21:G>A TGCAGGGGAATCGCCCATGGAGAGCTCCCG 21:G>A
2286159|F|0-44:C>T-44:C>T TGCAGCGCCTCCCACGAGCGGTCGTCGGCGTAGGCGCGCTCCCACGCCTCGAGGACGCGGCCGTCGCCG 44:C>T
3948849|F|0-27:G>A-27:G>A TGCAGCTGTTCCAGGAGTACAAGGAGAGGGTCGGGGGCAGCGTCCCG 27:G>A
1027875|F|0-65:C>G-65:C>G TGCAGCAGCTTCTCCACGATCATGATGATGTTGGCGTAGTGTGATGCCAGCGCCGAGCCGCCCACCGTC 65:C>G
2267352|F|0-15:T>C-15:T>C TGCAGCTCCCCGTCGTCCCCCTCTCGCCCACCCTCCCCTTCGCTCGCGATGGGGTCCGCGGCGTCGACC 15:T>C
14385650|F|0-14:G>C-14:G>C TGCAGCGCCGCGCGGGACGCCG 14:G>C
2257074|F|0-28:G>C-28:G>C TGCAGTACCTGCACGAGGAGTCCAACCTGCGCATCGTCCACCGCGACATCAAGGCCAGCAACATCCTCC 28:G>C
5992634|F|0-6:G>A-6:G>A TGCAGCGCCATATTATATGTCTGCTTCCG 6:G>A
1117458|F|0-25:A>C-25:A>C TGCAGGTGTCCACGTCGCTCCGGGAAGCGGACTTCTCGTACAACAACTTCTCCG 25:A>C
5324143|F|0-16:G>C-16:G>C TGCAGGGCGCCCTGGCGAGGCTCGGGTTCCCG 16:G>C
3030533|F|0-8:C>T-8:C>T TGCAGCGCCGACGGCGCCCGCGTCGTCGAGCTGCGCCTGCCG 8:C>T
6044092|F|0-8:G>C-8:G>C TGCAGGCCGAGCGCCTTCATGAAGCCGCCGTGGACGCGGCAGCCG 8:G>C
1008561|F|0-61:G>A-61:G>A TGCAGCGCCAACGTGTTCCTGGACAACGCCGAGTACCGCCAGTTCCTCCGCAAGCAGTTCGGGTGCACC 61:G>A
3024455|F|0-24:G>A-24:G>A TGCAGAAAGCACGTGATGGGGTTCGATGGAAACGATTGGTTTGACCG 24:G>A
984725|F|0-51:C>G-51:C>G TGCAGTTCATGTGCGCAGGCGCAGCTAGCTGCTGCTTAAACATGGTCAAATCCTGCTGGGCTGGCTGCA 51:C>G
4261071|F|0-26:G>A-26:G>A TGCAGGTGGAAAGGGAGCGGGAAATAGCTGCCCCCCGCCG 26:G>A
1092680|F|0-43:C>G-43:C>G TGCAGCAGCCTGGTGAGGCCTGGGTTCATGTCGGAGGGGTTCGCGGGCAGACGGTCGGTGAAGGAGGAG 43:C>G
4260627|F|0-5:G>C-5:G>C TGCAGGAGGCGTGGCTGGAGGAGTTCGTCGACGCGCGGCTGCGCGGCGACTTCAGCCACCCGCAGGCGG 5:G>C
1110041|F|0-50:G>C-50:G>C TGCAGAGGCGTCTCGTCCTCGCCGCCCTGGCTCAGCGTCTCCATCAGGTTGCCCCACTCCGTCCGCATC 50:G>C
1099099|F|0-20:C>G-20:C>G TGCAGGTTCCTCTTGCTCAGCGTGGTGTTCCAGTAGTTCTTGATCTCATTGTCTGTTCGCCCCG 20:C>G
1206583|F|0-11:G>C-11:G>C TGCAGCTACGCGCTCAGCTACCGCGACGGGTCCTACTCCCGCGGCGTGCTGGCGCACGACAGGCTGAGC 11:G>C
5579679|F|0-20:C>G-20:C>G TGCAGGCAGCCGCTGAGCATCGACGTCCACGACACCACGTTACGGCTAAGGATCCTGTCGAACACCTTG 20:C>G
1214622|F|0-25:C>T-25:C>T TGCAGCCGCTGCTCGGCCGCCCCTTCGGCTCCCTCTTCCGCGTCGGGCCCTCCG 25:C>T
1164189|F|0-41:G>C-41:G>C TGCAGCTTATGCCGGCCCTCGGGAGTCTTGAAGTAAGTCTTGAGTCCAGGCGCGCCCCCG 41:G>C
1228643|F|0-18:G>C-18:G>C TGCAGCGTTCCAGAAAGCGCCGAGCTGCCCAAACCATGAGAGCCAGTGGATGGGGAGGCTGTTGATGAG 18:G>C
1048831|F|0-22:G>A-22:G>A TGCAGTTCAACCTTCTGGGCCTGCACATACTCAAGAAGCTGGCCAGGGACCACGACAACTGCGGCAAGA 22:G>A
1114004|F|0-47:T>G-47:T>G TGCAGGGTGGAGACGAGCGCGCCGACCAGCGCCGCGCCATCCCTGTCTACGGCGCGCGCCAGGCACCGC 47:T>G
5567231|F|0-12:A>G-12:A>G TGCAGCGGATGAACGGCAGCTACGGGAGAGGTTTCCAAGGCGGCCG 12:A>G
1164189|F|0-17:C>T-17:C>T TGCAGCTTATGCCGGCCCTCGGGAGTCTTGAAGTAAGTCTTGAGTCCAGGCGCGCCCCCG 17:C>T
5580160|F|0-20:C>G-20:C>G TGCAGCACGCTGACGGTCTCCCCG 20:C>G
1023241|F|0-13:C>T-13:C>T TGCAGAACGGCTGCGAAAACGGCCCGAAGTTTTGATTAGGATCGTCTGCCATCTCTATGCCCAGCAACT 13:C>T
2248878|F|0-59:A>C-59:A>C TGCAGCACGTCGTCGTCCAGGTACTCGAACTCGATCACCCCGCTCTGCCCCTGCTGCTGATTCTGCTGC 59:A>C
12419289|F|0-22:T>C-22:T>C TGCAGGAGAAGCTTCTGGAGGCTAGGATGGATGATCGCAAGCCCTCGCCCAGCTCTGCTGCCAAGCACA 22:T>C
2248029|F|0-14:G>A-14:G>A TGCAGTGACGCGCCGAAGACGTAGGGACTGTAGTTCTCCCTGTCGTAGTAGTACCGCATGACCACCCGC 14:G>A
1368203|F|0-40:C>G-40:C>G TGCAGCCCAGCGACACCTTCCCGCGGAGGCACAACTCCGCCACCCCCGCCGAGCAGGCCGTCATGGCGT 40:C>G
2271197|F|0-20:C>A-20:C>A TGCAGCTGGGCCTACCAGGGCAACTACGTCGGCGCCATGCCGCTCTTCGACTCGCGGTGGCCG 20:C>A
4260627|F|0-22:G>T-22:G>T TGCAGGAGGCGTGGCTGGAGGAGTTCGTCGACGCGCGGCTGCGCGGCGACTTCAGCCACCCGCAGGCGG 22:G>T
1010039|F|0-20:C>G-20:C>G TGCAGCATCGCCGCGTCCCTCGGCGCCACGGGCTGCGCCGCCAGCTCCCCG 20:C>G
4006089|F|0-15:T>A-15:T>A TGCAGGAGCACGCCCTTGACCACGTCCTTCTTGGACGCCATGTTCTTGGTCTCCTCGTCCCTCCTCGAG 15:T>A
3030533|F|0-17:C>G-17:C>G TGCAGCGCCGACGGCGCCCGCGTCGTCGAGCTGCGCCTGCCG 17:C>G
2332200|F|0-20:C>T-20:C>T TGCAGGGACTGCTCGATCTCCCGGCACGCCATGACGGCCTCCACGGCGTGCACCCG 20:C>T
4985540|F|0-47:G>C-47:G>C TGCAGGCTTGCAAGCGCCGCCTCGACGCCGCGGAGACGGTCGACCCAGCGAAGCGCCGGGAAGAAGTCC 47:G>C
7165112|F|0-8:T>C-8:T>C TGCAGCCATAGGCTTCTTGGCTTTGCGCCTAGGCGCTGCCTGGCCG 8:T>C
3949303|F|0-38:C>G-38:C>G TGCAGCGCCATCTCCAGGAACCTCATCATCTCCGTCTCCTCGTCGCCG 38:C>G
5577942|F|0-10:C>T-10:C>T TGCAGCAGAGCACCATCAAGAAGTGGGCCG 10:C>T
1024581|F|0-10:C>T-10:C>T TGCAGGACGACCTGCACCACGGAGCCGAACCTGAGCTTGTACAGCTTGGTCGCCGGGATCGGCTGCCAC 10:C>T
1102451|F|0-18:G>A-18:G>A TGCAGCCGTGGCAACAGGGGTGTCCAGCGGTCTGGTCATGCTTCTGCGCAAGATGATGCGTGCAGACTG 18:G>A
1230090|F|0-25:T>C-25:T>C TGCAGGGGGTGTGGGTGTGGCGGCCTTCGTAGGTGGTGATCACCATGCGGCAGTCCTCCGACAGCCGCT 25:T>C
1240323|F|0-28:G>A-28:G>A TGCAGCGGGAGGTGCAGGAGCTGCGCGCGCTCAAGCTGCTCGCCCCGCCG 28:G>A

<table>
<tr><td>5578265|F|0-18:C>T-18:C>T</td><td>TGCAGGCCGTGCTCGTCTCCCCCGCCGCGCTGGCCG</td><td>18:C>T</td></tr>
<tr><td>1168705|F|0-35:T>A-35:T>A</td><td>TGCAGCTGGGGACGCCGCCGAAGCAGCCGTGCTGCTCGCTGATCCAGGGCCTCGCCGACCTGGAGGCGG</td><td>35:T>A</td></tr>
<tr><td>1227191|F|0-10:A>G-10:A>G</td><td>TGCAGCAGCCACCGCCAGGCGCACTCACCCTGCCCATGGCGTAACTGATGCACGGGGCCAGGTAGCTCA</td><td>10:A>G</td></tr>
<tr><td>12773432|F|0-40:C>G-40:C>G</td><td>TGCAGCGCAACGAGGTGTTCAACAGGGACAACATCCCGGCCTGGGTGACCTACGCCG</td><td>40:C>G</td></tr>
<tr><td>6042908|F|0-9:A>G-9:A>G</td><td>TGCAGCAGCAACATGGAGGAGAACAAGCAGCACAGCTGGAAGAAGATGTCAACTGCAAAGCAGTACAAG</td><td>9:A>G</td></tr>
<tr><td>3956224|F|0-40:C>G-40:C>G</td><td>TGCAGATGGGCAAGGCTGGCAGGTGGCTCAGAAGCTTCCTCACCG</td><td>40:C>G</td></tr>
<tr><td>3024455|F|0-16:T>C-16:T>C</td><td>TGCAGAAAGCACGTGATGGGGTTCGATGGAAACGATTGGTTTGACCG</td><td>16:T>C</td></tr>
<tr><td>1024631|F|0-25:G>A-25:G>A</td><td>TGCAGCCGACTGACTACGTCATGCCGCTGGTGCTAGTCACCCCGTCGGACGGCGCCGCTATCCAGAAGT</td><td>25:G>A</td></tr>
<tr><td>1023651|F|0-13:G>T-13:G>T</td><td>TGCAGGAAGCTACGGTGGAGCTCGGCCGTCCATTGCATCCTGGGAGCCCG</td><td>13:G>T</td></tr>
<tr><td>7346325|F|0-13:G>C-13:G>C</td><td>TGCAGGAGCTCCCGCCTCCCGCCG</td><td>13:G>C</td></tr>
<tr><td>3574740|F|0-7:G>T-7:G>T</td><td>TGCAGTTGACCCGCCACACGGCGTTGCCAATCGCCTGCGGGAGCAGGAACAGGTCCTGCATCAGCGCCA</td><td>7:G>T</td></tr>
<tr><td>1069344|F|0-25:C>T-25:C>T</td><td>TGCAGTTTCTGGGGTGCACGCTCGGCTGGAGAAGAAGGACGGGAACCTGGTGGTGACGGACATGGACAG</td><td>25:C>T</td></tr>
<tr><td>13871884|F|0-13:C>G-13:C>G</td><td>TGCAGGTCGAGGGCCGCGAGCGCCG</td><td>13:C>G</td></tr>
<tr><td>5360809|F|0-39:C>T-39:C>T</td><td>TGCAGCAGCCGCATCATCTCCTCCTCCACGCGCGGCTCGCTGCCGATGACGGAGTCGAACACCTCGGAC</td><td>39:C>T</td></tr>
<tr><td>5579682|F|0-67:A>G-67:A>G</td><td>TGCAGGCCAGGAAGCTCTCACTCGGAGGAGGCATCGGCGGCAAGCTCAAGGCTGTAGGGGAGATGGGAG</td><td>67:A>G</td></tr>
<tr><td>2255577|F|0-6:C>T-6:C>T</td><td>TGCAGGCGCTGGAGGGCGCGTGCAGGCAGAAGGGCGAGGAGTTGGGCAGCGCCGCCCTGCAAATCGACC</td><td>6:C>T</td></tr>
<tr><td>5001536|F|0-5:A>C-5:A>C</td><td>TGCAGAGGGCGCCGCCCAGCGCGGCCGCCAGCGCCTCCCCG</td><td>5:A>C</td></tr>
<tr><td>6047617|F|0-23:T>C-23:T>C</td><td>TGCAGGAACATGAGCTTCCGCGATGCCAGGAATGCGTTGTTCCG</td><td>23:T>C</td></tr>
<tr><td>2292322|F|0-34:G>A-34:G>A</td><td>TGCAGGGCCTGCGCGCCGTTGACGGCGAGGACCCGCCCAGGATACTGCACCTTAACCCCAGGCTGAGGG</td><td>34:G>A</td></tr>
<tr><td>3025819|F|0-26:A>G-26:A>G</td><td>TGCAGGAGGCTCCTGCGTATCTCGCCATGCATCCCGCCG</td><td>26:A>G</td></tr>
<tr><td>1863137|F|0-26:T>G-26:T>G</td><td>TGCAGTAATCTGATTGCGATCTTATATGAAAATATTGTTCTAGGTTCTCGAAGATCAATTGTCCTTTGT</td><td>26:T>G</td></tr>
<tr><td>1106803|F|0-10:G>C-10:G>C</td><td>TGCAGCGCTCGTGGATCGTGCTCTTCCTCTTCGCATTAGCGCTCACGCCCACCTACGTCTTCACCGAGG</td><td>10:G>C</td></tr>
<tr><td>1863340|F|0-29:G>A-29:G>A</td><td>TGCAGCTCAAAGAACCCGTGCTCGATCCCGGCGATCTTGAATGCCTCGGCAGCGGTCACCTTGCTGCCA</td><td>29:G>A</td></tr>
<tr><td>1696351|F|0-32:G>A-32:G>A</td><td>TGCAGTTTCATGTGCTACTTCCACCTTTTCGGGTTCGTGGAGCAGAGCTGGACCATGGTCGCTGCCTTC</td><td>32:G>A</td></tr>
<tr><td>4540156|F|0-8:T>C-8:T>C</td><td>TGCAGAGATGACACGGAGCGGAGGAAGCAAACTCTCAATGGCGGCCG</td><td>8:T>C</td></tr>
<tr><td>1014911|F|0-40:G>C-40:G>C</td><td>TGCAGCGCCGCACCGTGGCGCAGGAGGACGAGCTGTCGCGGGAGATGGCGCGGGTGCAGGAGGGCCACG</td><td>40:G>C</td></tr>
<tr><td>32016468|F|0-23:G>A-23:G>A</td><td>TGCAGCGCCTGCTGCATCAGGAGGGCCTGCTGCTGTTGCTGCTGCTGCTGCCG</td><td>23:G>A</td></tr>
<tr><td>1090705|F|0-36:T>C-36:T>C</td><td>TGCAGGATGATGAGCGGCAGCGCGTAGTCCAGGGGGTTCTCCCCCTGGAACGCGCCGTGCGACGCCACC</td><td>36:T>C</td></tr>
<tr><td>3946181|F|0-8:A>G-8:A>G</td><td>TGCAGGCCACCATCGATTAGGTGCACATGGGAGTCGATGAATCCCG</td><td>8:A>G</td></tr>
<tr><td>1162710|F|0-28:C>G-28:C>G</td><td>TGCAGCCGACCCTGGCCACCGAGTACGCCAGCCAGGCCGGCCGCTTCACCCTCAACATCACCCGCTCCA</td><td>28:C>G</td></tr>
<tr><td>5360809|F|0-8:C>T-8:C>T</td><td>TGCAGCAGCCGCATCATCTCCTCCTCCACGCGCGGCTCGCTGCCGATGACGGAGTCGAACACCTCGGAC</td><td>8:C>T</td></tr>
<tr><td>1090124|F|0-19:C>G-19:C>G</td><td>TGCAGGACGGCCACGGCCGCTACATCTGGCGCCACGGCCATGAGTACATCGGCACCTGGAAGGCCGAGA</td><td>19:C>G</td></tr>
<tr><td>1395477|F|0-49:C>T-49:C>T</td><td>TGCAGCTCGATCTCTGCTTTCGGCTGCACCATGCACACCTGCTTGGTGACGTCGTCGTTCCTCTCCTCC</td><td>49:C>T</td></tr>
<tr><td>1228643|F|0-66:G>C-66:G>C</td><td>TGCAGCGTTCCAGAAAGCGCCGAGCTGCCCAAACCATGAGAGCCAGTGGATGGGGAGGCTGTTGATGAG</td><td>66:G>C</td></tr>
<tr><td>1026037|F|0-17:C>G-17:C>G</td><td>TGCAGCTCCGACCCGATCAGCGACTGCATCCGCGCGCGCTCCCTGTCCCGCGGGTACGTGCGGTACAGC</td><td>17:C>G</td></tr>
<tr><td>1028221|F|0-5:A>G-5:A>G</td><td>TGCAGAGCGACATCGCCGTGGGGCTCGGGTTCGCGGCGTTCGCGTTCCTGGCGCTGGCGGCGCTCGCGA</td><td>5:A>G</td></tr>
<tr><td>988133|F|0-10:A>T-10:A>T</td><td>TGCAGATAAGATTAAAAAGTGTGCCTTCTTGCAGGAGGAGCATGAAGCAATTCTGAAGAAAAGAGAAGA</td><td>10:A>T</td></tr>
<tr><td>6043728|F|0-35:G>C-35:G>C</td><td>TGCAGCACCAGCCCCTCCCTGGGGTAGAACGCGTGGCCGTGCCGCGACGCGTACGCCACCG</td><td>35:G>C</td></tr>
<tr><td>5331558|F|0-18:C>T-18:C>T</td><td>TGCAGAGCCCGCTGCTGCCGCCGCCG</td><td>18:C>T</td></tr>
<tr><td>5581338|F|0-25:C>G-25:C>G</td><td>TGCAGCAGTCGGCGCTGGGGTCGGCCGCGCCG</td><td>25:C>G</td></tr>
<tr><td>1016328|F|0-56:G>A-56:G>A</td><td>TGCAGCACGGTCTTGAGCGCCCTCCCCGCCGTCGCGCCCACGCACATGATGAACCCGAACAGATGGAAG</td><td>56:G>A</td></tr>
<tr><td>4986116|F|0-13:C>T-13:C>T</td><td>TGCAGGTCCGCGACGACGTGCCGCTCATCGGGTTCATCGGGCGCCTGGACCACCAGAAGGGCGTGGACA</td><td>13:C>T</td></tr>
<tr><td>6048231|F|0-38:A>G-38:A>G</td><td>TGCAGGGCAGCGTCAGGCTCCAGCTCGTGTGCCTCGCCACCGTCCTCCTCGGCGCCG</td><td>38:A>G</td></tr>
<tr><td>5363489|F|0-17:A>G-17:A>G</td><td>TGCAGCGCCGCCGCCCTATGGAAGGCCG</td><td>17:A>G</td></tr>
<tr><td>986053|F|0-46:C>A-46:C>A</td><td>TGCAGATCCTAGAGCCCCATTGATTAAAAAAATAAACCAGTTGGCACGCTAAAGATCTTCCCAGATCTT</td><td>46:C>A</td></tr>
<tr><td>12855613|F|0-17:T>C-17:T>C</td><td>TGCAGCGTCAATGGCGTTCCCG</td><td>17:T>C</td></tr>
<tr><td>1168705|F|0-21:A>G-21:A>G</td><td>TGCAGCTGGGGACGCCGCCGAAGCAGCCGTGCTGCTCGCTGATCCAGGGCCTCGCCGACCTGGAGGCGG</td><td>21:A>G</td></tr>
<tr><td>977449|F|0-25:G>T-25:G>T</td><td>TGCAGGCGCTGCTCTCCCGTGGACCGCTCTCGGAGCGAGACTTCCACGCCCTCTTCACCGCCATCTCCG</td><td>25:G>T</td></tr>
<tr><td>1030407|F|0-17:C>T-17:C>T</td><td>TGCAGGAGAAGAGGGGGCTCCTGTTCTGCAAGGAGGACAGGGCGATCCTGTGCCG</td><td>17:C>T</td></tr>
<tr><td>1026848|F|0-61:C>T-61:C>T</td><td>TGCAGCTTCCTCCGTCCAGGCGTCCACCCGATGCCGCTCCTCGCCACCCCACCCTATATAACGCCGCGC</td><td>61:C>T</td></tr>
<tr><td>1107994|F|0-56:G>A-56:G>A</td><td>TGCAGAACCGCTCCAGCGCCGCGGGGTCGCTGCCGCCGCGCACGCGCATCAGGTTCGCCG</td><td>56:G>A</td></tr>
<tr><td>6045803|F|0-17:G>C-17:G>C</td><td>TGCAGGTTCGAAAGGAAGGAATCCCACGGGCTGGACGCGCCTTGCCG</td><td>17:G>C</td></tr>
<tr><td>1024870|F|0-17:C>A-17:C>A</td><td>TGCAGCAACTGCGGGGACGGCTTCTCGCAGTGGTGGGGCACCTGCCGCGGCTGCCAGGCCGTGGGGACC</td><td>17:C>A</td></tr>
<tr><td>1023247|F|0-23:A>C-23:A>C</td><td>TGCAGCACTACGACAAGAGGGACATCAAGTCCCCCACCTTCAGGACGTGGAACGACGACTCCGTCAGGA</td><td>23:A>C</td></tr>
<tr><td>3940720|F|0-5:A>C-5:A>C</td><td>TGCAGAGTGAGCTTCTTTGGAGCATGCTCCTCCGCCG</td><td>5:A>C</td></tr>
<tr><td>6044966|F|0-52:G>C-52:G>C</td><td>TGCAGCTGACGCCTTGCCACCCCTGGCACGGGGACACCGAGCGGTCCCACTTGAGATGGTTTCCCACGG</td><td>52:G>C</td></tr>
<tr><td>5324413|F|0-8:C>G-8:C>G</td><td>TGCAGCATCCGCATGAGCACGTCCTCCTCCGCGGCGGGCTCGGCGCCG</td><td>8:C>G</td></tr>
<tr><td>3023916|F|0-40:C>G-40:C>G</td><td>TGCAGAAGATCACCAGCCTGCTGCTCTGGGGCAATGCCCTCTCCG</td><td>40:C>G</td></tr>
<tr><td>12420054|F|0-41:G>A-41:G>A</td><td>TGCAGCACCCCCTCGAACGGGTACTTGACCAGCGACAGGTAGTGGAACCACAGCCAGTAGGCCGGTATC</td><td>41:G>A</td></tr>
</table>

4006226|F|0-14:G>A-14:G>A	TGCAGGTCGTAGACGGAGACATGCGTGGGGCGGGCATCGACGGTGCACCGCAAGCTCTCCTCCCACATC	14:G>A
4993489|F|0-37:G>C-37:G>C	TGCAGCCCATGGGCAGCTGCTACAACCCCAACACGATGCAGGCGCACGCCTCCTACGCCTTCAACGCCT	37:G>C
1001484|F|0-34:T>C-34:T>C	TGCAGCGGCACAACACCCTGAAAGGAGGCTCGGTTCGCAAGGACAGCAAGGAGCCGAGCTTCAGGGCGG	34:T>C
2246307|F|0-17:A>C-17:A>C	TGCAGAAACACGCCAATACACCAGTCGATCAGCGCCAGGAACCGCCAGGACAACGAGATCTGGGGAGGG	17:A>C
1028354|F|0-49:C>A-49:C>A	TGCAGGGCGAGGAGGACTACCCCTCGGCCGACTGCTGCGACGGCGCGTCCAGCCTCGTGGCGGCCGCGG	49:C>A
1127770|F|0-6:G>A-6:G>A	TGCAGGGGGCTCAACTTCCCGGCGATAATGGGGCTGATCATCTGCACGGCGTCGTTCGGCATCAACCTT	6:G>A
2335452|F|0-64:C>G-64:C>G	TGCAGGTGGCCGTCTTCCTCCTCGCCGAGCTCGCCTCCCGCGACGACGGCGTCGTGCAGACGCTCACGC	64:C>G
1024870|F|0-20:C>G-20:C>G	TGCAGCAACTGCGGGGACGGCTTCTCGCAGTGGTGGGGCACCTGCCGCGGCTGCCAGGCCGTGGGGACC	20:C>G
1021340|F|0-41:G>A-41:G>A	TGCAGCGAGGAGAGGCGCTGCCTGTTGGCGACGAGGTACATGTGGTTGGCGAGCACGAAGGTGAGCGGG	41:G>A
3936987|F|0-16:T>C-16:T>C	TGCAGGACCTCCCTGATGTTGCTCACGGTGAAGACGATGTCGGTGAGCTCCCGTCGAAGCAGCTTCCCG	16:T>C
2319654|F|0-59:T>G-59:T>G	TGCAGATCTCTGCGTTGGAGGGGCTGCGCACGCACACCTTGGCGTACCTGTGGCAGGCCTTGCAGTGGA	59:T>G
1221099|F|0-39:T>C-39:T>C	TGCAGGTTCTTGACCTGTCCAAGAACAACATCTCTGGAGTGATCAGCATCTCCACATCACAGCTGAAGA	39:T>C
1122653|F|0-7:T>C-7:T>C	TGCAGTGTGTTGTCAAGGAGACCCTTCGGATCTGCCCCGTAGGCCCGATCATCCCGGCGCACGAGGCCA	7:T>C
1041486|F|0-68:G>C-68:G>C	TGCAGGTTGGTTTCGTTGTCGGAGTACCACCCGTGCTTGGAGGAGTCGTACTTCTCGCCGTCGCCCCAG	68:G>C
12419279|F|0-23:C>A-23:C>A	TGCAGCTGGTGGAGTCGGAGAAGCGGAAGATCAGGCAGGAGTACGAGCGCAAGCAGAAGCAGGTGGACA	23:C>A
7478650|F|0-16:C>G-16:C>G	TGCAGGTCGAGGACGTCCCCG	16:C>G
2251815|F|0-47:C>T-47:C>T	TGCAGCTTCGAGGCGTACCAGGACACGCTCTACACGCACTCCCTCCGCCAGTTCTACCGCGGCTGCGAG	47:C>T
4393999|F|0-35:G>A-35:G>A	TGCAGGGAGACGACAGAGACCCTCCCGCTAGCGTCGCAGGAGACGCCCTCGAAGTAGTCCCCGCGGCCG	35:G>A
5580524|F|0-13:A>G-13:A>G	TGCAGGTGGCGTTATCGTAGCCGCTCCCG	13:A>G
994161|F|0-41:G>A-41:G>A	TGCAGGGCCCACGCGCCCGTGGCTCCCAAGGTGATGTTCGCGGATTGGCTCAACATGGACTACATCGGT	41:G>A
994899|F|0-20:A>G-20:A>G	TGCAGGGACCTGAGCAGGGGAAAAATAGAGCTCTCAAGTTCTTCTTCAATCTTCTTTTTTGCAGAGGTC	20:A>G
7912012|F|0-10:T>C-10:T>C	TGCAGTCCGTTCTTGTGAATTTCGTCAAGGGTGTTGGTAACCTTAGCCATGCAGAATGGCGGTCATTCT	10:T>C
1005055|F|0-44:C>G-44:C>G	TGCAGCAACGGGGACCTCTTCAAGTGGCAGCAGCTATGGCCGATCCTCGCCAACCAGTTCGGCGTGGCG	44:C>G
7927322|F|0-29:A>G-29:A>G	TGCAGCGACGTGATGGCGCCGACGCAGCCATAGATGGGGTCGCGCACGCGCGCGTTCGCCTCGTACACC	29:A>G
1245875|F|0-58:C>G-58:C>G	TGCAGACGGCTAACAGCTTCTGCATGAGGTAGTTCCCGAACGGGTTCACCATGAGGTCCACGGCGTGAC	58:C>G
7920119|F|0-7:A>G-7:A>G	TGCAGCCAAGGATGCGGCCG	7:A>G
32026643|F|0-6:G>A-6:G>A	TGCAGCGTCAGCGGGCACTTCACTGACAGCGTGCTCGCCGACGTCGTCATCACCCCGCCATACCTGTAC	6:G>A
1111624|F|0-7:A>G-7:A>G	TGCAGGAACTGGAGGACGAGCAGGCCAAGGGGTACGCGCCGTCGCTGCGCCACGCCG	7:A>G
32023728|F|0-49:G>C-49:G>C	TGCAGCTCGCCTGCGAGGAGTTCGGCCTCGGCTCCGACGACCTCAGCCTGGACAACATGATCAGAGAAG	49:G>C
5365877|F|0-8:C>T-8:C>T	TGCAGGTCCTCAGGGTTTACAATGCCGGCCAGGATTTTGAGCCTCTCTGTGTAGGGCACGTTGCTGTCC	8:C>T
7339030|F|0-37:T>C-37:T>C	TGCAGGCTGGCGTCGAGCAGCGCGGCCTGCTTGCCGATGTCGGCGTCGGCCACGAAGCCGCCGAACCGA	37:T>C
1047496|F|0-17:A>C-17:A>C	TGCAGTCTCAAGCCGACAAGAAGCGGTACGAGAAGGAATCTGCTGCCTACCGCGCTGCTGCCCCAGTGG	17:A>C
2249760|F|0-47:A>G-47:A>G	TGCAGGTGAGGCCATGCCGGGTCCATGACAGGCTCTTCCTCCTCCAAATCAAATGACTCGATTACCTTG	47:A>G
994899|F|0-54:T>C-54:T>C	TGCAGGGACCTGAGCAGGGGAAAAATAGAGCTCTCAAGTTCTTCTTCAATCTTCTTTTTTGCAGAGGTC	54:T>C
3028329|F|0-20:C>G-20:C>G	TGCAGCGCCCGCAGCACGTCCGACCCGCGCAGCTGCCCCCG	20:C>G
12772522|F|0-27:C>A-27:C>A	TGCAGGTGGCGCCGCTGGGCGTGCCGGCCGTGGCGCGCGGCTGCTACGAGAAGGCGACGACCATGATGG	27:C>A
1027810|F|0-51:C>G-51:C>G	TGCAGCGGCGTCGGGAAGAAGGCGTGGCCCTTGAGCTGGCTGGTGGCGTTGCTCAGCTGGAGCAGCCCG	51:C>G
993579|F|0-53:T>C-53:T>C	TGCAGCTGCTCGTCGCTCACCACCCACGCGCCCTGCGTCCGCTGGATCTCGTCTATGGCCGCGTTGAAC	53:T>C
1106658|F|0-26:G>A-26:G>A	TGCAGGCCACCGAGCAGCACCGTGACGAAGAAGAGCGGCATCATCTCCAGCGAGTTCTGGTGCCCCCTC	26:G>A
7911824|F|0-23:C>A-23:C>A	TGCAGCTGGGGACGCCGCCGAAGCAGCCGTGCTGCTCGCTAATCCAGGGGCTCGCCGACCTGGAGGCGG	23:C>A
2252532|F|0-47:C>G-47:C>G	TGCAGCATGCTCAGCTTCCACCTCCACTCGTCGATGTTCGCCACCGACGACGACGGCTGCGGTTAGCAT	47:C>G
2250763|F|0-61:C>T-61:C>T	TGCAGCTGCCTCACCGTGTCGAACCACTCCTGGAAATGGTACGTCATCTTGGTCGCGTTGGCCCCCTTG	61:C>T
1006043|F|0-59:A>G-59:A>G	TGCAGCGGAGGAGGACCTGGACGTTCACCGACTTCTCCTTGTCCACCCGCGACATGGCGACGGCGGCGG	59:A>G
986053|F|0-11:G>T-11:G>T	TGCAGATCCTAGAGCCCCATTGATTAAAAAAATAAACCAGTTGGCACGCTAAAGATCTTCCCAGATCTT	11:G>T
1091637|F|0-25:C>G-25:C>G	TGCAGGTGACCCCGTCGGTGCTGGCCGCAGGGCTGGCCGCCGACAACGTCATCTGCGCGCTCTACTTCA	25:C>G
1024581|F|0-50:C>T-50:C>T	TGCAGGACGACCTGCACCACGGAGCCGAACCTGAGCTTGTACAGCTTGGTCGCCGGGATCGGCTGCCAC	50:C>T
5357292|F|0-13:G>C-13:G>C	TGCAGACGCGGACGGAGACGACCTGGCCG	13:G>C
1130166|F|0-40:G>C-40:G>C	TGCAGTCGCTGGCGACGGCCGCGGACGCGCTCACCGCGGCGCGCATCCTGGTGCAGGCCAGCCAGGGCG	40:G>C
3943788|F|0-10:C>G-10:C>G	TGCAGCAGCGCAAGGAGGCCGCGCACGTCGTCGAGCTCGAGAGACGCTTCAAGGTGCTCGACGGCCTCA	10:C>G
1029743|F|0-12:G>C-12:G>C	TGCAGCGACCGCGGAGATGTACGACTCAGAGCTCAAGTTCTTGGCCGCCG	12:G>C
2249760|F|0-46:A>G-46:A>G	TGCAGGTGAGGCCATGCCGGGTCCATGACAGGCTCTTCCTCCTCCAAATCAAATGACTCGATTACCTTG	46:A>G
1024248|F|0-67:G>A-67:G>A	TGCAGCACGTGGTGTTCGGCATCGCGGCGTCGGCGCGCATGTGGGAGAAGCGGAAGGAGTACATTAAGA	67:G>A
7344206|F|0-24:G>C-24:G>C	TGCAGCTCGCCGCCGTGCTGCACGGCCG	24:G>C
1026294|F|0-10:T>G-10:T>G	TGCAGCTTCTTGGGCGTGAGCACCTTAGGAGAAGGTGGGGGTGTTGGGTGCGCGCGGTTGAGGACGCGC	10:T>G
5363033|F|0-8:C>T-8:C>T	TGCAGAAACGACGCGTGGCCCG	8:C>T
4262840|F|0-17:C>T-17:C>T	TGCAGCTCGGCAGTGTGCGCGCCATGCATGCAAAATATGGCCG	17:C>T
1230163|F|0-17:G>C-17:G>C	TGCAGGCGCCACCCCAGGCGCTCCGACAGCTCCTGCATCCGCTGCTTCTGCTCCGCGGTGAACTTGGTG	17:G>C
2248971|F|0-23:A>G-23:A>G	TGCAGGTACTCCGCGGGCTTCCCAAGGCCGAAGGGGTCGAAGCCGTAGTCGCCGACGAGCGTCCCGTCG	23:A>G
995833|F|0-46:C>G-46:C>G	TGCAGCTCGCCCTCGCGCTCCGCCTCTGCGCCGACGCCGCCTGCGCCGCCGACCCCG	46:C>G

5006976|F|0-13:A>C-13:A>C	TGCAGCAGCTGCTAAAGCGGTGGAAGCGGATGGCGGTGGCGCCG	13:A>C
1027619|F|0-23:A>G-23:A>G	TGCAGTCGCTCAACCGCAAGCTCACCGCCATGAACAAGCTGCTCATGGAGGAAAACGACCGCCTCCAGA	23:A>G
1008555|F|0-6:G>T-6:G>T	TGCAGCGCCGCGGCCACAGTTTCAGTTCCTGCACCAAGGAACTCGGAGCACATGCCCACGAGCTCGCCG	6:G>T
7163702|F|0-44:C>G-44:C>G	TGCAGCCGCGTGTGGCTGGTGCGGTGCCCGCCCAGCGCCTGGAACGACGTGAACCGCTTGCTGCACGTC	44:C>G
7492111|F|0-20:T>C-20:T>C	TGCAGCGGCGGTCCACGAGTTCACTGGACACCG	20:T>C
983675|F|0-25:A>G-25:A>G	TGCAGGACATGTACAACAGCCTGACAGCGTGCAAGGAGCTCGTCAGAGTCCTCGGCAATATCTGGGGGC	25:A>G
1021340|F|0-28:C>A-28:C>A	TGCAGCGAGGAGAGGCGCTGCCTGTTGGCGACGAGGTACATGTGGTTGGCGAGCACGAAGGTGAGCGGG	28:C>A
4993802|F|0-23:G>T-23:G>T	TGCAGGAAGCTGCCGAGCGCGTCGCCG	23:G>T
1126919|F|0-40:A>C-40:A>C	TGCAGGCCCCGCTCCTCGCCGTCGGCTTCGTGGCCTTCCTAGTCTCGCTCCCG	40:A>C
3026742|F|0-13:C>A-13:C>A	TGCAGGCAGGCTGCTACTCCTCCGTGCCGCCGCCATCATCCG	13:C>A
1075245|F|0-7:T>C-7:T>C	TGCAGGATCTCCCTGACGTTGCTCACAGTGAAGACGATTTCGGTGAGCTCCCGTCGGAGCAGCTTCCCG	7:T>C
1278807|F|0-37:C>G-37:C>G	TGCAGGAGGCGCTGGAGCGGCTGATGAAGGGGCGGACCACGGTGCTGGTGGCGCACCG	37:C>G
3941003|F|0-26:T>G-26:T>G	TGCAGTGGCAGGCCGAACACCCACCATGGGAGCTCGCCG	26:T>G
7171134|F|0-35:G>C-35:G>C	TGCAGCGCGGCGTAGCTGCTGCCGCTCATCTGGTCGCCGACGAAGGCGCCGTGCGACGGCGTCACGTTG	35:G>C
1667530|F|0-9:G>C-9:G>C	TGCAGCGTTGTCTGAGGTACTCCTCGAGCGGGCTGAGGTTGGTGTGCTCGGTGCTGGAGATGGACTCGG	9:G>C
1026025|F|0-24:C>G-24:C>G	TGCAGCTGCGGCGGAAGGGCGCGGCGACGATGTCGCAGGTGATGTCGCCG	24:C>G
1076531|F|0-68:G>A-68:G>A	TGCAGCTTGCGCTTGAGGTGCGTGTTCCAGTAGTTCTTGATGTCGTTGTCGGTGCGCTCGGGGAGGTAG	68:G>A
3942729|F|0-32:A>C-32:A>C	TGCAGGAGCTCGTGCAGCCTCCGTTCCAGCACACGGGCCG	32:A>C
1010809|F|0-42:C>G-42:C>G	TGCAGGGCGCACGACAAGTCGGTAGGCCCGCTGGAGATACGGCTGGTCGAGGCCG	42:C>G
7170884|F|0-8:G>C-8:G>C	TGCAGGTTGAGCCCGTAGCGCGGCAGGCCCG	8:G>C
12419757|F|0-26:G>A-26:G>A	TGCAGCTCCGCCACCTCGTCCTCCTCGCCGCGGTAGGCCGCCGCGGCGATCTCGTGCGGCAGGACGGCG	26:G>A
1079151|F|0-16:A>C-16:A>C	TGCAGGAGAAGACCGCAAAGAAGGCGGCGCAGGCCACGCAGGGGGCCGACGACGCCAAGAAGAAGAACG	16:A>C
1251758|F|0-25:C>G-25:C>G	TGCAGGTGCTGGCCATCCTGCCGTCCTACACGGGCAAAACGGTGCTGGAGCTCGGCGCCG	25:C>G
1127770|F|0-23:G>A-23:G>A	TGCAGGGGGCTCAACTTCCCGGCGATAATGGGGCTGATCATCTGCACGGCGTCGTTCGGCATCAACCTT	23:G>A
2284385|F|0-8:C>A-8:C>A	TGCAGGGCCGCCAGGCCATCCCCTTCCTCGAGTCCCTCGTCGTCGCCGACGTCGCCGCCCTCAAGGACG	8:C>A
1679384|F|0-56:A>T-56:A>T	TGCAGCACGGCTACACACTCGTGCTCGTTCCCTGGCTGCACCTCCCATATCCACACAGCCTTGTCCCGG	56:A>T
5340989|F|0-13:G>A-13:G>A	TGCAGGTTGGACAGGCTCGCGACCTGCGCCGGGATGCTGCCATGGATGGCATTGTGGCTCACGTCGACG	13:G>A
7169103|F|0-10:C>T-10:C>T	TGCAGTTGCACGCATACTTGCATAGCGGTGAAAGACGGTGAGCGAAAGAGGAGGAAAGCTCTCTCTCGG	10:C>T
2253805|F|0-41:T>G-41:T>G	TGCAGAGTTTGCGGCATGCTCGAACTGCGACCTGATCTCCCTTGCCACCTCAATATCATCGTTGTAGGA	41:T>G
1237715|F|0-37:G>T-37:G>T	TGCAGCGTGGCATTGACCCAGCTGAGGATCTCGCCCCGCCCCACGAAGTAGGCCTTGTCCATCAGCCCG	37:G>T
1064289|F|0-55:C>T-55:C>T	TGCAGGGCAACCGCCTGAACGGCCCCATCCCGGAGACGCTCGCCGACCTGAGGAGCCTCAACGTGCTGA	55:C>T
2350020|F|0-28:A>G-28:A>G	TGCAGCACCGCAACCTGGTGCGGCTGCTAGGCTGGTGCGCCGAGCGCGACGAGAAGCTGCTCGTCTACG	28:A>G
1106820|F|0-46:G>C-46:G>C	TGCAGATCGCGAACGACAGCGCCAGCGCCGCGCCGCCGTGCAGCACGGACATGCCGCCCCG	46:G>C
1087107|F|0-23:C>T-23:C>T	TGCAGCTTCGCCGCCGCCGACGACGCCGACGTGCCCTCGCCCGCCGGGAGCATCGCCG	23:C>T
5576944|F|0-68:A>C-68:A>C	TGCAGCAACTTCTCCAGAGCGGAGACCACAGCAGCATGGCTCTGGTCGCCCACAATCTCCG	68:A>C
4993802|F|0-17:C>G-17:C>G	TGCAGGAAGCTGCCGAGCGCGTCGCCG	17:C>G
6041492|F|0-20:A>G-20:A>G	TGCAGCGGCGGCAGCGTGTCAGCGATGGTGACCTGCACGGTGGCTGTCTGCCG	20:A>G
1100951|F|0-23:G>A-23:G>A	TGCAGGGGAGGGCAGTGGAACTCGGGAGGGAGATGCCATAGGGAGACCGAGCCGATATTCAACCACACG	23:G>A
1027504|F|0-7:A>G-7:A>G	TGCAGCGAGTGGCGTTCCTCAAGGCGTCGTCGGAGGAGGCGCCGTACGTGGGGGTAGAGACGGCGAGAC	7:A>G
1064289|F|0-23:C>A-23:C>A	TGCAGGGCAACCGCCTGAACGGCCCCATCCCGGAGACGCTCGCCGACCTGAGGAGCCTCAACGTGCTGA	23:C>A
5354875|F|0-41:C>T-41:C>T	TGCAGCTACGTGACCGAGGGCGGCGACGTCGAGGTCGACAACGTCGACGAGCCCCTCATCCTCGACGGC	41:C>T
5367451|F|0-7:G>C-7:G>C	TGCAGGTGGAGGAGATCGGGCAGTCGGCCGCCG	7:G>C
4993802|F|0-14:G>C-14:G>C	TGCAGGAAGCTGCCGAGGGCGTCGCCG	14:G>C
2260637|F|0-64:C>G-64:C>G	TGCAGCTCTACTCCAAGGAGGTCAGCCGCCGCCTCCTCGACTTCGTCAAGTCCCGCTCCGCCGCCGCCA	64:C>G
4005704|F|0-10:T>G-10:T>G	TGCAGTTGACTATGAGGATGCGCACGTCGCGGCGCGGGTCGATCCCG	10:T>G
5580390|F|0-14:A>C-14:A>C	TGCAGGATCCGCTTAACCGCCG	14:A>C
1092362|F|0-8:C>T-8:C>T	TGCAGGCGCTTCTCGTAGTCCTCGCGGCCGCCGAAGAGGCCGCGCCACATCCTCCCCAGCACCCCTCCT	8:C>T
1140543|F|0-17:T>G-17:T>G	TGCAGCAACACCGCCCTTGCCTCTGGAACTCTCTTCTCCATCATCAGCCACCTAGGGGACTCCG	17:T>G
1221473|F|0-37:C>T-37:C>T	TGCAGTGCCAGAAGGTGAGCGACTTCCTCATCGCGGCCTCCTACCTCTCCATCCCGCTCGAGCTGCTCT	37:C>T
5372911|F|0-10:A>C-10:A>C	TGCAGCTAGGAGACACGGTGCGGCTGCTGCCG	10:A>C
1091076|F|0-11:C>T-11:C>T	TGCAGCGACATCGCCGAGGGGGTGGCCTACCTGCACCACCACTCGCCAGTCAAGGTCATCCACTGTGAC	11:C>T
3940054|F|0-11:A>G-11:A>G	TGCAGCGCTGCAACGGCGCCACCTTCTACCG	11:A>G
984725|F|0-16:A>G-16:A>G	TGCAGTTCATGTGCGCAGGCGCAGCTAGCTGCTGCTTAAACATGGTCAAATCCTGCTGGGCTGGCTGCA	16:A>G
5357831|F|0-8:G>C-8:G>C	TGCAGCGCGGCCTCGACGCGCGGGCCG	8:G>C
1026254|F|0-56:G>C-56:G>C	TGCAGGCTGCCGAGCACCGACATCCCCCGCATGCTCACCATCCCGAGGCAGGCCACGCCG	56:G>C
992303|F|0-33:C>G-33:C>G	TGCAGCGAGTAGGACGCGCTGGTCTCCTTGGCGCACTTGGACACCAGGATCCCCACGCCCTGGCCCCTC	33:C>G
981263|F|0-43:A>G-43:A>G	TGCAGAACGTGGCTATCATGCTCAAGGCGCTCAATGTGACCGCAGACGAGGTGATCGGAGCACTCGTGC	43:A>G
1014714|F|0-8:G>C-8:G>C	TGCAGGACGGCGACGAGGTCGGCCTTGCGGATGGTGGAGAGCCTGTGCGCGATGACGAGGGTGGTGCGG	8:G>C
1219191|F|0-41:G>A-41:G>A	TGCAGTCCCCGCCGACGCAGCAGCAGCAGGACCTGCTGGGCGGGCTGGACACGGAGCTGAGCGACATGC	41:G>A

2245873|F|0-61:T>C-61:T>C	TGCAGGAGGACTCCCAAGTCGTGGTTGCTGTGCTCGACACTTTGTCAAACCTTAACCTCGATGCGCTGT	61:T>C
1229860|F|0-55:C>G-55:C>G	TGCAGCAGGAGCGCGCGCTCGGCCGCCCCATGTTCCCCAAGGCCATCGGCGAGCTCCACGGCCACTCCG	55:C>G
1025251|F|0-13:G>C-13:G>C	TGCAGGCGCAGGCGGCGCAGCAGTTCAGGATGCAGGACCTGAGCAAGGCGATGAGGCCCGACGCGGCGG	13:G>C
7171605|F|0-14:G>C-14:G>C	TGCAGGAAGTCGCCGACGTTCACCACCAGCGCGCCG	14:G>C
4910573|F|0-27:C>T-27:C>T	TGCAGATGGCTCTCAGGGCTTCGACACCGCGCACCCGCGAGGCGTCCCGCCAGGGCGAGACGTCGTCGT	27:C>T
4993547|F|0-34:C>G-34:C>G	TGCAGCTTCGAACTGTGGCAAAGGTGCAGCCCTACCTGATCTTGTCCTCATGCGAGAGAACTGGTCATG	34:C>G
1127770|F|0-68:T>C-68:T>C	TGCAGGGGGCTCAACTTCCCGGCGATAATGGGGCTGATCATCTGCACGGCGTCGTTCGGCATCAACCTT	68:T>C
992847|F|0-11:C>T-11:C>T	TGCAGGAGCCGCGCGAACAGCATGACGGTGAAGAGGGTGCCCAGCGCGATCCCG	11:C>T
1133693|F|0-25:T>C-25:T>C	TGCAGGCGTCCATATGGAGCAAGGATGTGGAGTTCTCCTTCGGCGAGATTCTGGCGGCGACGGAGCACT	25:T>C
6045457|F|0-23:A>G-23:A>G	TGCAGCATCTGCCTGGAGACGGCATCGGAGCCG	23:A>G
985286|F|0-14:G>A-14:G>A	TGCAGCATCTTCAGGCCACCCAACGCCGCCGGCACGCCGCCGCCAATGCTGTTCCAGCTCAGGTCCAGG	14:G>A
981116|F|0-33:C>G-33:C>G	TGCAGAGCCACCCAAGGGCGGCAACCCAGACTCCAACACGCTCATCAGCGACACGACGACGGTCATATG	33:C>G
1089663|F|0-16:C>G-16:C>G	TGCAGGACGCGCGGCGCTACTCCGTGTCCGACGCCGACGCCTACGGGCTGGGCCTCCG	16:C>G
997377|F|0-5:G>C-5:G>C	TGCAGGCCGACCATGGTGAACACGTCCGCCGCGCCGAACAGCACGTACTGCGGCACCATCCACCACAGG	5:G>C
2253158|F|0-20:C>G-20:C>G	TGCAGCAGCAGCCTGTCCTCCGCCGACGACTCGAACCCGTGCAGGAACGACATGTCCGCGCCGCCGCCA	20:C>G
2259485|F|0-7:A>G-7:A>G	TGCAGTTAAGGTCGCGGTGGATGATGCAGGGGTCGTGGGTGTGGAGGTGGTCGAGGCCCTCGAGGATCT	7:A>G
7156467|F|0-20:T>C-20:T>C	TGCAGTCCACGGAGCGGCAGTGCGCTCTCCCG	20:T>C
1091862|F|0-55:C>T-55:C>T	TGCAGTTCGGCTACGACGGGGAGGCGCCGCTCTTCGCGCGCTTCAACCTCCGCGTCGCCCCCG	55:C>T
2243350|F|0-43:T>C-43:T>C	TGCAGGAGAAGCTGGAGCGCCTGCCTGAGATCGAGCGCGCCTTTGTCCACCTCGACTACGAGTTCACCC	43:T>C
3940998|F|0-8:G>C-8:G>C	TGCAGCTCGCGCACGAGGTGCACCAGCCGCCGCGCCG	8:G>C
2283814|F|0-17:G>C-17:G>C	TGCAGGTGCTTGACGGCGACCGCCTCCTCGCCGTCGATGGCGATGGCCATCTCCATGTCGTCCTCGCCG	17:G>C
1152867|F|0-20:C>G-20:C>G	TGCAGAGCGCCCTGAATCTCCGCCATGTACTTGTTGTCTTCGTCTAACCATCGGGCGTACTCCATGTCG	20:C>G
32026717|F|0-28:C>T-28:C>T	TGCAGGCGGAGCGGATCAAGGGCCACCACGCGTGGCTCCGCGGGGCGGTCTCGATGTTCGGGAAGCCGA	28:C>T
1041702|F|0-31:C>T-31:C>T	TGCAGCCAGCTCTTGAAGGCGTCGCATTTCACAGGCCCTGAAGCGGTGAAGCGCACCAAAACTCAGAAC	31:C>T
1041702|F|0-43:C>T-43:C>T	TGCAGCCAGCTCTTGAAGGCGTCGCATTTCACAGGCCCTGAAGCGGTGAAGCGCACCAAAACTCAGAAC	43:C>T
1026294|F|0-38:G>C-38:G>C	TGCAGCTTCTTGGGCGTGAGCACCTTAGGAGAAGGTGGGGGTGTTGGGTGCGCGCGGTTGAGGACGCGC	38:G>C
986053|F|0-47:G>A-47:G>A	TGCAGATCCTAGAGCCCCATTGATTAAAAAAATAAACCAGTTGGCACGCTAAAGATCTTCCCAGATCTT	47:G>A
5411802|F|0-22:G>C-22:G>C	TGCAGGCGCGCGCGGCCACGCGGTGGCGACCG	22:G>C
1023819|F|0-10:T>C-10:T>C	TGCAGGACTTTGTCGCCAGCCACAGCCCCGACGCGGACGCCGCAAAGGCTGCCGCCGACGCCCGTAAGG	10:T>C
1041702|F|0-23:G>A-23:G>A	TGCAGCCAGCTCTTGAAGGCGTCGCATTTCACAGGCCCTGAAGCGGTGAAGCGCACCAAAACTCAGAAC	23:G>A
998786|F|0-50:G>A-50:G>A	TGCAGAGGGTGGAGAGGTCGTGGGGCTTCGTGTGCGCGGGGAGGTACGGGGCCGACCACCCGTTCATCA	50:G>A
1081186|F|0-39:A>T-39:A>T	TGCAGAATCCATTTGCAAGAATCTAATCTACGCTCAGATAGTCCTGGCACCG	39:A>T
1038958|F|0-48:A>G-48:A>G	TGCAGCCCGCTCCTCTGGCTCTACGTGATCGCCATGTTCCTGCTCGTCATCGGCATGTTCTTCGTCACC	48:A>G
986389|F|0-57:A>G-57:A>G	TGCAGCCCGATGATCGTGCACCGCGACGTCAAGTCCAACAACATCCTCCTCGGCGACAACCTGGAGGCC	57:A>G
7165352|F|0-13:C>G-13:C>G	TGCAGTGCGTTCCCTGGGAGTTCGCGGCGCGGCTCGCGGCCG	13:C>G
7167633|F|0-19:C>T-19:C>T	TGCAGCAGGCGGAGCCCGCCGTGATCGCGGCCACCG	19:C>T
5367403|F|0-12:A>C-12:A>C	TGCAGGGGTTCAACTGGAGCTTGCCGCCCG	12:A>C
1079022|F|0-46:G>A-46:G>A	TGCAGGCTGACATCTGCAAGAAAGACTCTGAAATCGGTTGGCTTCGGCCCGAGCTGGACGAGCTGGAAA	46:G>A
1138044|F|0-11:C>G-11:C>G	TGCAGGTTTGTCATCGTCGCAGCCGCTAATTTAGCTTTGCAAGACATTACAGATTCTGTCTAAATATAG	11:C>G
3938664|F|0-38:G>A-38:G>A	TGCAGGGAGCTTGAATCGCGCGTCGGCACTGGTAACGCGCCCAGCTTCCTTCCTCACTCCGCTCGAAAT	38:G>A
2343977|F|0-50:C>G-50:C>G	TGCAGCTGCTGGTAGCCGCCCATGCTCTCCTTCGTCCCCGTCGACGGCTCCGCCAGTCTGCGGATGTAA	50:C>G
1063953|F|0-11:C>T-11:C>T	TGCAGACACCACTAGATGTGTAGGCGGTAGACCCATGCTAACCGCAGCAGCCCCATGATGCCACCTTCT	11:C>T
988652|F|0-32:T>C-32:T>C	TGCAGAACGCCGTGAAAGGTAAGGCACGCTCATCACCTTTCGCTTTCATGAATGCTGCCATGCTGGTGC	32:T>C
5367352|F|0-17:C>A-17:C>A	TGCAGGCTGCGGTGGACCAACTACCTCAGACCCG	17:C>A
3944091|F|0-59:G>A-59:G>A	TGCAGCGGCGGCCGCTTGCTCGACGAGATGGCCAGGGACGGCACCTTCTCCGAGCAGCGGGCCGCCCTT	59:G>A
5363303|F|0-10:A>C-10:A>C	TGCAGCAGGGAAAAGCATTTACCG	10:A>C
1048892|F|0-55:G>T-55:G>T	TGCAGGAGAAGAGGGGCTTCTTGTTCTGCAAGGAGGACAGGGCGATCCTGTGCCGGGAGTGCGACGTGT	55:G>T
1203873|F|0-23:C>G-23:C>G	TGCAGCAGCGCGTGCTTCTCCTGCGCGCCCAGCTTCTCCACGTTCACCTCCTTGAGCTCCCCCTCCGAG	23:C>G
1081085|F|0-43:A>G-43:A>G	TGCAGAGAATGATTCAGAGACAAGTTACACCGAATTAATGCGCAGTGCACGGTGAGGAGCAAGGAACCG	43:A>G
1092142|F|0-7:G>C-7:G>C	TGCAGGTGGCGCCGCGGGGCTCGCACGCGCTGGTGGTGTCCACGGAGACCATCACGCCCAACTACTACG	7:G>C
1219191|F|0-10:G>A-10:G>A	TGCAGTCCCCGCCGACGCAGCAGCAGCAGGACCTGCTGGGCGGGCTGGACACGGAGCTGAGCGACATGC	10:G>A
32024578|F|0-39:G>A-39:G>A	TGCAGGGGTCGGCGGACCCCTCGATCCCGGCGTTCGCGGGCTCCGCCCCGCTGGCGCTGGTCTCCATGG	39:G>A
2330303|F|0-43:C>T-43:C>T	TGCAGGTCATCGAAGGCGTCGCCGCCTGCCACCGCGCCGGCCTCGTGCACCGCGACCTCAAGCCCGAGA	43:C>T
12692150|F|0-17:G>T-17:G>T	TGCAGCGGGTGGAGGTAGGTGCTGCCGAAGAGCGGGTTGTTCTTGGAGAAGAAGATCTCGAGGGAGCCC	17:G>T
1228382|F|0-25:T>C-25:T>C	TGCAGCTGATGCGGACGCGGACCGATAGGGAGCTGGGGCGCCTCCTCTCTTCGGCGTCGGCGCTCGCGG	25:T>C
3025302|F|0-5:C>T-5:C>T	TGCAGCCAGGTGCACATGGCCATGTACATCTCGCTGGCCGCCGCCG	5:C>T
3941906|F|0-37:T>C-37:T>C	TGCAGCACCGCAACCTGGTGCGGCTGCTGGGCTGGTGTGCCGAGCAGGACGAGAAGCTGCTTGTCTACG	37:T>C
3953240|F|0-12:C>T-12:C>T	TGCAGCCACTAGCAGAAGCCG	12:C>T
1091590|F|0-35:A>T-35:A>T	TGCAGGAGGTGTCCCGCGCCTTCTCCGTCTGCATCAACATCATGGGCGGCTCCGCCCCG	35:A>T

12419799|F|0-23:G>C-23:G>C	TGCAGCGCCCAGCCGTACTGGATGCCGCCG	23:G>C
1023241|F|0-7:C>T-7:C>T	TGCAGAACGGCTGCGAAAACGGCCCGAAGTTTTGATTAGGATCGTCTGCCATCTCTATGCCCAGCAACT	7:C>T
1248805|F|0-60:C>T-60:C>T	TGCAGGACATGTTCGCGGCGGGAACGGAGACGACCTACATCGCGCTGGAATGGGCCATGTCGGAGCTGA	60:C>T
6030814|F|0-7:C>G-7:C>G	TGCAGGGCAACCGCCTGAACGGCCCCATCCCG	7:C>G
1076647|F|0-30:C>G-30:C>G	TGCAGCGTCAGTTGCACATTGTACTATATTCGGTGCACGAATCATACTTGACATATGCCG	30:C>G
1113745|F|0-45:C>G-45:C>G	TGCAGCGGCGACGAGTGCGAGAAGGACGCGGAGCCACGGCCCCACCCCCGCTGGCCTCCATCAAGCCCC	45:C>G
7170909|F|0-16:T>C-16:T>C	TGCAGTCCCTGGACGCTAGCAACAACCGCCTCACCG	16:T>C
982967|F|0-31:G>C-31:G>C	TGCAGTTCGGGTGGTACCTGTCCTTCATGACGGTGTTCGTCCTCGCGGTCTACCTGTACGTGTGCAGCA	31:G>C
2286159|F|0-5:C>G-5:C>G	TGCAGCGCCTCCCACGAGCGGTCGTCGGCGTAGGCGCGCTCCCATGCCTCGAGGACGCGGCCGTCGCCG	5:C>G
5358496|F|0-25:C>G-25:C>G	TGCAGTACATCGTGAGGGGCGGGCGCAACCTCTTCCCGCTGCTCCCG	25:C>G
1164753|F|0-67:A>G-67:A>G	TGCAGTTACCTGGGCCATGCGGCGAACCCACGCCAGCTTGTTGTGATTCTCGTAGCCGATGACGGTGAC	67:A>G
6041401|F|0-21:T>C-21:T>C	TGCAGCCGCGGCCGCCGCGGCTGCCATCGGCGACCCG	21:T>C
6041401|F|0-15:C>G-15:C>G	TGCAGCCGCGGCCGCCGCGGCTGCCATCGGCGACCCG	15:C>G
1229860|F|0-19:C>G-19:C>G	TGCAGCAGGAGCGCGCGCTCGGCCGCCCCATGTTCCCCAAGGCCATCGGCGAGCTCCACGGCCACTCCG	19:C>G
990178|F|0-48:T>C-48:T>C	TGCAGACATCGGCGTGATGAGCGGAGCGTCGCTGTACATCCAGAAGGATCTGAAGATCAACGACACCCA	48:T>C
12772018|F|0-49:G>T-49:G>T	TGCAGATGCAGGAGGCGAGCCTTCGAACGAACTTAGTTCAGGCGAAGCAGAAGCGTGATCGCGACATGG	49:G>T
7352336|F|0-13:C>A-13:C>A	TGCAGCTGCCTCCCCCGCCG	13:C>A
3064839|F|0-22:C>G-22:C>G	TGCAGGCCCTGCTCGCGCACCTCTGGCGTGCCGTGTGCCGAGCCCG	22:C>G
5369519|F|0-19:C>T-19:C>T	TGCAGGAAGGGGTGGGGGTCGGGGTCGGGGTAGTCCG	19:C>T
5411755|F|0-21:C>T-21:C>T	TGCAGGTTCCCCGTCCGACGTCCCG	21:C>T
1092362|F|0-37:G>T-37:G>T	TGCAGGCGCTTCTCGTAGTCCTCGCGGCCGCCGAAGAGGCCGCGCCACATCCTCCCCAGCACCCCTCCT	37:G>T
7865065|F|0-16:G>C-16:G>C	TGCAGGGGCTCGTGAGGAAGGGCCTGTGGGAGGACGCCATCGACTACCTCGATGGCTTCCTGCCTGCCG	16:G>C
2294176|F|0-32:G>A-32:G>A	TGCAGACCATCCGCCACATGCTCCAGATTCACGTCCTCGTCGACTACTACGGCGGCAAGAAGCTCCACC	32:G>A
1094021|F|0-27:C>T-27:C>T	TGCAGGGAGGATGGCTCGGCGGAGGTGCTGCTCGGCGAGACGTGCGTGATGGGCTATGTGACCGCCCAA	27:C>T
986694|F|0-12:A>G-12:A>G	TGCAGCAATTCCACGATTGGTTATCCTGCAAAGCAAGGAAGAAGCCACGGAGCCCATCGTGGTCGTCTC	12:A>G
992847|F|0-41:C>G-41:C>G	TGCAGGAGCCGCGCGAACAGCATGACGGTGAAGAGGGTGCCCAGCGCGATCCCG	41:C>G
989848|F|0-22:G>C-22:G>C	TGCAGGCCGCGCTCTCCGACGCGTACCACCGCTTCAGCATCCGCACCGTCCGCCTCCTCATGTTCCTCT	22:G>C
1065412|F|0-25:G>A-25:G>A	TGCAGCAGCACATGGAGATGAGGCCGACCATGGCCATGGACAGCTTCTTGGCGTTCAACGAGAGCTGCA	25:G>A
3029316|F|0-17:T>A-17:T>A	TGCAGTAACGCAGTAAATCCAGCGCGCGCAGCAGTGGCGCCG	17:T>A
1714527|F|0-24:G>T-24:G>T	TGCAGCTCCCTGCCGTTCTTGCCGGCCGACATGGCGTCGGAGAGCATGGCCGTGAGGCTCCG	24:G>T
1236663|F|0-7:T>A-7:T>A	TGCAGCATGTCCGCCTCCTCCAGGGATTCCATGGCCTTGAGCACCTTGGCCTTGTAGCTCCGCAGGCTG	7:T>A
3955944|F|0-29:G>C-29:G>C	TGCAGCGGCGGCATGTGCGGGCACTACACGCAGATCGTGTGGCGCGGCAGCACCCGCGTGGGGTGCGCC	29:G>C
32019576|F|0-23:C>G-23:C>G	TGCAGCGTCCAGAGCGCCCAGTCCAGGTCGAGGTCGGCGGCGACGGCGGCGGCGCAGCCGTAGTAGCGG	23:C>G
32018389|F|0-17:G>C-17:G>C	TGCAGGCCGTTGTAGGGGGACGCCGCGTCGCTGCCGTACAGGTCCCACTGCCCCGTGGTGTTGGCGATG	17:G>C
1862637|F|0-29:G>A-29:G>A	TGCAGGGGCTCGTGAGGAAGGGCCTGTGGGAGGATGCCATCGACTACCTTGATGGCTTCCTGCCTGCCG	29:G>A
32023673|F|0-62:A>C-62:A>C	TGCAGCGACCTGCCCAGCTCTAAGTGCACCTCGGCCAAGTGCGGCTACACGTACACCTACGGAGACTCC	62:A>C
1696630|F|0-44:C>G-44:C>G	TGCAGCTAGACGGCAACGCCATCGCGGTCTCCACCAACACCGTCCTCCACGAGCCCATGTCCACTTCGT	44:C>G
5578330|F|0-9:C>T-9:C>T	TGCAGGGCGCGGGGGTTCCACGTGAAGCCG	9:C>T
12763916|F|0-29:A>G-29:A>G	TGCAGCCCTAAATATTACAATTTCCCACAAATTAGTTGCTGCACGAAGAGCGCTACATAGAAAAGCTGA	29:A>G
3570100|F|0-28:T>C-28:T>C	TGCAGGGCGACATGCGGGAGCTCCCGTTTACCCTGGCCG	28:T>C
1230536|F|0-31:G>C-31:G>C	TGCAGGTGCTGGCCATCCTGCCGTCGTACGCGGGCAAGACGGTGCTGGAGCTCGGCGCCGGCATCGGCC	31:G>C
6041360|F|0-9:G>C-9:G>C	TGCAGCCACGTGTGCTGCGCCGTGCCG	9:G>C
1024064|F|0-35:A>G-35:A>G	TGCAGCAGCAATCAACTGATGCAAGAGCCCATGGGATGGCGTATAAATAAAAGCAGGGAGCTAGCCTGG	35:A>G
3028916|F|0-68:G>A-68:G>A	TGCAGCAGCGCCACCATCTCCTCCTCGGCGCTCGCGCCGAGCCGCACCAGCTCGACGTCGAACACCTCG	68:G>A
2254188|F|0-44:G>A-44:G>A	TGCAGCTGCTCCGCCGTGGACATGATGAACGGCACGTACCGCTCGAGCGCCGCCTCCGTGTGCTCGGCG	44:G>A
2254188|F|0-47:C>G-47:C>G	TGCAGCTGCTCCGCCGTGGACATGATGAACGGCACGTACCGCTCGAGCGCCGCCTCCGTGTGCTCGGCG	47:C>G
980661|F|0-26:G>T-26:G>T	TGCAGCCTCTTCAACCCGACGCCGTCGCTGGCGTCCATGATCATCCACCATTACAAGATGCGGGAGGAC	26:G>T
3022522|F|0-27:T>A-27:T>A	TGCAGCCACACTCTTTGAGCTTGGGGCTCAGGTGCATGCCCG	27:T>A
1009966|F|0-23:G>C-23:G>C	TGCAGCCCGTGCACGATCCCCGTGGCGAACGTCGCGAAGCTGATCTCCTTCTTCTTGCCGCCG	23:G>C
2330303|F|0-10:C>T-10:C>T	TGCAGGTCATCGAAGGCGTCGCCGCCTGCCACCGCGCCGGCCTCGTGCACCGCGACCTCAAGCCCGAGA	10:C>T
1130006|F|0-40:C>T-40:C>T	TGCAGGGGGCACTGCTGCCGTCCGTCCAGAGCGCAGGAGCCCGCGCGGCCG	40:C>T
5580424|F|0-7:C>G-7:C>G	TGCAGGCCGTGCGGAGGTCGCTCGACGACATGCCG	7:C>G
1080531|F|0-38:A>C-38:A>C	TGCAGACCTTCTTCAACTGGTACTACACCGTGCTGGGGATCTCCCTCGTCGTTGCGGTCCTCGTCATCG	38:A>C
5325006|F|0-13:G>C-13:G>C	TGCAGTTCCCCGTGGGCCGCGTCGCCCG	13:G>C
12770272|F|0-12:G>C-12:G>C	TGCAGAGGAGCAGGTCGTGGCCG	12:G>C
1321523|F|0-10:T>C-10:T>C	TGCAGCACGATCTCCACCGTGGAGTTGTAGGGCAGGCTCCTCACCTTGGTGCCCTCCACCATGGCCGTG	10:T>C
3934442|F|0-66:A>G-66:A>G	TGCAGCGACCGCCCGCTCTCCTTCACCGCGCCGTCGGGCCAGACCGCCTGGAGCTACTACAAGATCATG	66:A>G
1264603|F|0-33:G>A-33:G>A	TGCAGCTCCTTGGTGGAGATCATGCGGACGTCGGCGGCGTCCTTGCTGGCGGCCG	33:G>A
1024801|F|0-14:C>G-14:C>G	TGCAGGAGCGGCGCCACGCTCGCCATCTCCTCCTTGGCCGCCGCGTACCG	14:C>G

991595|F|0-7:G>A-7:G>A	TGCAGGCGCCTACGCCGCTGCGGCGCACGGAGCTGTGCGAGCCCGCTTCCGCCCACCGCAGCTCCCGAG	7:G>A
1091785|F|0-53:G>C-53:G>C	TGCAGCGCGCGCACGCGCTTGATGGCGGCTTCCTCCCTGCTCCTCATCACGGCGCCCATCTCTTCCTTG	53:G>C
1021037|F|0-57:A>G-57:A>G	TGCAGGTTGTACCCTCTTCTTAGGCTCCCGTGGCTTTGACTCAGCCTTCTTGATGCCACCCTTGGCGGC	57:A>G
1092720|F|0-40:G>C-40:G>C	TGCAGTCCTGCGCGCCCCTGGCGGCGACCCCAAACCCGAGGGGCACGTCGGCCATGGACATGACGACGA	40:G>C
987511|F|0-50:A>G-50:A>G	TGCAGGAACTCGTTGGCGCAGTAGGCAGGCGACACGCCTCGCGGGAGGCCAAACGCCGCCGCTCCCGAT	50:A>G
9364972|F|0-6:A>G-6:A>G	TGCAGCAGCGCGCATGGCGCCG	6:A>G
996917|F|0-29:G>C-29:G>C	TGCAGAGCTTCCTCGGCCTCCGCCTCCTCGTCGTGGCCGCCCTCGCCGCCTTCCTCCTCCTCTTCTCCG	29:G>C
12692150|F|0-59:G>C-59:G>C	TGCAGCGGGTGGAGGTAGGTGCTGCCGAAGAGCGGGTTGTTCTTGGAGAAGAAGATCTCGAGGGAGCCC	59:G>C
1242990|F|0-7:C>G-7:C>G	TGCAGCTCCCGGCGGCGAAGGCGGCCATGAGGCCGAGCAGCTCCACCAGGACGCACACCG	7:C>G
1264603|F|0-45:C>T-45:C>T	TGCAGCTCCTTGGTGGAGATCATGCGGACGTCGGCGGCGTCCTTGCTGGCGGCCG	45:C>T
2255867|F|0-22:G>C-22:G>C	TGCAGAACCGCGTCCTCTACATGGTGCTCGGCCAGGCGTGGCGGACGATCGTGTTCGTGCGAGCGAACG	22:G>C
1210756|F|0-42:G>A-42:G>A	TGCAGCTGCCTCCTGGGAAATGCACATCACCGAAGCAGAATCGGGTTCAGTGTGGCTCCTGCTCAGAGG	42:G>A
986548|F|0-15:A>G-15:A>G	TGCAGTTGAGGACGCAGCAGCGGAGATGGCGGCGAGGAGGCGGGAGGTGGGAACGGGCAGGGGCAGGGG	15:A>G
1037030|F|0-8:G>A-8:G>A	TGCAGGTGGCTCTTGACCTCATCATTCGTGAGCCCATCCACCTTCATCATCTCCCTGATTTGCTTAGGA	8:G>A
2283814|F|0-20:C>G-20:C>G	TGCAGGTGCTTGACGGCGACCGCCTCCTCGCCGTCGATGGCGATGGCCATCTCCATGTCGTCCTCGCCG	20:C>G
1113733|F|0-43:T>C-43:T>C	TGCAGCCCTGCAAGCCGCCGCTGCACGAGACGGACCACCTCGGTGGCGCTGTGTTCACCG	43:T>C
1009790|F|0-6:T>A-6:T>A	TGCAGCTATTCTGAATGGGTATGGCAGCAATCAAAACCACTGATGCACAAGAACGCCGCTCTCCCGAGA	6:T>A
3936367|F|0-29:A>C-29:A>C	TGCAGGTACTGCACGCTCACGGTGCAGAGAACGGCGTCGAGGGAGGCGCTATCGAGCTCCAGCCGCTGC	29:A>C
988825|F|0-37:G>A-37:G>A	TGCAGGCGTCGGACCTCAACGACTTCATCGCCCCCTCGCAGGACTGCGTCGTCTCCCTCAACAAGAACT	37:G>A
994689|F|0-28:C>T-28:C>T	TGCAGTTCGGCAACGACTACGTGCTGGGCTACTGGCCGTCGTTCCTCTTCTCGTACCTGGCGGACAGCG	28:C>T
1008179|F|0-7:C>G-7:C>G	TGCAGGGCTCTGGGCGCTGCTGCTGGTGCCTCTCTGGAGCACGTCGCTGTCGGCTAAATAGCGCAGGGA	7:C>G
3941994|F|0-27:G>A-27:G>A	TGCAGACGTCTCCTCGCCGATGAACTTGTGGTCCGGGTAGAGCATCCG	27:G>A
3222308|F|0-20:T>C-20:T>C	TGCAGCGACCTGCCCAGCTCTAAGTGCACCTCGGCCAAGTGCGGCTACACGTACACCTACGGCGACTCC	20:T>C
1028057|F|0-22:C>G-22:C>G	TGCAGGCCCACGCCTTCCTCCTCCCCCGCGGCGGCCACCGCAACGCGCGACTCCTCTCGGCGCTCCTCC	22:C>G
993049|F|0-17:A>C-17:A>C	TGCAGCAGCATGGCCACAGAGTATGCCGTCATCCTCTCGGCCATGGCGATGCCCGCGCAGATCCTCCTC	17:A>C
7341777|F|0-14:G>C-14:G>C	TGCAGCGTCACCTTGGTGCCG	14:G>C
2253084|F|0-67:C>G-67:C>G	TGCAGCCACCAGTTGCCCTTCTTGGGGTCCTTCCAGATCATGAGCGTGATGTCGAACTGCCGGCCGCCG	67:C>G
992391|F|0-20:G>A-20:G>A	TGCAGCAGCCTATCCTCCGCGCGCCGCTTCCCGCGGTTGAGCTCCTCCAGCTGGCTCAGCAGGCCGAGA	20:G>A
2293741|F|0-11:T>C-11:T>C	TGCAGGTGGGGTGAGACGTATGATGGCAGCGGAGGCAGCGTGAAATACACGGACAAATGGGCCGAGCGG	11:T>C
987629|F|0-34:G>A-34:G>A	TGCAGTTTTGCTGAGCCAACTGTTCTACCAAAAGGCTTTCCATGCCGTACATAAGTGATGTCTTCTGCC	34:G>A
32026629|F|0-58:C>G-58:C>G	TGCAGAGCGTGCGCGATGAGGTGGTGATACGGCTGCTCCTCCACCTGCTCGTGCTTGGCTGCTGACGGC	58:C>G
3945931|F|0-35:G>C-35:G>C	TGCAGCGCCGCGATGCGGAAGATCATCTCCCGCATGGCCGCCG	35:G>C
1162710|F|0-10:C>G-10:C>G	TGCAGCCGACCCTGGCCACCGAGTACGCCAGCCAGGCCGGCCGCTTCACCCTCAACATCACCCGCTCCA	10:C>G
1091862|F|0-28:G>C-28:G>C	TGCAGTTCGGCTACGACGGGGAGGCGCCGCTCTTCGCGCGCTTCAACCTCCGCGTCGCCCCCG	28:G>C
2251549|F|0-26:G>A-26:G>A	TGCAGCACGTCCTCCCACACCCCGCCGTACCACACGTCGTCGACCACCAGCAGGAACCTCTTGTTCGCC	26:G>A
990479|F|0-54:C>T-54:C>T	TGCAGCTTCTGCTGGACCATGGCGTTGTGGCGCTGCGCGAAGTCGTTGTACGTCCTCAGGCACCCGACG	54:C>T
1029726|F|0-7:A>G-7:A>G	TGCAGCTATCGCCGCCGCTGCTCACCAGCGGCTGCACCGTGCCGCTGCTCCCGCCG	7:A>G
2267348|F|0-39:C>T-39:C>T	TGCAGCTCGCGGAAGTCGCGCTCGAAGCCCTCGACCCTCCTCTCGCCGCGCCG	39:C>T
12692150|F|0-14:G>A-14:G>A	TGCAGCGGGTGGAGGTAGGTGCTGCCGAAGAGCGGGTTGTTCTTGGAGAAGAAGATCTCGAGGGAGCCC	14:G>A
6046306|F|0-14:T>G-14:T>G	TGCAGCTGCATGCATAGCTGGATCCTCTCCATGGCCGACGCGCTCAGCGGCTGCTGCTCGCCGTCGTTG	14:T>G
3938967|F|0-14:A>C-14:A>C	TGCAGCGTCACCGCAGCCGCCTCGTGCTTGGGGCTCTCCG	14:A>C
6044178|F|0-14:T>C-14:T>C	TGCAGGTCCTGGAGTGTGCCGTCCAGCGCCACCGGGACGTCCTCCG	14:T>C
1717652|F|0-47:C>G-47:C>G	TGCAGCGTGCTGTAAAGCCCGATCCTCCCCACTAAGAAGAGCCTTTTCCGAGCTCCGGCGACGAGATAC	47:C>G
5582066|F|0-9:C>T-9:C>T	TGCAGGTCACGGCGAGCCGCCGCGTCGTGGCACCCG	9:C>T
2249753|F|0-15:G>T-15:G>T	TGCAGTCTGCGGTCTGGAACGAAAGCTCCCCCTTGCCGATGTCGTAGAGCAGGTGCAAGTCTTGCTGCT	15:G>T
999562|F|0-59:G>C-59:G>C	TGCAGCCGCGCCCGCACCGTGTGCGACGACAGCTGCGTCGGCACGTTCGTGCACTCCTTGGCCACCACC	59:G>C
3023667|F|0-13:T>G-13:T>G	TGCAGCTTCTCCCTGTACGAGTTGATCTTCTCCAGCTTCAGCCCG	13:T>G
6041680|F|0-9:C>T-9:C>T	TGCAGGATGCTCTCCGCGAGCCCGATGCCG	9:C>T
5332268|F|0-14:G>A-14:G>A	TGCAGGCACACCGAGCAGCAGAGCGCCTCCCCG	14:G>A
987600|F|0-27:A>G-27:A>G	TGCAGCCAGTACCTGAGCGCCTACATCATCGGCTTCGGGGTGGTGCAGGTCATCTTCTGCCAGGTGCCC	27:A>G
1204431|F|0-38:C>T-38:C>T	TGCAGCTTGGTGACAATCATTTTGAAGGAGCCATCCCACACAGCTTGGGCAATCTTCAGTACATCTCTA	38:C>T
1013484|F|0-22:G>T-22:G>T	TGCAGGCGCGCGGCGAGTTCGCGGGGGCGCTGGCGTCCTTCACGGCGGTGGTGAGCGGGTACAAGGAGC	22:G>T
4986915|F|0-10:G>C-10:G>C	TGCAGAGCGGGCGCGGGCTGCTGTCCTCCGACGAGGTGCTGCTGACGCACCCCAAGACGCGGCGGTTCG	10:G>C
1102595|F|0-40:C>G-40:C>G	TGCAGTCGCTGGTGACCGAGCACGGGCTCCCCGCTGCCGACGCCTCTCCCCCTTCCCCTCCTGTTGCCG	40:C>G
1240703|F|0-62:C>G-62:C>G	TGCAGCAGCGCCTTGAGCTGGTCCTGCGCCAGGGCCTTGGTCGGGAACACGTAGAGCGCGCACGCCGCC	62:C>G
1055791|F|0-26:C>T-26:C>T	TGCAGCTGCGCGGCGTGGATGGTGTGCGACACCGCCAGCGTCAGCCGCGCCGTCACGTCGCTGTGGGTG	26:C>T
3957717|F|0-22:A>G-22:A>G	TGCAGGAGCTGTGCGTGAGGGCAGCCCTGGCCG	22:A>G
5581330|F|0-14:G>C-14:G>C	TGCAGCAGCGGCATGCCGGCGGCGAGCCCCGCGGGTATCTCGCCG	14:G>C
1251314|F|0-32:C>A-32:C>A	TGCAGCTGGAGCTCCACCAGCTCCCGGCATTTCCCGATTTCTCGTGGGATCGTTCCG	32:C>A

992844|F|0-58:A>G-58:A>G	TGCAGGAGCTCCGCGCCATCAAGTTCGCCCCGCCGCCACCGCCCAACAACGCCAGCCAACACCCCGAGA	58:A>G
3949677|F|0-14:G>A-14:G>A	TGCAGCAGTCCGTAGGCGAAGAGACCCG	14:G>A
1028057|F|0-23:C>A-23:C>A	TGCAGGCCCACGCCTTCCTCCTCCCCCGCGGCGGCCACCGCAACGCGCGACTCCTCTCGGCGCTCCTCC	23:C>A
1863483|F|0-5:G>A-5:G>A	TGCAGGCGTGGTGCCCACCCAACGTGGACCCACGGACCGCCGTGCCCATGGACACTGTGACGCCGCGGG	5:G>A
3946150|F|0-20:C>G-20:C>G	TGCAGGAACGCGTCGTACACCACGGCGCGCACGGGCCG	20:C>G
996910|F|0-45:G>C-45:G>C	TGCAGAGGAGCGAGCAGCTGCGGGAGCTCTACGAGTCTCTCCTCTGCGGCCAGTGCGACCACCG	45:G>C
3948387|F|0-5:C>G-5:C>G	TGCAGCGGCACCCCCTTGGGCGCGCCG	5:C>G
991525|F|0-52:G>T-52:G>T	TGCAGGAGGGCGACGGCCTGGACTTCTCCGACGTGAGCAGCCTCAACATGGTGACAGAGCTCACGCCGC	52:G>T
29308142|F|0-58:C>A-58:C>A	TGCAGAGAAGGGACATCGTCACAGATTCAGTCAAAATCCTCGCGAAATAGTTTTGACTCGGCGGGTGCT	58:C>A
3939596|F|0-22:C>T-22:C>T	TGCAGCACCGCCTCAAGCCCACCGCCGTGCTCTGGGGCTGGCCG	22:C>T
1244951|F|0-35:G>C-35:G>C	TGCAGGATGTTGCGCCACGGGTGCTGCACCAGCTTGGACTCCTCGCTCGCCACCACCAGGTCCGCGTAC	35:G>C
1108119|F|0-5:C>G-5:C>G	TGCAGCCGCGCCCTGCTCTGCACCCTGGGCTTGCTGAAGTCCTCGGCCAGGATGTCCACCAGCGCCATG	5:C>G
7351424|F|0-16:G>T-16:G>T	TGCAGTCCGCGCCGCTGGCCG	16:G>T
7338780|F|0-10:C>G-10:C>G	TGCAGGAGAACCCCATGCCCG	10:C>G
2278984|F|0-10:G>C-10:G>C	TGCAGGCGCTGAGGGCCACCATCGACAGGTTCGGGTCCGAGAACGTGTGCCTCTGCGGCCACTCCCTGG	10:G>C
1211001|F|0-43:G>C-43:G>C	TGCAGCTGGATGAGAAGGAGGGCTTGGGCAGGCGCGCCGACCTGAAAGCCAGTGTTCTCATGAACCAGA	43:G>C
32025058|F|0-11:C>G-11:C>G	TGCAGCGCCGTCGCCAGCAGCTTGTTGGGGTACTCCTTGAGGAGGCGCGCCTGGAGGACGATCCAGAGC	11:C>G
2293741|F|0-39:G>A-39:G>A	TGCAGGTGGGGTGAGACGTATGATGGCAGCGGAGGCAGCGTGAAATACACGGACAAATGGGCCGAGCGG	39:G>A
2263784|F|0-24:G>A-24:G>A	TGCAGCAGGATAATGGCATCGAGGGAGAACTCGTCGGCGGGCAACAACGGAGGAGGCTGCGGCGCGAGC	24:G>A
983777|F|0-56:T>C-56:T>C	TGCAGCTGGCCATACAATGCCTCCAATATCTTGTCCCCCTTGGGCAGCTTCTCTGCTAGGTTGAAGGCC	56:T>C
1057884|F|0-47:G>C-47:G>C	TGCAGCTTGAGGGGTAGATATACCTCAGTCAGGTACTGGAACCCAAAGGAGCTGAGAGCCTGAATCTCA	47:G>C
5581535|F|0-9:G>C-9:G>C	TGCAGGTAGGTGAGGAAGGTGTGGAACGGGCCG	9:G>C
5363443|F|0-17:C>T-17:C>T	TGCAGCCTTCCTTTTCCCACCTCCG	17:C>T
2243690|F|0-10:T>C-10:T>C	TGCAGGTAAATGACTTCATATCCATCGCCGATAACTCCTACTCGAGGCAGCAGATCCTGTCAATGGAGA	10:T>C
1108119|F|0-47:C>G-47:C>G	TGCAGCCGCGCCCTGCTCTGCACCCTGGGCTTGCTGAAGTCCTCGGCCAGGATGTCCACCAGCGCCATG	47:C>G
3938426|F|0-45:G>T-45:G>T	TGCAGCTCCGTCGACGCGGGGGAGGCTCCGTCGCCGTCGCTGGCGGCGGGCATGGAGGGATCAGATCGA	45:G>T
5358629|F|0-17:C>T-17:C>T	TGCAGTGGGTACACGATCCCG	17:C>T
1108119|F|0-61:G>T-61:G>T	TGCAGCCGCGCCCTGCTCTGCACCCTGGGCTTGCTGAAGTCCTCGGCCAGGATGTCCACCAGCGCCATG	61:G>T
5970733|F|0-46:T>C-46:T>C	TGCAGCGCGATGCGCGCCAGGAAGAAGATGTCGAACAGGGTGCGGATCACGGCGGTGGAGATGGCGAGG	46:T>C
2259943|F|0-25:G>A-25:G>A	TGCAGTTGACGACGAGGACGCCGACGTCCTTGGGGCGGACGCGGCACTTGTCGAAGAGCTCGTCGAGCG	25:G>A
12772260|F|0-68:G>A-68:G>A	TGCAGCGTCCAGAGCGCCCAGTCGAGGTCGAGGTCGGCGGCGACGGCGGCGGCGCAGCCGTAGTAGCGG	68:G>A
5582055|F|0-16:C>G-16:C>G	TGCAGGGCGTGGTCGACCTGCTCCCG	16:C>G
3956756|F|0-41:G>C-41:G>C	TGCAGGTCCTCGCGCTTGATGTGGAGGCTGACCCCGAGGCGGTCGGAGAGCTTGGCGGCGCGCTCGAGC	41:G>C
1057947|F|0-21:A>G-21:A>G	TGCAGCCGATCTGCAAGCCACACCACATGAATCAGAATTCAGAAACACGAACAAACAGAACCGCAGATC	21:A>G
994188|F|0-49:T>A-49:T>A	TGCAGGCGCGCAACAAGGCGCCCAGCGGCGTCACGCAGAACGTCTAATTTATCATCGTCGCCG	49:T>A
1064179|F|0-38:A>G-38:A>G	TGCAGTACTCCTCATTGGACCAGCAGTGTTCGTCCAGGACGGGGCCCTCGGGGAGCTTGGGGTTGATGA	38:A>G
4004038|F|0-23:C>T-23:C>T	TGCAGGCCATGGACCGCAGGCTCCGCGCGGCGGAGAACCG	23:C>T
12766299|F|0-13:G>C-13:G>C	TGCAGTCGTCGCTGCTCGGGAAGCTCGGGCAGCCG	13:G>C
1027358|F|0-62:T>G-62:T>G	TGCAGGTTGGGCGCCTCGGGGGTGGAGGCCGCGCGGAAGAGGAACGGCAGCAGCTCGGCCCATGTGTTC	62:T>G
1055791|F|0-32:C>G-32:C>G	TGCAGCTGCGCGGCGTGGATGGTGTGCGACACCGCCAGCGTCAGCCGCGCCGTCACGTCGCTGTGGGTG	32:C>G
1042639|F|0-14:A>G-14:A>G	TGCAGCCCATGGGGACGGTCTGTGCCCAAAGAACTCCAGCTGGGAGATGCAGCTATACACCATTGCCGA	14:A>G
1078576|F|0-56:G>A-56:G>A	TGCAGGTTCTCGGCCCAGGGCGAGAGGCCCTCGCAGCTCTTGAGGACGACGATGGAGTCGCGCCAGGAG	56:G>A
3021526|F|0-16:T>C-16:T>C	TGCAGGAACCATTCGTTGAGATACCCGATGAGAAAATCCG	16:T>C
5411894|F|0-13:A>C-13:A>C	TGCAGATGCTCACACTCTCCTACAACAGGCTCGCCG	13:A>C
5358010|F|0-13:G>C-13:G>C	TGCAGCTCTCCGCGCGCGACGTCTATCCCG	13:G>C
1108119|F|0-59:C>G-59:C>G	TGCAGCCGCGCCCTGCTCTGCACCCTGGGCTTGCTGAAGTCCTCGGCCAGGATGTCCACCAGCGCCATG	59:C>G
1021798|F|0-18:C>G-18:C>G	TGCAGTTGCTCCTGATCTCGCCGTTGCGGCCAGTGATGGGCGAGATGTTGCCCATCTTCACCATGGACT	18:C>G
2258675|F|0-46:C>T-46:C>T	TGCAGGCAGCCGCTGAGCATGGACGTCCACGACACCACGTTACGGCCAAGGATCCTGTCGAACACCTTG	46:C>T
1154906|F|0-35:A>G-35:A>G	TGCAGGCTGCGGTGGCTCAACTACCTCAGGCCGGGACTGCGGCGCGGCAGCATATCGCCG	35:A>G
7338883|F|0-40:A>T-40:A>T	TGCAGCACCAGCGGGCACTTCACCGACAGCGTGCTCGCCGAGGTCGTCATCACACCGCCGTACCTGTAC	40:A>T
4003199|F|0-17:A>G-17:A>G	TGCAGGCTGGTGAGGCCAACGGAGCGGAGCCCCTCCAGGATCTCCG	17:A>G
1164189|F|0-23:A>G-23:A>G	TGCAGCTTATGCCGGCCCTCGGGAGTCTTGAAGTAAGTCTTGAGTCCAGGCGCGCCCCCG	23:A>G
1025251|F|0-64:G>C-64:G>C	TGCAGGCGCAGGCGGCGCAGCAGTTCAGGATGCAGGACCTGAGCAAGGCGATGAGGCCCGACGCGGCGG	64:G>C
1024863|F|0-65:G>C-65:G>C	TGCAGCAGCTGCTGCTGCTTCAGGAAGTTGTGCAGGGTAATGTGCTTCGTCGGCTCGGTGCTGCTGGCA	65:G>C
1013927|F|0-14:A>G-14:A>G	TGCAGCATCGCCGCATCCCTGGGGGCCACGGGCTGCGCCGCTAGCTCCCCG	14:A>G
1200500|F|0-59:C>G-59:C>G	TGCAGGGCGAGGTACAAGTGCTTGTCGTTGCAGGAGTCCATGGACAGCGACCTTCTCATCGGCTGCTGC	59:C>G
2255867|F|0-31:C>G-31:C>G	TGCAGAACCGCGTCCTCTACATGGTGCTCGGCCAGGCGTGGCGGACGATCGTGTTCGTGCGAGCGAACG	31:C>G
32026702|F|0-49:C>G-49:C>G	TGCAGGACCTCGGCCTGCGCCTGCCGTTCGCGCCCCTCGCCGCCGACTTCTCGGAGATGCTGGATTCGG	49:C>G
979207|F|0-41:G>A-41:G>A	TGCAGCAGGATGCTGGGAATGAAATACGAGTCGCAGAGGGGGTACATTGGACTGGAGTACTATGGACGG	41:G>A

1065412|F|0-10:C>T-10:C>T	TGCAGCAGCACATGGAGATGAGGCCGACCATGGCCATGGACAGCTTCTTGGCGTTCAACGAGAGCTGCA	10:C>T
4541700|F|0-13:C>G-13:C>G	TGCAGGAGGCGCTCGAGGAGAGCGAGAAGTCCG	13:C>G
7352622|F|0-22:A>C-22:A>C	TGCAGCTCGCGTACGCCGTCGGAGGCGGCTGCCCG	22:A>C
1393749|F|0-14:C>T-14:C>T	TGCAGCTCGAGGACCTGGTTCTGGAGCTTCTTGATGTGACCGATGGCGTCCGCCAGGGTGGACTCCTTG	14:C>T
4733756|F|0-5:C>G-5:C>G	TGCAGCAGCGCCTCGCCCAGGCCCTGCCTCCG	5:C>G
5578007|F|0-28:G>C-28:G>C	TGCAGCCGCAGCCGCTGTCGCAGCAGCCGCCG	28:G>C
999758|F|0-19:C>G-19:C>G	TGCAGAGGCAGCTGAAGAGCCCGCACGCCAGGCACTTGAACGGGCAGCACAGGCACGACGCCGCGCGGC	19:C>G
1231985|F|0-60:T>G-60:T>G	TGCAGCGGCGGGGGCGGCGGCGCGACGGCGTGGAACGGCACGAAGTGCGGCGCGAACTGGTGGGGCGGC	60:T>G
1126050|F|0-44:A>C-44:A>C	TGCAGCGCCGCGATGTGGAAGATCATCTCCCGCATCGCGTCCGAAGACGACGGGGAAGAGTACCCCTGC	44:A>C
1127770|F|0-39:A>C-39:A>C	TGCAGGGGGCTCAACTTCCCGGCGATAATGGGGCTGATCATCTGCACGGCGTCGTTCGGCATCAACCTT	39:A>C
1065531|F|0-25:C>G-25:C>G	TGCAGAGGCTCAAGGACAGCTGCATCAGGTTCCTCGTGGACTTCAGGAAGGTGTACGAGATGCAGGAGG	25:C>G
1099860|F|0-26:T>A-26:T>A	TGCAGCACAGCACCGACCGATCCATCTACTGCGGCGCGGCTCATCTTTCGCGTGCGTACGAGAGCGAGA	26:T>A
100253421|F|0-10:C>G-10:C>G	TGCAGAAGGCCATCCAGGACCAGGGGCGCGGGCAGGTGATGGACACCATCTCGGAGATCCAGGAGCGGA	10:C>G
1246170|F|0-50:A>G-50:A>G	TGCAGGTTCGACGCCCCCTGCTCCAGTATCCGCATCGCGTCCTCTGGCACACCAGCAGCAAAACAGCGG	50:A>G
3025456|F|0-29:C>G-29:C>G	TGCAGCAGAGGAACCGCTGCATGATCATGCCCCCCGTGCCG	29:C>G
1277418|F|0-37:C>G-37:C>G	TGCAGCTGGACGAGCGGCGCGCGGCCGACGCCAGGCTCAGCGGCTTCAACGGCTTCCGCGGGCTGCTCT	37:C>G
6046206|F|0-8:T>A-8:T>A	TGCAGATCTCCACGGCGGAGTGCGTCGGCAAGCTGACCGCGATCTCCCTCTGGGGATTGGCCG	8:T>A
2251092|F|0-68:A>G-68:A>G	TGCAGGTCACTCTGGCGCTGGAGCCGAGGTTCAAGAGAAGCGTGACCTACGACTACAGGGACGACTCCA	68:A>G
1239508|F|0-46:C>G-46:C>G	TGCAGTTCCTGGCCATGCTCCCTGACCTCCGCCCCAAAGTCAGGGTCATCGCGGAATGCGGCAACTGGT	46:C>G
1712215|F|0-7:T>C-7:T>C	TGCAGAATGAGTCTGTCGAAGCGTCTGATGAGCATGAGGCGTCGAGAGAGAACCCTGAGCGCAGTAGCA	7:T>C
1026430|F|0-45:G>T-45:G>T	TGCAGACGACGACCTGCTCGCTGGTTTCGGAACCAAGTCAGAGGCGAGGGAGGTGCCG	45:G>T
1035374|F|0-26:C>A-26:C>A	TGCAGGGGCAACCAGGTGTACATCCGCGGGGAGGACGTCTGCGTGGCCAACAGCATGTCCAGGTTCGGC	26:C>A
1168242|F|0-23:A>C-23:A>C	TGCAGGGGCTCATCGTGGCCCACAAGGGCGAGAGGCGGAGCCTGGTGAGGAAGCTGCTGCTGGAGCTCA	23:A>C
1117598|F|0-37:T>C-37:T>C	TGCAGAACACGCAGCCGCTGCCGACGTTCAGCAACTTTGGCGACGACGTCTACTTCGTGGCCGACCAGC	37:T>C
2279567|F|0-19:G>A-19:G>A	TGCAGTCCACCAACGAGGAGACCAAGACGGCGCACGACGCCCGCACCATCAAGGCCCTCCGCTCCCGCA	19:G>A
1081085|F|0-44:G>C-44:G>C	TGCAGAGAATGATTCAGAGACAAGTTACACCGAATTAATGCGCAGTGCACGGTGAGGAGCAAGGAACCG	44:G>C
1150703|F|0-29:G>A-29:G>A	TGCAGACGGTGGACGTGAGCGCCAAGTTCGTCAACGGGAAGCCGGCCAGGGTGGAGGCCAAGTACGTGA	29:G>A
999542|F|0-17:G>T-17:G>T	TGCAGCCTGAGGGTGGAGGCGACGACGATGGCGAGCACGGCGAAGCAGGCCG	17:G>T
1031759|F|0-8:A>G-8:A>G	TGCAGGATATCTGAACCTAGGTGATTGCCCAAACTAACCTGCGAGGAGGAGGATGGAAACGACATAGGA	8:A>G
7165035|F|0-23:G>A-23:G>A	TGCAGCAGCCGCGAGATGAGGTCGGTCAGCTCCG	23:G>A
1201330|F|0-9:T>C-9:T>C	TGCAGCTTGTCCATCACCCTCGGGCTCCGCATCAGCTCCGCCATGCCCCAGTCCAGCGACGACGATATC	9:T>C
995875|F|0-7:G>C-7:G>C	TGCAGCGGTCCTGGATCGTGCTCTTCCTCTTCGCGCTGGCGCTCACGCCCACCTACGTCTTCACCGAGC	7:G>C
1214220|F|0-46:C>G-46:C>G	TGCAGCAGCCCGAGTTCCGGCCGGACAAGAAGACGATGAATGCGCTCACCGTGCAGCTGCTCCG	46:C>G
12768836|F|0-59:C>G-59:C>G	TGCAGCTCCGCCTTGCGGCAGGTCTCCCCCAGCGCCTCGAGGAGGCGCGAGCATGGCCACGAGAAGGCG	59:C>G
1343244|F|0-14:A>G-14:A>G	TGCAGATGCGGCAGACGCTCTTCTACGAGGACATGCAGAGCGTGCTGGCGCGCGTCCACGCCG	14:A>G
4993586|F|0-22:T>C-22:T>C	TGCAGTGCTGGAACGGAGGCGGTGGCGCCG	22:T>C
1199979|F|0-68:G>A-68:G>A	TGCAGGCTGGTAAGATGCGTAAGGTGAACCACATCTACTTCTTTCTCCTTGCCCTTGAGTGAGTTTTCG	68:G>A
5331810|F|0-18:C>T-18:C>T	TGCAGCATTGCGTGCCACCCCG	18:C>T
14583260|F|0-12:A>T-12:A>T	TGCAGTTGTAGGAGGTACACCG	12:A>T
2263784|F|0-25:A>G-25:A>G	TGCAGCAGGATAATGGCATCGAGGGAGAACTCGTCGGCGGGCAACAACAGAGGAGGCTGCGGCGCGAGC	25:A>G
5361369|F|0-32:G>C-32:G>C	TGCAGGAGCGCGGCGTGGAGCGCGCGCAGCTCGGTGGCCTTGGCGATGGCCGCCTGGATTTCCTGGCGC	32:G>C
3029132|F|0-35:C>G-35:C>G	TGCAGCGCCGACTTGTCGAGCACCTGGTCGTCGGCCAGGGACCCCG	35:C>G
1338978|F|0-28:G>A-28:G>A	TGCAGGTGCCTGCACGGTCTCAGTCGGCGCGTTCCAACAGCAGCATCTCCACGGACCATGACGCGCGGC	28:G>A
2247790|F|0-33:A>C-33:A>C	TGCAGTGCCAAGATCCAGAGGGTCCTGTCCTCCATCCAGGACCGAGGCAAGTTTGTCATCGAGAAGATC	33:A>C
2251092|F|0-52:C>T-52:C>T	TGCAGGTCACTCTGGCGCTGGAGCCGAGGTTCAAGAGAAGCGTGACCTACGACTACAGGGACGACTCCA	52:C>T
1214365|F|0-31:G>C-31:G>C	TGCAGCTGCGCAAGAATGCCTTCTCTGGCGAGCTCCGGCCGGCGGGGCGGTTGCCTTCGGGCGCCACGG	31:G>C
1004571|F|0-22:A>G-22:A>G	TGCAGGCGTTCTTGGCAGAGGCAGAGGCGGCCAAGAACGAGATGGCCGCGCTGCGCAACGAGCTTTCCC	22:A>G
1168242|F|0-45:T>C-45:T>C	TGCAGGGGCTCATCGTGGCCCACAAGGGCGAGAGGCGGAGCCTGGTGAGGAAGCTGCTGCTGGAGCTCA	45:T>C
1021064|F|0-59:C>T-59:C>T	TGCAGCTCCTCCTCCATGCCCCTCCGCTCGTCCCGCAGCCTCTGCACCGCCTGGATCGTCCCCCGCACC	59:C>T
3935201|F|0-10:A>G-10:A>G	TGCAGCGGCGAGGCGGCGCGGTGCGGTTCAGGATGCCGCGGCGCCG	10:A>G
29308206|F|0-63:A>G-63:A>G	TGCAGGGCCAGTTCACGCAGGTCGAGCTCCGATCGCTCAAGGCCAAGGTGACAACCCGAATCCACGGCT	63:A>G
3947192|F|0-19:C>T-19:C>T	TGCAGCGCCACCCACTTGGCGCCGAACCGCGGGATCTGCCGCTGCCG	19:C>T
1695052|F|0-26:C>A-26:C>A	TGCAGATTTACCTCATCAAGGAGTACCACGTGGAGAGCATCGACGCGGCGCAGATCGCCAACATCGTCC	26:C>A
7907363|F|0-44:G>A-44:G>A	TGCAGCAGCTGGAGTTCACGGTGCACACCTTGAAGGTGAGAGACGCGACATCCACAGGAGCTCCGTTGA	44:G>A
32027290|F|0-42:T>G-42:T>G	TGCAGCTTCCCCCTGCTCTGCACCTTGGGGTTCATGAAGTCCTCGGCTAGGAGGTCGACGAGGGCCATG	42:T>G
1695026|F|0-14:C>T-14:C>T	TGCAGCTCTCCTACCCCATCCTCGACCAGGAAGGCCATGGCGAGGCCCCAGGTGATGTGCACATCCAGA	14:C>T
1714527|F|0-49:C>T-49:C>T	TGCAGCTCCCTGCCGTTCTTGCCGGCCGACATGGCGTCGGAGAGCATGGCCGTGAGGCTCCG	49:C>T
2249343|F|0-59:T>C-59:T>C	TGCAGGATGGATGGATGCAGCTAAGCCCAATTCGAGAAAGCCGCGGATCTGTCTGTCGTTGCTCCTGAT	59:T>C
12688710|F|0-11:A>G-11:A>G	TGCAGGAGAGAAGCCTTGCCG	11:A>G

1023841|F|0-55:G>C-55:G>C	TGCAGGACCTCGGGGTGTCGGCCTTCATCAACATCCTGGGGGCCTTCGTCTTCCTGCTGCTCTTCGCCG	55:G>C
3957579|F|0-27:A>G-27:A>G	TGCAGTGCCTTGGTCCTCTCCTCCTCGAAGCTGGGCTGCGCTGCCACGGCGGACATGCCGTCCACCCTC	27:A>G
2247790|F|0-24:C>A-24:C>A	TGCAGTGCCAAGATCCAGAGGGTCCTGTCCTCCATCCAGGACCGAGGCAAGTTTGTCATCGAGAAGATC	24:C>A
1027263|F|0-6:G>A-6:G>A	TGCAGAGGACTTCTCCAGAGCGGTACGCGCCATCTCAGAGCTCGCCGCCG	6:G>A
23889673|F|0-26:C>A-26:C>A	TGCAGTACGTGTACTGGTCCAACGGGCGGGAGACCGAGAGCCCCAGCGTGGACAACAAGCAGTGCCCAG	26:C>A
12765907|F|0-16:G>C-16:G>C	TGCAGCCACCTTCCTAGGTCCGCCCCG	16:G>C
998540|F|0-11:G>A-11:G>A	TGCAGCCTGGAGTGCAGGCAGCAGCAGATGAACCTGGACGAGCTCAAGGACAAGAAGTGCTTCCCGAGA	11:G>A
1101812|F|0-65:A>G-65:A>G	TGCAGCCTCTTCCTCCGCTCCATCGGCCTGCGAGAGTACTCCAGGTACCTCGCCTTCAACCACCCATGC	65:A>G
4993624|F|0-18:A>G-18:A>G	TGCAGGTGCCGCAGCGGCAGCACGCTGGCCTTGCCGAGGTGGCCCAGCTCCG	18:A>G
100423254|F|0-36:G>C-36:G>C	TGCAGAGGGCAGCGTCAAGTTCATGTGCAGCTACGGGGGTATGATCCTCCCACGCCACCCCGACGGCGC	36:G>C
1230160|F|0-34:G>A-34:G>A	TGCAGAAGGAGTTCGCGCGGCGGCTCGTGGCCATGCCAGGCGACTCGGAGTATAACCGCCTGGCAGCCA	34:G>A
7343208|F|0-64:T>C-64:T>C	TGCAGTGCCTTGGTCCTCTCCTCCTCAGAGCTGGGCTGCGCCGCCACGGCGGACATGCCGTCCATCCTC	64:T>C
5368535|F|0-16:G>C-16:G>C	TGCAGGCGATGAGGGGGTGGGTCGTGGACCACAAGCTGCGGGCCGTAGGTGCGCCGCGGGGAGCTCCCC	16:G>C
1200500|F|0-16:A>G-16:A>G	TGCAGGGCGAGGTACAAGTGCTTGTCGTTGCAGGAGTCCATGGACAGCGACCTTCTCATCGGCTGCTGC	16:A>G
992289|F|0-25:C>T-25:C>T	TGCAGCGGTAGTAGCCCCTCGGGTACGGCGACCCCTTGATGGGCTTCTGCCCGTACTTCCTCCACGCCC	25:C>T
1110107|F|0-53:G>C-53:G>C	TGCAGCGGGCGGTGCGTGTCCAGCCGCCAGATGGAGCCCCACGACTCCCTCATGGGCGTCCAGACCCCC	53:G>C
2266783|F|0-31:C>T-31:C>T	TGCAGACCGTGAGCGCGATCGCCCTCGGGAGCGAGACAGAGAGGGCCCCGTGGAAGGCGGTGTGAAGGA	31:C>T
1033013|F|0-29:C>T-29:C>T	TGCAGCTTGTCTGGGTTCTTCCCGCTCATCACAAGCTCCCTGTCTGCCTACACCAAGACAATAAGCAAG	29:C>T
6044207|F|0-13:C>G-13:C>G	TGCAGGTGTCGCACGAGCGGCAGGAGTCGATGAAGTAGCCCACGCCCACCGTGTCCCCG	13:C>G
1029272|F|0-19:G>C-19:G>C	TGCAGTCGCCGTCCGCGGCGAGGCGGCCGACCATGGAGCACGCCGTGACGGTGCTCTCGGCGGCCATCA	19:G>C
2263784|F|0-38:G>T-38:G>T	TGCAGCAGGATAATGGCATCGAGGGAGAACTCGTCGGCGGGCAACAACGGAGGAGGCTGCGGCGCGAGC	38:G>T
1182403|F|0-19:G>T-19:G>T	TGCAGGTCCTCCAGGCACCGCACCTCCGCCATGCACGCGAACGACTGCGACTTGCCCTGGTAGTACGCC	19:G>T
1234044|F|0-59:C>T-59:C>T	TGCAGGCAAGCGCTTATCCGATTTCCCCTTGTTAATTGTTGGGATTTGGTTCGTTGTTGCTCATTTCAA	59:C>T
3959956|F|0-26:G>C-26:G>C	TGCAGCTTCCTGCCGCAGAACACCTCGACCTCCGCCGCGTACGTCGCCGTCACCG	26:G>C
1121013|F|0-20:G>A-20:G>A	TGCAGAGCCTCGTGCTGTCCGGGAACTGGCTCAACGGCACCGTGCCGTCCCTCTCCG	20:G>A
5324401|F|0-23:A>G-23:A>G	TGCAGCACCTCGCGGCCTCGTCGACGGGCCCCGCCG	23:A>G
986801|F|0-56:A>G-56:A>G	TGCAGCAGGATGCTGGGAATGAAATACGAGTCGCAGAGGGGATACATTGGACTGGAATATTATGGACGG	56:A>G
32024058|F|0-32:G>A-32:G>A	TGCAGAAGATCGTGATCAGAGCTGATCTCATCGGCGAGAAGTGCAAGAGCAAGATCATGTCAATTGTTG	32:G>A
1026636|F|0-47:G>C-47:G>C	TGCAGCCGCCTCTCCCACTGGCCCTTGGAGGAGGAGGCGGGCCTCTGGGCCGCCG	47:G>C
1240354|F|0-29:C>G-29:C>G	TGCAGGTTGGACGAGCGGAGCACGGCCGTCAGGGACTCCGCCAGGTTGCCGTGCCTGGACACAACGCGG	29:C>G
2247790|F|0-15:C>A-15:C>A	TGCAGTGCCAAGATCCAGAGGGTCCTGTCCTCCATCCAGGACCGAGGCAAGTTTGTCATCGAGAAGATC	15:C>A
3956213|F|0-31:G>C-31:G>C	TGCAGCTGGTGATGTTGCTGGGCCGCGTGCTGGGCTGCCG	31:G>C
1245799|F|0-18:A>G-18:A>G	TGCAGGCACGGCGAGGCAAACACACGTGAGCTACCGCCCGCTGAGCGCCTACGGCGCCG	18:A>G
2279567|F|0-44:A>G-44:A>G	TGCAGTCCACCAACGAGGAGACCAAGACGGCGCACGACGCCCGCACCATCAAGGCCCTCCGCTCCCGCA	44:A>G
992847|F|0-26:G>A-26:G>A	TGCAGGAGCCGCGCGAACAGCATGACGGTGAAGAGGGTGCCCAGCGCGATCCCG	26:G>A
1266287|F|0-9:T>C-9:T>C	TGCAGCTGATCATTCTGAAGTGGTACTCTATGCCGGCCCGCTCCGCCAGCTTCTTGAGCCGCTCCCCGA	9:T>C
2249902|F|0-51:C>G-51:C>G	TGCAGCGCCGTCATCAGCGGGGTCTCCAGCCCGAACATCCTGCCCTCCATTCTCGATCGCTGATCGATC	51:C>G
1168242|F|0-33:G>A-33:G>A	TGCAGGGGCTCATCGTGGCCCACAAGGGCGAGAGGCGGAGCCTGGTGAGGAAGCTGCTGCTGGAGCTCA	33:G>A
3028384|F|0-35:C>G-35:C>G	TGCAGAGACTGTAGCTGCGCCTTGCCGCTGAGGCCCCGCTGCCG	35:C>G
1214924|F|0-8:G>C-8:G>C	TGCAGCACGAGGATGAGCGGCTGCGGCAGGATGACGCAGTAGCACACTGACGGGAACCCCATGGCCGAG	8:G>C
1094116|F|0-50:G>A-50:G>A	TGCAGCGTCGGCGAGCCCCCGCCGTTGGTGCTCCCCGTCACGAAGTTGAGGCACGGCGTGAACGTGCTG	50:G>A
997568|F|0-35:G>T-35:G>T	TGCAGCGGGCGGAGCCTGTCCAGGCGCCAGATGGAGCCCCACGACTCCCTCATCGGCGTCCAGTACCCC	35:G>T
999194|F|0-29:G>T-29:G>T	TGCAGGTAGGTCCTGAGCTCCTGCTGGACGACGATGACGATGGGGAAGAAGATGAGGAGGAGGAGCACG	29:G>T
1001090|F|0-34:T>C-34:T>C	TGCAGTACAGGAAGGAGTATATGGCGGCGCAGACTGTGAACGGTATCGCGATGGCCGTGTACAGCGCCT	34:T>C
5580389|F|0-11:A>C-11:A>C	TGCAGGATCCCACCGACGAGCGCGTGGAACAGCCGACGCGGCTCGCCG	11:A>C
2245232|F|0-26:G>A-26:G>A	TGCAGCAGCGCGTCGAACTCGTCCTGGTTGAGCTGCTTGTTAATCTCGGCGTTGAACAGCTGCTCCATC	26:G>A
5577979|F|0-8:A>G-8:A>G	TGCAGCCAATATGTATTTGGTTCACCG	8:A>G
3954986|F|0-31:G>A-31:G>A	TGCAGGAGTACGACAGCGGCGGCGGCGTCCGGGAGGCGTGCCG	31:G>A
1025770|F|0-20:G>A-20:G>A	TGCAGGAACCAGTCCTCGGAGGCGGCCGACGAGCAGTCGGCGTCGCCCGAGTCGGAGGAGGAGCAGAAG	20:G>A
2247211|F|0-27:C>T-27:C>T	TGCAGGATCTTGCTGGGCATTCGCCGCCGTGGGTGCAATCGAAGGACTACACAAGATCAAGAGAGGGAC	27:C>T
1221747|F|0-7:G>A-7:G>A	TGCAGATGAGCGCGATCAGGAAGAACTTGCCCCGCGTCATCCGCCGCTTCTCCTTCTCGTGCATCGCCC	7:G>A
3029511|F|0-20:T>G-20:T>G	TGCAGCAGCCCGTAGGCGAATATCGCCGGGATCATCCACCG	20:T>G
1020035|F|0-10:T>C-10:T>C	TGCAGAAATCTGTGACCTCGTGCGTCTCAGGGCTCTCCACAAGGTAGCTGTCCACCACATCCTCCTGGG	10:T>C
1020035|F|0-55:C>T-55:C>T	TGCAGAAATCTGTGACCTCGTGCGTCTCAGGGCTCTCCACAAGGTAGCTGTCCACCACATCCTCCTGGG	55:C>T
5363381|F|0-11:G>C-11:G>C	TGCAGCCCGCCGCGGGCCAGGATCTCCTTGCCG	11:G>C
1234044|F|0-30:G>C-30:G>C	TGCAGGCAAGCGCTTATCCGATTTCCCCTTGTTAATTGTTGGGATTTGGTTCGTTGTTGCTCATTTCAA	30:G>C
13590530|F|0-25:T>G-25:T>G	TGCAGCGTGGCCCGCGTCTTCTCCATGCCG	25:T>G
5360748|F|0-17:A>C-17:A>C	TGCAGCACATGGAGCTGATCGAGGAGGGTGGCGACGGCGACGAGCGGTTCGAACTCTTCGAAACGGAAG	17:A>C
985159|F|0-47:C>A-47:C>A	TGCAGGGGCTTCCACGTCACCTTCGTCAGCACCGAGTACAACCACCGCCGCCTCGTCCGCTCCCGCGGC	47:C>A

<table>
<tr><td>1093342|F|0-36:C>G-36:C>G</td><td>TGCAGATCCCCGCCGACCAGGCCGCGCTCGCGCTCGCGACGCTCGCCGCGGTGCTCCCCG</td><td>36:C>G</td></tr>
<tr><td>3027745|F|0-31:T>A-31:T>A</td><td>TGCAGGAACCCGAGGCACGGAGACAAGTCGGTGAGCATGCCG</td><td>31:T>A</td></tr>
<tr><td>1234044|F|0-10:C>T-10:C>T</td><td>TGCAGGCAAGCGCTTATCCGATTTCCCCTTGTTAATTGTTGGGATTTGGTTCGTTGTTGCTCATTTCAA</td><td>10:C>T</td></tr>
<tr><td>3959048|F|0-17:A>C-17:A>C</td><td>TGCAGCAGGCCGACGACAGGGAACACCCTGCCG</td><td>17:A>C</td></tr>
<tr><td>1078576|F|0-44:G>C-44:G>C</td><td>TGCAGGTTCTCGGCCCAGGGCGAGAGGCCCTCGCAGCTCTTGAGGACGACGATGGAGTCGCGCCAGGAG</td><td>44:G>C</td></tr>
<tr><td>1251116|F|0-29:T>G-29:T>G</td><td>TGCAGCTCCCGCTCCTTGCGCTTGCCCAGTGCGATACGCGCCGCTTCCAGCTTCTCGCTCAGCGACATA</td><td>29:T>G</td></tr>
<tr><td>2282092|F|0-55:C>G-55:C>G</td><td>TGCAGGGCGCCATCGACCGCGAGATCGACAAGCCCAAGTACCGCTGCGGCTGCGCCTGCGTCGACGCGG</td><td>55:C>G</td></tr>
<tr><td>5358015|F|0-16:G>C-16:G>C</td><td>TGCAGCTCTGCGTGGCGGAGGAGGCCGCCGCCG</td><td>16:G>C</td></tr>
<tr><td>999615|F|0-10:A>G-10:A>G</td><td>TGCAGCAGGGAAGGGCCGCGCCGAACCCGCCGCACGCCGAGCACGGGTCCG</td><td>10:A>G</td></tr>
<tr><td>3948107|F|0-20:G>C-20:G>C</td><td>TGCAGGCGCTCGTTGATGCGGAGCAGCGCCCCG</td><td>20:G>C</td></tr>
<tr><td>988729|F|0-66:C>G-66:C>G</td><td>TGCAGCACATGATCTCCGAGCGCAAGCGCCGGGAGAAGCTCAACGACAGCTTCCAGGCTCTCAAGACCG</td><td>66:C>G</td></tr>
<tr><td>2251099|F|0-14:A>G-14:A>G</td><td>TGCAGGCCCTTGAAATACTCCACGTCGAAGGCGTTGCTGGTGGCGTCGAGCTTGGCCGTGCCGCCGTCG</td><td>14:A>G</td></tr>
<tr><td>1164254|F|0-38:G>T-38:G>T</td><td>TGCAGCCAGGGGAGGATGCAGTCCTCGTGGTAGATGTGGGCGCAGGGCATCTCCCTGGCCTCGGCGCCG</td><td>38:G>T</td></tr>
<tr><td>7168999|F|0-62:C>G-62:C>G</td><td>TGCAGGTCGCGCCCGTACGGCGGGAAGTTGCTCTTCAGCACCGTGCCGATCACGTTGTTGTTCCCCGTG</td><td>62:C>G</td></tr>
<tr><td>997935|F|0-13:G>A-13:G>A</td><td>TGCAGAAGCCGCGGTGCGCCACATGCAGCGGGACGGGAAGGGTGGAGTGCCTGTGCTCCCG</td><td>13:G>A</td></tr>
<tr><td>2242991|F|0-20:G>A-20:G>A</td><td>TGCAGGGAGAATCAAAGCCAGGATAGATGTTAGTGCCATATGTTCATGACAAATTAATCGATCTAGCTA</td><td>20:G>A</td></tr>
<tr><td>2271197|F|0-32:T>C-32:T>C</td><td>TGCAGCTGGGCCTACCAGGGCAACTACGTCGGTGCCATGCCGCTCTTCGACTCGCGGTGGCCG</td><td>32:T>C</td></tr>
<tr><td>1025770|F|0-35:G>A-35:G>A</td><td>TGCAGGAACCAGTCCTCGGAGGCGGCCGACGAGCAGTCGGCGTCGCCCGAGTCGGAGGAGGAGCAGAAG</td><td>35:G>A</td></tr>
<tr><td>1084609|F|0-40:C>G-40:C>G</td><td>TGCAGGAGTTCTTCTACGACCAGGTGCCCGACGCGCTGCGCAGCCTGGGGCTGGCCTTCTTCCTCAGCA</td><td>40:C>G</td></tr>
<tr><td>4260717|F|0-25:G>C-25:G>C</td><td>TGCAGAAGCGGGGGTGGCGCGCGAGGGTGGCCTCGAGCCCG</td><td>25:G>C</td></tr>
<tr><td>990869|F|0-61:A>T-61:A>T</td><td>TGCAGTCGCTGGTGACCGAGCACGGGCTCCCCGCTGCCGAGGCCTCTCCCCCTTCCCCTCCAGTTGCTG</td><td>61:A>T</td></tr>
<tr><td>2253084|F|0-35:G>A-35:G>A</td><td>TGCAGCCACCAGTTGCCCTTCTTGGGGTCCTTCCAGATCATGAGCGTGATGTCGAACTGCCGGCCGCCG</td><td>35:G>A</td></tr>
<tr><td>1248566|F|0-16:G>C-16:G>C</td><td>TGCAGCAGCGCGCCGCGGCCGGGATGATGGAGGGGTCCTGGGTGGTCGGCGGCGCCAGCTACCCGCTCA</td><td>16:G>C</td></tr>
<tr><td>3025962|F|0-10:A>G-10:A>G</td><td>TGCAGCGTTTATGTTCGGCGATTCGATCGTCGATCCCG</td><td>10:A>G</td></tr>
<tr><td>1026250|F|0-52:A>G-52:A>G</td><td>TGCAGGGAGACGATGATGGAGTACAGGACGGAGCTGAAGAAGCTGGCGGAGAAGCTGCTGGGCGTCATG</td><td>52:A>G</td></tr>
<tr><td>3958280|F|0-38:C>G-38:C>G</td><td>TGCAGCGCCGACTTGTCGAGCACCTGGTCGTCGGCGAGCGAGCCCG</td><td>38:C>G</td></tr>
<tr><td>1164254|F|0-23:C>G-23:C>G</td><td>TGCAGCCAGGGGAGGATGCAGTCCTCGTGGTAGATGTGGGCGCAGGGCATCTCCCTGGCCTCGGCGCCG</td><td>23:C>G</td></tr>
<tr><td>5363862|F|0-19:C>G-19:C>G</td><td>TGCAGGGCGCCATGCCGCGCCCG</td><td>19:C>G</td></tr>
<tr><td>7170120|F|0-5:C>G-5:C>G</td><td>TGCAGCGACGACGTGGCAGCGGACGGGCGGTGGTGGCCGAGGTGGTCGAACGACATCACGGCCG</td><td>5:C>G</td></tr>
<tr><td>5363987|F|0-13:G>A-13:G>A</td><td>TGCAGGTTGGACAGGCTCGCGACCTGCGCCG</td><td>13:G>A</td></tr>
<tr><td>1092425|F|0-11:A>G-11:A>G</td><td>TGCAGCCTATCAAGGATGAAGACCAATAACTTTTTGTCAGGCAGGGGCGTCGTGGGGCCG</td><td>11:A>G</td></tr>
<tr><td>2306025|F|0-22:C>G-22:C>G</td><td>TGCAGCGCGCGCAGAGCGTCACCGACGACCAGCTCGACGAGCTGCGCGGCTCCCTCGATCTGGGCTTCC</td><td>22:C>G</td></tr>
<tr><td>7343309|F|0-14:T>C-14:T>C</td><td>TGCAGCCACTGGTGTCTACCCG</td><td>14:T>C</td></tr>
<tr><td>2306025|F|0-52:C>T-52:C>T</td><td>TGCAGCGCGCGCAGAGCGTCACCGACGACCAGCTCGACGAGCTGCGCGGCTCCCTCGATCTGGGCTTCC</td><td>52:C>T</td></tr>
<tr><td>5578049|F|0-21:G>T-21:G>T</td><td>TGCAGCACGCAGCGCGACGGCGTGGCCG</td><td>21:G>T</td></tr>
<tr><td>1078927|F|0-13:A>C-13:A>C</td><td>TGCAGGTCCCGCGATTTGCCTTCCTCCGTCAAAATCTCCATCAGGCAGCTCTCAAAACTCCG</td><td>13:A>C</td></tr>
<tr><td>1087892|F|0-7:A>G-7:A>G</td><td>TGCAGGTAGTGTGTACAAAGCAAGCGCATTGGCGTCTAGTGAGGTCCTCCTCGCCGTTGCCG</td><td>7:A>G</td></tr>
<tr><td>12735159|F|0-20:A>G-20:A>G</td><td>TGCAGGAGGTGGCGCGGGGAATCGAGTCGGTTGAGCAGGGAGAACGCGCGGTGTAGCGCTAGGGGCAGA</td><td>20:A>G</td></tr>
<tr><td>5363799|F|0-16:T>C-16:T>C</td><td>TGCAGGCGCTCCTCCATGCGCCGCCGCTCCG</td><td>16:T>C</td></tr>
<tr><td>990388|F|0-52:C>A-52:C>A</td><td>TGCAGGGGAAGGGCCTCTGCCTCATGCCCATCTCCCTCGCCTCCGCCATCTCCTCCGCCACCACCACCA</td><td>52:C>A</td></tr>
<tr><td>1033013|F|0-38:C>T-38:C>T</td><td>TGCAGCTTGTCTGGGTTCTTCCCGCTCATCACAAGCTCCCTGTCTGCCTACACCAAGACAATAAGCAAG</td><td>38:C>T</td></tr>
<tr><td>3030053|F|0-31:C>T-31:C>T</td><td>TGCAGACGCCGCGGCCCATGTACCAGTACTCCGACTTCCCGTGGCCG</td><td>31:C>T</td></tr>
<tr><td>1047138|F|0-56:A>G-56:A>G</td><td>TGCAGGTATGCCACAGACGGAAGAGATGTCCTTTCACAGATTAAGGCTGGAGATAAAATTCGCTCCGCC</td><td>56:A>G</td></tr>
<tr><td>5363381|F|0-14:C>G-14:C>G</td><td>TGCAGCCCGCCGCGCGCCAGGATCTCCTTGCCG</td><td>14:C>G</td></tr>
<tr><td>16530676|F|0-58:T>C-58:T>C</td><td>TGCAGTACCACCCCGACGTGTGCCCGCCGTCGCGCAGGGCCGAGTCCACGGAGCTCTTTGTCGAGCTCC</td><td>58:T>C</td></tr>
<tr><td>2260269|F|0-37:C>T-37:C>T</td><td>TGCAGGCGAGGACGTGGAGCGCGTACAGTGCCCACGACAACGTCGCCAGGAGCCTCCGCGCCGTGGAGG</td><td>37:C>T</td></tr>
<tr><td>7165805|F|0-14:A>T-14:A>T</td><td>TGCAGGCGGCGCACACGCCGTTCCTGCCG</td><td>14:A>T</td></tr>
<tr><td>984769|F|0-13:C>G-13:C>G</td><td>TGCAGTACTACGGCTTCTGGTGGGACAACACGACGGAGGCGTCGGAGCAGCATATGAGCTGGGTCAGGG</td><td>13:C>G</td></tr>
<tr><td>1208321|F|0-67:A>G-67:A>G</td><td>TGCAGCACATAAGTACTCGCACTAGTGCCGCCATGCACGCTAGCCTGGATTATTCAGGCTTGGGAGCAC</td><td>67:A>G</td></tr>
<tr><td>1025508|F|0-11:C>G-11:C>G</td><td>TGCAGCTTTGGCACGCTGATGACGGCGACGCTGGACTGGGAGGGCATCCCG</td><td>11:C>G</td></tr>
<tr><td>4261738|F|0-37:C>G-37:C>G</td><td>TGCAGCAGCTGTTCCTCGACGACAACAGCTTCGTCGGCGAGCTGCCG</td><td>37:C>G</td></tr>
<tr><td>3946150|F|0-17:C>G-17:C>G</td><td>TGCAGGAACGCGTCGTACACGACGGCGCGCACGGGCCG</td><td>17:C>G</td></tr>
<tr><td>3952674|F|0-20:G>C-20:G>C</td><td>TGCAGGATCCCGGTGACGCAGCCGATCTGGCGGTGCAGGTCCG</td><td>20:G>C</td></tr>
<tr><td>1020894|F|0-15:T>C-15:T>C</td><td>TGCAGGACTTCGTCGTCAGCCACAGCCCCGACGCGGACGCCGCAAAGGCCGCCGCCGACGCCCGTAAGG</td><td>15:T>C</td></tr>
<tr><td>5578265|F|0-7:C>G-7:C>G</td><td>TGCAGGCCGTGCTCGTCTCCCCCGCCGCTCTGGCCG</td><td>7:C>G</td></tr>
<tr><td>1203873|F|0-20:C>T-20:C>T</td><td>TGCAGCAGCGCGTGCTTCTCCTGCGCGCCCAGCTTCTCCACGTTCACCTCCTTGAGCTCCCCCTCCGAG</td><td>20:C>T</td></tr>
<tr><td>1087107|F|0-12:G>T-12:G>T</td><td>TGCAGCTTCGCCGCCGCCGACGACGCCGACGTGCCCTCGCCCGCCGGGAGCATCGCCG</td><td>12:G>T</td></tr>
<tr><td>3030529|F|0-20:C>G-20:C>G</td><td>TGCAGCGCGAGTTCCGCGGCCCCGTCAGGTACGCCATGGAGCGCCG</td><td>20:C>G</td></tr>
<tr><td>7351795|F|0-31:A>C-31:A>C</td><td>TGCAGCAGCGGGCACGGCGGGTAGTGGTTCAACCG</td><td>31:A>C</td></tr>
</table>

6043694|F|0-7:T>G-7:T>G TGCAGATTCTCATCGTTCGATATGGCCG 7:T>G

1278972|F|0-14:C>T-14:C>T TGCAGAGCCGTCTCCGAGACATTGGTTGAAGCAACACACCAAGTACCATTTGAACGGTTTCCTCCAGTC 14:C>T

7338943|F|0-44:G>C-44:G>C TGCAGCGCGCCGTGGAGCCTGGCGGAGCCGCGGAGGCTGTTGCAGGCCAGCAGGCGCAGCTCGTCCTCC 44:G>C

1024458|F|0-33:C>G-33:C>G TGCAGAGACCATGCCTGTGATCACTGCGTTCCACGAAAAGTTGTTCCTTTCCG 33:C>G

1091041|F|0-9:G>A-9:G>A TGCAGCAGGGCGGTGACGAAGGTGAAGAAGAAGCAGGTGACCAGGGCTTCCACGGCCG 9:G>A

32027290|F|0-8:C>T-8:C>T TGCAGCTTCCCCCTGCTCTGCACCTTGGGGTTCATGAAGTCCTCGGCTAGGAGGTCGACGAGGGCCATG 8:C>T

981818|F|0-8:G>C-8:G>C TGCAGGTAGACGCCGATGGCCGTGAACTTGATGAAGTTGCCCCCGATCTCCATCCCCCGCACGCCTGCA 8:G>C

1023984|F|0-24:T>C-24:T>C TGCAGGCTTTCCAGGCCGTGGAGGTGGCCGTGCGGAGGTTCTTCTTCGCGTACCG 24:T>C

1093935|F|0-46:G>T-46:G>T TGCAGTCTCCACCACACGGACTTGCCACCATGACGGCGGCTCCTCTGTTATCGTTGACGGATGCGGCGA 46:G>T

3028599|F|0-22:C>A-22:C>A TGCAGTGCTACCTCTACACCACCAGTGGGCCCATCGCCG 22:C>A

1129283|F|0-10:A>G-10:A>G TGCAGGGGGAAATGTGTCGCCTGGCGCTGCGCGAGCCCACCAAGGACGGTGCCGAGCACCTCCGCGCCG 10:A>G

1696929|F|0-65:G>C-65:G>C TGCAGGCACTCCGCCGCGTACTCCTTGGAGCCCAGCACGACGCCGCGGTCGAGCAGGCTGATCATGACG 65:G>C

1016328|F|0-59:C>G-59:C>G TGCAGCACGGTCTTGAGCGCCCTCCCCGCCGTCGCGCCCACGCACATGATGAACCCGAACAGATGGAAG 59:C>G

986634|F|0-28:A>G-28:A>G TGCAGGTGGGGGTGAAGGCGCCAGGGACACCGAAGAGGATGACCTTCTTGCCG 28:A>G

1022812|F|0-37:A>G-37:A>G TGCAGACGCACTACAAGCGCAGGCACTGTGCCAAGCCATTCCTCTGCCGCAAGTGCGGCAAGGCGCTCG 37:A>G

1049669|F|0-31:G>C-31:G>C TGCAGCTTCTGCGTGGTCTGGTCCAGCTCCTGCTTCAGAGAAGAAACCAAGGTGGCGTCCGAGGACACC 31:G>C

994381|F|0-14:G>C-14:G>C TGCAGCCTGCGCTGGAGCTGCTGGTTGGCGGCGCGCAGCTTCTTGACCTCCTCCTCCAGGTACGCGGCG 14:G>C

1055814|F|0-30:G>C-30:G>C TGCAGCTGCAAACGAGCTGTACAAGGTAGCGAAATCGCTGGGCCTCGCCGACCAGGACTTCTCCGCGGT 30:G>C

4261327|F|0-26:A>C-26:A>C TGCAGGCGGTGCTCAAGGAGACCCTGAGGCTGCGGCCGCCG 26:A>C

18732688|F|0-18:C>T-18:C>T TGCAGCCTTGAGGAGCAGCACTGATGCAGGGGGTGTCTTCAAGATAAAGGTGAAGCTCTTATCCTGCAA 18:C>T

996735|F|0-33:A>T-33:A>T TGCAGCATTTCATAGCGTTCCACCGCTTGACAAACATCAACCTTCAAGTAAATTTTATTCAGACATTAT 33:A>T

5580334|F|0-8:G>C-8:G>C TGCAGCTTGCCGTCCCTGGGCGGGCCCCG 8:G>C

7909216|F|0-10:C>G-10:C>G TGCAGGTGACCGACCTCCGCGGCTCCCCCG 10:C>G

1221747|F|0-10:C>G-10:C>G TGCAGATGAGCGCGATCAGGAAGAACTTGCCCCGCGTCATCCGCCGCTTCTCCTTCTCGTGCATCGCCC 10:C>G

3949563|F|0-12:A>G-12:A>G TGCAGGGCGGCCACGTGTTCGAGAAGGCCGCCGTGAACGTGTCCGTCGTCTACGGAGTCATGCCCCCAG 12:A>G

986053|F|0-17:C>A-17:C>A TGCAGATCCTAGAGCCCCATTGATTAAAAAAATAAACCAGTTGGCACGCTAAAGATCTTCCCAGATCTT 17:C>A

13871868|F|0-5:C>T-5:C>T TGCAGCAGCTCGATCTTGCCG 5:C>T

5324855|F|0-23:A>G-23:A>G TGCAGCCTCGGCAGCGCAATGCCATCGACCCCGCCG 23:A>G

1094822|F|0-46:C>G-46:C>G TGCAGGCCAGCACGCGCGGGCACTTGACGACCACGCGGCGGTAGGCCGCCTCCG 46:C>G

5363497|F|0-7:C>T-7:C>T TGCAGCGCCTGGCGGCCGAGCTGGGTGGCGCAGCGGTGCCG 7:C>T

1127471|F|0-26:T>C-26:T>C TGCAGCGGCGTCAAGAGACTCGCCGCTGCCACCCAGACCACTGTTGACAGGCGCGCCGTGTGCAACTGC 26:T>C

2349733|F|0-14:C>G-14:C>G TGCAGCGTGCAGGGCGAGGCGGCGACGAGCAGCAGGCCGCCGCTGGTGCACCTCGCCGCGCGGCACGTC 14:C>G

6043031|F|0-17:A>C-17:A>C TGCAGCGGCGTCAAGAGACTTGCCGCTGCCACCCAGACCACTGTTGACAGGCGCGCCGCGTGCAACTGC 17:A>C

1012259|F|0-23:T>G-23:T>G TGCAGGATGTTGTAGAGCACCCATCCGAGGGCGGGCGCCACCACGAAGAGCAGCAGCAGGCCCCGAGAT 23:T>G

991362|F|0-50:G>T-50:G>T TGCAGACATATTATCTGATGCAGTTTTCTGAGACGAAGGCACTAGGCGATGCTGTTGGATTTGGGTCCG 50:G>T

2264864|F|0-54:T>C-54:T>C TGCAGCGGCACGACGAGGCGGACTCGGCGCTCCGCGGCGCGCCCAGGTTCGGCGTCGACGAGTCCACCA 54:T>C

1863072|F|0-20:C>G-20:C>G TGCAGCTCCCTCCCCGACTCCGTGAGCGGCAAGGTGGTGCTCTGCGAGAGCCGCGGCTTCAAGGAGCAC 20:C>G

5581330|F|0-22:C>T-22:C>T TGCAGCAGCGGCATGCCGGCGGCGAGCCCCGCGGGTATCTCGCCG 22:C>T

1119431|F|0-13:A>C-13:A>C TGCAGCGCCTCATAGAGGCGCGCGCCACCGTGCCCGTCGCCGCCCAGCGCCTCTCCCTCGACCCGAGAT 13:A>C

1099860|F|0-51:G>T-51:G>T TGCAGCACAGCACCGACCGATCCATCTACTGCGGCGCGGCTCATCTTTCGCGTGCGTACGAGAGCGAGA 51:G>T

3028663|F|0-12:T>G-12:T>G TGCAGCTTCGCCTCCGCCGACGACGCCGACGTGCCCTCGCCCGCCG 12:T>G

1696577|F|0-10:G>C-10:G>C TGCAGTTCACGGTCCCCGCCCTCAAAGGCGGCACGCTCGACTCCCTCCTCGCCCTCAGCGACGACCTCA 10:G>C

1694889|F|0-45:T>C-45:T>C TGCAGGAGCGCGCGGGTGATCTCGTTGAGGCAGTCGCCGAGGACCTTCTCGTCGACGACCGTGGCGCTC 45:T>C

990388|F|0-37:C>G-37:C>G TGCAGGGGAAGGGCCTCTGCCTCATGCCCATCTCCCTCGCCTCCGCCATCTCCTCCGCCACCACCACCA 37:C>G

1210745|F|0-17:G>C-17:G>C TGCAGGCCGAGCAGCATGAGCTTCTCGGCCTGCGCGAACGGCCACTCCCCG 17:G>C

1026048|F|0-64:C>T-64:C>T TGCAGCGGCATTGCTCGTCGCCACGTCGCTGCTGGTCGCCACCCTCCTCACCTCGCCGCTGCCGCTGCT 64:C>T

986790|F|0-24:G>A-24:G>A TGCAGCCAACTCGGGTCCTGGCGAGGATGGGAGTTGAAGGCGAGGAAGGGAGGAGGAGGTGGACGCGAC 24:G>A

1668341|F|0-11:G>A-11:G>A TGCAGCAGGGGGTCGACGACCCTGGCGTTGAGCCACGCGAGGACCACCGCCATTTCCTCCCCCCTCCGC 11:G>A

1070572|F|0-56:A>G-56:A>G TGCAGTCGTGCAGGGACAGCTACGCGTCGCTGACGGACTCGCTGGGGAAGTCGCGGACGACGCTCAAGA 56:A>G

7906817|F|0-10:G>C-10:G>C TGCAGTTGCTGAGCACCGCCG 10:G>C

3949227|F|0-19:A>G-19:A>G TGCAGGCCTTGACGCGGGGACGCGCCTCGTCGGCGAGCTCCACGGTGACGTTCGAACCATCCTTTGCCA 19:A>G

1206915|F|0-24:A>T-24:A>T TGCAGCTTGAGCGGGCACTTGGCCAGGACCTTGCACTTGGTGAAGTGCGCCTGGTACCTGACCTCGCCC 24:A>T

3949260|F|0-40:C>T-40:C>T TGCAGCGCGTACAGCCCGCCGAGGCTGTGCGCCATGAGGACCGCCG 40:C>T

3959090|F|0-11:G>C-11:G>C TGCAGCGACGCGCACGGCGACGAGCCCTGGATCCTCGCGCTCGTCGACCTCATCCGCCGCCAGCTCGCC 11:G>C

2290593|F|0-10:G>A-10:G>A TGCAGTTCCTGCCGTCCTGCTCAATCGGGGAGGAGAGTCTGCAAGAGCTCCGCGAGTTCGAGTTGGCCG 10:G>A

2295453|F|0-54:T>C-54:T>C TGCAGAGCGCCAGCCTGAGCTGTGGGATGCTCCTCTTGCTGAACATCTCGAGGATGGCGGCCTCGTCGA 54:T>C

7913651|F|0-15:T>C-15:T>C TGCAGGCATCGCTGATGGCGGGTTTTGAGCTGCCG 15:T>C

1194119|F|0-47:C>T-47:C>T TGCAGCCCCACCAGCAGCCGCAGCAGCTGCCTCGGCCGCCGCCGGGACAGCATCCGCAGGTTAGCGTGG 47:C>T

1064289|F|0-7:C>G-7:C>G TGCAGGGCAACCGCCTGAACGGCCCCATCCCGGAGACGCTCGCCGACCTGAGGAGCCTCAACGTGCTGA 7:C>G

5367344|F|0-19:T>G-19:T>G TGCAGGCGGGCTCGGAGCGTGCGGCGCCGCTCCCG 19:T>G
5361008|F|0-55:A>G-55:A>G TGCAGCCTGAGCGCACCTGGAAGTCGCCGCAGCAGATGAGGAGGTCGATCTTGATACCCTCGGCCGCCT 55:A>G
1153577|F|0-14:C>G-14:C>G TGCAGCTGAGACTGCACAAGGTAGGCACCCAGTTTCTTCGCTGATGGATCATTCGGCTTGCTGCCGGCC 14:C>G
6043893|F|0-13:C>G-13:C>G TGCAGCGCCCCTCCAGCGGCCCCAAGAAGAGCGGGGCGGCGAGCAGGCCG 13:C>G
1231985|F|0-20:C>T-20:C>T TGCAGCGGCGGGGGCGGCGGCGCGACGGCGTGGAAGGGCACGAAGTGCGGCGCGAACTGGTGGGGCGGC 20:C>T
1030019|F|0-52:G>A-52:G>A TGCAGCGCGTGGGGCTCGGCCACGTCGTGAACATTGCGGGCATGGTGGTCGCGGCTCTGGTGGAGCGCC 52:G>A
987600|F|0-31:G>C-31:G>C TGCAGCCAGTACCTGAGCGCCTACATCATCGGCTTCGGGGTGGTGCAGGTCATCTTCTGCCAGGTGCCC 31:G>C
2248284|F|0-13:A>T-13:A>T TGCAGCCGTCCACACCTCTCCTCGATCTTCTATCCTCCCCTTCCATTAATAACCTACATGGACGAGCAG 13:A>T
1092362|F|0-38:G>A-38:G>A TGCAGGCGCTTCTCGTAGTCCTCGCGGCCGCCGAAGAGGCCGCGCCACATCCTCCCCAGCACCCCTCCT 38:G>A
1026235|F|0-11:C>T-11:C>T TGCAGGTACCTCCCGTGGTAGATGAGCCTGGACATGGCCTTGTACGCCGCCTGGGTCATGTGGATGCCG 11:C>T
1203721|F|0-42:A>T-42:A>T TGCAGTACTACTCCGCCATGGCGATCATGGAGATCACCCGCGAAGCCGAGCACAACCTCGCGCTGCGGC 42:A>T
1105547|F|0-45:A>G-45:A>G TGCAGCCTGCTCTTGAAGACGTAGCAATTCTCGACCCCTGCGCCAAAATCAAGCGCACCACGGCTCAGG 45:A>G
992289|F|0-64:C>T-64:C>T TGCAGCGGTAGTAGCCCCTCGGGTACGGCGACCCCTTGATGGGCTTCTGCCCGTACTTCCTCCACGCCC 64:C>T
2245232|F|0-56:C>T-56:C>T TGCAGCAGCGCGTCGAACTCGTCCTGGTTGAGCTGCTTGTTAATCTCGGCGTTGAACAGCTGCTCCATC 56:C>T
1135493|F|0-35:A>C-35:A>C TGCAGCTGCGCGCTTGCGACGGCATCGGAGATGGCACCTCCTCGGCTGCCTTCTCAGGATGCCTAGCCG 35:A>C
1000168|F|0-23:G>T-23:G>T TGCAGGGCGAACTCGTTGTGGTAGACGGCGACGAGCACGGCGGCGAGGAGCGCGAGGGGAAGCAGGAGC 23:G>T
4002940|F|0-7:A>G-7:A>G TGCAGGGAGCCAACCAGGCGCTGTTCGACGGGTTCGGGGCCGCCG 7:A>G
5358259|F|0-5:G>C-5:G>C TGCAGGCGCGGGAGGTCGCAGAGCGAGGCCG 5:G>C
1141761|F|0-65:T>C-65:T>C TGCAGTTGCAGATGCCCCTGCATTTGGGGCAGGTCCAGCCCGCCTCCTTGGTCATATCCTCGGCGTTCT 65:T>C
5580147|F|0-7:T>C-7:T>C TGCAGCATAGGCTGGCCCTCCCTGTCGAGTGCCG 7:T>C
1025770|F|0-40:C>T-40:C>T TGCAGGAACCAGTCCTCGGAGGCGGCCGACGAGCAGTCGGCGTCGCCCGAGTCGGAGGAGGAGCAGAAG 40:C>T
1091865|F|0-11:G>C-11:G>C TGCAGGTTGGCGACGACGTTGACGACGGCGTTCACGTTCTGCGGCGCCTCCGCCATGGCCAGGCACGCC 11:G>C
7918764|F|0-17:G>A-17:G>A TGCAGGCAGAGGCGGCCGCCGCTCACGGTGCGCGGGCGAGCGGCGAGGAAGCAAGGCCG 17:G>A
1096975|F|0-44:C>T-44:C>T TGCAGCGAGCGGCGGAGCACGAGGAGCGGGCGGCGGCCGTACACCCTGGCGATGCCG 44:C>T
1155225|F|0-35:G>A-35:G>A TGCAGGTCCACGGCCGCGCCGGCCGCCTCGGTCTCGGCCAGCACGGGCAGCAGCCGCCG 35:G>A
1058658|F|0-41:G>C-41:G>C TGCAGCATCTGCTGCGTGCTGGCCATCCCGCGCTGGAGCACGGCCATGTCGTCCCGCAGGTCGTGGATG 41:G>C
1150941|F|0-64:G>A-64:G>A TGCAGAGCATCACCACCATGCCCATCCTCGTCAAGGGAGTCATCACCGCGGAAGACGGTAACCCGTCTC 64:G>A
1121326|F|0-49:C>T-49:C>T TGCAGTTCTTTTCAAAGTATGGTGAAATTGAGGAGGGTCCTCTTGGGCTCGACAAGGCAACCG 49:C>T
1026250|F|0-18:G>C-18:G>C TGCAGGGAGACGATGATGGAGTACAGGACGGAGCTGAAGAAGCTGGCGGAGAAGCTGCTGGGCGTCATG 18:G>C
3943087|F|0-38:G>T-38:G>T TGCAGCTGCGCGCTTGCGACGGCATCGGAGATGGCCCCGCGTCGAATGCCTTCTCAGGATGCCTCGCCG 38:G>T
7352752|F|0-5:G>A-5:G>A TGCAGGAGCAGCAGCTCCCG 5:G>A
3934237|F|0-31:C>G-31:C>G TGCAGAGGCGGCGGCACACGCCGTTGAAGGGCCCGATGGCCG 31:C>G
1027882|F|0-59:T>G-59:T>G TGCAGCACGGCGATGTCCCAGGGCGCCTCCTTGACCGACTTCACGTAGAACACGGCGGCTGTCCCGTTC 59:T>G
7330910|F|0-13:G>A-13:G>A TGCAGCAGGACCCGCCCGCCG 13:G>A
2244173|F|0-35:T>C-35:T>C TGCAGCCATAGCAGAGCAAGCAACCATTATCTCGATAGCTTCCTACAATGCCAAAGGTGTTCGATGCCA 35:T>C
1204824|F|0-56:G>C-56:G>C TGCAGCGCGCCGTGGAGCCTGGCGGAGCCGCGGAGGCTGTTGCACGCCAGCAGGCGGAGCTCGTCCTCC 56:G>C
1002485|F|0-53:C>T-53:C>T TGCAGCTGTATGTTGCGGAAGCGCTCCTGCGCGGAGTTCATGGCGAAGGAGCGCTCCCG 53:C>T
1245624|F|0-17:G>C-17:G>C TGCAGCGTGCGGCAGCAGACCAGATCCGTGGGGTTGAAGCTCACCGCCGTCCGCCCCTGCGCCTTGGAG 17:G>C
2245452|F|0-35:T>C-35:T>C TGCAGGCCAATGCCTTGAAGGTGTTCGACGAAAGGTTTGATGATCTTCCCCACCCGCATGCCCAGCACC 35:T>C
2348881|F|0-45:A>G-45:A>G TGCAGCACCGCAACCTGGTGCGGCTGCTGGGCTGGTGCGCCGAGCACGGCGAGAAGCTGCTTGTCTACG 45:A>G
4006083|F|0-10:C>T-10:C>T TGCAGGCTTTCATAAGGGCGTGGATGAAGGCGTGCGCGTCGCCG 10:C>T
5579583|F|0-66:C>T-66:C>T TGCAGCCCTCGTCGCTGCCCGCGCCGTCGTCCTCCTCCGACTGGATGCCGCCCGCCACGGGGCCCACCA 66:C>T
2349733|F|0-38:G>A-38:G>A TGCAGCGTGCAGGGCGAGGCGGCGACGAGCAGCAGGCCGCCGCTGGTGCACCTCGCCGCGCGGCACGTC 38:G>A
1028066|F|0-35:C>G-35:C>G TGCAGGATGGAGAAGACGGCCGCGACGGTGCCGCGCAGGTAGTTGGCGTCCAGCGTCATGGCGATGTGC 35:C>G
1058658|F|0-29:G>C-29:G>C TGCAGCATCTGCTGCGTGCTGGCCATCCCGCGCTGGAGCACGGCCATGTCGTCCCGCAGGTCGTGGATG 29:G>C
2250333|F|0-18:G>C-18:G>C TGCAGGTGAACAATGCCGGGGTATCGTTCAACGAGATCGATACCAACTCGGTGGAGCATGCTGAGACAG 18:G>C
1208175|F|0-61:C>T-61:C>T TGCAGGCACAGGCTGACCTGTGGCGCCACAGCCTCTACTACCTCACGTCCATGGGGCTCCGCTGCGCCA 61:C>T
984867|F|0-20:C>T-20:C>T TGCAGCTGTTGGTAAAGGGGCAAGTCCATGGGGCTAGGCGAATCATAGAAAGGAAACATGCTTTAGTGA 20:C>T
3950266|F|0-40:G>C-40:G>C TGCAGCACGTCGAGTCCGTCGCCGACGAGATGGAGCTGCGGCGCCGCGAGCTGCGCCTCTACGCCAACA 40:G>C
1204744|F|0-31:A>C-31:A>C TGCAGGCGCTGGGACTCGCCGATGAGCTTGGAGAGGAGGGCGACGAAGGGGTCGCGGTCCAGGACTCCG 31:A>C
1030747|F|0-49:G>C-49:G>C TGCAGGCGGCGGTGGAGCGGGCGGCGAGGCTGCTGGCGCCCTACGGGGCGAGCATCGTGGAGTACACGA 49:G>C
2271235|F|0-61:C>T-61:C>T TGCAGCTCCTTACCTCGCCGTCGTCACACTCGTGGAACACGCTCCCTGAGCTGTCGGCGTCCGCGGCCT 61:C>T
2260269|F|0-31:C>G-31:C>G TGCAGGCGAGGACGTGGAGCGCGTACAGTGCCCACGACAACGTCGCCAGGAGCCTCCGCGCCGTGGAGG 31:C>G
3934632|F|0-14:G>A-14:G>A TGCAGTATGTGCGCGTACCG 14:G>A
5369274|F|0-6:T>C-6:T>C TGCAGATGGGACGAAGAAGACGAATCCG 6:T>C
12777613|F|0-7:G>C-7:G>C TGCAGCGGGTACCCACACGGGCCTTGAGGTCGGCGACGAACTCGTCCG 7:G>C
5372619|F|0-35:G>C-35:G>C TGCAGCTCGACGGCGGCGGCGAACTTGTGGAGCGTGGCCACCG 35:G>C
1695116|F|0-24:C>T-24:C>T TGCAGCGGAAGAAGTACCAGCCGTCCCCGTTCTTCCATGTCGACAGGGAGAAGAAGTGCCG 24:C>T
7170471|F|0-24:G>T-24:G>T TGCAGAGCGGCACACGGCAGGCCTGGTCCG 24:G>T

13880537|F|0-28:C>T-28:C>T	TGCAGAACACGTGCCTCAAGACGTGGGGCGGCCG	28:C>T
16795568|F|0-17:G>A-17:G>A	TGCAGCAGGTAGACAAGGTCCTCGTTGGCGCCG	17:G>A
1125398|F|0-31:C>G-31:C>G	TGCAGCACCTGTCGGCCACCCCGACGTTCATCGCCGCCGTGCAGGCGCAGGCCGGCATGGCGCCGAGAT	31:C>G
3943944|F|0-26:G>C-26:G>C	TGCAGCAGGTGGCTGGTGGCCTTGTCGACCCGCGACGCCGCCG	26:G>C
32027968|F|0-17:T>A-17:T>A	TGCAGCTCCCGGCGGCGTAGGCGGCCATGAGGCCCAACAGCTCCACCAGGACGCACACCG	17:T>A
1133246|F|0-31:G>C-31:G>C	TGCAGGAGGGCGTGATCCCGGCGCTGGTGTCGCTGACGGCGAACGGCACGGGCAAGGCCAAGGACAAGG	31:G>C
988825|F|0-47:G>A-47:G>A	TGCAGGCGTCGGACCTCAACGACTTCATCGCCCCCTCGCAGGACTGCGTCGTCTCCCTCAACAAGAACT	47:G>A
1010797|F|0-64:G>C-64:G>C	TGCAGGGGCGGCTGCGGAGCACGCAGCCGCTGACCAAGGGCGTGGTGGCGGAGTGCGTGCTGGGGGCGG	64:G>C
1005220|F|0-66:G>A-66:G>A	TGCAGACGGCGGTCCTGCTCGCCGTCATCTGGAACACGGACTGGGAGGACGAGGCGGCGCAGGCGAGCG	66:G>A
1019992|F|0-64:G>A-64:G>A	TGCAGGTAGATGCCCATCATGTGGTACTGCTTCGCCCCGTACGACTGCCCGCACAACGTCTCCAGCGCG	64:G>A
26673479|F|0-10:G>A-10:G>A	TGCAGCACCAGGACCATGAAGACCAGGAGCAGGAGGACGCCTCGTCCTGCGACCTCTCCTCCCCGCTGG	10:G>A
1396824|F|0-32:G>A-32:G>A	TGCAGCGCCATGAGCCTGCTGTAGGGGTTGCTGTCCACCACCTCCGCGCTCATGTCCTGCGCCACGCAA	32:G>A
2247907|F|0-44:C>G-44:C>G	TGCAGGAGCAAGCTCTCGAGCCCACGCTGGTGCCGAACACCATACGCCCACTGAGGGCTGTTCCATACC	44:C>G
1023347|F|0-14:C>G-14:C>G	TGCAGCTACTGTGCCTAGCTCTCGTGTAGGCAAGTTCCTAGGCTGGTCCTCCCG	14:C>G
3955357|F|0-26:C>G-26:C>G	TGCAGGCTCGTCACGCGCGGCGGCTCCGCGAACTCAAAGTCGTGCTGGTAGTAGTCGATCACCATGTCC	26:C>G
3935985|F|0-8:C>G-8:C>G	TGCAGCGCCGAGGCGCGCGGGCAGCTGGCGCCGTCGCACGCCG	8:C>G
5581080|F|0-44:G>C-44:G>C	TGCAGGTCGAGCGCCTCGCCGCGGAGCTTGTCGGGGAGGGCGTCGACGAGCGCGATCTCGTCCGCCAGG	44:G>C
4001992|F|0-42:A>T-42:A>T	TGCAGCAGCGCGGTGAGGTAGACGAGCTCGTCCTTGGCGCCAATGCCG	42:A>T
3028529|F|0-23:G>C-23:G>C	TGCAGCCAGTTGCCGAAGATGGGGACCGGGAAGGGGCCCG	23:G>C
6046077|F|0-5:T>C-5:T>C	TGCAGTAGCACCCCGACCTCCGCCAGCGACAGCACGTCCTCCAGTATGACCTTGGCGAATATCCGAGAT	5:T>C
1067755|F|0-57:T>C-57:T>C	TGCAGGCTGCCTGTCTTTTGTGGAGAGAGATGTGGGAGATGGCGTGTCATGTGTGGTTTGGTGCTCCCT	57:T>C
2258199|F|0-46:G>C-46:G>C	TGCAGTTCCGCACCGCCTTCATCGCGCCCTCCTCCAGGGTGTTCGGGCGCGGCGAGCTGGTGATCGACA	46:G>C
1094855|F|0-37:C>G-37:C>G	TGCAGTCGCCGTCCGCGGCCAGGCGGCCGACCATGGACCACGCCATGACGGTGCTCTCGGCGGCCATCA	37:C>G
6049600|F|0-16:A>G-16:A>G	TGCAGCCAGGTCATGGACTGGCAGTCGGCGCTCGTCATGTTGAGCTCCG	16:A>G
7158379|F|0-20:T>G-20:T>G	TGCAGCCACCGCGCCATGACTGCGTCGCTCGAGTCCCCCATGTCCGCGGCCTGCGCGGACGCCCCTCCG	20:T>G
32026917|F|0-5:G>C-5:G>C	TGCAGGGCCTGGAGGGCCTTGGCGGCCTGGTCCTCCTCCCTGAACTGCACGTAGACGTTGCCAATGAGG	5:G>C
2251979|F|0-8:T>C-8:T>C	TGCAGCCGTGCCTCGAGCGCCTCCTTGGGCGTGCCCCGCACGGGCTCGCACTCGAACTCCCACCGCAGC	8:T>C
12764284|F|0-36:A>C-36:A>C	TGCAGCTCGATCTTGGCAAATCTCTCAAATGGATGTAAAAAATGCTTTCCTTCATGGTGATCTACATGA	36:A>C
32020973|F|0-25:G>C-25:G>C	TGCAGTGGGACCTGGACGTGCTGGAGGACGATGGCAGGCACCGCGGCTCGCCGCTGAACTGGATCACGT	25:G>C
1023867|F|0-65:T>G-65:T>G	TGCAGCCCATGGGCAGCTGCTACAACCCCAACACGATCCAGGCGCACGCCTCCTACGCCTTCAACTCCT	65:T>G
100449442|F|0-17:A>G-17:A>G	TGCAGCCTCCGAATCTCATCGAGGTCGATGGCCTCCTCGGGGGACAGCTCGTCGGCGGGGGCGTCCACC	17:A>G
6046777|F|0-11:C>G-11:C>G	TGCAGCTTCTTCAAGCTCCCGATGCTCGCCG	11:C>G
4986425|F|0-8:T>C-8:T>C	TGCAGCAGTGCGGTGAGGTAGACGAGCTCGTCCTTGGCGCCGTTGCCG	8:T>C
5360809|F|0-43:T>C-43:T>C	TGCAGCAGCCGCATCATCTCCTCCTCCACGCGCGGCTCGCTGCTGATGACGGAGTCGAACACCTCGGAC	43:T>C
6038283|F|0-13:G>C-13:G>C	TGCAGTCCCTCTCGGAGATCACGGCGTCCCCCG	13:G>C
3023170|F|0-6:A>G-6:A>G	TGCAGCACCTGCTTTATCACACGGAGAACTCTTGCCG	6:A>G
3029106|F|0-22:A>G-22:A>G	TGCAGCTGCCGCACTCAGGCCTAACGTGGTCGCAGCAGCTGTGCCG	22:A>G
5373180|F|0-25:G>A-25:G>A	TGCAGCTCGCCGTCGGCCATGGCACGTCCTCGCCG	25:G>A
3947544|F|0-38:G>A-38:G>A	TGCAGCGGTTCCTCGAGAAGCGCAAGGCGAGGGCCAGCGCCGCGTCGCCGTACGTCGTCCACCG	38:G>A
4262503|F|0-26:T>C-26:T>C	TGCAGGCGGATGCGGAGCGTGTCCAGTGGGTGGCCCGCCAGCACGCCG	26:T>C
1001495|F|0-20:T>G-20:T>G	TGCAGCGCGGGGGGCGAGGATGAGGAGGCGACGGACTTGTAGAGGAAGGTGAGGAGATCGGGCCAGGAG	20:T>G
1028379|F|0-53:A>G-53:A>G	TGCAGGAGCAGCTGCTGCCGTTCGCGTCCACGGCGGCGGGCAAGCCGCTCCTCACCGCGCTCCAGGCCG	53:A>G
1023816|F|0-22:T>A-22:T>A	TGCAGGAGGCCGTCTGGGACGCTGACATTGTCATCAACGGCCTGCCGTCCACGGAGACGAGGGTTGTGT	22:T>A
2245285|F|0-49:C>T-49:C>T	TGCAGCATCTGCACCAGCTCATCCTCGATGTTGTGGTACTTCATCAGCGCGACGTCGAACACCTCGGCG	49:C>T
1133759|F|0-61:G>C-61:G>C	TGCAGCTCTCCGCCGCCGCCGTCGGCCTCTCCGTCGTCTCCAACTTCTCCTTCGGCTTCCTGCTCGGCA	61:G>C
992458|F|0-47:G>A-47:G>A	TGCAGATCCCGTCACTTCCTTCCTCTACCGCGCGCTCCACGTCAGGCGAGCACGTAGGTCCCTGTCAGG	47:G>A
3958962|F|0-5:G>C-5:G>C	TGCAGGGCGGCGTCGACGGAGGCGACGTAGCTGGAGACGGTGCTCTGGGCGGCGAGGCGGGGGAAGTGG	5:G>C
2373478|F|0-37:C>G-37:C>G	TGCAGGAGAGCAAGCTGCGGGAGGCGCTGGAGGAGGCCTCCGAGGACGGGTGCCTCACCAAGTCCCGCG	37:C>G
1220044|F|0-16:A>G-16:A>G	TGCAGGTCCATCACTAAGTCCTGATACTCCTGAATTCTAGAGCTTATATGAGACAGATACCTCGGGTCG	16:A>G
3959668|F|0-16:A>G-16:A>G	TGCAGGTGCGGATGGCAGCGTCAAACATGTCCTCGGCGGTGTACCG	16:A>G
5582007|F|0-5:C>T-5:C>T	TGCAGCTGCTGGAGACGAGGCCG	5:C>T
7168152|F|0-22:A>C-22:A>C	TGCAGGGACCTCAAGGAGCAAAACGAGGCCG	22:A>C
7906790|F|0-8:C>G-8:C>G	TGCAGCGGCGACGGCGGCATGCCG	8:C>G
12776192|F|0-6:A>C-6:A>C	TGCAGGAGGATCGTCCACAACACGGTGGGCAGCGACAAGGCGGTGGCGAAGCTCTTCAGCGGGATGTCG	6:A>C
7340651|F|0-9:C>T-9:C>T	TGCAGAGCGCGCCCGATGAGGTGGTGATACGGCTGCTCCTCCACCTGCTCGTGCTTGGCTGCTGACGGC	9:C>T
1104343|F|0-56:A>G-56:A>G	TGCAGAACGTGGCTATCATGCTCAAGGCGCTCAATGTGACCGCGGACGAGGTGATCAGAACACTCATGC	56:A>G
1320455|F|0-61:G>C-61:G>C	TGCAGTTTGGGAAGGTCGCGAGGGACATGTTCACCATGGACTACCGCTACCCTCTGTCGGCGTTCCAGG	61:G>C
1211048|F|0-32:T>C-32:T>C	TGCAGCGGTTCCTGTCGCTCAACTCGGCGCGCTACCACCACCTCATCCTCCGCGCCTTCGCCGACGTAT	32:T>C
5971197|F|0-37:C>G-37:C>G	TGCAGTGGTAGGCGTCGGAGGAGGAGACGACCCTGGCCGGCAGCCAGATGAGGAGGCAGCACCTGCCGA	37:C>G

1123953|F|0-22:G>C-22:G>C TGCAGCTGCACCTGCCGCTGGTGGAGGCCTCCAACGTGCTCACGAACTACCACGGCACCACCAACCTCA 22:G>C
1212373|F|0-5:C>G-5:C>G TGCAGCCCGAGCAGCATGAGCTTCTCGGCCTGCGCGAACGGCCACTCCCCG 5:C>G
4262728|F|0-13:A>G-13:A>G TGCAGACGCTGGCACGGGAGCTCCTGGACCGCCACGGCCG 13:A>G
4911215|F|0-32:C>G-32:C>G TGCAGGCCCAGCGCCTTCATGAAGCCGCCGTGCACGCGGCAGCCG 32:C>G
1168249|F|0-67:T>C-67:T>C TGCAGCAAGGGCTGCACGGGTACCACTTCGCCGACGGCGACATCGGCTCACAGCTGGCCGACGGGTTTC 67:T>C
1221887|F|0-14:A>G-14:A>G TGCAGCAGGCTTGCAGCGACGCGCCGTGCATGGTGGTGGCGTACGCTCCGATTGACGCGGGAGAGCTCA 14:A>G
3028116|F|0-29:T>G-29:T>G TGCAGGGGTGCTGCGACGAGCTGGACCGCTCCTTCGCGGACCCCG 29:T>G
1157385|F|0-68:C>T-68:C>T TGCAGCCTGGAGTGCCGGCAGCAGCACATAACGCACGAGGAGTGGAAGGACAAGTGCACGTCAAGGTCC 68:C>T
12469751|F|0-15:A>T-15:A>T TGCAGAGCACTCCCCATCTCCG 15:A>T
5581488|F|0-7:T>C-7:T>C TGCAGGCTGGCGGCGTGGAGCGGGTGTTCCG 7:T>C
1029449|F|0-26:A>T-26:A>T TGCAGCCCGCCCAGGTGGCCATCGGCAGCGCCGTAGTGCGCTGCATGGAAGCCGCCGCCG 26:A>T
1211417|F|0-11:T>A-11:T>A TGCAGGGCGGCTCGCAGATGCTGGTGAGCCAGCTGGTGGTGGGCACCCTGATCGCGGTCAAGTTCGGGA 11:T>A
1225917|F|0-40:G>C-40:G>C TGCAGCGGCTGGTGGAGGCGGAGGGCCGCCCCGTCGGCGGGACCGACGCCTCCG 40:G>C
986325|F|0-13:C>G-13:C>G TGCAGCTGCACCGCCGCGGCCACAACCTGCCATGGAAGCTGAAGCAGAAGAACCCGAACCAGGTGCAGC 13:C>G
1017082|F|0-14:A>G-14:A>G TGCAGCTCCATGATAGGCTTCATCTTGCGGTCCGTGATGGCGATGGCGATGTCGTTGGGGAAAAGGTCG 14:A>G
1094116|F|0-29:G>A-29:G>A TGCAGCGTCGGCGAGCCCCCGCCGTTGGTGCTCCCCGTCACGAAGTTGAGGCACGGCGTGAACGTGCTG 29:G>A
1158377|F|0-27:G>C-27:G>C TGCAGGGAGCGGGGCGAAGCTGAGATAGGAGTCGGGGATGAGGCGGCGGTCGAACCCG 27:G>C
32019379|F|0-31:G>T-31:G>T TGCAGAGGAGGCGGCGGAGCCCCAGAGGCCTGCGGCTGAGGCGCGTGCTGGCCGTTCATTGGCAGCTGG 31:G>T
1231223|F|0-67:T>C-67:T>C TGCAGCGACGCCCTGATCAGCCTGGCGGGGTGCCTGAGCTACGTGCAGGAGGGGAGCACGGTGGCGATG 67:T>C
12771981|F|0-23:T>G-23:T>G TGCAGAGGCGTCTCGTCCTCGCCTCCCTGGCTCAGCGTCTCCATCAGGTTCCCCCACTCCGTCCGCATC 23:T>G
1027696|F|0-25:G>C-25:G>C TGCAGGTGTACACGATCGTCGCGGCGGGCACCGTGTACGTCAAGTACGAGAAGGAGCGGATCAGCGTCT 25:G>C
2259555|F|0-29:G>T-29:G>T TGCAGGAGGCGCTGGAGGAGAGCGAGAAGGCCGGCAACGTGCCGCTCGACGTGTACGTCACCGTGCCGA 29:G>T
32025038|F|0-5:T>C-5:T>C TGCAGTTATGGGGAAGGAGTGCCTGCCTACCGTCGCCATGGTGCTAGTGCAGCTAGGCTTTGCCGGTAT 5:T>C
2259105|F|0-58:G>T-58:G>T TGCAGCTGGCGCGCGCTCCCGCGGGACGAGGATGTCGTGGTCGATTTCGACCCCTGGCGGTTCGAAGGA 58:G>T
1021826|F|0-62:G>C-62:G>C TGCAGGTGCGAGGGCACGTCCACCTATCCGCGGACCTACGATCTCATCCACGCCGACTCGGTGTTCACT 62:G>C
992869|F|0-11:T>G-11:T>G TGCAGGAAGCTTCTGTGGAGCTCGGCCGTCCATTGCATCCTGGGAGCCCG 11:T>G
2244313|F|0-20:G>C-20:G>C TGCAGCGACAGCTTCGACCTGTACAACGGCCTCTTCATGTACAACAAGAACTCGACGGAGCTGCTCCAC 20:G>C
2245285|F|0-11:C>G-11:C>G TGCAGCATCTGCACCAGCTCATCCTCGATGTTGTGGTACTTCATCAGCGCGACGTCGAACACCTCGGCG 11:C>G
3064550|F|0-38:A>G-38:A>G TGCAGCCTGTCCTCGAGAAAGTGCTTCCGCATGTGGCCACCCAGCGCCTGCCCG 38:A>G
994899|F|0-19:G>A-19:G>A TGCAGGGACCTGAGCAGGGGAAAAATAGAGCTCTCAAGTTCTTCTTCAATCTTCTTTTTTGCAGAGGTC 19:G>A
1093844|F|0-31:C>A-31:C>A TGCAGGGCGTCGGCGACGTTGACGAAGAAGGCGTCGGGGTCGGGCGGGACGGCGGCCCAGGAGCCGTCG 31:C>A
5372910|F|0-5:G>C-5:G>C TGCAGGTCGGCGAGACGGTGCGGCTGCTGCCG 5:G>C
7909664|F|0-16:T>C-16:T>C TGCAGTGCATACTCATTAAATTCCG 16:T>C
1863988|F|0-56:G>C-56:G>C TGCAGGATGCCCTTGGTCGTGAAGTCGAACGCCATGGCAGGGCCGCCGACCGCCTTGGCCCAGTTCACC 56:G>C
5363638|F|0-23:T>G-23:T>G TGCAGCTGAGCCCGTGCCCCATCTCCTCCG 23:T>G
5970727|F|0-21:C>T-21:C>T TGCAGCGACGCGGGCGCCTTCCTCTGGAGCCTGCTCGGCGGGCGGTCCTTGCAGCCTAGCACCTCGCCG 21:C>T
5581382|F|0-11:A>C-11:A>C TGCAGCGAGGCAGAAGACGACCCG 11:A>C
1219191|F|0-43:G>C-43:G>C TGCAGTCCCCGCCGACGCAGCAGCAGCAGGACCTGCTGGGCGGGCTGGACACGGAGCTGAGCGACATGC 43:G>C
1203614|F|0-15:A>G-15:A>G TGCAGCAACACGAAGAAAATCACCATCTTGTGGCATCAAAGTCTGATTTTGATCAGCAAAACGTGTGCA 15:A>G
5580081|F|0-14:G>A-14:G>A TGCAGACCATGGCGGCCAGCCCCG 14:G>A
1696797|F|0-35:C>G-35:C>G TGCAGCTCCGACGCCGAGATGAAGCCGTCGCCGTCCACGTCGAACACCTTGAAGGCCTCCCGCATCTCC 35:C>G
1200550|F|0-35:G>A-35:G>A TGCAGCAATGGGTTGATCTTGCTCGGTACACTGGAGTACAGAAGATTCAGGAACAGCTTCCTTCCGATG 35:G>A
23105021|F|0-34:G>C-34:G>C TGCAGCACTACGACAAGAGGGACCTCAAGTCCCCGACCTTCAGGACGTGGAACGACGACTCCGTCAGGA 34:G>C
1284172|F|0-22:G>A-22:G>A TGCAGGAGTCGAATCCATGGACGAAAATGATCTTGTACTTGGCCTTCTCCTTTGGGACGCCAGATTCCA 22:G>A
7353693|F|0-10:T>C-10:T>C TGCAGCGGCGTGCATGGTGCCG 10:T>C
32026974|F|0-55:A>C-55:A>C TGCAGGTTACTTGAGAAGGTCTGGCCGAACCTCCAGCTGGGCGGCACCACGTCGGAGAAGACGAGCGTC 55:A>C
1105544|F|0-37:T>C-37:T>C TGCAGCGCCATCCGCGCCCGTTCACGCGGCTGCACCGTGACCCCTCCATCAGCACCGCCG 37:T>C
2251060|F|0-7:C>T-7:C>T TGCAGCACGCCCTCCTCGTCGATCATGTAGAACCCCGTGATGTCCCCGAGCTCCGCCGACGAGTCGAAC 7:C>T
7902462|F|0-5:T>C-5:T>C TGCAGTTGAAGCCTGTGGAGTTGCGCATCGACCG 5:T>C
7340694|F|0-55:A>C-55:A>C TGCAGCATGTCGTTGTGGTGGCCGCGGGAGTTGATGGCCTGGTAGATGACGTCCGACTCCTTGAACGCG 55:A>C
1166440|F|0-25:C>G-25:C>G TGCAGGTGGTGTCGGAGGTGGTGTCCACGTTCCTGCTGGTGTTCGTGACGTGCGGGGCGGCGGCGATCA 25:C>G
1027722|F|0-5:G>C-5:G>C TGCAGGGCGTACTGGACGCCGTCCACGTCGCCTGCCGCCGCGAGCCTCCTGACGGTGGCCTCATGGGCC 5:G>C
5579630|F|0-32:A>G-32:A>G TGCAGCTAGGAGACACGGTGCGGCTGCTGCCGACGTGCCCGCACGTGTACCACGCGGAGTGCATCGACC 32:A>G
5347859|F|0-19:T>C-19:T>C TGCAGGAGGTGCGGCTCGCTCTGCCG 19:T>C
3028021|F|0-5:C>G-5:C>G TGCAGCCCAAGCGCGCCGAGCGCCTGGTACACGGACTTCATCGCCG 5:C>G
1215118|F|0-49:A>G-49:A>G TGCAGAGCTACTGCGACGTGTCGGTGGTGAGCGGCTGGTCCACCGTGGGATACGTCGGGCACGGCCTCG 49:A>G
5360214|F|0-22:A>C-22:A>C TGCAGCTCCACGCGCACCTCCTAACCGCCG 22:A>C
5580071|F|0-14:T>G-14:T>G TGCAGAAGCGGCGCTGAGGCGTTGCCGTCGCCG 14:T>G
990007|F|0-47:C>G-47:C>G TGCAGCCGAGACCGCACCGCCTCGCCCAGCGACACGTTCGGCGCCGTCTCCGCGTTCTTCAGCCACGGG 47:C>G

1338978|F|0-6:T>G-6:T>G	TGCAGGTGCCTGCACGGTCTCAGTCGGCGCGTTCCAACAGCAGCATCTCCACGGACCATGACGCGCGGC	6:T>G
2358193|F|0-58:G>T-58:G>T	TGCAGAACGAGGGGCGGCTGTTCGAGTGCAGCTACCCGCGGAGCATCGGCGGCATCCTGGGGAAATGGT	58:G>T
1028148|F|0-32:G>A-32:G>A	TGCAGCCAGCGGTCGGCAGCGGTGCGGATGGTGTCGTCGGGGTGGTGGTACAGCGCCGCGAGCGCCTCC	32:G>A
981900|F|0-68:C>G-68:C>G	TGCAGGCGGATCGTCCACAACACGGTGGGCAGCGACAAGGCGGTGGCGAAGCTCTTCAGCGGGATGTCC	68:C>G
3024767|F|0-21:T>A-21:T>A	TGCAGGGGTGAGATCCATCACTGGTTCAGTTACTGCGCATGCCCG	21:T>A
1090634|F|0-15:T>C-15:T>C	TGCAGAACTGGTCGGTGAAGCAGGGCCACGTCAGCGTCGGCACGCCGTGGGAGATGGCCTCCAGCGTCG	15:T>C
3023480|F|0-14:C>G-14:C>G	TGCAGCTTGGGGGCCGAAACAATTTTCACCGTCGCTCTGCCACTCGGCAAATGGATGCCCACCAGTTCT	14:C>G
981818|F|0-41:C>G-41:C>G	TGCAGGTAGACGCCGATGGCCGTGAACTTGATGAAGTTGCCCCCGATCTCCATCCCCCGCACGCCTGCA	41:C>G
3960588|F|0-23:G>C-23:G>C	TGCAGCGACTCCCCGATGGCGTGGGCTTCCCGCAGCCGCATGTCCTCCGAGAGCTCGATGTCGACCTGT	23:G>C
1022410|F|0-68:T>G-68:T>G	TGCAGTCCAACGCGGACGACGCCGTGGTGCACAAGATCTCCGACCGCATGGTGCGCAACGGCTCCATCT	68:T>G
1228787|F|0-49:G>C-49:G>C	TGCAGCTCACGGTGTAGCAGCTGTCGGGGCGGGAGAGCGCGTCCGAGAAGCGCACCGCGGCCTCCTGGT	49:G>C
1030747|F|0-25:G>C-25:G>C	TGCAGGCGGCGGTGGAGCGGGCGGCGAGGCTGCTGGCGCCCTACGGGGCGAGCATCGTGGAGTACACGA	25:G>C
2281920|F|0-17:T>A-17:T>A	TGCAGGCCCTCAGCGGCTCCAACATCGGCCTCATCATCGACGTGGCCAACGAGGACCTCGCCTCCCTCG	17:T>A
1080531|F|0-64:C>G-64:C>G	TGCAGACCTTCTTCAACTGGTACTACACCGTGCTGGGGATCTCCCTCGTCGTTGCGGTCCTCGTCATCG	64:C>G
7911664|F|0-39:C>T-39:C>T	TGCAGCAGCATCTTCTCCTCCAGCGGCAGCTCGAAGAACCTCTTGCTCTCGGCGAACAACGCCTCCAGC	39:C>T
6046401|F|0-13:G>T-13:G>T	TGCAGGGCTCTGGGCGCTGCTGGTGGTGCCTCTCTGGAGCACGTCGCTATCGGCTAAATAGCGCAGGGA	13:G>T
1099127|F|0-14:C>G-14:C>G	TGCAGGACGAGGAGCGAGCGCAGCGTGTCCCGGAGGCGGTTGGCGCGCCTCCG	14:C>G
1021826|F|0-29:G>A-29:G>A	TGCAGGTGCGAGGGCACGTCCACCTATCCGCGGACCTACGATCTCATCCACGCCGACTCGGTGTTCACT	29:G>A
3949947|F|0-34:C>G-34:C>G	TGCAGCGCCGCACCAGCCGCGCCATCTCCACGCTCGCGGTGGGGGTCGAGGTGCGCTCCATGTCGCTCG	34:C>G
5358138|F|0-16:G>C-16:G>C	TGCAGGAGAGGCGGCGGGCGGCGCCG	16:G>C
7940796|F|0-58:T>C-58:T>C	TGCAGGGGATGGTTGAGGCAGCAGTACATGCCGCCGACAAGGAGGTGGCTAGGGCTGCTAGGAGGATAC	58:T>C
1327349|F|0-10:C>G-10:C>G	TGCAGAAGCTCGGGGTGAAGAAGATCCTGGTGAACCAGCTGCACCCCGTCGGCTGCACGCCGTGGCTCA	10:C>G
5365204|F|0-29:C>G-29:C>G	TGCAGCACGACCTCCACCGTCGAGTTGTACGGCAGGCTCCTCACCTTGGTGCCCTCCACCATGGCCGTG	29:C>G
7344491|F|0-20:C>G-20:C>G	TGCAGCGGCGCCTACGGCACCCCG	20:C>G
3940413|F|0-50:T>C-50:T>C	TGCAGGAGCGTCTTCGCCCTCGACTCGCCGCCGCTCTGGGGGCTCGAGTCTGTATGCGGCCGCCGCCCG	50:T>C
32023729|F|0-32:G>T-32:G>T	TGCAGCTCGCCTGCGAGGAGTTCGGCCTCGGCGGCGACGACCTCAGCCTCGACAACATGATCAGAGAAG	32:G>T
1097915|F|0-8:C>G-8:C>G	TGCAGCCACGACACGAGCGTCCCGGCGACGAAGATGCCGAGGTTGATGGCGCCGAAGAACCAGGTGAAG	8:C>G
7865065|F|0-34:C>T-34:C>T	TGCAGGGGCTCGTGAGGAAGGGCCTGTGGGAGGACGCCATCGACTACCTCGATGGCTTCCTGCCTGCCG	34:C>T
5355051|F|0-44:C>G-44:C>G	TGCAGGACGATGGGGCCGTGGAGGTAGGAGAGCCGCTTCGTGGCCGTGGCGGCGGCGCGGTCGGGGAGG	44:C>G
2249099|F|0-23:A>C-23:A>C	TGCAGGAGCCCGACCCTATGGGGATCGATGAAGTACCTCCACGACAAGATCCACAAGGACTCCAACTGC	23:A>C
1127182|F|0-54:G>C-54:G>C	TGCAGCGCCTTGTCCCTGGCCATCCCGCCCTCCACGGCGACGAACAGCAGGATCGCCAGCGTGGCGGCC	54:G>C
1023288|F|0-40:C>G-40:C>G	TGCAGTCGTAGACGGTGGCCGAGGCCGAGGCCACCGTCGCCAGGAGGCGGGAGCCGTCGCGAGAGAAGG	40:C>G
1061784|F|0-8:G>A-8:G>A	TGCAGGCGGCGAGCCAGGTGAACGAGGTGCCACGTCAGGGCCATCAGGGACAGCACGTTGCACAGGGAC	8:G>A
5970989|F|0-19:C>A-19:C>A	TGCAGCGCGGCGTCGACGGCGGCGACGTAGCTGGAGACGGTGCTCTGGGCGGCGAGGCGGGGGAAGTGG	19:C>A
1695002|F|0-7:C>G-7:C>G	TGCAGGTCGCGCTCAACCACAGCAAACTCGTCGACCGCCAGCTCAGGTCGCTCTACGCCCCGCCACCGA	7:C>G
5581464|F|0-20:T>C-20:T>C	TGCAGGAGCAACACCGTCAATGCGGCTGCCG	20:T>C
32021781|F|0-68:A>G-68:A>G	TGCAGCAATCGAGGGGATGAAGAGCCAGATGGGCGGCGCCGCCAGCGAGGAGACGGTGAGCGACCACGA	68:A>G
1109699|F|0-23:G>C-23:G>C	TGCAGCGGGCGGTGCGTGTCCAGGCGCCAGATGGATCCCCACGACTCCCTCATCGGCGTCCAGACCCCC	23:G>C
1102872|F|0-21:A>G-21:A>G	TGCAGGGCCCACGCGCCCGTGATTCCCAAGGTGATGTTCGCAGATTGGCTCAACATGGACTACATCGGT	21:A>G
3953270|F|0-5:A>C-5:A>C	TGCAGAGGCGCGCTGCCGCGGCCGTCACTGTGCAGTCGGCGTTCCG	5:A>C
1082524|F|0-5:C>A-5:C>A	TGCAGCGGCTCCTGCTGCTCTTCGCGCTGCTCGGGACGTGCATGCTCATCGGCGACGGCGTGCTCACCC	5:C>A
4733507|F|0-6:T>C-6:T>C	TGCAGCTCCGACGACTTGGCGATCTCGTCGATGAGCGCCGTCTTCTCCTCGCGCGGCACGGTGGGGTTG	6:T>C
1095334|F|0-17:C>G-17:C>G	TGCAGCGAGGAGATCCTCAACCCGTCGGTGCCCATGGCGCTCAGGCTCTCCGGGATCCTCATGGGTGAG	17:C>G
14385162|F|0-18:G>A-18:G>A	TGCAGTGGTTTCTCCTCTGCCG	18:G>A
1126397|F|0-47:C>G-47:C>G	TGCAGTCGTAGTAGTAGTCCCCGACGGCGACGGGGTCGTCGTCGCTCCCTCCCCAGGGCTCGTTGTCGG	47:C>G
1025890|F|0-64:G>C-64:G>C	TGCAGAGAGCAGCCTGAGCTGTTTGTTCGGTGGCACTGTACTGCTTGCTGCCATGGCCACCATGGACGG	64:G>C
1125514|F|0-59:C>G-59:C>G	TGCAGCAACGCGATGACGGTGGACTTCCCCGACCCGCTGCCGCCGACGAGGCCGATGGTCGCGCCCTCG	59:C>G
13196441|F|0-35:C>T-35:C>T	TGCAGTGGAAGCATCCATATGTCATGAAAGTACCACGCATAGTCCCCCACCCGCTGCACATCGACCGCC	35:C>T
2268151|F|0-50:G>C-50:G>C	TGCAGGGCGCTGAGCCGCTTCACGGCCGCCACGATGCCGTCCTCGAGGACGGCCTTGTACGTGCTCCCG	50:G>C
12683485|F|0-11:T>C-11:T>C	TGCAGCAACGATTCCGCCGCCG	11:T>C
3944463|F|0-41:A>G-41:A>G	TGCAGTGGGCGACGGAGGTGGAGGCCGGCGGCAGGAAGGAGATCGCCG	41:A>G
2281626|F|0-39:C>T-39:C>T	TGCAGCTCGGCTTGCAAGTGCAACTCCACCTCCAGCTCACATCCTGAGCAGAGGCATCGGGTGGCAGGT	39:C>T
1049067|F|0-21:G>A-21:G>A	TGCAGACACCACTAGATGTGTGGGCGGTAGACCCATGCTAACCGCAGCAACCCCATGATGCCACCTTCT	21:G>A
5580070|F|0-16:G>C-16:G>C	TGCAGAAGCACTACGCGTCGCTGGACGTCCACCCCG	16:G>C
1083150|F|0-41:G>C-41:G>C	TGCAGGTGCCGCCACGCCCACTCCGCCAGCGAGCAGTGCTCGCCGCCGTTGCCGGCCTCCCTGCCGAGA	41:G>C
26673479|F|0-37:C>T-37:C>T	TGCAGCACCAGGACCATGAAGACCAGGAGCAGGAGGACGCCTCGTCCTGCGACCTCTCCTCCCCGCTGG	37:C>T
5577976|F|0-23:T>C-23:T>C	TGCAGCATGGTGGCGACCAGTATTATGACCG	23:T>C
1127954|F|0-43:T>C-43:T>C	TGCAGATCATCACGGCGCGGCCGCCCATGGGGCTGACGCACCATGTGGCGCGCGCGCTGGACCACGGCA	43:T>C
4544250|F|0-27:A>C-27:A>C	TGCAGCTTCGTCTTCGGCGTCGCGGAGATGGGCGTCCGCAGCCAGGAGTGCCGCGACCACACCAAGCTC	27:A>C

6047240|F|0-65:G>C-65:G>C	TGCAGCTGGAAGTCGAGGTTGAGGTGCTTGAAGTTGCGGGTCAGCGTGCCCCGCGGCCCCGTCACGGAG	65:G>C
2358193|F|0-28:C>T-28:C>T	TGCAGAACGAGGGGCGGCTGTTCGAGTGCAGCTACCCGCGGAGCATCGGCGGCATCCTGGGCAAATGGT	28:C>T
1199905|F|0-13:C>G-13:C>G	TGCAGTTGACGACCACGACGCCGATGTCCCTGGCCCGCACCCCTGTCTTGGCGAGCAGGCTGTCGATGG	13:C>G
1024266|F|0-55:T>C-55:T>C	TGCAGAGGTCGGAGAGCTCCTTGCGGCCCTCCACAATGTCGTCCAGGAAGTCGTCTAGCTGGCACGCCA	55:T>C
1204198|F|0-46:T>C-46:T>C	TGCAGCAGAACAGCTACGCCAAGCAGCAGCACGAGCCCGCCGACGATGACCACAACGGCAACGGCAACG	46:T>C
989443|F|0-8:G>C-8:G>C	TGCAGCCAGTTTTCTCCCGGAGTCCTCGGCCGCCGATCTTGTTCCTGAGCTTCCTTACAGCCTCCCATC	8:G>C
3027853|F|0-19:C>G-19:C>G	TGCAGGACCAGCTGCTGGGCTTCGCGCTCGTGCCGCTCCCG	19:C>G
1211015|F|0-20:C>G-20:C>G	TGCAGCTCGGCCAGCTCGGCCAGCAGCGCCGCATCGGCCGACGACTTGGACATGTCGCCG	20:C>G
5581519|F|0-11:C>T-11:C>T	TGCAGGGCCTTCTCTGGCGGTGGCGCCG	11:C>T
5411721|F|0-16:T>C-16:T>C	TGCAGCCCCCCTGCGCTGTCGCCG	16:T>C
12768272|F|0-7:G>C-7:G>C	TGCAGGGGAGGCGGCTCTCCG	7:G>C
4910350|F|0-59:G>C-59:G>C	TGCAGGCTCTTGACGTACACCCAGGACTCGCGGCTCGAGACGTCGGCCCACCAGCGGCTGACGTTCCTG	59:G>C
4992965|F|0-8:T>G-8:T>G	TGCAGTCCTACGAGGCGAGGCTACCG	8:T>G
7915346|F|0-41:C>G-41:C>G	TGCAGGACGATGGGGCCGTGGAGGTAGGAGAGCCGCTTCGTCGCGGTGGTGGCGGCGCGGTCGGGGAGG	41:C>G
2250143|F|0-40:T>C-40:T>C	TGCAGCGCCTCGACCCCGCCGTCGAAGACATCCTCATCACTGCCGCGCACGTCACGCTCTACGACTTCA	40:T>C
5366991|F|0-14:G>C-14:G>C	TGCAGCCCCACGAGGCCACCGATCCCCGCCG	14:G>C
3027624|F|0-5:G>A-5:G>A	TGCAGGTCAGGAAGGATGGGGAATGGTTCTCCGTGGACGCCCTGCCCG	5:G>A
5361287|F|0-44:C>G-44:C>G	TGCAGCTTCCTCTGGACGGCCGCCACGTCGCCAGCGCGCACGAGCCCGTGGATGTTCTCGGACTTGAGC	44:C>G
3027225|F|0-32:A>G-32:A>G	TGCAGTATCTCCGTGGCCCTCCTGTTGGCGTCAGTCTCGCCGTCGCCG	32:A>G
1001631|F|0-10:A>C-10:A>C	TGCAGCAGGCACCCCCGCCGCAGCAGTACTACCAGGCGCCGCCGCAGTACTACCAGCAGGGACCCCCGC	10:A>C
1863066|F|0-17:T>C-17:T>C	TGCAGCTGCCGCTGCTGTTGCTGGAGCAGGAGCACGGTGTGGTTGAAGGCGCCGTCGACGGCGTGGAGC	17:T>C
2251549|F|0-38:G>A-38:G>A	TGCAGCACGTCCTCCCACACCCCGCCGTACCACACGTCGTCGACCACCAGCAGGAACCTCTTGTTCGCC	38:G>A
5580180|F|0-17:G>C-17:G>C	TGCAGGAGCGCCTCGCCGAGGCCCTGCCTCCG	17:G>C
2251549|F|0-7:C>T-7:C>T	TGCAGCACGTCCTCCCACACCCCGCCGTACCACACGTCGTCGACCACCAGCAGGAACCTCTTGTTCGCC	7:C>T
5576949|F|0-7:C>T-7:C>T	TGCAGCACGCCCGCTTCCCGGCGCGCGCCCCGCCGTCGTCGTCGATCCTGTACCGCATGGCGCAGCCGA	7:C>T
7343582|F|0-30:G>A-30:G>A	TGCAGAGCGCGGCGTGGCCGGCGCGGCAGAGGCAGCACACCACGTGCCCG	30:G>A
7337227|F|0-20:G>C-20:G>C	TGCAGCGGCGAGAGGATGTCGACCCCCGGGATGGGCACGCAGCCG	20:G>C
3025735|F|0-10:A>G-10:A>G	TGCAGCCCCCACCAGCACCAATTGCGTCGCCCTCGCCG	10:A>G
1200500|F|0-46:G>A-46:G>A	TGCAGGGCGAGGTACAAGTGCTTGTCGTTGCAGGAGTCCATGGACAGCGACCTTCTCATGGGCTGCTGC	46:G>A
1090486|F|0-38:C>G-38:C>G	TGCAGGACGGTGTCGTTGCCCTTGAACGCCTCGTTGCCCACGGTAATGCAGGTGATGCGCGCCCCGAGA	38:C>G
3956776|F|0-11:G>C-11:G>C	TGCAGCTTGGAGAGCAGCTCGGAGATCTGCTCCTCCGAGATCGACGGCGAGCTCCCG	11:G>C
1167413|F|0-12:A>T-12:A>T	TGCAGCCCCACCAGCTTCGCCTCCATCTGCCGCGCGTTCGGCCCTTGGTCGCCGTAGATGGGCAGCATG	12:A>T
1026294|F|0-47:G>A-47:G>A	TGCAGCTTCTTGGGCGTGAGCACCTTAGGAGAAGGTGGGGGTGTTGGGTGCGCGCGGTTGAGGACGCGC	47:G>A
3064730|F|0-14:T>C-14:T>C	TGCAGCCAGGTGCATATGGCCATGTACATCTCGCTGGCCGCGGCCG	14:T>C
1105347|F|0-52:T>C-52:T>C	TGCAGTGCGTTGTCAAGGAGACTCTTCGGCTGTGCCCCGTCGGCCCAATCATTCCTGCGCACGAGGCCA	52:T>C
6044971|F|0-13:G>C-13:G>C	TGCAGCTGCCTCCGTTGCCGCAATGCCGCGAGCTGCGCGCACGCCTTGACAACGAACGGGAACGTGTAG	13:G>C
2261883|F|0-27:C>T-27:C>T	TGCAGGTGTGCTGGCCGTGGTAGACGACGTCGAAGAGCGCGGGGTCCTGGTCGGCGCGCTGCACCTGCT	27:C>T
2263309|F|0-29:T>G-29:T>G	TGCAGCAGTGCTCGATCTGACCGAGGGACTTGTCGTCGCGCCGCGACGGCGGCAGGTCGTGCACCACCG	29:T>G
7340694|F|0-19:G>A-19:G>A	TGCAGCATGTCGTTGTGGTGGCCGCGGGAGTTGATGGCCTGGTAGATGACGTCCGACTCCTTGAACGCG	19:G>A
994381|F|0-60:T>A-60:T>A	TGCAGCCTGCGCTGGAGCTGCTGGTTGGCGGCGCGCAGCTTCTTGACCTCCTCCTCCAGGTACGCGGCG	60:T>A
12695468|F|0-19:C>T-19:C>T	TGCAGATCATCATGGATACCCTGGTTGCCGCCAGGCAGGTCAACAAGGCCGTCCAACTGCTCCAGAGCG	19:C>T
3942782|F|0-24:G>A-24:G>A	TGCAGCTCGCCGACGACATGGCCGGCCTCTTCTGCGAGCCCTTCCCG	24:G>A
1105915|F|0-19:G>C-19:G>C	TGCAGTGCGTGACGCCGACGGTGTGCGCGCCCAGGAGAACGACCATGTCGTCCGAGGTGAGGCCGAGGT	19:G>C
32023370|F|0-17:C>G-17:C>G	TGCAGGATGCGCAGCGACGAGTTCCTCCCGCCGCCGAACGCCGCGGCCG	17:C>G
7911488|F|0-11:G>C-11:G>C	TGCAGCGGGCGGGTGTCCCG	11:G>C
5370850|F|0-29:C>G-29:C>G	TGCAGCTCCCGCTCCGACGCGGACGGCGACGCCG	29:C>G
4003845|F|0-8:A>G-8:A>G	TGCAGCACAAGCTCGTCCAGGCCGCCCCTGACCATGGCCG	8:A>G
1114947|F|0-8:A>G-8:A>G	TGCAGGGTAGTGTTGATCCAGGCGAGGATCTCGTTCCGCCCCACGAAGTAGGCGCCGTCCATCATCCCT	8:A>G
1022979|F|0-53:C>G-53:C>G	TGCAGTCCGTCCTTGTGAATTTCGTCAAGGGTGTTGGTAACCTTAGCCATGCACAATGGCGGTCATTCT	53:C>G
1087621|F|0-8:C>G-8:C>G	TGCAGAGCCCCGCGTGGCCGGCGCGGCAGAAGCAGCACACCACGTGCCCG	8:C>G
32023660|F|0-45:A>T-45:A>T	TGCAGCGGTGCTGTTACTCGCCGCATCGGCGGCCAGGCACGCGCTAAGCTCGTCGGCGGCGAGGAGGGC	45:A>T
5358325|F|0-5:C>G-5:C>G	TGCAGCGCCAGCACGTAGTGGTCGAACCCG	5:C>G
32025496|F|0-38:G>C-38:G>C	TGCAGCAGCGCCACCATCTCCTCCTCGGCACTCGCGCCGAGCCGCACCAGCTCGACGTCGAACACCTCG	38:G>C
1093353|F|0-53:C>G-53:C>G	TGCAGGGCGAACTCGTTGTGGTATACGGCGACGAGCACGGCGGCGAGGAGCGCCAGGGGAAGCAGGAGG	53:C>G
1133495|F|0-23:T>A-23:T>A	TGCAGGTGGGGTTCGCCGGGATGTACATCATCGCCGTGGCGTCCCTCAAGGCCG	23:T>A
1194889|F|0-59:C>T-59:C>T	TGCAGCTACGGCTACTGCTACTCCGGCATGAAGCTCGCCGTCACCGCCAAGAAGGGCCCCGCCTCTTCC	59:C>T
9721901|F|0-5:G>C-5:G>C	TGCAGGAGCGCGCGGGAGATCTCGTTGAGGCACTCCCCGAGCACCTTCTCGTCGATCACCGTGGCGTTG	5:G>C
4990067|F|0-20:C>G-20:C>G	TGCAGCGGCCCACAGGCGGTCGTGTACTGCCG	20:C>G
1096261|F|0-9:C>T-9:C>T	TGCAGCCGCCGCTCCCGGAGGGCTACTTTGGCAACGTCATCTTCACGGCCACGCCGCTCGCGGTGGCGG	9:C>T

13375760|F|0-6:T>C-6:T>C	TGCAGATGGAACCATAGCCG	6:T>C
1009951|F|0-31:T>C-31:T>C	TGCAGCCGCGCGCCGCCGCCGCCGCCACCGCTGCCGTGGCGCTTCTTCTGGATCTTGCCGCCG	31:T>C
2257769|F|0-28:C>A-28:C>A	TGCAGCAGCCTGAAGCCGACGTTGGAGGCGGCGAGCCGCGTGTCGACGGAGGCCTGCTCCAGCCCCTCC	28:C>A
3029176|F|0-35:T>C-35:T>C	TGCAGCAGCACGCGCGCCGCGCCCTCGCTGCTGGTTCCGCCG	35:T>C
5367134|F|0-11:A>T-11:A>T	TGCAGCTGGCAACCCACTCTGGCCG	11:A>T
7343958|F|0-25:T>C-25:T>C	TGCAGGCGGGAGAGCGCGGCGGGGGTGACGGCCG	25:T>C
3956325|F|0-34:G>C-34:G>C	TGCAGCAGTGGTTGTTGGGGCACGTCACGGCGTTGCCCCCG	34:G>C
1014714|F|0-56:G>C-56:G>C	TGCAGGACGGCGACGAGGTCGGCCTTGCGGATGGTGGAGAGCCTGTGCGCGATGACGAGGGTGGTGCGG	56:G>C
997337|F|0-32:G>A-32:G>A	TGCAGCCCATGGGGGCGGTCTGTGCCCAAAGAGCTCCAGCTGGGAGATGCAGCTATACACCATTGCCGA	32:G>A
12470472|F|0-16:G>C-16:G>C	TGCAGCGGCCCGACGCGCACGCCG	16:G>C
1026250|F|0-54:C>A-54:C>A	TGCAGGGAGACGATGATGGAGTACAGGACGGAGCTGAAGAAGCTGGCGGAGAAGCTGCTGGGCGTCATG	54:C>A
1209032|F|0-26:G>C-26:G>C	TGCAGCTTGACGGAGGCGTTCATGGGGCGCGACGTGTCGTGGGGCACGAGGTCCTGCGCCCCGATGGCG	26:G>C
1211398|F|0-41:C>G-41:C>G	TGCAGGTCGGCCGTGGTGGCCACCAGCTCGTCCATGGTGGTCTCCAGGAGCTCCATGCAGTCCGTGACG	41:C>G
5361352|F|0-37:C>G-37:C>G	TGCAGGACGTCCACCCCGACGTCGGCGACGCGCTCCTCGGCTTCGTCTACGACCCGCTCGACCCCGCCG	37:C>G
1696929|F|0-29:G>A-29:G>A	TGCAGGCACTCCGCCGCGTACTCCTTGGAGCCCAGCACGACGCCGCGGTCGAGCAGGCTGATCATCACG	29:G>A
5365929|F|0-55:G>C-55:G>C	TGCAGTACTACTCCGCCATGGCGATCATGGAGATCACCCGCGTCGCCGAGCACAAGCTCGCGCTACGGC	55:G>C
5370467|F|0-56:T>C-56:T>C	TGCAGCAACACGAAGAAAATCACCATCTTGTGGCATCGAAGTCTGATTTTGATCAGTAAAACGTGTGCA	56:T>C
1154956|F|0-60:G>A-60:G>A	TGCAGCTGCCGCCGAGGCGTGGTGGGGCGCCCCGACGGCGAGGTGGCGTCCATCCGCGGAGGCGGCGCC	60:G>A
5971101|F|0-38:G>C-38:G>C	TGCAGGATCCCCCCGACGAGCGCCTGGAACAGCCGACGGGGCTGGCCG	38:G>C
999261|F|0-32:T>C-32:T>C	TGCAGGCTCCAGTCCTTGGACCTCTCCGATAATCAGCTGGTCGGGGGCTTCCCG	32:T>C
995197|F|0-17:T>G-17:T>G	TGCAGCAGCAGAGAGAATGCCTCCTCCATGTTGAGCTTGTCGGGCGTCTGGCCCGCCG	17:T>G
1091812|F|0-27:G>A-27:G>A	TGCAGTGGGAGCCGAGGAGAGAGACACGATAGCACCCGCGCACTCATACAGAACCGCGGTGCTTGGAGA	27:G>A
1047177|F|0-8:T>C-8:T>C	TGCAGGAGTCACCAGAATAGGGTGAGAAAGCCTTTCCACCCATTCAAGGCGAACCCCTTCTAGCCCAGG	8:T>C
5371630|F|0-26:G>C-26:G>C	TGCAGCGCCGTCCCGCACAGCATCCCGGCCCAGATGCCCTGGGGCCCGAGCCG	26:G>C
32022726|F|0-54:C>T-54:C>T	TGCAGAGCCCGCACGCACGGTCCATGACTACTCCGTGCGTGCGTGCGGATTGACCCGCGCGCGCACACA	54:C>T
5357637|F|0-7:A>G-7:A>G	TGCAGCCACCCTACACCTCACCG	7:A>G
2266466|F|0-17:G>A-17:G>A	TGCAGCGTCTCGATGGCGTCCTGCTTGCGCCCGCGCACCAGCAGCCACCGCGGCGACTCTTGGACGAGG	17:G>A
3026632|F|0-6:A>C-6:A>C	TGCAGCAACACGCGCAGCCTCCGCACGGGCACGCTCCCG	6:A>C
9721901|F|0-56:C>G-56:C>G	TGCAGGAGCGCGCGGGAGATCTCGTTGAGGCACTCCCCGAGCACCTTCTCGTCGATCACCGTGGCGTTG	56:C>G
3948727|F|0-38:C>G-38:C>G	TGCAGCTTCATGACGTCGCCGCAGGCCGGCGCGCCGACCAGCCCG	38:C>G
1049650|F|0-31:C>T-31:C>T	TGCAGGAGAAGCTTCTGGAGGCCAGGATGGACGATCGCAAGCCCTCGCCCAGCTCTGCTGCCAAGCACA	31:C>T
988456|F|0-68:C>G-68:C>G	TGCAGCTGCGGCGAGAGCTTGAGAGCCGTCATGGAGGACTCGGCGTCCTGGCCGTAGATCTGGTCGCCC	68:C>G
1136320|F|0-26:T>C-26:T>C	TGCAGGTCCAGCATCTCGCCGCGGACTCTGTCAGCCTCGGCGTCCACGAGCGCGATCTCGTCGGCGAGG	26:T>C
1038385|F|0-62:A>G-62:A>G	TGCAGCTGACGAAGAATCCATGCCTGCGGTGGACGGTGGTGATCAAAGTGTGAGTTTCTTGCAGCTTGG	62:A>G
2252110|F|0-7:G>C-7:G>C	TGCAGGTGAGCAGCGCCACGTCCGCCGCCGTGTCGATCATGTTGTCCATGAAGAAGACGTAGGCGGTCA	7:G>C
1028057|F|0-7:C>G-7:C>G	TGCAGGCCCACGCCTTCCTCCTCCCCCGCGGCGGCCACCGCAACGCGCGACTCCTCTCGGCGCTCCTCC	7:C>G
985626|F|0-41:C>G-41:C>G	TGCAGCCGTGCACGAGCCACCGCATCGCCAGCAGGATGTTGCACTTGGTCGGCGTCCTGTACGGATCGC	41:C>G
1028057|F|0-39:G>A-39:G>A	TGCAGGCCCACGCCTTCCTCCTCCCCCGCGGCGGCCACCGCAACGCGCGACTCCTCTCGGCGCTCCTCC	39:G>A
12696206|F|0-19:C>T-19:C>T	TGCAGGCTCTTGACGTAGGCCCAGGCCTCCCGCTGCGAGATCTTGTCCCACCAGCGGCTGACGTTCTTG	19:C>T
1298945|F|0-11:A>G-11:A>G	TGCAGGGCGAGAACTCGTAGATTGCAGAACCCCCCTGCAATGACGTGCAGATCGTCGTCGATAACACCG	11:A>G
1135636|F|0-17:G>C-17:G>C	TGCAGCCCCGAGCGCCAGTCGCCGTCGTACTGGTCGCCGTTGGCGTAGCTCTTCCTGCCGTGGCCGTGC	17:G>C
1093857|F|0-8:G>C-8:G>C	TGCAGCAAGGCGATGACGGTGGACTTGCCCGACCCGCTGCCGCCGACGAGGCCGATGGTGGCGCCCTCG	8:G>C
7914712|F|0-9:C>T-9:C>T	TGCAGCGGCCCCGTCTTCCCG	9:C>T
1157378|F|0-7:C>G-7:C>G	TGCAGGTCGCCCCCCACGGCGCGCACGCGCTGGTCGTCTCCACGGAGACCATCACGCCCAACTACTACG	7:C>G
7353694|F|0-9:T>G-9:T>G	TGCAGCGGCTCGCATGGCGCCG	9:T>G
7165147|F|0-5:C>G-5:C>G	TGCAGCCCCAGCGGCCGGCCGCACACACTCTCCG	5:C>G
4544234|F|0-11:A>C-11:A>C	TGCAGCTTCGTATTCGGCGTCGCGGAGCTGGGCGTCCGCAGCCAGGAGTGCCGCGACCACACCAAGCTC	11:A>C
5971436|F|0-11:G>C-11:G>C	TGCAGCGTGAGGACGGCCTTCTCGCGGCCGACGAGGCTGCTGCCG	11:G>C
1279303|F|0-12:G>A-12:G>A	TGCAGCCACCTTGTCCAACTATTGTACCTATCTGGTCAGGAAGGCACTGGTGCCG	12:G>A
23919758|F|0-26:C>G-26:C>G	TGCAGCGTGCGGCCGGCCCTGGAGCTCCTCGTGTTCACGGTGCTCCTCTCGATCATCTGGAAGATCGGA	26:C>G
2329974|F|0-5:G>C-5:G>C	TGCAGGCGCTCCCTGGAGAAGGCGAACACGTCGTTCCACTCGGGGCCCGCGGTCTTCTCGATGTGGCGC	5:G>C
5358259|F|0-23:C>G-23:C>G	TGCAGGCGCGGGAGGTCGCAGAGCGAGGCCG	23:C>G
4393921|F|0-9:T>C-9:T>C	TGCAGAACGTCACGGGCCTCGCCACGCTCTACGACTTCGCCAAGCAGCGGCCGTACGCGTCGGCTGGCG	9:T>C
1212241|F|0-8:A>G-8:A>G	TGCAGCACATCAAGTCCATCGCCGACGAGATGGAGTTGCGCCGCCGCGAGCTGCGGCTCTACGCCAACA	8:A>G
3954197|F|0-37:G>C-37:G>C	TGCAGACGCTGGCGGTGTTCTTCCGGCCCAAGACGACGAACCG	37:G>C
2329974|F|0-48:G>A-48:G>A	TGCAGGCGCTCCCTGGAGAAGGCGAACACGTCGTTCCACTCGGGGCCCGCGGTCTTCTCGATGTGGCGC	48:G>A
1194889|F|0-56:C>T-56:C>T	TGCAGCTACGGCTACTGCTACTCCGGCATGAAGCTCGCCGTCACCGCCAAGAAGGGCCCCGCCTCTTCC	56:C>T
5367449|F|0-10:G>C-10:G>C	TGCAGGTGCTGGGCATGGTGCTCACCG	10:G>C
1157385|F|0-32:G>C-32:G>C	TGCAGCCTGGAGTGCCGGCAGCAGCACATAACGCACGAGGAGTGGAAGGACAAGTGCACGTCAAGGTCC	32:G>C

1007387|F|0-34:C>T-34:C>T	TGCAGCAGGGTTCCAGATTTCGTCGTCGGCAGGGCGAGGTATGGGCAGGTGAAGTTCTCTGGCAGCACC	34:C>T
25258792|F|0-16:T>C-16:T>C	TGCAGGGCCTGCGCGCTGTCGACGGCGAGGACCCGCCCAGGATACTGCACCTTAACCCCAGGCTGAGGG	16:T>C
32023579|F|0-67:G>A-67:G>A	TGCAGCAAACCAACACCTCCGACATGCTGGATCTCGCTGTCGACTACATCAAGGAGCTCCAGATGCAGG	67:G>A
12419631|F|0-14:C>G-14:C>G	TGCAGCATGCCCTTCAGCCTCGTGGCCCCCTCGCGCGCCTCGATCAGGTCGTTCACCGCCGCGGCCGCC	14:C>G
7340675|F|0-5:T>C-5:T>C	TGCAGTAGCCTGCTCACCGTCGACATGAGGGGCCTTCTCTTCCCGACCACGACCACCACTGTCGCCGCC	5:T>C
1216778|F|0-61:G>C-61:G>C	TGCAGGTGTACGGTGGAGTGACCATGAACAACACGCTCTGCCTGGCAGTGTTCTTGGCTCTGGTCTACG	61:G>C
1118189|F|0-64:A>G-64:A>G	TGCAGCACCGCCTGGTACGGGTACTTGACGAGCGAGATGTAGTGGAACCAGATCCAGTAGTCCG	64:A>G
1127296|F|0-32:T>G-32:T>G	TGCAGCACGTGGTGTTCGGCATCGCGGCCTCGTCGCGGTTCTGGGACAAGCGCAAGGAGTACATCAAGG	32:T>G
12770583|F|0-22:T>C-22:T>C	TGCAGGAACGACTTCCCTCCCGTCGCCG	22:T>C
1865118|F|0-16:G>C-16:G>C	TGCAGAGCAAGTACCCGTACGCCAAGATCATGTACGCCGACTTCTACTCCCACGTCTTCGACATGGTCA	16:G>C
4544253|F|0-50:T>C-50:T>C	TGCAGCTTCGTCTTCGGCGTCGCGGAGCTGGGCGTCCGCAGCCAGGAGTGTCGCGACCACACCAAGCTC	50:T>C
3027065|F|0-22:C>A-22:C>A	TGCAGGGACCTCAACGAGCAAACCGAGGCCGGGAAGCCGCCG	22:C>A
7869548|F|0-5:T>G-5:T>G	TGCAGTTGCCTCGGCGCCGCCG	5:T>G
1695029|F|0-41:C>T-41:C>T	TGCAGCTCCTGCTTGGTGAAGAGCGTGAAGGACATTCCCTGCTTGTTGGACTTCTTCATCTCCTCGAAC	41:C>T
5358608|F|0-29:C>G-29:C>G	TGCAGTGCGCGCCCTGCCTCGACTGCTTCCACCAGACCGGCCCCGTCTTCGACCCG	29:C>G
1001150|F|0-13:G>C-13:G>C	TGCAGGTGAAGGGGTTCGTCGGGGACGACCAGCTCGGGGAGGCGCTGGAGGGCGCCGACCTCGTCATCA	13:G>C
5367010|F|0-6:T>C-6:T>C	TGCAGCTGCCCTGCACGCCACCG	6:T>C
12763825|F|0-64:G>C-64:G>C	TGCAGAGGCGGTCGGCCGCGAGCCTGCCCTTGTGGGTGCTGCTCTTGTACTCCTTCCAGGTGAAGTCCC	64:G>C
1027406|F|0-17:G>C-17:G>C	TGCAGGCTCTTGACGTAGGCCCAGGCCTCCCGCTGCGAGACCTTGTCCCACCAGCGGCTGACGTTCTTG	17:G>C
1114730|F|0-22:A>G-22:A>G	TGCAGCGGGCGGTGCGTGTCCAACCGCCAGATGGAGCCCCACGACTCCCTCATCGGCGTCCAGACCCCA	22:A>G
1127963|F|0-40:G>C-40:G>C	TGCAGAAGATCACCAGCCTGCTGCTCTGGGGCAATGCCCTGTCCGGGAAGATCCCGCCG	40:G>C
1051205|F|0-38:G>A-38:G>A	TGCAGGGTGGAGAAGGCAATGTAGGCATGAGCTTCGTAGATGAAAACCCCTTGATATGGAACGAAACAG	38:G>A
1029925|F|0-7:G>A-7:G>A	TGCAGGTGGTGGTCCTCGGAGGGGGCTCGTTCGGAACGGCGATGGCGGCGCAGGTGGCAGCCAAGAAGC	7:G>A
1210919|F|0-40:G>C-40:G>C	TGCAGGGCGGCAGGGGCCTCCTGGGCTCCGACCAGGTGCTGTACACGGACCCGAGGTCACGGGGCACCG	40:G>C
1152867|F|0-56:A>G-56:A>G	TGCAGAGCGCCCTGAATCTCCGCCATGTACTTGTTGTCTTCGTCTAACCATCGGGCATACTCCATGTCG	56:A>G
5324107|F|0-20:A>C-20:A>C	TGCAGGAGCTCTACCAGCTGATGCTTGCCG	20:A>C
1215238|F|0-50:A>C-50:A>C	TGCAGCGCCTCGCGCGGCACGTTCTCCAGCAGCCACGTCGGCCACCCGTCAACCAGCTGCTCGCGGTCG	50:A>C
1669907|F|0-17:G>C-17:G>C	TGCAGGTCGCGCCCGTAGGGCGGGAAGTTGCTCTTCAGCACCGTGCCGATCACGTTGTTGTTGCCCGTG	17:G>C
1068668|F|0-5:G>A-5:G>A	TGCAGGGTCTGTAAATCAAGCGAGGCGTGCATGCTCTTGCTGCCCTGCCG	5:G>A
2259772|F|0-5:G>C-5:G>C	TGCAGGGACTGGACGTAGTTGATGATCTCGTCGAGCATGCCGGCCTTGCCG	5:G>C
1091890|F|0-44:C>G-44:C>G	TGCAGCACCCTCCGCATCCGCAGGCCGTCGCCCTTGGTGCCCTCCAGGAGATACGCCAGCCTGGGCGGC	44:C>G
4910545|F|0-24:A>C-24:A>C	TGCAGCGACGAGGCGTACCAGTCGACGTGCGCCGCGAACTCGCTGGGCATGGCCGCGTGCGAGCAGTGC	24:A>C
1001554|F|0-30:A>T-30:A>T	TGCAGCCCTTCCTCGGCGGCGACACGGCCGACCTCCTGCGCTTCTCGTCGCAGGTCGGGGAGTGGGTCA	30:A>T
12774626|F|0-29:A>G-29:A>G	TGCAGCTGCCGCTGCTGCTGCTGGAGCAGAAGCACGGTGTGGTTGAAGGCGCCGTCGACGGCGTGGAGC	29:A>G
3952423|F|0-40:A>G-40:A>G	TGCAGCGCCTCTGCTCCGACACGGAGGCCGACGAGCAGCTACCG	40:A>G
989217|F|0-6:C>T-6:C>T	TGCAGTCGCATGCATACTTGCATAGCGGTGAAAGACGGTGAGCGAAAGAGGAGGAAAGCTCTCTCTCGG	6:C>T
1206457|F|0-52:C>G-52:C>G	TGCAGGTGTTCTACGGCCTCATGGGCAGCTGGACCGCCTACCTCATCAGCGTCCTCTACGTCGAGTACC	52:C>G
1689110|F|0-54:A>G-54:A>G	TGCAGTCCACGGAGCGGCAGTGCGCTCTCCCGGCGTCCTCGCACTCCTCCAACAACACATCCCTGAAGT	54:A>G
1007448|F|0-45:T>C-45:T>C	TGCAGCACCGCGTTCCAGCATTCAGCCGCGGCCTCGGCCACCTCGTCCTCCCCCG	45:T>C
1132035|F|0-8:G>C-8:G>C	TGCAGCGCGGCGATGTGGAAGATCATCTCCCGCATCGCGTCCGACGACGACGGGGACGAGTACCCCTGC	8:G>C
1227011|F|0-61:G>A-61:G>A	TGCAGCGCTCGCGGCTGCGGCGGGCCCTGACGACGTGCCAGTGGTTCTTGACGGCGTTGTCGGTGCGGC	61:G>A
1094924|F|0-11:G>C-11:G>C	TGCAGCCGCGTGGAGGAGGGGTTCGGCGACAAGACCGACGGGCTCATGGGGCTCGGCGGCGACGCGCAG	11:G>C
1088981|F|0-32:G>C-32:G>C	TGCAGGTTGGCGACGACGTTGACGACGGAGTTGACGTTCTGCGGCGCCTCCGCCATGGCCAGGCACGCC	32:G>C
1147382|F|0-46:A>C-46:A>C	TGCAGTCCATGGCCGGCATCGGCTACCTCTTCGGCGCCATCTCGCCAATCCTCAAGGCCGCGCTCGGCT	46:A>C
994689|F|0-40:C>G-40:C>G	TGCAGTTCGGCAACGACTACGTGCTGGGCTACTGGCCGTCCTTCCTCTTCTCGTACCTGGCGGACAGCG	40:C>G
12768433|F|0-10:T>G-10:T>G	TGCAGGAGCTTGACCTGGCCG	10:T>G
1863483|F|0-55:T>A-55:T>A	TGCAGGCGTGGTGCCCACCCAACGTGGACCCACGGACCGCCGTGCCCATGGACACTGTGACGCCGCGGG	55:T>A
3222230|F|0-45:C>A-45:C>A	TGCAGCAACACGAAGGAAATCACCATCTTGTGGCATCGAAGTCTGCTTTTGATCAGCAAAACGTGTGCA	45:C>A
5971655|F|0-13:C>G-13:C>G	TGCAGCCGCTGCTCCCGGTGTCGCAGTCCACGAGCTGCTGCTCCGACAGCGACACCAGCTCGCCGAGAT	13:C>G
1129242|F|0-8:C>G-8:C>G	TGCAGCAGCCCCCCCCGCCGCCGCAGTACTACCAGGCGCCGCCGCAGTACTACCAGCAGGGACCCCCGC	8:C>G
4261428|F|0-19:T>G-19:T>G	TGCAGCTGGCGCGGATCGGTGAGCACGAGCCCGTGCCG	19:T>G
12772090|F|0-17:C>G-17:C>G	TGCAGCAGCAGGCGGAACAGCGAAGAGTAGGGCGCGTGTGAAAGTATGACAACGGACTTCTGCTCGCCG	17:C>G
1251525|F|0-64:T>C-64:T>C	TGCAGGCCATGAGTGCCAACCAGAGCAGCATGCTGATCCTTCAGGAAACGTGCACTGATGCATCTGGGT	64:T>C
3022763|F|0-22:T>C-22:T>C	TGCAGCTAAGCCAACACACATGTTGACAGATGTCTTTCAGCCCCCG	22:T>C
1022191|F|0-47:A>C-47:A>C	TGCAGGAACAAGGTGGTGGCCTGCGCAAAGCACTTCGTCGGCGACGGAGGCACGGTGAACGGCATCAAC	47:A>C
5582362|F|0-7:A>C-7:A>C	TGCAGCTAGGGGAGACGGTGCGGCTGCTGCCG	7:A>C
1266287|F|0-37:C>G-37:C>G	TGCAGCTGATCATTCTGAAGTGGTACTCTATGCCGGCCCGCTCCGCCAGCTTCTTGAGCCGCTCCCCGA	37:C>G
1712726|F|0-10:A>G-10:A>G	TGCAGCGCTCACGGCTGCGGCGGGCCCTGACGACGTGCCAGTGATTCTTGACGGCGTTGTCAGTGCGGC	10:A>G
1012397|F|0-22:C>G-22:C>G	TGCAGCTGCCGCCGCCGCTCGCCGCCGCGGCCTACGCCGCCCTGCTCTCCCCG	22:C>G

1235291|F|0-58:T>C-58:T>C	TGCAGCAGGAGCGCGCGCTGGGCCGCCCGATGTTCCCCAAGGCCATCGGCGAGCTCCATGGCCACTCCG	58:T>C
1219843|F|0-21:T>C-21:T>C	TGCAGTGTTCTTCCTGTAGTCTACCACAGTGCCACTACCCTGCGGCTCTGCTCTCTTTAAAGAAACAGC	21:T>C
1203913|F|0-5:A>G-5:A>G	TGCAGAGGCAAGGGCATCCAGCTGTGCGCCTACTCGCCGCTGGGAGCCAAGGGCACGCACTGGGGCAGC	5:A>G
3222308|F|0-27:G>A-27:G>A	TGCAGCGACCTGCCCAGCTCTAAGTGCGCCTCGGCCAAGTGCGGCTACACGTACACCTACGGCGACTCC	27:G>A
2251487|F|0-24:G>A-24:G>A	TGCAGCATGCACTCGCAATTGTGAGGGGTCGGCCCCAGCGCTACCCACTTCAGTTGTGTCATCGGCCTC	24:G>A
2263708|F|0-8:C>G-8:C>G	TGCAGCGTCCACCGCTACTCGGACAAGCACGACTGCGGATTCGACTACAAGACCGCCGGCCAGGAGCGA	8:C>G
26673479|F|0-43:G>A-43:G>A	TGCAGCACCAGGACCATGAAGACCAGGAGCAGGAGGACGCCTCGTCCTGCGACCTCTCCTCCCCGCTGG	43:G>A
1030555|F|0-62:G>A-62:G>A	TGCAGCTCCTCCTCGTCCTCCTCCTCCTTCTTCTTGTACTTCTCCACATGGTCAACCCTGATGATCCTC	62:G>A
2255334|F|0-9:G>A-9:G>A	TGCAGAGCCGCCACGTCCATAGATGGGTATAATGGCATCGTCAGCAACCTGGCCCTTGCAGACAGGGCA	9:G>A
2249368|F|0-48:A>G-48:A>G	TGCAGGTTTTCAGCTTTAAGAGCAGCTACACATTCCTGGGCATCGCGAAAATGGCCATACTGACCGTAA	48:A>G
3025841|F|0-40:T>C-40:T>C	TGCAGCACATCGCGGCCGCCGACTACGACATGCTCCTCCTTCCCG	40:T>C
5971308|F|0-34:A>G-34:A>G	TGCAGGAGGGCGTGATCCCGGCGCTGGTGTCCCTAACGGCGAACGGCACGGGCAAGGCCAAGGACAAGG	34:A>G
4910994|F|0-19:A>C-19:A>C	TGCAGCCACAGATACTCAGATTCCCG	19:A>C
1102636|F|0-15:C>G-15:C>G	TGCAGAAAATCCACTCGTAGCAGCGCAGCAAGATTCACATCCAGCAGAACCAAGGAATCAACAAACGAG	15:C>G
5578135|F|0-18:T>G-18:T>G	TGCAGCTCCGAGGACGCCTTGGTGGCCG	18:T>G
2330907|F|0-10:A>G-10:A>G	TGCAGAGATGACGGCCACCGGCCCAAGTCGACGTCTCTCCCCTTCCACCCACATATCCCCAATCTCCCT	10:A>G
3947227|F|0-55:A>G-55:A>G	TGCAGCAGTGCTCGATCTGGCCGAGGGACGTGTCGTCGCGCCGCGACGGCGGCAGATCGTGCACCACCG	55:A>G
2251206|F|0-64:T>C-64:T>C	TGCAGCAGCCTGCTCACCGTCGACATGAGGGGCCTCCTCTTCCCGACCACGACCACCACTGTCGTCGCC	64:T>C
11914930|F|0-7:G>C-7:G>C	TGCAGGTGCTGGAGCTGGCCG	7:G>C
4993479|F|0-18:C>T-18:C>T	TGCAGCTCCTCCTTGCCGCGCGGCCG	18:C>T
3025302|F|0-36:T>G-36:T>G	TGCAGCCAGGTGCACATGGCCATGTACATCTCGCTGTCCGCCGCCG	36:T>G
1019775|F|0-44:T>G-44:T>G	TGCAGCACCTGGTGGAGGTGTCGGTGGCCAAGCGTGGGCTGGCGTTGAGGACGTGGTGGAACGAGCAGA	44:T>G
4910795|F|0-5:T>A-5:T>A	TGCAGTGATCGTGCCAGCGCCACCTGCCGCCCCAGGGAAGAAGCCCTGTACCACGCAAACCCCACGGTT	5:T>A
1222137|F|0-7:G>C-7:G>C	TGCAGGTGCGCGACGGCGTGCCGCTCATCGGGTTCATCGGGCGGCTGGACCACCAGAAGGGCGTGGACA	7:G>C
26673916|F|0-7:A>G-7:A>G	TGCAGGGAGAGGTCTGCCCCTTCGGCCTCGCGCCCGTCACCTCCACCGCCATACAGATGGTCTTCGGCG	7:A>G
1129639|F|0-37:A>G-37:A>G	TGCAGGTGCCCGGAGGGTGCGCGAGCGCGTGCGGCAAATTCGGCGGCGACACCTATTGCTGCCG	37:A>G
6042911|F|0-11:T>C-11:T>C	TGCAGCAGCAGTCTGTCCTCCGCCGACGACTCGAACCCGTGCAGGAACGACATGTCCGCGCCGCCGCCG	11:T>C
2255584|F|0-23:C>T-23:C>T	TGCAGGATGTACGGCTGCCCCGTCCTGTTCCCCAAGAACTCGAAGTCGATCTCGTCGTGCTCCG	23:C>T
1197618|F|0-26:T>G-26:T>G	TGCAGCGGGAGCCTCTCGCCGCCGACTCCCAGCCCCGTCAAGCCCACGTAGTAGAAGCTCGGCTCCTGC	26:T>G
1002617|F|0-68:G>C-68:G>C	TGCAGCGCCGTGGCCAGCAGCTTGTTGGGGTACTCCTTGAGGAGGCGCGCCTGGAGGACGATCCAGAGG	68:G>C
1106996|F|0-17:T>C-17:T>C	TGCAGCCTCTTCCTTCGTTCCATCGGCCTGCGAGAGTACTCCAGGTACCTCGCCTTCAACCACCCATGC	17:T>C
1230258|F|0-19:C>G-19:C>G	TGCAGGCGGCGGTGGAGCGCGCGGCGAGGCTGCTGGCGCCCTACGGGGCCAGCATCGTGGAGTACACGA	19:C>G
2258953|F|0-14:G>C-14:G>C	TGCAGCACGACGGCGAGCGCCACCTTGGCCTCGATCAGCGCCAGGTTCTGCCCGATGCACACCCGCTCG	14:G>C
5970818|F|0-13:T>C-13:T>C	TGCAGACATTTGGTGTGCAGGGCCG	13:T>C
1153285|F|0-32:T>G-32:T>G	TGCAGCAGCCTGGTGAGGCCGGGGTTCATGTCTGAGGGGTTCGGGGGCAGGCGGTCGGTGAAAGAGGAG	32:T>G
5582052|F|0-19:T>C-19:T>C	TGCAGGGGCACTGCCCCACTGCCG	19:T>C
7931190|F|0-20:C>G-20:C>G	TGCAGCGGCGCGCACGGGACCCCG	20:C>G
2251280|F|0-29:T>C-29:T>C	TGCAGGTCCCGATCTCCATCCTCACTCCCTTCCTCTTCCTCGACATTGTTGTGCTCCTCAGCCTCGCGA	29:T>C
4993560|F|0-26:T>G-26:T>G	TGCAGGCCGTCGCCGTGCTTGACGAATGAGTCGAACTTGAGTGGCAGCCCCTCGCCG	26:T>G
17568894|F|0-9:A>G-9:A>G	TGCAGCCCCAAGATGCCGTTCTCCCTCCCG	9:A>G
5369547|F|0-13:T>C-13:T>C	TGCAGGATGGAGGTGAGAACGGATCGCGCCGACCGAGGGGCCGCCCG	13:T>C
2248291|F|0-7:A>G-7:A>G	TGCAGAAAATTGTGATCAGAGCCGATCTCGTCGGCAAGAAGTGCACGAGCGGGATCCTGTCAATCGTTT	7:A>G
1229000|F|0-5:C>G-5:C>G	TGCAGCCGCCACCCCAGCCGCTCCGACAGCTCCTGCATCCGCTGCTTCTGCTCCGCGGTGAACTTGGTG	5:C>G
2266496|F|0-14:G>C-14:G>C	TGCAGCGCCGAGGTGGACCGCCTCACCGACGCCGTCGACGCGCTCGAGCGCCTCGCCG	14:G>C
5366956|F|0-22:A>G-22:A>G	TGCAGCCACCCGTAGAAGAAGAACGCCG	22:A>G
4910712|F|0-48:C>G-48:C>G	TGCAGAGCGCGGCGGCGCCAAGCGCGCTGCGGGTGTCGAGACGGCCCACGATCTGGAGGATCAGGTCGT	48:C>G
5971082|F|0-5:C>G-5:C>G	TGCAGCGCGCCCAGCACGGCGTCCCG	5:C>G
1110223|F|0-28:A>G-28:A>G	TGCAGCCGAGACCGCACCGCCTCGCCCAAAGACACGTTCGGCGCCGTGTCCGCGTTCTTCAGCCACGGG	28:A>G
1019808|F|0-23:C>G-23:C>G	TGCAGGTACGCCGAGTCCTTGCCCAGGCAGATCCGCGGCCCCGCCTGGAACGCCGTGAACTTGAACGGC	23:C>G
1097275|F|0-7:G>C-7:G>C	TGCAGCCGTTCCTCGGCGGCGACACGGCCATCCTCCTGCGCTTCTCGTCGCAGGTCGGGGAGTGGGTCA	7:G>C
5324668|F|0-14:G>A-14:G>A	TGCAGATCGACGGCGCGATCGTGGCGCCGAGCGAGCCCACGACGTGGCCG	14:G>A
1238422|F|0-63:C>T-63:C>T	TGCAGCACGGCGCGGATGTTGCTGACCGTGAACACGATCTCCGCCACCTCTCGCCGCAGCTGTCTCCCT	63:C>T
32025555|F|0-20:C>T-20:C>T	TGCAGCGTCGATGGCAACAGCGGCTGCAATGGCGGCTTGATGGACTACGCCTTCTCCTACATCAAATCC	20:C>T
2281032|F|0-44:T>C-44:T>C	TGCAGGAGCCACTCGATGGTCTCGCCGTCCGTCTTGTGCCCCAGTTCCCGCGTCAGCTGGAACACCCGC	44:T>C
1153596|F|0-31:A>G-31:A>G	TGCAGCGCGGCTTGCCGCACTTGCGCGCGTGATGGCCCAGTAGCGCCCAGCCGAGCGCGGCGCCGAGAT	31:A>G
996013|F|0-46:A>C-46:A>C	TGCAGCAAAACAAAAACAAAGGGCAGCAGCTGCCGAGCGAGCAAACAACCACCAGCTCACTCGCGCACG	46:A>C
1717007|F|0-49:A>G-49:A>G	TGCAGGCGCATGCGGATGCGGGGGATCTCGTCGCGCGTCGGGTTGATCAAGCCGGAGTTGAAGAGCGTC	49:A>G
3030530|F|0-6:G>A-6:G>A	TGCAGCGCGAGCTCGTCCAGCCCGCCCCTGATCATGGCCG	6:G>A
6046226|F|0-49:C>T-49:C>T	TGCAGCACATAAGTACTCGCACTAGTGCCGCCATGCACGCTAGCCTGGACTATTCAGGCTTGGGAGCAC	49:C>T

3064384|F|0-32:A>G-32:A>G	TGCAGAAAGGCCAGGCCTTTGCTCGTGGAGAGATACCG	32:A>G
4993226|F|0-59:G>C-59:G>C	TGCAGGAACAAGGTGGTGGCCTGCGCGAAGCACTTCGTCGGCGACGGCGGCACGGTGAAGGGCATCAAC	59:G>C
7340702|F|0-38:C>G-38:C>G	TGCAGCCAGGACACGAGCGTCCCGGCGACGAAGATGCCCAGGTTGATGGCGCCGAAGAACCAGGTGAAG	38:C>G
1207849|F|0-35:A>G-35:A>G	TGCAGCCGCTCCGTGGGCGTGGCGAAGCTCTCCTCAACGTCACGCAGCACCGCCATGGCCCG	35:A>G
1009880|F|0-55:T>C-55:T>C	TGCAGCGCGCCGAGGCAAACCGAGCACGTCGCCGCCTCCTCCCCTCCGCCCGTCATGTTGTGCTTCACC	55:T>C
12770616|F|0-12:T>C-12:T>C	TGCAGGCACTTGTAATTACTCTGTGCCG	12:T>C
1165296|F|0-17:A>G-17:A>G	TGCAGGTGGCCCCCCACAGCGCGCACGCGCTCGTGGTCTCCACGGAGACCATCACGCCCAACTACTACC	17:A>G
1024890|F|0-13:G>T-13:G>T	TGCAGAGTAGATGGGACCTTGGGAGAGGAGGCGGCTGACCGCCTCGTCAGGGTCCATGCCGCAGTCGCG	13:G>T
5324067|F|0-21:C>T-21:C>T	TGCAGGCCGCCCAGCACGGCGCCCCG	21:C>T
1139869|F|0-29:T>G-29:T>G	TGCAGCCGCGCGCCGCCGCCGCCGCCACCTCCGCCGTGTCGCTTCTTCTGGATCTTGCCGCCG	29:T>G
2338492|F|0-49:G>C-49:G>C	TGCAGCTCCACTGGAAGGAGCTGGCCCGCGACATCGAGACCGGCACGCTGAGCGCCAAGGTGGCGGAGC	49:G>C
2291466|F|0-18:G>A-18:G>A	TGCAGGGTCAGTCTCTATGACTTTGAACTCCACGCTTGTCATCCCGCCTCTCACATGGAAAAGGTCCCC	18:G>A
1213121|F|0-32:G>C-32:G>C	TGCAGGCAGATGGGCTGAGGCAGCAGACCCTGGACAAGCTGCACGGCATGCTGACGGTCCGACAGGCCG	32:G>C
991630|F|0-16:T>C-16:T>C	TGCAGGAGCTCCTCAGTGAGAACAGGAGCCTGGACTGGGACACACGGTACAAGATCGCGGTCGGGGCGG	16:T>C
1139866|F|0-8:A>G-8:A>G	TGCAGCGCACCGAGGCACACCGAGCACGTCGCCGCCTCCTCCCCTCCGCCCGTCACGTTGTGCTTCACC	8:A>G
1083634|F|0-35:T>C-35:T>C	TGCAGCGAGGACTGCCGCTACGAGCAGATGCACTTTGACGCCGCCATGGCCAGGCAGCAGGCCAGCGCC	35:T>C
1096953|F|0-11:G>C-11:G>C	TGCAGGAACTCGCGGGCCATGGCGGGGGAGGAGACGACCACCGTGGTGACCGCACCCAGGCGGATGGAC	11:G>C
7338976|F|0-38:A>C-38:A>C	TGCAGCTCCTCCACCTCGTCGAGCGCTTCCCGTCCGAGACGTGCGCCGAAGCACATGTACAGGAGCAGC	38:A>C
12692206|F|0-26:C>T-26:C>T	TGCAGCTCCTCCACCTCGTCGAGCGCCTCCCGTCCGAGCCGTGCGCCGAAGCACATGTACACGAGCAGC	26:C>T
2249760|F|0-32:T>C-32:T>C	TGCAGGTGAGGCCATGCCGGGTCCATGACAGGTTCTTCCTCCTCCAAATCAAATGACTCGATTACCTTG	32:T>C
2307262|F|0-10:A>G-10:A>G	TGCAGGGGTCAGCGGACACCTCGATCCCGGCGTTCGCGGGCTCCGCCCCGCTGGCGCTGGTCTCCATGG	10:A>G
3958358|F|0-13:A>C-13:A>C	TGCAGCACCGCCTAAAGCCCACCGCCGTGCTCTGGGGCTGGCCG	13:A>C
4001992|F|0-23:A>G-23:A>G	TGCAGCAGCGCGGTGAGGTAGACAAGCTCGTCCTTGGCGCCAATGCCG	23:A>G
3935321|F|0-29:A>G-29:A>G	TGCAGCAGCGCGGTGAGGTAGACGAGCTCATCCTTGGCGCCATTGCCG	29:A>G
11314899|F|0-14:A>G-14:A>G	TGCAGTCGAGGGCCACGGGGTTCGGCTCCG	14:A>G
1004595|F|0-44:A>C-44:A>C	TGCAGGCCAGGAAGCTCTCACTCGGAGGAGGCATCGGCGGCAAGATCAAGGCTATAGGGGAGATGGGGG	44:A>C
9766799|F|0-11:G>C-11:G>C	TGCAGCATCCCGGCCGCCAGCTCCG	11:G>C
2248992|F|0-23:T>C-23:T>C	TGCAGACATGCCTCTTCAGCACATAGACTTCGGAATTCAGCAGTCATTTCATGGGCTAAATTGGACCAG	23:T>C
986053|F|0-14:T>C-14:T>C	TGCAGATCCTAGAGTCCCATTGATTAAAAAAATAAACCAGTTGGCACGCTAAAGATCTTCCCAGATCTT	14:T>C
1865036|F|0-14:T>C-14:T>C	TGCAGGTGCACCATTTTGGCGTACACGCCGCCTTCGCCGCCGTCCATGGCGACGAGCTCGTCGTGCGTG	14:T>C
983419|F|0-59:C>G-59:C>G	TGCAGGTCCGTGGACAGGATGAAGCTCATCCACGACGCCTGCGGCGAAGACCAGGCGGCCCTCTGGGGC	59:C>G
3957638|F|0-17:T>G-17:T>G	TGCAGGGCGACGCCGACTGCGGTGGGGCGCTCGTCGCCG	17:T>G
3955343|F|0-6:A>G-6:A>G	TGCAGCAGGGCGCCGACAGCGGCAACACAGGCGCCACCG	6:A>G
4262071|F|0-50:A>G-50:A>G	TGCAGCATGTCGTTGTGGTGGCCGCGGGAGTTGATGGCCTGGTAGATGACATCCGATTCCTTGAACGCG	50:A>G
1083258|F|0-10:T>C-10:T>C	TGCAGCAGAGTACCATCAAGAAGTGGGCCGGCAAGAAGGAGAACGTCGCCGACGCGCAGGCCACCTTCC	10:T>C
1229142|F|0-46:T>G-46:T>G	TGCAGGCTCGGGTCGCGGATCTCCGCGGCGTACTTGCCCCACGGCCTTTGCCGCACGCCACGGAACCGC	46:T>G
1022613|F|0-68:T>C-68:T>C	TGCAGCTCCATCGTCTCCATCCTCCTCGCCTCCGTCCGCCAGTCCGTGCTCGTCATCTCCACCGACCCT	68:T>C
5970872|F|0-14:G>C-14:G>C	TGCAGCTGGAGCTCGACCAGCTCCCG	14:G>C
3022874|F|0-36:C>T-36:C>T	TGCAGCTAAGCCAACACACATGCTGACAGATGTCTTCCAGCTCCCG	36:C>T
1140966|F|0-32:T>C-32:T>C	TGCAGAGACGGCCCCGCCCTCGCCCTCGCCCTTTTCAACCGTATCTTCCGAGAAGAAGCCGGCATGCGG	32:T>C
2252950|F|0-65:A>G-65:A>G	TGCAGGTACTTGATGTCGAACGTCGGCACGACGATGTTGGTGACGGTGTCGGCGACGGTCACGTCATTG	65:A>G
3942136|F|0-14:G>C-14:G>C	TGCAGGAGCCTCGTGAGGCTGTCGCAGATGGTGGAGGAGCCGATCCCG	14:G>C
1023816|F|0-46:A>G-46:A>G	TGCAGGAGGCCGTCTGGGACGCTGACATTGTCATCAACGGCCTGCCATCCACGGAGACGAGGGTTGTGT	46:A>G
3222525|F|0-8:A>G-8:A>G	TGCAGCAGATGGCTGGTGGCCTTGTCCACCCGCGACGCCGCCG	8:A>G
10984025|F|0-61:A>G-61:A>G	TGCAGGCGTGGTGCCCACCCAACGTGGACCCACGGACCGCCGTGCCCATGGACACAGTGACACCGCGGG	61:A>G
1209015|F|0-65:T>G-65:T>G	TGCAGGAACCAAGCTAAGCTCCAGCAGAACCTCATGTACCTCGCCGCCATCGCCGACAGCCAGCCTCCG	65:T>G
1028148|F|0-22:C>T-22:C>T	TGCAGCCAGCGGTCGGCAGCGGCGCGGATGGTGTCGTCGGGGTGGTGGTACAGCGCCGCGAGCGCCTCC	22:C>T
4910961|F|0-17:A>G-17:A>G	TGCAGCGCGGCGTCGACAGAGGCGACGTAGCTGGAGACGGTGCTCTGGGCGGCGAGGCGGGGGAAGTGG	17:A>G
10984281|F|0-6:T>C-6:T>C	TGCAGGTGCTCTTCAGCGTGGACATGCTGGTGCTGTTCGTGGCCACCATCTGCGGCGTGGGCGGCACGC	6:T>C
14385515|F|0-30:T>G-30:T>G	TGCAGGAGGCGGCGATGCCGTCGGCAGTGATGGAGGATCGTCGGATCTGATCACCGATGATCAACCGAG	30:T>G
1222924|F|0-25:G>C-25:G>C	TGCAGGAGGAGCTCGACGCCATCCCGAAGGCCAAGGTATCGTTGCCAGTTCTCGTGATCTATTGGGTTG	25:G>C
2242200|F|0-35:T>C-35:T>C	TGCAGGGCCGAGGGCTTCGACAACGGCCATTGTGATAACCTCTTTACATGCATCTGCTACAGAAACTGC	35:T>C
3946842|F|0-16:C>T-16:C>T	TGCAGTTACAATGGTACTGGTACCTGTGAGATCATTCCCGCAGCACCG	16:C>T
4990814|F|0-16:A>C-16:A>C	TGCAGTCCCACTGGCCAGCAGACCTGCGAGACGGTCCGTAGCTCCAACCTGAGCCTGCTCCTCCTGGGC	16:A>C
12775627|F|0-5:A>T-5:A>T	TGCAGAGCTGGGAGACCGACGCCGTCGTACCCGTCGGCGGCCG	5:A>T
6030685|F|0-10:A>C-10:A>C	TGCAGGAAATACGCCATCGCTCACCCG	10:A>C
12779825|F|0-17:C>G-17:C>G	TGCAGCGCGTTGTGGCTCAGGTCCAGGATGCTGAGCTGCTGGAACCTGAGGTTGGGCACCTGGCCGAGA	17:C>G
14385312|F|0-23:T>A-23:T>A	TGCAGCAGCTCGGCATCAGTGGGTACCTCGATCGCCG	23:T>A
7491751|F|0-13:A>G-13:A>G	TGCAGGTGAAGGTACTGGAGCTGTCGTCGTAGGCGTAGCTGTAGGCGCGCGGGCACGCCATCTTGAAGA	13:A>G

<table>
<tr><td>7157306|F|0-5:G>C-5:G>C</td><td>TGCAGGAGGAGCCCGAGCTTGCGGTCTTGGGGCGGGTGCCG</td><td>5:G>C</td></tr>
<tr><td>14385455|F|0-25:G>T-25:G>T</td><td>TGCAGCCTGCTGTTGTTCTCCCCACGGAAGATCCG</td><td>25:G>T</td></tr>
<tr><td>16796135|F|0-6:G>A-6:G>A</td><td>TGCAGCGGCGTAGTAGATCGCTCCG</td><td>6:G>A</td></tr>
<tr><td>2274655|F|0-22:T>G-22:T>G</td><td>TGCAGAGCTTCGCCGCCAAGGGTCTCGACGTCAACGACCTCGTCGCGCTCTCCG</td><td>22:T>G</td></tr>
<tr><td>100467188|F|0-51:T>C-51:T>C</td><td>TGCAGAGGATAGACGAGGAGCTGCTCAGGCGACCGCTTGACGGCGTCACGCTGGGACAGTGGTGCGCCG</td><td>51:T>C</td></tr>
<tr><td>6048513|F|0-40:T>C-40:T>C</td><td>TGCAGCACATATACCGCGAGGGCAACCAGGTCGCCCACGTTCTGTGCAAAGAAGCATATCAGCGGCCGA</td><td>40:T>C</td></tr>
<tr><td>7173830|F|0-10:G>T-10:G>T</td><td>TGCAGTGAGTGGATTTTGCTCCATGTCCCG</td><td>10:G>T</td></tr>
<tr><td>12780495|F|0-8:C>G-8:C>G</td><td>TGCAGTTTCGCTTTGTTCTGGCCCG</td><td>8:C>G</td></tr>
<tr><td>100481260|F|0-20:A>G-20:A>G</td><td>TGCAGGTACCGCATGAACGGAAGCTTGGCGTCGTCGGGGAAGGGGAGGCCCCG</td><td>20:A>G</td></tr>
<tr><td>100544766|F|0-24:T>G-24:T>G</td><td>TGCAGGCAACAGAGGTGATGTCAGTCCCGTAGGGGCGAGGCGTGTACATGCTCCCAGCCATGTCTGGCC</td><td>24:T>G</td></tr>
<tr><td>100547455|F|0-21:A>G-21:A>G</td><td>TGCAGACGCGTCGTTCAGGGGAAACCTGCCGCGGCAGGTGCGCGCGCCG</td><td>21:A>G</td></tr>
<tr><td>7172444|F|0-26:G>C-26:G>C</td><td>TGCAGGTGGGCAACGACCTCAACCACGGCTTCCTGGCGCGGCTGTCGGCGCGGTACGGCCCCGTGTTCC</td><td>26:G>C</td></tr>
<tr><td>7909118|F|0-12:A>G-12:A>G</td><td>TGCAGTGATATCAGTGCAAAAGTGGCCG</td><td>12:A>G</td></tr>
<tr><td>100450299|F|0-54:T>G-54:T>G</td><td>TGCAGCTCCTTGGTGGAGATCATGCGGACGTCGGCGGCGTCCTTGCTGGCGGCCTGCATTGCGTCGGCT</td><td>54:T>G</td></tr>
<tr><td>15328232|F|0-23:G>C-23:G>C</td><td>TGCAGCGCCACGTACGCCTTCTCGAGCCGCGAGTTGTTGGTCGCCG</td><td>23:G>C</td></tr>
<tr><td>100483563|F|0-10:G>C-10:G>C</td><td>TGCAGCGCACGCAGACGCCG</td><td>10:G>C</td></tr>
<tr><td>7908875|F|0-18:A>G-18:A>G</td><td>TGCAGCGTGATCAAGACTACTGCTCCCAAGGCCAAGGCCACAAGGAAGACCCG</td><td>18:A>G</td></tr>
<tr><td>4396514|F|0-27:G>C-27:G>C</td><td>TGCAGTGACCTCTCATCTTTTGAGAGGGCCCG</td><td>27:G>C</td></tr>
<tr><td>7338786|F|0-8:G>A-8:G>A</td><td>TGCAGGTGGGCGGCGCCGCCG</td><td>8:G>A</td></tr>
<tr><td>2241753|F|0-35:C>A-35:C>A</td><td>TGCAGGTTCGAATCCTGTCCCCGCCTAATCATAGTCAAAATCTGAGTTTGATCCTGGCTCAGAAGGAAC</td><td>35:C>A</td></tr>
<tr><td>1027619|F|0-52:G>A-52:G>A</td><td>TGCAGTCGCTCAACCGCAAGCTCACCGCCATGAACAAGCTGCTCATGGAGGAGAACGACCGCCTCCAGA</td><td>52:G>A</td></tr>
<tr><td>100303398|F|0-40:A>C-40:A>C</td><td>TGCAGGCTGCGCTGGTTGAGGCGCTTGACGGCGAGGACGAAGCGGCCGCCG</td><td>40:A>C</td></tr>
<tr><td>1319312|F|0-60:C>A-60:C>A</td><td>TGCAGCTGCTGGTACTTTATCTCTGTCGCTTTCAACTCCCACTCCTTCTCCCTTTGGTTTCTATAGGAA</td><td>60:C>A</td></tr>
<tr><td>100350396|F|0-19:T>C-19:T>C</td><td>TGCAGCACTGCAACAACAATAACAACGCCG</td><td>19:T>C</td></tr>
<tr><td>7333613|F|0-21:A>G-21:A>G</td><td>TGCAGCGAATCTGCCACTAGCACATACCTTCCCG</td><td>21:A>G</td></tr>
<tr><td>12855034|F|0-11:C>T-11:C>T</td><td>TGCAGCTTGAACACGGCCTTGGCCTTGCCG</td><td>11:C>T</td></tr>
<tr><td>1201162|F|0-58:G>T-58:G>T</td><td>TGCAGCGGGAGCTCAAGACGGTGAACGCCGACCTGGCCTTCTCCAAGGAGCGGTGCGCGCAGCTGGAGG</td><td>58:G>T</td></tr>
<tr><td>3934969|F|0-38:C>T-38:C>T</td><td>TGCAGCCCCACCAGCAGCCGCAGCAGCTGCCTCGGCCGCCGCCG</td><td>38:C>T</td></tr>
<tr><td>100387846|F|0-10:T>C-10:T>C</td><td>TGCAGGAGGTTGTCTCCCTCGCCGCCCCG</td><td>10:T>C</td></tr>
<tr><td>3948822|F|0-25:C>T-25:C>T</td><td>TGCAGGAGGTCGTCTCCCTCGCCGCCCCG</td><td>25:C>T</td></tr>
<tr><td>100388483|F|0-5:T>G-5:T>G</td><td>TGCAGTGGAATCGCCCATGGAGGGCTCCCG</td><td>5:T>G</td></tr>
<tr><td>5358509|F|0-8:A>T-8:A>T</td><td>TGCAGTAGAGGCGGCAAAGGGTGGACGGATCCG</td><td>8:A>T</td></tr>
<tr><td>4394534|F|0-17:G>T-17:G>T</td><td>TGCAGCGGCATATAGCAGCCG</td><td>17:G>T</td></tr>
<tr><td>100395152|F|0-64:T>C-64:T>C</td><td>TGCAGCGACGCCCTGATCAGCCTGGCGGGGTGCCTGAGCTACGTGCAGGAGGGGAGCACGGTGGTGACG</td><td>64:T>C</td></tr>
<tr><td>1080531|F|0-7:C>T-7:C>T</td><td>TGCAGACCTTCTTCAACTGGTACTACACCGTGCTGGGGATCTCCCTCGTCGTTGCGGTCCTCGTCATCG</td><td>7:C>T</td></tr>
<tr><td>1714231|F|0-31:G>A-31:G>A</td><td>TGCAGATCTCGACGCCATGAAGGCCCTGCTCGACAAGCTCGTGGTGCTCAAGCTCAACGGAGGCCTCGG</td><td>31:G>A</td></tr>
<tr><td>1023881|F|0-51:C>G-51:C>G</td><td>TGCAGCACGTCGCTGTCGGCATTGGCTAAATAGCGCAGGGAGGAGGAGGGGCCGCGGCTTGCGGGGGAA</td><td>51:C>G</td></tr>
<tr><td>1040984|F|0-43:C>T-43:C>T</td><td>TGCAGCCACAACGACGTGCTAACCCACACGCATCACCAAAGCGCCTATATAAACATTAGCTCCG</td><td>43:C>T</td></tr>
<tr><td>100398878|F|0-5:T>C-5:T>C</td><td>TGCAGTGAGGCTCTCTGGACGAGGCCTCGCGTGCCGCGGGAGTGGGCGCCGAGCAGGTTGGGGTTGGCC</td><td>5:T>C</td></tr>
<tr><td>2250536|F|0-41:T>C-41:T>C</td><td>TGCAGGACCAGTCGAAGGACGCCACGCACACGTTCCCGGCCTTCTTCTTCCACTCGCAGTCTGCAAAAC</td><td>41:T>C</td></tr>
<tr><td>100400786|F|0-41:G>T-41:G>T</td><td>TGCAGGAGGAGCTCGACGCCATCCCCAAGGCCAAGGTATCGGTGCCAGTTCTCGTGATCTATTGGGTTG</td><td>41:G>T</td></tr>
<tr><td>2289355|F|0-34:C>T-34:C>T</td><td>TGCAGTACTGGCCGTGGAACCGACTCGAATTCGACCCGCAGCCTGTCGATGCTGGCAAACAGATTTTTC</td><td>34:C>T</td></tr>
<tr><td>1028815|F|0-55:C>A-55:C>A</td><td>TGCAGTAGCGCATGCGTGGCTCCAATGCGTACTTGTCTCCGAGCATTTCCTTCTCCTGCGCTGCGCGCA</td><td>55:C>A</td></tr>
<tr><td>991902|F|0-27:G>C-27:G>C</td><td>TGCAGACGACGAAGCCGCACCTCCTCCGCGAGCACCAGGTGCATCCGCAGCCG</td><td>27:G>C</td></tr>
<tr><td>7153469|F|0-36:G>C-36:G>C</td><td>TGCAGAGCCCGAGAGCTGATTGGAAGCGTGGATCCAGTGGACCGCCG</td><td>36:G>C</td></tr>
<tr><td>3959124|F|0-12:G>A-12:G>A</td><td>TGCAGCAGGTGCGCCGACGAGCAGAGCACGTACCG</td><td>12:G>A</td></tr>
<tr><td>1063684|F|0-10:T>C-10:T>C</td><td>TGCAGCGTAGTTTAGGTGTACATGAGCTATGCTTTTCATGGTATTTGCTGCACTGCCG</td><td>10:T>C</td></tr>
<tr><td>100418166|F|0-14:C>G-14:C>G</td><td>TGCAGGTGGGCATTCGTGAAGGACCG</td><td>14:C>G</td></tr>
<tr><td>3945150|F|0-16:C>A-16:C>A</td><td>TGCAGCCGCGCCACGGCGCCG</td><td>16:C>A</td></tr>
<tr><td>2338810|F|0-20:C>G-20:C>G</td><td>TGCAGCGCCACCAGCTGCTGCTCCAGCTGCTTCACGTAGTCGATGGCTCCCCCCACCACCGTCGCTTGG</td><td>20:C>G</td></tr>
<tr><td>1213145|F|0-58:G>A-58:G>A</td><td>TGCAGGAGCACTTCTCCAACTTCGGCGAGGTCTCCCAGGCCGCCGTCATGCGCGACAAGCTCACCGAGA</td><td>58:G>A</td></tr>
<tr><td>2250844|F|0-34:G>T-34:G>T</td><td>TGCAGGCGCCGAGCCAGTCGATGGGGATCGGGACGACCAACAGCCGCAACCTGAGCCAGATGCTGAGCC</td><td>34:G>T</td></tr>
<tr><td>100431167|F|0-25:A>G-25:A>G</td><td>TGCAGTTCCTCTCGATGAAGCTCTCAGCGGTCAGTCCCGAGCTGAACTGCGATCTCGATCTCCAAGACG</td><td>25:A>G</td></tr>
<tr><td>3024371|F|0-11:C>T-11:C>T</td><td>TGCAGAAAGAACGATGTGAGGAAGGTGGTGTGCATCCCG</td><td>11:C>T</td></tr>
<tr><td>5357283|F|0-5:A>C-5:A>C</td><td>TGCAGACGACTTGCCAGCAAGGAGGCCG</td><td>5:A>C</td></tr>
<tr><td>4261355|F|0-12:A>G-12:A>G</td><td>TGCAGAGCACATACCCTGTAAGTGCAATATTATTACCG</td><td>12:A>G</td></tr>
<tr><td>4261355|F|0-23:G>A-23:G>A</td><td>TGCAGAGCACATACCCTGTAAGTGCAATATTATTACCG</td><td>23:G>A</td></tr>
<tr><td>1023404|F|0-39:G>T-39:G>T</td><td>TGCAGAGGGATGGGAGCAGCAGGTGGCGACGGCAAAGGTGTGCGGGGATGACCG</td><td>39:G>T</td></tr>
<tr><td>100439569|F|0-47:T>A-47:T>A</td><td>TGCAGCAGCACCTGGCCGACCCATGGCACACCACCATCATCCAGACCTTCTCCG</td><td>47:T>A</td></tr>
</table>

<table>
<tr><td>100440109|F|0-7:A>G-7:A>G</td><td>TGCAGCCACACCGCGCGCGGCAGCGCCG</td><td>7:A>G</td></tr>
<tr><td>100441225|F|0-17:A>G-17:A>G</td><td>TGCAGCTTGGACATCGAATATGATCAGGGCGAGGCCG</td><td>17:A>G</td></tr>
<tr><td>4262411|F|0-24:C>A-24:C>A</td><td>TGCAGCTTGGACATCGAGTATGATCAGGGCGAGGCCG</td><td>24:C>A</td></tr>
<tr><td>4261199|F|0-23:G>T-23:G>T</td><td>TGCAGGAGTGCCAGGGCTTGGTGGCGGCCATGGAGCAGGCCG</td><td>23:G>T</td></tr>
<tr><td>4734103|F|0-17:T>G-17:T>G</td><td>TGCAGGAGTTCATCCTGTCGTCCAACCAGCTGTCGGGCGCGATCCCG</td><td>17:T>G</td></tr>
<tr><td>4734103|F|0-37:C>T-37:C>T</td><td>TGCAGGAGTTCATCCTGTCGTCCAACCAGCTGTCGGGCGCGATCCCG</td><td>37:C>T</td></tr>
<tr><td>4538642|F|0-8:T>A-8:T>A</td><td>TGCAGGTGTCCACGTCGCTCCG</td><td>8:T>A</td></tr>
<tr><td>986912|F|0-46:C>T-46:C>T</td><td>TGCAGAAACTCACCTTGGGTGATCTTGTTGAGGCCACCAATGGCTTCCACAATGAGAGCTTGATTGGGT</td><td>46:C>T</td></tr>
<tr><td>996653|F|0-7:G>A-7:G>A</td><td>TGCAGAAGGAACTGAACAGATACATCCCTACTGCCGCTGCATTTGGTGGAGTGTGCATCGGCGCATTGA</td><td>7:G>A</td></tr>
<tr><td>2249272|F|0-15:C>T-15:C>T</td><td>TGCAGCCGTGCTCCTCCTCGCCGCAGCCTACTCCCTCGACTCGGCCTACCAGGTCTTCGCCTCCATTCT</td><td>15:C>T</td></tr>
<tr><td>2249272|F|0-60:C>T-60:C>T</td><td>TGCAGCCGTGCTCCTCCTCGCCGCAGCCTACTCCCTCGACTCGGCCTACCAGGTCTTCGCCTCCATTCT</td><td>60:C>T</td></tr>
<tr><td>1001439|F|0-21:G>A-21:G>A</td><td>TGCAGCTCACCGACCTCCAGCGGCAGGGCCTCGGCGGCGAGGAGAGCGGCGGCACGGGGTTCTCGCCGC</td><td>21:G>A</td></tr>
<tr><td>7168797|F|0-40:G>C-40:G>C</td><td>TGCAGCTCCACTGGAAGGAGCTGGCCCGCGACATCGAGACGGGCACGCTCAGCGCCAAGGTGGCGGAGC</td><td>40:G>C</td></tr>
<tr><td>2278631|F|0-55:G>T-55:G>T</td><td>TGCAGCTCCCCTCCGACTGCAACGTCGACGGCATCCGCGCCAAGTTCGAGAACGAGGCGCTCACCATCA</td><td>55:G>T</td></tr>
<tr><td>2242355|F|0-17:G>A-17:G>A</td><td>TGCAGCTGCACATTACGGTCCTTGAACGCTGAATCATGGCCCAAGAAGTCGAAATCCTGACATGAAGCA</td><td>17:G>A</td></tr>
<tr><td>100452202|F|0-22:A>T-22:A>T</td><td>TGCAGGGCAGAGAATCCTGTGCAGTTTTTTTACCATCGCCAGTCACCATGCCCGCTTTTTACAGTCTTT</td><td>22:A>T</td></tr>
<tr><td>12419879|F|0-47:G>A-47:G>A</td><td>TGCAGGGGGCGCTCGCGGAACGGCGGGCGCGTGGTGAGCTCGGGCCTGTCGATGGAGCGCCTGCTGGAG</td><td>47:G>A</td></tr>
<tr><td>1001758|F|0-22:A>G-22:A>G</td><td>TGCAGATGAGCAGGGCGAGGGAACTCTTGGAGGAGATGACGACGGACGCCG</td><td>22:A>G</td></tr>
<tr><td>1019670|F|0-16:T>C-16:T>C</td><td>TGCAGCAAGGACATGCTCACCCTTTTCTGATATGGAATACTCAACAAGTGGGTCTGCCCG</td><td>16:T>C</td></tr>
<tr><td>986811|F|0-6:A>T-6:A>T</td><td>TGCAGCACCGCCGTGTGCTCCTTCTTCTTCTCGCGGTACTTGCGCACGGCCGCCCG</td><td>6:A>T</td></tr>
<tr><td>986811|F|0-32:G>T-32:G>T</td><td>TGCAGCACCGCCGTGTGCTCCTTCTTCTTCTCGCGGTACTTGCGCACGGCCGCCCG</td><td>32:G>T</td></tr>
<tr><td>3948986|F|0-5:C>T-5:C>T</td><td>TGCAGCAGCAGAGAAAATCTCTCCTCCATGTCCG</td><td>5:C>T</td></tr>
<tr><td>3940734|F|0-8:C>G-8:C>G</td><td>TGCAGCAGCAGAGAAAATGTCTCCTCCATGTCCG</td><td>8:C>G</td></tr>
<tr><td>3022240|F|0-8:T>A-8:T>A</td><td>TGCAGTGGTTTCCCTGTTTGGCTCAAGTATGTCCCCG</td><td>8:T>A</td></tr>
<tr><td>3954390|F|0-5:G>A-5:G>A</td><td>TGCAGGATGGCGCGCTCCAGCGCGGCGCCGACGAGGCCG</td><td>5:G>A</td></tr>
<tr><td>4397615|F|0-24:C>A-24:C>A</td><td>TGCAGGCGGTGGGGTCGCCTCAGCCCCGCCCCGATCCG</td><td>24:C>A</td></tr>
<tr><td>3937793|F|0-31:A>G-31:A>G</td><td>TGCAGGCTGCCATCTGATGCATTCAATGACAAAAAGGGCCACAATCCG</td><td>31:A>G</td></tr>
<tr><td>4539547|F|0-14:C>T-14:C>T</td><td>TGCAGGGACGCGTGCGTCGGGGACTTGCCG</td><td>14:C>T</td></tr>
<tr><td>6038140|F|0-10:C>T-10:C>T</td><td>TGCAGGGGTACGTCAAGGACGCCCG</td><td>10:C>T</td></tr>
<tr><td>6038140|F|0-19:C>T-19:C>T</td><td>TGCAGGGGTACGTCAAGGACGCCCG</td><td>19:C>T</td></tr>
<tr><td>7350355|F|0-15:G>A-15:G>A</td><td>TGCAGAATGTCTCAGGCTACCG</td><td>15:G>A</td></tr>
<tr><td>1250878|F|0-31:C>T-31:C>T</td><td>TGCAGAAGGCGCACAGGCGGCTGTCGCAGGGCAGCGTGGACAGCGCCATGACCATGGAGATCCAGAAGT</td><td>31:C>T</td></tr>
<tr><td>1063953|F|0-7:A>T-7:A>T</td><td>TGCAGACACCACTAGATGTGTAGGCGGTAGACCCATGCTAACCGCAGCAGCCCCATGATGCCACCTTCT</td><td>7:A>T</td></tr>
<tr><td>100466860|F|0-48:A>C-48:A>C</td><td>TGCAGACGGGGCAGGAGCTGTTCGCCTCCAGCCACGGCAGGAGGCACAAGTCGTGGAAGCGGTGCTTGC</td><td>48:A>C</td></tr>
<tr><td>100467109|F|0-43:C>T-43:C>T</td><td>TGCAGAGCCCGCACGCACGGTCCATGACTACTCCGTGCGTGCGCGCGGATTGACTCGCGCGCGCACACA</td><td>43:C>T</td></tr>
<tr><td>1042888|F|0-44:G>T-44:G>T</td><td>TGCAGATATAAGTTGGATGTTGTTGCTGCTGGGAAGAACTTCACGAAAATAAGGAAAGCAATCACTGCC</td><td>44:G>T</td></tr>
<tr><td>1022912|F|0-25:G>T-25:G>T</td><td>TGCAGCCGCGAGTGCGTCGGCGTGGGCCGAGACAGGGAGGCGGCGGTCGGCCAGGCGTTCGCGGGCGCG</td><td>25:G>T</td></tr>
<tr><td>100470432|F|0-7:A>G-7:A>G</td><td>TGCAGCTAGACGAGCGGCGCGCGGCCGACGCCAGGCTCAGCGGCTTCAACGGCTTCCGCGGGCTGCTCT</td><td>7:A>G</td></tr>
<tr><td>1025770|F|0-22:C>T-22:C>T</td><td>TGCAGGAACCAGTCCTCGGAGGCGGCCGACGAGCAGTCGGCGTCGCCCGAGTCGGAGGAGGAGCAGAAG</td><td>22:C>T</td></tr>
<tr><td>100471182|F|0-47:A>C-47:A>C</td><td>TGCAGGAGTTCCCCAGCGAGTCTCTGGAGGTGCTGCGGGCGGCGGTGAGGAGCATGGTCGATGCTCCGA</td><td>47:A>C</td></tr>
<tr><td>1025480|F|0-17:G>A-17:G>A</td><td>TGCAGGCGTCCTTCACGGGCGCGCTCCACTCCCGCGTGGACGGCGGCGGGAGCGTGCTGGTGGCGCTGT</td><td>17:G>A</td></tr>
<tr><td>5579734|F|0-28:T>C-28:T>C</td><td>TGCAGGTTCGAGAGGCACAAGCTGCGGATGGCGGAGGCGAGGCGGCGGGCCGTGTACGAGGCGGAGGGG</td><td>28:T>C</td></tr>
<tr><td>1006631|F|0-34:C>G-34:C>G</td><td>TGCAGTCTGATGGATTCTTCTGCCACTGCTGGAACAGAGCCCCGTACTTCTCCTTCCCTTCATGCTTCA</td><td>34:C>G</td></tr>
<tr><td>3939330|F|0-7:G>T-7:G>T</td><td>TGCAGACGCAGACCTTCCTCGCCCTGCTGTGCCG</td><td>7:G>T</td></tr>
<tr><td>100478271|F|0-26:T>C-26:T>C</td><td>TGCAGACGCAGACCTTCCTCGCCCTGTTGTGCCG</td><td>26:T>C</td></tr>
<tr><td>1087713|F|0-31:G>T-31:G>T</td><td>TGCAGCACATGCGTGGCCATCGCACAGCAGCGATTCGTTGGCAAGGCGTCCG</td><td>31:G>T</td></tr>
<tr><td>100479305|F|0-19:T>C-19:T>C</td><td>TGCAGCGCGATCTCGCCCGTGGCATGGCTCCCCGCGCCTCAGCCG</td><td>19:T>C</td></tr>
<tr><td>2257132|F|0-38:C>T-38:C>T</td><td>TGCAGCCGCAGCCTCCTCGACAACTTCCGCTTCTGCTCCCTCGGCTGCAAGGTCGCCG</td><td>38:C>T</td></tr>
<tr><td>987584|F|0-24:G>A-24:G>A</td><td>TGCAGCCTCAGGTCGTCCAGGACGGGCGCGGCCGCGGCCACCTTGGAGCACCG</td><td>24:G>A</td></tr>
<tr><td>3021510|F|0-6:T>C-6:T>C</td><td>TGCAGCTAAATCATCTCAGCCAAATCGTAATGTTTAGGCCG</td><td>6:T>C</td></tr>
<tr><td>4004038|F|0-14:C>A-14:C>A</td><td>TGCAGGCCATGGACCGCAGGCTCCGCGCGGCGGAGAACCG</td><td>14:C>A</td></tr>
<tr><td>1719106|F|0-6:C>A-6:C>A</td><td>TGCAGGCCTTCAGGGAGACCCACTTCCCGGCCCTTGTGGCGCGCCTCCG</td><td>6:C>A</td></tr>
<tr><td>1089646|F|0-5:G>A-5:G>A</td><td>TGCAGGCGGCCCCGCGCCTCAGCCTCCACCTGACCCCGTCCGAACACCGTGGCCG</td><td>5:G>A</td></tr>
<tr><td>100481262|F|0-48:T>C-48:T>C</td><td>TGCAGGTACCGCATGAACGGGAGCTTGGCGTCGTCGGGGAAGGGGAGGTCCCG</td><td>48:T>C</td></tr>
<tr><td>5350074|F|0-15:T>G-15:T>G</td><td>TGCAGCAGCGTACCCTCCGCCG</td><td>15:T>G</td></tr>
<tr><td>3940040|F|0-10:G>A-10:G>A</td><td>TGCAGCACGCGGTGCGCGCCG</td><td>10:G>A</td></tr>
<tr><td>1009966|F|0-59:G>C-59:G>C</td><td>TGCAGCCCGTGCACGATCCCCGTCGCGAACGTCGCGAAGCTGATCTCCTTCTTCTTGCCGCCG</td><td>59:G>C</td></tr>
<tr><td>1023921|F|0-9:C>G-9:C>G</td><td>TGCAGCTCGCCCTGCCTCTCCTGCTGGTGGCCATGGCCTTCGCGCTTCAGAACTTCGCCAAAACCAAAA</td><td>9:C>G</td></tr>
<tr><td>1026848|F|0-27:C>T-27:C>T</td><td>TGCAGCTTCCTCCGTCCAGGCGTCCACCCGATGCCGCTCCTCGCCACCCCACCCTATATAACGCCGCGC</td><td>27:C>T</td></tr>
</table>

2253507|F|0-53:G>A-53:G>A	TGCAGCTTGGGCAGGTCCTCCTCGAGGAGCTTGGCCTTGGCGCGGCGGATCTCGGCGTTGAGCGCGACG	53:G>A
2249343|F|0-17:C>T-17:C>T	TGCAGGATGGATGGATGCAGCTAAGCCCAATTCGAGAAAGCCGCGGATCTGTCTGTCGTTGCTCCTGAT	17:C>T
2244688|F|0-25:T>C-25:T>C	TGCAGTAGCCAAGTCTCCCGCCATGTCCAGCAGGTTGCAACTGCTCTCGAGGGACGCCGTCGTCGTCTG	25:T>C
2244688|F|0-26:C>T-26:C>T	TGCAGTAGCCAAGTCTCCCGCCATGTCCAGCAGGTTGCAACTGCTCTCGAGGGACGCCGTCGTCGTCTG	26:C>T
100490533|F|0-5:C>T-5:C>T	TGCAGCGGCGTACGTGCTTACCTTCCAGAGGCCGACCTCGAGGCCGTACTTCTCGGTGATCTCCTCGGC	5:C>T
1070952|F|0-6:G>A-6:G>A	TGCAGCGTGATCAAGACTGCTGCTCCCAAGACCAAGGCCACAAGGAAGACCCG	6:G>A
100496080|F|0-34:T>C-34:T>C	TGCAGGAACCTGGAGACGTCCGCGTACGTGATCGTGGTCGTGGCCG	34:T>C
100496130|F|0-29:T>C-29:T>C	TGCAGGAAGGTGTTCCAGTCGCGGCGCTTTTGCGACTCGTACCG	29:T>C
7172661|F|0-23:A>G-23:A>G	TGCAGGTGACCGTCGGCGGCCTTAGCCCCG	23:A>G
3024793|F|0-17:C>A-17:C>A	TGCAGGTTCCCAGGCCACGATTTCTGGCCGTCAATTCTTGACCG	17:C>A
5970911|F|0-17:G>C-17:G>C	TGCAGTTCATGACGCCAGCCACGCCG	17:G>C
4397616|F|0-6:G>T-6:G>T	TGCAGTGCAGCGCAGGCTCCG	6:G>T
2294962|F|0-58:T>C-58:T>C	TGCAGCCGCCGCTGCATCCACGTCATCCCCCTCGCCGATGGGGACGAGGCGGAGGACCTCAGAAATGGA	58:T>C
1864063|F|0-28:A>G-28:A>G	TGCAGCGGAAGAGAATGAGAAACGAGTCAAAACTTGTTCGAAAAGTCAATGATTTTGCCCCCGCTCTTT	28:A>G
1864063|F|0-45:T>C-45:T>C	TGCAGCGGAAGAGAATGAGAAACGAGTCAAAACTTGTTCGAAAAGTCAATGATTTTGCCCCCGCTCTTT	45:T>C
1088506|F|0-6:G>A-6:G>A	TGCAGCGGGAACGAGGGCATCCCCACTGTGTGGCACCAGTCGCCGCCGTAGGCAAAAGGCCACCCGTGC	6:G>A
995805|F|0-19:G>A-19:G>A	TGCAGCTGGATGAGAAGGAGGGCTTGGGCAGGCGCGCCGACCTCAAGGCCAGTGTTCTCATGAACCATA	19:G>A
100502535|F|0-36:C>G-36:C>G	TGCAGCTTGCAAGTAGTGACAACATATGTGTATACGCCCTTGCTGTGTGTATCAGATGTATATTTGCTG	36:C>G
1244642|F|0-19:T>C-19:T>C	TGCAGGACGCCGTGGTCATTGGCGGCAGCCCAACCCCAACAGGGGCGAGCACCACAGGGACGTTGCCGC	19:T>C
12768972|F|0-23:G>A-23:G>A	TGCAGGCCAAGATAATCAAGTCGGTCGACGTGTCGTACGGCGGCGAGAGCGGCTTCAACCAGGCCATTG	23:G>A
1015237|F|0-25:C>T-25:C>T	TGCAGGTGGCTTGAATGGAAGTGTCCCACGGGCGTTTGTTTCGCCGCGTTTACTCTCCCGTTCCATCCA	25:C>T
1051971|F|0-55:G>A-55:G>A	TGCAGTCCGATTCCGTATCTCCTCCCCATACGGATCCACTAACTCCGCGAGTCTAGCCAAATCAACAGC	55:G>A
1081341|F|0-14:G>T-14:G>T	TGCAGTCTGCCCCTGCTGCCACTGCCACCGCGGCGCCTCCTCCTCGTCGTCCTCGTCGCCATGCTCACC	14:G>T
996241|F|0-58:C>A-58:C>A	TGCAGTTACCCAGGCACAGCTGCCTGCACTCGGCCAGAGTCATGTCGGGGCGCACCGTCGCTTCCGTCG	58:C>A
100507826|F|0-12:G>A-12:G>A	TGCAGAGATGACGCGGAGCGGAGGAAGCAAACTCTCAATGGCGGCCG	12:G>A
3954408|F|0-40:A>G-40:A>G	TGCAGAGCAGCAGAGATTGAGGCATCCAAGCTGGGGTGCGAGTCCG	40:A>G
3936848|F|0-22:G>A-22:G>A	TGCAGCAGCTGGAGTCGTCGACGCGGCAGAGCAAGCTGCGGCCG	22:G>A
5367078|F|0-13:C>T-13:C>T	TGCAGCGCCTGCGCCGGTATGTCCGTGGGGATCCCG	13:C>T
1153255|F|0-14:G>A-14:G>A	TGCAGCTGTCGAGGGCCGTGACGGTGGACGAGTCGGCCGACGCGTTCCCGAACCCG	14:G>A
100509262|F|0-46:A>C-46:A>C	TGCAGCTGTCGAGGGCCGTGACGGTGGACGAGTCGGCCGACGCGTTACCGAACCCG	46:A>C
3936124|F|0-26:C>T-26:C>T	TGCAGGTGGGCCTCGATGCGGCACGGCGTGGCCG	26:C>T
1049522|F|0-26:T>C-26:T>C	TGCAGTGCTTCCTGATTGAAGTTGTGTGCGCGTGCATCTCAGGCTCGTTGTTTCCCCCG	26:T>C
1045011|F|0-5:C>T-5:C>T	TGCAGCAATGGCAACAGAATAGTACAGATCGGGTAGGTGCCCTGCCTGCCTGATGCCTTCTCCGACACG	5:C>T
12850977|F|0-64:G>A-64:G>A	TGCAGCCGATCACGTCGTCGAAGAGCTGGACGGCGAGCTCGGCCGACCCGCTATCCACGTTGTGGAGTT	64:G>A
1044779|F|0-18:C>T-18:C>T	TGCAGCCTCGCCAGACGACTCCAAAGGGCTGGAGACGATGACCTTGTGCGCATGATGCTCGCCTTCAGC	18:C>T
100513829|F|0-26:T>C-26:T>C	TGCAGCGACCCATCTACCCACCTCTTTTGGGACGGCATCCACTACACCGAGGCCGCCAACAAGTACAGC	26:T>C
5341583|F|0-7:G>A-7:G>A	TGCAGCGGCAGGGCACGAAGAACTTCATCGGCCGCGGCCTGCTCATGGCCAACGGCGCCAGGTGGTCGC	7:G>A
7168827|F|0-35:C>T-35:C>T	TGCAGCTGGAGTGACGCCCTTCGTCGTTGCAGGGGCCGAGTTCGGCAAAAGAATTGTGAGTTCCGAGAT	35:C>T
5579659|F|0-53:G>T-53:G>T	TGCAGCTTCGGCGTCGACGCGGCCGCAGACGAAGGCTCGCGCTTGGCGTGGTGGCCAGCAGCGGAGGCG	53:G>T
995773|F|0-9:C>T-9:C>T	TGCAGGAAGCCCATTGCTGTCTGGTCCGCCCCTATGTAGACGCCCTGGGCCCGTCCCACGGCCGCTGAC	9:C>T
7166994|F|0-18:C>G-18:C>G	TGCAGGAAGCCCATTGCTCTCTGGTCCGCCCCTATGTAGACGCCCTGGGCCCGTCCCACGGCCGCCGAC	18:C>G
1217398|F|0-64:G>A-64:G>A	TGCAGGAGCTGCTGTTTGCCCGTACCATCCTCGCAGACAAGTTCGGCGGCGACTTTGCTGTCGCGGCCA	64:G>A
2251092|F|0-13:G>A-13:G>A	TGCAGGTCACTCTGGCGCTGGAGCCGAGGTTCAAGAGAAGCGTGACCTACGACTACAGGGACGACTCCA	13:G>A
994831|F|0-25:G>T-25:G>T	TGCAGGTGGGGGCGGCGCTGGAGACGTCCATCAACGAAACGCTGGCGGAGCTCAGCGCCCCGCTGCTGG	25:G>T
993908|F|0-8:C>T-8:C>T	TGCAGTTCCAGAGGCCAAGAAAGGCACAAAAGTTACCAACCAAGCTCCCTCCAACAAAACCCTAACCAC	8:C>T
1088357|F|0-52:C>A-52:C>A	TGCAGCACCATCGTGGGAGGCTAGGGTTTGCTCGGGCACCGCGCTCTCTGGTCTCCG	52:C>A
5580186|F|0-29:G>A-29:G>A	TGCAGCAGCTTCCTTCCCTCCTCCCCGACGAACCG	29:G>A
3936493|F|0-42:A>G-42:A>G	TGCAGCCACAGGCTTCTTGGCCTTGCGCTTAGGCGCTGCCTGACCG	42:A>G
1667327|F|0-14:G>T-14:G>T	TGCAGCCTCCCGCCGTCGAGTCCCAGCCCCTTGGACAGCACCTTTGATACCCTCCG	14:G>T
1044692|F|0-26:G>T-26:G>T	TGCAGCTCATGAGGCCATTAAAAGATGCAGCCTGACAGAGCTCTGGACGGCCG	26:G>T
5363660|F|0-10:G>C-10:G>C	TGCAGCTGGCGGCGGAGTTCGCCGCGTCGTGGGTGCCGTTCTGCCG	10:G>C
3956937|F|0-14:G>A-14:G>A	TGCAGCTGGCGGCGGAGTTCGCCGCGTCCTGGGTCCCCTTCTGCCG	14:G>A
5582533|F|0-10:G>A-10:G>A	TGCAGGCGCTGGTGCGGGGTCAGCTGGTGAGGAAGCAGGCCAAGGCCACGCTCCG	10:G>A
100521543|F|0-27:A>G-27:A>G	TGCAGGTAACCCAATCCCCTCTACTACATCGTTTTGACCG	27:A>G
100521927|F|0-20:A>G-20:A>G	TGCAGTGCTGGGAGACCGACACCGTCGTACCCGTCGGCGGCCG	20:A>G
1036388|F|0-63:G>A-63:G>A	TGCAGATCATACATACACGTAATATAATGGCGTAAAGTTGTTGCAAACCCAACGAGCTCCCCTGGAATG	63:G>A
2249417|F|0-61:G>T-61:G>T	TGCAGATGTCCAGCTTCAAGGACGACTCGGGCCACCTGGCCTGGGACTGCTCCGCGTGGGTGCGCACCT	61:G>T
100524698|F|0-53:A>C-53:A>C	TGCAGCCACCGCGCCATGACGGCGTCGCTCGAGTCCCCCATGTCCGCGGCCTGAGCGGACGCCCCTCCG	53:A>C
100524700|F|0-61:A>C-61:A>C	TGCAGCCACCGCGCCATGACGGCGTCGCTCGAGTCCCCCATGTCGGCGGCCTGCGCGGACGACCCTCCG	61:A>C

1103721|F|0-5:C>T-5:C>T	TGCAGCCTGCATGCCGCCAGCCCCAGTGGCGCCACCTTGTGCTCCTGGGAGTTGAGCAGACATCACACC	5:C>T
7940657|F|0-41:C>G-41:C>G	TGCAGCTCCCGCACGTGCCGCACCACCTCGCCCAGCAACGCCGCCTTGTCCATCTGCACGCACGAACGT	41:C>G
100525953|F|0-25:A>T-25:A>T	TGCAGCTGCATCGACAACTCTAGGTACCATAGCACGTCGCCCATGGCTGGCCGCTGCGTGCCTTCGTGC	25:A>T
1173088|F|0-56:C>T-56:C>T	TGCAGCTGCATCGACAACTCTAGGTTCCATAGCACGTCGCCCATGGCCGGCCGCTCCGTGCCTTGGTGC	56:C>T
2242955|F|0-17:C>A-17:C>A	TGCAGCTTTGTACGAGCCGAGCGCTCTGGCCGTCGATCAAACGGATTCATCCTTTATCAGGAGCAGCAA	17:C>A
1247342|F|0-43:G>A-43:G>A	TGCAGGACATGTTCGCGGCGGGAACGGAAACGACATACATCGCGCTGGAATGGGCCATGTCGGAGCTGA	43:G>A
100526584|F|0-6:T>A-6:T>A	TGCAGGTGTGTCGGCTGGCCGTCTCATGAATAACCTTTGCTTGTCGGATAGGGAGGGAATAAAGAGAGA	6:T>A
2252818|F|0-12:T>G-12:T>G	TGCAGGCGAGCATGTAGTCGAGGCCACGGTCGGTGATGGTGATTAGGAACATGTAGAGCTCCCG	12:T>G
100526849|F|0-57:A>G-57:A>G	TGCAGGCGAGCATGTAGTCGAGGCCACGGTCGGTGATGGTGATCAGGAACATGTAGAACTCCCG	57:A>G
1051500|F|0-32:G>C-32:G>C	TGCAGGGGGCTCAACTTCTTGGCGATCATCGGGCTGATCCTCTGCACGGCGTCGTTCGGCATCAACCTT	32:G>C
3946644|F|0-41:G>A-41:G>A	TGCAGGTACTCCGCGGGCTTCCCGAGGCCGAAGGGGTCGAAGCCGTAGTCGCCGACGAGCGTGCCGTCG	41:G>A
1016715|F|0-64:C>T-64:C>T	TGCAGTCGGTTGGTGCGGCATCCTCTGTCCCTGACCACCACAACATGCTCATCGGCACACTGTTCTTAG	64:C>T
1053258|F|0-24:A>G-24:A>G	TGCAGTTGTCCAACTCCTTTACAAAAACTTCTACAGGCTCTACTACTCAGCCTATAAGCTGCCTCCTGG	24:A>G
1397091|F|0-31:G>T-31:G>T	TGCAGACGGTGGTGGTGACCACCAAGTGGGAGACGTTCGACAGCAGCCCCCCG	31:G>T
4261219|F|0-7:C>A-7:C>A	TGCAGATCAAGAGGAACGGCAGCTGGCTCCCTGTCAAGCCTGTCCCG	7:C>A
1026406|F|0-29:G>A-29:G>A	TGCAGCTGCCACTCGGCGGCCGTGGACGGGAGGTTGGTGCCGTCGCCGTCGTTATTGCCCG	29:G>A
4010156|F|0-10:A>G-10:A>G	TGCAGCTTGAAGGCCGCGCGGCTCCG	10:A>G
4010156|F|0-14:C>A-14:C>A	TGCAGCTTGAAGGCCGCGCGGCTCCG	14:C>A
1023480|F|0-20:C>T-20:C>T	TGCAGGTCGGCCGCCGCGAGCGCGGGGAAGAAGTCGGACACGTTCGGGCTGCCG	20:C>T
4261690|F|0-23:G>A-23:G>A	TGCAGGTGGCAAATATCCTTTTCGTTGATACACCG	23:G>A
2255867|F|0-36:C>T-36:C>T	TGCAGAACCGCGTCCTCTACATGGTGCTCGGCCAGGCGTGGCGGACGATCGTGTTCGTGCGAGCGAACG	36:C>T
1026702|F|0-14:G>A-14:G>A	TGCAGAGGAGGATCGGCGGCGAGCTGGAGGCGGTGAGCATGGCGAGTCCTGCGGCTCATCGGAGTCAGA	14:G>A
12778480|F|0-20:T>C-20:T>C	TGCAGGTCCCCTAACCAGAGTGTCCTGACCTCGTTGGGCCCAGCGGGCGCCTGCGTCGGGGCAGCCGAG	20:T>C
100534060|F|0-9:G>C-9:G>C	TGCAGATGAGATGGTGATGAGCGTAGACTGTAAGGCCACCATGAGCAGCTGCACGCATGTCAGCTTCAC	9:G>C
997610|F|0-9:G>A-9:G>A	TGCAGCCTCGGTGCCATGGAGGCGGAGCCTCCTCCTGCTCCCGACCCTTGTGCTCCTGTGGCAGCTCTG	9:G>A
977536|F|0-62:C>T-62:C>T	TGCAGCGAGGTGGCGGACATCATGCGGTGCGTCAAGGTGGCGCTGCTTTGCGTGCAGGACAACGCCATG	62:C>T
100535313|F|0-7:C>T-7:C>T	TGCAGCGCCTCATATTGGGAAGTGATCACACACTAAAGTGATTTGCCTATCGAGGAGAAAAAGGCTATG	7:C>T
100535316|F|0-55:G>A-55:G>A	TGCAGCGTCTCATATTGGGAAGTGATCACACACTAAAGTGATTTGCCTATCGAGGGGAAAAAGGCTATG	55:G>A
100535971|F|0-35:A>T-35:A>T	TGCAGCTTGTGAAGAACAGGAGATACGCAGCTAGGAAGGACTGTAGCGGAGAACGAATCTCACCCCCAA	35:A>T
1005852|F|0-43:G>T-43:G>T	TGCAGGACGATGGAGGCGCCCTCGGCCTCGGGGGAGGCGGCGAGGGGGATGGATGCCGTGCCGTTGCCG	43:G>T
3030558|F|0-31:G>A-31:G>A	TGCAGCCGCAGACGCAGAGGATCCGCGCCGCGCACCG	31:G>A
25708077|F|0-38:A>C-38:A>C	TGCAGCCGCGCGCCCACCACGGCATGACGCCGCCGATGATCGGCGCCG	38:A>C
100540108|F|0-26:A>G-26:A>G	TGCAGCGACGCCGTCCACAGGATGCTAAGGGAGACGCCG	26:A>G
1086644|F|0-6:G>A-6:G>A	TGCAGCGGAGCGAGATGGCCATGCTCTCGCCCTCCGCGTCGTCTTTGCTCCCCG	6:G>A
995691|F|0-32:C>T-32:C>T	TGCAGGGACGACGAGGAGATGCGCGCCATCCTCGGCGACAGCATCGGGAACCCG	32:C>T
995691|F|0-50:C>T-50:C>T	TGCAGGGACGACGAGGAGATGCGCGCCATCCTCGGCGACAGCATCGGGAACCCG	50:C>T
14385546|F|0-10:C>T-10:C>T	TGCAGGTGTGCGACGCCAAGCTGCTGGGCGGGCCG	10:C>T
1000127|F|0-14:G>A-14:G>A	TGCAGGTGAGCGACGCCATCTCGTCCTTCACCGCCGAGGCGAAGGTGCCCACGCCGCCG	14:G>A
100541822|F|0-11:T>C-11:T>C	TGCAGCTCGCTTTGCGCCTCCG	11:T>C
1062548|F|0-5:A>G-5:A>G	TGCAGATCAGCAAGGTCACCTACCAGTCAGTACCAGGCAATCGCGAGCGAAAGCGGCGAATTCCGAGAT	5:A>G
100543358|F|0-16:T>C-16:T>C	TGCAGCCCGTCCATGGTGTGCATGGCGACATCGAAGAAGACGAGATTGTGGTTGGCGTACCG	16:T>C
100544030|F|0-48:A>T-48:A>T	TGCAGGTCCTGCTTCTTCTTCTGCTTCCTATCCTGAGCCCCCTTCATCAGCACGGAGGACGGCATTAAC	48:A>T
1024595|F|0-10:G>A-10:G>A	TGCAGCTGGTGTGCTTCGGCTGCACCACGCTCCCGACGTCCCGCTATGGCCCCTACTGGCGCAACCTCC	10:G>A
1727742|F|0-52:C>T-52:C>T	TGCAGGCGCAGAGCAAGAACATGATCATCACCTACCTCGCGTTCGTCTCCCTCGGCGTCCATCTCTTCC	52:C>T
1267399|F|0-27:A>T-27:A>T	TGCAGGGGCGACAGGGCTGCGTAGTTGATGGCAGCATGCATGGTGTGGTGGCTCACGGCTCCCGCCGAG	27:A>T
1073731|F|0-43:C>A-43:C>A	TGCAGTCGCTCTCCCGCCAGCGCAGCTGCACCGAGCTCCCGCGCTTCGCGGAGGCCGACGCCGACGCCG	43:C>A
1150469|F|0-55:C>G-55:C>G	TGCAGTCGCTCTGGAACGGCGCCGCGTACCACGGCGTGCACCACCTGCCGCGCGGCGTCAGGTACAACT	55:C>G
100546540|F|0-23:T>C-23:T>C	TGCAGGTTACACTCGCTTACTGGTTCAGACAGAGTCCAGACAGTTTCATGTCATATTCAGCAGTATAAT	23:T>C
100547244|F|0-9:A>C-9:A>C	TGCAGGAGGATGTCTCCTCTGGCGAGGAGGGCGCCGAGCACGACGAGCTGGAAGATGCTCTGGCCGAGA	9:A>C
5363035|F|0-10:C>G-10:C>G	TGCAGGTAGACGGAGACCCAGCCG	10:C>G
100547456|F|0-33:C>G-33:C>G	TGCAGACGCGTCGTTCAGGGGGAACCTGCCGCGCCAGGTGCGCGCGCCG	33:C>G
1003778|F|0-17:C>T-17:C>T	TGCAGCCCCGCGCAGCGCGGCCGCCAGCGGCGCCAGCTCCAGCGCCACCCG	17:C>T
1042706|F|0-63:C>G-63:C>G	TGCAGAAACGATAATTACCTAACCCGACAAGCACTGAGGCACCGACGAGAGTGCACGGTGATTCAACCA	63:C>G
2243154|F|0-23:G>T-23:G>T	TGCAGAACACGTATCCACAAGTAGTCGACCGTGTCTGTCGGGTCGCCATCCATCCTTCCACATTTGAGC	23:G>T
1095225|F|0-51:C>G-51:C>G	TGCAGCACCGTGGACGCCAAGGCGTGGACGCACGGCGGCTGCGTCGCCGCCCTGGTGTTCGTCCGCGAC	51:C>G
1091961|F|0-32:G>A-32:G>A	TGCAGCCGCGACTTGTGCCATGACAGGTAGGAGCGGCGCAGGTTCTTCTGGACCAAGAAGGCCAAGAGG	32:G>A
983787|F|0-20:C>G-20:C>G	TGCAGCTCCTTATACGATCGCAGCCGCACCGCGTACCTGTCCGCCGACGTCGCCGACGACGCCTTCGCC	20:C>G
983787|F|0-41:C>A-41:C>A	TGCAGCTCCTTATACGATCGCAGCCGCACCGCGTACCTGTCCGCCGACGTCGCCGACGACGCCTTCGCC	41:C>A
983787|F|0-49:T>A-49:T>A	TGCAGCTCCTTATACGATCGCAGCCGCACCGCGTACCTGTCCGCCGACGTCGCCGACGACGCCTTCGCC	49:T>A

2252771|F|0-7:G>C-7:G>C	TGCAGCTGCGCCGATCGTCTCCTCGATATTTCAATCATGTCGATGTCGGCGCTCGTATCTGCCCTCCGC	7:G>C
2252771|F|0-40:C>G-40:C>G	TGCAGCTGCGCCGATCGTCTCCTCGATATTTCAATCATGTCGATGTCGGCGCTCGTATCTGCCCTCCGC	40:C>G
990367|F|0-30:C>G-30:C>G	TGCAGGTCGTTGCGGTGCTCGTGGAGGATCCTGGCGGCGCCGAGCAGCATCGCCGTGTGCGCGTCGTGG	30:C>G
1023484|F|0-22:G>T-22:G>T	TGCAGGGCGCGATCCAGAGCATGGCAGGAAGCATCATGTCGGCGTGCGCGATGGCGCTGGGCCTCCTCG	22:G>T
1075099|F|0-6:T>C-6:T>C	TGCAGGTCCATTCCAGTTCGTCGCAGAGCCCTACAACGGCACCATACCGCGCCTGCAAATCAACCCCTA	6:T>C
100552713|F|0-37:A>C-37:A>C	TGCAGTATCCAGTGGCAGTGCCTGTCGGAGAGCCTGGAGCGGTTGTCGCGGAGCGGCCGCGGCGACGAG	37:A>C
5577181|F|0-29:A>G-29:A>G	TGCAGTGCAGGACACGGCTCGTTTGGTCGATGGTCACGGTCAGGGGTTACCCGTCGTGACACCGATCTG	29:A>G
1039421|F|0-17:C>G-17:C>G	TGCAGTTCCTCGGCCATCCTGCCCATGTTCCCATGATCGGAAGCAGCAGGGGTAGCCAGAGCTGGTGCA	17:C>G
2242751|F|0-17:C>G-17:C>G	TGCAGGACGTGTTCATGCGGCTGACGTTCGACCTCACGGCGATGTTCGTGTTCGGTGTCGACCCCGAGA	17:C>G
2242751|F|0-18:G>A-18:G>A	TGCAGGACGTGTTCATGCGGCTGACGTTCGACCTCACGGCGATGTTCGTGTTCGGTGTCGACCCCGAGA	18:G>A
5000878|F|0-13:C>G-13:C>G	TGCAGATTGGCTACTCGGCACCAGCTGAGGCCGGGATCAGCCG	13:C>G
3938134|F|0-10:C>G-10:C>G	TGCAGCATGGCGTTGCTCCACGGGAACCG	10:C>G
22840571|F|0-40:G>C-40:G>C	TGCAGGCGATGCCGTACCTGAAGGCCGTCGTCCTCGAGGGGCTGCGGCTCCACCCGCCG	40:G>C
5364061|F|0-55:T>G-55:T>G	TGCAGTCGAGGGCGCAGGCCGTGCGGCTCGTGAGGTCGCTGGCCGACGTCTCCGTTCCG	55:T>G
100555272|F|0-12:T>C-12:T>C	TGCAGTGATGCGTATGCATTGGCCGCCG	12:T>C
3947417|F|0-8:C>T-8:C>T	TGCAGGGTCGAGTGGCCGAAGCTCACCTCCCTGAACGCAGAGTAGTCCG	8:C>T
990138|F|0-42:G>A-42:G>A	TGCAGAGGGGATGGCTTCTTAGCCTTTCTTTGTCATGAACATGTTAGATTTGCTGGATTAGCATGCCTG	42:G>A
1002827|F|0-38:C>G-38:C>G	TGCAGCAACGGCATGAAGCTCGTGGTCAACGTCGCCGCCGCCACCGCCGCCTCGCCGCCCAAGGCGTCC	38:C>G
2246882|F|0-46:A>G-46:A>G	TGCAGCGACAGAGATGGCCTGCGCCTGCCTCGTCTTGAACTCGACCATCCTGTTAACGTCCG	46:A>G
28620388|F|0-5:T>C-5:T>C	TGCAGTTGCCGCCCTCCTACCCCGCCGTGCCACCCCAGGTGTACTATCGCTCCTTCGGCCTTCGCCTGA	5:T>C
1046618|F|0-26:G>C-26:G>C	TGCAGGAACTCGGACGACGGCCCGTTGCGCACCGTGGCGTTGAACCCGCAGAGGTGCTGTTGGAGCTCC	26:G>C
6047251|F|0-8:C>G-8:C>G	TGCAGGAACTGCACTGTGCGGGAATAATGGGCTCTTTTAATGAGGACGGAGACGTTCACCCGTGTGATC	8:C>G
989821|F|0-41:C>G-41:C>G	TGCAGGGACGAGATGCGCAGGGTGCTCGGCGTCGCGCTGCTCTGCGCCTCCAGCCTCCCGATCAACCGA	41:C>G
1691958|F|0-23:G>T-23:G>T	TGCAGGTTCTGCGTGGGGAGGTAGGCGGAGAAGACGAAGAGGAAGGCGGCCGACAGGAGGTGGACGTCG	23:G>T
1044204|F|0-56:A>C-56:A>C	TGCAGTCGCTCGTGAATCTGTTAAAGATGATCTCGGAGCAGAAGTCTCCTATAACCAGGAGGAAAAGCC	56:A>C
1700366|F|0-32:C>T-32:C>T	TGCAGCGTCACCACGCGCCATGCGCCGACGACCGCGCTGGCCGACGCGACATGCCCGTCTTCCAGGATG	32:C>T
992871|F|0-17:C>G-17:C>G	TGCAGGAAGCCCAGCTCCGCCTGGTCCGCCCCCATGTACAGGCCCTGGGCGCGGCCGACGGCCGCCGAG	17:C>G
100561589|F|0-37:G>C-37:G>C	TGCAGAGATCAACCAAGGGGTTCAAGGAGAAGCTGGGGGCGGGCGGGTTCGGCGCCGTGTACCGCGGCG	37:G>C
1214870|F|0-62:C>G-62:C>G	TGCAGAGTGGAACTGAATGACCATGACAGTATCTGGTGCAGCTCGGCGGCCCAGCCAGTGGCCGCCGAG	62:C>G
1091419|F|0-8:G>A-8:G>A	TGCAGCCAGCGGAAGTCCTCCTCCCCCGCGCGCGGCGCCATCGGGTTCGCCGTCGCCACAACCG	8:G>A
1091420|F|0-30:C>G-30:C>G	TGCAGCCAGCGGAAGTCCTCCTCCCCCGCGCGCGGCGCCATCGGGTTCGCCGTCGCCGCCACCG	30:C>G
3952704|F|0-22:G>A-22:G>A	TGCAGCGTGGGTACAACATCGAGTCGTCGACCTCGGGAAGCTGCTCCCCTCTCGCGCCCTCATGATCAT	22:G>A
1023665|F|0-59:G>T-59:G>T	TGCAGCTGCCCACTGGAGCTGGAGACGCTGGCTCCTAGCACCACCAGCAGCAGGGCCACGGCACCGCTG	59:G>T
6047259|F|0-51:C>G-51:C>G	TGCAGGAGCTCCGCGCCATCAAGTTCGCCCCGCCGCCACCGCCCAACAGCGCCAGCCAACACCCCGAGA	51:C>G
6047259|F|0-64:C>T-64:C>T	TGCAGGAGCTCCGCGCCATCAAGTTCGCCCCGCCGCCACCGCCCAACAGCGCCAGCCAACACCCCGAGA	64:C>T
13196538|F|0-68:G>A-68:G>A	TGCAGGGCGTGCAGGCGAAGCTGCGGAAGATCCACCTCCGCTTCGACGACATGATCACCAGCCTCCTGG	68:G>A
4261942|F|0-7:G>C-7:G>C	TGCAGCGGTTCTCGGCGGGAGCAGAGTCCG	7:G>C
32027204|F|0-16:A>G-16:A>G	TGCAGCCCTGCAAGCCACCGCTGCACGAGACGGACCACCTCGGCGGCGCCGTGTTCACCG	16:A>G
1228871|F|0-13:A>C-13:A>C	TGCAGCCGCAACAACTCGTCACGCAAGAGCTGTTCCCAGCCGCGGCCGCCGCCG	13:A>C
1228871|F|0-19:C>T-19:C>T	TGCAGCCGCAACAACTCGTCACGCAAGAGCTGTTCCCAGCCGCGGCCGCCGCCG	19:C>T
1096951|F|0-43:C>G-43:C>G	TGCAGGACAGCATCCCCAAGGTGTGCCTCGCCGCCGCGGCCGCCGCGCTGTACGCGCGCGCGTCGTCGT	43:C>G
100567535|F|0-42:T>C-42:T>C	TGCAGGCTCGATCCATCATGGCGGCCTCCTCGGCCGTGCGGGTGATCTCCTTGAGCGCGGCGAGCACCG	42:T>C
3023760|F|0-22:G>C-22:G>C	TGCAGATGTACACCCGAAATGTGGGTTTCAGACCTGACCAAGCCG	22:G>C
1863780|F|0-6:G>A-6:G>A	TGCAGCGACACCGATGGACGTCGAGGCAGAAGCCCAACAACACCAGCCATGCCG	6:G>A
5001015|F|0-14:G>A-14:G>A	TGCAGCTCCGAGATGCCCTTCTCCCTCCCGGTGAGCCTCCTGGCAGCCG	14:G>A
2258576|F|0-13:C>G-13:C>G	TGCAGAGCACCGTCGCGCTGCTGCCGTGGTCCGAGGACCTCAGGCTCGTCGCGCGCTGCGTCGACTCCA	13:C>G
2258576|F|0-22:G>T-22:G>T	TGCAGAGCACCGTCGCGCTGCTGCCGTGGTCCGAGGACCTCAGGCTCGTCGCGCGCTGCGTCGACTCCA	22:G>T
1228787|F|0-25:G>A-25:G>A	TGCAGCTCACGGTGTAGCAGCTGTCGGGGCGGGAGAGCGCGTCCGAGAAGCGCACCGCGGCCTCCTGGT	25:G>A
100570589|F|0-36:T>C-36:T>C	TGCAGGAACGCGTCGTACACCACGGCGCGCACGGGCTGGCCCTTCTCCGCCTCGGATAGCAGCAGCTCG	36:T>C
2269540|F|0-16:G>A-16:G>A	TGCAGGCGGCGAGGGAGTCCCCCACGCTGGCGCTGCTGGACTGGTTGGACGGCTTCGAGGATGCCGCCG	16:G>A
1025311|F|0-35:G>T-35:G>T	TGCAGCCGCATGGGCGCGTACCAGGCCCAGCTCAAGCGCTGGCTCCGTCTCCTCGCCGCCAGGGGCGTC	35:G>T
1696733|F|0-23:A>G-23:A>G	TGCAGCACGCACGCCAACGGATGAGGCGCTCGACTCAAGCTGTCGGCCGAGGTGAAAACCG	23:A>G
4909658|F|0-28:C>A-28:C>A	TGCAGCGCCTGCTTTATCTCACAGAGAACTCTTGCCG	28:C>A
100571966|F|0-16:G>C-16:G>C	TGCAGCAGCAGGCCAGGAAACAAGAAATCCACCGTACGGCTGCGCCCG	16:G>C
1125284|F|0-37:G>A-37:G>A	TGCAGGGGCCTGACGCGGCGCCGCCTGAGTACCTGACGCTGTCGCTCACGCCCGCCG	37:G>A
100572484|F|0-34:C>G-34:C>G	TGCAGTTGACGATGAGTATGTCGATGGCGTCTGGCGCGGTGCCG	34:C>G
6042717|F|0-54:C>A-54:C>A	TGCAGCCCATATTCGGCCTGATAAGCGTTGCCACCGACATCCTCGCCTTGGCCGCGTTGGTCACCACCC	54:C>A
6048417|F|0-50:G>A-50:G>A	TGCAGCCGCACCGCGTGCGCCACGGGCCTCACGTCGAACCCTTCGGCGTCGGAGTCGCCGCCCTCGTCC	50:G>A
100573658|F|0-41:T>A-41:T>A	TGCAGCGCGTCCCCGTATCTGCCCTCCTCGTCGACAAGGCGTTGGCAATGGCAATGCCAACGGTGGCGA	41:T>A

4910945|F|0-62:G>A-62:G>A TGCAGCGCGTCCCCGTATCTGCCCTCCTCGTCGACAAGGCAATGGCAATGGCAATGCCAACGGTGGCAA 62:G>A

1121459|F|0-65:A>T-65:A>T TGCAGCGGCACCGCCTTGGGGAGCGTCAGCCCACCTCCTTCACCCATGTCAACCTCAGCATCGCCAACC 65:A>T

1132390|F|0-32:C>T-32:C>T TGCAGCTGCCCGTCGGACATTATCCTCGGCGTCCGTCGTAGGGCTCCGACCTGCTGCGGCGGCGCCGCT 32:C>T

978877|F|0-68:C>G-68:C>G TGCAGCTTGATGCATGGTATATTTTGTTGCTCTGGAAAGAAGAAAGAAAATTTTACAGGTACGTGTATC 68:C>G

1036908|F|0-26:C>A-26:C>A TGCAGCTTGTCGGCCAAGCTCTTGGCCCCCTTGAGCGAGAGCACGAGCCAGACGAAGGAGAGGAACTCC 26:C>A

1036908|F|0-50:G>C-50:G>C TGCAGCTTGTCGGCCAAGCTCTTGGCCCCCTTGAGCGAGAGCACGAGCCAGACGAAGGAGAGGAACTCC 50:G>C

100574291|F|0-26:C>G-26:C>G TGCAGGGGGAACTCTTTGTCGTCGTCCTCGGTGCGCCCCTATGTTCGCTCCAAGAACCCGCGCCTCCGC 26:C>G

5411644|F|0-7:C>T-7:C>T TGCAGGTCTGCTGGTGAGGCTGGACAGCGGCAGGGGTCGCCTCAAGGAGACGGTAGAGGCGCTGAGGGT 7:C>T

4910267|F|0-14:G>T-14:G>T TGCAGTAGGCGGGCGCGGAGAGGGACACGGCGAGCGCCATCCCCACGACCGCGCGGCGGCCGAGGCCGA 14:G>T

3954453|F|0-5:A>G-5:A>G TGCAGAACGGGAGCGGGGGCAGCGTCACCCCGACCGTCCAGTGGCCG 5:A>G

1075816|F|0-22:C>G-22:C>G TGCAGACGACAGGCGGCACCATCCTACTGCTCAGCACTGTCCTTGGAATTAAAATCTCCG 22:C>G

1075816|F|0-33:G>C-33:G>C TGCAGACGACAGGCGGCACCATCCTACTGCTCAGCACTGTCCTTGGAATTAAAATCTCCG 33:G>C

5581288|F|0-23:C>T-23:C>T TGCAGAGCAGCAGCAGGATCCATCTCGTATGGCTGGCTGTTGCCGTCACCG 23:C>T

100576393|F|0-40:C>G-40:C>G TGCAGCGACGCGGGGTGGGAGGCCAGCGCCTTGGAGGCGACGGGCCACATGTTGAGCGGCGAGCCGCCG 40:C>G

1028131|F|0-43:G>A-43:G>A TGCAGCGACGCGGGGTTGGAGGCCAGCGCCTTGGAGGCGAGGGGCCACATGTTGAGCGGCGAGCCGCCG 43:G>A

1095241|F|0-52:C>T-52:C>T TGCAGGGCGGCGTCGCGGCGGACGGGGAGGTCAAGCAGGGGGTCGTAGAGGACGGAGACGACGGACAGG 52:C>T

1206102|F|0-17:C>T-17:C>T TGCAGTACCCACAAGGGCACTGCCTCTCTGCGGGGGAGCTCGCCGCCATCCTGGGCGCACAGTACATGC 17:C>T

5411999|F|0-65:A>G-65:A>G TGCAGTAGTCGAGGTCGTCGACCGCATCGTCGGCTTCGTAGAGCAGGTCCTTGAGGCGAGCGAGAAACC 65:A>G

1095546|F|0-21:G>A-21:G>A TGCAGCAGCGCGATGGGGACGGGATCGTGTTCGCGTCGTCGGTGGTGACCG 21:G>A

3940936|F|0-8:G>A-8:G>A TGCAGGCCGTTGTCGTAAGGCTTGTCGCGGTGGCCGATGCCG 8:G>A

100578228|F|0-25:A>G-25:A>G TGCAGCGGTCAAGGCATCAACCTCGAACTGCGCGAGCCG 25:A>G

1022539|F|0-6:T>A-6:T>A TGCAGTTCTTCAACGTCAGCTACAACAAGCTCTGCGGCCCCGTGCCGACCG 6:T>A

3946266|F|0-25:C>T-25:C>T TGCAGGTCATGGCGAGCCGCCGCGTCGTGGCAACCG 25:C>T

3939446|F|0-58:G>A-58:G>A TGCAGCTTCTCGACCTCAAGAGGGGCCACCACCATCTCGGCTCCCCCGTCGCCCCGCCGTTGGCTCTCC 58:G>A

1003777|F|0-24:G>A-24:G>A TGCAGCCCGAACACGGCGGCGATGGCGACGACGGTGAGGTACGCGACGCCCTCGGCGGCGCCGAGCAGG 24:G>A

1003777|F|0-26:G>A-26:G>A TGCAGCCCGAACACGGCGGCGATGGCGACGACGGTGAGGTACGCGACGCCCTCGGCGGCGCCGAGGAGG 26:G>A

994244|F|0-36:G>A-36:G>A TGCAGGAGCGTCGCCGCGAGCAGGGACACCAGCGCGGAGGCGACCACGGCCACGGCGGAGACCG 36:G>A

1696537|F|0-17:C>T-17:C>T TGCAGGTGCACGCGGCACGGATGAGGTGGGCAGGCGAGGGGAGGCGCAGGAACACCAGCTCGAGAAGCT 17:C>T

100579391|F|0-25:T>G-25:T>G TGCAGTCCGTGCGCCGACGCCGAGCTCCCGCCGCCACGGCGGAGGACGAGCGGCAACGTGAAGGACGGA 25:T>G

4003819|F|0-21:G>C-21:G>C TGCAGCAGGAGCATCAGCAGAGCACATGCTTGGAATGGAAGGCCG 21:G>C

992762|F|0-14:C>A-14:C>A TGCAGGGGCCTGGGCGATGAAGACTCCGACGAGCAACGCCAGTGCAAGCACCG 14:C>A

4262551|F|0-11:G>A-11:G>A TGCAGTCTGCCGTGGCCGCGGCCGTGGCGGTCGTCCTCGCCCCCGCCG 11:G>A

3947374|F|0-12:G>T-12:G>T TGCAGCGGGACGGCGTTGATGGGTTCCGTGGCCATTGCTTCCG 12:G>T

100581131|F|0-8:T>C-8:T>C TGCAGCTGTTCGTCACCGACTATGCGCCGCACGGCAGCCTGGAGGCGCGGCTCCACGGCAAGGACGGCG 8:T>C

1228561|F|0-23:A>G-23:A>G TGCAGGCGGCAACGGGGCTTTGCAGGAGCTTCCACGGGTGACGGCGGCGACTACGCCCGCTCTAGGCCC 23:A>G

4262607|F|0-36:A>G-36:A>G TGCAGGCAGTACTCCAGGTTCCACAGCACGTCGCCCATGGACGGCCG 36:A>G

3958841|F|0-23:T>G-23:T>G TGCAGCTGGATGGCGCCGTCGTCTCCCCGCGAGATGGGCGCCG 23:T>G

5340989|F|0-56:G>A-56:G>A TGCAGGTTGGACAAGCTCGCGACCTGCGCCGGGATGCTGCCATGGATGGCATTGTGGCTCACGTCGACG 56:G>A

999513|F|0-5:C>T-5:C>T TGCAGCGCCCAGGCGGTGTCCGCCACGTACCCTTGGCCGCGGATCCTCTCCGTCATCTCCTCCGCCTTC 5:C>T

100583205|F|0-44:A>G-44:A>G TGCAGGTGGCCTTCCCGCTGGATCCTCAGGAGCACGTCGAGCAAATCCTCCTTGTCGTCATCGGCTGCT 44:A>G

100583546|F|0-54:A>C-54:A>C TGCAGCTTGGACAGCAGCTCGGAGATCTGCTCCTCCGAGATCGACGGCGAGCTCACGGAGCGCCGCATG 54:A>C

1075269|F|0-15:C>T-15:C>T TGCAGGAGCACACCGCCGTCTTCTTCAGGGTGTTCAGGCACCATGACAAGTACAGAGTGCTCATGTGCA 15:C>T

1075269|F|0-16:C>A-16:C>A TGCAGGAGCACACCGCCGTCTTCTTCAGGGTGTTCAGGCACCATGACAAGTACAGAGTCCTCATGTGCA 16:C>A

7925282|F|0-6:T>C-6:T>C TGCAGATTAGCGGCTCGTTGCCTCCCCG 6:T>C

4002965|F|0-30:G>C-30:G>C TGCAGCGCAGAGCTACCGTCGAGGCCCTTGGCCCTGTTGTGCCG 30:G>C

100584045|F|0-23:A>C-23:A>C TGCAGCGGGAGGCCATCCAGGGCATCGTCGCCGCTTTGGGGAAGGAACTGCCG 23:A>C

3956426|F|0-37:G>A-37:G>A TGCAGCTGCGCCCACGCCTCCTCCGTGTCGATCAGGCGGCGCAACCACGCCG 37:G>A

1667095|F|0-29:C>T-29:C>T TGCAGTGCGGCAACTTCCGCTCCGTCATCCCCGTCGACCCCAAGGTGCTCCGCGTCGTCGTCCCCGAGA 29:C>T

1667095|F|0-52:C>T-52:C>T TGCAGTGCGGCAACTTCCGCTCCGTCATCCCCGTCGACCCCAAGGTGCTCCGCGTCGTCGTCCCCGAGA 52:C>T

1091690|F|0-22:G>T-22:G>T TGCAGAGTACGCTGGACTACGCGTGCGGCCACGGCGCCGACTGCGCCCCGCTGCTCCCCG 22:G>T

100585541|F|0-8:T>A-8:T>A TGCAGGTATACATGTCTCGTGTGGGCAGGGCCTCGCGTAGAACTTGGCGATGTTATCCCCG 8:T>A

983897|F|0-17:C>G-17:C>G TGCAGCTAAACACGCCTCGTGCGGGCAGGGCCTCGCGTAGAACTTCGCGATGTTATCCCCG 17:C>G

3025367|F|0-8:G>C-8:G>C TGCAGGGAGCTGCTCCCTTCCGATGATCCCAAGCTCGCCG 8:G>C

3948012|F|0-35:T>A-35:T>A TGCAGGCCGCGCCCGACGGGTACAGGGTGGGCAGCTCCG 35:T>A

1092571|F|0-40:G>C-40:G>C TGCAGCGTAGGGGCTCTGGTGGGTGTAGCCGTACATTGGCGTTGCTGCCTCTGCCTCTGGCTCCGAGAT 40:G>C

1241343|F|0-7:C>T-7:C>T TGCAGCTCGGACACGTCGTCGTCCTCGCCGCGGTAGGCGGACGCCGCGATCTCGTGGGGCAGCAGCGCC 7:C>T

1111754|F|0-40:C>T-40:C>T TGCAGCCCCTGCCGCGATCCGTGCGAGACCACCTTGACGGCGACCTCGGCGCCG 40:C>T

2257474|F|0-51:G>A-51:G>A TGCAGAAGCGTTGAATCGATTCCTTGGTGCGCGGCGATTGCCGAGGGGAGCGAGATGGTACGGAAGCGC 51:G>A

1015137|F|0-22:C>T-22:C>T TGCAGCAAGACGACCGCGCCGACGTCGGCTTCGCCACGCCGCTGGCCACGGGTGGAGTAATTGGGCCGC 22:C>T

1015137|F|0-26:G>A-26:G>A	TGCAGCAAGACGACCGCGCCGACGTCGGCTTCGCCACGCCGCTGGCCACGGGTGGAGTAATTGGGCCGC	26:G>A
1015137|F|0-42:T>A-42:T>A	TGCAGCAAGACGACCGCGCCGACGTCGGCTTCGCCACGCCGCTGGCCACGGGTGGAGTAATTGGGCCGC	42:T>A
1015137|F|0-65:C>A-65:C>A	TGCAGCAAGACGACCGCGCCGACGTCGGCTTCGCCACGCCGCTGGCCACGGGTGGAGTAATTGGGCCGC	65:C>A
1322742|F|0-19:G>C-19:G>C	TGCAGGAGTCTTCGCCTGAGGCGACCTTGGAGGCGGGGGAGCTGGTGGACGGGATGATGGCCAAGCTGT	19:G>C
1053302|F|0-24:G>T-24:G>T	TGCAGTGCCTCAATGATCAGATGGGGGAAGACGACGTCAGCCTGGTGGCAATGAGCACCAACGACGGCG	24:G>T
1695408|F|0-27:A>T-27:A>T	TGCAGTTCCACGGGTTCCACGGCGTGAAGCAGGAGGAGAAGAAGCTCGGCCAGAAGTTCGTCATTGACG	27:A>T
7343170|F|0-37:G>A-37:G>A	TGCAGCAGGTGGCTGGGGTCTACAAGATGCTTCCGCCGCTTCTGTCGACGGTGCACGAGCAGACGGGGA	37:G>A
1013536|F|0-28:G>A-28:G>A	TGCAGAACCATTGTGCGATGCAGCTTTCGTGGAAGGAGTGCAAGCAGCCG	28:G>A
4261842|F|0-37:G>T-37:G>T	TGCAGGATCTCAACGTGTCCAACAATCTCCTCTCGGGGAGGATTCCG	37:G>T
983247|F|0-16:G>A-16:G>A	TGCAGTAGGGGAGCACGTTGCCGTCCACCACCTCCTCCTCCTGCTGCTGCACCTGCCCTTGCTGCTCGG	16:G>A
100590234|F|0-67:A>T-67:A>T	TGCAGCCCCACCTTCTTCGCCTCCATTTGCCGCGCGTTCGGCCCCTGGTCACCGAAGATGGGCAGCAAG	67:A>T
1136868|F|0-12:T>G-12:T>G	TGCAGCACGGCCTGAAAGTGCAACAACAAACCTTGATGCAGCCACAATGGCCGCGCAAGAGCGTGACCG	12:T>G
100591481|F|0-13:T>A-13:T>A	TGCAGCTCCCTCCTCATTTTCACATCCG	13:T>A
1094056|F|0-25:C>T-25:C>T	TGCAGCGGCGCGATGGGCAGCATGGCCATGGAGCCGCTTGCCTTGGGCGTGACGCAGCCG	25:C>T
1244263|F|0-5:C>T-5:C>T	TGCAGCGAGAGCGGCAGCTGCACGAACATCCACGTCAACTCCATGGCGATCACAGCTGCGTACGTTGGA	5:C>T
7173978|F|0-6:A>G-6:A>G	TGCAGCAGCTGGTCCAGCGTCGTGCGGAGGCCG	6:A>G
2262799|F|0-42:T>C-42:T>C	TGCAGCGCCCCTACGACGAGGCGAGCGGCGCACGCTACAGCTTCGACGGCACCGTGCGGAAGCTCTGGG	42:T>C
1230965|F|0-18:C>T-18:C>T	TGCAGCGCCTCCAGCTCGCCACGCTGCCGCGCGTTGACGAGCCCCGTGAAGTAGGCCGAGTCGGCGTGC	18:C>T
1050910|F|0-7:G>T-7:G>T	TGCAGCCGCTGGATTCAGCTTGGCGCATTCTACCCGTTCGCCAGGGCCCACGCAGAGAAGACAACCCTC	7:G>T
5579661|F|0-9:C>T-9:C>T	TGCAGCTTGCTGGCAGGGTTGGTCGGGGCAGGCTGGGGAGTCGGCGCGGCGGTGTCGAGGACGGTGATC	9:C>T
4261276|F|0-8:A>G-8:A>G	TGCAGCTCATCACTCGATTCACCCG	8:A>G
5578296|F|0-9:C>A-9:C>A	TGCAGGCGTCCCGCGAGGGCGGCCG	9:C>A
4734045|F|0-28:G>C-28:G>C	TGCAGGTGCACCCGAACATCAAGGAGGCGGAGAGGTACCG	28:G>C
5580213|F|0-10:G>A-10:G>A	TGCAGCCCAAGAGGCAGCTCCCGCCCG	10:G>A
4989846|F|0-5:C>T-5:C>T	TGCAGCGAGTTTCTGAAACATCCG	5:C>T
3942614|F|0-6:G>A-6:G>A	TGCAGCGAGTTCCTGAAACATCCG	6:G>A
5332157|F|0-13:C>T-13:C>T	TGCAGCTTCGTGGCCCTGTACGACCG	13:C>T
7346454|F|0-15:C>T-15:C>T	TGCAGGCGGACTCGTCCGACCCG	15:C>T
1201162|F|0-19:G>A-19:G>A	TGCAGCGGGAGCTCAAGACGGTGAACGCCGACCTGGCCTTCTCCAAGGAGCGGTGCGCGCAGCTGGAGG	19:G>A
4407908|F|0-20:G>A-20:G>A	TGCAGGGCGTTCACCTCGTCGTAGTCGTAGTAGACGGTGGCGCCG	20:G>A
100397560|F|0-31:C>A-31:C>A	TGCAGCAGAGGAACCGCTGCATGATCATGCCCCCCGTGCCGGCCGAGCGGCGGTTAGAGCCG	31:C>A
992114|F|0-39:A>G-39:A>G	TGCAGGTCGCAGACCATCTTGAAGCGGACGTCGGCCTGCATGAGCGCGCGCGCGATCTCGTTGAGGCAC	39:A>G
2243988|F|0-44:G>A-44:G>A	TGCAGTATGCACATGCCAAATGTGTTGCCGAGGACCCTCTCCGAGTCCTTGTACATGCAGTAGGTGATG	44:G>A
4535958|F|0-20:G>T-20:G>T	TGCAGAGATAGAGCCATCAGGAGTTTCGCCG	20:G>T
1025508|F|0-10:G>A-10:G>A	TGCAGCTTTGGCACGCTGATGACGGCGACGCTGGACTGGGAGGGCATCCCG	10:G>A
4995775|F|0-10:G>A-10:G>A	TGCAGGAACAGCTTCCGCCGCCCCAGCCG	10:G>A
7913651|F|0-5:G>T-5:G>T	TGCAGGCATCGCTGATGGCGGGTTTTGAGCTGCCG	5:G>T
5363761|F|0-20:G>A-20:G>A	TGCAGGCATCGCTGACGGCGGGTTTTGAGCTTCCG	20:G>A
1089503|F|0-16:G>A-16:G>A	TGCAGTACCGTTGGCAGCCACGAGCAGGTCCTGGAGCATGCTGCTGCTGCTCCG	16:G>A
100422729|F|0-7:G>C-7:G>C	TGCAGCCGCGCCACGGAGCCG	7:G>C
3945150|F|0-11:C>G-11:C>G	TGCAGCCGCGCCACGGCGCCG	11:C>G
12735261|F|0-11:C>T-11:C>T	TGCAGCGTCAGCTCGAGCCG	11:C>T
12469789|F|0-14:G>A-14:G>A	TGCAGCTGGTGCCCGCCGCCCCG	14:G>A
997575|F|0-38:C>A-38:C>A	TGCAGCGGAGCTGCTCCGCGTCGCCGTCCTACGACGAGCGCCTCTCCTTCAGCCGCTGCGGGTGGGGGG	38:C>A
1104410|F|0-18:T>C-18:T>C	TGCAGGGGCGCTACGAGATGGGCCGCGTGCTCGGCCACGGCAACTTCGGGCGGGTGCACATCGCGCGGG	18:T>C
3021702|F|0-21:G>A-21:G>A	TGCAGAAGAGAACAAGCACAAGAGCTCTAACTCGACCTCCG	21:G>A
5567238|F|0-13:G>A-13:G>A	TGCAGCGGCTCCCGCCGAGCAGCCGCCGCAGGGCGCGCCTCGCCTGCCG	13:G>A
1025480|F|0-7:G>A-7:G>A	TGCAGGCGTCCTTCACGGGCGCGCTCCACTCCCGCGTGGACGGCGGCGGGAGCGTGCTGGTGGCACTGT	7:G>A
6045402|F|0-28:G>A-28:G>A	TGCAGCAAGACGAGCAGCGGAACAGCGCGCCACCGCCG	28:G>A
4538175|F|0-42:C>T-42:C>T	TGCAGCTTGGACAGGTCCTCCTCGAGGAGCTTGGCCTTGGTGCGGCGGATCTCGGTGTTGAGCGCAACG	42:C>T
2242200|F|0-14:C>T-14:C>T	TGCAGGGCCGAGGGCTTCGACAACGGCCATTGTGATAACCTCTTTACATGCATCTGCTACAGAAACTGC	14:C>T
1027333|F|0-36:G>T-36:G>T	TGCAGTGAAGTGTGATGAACTGAGGTGGCTAGGAGCGCTCGCGTATATGTACTGGTCCGTCGAGCACCG	36:G>T
100496778|F|0-11:G>T-11:G>T	TGCAGGGCCTGGGCGCGCCAGCCAACGCCTGCGACTTGAGCCTGGAGATGAACTCCCG	11:G>T
1862658|F|0-28:C>T-28:C>T	TGCAGCAAGTCCATGAGAACGCCGCAGCCGAGTGGGCGCAGGGAAGACTTGTTCGAGATAGAGGACGTT	28:C>T
1149419|F|0-10:A>G-10:A>G	TGCAGGAGCGAAAACACGAGAACATTGAGATTAAGAGAGATCTAGCGGGAAACGGAACAAATCAAGCGC	10:A>G
3940344|F|0-6:G>A-6:G>A	TGCAGCGACGACGAGAAACAACCTTGGGCAGCCG	6:G>A
5367078|F|0-11:C>G-11:C>G	TGCAGCGCCTGCGCCGGTATGTCCGTGGGGATCCCG	11:C>G
6047576|F|0-23:G>T-23:G>T	TGCAGCGTCACCGCCGCCGCCTCGTGCTTCGGGCTCTCCG	23:G>T
1694889|F|0-29:G>A-29:G>A	TGCAGGAGCGCGCGGGTGATCTCGTTGAGGCAGTCGCCGAGGACCTTCTCGTCGACGACCGTGGCGCTC	29:G>A

1694889|F|0-43:C>T-43:C>T	TGCAGGAGCGCGCGGGTGATCTCGTTGAGGCAGTCGCCGAGGACCTTCTCGTCGACGACCGTGGCGCTC	43:C>T
1018625|F|0-19:T>C-19:T>C	TGCAGCCCAAGGAAGGGCTTGCCATGGTGAACGGCACAGCAGTGGGCTCCG	19:T>C
1093237|F|0-13:G>A-13:G>A	TGCAGGCAGCAGCGCGCACGCCATGGCTGGACTCTGCCTCCCAAGGGTTTGCGCCAAGAGCGCCGAGAT	13:G>A
1065940|F|0-5:G>A-5:G>A	TGCAGGGCAGCATGTCCAAGCTCAAATCGCAACTGAAGCCTTTCTGGCACAAGTACCCGTATGAGGAGG	5:G>A
5002132|F|0-10:G>A-10:G>A	TGCAGGAGGGGAACCACATGCTCCG	10:G>A
983787|F|0-54:G>A-54:G>A	TGCAGCTCCTTATACGATCGCAGCCGCACCGCGTACCTGTCCGCCGACGTCGCCGACGACGCCTTCGCC	54:G>A
2252771|F|0-31:C>A-31:C>A	TGCAGTTGCGCCGATCGTCTCCTCGATATTTCAATCATGTCGATGTCGGCGCTCGTATCTGCCCTCCGC	31:C>A
1133587|F|0-28:G>A-28:G>A	TGCAGCTGCTGCTCAACGAGAAGAACGAGGCACGGATCGCCGCCTTTCTCGTGCTCCTCCG	28:G>A
4261325|F|0-16:T>A-16:T>A	TGCAGGTGCTCGCGCATGCGGAGCCAGTCGTGGACGAAGTCCG	16:T>A
100555827|F|0-12:G>C-12:G>C	TGCAGCGGCCGCGACGCCGCCG	12:G>C
1000446|F|0-62:C>G-62:C>G	TGCAGCGCCTCCCTCATGCTGCTCCGCGGCTGCGGCAACGGCTGCTCCGACATTGCTCCCGCCCTCCTC	62:C>G
1027166|F|0-40:G>A-40:G>A	TGCAGCTCACGCGGTTCCGCTGCGGCTCCCTCGCCGTCGGGTTCACGTCCAAGCACGTCGTCGCCGACG	40:G>A
5325120|F|0-21:C>T-21:C>T	TGCAGAGGGTCTCGGCGGGAGCAGAGTCCG	21:C>T
5971564|F|0-8:C>T-8:C>T	TGCAGCCGCCCGCAGCAGTACACGGCCCGCGGAACGCTCTACGGCTGCCG	8:C>T
1018396|F|0-12:C>T-12:C>T	TGCAGCTAACCTCGGTTGGACTGTCAAAATCAGGTGTTCATATCTGACGTGATTTGGACCTAGCTAGAA	12:C>T
1694837|F|0-15:C>G-15:C>G	TGCAGGTGCAGCACGCGTGCGTGAACCGCGTGGAGGGCGTGACCGTGCAGGACGTCGTCGTCGACGTGC	15:C>G
1278385|F|0-53:G>A-53:G>A	TGCAGGCGGTCGTACTCGTCGCGCAGGTGGGCGAGCTCCTCGTCGCAGGTGACGGACACGAGCACTTCC	53:G>A
1214879|F|0-12:G>T-12:G>T	TGCAGTGGCAGCGCATGGTTGACGACATGCGCGCCCTCGGCAAGCTCAACAGCTGCGTGGCCGTGTGCG	12:G>T
5370763|F|0-17:G>A-17:G>A	TGCAGCGGAGCGAGATCGTCAGGCCCG	17:G>A
2253706|F|0-10:C>T-10:C>T	TGCAGGTGGCCTTCCCGCTGGATCCTCAGGAGCACGTCGAGCAAGCCCTCCTTGTCGTCGTCGGCCGCT	10:C>T
2253706|F|0-63:G>A-63:G>A	TGCAGGTGGCCTTCCCGCTGGATCCTCAGGAGCACGTCGAGCAAGCCCTCCTTGTCGTCGTCGGCCGCT	63:G>A
3027570|F|0-27:G>C-27:G>C	TGCAGCGCAACGGGTGTCAGTCTTTCTGGGCACCATCCG	27:G>C
4536126|F|0-6:G>C-6:G>C	TGCAGCGCAGAGCTGCCGTCGAGGCCCTTGGCCCTGTTGTGCCG	6:G>C
1014038|F|0-5:C>T-5:C>T	TGCAGCTACCTAGAGTCAACTCTTAATTTGTACTTAGCATAGGATCAAGTACGTTTCCG	5:C>T
7353366|F|0-57:C>A-57:C>A	TGCAGACGCAGGCGGCGCGGACGAGGCTGGTGGGGAAGCCGACGCGGACGATGATCTCCGTCAGGAGGT	57:C>A
1050313|F|0-38:C>G-38:C>G	TGCAGGTTCAGCCCGTCCGACGCCGACCTCATCTCCTTCTACCTCCGCCCCATGATCGCGGGCGAGCCC	38:C>G
3946971|F|0-22:T>C-22:T>C	TGCAGTTTCCCCGCTCCGCAGATTCCG	22:T>C
3954530|F|0-37:C>A-37:C>A	TGCAGGCGCTCGCCATGGACCGCGCCGTCGGCCGCTTCACCGTGCCG	37:C>A
3937614|F|0-5:C>T-5:C>T	TGCAGCATGTGCTGTCCTTCTTGCCG	5:C>T
1698915|F|0-41:G>A-41:G>A	TGCAGCCAGCTCATCTCCTTGCAGTCAGCGATCGTCACCCCGAGCTCTGGGAACCG	41:G>A
3934265|F|0-21:A>G-21:A>G	TGCAGGGCCTTGACGCGCGACATGATGGCGTGGAGGAGATCGTCCG	21:A>G
2266625|F|0-26:A>G-26:A>G	TGCAGCAGCAGGATCCCGTCGACCGAATCGAGGACGCAGTGGTTGCTGAAAAGCGGGAGCCG	26:A>G
12835816|F|0-16:T>C-16:T>C	TGCAGCGCTCCTGGATTGTCCTCGCCG	16:T>C
4539385|F|0-5:G>C-5:G>C	TGCAGGTGTTGCCAAGAGACAAAGAACTCACCACCCACTCCTTGGTCTCGGCCACGTACGTCTCCCCGA	5:G>C
7172063|F|0-5:A>C-5:A>C	TGCAGATGGCCGTGCCGTTGATGGTCCACCCG	5:A>C
7173859|F|0-16:G>A-16:G>A	TGCAGCTTCGGGACGGGGGCGCCGTGGGCAGCCG	16:G>A
14822711|F|0-18:G>C-18:G>C	TGCAGCTCGTAGGAGGTGGTGGTGATGAGCGCGTCGGCGCCG	18:G>C
100582911|F|0-26:A>G-26:A>G	TGCAGCAGCTGGAACTTGGCGGCGTCAACCTGGAGCTTGAGCGCGTTCATGTCCCAGGCGGCCGTGGCC	26:A>G
100361187|F|0-13:C>G-13:C>G	TGCAGCGCACCGACCGCGTCCG	13:C>G
7173105|F|0-21:T>C-21:T>C	TGCAGCAGGCGCCGCCCAGCGTGGCCGCCAGCGCTTCCCCG	21:T>C
7172050|F|0-23:C>A-23:C>A	TGCAGAGCCCAAGCCCCACGACCCGATTCCCAGGCCCACCCG	23:C>A
100584514|F|0-16:C>T-16:C>T	TGCAGAACGTGGTCCCCCTCGCCACCTTCCGCGACTTCGGGGACGACGTCTACTTCGTCGCCGACCAGC	16:C>T
100301181|F|0-37:T>C-37:T>C	TGCAGAGGTTGTCGATGCTCATGGCCTCGTCCTCGTCTACCTCCG	37:T>C
7352674|F|0-5:A>G-5:A>G	TGCAGAGCATCGCCGATACTGCGCAGCAGCTCGGGCAGCTTATCCTTCTTCTCCACCTCCG	5:A>G
7340772|F|0-45:T>C-45:T>C	TGCAGGAGCGAAAACACGAGAACATTGAGATTAAGAGAGATCTAGTGGGAAACGGAACAAATCAAGCGC	45:T>C
100557834|F|0-32:T>C-32:T>C	TGCAGGAACTCGTTGGCGCAGTAGGCAGGCGATACGCCTCGCGGGAGGCCGAACGCCGCCGCTCCCGAT	32:T>C
1075422|F|0-31:C>G-31:C>G	TGCAGCGGTTCTCTTTTGAGCTGTCGCCGACCTACGTGCACGCACCCTATGCCGTCCTTACACTGCACC	31:C>G
100575430|F|0-25:A>C-25:A>C	TGCAGGATCTTCAGGAGGACCATCAACTACCG	25:A>C
100575574|F|0-21:G>C-21:G>C	TGCAGTAGTTGCCCAAGCTAGGTCCATATGATTAGTTAGGCCG	21:G>C
4396657|F|0-5:C>T-5:C>T	TGCAGCGCGCCCACAAGCTCCCCG	5:C>T
100303194|F|0-15:T>G-15:T>G	TGCAGGCAGCGTGCTTAGCGGCGGGGGCAGCGTCCCG	15:T>G
3021365|F|0-5:G>A-5:G>A	TGCAGGCCTCACTTTGACCAGCTCTTCAAGAGGACCG	5:G>A
3021365|F|0-23:C>A-23:C>A	TGCAGGCCTCACTTTGACCAGCTCTTCAAGAGGACCG	23:C>A
3020326|F|0-34:A>G-34:A>G	TGCAGTCAAAGATGTGTAGTGTAGAGAAAACAAGAAACTCACCG	34:A>G
1074085|F|0-5:G>A-5:G>A	TGCAGGAGATCATACACAGCCCTGTGTCTCTTGAGCATGCTCTTCCCCTCAAACTCCTTGGACACCACC	5:G>A
100349617|F|0-9:G>A-9:G>A	TGCAGACAGGTTTGGCCACACCG	9:G>A
5358090|F|0-11:C>G-11:C>G	TGCAGCTTGTTCATGGCCGCGCCCG	11:C>G
5359215|F|0-22:A>G-22:A>G	TGCAGGACGCGCTGAACAAGCTACTCCG	22:A>G
100352565|F|0-7:A>C-7:A>C	TGCAGGGAGACATGCGGGACCTCCCGTTCACCCTGGCCG	7:A>C

3027710|F|0-7:C>T-7:C>T	TGCAGTGCTGTGTCCTCTGTACTACTACTACCAGCGGCACCCG	7:C>T
3027710|F|0-31:C>A-31:C>A	TGCAGTGCTGTGTCCTCTGTACTACTACTACCAGCGGCACCCG	31:C>A
3944003|F|0-30:C>T-30:C>T	TGCAGAACCTTGGGCGGGCGCTCATCTTTGCGGCCG	30:C>T
1127949|F|0-38:C>G-38:C>G	TGCAGCAGCCGCCCCACCACCTGCTTCCGCCACCGCGTCACGCCGCCCCGCCG	38:C>G
100386564|F|0-12:A>G-12:A>G	TGCAGCCTGAGCAAAAGCCACCG	12:A>G
5971350|F|0-14:C>T-14:C>T	TGCAGCGGCAGCGGCGCGGTACCCG	14:C>T
100388353|F|0-34:A>G-34:A>G	TGCAGGGACCCGAGGCGCGAGAGGATGTCCTCCAAGAGGCAGTCCG	34:A>G
4261178|F|0-7:C>T-7:C>T	TGCAGTGCAGTTATGCTTTTGCAGATCAGGACCATGTTGCTCCG	7:C>T
100396200|F|0-61:C>G-61:C>G	TGCAGAGCAAGTCCTTCGTGAAGAAGACGAAGCAGGGCCGCGTCGTCAAGGTCGCCGCTCGCCACCACA	61:C>G
100402009|F|0-38:A>G-38:A>G	TGCAGGGTCTCGAGATGCATCTTCCTCCTCATGCTGGCAATGAACTCTTCGAATTTCTTGTTCATCTCG	38:A>G
4262238|F|0-22:C>T-22:C>T	TGCAGACGTTCGTACGTACCGTCGGTGCATGCCTGTGACCG	22:C>T
5363342|F|0-5:C>A-5:C>A	TGCAGCCTCAGGAATTCGCGGCCG	5:C>A
4734188|F|0-25:C>G-25:C>G	TGCAGGCCCTGGGCAACGCCGCGCTCGGCGGGGACCG	25:C>G
5332338|F|0-11:C>A-11:C>A	TGCAGGCGCTCCCCGACGCGCGACGCGCCG	11:C>A
5364489|F|0-7:G>A-7:G>A	TGCAGGGGAACGCGCTCCGCGGCATCCTGCCG	7:G>A
4995508|F|0-17:T>A-17:T>A	TGCAGTGAAAAGATGGATAAATTTCTGAAGAGATGCTTCTATCAGTCCG	17:T>A
7349727|F|0-5:C>A-5:C>A	TGCAGCGGACGATGTACACCG	5:C>A
7349727|F|0-10:G>C-10:G>C	TGCAGCGGACGATGTACACCG	10:G>C
7349727|F|0-15:A>G-15:A>G	TGCAGCGGACGATGTACACCG	15:A>G
2252238|F|0-34:G>A-34:G>A	TGCAGCCCGAACTCGCCGCTCGACTTGCTCATCAGGACGAACTCGTCCATCCGCTTGCCGCCG	34:G>A
986389|F|0-32:A>G-32:A>G	TGCAGCCCGATGATCGTGCACCGCGACGTCAAATCCAACAACATCCTCCTCGGCGACAACCTGGAGGCC	32:A>G
2248259|F|0-41:C>T-41:C>T	TGCAGCTGGAGGAACTCCAGGACCTTCACCAGCAGCTGGTCCTCGAAGTTGAAGTGGTCCCACCGAGAT	41:C>T
100438737|F|0-15:C>G-15:C>G	TGCAGACGACCTGGTCCGTGGCGTTCCG	15:C>G
6037509|F|0-6:A>G-6:A>G	TGCAGAAGCGAGGCACTGAAACGAACTAGGGCCG	6:A>G
4993887|F|0-22:A>T-22:A>T	TGCAGAGTCGTGGTGGCTTGGAAACTTCCG	22:A>T
100440628|F|0-12:A>G-12:A>G	TGCAGCGGCGGGATCGAGGGCGGCGACGACCAGCGCGTTGGGCACGCCGCTCCG	12:A>G
5346555|F|0-11:T>G-11:T>G	TGCAGGCTGGCTGGTGGACGCCG	11:T>G
1041672|F|0-5:T>A-5:T>A	TGCAGTCCGACATCAGGGAGGTCGCAAGCAAGTGCGCCGACGCCGCCAAGATGGCCG	5:T>A
1041672|F|0-7:C>G-7:C>G	TGCAGTCCGACATCAGGGAGGTCGCAAGCAAGTGCGCCGACGCCGCCAAGATGGCCG	7:C>G
5411684|F|0-6:C>T-6:C>T	TGCAGTCTCCATGCCCGCCGCCCCGCCG	6:C>T
5411684|F|0-19:G>A-19:G>A	TGCAGTCTCCATGCCCGCCGCCCCGCCG	19:G>A
1018240|F|0-18:G>T-18:G>T	TGCAGAGTATAGCCGCGTGTGGTGTGGGTTTTATAGGACGGCAGGAGGTGGGGAAATGTGGCGGCACGC	18:G>T
1030008|F|0-65:G>C-65:G>C	TGCAGCGTCGTGCGGGCGCAGCGGCGGTGAAACTGGCGATGTGCAGAGGAAGGGGATTTGGGGTGGTGG	65:G>C
2278631|F|0-54:A>G-54:A>G	TGCAGCTCCCCTCCGACTGCAACGTCGACGGCATCCGCGCCAAGTTCGAGAACGAGGCGCTCACCATCA	54:A>G
29308187|F|0-47:C>T-47:C>T	TGCAGCTGGGGCGCCGTCTTCTTCCCCAAGCACTGGCGCGAGTTCTACGCCTACATGGCCGCGCGCTTC	47:C>T
5324801|F|0-38:A>T-38:A>T	TGCAGGTGCTGCCGCGCGCGCTTGTGGCAGGCTTCGTCAAGACAGATATTGACTTCCAGCGCAAGGGTG	38:A>T
992102|F|0-49:G>T-49:G>T	TGCAGGTGCTTCACGCGCTGGACGTGGCGAGGACGCAGAGGTACCACGCGTGGGCGGTGGTGATCGCCG	49:G>T
996034|F|0-8:G>A-8:G>A	TGCAGATGGCGTCCTCTGCTGGTAATGGCAAGGAGATGGCCGCGCTCGAGTCCG	8:G>A
1100114|F|0-13:T>C-13:T>C	TGCAGATTTACGGTGTATTTTGGACGATGGCATTGGAAATTTAGAATGCTCTTGGCCG	13:T>C
4734172|F|0-25:C>A-25:C>A	TGCAGCCTCCGCGGCAGGAAGGAGCCCGCGCTGGCCTCCCCG	25:C>A
4261686|F|0-8:T>A-8:T>A	TGCAGGAATTATTCTTGGCCCAGCATGGAAGTGGGCAACACAGCCCCG	8:T>A
1030286|F|0-31:C>G-31:C>G	TGCAGGAGTGGGAGCTCCTCCTCGCCTCCCCCACCGCCGCGGCGGCGCCCG	31:C>G
4262467|F|0-28:C>T-28:C>T	TGCAGGATTCTCGCCGACGCCCGCCGCTCCCGCCCCTACCCCCG	28:C>T
1097614|F|0-28:G>A-28:G>A	TGCAGGCGATGGCGCAACGTAATCGCGGGCGACGGCGGCGGCTTCCTCCGCCG	28:G>A
1249783|F|0-17:C>T-17:C>T	TGCAGGTTGATGACCCGCAGCTTGACGCAGGAGGCCAGGTCGGCCGGCAGCGGGCCG	17:C>T
3939391|F|0-10:G>A-10:G>A	TGCAGCTCGAGCGCCGCCCG	10:G>A
1055233|F|0-34:G>C-34:G>C	TGCAGAGCATTCCAGAGTCAATGATCGCGCGGCTGCTGAACGTGGTGGTGCTGGACGTGCGCTCCAACC	34:G>C
978727|F|0-10:C>G-10:C>G	TGCAGAGGAACAATAGCCTGAAGCTCAAACAAAAGGCGAATGGGAAGAGAAGAATAACCTGAAGTGAGA	10:C>G
100467251|F|0-16:C>G-16:C>G	TGCAGAGGGTCATCAACCAGGAGTTCTCAGGGTGCACGGTGATCACCATAGCGCACAGGGTTCCCACCG	16:C>G
100468603|F|0-36:C>T-36:C>T	TGCAGCCACTTTTCTCCCGGAGTCCTCGGCCGCCGACCTTGTTCCTGAGCTTCCTTACAGCCTCCCATC	36:C>T
100471603|F|0-11:T>C-11:T>C	TGCAGGCCTTCTTGGCCTCCTGCGGCGATCCAATCGGCACATCAGATTCAGAGGCAACGGACAAAGGAA	11:T>C
2247444|F|0-10:C>T-10:C>T	TGCAGGGAACCCATCCTCGAGTCCAAGATCGCCAGCTGGGAGACGACCCAGTTGGGGTTGTTCCTCCCC	10:C>T
1036705|F|0-23:G>T-23:G>T	TGCAGGGATCTTTTCACCTTTTGGATGCTGCATGATGCAAACAGGGACAAGATGCCATGAGGAGACACA	23:G>T
1036705|F|0-39:A>T-39:A>T	TGCAGGGATCTTTTCACCTTTTGGATGCTGCATGATGCAAACAGGGACAAGATGCCATGAGGAGACACA	39:A>T
2299581|F|0-39:A>G-39:A>G	TGCAGGGCGTGCAGAGCATGTACGCCGTCGGGAAGGTGAAGATGTGCGCGTCCGAGTTCCACCTCGGCG	39:A>G
100472529|F|0-13:T>C-13:T>C	TGCAGGTGCTCGATGTCAGCTCCAACCTGCTCAACGGCACGCTCCCGGCGGACTTCGGAGGCGCCGCGC	13:T>C
100473185|F|0-28:A>G-28:A>G	TGCAGTCCGTCCTTGTGAATTTCGTCAAAGGTGTTGGTAACCTTAGCCATGCAGAATGGCGATCATTCT	28:A>G
1087713|F|0-43:A>C-43:A>C	TGCAGCACATGCGTGGCCATCGCACAGCAGCGATTCGTTGGCAAGGCGTCCG	43:A>C
5580334|F|0-5:C>A-5:C>A	TGCAGCTTGCCGTCCCTGGGCGGGCCCCG	5:C>A

<table>
<tr><td>4398082|F|0-21:C>G-21:C>G</td><td>TGCAGGCCCCGAGGCCCTGCTCCCCG</td><td>21:C>G</td></tr>
<tr><td>3032866|F|0-31:C>A-31:C>A</td><td>TGCAGGCCGTGCTCCTGGTCCGCCGCCGTCCCCCCGCCGTCACCG</td><td>31:C>A</td></tr>
<tr><td>100481732|F|0-6:T>C-6:T>C</td><td>TGCAGTTCGCTCGCTACAGCTCCACCG</td><td>6:T>C</td></tr>
<tr><td>100481733|F|0-12:A>G-12:A>G</td><td>TGCAGTCCGCTCACTACAGCTCCACCG</td><td>12:A>G</td></tr>
<tr><td>4261582|F|0-6:G>A-6:G>A</td><td>TGCAGCGCGGCGTAGTGGTACCG</td><td>6:G>A</td></tr>
<tr><td>1089655|F|0-56:G>C-56:G>C</td><td>TGCAGAACGACACGCACTACACACTGTCAGCGTGGTTGCAGGTGAGCCATGGTGTCGCCGCCGACGTGT</td><td>56:G>C</td></tr>
<tr><td>991901|F|0-42:T>C-42:T>C</td><td>TGCAGACGAGACCCTCCGCTTCTGGAACGTGTTTGGCACTCCTGAGGTAGCCAAGCCTGCACCCAAGGC</td><td>42:T>C</td></tr>
<tr><td>1041702|F|0-30:A>G-30:A>G</td><td>TGCAGCCAGCTCTTGAAGGCGTCGCATTTCACAGGCCCTGAAGCGGTGAAGCGCACCAAAACTCAGAAC</td><td>30:A>G</td></tr>
<tr><td>1001496|F|0-42:T>C-42:T>C</td><td>TGCAGCGCGCGCGTGCCGACGGGTCGCTCGCGGCGCGGGGTGTTTCTGCTGCCGCCGCGGCCCAGAAGA</td><td>42:T>C</td></tr>
<tr><td>100486952|F|0-44:T>G-44:T>G</td><td>TGCAGCGGCGCAGGCGGCGACGTGGGGGAGAGAGCGGGACGCGGTATTCGCAGTGGGGGATGAGGAATT</td><td>44:T>G</td></tr>
<tr><td>1202403|F|0-10:G>C-10:G>C</td><td>TGCAGCTGGGGCCAGTGTGTGCTGCTGGTCGGCCATTGCTTGGAAGAACGGGAAGCCTTGCATCTGCTG</td><td>10:G>C</td></tr>
<tr><td>1000439|F|0-67:C>T-67:C>T</td><td>TGCAGCTTTAGCAAGGTCAGTTGACAAGCAAGGCGTGTACACGGCATGCACACGCTTGCCTTCATCTCA</td><td>67:C>T</td></tr>
<tr><td>1005761|F|0-58:C>T-58:C>T</td><td>TGCAGGCGGCGGCGGATGGCCTGCACCTGTGCCTCCATGGCCGTCACCGACGTGTCCACGGCGGTGAGG</td><td>58:C>T</td></tr>
<tr><td>2282092|F|0-61:C>T-61:C>T</td><td>TGCAGGGCGCCATCGACCGCGAGATCGACAAGCCCAAGTACCGCTGCGGCTGCGCCTGCGTCGACGCGG</td><td>61:C>T</td></tr>
<tr><td>2247426|F|0-31:C>T-31:C>T</td><td>TGCAGGTGTCGTTCGACGAGTACCTCCAGGACCGCCCCCGCGTGTTCCGCGCCATGTTCCCCGACGAGA</td><td>31:C>T</td></tr>
<tr><td>988814|F|0-46:T>C-46:T>C</td><td>TGCAGTTTGGCTACATCGTCCTGACAACCTCTGCTGGCATCATGGATCACGAGGAAGCCAGGAGGAAGA</td><td>46:T>C</td></tr>
<tr><td>3940720|F|0-17:T>C-17:T>C</td><td>TGCAGAGTGAGCTTCTTTGGAGCATGCTCCTCCGCCG</td><td>17:T>C</td></tr>
<tr><td>100494417|F|0-26:T>C-26:T>C</td><td>TGCAGAGTGAGCTTCTTTGGAGCAAGTTCCTCCGCCG</td><td>26:T>C</td></tr>
<tr><td>985294|F|0-38:G>A-38:G>A</td><td>TGCAGCAGCGTGTCAATGGGCCATGGACAAGTGTTGCAGAACCTCGCCATCTCCG</td><td>38:G>A</td></tr>
<tr><td>7169370|F|0-25:A>T-25:A>T</td><td>TGCAGCATGCGCGAGCTCCTCCAGAAGGCCCG</td><td>25:A>T</td></tr>
<tr><td>3028310|F|0-11:G>C-11:G>C</td><td>TGCAGCTCCTGGGAGCCCCCAGCCTGCTCCGCCGCCGCGTCCG</td><td>11:G>C</td></tr>
<tr><td>3023563|F|0-32:C>T-32:C>T</td><td>TGCAGGAGGTTTCTGCGTATGTCGCCGTGCATCCCACCG</td><td>32:C>T</td></tr>
<tr><td>5363832|F|0-6:C>T-6:C>T</td><td>TGCAGGCTCGGTCTCCGCGGCGGGCTCCG</td><td>6:C>T</td></tr>
<tr><td>100497465|F|0-7:A>G-7:A>G</td><td>TGCAGTTACGTCTGATCTCGGTGGGGGTTCCG</td><td>7:A>G</td></tr>
<tr><td>5011056|F|0-22:G>A-22:G>A</td><td>TGCAGTTGCGCCTGATCTGGGTGACGGCTCCG</td><td>22:G>A</td></tr>
<tr><td>12341528|F|0-61:C>G-61:C>G</td><td>TGCAGCTGAGGACGTCGATCGCGGGGTGCCTGCTGGTGTCGGACAACCTGGAAGGGTCGTCCAGGCCGC</td><td>61:C>G</td></tr>
<tr><td>100501075|F|0-26:T>G-26:T>G</td><td>TGCAGCCGCGCCTCGAGCGCCTCCTTTGGCGTGCCCCGCACGGGCTCGCACTCGAACTCCCACCGCAGC</td><td>26:T>G</td></tr>
<tr><td>100501077|F|0-67:T>G-67:T>G</td><td>TGCAGCCGCGCCTCGAGCGCCTCCTTGGGCGTGCCCCGCACGGGCTCGCACTCGAACTCCCACCGCATC</td><td>67:T>G</td></tr>
<tr><td>1094116|F|0-53:C>T-53:C>T</td><td>TGCAGCGTCGGCGAGCCCCCGCCGTTGGTGCTCCCCGTCACGAAGTTGAGGCACGGCGTGAACGTGCTG</td><td>53:C>T</td></tr>
<tr><td>1018968|F|0-40:C>G-40:C>G</td><td>TGCAGCTGGAGGCCGTCCGAGCGGGTGCCATGAGTCAGCTCCTCATGATGGTGCAAGGCGGCTGCTCGG</td><td>40:C>G</td></tr>
<tr><td>1024384|F|0-44:G>A-44:G>A</td><td>TGCAGCTGTTGCCAAGAGACAGAGAACTCACCACCCACTCCTTGGTCTCGGCCACGTACGTCTCCCCGA</td><td>44:G>A</td></tr>
<tr><td>1397609|F|0-14:C>T-14:C>T</td><td>TGCAGGAAGGACTCCTTGGTGCCTTCGTCCTCGCCCAGAATGCCCACGGGCACGGCGCCCACGGACTTG</td><td>14:C>T</td></tr>
<tr><td>1397609|F|0-19:T>C-19:T>C</td><td>TGCAGGAAGGACTCCTTGGTGCCTTCGTCCTCGCCCAGAATGCCCACGGGCACGGCGCCCACGGACTTG</td><td>19:T>C</td></tr>
<tr><td>1017210|F|0-48:G>T-48:G>T</td><td>TGCAGGAGCGAAAACACGAGAACATTGAGATTAAGAGAGATCTAGCGGGACACGGAACAAATCAAGCGC</td><td>48:G>T</td></tr>
<tr><td>1075616|F|0-41:T>G-41:T>G</td><td>TGCAGTAGAGGTAGATAGAGAGATACTTGCCATTGTTGGCTTTCACCCACCCACGCGGGTAGCAATGTA</td><td>41:T>G</td></tr>
<tr><td>1096346|F|0-11:G>T-11:G>T</td><td>TGCAGTTGAGAGATAAATGACGCACCTGCTCGCTGTGGCCCTGTGGAAAGTAGACGACAAGGCTGCCCA</td><td>11:G>T</td></tr>
<tr><td>3027821|F|0-9:A>G-9:A>G</td><td>TGCAGTGCAAGCTCGTCTTCCTCGCGCAGCCACCGCTCCAATCCCCCG</td><td>9:A>G</td></tr>
<tr><td>7349698|F|0-16:A>G-16:A>G</td><td>TGCAGCCGAGGTTAGTACCG</td><td>16:A>G</td></tr>
<tr><td>7169940|F|0-22:C>A-22:C>A</td><td>TGCAGAACACCACCCAGTTCGGCCTCATGGCCGTCAGCCTCGGCTTCTACGACACGCTCATGTCCTGCT</td><td>22:C>A</td></tr>
<tr><td>987258|F|0-16:G>T-16:G>T</td><td>TGCAGAGCGGGCAAGTGCTGCTGGTGCGCAGCCACGTCCTGAGGCAGCGCCTGTGGAACATGTGGCCGC</td><td>16:G>T</td></tr>
<tr><td>1046799|F|0-40:G>T-40:G>T</td><td>TGCAGCAGTATCAGTACCAGCAGATGGGCGGCCAGACAGGGGCCAGGGAGCCGCTCCAGTTCACCAACG</td><td>40:G>T</td></tr>
<tr><td>1046799|F|0-55:C>T-55:C>T</td><td>TGCAGCAGTATCAGTACCAGCAGATGGGCGGCCAGACAGGGGCCAGGGAGCCGCTCCAGTTCACCAACG</td><td>55:C>T</td></tr>
<tr><td>1237462|F|0-29:C>G-29:C>G</td><td>TGCAGCCGCACGATCCCCCGCAGCGCCCTCAGCGCCCTCCTGGCCTGCACGAGCACCCAAAAGAAGCGC</td><td>29:C>G</td></tr>
<tr><td>2268541|F|0-51:A>G-51:A>G</td><td>TGCAGCCTGAGCGACAGCCGCGACGAGGCGGAGCAGGTCATCTTTGCAGCCATCGACGACCTGCTCGCC</td><td>51:A>G</td></tr>
<tr><td>1020945|F|0-26:C>T-26:C>T</td><td>TGCAGTGCACGCTCCAGCAATCCGAACCCACCCAACCACCGCACGCTCCGCGGATCCTCCTCGTGCTCC</td><td>26:C>T</td></tr>
<tr><td>1220240|F|0-32:G>A-32:G>A</td><td>TGCAGCGGCACCTCCAGCTCGTGCGGCGCCACGGCCCACGGGTAGAGCTGCACCGTGTAGCCCCACGCG</td><td>32:G>A</td></tr>
<tr><td>3959631|F|0-58:C>T-58:C>T</td><td>TGCAGGCCGTAGACGCAGTCGACGATGCGCTTCTGCTCGGCGGTGCTGAACACGCCGGCGTGGAGCTCC</td><td>58:C>T</td></tr>
<tr><td>1094021|F|0-41:G>T-41:G>T</td><td>TGCAGGGAGGATGGCTCGGCGGAGGTGCTGCTCGGCGAGACGTGCGTGATGGGCTATGTGACCGCCCAA</td><td>41:G>T</td></tr>
<tr><td>1063492|F|0-19:G>T-19:G>T</td><td>TGCAGGTGAAGGTGCTGGTGCTGTCGTCGTAGGCGTAGCTGTAGGCGCGCGGGCACGCCATCTTGAAGA</td><td>19:G>T</td></tr>
<tr><td>1078576|F|0-33:C>G-33:C>G</td><td>TGCAGGTTCTCGGCCCAGGGCGAGAGGCCCTCGCAGCTCTTGAGGACGACGATGGAGTCGCGCCAGGAG</td><td>33:C>G</td></tr>
<tr><td>993908|F|0-36:C>T-36:C>T</td><td>TGCAGTTCCAGAGGCCAAGAAAGGCACAAAGGTTACCAACCAAGCTCCCTCCAACAAAACCCTAACCAC</td><td>36:C>T</td></tr>
<tr><td>3574740|F|0-13:A>C-13:A>C</td><td>TGCAGTTGACCCGACACACGGCGTTGCCAATCGCCTGCGGGAGCAGGAACAGGTCCTGCATCAACGCCA</td><td>13:A>C</td></tr>
<tr><td>100517521|F|0-16:A>C-16:A>C</td><td>TGCAGTTGACCCGCCAAACGGCGTTGCCAATCGCCTGCGGGAGCAGGAACAGGTCCTGCATCAGCGCCA</td><td>16:A>C</td></tr>
<tr><td>1114012|F|0-14:T>C-14:T>C</td><td>TGCAGCACCATCACTCCACCAGCCCACCCCCGTGATCTACCTGCTGTCGCCG</td><td>14:T>C</td></tr>
<tr><td>1114012|F|0-21:G>C-21:G>C</td><td>TGCAGCACCATCACTCCACCAGCCCACCCCCGTGATCTACCTGCTGTCGCCG</td><td>21:G>C</td></tr>
<tr><td>5357587|F|0-6:A>G-6:A>G</td><td>TGCAGCAGGCAGCAGGTGACCTCCTCCCCG</td><td>6:A>G</td></tr>
<tr><td>1759505|F|0-32:G>T-32:G>T</td><td>TGCAGCAGGTAGACCAGGTCCTCGTTGGCGCCGGCCTCGGCGCGCGCCAGCGCGAAGTCCG</td><td>32:G>T</td></tr>
<tr><td>100520550|F|0-5:T>C-5:T>C</td><td>TGCAGTGCCTCGGCGGCCTGCCTGATCGCCG</td><td>5:T>C</td></tr>
<tr><td>100520551|F|0-13:T>C-13:T>C</td><td>TGCAGCGCCTCGGTGGCCTGCCTTATCGCCG</td><td>13:T>C</td></tr>
</table>

4733950|F|0-7:C>T-7:C>T	TGCAGCTCCACCGCCGAGGCTCGCTGCTGCGAGTGCGAGCTCATGCCG	7:C>T
100521096|F|0-29:C>G-29:C>G	TGCAGGAGACGCCGACCCAAGAGCAGAAGCGTGTGCCG	29:C>G
1043272|F|0-23:C>T-23:C>T	TGCAGGCACGAGTTGTCGTACACCGAGGAGAGATCAAGGTCGTAGAGGTTGAACCG	23:C>T
1049979|F|0-17:C>G-17:C>G	TGCAGGGCTTACTGCTACCAGCGAGGCTACGACCTGTTGAAAAGCTATTGCAAGCCG	17:C>G
1862414|F|0-7:C>T-7:C>T	TGCAGGTCAAGATCGACGGCCGCGTCAAGTGGAAGGTCGGGACCTGGGTCTCCG	7:C>T
3028790|F|0-15:C>T-15:C>T	TGCAGTGTACCTGAACGGGAACAAGTTTGCTGGCGCGGTGCCG	15:C>T
3028790|F|0-36:G>A-36:G>A	TGCAGTGTACCTGAACGGGAACAAGTTTGCTGGCGCGGTGCCG	36:G>A
994650|F|0-10:A>T-10:A>T	TGCAGAAACGAGAACACCAAAAAGACCTACCAGGTTGTGGTAGTTGGCGAGCTTGGCCATGGTGGTGCT	10:A>T
1047086|F|0-26:A>G-26:A>G	TGCAGACCGCTCAGCCAAACCATTCAAGACAGTGGCAAAGGTGAACTCATCAGGCCGAACCCCGCAGCC	26:A>G
1065076|F|0-56:A>T-56:A>T	TGCAGAGAGCCAAAGCCAAGAAACCAAGAACCAAGCAGATGATGCCAGCAAGCCTAACGAGGCCGCCGA	56:A>T
2253805|F|0-39:C>T-39:C>T	TGCAGAGTTTGCGGCATGCTCGAACTGCGACCTGATCTCCCTTGCCACCTCAATATCATCGTTGTAGGA	39:C>T
1058412|F|0-33:G>C-33:G>C	TGCAGATATGCCTGCCGCTGTACGTGCTCGGAGGAGCCCTGGCCGCGGCCGTCCACGTGATCAACGTCC	33:G>C
32025475|F|0-57:T>C-57:T>C	TGCAGATCTCTGCGTTGGAGGGGCTGCGCACGCACACCTTGGCGTACCTGTGGCAGGTCGCGCAGTTGA	57:T>C
2263784|F|0-59:C>T-59:C>T	TGCAGCAGGATAATGGCATCGAGGGAGAACTCGTCGGCGGGCAACAACGGAGGAGGCTGCGGCGCGAGC	59:C>T
32025505|F|0-32:A>G-32:A>G	TGCAGCAGTTCTTTGGGGCATTTCTTCGAACAACCTCCTTGCACTCTCCAGGTCCCCGCACTTCACGCA	32:A>G
1178758|F|0-19:G>C-19:G>C	TGCAGCGGTCGCGGCTCGGGTTCAGCTCCTCCCTGCTCTTCCGCCAGACCACCTGCAACAACTTCCGCC	19:G>C
1067955|F|0-15:G>C-15:G>C	TGCAGCGTGTCCGCGGCCAGGAAGCGGAGGTTCATCTCGTAGGGGTCGTTGGTGGAGATGAAGTGGAAG	15:G>C
2242955|F|0-24:T>C-24:T>C	TGCAGCTTTGTACGAGCCGAGCGCTCTGGCCGTCGATCAAACGGATTCATCCTTTATCAGGAGCAGCAA	24:T>C
3934197|F|0-13:C>T-13:C>T	TGCAGACAAAGATCGTCGAGGCGCTGCACCGCGAGGTGTCCG	13:C>T
5580225|F|0-13:G>A-13:G>A	TGCAGCCGCAACTGCCTGATGGTCCACTCGCCG	13:G>A
4909919|F|0-23:G>A-23:G>A	TGCAGCACCTGAGCCGCGGCCGCGCCGCCGCCG	23:G>A
3936465|F|0-8:T>C-8:T>C	TGCAGCAGTGGTATGAATGCAAAGTGCACCG	8:T>C
3026915|F|0-23:A>T-23:A>T	TGCAGCGACGGGAAGGCGGCACCAGCACTGCTGCCACCG	23:A>T
993630|F|0-7:G>T-7:G>T	TGCAGCGGTTCCCGCGCGGCCAGGCGGCGTCCTCGTCCGCCGACGCGCACGAGGTCCG	7:G>T
2267244|F|0-8:G>A-8:G>A	TGCAGGACGGGCTCTTCGACCTCACCGAGGACGGCGGCGGCGGCGTGGTGATGGACACCG	8:G>A
3938882|F|0-26:C>G-26:C>G	TGCAGGTAGAAGGACACCCCCGTGACCACGAACCCG	26:C>G
2251892|F|0-52:C>T-52:C>T	TGCAGAATCCACCACCACGAGATGTTTTCGAAACCCCAGAACGTCACAGCGGCGGAAGCCGATAAACCA	52:C>T
1125497|F|0-46:G>T-46:G>T	TGCAGACCCAGCAGCGCGACATCCTCGGCTCCGCGGCCGTCTCTCCGCAGCGCCTCTTCGCCGACGCCT	46:G>T
1165550|F|0-51:G>T-51:G>T	TGCAGCATGCTGCCGAGCTTCCTCACCGACCCGTTCCCCGCGGACCCCCTGGTGGAGGACTGCGACGGC	51:G>T
4910997|F|0-45:A>T-45:A>T	TGCAGCGACAGCGGTGGAGGTGGCTGGAATTTCTCGGGGGTGTTAAAACTAGGCGGGCGGGGATTGGTG	45:A>T
1863557|F|0-37:C>T-37:C>T	TGCAGCGATGAAGACGGCGACAGCGGCGATCGAGCAGCTGGTTTCAGCACGGAAGAGGTCGGGGCTTTC	37:C>T
1863557|F|0-45:A>G-45:A>G	TGCAGCGATGAAGACGGCGACAGCGGCGATCGAGCAGCTGGTTTCAGCACGGAAGAGGTCGGGGCTTTC	45:A>G
987148|F|0-64:C>T-64:C>T	TGCAGCGCACACGTCCGTCCACTCATCATCGCACCCGAACGGATTAGACAAACGGCTCAAAAGGCTTTC	64:C>T
1069859|F|0-43:C>T-43:C>T	TGCAGCTCCGCTGAGGCCTCAGAATGAGCTCAAGCACGCAAGACCCTGCGCGACCACGGACAACGACCG	43:C>T
100535469|F|0-37:G>T-37:G>T	TGCAGCTCCTCAGCCCGCTCGACGACGCCATCGCCATGGTTGCTCTCTCGATGCTGGCTGCTCCTTGCT	37:G>T
100535468|F|0-41:G>A-41:G>A	TGCAGCTCCTCAGCCCGCTCGACGACGCCATCGCCATTGCTGCTCTCTCGATGCTGGCTGCTCCTTGCT	41:G>A
983392|F|0-35:C>A-35:C>A	TGCAGCTTTTGCTTTCGGTACATAAATGATTTTGTCTTCAAAATGAAAGCCTACAGCCATGTGGACCGA	35:C>A
1025479|F|0-64:G>A-64:G>A	TGCAGGCGTCGGTGATCCGCGACCTGGTCCTCCTCTCCTGCGTCGGCCTGCGCCCCGTGCTCGTGCACG	64:G>A
6048003|F|0-23:C>T-23:C>T	TGCAGGCTCCTCGCCAAGGCCGCCTCGTCCACCTTCCCGTCCTACTCCTACAGCAGCGGCGCCCAGCTC	23:C>T
1250602|F|0-8:C>T-8:C>T	TGCAGTGTCTTGGAAGCTCTTGATGAGATCTTGAGCGGCGCGCAGGTTCTCGCCGTCCCGCTCCGCTCG	8:C>T
1302338|F|0-25:A>C-25:A>C	TGCAGTGTCTTGGAAGCTCTTGATGAGATCTTGAGCGGCGCACAGGTGCTCGCCGTCCCGCTCCGCTCG	25:A>C
7911885|F|0-67:G>A-67:G>A	TGCAGGAGCTCGAGCTCGCCGGGAACGGCCTCGGCGGCAGGCTGCCGTCGTTCATCGGCGAGCTCTCGC	67:G>A
1220051|F|0-43:C>G-43:C>G	TGCAGGGCTTCGTGATCTCAGATTGGATGGGCATTGACAAGCTCACAACCCCTTATGGGGCGGACTACC	43:C>G
3023698|F|0-32:T>C-32:T>C	TGCAGATGGTTCGAGAACATGATCTAATGTTTTCCCG	32:T>C
4005867|F|0-20:C>G-20:C>G	TGCAGCAGCGTCGCCTCCGCCGACTTCGAGGGCCTCTTCTCCCCCTCCG	20:C>G
998600|F|0-5:C>G-5:C>G	TGCAGCATGTCCGCCTGCGACGCGGCGACTCTGGCGCGCACCATCTCCGCGCCCG	5:C>G
1004688|F|0-26:G>A-26:G>A	TGCAGCTTGTGCGGCCCCTTCGCCTCGGCCGCACCCGCGTCGCCCTCGGCCGCGACCTCCG	26:G>A
1004688|F|0-32:A>T-32:A>T	TGCAGCTTGTGCGGCCCCTTCGCCTCGGCCGCACCCGCGTCGCCCTCGGCCGCGACCTCCG	32:A>T
1097708|F|0-30:A>G-30:A>G	TGCAGGTCCATCCACGCCACGGCCGGCAGGAAGTCGCCGAGGTTCACCAGCCCG	30:A>G
3940941|F|0-21:G>A-21:G>A	TGCAGTGCAGAACCATGACGCGGCGCGGTTCCCTTGATGTGGCCG	21:G>A
1027942|F|0-19:C>T-19:C>T	TGCAGAAGCTCCTGCACGCCGCCTACAAGCGCGGGGACACGTCCGTGGAGGTGCGCGTGCGGCGCCCCG	19:C>T
100542515|F|0-15:T>C-15:T>C	TGCAGAGGAGGAGGCTGCCGCAGCACTGCTGGATCTCGACGCGCTCGTAGCGCCCTCGCAGGAACGGGA	15:T>C
100542684|F|0-28:T>C-28:T>C	TGCAGATCATCATGGATACTCTGGTTGCTGCCAGGCAAGTCAACAAGGCCGTCCAACTGCTCGAGAGCG	28:T>C
3943304|F|0-10:G>A-10:G>A	TGCAGATGGGGACCAAGTTCAGGGTGCTCGAGGCGGACTTGGCGCGGTCGCGGTGGGCGAGGCTGAGGA	10:G>A
1167345|F|0-56:T>C-56:T>C	TGCAGCGCCGTCATCAGCGGGGTCTCCAGCCCGAACATCCTGCCCTCCATTCTCGCTCGCTGCTGATCT	56:T>C
1106813|F|0-15:G>T-15:G>T	TGCAGCAGAGACAGTGCATGGCCGCGGCACCACATCACCTGCTCTATGAATTCGCCAAGGCTGCACTCA	15:G>T
1766794|F|0-6:C>A-6:C>A	TGCAGCCGCTGGATCCAGCTCGGTGCATTCTACCCGTTCGCGAGGGCGCACATGGAGAGAACCACCGAA	6:C>A
1092000|F|0-50:C>G-50:C>G	TGCAGCGCGTACGCCAGCCGCACGATGTCCCGCCGCTCGCGCCGCTTGGGCGTGGTCAGCACCCCGACG	50:C>G
1046380|F|0-50:A>G-50:A>G	TGCAGCTTGTTCCAGGACTCCTCCCTCTGCATCTTGAGCTGCTTGTAGAAATTGCCGATGAAGGCGTCG	50:A>G

998377|F|0-6:A>G-6:A>G	TGCAGGAACGACGGCGGCGAGCGTGAGGGGCGAGGTGGACAAGAGCAGTGCTCTCCTGGTTGTGGCCGA	6:A>G
998377|F|0-7:A>G-7:A>G	TGCAGGAACGACGGCGGCGAGCGTGAGGGGCGAGGTGGACAAGAGCAGTGCTCTCCTGGTTGTGGCCGA	7:A>G
998377|F|0-17:C>T-17:C>T	TGCAGGAACGACGGCGGCGAGCGTGAGGGGCGAGGTGGACAAGAGCAGTGCTCTCCTGGTTGTGGCCGA	17:C>T
998377|F|0-20:G>A-20:G>A	TGCAGGAACGACGGCGGCGAGCGTGAGGGGCGAGGTGGACAAGAGCAGTGCTCTCCTGGTTGTGGCCGA	20:G>A
998377|F|0-23:T>C-23:T>C	TGCAGGAACGACGGCGGCGAGCGTGAGGGGCGAGGTGGACAAGAGCAGTGCTCTCCTGGTTGTGGCCGA	23:T>C
1766608|F|0-19:G>A-19:G>A	TGCAGTCCTCGAGGCGGTCGAGGAGGTTATCGGCGACCGCGTCTACCAGTGGGTCAGTTTCCCGCATCG	19:G>A
100547369|F|0-11:C>G-11:C>G	TGCAGATGCTGCTGGTAGCTGCACGACTCGTTGTGGTTGGGCGTCTTGCACGCAACGTGCGGGAAGCGG	11:C>G
5367573|F|0-7:G>C-7:G>C	TGCAGAAGAAGGACAGCTTGAACTTGGGTAGCCG	7:G>C
5367573|F|0-25:G>A-25:G>A	TGCAGAAGAAGGACAGCTTGAACTTGGGTAGCCG	25:G>A
4540861|F|0-5:C>A-5:C>A	TGCAGCGAGCGGCGGAGCACGAGGAGCGGGCGGCGGCCGTACACTCTAGCGATGCCG	5:C>A
7173292|F|0-12:G>C-12:G>C	TGCAGCGCCCCTGGAGCGACTCCAGGAAGAGCGGGACCGCGAGCAGGCCG	12:G>C
993527|F|0-13:G>A-13:G>A	TGCAGGACGTGACGGTGGCCGCGTACGCCCTGGAGGCCTGGGTCGCGGCGAACCG	13:G>A
100549692|F|0-15:T>C-15:T>C	TGCAGGCGGCGGCGGTGGACGTCCGACGTGGCCGTGCTGCCGCTGCACGGCGCTAGGCCGTTGTTAGGT	15:T>C
3959006|F|0-23:A>G-23:A>G	TGCAGATCAGCAACGGTAATAAGATGTGAGCAATCGATTCAATTCAAGGATTCTGTAACTGTAATCTGC	23:A>G
1146832|F|0-40:A>G-40:A>G	TGCAGATGCATTAATTGGCGTTATTATGGCAGGCATGAGTACACACGTAGAGTGTATTTGTTGCCGCCT	40:A>G
100550064|F|0-53:C>T-53:C>T	TGCAGGTTCATGAACTCGATGGGGATCCCCGAGTCCTGCGAGGAGTAGAGCCACGTGTTGGCCCGAGCC	53:C>T
1120742|F|0-61:C>A-61:C>A	TGCAGCACGCACGCATCCATGATGCAACACACATCAGCCAGAAACCATGCAAGACCAACACCATTTCCA	61:C>A
2252132|F|0-20:C>T-20:C>T	TGCAGCGACGAGATCGAGAGCCTCGAGCGGGAGATCGGCGACCGAGCGTCCGAGAGATGGACTGCCGCC	20:C>T
2252132|F|0-23:C>G-23:C>G	TGCAGCGACGAGATCGAGAGCCTCGAGCGGGAGATCGGCGACCGAGCGTCCGAGAGATGGACTGCCGCC	23:C>G
100551082|F|0-52:T>G-52:T>G	TGCAGCTCAAGGAGTTCGCGCCGCCGTTCCTGGACAGAAGCCTGTCCGACAATGACATCGTGACGGGGG	52:T>G
1095280|F|0-22:C>G-22:C>G	TGCAGGCGCCGCCGCGGCAGGACACGTGGGGGTTCTCGCCGCTGGATGCGCAGGCCGCGTACATGCCGA	22:C>G
1075099|F|0-15:G>T-15:G>T	TGCAGGTCCATTCCAGTTCGTCGCAGAGCCCTACAACGGCACCATACCGCGCCTGCAAATCAACCCCTA	15:G>T
3020650|F|0-14:G>T-14:G>T	TGCAGAGATGACACGTGGGAAAGATGCAACCTTTTGAGCCG	14:G>T
1207327|F|0-17:C>T-17:C>T	TGCAGCTGGTAGTAGAGCTGGCACGCGGACAGCCCAGGCGACGGTGTCAGTGGTGGTGCCG	17:C>T
3031590|F|0-25:C>G-25:C>G	TGCAGCTGCATGGCGGCGTTGTCTCCCTGCGAGATGGGCGCCG	25:C>G
100555769|F|0-35:A>G-35:A>G	TGCAGCTGCATGGCGGCGTCGTCTCCCTGCGAGATAGGCACCG	35:A>G
3934986|F|0-16:G>T-16:G>T	TGCAGGCCCTGCTCGCGCACCTGTGGCGCGCGGTGTGCCGAGCCCG	16:G>T
990138|F|0-39:C>G-39:C>G	TGCAGAGGGGATGGCTTCTTAGCCTTTCTTTGTCATGAACATGTTAGATTTGCTGGATTAGCATGCCTG	39:C>G
990138|F|0-44:T>C-44:T>C	TGCAGAGGGGATGGCTTCTTAGCCTTTCTTTGTCATGAACATGTTAGATTTGCTGGATTAGCATGCCTG	44:T>C
990138|F|0-68:G>C-68:G>C	TGCAGAGGGGATGGCTTCTTAGCCTTTCTTTGTCATGAACATGTTAGATTTGCTGGATTAGCATGCCTG	68:G>C
1066193|F|0-55:C>T-55:C>T	TGCAGCCACCGCGACATGGTCTTCTCGGTGGGGTTGAGGGAGCTCGCCGCATCAGCACACTGCATGTCG	55:C>T
1066193|F|0-59:C>G-59:C>G	TGCAGCCACCGCGACATGGTCTTCTCGGTGGGGTTGAGGGAGCTCGCCGCATCAGCACACTGCATGTCG	59:C>G
100557047|F|0-16:T>C-16:T>C	TGCAGCCTGGGGTTGATGACGGCGATCGAGCCCCACGAGTGGCGGCCCGTGGCGGGCCATGCCGCCGCC	16:T>C
1060660|F|0-26:A>G-26:A>G	TGCAGCGACCTCTTGAGCTCGGTGAAAAGGTCACCCCAGCCATCGGTGCCCGTGTCGCTGACCACGGTC	26:A>G
1060660|F|0-28:G>T-28:G>T	TGCAGCGACCTCTTGAGCTCGGTGAAAAGGTCACCCCAGCCATCGGTGCCCGTGTCGCTGACCACGGTC	28:G>T
15306706|F|0-35:G>A-35:G>A	TGCAGCTACATGTGTTTTCAATTGCTTTCTCCGCTGCCCCTTTTGCCTGCTACGAGCAGTAGTAGTATA	35:G>A
1156230|F|0-16:G>A-16:G>A	TGCAGCTGCAAGTCAAGGTGCTGGCGACGGACGAGTTCTGGCCGGCGCAAGGGGGAACGGCGCCGAGAT	16:G>A
980509|F|0-59:A>G-59:A>G	TGCAGCTGCTCCTAGCAGTATGTACATTCACTAATGTGCAGAGTGAAATGGGACGATCAAGAACACATC	59:A>G
1046618|F|0-12:G>A-12:G>A	TGCAGGAACTCGGACGACGGCCCGTTGCGCACCGTGGCGTTGAACCCGCAGAGGTGCTGTTGGAGCTCC	12:G>A
1046618|F|0-50:G>C-50:G>C	TGCAGGAACTCGGACGACGGCCCGTTGCGCACCGTGGCGTTGAACCCGCAGAGGTGCTGTTGGAGCTCC	50:G>C
1046618|F|0-59:T>C-59:T>C	TGCAGGAACTCGGACGACGGCCCGTTGCGCACCGTGGCGTTGAACCCGCAGAGGTGCTGTTGGAGCTCC	59:T>C
100557833|F|0-29:T>C-29:T>C	TGCAGGAACTCGTTGGCGCAGTAGGCAGGTGATACGCCTCGCGGGAGGCCAAACGCCGCCGCTCCCGAT	29:T>C
5368734|F|0-27:G>A-27:G>A	TGCAGTCGCTTGAGGCCCAGAAGCGGCGGCGCGCGCAGCTGGCGTCGACGCCGCCGTTCGCCG	27:G>A
7170394|F|0-10:G>A-10:G>A	TGCAGTGGCGGGACGGCTGCCCCGTGGAGCAGCCCGTTCTCGACCAGCGGAGCAGCGACAGCGACGACG	10:G>A
12764166|F|0-23:G>A-23:G>A	TGCAGTGGCGGGACGGCTGCCCCGTGGAGCAGCCCGTTCTCGATCAGCGGAGCAGCGACAATGACGACG	23:G>A
1214689|F|0-61:C>A-61:C>A	TGCAGTGGGCGGCGCCGAGGGAGCCGCTCTGCCAGACGCAGGCGGACTGCGAGGACGGCGCCAACGCCA	61:C>A
2265324|F|0-14:G>A-14:G>A	TGCAGGTTGCCGGCGCTGGCGTCGAGGGACTGGTCGTCGAGGCCGACCAGCGTGGCCG	14:G>A
2260116|F|0-8:G>C-8:G>C	TGCAGCAAGCACACTGAGGGCAAGCGTCCGCTCTACGCCATCAGCTTCAACTTCATCGACGCCCGCTAC	8:G>C
991791|F|0-43:C>A-43:C>A	TGCAGCAGCACCATCGCCAGGTATGGCTTCACATCGCTCACAACTTTCCACCCCATACCCATACTTCTA	43:C>A
2277639|F|0-8:G>A-8:G>A	TGCAGCATGCGCGTCATGAAACGGAAGGTGCCGTCGTCGATGTACGGCGACAGACGGGCGCTCTCGATG	8:G>A
1090765|F|0-10:G>A-10:G>A	TGCAGCCTGCGCGACTTCGCCGACGGCCTGCTGGACGTGACCTGCGGCAGCACGGACGTGACCGTCTCC	10:G>A
1090765|F|0-14:C>A-14:C>A	TGCAGCCTGCGCGACTTCGCCGACGGCCTGCTGGACGTGACCTGCGGCAGCACGGACGTGACCGTCTCC	14:C>A
1031593|F|0-52:G>A-52:G>A	TGCAGGGCAAAGACACACCGCACAAGGATGTGCTGAATTGACTCCTCAACTCGGTCACAGAAGGGGAAA	52:G>A
1031593|F|0-65:G>A-65:G>A	TGCAGGGCAAAGACACACCGCACAAGGATGTGCTGAATTGACTCCTCAACTCGGTCACAGAAGGGGAAA	65:G>A
2246647|F|0-40:C>T-40:C>T	TGCAGGGTGTTCAGGCGAAGCTGCGGAAGATCCACCTCCGCTTCGACGACATGATCACCAGCCTCCTGG	40:C>T
1090680|F|0-11:T>C-11:T>C	TGCAGGTGCTGTTGCTGCTGCTGCTGGTAGTGCATGGCGAGGAGGGACCAGTAGTCCTTCTCCGCCGAG	11:T>C
1066648|F|0-19:T>G-19:T>G	TGCAGTCGTTCATCTGCCTTACCATCATCACCTTCGTCCCTGTGTACGACCG	19:T>G
4398235|F|0-28:G>A-28:G>A	TGCAGGCTGGTTAGTTGATCGGGCGATCGGCCGACGTACGTGGTCCG	28:G>A
7172278|F|0-7:G>C-7:G>C	TGCAGTTGGCCCTGATCTCGGCGTGGCGGCCG	7:G>C

1092578|F|0-30:G>A-30:G>A	TGCAGATGGCCCACCACCACCGTGTACGTCGTTTCCCTCGTCGGCGCCGTGCTCCTCTACATTGTGCTG	30:G>A
1092578|F|0-53:C>T-53:C>T	TGCAGATGGCCCACCACCACCGTGTACGTCGTTTCCCTCGTCGGCGCCGTGCTCCTCTACATTGTGCTG	53:C>T
981918|F|0-52:C>G-52:C>G	TGCAGGCCGCGGAGGCCGAGCTGTCCATGGCGAAGAACGAGCTGGAGAGCATCAAGGCGGGGGCGTTCC	52:C>G
1696589|F|0-14:G>A-14:G>A	TGCAGGTACTACTCGACCTGGGGACACGTCGCGACGCTGGCGGAGGAGATAAAGAAGGGCGCCGACTCC	14:G>A
993425|F|0-33:G>A-33:G>A	TGCAGGTACTCGGGCTCCAGCGCGAACAGCACGGCGATGCCGTCGTCCCTGTCGGCGCCGCGCTGCGTC	33:G>A
993425|F|0-35:G>A-35:G>A	TGCAGGTACTCGGGCTCCAGCGCGAACAACACGGCGATGCCGTCGTCCCTGTCGGCGCCGCGCTGCGTC	35:G>A
22765217|F|0-13:C>G-13:C>G	TGCAGTGGCTGTCCGCGCGGCCGCAGTGGGCGGCCTTCGGCGGCCGCGACCACTTCATGGTCGTCGGCA	13:C>G
100567888|F|0-18:A>G-18:A>G	TGCAGTGGCTATCGGCGCAGCCGCAGTGGGCGGCCTTCGGTGGCCGCGATCACTTCATGGTCGTCGGCA	18:A>G
100568471|F|0-23:A>C-23:A>C	TGCAGAAGATGTTCGAGGACCAGATCAAGGCCAGCCGCAACGGCGCGCCG	23:A>C
5971324|F|0-9:G>A-9:G>A	TGCAGAATGGCCCGATCTCGTGCCCG	9:G>A
989564|F|0-8:G>A-8:G>A	TGCAGAGCGAGCCGACGGACGCAGAGGTCGCCGAGGCCATGAACATGCCG	8:G>A
3023760|F|0-20:G>T-20:G>T	TGCAGATGTACACCCGAAATGTGGGTTTCAGACCTGACCAAGCCG	20:G>T
4404352|F|0-8:G>T-8:G>T	TGCAGGACGGGACCGTGGCGTGGGGCAAGGCCGCCACCTTCCGCGCGTCCCCCTACCCG	8:G>T
1025762|F|0-41:C>T-41:C>T	TGCAGGACGGGACGGTGGCGTGGGGCAAGTCCGCCACCTTCCGCGCGTCCCCCTACCCG	41:C>T
1025762|F|0-42:G>T-42:G>T	TGCAGGACGGGACGGTGGCGTGGGGCAAGTCCGCCACCTTCCGCGCGTCCCCCTACCCG	42:G>T
7170071|F|0-17:A>G-17:A>G	TGCAGCCACTCCCCTCCACTTGCGCTCTGCACCACCGTGACGAGCAGCGTTCCCAGGTAGTTCCCGAGC	17:A>G
1117334|F|0-44:C>T-44:C>T	TGCAGCGATATCAATTCAGCGGAACAATGTGAGAGAAGCGAGCACTGTGCGGAGGGAGAAAAGCTGAGG	44:C>T
1190272|F|0-25:G>A-25:G>A	TGCAGCTCCCTGAGAACCCTGCCGAGTTATACGAGATGAGCAGAAGCAAAGCTTCTCCCGAGGAGGAGT	25:G>A
1008453|F|0-7:C>A-7:C>A	TGCAGCTCCTGACGGGGCGCAGGCCCTACGACAATGTGCGGCCGCGAGGGGAGAGGCTCCTGGTGCCGT	7:C>A
5350155|F|0-19:G>A-19:G>A	TGCAGCTGCTCGTCTCCGCGGGGCCGCCG	19:G>A
1220649|F|0-24:G>A-24:G>A	TGCAGACACGGTGTTCTTCCTGGCGTCCAAGCTCCTCAAGACGGTCCCCCAGGTCAGTCACTCTCACTT	24:G>A
1024841|F|0-9:G>A-9:G>A	TGCAGCGCAGCGTTTGCTGTGCATGCACCGCGCATGTGCCTGGTCTCTATCTGTTCGCCTGTGCCCTCC	9:G>A
1024841|F|0-19:T>C-19:T>C	TGCAGCGCAGCGTTTGCTGTGCATGCACCGCGCATGTGCCTGGTCTCTATCTGTTCGCCTGTGCCCTCC	19:T>C
1024841|F|0-48:A>G-48:A>G	TGCAGCGCAGCGTTTGCTGTGCATGCACCGCGCATGTGCCTGGTCTCTATCTGTTCGCCTGTGCCCTCC	48:A>G
1040610|F|0-11:C>T-11:C>T	TGCAGCGGGTTCGGCCCTGCCCCGTCGCTCACCGCCATCATCGCCAACCGCTACCGCATGCCCAGCGAC	11:C>T
3945020|F|0-34:C>T-34:C>T	TGCAGCTCATCGACCTCTACTACGACTGGCCCCGCGGCCGCAACCTGAACCTCATCCACAACCAGCTCT	34:C>T
100573837|F|0-9:C>G-9:C>G	TGCAGCTCCCCCACCTCGTCCTCCTCGCCGCGGTAGGCCGCGGCGGCGATCTCGTGCGGCAGCACCGCG	9:C>G
4909970|F|0-50:G>A-50:G>A	TGCAGGACGACGGGGCCGTGGAGGTAGGAGAGCCGCTTCGTGGCGGTGGCGGCGGCGCGGTCGGGGAGG	50:G>A
1696624|F|0-34:T>C-34:T>C	TGCAGGCACGACGCCGCCGAGTGCGCCAGGCTCCTCCTCGACGGGGAGTACGGGATCACGCCG	34:T>C
1696624|F|0-39:G>C-39:G>C	TGCAGGCACGACGCCGCCGAGTGCGCCAGGCTCCTCCTCGACGGTGAGTACGGGATCACGCCG	39:G>C
1118336|F|0-24:C>T-24:C>T	TGCAGGCGACGGTAAGCAAAGCTTCGTTGTGGTGTCAACCAGGTCATTTCAGACACAAGATGTCTGCTC	24:C>T
3222334|F|0-32:C>T-32:C>T	TGCAGCAGCAAGAACACCAGCGACAGCAGCGCCGCGGTTCCG	32:C>T
5011113|F|0-13:C>G-13:C>G	TGCAGCTTCGGGACGGAGGCGCCGTGGGCAGCCG	13:C>G
3948519|F|0-20:C>T-20:C>T	TGCAGTAGCAATTCGTCGAACAGGTGGTGTGCGTCTTCAGGCCTGAGCGTCCCG	20:C>T
1103078|F|0-7:C>G-7:C>G	TGCAGAGCACCGAGGAGAAGTGCAGCACTTGGGAGAAGCGCTCGCGGTTCTTCATGGACGAGTATATCG	7:C>G
1028131|F|0-56:C>G-56:C>G	TGCAGCGACGTGGGGTTGGCGGCCAGCGCCTTGGAGGCGAGGGGCCACATGTTGAGCGGCGAGCCGCCG	56:C>G
1863482|F|0-10:G>A-10:G>A	TGCAGGCGTTGTCCTCCTGCCGCCGCCTCGACCACGTCCGCCACGTCACGGCGAGGCCCACGGCGACCA	10:G>A
1863482|F|0-21:G>C-21:G>C	TGCAGGCGTTGTCCTCCTGCCGCCGCCTCGACCACGTCCGCCACGTCACGGCAAGGCCCACGGCGACCA	21:G>C
1001188|F|0-42:A>C-42:A>C	TGCAGGGTGTTCCAGAAGCAGAAACTGGACGGCGAGCAAGACAACGCCCGCTCCTCCTCGCCGACCTTC	42:A>C
100578178|F|0-6:C>T-6:C>T	TGCAGGCGATCCTGCACCAGCAGCTGCAAGTGCTGGTCGAGAGGTACCG	6:C>T
15306753|F|0-20:A>G-20:A>G	TGCAGTCCCTGGTCCTCGTCAACAACAACCTCACCG	20:A>G
6047715|F|0-27:G>A-27:G>A	TGCAGTCACTGGTCCTCGTCGACAACAGCCTCACCG	27:G>A
5368032|F|0-34:C>T-34:C>T	TGCAGACACGCGCTGATCACGATGTTCCAGGAGACGGCGTCGACCTCGGCCATTGCTCTGAATGCCCGC	34:C>T
1221211|F|0-52:T>C-52:T>C	TGCAGCAAGTCGGGAGCCATCATCCCCTGGAACACGTCCTCCTCCAAGAAGCTCAGGAAGCCCCCGAGA	52:T>C
1202706|F|0-66:G>C-66:G>C	TGCAGCGAGCCCTCCGAGATTTTGACCTCCACCAGCGCAAGCTTCTTGAGCAGGGGAAACTGAAGCGTC	66:G>C
1169049|F|0-22:C>A-22:C>A	TGCAGCCTTCCAAAAATACTGCCCAACTGCCTGTGATGGATCACCAGCTGTACGGGGACCCGTCCGCGA	22:C>A
1149870|F|0-52:G>T-52:G>T	TGCAGCTCATGCCGCCGTCCAGCAGCAGCACGGGTGGTGCGTCTGCGCCTCTGGGCGTGGCGCCGTCGC	52:G>T
1041718|F|0-5:G>A-5:G>A	TGCAGGAGGCTCGTGAGGCACAAGAGCACGACAAGGTATGGCGTGGAAGAAGCCATCTTGATTACTGTA	5:G>A
5365560|F|0-34:G>A-34:G>A	TGCAGCTGCTCGACCTGTCGTCCAACTCCCTCACGGGCGCCATCCCGCCTGAGCTCGGCAAGCTCCAGG	34:G>A
100580626|F|0-8:C>T-8:C>T	TGCAGAACCATGAGAATTGTCCAAAGTCATGACTTACAGGCCTCCGACGATTGCGGAGAAGATGGCCGT	8:C>T
100580627|F|0-17:C>T-17:C>T	TGCAGAACTGTGAGAATCGTCCAAAGTCATGACTTACAGGCCACCGACGATTGCGGAGAAGATGGCCGT	17:C>T
3222474|F|0-37:C>T-37:C>T	TGCAGTGCCGACCGCAGCTTGGCGGCTTCCTCCGCGTCGTTGGATGCAGACAGCAGGCCGAGATCGATT	37:C>T
7171134|F|0-5:C>G-5:C>G	TGCAGCGCGGCGTAGCTGCTGCCGCTCATCTGGTCGCCGACGAAGGCGCCGTGCGACGGCGTCACGTTG	5:C>G
1022468|F|0-43:A>T-43:A>T	TGCAGCTCGTCCAGCACGCCTCGCCCCCCTGCGTGGATGCAGAAGTGCTCCAGCGCCAGAGTGAAGTCG	43:A>T
1151616|F|0-17:C>A-17:C>A	TGCAGGACGTTGCCGACCACGCTGAGCCCGTCGTCCGTCGCCTCCAGCGCCAGGCAGTGGTACCTCCCG	17:C>A
1136234|F|0-26:C>T-26:C>T	TGCAGCCATGGACGGCGTGTACCAAGCGCTGGTCGTGACCGTGGCTGCCTCCGTAGGCATCTCCTTCCC	26:C>T
3934798|F|0-14:C>T-14:C>T	TGCAGCCACGCGCGCAGCCTCCGCACCGACACGCTCCCG	14:C>T
4002189|F|0-19:G>A-19:G>A	TGCAGCTGGGCGGCGACGTGGGCGGCGGCCGCCTCAGCACCGACCCG	19:G>A
7340709|F|0-29:G>T-29:G>T	TGCAGCCCTGGAGAGCAGAAACCTTGCAAGCATCTTTTCGAGAGGCCGCCCTTGGAAGTTTGCAATGTT	29:G>T

999513|F|0-47:C>A-47:C>A	TGCAGCGCCCAGGCGGTGTCCGCCACGTACCCTTGGCCGCGGATCCTCTCCGTCATCTCCTCCGCCTTC	47:C>A
992804|F|0-8:G>A-8:G>A	TGCAGGCCGCGCTGCTCAAGGAGTACCCCAACAAGCTGCTCGTGACGGCGACGCAGTGCGTCTTCAGCG	8:G>A
1103950|F|0-12:T>C-12:T>C	TGCAGTGCGTTGTCAAGGAGACTCTTCGGCTCTGCCCCGTCGGCCCAATCATCCCTGCGCACGAGGCCA	12:T>C
1022281|F|0-34:G>A-34:G>A	TGCAGTCGCAGAGCCTCATCATGCCGCTGCTCCTGTCGTCCGTGGCCACGCTGGCGCTCCACGTCCCCC	34:G>A
3024696|F|0-20:A>C-20:A>C	TGCAGCAGCTGCTCCAGGCCATCGGCTCTAACAACAGCACCACCG	20:A>C
3024696|F|0-25:C>T-25:C>T	TGCAGCAGCTGCTCCAGGCCATCGGCTCTAACAACAGCACCACCG	25:C>T
1014038|F|0-47:A>C-47:A>C	TGCAGCTACCTAGAGTCAACTCTTAATTTGTACTTAGCATAGGATCAAGTACGTTTCCG	47:A>C
100584493|F|0-41:C>G-41:C>G	TGCAGCCACTGGGGCTCGGCCACGACGCCGTCCCCGAGGTACTCCGCCGCCACCACGCGGTACCTGACG	41:C>G
3935706|F|0-60:T>C-60:T>C	TGCAGCCACTGGGGCTCGGCCACGATGCCGTCTCCCAGGTACTCCGCCGCCACCACGCGGTACCTGACG	60:T>C
32027774|F|0-51:A>G-51:A>G	TGCAGAACGTGGTCCCTCTCGCCACCTTCCGCGACTTCGGGGACGACGTCTACTTCGTCGCCGACCAGC	51:A>G
1863229|F|0-6:G>T-6:G>T	TGCAGAGCGCGGCGGCGCCGAGCGCGCTGCGGGTGTCGAGACGGCCCAGGATCTGGAGGATCAGGTCGT	6:G>T
100584571|F|0-37:A>G-37:A>G	TGCAGAGCGCGGCGGCGCCAAGCGCGCTGCGGGTGTCAAGACGGCCCAGGATCTGGAGGATCAGGTCGT	37:A>G
100584573|F|0-46:T>C-46:T>C	TGCAGAGCGCGGCGGCGCCAAGCGCGCTGCGGGTGTCGAGACGGCCTAGGATCTGGAGGATCAGGTCGT	46:T>C
1862907|F|0-47:T>C-47:T>C	TGCAGCCTCCCTGCACACAACCTGTTCGACGAAACGCCTCCGAGCTCTGACCCCATGGCGGAGGCGTCG	47:T>C
1696446|F|0-20:T>C-20:T>C	TGCAGTTTGTGACATACTTCTGCTGCTGATTGACTGGCCTGAAGTCTGAACCTCATGGAGACCGTTTCG	20:T>C
3938852|F|0-35:C>T-35:C>T	TGCAGGACGATCATGCCGATCATCACGCCGATCGACATGAACAACAGTATGCCG	35:C>T
1090179|F|0-22:T>C-22:T>C	TGCAGAGAAGACTGAGGTAGTGTCTACCTTTCGGAGCGACGGGAGGTGGTCCCCCCACACCACGAGGTC	22:T>C
1092571|F|0-20:G>C-20:G>C	TGCAGCGTAGGGGCTCTGGTGGGTGTAGCCGTACATTGGCGTTGCTGCCTCTGCCTCTGGCTCCGAGAT	20:G>C
1073903|F|0-65:G>C-65:G>C	TGCAGGTGAGCATCATCAGCCAGGCGCTCATCTTCGTGACGCGGTCGCGGGGGTGGTCGTTCCG	65:G>C
1236203|F|0-30:T>A-30:T>A	TGCAGTTCTTGATCAGCTGGATGTCGCGGTTCCCGCAGTACCGGCACCGGGATTTCTACATCGCCGAGA	30:T>A
4262953|F|0-21:G>A-21:G>A	TGCAGAAGGTCGACGAGGAGGGCGCTGCCGCCGCCGTCATCAGGCCG	21:G>A
2257474|F|0-15:T>A-15:T>A	TGCAGAAGCGTTGAATCGATTCCTTGGTGCGCGGCGATTGCCGAGGGGAGCGAGATGGTACGGAAGCGC	15:T>A
4993562|F|0-10:G>A-10:G>A	TGCAGCGGGCGGCGACGGCGAGGCAGGGCTGACCCG	10:G>A
1017445|F|0-39:T>C-39:T>C	TGCAGCACATATACCGCGAGGGCAACCAGGTCGCCCACGTCCTGTGCAAAGAAGCATACCAGCGACCGA	39:T>C
100589787|F|0-7:T>C-7:T>C	TGCAGAATTTTCCTCTCGTCCAGTCTCTTCTCGGCAAGGACCAGGAAGGAGATGACAAAGGCAAGAAGA	7:T>C
1109496|F|0-31:T>C-31:T>C	TGCAGCACGCCGTCCCCGTCGCAAGGCATGCTTCGGCCGACTTCTTGGAGCAATCATACGCTGGTAGGG	31:T>C
983247|F|0-20:C>T-20:C>T	TGCAGTAGGGGAGCACGTTGCCGTCCACCACCTCCTCCTCCTGCTGCTGCACCTGCCCTTGCTGCTCGG	20:C>T
1127120|F|0-19:G>A-19:G>A	TGCAGGTGCTTCCCTGGTTGGTGGCAGTGCTGCTGTGCCCCTGTTCGTCCACGACGGCGCTAGCCGAGA	19:G>A
6048473|F|0-5:G>A-5:G>A	TGCAGGGCGTCGAACTTGTATTCCTCGCTGATGCTGCATGGGATGTGCACCACCTTGTACTGCCCGAGA	5:G>A
2258098|F|0-18:T>C-18:T>C	TGCAGCGTGCACCGCTACTCGGAGAAGCACGACTGTGGATTCGACTACAAGGCCGCCGGCCAGGAGCAG	18:T>C
100594290|F|0-37:A>C-37:A>C	TGCAGCGCTTCCCTAAACATCCCAGAGCCGCGGGGCAAGCCTACACCTTCGGCACACGCTGCCATCGCC	37:A>C
1008893|F|0-33:A>C-33:A>C	TGCAGGAGGCAGGCGATGCCCAAGAGTAGGACAAGGCAAGGCATGGAAGAAGTCATGTTGGTTGATTAA	33:A>C
1084947|F|0-8:C>G-8:C>G	TGCAGCGCCGCGGGCGGCACGACGATGCCCGTGGCCGCGCCCAGCCGCCG	8:C>G
5579661|F|0-51:G>T-51:G>T	TGCAGCTTGCTGGCAGGGTTGGTCGGGGCAGGCTGGGGAGTCGGCGCGGCGGTGTCGAGGACGGTGATC	51:G>T
7354294|F|0-32:G>T-32:G>T	TGCAGATCCAGCTCAACAGGTACAGGTGCGGTGGTCTCGTGGTCGGCATATGCTCCCACCATCAAGCCG	32:G>T
1327474|F|0-14:G>A-14:G>A	TGCAGTCGTAGGTGGAAGCCTTTCTTTCTTCTTGCACGTATTGAGCTTGGCGCTCCTTGAGATCTCGGA	14:G>A
5577861|F|0-8:A>G-8:A>G	TGCAGAGAATCTTCAGCGGCGCCACCG	8:A>G
7162113|F|0-25:C>T-25:C>T	TGCAGGGCAAGCGGAAGGCCGCGTTCCCG	25:C>T
4734163|F|0-5:G>T-5:G>T	TGCAGGCGTCCTTCGACGGCGTCCCG	5:G>T
5580147|F|0-9:G>T-9:G>T	TGCAGCATAGGCTGGCCCTCCCTGTCGAGTGCCG	9:G>T
4397159|F|0-8:G>A-8:G>A	TGCAGCCAGTTGCCGACGATGGGCGCGCCG	8:G>A
4397159|F|0-17:G>A-17:G>A	TGCAGCCAGTTGCCGACGATGGGCGCGCCG	17:G>A
3940639|F|0-29:C>T-29:C>T	TGCAGGGACCCGAGGCGCGAGAGGATGTCCTCCAGGAGGCAGTCCG	29:C>T
5578343|F|0-10:C>A-10:C>A	TGCAGGGGCCCTTCTACATGGTCAACCTGACCG	10:C>A
4261178|F|0-31:C>T-31:C>T	TGCAGTGCAGTTATGCTTTTGCAGATCAGGACCATGTTGCTCCG	31:C>T
1090832|F|0-17:G>C-17:G>C	TGCAGTTCCATGATCGCGGCGGCGAGCGCCTCGTCCAGCACCAGCCTCTCCAGCACCGTCATGCCGCTC	17:G>C
3029176|F|0-9:A>C-9:A>C	TGCAGCAGCACGCGCGCCGCGCCCTCGCTGCTGGTTCCGCCG	9:A>C
5332338|F|0-17:G>A-17:G>A	TGCAGGCGCTCCCCGACGCGCGACGCGCCG	17:G>A
7162123|F|0-19:C>G-19:C>G	TGCAGGGCCACCACCAGAGCTACACCG	19:C>G
3936945|F|0-36:G>A-36:G>A	TGCAGGGGAGCCACCTCGCGGTCTGCAAGAACGGGAGGAGGTGCGCCG	36:G>A
4404705|F|0-23:C>A-23:C>A	TGCAGCTTCCTCAGGCAGCCCACCACGCAGCCG	23:C>A
3934237|F|0-38:G>T-38:G>T	TGCAGAGGCGGCGGCACACGCCGTTGAAGGGCCCGATGGCCG	38:G>T
996034|F|0-11:T>C-11:T>C	TGCAGATGGCGTCCTCTGCTGGTAATGGCAAGGAGACGGCCGCGCTCGAGTCCG	11:T>C
4262467|F|0-39:C>G-39:C>G	TGCAGGATTCTCGCCGACGCCCGCCGCTCCCGCCCCTACCCCCG	39:C>G
100462933|F|0-10:C>A-10:C>A	TGCAGGGCTCCTACAACGAACACATCTCCG	10:C>A
2241528|F|0-6:A>T-6:A>T	TGCAGAAATTCCTTACATTCACTCTCTCGACTCTGCGCATTTCATCCTCGAGGAGTGTAACTTGTGCTG	6:A>T
2249622|F|0-16:C>G-16:C>G	TGCAGCGCGTCATCGTCTTCGGCGACATGGGGAAGGCGGAGAGGGACGGGTCGAACGAGTACGCCAACT	16:C>G
2330303|F|0-20:G>A-20:G>A	TGCAGGTCATCGAAGGCGTCGCCGCCTGCCACCGCGCCGGCCTCGTGCACCGCGACCTCAAGCCCGAGA	20:G>A
2258232|F|0-46:G>A-46:G>A	TGCAGGTGCTCGACGTCAGCTCCAACATGCTCAACGGCACGCTCCCGGTGGACTTCGGAGGCGCCGCGC	46:G>A

<table>
<tr><td>4398082|F|0-8:C>A-8:C>A</td><td>TGCAGGCCCCGAGGCCCTGCTCCCCG</td><td>8:C>A</td></tr>
<tr><td>2249398|F|0-6:G>A-6:G>A</td><td>TGCAGGGCCACATCAACTCGATGCTTCATTTCGCCGCGGGGCTCCTCGACGCCG</td><td>6:G>A</td></tr>
<tr><td>1000439|F|0-32:G>A-32:G>A</td><td>TGCAGCTTTAGCAAGGTCAGTTGACAAGCAAGGCGTGTACACGGCATGCACACGCTTGCCTTCATCTCA</td><td>32:G>A</td></tr>
<tr><td>1000439|F|0-64:T>G-64:T>G</td><td>TGCAGCTTTAGCAAGGTCAGTTGACAAGCAAGGCGTGTACACGGCATGCACACGCTTGCCTTCATCTCA</td><td>64:T>G</td></tr>
<tr><td>1864490|F|0-27:G>C-27:G>C</td><td>TGCAGCACAGGAATCGGGGGAGAAAGAGGAAAGGGAAAGGAGGGATCGATCAAAAGGAGAGAAAGGCCC</td><td>27:G>C</td></tr>
<tr><td>1128374|F|0-27:A>T-27:A>T</td><td>TGCAGCCCTTCTCCTTCCCCGAGTGGGACGAGCAACGCCGTCTGTCGGCCGGGAAGACCCGCCG</td><td>27:A>T</td></tr>
<tr><td>1397609|F|0-30:T>A-30:T>A</td><td>TGCAGGAAGGACTCCTTGGTGCCTTCGTCCTCGCCCAGAATGCCCACGGGCACGGCGCCCACGGACTTG</td><td>30:T>A</td></tr>
<tr><td>1397609|F|0-56:G>A-56:G>A</td><td>TGCAGGAAGGACTCCTTGGTGCCTTCGTCCTCGCCCAGAATGCCCACGGGCACGGCGCCCACGGACTTG</td><td>56:G>A</td></tr>
<tr><td>1263957|F|0-11:G>A-11:G>A</td><td>TGCAGGCCATCGTGCGCGGCCGGCAGGTGCGGAAGCAGGCGGCCGTGACGCTCCG</td><td>11:G>A</td></tr>
<tr><td>1046799|F|0-28:C>T-28:C>T</td><td>TGCAGCAGTATCAGTACCAGCAGATGGGCGGGCAGACAGGGGCCAGGGAGCCGCTCCAGTTCACCAACG</td><td>28:C>T</td></tr>
<tr><td>1347849|F|0-60:C>A-60:C>A</td><td>TGCAGCGGAGAGGAGGGAGGCGAGAGCGTGAAACGCGGGGAAGCAGTGTCGGAAGCCCTTCTGGCGGCC</td><td>60:C>A</td></tr>
<tr><td>3959631|F|0-20:G>A-20:G>A</td><td>TGCAGGCCGTAGACGCAGTCGACGATGCGCTTCTGCTCAGCGGTGCTGAACACGCCGGCGTGGAGCTCC</td><td>20:G>A</td></tr>
<tr><td>2256906|F|0-11:G>T-11:G>T</td><td>TGCAGTCGGCGGCGCGCACCTGGAGCTTCATCTGCGCGGGCAAGTACCCCATCGACCACCCGTACGCGA</td><td>11:G>T</td></tr>
<tr><td>2256906|F|0-52:C>T-52:C>T</td><td>TGCAGTCGGCGGCGCGCACCTGGAGCTTCATCTGCGCGGGCAAGTACCCCATCGACCACCCATACGCGA</td><td>52:C>T</td></tr>
<tr><td>3574740|F|0-61:C>G-61:C>G</td><td>TGCAGTTGACCCGCCACACGGCGTTGCCAATCGCCTGCGGGAGCAGGAACAGGTCCTGCATCAGCGCCA</td><td>61:C>G</td></tr>
<tr><td>100519849|F|0-37:G>C-37:G>C</td><td>TGCAGATTCTGGTTGTGCTGACCTCGCCCGATCCTCCGTCTCCATCCACAGCCG</td><td>37:G>C</td></tr>
<tr><td>3026500|F|0-34:C>T-34:C>T</td><td>TGCAGTCAGGGCAGTGCTCCTTGGGAGCCACATGCCCTACCG</td><td>34:C>T</td></tr>
<tr><td>2252023|F|0-67:T>A-67:T>A</td><td>TGCAGCTCCCGCCGAGGCACCGCCTGTAAGGAGAGGAACCTGTCGATGACGTACATTGTCAAGAACATG</td><td>67:T>A</td></tr>
<tr><td>5361383|F|0-64:C>T-64:C>T</td><td>TGCAGGAGGTTGGGCGGGATAGAGGTGGGGCGGCCGCCGCGGCTGCCGCCACGGAGGCGCAGCACCAGG</td><td>64:C>T</td></tr>
<tr><td>2251892|F|0-9:C>G-9:C>G</td><td>TGCAGAATCCACCACCACGAGATGTTTTCGAAACCCCAGAACGTCACAGCGGCGGAAGCCAATAAACCA</td><td>9:C>G</td></tr>
<tr><td>1026702|F|0-34:G>T-34:G>T</td><td>TGCAGAGGAGGATCGGCGGCGAGCTGGAGGCGGTGAGCATGGCGAGTCCTGCGGCTCATCGGAGTCAGA</td><td>34:G>T</td></tr>
<tr><td>4005867|F|0-14:C>G-14:C>G</td><td>TGCAGCAGCGTCGCCTCTGCCGACTTCGAGGGCCTCTTCTCCCCCTCCG</td><td>14:C>G</td></tr>
<tr><td>3943304|F|0-15:A>T-15:A>T</td><td>TGCAGATGGGGACCAAGTTCAGGGTGCTTGAGGCGGACTTGGCGCGGTCGCGGTGGGCGAGGCTGAGGA</td><td>15:A>T</td></tr>
<tr><td>1042028|F|0-46:C>G-46:C>G</td><td>TGCAGAACCTCGTGCTCTCTGCTTTCAACCTGTCGTATAACAAGCTCAGTGGACCTCTGCCTCTCTTCT</td><td>46:C>G</td></tr>
<tr><td>3570190|F|0-63:G>A-63:G>A</td><td>TGCAGCGTGCTCAGCGCGCTGCTCTTCGAGATGGGCCCGCTGAGGTTCGTGATCGAGCCCTACGACGGC</td><td>63:G>A</td></tr>
<tr><td>1020888|F|0-28:C>T-28:C>T</td><td>TGCAGGCAGAAGCGCGCCCCTTGGAAGACGCGCATGAAGGTGCTGGGCACGAGGCAGTTCTTCTCGCTC</td><td>28:C>T</td></tr>
<tr><td>4002601|F|0-23:C>A-23:C>A</td><td>TGCAGGCGCTGCTCGCCATCGCGCTGTCGGAGAAGCCG</td><td>23:C>A</td></tr>
<tr><td>977165|F|0-22:C>T-22:C>T</td><td>TGCAGGGCATCGCGCAGAGCGGCAGCGTCGTGATCATGTCCATCCACCAGCCGAGCTACCGCATCCTCG</td><td>22:C>T</td></tr>
<tr><td>1001920|F|0-23:G>C-23:G>C</td><td>TGCAGAACGGCGTCGTCTGGTCCGTCTCCAAGGACGTCACCAGGCACGCGTCCCTCCTCGCCGCCGAGA</td><td>23:G>C</td></tr>
<tr><td>100567849|F|0-18:C>T-18:C>T</td><td>TGCAGTCCGCCCCGTGGCCACACGCCCAGTCCAGCGCCGCCTGGAGGCGTGCGTCCCCGACCGCTGCGT</td><td>18:C>T</td></tr>
<tr><td>1018027|F|0-50:G>C-50:G>C</td><td>TGCAGGTGCAAATGGCGAACGTGACGAGGCTGTGCGCCAGCAAGAGCATCGTGACGGTGAACAGGGAGT</td><td>50:G>C</td></tr>
<tr><td>977602|F|0-17:C>A-17:C>A</td><td>TGCAGCCTAAGTTTGGCCTCCCCGTCGTTATGAAGCTGCTGGATGTATGAAACGGAACCG</td><td>17:C>A</td></tr>
<tr><td>1115309|F|0-27:G>A-27:G>A</td><td>TGCAGGCACTGCGCTCCAGGCGCTGAGGTCCATTGGAGCCAAGTTTGACAGCACCG</td><td>27:G>A</td></tr>
<tr><td>1695054|F|0-37:G>C-37:G>C</td><td>TGCAGATGCTCCCCGTCATGCCGCTCGACGCCGACGTGTTCCGGCAGCCGCCTGGCCACAATGCGCCGA</td><td>37:G>C</td></tr>
<tr><td>1026243|F|0-28:G>T-28:G>T</td><td>TGCAGGCGTCGGTGAGGGACGAGGCGGCGGAGCCGAGGTTGGTGAGCACGCGGCGGAAGTCGGCGGCGA</td><td>28:G>T</td></tr>
<tr><td>12779633|F|0-16:C>A-16:C>A</td><td>TGCAGCCCAGTGCTCCCGCCG</td><td>16:C>A</td></tr>
<tr><td>991667|F|0-30:C>A-30:C>A</td><td>TGCAGATCCAGAGAAGCGGCAGAGAGTTCCCTCGGCATACAACCGATTCATCAAGTGAGTGATCAGTGG</td><td>30:C>A</td></tr>
<tr><td>1025047|F|0-37:C>T-37:C>T</td><td>TGCAGCGGGGTGAGGACCCTGAACGGCAAGGCGTCCACCTCGGCCGACCG</td><td>37:C>T</td></tr>
<tr><td>5582334|F|0-6:A>G-6:A>G</td><td>TGCAGCACGGGAGACGGCGGGGCATCGTCTACTACCG</td><td>6:A>G</td></tr>
<tr><td>3020754|F|0-24:G>A-24:G>A</td><td>TGCAGAACTATGAAAGTGAGATTTGGGTCTTAAAGTACCG</td><td>24:G>A</td></tr>
<tr><td>7353366|F|0-66:G>C-66:G>C</td><td>TGCAGACGCAGGCGGCGCGGACGAGGCTGGTGGGGAAGCCGACGCGGACGATGATCTCCGTCAGGAGGT</td><td>66:G>C</td></tr>
<tr><td>1219453|F|0-8:G>C-8:G>C</td><td>TGCAGAGCGTCATCCAGATGATCTCCGTCATGTTCGTCGGGCACCTGGGCGAGCTCGCGCTGGCCAGCG</td><td>8:G>C</td></tr>
<tr><td>1017445|F|0-30:T>A-30:T>A</td><td>TGCAGCACATATACCGCGAGGGCAACCAGGTCGCCCACGTCCTGTGCAAAGAAGCATACCAGCGACCGA</td><td>30:T>A</td></tr>
<tr><td>1694954|F|0-37:G>A-37:G>A</td><td>TGCAGGGCGTGGCGCGACACCGTCGACGCCCGCCTGCGCGGCCACCTGCTCTCGCGCTCCGTGCGCGGC</td><td>37:G>A</td></tr>
<tr><td>7352523|F|0-8:C>A-8:C>A</td><td>TGCAGCACCTCGCCGAGTTCCG</td><td>8:C>A</td></tr>
<tr><td>3027903|F|0-25:A>G-25:A>G</td><td>TGCAGCACGCCGCGGCGTTCCTCCCACGCGTCCGCATCGTCGCCG</td><td>25:A>G</td></tr>
<tr><td>7478659|F|0-13:C>T-13:C>T</td><td>TGCAGATCCAAGGCGAGGAAGCCAGGTTCGTGAACGTCCTCACCG</td><td>13:C>T</td></tr>
<tr><td>6047060|F|0-66:A>C-66:A>C</td><td>TGCAGGGCGCCATCGACCGCGAGATCGACAAGCCCAAGTACCGCTGCGGCTGCGCGTGCGTCGACGAGG</td><td>66:A>C</td></tr>
<tr><td>7351749|F|0-20:A>G-20:A>G</td><td>TGCAGATCGGCAGGCACCGAATCGAAGCCGAGCCG</td><td>20:A>G</td></tr>
<tr><td>7165795|F|0-31:T>G-31:T>G</td><td>TGCAGGCGCTCCACCACGCCAAGAGCGGCGTTGACGTGGTGTCCG</td><td>31:T>G</td></tr>
<tr><td>32025365|F|0-51:C>T-51:C>T</td><td>TGCAGGACGCGAGGGTCGAGGAGGCGTGGGCGCTGGAGGAGGCTATGCGGCCGCCG</td><td>51:C>T</td></tr>
<tr><td>12776664|F|0-19:A>G-19:A>G</td><td>TGCAGCCCCTCCACGTAGCACCGCCG</td><td>19:A>G</td></tr>
<tr><td>12775287|F|0-34:A>G-34:A>G</td><td>TGCAGATCAGGCCGAAGGCGAAGAGCCTGTGGCTACTGGTGCGCCG</td><td>34:A>G</td></tr>
<tr><td>12774391|F|0-30:G>A-30:G>A</td><td>TGCAGCTTCTCGACCTCAAGAGGGGCCACCGCCATCTCGGCTCCCCCGTCGCCCCGCCGTTGGCTCTCC</td><td>30:G>A</td></tr>
<tr><td>2253859|F|0-22:T>C-22:T>C</td><td>TGCAGCATCCCGCCTCCGTCCATGATCCTCCTGAGGGAGAGCGTCTCCAGCGACGCCATGTCGACGCTC</td><td>22:T>C</td></tr>
<tr><td>12776020|F|0-43:C>G-43:C>G</td><td>TGCAGCACGCCCTTGCGAGGTCCATCCAAGCACGTGCGGCTGCCTGCATGTCGATCGAAGGCGGGGGAG</td><td>43:C>G</td></tr>
<tr><td>6046312|F|0-43:T>G-43:T>G</td><td>TGCAGCTGCGCGCTTGCGACGGCATCGGAGATGGCCCCTCCTCTGCTGCCTTCTCAGGATGCCTAGCCG</td><td>43:T>G</td></tr>
<tr><td>100302317|F|0-5:A>C-5:A>C</td><td>TGCAGAGCCTCGCGAGCCGCCTTCCTGAGCCCG</td><td>5:A>C</td></tr>
<tr><td>11863134|F|0-27:G>A-27:G>A</td><td>TGCAGGTTGCACCGCCAGCCGAGCCACGGGAAGAGCCGCAACCAGAAGCAGCACCACGACCG</td><td>27:G>A</td></tr>
</table>

<table>
<tr><td>5578132|F|0-16:G>A-16:G>A</td><td>TGCAGCTCCCTCGTTAGCAGGTCGCTCGTCGTCTCCACAGGCCCCG</td><td>16:G>A</td></tr>
<tr><td>7170796|F|0-6:T>C-6:T>C</td><td>TGCAGGTAAGATTGCCTCTGGCTCTAGCCG</td><td>6:T>C</td></tr>
<tr><td>12856435|F|0-7:C>G-7:C>G</td><td>TGCAGCCCAGCTCCCAGACGTACTGCCG</td><td>7:C>G</td></tr>
<tr><td>100540107|F|0-6:T>G-6:T>G</td><td>TGCAGCTACGCCGTCCACAGGATGCTGAGGGAGACGCCG</td><td>6:T>G</td></tr>
<tr><td>100534067|F|0-59:C>T-59:C>T</td><td>TGCAGATGACATGGTGATGAGCGTAGCCTGGAAGGCCACCATGAGCAGCTGCACGCATGCCAGCTTCAC</td><td>59:C>T</td></tr>
<tr><td>100536826|F|0-61:A>G-61:A>G</td><td>TGCAGGGCGAGGAGGACTACCCCTCGGCCGACTGCTGCGACGGCGCGTCCAGCCTCGTGGCAGCGGCGG</td><td>61:A>G</td></tr>
<tr><td>7173647|F|0-5:G>A-5:G>A</td><td>TGCAGGTTAGCCGCTCGTTGCCTCCCCG</td><td>5:G>A</td></tr>
<tr><td>100441842|F|0-28:A>G-28:A>G</td><td>TGCAGGCGGCGTCGTCGAAGGTGCTGCCACCGCCG</td><td>28:A>G</td></tr>
<tr><td>100469576|F|0-38:T>G-38:T>G</td><td>TGCAGCGGCGGGTCGTGCCTGAGCTGCCCGAAGCCGTCTTGCCCCGAGTGCTCCTGCGGGTGCGTGTGG</td><td>38:T>G</td></tr>
<tr><td>7913053|F|0-20:T>C-20:T>C</td><td>TGCAGCTCGGTGTCGACCAGTGACTCGTCGTACGACCCCG</td><td>20:T>C</td></tr>
<tr><td>7339606|F|0-7:T>C-7:T>C</td><td>TGCAGCGTGTAGACGGTCCGCAGCAGGTGCCGCCGCTCGTACTTGCCCG</td><td>7:T>C</td></tr>
<tr><td>100499971|F|0-46:C>T-46:C>T</td><td>TGCAGCAGCATCTTCTCCTCCAGCGGCAGCTCGAAGAACTTCTTGCCCTCGGAGAACAAGGCCTCCAGC</td><td>46:C>T</td></tr>
<tr><td>100512510|F|0-26:T>C-26:T>C</td><td>TGCAGCAAAACAAAAACAAAGGGCAGTAGCTGCCGAGCGAGCAAACCACCACCAGCTCACTCGCGCACG</td><td>26:T>C</td></tr>
<tr><td>100526461|F|0-55:A>C-55:A>C</td><td>TGCAGGAGATGCGGCTGCGCGGGGTGGCGTGCCCCGAGGGGGCCCTCGTCGCCGCAATCTGCGCCTTCG</td><td>55:A>C</td></tr>
<tr><td>100583719|F|0-33:A>C-33:A>C</td><td>TGCAGAGTAGAAGGCATGAAGCGTACCATGGAGACCCGTGTTATGAAGTTGGAGCTCGGTAACATGTCC</td><td>33:A>C</td></tr>
<tr><td>100302030|F|0-41:A>G-41:A>G</td><td>TGCAGCCGCACCGCATCCTCGACACGCTCGACGAGGTCCAGATCTCCG</td><td>41:A>G</td></tr>
<tr><td>100317433|F|0-16:T>A-16:T>A</td><td>TGCAGGAGATGGGCATTCCG</td><td>16:T>A</td></tr>
<tr><td>1055251|F|0-17:G>A-17:G>A</td><td>TGCAGCCTCTTCTCCCCGACGCCCTCCCTCTCCGCCATGATCATCAACAAGTACAAGCTCCGCAGCAAC</td><td>17:G>A</td></tr>
<tr><td>1036616|F|0-6:G>A-6:G>A</td><td>TGCAGGGTTGCTTGGTGGGGGGAATTCTGTTGTTCAGGTCTCAACATCCCTCTGATGTTTCAAACCTAC</td><td>6:G>A</td></tr>
<tr><td>1215801|F|0-5:C>T-5:C>T</td><td>TGCAGCTCTACAAGGCTCACTCCGACAGCTACATCTGCTCGCTCGTCCCCG</td><td>5:C>T</td></tr>
<tr><td>100352131|F|0-5:A>G-5:A>G</td><td>TGCAGAATCCGCGGAGCGACAGGCCG</td><td>5:A>G</td></tr>
<tr><td>4262332|F|0-7:T>C-7:T>C</td><td>TGCAGGATCCGCGGAGCGACAGGCCG</td><td>7:T>C</td></tr>
<tr><td>100352133|F|0-9:G>C-9:G>C</td><td>TGCAGGATCGGCGGAGCGACAGGCCG</td><td>9:G>C</td></tr>
<tr><td>100352519|F|0-11:T>C-11:T>C</td><td>TGCAGGGAGGTTGGCCAGCAGGACCG</td><td>11:T>C</td></tr>
<tr><td>3960380|F|0-7:G>T-7:G>T</td><td>TGCAGGGGTGGGACAACGCCACCATCGCCG</td><td>7:G>T</td></tr>
<tr><td>5358049|F|0-5:C>T-5:C>T</td><td>TGCAGCTGCCGCTTGCTGATCCG</td><td>5:C>T</td></tr>
<tr><td>2247078|F|0-34:G>C-34:G>C</td><td>TGCAGCAATTCTTCAAGAAGGGCAAATCATCTGGGACTAGCAAGTCAAGCCAGAAAGACGAAGCAAGCA</td><td>34:G>C</td></tr>
<tr><td>7337227|F|0-8:C>G-8:C>G</td><td>TGCAGCGGCGAGAGGATGTCGACCCCCGGGATGGGCACGCAGCCG</td><td>8:C>G</td></tr>
<tr><td>6050212|F|0-9:C>G-9:C>G</td><td>TGCAGAGCTCGACGATGCCGCGGACGACGCCG</td><td>9:C>G</td></tr>
<tr><td>10984571|F|0-15:G>C-15:G>C</td><td>TGCAGCCTAATTCGAGCGCCG</td><td>15:G>C</td></tr>
<tr><td>12010231|F|0-15:T>C-15:T>C</td><td>TGCAGCTCGGTGGTCTGCCG</td><td>15:T>C</td></tr>
<tr><td>7338616|F|0-8:G>A-8:G>A</td><td>TGCAGCAGGAGCGGTATCCCG</td><td>8:G>A</td></tr>
<tr><td>100395234|F|0-26:G>A-26:G>A</td><td>TGCAGAAAATCCACTGGTAGCAGCGCGGCAAGATTCACATCCAGCAGAACCAAGGAATCAATAAACGAG</td><td>26:G>A</td></tr>
<tr><td>1022812|F|0-7:G>C-7:G>C</td><td>TGCAGACGCACTACAAGCGCAGGCACTGTGCCAAGCCATTCCTCTGCCGCAAGTGCGGCAAGGCGCTCG</td><td>7:G>C</td></tr>
<tr><td>998551|F|0-49:G>C-49:G>C</td><td>TGCAGCCGCTCGCCATCACCCGCCGCAGCCTGGACGAGTGGCCCAAGGCGGGCTCCGACGACGTGGGCG</td><td>49:G>C</td></tr>
<tr><td>100415358|F|0-14:C>G-14:C>G</td><td>TGCAGCAGGGCAAACCATTTACCG</td><td>14:C>G</td></tr>
<tr><td>100416334|F|0-26:G>C-26:G>C</td><td>TGCAGCGTGCCGTAGAGCCGCACCTTGCCGCCG</td><td>26:G>C</td></tr>
<tr><td>6045606|F|0-17:T>G-17:T>G</td><td>TGCAGCTGCCCTTCAAGTGCCTCGCCG</td><td>17:T>G</td></tr>
<tr><td>2286159|F|0-14:C>T-14:C>T</td><td>TGCAGCGCCTCCCACGAGCGGTCGTCGGCGTAGGCGCGCTCCCACGCCTCGAGGACGCGGCCGTCGCCG</td><td>14:C>T</td></tr>
<tr><td>2286159|F|0-35:G>A-35:G>A</td><td>TGCAGCGCCTCCCACGAGCGGTCGTCGGCGTAGGCGCGCTCCCACGCCTCGAGGACGCGGCCGTCGCCG</td><td>35:G>A</td></tr>
<tr><td>2251815|F|0-29:C>G-29:C>G</td><td>TGCAGCTTCGAGGCGTACCAGGACACGCTCTACACGCACTCCCTCCGCCAGTTCTACCGCGGCTGCGAG</td><td>29:C>G</td></tr>
<tr><td>1215068|F|0-63:C>T-63:C>T</td><td>TGCAGGAGGAGGACGCGGCGGGTGAGCAGCGCGTAGAGGAAGGCGGACGCGATGGAGAGCACGCGGTCG</td><td>63:C>T</td></tr>
<tr><td>1043083|F|0-8:G>T-8:G>T</td><td>TGCAGGCCGAGGTTGCCTTTCATCGTCTCATAGAAGCCCCAATTCCGAATGCTCAAGCTGCCGTCGTCG</td><td>8:G>T</td></tr>
<tr><td>1043083|F|0-35:G>C-35:G>C</td><td>TGCAGGCCGAGGTTGCCTTTCATCGTCTCATAGAAGCCCCAATTCCGAATGCTCAAGCTGCCGTCGTCG</td><td>35:G>C</td></tr>
<tr><td>4262594|F|0-27:C>A-27:C>A</td><td>TGCAGAAGCTGCAAGAGAAATGGAAGTCCACGGCGGCGCCGTACCCG</td><td>27:C>A</td></tr>
<tr><td>3940659|F|0-13:C>T-13:C>T</td><td>TGCAGAAGCTGGACCTGAGCTCCAACAACCTCACCG</td><td>13:C>T</td></tr>
<tr><td>1020000|F|0-12:T>C-12:T>C</td><td>TGCAGAGGCCAATGTTGTGTTCTCATCTCTTATGGGTGCTCGGGTAAGCAACCG</td><td>12:T>C</td></tr>
<tr><td>4261660|F|0-12:A>G-12:A>G</td><td>TGCAGCAGTAGCATGAGCCAGTAGCGGCACGTAGCGTCCG</td><td>12:A>G</td></tr>
<tr><td>1395478|F|0-24:T>G-24:T>G</td><td>TGCAGCTCGATCTCCGCCGCCTCGTCCTCCTCCCCCTCGCCCGCCATGGCTCACCG</td><td>24:T>G</td></tr>
<tr><td>1023842|F|0-48:G>T-48:G>T</td><td>TGCAGCTCGTGCACGGCCGTGTTCATGTCTGCCACGGCGAAGTCCAGCGCCG</td><td>48:G>T</td></tr>
<tr><td>4405682|F|0-17:C>T-17:C>T</td><td>TGCAGGCCCCATTCCGACCGAACGACACGACTCCG</td><td>17:C>T</td></tr>
<tr><td>100442377|F|0-22:T>C-22:T>C</td><td>TGCAGGTGGCCAACAAGCTGAATAAGACCCCG</td><td>22:T>C</td></tr>
<tr><td>4396339|F|0-7:G>A-7:G>A</td><td>TGCAGCCGCGGTCGAGCCGCCCG</td><td>7:G>A</td></tr>
<tr><td>1319093|F|0-28:G>T-28:G>T</td><td>TGCAGAAGCTGACGCCATGGGCGGACGGGCCGAGCATGGTGACGCAGTGCCCGATCCAGCCCAACGGCT</td><td>28:G>T</td></tr>
<tr><td>1030388|F|0-22:C>G-22:C>G</td><td>TGCAGCAGCTGCGGCGCGCCGACCCGTCCGTCTACCTCTCCCCGTCCGCCGACCTCGCCGCCGCCGCCC</td><td>22:C>G</td></tr>
<tr><td>1056824|F|0-27:G>A-27:G>A</td><td>TGCAGCATCACCACCATGGCCGACCGCGCGTCGCCTTCCTCGTACACGCTGGTGCAGTGGTTGTGCAGC</td><td>27:G>A</td></tr>
<tr><td>1215014|F|0-43:C>A-43:C>A</td><td>TGCAGGCCGTACGTCGTCATGCCTGACATGGTTGACGGCCGCGCGCGGCGGGGTGAACTGGGAAGGGGT</td><td>43:C>A</td></tr>
<tr><td>1041297|F|0-7:T>C-7:T>C</td><td>TGCAGGTTGCACCGCCAGCCGAGCCACAAGAAGAGCCGCAACCAGAAGCAGCACCACGACCG</td><td>7:T>C</td></tr>
<tr><td>990314|F|0-16:G>A-16:G>A</td><td>TGCAGTAGGTCTTGCAGCCTGGGCACATGACGATGCACTCGTTGGGGCAGCGGACCTTGTCGCAGGTGG</td><td>16:G>A</td></tr>
</table>

2249364|F|0-56:G>A-56:G>A	TGCAGTGGCTGCTCGTCACCATGTCCGACAAGTCCATCCTTGTCATCGAGGACATCGACTGCTGCTGCG	56:G>A
1035262|F|0-9:T>A-9:T>A	TGCAGAATCTCCACCAGATAATTTAAACCAATTCAAAATCCCTAAATTACCCG	9:T>A
4261401|F|0-10:G>A-10:G>A	TGCAGCCCCCGAACGTGGTCGTCGACGAGCAGCATGGTCATGCCGCCG	10:G>A
100461480|F|0-5:G>C-5:G>C	TGCAGGGCCTTGTTGTACATAAGCGCGGCGTAGTTCCG	5:G>C
5011091|F|0-26:G>A-26:G>A	TGCAGCTTGTCGATGGCCTGGAGCAGGAAGGGCCG	26:G>A
3025223|F|0-10:C>T-10:C>T	TGCAGGCTGGCTGCATGATCACTGACCACCTGGAACTGCACCG	10:C>T
3025223|F|0-16:G>A-16:G>A	TGCAGGCTGGCTGCATGATCACTGACCACCTGGAACTGCACCG	16:G>A
3937291|F|0-11:C>T-11:C>T	TGCAGGGCGCCCTGGCCAGGCTCGGGTTCCCG	11:C>T
3937291|F|0-20:C>T-20:C>T	TGCAGGGCGCCCTGGCCAGGCTCGGGTTCCCG	20:C>T
1150172|F|0-67:C>T-67:C>T	TGCAGCGCGTACGCCAGCCGCACGATGTCGCGCCGCTCCCGCCGCTTCGGCATCGTCAGGATGCCGACC	67:C>T
2241677|F|0-26:C>T-26:C>T	TGCAGGAACCAAATTGCAGTATCAAGCTCTCACCTGCATTCGAGATCATTACATGAGAACCTGGCTCCG	26:C>T
100471299|F|0-59:A>G-59:A>G	TGCAGGATGTACGTCGTGCCCATCGGCGTGTACAGCACCTTCTGGCTGTTGAGGCAGGGATGCGTGCGC	59:A>G
1248967|F|0-48:G>A-48:G>A	TGCAGGCACGTGTTGTTCTGCTAGCTGAAGCAAAGCAGCAGCACTCACGGCAATGGTGGGAAGCAAAAG	48:G>A
12692539|F|0-41:A>G-41:A>G	TGCAGGTCGCGGACCTTCGCCCCGTCGTCGCCAAGAACACGACCGCGGCGGCGGCCGCGGCGCAGCACT	41:A>G
100478342|F|0-15:T>A-15:T>A	TGCAGAGAGCACCACTGCGGGGAGCTCGCCG	15:T>A
7170575|F|0-31:C>T-31:C>T	TGCAGCAGGCCAGCCTCTGTCAACAAATCGCCGCCG	31:C>T
7169369|F|0-18:C>T-18:C>T	TGCAGCATGAAGCGCATTCTGTCCATCTGGCCCG	18:C>T
100479612|F|0-13:C>G-13:C>G	TGCAGCGCACCGCCGAGGAGATCGGGACCGCTGCCG	13:C>G
100480091|F|0-8:A>G-8:A>G	TGCAGCTCAGTGTCGACCAGCGACTCGTCGTACGACCCCG	8:A>G
6044005|F|0-17:C>A-17:C>A	TGCAGCTGGGCTGCGAACCGCGGGCTCAGCGTCGGGTCGGCTGTCGGCCGCAGCCGCCCG	17:C>A
100480647|F|0-18:G>T-18:G>T	TGCAGGCAAGATTGCCTCGGGCTCTGGCCG	18:G>T
7169690|F|0-10:G>A-10:G>A	TGCAGGCGCCGCCCTCGTGGGTGCCCG	10:G>A
3947093|F|0-47:A>G-47:A>G	TGCAGGTAAGCCACTCTCGAGCACTACAAGCTCCTGAAACACGATTGATACCG	47:A>G
7345550|F|0-5:A>C-5:A>C	TGCAGATTGTGGTGCACCGACCG	5:A>C
1166826|F|0-22:C>T-22:C>T	TGCAGATCCAAGAAGGCGCGACCGTGACCGTGGCGGAGGAGGACGGGGAGTTCATGGTCATTGGGTCCC	22:C>T
100485126|F|0-47:T>C-47:T>C	TGCAGCAAGCGCGGCCGCGGGCCCATAATGGCCAGGACCGTCGCGGCTACCATGTTCGTCGTGCTCGGC	47:T>C
2251743|F|0-45:G>A-45:G>A	TGCAGCAGCTCCGACTGGGACTCCGACTCCTCCTCCCCCGTCCTCGACCGCGCGCCGCACCGCAACACC	45:G>A
100486513|F|0-53:G>A-53:G>A	TGCAGCCTGAGCGCACCTGGAAGTCGCCGCAGCAGATGAGGAGGTCGATCTTGGTGCCCTCGACCGCCT	53:G>A
1004991|F|0-6:C>T-6:C>T	TGCAGGCTTGTCAGCAGTGCGGATTTAAAGTAGCATGGAGACGGCGACATGGCGTGCCACTGTGAACGG	6:C>T
1009326|F|0-52:C>T-52:C>T	TGCAGTAGTGGCGGCGCCTACCCCCGCGCGACTCCTCCTCGTCTCCGCCCGCCGCGCTGCTCCGCGATT	52:C>T
1015319|F|0-13:C>T-13:C>T	TGCAGAACGGCGGCCACTTCGCAGTCGGGGACGCGTGTCAGCCGTCGGTGGGCCTGACCG	13:C>T
5580158|F|0-23:C>T-23:C>T	TGCAGGACGCGCTTCTCGAACTCCTCGTCGGCGGCGGGGGCGGTGGCGACCG	23:C>T
1020233|F|0-42:G>T-42:G>T	TGCAGCTGGAGATGGCGGCGGCGTAGGTGGTTGTGAGGAGTGGGGAGGGGGAGAGCCG	42:G>T
1012307|F|0-17:G>T-17:G>T	TGCAGGAATCTCCAGATGGAGTACGTGGACCTGTACCTTGTCCACTGGCCG	17:G>T
1228456|F|0-26:T>C-26:T>C	TGCAGGTGGTCGGACAGTGCAGCTGCTGCATACAGCGCCCGCAGTCCCCG	26:T>C
3960595|F|0-26:G>A-26:G>A	TGCAGGCTCGACACGCTCAGGTTCCTGAACACGCCG	26:G>A
995666|F|0-21:G>A-21:G>A	TGCAGGGGTCGTGCGGGCTGAGCAAGGAGCGAGCACGACGCAGAGACGGCCTTCCG	21:G>A
3938324|F|0-7:C>G-7:C>G	TGCAGTTCAGGCATCGTGTACAGGTCACCCG	7:C>G
3938324|F|0-14:C>G-14:C>G	TGCAGTTCAGGCATCGTGTACAGGTCACCCG	14:C>G
1062105|F|0-26:T>G-26:T>G	TGCAGCTCTGCAAGGAGGAGTAGCTCTTGTGTCGTAGCTTATGAGTGAGCAGTGAACCTCCATGTTTCA	26:T>G
1070366|F|0-60:T>A-60:T>A	TGCAGTTGCCTCTGGTTTTATCTGTGGAGATGGTATATGGATGTTCCCTTCTTCCTTGCTTTCCCTTGC	60:T>A
1695414|F|0-66:A>G-66:A>G	TGCAGCACGCAGGCGAGGGACGACACGAGCGCCTGCCGCTCGAGCTCCTCCTTGGAGCCGCGGTCCAGG	66:A>G
1321129|F|0-29:T>C-29:T>C	TGCAGCATGCACATTGCTATCTCTTTCTGTGCCCATTACAGCAACTTTTTCTGGTGTATTATTGCCGAG	29:T>C
3951898|F|0-26:C>A-26:C>A	TGCAGCCCTACCGTGTCCAGCCCCAGCGCCAGCGGCACGCTCCCTGGCATTGGCGAAACTCGACTGCTC	26:C>A
3954408|F|0-14:G>T-14:G>T	TGCAGAGCAGCAGAGATTGAGGCATCCAAGCTGGGGTGCGAGTCCG	14:G>T
100508263|F|0-19:T>C-19:T>C	TGCAGCAGCTCGACCTCTCTTCCAACGCTTTCAACGGCTCCATCCCGCCG	19:T>C
100509580|F|0-26:T>C-26:T>C	TGCAGGCACCACCGAGTCGACAACCATTCACGAGAACAACGGCTCCG	26:T>C
3937257|F|0-32:C>T-32:C>T	TGCAGTACAACGGGAGCGGACGGCGTTGAAAACGGACCG	32:C>T
100512512|F|0-47:G>A-47:G>A	TGCAGCAAAACAAAAACAAAGGGCAGCAGCTGCCGAGCGAGCAAACCGCCACCAGCTCACTCGCGCACG	47:G>A
100512976|F|0-17:T>C-17:T>C	TGCAGCAGCCTGCTCACTGTCGACATGAGGGGCCTCCTCTTCCCGACCACGACCACCACTGTCGCCGCC	17:T>C
2251206|F|0-40:T>C-40:T>C	TGCAGCAGCCTGCTCACCGTCGACATGAGGGGCCTCCTCTTCCCGACCACGACCACCACTGTCGCCGCC	40:T>C
7487707|F|0-41:T>C-41:T>C	TGCAGCAGCCTGCTCACCGTCGACATGAGGGGCCTCCTCTTTCCGACCACGACCACCACTGTCGCCGCC	41:T>C
1027504|F|0-6:G>A-6:G>A	TGCAGCGAGTGGCGTTCCTCAAGGCGTCGTCGGAGGAGGCGCCGTACGTGGGGGTGGAGACGGCGAGAC	6:G>A
1095342|F|0-56:G>A-56:G>A	TGCAGCGCGTCGGCGTCGGCCTCGTGCTCTCCGTCGTCTCCATGTGCATCGCCGCCGTCGTGGAGATGC	56:G>A
2248204|F|0-12:C>A-12:C>A	TGCAGCGGATACCTGGCGCCTGAATACGTGGTGCACGGGCAGCTGACCAAGAAGGCCGACGTCTACAGC	12:C>A
1266287|F|0-35:G>A-35:G>A	TGCAGCTGATCATTCTGAAGTGGTACTCTATGCCGGCCCGCTCCGCCAGCTTCTTGAGCCGCTCCCCGA	35:G>A
2350626|F|0-7:G>T-7:G>T	TGCAGGCGCTGGCCAAGGCCGGCGGCATGTCGGCCAGGTGCTTCGAGAGCCACTCCCGCCTCCGCCGCT	7:G>T
1046581|F|0-27:C>T-27:C>T	TGCAGGGCGTGCACATGGTGAACTGCTCGTACAACAGCCTGTGGATGGGCCAGTTCGTGCGGCCGAGAT	27:C>T
1306480|F|0-44:G>A-44:G>A	TGCAGGTATCGATTATGAGTACAGGGCGGTGAACCTCCTCAAGGGCGAGCAGTCTGATCCCG	44:G>A

1045054|F|0-18:G>A-18:G>A	TGCAGTCCACATCCGACAGCACAGCACCGACACAAATCTCCTCTCACGCACTCACACACACCG	18:G>A
982614|F|0-28:C>T-28:C>T	TGCAGTGCTTCATGGGCATCAGCATGATCACCTGCGTAGCGCTGTACGACCGCGTCCTGGTGCCCGTGG	28:C>T
6046663|F|0-14:T>C-14:T>C	TGCAGCAGCCAGTTTGCCAGGACGCTGAAGAGCGCCGTCTGGTGCTTGACCTCGCCG	14:T>C
2247100|F|0-19:G>A-19:G>A	TGCAGCCCTTGGTGGTTTCGACGGTGCACAGGGTGTCGAGGGCATCGTCGCTCCG	19:G>A
3024886|F|0-19:C>G-19:C>G	TGCAGGACAACAAGGCCACCGCCGCCATGGAGCTCCCCG	19:C>G
7171634|F|0-52:T>C-52:T>C	TGCAGGCCGTCGCCACGCCGCGCCTGGAGAATGAGGAGCTGTACAAGAGGGTTCCG	52:T>C
7171687|F|0-16:G>A-16:G>A	TGCAGGTGGAGGGGGTGAGCACCAAGGAGGACGTGTCCTACTACGCCG	16:G>A
2242168|F|0-34:C>G-34:C>G	TGCAGTCCACCCAGGAGGAGGAGCAGATCGACACCCAGCTCAAGGGCAAGGCCTCATCCG	34:C>G
3938835|F|0-25:G>C-25:G>C	TGCAGTTCGATGAGGCAGAGAAACTGTGCCGCAAAGCCCTCGAGATCCACCG	25:G>C
994650|F|0-27:T>C-27:T>C	TGCAGAAACGAGAACACCAAAAAGACCTACCAGGTTGTGGTAGTTGGCGAGCTTGGCCATGGTGGTGCT	27:T>C
5360556|F|0-45:C>T-45:C>T	TGCAGACCTTCGGCGAGCCACTGGCGGAGGGTGGCGCGGTCGAGGCGGATCTCGACGGCGGCGCGGTCC	45:C>T
1242815|F|0-22:C>G-22:C>G	TGCAGACGGGCAGGACCATGACCTACGACATTCATGGCGACGGCGGCCAGCTGACATTCGAGCCGAGAT	22:C>G
1242815|F|0-43:C>T-43:C>T	TGCAGACGGGCAGGACCATGACCTACGACATTCATGGCGACGGCGGCCAGCTGACATTCGAGCCGAGAT	43:C>T
1089638|F|0-10:G>T-10:G>T	TGCAGATGCAGGAGGCGAGCCTTCGAACGAACTTAGTTCAGGCGAAGCAGAAGCGTGATCACGACATGG	10:G>T
1046695|F|0-55:G>C-55:G>C	TGCAGATTCGTCTAGAAAATCTAACTCGTGAAAAGCAGCTCGCAAATTCCCTGGAGTTAGGCAGGCCCA	55:G>C
1024066|F|0-43:C>A-43:C>A	TGCAGCACTGCGACTGCACCGCGCGCATCGTCGCCTTGAACTCCTCGCTGCGGTACACGTTGGTGGTCG	43:C>A
7170182|F|0-37:C>T-37:C>T	TGCAGCTCCGCCATGCCGCTCTGCTGCCACGCGACCGCCTCACGCGGCGCGGCCGCCTCGTCACCGAGA	37:C>T
1714527|F|0-17:C>T-17:C>T	TGCAGCTCCCTGCCGTTCTTGCCGGCCGACATGGCGTCGGAGAGCATGGCCGTGAGGCTCCG	17:C>T
100526459|F|0-49:T>C-49:T>C	TGCAGGAGATGCGGCTGCGCGGGGTGGCGTGCCCCGAGGGGGCCCTCGTTGCCGCCATCTGCGCCTTCG	49:T>C
2244008|F|0-67:A>C-67:A>C	TGCAGGCAGCCAGAAGAAAAAAGTCACTGGTCTTTCCATAGATTAGGACAGCTACACAAAGCAGCACAG	67:A>C
100527661|F|0-39:C>T-39:C>T	TGCAGGTTTTGGTCAGATCCAAGGAGATTAGTTGGAAGTCGGGAAGCAGGAGAGTGCCTTGTCCGAGAT	39:C>T
7350377|F|0-19:G>C-19:G>C	TGCAGACTCTCGCAAGATTGCTCTCCGACATGTTCATGGAGCCG	19:G>C
3022287|F|0-25:C>A-25:C>A	TGCAGACTCTCGCAAGATTCCTCTCCGACATGTTCATGGATCCG	25:C>A
4993734|F|0-14:C>T-14:C>T	TGCAGCGACTCCACCGCGCGGGCCACCTCCG	14:C>T
3954141|F|0-29:G>C-29:G>C	TGCAGCTGGAGAGGGCGCAGGTCAGGGCTGGACCG	29:G>C
4405698|F|0-32:G>C-32:G>C	TGCAGGTAGAGCACCTCGAGCTTGGACTTGGAGCCG	32:G>C
100532270|F|0-18:G>A-18:G>A	TGCAGGTTCGAAATCTGAGTACTCTCGTCATGTTGTTTGGCGCAGGTATGAGCCG	18:G>A
7170927|F|0-23:C>T-23:C>T	TGCAGTGCGGCGGCGTGAGGCTGCCGCCG	23:C>T
7170823|F|0-23:G>T-23:G>T	TGCAGGCGCTTCTTCAGGCGCTCGGTGGAGACCGCCGCGGCGGCCGCGGTGTCCCCCG	23:G>T
5341347|F|0-63:A>G-63:A>G	TGCAGAACCAGCATCTTGGAAGACATCACGTCGCCGACAGTTGCAATCTCCTTCTGAATGCCCACGGCC	63:A>G
992992|F|0-15:C>G-15:C>G	TGCAGCCCTCAGCGCCGAGGACCGCGTCGGCCTCGTCAATGCCCTGAAGGTACGGTTACTCGTTGTTCG	15:C>G
1338752|F|0-8:C>T-8:C>T	TGCAGCGGCCAAGCAGAAGGTGAAGAAAAATGCCAAGAAGGAGGTGGTGGCGGCCGCGGTAGATGATGG	8:C>T
1338752|F|0-35:A>T-35:A>T	TGCAGCGGCCAAGCAGAAGGTGAAGAAAAATGCCAAGAAGGAGGTGGTGGCGGCCGCGGTAGATGATGG	35:A>T
1046898|F|0-5:C>T-5:C>T	TGCAGCTCTCTCCACGGTGGTATTACAAATCTCATGTATTTTAGCTCAGCGCTGGAAAACGAAACAATA	5:C>T
1070912|F|0-19:G>T-19:G>T	TGCAGCTCTGGGTCCGCGGGCAGGCCAGAGGGAGCGACAATGGGTGGCTTCTGGAGAGGGAGATGCTCA	19:G>T
2256826|F|0-6:T>C-6:T>C	TGCAGCTGAAACGCTCGGCTTCGAGGGCTACGCGCCGCCGTACCTGAGCCCGCTGGCGTCGGGGAAGAA	6:T>C
2256826|F|0-9:A>G-9:A>G	TGCAGCTGAAACGCTCGGCTTCGAGGGCTACGCGCCGCCGTACCTGAGCCCGCTGGCGTCGGGGAAGAA	9:A>G
2256826|F|0-27:C>T-27:C>T	TGCAGCTGAAACGCTCGGCTTCGAGGGCTACGCGCCGCCGTACCTGAGCCCGCTGGCGTCGGGGAAGAA	27:C>T
1224753|F|0-26:C>G-26:C>G	TGCAGCTGGTCGGCGGCCTGGAGCTGCTCCGCGGAGAGGCCGCCCAGGTCGCCGTCCCCGAGGCTGCCG	26:C>G
1004571|F|0-8:T>C-8:T>C	TGCAGGCGTTCTTGGCAGAGGCAGAGGCGGCCAAGAACGAGATGGCCGCGCTGCGCAACGAGCTTTCCC	8:T>C
1156567|F|0-9:G>A-9:G>A	TGCAGGTGTGGTGGTGAGTGGCAAGTGTGGCTGCCTTGGTCCGATGCTGCCGCCACCACCACCGTGGCG	9:G>A
4993665|F|0-20:C>T-20:C>T	TGCAGAAGCATGGCCATTGCCGTTCTGCTCTCCG	20:C>T
100539444|F|0-26:A>G-26:A>G	TGCAGAGAACGTCCAGCGGCGGCGTCATCCATGGCGCGCCCGCGGCCGCGCCG	26:A>G
5363906|F|0-7:T>C-7:T>C	TGCAGGGTTCCGCCGCGCGTCCTTGCCG	7:T>C
1694930|F|0-62:C>T-62:C>T	TGCAGCAGCCTTCGAAGAGCATTCTCCAAGGTCTGGTTCTCCTCTAGCACCAGCACCGTTTGCTTCTGC	62:C>T
1217506|F|0-24:C>A-24:C>A	TGCAGCCAGGAGTGCAGGGAGCATCAGATCAAGAAAGACGAGCTCATGGAACAGAACTGCACCATCACA	24:C>A
993664|F|0-17:G>A-17:G>A	TGCAGCCTGTCCAGCACGTTCTCCACGCCCACGTCCTCCCTCAGGCTCTCCTCGCAGGCCTTCTTCAGG	17:G>A
100543735|F|0-36:G>T-36:G>T	TGCAGCGCGTACGCCAGCCGCACGATGTCCCGCCGCGCGCGCCGCTTGGGCGTGGTCAGCACCCCGACG	36:G>T
988005|F|0-56:G>A-56:G>A	TGCAGCGGCATCAGCCACAGGTTGTGGAGCTCGTGCGTGACATTGGACACCTCCTCGGCATCCACCTCC	56:G>A
1026038|F|0-26:A>G-26:A>G	TGCAGCTCGCGCGCCGCGCCAAGATGATCGGGGACCTGCTGCACCAGCTCCACGCCGCGCAGCTCATGC	26:A>G
2254110|F|0-19:G>C-19:G>C	TGCAGCTGTCGCCGTCGTCGTTGAGGGAGGCGCTCTCCTGCACCTCCTCCTTGTCCCTGAGAGAGCAGC	19:G>C
5579679|F|0-16:G>T-16:G>T	TGCAGGCAGCCGCTGAGCATCGACGTCCACGACACCACGTTACGGCTAAGGATCCTGTCGAACACCTTG	16:G>T
1059098|F|0-56:A>G-56:A>G	TGCAGGTAAGTGTAGGTAACTTCAGTGTACTGTAGCACATAACATTTTGTCAGGAGATCACGCTCAAGA	56:A>G
1745081|F|0-15:T>G-15:T>G	TGCAGGTCGAGCAGCTTCTCCTCGCCACCCTGTCCAGCGGAGGCAGAGTGGAGCAGAGGAATGAGGCGG	15:T>G
6048512|F|0-43:A>C-43:A>C	TGCAGAGAGAGATGCGTCAGATAGATTGAATTGCTGACGAGGAACAGCGCTGCACGGGGCCCGCACCGC	43:A>C
3943256|F|0-17:T>C-17:T>C	TGCAGCAGCGGCTCTTCTATCCG	17:T>C
100547884|F|0-9:T>C-9:T>C	TGCAGCCTTTGGCAGCGACGGTGGCATCGGCAGCACGGTGCCCG	9:T>C
1134607|F|0-14:G>T-14:G>T	TGCAGGACCTCGGCGATGGCGCGGTCCACGCGGTCGGCGTCCTCGCGGAGCGCCG	14:G>T
6047648|F|0-24:C>T-24:C>T	TGCAGGCGGCGGCCACGTCGGGGGCGAGCGCGGCCG	24:C>T

1390111|F|0-60:C>T-60:C>T	TGCAGACTGTGGGTCTTGGACGATGCTGGAGACGAGCTCGTAGGCGCTGCCAATGGCGTACTCCATGCC	60:C>T
5360641|F|0-62:C>T-62:C>T	TGCAGAGGTTCTACCGCCCAGACAGCGGGGAGATACTGCTCGACGGCGACGACATCGGCTCGCTCAACG	62:C>T
1014880|F|0-53:A>C-53:A>C	TGCAGATCGCCTCCGAGGCGCGCCTCTCCGTGAAGCAGCTCTGCAAGGCGCTGATCCAGCAGGTGGAGG	53:A>C
12692035|F|0-5:G>C-5:G>C	TGCAGGCGGCGCTGGAGCTGCTGGTTGGCGGCGCGCAGCTTCTTGACCTCCTCCTCCAGGAACGCGGCG	5:G>C
100551203|F|0-18:A>C-18:A>C	TGCAGCTGACGAAGAATCAATGCCTGCGGTGGACGGTAGTGATCAAAGTGTGAGTTTCTTGCGGCTTGG	18:A>C
1108898|F|0-37:G>T-37:G>T	TGCAGGAAGACGTGCGTGCCACGGATGAGGAGAGCCAGCCTCACTTCCTGATCGTGACGTACCCGAGAT	37:G>T
981978|F|0-7:G>A-7:G>A	TGCAGGAGCTGGAGGAATGCTTCATGCGCCCCAGGCCGCCGCCCCCGCACATGTCCGCCGAGATGTACG	7:G>A
1019879|F|0-55:C>G-55:C>G	TGCAGGGACAAGGACGAAGGCGATGCGATCAAGGGACTCCTCGCAACGGCGTCGGCGGCCACGTACGAC	55:C>G
6043294|F|0-31:C>G-31:C>G	TGCAGGTGCTGTTCGCGTGGCAGAACCGCCTCGAGGGCGCCATCCCGGCCACCCTTGCGTCGATGGCGA	31:C>G
3574722|F|0-17:C>T-17:C>T	TGCAGCATGCTCTCGCGCTGCTCGAGGAACTGCTCCG	17:C>T
1231549|F|0-26:G>A-26:G>A	TGCAGCGCGCGCAGAGGAAGCTGCGGGACAAGGAGCGCGAGGTGTCCCG	26:G>A
100554654|F|0-19:T>C-19:T>C	TGCAGCTCCGAGGCGCGCGTCGCCGTGCTCGTGCCACGTGCCG	19:T>C
2256296|F|0-9:C>T-9:C>T	TGCAGGCCACCTCGGCGGCGTCCAGGGCGCTCGACCGCTTCGACGTCCTCGACTGCCG	9:C>T
1238476|F|0-6:A>C-6:A>C	TGCAGGATCCAGTCCCTGGACCTCTCCGCGAACCAGCTGGTCGGGGGCTTCCCG	6:A>C
1094041|F|0-24:G>T-24:G>T	TGCAGAAATCGGCGGCGCTCTCCTGTTCGCCCTCGCCGCCGCCGCGGCCCTCTTCGCCGCCGTCTCCAC	24:G>T
1094041|F|0-30:C>G-30:C>G	TGCAGAAATCGGCGGCGCTCTCCTGTTCGCCCTCGCCGCCGCCGCGGCCCTCTTCGCCGCCGTCTCCAC	30:C>G
1094041|F|0-35:C>T-35:C>T	TGCAGAAATCGGCGGCGCTCTCCTGTTCGCCCTCGCCGCCGCCGCGGCCCTCTTCGCCGCCGTCTCCAC	35:C>T
100556099|F|0-49:A>C-49:A>C	TGCAGAGCGCGTGAACGACAGCATTGTACGATGAGCACACAGGCACCGTATCTGCCACGACCCTCCTGG	49:A>C
1002827|F|0-44:C>A-44:C>A	TGCAGCAACGGCATGAAGCTCGTGGTCAACGTCGCCGCCGCCACCGCCGCCTCGCCGCCCAAGGCGTCC	44:C>A
2283560|F|0-67:T>C-67:T>C	TGCAGCAGCCCCGTCGAGCCTTCCACGACGAGCCGCGTCAGCAGCGTCGGGGAGCTGATGTAGTAGGTG	67:T>C
2362796|F|0-64:C>G-64:C>G	TGCAGCAGTACTGCGCCGTCCCGAACGCCTCGCACGCGCTCTTGCACGCCACGCCGCCCGCCCCCGCCG	64:C>G
7166763|F|0-7:C>G-7:C>G	TGCAGCCCACGGGCCACCGACAGGGTACCAACTCCTCAAAAGTAGCAGCAAATCGGCCCCTGACGCCGA	7:C>G
1096650|F|0-42:C>T-42:C>T	TGCAGCCGACGGGCCACCGACAGGGTACCAACTCCTCAAAAGCAGCAGCAAATCGGCCCCCGACGCCGA	42:C>T
1014875|F|0-7:G>A-7:G>A	TGCAGCGGTAGAAGGCCGACAGGTCCGCGTTCACCCGCAGCGCCACCGCCTGGTGCTTGGCCG	7:G>A
996638|F|0-67:G>A-67:G>A	TGCAGCGTCGCCTCCGCCTCGTCCTCCGTGAAGTCGGCCCCGCCGATCAGCTGCTCCAGCACCTGGCGC	67:G>A
3024910|F|0-22:C>T-22:C>T	TGCAGTCCCCTCCACATTTTCTCATCCGGGCTTGGGACCG	22:C>T
3955058|F|0-5:G>A-5:G>A	TGCAGGCCCTGCTCGCGCACCTGTGGCGTGCGGTGTGCCG	5:G>A
100560513|F|0-45:C>T-45:C>T	TGCAGGCCGCGGGGCACGACGTAGAGCTCGTTCTTCTTCACGACCCTGGAGTAGAGCCG	45:C>T
6041725|F|0-47:A>G-47:A>G	TGCAGGCCGCGGGGCACGACGTAGAGCTCGTTCTTCCTCACGACCTTAGAGTAGAGCCG	47:A>G
3023267|F|0-7:G>A-7:G>A	TGCAGTCGGAGCACTGCGTCCAGATGAGGTCACTGCCG	7:G>A
1212403|F|0-8:T>C-8:T>C	TGCAGTGTTTGTGGAGCTCCCGCTGATGGATGCGATGGATTCGGTCGCCGTGATGGAGGTGGCGCCGAG	8:T>C
7170000|F|0-40:A>G-40:A>G	TGCAGATGAAGTAGTTCGTCCCAGGGACGAGCGTCACGCGATCGCTACCCGAGGTGTAGGTGTGCGTCG	40:A>G
997705|F|0-54:G>A-54:G>A	TGCAGCAGGCGGCGGCGATGCACGGGCTCATGCTGCCTTGCGATGCGCAGAGCAGGGCGGTCGGCTCCG	54:G>A
1091419|F|0-28:C>A-28:C>A	TGCAGCCAGCGGAAGTCCTCCTCCCCCGCGCGCGGCGCCATCGGGTTCGCCGTCGCCACAACCG	28:C>A
1091419|F|0-54:G>A-54:G>A	TGCAGCCAGCGGAAGTCCTCCTCCCCCGCGCGCGGCGCCATCGGGTTCGCCGTCGCCACAACCG	54:G>A
1090646|F|0-22:C>A-22:C>A	TGCAGCGCGTGCAGGCGCACCTCCCGAACGCGACGCAGGTGGTGGGCCTGCTGACCCTGCTGCTCGCCG	22:C>A
1090646|F|0-47:C>G-47:C>G	TGCAGCGCGTGCAGGCGCACCTCCCGAACGCGACGCAGGTGGTGGGCCTGCTGACCCTGCTGCTCGCCG	47:C>G
2256569|F|0-57:C>A-57:C>A	TGCAGCTCCGCGTCGCCGTCCACCTCCTTGTCGCTGTGGTAGAAGTGGGCGCAGTAGCTGCGCACCCAC	57:C>A
1004730|F|0-45:G>A-45:G>A	TGCAGCTGCCCCGTCGCGCCGTCCAGGATCAGCCGCGACAGCGCCGACGGGTCGCTGACGTAGTAGGCG	45:G>A
2242659|F|0-38:G>C-38:G>C	TGCAGCTTGAGAGGGGCGATACCCGACTCCGTCTTCGAGCTGACCCTGCTGGAGACGCTGGATTTGTCC	38:G>C
1092126|F|0-29:G>C-29:G>C	TGCAGGGCCACCGTCGGCAAGCCCGACGCGTCGCAGGCGCTTACACCGACGAGTCTCCCTTCCTTGCCA	29:G>C
100563778|F|0-24:A>G-24:A>G	TGCAGTTCGGCACGGCGTTCGAACAGCTTGTCAATGCTCACCAGGACTCAAAAACAACACTGGACTCCA	24:A>G
1088479|F|0-22:G>T-22:G>T	TGCAGCAAATGGTGAGCGGACAGATCCATCCTTGGCATTCGTCTCCGATGTTTCCTCCCCG	22:G>T
989810|F|0-7:G>A-7:G>A	TGCAGGGGCTCGGGGTCAACCCCGCCGTGCACGAGGTCGCCGACGAGGCCGCGCTCGCCG	7:G>A
1024305|F|0-16:C>T-16:C>T	TGCAGTCCTGCTTGGTCGCCACCGCCGCCATCCGCTCCTCCTCCGTGTCGTAGCGCACCCG	16:C>T
12419870|F|0-41:C>A-41:C>A	TGCAGCTGCTGGATGTACTCGACGGCGTCCCTGAGGATGGACACCTTATCCATCTGCCAGGAAGAATGG	41:C>A
12419870|F|0-63:G>C-63:G>C	TGCAGCTGCTGGATGTACTCGACGGCGTCCCTGAGGATGGACACCTTATCCATCTGCCAGGAAGAATGG	63:G>C
1114733|F|0-11:G>A-11:G>A	TGCAGATGTCGGCCCCTCATCTTGTCCCCGACCCCGAGGGCGTCAAGGGCGCGGAAGGCGTTGGTCCAT	11:G>A
7171026|F|0-33:T>C-33:T>C	TGCAGATGTCGGCCCCTCATCTTGTCCCCGACCTCGAGGGCGTCAAGCGCGCGGAAGGCGTTGGTCCAT	33:T>C
1210668|F|0-38:C>T-38:C>T	TGCAGCACCACGCAAGAACGAAACGCCAGCGTCGGCTACTCGCCCTCCCGTCACCTGAAGAACTACAAC	38:C>T
7168819|F|0-23:C>G-23:C>G	TGCAGCTGCGGTGGAGGAGGGGTCGACGGCGGGGCCGCAGGCGGGGGGCGCCGAGATCGCCTTCTTCGA	23:C>G
1029912|F|0-58:G>A-58:G>A	TGCAGCTCCACGCCAATGCCTCTCAGGAGGCCCAGCTGAGAGAGTTCATCTCGTCCAGGAAGAACAGCG	58:G>A
100567177|F|0-56:T>C-56:T>C	TGCAGCTGCTGGGACGCGTCGGGCACGCAGAGCGCTCCGTGGCCGATCCACCGCGCTGCGTCCCGAGAT	56:T>C
1038665|F|0-27:G>A-27:G>A	TGCAGGACAGGAGAGGGGCCCGCGCGCGGCATGCACGTGAAAGCGAGTACGATGCACAATTATGATGTT	27:G>A
3935268|F|0-13:C>A-13:C>A	TGCAGAAGATGTTCGAGGACCAGCTCAAGGCCAGCTGCAACGGCGCGCCG	13:C>A
3935268|F|0-19:C>G-19:C>G	TGCAGAAGATGTTCGAGGACCAGCTCAAGGCCAGCTGCAACGGCGCGCCG	19:C>G
1695138|F|0-43:C>G-43:C>G	TGCAGAAGATGTTCGAGGACCAGCTCAAGGCCAGCGGCAACACCGCGCCG	43:C>G
1100956|F|0-50:G>A-50:G>A	TGCAGCGACGCCGAGTTGACCCTCAGGTCGCCTTGGTCAAACAAGAAGCCGCCGAGCCG	50:G>A
4262656|F|0-15:T>C-15:T>C	TGCAGCTTCCCAGCGTCGCCTATCGACGACGGTATCGCGCCG	15:T>C

3938950|F|0-14:G>A-14:G>A	TGCAGGCGGGAGGAGTTGAGGAGGAACCTGGAGGAGGTGGCCG	14:G>A
3030025|F|0-16:G>A-16:G>A	TGCAGAGGAGCGAGCAGCTGCGGGAGCTCTACGAGTCCCTCCTCTCCG	16:G>A
4911118|F|0-41:A>T-41:A>T	TGCAGAATGTCCGCACAAAACTTAACCAAGTTGCTTCTGTAAGTCCACAAGCTTTGATTTCGTGCTCTT	41:A>T
100569607|F|0-48:G>A-48:G>A	TGCAGAATGTCCGCACAAAACTTAACCAAGTTGCTTCTGTAAGTCCAAGAGCTTTGTTTTCCTGCTCAT	48:G>A
987197|F|0-31:G>A-31:G>A	TGCAGCAGGACCTTGAGAATCCTATCTGCCAGCCTCAATTCCTCACGGGGCCTCCGCCTGGAACCGAGA	31:G>A
100570370|F|0-45:T>G-45:T>G	TGCAGCGTTCCAGAAAGCGCCGAGCTGCCCGAACCAGGACAGCCATTGGATGGGGAGGCTGTTGATGAC	45:T>G
977494|F|0-29:C>A-29:C>A	TGCAGCTACGGGGACGAAGAGGGGAAGCTCAATCTTGGGTACGATGCCATGGCGATGGCGAGCACCGAG	29:C>A
1228787|F|0-8:A>T-8:A>T	TGCAGCTCACGGTGTAGCAGCTGTCGGGGCGGGAGAGCGCGTCCGAGAAGCGCACCGCGGCCTCCTGGT	8:A>T
100570434|F|0-39:A>G-39:A>G	TGCAGCTCCTGACGGGGCGCAGGCCCTACGACAATGCGCAGCCGCGAGGGGAGAGGCTGCTGGTGCCGT	39:A>G
996459|F|0-10:G>C-10:G>C	TGCAGGCGGCGGCGGACATGACCACGCTGCTCAAGGCCCTCGTCAAGGAGCTGACGACCTTGCGGAGCA	10:G>C
1069454|F|0-42:C>A-42:C>A	TGCAGTGCGGCCACGAGTTCCACCTCGGTAAGGGGCTATGGTCCGATCTGTTGATGCGTTATTGCACTG	42:C>A
1069454|F|0-58:T>A-58:T>A	TGCAGTGCGGCCACGAGTTCCACCTCGGTAAGGGGCTATGGTCCGATCTGTTGATGCGTTATTGCACTG	58:T>A
6048112|F|0-12:C>T-12:C>T	TGCAGCACTCCGCGTCGTGCGTCTTTAGAGCCTCCCGCGCGGCCTCCG	12:C>T
3030447|F|0-20:G>A-20:G>A	TGCAGTTTCGACCAAACGAAGGGGCTTTATTTAGCCG	20:G>A
999542|F|0-23:G>A-23:G>A	TGCAGCCTGAGGGTGGAGGCGACGACGATGGCGAGCACGGCGAAGCAGGCCG	23:G>A
1203614|F|0-29:G>C-29:G>C	TGCAGCAACACGAAGAAAATCACCATCTTGTGGCATCGAAGTCTGATTTTGATCAGCAAAACGTGTGCA	29:G>C
1218327|F|0-15:G>C-15:G>C	TGCAGCATCTCGTGAGCAACCTTAAGGCCTTCCTTGTACCTGCCCTCTCTGAAGAACCCGCGCATAAGA	15:G>C
6048417|F|0-14:G>A-14:G>A	TGCAGCCGCACCGCGTGCGCCACGGGCCTCACGTCGAACCCTTCGGCGTCGGAGTCGCCGCCCTCGTCC	14:G>A
1110416|F|0-37:C>A-37:C>A	TGCAGTGATAAACGCCATGGCAGGTTGCTGTCCTCGGCAGAGTAAGGCACCCGAACCTCGTTGCGCTGA	37:C>A
7171123|F|0-17:G>T-17:G>T	TGCAGCGCAGCGTTTGCGGTGCATGCACCGCGCATGTGCCTGGTCTCTATCTGTTCGCCTGTGCCCTCC	17:G>T
24505227|F|0-62:C>A-62:C>A	TGCAGGCCATGCAGGCGATGCTGTTCCGCTCCAACCGCCTCGTCGGCGTGCCGCTGGCCAGGCTGCTCC	62:C>A
1092599|F|0-23:G>A-23:G>A	TGCAGGTCGGCGCGGAGCACGTTGGCGGCGGTGTCGTGGGACTGCGCGACGTCGCGCCACATCTGAGCC	23:G>A
1078167|F|0-20:A>G-20:A>G	TGCAGTGCGACAGCAGGTGCAAGTACGCGCTCGTGGTGTCCACGGAGATTCTCACCTCGGATTACTACT	20:A>G
6047501|F|0-28:G>T-28:G>T	TGCAGCAGCAAGAACACCAGCGCCAGCAGTGCCGCGGTTCCG	28:G>T
4396519|F|0-13:G>A-13:G>A	TGCAGGAGCTGGCGCGCCATCCTCTCCGACCCCG	13:G>A
3947593|F|0-15:A>C-15:A>C	TGCAGGATCTTCAGGAGGGCCATCACCTACCG	15:A>C
1203622|F|0-57:A>T-57:A>T	TGCAGATGAAAGGCATCGGCTACGAGTACATGGAGCAGGACCTGTTCACCAAGGGCGAGCTCCTCCGCA	57:A>T
1079174|F|0-39:G>T-39:G>T	TGCAGCTTCGGCGGCGACACCTTCAAAGGCTTTTGCGGGGGCGGGGGCGACAGCTTTGAATGCTTCTGC	39:G>T
7172800|F|0-28:A>C-28:A>C	TGCAGGCGTTGTCCTCCTGCCGCCGCCTAGACCACGTCCGCCACGTCGCCGCAAGGCCCACGGCGACCA	28:A>C
2251044|F|0-30:C>G-30:C>G	TGCAGGTACCCAGCGTACGACAGGCTTCTGCCCTGCCCGTCGAAGGACGACCCTCCGAGGATCGAGCAC	30:C>G
2251044|F|0-56:G>A-56:G>A	TGCAGGTACCCAGCGTACGACAGGCTTCTGCCCTGCCCGTCGAAGGACGACCCTCCGAGGATCGAGCAC	56:G>A
1023809|F|0-26:G>A-26:G>A	TGCAGGCACTGCGCTCCAGGCACTGAGGTCCATTGGTGCCAAGTTTGACGGCACCG	26:G>A
1207119|F|0-41:C>A-41:C>A	TGCAGAAGGTTGTGCTCAAGCTGGACGTGCACGACGACAGGCACAAGGCCAAGGCGCTCAAGGCCGTCT	41:C>A
1741671|F|0-40:C>G-40:C>G	TGCAGAGCGCGGTAGGGGTGCAGTTGCGGCTCCATATGGGCAGGCACGTGGCGCTGGAGCAGGGCACAG	40:C>G
1024669|F|0-12:A>T-12:A>T	TGCAGATGGGGCAGGTCGGCCTCGTCGTCGTCTCGTCCCGCGAGGCGGCCAGGGAGGTCATGAAGGTGC	12:A>T
1202706|F|0-12:T>G-12:T>G	TGCAGCGAGCCCTCCGAGATTTTGACCTCCACCAGCGCAAGCTTCTTGAGCAGGGGAAACTGAAGCGTC	12:T>G
1074139|F|0-5:C>A-5:C>A	TGCAGCCCCACGTCGCTAAACACCACCGTGAGGAAAACCACGGCGGCGAAGAAGGAGTGCACGAAGTCC	5:C>A
3027174|F|0-16:C>A-16:C>A	TGCAGCGCCTTACGCCCGTCCCCGTGCAACGCCAAGCCG	16:C>A
1070948|F|0-16:C>T-16:C>T	TGCAGGTCCAGGTGAACGGGGCGACGAGGCGTGTGGGCGGGGTCTTCGGGACGCCG	16:C>T
5970951|F|0-12:G>A-12:G>A	TGCAGGACCGTCGGGCAGGATGGCGAAGCCTGATGGCAGGAGCGCGACGTAGTCTGGGTCGCCGCCGTT	12:G>A
991667|F|0-15:G>A-15:G>A	TGCAGATCCAGAGAAGCGGCAGAGAGTTCCCTCGGCATACAACCGATTCATCAAGTGAGTGATCAGTGG	15:G>A
1117759|F|0-31:G>A-31:G>A	TGCAGCAATCACGCGTGTGCCGAAGAACGTCGTCGTCCATCCCTCACTTCAACGGCCTGAGGATCCTGC	31:G>A
1025047|F|0-17:C>A-17:C>A	TGCAGCGGGGTGAGGACCCTGAACGGCAAGGCGTCCACCTCGGCCGACCG	17:C>A
1025047|F|0-20:G>A-20:G>A	TGCAGCGGGGTGAGGACCCTGAACGGCAAGGCGTCCACCTCGGCCGACCG	20:G>A
1025047|F|0-28:A>T-28:A>T	TGCAGCGGGGTGAGGACCCTGAACGGCAAGGCGTCCACCTCGGCCGACCG	28:A>T
4002189|F|0-16:C>T-16:C>T	TGCAGCTGGGCGGCGACGTGGGCGGCGGCCGCCTCAGCACCGACCCG	16:C>T
4002189|F|0-17:G>A-17:G>A	TGCAGCTGGGCGGCGACGTGGGCGGCGGCCGCCTCAGCACCGACCCG	17:G>A
3064641|F|0-21:G>C-21:G>C	TGCAGGCCCTCAAATCCTCGGGCGACGTCTATCTGCTGCCG	21:G>C
3064808|F|0-25:C>T-25:C>T	TGCAGCACGACGCCGTCGACTTAGTCCGCTTCCACGCCGTCTGCAAGCCATGGCGGAACTCCCG	25:C>T
3064808|F|0-58:C>T-58:C>T	TGCAGCACGACGCCGTCGACTTAGTCCGCTTCCACGCCGTCTGCAAGCCATGGCGGAACTCCCG	58:C>T
7171109|F|0-8:T>C-8:T>C	TGCAGCGATCTTGCCCCCTCCTCGTCTTCGGCGCCATCGTCCCCTCCTCCCACGCCACCGCTGCCTTCG	8:T>C
2254739|F|0-11:T>A-11:T>A	TGCAGCGACCTTGCCCCCTCGTCGTCTTCGGCGCCCTCGTCCCCTCCTCCCACGCCACCGCTGCCTTCG	11:T>A
1719641|F|0-57:G>T-57:G>T	TGCAGCTCTACAACAACAGCCTGAGCGGAGAGGTTCCTGCCGGGATCACGCAGTCGAGGAAGCTCAGAG	57:G>T
1231770|F|0-6:G>T-6:G>T	TGCAGGGGCGGCTGCGGAGCACGCAGCCGCTGACCAAGGGCGTGGTGGCGGAGTGCGTGCTGGGCGCCG	6:G>T
1029735|F|0-38:C>A-38:C>A	TGCAGCGTGGCGGTGGAGGAGCCGATGGAGTGCCTGGGCACGGGGCTGGTGAGCCCG	38:C>A
5361199|F|0-41:G>T-41:G>T	TGCAGCTCGGCGACGGCGAACCTGTCAGGCGGCCCGTGCCCGCGATCGGCCTGGGTGAGGAGGCCCATG	41:G>T
7168869|F|0-25:C>T-25:C>T	TGCAGGAGCGCGACGGGGTTCTGCGCGCGGCTGTAGCTGGGGCGCCACTCGACGTCCAGCCCCACGATG	25:C>T
988349|F|0-24:G>C-24:G>C	TGCAGGTACGTGTCGGCCACGATAGCGTCTGGCCTCCGCCCCAGCTCCAGCGCCAGCCG	24:G>C
1001684|F|0-56:C>A-56:C>A	TGCAGCACCGAGAGCTGCACGATGATGTCGCGCGCCCACTTGCCGTACATGGCGTCCAGCAGCGGCACG	56:C>A

1098289|F|0-7:C>T-7:C>T	TGCAGGTCGACTGCGGCGGCGAGGGCAGCGGCGTCTGGTTCACGGAGGCCGCGGCCG	7:C>T
1022225|F|0-12:C>T-12:C>T	TGCAGCAATGGCCGCCGCGGTTGGAGCCCGAGCCAGCCCCGATGATGTCGGCCG	12:C>T
5971693|F|0-8:G>A-8:G>A	TGCAGCGGGATGAAGCTCAGGAACATCAGGCTGAGGTACCG	8:G>A
4993562|F|0-19:G>A-19:G>A	TGCAGCGGGCGGCGACGGCGAGGCAGGGCTGACCCG	19:G>A
100589959|F|0-17:G>T-17:G>T	TGCAGCGCGAGCACCGCGCCCCCCTTGGAAAGAGGCGGTGCGAGCGGCGCGATCTTGCGGACCTCCCTC	17:G>T
5371894|F|0-25:G>A-25:G>A	TGCAGCACGACGGGCGACGGCTTGGGCGGCGCGAACTCGACCAGTGTGGCCCCGTCCACACGCGCCTCG	25:G>A
989664|F|0-9:C>T-9:C>T	TGCAGTTGGCCAGGCACAGCTGTCTGCACTCGTCCAGCGTCATGTCGGCGTGCACCGTCGCGTTAGTCG	9:C>T
989664|F|0-41:T>A-41:T>A	TGCAGTTGGCCAGGCACAACTGTCTGCACTCGTCCAGCGTCTTGTCGGCGTGCACCGTCGCGTTAGTCG	41:T>A
1122854|F|0-9:T>C-9:T>C	TGCAGATTCTTGCTGTGGCATGCGCTTGTATATCTTTGTTCTCTTGTGTGCCG	9:T>C
2246456|F|0-37:T>A-37:T>A	TGCAGCTGCCCGTCCACCAGCTTGAACCTGCCTGCCATCCCGTCGAACGTCGTGTTGAGAATCGCCCTA	37:T>A
995959|F|0-50:G>A-50:G>A	TGCAGCAGGTTCCAGTGCCTGCTCGCCCACGTCTGGAAGAAAATCACGGCGGCGCGGGACCTGAAGCCG	50:G>A
1024792|F|0-6:C>T-6:C>T	TGCAGGCCACCGTGATGCGCCGTGGCCTCGCCATCGGCATGGCCGTGCACCACACCGCCTGCGACGGGG	6:C>T
3950856|F|0-21:C>G-21:C>G	TGCAGAGCGTACCCTGCGACACTGCCCG	21:C>G
4395242|F|0-7:G>A-7:G>A	TGCAGCGGGAGAAGCGGAAGCCG	7:G>A
5332312|F|0-14:G>C-14:G>C	TGCAGGCCGTCGGGGCGGGTCACGTACTCCCG	14:G>C
3940310|F|0-12:G>C-12:G>C	TGCAGGAGATGGGCATACCG	12:G>C
4261474|F|0-5:C>T-5:C>T	TGCAGCTTCTTCACCTCCTCGATGGTGAAGTCCG	5:C>T
4393753|F|0-39:G>C-39:G>C	TGCAGGAGACCATCGCGTCCGTCTGCAAGTCCATCTTCAGCCGCCCCG	39:G>C
3959118|F|0-12:G>C-12:G>C	TGCAGGTTTCGGGCGTGGATAGGCCG	12:G>C
3959118|F|0-17:G>C-17:G>C	TGCAGGTTTCGGGCGTGGATAGGCCG	17:G>C
3956890|F|0-5:G>T-5:G>T	TGCAGGCTGTGCCGACGACGAGGATCGCGGTGGAGATGACCG	5:G>T
7337096|F|0-18:C>G-18:C>G	TGCAGCAGCACCTGGGTTCGCTGGCCAGCTCCG	18:C>G
990539|F|0-24:G>T-24:G>T	TGCAGCGGCCACCCCTCCCTCGTTGCTCTGCGTGGCGTCGTGCGCACGCCCG	24:G>T
100416285|F|0-21:G>A-21:G>A	TGCAGCGTCAGGCAGTACCCCGTGGCGCGCCG	21:G>A
3950580|F|0-19:C>G-19:C>G	TGCAGGCGGCGGCGCCTTCCGATGACTCCGCCGTGCTGCCG	19:C>G
4261062|F|0-20:C>T-20:C>T	TGCAGGGACGGCGCGTCCAGCACCTGCACGCCG	20:C>T
4261062|F|0-23:C>G-23:C>G	TGCAGGGACGGCGCGTCCAGCACCTGCACGCCG	23:C>G
3940813|F|0-8:T>A-8:T>A	TGCAGGGCTCCATCAACATCTTCTCCGACGCCGGCCCCGACATCCG	8:T>A
5580524|F|0-10:G>A-10:G>A	TGCAGGTGGCGTTATCGTAGCCGCTCCCG	10:G>A
1032506|F|0-10:G>A-10:G>A	TGCAGAAGCCGCAGAGATGACAGCAGAAGATGAGCCACTAAACCCTTGAAACACACCAGCAGCCATTGA	10:G>A
1215068|F|0-8:G>A-8:G>A	TGCAGGAGGAGGACGCGGCGGGTGAGCAGCGCGTAGAGGAAGGCGGACGCGATGGAGAGCACGCGGTCG	8:G>A
1070571|F|0-34:C>T-34:C>T	TGCAGTACGCGTGCAGGAAAACCTTGGCGGACAGCAGGCCGCGCGTCCGCGGCAGGTTCGCCAAGAACG	34:C>T
4261660|F|0-29:C>G-29:C>G	TGCAGCAGTAGCATGATCCAGTAGCGGCACGTAGCGTCCG	29:C>G
4397718|F|0-8:C>A-8:C>A	TGCAGCGTCTGGGTGCACACGCGGTGCGCGCGCTACGTCCG	8:C>A
3937529|F|0-9:G>T-9:G>T	TGCAGCATAGGCTGGCCCTCCCTGTCGAGTGCCGGGACAACGGGTACCG	9:G>T
4999155|F|0-40:G>A-40:G>A	TGCAGCTCCCTCGTTAACAGGTCGCTCGTCTTCTCCACGGGCCCCG	40:G>A
1150172|F|0-5:C>A-5:C>A	TGCAGCGCGTACGCCAGCCGCACGATGTCGCGCCGCTCCCGCCGCTTCGGCATCGTCAGGATGCCGACC	5:C>A
988825|F|0-7:G>T-7:G>T	TGCAGGCGTCGGACCTCAACGACTTCATCGCCCCCTCGCAGGACTGCGTCGTCTCCCTCAACAAGAACT	7:G>T
5357321|F|0-27:C>T-27:C>T	TGCAGAGACCGCTACGCGCCACCTCGGCGGCGCCG	27:C>T
6044005|F|0-52:G>A-52:G>A	TGCAGCTGGGCTGCGAACCGCGGGCTCAGCGTCGGGTCGGCTGTCGGCCGCAGCCGTCCG	52:G>A
5582368|F|0-8:G>C-8:G>C	TGCAGCTCGTCCCCGCCGTCGCCG	8:G>C
11914914|F|0-14:C>G-14:C>G	TGCAGCCTTGAGGACTGCCG	14:C>G
1695356|F|0-23:C>A-23:C>A	TGCAGGCTCCAGATATGGAAGCTCGCCACCCGCCTCTGGAACGCCGTCGTCGACCGCGCCAACTCCGCC	23:C>A
987258|F|0-64:G>A-64:G>A	TGCAGAGCGGGCAAGTGCTGCTGGTGCGCAGCCACGCCCTGAGGCAGCGCCTGTGGAACATGTGGCCGC	64:G>A
1053137|F|0-67:G>C-67:G>C	TGCAGATCTCCGACCATCTTCATCAGGATCTCCCCCACATCAGACTGCTTCGCCGTGAGGGACTCGTGG	67:G>C
1132525|F|0-52:G>T-52:G>T	TGCAGCTCCATCTCCCCGCGGCCACGGATGTCGCGCACCGACACGTACCTGTGCACGCTCGGCGCGTCC	52:G>T
985159|F|0-11:C>T-11:C>T	TGCAGGGGCTTCCACGTCACCTTTGTCAGCACCGAGTACAACCACCGCCGCCTCGTCCGCTCCCGCGGC	11:C>T
6046608|F|0-20:G>C-20:G>C	TGCAGAGCATTCCCGTTGATGGTGGCGTTGCCCAACACGGTGAGGTTACCG	20:G>C
5369378|F|0-7:A>T-7:A>T	TGCAGCCAGGTTAGCTATGAGGCCG	7:A>T
1002383|F|0-37:G>T-37:G>T	TGCAGGCAGAGGCGGCCACCGCTCACGGTGCGCGGGCGAGCGGCGAGGAAGCAAGGCCG	37:G>T
7352622|F|0-18:T>G-18:T>G	TGCAGCTCGCGTACGCCGTCGGAGGCGGCTGCCCG	18:T>G
4262413|F|0-14:T>C-14:T>C	TGCAGGGCTACGACTACAGCTCCG	14:T>C
7170927|F|0-8:G>A-8:G>A	TGCAGTGCGGCGGCGTGAGGCTGCCGCCG	8:G>A
988652|F|0-40:C>G-40:C>G	TGCAGAACGCCGTGAAAGGTAAGGCACGCTCATCACCTTTCGCTTTCATGAATGCTGCCATGCTGGTGC	40:C>G
981258|F|0-44:G>A-44:G>A	TGCAGCCCAGAAGGATTTATCTTGTGATGAAAGCGGAGTGCGAGGAGCAGAAGAGAAAATATTTTCAGG	44:G>A
4262166|F|0-9:C>G-9:C>G	TGCAGGTCGCCCTCGCTTAGCTCCG	9:C>G
3020320|F|0-22:C>A-22:C>A	TGCAGGTGGAGATATGTTTAACCTCCAATGTTATGACCG	22:C>A
1027297|F|0-11:C>T-11:C>T	TGCAGTTTTTGCTGCCCCAGTCGTGTCCGACCTGGCACTTCCCTTTCCACCTTGATGGCACCG	11:C>T
7164893|F|0-13:G>A-13:G>A	TGCAGATGCTGCGGGTGCAACCGAGGTGGGCGGATCAGGCGGACGCGGAGGCGGCCG	13:G>A

1096626|F|0-20:G>A-20:G>A	TGCAGGCGCGCGATGACGGCGTGCATCAGGCCGATGGTCTGCTCCTGCCTCATCGCGGTGGCCTCCATC	20:G>A
3934914|F|0-12:C>A-12:C>A	TGCAGGAACATCCACACCGACCG	12:C>A
986352|F|0-44:C>T-44:C>T	TGCAGCGGCTGCGCCATGATCTCGCTCATCACGCTCTCGTTGTACCCGAGGCCGCACACGTAGGGGATG	44:C>T
1230460|F|0-27:G>A-27:G>A	TGCAGCTGACGTGGAACTACAACTACGGGGCGGCGGGGCAGAAGCTCGGCTTCAACGGGCTCGACAACC	27:G>A
992506|F|0-34:C>G-34:C>G	TGCAGACGTTCGCCGCCGCGGACAACGAGCTGATCGGGGGTGTGCCTGATGAGCTCGGCGATTGCCGAG	34:C>G
996761|F|0-6:A>C-6:A>C	TGCAGCAGCCGCCGCCGCTGCACCTGCTCCCCGCCGCCGCCACGTGCTACGGGAAACCCATGGCCGAGA	6:A>C
1001408|F|0-19:C>T-19:C>T	TGCAGCTGCCCCGTCGCGCCGTCCAGGATCAGCCGCGACAGAGCCGACGGGTCGCTGACGTAGTAGGCA	19:C>T
986239|F|0-41:G>A-41:G>A	TGCAGGGCCAGGTCCTGGTGCTCCTTCTTGCACACCTGGTCGGCGCCGATTTTGCCGTACCG	41:G>A
5364341|F|0-26:T>C-26:T>C	TGCAGCGGACGAGGGGAAAGCGGGGCTGCTGCGGCGGCTCGTCCCCG	26:T>C
1005220|F|0-21:C>T-21:C>T	TGCAGACGGCGGTCCTGCTCGCCGTCATCTGGAACACGGACTGGGAGGACGAGGCGGCGCAGGCGAGCG	21:C>T
3029371|F|0-5:G>T-5:G>T	TGCAGGCCGTGACCCTCCAGGAACACCCCGTCCGCCG	5:G>T
1021829|F|0-20:G>T-20:G>T	TGCAGGGGGAACTCTTTGTCGTCCTCGTCGGTGCGCCCCTACGTCCGCTCCAAGAACCCGCGCCTCCGC	20:G>T
5323908|F|0-33:G>A-33:G>A	TGCAGGGTTCAGGTGGGATCGGCAGTGGTTGGTGGTGAACGCCAAGGGCAGGGCGCTCACGCAGAGGGT	33:G>A
3934554|F|0-20:G>A-20:G>A	TGCAGGCCTCCGTCCCCAATGCCG	20:G>A
3946816|F|0-12:T>A-12:T>A	TGCAGGCGTCCGTCAACGCCGACGGGACCTTCGGCCAGGTCTTCGCCG	12:T>A
7492291|F|0-31:C>T-31:C>T	TGCAGGCGTCCGTCAACGCCGACGGGACCTTCGGCGAGGTTTTCGCCG	31:C>T
1408775|F|0-6:T>C-6:T>C	TGCAGGTCGGGTTCCCCCCTAAGCCGCTCGACGACGCGACCAAGACCATTGAGGAAGCCG	6:T>C
6042751|F|0-31:C>A-31:C>A	TGCAGTCGCAGAGCCTCACCACGCCGCTGCTCCTGTCGTCCGTGGCCACGCTGGCGCTGCACGTCCCGC	31:C>A
4002569|F|0-30:G>A-30:G>A	TGCAGTCCAAGAGCGTGTGCGTGGTGGGGGGCGGCGTGGCCG	30:G>A
4539060|F|0-5:C>G-5:C>G	TGCAGCTGGTGTCCGTGATGTTCGTGGGCCACCTCGGCGAGCTTACCCTCGCCG	5:C>G
1666712|F|0-8:G>A-8:G>A	TGCAGGACGTGTTCATGCGCCTGACGTTCGACCTCACGGCGATGTTCATATTCGGTACGGACCCCGAGA	8:G>A
1694954|F|0-12:C>T-12:C>T	TGCAGGGCGTGGCGCGACACCGTCGACGCCCGCCTGCGCGGCCACCTGCTCTCGCGCTCCGTGCGCGGC	12:C>T
1020019|F|0-8:C>G-8:C>G	TGCAGCATCGGGTTGATGTGGCCCTGGAACGGCAGCGGAAACAACAGCACGCGCCG	8:C>G
1050910|F|0-59:G>A-59:G>A	TGCAGCCGCTGGATTCAGCTTGGCGCATTCTACCCGTTCGCCAGGGCCCACGCAGAGAAGACAACCCTC	59:G>A
3026331|F|0-23:A>G-23:A>G	TGCAGGAAGCGCAGCCAACACCAAAGGCTGAAACAGCCG	23:A>G
7487642|F|0-51:C>G-51:C>G	TGCAGTACGCGGGGTACCACGAGCGGCTGCTGCGCCTGGTGCGCGTCGGCGCCGCCATCGCCTACGACA	51:C>G
997849|F|0-16:T>G-16:T>G	TGCAGAGCTGCGAGCATCGGTTCATCTGGGTGCTGCGCGACGCCGACCGCGCCGACATCTTCGCGGAGG	16:T>G
1206894|F|0-35:G>C-35:G>C	TGCAGCTCGTAGTCGTCCTGCACGGCCTCGTCGGAGGGGTAGTAGGCCGCCACGTAGGCCTCGCACCAC	35:G>C
3030984|F|0-14:A>G-14:A>G	TGCAGTGCCCTACGAGGTCACGGCCAAGGCGTCGGCGCCG	14:A>G
4262864|F|0-20:A>G-20:A>G	TGCAGCACGCGGAGGCGGTCACTCACGCGCTCCCGCCG	20:A>G
7492112|F|0-49:G>C-49:G>C	TGCAGCGGCTGGTGGAGGCGGAGGGCCGCCCCGTCGGCGGCACCGACGCGTCCG	49:G>C
1023927|F|0-5:T>G-5:T>G	TGCAGTGTGTAGGTGCCCTCCGCCTCCACCAGCAGCAGCTTGTGGCCCTGGAGCCG	5:T>G
4003300|F|0-29:G>C-29:G>C	TGCAGCTGCTGGGGCGCGGTGTACAACGGGGGGAGCGGCTACTGCTACCTCATCGACTTTCCCGTGGAG	29:G>C
7349227|F|0-17:T>G-17:T>G	TGCAGCCTCCATGCGCCTCTGCCG	17:T>G
6044036|F|0-20:A>C-20:A>C	TGCAGGAAGTTGCGCGCGAGACCCACGGAGACGAGCCCG	20:A>C
16795586|F|0-23:C>G-23:C>G	TGCAGGGCGCTCTTCCCGTGCTTCACCTCTCCG	23:C>G
7173274|F|0-31:T>C-31:T>C	TGCAGCAGGGCGTGGACCTCGTGGGCGGGTATTACGACGCCG	31:T>C
7173856|F|0-13:T>G-13:T>G	TGCAGCACGCCGCTGCGTTCCTCCCGCGCGTCCGCATCGTCGCCG	13:T>G
6045781|F|0-21:T>G-21:T>G	TGCAGGTCGTCGCGGAGCAACTTATGCTCCAAGTACCG	21:T>G
7167784|F|0-30:A>G-30:A>G	TGCAGCGCAGGTAGGAATCTTTTGGCTAGGACCCG	30:A>G
11315174|F|0-8:A>C-8:A>C	TGCAGATCATCCTCCTCTCGCTCGCCG	8:A>C
6037500|F|0-10:A>G-10:A>G	TGCAGAGCGGACGGCGCAGTGGCAGTTCCG	10:A>G
100366812|F|0-19:A>G-19:A>G	TGCAGCTGCTGCTCAAGGCATCGCAGGACAAGCGGTTCGTGTGCGAGGAGGCCGAGAAGGCGATGCGCG	19:A>G
100501698|F|0-52:A>G-52:A>G	TGCAGCGGCTCGTGGGCCGCCACGGCGCGCGCAACTGGTCCCTCATCAGCAAATCCATCCCG	52:A>G
100562459|F|0-64:T>C-64:T>C	TGCAGCGCGTGGACGACACGCTGGGCGTGTTCCACACCCACGCCGTGGCGGGGTTCCTCGGCGGTGTGA	64:T>C
6047423|F|0-28:A>C-28:A>C	TGCAGAACGGGAGCGGGGGCAGCGTCACACAGACCGTCCAGTGGCCG	28:A>C
3938927|F|0-27:G>C-27:G>C	TGCAGGAGATCGCGCAGAACAGGGCCAGCACGCCG	27:G>C
7170322|F|0-23:A>G-23:A>G	TGCAGGGTGGAGACGAGCGCGCCAACCAGCGCCGCGCCATCCCTGTCGACGGCGCGCGCCAGGCACCGC	23:A>G
7165959|F|0-23:A>G-23:A>G	TGCAGGTCGCGCTCGAGGTCCTCAAAGGTGGGGCCCGCCG	23:A>G
100418887|F|0-24:T>C-24:T>C	TGCAGTTCTTCCGCGCGGACAAGGTGGCCG	24:T>C
100449491|F|0-39:A>G-39:A>G	TGCAGCCTGCTCTTGAAGACGTAGCAATTCTCGACCCCTAAGCCAAAATCAAGCGCACCACCGCTCAGG	39:A>G
100496877|F|0-5:A>G-5:A>G	TGCAGAGGTTCAGCGAGCAGCAGGTCCG	5:A>G
7172978|F|0-22:A>C-22:A>C	TGCAGTTCCCGTCGGACATAGCAACCGTTCTTCTTGCCGCCG	22:A>C
1184439|F|0-66:G>A-66:G>A	TGCAGACTCGAGCTTCGTGCCGCCGCTGGTGTACGCGATGATGGGGAGCTCCAAGGACCTGGCGGTGGG	66:G>A
2244173|F|0-57:T>C-57:T>C	TGCAGCCATAGCAGAGCAAGCAACCATTATCTCGATAGCTTCCTACAATGCCAAAGGTGTTCGATGCCA	57:T>C
100324051|F|0-42:T>C-42:T>C	TGCAGGACATGCGAGAGGTGACGAGGCCATCGATCTTGATGATGCTGGGGTGGTCGAGGCGGCGGAGGA	42:T>C
5970818|F|0-14:G>A-14:G>A	TGCAGACATTTGGCGTGCAGGGCCG	14:G>A
3023795|F|0-24:C>T-24:C>T	TGCAGCATTCTCAAGATCTGGAACCTGAACGCGCACTCCG	24:C>T
3951133|F|0-9:C>G-9:C>G	TGCAGCCGTCCATGCCAAGGCGGCAGTCACCCG	9:C>G

100351192|F|0-5:T>C-5:T>C	TGCAGTGCGGCGGCCGACCTCTTGCCCTTCCG	5:T>C
4404381|F|0-23:C>T-23:C>T	TGCAGGACGAGGAGCGAGCGCAGCGTGTCCCG	23:C>T
6038041|F|0-7:C>T-7:C>T	TGCAGGCCACAAACACCTACCCG	7:C>T
7167794|F|0-17:G>C-17:G>C	TGCAGCGCCAGGTGCACGCGTCCCCG	17:G>C
3029480|F|0-25:A>T-25:A>T	TGCAGCCGCGCCCGCAGCGCATCAAACAGCGAGCAACCG	25:A>T
4002743|F|0-27:G>T-27:G>T	TGCAGCGGCGGCGAAGCTGCGGAAGGGGCTGTGGTCGCCG	27:G>T
3948727|F|0-20:G>A-20:G>A	TGCAGCTTCATGACGTCGCCGCAGGCCGGCGCGCCGACCAGCCCG	20:G>A
7168093|F|0-20:C>A-20:C>A	TGCAGGCAGGTGGCTCTGTTCCTCCG	20:C>A
100388091|F|0-11:T>C-11:T>C	TGCAGGCCCTGTGGCGTGACGAAGCGAAAGTCGCCG	11:T>C
5578361|F|0-22:A>T-22:A>T	TGCAGGTACTTTTGGTGGCTGGACTGCGCTCCG	22:A>T
1062099|F|0-6:C>T-6:C>T	TGCAGCCTGCTGGAGGAGACCGCCGACTACGTCCGCTGCCTCCGCGCGCAGGTGCAGCTCATGCAGGGC	6:C>T
1118792|F|0-8:C>T-8:C>T	TGCAGCTGCCCAAGGGCATGGAGTCCGTGAACCACATCTGGCAGGTGGGGTCCGCGGTGGCCAACGGGG	8:C>T
1118792|F|0-28:G>C-28:G>C	TGCAGCTGCCCAAGGGCATGGAGTCCGTGAACCACATCTGGCAGGTGGGGTCCGCGGTGGCCAACGGGG	28:G>C
1395408|F|0-59:G>A-59:G>A	TGCAGGCCGTTCATGGCGTACAGGGACGCCGAGATCCGCCGCTCCACCTTCATCCGCTCGAACTCCACC	59:G>A
991595|F|0-23:C>T-23:C>T	TGCAGGCGCCTACGCCGCTGCGGCGCACGGAGCTGTGCGAGCCCGCTTCCGCCCACCGCAGCTCCCGAG	23:C>T
4262798|F|0-19:G>T-19:G>T	TGCAGACGTTCGCGCAGGAGCTGGGGAAGCTGGAGCCG	19:G>T
3949483|F|0-17:C>G-17:C>G	TGCAGAGCCGAGATGCTCGGTCCG	17:C>G
100414687|F|0-16:T>C-16:T>C	TGCAGAGGAGGTCGTATAACCCCCG	16:T>C
6037684|F|0-25:G>A-25:G>A	TGCAGCCACGTCGGCCGCGATGGAAGCGGCCG	25:G>A
1127963|F|0-19:G>C-19:G>C	TGCAGAAGATCACCAGCCTGCTGCTCTGGGGCAATGCCCTCTCCGGGAAGATCCCGCCG	19:G>C
7487970|F|0-14:C>T-14:C>T	TGCAGCACAACCGCCTCACCG	14:C>T
1021051|F|0-58:C>A-58:C>A	TGCAGGATGTACTCTACCGAGTCCTTGGAGCGCGCGCGGGCCAGCAGCGGGAGGTGCTCGTGCCCCCAG	58:C>A
1018924|F|0-13:G>A-13:G>A	TGCAGGCCAGCAGGGCTGAGCATCTCGCGGACGCAGACGCGGCGGAGCAGACGCCAGGTGGGACCGACG	13:G>A
2242439|F|0-29:A>G-29:A>G	TGCAGGCCATCATCGAGGGCTCCAGGGAGACCTGGACCTACGCCATCTTCTGGCAGTCCTCCACCGACG	29:A>G
5331507|F|0-5:A>G-5:A>G	TGCAGACGAAGAGGGACTTGAGGCCG	5:A>G
7164971|F|0-6:A>G-6:A>G	TGCAGCACCGCCGTCGGCTGCTCCGACCG	6:A>G
100439629|F|0-17:A>C-17:A>C	TGCAGCAGCGCCGTCGCAGACATGTCGGTGGGCGCCGCGGCCG	17:A>C
100440387|F|0-11:T>C-11:T>C	TGCAGCGATTGTCTTCACCAGGAACCG	11:T>C
3028329|F|0-28:G>T-28:G>T	TGCAGCGCCCGCAGCACGTCCGACCCGCGCAGCTGCCCCCCG	28:G>T
4262292|F|0-10:C>T-10:C>T	TGCAGGAAGTCGCTGGCGCCGATGCTGAAGATGTAGAGGGCGCCG	10:C>T
5332206|F|0-7:C>T-7:C>T	TGCAGGACGGACGTCTGACCGAACCG	7:C>T
1071840|F|0-25:C>A-25:C>A	TGCAGGAGTTCAAGAACGCCGTGCGCAAGATGGGCCGCGTCGGCGTGCTCACCG	25:C>A
1071840|F|0-37:C>T-37:C>T	TGCAGGAGTTCAAGAACGCCGTGCGCAAGATGGGCCGCGTCGGCGTGCTCACCG	37:C>T
100442053|F|0-23:A>G-23:A>G	TGCAGGGCGTTGCCCGTGACATCAGCGAGCGGCCG	23:A>G
4261236|F|0-30:C>G-30:C>G	TGCAGGTACAGTTGCAATTCCAGGCCATGCCTAGCCG	30:C>G
1075878|F|0-64:C>G-64:C>G	TGCAGAAGCCGCCGAACACGCCGCACGCCTCGCTCCACGTCACCTCCTTGTCGCCGTTGTACGCCAGGT	64:C>G
5324914|F|0-61:G>A-61:G>A	TGCAGATCTGGTGCATCGACCGCGGCGGCCCGCTCTTCACCGCCGTGTTCCAGCCCGTGCAGACCGTCG	61:G>A
1221747|F|0-28:G>A-28:G>A	TGCAGATGAGCGCGATCAGGAAGAACTTGCCCCGCGTCATCCGCCGCTTCTCCTTCTCGTGCATCGCCC	28:G>A
1194806|F|0-46:G>A-46:G>A	TGCAGATGATGCAGCCCCCGCCGATGGGCTGCGGCGGGCCGGCGTCGTCGTGCAGAGGGTGCAAGGGCT	46:G>A
100450341|F|0-10:T>C-10:T>C	TGCAGCTCGGTCACGCCTACCGCCTCATCGCCGTCGACGAGGTCACCAGGGCCGTGCAGGCCAGGAAGG	10:T>C
1019650|F|0-7:A>G-7:A>G	TGCAGGCAGCCTGGTCTTCCTCTTCTTGACGGACGCCACCACCACCCTGAGGATGTCCCTGAGCGGGCT	7:A>G
1092362|F|0-54:C>G-54:C>G	TGCAGGCGCTTCTCGTAGTCCTCGCGGCCGCCGAAGAGGCCGCGCCACATCCTCCCCAGCACCCCTCCT	54:C>G
1036775|F|0-37:G>A-37:G>A	TGCAGGTCTCGGCCAGGGTGCCCTCGTTGCCGTTCTGGCCCCAGTAGATGGCGATGCTGCCGCCGTGGC	37:G>A
1091637|F|0-58:G>A-58:G>A	TGCAGGTGACCCCGTCGGTGCTGGCCGCAGGGCTGGCCGCCGACAACGTCATCTGCGCGCTCTACTTCA	58:G>A
992207|F|0-64:G>A-64:G>A	TGCAGTTCTGCGACGAGGTGGGCGTCAAGCGCCACGTCCTCAAGGTGTGGATGCACAACAACAAGCACA	64:G>A
1026036|F|0-11:C>G-11:C>G	TGCAGCTCCGCCAGCATCGGCAGCGTGCTCACCGTCAGCATGGCCACCGCCG	11:C>G
1014663|F|0-25:G>A-25:G>A	TGCAGGCAGGCTACAGGCTGGTCAGGGCGACCCAGTTCAGGAGGTTCACGTACCG	25:G>A
3020870|F|0-33:T>A-33:T>A	TGCAGTGGATATTTGGGTTCGTGACGTTCTTCTTCCCCG	33:T>A
2241528|F|0-49:G>C-49:G>C	TGCAGAAATTCCTTACATTCACTCTCTCGACTCTGCGCATTTCATCCTCGAGGAGTGTAACTTGTGCTG	49:G>C
100468094|F|0-47:A>G-47:A>G	TGCAGCACGACCTCCACCGTCGAGTTGTAGGGCAGGCTCCTCACCTTAGTGCCCTCCACCATGGCCGTG	47:A>G
2242338|F|0-29:C>T-29:C>T	TGCAGCAGTCGTGGTGTCTGCATTAGCACCATTCTCGTCGATCCTCATCTGGGTCTTGGCTGGCACGAA	29:C>T
2242338|F|0-63:C>T-63:C>T	TGCAGCAGTCGTGGTGTCTGCATTAGCACCATTCTCGTCGATCCTCATCTGGGTCTTGGCTGGCACGAA	63:C>T
1064449|F|0-27:T>C-27:T>C	TGCAGCCTGCACGCGTCGCGCCGCCGCTTGCCGACGCCGAACACGAAGCCCTTGAACACGCGCAGCTCC	27:T>C
1244951|F|0-20:G>A-20:G>A	TGCAGGATGTTGCGCCACGGGTGCTGCACCAGCTTCGACTCCTCGCTCGCCACCACCAGGTCCGCGTAC	20:G>A
1863483|F|0-16:A>G-16:A>G	TGCAGGCGTGGTGCCCACCCAACGTGGACCCACGGACCGCCGTGCCCATGGACACTGTGACGCCGCGGG	16:A>G
1863483|F|0-31:A>G-31:A>G	TGCAGGCGTGGTGCCCACCCAACGTGGACCCACGGACCGCCGTGCCCATGGACACTGTGACGCCGCGGG	31:A>G
1006631|F|0-13:A>G-13:A>G	TGCAGTCTGATGGATTCTTCTGCCACTGCTGGAACAGAGCCCCGTACTTCTCCTTCCCTTCATGCTTCA	13:A>G
985768|F|0-7:A>G-7:A>G	TGCAGTTAAAGAACACCGACATGTCGCCCTGCACGTGCAGCGCCACCGCCTGGTGCCCGTCCG	7:A>G
985768|F|0-40:C>A-40:C>A	TGCAGTTGAAGAACACCGACATGTCGCCCTGCACGTGCAGCGCCACCGCCTGGTGCCCGTCCG	40:C>A

1102322|F|0-32:C>T-32:C>T	TGCAGCACGTTGCCGTCGTCCACCTCCACCTTCACCTCCTCCTTCCTCAGCCCCG	32:C>T
3025836|F|0-8:C>A-8:C>A	TGCAGCCCCGTCCGCCACACGTCGCAGATGTAGCCCCG	8:C>A
1865366|F|0-13:C>T-13:C>T	TGCAGCCCTGCGACGGCGTCGAACGCGCACGGGCCAGCCGCCTGCCCGTGCCCG	13:C>T
4394733|F|0-8:G>C-8:G>C	TGCAGCGCGACGCAGCGGCCGATCTCCG	8:G>C
4261708|F|0-29:G>T-29:G>T	TGCAGCGCGGGCTTGACCAGGCCCTCGTCGGGCCAGCCCG	29:G>T
998498|F|0-20:C>G-20:C>G	TGCAGCGGCAGCACCGCCGTCACCGTCCTCAAGCTCGTACGTGGCACTGCCGCCG	20:C>G
998498|F|0-44:C>A-44:C>A	TGCAGCGGCAGCACCGCCGTCACCGTCCTCAAGCTCGTACGTGGCACTGGCGCCG	44:C>A
100480067|F|0-21:T>C-21:T>C	TGCAGCTCGCAGCGCAGAAGGTAGCCCTTCTCCAGCAGCGACCTCCCCG	21:T>C
6030669|F|0-10:C>T-10:C>T	TGCAGCTTCCCGCTCTTGGCAATGGCGGGCGGTATTCTCCCG	10:C>T
6030669|F|0-29:C>A-29:C>A	TGCAGCTTCCCGCTCTTGGCAATGGCGGGCGGTATTCTCCCG	29:C>A
1058658|F|0-10:G>C-10:G>C	TGCAGCATCTGCTGCGTGCTGGCCATCCCGCGCTGGAGCACGGCCATGTCGTCCCGCAGGTCGTGGATG	10:G>C
1058658|F|0-26:C>A-26:C>A	TGCAGCATCTGCTGCGTGCTGGCCATCCCGCGCTGGAGCACGGCCATGTCGTCCCGCAGGTCGTGGATG	26:C>A
12763892|F|0-11:G>A-11:G>A	TGCAGCATGACGAGGTTGGAGACAGGCTCGCCGATGCAGCTGTAGCGCGAGTAGCAGGCGCTCAGCTCC	11:G>A
100488260|F|0-26:T>G-26:T>G	TGCAGGAGCGCGACGGGGTTCTGGGATGGGCTGTAGCTGGGGCGCCACTCGACGTCGATCCCGACGACG	26:T>G
1019516|F|0-28:C>A-28:C>A	TGCAGGCAGCGTTCGGCGGGGAGGGCGGCGAGGGGGCATGCATGAGGGTTAACTTCTACCCGAGGTGCC	28:C>A
4262304|F|0-28:C>T-28:C>T	TGCAGACGCAGATGCTCATGGGAGACGGCGGCGCGTCAGCCG	28:C>T
3937200|F|0-16:C>G-16:C>G	TGCAGCCCGAATAGCTCCTTCGGGATCGGCCCG	16:C>G
100495138|F|0-25:T>C-25:T>C	TGCAGCCCGCGCCGCACCTCCCCGATATGGCCGACGCCGCGCCG	25:T>C
1025054|F|0-10:C>T-10:C>T	TGCAGCGCCACCCGCGCCCGTTGACGCGGCTGCACCGCGACCCTTCCATCAGCACCG	10:C>T
1025054|F|0-19:G>A-19:G>A	TGCAGCGCCACCCGCGCCCGTTGACGCGGCTGCACCGCGACCCTTCCATCAGCACCG	19:G>A
7167790|F|0-36:T>G-36:T>G	TGCAGCGCCACCCGCGCCCGTTCACGCGGCTGCACCTCGACCCTTCCATCAGCACCG	36:T>G
100495494|F|0-19:T>G-19:T>G	TGCAGCGCGCGCGCGAACCTCGCCTCCCATCGCCCG	19:T>G
100495516|F|0-26:A>C-26:A>C	TGCAGCGCGGACCAAAAGGATTGCATACCG	26:A>C
1012307|F|0-32:G>C-32:G>C	TGCAGGAATCTCCAGATGGAGTACGTGGACCTGTACCTTGTCCACTGGCCG	32:G>C
4262820|F|0-25:C>T-25:C>T	TGCAGGAGGTCGCCGAGCTGGCGTCCCCGTTCGGGTCGTCCCCG	25:C>T
100496380|F|0-21:A>C-21:A>C	TGCAGGCCACCCAAATCCGCAACAATTTGGGCGAAAAATTGCACCG	21:A>C
100496415|F|0-22:G>C-22:G>C	TGCAGGCCCCGACGGCCGAGCCGCACGGCGCCG	22:G>C
1026254|F|0-6:C>T-6:C>T	TGCAGGCTGCCGAGCACCGACATCCCCCGCATGCTCACCATCCCGAGGCAGGCCACGCCG	6:C>T
1013852|F|0-41:A>G-41:A>G	TGCAGGGCAGTGGCACTGGCATCTAGCCAGCTATGGCAATTATATATCGCCG	41:A>G
3028633|F|0-32:C>T-32:C>T	TGCAGGGCGTCGGCGTCCGGGAAGTAGATGCGCATGCCG	32:C>T
1089514|F|0-13:G>A-13:G>A	TGCAGGGGACCAGGTGGGCCGCCGCCGTGTCCGACGGCCGCAAGGCCGACCCG	13:G>A
3948953|F|0-10:C>T-10:C>T	TGCAGGGGTTCAGCGAGCAGCAGGTCCG	10:C>T
100497358|F|0-32:T>A-32:T>A	TGCAGTGGGATTCCGTGACAAGCAGCTACATGTCATTTGGGGCCCG	32:T>A
100498707|F|0-55:G>T-55:G>T	TGCAGTTGCCTCTGGTTTTATCTGTGGAGATGGTATATGGATGTTCCCTTCTTCCGTGCTTTCCCTTGC	55:G>T
1116802|F|0-67:G>C-67:G>C	TGCAGAGCATCGGTAGGAACGACTGCGTCTGCACGAACGTGAGGCCGAGGCCGATGGACACGTTCATGG	67:G>C
100499639|F|0-33:T>A-33:T>A	TGCAGATCCTAGAGCCCCATTGATTAAAAAAATTAACCAGTTGGCACACTAAAGATCTTCCCAGATCTT	33:T>A
1025335|F|0-67:G>A-67:G>A	TGCAGCAGCAGCAGCCAATGGAGAGCTTCTTCGCGTGCAGCGATGGCGGCGGCATTGGGTCGTTGGGGA	67:G>A
1035638|F|0-65:G>A-65:G>A	TGCAGCTGAGACTGCACAAGGTAGGCACCCAGTTTCTTCGCTGATGGATCATTCGGCTTGCTGCCGAGA	65:G>A
2262698|F|0-7:G>A-7:G>A	TGCAGGCGCCCGACGTCGACGCCTTGCGGGCCCCCGTCGACGAGTTCGGCCCCGTCGACACCG	7:G>A
1863286|F|0-11:T>G-11:T>G	TGCAGGCGGCGTCGGACGCGGCCAAGAAGCGGCACTCGGACCTCAAGCTCGTCGTCTTCGACATCTACA	11:T>G
1695356|F|0-62:C>T-62:C>T	TGCAGGCTCCAGATATGGAAGCTCGCCACCCGCCTCTGGAACGCCGTCGTCGACCGCGCCAACTCCGCC	62:C>T
2259295|F|0-14:G>A-14:G>A	TGCAGGTCGGCCGTGGTGGCCACCAGCTCGTCCATGGTGGTGTCGAGGAGCTCCATGCAGTCCATGACG	14:G>A
1667947|F|0-13:C>A-13:C>A	TGCAGTGGTTCAGCAAGAGGAAGAAGAAGGCGCTGTTCGACGCCCTGGAGAAGGTTAAATCCG	13:C>A
4262464|F|0-36:G>C-36:G>C	TGCAGCACACCTTCCCCATGCTAGCCTGAGCAGAGAGGTTGCTTCCCG	36:G>C
1001639|F|0-14:G>A-14:G>A	TGCAGCAGCGGGCCGAGGACCAAGGGCGGGCACACGGTGACCACGTCCAGGCCG	14:G>A
6030374|F|0-20:C>G-20:C>G	TGCAGCAGTAACTCATCATGCGTGCAGCAGGCCG	20:C>G
1130405|F|0-6:G>A-6:G>A	TGCAGCGACGACACCAAGTACTCCAACGGCCGCGGCCACAGGGACACCG	6:G>A
3944757|F|0-30:C>T-30:C>T	TGCAGCGACGACACCAAGTACTCCAACGGCCGCGGCCACAGAGACACCG	30:C>T
7167765|F|0-40:A>G-40:A>G	TGCAGCGACGACACCAAGTACTCCAACGGCCGCGGCCACAAAGACACCG	40:A>G
7339594|F|0-11:G>C-11:G>C	TGCAGCGAGTCGGCTTCTTTCTCGTCGGCCATGAGGAGTTTCCG	11:G>C
1077776|F|0-12:C>A-12:C>A	TGCAGCTTTTCTCCGTGGTTACTGCCCTGCTACTCCACGGACTTTATTTCTCACCG	12:C>A
1024793|F|0-11:C>T-11:C>T	TGCAGGCACAGCTCGCCGTCGCCGTTCGCCGCCATGGCCGAGAACATCTGCCG	11:C>T
1026114|F|0-33:G>C-33:G>C	TGCAGCCAGAGGACGGTCGGCCATGGCTTGGTGGGCGTGGACACCCTCTGGGCCTGGGGGCTCTTGTAG	33:G>C
100513452|F|0-38:T>C-38:T>C	TGCAGCCCCACCAGCAGCCGCAGCAGCTGCCTCGGCCGTCGCCGGGACAGCATTCGCAGGTTCGCGTGG	38:T>C
1215238|F|0-63:C>T-63:C>T	TGCAGCGCCTCGCGCGGCACGTTCTCCAGCAGCCACGTCGGCCACCCGTCCACCAGCTGCTCGCGGTCG	63:C>T
1264244|F|0-10:G>A-10:G>A	TGCAGCTGGCGTTCCAGAGCATCGGCGTGGTGTACGGCGACGTGGGGACGTCGCCGCTGTACGTGTACT	10:G>A
1025510|F|0-11:G>C-11:G>C	TGCAGCTTGTTGACGATCGGGATCACCGAGGAGCCCACCACCGCGGCGGCCTCGGCCGCCGCCGCCGCC	11:G>C
5365639|F|0-46:G>A-46:G>A	TGCAGGACTGGCAGCGGCGGTACGCCGACGTGCTGCGGCGGAAGGGGAAGACCGTGGAGGTGGTGGAGT	46:G>A
2247504|F|0-41:G>A-41:G>A	TGCAGGAGGAGGTGCTCGAGGTACCACATCAGGGGCGACAAGGCAGGCACGGTGGAGAGCTTCATCGAC	41:G>A

999128|F|0-41:G>T-41:G>T	TGCAGTACCTCCGCCGCATGGCCCTGGACCGCCCGCTGCCCGCCGCCGTCATCCCCGTCGTCCACGCCG	41:G>T
100519469|F|0-29:T>A-29:T>A	TGCAGAAAGCACGTGATGGGGTTCGATGGTAACGATTGGTTTGACCG	29:T>A
1095052|F|0-31:C>T-31:C>T	TGCAGATGCCCTGCGACCGCGAGAACTTCTACCCGTTCCCGCCCTTCATGTCCG	31:C>T
1088798|F|0-27:C>T-27:C>T	TGCAGCACAAGATGAGCGCCAAGGAGTCCGACACCTGGTCCAAGGTGCTCCG	27:C>T
4984856|F|0-22:G>C-22:G>C	TGCAGCGCCGCGGGCGGTATCTGGAACCCCGACGACCCGACCCTCCG	22:G>C
100521019|F|0-7:G>A-7:G>A	TGCAGGAGGTACGGCGTGCGGTGCCCGTGCCCCGACAGGTTCTTCGCCG	7:G>A
1023241|F|0-20:G>A-20:G>A	TGCAGAACGGCTGCGAAAACGGCCCGAAGTTTTGATTAGGATCGTCTGCCATCTCTATGCCCAGCAACT	20:G>A
993195|F|0-49:G>A-49:G>A	TGCAGAATGTGCACGGCCTCCGCCGCCGCGAGTTCAGGGCCACGCTCGCGCACCTGCACTCCATGGCCA	49:G>A
7168520|F|0-66:G>C-66:G>C	TGCAGAATGTGCACGGCCTCCGCCGCCGCGAGTTCAGGGCCACGCTCGCGCACCTTCACTCCATGGGCA	66:G>C
1081085|F|0-10:G>A-10:G>A	TGCAGAGAATGATTCAGAGACAAGTTACACCGAATTAATGCGCAGTGCACGGTGAGGAGCAAGGAACCG	10:G>A
1047019|F|0-66:A>T-66:A>T	TGCAGAGATTCCATCTGATCCGATCAGGTTCTCTGCATGCTCGGCGCAAAAGTCTTCTTCTGATGCAGC	66:A>T
1018977|F|0-14:A>G-14:A>G	TGCAGAGGCTTCTGATTCCACACCTGACCAATGCATCATGATGTTGCTTAATCATTTTATCTCCACGAT	14:A>G
1018977|F|0-65:C>T-65:C>T	TGCAGAGGCTTCTGATTCCACACCTGACCAATGCATCATGATGTTGCTTAATCATTTTATCTCCACGAT	65:C>T
1025585|F|0-26:C>A-26:C>A	TGCAGCAGCGACGACGACGCCATCGTCGGCATGCTGACCTGCGTCTGGAGGAACGGCTGCTGGAGCTGG	26:C>A
1128488|F|0-8:G>T-8:G>T	TGCAGCAGGAGCCACCCGTGCAGCGGGATCAGCATGCAGGACGTGAAGTTGATGTACGACAGGCGCGTC	8:G>T
1128488|F|0-23:C>T-23:C>T	TGCAGCAGGAGCCACCCGTGCAGCGGGATCAGCATGCAGGACGTGAAGTTGATGTACGACAGGCGCGTC	23:C>T
2243720|F|0-54:A>G-54:A>G	TGCAGCCTTCATTCCAATATCGAACACGTCCCCAATCTCTCCTGCAACGCACCACTCTGAGCATCAGA	54:A>G
100525284|F|0-45:C>T-45:C>T	TGCAGCGCGAGGCTGGTGAGCACAAGCACGCGGATTTCCCACTCGCTCCACACAGTGAGTAGGCCTTCC	45:C>T
7166937|F|0-23:A>C-23:A>C	TGCAGCTGCTGCGGCGGGCGGCGAGGCGGAGCCGCCACCGCATGTCCAGGCTCGTCGCGCGGACCACCG	23:A>C
7166937|F|0-43:G>C-43:G>C	TGCAGCTGCTGCGGCGGGCGGCGAGGCGGAGCCGCCACCGCATGTCCAGGCTCGTCGCGCGGACCACCG	43:G>C
1044289|F|0-17:C>G-17:C>G	TGCAGCTTCGGGTCGGCCCAGGAGACCCCGCAGCTCAACCTGGCGGGCCACTGCGACCCCTACTCCAAC	17:C>G
1041280|F|0-14:C>A-14:C>A	TGCAGGCCCAGCACCGCCAACTGCTCCGCCACCGCCAAGAACGTCTGCCCGCCCTACCTCGTCGTCTAC	14:C>A
1025726|F|0-52:G>C-52:G>C	TGCAGGGGGCTCCGCGCCGCGGCGGAGGGGCACGACCAGCAGCAAGAGGGGGGCGACGACGCGGCGGGA	52:G>C
994295|F|0-12:A>G-12:A>G	TGCAGTGGAACCACCAAGAACAAGCCTGTCCAAGAGGCCATCGCTTGGAGCTTCTGCAAAGGTCCAGTC	12:A>G
1027592|F|0-54:A>T-54:A>T	TGCAGTGGATCATGGCCGAACTGGTCAAGAACCCAGAAATCCAGGAGAAGCTCTACGAGGAGATCAAAG	54:A>T
1027581|F|0-48:C>T-48:C>T	TGCAGTGGATCATGGCGAACCTGGTGAAGCGCCCACACATGCAAGAGGCTGTCCGTAGGGAGATCAACG	48:C>T
3936383|F|0-13:C>G-13:C>G	TGCAGATCAAACGCGCGGATGATTCAGCGAGCGACCG	13:C>G
3938575|F|0-27:C>G-27:C>G	TGCAGATCAAACGCGCGGATGATTCAGCGAGCGGCCG	27:C>G
1128975|F|0-33:C>G-33:C>G	TGCAGCCGCCGTCCAAGCAGCCCCGCCTCTCCTCCGCCGACTCGAACGCCG	33:C>G
2267348|F|0-34:C>T-34:C>T	TGCAGCTCGCGGAAGTCGCGCTCGAAGCCCTCGACCCTCCTCTCGCCGCGCCG	34:C>T
3020583|F|0-35:A>T-35:A>T	TGCAGTGCACCTGTCTAATTTGACTAATTGCCACCAGCATGGACCG	35:A>T
1075930|F|0-51:C>A-51:C>A	TGCAGAAACAGGCGCTGGAGCGGGTGCAGGCGGAGGCGTAACATTGGAAGGCTTCGTGGCACCAGAACT	51:C>A
5325262|F|0-63:G>A-63:G>A	TGCAGCAATTGCAGAGAAACTGACCTGGATCTCGCGCTTGATGTGTTCGAAATCGTCGAATCGGCGGCC	63:G>A
6042942|F|0-20:C>T-20:C>T	TGCAGCATCTTCAGGCCACCCAACGCCGCAGGCACGCCGCCGCCAATGCTGTTCCAGCTCAGGTCCAGG	20:C>T
6042942|F|0-37:C>T-37:C>T	TGCAGCATCTTCAGGCCACCCAACGCCGCAGGCACGCCGCCGCCAATGCTGTTCCAGCTCAGGTCCAGG	37:C>T
1001583|F|0-26:T>G-26:T>G	TGCAGCCACCAGGTCGCTCACTTCCCTGCCACCGCCTCCCCCATTGGCTTGTAGCGCTTTGCAATTGCA	26:T>G
5579583|F|0-22:G>T-22:G>T	TGCAGCCCTCGTCGCTGCCCGCGCCGTCGTCCTCCTCCGACTGGATGCCGCCCGCCACGGGGCCCATCA	22:G>T
1020984|F|0-9:G>A-9:G>A	TGCAGCCTCGGCGGCGCAGAAGGAGCCTCCGCGCAGAGCATCTCGGACTGGAGCACGGCGCAGTAGTGG	9:G>A
1020984|F|0-56:G>A-56:G>A	TGCAGCCTCGGCGGCGCAGAAGGAGCCTCCGCGCAGAGCATCTCGGACTGGAGCACGGCGCAGTAGTGG	56:G>A
2246569|F|0-37:C>T-37:C>T	TGCAGCGGGTCGTCAACTCCCTGGCTGTCGAGATGTTCGCTGGACTCGCGTCTGAGGACGCCAGCAAGG	37:C>T
1025502|F|0-29:G>C-29:G>C	TGCAGGATCTCCCGTGGCGTGCGGATGCTGGCGGACCAGTTCACCGCCGAGCCGCCGCCGACGGTGGTG	29:G>C
1220707|F|0-23:C>T-23:C>T	TGCAGGCTCGCCGCGGCCGCGCGCAGGATGGCGCAGGAGCGGGACGAGGCCTGGAACCAGCGGAACGCG	23:C>T
100536702|F|0-9:G>C-9:G>C	TGCAGGCTTGGATGATTAGTTCATTGATTGGTTAGCCTCGCACGGGCGCGGGATAATTGTTTCTCCGAG	9:G>C
1696089|F|0-27:T>C-27:T>C	TGCAGGTCACCGTGCCGCTGAAGAAGGTCGCCAAGACGGAAGCGCTGGCCATCGGCGACCCTACCCGTC	27:T>C
1064241|F|0-52:T>C-52:T>C	TGCAGGTGATTGAGCATGTGGAGGAGGACCAAGCAAGTTTATTCGGCAACGTTGCATTGAGTTCATTCT	52:T>C
100537243|F|0-10:A>G-10:A>G	TGCAGGTTGGATCACCGTGGTTCACGATCCTGTTCGCCCGCAAGACTGAGGAGAAGATGGAGGCGGCAG	10:A>G
7172870|F|0-17:C>A-17:C>A	TGCAGCAGACGGCGGCCCTCCGCGGCGCCGCGCGGGTGGACGTCCG	17:C>A
6045637|F|0-31:T>G-31:T>G	TGCAGCTTGGCCTTCATGGCGACTAGGTGGATCGCCTTCCG	31:T>G
2242135|F|0-17:C>T-17:C>T	TGCAGACAAAACATTCCCGAACCATATCACCGTGTTCAGATCTTCAGTTGACCACTCAACATCGACAGC	17:C>T
100542531|F|0-19:T>C-19:T>C	TGCAGAGGATCAGGGACTGTGTCCAGAGGTGGTACCACGACGAGGGGTTCGTGTGCGCTCAGGTGATCA	19:T>C
1233858|F|0-55:C>G-55:C>G	TGCAGCACCTCGCCAAGCTGAAGAAGCTCTGGAAGCTCAGCGTCAACGTCACCACCGACGCCAACGTGG	55:C>G
2257770|F|0-46:C>A-46:C>A	TGCAGCAGCCGCCGTCGAAGCACGGGTACGGCGCCGTCAGGAACTGCGCCACGATCTCCGACTCCTCCT	46:C>A
1862984|F|0-57:C>G-57:C>G	TGCAGCCTGGACGGGAAGTAGACGAAGTGCCAGGGGGACGGGTGGGCGACGTCGAGGCGGCCGCCGAAG	57:C>G
1001484|F|0-40:G>A-40:G>A	TGCAGCGGCACAACACCCTGAAAGGAGGCTCGGTTCGCAAGGACAGCAAGGAGCCGAGCTTCAGGGCGG	40:G>A
3946379|F|0-17:C>A-17:C>A	TGCAGCTACGACTTCCTCGGCGAGCCCGACGTGTCGCTCGGGATCAACACGCTCTCCTACTGCGGCAGC	17:C>A
2252070|F|0-47:C>A-47:C>A	TGCAGCTCGTCCTGCACCCCCGCGAACAGACAAACCAGATCAGGCAACCCTGGACCGACGGGACGGGAT	47:C>A
2252070|F|0-61:G>A-61:G>A	TGCAGCTCGTCCTGCACCCCCGCGAACAGACAAACCAGATCAGGCAACCCTGGACCGACGGGACGGGAT	61:G>A
100544176|F|0-65:T>C-65:T>C	TGCAGCTGCCCGCCGTCCACCTCCCCGTCGTGCTGCTGCTGCTGGTGGTCTGGTACGGATCCCCCTATG	65:T>C
1864692|F|0-50:C>A-50:C>A	TGCAGGACATGTACAACAGCCTGACGGCGTGCAAGGAGCTCGTCAGAGTCCTCGGCAACATCTGGGGGC	50:C>A

2260271|F|0-35:A>G-35:A>G	TGCAGGCCCAGGTCCTCGACGCGAACCTCGGCACCATTGTCGAAGACTGCATGCGGCACCACGACGAGC	35:A>G
1010818|F|0-6:G>C-6:G>C	TGCAGGGATGGGCGGCCTGGGCGTGGAGGAGACGGCGCTGGTGTCGGCGCTGGGGCGGTGGAGGAAGCA	6:G>C
998211|F|0-28:C>G-28:C>G	TGCAGGTCCGCGCCGACGTGCCGCTGCTCGGCTTCATCGGCCGCCTGGACGGGCAGAAGGGCGTGGAGA	28:C>G
992043|F|0-65:G>A-65:G>A	TGCAGTCGCACTCACGCTTATCCCATGGGAGACATAATGTCTTGCTTGCACGGCAGGAACAATTCGGGC	65:G>A
1694835|F|0-17:C>A-17:C>A	TGCAGTCGGGGCTCCCCCTCGACGACCGCCCTGAGGGCGCCCGCTCGCCGTCCCCTGAGCCAATATACG	17:C>A
1021367|F|0-16:G>A-16:G>A	TGCAGACTCTATTGGCGATGAACCAGGTGGCGGAGCACATGGTCCTCGCCG	16:G>A
1021367|F|0-22:C>T-22:C>T	TGCAGACTCTATTGGCGATGAACCAGGTGGCGGAGCACATGGTCCTCGCCG	22:C>T
1004000|F|0-28:C>G-28:C>G	TGCAGATAATGATGGCGGCCACGGCGCTCTACGCCTTCCCGTCGCTGCGGCACCTCCCG	28:C>G
3028464|F|0-19:G>T-19:G>T	TGCAGGAACATGAGCTTCCGCGATGCCAGGAACGCGTTGTTCCG	19:G>T
4005848|F|0-31:G>A-31:G>A	TGCAGGAGATGCGGCCGTTCTGCGAGGAGGAGCTCAGGAGGCGCCG	31:G>A
1027759|F|0-22:G>A-22:G>A	TGCAGGAGCCATGACGGAAGTCGTGGTGCGGGGTGATCCTGAGGCGGCCGCCG	22:G>A
1037736|F|0-15:T>C-15:T>C	TGCAGATTTGTGGTATTGCTTCAAAGCTTGCAGGGTCTTCAAGAACCTTGCAAGACAAGGTATCTCTCC	15:T>C
100550188|F|0-11:A>G-11:A>G	TGCAGCACCCTAGCACGCAGCACACGGTACCTCCTCTCTGACACGCCCCGCAGGATGGCCTTCAGCTCC	11:A>G
1129678|F|0-13:C>A-13:C>A	TGCAGCACCCTGGCACGCAGCACACGGTACCTCCTCTCTGACACGCCCCGCAGGATGGCCTTCAGCTCC	13:C>A
1040306|F|0-56:A>G-56:A>G	TGCAGCATGATTCAATTGAAGCTGTTGTAATTGGAGTTGTAATTTTGAGCCCTGAAAAAGGGGATTTTG	56:A>G
1091228|F|0-18:G>C-18:G>C	TGCAGCGCCGCTCTTCTGGACGGTGAAGGAGACGGGTGCTGGCGGCGACGTGCTCGGGTCGGACCCGTT	18:G>C
1091228|F|0-29:A>G-29:A>G	TGCAGCGCCGCTCTTCTGGACGGTGAAGGAGACGGGTGCTGGCGGCGACGTGCTCGGGTCGGACCCGTT	29:A>G
986306|F|0-13:G>A-13:G>A	TGCAGCTTGGACAGCCCGCCGAAGCTTCTCGGGATGGGGCCCGTGAGCATGTTGAGCGAGAGGTCAATG	13:G>A
986306|F|0-20:G>A-20:G>A	TGCAGCTTGGACAGCCCGCCGAAGCTTCTCGGGATGGGGCCCGTGAGCATGTTGAGCGAGAGGTCAATG	20:G>A
100551589|F|0-57:C>T-57:C>T	TGCAGGAGAACCAGAAGCTGAAGAAGCAGGTGGAGAAGATGAGGAAGAAGCACGAGGCGGAGATCGAGA	57:C>T
1215126|F|0-18:T>C-18:T>C	TGCAGGAGGGCGCTTGGATGATGGGAGGAGTGGTTGGCCATGGCGGGGAGGGGTTGGGGACGCCGAGGC	18:T>C
5365797|F|0-41:G>A-41:G>A	TGCAGGGACGACGTGGCGGCGGACGGGCGGTGGTGGCCGAGGTGGTCGAACGACATCACGGCCG	41:G>A
995538|F|0-11:G>T-11:G>T	TGCAGTCGCGCGCCCGCGTCCGCCTCGTCTCTTCCCGCCTCCTGCCGTCCG	11:G>T
100556988|F|0-65:G>C-65:G>C	TGCAGCCGCTCCAGGACAGGACCAGCGACGCCTACCGCTGGATGGTGCACCACGGCGAGAGGTACGGCG	65:G>C
2264864|F|0-8:C>T-8:C>T	TGCAGCGGCACGACGAGGCGGACTCGGCGCTCCGCGGCGCGCCCAGGTTCGGCGTCGACGAGTCCACCA	8:C>T
2261626|F|0-35:G>C-35:G>C	TGCAGCTCCGTCACCGCCTCCGTCTCCGCCAGCACGCCGCCGAGCACGGCGAAGAAGTCGTGGTCGCGG	35:G>C
1864042|F|0-31:C>T-31:C>T	TGCAGCTCCTTGTCGTCGATGGTCCCGCTGCCGTCGCGGTCGGCGGCCTGGAAGCACGCCACCACGTTG	31:C>T
2265552|F|0-32:C>G-32:C>G	TGCAGCTGGAGGTGGAGCCGTCGACGCGCGACCCGCGCGACGAGGGCACGGCGGACGCCTGGATCGACC	32:C>G
1105599|F|0-49:G>A-49:G>A	TGCAGGATCTTGGCCTCCAGTTCACTCCTGTCAACGACTCTCTGTACGAGACGGTGAAGAGCCTCCAGG	49:G>A
7172806|F|0-24:T>C-24:T>C	TGCAGGGACGAGATGCGCAGGGTATTCGGCGTCGCGCTGCTCTGCGCGTCCAGCCTCCCGATCAACCGA	24:T>C
3936610|F|0-54:G>T-54:G>T	TGCAGGTTGTCGGCCAGCTCCACGATGTCGACGGCGATGCTCTCCTCCGTCCGCGCCG	54:G>T
1003085|F|0-30:T>G-30:T>G	TGCAGAAAAGGGAGAGCAAGACCAATATCATCTCCAGGACTAAGCAAAGACCCACTCCATCTCCGAAAC	30:T>G
3222134|F|0-31:G>T-31:G>T	TGCAGCACAAAGGCCCCTAACCCCTAAATCCGCGCAAAGCATGCCAGATTCTCTCGACTTAATAACCAA	31:G>T
100561857|F|0-63:T>A-63:T>A	TGCAGCACAAAGGCCCCTAACCCCTAAATCCGCGCAAAGCATGCCAGATTCTCTCGACCTAATTACCAA	63:T>A
1095481|F|0-46:G>T-46:G>T	TGCAGCGGAGGACGGAGGGGCGGTACCCCGAGCCGAGGCGCGGCACGGCGAGGGCGCTGTTCGTCGTCA	46:G>T
3952704|F|0-24:C>T-24:C>T	TGCAGCGTGGGTACAACATCGAGTCGTCGACCTCGGGAAGCTGCTCCCCTCTCGCGCCCTCATGATCAT	24:C>T
3952704|F|0-67:A>T-67:A>T	TGCAGCGTGGGTACAACATCGAGTCGTCGACCTCGGGAAGCTGCTCCCCTCTCGCGCCCTCATGATCAT	67:A>T
2256569|F|0-66:C>A-66:C>A	TGCAGCTCCGCGTCGCCGTCCACCTCCTTGTCGCTGTGGTAGAAGTGGGCGCAGTAGCTGCGCACCCAC	66:C>A
1106349|F|0-17:G>A-17:G>A	TGCAGGAGGCGGAGGCCGACCCCGCCGAGCGTCCCCGCCGACCCCTGCGGGCTATTCATCCACACTCCG	17:G>A
1213790|F|0-8:G>A-8:G>A	TGCAGGCCGTGGCCAAGGAGACGCTGCGGCTTCACCCGACGGGCCCGCTGGTGGTGCGGCAGTCCCCGA	8:G>A
1002300|F|0-38:G>T-38:G>T	TGCAGGGCGTCGGCGACGTTGACGAAGAAGGAGTCGGGGTCGGGCGGCACGGCGGCCCAGGAGCCATCG	38:G>T
1002300|F|0-53:G>T-53:G>T	TGCAGGGCGTCGGCGACGTTGACGAAGAAGGAGTCGGGGTCGGGCGGCACGGCGGCCCAGGAGCCATCG	53:G>T
1253974|F|0-24:G>A-24:G>A	TGCAGGGGAAGGCAGCGGCGGCATGCGCGCGCGGCGCAGAGAGCGGCAGCTCCATGGCGAGAGCTAGCC	24:G>A
7487642|F|0-9:C>T-9:C>T	TGCAGTACGCGGGGTACCACGAGCGGCTGCTGCGCCTGGTGCGCGTCGGCGCCGCCATCGCCTACGACA	9:C>T
7173077|F|0-34:G>C-34:G>C	TGCAGAGGCTCATCTGGGTGGAGAGGCCAGATCTGTTCCAGCTGCCACCG	34:G>C
100566068|F|0-10:A>G-10:A>G	TGCAGAAGGCAGCCCAGATGGGCGCGACGACGTCGAGCGGCTACGGCGTGAGCTTCACCCCTGGCCACC	10:A>G
1740851|F|0-50:G>A-50:G>A	TGCAGAAGGCGGCCCAGATGGGCGCGACGACGTCGAGCGGCTACGGCGTGGGCTTCACCCCTGGCCACC	50:G>A
1864986|F|0-34:G>A-34:G>A	TGCAGAGCGCCACGAGCGCCGTGTTGACCGTGACGGCGTCGAACGGGACGCCGCGCTTGCACATCTCGG	34:G>A
1020082|F|0-58:C>T-58:C>T	TGCAGATCTCCAAGATGCCTGAGCACAGGGAACTCAAGAGGACGGCCCTCACCGACCTCTCTGGCGGCG	58:C>T
1210668|F|0-24:G>A-24:G>A	TGCAGCACCACGCAAGAACGAAACGCCAGCGTCGGCTACTCGCCCTCCCGTCACCTGAAGAACTACAAC	24:G>A
1210668|F|0-62:C>T-62:C>T	TGCAGCACCACGCAAGAACGAAACGCCAGCGTCGGCTACTCGCCCTCCCGTCACCTGAAGAACTACAAC	62:C>T
100566525|F|0-59:A>G-59:A>G	TGCAGCAGCGCCTTGAGCTGGTCCTGCGCCAGGGCCTTGGTCGGGAACACGTAGAGCGCACAGGCCGCC	59:A>G
1002709|F|0-8:C>G-8:C>G	TGCAGCCACACGGCCGCCCCGAGCCCCAGCCACGGCGGCCTGCTCCCCGCCTTCACCGCGCCCG	8:C>G
100567169|F|0-46:T>G-46:T>G	TGCAGCTGCTGCGCGCCGACCTGCTGGTGGAGGGCTCCTTCGACGCTGCGGTGAGCGGCGTGGACGGCG	46:T>G
100567170|F|0-53:T>A-53:T>A	TGCAGCTGCTGCGCGCCGACCTGCTGGTGGAGGGGTCCTTCGACGCGGCCGTCTGCGGCGTCGACGGCG	53:T>A
1038665|F|0-22:C>A-22:C>A	TGCAGGACAGGAGAGGGGCCCGCGCGCGGCATGCACGTGAAAGCGAGTACGATGCACAATTATGATGTT	22:C>A
986239|F|0-42:G>C-42:G>C	TGCAGGGCCAGGTCCTGGTGCTCCTTCTTGCACACCTGGTCGGCGCCGATGTTGCCGTACCG	42:G>C
1214019|F|0-24:C>T-24:C>T	TGCAGGTTCTTCTCGGCAAGGAAGCACGGCTTGGTGGATGCTGTAGTGCGCAACAGCTCCGCTGATAAA	24:C>T
100567838|F|0-46:G>C-46:G>C	TGCAGTCGGACGAGCCGTCGACGGACGCAAGCACCACCACGACGACGACGACCACCATGAGCATGACGG	46:G>C

<table>
<tr><td>1049669|F|0-12:G>A-12:G>A</td><td>TGCAGCTTCTGCGTGGTCTGGTCCAGCTCCTGCTTCAGAGAAGAAACCAAGGTGGCGTCCGAGGACACC</td><td>12:G>A</td></tr>
<tr><td>1095082|F|0-7:C>T-7:C>T</td><td>TGCAGCGCGCCGTGGAGCGCGCGTCGGCGCTGCTCCGCCCGCACGGCGCGTCCGTGGCGGAGTACACCA</td><td>7:C>T</td></tr>
<tr><td>1717915|F|0-18:G>A-18:G>A</td><td>TGCAGCGGTGGAGTTCGTGGAGCGCGTCGGCTTCTACGGCGTGCAGGGCAACCTGATCATGTACCTCAC</td><td>18:G>A</td></tr>
<tr><td>999296|F|0-38:C>T-38:C>T</td><td>TGCAGGCATCGCTGACCGCGGCGGCCCCGCCGCCGCCGCCATCGTGGGAGCTGCCG</td><td>38:C>T</td></tr>
<tr><td>3947115|F|0-7:C>T-7:C>T</td><td>TGCAGAGCATCAAAGAAGCGGTCGCCG</td><td>7:C>T</td></tr>
<tr><td>999542|F|0-8:G>T-8:G>T</td><td>TGCAGCCTGAGGGTGGAGGCGACGACGATGGCGAGCACGGCGAAGCAGGCCG</td><td>8:G>T</td></tr>
<tr><td>12775938|F|0-34:C>G-34:C>G</td><td>TGCAGAAGGAGCACGAGCAGTCGCCGCCGCGGCCCTCGAACCGCAGGTCGCAGTTCGAGGAGATGACGA</td><td>34:C>G</td></tr>
<tr><td>981730|F|0-27:A>G-27:A>G</td><td>TGCAGATCCTCGTCCAGCACCACCCGCAACCGTGTCATGTCGTCCTCAAGCAGATGGTCAGCTCGCCGC</td><td>27:A>G</td></tr>
<tr><td>7171155|F|0-28:A>G-28:A>G</td><td>TGCAGCGGTTCTCTTTTGAGCTGTCGCCAACGTACGTGCATGCGCCCTATGCCGTCCTTACACTGCACC</td><td>28:A>G</td></tr>
<tr><td>1667027|F|0-7:G>T-7:G>T</td><td>TGCAGCTGCCCAAGGGCATGGAGGCCGTGAACCACATCTGGCAGGTAGGGTCGACGGTGGCCAATGGGG</td><td>7:G>T</td></tr>
<tr><td>1092201|F|0-8:G>T-8:G>T</td><td>TGCAGGCAGTCGGCGGCGTGCTCCTTGGAGCCTAGCACGGTGCCGTAGTCCAGCAGGCTGACCATGACG</td><td>8:G>T</td></tr>
<tr><td>1092201|F|0-30:C>A-30:C>A</td><td>TGCAGGCAGTCGGCGGCGTGCTCCTTGGAGCCTAGCACGGTGCCGTAGTCCAGCAGGCTGACCATGACG</td><td>30:C>A</td></tr>
<tr><td>1085168|F|0-32:A>G-32:A>G</td><td>TGCAGTACGTGTACTGGTCCAACGGGCGGGAGACCGAGAGCCCCAGCGTGGACAACAAGCAGTGCCCCG</td><td>32:A>G</td></tr>
<tr><td>1863429|F|0-46:C>G-46:C>G</td><td>TGCAGTCTTCATGTGACATATCGACTGTGACATAGGTATGTCTAGCCCCAAACAGGCTGAAGCCGAGAT</td><td>46:C>G</td></tr>
<tr><td>7173434|F|0-12:A>C-12:A>C</td><td>TGCAGGTTGTCGACAAGACGGCACCGCTGCCG</td><td>12:A>C</td></tr>
<tr><td>3027903|F|0-40:C>T-40:C>T</td><td>TGCAGCACGCCGCGGCGTTCCTCCCGCGCGTCCGCATCGTCGCCG</td><td>40:C>T</td></tr>
<tr><td>977990|F|0-49:G>A-49:G>A</td><td>TGCAGCTCACGCCCTCCAACTCCAACCTCACCCTCCTCTACAACTGCAAGGCCG</td><td>49:G>A</td></tr>
<tr><td>1038614|F|0-43:C>T-43:C>T</td><td>TGCAGCTGATCGGCACGTGCAATGGTCTTCTCTGCCTGTGCGACAACAAGGAGGCACCCG</td><td>43:C>T</td></tr>
<tr><td>3934803|F|0-28:C>T-28:C>T</td><td>TGCAGGATTTCTTTGACGGGAAGAAGCTCTGCAAGAGAATCAATCCG</td><td>28:C>T</td></tr>
<tr><td>996868|F|0-33:G>T-33:G>T</td><td>TGCAGATCTCCACGGCGGAGTGCGTCGGCAAGCGGACGGCGATCTCGCTGTGGGGATTGGCCGGGATCA</td><td>33:G>T</td></tr>
<tr><td>979360|F|0-62:A>C-62:A>C</td><td>TGCAGATGTATAGGACGGTGAAGGGCACAGACAGATCGTCGCACGTCGCCACAGGTACACACACATGCA</td><td>62:A>C</td></tr>
<tr><td>990605|F|0-7:G>A-7:G>A</td><td>TGCAGCAGGGAGACGGTGTTGGGGAGCTGGAACGACACGTTGTTCATGCTCGCGCCGAACCG</td><td>7:G>A</td></tr>
<tr><td>6048426|F|0-11:C>G-11:C>G</td><td>TGCAGCGTGTCCGCGGCCACGAGGCGGGTGTTCATGGCGTAGGGGTCGTTGGTGGAGACCATGTCGAAG</td><td>11:C>G</td></tr>
<tr><td>979441|F|0-31:A>C-31:A>C</td><td>TGCAGAGGTTCTTCCTCGGAGGGAGATTGATATTCGGGCCCGATGCCAAGTCCCTGCTCGTCTCGGTTG</td><td>31:A>C</td></tr>
<tr><td>3573393|F|0-5:G>A-5:G>A</td><td>TGCAGGTACGCCGACGGCGTCGCCTTCGACCCCGAGCCG</td><td>5:G>A</td></tr>
<tr><td>7168665|F|0-34:G>A-34:G>A</td><td>TGCAGCATCACCACACCGAAGCAGTACACGTCGCGGCCTGTGGCCGCCTCCGTCGTTGTGGCTTCTTCC</td><td>34:G>A</td></tr>
<tr><td>7168665|F|0-43:C>T-43:C>T</td><td>TGCAGCATCACCACACCGAAGCAGTACACGTCGCGGCCTGTGGCCGCCTCCGTCGTTGTGGCTTCTTCC</td><td>43:C>T</td></tr>
<tr><td>1095870|F|0-15:G>T-15:G>T</td><td>TGCAGCGACGGCGACGTCCGATGCACCAACGACGCAAAGAGGAACGTGCCGCCGCACGTCCTGGCCGAG</td><td>15:G>T</td></tr>
<tr><td>7172389|F|0-21:G>C-21:G>C</td><td>TGCAGCTGCCGTCGGTGATTGGATTCCACGATGGAGCGGTGACGACAACGGCGGCCACGCACCAGATGT</td><td>21:G>C</td></tr>
<tr><td>100581281|F|0-42:T>C-42:T>C</td><td>TGCAGGGCAAGCTGGACCCCAGCCACCCCAGTTTTGTCAAGCTCATGACCCAGTCTCACGTCACCGAGA</td><td>42:T>C</td></tr>
<tr><td>2256421|F|0-65:A>G-65:A>G</td><td>TGCAGGGGATGGTCGAGGCAGCAGTCCATGCCGCTGACAAGGAGGTGGCTAAGGCTGCCAGGAGGATAA</td><td>65:A>G</td></tr>
<tr><td>1200118|F|0-44:G>A-44:G>A</td><td>TGCAGAGGATGCACCCCAACGGGTCGTGGGAGCAGTGGCGGTTCGTCGGCGGCACGGGCCTGCTCGGCA</td><td>44:G>A</td></tr>
<tr><td>1125573|F|0-56:C>T-56:C>T</td><td>TGCAGCATCAACGACACGGCGACGGCGAGCAGGATCAGGTGCGCCGTCGTTGCCATCGTGAGGTGTCAA</td><td>56:C>T</td></tr>
<tr><td>1033462|F|0-23:G>A-23:G>A</td><td>TGCAGCCATGCGACCTGCTCGGCGTACTTGTCGGGGGTCGCGTTGGGGTCGTTGCCGCACTCCACGTTG</td><td>23:G>A</td></tr>
<tr><td>100583451|F|0-10:C>G-10:C>G</td><td>TGCAGTTCGGCCGCGCGCGCCGGGACTTCGTGCTGCGCTCCCACGGCGGGGAGGGCTTCTCCGTCGCCG</td><td>10:C>G</td></tr>
<tr><td>1220449|F|0-5:A>T-5:A>T</td><td>TGCAGAGTAGAAGGCATGAAGCGTACCATGGAGCCCCGCGTTATGAAGTTGGAGCTCGGTAACATGTCC</td><td>5:A>T</td></tr>
<tr><td>1025833|F|0-26:G>C-26:G>C</td><td>TGCAGCCTCGCCGCGTGAGCGGCCGCGAGGGCGACGCGCAAGGTATAGGTGCCGCCG</td><td>26:G>C</td></tr>
<tr><td>1025833|F|0-35:G>A-35:G>A</td><td>TGCAGCCTCGCCGCGTGAGCGGCCGCGAGGGCGACGCGCAAGGTGTAGGTGCCGCCG</td><td>35:G>A</td></tr>
<tr><td>6049143|F|0-5:A>C-5:A>C</td><td>TGCAGAGAGAGGCGGCACGCGCGTGGTCGCCG</td><td>5:A>C</td></tr>
<tr><td>992517|F|0-52:C>T-52:C>T</td><td>TGCAGACCAAGATGATGAGGATGAAGGGGGTGGTCAGCGCCCAGACGGACCTCGCCGCCG</td><td>52:C>T</td></tr>
<tr><td>3940017|F|0-14:G>A-14:G>A</td><td>TGCAGCATCTTGTAGAAGCTGTTGTCCAGCTTGGACGCGCTGGACCG</td><td>14:G>A</td></tr>
<tr><td>1022225|F|0-19:G>C-19:G>C</td><td>TGCAGCAATGGCCGCCGCGGTTGGAGCCCGAGCCAGCCCCGATGATGTCGGCCG</td><td>19:G>C</td></tr>
<tr><td>5582524|F|0-16:G>C-16:G>C</td><td>TGCAGCAGGAGGTTGTGGCTACGGAAGTGAACCCCG</td><td>16:G>C</td></tr>
<tr><td>3023143|F|0-8:C>T-8:C>T</td><td>TGCAGCAGCATGTTGTACACGTCCCTGTGCGCCTTCTCGACCTCGTCGAACAGCACCACCGCGTACGGC</td><td>8:C>T</td></tr>
<tr><td>3020656|F|0-9:T>C-9:T>C</td><td>TGCAGGCATTATTATGTCACCTTCAGCATTGGGGATCCG</td><td>9:T>C</td></tr>
<tr><td>3948116|F|0-23:C>T-23:C>T</td><td>TGCAGGAAGTCCCCCACGTTCACCACCAGCGCGCCCGCCACCG</td><td>23:C>T</td></tr>
<tr><td>1090065|F|0-50:C>T-50:C>T</td><td>TGCAGGCCCCGCACGAACGCCACGGCGTACTTGCTCGCCGTGGTGGGGTCCTCGCCG</td><td>50:C>T</td></tr>
<tr><td>4544199|F|0-32:T>C-32:T>C</td><td>TGCAGGTAAGGGAGGGAAGGGTCGAACGGGGCTGCGCCGCAGGAGACGCTGGCGAAGTGGTGGCGATTC</td><td>32:T>C</td></tr>
<tr><td>2336178|F|0-23:G>C-23:G>C</td><td>TGCAGCTCATGGAAGGAGTTGACGAGCACGTGGTCGGCGGCGTCGAGGCCCACGAACTGTCTCACCAGT</td><td>23:G>C</td></tr>
<tr><td>2255238|F|0-45:C>G-45:C>G</td><td>TGCAGACCGCCCCGTCATCGAACAGCACATCCACGTCGATGGACACCTCGACAACCCG</td><td>45:C>G</td></tr>
<tr><td>1231832|F|0-10:C>T-10:C>T</td><td>TGCAGCCGTACTTCGGCGGCGAGGAGCGCACCGAAGCAGAGCTGAGGCTGGAGGGCGTGGGGCCGAGAT</td><td>10:C>T</td></tr>
<tr><td>1690848|F|0-13:G>A-13:G>A</td><td>TGCAGGACTGGCAGCGGCGGTACGGCGCCATGCTGCGGCGGAAGGGGAAGGCGGTCAAGGTGCTCGAGT</td><td>13:G>A</td></tr>
<tr><td>1093855|F|0-10:G>A-10:G>A</td><td>TGCAGCATCGGGTTGAGGTGTCCCTGAAACGGCAGCGGGAACATCAGCACGCGCCG</td><td>10:G>A</td></tr>
<tr><td>7173883|F|0-5:C>T-5:C>T</td><td>TGCAGCGGCGCACGCTTCCTCGTTGGCGCCG</td><td>5:C>T</td></tr>
<tr><td>3571299|F|0-9:C>G-9:C>G</td><td>TGCAGATGCCGCCGATGGCCATGCACGGTCACGGTGACGTGTGCCCG</td><td>9:C>G</td></tr>
<tr><td>5411961|F|0-14:T>G-14:T>G</td><td>TGCAGGTATAGTCCTAGCATGCGGTATAGTCGTGCGCCGTAGGCCTGCCCGCATAGAGTCTCCAGGGCG</td><td>14:T>G</td></tr>
<tr><td>1695012|F|0-6:C>T-6:C>T</td><td>TGCAGGCCAAGAAGATCGACGCCGACCCCAGGGACTACCTTACCTTCTTCTGCTTAGGGAACCG</td><td>6:C>T</td></tr>
<tr><td>1012387|F|0-53:C>G-53:C>G</td><td>TGCAGCTGCTGCGCCACCTCGGCCACGGCGGCCACGAACTCCCGCAGCGGCCGCCCCGAGCGCTTCACC</td><td>53:C>G</td></tr>
<tr><td>3941966|F|0-32:C>A-32:C>A</td><td>TGCAGCCACTTCGGGTCCAACGTCTCGGCATCCTCCAGGTTCGGGACCTCGAAGACCGCCTGCACGTCC</td><td>32:C>A</td></tr>
</table>

2251142|F|0-38:G>A-38:G>A	TGCAGGATCTGCTCGATGTTGGTGGTGCGGCCGAGCGAGGGCGGCACGGCGGAGGCGAGCCCG	38:G>A
3958945|F|0-13:G>A-13:G>A	TGCAGCAGATGAAGCACATCTGCGGGATGACCG	13:G>A
7349741|F|0-17:C>T-17:C>T	TGCAGCTACCTGGGCAGCCCCG	17:C>T
7349753|F|0-14:A>C-14:A>C	TGCAGCTGCGCCAGATGCGCCG	14:A>C
2295883|F|0-21:C>T-21:C>T	TGCAGCATGGACAGCACGTTCCTCATCGACGGCCTGTCCTGCGGGCACCG	21:C>T
3948818|F|0-14:C>A-14:C>A	TGCAGCGCCGCGAGCGTGCGCACCAGAGCCGTGGACGGCTCCCCG	14:C>A
5332228|F|0-17:C>T-17:C>T	TGCAGGAGGAGGCCGAGCTGGCCGCGCCG	17:C>T
3943756|F|0-23:C>A-23:C>A	TGCAGGCCGTCTTCAACAAGATCCTGAACGTGGCGGTGGCCG	23:C>A
5366753|F|0-13:C>T-13:C>T	TGCAGAACCAGGCCGAGATGCTGCGCCG	13:C>T
3949035|F|0-9:G>A-9:G>A	TGCAGCGCAGGAGTACGTCGAAAAACGACGGCCG	9:G>A
13871885|F|0-13:G>T-13:G>T	TGCAGGTTGCCGAGGCGGCCG	13:G>T
2243185|F|0-31:G>A-31:G>A	TGCAGGTTCATGTACTTGCTCCGCGCCGCCAGCGCCTTCACCTCCTCGATGGCCTTGTTGTCCACCCGC	31:G>A
4261819|F|0-14:G>A-14:G>A	TGCAGAGCCCTGTTGCCGCCGTGCTGCCAGCGGACATGGCCG	14:G>A
6037684|F|0-12:G>A-12:G>A	TGCAGCCACGTCGGCCGCGATGGAAGCGGCCG	12:G>A
1028626|F|0-19:G>C-19:G>C	TGCAGTAGTAGAGGAAGGCGGAGGCGTTGCCGAGGCAGTTGGCGGTGGAGCCGTCCG	19:G>C
2250762|F|0-13:C>T-13:C>T	TGCAGCTGCTGGACTTCGTGCTCTTCGACTGGGACCGCGTGCTCCGCCCCG	13:C>T
1050193|F|0-6:A>T-6:A>T	TGCAGAAGGTCACCAACCCCAAGACGATCCAGAAGATCGCCAAGTCCAAGAACCGAGGTCAGCTCAGGG	6:A>T
1863053|F|0-33:T>A-33:T>A	TGCAGGACGACGTGGGCGGGCTGGAGGTCCTCGTCGACGGCGACTGGCGCCCCGTCCGCCCCGTCCCCG	33:T>A
1051596|F|0-53:G>C-53:G>C	TGCAGTACCAGGACTTCGCCAACCGCTACCAGCTCTGCCTCTCCCACCTCGCCGACGCCGCGCGCGAGG	53:G>C
3935850|F|0-16:C>T-16:C>T	TGCAGAGCGACTGCCTCCACTTCTGCCTCCCCG	16:C>T
3947063|F|0-18:C>T-18:C>T	TGCAGCAGCAGCTTGACGCGCTCCACCG	18:C>T
4998218|F|0-22:C>A-22:C>A	TGCAGCCCGAGCGCTGCCTCCTCCCCCACGCCG	22:C>A
3031511|F|0-14:G>A-14:G>A	TGCAGGAACAGCGTGTCCAGCTTCGCGAGATTCCCGAGCTCCG	14:G>A
4394530|F|0-14:G>A-14:G>A	TGCAGGACAAGGTGGTGTACGGCACGGCAGACCG	14:G>A
3020870|F|0-31:C>T-31:C>T	TGCAGTGGATATTTGGGTTCGTGACGTTCTTCTTCCCCG	31:C>T
5357481|F|0-9:C>A-9:C>A	TGCAGCAAGCCGAGCGAGAAGAACCCCTCCG	9:C>A
3025836|F|0-11:C>T-11:C>T	TGCAGCCCCGTCCGCCACACGTCGCAGATGTAGCCCCG	11:C>T
1021968|F|0-10:C>A-10:C>A	TGCAGCCGACCAACGAGGTGCTGGAGGGCCTCCTGTCGGACCCCAAGGCCG	10:C>A
1206894|F|0-5:C>T-5:C>T	TGCAGCTCGTAGTCGTCCTGCACGGCCTCGTCGGAGGGGTAGTAGGCCGCCACGTAGGCCTCGCACCAC	5:C>T
100495137|F|0-10:G>A-10:G>A	TGCAGCCCGCGCCGCATCTCCCCGACATGGCCGACGCCGCGCCG	10:G>A
3956879|F|0-16:C>T-16:C>T	TGCAGCCCGCGCCGCACCTCCCCGACATGGCCGACGCCGCGCCG	16:C>T
1027515|F|0-17:C>T-17:C>T	TGCAGCCGTTGGTCCGACGGCGACGTCGGCTGCGGGACGTGCTCTGGGTCCG	17:C>T
2266482|F|0-19:T>G-19:T>G	TGCAGCGGAAGCTGCCTCCTCGCGAACTTGCACTCGAAGGGCGGCAGGACCG	19:T>G
3948962|F|0-5:C>A-5:C>A	TGCAGCGCTCCTGGATCATCCTGACCG	5:C>A
991658|F|0-7:G>T-7:G>T	TGCAGCTGGCGATTGATGAGGAGGACGACGCCACCAGCAAGCTGGCGCAGCCGATGCAGCCCTCCCGAG	7:G>T
4262986|F|0-18:C>G-18:C>G	TGCAGGAGGGCTTCTCGGCAGCGTCCTCGATCACGCCG	18:C>G
4261634|F|0-15:T>A-15:T>A	TGCAGTCAAGGAAGTTCAGGATGCCG	15:T>A
1026114|F|0-13:C>T-13:C>T	TGCAGCCAGAGGACGGTCGGCCATGGCTTGGTGGGCGTGGACACCCTCTGGGCCTGGGGGCTCTTGTAG	13:C>T
3942054|F|0-20:C>T-20:C>T	TGCAGCGGAGAGGAGGGAGGCGAGAGCGTGAAACGCAGGGAAGCAGTGTCGGAAGCCCTTCTGGCGGCC	20:C>T
977117|F|0-25:C>A-25:C>A	TGCAGGTGAGCGCGCCACGCCTGCGCGCGCTCCGCGTCACCGAGTGCTTCAGGCTGGAGTCCAGCGCAG	25:C>A
7169197|F|0-32:G>C-32:G>C	TGCAGACGTAGGGCGCGTCACGGCCGTGGTACGTGCCG	32:G>C
6037784|F|0-8:C>A-8:C>A	TGCAGCGCCAAGGGCACAATCAGGAACCGCAAGCCG	8:C>A
3958593|F|0-33:T>C-33:T>C	TGCAGTTGAGTTATGGTGGCTTCTGTGACGCTGTCCTGCCTTTCCG	33:T>C
1027219|F|0-35:C>A-35:C>A	TGCAGCACCTGCGCGCTGACGGAGTTGAAGAGGATCTCCACGAAGCCGTTGATGACGGCGGGCTTGGCC	35:C>A
1143356|F|0-32:G>A-32:G>A	TGCAGCTGGAAGTCGAGGTTGAGGTGCTTGAAGTTGCGGGTCAGCGTGCCCCGCGGCCCCGTCACCGAG	32:G>A
2247422|F|0-11:A>G-11:A>G	TGCAGTGATCCATGCCGACGACATCGAGATATCGCTCTGCGACGGCAACTCGGAGGACGAGCGCCGCCG	11:A>G
1027581|F|0-56:A>T-56:A>T	TGCAGTGGATCATGGCGAACCTGGTGAAGCGCCCACACATGCAGGAGGCTGTCCGTAGGGAGATCAACG	56:A>T
997849|F|0-48:G>A-48:G>A	TGCAGAGCTGCGAGCAGCGGTTCATCTGGGTGCTGCGCGACGCCGACCGCGCCGACATCTTCGCGGAGG	48:G>A
1020984|F|0-31:C>T-31:C>T	TGCAGCCTCGGCGGCGCAGAAGCAGCCTCCGCGCAGAGCATCTCGGACTGGAGCACGGCGCAGTAGTGG	31:C>T
1667164|F|0-14:G>A-14:G>A	TGCAGCCCGTCCATGGCGCGCATGGCCACGTCGAAGAAGACGAGGTTGTGGTTGGCGTACCG	14:G>A
992043|F|0-56:G>C-56:G>C	TGCAGTCGCACTCACGCTTATCCCATGGGAGACATAATGTCTTGCTTGCACGGCAGGAACAATTCGGGC	56:G>C
3025891|F|0-22:C>A-22:C>A	TGCAGTGTCCTGAGCACCACGTCCATCGTGTTGCCATGCGCCG	22:C>A
3025891|F|0-29:G>C-29:G>C	TGCAGTGTCCTGAGCACCACGTCCATCGTGTTGCCATGCGCCG	29:G>C
1120791|F|0-51:C>T-51:C>T	TGCAGACAGGGCAAAGACCATCCTTGCTCTAGCCTCCCCTGACTCCAAGCCCAGTGACCTCAGAAGGCC	51:C>T
1096626|F|0-28:G>A-28:G>A	TGCAGGCGCGCGATGACGGCGTGCAGCAGGCCGATGGTCTGCTCCTGCCTCATCGCGGTGGCCTCCATC	28:G>A
3944995|F|0-14:T>A-14:T>A	TGCAGATTGGCTACTCGGCACCAGCTGAGGCCAGGATCAGCCG	14:T>A
3027951|F|0-28:G>A-28:G>A	TGCAGTCTATCGGGACCTGGAACGGACCGTCCAAGCCG	28:G>A
996761|F|0-23:C>T-23:C>T	TGCAGCAGCCGCCGCCGCTGCACCTGCTCCCCGCCGCCGCCACGTGCTACGGGAAACCCATGGCCGAGA	23:C>T
1029081|F|0-49:G>T-49:G>T	TGCAGCGCGTGGACGACACGCTGGGCGTGTTCCACACCCACGCCGTGGCGGGGTTCCTCGGCGGCGTGA	49:G>T

1862571|F|0-34:C>A-34:C>A	TGCAGCTCCAGTCAGGGAACTCGACCAAGAGCTTCAGCCGCGAGTGCCAGGTCCTGAAGCGCATCCGCC	34:C>A
1133314|F|0-45:G>A-45:G>A	TGCAGGAAGTCATACATCTTCTGCGGGACGAACCGCTCCCGCGCGGCCGTCACCTGTTCGGGGAGGTAC	45:G>A
1129167|F|0-10:G>A-10:G>A	TGCAGGACACGGACGCCACCACCGTCGTGCACCGCATCATCTTCAAGGCCTTCGACGAGGCCCG	10:G>A
989810|F|0-37:C>A-37:C>A	TGCAGGGGCTCGGGGTCAACCCCGCCGTGCACGAGGTCGCCGACGAGGCCGCGCTCGCCG	37:C>A
32027290|F|0-44:G>C-44:G>C	TGCAGCTTCCCCCTGCTCTGCACCTTGGGGTTCATGAAGTCCTCGGCTAGGAGGTCGACGAGGGCCATG	44:G>C
1696572|F|0-58:C>G-58:C>G	TGCAGAGGTTCGAGAAGGCGGACGCCGCGGGCGAGGCGCGGCGCATGGTCTGCGCCGCCGACAGCAACA	58:C>G
1667027|F|0-26:G>T-26:G>T	TGCAGCTGCCCAAGGGCATGGAAGCCGTGAACCACATCTGGCAGGTAGGGTCGACGGTGGCCAATGGGG	26:G>T
1090123|F|0-37:C>T-37:C>T	TGCAGGCAGTCGGCGGCGTGCTCCTTGGAGCCCAGCACGGTGCCGTAGTCCAGCAGGCTGACCATGACG	37:C>T
3027903|F|0-21:T>A-21:T>A	TGCAGCACGCCGCGGCGTTCCTCCCGCGCGTCCGCATCGTCGCCG	21:T>A
5367812|F|0-8:C>A-8:C>A	TGCAGTGGCTCGACGGCCAGGCAACCG	8:C>A
4396622|F|0-21:G>A-21:G>A	TGCAGCAAGCTCCTCCCCGTAGATCCG	21:G>A
4411296|F|0-8:C>A-8:C>A	TGCAGGCACCGCGCCAACACCCGCTTGGCCTCCTCCTGGCGGTGCTCCG	8:C>A
1084594|F|0-54:T>A-54:T>A	TGCAGGCCGTCGGCGGCACGGGCGTCAACGTCGTCGTCGGGGCGCCTAACGACGTGCTCTCCAACCTCG	54:T>A
1125013|F|0-11:G>A-11:G>A	TGCAGGTCCCAGCGCAGCGTCCACGTGCTCGCCCATCCATGTCCGTTGCCG	11:G>A
3961328|F|0-34:G>A-34:G>A	TGCAGTTGGCCGCCAGCTTCCTCGAGAAGGTGTCGTCGGCGCGCTTGGACCG	34:G>A
3941966|F|0-15:T>G-15:T>G	TGCAGCCACTTCGGGTCCAACGTCTCGGCATCCTCCAGGTTCGGGACCTCGAAGACCGCCTGCACGTCC	15:T>G
5971434|F|0-10:G>A-10:G>A	TGCAGCGGCGGCATGGGCGGCGGCGGCGCAGCCG	10:G>A
3026787|F|0-28:T>C-28:T>C	TGCAGATGGTGATCAACGAGACGCTGCGTCTGTACCCGCCG	28:T>C
12772280|F|0-37:G>C-37:G>C	TGCAGCTCATCCACGAGAACCTCGCCAACTTCGCCGCGGAGCTCGGGGTCCCCTTCCAGTTCGTCGTGT	37:G>C
2251021|F|0-13:C>G-13:C>G	TGCAGATGGGCGTCATGCTCACCACGGCGACCGTGGTGGTCACCGCGGGCATCGTCGTCGTGAGCTTGT	13:C>G
7908984|F|0-29:A>G-29:A>G	TGCAGGCCATCAAGTCGGCCCTCGGCTCCACGCCG	29:A>G
1245875|F|0-12:A>G-12:A>G	TGCAGACGGCTAACAGCTTCTGCATGAGGTAGTTCCCGAACGGGTTCACCATGAGGTCCACGGCGTGAC	12:A>G
1003509|F|0-62:A>G-62:A>G	TGCAGGAGGAAGGTGATGAAGGCCATGAACCTCAAGGGCGTGCTGAGGACGGAGATCCACCCATCGCTC	62:A>G
1286530|F|0-26:A>G-26:A>G	TGCAGCTCCCGCCACGACCTGGCGTCAATGAACTGCTTGACGCGCACCAGGTTCCTCGCGTGCGTCCTC	26:A>G
5580135|F|0-10:C>A-10:C>A	TGCAGCAGCCCAGCCATTGAATCCG	10:C>A
7352621|F|0-39:G>A-39:G>A	TGCAGCGTTAATGAATCGTGCACCAAAATCTGAATTATCGACCG	39:G>A
6041602|F|0-23:T>C-23:T>C	TGCAGCTTCGCCGCCGCCGACGATGCAGACGTGCCCTCGCCCGCCG	23:T>C
6048641|F|0-8:C>G-8:C>G	TGCAGGTTCGTGAGCTGCGCCAGCTCTGGTGGGATCGCGCCG	8:C>G
7170808|F|0-7:C>T-7:C>T	TGCAGGCCCATCTGTTCTTGGAGATTCCG	7:C>T
7172031|F|0-17:A>G-17:A>G	TGCAGACCAGCACGCCCACCGCCGCCGCCGTGGTGACGGCGCCG	17:A>G
5338368|F|0-58:A>C-58:A>C	TGCAGCACATTCAAAGGTTGAGCGGCAAAGTTTGGCTGAAAGAATTGGTATACATATCAGAATATCGCA	58:A>C
6045089|F|0-31:T>C-31:T>C	TGCAGGTACTACTCGACCTGGGGACACGTCGTGACGCTGGCGGAGGAGATGAAGAAGGGCGCCGACTCC	31:T>C
12768671|F|0-20:T>C-20:T>C	TGCAGCAGCGCAGCCTCTTGTGGCGCCGTGGCGTCGTTGAGGAGGAGCTGCTGGTTGCGGGCCTTGAGG	20:T>C
6049980|F|0-17:T>G-17:T>G	TGCAGCGCACGGCGGCATCCACCG	17:T>G
14822559|F|0-33:C>A-33:C>A	TGCAGCGCCGCCAGGCGTTCGACGCGTACGCCGCGTACCAGCCCG	33:C>A
7168192|F|0-37:G>C-37:G>C	TGCAGGGGGCTCGCCGACATCAGGGACATGGCGCCGAGCTTTGACCG	37:G>C
6049603|F|0-20:A>G-20:A>G	TGCAGCTTCATGACGTCGCCACAGGCCG	20:A>G
100463116|F|0-14:T>A-14:T>A	TGCAGGGTCGCTCCTTCGGTGCTGCCG	14:T>A
100509659|F|0-14:G>C-14:G>C	TGCAGGCCCAGGTAGGCGTCCTCCCGCGCCGCGCCG	14:G>C
100525746|F|0-23:T>C-23:T>C	TGCAGCTCCGCCATGCCGCTCTGTTGCCACGCGACCGCCTCACGCGGCGCGGCCGCCTCGTCACCGAGA	23:T>C
100569901|F|0-5:T>C-5:T>C	TGCAGTACCATCTGCTTCGCGGAAGCGGCGGCGCCGCCAGGGCCGCCGCTAATCTGGTGCTGCCGAGAT	5:T>C
100578167|F|0-7:A>G-7:A>G	TGCAGGTAACGTGGTGATAAATCTGTGGTTCAGCATCCCG	7:A>G
100469997|F|0-25:T>C-25:T>C	TGCAGCTCCCGCCACGACCTGGCGTTGATGAACTGCTTGACGCGCACCAGGTTCCTCGCGTGCATCCTC	25:T>C
23919785|F|0-25:C>T-25:C>T	TGCAGCGCGTCCTGCGCCACCGACGCCGACGCCGCCATCGACACGGCGACCAGCACCGCCGCCAGCTGA	25:C>T
100500680|F|0-5:G>C-5:G>C	TGCAGGATCGTGCGCGCGGGATTGCGCGGATTGGTGCTGGGGCACCGTGCGCGCGGGCGTGTGGCTCGC	5:G>C
6043278|F|0-9:T>C-9:T>C	TGCAGGTCGTGCGCCATGTACACGTTGCTGTACGTTCCCTGCCCGATCTTGTCCAACCTCTCGAAGGTG	9:T>C
7170485|F|0-34:G>C-34:G>C	TGCAGAGGTTCACGGCGGCGACGCTGGAGCACGGGATGAACCCGCCG	34:G>C
7168570|F|0-46:T>C-46:T>C	TGCAGAGGGTGGAGAGGTCGTGGGGCTTCGTGTGCGCGGGGAGGTATGGACCGACCACCCGTTCATCA	46:T>C
100567053|F|0-19:T>C-19:T>C	TGCAGCTCAAGCTCCACGCTTTCCTCCTCTGGTCGTCCGTCGGCTTCCTGATGCCGATCGGCGTGCTGC	19:T>C
7353307|F|0-6:C>T-6:C>T	TGCAGCCAATGACCACGCACACGCCG	6:C>T
100416069|F|0-28:G>C-28:G>C	TGCAGCGCGCCTTCCTGGAGATGACCGCGGCCG	28:G>C
100460017|F|0-9:A>G-9:A>G	TGCAGACCAACACGCCCGCCGCCGCCGCCGTGGTGACGGCGCCG	9:A>G
7916160|F|0-17:T>C-17:T>C	TGCAGCATAAACTCCTCTTCATCGGCGCCTCCG	17:T>C
100463168|F|0-17:T>C-17:T>C	TGCAGGTACGGCAGCGCTGGCAGGGACGACTCGGCGACCG	17:T>C
7916308|F|0-22:T>C-22:T>C	TGCAGGATCCTGTTCCTGCTCATCCTCTCCTTCCCCCG	22:T>C
7478586|F|0-8:A>G-8:A>G	TGCAGTTGAGACGCTACCTGGTCCCGCAGCCTCGTCCG	8:A>G
100552606|F|0-38:G>C-38:G>C	TGCAGGTTGGACGAGCGGAGCACGGCCGTGAGGGACTCGGCCAGGTTGCCGTGCCTGGACACGACGCGG	38:G>C
6037519|F|0-15:G>A-15:G>A	TGCAGAGGTGGTGGTGGAGCCCGTGGCGGCCCG	15:G>A
100301409|F|0-12:A>G-12:A>G	TGCAGATTGGTGATGAAAACCAAGGTGCCG	12:A>G

100302837|F|0-20:T>C-20:T>C	TGCAGCTTCAGCTCGACGCCTACCCG	20:T>C
100303980|F|0-13:T>C-13:T>C	TGCAGTACGCGCGTCCCGAAGGGATCGACGTGCTCCTCCG	13:T>C
7868783|F|0-7:G>C-7:G>C	TGCAGGGGCGCCGCGATGCCG	7:G>C
1036613|F|0-44:C>G-44:C>G	TGCAGGTTCTGGTCCCTCTGGAAGCCCTTGCCGCACACCTCGCACACGAACCG	44:C>G
1076801|F|0-17:C>T-17:C>T	TGCAGCAGCAGCAGCCACGAGATCCAGGGCGCGAACAGGTAGTTCATGCCGACGTGGTGCCG	17:C>T
987969|F|0-36:C>G-36:C>G	TGCAGCCGCGGTGGGGCTCTCCTTCTTGCAGTTCACCAACATGAACTCCATGCGCAACCTGTTCATCGT	36:C>G
1029041|F|0-24:T>G-24:T>G	TGCAGGGAATTATTCTGTTAGTTATGCACACACTTGGTGGCTACGAGAACAAATCATCATTTGACATTC	24:T>G
1019808|F|0-68:C>A-68:C>A	TGCAGGTACGCCGAGTCCTTGCCCAGGCAGATCCGCGGCCCCGCCTGGAACGCCGTGAACTTGAACGGC	68:C>A
1072661|F|0-15:C>A-15:C>A	TGCAGACCTCCTTCGCATATGGCGCATCACGGACGACGACGCCGCTGCCCCG	15:C>A
7167784|F|0-24:G>C-24:G>C	TGCAGCGCAGGTAGGAATCTTTTGGCTAGGGCCCG	24:G>C
100387487|F|0-17:G>C-17:G>C	TGCAGCTTCCTCCCCACGACCGCCG	17:G>C
12766093|F|0-20:A>G-20:A>G	TGCAGCTTCTTTATTGTGTCAATCTGCTTGGCCG	20:A>G
4261643|F|0-7:A>T-7:A>T	TGCAGTCATGGGAGGAGAAGTTCCG	7:A>T
1141772|F|0-44:G>A-44:G>A	TGCAGTTCTTCGCCCCCAACCCGACCTGCGGCCTCTCCCCCTTCGTCCG	44:G>A
7352269|F|0-8:G>A-8:G>A	TGCAGCATGTCCCGCGCCTCCG	8:G>A
1217150|F|0-17:G>C-17:G>C	TGCAGCGACCTGAGCAGGAGCAGCAGCAGCTTCCTCCTCCGCTTCCCCTTGTTCTTGCCGCCCGCCTCC	17:G>C
1217150|F|0-57:C>T-57:C>T	TGCAGCGACCTGAGCAGGAGCAGCAGCAGCTTCCTCCTCCGCTTCCCCTTGTTCTTGCCGCCCGCCTCC	57:C>T
1092419|F|0-13:C>A-13:C>A	TGCAGGACCTGCCCGAGGAGCAGCGCGCGGACGCGGTGAGCAGCATGGTGTACGAGGCGGCGGCGCGCG	13:C>A
2257074|F|0-31:C>G-31:C>G	TGCAGTACCTGCACGAGGAGTCCAACCTGCGCATCGTCCACCGCGACATCAAGGCCAGCAACATCCTCC	31:C>G
984670|F|0-37:C>T-37:C>T	TGCAGAGCGGCAAGATCGTGGCGCTGAAGCGGGTGCGCTTCGTCAACATGGACCCG	37:C>T
3027023|F|0-32:C>T-32:C>T	TGCAGCAACAACGGCCACAACGCGCGGACCTGCCCCGCCCGCTCCG	32:C>T
100415177|F|0-8:C>T-8:C>T	TGCAGCACCTGGTTGCCGACGCCAGCACCG	8:C>T
3030539|F|0-25:G>A-25:G>A	TGCAGCGAGGATGTTAGGGCGCCTCGTAGGCCCTCCG	25:G>A
5411830|F|0-25:G>A-25:G>A	TGCAGCGCAATGCGGAGGAGCAGGAGATCATCTCCAGGGACCG	25:G>A
100417240|F|0-22:A>G-22:A>G	TGCAGGATGTCCAAGGGCGTGCAGGAGCTGCCG	22:A>G
100417836|F|0-17:A>G-17:A>G	TGCAGGGGCGGCCTCAAAAAAGCAGGGGTCAACGGCACCG	17:A>G
7911484|F|0-10:C>T-10:C>T	TGCAGCCATGCCCATCACCG	10:C>T
7911484|F|0-14:T>A-14:T>A	TGCAGCCATGCCCATCACCG	14:T>A
990385|F|0-19:G>A-19:G>A	TGCAGGGGTGGGCCAAGAGGAAGCGATCGCGCCGCCAGCGCTCCGAGGAGGAGAACCTCGCGCTCTGCC	19:G>A
1089540|F|0-46:C>G-46:C>G	TGCAGAGGCGGAGCAGGTCGCAGGAGGAGAGCACGAGCGAGACCATCGCGAGGCCGTTGGTGATCCGCA	46:C>G
1322100|F|0-27:C>T-27:C>T	TGCAGGAATAAGAAAACTCGCTATTCACTCAGTTTATTTTCCATAATAAGTTATGTAGGAGAGATGGCC	27:C>T
1695009|F|0-28:G>C-28:G>C	TGCAGGCGTCGTCGGCGTAGCTGAACACGGCCTCGCGGTCGATGGCGGTGATGAGGCTCTTCTCGCTGA	28:G>C
1182403|F|0-53:G>A-53:G>A	TGCAGGTCCTCCAGGCACCGCACCTCCGCCATGCACGCGAACGACTGCGACTTGCCCTGGTAGTACGCC	53:G>A
2252453|F|0-31:G>T-31:G>T	TGCAGTTGACGACGAGGATGTCGATGTCCTTGGGCTTGAGGCCCGTGCGGCGGACGAGGTCGTCGATGG	31:G>T
3027900|F|0-20:G>A-20:G>A	TGCAGCAGCATCGCGTAGAGGATGTGGTTCCACGCGTCCG	20:G>A
3027900|F|0-25:G>T-25:G>T	TGCAGCAGCATCGCGTAGAGGATGTGGTTCCACGCGTCCG	25:G>T
4262602|F|0-14:C>T-14:C>T	TGCAGCGAGCCCGTCACGATGCCCG	14:C>T
100440579|F|0-5:T>C-5:T>C	TGCAGTGGAGTCGGCCCAAACATGTTGCGCGGCCCG	5:T>C
1018433|F|0-27:G>A-27:G>A	TGCAGTCAGCATACCAGGGGCATCGGCGTCAGGGTTGCGGCGGGCTACAACATACTCGCCG	27:G>A
4993737|F|0-10:G>A-10:G>A	TGCAGACGTAGGTGCTGAAGCCG	10:G>A
1368203|F|0-16:C>G-16:C>G	TGCAGCCCAGCGACACCTTCCCGCGGAGGCACAACTCCGCCACCCCCGCCGAGCAGGCCGTCATGGCGT	16:C>G
2256197|F|0-8:G>A-8:G>A	TGCAGCGCGAACTCGAGGCACCAGAGCACGTCCCCCATGGTGGGCCGCTCCACCCCGTACTCGGCGAGG	8:G>A
1096523|F|0-48:G>C-48:G>C	TGCAGGTGATGGCGGACGTGATCCACTGGTGCACCCACAAGGACGGCGGGGCGCGGCCGTCGATGAAGC	48:G>C
4006123|F|0-23:T>C-23:T>C	TGCAGGAGAGATTTGGAGCCGCGTTGCTAGGTAAGGACTCCG	23:T>C
5363292|F|0-20:G>C-20:G>C	TGCAGCAGCTCGCCCAGCTTGCCCG	20:G>C
100461432|F|0-25:C>G-25:C>G	TGCAGCGCAGCAACCCGCGAAAGCGCCCACCACCG	25:C>G
4004589|F|0-11:C>T-11:C>T	TGCAGGCCCAGCGCCTTCATGAAGCCGCCGTGGACGCGGCAGCCG	11:C>T
1141147|F|0-40:C>A-40:C>A	TGCAGGCGACGATCGCCGCCCGCAACCCCAACTCCCGCGTCGGCATCTACTTCGACCG	40:C>A
3027337|F|0-41:C>A-41:C>A	TGCAGGGCCGAGAAGATGAACTCCACCACCACCATGGCCGCCGCCG	41:C>A
5363908|F|0-9:G>T-9:G>T	TGCAGGGTCGCTCCATCGGTGCTGCCG	9:G>T
4733836|F|0-6:A>C-6:A>C	TGCAGCAGCGGGATCAGCCG	6:A>C
981038|F|0-5:A>G-5:A>G	TGCAGATATTCAACAAGAGGTGGAAGGAGGCGGTGGCGGAGGACATGTGCTGGACGGACCCTTACCCGC	5:A>G
1105546|F|0-33:C>A-33:C>A	TGCAGCGACCTGCCCATCACGCAGTACTTCGACCTCGCCATGCAGGCCAGGCTGCTCAAGCGCCACCGC	33:C>A
100469996|F|0-14:A>C-14:A>C	TGCAGCTCCCGCCAAGACCTGGCGTCGATGTACTGCTTGACGCGCACCAGGTTCCTCGCGTGCGTCCTC	14:A>C
1342351|F|0-55:G>T-55:G>T	TGCAGGCGCGGCAGGCGGCGTGCGCGTCGTCCCCGCAGGCCGCGCAGGGAGGATGGGTCCCCG	55:G>T
2243220|F|0-67:C>T-67:C>T	TGCAGGGGCCCTACGTGTACTACCTCTACAGCCAGTACGGGTTCGACAAGGGCGACATCGGCCGCCTCT	67:C>T
995643|F|0-19:G>A-19:G>A	TGCAGGTCCGCGACGACGTGCCGCTCATCGGGTTCATCGGGCGGCTGGACCACCAGAAGGGCGTGGACA	19:G>A
100472525|F|0-20:A>G-20:A>G	TGCAGGTGCTCCAGCACGTCACGGCTGGCCGCGTGCATGCAGAAGTGCTCGAACGCGCGCTTGAAGTCC	20:A>G
1055414|F|0-28:G>A-28:G>A	TGCAGTCCATCACCACGATGCCGATCCTGGTGAAAGGAGTGATCACCGCAGAGGACAGCAGGCTCGCCG	28:G>A

<table>
<tr><td>100478294|F|0-20:T>C-20:T>C</td><td>TGCAGACGGTCCGATACTTCTTGGATAAGCTCCGCCG</td><td>20:T>C</td></tr>
<tr><td>7171540|F|0-16:T>C-16:T>C</td><td>TGCAGCGACGCCGACGTCGTCAGGCCCG</td><td>16:T>C</td></tr>
<tr><td>7161321|F|0-6:G>T-6:G>T</td><td>TGCAGCGGCATGCGGAACTCGCCAAGCGCGAATCCCGCGGCTGCGTACTCCG</td><td>6:G>T</td></tr>
<tr><td>3954317|F|0-30:C>T-30:C>T</td><td>TGCAGCGTCGCGTTGGTGAGGCCGAGGAACCCGCCGTGGCTGCCG</td><td>30:C>T</td></tr>
<tr><td>4261849|F|0-38:C>T-38:C>T</td><td>TGCAGCTCCCTGAGCACGGCGTCGCGGCTCTTGAGGAACCCG</td><td>38:C>T</td></tr>
<tr><td>1862895|F|0-47:C>G-47:C>G</td><td>TGCAGCTCGCTGGTGTTTGTGCTCGAGTCTGGCTCGGAGGGCTCCGTCTCCG</td><td>47:C>G</td></tr>
<tr><td>5582011|F|0-23:C>T-23:C>T</td><td>TGCAGCTTCGGCCGCCTCATCGGCACCACCG</td><td>23:C>T</td></tr>
<tr><td>4262705|F|0-11:G>A-11:G>A</td><td>TGCAGGGAGGGGTCGGAGACGACGACCCCGACGAAGCCG</td><td>11:G>A</td></tr>
<tr><td>3954902|F|0-20:G>T-20:G>T</td><td>TGCAGGTAGGACATGAGCTCGTGCGGCTCGATCGCCG</td><td>20:G>T</td></tr>
<tr><td>3951089|F|0-8:C>A-8:C>A</td><td>TGCAGGTGCTGTGCTCCCACAAGTCCG</td><td>8:C>A</td></tr>
<tr><td>3951089|F|0-19:C>G-19:C>G</td><td>TGCAGGTGCTGTGCTCCCACAAGTCCG</td><td>19:C>G</td></tr>
<tr><td>100481728|F|0-15:G>C-15:G>C</td><td>TGCAGTCCTTGGACGGCAGCAACAACCGCCTCACCG</td><td>15:G>C</td></tr>
<tr><td>100481859|F|0-29:C>G-29:C>G</td><td>TGCAGTGGAGCAGTCAAGAAGTATCCCAACAACAGAATGCTCGGCCG</td><td>29:C>G</td></tr>
<tr><td>3942117|F|0-5:C>T-5:C>T</td><td>TGCAGCAGGAGCGTTGCTGTCCG</td><td>5:C>T</td></tr>
<tr><td>100484532|F|0-56:A>G-56:A>G</td><td>TGCAGAGTCCAGAAGCGCACCTGGTGGACGAGGTGCACGGTGGGCGCGAGGAAGACAACGATCCGAGAT</td><td>56:A>G</td></tr>
<tr><td>1214220|F|0-49:C>G-49:C>G</td><td>TGCAGCAGCCCGAGTTCCGGCCGGACAAGAAGACGATGAATGCGCTCACCGTGCAGCTGCTCCG</td><td>49:C>G</td></tr>
<tr><td>1229860|F|0-46:C>A-46:C>A</td><td>TGCAGCAGGAGCGCGCGCTCGGCCGCCCCATGTTCCCCAAGGCCATCGGCGAGCTCCACGGCCACTCCG</td><td>46:C>A</td></tr>
<tr><td>997568|F|0-65:C>G-65:C>G</td><td>TGCAGCGGGCGGAGCCTGTCCAGGCGCCAGATGGAGCCCCACGACTCCCTCATCGGCGTCCAGTACCCC</td><td>65:C>G</td></tr>
<tr><td>1253585|F|0-38:G>C-38:G>C</td><td>TGCAGCGTCTGGTTCGGCGGCAGGTCCCGCTCGCTGTTGGGTCCCGTCAGAAGGACGGACGCGTCGCAG</td><td>38:G>C</td></tr>
<tr><td>1023921|F|0-40:C>T-40:C>T</td><td>TGCAGCTCGCCCTGCCCCTCCTGCTGGTGGCCATGGCCTTCGCGCTTCAGAACTTCGCCAAAACCAAAA</td><td>40:C>T</td></tr>
<tr><td>995799|F|0-5:T>C-5:T>C</td><td>TGCAGTTGTAGCGGCACCAAGACGGAGGCAGAACGTGGGGACACGAGGCTCGTGGTGATCGTGGGGGCA</td><td>5:T>C</td></tr>
<tr><td>995799|F|0-29:A>G-29:A>G</td><td>TGCAGCTGTAGCGGCACCAAGACGGAGGCAGAACGTGGGGACACGAGGCTCGTGGTGATCGTGGGGGCA</td><td>29:A>G</td></tr>
<tr><td>7168845|F|0-11:G>T-11:G>T</td><td>TGCAGCTTGCGGCGGCGCTTGGCGGAGGCCTCGTCGTCGACGACGGCGGAGGAGGCGGAGGAGGAGGAC</td><td>11:G>T</td></tr>
<tr><td>2247634|F|0-59:G>T-59:G>T</td><td>TGCAGGAACAGCATGGGCTCCGCCGACGGGCTCACCGCCTGCCGCAGCATCGACCCCATGATCAGCGAC</td><td>59:G>T</td></tr>
<tr><td>2247936|F|0-62:C>T-62:C>T</td><td>TGCAGGAACGTCTCGCTCTCCCCCGACGAGATGAGCGCATCGATCGTGCTCTCCTCCGCCGTCTCCCCC</td><td>62:C>T</td></tr>
<tr><td>1019160|F|0-38:G>A-38:G>A</td><td>TGCAGGACCTTGCCGCACGAGTCGCAGGAGACGAACTCGCCCTCCATCCTGACGCGGCGGAGGCCGCCG</td><td>38:G>A</td></tr>
<tr><td>2249099|F|0-39:C>T-39:C>T</td><td>TGCAGGAGCCCGACCCTATGGGGATCGATGAAGTACCTCCACGACAAGATCCACAAGGACTCCAACTGC</td><td>39:C>T</td></tr>
<tr><td>1698386|F|0-13:C>A-13:C>A</td><td>TGCAGGATGTTGGCCGATGTCCACGTGGTGGCGTAGAGACGGCCGCGGAAAAGGAGGACGCTCTGGAGC</td><td>13:C>A</td></tr>
<tr><td>100488509|F|0-37:T>G-37:T>G</td><td>TGCAGGATGTTGGCCGATGTCCACGTGGTGGCGTAGATACGGCCGCGGAAAAGGAGGACGCTCTGGAGC</td><td>37:T>G</td></tr>
<tr><td>1029006|F|0-49:G>A-49:G>A</td><td>TGCAGGCGGCGTCCAGGGGCCGTCCACGCGGAGGCTGACGAGCAGCGACGAGAGGCTGGAGGCGGCGGC</td><td>49:G>A</td></tr>
<tr><td>1120263|F|0-14:C>T-14:C>T</td><td>TGCAGGTATCCGAGCGACGTGGACAAGCACATCCTGGCTCGCCAGACGGGCCTCTCACGGAGCCAGGTA</td><td>14:C>T</td></tr>
<tr><td>1120263|F|0-68:A>G-68:A>G</td><td>TGCAGGTATCCGAGCGACGTGGACAAGCACATCCTGGCTCGCCAGACGGGCCTCTCACGGAGCCAGGTA</td><td>68:A>G</td></tr>
<tr><td>100494246|F|0-12:T>G-12:T>G</td><td>TGCAGACTATCGTCGCTCATGGCAGAGACCG</td><td>12:T>G</td></tr>
<tr><td>7167565|F|0-18:G>C-18:G>C</td><td>TGCAGCAACCTCCGCGGCGGCGGGTGCAGGTCCATGTCGCCG</td><td>18:G>C</td></tr>
<tr><td>1011276|F|0-26:G>C-26:G>C</td><td>TGCAGCCGCTCGCGCCGAGGGTCGTCGGCTCGGAGGTCACCACGCTGGGCGGGCGCGTCCG</td><td>26:G>C</td></tr>
<tr><td>1694878|F|0-44:C>T-44:C>T</td><td>TGCAGCGAGTCGGACCGCAGGTTGAAGCCGTTGAGGTCGACGGACGCGACGGTCCG</td><td>44:C>T</td></tr>
<tr><td>4261737|F|0-19:T>C-19:T>C</td><td>TGCAGCTGCATGCATGTTGTCTGTTTGCAAATTCCAACCG</td><td>19:T>C</td></tr>
<tr><td>100495932|F|0-20:A>G-20:A>G</td><td>TGCAGCTGTTTCTCGCCGTCAGTCACCAAGCAAAGTCGCCCATCCG</td><td>20:A>G</td></tr>
<tr><td>100496231|F|0-27:T>C-27:T>C</td><td>TGCAGGAGCAGTGGCGCTGCCCACTGTTCATCCTGCTGCTTACTACTTGTGCGCTTACCCG</td><td>27:T>C</td></tr>
<tr><td>100496404|F|0-16:G>A-16:G>A</td><td>TGCAGGCCCTCCAGATGAGCTTGATCCG</td><td>16:G>A</td></tr>
<tr><td>4262503|F|0-29:G>A-29:G>A</td><td>TGCAGGCGGATGCGGAGCGTGTCCAGCGGGTGGCCCGCCAGCACGCCG</td><td>29:G>A</td></tr>
<tr><td>4910031|F|0-6:G>A-6:G>A</td><td>TGCAGGGTGGCCGAGTCGCTCCG</td><td>6:G>A</td></tr>
<tr><td>5357473|F|0-14:G>T-14:G>T</td><td>TGCAGCAACATGCGGGGTGCCG</td><td>14:G>T</td></tr>
<tr><td>1094579|F|0-31:G>A-31:G>A</td><td>TGCAGCCGTTGGGGAAGGCGCAGAGGAGGCCGTCGCCGCGGGGGTGTACGGCCATCCTGTATGGTACCT</td><td>31:G>A</td></tr>
<tr><td>1081801|F|0-26:C>A-26:C>A</td><td>TGCAGCGCCTTCATCTTCTCGTTGATCTTGCACCGCCGCCTCTTCTCGGAGAGGTTGTGCACCTCGGCG</td><td>26:C>A</td></tr>
<tr><td>5970733|F|0-11:C>G-11:C>G</td><td>TGCAGCGCGATCCGCGCCAGGAAGAAGATGTCGAACAGGGTGCGGATCACGGCGGTGGAGATGGCGAGG</td><td>11:C>G</td></tr>
<tr><td>2278806|F|0-37:C>T-37:C>T</td><td>TGCAGCTCCTCTTCGACGCCTGCGTCGAGGCCTGCATCCGCTTCCTCGAGGCCGTGCCCTGGTCCGAGG</td><td>37:C>T</td></tr>
<tr><td>2271197|F|0-5:C>T-5:C>T</td><td>TGCAGCTGGGCCTACCAGGGCAACTACGTCGGCGCCATGCCGCTCTTCGACTCGCGGTGGCCG</td><td>5:C>T</td></tr>
<tr><td>5579682|F|0-39:G>T-39:G>T</td><td>TGCAGGCCAGGAAGCTCTCACTCGGAGGAGGCATCGGCGGCAAGCTCAAGGCTGTAGGGGAGATGGGAG</td><td>39:G>T</td></tr>
<tr><td>100503317|F|0-29:T>C-29:T>C</td><td>TGCAGGTCCCTCGTCTCCTCCCTCAGCAGTGCGACGAGCCGCGGGAAGTCCATGCGCAGCGCCTCCCCT</td><td>29:T>C</td></tr>
<tr><td>1005763|F|0-19:G>A-19:G>A</td><td>TGCAGGCGGAGCTGAGGCAGAGGAGCTGGAGCTCCTGGGAGCCGTAGGTGAAGGTCTTCATCTCGTAGC</td><td>19:G>A</td></tr>
<tr><td>100504107|F|0-5:A>G-5:A>G</td><td>TGCAGATCCCGCGCTTTGCCTTCCTCCGTCAAAATCTCCATCAGGCAGCTCTCAAAACTCCG</td><td>5:A>G</td></tr>
<tr><td>1105855|F|0-41:C>T-41:C>T</td><td>TGCAGGTCGCGCGCCATGTACACGTTGCTGTACGTGCCCTGCCCGATCTTGTCCAACCTCTCGAAGGTG</td><td>41:C>T</td></tr>
<tr><td>1017783|F|0-7:G>C-7:G>C</td><td>TGCAGTTGGTCTGCTGCTCCGCGAAGAAGGGCCAGCTGAGCATCGGCACGCCGTTAGAGATGCTCTCCA</td><td>7:G>C</td></tr>
<tr><td>3941020|F|0-11:C>T-11:C>T</td><td>TGCAGACGGACCCGCGATCGGCGCCG</td><td>11:C>T</td></tr>
<tr><td>7164908|F|0-18:T>C-18:T>C</td><td>TGCAGATTCTGCTCTGCGTCCTGCCCCCG</td><td>18:T>C</td></tr>
<tr><td>5581330|F|0-8:C>T-8:C>T</td><td>TGCAGCAGCGGCATGCCGGCGGCGAGCCCCGCGGGTATCTCGCCG</td><td>8:C>T</td></tr>
<tr><td>4262475|F|0-8:C>G-8:C>G</td><td>TGCAGCGACGCCTTCCTCGCGATCGGCATGTCGACGAACGCCG</td><td>8:C>G</td></tr>
<tr><td>4262825|F|0-11:G>C-11:G>C</td><td>TGCAGCTTGTCGAGGTCCTTGGCGAGGTCCACGGGGGCCG</td><td>11:G>C</td></tr>
</table>

<table>
<tr><td>5000505|F|0-13:C>G-13:C>G</td><td>TGCAGGAGGTCCTCGACGGCCCAGCCG</td><td>13:C>G</td></tr>
<tr><td>1024793|F|0-25:T>A-25:T>A</td><td>TGCAGGCACAGCTCGCCGTCGCCGTTCGCCGCCATGGCCGAGAACATCTGCCG</td><td>25:T>A</td></tr>
<tr><td>5005882|F|0-8:C>G-8:C>G</td><td>TGCAGGCCCAGGTACGCGTCCTCCCGCGCCGCGCCG</td><td>8:C>G</td></tr>
<tr><td>2260887|F|0-37:G>T-37:G>T</td><td>TGCAGGGGCAGTTCGAGATCCTGTCGCTCACGGGCACGGTGCTGCCGCCGCCG</td><td>37:G>T</td></tr>
<tr><td>1020713|F|0-22:C>T-22:C>T</td><td>TGCAGGTGAGCTCCGACGGCAGCTTGCTCAGGAACGTGACGGTGTACACCG</td><td>22:C>T</td></tr>
<tr><td>5581552|F|0-37:G>C-37:G>C</td><td>TGCAGGTGCGGATGGCGGCGTCGAACATGTCCTCGGCGGTGTACCG</td><td>37:G>C</td></tr>
<tr><td>1029079|F|0-11:C>A-11:C>A</td><td>TGCAGGCCGTCCTGGCGCGCGCCCACGGCGAGATGCAGGACTCGTTCGTCTGGCTCTTCCAGCACATCT</td><td>11:C>A</td></tr>
<tr><td>1021890|F|0-38:G>A-38:G>A</td><td>TGCAGAGATGGGGCGGCATCAAGAACATCACCGTGGATGAGGCCAAGGCCAGGATCGCCGACATCCTGG</td><td>38:G>A</td></tr>
<tr><td>100513008|F|0-25:C>T-25:C>T</td><td>TGCAGCAGCTTCTCCACGATCATGACGATGTTGGCGTAGTGTGATGCCAGCGCCGAGCCGCCCACGGTC</td><td>25:C>T</td></tr>
<tr><td>985642|F|0-36:C>T-36:C>T</td><td>TGCAGCCAGGAGGAGGACGAGGCGGGGTTCAACCTGCTGCCGCTGGTGATGGCCGCCTTCGTCATCATG</td><td>36:C>T</td></tr>
<tr><td>1167398|F|0-16:G>A-16:G>A</td><td>TGCAGCCGCTGTTCAAGCGGTCGGTGACGTTCGTGACGCGCGACGACTCGGACTTCGCGGACCAGGTGG</td><td>16:G>A</td></tr>
<tr><td>1167398|F|0-41:G>A-41:G>A</td><td>TGCAGCCGCTGTTCAAGCGGTCGGTGACGTTCGTGACGCGCGACGACTCGGACTTCGCGGACCAGGTGG</td><td>41:G>A</td></tr>
<tr><td>985222|F|0-58:C>A-58:C>A</td><td>TGCAGCTGCCTCAACGTGGAGAAAATAGCGGGCTGCTCCAGAGAAAAATCTCCGCCATCGTCATCGCGT</td><td>58:C>A</td></tr>
<tr><td>1863316|F|0-62:G>A-62:G>A</td><td>TGCAGCTTCGTGTAGTTGCCGTGCGCGTCGACGTGCGTCAGGGCCACGCGCAGGCCCTCCAGGCGGCCC</td><td>62:G>A</td></tr>
<tr><td>5365639|F|0-18:G>T-18:G>T</td><td>TGCAGGACTGGCAGCGGCGGTACGCCGACGTGCTGCGGCGGAAGGGGAAGACCGTGGAGGTGGTGGAGT</td><td>18:G>T</td></tr>
<tr><td>1064334|F|0-30:C>G-30:C>G</td><td>TGCAGGCACCAGGCGGACACAGCGCCGCGACGGCCGACGCCCCAACCACGCCGTAACAGCGAGATGCCA</td><td>30:C>G</td></tr>
<tr><td>1034779|F|0-37:C>A-37:C>A</td><td>TGCAGGTGTATTACTTCCAAATGTACCTGGCACTCGTCCTCATTGGCTTTCTGCATGGGCTTATCTTCC</td><td>37:C>A</td></tr>
<tr><td>1047496|F|0-13:C>G-13:C>G</td><td>TGCAGTCTCAAGCCGACAAGAAGCGGTACGAGAAGGAATCTGCTGCCTACCGCGCTGCTGCCCCAGTGG</td><td>13:C>G</td></tr>
<tr><td>1069344|F|0-42:G>T-42:G>T</td><td>TGCAGTTTCTGGGGTGCACGCTCGGCTGGAGAAGAAGGACGGGAACCTGGTGGTCACGGACATGGACAG</td><td>42:G>T</td></tr>
<tr><td>3028384|F|0-18:G>A-18:G>A</td><td>TGCAGAGACTGTAGCTGCGCCTTGCCGCTGAGGCCGCGCTGCCG</td><td>18:G>A</td></tr>
<tr><td>3959624|F|0-41:C>A-41:C>A</td><td>TGCAGATGGGCCAGGCTCCCTTGTCGCTGTCGCTCCACGGGCCGCCG</td><td>41:C>A</td></tr>
<tr><td>5580153|F|0-17:C>A-17:C>A</td><td>TGCAGCACCGTCTTCAGCCCCCCGCAGCAGTCCG</td><td>17:C>A</td></tr>
<tr><td>1230357|F|0-41:G>A-41:G>A</td><td>TGCAGCGTCTCGGCGGCCAGCCGCAGCTGCTGCCCGACGGCGCCCATGCGGAACACGCCG</td><td>41:G>A</td></tr>
<tr><td>5581467|F|0-10:G>C-10:G>C</td><td>TGCAGGAGCGGGCGACGAGGCGGAGCGCGTCGGTGGAGAAGCCG</td><td>10:G>C</td></tr>
<tr><td>7354026|F|0-39:G>T-39:G>T</td><td>TGCAGGTTCGCGCCCTTCTGGGCCCTCACGGCGGCCAGCGTCCG</td><td>39:G>T</td></tr>
<tr><td>100521894|F|0-12:G>C-12:G>C</td><td>TGCAGTGCCTCAGCATGCTGTCCACGCCG</td><td>12:G>C</td></tr>
<tr><td>7166726|F|0-57:A>G-57:A>G</td><td>TGCAGCAGGATGGCGAGGATCTCGGAGGCGTACTGCTTGTTGGCCTCGAAGTCGCGGACCTTGACGCGG</td><td>57:A>G</td></tr>
<tr><td>1083484|F|0-34:G>T-34:G>T</td><td>TGCAGACCTCGACCTCCTCGGCGTCGGAGGACTCGTCCATGTCGGAGGCCGTGGAGGCGCTCCCGTCGT</td><td>34:G>T</td></tr>
<tr><td>5360556|F|0-14:G>T-14:G>T</td><td>TGCAGACCTTCGGCGAGCCACTGGTGGAGGGTGGCGCGGTCGAGGCGGATCTCGACGGCGGCGCGGTCC</td><td>14:G>T</td></tr>
<tr><td>5360556|F|0-23:G>T-23:G>T</td><td>TGCAGACCTTCGGCGAGCCACTGGTGGAGGGTGGCGCGGTCGAGGCGGATCTCGACGGCGGCGCGGTCC</td><td>23:G>T</td></tr>
<tr><td>1042145|F|0-42:G>C-42:G>C</td><td>TGCAGAGCTCGCCTACTTGAGGGTGACGACCAAGTGCGACGTGTACAGCTTCGGCGTGGTGGCCATGGA</td><td>42:G>C</td></tr>
<tr><td>1865587|F|0-29:G>A-29:G>A</td><td>TGCAGGTTACCATTGGGGACGTAGTTGTAGAGCAAGAGCTTGACAGACTTGTTCGAGCAGTAACCAAGC</td><td>29:G>A</td></tr>
<tr><td>1089129|F|0-18:T>C-18:T>C</td><td>TGCAGCACATTCAAAGGTTGAGCGGCAAAGTTTGGCTGAAAGAATTGGTATACATATCCGAACATCGCA</td><td>18:T>C</td></tr>
<tr><td>1089129|F|0-24:G>A-24:G>A</td><td>TGCAGCACATTCAAAGGTTGAGCGGCAAAGTTTGGCTGAAAGAATTGGTATACATATCCGAATATCGCA</td><td>24:G>A</td></tr>
<tr><td>1027219|F|0-10:G>C-10:G>C</td><td>TGCAGCACCTGCGCGCTGACGGAGTTGAAGAGGATCTCCACGAAGCCGTTGATGACGGCGGGCTTGGCC</td><td>10:G>C</td></tr>
<tr><td>1023880|F|0-33:A>G-33:A>G</td><td>TGCAGCAGCCAGTGGAACTGCCCCTCCACGCCCATGTGGTCCGTGGAGTGGAGGTGGAAGACGAGCAGC</td><td>33:A>G</td></tr>
<tr><td>1025585|F|0-50:G>A-50:G>A</td><td>TGCAGCAGCGACGACGACGCCATCGTCGGCATGCTGACCTGCGTCTGGAGGAACGGCTGCTGGAGCTGG</td><td>50:G>A</td></tr>
<tr><td>977583|F|0-51:A>G-51:A>G</td><td>TGCAGCCTCTTGTCCATGTACTCCCTCACCGCTCCGACGCCCTTGGCCACCACCTCCAGGGGCGGCACG</td><td>51:A>G</td></tr>
<tr><td>1204117|F|0-7:C>G-7:C>G</td><td>TGCAGCTCGGTGGCCTCTTCATATGGACGTACACCTACAGTCTGATGCAGAAGTCTGGCAAGCTGTACC</td><td>7:C>G</td></tr>
<tr><td>100525972|F|0-41:A>G-41:A>G</td><td>TGCAGCTGCGGCGAGAGCTTGAGAGCCGTCATGGAGGACTCAGCGTCCTGGCCGTAGATCTGGTCGCCG</td><td>41:A>G</td></tr>
<tr><td>993344|F|0-28:C>T-28:C>T</td><td>TGCAGCTTGGCCATTACCCGCGGGTTTCCGATGAGCTCCGCCATGACCCATTCCAGCGTGATAGATGAC</td><td>28:C>T</td></tr>
<tr><td>1007011|F|0-20:C>G-20:C>G</td><td>TGCAGGACTGCGCCGTCCGCCGAGCGCTCCGCTGCGGACAGGAGCTCCACGCGCGGCTGCTCCGCTCCG</td><td>20:C>G</td></tr>
<tr><td>1007011|F|0-28:C>A-28:C>A</td><td>TGCAGGACTGCGCCGTCCGCCGAGCGCTCCGCTGCGGACAGGAGCTCCACGCGCGGCTGCTCCGCTCCG</td><td>28:C>A</td></tr>
<tr><td>1696929|F|0-14:C>A-14:C>A</td><td>TGCAGGCACTCCGCCGCGTACTCCTTGGAGCCCAGCACGACGCCGCGGTCGAGCAGGCTGATCATCACG</td><td>14:C>A</td></tr>
<tr><td>5361474|F|0-59:C>T-59:C>T</td><td>TGCAGGCGGAAGTAGAGCAACCCCTCGACGGCGTCGGTGCGGAGCGTGGCGACGCCGCGCGCCATGAAG</td><td>59:C>T</td></tr>
<tr><td>1125324|F|0-49:C>G-49:C>G</td><td>TGCAGGGCCGCGTGAACGTGACCACCGCGCACTCGCTGGACATCATGGGCGTCTCCTACCCGCCCTCCG</td><td>49:C>G</td></tr>
<tr><td>1125324|F|0-58:C>T-58:C>T</td><td>TGCAGGGCCGCGTGAACGTGACCACCGCGCACTCGCTGGACATCATGGGCGTCTCCTACCCGCCCTCCG</td><td>58:C>T</td></tr>
<tr><td>100527589|F|0-6:C>T-6:C>T</td><td>TGCAGGCTCGCGGCATGTCGCAGGACCTCAGCTGGGACCACGCCGCCGAGCTCTACGAGGACGTCCTCG</td><td>6:C>T</td></tr>
<tr><td>1046837|F|0-7:G>C-7:G>C</td><td>TGCAGGTGTGCAAGGTGTATGTGTCCGTGTTCGGGGACGAGAGGGGGAAGAAGGTGGCCATGGCCGAGA</td><td>7:G>C</td></tr>
<tr><td>1232096|F|0-59:G>T-59:G>T</td><td>TGCAGGTTCACCAGCTTGCTTATCGTGCTCGGCAGCCTCTCCAGCTGCCCGCACTGGAGGCCCAGGTAC</td><td>59:G>T</td></tr>
<tr><td>1027358|F|0-5:G>A-5:G>A</td><td>TGCAGGTTGGGCGCCTCGGGGGTGGAGGCCGCGCGGAAGAGGAACGGCAGCAGCTCGGCCCAGGTGTTC</td><td>5:G>A</td></tr>
<tr><td>1041292|F|0-26:G>A-26:G>A</td><td>TGCAGTAATGGGACAGGAACTCCATGGTGAACCGCTTCATCTCCAGGTCGTCCCCGTAGTCCG</td><td>26:G>A</td></tr>
<tr><td>987629|F|0-11:T>C-11:T>C</td><td>TGCAGTTTTGCTGAGCCAACTGTTCTACCAAAAGACTTTCCATGCCGTACATAAGTGATGTCTTCTGCC</td><td>11:T>C</td></tr>
<tr><td>987629|F|0-18:A>G-18:A>G</td><td>TGCAGTTTTGCTGAGCCAACTGTTCTACCAAAAGACTTTCCATGCCGTACATAAGTGATGTCTTCTGCC</td><td>18:A>G</td></tr>
<tr><td>4993734|F|0-13:C>T-13:C>T</td><td>TGCAGCGACTCCACCGCGCGGGCCACCTCCG</td><td>13:C>T</td></tr>
<tr><td>4993734|F|0-18:C>T-18:C>T</td><td>TGCAGCGACTCCACCGCGCGGGCCACCTCCG</td><td>18:C>T</td></tr>
<tr><td>6043890|F|0-48:T>G-48:T>G</td><td>TGCAGCGCCATCCGCGGCCGTTCACGCGGCTGCACCGCGACCCCTCCATCAGCACCGCCG</td><td>48:T>G</td></tr>
<tr><td>4987790|F|0-13:G>A-13:G>A</td><td>TGCAGCGCCGCCAGGCGTTCGACGCGTACGCCGAGTACCAGCCCG</td><td>13:G>A</td></tr>
<tr><td>990516|F|0-18:T>C-18:T>C</td><td>TGCAGCTCAAGTTCGGGGTGGATGAATCGTACAAGCTGACCGTGCCTGCAACCG</td><td>18:T>C</td></tr>
</table>

988829|F|0-20:C>T-20:C>T	TGCAGGCGCCCAGCAGCGCGCCCCAGACCACCGAGTGCTCCTGGCAGTCCG	20:C>T
1026620|F|0-64:C>T-64:C>T	TGCAGCGGCTGGCCTACGTGAACTGCGTCGCCTACCCGCTCACCTCCATCCCGCTCACCGTCTACTGCG	64:C>T
1063793|F|0-10:C>A-10:C>A	TGCAGCAGCACCCCGACCTCCGTCAGCGACAGCACGTCCTCCAGTATGACCTTGGCGAATATCCGAGAT	10:C>A
12768836|F|0-10:C>A-10:C>A	TGCAGCTCCGCCTTGCGGCAGGTCTCCCCCAGCGCCTCGAGGAGGCGCGAGCATGGCCACGAGAAGGCG	10:C>A
100535479|F|0-11:T>C-11:T>C	TGCAGCTCCTCTACCTCGTCGAGCGCTTCCCGTCCGAGCCGTGCGCCGAAGCACATGTACACGAGCAGC	11:T>C
100535722|F|0-51:C>G-51:C>G	TGCAGCTGCTGCGCGCAGGCCTCGCAGTGCATGTTGACAAGCAGCTCCACGCTGCGGGCCTCGCTGACG	51:C>G
5970348|F|0-31:C>T-31:C>T	TGCAGGACCAGCTCGGCTTCGACAAGGCCAGCAAGACGGTGGACTGGCTGCTCACCCAGTCCAAGCCAG	31:C>T
1084609|F|0-19:C>T-19:C>T	TGCAGGAGTTCTTCTACGACCAGGTGCCCGACGCGCTGCGCAGCCTGGGGCTGGCCTTCTTCCTCAGCA	19:C>T
100536347|F|0-31:T>C-31:T>C	TGCAGGAGTTCTTCTACGACCAGGTCCCCGATGCGCTGTGCAGCCTGGGACTGGCCTTCTTCCTCAGCA	31:T>C
100536746|F|0-22:T>C-22:T>C	TGCAGGGGCGGTGGAGGCTGGTTTACAGCAGCGCCTTCTCGTCGCGGACGCTCGGCGGCAGCCGCCCCG	22:T>C
1696089|F|0-29:G>T-29:G>T	TGCAGGTCACCGTGCCGCTGAAGAAGGTCGCCAAGACGGAAGCGCTGGCCATCGGCGACCCTACCCGTC	29:G>T
1696089|F|0-34:G>A-34:G>A	TGCAGGTCACCGTGCCGCTGAAGAAGGTCGCCAAGACGGAAGCGCTGGCCATCGGCGACCCTACCCGTC	34:G>A
100540067|F|0-14:T>C-14:T>C	TGCAGCCTGCCGCTTGCAGCCGACGCCTTCTTCTCCG	14:T>C
7169586|F|0-20:A>G-20:A>G	TGCAGCTGCGTCTTGACAACATCGAGTGGTGTGGTCAAGGCGGCTGCTAGAGCACCG	20:A>G
1151484|F|0-26:G>A-26:G>A	TGCAGCTTGAGGAGGACGCCGAGCAGGTCCTCATCTCTCTCTGGCTCCG	26:G>A
4262795|F|0-39:T>C-39:T>C	TGCAGGACTTGACGGGGTATATGAGGATGGACGCCACCGTCGCCGCCG	39:T>C
5578236|F|0-31:A>G-31:A>G	TGCAGGATGGACAGCTCGCTCCCAGCCGCGGACGGAGCCG	31:A>G
6041755|F|0-24:G>C-24:G>C	TGCAGGCGTGCATGCACAGAGTGGGCACCGAGCCAGCGGCGCGAGGCTCGCGGTGGCCG	24:G>C
1215372|F|0-25:C>A-25:C>A	TGCAGGCGTGCATGCACAGAGTGGCCACCGAGCCAGCGGCGCGAGGCTCGCGGTGGCCG	25:C>A
1215372|F|0-26:A>T-26:A>T	TGCAGGCGTGCATGCACAGAGTGGCCACCGAGCCAGCGGCGCGAGGCTCGCGGTGGCCG	26:A>T
1005371|F|0-19:C>A-19:C>A	TGCAGTTGATGTCCGCGGGCCCGCTGGTGGACGTGGGCCTCGCGGCGCTGCGGTCTCCG	19:C>A
1093292|F|0-52:C>T-52:C>T	TGCAGGCGCTCTCGTCCGAAAACCCCTCCGCCTCCGCCGCGGCGTCGCTCCTCGCCGCCGCCGCGCGCG	52:C>T
1863399|F|0-24:C>T-24:C>T	TGCAGAGCCTCTCCAGCTCCTTCACGTCGGCCTCCTTGGCCAGCCTCTCGTCGAGCAGCCCGACCACGT	24:C>T
1053162|F|0-56:T>C-56:T>C	TGCAGAGGCGAGCGGGTGGTTGTGCTCGGCGACAAATTCAGTGACACGGAAGGCATTGGCCGAAGGGAG	56:T>C
1233858|F|0-41:G>C-41:G>C	TGCAGCACCTCGCCAAGCTGAAGAAGCTCTGGAAGCTCAGCGTCAACGTCACCACCGACGCCAACGTCG	41:G>C
1204198|F|0-44:G>A-44:G>A	TGCAGCAGAACAGCTACGCCAAGCAGCAGCACGAGCCCGCCGACGACGACCACAACGGCAACGGCAACG	44:G>A
1029403|F|0-52:G>A-52:G>A	TGCAGCAGTGAGGTTGGTATCTGCCCATGGAGCTGGTTGGAGGACAGGTTCAGCCGCTCCAGCTTCACG	52:G>A
6046246|F|0-18:C>G-18:C>G	TGCAGCCCATGATCAATGCGGCAGCATCGTTTGGGGATCCAGAGCTGCTCGGCGACATCCAGATCGGCC	18:C>G
2259386|F|0-5:C>T-5:C>T	TGCAGCCCGCCGTACCTGCCCTGGCCGACGCACAGCTCCTCGTTGCCGCGCACGTCGAGCCGCCTGCCG	5:C>T
2259386|F|0-14:C>T-14:C>T	TGCAGCCCGCCGTACCTGCCCTGGCCGACGCACAGCTCCTCGTTGCCGCGCACGTCGAGCCGCCTGCCG	14:C>T
1214869|F|0-10:T>A-10:T>A	TGCAGCCGCTTTGGCTGGTGCGGCTCCACCTCGGACTACTGCGGAGACGGATGCCAGAGCCAGTGCTCC	10:T>A
3937786|F|0-56:C>T-56:C>T	TGCAGCGCCAGCTCGAGCTCGCCGAACTGGCCGTGGTGAACCGAGCTCGGATGGACCTCGCTCATGACC	56:C>T
1234044|F|0-33:A>T-33:A>T	TGCAGGCAAGCGCTTATCCGATTTCCCCTTGTTAATTGTTGGGATTTGGTTCGTTGTTGCTCATTTCAA	33:A>T
1234044|F|0-37:G>A-37:G>A	TGCAGGCAAGCGCTTATCCGATTTCCCCTTGTTAATTGTTGGGATTTGGTTCGTTGTTGCTCATTTCAA	37:G>A
1040382|F|0-10:T>G-10:T>G	TGCAGGGTGTTGTGGTAGTGCTGAACACCCCTGGACCATGGAGATGGAGAGGACATTGCCAATTGGAAC	10:T>G
999194|F|0-26:G>A-26:G>A	TGCAGGTAGGTCCTGAGCTCCTGCTGGACGACGATGACGATGGGGAAGAAGATGAGGAGGAGGAGCACG	26:G>A
3026943|F|0-6:C>G-6:C>G	TGCAGACTCCCCTAATAGACCTCTCCGCCATATTCACGCGCCG	6:C>G
4262786|F|0-14:T>C-14:T>C	TGCAGCGGCGGCGCTTGGTCGCCATCTCCTACCTCCG	14:T>C
1021231|F|0-15:A>G-15:A>G	TGCAGGCCATCGCGTACGCCTGCTCCTCGCCGCAGCACCGCGGCCACCCCG	15:A>G
100549398|F|0-59:A>T-59:A>T	TGCAGAAGATTGTGATCAGAGCCGATCTCGTCGGCAAGAAGTGCACGAGTGGGATCCTGACAATTGTTT	59:A>T
1107737|F|0-45:C>T-45:C>T	TGCAGACAGGGCAAAGACCATCCTTGCTCTGGCCTCCCCTGACTCCAAGCCCAGTGATCTCAGAAGGCC	45:C>T
2253158|F|0-47:C>T-47:C>T	TGCAGCAGCAGCCTGTCCTCCGCCGACGACTCGAACCCGTGCAGGAACGACATGTCCGCGCCGCCGCCC	47:C>T
1029272|F|0-17:G>T-17:G>T	TGCAGTCGCCGTCCGCGGCCAGGCGGCCGACCATGGAGCACGCCGTGACGGTGCTCTCGGCGGCCATCA	17:G>T
100552815|F|0-58:T>G-58:T>G	TGCAGTCGCCGTCCGCGGCCAGGCGGCCGACCATGGAGCACGCCGTGACGGTGCTCTCTGCGGCTATCA	58:T>G
3025393|F|0-8:C>A-8:C>A	TGCAGTCTCCAGCCCCATACCCCTGTCACCGCCGATCTTTGACCG	8:C>A
3222237|F|0-40:T>C-40:T>C	TGCAGCACCGCCCACACCCTTGCTTCCATGCTCCTCCCGATCGAAACCAGGATTCTGCGGGAACAGCGA	40:T>C
2247175|F|0-44:T>C-44:T>C	TGCAGCATCATCTCGATCTCCTTGACGACGTCGCTCATGGACGGTCGGGCGGCAGCGCCCTCCTCCACG	44:T>C
2246640|F|0-55:T>C-55:T>C	TGCAGCATCCTCCTGAGCAGCAAGATCCCTTCCTCGGTCCTCCCCTCCTCGAAGATCCACAGCGCCAGC	55:T>C
2264864|F|0-59:G>A-59:G>A	TGCAGCGGCACGACGAGGCGGACTCGGCGCTCCGCGGCGCGCCCAGGTTCGGCGTCGACGAGTCCACCA	59:G>A
2271235|F|0-50:C>T-50:C>T	TGCAGCTCCTTACCTCGCCGTCGTCACACTCGTGGAACACGCTCCCTGAGCTGTCGGCGTCCGCGGCCT	50:C>T
1212200|F|0-34:T>C-34:T>C	TGCAGCTGGGCCTGGGCCTCGGCGCGGCGGAGGCTGTCGCGGAAGCACTTGGAGCAGAGGCCGCCGAGA	34:T>C
1061595|F|0-52:C>T-52:C>T	TGCAGGTAGGGTGCTCTTAGGCTGAAGACTTGTCGCCGCCAAAGGCATTCTCCAGAACACCG	52:C>T
981818|F|0-20:C>T-20:C>T	TGCAGGTAGACGCCGATGGCCGTGAACTTGATGAAGTTGCCCCCGATCTCCATCCCCCGCACGCCTGCA	20:C>T
1213799|F|0-27:C>A-27:C>A	TGCAGGTTCACGTCGGAGCTCGGGGGCCAGTTTGGCGATTTCTCGGCGTCCCTCGGAGGAGCGGCTGCC	27:C>A
1213799|F|0-55:G>A-55:G>A	TGCAGGTTCACGTCGGAGCTCGGGGGCCAGTTTGGCGATTTCTCGGCGTCCCTCGGAGGAGCGGCTGCC	55:G>A
4995495|F|0-10:C>T-10:C>T	TGCAGTGAGGCGTCCAGCTCGACTTCGCCATGCGGGTCGGGCTCCACGTTCGTCCCAGCGCCG	10:C>T
2244710|F|0-21:A>T-21:A>T	TGCAGTGAGGCATCCAGCTCGACTTCGCCATGCGGGTCGCGCTCCACGTTCGTCCCATCGCCG	21:A>T
2248959|F|0-25:G>T-25:G>T	TGCAGCGAGGGCAGCGGAGCTGGCCGTTGGGATTATTCGAATGGCCTCCTTCAGCACCACGTCCGAGAT	25:G>T
100559659|F|0-67:C>A-67:C>A	TGCAGTGGGTGAGCCTGGTGATCATCATCGGGGCACTGGCGTGCAGCCTGGCAATAAGGCCCCTGTCCC	67:C>A

3940093|F|0-16:C>G-16:C>G	TGCAGACCCTGCGGACCTCTCCCTGTGTGCCG	16:C>G
1063814|F|0-28:C>G-28:C>G	TGCAGCAAGCTGGAGGCGCTCTTGCTCTCCTCCAACCTGCTGGATGACGTCATCCCGCCG	28:C>G
1134068|F|0-36:C>T-36:C>T	TGCAGCACATCGAGCCCTCCCGCTTCATCTCCTTCTCCTTCCCCAACCCTTTCCTCCACGACGCCTCCA	36:C>T
4544261|F|0-56:C>T-56:C>T	TGCAGGGCGTACGCGGCGGCGGACGTCAGCGGGGGCATCAACCGCGGGTGCGTCATCTGGGCCGTGGAT	56:C>T
1023799|F|0-19:A>G-19:A>G	TGCAGGGGAGGCAGGAGCCACCTGAAGGAGGACGCGAGGAGGAGGGCGCCGCAGGCGACGATGTACGGG	19:A>G
100563728|F|0-41:A>C-41:A>C	TGCAGTGGGTAATGGCGGAGCTCATGCGGAACCCGAGGGTTAGGCATAAGGCGCAGGAGGAGGTCCGAG	41:A>C
1025842|F|0-35:C>G-35:C>G	TGCAGCCCTGCAAGCCGCCGCCGCACGAGACGGACCACCTCGGCGACGCCGTGTTCACCG	35:C>G
992772|F|0-22:C>G-22:C>G	TGCAGGGCGGGCACGCCGAAGCCACAGGCGTACCGAGGCGGCAGCAAGACGGTCGGCCG	22:C>G
1245609|F|0-17:C>T-17:C>T	TGCAGCTCCCCGTGCAGCCTCCACGCGGCGGCCAGGTCGCGGTCCATGGCCCGCGCCGTCGACCGAGAT	17:C>T
991352|F|0-24:C>A-24:C>A	TGCAGACGGCAGCTTATTAAGGGGCTAGTTTAAAGCGGGGGCGGCAATGCGCTGGGTCCGCGAAGGCCG	24:C>A
992942|F|0-51:T>A-51:T>A	TGCAGCGTGGGCCACCAGCAGAAAGGAGCAGCTCAACTACCCACACCGACCTGGACCCACCCGCCAGGG	51:T>A
1695397|F|0-26:G>A-26:G>A	TGCAGCTTCCCCCTGCTCTGCACCTTGGGGTTCATGAAGTCCTCGGCGAGGAGGTCGACGAGGGCCATG	26:G>A
1039946|F|0-52:G>C-52:G>C	TGCAGGAAAAATGAGAAACTACAGTCATCATCTAAAATTTGTCCTTTGTCTAGTCCTCGGAAGGCGGCG	52:G>C
1716053|F|0-56:C>G-56:C>G	TGCAGGTCTTCAGGGTTTACAATGCCGGCCAGGATTTTCAGCCTCTCTGTGTAGGGCACGTTGCTGTCC	56:C>G
1696572|F|0-55:C>G-55:C>G	TGCAGAGGTTCGAGAAGGCGGACGCCGCGGGCGAGGCGCGGCGGATGGTCTGCGCCGCCGACAGCAACA	55:C>G
1696572|F|0-64:C>T-64:C>T	TGCAGAGGTTCGAGAAGGCGGACGCCGCGGGCGAGGCGCGGCGGATGGTCTGCGCCGCCGACAGCAACA	64:C>T
6046254|F|0-11:A>G-11:A>G	TGCAGCCGCGGATTGGGCTCCACCCGCAGCGAGCCGCCGAGCTGGTTCCCCGACACGTCGACGGCGCGG	11:A>G
1093922|F|0-7:T>G-7:T>G	TGCAGCCTGAGGGTGGAGGCGACGACGATGGCGAGCACGGCGAAGCAGGCCGGGATGAGCCCG	7:T>G
1093922|F|0-32:G>A-32:G>A	TGCAGCCTGAGGGTGGAGGCGACGACGATGGCGAGCACGGCGAAGCAGGCCGGGATGAGCCCG	32:G>A
999492|F|0-5:C>T-5:C>T	TGCAGCGGCGTGACGTGCCCGAGGCGCGACGGGTGGAGGGTCTCGGAGGTGATGTTGCACCG	5:C>T
999492|F|0-10:T>A-10:T>A	TGCAGCGGCGTGACGTGCCCGAGGCGCGACGGGTGGAGGGTCTCGGAGGTGATGTTGCACCG	10:T>A
1387326|F|0-34:C>A-34:C>A	TGCAGGACGAGATTCCCAGTGTTCAGGAGGACAGCAATGGTGTCAGTGGTTGTGACTCTTGCTTGAGAA	34:C>A
100570802|F|0-63:T>C-63:T>C	TGCAGGCGCAGAGCAAGCTGATGGCCATGGCGGCGGTGTCGGCGGCGGCGCTGGCGTTCCACGTCGCGC	63:T>C
1091000|F|0-6:T>C-6:T>C	TGCAGGTGGGCAACGACCTCAACCACCGCTTCCTAGCGCGGCTGTCGGCGCGGTACGGCCCCGTGTTCC	6:T>C
1695188|F|0-7:T>C-7:T>C	TGCAGGCTCTTGAGCTCTGAATCAAAATCTTTCTGCATGGACACGGCGCGGCGGCTGAACTGCTCGTCG	7:T>C
1019389|F|0-13:C>A-13:C>A	TGCAGACCATCCGCAAGCTCTCGCCCAAGGCGTTCGTGCTCGTGGAGCAGGACGCCG	13:C>A
1019389|F|0-22:G>C-22:G>C	TGCAGACCATCCGCAAGCTCTCGCCGAAGGCGTTCGTGCTCGTGGAGCAGGACGCCG	22:G>C
4261744|F|0-13:C>G-13:C>G	TGCAGACCGACGGCGAAACGACGACCCATCGCTCTCCG	13:C>G
100572253|F|0-14:T>C-14:T>C	TGCAGGCAGTGCAGTGCCAGCGTCGCCGCGCCGCCCG	14:T>C
3943268|F|0-19:G>A-19:G>A	TGCAGGCAGTGCAGCGCCAGCGTCGCCGCGCCGCCCG	19:G>A
100572345|F|0-12:A>G-12:A>G	TGCAGGGGGCTCACCGACATCAGGGACATGGCGCCGACCTTTGACCG	12:A>G
100572348|F|0-33:G>C-33:G>C	TGCAGGGGGCTCGCCGACATCAGGGACATGGCGGCGACCTTTGACCG	33:G>C
3027422|F|0-26:G>A-26:G>A	TGCAGTTCCCGTCGGACATAGCCACCGTTCTTCTTGCCGCCG	26:G>A
1014251|F|0-21:A>G-21:A>G	TGCAGAACCGCCGCCGCCGTCAGCAGGAGCCCCGCGGCTGCGGCGAGGCTCATCCACGGCGGCGCCGAG	21:A>G
981730|F|0-10:C>T-10:C>T	TGCAGATCCTCGTCCAGCACCACCCGCAACCGTGTCATGTCGTCCTCAAGCAGATGGTCAGCTCGCCGC	10:C>T
100573868|F|0-12:C>T-12:C>T	TGCAGCTCGAGGCGACCAGGCTGAGCACGCCGTGGGGCTACCTGAGCGCCGTGGCCTTGGCCGTCACGA	12:C>T
100573870|F|0-59:A>G-59:A>G	TGCAGCTCGAGGTGACCAGGCTGAGCACGCCGTGGGGCTACCTGAGCGCCGTGGCCTTGACCGTCACGA	59:A>G
6044286|F|0-24:G>A-24:G>A	TGCAGTTATGCCGAGAAAGATACTGGCCACAGTGCGCATTCATTTCTAGCCG	24:G>A
4992336|F|0-7:G>T-7:G>T	TGCAGGTGTCGTGCGCCGTTTCCG	7:G>T
100576307|F|0-16:C>G-16:C>G	TGCAGCGACAGGCGCACCAGCAGGAGTAGGAGACACCTTTGGTTTTGGCTGGATGCCAGCGGCTGCCTC	16:C>G
1242275|F|0-23:G>C-23:G>C	TGCAGCTCCCGCAGCTCCCTCTTGACGCGGTCCAGCTCGCGCATCACCTCGGCGTACGTCGCGCCGCCG	23:G>C
1081828|F|0-29:T>A-29:T>A	TGCAGTTCGACAGGTACAGATACGTCAGGTTCTTGAGGTTCCCAATGCTGGGAGGCATCTCCCCTTGAT	29:T>A
4412235|F|0-12:T>C-12:T>C	TGCAGGAGCGGGTGCTGAGTTGCCATGAAGCGATGATCCAGGCCACCG	12:T>C
6040084|F|0-6:A>G-6:A>G	TGCAGCAAGTCAGGAGCCATCATCCCCTGGAAGACGTCCTCCTCGAAGAAGCTCAGGAATCCCCCGAGA	6:A>G
100579101|F|0-22:T>C-22:T>C	TGCAGGACGGCGGGGGGCTGCTTGCCTCCGACCAGCTCCTCTACAACGACAACCGCACGCGGCCCCTCG	22:T>C
1088965|F|0-32:G>T-32:G>T	TGCAGGACGGTGATGCCACGAGGGTTCCCTGTGTGCTTTCTAGCGAGCGGGACGAGGATCCG	32:G>T
5579748|F|0-16:C>T-16:C>T	TGCAGTTCTTCCTGATCTCGAAACGGCTGCCCTCGGCGGGGATCTCGCTGAGCTTGGCCATGGCCTTGC	16:C>T
993679|F|0-39:C>T-39:C>T	TGCAGCCGCTGGCGCCGCCTCCTCTCCGACCCCGCCTTCCGCCGCCGCTTCCGCATTCACCACCGCCGC	39:C>T
1070948|F|0-32:G>A-32:G>A	TGCAGGTCCAGGTGAACGGGGCGACGAGGCGTGTGGGCGGGGTCTTCGGGACGCCG	32:G>A
3935723|F|0-5:A>C-5:A>C	TGCAGATCGGCCTCCCCGTGGTGCCCTTCGCGCGCGCCATGGACGACGTGCTCGAGACGCTCTTCCTCC	5:A>C
1159349|F|0-21:C>T-21:C>T	TGCAGCGCGTGCCCACGCGGGCCTTGAGGTCGGCGACGAACTCGTCCGGGAAGTGCACGGCCAGGTTCT	21:C>T
1093143|F|0-17:G>A-17:G>A	TGCAGCGGGAGCGAGGCGCGGAGGTAGGCGCGCATGACGGTGCTGACGACGAACGGGTCCGCGTCGGCC	17:G>A
1093143|F|0-49:C>A-49:C>A	TGCAGCGGGAGCGAGGCGCGGAGGTAGGCGCGCATGACGGTGCTGACGACGAACGGGTCCGCGTCGGCC	49:C>A
1036446|F|0-22:C>T-22:C>T	TGCAGTGGAGGATCTTGGCCAGCTTCAGCATCGGCGTGACGTGCCCCTGCGCCG	22:C>T
5372260|F|0-11:C>T-11:C>T	TGCAGGCTGCCCGCGGCGCACGTGAGCGCCGTGGCCCAGTACCG	11:C>T
1059990|F|0-29:G>A-29:G>A	TGCAGCGCTTGCCTAAACATCCCAGAGCCGCGGGGCACGCCTAGAGCTTCGGCGCATGCCACCATCTCC	29:G>A
1230844|F|0-56:G>A-56:G>A	TGCAGGGGAGGCTGCGGAGCACGCAGCCGCTGACCAAGGGCGTGGTGGCGGAGTGCGTGCTGGGCGCGA	56:G>A
3027505|F|0-24:G>A-24:G>A	TGCAGACATGCGACGGATGGAACCGCCAGTCAGATAGAGCCG	24:G>A
1025010|F|0-43:C>T-43:C>T	TGCAGGCCATCAAGGGGGACCTCTCCGACCCCCGCGGCTTCCTCCGCTCCTGGAACGCCACCG	43:C>T

100586314|F|0-32:A>C-32:A>C	TGCAGGAGAAGGCCACGCTCCTGGCGTTGAAAAGCTCGCTCACCTTGCTGTCGCCGTCGGATCTGGCCG	32:A>C
5582750|F|0-21:A>G-21:A>G	TGCAGCTGGCGGACGTGCTGCACGCCCG	21:A>G
4537376|F|0-13:T>C-13:T>C	TGCAGCTCCTCCTTGCCAGGCAGCCG	13:T>C
100592508|F|0-11:G>A-11:G>A	TGCAGGAGATCGTCGGCGTGGCGTTGCCGTCCGTGGAGATGCGCAACCG	11:G>A
1111616|F|0-47:G>A-47:G>A	TGCAGGAGCACGGGGTCACGGCGCTGCTCAACCTCTCCATCTGCGACGAGAACAAGGCCATCATGGTCG	47:G>A
6047996|F|0-34:C>T-34:C>T	TGCAGGACGCCACGGGCCTCGCCACGCTCTTCGACGTGGTGACCCAGGGGTGGCTGGACGCCGACGCGC	34:C>T
6048756|F|0-29:G>A-29:G>A	TGCAGGACGATCCACAGGGACCACGTCACGTTGACGAGCACCATGAGGAGCGTGCCCATGATCCACGTC	29:G>A
3943830|F|0-7:T>C-7:T>C	TGCAGGCTCTTGAGATGCTCGTACGTGGACGGCTTGTCGCCGCCG	7:T>C
1009527|F|0-20:G>A-20:G>A	TGCAGGCGTGGTGGAGCGAGGTGCGAAACGTGGGCCACGGCGACCTGCGCGACGCGCCGTGGTGGCCGA	20:G>A
2290689|F|0-16:C>G-16:C>G	TGCAGGTCTGCTTCTCCAAGCGCCGCAAGGGCCTCGTCAAGAAGGCCTTCGAGCTCTCCGTGCTCTGCG	16:C>G
3946683|F|0-14:C>A-14:C>A	TGCAGCCTCGCCCCCGAGGAGCCG	14:C>A
4262471|F|0-16:C>T-16:C>T	TGCAGCTTCAGCTCGACGCCCACCCG	16:C>T
6031042|F|0-19:C>A-19:C>A	TGCAGTTGAAGGTCACGGCCACCG	19:C>A
7349724|F|0-11:C>T-11:C>T	TGCAGCGCGTACGCCATCCG	11:C>T
2247474|F|0-61:C>T-61:C>T	TGCAGCTCCTCCATCGTGCTCTCCTCCGACGACGTCGACACCTTCACCAGCGACCCGCCCACCGACAGG	61:C>T
3941654|F|0-6:C>T-6:C>T	TGCAGCCTCTTTGGGTCGAGCAGCCG	6:C>T
1092547|F|0-32:C>T-32:C>T	TGCAGGGCCATCCAGTACGGCTCCAAGTTCATCAGCAACGGCCAGCCCG	32:C>T
4987620|F|0-7:C>G-7:C>G	TGCAGAACGCTCCAAGGGCGACTCCG	7:C>G
7153206|F|0-38:G>T-38:G>T	TGCAGAAGGAGCTCGCCGAGTGGCAGGTCGGCCCGCCCGCCG	38:G>T
4261826|F|0-17:C>T-17:C>T	TGCAGCTCCCTGCCGTTCTTGCCG	17:C>T
3960764|F|0-23:G>C-23:G>C	TGCAGGATCTCGTCGCCGATGCCGCCCG	23:G>C
3947972|F|0-8:T>A-8:T>A	TGCAGGGCTCCATCAACATCTTCTCCGACGCCG	8:T>A
2248034|F|0-29:G>A-29:G>A	TGCAGCTTCCAGTGGAGGTCCTGGTTGATGAAGCTGAGCGACTCGATGGACGACGCGATGGCGCTGCTC	29:G>A
1065215|F|0-55:G>T-55:G>T	TGCAGGAACTTCTGGATGGGGAAGTTGGCCGCGTACGCGAAGATCTGCGGGATGAGGCCGTAGGCGAAC	55:G>T
3028230|F|0-13:G>A-13:G>A	TGCAGAAAATCCCGGTGAAGATGTGCCCCAGGAGGCCG	13:G>A
1026916|F|0-44:G>T-44:G>T	TGCAGCACTGCGTGGAGACGGCGGCGCTGCTCGCGGAGCTCGGCGCAGGCCCTCCG	44:G>T
6030399|F|0-14:C>T-14:C>T	TGCAGCCACAGCAGCGACGACGAGCCG	14:C>T
5000238|F|0-28:G>A-28:G>A	TGCAGCGCGCGAGCCTTGGGCGCCTGGCGCCGAACGCCG	28:G>A
3960968|F|0-24:G>A-24:G>A	TGCAGCTTCCGACTGGCCCCCATGGCATCCG	24:G>A
1039167|F|0-37:C>A-37:C>A	TGCAGAGCTCGCGGTCGCAGTTGCCGTTCTGCCACAGCGCGCACATCTTGGTCTTGTACGCCG	37:C>A
3023963|F|0-8:C>T-8:C>T	TGCAGCGCCACTTCGAGTCCTCTGTGTTCCTCTTCCG	8:C>T
3023963|F|0-28:C>T-28:C>T	TGCAGCGCCACTTCGAGTCCTCGGTGTTCCTCTTCCG	28:C>T
5580325|F|0-14:C>A-14:C>A	TGCAGCTGCGTCTTCAGCAGCGGCGGGAACGACGCCG	14:C>A
3944441|F|0-23:C>T-23:C>T	TGCAGCTGCGTCTTCAACAGCGGCGGGAACGACGCCG	23:C>T
6041655|F|0-8:C>G-8:C>G	TGCAGGAGCTCGACGTCTCGCACAACCAGCTCACCG	8:C>G
3937911|F|0-9:C>T-9:C>T	TGCAGGGAGCGGTTGGAGATGGTGGCGCCCG	9:C>T
100445823|F|0-6:C>A-6:C>A	TGCAGTCCCTTAACGTGTCCG	6:C>A
2253117|F|0-60:G>A-60:G>A	TGCAGTCCTCGTCGAAGGGCACCTCCTTGTGGAAGATGCAGCACTTTTTGGAGCTCTTGCGGCCGAGGG	60:G>A
5347251|F|0-24:C>G-24:C>G	TGCAGATCGCGCACGGGGTCCCGTCCCCG	24:C>G
3956224|F|0-16:T>C-16:T>C	TGCAGATGGGCAAGGCTGGCAGGTGGCTCAGAAGCTTCCTGACCG	16:T>C
4261201|F|0-32:C>A-32:C>A	TGCAGCATGGAGATCGCGCCCATGCAGCCGTACACCG	32:C>A
4022587|F|0-28:C>A-28:C>A	TGCAGGGAGCAGGTGATCCTGGCGCCAGCGTCAACGCCGAACGCCCG	28:C>A
3938302|F|0-10:C>G-10:C>G	TGCAGGGCCTCGACTCGCTCATCATCCCCG	10:C>G
4262338|F|0-22:A>G-22:A>G	TGCAGGTACCAGCTGCCCACGGACGAGACCACGGAGCCG	22:A>G
3942506|F|0-11:G>C-11:G>C	TGCAGGTGGTGGCTGCCAAACTCCG	11:G>C
1015449|F|0-46:C>T-46:C>T	TGCAGGTCGAGGCCTTCTGGCGGCGCAACTACCTGCTGCTCGTCGGCGCCG	46:C>T
7333805|F|0-5:G>T-5:G>T	TGCAGGAGGTTGCAGAGCGCCG	5:G>T
7914816|F|0-5:G>A-5:G>A	TGCAGGTTGGGCAGGCTCCG	5:G>A
2279513|F|0-14:G>A-14:G>A	TGCAGCTCCGCCCCGTCGACCACAAGGCCGTCGCGGAGGAGTGCTCCCTCGCGCTCCAGGCCGAGCCGC	14:G>A
1696660|F|0-16:G>A-16:G>A	TGCAGGACACCACGCTGGGCTCGCTGCTCTCGGCGCTCATGCAGCACTGCGATCCCCCGCAGCGAAGGT	16:G>A
1342351|F|0-34:G>A-34:G>A	TGCAGGCGCGGCAGGCGGCGTGCGCGTCGTCCCCGCAGGCCGCGCAGGGAGGATGGGTCCCCG	34:G>A
5009032|F|0-16:G>A-16:G>A	TGCAGACGTGGCAGACGAACTGCACCGCGCCGATGAGTATCTGCGCCG	16:G>A
3021100|F|0-8:T>G-8:T>G	TGCAGAGATGAGCATCTGCACTACATTCTTCATACCG	8:T>G
1021968|F|0-43:C>T-43:C>T	TGCAGCCGACCAACGAGGTCCTGGAGGGCCTCCTGTCGGACCCCAAGGCCG	43:C>T
5581411|F|0-37:G>T-37:G>T	TGCAGCGTCGCGTTGGTGAGGCCGAGGAAGCCGCCGTGGCTGCCG	37:G>T
3949695|F|0-16:G>C-16:G>C	TGCAGGATGGACGTGAGCGCGGCGCCGACCCCCG	16:G>C
100480737|F|0-5:G>C-5:G>C	TGCAGGCCATGTACGAGTCGGAGTCCCCG	5:G>C
4398082|F|0-6:C>G-6:C>G	TGCAGGCCCCGAGGCCCTGCTCCCCG	6:C>G
979959|F|0-29:C>G-29:C>G	TGCAGTACAGGTGGCACCTCCCCGAGGACCACGCCAAGTACTCGGTGTTCATCCCG	29:C>G

4404916|F|0-10:G>A-10:G>A	TGCAGGTCCTGGAGATGGCCG	10:G>A
1017604|F|0-18:C>G-18:C>G	TGCAGGCCTCCCTGCTGCCGCCGATCAGCTTCCCGACCTCATTCGTTCCCACGGCAAGCCCGTCTCTCA	18:C>G
1017604|F|0-24:T>G-24:T>G	TGCAGGCCTCCCTGCTGCCGCCGATCAGCTTCCCGACCTCATTCGCTCCCACGGGAAGCCCGTCTCTCA	24:T>G
3938753|F|0-28:G>T-28:G>T	TGCAGAACGACATCGACCTGCTGAACCCGCCG	28:G>T
4394808|F|0-15:C>T-15:C>T	TGCAGATCCTCCACACAAAGAGGAACCCG	15:C>T
7169631|F|0-7:C>A-7:C>A	TGCAGGACCTCACCCTCCGCCACCTCCCG	7:C>A
100496222|F|0-5:G>C-5:G>C	TGCAGGAGCAGCAGGTGAACGTGCCG	5:G>C
3955177|F|0-7:A>C-7:A>C	TGCAGGTACACCTTCTGGCTGACGCTGTGGTACGGCCGCGTCCG	7:A>C
5582088|F|0-29:G>A-29:G>A	TGCAGTGGGCGCTCCTCCTCGCGTCGCTGGCCGCGGCGGGGCACCG	29:G>A
5582088|F|0-31:C>A-31:C>A	TGCAGTGGGCGCTCCTCCTCGCGTCGCTGGCCGCGGCGGGGCACCG	31:C>A
1666874|F|0-64:C>A-64:C>A	TGCAGACGGACATCAACATGGCGCGCCGCGCGCTGCGCGAGGCGCTCACCACGCTCGACGACATCAAGC	64:C>A
1264683|F|0-8:C>G-8:C>G	TGCAGCAGCTCGACGGCGAGCACGTCGGACGGTGCGGTGGCCATGGAGATGACGTAGGCCCCGAAGCTG	8:C>G
1055814|F|0-18:G>A-18:G>A	TGCAGCTGCAAACGAGCTGTACAAGGTAGCCAAATCGCTGGGCCTCGCCGACCAGGACTTCTCCGCGGT	18:G>A
988449|F|0-25:C>G-25:C>G	TGCAGCTGGCCGTGGACGCCGCCGCCGCCAAGTGCCTCGCCGCCGAGAAGCTGGAGGTGGTGGACTACA	25:C>G
1089095|F|0-21:A>T-21:A>T	TGCAGGAGGGGCACCTGGCCCAGGAACTTCGTCACTGCACATCAACGGGAATCGTCCCCAGAATTAGCC	21:A>T
1094226|F|0-8:G>T-8:G>T	TGCAGGCCGTCGGCGCACGTCTGCTGGTTGGTCAGGATGGCCGACAGCAGCGTCTGCACGTCCTCGGCC	8:G>T
3936848|F|0-10:G>C-10:G>C	TGCAGCAGCTGGAGTCGTCGACGCGGCAGAGCAAGCTGCGGCCG	10:G>C
7165379|F|0-32:C>G-32:C>G	TGCAGCGGCAGGCGTCGTCGGGGTCCCTGACGCAGGGCCCGTCGGGGCAGCTCCG	32:C>G
100509495|F|0-8:G>A-8:G>A	TGCAGGAGGTCCACGACGGCCCAGCCG	8:G>A
1024793|F|0-13:C>T-13:C>T	TGCAGGCACAGCTCGCCGTCGCCGTTGGCCGCCATGGCCGAGAACATCTGCCG	13:C>T
7341303|F|0-6:G>A-6:G>A	TGCAGGGCCCTCTTCCTGGCCG	6:G>A
1167398|F|0-43:C>T-43:C>T	TGCAGCCGCTGTTCAAGCGGTCGGTGACGTTCGTGACGCGCGACGACTCGGACTTCGCGGACCAGGTGG	43:C>T
1026265|F|0-33:G>T-33:G>T	TGCAGGCCGCCATGAAGCAGGCCTCCCGAAGGAGCTCACCGACCGATTCTTGGGTGGGCAAGCTGCGGA	33:G>T
3954098|F|0-14:G>C-14:G>C	TGCAGCATCTCGGCGGTGTTCAGCCTGTGGCCGCCG	14:G>C
3954098|F|0-28:G>A-28:G>A	TGCAGCATCTCGGCGGTGTTCAGCCTGTGGCCGCCG	28:G>A
4005686|F|0-17:C>G-17:C>G	TGCAGGCACGCCCTGGCCACGCCCATGACGGTGGCCACGCTCCCCG	17:C>G
5360556|F|0-16:G>T-16:G>T	TGCAGACCTTCGGCGAGCCACTGGCGGAGGGTGGCGCGGTCGAGGCGGATCTCGACGGCGGCGCGGTCC	16:G>T
1027219|F|0-14:G>C-14:G>C	TGCAGCACCTGCGCGCTGACGGAGTTGAAGAGGATCTCCACGAAGCCGTTGATGACGGCGGGCTTGGCC	14:G>C
1027219|F|0-56:G>A-56:G>A	TGCAGCACCTGCGCGCTGACGGAGTTGAAGAGGATCTCCACGAAGCCGTTGATGACGGCGGGCTTGGCC	56:G>A
32025821|F|0-13:G>A-13:G>A	TGCAGCCGCTGGCGCCAGCGCAGGACCGACCCG	13:G>A
4993734|F|0-12:A>T-12:A>T	TGCAGAGACTCCACCGCGCGGGCCACCTCCG	12:A>T
2267244|F|0-7:C>A-7:C>A	TGCAGGACGGGCTCTTCGACCTCACCGAGGACGGCGGCGGCGGCGTGGTGATGGACACCG	7:C>A
2259386|F|0-6:C>A-6:C>A	TGCAGCCCGCCGTACCTGCCCTGGCCGACGCACAGCTCCTCGTTGCCGCGCACGTCGAGCCGCCTGCCG	6:C>A
1051808|F|0-47:C>G-47:C>G	TGCAGGAACGGCACCATCCTGCCCAAGACCATGGACGAGGCGCAGTCCAGGTCGGCGTTCCAGATGGAG	47:C>G
3027026|F|0-37:G>C-37:G>C	TGCAGATGCTCCCCGTCATGCCGCTCGACGCCGACGTGTTCCG	37:G>C
3959001|F|0-8:C>T-8:C>T	TGCAGCCACAGTAGATGGCCTGCAATCCAGCACACTGCCG	8:C>T
1021231|F|0-29:C>T-29:C>T	TGCAGGCCATCGCGTACGCCTGCTCCTCGCCGCAGCACCGCGGCCACCCCG	29:C>T
981402|F|0-64:C>T-64:C>T	TGCAGCAGCAGCACGATGACCACCACCGACGCCACGAAGGAGGTGGAGTTGCAGTAGAAGAAGGCCCGA	64:C>T
3953003|F|0-23:C>T-23:C>T	TGCAGGAGCCTGGCGCACGCGTGCGAGTCGACGCCG	23:C>T
2279165|F|0-39:G>T-39:G>T	TGCAGCACGTACTTCATGGTCGGCCGCGACGACGGAGACGCGCTGGTGCACATCACGCCTAGCCTGAAC	39:G>T
1667842|F|0-54:C>G-54:C>G	TGCAGCAGCCACTGGTGATCCACGTCGACGAGGAAGTCCTTGTGGTAGTGCTCCCTCACCTTCTCCCTC	54:C>G
981818|F|0-42:C>T-42:C>T	TGCAGGTAGACGCCGATGGCCGTGAACTTGATGAAGTTGCCCCCGATCTCCATCCCCCGCACGCCTGCA	42:C>T
3940093|F|0-13:G>A-13:G>A	TGCAGACCCTGCGGACCTCTCCCTGTGTGCCG	13:G>A
1011518|F|0-11:C>G-11:C>G	TGCAGATGGTGCCGTGGACGGCGCGGCAGTGGTAGCACACGACGTGGCCG	11:C>G
982414|F|0-11:C>T-11:C>T	TGCAGCAGAGGCCGTTGCTTAAGCCACCAGCACATACACCGCGTAGATGATCCCCG	11:C>T
997705|F|0-57:C>T-57:C>T	TGCAGCAGGCGGCGGCGATGCACGGGCTCATGCTGCCTTGCGATGCGCAGAGCAGGGCGGTCGGCTCCG	57:C>T
100564955|F|0-23:G>C-23:G>C	TGCAGCCTCGTGAGCATCGGCTCGGTGACGTGGAAGGAGTAACACGCCATGTCGACGCCG	23:G>C
1020063|F|0-41:C>T-41:C>T	TGCAGGAGCTCCCTGAGGATCTCCAGGTCGCCGCCCCTCGCCGCGGCGTGCATTGCCCG	41:C>T
1064061|F|0-54:T>A-54:T>A	TGCAGGAACGCCATCCTCCTCATCCGCCGCAGCTGCTCCGTGGTGGTGGCGTCGTGCGCCTCGCTCTGC	54:T>A
1295159|F|0-17:C>T-17:C>T	TGCAGCTACAAGCGCTTCTTCGCGTTCGGTGACTCCCTCATCGACACCG	17:C>T
3947544|F|0-33:C>G-33:C>G	TGCAGCGGTTCCTCGAGAAGCGCAAGGCGAGGGCCAGCACCGCGTCGCCGTACGTCGTCCACCG	33:C>G
4440348|F|0-11:G>C-11:G>C	TGCAGGTACGGGCGGCTGTTCCG	11:G>C
1667015|F|0-13:G>T-13:G>T	TGCAGGCGAGGTGGAGGAGCCTGTTGGACATCTCCTGGTCGAGGATCCTGGCGGCGTCGGAGCCGTTGC	13:G>T
1106013|F|0-64:G>A-64:G>A	TGCAGAAGACGTTGTCCTGGGTCCTGAGCACCGCCTCGGCGAGCGAGGCCGAGGAGACCACGATGGCCC	64:G>A
4261086|F|0-10:C>A-10:C>A	TGCAGAGCCTCGCCGACCTGGCGTGGCGCACGGGGCCG	10:C>A
3960371|F|0-14:G>C-14:G>C	TGCAGATCCAAGGTGAGCAAGCCAGGTTCGTGAACGTCCTCACCG	14:G>C
993679|F|0-20:C>T-20:C>T	TGCAGGCGCTGGCGCCGCCTCCTCTCCGACCCCGCCTTCCGCCGCCGCTTCCGCATTCACCACCGCCGC	20:C>T
3957016|F|0-9:A>G-9:A>G	TGCAGATCAATAGGTCACTGGTTCGAACCCG	9:A>G
3957016|F|0-12:G>T-12:G>T	TGCAGATCAATAGGTCACTGGTTCGAACCCG	12:G>T

1000275|F|0-11:C>T-11:C>T	TGCAGGACTTCCGTCGCTGGCTCTCTTCCCAGGGAGCGGAGACGGGCTCGGTGGCTCCG	11:C>T
1141055|F|0-8:C>G-8:C>G	TGCAGTACCAAGGGATCTCGCAGAACGAAAAGGCCGCCATCCACCTCGGCAACACCAAGTCCTGCATCG	8:C>G
1863505|F|0-9:C>A-9:C>A	TGCAGGCACCGCGCCAGCACGCGCTTGGCCTCCTCCTGGCGGTGCTCCG	9:C>A
3938308|F|0-59:C>G-59:C>G	TGCAGGAGTTCTTCGCCATGATCGCCAAGGACTCGGCACGGGCTTGCTACGGGCCCAAGCACGTCGAGG	59:C>G
3025822|F|0-29:C>A-29:C>A	TGCAGGAAGTCGCCGACGTTCACCACCAGCGCCCCCGCCACCG	29:C>A
1022719|F|0-43:G>C-43:G>C	TGCAGGGCACCGTCACGCCGTCGCGGGAGCGCGAGCTCGCCGCGTCCAAGCCG	43:G>C
5371959|F|0-50:G>A-50:G>A	TGCAGCTTGCCAGCAGGGTTGGTCGGGGCTGGCTGGGGAGTCGGCGGGGCGGTCTCGAGGATGGTGATC	50:G>A
1092011|F|0-10:T>C-10:T>C	TGCAGGTGGGTTCCATCAGACGGCCGCAGTTGACCTTCCAGGCCCCGCCTCCGCGGCCCGCCG	10:T>C
5971223|F|0-20:T>C-20:T>C	TGCAGCATGCCGAGCTCTGGTGGGATCGGCCCG	20:T>C
5582759|F|0-34:A>G-34:A>G	TGCAGTCGCGCGCCCTCCGCGGCGGGAGCGACACAAAATGGCGACCCG	34:A>G
13026141|F|0-11:C>G-11:C>G	TGCAGCACGCGCAGGAGCACGGCCTCCTCGGCGCCG	11:C>G
3024405|F|0-25:A>G-25:A>G	TGCAGATCGCCACCACCTCCTCCGCAACCAAGAGAAAGCGCCG	25:A>G
5338360|F|0-11:G>A-11:G>A	TGCAGAGGCTCGTCTCGGTGGAGAATCCG	11:G>A
5412004|F|0-10:G>C-10:G>C	TGCAGCTGCTGAACCTCATGTGCAACCG	10:G>C
4993450|F|0-17:T>C-17:T>C	TGCAGCCATGCGACCTGTTCGGCGTACTTGTCCG	17:T>C
7343987|F|0-15:C>A-15:C>A	TGCAGGAACTGCGTGCTGCCCG	15:C>A
11914853|F|0-20:G>T-20:G>T	TGCAGTTCCTCCCCCCGCCCGTCTTCAACCTCACGCAGCTCGTCGACAGCTTCAAGGCCAAGAACATGA	20:G>T
2251731|F|0-47:A>C-47:A>C	TGCAGGAGGAGCAGGATGGTGGGCACGCTGTTCCCCCGCGCGTCGTGAGCGCACTGCATCGACTCCGTC	47:A>C
7165606|F|0-20:A>C-20:A>C	TGCAGCTTGGTGATGATGTCAACCTCCCG	20:A>C
7167942|F|0-17:G>T-17:G>T	TGCAGCTGCATCAAATGGATCCCCACCG	17:G>T
7172600|F|0-34:T>G-34:T>G	TGCAGCTCCAGCAGGTTGAGCCGCGGGAGGTAGATGAAGCCG	34:T>G
6044158|F|0-17:A>G-17:A>G	TGCAGGGGTAGCGCCACAGTTGAATGGTCTCCGCTCTACGACCCG	17:A>G
5411832|F|0-10:G>C-10:G>C	TGCAGGAAGTGGCCGACGTTCACCACCAGCGACGCCTCCCCCATCGCCG	10:G>C
4910546|F|0-37:A>C-37:A>C	TGCAGGGCAGCGTCAGGCTCCAGCTCATCTGCCTCACAGCCGTCCTCCTCGGCGCCG	37:A>C
7350989|F|0-16:A>G-16:A>G	TGCAGTGTGCCTCGACAGGTGCCCGCCG	16:A>G
7174041|F|0-21:G>A-21:G>A	TGCAGCAGGAGAAGCCCTTCCGCCG	21:G>A
12770509|F|0-20:A>G-20:A>G	TGCAGCGGCGTCGTGGTCTCACAGGGAGGGCCGAAGTAGTTGCCGTTGAGCGCGCCG	20:A>G
7170677|F|0-32:G>C-32:G>C	TGCAGCGGGTCATAGCCGCCGATGACGAGCAGGACCG	32:G>C
7916389|F|0-13:A>G-13:A>G	TGCAGTACCTGAAACTGGTGATCAAGGAGGCGCTCCG	13:A>G
5581904|F|0-40:T>C-40:T>C	TGCAGAACCTGGTGTTCCTGGACCTGTACTCGAACAGGTTTACCG	40:T>C
100424851|F|0-57:G>T-57:G>T	TGCAGCAACTTCTCCAGAGCGGAGACCACAGCAGCATGGCTCTGGTCGCCCACAATCGCCGGGATCGAC	57:G>T
22841129|F|0-8:G>C-8:G>C	TGCAGCGTGAGGAACTCGGCCG	8:G>C
4002650|F|0-31:A>T-31:A>T	TGCAGGAGGAGCAGGAAGAGACGGCAGCGGCAGCCGCGCCG	31:A>T
14822700|F|0-11:G>C-11:G>C	TGCAGCTCCGCGACGGTATCCGCCG	11:G>C
100353248|F|0-18:A>G-18:A>G	TGCAGTCGTCGATGCACAACTGCCG	18:A>G
100429637|F|0-52:A>G-52:A>G	TGCAGGTCAGCAGCGCCACGTCCGCCGCCGTGTCGATCATGTTGTCCATGAAAAAGACGTAGGCGGTCA	52:A>G
7908899|F|0-12:G>C-12:G>C	TGCAGCTCCTTGGCCTCGTCTCCGATCTCCG	12:G>C
100441567|F|0-8:A>G-8:A>G	TGCAGGATATTCTCCGCCAGCCCGATGCCG	8:A>G
7914904|F|0-10:T>G-10:T>G	TGCAGCATCCTCTCCACGGCGTCGATGCCGTACACCTCGAGGTAGCTGAGGATGCAGTAGAACCCGAGA	10:T>G
6047576|F|0-19:A>C-19:A>C	TGCAGCGTCACCGCCGCCGACTCGTGCTTCGGGCTCTCCG	19:A>C
100560345|F|0-17:G>C-17:G>C	TGCAGCTCCGACTTGCTGCGTGTGGCGCGCACGAACCCCG	17:G>C
100583347|F|0-38:G>C-38:G>C	TGCAGGTCGAGCGCCTCGCCGCGGAGCTTGTCGGGGAGGGCGTCCACGAGCGCGATCTCGTCCGCCCGA	38:G>C
14583286|F|0-17:A>G-17:A>G	TGCAGAGGCATGATTCCATGCTCGCCACCCG	17:A>G
4262455|F|0-17:A>T-17:A>T	TGCAGTTCCTCACGGGAAATTTACCG	17:A>T
3952057|F|0-12:C>G-12:C>G	TGCAGAGCGAGCCCTGGCGCGAGATGGCCCCACCG	12:C>G
3385081|F|0-17:G>A-17:G>A	TGCAGCGCCGTCACGGAGGCGAACACCACCATCATGCCCG	17:G>A
100351223|F|0-31:A>G-31:A>G	TGCAGCGCTGGAAGGAGGAGGAGCAGCGGTCACAGGACGGCCG	31:A>G
100352768|F|0-23:A>G-23:A>G	TGCAGGTCCTTCTCCTCCAGGAAAAGCGAGAAGCCG	23:A>G
1021455|F|0-5:G>A-5:G>A	TGCAGGTCGTAGAGCTGGATCGCCGTCTGCACGGACAGCCGCACGCGCCG	5:G>A
7349756|F|0-8:C>T-8:C>T	TGCAGCTGCTCCATCTTCACCG	8:C>T
100361659|F|0-13:T>C-13:T>C	TGCAGAGGCGGTGTGGTCCG	13:T>C
1074290|F|0-28:G>A-28:G>A	TGCAGCCGACGGTGAACGCGAGCACGGAGCACGTGATGAAGTACCTGACGGGGTCGGCCAAGACGTACG	28:G>A
5411854|F|0-22:C>A-22:C>A	TGCAGAGCCTGAGCTCCAGCGGCGGATCCCGTCCG	22:C>A
3026611|F|0-37:C>G-37:C>G	TGCAGCGCGCGCTCCACCTGCGCCATGTCCACCTCCTCCCCG	37:C>G
5580347|F|0-29:G>C-29:G>C	TGCAGCTTGGTGACGGAGACGATCCAGAAGGTGTCGCCG	29:G>C
1052520|F|0-22:G>T-22:G>T	TGCAGGACAAGAAGCACATCGTGGGGATGACGGGCGACGGCGTGAACGACGCGCCG	22:G>T
100388407|F|0-31:C>G-31:C>G	TGCAGGGCATCGTGTCGGAGGCCAGGGCGGTCTTCCG	31:C>G
4262403|F|0-21:G>T-21:G>T	TGCAGGTCTGTCGCCAATGCTGGGGAAGCGCCCG	21:G>T
2249414|F|0-65:C>T-65:C>T	TGCAGCACCACCACGTGGAACAGCTCGCTGTTCATCGACCTCAGCCCGTCCACCACCACGTCCCTCGAG	65:C>T

1041969|F|0-20:G>A-20:G>A	TGCAGCCTCATCTCCTCCCTGAACTTCCTGATGAACATCTCCGCCCTCACGTCCACCTCCCCCATCCCA	20:G>A
2246877|F|0-38:G>A-38:G>A	TGCAGGGCGCGGATCTTGTGCACGTAGGGCAGGAACAGGTCGTGGAAGTCGAGCATGAAGAGCCTCTTG	38:G>A
1050789|F|0-32:C>T-32:C>T	TGCAGGTACTGCATGGCGGCGCCCATGTCCTCCTCCATCAGCTTGGCCACCTGCTGCTCCGTCACCGTC	32:C>T
1050789|F|0-41:C>T-41:C>T	TGCAGGTACTGCATGGCGGCGCCCATGTCCTCCTCCATCAGCTTGGCCACCTGCTGCTCCGTCACCGTC	41:C>T
1059062|F|0-28:G>A-28:G>A	TGCAGTCGCTGCCACCAAACCCCGACGAGGACGCGCTCTTCGCGGAGTACTGCCGATTGGCGAGCCTCT	28:G>A
7167525|F|0-9:A>C-9:A>C	TGCAGATCAAGCTGGGTGTCCCGAGGCCG	9:A>C
991279|F|0-35:T>G-35:T>G	TGCAGATGCCCTGGGTCTCCACCCGCACCACGCCCTTCCTGAACGTGAAGAGCTCCG	35:T>G
1093065|F|0-19:G>T-19:G>T	TGCAGCGCAGCCTGACGCTGTCGGACGAGCAGGTGGAGGCGTCGCGGATGACGCTGCACCG	19:G>T
5332167|F|0-14:C>A-14:C>A	TGCAGCTTGCGCAGCTGGCCGAGCTGCGGCGGCACCG	14:C>A
4262144|F|0-19:C>T-19:C>T	TGCAGTACCTCCACGAGGACTCCCAGCTGAGGATCGTCCACCG	19:C>T
1039167|F|0-16:G>A-16:G>A	TGCAGAGCTCGCGGTCGCAGTTGCCGTTCTGCCACAGCGCGCACATCTTGGTCTTGTACGCCG	16:G>A
1043522|F|0-35:G>A-35:G>A	TGCAGCATCCTGTACAGGAGCAGAGCGGACATGCGGGCCTGGTCGCGCACCGCCTCCAGGTCGCGGTGC	35:G>A
1043522|F|0-50:C>T-50:C>T	TGCAGCATCCTGTACAGGAGCAGAGCGGACATGCGGGCCTGGTCGCGCACCGCCTCCAGGTCGCGGTGC	50:C>T
2252532|F|0-64:A>G-64:A>G	TGCAGCATGCTCAGCTTCCACCTCCACTCGTCGATGTTCGCCACCGAGGACGACGGCTGCGGTTAGCAT	64:A>G
986348|F|0-5:C>T-5:C>T	TGCAGCGTCTTGACGTACTTGATCGCGTCGCCCAGCACCGACGCCTTGTCCATCTTCTTGAGGCCCGAG	5:C>T
1051874|F|0-50:G>A-50:G>A	TGCAGCTTCTCGTCGCCGATGAGCGGGCGGGCATGGACCGCCACCTTGACGCAGCAATCCTCGCCGTGC	50:G>A
1055548|F|0-68:G>C-68:G>C	TGCAGGAGCTTGATGATGTTGCTGGCGCCGAAGACGCGGTGCGCGGTGGCGAACTTGTGGGGGTCCGTG	68:G>C
3028947|F|0-13:G>A-13:G>A	TGCAGAGGAAGACGTGCGGGACGTCCTCCGTGTCCGCGACCCCG	13:G>A
994451|F|0-19:G>T-19:G>T	TGCAGCAGCAGAAGGGCCTGCTGTCGTCGGACCAGGTGCTCTTCGCCGACCGCCGCTCCCG	19:G>T
989471|F|0-20:G>C-20:G>C	TGCAGCAGTCCTCGGGCCGCGCGAACGCCTCGCACGCGCTCTTGCACGCGATGCCG	20:G>C
6045490|F|0-13:G>C-13:G>C	TGCAGCCGACGAAGGAGAACCCCGTGTCCTCCAGCATGCTCTGCCG	13:G>C
3029131|F|0-23:C>T-23:C>T	TGCAGCGCCGCCTCCACGATGGCCTCCTCGCAGGCCG	23:C>T
5357855|F|0-5:C>A-5:C>A	TGCAGCGCGTGGCACGGCGGGTAGTGGTTCACCCG	5:C>A
3030373|F|0-22:G>A-22:G>A	TGCAGGCCGAGGTCGCGCGCCAGCCGCGCCTTGCGCTCCG	22:G>A
5324118|F|0-21:C>T-21:C>T	TGCAGGCCGCGCTGTCGGTTGCGCGCCCG	21:C>T
7166096|F|0-17:G>A-17:G>A	TGCAGTCCATGTCCGTGGGAGTCCCG	17:G>A
5373570|F|0-14:G>A-14:G>A	TGCAGGTGCGGCACGGCGCCCG	14:G>A
1075878|F|0-58:G>A-58:G>A	TGCAGAAGCCGCCGAACACGCCGCACGCCTCGCTCCACGTCACCTCCTTGTCGCCGTTGTACGCCAGGT	58:G>A
993148|F|0-57:G>A-57:G>A	TGCAGAGTACACAGCGGCAGTGGCACCCACACGGCCATTAGCCGAGACCCTCTGCTTGAGCTGCCTGTG	57:G>A
2251741|F|0-41:C>A-41:C>A	TGCAGCGAGGACTGGAAATTGGAGAGCAGGCGCGTGAGGTCCCGTGGCCGTATGGCGAGGCGGCTGGGG	41:C>A
2256197|F|0-40:T>C-40:T>C	TGCAGCGCGAACTCGAGGCACCAGAGCACGTCCCCCATGGTGGGCCGCTCCACCCCGTACTCGGCGAGG	40:T>C
2299791|F|0-61:C>T-61:C>T	TGCAGCGCGTCGGCGTCGAGCCGCAGTTCCGCAACGGCCTCCGCGTCACCGACGCGCTCACCATGGAGG	61:C>T
1092636|F|0-35:C>G-35:C>G	TGCAGAAGCATGGAGGTGAGCCCGACGATCTGTGCCCGCACGGCCCGCGGCGCCG	35:C>G
3023337|F|0-29:A>T-29:A>T	TGCAGAGCCTCATGGACTTCTACTACGCCAGCGTGCCG	29:A>T
7169366|F|0-20:C>T-20:C>T	TGCAGCATCTTCAGGCCACCCAACGCCGCCG	20:C>T
100461914|F|0-33:G>A-33:G>A	TGCAGCTCCTCCTCCTGCGGGTGCTCCCGCGGCGCCG	33:G>A
3020490|F|0-28:G>T-28:G>T	TGCAGCTTCTGTTCGAGAAGTGGCACCTGCTACTTGCCG	28:G>T
4005821|F|0-26:G>A-26:G>A	TGCAGCTTGATGATCTTCATGCTGTTGAGCACCTCGGACGTGGACCG	26:G>A
1141147|F|0-19:C>G-19:C>G	TGCAGGCGACGATCGCCGCCCGCAACCCCAACTCCCGCGTCGGCATCTACTTCGACCG	19:C>G
4262675|F|0-17:C>T-17:C>T	TGCAGGTTGATGACCCGCAGCTTGACGCAGGAGGCCAGGTCGGCCG	17:C>T
100463862|F|0-17:C>G-17:C>G	TGCAGTGTGCCTCGACGCGAGCGCGCCG	17:C>G
100463904|F|0-25:A>G-25:A>G	TGCAGTTCGCCAGCGCGGATGACCTACCGCTGCCG	25:A>G
7911378|F|0-7:G>A-7:G>A	TGCAGCCGCCGCGCAGGCCCG	7:G>A
100466641|F|0-7:G>A-7:G>A	TGCAGACGGAAGAGAAGATCAAAAGAGATCGGGCGATGCAGCACAAGATTGGAGGGCAACAACAAGCGC	7:G>A
4262646|F|0-34:C>T-34:C>T	TGCAGACGGCGGCGGCCTGGCGCTCGGAGTAGCTCCCCTTGGCGGTGATGCGGTCGAAGAGCTCCCCGC	34:C>T
990006|F|0-37:C>G-37:C>G	TGCAGCCGCCGATGCGGCAGCTCTCTGCGGCGTCATCCACCAACCTCGCGCCCGACTACGCCG	37:C>G
1007223|F|0-12:C>T-12:C>T	TGCAGCGCGCGCCTCACGTCGGCGAAGAGGTCCGCGCAGCCGTCGCCGCAGCTGAGCGTCGCCTTGACG	12:C>T
2281139|F|0-14:C>T-14:C>T	TGCAGGATCCTCGACCTCCTCTCCTTGGGGTGCCGGGAGAACTGGTCCGCGCCGAAGGCCATGGAGCAC	14:C>T
1025480|F|0-46:C>T-46:C>T	TGCAGGCGTCCTTCACGGGCGCGCTCCACTCCCGCGTGGACGGCGGCGGGAGCGTGCTGGTGGCGCTGT	46:C>T
1252133|F|0-62:G>A-62:G>A	TGCAGGCTGAGGTGGACCAACTACCTGAGGCCCGACCTCAAGCGGGGGCTCCTCTCCGACGAGGAGCAG	62:G>A
1037030|F|0-29:G>A-29:G>A	TGCAGGTGGCTCTTGACCTCATCATTCGTGAGCCCATCCACCTTCATCATCTCCCTGATTTGCTTAGGA	29:G>A
2271923|F|0-10:C>A-10:C>A	TGCAGGTTGGCGTAGTAGGCGTGGTCGAACTGGAACGGCGTGCTCACGTCGAACGGCACCACCGCGCGG	10:C>A
986877|F|0-19:C>T-19:C>T	TGCAGACCAGCCCCAAGTGCCTGTGCGTGCTCGTCAAGGACCGCGACGACCCCG	19:C>T
993076|F|0-43:C>T-43:C>T	TGCAGCACAACTACTTCACCAACCCGTGGGCCATCGTCTCCGTCGTCGCCG	43:C>T
100478939|F|0-28:C>G-28:C>G	TGCAGCACTACGCCGCGGCGTTCGACGCCGTGGGCGCGGCCG	28:C>G
100479082|F|0-20:A>C-20:A>C	TGCAGCAGCTGGTCGGCGAGAGCGTAGTCCTCCTTCCCCG	20:A>C
1143830|F|0-39:G>A-39:G>A	TGCAGCCGCGACGGCGACGAGCACGACTTCTTCCGCGCCGCGCAGGCCG	39:G>A
5411793|F|0-17:C>A-17:C>A	TGCAGCGACAAGAGTTTCCCGAGCGACGCCG	17:C>A
4002562|F|0-39:A>G-39:A>G	TGCAGCGCCAGCTCGTCTCCGCCGCCTGCGCCGAGGACAAGTCCG	39:A>G

<table>
<tr><td>3945381|F|0-5:C>A-5:C>A</td><td>TGCAGCGGTGCGGCAAGAGCTGCCGCCTCCG</td><td>5:C>A</td></tr>
<tr><td>4261907|F|0-19:C>T-19:C>T</td><td>TGCAGGAGACGTGCACCGACGCGTCCG</td><td>19:C>T</td></tr>
<tr><td>6045666|F|0-28:C>A-28:C>A</td><td>TGCAGGAGGTGGAGGTCGGGGGACTTGGCGCGGAGCGCGCCG</td><td>28:C>A</td></tr>
<tr><td>7169705|F|0-26:G>T-26:G>T</td><td>TGCAGGCTGCTGTTCGGGTACGCGATGCCG</td><td>26:G>T</td></tr>
<tr><td>4262404|F|0-23:A>G-23:A>G</td><td>TGCAGGGGTAGCGCCACGGTCGAATGGTCTCCGCTCTACGACCCG</td><td>23:A>G</td></tr>
<tr><td>4262170|F|0-15:G>C-15:G>C</td><td>TGCAGGTCGATGCGCGCGTAGAGGTACGTGTCCCTGGACCG</td><td>15:G>C</td></tr>
<tr><td>1062296|F|0-46:G>T-46:G>T</td><td>TGCAGAGGAGCGGGTGGGGCGGGTACGAGGTGAGGCACGACGCCGAGGGGCACTGCTTCTTCCTCTGCT</td><td>46:G>T</td></tr>
<tr><td>100486326|F|0-20:T>C-20:T>C</td><td>TGCAGCCGCAGGATCATGGCTAGCGCCTCGTCGGCCCCCGACGACGCCGCCTGCCTCTCCTCCTCCAGC</td><td>20:T>C</td></tr>
<tr><td>100486327|F|0-32:T>G-32:T>G</td><td>TGCAGCCGCAGGATCATGGCCAGCGCCTCGTCTGCCCCCGACGACGCCGCCTGCCTCTCCTCCTCCAGC</td><td>32:T>G</td></tr>
<tr><td>1218352|F|0-20:G>T-20:G>T</td><td>TGCAGCGCGCTGATGACGGTGCGGAGCGCGCGGCTCGGGGACCCGCGGTTGGCGAGCATGATCTCGCCG</td><td>20:G>T</td></tr>
<tr><td>2259595|F|0-11:G>T-11:G>T</td><td>TGCAGCGGCGCGTTCGCCAACCCCAACACCTGCCGCCCCACCGCCTACTCGCAGGCGTTCAAGCTGGCG</td><td>11:G>T</td></tr>
<tr><td>2259595|F|0-35:C>G-35:C>G</td><td>TGCAGCGGCGCGTTCGCCAACCCCAACACCTGCCGCCCCACCGCCTACTCGCAGGCGTTCAAGCTGGCG</td><td>35:C>G</td></tr>
<tr><td>2254188|F|0-62:C>G-62:C>G</td><td>TGCAGCTGCTCCGCCGTGGACATGATGAACGGCACGTACCGCTCGAGCGCCGCCTCCGTGTGCTCGGCG</td><td>62:C>G</td></tr>
<tr><td>2247634|F|0-9:A>G-9:A>G</td><td>TGCAGGAACAGCATGGGCTCCGCCGACGGGCTCACCGCCTGCCGCAGCATCGACCCCATGATCAGCGAC</td><td>9:A>G</td></tr>
<tr><td>2247634|F|0-20:C>G-20:C>G</td><td>TGCAGGAACAGCATGGGCTCCGCCGACGGGCTCACCGCCTGCCGCAGCATCGACCCCATGATCAGCGAC</td><td>20:C>G</td></tr>
<tr><td>1002433|F|0-43:G>A-43:G>A</td><td>TGCAGGAGACGATGTGGCGGAGGAGGTGGCGGCCGACGCCGCCGCAGCCGAGGAGCACCACCG</td><td>43:G>A</td></tr>
<tr><td>2255388|F|0-44:G>A-44:G>A</td><td>TGCAGGCCCAGGGCACGGCGGAGCCCGCCTGCGATCTCGTTGACGAAATCAGTGGGCGGCATGACGGCG</td><td>44:G>A</td></tr>
<tr><td>2279291|F|0-11:G>A-11:G>A</td><td>TGCAGGCTGGTGACGCGCGGCGGCTCGGCGAACTCGTAGTCGTACCTGAAGTAGTCGAGCACCATGTCC</td><td>11:G>A</td></tr>
<tr><td>2250961|F|0-10:C>T-10:C>T</td><td>TGCAGGGCCACCCCAACGTGATCGGCCTCCATGGCCTCATCACCTCCCGCTCCTCCGCCTGCATCTACC</td><td>10:C>T</td></tr>
<tr><td>2250961|F|0-22:C>T-22:C>T</td><td>TGCAGGGCCACCCCAACGTGATCGGCCTCCATGGCCTCATCACCTCCCGCTCCTCCGCCTGCATCTACC</td><td>22:C>T</td></tr>
<tr><td>1040926|F|0-11:G>A-11:G>A</td><td>TGCAGGTGCTCGGCCGCGATGGCCGCTGGGCCGACGTCCAGCCCGTGGAGAACGCCATCGTCATCAACA</td><td>11:G>A</td></tr>
<tr><td>1040926|F|0-13:C>T-13:C>T</td><td>TGCAGGTGCTCGGCCGCGATGGCCGCTGGGCCGACGTCCAGCCCGTGGAGAACGCCATCGTCATCAACA</td><td>13:C>T</td></tr>
<tr><td>1040926|F|0-23:C>A-23:C>A</td><td>TGCAGGTGCTCGGCCGCGATGGCCGCTGGGCCGACGTCCAGCCCGTGGAGAACGCCATCGTCATCAACA</td><td>23:C>A</td></tr>
<tr><td>2283814|F|0-15:G>A-15:G>A</td><td>TGCAGGTGCTTGACGGCGACCGCCTCCTCGCCGTCGATGGCGATGGCCATCTCCATGTCGTCCTCGCCG</td><td>15:G>A</td></tr>
<tr><td>1075340|F|0-9:T>C-9:T>C</td><td>TGCAGTGGGTGGTGACCGCCTTCTCCGTGGCGTCGCTGCCCAACACCATCATCATGGGCGTGCCGCTCC</td><td>9:T>C</td></tr>
<tr><td>3952729|F|0-5:A>C-5:A>C</td><td>TGCAGACGCCGTCCCCGACGGCGCAGGACCCGACCATGGACCCCG</td><td>5:A>C</td></tr>
<tr><td>1028793|F|0-38:G>C-38:G>C</td><td>TGCAGCCGCTGGACGCGGGTGTCGGCGGCGCGTCGGCGGCGCCGCCAATCCG</td><td>38:G>C</td></tr>
<tr><td>7171531|F|0-23:G>C-23:G>C</td><td>TGCAGCCTCTACCTCGTCTTCGAGTACATGGAGCACGACCTCGCCG</td><td>23:G>C</td></tr>
<tr><td>4262820|F|0-10:C>T-10:C>T</td><td>TGCAGGAGGTCGCCGAGCTGGCGTCCCCGTTCGGGTCGTCCCCG</td><td>10:C>T</td></tr>
<tr><td>1020517|F|0-28:C>G-28:C>G</td><td>TGCAGGCCAGCATCATCTCGCCCTAGCTCCATGGCTAGCGGGCATTTGACATGGATTCCG</td><td>28:C>G</td></tr>
<tr><td>6048233|F|0-29:G>C-29:G>C</td><td>TGCAGGGCGATGGGCGACCAGCTGGAGGGGGACGAGGGGCAGCACATGAAGTACCG</td><td>29:G>C</td></tr>
<tr><td>1026423|F|0-10:T>G-10:T>G</td><td>TGCAGAAGGCTACCAACAACTTCACCACGATTCTGGGGCAAGGGTCGTTTGGTCCTGTGTACAAGGCTG</td><td>10:T>G</td></tr>
<tr><td>1026423|F|0-67:T>C-67:T>C</td><td>TGCAGAAGGCTACCAACAACTTCACCACGATTCTGGGGCAAGGGTCGTTTGGTCCTGTGTACAAGGCTG</td><td>67:T>C</td></tr>
<tr><td>100500228|F|0-68:C>G-68:C>G</td><td>TGCAGCACGACGGCCAGCGCCACCTTGGCCTCGATCAGCGCCAGGTTCTGCCCGATGCACACCCGCTCC</td><td>68:C>G</td></tr>
<tr><td>1078072|F|0-31:G>A-31:G>A</td><td>TGCAGCAGGTGAAGCTCGCCGTGGTGCACCTGTACCGACGGTACGTCTTCCGCCACTCGCCG</td><td>31:G>A</td></tr>
<tr><td>1164942|F|0-60:C>G-60:C>G</td><td>TGCAGCTCCTTGAAGTCGAGCCACGGCTTGACCCAGACCTTGGCCTCGTACACCTTCTTCCGCCCGAGA</td><td>60:C>G</td></tr>
<tr><td>1283131|F|0-37:C>T-37:C>T</td><td>TGCAGCTCGACATCGTCAGCCCACCACTGCTCCAGCCCGTGCGCGGCCAGGAACTTGCTCGGCCCCTTG</td><td>37:C>T</td></tr>
<tr><td>1863313|F|0-12:G>A-12:G>A</td><td>TGCAGGACACGAGCATCCAGGGCGCCGAGAGCCACCCGCTGCACCTGCACGGGTTCGACTTCTTTGTCG</td><td>12:G>A</td></tr>
<tr><td>1214567|F|0-31:C>T-31:C>T</td><td>TGCAGGAGAGCTGCACGGACACGTCGGGGTGCTCCATGGTGGTTCACTCGCCCATCGACATCCCGAGAT</td><td>31:C>T</td></tr>
<tr><td>1694959|F|0-26:C>T-26:C>T</td><td>TGCAGGAGGATTAGCCACAGGACGCACCACCAGAACCACGGCCACGTCGAGAATGACGAGTCCTGGCAT</td><td>26:C>T</td></tr>
<tr><td>991525|F|0-9:G>A-9:G>A</td><td>TGCAGGAGGGCGACGGCCTGGACTTCTCCGACGTGAGCAGCCTCAACATGGTGACAGAGCTCACGCCGC</td><td>9:G>A</td></tr>
<tr><td>7865065|F|0-42:A>G-42:A>G</td><td>TGCAGGGGCTCGTGAGGAAGGGCCTGTGGGAGGACGCCATCGACTACCTCGATGGCTTCCTGCCTGCCG</td><td>42:A>G</td></tr>
<tr><td>1231121|F|0-43:C>T-43:C>T</td><td>TGCAGGTGGCTTCTAGGAACATTGGCGGCTGCTCGCCCAATTCCGACGGCCGCGCCGTGGACGCCGAGA</td><td>43:C>T</td></tr>
<tr><td>1064108|F|0-52:G>A-52:G>A</td><td>TGCAGTGCTTGGCGTAGATGAGGAAGAGGAAGAGCCCCAGGAGGACGCCCGTGAGCACGCCCACGATGA</td><td>52:G>A</td></tr>
<tr><td>1017783|F|0-58:G>A-58:G>A</td><td>TGCAGTTGGTCTGCTGCTCCGCGAAGAAGGGCCAGCTGAGCATCGGCACGCCGTTGGAGATGCTCTCCA</td><td>58:G>A</td></tr>
<tr><td>996110|F|0-8:G>T-8:G>T</td><td>TGCAGACGGCGCGCTGGACGTCGCGGAGGATGGACTCCTTGAGGCTCTCCG</td><td>8:G>T</td></tr>
<tr><td>1862697|F|0-37:C>T-37:C>T</td><td>TGCAGCAAGCGCTCTACTGGCACATCGAGGCGCGCGTCGTGCTGGATCTCCG</td><td>37:C>T</td></tr>
<tr><td>100508396|F|0-15:T>A-15:T>A</td><td>TGCAGCCAGGTGCACTTGGCCATGTACATCTCGCTGGCCGCCGCCG</td><td>15:T>A</td></tr>
<tr><td>3027178|F|0-18:G>A-18:G>A</td><td>TGCAGCGACGTGCTCGTCGACGGCCTCTTCATCAGGTCCCCG</td><td>18:G>A</td></tr>
<tr><td>100509089|F|0-16:A>G-16:A>G</td><td>TGCAGCTCCTGCTCCAACTGCGCCAGCTTTATCCG</td><td>16:A>G</td></tr>
<tr><td>100509420|F|0-7:T>C-7:T>C</td><td>TGCAGGATCGTGACGACGGCGGCGGCGATGTAGTCCGCCTCCG</td><td>7:T>C</td></tr>
<tr><td>1024350|F|0-7:G>A-7:G>A</td><td>TGCAGGCGCAGTTCGGCAACAAGTGGGCGAGGATCGCCACCTACCTGCCG</td><td>7:G>A</td></tr>
<tr><td>1024350|F|0-31:G>A-31:G>A</td><td>TGCAGGCGCAGTTCGGCAACAAGTGGGCGAGGATCGCCACCTACCTGCCG</td><td>31:G>A</td></tr>
<tr><td>1230214|F|0-5:G>A-5:G>A</td><td>TGCAGGCCCTGCACGCTGCCGTTGCCGTGGCAGGAGACGCCGTACCAGGAGGAGTGGTTGCCGTTGCAC</td><td>5:G>A</td></tr>
<tr><td>1230214|F|0-32:G>A-32:G>A</td><td>TGCAGGCCCTGCACGCTGCCGTTGCCGTGGCAGGAGACGCCGTACCAGGAGGAGTGGTTGCCGTTGCAC</td><td>32:G>A</td></tr>
<tr><td>1031533|F|0-14:G>T-14:G>T</td><td>TGCAGGTTCTCGTTGCCCACCGAGATGATGAACTCCACGCCCGTGTTGGCGAACGCCGTCAGCACCTTG</td><td>14:G>T</td></tr>
<tr><td>1029079|F|0-46:C>T-46:C>T</td><td>TGCAGGCCGTCCTGGCGCGCGCCCACGGCGAGATGCAGGACTCGTTCGTCTGGCTCTTCCAGCACATCT</td><td>46:C>T</td></tr>
<tr><td>1085795|F|0-5:C>G-5:C>G</td><td>TGCAGCACCACCTCCACCGTCGTGTTGAAGCGCAGCCTCTTCACCCTGGTGCCCTTGGACGTCAGCATC</td><td>5:C>G</td></tr>
<tr><td>1095342|F|0-7:C>T-7:C>T</td><td>TGCAGCGCGTCGGCGTCGGCCTCGTGCTCTCCGTCGTCTCCATGTGCATCGCCGCCGTCGTGGAGATGC</td><td>7:C>T</td></tr>
</table>

100514321|F|0-55:A>G-55:A>G	TGCAGCTAGCGTCCGCCACGGCCTGCGTCACGCTGTCGGCGGTGCGGCTCGTCAGACAGGACTTCGGCG	55:A>G
1019894|F|0-14:A>T-14:A>T	TGCAGCTCGGAGAGAGGGGAAACGGGGAGGAGGAGGCGGACGAGACGGATGGGGAATTAAGGAGAGAAT	14:A>T
1156192|F|0-5:G>A-5:G>A	TGCAGGCACACCACCGTGGCGGCGCCGCCCATGAACCCGACGATGGCGGCCTGCGACAGAAAGTCCACC	5:G>A
1202071|F|0-23:C>G-23:C>G	TGCAGGCAGATGGGCTGAGGCAGCAGACCCTGCACAAGCTGCACGGCATGCTGACGGTCCGACAGACCG	23:C>G
1028367|F|0-53:C>A-53:C>A	TGCAGGCCGTCGCGCCAGTGGCCGTCGTAGACGTCGCCGTTGGCGTAGGCCTGCGTGCCGCGGCCGTGC	53:C>A
100516267|F|0-8:T>G-8:T>G	TGCAGGTCTCGCCCGTACGGCGGGAAGTTGCTCTTCAGCACCGTGCCGATCACGTTGTTGTTGCCCGTG	8:T>G
1092142|F|0-22:G>A-22:G>A	TGCAGGTGGCGCCGCGGGGCTCGCACGCGCTGGTGGTGTCCACGGAGACCATCACGCCCAACTACTACG	22:G>A
1130166|F|0-20:G>T-20:G>T	TGCAGTCGCTGGCGACGGCCGCGGACGCGCTCACCGCGGCGCGCATCCTGGTGCAGGCCAGCCAGGGCG	20:G>T
1130166|F|0-28:G>C-28:G>C	TGCAGTCGCTGGCGACGGCCGCGGACGCGCTCACCGCGGCGCGCATCCTGGTGCAGGCCAGCCAGGGCG	28:G>C
3024682|F|0-29:C>T-29:C>T	TGCAGAACAAGAACACCCAGATGATCAACCTGCGGATCAACGCACCG	29:C>T
100520200|F|0-11:T>C-11:T>C	TGCAGCATCGCTGCGTCCCTGGGCGCCACGGGCTGCGCCGCCAGGTCGCCG	11:T>C
984861|F|0-5:C>A-5:C>A	TGCAGCTTAGGAATCAGTTCCTCCTGCTCCAGGACGCAGGGCAGCACGGTCCG	5:C>A
984861|F|0-44:C>T-44:C>T	TGCAGCTTAGGAATCAGTTCCTCCTGCTCCAGGACGCAGGGCAGCACGGTCCG	44:C>T
1030427|F|0-30:A>C-30:A>C	TGCAGGACCAGATCACGTACAAGGTTGGCAAGCACTACGCCGACTGCCCCAGCTTCCG	30:A>C
5582039|F|0-13:C>T-13:C>T	TGCAGGCCACCTCCGACGACATGGCGGCGGACGGCCTCACCGCCCG	13:C>T
985874|F|0-11:A>T-11:A>T	TGCAGGGGAGCAGCGGGAGGGAGCTCGTGGCGGCGCTCATGGGGAACCCCG	11:A>T
985874|F|0-30:C>T-30:C>T	TGCAGGGGAGCAGCGGGAGGGAGCTCGTGGCGGCGCTCATGGGGAACCCCG	30:C>T
5580593|F|0-16:G>A-16:G>A	TGCAGTGGTCGACGTGGTGGTGCACCTCCCCG	16:G>A
1042145|F|0-30:C>A-30:C>A	TGCAGAGCTCGCCTACTTGAGGGTGACGACCAAGTGCGACGTGTACAGCTTCGGCGTGGTGGCCATGGA	30:C>A
100524046|F|0-8:A>C-8:A>C	TGCAGCAAAGCACTCACGTCTTTGTTGTCTTCGCTGCTCGGCGCCGCTCCGTACTCTGTAACGTCATAA	8:A>C
1077177|F|0-42:A>G-42:A>G	TGCAGCAACGCACTCACGTCTTTGTTGTCTTCGCTGCTCGGCACCGCTCCATACTCTGTAACGTCATAA	42:A>G
1024066|F|0-49:G>T-49:G>T	TGCAGCACTGCGACTGCACCGCGCGCATCGTCGCCTTGAACTCCTCGCTGCGGTACACGTTGGTGGTCG	49:G>T
1026371|F|0-60:C>T-60:C>T	TGCAGCAGCGCCGTGTTGCTGCGCCGCAGCCGCTCGTTCTCGTCCATCAGCACCGCCGCGCTGGACGCG	60:C>T
1028471|F|0-53:C>G-53:C>G	TGCAGCATGGACGGCACGACGAAAATTGCGGCCGCCGCGAGAAGCGAGACGGCCACCCACGGCGACGGG	53:C>G
2254058|F|0-29:C>T-29:C>T	TGCAGCCAGTAGAAGGCCCTCCCGACGTGCCTCGACAGGAAGCCCCAGCTCTTCAATCTCCAGCTCCAT	29:C>T
32025517|F|0-32:T>C-32:T>C	TGCAGCCAGTAGAAGGCCCTCCCGACGTGCCTTGACAGGAAGCCCCAGCTCTTGAATCTCCAGCTCCAT	32:T>C
100524820|F|0-11:A>G-11:A>G	TGCAGCCCATGAGCAGCTGCTACAACCCCAACACGATCAAGGCGCACGCCTCCTACGCCTTCAACGCCT	11:A>G
23889613|F|0-12:T>G-12:T>G	TGCAGCCCATGGTCAGCTGCTACAACCCCAACACGATCCAGGCGCACGCCTCCTACGCCTTCAACGCCT	12:T>G
100525277|F|0-16:C>G-16:C>G	TGCAGCGCAAGCGGCCCCCGAGGCTGACGGTGAGCAAGAACCGTCTGGACAAGGGCGCGGCGGAGCAGG	16:C>G
7166826|F|0-58:A>G-58:A>G	TGCAGCGCAAGCGGCCGCCGAGGCTGACGGTGAGCAAGAATCGTCTGGACAAGGGCGCAGCGGAGCAGG	58:A>G
1019996|F|0-6:C>T-6:C>T	TGCAGGCCGTGATCAAGGAGACGCTGCGGCTGCACTCCCCGATCCCGCTCCTCGTCCCCCACATGAACC	6:C>T
1140846|F|0-61:G>A-61:G>A	TGCAGGCGTCCATATGGAGCAAGGACGTGGAGTTCTCCTTCGGCGAGATCCTGGCGGCGACGGAGCACT	61:G>A
1075847|F|0-56:G>A-56:G>A	TGCAGGTAGTACTGGCGCAGCCCCTCGCCTCCGCCACGCGCGCCCTTGGCCGACTCGTCGCCG	56:G>A
2249233|F|0-50:G>A-50:G>A	TGCAGGTGTTCGACGCGATGATCAGGGAGGGACACCAGCCCGACGAGGCCGTGTACGTCGGCGTGCTGA	50:G>A
2249233|F|0-64:G>T-64:G>T	TGCAGGTGTTCGACGCGATGATCAGGGAGGGACACCAGCCCGACGAGGCCGTGTACGTCGGCGTGCTCA	64:G>T
1126720|F|0-19:G>A-19:G>A	TGCAGATCATCACGGCGCGGCCGCCCATGGGGCTGACGCACCACGTGGCGCGCGCGCTGGACCATGGCA	19:G>A
3025796|F|0-14:C>A-14:C>A	TGCAGAAGCTATCCCTTGATGCCAAGACTGAAGCCATCGACGCGCCG	14:C>A
3945168|F|0-5:C>T-5:C>T	TGCAGCCTGCCATGCCCGAAGCTGGCCTCCCG	5:C>T
7172136|F|0-12:T>C-12:T>C	TGCAGCGCGGGCTTGAGCCTGTCCAGGAGGAGGGTGACGTCGCCG	12:T>C
7172136|F|0-14:G>C-14:G>C	TGCAGCGCGGGCTTGAGCCTGTCCAGGAGGAGGGTGACGTCGCCG	14:G>C
7172136|F|0-32:G>C-32:G>C	TGCAGCGCGGGCTTGAGCCTGTCCAGGAGGAGGGTGACGTCGCCG	32:G>C
992184|F|0-42:G>A-42:G>A	TGCAGGCACGACCGCATGCCCTTCCTGGCGTCGGCGTCCGCCGCCGAGGCCG	42:G>A
988829|F|0-16:C>G-16:C>G	TGCAGGCGCCCAGCAGCGCGCCCCAGACCACCGAGTGCTCCTGGCAGTCCG	16:C>G
100532032|F|0-25:A>G-25:A>G	TGCAGGGGTTCCAGTCACGGGGCGTATCGAGGGAGCAGGGCGAGGCGCTGCTCCG	25:A>G
3941489|F|0-20:C>G-20:C>G	TGCAGGTAGGGGAGCCTGTGCGTGTCCG	20:C>G
100532185|F|0-44:A>C-44:A>C	TGCAGGTGCCGCAGCGGCGGCGCGCTGGCCTTGCCGAGGTGGCCAAGCTCCG	44:A>C
32025930|F|0-25:C>T-25:C>T	TGCAGAAGACGAAGGTGATCGAGACCTTCCAGGCCCAGGCCAACAAGACCGACGAGGGCATCACCATCT	25:C>T
998786|F|0-5:A>C-5:A>C	TGCAGAGGGTGGAGAGGTCGTGGGGCTTCGTGTGCGCGGGGAGGTACGGGACCGACCACCCGTTCATCA	5:A>C
100533894|F|0-25:T>C-25:T>C	TGCAGAGGGTGGAGAGGTCGTGGGGTTTCGTGTGCGCGGGGAGGTACGGGACCGACCACCCGTTCATTA	25:T>C
100534789|F|0-47:A>G-47:A>G	TGCAGCCGCGTCGAGGAGGGCTTCGGCGACAAGACCGACGGGCTCATAGGACTCGGCGGCGACGCGCAG	47:A>G
1713534|F|0-19:C>T-19:C>T	TGCAGCGCGAGGTACTGATCGTAGAGCTGGAGGACGACGTCGAGGTTGGCGGCGGAGTTCCGATTCCGA	19:C>T
1110107|F|0-5:C>T-5:C>T	TGCAGCGGGCGGTGCGTGTCCAGCCGCCAGATGGAGCCCCACGACTCCCTCATGGGCGTCCAGACCCCC	5:C>T
999482|F|0-18:G>C-18:G>C	TGCAGCGTCACCTCCCCAGGCCCAGGCGTCTCCTTCTCCACAATCTCCACCTTCCTCCCCTCGTCCTCC	18:G>C
1021154|F|0-29:G>T-29:G>T	TGCAGCTCGGCATTGGGCGGCAGGATGACGGGCTTCTGCACGACGATGCCGCCGCCGCCGTCGGCGGGG	29:G>T
2242683|F|0-58:A>C-58:A>C	TGCAGCTGCTGGATGTACTCGATCGCGTCCTTGATGATCGAGGCCTTGTCCATCTGCAACGACGAGGGA	58:A>C
990479|F|0-26:G>A-26:G>A	TGCAGCTTCTGCTGGACCATGGCGTTGTGGCGCTGCGCGAAGTCGTTGTACGTCTTCAGGCACCCGACG	26:G>A
100536043|F|0-20:T>C-20:T>C	TGCAGGAACGTGTTCCAGTCTCTGCGCTTCTGCGCCTCGTACCTGCTCAGCTGCGCAGGCGGCTTCGGC	20:T>C
1055670|F|0-50:G>A-50:G>A	TGCAGGAACTTCTGGATGGGGAAGTTGAGCGCGTAGGCGAAGAGCTGCGGGATCATGTAGAGCGAGAAC	50:G>A
2251238|F|0-24:T>C-24:T>C	TGCAGGATCTCCACCTGCGGGTACTTCTCCCTGAAGCCTTCGATGAGCTCCTTGCTGAGCGGCGCGCCG	24:T>C

<table>
<tr><td>1028366|F|0-31:C>A-31:C>A</td><td>TGCAGGCGCTCGTGTCGTGGCGCGACCCGCGCGCCACAGGGATGTTCGTGGTGCTCTGCGTCGTGGTGG</td><td>31:C>A</td></tr>
<tr><td>1028366|F|0-43:G>T-43:G>T</td><td>TGCAGGCGCTCGTGTCGTGGCGCGACCCGCGCGCCACAGGGATGTTCGTGGTGCTCTGCGTCGTGGTGG</td><td>43:G>T</td></tr>
<tr><td>12690891|F|0-16:C>T-16:C>T</td><td>TGCAGGCGGAGGCCTTCAAGCACAGCACGGAGCGGCTGGTGAACGACCTCACGCGCTCCTCCAAGTCGG</td><td>16:C>T</td></tr>
<tr><td>12690891|F|0-45:A>G-45:A>G</td><td>TGCAGGCGGAGGCCTTCAAGCACAGCACGGAGCGGCTGGTGAACGACCTCACGCGCTCCTCCAAGTCGG</td><td>45:A>G</td></tr>
<tr><td>1863287|F|0-50:G>T-50:G>T</td><td>TGCAGGCGGCGGAGCTTGAAGGAGGAGAGCTTCTGCGGGCCGTCCTTGACGGAGGGCTCCTCCTCGAAG</td><td>50:G>T</td></tr>
<tr><td>1020717|F|0-41:G>C-41:G>C</td><td>TGCAGGTAGTCCAGCTTCGCCTTCTCCAGATCCGCCATCACGCGATCCGCCTTGTATATCCG</td><td>41:G>C</td></tr>
<tr><td>1124006|F|0-13:C>G-13:C>G</td><td>TGCAGGTGGGCTCCATCCGACGGCCGCAGTTGACCTTCCAGGCCCCGCCTCCGCGGCCCGCCG</td><td>13:C>G</td></tr>
<tr><td>1124006|F|0-27:A>G-27:A>G</td><td>TGCAGGTGGGCTCCATCCGACGGCCGCAGTTGACCTTCCAGGCCCCGCCTCCGCGGCCCGCCG</td><td>27:A>G</td></tr>
<tr><td>1696689|F|0-59:C>T-59:C>T</td><td>TGCAGTCCAGCCGACGTCGCCTACACGCTGATCCCGACAATGTGCTACTTCGAGTTCCTCCCCGTCCAA</td><td>59:C>T</td></tr>
<tr><td>100539430|F|0-48:C>G-48:C>G</td><td>TGCAGACTTGGATCCAGCCTTGGTCGGGGTCCCAGTCGGGCAACGGCACGCCG</td><td>48:C>G</td></tr>
<tr><td>993067|F|0-52:G>A-52:G>A</td><td>TGCAGCACGCGCACAGGATGCACTCGTACAGCCCGTCCAGCTTCCGCCGCTCGCCCG</td><td>52:G>A</td></tr>
<tr><td>999506|F|0-16:G>A-16:G>A</td><td>TGCAGCGCGGCCTCGGGCTGTCGGACAAGCAGGTGGAGCCGTCGCGCATGGCGCTGCACCG</td><td>16:G>A</td></tr>
<tr><td>1154341|F|0-14:A>G-14:A>G</td><td>TGCAGGACCCCCGCATCGCGGCGGCCGCCCCCGCCCTCATACGCGCCGCCACAGCCTCCG</td><td>14:A>G</td></tr>
<tr><td>1053893|F|0-29:C>G-29:C>G</td><td>TGCAGGTTCTGCCACACCACCGTCCACAGCACCGCGATGATGCTGAGCACCACCCCG</td><td>29:C>G</td></tr>
<tr><td>4261611|F|0-23:G>T-23:G>T</td><td>TGCAGGTACGGCATCTGCGCCACGTCCG</td><td>23:G>T</td></tr>
<tr><td>1249581|F|0-10:C>T-10:C>T</td><td>TGCAGAACAACAACATCAATGGCCGAATCCCGGCAGAGATTGGCAGGCTCACGAAGCTTAGGACGCTTG</td><td>10:C>T</td></tr>
<tr><td>1249581|F|0-13:C>T-13:C>T</td><td>TGCAGAACAACAACATCAATGGCCGAATCCCGGCAGAGATTGGCAGGCTCACGAAGCTTAGGACGCTTG</td><td>13:C>T</td></tr>
<tr><td>1089113|F|0-19:G>T-19:G>T</td><td>TGCAGAACTGGTCGGCGATGGTGGGCCACGTCAGAGTCGGCACGCCGTAGGAGACGGCCTCCAGCGTCG</td><td>19:G>T</td></tr>
<tr><td>4989526|F|0-16:C>T-16:C>T</td><td>TGCAGCAGAAGTACACCCAGAGCGGCGGAGTCCGCCCCTTCGGCCTCTCCACCCTCATCGTCGGCTTCG</td><td>16:C>T</td></tr>
<tr><td>12772134|F|0-50:G>A-50:G>A</td><td>TGCAGCCAGCAGCACTACGACGGTGACGGTGCTCACCGCGAGCTACTCCCGCTCAACCCTCACCTCTCT</td><td>50:G>A</td></tr>
<tr><td>1265881|F|0-8:C>G-8:C>G</td><td>TGCAGCGCCGACACGAAGGCGGAGGACGGCGGCGACGGCGAGTAGAGGTGCGGGGTGAAGGAGATGGCC</td><td>8:C>G</td></tr>
<tr><td>3944091|F|0-26:G>A-26:G>A</td><td>TGCAGCGGCGGCCGCTTGCTCGACGAGATGGCCAGGGACGGCACCTTCTCCGAGCAGCGGGCCGCCCTT</td><td>26:G>A</td></tr>
<tr><td>100544608|F|0-58:A>G-58:A>G</td><td>TGCAGGACTGGCAGCGGCGGTACGCCGACGTGCTGCGGCGGAAGGGGGTGAAGGTCCAAGTGGCGGAGT</td><td>58:A>G</td></tr>
<tr><td>6040521|F|0-55:G>A-55:G>A</td><td>TGCAGGGAGGAGGCGGAGGCGGGGCTGGGCATAAGAGGAGCGGCTCCATGGATGGGGCGAGCTCGCCGT</td><td>55:G>A</td></tr>
<tr><td>1865036|F|0-50:G>A-50:G>A</td><td>TGCAGGTGCACCATCTTGGCGTACACGCCGCCTTCGCCGCCGTCCATGGCGACGAGCTCGTCGTGCGTG</td><td>50:G>A</td></tr>
<tr><td>7352454|F|0-22:C>T-22:C>T</td><td>TGCAGTCGGGGCTCCCCCTCGACGACCGTCCTGAGGGCGCGCGCTCGCCGTCCCCTGAGCCAATATACG</td><td>22:C>T</td></tr>
<tr><td>100546193|F|0-21:A>C-21:A>C</td><td>TGCAGTTGCTCATGACATCTGATGAAGGCCACTGTCCATGGCCATTGTGGCAGCTCCCCTTCCG</td><td>21:A>C</td></tr>
<tr><td>1022735|F|0-38:G>A-38:G>A</td><td>TGCAGCTTGGGGAGCAGGTAGTACCTGATCAGCTCGTCGTCGCTCGGCCTGAACCG</td><td>38:G>A</td></tr>
<tr><td>1023983|F|0-9:C>T-9:C>T</td><td>TGCAGGGATCATCAGGCGCGTGCCTGGGGACGCCGCGGCGGAGGAGGTGAACCCG</td><td>9:C>T</td></tr>
<tr><td>1023983|F|0-27:G>C-27:G>C</td><td>TGCAGGGATCATCAGGCGCGTGCCTGGGGACGCCGCGGCGGAGGAGGTGAACCCG</td><td>27:G>C</td></tr>
<tr><td>1023297|F|0-7:C>T-7:C>T</td><td>TGCAGTACCTGTCCAAGGAGGAGGCCGCCGTCCACGCGCGGATGCGCCGCCG</td><td>7:C>T</td></tr>
<tr><td>100550233|F|0-11:G>C-11:G>C</td><td>TGCAGCACGCAGCCATCCACGACGCAACACACATCAGCCAGAAACCATGCAAAACCAACACCATTTCGA</td><td>11:G>C</td></tr>
<tr><td>100550237|F|0-39:C>A-39:C>A</td><td>TGCAGCACGCACCCATCCATGACGCAACACACATCAGCCCGAAACCATGGAAAACCAACACCATTTCGA</td><td>39:C>A</td></tr>
<tr><td>1080483|F|0-12:C>T-12:C>T</td><td>TGCAGGACCTCGCCGACGGCCTCGGCGCCGTCTTCCAGGACTACGTCTCCTTCCTCGCCTCCTGCGGCA</td><td>12:C>T</td></tr>
<tr><td>1022730|F|0-5:G>C-5:G>C</td><td>TGCAGGCCCTGGCTGCATGGCGCTGCTGATGGACTGCACCTGCGTCTTGCCGCTGGGGCCCTTGGTGCG</td><td>5:G>C</td></tr>
<tr><td>1130186|F|0-32:C>G-32:C>G</td><td>TGCAGGCGCAGACGCAGACGCACCCGCCGTCGCCGAGGTACCGCGGCGTCGTCGACTGCTTCCG</td><td>32:C>G</td></tr>
<tr><td>991920|F|0-8:G>A-8:G>A</td><td>TGCAGGTTGGTTTCAACAATGGAGTCCCCATCACCGAGGAGTTCTACAGCGCCAACATCAGCGGGTGGC</td><td>8:G>A</td></tr>
<tr><td>991920|F|0-50:G>A-50:G>A</td><td>TGCAGGTTGGTTTCAACAATGGAGTCCCCATCACCGAGGAGTTCTACAGCGCCAACATCAGCGGGTGGC</td><td>50:G>A</td></tr>
<tr><td>1022067|F|0-29:C>A-29:C>A</td><td>TGCAGGAACACTGGTGATGACGACGTGGCCATAAAGGTGCTCTACTGCGGCATCTGCCACTCCGACCTG</td><td>29:C>A</td></tr>
<tr><td>3025416|F|0-21:A>C-21:A>C</td><td>TGCAGCAGCCGCTTCTCCTGGAGCTCCTGGGACAGGACCACCG</td><td>21:A>C</td></tr>
<tr><td>3937339|F|0-17:C>A-17:C>A</td><td>TGCAGCGACAAGAGTTTCCCGAGCGACGCCGGGATGTGGCCG</td><td>17:C>A</td></tr>
<tr><td>3942805|F|0-14:G>A-14:G>A</td><td>TGCAGCTCGCCGTCGGCCATGGCACATCCACGCCG</td><td>14:G>A</td></tr>
<tr><td>1022995|F|0-35:G>T-35:G>T</td><td>TGCAGGTTGGACGCGCCGTGCATCGCGAAGTACTCGCGCACCATCTCCCG</td><td>35:G>T</td></tr>
<tr><td>4262307|F|0-62:C>A-62:C>A</td><td>TGCAGCAGCCACTGGTGATCCACGTCGATGAGGAAGTCCTTGTGGTAGTGCTCCCTCACCTTCTCCCTC</td><td>62:C>A</td></tr>
<tr><td>989982|F|0-46:C>G-46:C>G</td><td>TGCAGCGGGTCCTGCTCGTGCTCGCGCTCGTCGGCACCTGCATGGTCATCGGCGACGGCGTCCTCACGC</td><td>46:C>G</td></tr>
<tr><td>1129014|F|0-13:T>G-13:T>G</td><td>TGCAGGTCGGCGGTCGCCGAGTCCCGCACCTCGCGCCAGCCCTTGGAGATGAAGGCGAACGCGCCGTCG</td><td>13:T>G</td></tr>
<tr><td>100558440|F|0-10:A>C-10:A>C</td><td>TGCAGGTGCTAATCCTCCGCCTCCGTTCCCGCGGGAAGGAGGTCATTTCGTCGCACATCTCCACCTACT</td><td>10:A>C</td></tr>
<tr><td>1053022|F|0-51:G>C-51:G>C</td><td>TGCAGCTCCAAGAACTCCAGCTAGTACATGTTTCCACCTGACGCCTTTCTTGCCCG</td><td>51:G>C</td></tr>
<tr><td>3940036|F|0-7:G>T-7:G>T</td><td>TGCAGGTGTACCATCCCAACATCGGCCCG</td><td>7:G>T</td></tr>
<tr><td>3951439|F|0-30:C>G-30:C>G</td><td>TGCAGGTTCGAAAGGAACGAATCCCACGGGCTGGACACGCCTTGCCG</td><td>30:C>G</td></tr>
<tr><td>1021565|F|0-52:C>A-52:C>A</td><td>TGCAGACGCTCATGGACAAGGGCTGCGACGTCAACGCGAGGGCGGAGGCTGGCGATGTCGCCGTCATCC</td><td>52:C>A</td></tr>
<tr><td>1021430|F|0-25:C>T-25:C>T</td><td>TGCAGCCTCTTCGGCAAGCAGGCGCCCCAGCCGCAGCACCGCGCGGGCGGCGGCAGCGCCAAGGGGAAG</td><td>25:C>T</td></tr>
<tr><td>987463|F|0-12:G>A-12:G>A</td><td>TGCAGGCTGGTCGCCCGCGGCGGCTCGGCGAACTCGTAGTCGTGCTTGAAGTAGTCCAGGATCATGTCC</td><td>12:G>A</td></tr>
<tr><td>987463|F|0-17:C>T-17:C>T</td><td>TGCAGGCTGGTCACCCGCGGCGGCTCCGCGAACTCGTAGTCGTGCTTGAAGTAGTCCAGGATCATGTCC</td><td>17:C>T</td></tr>
<tr><td>1696774|F|0-22:G>A-22:G>A</td><td>TGCAGGGGAGCTGGTGGAGGTAGGGGGTGTTGGCGCCGTGCTTGGTGGCGCCGAAGAAGGCGGTGGCGT</td><td>22:G>A</td></tr>
<tr><td>1024561|F|0-12:C>G-12:C>G</td><td>TGCAGGCGTTCGCCCTCGGCCTGAACAAGCGCCTCGCCGACGACGACGCCG</td><td>12:C>G</td></tr>
<tr><td>1024561|F|0-38:G>A-38:G>A</td><td>TGCAGGCGTTCGCCCTCGGCCTGAACAAGCGCCTCGCCGACGACGACGCCG</td><td>38:G>A</td></tr>
<tr><td>2248179|F|0-48:C>A-48:C>A</td><td>TGCAGGGGAGGCGATTAAGCAGTCGCCGTCGATGCCGACGACCCCGACCACGCCG</td><td>48:C>A</td></tr>
<tr><td>100565317|F|0-29:T>C-29:T>C</td><td>TGCAGGGGCGTCTCCGTGCCGCAGGCCCCTGTCACCTCCACCG</td><td>29:T>C</td></tr>
</table>

989810|F|0-22:C>T-22:C>T	TGCAGGGGCTCGGGGTCAACCCCGCCGTGCACGAGGTCGCCGACGAGGCCGCGCTCGCCG	22:C>T
100566124|F|0-33:A>C-33:A>C	TGCAGACCAAGATTTCACAGAGCTCGTGCAGAAAGTCCCTGATGGCTGCCTGTTCACCATAGTCTCCGA	33:A>C
1037648|F|0-14:G>A-14:G>A	TGCAGACCATCAACGGCCCCAACTCCGACTTCCCCGTCTACTCGTCGCAGCTCAAGGAGCTCAAGGGCA	14:G>A
1025886|F|0-11:T>C-11:T>C	TGCAGAGGCTCTGCCTTTCGCGCTGCAAGCCCATGACGGACATGGGGCTCGGCTGCATCGCCGTCGGGT	11:T>C
100566295|F|0-28:A>G-28:A>G	TGCAGAGGCTCTGCCTTTCACGCTGCAAACCCATGACGGACATGGGGCTCGGCTGCATCGCCGTTGGCT	28:A>G
1020082|F|0-19:T>C-19:T>C	TGCAGATCTCCAAGATGCCTGAGCATAGGGAACTCAAGAGGACGGCCCTCACCGACCTCTCTGGCGGCG	19:T>C
1020082|F|0-46:C>A-46:C>A	TGCAGATCTCCAAGATGCCTGAGCATAGGGAACTCAAGAGGACGGCCCTCACCGACCTCTCTGGCGGCG	46:C>A
1067927|F|0-5:C>T-5:C>T	TGCAGCTCCTTCAGCGCGCGGCTGCACGGGTACAACGCCACCACGGGCACGGGCCAGGACCCG	5:C>T
1067927|F|0-29:G>A-29:G>A	TGCAGCTCCTTCAGCGCGCGGCTGCACGGGTACAACGCCACCACGGGCACGGGCCAGGACCCG	29:G>A
1695397|F|0-17:C>T-17:C>T	TGCAGCTTCCCCTGCTCTGCACCTTGGGGTTCATGAAGTCCTCGGCGAGGAGGTCGACGAGGGCCATG	17:C>T
1378383|F|0-43:C>G-43:C>G	TGCAGGCCATGGACTCCTCCCACGTCGCGCTCGTGGCCCTCCTCCTCCGCTCCGAGGGCTTCGAGCACT	43:C>G
986239|F|0-38:G>A-38:G>A	TGCAGGGCCAGGTCCTGGTGCTCCTTCTTGCACACCTGGTCGGCGCCGATGTTGCCGTACCG	38:G>A
986239|F|0-44:G>A-44:G>A	TGCAGGGCCAGGTCCTGGTGCTCCTTCTTGCACACCTGGTCGGCGCCGATGTTGCCGTACCG	44:G>A
1090564|F|0-42:A>C-42:A>C	TGCAGGGGAGAGAGGGGAAGGGTATCACGGAGAGTGAGACAGAGGAGGATACTGGTGGGATTTAGGGTT	42:A>C
2254889|F|0-10:G>C-10:G>C	TGCAGGTCCTGGCGGCGGAGCTGCCGACGGGCACCATCCGCTACTCCTCCAAGATCGTCTCCATCGACG	10:G>C
100567635|F|0-56:A>G-56:A>G	TGCAGGTCCTGGCGGCGGAGCTGCCGACGGGCACCATCCGCTACTCCTCCAAGATCATCTCCATCGACG	56:A>G
1019320|F|0-31:C>T-31:C>T	TGCAGTGGGCGATGGCGGAGCTGATACGGAACCCGCGGGTTATGCGGAAGGCGCAAGACGAGGTCCGAG	31:C>T
32027320|F|0-47:C>T-47:C>T	TGCAGCCGTTCTTCGGCGGCGAGGCGGCGACCGCGTCGGAGCAGCGGCTCCGCCACGCGCCGTTCGGGG	47:C>T
1041520|F|0-16:G>C-16:G>C	TGCAGGAGACGACGAGGGAGGAGGAGGAGCTCTTGGGGTGGAAGACGGGGAAGGAGCCG	16:G>C
100570395|F|0-13:A>G-13:A>G	TGCAGCTCACGGTATAGCAGCTGTCGGGGCGGGAGAGCGCGTCCGAGAACCGCACCGCGGCCTCCTGGT	13:A>G
6045050|F|0-26:C>G-26:C>G	TGCAGGCCCTCCATCCGCGCCGCCATCGCGTCGCCGACGGCCCTGACCTCCCTCTCGATCCCCGCCGCC	26:C>G
100572004|F|0-22:A>G-22:A>G	TGCAGCAGGCGGCGGCGCGCGCATGGTCACCCGATGGGTCGTCGCGCCG	22:A>G
992330|F|0-9:G>A-9:G>A	TGCAGCCGCGCCGTCACCTGCTGGATCTGCTCCGCGGTGGGGGTGCGCAGCCCCTGCCG	9:G>A
1104983|F|0-5:A>G-5:A>G	TGCAGAGCCAGGCCGACAAGTCGCTGGAGCGCCTGCACGAGTGCGCCGAGAAGGACAGGACGAGCCTCG	5:A>G
2266494|F|0-24:C>A-24:C>A	TGCAGCGCCTCCTGCTCCCGCAGCCGCTTCACGAGGTGTGTCGACGGCGAGCGCGCCAGCTTCTTGAGG	24:C>A
1044033|F|0-35:G>T-35:G>T	TGCAGCTCCAACTTCGCTGTCACAGAGAGGACACAGGACGCAGGGCGCACGGTTTCCGTGGGCTACAAC	35:G>T
1090123|F|0-53:C>A-53:C>A	TGCAGGCAGTCGGCGGCGTGCTCCTTGGAGCCCAGCACGGTGCCGTAGTCCAGCAGGCTGACCATGACG	53:C>A
1058456|F|0-58:G>C-58:G>C	TGCAGTTCGCGGTGCCCATGGTCCTCACCAACATGTCCTACTACGGCATCCCGCTGGTGTCCGTCATGT	58:G>C
1058456|F|0-61:C>A-61:C>A	TGCAGTTCGCGGTGCCCATGGTCCTCACCAACATGTCCTACTACGGCATCCCGCTGGTGTCCGTCATGT	61:C>A
4396519|F|0-7:G>C-7:G>C	TGCAGGAGCTGGCGCGCCATCCTCTCCGACCCCG	7:G>C
3939145|F|0-8:T>A-8:T>A	TGCAGAGCTTGACGATGTCGTCGACGACGCCG	8:T>A
986367|F|0-5:C>G-5:C>G	TGCAGCGACGCCGAGTACCGCACCGCCTCCCGCACCGTCAGCGTCGTCATCAGCACGTCGTCCTGCGTC	5:C>G
3934803|F|0-17:G>T-17:G>T	TGCAGGATTTCTTTGACGGGAAGAAGCTCTGCAAGAGAATCAATCCG	17:G>T
7338872|F|0-49:T>C-49:T>C	TGCAGATGTACGCCCGCTGCGCAGACGCCGCATGTGCTCGCAGGGTGTTTGACGCGATGCCCCACAGGG	49:T>C
1001554|F|0-19:C>T-19:C>T	TGCAGCCCTTCCTCGGCGGCGACACGGCCTACCTCCTGCGCTTCTCGTCGCAGGTCGGGGAGTGGGTCA	19:C>T
1001554|F|0-50:C>G-50:C>G	TGCAGCCCTTCCTCGGCGGCGACACGGCCTACCTCCTGCGCTTCTCGTCGCAGGTCGGGGAGTGGGTCA	50:C>G
1079000|F|0-18:T>C-18:T>C	TGCAGGGCCAGAGTCACCTGGGAGACAACGCCGAGGACCCCAAGGGAGACCTTGGCGGCGTCCAGGTCC	18:T>C
1079000|F|0-58:C>A-58:C>A	TGCAGGGCCAGAGTCACCTGGGAGACAACGCCGAGGACCCCAAGGGAGACCTTGGCGGCGTCCAGGTCC	58:C>A
1079000|F|0-59:G>A-59:G>A	TGCAGGGCCAGAGTCACCTGGGAGACAACGCCGAGGACCCCAAGGGAGACCTTGGCGGCGTCCAGGTCC	59:G>A
1263850|F|0-22:C>A-22:C>A	TGCAGAATGCAATGGCCTGCCTCTCTTCCTCGCCACCGTCGGCCGTGCCATGTCCAACAAACGTACAGC	22:C>A
100580200|F|0-50:T>C-50:T>C	TGCAGCGTGCTCCTCGACATCAACGACGGCGACCGCCTCGCCTTCTTCCGTCTCACCGCCG	50:T>C
5371661|F|0-12:T>G-12:T>G	TGCAGCTGGAGCTCCACCAGCTCCCGGCACTTCCCGATTTCTGGTGGGATTGTTCCG	12:T>G
993594|F|0-7:C>T-7:C>T	TGCAGCTCGGCTCTACTCTCCTCCACAGGCTCTGCTCATCTTGAGGAGCGCCGAGAGCACGCCGTTCAT	7:C>T
993594|F|0-39:C>T-39:C>T	TGCAGCTCGGCTCTACTCTCCTCCACAGGCTCTGCTCATCTTGAGGAGCGCCGAGAGCACGCCGTTCAT	39:C>T
993594|F|0-47:G>C-47:G>C	TGCAGCTCGGCTCTACTCTCCTCCACAGGCTCTGCTCATCTTGAGGAGCGCCGAGAGCACGCCGTTCAT	47:G>C
993594|F|0-60:G>A-60:G>A	TGCAGCTCGGCTCTACTCTCCTCCACAGGCTCTGCTCATCTTGAGGAGCGCCGAGAGCACGCCGTTCAT	60:G>A
100581280|F|0-21:A>G-21:A>G	TGCAGGCCAAGCTGGACCCCCAGCACCCCAGTTTTGTCAAGCCCATGACCCAGTCTCATGTCACCGAGA	21:A>G
985521|F|0-34:G>T-34:G>T	TGCAGGGCCTGTGGTTCTACCAGACGGCCTTCACGCTCTACGGGCCGATGCTCCCCGAGGGGTGCCACC	34:G>T
1695126|F|0-22:G>A-22:G>A	TGCAGCAGCATGTACTCCAGCAGGGGCATCATCGCCAACTCCTTCAGCGAGCTCGAGTCGGTCTACATC	22:G>A
4002646|F|0-64:C>G-64:C>G	TGCAGGGACACGCCGTGAAGCTCGGGTTCCTCGAGCACGACGAGCACGCGCAGAACAGCCTCATCAGCT	64:C>G
5582258|F|0-41:G>A-41:G>A	TGCAGTTCCTTCCCCCGCCCTTCTTCAACCTCACGCAGCTCGTCGACAGCTTCAAGGCCAAGAACATGA	41:G>A
3024405|F|0-7:C>T-7:C>T	TGCAGATCGCCACCACCTCCTCCGCAACCAAGAGAAAGCGCCG	7:C>T
7173085|F|0-33:A>G-33:A>G	TGCAGATCGCCGCCACCTCCTCCGCGACCAAGAAAAAGCGCCG	33:A>G
1025833|F|0-38:C>G-38:C>G	TGCAGCCTCGCCGCGTGAGCGGCCGCGAGGGCGACGCGCAAGGTATAGGTGCCGCCG	38:C>G
1024214|F|0-13:C>G-13:C>G	TGCAGCGGCCTCTCCGCCGAGGAGGTGGAGTCTCCCTACGTCAACTTCGGTCGCCG	13:C>G
2247862|F|0-52:T>C-52:T>C	TGCAGTGCGCGCCCTGCCTCGACTGCTTCGAGCAGAGCGGCCCATCTTCGATCCG	52:T>C
1078289|F|0-10:C>T-10:C>T	TGCAGTCCTGCGCGCCCCTGGCGGCGACCCCGAACCCGAGCGGCACGTCGGCCATGGACATGACGACGA	10:C>T
100586744|F|0-21:G>A-21:G>A	TGCAGCAGCGTGGTGTCGTCCGCCAGCAGGATGCGCATCCCCTCCAGCGGCTTGCCTTCTCCAGCATTG	21:G>A
1112302|F|0-52:G>T-52:G>T	TGCAGGCGAGCGGCCACGTGTGCGGGATGACGGGCGACGGCGTGAACGACGCGCCG	52:G>T

1007231|F|0-6:G>A-6:G>A	TGCAGCGCCGCCTGGTCGGGAGCGTACAGCCGCATGGAGTTGATGCCGTTGGACTTGAACATGCTGACG	6:G>A
1007231|F|0-14:G>A-14:G>A	TGCAGCGCCGCCTGGTCGGGAGCGTACAGCCGCATGGAGTTGATGCCGTTGGACTTGAACATGCTGACG	14:G>A
1696722|F|0-66:G>A-66:G>A	TGCAGCGCCTTGCCGAGGAGGAGCTCGTTGGTGTGCGGGCCGTAGATGTCGGAGGTGTCGAGGAGGGTG	66:G>A
1695220|F|0-40:C>T-40:C>T	TGCAGCCGCTTGGACTGCCACACGAGCTTGCGCATCTGGTCGAAGCGCGGGCTCATGTGCTTGATCACC	40:C>T
1089751|F|0-31:C>T-31:C>T	TGCAGCGCTCCTGGATCATCCTCGCCGTCGCCGCGCTCCTCATGGTGCCCTTCTACGTCTTCGCCGAGC	31:C>T
2243250|F|0-38:G>A-38:G>A	TGCAGTATGGTGAGAGCGTCCAGCTTCCCGCGGTTGAAGTCGTCGGGGATGTCGTCGTCGATGAACGGG	38:G>A
2245281|F|0-14:G>A-14:G>A	TGCAGGCGGCTCTTGAAGCTGGGGTCTGAAATTTGATCCCAGAACTCACGCAGCTCCGCCTTGTCGATG	14:G>A
3952155|F|0-22:C>G-22:C>G	TGCAGCCGCTCCTCCTCCGCGTCCTCGCCGCCG	22:C>G
5348032|F|0-7:G>A-7:G>A	TGCAGGGGAAGGTGAAGGTGTACCCG	7:G>A
3952057|F|0-29:C>T-29:C>T	TGCAGAGCGAGCCCTGGCGCGAGATGGCCCCACCG	29:C>T
5331962|F|0-6:G>T-6:G>T	TGCAGCGCCGCGTCGACACGGACGCCG	6:G>T
3022187|F|0-5:C>T-5:C>T	TGCAGCGCCGTCCAGGTCTCCTTCATCCACTACCTGCCCG	5:C>T
3022187|F|0-11:C>T-11:C>T	TGCAGCGCCGTCCAGGTCTCCTTCATCCACTACCTGCCCG	11:C>T
3943849|F|0-23:C>A-23:C>A	TGCAGCGTGGGGAAGAAGGGCGGCGCCG	23:C>A
5332148|F|0-17:C>G-17:C>G	TGCAGCTTACCCGCAAGCCTTTTCCG	17:C>G
3945997|F|0-10:G>C-10:G>C	TGCAGTCGCTGCGCCCGCCCCCG	10:G>C
4006132|F|0-36:G>C-36:G>C	TGCAGAACGAGGGCAACGTCGTCACCATCAAGACGGGCGCCTCCG	36:G>C
5581450|F|0-29:C>T-29:C>T	TGCAGGAACACGGCCCCCGTGGGCAGGTACACGCAGTTTGTCAGCCCG	29:C>T
4262277|F|0-10:G>A-10:G>A	TGCAGGTCGGGTACTACGACACGCTGTGCCCG	10:G>A
7353627|F|0-10:T>C-10:T>C	TGCAGCTGGATGGGCCCCCG	10:T>C
1059725|F|0-42:C>T-42:C>T	TGCAGTAGTAGATGACGGCCTGCGGGTCCTGGTAGATCATGGCGCGGCAGTAGGGGCACCGCCCGAACC	42:C>T
3944620|F|0-18:C>T-18:C>T	TGCAGCAGCTGCACCAGTCGCTCGCCGACACCGTCGCCGCCG	18:C>T
1038399|F|0-6:C>T-6:C>T	TGCAGCCTGATCTCCCTGTTGAACCTGGTGATGAAGTCCTCCACCCTCCG	6:C>T
5332167|F|0-28:G>A-28:G>A	TGCAGCTTGCGCAGCTGGCCGAGCTGCGGCGGTACCG	28:G>A
4261797|F|0-8:C>T-8:C>T	TGCAGGATCGCCTGCTGGTGCATCCAGGACGAGGAGGCCGACCG	8:C>T
986253|F|0-19:C>T-19:C>T	TGCAGGCGCAGGGGTAGCGCAGCGGGTGCTCGTCGTCGCCGCGGTTGCGGCAGATCCG	19:C>T
3025752|F|0-25:A>T-25:A>T	TGCAGTTGGTGTCCTGGCTGATCAAATTCAGGGTGGTTCCG	25:A>T
999758|F|0-15:A>C-15:A>C	TGCAGAGGCAGCTGAAGAGCCCGCACGCCAGGCACTTGAACGGGCAGCACAGGCACGACGCCGCGCGGC	15:A>C
2252110|F|0-29:G>C-29:G>C	TGCAGGTGAGCAGCGCCACGTCCGCCGCCGTGTCGATCATGTTGTCCATGAAGAAGACGTAGGCGGTCA	29:G>C
3020464|F|0-8:A>G-8:A>G	TGCAGAGGATTGTGCTGGTCATCATGAGCAAGGCCACCG	8:A>G
4261096|F|0-15:G>T-15:G>T	TGCAGAGTGCCACCAGAAGCTTTTGCAGCTTGGAGTCGTAGGCCG	15:G>T
5363323|F|0-23:G>T-23:G>T	TGCAGCATCCTGGTGAGCGGGCTGCCGCCG	23:G>T
1713347|F|0-19:G>A-19:G>A	TGCAGCCCATGCCGCTGAGGTTGTGGCTGACGACGTCGTGGCGGAGGCGGTACCG	19:G>A
5357855|F|0-23:G>A-23:G>A	TGCAGCGCGTGGCACGGCGGGTAGTGGTTCACCCG	23:G>A
994321|F|0-35:A>G-35:A>G	TGCAGCTCGCCGTGCTCTACGTCTCGCTGCTCTTCACGTCCCTCGGCTCCG	35:A>G
4262316|F|0-36:G>A-36:G>A	TGCAGCTGCGATGAACTGCCCCGTTGCTGCATGTGCGCGCTCATGCCG	36:G>A
5346063|F|0-5:C>A-5:C>A	TGCAGCGGCGGGAGGCAGGCCCG	5:C>A
7350670|F|0-8:C>T-8:C>T	TGCAGCTGCACGCCCAGGCGCCG	8:C>T
984247|F|0-8:C>T-8:C>T	TGCAGCATCTCCCACATGACCTGCACGTCCTCGTACGCGCACGTCTGCACGTCGTCGTGGAGCTTCACC	8:C>T
993681|F|0-64:G>C-64:G>C	TGCAGCCGCGGATCATCGCGTGCGGGAACAAGCGGGCGACGTTCGCCATGGCCGTGCGGTTCCTGACGG	64:G>C
2256197|F|0-32:C>T-32:C>T	TGCAGCGCGAACTCGAGGCACCAGAGCACGTCCCCCATGGTGGGCCGCTCCACCCCGTACTCGGCGAGG	32:C>T
1020656|F|0-48:C>G-48:C>G	TGCAGCTGTGGAAGACGGCCTTCTACGACCAGCAGGCGATGCAGGCCACCGAGTTCCTCCTCAGCGATG	48:C>G
1035766|F|0-61:C>T-61:C>T	TGCAGGAGTTCGTCAACCACGGGGGCGTCATCTTCAAGGTTTACGTCGTCGGCGGCCACGTCACCTGCG	61:C>T
1696470|F|0-8:G>A-8:G>A	TGCAGGTCGAGCGTGACCTCGACGAGCTCCTCGCCGTCGTCGGAGCTGCGGTCGGAGCCGCGCGGCGAG	8:G>A
2281902|F|0-10:G>C-10:G>C	TGCAGGTCTCGGCGACGCGGGGATCGCGGTCGCCGAGCGCGGCCATCTGGATCTCGAAGCCCTTGCCGT	10:G>C
100452741|F|0-13:G>A-13:G>A	TGCAGGTCTCGGCGACGCGGGGGTCGCGGTCGCCGAGCGCGGCCATCTGGATCTCGAAACCTTTGCCGT	13:G>A
9721822|F|0-17:G>T-17:G>T	TGCAGAGCTCGGCGTCCGTGCACCG	17:G>T
5366914|F|0-29:C>T-29:C>T	TGCAGCAGCTTCGCCCCGCTCGGCGCGCCCCCCACCATCGCCTCGCCG	29:C>T
3032806|F|0-33:A>C-33:A>C	TGCAGGGCACGACCTCGAAGCCGACGACCATCCAGCCG	33:A>C
100463902|F|0-14:G>A-14:G>A	TGCAGTTCGCCAGCGTGGACGACCTGCCGCTGCCG	14:G>A
1015989|F|0-68:C>T-68:C>T	TGCAGACAGAAGAGAAGATCAAAAGAGATCGGGCGATGCAGCACAAGATTGGAGGGCAACAACAAGCGC	68:C>T
994237|F|0-35:G>A-35:G>A	TGCAGGAGGCAGGCGGCGCGGTTGCTGTGGAAGACGGCGCGGTCGGGATGGCCACGCGGGGCAAGGCGG	35:G>A
1090847|F|0-43:C>T-43:C>T	TGCAGCACGTCCTCCAGACGGCGCTCGTGCTCTGGCTCGTCCTCAACGCCACCG	43:C>T
3945381|F|0-23:C>G-23:C>G	TGCAGCGGTGCGGCAAGAGCTGCCGCCTCCG	23:C>G
1092909|F|0-23:G>A-23:G>A	TGCAGCGTATCGGCGGCGGCGAAGCGCAGGTCCATGGCGTACAGGTTGCTGCCG	23:G>A
1164941|F|0-29:C>A-29:C>A	TGCAGCTCGACGCCATCATCGGCCACCACCAGAGCACCAGCCTAGCCCTGCCG	29:C>A
1130281|F|0-41:C>T-41:C>T	TGCAGGCCCTGCATCCTGCACATGCTGGAGCACGTGAGCGCCTCGAGCCCCG	41:C>T
100483486|F|0-15:G>A-15:G>A	TGCAGCGGCGTCGGCGTTTGGCCG	15:G>A
2252543|F|0-31:C>T-31:C>T	TGCAGATGATGAGCCTCGACGCGCGCAAGTCCGTCGAGCGCTTCATCGAGACGCACGGGCCCG	31:C>T

2259595|F|0-50:G>A-50:G>A	TGCAGCGGCGCGTTCGCCAACCCCAACACCTGCCACCCCACCGCCTACTCGCAGGCGTTCAAGCTGGCG	50:G>A
2250961|F|0-42:C>T-42:C>T	TGCAGGGCCACCCCAACGTGATCGGCCTCCATGGCCTCATCACCTCCCGCTCCTCCGCCTGCATCTACC	42:C>T
1022410|F|0-43:C>T-43:C>T	TGCAGTCCAACGCGGACGACGCCGTGGTGCACAAGATCTCCGACCGCATGGTGCGCAACGGCTCCATCT	43:C>T
4005999|F|0-11:G>T-11:G>T	TGCAGCGCGGCGGTTAGCATGATCGGGTCCACCAGTGTCCG	11:G>T
1025293|F|0-43:C>A-43:C>A	TGCAGCGTCGCCGTGCGGCGTTTCCTGTACATGATGTCGGGCACGGCCCG	43:C>A
1707920|F|0-44:G>C-44:G>C	TGCAGGCAGAGGAGCGGCAGATGGAGGCGGAGGTGTCCGCGCTCGAGTCCGCCACCG	44:G>C
7173428|F|0-5:G>A-5:G>A	TGCAGGCGGCGACGACGACCTGGCAGAGGCTCCTGAGCGGCGAGCCG	5:G>A
3028103|F|0-25:C>T-25:C>T	TGCAGGTGTACGTGTTCGCGCTCTTCAACGAGGACCAGAAGCCCG	25:C>T
4396666|F|0-5:C>A-5:C>A	TGCAGCGGTACAGCGCGAGCACCG	5:C>A
988576|F|0-52:C>G-52:C>G	TGCAGATCATCACGGCGAAGCCGCCCATGGGGCTGACGCACCACATCGGGCGCGCGCTGGAGCGCGGCA	52:C>G
1019857|F|0-17:G>A-17:G>A	TGCAGCACGCTGAGGTTGTTGTTAGCCTTCACCGCGTACCCCACGATTGAGCGGAGCCCCTGGAGCGCC	17:G>A
1863313|F|0-22:C>A-22:C>A	TGCAGGACACGAGCATCCAGGGCGCCGAGAGCCACCCGCTGCACCTGCACGGGTTCGACTTCTTTGTCG	22:C>A
987045|F|0-13:C>T-13:C>T	TGCAGGCGGAGGCCGAGGTCGGGGAGGACGGCAGGATGCGGGTCACCGTGCGCAAGTCCACCAGCTCCC	13:C>T
1044828|F|0-19:A>C-19:A>C	TGCAGGGAGAACTGGACGGAGATGTGCGCGGTGCAGCGCGGGCAATTGGCGTCGGCGTGGATCCGAGAT	19:A>C
1862637|F|0-54:G>A-54:G>A	TGCAGGGGCTCGTGAGGAAGGGCCTGTGGGAGGATGCCATCGACTACCTTGATGGCTTCCTGCCTGCCG	54:G>A
996110|F|0-24:G>C-24:G>C	TGCAGACGGCGCGCTGGACGTCGCGGAGGATGGACTCCTTGAGGCTCTCCG	24:G>C
1025619|F|0-38:C>A-38:C>A	TGCAGAGGTCCACGGTGGAGGTGGTCGGGCACAAGGCGCACGGAGCCGTGCCGCAGCCG	38:C>A
3937001|F|0-31:G>T-31:G>T	TGCAGCGGGAGAAGGCTGAGGCCATGATGGAGGCGCGCCAGTTCCG	31:G>T
3937845|F|0-15:C>A-15:C>A	TGCAGGGTCTTCGTCCTGCTGCTGCTCCG	15:C>A
997667|F|0-15:C>A-15:C>A	TGCAGGTACTCCAAGCAGGGCAGGTTCTGAAACTCCATCGGAATTGCGCCG	15:C>A
1061569|F|0-58:C>T-58:C>T	TGCAGACGGCGCTGCGGGCGAGCGCCGCCAACCTCAAGCAGGACCTGTACGCCATCATCCAGGCGAGGC	58:C>T
999511|F|0-67:T>C-67:T>C	TGCAGCAACTCACCCTCTGACAGCCCGAAGCTCTGGTAAAATGCCATTTTCCGCAGCCATGAGTCCCTC	67:T>C
985942|F|0-46:C>T-46:C>T	TGCAGCTGTTGATGCAGCTCATCTCCACCGTCATGGTGGGGCACCTCGGCGAGGTCGCGCTCGCTGGTG	46:C>T
1020374|F|0-37:C>A-37:C>A	TGCAGTACGCCGTCGCCAACTCCTTCCTCGCCGCGCTCTACGCCGACTACATGGAGTCCGTCAACGTCC	37:C>A
3029232|F|0-6:A>G-6:A>G	TGCAGAAGGTCGGGAATGCCCTCAAGCGGTCCGCGGGCTCCG	6:A>G
987623|F|0-8:C>T-8:C>T	TGCAGCAGCGGGTGGTGGACGTCGTCGTAGGGCATGGTTGGCCACGGCTGCATCTCCG	8:C>T
1027722|F|0-66:G>A-66:G>A	TGCAGCGCGTACTGGACGCCGTCCACGTCGCCTGCCGCCGCGAGCCTCCTGACGGTGGCCTCATGGGCC	66:G>A
984858|F|0-14:G>C-14:G>C	TGCAGCTTGCCGCCGAGGCCCCTGGCGAAGGCCTGGGCGATGAAGAAGCGCGCGAAGCGGTGGATGCGC	14:G>C
4407215|F|0-8:G>T-8:G>T	TGCAGGCGGATCGTCCACAACACGGTGGGCAGCGACAAGGCGGTGGCGAAGCTCTTCAACGGGATGTCG	8:G>T
1023501|F|0-67:C>A-67:C>A	TGCAGGAGGTGGACAACATGACGCTGCGGAGGTTCCTGCGCGCGCGCGACCACGACGTAGGCAAGGCCT	67:C>A
1166299|F|0-48:C>T-48:C>T	TGCAGTTCATGGACCTCTCCAACAACTCCTTCTCGGGCCAAATCCCCGCTGCCAAGGCAAGTCACAACT	48:C>T
3940807|F|0-8:G>A-8:G>A	TGCAGCGCGTACGTTGACTAATCGACCCG	8:G>A
3945503|F|0-11:G>A-11:G>A	TGCAGGTCGGTGATCAGCGCCACGTACTTCTCCG	11:G>A
2246609|F|0-6:C>A-6:C>A	TGCAGCCCCACCGCGCCCTCTCAGACAAGGTGGAGCTGATCGAGATCGACCTCTCCGAGGAATCCGCCT	6:C>A
999482|F|0-50:C>T-50:C>T	TGCAGCGTCACCTCCCCAGGCCCAGGCGTCTCCTTCTCCACAATCTCCACCTTCCTCCCTTCGTCCTCC	50:C>T
1031695|F|0-19:C>T-19:C>T	TGCAGGCCTACTTCAACGCCTACTACGCGTCCATCGGGGTGGCCTTCACGGTCGCCGTCACCG	19:C>T
999252|F|0-35:C>A-35:C>A	TGCAGGCTGCTCAGCATCCGCGCCTCCCGCACCTCCCGCCGTCGCACGCGCACCGCCACCGCCG	35:C>A
7343309|F|0-5:C>T-5:C>T	TGCAGCCACTGGTGTCTACCCG	5:C>T
990582|F|0-26:C>T-26:C>T	TGCAGCCAGCCGACGCTGGTCGGCTACTCCCTCTGCGAGCACCACCTCGGCAAGGGCCG	26:C>T
999506|F|0-8:G>C-8:G>C	TGCAGCGCGGCCTCGGGCTGTCGGACAAGCAGGTGGAGGCGTCGCGCATGGCGCTGCACCG	8:G>C
1154341|F|0-26:G>A-26:G>A	TGCAGGACCCCCGCATCGCGGCGGCCGCCCCCGCCCTCATACGCGCCGCCACAGCCTCCG	26:G>A
100543575|F|0-52:A>G-52:A>G	TGCAGCGACGCGGGCGCCTTCTTCTGGAGCCTGCTCGGCGGGCGGTTCTTGCAGGCTAGCACCTCGCCG	52:A>G
3956546|F|0-22:C>T-22:C>T	TGCAGGCCAACATCAAGAAGTCCGTCAACCTCGAGGCCCTCGCCACCG	22:C>T
5562969|F|0-22:G>C-22:G>C	TGCAGAGCGGCAGGTCGGCGGGGAGCGCCTCCAGGCTGAGGTTGGCGTCCCTCAGCGCGAACACCACCT	22:G>C
5562969|F|0-33:G>A-33:G>A	TGCAGAGCGGCAGGTCGGCGGGGAGCGCCTCCAGGCTGAGGTTGGCGTCCCTGAGCGCGAACACCACCT	33:G>A
1080483|F|0-17:G>A-17:G>A	TGCAGGACCTCGCCGACGGCCTCGGCGCCGTCTTCCAGGACTACGTCTCCTTCCTCGCCTCCTGTGGCA	17:G>A
5578282|F|0-25:G>A-25:G>A	TGCAGGCGGCGGCGCAGCGGACGAGGTCCCCG	25:G>A
1050113|F|0-39:C>G-39:C>G	TGCAGCACTCCACCGTGCTCTACCTCAACCACGCCATCGCAGACTTCGCCGAGGCGCTGGCCGCCAAGA	39:C>G
1029774|F|0-20:G>C-20:G>C	TGCAGCCACCCAAGGACGGCGAGTGGTACGAGCGGCTCGCCTACGGCCTGCCCAAAGCCAGGAAGAGCG	20:G>C
987465|F|0-5:G>T-5:G>T	TGCAGGCTCTCGCCATGGACCGCTCCCTCGGACCGCGCTTCAAGATCCCAGCAGGGTCCATCACTGTCT	5:G>T
1025720|F|0-57:A>T-57:A>T	TGCAGGTGCTGTCGCCGCTGGTGCCGATCAGGGAGGTGACGTTCCTGCGCTTCTGCAAGCAGCACGCCG	57:A>T
1021222|F|0-9:C>T-9:C>T	TGCAGGTGACGAAGCGCGACGGCGACCCGCCCAAGAGCGAGTGGCAGAAGTGGAACTGGATATCCGACG	9:C>T
1095686|F|0-10:G>T-10:G>T	TGCAGGTGACGAAGCGCGACGGCGACCCGCCGAAGAGCGAGTGGCAGAAGTGGAACTGGGTATCCGACG	10:G>T
1004340|F|0-15:T>A-15:T>A	TGCAGTATCCTAGCCTGTTGCTGGAGTTCATGGGCAGCTATCTCACCGAGCTGAGCCTTTTGGATTATG	15:T>A
1251843|F|0-53:A>C-53:A>C	TGCAGCTGTTGTCGCCGCCTCGCGCGACGGCTTCTTCGGCCTGGGCGAGTGCAAGGGCCAGGTGGAGAT	53:A>C
2252536|F|0-47:C>A-47:C>A	TGCAGCACCCGCAGCCTCTCCTTGTCGATGTACATAGGAGACTTCTCCTCCTGCTCCTCTTCCTCGGCG	47:C>A
1862571|F|0-59:C>T-59:C>T	TGCAGCTCCAGTCAGGGAACTCGACCAAGAGCTTCAGCCGCGAGTGCCAGGTCCTGAAGCGCATCCGCC	59:C>T
1022323|F|0-17:G>A-17:G>A	TGCAGGAACTCTCTGGTGACCTCCTCCTCGTCGGCCTCCAGCCCCTCCACCGCCGCCTCGTCCTCCTGC	17:G>A
1097879|F|0-65:G>A-65:G>A	TGCAGGACTGGCAGAGGCGGTACTGCGAGATGCTGAAGGACAGCGGCAAGGACGTGAGGGCGTTGGAGT	65:G>A

1024561|F|0-33:T>A-33:T>A	TGCAGGCGTTCGCCCTCGTCCTGAACAAGCGCCTCGCCGACGACGACGCCG	33:T>A
7353656|F|0-6:A>G-6:A>G	TGCAGCACACGATGGACCCG	6:A>G
1695238|F|0-13:C>T-13:C>T	TGCAGATGCCTCGCGCCACGGTGGAGGAGAGGAACCTGGACGGCGGCATCGAGGAGGAGAGCATCGCGC	13:C>T
1378383|F|0-19:C>G-19:C>G	TGCAGGCCATGGACTCCTCCCACGTCGCGCTCGTGGCCCTCCTCCTCCGCTCCGAGGGCTTCGAGCACT	19:C>G
1246053|F|0-31:T>C-31:T>C	TGCAGGCGGCGCGTGCGGGTACGGTAACCTCTACAGTACGGGGTACGGCACGAACACGGCGGCGCTGAG	31:T>C
986643|F|0-8:C>T-8:C>T	TGCAGGTACGAGGACGAATGGGCGGCGCCGTACAGCTGCTCCGCCCTCGTCGTCGCCATGCCCAGGCCG	8:C>T
3951950|F|0-8:C>T-8:C>T	TGCAGCGCCTGCTTGGAGCCATCGAGCCCG	8:C>T
1862305|F|0-59:A>T-59:A>T	TGCAGCCGAGGGCGAGCGGGTAGGCGGCGAGGTCGTCGAGGCCGAGGCCGAGGGAGCGAAGGAACTCAG	59:A>T
1862305|F|0-64:C>T-64:C>T	TGCAGCCGAGGGCGAGCGGGTAGGCGGCGAGGTCGTCGAGGCCGAGGCCGAGGGAGCGAAGGAACTCAG	64:C>T
5371683|F|0-14:C>T-14:C>T	TGCAGGAGCTGGCGCGCCATCCTCACCGACCCCG	14:C>T
3947227|F|0-34:G>A-34:G>A	TGCAGCAGTGCTCGATCTGGCCGAGGGACGTGTCGTCGCGCCGCGACGGCGGCAGGTCGTGCACCACCG	34:G>A
986367|F|0-45:G>A-45:G>A	TGCAGCGACGCCGAGTACCGCACCGCCTCCCGCACCGTCAGCGTCGTCATCAGCACGTCGTCCTGCGTC	45:G>A
1056056|F|0-46:G>C-46:G>C	TGCAGGTGGTGGCTCCTCATCTTGTCGCCGACGCCGAGGGCGTCAAGCGCGCGGAAGGCGTTGGTCCAT	46:G>C
1021879|F|0-41:C>T-41:C>T	TGCAGCAGCCGCTCCACCACGTGGCTGAGGCAGCACCCGCGCCTCCCCACCACCAGCACCG	41:C>T
1126648|F|0-10:G>A-10:G>A	TGCAGCGCCTGCCGCGGATCGTCGGCTACGGGAACGCCGCTGACCTCGCCCTCACCG	10:G>A
5363838|F|0-8:G>A-8:G>A	TGCAGGCTGGCGCACTTCCCGAGCTCCG	8:G>A
32027727|F|0-9:G>A-9:G>A	TGCAGCGGAGGACCGTGGCCAGCTTCATCATCGGCGTGATGTGCCCCTGCGCCG	9:G>A
1223620|F|0-11:C>A-11:C>A	TGCAGGTCGAGCGCCTCGCCGCGGAGCTTGTCGGGGAGGGCGTCCACGAGCGCGATCTCGTCCGCCAGG	11:C>A
100586575|F|0-53:G>T-53:G>T	TGCAGGATCCCCGTGGCCACCGGCATCATCATGACGGTGCAGGGCGTGTTGTGGATCCACATGCTGACG	53:G>T
2243750|F|0-46:C>G-46:C>G	TGCAGTGCTCCTGCACCTCCTCGATGTCGTACTGCGTGAGCATCGACGACGCGTTCCCCATCGCCGAGA	46:C>G
3946185|F|0-36:A>C-36:A>C	TGCAGGAGATCATCGGCGTGGCGTTGCCGTCCGTGGAGATGCGTAACCG	36:A>C
3938551|F|0-29:C>A-29:C>A	TGCAGTGGGTCAAACCCACCGACCACCACCATCGCCG	29:C>A
2243250|F|0-20:C>T-20:C>T	TGCAGTATGGTGAGAGCGTCCAGCTTCCCGCGGTTGAAGTCGTCGGGGATGTCGTCGTCGATGAACGGG	20:C>T
14583246|F|0-18:G>T-18:G>T	TGCAGCCGCTCCGCCGCCGCCG	18:G>T
1864156|F|0-49:G>C-49:G>C	TGCAGAGCAACGCGCTCGCCGTCCTCGAGGCCGTCGACATGCCCGCCGCGGACCAGATCATGGACGCCG	49:G>C
7352112|F|0-7:A>G-7:A>G	TGCAGTCAATAAACCCGCACCG	7:A>G
1667137|F|0-22:G>C-22:G>C	TGCAGGCCGAGTCCGACTTCGTGTACGTCCTCGACGACGACATGATCCCCG	22:G>C
5971117|F|0-7:T>G-7:T>G	TGCAGGTTGTTTCCGTCATGTTCGCCG	7:T>G
3028034|F|0-37:T>C-37:T>C	TGCAGCACGCCGCCGCCTTCCTCCCGCGCGTCCGCATTGTCTCCG	37:T>C
1865271|F|0-41:T>G-41:T>G	TGCAGGTGGCTCTCGGAGAGGGAGCCGATGTAGTAGGGCTGTCGGTGGTCGAACTTGTTGAGGACGGTG	41:T>G
4005907|F|0-38:A>G-38:A>G	TGCAGCAGCCTGATGTGGCTGGACCTCAACAGCAACAGACTCACCG	38:A>G
3945603|F|0-37:T>C-37:T>C	TGCAGTTCATGGACCTCTCCAACAACTCCTTCTCGGGTCAAATCCCCGCTGCCGAGGCAAGTCACAACT	37:T>C
1863083|F|0-16:G>C-16:G>C	TGCAGCGCGAGACGTTGCCTGATGTGATGAGGCTCATGGACAAGCACGAGCAGATCGACCGCATCCTTT	16:G>C
11314403|F|0-19:C>G-19:C>G	TGCAGCATGCCGAGCTCTGCCGGGATCGGCCCG	19:C>G
7342612|F|0-17:A>G-17:A>G	TGCAGGTCGACGGCCTCACCG	17:A>G
3027093|F|0-6:C>G-6:C>G	TGCAGCCCGAGCTTGAGCGTGTCCATGAGCTCGTCCCCCTCCG	6:C>G
1100627|F|0-14:T>C-14:T>C	TGCAGCCTCTGCATTCTGCCACGCTTAGCCTCCCTCAGCCTCTCCGCCTCCGATTCCAGCCTCGCGATC	14:T>C
3022332|F|0-17:A>G-17:A>G	TGCAGGGTAACCATCTCAAAGAAGGAGTCCTCTGGCCCG	17:A>G
1024822|F|0-26:A>G-26:A>G	TGCAGCTGCTCTTCCTCCACCGTCTTAACCACGGCGTGGGTCACGGCGACGCTGCCGCAGCCG	26:A>G
6050253|F|0-17:A>G-17:A>G	TGCAGCACCTCCTGTGCATACTCCG	17:A>G
21311350|F|0-9:A>T-9:A>T	TGCAGGATCATCAGGAGGACCATCACCTACCG	9:A>T
6035530|F|0-13:G>C-13:G>C	TGCAGAACGTCCTGCCCACGCAGCTCCTCTCCCTCCTCCACTCGCTCTGGCAGTCGCTCCAGGACTCGC	13:G>C
7169645|F|0-28:A>G-28:A>G	TGCAGGAGCGGGCGACGAGGCGGAGGGCATCGGTGGAGAAGCCG	28:A>G
7170761|F|0-8:T>C-8:T>C	TGCAGCTTTTCCTCGGGGCTTGCCTGGAGGAGGTAGGATGTGACGCCG	8:T>C
6046886|F|0-7:C>G-7:C>G	TGCAGGTCAGCATCACCAGCCAGGCGCTGATATTCGTGACCCGCAGCCG	7:C>G
7492233|F|0-31:T>G-31:T>G	TGCAGGACATGCGGGAGGTGACGAGGCCGTATAGCTTCATGACGTTGGGGTGGTCGAGGCGGCGGAGGA	31:T>G
6041609|F|0-17:G>C-17:G>C	TGCAGCTTCAGGAGGTGGATCCG	17:G>C
14385281|F|0-62:A>G-62:A>G	TGCAGGCGCCAGCCAGGGACGTCGTCGCCGTGCTCTCCGCCTACGTGCGCAAGCTCGAGGAGAAGTCCG	62:A>G
21311046|F|0-23:A>G-23:A>G	TGCAGCGGATGGGCGTGGGGAGGATCCTGGTGGTTCTGGCCATGGCGACGGCGGCGCTGGTGGAGAGCC	23:A>G
6045791|F|0-8:G>C-8:G>C	TGCAGGTGGCCCCGCACGATGAACAGCATCCTCTGCACCG	8:G>C
16796508|F|0-35:T>C-35:T>C	TGCAGGCACCGCGCCAGCACCCGCTTGGCCTCCTCTTGGCGGTGCTCCG	35:T>C
32020797|F|0-58:A>C-58:A>C	TGCAGGACCAGATCTCGTCGGCGGACGACGTGACGTCGGCGGAGCGGAAGGACGCGGAAGGGCAGGTGT	58:A>C
5372873|F|0-7:A>C-7:A>C	TGCAGCAACTGCTGGACATCCCG	7:A>C
100472288|F|0-32:T>C-32:T>C	TGCAGGTACGAGAAGAGTTATATGCACAGGGATGTCGTCACGCACGTCGCCGTTTCCCCTGCTGACTTC	32:T>C
100514232|F|0-27:T>C-27:T>C	TGCAGCGGTTCCTGTCGCTCAACTCGGTGCGCCACCACCACCTCATCCTCCGCGCCTTCGCCGACGTCT	27:T>C
7170248|F|0-64:T>C-64:T>C	TGCAGGAGCACCGCCTGCCGCTCTCCAACCTCGACCTCATCCTGCCCCCCATCGACGTCAGCGTTTTCT	64:T>C
100520262|F|0-17:G>C-17:G>C	TGCAGCCACCTGTGCTGGGCCGTGCCG	17:G>C
1022874|F|0-56:G>C-56:G>C	TGCAGGGGATTTGGACACCGCCCACAGTCTGCTGGACGAGATGCCCGTCAAGGATGGTGTGGCTGTCAA	56:G>C
100417401|F|0-13:C>T-13:C>T	TGCAGGCCGAGCACATGGAGAAGTTCCG	13:C>T

7916331|F|0-28:C>G-28:C>G	TGCAGGCGCAGCTCGCCTCGCTCAAGGCCCACGCCCCGCCG	28:C>G
7918704|F|0-14:G>C-14:G>C	TGCAGCTCGTCCGCGGCCAGCTCCGCGTCGCCGTCCG	14:G>C
1281739|F|0-29:T>C-29:T>C	TGCAGGGTGCTGCTGATGCTGCTGGCGGATGGGCTGCGCGGTCACCTGGCCGACCTGGCCGAGCTGACC	29:T>C
5332174|F|0-5:C>T-5:C>T	TGCAGCTTGTCAAGAATCATATCCAGCGCCTGCCG	5:C>T
7167403|F|0-22:G>C-22:G>C	TGCAGAAGATGAACTCCATCTGGGAGACCCG	22:G>C
100303012|F|0-10:A>G-10:A>G	TGCAGGAGACATACGCGTGCTCGCCG	10:A>G
100303076|F|0-10:A>G-10:A>G	TGCAGGAGGCACGCGCGCTCCTCGAGTACAACCCCCG	10:A>G
3959896|F|0-19:G>C-19:G>C	TGCAGGTACCAAAGGCTCAGCCCG	19:G>C
100303747|F|0-19:T>C-19:T>C	TGCAGGTCTTGGGCGCGTGTTTGTGGTACATCTGCCG	19:T>C
5578451|F|0-13:C>T-13:C>T	TGCAGTGCGAGCACCGCCTCGTCATCCCCG	13:C>T
2246615|F|0-28:G>A-28:G>A	TGCAGAAGGTGTTCTACCGCTCCAACGCGGCGCGGGAGGCGTTCGTGGAGATGTGCGCCGACGACTACG	28:G>A
1065387|F|0-32:C>G-32:C>G	TGCAGCATCTTGATCACGTCCACCGTGTCGTCCAGGTAGTACTTGGCCATGCTCGGCTTCTTCCCCACG	32:C>G
1055791|F|0-50:C>G-50:C>G	TGCAGCTGCGCGGCGTGGATGGTGTGCGACACCGCCAGCGTCAGCCGCGCCGTCACGTCGCTGTGGGTG	50:C>G
3023916|F|0-19:G>C-19:G>C	TGCAGAAGATCACCAGCCTGCTGCTCTGGGGCAATGCCCTCTCCG	19:G>C
100350486|F|0-7:A>G-7:A>G	TGCAGCAACATCGCGCGCTCGTTGCCG	7:A>G
1023632|F|0-25:G>A-25:G>A	TGCAGGCGCTCAAGGCCAAGGAGGCGCAGAAGAAGGTGGCGCAGGCGGACGAGGAGGCCG	25:G>A
1020188|F|0-29:G>C-29:G>C	TGCAGCTTGAGGGAGATGCCGCGCACGGGGCCGCGCTGGATGCGGCGCATCAGATGGGTGGTGAAGCCG	29:G>C
1030791|F|0-28:A>T-28:A>T	TGCAGCTTGCGGAGGTCCTCGGTGGGGAAGTTGTCGTCGCCCATGTAGTTGGACAGCCATTCCAGCTCC	28:A>T
5577874|F|0-10:A>C-10:A>C	TGCAGAGCATACAGGCGGCGCAGATGCTGCCCCG	10:A>C
12775319|F|0-11:G>C-11:G>C	TGCAGCAGCTCGCCCAGCGTCACCACCG	11:G>C
5578080|F|0-6:G>C-6:G>C	TGCAGCGGATGGGCTTGTCGGTGCCG	6:G>C
100387367|F|0-13:C>G-13:C>G	TGCAGCTGCGGGTCAACGGGGCGCCG	13:C>G
3959764|F|0-32:T>C-32:T>C	TGCAGGAAGGAGATGGCCCCCACGCAGCCGTATACCG	32:T>C
1024349|F|0-11:T>C-11:T>C	TGCAGGCGCAGTTCGGGAACAAGTGGGCGAGGATCGCGACGTACCTGCCCG	11:T>C
100388413|F|0-13:T>C-13:T>C	TGCAGGGCCACGTTACCAACCCCGCCG	13:T>C
6041847|F|0-22:G>A-22:G>A	TGCAGGTGACCACGCAGGCGCCGCCGCCGCCCATGTCGGTGGCCTCCG	22:G>A
4262614|F|0-10:G>C-10:G>C	TGCAGTCGCCGTTCACGTTCCGCAAGGACGGGCAGGCCAGCCG	10:G>C
6030590|F|0-7:C>T-7:C>T	TGCAGCTCACCGTGTCGAGCCG	7:C>T
1041707|F|0-16:C>T-16:C>T	TGCAGGGCATCTGCTTCGCCTTGGGGCTCCTCGTCTCGCAGGTCCTCAACGAGCTCATCAAGCACTCCG	16:C>T
1019104|F|0-34:G>T-34:G>T	TGCAGACCTCGGAGATGGCCAGGATGGCCAAGACGTACGGCAAGTTCTGGTGCACGTGGCAGGTGGACC	34:G>T
1068079|F|0-13:G>A-13:G>A	TGCAGCAGAAGGAGTCGGCGTTCGCGCTGGCGGCGCTCATGGAGGTGCCCATCCAGTACAAGGCCACCA	13:G>A
1032582|F|0-24:A>T-24:A>T	TGCAGCCCCTATTTAGTGATCATCAGCTAGCCGTCGCATCCCTCCCTCTCGTGTCTGCCTCCTGCCCAA	24:A>T
1278033|F|0-11:G>A-11:G>A	TGCAGCTCGGCGATGGTGTCGACGCGAACGGCGCGGCAGAGCTCGCGAAGCTGGATGCGGCGGCGCTCA	11:G>A
1235667|F|0-28:G>T-28:G>T	TGCAGGAGAGCTGCGCGGACGCGTCGGGGTCGCTGGTGGTGTACGCGCCGATCGACCTCCCG	28:G>T
3385186|F|0-5:C>G-5:C>G	TGCAGCGCCAGCATCGCCACGTGGAGCTGCGCCTTCTCCG	5:C>G
4396686|F|0-7:G>T-7:G>T	TGCAGCTGGCGCTGCTGCTGTTTGCAATGGCCCG	7:G>T
3954211|F|0-14:C>T-14:C>T	TGCAGGATCCTCGACCTCCTCTCCTTGGGGTGCCG	14:C>T
1383897|F|0-19:G>A-19:G>A	TGCAGGTGATGGAGCGCAAGATGCGGTCCAGCGGCATCGAGCGGCTCATCAAGCGCCG	19:G>A
3028272|F|0-13:C>T-13:C>T	TGCAGGTGCTCGACGTCAGCTCCAACATGCTCAACGGCACGCTCCCG	13:C>T
5363999|F|0-13:T>C-13:T>C	TGCAGTACATGGATCTCGCCAACAACTCGCTCGCCG	13:T>C
7909250|F|0-19:C>A-19:C>A	TGCAGTCCTTCCCGCCCGCCGAGGCCATCCG	19:C>A
4262739|F|0-25:C>G-25:C>G	TGCAGTGCGTCGTGCACGTCTCCCTCGTCTGCTCCG	25:C>G
3944781|F|0-14:A>G-14:A>G	TGCAGTTGCAGCGGATCTCGCCG	14:A>G
1014406|F|0-41:G>A-41:G>A	TGCAGTTTCACATACGGGTCGCTGAACCCGTTGAAATCCATGGCGCGCAGGTTGCGCGCTCCGATCACA	41:G>A
1014406|F|0-44:G>A-44:G>A	TGCAGTTTCACATACGGGTCGCTGAACCCGTTGAAATCCATGGCGCGCAGGTTGCGCGCTCCGATCACA	44:G>A
2262365|F|0-68:G>C-68:G>C	TGCAGCTCGGCGACGGTCTCGAAGAGCTGCGTCTGGAGGGAGGTGAGCATCGACATGGCCTCCTTGGTG	68:G>C
6040394|F|0-46:G>C-46:G>C	TGCAGGACCAGATCTCGTCGGCGGACGACGTGACGTCGGCGGAGCGGAAGGACGCGGACGGGCAGGTGT	46:G>C
1164510|F|0-67:C>T-67:C>T	TGCAGGCCTACTACTTCAGCAAGGACGAGCGGCTCATGGTGTACGAGTTCGTCGCCACGGGCAGCCTCT	67:C>T
1694672|F|0-34:G>C-34:G>C	TGCAGGTGCCCTCGACGTCCTTCATGAGCTTGGCGACGAGCTTGTCGCGGAGGTGCGGGCCGAAGTGCC	34:G>C
6037648|F|0-5:C>T-5:C>T	TGCAGCAGCTTGCTTCCCTCCCCGACAAACCG	5:C>T
989467|F|0-32:C>G-32:C>G	TGCAGCATCACCTCCGACACCCACACCGCATACGCCCTCTTCTCTCTGCCCTCGCGCCCCG	32:C>G
7167685|F|0-16:G>A-16:G>A	TGCAGCCAGAGCGAGCGCGAGCCCGAGGCCG	16:G>A
100440225|F|0-29:A>G-29:A>G	TGCAGCCTCTGCTGCTGCTCCCTGTCGTGATCGAACCGCCG	29:A>G
100440315|F|0-19:C>G-19:C>G	TGCAGCGAGAAGAGCTCGACCGACCCG	19:C>G
100440900|F|0-13:T>C-13:T>C	TGCAGCTCGCAAGTTGCATCGGGCTCATCGCCG	13:T>C
7909245|F|0-19:G>T-19:G>T	TGCAGTCCACGCTGTGGCCGCTGGGCCACCCG	19:G>T
6044249|F|0-19:G>T-19:G>T	TGCAGTCGCCTCTGAGGGGGGAGTCTGCACCG	19:G>T
988194|F|0-16:G>C-16:G>C	TGCAGAAGGAGCTGGCGGAGCTGCGCGCGCTCAAGACGGTGCACCCCTTCTACATGCACCTCCCGAGAT	16:G>C
1052648|F|0-41:C>A-41:C>A	TGCAGCAGGCTCACGAGCTCCTCCTCCGACTGTGACGAAACCATACAAAGGTACAGTTTGCAACAGAAG	41:C>A

<table>
<tr><td>1250733|F|0-56:G>A-56:G>A</td><td>TGCAGCATGTACCGCCCGTTGTTCATCAGGAAGATGCTGCTCAGCGCCAGGTCCTTGTACAGCTTCGAC</td><td>56:G>A</td></tr>
<tr><td>1006013|F|0-11:G>A-11:G>A</td><td>TGCAGCGTGGCGACGGAGCGCGCGCGGTGGTAGTCCTCGTGGGTGCGGCACTTGATGGTGTAGTCCGCC</td><td>11:G>A</td></tr>
<tr><td>1020101|F|0-12:T>A-12:T>A</td><td>TGCAGGTGGGGTTCTACTCCAAGTCGTGCCCCAGCGCCGAGTCCACCGTCGCGTCCGTCGTCCG</td><td>12:T>A</td></tr>
<tr><td>1065426|F|0-50:C>A-50:C>A</td><td>TGCAGCACCTTCCGCACCCCCGCCTCGCCGTCCACCGCCAGCGAGTACAGCACCG</td><td>50:C>A</td></tr>
<tr><td>1862986|F|0-34:C>G-34:C>G</td><td>TGCAGCCGCGCGGGGACAGCTACACGCGGCGGTCCCTGTTCGACTGCATGGTGGCCG</td><td>34:C>G</td></tr>
<tr><td>100461469|F|0-8:T>C-8:T>C</td><td>TGCAGCGCTGCGCTGCTGTGCACGCGGTTGTTCGCCG</td><td>8:T>C</td></tr>
<tr><td>4990067|F|0-6:G>A-6:G>A</td><td>TGCAGCGGCCCACAGGCGGTGGTGTACTGCCG</td><td>6:G>A</td></tr>
<tr><td>3958507|F|0-26:C>T-26:C>T</td><td>TGCAGCTCGCGCTCCTCCTCCGCCGTCAGCGCCGCCG</td><td>26:C>T</td></tr>
<tr><td>7167999|F|0-13:A>T-13:A>T</td><td>TGCAGCTTGGGCGACTTGGGCGTCGGGGGCGGCGAGGACGCGCCG</td><td>13:A>T</td></tr>
<tr><td>1021132|F|0-13:C>T-13:C>T</td><td>TGCAGGCGTACACCAGGGCACGCTACGGCGAGGACGCGATGATCGTGTACCG</td><td>13:C>T</td></tr>
<tr><td>1061650|F|0-17:G>A-17:G>A</td><td>TGCAGGGTGAGCACGTCGGTCTTCATGTTGGGCTCGTTCTCCGCCACGCCCG</td><td>17:G>A</td></tr>
<tr><td>2249021|F|0-15:A>T-15:A>T</td><td>TGCAGAACAGCGCCAAGGGGAGGGAGGTCGTCGAGCGGATCTGCCGCTACCTCGAGGCCGAGGCCGCCG</td><td>15:A>T</td></tr>
<tr><td>2246126|F|0-53:C>T-53:C>T</td><td>TGCAGACCGCACCAGATGTCGTTCTGGTTGGACTCGGTGATGCCACGGTCGATCACAGCACTGGCCTCC</td><td>53:C>T</td></tr>
<tr><td>1018334|F|0-49:G>C-49:G>C</td><td>TGCAGCAGTTCTCGGCGATGAACAAGTTCAAGAAGAAGGCGCTGGGCGTGGTGGCGCGGAACATGCCGA</td><td>49:G>C</td></tr>
<tr><td>987498|F|0-43:T>C-43:T>C</td><td>TGCAGCTTCACGTGGTTGTAGTAGCCCACGCCCAGGAAGGCGATCCCGTAGCCGAAGAGGTTGATCGGG</td><td>43:T>C</td></tr>
<tr><td>1020327|F|0-34:G>C-34:G>C</td><td>TGCAGGCGGAGACGCCGTCGCTGGGGGACTTCGCGTACTCGGTGGAGGTGGGGACGCGGAAGAAGCCGC</td><td>34:G>C</td></tr>
<tr><td>1043238|F|0-68:C>A-68:C>A</td><td>TGCAGGTGGCTCTTGACGTGCGCCAGCGTCAGATCCTTGACGTTCATCAGCTCCAGCACCGACTTCGGC</td><td>68:C>A</td></tr>
<tr><td>4002486|F|0-13:G>A-13:G>A</td><td>TGCAGCAGAGCGCGCTGACGACCTCGCGCGCGCGCCG</td><td>13:G>A</td></tr>
<tr><td>4002486|F|0-28:C>T-28:C>T</td><td>TGCAGCAGAGCGCGCTGACGACGTCGCGCGCGCGCCG</td><td>28:C>T</td></tr>
<tr><td>100479513|F|0-22:A>G-22:A>G</td><td>TGCAGCGACGCCGATGCCGTCAAGTCCG</td><td>22:A>G</td></tr>
<tr><td>100480301|F|0-17:A>G-17:A>G</td><td>TGCAGCTTCGAGAGGTTAGCAAGAGAGGCCG</td><td>17:A>G</td></tr>
<tr><td>6030670|F|0-25:T>C-25:T>C</td><td>TGCAGCTTCTACGCCTCCACGTCCCTCCCG</td><td>25:T>C</td></tr>
<tr><td>988439|F|0-29:G>C-29:G>C</td><td>TGCAGCTTGAGGACGGTGGTCTTCTGCGTGTCGGTGAGCACGCTTCCGCCCCCG</td><td>29:G>C</td></tr>
<tr><td>3938865|F|0-5:G>C-5:G>C</td><td>TGCAGGATCTGCGCGGCGCTCCAGCTGGCCCCG</td><td>5:G>C</td></tr>
<tr><td>1023984|F|0-43:C>T-43:C>T</td><td>TGCAGGCTTTCCAGGCCGTGGAGGCGGCCGTGCGGAGGTTCTTCTTCGCGTACCG</td><td>43:C>T</td></tr>
<tr><td>1667127|F|0-22:G>A-22:G>A</td><td>TGCAGGGCGGGATCCCGTTCGAGAAGTCCAAGGGCCAGCACATCCTGCGGAACCCG</td><td>22:G>A</td></tr>
<tr><td>1067716|F|0-41:C>T-41:C>T</td><td>TGCAGGGGATGCCGTACCTCAAGGCCGTGGTGCTGGAGGGGCTGAGGCGGCACCCGCCG</td><td>41:C>T</td></tr>
<tr><td>1088261|F|0-31:C>A-31:C>A</td><td>TGCAGGTCGCGGAGAAGCCCAGCATCGCGCTCTCCATCGCCTCCGCCCTCTCCG</td><td>31:C>A</td></tr>
<tr><td>3935771|F|0-13:C>G-13:C>G</td><td>TGCAGTCGATAAACCCGCACCG</td><td>13:C>G</td></tr>
<tr><td>5358687|F|0-7:G>A-7:G>A</td><td>TGCAGTTGACGACGAGGATGCCGACGTCCTTGGGCTTGACGCCG</td><td>7:G>A</td></tr>
<tr><td>5367153|F|0-15:C>A-15:C>A</td><td>TGCAGCTCCAGGATGCCCCCGCCG</td><td>15:C>A</td></tr>
<tr><td>1082056|F|0-52:C>T-52:C>T</td><td>TGCAGATCTCCACCATGTTCACGCACCACCAGAAGACCGTCATCGTGGACCACGACATGCCCCAGAGCG</td><td>52:C>T</td></tr>
<tr><td>1862657|F|0-34:C>T-34:C>T</td><td>TGCAGCACGGGAAGACGGTGTTCATGGACATGCTCGTGGAGCAGACCCACGAGGTGGACACGTTCGACT</td><td>34:C>T</td></tr>
<tr><td>2247355|F|0-14:C>A-14:C>A</td><td>TGCAGCATCCTCATCTGCATCTGCTTCGGCGCCAAGATCCCCGACGAGCTCATCGTGGAGATCGAGGAG</td><td>14:C>A</td></tr>
<tr><td>2247355|F|0-44:C>T-44:C>T</td><td>TGCAGCATCCTCATCTGCATCTGCTTCGGCGCCAAGATCCCCGACGAGCTCATCGTGGAGATCGAGGAG</td><td>44:C>T</td></tr>
<tr><td>1863083|F|0-32:C>T-32:C>T</td><td>TGCAGCGCGAGACGTTCCCTGATGTGATGAGGCTCATGGACAAGCACGAGCAGATCGACCGCATCCTTT</td><td>32:C>T</td></tr>
<tr><td>100486823|F|0-58:T>C-58:T>C</td><td>TGCAGCGCGAGACGTTCCCTGATGTGATGAGGCTCATGGACAAGCACGAGCAGATCGATCGCATCCTTT</td><td>58:T>C</td></tr>
<tr><td>1218352|F|0-48:T>C-48:T>C</td><td>TGCAGCGCGCTGATGACGGTGCGGAGCGCGCGGCTCGGGGACCCGCGGTTGGCGAGCATGATCTCGCCG</td><td>48:T>C</td></tr>
<tr><td>100487158|F|0-49:A>G-49:A>G</td><td>TGCAGCGTTCTCTGAGGTACTCCTCGAGCGGGCTGAGGTTGGTGTGCTCAGTGCTGGAGATGGACTCGG</td><td>49:A>G</td></tr>
<tr><td>2243289|F|0-62:C>G-62:C>G</td><td>TGCAGCTGCGAGCTCAGGTCCTCGATCTTGGCCTCCCCGAAGATCACGTACGTCTCCGACGTCGGGCTC</td><td>62:C>G</td></tr>
<tr><td>23919584|F|0-61:C>T-61:C>T</td><td>TGCAGGAGCTGGTGCCCAACGGCAACAAGGTGGACATGGTCACCATGCTCGACAAGGCCATCACTTACG</td><td>61:C>T</td></tr>
<tr><td>2276886|F|0-28:C>T-28:C>T</td><td>TGCAGGCATCTCCGCTGCTGACTGAAGACAGAGCTCAGGAATGCATCGACCCGAGGCTCGGCGACAAGT</td><td>28:C>T</td></tr>
<tr><td>2276886|F|0-49:C>T-49:C>T</td><td>TGCAGGCATCTCCGCTGCTGACTGAAGACAGAGCTCAGGAATGCATCGACCCGAGGCTCGGCGACAAGT</td><td>49:C>T</td></tr>
<tr><td>2276886|F|0-61:C>A-61:C>A</td><td>TGCAGGCATCTCCGCTGCTGACTGAAGACAGAGCTCAGGAATGCATCGACCCGAGGCTCGGCGACAAGT</td><td>61:C>A</td></tr>
<tr><td>3947066|F|0-14:G>A-14:G>A</td><td>TGCAGGTTCCTCTCGAACTCCCTCAGGTAGTCCAGGAGCAAAACGATCACGAAGAGGATGAACCCACCG</td><td>14:G>A</td></tr>
<tr><td>3947066|F|0-40:A>G-40:A>G</td><td>TGCAGGTTCCTCTCGAACTCCCTCAGGTAGTCCAGGAGCAAAACGATCACGAAGAGGATGAACCCACCG</td><td>40:A>G</td></tr>
<tr><td>1019445|F|0-52:C>G-52:C>G</td><td>TGCAGTCCCTCCTGGCTCTGTCCGACAGGGAGAGCGTCGTGTACAGCTACGGCATGGTGCTGCTGGAGA</td><td>52:C>G</td></tr>
<tr><td>3937142|F|0-31:G>T-31:G>T</td><td>TGCAGAAGCTTCTGTTGGCCGACAACAAGCTGTCCG</td><td>31:G>T</td></tr>
<tr><td>7164783|F|0-6:C>T-6:C>T</td><td>TGCAGACGGCTCGGAGTCTCGACATCCACCG</td><td>6:C>T</td></tr>
<tr><td>1152398|F|0-8:C>T-8:C>T</td><td>TGCAGCGACGACACCAAGTTCTCCGACGGCACGGGCCACCGTGACACCG</td><td>8:C>T</td></tr>
<tr><td>5582352|F|0-6:G>A-6:G>A</td><td>TGCAGCGCCTCAGCGCCGAGGTGCTCACCCG</td><td>6:G>A</td></tr>
<tr><td>985964|F|0-34:C>T-34:C>T</td><td>TGCAGCGTTACCAGCCGTTCGGGTCGCCGCCTGGCGAGTACCACCATTTCGGCCCG</td><td>34:C>T</td></tr>
<tr><td>1068826|F|0-29:G>C-29:G>C</td><td>TGCAGGAAAGAGGAGTAGATGGCGCAGAAGGGCTTGAGCCCCTCGCAGGCCAGGCCG</td><td>29:G>C</td></tr>
<tr><td>4262345|F|0-5:G>A-5:G>A</td><td>TGCAGGTGCCCACCCTCAGGAGCTGCGACTTCACGCGGCTCTACTCCG</td><td>5:G>A</td></tr>
<tr><td>986473|F|0-7:G>T-7:G>T</td><td>TGCAGACGTCGCGGGAGCCCACGCCGCGGCAGTCCAGCTTCAACCAGTCCGACTTCTACTCCATGTTCA</td><td>7:G>T</td></tr>
<tr><td>1025864|F|0-23:G>A-23:G>A</td><td>TGCAGGGACATGCCGAGGAAGGCGAGCATGGCGAGGCGGCCGTTCTTGATCTCCTTGAGCTTGAGCTCC</td><td>23:G>A</td></tr>
<tr><td>1150941|F|0-63:C>G-63:C>G</td><td>TGCAGAGCATCACCACCATGCCCATCCTCGTCAAGGGAGTCATCACCGCGGAAGACGGTAACCCGTCTC</td><td>63:C>G</td></tr>
<tr><td>1215118|F|0-7:C>T-7:C>T</td><td>TGCAGAGCTACTGCGACGTGTCGGTGGTGAGCGGCTGGTCCACCGTGGGGTACGTCGGGCACGGCCTCG</td><td>7:C>T</td></tr>
<tr><td>1343244|F|0-48:C>A-48:C>A</td><td>TGCAGATGCGGCAGGCGCTCTTCTACGAGGACATGCAGAGCGTGCTGGCGCGCGTCCACGCCG</td><td>48:C>A</td></tr>
</table>

2256219|F|0-53:C>A-53:C>A	TGCAGCAGCAGCCACCCCGCGAACCCCACCACGTTCGCCAGCATCGACGCCGACCCCAGCACCCACCGC	53:C>A
1694774|F|0-50:G>C-50:G>C	TGCAGCATGGGCGACAGGTCGGAGAAGGACTTGTAGGCCGCCAGCTTGAGGGCGACGTCGAGCGGTGAG	50:G>C
100501329|F|0-58:T>A-58:T>A	TGCAGCGAGGAGTGCAGGACGGAGCAGATGGAGGCGGACGAGGAGATGGAGAGGAAGGTGAAGAGCGCG	58:T>A
1021956|F|0-56:C>A-56:C>A	TGCAGCTGCTCTTCCAACTGCCGCTTGTTCCCGCGGCGCCGTGACCAGAGGGTGCTCCCCTCGGGCTTC	56:C>A
1864938|F|0-11:G>A-11:G>A	TGCAGGACAACGGCGTCTTCTTCGACCACTTCAAGAACTCCATCAAGCGCATGGGCCAGATCGGCGTCC	11:G>A
25949778|F|0-43:C>G-43:C>G	TGCAGGCGATGAGGGACACCTTCGACGGGACGCTGATGGTCGGCGGAGGGTACGGCAGGGAGGAGGGGA	43:C>G
1696481|F|0-55:G>A-55:G>A	TGCAGGTACTCTGGCTCGAGCGCGAACAGCACGGACACGCCGTCGTCCTTGTCGGGCGCCTGCGTGACG	55:G>A
5971217|F|0-10:G>T-10:G>T	TGCAGCAACAGAAGCAGCCGCGTGAGCAGCCCG	10:G>T
6045398|F|0-23:G>A-23:G>A	TGCAGCAACAGCAGCAGCCGCGTGAGAAGGCCG	23:G>A
100508715|F|0-6:C>G-6:C>G	TGCAGCCCCGCTGCGCCCAGCGCCGCCGATCCG	6:C>G
3030529|F|0-27:G>A-27:G>A	TGCAGCGCGAGTTCCGCGGCGCCGTCAGGTACGCCATGGAGCGCCG	27:G>A
12776768|F|0-36:C>G-36:C>G	TGCAGGAACTCGTCGTCGCAGAGGAGGCGGCCCATGCTGTCCG	36:C>G
5580569|F|0-37:C>A-37:C>A	TGCAGTCGTCGTTGCTGGCGAAGGAGACGTCGGGGGCCACGGCGGCGGCCCG	37:C>A
4261722|F|0-20:T>G-20:T>G	TGCAGTTCATCGGGCAGGGCTTCCGCTCCG	20:T>G
1031533|F|0-26:G>T-26:G>T	TGCAGGTTCTCGTTGCCCACCGAGATGATGAACTCCACGCCCGTGTTGGCGAACGCCGTCAGCACCTTG	26:G>T
2277895|F|0-37:G>A-37:G>A	TGCAGAGCGGGCAGCTGGTGTGGGAGGCAAGCCACATGTCGACGCACTCGGTGTGGAAGCCGTGGCCGC	37:G>A
2262079|F|0-8:C>T-8:C>T	TGCAGCAACTGCGTCACCCAGGCCGTGCAGCGCGTCGCCGTCGAGTGCGGCGGCGCCCCCTCCG	8:C>T
1168249|F|0-61:C>T-61:C>T	TGCAGCAAGGGCTGCACGGGTACCACTTCGCCGACGGCGACATCGGCTCACAGCTGGCCGACGGGTTCC	61:C>T
1236663|F|0-15:T>G-15:T>G	TGCAGCATGTCCGCCTCCTCCAGGGATTCCATGGCCTTGAGCACCTTGGCCTTGTAGCTCCGCAGGCTG	15:T>G
1236663|F|0-50:C>T-50:C>T	TGCAGCATGTCCGCCTCCTCCAGGGATTCCATGGCCTTGAGCACCTTGGCCTTGTAGCTCCGCAGGCTG	50:C>T
1024657|F|0-22:C>T-22:C>T	TGCAGCACCTGCCTCCTCTCCTCCTCCGCGCCAGGGGGAGCGCGCGCGTGCGCCAGCCAAGGTGAGAGC	22:C>T
100513181|F|0-35:A>G-35:A>G	TGCAGCATCTCCTGCACCTGCGCCTCCTGCTTCCCACGCAGCCGCGGGTCCAGCAGCTCCGCCACGCCC	35:A>G
5579587|F|0-8:C>T-8:C>T	TGCAGCCGCACACCGTCAACTTCCTCAACGTGGTCATCGACAACACCCGCGCCAGCCTGATGCCGCAGA	8:C>T
2248798|F|0-13:C>T-13:C>T	TGCAGCCGCACACCGTCAACTTCCTCAACGTGGTCATCGACAACACCCGCGCGGGCCTGATGCCGCAGA	13:C>T
100514752|F|0-49:T>C-49:T>C	TGCAGCTGGCCGCGCACGACTGCTCGCACTCCGCCGCCGTCTTGTACGTTTCGAACGGCAGCTGCTCCT	49:T>C
2258071|F|0-68:C>A-68:C>A	TGCAGGAAGGTGAGCTTCTCGAGGAAGCCGAGCTGCGCGTTGCCCACGGCGAGCGCGAAGACGAGGAGC	68:C>A
1055033|F|0-13:G>A-13:G>A	TGCAGGAGTGGAAGATTGCGCCGACGGTGCTCACCCTGTGCGAGCACCTGTTTGTGAGCTTCGCGGTGC	13:G>A
1079033|F|0-20:G>A-20:G>A	TGCAGGCGGCGGGACGGCGCGCGCTTGCCGAGGAGCTTCTTCTTGAGCTTGGTGTTCCAGTAGTTCTTG	20:G>A
1094021|F|0-59:G>C-59:G>C	TGCAGGGAGGATGGCTCGGCGGAGGTGCTGCTCGGCGAGACGTGCGTGATGGGCTATGTGACCGCCCAA	59:G>C
1095573|F|0-7:G>T-7:G>T	TGCAGGGGGAGATGTGCCGCCTGGCGCTGCGCGAGCCCACCAAGGACGGCGCCGAGCACCTCCGCGCCG	7:G>T
1090093|F|0-61:G>A-61:G>A	TGCAGGGGTACGGGCTGACGGAGAGCACGGCCATCGGCGCGTCCACGGACTCGGCCGAGGAGAGCCGCC	61:G>A
2253831|F|0-41:G>C-41:G>C	TGCAGGTCCTCGACGCCGCAGGCCGCGGCGTAGTCGACGCAGCGCTTGAGGTCGGCCTGCGTGAGCTGG	41:G>C
1021225|F|0-11:C>T-11:C>T	TGCAGGTCCTGCAAGTAGAAGGAAGGCTTGGTGGGGCGGAGCGCAAGGTAGACGACGAGCGCGATGAAT	11:C>T
3026535|F|0-11:C>T-11:C>T	TGCAGCAGCTGCTCCAGCTCCTTGACGAAGTTGATGGCCCCG	11:C>T
3953808|F|0-32:C>G-32:C>G	TGCAGCATGATACACCACCACCTGGTCCTGCACCCG	32:C>G
2248694|F|0-28:T>C-28:T>C	TGCAGCCTCATCGAGATCGACGGCGTGGTGCACGAGTTCCAAGCAGTTCCG	28:T>C
4395684|F|0-5:C>G-5:C>G	TGCAGCGCCGCGACCCAGATCCCGCCG	5:C>G
3024533|F|0-28:G>A-28:G>A	TGCAGGTGCACATTGTGTACATGGGGGAGCTGCACCCG	28:G>A
4910610|F|0-19:G>C-19:G>C	TGCAGTGGGCGACGGAGGTGGAGGCCG	19:G>C
1017118|F|0-23:C>T-23:C>T	TGCAGGTTCTGCTCCCGCTGGAACCCCTTGCCGCACACCTCGCACACGAACCG	23:C>T
12768489|F|0-13:C>G-13:C>G	TGCAGAAGCTCATCGTGCTCGCGCTCCTCACCCTCTGGAGCCACCTCTCCCGCAACGGCTCCCTCGAGT	13:C>G
1863625|F|0-10:G>A-10:G>A	TGCAGAAGGCGATCGACTACGCCTGCGGGCAGGGCGCGGACTGCACGGCCATCGCGCAGAGCGGGGCGT	10:G>A
1863625|F|0-34:C>G-34:C>G	TGCAGAAGGCGATCGACTACGCGTGCGGGCAGGGCGCGGACTGCACGGCCATCGCGCAGAGCGGGGCGT	34:C>G
2248101|F|0-19:C>G-19:C>G	TGCAGATCCTTGGCCCCCTCTGCTTCGACATAGGCCACACTGCGCTGACAATGGTGGCGTCGCACGTTG	19:C>G
989503|F|0-7:C>T-7:C>T	TGCAGCACAACAACCTGAGTGGCGAGATCCCAGCGGTGCTCGCGGCGCTGCCGTCGGTCACCGAGGTGG	7:C>T
989503|F|0-21:G>A-21:G>A	TGCAGCACAACAACCTGAGTGGCGAGATCCCAGCGGTGCTCGCGGCGCTGCCGTCGGTCACCGAGGTGG	21:G>A
100524139|F|0-52:C>T-52:C>T	TGCAGCACATAAGTACTCGCACTAGTGCCGCCATGCACGCTAGCCTGGATTACTCAGGCTTGGGAGCAC	52:C>T
1040206|F|0-22:G>A-22:G>A	TGCAGCCGATGACGTAGACGACGAGGAGCGCGACGGTGGCGGCGACGAGCGCCACGTTGGCCCG	22:G>A
1128688|F|0-46:C>T-46:C>T	TGCAGCGCATCGGGGTCGGCCTCGGCACCGTCGCCTTCTCCATGGTCGCCGCCGCCACCGTCGAGCGCC	46:C>T
2246123|F|0-8:G>A-8:G>A	TGCAGCTTGAGCGGGCAGACGGCCTCGATCTTGCACTTGGTGTAGCGCGCCGTGTAGCTCACCTTGCGG	8:G>A
1005839|F|0-5:G>C-5:G>C	TGCAGGAGCTCCTGTACGGCGCGCTCGTGGCGCTCCCGCGCGGCGCGGTGGTCGTCGGAGACCTCGTAG	5:G>C
2254173|F|0-55:C>T-55:C>T	TGCAGGCCGAGATGGAGTCGTTCCTGCGGAAAGACGGCAAGAGCATCGACGCCAACAACTCCACGTTGC	55:C>T
1051522|F|0-19:C>T-19:C>T	TGCAGGGCCAGCTCAAGGACCTCAAGGCGGAGCTCTCCCTCCTCCGCGTCGCCAAGGTCACCG	19:C>T
1089209|F|0-59:C>T-59:C>T	TGCAGGGTCTCCTCCGCGTCCTTGTCCTCGTGCCCCTTCACGTACGTGAAGTTCACCGTCGCGCCCTCC	59:C>T
1075847|F|0-30:C>G-30:C>G	TGCAGGTAGTACTGGCGCAGCCCCTCGCCTCCGCCACGCGCGCCCTTGGCCGACTCGTCGCCG	30:C>G
1132535|F|0-20:G>A-20:G>A	TGCAGCCTCCCCTGCGTGCAGGCCCACAAGCGCCGCACCGCCTGCTCCG	20:G>A
100531358|F|0-30:G>C-30:G>C	TGCAGCTCGTCCGCCGCCAGCTCCGCGTCGGCGTCCG	30:G>C
3941056|F|0-14:G>A-14:G>A	TGCAGCTTCCGCCCGCAGAACACCTCCACCTCCGCCGCGTACGTCGCCGTCACCG	14:G>A
1049393|F|0-17:G>A-17:G>A	TGCAGGATGATCACCTCGCTCAGCGGCACCCTCTTCCGCACCGCGCCCTCCTCCG	17:G>A

<table>
<tr><td>100532006|F|0-27:C>A-27:C>A</td><td>TGCAGGGGGCACTGCTGCCGTCCGTCCCGAGCGCAGGAGCCCGCGCGGCCG</td><td>27:C>A</td></tr>
<tr><td>1062823|F|0-25:G>A-25:G>A</td><td>TGCAGAAGAGCCTGCGGAACTGCGGGCTGGCGAGGAGGTACCTCCACTCCGAGGTCTTGACCGCGGCAG</td><td>25:G>A</td></tr>
<tr><td>989113|F|0-31:C>T-31:C>T</td><td>TGCAGAAGGCCATCGGCGAGCACGACGCGGCCGCCGTGACGCGGAGGCTGGACGAGTACGAGGCGGCGC</td><td>31:C>T</td></tr>
<tr><td>989113|F|0-58:C>T-58:C>T</td><td>TGCAGAAGGCCATCGGCGAGCACGACGCGGCCGCCGTGACGCGGAGGCTGGACGAGTACGAGGCGGCGC</td><td>58:C>T</td></tr>
<tr><td>985696|F|0-11:C>T-11:C>T</td><td>TGCAGAGCAACCTGGCCGTGGCGGAGATCTCCAAGATCGCGTCCTCGCTCCTGCCGTTCGGGGAGGAGG</td><td>11:C>T</td></tr>
<tr><td>1087731|F|0-19:C>T-19:C>T</td><td>TGCAGCAGACGTTCGTCGTCTCCGACGCCACCCGCCCCGACATCCCCATCATCTACGCCAGCGCCGAGA</td><td>19:C>T</td></tr>
<tr><td>1018656|F|0-42:G>A-42:G>A</td><td>TGCAGCGAAAGAGGAAGAGAAATCGCGTGAAAGTACGTCGCCGAGAAGCAGGTGGCAGAAATGGAGGCG</td><td>42:G>A</td></tr>
<tr><td>2243824|F|0-65:C>A-65:C>A</td><td>TGCAGCGGCGTCTGCAACCAAATACAACCAACCCCATCAACACCCTGCATGTCGACCACATAGATCGCG</td><td>65:C>A</td></tr>
<tr><td>1069006|F|0-13:C>T-13:C>T</td><td>TGCAGCTCTGCTGCATCTCGCTCGGCCTTCTCCTGATGGCCGCCGTCTGCCCGCACGTCCCGCTGCTCA</td><td>13:C>T</td></tr>
<tr><td>1055670|F|0-43:G>A-43:G>A</td><td>TGCAGGAACTTCTGGATGGGGAAGTTGAGCGCGTAGGCGAAGAGCTGCGGGATCATGTAGAGCGAGAAC</td><td>43:G>A</td></tr>
<tr><td>4002549|F|0-5:G>A-5:G>A</td><td>TGCAGGTCCTGGTCGAAGGACCGCGCCCAGTAGGCCACATCATGGGTGCTCACATACTTGTAATGCTTC</td><td>5:G>A</td></tr>
<tr><td>1021826|F|0-66:A>T-66:A>T</td><td>TGCAGGTGCGAGGGCACGTCCACCTATCCGCGGACCTACGATCTCATCCACGCCGACTCGGTGTTCACT</td><td>66:A>T</td></tr>
<tr><td>100539976|F|0-25:T>C-25:T>C</td><td>TGCAGCGGAGGCGGCAAGAGGCGGGTGGATCCG</td><td>25:T>C</td></tr>
<tr><td>3940182|F|0-32:C>A-32:C>A</td><td>TGCAGGAATTCCTTCATGTTGCTGGCGGCGATCAGCCCGCGGCCGCCG</td><td>32:C>A</td></tr>
<tr><td>1862865|F|0-32:G>C-32:G>C</td><td>TGCAGGTCTTCTCCACCGACTTCATCGACGGCGACTTCGTCGACGCCTGCGCCG</td><td>32:G>C</td></tr>
<tr><td>995441|F|0-64:G>A-64:G>A</td><td>TGCAGTTGTTGGACCTTGGCGAGGAGCTGCTCCAGCGACATCTCGCTCGTCGGCGTCTCCCTCGGCACG</td><td>64:G>A</td></tr>
<tr><td>993046|F|0-67:G>T-67:G>T</td><td>TGCAGCAGCTCGGGGCTGATGCTCAGGTGCACGGCGCCGCTGCCTTTCTTGGACCTCCCACGCCTCCGA</td><td>67:G>T</td></tr>
<tr><td>1023546|F|0-23:G>C-23:G>C</td><td>TGCAGCAGGGCGTTGTGCACCCAGGAGAGCGTGTTAAGGTCCTTGATGCAGCCGCTGCGCGGGCCGTAC</td><td>23:G>C</td></tr>
<tr><td>1154987|F|0-9:G>T-9:G>T</td><td>TGCAGCGCCGTGAGCGACGGGGGCGCGGCGGTGGTGTGGTTCAAGCATGACCTCCGCCTCGACGACCAC</td><td>9:G>T</td></tr>
<tr><td>100543713|F|0-5:A>C-5:A>C</td><td>TGCAGAGCGGAGACGAAGGCGGAGGACGGCGGCGAGGGCGAGCCGAGGTAGGGCGAGGCGGGGCTCGGC</td><td>5:A>C</td></tr>
<tr><td>1230187|F|0-12:A>G-12:A>G</td><td>TGCAGCGCGGAGACGAAGGCGGAGGACGGCGGCGAGGGCGAGCCGAGGTAGGGCGAGGCGGGGCTCGGC</td><td>12:A>G</td></tr>
<tr><td>1230187|F|0-20:G>A-20:G>A</td><td>TGCAGCGCGGAGACGAAGGCGGAGGACGGCGGCGAGGGCGAGCCGAGGTAGGGCGAGGCGGGGCTCGGC</td><td>20:G>A</td></tr>
<tr><td>3935290|F|0-44:G>A-44:G>A</td><td>TGCAGCTGGTGGCACAGCGTGTTGGTCTCCAGGAAGAACTCGAGGAACACCTTGCTCTCGGAGTCGCCG</td><td>44:G>A</td></tr>
<tr><td>1091482|F|0-65:G>A-65:G>A</td><td>TGCAGGGAGTCGTCGGAGTAGGTCCCCTTGCGCATGAAGCGCGACAGGAGGTCGTCGTGCGGCGTGGAC</td><td>65:G>A</td></tr>
<tr><td>1096095|F|0-12:A>G-12:A>G</td><td>TGCAGGGCGAGGACTCGCAGATTGCAGAACCCCCCTGCAATGACGTCCAGATCGTCGTCGATAACACCG</td><td>12:A>G</td></tr>
<tr><td>1013635|F|0-64:G>A-64:G>A</td><td>TGCAGTTCACGGTGAAGGACTCTGTGCGGGAGAGCGTGTCCAGCGCGTCCGAGAACCTCTCCTCGTCAT</td><td>64:G>A</td></tr>
<tr><td>100547312|F|0-47:G>A-47:G>A</td><td>TGCAGTTCATGCGGGAGTGGGGGCCGACGGTGATCTACAGGTCGAGGGCGGGGACGGAGCGGATGGTCA</td><td>47:G>A</td></tr>
<tr><td>4261494|F|0-11:C>T-11:C>T</td><td>TGCAGCACCTGCTGGACGTCCCG</td><td>11:C>T</td></tr>
<tr><td>1022735|F|0-32:C>T-32:C>T</td><td>TGCAGCTTGGGGAGCAGGTAGTACCTGATCAGCTCGTCGTCGCTCGGCCTGAACCG</td><td>32:C>T</td></tr>
<tr><td>1081143|F|0-10:T>C-10:T>C</td><td>TGCAGACACTTGATCTGTCATCCAACAACATCACAGGCAGCATCCCTGTTTCATTGGCTCAGATTCCAA</td><td>10:T>C</td></tr>
<tr><td>1024248|F|0-7:C>A-7:C>A</td><td>TGCAGCACGTGGTGTTCGGCATCGCGGCGTCGGCGCGCATGTGGGAGAAGCGGAAGGAGTACATTAAGA</td><td>7:C>A</td></tr>
<tr><td>1022310|F|0-41:G>A-41:G>A</td><td>TGCAGCGACACCTGCCGCTCCATCTGGATGTGCTTGTAGAAGTTGGCGATGAACTGCTCCGCGCGCCGA</td><td>41:G>A</td></tr>
<tr><td>994161|F|0-51:A>G-51:A>G</td><td>TGCAGGGCCCACGCGCCCGTGGCTCCCAAGGTGATGTTCGCAGATTGGCTCAACATGGACTACATCGGT</td><td>51:A>G</td></tr>
<tr><td>1161775|F|0-9:C>T-9:C>T</td><td>TGCAGGTGGCCCCCCACGGCGCGCACGCGCTGGTCGTCTCCACGGAGACCATCACGCCCAACTACTACC</td><td>9:C>T</td></tr>
<tr><td>1129014|F|0-32:G>C-32:G>C</td><td>TGCAGGTCGGCGGTCGCCGAGTCCCGCACCTCGCGCCAGCCCTTGGAGATGAAGGCGAACGCGCCGTCG</td><td>32:G>C</td></tr>
<tr><td>1004340|F|0-13:C>T-13:C>T</td><td>TGCAGTATCCTAGCCTGTTGCTGGAGTTCATGGGGAGCTATCTCACCGAGCTGAGCCTTTTGGATTATG</td><td>13:C>T</td></tr>
<tr><td>1004340|F|0-58:T>C-58:T>C</td><td>TGCAGTATCCTAGCCTGTTGCTGGAGTTCATGGGGAGCTATCTCACCGAGCTGAGCCTTTTGGATTATG</td><td>58:T>C</td></tr>
<tr><td>3952773|F|0-7:G>A-7:G>A</td><td>TGCAGCAGGGCGTGGACCTGGTGGGCGGGTACTACGACGCCG</td><td>7:G>A</td></tr>
<tr><td>4993716|F|0-13:C>T-13:C>T</td><td>TGCAGCGCGTGTTCTACGGCGACAACGCCG</td><td>13:C>T</td></tr>
<tr><td>3951439|F|0-21:T>C-21:T>C</td><td>TGCAGGTTCGAAAGGAACGAATCCCACGGGCTGGACACGCCTTGCCG</td><td>21:T>C</td></tr>
<tr><td>2300078|F|0-23:G>T-23:G>T</td><td>TGCAGAAGCCACTCGACGGTCTCGCCGTCGGTCTTGTGGCCGAGCTCCCGCGTGAGCTGGAACACCCGC</td><td>23:G>T</td></tr>
<tr><td>1120978|F|0-17:G>T-17:G>T</td><td>TGCAGCGGCGACCTGCCGTTCGCGCTCGTCGGCAACTACTCTGGCCTCGCCTGCCAGCCCGTCTGGAAC</td><td>17:G>T</td></tr>
<tr><td>1120978|F|0-20:C>A-20:C>A</td><td>TGCAGCGGCGACCTGCCGTTCGCGCTCGTCGGCAACTACTCTGGCCTCGCCTGCCAGCCCGTCTGGAAC</td><td>20:C>A</td></tr>
<tr><td>1120978|F|0-38:C>T-38:C>T</td><td>TGCAGCGGCGACCTGCCGTTCGCGCTCGTCGGCAACTACTCTGGCCTCGCCTGCCAGCCCGTCTGGAAC</td><td>38:C>T</td></tr>
<tr><td>2245452|F|0-19:G>C-19:G>C</td><td>TGCAGGCCAATGCCTTGAAGGTGTTCGACGAAAGGTTTAATGATCTTCCCCACCCGCATGCCCAGCACC</td><td>19:G>C</td></tr>
<tr><td>1002042|F|0-48:G>T-48:G>T</td><td>TGCAGGGTGCCACGACGGAGGAGATGCATGGAGAAGAGGAAGAGGAAAGGAGACAGCTCCTCGTGCCTC</td><td>48:G>T</td></tr>
<tr><td>1129938|F|0-22:C>A-22:C>A</td><td>TGCAGCGCATCGCCTGGGTCGGCGACGACCACGAGCGCTTCCTCTCCAAGTTCAAGGACCGCGTCGACC</td><td>22:C>A</td></tr>
<tr><td>1017267|F|0-8:G>A-8:G>A</td><td>TGCAGGTTGAACGTGCCCGTGACCACGGCCGTCTCCGCGACCACGCTCCGCATCTCCTCGGTGTCGTTG</td><td>8:G>A</td></tr>
<tr><td>1017267|F|0-61:T>C-61:T>C</td><td>TGCAGGTTGAACGTGCCCGTGACCACGGCCGTCTCCGCGACCACGCTCCGCATCTCCTCGGTGTCGTTG</td><td>61:T>C</td></tr>
<tr><td>3953982|F|0-52:G>A-52:G>A</td><td>TGCAGAGCACGGACGACGCAGAGGTCCACATGTCGTCGGCCTCGACGGCGACGACGTCAGGGCGCGGCT</td><td>52:G>A</td></tr>
<tr><td>3021295|F|0-29:C>T-29:C>T</td><td>TGCAGCGACAGCTTCGACCTCTACAACGGCCTCTTCATGTACAACAAGAACTCCACCGACCTGCTCAAC</td><td>29:C>T</td></tr>
<tr><td>100571202|F|0-17:A>C-17:A>C</td><td>TGCAGTGCCTTGGTCCTATCCTCCTCAGAGCTGGGCTGCGCCGCCACGGCAGACATGCCGTCCACCCTC</td><td>17:A>C</td></tr>
<tr><td>3948841|F|0-10:C>G-10:C>G</td><td>TGCAGGCGCTCTACACCGCCG</td><td>10:C>G</td></tr>
<tr><td>12735246|F|0-5:C>A-5:C>A</td><td>TGCAGCCTGTCCATGGTGTCCG</td><td>5:C>A</td></tr>
<tr><td>10592804|F|0-10:C>T-10:C>T</td><td>TGCAGGCGTTCGGCGTGGCGCCG</td><td>10:C>T</td></tr>
<tr><td>1026052|F|0-14:G>A-14:G>A</td><td>TGCAGCGCGAAGTTGTGCGGAAGAGAAGGCACCTGCATGGAGAAGCCCTGCATCTGCGCCGCCGCCGAG</td><td>14:G>A</td></tr>
<tr><td>988898|F|0-65:G>A-65:G>A</td><td>TGCAGCTGCTCGTCGCTCACCACCCACGCCCCCTGCGTCTTCTGGATATCGTCCATGGCGGAGTTGAAC</td><td>65:G>A</td></tr>
<tr><td>100574528|F|0-18:C>G-18:C>G</td><td>TGCAGTGCGACAGCAGGTCCACGTACGCGCTCGTGGTGTCCACGGAGATTCTCACCTCGGATTACTACT</td><td>18:C>G</td></tr>
<tr><td>13881820|F|0-6:T>C-6:T>C</td><td>TGCAGCTCCTTGGCCTTCGCCCG</td><td>6:T>C</td></tr>
</table>

1052650|F|0-65:C>T-65:C>T	TGCAGCAGCATCACGCCCAGCGAGTAGACGTCCGACTTGACGCCCAGCATCCCCGTCTGCTGGTACTCC	65:C>T
1863581|F|0-5:C>A-5:C>A	TGCAGCAGCGCCGTGTTGTAGAGCAGCTCGATCCCGTGGAGGCGCGCGTAGTCGACCTTGTTCTTGAGG	5:C>A
992922|F|0-22:A>G-22:A>G	TGCAGCTCCCACTGGCATCTAAATGCGGCTACATAAGGAAGCTGGTGTCGGGGGCCAACGGGTCCAGGG	22:A>G
992922|F|0-55:C>T-55:C>T	TGCAGCTCCCACTGGCATCTAAATGCGGCTACATAAGGAAGCTGGTGTCGGGGGCCAACGGGTCCAGGG	55:C>T
3939963|F|0-12:G>A-12:G>A	TGCAGCGCCCTCGCCGAAAGCGTGAAGCGCTGGGCGCCG	12:G>A
3960009|F|0-6:G>A-6:G>A	TGCAGCGGCCAGGGGAACACCAGCACGTGCACCG	6:G>A
991105|F|0-7:C>T-7:C>T	TGCAGCTCGCGCAGTTCCCCTACGACGCGGAGGCCATTCAGTCCGCGGACGCCGCCGTCGTCGCGGAGC	7:C>T
1062282|F|0-33:G>A-33:G>A	TGCAGCCACCCGTCCCTCGTCGCCGTGTACAGCGTCCCTCCCGCCGCCGCGTCCACGTACACGTCCTCC	33:G>A
1001059|F|0-37:G>C-37:G>C	TGCAGTATCACGGGGGAGGCCAGGTGGAAGACGCCGTGGACGCCCTCGACCGCCG	37:G>C
1001059|F|0-42:C>G-42:C>G	TGCAGTATCACGGGGGAGGCCAGGTGGAAGACGCCGTGGACGCCCTCGACCGCCG	42:C>G
6048922|F|0-23:C>A-23:C>A	TGCAGGCACCCATATCGTTCCGCCTCACCTCCG	23:C>A
1004847|F|0-47:C>T-47:C>T	TGCAGCGCCGTCATGAGCAGCAGCACGACGGCGGCGAGCAGCGTGAGCAGCCTCCACGACCTGAACACG	47:C>T
1000580|F|0-21:C>T-21:C>T	TGCAGCAGGTTCCTCGCGAGCCCCACGGCGATGAGCCCCGCGCTGCACCCCATGCCG	21:C>T
5372263|F|0-11:C>T-11:C>T	TGCAGTCTGCGCCGAAGTACCG	11:C>T
3961174|F|0-7:G>A-7:G>A	TGCAGCGGGTCGAACCCGCCGACCAGCACCATCGCCG	7:G>A
4396424|F|0-26:C>G-26:C>G	TGCAGCGGGTCCAGCCCGCCGACGGCCACCATCGCCG	26:C>G
1091486|F|0-29:G>C-29:G>C	TGCAGGCGCTCTTCAGCGTGGACATGCTGGTGCTGTTCGTGGCCACCATCTGCGGCGTGGGCGGCACGC	29:G>C
1862682|F|0-21:T>A-21:T>A	TGCAGGACGTGTTCCTCAGGCTGACGTTCGACACGACGACCACGCTGGTGTTCGGCGTCGACCCGAGAT	21:T>A
4262880|F|0-19:G>T-19:G>T	TGCAGGTGCACCCCTACACGTACCG	19:G>T
7353541|F|0-7:G>T-7:G>T	TGCAGCTGCCCGCCGAGACCG	7:G>T
4262756|F|0-11:C>A-11:C>A	TGCAGCAGCTCCAAGGGGTGCCCG	11:C>A
4262198|F|0-15:T>C-15:T>C	TGCAGCGTCTGCTCGTTGGGGATCTCGATGTCCG	15:T>C
4261984|F|0-20:A>G-20:A>G	TGCAGCTCATGATGTACAACAACATGTACCGCATCATGTTCGACCG	20:A>G
32016496|F|0-41:G>A-41:G>A	TGCAGCTCATGATGTACAACGACATGTTCCGCATCATGTTCGACCG	41:G>A
13871879|F|0-6:A>C-6:A>C	TGCAGGAACGACTCGCTCTCCCCG	6:A>C
13880485|F|0-13:C>T-13:C>T	TGCAGAAGAAGGCCATCGCCG	13:C>T
1207911|F|0-46:G>A-46:G>A	TGCAGCACCTGACGGACAAGAGCGACGTGTACAGCTTCGGGGTGCTGCTGCTGGAGCTGCTGTCCGAGA	46:G>A
1018181|F|0-57:C>T-57:C>T	TGCAGCTGGTGATGAAGAGCGGCAAGTACACGCTCGGCTACAAGACCGTCCTCAAGACGCTCCG	57:C>T
7170554|F|0-7:C>T-7:C>T	TGCAGCACTCGCTCTGCGGCGTCGGGGTGTCCG	7:C>T
3026621|F|0-26:C>T-26:C>T	TGCAGCCCGTTGCTGGAGCTCAGCTGCCTCAGGAAGTGCTCCCCG	26:C>T
1046609|F|0-20:C>T-20:C>T	TGCAGGAGGTGCTCAAGAAGCTGGGCATGCCCGAGGCCAGCAGCCTGGCCAACGTCCG	20:C>T
2340595|F|0-6:G>A-6:G>A	TGCAGCGGTCGAAGCACGACGCGGAGCTGCGCATGCTGGCGTGCGTGCACACCACCCGCAACGTGCCGT	6:G>A
1062819|F|0-58:G>C-58:G>C	TGCAGGACGGGACGCGGACGGCGACGACGTGGAAGCTGGGCAAGGCGGAGAAGGAGGCGCTGAGCATCA	58:G>C
5577874|F|0-19:G>T-19:G>T	TGCAGAGCATACAGGCGGCGCAGATGCTGCCCCG	19:G>T
7153661|F|0-7:C>A-7:C>A	TGCAGATCGTGTCCAAGAAGGCGCCGCCGCCG	7:C>A
4262968|F|0-8:C>A-8:C>A	TGCAGATTCTGGGGAAGGCCGAATTTCTGAACCCG	8:C>A
3952199|F|0-7:T>A-7:T>A	TGCAGCATCCATCTCTTGAGCTGGCCG	7:T>A
3938457|F|0-7:C>T-7:C>T	TGCAGCCCAGGACGCGCCGCCGCGACAGGGCCG	7:C>T
4397292|F|0-5:C>T-5:C>T	TGCAGCGCCTGCTTGATCACCGCCTCCG	5:C>T
4397292|F|0-11:C>T-11:C>T	TGCAGCGCCTGCTTGATCACCGCCTCCG	11:C>T
5578080|F|0-12:G>A-12:G>A	TGCAGCGGATGGGCTTGTCCGTGCCG	12:G>A
10984254|F|0-15:A>T-15:A>T	TGCAGTTGGCGTGGGAGGAGGCGAATCCG	15:A>T
6030590|F|0-11:G>A-11:G>A	TGCAGCTCACCGTGTCGAGCCG	11:G>A
4543858|F|0-15:T>C-15:T>C	TGCAGAACCTGCTCGTCGCCG	15:T>C
1020967|F|0-14:C>G-14:C>G	TGCAGATCTGCTCACCAGGGACTACACCTACGACCAGAAGCTGTCCGTCTCCACCGTCAGCTCCTCCGA	14:C>G
1235667|F|0-37:G>A-37:G>A	TGCAGGAGAGCTGCGCGGACGCGTCGGGGTCGCTGGTGGTGTACGCGCCGATCGACCTCCCG	37:G>A
1235667|F|0-40:G>C-40:G>C	TGCAGGAGAGCTGCGCGGACGCGTCGGGGTCGCTGGTGGTGTACGCGCCGATCGACCTCCCG	40:G>C
100400800|F|0-20:C>T-20:C>T	TGCAGGAGGCCATGGAGTTCCTGCGGAGGGTGGAGACGCAGCTCAACTCCCTCTCCATCTCCG	20:C>T
1044824|F|0-49:C>T-49:C>T	TGCAGGGCGGGACGCAGATGATCATCTCGCAGATCCTGGTGGGCACCTTCATCGGGCTCCAGTTCGGGC	49:C>T
1017897|F|0-22:C>A-22:C>A	TGCAGACGTGGAACAACGGGGCCAACAACACGAAGCTGATCGTGCAGGCCG	22:C>A
4396875|F|0-8:G>A-8:G>A	TGCAGCGAGTCCACGGCCAGCGGCATGTTCTCCTGGTCCG	8:G>A
1022209|F|0-16:G>T-16:G>T	TGCAGCGGGTGATGGAGGAGCAGCCGCGGGAGTAGGGGTTGGCCTGCCCGCCG	16:G>T
1022209|F|0-40:G>C-40:G>C	TGCAGCGGGTGATGGAGGAGCAGCCGCGGGAGTAGGGGTTGGCCTGCCCGCCG	40:G>C
4261770|F|0-5:C>T-5:C>T	TGCAGCGTAAAGCAACGGCGAGTGGGAGGAGCCCG	5:C>T
4261770|F|0-11:G>T-11:G>T	TGCAGCGTAAAGCAACGGCGAGTGGGAGGAGCCCG	11:G>T
990511|F|0-8:C>A-8:C>A	TGCAGCTCCTCCGCGACGCGCATGGCACGCCTTTCATCCGCGACGTTGCCGCCG	8:C>A
1096054|F|0-38:G>T-38:G>T	TGCAGCTGGATGGCCTCCAGCGGCGACGCGCCGCCCATGATGAGCCCCGTCATGGCCCCCG	38:G>T
4261577|F|0-31:G>A-31:G>A	TGCAGCTGGGCCTGCTGCTGCTGGAGCAGGTGGTGGTGTGGCACCG	31:G>A
4262086|F|0-16:C>T-16:C>T	TGCAGCTTGTACCTCCCCTCGGGCGTCTTGAAGTAGGTCTTGAGCCCG	16:C>T

3944781|F|0-8:C>G-8:C>G	TGCAGTTGCAGCGGATCTCGCCG	8:C>G
4734196|F|0-6:C>G-6:C>G	TGCAGGCGCCGCAGCTGCCG	6:C>G
100422759|F|0-8:C>G-8:C>G	TGCAGGCGCCACAGCTGCCG	8:C>G
5364986|F|0-25:C>T-25:C>T	TGCAGAACGAGTTCGCGCGCGGCGGCGAGTGCTTCGTGCGCGGCGCGCAGATCTTCGACAACTCGCCGC	25:C>T
2242439|F|0-7:C>T-7:C>T	TGCAGGCCATCATCGAGGGCTCCAGGGAGACCTGGACCTACGCCATCTTCTGGCAGTCCTCCACCGACG	7:C>T
2256019|F|0-59:G>A-59:G>A	TGCAGGTGTCGGCGCTGATCGACCACGTCGCACGCGTCGACTGGTCCCTCCTCGACCGCGTCCCCGAGA	59:G>A
4262160|F|0-10:G>A-10:G>A	TGCAGAAGGGGCGGCAGCTGGAGACGGTGGAGATGAAGGTCCG	10:G>A
5000611|F|0-10:C>A-10:C>A	TGCAGCAGCGCGCACAAGACGCTCCCCCAGGCCCCCGCGCCCACGCCG	10:C>A
3944131|F|0-8:G>A-8:G>A	TGCAGCATGCGCTTGCACCGCGCGCAGCCGCCCG	8:G>A
3944131|F|0-23:G>A-23:G>A	TGCAGCATGCGCTTGCACCGCGCGCAGCCGCCCG	23:G>A
3940115|F|0-7:C>T-7:C>T	TGCAGGACGGGTACGCCGTGGTGGCCGCCGACGGGCCCG	7:C>T
2246387|F|0-45:C>A-45:C>A	TGCAGGAGTATTACATCAGGCACCTCGACGACGGCGCCGCCGCCTCCGACCAAGACCG	45:C>A
3944416|F|0-11:G>A-11:G>A	TGCAGGCGTCGGCGCTGGCGTTCCG	11:G>A
4993768|F|0-8:C>T-8:C>T	TGCAGGGGCTGGGGTTCGGGGAGCCG	8:C>T
1083636|F|0-33:G>A-33:G>A	TGCAGCGACATGTCCCTGGACGAGATGGACGCCGCGGTCAGGAGGATGGAGTTCGTCGTCTTCGACCGA	33:G>A
1204824|F|0-53:G>C-53:G>C	TGCAGCGCGCCGTGGAGCCTGGCGGAGCCGCGGAGGCTGTTGCACGCCAGCAGGCGCAGCTCGTCCTCC	53:G>C
1080576|F|0-26:G>A-26:G>A	TGCAGCTGCTCGGCTTCGCGGACGCCGTCGCCATGTCTGCGCGCGCCACCGAGAAGCTCTACCGCACGC	26:G>A
12764043|F|0-65:G>A-65:G>A	TGCAGGATCCGCGACGACGGCTGGTGGATGGTCAGCACCACGGTGCTCCCTGCGCACGCGATGTCGTGC	65:G>A
1068434|F|0-40:A>T-40:A>T	TGCAGGTAGACGCCGAGCATGGGGAGCTTCTTGGCGCCGTAGGCCTGCCCGCACAGCGTCTCCAGCGCG	40:A>T
987633|F|0-36:G>T-36:G>T	TGCAGCACGCGGAGGACATGCACGAGTGGATCGTCGGCAACCTGCGCCGCACCG	36:G>T
1021854|F|0-44:T>A-44:T>A	TGCAGCTGTCCGAGTCGGAGTCCGACGTCCAGGGCAGGCCCATCTCTTCCG	44:T>A
7167999|F|0-24:G>T-24:G>T	TGCAGCTTGGGCGACTTGGGCGTCGGGGGCGGCGAGGACGCGCCG	24:G>T
3940238|F|0-11:G>A-11:G>A	TGCAGGGTGAGGAGCGTCAGGCAGAGCGCTACCTTACGCCG	11:G>A
12856456|F|0-10:C>G-10:C>G	TGCAGGTCTTCAGGGTTTATAATGCCG	10:C>G
3032674|F|0-25:G>T-25:G>T	TGCAGGTGGAGGAGATGAAGGGCTTGGGCGCGAGGATCCG	25:G>T
1863668|F|0-22:C>T-22:C>T	TGCAGTACCTGGCCATGGACATCTCCACCGACTACATCGACCACTTCAAGGCCCG	22:C>T
2251556|F|0-37:C>A-37:C>A	TGCAGACCTCGCGCAAGAGGTACTTCGTGCTCGAGGACTCCGCGCTCCGCTGCTTCAAGGCCGCCCCGA	37:C>A
1067301|F|0-9:T>C-9:T>C	TGCAGAGCGTGTACGTGGAGCACGAGGTGGAGGCGCTGATCCAGGTGGCGCTGCTGTGCACGCAGGGGT	9:T>C
1067301|F|0-33:C>A-33:C>A	TGCAGAGCGTGTACGTGGAGCACGAGGTGGAGGCGCTGATCCAGGTGGCGCTGCTGTGCACGCAGGGGT	33:C>A
1018334|F|0-34:G>C-34:G>C	TGCAGCAGTTCTCGGCGATGAACAAGTTCAAGAAGAAGGCGCTGGGCGTGGTGGCGCGGAACATGCCGA	34:G>C
1075544|F|0-22:C>A-22:C>A	TGCAGCATCCGCCTACCTACACCCTCGGCAAGCGGCGCACCGACCACAACCTGCTCGTGCCG	22:C>A
2277878|F|0-42:C>T-42:C>T	TGCAGCGACTCGTCAGGCAGCTTCCCGCTCTCCGTGAACATGCTGCCGATCTGGCATTGCTGGATGATC	42:C>T
1046610|F|0-16:C>T-16:C>T	TGCAGCGCATGTCGTACATCAACGTCGGCATCTACCCGTTCACGTCCCTCTTCCTCATCATGTACTGCC	16:C>T
2269789|F|0-54:C>T-54:C>T	TGCAGCTGCCCGCGGCATAGGCCCCCATGAGACCGAACTGGTCGAGCAGCATGGCGGCCTGGAGCGCGC	54:C>T
987498|F|0-41:G>A-41:G>A	TGCAGCTTCACGTGGTTGTAGTAGCCCACGCCCAGGAAGGCGATCCCGTAGCCGAAGAGGTTGATCGGG	41:G>A
1120914|F|0-55:G>T-55:G>T	TGCAGGAGAAGGACAAGTTGGTGGCCGAGGTAATGCGCTACGCGCTCTTCAAGACGCACCAGAACTCCG	55:G>T
1020327|F|0-52:G>A-52:G>A	TGCAGGCGGAGACGCCGTCGCTGGGGGACTTCGCGTACTCGGTGGAGGTGGGGACGCGGAAGAAGCCGC	52:G>A
989478|F|0-11:C>G-11:C>G	TGCAGCAGCCCCACGGCCACCACCACGTCCATCCCCGCCTTCACCAGCGCCAGCAGCCG	11:C>G
3026681|F|0-30:A>T-30:A>T	TGCAGCTGATCCAGATACAGAAGCAGCACAAGCTCATCGCCCCG	30:A>T
6030670|F|0-5:C>T-5:C>T	TGCAGCTTCTACGCCTCCACGTCCCTCCCG	5:C>T
4262581|F|0-5:C>T-5:C>T	TGCAGCTTGTCGAGGTACTGAGCCCCATGGCCCTTCTGGATGGTGCCG	5:C>T
1023984|F|0-40:C>T-40:C>T	TGCAGGCTTTCCAGGCCGTGGAGGTGGCCGTGCGGAGGTTCTTCTTCGCGTACCG	40:C>T
4993510|F|0-28:C>A-28:C>A	TGCAGGGCGACAACATGAGCAAGAACATCCGCGCCG	28:C>A
5367441|F|0-5:G>A-5:G>A	TGCAGGTTAGGCGGGAGCAGCCCG	5:G>A
100481738|F|0-14:C>G-14:C>G	TGCAGTCCGTGGAGCCAGCGTCTTATCCG	14:C>G
5332656|F|0-16:C>G-16:C>G	TGCAGTCGAACCGCAACGCCACCG	16:C>G
3940943|F|0-8:C>T-8:C>T	TGCAGAAGCGGAGCCACCCG	8:C>T
2247355|F|0-53:C>T-53:C>T	TGCAGCATCCTCATCTGCATCTGCTTCGGCGCCAAGATCCCCGACGAGCTCATCGTGGAGATCGAGGAG	53:C>T
1018472|F|0-17:G>C-17:G>C	TGCAGCCCGATCATGGCGAACACGTCCGACAGCCCGAACATCACGTACTGCGGCACCATCCACCACAGG	17:G>C
985174|F|0-10:C>T-10:C>T	TGCAGGCGCACCTGGCCGACAGCAACCTGGGCGCCGTGGTGGAGGAGTGCATGCAGCACTACGACGAGC	10:C>T
985174|F|0-13:G>T-13:G>T	TGCAGGCGCACCTGGCCGACAGCAACCTGGGCGCCGCGGTGGAGGAGTGCATGCAGCACTACGACGAGC	13:G>T
1297395|F|0-6:C>A-6:C>A	TGCAGTCCCTCCTGGCGCTGTCCGACAGGGAGAGCGTCGTGTACAGCTACGGCATGGTGCTGCTGGAGA	6:C>A
4261457|F|0-13:G>C-13:G>C	TGCAGAAGCTTCTGTTGGCCGACAACAAGTTGTCCG	13:G>C
5357788|F|0-14:C>G-14:C>G	TGCAGCGACGTGGGCGGCGGGCCG	14:C>G
985964|F|0-38:T>C-38:T>C	TGCAGCGTTACCAGCCGTTCGGGTCGCCGCCTGGCGACTACCACCATTTCGGCCCG	38:T>C
1088080|F|0-7:G>T-7:G>T	TGCAGGCGCTCCAGGCCGAGGCGGCCACCGCCTCCTCCATGCTCTCCTCCG	7:G>T
4262345|F|0-40:C>T-40:C>T	TGCAGGTGCCCACCCTCAGGAGCTGCGACTTCACGCGGCTCTACTCCG	40:C>T
1091381|F|0-64:G>T-64:G>T	TGCAGCACAGCATCGAGTACTGGCTCACGCTGGACCTCCTCGCCTCCGAGCAGGGCGCGCCCACGCCGT	64:G>T
1048955|F|0-42:C>A-42:C>A	TGCAGCCTCTTCAGCTGGGCCTTCAGGGCCAACCACGGGCCTCCCTATCTCATGCACTCCCAGCCACTC	42:C>A

1119431|F|0-47:C>A-47:C>A	TGCAGCGCCTCATCGAGGCGCGCGCCACCGTGCCCGTCGCCGCCCAGCGCCTCTCCCTCGACCCGAGAT	47:C>A
2258923|F|0-36:G>T-36:G>T	TGCAGCGCGTGCAGTCGGAGGCGCTGCGCGCGCTGGGGCCGCGCGACTTCGACCCCAAGCTCTACGTCG	36:G>T
992863|F|0-49:C>T-49:C>T	TGCAGGACACCAGCATCCAGGGCGCCGAGAGCCACCCGCTGCACCTGCACGGCTACGACTTCCACGTCG	49:C>T
3946717|F|0-12:T>C-12:T>C	TGCAGCGCGAGTTCCGCGGCGCCGTCAGGTACGCCATGGAACGCCG	12:T>C
4261788|F|0-5:G>A-5:G>A	TGCAGGCCGCGCCGCCCTCGCCTCGGCGCGAGATCCCCG	5:G>A
3955279|F|0-21:T>A-21:T>A	TGCAGGCGCACTACCACCGCATCCCCG	21:T>A
5580569|F|0-27:C>A-27:C>A	TGCAGTCGTCGTTGCTGGCGAAGGAGACGTCGGGGGCCACGGCGGCGGCCCG	27:C>A
1016339|F|0-49:C>A-49:C>A	TGCAGCACACCATGTTCCGCGACACGCCCGCCCCCTTCCACTGCCAGTCCTGCGGCGACGCCGCCGTCA	49:C>A
2321212|F|0-60:G>C-60:G>C	TGCAGCTGGCCGCGCACGACTGCTCGCACTCCGCCGCCGTCTTGTACGTCTCGAACGGCAGCTGCTCCT	60:G>C
1055033|F|0-22:G>A-22:G>A	TGCAGGAGTGGAAGATTGCGCCGACGGTGCTCACCCTGTGCGAGCACCTGTTTGTGAGCTTCGCGGTGC	22:G>A
1071014|F|0-35:C>T-35:C>T	TGCAGGTACAGCAGCACAGGCGGCGGCAGGGGCAGCGCGACCGCTCCGTTATTATGCGCGCGGCCGAGA	35:C>T
1013738|F|0-10:G>C-10:G>C	TGCAGTCGCTGCTCAGCTGGCGCGACCCGAGGGCGTCCTGCCTCTTCGTCTTCTTCTGCCTCATCGCCG	10:G>C
7172131|F|0-15:C>A-15:C>A	TGCAGCGCCGCGACCCAGATGCCGCCG	15:C>A
1096712|F|0-15:A>C-15:A>C	TGCAGGAACTTGGTGATGGGCTGGATCACCGCGTTGGCGAAGAGCCCCG	15:A>C
1696543|F|0-16:C>T-16:C>T	TGCAGGAGAACATCGACTTCGTGTGCGGAGGGGGCATGGACTGTGGTCCGATACGGCCG	16:C>T
1696543|F|0-50:A>T-50:A>T	TGCAGGAGAACATCGACTTCGTGTGCGGAGGGGGCATGGACTGTGGTCCGATACGGCCG	50:A>T
3946919|F|0-19:G>A-19:G>A	TGCAGGAGCGGGCGACGAGGCGGAGGGCGTCGGTGGAGAAGCCG	19:G>A
1023497|F|0-36:C>T-36:C>T	TGCAGGCCGCGGGCAACGGGTCCTCGCAGAGCGCCGCGGCGAGGGAGCGCCG	36:C>T
1017354|F|0-20:G>A-20:G>A	TGCAGGTTCTGCTCGCGCTGGAACCCCTTGTTGCACACCTCGCACACGAACCG	20:G>A
7171889|F|0-32:G>A-32:G>A	TGCAGCTTGGAGATGATGGTGGGGCGGCGGAAGCGGGCGGGGTCGACCAGGACGAGGCGGGCGATCTGG	32:G>A
1089209|F|0-11:C>G-11:C>G	TGCAGGGTCTCCTCCGCGTCCTTGTCCTCGTGCCCCTTCACGTACGTGAAGTTCACCGTCGCGCCCTCC	11:C>G
1004893|F|0-26:C>T-26:C>T	TGCAGTACAGCCACGCCGATCCCAACCGCACGGTGGTGAACCCGTACGTGCACTTCGGGCAGCCGTCGG	26:C>T
1696212|F|0-16:C>T-16:C>T	TGCAGTTCCTCACCAACTCCTGCGTCGTGCTCTTCATGATCCAGAGCGTCGACCGCCTCGTCCTCTGCC	16:C>T
1082678|F|0-23:G>T-23:G>T	TGCAGCGCAACGCCACGCGCTACGGCGCAGCGGCCACGGCCGTGATCCG	23:G>T
1020648|F|0-44:C>A-44:C>A	TGCAGCTCCCGCACGATGAGCACCATGGAGGAGAAGGCGCGGCCCACCGACTCGCCG	44:C>A
4395246|F|0-17:C>G-17:C>G	TGCAGGCTGGTGCAGGGCATCAGAGACGCCG	17:C>G
1080223|F|0-51:G>A-51:G>A	TGCAGATCGCGCGCGGCATGGAGCACCTCCACTCCAAGAAGATCTACCACGGCGAGCTCAACCCGTCCA	51:G>A
100536233|F|0-21:G>A-21:G>A	TGCAGGACGGCACGAACACCCGCGGCATCTTCGGGCGCGAGACGGCGACGGTGGCGGTGTCGGGCGGGA	21:G>A
1046269|F|0-24:T>A-24:T>A	TGCAGGACGTCTACGGCGAGGTGTTCCGCGTGCTCAAGCCAGGGGGCCTCTACGTCTCCTACGAATGGG	24:T>A
1016778|F|0-49:C>A-49:C>A	TGCAGTCGTCGCGGAACCCCACGCCCAGGGGCTCCAGCTTCAACCACACCGACTTCTACTCCATGGTCG	49:C>A
5581358|F|0-26:G>A-26:G>A	TGCAGCGGAGGCGGCAAGAGGCGGGCGGATCCG	26:G>A
6041457|F|0-14:G>A-14:G>A	TGCAGCGAGGCGTCGGGGGTCGCCG	14:G>A
4262240|F|0-19:G>C-19:G>C	TGCAGACGCAGAACGTGGTGGTGCCCGTGATGCTCAGCTCCG	19:G>C
1029559|F|0-28:C>T-28:C>T	TGCAGATCATGGCCCTGCACTCCTCCAGCCCCATCTTCTCGGCCCCCAGCAGCTTGCCCAGCATCCGCC	28:C>T
3944091|F|0-35:G>A-35:G>A	TGCAGCGGCGGCCGCTTGCTCGACGAGATGGCCAGGGACGGCACCTTCTCCGAGCAGCGAGCCGCCCTT	35:G>A
4544309|F|0-38:G>T-38:G>T	TGCAGCGTCCGCGGCATTGGCGGCATGTCGACGTGCACGACGCCCTCCAGCGCCTGCACGGCCTGCGCC	38:G>T
1863289|F|0-31:C>A-31:C>A	TGCAGGCGCTCCGTCTCGGTGAAGCGGGAGGCGTACGTCTCGGCGACGCCGTTGCGGATCCGCAGGGCG	31:C>A
1091482|F|0-14:G>A-14:G>A	TGCAGGGAGTCGTCGGAGTAGGTCCCCTTGCGCATGAAGCGCGACAGGAGGTCGTCATGCGGCGTGGAC	14:G>A
1012889|F|0-30:G>T-30:G>T	TGCAGTACTCGCTCTACTTCGTCTACCTCGGCCTCGTCGTCTGCGCATCCTCGTACCTGGGTGAGTTCA	30:G>T
3027494|F|0-7:C>T-7:C>T	TGCAGCACATCGCCGCCGCCGACTACGACATGCTGCTCCTCCCCG	7:C>T
1022735|F|0-47:C>G-47:C>G	TGCAGCTTGGGGAGCAGGTAGTACCTGATGAGCTCGTCGTCGCTCGGCCTGAACCG	47:C>G
1030407|F|0-10:G>T-10:G>T	TGCAGGAGAAGAGGGGGTTCCTGTTCTGCAAGGAGGACAGGGCGATCCTGTGCCG	10:G>T
7340523|F|0-7:C>T-7:C>T	TGCAGCACGGCGGCGTGCGCGCCG	7:C>T
6047053|F|0-27:T>A-27:T>A	TGCAGGACGTGAGCCAGAGCGAGGAGGTGTTCCAGGCCGAGCTGGGCGCGATCGGCAGGATCTACCACA	27:T>A
981764|F|0-16:G>T-16:G>T	TGCAGATCACGGACGTGCAGCTGGAGATCATGATGGGCATCCTGAGCGTGTACGCGCTCATCGGGTCCT	16:G>T
3940903|F|0-31:G>T-31:G>T	TGCAGCATGCCCTCGCGCTCCTCCAGGAACTGCTCCG	31:G>T
5581387|F|0-12:C>A-12:C>A	TGCAGCGCCGCACCGAGCCCAAGTTGCCG	12:C>A
4405729|F|0-9:G>C-9:G>C	TGCAGGATCGTGTAGTCCTCCCCCCTTGCCG	9:G>C
1111533|F|0-12:G>A-12:G>A	TGCAGCAGCGCCGTCGTCGGGAGCACGAAGGAGACGTTGTTCACCGCCGCCGCGAACCG	12:G>A
1141256|F|0-5:G>A-5:G>A	TGCAGGAGATCGCCGCGTCCAGGGGCAAGAGTGTGGCGCAGGTGTGCCTGAGGTGGGTGTACGAGCAGG	5:G>A
3028898|F|0-14:C>T-14:C>T	TGCAGCCCCTACCGCGCCGACCTCTCCGACCGCCCCTGCGCCGCCG	14:C>T
2300078|F|0-14:G>A-14:G>A	TGCAGAAGCCACTCGACGGTCTCGCCGTCGGTCTTGTGGCCGAGCTCCCGCGTGAGCTGGAACACCCGC	14:G>A
1215433|F|0-19:G>C-19:G>C	TGCAGATCGAGCGGCTGCTGTTCAAGGACCTGGTGGCCGACGCCATCTGCGAGCTCGCCGAGGCCGACC	19:G>C
4993523|F|0-13:G>A-13:G>A	TGCAGTTGAGCCAGACGAGGTCGCTGCCG	13:G>A
7491685|F|0-15:T>C-15:T>C	TGCAGCTCGAGCCGCTTGCCG	15:T>C
2252024|F|0-6:G>A-6:G>A	TGCAGCGTCCGCAAGAACATGGTCCCCGTCCTCGACTACCTCGGCAAGATCGGCGTCCGCCGCGACGAG	6:G>A
2252024|F|0-10:G>A-10:G>A	TGCAGCGTCCGCAAGAACATGGTCCCCGTCCTCGACTACCTCGGCAAGATCGGCGTCCGTCGCGACGAG	10:G>A
1017267|F|0-12:G>A-12:G>A	TGCAGGTTGAACGTGCCCGTGACCACGGCCGTCTCCGCGACCACGCTCCGCATCTCCTCGGTGTCGTTG	12:G>A
3953982|F|0-9:C>T-9:C>T	TGCAGAGCACGGACGACGCAGAGGTCCACATGTCGTCGGCCTCGACGGCGACGACGTCAGGGCGCGGCT	9:C>T

1271508|F|0-21:C>A-21:C>A	TGCAGCTCCCAGCGGCGTAGGCCCCCATGAGCCCCAGCAAGCCCAGGATCATGGCGAACTGAAGCGCGC	21:C>A
1024769|F|0-40:G>A-40:G>A	TGCAGGTCGGGTTCTACAATGCGACGTGCCCGATCGCGGAGGGGGTGGTGTTCGCGGAGATGCACGCCA	40:G>A
1049074|F|0-31:G>T-31:G>T	TGCAGTACGTGTTCCTCAAGTGGGCGCTGGCGTTCCTGGTGGGGCTCCTCACCG	31:G>T
1057410|F|0-19:C>G-19:C>G	TGCAGCAGAAGATGAGCGCCAAGGAGTCCGAGACCTGGTCCAAGGTGCTTAGGCAGGAGGAGAAGCTTA	19:C>G
3958586|F|0-20:G>A-20:G>A	TGCAGCACCTTGAACGTGCCGCCCTCCCGCCG	20:G>A
1056056|F|0-37:G>T-37:G>T	TGCAGGTGGTGGCTCCTCATCTTGTCGCCGACGCCGAGGGCGTCAAGCGCGCGGAAGGCGTTGGTCCAT	37:G>T
1145044|F|0-46:G>A-46:G>A	TGCAGCAGCATCACGCCCAGCGAGTACACGTCCGACTTGACCCCCAGCATCCCCGTCTGCTGGTACTCC	46:G>A
992922|F|0-46:G>A-46:G>A	TGCAGCTCCCACTGGCATCTAAATGCGGCTACATAAGGAAGCTGGTGTCGGGGGCCAACGGGTCCAGGG	46:G>A
3027571|F|0-27:A>T-27:A>T	TGCAGCGACAGGTGCAGCAGCTGGTTGAGGTGGCCCTGCGCCG	27:A>T
3027571|F|0-29:G>A-29:G>A	TGCAGCGACAGGTGCAGCAGCTGGTTGAGGTGCCCCTGCGCCG	29:G>A
5368388|F|0-32:G>T-32:G>T	TGCAGCTCCGCCTCGTCGGTCTTGTCGGACTCGATGGACAGCAGGACGTTCTTGCGGCGGACGAACTGG	32:G>T
4397728|F|0-5:T>C-5:T>C	TGCAGTGGTAGTATGTTGCCGCCG	5:T>C
980135|F|0-6:T>C-6:T>C	TGCAGGTGGCCGTCTGGGCGTGCATGGTGATGTCGGTGATGCTGGTGGTGGAGGCCCTGTACAACTGCG	6:T>C
4733790|F|0-13:C>T-13:C>T	TGCAGGATCTCGACGACGAGGCCG	13:C>T
1097222|F|0-50:G>A-50:G>A	TGCAGGGTGCGGAAGTCCTTGAGCGGCTTGGCGCGCGGGTGGTCGACGTTGTTCCG	50:G>A
5371676|F|0-18:A>G-18:A>G	TGCAGGAGATGCGCAGGCACGTGTCGAACCG	18:A>G
7331308|F|0-11:C>G-11:C>G	TGCAGGAGTACCCGCGCCCG	11:C>G
4001970|F|0-16:T>C-16:T>C	TGCAGACCATGGCGCTTGACAGGGTGCTGGAGGAGTGCAGGAGCACCG	16:T>C
4262110|F|0-11:T>C-11:T>C	TGCAGACGGTCTGATACTTCCTGGATAAGCTCCGCCG	11:T>C
16311331|F|0-11:A>G-11:A>G	TGCAGTGCCAGACTCCGACCG	11:A>G
7492474|F|0-23:T>G-23:T>G	TGCAGTACATCGACGAGGCCTTCTCCG	23:T>G
5582073|F|0-10:C>T-10:C>T	TGCAGGTTAACCGTACGAGTAGAAGGACCG	10:C>T
5581600|F|0-19:C>G-19:C>G	TGCAGTTGACGATGAGGATCCGCACGTCGCGGCGCGGGTCGATCCCG	19:C>G
5372224|F|0-16:T>C-16:T>C	TGCAGCTCGTCGATGATTGCGCGCCCGCCG	16:T>C
5371677|F|0-19:T>C-19:T>C	TGCAGGAGATGCGCGGGCTTGTGTCGAACCG	19:T>C
5578420|F|0-10:T>C-10:T>C	TGCAGTCCATTTACCATATAAGGACCG	10:T>C
2261504|F|0-34:A>G-34:A>G	TGCAGTACCTGCGCAACCACAAGCCGCCGCTGACACTGGCGCGGTGCAGCGGCGCGCACGTCATCGAGT	34:A>G
5582991|F|0-8:A>C-8:A>C	TGCAGGAAAGCATCGAGGTCACCG	8:A>C
15306874|F|0-17:A>G-17:A>G	TGCAGGCCGTGCGGCTCAATCCG	17:A>G
7173892|F|0-34:G>C-34:G>C	TGCAGCAGCAGTTCGTGTCCTTGGCCACCATCGTGCCCG	34:G>C
5582815|F|0-22:C>A-22:C>A	TGCAGGTACACGCCCAGCATGGCCACCTGGCCG	22:C>A
32027902|F|0-26:G>C-26:G>C	TGCAGGCTGCGGTGGCTCAACTACCTGAGGCCG	26:G>C
15306868|F|0-64:G>C-64:G>C	TGCAGTGCTCCTGCACCTCCTCGATGTCGTACTGCGTGAGCATCGACGACGCGTTCCCCATCGCGCCGC	64:G>C
12779914|F|0-15:G>A-15:G>A	TGCAGTAATCGTAGAGCTCGCCG	15:G>A
6040233|F|0-44:G>C-44:G>C	TGCAGCGAGTAGATCTCGGCGTTGGTGAGGTTGCTGGGGCGCGGGGTGGTGCTGGAGAACCCCATGGAC	44:G>C
6047668|F|0-12:A>G-12:A>G	TGCAGGCGGCCAAGTACCTGCTGGGCTCCCCG	12:A>G
15306741|F|0-17:A>G-17:A>G	TGCAGGAAGATCCACATACTGGCGCTCGTGTCCG	17:A>G
32020491|F|0-12:A>C-12:A>C	TGCAGCCGAACAACAAGCCG	12:A>C
32021646|F|0-10:G>A-10:G>A	TGCAGGGGCGGGTCGATCCG	10:G>A
7161587|F|0-21:C>T-21:C>T	TGCAGCTGCCCGTTGACAAGCCCGAACCTTCCG	21:C>T
32018987|F|0-6:T>G-6:T>G	TGCAGCTTCCGTGTCCACCACCG	6:T>G
5331973|F|0-8:G>C-8:G>C	TGCAGCGCGGCGGCGTCGGCGTCGCCG	8:G>C
5331973|F|0-9:T>G-9:T>G	TGCAGCGCATCGGCGTCGGCGTCGCCG	9:T>G
4909672|F|0-10:A>G-10:A>G	TGCAGATCCCATCACTTCCTTCCTCTACCGCGCGCTCCACGTCAGGCAAGCACGTAGGTCCCTGTCAGG	10:A>G
4910702|F|0-43:G>C-43:G>C	TGCAGATGAGCCCCACGGCGAAACTCGGGGAGGTGATGTCGCGGCCG	43:G>C
2256419|F|0-45:C>G-45:C>G	TGCAGGGTTCTTCGAGTCTCGGCGCGCGTGCTGCGGGACGGGGACCATCGAGACGTCGGTGCTGTGCCA	45:C>G
7926412|F|0-10:A>G-10:A>G	TGCAGCGACGATGAGTGAGGGCGAGCGCCG	10:A>G
2245644|F|0-39:T>G-39:T>G	TGCAGGCTTCGATGATTAGTTCATTGATTGGTTGGCCTCTCACGGGCGCGGGATAATTGTTTCTCCGAG	39:T>G
6040331|F|0-22:C>T-22:C>T	TGCAGCTGCTGGATGTACTCGACCGCGTCCTTGATGATCGAGGCCTTGTCCATCTGCAAAGACGAGGGA	22:C>T
5984482|F|0-18:A>G-18:A>G	TGCAGCGAGAGCGGCCGCACGCTGGTGCCGCACCACTCGGTCCTCATCTTCTAGGCCTTCTCCGCGAGG	18:A>G
16796597|F|0-13:A>G-13:A>G	TGCAGAAGAAGTCAACGACGAGGACGGCCG	13:A>G
6047678|F|0-6:A>G-6:A>G	TGCAGGATCAACACGGTGAGGCGGCGCTCCG	6:A>G
7916312|F|0-39:T>C-39:T>C	TGCAGGCACCACCGAGTCGACAACCACTCACGAGAACAATGGCGCCG	39:T>C
7344436|F|0-17:T>C-17:T>C	TGCAGATCAATAGGTCATCGGTTCGAACCCG	17:T>C
7912984|F|0-26:G>C-26:G>C	TGCAGCCGCTGGCGCCACCGCAGGACGGACCCG	26:G>C
100484250|F|0-34:A>C-34:A>C	TGCAGACGGCTAGCAGCTTCTGCATGAGGTAGTTACCGAATGGGTTCACCATGAGGTCGACGGCGTGAC	34:A>C
7928732|F|0-7:A>G-7:A>G	TGCAGTTACAAGTGAGCTTGTGAGCCG	7:A>G
12771784|F|0-8:C>G-8:C>G	TGCAGCTGCTCAACGGCTCCG	8:C>G
1242814|F|0-5:T>C-5:T>C	TGCAGTCAGCTCATCTCCTTGCGGTCAGCGATCGTCACCCCCAGCTCTGGGAACCG	5:T>C

6043221|F|0-49:C>G-49:C>G	TGCAGGCGTCGGTGATCCGCGACCTGGTCCTCCTCTCCTGCGTCGGCCTCCGCCCCGTGCTCGTGCACG	49:C>G
12775334|F|0-14:T>C-14:T>C	TGCAGCATAAACTCTTCCTCGTCGGCGCCTCCG	14:T>C
6045763|F|0-32:T>C-32:T>C	TGCAGGGGGTTCCATGTCACCTTCGTCAACTCTGAGTTCAACCACCG	32:T>C
3031666|F|0-32:A>G-32:A>G	TGCAGCGTCCGCGGCATCGGCGGCATGTCGACATGCACGACGCCCTCCAGCGCCTGCACGGCCTGCGCC	32:A>G
12776700|F|0-14:T>G-14:T>G	TGCAGCGCCTCCCCTATGTCGTGCGCCTGGCTGAGCGGCATGTCGCCG	14:T>G
12775410|F|0-25:G>C-25:G>C	TGCAGCGGGTGCACCACTCCGCCGAGGCCGCCGACCGCGCCG	25:G>C
12766048|F|0-13:T>G-13:T>G	TGCAGCTCATCGTTTACGGGCCCG	13:T>G
6017750|F|0-5:G>A-5:G>A	TGCAGGAAATGGGGTGCAAGACCG	5:G>A
12776896|F|0-16:T>G-16:T>G	TGCAGTGGGCGACGGATGTGGAGGCCG	16:T>G
4910829|F|0-14:T>A-14:T>A	TGCAGATGAGTATTTCTTCTCGGCGCTGCCG	14:T>A
12775476|F|0-6:T>A-6:T>A	TGCAGGTAGTCGCCGATGTTGACCACCAGCGCCCCG	6:T>A
7171792|F|0-7:T>C-7:T>C	TGCAGAGTAACCTGGCCGTGGCGGAGATCTCCAAGATCGCGTCCTCGCTCCTGCCGTTCGGGGAGGACG	7:T>C
7174073|F|0-14:T>A-14:T>A	TGCAGTTGAACCAGTCGAGGTCGCTGCCG	14:T>A
7171044|F|0-38:G>C-38:G>C	TGCAGCACCTTGCGGCCCGTCATGCCCAGCAGGAACGGGTCGGTGGAGGCGGCCAGGTAGCGGCCGTAC	38:G>C
6030864|F|0-12:T>C-12:T>C	TGCAGGTCCTCGTTCGACATGGCCG	12:T>C
6038365|F|0-18:A>G-18:A>G	TGCAGTGTGCCTCGACGGAAGCGCGCCG	18:A>G
100531440|F|0-18:C>G-18:C>G	TGCAGCTGCTCGAGCGCGCTCACCACCTCCGCCATACCG	18:C>G
100502744|F|0-13:T>G-13:T>G	TGCAGGACGCCGTTGTCATCGGCGGCAGCCCAACCCCGACAGGGGCGAGCACCACAGGGACGTTGCCGC	13:T>G
100508750|F|0-36:A>G-36:A>G	TGCAGCGCGAGCTCCTCCGCCTCCGCGTGGCGCTCCACTGGCGCGGCCG	36:A>G
4910641|F|0-11:T>C-11:T>C	TGCAGCTCACGTGGCCGACGTCGGCGTCGCCCG	11:T>C
5580079|F|0-16:C>T-16:C>T	TGCAGACCAATCAATACTGGTCGCCG	16:C>T
5579599|F|0-29:T>C-29:T>C	TGCAGCCTTCCAAAAATACTGCCCAACTGTCTGTGATGGATCACCAGCTATACGGGGACCCGTCCGCGA	29:T>C
100484212|F|0-57:A>C-57:A>C	TGCAGACGCATGTGAAAAGCCACCGTGGTCCTCGTGGAACTCCATCTATCCATCTATACGCACCGAGAT	57:A>C
100533866|F|0-8:T>A-8:T>A	TGCAGAGGTGGATCGGCGGCGAGCTGGAGGCGGTGAGCATGGCGAGTCCTGCGGCTCATCGGAGTCAGA	8:T>A
6042997|F|0-43:C>G-43:C>G	TGCAGCCTGGGGTTGACGACGGCGATCGAGCCCCACGAGTGGCCGTCCGTGGCGGGCCATGCCGCCGCC	43:C>G
6043071|F|0-19:A>G-19:A>G	TGCAGCTCTGCAAAGAGGAATAGCTCTTGTGTCGTAGCTTATGAGTGAGCAGTGAACCTCCATGTTTCA	19:A>G
7922040|F|0-36:T>C-36:T>C	TGCAGGTCGGGTTCCCCCCTAAGCCGCTCGACGACGTGACCAAGACCATTGAGGAAGCCG	36:T>C
7167955|F|0-34:T>C-34:T>C	TGCAGCTGCTGAGGATCGAGGAGGAGCTCGGCGATGCGGCGGTGTACGCCG	34:T>C
100576792|F|0-50:T>G-50:T>G	TGCAGGCCGTGACCCTCCAGGAACACCCCGTCCGCCGGCACCGAGTCCCCTATCTTGAGTATCACCACG	50:T>G
5371987|F|0-49:T>C-49:T>C	TGCAGGCCACCGTGATGCGCCGTGGCCTCGCCATCGGCATGGCCGTGCATCACACCGCCTGCGACGGGG	49:T>C
100419002|F|0-52:A>C-52:A>C	TGCAGAAGATCACCAGCCTGCTGCTCTGGGGCAATGCCCTCTCCGGGAAGATACCGCCG	52:A>C
7171530|F|0-8:T>C-8:T>C	TGCAGCCTTCCCCAGTGTATCTCCCACATGTCGGTGCTGAGCCCG	8:T>C
7169005|F|0-25:G>C-25:G>C	TGCAGGTGATGGCGGACGTGATCCAGTGGTGCACCCACAAGGACGGCGGGGCTCGGCCGTCGATGAAGC	25:G>C
32025957|F|0-64:A>G-64:A>G	TGCAGATCAGAGGCGAGCGGAACAAGGAGCAGGAGGAGAAGACCGACACCTGGCACCGCGTGGAACGCA	64:A>G
100536349|F|0-49:A>G-49:A>G	TGCAGGAGTTCTTCTACGACCAGGTGCCCGACGCGCTGCGCAGCCTGGGACTGGCCTTCTTCCTCAGCA	49:A>G
100549643|F|0-23:A>G-23:A>G	TGCAGACCTGGGCACGAACAGAGAGCTGTCGTACATCGACATGAGCGACAACCTTCTTGAAGGAAGGAT	23:A>G
32017566|F|0-31:T>G-31:T>G	TGCAGACGACGGCGTCGACCCAGAACCACCATCCG	31:T>G
6045093|F|0-10:G>C-10:G>C	TGCAGGTCAGGAGCGCCACGTCCGCCGCCGTGTCGATCATGTTGTCCATGAAGAAGACGTAGGCGGTCA	10:G>C
100414901|F|0-13:A>G-13:A>G	TGCAGATGTGGAGAATTTCTTCCG	13:A>G
100494865|F|0-19:A>G-19:A>G	TGCAGCAGCGGGCACGGCGAGTAGTGGTTCACCCG	19:A>G
7354198|F|0-49:G>C-49:G>C	TGCAGGCCTTGCAGAGCGTGCCGAGGGAGTTGGCGAGGGAGGGCGCCTCGTCCGCTCCG	49:G>C
100530904|F|0-30:T>C-30:T>C	TGCAGCCTCCCCTGCGTGCAGGCCCACAAGTGCCGCGCCGCCTGCTCCG	30:T>C
5361385|F|0-16:T>C-16:T>C	TGCAGGAGTTCTTCTATGACCAGGTGCCCGACGCGCTGCGCAGCCTGGGGCTGGCCTTCTTCCTCAGCA	16:T>C
7352482|F|0-9:A>G-9:A>G	TGCAGAAGGACCAAGAGCCCATCTTCGGCGCCG	9:A>G
6044648|F|0-20:C>G-20:C>G	TGCAGCTTCTCCCCCATCATCCCCTGGCTCACGTCCAGCACCGTCACGAGGTCGATGGGCGCGCGCCGC	20:C>G
3026282|F|0-11:T>G-11:T>G	TGCAGCTGCTCTAGCGCGGTCACCACCTCCGCCATGCCG	11:T>G
7353270|F|0-29:A>G-29:A>G	TGCAGACCACCAAGGCCGTGCTCGGCACCATCGGCTACATCGACCCG	29:A>G
7352694|F|0-32:T>C-32:T>C	TGCAGGTGGCGGACATGCACTACGCGGACGGGTGGAGCACCG	32:T>C
100462687|F|0-8:A>G-8:A>G	TGCAGGCCACGGACGTGCTCGTCGGCGAGGAGCACGGTGAGGACGCCCG	8:A>G
23919932|F|0-37:T>C-37:T>C	TGCAGGAGGACGGTGATGGCGAGGTCGGGGCTGAGCATGCCGCTGCTCACCATCTCGTCCAGCGTCTCC	37:T>C
6044137|F|0-29:A>G-29:A>G	TGCAGGGACTGGACGTAGTTGATGATCTCATCGAGCATGCCG	29:A>G
7351812|F|0-39:T>C-39:T>C	TGCAGCATCCAGTTATGGTACTGCGAGAGGCCGTTGAGCTGCCGCAGGCACCCCGTCCG	39:T>C
100570411|F|0-19:T>G-19:T>G	TGCAGCTCCCAGCGGCGTATGCCCCCATGAGCCCCAGCAAGCCCAGGATCATGGCGAACTGAAGCGCGC	19:T>G
100521892|F|0-10:T>C-10:T>C	TGCAGTGCCTTACCATGCTGCCCACGCCG	10:T>C
4992136|F|0-22:T>C-22:T>C	TGCAGAAGCTGCGGTGGTACTGTCAGATGTGCGAGAAGCAGTGCCGCGACGAGAACGGCTTCAAGTGCC	22:T>C
7354311|F|0-8:T>C-8:T>C	TGCAGCAGTGGAAGGGCCTGCTGAGCTCGGACCAGACGTTGTTCGAGTCGCCG	8:T>C
100440452|F|0-38:T>C-38:T>C	TGCAGCGCCCGCAGCACGTCGGACCCGCGCAGCTGCCCTCCG	38:T>C
7172494|F|0-24:A>G-24:A>G	TGCAGAACAACAACATCACGGGGCAGTTGCCGCCG	24:A>G
100318119|F|0-5:G>C-5:G>C	TGCAGGGCGCCACCTCGCCG	5:G>C

<table>
<tr><td>100561264|F|0-14:A>G-14:A>G</td><td>TGCAGGTGTGGTGCACCGCCG</td><td>14:A>G</td></tr>
<tr><td>100575425|F|0-26:G>C-26:G>C</td><td>TGCAGGAGCTGGCGCGCCATCCTCACGGACCCCG</td><td>26:G>C</td></tr>
<tr><td>6050161|F|0-15:T>C-15:T>C</td><td>TGCAGCTCGAAGAAGTCATGTTGGATGCCG</td><td>15:T>C</td></tr>
<tr><td>100461036|F|0-5:G>C-5:G>C</td><td>TGCAGGCACGACGGGCTGCACGGGACGAGCCG</td><td>5:G>C</td></tr>
<tr><td>100583428|F|0-8:A>G-8:A>G</td><td>TGCAGTGCATTGTCAAGGAGACCCTTCGGATCTGCCCCGTCGGCCCAATCATCCCGGCGCACGAGGCCA</td><td>8:A>G</td></tr>
<tr><td>6048575|F|0-11:A>G-11:A>G</td><td>TGCAGCGCAGAACTGCCGTCGAGGCCCTTAGCCCTGTTGTGCCG</td><td>11:A>G</td></tr>
<tr><td>7173070|F|0-17:A>T-17:A>T</td><td>TGCAGAACGACTTCTACATCGTGCCG</td><td>17:A>T</td></tr>
<tr><td>7171740|F|0-33:C>T-33:C>T</td><td>TGCAGTTCAACGGCCACCTGGCGCTGCTCGTGCCCGCCG</td><td>33:C>T</td></tr>
<tr><td>100514110|F|0-56:G>C-56:G>C</td><td>TGCAGCGGAGAGGAGGGAGGCGAGAGCGTGAAACGCAGGGAAGCAGTGTCGGAAGCGCTTCTGGCGGCC</td><td>56:G>C</td></tr>
<tr><td>7166778|F|0-5:T>C-5:T>C</td><td>TGCAGTCGATGACGTAGACGACGAGCAGCGCGACGGTGGCGGCGACGAGCGCCACGTTGGCCCG</td><td>5:T>C</td></tr>
<tr><td>6039840|F|0-28:T>C-28:T>C</td><td>TGCAGTAGGGGAGCACGTTGCCGTCCACTACCTCGTCCTCCTGCTGCTGCACCTGTACTTGCTGCTCGG</td><td>28:T>C</td></tr>
<tr><td>100598329|F|0-8:A>G-8:A>G</td><td>TGCAGGTCATCGTGTGGTTCCCGCTGATGCTCATCGTCTTCGAGGCGAGGCAGGCCTGGCTGGAGGTGA</td><td>8:A>G</td></tr>
<tr><td>1089789|F|0-25:A>G-25:A>G</td><td>TGCAGGGCGCGTGCGTGATGAACACACTGGACTCCGTGGTGGACGCGCTGGCCAGGGACCCCG</td><td>25:A>G</td></tr>
<tr><td>100439717|F|0-10:T>C-10:T>C</td><td>TGCAGCAGGCTGACGACAGGGAACACCCTGCCG</td><td>10:T>C</td></tr>
<tr><td>100462242|F|0-19:T>C-19:T>C</td><td>TGCAGGAACGCCCTCAGGGTCTCCG</td><td>19:T>C</td></tr>
<tr><td>100531448|F|0-43:C>G-43:C>G</td><td>TGCAGCTGCTGAGGATCGAGGAGGAGCTCGGCGACGCGGCGGTCTACGCCG</td><td>43:C>G</td></tr>
<tr><td>100422765|F|0-14:T>G-14:T>G</td><td>TGCAGGGAGCCGCCTCGCCG</td><td>14:T>G</td></tr>
<tr><td>100440850|F|0-27:A>G-27:A>G</td><td>TGCAGCTCCCAATCTGTGCCGGGATGCAGCCG</td><td>27:A>G</td></tr>
<tr><td>100447717|F|0-13:A>G-13:A>G</td><td>TGCAGATGATGCAACCCCCGCCGATGGGCTGCGGCGGGCCAGCGTCGTCGTGCAGAGGGTGCAAGGGCT</td><td>13:A>G</td></tr>
<tr><td>4393720|F|0-26:T>G-26:T>G</td><td>TGCAGACTGCCCAACTCTGTAGGAATTCCACCGGAGATGGAATTCCCG</td><td>26:T>G</td></tr>
<tr><td>100515253|F|0-11:T>G-11:T>G</td><td>TGCAGGAGAGGTAGCCTCTCCGCGGCCTCGAACAGCACAAGCACGTCCGACGCCGACGTGTAGCACAGC</td><td>11:T>G</td></tr>
<tr><td>100520956|F|0-7:C>T-7:C>T</td><td>TGCAGTTCGCCTCTGCCCCACCG</td><td>7:C>T</td></tr>
<tr><td>100521433|F|0-22:G>C-22:G>C</td><td>TGCAGGGAGGGGGCTGCTTCTCGCCG</td><td>22:G>C</td></tr>
<tr><td>100540626|F|0-13:T>G-13:T>G</td><td>TGCAGGAGCTGGATGACGAGCAGGCCAAGGGGTACGCGCCGTCGCTGCGCCACGCCG</td><td>13:T>G</td></tr>
<tr><td>100554245|F|0-28:A>G-28:A>G</td><td>TGCAGATTGGCTACTCGGCACCAGCTGAAGCCAGGATCAGCCG</td><td>28:A>G</td></tr>
<tr><td>100556467|F|0-42:C>G-42:C>G</td><td>TGCAGCACCGCCCACACCCTTGCTTCCATCCTCCTCCCGATCCAAACCAGGATTCTGCGGGAACAGCGA</td><td>42:C>G</td></tr>
<tr><td>6046669|F|0-31:A>G-31:A>G</td><td>TGCAGCAGGAGCCCGAGCTCGCCGTCTTGGGACGGGCGCCG</td><td>31:A>G</td></tr>
<tr><td>3032705|F|0-23:T>C-23:T>C</td><td>TGCAGGTCGGGGGGCATCGGTCGTCGAGGCCGAGAAGACCG</td><td>23:T>C</td></tr>
<tr><td>7161119|F|0-22:T>G-22:T>G</td><td>TGCAGCCTATGCGCCGCCGACGTCACGAGCGGCCCG</td><td>22:T>G</td></tr>
<tr><td>100580147|F|0-8:A>C-8:A>C</td><td>TGCAGCGCATCCGCGAGCACCACGACACCCACGCGCCCG</td><td>8:A>C</td></tr>
<tr><td>7173520|F|0-22:A>C-22:A>C</td><td>TGCAGGCCAAGGGGAGCTGCCCAATGTGCCG</td><td>22:A>C</td></tr>
<tr><td>1092419|F|0-22:A>G-22:A>G</td><td>TGCAGGACCTGCCCGAGGAGCAACGCGCGGACGCGGTGAGCAGCATGGTGTACGAGGCGGCGGCGCGCG</td><td>22:A>G</td></tr>
<tr><td>6018415|F|0-12:G>A-12:G>A</td><td>TGCAGGCAAGATGTAGGGTGCCCG</td><td>12:G>A</td></tr>
<tr><td>7170864|F|0-22:A>G-22:A>G</td><td>TGCAGGTCGAGGCCTTCTGGCGACGCAACTACCTTCTGCTCGTCGGCGCCG</td><td>22:A>G</td></tr>
<tr><td>22839811|F|0-31:T>C-31:T>C</td><td>TGCAGGGGCGGTGGAGGCTGGTCTACAGCAGTGCCTTCTCGTCGCGGACGCTCGGCGGCAGCCGCCCCG</td><td>31:T>C</td></tr>
<tr><td>7168808|F|0-64:A>C-64:A>C</td><td>TGCAGCTCGCCAACGTGAAGACGGCGCTGGGGAGGCGGGAGGAGGCGCTCGCCGACCTGCGCGCAAGTG</td><td>64:A>C</td></tr>
<tr><td>6046084|F|0-41:A>G-41:A>G</td><td>TGCAGCCGCGACTTGTGCCATGACAGGTAGGAGCGGCGCAGATTCTTGTGCACCAAGAAGGCCAAGAGG</td><td>41:A>G</td></tr>
<tr><td>7170284|F|0-17:G>A-17:G>A</td><td>TGCAGGCGCAGACGCAGGCGCACCCGCCGTCGCTGAGGTACCGCGGCGTCGTCGACTGCTTCCG</td><td>17:G>A</td></tr>
<tr><td>100557995|F|0-8:A>C-8:A>C</td><td>TGCAGGATAAAAAAAGCTTGTATTAAAGTTTCGATCGGTTAATAACATTGTAATTGCCCCCGCCAGTAC</td><td>8:A>C</td></tr>
<tr><td>100414579|F|0-6:A>G-6:A>G</td><td>TGCAGAACATAATCCTGCTCTGCACCG</td><td>6:A>G</td></tr>
<tr><td>100469081|F|0-56:A>C-56:A>C</td><td>TGCAGCCTGCGTGTTCCTCTGCTCGGAATGGCAAAATCGGTGGGCAGCTGGACGAGACTCAGACAGTTC</td><td>56:A>C</td></tr>
<tr><td>100549345|F|0-8:A>G-8:A>G</td><td>TGCAGAGCAGCAGGTCGGCGGGGAGCGCCTCCAGGCTGAGGTTGGCGTCCCTCAGCGCGAAAACCACCT</td><td>8:A>G</td></tr>
<tr><td>100561212|F|0-16:A>C-16:A>C</td><td>TGCAGGGGCACTGCCCAACCGCCG</td><td>16:A>C</td></tr>
<tr><td>7173698|F|0-27:A>T-27:A>T</td><td>TGCAGGAGCCGCACTATCTCATGCTTGAGCTCCG</td><td>27:A>T</td></tr>
<tr><td>100570748|F|0-38:T>C-38:T>C</td><td>TGCAGGCCCTACCTCGCCAGGTCCCTCTTCCTCCTCGGTTCCATCGGCGGCAACGACTACAACGCCATG</td><td>38:T>C</td></tr>
<tr><td>100586574|F|0-30:T>A-30:T>A</td><td>TGCAGGATCCCCGTGGCCACGGGCATCATCTTGACGGTGCAGGGCGTGTTGTGGATCCACATGCTGACG</td><td>30:T>A</td></tr>
<tr><td>7913082|F|0-7:A>G-7:A>G</td><td>TGCAGCTAGTGGAGCTCCGTCTCGTACCCCTCCG</td><td>7:A>G</td></tr>
<tr><td>1753649|F|0-26:T>C-26:T>C</td><td>TGCAGCTCCACCTGCACCAGCAACTCTTCAAAAATCTCAACAGAACCATCCACCACCCTCTTCACAAGC</td><td>26:T>C</td></tr>
<tr><td>100483551|F|0-12:A>C-12:A>C</td><td>TGCAGTTGGCCGACGCCTTCCG</td><td>12:A>C</td></tr>
<tr><td>7167111|F|0-56:T>C-56:T>C</td><td>TGCAGGGAGACCGTCATGACCATCTGGCACTACCACGGCGGGTGCCACGTCGGCGCTGTCGTCGACGAT</td><td>56:T>C</td></tr>
<tr><td>7171812|F|0-42:T>C-42:T>C</td><td>TGCAGCACAACAACCTGAGTGGCGAGATCCCAGCGGTGCTCGTGGCGCTGCCGTCGGTCACCG</td><td>42:T>C</td></tr>
<tr><td>7172024|F|0-11:T>C-11:T>C</td><td>TGCAGAAGGGCTAAGAGCCCATCTTCGGCGCCG</td><td>11:T>C</td></tr>
<tr><td>23919947|F|0-17:C>G-17:C>G</td><td>TGCAGCCCCTCCACGTACCGCCGCCG</td><td>17:C>G</td></tr>
<tr><td>100554580|F|0-16:A>G-16:A>G</td><td>TGCAGCGCGACACCGAACCCAAGCTGCCG</td><td>16:A>G</td></tr>
<tr><td>7171557|F|0-12:A>G-12:A>G</td><td>TGCAGCGGGGTGAAGACGAGGAAGACGAACTCGCAGCCCTGGATCGCCG</td><td>12:A>G</td></tr>
<tr><td>7171305|F|0-18:A>C-18:A>C</td><td>TGCAGGTACGGCAGCTTGATGATGCTGGACTCGGTGATGGGCTCGTCGTCGCCGAGCACGTCCCTGATC</td><td>18:A>C</td></tr>
<tr><td>15306913|F|0-22:A>G-22:A>G</td><td>TGCAGGGCGTGCAGGCCAAGCTACTCCG</td><td>22:A>G</td></tr>
<tr><td>100589068|F|0-31:T>C-31:T>C</td><td>TGCAGTGCTCCTGCACCTCCTCGATGTCGTATTGCGTAAGCATCGACGACGCGTTCCCCATCGCCGCCG</td><td>31:T>C</td></tr>
</table>

Table S2 The 66, 434 SNP markers applied to genotype the six polyploid *Triticum* species

<table>
<tr><th>Allele ID</th><th>Trimmed sequence</th><th>SNP</th></tr>
<tr><td>1342318|F|0-37:C>A-37:C>A</td><td>TGCAGACTCTACTGGTGGTATTTCCCGAGGCGAGGGTCAATATGTGCATTCATATCGGTCAGGAAACGA</td><td>37:C>A</td></tr>
<tr><td>1329644|F|0-29:A>T-29:A>T</td><td>TGCAGTAGCTAGAAAAGGTCTCCCCAAAAAAAAATAATAGGAAAGCTCTTATCCTACATCTCCAAGACA</td><td>29:A>T</td></tr>
<tr><td>3944304|F|0-9:T>G-9:T>G</td><td>TGCAGCTAGTGCGTGTGTGGAAGAGAATTGATCCG</td><td>9:T>G</td></tr>
<tr><td>5369712|F|0-16:C>T-16:C>T</td><td>TGCAGTTCTTCCTGATCTCGCCCTGCGACCCG</td><td>16:C>T</td></tr>
<tr><td>4261115|F|0-24:T>C-24:T>C</td><td>TGCAGCCTGCCTCTTCTTTAATTGTAGTATCATGCACTGACCG</td><td>24:T>C</td></tr>
<tr><td>4261115|F|0-12:C>T-12:C>T</td><td>TGCAGCCTGCCTCTTCTTTAATTGTAGTATCATGCACTGACCG</td><td>12:C>T</td></tr>
<tr><td>100560011|F|0-15:A>T-15:A>T</td><td>TGCAGCATCCTCTCCACATGGCGCTCTGTGTGTTTCTAGCTCTCTGCTTCATTCCAGCCG</td><td>15:A>T</td></tr>
<tr><td>3947593|F|0-17:G>A-17:G>A</td><td>TGCAGGATCTTCAGGAGGGCCATCACCTACCG</td><td>17:G>A</td></tr>
<tr><td>1749610|F|0-7:T>G-7:T>G</td><td>TGCAGCATCACAACCTCCTGGTGCTCCGCCTCCAGGTCTGGGTGCAGCTTGAGCAGCTCCG</td><td>7:T>G</td></tr>
<tr><td>1016230|F|0-11:A>T-11:A>T</td><td>TGCAGGTCTTCATGCGGCTGAAGTACGTAGAGCTCGGCGGCGACGTGCGAAAATACCG</td><td>11:A>T</td></tr>
<tr><td>3955947|F|0-6:T>A-6:T>A</td><td>TGCAGCTATTGCGCGTGTGTGTGTGGAGGAGAATGGCTCCG</td><td>6:T>A</td></tr>
<tr><td>1016230|F|0-35:A>G-35:A>G</td><td>TGCAGGTCTTCATGCGGCTGAAGTACGTAGAGCTCAGCGGCGACGTGCGAAAATACCG</td><td>35:A>G</td></tr>
<tr><td>1306353|F|0-7:G>C-7:G>C</td><td>TGCAGGGGTACATCAAGAGGATGAAGAAGCTGAGCAAGATGTTCGACCG</td><td>7:G>C</td></tr>
<tr><td>1313915|F|0-5:G>A-5:G>A</td><td>TGCAGGCCTGCGGTGCGCGTTCTTTGCTTCTTCCTCTGCTCCTCCGATCAGACCCGCTTGGCTTGGCTT</td><td>5:G>A</td></tr>
<tr><td>986760|F|0-7:T>C-7:T>C</td><td>TGCAGTGTTGCGTGAGGCAGCAAAGTTGTGTGATGCGACAAGCGCATGGGCCGCAAAGCGAACCGAGAT</td><td>7:T>C</td></tr>
<tr><td>1016230|F|0-26:G>A-26:G>A</td><td>TGCAGGTCTTCATGCGGCTGAAGTACGTAGAGCTCGGCGGCGACGTGCGAAAATACCG</td><td>26:G>A</td></tr>
<tr><td>100569269|F|0-29:G>A-29:G>A</td><td>TGCAGTTTTGGAAGTGGATGAAGCCAGGGGATCAGTTTCTGCCTCCGCCTCAGCCGCCG</td><td>29:G>A</td></tr>
<tr><td>3957382|F|0-12:A>C-12:A>C</td><td>TGCAGTGCGAAGAAACAATGTAATCAGCCG</td><td>12:A>C</td></tr>
<tr><td>4994940|F|0-19:G>A-19:G>A</td><td>TGCAGACAGATCCCACGAGGCCAGGCTAGTTTACATGTGAATCGATGGAACGTTCTCCG</td><td>19:G>A</td></tr>
<tr><td>4261260|F|0-21:T>A-21:T>A</td><td>TGCAGGATAAGCAATTATGCGTACGCGTATTCGCCCG</td><td>21:T>A</td></tr>
<tr><td>100549528|F|0-46:A>C-46:A>C</td><td>TGCAGAAGATGAAGATGACGCTGGAGTCGGTGGAGGCCGCGCTCAGAGACGCCGAGAGGCGGTCCATCA</td><td>46:A>C</td></tr>
<tr><td>100575959|F|0-32:G>C-32:G>C</td><td>TGCAGACATCGGCCTTGCGGTAGCCCATCTGGGCGAAGTGGTCGACGGCGGCGTCCATCCGCCG</td><td>32:G>C</td></tr>
<tr><td>3937560|F|0-8:A>G-8:A>G</td><td>TGCAGTGCACGAGAACGGGTTGGCCTGCAAGGACCCG</td><td>8:A>G</td></tr>
<tr><td>5007514|F|0-7:G>A-7:G>A</td><td>TGCAGTGGCTTGGACTCTTGACAATTTCTTCACCACCTGGCCCTGCAACAGCCACAACTGGACTGAACG</td><td>7:G>A</td></tr>
<tr><td>5357395|F|0-28:G>A-28:G>A</td><td>TGCAGAGTAGCAGTGATCGAGAGCAGAAGATAACCG</td><td>28:G>A</td></tr>
<tr><td>100303162|F|0-16:T>G-16:T>G</td><td>TGCAGCCAAGTTGTTGTGGGTAGGGACAAGGAATAGAGAAACAACCG</td><td>16:T>G</td></tr>
<tr><td>1389385|F|0-24:G>A-24:G>A</td><td>TGCAGTGCTCTGTGCCAAGGATGGGTGTGACCACCGTGATTGCCATGGCGGCCCTTTCCTCGTGGCCTT</td><td>24:G>A</td></tr>
<tr><td>4405250|F|0-20:C>T-20:C>T</td><td>TGCAGGGCGTGCAGGCCAAGCTGCGCCG</td><td>20:C>T</td></tr>
<tr><td>3940229|F|0-25:C>T-25:C>T</td><td>TGCAGTCCTTCCTGACCTCCCCCTGCCCGCCG</td><td>25:C>T</td></tr>
<tr><td>986760|F|0-35:C>T-35:C>T</td><td>TGCAGTGTTGCGTGAGGCAGCAAAGTCGTGTGATGCGACAAGCGCATGGGCCGCAAAGCGAACCGAGAT</td><td>35:C>T</td></tr>
<tr><td>5581467|F|0-20:C>A-20:C>A</td><td>TGCAGGAGCGGGCGACGAGGCGGAGCGCGTCGGTGGAGAAGCCG</td><td>20:C>A</td></tr>
<tr><td>1013536|F|0-40:C>G-40:C>G</td><td>TGCAGAACCATTGTGCGATGCAGCTTTCGTGGAAGGAGTGCAAGCAGCCG</td><td>40:C>G</td></tr>
<tr><td>100575958|F|0-11:C>G-11:C>G</td><td>TGCAGACATCGCCCTTGCGGTAGCCCATCTGGCCGAAGTGGTCGATCGCGGCGTCCATCCGCCG</td><td>11:C>G</td></tr>
<tr><td>4262112|F|0-19:G>T-19:G>T</td><td>TGCAGTGCACGAGAACGGGGTGGCGTGCAAGGACCCG</td><td>19:G>T</td></tr>
<tr><td>4261148|F|0-5:C>T-5:C>T</td><td>TGCAGCGCCATTGGCTTTTCTTATTTGACCG</td><td>5:C>T</td></tr>
<tr><td>3935466|F|0-32:C>G-32:C>G</td><td>TGCAGGTACACCTTCTGGCTGACGCTCTGGTACGGCCGCGTCCG</td><td>32:C>G</td></tr>
<tr><td>1342328|F|0-15:A>G-15:A>G</td><td>TGCAGGGTAGGGAGAAGCCTTGCGCCTTCTTTTTTGAAGGCAAGTGACTTCGCAAACTGCTCAATTTTG</td><td>15:A>G</td></tr>
<tr><td>5363428|F|0-38:C>T-38:C>T</td><td>TGCAGCCTCCACAGCCCCACGCCGTCCTTCACCTCGCTCGTCGTGAACCG</td><td>38:C>T</td></tr>
<tr><td>5372433|F|0-48:T>C-48:T>C</td><td>TGCAGCTTCGAGTGCGCTGCTGTGCCTTGCTGCTCCTGCTTCGTACGATAGCGGATGACCTCCGTCGAT</td><td>48:T>C</td></tr>
<tr><td>22838966|F|0-40:A>G-40:A>G</td><td>TGCAGCTACTGGGCCATTGGCTTGCTGTTTTCTACACTCGACAACTCCG</td><td>40:A>G</td></tr>
<tr><td>4439683|F|0-13:A>G-13:A>G</td><td>TGCAGTGCCAAGAAACAGTATAATCACCCG</td><td>13:A>G</td></tr>
<tr><td>4261611|F|0-14:C>A-14:C>A</td><td>TGCAGGTACGGCATCTGCGCCACGTCCG</td><td>14:C>A</td></tr>
<tr><td>1083646|F|0-28:A>C-28:A>C</td><td>TGCAGTGTCACCCCGAAGAAACTATTCTATGGTGGCGGAGACACCACGAAGAATCTACCG</td><td>28:A>C</td></tr>
<tr><td>6044988|F|0-11:G>A-11:G>A</td><td>TGCAGCTTATTGAGATGGATCGAAGCAGCAGCTATATATGCATTGTAATACATGAATATGTAGTCCGAG</td><td>11:G>A</td></tr>
<tr><td>1269998|F|0-16:T>C-16:T>C</td><td>TGCAGGTATAGAGGCCTTTCTGGAGATTGGGGAGTTTACAGAGGTGTACTGGAGTCTGAGATTCTTCCG</td><td>16:T>C</td></tr>
<tr><td>1018458|F|0-40:C>G-40:C>G</td><td>TGCAGTCCGTGCACCTCAGGCGTTGCCGCGCCAAAACCCACAGCACCATACCCAGACCCAGAGTCATGG</td><td>40:C>G</td></tr>
<tr><td>3944277|F|0-9:C>G-9:C>G</td><td>TGCAGTTGACGACGAGGATGTCGATGTCCTCCG</td><td>9:C>G</td></tr>
<tr><td>7928031|F|0-20:C>T-20:C>T</td><td>TGCAGCAACCAAACAGGCCACGTCGTCAAGCTTCACCTTCGCAATACAAGTCCG</td><td>20:C>T</td></tr>
<tr><td>7913248|F|0-37:C>T-37:C>T</td><td>TGCAGTGAGATGAATCGGACTGTCAGATTGATGCTCGCGCCGCCG</td><td>37:C>T</td></tr>
<tr><td>1021726|F|0-30:C>T-30:C>T</td><td>TGCAGGAGGCTTCGCGCGAGCACCCGATCTCCCGTGTGTCCACCCCTGGGTTCTCGTCATCGCGCCGAG</td><td>30:C>T</td></tr>
<tr><td>1016277|F|0-42:G>A-42:G>A</td><td>TGCAGGAGACAAAGACGAGCTACACGAAGCATCAGGCAGCAAGGTCATGTCATATTTGCGCATGCGATC</td><td>42:G>A</td></tr>
<tr><td>1214075|F|0-34:G>A-34:G>A</td><td>TGCAGACGAGCTTGCAGCTGGGGCAGTGCACGTAGCAGAGGTCGCGGCAGCCG</td><td>34:G>A</td></tr>
<tr><td>1016277|F|0-21:A>G-21:A>G</td><td>TGCAGGAGACAAAGACGAGCTACACGAAGCATCAGGCAGCAAGGTCATGTCATATTTGCGCATGCGATC</td><td>21:A>G</td></tr>
<tr><td>3020926|F|0-19:G>A-19:G>A</td><td>TGCAGGATTTCTTTGACGGGAAGAAGCTCTGCAAGAAAATTAATCCG</td><td>19:G>A</td></tr>
<tr><td>3572094|F|0-20:G>C-20:G>C</td><td>TGCAGTACGTGTCCAAGCCTGGTTGCAGGCTGTTGCAACGTCCG</td><td>20:G>C</td></tr>
</table>

3941970|F|0-20:G>C-20:G>C	TGCAGCCACGCGACCTGCTCGGCGTACTTCTCCG	20:G>C
3944277|F|0-12:C>T-12:C>T	TGCAGTTGACGACGAGGATGTCGATGTCCTCCG	12:C>T
4990868|F|0-28:A>C-28:A>C	TGCAGGTATGTGTGGTAGGGCAGCAGGGACCG	28:A>C
32027719|F|0-10:G>A-10:G>A	TGCAGTTACTGTGCTTATAGATGATTCTAGTGTTCTTTGAATCTCCGTCAAGCACTACTAATTTTACTA	10:G>A
1233606|F|0-19:G>A-19:G>A	TGCAGTTGTTTCATTTTCTGGAGTTGCTTCAGTCCTCTCGGCCCCTTGATTCCCTGCTAAAACTCTGGG	19:G>A
3020926|F|0-37:A>T-37:A>T	TGCAGGATTTCTTTGACGGGAAGAAGCTCTGCAAGAAAATTAATCCG	37:A>T
4261297|F|0-29:C>T-29:C>T	TGCAGTAAATCTGACACTGACTAGGTTTTCCATTCACCG	29:C>T
1019196|F|0-31:C>T-31:C>T	TGCAGGCAGACGTGTGATGCTCGCGACAGCGCACGGGAGGCGGAGGCATGCGAGTGAGAGCCG	31:C>T
100576638|F|0-48:C>T-48:C>T	TGCAGCTTCGGGCACCTCAAGAGCCTGGAGGCCAACAAAACTGGGCACCTGGATCCCTCCTCCCCATC	48:C>T
3024791|F|0-9:G>C-9:G>C	TGCAGTCTCGAACCCCATACCCCTGGCACCGCCGATCTTTGACCG	9:G>C
4990868|F|0-25:G>A-25:G>A	TGCAGGTATGTGTGGTAGGGCAGCAGGGACCG	25:G>A
3572094|F|0-17:C>T-17:C>T	TGCAGTACGTGTCCAAGCCTGGTTGCAGGCTGTTGCAACGTCCG	17:C>T
4261638|F|0-8:G>C-8:G>C	TGCAGCTCGTCATCAAGGAGACGCTCCG	8:G>C
3939871|F|0-6:C>A-6:C>A	TGCAGCCACAGTAGATGGCCTGTAATCCAGCACACTGCCG	6:C>A
4005821|F|0-21:C>G-21:C>G	TGCAGCTTGATGATCTTCATGCTGTTGAGCACCTCGGACGTGGACCG	21:C>G
2245281|F|0-5:G>A-5:G>A	TGCAGGCGGCTCTTGAAGCTGGGGTCTGAAATTTGATCCCAGAACTCACGCAGCTCCGCCTTGTCGATG	5:G>A
1153800|F|0-33:C>T-33:C>T	TGCAGCAGAGTCCTACGGGTTCGTCGCTCTCTGCATTTCATGCCATCCG	33:C>T
5582524|F|0-10:G>C-10:G>C	TGCAGCAGGAGGTTGTGGCTACGGAAGTGAACCCCG	10:G>C
100463732|F|0-5:G>T-5:G>T	TGCAGGGCATGCTCTCCCCATCATCGTCATCAGCTCACGCATGCATGCATCTTTCCTCCG	5:G>T
22841141|F|0-64:T>C-64:T>C	TGCAGGCCGCCTTGGCCGCCGCCGCGCTCGCCGTCATGGTGTCCACCGACGACTTCTCCTCCGTTACCA	64:T>C
3935583|F|0-10:C>A-10:C>A	TGCAGTATGACAACTTGCAATCAGCCGTAGTATTTGATCCCGCCG	10:C>A
1686962|F|0-8:G>A-8:G>A	TGCAGTGAGAAGATGGATCAATTTCTGAAGAGATGCTTCTATCAGTCCG	8:G>A
1052650|F|0-11:C>G-11:C>G	TGCAGCAGCATCACGCCCAGCGAGTAGACGTCCGACTTGACGCCCAGCATCCCCGTCTGCTGGTACTCC	11:C>G
4995081|F|0-11:C>T-11:C>T	TGCAGTGCATGCTCTCCCCGTCATCGTCATCAGCTCACGCATGCATGCATCTTTCCTCCG	11:C>T
5002548|F|0-17:A>G-17:A>G	TGCAGCTGCGGGCTGCTAAACAGCTTCATCGGCACCGTGCCGTAGAGTCTTCCG	17:A>G
7166530|F|0-5:A>G-5:A>G	TGCAGAATAACGTCGCAGCTCCAGTCTTCTCCCCGCGGTGGTCCATGGCGCTATCACTGTCGTCCTCAG	5:A>G
3948597|F|0-10:C>A-10:C>A	TGCAGCTGCTCCGCGGGGTGACCCCCAGGCTCCG	10:C>A
7920043|F|0-10:T>C-10:T>C	TGCAGAAAAATGAAGATGGCGATGACATGGTCATGGCATACCCGAAGCTGGTCGAGCTATGTTCCATGA	10:T>C
100386325|F|0-8:G>A-8:G>A	TGCAGCCCGTCTGATGGGGAAGGGTTGCCTTGGTTCCGAGACCG	8:G>A
4995395|F|0-6:T>C-6:T>C	TGCAGGTAGGGGATTGATTACTCGTAAGGAAGAGGAGGAACGGGACGGACCAGCTTGGACGCGTTCCGA	6:T>C
22840613|F|0-17:G>A-17:G>A	TGCAGGCTCTCATTCTCGTCGTCGTCCATAGTGACACCAAGGTTGACGCGCACCG	17:G>A
32027750|F|0-10:T>C-10:T>C	TGCAGCTACGTAGCGCTCACGTTTCCTGCGCATACATGCATGTAGCGGAGCGAGCGATCCGCCCGAGAT	10:T>C
3934124|F|0-19:C>T-19:C>T	TGCAGTGCATGCGATTCTCCCATTATAGAAGTCCG	19:C>T
1002389|F|0-36:C>T-36:C>T	TGCAGTTGTTTCATTTTCTGGAGTTGCTTCAGTCCTCTTGGCCCCTTGAATCCCTGCTAAAACTCCGAG	36:C>T
3934124|F|0-5:T>A-5:T>A	TGCAGTGCATGCGATTCTCCCATTATAGAAGTCCG	5:T>A
4999666|F|0-5:T>C-5:T>C	TGCAGTTGTTTCATTTTCTGGAGTTGCTTCAGTCCTTGCGGCCCCTTGATTCCCTACTAAAACTCCGAG	5:T>C
4999044|F|0-37:T>C-37:T>C	TGCAGGTTTACATGATGCAGGATGTCCGTCTTTTTCATATGAGAATCCG	37:T>C
3572391|F|0-57:T>C-57:T>C	TGCAGGGATGAGACTCAGAAAAGTTACATTGAGGAAGCAGAGGAGGGAGCCCGCCAGTGATCCG	57:T>C
5008037|F|0-42:C>G-42:C>G	TGCAGCTGACGTGGAACTACAACTACGGGGCGGCGGGGAAGACCGTCGGGTTCGACGGGCTCAACAACC	42:C>G
32022089|F|0-32:C>T-32:C>T	TGCAGTACGACGCCCTTACTTTATTTGCTCGACGGATTTGGCTTCGGAGCAAGTAGAAGCTCCG	32:C>T
3936453|F|0-12:G>T-12:G>T	TGCAGCTAGTGCGTTTGTGTGGAGGAGAATGGCTCCG	12:G>T
3940017|F|0-18:C>T-18:C>T	TGCAGCATCTTGTAGAAGCTGTTGTCCAGCTTGGACGCGCTGGACCG	18:C>T
1052650|F|0-38:G>C-38:G>C	TGCAGCAGCATCACGCCCAGCGAGTAGACGTCCGACTTGACGCCCAGCATCCCCGTCTGCTGGTACTCC	38:G>C
3934124|F|0-18:C>T-18:C>T	TGCAGTGCATGCGATTCTCCCATTATAGAAGTCCG	18:C>T
3950735|F|0-19:C>G-19:C>G	TGCAGCCACGCCAGGTGGCCGTCTGGTTCCAGAACCGCCGCGCCCG	19:C>G
22836058|F|0-8:A>C-8:A>C	TGCAGAACAAAGAATCAATGCCATCCAATCCAAGAATCAGAGTCCG	8:A>C
5002740|F|0-41:C>T-41:C>T	TGCAGTTGGAGAGAGGATATCATCGATCTGGCGTAGGAAAACCGACCCG	41:C>T
100461908|F|0-30:T>C-30:T>C	TGCAGCTTCTCCTCACCGCCGTCGTCGCGGTCGTCGTCGTCAGGGTCCG	30:T>C
100463719|F|0-13:A>T-13:A>T	TGCAGTGCACTGTAAAGAACAAGGAGCCTGTGTATCTCTGCGCCCTGATTCCG	13:A>T
7173895|F|0-33:G>A-33:G>A	TGCAGCGAGCGGCTGCTCGTGGACGTGCTCCGCGTAGGCGTCAGGTCCG	33:G>A
5369694|F|0-8:G>C-8:G>C	TGCAGTGCGAAGAAACAGTGTAATCACCCG	8:G>C
5007351|F|0-7:T>C-7:T>C	TGCAGGATCTGCCCCAGTACGCCAAGATCACCGAGTGCCACCAGGACGAGGTCTGGGAGGCGCCGCTGG	7:T>C
3941656|F|0-7:T>C-7:T>C	TGCAGCATTAGAAAGTGATCAAGAGGGTGACCCG	7:T>C
4262003|F|0-6:G>A-6:G>A	TGCAGGGAAGGATGGTGGCAAGAGGATGGGGCTCAAGTTCGACCG	6:G>A
1252690|F|0-19:C>T-19:C>T	TGCAGGATTACCCCTACGACCACGACCAAGAGGCGAAGCTGTACGACGTGGGCCGCGACGGCCGCGCGC	19:C>T
3936453|F|0-8:G>T-8:G>T	TGCAGCTAGTGCGTTTGTGTGGAGGAGAATGGCTCCG	8:G>T
3941580|F|0-26:A>G-26:A>G	TGCAGGCCCTGCGGCGTGACGAAGCGAAAGTCGCCG	26:A>G
3936453|F|0-7:A>G-7:A>G	TGCAGCTAGTGCGTTTGTGTGGAGGAGAATGGCTCCG	7:A>G
1018402|F|0-41:C>A-41:C>A	TGCAGAATCGTTGCCGTACAGCATCATTCAGCAGCTCCACACAGCCTCCG	41:C>A

5373857|F|0-5:C>T-5:C>T	TGCAGCCATCTCGCTCGCGCCCTTCCTCCTCGCCG	5:C>T
100487568|F|0-45:G>A-45:G>A	TGCAGCTCTTCCCGCACCTCAGCAGCCCTGCGCACACAGTGCAACGTATCCATGTCAAGTGAGAACGTC	45:G>A
4997787|F|0-49:G>T-49:G>T	TGCAGATGCCGTGGGAGACGGTCATTTGCGAATTGCGACTGGGTTCTTCGTCCG	49:G>T
1016466|F|0-37:C>T-37:C>T	TGCAGTAATCATCCGTACATGCATCTAGCTGCTTGCCCTCAGTTGATAAGCATGCATCCAGCTAATCGT	37:C>T
1252690|F|0-15:A>G-15:A>G	TGCAGGATTACCCCTACGACCACGACCAAGAGGCGAAGCTGTACGACGTGGGCGGCGACGGCCGCGCGC	15:A>G
1865587|F|0-53:C>G-53:C>G	TGCAGGTTACCATTGGGGACGTAGTTGTAGAGCAAGAGCTTGACAGACTTGTTCGAGCAGTAACCAAGC	53:C>G
3938526|F|0-30:G>T-30:G>T	TGCAGTCGGTGAGGAGGACGGCGCGGAAGGGGCCCG	30:G>T
4993329|F|0-13:C>T-13:C>T	TGCAGTGGCTCGACGTGTCGACGAACGCGCTCTCCG	13:C>T
5361445|F|0-35:A>G-35:A>G	TGCAGGCCGCTTCGGCTTCTTGGCGGCGGCGGCGGACTTGGCGACGCAGGCGGGGTTGATCCG	35:A>G
1025815|F|0-38:G>C-38:G>C	TGCAGCGGTGAGGCCCCGCGGAAAGTGGTGGCGCGCGGGCGCTCGTGCTCGGGCCGCACGAACTCCGAG	38:G>C
100574823|F|0-26:T>G-26:T>G	TGCAGGCCGAACAGTGACTTCGGAATTGGGCCTACGAGCAGGTTGTCGTCGAGGGCGAGGGAGCTCAGA	26:T>G
1018944|F|0-29:A>G-29:A>G	TGCAGAAGCACGAGTCCGAGCGGGTCCGCATCCTCGCCAGAGGCATCGTGAGCGGGTGGAGGGCGTCCG	29:A>G
1865587|F|0-14:G>A-14:G>A	TGCAGGTTACCATTGGGGACGTAGTTGTAGAGCAAGAGCTTGACAGACTTGTTCGAGCAGTAACCAAGC	14:G>A
3575470|F|0-17:G>C-17:G>C	TGCAGGGCCATGGCCGTGGACGATGAGATGTCGCGCCG	17:G>C
977010|F|0-9:C>G-9:C>G	TGCAGTAATCATCCGTACATGCATCTAACTGCTTGCCCTCAGTTAATAAGCATGCATCCAGCTAATCGT	9:C>G
7172204|F|0-18:G>T-18:G>T	TGCAGGCGACGGAGCGGCGGAGTGACCG	18:G>T
4262003|F|0-15:T>C-15:T>C	TGCAGGGAAGGATGGTGGCAAGAGGATGGGGCTCAAGTTCGACCG	15:T>C
3943092|F|0-5:G>A-5:G>A	TGCAGGCGCCGCCGTATTTGCTCGTTGCACACACTGCCG	5:G>A
7172743|F|0-5:C>G-5:C>G	TGCAGCATGGAGTCCAGCTCCCTGTCCCACAGCCAGGTGCAGAGCACCTCATCCTCTAGCTTCTTTCTC	5:C>G
4262414|F|0-28:C>T-28:C>T	TGCAGCTCCTCCTTGAAGCTCTTCGCCGCAGTGGTCCG	28:C>T
5361645|F|0-11:A>G-11:A>G	TGCAGGTGCAGAAAGAAAAGAAAGTACGCGCAGCCGTCGCATGTCTTCGGAGATGGAGTCCACTGGATC	11:A>G
100350228|F|0-46:A>C-46:A>C	TGCAGCAACAGTTGGTTCTGAAAATCCGTCGAGTTTCTGACACTTCAGATATGACCG	46:A>C
4995566|F|0-47:T>A-47:T>A	TGCAGGAGTCGTCTTCAAAGCGCATCAAAAATCCATCTGGAGCTACGTGCCATGGCGTCCG	47:T>A
1042084|F|0-11:A>G-11:A>G	TGCAGTCTAACAATGGTGATGGTTGATGCATCCTAGGATGAGCCAATGAGGTGTTCCG	11:A>G
3936453|F|0-16:G>C-16:G>C	TGCAGCTAGTGCGTTTGTGTGGAGGAGAATGGCTCCG	16:G>C
4262003|F|0-39:C>T-39:C>T	TGCAGGGAAGGATGGTGGCAAGAGGATGGGGCTCAAGTTCGACCG	39:C>T
3935071|F|0-11:C>A-11:C>A	TGCAGCAACCCCGTGTCGTGGTCGACGAGCCG	11:C>A
32027657|F|0-23:C>G-23:C>G	TGCAGGAGGACGGTGATGGCGAGCTCGGGGCTGAGCGCGCCGCTGCCCACCATCTCGTTCAGCGTCTCT	23:C>G
1019046|F|0-11:A>G-11:A>G	TGCAGCTATCTACGAATGGAAGGAGAAGTAGCTAGTGTGTGTGGCACTACCG	11:A>G
980168|F|0-42:C>G-42:C>G	TGCAGGTGAGGTGGACGCAGCGGAAGGGCCCCTGGTGCGCGGCGAGTATGCGGGAGACGGCCGCGGTGA	42:C>G
7921334|F|0-21:C>T-21:C>T	TGCAGATGCCGATGCATCAGACACAGTGCCCATAAACACGCCAAAATTTGGGCTCTGTCCGTCTTTCTT	21:C>T
3571727|F|0-30:G>A-30:G>A	TGCAGAGCAAACGGCATGCTGACATGTACTGTGTCACATTGTCGCTCGCGTTCCG	30:G>A
3025884|F|0-5:G>A-5:G>A	TGCAGGTCCTGTGCGCACATGCAACCAAAAGCGAACCG	5:G>A
1023071|F|0-6:A>T-6:A>T	TGCAGAAGGCACCTAATTCAGTTGAGCTATGGTTTATTGTATGTGATCCG	6:A>T
3575470|F|0-30:T>C-30:T>C	TGCAGGGCCATGGCCGTGGACGATGAGATGTCGCGCCG	30:T>C
3936013|F|0-23:A>G-23:A>G	TGCAGGACCATCCATGGCCCCCAACCATGCACCG	23:A>G
3033091|F|0-5:C>T-5:C>T	TGCAGCTCCTTGAAGTAGACGCCGTCGAAGGCCGTGCTCGTCCG	5:C>T
1018447|F|0-24:T>C-24:T>C	TGCAGATGTCGCGGTCTTCCATGGTAGGGCATGCCACAGCCGTGTCGTCGCAGAAGCGGAGCAGCTCAT	24:T>C
3938338|F|0-17:G>A-17:G>A	TGCAGTTTCAGAGCGACGAGAGAGTGCGAGAACACCG	17:G>A
3022203|F|0-33:G>A-33:G>A	TGCAGGTGCAGGCCAAAGATTGATATGTCGGATGGAGCACCG	33:G>A
5369498|F|0-23:G>A-23:G>A	TGCAGGTGGAGGTTGTGGCTACGGAAGTGAACCCCG	23:G>A
3939548|F|0-12:C>A-12:C>A	TGCAGGTGCAGGCCAAAGATTGATATGTCGGACGGAGCACCG	12:C>A
3934156|F|0-7:C>T-7:C>T	TGCAGGCCGTGGTGAAGGAGGTGATGCGGCTGCACCCG	7:C>T
5363827|F|0-16:T>G-16:T>G	TGCAGGCTAGCTAGCATTACCATTAGCCTCTACCGTGCAAGTCCACGGCAACTCCG	16:T>G
100366262|F|0-6:A>T-6:A>T	TGCAGCAAGTGACCCATGGAATTAATGCAGGTGCTGGGTGGTGCGTGTGATGGTGACGTACCG	6:A>T
1222941|F|0-21:G>T-21:G>T	TGCAGGAAACAATTGACCACCGCTTAGTAGCCTGTGTCTTCTCCAGATCGGCTTGGCACACGGTGCTGG	21:G>T
100463690|F|0-6:A>C-6:A>C	TGCAGTATTCTTCAGAGATCAGGAGGGTTCGATTCAATGCAACTCCG	6:A>C
5369656|F|0-39:A>G-39:A>G	TGCAGGTTGAAACATCCTCGCTAGTTCGTTGCTCCTACCATAGCCGTCACCGTGGCCG	39:A>G
5369377|F|0-30:C>G-30:C>G	TGCAGCCACGGCGGGCGCTGGGCGTTTGGACCCTAATGGTGGATGGCGTCGACGAGTCCG	30:C>G
100516740|F|0-15:C>G-15:C>G	TGCAGTAGCAGGCCGCACGGTGTTCTGCAAGTGGCTACTGCTTATGCGTATAACCTGGGTTAGGGTTCG	15:C>G
22838442|F|0-40:G>C-40:G>C	TGCAGGAAGTGCAGCGCTGCCTGCACGCCTTCAAAGAGTCGTGCGACCGCGCCGAGGACCTCGTCCGAG	40:G>C
100515770|F|0-26:G>C-26:G>C	TGCAGGCTCGGCGCTCCACGAGCCATGGGGTTCAACCTCGGGTCGTCCATCCCGTCTGCACTTTCCGAG	26:G>C
1745947|F|0-59:A>G-59:A>G	TGCAGGGTTTCGGTCAGGGAGAAGGAATCTCCATTCGGTTTGTTTCCCGTACTGTTGTAATCAGACCGA	59:A>G
1131975|F|0-22:G>A-22:G>A	TGCAGGTCTTGGCGCTCTGGCCGTTGTGGGCTCGGAGGGTAAGGTCGAACGCCG	22:G>A
3938309|F|0-17:T>C-17:T>C	TGCAGATATACATTTGTTAAGCAATAGGAGCCG	17:T>C
7337050|F|0-10:C>A-10:C>A	TGCAGATATACATTTGTTAAGCCATAGGAGCCG	10:C>A
1018944|F|0-14:T>A-14:T>A	TGCAGAAGCACGAGTCCGAGCGCGTCCGCATCCTCGCCAGAGGCATCGTGAGCGGGTGGAGGGCGTCCG	14:T>A
987992|F|0-25:C>G-25:C>G	TGCAGACCATGTATCGGCTCAACGTCGGAGGCCAGGCGATCTCCCCTTCCAAGGACTCCG	25:C>G
4261848|F|0-20:G>C-20:G>C	TGCAGTTTATTCTTTTAGATGACAATTCATCTGTCAGTTAGTTAACCG	20:G>C

7171683|F|0-7:G>A-7:G>A	TGCAGGTGAAGAAGGATGGCCAGTGGATCACCGTCGACCCG	7:G>A
3575470|F|0-6:G>A-6:G>A	TGCAGGGCCATGGCCGTGGACGATGAGATGTCGCGCCG	6:G>A
5002540|F|0-5:C>T-5:C>T	TGCAGCTGGTCTCGTCGGAAACCAGCCAGGGAAGGTTTAACACAAGAGGGAAGGTCAAAAGAATGGCCG	5:C>T
5355006|F|0-14:A>C-14:A>C	TGCAGCTTGAGGACATTGAACCCATCGTGCACGGCGATGAAGTGGCGGGCAGGGTTGCACATGAAGGGC	14:A>C
7171683|F|0-22:G>A-22:G>A	TGCAGGTGAAGAAGGATGGCCAGTGGATCACCGTCGACCCG	22:G>A
5369694|F|0-5:T>C-5:T>C	TGCAGTGCGAAGAAACAGTGTAATCACCCG	5:T>C
3934168|F|0-25:G>A-25:G>A	TGCAGCTATGTTCAGTGGTGCTCATGTCCG	25:G>A
4993888|F|0-12:A>G-12:A>G	TGCAGCTCGTCCACGGGGAGAAAGTCGCCGTTGTTGGCGCCGTCGTCGGCCG	12:A>G
3942423|F|0-18:G>A-18:G>A	TGCAGGTATTCAAGCCAGGTGGCATAAGTTGACCG	18:G>A
1159063|F|0-23:T>A-23:T>A	TGCAGAATCGCCCAGGAATGAGATTCCTCTCCAACATATTCATGCACCG	23:T>A
100520682|F|0-29:C>A-29:C>A	TGCAGCGGTGGTCGTCGTCGATGTGGGGGCTGAGCGGCAGCGACCG	29:C>A
5366077|F|0-39:T>C-39:T>C	TGCAGTTGACGACGACGAGGCAAACTGCCTGGACGTGGATGGACGTTGTGAGCACGTGAAAATCACGCC	39:T>C
7907979|F|0-11:A>C-11:A>C	TGCAGCTTTGGAGTGTGCATGCTGAGGCTTTGAGCGTGCGGATGGCGGACCCTGCGGCGGCGGCGGCGC	11:A>C
5374225|F|0-9:A>T-9:A>T	TGCAGCAACAGGAGGTCGATGGCGGAGCAGCAGCAAGACGAAGAGCTCAGCGGCCTGGACTCGCCGAGA	9:A>T
13879551|F|0-5:T>C-5:T>C	TGCAGTCGATCGTTTCCTTGATGGCAGCGTGCACATCTCCGCCG	5:T>C
1246734|F|0-49:T>C-49:T>C	TGCAGAAGAAATTGGCTTGTTTGCATTCCGACCGACATGCTGATCAACATCCAAGGTAGGGGGATCTGT	49:T>C
5973101|F|0-20:A>G-20:A>G	TGCAGTCGCAGTGCACATTCATTCGATGAGACCAGACGCAGAGAGATTCCTTGATAGTGGTACCATGCC	20:A>G
5358829|F|0-17:T>C-17:T>C	TGCAGATCGATGTAGCGTGCGTACCAGATCCGAGGAGGAATGCGAGGAGGAGGGCGCCG	17:T>C
1018927|F|0-23:G>T-23:G>T	TGCAGCTGCTCACCACCATGCTCGGCTTCAACCGTCACCAAGAAAGGATCCACATCCTCAAAGATGTCA	23:G>T
3939599|F|0-5:G>C-5:G>C	TGCAGGAGAAGATCAAGGCCCTGGAGGCAGCCGCCG	5:G>C
3024314|F|0-19:G>C-19:G>C	TGCAGCTTTTGCTTCGCGCGGATGTGATAAGATGACCCG	19:G>C
3021825|F|0-6:C>T-6:C>T	TGCAGACCGTAAGCTGCTCATCAGCCTCATATTGGCCG	6:C>T
4993329|F|0-19:G>C-19:G>C	TGCAGTGGCTCGACGTGTCGACGAACGCGCTCTCCG	19:G>C
4008019|F|0-25:A>G-25:A>G	TGCAGCTAGTGCGTGTGTGGATGAGAATGGCTCCG	25:A>G
3934803|F|0-25:G>A-25:G>A	TGCAGGATTTCTTTGACGGGAAGAAGCTCTGCAAGAGAATCAATCCG	25:G>A
5369460|F|0-37:C>T-37:C>T	TGCAGCGTTCATAAATTGTTACAGCTCAGTTCTTGTTCATTTGACCG	37:C>T
32025988|F|0-42:A>G-42:A>G	TGCAGCCTCTTCAACCCCACGCCCTCGCTCTCCGCCATGGTCATCAACAACTACAAGCTCCG	42:A>G
1080311|F|0-16:C>T-16:C>T	TGCAGAAACTTGGAAGCAACTCAAGCTGGAACGTGTGTGTGCTGTTAGTCAGTTGAGTAATAACCGAGA	16:C>T
1080311|F|0-52:T>G-52:T>G	TGCAGAAACTTGGAAGCGACTCAAGCTGGAACGTGTGTGTGCTGTTAGTCAGTTGAGTAATAACCGAGA	52:T>G
1080311|F|0-28:G>A-28:G>A	TGCAGAAACTTGGAAGCGACTCAAGCTGGAACGTGTGTGTGCTGTTAGTCAGTTGAGTAATAACCGAGA	28:G>A
4262607|F|0-10:A>G-10:A>G	TGCAGGCAGTACTCGAGGTTCCACAGCACGTCGCCCATGGACGGCCG	10:A>G
5002348|F|0-63:G>C-63:G>C	TGCAGTCTACGGGCGGTCTTATCATAGCCGCGCGTACGTGCACTCGCTAGCAGTAGCATGTAGGCTGTA	63:G>C
32017802|F|0-45:C>G-45:C>G	TGCAGGTGGGGCTCACTGCGTCCATGAGCGGCGTTGGTTGGATCGCCCG	45:C>G
1762370|F|0-17:C>G-17:C>G	TGCAGCTCGAAGACAGCCAGGCGTCGTCTGCGCAGCCTCCTCGTCCTCGCCGCGGACTACCTCAAGTAC	17:C>G
5001968|F|0-7:T>C-7:T>C	TGCAGGTTTTGAAGCTCTGGCTGTTGTGGGCTCGGAGGGTAAGGTCGAACGCCG	7:T>C
4262268|F|0-29:G>C-29:G>C	TGCAGGTTGCCCTTGGGCATGAACTCGTAGACCACGAACCG	29:G>C
3021825|F|0-29:T>C-29:T>C	TGCAGACCGTAAGCTGCTCATCAGCCTCATATTGGCCG	29:T>C
989804|F|0-43:C>A-43:C>A	TGCAGGGTGCGGAAGTCCTTGAGCGGCCGCGCGCGCGGGTGGGCCACGCTGTTCCG	43:C>A
32017071|F|0-25:C>T-25:C>T	TGCAGGACCGCTGGATCACGCCCGACGAGAAGAGCAACTAGTATCTTGGTCAGAACTCTTGACACAAAG	25:C>T
3958586|F|0-11:G>A-11:G>A	TGCAGCACCTTGAACGTGCCGCCCTCCCGCCG	11:G>A
1091990|F|0-41:G>A-41:G>A	TGCAGTGGGCGGCGCGGTCGGTCGCTGCGGGGCGATATAGCGGCGTTCCG	41:G>A
3030750|F|0-12:A>C-12:A>C	TGCAGAGATCACACTCCTGAATGTGAAGTGCGTCGATAAGAAGACCG	12:A>C
1017897|F|0-31:G>C-31:G>C	TGCAGACGTGGAACAACGGGGCCAACAACACGAAGCTGATCGTGCAGGCCG	31:G>C
3937779|F|0-17:A>G-17:A>G	TGCAGAACCAGCCGAGTAATGGTTACATCTCACCG	17:A>G
3028271|F|0-11:A>G-11:A>G	TGCAGGTGGTCATGGTGGAGGTGCTCACCAGGTTCGCCG	11:A>G
5007068|F|0-35:A>G-35:A>G	TGCAGGATGGACTCGACGGGATCAAAACCATCGGCACCGTCGCTCTTCAAGAATGGACTCCG	35:A>G
1074197|F|0-9:C>T-9:C>T	TGCAGCTCCCGAAGTCCGAATGCACGAGTTGGTGTGGTCAAAGAATTGGCCG	9:C>T
6046664|F|0-19:A>G-19:A>G	TGCAGCAGCGTGTCAATGGATCATGGACAAGTGTTGCAGAACCTCGCCATCTCCG	19:A>G
1387386|F|0-6:C>T-6:C>T	TGCAGCCCCTGTCGGTGCTGTTACTGTGGCCTGCCCGCATGCATGCATGCACGCCTATGCCTGATCTGA	6:C>T
5364112|F|0-30:G>C-30:G>C	TGCAGTGTCTGAAGAACATGACGAGCATGAGCAAACGAGCAGCACGCCG	30:G>C
15306830|F|0-30:G>A-30:G>A	TGCAGCCCATCTAACATTAACAGGCCGAAAGAAAAACCCG	30:G>A
7925376|F|0-16:T>C-16:T>C	TGCAGTTGATGTCGACTTCAGTAAAAATAGTCTACTCTACTATTCGCCG	16:T>C
5365309|F|0-25:C>G-25:C>G	TGCAGCCATCCTCGTCTGCTGCGCTCGTAGTTGGCGGCGTCTCCATGGAGGTGCCTCCAGCCAAGCCTG	25:C>G
7917744|F|0-5:A>G-5:A>G	TGCAGAGAAGCTAGCTGTATAGACGCCACGTGATTGGCTAGGTGTTACTTATTGTTTGGCCG	5:A>G
5006963|F|0-30:T>C-30:T>C	TGCAGCCACGACGGAGTGCCACGACCTGGATGACAGCCG	30:T>C
5368285|F|0-31:T>C-31:T>C	TGCAGCCTCAACACCACCGTCGTGGAGCTAGTCCGCTTCTTGGCGGCCAAGTACCCGCACTACAACGTC	31:T>C
5004132|F|0-30:T>G-30:T>G	TGCAGAAGGAGGGGAAACCAAACAAACGGTTAAAAATCGCTACGAATCGAAGCTTGGCGGAGGCGATCT	30:T>G
22838989|F|0-36:T>C-36:T>C	TGCAGCTGCTTCCTGAGGCTGCTAGATTCTCCATTGTTTGCTTCAGGGTGTCGGACCTCCG	36:T>C
3953491|F|0-20:G>A-20:G>A	TGCAGACCCCCGTCCTGGCAGTAGCCGATGAGGGTCACCACGTTCCG	20:G>A

4261876|F|0-26:C>T-26:C>T	TGCAGGTACGGCAGGTTTGGGATGTCCGACTCGTCGGCGAGCCG	26:C>T
3955008|F|0-6:T>G-6:T>G	TGCAGCTGCGTCGGGATGGCGGAGGACTTGAGCCG	6:T>G
1018672|F|0-28:G>T-28:G>T	TGCAGGTGTTCGGGCACAGCGACTACGTGACGTGCATCCAGTTCAACCCG	28:G>T
4993329|F|0-27:C>G-27:C>G	TGCAGTGGCTCGACGTGTCGACGAACGCGCTCTCCG	27:C>G
1069659|F|0-11:G>A-11:G>A	TGCAGGTACCAGTGCAACGAGTGGGGCGTCGGCATGGAGATCAATAACAACGTCCG	11:G>A
4539886|F|0-7:G>A-7:G>A	TGCAGGCGCGTGGCTATGGAGCTGGAGACGCGCCG	7:G>A
4261876|F|0-18:G>C-18:G>C	TGCAGGTACGGCAGGTTTGGGATGTCCGACTCGTCGGCGAGCCG	18:G>C
1148777|F|0-32:A>G-32:A>G	TGCAGACTCGCCCAGGAATGAGATTCCTCTCCAACATATTCATGCACCG	32:A>G
1032579|F|0-32:C>A-32:C>A	TGCAGCTCAAAGAAGCGCTCAGAGGATTGTCCCTTGATGATCCATTCTGGTACAGGGTGAAGCTTTGGC	32:C>A
7173895|F|0-20:G>C-20:G>C	TGCAGCGAGCGGCTGCTCGTGGACGTGCTCCGCGTAGGCGTCAGGTCCG	20:G>C
1022649|F|0-11:C>T-11:C>T	TGCAGATCGTGCCGTGGGGGAACATGGACTGGATGGCGACCAACTTCTACGGGCAGCCG	11:C>T
5008587|F|0-5:A>G-5:A>G	TGCAGAAGCAGCCATGGACGTCGTGTCGCAAGCAGCAGCCGCCGCCG	5:A>G
100479182|F|0-28:A>G-28:A>G	TGCAGCATGTGTCGCTCGCCGACAACTCATTCTCCG	28:A>G
1383897|F|0-37:G>C-37:G>C	TGCAGGTGATGGAGCGCAAGATGCGGTCCAGCGGCATGGAGCGGCTCATCAAGCGCCG	37:G>C
100451536|F|0-50:G>A-50:G>A	TGCAGGCAGACAGGTAGGTGGGTAGGTGTTTCTGTGGGCGTGCGCGTGGCGGGAGCTGCCACGTTGTTG	50:G>A
1015998|F|0-33:C>A-33:C>A	TGCAGCCTCATAGAAGTCGCGCGCATCAGCATACTCTGCCACCTTGAGCTCGTCCATCTCCTCTTCCGA	33:C>A
3940370|F|0-17:A>T-17:A>T	TGCAGTCTGCTGTTTACAGTTCCTTTTTCCG	17:A>T
3020926|F|0-16:C>A-16:C>A	TGCAGGATTTCTTTGACGGGAAGAAGCTCTGCAAGAAAATTAATCCG	16:C>A
987394|F|0-41:G>T-41:G>T	TGCAGTATCAGTACGCCAACTACTCGGCCAACTACCTCTACGGGGGCAAGGGCGCCATCCG	41:G>T
3020885|F|0-24:A>G-24:A>G	TGCAGGAGATCGATACTGTCGTTCAAAGTTCAGAAACCCG	24:A>G
3575470|F|0-7:C>T-7:C>T	TGCAGGGCCATGGCCGTGGACGATGAGATGTCGCGCCG	7:C>T
1147988|F|0-23:C>G-23:C>G	TGCAGGACGATCATGCCGATCATCACGCCGATCGACATGAACAGCAGTATTCCG	23:C>G
1055097|F|0-46:C>A-46:C>A	TGCAGTATCAGTACGCCAACTACTCGGCGAACTACCTCAAAGGTGGCAAGGGCACCATCCG	46:C>A
4003288|F|0-10:A>G-10:A>G	TGCAGTCTACATGTTACCTCTCAGTTCGTGCGATCTCCG	10:A>G
4003288|F|0-5:T>C-5:T>C	TGCAGTCTACATGTTACCTCTCAGTTCGTGCGATATCCG	5:T>C
7903644|F|0-18:A>G-18:A>G	TGCAGTTGCTCCCGAGGCAGTGGACTAAGACAGCCCCTGTACATTGTGTCTTGTCCG	18:A>G
100520207|F|0-29:T>C-29:T>C	TGCAGCATCGGGTTGATGTGGCCCTGGAATGGCAGCGGGAACATCAGTACCCG	29:T>C
4997209|F|0-7:A>C-7:A>C	TGCAGCGATGGAAGGAGGAGGAGCAGCGGTCGCAGGACGGCCG	7:A>C
5367423|F|0-32:A>G-32:A>G	TGCAGGTACGGCAGGTTTGGGATGTCCGACTCATCGGCGAGCCG	32:A>G
5361331|F|0-34:G>A-34:G>A	TGCAGGAATTGGTTGCGCTGCCAAGAAATGTTTGGGAGGATTGATCATACAGGGATTACTCAGCCGAGA	34:G>A
5370614|F|0-43:G>A-43:G>A	TGCAGTGCAGGCCACCGCAGTACCGCCCCAACCTCCATGACCAGCGCCTCATCCCTACCTCCG	43:G>A
5373889|F|0-18:A>G-18:A>G	TGCAGATCGACGGAACTTAGTGCCTCGATGAAAAGCAACAGCAGCGAGCGGCTCCAGGAGAGGGACTGC	18:A>G
100386576|F|0-48:G>C-48:G>C	TGCAGCCTGGCACTAGCAGGGCTGGATTTGGCTGGTTTCCAATCAAAGGTTAGTACTCCG	48:G>C
5361157|F|0-11:G>A-11:G>A	TGCAGCTCAAGGCGCTCACGGAAGCCATGTACCAGGGGTACGACGCCCTGGACACCCATGGCCCCCTGG	11:G>A
22838455|F|0-17:C>G-17:C>G	TGCAGGTGGCGAGGAACCAGGACGACGGGTCGTTCGACCGCCATGGCGTCTCGAGCGCCGTCCG	17:C>G
5009372|F|0-11:T>A-11:T>A	TGCAGTGTAATTAGATATGACATAACCATAATTCTCGAATTAGTCCCG	11:T>A
100554077|F|0-39:C>A-39:C>A	TGCAGAATCCCCCATACGGATACCTCCTCGGGATCATCTCCCTGGAGCCG	39:C>A
22838880|F|0-39:A>G-39:A>G	TGCAGATCCGTGAGCTCCCGAGCTTAGCATGGCGTGCTGATTTTCCG	39:A>G
100494639|F|0-42:C>T-42:C>T	TGCAGCAACGTCCTTGACGGCGGCGAACACCCTGAGCTCCACCATCTCCG	42:C>T
3952194|F|0-24:G>A-24:G>A	TGCAGCCGCGGCAGCGTCGAGACCGCTCACGACCGCAAGCTTTCCCACTCCGACTCCTTCTGGACGCCG	24:G>A
4262800|F|0-5:G>A-5:G>A	TGCAGGCCTTGTACATGGCCAAGTGTGCGCCG	5:G>A
3946889|F|0-19:G>A-19:G>A	TGCAGTGCATCGTGAGCGAGACGCTGCGGCTGTACCCG	19:G>A
3946889|F|0-22:G>A-22:G>A	TGCAGTGCATCGTGAGCGAGACGCTGCGGCTGTACCCG	22:G>A
1069659|F|0-26:C>T-26:C>T	TGCAGGTACCAGTGCAACGAGTGGGGCGTCGGCATGGAGATCAATAACAACGTCCG	26:C>T
5369694|F|0-17:G>A-17:G>A	TGCAGTGCGAAGAAACAGTGTAATCACCCG	17:G>A
3935173|F|0-11:T>C-11:T>C	TGCAGGGGACGTGCACCACCTTATACTGACCG	11:T>C
1302667|F|0-41:G>A-41:G>A	TGCAGGTTCTGGTCGCGCTGGAATCCCTTGCCGCAGATCTCGCACACGAACCG	41:G>A
3022215|F|0-5:A>C-5:A>C	TGCAGAAATGAAATCACCAGGAATGGAAAAACAACCG	5:A>C
1044294|F|0-25:C>G-25:C>G	TGCAGCTGCTGTCGTCGCTGGACACCACCAAGGTCAAGGACAAGAAGGACCG	25:C>G
3939651|F|0-17:G>A-17:G>A	TGCAGGACGATCATGCCGATCATCACGCCGATCGACATGAACAACAGTATACCG	17:G>A
3954751|F|0-24:A>G-24:A>G	TGCAGGCGACGGTGCAGATGATGAACCG	24:A>G
4993621|F|0-17:G>T-17:G>T	TGCAGCTGCATCGGGATGGCGGAGGACTTGAGCCG	17:G>T
3025705|F|0-34:C>T-34:C>T	TGCAGAGCTCCAAGAATCTAAAGAACGACTCGTTCACTGTCCG	34:C>T
6040361|F|0-44:C>G-44:C>G	TGCAGCTTCAGTCCCTTGCTCGTTTACCTCAATGACAGCCTTATCAAAAACGTCATTCTCAACCAGATG	44:C>G
4261249|F|0-14:G>A-14:G>A	TGCAGGCCCAGGTAGCAGAGCGAGGAGGCGTCCG	14:G>A
3953727|F|0-9:T>C-9:T>C	TGCAGGCCGTGGTCAAGGAGGCGCTCCG	9:T>C
4404711|F|0-26:G>A-26:G>A	TGCAGGATAGCACCCCATCCGCACGCGGGCTGGTGGTCGCCG	26:G>A
3024314|F|0-35:C>G-35:C>G	TGCAGCTTTTGCTTCGCGCGGATGTGATAAGATGACCCG	35:C>G
3949564|F|0-22:C>T-22:C>T	TGCAGAAGTACCTCGGCAACTACGTCCG	22:C>T

4439874|F|0-19:T>C-19:T>C	TGCAGCGGCGTTGGCATGGTGGAGGATTTGAGCCG	19:T>C
1216100|F|0-19:T>A-19:T>A	TGCAGCATCTGTAACTTCCTTCTCTCCTTCGTCCACCTTCCGCGGCTGATTAATGGCAACCATGATGAC	19:T>A
32027723|F|0-38:T>G-38:T>G	TGCAGACGACGGGTCGGCGTCGTTGCGTGTATTCTACGTCCACCG	38:T>G
5007846|F|0-47:T>C-47:T>C	TGCAGAGTCATGAGCGGAGCAGGATGATGAGGACCTGCGGCGAGCTATAAGTAATAATTTAGAATTCGG	47:T>C
5363986|F|0-6:C>T-6:C>T	TGCAGGCTCTTGACCTGTCGGACAACCAGCTCGCCG	6:C>T
1112033|F|0-16:A>G-16:A>G	TGCAGACGCTATGCGCAATGAGCCAAGTGTCGCAGAACATCATCGTGGGGGACGGCGAGTTCATCGTGG	16:A>G
22840091|F|0-29:G>C-29:G>C	TGCAGGCTCACCGCGCTCTCCTTGGACTCGCCG	29:G>C
1091322|F|0-19:G>A-19:G>A	TGCAGCGTGGAGTCCCCTGGCAAGGCCGCGGCTTCGGCGATGGCCAACATGTGCACCG	19:G>A
6044173|F|0-31:G>C-31:G>C	TGCAGGTCATGGCCAACATCGCCGACGCGGTGATTGGGGAGACCG	31:G>C
981742|F|0-9:T>G-9:T>G	TGCAGATCATAGGAGGTGGGCTCTGCGCGGTGGAGACGGAGAGTGGGTTCTTGGTTTCTTGGCGCTCCG	9:T>G
980168|F|0-20:C>T-20:C>T	TGCAGGTGAGGTGGACGCAGCGGAAGGGCCCCTGGTGCGCGGCGAGTATGCGGGAGACGGCCGCGGTGA	20:C>T
3024963|F|0-23:T>G-23:T>G	TGCAGTTCACGGCGTACATATACTTCGGCGACGCCACCATCAACCG	23:T>G
988300|F|0-20:A>G-20:A>G	TGCAGTACTACAAAGTGCCTAAAGAAGCCACTAAGCCGTTCCTTCAAGATCCG	20:A>G
3945302|F|0-9:G>T-9:G>T	TGCAGAAAAGGGGGTCGCAGTTTCTTTGCACTGGAGAAAGCAACCG	9:G>T
1054309|F|0-46:G>C-46:G>C	TGCAGTTCCGTGGAGTCGTGTGATCAACAACTTCTTCTAGATGAGAGCCG	46:G>C
1216100|F|0-53:T>C-53:T>C	TGCAGCATCTGTAACTTCCTTCTCTCCTTCGTCCACCTTCCGCGGCTGATTAATGGCAACCATGATGAC	53:T>C
5371354|F|0-27:C>G-27:C>G	TGCAGTAGAAAACGTACCAGAAATTACCACGCTAGCGGCGGCTATTCGAGAACCACACCATGTCTGAAA	27:C>G
3936829|F|0-38:T>C-38:T>C	TGCAGTGAGCGGCGAGTTGGGGGAGAACAGTGACGTGATGACCG	38:T>C
1068798|F|0-24:A>G-24:A>G	TGCAGGACAATGACGCTGTGCGCTACAAAGTTTGCAGCGATTGCTGGTTTACTGCTACACGTTGGTGGA	24:A>G
22838894|F|0-24:A>G-24:A>G	TGCAGCGCGCGGTTGCTATCAGGCACATGTGTCGACGGCACATTTTTCGTCCG	24:A>G
5004132|F|0-32:A>G-32:A>G	TGCAGAAGGAGGGGAAACCAAACAAACGGTGAAAAATCGCTACGAATCGAAGCTTGGCGGAGGCGATCT	32:A>G
4996987|F|0-21:T>C-21:T>C	TGCAGCCAAACAAGAAATCATTAATGCTGAAGTTGTTTCCTAAAGTGAGCTTGTCCTTGGCCAAGCTTC	21:T>C
5370406|F|0-37:C>A-37:C>A	TGCAGTTACGAAGGCATGTTACTCACTACCAAGTTTCCGCACAGACAGTACACAAAGCTGCAATGTGCC	37:C>A
7908059|F|0-45:T>G-45:T>G	TGCAGTATCACAGACAGTGCCTCGGGGTGTTGCCTCGGGGATTCATGTAGTTGCTCTCAGCCAGACCGA	45:T>G
1387254|F|0-22:T>C-22:T>C	TGCAGGTTTACCCGCTGATCGGTGCGATGTCGCTGGTGACGGGGATGTGCGTCTTCCAGCTCGCCAGGA	22:T>C
100581773|F|0-8:T>C-8:T>C	TGCAGTGCTTTCTTTGAATCCCACTGCGCGGCTACTGTAGCTCCCTGCCGTGCCTTCAGCACGGTAGAG	8:T>C
12469554|F|0-53:A>C-53:A>C	TGCAGAATACGATGTCCAGGAGTTTTAGTGTATCACATTGTGTTGTCGTCCACACGCTCTCAGGAAAAC	53:A>C
100451058|F|0-48:A>C-48:A>C	TGCAGGACCTCGTCGGCGGCCTCCAGGCCACCCGCGACGGCGGCAAGAACTGGATCACCGTCCAGCCCA	48:A>C
1862642|F|0-35:G>A-35:G>A	TGCAGGCCATGCATGCATGCGTCAAGAAGGTCTCGGATTGATCGATGCATCTGGTGGAATACATCGTAC	35:G>A
5357200|F|0-32:G>C-32:G>C	TGCAGAAAATCCAAAACAAACTATGCAGCATAGTCGCTCTCAATCACCG	32:G>C
100570545|F|0-20:A>C-20:A>C	TGCAGCTTCCCCTCCTCGTCATCCACCTCCTCCTCGTTCCGTTCCGCCTCGCTCTCTTGCAGCCCGAGA	20:A>C
977751|F|0-48:C>A-48:C>A	TGCAGCACACACGAGAGAAGACACAGGATGAGCAATGGACAGGTTCCACTTAAAACAAGCTTGTCCGAG	48:C>A
3934741|F|0-22:A>C-22:A>C	TGCAGCTATGTTCAGTGGTGCTAATGTCCG	22:A>C
1018447|F|0-5:A>T-5:A>T	TGCAGATGTCGCGGTCTTCCATGGTAGGGCATGCCACAGCCGTGTCGTCGCAGAAGCGGAGCAGCTCAT	5:A>T
3020443|F|0-27:T>C-27:T>C	TGCAGCCTGCCTCTTCTTTAATTGTAGTATCCTCATGCACTGACCG	27:T>C
3020443|F|0-11:T>C-11:T>C	TGCAGCCTGCCTCTTCTTTAATTGTAGTATCATCATGCACTGACCG	11:T>C
1047582|F|0-7:A>C-7:A>C	TGCAGCCAGTCTACGGTTGACTTCTGCCCTCTATCCTTAGGATCACGCGGCGCAGGAGCGTACTGCATA	7:A>C
1100145|F|0-65:C>A-65:C>A	TGCAGCATGTCGTCCAGCATGCCTTGGTCCAAGGTATCCAGGTACAAAGGTTCCTGTTGGCCCATCTCC	65:C>A
1667164|F|0-31:C>A-31:C>A	TGCAGCCCGTCCATGGCGCGCATGGCGACGTCGAAGAAGACGAGGTTGTGGTTGGCGTACCG	31:C>A
5971695|F|0-20:T>A-20:T>A	TGCAGGTGTTTGAGGCTGACTTGCAGAAGGGCCG	20:T>A
12779615|F|0-23:A>G-23:A>G	TGCAGGGTTTGGTTTGGTGTAGCATGATCGCCCG	23:A>G
1062282|F|0-28:A>G-28:A>G	TGCAGCCACCCGTCCCTCGTCGCCGTGTACAGCGTCCCTCCCGCCGCCGCGTCCACGTACACGTCCTCC	28:A>G
4004344|F|0-14:T>G-14:T>G	TGCAGGTTGCAGTGTTGCCTTGTTCACCG	14:T>G
1011276|F|0-29:T>G-29:T>G	TGCAGCCGCTCGCGCCGAGGGTCGTCGGCTCGGAGGTCACCACGCTGGGCGGGCGCGTCCG	29:T>G
3033223|F|0-5:C>T-5:C>T	TGCAGCCTGCCGCTCGCAGCCGACGACTTCTTCTCCG	5:C>T
3570084|F|0-5:C>A-5:C>A	TGCAGCTACAAGCGCATCTTCGCGTTCGGTGACTCCCTCATCGACACCG	5:C>A
7164286|F|0-58:T>A-58:T>A	TGCAGGTATAGAGGCCCTTCTGGAGACTGGGGAGTTTACAGAGGTGTACTGGAGTCTGTGATTCTTCCG	58:T>A
1862331|F|0-12:A>G-12:A>G	TGCAGTCTCGAGACTGCACTGAGTCGAAGTAGCGTGAAGGACAGGAGGAGCTTCCG	12:A>G
7172204|F|0-7:G>C-7:G>C	TGCAGGCGACGGAGCGGCGGAGTGACCG	7:G>C
32025813|F|0-16:G>T-16:G>T	TGCAGCATATATGCATGTTGTACTTACTTTTATCTTCGAATCCACCGCACTGACCCG	16:G>T
32020286|F|0-31:T>C-31:T>C	TGCAGGATGGGGGCAGCGAGAGGGCCATGGGTTTTCCTCCG	31:T>C
3025884|F|0-10:G>A-10:G>A	TGCAGATCCTGTGCGCACATGCAACCAAAAGCGAACCG	10:G>A
16796131|F|0-30:C>T-30:C>T	TGCAGATGGAGTACGTGGACCTGTACCTGACCCACTGGCCGATCAGCCTGAAGCCCG	30:C>T
5003862|F|0-16:C>T-16:C>T	TGCAGCTCCCGTTGAACCGTCCAACTTCCGAGGTCAAAGCCCCGTCCAACCATGGCGAGACGAGAGCCC	16:C>T
5341490|F|0-16:G>A-16:G>A	TGCAGCAGCGTTGGCTGCGGTGGACGTACGGCGGAGGACGGCGGCCTGGGGGGAGAACGAAGGGAGGAT	16:G>A
100527862|F|0-30:A>C-30:A>C	TGCAGGTTCGACGCTCGGCTGCTGCTGGAGAGGCTGCGCAACAAGCGGCTGATGTTCGTGGGGGATTCG	30:A>C
100548058|F|0-31:T>C-31:T>C	TGCAGCTAGCACGCTCGGCCTCGACGCGCCGTTCCCTCCGCCG	31:T>C
3222210|F|0-32:C>T-32:C>T	TGCAGAGAATGAAGTTATTGTCATCGCTATCACCTTTAGTACTGTAACTAGCAAGACGATTAGCTACCG	32:C>T
4002132|F|0-8:C>T-8:C>T	TGCAGATGCTTCTCTAACTCTTGCTCCAAGTGATCGGCCG	8:C>T

7169915|F|0-7:C>T-7:C>T	TGCAGTACGAACAGTTGAACATGATTCCTTTCGGTTTGCACGACCCG	7:C>T
5581505|F|0-32:G>C-32:G>C	TGCAGGCTGAGGTGGTTCAACCAGCTGGACCCGAGGATCAACCG	32:G>C
2241554|F|0-50:G>A-50:G>A	TGCAGTGTGTGCATATGATTATCATCTCGACATGCATTTCAGTGAAAGTTGGATCGATCGACCG	50:G>A
1018447|F|0-38:G>A-38:G>A	TGCAGATGTCGCGGTCTTCCATGGTAGGGCATGCCACAGCCGTGTCGTCGCAGAAGCGGAGCAGCTCAT	38:G>A
3953508|F|0-16:C>G-16:C>G	TGCAGAAGAAGTCCACCACGAGGACGTCCG	16:C>G
3942165|F|0-22:G>A-22:G>A	TGCAGAAGAAGTCCACCACGAGGAAGTCCG	22:G>A
1673680|F|0-34:G>C-34:G>C	TGCAGTGCGTGTCGACGATCTTGGCGAGGTTGGAGGTCTCCTGCATGGCCCG	34:G>C
1667164|F|0-26:G>C-26:G>C	TGCAGCCCGTCCATGGCGCGCATGGCGACGTCGAAGAAGACGAGGTTGTGGTTGGCGTACCG	26:G>C
1068798|F|0-40:T>C-40:T>C	TGCAGGACAATGACGCTGTGCGCTACAAAGTTTGCAGCGATTGCTGGTTTACTGCTACACGTTGGTGGA	40:T>C
1090477|F|0-19:G>T-19:G>T	TGCAGGTGAGGTGGACGCAGCGGAAGGGCCCCTGGTGCGCGGCGAGGACGCGGGAGACTGCCGCGATGA	19:G>T
1087804|F|0-10:C>T-10:C>T	TGCAGTCTAGCAAACTAGTGCCCAGATACTAAACTAGGAACTGGCTGGCCTTAAGAACGATTGCCGATT	10:C>T
3032772|F|0-21:C>T-21:C>T	TGCAGGGTCGTACACAGCCAGCGTCCGTTTGGAGCCTGCGTAGCCCG	21:C>T
1080817|F|0-14:G>T-14:G>T	TGCAGCAGTGGGATGGAGCCTGGCGGAGGGGAGGAGCGATGGAGTAAGACGAAGATCCCCG	14:G>T
1864501|F|0-10:G>C-10:G>C	TGCAGCAACTGGAGGTCGATGGCGGAGCAGCAGCAAGACGAAGAGCTCGGCGGTCTGGACTCGCCGAGA	10:G>C
1092679|F|0-24:T>C-24:T>C	TGCAGCCACATGTACCACCAGGACTGCATCCTGCCGTGGCTCGCGCTGCGTAACTCGTGTCCTGTGTGC	24:T>C
3935173|F|0-10:G>A-10:G>A	TGCAGGGGACGTGCACCACCTTATACTGACCG	10:G>A
1032579|F|0-15:C>T-15:C>T	TGCAGCTCAAAGAAGCGCTCAGAGGATTGTCCCTTGATGATCCATTCTGGTACAGGGTGAAGCTTTGGC	15:C>T
1032679|F|0-27:A>C-27:A>C	TGCAGTACTTGCATAAATTCGTGAACAAATATCTGCATGCAACGTACGTGAAGATAAACATGCATGCTA	27:A>C
1215300|F|0-17:G>C-17:G>C	TGCAGTGGCGGTGCAGGGAGGGGTCTGATCACAGGTGAACAGTGGCGCGAACCG	17:G>C
1080817|F|0-50:G>A-50:G>A	TGCAGCAGTGGGATGGAGCCTGGCGGAGGGGAGGAGCGATGGAGTAAGACGAAGATCCCCG	50:G>A
1059801|F|0-13:G>A-13:G>A	TGCAGGTCGTCCCGTGGGGCGACATGAGGTGGGCGTCCTTCAGTGCCTTCGGCGCGCCGACGGCCGACA	13:G>A
5358617|F|0-26:C>T-26:C>T	TGCAGTGGAGCAGCGGGGGAGATTGGCGGGAAGCTCGTGGCCGTGAAATAGAACCG	26:C>T
5008001|F|0-36:A>C-36:A>C	TGCAGGTTCCTCGACGTCGACCAGTTCACCGCCGAGATGCGCCG	36:A>C
1313859|F|0-8:G>A-8:G>A	TGCAGAGCGGTTGAGCACAAGGGCTGCTTGAGCTACTAGGGCTGTTGCACCG	8:G>A
32026099|F|0-40:G>C-40:G>C	TGCAGTTGCCTTTAGGTATTTGTGCAGAAGCCCTTTTTGTGTGGTCAAAGTCTATGGATGGAAGCGTCG	40:G>C
1862585|F|0-46:C>G-46:C>G	TGCAGCCCGCTTGAGTGATACCCGACCTGACAAATCTCGAAAGATACAACAAGACAGATGGGCTGTGCA	46:C>G
5361926|F|0-45:C>G-45:C>G	TGCAGCTTCGGCGTCGACGCGGCCGCGGACGAAGGCTCGCGCTTGCCGTGGTGGCCAGCAGCGGAGGCG	45:C>G
5363118|F|0-13:G>A-13:G>A	TGCAGAGAGCGACGGGTACTTAAAGGGCTGGCCCCGCCTGAGCCG	13:G>A
5363118|F|0-34:C>T-34:C>T	TGCAGAGAGCGACGGGTACTTAAAGGGCTGGCCCCGCCTGAGCCG	34:C>T
5363910|F|0-7:C>T-7:C>T	TGCAGGGCTGGGTTGCACGTTCGTGCGCATTCAGATTCCGTTGGAAGGCGTGTCCG	7:C>T
1138131|F|0-8:G>A-8:G>A	TGCAGTAGGCGCGATGTTCCGCGTGGCGATTTTACAGAGAAGAGAAACCTACTGCATGCAACACGCATG	8:G>A
100506756|F|0-42:T>C-42:T>C	TGCAGTCTTCTCTGAATTCACGAGTTATTATTCAGATATTCTTCACCCTCTCTGAACTCAGCAGACCGA	42:T>C
3572460|F|0-33:G>C-33:G>C	TGCAGCAGAACCACCAGTCACTCCCTTTTAAGCGCCG	33:G>C
1382737|F|0-13:C>T-13:C>T	TGCAGCTTGATTGCTGGTGAGCGACATTAATACGGGCTGGCTAAGTGGGGGAGGGGATTATTGACCGAG	13:C>T
100552532|F|0-29:C>G-29:C>G	TGCAGGTGGCCGCGCCCGTGCTGGAGTCCCTCGCCTACCACGGCGACGTCCTCATCTGGAGAGAGGACG	29:C>G
1277932|F|0-33:A>T-33:A>T	TGCAGCATGCTACCTAGGATGGAGCCTAAAGGAAAATCCTTGGGCATTGGCAGCATGGTGAGGCACTGG	33:A>T
5368531|F|0-61:A>G-61:A>G	TGCAGGTGAGAGCAAGATAACCTTGCAATTTTGCATCCGCCGTTTCTTTTGTCATTTGTCAAATGGGTA	61:A>G
1075785|F|0-13:C>T-13:C>T	TGCAGAGACACTCCGCGAAATTTGTCGGTTTGGCCGAAGCTGGAGATATCCAATGGAGTCATGTAGCGT	13:C>T
4982755|F|0-41:A>C-41:A>C	TGCAGCGGCACCACGTCCTCGGGCCACGTCTTGGTCACGTCAAGCGGGTCGAAGTCGAAGCGGTCCTCG	41:A>C
100559929|F|0-18:A>C-18:A>C	TGCAGATGCCGTCGGAGAAGGTCATTTGCGAATTGCGACTGGGTTCTTCTTCCG	18:A>C
100414752|F|0-37:G>A-37:G>A	TGCAGAGTCTCCTGACCAAGAAACAAGCAAAGGCGTCGGTATCGCCG	37:G>A
1384396|F|0-36:T>G-36:T>G	TGCAGATCATACATACACGTAATATAATGGCGTAAATTTGTTGCAAACCAAACGAACTCCCCTGGAATG	36:T>G
3032772|F|0-12:C>T-12:C>T	TGCAGGGTCGTACACAGCCAGCGTCCGTTTGGAGCCTGCGTAGCCCG	12:C>T
4262160|F|0-16:G>A-16:G>A	TGCAGAAGGGGCGGCAGCTGGAGACGGTGGAGATGAAGGTCCG	16:G>A
1011276|F|0-16:G>C-16:G>C	TGCAGCCGCTCGCGCCGAGGGTCGTCGGCTCGGAGGTCACCACGCTGGGCGGGCGCGTCCG	16:G>C
5372638|F|0-5:G>A-5:G>A	TGCAGGCGCGTGGCCACGGAGCTGGAGACGCGCCG	5:G>A
15306785|F|0-21:A>G-21:A>G	TGCAGATGGCATGACTAGCTAAGTTCATAACAGTTGTTGTAACTATCCGTCCG	21:A>G
1666899|F|0-7:T>C-7:T>C	TGCAGTATACCCACGCGGTGCTGTTCGAGAACATGCGGCTGTTCCCGCCG	7:T>C
4261134|F|0-17:C>T-17:C>T	TGCAGGATCATCGAGAACGATCTGTTCAAGCACGACTGGAGGAGCCG	17:C>T
5364137|F|0-42:T>C-42:T>C	TGCAGTTGAGTTATGGTGGCTTCTGTGATGCTGTCCTGCCTTTCCG	42:T>C
7164286|F|0-7:A>G-7:A>G	TGCAGGTATAGAGGCCCTTCTGGAGACTGGGGAGTTTACAGAGGTGTACTGGAGTCTGTGATTCTTCCG	7:A>G
6041266|F|0-11:C>T-11:C>T	TGCAGATTTAACTGAGAACTCAGTTATGTTGTAGTGGGCCAAGGTATTTGCAATGGCGCCG	11:C>T
4002132|F|0-7:G>A-7:G>A	TGCAGATGCTTCTCTAACTCTTGCTCCAAGTGATCGGCCG	7:G>A
2241554|F|0-27:C>T-27:C>T	TGCAGTGTGTGCATATGATTATCATCTCGACATGCATTTCAGTGAAAGTTGGATCGATCGACCG	27:C>T
3027499|F|0-26:T>G-26:T>G	TGCAGATCTACGCCGCCGCCGAAGACTGTGGCAAGCCG	26:T>G
1036575|F|0-11:G>T-11:G>T	TGCAGAATTGCGTGAGAGGGAAAGCATGGAATATGAAAGAAGACATAGGGACCG	11:G>T
5001594|F|0-15:A>T-15:A>T	TGCAGGAAGTATACCACCAGGCAGCTGAAGGTGGATACACATACGGCCG	15:A>T
1762370|F|0-53:G>A-53:G>A	TGCAGCTCGAAGACAGCGAGGCGTCGTCTGCGCAGCCTCCTCGTCCTCGCCGCGGACTACCTCAAGTAC	53:G>A
4999436|F|0-15:G>C-15:G>C	TGCAGCGAGCAGCTGGTCGTGGACGTGCTCGGCGTGGGCGTCAGGTCCG	15:G>C

992985|F|0-53:G>C-53:G>C	TGCAGGTCACACGGCCATTGGCCTCAAGTGGGTATTTAAGCTGAAGAAAGATGGCGCCG	53:G>C
100369880|F|0-38:A>C-38:A>C	TGCAGTCGCTGGTGCGCCGTCCACAGCTGCCTTGGGTGACAGTGCACCAAGGGCCCTCTCGCCCAAGTC	38:A>C
5358449|F|0-22:T>C-22:T>C	TGCAGGTGAGATAAACTCAGGTTTTCAGTATGATCCG	22:T>C
3947992|F|0-21:G>C-21:G>C	TGCAGCCTTTCGAGTGCACATGAAAATGCACCG	21:G>C
1022729|F|0-66:C>G-66:C>G	TGCAGGCCGCCTTCGCGGCCGCCGCGCTCGCCGTCATGGCGTCCACCGACGACTTCTCCTCCGTCACCA	66:C>G
3958329|F|0-32:C>G-32:C>G	TGCAGCGGGAAGTGCTTCTCGGCGTGCTGCTTCGGGATCACCAGCCG	32:C>G
1667164|F|0-32:G>A-32:G>A	TGCAGCCCGTCCATGGCGCGCATGGCGACGTCGAAGAAGACGAGGTTGTGGTTGGCGTACCG	32:G>A
1019249|F|0-5:C>T-5:C>T	TGCAGCAGTGGGGTGGAGCCTGGCGGAGGGGAGGAGCGATGGAGGAAGACGAAGATCCCCG	5:C>T
5971571|F|0-10:G>T-10:G>T	TGCAGGCCACGCGGCCGTCGGCCACCTTCCG	10:G>T
1022729|F|0-21:C>T-21:C>T	TGCAGGCCGCCTTCGCGGCCGCCGCGCTCGCCGTCATGGCGTCCACCGACGACTTCTCCTCCGTCACCA	21:C>T
5369498|F|0-17:G>A-17:G>A	TGCAGGTGGAGGTTGTGGCTACGGAAGTGAACCCCG	17:G>A
3028271|F|0-25:C>G-25:C>G	TGCAGGTGGTCATGGTGGAGGTGCTCACCAGGTTCGCCG	25:C>G
1040349|F|0-25:C>T-25:C>T	TGCAGTGGGTGCGCAACATGGGCTACGTCATCTACGACTACTGCACCGACATGAGCAACCG	25:C>T
1388519|F|0-5:A>G-5:A>G	TGCAGACGCTGGGGTGGAAGTTCAGGGCGGTGGAGGAGTGTCTCAGGGACAGCGTGGAGTCGTACAAGG	5:A>G
3023394|F|0-41:A>G-41:A>G	TGCAGAACCTGGTGTTCCTGGACCTGTACTCGAACAGGTTCACCG	41:A>G
3949863|F|0-40:G>A-40:G>A	TGCAGGGTTGGCGTCGCTGGGCGGGGGCGCGCTACCAGAAGGTCTTCGGCATACTTGACGGAATTATCG	40:G>A
2245453|F|0-56:G>A-56:G>A	TGCAGGATGTCCCTGATCTGGTCGATAGCGTACCCTGCCCTGGGGTCTTCCCTCTCGACGCCCTTGTGG	56:G>A
1727366|F|0-21:C>G-21:C>G	TGCAGCACCAGCACCGACGCGCAGCACCCCAAGAAGTCGTCCGACGCCAGCAGCTCCCCGACGGCGCCC	21:C>G
1093181|F|0-45:A>C-45:A>C	TGCAGCACAACATGTCTCCTTCGTGCTGCTGCTGCTCCTTGTGTCAATGGCCGCAGTTAGATCGACCGC	45:A>C
3946032|F|0-11:C>A-11:C>A	TGCAGGCCACGCGGCCGTCGGCGACCTTCCG	11:C>A
3020366|F|0-23:T>C-23:T>C	TGCAGATATCGCCTGATTGCAGATGGAAATAATGAACCG	23:T>C
1023263|F|0-10:G>C-10:G>C	TGCAGAGGCGGCTGTGCGAAGCCGAGCGACCTCGCGATCTTGCTCACTGGTGCAGCAGTGTCCG	10:G>C
1666899|F|0-10:C>T-10:C>T	TGCAGTATACCCACGCGGTGCTGTTCGAGAACATGCGGCTGTTCCCGCCG	10:C>T
3022508|F|0-13:C>T-13:C>T	TGCAGTACCTCACCGAACTGGACCTGAGCAACAACCTGTTCTCCG	13:C>T
3946032|F|0-23:A>T-23:A>T	TGCAGGCCACGCGGCCGTCGGCGACCTTCCG	23:A>T
2259573|F|0-22:G>A-22:G>A	TGCAGCTGGATATGCATGGGTCGATCAGTACTGCGGCAGGCTGCACTCGGGGCCG	22:G>A
5371307|F|0-27:A>C-27:A>C	TGCAGGCGCACAACGACGCGCGCCGCGAGGTCGGCGTGGCACCCCTGACGTGGAACTCGACGCTGGAGC	27:A>C
3021013|F|0-9:C>T-9:C>T	TGCAGTGCTCGTTCGATTTCAGTTGCTGTCTTCATGGGCCG	9:C>T
3957710|F|0-13:C>G-13:C>G	TGCAGGCGCTGGTCCGCGGCAACATCGTCCG	13:C>G
1038026|F|0-59:T>C-59:T>C	TGCAGTTTTGTATGTACGATATGATCCACCGTACCAGTGGCAGATGGGGACGCCTGTTTTTCCG	59:T>C
1215401|F|0-33:C>A-33:C>A	TGCAGTGGGTAACGGCGGAGCTCATGCGGAACCCGAGGGTTCGGCATAAGGCGCAGGAGGAGGTCCGAG	33:C>A
1215401|F|0-44:C>T-44:C>T	TGCAGTGGGTAACGGCGGAGCTCATGCGGAACCCGAGGGTTCGGCATAAGGCGCAGGAGGAGGTCCGAG	44:C>T
3022091|F|0-34:G>A-34:G>A	TGCAGTTTTCTAACACCACGAACTGAAAACCATCGCCCG	34:G>A
4004170|F|0-15:C>G-15:C>G	TGCAGTTTTCTAACACCACGAACTGAAAACCGTCGTCCG	15:C>G
3022091|F|0-29:C>G-29:C>G	TGCAGTTTTCTAACACCACGAACTGAAAACCATCGCCCG	29:C>G
5371688|F|0-39:G>C-39:G>C	TGCAGGAGGTGTTCTTGCGGCTGCCGTCAAAGTCTCTCGGCCGCCTCCG	39:G>C
2241554|F|0-25:C>G-25:C>G	TGCAGTGTGTGCATATGATTATCATCTCGACATGCATTTCAGTGAAAGTTGGATCGATCGACCG	25:C>G
7903066|F|0-10:G>A-10:G>A	TGCAGCTACAGTACTTGCTCGGCCCACTAGCGCCGCGCCAGAGGTAATCCG	10:G>A
4997124|F|0-41:A>G-41:A>G	TGCAGGTAACATGCACACCATGCAACTTGTATCATGTATGGATGATCCG	41:A>G
4999470|F|0-28:G>A-28:G>A	TGCAGTGGTAATAAAAGGGCGGGAAGCGGCCG	28:G>A
5004805|F|0-36:C>G-36:C>G	TGCAGGGGAGGAGGCGGAGCGTGGTGGTAGGTGGCGCGGTAGGGCTCCG	36:C>G
5358911|F|0-14:T>C-14:T>C	TGCAGCAGGCTGCTTCGCCTCGCCGTACTGCCACCG	14:T>C
5358619|F|0-7:A>G-7:A>G	TGCAGTGATAACCACTAGGAGTTGTTGGTGTGTAAGGCGTAGCCG	7:A>G
5366748|F|0-18:T>A-18:T>A	TGCAGAAACTGAGAGCCATAGCCATGCTTAATGGAGAAGCTTGAGCCG	18:T>A
5365316|F|0-51:C>T-51:C>T	TGCAGCCCCCGTCAATGCGTAAAGGGATCCTATGTCTCGACCTAGCTGCGCCGCGCTCGCTGCTGCCTC	51:C>T
100303740|F|0-14:T>C-14:T>C	TGCAGGTCTCGGAGTTCTTCCGTCCGTCGACCAGGACCGCGGTCTCCG	14:T>C
5367492|F|0-22:A>G-22:A>G	TGCAGTCATGGGGTGCTCCCTCATGGTGTACCTCGCGGGGTGGCGGACGTACCG	22:A>G
6041253|F|0-48:G>T-48:G>T	TGCAGATGTTGCTTGTGACCGCAACGAGATCGTCGGGTGTCGACCGCAGGAGAACCTTCCG	48:G>T
5363044|F|0-29:A>C-29:A>C	TGCAGAACCCTGGTGGAAACGCAAGGCGGAGCTGGTGGCCG	29:A>C
100426893|F|0-19:A>T-19:A>T	TGCAGCGTATCCGCGGAGAAGACACCAACATCGATGCCGAGTTCAAGGACATCGTCCGCGCCGTGGACG	19:A>T
1740413|F|0-68:C>G-68:C>G	TGCAGCTCGTTCTTGGCTACGGGGATGACGTGGGAAGCCAAGGTGACGTCCGTGACGATGGCTGACGCC	68:C>G
5341725|F|0-9:C>A-9:C>A	TGCAGGCGACCAGAGGCGGGCGCACGGGATGGCAGCGAGGGCGAGCACAAAGAGCAGGGCGGCTTGCAG	9:C>A
4994585|F|0-31:A>G-31:A>G	TGCAGTACGTGTCCAAGCCTGGTTGCAGGCTATTGCAATGTCCG	31:A>G
6038129|F|0-5:C>G-5:C>G	TGCAGCCGAGAAGGATTTCGGCCTGGAAGGCCCAACACCG	5:C>G
100516467|F|0-16:T>C-16:T>C	TGCAGGTGTTTGGTATTGTTGCTGCGCGTGATTACTTGGATCGTAAGCGCAATATTATTTTCCACCGAG	16:T>C
100515609|F|0-40:A>G-40:A>G	TGCAGGCCTAGACCATAAAGGTCCAATGTTTTCAGGTACCAAGAGGCGAGCGGCAGGTCGTCCG	40:A>G
7910188|F|0-5:C>T-5:C>T	TGCAGCCCTGCACCCAAATATTGCGAAGCAGCCG	5:C>T
100574400|F|0-38:G>C-38:G>C	TGCAGGTGCAACCGCGGCACGTCCTTGGACCAGCCCGCGGCGCCACTGCACGGGAGCTTCACCGCGACG	38:G>C
1207029|F|0-5:C>T-5:C>T	TGCAGCAGCAGCCAGTTCACCTTGGCCAGCACCAGCTGGTCGTCGTTGCAGTAGAAGAGCCG	5:C>T

1079943|F|0-7:C>T-7:C>T	TGCAGTTCCCTGGACTTGTGTGATCAACAACTTCTTCTAGATGACAGCCG	7:C>T
4006110|F|0-10:C>T-10:C>T	TGCAGTTGGTCTGCTGGTCTGCGAAGAATGGCCAGCTGATCACCG	10:C>T
4008246|F|0-14:G>A-14:G>A	TGCAGGTTGTCTATGATTGATTGCAAAACGATCCG	14:G>A
4002132|F|0-19:C>T-19:C>T	TGCAGATGCTTCTCTAACTCTTGCTCCAAGTGATCGGCCG	19:C>T
3943230|F|0-15:C>A-15:C>A	TGCAGAGGCATGACTCCGTGCTCGCCACCCG	15:C>A
3021013|F|0-10:G>A-10:G>A	TGCAGTGCTCGTTCGATTTCAGTTGCTGTCTTCATGGGCCG	10:G>A
1023263|F|0-28:A>G-28:A>G	TGCAGAGGCGGCTGTGCGAAGCCGAGCGACCTCGCGATCTTGCTCACTGGTGCAGCAGTGTCCG	28:A>G
3027758|F|0-10:C>T-10:C>T	TGCAGCTCGCCATCCTCGCCGCCACCGTCACCTACCAGACCG	10:C>T
1019367|F|0-22:C>T-22:C>T	TGCAGTCGTAGCACTAGCACAACGGTAACGTGCCTTCATTTAGAAAATAAGACCCG	22:C>T
1017609|F|0-17:A>G-17:A>G	TGCAGATCCGAGAACTGAGATAGATACAACCCCAATCCCAATCCCAATCCCAATACAATGGAAGACCGA	17:A>G
3934928|F|0-15:A>C-15:A>C	TGCAGGTAGCACATGATGCACGAGTGTCCG	15:A>C
6046651|F|0-37:C>G-37:C>G	TGCAGCACATCAGCCCATACTTCCTGATCAGTCCATGCGAGTTTCCACAGACCCG	37:C>G
5582750|F|0-9:C>T-9:C>T	TGCAGCTGGCGGACGTGCTGCACGCCCG	9:C>T
3947992|F|0-10:C>T-10:C>T	TGCAGCCTTTCGAGTGCACATGAAAATGCACCG	10:C>T
4261275|F|0-10:G>C-10:G>C	TGCAGATTTGGGTGACTTGATTAGTCCG	10:G>C
1673680|F|0-25:G>A-25:G>A	TGCAGTGCGTGTCGACGATCTTGGCGAGGTTGGAGGTCTCCTGCATGGCCCG	25:G>A
3024121|F|0-31:G>A-31:G>A	TGCAGATCGCCAGGACTCTCGGCAGCCTCGTGGTGCCG	31:G>A
4261134|F|0-20:T>C-20:T>C	TGCAGGATCATCGAGAACGATCTGTTCAAGCACGACTGGAGGAGCCG	20:T>C
3022508|F|0-19:G>C-19:G>C	TGCAGTACCTCACCGAACTGGACCTGAGCAACAACCTGTTCTCCG	19:G>C
5358449|F|0-9:G>A-9:G>A	TGCAGGTGAGATAAACTCAGGTTTTCAGTATGATCCG	9:G>A
3946001|F|0-25:C>T-25:C>T	TGCAGCGGCCACGGGAACACGAGAACATGCACCG	25:C>T
1207029|F|0-24:G>C-24:G>C	TGCAGCAGCAGCCAGTTCACCTTGGCCAGCACCAGCTGGTCGTCGTTGCAGTAGAAGCGCCG	24:G>C
1862959|F|0-56:C>T-56:C>T	TGCAGGCGGCTGTCGAAGCTGGTGTCTGAAATTTGATCCCAGAACTCACGCAGCTCCGACTTGTTGATG	56:C>T
7931175|F|0-5:C>T-5:C>T	TGCAGCCTTTCGGGTGCACATGAAAATGCACCG	5:C>T
4261546|F|0-18:A>G-18:A>G	TGCAGCAATGTCTGATCGATCTTGATTGTTCCTCGTCGTGGAATGTCCG	18:A>G
3934928|F|0-13:T>C-13:T>C	TGCAGGTAGCACATGATGCACGAGTGTCCG	13:T>C
3022472|F|0-7:A>C-7:A>C	TGCAGAGATAACGGTTTTGTCTTAGAGAAAGGCAAATGTGCACCG	7:A>C
1023411|F|0-10:C>T-10:C>T	TGCAGACCTGCGGTGCGCGTGCTTTGCTTCTTCCTCTGCTCCTCCGATCAGACCCGCTTGGCTTGGCTT	10:C>T
1023411|F|0-19:T>C-19:T>C	TGCAGACCTGCGGTGCGCGTGCTTTGCTTCTTCCTCTGCTCCTCCGATCAGACCCGCTTGGCTTGGCTT	19:T>C
3960371|F|0-16:G>A-16:G>A	TGCAGATCCAAGGTGAGCAAGCCAGGTTCGTGAACGTCCTCACCG	16:G>A
1021367|F|0-7:T>G-7:T>G	TGCAGACTCTATTGGCGATGAACCAGGTGGCGGAGCACATGGTCCTCGCCG	7:T>G
4006221|F|0-8:G>C-8:G>C	TGCAGCTAGAAATGGATGGATGTGTTACAGGAAGAAGAATTGTCCG	8:G>C
4002116|F|0-9:C>T-9:C>T	TGCAGCCTCCGATCCCTGCCCTAGAGAACCTCGTCGACATACGCCG	9:C>T
4538357|F|0-32:C>A-32:C>A	TGCAGCTGGAGGTTGTGGCTACGGAAGTGAACCCCG	32:C>A
3022472|F|0-24:G>A-24:G>A	TGCAGAGATAACGGTTTTGTCTTAGAGAAAGGCAAATGTGCACCG	24:G>A
3947992|F|0-6:C>A-6:C>A	TGCAGCCTTTCGAGTGCACATGAAAATGCACCG	6:C>A
2259573|F|0-17:G>A-17:G>A	TGCAGCTGGATATGCATGGGTCGATCAGTACTGCGGCAGGCTGCACTCGGGGCCG	17:G>A
3940475|F|0-11:C>T-11:C>T	TGCAGCGAGTACATGCTGGTGGTGCTGACCG	11:C>T
1018927|F|0-14:A>T-14:A>T	TGCAGCTGCTCACCACCATGCTCGGCTTCAACCGTAACCAAGAAAGGATCCACATCCTCAAAGATGTCA	14:A>T
990523|F|0-37:T>A-37:T>A	TGCAGCTACTGGGCCATTGGCTTGCTGTTTTCTACACTCGGCAACTCCG	37:T>A
3022472|F|0-35:A>G-35:A>G	TGCAGAGATAACGGTTTTGTCTTAGAGAAAGGCAAATGTGCACCG	35:A>G
5001808|F|0-9:C>A-9:C>A	TGCAGTTTCCATCTGATTGATTGTTTCCTTTGCTCGCGATCGATGGATGCAGGTGACAGTGACCATGGC	9:C>A
4995325|F|0-40:C>A-40:C>A	TGCAGGCTGAAGCTCGTGCCGTGAGCTAGCAGCACAAAGGCCTCGTCCCAAATGGACGGGCGCGGCAGC	40:C>A
5000361|F|0-9:T>C-9:T>C	TGCAGACTGTGTGAGTGCGACTGGCCGTGACGGCGCGGCCGCTTCTCAGCGGATGACGAGGCCCCCGAG	9:T>C
100526883|F|0-45:A>G-45:A>G	TGCAGGCGCGATGTAAGCCCCCGCCTTTTCCCCTCTTTCTCACGCATGGTTTCGTCCCCGTAGCCGTCG	45:A>G
100396628|F|0-61:G>T-61:G>T	TGCAGATCAGATGCTTGTGTCTGTGCATGTTCCGTACGGATGTCATGTCAGGATTGGATTTGGTGCGGC	61:G>T
100453525|F|0-11:A>C-11:A>C	TGCAGTCGAATAGCTCGCTCATGATCCGTTTCCACTTTCCATAAAACAAGCCAACCTTCCACCG	11:A>C
4262404|F|0-5:G>A-5:G>A	TGCAGGGGTAGCGCCACGGTCGAATGGTCTCCGCTCTACGACCCG	5:G>A
1038026|F|0-34:C>T-34:C>T	TGCAGTTTTGTATGTACGATATGATCCACCGTACCAGTGGCAGATGGGGACGCCTGTTTTTCCG	34:C>T
1038026|F|0-45:G>T-45:G>T	TGCAGTTTTGTATGTACGATATGATCCACCGTACCAGTGGCAGATGGGGACGCCTGTTTTTCCG	45:G>T
2259573|F|0-12:T>C-12:T>C	TGCAGCTGGATATGCATGGGTCGATCAGTACTGCGGCAGGCTGCACTCGGGGCCG	12:T>C
1023469|F|0-13:G>A-13:G>A	TGCAGTACATGCAGTTTTGGTGCAGAGAGAGCGGCGGCGACGAGGACCCG	13:G>A
3954378|F|0-14:C>G-14:C>G	TGCAGCTGCGGGCACGAGGCGAAGACGCCCTCGTAGGCGGCCG	14:C>G
4002705|F|0-20:A>G-20:A>G	TGCAGTACTAGCTCATGGTTAGCTGCATTTTCTTTAACCACAGGCCG	20:A>G
1151429|F|0-52:G>A-52:G>A	TGCAGGTGATCAGTGTAAGAAGCGAAGAGCTGGCCGCACTGCACTGTGGATAGGACCG	52:G>A
7166695|F|0-14:C>T-14:C>T	TGCAGCAGAACAACCCCAGAGCAAAAGCAAAGCAGCCAAGTTGTACTCTGCAATCCAGTACGGGTAGTA	14:C>T
3960371|F|0-19:A>G-19:A>G	TGCAGATCCAAGGTGAGCAAGCCAGGTTCGTGAACGTCCTCACCG	19:A>G
7167761|F|0-16:C>T-16:C>T	TGCAGCCTTTCGAGTGCACATCAAAATGCACCG	16:C>T
1043401|F|0-17:G>C-17:G>C	TGCAGCCTCTTCAACCCGACGCCCTCGCTCTCCGCCATGATCGTCAACCG	17:G>C

3935831|F|0-13:G>A-13:G>A	TGCAGCATGTGTCGCTCGCCGACAACTCGTTCTCCG	13:G>A
2241528|F|0-28:G>A-28:G>A	TGCAGAAATTCCTTACATTCACTCTCTCGACTCTGCGCATTTCATCCTCGAGGAGTGTAACTTGTGCTG	28:G>A
3960371|F|0-30:T>C-30:T>C	TGCAGATCCAAGGTGAGCAAGCCAGGTTCGTGAACGTCCTCACCG	30:T>C
1148777|F|0-6:C>A-6:C>A	TGCAGACTCGCCCAGGAATGAGATTCCTCTCCAACATATTCATGCACCG	6:C>A
1023470|F|0-7:C>T-7:C>T	TGCAGTACATGCAGTTTTGGTGCCGAGAGAGCGGCGGCGACGAGGACCCG	7:C>T
3952428|F|0-13:G>A-13:G>A	TGCAGAGACGCACGTGGCGCGTGACATCCG	13:G>A
4002705|F|0-39:A>T-39:A>T	TGCAGTACTAGCTCATGGTTAGCTGCATTTTCTTTAACCACAGGCCG	39:A>T
983408|F|0-21:C>T-21:C>T	TGCAGGTCTTGAAGCTCTGGCCGTTGTGGGCTCGGAGGGTAAGGCCGAACGCCG	21:C>T
1020827|F|0-10:C>T-10:C>T	TGCAGCCCCACTCCTTTCACGTGTCGTCATCTTTGTTTGACCGCTACTAATCCG	10:C>T
3020442|F|0-26:G>A-26:G>A	TGCAGTGCTATTGGCCAAGTACTGCTGTACTTTGCTATGTCCG	26:G>A
4262404|F|0-12:G>T-12:G>T	TGCAGGGGTAGCGCCACGGTCGAATGGTCTCCGCTCTACGACCCG	12:G>T
4705829|F|0-25:C>G-25:C>G	TGCAGGTCATCTCCACCAACGTCGCCTGGTCCACATCGCTGGATTTAAACGGGCAGTGGATCTCCCACG	25:C>G
7167034|F|0-22:A>C-22:A>C	TGCAGGAGGAGCACCCTGGGGCAGCCGTGACGGTTGGCTAGGCGAAGGTGGAGGTCGGAGAAGTCATCG	22:A>C
3020337|F|0-29:T>C-29:T>C	TGCAGTACTAGCGCTTATGGGCATATGTGTGTACATGCTCCG	29:T>C
2259573|F|0-19:G>T-19:G>T	TGCAGCTGGATATGCATGGGTCGATCAGTACTGCGGCAGGCTGCACTCGGGGCCG	19:G>T
4261388|F|0-23:C>G-23:C>G	TGCAGGATGTCCAGCACCTTGTACGTCGGCCAGTAGAGCCG	23:C>G
3020443|F|0-13:T>G-13:T>G	TGCAGCCTGCCTCTTCTTTAATTGTAGTATCATCATGCACTGACCG	13:T>G
6041365|F|0-39:T>C-39:T>C	TGCAGCCACGGCGGGCGCTGGGCGTTTGGAGCCTAATGGTGGATCGCGTCGACGAGTCCG	39:T>C
3570097|F|0-36:T>C-36:T>C	TGCAGGAACCTCGACGACGCCACCTACTCGACCTGCTGGCGACGACCG	36:T>C
4406156|F|0-11:C>T-11:C>T	TGCAGGAACCTCAACGACGCCACCTACTCGACCTGCTGGCGACGACCG	11:C>T
4993334|F|0-6:T>C-6:T>C	TGCAGGTCGTATCATGTGCTGTCGCCCG	6:T>C
3033596|F|0-12:C>T-12:C>T	TGCAGAGCCGTCCCACCAGGTCTCCCCTCGCGCGCCG	12:C>T
7173999|F|0-13:A>G-13:A>G	TGCAGTGGGCGGCACGGTCGGCTGCTGCTGGGCGATATAGCGGTGTACCG	13:A>G
32027149|F|0-32:A>G-32:A>G	TGCAGGGCGTGAGGCACGTGATCATCCACAACATCCACATCCACCACTCCGTGCTGCACTCGGGCGGCA	32:A>G
5367354|F|0-31:A>G-31:A>G	TGCAGGCTCGCGCGGCGCTGGCGCCACCTCTAGAAATCCACCCG	31:A>G
4995261|F|0-10:T>C-10:T>C	TGCAGGTGATTCAGGAGGTGTTCAGGGCGGTCCG	10:T>C
5361936|F|0-28:A>C-28:A>C	TGCAGCTCGCCCAGCACCCCTACGACGCAGAGGCGATACAGTCGGCGGACGCCGCGTTGGTGGTGGAGC	28:A>C
5002244|F|0-29:T>C-29:T>C	TGCAGCTCCTCCTCCTGCGGGTGCTCCCGTGGAACCG	29:T>C
2244096|F|0-12:C>G-12:C>G	TGCAGTCGTCGACGCTAGTCGTCGAGGCACCAGCAGTGATAGTCGGGCTTGGACATGCTGAGGAGCTCC	12:C>G
5411101|F|0-6:C>A-6:C>A	TGCAGACCATCTTCCACTGTTACGCACCCGATTAATGACGGCCGCTCGAACCCCAGAAGGATGATGTCG	6:C>A
5366098|F|0-18:C>T-18:C>T	TGCAGTTTGATCTTGGACCCCTCTAGTTCCAGGGATCGCCAAGATTGTTACAAACTTGTTGACATATCA	18:C>T
5361214|F|0-55:A>G-55:A>G	TGCAGCTCTAACTCCGAAATCAACCACACTAAACTCAGGTTTCAGAGATGGCAACAGTGAATACACAGT	55:A>G
13879642|F|0-38:G>C-38:G>C	TGCAGGTGCAGCGGTTCTTGACACTAGTACCTGGTCTAGGAACACCG	38:G>C
1071336|F|0-20:G>C-20:G>C	TGCAGAACACGCAACCCCTGGGATCCACCCACACTCCTTATATATAGTGCGTTCCG	20:G>C
100440223|F|0-11:A>C-11:A>C	TGCAGCCTCTGATGCTGCTCCCTGTCGTGGTCGAACCGCCG	11:A>C
990866|F|0-61:T>G-61:T>G	TGCAGGATTTGCAAGGAGGTGATGGCCGACGCGGTGGTGGCGAGCAAGTGCTGCTTCAACATCTTCTGC	61:T>G
3941830|F|0-8:G>A-8:G>A	TGCAGTCCGTACCGTATCCAAGGCCATCGTGTTCCG	8:G>A
5362342|F|0-26:A>G-26:A>G	TGCAGCTTCGAGCACCTCAAGGGCCTAGACGCCAACAAAACTGGGCACTTGGATCCCCTCCTCCCCATC	26:A>G
5360879|F|0-18:T>A-18:T>A	TGCAGCATGTCTTTGAGGTGGGCGCGGCCGTGGCGGTACCCGAGGAAATAGATGTCGTAGCGGCGGTCG	18:T>A
5004242|F|0-12:G>A-12:G>A	TGCAGGCCGTAGGGTTGGAGTTTTGCTCTTGTTGTTTGGTGGTATTAGACTCTTGGAAATGAACTTGCC	12:G>A
100429817|F|0-13:T>C-13:T>C	TGCAGGTGCAGGCTGCGCATGGCACCAAACCATTATCAACACCTTTAGGTAGCGTAACCTCGAGTAAAG	13:T>C
100542591|F|0-21:A>C-21:A>C	TGCAGAGTGGATAGGAGTCGAACTTCACTCCAACGCTCAGGAGGTGCCGAGCGTGACGGAGGATGCCGA	21:A>C
4009093|F|0-15:T>C-15:T>C	TGCAGGTGGGCATTTTCAGCCCACGAGGGAGGAGCACAAGGCCG	15:T>C
100520036|F|0-5:A>C-5:A>C	TGCAGAAGCAGCTGCAAGTGCTGGTGGAGAGGTACCG	5:A>C
1390035|F|0-29:G>C-29:G>C	TGCAGCAGGAGAGAGACGAGGTGCTCCATGGAACGTCGCCGTGGGAACGACATGGTGGTAGCCAGATCC	29:G>C
22839813|F|0-14:A>C-14:A>C	TGCAGGGTTGTCGTAGACAATGAGACGCGCGAGGACTGCTCGCTCGTCAAGGTCGACAGCGTGAACCGA	14:A>C
5370325|F|0-55:A>G-55:A>G	TGCAGGGGCGAAGCACAAGGCAAGGGCCATGAAAGTGGCAGTGATCTGTGACATGACGATGGTGGAGCG	55:A>G
4996194|F|0-41:T>G-41:T>G	TGCAGGCCGAGGCCTCCTCCATCTGCAAGTGGTGGCGAGCTTCCACGATGGACGGCGATGGGGTGCTCC	41:T>G
1383796|F|0-40:C>T-40:C>T	TGCAGCCTATCCTGCTCTCCGCGGACGTACACCACGTACACGCACATGCCCATTCGGTGGATCCAACAC	40:C>T
3023679|F|0-38:C>T-38:C>T	TGCAGAAGGCATCCAGCGTGATCTCCAGGTGGATGATGCCCCG	38:C>T
100439028|F|0-5:G>A-5:G>A	TGCAGGGTCGCTGACAGAGAGCAAAAGCTGGCCG	5:G>A
100498915|F|0-40:C>T-40:C>T	TGCAGAAGGAGGGGAAACCAAACAAACGGTGAGAAATCGCCAGGAATCGAAGCTTGGCGGAGGCGATCT	40:C>T
1726482|F|0-10:T>C-10:T>C	TGCAGAGGAATTGGAGAGCCAGCCCCAGCACGACCTTCATGATGCTCAGCCCCATGTGCATATGGAAGC	10:T>C
100573686|F|0-34:T>G-34:T>G	TGCAGCGGTGAGGCCCCGCGGAAAGTGGTGGCGCTCGGGCGCTCGTGCTCGGGCCGCACGAACTCCGAG	34:T>G
3020764|F|0-25:T>C-25:T>C	TGCAGGTGTATCCAGTTCCTCATGATCCACACGCCAATCCG	25:T>C
5412069|F|0-12:T>C-12:T>C	TGCAGTGTTCGTTAAGCCGCGGAGGAATCAGCCG	12:T>C
1694808|F|0-6:G>A-6:G>A	TGCAGAGACACCCGTCCGAGCTCGACGACGCGGAGCCACACACTCGGTGGCGGCGATGAGTGCGCCGAG	6:G>A
3942531|F|0-8:G>T-8:G>T	TGCAGATCGCCTGCGGCAAGTCCATTCTCCG	8:G>T
4261588|F|0-33:T>C-33:T>C	TGCAGTCGAGCTGGGCGCGGGAGATCTCGTAGGTTATGACCCG	33:T>C

3026027|F|0-20:T>C-20:T>C	TGCAGAGCGACGACTTAATTTGATGGGAGTAATTTAACCG	20:T>C
2259573|F|0-16:T>C-16:T>C	TGCAGCTGGATATGCATGGGTCGATCAGTACTGCGGCAGGCTGCACTCGGGGCCG	16:T>C
4005207|F|0-25:C>A-25:C>A	TGCAGGGTCTGTGTCGAGGTTGCTTCGGTATGCTCCG	25:C>A
1092441|F|0-20:G>C-20:G>C	TGCAGTCTGATCGTGGTCGTGGCACGCACCAAGCGGCAATGGTTGGCCG	20:G>C
1096148|F|0-15:T>A-15:T>A	TGCAGAGCGCCGCCGTCGTGGACGTGGACGATCTCGTGCTCCTCGACGAGCCCG	15:T>A
1021367|F|0-47:G>A-47:G>A	TGCAGACTCTATTGGCGATGAACCAGGTGGCGGAGCACATGGTCCTCGCCG	47:G>A
1033901|F|0-48:T>C-48:T>C	TGCAGCTCTAGTGGGGAATTTAGGTACAGGGAAACAGTGGCATTATCTTTCATTACATCCG	48:T>C
5358070|F|0-6:G>T-6:G>T	TGCAGCGGTTTTTATAGCTAGTGCCAGGATTAGCCG	6:G>T
1707771|F|0-62:A>G-62:A>G	TGCAGGTTCGACGCTCGGCTGCTGCTGGAGCGGCTGCGCAACAAGCGGCTGATGTTCGTGGGAGATTCG	62:A>G
3941830|F|0-13:G>A-13:G>A	TGCAGTCCGTACCGTATCCAAGGCCATCGTGTTCCG	13:G>A
1004021|F|0-19:G>A-19:G>A	TGCAGCTCATGCCGAACCTGCACAATCCAGTCCTGAGGTAGTAGGTGCAGTCCG	19:G>A
3939376|F|0-23:C>G-23:C>G	TGCAGAAAAGGCGGTCGCAGTTTCTTGGCACTGGAGAAAGCGACCG	23:C>G
2245631|F|0-14:A>C-14:A>C	TGCAGACGCGTTGAATCGATTCCTTGGTGCGCGGTGATTGCCGAGGGGAGCGAGATGGTAGAGAAGCGC	14:A>C
1019367|F|0-9:A>T-9:A>T	TGCAGTCGTAGCACTAGCACAACGGTAACGTGCCTTCATTTAGAAAATAAGACCCG	9:A>T
1004021|F|0-43:G>A-43:G>A	TGCAGCTCATGCCGAACCTGCACAATCCAGTCCTGAGGTAGTAGGTGCAGTCCG	43:G>A
1022729|F|0-8:G>C-8:G>C	TGCAGGCCGCCTTCGCGGCCGCCGCGCTCGCCGTCATGGCGTCCACCGACGACTTCTCCTCCGTCACCA	8:G>C
5582091|F|0-22:G>A-22:G>A	TGCAGTTGGTCTGCTGGTCGGCGAAGAACGGCCAGCTGATCACCG	22:G>A
1371147|F|0-11:A>C-11:A>C	TGCAGGCATACACATGCATATTCATGTCGTCTCGCCATCTTCAGCGTCCG	11:A>C
4261588|F|0-18:G>A-18:G>A	TGCAGTCGAGCTGGGCGCGGGAGATCTCGTAGGTTATGACCCG	18:G>A
3026027|F|0-6:G>A-6:G>A	TGCAGAGCGACGACTTAATTTGATGGGAGTAATTTAACCG	6:G>A
1092441|F|0-35:G>A-35:G>A	TGCAGTCTGATCGTGGTCGTGGCACGCACCAAGCGGCAATGGTTGGCCG	35:G>A
2243750|F|0-63:C>G-63:C>G	TGCAGTGCTCCTGCACCTCCTCGATGTCGTACTGCGTCAGCATCGACGACGCGTTCCCCATCGCCGAGA	63:C>G
1671457|F|0-41:C>A-41:C>A	TGCAGCTGCCATCGACGGTAGTAGTCTTCATATGCATCATGCAAAGAACCCG	41:C>A
3023448|F|0-5:T>C-5:T>C	TGCAGTGTCCTCCACGAGCCCCACGCATGCCGCATCCG	5:T>C
2259573|F|0-48:G>T-48:G>T	TGCAGCTGGATATGCATGGGTCGATCAGTACTGCGGCAGGCTGCACTCGGGGCCG	48:G>T
3940299|F|0-30:G>T-30:G>T	TGCAGTCCGTACCGTATCCAGCGCCATCGTGTTCCG	30:G>T
4261388|F|0-26:C>G-26:C>G	TGCAGGATGTCCAGCACCTTGTACGTCGGCCAGTAGAGCCG	26:C>G
3959134|F|0-33:G>A-33:G>A	TGCAGTGTGGGGAATGCGCCGATGAAGCTCGACGGCGAAGTCTGTGCCGCTTAGGTTGGTAGCGCGTCT	33:G>A
5004271|F|0-23:T>C-23:T>C	TGCAGGCCATGGCAGGCCGACGATAAGAGCAATGCGCATGTCAGCGCTACGGAGAGCTTAAGCCAGCCG	23:T>C
4997538|F|0-31:C>G-31:C>G	TGCAGCCAAATCAAGAAATCGAATAAGAAATCCTGCCCCTTTCCCGCTCCG	31:C>G
1020654|F|0-19:A>C-19:A>C	TGCAGCTTGAGGTTCTGAGAAAGATACGCCACCCACACTTGCTGATGCTCCTGGGAGCCTGTCCGAGAT	19:A>C
4994785|F|0-42:A>G-42:A>G	TGCAGGAGGCGGCGATGCCGTCGGCAGTGAGGGAGGATCGTCAGATCTGATCACCGATGATCAGCCGAG	42:A>G
1314482|F|0-14:T>C-14:T>C	TGCAGCTGCAACTCTTGGGCTGCCTCGGCGGGGTGGAGATGGTGCTCAAGGACATCGGGTACCCGAGAT	14:T>C
100324609|F|0-29:G>C-29:G>C	TGCAGGCCACCCTCCTCCGCCTGCCACACGACGCACACGGAGATCACGCATGTATTGCGAGTGATGGAA	29:G>C
100495036|F|0-37:T>G-37:T>G	TGCAGCCACGGCGGGCGCTGGGCGTTTGGAGCCTAATTGTGGATGGCGTCGACGAGTCCG	37:T>G
100517047|F|0-9:C>T-9:C>T	TGCAGTGATCTGATGTTGCTCCATATCCCAGTCTCGGATGAGTAAAGGCATACGGAGACTCTCGTACAT	9:C>T
1266593|F|0-7:C>A-7:C>A	TGCAGGGCGAGTCACCGACCGATCTGATCACGAGCTACACGGGGTCGACCG	7:C>A
1016408|F|0-17:G>A-17:G>A	TGCAGCCTCTTCAACCCGACGCCGTCGCTCTCCGCCATGGTCGTCAACCACTATAAGCTCCGCGGTAAC	17:G>A
1016408|F|0-14:C>T-14:C>T	TGCAGCCTCTTCAACCCGACGCCGTCGCTCTCCGCCATGGTCGTCAACCACTATAAGCTCCGCGGTAAC	14:C>T
1033901|F|0-42:T>C-42:T>C	TGCAGCTCTAGTGGGGAATTTAGGTACAGGGAAACAGTGGCATTATCTTTCATTACATCCG	42:T>C
1023470|F|0-16:T>C-16:T>C	TGCAGTACATGCAGTTTTGGTGCCGAGAGAGCGGCGGCGACGAGGACCCG	16:T>C
6048317|F|0-23:C>G-23:C>G	TGCAGCAGCGCCGTCGTGGGGAGCACGAAGGAGACGTTGTTCACGGCCGCCGCGAACCG	23:C>G
3940337|F|0-12:C>A-12:C>A	TGCAGCGGCTTTCGTTACGGATAATTTTGACCG	12:C>A
3020403|F|0-19:T>C-19:T>C	TGCAGGTGTATCCAGTTCCTCATGATCCGCATGCCAATCCG	19:T>C
3937257|F|0-10:C>G-10:C>G	TGCAGTACAACGGGAGCGGACGGCGTTGAAAACGGACCG	10:C>G
2242191|F|0-31:T>C-31:T>C	TGCAGTGGCCGCACGCAGCATCTCGATGTCCTCATCTGAGGAGGGTTTGTCTGCCTCTGTTTGCCCCCG	31:T>C
3032649|F|0-5:G>A-5:G>A	TGCAGGTCGAGGGAGAGGAAGATGGCGCCCTTGAGGTCCCAGCCG	5:G>A
1266994|F|0-21:T>C-21:T>C	TGCAGCAGCAGCCAGTTCACCTTGGCCAGTACGAGCTGGTCGTCGTTGCAGTAGAAGAGCCG	21:T>C
1695171|F|0-43:T>C-43:T>C	TGCAGGTGAGAGCAAGATAACCTTGCAATTTTGCATCCGCCGTTTCTTTTGTCATTTGTCAGATGGGTA	43:T>C
4002338|F|0-19:C>G-19:C>G	TGCAGAGAAGCATGCATTACTTCACAAAGTGGCCAACAACCG	19:C>G
1863707|F|0-26:G>A-26:G>A	TGCAGGCGGCTGTCGAAGCTGGTGTCGGAGATCTGGTCCCAGAACTCGAGCAGCTCCGCCTTGCTGATG	26:G>A
7160412|F|0-7:A>G-7:A>G	TGCAGAGACTTCTCCCTCAAGCCACGAGGCACACCTGTCGCCCG	7:A>G
1226885|F|0-38:T>C-38:T>C	TGCAGCTGAAAGCCTGAAAGTGGCAGAATTCGCAATCGTCGAAAGCAATTCAGCAGAGAAGCGGGTCAT	38:T>C
1863300|F|0-17:C>T-17:C>T	TGCAGGCAACAAAATGGCTCGATTTATATGTGAAAATAACACATCATATACTACCG	17:C>T
2259573|F|0-27:G>A-27:G>A	TGCAGCTGGATATGCATGGGTCGATCAGTACTGCGGCAGGCTGCACTCGGGGCCG	27:G>A
1044756|F|0-30:C>T-30:C>T	TGCAGCGAGTCTACGGTTGACTTTTGCCCTCTATCCTTAGGATCATGCGGCGCGGGAGCGTACTGCATA	30:C>T
1221582|F|0-26:G>A-26:G>A	TGCAGCATGGGGCTCCCCAGGAGCACGGAGTGCAAGTGCAGCAGCTTCATGGGGCTCCG	26:G>A
1020061|F|0-12:C>A-12:C>A	TGCAGGGACATGCAGGTCTCCGAATCGGGCGGGACGCCGTAGCGCGACAGCCG	12:C>A
1016408|F|0-65:T>C-65:T>C	TGCAGCCTCTTCAACCCGACGCCGTCGCTCTCCGCCATGGTCGTCAACCACTATAAGCTCCGCGGTAAC	65:T>C

<table>
<tr><td>6045805|F|0-12:A>G-12:A>G</td><td>TGCAGGTTCTTCAGCCTTCTTCTCTGCCGCCG</td><td>12:A>G</td></tr>
<tr><td>1018458|F|0-37:C>A-37:C>A</td><td>TGCAGTCCGTGCACCTCAGGCGTTGCCGCGCCAAAACCAACAGCACCATACCCAGACCCAGAGTCATGG</td><td>37:C>A</td></tr>
<tr><td>2242191|F|0-20:T>C-20:T>C</td><td>TGCAGTGGCCGCACGCAGCATCTCGATGTCCTCATCTGAGGAGGGTTTGTCTGCCTCTGTTTGCCCCCG</td><td>20:T>C</td></tr>
<tr><td>2242191|F|0-42:G>T-42:G>T</td><td>TGCAGTGGCCGCACGCAGCATCTCGATGTCCTCATCTGAGGAGGGTTTGTCTGCCTCTGTTTGCCCCCG</td><td>42:G>T</td></tr>
<tr><td>3024045|F|0-25:C>T-25:C>T</td><td>TGCAGGATCCTCGGGCCATTCGTTTCTTTGATCGCCG</td><td>25:C>T</td></tr>
<tr><td>1713513|F|0-24:A>G-24:A>G</td><td>TGCAGGTAGATCAGCTCGATGTCTAGTGGCTCCAGCTTGCAGCCGATGCTGAGCCG</td><td>24:A>G</td></tr>
<tr><td>1713513|F|0-44:G>T-44:G>T</td><td>TGCAGGTAGATCAGCTCGATGTCTAGTGGCTCCAGCTTGCAGCCGATGCTGAGCCG</td><td>44:G>T</td></tr>
<tr><td>5011530|F|0-19:G>T-19:G>T</td><td>TGCAGTGGCTATGATCACAGGAGTGGAGACAGACCG</td><td>19:G>T</td></tr>
<tr><td>5011530|F|0-11:T>C-11:T>C</td><td>TGCAGTGGCTATGATCACAGGAGTGGAGACAGACCG</td><td>11:T>C</td></tr>
<tr><td>4262243|F|0-7:T>C-7:T>C</td><td>TGCAGGTTGCACAGAGCAGCAGGAGCACTAGCCCG</td><td>7:T>C</td></tr>
<tr><td>1016408|F|0-62:C>T-62:C>T</td><td>TGCAGCCTCTTCAACCCGACGCCGTCGCTCTCCGCCATGGTCGTCAACCACTATAAGCTCCGCGGTAAC</td><td>62:C>T</td></tr>
<tr><td>3020764|F|0-16:T>C-16:T>C</td><td>TGCAGGTGTATCCAGTTCCTCATGATCCACACGCCAATCCG</td><td>16:T>C</td></tr>
<tr><td>1018692|F|0-29:C>T-29:C>T</td><td>TGCAGGTCACGGCCACCGTTACGGCGTCGCGTGGAGCTCAGAGGTGCTGTTAGCGATGCGTGGCTGTGT</td><td>29:C>T</td></tr>
<tr><td>3936319|F|0-8:T>C-8:T>C</td><td>TGCAGCAGTTCCGCTCCTACTTCCAAAGGGTAAACAGGAGTGCACCG</td><td>8:T>C</td></tr>
<tr><td>1016721|F|0-41:T>C-41:T>C</td><td>TGCAGTTCTGTCAAGACTGTTAAATTCAGTTAACACATTTTTCACATGAGCAACACATCTATATGTAGC</td><td>41:T>C</td></tr>
<tr><td>1207029|F|0-10:G>A-10:G>A</td><td>TGCAGCAGCAGCCAGTTCACCTTGGCCAGCACCAGCTGGTCGTCGTTGCAGTAGAAGAGCCG</td><td>10:G>A</td></tr>
<tr><td>1686609|F|0-13:T>C-13:T>C</td><td>TGCAGTTCGTGTTTCCAGGGATGTGGCTCTCGGATTTGACCAAGGGCCG</td><td>13:T>C</td></tr>
<tr><td>3940667|F|0-19:G>C-19:G>C</td><td>TGCAGGGAGTGCAGGCCAAGCTGCGCCG</td><td>19:G>C</td></tr>
<tr><td>3941360|F|0-15:A>G-15:A>G</td><td>TGCAGCCACGTCACCACGATGGGGCTCCG</td><td>15:A>G</td></tr>
<tr><td>1018962|F|0-32:G>C-32:G>C</td><td>TGCAGCCGCTGTGCCATGATCACGTTCCCGCCGACAGTCGCCGTGTTCCG</td><td>32:G>C</td></tr>
<tr><td>1029403|F|0-32:C>T-32:C>T</td><td>TGCAGCAGTGAGGTTGGTATCTGCCCATGGAGCTGGTTGGAGGACAGGTTCAGCCGCTCCAGCTTCACG</td><td>32:C>T</td></tr>
<tr><td>1036575|F|0-17:G>A-17:G>A</td><td>TGCAGAATTGCGTGAGAGGGAAAGCATGGAATATGAAAGAAGACATAGGGACCG</td><td>17:G>A</td></tr>
<tr><td>1047582|F|0-57:G>A-57:G>A</td><td>TGCAGCCAGTCTACGGTTGACTTCTGCCCTCTATCCTTAGGATCACGCGGCGCAGGAGCGTACTGCATA</td><td>57:G>A</td></tr>
<tr><td>32022945|F|0-39:A>C-39:A>C</td><td>TGCAGGCAGAGATATGAATGCAGGTGCATGTGCAGTGGCAGCACTCATGAGCTCCACATCTGCGTCGCG</td><td>39:A>C</td></tr>
<tr><td>5355973|F|0-28:C>T-28:C>T</td><td>TGCAGCGCATGCATGCACGTTCCGCGTCCGAGCAGTACGCCGTTGGCGGCTTGCACCGCCACCCGCCAC</td><td>28:C>T</td></tr>
<tr><td>32019399|F|0-8:A>C-8:A>C</td><td>TGCAGATCAACAGGGCGATTGGTCCACAGAACATTGTTAACAACAAATGTAGCCATCGGTGAGTGAGTG</td><td>8:A>C</td></tr>
<tr><td>3957535|F|0-5:A>G-5:A>G</td><td>TGCAGATCATGTGCCGCCTCGTGGCTGCTCCAGGAGAGACCG</td><td>5:A>G</td></tr>
<tr><td>4998764|F|0-15:G>A-15:G>A</td><td>TGCAGAATTGGTTGGGGATGCACCCACCTTGTTGGTGTAGATGGTCCAGTTTGTGAAGAGCTCCGAGAG</td><td>15:G>A</td></tr>
<tr><td>3572758|F|0-34:C>T-34:C>T</td><td>TGCAGAGTGGCCGATCTTGCTTGGGTGAGATAATCTACCG</td><td>34:C>T</td></tr>
<tr><td>1046321|F|0-37:G>A-37:G>A</td><td>TGCAGCGAGACTCGGATTTGGCTGCCCGCGGCACAGAGCATGGAAACGAATCAGGGGAAAGTGAAATCG</td><td>37:G>A</td></tr>
<tr><td>32025073|F|0-22:A>C-22:A>C</td><td>TGCAGCTAGCGTCCGCCACGGCATGCGTCACGCTGTCGGCGGTGCGGCTCGTCAGGCAGGACTTCGGCG</td><td>22:A>C</td></tr>
<tr><td>5006836|F|0-11:T>C-11:T>C</td><td>TGCAGGAGGAGTAGGATGGTGGGCACGCTGTTGCCCCGCGCGTCGTGCGCGCACTGCATCGACTCCGTC</td><td>11:T>C</td></tr>
<tr><td>1385349|F|0-17:T>G-17:T>G</td><td>TGCAGAACTCCATCCGTTTTGGGTAAAAATTCCAGGACTGCTTAGTCCAACCACAAACATGCCACTGTA</td><td>17:T>G</td></tr>
<tr><td>4998694|F|0-16:T>C-16:T>C</td><td>TGCAGCTGCTCAGTCTTATTGCTTCCTTTTGCAGGTAGATTCATGAGCTACAACCACACCTGTCCATAA</td><td>16:T>C</td></tr>
<tr><td>3575772|F|0-11:A>G-11:A>G</td><td>TGCAGATTGGCAGAGTAAGTCTTGGAAGAGTGGTGCACCG</td><td>11:A>G</td></tr>
<tr><td>5371147|F|0-11:C>A-11:C>A</td><td>TGCAGATCCGTCGGGCGATTGGTCCACGGAACATTGTTAACAACAAATGTAGCCATCGCTGAGTGAGTG</td><td>11:C>A</td></tr>
<tr><td>5361923|F|0-29:A>G-29:A>G</td><td>TGCAGATTCATGCAAGCCAGGATCGATCAATCGAGAGACGCTTCCGTAAAGAAGATGATCGGTGGCATG</td><td>29:A>G</td></tr>
<tr><td>3574511|F|0-11:T>C-11:T>C</td><td>TGCAGCTACTGTATGAAATCCTCGAGCAAAAACATAAACATCAATTCACTGTATTTAAACAGAATTTCC</td><td>11:T>C</td></tr>
<tr><td>100550640|F|0-28:C>T-28:C>T</td><td>TGCAGCCTGAACCTGGCGAGGCCATCGTCCAGAGCCATCTGCAAAGAAGGGAGGCGACCGCCG</td><td>28:C>T</td></tr>
<tr><td>1269998|F|0-5:A>G-5:A>G</td><td>TGCAGATATAGAGGCCTTTCTGGAGATTGGGGAGTTTACAGAGGTGTACTGGAGTCTGAGATTCTTCCG</td><td>5:A>G</td></tr>
<tr><td>100327263|F|0-19:A>G-19:A>G</td><td>TGCAGTGGCCACTGCCAGCAGAGTGCCGTGAGTCGTTTTCACGTCAGCACATGGGCGCTGGGGCCCACG</td><td>19:A>G</td></tr>
<tr><td>985711|F|0-53:C>T-53:C>T</td><td>TGCAGACACTATGGCCATGGCGGAGAGGATCAAGATGGGGACAGCTGCTGCCACGGGGGAGCTCCGAGA</td><td>53:C>T</td></tr>
<tr><td>100537150|F|0-15:A>G-15:A>G</td><td>TGCAGGTGCATCACAAGTAGGCACGGTGGACATCGGGTGGTGGCAGCAGTAGGGACAGCAAGGCTCACC</td><td>15:A>G</td></tr>
<tr><td>100554801|F|0-5:G>C-5:G>C</td><td>TGCAGGTTGTTTACGAGCGAGATCAGGTTCTCCATGGCCGCCG</td><td>5:G>C</td></tr>
<tr><td>5354297|F|0-29:C>T-29:C>T</td><td>TGCAGAGACGGAGACGAGTCGGCCATGGCCCGACCCGAGGCTCGCGCAGGTCATCAGCTCACAGAGGCC</td><td>29:C>T</td></tr>
<tr><td>1863019|F|0-36:A>T-36:A>T</td><td>TGCAGTGAGATGAACATGGTGAATCGAACTGTCAGAATGATGCTCATGCCG</td><td>36:A>T</td></tr>
<tr><td>100544894|F|0-23:A>G-23:A>G</td><td>TGCAGGCCCACCACCGTCAGCACATCCGCCACCCCCATCATCGCGTACTGCGGCACCAGCCACGCCCAG</td><td>23:A>G</td></tr>
<tr><td>1757391|F|0-29:C>T-29:C>T</td><td>TGCAGACCACTACGAACTAGCAGATCTGACCGAGTACAGCGAGTGAGAAGGGTAGGCAGCTGTGCTCAC</td><td>29:C>T</td></tr>
<tr><td>2253121|F|0-37:A>G-37:A>G</td><td>TGCAGTACTCGTCGACCCAAGTCTCGATGGCCGACCAAACGGCCAACCCATCCACCGCGTACGGATAGT</td><td>37:A>G</td></tr>
<tr><td>7925111|F|0-54:T>C-54:T>C</td><td>TGCAGTAGATGACGAGGTAGAGCGGCGAAGAGCCCGAGACGGCGGCCGCCATCATGTTGGGACGCATGC</td><td>54:T>C</td></tr>
<tr><td>6035483|F|0-22:G>A-22:G>A</td><td>TGCAGGTGCTCCAAAATGCAAAGCCATTAACCAGATTGCCACTTGTCGGCCCCATGCTAACTCCTCCAG</td><td>22:G>A</td></tr>
<tr><td>1382850|F|0-18:A>T-18:A>T</td><td>TGCAGATGTATCAGTTGGATCATTTGCTAAACGAAAGCGCTCGCAAGTGGATGTTTCTCAAACTGGCAA</td><td>18:A>T</td></tr>
<tr><td>1747451|F|0-28:C>G-28:C>G</td><td>TGCAGTCGTGCGAGCTGGACCTCTGCCTCACGCCCCTGTCGTCGCCGCTCGCCG</td><td>28:C>G</td></tr>
<tr><td>100470341|F|0-56:G>C-56:G>C</td><td>TGCAGCTGCGGCTACTCGTCGAGCACATTGTCCTCTGGATCTACCGCGTCCACGTCGCTGTTCTCGCTA</td><td>56:G>C</td></tr>
<tr><td>991813|F|0-38:T>C-38:T>C</td><td>TGCAGCAACGTCCTTGACGGCGGCGAACACCCTGAGCTTCACTATATCCG</td><td>38:T>C</td></tr>
<tr><td>5365174|F|0-49:T>C-49:T>C</td><td>TGCAGCAAGAACTTACGCTTCTTCTCGAGGTCCAGTTTGGGCTGGTTGTTGCCCGAGAGCTGGAGCCAC</td><td>49:T>C</td></tr>
<tr><td>1863300|F|0-36:T>C-36:T>C</td><td>TGCAGGCAACAAAATGGCTCGATTTATATGTGAAAATAACACATCATATACTACCG</td><td>36:T>C</td></tr>
<tr><td>1018672|F|0-12:G>C-12:G>C</td><td>TGCAGGTGTTCGGGCACAGCGACTACGTGACGTGCATCCAGTTCAACCCG</td><td>12:G>C</td></tr>
<tr><td>1092441|F|0-19:T>A-19:T>A</td><td>TGCAGTCTGATCGTGGTCGTGGCACGCACCAAGCGGCAATGGTTGGCCG</td><td>19:T>A</td></tr>
</table>

1092441|F|0-17:C>A-17:C>A	TGCAGTCTGATCGTGGTCGTGGCACGCACCAAGCGGCAATGGTTGGCCG	17:C>A
1018692|F|0-17:G>A-17:G>A	TGCAGGTCACGGCCACCGTTACGGCGTCGCGTGGAGCTCAGAGGTGCTGTTAGCGATGCGTGGCTGTGT	17:G>A
3572738|F|0-7:G>C-7:G>C	TGCAGGTGTGCGTCTTCTACCTCGTGCTCCG	7:G>C
1209156|F|0-5:C>G-5:C>G	TGCAGCAGCACAACCTCCTCGTGCTCCGCCTCCAGGTCAGGATGCAGCTTGAGCAGCTCCG	5:C>G
1686609|F|0-31:G>C-31:G>C	TGCAGTTCGTGTTTCCAGGGATGTGGCTCTCGGATTTGACCAAGGGCCG	31:G>C
4261595|F|0-23:G>A-23:G>A	TGCAGGAGTTCGTGTCGGAGATCGTGAGCATGAGCCG	23:G>A
32021480|F|0-7:T>C-7:T>C	TGCAGGGTACGCAGTTTCGTAATTCGGCTCGTGCCGTTTACACACAGACCGTGGACCG	7:T>C
993875|F|0-40:C>A-40:C>A	TGCAGAAACTCTTGTATGCCCCAATGGGGGAAGTCCTCATCTTGCAGCCG	40:C>A
1020061|F|0-13:A>T-13:A>T	TGCAGGGACATGCAGGTCTCCGAATCGGGCGGGACGCCGTAGCGCGACAGCCG	13:A>T
32017691|F|0-29:C>G-29:C>G	TGCAGCGTCTTCATGTTGAGCGACATCTCCTCCGCCG	29:C>G
6048864|F|0-10:G>T-10:G>T	TGCAGAGTTAGGTGCAGGGCATGGGGTTGCTCGCCG	10:G>T
3940307|F|0-20:G>C-20:G>C	TGCAGCAGCATGCGGCGGTTGAGCTCCG	20:G>C
4993450|F|0-10:C>T-10:C>T	TGCAGCCATGCGACCTGCTCGGCGTACTTGTCCG	10:C>T
3949194|F|0-10:C>A-10:C>A	TGCAGCAACTCAAAGGCTGAGGGCATCTTCCG	10:C>A
1671457|F|0-9:C>T-9:C>T	TGCAGCTGCCATCGACGGTAGTAGTCTTCATATGCATCATGCAAAGAACCCG	9:C>T
4262492|F|0-33:G>T-33:G>T	TGCAGGACAAAGAGAATATAAAAGACTGGATGAGCAGATTAGCCG	33:G>T
1026641|F|0-67:A>G-67:A>G	TGCAGTGACGATGATCTAACTGTGCTCGTATTCTAACGAGAGATCCAAGTCGCGTATCAGATTCGGAAG	67:A>G
4261363|F|0-9:G>C-9:G>C	TGCAGCACGGTCGCCCACGAGCCCACCCG	9:G>C
1278118|F|0-35:G>A-35:G>A	TGCAGCAGCACGACCTCCTGGTGCTCCGCCTCCAGGTCAGGATGCAGCTTGAGCAGCTCCG	35:G>A
3024920|F|0-27:A>T-27:A>T	TGCAGCTCTTCTCCATGATGCTGCGCCAAGGTGTTCGACCG	27:A>T
3023743|F|0-39:G>A-39:G>A	TGCAGGATCTCAACGTGTCCAACAATCTCTTGTCTGGGAGGATTCCG	39:G>A
1006587|F|0-10:T>C-10:T>C	TGCAGTTGCCTTTAGGTATTTGTGCAGAAGCCCTTTTTGTCTGGTCAAAGTCTATGGATGGAAGCGTCG	10:T>C
3026027|F|0-8:G>C-8:G>C	TGCAGAGCGACGACTTAATTTGATGGGAGTAATTTAACCG	8:G>C
3937257|F|0-33:G>C-33:G>C	TGCAGTACAACGGGAGCGGACGGCGTTGAAAACGGACCG	33:G>C
1019765|F|0-18:T>C-18:T>C	TGCAGCTCCCGTCGTCGTTGCCACCATGGCCGCCGCTACAGCCTCTCGGACGTGAACCCTGCTGCATCT	18:T>C
1141725|F|0-5:A>T-5:A>T	TGCAGAAAACTGCTGCATGTACTGCTGTTCATGAGTTGTTATGTAGCGAACCATCCG	5:A>T
7173620|F|0-8:T>C-8:T>C	TGCAGCAGTAGCCAGTTCACCTTGGCCAGTACGAGCTGGTCGTCGTTGCAGTAGAAGAGCCG	8:T>C
4002338|F|0-16:T>A-16:T>A	TGCAGAGAAGCATGCATTACTTCACAAAGTGGCCAACAACCG	16:T>A
979628|F|0-13:G>A-13:G>A	TGCAGAACTTCACGGCGAACCACAACAACTTCAGTGGCACGCTGCCGCCGTGCCTCAAGAACTGCACGG	13:G>A
3940025|F|0-21:T>A-21:T>A	TGCAGTTGCGGAGGCCCATGCTGCTCCG	21:T>A
3032649|F|0-26:G>A-26:G>A	TGCAGGTCGAGGGAGAGGAAGATGGCGCCCTTGAGGTCCCAGCCG	26:G>A
1694890|F|0-65:A>G-65:A>G	TGCAGGACGTGTTCCGTCGCTTCGCCTTCGACTGCATATGCAAGATCTCCTTCGGCCTCGACCCGAGAT	65:A>G
3943527|F|0-11:G>A-11:G>A	TGCAGCTCCACGTCGCCCTGCACCACGCCGTCGTCCG	11:G>A
1016721|F|0-9:G>T-9:G>T	TGCAGTTCTGTCAAGACTGTTAAATTCAGTTAACACATTTTTCACATGAGCAACACATCTATATGTAGC	9:G>T
1023071|F|0-26:C>G-26:C>G	TGCAGATGGCACCTAATTCAGTTGAGCTATGGTTTATTGTATGTGATCCG	26:C>G
1166676|F|0-27:G>A-27:G>A	TGCAGCCGTTTCGGGTTCTGCGGCAGCGGCTCTGAGTGGTGCGGAGCCG	27:G>A
2245263|F|0-17:C>A-17:C>A	TGCAGCAGCAGCGTCTCCAGGTTGGACAGCTCGATGTAGCCGAGGTTGTCGGGGTCGAGCTCCTCCATG	17:C>A
1019349|F|0-23:G>A-23:G>A	TGCAGAAAACTGCTGCATGTACTGCTGTTCAAGAGTTGTTATGTAGCGAATCATCCG	23:G>A
2245263|F|0-24:G>T-24:G>T	TGCAGCAGCAGCGTCTCCAGGTTGGACAGCTCGATGTAGCCGAGGTTGTCGGGGTCGAGCTCCTCCATG	24:G>T
32017752|F|0-11:T>C-11:T>C	TGCAGGATCGTTTTGAACTGGTCGCGCAGGAGCGCCTCGCCG	11:T>C
3937257|F|0-15:G>A-15:G>A	TGCAGTACAACGGGAGCGGACGGCGTTGAAAACGGACCG	15:G>A
2242191|F|0-23:C>A-23:C>A	TGCAGTGGCCGCACGCAGCATCTCGATGTCCTCATCTGAGGAGGGTTTGTCTGCCTCTGTTTGCCCCCG	23:C>A
1018962|F|0-5:C>T-5:C>T	TGCAGCCGCTGTGCCATGATCACGTTCCCGCCGACAGTCGCCGTGTTCCG	5:C>T
3941709|F|0-29:G>C-29:G>C	TGCAGAGTCTCACTGTCTCAGTGGCTACTGACCG	29:G>C
1018962|F|0-29:G>T-29:G>T	TGCAGCCGCTGTGCCATGATCACGTTCCCGCCGACAGTCGCCGTGTTCCG	29:G>T
1026641|F|0-6:G>C-6:G>C	TGCAGTGACGATGATCTAACTGTGCTCGTATTCTAACGAGAGATCCAAGTCGCGTATCAGATTCGGAAG	6:G>C
3024045|F|0-16:C>T-16:C>T	TGCAGGATCCTCGGGCCATTCGTTTCTTTGATCGCCG	16:C>T
1026641|F|0-10:A>G-10:A>G	TGCAGTGACGATGATCTAACTGTGCTCGTATTCTAACGAGAGATCCAAGTCGCGTATCAGATTCGGAAG	10:A>G
4002116|F|0-25:G>C-25:G>C	TGCAGCCTCCGATCCCTGCCCTAGAGAACCTCGTCGACATACGCCG	25:G>C
3020394|F|0-18:T>G-18:T>G	TGCAGCAGGTTCCATTCATTTTACCACTTATCATTGCGCCG	18:T>G
3021573|F|0-6:T>A-6:T>A	TGCAGGTGCAACAGCTCTGAGGGTGCTGGTCTCCCAACCTTCCG	6:T>A
1062282|F|0-20:C>G-20:C>G	TGCAGCCACCCGTCCCTCGTCGCCGTGTACAGCGTCCCTCCCGCCGCCGCGTCCACGTACACGTCCTCC	20:C>G
985682|F|0-33:G>A-33:G>A	TGCAGGTCCTGAGATGATGATGAAGATGCAGCAGCGCAAGTTCATCCAGGACCG	33:G>A
1020912|F|0-29:G>A-29:G>A	TGCAGCCCACATGAGAGAAGACACAGGATGAGCAATGGACAGGTTCCACTTAAAACAAGTTTGTCCGAG	29:G>A
3959280|F|0-15:C>T-15:C>T	TGCAGATATATTATGCTTATAAAGAGTGATTGAAGAATACCG	15:C>T
1694890|F|0-55:C>T-55:C>T	TGCAGGACGTGTTCCGTCGCTTCGCCTTCGACTGCATATGCAAGATCTCCTTCGGCCTCGACCCGAGAT	55:C>T
1006653|F|0-25:T>C-25:T>C	TGCAGTATCTCCTCAAAGCCTATCATGAAGATCAGGGACCATAATCCACCG	25:T>C
4005981|F|0-7:A>G-7:A>G	TGCAGCAATGAGACTTTGGGAACTCGTCGATAAAAATCTGTACCG	7:A>G
1063013|F|0-5:C>T-5:C>T	TGCAGCAGAAGAAGAAGAGGGAGGCGCGACAAAGGCGATGGCACGCGGGTCCG	5:C>T

<table>
<tr><td>1063013|F|0-25:G>A-25:G>A</td><td>TGCAGCAGAAGAAGAAGAGGGAGGCGCGACAAAGGCGATGGCACGCGGGTCCG</td><td>25:G>A</td></tr>
<tr><td>32018180|F|0-13:A>G-13:A>G</td><td>TGCAGCCCTTCACATGTATATATACATGCAAGGACCAACCGACTAATACTCTTGAGTCCAAACCGAGAT</td><td>13:A>G</td></tr>
<tr><td>100303693|F|0-29:T>C-29:T>C</td><td>TGCAGGTCAGAGAGGTCGGAGTTCGGGAGTCGATGGCCG</td><td>29:T>C</td></tr>
<tr><td>100513494|F|0-28:A>C-28:A>C</td><td>TGCAGCCCCTGCTGAACGGGAACGGCCGACCCTTCCACTCTGAACCCGAACCAAAATTCAGACATCCCA</td><td>28:A>C</td></tr>
<tr><td>100441997|F|0-28:T>C-28:T>C</td><td>TGCAGGGCAGATCAGGCAAGAACAGGGGTATCACCG</td><td>28:T>C</td></tr>
<tr><td>1269338|F|0-19:G>A-19:G>A</td><td>TGCAGCGAAGCTCGTGGGGGACCCAAGGAACATGACATGATCACCCAAACTCCG</td><td>19:G>A</td></tr>
<tr><td>100442411|F|0-31:A>C-31:A>C</td><td>TGCAGGTTAATGCATGAGCGAGTCAGACGTGAACAGCCG</td><td>31:A>C</td></tr>
<tr><td>100453526|F|0-46:G>C-46:G>C</td><td>TGCAGTCGAATCGCTCGCTCATGATCCGTTTCCACTTTCCATAAAAGAAGCCAATCTTCCACCG</td><td>46:G>C</td></tr>
<tr><td>4989030|F|0-8:T>C-8:T>C</td><td>TGCAGCCGTTTCGGGTTCTGCGGCAGCGGCTCCGACTGGTGCGGAGCCG</td><td>8:T>C</td></tr>
<tr><td>3947551|F|0-24:G>A-24:G>A</td><td>TGCAGACCAACACGGGATCTTGCAGAGGAGCCG</td><td>24:G>A</td></tr>
<tr><td>3956466|F|0-9:G>T-9:G>T</td><td>TGCAGCTGGGGCGATGCTTTCTCTGAAGTTCTCTGCAATGGCCG</td><td>9:G>T</td></tr>
<tr><td>1019134|F|0-31:C>T-31:C>T</td><td>TGCAGATGTACATGCATGTCACCGAGGCTGACGCATTAGTCAAAGCACGGTCACAACGAAGCACATGCA</td><td>31:C>T</td></tr>
<tr><td>3943684|F|0-5:G>T-5:G>T</td><td>TGCAGGTGTTCTGGTCCGTGGTGCAGCCG</td><td>5:G>T</td></tr>
<tr><td>1019765|F|0-16:G>T-16:G>T</td><td>TGCAGCTCCCGTCGTCGTTGCCACCATGGCCGCCGCTACAGCCTCTCAGACGTGAACCCTGCTGCATCT</td><td>16:G>T</td></tr>
<tr><td>5358687|F|0-10:G>C-10:G>C</td><td>TGCAGTTGACGACGAGGATGCCGACGTCCTTGGGCTTGACGCCG</td><td>10:G>C</td></tr>
<tr><td>1002448|F|0-54:G>T-54:G>T</td><td>TGCAGGAAGGCCGCCCTGGGCCGCATGGATCTCGCCACCTCGGGGGTGCTGATGGCCG</td><td>54:G>T</td></tr>
<tr><td>4005981|F|0-5:C>A-5:C>A</td><td>TGCAGCAATGAGACTTTGGGAACTCGTCGATAAAAATCTGTACCG</td><td>5:C>A</td></tr>
<tr><td>1151429|F|0-10:C>G-10:C>G</td><td>TGCAGGTGATCAGTGTAAGAAGCAAAGAGCTGGCCGCACTGCACTGTGGATAGGACCG</td><td>10:C>G</td></tr>
<tr><td>5367573|F|0-13:C>G-13:C>G</td><td>TGCAGAAGAAGGACAGCTTGAACTTGGGTAGCCG</td><td>13:C>G</td></tr>
<tr><td>3020843|F|0-9:T>C-9:T>C</td><td>TGCAGCAAATCATCAGCTGACGTCGATTTTTCCTTTCCG</td><td>9:T>C</td></tr>
<tr><td>3028258|F|0-14:G>A-14:G>A</td><td>TGCAGTATGCTTGCGCCGACGCCTTTGCATCGTTCGAGGTTGGCCG</td><td>14:G>A</td></tr>
<tr><td>3020379|F|0-11:T>C-11:T>C</td><td>TGCAGCAGAGCTTATGCAATGTAGTAGATGCCTAGCCG</td><td>11:T>C</td></tr>
<tr><td>992659|F|0-60:T>C-60:T>C</td><td>TGCAGTATATGCACGGCCTGGTGCAGCATAATGATGGTTCTATCAATGCAGAATTCGCATTGTTCGCGA</td><td>60:T>C</td></tr>
<tr><td>1010782|F|0-45:A>G-45:A>G</td><td>TGCAGCGTTTTCTTCTCTTCTACCTATTTGCTGTTACAGCAACACATTCTTTCTAGCCTACCG</td><td>45:A>G</td></tr>
<tr><td>6045421|F|0-26:G>A-26:G>A</td><td>TGCAGCACCTTCACCACCATGAAGGCGCTGGTGGAATCGAGCCCG</td><td>26:G>A</td></tr>
<tr><td>1268005|F|0-40:T>C-40:T>C</td><td>TGCAGTATATGCACAGCCTGGTGCAGTATAATGATGGTTCTATCGATGCAGAATTTGCCTTGTTCGCGA</td><td>40:T>C</td></tr>
<tr><td>1022726|F|0-41:T>G-41:T>G</td><td>TGCAGCTTTCATTCATGGTGTTGCATTTTCTTTCCCGATTCTAGGCCTCTTCGTCCG</td><td>41:T>G</td></tr>
<tr><td>1020101|F|0-34:C>T-34:C>T</td><td>TGCAGGTGGGGTTCTACTCCAAGTCGTGCCCCAGCGCCGAGTCCACCGTCGCGTCCGTCGTCCG</td><td>34:C>T</td></tr>
<tr><td>3943527|F|0-25:C>T-25:C>T</td><td>TGCAGCTCCACGTCGCCCTGCACCACGCCGTCGTCCG</td><td>25:C>T</td></tr>
<tr><td>1245096|F|0-13:C>G-13:C>G</td><td>TGCAGCCGCTGACCATGAAGTACAGGAAGGAGAAGGAGCGGATCGACGAGATCCG</td><td>13:C>G</td></tr>
<tr><td>3937290|F|0-7:G>C-7:G>C</td><td>TGCAGCCGCTGACCATGAAGTACCGCAAGGAGAAGGAGCGGATCGACGAGATCCG</td><td>7:G>C</td></tr>
<tr><td>3946839|F|0-11:C>T-11:C>T</td><td>TGCAGGCCGACCAGGTAGTAGGCCATCGCGCCCG</td><td>11:C>T</td></tr>
<tr><td>1316578|F|0-48:T>C-48:T>C</td><td>TGCAGGTCAGGTGCGGGCGACTCGGAGAGGAGCTTTGGCCGTAAGGACTATACAGCTTAGTTTCTCCTG</td><td>48:T>C</td></tr>
<tr><td>5373593|F|0-13:A>C-13:A>C</td><td>TGCAGCTCTAGCGACCTGAGCGCGCCCATCAGCCGCGCCG</td><td>13:A>C</td></tr>
<tr><td>1666899|F|0-40:G>A-40:G>A</td><td>TGCAGTATACCCACGCGGTGCTGTTCGAGAACATGCGGCTGTTCCCGCCG</td><td>40:G>A</td></tr>
<tr><td>5363839|F|0-13:A>G-13:A>G</td><td>TGCAGGCTGGCGCATTTCCCGAGCTCCG</td><td>13:A>G</td></tr>
<tr><td>3949194|F|0-19:A>C-19:A>C</td><td>TGCAGCAACTCAAAGGCTGAGGGCATCTTCCG</td><td>19:A>C</td></tr>
<tr><td>3023902|F|0-10:C>T-10:C>T</td><td>TGCAGGGAACCCAAGGACATGGTCGTCTCGTTGTGGCACTTCCTCCG</td><td>10:C>T</td></tr>
<tr><td>2252217|F|0-31:G>A-31:G>A</td><td>TGCAGGTCACGGTCTCCTCGAGTTGCATGCCGTCTAAATCTGGAGTTATGGACTGTCTGATCTACCGAG</td><td>31:G>A</td></tr>
<tr><td>3020399|F|0-19:T>C-19:T>C</td><td>TGCAGCTGCTCTATCCCCTTGCCGTGCCACAGTATCCG</td><td>19:T>C</td></tr>
<tr><td>4439426|F|0-14:C>G-14:C>G</td><td>TGCAGCCTGCCGTTCCCGCTGAAGATCCG</td><td>14:C>G</td></tr>
<tr><td>3533791|F|0-33:G>T-33:G>T</td><td>TGCAGAAAAGGGGGTCGCAGTTTCTTGGCATTGGAGAAAGCGACCG</td><td>33:G>T</td></tr>
<tr><td>998862|F|0-39:T>G-39:T>G</td><td>TGCAGCTCTTCCCGCACCTCAGCAGCCCTGCGCACACAGTGCAACATATCCATGCCAAGTGAGAACGTC</td><td>39:T>G</td></tr>
<tr><td>4705943|F|0-31:C>T-31:C>T</td><td>TGCAGTCCGCGAGGCCGTCGTCGGGGGAGGCCGCGGCTTGGTGCGCCG</td><td>31:C>T</td></tr>
<tr><td>3937379|F|0-26:G>C-26:G>C</td><td>TGCAGCGGCGTGCCCATGAAGGTGACGAGCCCG</td><td>26:G>C</td></tr>
<tr><td>1275879|F|0-8:C>G-8:C>G</td><td>TGCAGTCTCGAGACTCCACTGAATTGAAGTAGCATGAAGGACAGGAGGAGCTTCCG</td><td>8:C>G</td></tr>
<tr><td>1041292|F|0-25:G>C-25:G>C</td><td>TGCAGTAATGGGACAGGAACTCCATGGTGAACCGCTTCATCTCCAGGTCGTCCCCGTAGTCCG</td><td>25:G>C</td></tr>
<tr><td>1020101|F|0-56:G>A-56:G>A</td><td>TGCAGGTGGGGTTCTACTCCAAGTCGTGCCCCAGCGCCGAGTCCACCGTCGCGTCCGTCGTCCG</td><td>56:G>A</td></tr>
<tr><td>3956466|F|0-27:G>A-27:G>A</td><td>TGCAGCTGGGGCGATGCTTTCTCTGAAGTTCTCTGCAATGGCCG</td><td>27:G>A</td></tr>
<tr><td>3023743|F|0-13:C>T-13:C>T</td><td>TGCAGGACCTCAACGTGTCCAACAATCTCTTGTCTGGGAGGATTCCG</td><td>13:C>T</td></tr>
<tr><td>4262831|F|0-5:G>A-5:G>A</td><td>TGCAGGTTCCACTTAGTTTAGAGCAGCCG</td><td>5:G>A</td></tr>
<tr><td>1707771|F|0-27:G>A-27:G>A</td><td>TGCAGGTTCGACGCTCGCCTGCTGCTGGAGCGGCTGCGCAACAAGCGGCTGATGTTCGTGGGAGATTCG</td><td>27:G>A</td></tr>
<tr><td>3025752|F|0-28:C>G-28:C>G</td><td>TGCAGTTGGTGTCCTGGCTGATCAAATTCAGGGTGGTTCCG</td><td>28:C>G</td></tr>
<tr><td>1120100|F|0-15:G>T-15:G>T</td><td>TGCAGTCAAGATCATGAAAACGAAAGAAAGGAACACCATCATCACGAGTTCACGGCTCGACCG</td><td>15:G>T</td></tr>
<tr><td>1322187|F|0-29:T>G-29:T>G</td><td>TGCAGGATTGCCCCCCATCATTCCAGCTTTAACTGCCTATTCGGCTTCAGCAGTGGGTTTCCAAAGGCG</td><td>29:T>G</td></tr>
<tr><td>7492634|F|0-7:C>T-7:C>T</td><td>TGCAGACCGTCGCATGTCCAACGCCAGCACGACCCG</td><td>7:C>T</td></tr>
<tr><td>3020431|F|0-28:A>G-28:A>G</td><td>TGCAGTAGATGAGAACAGCAAAGTTCAGACAATGCATAATCCG</td><td>28:A>G</td></tr>
<tr><td>3025752|F|0-32:G>T-32:G>T</td><td>TGCAGTTGGTGTCCTGGCTGATCAAATTCAGGGTGGTTCCG</td><td>32:G>T</td></tr>
<tr><td>7171077|F|0-14:G>A-14:G>A</td><td>TGCAGCATTGTGATGCCATGAGGGTTCCCCGTTCGCTTTCTAGCTAGCGGAACGAGGATCCG</td><td>14:G>A</td></tr>
</table>

984205|F|0-20:A>T-20:A>T	TGCAGCATTGTGATGCCATGAGGGTTCCCCGTGTGCTTTCTAGCCAGCGGAACGAGGATCCG	20:A>T
1024013|F|0-14:G>C-14:G>C	TGCAGCTTGTACGTGGACGGCCTCATCACGGCGGCGGCGACGCCGTCCG	14:G>C
1024013|F|0-11:C>T-11:C>T	TGCAGCTTGTACGTGGACGGCCTCATCACGGCGGCGGCGACGCCGTCCG	11:C>T
4261737|F|0-17:T>C-17:T>C	TGCAGCTGCATGCATGTTGTCTGTTTGCAAATTCCAACCG	17:T>C
984205|F|0-17:A>G-17:A>G	TGCAGCATTGTGATGCCATGAGGGTTCCCCGTGTGCTTTCTAGCCAGCGGAACGAGGATCCG	17:A>G
3020394|F|0-14:T>C-14:T>C	TGCAGCAGGTTCCATTCATTTTACCACTTATCATTGCGCCG	14:T>C
4405420|F|0-18:T>C-18:T>C	TGCAGACAAGGTTTTGTGTAGAGAAAGGATTGGCCG	18:T>C
1033299|F|0-10:T>C-10:T>C	TGCAGCTCGCTCCACCCAGGGAGGAAGGATACCTTCCTGAATCCATCCATCCATGCTTGAAGCTTAGAC	10:T>C
1033299|F|0-22:G>A-22:G>A	TGCAGCTCGCTCCACCCAGGGAGGAAGGATACCTTCCTGAATCCATCCATCCATGCTTGAAGCTTAGAC	22:G>A
3020418|F|0-13:A>C-13:A>C	TGCAGCTGGGGCTATGCTTTCTCTGAAGTTCTCTGCAATGCCCG	13:A>C
3939586|F|0-15:G>C-15:G>C	TGCAGGTGCCACTTAGTTTAGAGCAGCCG	15:G>C
1010970|F|0-5:G>A-5:G>A	TGCAGGAAGTCGTACAGCTTCACCTGGTTCCACGCCACCTCCATCCCCTGGTACTCGTCGAACGCCCGA	5:G>A
1036613|F|0-5:G>A-5:G>A	TGCAGGTTCTGGTCCCTCTGGAAGCCCTTGCCGCACACCTCGCACACGAACCG	5:G>A
32023781|F|0-34:T>C-34:T>C	TGCAGGATGGACTCGACGGGATCAAAACCATCGGTGCCGCCGCTCTTCAAGAACGGACTCCG	34:T>C
32027624|F|0-47:T>C-47:T>C	TGCAGGACGGTGATGCCACGAGGGTTCCCTGTGTGTTTTTTAGCGAGTGGGACGAGGATCCG	47:T>C
3954940|F|0-13:A>G-13:A>G	TGCAGTCAAGATCATGAAAACGAAAGAAAGAAACACCATCATCACGAGTTCACAGCTCGACCG	13:A>G
4999015|F|0-41:G>C-41:G>C	TGCAGCTCTCCCTCAACAAGGTCGAGGAGGTCAGTCGCCCAGGCTTCCTTCCGATCCCCCATTCCGAGA	41:G>C
1069558|F|0-6:G>A-6:G>A	TGCAGTGCTGCACGATGGCAATATGAATATCACGCGGATGAGCTCTACTGAACCG	6:G>A
1160898|F|0-52:T>C-52:T>C	TGCAGCTTGTCATAGCCGCGCTTCTTGCGCTGTCCCTGTCCACGACAGCGTCTTCGTCGCCTGCGCCGG	52:T>C
5004792|F|0-27:T>C-27:T>C	TGCAGGGGCAGGGCCAACCCACCCGTGTCAATGGACTTCTTCGGGAACATGGTGCTCTGGGCTTTCCCA	27:T>C
100466643|F|0-44:G>A-44:G>A	TGCAGACAGAAGAGAAGATCAAAAGAGATCGGGCGATGCAGCACGAGATTGGAGGGCAACAACAAGCGC	44:G>A
1001137|F|0-53:A>G-53:A>G	TGCAGTGCACCGCGAAGGAGCGGACAGAAAGACCCACCATGTCGGATGTTATTAACTTGCTAGACGGCA	53:A>G
12768842|F|0-46:G>A-46:G>A	TGCAGCTCCTGTCGGTGCTGTTACTGTGGCCTGCCTGCATGCATGCGGGCACGCCTATGCCTGCTCTGA	46:G>A
5367486|F|0-16:C>G-16:C>G	TGCAGTATGCCAGAAACTATGGTTTTTTGCAATATATATCTAAAAAAATGGTCCG	16:C>G
7907763|F|0-18:C>A-18:C>A	TGCAGCCCAACACCAAAACAAGCTTCAGTCGTCTCCATTGAGGCAAAAGATCGAACAGATAGCCGAGAT	18:C>A
4998915|F|0-28:T>G-28:T>G	TGCAGCAAAGGATTTCCGTGGAGGCGACTACAAGGCGGCTCATGAACCG	28:T>G
5360508|F|0-67:C>G-67:C>G	TGCAGAAGCTGCTCAGGTGCTCCGAGCAGACGCACAAGAAGGGCAAGGTGACTCCGCTCTTGCAGACCT	67:C>G
100469874|F|0-28:A>T-28:A>T	TGCAGCTCAAAGCCTCAAAGAGCAGAGTATCTAACAAGCAGACCGTCAGCATGACAAGTCCG	28:A>T
4002312|F|0-28:A>G-28:A>G	TGCAGCTGCGAAGACCAAGGCCCTGCAAAGAACCGAGCCG	28:A>G
12698284|F|0-11:A>G-11:A>G	TGCAGTGCACCACAGCAAGCAGGAAACCTGCAATTGATGGCATATCAATGGCCG	11:A>G
29308134|F|0-45:C>T-45:C>T	TGCAGAATGGTTGGTTGGCCAACATAAGGAGGTTAAGGACAATAACCCCAGACCAAGATACTCAACCAT	45:C>T
1100734|F|0-53:G>C-53:G>C	TGCAGCAAACAAACCACGAGCGCGCGTCAGTCATCCACGCCATGATTGATCGTGTGCCCG	53:G>C
100555180|F|0-32:C>T-32:C>T	TGCAGTAAGGATCTGGCCGCAGCGCTTCCTCACAGCCGCCCG	32:C>T
100557017|F|0-53:T>C-53:T>C	TGCAGCCTCAACACCACCGTCGTGGAGCTAGCCCGCTTCTTGGCGGCCAAGTATCCGCACTACAACGTC	53:T>C
7913009|F|0-16:T>C-16:T>C	TGCAGCGCTGCCTGCATGCCTTCAACGAGTCCTGCGACCGCGCCGAGGACCTCGTCCG	16:T>C
1768657|F|0-11:C>T-11:C>T	TGCAGAAACTTCCTTTTTCCTCTGAAAACTTGCATCTGTTGGGATCACTCACAGCCATCAGCAGAATGA	11:C>T
100520545|F|0-34:C>T-34:C>T	TGCAGCGCCTCCCCGATGTTTCTGGGCGCGACTACGCAAATGGGCCG	34:C>T
100539857|F|0-5:A>C-5:A>C	TGCAGAAGTGGTCACGAAGATGACATTTGCTCCCTCGGCAAAGCCCAACACATGCACCG	5:A>C
100540243|F|0-30:T>C-30:T>C	TGCAGCGGCACAGCAACGGGCCGATGCGGTTAGACTCTCTGCGGACGCCG	30:T>C
1753533|F|0-37:T>C-37:T>C	TGCAGCAGGTCAGTTCCCAACTACTCCACGAGTGAACTAAGAGATGCAATTGAAGTGCCG	37:T>C
100423977|F|0-45:A>G-45:A>G	TGCAGAGCTCTCCTCCAGGAGGCGAAGCTCACGCCAGACGGCATCACCGACCCTTAGAATCCG	45:A>G
100494469|F|0-12:G>C-12:G>C	TGCAGATCCGACGACCATATCCATGATTATCACCTTCCCTCCG	12:G>C
100556484|F|0-60:G>T-60:G>T	TGCAGCACGAGCGGATCTCAACTACTCCTATTAAATGATGATGACGAGTGCAGCTTCCCCGCCTGGGCC	60:G>T
1392593|F|0-53:T>C-53:T>C	TGCAGATGATTGCTGGGCAGAACCCGAACGTGATGCAAAGGTTTGTGCAGAATTTGCCCTCCCACCGAG	53:T>C
100470944|F|0-8:A>G-8:A>G	TGCAGGACAGATAAACAGTTGCTATCCAGGTGACGAAAATTGAACAGGAGTTGAAGAAAAAGAGAGGCC	8:A>G
5360729|F|0-19:G>T-19:G>T	TGCAGCAAGAACTTGCGCCGCCTCTCGAGCCTTTTGCGATCTTCTTCACTGCCCTCGGGCTCGGACCCA	19:G>T
22836060|F|0-8:A>C-8:A>C	TGCAGAAGAGGATGGCGGAGGAGGCCGCGGGCGACGACGGCGACGGCGCGCCG	8:A>C
100463970|F|0-7:C>T-7:C>T	TGCAGTTCACACGACACACATCCCATGTTATCACCG	7:C>T
100505240|F|0-9:T>C-9:T>C	TGCAGTTTTTCGATCAACCCCTGACCGACCACATATTTGGAAAAGCTTTCCCACTCTCGTGCTATCCCT	9:T>C
3024920|F|0-13:C>T-13:C>T	TGCAGCTCTTCTCCATGATGCTGCGCCAAGGTGTTCGACCG	13:C>T
3954757|F|0-30:G>C-30:G>C	TGCAGGGTCGTACACAGCCAGCGTCCGCTTGGAGCCTGCGTAGCCCG	30:G>C
1034740|F|0-15:A>T-15:A>T	TGCAGGAGGAAGAACATGGCCTTCTTTCTTCTTTGCCCACAGAACTGCATAAAGGCCG	15:A>T
3021237|F|0-11:A>C-11:A>C	TGCAGGTGGCCAATATTCTGAACAACCCTTTCTCGCTCGTCCG	11:A>C
3021237|F|0-30:T>C-30:T>C	TGCAGGTGGCCAATATTCTGAACAACCCTTTCTCGCTCGTCCG	30:T>C
5359239|F|0-15:G>A-15:G>A	TGCAGGAGGCCGAGGGGAGGCTCGAGAAGGTCTACCG	15:G>A
3021573|F|0-23:T>G-23:T>G	TGCAGGTGCAACAGCTCTGAGGGTGCTGGTCTCCCAACCTTCCG	23:T>G
1066973|F|0-9:G>T-9:G>T	TGCAGCTCGGCGCCATGATCCTCGTCTTGTGCGTCCTGAACATCTACTTCAATATTTACAACAAGCCGA	9:G>T
1219523|F|0-32:T>C-32:T>C	TGCAGGGAGCTCAAACGGTGTGCCTTCCTCCGTCCCATATGGCCCAAAAGTGCGCCG	32:T>C
4002338|F|0-31:G>A-31:G>A	TGCAGAGAAGCATGCATTACTTCACAAAGTGGCCAACAACCG	31:G>A

3021237|F|0-28:T>C-28:T>C	TGCAGGTGGCCAATATTCTGAACAACCCTTTCTCGCTCGTCCG	28:T>C
1229120|F|0-10:C>T-10:C>T	TGCAGGAGAGCCGCATGCCGCCCCGCACGCTCGTTGAGTAGCTTCGATCCG	10:C>T
1166676|F|0-32:T>C-32:T>C	TGCAGCCGTTTCGGGTTCTGCGGCAGCGGCTCTGAGTGGTGCGGAGCCG	32:T>C
1022118|F|0-8:C>T-8:C>T	TGCAGACACAGGAGGAGGAAGGCCATGGCAGAGGAGGGGCTTGTGCACGGGAGGGAGCAGCAAGGCGGC	8:C>T
4004713|F|0-11:G>A-11:G>A	TGCAGACATGAGCAAACAGTCGAATTAGCAGTGACCG	11:G>A
1034740|F|0-18:G>A-18:G>A	TGCAGGAGGAAGAACATGGCCTTCTTTCTTCTTTGCCCACAGAACTGCATAAAGGCCG	18:G>A
2278414|F|0-33:A>T-33:A>T	TGCAGGAGAGGGCGGAGTTCCCCTCGATCGCGCAGCTGGACTGGGTGGACTACATCGAGGACACCGCCG	33:A>T
1322187|F|0-12:C>A-12:C>A	TGCAGGATTGCCCCCCATCATTCCAGCTTTAACTGCCTATTCGGCTTCAGCAGTGGGTTTCCAAAGGCG	12:C>A
1864501|F|0-12:A>G-12:A>G	TGCAGCAACTGGAGGTCGATGGCGGAGCAGCAGCAAGACGAAGAGCTCGGCGGTCTGGACTCGCCGAGA	12:A>G
1022729|F|0-13:C>T-13:C>T	TGCAGGCCGCCTTCGCGGCCGCCGCGCTCGCCGTCATGGCGTCCACCGACGACTTCTCCTCCGTCACCA	13:C>T
32027819|F|0-25:T>C-25:T>C	TGCAGAGCATTGTCGACATATGGCGTGTCCACGACATGCCTGCGCAAGAAGACAAGCACGATCCCTATG	25:T>C
3958635|F|0-19:G>C-19:G>C	TGCAGTCTAGAGCCCAGTTGTGGCCGCCGACCTTAAAGGCGCCG	19:G>C
1122854|F|0-10:T>A-10:T>A	TGCAGATTCTTGCTGTGGCATGCGCTTGTATATCTTTGTTCTCTTGTGTGCCG	10:T>A
1010970|F|0-35:C>T-35:C>T	TGCAGGAAGTCGTACAGCTTCACCTGGTTCCACGCCACCTCCATCCCCTGGTACTCGTCGAACGCCCGA	35:C>T
3941709|F|0-19:A>G-19:A>G	TGCAGAGTCTCACTGTCTCAGTGGCTACTGACCG	19:A>G
3021573|F|0-15:T>C-15:T>C	TGCAGGTGCAACAGCTCTGAGGGTGCTGGTCTCCCAACCTTCCG	15:T>C
1020063|F|0-44:G>T-44:G>T	TGCAGGAGCTCCCTGAGGATCTCCAGGTCGCCGCCCCTCGCCGCGGCGTGCATTGCCCG	44:G>T
3951141|F|0-7:G>C-7:G>C	TGCAGAGGATGATGGCCAGGCTCTTGTTCCG	7:G>C
5582078|F|0-5:T>C-5:T>C	TGCAGTACGTACGTAACGGAGAGAGACCG	5:T>C
3947951|F|0-21:A>G-21:A>G	TGCAGGCTCTGGATGGCGCGGACACGCGGCTCCG	21:A>G
3949250|F|0-6:C>T-6:C>T	TGCAGGCCGTTTCTCGTCGGCGAGGCCACCG	6:C>T
1019010|F|0-54:C>T-54:C>T	TGCAGCCTTCGCGTGCACTCCTCTTATTATACGCTTGCATGCTTCGCAACAGTGCGCCGTAACAAAAGG	54:C>T
32021322|F|0-5:T>C-5:T>C	TGCAGTACAGCTATTTTAAGTGCACGACGACAGGAGATGCCTAGGAAACTCCG	5:T>C
983145|F|0-45:G>A-45:G>A	TGCAGTCGTCGTTGCAGATCTGTGCGTGATTCTGTGAGACCAGGCGTGGTCAACCGATCCG	45:G>A
3961231|F|0-27:G>C-27:G>C	TGCAGCTGGAGATGGATTTGATTTGGAGTTACTCCG	27:G>C
1862737|F|0-8:G>A-8:G>A	TGCAGCTCGAAAATCCTTCTTCCTTCGAGGTAGCTGCTGCCGAACGCGGTGCGTGAGATGACATCTCCA	8:G>A
1862682|F|0-25:G>A-25:G>A	TGCAGGACGTGTTCCTCAGGCTGACGTTCGACACGACGACCACGCTGGTGTTCGGCGTCGACCCGAGAT	25:G>A
1129747|F|0-25:A>T-25:A>T	TGCAGCGTTTTCTTCTCTTCTACTTATTTGCTGTTACAGCAACATATTCTTCCTAGTCTACCG	25:A>T
3020710|F|0-30:C>G-30:C>G	TGCAGGTTGTTGTATCTGCTGAAGGTCTTGCAGCACACCG	30:C>G
1862737|F|0-38:G>A-38:G>A	TGCAGCTCGAAAATCCTTCTTCCTTCGAGGTAGCTGCTGCCGAACGCGGTGCGTGAGATGACATCTCCA	38:G>A
2245631|F|0-9:G>A-9:G>A	TGCAGACGCGTTGAATCGATTCCTTGGTGCGCGGTGATTGCCGAGGGGAGCGAGATGGTAGAGAAGCGC	9:G>A
7915621|F|0-62:A>C-62:A>C	TGCAGGTAGCCGCCACCGAACGTGGGAGGAGAAGGCGTGGGAGACGCGTACAGCGTTGGGGGAGGTGAG	62:A>C
1065465|F|0-18:T>C-18:T>C	TGCAGATTTCATACCCAGTAGACAGTGACGGAAATCGGACTCATCGCTGTCCG	18:T>C
2241817|F|0-16:G>A-16:G>A	TGCAGAAGAAGTGATGGTGATACGTACCAGCCGACCACATGGCAGATAAGGTTCAGCCTCGACGCCGTC	16:G>A
983145|F|0-12:T>C-12:T>C	TGCAGTCGTCGTTGCAGATCTGTGCGTGATTCTGTGAGACCAGGCGTGGTCAACCGATCCG	12:T>C
3027505|F|0-11:G>A-11:G>A	TGCAGACATGCGACGGATGGAACCGCCAGTCAGATAGAGCCG	11:G>A
4397689|F|0-20:G>A-20:G>A	TGCAGGTAGACGCCGAGCATGGACACCTGGCCG	20:G>A
1088390|F|0-38:G>A-38:G>A	TGCAGCATCGCCAAGGCGACATTCGTGATCCTGGGCGAGAAGATGGACATCCAGTGCACTGACGAGGAC	38:G>A
32024147|F|0-11:G>A-11:G>A	TGCAGCTATCAGGATCTCGACGCTCCTGGAGATGAAGTGACAGTTTTGGACTACCG	11:G>A
3935007|F|0-68:C>G-68:C>G	TGCAGAAGAAGTGATGGGGATACGTACCAGCCCACCACATGGCAGATAAGGTTCAGCCTCGACGCCGTC	68:C>G
5581545|F|0-19:T>C-19:T>C	TGCAGGTGAAGCTTGTGGGTTACAACCCG	19:T>C
3030298|F|0-12:A>G-12:A>G	TGCAGGTCGCACACCAGGTGGATTCGACGTGGCCTCCG	12:A>G
3945781|F|0-16:T>C-16:T>C	TGCAGGTAGGGCAGGTTGGGGAAGTCCGTCTCCGACAGCACCCG	16:T>C
1244892|F|0-7:G>C-7:G>C	TGCAGCAGCTGGTGGCCTGCGGCCAGTGGAGGCAAGCCCTCGACTACATCCG	7:G>C
1143285|F|0-20:A>G-20:A>G	TGCAGGACGGTGATGCCACGAGGGTTCCCTGTGCGTTTTCTAGCGAGCGGGACGAGGATCCG	20:A>G
6045203|F|0-14:G>A-14:G>A	TGCAGTTTGGTGCGGTGACCTAATAATCCTGGCTTAGCTAGCAGCACTTCACTCCCTCCCTTGCGCCCT	14:G>A
3031511|F|0-8:C>T-8:C>T	TGCAGGAACAGCGTGTCCAGCTTCGCGAGATTCCCGAGCTCCG	8:C>T
4261737|F|0-9:A>G-9:A>G	TGCAGCTGCATGCATGTTGTCTGTTTGCAAATTCCAACCG	9:A>G
1069335|F|0-10:A>G-10:A>G	TGCAGTTTTAATAAATCAAGAAACATGGAAGCAATCGTATAACAGTAAAACTCCG	10:A>G
1065465|F|0-9:C>T-9:C>T	TGCAGATTTCATACCCAGTAGACAGTGACGGAAATCGGACTCATCGCTGTCCG	9:C>T
4008865|F|0-15:T>C-15:T>C	TGCAGGCCGTAGACGTAGTCGACGATGCACTTCTGCTAGGCGCTGCTGAACACGCCG	15:T>C
3956050|F|0-22:T>G-22:T>G	TGCAGGGCCTTGAGGCGCGACATGATGGCGTGGAGGAGGTCGTCCG	22:T>G
3941913|F|0-22:G>T-22:G>T	TGCAGGGCCCTTTGAAGGAGAAGTTGCAAGGCCG	22:G>T
4262543|F|0-16:G>A-16:G>A	TGCAGTACCTGTACCAGCAGCAGTATGCCATGTCCAACCAATCCG	16:G>A
1009308|F|0-37:C>A-37:C>A	TGCAGTTCACTCCGTTGGAGAAGATCATCAGGGACGCCGTGGAGAGCCTCCG	37:C>A
3943089|F|0-22:G>A-22:G>A	TGCAGGTATGTGTGGTAAGGCAGCAGGGACCG	22:G>A
1066973|F|0-26:T>C-26:T>C	TGCAGCTCGGCGCCATGATCCTCGTCTTGTGCGTCCTGAACATCTACTTCAATATTTACAACAAGCCGA	26:T>C
3030298|F|0-24:C>A-24:C>A	TGCAGATCGCACACCAGGTGGATTCGACGTGGCCTCCG	24:C>A
998862|F|0-10:C>G-10:C>G	TGCAGCTCTTCCCGCACCTCAGCAGCCCTGCGCACACAGTGCAACATATCCATGCCAAGTGAGAACGTC	10:C>G

<table>
<tr><td>4993927|F|0-42:A>G-42:A>G</td><td>TGCAGCAGCCTCTGGGGGATTATCAGTTGGCTGTCGGGATATATTACGTGATCAGATGGCGGTGGCGGA</td><td>42:A>G</td></tr>
<tr><td>6047355|F|0-19:T>C-19:T>C</td><td>TGCAGTGTGGGGAATGCGCTCATGAAGCTCGACGGCGAAGTCTGTGCCGCTTAGGTTGGTAGCGCGTCT</td><td>19:T>C</td></tr>
<tr><td>100323137|F|0-8:G>A-8:G>A</td><td>TGCAGCTCGACGGCGCCGCCAACTACACGGTGACGAGCTTCGACGGCGCGCTCGCAACGCTCTATCTAG</td><td>8:G>A</td></tr>
<tr><td>100387242|F|0-29:A>G-29:A>G</td><td>TGCAGCTCGGCGCGAACCTTTGACATGACAGCCG</td><td>29:A>G</td></tr>
<tr><td>100504218|F|0-10:T>C-10:T>C</td><td>TGCAGGTGCATACCAACAGCAAGGCGTGCGTGGCGTCCTATCCGTGCGTGGTGCAGAACAAGCTCCTCT</td><td>10:T>C</td></tr>
<tr><td>5341264|F|0-65:G>C-65:G>C</td><td>TGCAGTTCCTCGACCAGGTACCTGCAAACCTCCATCCTCCCTCGGTTAGCGGCGACGTGCAGCGCGCCA</td><td>65:G>C</td></tr>
<tr><td>100478776|F|0-45:T>G-45:T>G</td><td>TGCAGCAAGCGTACGTGAACAATCTAAATGGCTGAGTTATCTTCTTCCG</td><td>45:T>G</td></tr>
<tr><td>100542617|F|0-43:G>C-43:G>C</td><td>TGCAGATAAAGGAGATGAGGCTGATCTTCCACTCGAATCAGGAGGCTGTGAGGCAGTACATGGCCCGCA</td><td>43:G>C</td></tr>
<tr><td>4538277|F|0-8:G>A-8:G>A</td><td>TGCAGGTAGAAAAACCCCCGATGGGCTGCTGTACCG</td><td>8:G>A</td></tr>
<tr><td>3020442|F|0-39:T>C-39:T>C</td><td>TGCAGTGCTATTGGCCAAGTACTGCTGTACTTTGCTATGTCCG</td><td>39:T>C</td></tr>
<tr><td>3020588|F|0-22:G>C-22:G>C</td><td>TGCAGTACCTGTACCAGCAGCAGTATGCCATGTTCAACCAATCCG</td><td>22:G>C</td></tr>
<tr><td>3020588|F|0-5:T>C-5:T>C</td><td>TGCAGTACCTGTACCAGCAGCAGTATGCCATGTTCAACCAATCCG</td><td>5:T>C</td></tr>
<tr><td>1151429|F|0-23:A>G-23:A>G</td><td>TGCAGGTGATCAGTGTAAGAAGCAAAGAGCTGGCCGCACTGCACTGTGGATAGGACCG</td><td>23:A>G</td></tr>
<tr><td>1041292|F|0-13:C>G-13:C>G</td><td>TGCAGTAATGGGACAGGAACTCCATGGTGAACCGCTTCATCTCCAGGTCGTCCCCGTAGTCCG</td><td>13:C>G</td></tr>
<tr><td>5371104|F|0-22:G>C-22:G>C</td><td>TGCAGGCGGTGATCAAGGAGACGCTCCG</td><td>22:G>C</td></tr>
<tr><td>1006587|F|0-5:T>C-5:T>C</td><td>TGCAGTTGCCTTTAGGTATTTGTGCAGAAGCCCTTTTTGTCTGGTCAAAGTCTATGGATGGAAGCGTCG</td><td>5:T>C</td></tr>
<tr><td>3957199|F|0-15:G>A-15:G>A</td><td>TGCAGACGATGAAGCGGTGCGCGCCCCG</td><td>15:G>A</td></tr>
<tr><td>1139101|F|0-53:C>A-53:C>A</td><td>TGCAGCTGCTCTTCCTACGCGCCGCCTCCCTCGCTGAGCCCCGTGGCGTGGCCCTTCTGCGGCAACTAG</td><td>53:C>A</td></tr>
<tr><td>1041510|F|0-10:G>T-10:G>T</td><td>TGCAGGCAAGGGTTAGGTGGGCTTGGGGGCGACCCAGTAGAGAGGAGTGGGTCAGCCG</td><td>10:G>T</td></tr>
<tr><td>6049333|F|0-13:T>C-13:T>C</td><td>TGCAGCAGGAGCTTGCCGACACAGTGGGTCTCGACCG</td><td>13:T>C</td></tr>
<tr><td>13590222|F|0-5:A>G-5:A>G</td><td>TGCAGACGATGAAGCGATGCGCGCCCCG</td><td>5:A>G</td></tr>
<tr><td>3031511|F|0-26:G>A-26:G>A</td><td>TGCAGGAACAGCGTGTCCAGCTTCGCGAGATTCCCGAGCTCCG</td><td>26:G>A</td></tr>
<tr><td>3021021|F|0-5:T>C-5:T>C</td><td>TGCAGTGGGCTATTTCACATGCACACTCCAACCGAACCG</td><td>5:T>C</td></tr>
<tr><td>991503|F|0-55:T>C-55:T>C</td><td>TGCAGGTAGCACAAGGGTGCAGGATGCGTGGGGCTAAGCAGATTATTGGAGTGGATCTGAACCCGAGAT</td><td>55:T>C</td></tr>
<tr><td>7170931|F|0-13:C>T-13:C>T</td><td>TGCAGTGCTCCTACCACGGCTGGTCCTTCGACGGCTCCG</td><td>13:C>T</td></tr>
<tr><td>986029|F|0-19:C>T-19:C>T</td><td>TGCAGCACATCAGCCCATACTTCCTGATCAGTCCATGGGAGTTCCCACAGACCCG</td><td>19:C>T</td></tr>
<tr><td>32015326|F|0-31:T>C-31:T>C</td><td>TGCAGATGCCAAGCTCGCGGAGGTGGGGAAATGTCCG</td><td>31:T>C</td></tr>
<tr><td>982123|F|0-16:G>C-16:G>C</td><td>TGCAGCTCGGCGCCATGATCCTCGTTTTGTGCCTCCTGAATATCTACTTCAATATTTACAACAAGCCGA</td><td>16:G>C</td></tr>
<tr><td>1122854|F|0-49:G>A-49:G>A</td><td>TGCAGATTCTTGCTGTGGCATGCGCTTGTATATCTTTGTTCTCTTGTGTGCCG</td><td>49:G>A</td></tr>
<tr><td>1017450|F|0-32:A>G-32:A>G</td><td>TGCAGGATCTCGTGTGAAGACAGGGTGGTGGCATAGAGCTTTCCTTGGAAGGACAAGGTATTCCAAACA</td><td>32:A>G</td></tr>
<tr><td>3026027|F|0-21:G>C-21:G>C</td><td>TGCAGAGCGACGACTTAATTTGATGGGAGTAATTTAACCG</td><td>21:G>C</td></tr>
<tr><td>1052623|F|0-28:T>G-28:T>G</td><td>TGCAGCCACGTCCACTGCGGCGTGTACTTGCCGTAGGCAGAGTAGGAGGAGAGCATGATGACGTGCGCC</td><td>28:T>G</td></tr>
<tr><td>1049351|F|0-13:C>A-13:C>A</td><td>TGCAGGCCATGAGCCCGTACAGCGAGTTGTGGGCATAGTTGATGAGGATCCG</td><td>13:C>A</td></tr>
<tr><td>4262492|F|0-12:A>G-12:A>G</td><td>TGCAGGACAAAGAGAATATAAAAGACTGGATGAGCAGATTAGCCG</td><td>12:A>G</td></tr>
<tr><td>1041292|F|0-48:C>T-48:C>T</td><td>TGCAGTAATGGGACAGGAACTCCATGGTGAACCGCTTCATCTCCAGGTCGTCCCCGTAGTCCG</td><td>48:C>T</td></tr>
<tr><td>1041292|F|0-58:G>T-58:G>T</td><td>TGCAGTAATGGGACAGGAACTCCATGGTGAACCGCTTCATCTCCAGGTCGTCCCCGTAGTCCG</td><td>58:G>T</td></tr>
<tr><td>4262243|F|0-30:G>A-30:G>A</td><td>TGCAGGTTGCACAGAGCAGCAGGAGCACTAGCCCG</td><td>30:G>A</td></tr>
<tr><td>1069335|F|0-7:T>A-7:T>A</td><td>TGCAGTTTTAATAAATCAAGAAACATGGAAGCAATCGTATAACAGTAAAACTCCG</td><td>7:T>A</td></tr>
<tr><td>4008040|F|0-7:C>T-7:C>T</td><td>TGCAGGCCGTCTACTCCAGCATGCGCGGCCTCGTCCG</td><td>7:C>T</td></tr>
<tr><td>1017643|F|0-33:T>C-33:T>C</td><td>TGCAGGTGGGCAAGGCGCTGGCCAAGGAGGGCGTCTTCGTGGAGTGCCACGTGTTCAACCCGCCGTCCG</td><td>33:T>C</td></tr>
<tr><td>3025720|F|0-7:C>A-7:C>A</td><td>TGCAGGGCATCAGCCGCAGGCAGAAAGGACAAGACGTGCCG</td><td>7:C>A</td></tr>
<tr><td>6037725|F|0-16:T>A-16:T>A</td><td>TGCAGCCGCAGTCTTCTCGATCTTCTCCG</td><td>16:T>A</td></tr>
<tr><td>5363839|F|0-22:G>A-22:G>A</td><td>TGCAGGCTGGCGCATTTCCCGAGCTCCG</td><td>22:G>A</td></tr>
<tr><td>3940903|F|0-14:G>A-14:G>A</td><td>TGCAGCATGCCCTCGCGCTCCTCCAGGAACTGCTCCG</td><td>14:G>A</td></tr>
<tr><td>6043333|F|0-41:G>A-41:G>A</td><td>TGCAGGCCCTGAAGCAGGCCTCGGAACCTCTCCACGTTGCCGCCCAACTGCCCTTGCTTCAAAATCCTG</td><td>41:G>A</td></tr>
<tr><td>5371316|F|0-31:T>C-31:T>C</td><td>TGCAGGCTTCGAGGCAATGAGACTCGAGTGGTGAGAAAACGAAGGAGAAAGTGCTGGTTGCCG</td><td>31:T>C</td></tr>
<tr><td>32027343|F|0-52:A>G-52:A>G</td><td>TGCAGTGCATCGCGCAGTTCCAGGCGACCACGTCCCGCGTCGCCATTTCGTCAAACACCCG</td><td>52:A>G</td></tr>
<tr><td>3027208|F|0-8:A>G-8:A>G</td><td>TGCAGAGCATCAGCCGCAGGCAGAAAGGACAGCACGTGCCG</td><td>8:A>G</td></tr>
<tr><td>2245453|F|0-44:G>A-44:G>A</td><td>TGCAGGATGTCCCTGATCTGGTCGATAGCGTACCCTGCCCTGGGGTCTTCCCTCTCGACGCCCTTGTGG</td><td>44:G>A</td></tr>
<tr><td>5580186|F|0-11:C>G-11:C>G</td><td>TGCAGCAGCTTCCTTCCCTCCTCCCCGACGAACCG</td><td>11:C>G</td></tr>
<tr><td>3938711|F|0-30:C>T-30:C>T</td><td>TGCAGTAGTGCACTGCTGTGGTCGTGATCTCTCCG</td><td>30:C>T</td></tr>
<tr><td>32026214|F|0-5:A>C-5:A>C</td><td>TGCAGAGGCACAGCAACGGGCCGATGCGGTCAGACTGTCTGCGGACGCCG</td><td>5:A>C</td></tr>
<tr><td>983145|F|0-26:T>C-26:T>C</td><td>TGCAGTCGTCGTTGCAGATCTGTGCGTGATTCTGTGAGACCAGGCGTGGTCAACCGATCCG</td><td>26:T>C</td></tr>
<tr><td>1200977|F|0-11:G>C-11:G>C</td><td>TGCAGCCTCGCGCGCCGTGCTCCCGAGCCCCCTCGTCCAGGAGCTCCACTAGGAGAGGAGCAACAACGC</td><td>11:G>C</td></tr>
<tr><td>978660|F|0-30:T>A-30:T>A</td><td>TGCAGTCAAAATATGCACATGCGTTGAGTTTTTTGACTTCATATACTCCTATATTCTACCG</td><td>30:T>A</td></tr>
<tr><td>2246920|F|0-40:T>G-40:T>G</td><td>TGCAGGTAGTAACATCTTTGATCACTGTAATTAAAATGCCTTCTTCGATCAGTCAAGCATAGCTGCAAT</td><td>40:T>G</td></tr>
<tr><td>1034740|F|0-54:G>A-54:G>A</td><td>TGCAGGAGGAAGAACATGGCCTTCTTTCTTCTTTGCCCACAGAACTGCATAAAGGCCG</td><td>54:G>A</td></tr>
<tr><td>1014615|F|0-43:G>T-43:G>T</td><td>TGCAGGCAGTGCTGCGGCAGCGCCGAGAAGCCCTCCACGCCGAGGATGGCCATGTTGCGGTAGATGAGC</td><td>43:G>T</td></tr>
<tr><td>2275611|F|0-25:A>G-25:A>G</td><td>TGCAGCGCTAGTCCTAGCTGGAAACATAGAGAGGGAGGAGCGCTGACCAGAGAAGACAGCTTCCGCGGA</td><td>25:A>G</td></tr>
</table>

5582078|F|0-14:A>G-14:A>G TGCAGTACGTACGTAACGGAGAGAGACCG 14:A>G

3951673|F|0-10:A>G-10:A>G TGCAGCTCTCAGGATTATTTCCTGTCCG 10:A>G

1041292|F|0-31:C>G-31:C>G TGCAGTAATGGGACAGGAACTCCATGGTGAACCGCTTCATCTCCAGGTCGTCCCCGTAGTCCG 31:C>G

3955058|F|0-10:G>C-10:G>C TGCAGGCCCTGCTCGCGCACCTGTGGCGTGCGGTGTGCCG 10:G>C

1017579|F|0-8:A>G-8:A>G TGCAGCCAAAACTCATGCTATTCACGCACCAATAATTGGTGCATGCATGCATGCATGTGCAACCGCCCG 8:A>G

1017579|F|0-7:A>C-7:A>C TGCAGCCAAAACTCATGCTATTCACGCACCAATAATTGGTGCATGCATGCATGCATGTGCAACCGCCCG 7:A>C

1061265|F|0-11:G>A-11:G>A TGCAGCTTGCTGAAAGGCAGGGGGATCGGCACGGGGCTGGCATCAGTGAACCACCTCCG 11:G>A

3026157|F|0-29:C>T-29:C>T TGCAGGGCATCAGCAACAGGCAGAAAGGACAGGACGTGCCG 29:C>T

7172035|F|0-41:T>A-41:T>A TGCAGACGACGGGTCGGCGTCGTTGCGTGTATTCTACGGCCTCCG 41:T>A

12773303|F|0-27:G>A-27:G>A TGCAGATTCCGCGGTGCAAGGGCTCGCGCCG 27:G>A

4998504|F|0-17:T>C-17:T>C TGCAGCACACGTACGTATAGCATGTTAGCAAAGACCTGGATCGGCTCCGTTTACCG 17:T>C

3573346|F|0-7:A>G-7:A>G TGCAGCTAGCCAGGTCGAGATCCATGGCCGACCTCATCCTTTCTACGACAAACCG 7:A>G

7157934|F|0-58:C>T-58:C>T TGCAGAGCTGATGTCCGTAGGGGAAGTACAAAGACGTACTGATGCACAACACCTTGTACGCTCCGAGAT 58:C>T

5003155|F|0-38:C>G-38:C>G TGCAGTTCAGCCCGACGGAGAAAACACAGGAGTACGTGCTCACCTTTGCGCAACGAACCACCG 38:C>G

7173412|F|0-7:A>C-7:A>C TGCAGCCACCCTACGCCGTCGCCGTGGGTCACCTGCGTTCTGCTGCTCCG 7:A>C

32025081|F|0-51:A>G-51:A>G TGCAGCTCCTCCCTTCGAATCGCCGTCACGCCCACTTGCAGCACGCGAGCGAGCTGTTGAGCCAACAGG 51:A>G

32019176|F|0-5:C>T-5:C>T TGCAGCTGCTGGCCATGGGCACCAGGCCCG 5:C>T

32023250|F|0-12:G>C-12:G>C TGCAGACAGATCGCACGAGACCAGGCTAGTTTACATGTGAATCGACGGAACGTTCTCCG 12:G>C

5341805|F|0-38:G>A-38:G>A TGCAGGTCCAGCACCTTGCCACCGTCGTCGTCGTGGGAGCAGGCAGAGTCAAGCAGGGGAACGAGACGG 38:G>A

7919814|F|0-9:A>G-9:A>G TGCAGTACCAAGCTCAGATCCCAGCCACCAGCAGCGCCCTACCACCCTATAGAGGCCCG 9:A>G

5366281|F|0-31:A>T-31:A>T TGCAGCGAGCTTATTTACTGTAGCATCTGCGACGAGGCATGGTCGCTGGTCAGGTCTAGATGGATGGCG 31:A>T

5368297|F|0-12:A>G-12:A>G TGCAGCCTGGCTAGGAAGTAGACGAAGTGCCAGGGGGACGGGATGGCGACGTCGACGCGGCCG 12:A>G

5370413|F|0-36:T>C-36:T>C TGCAGTTCGGAATGAACAGAGGGATGTTAATGACGATTAAGTTTCAGCGCAGACAGTACAGAAAGCTAG 36:T>C

5366057|F|0-43:C>A-43:C>A TGCAGTTAGTTGTTGCTGTTGTGCAAGTGAACTTGATCGACCCCAAAGAAATGACCGCGGCAATTGGCC 43:C>A

4995120|F|0-44:C>G-44:C>G TGCAGGGTCTCCAGGCCCAGCGACGGCCACTGTGCGAGGCAGAGCGCGCGCCACGTCGCCG 44:C>G

4998031|F|0-50:A>G-50:A>G TGCAGATTTGGAAAGGCAAGCTAATGTCCAGCGATGCAAGGCTGCGGCTCATCAACTCCATTCTTTTTG 50:A>G

7911693|F|0-8:A>G-8:A>G TGCAGGACACGATCATACTGTCCTCCCTTCCGTAGGTACGGGCAGACGGACTTAAAGAAGCCGAGCGTC 8:A>G

22836168|F|0-7:T>C-7:T>C TGCAGGATGAGGTGGAGCTAATCGAGTCGCTCCTGCCGCGCTTCCG 7:T>C

100323022|F|0-21:C>G-21:C>G TGCAGCTACCTGTTGCGTTCGCTGATGTGTGAACCCATTTAGACACATAGAGATTCCAGCATCACGTCC 21:C>G

15306661|F|0-48:C>T-48:C>T TGCAGTGTATTTGGGTTGGAATCTTGAATTTTCTCAGACTGGATTTTGCACCG 48:C>T

7918515|F|0-13:T>C-13:T>C TGCAGAATGCAAGTGAAGCCCAAGATGACGTAGGAAGTCACAGGAGCATCCG 13:T>C

100503712|F|0-66:C>G-66:C>G TGCAGGGAAGTGCACCGTGATGTTCTTGATCTTGTCCATGGGGAGAACGGCATGGGACTGGCTGGACCC 66:C>G

4996856|F|0-29:G>C-29:G>C TGCAGCGTGTGGAGATCAAGCGAGAAGCAGACCATATGCGGCCTGGAGACAGTGTACAAATCCCAGTCT 29:G>C

13881050|F|0-36:G>T-36:G>T TGCAGGGAGAGGTTGAGGTCGATGTTGCCCTCTCCCGCGCCACCGTTGGACTCCATATGTGGGCCGAGA 36:G>T

5006686|F|0-34:G>T-34:G>T TGCAGTAATGTTTTATAACAGAAGATTGCAATCAGGTTGCAGTACTCACCTGAAGAACTGGAAGGTAGA 34:G>T

100480782|F|0-34:T>G-34:T>G TGCAGGCCGTCGCGCTCGTCGGGGAGGTAGATGTTCATGCTGAACCG 34:T>G

3934809|F|0-9:C>A-9:C>A TGCAGATTGCTAGACATTAGCCGCTTTTTTGATAGACCG 9:C>A

3934809|F|0-18:A>T-18:A>T TGCAGATTGCTAGACATTAGCCGCTTTTTTGATAGACCG 18:A>T

100520580|F|0-28:A>G-28:A>G TGCAGCGCTGCCTGCACGCCTTCAAAGAATCCTGCGACCGCGCCGAGGACCTCGTCCG 28:A>G

12779672|F|0-40:T>A-40:T>A TGCAGCCCCGCTACATGCACTTGTATATCCAGCCATTCGATTACCTGGAGATGGGTGCGCTGGCCTGGT 40:T>A

100470987|F|0-44:C>A-44:C>A TGCAGGAGAATTCCCAATATCCAGCTCCTTGAGGGATGAGTATCCACTTGACTCGCGTGGTGAAGAAGA 44:C>A

100568809|F|0-6:A>G-6:A>G TGCAGCACGTTGACGGCCTCCAGCCGCGCGCGGCCG 6:A>G

6046345|F|0-26:G>C-26:G>C TGCAGGACGATCACGGAGCCCATCTCGAGCGACTCGTCCGACGCGAGGATGTCCCCGATTGGGCCCATC 26:G>C

3570422|F|0-10:T>C-10:T>C TGCAGTCGCATTTGTCTGCATCGCAACTCGCAGCAGTTCAACACTTACACAGCCATACATATCACCTGT 10:T>C

1387382|F|0-26:T>C-26:T>C TGCAGCCGCTGCTTCACAGCAAGGAGTTCTTGGTGCTGCATAAGCATATTCTCTTCCATCATCCTCAGC 26:T>C

100503136|F|0-43:T>C-43:T>C TGCAGGCACACAGGTCAGTGATTCTATATTTGGCAACTATTATTTCTGCTTGTTTGCGCCACACCAGAT 43:T>C

100494082|F|0-7:A>G-7:A>G TGCAGAAAAGAAATTGCAAACACGAAACCAATGATTAGAAATGGTTCCAGTAACCCG 7:A>G

100513164|F|0-35:A>C-35:A>C TGCAGCATCGCCTCGAGGTTCTGCTGATCCTCGCCAAGGCCGCCGCCGTTGCTGTCCGTGGCCGCGGCT 35:A>C

1390380|F|0-59:A>C-59:A>C TGCAGCTCCAGCTAGCCGCCATGGCCGCACCACCATGCAAGGCACAACAAACTCTATGTAATCGCGTGT 59:A>C

1016277|F|0-16:A>G-16:A>G TGCAGGAGACAAAGACAAGCTACACGAAGCATCAGGCAGCAAGGTCATGTCATATTTGCGCATGCGATC 16:A>G

3021582|F|0-40:C>T-40:C>T TGCAGGTCGTAGATAGGAGCCTGATGGATCCATTCAAACACTTCCG 40:C>T

6046677|F|0-32:A>G-32:A>G TGCAGCATGGCTGCTGTGCTCTTCTGCCTTTTAGTTGCTGCTGCCTCTGACCGCCG 32:A>G

100480029|F|0-14:C>T-14:C>T TGCAGCTCATAGGGCTGTTACTTACCATAAAGAGGCCCCAGGACTCGATGGCTGACCG 14:C>T

100586680|F|0-23:A>C-23:A>C TGCAGAGCATTGTCGACATATGGAGTGTCCACGACATGCCTGCGCAAGAAGACAATCACGATCCCTATG 23:A>C

100534113|F|0-14:A>C-14:A>C TGCAGATGGGGCACATGTCAATCTTCTCGAGTGCGAGCTTCAAGCACGCACCATGGAAGATGTGGGGCC 14:A>C

32027056|F|0-7:C>G-7:C>G TGCAGGGCCTCGCCAACGCGCCGTCGCAGCAGCGGCCGTGGCCCGTGCAGCTCCG 7:C>G

3936463|F|0-29:C>T-29:C>T TGCAGGACGTGCATTGCCTGGGCGACAGACGGCCGCTCGCTCCGTTCCG 29:C>T

4397842|F|0-7:C>G-7:C>G TGCAGGACACGGGCACCACTAGGACTTCCTCGCCG 7:C>G

1139101|F|0-6:T>A-6:T>A TGCAGCTGCTCTTCCTACGCGCCGCCTCCCTCGCTGAGCCCCGTGGCGTGGCCCTTCTGCGGCAACTAG 6:T>A

1139101|F|0-60:G>A-60:G>A	TGCAGCTGCTCTTCCTACGCGCCGCCTCCCTCGCTGAGCCCCGTGGCGTGGCCCTTCTGCGGCAACTAG	60:G>A
100440224|F|0-14:C>T-14:C>T	TGCAGCCTCTGCTGCTGCTCCCTATCGTGGTCGAACCGCCG	14:C>T
1018914|F|0-17:A>G-17:A>G	TGCAGTGGATCATGGCCAACTTGGTGAAGCGTCCCGACATACAGGAGGCCGTCCG	17:A>G
3951176|F|0-43:C>G-43:C>G	TGCAGGTATTGCTTAATCGTTCCCGTTCGATTCGTCCAGTTTCCTGGAGTTGCTTGTTCTTGGGGAGCT	43:C>G
4993450|F|0-8:T>C-8:T>C	TGCAGCCATGCGACCTGCTCGGCGTACTTGTCCG	8:T>C
3020934|F|0-32:G>A-32:G>A	TGCAGCAACACGGTCTTGAGCAGGAAGTTCTTGACAATCTCCG	32:G>A
6042067|F|0-29:T>C-29:T>C	TGCAGTGTTGTAGTGAGCAGGAACTTGCTTTAGCACAAGACATCCG	29:T>C
991492|F|0-12:G>C-12:G>C	TGCAGGTGCTGAGCAACAAGAAGTTCAAGAGCGCCACGCACAGGGTGGTGAGGAGGCCG	12:G>C
3949012|F|0-41:G>C-41:G>C	TGCAGTAGAATTGTCGACGAGGGCGATGAGCGGGATGAGGCGGATGAACATGTCGATCTCGTGCTGCGT	41:G>C
1004660|F|0-5:G>C-5:G>C	TGCAGGACGTGCATTGCCTGGGCGACAGACGGCCGCTCGCTTCGGTCCG	5:G>C
1021258|F|0-8:G>C-8:G>C	TGCAGCATGGAGATGCCGCTCCTCACCCTGCCCCCGATCTCCGCCAGATCGCTCCG	8:G>C
1038581|F|0-10:C>T-10:C>T	TGCAGCTGGGCGCCTGGCCGCTCTACTGGGTCGTGCAGGGCTGCGTCATGACCG	10:C>T
1026860|F|0-13:A>G-13:A>G	TGCAGGGCCCATCAGAAGTGTGGCTGACACTGATGCTGTTGTTGGGCTGGTTCTGGTCCG	13:A>G
3020972|F|0-10:G>A-10:G>A	TGCAGAACGAGTACCCATCGGACCTACACCTTCTTGTGACCG	10:G>A
3024805|F|0-30:C>A-30:C>A	TGCAGAGCCCAAGCCCCACGACCAGATTCCCAGGCCCACCCG	30:C>A
1863632|F|0-41:A>G-41:A>G	TGCAGTTCTGAATGTATGATATGATCGACCGTACCAGTGGCAGATGGGGACGCTTGTTTTTCCG	41:A>G
1038581|F|0-7:G>T-7:G>T	TGCAGCTGGGCGCCTGGCCGCTCTACTGGGTCGTGCAGGGCTGCGTCATGACCG	7:G>T
1009308|F|0-14:T>C-14:T>C	TGCAGTTCACTCCGTTGGAGAAGATCATCAGGGACGCCGTGGAGAGCCTCCG	14:T>C
980168|F|0-46:T>G-46:T>G	TGCAGGTGAGGTGGACGCAGCGGAAGGGCCCCTGGTGCGCGGCGAGTATGCGGGAGACGGCCGCGGTGA	46:T>G
3022543|F|0-16:A>T-16:A>T	TGCAGCACATACATACACGATCGTGCTTGGGTTATCCG	16:A>T
3022543|F|0-11:C>G-11:C>G	TGCAGCACATACATACACGATCGTGCTTGGGTTATCCG	11:C>G
1713915|F|0-7:T>G-7:T>G	TGCAGGTTGTCGGCCAGCTCCGCGATGTCGACGGCGATGCTCTCCTCCGTCCGCGCCG	7:T>G
1050374|F|0-22:C>G-22:C>G	TGCAGAGCTCGGACACGTTCCTCACCATGCTGCTGCTGCTGTCCAAGTACCG	22:C>G
3952249|F|0-19:G>T-19:G>T	TGCAGCACTGGTCTGTACAGTCAATAAGCCG	19:G>T
2278414|F|0-62:A>C-62:A>C	TGCAGGAGAGGGCGGAGTTCCCCTCGATCGCGCAGCTGGACTGGGTGGACTACATCGAGGAAACCGCCG	62:A>C
7170761|F|0-20:T>G-20:T>G	TGCAGCTTCTCCTCGGGGCTTGCCTGGAGGAGGTAGGATGTGACGCCG	20:T>G
5581941|F|0-23:C>G-23:C>G	TGCAGCAGCGGCCCGACCACCAGCACCG	23:C>G
1057678|F|0-20:C>A-20:C>A	TGCAGTCTAGCAAACTAGTGCCCAGATACTAAATTAGGAACTGGCTGGCCTTAAGAGCGATTGCAGATT	20:C>A
3024373|F|0-25:G>A-25:G>A	TGCAGTGTTGTAGATCTCTTGCGAGGCTGGTACCTATGTCCG	25:G>A
7168589|F|0-60:T>C-60:T>C	TGCAGATGCATAGCTTACACCTCTAAGCAGAGCAGTGCCGATCAGACGACGATGAGCTTTTAGCCACAG	60:T>C
7170931|F|0-19:C>A-19:C>A	TGCAGTGCTCCTACCACGGCTGGTCCTTCGACGGCTCCG	19:C>A
3571381|F|0-26:C>G-26:C>G	TGCAGGTGGGACAAGGCGTACAGCTTCGCCTACCACCCGACGTCCG	26:C>G
3951176|F|0-31:T>C-31:T>C	TGCAGGTATTGCTTAATCGTTCCCGTTCGATTCGTCCAGTTTCCTGGAGTTGCTTGTTCTTGGGGAGCT	31:T>C
32023383|F|0-29:A>G-29:A>G	TGCAGGCTGTGCGCGGCCGCGATCACCGCATGGTCCG	29:A>G
32026911|F|0-11:T>C-11:T>C	TGCAGCCTCTATGGGGACGCGCTGGTGTCGCCCTCGCTGGAGCGCCTCGCCATCGTCGACTGCCACACT	11:T>C
100350944|F|0-10:T>A-10:T>A	TGCAGCCTCATACTGGTTCCGAAGGAGACGCGTGTGATAAGCAGCCCG	10:T>A
3574722|F|0-9:C>T-9:C>T	TGCAGCATGCTCTCGCGCTGCTCGAGGAACTGCTCCG	9:C>T
1019171|F|0-13:C>T-13:C>T	TGCAGCACGTGTACCTGAACCTGGGCGGGGAGGGCAGCGGCGACATCCTGGGCCACACGCTCCAGCTCT	13:C>T
3943264|F|0-5:A>C-5:A>C	TGCAGATCAGCCCTCCCTCGCCAAAGGCGCCG	5:A>C
3945070|F|0-14:G>C-14:G>C	TGCAGCGAGTTCTGGACCCCTCCGACGACGCACCG	14:G>C
3027354|F|0-5:C>T-5:C>T	TGCAGCTGCTGTCAGATGAGAATAGAGAGAAGCGAGCCAGCGTCCG	5:C>T
4261261|F|0-19:A>G-19:A>G	TGCAGAGGAGAAATCGAAAAGCACGAACCTTGCCG	19:A>G
3952560|F|0-5:T>C-5:T>C	TGCAGTGGCGCCGTTGGGTAGATCGGGGGCGAGACCG	5:T>C
7170761|F|0-35:G>A-35:G>A	TGCAGCTTCTCCTCGGGGCTTGCCTGGAGGAGGTAGGATGTGACGCCG	35:G>A
6043670|F|0-19:G>C-19:G>C	TGCAGAGGTTCGAGGTGCAGGGCAACGCCTTCACCG	19:G>C
13879602|F|0-11:A>G-11:A>G	TGCAGCTTGTCATCGTCGTCGTCGGGACGGCGGTCCG	11:A>G
5366004|F|0-10:A>G-10:A>G	TGCAGTGCAGACCATTCTGTGCAGAGCGGCAGAGCTATGCCTGCGTCGCTTGTATGGACGGAGAGATTT	10:A>G
3949541|F|0-5:C>G-5:C>G	TGCAGCCGCAGCCCCATGCCTCCCAGTACGCAGCCG	5:C>G
1018692|F|0-23:C>G-23:C>G	TGCAGGTCACGGCCACCGTTACGCCGTCGCGTGGAGCTCAGAGGTGCTGTTAGCGATGCGTGGCTGTGT	23:C>G
1010782|F|0-19:C>T-19:C>T	TGCAGCGTTTTCTTCTCTTCTACCTATTTGCTGTTACAGCAACACATTCTTTCTAGCCTACCG	19:C>T
3030959|F|0-24:T>G-24:T>G	TGCAGTGGGGGTAGAATCACAAACTGTCAGGGAGTACCG	24:T>G
1069052|F|0-8:C>G-8:C>G	TGCAGCACCGACAGCTGCACGACGATGTCGCGCGCCCACTTGCCGTACATGGCGTCCAGCAGCGGCACG	8:C>G
4998348|F|0-23:G>A-23:G>A	TGCAGGGAGTTTCTATATGAAACGCAAACAGAACCG	23:G>A
998972|F|0-10:C>T-10:C>T	TGCAGCAATGCAAATACGATACTACTGTACGTATCCGTGTGCAGGCACAAGGGGTGCATGCCGCCTAAC	10:C>T
1018914|F|0-37:C>T-37:C>T	TGCAGTGGATCATGGCCAACTTGGTGAAGCGTCCTGACATACAGGAGGCCGTCCG	37:C>T
1005369|F|0-61:G>A-61:G>A	TGCAGACCAAAGGTGATGCTTGCAGAAGAGCCAGCCTCCTCATGTGAAAAGCAAAGAGAGAGAAGAGAA	61:G>A
3030959|F|0-21:A>G-21:A>G	TGCAGTGGGGGTAGAATCACAAAATGTCAGGGAGTACCG	21:A>G
3025161|F|0-15:G>C-15:G>C	TGCAGCAGAGACGTCGTTTTCGCAGTCTTCAGAAAACGTTCCG	15:G>C
3951673|F|0-15:T>C-15:T>C	TGCAGCTCTCAGGATTATTTCCTGTCCG	15:T>C

3024805|F|0-20:A>C-20:A>C	TGCAGAGCCCAAGCCCCACGACCAGATTCCCAGGCCCACCCG	20:A>C
1004340|F|0-10:T>C-10:T>C	TGCAGTATCCTAGCCTGTTGCTGGAGTTCATGGGGAGCTATCTCACCGAGCTGAGCCTTTTGGATTATG	10:T>C
5360556|F|0-11:G>T-11:G>T	TGCAGACCTTCGGCGAGCCACTGGTGGAGGGTGGCGCGGTCGAGGCGGATCTCGACGGCGGCGCGGTCC	11:G>T
3024373|F|0-11:A>G-11:A>G	TGCAGTGTTGTAGATCTCTTGCGAGGCTGGTACCTATGTCCG	11:A>G
3028678|F|0-30:T>C-30:T>C	TGCAGCGTCTTCATGTTGAGCGACATCTCGTCCGCCG	30:T>C
1026860|F|0-10:A>G-10:A>G	TGCAGGGCCCATCAGAAGTGTGGCTGACACTGATGCTGTTGTTGGGCTGATTCTGGTCCG	10:A>G
3934052|F|0-42:C>T-42:C>T	TGCAGCGTTTTCTTCTCTTCTACTTATTTGCTGTTACAGCAACACATTCTTCCTAGCCTACCG	42:C>T
3021573|F|0-8:C>T-8:C>T	TGCAGGTGCAACAGCTCTGAGGGTGCTGGTCTCCCAACCTTCCG	8:C>T
5363676|F|0-7:G>C-7:G>C	TGCAGCTGCTGCTCGCCGTCGGTCACCAAGCAAAGCCGCCCATCCG	7:G>C
4261204|F|0-9:A>G-9:A>G	TGCAGAGGAACCTCCCTCTTACGCTTCAGATGGGCTGATCCG	9:A>G
3956602|F|0-37:A>T-37:A>T	TGCAGAGTATGGATTGGGAAGCGATTGGAAAGGCAGAACCG	37:A>T
4004295|F|0-6:A>G-6:A>G	TGCAGAATTCCAACCTCAAGATGCAAATCTGTCGGCGCAAACAATCCG	6:A>G
5582047|F|0-6:C>T-6:C>T	TGCAGGCGGCCAGGTACCTGCTCGGCTCCCCG	6:C>T
3957713|F|0-10:C>T-10:C>T	TGCAGCAGTGCCCTACGGGTTCGTCGCTCTCTGCATTTCATGCTACCCG	10:C>T
2245768|F|0-44:G>A-44:G>A	TGCAGCGAGGAGTGTAGGAGGAAGCAAATCGAGACGGAGAAGGCGAGACACCG	44:G>A
3020322|F|0-11:C>T-11:C>T	TGCAGACCTTCCTCGACATGTAAAAGCTCATTAAACTCCG	11:C>T
1017811|F|0-26:C>T-26:C>T	TGCAGAATTTCTTGAGTGGAGGGAAACCTTGCAGTTGAGGCAAAAGTCTGAAGACTAGAGAGCACAACT	26:C>T
3959679|F|0-9:A>G-9:A>G	TGCAGACGCACCACTTCGCCTACGAGGAGCTGGAGGAGGCCACCG	9:A>G
1031053|F|0-8:A>G-8:A>G	TGCAGACGATCCGCAAGGCTGCCGTTCTCCATGTACTCGTACACCAAGCAGCCCTTGTCCG	8:A>G
3954468|F|0-6:C>T-6:C>T	TGCAGCCGCGCCTTGTCCTCGGGCGGTGCCAACCTGCTCGGCTCCG	6:C>T
4003819|F|0-6:A>T-6:A>T	TGCAGCAGGAGCATCAGCAGAGCACATGCTTGGAATGGAAGGCCG	6:A>T
997667|F|0-25:T>C-25:T>C	TGCAGGTACTCCAAGCAGGGCAGGTTCTGAAACTCCATCGGAATTGCGCCG	25:T>C
3938196|F|0-10:C>T-10:C>T	TGCAGCACCTCCGCCGCCCGCCTCTGTTTGCCG	10:C>T
1667453|F|0-15:T>C-15:T>C	TGCAGTCCATGACGGTCGATGGATCGCGTTGTTTGCATGCGGCCAAACACATCCG	15:T>C
4003791|F|0-22:G>A-22:G>A	TGCAGGTTACAACAATGAAGTCGGGCGGCCG	22:G>A
32020228|F|0-6:A>G-6:A>G	TGCAGCATAAAGCAACGGCGAGTGGGAGGAGCCCG	6:A>G
999276|F|0-20:G>A-20:G>A	TGCAGGCCTGCGGGGTGAGGGCAGCCTTGCGACTTGCGAGACGGGCGACGACGGGGCTGACCCGTCGGA	20:G>A
4999182|F|0-5:G>A-5:G>A	TGCAGGTACACGAAGGAGCCCGCACAACAGGCCCTTGCACTCAAGGCGGCCGCGGGCGCCACCCATGGT	5:G>A
3574207|F|0-66:G>T-66:G>T	TGCAGGAAGTGGTTTGCCTCTGCAAGCATCACCTTTAGCTGAGTTGGACTCTGTGAGCCTACGTGGGAT	66:G>T
5005497|F|0-34:T>C-34:T>C	TGCAGCAGAAATGTCTGGAACTCAAATAGGAGGATAGGTGATGGAAATCCG	34:T>C
5354445|F|0-61:C>T-61:C>T	TGCAGCAAATTCAGTCCAAGTAGCCTTGAAATGGCTTGCTTGCCCTGGTAAACATGCACACCCGAAAGG	61:C>T
5372116|F|0-5:T>C-5:T>C	TGCAGTGGTGCTCGAGCCTGACCAGCACGGCCGCGGCGAGCGTCCCCCGTCGATGGAGAGCACGTGCAC	5:T>C
3572059|F|0-49:G>C-49:G>C	TGCAGCTCCACTCGGTCCTCTTACTTACATGAACGAGTCGAACTTTACTGGCGACCCG	49:G>C
32027670|F|0-13:A>G-13:A>G	TGCAGGAGGACGCACGCGAGCACGCGACGCACGCACAAGGCGAGGCTGCGCCCG	13:A>G
1308648|F|0-24:G>A-24:G>A	TGCAGCAGCCGCCTCCTGGACCGAGCCCCCACTGTCGCGTACCAATGGTTGGAGCAGCACGTCGCGGAT	24:G>A
100488262|F|0-68:A>G-68:A>G	TGCAGGAGCGCGACGGGGTTCTGGGAGGGGCTGTAGCTGGGGCGCCACTCGACGTCGATCCCGACGACA	68:A>G
4998077|F|0-65:A>C-65:A>C	TGCAGCCGCTCCTCGGCGGCTCTACCGTTGATGAGCAGGCGCGTGGGAATGGAGCGGCCAAGGAGAGAG	65:A>C
100367883|F|0-34:C>T-34:C>T	TGCAGGCAGATCGGTGAGCGTCCAGGTGCCATTTCCTTCGATAGACCCCAGCTCTTCCAGCATCGCGCT	34:C>T
1090023|F|0-8:A>T-8:A>T	TGCAGCAGATTCATCCACGTTATGCTCGCCGAGGCGCTGGCGACCAGCGAGTACGGCAACTGGGAGGAT	8:A>T
3020710|F|0-17:G>A-17:G>A	TGCAGGTTGTTGTATCTGCTGAAGGTCTTGCAGCACACCG	17:G>A
4539826|F|0-21:T>C-21:T>C	TGCAGTGGGTAATGGCGGAGCTGATGCGGAACCCGAGGGTTCGGCATAAGGCGCAGGAGGAGGTCCGAG	21:T>C
4002146|F|0-26:C>T-26:C>T	TGCAGCCTCCCGAAGGTGCTGATCGACGGGCCGTACGGGGCGCCG	26:C>T
3572070|F|0-5:A>G-5:A>G	TGCAGAGCCTTGAGATTGCACCAGCAGTCCG	5:A>G
4008040|F|0-25:C>T-25:C>T	TGCAGGCCGTCTACTCCAGCATGCGCGGCCTCGTCCG	25:C>T
6045849|F|0-7:C>G-7:C>G	TGCAGTGCCTTGATGACCTGAAAACGGCAACCTGAGCCG	7:C>G
1053523|F|0-40:C>T-40:C>T	TGCAGCTGGATCAGCGATGAGTAGGCGCTGAGAGGGTTGGCCATGAGCTGCTCGTGCGTGCCG	40:C>T
4261261|F|0-9:G>A-9:G>A	TGCAGAGGAGAAATCGAAAAGCACGAACCTTGCCG	9:G>A
4261261|F|0-29:T>A-29:T>A	TGCAGAGGAGAAATCGAAAAGCACGAACCTTGCCG	29:T>A
979472|F|0-29:G>A-29:G>A	TGCAGAGCCCCCACGAAGAGGGGCGGAATGCTCTGGGCATTGCGGAACACGTTCTCCG	29:G>A
1020441|F|0-26:G>A-26:G>A	TGCAGGTGGCGGACATGCACTACGCGGACGGGCGGAGCACCGCGTGCGAGGACGTGATGCCCGAGCAGG	26:G>A
3030313|F|0-11:C>T-11:C>T	TGCAGGTAGTGCCCAATCTGCACATGGGCTGGTGGTCGCCG	11:C>T
3026027|F|0-7:C>T-7:C>T	TGCAGAGCGACGACTTAATTTGATGGGAGTAATTTAACCG	7:C>T
1019150|F|0-13:C>G-13:C>G	TGCAGTCGTGGTGCAGGTAGCAGAGCCCGCGCGCCGCCTCCAGCGCGATCCG	13:C>G
1058456|F|0-39:A>T-39:A>T	TGCAGTTCGCGGTGCCCATGGTCCTCACCAACATGTCCTACTACGGCATCCCGCTGGTGTCCGTCATGT	39:A>T
1026249|F|0-41:A>C-41:A>C	TGCAGATGCATCTCGTCGCGCTCATGCAGCAAAGGACCCAGATGCCTCCG	41:A>C
4734058|F|0-6:C>G-6:C>G	TGCAGTCAAGTGTCCATGTTGGATGGCTCTCCTGATAACCCG	6:C>G
5363676|F|0-28:A>T-28:A>T	TGCAGCTGCTGCTCGCCGTCGGTCACCAAGCAAAGCCGCCCATCCG	28:A>T
1041520|F|0-6:A>C-6:A>C	TGCAGGAGACGACGAGGGAGGAGGAGGAGCTCTTGGGGTGGAAGACGGGGAAGGAGCCG	6:A>C
999830|F|0-29:C>A-29:C>A	TGCAGGAGGTCTGGAAGCATTATCAACTTCATCATCTAAATCCATTATTGCCTGGTCCG	29:C>A

5365128|F|0-8:C>G-8:C>G	TGCAGATCCAAGGCGCGATGAGGGTGGTCGAGGCCACCAACGACGACAGGGAGCTGAGGATATACATGT	8:C>G
1005369|F|0-35:C>T-35:C>T	TGCAGACCAAAGGTGATGCTTGCAGAAGAGCCAGCCTTCTCATGTGAAAAGCAAAGAGAGAGAAGAGAA	35:C>T
3934238|F|0-20:C>T-20:C>T	TGCAGCGCGCGCGCGTCGGCCAGGAAATGGCCCG	20:C>T
991492|F|0-37:G>A-37:G>A	TGCAGGTGCTGAGCAACAAGAAGTTCAAGAGCGCCACGCACAGGGTGGTGAGGAGGCCG	37:G>A
1021132|F|0-34:C>T-34:C>T	TGCAGGCGTACACCAGGGCACGCTACGGCGAGGACGCGATGATCGTGTACCG	34:C>T
1046384|F|0-5:C>T-5:C>T	TGCAGCAAACGTGTAGCGTTTTACACTGCTATTCCAGGCGTGGAGCAAATTGAATTATACTGTACACCA	5:C>T
1018396|F|0-22:G>A-22:G>A	TGCAGCTAACCTCGGTTGGACTGTCAAAATCAAGTGTTCATATCTGACGTGATTTGGACCTAGCTAGAA	22:G>A
3935248|F|0-40:G>A-40:G>A	TGCAGGTCATGGCCAACATCGCCGACGCGGTCATCGGGGAGACCG	40:G>A
4002338|F|0-24:C>T-24:C>T	TGCAGAGAAGCATGCATTACTTCACAAAGTGGCCAACAACCG	24:C>T
7164225|F|0-17:G>C-17:G>C	TGCAGGGCATTGATGATGAATGGCCCGATCGAAGTCGTGAGATGTACGAACGGATCAAGCACGTCGTAT	17:G>C
1399750|F|0-10:G>C-10:G>C	TGCAGACCTTGGTTCTGAGGGTGTCCATCCACTGCCATGGCTGCAAGAAGAAGGTCCG	10:G>C
100543028|F|0-67:T>C-67:T>C	TGCAGCAGCAGCCAGGCGGCGCCACGGCCACAAACTCCTTCGACGTCAATGGCGCCCGTCACGCGGCTG	67:T>C
1021258|F|0-35:G>T-35:G>T	TGCAGCATGGAGATGCCGCTCCTCACCCTGCCCCCGATCTCCGCCAGATCGCTCCG	35:G>T
3949541|F|0-7:G>A-7:G>A	TGCAGCCGCAGCCCCATGCCTCCCAGTACGCAGCCG	7:G>A
3940025|F|0-10:G>A-10:G>A	TGCAGTTGCGGAGGCCCATGCTGCTCCG	10:G>A
1020803|F|0-40:G>T-40:G>T	TGCAGGCGCCAATTAGGTAAGCTGGCGCTCACAGCCTGGAGCTCGTGGGCCG	40:G>T
1043700|F|0-50:C>T-50:C>T	TGCAGTCGGAGCGCCTCCTTATGCAGCAAGCTATTGTTGTGCTCCTCTAGCGCACTCATCTGCATGTTC	50:C>T
5360556|F|0-29:G>A-29:G>A	TGCAGACCTTCGGCGAGCCACTGGTGGAGGGTGGCGCGGTCGAGGCGGATCTCGACGGCGGCGCGGTCC	29:G>A
1091000|F|0-48:C>A-48:C>A	TGCAGGTGGGCAACGACCTCAACCACCGCTTCCTAGCGCGGCTGTCGGCGCGGTACGGCCCCGTGTTCC	48:C>A
1043700|F|0-20:A>G-20:A>G	TGCAGTCGGAGCGCCTCCTTATGCAGCAAGCTATTGTTGTGCTCCTCTAGCGCACTCATCTGCATGTTC	20:A>G
3959034|F|0-9:T>C-9:T>C	TGCAGAGCCTTGAGCTTGCACCAACAGTCCG	9:T>C
7170761|F|0-12:T>G-12:T>G	TGCAGCTTCTCCTCGGGGCTTGCCTGGAGGAGGTAGGATGTGACGCCG	12:T>G
3573862|F|0-9:C>A-9:C>A	TGCAGCCATCCAGATGCAGGAGATGAGTTCAACCG	9:C>A
2242910|F|0-9:C>G-9:C>G	TGCAGCACGCGGTTGCTATCAGGGGCATGTGTCGACGGCACATTTTTCGTCCG	9:C>G
1014615|F|0-35:C>G-35:C>G	TGCAGGCAGTGCTGCGGCAGCGCCGAGAAGCCCTCCACGCCGAGGATGGCCATGTTGCGGTAGATGAGC	35:C>G
1006653|F|0-22:T>C-22:T>C	TGCAGTATCTCCTCAAAGCCTATCATGAAGATCAGGGACCATAATCCACCG	22:T>C
1017811|F|0-54:C>G-54:C>G	TGCAGAATTTCTTGAGTGGAGGGAAACCTTGCAGTTGAGGCAAAAGTCTGAAGACTAGAGAGCACAACT	54:C>G
4261851|F|0-13:C>G-13:C>G	TGCAGCTGTTGGTCAAGGACGGCGAGAGCCGCGTCACCATCGCCG	13:C>G
3953384|F|0-13:T>C-13:T>C	TGCAGGTGTACCATCCCAACATCGGGCCG	13:T>C
1082662|F|0-5:C>T-5:C>T	TGCAGCTGAAGTGGACGGAAAAGGACGCGTCTTGAAAAGAAATCCTGGGCTTCTAGATCGACCGACCGA	5:C>T
1020803|F|0-20:G>A-20:G>A	TGCAGGCGCCAATTAGGTAAGCTGGCGCTCACAGCCTGGAGCTCGTGGGCCG	20:G>A
3020327|F|0-9:T>A-9:T>A	TGCAGTCTATCCCTTTCATAGCAAACTTCGATTGATTTACCG	9:T>A
22839976|F|0-9:A>G-9:A>G	TGCAGCAGAATCGACAGAGAAATCAAGAAACCAAGATGAGACGACTCGCCG	9:A>G
1071245|F|0-7:C>T-7:C>T	TGCAGGCCATGAGTGCCAACCAGAGCAGCATGCTGATCCTTCAGGAAACATGCACTGATGCATCCGAGA	7:C>T
3571381|F|0-17:G>C-17:G>C	TGCAGGTGGGACAAGGCGTACAGCTTCGCCTACCACCCGACGTCCG	17:G>C
2242910|F|0-6:A>T-6:A>T	TGCAGCACGCGGTTGCTATCAGGGGCATGTGTCGACGGCACATTTTTCGTCCG	6:A>T
4261851|F|0-7:G>A-7:G>A	TGCAGCTGTTGGTCAAGGACGGCGAGAGCCGCGTCACCATCGCCG	7:G>A
4008494|F|0-20:T>G-20:T>G	TGCAGTGGCAATGATAATTTTCAGCAAACGAACGACCG	20:T>G
4262704|F|0-31:C>G-31:C>G	TGCAGGCGCAGGCGGACGACAAGTTCTTCGCCGAGTTCTACCG	31:C>G
3934492|F|0-5:G>A-5:G>A	TGCAGGGCCAGCACGGTGAGGCGGTGCTCCG	5:G>A
3942787|F|0-9:C>T-9:C>T	TGCAGCTGGCGGAGCTCCGTCTCGTACCCCTCCG	9:C>T
1046384|F|0-12:G>T-12:G>T	TGCAGCAAACGTGTAGCGTTTTACACTGCTATTCCAGGCGTGGAGCAAATTGAATTATACTGTACACCA	12:G>T
7908938|F|0-10:A>C-10:A>C	TGCAGCTTGGATAGCCGCGAGATCAGGCCCG	10:A>C
1017811|F|0-29:T>A-29:T>A	TGCAGAATTTCTTGAGTGGAGGGAAACCTTGCAGTTGAGGCAAAAGTCTGAAGACTAGAGAGCACAACT	29:T>A
1018396|F|0-18:G>C-18:G>C	TGCAGCTAACCTCGGTTGGACTGTCAAAATCAAGTGTTCATATCTGACGTGATTTGGACCTAGCTAGAA	18:G>C
1666712|F|0-45:T>C-45:T>C	TGCAGGACGTGTTCATGCGCCTGACGTTCGACCTCACGGCGATGTTCATATTCGGTACGGACCCCGAGA	45:T>C
3939482|F|0-18:G>A-18:G>A	TGCAGGCTACCCATCGTCGTTGATGCGTCCG	18:G>A
32024084|F|0-6:C>T-6:C>T	TGCAGACTGTGGTTGTCGGGATCCTGGGGTTGTTATCCGTTCCCGAGCGCCG	6:C>T
1004340|F|0-22:G>A-22:G>A	TGCAGTATCCTAGCCTGTTGCTGGAGTTCATGGGGAGCTATCTCACCGAGCTGAGCCTTTTGGATTATG	22:G>A
998972|F|0-43:G>A-43:G>A	TGCAGCAATGCAAATACGATACTACTGTACGTATCCGTGTGCAGGCACAAGGGGTGCATGCCGCCTAAC	43:G>A
1262989|F|0-29:G>C-29:G>C	TGCAGGGTGCGGAAGTCCTTGAGCGGCTTGGCCCGCGGGTGGTCGATGTTGTTCCG	29:G>C
1046384|F|0-11:T>C-11:T>C	TGCAGCAAACGTGTAGCGTTTTACACTGCTATTCCAGGCGTGGAGCAAATTGAATTATACTGTACACCA	11:T>C
3020326|F|0-11:A>G-11:A>G	TGCAGTCAAAGATGTGTAGTGTAGAGAAAACAAGAAACTCACCG	11:A>G
3947537|F|0-8:T>C-8:T>C	TGCAGCCATCCAGATGCAGGAGATCAGTTCAACCG	8:T>C
4261205|F|0-8:G>T-8:G>T	TGCAGTATGCAAGGCAAGAAAATTAGCGGCCG	8:G>T
1020841|F|0-35:T>C-35:T>C	TGCAGAGTCATGAGCGGAGCAGGATGATGAGGACCTGCGGCGAGCTACAAGTAGTAGTTCAGAATTCGG	35:T>C
6045805|F|0-15:C>T-15:C>T	TGCAGGTTCTTCAGCCTTCTTCTCTGCCGCCG	15:C>T
1032506|F|0-39:A>G-39:A>G	TGCAGAAGCCGCAGAGATGACAGCAGAAGATGAGCCACTAAACCCTTGAAACACACCAGCAGCCATTGA	39:A>G
1132668|F|0-35:G>A-35:G>A	TGCAGATGCTGACCTACCTCGCCATGAAGCACGACGCGCGCGTGCAGCGGGTCGACACGGTCCG	35:G>A

<table>
<tr><td>32025125|F|0-23:G>A-23:G>A</td><td>TGCAGGCCCCCGACCTGGTCCTGGCAAAGGATGGTGAGGAAGTCGCTGTCCGTGTGCGGCACCAGGCCG</td><td>23:G>A</td></tr>
<tr><td>1666899|F|0-19:G>T-19:G>T</td><td>TGCAGTATACCCACGCGGTGCTGTTCGAGAACATGCGGCTGTTCCCGCCG</td><td>19:G>T</td></tr>
<tr><td>3936319|F|0-21:T>A-21:T>A</td><td>TGCAGCATTTCCGCTCCTACTTCCAAAGGGTAAACAGGAGTGCACCG</td><td>21:T>A</td></tr>
<tr><td>3032674|F|0-34:G>A-34:G>A</td><td>TGCAGGTGGAGGAGATGAAGGGCTTGGGCGCGAGGATCCG</td><td>34:G>A</td></tr>
<tr><td>1019010|F|0-68:G>A-68:G>A</td><td>TGCAGCCTTCGCGTGCACTCCTCTTATTATACGCTTGCATGCTTCGCAACAGTGCGCCGTAACAAAAGG</td><td>68:G>A</td></tr>
<tr><td>4262536|F|0-20:C>G-20:C>G</td><td>TGCAGCTCGTCCGCCGCCAGCTCCGCGTCGCCGTCCG</td><td>20:C>G</td></tr>
<tr><td>4008040|F|0-16:C>T-16:C>T</td><td>TGCAGGCCGTCTACTCCAGCATGCGCGGCCTCGTCCG</td><td>16:C>T</td></tr>
<tr><td>1019241|F|0-8:C>T-8:C>T</td><td>TGCAGCTTCTTGGTGTCCGTGGTGCTCCAGGAGATGAGGTGGAACAGCCG</td><td>8:C>T</td></tr>
<tr><td>4003599|F|0-8:C>T-8:C>T</td><td>TGCAGATCCTGAAGGCCTGCTCCGATTGGCAGGAGACAACAATCTCCG</td><td>8:C>T</td></tr>
<tr><td>1014615|F|0-12:T>C-12:T>C</td><td>TGCAGGCAGTGCTGCGGCAGCGCCGAGAAGCCCTCCACGCCGAGGATGGCCATGTTGCGGTAGATGAGC</td><td>12:T>C</td></tr>
<tr><td>3956664|F|0-25:G>A-25:G>A</td><td>TGCAGCTGTAGAAGGTCGACAGGTCGGCGCTCGACCG</td><td>25:G>A</td></tr>
<tr><td>1094073|F|0-32:C>T-32:C>T</td><td>TGCAGGTTGGCGAACACGATGCTCACAGCGGCCAGCACGAGTATCCTCATCCG</td><td>32:C>T</td></tr>
<tr><td>997408|F|0-40:T>G-40:T>G</td><td>TGCAGGATGGCGTCTACCGTCTTGTGGATGTCCTGGAGGCTGCGCTTCATGCCG</td><td>40:T>G</td></tr>
<tr><td>2242191|F|0-5:T>G-5:T>G</td><td>TGCAGTGGCCGCACGCAGCATCTCGATGTCCTCATCTGAGGAGGGTTTGTCTGCCTCTGTTTGCCCCCG</td><td>5:T>G</td></tr>
<tr><td>1695387|F|0-26:T>C-26:T>C</td><td>TGCAGAACACCAACGCCGAGATCTCCTTCTCGTCGGGGCACCGTCCGAAGCCTGATGCGTTCGAGCTCT</td><td>26:T>C</td></tr>
<tr><td>7169369|F|0-9:A>G-9:A>G</td><td>TGCAGCATGAAGCGCATTCTGTCCATCTGGCCCG</td><td>9:A>G</td></tr>
<tr><td>1051309|F|0-39:G>C-39:G>C</td><td>TGCAGCGTGCTGCTCGCCCTCATCCTCGTCCTCGCCATCGTCTTCGTCGCGCTCTACTTCACCGTGTTC</td><td>39:G>C</td></tr>
<tr><td>1005369|F|0-6:C>T-6:C>T</td><td>TGCAGACCAAAGGTGATGCTTGCAGAAGAGCCAGCCTTCTCATGTGAAAAGCAAAGAGAGAGAAGAGAA</td><td>6:C>T</td></tr>
<tr><td>989318|F|0-44:C>A-44:C>A</td><td>TGCAGGATGGCGTCCACCGTCTTGTGGATGTCCTGGAGTCTGCGCTTCATGCCG</td><td>44:C>A</td></tr>
<tr><td>4262876|F|0-12:G>C-12:G>C</td><td>TGCAGAAGCTGCGCCTGCCTGAGTGCGCCG</td><td>12:G>C</td></tr>
<tr><td>1083636|F|0-15:C>T-15:C>T</td><td>TGCAGCGACATGTCCCTGGACGAGATGGACGCCGCGGTGAGGAGGATGGAGTTCGTCGTCTTCGACCGA</td><td>15:C>T</td></tr>
<tr><td>7171352|F|0-8:G>T-8:G>T</td><td>TGCAGTTTGGCGGCGCGTACACAAAGTTCGTCGTTGGCCTGTGGCATCATCCTGCTGGTCAGCGACCAA</td><td>8:G>T</td></tr>
<tr><td>32022470|F|0-31:G>C-31:G>C</td><td>TGCAGCTGTAGAAGGTCGACAGGTCGGCGCTGGACCG</td><td>31:G>C</td></tr>
<tr><td>5365961|F|0-5:A>T-5:A>T</td><td>TGCAGACTAAGTACTAGTCTGGCGGTGTGGTAACGGAGAATGAAGACGAATGGAGGGTTTTGTAACGGA</td><td>5:A>T</td></tr>
<tr><td>4996014|F|0-33:T>C-33:T>C</td><td>TGCAGGTTAACCCGAGGTGTCAAACGAGTGCGGTGTCGAGAGGGGGTTCGAACCGTGACAAGTGGAGCG</td><td>33:T>C</td></tr>
<tr><td>4995293|F|0-57:C>G-57:C>G</td><td>TGCAGCACTATAGGTCGATCTAGTGGCCTATTTGATCTCCTCTTCATGCTCGATGTTCAACAGTACGAA</td><td>57:C>G</td></tr>
<tr><td>5355818|F|0-45:T>C-45:T>C</td><td>TGCAGCAATCAACCGAAAACGGAACGAAATCAATTTGACAATTTCTATGTGAGCATTCATTCATGCATG</td><td>45:T>C</td></tr>
<tr><td>5365656|F|0-37:G>C-37:G>C</td><td>TGCAGGAGCTGCTCTAGCTGGCTTTCTGCCCTTGTTGGACGGTTGGTCGAGAGGGCTCGGTGACAACTG</td><td>37:G>C</td></tr>
<tr><td>4993981|F|0-55:G>C-55:G>C</td><td>TGCAGGACGTGGAGGCTTCGCTGAGGCAGTCGAGGGCGCCGTGGAAGATCGTCGTGGGGCACCACCCCA</td><td>55:G>C</td></tr>
<tr><td>32025523|F|0-50:T>C-50:T>C</td><td>TGCAGCCCAACATGAGGGAGATTGAGTCCGCCCAGGACCTCGTCGACTCGTTGGCTAACGCCG</td><td>50:T>C</td></tr>
<tr><td>100222165|F|0-32:G>C-32:G>C</td><td>TGCAGGTTGCCCACGAGGTCTGAGAGGCAGGAGGGCCG</td><td>32:G>C</td></tr>
<tr><td>5005129|F|0-31:C>T-31:C>T</td><td>TGCAGCGGCGTACTGTAGATAGTGCATGCAGCGCGTGATATTCCCCTTACGTGGCCAGGTGGGTACGGA</td><td>31:C>T</td></tr>
<tr><td>5355370|F|0-12:G>T-12:G>T</td><td>TGCAGTAAATTCGGAATCGGAGCGTCGAAGTATCAGGCAAAGATCTCCCACAAGTATAAGAAACGCCGA</td><td>12:G>T</td></tr>
<tr><td>5354362|F|0-22:C>T-22:C>T</td><td>TGCAGAGTCTCATTCAGCTATGCTTTATGTCTTAGTGGAGCTGAACCAATGTATATTTTGTTTCCCATT</td><td>22:C>T</td></tr>
<tr><td>1667169|F|0-31:T>C-31:T>C</td><td>TGCAGCCACTGCTCCGTCGTGCTGCTGTCGTTGACGCTGTTCCTCATCTCTAACCCGAGCACCTCACTG</td><td>31:T>C</td></tr>
<tr><td>7913473|F|0-14:G>A-14:G>A</td><td>TGCAGAGACATGGGGGAATCTGTTTCGGCGGAAAGGCCATTCTAGCTATGTTTCGCCCG</td><td>14:G>A</td></tr>
<tr><td>5368443|F|0-14:C>T-14:C>T</td><td>TGCAGCTTCACATCCGAACAATGCAGGCGTGCATGCATGCCACGGGATCGTCCTCGGCCCTCTTGCGCC</td><td>14:C>T</td></tr>
<tr><td>1387276|F|0-29:C>T-29:C>T</td><td>TGCAGGTACACAGGCTGGCGAGGTGAGGGCGATTAGAGAAACAGGGGCGAACGACGTGCATGCTACAGC</td><td>29:C>T</td></tr>
<tr><td>5365975|F|0-6:C>G-6:C>G</td><td>TGCAGTCGCGCGATGGCTACCCGAACCAGAGGCCGAGGCTGTCGAGGATGCGGACGCCGAAGAAGGTGA</td><td>6:C>G</td></tr>
<tr><td>100508395|F|0-6:A>C-6:A>C</td><td>TGCAGCAAGGTGCACATGGCCATGTACATCTCGCTGGCCGCCGCCG</td><td>6:A>C</td></tr>
<tr><td>5365975|F|0-48:T>C-48:T>C</td><td>TGCAGTCGCGCGATGGCTACCCGAACCAGAGGCCGAGGCTGTCGAGGATGCGGACGCCGAAGAAGGTGA</td><td>48:T>C</td></tr>
<tr><td>5340906|F|0-64:T>C-64:T>C</td><td>TGCAGGTAGTCGTCCATGCCAGGCATGTCCAGAGACGAGCCAAACTGAATTTGGAGCAAGGTGTTAGAA</td><td>64:T>C</td></tr>
<tr><td>5370180|F|0-11:C>T-11:C>T</td><td>TGCAGCGCCGCCATGCAAAACACGACGGACTTCGCCTGCCGAGGCGCTCATAGCGAGTGCTTCAATGTC</td><td>11:C>T</td></tr>
<tr><td>100386574|F|0-12:T>C-12:T>C</td><td>TGCAGCCTGGCATTGGCAGGGCTGGATTTGGCTGGTTTCCAATGAAAGCTTGCTACTCCG</td><td>12:T>C</td></tr>
<tr><td>1758823|F|0-6:T>A-6:T>A</td><td>TGCAGCTAACGTGTAGCGTTTTACACTGCTATTCCAGACGTGGAGCAAACTGAATTATACTGTACACCA</td><td>6:T>A</td></tr>
<tr><td>100574639|F|0-7:C>T-7:C>T</td><td>TGCAGTTCTGTTTTCTTACTTCTGAGTTCAAGATCTGATCTGGAGCTTGTTTTTCTCAAAATGCCACCG</td><td>7:C>T</td></tr>
<tr><td>1388334|F|0-62:G>A-62:G>A</td><td>TGCAGGCATTAATTAGTTCGCTTTTCCTCTTTCGGTATGTTGCCGCCAACGGAATTTCCACTGACCGCA</td><td>62:G>A</td></tr>
<tr><td>4407107|F|0-14:G>C-14:G>C</td><td>TGCAGATTGTTGATGTAGAGGATGTATCAAGTTTGAGATGCAGCCG</td><td>14:G>C</td></tr>
<tr><td>100385848|F|0-6:T>A-6:T>A</td><td>TGCAGCTCCCCAACAAGTCGTCCGACGCCAGCACCTCCCCCACCGCGCCCAGCTCCG</td><td>6:T>A</td></tr>
<tr><td>100469967|F|0-63:T>C-63:T>C</td><td>TGCAGCTCCAGTAGATAATGTCGCTGACGATGATCTATAGGACGTCGCAGAGCGCGTGGATGTTGGCAA</td><td>63:T>C</td></tr>
<tr><td>100558138|F|0-47:C>G-47:C>G</td><td>TGCAGGCGCTCCCATCAGAATTAAGAAAACAAACAAGCTCAGTCGTCCGTGTATTCAACCCCG</td><td>47:C>G</td></tr>
<tr><td>5370650|F|0-12:A>G-12:A>G</td><td>TGCAGAGGGCAAACAGGGCGGGAGCTCGTCCAAGTGTGGCGACAACGACGAGGTGGACTTGTCTCTCCG</td><td>12:A>G</td></tr>
<tr><td>32019630|F|0-50:G>A-50:G>A</td><td>TGCAGCTGGGACATGTTGAAGATAGCAGGAGGCATGGGGCCTGAGAGAAGGTTGCCTTCCAGGACCAGC</td><td>50:G>A</td></tr>
<tr><td>5368229|F|0-20:T>G-20:T>G</td><td>TGCAGCCACATGTACCACCATGACTGCATCCTGCCGTGGCTCGCGCTGCGTAACTCGTGTCCCCTGTGC</td><td>20:T>G</td></tr>
<tr><td>5000355|F|0-14:T>C-14:T>C</td><td>TGCAGCCACCCACGTCACAAGATTACGAGGTCCAGGAGGAGGCCGAACCCGCGCCGCCTGTGCCGCCCG</td><td>14:T>C</td></tr>
<tr><td>100384970|F|0-11:A>G-11:A>G</td><td>TGCAGAAGATTATGTCGGCCAAATCTTGAAGTAGGGACGCCCG</td><td>11:A>G</td></tr>
<tr><td>1746011|F|0-37:G>A-37:G>A</td><td>TGCAGGAGTGCCTTAATTGTGCAAGCACAAGGTACCAGCAGAGGAGTAATTTGCACATGTGGCCCGAGA</td><td>37:G>A</td></tr>
<tr><td>100558563|F|0-25:T>C-25:T>C</td><td>TGCAGTACATGGAGCTGACCAAGACTTTCTGTGTCCGCGTGGGATGGGCCGAGTCGTGCACCGCCGAGC</td><td>25:T>C</td></tr>
<tr><td>100579494|F|0-7:C>T-7:C>T</td><td>TGCAGAGCATAGCCGCGTGTGGTGTGGGTTTTATAGGACGACAAGATGCGTGGGGAAATGTGGCGGCAC</td><td>7:C>T</td></tr>
</table>

100562050|F|0-29:T>C-29:T>C	TGCAGCATCAGCGAAAAGCTTTTGCGGCCTCTCTGGTTCCAAGTAGAAATTCTGTTCGATGTTGTTAGT	29:T>C
100569829|F|0-14:T>C-14:T>C	TGCAGATGTGTCGTTCCTCAAGCAGTCGCTGAGCGCTGCAACACTGGAGGAGAAATCGCCCGCCGTTTC	14:T>C
100533918|F|0-27:C>G-27:C>G	TGCAGATAGCAAGGCTAACAGCGACGACGACGAGGAGGAGGAAGCAGCTGAGCTTCTTGTGAGCCTGAG	27:C>G
2257575|F|0-29:T>C-29:T>C	TGCAGCGTGGAGCGGTTCCAGAGGGCGGTTGACGCGGCGAGGGCCCAAGAACGGCACCATCATCATCAC	29:T>C
1094077|F|0-9:G>A-9:G>A	TGCAGGCGTGGAGGCATCAATGCTGGCTGGGCTGGGCTTTATCTTTGGAGGAAAATGAATGCCG	9:G>A
1739761|F|0-6:A>G-6:A>G	TGCAGAACAGATTCACCTCTCCACGACAGGGGGACGCGCGCAGGCGAAGCACTCTCCTCGTGGTCTCGC	6:A>G
100460959|F|0-42:A>T-42:A>T	TGCAGCATCTCGCTAGGGATCTCCCTGCTGCTGCGATCAGGCACCG	42:A>T
1008666|F|0-16:A>G-16:A>G	TGCAGCCCACGGCGAAATTGCGCCTGGCGGCGCCCCGTGGCGACATGCGCAGCGTGTCGGAGACGAGGA	16:A>G
100582919|F|0-27:G>A-27:G>A	TGCAGCAGCTGGTCTCGAGCTGCCCGCGCCTCGCCGAACTGACGCTCGAGGAGTGCCCGACCGCCACCA	27:G>A
100463703|F|0-8:A>G-8:A>G	TGCAGTGAAATGAATCGGACTGTCAGATTGATGCTCGTGCCGCCG	8:A>G
100527572|F|0-68:G>C-68:G>C	TGCAGGTCCCGAGGCCACCTAGCCATTGCAGGACACGCGGTGGCAAGCGGAGGGCGCGGCCACGTCTAG	68:G>C
7155201|F|0-16:C>T-16:C>T	TGCAGCTGGATGTTTACCAAGAACCACCG	16:C>T
1667357|F|0-55:A>G-55:A>G	TGCAGCAACAATGCTAAACATCGAACTACACTCCTCTGCTACATGCTACTGGTTCATTCCG	55:A>G
3943589|F|0-10:C>T-10:C>T	TGCAGAGCAGCAGCGCCAAGGGGGAACTGAGCCG	10:C>T
6041218|F|0-22:C>G-22:C>G	TGCAGATCAAGGAATCCAGGCCCTAATTATCAAAAAGGGCTATTCCATTTCTTCCG	22:C>G
1024232|F|0-40:C>T-40:C>T	TGCAGCCAGCAATTCCAGCGTCTTGTGTCTGCGAGGATCCCCAACACGCAGCAAGCATCCG	40:C>T
1044294|F|0-16:G>C-16:G>C	TGCAGCTGCTGTCGTCGCTGGACACCACCAAGGTCAAGGACAAGAAGGACCG	16:G>C
4003599|F|0-24:A>G-24:A>G	TGCAGATCCTGAAGGCCTGCTCCGATTGGCAGGAGACAACAATCTCCG	24:A>G
4539854|F|0-14:G>A-14:G>A	TGCAGGTGTTTGAGGCGGACTTGCAGAAGGGCCG	14:G>A
3025086|F|0-26:A>C-26:A>C	TGCAGCAAGTGCCGCACGCCCATCCGAGGCAAGAACCCG	26:A>C
1051488|F|0-22:C>A-22:C>A	TGCAGGGGTGGGACAATGCCACCATCGCTGGTAAGTAGCCCAGATTTGTTTTACCACCTTGTTAGATCA	22:C>A
1695415|F|0-28:G>C-28:G>C	TGCAGATCTCGTGGAACTACAACTACGGGGCGGCAGGGCAGAGCCTCGGCTTCGACGGGCTCAACGACC	28:G>C
3946113|F|0-9:C>G-9:C>G	TGCAGCATGCAAGGCAAGAAAATTAGCGGCCG	9:C>G
3935617|F|0-13:C>G-13:C>G	TGCAGAGCCCAAGCCCCACGACCAGCTTCCCAGGCCCACCCG	13:C>G
32027922|F|0-8:G>A-8:G>A	TGCAGTGGGCCATGGCGGAGCTGATGCGGAGCCCG	8:G>A
4262794|F|0-9:G>C-9:G>C	TGCAGCCATGGCCGCGGTAGTCACCGTAGATGTCCG	9:G>C
1090023|F|0-43:C>A-43:C>A	TGCAGCAGATTCATCCACGTTATGCTCGCCGAGGCGCTGGCGACCAGCGAGTACGGCAACTGGGAGGAT	43:C>A
1079000|F|0-5:G>T-5:G>T	TGCAGGGCCAGAGTCACCTGGGAGACAACGCCGAGGACCCCAAGGGAGACCTTGGCGGCGTCCAGGTCC	5:G>T
3021148|F|0-25:G>C-25:G>C	TGCAGCATTCAGACCACTATGGCCAGTACATTAGTGAACACATACCG	25:G>C
1061949|F|0-10:T>C-10:T>C	TGCAGGCATCTGTAGTCCACCAGCGATGTTTCGGCGAGCTGATGCGCCAACCTCTGAACCTCCAGCTCG	10:T>C
4261443|F|0-10:A>T-10:A>T	TGCAGTGCTCACTCATCCGAAAGTGCTACTCTTTCCG	10:A>T
4262296|F|0-7:G>T-7:G>T	TGCAGCCGCCTCCTCCTCCGCTGCATCAACCG	7:G>T
3572070|F|0-26:G>A-26:G>A	TGCAGAGCCTTGAGATTGCACCAGCAGTCCG	26:G>A
1063727|F|0-43:C>T-43:C>T	TGCAGGGTTGGAGCTACGTTGGTGACAGGCTATTGTCGGCAGTCGTGCCGTATGAAGAAACCG	43:C>T
1041520|F|0-28:G>C-28:G>C	TGCAGGAGACGACGAGGGAGGAGGAGGAGCTCTTGGGGTGGAAGACGGGGAAGGAGCCG	28:G>C
1060195|F|0-29:C>G-29:C>G	TGCAGACGCTATGGGGTGTAAAACCTGCCCCAGCAGGAGAATGGGTTCAAGGGAATTGCCCACTGCACA	29:C>G
1043700|F|0-6:C>T-6:C>T	TGCAGTCGGAGCGCCTCCTTATGCAGCAAGCTATTGTTGTGCTCCTCCAGCGCACTCATCTGCATGTTC	6:C>T
5358200|F|0-29:G>A-29:G>A	TGCAGGCACCATGACATGAGCGGGAAGGCGGACCCG	29:G>A
3030313|F|0-22:C>A-22:C>A	TGCAGGTAGTGCCCAATCTGCACATGGGCTGGTGGTCGCCG	22:C>A
1075816|F|0-13:C>T-13:C>T	TGCAGACGACAGGCGGCACCATCCTACTGCTCAGCACTGTCCTTGGAATTAAAATCTCCG	13:C>T
3946372|F|0-5:C>T-5:C>T	TGCAGCCTCCCGAGCTCGGGCGGTATCGGCCCG	5:C>T
4005771|F|0-9:G>A-9:G>A	TGCAGGTTGGGGACGGAGGGGATGTGCTCGCTGGGACGGACCATCCG	9:G>A
1079000|F|0-8:C>G-8:C>G	TGCAGGGCCAGAGTCACCTGGGAGACAACGCCGAGGACCCCAAGGGAGACCTTGGCGGCGTCCAGGTCC	8:C>G
3031017|F|0-38:T>C-38:T>C	TGCAGTCTGAAGCCGAAGCTCAGCGCCGCCAGTGCCTCTTTCCG	38:T>C
3937731|F|0-17:G>A-17:G>A	TGCAGGTCGATTGCGCCGCCGCACAAAGTTCACCG	17:G>A
1019567|F|0-19:T>C-19:T>C	TGCAGCTTTCCTGGCTTTCTTGGTGATGCTGACGTTCCAGTGACAGTGCGACGGCCG	19:T>C
3935746|F|0-7:G>T-7:G>T	TGCAGAAGCGAGGTCATGGCCGAAAGCATTGCGTCAGGGACCG	7:G>T
3025416|F|0-5:C>G-5:C>G	TGCAGCAGCCGCTTCTCCTGGAGCTCCTGGGACAGGACCACCG	5:C>G
1053754|F|0-32:T>C-32:T>C	TGCAGGCCATTTACGCCGACTGGACATTACGGTGAAGAGGGTGCCGCGTTCGGGGTTGCAAGCAAGTGA	32:T>C
989036|F|0-31:A>C-31:A>C	TGCAGATGCCGTGGGAGACGGTCATTTGCGAATTGCGACTGGGTTGTTCTTCCG	31:A>C
1019161|F|0-5:G>A-5:G>A	TGCAGGAACACGCGGAAGCTGCGCAGCTCGTCGTGGATGACCGACTTGCTCCGCGCGTAGGCCG	5:G>A
3936028|F|0-26:C>G-26:C>G	TGCAGTTGGTGTCCTGGGCGACGAGGCTGAGCCG	26:C>G
1019150|F|0-25:C>G-25:C>G	TGCAGTCGTGGTGCAGGTAGCAGAGCCCGCGCGCCGCCTCCAGCGCGATCCG	25:C>G
3027505|F|0-20:A>T-20:A>T	TGCAGACATGCGACGGATGGAACCGCCAGTCAGATAGAGCCG	20:A>T
6045673|F|0-15:C>T-15:C>T	TGCAGGATGAATGTGCCGCTATTGCTTCAAGATGCTTCGAACCG	15:C>T
32021988|F|0-36:T>G-36:T>G	TGCAGGATGTGGCTGTTGGTTGGCCAGCGCGTCCATTCTCAGTGATCCATTTCATGTCCATTGTTTTTT	36:T>G
6041214|F|0-30:A>G-30:A>G	TGCAGAGTTCAGTCGCAAAACTACACCAACATCACCTTCAGTTCTCCG	30:A>G
7164296|F|0-17:A>G-17:A>G	TGCAGGTCCTTCTCGGGAGTCGACGTCTGCGACGACGCCCACCCCCAGTGCCCCAAGGGCTGCTCGGCC	17:A>G
3955155|F|0-7:C>T-7:C>T	TGCAGGACACGTACCTCCCTCGCAATCCG	7:C>T

3945669|F|0-27:A>G-27:A>G	TGCAGACTCACCCCGATCGAGTTCCGTAATCCGCCG	27:A>G
4993340|F|0-5:C>A-5:C>A	TGCAGCACGGGGAGCAGGAACGGCTTGGACGCGTTGGTGAACTCGCCG	5:C>A
1035262|F|0-5:A>G-5:A>G	TGCAGAATCTCCACCAGATAATTTAAACCAATTCAAAATCCCTAAATTACCCG	5:A>G
1035262|F|0-21:T>C-21:T>C	TGCAGAATCTCCACCAGATAATTTAAACCAATTCAAAATCCCTAAATTACCCG	21:T>C
1035262|F|0-24:A>C-24:A>C	TGCAGAATCTCCACCAGATAATTTAAACCAATTCAAAATCCCTAAATTACCCG	24:A>C
3028857|F|0-30:C>G-30:C>G	TGCAGCTGCCATGATCGGCCGATCTATCCTCGTCTCCCTCCTCCG	30:C>G
3022428|F|0-33:G>T-33:G>T	TGCAGCAGGTTCTTCCACGGGGATCTGAAGTCTGGTCCG	33:G>T
4261116|F|0-23:T>C-23:T>C	TGCAGCGTCCTAGTCTTCGACCATGCGAGCCCG	23:T>C
4008494|F|0-21:C>T-21:C>T	TGCAGTGGCAATGATAATTTTCAGCAAACGAACGACCG	21:C>T
1094574|F|0-11:G>A-11:G>A	TGCAGGAGCCCGCCAAGGAGGCGATCGGGCCGTGCACGGCGGTCCCGTCCCTAGACTTGGCCGCCGAGA	11:G>A
1018458|F|0-64:C>G-64:C>G	TGCAGTCCGTGCACCTCAGGCGTTGCCGCGCCAAAACCAACAGCACCATACCCAGACCCAGAGTCATGG	64:C>G
1062799|F|0-42:T>C-42:T>C	TGCAGGCAAATCATTCTTGGCTCTACTGCATTGGGGTGTCCTTCCACATCGCACTACCTCTTCCCTGAA	42:T>C
979472|F|0-8:C>A-8:C>A	TGCAGAGCCCCCACGAAGAGGGGCGGAATGCTCTGGGCATTGCGGAACACGTTCTCCG	8:C>A
1047199|F|0-48:T>C-48:T>C	TGCAGCTTCTGCGTCGTCGCCTGCTCCGCCGAGAGATGATGGTGGTGGTCCACGACCG	48:T>C
1083636|F|0-14:C>G-14:C>G	TGCAGCGACATGTCCCTGGACGAGATGGACGCCGCGGTGAGGAGGATGGAGTTCGTCGTCTTCGACCGA	14:C>G
990866|F|0-8:T>C-8:T>C	TGCAGGATTTGCAAGGAGGTGATGGCCGACGCGGTGGTGGCGAGCAAGTGCTGCTTCAACAGCTTCTGC	8:T>C
4262423|F|0-10:G>C-10:G>C	TGCAGGCGGCGACGACGACCTGGCAGAGGCTCTTGAGCGGCGAGCCG	10:G>C
32019479|F|0-9:C>G-9:C>G	TGCAGCAGTCCAAGAGGGGTGTTATTGTTGGCGCAGGATCTCCTGCCGTGCAGAAGGGCCAAAGGACAC	9:C>G
32027283|F|0-16:A>G-16:A>G	TGCAGCTAATGTCAAAACCAACCTCCACGAGATCGTCACTGCACATGACACGGCTCTCGCGCATGATCC	16:A>G
100497199|F|0-36:A>G-36:A>G	TGCAGTTAGCTCCCCGACCGTCACCGTCTTGAGCTCACCGTCGGCCG	36:A>G
1019161|F|0-50:C>T-50:C>T	TGCAGGAACACGCGGAAGCTGCGCAGCTCGTCGTGGATGACCGACTTGCTCCGCGCGTAGGCCG	50:C>T
3934835|F|0-44:C>G-44:C>G	TGCAGGCACCGCGCCAGCACCCGCTCCGCCTCCTCCTGCCTGTGCTCCG	44:C>G
4006219|F|0-12:T>C-12:T>C	TGCAGTAATTTATCATTTTAATCCGTGGTTGACAAATGGGCCG	12:T>C
989036|F|0-29:G>A-29:G>A	TGCAGATGCCGTGGGAGACGGTCATTTGCGAATTGCGACTGGGTTCTTCTTCCG	29:G>A
4406723|F|0-14:G>A-14:G>A	TGCAGAACGACGTCGGCGGCCAATGATCCG	14:G>A
1014447|F|0-28:A>G-28:A>G	TGCAGTAGTAGTATTTTCTACAGCCATAACAGTTTCTACAGCCATAACAGGAAACTGAGATGGGCCTCC	28:A>G
1018914|F|0-22:G>A-22:G>A	TGCAGTGGATCATGGCCAACTTGGTGAAGCGTCCTGACATACAGGAGGCCGTCCG	22:G>A
7173562|F|0-21:G>A-21:G>A	TGCAGCCTCCCCAGCTCGTGCGTGCCACTGAGCGACCTTGTTCATGGACTGAAGAGAAGCCACCGAGAT	21:G>A
978166|F|0-11:T>C-11:T>C	TGCAGTTTTAATAAGTCAAGAAACATGGAAGCAATCGTATAACAGTAAAACTCCG	11:T>C
32017739|F|0-9:G>T-9:G>T	TGCAGGAAGGCACATACAGTTGAAGCATTGAGGCAGCAGTCCG	9:G>T
1019567|F|0-28:C>G-28:C>G	TGCAGCTTTCCTGGCTTTCTTGGTGATGCTGACGTTCCAGTGACAGTGCGACGGCCG	28:C>G
998972|F|0-23:A>G-23:A>G	TGCAGCAATGCAAATACGATACTACTGTACGTATCCGTGTGCAGGCACAAGGGGTGCATGCCGCCTAAC	23:A>G
1167183|F|0-7:G>T-7:G>T	TGCAGGCGGTTCAGGATTAGCTGCGTGTCCACTGGACGATTCCGCTGCGGTCTTCGAGCCG	7:G>T
1242975|F|0-42:C>T-42:C>T	TGCAGGCGGGTCAGGATTAGCTGCGTGTGCACTGTACGATTCCGCCGCGGTCTTCGAGCCG	42:C>T
1035262|F|0-16:G>A-16:G>A	TGCAGAATCTCCACCAGATAATTTAAACCAATTCAAAATCCCTAAATTACCCG	16:G>A
4538893|F|0-18:G>A-18:G>A	TGCAGACGGGAGGAAGAAGACGAATCCG	18:G>A
1028993|F|0-8:A>G-8:A>G	TGCAGGGGAAGGGGAGTCGGTGCCGTGGCAGTAGGAGTCGTTGGCGCGGTCGACGGCCG	8:A>G
6038439|F|0-7:C>T-7:C>T	TGCAGATCTCTAGAGAGGATAGGTGCACTAACCG	7:C>T
4003599|F|0-23:G>A-23:G>A	TGCAGATCCTGAAGGCCTGCTCCGATTGGCAGGAGACAACAATCTCCG	23:G>A
1044828|F|0-25:G>T-25:G>T	TGCAGGGAGAACTGGACGGAGATGTGCGCGGTGCAGCGCGGGCAATTGGCGTCGGCGTGGATCCGAGAT	25:G>T
1020967|F|0-26:C>G-26:C>G	TGCAGATCTGCTCACCAGGGACTACACCTACGACCAGAAGCTGTCCGTCTCCACCGTCAGCTCCTCCGA	26:C>G
3952211|F|0-13:G>T-13:G>T	TGCAGAGCCTGCTGCACAGGCTGGGGTCCGACCCG	13:G>T
3032674|F|0-22:C>G-22:C>G	TGCAGGTGGAGGAGATGAAGGGCTTGGGCGCGAGGATCCG	22:C>G
7173646|F|0-21:C>G-21:C>G	TGCAGATTAGCCGCTCGTTGCCTCCCCG	21:C>G
3020885|F|0-36:C>G-36:C>G	TGCAGGAGATCGATACTGTCGTTCAAAGTTCAGAAACCCG	36:C>G
1028993|F|0-50:C>G-50:C>G	TGCAGGGGAAGGGGAGTCGGTGCCGTGGCAGTTGGAGTCGTTGGCGCGGTCGACGGCCG	50:C>G
4261851|F|0-10:G>T-10:G>T	TGCAGCTGTTGGTCAAGGACGGCGAGAGCCGCGTCACCATCGCCG	10:G>T
1021039|F|0-53:G>T-53:G>T	TGCAGACTGAAGTTGTACCTTGACATGAAGTACTTGGGGTTGAGCGCAATAGCGCCAAAAACGGCAAAG	53:G>T
4006126|F|0-19:G>A-19:G>A	TGCAGCCGCGGATGGTGATGCCGTGGATGATCACGTTGCTCCG	19:G>A
1203846|F|0-21:C>A-21:C>A	TGCAGCGAAGCTCGTGGGGAACCCAAGGAACAGGACATGATCACCCAAACTCCG	21:C>A
1314324|F|0-9:G>T-9:G>T	TGCAGACCTGCGGTGCGCGTGCTTTGCTTCTTCCTCTGCTCCTCCGATCAGAGCCGCTTGGCTTGGCTT	9:G>T
1059484|F|0-12:G>A-12:G>A	TGCAGACGCTATGGGGTGTAAAACCAGCCCTAGAAGGAGAATGGGATCAAGGGAGTTGCCCACTGCACA	12:G>A
1059484|F|0-65:C>A-65:C>A	TGCAGACGCTATGGGGTGTAAAACCAGCCCTAGAAGGAGAATGGGATCAAGGGAGTTGCCCACTGCACA	65:C>A
1090217|F|0-67:C>G-67:C>G	TGCAGGTAATACGGCCGTTGCGGAGACCATCAAAGTGGAGGATCCGAAGCCCGTGGTGGTGAGCAAGCC	67:C>G
1019567|F|0-22:G>A-22:G>A	TGCAGCTTTCCTGGCTTTCTTGGTGATGCTCACGTTCCAGTGACAGTGCGACGGCCG	22:G>A
5360641|F|0-40:C>G-40:C>G	TGCAGAGGTTCTACCGCCCAGACAGCGGGGAGATACTGCTCGACGGCGACGACATCGGCTCGCTCAACG	40:C>G
1021742|F|0-10:G>A-10:G>A	TGCAGCTACAGTAACTGAATGTTACTGTAGCTAGCAGTCAGTAGGAGCGGCAGCAGTATTAGCGCAGCA	10:G>A
4995494|F|0-16:C>T-16:C>T	TGCAGGCGGAGAGGGACGGATCAAACGAGTTCGCCAACTACCAGCCG	16:C>T
4995694|F|0-29:C>T-29:C>T	TGCAGCCAAGTCGCTGGTGCATTTCTGGGCCTGAACAGCAAGGTTTTTGTGGCCAGTAGCTCGGTATCC	29:C>T

<table>
<tr><td>5004390|F|0-35:A>G-35:A>G</td><td>TGCAGGAGGAAGTCGACGATGCGCTCCGTCTCGACATGCCGTCCGAACAAGCAGCGGTCCATGTACAGC</td><td>35:A>G</td></tr>
<tr><td>4994653|F|0-20:T>G-20:T>G</td><td>TGCAGCAGCGTGCTGATGGTTGCGTTCACCAGCGCGCTGAGCGGCGACGGCCTGAAGCCG</td><td>20:T>G</td></tr>
<tr><td>5002107|F|0-31:G>A-31:G>A</td><td>TGCAGGTCCCGCGCTTTGCCTTCCTCCGTCAGAATCTCCATCAGGCAGCTCTCAAAACTCCG</td><td>31:G>A</td></tr>
<tr><td>5365709|F|0-11:A>C-11:A>C</td><td>TGCAGGCCCAGAGCCGACTCCTCGGCCGCCTCCTTCTCCATCGCCAGCCGCGCCGCGGCCGCCTCCAGC</td><td>11:A>C</td></tr>
<tr><td>5003350|F|0-44:T>C-44:T>C</td><td>TGCAGCACTTCGAGGTTCGGCAGGTCGCCGATAAATTCGGGAATTTCGCCG</td><td>44:T>C</td></tr>
<tr><td>100255343|F|0-13:A>G-13:A>G</td><td>TGCAGCAGGGATCAAGCAAAGCATGAACAAATCGAGCAAAACATGAAGCAGAACGAATGAAGCAAACCA</td><td>13:A>G</td></tr>
<tr><td>7927078|F|0-5:T>C-5:T>C</td><td>TGCAGTGACGCCGATGAAGAAGTATCTACCGAGCAGGAGGGAGATGTCCTTACAGATGGAGCGCCCGAG</td><td>5:T>C</td></tr>
<tr><td>7164202|F|0-18:G>A-18:G>A</td><td>TGCAGGGACGTAGAGTAGGGACGGATCAGCCACTGGCTCGCCTCGCCTTTGGTCTTGGTGGTGAACCGA</td><td>18:G>A</td></tr>
<tr><td>1017811|F|0-33:G>A-33:G>A</td><td>TGCAGAATTTCTTGAGTGGAGGGAAACCTTGCAGTTGAGGCAAAAGTCTGAAGACTAGAGAGCACAACT</td><td>33:G>A</td></tr>
<tr><td>32019128|F|0-41:G>A-41:G>A</td><td>TGCAGGTTCCCGATCGAGCTCATGTCATGGCATGGCATGGCGTCATCCG</td><td>41:G>A</td></tr>
<tr><td>1023575|F|0-37:T>G-37:T>G</td><td>TGCAGAATCTTCTAAACAAAGTCGCAACTGATCAGCATCAGCCGCAACAACAAGGGCAGCAGCACCGCC</td><td>37:T>G</td></tr>
<tr><td>100499695|F|0-50:A>C-50:A>C</td><td>TGCAGATCGACAGTCATGTGGTGAGCAATCAAGTGGTGCAGATGGTAACCAAGAGTAGTGGCGCTAATA</td><td>50:A>C</td></tr>
<tr><td>100454065|F|0-41:G>A-41:G>A</td><td>TGCAGTGTCCGTCCCTCGGGCTCTGCGCTGCGGCGGGCAACGGCTCGACCTGCACGTGCCCTCCGCTCT</td><td>41:G>A</td></tr>
<tr><td>100461744|F|0-37:T>G-37:T>G</td><td>TGCAGCGTGGACACGTGTTCCGCGCCGCGGCAGGACATGTATCGTGCGCCG</td><td>37:T>G</td></tr>
<tr><td>1079000|F|0-35:G>T-35:G>T</td><td>TGCAGGGCCAGAGTCACCTGGGAGACAACGCCGAGGACCCCAAGGGAGACCTTGGCGGCGTCCAGGTCC</td><td>35:G>T</td></tr>
<tr><td>3026919|F|0-16:G>C-16:G>C</td><td>TGCAGCAGCTGTTCGTGGACATGTTCCAGGCCGACCTCGTCGCCG</td><td>16:G>C</td></tr>
<tr><td>1020841|F|0-48:A>C-48:A>C</td><td>TGCAGAGTCATGAGCGGAGCAGGATGATGAGGACCTGCGGCGAGCTACAAGTAGTAGTTCAGAATTCGG</td><td>48:A>C</td></tr>
<tr><td>3952211|F|0-16:C>T-16:C>T</td><td>TGCAGAGCCTGCTGCACAGGCTGGGGTCCGACCCG</td><td>16:C>T</td></tr>
<tr><td>1042898|F|0-50:G>A-50:G>A</td><td>TGCAGACTCTATGGGGTGTAAAACCTGCCCCAGGAGGAGAATGGGATCAAGGGAATTGCCCACTGCACA</td><td>50:G>A</td></tr>
<tr><td>7155201|F|0-8:G>A-8:G>A</td><td>TGCAGCTGGATGTTTACCAAGAACCACCG</td><td>8:G>A</td></tr>
<tr><td>3949250|F|0-15:G>C-15:G>C</td><td>TGCAGGCCGTTTCTCGTCGGCGAGGCCACCG</td><td>15:G>C</td></tr>
<tr><td>1047199|F|0-38:A>G-38:A>G</td><td>TGCAGCTTCTGCGTCGTCGCCTGCTCCGCCGAGAGATGATGGTGGTGGTCCACGACCG</td><td>38:A>G</td></tr>
<tr><td>4261115|F|0-15:G>C-15:G>C</td><td>TGCAGCCTGCCTCTTGTTTAATTGTAGTATCATGCACTGACCG</td><td>15:G>C</td></tr>
<tr><td>3950334|F|0-15:C>T-15:C>T</td><td>TGCAGGTAAAATAGCCGACGGCAGCAACCCG</td><td>15:C>T</td></tr>
<tr><td>1082662|F|0-22:G>T-22:G>T</td><td>TGCAGCTGAAGTGGACGGAAAAGGACGCGTCTTGAAAAGAAATCCTGGGCTTCTAGATCGACCGACCGA</td><td>22:G>T</td></tr>
<tr><td>978660|F|0-33:T>C-33:T>C</td><td>TGCAGTCAAAATATGCACATGCGTTGAGTTTTTTGACTTCATATTCTCCTATATTCTACCG</td><td>33:T>C</td></tr>
<tr><td>3940059|F|0-28:G>A-28:G>A</td><td>TGCAGGGCCTGTTCCTTGCGGACAATCAGCTGTCCG</td><td>28:G>A</td></tr>
<tr><td>1053523|F|0-38:G>A-38:G>A</td><td>TGCAGCTGGATCAGCGACGAGTAGGCGCTGAGAGGGTTGGCCATGAGCTGCTCGTGCGTGCCG</td><td>38:G>A</td></tr>
<tr><td>1053523|F|0-8:G>T-8:G>T</td><td>TGCAGCTGGATCAGCGACGAGTAGGCGCTGAGAGGGTTGGCCATGAGCTGCTCGTGCGTGCCG</td><td>8:G>T</td></tr>
<tr><td>1021039|F|0-12:T>C-12:T>C</td><td>TGCAGACTGAAGTTGTACCTTGACATGAAGTACTTGGGGTTGAGCGCAATAGCGCCAAAAACGGCAAAG</td><td>12:T>C</td></tr>
<tr><td>3952211|F|0-5:A>G-5:A>G</td><td>TGCAGAGCCTGCTGCACAGGCTGGGGTCCGACCCG</td><td>5:A>G</td></tr>
<tr><td>4261086|F|0-24:G>C-24:G>C</td><td>TGCAGAGCCTCGCCGACCTGGCGTGGCGCATGGGGCCG</td><td>24:G>C</td></tr>
<tr><td>4262100|F|0-16:G>C-16:G>C</td><td>TGCAGCAGAGTGCCTGGACGATGCGCGTGCGGTGCTCCG</td><td>16:G>C</td></tr>
<tr><td>3025079|F|0-9:G>T-9:G>T</td><td>TGCAGCTGGGGGCAGTGTCCATCCAGCATCAGGTCTCCG</td><td>9:G>T</td></tr>
<tr><td>1017338|F|0-13:T>G-13:T>G</td><td>TGCAGCCGAGGAGTATTTCATATTGGACTTGTGCCACTCACCTGAAACTCATTAGCAAGAGCAGCGAGA</td><td>13:T>G</td></tr>
<tr><td>1017338|F|0-7:G>A-7:G>A</td><td>TGCAGCCGAGGAGTATTTCATATTGGACTTGTGCCACTCACCTGAAACTCATTAGCAAGAGCAGCGAGA</td><td>7:G>A</td></tr>
<tr><td>1020841|F|0-39:G>T-39:G>T</td><td>TGCAGAGTCATGAGCGGAGCAGGATGATGAGGACCTGCGGCGAGCTACAAGTAGTAGTTCAGAATTCGG</td><td>39:G>T</td></tr>
<tr><td>3023022|F|0-31:G>A-31:G>A</td><td>TGCAGCTGCTCGATGCCGTCGCCGTGCCACAGTATCCG</td><td>31:G>A</td></tr>
<tr><td>6045752|F|0-20:G>A-20:G>A</td><td>TGCAGGGCGAGCAGTGGCTCGTCTTCTCCG</td><td>20:G>A</td></tr>
<tr><td>5369511|F|0-26:G>T-26:G>T</td><td>TGCAGCTTGCCCCTCCTCCATGGATGGCCTGCACCG</td><td>26:G>T</td></tr>
<tr><td>3029016|F|0-9:G>A-9:G>A</td><td>TGCAGTCGAGGTCGAACCCTCCGTGGCGTCCGCGAAGCTGCCG</td><td>9:G>A</td></tr>
<tr><td>3031017|F|0-13:C>T-13:C>T</td><td>TGCAGTCTGAAGCCGAAGCTCAGCGCCGCCAGTGCCTCTTTCCG</td><td>13:C>T</td></tr>
<tr><td>4262100|F|0-19:G>A-19:G>A</td><td>TGCAGCAGAGTGCCTGGACGATGCGCGTGCGGTGCTCCG</td><td>19:G>A</td></tr>
<tr><td>1014038|F|0-8:C>G-8:C>G</td><td>TGCAGCTACCTAGAGTCAACTCTTAATTTGTACTTAGCATAGGATCAAGTACGTTTCCG</td><td>8:C>G</td></tr>
<tr><td>1019198|F|0-47:G>T-47:G>T</td><td>TGCAGGCAATCGCCTTTGGGTGGACTGTGTGAGGGAGAAAGGGAAGAGCGGAAAGGTTGCCG</td><td>47:G>T</td></tr>
<tr><td>3020583|F|0-5:T>C-5:T>C</td><td>TGCAGTGCACCTGTCTAATTTGACTAATTGCCACCAGCATGGACCG</td><td>5:T>C</td></tr>
<tr><td>7167968|F|0-8:G>C-8:G>C</td><td>TGCAGCTGGCTCGCTTTTGCTTGCGCCG</td><td>8:G>C</td></tr>
<tr><td>1026148|F|0-31:G>C-31:G>C</td><td>TGCAGAACAAGGTGACCCTGGAGTGGCTTCTGGCCCTGGGTGACAAGCTCGGCATGGTCCGTGTGCCGA</td><td>31:G>C</td></tr>
<tr><td>15306648|F|0-25:T>C-25:T>C</td><td>TGCAGGATGCGCAGCGTGATTTCATTAGAGCATCGGTAGGAGTGAGCCG</td><td>25:T>C</td></tr>
<tr><td>982446|F|0-13:T>C-13:T>C</td><td>TGCAGCAATAGCATCATGCCTTCACATCATCGGCGAATTACCACCCAATAATTCGTCCG</td><td>13:T>C</td></tr>
<tr><td>3030313|F|0-17:C>A-17:C>A</td><td>TGCAGGTAGTGCCCAATCTGCACATGGGCTGGTGGTCGCCG</td><td>17:C>A</td></tr>
<tr><td>3941715|F|0-20:C>G-20:C>G</td><td>TGCAGAAGCAGAGGGGGTGGCGAATTGGAGCAGACAGCCG</td><td>20:C>G</td></tr>
<tr><td>1042371|F|0-16:T>C-16:T>C</td><td>TGCAGCTGCATCATCGTTCATTCATTCATTATTGAAGAACTAATCAGCCG</td><td>16:T>C</td></tr>
<tr><td>1131975|F|0-5:G>T-5:G>T</td><td>TGCAGGCCTTGGCGCTCTGGCCGTTGTGGGCTCGGAGGGTAAGGTCGAACGCCG</td><td>5:G>T</td></tr>
<tr><td>1082662|F|0-42:T>C-42:T>C</td><td>TGCAGCTGAAGTGGACGGAAAAGGACGCGTCTTGAAAAGAAATCCTGGGCTTCTAGATCGACCGACCGA</td><td>42:T>C</td></tr>
<tr><td>1019150|F|0-28:G>C-28:G>C</td><td>TGCAGTCGTGGTGCAGGTAGCAGAGCCCGCGCGCCGCCTCCAGCGCGATCCG</td><td>28:G>C</td></tr>
<tr><td>1862764|F|0-39:C>T-39:C>T</td><td>TGCAGACGCACTTGCAGCAACGGGTGGCCTTGAAGGGCACGCGCGAGATGATCTCGACGAGGAGGTCGT</td><td>39:C>T</td></tr>
<tr><td>1019161|F|0-44:C>T-44:C>T</td><td>TGCAGGAACACGCGGAAGCTGCGCAGCTCGTCGTGGATGACCGACTTGCTCCGCGCGTAGGCCG</td><td>44:C>T</td></tr>
<tr><td>3020664|F|0-33:C>T-33:C>T</td><td>TGCAGAACTTTGCGTACTCTTCGTGTTGAATTCCGTCCG</td><td>33:C>T</td></tr>
</table>

996255|F|0-39:G>A-39:G>A	TGCAGTGGGCGGCGCGGTCGGTTGCTGCTGGGCGATATAGTGGCGTACCG	39:G>A
1055737|F|0-42:C>T-42:C>T	TGCAGCTTCTGCCCCTTCTCCTCCGCAGCCTCCTTCGCTTTGCTAAGCTCACTACGTAGTTTGTCCGAG	42:C>T
1055737|F|0-29:C>T-29:C>T	TGCAGCTTCTGCCCCTTCTCCTCCGCAGCCTCCTTCGCTTTGCTAAGCTCACTACGTAGTTTGTCCGAG	29:C>T
3028428|F|0-12:A>G-12:A>G	TGCAGGTTCAAGAAGCACGGCTGCACCGAGACCGTCCG	12:A>G
3028428|F|0-13:A>T-13:A>T	TGCAGGTTCAAGAAGCACGGCTGCACGGAGACCGTCCG	13:A>T
1022232|F|0-58:G>A-58:G>A	TGCAGATGACCCTGTACGAGCTGGTGATGATCTGCCACGACGAGACGGGTCGGCTGCTGAACCG	58:G>A
3031451|F|0-8:A>T-8:A>T	TGCAGGATAAGCAATGATGCGTACGCGTATTCAACCG	8:A>T
3024533|F|0-13:T>C-13:T>C	TGCAGGTGCACATTGTGTACATGGGGGAGCTGCACCCG	13:T>C
1075505|F|0-44:A>C-44:A>C	TGCAGTCGGTCTGGAACGGCGCCGCGTACCACGGCGTGCACCACATGCCGCGCGGCGTCCG	44:A>C
1043008|F|0-31:A>G-31:A>G	TGCAGGTGCGGTATACCTCACCGCGCATCACATCAACTGGCCCATGATCATGTGCATAGCGATCCGAGA	31:A>G
1014880|F|0-19:G>C-19:G>C	TGCAGATCGCCTCCGAGGCGCGCCTCTCCGTGAAGCAGCTCTGCAAGGCGCTGATCCAGCAGGTGGAGG	19:G>C
1080336|F|0-22:G>A-22:G>A	TGCAGGGCGAAAGAACAGAGGAGTAGGACAGAGCAATTATCTGTCAACCG	22:G>A
4733892|F|0-39:C>T-39:C>T	TGCAGGATGACTGGAACTGAACATTTTGATATATGGATACTCCG	39:C>T
1075974|F|0-13:T>C-13:T>C	TGCAGTTTCCAAATCATTTTCGGTGTACTCCTGCGCGCCGTTCAGGAACAGGTCGCCTTCAGACCGAGA	13:T>C
2243720|F|0-27:A>G-27:A>G	TGCAGCCTTCATTCCAATATCGAACACATCCCCCAATCTCTCCTGCAACGCACCACTCTGAGCATCAGA	27:A>G
1020392|F|0-11:C>T-11:C>T	TGCAGCTCCAGCTCCGCCTTGAGCCACTTGCAGAACCTGCCGAGGTTGCCGTTGTTGTGGTAGCCAATG	11:C>T
1079188|F|0-68:C>T-68:C>T	TGCAGCTGGTGAACTCTCGGCTCCAAGTAACCCAGCACCCGCAGCTTCGGCGCGTCAGCAATGCTGACC	68:C>T
1020063|F|0-33:C>A-33:C>A	TGCAGGAGCTCCCTGAGGATCTCCAGGTCGCCGCCCCTCGCCGCGGCGTGCATTGCCCG	33:C>A
3946249|F|0-11:G>A-11:G>A	TGCAGAGGAGGGAGGAGATCCGCCGCCACTGCACCG	11:G>A
1082662|F|0-33:G>C-33:G>C	TGCAGCTGAAGTGGACGGAAAAGGACGCGTCTTGAAAAGAAATCCTGGGCTTCTAGATCGACCGACCGA	33:G>C
1082662|F|0-14:A>G-14:A>G	TGCAGCTGAAGTGGACGGAAAAGGACGCGTCTTGAAAAGAAATCCTGGGCTTCTAGATCGACCGACCGA	14:A>G
1020841|F|0-56:G>A-56:G>A	TGCAGAGTCATGAGCGGAGCAGGATGATGAGGACCTGCGGCGAGCTACAAGTAGTAGTTCAGAATTCGG	56:G>A
1024820|F|0-51:A>G-51:A>G	TGCAGCTGGGGAGGCAGCGGGCCAAGACCAAGGTGATCAGGAAGTCGGCCAACCCCGTGTGGGACGAGG	51:A>G
1016849|F|0-17:C>T-17:C>T	TGCAGTACCAGATACGGCTTCCCTTCAGTTGTTCGGCATATGTGAATTTGGTGGAATGGGATGCCAAGA	17:C>T
3936511|F|0-11:C>T-11:C>T	TGCAGGCTCACCCCGATCGAGTTCCGTAATCCGCCG	11:C>T
4262185|F|0-6:A>T-6:A>T	TGCAGAATTGCAAGGTCGTCTTTCATCCG	6:A>T
6026367|F|0-18:C>T-18:C>T	TGCAGACAAAATACACGACGACAGTATGAGACAGACGCAGCAACAACACTACTACTACGTGGCTACATG	18:C>T
5369353|F|0-37:C>T-37:C>T	TGCAGCAGCCGCTTCTCCTGGAGCTCCTGGGGCAGGACGGCCG	37:C>T
4411296|F|0-22:G>T-22:G>T	TGCAGGCACCGCGCCAACACCCGCTTGGCCTCCTCCTGGCGGTGCTCCG	22:G>T
3027174|F|0-9:T>G-9:T>G	TGCAGCGCCTTACGCCCGTCCCCGTGCAACGCCAAGCCG	9:T>G
1059315|F|0-49:C>T-49:C>T	TGCAGGTGGCGGAGCAGCGAGTCCTCGTCCCAGGAGAAGGTCCAGGGCTCCTCCG	49:C>T
2249018|F|0-5:C>G-5:C>G	TGCAGCAGCTTGGTGGTGTCGATGGCGCCCAGCTTCTCGATGTAGATTGGCCG	5:C>G
1043008|F|0-46:A>G-46:A>G	TGCAGGTGCGGTATACCTCACCGCGCATCACATCAACTGGCCCATGATCATGTGCATAGCGATCCGAGA	46:A>G
3946415|F|0-20:C>T-20:C>T	TGCAGTTCATCGCAAGTTTCCAACGCCG	20:C>T
3025367|F|0-17:T>A-17:T>A	TGCAGGGAGCTGCTCCCTTCCGATGATCCCAAGCTCGCCG	17:T>A
5582634|F|0-22:G>C-22:G>C	TGCAGAAGAAGTCGACGACGAGGACAGCCG	22:G>C
1019567|F|0-44:A>T-44:A>T	TGCAGCTTTCCTGGCTTTCTTGGTGATGCTGACGTTCCAGTGACAGTGCGACGGCCG	44:A>T
990605|F|0-53:G>C-53:G>C	TGCAGCAGGGAGACGGTGTTGGGGAGCTGGAACGACACGTTGTTCATGCTCGCGCCGAACCG	53:G>C
4262347|F|0-40:C>G-40:C>G	TGCAGCTCTCGCTGGACCTGATCGAGGAGTGGAGCAAGGCCCACCCG	40:C>G
4261116|F|0-6:G>A-6:G>A	TGCAGCGTCCTAGTCTTCGACCATGCGAGCCCG	6:G>A
1003407|F|0-55:G>A-55:G>A	TGCAGGGCAGCAGTTACGTGGAGTTGCGTTGACGCGACCCGTCGATTGGCGATGGGGCTTGGCGTCCGA	55:G>A
6049285|F|0-8:T>C-8:T>C	TGCAGGTATGGGAGGCTGTTCAGGACGCACATCCTTGGCTGCCCCACGGTGGTGTGCATGGACCCGAGA	8:T>C
3937731|F|0-14:G>A-14:G>A	TGCAGGTCGATTGCGCCGCCGCACAAAGTTCACCG	14:G>A
3947392|F|0-16:G>C-16:G>C	TGCAGAATTCCAACCTGAAGATGCAAATCTGTCGGCGCAAACAATCCG	16:G>C
1075505|F|0-5:T>G-5:T>G	TGCAGTCGGTCTGGAACGGCGCCGCGTACCACGGCGTGCACCACATGCCGCGCGGCGTCCG	5:T>G
4411242|F|0-18:A>G-18:A>G	TGCAGGTCGCGGGTTCGAATCGCGCTCGCGCGCGTTTTTTAGTTCCG	18:A>G
1003806|F|0-35:G>A-35:G>A	TGCAGCCACATGTACCACCAGGACTGCATCCTGCCGTGGCTCGCGCTGCGCAACTCGTGCCCCGTGTGC	35:G>A
1018334|F|0-28:C>G-28:C>G	TGCAGCAGTTCTCGGCGATGAACAAGTTCAAGAAGAAGGCGCTGGGCGTGGTGGCGCGGAACATGCCGA	28:C>G
3955155|F|0-10:G>T-10:G>T	TGCAGGACACGTACCTCCCTCGCAATCCG	10:G>T
4540079|F|0-11:G>C-11:G>C	TGCAGCTGGCCGTGCTTGAGAAGGCGGCGAGCCG	11:G>C
3956664|F|0-13:G>T-13:G>T	TGCAGCTGTAGAAGGTCGACAGGTCGGCGCTCGACCG	13:G>T
3028630|F|0-9:C>G-9:C>G	TGCAGGTAACCGAGGCCTCGAGCGCACTGGTCGTGTACGAAGACTGGGACGACGCCG	9:C>G
3958635|F|0-21:G>A-21:G>A	TGCAGTCTAGAGCCCAGTTGTGGCCGCCGACCTTAAAGGCGCCG	21:G>A
3026562|F|0-14:T>C-14:T>C	TGCAGTTTGCAATGTGTTTTCCGCGGGGCAACTCGGTCCG	14:T>C
4261836|F|0-22:C>T-22:C>T	TGCAGCGCATCAGCAGCGTCGACAGCCCG	22:C>T
3934581|F|0-24:A>G-24:A>G	TGCAGCTCCTGTATCTCGTCCAGCACCTCCG	24:A>G
1042371|F|0-23:A>G-23:A>G	TGCAGCTGCATCATCGTTCATTCATTCATTATTGAAGAACTAATCAGCCG	23:A>G
1019161|F|0-22:G>T-22:G>T	TGCAGGAACACGCGGAAGCTGCGCAGCTCGTCGTGGATGACCGACTTGCTCCGCGCGTAGGCCG	22:G>T
4405420|F|0-16:T>G-16:T>G	TGCAGACAAGGTTTTGTGTAGAGAAAGGATTGGCCG	16:T>G

2246456|F|0-33:G>A-33:G>A TGCAGCTGCCCGTCCACCAGCTTGAACCTGCCTGCCATCCCGTCGAACGTCGTGTTGAGAATCGCCCTA 33:G>A

3026562|F|0-31:C>T-31:C>T TGCAGTTTGCAATGTGTTTTCCGCGGGGCAACTCGGTCCG 31:C>T

1047903|F|0-11:G>A-11:G>A TGCAGCATTATGTCCGCGATCTCTTTGACTTCCAGCTTGGTCAGCTGTCTGAAGACGATCATCTCGTCC 11:G>A

4405248|F|0-6:G>A-6:G>A TGCAGCGGACGGAACGGTAAACATCTCGGCCG 6:G>A

3026521|F|0-31:T>C-31:T>C TGCAGCTAGCGAGCACATGCATGCGCCGAACTAACAACAGCCG 31:T>C

1079188|F|0-7:G>C-7:G>C TGCAGCTGGTGAACTCTCGGCTCCAAGTAACCCAGCACCCGCAGCTTCGGCGCGTCAGCAATGCTGACC 7:G>C

1083636|F|0-5:C>T-5:C>T TGCAGCGACATGTCCCTGGACGAGATGGACGCCGCGGTGAGGAGGATGGAGTTCGTCGTCTTCGACCGA 5:C>T

3955925|F|0-41:G>C-41:G>C TGCAGCTCCTCCACCTCGGCGCAGATGGCGTTCTTCTTGAAGACCCG 41:G>C

1024820|F|0-7:G>C-7:G>C TGCAGCTGGGGAGGCAGCGGGCCAAGACCAAGGTGATCAGGAAGTCGGCCAACCCCGTGTGGGACGAGG 7:G>C

999436|F|0-33:G>A-33:G>A TGCAGCCGTTCCTCAAGTTCCTGTCGGCGACGAGGGCCCCGTTCCTCATCAACTGCTACCCGTTCTTCG 33:G>A

3940094|F|0-12:G>C-12:G>C TGCAGACGACGGGTCGGCGTCGTTGCGTGTATTCTACGGCCACCG 12:G>C

4261131|F|0-19:C>T-19:C>T TGCAGAGAAACAGAGAATTCAGCATGAGACCG 19:C>T

1016849|F|0-29:T>C-29:T>C TGCAGTACCAGATACGGCTTCCCTTCAGTTGTTCGGCATATGTGAATTTGGTGGAATGGGATGCCAAGA 29:T>C

4397115|F|0-9:T>G-9:T>G TGCAGCTCGTAGGAGGTGCTGGTGATGAGCGCGTCGGCACCG 9:T>G

1017445|F|0-31:C>T-31:C>T TGCAGCACATATACCGCGAGGGCAACCAGGTCGCCCACGTCCTGTGCAAAGAAGCATACCAGCGACCGA 31:C>T

3026562|F|0-12:T>C-12:T>C TGCAGTTTGCAATGTGTTTTCCGCGGGGCAACTCGGTCCG 12:T>C

1022281|F|0-16:C>G-16:C>G TGCAGTCGCAGAGCCTCATCATGCCGCTGCTCCTGTCGTCCGTGGCCACGCTGGCGCTCCACGTCCCCC 16:C>G

1023336|F|0-26:C>T-26:C>T TGCAGCTGCGGGCTGCTGAACAGCTTCATCGGCACCGTGCCGTAGAGTCTTCCG 26:C>T

1711331|F|0-12:A>T-12:A>T TGCAGGGCACGCAGCTTCGTAATTCGGCTCGTGCCGTTTACATGCAGACCGTGGACCG 12:A>T

7172245|F|0-14:G>C-14:G>C TGCAGGTTGTCGGCGAGCTCAGCGATGTCGACGGCGATGCTCTCCTCCGTGCGCGCCG 14:G>C

32025870|F|0-41:C>G-41:C>G TGCAGGTGCCGCAGCGGCGGCGCGCTGGCCTTGCCGAGGTGCCCCAGCTCCG 41:C>G

5004074|F|0-8:G>C-8:G>C TGCAGCGCGGAGCTGGACGACAAAGGGAAGCAGCAGCACAAGGTGACCG 8:G>C

1024183|F|0-16:A>G-16:A>G TGCAGGAGCCTGGTGCACTGTTGTTTGGGCCCTCCACGGGAAGGGGAAGCCG 16:A>G

1020850|F|0-41:A>G-41:A>G TGCAGAACGGGCCGCAGTTCCACCTGTCCTTCACGCGGTGCATGGACGTGAAGGCCAACTTCCTCCGAG 41:A>G

100504565|F|0-16:G>A-16:G>A TGCAGTAGAGGTAGATGGAGAGATACTTGCCATTGTTGGCTTTCACCCACCCACGCGGGTAGCTATGTA 16:G>A

5008993|F|0-18:T>C-18:T>C TGCAGGCTTATTCGGCCGTTGCGCCCTACTACGCCCAGGGCTACCAGGCCGTGCAAGGTACGTGAGAAA 18:T>C

5361531|F|0-40:T>C-40:T>C TGCAGGGATTCAGGAAGATAGTGGCCGACAGATGGGAGTTTGCCAATGAGTTCTTCAGGAAGGGAGCAA 40:T>C

5361492|F|0-30:A>G-30:A>G TGCAGGCTCCGAGGTTGTTGGCCCAGACCCAACGCCGAGTACTTTCCGACGACGATGATATGACCGACG 30:A>G

5340921|F|0-16:A>C-16:A>C TGCAGGTCCGTTTATCATCCAAACAGACCGTTCCAATGTGATAAAATTGTTGAAGGATACAAAGCCGAG 16:A>C

7911721|F|0-8:T>C-8:T>C TGCAGGCATTTTTCGCTCAGAGAGCGACTAGCCATGTCACATCATTCATTTGATTTGAAATTAAAGCAA 8:T>C

7919618|F|0-13:G>A-13:G>A TGCAGACACGCGCGCAAGCGGCGTGGCACTGGCACTGAACCAACGAAGCAAACGTCGCAGTTCGGTGGT 13:G>A

5358377|F|0-20:A>G-20:A>G TGCAGGGGTCAAATCCATCCATGTTGTGTTGGTGGTGAGTGACCGACCG 20:A>G

1204226|F|0-9:A>G-9:A>G TGCAGATCCATAGTCTCCAAGCTGCACTACTACCTGCCCCTCTTGGGCTCCTTCCACAACTTCCTCCGA 9:A>G

6031044|F|0-9:G>C-9:G>C TGCAGTTGCGAAATGCAAGCTGTGAGCCAGCCGAGCCAGGGAAACCG 9:G>C

5361278|F|0-40:G>C-40:G>C TGCAGCTTACAAAGCGAGCGTGGAAGGTTTCGTCTCCATGGATGATGCATGATGTCAGCTCAGTAGCGC 40:G>C

5007530|F|0-63:A>G-63:A>G TGCAGTGACCACCCCACCACCGACTGACCAGCCAACACCAGCCCGAGGAAAGGGAAGGCAATGAAGAAA 63:A>G

7916093|F|0-37:A>C-37:A>C TGCAGAGGGAGGGCTGTGTAGTACGTGCTGGAGCATTATGTACCG 37:A>C

12774909|F|0-10:T>C-10:T>C TGCAGTTTCATACTGAAAATCTTCATGGGATCATTACTTGAGACTTGTTCCTCCCAGGCCGCCTTCACT 10:T>C

5005709|F|0-8:T>C-8:T>C TGCAGCGGTAGCAAGATGGAGCGCAAGGACGTGGAGAAGAACCG 8:T>C

16796240|F|0-7:A>G-7:A>G TGCAGGAAGAGGACGACTGCGACCCCACCGCCAGTCGTCGTGGTCTTCTCAGCAACGGCCAGCTCGCGG 7:A>G

3571391|F|0-33:T>C-33:T>C TGCAGCCATATATGTCTCCTCCTGCTTTGTTGTTGCGTGCCG 33:T>C

1018066|F|0-27:T>G-27:T>G TGCAGGCCAAGGATTTTGCCGACCTGTTCAACCGCAATAGGCCGATAGGCGGAGGAAGGGGCAGCCAAC 27:T>G

1728727|F|0-14:G>A-14:G>A TGCAGCCCTCCGCCGTGACCCGCGTGAGGTCTCCTACGTTGGGGACACTGCAGCCG 14:G>A

1059512|F|0-19:T>A-19:T>A TGCAGAATCATCCTTGCTTTAACTTTCATTGTAGAACCCATTTCTTCCCCCAGACAAGCAAACCAATTC 19:T>A

100399363|F|0-11:A>C-11:A>C TGCAGCGTGAGAGGGCAGCTGAGCTCCAGCCCACGGTTGTTGCCGAGGCCGAGGGAGATAGCCCCGAGA 11:A>C

5361834|F|0-14:C>T-14:C>T TGCAGTGTCCTGGACGAGGTACCTAAAGAACAAATTGCGAGTTGTTTATGAACAAATTGAAGAGCAATC 14:C>T

100558139|F|0-48:T>G-48:T>G TGCAGGCGCTCCCATCAGAATTAAGAAAACAAACAAGCTCAGTCGTCGTCGTATTCAACTCCG 48:T>G

100363102|F|0-54:A>C-54:A>C TGCAGAGGCTCAAGGCCAAGCTCTACACCATGATCGAAGAGGTTTGCATAGCTCAATGGCTAATTAACA 54:A>C

100487904|F|0-23:G>A-23:G>A TGCAGCTTGATTGTGGGTGAGCGGCATTAATACTGACTGGCTAAGTGGGGGAGGGGATTATTGACCGAG 23:G>A

1728781|F|0-42:G>C-42:G>C TGCAGCAACATCCTTCTTCAGTCTGGTGGAACACTCATGCATGGTATGCTCAGGTTGCCACGCAAATGG 42:G>C

100542089|F|0-12:G>A-12:G>A TGCAGAAGCAGAGTCCATGGCGTCGTCGTCGCAGCCGCAGCGGGGTGATGCCGTCGACGGTAAGGTCCG 12:G>A

6049592|F|0-6:T>A-6:T>A TGCAGCTGTATCCTACGGGTTCGTCGCTCTCTGCATTTCGTGCCATCCG 6:T>A

1726646|F|0-9:A>G-9:A>G TGCAGCTCCAACCCGTCGCGGGAGTTCTCCTTCCGAACTCCCCCGTCAGGCCTCCCCTTCACCTTCGCC 9:A>G

100601631|F|0-29:G>C-29:G>C TGCAGCTACGCGGTGCTGATGGACTCGTCGAACTTCACCTTCTCGACGACCTACGCGACCTCGCCGAGA 29:G>C

100539017|F|0-6:A>G-6:A>G TGCAGCAGGACGGTGCGCCCTGCCCGAGAAGCTTCGTGAGTAAGCGAAGGCTTTCGCTGCTGTTGGTGT 6:A>G

100550458|F|0-25:A>T-25:A>T TGCAGCCAAGTCGCTGGTGCATTTCAGGATCTCAACAGTAAGGTTTTTGTGGCCAGTAGCTCGGTTTGC 25:A>T

22839762|F|0-53:G>A-53:G>A TGCAGAAGAGAAAGAAGTTCAGTGGCGGCCATGGAAAAACCCTAATCTCTTCAGGAATCGAGACGGCCG 53:G>A

3026955|F|0-28:A>C-28:A>C TGCAGTTCTTGAGATCATGGTCCCCAAAAGGGGAAACGAGCCG 28:A>C

100426449|F|0-38:A>G-38:A>G TGCAGTGAGGCCAAAAGCAAGGCTACTCGCTAAGAGTAAAACCCGCAATGGAGCTGACATATAAGCAAG 38:A>G

<table>
<tr><td>100526756|F|0-7:G>C-7:G>C</td><td>TGCAGGCGCTCATGTACGGGGGCATCATGTTCCTCATCTACAGCATCCTTGTGCTGGTCGCGCTTCTCG</td><td>7:G>C</td></tr>
<tr><td>4996644|F|0-65:T>G-65:T>G</td><td>TGCAGAAACCAGTCGAACCAGTAGCTAAAACCATCGCATAGAGCTCCCCGCAAGAAACAGCAACGTCTG</td><td>65:T>G</td></tr>
<tr><td>100558179|F|0-40:A>G-40:A>G</td><td>TGCAGGCGGTCGAGCTCCAGCAGCGCGGCGGCAGGTGGGCACCGATGCAGCAGTCGTGGGGCGCGGTGT</td><td>40:A>G</td></tr>
<tr><td>4989271|F|0-14:A>G-14:A>G</td><td>TGCAGCCTTTTTCAATTGCTGAGACCGTCATCGTATGCATGCCCG</td><td>14:A>G</td></tr>
<tr><td>100496104|F|0-6:G>A-6:G>A</td><td>TGCAGGGAGCGGTGGGCGGAGTCGGACCAGCTTTTGGCCCG</td><td>6:G>A</td></tr>
<tr><td>5003755|F|0-41:G>A-41:G>A</td><td>TGCAGAATATGTTTGTTCCTTGTAGCCTGCCCAGACCCTCCGCCTGAGGCCG</td><td>41:G>A</td></tr>
<tr><td>4989952|F|0-32:G>A-32:G>A</td><td>TGCAGAGTTGGGGAGGAGTTGTCGACGGCGTCGGCGGCAGCCG</td><td>32:G>A</td></tr>
<tr><td>100576108|F|0-43:T>C-43:T>C</td><td>TGCAGATGCTGACAGAGGGGCCATGGGAAGAACACGATGAGATTGACCTCGAATTCCTGGGGAACAGCT</td><td>43:T>C</td></tr>
<tr><td>100577620|F|0-9:C>A-9:C>A</td><td>TGCAGAAGCCGAACATGGAGGAGTCTCACTCTCGCGCCGAGGTAGATTAATCCGACCG</td><td>9:C>A</td></tr>
<tr><td>1019198|F|0-11:G>A-11:G>A</td><td>TGCAGGCAATCGCCTTTGGGTGGACTGTGTGAGGGAGAAAGGGAAGAGCGGAAAGGTTGCCG</td><td>11:G>A</td></tr>
<tr><td>3020664|F|0-18:C>A-18:C>A</td><td>TGCAGAACTTTGCGTACTCTTCGTGCTGAATTCCGTCCG</td><td>18:C>A</td></tr>
<tr><td>1697029|F|0-9:G>A-9:G>A</td><td>TGCAGTGTAGAGACTCTTTCTTCCATCATAGGCAGGCAATTTGCCCCCGAGGGATGTCTTCCCGTGCAA</td><td>9:G>A</td></tr>
<tr><td>992807|F|0-17:C>T-17:C>T</td><td>TGCAGGCCCCGCGTTGGCGGCGGCGGAGGCGGGGGAGGAGCGGCGCTGATCCGCCG</td><td>17:C>T</td></tr>
<tr><td>1667357|F|0-33:C>A-33:C>A</td><td>TGCAGCAACAATGCTAAACATCGAACTACACTCCTCTGCTACATGCTACTGGTTCATTCCG</td><td>33:C>A</td></tr>
<tr><td>1396505|F|0-63:A>T-63:A>T</td><td>TGCAGTCGCACTTGGCTGCATCGCAACCCGCAGCAGTTCAACACTTACACAGCCATACACATCACCTGT</td><td>63:A>T</td></tr>
<tr><td>1666763|F|0-10:G>A-10:G>A</td><td>TGCAGGTGTTGGCGCCGCCCGTGCAGTCGAGGACTTGTTGCTCCGACATGGAGATGAGGTTGCCGAGAT</td><td>10:G>A</td></tr>
<tr><td>1023336|F|0-24:T>C-24:T>C</td><td>TGCAGCTGCGGGCTGCTGAACAGCTTCATCGGCACCGTGCCGTAGAGTCTTCCG</td><td>24:T>C</td></tr>
<tr><td>5363344|F|0-14:C>G-14:C>G</td><td>TGCAGCCAAGAGAGCTATCGCCGTTTTGGCCCG</td><td>14:C>G</td></tr>
<tr><td>4396872|F|0-13:C>G-13:C>G</td><td>TGCAGAGGCTCATCTGGGTGAAGAGGCCG</td><td>13:C>G</td></tr>
<tr><td>1713513|F|0-41:G>C-41:G>C</td><td>TGCAGGTAGATCAGCTCGATGTCTAGTGGCTCCAGCTTGCAGCCGATGCTGAGCCG</td><td>41:G>C</td></tr>
<tr><td>3940094|F|0-7:G>A-7:G>A</td><td>TGCAGACGACGGGTCGGCGTCGTTGCGTGTATTCTACGGCCACCG</td><td>7:G>A</td></tr>
<tr><td>7173327|F|0-17:C>T-17:C>T</td><td>TGCAGTCCGCGAGGCCGCCGTCGGGGGAGGCCGCGGCGTGGTGCGCCG</td><td>17:C>T</td></tr>
<tr><td>4262296|F|0-22:G>A-22:G>A</td><td>TGCAGCCGCCTCCTCCTCCGCTGCATCAACCG</td><td>22:G>A</td></tr>
<tr><td>3957157|F|0-29:G>C-29:G>C</td><td>TGCAGAATTCGCTGGAGGTGGACCTCCTCGACCG</td><td>29:G>C</td></tr>
<tr><td>3021131|F|0-18:T>A-18:T>A</td><td>TGCAGATGTTTCCTCTCCTGTCAGTCCTCTTGATGTGCTCCG</td><td>18:T>A</td></tr>
<tr><td>2246456|F|0-20:C>T-20:C>T</td><td>TGCAGCTGCCCGTCCACCAGCTTGAACCTGCCTGCCATCCCGTCGAACGTCGTGTTGAGAATCGCCCTA</td><td>20:C>T</td></tr>
<tr><td>1018962|F|0-11:T>C-11:T>C</td><td>TGCAGCCGCTGTGCCATGATCACGTTCCCGCCGACAGTCGCCGTGTTCCG</td><td>11:T>C</td></tr>
<tr><td>3953965|F|0-44:A>G-44:A>G</td><td>TGCAGACGTTCGACGCGGCGGTGGCAGACATCACCATGACGGCGAACCG</td><td>44:A>G</td></tr>
<tr><td>1089029|F|0-22:A>G-22:A>G</td><td>TGCAGGAATTCCGAGTCACGCAAGCAAGGGCGAAAGGAAGAGCACTGACCG</td><td>22:A>G</td></tr>
<tr><td>1149490|F|0-20:G>A-20:G>A</td><td>TGCAGCTCGTCGCGTGGGTCGGCGACGACCACGAGCGCTTCCTCTCCAAGTTCAAGGACCGCCTCGACC</td><td>20:G>A</td></tr>
<tr><td>5580340|F|0-10:C>G-10:C>G</td><td>TGCAGCTTCTCGCAAGAACCGAGCTCCG</td><td>10:C>G</td></tr>
<tr><td>3946372|F|0-11:G>C-11:G>C</td><td>TGCAGCCTCCCGAGCTCGGGCGGTATCGGCCCG</td><td>11:G>C</td></tr>
<tr><td>5580153|F|0-21:C>G-21:C>G</td><td>TGCAGCACCGTCTTCAGCCCCCCGCAGCAGTCCG</td><td>21:C>G</td></tr>
<tr><td>5581548|F|0-6:T>A-6:T>A</td><td>TGCAGGTGCCACATAGTTTAGAGCAGCCG</td><td>6:T>A</td></tr>
<tr><td>1017398|F|0-27:C>A-27:C>A</td><td>TGCAGTGTTATGGCCTATGGTGCAGGGCAAAGACGACAAGGACGACACGCGGCGGCGACGTGGCCATGA</td><td>27:C>A</td></tr>
<tr><td>1059094|F|0-46:G>C-46:G>C</td><td>TGCAGTCCAAAAGGAGAGCGATGTTGTATACAAGAGCAGTGGTCAAGATCCG</td><td>46:G>C</td></tr>
<tr><td>1117513|F|0-18:A>T-18:A>T</td><td>TGCAGTGCCTCAATGATCAGATGGGGGAAGACGACGTAAGCCTGGTGGCCATGAGCACCAGCGACGGCG</td><td>18:A>T</td></tr>
<tr><td>1202456|F|0-24:A>G-24:A>G</td><td>TGCAGCCGAGGCAAACGGCGGCTGACGGACCTCTTGTGATACGGCCAACAAGGCTGTTCACGAAGACGC</td><td>24:A>G</td></tr>
<tr><td>3064722|F|0-15:C>A-15:C>A</td><td>TGCAGAACACCTTCACCCCGTCGCTGACGGTGGCCTCCG</td><td>15:C>A</td></tr>
<tr><td>3020372|F|0-12:C>T-12:C>T</td><td>TGCAGGTGTTGTCACACATGATACATGACATATACCCTACCCG</td><td>12:C>T</td></tr>
<tr><td>3024831|F|0-28:A>T-28:A>T</td><td>TGCAGCCACTGTGAAACGGCGCTGTGGGAACCATTAAACTCCG</td><td>28:A>T</td></tr>
<tr><td>1014880|F|0-13:C>G-13:C>G</td><td>TGCAGATCGCCTCCGAGGCGCGCCTCTCCGTGAAGCAGCTCTGCAAGGCGCTGATCCAGCAGGTGGAGG</td><td>13:C>G</td></tr>
<tr><td>985213|F|0-14:G>A-14:G>A</td><td>TGCAGCTTGGAGACGATGGATGTGAGATGTAAGTTGCCTTGGCAGTAGGAGATGAGGCGCGCGATCTGA</td><td>14:G>A</td></tr>
<tr><td>1018832|F|0-5:A>G-5:A>G</td><td>TGCAGACTTGCGTGATGAATGAATGGAGTGCTGCGTGGTGGATGGATGGGTGCTGGAGGTGTTATGCGA</td><td>5:A>G</td></tr>
<tr><td>3955925|F|0-29:G>A-29:G>A</td><td>TGCAGCTCCTCCACCTCGGCGCAGATGGCGTTCTTCTTGAAGACCCG</td><td>29:G>A</td></tr>
<tr><td>1022232|F|0-13:G>T-13:G>T</td><td>TGCAGATGACCCTGTACGAGCTGGTGATGATCTGCCACGACGAGACGGGTCGGCTGCTGAACCG</td><td>13:G>T</td></tr>
<tr><td>1022232|F|0-11:C>T-11:C>T</td><td>TGCAGATGACCCTGTACGAGCTGGTGATGATCTGCCACGACGAGACGGGTCGGCTGCTGAACCG</td><td>11:C>T</td></tr>
<tr><td>3934101|F|0-25:T>A-25:T>A</td><td>TGCAGGCCACCCAAATCCGCACCAATTTGGGCGGAAAATTGCACCG</td><td>25:T>A</td></tr>
<tr><td>3952914|F|0-18:A>T-18:A>T</td><td>TGCAGGGGCGGACCCAGGACAAAAATGACCCG</td><td>18:A>T</td></tr>
<tr><td>1063533|F|0-12:G>A-12:G>A</td><td>TGCAGGGATTACGACGTGCTGAACCACGAGCTGCTCCAGACGCTGGTGGAGAAAGTCCG</td><td>12:G>A</td></tr>
<tr><td>3024780|F|0-6:G>A-6:G>A</td><td>TGCAGCGATTTGAATTCCCGATGATACGGGTGGTGTTCATCCG</td><td>6:G>A</td></tr>
<tr><td>3957157|F|0-19:G>C-19:G>C</td><td>TGCAGAATTCGCTGGAGGTGGACCTCCTCGACCG</td><td>19:G>C</td></tr>
<tr><td>1039493|F|0-68:A>T-68:A>T</td><td>TGCAGCTTTTTGACTGATAACTTTACTACATGGCCAAATCAATCAGCAGTGCTAACTCAGCTACACATA</td><td>68:A>T</td></tr>
<tr><td>1019241|F|0-20:G>T-20:G>T</td><td>TGCAGCTTCTTGGTGTCCGTGGTGCTCCAGGAGATGAGGTGGAACAGCCG</td><td>20:G>T</td></tr>
<tr><td>3942260|F|0-17:G>A-17:G>A</td><td>TGCAGCCCGATCTTCGCGGAGATCGTGCCG</td><td>17:G>A</td></tr>
<tr><td>1079000|F|0-30:C>G-30:C>G</td><td>TGCAGGGCCAGAGTCACCTGGGAGACAACGCCGAGGACCCCAAGGGAGACCTTGGCGGCGTCCAGGTCC</td><td>30:C>G</td></tr>
<tr><td>1065045|F|0-53:G>T-53:G>T</td><td>TGCAGGTGATTGAGCATGTGGAGGAGGACCAAGCAAGTTTATTCGGCAACGTTGTATTGAGCTCATTCT</td><td>53:G>T</td></tr>
<tr><td>1020841|F|0-14:C>T-14:C>T</td><td>TGCAGAGTCATGAGCGGAGCAGGATGATGAGGACCTGCGGCGAGCTACAAGTAGTAGTTCAGAATTCGG</td><td>14:C>T</td></tr>
<tr><td>1667357|F|0-16:A>T-16:A>T</td><td>TGCAGCAACAATGCTAACCATCGAACTACACTCCTCTGCTACATGCTACTGGTTCATTCCG</td><td>16:A>T</td></tr>
</table>

3020372|F|0-19:G>T-19:G>T	TGCAGGTGTTGTCACACATGATACATGACATTTACCCTACCCG	19:G>T
3572070|F|0-18:C>G-18:C>G	TGCAGAGCCTTGAGATTGCACCAGCAGTCCG	18:C>G
3020664|F|0-15:A>G-15:A>G	TGCAGAACTTTGCGTACTCTTCGTGTTGAATTCCGTCCG	15:A>G
3020784|F|0-32:A>T-32:A>T	TGCAGCTCAATCTCCGATGCAATACAGCCAGCATGTAGCCG	32:A>T
2242013|F|0-17:G>C-17:G>C	TGCAGCTAGTGTTGCGTGCTCAGTCAGTCAGTCAGCCATCGGCGCCGTCGATGCCTTGCCTAGCTTATA	17:G>C
6048820|F|0-57:T>C-57:T>C	TGCAGTGTCTACACACCACATGTGGCTGAATGCAATCGTTTTCTAGGAGACGTGAGTTGACCG	57:T>C
3941931|F|0-33:T>C-33:T>C	TGCAGGCAACGCTGGCCAGTGGCTACGTTTAATTTGTACACCG	33:T>C
2245281|F|0-37:C>T-37:C>T	TGCAGGCGGCTCTTGAAGCTGGGGTCTGAAATTTGATCCCAGAACTCACGCAGCTCCGCCTTGTCGATG	37:C>T
1862465|F|0-24:T>C-24:T>C	TGCAGAAGCTCCTCCTGGAAGCCGTCGGCTGGGAAGTCGACGATGGACCAGGTGCCTTCGTCGGCGTTG	24:T>C
2242013|F|0-9:T>C-9:T>C	TGCAGCTAGTGTTGCGTGCTCAGTCAGTCAGTCAGCCATCGGCGCCGTCGATGCCTTGCCTAGCTTATA	9:T>C
3024014|F|0-24:C>T-24:C>T	TGCAGTGTCGTACCAGTACTATAGCTCGGTCCTCGCTCCG	24:C>T
1044828|F|0-53:G>C-53:G>C	TGCAGGGAGAACTGGACGGAGATGTGCGCGGTGCAGCGCGGGCAATTGGCGTCGGCGTGGATCCGAGAT	53:G>C
2249018|F|0-29:C>A-29:C>A	TGCAGCAGCTTGGTGGTGTCGATGGCGCCCAGCTTCTCGATGTAGATTGGCCG	29:C>A
7169765|F|0-11:G>C-11:G>C	TGCAGGTCGCGGCCCTCCATCACCGTCTGGTTGCTCCG	11:G>C
1127437|F|0-35:C>T-35:C>T	TGCAGCCCGCCTGCCTGACATGGAGACCTGCACCACGCAACCCGTAGAAATGAACCACTGGTCGAAACC	35:C>T
3020324|F|0-35:A>C-35:A>C	TGCAGCCATAACATTTCTTCCCTATATAAAATCTCATTCATCCG	35:A>C
1043228|F|0-43:A>G-43:A>G	TGCAGTAATTGACAAGAACAAGAACCGTAGCAAAGAATGGGGAAGAAAGATCTCACTCACAGGCGACGT	43:A>G
1059094|F|0-40:G>A-40:G>A	TGCAGTCCAAAAGGAGAGCGACGTTGTATACAAGAGCAGTGGTCAAGATCCG	40:G>A
3020342|F|0-11:A>C-11:A>C	TGCAGATTTGAATCATATATAGTTTGTGTACATGTTTCCG	11:A>C
977394|F|0-6:T>G-6:T>G	TGCAGCTTGGTCCGTCAGCCATGACGCGAATCGTGACCTCGCCCCCTTCAATAATGGATGACGTAACCG	6:T>G
1023767|F|0-12:T>C-12:T>C	TGCAGTGGGTAATGGCGGAGCTCATGCGGAACCCGAGGGTTCGGCATAAGGCGCAGGAGGAGGTCCGAG	12:T>C
1020364|F|0-45:G>A-45:G>A	TGCAGTGAGCTCAGCGGCGGTGAAGGTGTAGGGCAGGTTGCCGACGTAGATTCGCCCG	45:G>A
3957157|F|0-20:G>T-20:G>T	TGCAGAATTCGCTGGAGGTGGACCTCCTCGACCG	20:G>T
3021598|F|0-6:A>G-6:A>G	TGCAGGATGAATGTGTCGCTATTTCTTCAAGATGCTTCGAACCG	6:A>G
1132668|F|0-58:G>C-58:G>C	TGCAGATGCTGACCTACCTCGCCATGAAGCACGACGCGCGCGTGCAGCGGGTCGACACGGTCCG	58:G>C
3959134|F|0-63:G>A-63:G>A	TGCAGTGTGGGGAATGCGCCGCTGAAGCTCGACGGCGAAGTCTGTGCCGCTTAGGTTGGTAGCGCGTCT	63:G>A
1019150|F|0-19:G>A-19:G>A	TGCAGTCGTGGTGCAGGTAGCAGAGCCCGCGCGCCGCCTCCAGCGCGATCCG	19:G>A
2243012|F|0-54:G>T-54:G>T	TGCAGTAGAATTGTCGACGAGGGCGATGAGCGGGATGAGGCGGATGAACATGTCGATCTCATGCTGTGC	54:G>T
1006604|F|0-43:A>G-43:A>G	TGCAGTTCGTGTTTCCAGGGATGTGGCTCTCGGATTTGACCAAAGGCCG	43:A>G
3957912|F|0-7:A>G-7:A>G	TGCAGCAACACAATAATAACACCACAAAACCGTCAGACATTTGAACCG	7:A>G
1076658|F|0-11:A>G-11:A>G	TGCAGCGATAAACAACAGGAGACGTCTAGTTTACTTCGCTTTGTTCCCTGTTAGCCG	11:A>G
4261502|F|0-5:C>T-5:C>T	TGCAGCTTCGTGGACAGCATGACCATCACCATGATCGAGGGCCG	5:C>T
1016849|F|0-18:T>C-18:T>C	TGCAGTACCAGATACGGCTTCCCTTCAGTTGTTCGGCATATGTGAATTTGGTGGAATGGGATGCCAAGA	18:T>C
1018231|F|0-33:C>T-33:C>T	TGCAGGTGCATCACAGGTAGGCACGGTGGACATCGGGTGGTGGCAGCAGTAGGGACAGCAAGGCTCGCC	33:C>T
3570345|F|0-10:G>C-10:G>C	TGCAGCCACTGTGAAACGGCGGTGCGGGAACCATTTAACTCCG	10:G>C
989036|F|0-24:T>G-24:T>G	TGCAGATGCCGTGGGAGACGGTCATTTGCGAATTGCGACTGGGTTGTTCTTCCG	24:T>G
7903376|F|0-14:A>T-14:A>T	TGCAGGGGAAGGAAAGGAAGGAGGATCGATCGACACGGCCG	14:A>T
7350852|F|0-6:A>G-6:A>G	TGCAGGAACCTCCCGCATCACCCGACCG	6:A>G
3950034|F|0-17:G>C-17:G>C	TGCAGGAGGCAGCCGACGACCAGAGGCCCG	17:G>C
3934424|F|0-24:T>C-24:T>C	TGCAGCGACTTGAATTTCCGATGATACGGGTGGTGTTCATCCG	24:T>C
5581548|F|0-7:G>T-7:G>T	TGCAGGTGCCACATAGTTTAGAGCAGCCG	7:G>T
4539686|F|0-8:C>T-8:C>T	TGCAGGCGCAGGGGGGAATGGAAGGGAAGATTCCG	8:C>T
3024930|F|0-22:C>T-22:C>T	TGCAGAGGAAAAACTCGCGCCACAGCCACAGTAATGGACCG	22:C>T
4993888|F|0-21:A>G-21:A>G	TGCAGCTCGTCCGCGGGGAGAAAGTCGCCGTTGTTGGCGCCGTCGTCGGCCG	21:A>G
7163290|F|0-5:A>T-5:A>T	TGCAGAAGCCACGGTCAGCTTCCATCCGACTCTCATCCTCGTCACTACCAAGAGCCACATCGCCGAGAT	5:A>T
1020364|F|0-15:G>T-15:G>T	TGCAGTGAGCTCAGCGGCGGTGAAGGTGTAGGGCAGGTTGCCGACGTAGATTCGCCCG	15:G>T
1022092|F|0-40:C>G-40:C>G	TGCAGCCACGCCGTGATCCAGCCGAGGTAGATCAGCTCCTCCATGTCCGCCGCCCACCG	40:C>G
6042715|F|0-13:C>T-13:C>T	TGCAGCCCATATTCGGCCTGATAAGCGTTGCCACAGACATCCTTGCCTTGGCCGAGTTCGTCACCACCC	13:C>T
1038822|F|0-14:T>A-14:T>A	TGCAGCTCAGAACGTGGCACCCCAGCCACACCCTGCACCTTGAGGCAGAGCCTGAGCAGGAAGGCCTCC	14:T>A
1040984|F|0-32:T>G-32:T>G	TGCAGCCACAACGACGTGCTAACCCACACGCATCACCAAAGCGCCTATATAAACATTAGCTCCG	32:T>G
997479|F|0-41:C>T-41:C>T	TGCAGCTTCTCCCGCCGCTTGCGCTCGGCGATGACGTGCTCCTGCGCGTTCGCCG	41:C>T
1033299|F|0-68:C>T-68:C>T	TGCAGCTCGCTCCACCCAGGGAGGAAGGATACCTTCCTGAATCCATCCATCCATGCTTGAAGCTTAGAC	68:C>T
1862764|F|0-21:G>A-21:G>A	TGCAGACGCACTTGCAGCAACGGGTGGCCTTGAAGGGCACGCGCGAGATGATCTCGACGAGGAGGTCGT	21:G>A
1055285|F|0-23:C>T-23:C>T	TGCAGGTCCGTGATCAAAGTGTACTCTTCACATCCTGTCATTCCCCACACCAGATCACCG	23:C>T
4262347|F|0-7:C>T-7:C>T	TGCAGCTCTCGCTGGACCTGATCGAGGAGTGGAGCAAGGCCCACCCG	7:C>T
1063013|F|0-8:A>C-8:A>C	TGCAGCAGAAGAAGAAGAGGGAGGCGCGACAAAGGCGATGGCACGCGGGTCCG	8:A>C
3938743|F|0-37:C>T-37:C>T	TGCAGCACATATACCGCGAGGGAAACCAGGTCGCCCACGTCCTGTGCAAAGAAGCATACCAGCGACCGA	37:C>T
3064368|F|0-19:A>G-19:A>G	TGCAGCATATATAAGTCCTATGCAGGGGCGCCATTCTTTGACAATCCG	19:A>G
1017803|F|0-21:G>A-21:G>A	TGCAGCTCCTAAGCCACCAACGGGCCTCCGTGTGCCTCCGCGGTGAGCTCCTCGTCCGATTCCCCCTCT	21:G>A

1079188|F|0-35:C>T-35:C>T	TGCAGCTGGTGAACTCTCGGCTCCAAGTAACCCAGCACCCGCAGCTTCGGCGCGTCAGCAATGCTGACC	35:C>T
5580153|F|0-14:C>G-14:C>G	TGCAGCACCGTCTTCAGCCCCCCGCAGCAGTCCG	14:C>G
4261551|F|0-10:G>C-10:G>C	TGCAGCCCATGCCGCCGAGGTTGAAGCTCCG	10:G>C
3023755|F|0-10:C>A-10:C>A	TGCAGGGGCCCTTCTCCATGCGTATCACCAGCGACTCCG	10:C>A
5368388|F|0-8:C>G-8:C>G	TGCAGCTCCGCCTCGTCGGTCTTGTCGGACTCGATGGACAGCAGCACGTTCTTGCGGCGGACGAACTGG	8:C>G
3940094|F|0-6:C>T-6:C>T	TGCAGACGACGGGTCGGCGTCGTTGCGTGTATTCTACGGCCACCG	6:C>T
3940094|F|0-9:C>T-9:C>T	TGCAGACGACGGGTCGGCGTCGTTGCGTGTATTCTACGGCCACCG	9:C>T
3940094|F|0-16:G>A-16:G>A	TGCAGACGACGGGTCGGCGTCGTTGCGTGTATTCTACGGCCACCG	16:G>A
1018625|F|0-40:A>G-40:A>G	TGCAGCCCAAGGAAGGGCTTGCCATGGTGAACGGCACAGCAGTGGGCTCCG	40:A>G
2242013|F|0-56:T>C-56:T>C	TGCAGCTAGTGTTGCGTGCTCAGTCAGTCAGTCAGCCATCGGCGCCGTCGATGCCTTGCCTAGCTTATA	56:T>C
6042977|F|0-44:C>G-44:C>G	TGCAGCCGCTGCACAGCGCAATCTCGGCGGCGAGGCTAACGTCACGGCTTGGGGCAGAGGTGGCCCGAG	44:C>G
4262614|F|0-17:T>G-17:T>G	TGCAGTCGCCGTTCACGTTCCGCAAGGACGGGCAGGCGAGCCG	17:T>G
1038080|F|0-17:A>G-17:A>G	TGCAGGTCAGGTAATGCAACGACAAGAGCTAACCGCTCGCCAAAGTACAGCCG	17:A>G
986029|F|0-10:C>G-10:C>G	TGCAGCACATCAGCCCATACTTCCTGATCAGTCCATGGGAGTTCCCACAGACCCG	10:C>G
4262195|F|0-15:C>T-15:C>T	TGCAGAGAAACAAGACTGCTTTGGTCTCAAATTCCG	15:C>T
3024696|F|0-8:C>T-8:C>T	TGCAGCAGCTGCTCCAGGCCATCGGCTCTAACAACAGCACCACCG	8:C>T
3936994|F|0-10:C>A-10:C>A	TGCAGCGCAGCCATGCATGAATGGACCG	10:C>A
1038822|F|0-17:C>T-17:C>T	TGCAGCTCAGAACGTGGCACCCCAGCCACACCCTGCACCTTGAGGCAGAGCCTGAGCAGGAAGGCCTCC	17:C>T
5580387|F|0-10:C>T-10:C>T	TGCAGGAGTTCGACACGATCCACGGCCCG	10:C>T
4006063|F|0-9:A>G-9:A>G	TGCAGTACTATGGGACTTAATGCGTAGGATCTGGCCGCTGCCCG	9:A>G
986016|F|0-39:A>T-39:A>T	TGCAGCAGCTCTAGTCCCGTCGCCACGTTCCCGAGCGGCAACGACGTCATCCCGCTCGCCGCCGTCGGG	39:A>T
3935542|F|0-7:G>A-7:G>A	TGCAGCTGGCCGTGCCGTTGATGGTCCACCCG	7:G>A
1046721|F|0-31:G>A-31:G>A	TGCAGCCCTTCACGTGTAGATATACACGCAAGGACCAACCGACTAATGCTCTTGAGTCCAAACCGAGAT	31:G>A
6013568|F|0-14:A>G-14:A>G	TGCAGCAACGCATCAGTCAGAAGTTCAAAAACCAAGGTCCG	14:A>G
6030873|F|0-15:G>T-15:G>T	TGCAGGTGAGCGTCTGTGCATATTGGGATCCG	15:G>T
32021337|F|0-30:T>C-30:T>C	TGCAGCAGCAGTATTTTAGGCATAGAAAAGTTTCCG	30:T>C
1047903|F|0-43:G>A-43:G>A	TGCAGCATTATGTCCGCGATCTCTTTGACTTCCAGCTTGGTCAGCTGTCTGAAGACGATCATCTCGTCC	43:G>A
1014880|F|0-28:C>G-28:C>G	TGCAGATCGCCTCCGAGGCGCGCCTCTCCGTGAAGCAGCTCTGCAAGGCGCTGATCCAGCAGGTGGAGG	28:C>G
1016849|F|0-30:G>A-30:G>A	TGCAGTACCAGATACGGCTTCCCTTCAGTTGTTCGGTATATGTGAATTTGGTGGAATGGGATGCCAAGA	30:G>A
3029281|F|0-11:T>C-11:T>C	TGCAGTCTTAATACGGGCGCTCTTATGTTCACGGTGCCATCCG	11:T>C
6036527|F|0-28:G>T-28:G>T	TGCAGTTGGGCGTCAGTTAACCTCCCAAGCGCTGAGCTTCTGAATTCACACTTGTTTCCTCCG	28:G>T
3533153|F|0-40:C>T-40:C>T	TGCAGGGTAGTTAGGTCACTGGTGAGCCCAAACAGATCGACATCGACCG	40:C>T
5365375|F|0-39:A>G-39:A>G	TGCAGCGAAAAGCCTGCGCCAACAAGGGCGCTCTCCCACACTACTAATTCTGGCTTTTCAAGGACCGAG	39:A>G
32022775|F|0-56:G>A-56:G>A	TGCAGCAATGGTAGCTTTGTTAACCAGGTGATGTCTGCCGCGTACATCTCGCTATAGCCCTGACCAAGG	56:G>A
4997588|F|0-21:G>T-21:G>T	TGCAGCAAGTCAGTTCCCAACGACTCCTCGAGTGAACCAAGAGATGCAATTGAAGCGCCG	21:G>T
5004635|F|0-6:T>A-6:T>A	TGCAGCTGTAGACAAGGAGAATAATACTCACCATCTCCAGCACTCGAGGCCGTACCAGCCAAAGGATCC	6:T>A
100513386|F|0-34:C>G-34:C>G	TGCAGCCATCCTCCTGGTCGAGGCGCCGCAGAAGCTCCTCCAGCTGCTTCCTCGTGATCCTGATCTTCA	34:C>G
100486809|F|0-45:T>A-45:T>A	TGCAGCGCCTGGAACGGGACCATGGCCTGATCCATCCATCCATGGTCCCAGGAAAGAAACCGTCCGAGA	45:T>A
2241720|F|0-54:A>T-54:A>T	TGCAGGTCTGAAACATGTGCCTTGTTCGACTGAACTTTCATAGTTTCATGCTCAAGCATGTACCTATCT	54:A>T
100558133|F|0-15:T>C-15:T>C	TGCAGGCGCTCCCATTAGAATTAAGAAAACAAACAAGCTCAGTCGTCGGTGTATTCAACTCCG	15:T>C
100461709|F|0-27:C>T-27:C>T	TGCAGCGTCAATGCAGTGCATGATCGGCCCAGTTGACTTTGCGTCCG	27:C>T
100512370|F|0-40:C>G-40:C>G	TGCAGATCTTCCTGGTTCATGTCAACCATGATCATCCACGCCTGTCGAGAAAGTTCATCTTTCCTCAAC	40:C>G
7172405|F|0-20:G>C-20:G>C	TGCAGGCAGGGGATTGATTAGTCGTAAGGAAGAGGAGGAACGGGACGGACCAGCTTGGACGCGTTCCGA	20:G>C
100526378|F|0-33:C>G-33:C>G	TGCAGGACGCGGTGCTGCGGCACGGCGTGTTCGCCGCGGTGGAGGCGCGGCTCGGGGACCCG	33:C>G
1047518|F|0-9:G>A-9:G>A	TGCAGTCACGTCGGCGACGTGTACGCGACGCCACGTCCAAGTGGCCCGTTCCTGTCTCGCCATGTGTCC	9:G>A
1024820|F|0-57:T>C-57:T>C	TGCAGCTGGGGAGGCAGCGGGCCAAGACCAAGGTGATCAGGAAGTCGGCCAACCCCGTGTGGGACGAGG	57:T>C
4002809|F|0-24:A>G-24:A>G	TGCAGCGTACGTACCGCGGTCTTGACGAGCTCATCGTCGTAGGCGCCG	24:A>G
1033696|F|0-57:G>C-57:G>C	TGCAGACTATGTGACAATGATAGGTGAGTTGACCGAGATGGTTGACGTGTACAGCTTGGGCGTGGTGCT	57:G>C
1014880|F|0-67:G>A-67:G>A	TGCAGATCGCCTCCGAGGCGCGCCTCTCCGTGAAGCAGCTCTGCAAGGCGCTGATCCAGCAGGTGGAGG	67:G>A
1048163|F|0-22:C>G-22:C>G	TGCAGGCCTGAACAGTCTAGGACCTGATCAAGGTGTGAGAAGAATTGCTATGCTCTGCACGTGCGTAAG	22:C>G
1039052|F|0-6:T>C-6:T>C	TGCAGGTGTATTTGAAGAGCTTGCTCATCGTCTACGGGTTGTGGGCATGTTGGTAATGGAGGCGGAGCC	6:T>C
985096|F|0-41:T>A-41:T>A	TGCAGTTCTGCGCGAGACGACAAGGTCGTGCGATACGGCGATGTTGTGTGAAGCAACAAGACGCGTGAT	41:T>A
1062469|F|0-29:C>T-29:C>T	TGCAGTTCTTGCAGCACACCCAGCCCATCCTCCACGAGGTGCAGGACCTGGCGGCGATGAAGCCCATCC	29:C>T
3938799|F|0-17:G>C-17:G>C	TGCAGCGGGACACGAAGGCTCGGAACCGAGGCCCG	17:G>C
1026038|F|0-52:C>T-52:C>T	TGCAGCTCGCGCGCCGCGCCAAGATGATCGGGGACCTGCTGCACCAGCTCCACGCCGCGCAGCTCATGC	52:C>T
1862764|F|0-20:C>T-20:C>T	TGCAGACGCACTTGCAGCAACGGGTGGCCTTGAAGGGCACGCGCGAGATGATCTCGACGAGGAGGTCGT	20:C>T
4262307|F|0-7:G>A-7:G>A	TGCAGCAGCCACTGGTGATCCACGTCGATGAGGAAGTCCTTGTGGTAGTGCTCCCTCACCTTCTCCCTC	7:G>A
1694713|F|0-34:A>T-34:A>T	TGCAGATGGCGCGGTCGACCCAGCTAGCTCGTGCATACTTACACGCACCG	34:A>T
3950953|F|0-5:G>A-5:G>A	TGCAGGTGGAGTCCCTAGCCCATGGGGCTATCTCCG	5:G>A

5411947|F|0-5:T>C-5:T>C	TGCAGTTCGATTTATCGTTTTGGTTGTCCG	5:T>C
992807|F|0-26:A>C-26:A>C	TGCAGGCCCCGCGTTGGCGGCGGCGGAGGCGGGGGAGGAGCGGCGCTGATCCGCCG	26:A>C
3951245|F|0-12:A>G-12:A>G	TGCAGTCGGAGCATCCCGAGAGCTTACCG	12:A>G
1026517|F|0-48:C>G-48:C>G	TGCAGTTTGGAAAATGGAGGGTGCAATCATCAGCTTGACCGAGGGCGCCGTGCGAGGCCTCCTATGCAA	48:C>G
5367273|F|0-14:G>A-14:G>A	TGCAGGATCGTGTAGTCCTCCCCCTGCGCCG	14:G>A
3024014|F|0-6:G>C-6:G>C	TGCAGTGTCGTACGAGTACTATAGCTCGGTCCTCGCTCCG	6:G>C
1020841|F|0-6:G>C-6:G>C	TGCAGAGTCATGAGCGGAGCAGGATGATGAGGACCTGCGGCGAGCTACAAGTAGTAGTTCAGAATTCGG	6:G>C
3024014|F|0-11:A>G-11:A>G	TGCAGTGTCGTACCAGTACTATAGCTCGGTCCTCGCTCCG	11:A>G
1019198|F|0-7:A>G-7:A>G	TGCAGGCAATCGCCTTTGGGTGGACTGTGTGAGGGAGAAAGGGAAGAGCGGAAAGGTTGCCG	7:A>G
5580153|F|0-8:C>T-8:C>T	TGCAGCACCGTCTTCAGCCCCCCGCAGCAGTCCG	8:C>T
1048163|F|0-46:G>A-46:G>A	TGCAGGCCTGAACAGTCTAGGACCTGATCAAGGTGTGAGAAGAATTGCTATGCTCTGCACGTGCGTAAG	46:G>A
3941385|F|0-5:G>A-5:G>A	TGCAGGCAGAGGACGACGTAAACCTTCCG	5:G>A
1051309|F|0-14:C>G-14:C>G	TGCAGCGTGCTGCTCGCCCTCATCCTCGTCCTCGCCATCGTCTTCGTCGCGCTCTACTTCACCGTGTTC	14:C>G
3957157|F|0-28:C>G-28:C>G	TGCAGAATTCGCTGGAGGTGGACCTCCTCGACCG	28:C>G
3384631|F|0-21:C>T-21:C>T	TGCAGCTAGTACAGAGAAGTTCGATCCCTTCCTTGCTGGTCAGAAGGTAATCCACACTTATCAGATGCA	21:C>T
1022127|F|0-52:T>C-52:T>C	TGCAGAACTCCACCAACGGGTGGATCGCGGACTCAGGCGCTTCTTCTCACATTACCTCGGACCCAGGTA	52:T>C
3943023|F|0-23:C>T-23:C>T	TGCAGCACGGGGAGCAGGAACGGCTTGGACTCGTCAGCGAACTCGCCG	23:C>T
1021176|F|0-56:T>C-56:T>C	TGCAGAGTTTCCCTCTAGATAGCAGCACAATCCCAAGCGCCAATCAAAGGCCATCGTCACTAGAGCCAC	56:T>C
3026191|F|0-19:G>C-19:G>C	TGCAGCACCACCACCTCCTGGTGCTCCGCGTCCAGGTCCG	19:G>C
1023470|F|0-28:G>A-28:G>A	TGCAGTACATGCAGTTTTGGTGCCGAGAGAGCGGCGGCGACGAGGACCCG	28:G>A
3024014|F|0-16:T>C-16:T>C	TGCAGTGTCGTACCAGTACTATAGCTCGGTCCTCGCTCCG	16:T>C
3026657|F|0-18:G>T-18:G>T	TGCAGTAGGATGAGGAATGGCTCCATCAGGAACACCGCCG	18:G>T
1038080|F|0-5:G>A-5:G>A	TGCAGGTCAGGTAATGCAACGACAAGAGCTAACCGCTCGCCAAAGTACAGCCG	5:G>A
4261189|F|0-28:C>G-28:C>G	TGCAGGCTGCTGGAGAAAGAGCAGGCAGCTGTCGACCG	28:C>G
1020841|F|0-10:T>G-10:T>G	TGCAGAGTCATGAGCGGAGCAGGATGATGAGGACCTGCGGCGAGCTACAAGTAGTAGTTCAGAATTCGG	10:T>G
3939712|F|0-10:C>G-10:C>G	TGCAGCCGCACTCGTAGCTGCCCCAGGTGTTCTTGCAGTGGCAGTCCG	10:C>G
3935387|F|0-14:C>T-14:C>T	TGCAGATATTGCCACCGAATGCCTGTCTTCCCCTTTCGAGAAAGGAGGAGCAAATCTCCTTGGATGTCC	14:C>T
4002976|F|0-28:T>A-28:T>A	TGCAGACCAGACCGCGCGCCGCACGATATGCATACCG	28:T>A
4261315|F|0-28:G>T-28:G>T	TGCAGCACATGCAGGGGGAGGAGGAGACGGCGATGCACCG	28:G>T
1043004|F|0-5:G>C-5:G>C	TGCAGGTGTTCGGTGAATTGCCCCACTGGGAAGATTGCGCCGCTGGCGTCGGATAAGCCAGGCTCCGAG	5:G>C
1016849|F|0-34:G>T-34:G>T	TGCAGTACCAGATACGGCTTCCCTTCAGTTGTTCGGCATATGTGAATTTGGTGGAATGGGATGCCAAGA	34:G>T
3955925|F|0-17:G>A-17:G>A	TGCAGCTCCTCCACCTCGGCGCAGATGGCGTTCTTCTTGAAGACCCG	17:G>A
3942527|F|0-30:A>G-30:A>G	TGCAGTAGTTGCCGAAGCTAGCTGCATGTGATTAGTTAGGCCG	30:A>G
6042750|F|0-12:G>A-12:G>A	TGCAGTCCAGCAGCAAATGACTTGGTTGTATAGTCCGTAGGCACATCGGGAGTTGTTTCTCGTTGTAGT	12:G>A
1148079|F|0-36:A>G-36:A>G	TGCAGCTCGACCCCGCGACCAAGCCGCCGCCCTGGAACTTCTCTGCTGCCG	36:A>G
989962|F|0-7:G>A-7:G>A	TGCAGCTGGCACAAAGAAGTCAATCTAGATGAGAGTTAAGAACCTGATCCTGACCG	7:G>A
5580368|F|0-7:G>T-7:G>T	TGCAGGAGATTCTGGTCCAGAACGACCACCG	7:G>T
1696212|F|0-58:C>T-58:C>T	TGCAGTTCCTCACCAACTCCTGCGTCGTGCTCTTCATGATCCAGAGCGTCGACCGCCTCGTCCTCTGCC	58:C>T
1020503|F|0-8:C>T-8:C>T	TGCAGGTTCGTGGGTAACAGGCGCGCCGCCCAGCGCACACCTCGCCCACCTTGCCGCCG	8:C>T
4261933|F|0-11:A>G-11:A>G	TGCAGGAGATGATTCCAAGCAACCTAATTGACCG	11:A>G
5002652|F|0-39:G>T-39:G>T	TGCAGACCATGGCGCTCGACAGGGTGCTCGAGGAGTGCAGGAGCACCG	39:G>T
4003062|F|0-14:G>A-14:G>A	TGCAGCGAGCGGCGGCACGCGCGTGGTCGCCG	14:G>A
981522|F|0-6:C>T-6:C>T	TGCAGCCCGCTCCGTCTTTGCTGTGATAACGAGGTAAAGTGGCATTTCTTTGTTACATGAGTCCGAGAT	6:C>T
7352087|F|0-6:T>C-6:T>C	TGCAGGTGGAGTCCCTAGCCCATGGGACTATCTCCG	6:T>C
3025705|F|0-6:G>A-6:G>A	TGCAGAGCTCCAAGAATCTAAAGAACGACTCGTTCACTGTCCG	6:G>A
1022127|F|0-61:C>T-61:C>T	TGCAGAACTCCACCAACGGGTGGATCGCGGACTCAGGCGCTTCTTCTCACATTACCTCGGACCCAGGTA	61:C>T
1040984|F|0-21:A>G-21:A>G	TGCAGCCACAACGACGTGCTAACCCACACGCATCACCAAAGCGCCTATATAAACATTAGCTCCG	21:A>G
6042977|F|0-26:G>A-26:G>A	TGCAGCCGCTGCACAGCGCAATCTCGGCGGCGAGGCTAACGTCACGGCTTGGGGCAGAGGTGGCCCGAG	26:G>A
1055285|F|0-7:C>T-7:C>T	TGCAGGTCCGTGATCAAAGTGTACTCTTCACATCCTGTCATTCCCCACACCAGATCACCG	7:C>T
4005981|F|0-38:T>C-38:T>C	TGCAGCAATGAGACTTTGGGAACTCGTCGATAAAAATCTGTACCG	38:T>C
1046126|F|0-6:C>G-6:C>G	TGCAGGCTGCTCCCGCCAGAGCTCTTGATGCTGATCATCTTGCCACACCG	6:C>G
1065256|F|0-25:C>T-25:C>T	TGCAGCTCTTCTGTGGCGACACCGTCCTGCTCAAGGGCAAGAAGCGGAAAGACACGATCTGCATTATGC	25:C>T
3021984|F|0-20:T>C-20:T>C	TGCAGCAGCAGTTCGTGTCCTTGGCCACCATCGTCCCCG	20:T>C
1074888|F|0-11:G>A-11:G>A	TGCAGTGCACTGCACGTGAGGCCTCTTTGTTTGTGTAGATATGAACAGATCAACACTTTTCAAGACCTG	11:G>A
4396433|F|0-12:G>T-12:G>T	TGCAGCTGCTGCGTGTTCGGGTGGAACGCCG	12:G>T
4262349|F|0-23:G>C-23:G>C	TGCAGAAGCACTGCACGGCGATGGCGAAAGAGTGGGCGTCCG	23:G>C
5004423|F|0-28:G>C-28:G>C	TGCAGCACCAGCTGCGGGGCACGGTGCGGCTCGACCCCTCCG	28:G>C
7172820|F|0-40:C>T-40:C>T	TGCAGGTGCATCACAGGTAGGCACGGTGGACATCGGGTGGCGGCAGCAGTAGGGACAACAAGGCTCGCC	40:C>T
1042871|F|0-5:C>A-5:C>A	TGCAGCAAGTGTCTAACTGACGAAGACTGAAGATACCACTTCGTCAGTTATCCTCCG	5:C>A

<table>
<tr><td>1223858|F|0-31:G>A-31:G>A</td><td>TGCAGCTCCTGACATCCCAGATAAGAACAACGTTTGACCCAGCAAGCCAATCCCAAAGAGATCCTAGCA</td><td>31:G>A</td></tr>
<tr><td>1223858|F|0-24:G>A-24:G>A</td><td>TGCAGCTCCTGACATCCCAGATAAGAACAACGTTTGACCCAGCAAGCCAATCCCAAAGAGATCCTAGCA</td><td>24:G>A</td></tr>
<tr><td>5578092|F|0-28:A>T-28:A>T</td><td>TGCAGCTGGATACGAAGGCTCGGAACCGAGGCCCG</td><td>28:A>T</td></tr>
<tr><td>4261565|F|0-29:T>G-29:T>G</td><td>TGCAGAGTCCAGAAGCTGCTGGTGTCTTCTCCACGACACCG</td><td>29:T>G</td></tr>
<tr><td>1069516|F|0-6:C>T-6:C>T</td><td>TGCAGTCCAAGTCGTGCCATAGCAGCAGGGCGACTCTTCCAGGATGCATCTCACGCTCAACCG</td><td>6:C>T</td></tr>
<tr><td>3064368|F|0-27:G>A-27:G>A</td><td>TGCAGCATATATAAGTCCTATGCAGGGGCGCCATTCTTTGACAATCCG</td><td>27:G>A</td></tr>
<tr><td>1708229|F|0-29:C>T-29:C>T</td><td>TGCAGGTTTACATGATGCAGGTTGTCCGTCTTTTTCACATGGGAATCCG</td><td>29:C>T</td></tr>
<tr><td>4396559|F|0-26:G>A-26:G>A</td><td>TGCAGACAAGGTTTTGTGTCGAGGAAGGATTGGCCG</td><td>26:G>A</td></tr>
<tr><td>1032655|F|0-32:C>T-32:C>T</td><td>TGCAGGCACCATCGCCGACATGCAGCAATATACAGAGTTCCAACGCCGTTACACCGTCTACTGGAAAGG</td><td>32:C>T</td></tr>
<tr><td>5363839|F|0-14:T>C-14:T>C</td><td>TGCAGGCTGGCGCATTTCCCGAGCTCCG</td><td>14:T>C</td></tr>
<tr><td>4261086|F|0-12:C>A-12:C>A</td><td>TGCAGAGCCTCGCCGACCTGGCGTGGCGCCTGGGGCCG</td><td>12:C>A</td></tr>
<tr><td>1286660|F|0-5:T>C-5:T>C</td><td>TGCAGTTTCTCGAGATGAAGCTCGGCTAGCAAGGCACCCTGTGGGATCGCCTGGAGAACCG</td><td>5:T>C</td></tr>
<tr><td>7164064|F|0-23:G>A-23:G>A</td><td>TGCAGGAGCTTGGGCATGCCCATGAGGTCCGAGAGGCAGGCGAGCGAGAACTCATAGGTGGAGTTCCGA</td><td>23:G>A</td></tr>
<tr><td>3064870|F|0-11:T>C-11:T>C</td><td>TGCAGGAGGGGTGCCGTTTCGGGGAGCAGCGGTTCTTGGTCCG</td><td>11:T>C</td></tr>
<tr><td>996598|F|0-24:C>G-24:C>G</td><td>TGCAGATTCTTGCTGTTTCATGCACTTGTATATCTTGGTTCTCTTGTGTGCCG</td><td>24:C>G</td></tr>
<tr><td>1062505|F|0-65:T>C-65:T>C</td><td>TGCAGTGTGAAATGAACGGTGTGGTTACTCGCAATGGGGGGGACAAACAAATGAGCTGGGGAAGATGAA</td><td>65:T>C</td></tr>
<tr><td>1046126|F|0-24:T>C-24:T>C</td><td>TGCAGGCTGCTCCCGCCAGAGCTCTTGATGCTGATCATCTTGCCACACCG</td><td>24:T>C</td></tr>
<tr><td>1040349|F|0-14:A>G-14:A>G</td><td>TGCAGTGGGTGCGCAACATGGGCTACGTCATCTACGACTACTGCACCGACATGAGCAACCG</td><td>14:A>G</td></tr>
<tr><td>990866|F|0-6:A>C-6:A>C</td><td>TGCAGGATTTGCAAGGAGGTGATGGCCGACGCGGTGGTGGCGAGCAAGTGCTGCTTCAACAGCTTCTGC</td><td>6:A>C</td></tr>
<tr><td>1007779|F|0-9:T>C-9:T>C</td><td>TGCAGAAGGTTGGCTTGTGCATTCCTCACGACTTGGGGAAACTCTGCTACGCGCACTGGTTGGATCTGG</td><td>9:T>C</td></tr>
<tr><td>4261355|F|0-7:C>T-7:C>T</td><td>TGCAGAGCACATACCCTGTAAGTGCAATATTATTACCG</td><td>7:C>T</td></tr>
<tr><td>1043004|F|0-40:C>T-40:C>T</td><td>TGCAGGTGTTCGGTGAATTGCCCCACTGGGAAGATTGCGCCGCTGGCGTCGGATAAGCCAGGCTCCGAG</td><td>40:C>T</td></tr>
<tr><td>1087304|F|0-6:G>C-6:G>C</td><td>TGCAGTGACGCGTCTATTTCGTTGGGCGCTCCCGTTCCAAAACAAGTCCCCG</td><td>6:G>C</td></tr>
<tr><td>1022674|F|0-50:A>G-50:A>G</td><td>TGCAGGCCAAGAAGGGTAAGTAAATCCAACTTTTGCGTTCGCTCCCTCCTACGTCCCGCGAGGCTCGTA</td><td>50:A>G</td></tr>
<tr><td>1022127|F|0-55:C>A-55:C>A</td><td>TGCAGAACTCCACCAACGGGTGGATCGCGGACTCAGGCGCTTCTTCTCACATTACCTCGGACCCAGGTA</td><td>55:C>A</td></tr>
<tr><td>1022232|F|0-46:G>A-46:G>A</td><td>TGCAGATGACCCTGTACGAGCTGGTGATGATCTGCCACGAGGAGACGGGTCGGCTGCTGAACCG</td><td>46:G>A</td></tr>
<tr><td>1047582|F|0-29:T>A-29:T>A</td><td>TGCAGCCAGTCTACGGTTGACTTCTGCCCTCTATCCTTAGGATCACGCGGCGCAGGAGCGTACTGCATA</td><td>29:T>A</td></tr>
<tr><td>1059094|F|0-33:G>T-33:G>T</td><td>TGCAGTCCAAAAGGAGAGCGACGTTGTATACAAGAGCAGTGGTCAAGATCCG</td><td>33:G>T</td></tr>
<tr><td>6042717|F|0-10:A>G-10:A>G</td><td>TGCAGCCCATATTCGGCCTGATAAGCGTTGCCACCGACATCCTCGCCTTGGCCGAGTTGGTCACCACCC</td><td>10:A>G</td></tr>
<tr><td>1019198|F|0-29:T>C-29:T>C</td><td>TGCAGGCAATCGCCTTTGGGTGGACTGTGTGAGGGAGAAAGGGAAGAGCGGAAAGGTTGCCG</td><td>29:T>C</td></tr>
<tr><td>3020395|F|0-10:T>C-10:T>C</td><td>TGCAGTGAGTTCAGTGTTCAGCAATTTATAAGCGATGTTCCG</td><td>10:T>C</td></tr>
<tr><td>1057966|F|0-22:A>T-22:A>T</td><td>TGCAGCCCATATTTGGCCTGATAAGCGTTGCCACCGACATCCTCGCCTTGGCCGAGTTGGTCACCACCC</td><td>22:A>T</td></tr>
<tr><td>1019940|F|0-10:G>C-10:G>C</td><td>TGCAGGTGCTGAGCGCGCTGGACGCGGCCAAGACGCAGTGGTACCACTTCACGGCCATCGTCGTCGCCG</td><td>10:G>C</td></tr>
<tr><td>1075974|F|0-18:T>C-18:T>C</td><td>TGCAGTTTCCAAATCATTTTCGGTGTACTCCTGCGCGCCGTTCAGGAACAGGTCGCCTTCAGACCGAGA</td><td>18:T>C</td></tr>
<tr><td>1022660|F|0-64:G>A-64:G>A</td><td>TGCAGACCATCAACGGCCCCAACTCCGACTTCCCCGTCTACTCGTCGGAGCTCAAGACGGTCAAGGGCA</td><td>64:G>A</td></tr>
<tr><td>7170865|F|0-7:C>T-7:C>T</td><td>TGCAGGTCGGCGCCCCTGGCACAGCAAATTTCGGTGTTCTTCCG</td><td>7:C>T</td></tr>
<tr><td>3956655|F|0-23:T>C-23:T>C</td><td>TGCAGATGCGAGGCAGGAGAGGGTGATCCG</td><td>23:T>C</td></tr>
<tr><td>985416|F|0-8:A>G-8:A>G</td><td>TGCAGTGGATCATGGCGAACTTGGTGAAGCATCTGGACGTGCAGGAGGCCGTCCG</td><td>8:A>G</td></tr>
<tr><td>1018579|F|0-64:T>C-64:T>C</td><td>TGCAGGTGTAGAGGCCCTTCTGGAGACTGGGGAGTTTACAGAGGTGTACTGGAGTCTGAGATTCTTCCG</td><td>64:T>C</td></tr>
<tr><td>3959873|F|0-19:C>G-19:C>G</td><td>TGCAGGGCGACAAGACAAGCAAGAACATCCGCGCCG</td><td>19:C>G</td></tr>
<tr><td>1059315|F|0-16:G>A-16:G>A</td><td>TGCAGGTGGCGGAGCAGCGAGTCCTCGTCCCAGGAGAAGGTCCACGGCTCCTCCG</td><td>16:G>A</td></tr>
<tr><td>4412235|F|0-27:A>C-27:A>C</td><td>TGCAGGAGCGGGTGCTGAGTTGCCATGAAGCGATGATCCAGGCCACCG</td><td>27:A>C</td></tr>
<tr><td>3570106|F|0-38:T>C-38:T>C</td><td>TGCAGCCCTCCGAGGTTGCTTGTCGACGGCCCGTATGGTGCTCCG</td><td>38:T>C</td></tr>
<tr><td>3955397|F|0-14:C>T-14:C>T</td><td>TGCAGCGGTGGTCACGAAAATGACATTTGCTCCCTCGGCAAAGCCCAACACATGCACCG</td><td>14:C>T</td></tr>
<tr><td>3943089|F|0-18:G>T-18:G>T</td><td>TGCAGGTATGTGTGGTAAGGCAGCAGGGACCG</td><td>18:G>T</td></tr>
<tr><td>1149858|F|0-12:C>G-12:C>G</td><td>TGCAGATCCACGCATCATGGGCCAGTAATTGTGCTGTTATGCATGTCCG</td><td>12:C>G</td></tr>
<tr><td>1149858|F|0-13:A>T-13:A>T</td><td>TGCAGATCCACGCATCATGGGCCAGTAATTGTGCTGTTATGCATGTCCG</td><td>13:A>T</td></tr>
<tr><td>3027302|F|0-16:T>G-16:T>G</td><td>TGCAGAGCACCGTATCTGAGATCAGCCACGCGGGCCAAATCCATCCG</td><td>16:T>G</td></tr>
<tr><td>1042871|F|0-21:G>A-21:G>A</td><td>TGCAGCAAGTGTCTAACTGACGAAGACTGAAGATACCACTTCGTCAGTTATCCTCCG</td><td>21:G>A</td></tr>
<tr><td>1042871|F|0-31:G>A-31:G>A</td><td>TGCAGCAAGTGTCTAACTGACGAAGACTGAAGATACCACTTCGTCAGTTATCCTCCG</td><td>31:G>A</td></tr>
<tr><td>1141147|F|0-28:C>A-28:C>A</td><td>TGCAGGCGACGATCGCCGCCCGCAACCCCAACTCCCGCGTCGGCATCTACTTCGACCG</td><td>28:C>A</td></tr>
<tr><td>3936994|F|0-21:T>G-21:T>G</td><td>TGCAGCGCAGCCATGCATGAATGGACCG</td><td>21:T>G</td></tr>
<tr><td>4005079|F|0-5:C>T-5:C>T</td><td>TGCAGCGTATATAAGCCCTATGCGAGGGCGCCATTCTTTGACAATCCG</td><td>5:C>T</td></tr>
<tr><td>3032129|F|0-7:G>A-7:G>A</td><td>TGCAGACGCACGCTGGAGAGGCTCAGCTCTGTCAACCGCCG</td><td>7:G>A</td></tr>
<tr><td>4005878|F|0-16:C>G-16:C>G</td><td>TGCAGGAGGCCCGCGCCCTCCTCGCCGCCAGGTTCGGCCG</td><td>16:C>G</td></tr>
<tr><td>6043333|F|0-13:G>A-13:G>A</td><td>TGCAGGCCCTGAAGCAGGCCTCGGAACCTCTCCACGTTGCCGCCCAACTGCCCTTGCTTCAAAATCCTG</td><td>13:G>A</td></tr>
<tr><td>1020364|F|0-54:C>G-54:C>G</td><td>TGCAGTGAGCTCAGCGGCGGTGAAGGTGTAGGGCAGGTTGCCGACGTAGATTCGCCCG</td><td>54:C>G</td></tr>
<tr><td>6047679|F|0-39:C>G-39:C>G</td><td>TGCAGGGCCGCGCGCTCACCAACGCCGTCGGCAACCTCGCCATCACCG</td><td>39:C>G</td></tr>
<tr><td>1013891|F|0-44:C>T-44:C>T</td><td>TGCAGGCCCTGGTTGTCGTACTTGCAGTAGTTGCTCTCCTTGTCCTGGTTCTTCCG</td><td>44:C>T</td></tr>
</table>

32026834|F|0-32:G>A-32:G>A TGCAGCTGGGACTATATCCAGGAGGGCAGCCAGGTGTCCAAGGACATGGACCG 32:G>A
6030873|F|0-13:C>T-13:C>T TGCAGGTGAGCGTCTGTGCATATTGGGATCCG 13:C>T
5367542|F|0-37:C>A-37:C>A TGCAGTGGCTCGACGACGAGCTCACCTCGCTCGCCCTCCCCGAACCCG 37:C>A
4407343|F|0-23:C>T-23:C>T TGCAGCTCCTGTATCTCGTTCAGCACCTCCG 23:C>T
3956655|F|0-11:G>T-11:G>T TGCAGATGCGAGGCAGGAGAGGGTGATCCG 11:G>T
3023924|F|0-26:C>T-26:C>T TGCAGGACCAGAAGGACATTGCCAATCGGAGCATTTTCCACCG 26:C>T
3025806|F|0-5:T>C-5:T>C TGCAGTAGTCCATAGCCGAAGAGGCCAGGGATCATCCACCG 5:T>C
3934624|F|0-24:G>A-24:G>A TGCAGGTTTTGAGGGAGATTGAGGGCGATCTCTCCG 24:G>A
1039551|F|0-45:C>T-45:C>T TGCAGAATATATTGCTCAGCCAGTAGTATGTGTTATCCTGGACCGCGCTATCTTATCCG 45:C>T
5971588|F|0-9:G>A-9:G>A TGCAGCACGGTGGTGGCCACGATGCGCAGCCG 9:G>A
6026502|F|0-31:C>T-31:C>T TGCAGATCCATCGTCAGTTACCTACCAACGCCGCTTTGTAAACATTCAACGGTTCCTTGGTCTCGACGT 31:C>T
1016260|F|0-26:G>C-26:G>C TGCAGCATCCAGTTATGGTACTGCGAGAGGCCGTTGAGCCGCCGCAGGCATCCCGTCCG 26:G>C
32020266|F|0-29:C>A-29:C>A TGCAGGACATCGAGGCCGTATCCGCCTCCCTGGCCCG 29:C>A
6031046|F|0-33:C>G-33:C>G TGCAGTTGGCGCACTAGAGAAGTGGTTGGATAACATGGCTTTGGAGCAGCGGGATCCG 33:C>G
1044960|F|0-41:A>G-41:A>G TGCAGAGAAGACAGGGGACGCCATCAAAGCAGAGGAGCGGCACGGGATGATCAAGGGAAGCGTCAAGAT 41:A>G
7354011|F|0-25:C>G-25:C>G TGCAGCTTCTCTTGGTGAAGCATCGCTGACCG 25:C>G
3022815|F|0-12:G>A-12:G>A TGCAGTGCCGTCGTCGTCTTCTTCCATATTCAATCCG 12:G>A
3959134|F|0-16:C>T-16:C>T TGCAGTGTGGGGAATGCGCCGCTGAAGCTCGACGGCGAAGTCTGTGCCGCTTAGGTTGGTAGCGCGTCT 16:C>T
7354276|F|0-52:G>C-52:G>C TGCAGTGCACCTCCAAGTGCGTCGCCACCTGCATCCGCGGCGGCGAGGGGCCGCTCAACGTCCG 52:G>C
4999646|F|0-66:C>T-66:C>T TGCAGATTCTTGGGATCCGTTTTGGCTATAGACGGCAGGGGGTCGCCGTGAGATCCCATCCCATCCCAT 66:C>T
5354240|F|0-44:G>T-44:G>T TGCAGAATCTGCTTCTTCCCGTGCTTGCAGTTGAGGTACCGCTTGGACCTTGGGTCGAGATCCAGGGCG 44:G>T
5412106|F|0-22:C>T-22:C>T TGCAGAGCATAACCTCACGCGTCGGGGACGGCTCCG 22:C>T
5001948|F|0-39:C>A-39:C>A TGCAGGTACCGACCTTCACCCCATTGATCAGTGCTTTCCCGAGAAACCG 39:C>A
1015484|F|0-15:A>G-15:A>G TGCAGCTAGTGACCCATGGAATTAATGCAGGTGCTGGGTGGTGTGTGTGATGGTGGCGTACCG 15:A>G
7168901|F|0-6:C>T-6:C>T TGCAGGCATGTCTCCAAGGGCCAGGCTTCCTCGGTTACGTCGTGTTCCCTCGTCGCCTTCGCCGTCCGA 6:C>T
7336276|F|0-5:C>T-5:C>T TGCAGCGACTGCCTCCGTGTGCGGGTAGTGTCTGAGAGCGGGGATGGCGACGCACTTCGTGCTCAACAC 5:C>T
1101462|F|0-33:G>A-33:G>A TGCAGATGTACATGTCACCGAGGCTGACGCATTGGTCAAAGCACGGTCACAACAAAGCACATGCATGAA 33:G>A
1018641|F|0-68:C>T-68:C>T TGCAGTCCAGCAACAAATGACTTGGTTGTATAGTCCGTAGGCACATCGGGAGTTGTTTCTTGTTGTAGC 68:C>T
1862983|F|0-63:C>G-63:C>G TGCAGCGAACAAGGTCTTGCACTCACCGACCATCTCCTCAGTGCTCAGTGGCTCGACTTCTTTCGACAT 63:C>G
5341431|F|0-5:A>G-5:A>G TGCAGATGCAGGCCACGCATGGCGCGAAACCATTATCAACGCCTTTAGGTAGCGTAACCTCGAGTAAAG 5:A>G
5001840|F|0-39:A>G-39:A>G TGCAGGTCGGCGCGCCCGTGCACGGCACGTCGTTCTGGCACGTCGCGTCCTGCTTCGTGCAGGGCCTGG 39:A>G
32026560|F|0-22:G>C-22:G>C TGCAGGACGAGTGGGACGCGGTGACGCTGAACTATACATCCG 22:G>C
5370080|F|0-27:C>A-27:C>A TGCAGCAGGAGAGAGACGAGGTGCTCCCTCGAACGTCACCGTGGGAACGACATGGTGGTAGCCAGATCC 27:C>A
6044620|F|0-6:T>C-6:T>C TGCAGATCGTGAAAGGGCCACATGTAGCATGGACATCGCACTGAGATTTCGGCACCGTGTAGACGGCTT 6:T>C
3572626|F|0-14:T>C-14:T>C TGCAGAACCAGTGGTCTCCTCGTCACGATCCCTTCGAACCACAACGCGCAACCCCGAAATTAAACAACC 14:T>C
1090620|F|0-34:A>G-34:A>G TGCAGCACCAAGACGGCGAGGCGTCGTCTGCGCAACCTCCTCGTCCTCGCCGCAGACTACCTCAAGTAC 34:A>G
5009089|F|0-41:A>G-41:A>G TGCAGGCTGCTGTAGTTCCAGAAGCGGACGTGGGTGAAGCCAACGACGCAGGACGCCGCCCCGACGACG 41:A>G
100579925|F|0-32:C>A-32:C>A TGCAGGAGGCTCGTGAGGCACAAGAGCACGACCAGGTAAGGCGCGGAAGAAGCCATCTCGATTAGCTTA 32:C>A
11314369|F|0-40:G>C-40:G>C TGCAGACGGTGCTCCTGTGGCAGAACCAGCTCGTCGGCACGATACCTCCG 40:G>C
1126306|F|0-42:A>C-42:A>C TGCAGCAGCGCGAGCAGCGCGCGGGCGCCCTCGTCGCGATCGACGTCGCCG 42:A>C
100463319|F|0-44:C>G-44:C>G TGCAGGTGGAAGCTGCGGCGGCCGCAGCGGATGCACAGCGTGTGCGTCTTGTTCCG 44:C>G
7926100|F|0-30:T>C-30:T>C TGCAGGTGCGCCAGGCTCGGTCGCTCGTTCTCGAGCAGAACGACAATAGCGGGCGGCGCTGGGACTCCG 30:T>C
5364603|F|0-39:A>T-39:A>T TGCAGACCAGCTCGGCTAGACTAGCGGATGTGAATAAGCACGGCACCG 39:A>T
5374032|F|0-25:A>G-25:A>G TGCAGCCACAGAAGAGGACGAGGCAAGGGCGTCGAGCTCCAAGAGGTGGGCGCTGGTCGTGGTCGCCGA 25:A>G
5002827|F|0-8:A>G-8:A>G TGCAGTACAGTGGGAACTAGTTGATGCATGCCTACGCTGCACTGTACAGAGCCGCCCG 8:A>G
1390221|F|0-60:G>A-60:G>A TGCAGTAGTACTGCACACGGAAGGAAGGAGGGATTCGTTCGGCTTTGGTTGGGGCCCTGCGCCTGCTGC 60:G>A
5361162|F|0-67:G>C-67:G>C TGCAGCTCAGATTTTTTGTTTCTTTTCTTTCTCTCATTTCGGGACAGAAGTGAAAGAAACGGGAGCAGT 67:G>C
5361132|F|0-55:A>G-55:A>G TGCAGCGTGGGTCCAGCTTGCGCTCATTGAACGCCCCGATCCAACGGCCCGCGAGACGGTTGATTTTGG 55:A>G
5368670|F|0-55:C>G-55:C>G TGCAGGTGGTGCTGGAGGGCCGAGGGGACAGACGGGCAGTGGCCGCATGTGACTTCTTGTTGCCGAGAT 55:C>G
5000642|F|0-59:G>T-59:G>T TGCAGCGGGCATTTGGGGCACTTAGCATCATGAAAAGCTTGAGTTTTAGTTGTGCCAAAGCTTAGACGA 59:G>T
7900053|F|0-30:T>A-30:T>A TGCAGACGTTTTCTTTCTTTCTTATTCCAGTCCTGACGGCTCATGGCTGACGGACAGAGCCG 30:T>A
5577128|F|0-50:G>A-50:G>A TGCAGGGATCACGCCATAATCACCATCATCTCACCATGCAACTGGCGCAAGGCTCGGGCCACTTGCAGG 50:G>A
7918673|F|0-43:A>G-43:A>G TGCAGCGGCTGCTCTCGAGCTGCCCGCGCCTTGCCAGCCTGACACTGGAGGAGTGCCCG 43:A>G
22834941|F|0-31:A>G-31:A>G TGCAGGTTGGTTAGCTCTTCAGGGATGCACCAAGACAAGAGATTGCCTGACAGATCGATGCCG 31:A>G
5361744|F|0-58:C>T-58:C>T TGCAGTCCACCCTTATCCTGAGACACACCGCACTCCACCAGCGCTCCCCAAGGACGCACCTCTCCTCCA 58:C>T
5365108|F|0-18:A>G-18:A>G TGCAGAGGTGACTCCGAAACGCATTCTCGAGCTGATGGATGTCTCAGGGCTCACCATATCCCACGTCAA 18:A>G
100350264|F|0-15:A>G-15:A>G TGCAGCAAGAACAGAATAGGTGGAGGCCAGCATGCATACCTGAACCCCG 15:A>G
100453677|F|0-34:G>T-34:G>T TGCAGTCTCTCAAGATGTACCTCCATGGTATAATGCACAACTTGTACATCAAGGCACTCAAGTTGCTGC 34:G>T
100363111|F|0-38:G>C-38:G>C TGCAGAGGCTGTCCTACCTCTACTTACACACATTAGTTGCAGACTCGAGACGAAAACCGCAGTCAGCCC 38:G>C

1758440|F|0-16:C>G-16:C>G	TGCAGGCCAAGGTCACCAGCCTCCAGGCCAAGTACAAGTCGGCGCGGATCATGTACGCCGACTTCTACG	16:C>G
1027878|F|0-6:G>A-6:G>A	TGCAGGGACTGCATCACTGGAAAATAATCCTTATACTTTGCTGCTTCATCTGCTGCTTCAAGTGCTGCC	6:G>A
100368175|F|0-29:G>T-29:G>T	TGCAGGCGACATTGATCCTCGGTCCTACAGAGTAGCATACTCGCGTCGCCTTTGTGCATGCTGTCCCCG	29:G>T
1726493|F|0-33:A>G-33:A>G	TGCAGATTGATCCAGGTGCGTTCTGATGAACTGAGCCAACAAAGGAGCAACATGACCCG	33:A>G
100503710|F|0-39:C>G-39:C>G	TGCAGGGAAGTGCACCGTGATGTTCTTGATCTTGTCCATCGGGAGAACGGCATGGGGCTGGCTGGAGCC	39:C>G
1006675|F|0-52:G>A-52:G>A	TGCAGTAGGTCGTCGGACCATCCAATCAGATCTCCACTAAACGGACGGAACTGGCAGCAAGAGGAGGGG	52:G>A
7169855|F|0-21:T>G-21:T>G	TGCAGTGGCTGTCAGAAGGGGTTCAAGGGTGTCCTCACCTGGGGAGTCGACCTCCG	21:T>G
3029018|F|0-31:A>C-31:A>C	TGCAGTCCGAGTCGGTGACGCTCGACGTGCCAAGGGCCG	31:A>C
100501823|F|0-16:C>T-16:C>T	TGCAGCGTGCACAGCGCGAGTACAAGCTTCTCTTCGTGTACCTCCACTCCCCTGACCATCCG	16:C>T
6041450|F|0-7:G>A-7:G>A	TGCAGCGGCGATCATCAGTACTCCCACAGGAGTGGCTCCTCGAGGCGGAACACGCCG	7:G>A
100508369|F|0-8:G>C-8:G>C	TGCAGCCAGCGCGAGTGCACGGTGAGTTTCTCCATGCCTGCCACACCG	8:G>C
4996967|F|0-15:A>G-15:A>G	TGCAGCCTGGTTGTCATGGTCTGCCGTGGATGTCGCTCGCACTGAATGTCGCTCTGTCTTGACTGCATG	15:A>G
100523083|F|0-60:G>C-60:G>C	TGCAGACATGAAAATAACAGCTGCGAGGTGCAACCAAAGATCACAGTAAGTAAAACCATCGAATCCAAA	60:G>C
100552614|F|0-54:T>G-54:T>G	TGCAGGTTGGCGTACGCGTACGCCTCGCACGAGCAGTTGGCGGCGCACCTGGCCTCGCACTCCTCCGCG	54:T>G
100470783|F|0-21:A>G-21:A>G	TGCAGGAAGCAAGCAATCATGATCACCTGACTTATCGGTGACGAGCGCAACCACATCCCCGTTCACCTT	21:A>G
4997127|F|0-11:T>C-11:T>C	TGCAGGCGCTGTCGTATCTGAACGCCGTGGTGCTGGAGGCGCTGCGGCTGCATCCGCCG	11:T>C
100540688|F|0-34:C>G-34:C>G	TGCAGGCAGAGAGGGACAACATCTCGAGCGTAGACAAGTTGTGATCCG	34:C>G
100558567|F|0-55:A>G-55:A>G	TGCAGTACATGGAGCTGACCAAGACCTTCTGTGCCAGCGTGGGATGGGCCGAGTCATGCACCGCCGAGC	55:A>G
7914840|F|0-5:G>A-5:G>A	TGCAGGAGAGAAAGAAGTTCAGTGGCGGCCATGGAAAAACCCTAATCTCTTCAAGAATCGAGACGGCCG	5:G>A
100486476|F|0-56:T>C-56:T>C	TGCAGCTTATACCTCGAGACAGTGCCCTACACCAACCGCAAGCACTTGATCGTCCGTTCTATCGAGTAC	56:T>C
100575351|F|0-14:C>T-14:C>T	TGCAGCTAAGCTGCCACCACAGGCAGAGGGTTTGGAAACCACGTTGAGCAGACAGCCG	14:C>T
5004284|F|0-5:C>G-5:C>G	TGCAGCCCCGTGTCGACGGCCATCTGGCGGTAGATGACGGAGAGGTTGTTCTCCTTCACGCAGGCATCG	5:C>G
100544134|F|0-49:C>G-49:C>G	TGCAGCTGATCCGCCACAGCACGTTGCCCACCACCTGGGGGAGGAGGAACAAGTCTTGGGCGAGGCCGA	49:C>G
100589936|F|0-5:T>C-5:T>C	TGCAGTGCCGCCGACGACGACGAGGCGGAGCGTCTCTGCGGGATTCTCGACGACGTCATGGCAGAGTCT	5:T>C
100472034|F|0-44:G>C-44:G>C	TGCAGGGCCTCGTCGATCTCCTGCTTCTCGTTCTCGGACACGCCGTCCACGCACAGCTGCGTCAGGCAC	44:G>C
100505104|F|0-49:A>C-49:A>C	TGCAGTTCCTGAGGTACAGGCTCGTCAGCTTCTCCATCTTCACGCCCAGACAGCTCGGCGACGTCGCCC	49:A>C
100303861|F|0-36:T>C-36:T>C	TGCAGGTTAGCCGTATGTTGTTGATTGCAGGCCAGGTACGGCCG	36:T>C
7917611|F|0-12:A>G-12:A>G	TGCAGCATGTCCAAAACCTCACTCCAATCCCCAGGAAGCTCCTCTGCGGAAGTAAAATTGTGAATCAGT	12:A>G
100524062|F|0-57:A>G-57:A>G	TGCAGCAAGAACTTGCGCCTCCTCTCGAGCTTTTTGCGATCTTCTTCACTGCCCTCGAGCTCGGACCCG	57:A>G
100452898|F|0-68:A>G-68:A>G	TGCAGGTGGCACTCAACCACAGCAAGCTAATCGACCGCCAGCTCAGGTCGCTCTACGCAGCGCCGCCGA	68:A>G
1763093|F|0-39:A>G-39:A>G	TGCAGACCCACGGCCTCCCGCGCGGGCTGCTCCCCTCGGAGATCGCGGCGTTCACGCACGACCCGAGAT	39:A>G
100549663|F|0-60:A>T-60:A>T	TGCAGACGCAACGGCTCCGCCAGCACGAGGCTCGCCTCCGCCTCGCGCAGCTCGACTCCGAGCACGCCG	60:A>T
1739634|F|0-5:G>A-5:G>A	TGCAGGGATGGGATTGAGGAAAGTTACATTGAGGAAGCAGAGGAGGGAGCCCGCCAGCGATCCG	5:G>A
1755542|F|0-59:C>A-59:C>A	TGCAGCCCAAGCCGCTCCGCCGCGTGCTCCGCTACCTGGAGGAGGACAAGATAGTGTCGCGGGACCACC	59:C>A
100490041|F|0-52:G>A-52:G>A	TGCAGTAGATCGCACATGCAAACCCTAGCAACTAGGCTACAGGATAGTACAAGAGGCGAGGGTAAACGT	52:G>A
1022633|F|0-14:T>C-14:T>C	TGCAGCATCCTCCATGCAGAAGATCGCTCGTCCAAGTATAACGAAAGCTGACAAGACAGCAGACGTGGC	14:T>C
100564729|F|0-42:C>T-42:C>T	TGCAGCACAGCGTGACACATGCACGGCCATTTGGCACCTGGACGGTATTGATTGCACCG	42:C>T
1019171|F|0-43:C>T-43:C>T	TGCAGCACGTGTACCTGAACCTGGGCGGGGAGGGCAGCGGCGACATCCTGGGCCACACGCTCCAGCTCT	43:C>T
4008171|F|0-38:G>A-38:G>A	TGCAGGCATCGCCTCACGCGGGCCGAGGAACCCTCGACGGAGCGCTCGTCCGCGGCCGCCGCCTTCCTC	38:G>A
4008171|F|0-48:T>C-48:T>C	TGCAGGCATCGCCTCACGCGGGCCGAGGAACCCTCGACGGAGCGCTCGTCCGCGGCCGCCGCCTTCCTC	48:T>C
4396098|F|0-26:C>T-26:C>T	TGCAGCCAGACCGCCACCCCTACCCTCGCGCTCCG	26:C>T
1069301|F|0-9:C>T-9:C>T	TGCAGAACACGGTGACCCGAAGCACCAGCAACCCATCATGTACATTGTCCCCGAGGAACTTAAAAACTT	9:C>T
3954468|F|0-8:C>T-8:C>T	TGCAGCCGCGCCTTGTCCTCGGGCGGTGCCAACCTGCTCGGCTCCG	8:C>T
3956602|F|0-33:C>A-33:C>A	TGCAGAGTATGGATTGGGAAGCGATTGGAAAGGCAGAACCG	33:C>A
3957912|F|0-19:C>T-19:C>T	TGCAGCAACACAATAATAACACCACAAAACCGTCAGACATTTGAACCG	19:C>T
1038399|F|0-11:C>T-11:C>T	TGCAGCCTGATCTCCCTGTTGAACCTGGTGATGAAGTCCTCCACCCTCCG	11:C>T
7171389|F|0-9:C>A-9:C>A	TGCAGACCACACCTGTCGGCCGCTTCGACGATTCCG	9:C>A
5370960|F|0-27:A>G-27:A>G	TGCAGTCGCGCGCCCTCCGCGGCGGGAACGACGCGAAATGGCGACCCG	27:A>G
3021240|F|0-14:T>C-14:T>C	TGCAGTTGCACCGTTTGACCCTGTAATTGTGAGATTGACCCG	14:T>C
3024534|F|0-13:C>T-13:C>T	TGCAGGGATCCATCCATCCCATTATTATTGTAAGCGTAAGCGGCCG	13:C>T
1022321|F|0-47:G>A-47:G>A	TGCAGGATATGAAGGAGGGAGGCGACGACGTGCTTGAGGGAGGGGACGAGGCCG	47:G>A
5369680|F|0-6:C>T-6:C>T	TGCAGTCGTAGAGGATGACGGAGCGGAAGCGGCCCG	6:C>T
1098230|F|0-47:G>A-47:G>A	TGCAGTTACGGATGGCAGTCATCTGGTCCATGAATCATGACAGAGGCGCCTGCTCCATGAACAAATTCA	47:G>A
1069052|F|0-53:G>A-53:G>A	TGCAGCACCGACAGCTGCACGACGATGTCGCGCGCCCACTTGCCGTACATGGCGTCCAGCAGCGGCACG	53:G>A
1063013|F|0-27:G>A-27:G>A	TGCAGCAGAAGAAGAAGAGGGAGGCGCGACAAAGGCGATGGCACGCGGGTCCG	27:G>A
1063013|F|0-45:C>A-45:C>A	TGCAGCAGAAGAAGAAGAGGGAGGCGCGACAAAGGCGATGGCACGCGGGTCCG	45:C>A
1035719|F|0-46:C>T-46:C>T	TGCAGAAAAGGGTTTTCCTCGCACAAGGTCATGGCCTTTATTTCTTCTCCG	46:C>T
985416|F|0-40:G>C-40:G>C	TGCAGTGGATCATGGCGAACTTGGTGAAGCATCTGGACGTGCAGGAGGCCGTCCG	40:G>C
992840|F|0-27:G>C-27:G>C	TGCAGGAGGGTCAACCACTTCTAAACAGACCGAGGGACTGTTCGGAGCCG	27:G>C

1023306|F|0-12:C>T-12:C>T	TGCAGGTGAGGACGAGCAAGCTCAAGGCACAGAATCAAGGAAGAAAGCTGATGGGAGCTATTCGGCGCC	12:C>T
1130405|F|0-26:C>T-26:C>T	TGCAGCGACGACACCAAGTACTCCAACGGCCGCGGCCACAGGGACACCG	26:C>T
1077420|F|0-48:G>A-48:G>A	TGCAGAGTGCAGGGACTACTTCAGCAAGTCCCTGTTTGTGGTGGGAGAGTTTGGGGGGAACGACTACAA	48:G>A
1014038|F|0-53:T>C-53:T>C	TGCAGCTACCTAGAGTCAACTCTTAATTTGTACTTAGCATAGGATCAAGTACGTTTCCG	53:T>C
1020247|F|0-31:A>G-31:A>G	TGCAGATCCATCGTCGCCAAGCTGCACTACTACCTGCCCCTCTTCGGCGGCTCCTTGGAGAAGCTGCTC	31:A>G
3022154|F|0-9:G>C-9:G>C	TGCAGCTTGGCAGTCATGGCCACAGATGTCTCAAGCGCCCG	9:G>C
3022154|F|0-12:G>A-12:G>A	TGCAGCTTGGCAGTCATGGCCACAGATGTCTCAAGCGCCCG	12:G>A
6047970|F|0-16:A>G-16:A>G	TGCAGGACTGGCAGAGACGGTACTGCGAGATGCTGAAGGACAGCGGCAAGGACGTGAGGGTGTTGGAGT	16:A>G
1022726|F|0-16:G>A-16:G>A	TGCAGCTTTCATTCATGGTGTTGCATTTTCTTTCCTGATTCTAGGCCTCTTCGTCCG	16:G>A
3027302|F|0-5:A>G-5:A>G	TGCAGAGCACCGTATCTGAGATCAGCCACGCGGGCCAAATCCATCCG	5:A>G
1157139|F|0-14:A>C-14:A>C	TGCAGAGTAACCTGAGCAAGCATGACACACAACACACATAAGGATGGGATTACCTTGTCTAGTGACATT	14:A>C
4261207|F|0-26:G>T-26:G>T	TGCAGGAAGAGCAGAGGCTTGCCAATGACATCTCTCGTGTTGCCG	26:G>T
6046794|F|0-19:A>C-19:A>C	TGCAGGAGAGGCTGCGGTGACCTCTGGATCGGAGCCG	19:A>C
6046794|F|0-32:A>G-32:A>G	TGCAGGAGAGGCTGCGGTGACCTCTGGATCGGAGCCG	32:A>G
3022355|F|0-14:G>A-14:G>A	TGCAGCACGTACTGGTGGAACAGGCTCTCCTGGATCCG	14:G>A
1020008|F|0-35:C>T-35:C>T	TGCAGCTCCACCTGCACCAGCAACTCCTCAAAAATCTCAACAGAGCCATCCACCACCCTCTTCACAAGC	35:C>T
1200957|F|0-28:C>T-28:C>T	TGCAGCTAACTAGGAGCCAAATAACTAACTAGGACCTAAACAACAGCTAGCAGAACTAGTGGCATCGAT	28:C>T
4262946|F|0-14:G>C-14:G>C	TGCAGCCAGCGGTGGAGGGAGCTGCCCCGCACCGCCGCCCG	14:G>C
1020653|F|0-5:C>A-5:C>A	TGCAGCTTGGCCATTGCCCGTGGGTTCCCGACCAGCTCCGCCATGGCCCATTCCAGCGTGACGGACGAT	5:C>A
1023701|F|0-46:C>G-46:C>G	TGCAGCACGTGGTGGCGCCGCGGACGAGCTCCGCCGCGCGGCCGCCCAGGCTGACCTCAAGATCGTCCG	46:C>G
1026038|F|0-64:C>G-64:C>G	TGCAGCTCGCGCGCCGCGCCAAGATGATCGGGGACCTGCTGCACCAGCTCCACGCCGCGCAGCTCATGC	64:C>G
1032506|F|0-65:T>C-65:T>C	TGCAGAAGCCGCAGAGATGACAGCAGAAGATGAGCCACTAAACCCTTGAAACACACCAGCAGCCATTGA	65:T>C
1039551|F|0-15:T>C-15:T>C	TGCAGAATATATTGCTCAGCCAGTAGTATGTGTTATCCTGGACCGCGCTATCTTATCCG	15:T>C
5361271|F|0-37:A>G-37:A>G	TGCAGCTGGTGTGCTTCGGCTGCACCACGCTCCCGACATCCCGCTACGGCCCCTACTGGCGCAACCTCC	37:A>G
15328418|F|0-23:A>G-23:A>G	TGCAGGGCGCATCGTACACGACGAGCATACCG	23:A>G
3935387|F|0-49:G>T-49:G>T	TGCAGATATTGCCACCGAATGCCTGTCTTCCCCTTTCGAGAAAGGAGGAGCAAATCTCCTTGGATGTCC	49:G>T
5369920|F|0-5:C>G-5:C>G	TGCAGCTCAAACAACTGGAGGAGATGGCCG	5:C>G
1038080|F|0-26:A>T-26:A>T	TGCAGGTCAGGTAATGCAACGACAAGAGCTAACCGCTCGCCAAAGTACAGCCG	26:A>T
1031914|F|0-14:C>G-14:C>G	TGCAGCCTCTTCGCCCCGACGCCGTCTTTCGCCGACATGGTCGTCAGCCG	14:C>G
1087304|F|0-5:T>C-5:T>C	TGCAGTGACGCGTCTATTTCGTTGGGCGCTCCCGTTCCAAAACAAGTCCCCG	5:T>C
1667947|F|0-37:C>T-37:C>T	TGCAGTGGTTCAGCAAGAGGAAGAAGAAGGCGCTGTTCGACGCCCTGGAGAAGGTTAAATCCG	37:C>T
3020345|F|0-14:C>T-14:C>T	TGCAGTGATGTGGACTCTGAAACCATTTGGTGCCAGCCG	14:C>T
1043004|F|0-37:C>T-37:C>T	TGCAGGTGTTCGGTGAATTGCCCCACTGGGAAGATTGCGCCGCTGGCGTCGGATAAGCCAGGCTCCGAG	37:C>T
1022674|F|0-52:G>A-52:G>A	TGCAGGCCAAGAAGGGTAAGTAAATCCAACTTTTGCGTTCGCTCCCTCCTACGTCCCGCGAGGCTCGTA	52:G>A
996598|F|0-26:T>C-26:T>C	TGCAGATTCTTGCTGTTTCATGCACTTGTATATCTTGGTTCTCTTGTGTGCCG	26:T>C
981048|F|0-11:C>T-11:C>T	TGCAGATATAACCTGACGAGAAAATTCAGGATTACTTTTCTGAACAATACACCG	11:C>T
4406158|F|0-21:G>A-21:G>A	TGCAGATGCCCAGCCGCCCGCGCTTGTGCCCG	21:G>A
3027282|F|0-20:A>G-20:A>G	TGCAGCCGCGCCAGGTGGCCATCTGGTTCCAGAACCGCCGCGCCCG	20:A>G
1062505|F|0-28:T>C-28:T>C	TGCAGTGTGAAATGAACGGTGTGGTTACTCGCAATGGGGGGGACAAACAAATGAGCTGGGGAAGATGAA	28:T>C
1033696|F|0-46:G>A-46:G>A	TGCAGACTATGTGACAATGATAGGTGAGTTGACCGAGATGGTTGACGTGTACAGCTTGGGCGTGGTGCT	46:G>A
2246456|F|0-13:C>T-13:C>T	TGCAGCTGCCCGTCCACCAGCTTGAACCTGCCTGCCATCCCGTCGAACGTCGTGTTGAGAATCGCCCTA	13:C>T
1126612|F|0-13:A>G-13:A>G	TGCAGATTAGAGAACGCGGTTCTGCCAAGTCGCCAAACCTAACGCCGCAAACCGACCG	13:A>G
4261355|F|0-29:T>A-29:T>A	TGCAGAGCACATACCCTGTAAGTGCAATATTATTACCG	29:T>A
1038399|F|0-24:C>T-24:C>T	TGCAGCCTGATCTCCCTGTTGAACCTGGTGATGAAGTCCTCCACCCTCCG	24:C>T
1383267|F|0-64:C>T-64:C>T	TGCAGAGCGCGATGCCGCAGTTGTATGTTAAGGCGGCAAGGATACACTCGCCTGAGGTGGGCGGCCCCG	64:C>T
1003509|F|0-56:C>T-56:C>T	TGCAGGAGGAAGGTGATGAAGGCCATGAACCTCAAGGGCGTGCTGAGGACGGAGATCCACCCGTCGCTC	56:C>T
987292|F|0-5:A>T-5:A>T	TGCAGAACAGAGGAGGTGGGCTCTGCGCGGTGGAGAGGGGGAGTGGGTTCTTGGTTTCTTGGGGCTCCG	5:A>T
1022674|F|0-40:G>T-40:G>T	TGCAGGCCAAGAAGGGTAAGTAAATCCAACTTTTGCGTTCGCTCCCTCCTACGTCCCGCGAGGCTCGTA	40:G>T
4406158|F|0-5:A>G-5:A>G	TGCAGATGCCCAGCCGCCCGCGCTTGTGCCCG	5:A>G
1046384|F|0-37:G>A-37:G>A	TGCAGCAAACGTGTAGCGTTTTACACTGCTATTCCAGGCGTGGAGCAAATTGAATTATACTGTACACCA	37:G>A
100505022|F|0-10:C>G-10:C>G	TGCAGTGGTTCAGAAAGAGGAAGAAGAAGGCGCTGTTCGACGCCCTGGAGAAGGTTAAATCCG	10:C>G
4003335|F|0-29:C>T-29:C>T	TGCAGGTACCAGTGCAACGAGTGGGGCGTCGGCATGGAGATCGACAGCAACGTCCG	29:C>T
5577965|F|0-24:G>C-24:G>C	TGCAGCATCACCAGCTGCTTCGTCGGAGGAAACCG	24:G>C
7167467|F|0-7:T>C-7:T>C	TGCAGACTGTTGCTCCAAGGAACAACAGCAGCAGCAGCAGCCG	7:T>C
7918905|F|0-27:C>G-27:C>G	TGCAGTTCAGTTTTGGTGGGCGCCACTCTCCTCTGACGGGCGTGTGTGCAGTGCGGTCCG	27:C>G
3949931|F|0-5:C>A-5:C>A	TGCAGCGTACGGCCATCATATGGAACGTCGTCCG	5:C>A
1062536|F|0-12:T>C-12:T>C	TGCAGATCCAGATGCTAGTGCCAGATCCAGGCAAATTTCTCAGTCCAAGAACTAAAAACACTGCAAAGT	12:T>C
1072588|F|0-37:G>C-37:G>C	TGCAGTCCGTGTTGGGCATGAACATGCGGTACATCCAGTACAACATCACGGCGGAGGAGAGCACGCTGT	37:G>C
6048448|F|0-65:A>G-65:A>G	TGCAGCTTGTTCCGTCAGCCATGACGCGAATCGTGACCTCGCCCCCTTCAATAATGCATGACGTAACCG	65:A>G

3934452|F|0-16:G>C-16:G>C	TGCAGGGGGAGAAGGCGGCGGTGCGGATGGAGGCCGACCAGTTCCG	16:G>C
3937877|F|0-7:C>G-7:C>G	TGCAGCGCGTCGTGCTGGTGACGGGCGGCCGCGTCGACATCAGGGGGCTCTGCCGCGGCTGGGATGTGG	7:C>G
1019940|F|0-52:G>C-52:G>C	TGCAGGTGCTGAGCGCGCTGGACGCGGCCAAGACGCAGTGGTACCACTTCACGGCCATCGTCGTCGCCG	52:G>C
3952675|F|0-7:C>T-7:C>T	TGCAGATCAAAACGGATGCTTGCAGAGGAGGCCG	7:C>T
1119917|F|0-49:G>A-49:G>A	TGCAGCTTACCCTCCCCACCGCCACCATGTTGCTGTCGCGGGAGATCGTGTACATTTGGCGGGACATCC	49:G>A
1089243|F|0-13:G>A-13:G>A	TGCAGAGCGTGCTGCACCAGGCGTCGGGTGCTGAGGCGGTGGACCTCACCGCCGCGCTCATCCG	13:G>A
7159089|F|0-10:G>C-10:G>C	TGCAGGATGAGGCCGCAGCTCCAGTCTTCTCCCCGCAGTGGTCCAGGGCGCTATCACTGTCGTCCTCAG	10:G>C
1089586|F|0-32:A>T-32:A>T	TGCAGCACGAGTTTGGACCGTGCCCAGCGCGTACGGGCGCACAACCGCGGCTGCCGTCACCGACGCCGT	32:A>T
1021176|F|0-47:A>C-47:A>C	TGCAGAGTTTCCCTCTAGATAGCAGCACAATCCCAAGCGCCAATCAAAGGCCATCATCACTAGAGCCAC	47:A>C
1017299|F|0-22:T>G-22:T>G	TGCAGGTGCAGGGCATGCATGGTCTTACAGATTCTCTTGAGCTTGTCTTTGAGGTCGGCGTGGTGCTCG	22:T>G
3027258|F|0-11:C>A-11:C>A	TGCAGCTGGGACGCTACCTGGTCCCGCAGCCTCGTCCG	11:C>A
3952675|F|0-18:C>G-18:C>G	TGCAGATCAAAACGGATGCTTGCAGAGGAGGCCG	18:C>G
4009325|F|0-6:C>A-6:C>A	TGCAGGCAGAGAGGGACAACATCTCGAGTGCAGAGAAGTTTTGATCCG	6:C>A
4261872|F|0-16:A>G-16:A>G	TGCAGACCGCATCTGGAAAGGCAGTCTCCATCTGCTTTGATTCATCCG	16:A>G
6049030|F|0-18:C>A-18:C>A	TGCAGACGAGAGGAGCAGCTGATTGTCGACCATAGCAGTGGAACGCAGATTGTGGGAAACCG	18:C>A
3939712|F|0-38:T>C-38:T>C	TGCAGCCGCACTCGTAGCTGCCCCAGGTGTTCTTGCAGTGGCAGTCCG	38:T>C
7917013|F|0-17:G>A-17:G>A	TGCAGCGCAAACAACTGGAGGAAATGGCCG	17:G>A
32027120|F|0-6:A>G-6:A>G	TGCAGCACGTGGACGACACGCTGGGCGTGTTCCACACCCACGCCGTGGCGGGGTTCCTCGGCGGCGTGA	6:A>G
32016810|F|0-19:C>A-19:C>A	TGCAGACGCAGCGCGCGGCCAGGAGCGAGGCCAGCGGCAGGCGGCTCAGCACCATCTCCAGCACATCGT	19:C>A
4002514|F|0-24:T>A-24:T>A	TGCAGAGGACGGGAGGTACGCCCGTGCAGTTGGCAATAATGACCCG	24:T>A
2242245|F|0-25:G>A-25:G>A	TGCAGCCAAGCATCGATGACCACCAGTTATGTTTCTCAAGATACCGTATGCCCATCAGTTGGGTGGCAA	25:G>A
1030892|F|0-29:C>T-29:C>T	TGCAGATTTCGGTGTGGAAGAAATTCAACCTGATCTTCCTCTTCTTCCTACTCCG	29:C>T
1074143|F|0-22:A>T-22:A>T	TGCAGCTTAGATGGATCTGTATATTGCAGCTTTTGCACATACTTCATTTAATTCTCCG	22:A>T
1287396|F|0-56:C>G-56:C>G	TGCAGCTTCGGCTGCTACTTCACGCACAGCAAGGGGAGCTTCATATACTTCAAGCTCGAGTCGCTCCGA	56:C>G
990141|F|0-30:G>C-30:G>C	TGCAGCTTCACGCCTCCCGTACAGGATCAAGTCCCGAGGAGGTCCAGTACTACTTACGTGAGATTACAA	30:G>C
3942400|F|0-19:G>A-19:G>A	TGCAGCTGCTGCGCCTCGAGGCCGCCACGCCG	19:G>A
1062469|F|0-40:G>T-40:G>T	TGCAGTTCTTGCAGCACACCCAGCCCATCCTCCACGAGGTGCAGGACCTGGCGGCGATGAAGCCCATCC	40:G>T
4262946|F|0-26:C>T-26:C>T	TGCAGCCAGCGGTGGAGGGAGCTGCCCCGCACCGCGGCCCG	26:C>T
7170533|F|0-47:C>T-47:C>T	TGCAGCACCACCTCCACCACGTCGCTGTGGTTCACCCTCTTCACCGACGTGGACTTCCG	47:C>T
1071743|F|0-7:C>T-7:C>T	TGCAGGGCATCGCCGATACTGCGCAGCAGCTCGGGCAGCTTCTCCTTCTTCCCCACCTCCG	7:C>T
1023701|F|0-19:G>A-19:G>A	TGCAGCACGTGGTGGCGCCGCGGACGAGCTCCGCGGCGCGGCCGCCCAGGCTGACCTCAAGATCGTCCG	19:G>A
3945602|F|0-16:C>A-16:C>A	TGCAGTCCGTGGAGCCCGCGTCTTATCCG	16:C>A
4009325|F|0-8:G>A-8:G>A	TGCAGGCAGAGAGGGACAACATCTCGAGTGTAGAGAAGTTTTGATCCG	8:G>A
1062759|F|0-39:G>C-39:G>C	TGCAGGGAAAAGCTTCGGAATCTCCTCCTTCGCGATGTTGTCATGGACGCACCTGCGGATCAGTATCCG	39:G>C
3028034|F|0-7:C>T-7:C>T	TGCAGCACGCCGCCGCCTTCCTCCCGCGCGTCCGCATCGTCTCCG	7:C>T
2241921|F|0-8:A>G-8:A>G	TGCAGATCATTGAGAGTGCGGTCGATTGCCGACGGAACGTCTTCGGGCGCGAGATCGCTGCACCTGCAT	8:A>G
1007196|F|0-12:C>T-12:C>T	TGCAGCGGCTGCCATGTTTTGTTGATGCATATGTGAGGAGGAGGTGAGCAGCAGGACGGGCCG	12:C>T
5370994|F|0-14:C>A-14:C>A	TGCAGAGGAGGAGGCGGCCGTTGCGGAAGTCCCG	14:C>A
4404458|F|0-48:C>T-48:C>T	TGCAGGCTTCCTCAGCATCGACTGCGGCGGCGGCGCCAACTACACGGACGCCCG	48:C>T
4001987|F|0-37:G>C-37:G>C	TGCAGACAGCCAATATTTCCAAAGCAGACAGTCACTGGGTACGCCCG	37:G>C
1230605|F|0-50:A>G-50:A>G	TGCAGCAGCTTTGCAGAGTGATTGATCTGCAAGACCACCACCACTGCGTTAATCATCACGGCGGCCAGC	50:A>G
1043028|F|0-54:T>C-54:T>C	TGCAGGCTAAGCACGGGATGCACCGTACTTTGTCACACGTGTGCTGGCAGCATTTGTGTGGTTCCTGAC	54:T>C
2249018|F|0-20:G>A-20:G>A	TGCAGCAGCTTGGTGGTGTCGATGGCGCCCAGCTTCTCGATGTAGATCGGCCG	20:G>A
4001987|F|0-19:C>T-19:C>T	TGCAGACAGCCAATATTTCCAAAGCAGACAGTCACTGGGTACGCCCG	19:C>T
1021485|F|0-64:A>T-64:A>T	TGCAGGGATTCAGGAAGATAGTGGCCGACAGATGGGAGTTCGCCAACGAGTTCTTCAGGAAGGGAGCAA	64:A>T
5371104|F|0-20:A>T-20:A>T	TGCAGGCGGTGATCAAGGAGACGCTCCG	20:A>T
1124651|F|0-39:G>A-39:G>A	TGCAGCTCGGAGAAGTGCATGATGGCGATGTACCCTGGCGAGGGGTCGTTGGGCTGGGGCTCCG	39:G>A
3937448|F|0-12:G>T-12:G>T	TGCAGTGAAGTGGACTCTGAAGCCATTTGGTGCCAGCCG	12:G>T
3020885|F|0-5:G>A-5:G>A	TGCAGGAGATCGATACTGTCGTTCAAAGTTCAGAAAGCCG	5:G>A
1020364|F|0-9:C>G-9:C>G	TGCAGTGAGCTCAGCGGCGGTGAAGGTGTAGGGCAGGTTGCCGACGTAGATTCGCCCG	9:C>G
1043028|F|0-6:C>A-6:C>A	TGCAGGCTAAGCACGGGATGCACCGTACTTTGTCACACGTGTGCTGGCAGCATTTGTGTGGTTCCTAAC	6:C>A
7171635|F|0-27:C>T-27:C>T	TGCAGGCCGTCGCCGAGCCGCTGCGGCCTCCTTATGTCCG	27:C>T
4261201|F|0-17:G>A-17:G>A	TGCAGCATGGAGATCGCGCCCATGCAGCCGTACACCG	17:G>A
4262403|F|0-30:C>G-30:C>G	TGCAGGTCTGTCGCCAATGCTGGGGAAGCGCCCG	30:C>G
1042871|F|0-8:G>C-8:G>C	TGCAGCAAGTGTCTAACTGACGAAGACTGAAGATACCACTTCGTCAGTTATCCTCCG	8:G>C
1019016|F|0-30:A>T-30:A>T	TGCAGATTGGTCTGCGCCTCTGCGGCGGTGAGGAGAGCACAGGACAGCGTTTGTGGTTCCG	30:A>T
977961|F|0-53:T>C-53:T>C	TGCAGAGCATCGTCTCTACAATTAAGCCACCATGTGACAGGTTGAATCTTACATCAGTAGCACCCGACA	53:T>C
5367542|F|0-24:C>A-24:C>A	TGCAGTGGCTCGACGACGAGCTCACCTCGCTCGCCCTCCCCGAACCCG	24:C>A
1020247|F|0-47:C>G-47:C>G	TGCAGATCCATCGTCGCCAAGCTGCACTACTACCTGCCCCTCTTCGGCGGCTCCTCGGAGAAGCTGCTC	47:C>G

1019056|F|0-6:A>G-6:A>G	TGCAGCAGCACCTGCGCAGGGTCACGGTGCCGCTGCTGGTGCTGCACGGCGCCGACGACATGGTGACCG	6:A>G
4542355|F|0-18:G>C-18:G>C	TGCAGCGACGGTGAGCGAGGGCGCGCGCCG	18:G>C
3024533|F|0-10:C>A-10:C>A	TGCAGGTGCACATTGTGTACATGGGGGAGCTGCACCCG	10:C>A
3024533|F|0-7:G>C-7:G>C	TGCAGGTGCACATTGTGTACATGGGGGAGCTGCACCCG	7:G>C
1035719|F|0-16:C>A-16:C>A	TGCAGAAAAGGGTTTTCCTCGCACAAGGTCATGGCCTTTATTTCTTCTCCG	16:C>A
3956790|F|0-44:T>C-44:T>C	TGCAGCGCGTGCCCACACGGGCCTTGAGGTCGGCGACGAACTCGTCCG	44:T>C
995078|F|0-29:C>G-29:C>G	TGCAGCGCCTTCACCAGCCGCTCGCCCTTCTCGGCCGAGAAGGCCTGCCG	29:C>G
3021712|F|0-12:G>A-12:G>A	TGCAGCAAAACAGAGCGAACAATCCCTTAAGAATCAGCCG	12:G>A
2275545|F|0-68:G>T-68:G>T	TGCAGGACCCTCCTCATCGTGCACCGTCGCAGTTCGGTTTCTTCTCTCTCAAGGTCGAAGGACGCCAGG	68:G>T
2275545|F|0-28:G>A-28:G>A	TGCAGGACCCTCCTCATCGTGCACCGTCGCAGTTCGGTTTCTTCTCTCTCAAGGTCGAAGGACGCCAGG	28:G>A
1145549|F|0-39:G>A-39:G>A	TGCAGTTCATGCGGGAGTGGGGGCCGACGGTGATCTACAGGTCGAGGACGGGGACGGAGCGGATGGTCA	39:G>A
12469787|F|0-14:A>G-14:A>G	TGCAGCTCTGCCTGATGATTATCTTTCTCCG	14:A>G
1129167|F|0-49:C>A-49:C>A	TGCAGGACACGGACGCCACCAGCGTCGTGCACCGCATCATCTTCAAGGCCTTCGACGAGGCCCG	49:C>A
1043028|F|0-7:T>C-7:T>C	TGCAGGCTAAGCACGGGATGCACCGTACTTTGTCACACGTGTGCTGGCAGCATTTGTGTGGTTCCTAAC	7:T>C
1046301|F|0-11:A>G-11:A>G	TGCAGCTGAAAAATCCTTCTCCCTTCCAGGTAACTGCTGCCGAACGCCGTCTGCGAGATGACATCTCCG	11:A>G
1089586|F|0-41:C>T-41:C>T	TGCAGCACGAGTTTGGACCGTGCCCAGCGCGTACGGGCGCACAACCGCGGCTGCCGTCACCGACGCCGT	41:C>T
3949931|F|0-8:A>G-8:A>G	TGCAGCGTACGGCCATCATATGGAACGTCGTCCG	8:A>G
1070948|F|0-7:C>G-7:C>G	TGCAGGTCCAGGTGAACGGGGCGACGAGGCGTGTGGGCGGGGTCTTCGGGACGCCG	7:C>G
3936251|F|0-23:G>C-23:G>C	TGCAGGTACACGGGGAAGTCGGAGTTGTTCGTCACGAGCACTGCGCCG	23:G>C
2241921|F|0-53:A>G-53:A>G	TGCAGATCATTGAGAGTGCGGTCGATTGCCGACGGAACGTCTTCGGGCGCGAGATCGCTGCACCTGCAT	53:A>G
1028023|F|0-25:A>G-25:A>G	TGCAGGTCATGACTCTTGACCCCGAATGCGCGACGGTCGACACGCCGATACTGGATGCTCTCCG	25:A>G
1145549|F|0-40:G>A-40:G>A	TGCAGTTCATGCGGGAGTGGGGGCCGACGGTGATCTACAGGTCGAGGACGGGGACGGAGCGGATGGTCA	40:G>A
6048444|F|0-31:A>G-31:A>G	TGCAGCTTGGAGACGATGGATGTGAGATGTAAGTTGCCGTGGCAGTAGGAGACGAGGCGCGCGATCTGA	31:A>G
3385512|F|0-41:T>A-41:T>A	TGCAGGTACACGGGGAAGTCGGAGTTGTTTGTCACCAGCACTGCGCCG	41:T>A
5369354|F|0-21:T>C-21:T>C	TGCAGGAGCCTGGCGCACGCGTGCGAGTCGACCCCG	21:T>C
5367362|F|0-19:C>T-19:C>T	TGCAGGGGCTGCGGCTGTACGATGCGGACAAATCCATTCTCCG	19:C>T
1019608|F|0-51:G>A-51:G>A	TGCAGGAGCTTCAGAAGAGATAGATGCCGAGGTCAAAAGGTTAGCATACCAGGAAGCTTAAGTTTCTGA	51:G>A
4262946|F|0-33:G>A-33:G>A	TGCAGCCAGCGGTGGAGGGAGCTGCCCCGCACCGCCGCCCG	33:G>A
7900110|F|0-13:G>A-13:G>A	TGCAGCAGCTGTCGCGCGGAGCGCGGAGTTCGAGCGCGGATAGGCGCGTGGAAGCGGCACCG	13:G>A
32020248|F|0-28:A>T-28:A>T	TGCAGCTGCTGCGCTCAAGGGGTGGTGTACGACGCCAACGTGATCCG	28:A>T
7353871|F|0-18:C>G-18:C>G	TGCAGCCAAGCAACAATTCTAGCTAAAAAGAGTAACTGAATACCAACCG	18:C>G
5372013|F|0-28:A>C-28:A>C	TGCAGGTCGGCATCGCGCTCTACGACAGACACGGCGGGTACATCGCCGCCTGGGAGTTCAACCTCCGCG	28:A>C
3572086|F|0-15:G>C-15:G>C	TGCAGAAACTGAGAGGCAAAGCCATGCTTAATGGAGAAGCTTGAGCCG	15:G>C
7911454|F|0-16:C>G-16:C>G	TGCAGAAGTCGCTCTCCATGCCTGCTGCGTACGACAATGCCGCTGCCGCCGCCGCCATCACCTGCACGT	16:C>G
5355669|F|0-22:C>G-22:C>G	TGCAGAAAATCTATACGATCGGCATCCGAGATCGGCGTAACGCGATTACGCGAGTGGCACCGAGCCCAT	22:C>G
5004653|F|0-41:T>A-41:T>A	TGCAGCTTTGAGACACACGAGTTTCACTGGAAACGTGGAGGTCGATCGTACCTGCGTTTATGTAGTCGA	41:T>A
5001302|F|0-15:T>G-15:T>G	TGCAGTGGGTGCAGCTGCGACTCTCTCGGCGTGGCGTCCGCTGCGATGCAACCACACGCTGGTCTCGGC	15:T>G
5361310|F|0-19:A>G-19:A>G	TGCAGAAACCCGCTCCGCCAATCCCCATCGCTCACGAAATCTCTCTTGCTCTCCTGCTCGCATCCCCAC	19:A>G
13880497|F|0-32:C>G-32:C>G	TGCAGCGCGTCGGCGCTGGCGTCGGCCGCGGCCGCGTTGAGCTCGACGAAGACCACGTAGTGCCCCGAG	32:C>G
7911745|F|0-35:C>G-35:C>G	TGCAGGCTGGCGTCGAGCAGCGCGGCCTGCTTGCCCACGTCGGCGTCGGCCACGAAGCCGCCGAACCGA	35:C>G
100327731|F|0-5:C>T-5:C>T	TGCAGCTGCACTGGTTTCGGTGCTGCTGCTGAGCCTCGCGCACGGCTCCCTGGGCGCTCATGGAGCCAG	5:C>T
100349879|F|0-17:A>G-17:A>G	TGCAGAGGATCGAGTCGAGTTCGAGACGCCGCGCAACTGGCGTTGCGAGTTGCGAGCCG	17:A>G
100351911|F|0-5:A>G-5:A>G	TGCAGAAATGTAGTTGGCAGACTTGATAACACAATGAAAGCACAAACCG	5:A>G
100480755|F|0-12:A>G-12:A>G	TGCAGGCCCGTCAGGCTGTGGATGCCGCTCCCTTCTCGCCG	12:A>G
100580278|F|0-31:T>C-31:T>C	TGCAGGAGGACGCGCGCGAGCACGCGACGCGTGCACAAGGCGAGACCGCGGCCG	31:T>C
14385278|F|0-57:C>G-57:C>G	TGCAGCCTCGTGGGGGAAGAGGAGGAGCAGGCGCTGCATCTCGGCAAGTTCGTGGGACACCTGCCGAGA	57:C>G
100503777|F|0-9:T>G-9:T>G	TGCAGGGCATCAGTTACATGGAGTTGCGTTGACGCGACCCGTCGATTGGCGATGGGGCTTGGCGTCCGA	9:T>G
100583591|F|0-31:C>G-31:C>G	TGCAGTGTCCGCGTCGACGACCACATCGCTGCCTTCGGCTTCAACAACCGCAGGGGTTGGAGACGGCAG	31:C>G
100471743|F|0-5:A>G-5:A>G	TGCAGACGTACAGATCGAGGCGGTCTCCCTTGCACATGCCGCTCTCCTGCACGAGCCGCCTCCAGTCGG	5:A>G
5978970|F|0-14:T>G-14:T>G	TGCAGCACGGAGGATAGAAAGGGCATCATCACGACCACGGTGAGTTCGCATGTGGTGCAGAACTCGTAC	14:T>G
100304097|F|0-29:C>A-29:C>A	TGCAGTCCAAGGCCGCGGAGCCGTACCTGCGGCTGGTCGGCATGGACCG	29:C>A
100557028|F|0-29:A>G-29:A>G	TGCAGCCTCTACGGGGACGCACTGGCGTCACCCTCGCTGGAGCGCCTCGCCATCGTCGACTGTCACACC	29:A>G
100472867|F|0-29:A>G-29:A>G	TGCAGTACACGATATGCTCCTCCTCTTGAAAACACAATTTGCATTCCTGTTAGCAGTAGTAAATTGGGT	29:A>G
100494832|F|0-33:T>G-33:T>G	TGCAGCAGCCAGATTTCGTGCGATTTGACGGACTCCG	33:T>G
100504359|F|0-24:T>A-24:T>A	TGCAGGTGTTGGTGCCGCCCGTGCTGTCGAGGACCTGTTGCTCCGACATGGAGATGAGGTTGCCGAGAT	24:T>A
100525767|F|0-37:G>A-37:G>A	TGCAGCTCCCCGCGGCCGCCACCGCCACCGCGCTGGGGGGAGGACGCTGAGTGCTCCTCCATGATCGAT	37:G>A
4993411|F|0-17:C>G-17:C>G	TGCAGCTCGTCGATGATCGCGCGGCCGCCG	17:C>G
1128975|F|0-46:C>T-46:C>T	TGCAGCCGCCGTCCAAGCAGCCCCGCCTCTCCTCCGCCGACTCGAACGCCG	46:C>T
992498|F|0-17:A>G-17:A>G	TGCAGAGACACTCTGCGAAATTTGTCAGTTTGGCCGAAGCTGAAGATATCCAATGGAGTCATGTAGTGT	17:A>G

3024222|F|0-6:G>T-6:G>T	TGCAGAGAGCATCACCGCCGAGGAATCAACAAGCCACCG	6:G>T
1028023|F|0-16:T>C-16:T>C	TGCAGGTCATGACTCTTGACCCCGAATGCGCGACGGTGGACACGCCGATACTGGATGCTCTCCG	16:T>C
992374|F|0-6:A>G-6:A>G	TGCAGCATCATCGTCATGGCGGTCGCAGCCAGGCCAGCCGCGACACTCGGATCTCGCCACGGATGTCAC	6:A>G
6037652|F|0-10:A>G-10:A>G	TGCAGCAGGAACGGTAACATCATTGGTTAGCTGTGATCTACAAAGATCCG	10:A>G
1028158|F|0-6:A>G-6:A>G	TGCAGCATCGGCTGACGCGACGGAGACGGCCATCTTGGCCTTCTTCTCCG	6:A>G
1233721|F|0-44:G>A-44:G>A	TGCAGCTACGAGCTTGTTTTGCCCAAGGTTCTGTCGTCCTCGCGGGCCAGCGTCACCGTCACCATCACC	44:G>A
4537810|F|0-15:T>C-15:T>C	TGCAGAGGCTGCTGTTCTCGTCGACTTCCG	15:T>C
1032068|F|0-58:T>C-58:T>C	TGCAGCACATCGGCACGTCCACGTGGCCTGGTTAAGCACCTTCCCTATGGTACATTTTTGCCCCGAGAT	58:T>C
4439521|F|0-7:C>T-7:C>T	TGCAGGGCGCGTCGTACACGACGAGCATACCG	7:C>T
6048761|F|0-6:G>A-6:G>A	TGCAGCGGCTGCCATATTTTGTTGATGCATATGCGAGGAGGAGGTGAGCAGCAGGATGGGCCG	6:G>A
1018967|F|0-12:G>A-12:G>A	TGCAGCTTCGTCGTTGCCGCACAAGCTCATATCTTTTTAGGTGTACCATCTCCTCAGCTCTGCATCTAT	12:G>A
1036296|F|0-10:G>C-10:G>C	TGCAGCATGAGAGGAAATGCCTGGCATAATCAAATGTGTGGAACCCAATCAAGTCGGCATTCAGCAGCG	10:G>C
1019083|F|0-12:C>A-12:C>A	TGCAGGCATACCCATGCATATTCATGTCGTCTCGCCATCTTCAGCGTCCG	12:C>A
4262532|F|0-20:C>G-20:C>G	TGCAGCCGCTGCGCAGATTCCTCACCTCCCTTGAACAGGGCCG	20:C>G
3937001|F|0-16:T>A-16:T>A	TGCAGCGGGAGAAGGCTGAGGCCATGATGGAGGCGCGCCAGTTCCG	16:T>A
3946276|F|0-29:G>A-29:G>A	TGCAGCCGTACCTGTCGCGCGGGAAGGACGTCCG	29:G>A
3020885|F|0-15:C>T-15:C>T	TGCAGGAGATCGATACTGTCGTTCAAAGTTCAGAAAGCCG	15:C>T
3026879|F|0-9:C>G-9:C>G	TGCAGTGGACTCGTAGTCACGTGGACTCGGTCAGGATAGCAGCCG	9:C>G
1667327|F|0-8:C>A-8:C>A	TGCAGCCTCCCGCCGTCGAGTCCCAGCCCCTTGGACAGCACCTTTGATACCCTCCG	8:C>A
1053743|F|0-38:C>T-38:C>T	TGCAGGCCGTGAACGGCGTTGTGCTTGCGCGCGAGACCCGTGTCTGATTTGGAAAGAATCAATCCGAGA	38:C>T
1019608|F|0-31:G>A-31:G>A	TGCAGGAGCTTCAGAAGAGATAGATGCCGAGGTCAAAAGGTTAGCATACCAGGAAGCTTAAGTTTCTGA	31:G>A
3949931|F|0-6:G>C-6:G>C	TGCAGCGTACGGCCATCATATGGAACGTCGTCCG	6:G>C
1014038|F|0-29:G>C-29:G>C	TGCAGCTACCTAGAGTCAACTCTTAATTTGTACTTAGCATAGGATCAAGTACGTTTCCG	29:G>C
3956790|F|0-30:C>T-30:C>T	TGCAGCGCGTGCCCACACGGGCCTTGAGGTCGGCGACGAACTCGTCCG	30:C>T
3031436|F|0-5:G>A-5:G>A	TGCAGGATGGCCAGCTTCTGGAGCTTGGAGAAGCCCG	5:G>A
12010150|F|0-10:C>T-10:C>T	TGCAGCGGTTCTCGTCGGGAGCAGAGTCCG	10:C>T
978884|F|0-9:C>T-9:C>T	TGCAGCTTCCCCTCCTCGTCCTCCACCTCCTCGTCGTTCCGTTCCGCCTCGCTCTCTTGCAGCCCGAGA	9:C>T
1025266|F|0-16:G>A-16:G>A	TGCAGGACCTGAGGGAGGCGATCGTCAAGGGGCTGGGGCTCCAGTCGGAGGAGCTCAAGGTGTCCGAGA	16:G>A
1102424|F|0-35:T>G-35:T>G	TGCAGGTCCCAGCGCAGCGTCCACGTGCGCGCCCATCCATGTCCGTCGCCG	35:T>G
1030427|F|0-25:T>C-25:T>C	TGCAGGACCAGATCACGTACAAGGTTGGCAAGCACTACGCCGACTGCCCCAGCTTCCG	25:T>C
4262843|F|0-6:C>T-6:C>T	TGCAGACTATCGGCGCTCATGGCAGAGACCG	6:C>T
977961|F|0-28:A>G-28:A>G	TGCAGAGCATCGTCTCTACAATTAAGCCACCATGTGACAGGTTGAATCTTACATCAGTAGCACCCGACA	28:A>G
977961|F|0-50:A>G-50:A>G	TGCAGAGCATCGTCTCTACAATTAAGCCACCATGTGACAGGTTGAATCTTACATCAGTAGCACCCGACA	50:A>G
3941873|F|0-14:G>C-14:G>C	TGCAGAGAGACTCTGCGAAATTTGTCGGTTTGGCCGAAGCTGAAGATATCCAATGGAGTCATGTAGCGT	14:G>C
1667327|F|0-42:T>C-42:T>C	TGCAGCCTCCCGCCGTCGAGTCCCAGCCCCTTGGACAGCACCTTTGATACCCTCCG	42:T>C
3938196|F|0-16:C>T-16:C>T	TGCAGCACCTCCGCCGCCCGCCTCTGTTTGCCG	16:C>T
3934624|F|0-7:T>A-7:T>A	TGCAGGTTTTGAGGGAGATTGAGGGCGATCTCTCCG	7:T>A
1035719|F|0-33:G>T-33:G>T	TGCAGAAAAGGGTTTTCCTCGCACAAGGTCATGGCCTTTATTTCTTCTCCG	33:G>T
3950290|F|0-15:A>T-15:A>T	TGCAGATCAACTAAGAGTTCACGCAGCCG	15:A>T
1068414|F|0-44:T>C-44:T>C	TGCAGTTAGTTCATTGCGAGACTATGACTTCTCTTGCATAGAAGTTAAGTCACCG	44:T>C
1017110|F|0-64:G>A-64:G>A	TGCAGGCGGAGGATATCTTCTACAAGAGACGTTGTGCTTCGCTGGAGCAGGAGATACTGTCAGCGTCGC	64:G>A
4261249|F|0-25:A>C-25:A>C	TGCAGGCCCAGGTAGCAGAGCGAGGAGGCGTCCG	25:A>C
3937331|F|0-14:C>A-14:C>A	TGCAGGCTCCGTATCGCGGTTATCCCCGCAGGGATCTCGCCG	14:C>A
3021712|F|0-16:G>C-16:G>C	TGCAGCAAAACAGAGCGAACAATCCCTTAAGAATCAGCCG	16:G>C
1079893|F|0-7:T>G-7:T>G	TGCAGCATCCGCCACCATCGTCCATGTTCCGCTTCTCGGCCACGCTCTGTGTTGCCACCATCGGAAATT	7:T>G
1024202|F|0-53:C>G-53:C>G	TGCAGCTCGTAGAGCACGGCGCCGTTGGCTGGCTCGGCCGAATTGTACACGTACCCTCCG	53:C>G
1141069|F|0-10:G>A-10:G>A	TGCAGTAAAAGATTTTCCTTGTACATACAGGTGAGAAGTAAAAGAGATCAATCATACTACCTTGTTCTC	10:G>A
3023776|F|0-31:C>A-31:C>A	TGCAGGCCACCCAAATCCGCACCAATTTGGGCGAAAAATTGCACCG	31:C>A
3942950|F|0-6:T>C-6:T>C	TGCAGATGATTCCAGCAATCTAACAACCG	6:T>C
12469787|F|0-20:A>C-20:A>C	TGCAGCTCTGCCTGATGATTATCTTTCTCCG	20:A>C
1217418|F|0-30:G>A-30:G>A	TGCAGCACCCCATCGCCGCGGAGCTGTCCCGAACTTTCTTGCGCTTCTTCGCGCCG	30:G>A
1277404|F|0-48:G>A-48:G>A	TGCAGCTTCCCTGGTAGCACATCGTTGCATTTGGCCGCTCGCAGCGGGGACTTGGATTGCATCCGAGAT	48:G>A
980435|F|0-23:C>G-23:C>G	TGCAGGGAAAAGCTTCGGAATCTCCTCCTTCGCGATGTTGTCATGGACGCACCTGCGGGTCAGTATCCG	23:C>G
1025266|F|0-31:G>A-31:G>A	TGCAGGACCTGAGGGAGGCGATCGTCAAGGGGCTGGGGCTCCAGTCGGAGGAGCTCAAGGTGTCCGAGA	31:G>A
1067490|F|0-21:C>G-21:C>G	TGCAGTGCACTGCACGTGAGCCCTCTTTGTTTGTGTAGATATGAACAGATCAACATTTTTCAAGACCTG	21:C>G
1667327|F|0-32:G>T-32:G>T	TGCAGCCTCCCGCCGTCGAGTCCCAGCCCCTTGGACAGCACCTTTGATACCCTCCG	32:G>T
1024410|F|0-20:C>T-20:C>T	TGCAGCGCCTTCACCAGCCGCTCGCCCTTCTCGGCCGTGAACGCCTTCCG	20:C>T
1046126|F|0-14:G>A-14:G>A	TGCAGGCTGCTCCCGCCAGAGCTCTTGATGCTGATCATCTTGCCACACCG	14:G>A
3024021|F|0-25:G>C-25:G>C	TGCAGGCACAACTCTACATCAGCAAGAAGCCTCAGCCG	25:G>C

1202486|F|0-40:G>A-40:G>A TGCAGCACCCCATCGCCGCGGAGCTGTACCGAACGTTCTTGCGCTTCTTCGCGCCG 40:G>A
3023160|F|0-7:C>T-7:C>T TGCAGGCCCAACCGTTGTTTAATTTTCGAAATTTGAAGCACCG 7:C>T
1264929|F|0-8:C>T-8:C>T TGCAGCTTCGTCGTTGCCGCACAAGCTCATGTCTTTTTAGGTGTACCATCTCCTCAGCTCTGCATCTAC 8:C>T
1232096|F|0-32:C>T-32:C>T TGCAGGTTCACCAGCTTGCTTATCGTGCTCGGCAGCCTCTCCAGCTGCCCGCACTGGAGGCCCAGGTAC 32:C>T
3030298|F|0-32:C>T-32:C>T TGCAGATCGCACACCAGGTGGATTCGACGTGGCCTCCG 32:C>T
3945781|F|0-17:G>T-17:G>T TGCAGGTAGGGCAGGTTGGGGAAGTCCGTCTCCGACAGCACCCG 17:G>T
12419861|F|0-5:C>G-5:C>G TGCAGCGCGTCGTGCTGGTGAAGGGCGGCCGCGTCGACGTCAGGCGGCTCTGCCGCGGCTGGGATGTGG 5:C>G
100503332|F|0-7:T>C-7:T>C TGCAGGCTGAGCAGCCCTTTGCCGAGATCCTTGCCGATACCTCATCCGACGCCCACGAGTCCTTTGCCT 7:T>C
1011384|F|0-31:T>C-31:T>C TGCAGCAGCTGCCTCGCCGCCTTCCAGCCGCTCGTGTCCCCGTCGTCGCAGCCGTGGTGTTGCTTCCCG 31:T>C
2242776|F|0-32:C>A-32:C>A TGCAGACATAGCCATGGCGTCGATGTTTTTCACCTCATGTACGGGTAAAGGTCCGTACGACATGGCTCC 32:C>A
1069516|F|0-38:C>A-38:C>A TGCAGTCCAAGTCGTGCCATAGCAGCAGGGCGACTCTTCCAGGATGCATCTCACGCTCAACCG 38:C>A
3955493|F|0-22:C>A-22:C>A TGCAGAACCTCACATCGGGCGGCGACAGCTTCCG 22:C>A
6037762|F|0-5:T>C-5:T>C TGCAGTGACATCGATCAATCCTCTGTACCCG 5:T>C
1039052|F|0-27:T>C-27:T>C TGCAGGTGTATTTGAAGAGCTTGCTCATCGTCTACGGGTTGTGGGCATGTTGGTAATGGAGGCGGAGCC 27:T>C
1024202|F|0-13:G>A-13:G>A TGCAGCTCGTAGAGCACGGCGCCGTTGGCTGGCTCGGCCGAATTGTACACGTACCCTCCG 13:G>A
1128975|F|0-17:C>G-17:C>G TGCAGCCGCCGTCCAAGCAGCCCCGCCTCTCCTCCGCCGACTCGAACGCCG 17:C>G
1311260|F|0-7:G>A-7:G>A TGCAGGAGCTTCAGAAGAGATAGATGCCGAGGTCAAAAGGTTAGTATACCAGGAAGCTGAAGTTTCTGA 7:G>A
1019608|F|0-52:G>A-52:G>A TGCAGGAGCTTCAGAAGAGATAGATGCCGAGGTCAAAAGGTTAGCATACCAGGAAGCTTAAGTTTCTGA 52:G>A
1021754|F|0-7:G>C-7:G>C TGCAGCCGAGCATGAACACCAGCACCAGGCACATACTCAAGTACCTCACCG 7:G>C
1089586|F|0-61:G>T-61:G>T TGCAGCACGAGTTTGGACCGTGCCCAGCGCGTACGGGCGCACAACCGCGGCTGCCGTCACCGACGCCGT 61:G>T
1030883|F|0-20:A>G-20:A>G TGCAGCCATGCTGCGGTGCGACGGCGACCGCGTCGTCATTGTCGGCGGTGGCATCGCCG 20:A>G
5370994|F|0-24:G>C-24:G>C TGCAGAGGAGGAGGCGGCCGTTGCGGAAGTCCCG 24:G>C
1120978|F|0-30:G>C-30:G>C TGCAGCGGCGACCTGCCGTTCGCGCTCGTCGGCAACTACTCTGGCCTCGCCTGCCAGCCCGTCTGGAAC 30:G>C
2245367|F|0-42:G>T-42:G>T TGCAGTAAGGGCACTGCAAAGGCACCGCTCAGATTTCGACGAGAGAAACGGTGGTGATATGTGACCGAG 42:G>T
1057314|F|0-30:C>A-30:C>A TGCAGCTAGTTCATTGCGAGACTATGACTTCTCTTCCACAGAAGTTAAGTCACCG 30:C>A
1128975|F|0-41:T>G-41:T>G TGCAGCCGCCGTCCAAGCAGCCCCGCCTCTCCTCCGCCGACTCGAACGCCG 41:T>G
2242245|F|0-23:C>T-23:C>T TGCAGCCAAGCATCGATGACCACCAGTTATGTTTCTCAAGATACCGTATGCCCATCAGTTGGGTGGCAA 23:C>T
1089877|F|0-30:T>C-30:T>C TGCAGGCGGAGGAGGACGGCGACGAACATGTCGTTTTTGCATCCAAGCCTGCGTTCTCTCCG 30:T>C
1667327|F|0-44:T>C-44:T>C TGCAGCCTCCCGCCGTCGAGTCCCAGCCCCTTGGACAGCACCTTTGATACCCTCCG 44:T>C
1287396|F|0-11:C>A-11:C>A TGCAGCTTCGGCTGCTACTTCACGCACAGCAAGGGGAGCTTCATATACTTCAAGCTCGAGTCGCTCCGA 11:C>A
1089877|F|0-48:C>A-48:C>A TGCAGGCGGAGGAGGACGGCGACGAACATGTCGTTTTTGCATCCAAGCCTGCGTTCTCTCCG 48:C>A
7149529|F|0-22:A>G-22:A>G TGCAGACACAGATGGAGAAGAGATCTCAGGGGATCGGTGAAGAAGGTCGCGACGATTCATGGAGATGAC 22:A>G
5324450|F|0-5:C>G-5:C>G TGCAGCTCCTTGCCCATGGGCGCGGCACCG 5:C>G
6037648|F|0-12:C>T-12:C>T TGCAGCAGCTTGCTTCCCTCCCCGACAAACCG 12:C>T
1396505|F|0-14:G>T-14:G>T TGCAGTCGCACTTGGCTGCATCGCAACCCGCAGCAGTTCAACACTTACACAGCCATACACATCACCTGT 14:G>T
7170899|F|0-23:T>C-23:T>C TGCAGTACGTGTCCAAGCCTGGTTGCAGACTGTTGCAACGTCCG 23:T>C
1038399|F|0-26:G>T-26:G>T TGCAGCCTGATCTCCCTGTTGAACCTGGTGATGAAGTCCTCCACCCTCCG 26:G>T
1141147|F|0-7:G>A-7:G>A TGCAGGCGACGATCGCCGCCCGCAACCCCAACTCCCGCGTCGGCATCTACTTCGACCG 7:G>A
1020000|F|0-24:A>G-24:A>G TGCAGAGGCCAATGTTGTGTTCTCATCTCTTATGGGTGCTCGGGTAAGCAACCG 24:A>G
5369342|F|0-7:C>T-7:C>T TGCAGCACTGATTCCGACCTCAAGTCTCCAAACCG 7:C>T
6045527|F|0-5:G>C-5:G>C TGCAGGGAGTCGGACCGCAGGTTGTAGCCGTTGAGGTCGACGGACGCGACGGTCCG 5:G>C
32027102|F|0-39:G>C-39:G>C TGCAGCAGGGGAGGCAAAGCCGTGCGCGAGGCGGACGGGGGCGCGTATGCGCGGGCGCAGCCG 39:G>C
978845|F|0-21:C>T-21:C>T TGCAGAGAAGACAGGGGACGCCATCAAAGCAGAGGAGAGGCAGGGGATGAACAAGGGAAGCGTCAAGAT 21:C>T
1062759|F|0-16:G>A-16:G>A TGCAGGGAAAAGCTTCGGAATCTCCTCCTTCGCGATGTTGTCATGGACGCACCTGCGGATCAGTATCCG 16:G>A
3938591|F|0-16:A>C-16:A>C TGCAGCAGTAATATGCAACTCTCTGAGCCTCCG 16:A>C
987177|F|0-14:C>T-14:C>T TGCAGCTTGTCCCTCAGGTCGTCCAGCTCCTTCCTCACCTCCACCAGCCG 14:C>T
3028857|F|0-33:C>T-33:C>T TGCAGCTGCCATGATCGGCCGATCTATCCTCGTCTCCCTCCTCCG 33:C>T
1043833|F|0-29:C>A-29:C>A TGCAGCAGCAGGTTGTGGTGCATCCCTAGCTTGTTGATATCTTCCAGGCAACCG 29:C>A
4261809|F|0-35:G>A-35:G>A TGCAGGCTGGCCACCCAGGTGCTCCACTTGTGGTCGTAGTACCG 35:G>A
1217579|F|0-5:G>A-5:G>A TGCAGGAGATGTCGCAGGACGGCGTCGTGGGCAAGCTTGGCCGCAGCACGATGAACCG 5:G>A
4002656|F|0-19:C>T-19:C>T TGCAGCTCGCCATCCTCGCCGCCACCGTCACGTACCAGACCG 19:C>T
1020000|F|0-18:G>A-18:G>A TGCAGAGGCCAATGTTGTGTTCTCATCTCTTATGGGTGCTCGGGTAAGCAACCG 18:G>A
2299065|F|0-47:C>G-47:C>G TGCAGCGTCATCACCTCCTCGATCCGCCGCCGCAGCGTGTAGAAGCTCGCGTTCGAGAAGGCCCGAGAT 47:C>G
3954031|F|0-10:T>C-10:T>C TGCAGCAGCTTTCTCACGCGTCTAGATGGGCCG 10:T>C
6035459|F|0-18:G>T-18:G>T TGCAGCTCCCCGTACGGCGCGATCTGAGCCGTCGCTGCAACGCGACCAGAAGAAGAGAAACGCGGTGAA 18:G>T
32017617|F|0-17:G>C-17:G>C TGCAGCAGCTATGCACCGTAGACGGATAAATCCAGCAGCAACTAGCACCG 17:G>C
1033841|F|0-10:G>A-10:G>A TGCAGTTCCAGGAGCATGGCCATCAATTCGCATGGTTGAGACGGGTGAATCACTTGTACCG 10:G>A
1215664|F|0-28:T>C-28:T>C TGCAGTGGCGGTGCAGGGAGGGGTCTGATCACAGGTGAACAGTGGAGCAAACCG 28:T>C
1021421|F|0-14:T>C-14:T>C TGCAGCTGGGACTATATCCAGGAGGGCAGCCAGGTGGCCAAGGACATGGACCG 14:T>C

1128975|F|0-18:A>T-18:A>T	TGCAGCCGCCGTCCAAGCAGCCCCGCCTCTCCTCCGCCGACTCGAACGCCG	18:A>T
1062505|F|0-13:G>C-13:G>C	TGCAGTGTGAAATGAACGGTGTGGTTACTCGCAATGGGGGGGACAAACAAATGAGCTGGGGAAGATGAA	13:G>C
3946153|F|0-9:G>A-9:G>A	TGCAGCTACGCAGCGCTCACGTCTCCTGCGCATACATGCACGTAGCGGAGCGAGCGATCCGTCCGAGAT	9:G>A
3957713|F|0-9:G>A-9:G>A	TGCAGCAGTGCCCTACGGGTTCGTCGCTCTCTGCATTTCATGCTATCCG	9:G>A
994295|F|0-22:A>G-22:A>G	TGCAGTGGAACCACCAAGAACAAGCCTGTCCAAGAGGCCATCGCTTGGAGCTTCTGCAAAGGTCCAGTC	22:A>G
1030892|F|0-10:G>A-10:G>A	TGCAGATTTCGGTGTGGAAGAAATTCAACCTGATCTTCCTCTTCTTCCTGCTCCG	10:G>A
1069335|F|0-14:A>G-14:A>G	TGCAGTTTTAATAAATCAAGAAACATGGAAGCAATCGTATAACAGTAAAACTCCG	14:A>G
1019016|F|0-14:C>T-14:C>T	TGCAGATTGGTGTGCGCCTCTGCGGCGGTGAGGAGAGCACAGGACAGCGTTTGTGGTTCCG	14:C>T
1091206|F|0-28:C>T-28:C>T	TGCAGTGGAGGCGCGACGCGAGGCAAGGCAGCAAGGTCAACGCTTGGACCTGGTTTTGTTCCG	28:C>T
1128975|F|0-13:C>G-13:C>G	TGCAGCCGCCGTCCAAGCAGCCCCGCCTCTCCTCCGCCGACTCGAACGCCG	13:C>G
7171888|F|0-14:C>A-14:C>A	TGCAGCTTCCCCTCCTTGTCCTCCACCTCCTCCTCGTTCCGTTCCGCCTCGCTCTCTTGCAGCCCGAGA	14:C>A
987437|F|0-10:G>C-10:G>C	TGCAGGTCCAGGGCCCTGGTTCTACTTATCGTCGTCGCCACCAACACAGCCACCTTCCTCCTCTTCTCC	10:G>C
3021119|F|0-8:C>G-8:C>G	TGCAGGCCCACATGTTTCTGTATGTGTAAGACAGATGGGCTACCG	8:C>G
7917779|F|0-18:A>G-18:A>G	TGCAGTACGCTGGGAGAGAGCTAGTGGTCACTCACCGACGCCACGCAGCTTGTTCCACGCGGAATCGAG	18:A>G
1217418|F|0-16:C>T-16:C>T	TGCAGCACCCCATCGCCGCGGAGCTGTCCCGAACTTTCTTGCGCTTCTTCGCGCCG	16:C>T
2299065|F|0-5:C>T-5:C>T	TGCAGCGTCATCACCTCCTCGATCCGCCGCCGCAGCGTGTAGAAGCTCGCGTTCGAGAAGGCCCGAGAT	5:C>T
4406642|F|0-19:C>G-19:C>G	TGCAGCTCCATAAGGTAGACCGAACCCTCCG	19:C>G
1062759|F|0-35:T>G-35:T>G	TGCAGGGAAAAGCTTCGGAATCTCCTCCTTCGCGATGTTGTCATGGACGCACCTGCGGATCAGTATCCG	35:T>G
3384979|F|0-28:C>A-28:C>A	TGCAGCAAAACAGAGCGAACAATCCCAACAGAATCAGCCG	28:C>A
3029038|F|0-8:G>C-8:G>C	TGCAGGTCGGCCTCCGTCACGGCCGCCTTCCCCTTGAACGCCTCCCG	8:G>C
3945288|F|0-14:A>T-14:A>T	TGCAGGTAGTGCCCAATCTGCACGTGGGCTGGTGGTCGCCG	14:A>T
1020850|F|0-28:C>G-28:C>G	TGCAGAACGGGCCGCAGTTCCACCTGTCCTTCACGCGGTGCGTGGACGTGAAGGCCAACTTCCTCCGAG	28:C>G
3021237|F|0-15:T>A-15:T>A	TGCAGGTGGCCAATATTCTGAACAACCCTTTCTCGCTCGTCCG	15:T>A
1026423|F|0-28:G>A-28:G>A	TGCAGAAGGCTACCAACAACTTCACCACGATTCTGGGGCAAGGGTCGTTTGGTCCTGTGTACAAGGCTG	28:G>A
997436|F|0-39:C>T-39:C>T	TGCAGCACCAATGTTCTGGCCAACCTTCATCGTTGCCATCCTTGCTGCCATCATAGCAAGCCAAGCTAT	39:C>T
4008171|F|0-46:C>T-46:C>T	TGCAGGCATCGCCTCACGCGGGCCGAGGAACCCTCGACGGAGCGCTCATCCGCGGCCGCCGCCTTCCTC	46:C>T
1083842|F|0-14:G>C-14:G>C	TGCAGCTTCCTCCAGAGGCAGATCATGTGCTGCGGCGACTGGTAGTGCGCGGTGTAGTAGCCGTCGAAG	14:G>C
4002146|F|0-5:C>G-5:C>G	TGCAGCCTCCCGAAGGTGCTGATCGACGGGCCGTACGGGGCGCCG	5:C>G
3021511|F|0-16:T>C-16:T>C	TGCAGATGTACAGGACTGTGAAGAATACCGAAAGGCCG	16:T>C
4410100|F|0-14:A>C-14:A>C	TGCAGGCGAAGGCCAGATTCACAACCCG	14:A>C
1020441|F|0-64:G>A-64:G>A	TGCAGGTGGCGGACATGCACTACGCGGACGGGCGGAGCACCGCGTGCGAGGACGTGATGCCCGAGCAGG	64:G>A
3946276|F|0-28:C>T-28:C>T	TGCAGCCGTACCTGTCGCGCGGGAAGGACGTCCG	28:C>T
991226|F|0-16:G>T-16:G>T	TGCAGACCGTTGATCAGTCTGAACTGGAGAAATGGTGGAAGAAAAACAAACGCATGTGTCCAGTTACCG	16:G>T
1026038|F|0-31:A>G-31:A>G	TGCAGCTCGCGCGCCGCGCCAAGATGATCGGAGACCTGCTGCACCAGCTCCACGCCGCGCAGCTCATGC	31:A>G
6046794|F|0-27:A>G-27:A>G	TGCAGGAGAGGCTGCGGTGACCTCTGGATCGGAGCCG	27:A>G
1030883|F|0-21:C>T-21:C>T	TGCAGCCATGCTGCGGTGCGACGGCGACCGCGTCGTCATTGTCGGCGGTGGCATCGCCG	21:C>T
1030883|F|0-5:C>T-5:C>T	TGCAGCCATGCTGCGGTGCGACGGCGACCGCGTCGTCATTGTCGGCGGTGGCATCGCCG	5:C>T
981351|F|0-12:C>T-12:C>T	TGCAGTTTCACACTGCCATCGGCATCTCGTCGTGCTCGCGGTATCACACGGCAGCTAGTATCTCATCTT	12:C>T
4261528|F|0-17:A>G-17:A>G	TGCAGGTGCAGCAGCGCAGCCCTCCTCCACGATCGGCACGACCG	17:A>G
3954468|F|0-33:C>T-33:C>T	TGCAGCCGCGCCTTGTCCTCGGGCGGTGCCAACCTGCTCGGCTCCG	33:C>T
1022118|F|0-20:G>A-20:G>A	TGCAGACACAGGAGGAGGAAGGCCATGGCAGAGGAGGGGCTTGTGCACGGGAGGGAGCAGCAAGGCGGC	20:G>A
3941443|F|0-5:A>G-5:A>G	TGCAGAAAAACGTCCATCGTGATTGCCACGCTCCG	5:A>G
1003364|F|0-65:T>C-65:T>C	TGCAGGAGGCAGGCGATGCCCAAGAGTAGGACAAGGCAAGCCATGGAAGAAGTCATTTTGATTGATTAA	65:T>C
1038665|F|0-17:G>C-17:G>C	TGCAGGACAGGAGAGGGGCCCGCGCGCGGCATGCACGTGAAAGCGAGTACGATGCACAATTATGATGTT	17:G>C
5363515|F|0-13:C>T-13:C>T	TGCAGCGGATCTGCGATGGAGTGTACCTGAACCG	13:C>T
1089586|F|0-29:C>T-29:C>T	TGCAGCACGAGTTTGGACCGTGCCCAGCGCGTACGGGCGCACAACCGCGGCTGCCGTCACCGACGCCGT	29:C>T
5580352|F|0-11:C>G-11:C>G	TGCAGGAAAAGCAGAGGCTTGGCAAGCACATCCCTGGTGTCACCG	11:C>G
6048900|F|0-13:G>A-13:G>A	TGCAGCCTTCCTGGAACATGGTGACCGCCTTTTCTCCGTCAGATTCCCCGCCG	13:G>A
1218587|F|0-35:C>A-35:C>A	TGCAGCCTCCCCAGCTCGTGCATCCCACTGAGCAACCTCGTTCATGGACTGAAGAGAAGCCACCGAGAT	35:C>A
1012031|F|0-62:G>C-62:G>C	TGCAGGTTGTCCAGCGCGTCGCGGAGCAGGTCGGCGGCGCCGCCGTACTGCTCCAGGCAGTGGTCGAAC	62:G>C
1079926|F|0-29:G>A-29:G>A	TGCAGCAGTTGCCTCGCCGCCTTCCAGCCGCTCGTGTCCCCGTCGTCGCCGCCGTGGAGTTGCTTCCCG	29:G>A
12765776|F|0-7:T>C-7:T>C	TGCAGACTATCGGCGCTCATGGCAGAGGCCG	7:T>C
1017299|F|0-13:C>T-13:C>T	TGCAGGTGCAGGGCATGCATGGTCTTACAGATTCTCTTGAGCTTGTCCTTGAGGTCGGCGTGGTGCTCG	13:C>T
4909330|F|0-29:G>C-29:G>C	TGCAGGTTGGGGTCCTTCTTGTACTTGCAGAGGCAGCTCGCCG	29:G>C
1023306|F|0-49:G>A-49:G>A	TGCAGGTGAGGACGAGCAAGCTCAAGGCACAGAATCAAGGAAGAAAGCTGATGGGAGCTATTCGGCGCC	49:G>A
4262532|F|0-27:C>T-27:C>T	TGCAGCCGCTGCGCAGATTCCTCACCTCCCTTGAACAGGGCCG	27:C>T
3939843|F|0-5:G>A-5:G>A	TGCAGGTCGAAGGAGACGAAGCCGAGGTGCGCGTCCG	5:G>A
6045729|F|0-17:T>C-17:T>C	TGCAGGCTTAGTATGGGTGACCTTGCTCATGTGCGTTGCGCTCGGCGCCG	17:T>C
4001987|F|0-29:C>A-29:C>A	TGCAGACAGCCAATATTTCCAAAGCAGACCGTCACTGGGTACGCCCG	29:C>A

2299065|F|0-35:C>A-35:C>A	TGCAGCGTCATCACCTCCTCGATCCGCCGCCGCAGCGTGTAGAAGCTCGCGTTCGAGAAGGCCCGAGAT	35:C>A
1017038|F|0-32:T>C-32:T>C	TGCAGTGCGTTCGTTTCACAGAAATCTGGTATTCTGTTCCAGTGGAAAGTCACGTGAACCTGTACTTTT	32:T>C
5371643|F|0-10:T>C-10:T>C	TGCAGCGGCTTGGCTGCGTCTGAAAGTGGTAGCAATTCCG	10:T>C
3020966|F|0-8:G>A-8:G>A	TGCAGGGTGCATGCTCACTACTGAGCTGCTGCAATCATACTCCG	8:G>A
5369498|F|0-5:G>C-5:G>C	TGCAGGTGGAGGTTGTGGCTACGGAAGTGAACCCCG	5:G>C
986139|F|0-11:A>G-11:A>G	TGCAGTTCTTCAACGTCAGCTACAATAGGCTCTGCGGCCCAGTGCCGACCG	11:A>G
1057966|F|0-19:G>C-19:G>C	TGCAGCCCATATTTGGCCTGATAAGCGTTGCCACCGACATCCTCGCCTTGGCCGAGTTGGTCACCACCC	19:G>C
990141|F|0-10:C>T-10:C>T	TGCAGCTTCACGCCTCCCGTACAGGATCAAGTCCTGAGGAGGTCCAGTACTACTTACGTGAGATTACAA	10:C>T
1036280|F|0-66:T>C-66:T>C	TGCAGGATGACACCAAGATCTTCATGGCCAAGTATTACTGCGTGCGGCTGCACGCCTTGCTCGGGATGG	66:T>C
1044960|F|0-27:A>G-27:A>G	TGCAGAGAAGACAGGGGACGCCATCAAAGCAGAGGAGCGGCACGGGATGATCAAGGGAAGCGTCAAGAT	27:A>G
3023142|F|0-18:C>G-18:C>G	TGCAGCATGAAGAAAGCTCTGGAGAGCCTGGGGCTCCG	18:C>G
1067795|F|0-10:A>G-10:A>G	TGCAGGCACCAGTTCGTATCATCCTGGTGCTGTGTATGGTTAATCTGATGGCAACAGTCGTGATCATCA	10:A>G
7171399|F|0-13:T>C-13:T>C	TGCAGACTCGAATTTCGGGTCGCGGGGGTTTCGATCGAGAGGAATCCG	13:T>C
1079893|F|0-34:C>T-34:C>T	TGCAGCATCCGCCACCATCGTCCATGTTCCGCTTCTCGGCCACGCTCTGTGTTGCCACCATCGGAAACT	34:C>T
2244037|F|0-36:G>T-36:G>T	TGCAGGTGAGCTGGGCAGCTCGTGCATCGATGAAACGTCGGAAGCAAGGCAAACCAGCTTTGGCAGTGG	36:G>T
3020451|F|0-27:A>C-27:A>C	TGCAGATTCCCAACAGTGCTTAAGCTCATCGAGGCCACTGGATGCTGACCAACGGGCAACTTCAGATTC	27:A>C
7165300|F|0-35:C>T-35:C>T	TGCAGCGATCAACAACAGGAGACGTATGGTTTACTCCGCTTTGTTCCCTGTTAGCCG	35:C>T
32026543|F|0-14:G>A-14:G>A	TGCAGATCAGTTGTGCTTGAACAGACGGGGACGGAGCAGGCCTTCCG	14:G>A
32027056|F|0-13:C>G-13:C>G	TGCAGGGGCTCGCCAACGCGCCGTCGCAGCAGCGGCCGTGGCCCGTGCAGCTCCG	13:C>G
5354314|F|0-24:C>G-24:C>G	TGCAGAGCCGCCGCGCCCGAGCTGCGTGAACTGCGAGCGCAACCTCAAGCTGGGCGAGCACGCCCTCAC	24:C>G
5371145|F|0-49:C>G-49:C>G	TGCAGATCAAGAATGGGTCAGAGTCGTCGTGGAAGGAAGCTGGGCTACTCTTGCTCAAGAGGCCGGCAA	49:C>G
4998641|F|0-32:A>C-32:A>C	TGCAGGTGTCCAAGGGCATCTCTACCCTCAAAACTTTCGTATACATCCG	32:A>C
32025161|F|0-13:A>T-13:A>T	TGCAGGTTATAATAGCCTCATTTGGTCATTGGGTTCGTCAACACGGTCGTAACCGCGTGGTCTCACGGG	13:A>T
5010547|F|0-9:A>G-9:A>G	TGCAGCGGCAGCGCTTTTCCCTCGAACGACAACTCGATTGAGCGGCGATCTTTCACGTGACGGCGGCTA	9:A>G
3021131|F|0-19:G>A-19:G>A	TGCAGATGTTTCCTCTCCTGTCAGTCCTCTTGATGTGCTCCG	19:G>A
32023249|F|0-29:C>T-29:C>T	TGCAGACAGATCCCACGAGACCAGGCTAGCTTACATGTGAATCGATGGAACGTTCTCCG	29:C>T
5354381|F|0-43:G>C-43:G>C	TGCAGATATTCACCAACTCTGACGAGGCTCACGCGGCGACCGTGCTGGAGAAGATGGGGCTGGAGGGGT	43:G>C
5364991|F|0-28:T>C-28:T>C	TGCAGAACGGGCTAAGGGGTGGATTCAGTAACGTCTCAGCGGGGTATCCACCTGCTATGAAGCCTGCCG	28:T>C
5001594|F|0-12:G>A-12:G>A	TGCAGGAAGTATGCCTCCAGGCAGCTGAAGGTGGATACACATACGGCCG	12:G>A
5370541|F|0-8:C>G-8:C>G	TGCAGCTCCACCTCGTCAGTCTTGTCGGACTCGACGGAGAGCAGCACGTTCTTGCGGCGGACGAACGTG	8:C>G
7165476|F|0-34:A>C-34:A>C	TGCAGCTCATGTACCACTTCGACTGGAGGCTGCCAGCCG	34:A>C
5364476|F|0-8:A>C-8:A>C	TGCAGGGCATCGTACTTGGAGGCCACCTCCCTGAGCATCTCGCCCAGCCGCCG	8:A>C
5361232|F|0-23:T>G-23:T>G	TGCAGCTGCACGGCGGGATGAAGTCCGTGAACCACATATGGCAGGTCGGCACCTCCGTCATCGCCGAGA	23:T>G
5366354|F|0-53:T>C-53:T>C	TGCAGGACGACGTGGGGAGGCTTATCGTTGCTCAGTTCCGCTGGCTTGATTTCTTTGTCGATGCCGACA	53:T>C
12688482|F|0-8:T>C-8:T>C	TGCAGCAATAAAATCAGACAGCATTAGCAATTCAAGAACGAGTAACAGACATAAAAGCCATGGTGATTG	8:T>C
5359374|F|0-36:T>G-36:T>G	TGCAGGTAGCCCGCGCGGGGCCGTGGTCCCACCTCTTGGGCGGGATTCGTGGCCG	36:T>G
7907793|F|0-23:A>G-23:A>G	TGCAGAAATCGACTGATCTGACGAGAGGCTGAGCATCACTAGTCTGATGCCTAGTGGTTCCCTACGTTA	23:A>G
5372448|F|0-13:A>G-13:A>G	TGCAGGAGTCGAGATGTCGCCTCCTTCAGTTCGTCAGCCGACATCGCGGCCTCGACCTCCATGGATGGC	13:A>G
5372770|F|0-59:A>G-59:A>G	TGCAGGGGGCCGTGGGCGTGCGGGGTGGCTGGGGCAGGCGCACGCAGGAGCGTGGGTGCAGCTACGGCG	59:A>G
5366856|F|0-49:C>G-49:C>G	TGCAGATTCCTTTGCCTTCTCGCGGCGACGAGGGCCGTTTCGAGGGACGCGCACGCCG	49:C>G
7919617|F|0-14:T>C-14:T>C	TGCAGACACGATGATGATTCAATCACGCAGAGGCGCAGTTCGCAGGATGACTCACCCTTCGTGGCGCCC	14:T>C
4994960|F|0-10:C>G-10:C>G	TGCAGGTGCACGAAGAAAAGAAAGTACGCGCAGCCGTCGCATGTCTTCGGAGATGGAGTCCACTGGATC	10:C>G
5005951|F|0-39:C>T-39:C>T	TGCAGGTCAACCCCATACGCCAGGTGGTTGTCATACTCGCACCAGCCCG	39:C>T
5361446|F|0-19:A>G-19:A>G	TGCAGGCCGTAGCAGCATAACATGGGTGGAATGACAGGGAATACGGTCAGCGTCAACGAGCTCCTGCTG	19:A>G
5009201|F|0-64:A>G-64:A>G	TGCAGTCCATCGGCCGCCGCCATCAGATCAGAGCTCAGCACGACGCAGGAAAATGATTGCTTTCAGTGA	64:A>G
7916861|F|0-5:T>C-5:T>C	TGCAGTGGTGCGGGAACGACGGCTCCAGCGCGGGGCTGCCCAAATCTCTCCG	5:T>C
1747022|F|0-18:C>T-18:C>T	TGCAGCCAGCAGAGCAAGCTATTCATATGGTGCAGGAAACCTTCATTGTCAAATATTACTCAAATGTCG	18:C>T
5361001|F|0-23:G>A-23:G>A	TGCAGCCTATTTACCAGCAGGACGTGGTTAGAGTCCACCCATGTGGTTGCTGATGATGTTGATGTGCAA	23:G>A
1694808|F|0-29:A>G-29:A>G	TGCAGAGACACCCGTCCGAGCTCGACGACACGGAGCCACACACTCGGTGGCGGCGATGAGTGCGCCGAG	29:A>G
100395164|F|0-28:C>T-28:C>T	TGCAGGGCTGGTAGCGGTCTTATTGCACCTCACTGTGCAAGGTAATGGATGGGTACTAAATTACAGAGG	28:C>T
100396518|F|0-9:G>A-9:G>A	TGCAGAGTTGCAGCCAAACCTACAAATTCGACTCGGATCGGATTGGGAGAATTTTTCGTCGCCCCGAGA	9:G>A
3573978|F|0-5:T>C-5:T>C	TGCAGTGGATAGGCCAGCAGTCGCAGCTTACTGAATTTCGCCG	5:T>C
7160345|F|0-31:G>A-31:G>A	TGCAGACGCCATTGTTGCCGCCGAACGGCACGCGTAAATAATGTGATTCTGGTCCG	31:G>A
100364448|F|0-7:A>G-7:A>G	TGCAGCAACGACATGCATCAGCACGCGGCACTAGATCTTCTCCAACTGTAATCAGATACCACCGCACAT	7:A>G
5365360|F|0-62:A>T-62:A>T	TGCAGCCTCAAGCTCCAAAAAAACATCACCTGTTCACAGCCAGATCTATAGCCTGCATCTGCATTTTCT	62:A>T
5004800|F|0-61:A>G-61:A>G	TGCAGCAGATAGAGGAGCGGCACTGGATCTGGATGCTCAAGGCTGTCAGCAAATTCCTCCCATACGGGA	61:A>G
100352539|F|0-29:T>C-29:T>C	TGCAGGGCAGGGACGCCCGCACGCTCATGTTGTTCCG	29:T>C
100579651|F|0-38:A>C-38:A>C	TGCAGATGGTGCCGTACGACGGCGGGAACAACGACGGCATACTCGAGCGATTCCCACGGGAACATGGCT	38:A>C
990866|F|0-14:A>G-14:A>G	TGCAGGATTTGCAAAGAGGTGATGGCCGACGCGGTGGTGGCGAGCAAGTGCTGCTTCAACAGCTTCTGC	14:A>G

100439233|F|0-25:A>G-25:A>G	TGCAGATGTTGCTTGTGACCGCAACAAGATCGTCGGGTGTCGACCGCATGAGAACCTTCCG	25:A>G
100486126|F|0-10:C>T-10:C>T	TGCAGCCATCCTCCATGTTTTATTGGCAATTGGAAAATTGCTTTCATGGAATCTTCAAAGTTGGCAACT	10:C>T
100327265|F|0-62:A>G-62:A>G	TGCAGTGGCCACTGCCAGCGGAGTGCCGTGAGTCGTTTTCACGTCAGCACATGGGCGCTGGGATCCACG	62:A>G
100416131|F|0-28:G>A-28:G>A	TGCAGCGGACATGTGTTCTGGACAAGTTGCCCG	28:G>A
100496754|F|0-51:T>A-51:T>A	TGCAGGGCCCTTTGCTTCATGCTGCTTTTTTGCCTCTCTACCGAATCATGATACACCG	51:T>A
5371526|F|0-40:C>T-40:C>T	TGCAGCGCTGGATCTGATCCTCCCACAAACTATCGCGCCACGACCAAGCAGAAGACACCACAAACTTCG	40:C>T
100398858|F|0-15:G>C-15:G>C	TGCAGCGAGCAGCACGGTGGCGGCGCCCCATCCGACCCGCTCCTGGACGTACACGACGACGGTGACCCC	15:G>C
100425420|F|0-53:C>G-53:C>G	TGCAGCAGTGCACCTGACGACTTGTCCTCAGCGGCTCTCTGTTCCCAGTACAGCTTCGCGCGATATCGA	53:C>G
6040698|F|0-39:T>C-39:T>C	TGCAGTTAGAACCTGCTTGCCTGACCTTGGGCCGTGCTTTGGATGCAGTGTTGGCTGAAAGTTTGCATG	39:T>C
100502842|F|0-54:T>C-54:T>C	TGCAGGACTTCTTTCCCATGCCAACGAGGCGCTCCGATCTGTCGACGCTGATGGTGAAGATGCGACCGA	54:T>C
100414490|F|0-12:G>A-12:G>A	TGCAGAGACAACGCACACATCCTGGGGCTCAGGGTCCGCGGATCCG	12:G>A
1276350|F|0-10:G>A-10:G>A	TGCAGAATTGGTCACGCTGGTCGTTCATTCGGCATGTAAGCGCCGTCCTGCTCCATAGACCACATACCA	10:G>A
100561983|F|0-16:C>T-16:C>T	TGCAGCGGCTGCCATGCTTTGTTGATGCATATGTGAGGAGGAGGTGAGCAGCAGGACGGGCCG	16:C>T
5354494|F|0-21:T>C-21:T>C	TGCAGCACCGTCTCCAAGAAGTCAAGACTCGGGGCGGGTCGTTGTGGTCCGCTCCCCCGACGATCTTCT	21:T>C
1079893|F|0-37:G>T-37:G>T	TGCAGCATCCGCCACCATCGTCCATGTTCCGCTTCTCGGCCACGCTCTGTGTTGCCACCATCGGAAACT	37:G>T
2246704|F|0-47:G>T-47:G>T	TGCAGGATGCCGACCCAATGAGCTGTGCAATCATCGAGTGCTGCTCCGTGAGCTCTGACTGTGCCAAGA	47:G>T
100553157|F|0-9:T>C-9:T>C	TGCAGCGCGTTGCCGGCGGCAGCCTCTCCGCCTGCGTCGCCGCCGTTGCTCCAGATGCTGATCTTGGGC	9:T>C
4983128|F|0-10:C>T-10:C>T	TGCAGCAGCGCCCATGGGGGACTGCGCCGTGGAGAACGGCCACGCCCACCCGAAGGAAGAGGACGCTCC	10:C>T
5002436|F|0-26:T>C-26:T>C	TGCAGCAGCTGGTCTCGAGCTGCCCGTACCTCGCCGAACTGACGCTCGAGGAGTGCCCGACCGCCACCA	26:T>C
100500831|F|0-29:T>C-29:T>C	TGCAGCCAGTGCCCGAGCACCTCGAACGGTAGGACGAGCAGGTGAGCCACACTCGCGAACAGTCCGAGA	29:T>C
32022673|F|0-55:C>T-55:C>T	TGCAGCTACTTATACATAACAGTGCAATAAAATTTGAACATATTAGGATTACAGTCCTACCTTAGTCCG	55:C>T
100595346|F|0-17:G>A-17:G>A	TGCAGAACGTCCCTGACGCGCTCCTGGAGCCGATCCTCGCGCGCCTCATGTCGTCGCCTCTCTGCCTCG	17:G>A
989804|F|0-41:G>C-41:G>C	TGCAGGGTGCGGAAGTCCTTGAGCGGCCGCGCGCGCGGGTGGGCCACGCTGTTCCG	41:G>C
3023918|F|0-22:C>G-22:C>G	TGCAGAACAAGATGAGAAGCAGCCGATCACGCTTCCG	22:C>G
3944715|F|0-12:G>C-12:G>C	TGCAGCCAAGGTGCGTGGAACTTTAACCG	12:G>C
978046|F|0-41:G>A-41:G>A	TGCAGCTAACTATGCCTGATTCCCACCGTTCGCCTCCTCCTGTATCGGAGGAGGATTTTCCG	41:G>A
1012031|F|0-34:C>A-34:C>A	TGCAGGTTGTCCAGCGCGTCGCGGAGCAGGTCGGCCGCGCCGCCGTACTGCTCCAGGCAGTGGTCGAAC	34:C>A
7171389|F|0-5:A>G-5:A>G	TGCAGACCACACCTGTCGGCCGCTTCGACGATTCCG	5:A>G
6045448|F|0-9:G>C-9:G>C	TGCAGCAGGGAAGTTGCATTGCAGAGAGTAATCTCCATTGGAAAAGGAAACCG	9:G>C
1232096|F|0-41:C>T-41:C>T	TGCAGGTTCACCAGCTTGCTTATCGTGCTCGGCAGCCTCTCCAGCTGCCCGCACTGGAGGCCCAGGTAC	41:C>T
1089243|F|0-32:G>C-32:G>C	TGCAGAGCGTGCTGCACCAGGCGTCGGGTGCTGAGGCGGTGGACCTCACCGCCGCGCTCATCCG	32:G>C
1083020|F|0-17:C>A-17:C>A	TGCAGCTCGTCCCGCCACGTCGGCTGTACGCCCGCCTCTTCGCCGTCCGCGGGCTACTGCGCCCGCCAC	17:C>A
7171888|F|0-8:C>T-8:C>T	TGCAGCTTCCCCTCCTTGTCCTCCACCTCCTCCTCGTTCCGTTCCGCCTCGCTCTCTTGCAGCCCGAGA	8:C>T
3064595|F|0-49:C>G-49:C>G	TGCAGTATTACAAGTTCGAGTTCGTGGTGATGTGCACGGGGAAGTACGGCGACGTGCCACGGATGCCTG	49:C>G
5581268|F|0-7:C>T-7:C>T	TGCAGAACCTCACGACGGGCGGCGACAGCTTCCG	7:C>T
4262689|F|0-31:G>T-31:G>T	TGCAGTATCTCGGACTCGATGGTGGTGACCCGCTCCAGGACCTCCG	31:G>T
5577872|F|0-25:C>A-25:C>A	TGCAGAGCAGTGGTGTGGAGTTTCGCCCCG	25:C>A
1056040|F|0-48:T>C-48:T>C	TGCAGGTTTGAAATCTGAATACTCTCGTCATGTTGTTTGGTGCAGGTATGAGCCG	48:T>C
4262383|F|0-29:G>C-29:G>C	TGCAGAGGAAGGGCCAGATCTGCTGGCTGGTGGCTCCG	29:G>C
3946734|F|0-15:C>G-15:C>G	TGCAGAGCCGTCCCACCAGGTCGACCCTCGCGCGCCG	15:C>G
3960832|F|0-7:C>G-7:C>G	TGCAGCTCAAGCTGGTGGAGGCCATCAAAGAAGCCG	7:C>G
1164713|F|0-44:A>C-44:A>C	TGCAGAAGATGAAGATGACGCTGGAGTCGGTGGAGGCCGCGCTCAGCGACGCCGAGAGGCGCTCCATCA	44:A>C
1010553|F|0-10:C>G-10:C>G	TGCAGTGCATCGCGCAGTTCCATGCGACCACGTCCCGCGTCGCCATTTCGTCGAACACCCG	10:C>G
1089586|F|0-35:G>A-35:G>A	TGCAGCACGAGTTTGGACCGTGCCCAGCGCGTACGGGCGCACAACCGCGGCTGCCGTCACCGACGCCGT	35:G>A
1208994|F|0-14:C>T-14:C>T	TGCAGGAGATGTCGCAGGACGGCGTCATGGGCAAGCTTGGCCGCGGCACGACGAACCG	14:C>T
3020711|F|0-34:A>G-34:A>G	TGCAGTAAAACTCATTTCCCAACTCTCTAGATCGACCG	34:A>G
3940307|F|0-11:G>C-11:G>C	TGCAGCAGCATGCGGCGGTTGAGCTCCG	11:G>C
3952055|F|0-14:G>C-14:G>C	TGCAGCTGCTCGCTGGACAGGGTGAACGTGGCGAGGAGCTGGTCCG	14:G>C
3939843|F|0-29:C>T-29:C>T	TGCAGGTCGAAGGAGACGAAGCCGAGGTGCGCGTCCG	29:C>T
3940626|F|0-5:C>G-5:C>G	TGCAGCTCTAAAAGGTAGACCGAACCCTCCG	5:C>G
1079926|F|0-52:C>A-52:C>A	TGCAGCAGTTGCCTCGCCGCCTTCCAGCCGCTCGTGTCCCCGTCGTCGCCGCCGTGGAGTTGCTTCCCG	52:C>A
5363219|F|0-26:C>T-26:C>T	TGCAGCAACCAGACCTCGTGCTCTGCCTACGCCG	26:C>T
3024534|F|0-30:T>C-30:T>C	TGCAGGGATCCATCCATCCCTTTATTATTGTAAGCGTAAGCGGCCG	30:T>C
4261618|F|0-8:C>A-8:C>A	TGCAGGCACGACAGGCGCCGCTGCCTCGCCATCCTCTTCTTCCG	8:C>A
1003364|F|0-50:A>T-50:A>T	TGCAGGAGGCAGGCGATGCCCAAGAGTAGGACAAGGCAAGCCATGGAAGAAGTCATTTTGATTGATTAA	50:A>T
1016289|F|0-28:G>C-28:G>C	TGCAGGATGCAGGCAATGCCCAAGAGCAGGACAAGGCAAGGCATGGAAGAAGTCATATTGATTGATTAA	28:G>C
3020934|F|0-8:C>A-8:C>A	TGCAGCAACACGGTCTTGAGCAGGAAGTTCTTGACAATCTCCG	8:C>A
4006298|F|0-15:G>C-15:G>C	TGCAGCACCGACGGCGCCATCAACACCTTAACCTCCG	15:G>C
3064424|F|0-14:T>C-14:T>C	TGCAGCGGCTTGGCTGCCTCTGAAAGTGGTAGCAATTCCG	14:T>C

<table>
<tr><td>1038822|F|0-29:A>G-29:A>G</td><td>TGCAGCTCAGAACGTGGCACCCCAGCCACACCCTGCACCTTGAGGCAGAGCCTGAGCAGGAAGGCCTCC</td><td>29:A>G</td></tr>
<tr><td>1038822|F|0-8:A>G-8:A>G</td><td>TGCAGCTCAGAACGTGGCACCCCAGCCACACCCTGCACCTTGAGGCAGAGCCTGAGCAGGAAGGCCTCC</td><td>8:A>G</td></tr>
<tr><td>1061219|F|0-28:T>C-28:T>C</td><td>TGCAGTCCGAAATAGACGATTCCCACAATGATCAGCATGAACCCAACCAGGAACAGAAGCCCG</td><td>28:T>C</td></tr>
<tr><td>3021843|F|0-6:G>A-6:G>A</td><td>TGCAGCGTCCACTTCTATTTTTGGGGCATCAGATTCTACCTCATCCG</td><td>6:G>A</td></tr>
<tr><td>1017038|F|0-18:C>T-18:C>T</td><td>TGCAGTGCGTTCGTTTCACAGAAATCTGGTATTCTGTTCCAGTGGAAAGTCACGTGAACCTGTACTTTT</td><td>18:C>T</td></tr>
<tr><td>3937142|F|0-29:C>T-29:C>T</td><td>TGCAGAAGCTTCTGTTGGCCGACAACAAGCTGTCCG</td><td>29:C>T</td></tr>
<tr><td>1026982|F|0-25:C>T-25:C>T</td><td>TGCAGGAGACGCTGGACTACGCGTGCGGGCAGGGCGCGGACTGCGCGCCG</td><td>25:C>T</td></tr>
<tr><td>2242191|F|0-56:C>A-56:C>A</td><td>TGCAGTGGCCGCACGCAGCATCTCGATGTCCTCATCTGAGGAGGGTTTGTCTGCCTCTGTTTGCCCCCG</td><td>56:C>A</td></tr>
<tr><td>3942666|F|0-23:C>A-23:C>A</td><td>TGCAGCGAGAAGACCATCCTCACCGTGAACGGCGAGTTCCCCG</td><td>23:C>A</td></tr>
<tr><td>1217418|F|0-7:C>T-7:C>T</td><td>TGCAGCACCCCATCGCCGCGGAGCTGTCCCGAACTTTCTTGCGCTTCTTCGCGCCG</td><td>7:C>T</td></tr>
<tr><td>1217998|F|0-15:T>C-15:T>C</td><td>TGCAGATGCATAGCTTACACCTCTAAGCAGAGCAGTGCCGATCAGACGACGATGAGCTTTCAGCGACAG</td><td>15:T>C</td></tr>
<tr><td>1029152|F|0-11:C>T-11:C>T</td><td>TGCAGGATTTCCTCAACATGTTCTTCAGGGAGCAGTACAAGCCGATCCCGCTGGTCTACAACCTTGTGC</td><td>11:C>T</td></tr>
<tr><td>1027276|F|0-37:G>C-37:G>C</td><td>TGCAGACGTGGAACAACGGGGCCGAGAACACGAAGCGGATCGTGGCCGCCG</td><td>37:G>C</td></tr>
<tr><td>3954921|F|0-7:G>C-7:G>C</td><td>TGCAGGGGAAGCCCATCACGGTGTACCG</td><td>7:G>C</td></tr>
<tr><td>5582091|F|0-28:C>T-28:C>T</td><td>TGCAGTTGGTCTGCTGGTCGGCGAAGAACGGCCAGCTGATCACCG</td><td>28:C>T</td></tr>
<tr><td>3937809|F|0-10:G>C-10:G>C</td><td>TGCAGTAGTGGACTGCTATGGTCGTGATCTCTCCG</td><td>10:G>C</td></tr>
<tr><td>5371884|F|0-23:G>A-23:G>A</td><td>TGCAGATGGCGAAGCTCGAAGGCGGCAGCAAGGATGTTGACGAAGCCATGGAGAAGTGGCACAACGGTT</td><td>23:G>A</td></tr>
<tr><td>1739623|F|0-43:T>C-43:T>C</td><td>TGCAGGCACAGGCTGACCTGTGGCGCCACAGCCTCTATTACCTTACCTCAATGGCGCTCCG</td><td>43:T>C</td></tr>
<tr><td>3020598|F|0-32:T>C-32:T>C</td><td>TGCAGGTCACCATTAGTCCATTACTTTATGGATGTCCG</td><td>32:T>C</td></tr>
<tr><td>990523|F|0-21:T>G-21:T>G</td><td>TGCAGCTACTGGGCCATTGGCTTGCTGTTTTCTACACTCGGCAACTCCG</td><td>21:T>G</td></tr>
<tr><td>4540733|F|0-5:G>T-5:G>T</td><td>TGCAGGTTACAACAATAAAGTCGAGCGGCCG</td><td>5:G>T</td></tr>
<tr><td>3034149|F|0-5:C>A-5:C>A</td><td>TGCAGCGAGAGCATCACGCCTATCATGCTCTTCGACTCCG</td><td>5:C>A</td></tr>
<tr><td>5580134|F|0-17:G>A-17:G>A</td><td>TGCAGCAACCAGACCTCGTGCTGTGCCTGCGCCG</td><td>17:G>A</td></tr>
<tr><td>4009170|F|0-58:T>G-58:T>G</td><td>TGCAGCGGCGCGTCCCCAACTTCTTCATCGTCGATGACGACGGTTCCCTCCTCCTGAGTCGCCGCGACG</td><td>58:T>G</td></tr>
<tr><td>3938702|F|0-5:T>C-5:T>C</td><td>TGCAGTGTAGATCTGCGCACTGACGTGTCCG</td><td>5:T>C</td></tr>
<tr><td>4001987|F|0-31:T>C-31:T>C</td><td>TGCAGACAGCCAATATTTCCAAAGCAGACAGTCACTGGGTACGCCCG</td><td>31:T>C</td></tr>
<tr><td>1034182|F|0-44:G>A-44:G>A</td><td>TGCAGGCCCCCGACCTGGTCCTGGCAGAGGATGGTGAGGAAGTCGCTGTCCGTGTGCGGCACCAGCCCG</td><td>44:G>A</td></tr>
<tr><td>1062168|F|0-18:T>C-18:T>C</td><td>TGCAGCAGCTTTGCAGAGTGATTGATCTCCAAGACCACCACCACTGCGTTAATCATCACGGCGGCGAGC</td><td>18:T>C</td></tr>
<tr><td>4009201|F|0-28:T>A-28:T>A</td><td>TGCAGAAAGCAGAAGAGGGGGTCGCAGTTTCATGGCACCG</td><td>28:T>A</td></tr>
<tr><td>3938866|F|0-15:T>C-15:T>C</td><td>TGCAGTTGCCGTAAATCTTGCTGCGAGATTCGCCTCCATGCCTCGAGCGATGGCTTCCTCATCGTCTCG</td><td>15:T>C</td></tr>
<tr><td>6047702|F|0-13:T>C-13:T>C</td><td>TGCAGGTTCTGAATCGACTTGGGCAGTGGCCCG</td><td>13:T>C</td></tr>
<tr><td>5373475|F|0-20:G>A-20:G>A</td><td>TGCAGCAGCAGCAAGGCACGGAACGGAGTCACCG</td><td>20:G>A</td></tr>
<tr><td>3023755|F|0-7:G>A-7:G>A</td><td>TGCAGGGGCCCTTCTCCATGCGTATCACCAGCGACTCCG</td><td>7:G>A</td></tr>
<tr><td>3533170|F|0-16:C>T-16:C>T</td><td>TGCAGGCCCTGAAATCCTCGGGTGACGTCTATCTGCTACCG</td><td>16:C>T</td></tr>
<tr><td>3030515|F|0-22:C>T-22:C>T</td><td>TGCAGCGTCGCCCTTCGGCACGCGTGTGGCGACGAAACCGACGGCCG</td><td>22:C>T</td></tr>
<tr><td>4002388|F|0-34:C>A-34:C>A</td><td>TGCAGGTACGTCGGCTTGCCCATGTCCAGCGACACGCACCG</td><td>34:C>A</td></tr>
<tr><td>3936661|F|0-13:T>C-13:T>C</td><td>TGCAGCTTTCTTTTCTAGTCGGGTCAACCG</td><td>13:T>C</td></tr>
<tr><td>3940556|F|0-15:G>C-15:G>C</td><td>TGCAGAATGAAACGCGATATGATAATGCTCCG</td><td>15:G>C</td></tr>
<tr><td>3020342|F|0-30:C>A-30:C>A</td><td>TGCAGATTTGAATCATATATAGTTTGTGTACATGTTTCCG</td><td>30:C>A</td></tr>
<tr><td>1020599|F|0-28:A>G-28:A>G</td><td>TGCAGAGAATGAAGTTATTGTCATCGCTATCATCTTTAGTACCGTAACTAGCAAGACGATTAGCTACCG</td><td>28:A>G</td></tr>
<tr><td>3951142|F|0-15:A>G-15:A>G</td><td>TGCAGAGGTGGGATCACGTCGAGAGGCACCG</td><td>15:A>G</td></tr>
<tr><td>1020648|F|0-35:G>C-35:G>C</td><td>TGCAGCTCCCGCACGATGAGCACCATGGAGGAGAAGGCGCGGCCCACCGACTCGCCG</td><td>35:G>C</td></tr>
<tr><td>4261140|F|0-17:C>G-17:C>G</td><td>TGCAGGTCCTTCTCCTCCAGGAAGAGCGAGAAGCCG</td><td>17:C>G</td></tr>
<tr><td>7168915|F|0-6:A>C-6:A>C</td><td>TGCAGGACGCTTCGGCTTCTTGGAGGCGGCGGTGGGCTTGGCGACGCAGGCGGGGTTGATCCG</td><td>6:A>C</td></tr>
<tr><td>3959653|F|0-7:C>T-7:C>T</td><td>TGCAGGACGGGGAATCTCGATCCACAAATGCACGGTGTTGGTGGACCG</td><td>7:C>T</td></tr>
<tr><td>3947831|F|0-8:G>A-8:G>A</td><td>TGCAGCGTGGAGTCGAAGGTGCGCACCTCCG</td><td>8:G>A</td></tr>
<tr><td>1091953|F|0-40:A>G-40:A>G</td><td>TGCAGGCGGAGGAGGACGGCGACGACCATGTCGTTTTCGCATCCAGGCCTGCGTTCTCCCCG</td><td>40:A>G</td></tr>
<tr><td>1023469|F|0-11:C>T-11:C>T</td><td>TGCAGTACATGCAGTTTTGGTGCAGAGAAAGCGGCGGCGACGAGGACCCG</td><td>11:C>T</td></tr>
<tr><td>3960559|F|0-30:G>A-30:G>A</td><td>TGCAGAAACTCTAGTCGGATTCGACGGATCGGCCG</td><td>30:G>A</td></tr>
<tr><td>1099470|F|0-16:C>T-16:C>T</td><td>TGCAGCACTTTGCCAACGCCTCCTCCCTTCCTCCAGCAACCCAATCCCCG</td><td>16:C>T</td></tr>
<tr><td>978046|F|0-30:C>T-30:C>T</td><td>TGCAGCTAACTATGCCTGATTCCCACCGTTCGCCTCCTCCTGTATCGGAGGAGGATTTTCCG</td><td>30:C>T</td></tr>
<tr><td>1132853|F|0-13:T>G-13:T>G</td><td>TGCAGGAACGCGATTGCTCCTGTCTACTCGCTCGCCTTGTGGTGTCATGGACAGAAGGTGTCCTCGAAG</td><td>13:T>G</td></tr>
<tr><td>3946135|F|0-21:T>C-21:T>C</td><td>TGCAGCGCGTACGTTGACTAATGGAGCCG</td><td>21:T>C</td></tr>
<tr><td>994295|F|0-54:T>C-54:T>C</td><td>TGCAGTGGAACCACCAAGAACAAGCCTGTCCAAGAGGCCATCGCTTGGAGCTTCTGCAAAGGTCCAGTC</td><td>54:T>C</td></tr>
<tr><td>4261822|F|0-23:G>A-23:G>A</td><td>TGCAGGCCGTAGGAGAACCTGGCGGCCGCGTAGAGCGCCG</td><td>23:G>A</td></tr>
<tr><td>4993746|F|0-29:C>T-29:C>T</td><td>TGCAGTTGCAAGGTCCTTGGAGTTCGAGTCACGCCG</td><td>29:C>T</td></tr>
<tr><td>1234784|F|0-59:G>A-59:G>A</td><td>TGCAGCTTGGCCATTGCCTGTGGGTTCCCGACCAGCTCCGCCATGGCCCATTCCAGCGTGAGGGACGAT</td><td>59:G>A</td></tr>
<tr><td>4262770|F|0-27:G>C-27:G>C</td><td>TGCAGGTTGGTGAGCTGTGCCAGCTCTGGCGGGATCGCGCCG</td><td>27:G>C</td></tr>
<tr><td>3020711|F|0-23:T>G-23:T>G</td><td>TGCAGTAAAACTCATTTCCCAACTCTCTAGATCGACCG</td><td>23:T>G</td></tr>
</table>

1020174|F|0-10:G>T-10:G>T	TGCAGGCCCTGAAGCAGGCCTCGGAACCTCTCCACGTTGCCACCGAACCGCCCGTCCTTCAAAATCCTG	10:G>T
988349|F|0-35:C>A-35:C>A	TGCAGGTACGTGTCGGCCACGATAGCGTCTGGCCTCCGCCCCAGCTCCAGCGCCAGCCG	35:C>A
2242643|F|0-18:C>G-18:C>G	TGCAGCTGGAGTGACGCCCTTCGTCGTTGCAGGGGTCGAGTTCGGCAAAAGAATTGTGAGTTCCGAGAT	18:C>G
1232096|F|0-9:A>T-9:A>T	TGCAGGTTCACCAGCTTGCTTATCGTGCTCGGCAGCCTCTCCAGCTGCCCGCACTGGAGGCCCAGGTAC	9:A>T
5411955|F|0-24:C>A-24:C>A	TGCAGCGTGTCGGCGGCCACGAGGCGGCCGTTCATGGCGTAGGGATCGTTGGTGGAGACCATGTCGAAG	24:C>A
1023306|F|0-6:T>C-6:T>C	TGCAGGTGAGGACGAGCAAGCTCAAGGCACAGAATCAAGGAAGAAAGCTGATGGGAGCTATTCGGCGCC	6:T>C
1149760|F|0-32:A>G-32:A>G	TGCAGCACGCTCATGTTGTGGTGCTGGTGATCATCCGTCGTCCCCTCCG	32:A>G
1026959|F|0-46:A>G-46:A>G	TGCAGAGGCTCATCTGGGTGGTGAGGCCAGATCTCTTCCAGCTGCCACCG	46:A>G
7169045|F|0-41:C>G-41:C>G	TGCAGTCCAAGTCGTGCCATAGCAGCAGGGCGACTCTTCCACGACGCATCTCACGCTCAACCG	41:C>G
7173446|F|0-57:A>G-57:A>G	TGCAGAAGCGTTGAATCGATTCCTTGGTGCGCGGCGATTGCCGAGGGGAGCGAGATGATACAGAAGCGC	57:A>G
997436|F|0-54:A>T-54:A>T	TGCAGCACCAATGTTCTGGCCAACCTTCATCGTTGCCATCCTTGCTGCCATCATAGCAAGCCAAGCTAT	54:A>T
3935896|F|0-13:T>A-13:T>A	TGCAGCGTCGCCCTTTGGCATGCGTGTGGCGACGAAACCGACGGCCG	13:T>A
3941648|F|0-18:C>T-18:C>T	TGCAGAGCAGAGTGGGCGCGGTAGATGGGCCG	18:C>T
3934144|F|0-11:G>T-11:G>T	TGCAGCAGTGCGTGCAGCGGTGCCAGCAGGACCG	11:G>T
1707968|F|0-25:C>T-25:C>T	TGCAGCTAGCGTCCGCCACGGCCTGCGTCACGCTGTCGGCGGTGCGGCTCGTCAGGCAGGACTTCGGCG	25:C>T
1136165|F|0-41:G>C-41:G>C	TGCAGGGTGTGCACGTCGGCGACGTGGGAGAGCGCCGTGCGGGAGGCCG	41:G>C
1022735|F|0-8:G>C-8:G>C	TGCAGCTTGGGGAGCAGGTAGTACCTGATCAGCTCGTCGTCGCTCGGCCTGAACCG	8:G>C
3021712|F|0-21:A>T-21:A>T	TGCAGCAAAACAGAGCGAACAATCCCTTAAGAATCAGCCG	21:A>T
3936669|F|0-26:C>T-26:C>T	TGCAGCTGCTGCGGCGTCGGCTGCTGCGAGAAGAAGTGGGGCACGCCG	26:C>T
992145|F|0-22:G>A-22:G>A	TGCAGGGCGCTGGAGCGCCTGAGGATCACTTCCATCAGCAAGTGCGACCGCCTCCATGTCCG	22:G>A
3952055|F|0-31:C>T-31:C>T	TGCAGCTGCTCGCTGGACAGGGTGAACGTGGCGAGGAGCTGGTCCG	31:C>T
1038665|F|0-56:C>T-56:C>T	TGCAGGACAGGAGAGGGGCCCGCGCGCGGCATGCACGTGAAAGCGAGTACGATGCACAATTATGATGTT	56:C>T
1089877|F|0-11:G>A-11:G>A	TGCAGGCGGAGGAGGACGGCGACGAACATGTCGTTTTTGCATCCAAGCCTGCGTTCTCTCCG	11:G>A
4261207|F|0-10:G>T-10:G>T	TGCAGGAAGAGCAGAGGCTTGCCAATGACATCTCTCGTGTTGCCG	10:G>T
32023405|F|0-6:G>T-6:G>T	TGCAGGGCTACCACGATCACAGCAATGGTGAGTCAGGCCACCG	6:G>T
1067877|F|0-33:G>T-33:G>T	TGCAGCTTGGACAGCCACGACGGTATCTGGCCCGTCAGACCGCACTCCTCCATGACGATCAGCCGAGAT	33:G>T
4406780|F|0-20:G>A-20:G>A	TGCAGGTCAATTGCGCCGCCGCACGAAGTTCACCG	20:G>A
1067877|F|0-5:C>T-5:C>T	TGCAGCTTGGACAGCCACGACGGTATCTGGCCCGTCAGACCGCACTCCTCCATGACGATCAGCCGAGAT	5:C>T
3385533|F|0-13:G>C-13:G>C	TGCAGGAGAGCGTGAGGCGCACGCGTGTGAAGTACCCG	13:G>C
1024410|F|0-32:G>C-32:G>C	TGCAGCGCCTTCACCAGCCGCTCGCCCTTCTCGGCCGTGAAGGCCTTCCG	32:G>C
1164713|F|0-22:G>C-22:G>C	TGCAGAAGATGAAGATGACGCTGGAGTCGGTGGAGGCCGCGCTCAGCGACGCCGAGAGGCGCTCCATCA	22:G>C
3570851|F|0-15:A>C-15:A>C	TGCAGGACAATGAGAAGGAGCTTTCGACCCAAAGCCAAATTCGATGGATCCG	15:A>C
1079655|F|0-42:G>A-42:G>A	TGCAGAGGATGCACGGAGAGATGTAGTGCCGTGACACGCAGCGTACACGTGAGATGCCG	42:G>A
4262532|F|0-38:G>A-38:G>A	TGCAGCCGCTGCGCAGATTCCTCACCTCCCTTGAACAGGGCCG	38:G>A
4262532|F|0-35:C>G-35:C>G	TGCAGCCGCTGCGCAGATTCCTCACCTCCCTTGAACAGGGCCG	35:C>G
3947529|F|0-16:C>T-16:C>T	TGCAGCTTGGGGACGACGGACTTGAACCG	16:C>T
3947529|F|0-11:G>C-11:G>C	TGCAGCTTGGGGACGACGGACTTGAACCG	11:G>C
5324461|F|0-13:C>T-13:C>T	TGCAGGAGATGAGCACCCGCGTAGCGGACCG	13:C>T
5361353|F|0-47:T>C-47:T>C	TGCAGGACGTCCCTGATCTGGTCGATCGCGTACCCTCCGCTGGGGTCTTCCCTCTCGACGCCATTGTGG	47:T>C
1020648|F|0-8:C>G-8:C>G	TGCAGCTCCCGCACGATGAGCACCATGGAGGAGAAGGCGCGGCCCACCGACTCGCCG	8:C>G
1233606|F|0-45:T>C-45:T>C	TGCAGTTGTTTCATTTTCTGGAGTTGCTTCAGTCCTCTCGGCCCCTTGATTCCCTGCTAAAACTCTGGG	45:T>C
1696973|F|0-47:T>C-47:T>C	TGCAGATGTTCTCGCCGCCGCTGATGATCACGTCCTTTGACCTGTCCTTTACCTCGATGTACCCATCCG	47:T>C
1045986|F|0-19:T>C-19:T>C	TGCAGCTTCAGTCCCTTGCTCGTTTACCTCAATGACAGCCTTATGAAAAACGTCATTCACAACCAGAGG	19:T>C
4733803|F|0-16:C>G-16:C>G	TGCAGATGCCGCTGTGCCAGGCACACCG	16:C>G
1088182|F|0-28:G>A-28:G>A	TGCAGACGCTATGCGCGATGAGCCAAGTGTCGCAGGACATCATCGTGGGGGACGGCGAGTTCTTCGTGG	28:G>A
3023918|F|0-6:A>C-6:A>C	TGCAGAACAAGATGAGAAGCAGCCGATCACGCTTCCG	6:A>C
7173327|F|0-16:G>A-16:G>A	TGCAGTCCGCGAGGCCGCCGTCGGGGGAGGCCGCGGCGTGGTGCGCCG	16:G>A
6026469|F|0-22:T>C-22:T>C	TGCAGAGGGATCGTGGACCCGATCAGGCCATGACACTACCATAGACTGCTCGAGTCTGTGGCCGTGTTG	22:T>C
4262532|F|0-5:C>T-5:C>T	TGCAGCCGCTGCGCAGATTCCTCACCTCCCTTGAACAGGGCCG	5:C>T
1022386|F|0-47:G>A-47:G>A	TGCAGAAGATGAAGATGACGCTGGAGGCGGTGGAGGCCGCGCTCAGCGACGCCGAGAGGCGGTCCATCA	47:G>A
3020862|F|0-15:A>T-15:A>T	TGCAGAGCAAACCAGAATAAGCCCCCAAAAAGTCAATGTTCCG	15:A>T
5008303|F|0-17:T>G-17:T>G	TGCAGGCCAGGATGCAGTGCAGCAACCG	17:T>G
1037648|F|0-34:C>G-34:C>G	TGCAGACCATCAACGGCCCCAACTCCGACTTCCCCGTCTACTCGTCGCAGCTCAAGGAGCTCAAGGGCA	34:C>G
3021100|F|0-13:G>A-13:G>A	TGCAGAGATGAGCGTCTGCACTACATTCTTCATACCG	13:G>A
4262713|F|0-8:G>C-8:G>C	TGCAGCGAGCAGAGCAGAGCAAGGAGAGGAGATTACCG	8:G>C
1141725|F|0-31:T>A-31:T>A	TGCAGAAAACTGCTGCATGTACTGCTGTTCATGAGTTGTTATGTAGCGAACCATCCG	31:T>A
4539437|F|0-7:C>A-7:C>A	TGCAGCACTTTGGCGGGTCGACGGGGTACGGCCACGACGACGCCG	7:C>A
1387412|F|0-48:C>G-48:C>G	TGCAGCAAGAACTTGCGCCTCCTCTCGAGCTTTTTGCGATCTTCTTCACTGCCCTCGGGCTCGGACCCG	48:C>G
1157517|F|0-9:G>A-9:G>A	TGCAGCTGCGCCCACGCCTCCTCCGTGTCGATCAGGCGGCGCAGCCACGCCG	9:G>A

<table>
<tr><td>3941908|F|0-41:A>T-41:A>T</td><td>TGCAGTCTTTGCAGACACATCACGGGATCACGGCACGATTCAAGCCG</td><td>41:A>T</td></tr>
<tr><td>1046301|F|0-41:G>C-41:G>C</td><td>TGCAGCTGAAAAATCCTTCTCCCTTCCAGGTAACTGCTGCCGAACGCCGTCTGCGAGATGACATCTCCG</td><td>41:G>C</td></tr>
<tr><td>1019879|F|0-21:G>A-21:G>A</td><td>TGCAGGGACAAGGACGAAGGCGACGCGATCAAGGGACTCCTCGCAACGGCGTCGGCGGCCACGTACGAC</td><td>21:G>A</td></tr>
<tr><td>1024063|F|0-10:G>A-10:G>A</td><td>TGCAGCAGCGGTTCCCCGTGGGGGACGGGACGATGGACCTGGTGCGGGCGGGGCACGCGGTGAACCGAG</td><td>10:G>A</td></tr>
<tr><td>1036280|F|0-57:T>C-57:T>C</td><td>TGCAGGATGACACCAAGATCTTCATGGCCAAGTATTACTGCGTGCGGCTGCACGCCTTGCTCGGGATGG</td><td>57:T>C</td></tr>
<tr><td>1769340|F|0-48:C>A-48:C>A</td><td>TGCAGTACATGTCTAACCTCTTGCTTGCCCATCAACACTTGATCGATTCTGCCCCATGCTACCCCAAGC</td><td>48:C>A</td></tr>
<tr><td>32024217|F|0-14:A>C-14:A>C</td><td>TGCAGGTGCCTGCAAAGACTGTCGGTATGTCTAGCCACGTCCGTGGCCG</td><td>14:A>C</td></tr>
<tr><td>1862723|F|0-67:C>G-67:C>G</td><td>TGCAGGGGCTGGCTTTGGGTCGGGCACTTCGAACGACCTTGTCAGCACGGAGCCTGACCGCTCCAGGCT</td><td>67:C>G</td></tr>
<tr><td>5365124|F|0-23:G>A-23:G>A</td><td>TGCAGATACGGGATGCTACCTGCGTGTAAATGAAGTTGTGCGGCTCGATGGTGCGGCTGAAGGCCTTGT</td><td>23:G>A</td></tr>
<tr><td>1020428|F|0-51:G>T-51:G>T</td><td>TGCAGTGGATTGCATTAGCAGCTGAACCCCCGCCAATATTCAGTGTCAGAAGCCG</td><td>51:G>T</td></tr>
<tr><td>3936835|F|0-7:C>A-7:C>A</td><td>TGCAGCCCTCGTTTCACCTGATGGGGAGAGGATGGGATCCACCG</td><td>7:C>A</td></tr>
<tr><td>1057318|F|0-17:G>A-17:G>A</td><td>TGCAGGTAATCGCATGCGTCGCCGCCGCCACATGGTGTCTTGAAATTGTGTTTCCG</td><td>17:G>A</td></tr>
<tr><td>1051518|F|0-6:T>C-6:T>C</td><td>TGCAGTTCAAGCTGATTCGTTCAAACAAAAGTCCGAAGTAATTAACAACCG</td><td>6:T>C</td></tr>
<tr><td>4261367|F|0-13:A>C-13:A>C</td><td>TGCAGTCTACAAGAGACAAAAACTCTACACATATACGTACCG</td><td>13:A>C</td></tr>
<tr><td>4262607|F|0-8:G>A-8:G>A</td><td>TGCAGGCAGTACTCGAGGTTCCACAGCACGTCGCCCATGGACGGCCG</td><td>8:G>A</td></tr>
<tr><td>6039809|F|0-40:T>C-40:T>C</td><td>TGCAGCCCTCATCCATTGATCGCGCCGCGGGCGGCCGCAATGCAACAAAAACGGCCTCTATCCTGAGGA</td><td>40:T>C</td></tr>
<tr><td>7917985|F|0-22:A>G-22:A>G</td><td>TGCAGCAGGGAAGGAGGGGGGAAGGAGCTCGAGGAGACGGAGGGGTGGTTCCCGTGGAAGCACGACGAC</td><td>22:A>G</td></tr>
<tr><td>3935731|F|0-5:G>A-5:G>A</td><td>TGCAGGATGTCCCTGATCTGGTCGATCGAGTACCCTCCGCTGGGGTCTTCCCTCTCGACGCCATTGTGG</td><td>5:G>A</td></tr>
<tr><td>3941397|F|0-26:G>T-26:G>T</td><td>TGCAGTTCCTGTCGGAGGGATCATGGGCGAACCATGACGAGATCGACCTGGAGTTCCTGGGCAACTCCA</td><td>26:G>T</td></tr>
<tr><td>4396766|F|0-6:A>G-6:A>G</td><td>TGCAGGAAGTTTCTATATGAAACGCGAACAGAACCG</td><td>6:A>G</td></tr>
<tr><td>1863668|F|0-25:C>G-25:C>G</td><td>TGCAGTACCTGGCCATGGACATCTCCACCGACTACATCGACCACTTCAAGGCCCG</td><td>25:C>G</td></tr>
<tr><td>1020803|F|0-7:G>A-7:G>A</td><td>TGCAGGCGCTAATTAGGTAAGCTGGCGCTCACAGCCTGGAGCTCGTGGGCCG</td><td>7:G>A</td></tr>
<tr><td>7172934|F|0-11:C>A-11:C>A</td><td>TGCAGGAGATGCGCGGGCGCGTGTCGAACCG</td><td>11:C>A</td></tr>
<tr><td>6044921|F|0-15:T>C-15:T>C</td><td>TGCAGCGGATCGGGGTCGGGATGCTGCTCCAGGTGGTGACCATGGTGATCACGGCGGGGATCGAGAACC</td><td>15:T>C</td></tr>
<tr><td>985423|F|0-28:T>C-28:T>C</td><td>TGCAGTGCAATCAATAACTGGCTCTGGCTGGCCGAAGTAGGCAAGGCGCGAGCTTTCCTGTTATGTACG</td><td>28:T>C</td></tr>
<tr><td>978660|F|0-17:C>T-17:C>T</td><td>TGCAGTCAAAATATGCACATGCGTTGAGTTTTTTGACTTCATATACTCCTATATTCTACCG</td><td>17:C>T</td></tr>
<tr><td>4262345|F|0-37:G>A-37:G>A</td><td>TGCAGGTGCCCACCCTCAGGAGCTGCGACTTCACGCGGCTCTACTCCG</td><td>37:G>A</td></tr>
<tr><td>3935751|F|0-26:G>A-26:G>A</td><td>TGCAGCCAGAATGTGCCGTTGGGGTCGCCG</td><td>26:G>A</td></tr>
<tr><td>2254227|F|0-48:G>C-48:G>C</td><td>TGCAGTGTGGGGAATGCGCCGATGAAGCTCGACAGTGAAGTCTGTGCCGCTTAGGTTGGTAGCGCGTCT</td><td>48:G>C</td></tr>
<tr><td>1033696|F|0-24:T>C-24:T>C</td><td>TGCAGACTATGTGACAATGATAGGTGAGTTGACCGAGATGGTTGACGTGTACAGCTTGGGCGTGGTGCT</td><td>24:T>C</td></tr>
<tr><td>4397822|F|0-23:C>G-23:C>G</td><td>TGCAGCGTCATCTGGGTAGCGAACAACCACAACCG</td><td>23:C>G</td></tr>
<tr><td>4993811|F|0-8:C>A-8:C>A</td><td>TGCAGCACCTGCTGGACATCCCAGATGGCGAAACCG</td><td>8:C>A</td></tr>
<tr><td>5581347|F|0-16:G>A-16:G>A</td><td>TGCAGCATTGCGTCCAGGGCCCTAGCCG</td><td>16:G>A</td></tr>
<tr><td>1089132|F|0-17:C>T-17:C>T</td><td>TGCAGGTTCGGCCACGACGGATGCCAGGCCGTTCACGTCAACGCCGACGTGGTTGCTGCTGATGTCCCG</td><td>17:C>T</td></tr>
<tr><td>2299403|F|0-6:T>C-6:T>C</td><td>TGCAGTTGGAGAGAGGATATCATCGATCTGGCGTAGGAAAATCGACCCG</td><td>6:T>C</td></tr>
<tr><td>3946296|F|0-25:T>A-25:T>A</td><td>TGCAGGTTGAGCATGAGGTGGTAGTTCCG</td><td>25:T>A</td></tr>
<tr><td>1045986|F|0-30:A>G-30:A>G</td><td>TGCAGCTTCAGTCCCTTGCTCGTTTACCTCAATGACAGCCTTATGAAAAACGTCATTCACAACCAGAGG</td><td>30:A>G</td></tr>
<tr><td>5371884|F|0-67:T>C-67:T>C</td><td>TGCAGATGGCGAAGCTCGAAGGCGGCAGCAAGGATGTTGACGAAGCCATGGAGAAGTGGCACAACGGTT</td><td>67:T>C</td></tr>
<tr><td>5371884|F|0-20:G>C-20:G>C</td><td>TGCAGATGGCGAAGCTCGAAGGCGGCAGCAAGGATGTTGACGAAGCCATGGAGAAGTGGCACAACGGTT</td><td>20:G>C</td></tr>
<tr><td>4262295|F|0-13:A>G-13:A>G</td><td>TGCAGACACTGACACTCCACGCCAACCG</td><td>13:A>G</td></tr>
<tr><td>7169758|F|0-35:G>C-35:G>C</td><td>TGCAGGTAGGGCAGGTTGGGGAAGTCCGTCTCCGAGAGGACCCG</td><td>35:G>C</td></tr>
<tr><td>6036195|F|0-44:C>T-44:C>T</td><td>TGCAGGCGCTGAACGTCGCCGTTGAGAGGAGCCTTGCGGCTGACCTCGTCGTCCGTCTCTGCGGGATCA</td><td>44:C>T</td></tr>
<tr><td>5582719|F|0-57:T>C-57:T>C</td><td>TGCAGCTCGTCCCGCCACGTCGGCTGTACGCCCGCCTCTTCGCCGTCCGCGGACTACTGCGTCCGCCAC</td><td>57:T>C</td></tr>
<tr><td>1157517|F|0-35:G>T-35:G>T</td><td>TGCAGCTGCGCCCACGCCTCCTCCGTGTCGATCAGGCGGCGCAGCCACGCCG</td><td>35:G>T</td></tr>
<tr><td>3956426|F|0-42:A>G-42:A>G</td><td>TGCAGCTGCGCCCACGCCTCCTCCGTGTCGATCAGGCGGCGCAACCACGCCG</td><td>42:A>G</td></tr>
<tr><td>3937159|F|0-35:A>C-35:A>C</td><td>TGCAGTCCAACATCTTCATCGAGGGCGTCTCCCACAAGATCCGCCG</td><td>35:A>C</td></tr>
<tr><td>4262383|F|0-30:T>C-30:T>C</td><td>TGCAGAGGAAGGGCCAGATCTGCTGGCTGGTGGCTCCG</td><td>30:T>C</td></tr>
<tr><td>4262383|F|0-18:T>G-18:T>G</td><td>TGCAGAGGAAGGGCCAGATCTGCTGGCTGGTGGCTCCG</td><td>18:T>G</td></tr>
<tr><td>1019765|F|0-20:C>T-20:C>T</td><td>TGCAGCTCCCGTCGTCGTTGCCACCATGGCCGCCGCTACAGCCTCTCAGACGTGAACCCTGCTGCATCT</td><td>20:C>T</td></tr>
<tr><td>1034182|F|0-26:G>C-26:G>C</td><td>TGCAGGCCCCCGACCTGGTCCTGGCAGAGGATGGTGAGGAAGTCGCTGTCCGTGTGCGGCACCAGCCCG</td><td>26:G>C</td></tr>
<tr><td>2243289|F|0-8:C>G-8:C>G</td><td>TGCAGCTGCGAGCTCAGGTCCTCGATCTTGGCCTCCCCGAAGATCACGTACGTCTCCGACGTCGGGCTC</td><td>8:C>G</td></tr>
<tr><td>4005981|F|0-39:G>A-39:G>A</td><td>TGCAGCAATGAGACTTTGGGAACTCGTCGATAAAAATCTGTACCG</td><td>39:G>A</td></tr>
<tr><td>1394726|F|0-51:A>T-51:A>T</td><td>TGCAGCCTGCATCATTCGTGATCAACACGAGAGAGAAACAGGAGTTCAGATAAAATGTGCTCGACTGCA</td><td>51:A>T</td></tr>
<tr><td>4005209|F|0-27:T>A-27:T>A</td><td>TGCAGGTAGTCGAACAGCTCGCGCTTCTTGCTGTCGCGCTCCG</td><td>27:T>A</td></tr>
<tr><td>4440229|F|0-28:A>G-28:A>G</td><td>TGCAGATACTGGCCCTGTTCGCCATGGAAACTCCG</td><td>28:A>G</td></tr>
<tr><td>2299403|F|0-32:G>T-32:G>T</td><td>TGCAGTTGGAGAGAGGATATCATCGATCTGGCGTAGGAAAATCGACCCG</td><td>32:G>T</td></tr>
<tr><td>1035719|F|0-23:C>G-23:C>G</td><td>TGCAGAAAAGGGTTTTCCTCGCACAAGGTCATGGCCTTTATTTCTTCTCCG</td><td>23:C>G</td></tr>
<tr><td>979515|F|0-14:T>C-14:T>C</td><td>TGCAGACTGGATGTTGGAGTGTGACGGAAGAAGTTGTTCTCCTTGAGGACATCCG</td><td>14:T>C</td></tr>
<tr><td>3940094|F|0-20:C>T-20:C>T</td><td>TGCAGACGACGGGTCGGCGTCGTTGCGTGTATTCTACGGCCACCG</td><td>20:C>T</td></tr>
</table>

3940807|F|0-23:G>A-23:G>A	TGCAGCGCGTACGTTGACTAATCGACCCG	23:G>A
4394235|F|0-24:G>C-24:G>C	TGCAGATCGTGATCCATGTTCCATGACGCATCACCG	24:G>C
1018967|F|0-40:G>A-40:G>A	TGCAGCTTCGTCGTTGCCGCACAAGCTCATATCTTTTTAGGTGTACCATCTCCTCAGCTCTGCATCTAT	40:G>A
3941908|F|0-17:C>G-17:C>G	TGCAGTCTTTGCAGACACATCACGGGATCACGGCACGATTCAAGCCG	17:C>G
1054953|F|0-17:G>A-17:G>A	TGCAGTGAGGCGGAGATGAGAAGAAAGGCTTGGAGTTTTTGCAGTGGACCTGAACTCCG	17:G>A
3533340|F|0-10:C>T-10:C>T	TGCAGATCACCAGAACGATGCCGCCGCCGACGCCCAACTTCGCCG	10:C>T
1056040|F|0-20:A>G-20:A>G	TGCAGGTTTGAAATCTGAATACTCTCGTCATGTTGTTTGGTGCAGGTATGAGCCG	20:A>G
6030257|F|0-6:A>G-6:A>G	TGCAGAAGTGGTGTGATCCTCGTCAGACCCG	6:A>G
4998006|F|0-18:C>T-18:C>T	TGCAGCAAGAACTTGCGCCTCCTCTCGAGCCTTTTGCGATCTTCTTCACTGCCCTCGGGCTCGGACCCG	18:C>T
1387412|F|0-20:C>T-20:C>T	TGCAGCAAGAACTTGCGCCTCCTCTCGAGCTTTTTGCGATCTTCTTCACTGCCCTCGGGCTCGGACCCG	20:C>T
3025755|F|0-35:C>T-35:C>T	TGCAGTTACGATTGGGATTCGTCCCTGATAATCATCGCCGCCCG	35:C>T
3571253|F|0-30:G>A-30:G>A	TGCAGCTGCTGCGCTCAAGGGGTGGTGTTCGACGCCAACGTGATCCG	30:G>A
4009197|F|0-44:T>A-44:T>A	TGCAGAATTTAAATTTCAGTTCGGTTGTCTGGTTTGGTCCAAGCTCCG	44:T>A
1020753|F|0-8:G>A-8:G>A	TGCAGATTGCCCAATGGATAAAGCAATATCTTGAAGGTTTTTGCCACTGCCG	8:G>A
7157715|F|0-67:T>A-67:T>A	TGCAGAAATTGTATGAAATGCACATCCATTCTCACTAGATACAGCGTTCCATAGATAGATATTTGGATT	67:T>A
4994552|F|0-30:G>A-30:G>A	TGCAGCATAACCTGCCGCCTCCAGCGCCTCGCCGATCGTCATCTTCGTATCGTCGTCAGCGGCTCCCCT	30:G>A
5360941|F|0-37:C>A-37:C>A	TGCAGCCCGCGTGGGTTCCAACGCATACCTATAAATACGCACACACACGAGCACGACACTAAGTAACAC	37:C>A
4996609|F|0-55:A>G-55:A>G	TGCAGCTGAGCTGGTCCTCGGCGTTCCGTGCACACGTGCTTGTTTATTCATTTTCAGTTTTGTATTGCG	55:A>G
4995270|F|0-38:C>G-38:C>G	TGCAGCCGAGCCCCACGGGGTGCAGGGTACAGGCCACACAGGCGAGTGAAGTGGAGTGGCCACCACACA	38:C>G
5356067|F|0-25:G>C-25:G>C	TGCAGCTCTTCCCGTTCCGCTGAAGGCCTGCAATTCACGGCAAAATTTAGCCCCCACCCGACCTGCCAA	25:G>C
32020237|F|0-13:C>T-13:C>T	TGCAGCTCCCGTTCATAACGTCCCTTAACCTCTCGTACAACGGCTTCCG	13:C>T
7918904|F|0-24:G>A-24:G>A	TGCAGTTCAGTTTTGGTGGGCGCCGCTGTCCTCTGACAGGCGTGTGTGCAGTGCGGTCCG	24:G>A
1013502|F|0-8:A>G-8:A>G	TGCAGGCCAACGAGTGACGCTGGTGCAGGCTGGTCGTCAGGGGGCGGGTGCTGCAAGTCGGCGTTGGCT	8:A>G
4996469|F|0-13:C>T-13:C>T	TGCAGTGTCCATCCGCTCACCTGCTTGTGAATGCTAATCACCGTGGATTAACTAAACAAATGGCGATCG	13:C>T
5363722|F|0-28:T>C-28:T>C	TGCAGGAGATCACGGAGGACCCGAACATTGACCCACCG	28:T>C
4994202|F|0-20:G>A-20:G>A	TGCAGCATGCATGTGACTGCGCACCATGCCAAGAAGCCCGCGCGTATCGACAACGCCCG	20:G>A
5006689|F|0-13:T>G-13:T>G	TGCAGCAAGCAAATAGGAAGACGTGGATTGCCCAATTAATGGCTGGAGCCG	13:T>G
5357540|F|0-21:G>C-21:G>C	TGCAGCAGAGCATGTGTGTGAGTAACCCGCCG	21:G>C
5004496|F|0-39:C>G-39:C>G	TGCAGATGTGATTAGCCAGCGGCAAGAGCCGATTCTGGTCAATAAACATAGCTTTTCCG	39:C>G
5354578|F|0-43:A>G-43:A>G	TGCAGCAGTCGCACGCGCTGTCGACGCCGTCGTCGACGCCGCCACTGATGCACTCGTGGGCGCAGAGCA	43:A>G
32025865|F|0-7:T>G-7:T>G	TGCAGGGTTTCCAGTCACGGGGCGTGTCGAGGGAGCAGGGCGAGGCGCTGCTCCG	7:T>G
6026411|F|0-41:G>A-41:G>A	TGCAGAGACATGCGAGCTACGTGCAAATGGATCTATGAGTAGCTCTTTTGGAGCAATCAACATGTCCGA	41:G>A
100221083|F|0-15:C>T-15:C>T	TGCAGCCTTTGCAGGCGCAGCTTGTAGCGGCCAGCCAATGCGTACGCGTATGCGTTGCCCG	15:C>T
100323414|F|0-10:T>G-10:T>G	TGCAGCTCTTTCCGCACCTCTCAATGCCTATTGCAGTTGCACAACCAGAGCATGATAAAGTAGAAATAG	10:T>G
1694748|F|0-58:G>C-58:G>C	TGCAGCCGCGACTCCTCCGAATATGGTGACGCGATGGAGAAGGAAGTGTGGCAGGGGGGGCATCGACCT	58:G>C
32021872|F|0-10:T>C-10:T>C	TGCAGCGAGATGTCGATGACCTCCACGTCGTAATAAAACGGCCTGCGGCGCCCGCCATTCACATCGGTG	10:T>C
100507737|F|0-18:C>T-18:C>T	TGCAGACCACTTCAGAGACTTCACCATCAGTAGTAACTTGTCTTTGGGTCTAGGCCAACCG	18:C>T
7169960|F|0-18:G>C-18:G>C	TGCAGCCCACGGCGGAAAGGAAGGCACGACACGCGCACGACCCCCTTCGGCCCTACCCACGCGTCCCAC	18:G>C
100512257|F|0-59:G>A-59:G>A	TGCAGAGTGATCGCTCGCTACCTGGCTCCATGCAAGAAGAGGAACAACTGAAGAAGACGGTGATGAATA	59:G>A
100452945|F|0-30:A>C-30:A>C	TGCAGGTGGTCCAAATTTCAAGTTCAAGTTATGGACTCATTCAGTCATTCTTCAACGAATATTCTCGTG	30:A>C
991533|F|0-43:T>G-43:T>G	TGCAGGTGCACACCAACAGCAAGGCGTGCGTGGCGTCGTACCCTTGCGTGGTGCAGAACAAGCTCCTCT	43:T>G
6045369|F|0-29:T>G-29:T>G	TGCAGATCCTGGATCAAAATGCCCGATTTTTCGCCG	29:T>G
4262345|F|0-13:C>G-13:C>G	TGCAGGTGCCCACCCTCAGGAGCTGCGACTTCACGCGGCTCTACTCCG	13:C>G
4261876|F|0-38:G>C-38:G>C	TGCAGGTACGGCAGGTTTGGGATGTCCGACTCGTCGGCGAGCCG	38:G>C
1666937|F|0-17:C>T-17:C>T	TGCAGCGAGAGCGGCCGCGCGGTGGTGTCGCACCACTCGGTCCTCATCTTCGAGGCCTTCTCCGCGACG	17:C>T
1023842|F|0-29:T>G-29:T>G	TGCAGCTCGTGCACGGCCGTGTTCATGTCTGCCACGGCGAAGTCCAGCGCCG	29:T>G
3533768|F|0-5:C>A-5:C>A	TGCAGCTCCACATGGACGTACCACGACGTCGACAAACATCCGCCG	5:C>A
1013852|F|0-33:T>G-33:T>G	TGCAGGGCAGTGGCACTGGCATCTAGCCAGCTATGGCAATTATATATCGCCG	33:T>G
1067877|F|0-39:C>T-39:C>T	TGCAGCTTGGACAGCCACGACGGTATCTGGCCCGTCAGACCGCACTCCTCCATGACGATCAGCCGAGAT	39:C>T
1045986|F|0-40:T>A-40:T>A	TGCAGCTTCAGTCCCTTGCTCGTTTACCTCAATGACAGCCTTATGAAAAACGTCATTCACAACCAGAGG	40:T>A
3949067|F|0-27:A>C-27:A>C	TGCAGTGCAGTGGCTACAGGCCTATGCATGCTGATGCAGGGCGGGCCG	27:A>C
3936835|F|0-9:C>A-9:C>A	TGCAGCCCTCGTTTCACCTGATGGGGAGAGGATGGGATCCACCG	9:C>A
1051518|F|0-27:A>G-27:A>G	TGCAGTTCAAGCTGATTCGTTCAAACAAAAGTCCGAAGTAATTAACAACCG	27:A>G
1045986|F|0-48:A>G-48:A>G	TGCAGCTTCAGTCCCTTGCTCGTTTACCTCAATGACAGCCTTATGAAAAACGTCATTCACAACCAGAGG	48:A>G
1157517|F|0-23:C>T-23:C>T	TGCAGCTGCGCCCACGCCTCCTCCGTGTCGATCAGGCGGCGCAGCCACGCCG	23:C>T
32027939|F|0-21:G>A-21:G>A	TGCAGGTCGCGGGTTCGAATCGCGCTCGCGTGCGTTTTTTCGTTCCG	21:G>A
999325|F|0-25:T>A-25:T>A	TGCAGGAAATGCAAACTGAACTACCTTGTTTTCAGGGATTTGAGCTTCGGTATGTTAGCCG	25:T>A
1022735|F|0-26:G>C-26:G>C	TGCAGCTTGGGGAGCAGGTAGTACCTGATCAGCTCGTCGTCGCTCGGCCTGAACCG	26:G>C
3023200|F|0-26:G>A-26:G>A	TGCAGTACCTGAAGATGGTGATCAAGGAGGCGCTCCG	26:G>A

3028857|F|0-32:T>C-32:T>C	TGCAGCTGCCATGATCGGCCGATCTATCCTCGTCTCCCTCCTCCG	32:T>C
1054953|F|0-10:C>T-10:C>T	TGCAGTGAGGCGGAGATGAAAAGAAAGGCTTGGAGTTTTTGCAGTGGACCTGAACTCCG	10:C>T
1277404|F|0-17:C>T-17:C>T	TGCAGCTTCCCTGGTAGCACATCGTTGCATTTGGCCGCTCGCAGCGGGGACTTGGATTGCATCCGAGAT	17:C>T
3020583|F|0-15:T>G-15:T>G	TGCAGTGCACCTGTCTAATTTGACTAATTGCCACCAGCATGGACCG	15:T>G
3934181|F|0-20:C>G-20:C>G	TGCAGAAACCACCAAAACGCCCAGCCGAGGAACGTGGCCCG	20:C>G
3021637|F|0-5:C>A-5:C>A	TGCAGCATGTAGCCGTGTCTCCGATCATGGACCTGTGTAGTACTCCG	5:C>A
2245367|F|0-60:G>A-60:G>A	TGCAGTAAGGGCACTGCAAAGGCACCGCTCAGATTTCGACGAGAGAAACGGTGGTGATATGTGACCGAG	60:G>A
3033027|F|0-7:G>C-7:G>C	TGCAGCTGTTGAACCAGTCATCGGGGCACGGAGCACCG	7:G>C
1067877|F|0-46:C>T-46:C>T	TGCAGCTTGGACAGCCACGACGGTATCTGGCCCGTCAGACCGCACTCCTCCATGACGATCAGCCGAGAT	46:C>T
1864490|F|0-19:G>T-19:G>T	TGCAGCACAGGAATCGGGGGAGAAAGAGGAAAGGGAAAGGAGGGATCGATCAAAAGGAGAGAAAGGCCC	19:G>T
990141|F|0-57:G>T-57:G>T	TGCAGCTTCACGCCTCCCGTACAGGATCAAGTCCCGAGGAGGTCCAGTACTACTTACGTGAGATTACAA	57:G>T
1040306|F|0-14:A>G-14:A>G	TGCAGCATGATTCAATTGAAGCTGTTGTAATTGGAGTTGTAATTTTGAGCCCTGAAAAAGGGGATTTTG	14:A>G
1264077|F|0-41:C>A-41:C>A	TGCAGACGAAGCAGCAGTTCATCTACGGCAGCGTCTCCACCCGCATCAAGCTCGTCCCG	41:C>A
5580134|F|0-19:G>A-19:G>A	TGCAGCAACCAGACCTCGTGCTGTGCCTGCGCCG	19:G>A
1028890|F|0-62:C>T-62:C>T	TGCAGGTGGAGGTGGTGCTCCTGCTGCTGCCTCCTGCTCCCCGCCGCCAACTCCTCCATGGCCGCGGCG	62:C>T
1686218|F|0-38:G>A-38:G>A	TGCAGTGTTTTCTTCTCTTCTACTTATTTGCTGTTACAGCAACACATTCTTCCTGGCCTACCG	38:G>A
3021021|F|0-32:C>A-32:C>A	TGCAGTGGGCTATTTCACATGCACACTCCAACCGAACCG	32:C>A
3937970|F|0-15:A>T-15:A>T	TGCAGTTTACACGACACACATCCCATGTTATCACCG	15:A>T
1026249|F|0-22:C>A-22:C>A	TGCAGATGCATCTCGTCGCGCTCATGCAGCAAAGGACCCAGATGCCTCCG	22:C>A
5009032|F|0-7:G>A-7:G>A	TGCAGACGTGGCAGACGAACTGCACCGCGCCGATGAGTATCTGCGCCG	7:G>A
5581044|F|0-19:G>A-19:G>A	TGCAGGAGAAGGGTGAGTAGGTGCTCGACGAGCTGGGGTCGAAGAGCGAGTCCGCCTGCGAGTGGCACT	19:G>A
1089243|F|0-31:T>C-31:T>C	TGCAGAGCGTGCTGCACCAGGCGTCGGGTGCTGAGGCGGTGGACCTCACCGCCGCGCTCATCCG	31:T>C
1099536|F|0-7:G>A-7:G>A	TGCAGCAGTGTCGCGTGCATTTCCCCGTGTTGTTAATTGAAGCGCTGTAGTTGGATTGCTGTTGCGATA	7:G>A
1021567|F|0-65:A>C-65:A>C	TGCAGACCTTCTGCACCGTCTACGACTGCAAGCGCTGGTTCGGCGGCATACTGGAACTGCCGCAGATAA	65:A>C
4262345|F|0-18:G>A-18:G>A	TGCAGGTGCCCACCCTCAGGAGCTGCGACTTCACGCGGCTCTACTCCG	18:G>A
1074197|F|0-42:G>C-42:G>C	TGCAGCTCCCGAAGTCCAAATGCACGAGTTGGTGTGGTCAAAGAATTGGCCG	42:G>C
1219004|F|0-22:C>T-22:C>T	TGCAGCAGCATTGCGCACACCACGAGCGCCACCGCGATAGAACCGAAGGACGCCATTGCCGCTGCTCTC	22:C>T
1111995|F|0-9:G>C-9:G>C	TGCAGGTCGGGGAAGAATCGGAGCAAGAGGAATCAGTTCAGAGAGACATCCG	9:G>C
989901|F|0-26:A>G-26:A>G	TGCAGGGAACCCCCGTTGACGAGGGCATGTGCAGTGCGACGACGGGAGCCG	26:A>G
4262170|F|0-9:A>G-9:A>G	TGCAGGTCGATGCGCGCGTAGAGGTACGTGTCCCTGGACCG	9:A>G
6046910|F|0-11:G>A-11:G>A	TGCAGTCCCACGCCGACGAGACGGTCCG	11:G>A
1038665|F|0-6:A>T-6:A>T	TGCAGGACAGGAGAGGGGCCCGCGCGCGGCATGCACGTGAAAGCGAGTACGATGCACAATTATGATGTT	6:A>T
3935557|F|0-6:C>G-6:C>G	TGCAGGCAGGAAAGATGCAACTTTCTGAGCCG	6:C>G
1034182|F|0-68:G>A-68:G>A	TGCAGGCCCCCGACCTGGTCCTGACAGAGGATGGTGAGGAAGTCGCTGTCCGTGTGCGGCACCAGCCCG	68:G>A
5325071|F|0-12:G>C-12:G>C	TGCAGGAGGGAGGGAGCGCCGCCTCCGCCG	12:G>C
1068691|F|0-7:C>T-7:C>T	TGCAGGGCGGGGCCATGGGAGGCAACTGGGACACCACGAACGCGGCGTCCTTCATCCG	7:C>T
6039841|F|0-47:G>A-47:G>A	TGCAGTCGCTCGGGACGAACACCGACGTCTGCCATGTCCTGCCTCCTGGACGCCGACCTCGATCAGGCC	47:G>A
12771904|F|0-46:C>G-46:C>G	TGCAGAAGAGCTTGGCGGGGTCGTACTCGTAGCCGATGTTCTTGGACGCGTTGCGGCGGTCGAGCCGCG	46:C>G
1065865|F|0-44:A>G-44:A>G	TGCAGGTTAATTTGGATCCTGCTAACATTACAGAAGAAGCTTCCATTTCCTCTCTTAGATCCGTTCGTC	44:A>G
4998006|F|0-17:C>T-17:C>T	TGCAGCAAGAACTTGCGCCTCCTCTCGAGCCTTTTGCGATCTTCTTCACTGCCCTCGGGCTCGGACCCG	17:C>T
1667164|F|0-34:A>G-34:A>G	TGCAGCCCGTCCATGGCGCGCATGGCGACGTCGAAGAAGACGAGGTTGTGGTTGGCGTACCG	34:A>G
3026393|F|0-25:G>C-25:G>C	TGCAGTCGTAATGGAGGGCAGGGCAGTTCGTGAGGTACCG	25:G>C
3951898|F|0-11:C>T-11:C>T	TGCAGCCCTACCGTGTCCAGCCCCAGCGCCAGCGGCACGCTCCCTGGCATTGGCGAAACTCGACTGCTC	11:C>T
3026532|F|0-33:T>C-33:T>C	TGCAGCCACAAGCAAGAACGCACGCACCCTGAGTGAAAATCGCCG	33:T>C
7911254|F|0-23:A>G-23:A>G	TGCAGCGTCAGATTGTTTCGTCCATATCTCAACGCCCG	23:A>G
3935402|F|0-12:C>A-12:C>A	TGCAGATTACATCGTTCTGATGGATTGAGAGGCCCG	12:C>A
6039809|F|0-32:G>T-32:G>T	TGCAGCCCTCATCCATTGATCGCGCCGCGGGCGGCCGCAATGCAACAAAAACGGCCTCTATCCTGAGGA	32:G>T
3947512|F|0-16:G>A-16:G>A	TGCAGGTGGCCAACAAGCTGAACAAGACCCCG	16:G>A
1051518|F|0-35:A>C-35:A>C	TGCAGTTCAAGCTGATTCGTTCAAACAAAAGTCCGAAGTAATTAACAACCG	35:A>C
3937970|F|0-32:A>G-32:A>G	TGCAGTTTACACGACACACATCCCATGTTATCACCG	32:A>G
5980409|F|0-11:G>T-11:G>T	TGCAGAAGACAGAGACGGAAACGGGAGGGGTCAATTAGGGATTCGGTGACGGCGGAGAGCCG	11:G>T
1167183|F|0-41:C>G-41:C>G	TGCAGGCGGTTCAGGATTAGCTGCGTGTCCACTGGACGATTCCGCTGCGGTCTTCGAGCCG	41:C>G
4261523|F|0-10:C>G-10:C>G	TGCAGTGGGCCGTGCGCAACTTCATCCG	10:C>G
1707934|F|0-11:G>A-11:G>A	TGCAGCACCTCGAGGCTTGGCAGGTCGCCGACGAAGTCGGGGATGTCGCCGCGCAGCTTGTTCCGAGAT	11:G>A
1059801|F|0-37:C>T-37:C>T	TGCAGGTCGTCCCGTGGGGCGACATGAGGTGGGCGTCCTTCAGTGCCTTCGGCGCGCCGACGGCCGACA	37:C>T
7912108|F|0-58:G>A-58:G>A	TGCAGAATTGGCCTCATTCGGGTCGTCGTGTTGCTTTCCTTCCGCTCGAGTGGCACTAGTTCAGCGGCT	58:G>A
32027521|F|0-34:T>C-34:T>C	TGCAGGCCGAACAGTGACTTCGGAATGGAGCCTATGAGCAGGTTGTGGTCGAGGGCGAGAGAACTCAGA	34:T>C
3020395|F|0-28:T>C-28:T>C	TGCAGTGAGTTCAGTGTTCAGCAATTTATAAGCGATGTTCCG	28:T>C
7168446|F|0-33:A>T-33:A>T	TGCAGCCTGGCATACTGCCGACCCCAAGCACAAAGCTTCCG	33:A>T

3020544|F|0-31:C>G-31:C>G	TGCAGTAGAAAAGCTATGCCACCACCAAATGCGTTTGTCCG	31:C>G
3020598|F|0-11:A>T-11:A>T	TGCAGGTCACCATTAGTCCATTACTTTATGGATGTCCG	11:A>T
1410187|F|0-33:A>G-33:A>G	TGCAGAAAGAATAAAAGGAAGCCGACGAAGATGATATGCATATGTCTTACCATGGCGTAGTGGTTGGCG	33:A>G
3955493|F|0-6:A>G-6:A>G	TGCAGAACCTCACATCGGGCGGCGACAGCTTCCG	6:A>G
3023558|F|0-11:C>T-11:C>T	TGCAGTGCTGCCCTGGCCAAAGGTGAAAGGCGTGTTCAATCCG	11:C>T
4005082|F|0-16:C>T-16:C>T	TGCAGCTGGATGGCGTCGTCGTCTCCCCGCGAGATGGGCGCCG	16:C>T
1019998|F|0-12:C>A-12:C>A	TGCAGGATGAAGCGGGGGAGAAGGCTATGGCTAGCTGCCTTGGGACTAGCAACCG	12:C>A
3020711|F|0-27:T>C-27:T>C	TGCAGTAAAACTCATTTCCCAACTCTCTAGATCGACCG	27:T>C
1078905|F|0-31:C>G-31:C>G	TGCAGGTGCTGAGCAACAAGAAGTTCAAGAGCGCCACGCATAGGGTGGTGAGAAGGCCG	31:C>G
2241744|F|0-6:G>A-6:G>A	TGCAGTGCCATATCGATCATGTAGTAGCCATTGGCTATAGCAACCATAGACTCAATAAATTGATCGCCC	6:G>A
2241744|F|0-45:A>G-45:A>G	TGCAGTGCCATATCGATCATGTAGTAGCCATTGGCTATAGCAACCATAGACTCAATAAATTGATCGCCC	45:A>G
1059801|F|0-62:G>A-62:G>A	TGCAGGTCGTCCCGTGGGGCGACATGAGGTGGGCGTCCTTCAGTGCCTTCGGCGCGCCGACGGCCGACA	62:G>A
3023558|F|0-21:G>A-21:G>A	TGCAGTGCTGCCCTGGCCAAAGGTGAAAGGCGTGTTCAATCCG	21:G>A
4002083|F|0-14:G>C-14:G>C	TGCAGGTGAGGAGGGGCGGCAGGTGGTTCCCGCTGGACCCGCTGCCG	14:G>C
3020909|F|0-40:T>C-40:T>C	TGCAGGGAGCATGCTCACTACTGAGCTGCTGTGATCATACTCCG	40:T>C
1242815|F|0-24:A>G-24:A>G	TGCAGACGGGCAGGACCATGACCTACGACATTCATGGCGACGGCGGCCAGCTGACATTCGAGCCGAGAT	24:A>G
1023842|F|0-14:G>C-14:G>C	TGCAGCTCGTGCACGGCCGTGTTCATGTCTGCCACGGCGAAGTCCAGCGCCG	14:G>C
3026056|F|0-28:G>T-28:G>T	TGCAGGAGACGCCGACCCAAGAGCAGAAGGATGTGCCG	28:G>T
1027099|F|0-37:T>C-37:T>C	TGCAGGCGGTGGTGTCCGAAGCTGATCTGCGCGGCGGTGCTGCCGCTCGCGGCCG	37:T>C
1027099|F|0-23:G>A-23:G>A	TGCAGGCGGTGGTGTCCGAAGCTGATCTGCGCAGCGGTGCTGCCGCTCGCGGCCG	23:G>A
1059801|F|0-26:A>G-26:A>G	TGCAGGTCGTCCCGTGGGGCGACATGAGGTGGGCGTCCTTCAGTGCCTTCGGCGCGCCGACGGCCGACA	26:A>G
3570184|F|0-13:C>G-13:C>G	TGCAGTGCAGTATCGATCATGTAGTAGACATTGGCTATAGCAACCATAGACTCAATAAATTGATCGCCC	13:C>G
3939367|F|0-25:A>G-25:A>G	TGCAGAGCAGCAGCGCCAAGGGCGAACTGAGCCG	25:A>G
1061532|F|0-40:G>T-40:G>T	TGCAGAGGAGGTACTGCCTGGTGGCGTGCAAGCAGATGGTGGGTCTCTTCGCAACGGTGTGGGCGCGGC	40:G>T
1036280|F|0-56:T>G-56:T>G	TGCAGGATGACACCAAGATCTTCATGGCCAAGTATTACTGCGTGCGGCTGCACGCCTTGCTCGGGATGG	56:T>G
1020985|F|0-24:G>C-24:G>C	TGCAGCCTATGCAGGCTGGCTAGCGAGCAGTGACAGCGGGTCAGCGAGTGCCACAGAGACCG	24:G>C
3953788|F|0-17:C>T-17:C>T	TGCAGTCAGCTCCCCGACAGTCACCGTCTTGAGCTCGCCGTCGGCCG	17:C>T
1208994|F|0-8:A>G-8:A>G	TGCAGGAGATGTCGCAGGACGGCGTCATGGGCAAGCTTGGCCGCGGCACGACGAACCG	8:A>G
1208994|F|0-10:G>A-10:G>A	TGCAGGAGATGTCGCAGGACGGCGTCATGGGCAAGCTTGGCCGCGGCACGACGAACCG	10:G>A
1217579|F|0-25:C>T-25:C>T	TGCAGGAGATGTCGCAGGACGGCGTCGTGGGCAAGCTTGGCCGCAGCACGATGAACCG	25:C>T
1087814|F|0-9:G>A-9:G>A	TGCAGGTCGGGTGGTGTTGCAGCCGTCGTTGCAAACACTGACGACAGCGACGCAGCCG	9:G>A
1018559|F|0-31:C>G-31:C>G	TGCAGCCACACCAAACACCACGAATCAATCCCCATTGCCGAAATTTTGCTCTTACCCGCTCCATCTCAA	31:C>G
5371744|F|0-26:G>A-26:G>A	TGCAGTTCAGCCTGACCTCCGTCTGCGTCCCG	26:G>A
1087011|F|0-12:G>A-12:G>A	TGCAGGGGAAGGGGAGTCGGTGCCGTGGCAGTTGGAGTCGTTGGCGCAGTCGACGGCCG	12:G>A
1399786|F|0-28:C>A-28:C>A	TGCAGAGCCAACTGAAAGATCAAGATTGCCTTGCTACACGTCGGTTCAGTAACGACCG	28:C>A
12854989|F|0-16:T>C-16:T>C	TGCAGATTCGGGTACGTGCGCGAGATCGACCCG	16:T>C
1036280|F|0-43:G>T-43:G>T	TGCAGGATGACACCAAGATCTTCATGGCCAAGTATTACTGCGTGCGGCTGCACGCCGTGCTCGGGATGG	43:G>T
1122234|F|0-21:T>C-21:T>C	TGCAGCTCTGTGATGCTGGCATCAGAGATCAAATGGCGCGGCCCTTTGCCTTCTCCG	21:T>C
3942636|F|0-7:A>G-7:A>G	TGCAGCCAGGGTGCGTGGAACTTTAACCG	7:A>G
987456|F|0-59:G>C-59:G>C	TGCAGGGATAGAGTCATTTTCCAAGCTTGGGGACTCCATCTACTTTGAGGAGAAAGGCGGCAAACCGAG	59:G>C
1019998|F|0-18:A>C-18:A>C	TGCAGGATGAAGCGGGGGAGAAGGCTATGGCTAGCTGCCTTGGGACTAGCAACCG	18:A>C
1012031|F|0-35:G>C-35:G>C	TGCAGGTTGTCCAGCGCGTCGCGGAGCAGGTCGGCGGCGCCGCCGTACTGCTCCAGGCAGTGGTCGAAC	35:G>C
1091381|F|0-60:C>T-60:C>T	TGCAGCACAGCATCGAGTACTGGCTCACGCTGGACCTCCTCGCCTCCGAGCAGGGCGCGCCCACGCCGT	60:C>T
1031914|F|0-26:T>C-26:T>C	TGCAGCCTCTTCGCCCCGACGCCGTCTTTCGCCGACATGGTCGTCAGCCG	26:T>C
3023743|F|0-25:T>C-25:T>C	TGCAGGACCTCAACGTGTCCAACAATCTCTTGTCTGGGAGGATTCCG	25:T>C
7164265|F|0-8:A>G-8:A>G	TGCAGGGTACCGCCCAAAACGGACCGAACAAGAATCATTCGCCCATGCATCAGGTCGTTTGGAAGAGCA	8:A>G
998918|F|0-33:G>A-33:G>A	TGCAGAAAGAAATGCTGACCTAGGATCAGCCCAGAATGAAGTTTTATCCAACTCCG	33:G>A
100536452|F|0-25:A>G-25:A>G	TGCAGGCAGCAGCGCGCACGCCATGACTGGACTCTGCCTCCCAAGGGTTTGCGCCAAGAGCGCCGAGAT	25:A>G
100497347|F|0-22:T>G-22:T>G	TGCAGTGGCGGCCTTCACCATTTTCAAGCCCTGAGGTTCATGCTCGTCTCTTTGTCCG	22:T>G
2242245|F|0-45:G>A-45:G>A	TGCAGCCAAGCATCGATGACCACCAGTTATGTTTCTCAAGATACCGTATGCCCATCAGTTGGGTGGCAA	45:G>A
1022595|F|0-48:A>G-48:A>G	TGCAGGCAACAAATTCCTGTGTGGCAAGCCAACGGACGTGGTCTGCGAATCCGCGTCGTCGCCCGCCGA	48:A>G
1017487|F|0-8:G>A-8:G>A	TGCAGGATGTATTGATAAAGGTCTCACCACCCATGGAAACTGAAACTGTGGTGAGAAATTTGCCGAGAT	8:G>A
1020648|F|0-29:G>C-29:G>C	TGCAGCTCCCGCACGATGAGCACCATGGAGGAGAAGGCGCGGCCCACCGACTCGCCG	29:G>C
1082538|F|0-38:T>C-38:T>C	TGCAGTTGCCGTAAATCTTGCTGCGAGATTCGCCTCCATGCCTCCAGCGACGGCTTCCTCATCGTCTAG	38:T>C
1149760|F|0-5:C>T-5:C>T	TGCAGCACGCTCATGTTGTGGTGCTGGTGATCATCCGTCGTCCCCTCCG	5:C>T
4261295|F|0-9:G>A-9:G>A	TGCAGTTCCGTCCGCCTCCTGCCGCACCAAATCCGCCCGCACTGTTTGCTACATCGGGGATTCAAGAAG	9:G>A
3064870|F|0-19:C>T-19:C>T	TGCAGGAGGGGTGCCGTTTCGGGGAGCAGCGGCTCTTGGTCCG	19:C>T
1034124|F|0-38:G>C-38:G>C	TGCAGAGCAGATTCCACTCGCTACAGTGTCATCCTGTTGTGTTTTCTGTTCATTGCGTTCAAATGGGCC	38:G>C
4261736|F|0-20:C>G-20:C>G	TGCAGGCTGATGATGGTGGACGAGGCGGCCAGGACCG	20:C>G

1041280|F|0-34:C>A-34:C>A	TGCAGGCCCAGCACCGCCAACTGCTCCGCCACCGCCAAGAACGTCTGCCCGCCCTACCTCGTCGTCTAC	34:C>A
1089281|F|0-14:G>A-14:G>A	TGCAGGATGGACTCGACGGGATCAAAACCATCGGCGCCGCCGCTCTTCAAGAATGGACTCCG	14:G>A
1069558|F|0-31:A>G-31:A>G	TGCAGTACTGCACGATGGCAATATGAATATCACGCGGATGAGCTCTACTGAACCG	31:A>G
1015989|F|0-59:C>A-59:C>A	TGCAGACAGAAGAGAAGATCAAAAGAGATCGGGCGATGCAGCACAAGATTGGAGGGCAACAACAAGCGC	59:C>A
1023306|F|0-31:G>A-31:G>A	TGCAGGTGAGGACGAGCAAGCTCAAGGCACAGAATCAAGGAAGAAAGCTGATGGGAGCTATTCGGCGCC	31:G>A
3022157|F|0-14:C>T-14:C>T	TGCAGCGCATTAGGCAGCGGAAACGTATTTAATTGCTCCG	14:C>T
3959134|F|0-41:C>A-41:C>A	TGCAGTGTGGGGAATGCGCCGCTGAAGCTCGACGGCGAAGTCTGTGCCGCTTAGGTTGGTAGCGCGTCT	41:C>A
32026271|F|0-40:C>G-40:C>G	TGCAGAACGACTTCTACTTCGTGCCGGCGCTGTACGACATCGCCCAGTTCGAGGGCGAGCTCCG	40:C>G
986255|F|0-44:C>T-44:C>T	TGCAGGCGATGCTTGCTCATTGCCCATCCCTGGAGTGCGCAAAGCTCAAGAACATCACCG	44:C>T
1048650|F|0-31:G>A-31:G>A	TGCAGAGTCCACACACGTCAAAACTAAACAAGCTCGCAGTCACAAGTAAACAACAACGCCAGTCCGAGA	31:G>A
4261876|F|0-15:T>C-15:T>C	TGCAGGTACGGCAGGTTTGGGATGTCCGACTCGTCGGCGAGCCG	15:T>C
4262375|F|0-6:G>C-6:G>C	TGCAGCGGATGATGAGCTGATATACCCAGATGTGATGGCACCG	6:G>C
2242245|F|0-55:C>A-55:C>A	TGCAGCCAAGCATCGATGACCACCAGTTATGTTTCTCAAGATACCGTATGCCCATCAGTTGGGTGGCAA	55:C>A
7170533|F|0-38:C>T-38:C>T	TGCAGCACCACCTCCACCACGTCGCTGTGGTTCACCCTCTTCACCGACGTGGACTTCCG	38:C>T
5971456|F|0-10:G>C-10:G>C	TGCAGATGGCGAAGCTCGAAGGCGGCAGCAAGGATGTTTACGAAGCCATGCAGAGGTGGCACAACCGTT	10:G>C
5371884|F|0-24:G>T-24:G>T	TGCAGATGGCGAAGCTCGAAGGCGGCAGCAAGGATGTTGACGAAGCCATGGAGAAGTGGCACAACGGTT	24:G>T
3022407|F|0-18:T>C-18:T>C	TGCAGGGTGCATGCTCACTGCTGAGCTGCTGCGATCATACTCCG	18:T>C
3947178|F|0-52:C>A-52:C>A	TGCAGTACATGTCTAACCTCTTGCTTGCCCATCAACACTTGATCGATTCTGCCCCATGCTACCCCAGGC	52:C>A
1046119|F|0-14:G>A-14:G>A	TGCAGACCTGACTGGCACTAGCTAGGTTGGACTAGACGCCGCGTCACTCATCCACGACACCAACCCGAG	14:G>A
1061532|F|0-63:C>T-63:C>T	TGCAGAGGAGGTACTGCCTGGTGGCGTGCAAGCAGATGGTGGGTCTCTTCGCAACGGTGTGGGCGCGGC	63:C>T
3957132|F|0-5:A>T-5:A>T	TGCAGAGGACGGAGCTTTCGAGTGGACGAGGAAATGCCG	5:A>T
1041523|F|0-28:G>T-28:G>T	TGCAGCTCTTCTGCGGCGACACCCTCCTGCTCAAGGGCAAGAAGCGGAAAGACACGGTCTGCATTGCGC	28:G>T
3033596|F|0-22:T>C-22:T>C	TGCAGAGCCGTCCCAGCAGGTCTCCCCTCGCGCGCCG	22:T>C
5582355|F|0-22:G>A-22:G>A	TGCAGCGCGCGCGCGTCGGCCAGAAAATGGCCCG	22:G>A
7922943|F|0-49:T>C-49:T>C	TGCAGTTGCCACGACCCACTCGTCACTCGAGACTACCACGATTCTCGTGTGACGAATGGGCATCCGAGA	49:T>C
3574170|F|0-42:C>T-42:C>T	TGCAGCCCCCTCCCACGGCTTTCCTCTTATATGCAAAGCGCGCGGCCCG	42:C>T
7169778|F|0-6:T>A-6:T>A	TGCAGGTGGAGCAATGGTACAATTTGCAGCTCCTCGAGCACAACCG	6:T>A
3029961|F|0-5:C>A-5:C>A	TGCAGCAGCTCGGCATCAGTGGGAACCTCGATCGCCG	5:C>A
1253255|F|0-58:G>A-58:G>A	TGCAGAAGAGCTTGGCGGGGTCGTACTCGTAGCCGATGTTCTTGGACGCGTTGCGGCGGTCCAGCCGCG	58:G>A
4261605|F|0-23:C>T-23:C>T	TGCAGCTGGAGGTCAACGTCAAGCGTGGAGACCTCCG	23:C>T
1046261|F|0-14:A>C-14:A>C	TGCAGGCCATCACGAGAAAGTCCCTATCAGCTGCCCAGGTGAACCAAACGGCCG	14:A>C
1010893|F|0-20:G>T-20:G>T	TGCAGGCACGGGAGGAGGAAGGCCCTGGCAGAGGCAGGCGCTGGGCACTTGGGGCGCCG	20:G>T
4396276|F|0-15:C>T-15:C>T	TGCAGCAGATCCTGCCTTGGTCAGTGCGTTCCG	15:C>T
4396276|F|0-13:G>A-13:G>A	TGCAGCAGATCCTGCCTTGGTCAGTGCGTTCCG	13:G>A
1013852|F|0-47:C>G-47:C>G	TGCAGGGCAGTGGCACTGGCATCTAGCCAGCTATGGCAATTATATATCGCCG	47:C>G
1055502|F|0-14:T>G-14:T>G	TGCAGGCCGCCGACTTGGTCCTGGCAGAGCACGGTGAGGAAGTCGCTGTCCGTGTGCGGCACCAGGCCG	14:T>G
1383267|F|0-12:T>C-12:T>C	TGCAGAGCGCGATGCCGCAATTGTATGTTAAGGCGGCAAGGATACACTCGCCTGAGGTGGGCGGCCCCG	12:T>C
3939797|F|0-39:T>G-39:T>G	TGCAGGAGGGGCGCCGTTTCGGGGAGCAGCGGATCTTGGTCCG	39:T>G
991920|F|0-22:A>G-22:A>G	TGCAGGTTGGTTTCAACAATGGAGTCCCCATCACCGAGGAGTTCTACAGCGCCAACATCAGCGGGTGGC	22:A>G
5358459|F|0-31:G>T-31:G>T	TGCAGGTGCTGTTCCTCTGGGGCAATGCGCTGTCCG	31:G>T
1045986|F|0-12:C>T-12:C>T	TGCAGCTTCAGTCCCTTGCTCGTTTACCTCAATGACAGCCTTATGAAAAACGTCATTCACAACCAGAGG	12:C>T
1004991|F|0-52:C>T-52:C>T	TGCAGGCTTGTCAGCAGTGCGGATTTAAAGTAGCATGGAGACGGCGACATGGCGTGCCACTGTGAACGG	52:C>T
992043|F|0-10:C>A-10:C>A	TGCAGTCGCACTCACGCTTATCCCATGGGAGACATAATGTCTTGCTTGCACGGCAGGAACAATTCGGGC	10:C>A
1041034|F|0-25:C>T-25:C>T	TGCAGAACTTCACGGCGAACCACAACAACTTCAGCGGCACGCTACCACCGTGCCTCAAGAACTGCACAA	25:C>T
12854989|F|0-13:A>T-13:A>T	TGCAGATTCGGGTACGTGCGCGAGATCGACCCG	13:A>T
1089243|F|0-15:A>G-15:A>G	TGCAGAGCGTGCTGCACCAGGCGTCGGGTGCTGAGGCGGTGGACCTCACCGCCGCGCTCATCCG	15:A>G
1667216|F|0-48:A>T-48:A>T	TGCAGAAAATCTTAAAAATATTCCAGGCATACAGTAATCTTCGATGTAAATTGTGAGAAACATTCCCGA	48:A>T
1089029|F|0-47:A>G-47:A>G	TGCAGGAATTCCGAGTCACGCAGGCAAGGGCGAACGGAAGAGCACTGACCG	47:A>G
4261612|F|0-32:C>T-32:C>T	TGCAGCTGGGCACGGAGTGCACCGAGCGGCGGCCCGACCG	32:C>T
1242815|F|0-28:C>G-28:C>G	TGCAGACGGGCAGGACCATGACCTACGACATTCATGGCGACGGCGGCCAGCTGACATTCGAGCCGAGAT	28:C>G
3023834|F|0-31:C>G-31:C>G	TGCAGAATTCGTTCAGCACAGCAGAACACATCGGGAGCCG	31:C>G
985496|F|0-5:G>C-5:G>C	TGCAGGTCGGCGCGCCCGTGCACGGCACGGCGTTCCAGCGCGTCGCGTCGTGCTTCGTGCAGGGCCTGG	5:G>C
1056056|F|0-20:C>T-20:C>T	TGCAGGTGGTGGCTCCTCATCTTGTCGCCGACGCCGAGGGCGTCAAGCGCGCGGAAGGCGTTGGTCCAT	20:C>T
987437|F|0-14:C>G-14:C>G	TGCAGGTCCAGGGCCCTGGTTCTACTTATCGTCGTCGCCACCAACACAGCCATCTTCCTCCTCTTCTCC	14:C>G
1043022|F|0-40:C>A-40:C>A	TGCAGGCGTTTCAATGAACCACACGCAAGGCATCAGTGATCACCAGCTCACTGCCATACTGAAACCGAG	40:C>A
3947373|F|0-7:G>C-7:G>C	TGCAGCAGTAATATGCAATTCTCTGACCCTCCG	7:G>C
1089029|F|0-18:C>G-18:C>G	TGCAGGAATTCCGAGTCACGCAGGCAAGGGCGAACGGAAGAGCACTGACCG	18:C>G
3022157|F|0-17:C>G-17:C>G	TGCAGCGCATTAGGCAGCGGAAACGTATTTAATTGCTCCG	17:C>G
3938702|F|0-18:A>G-18:A>G	TGCAGTGTAGATCTGCGCACTGACGTGTCCG	18:A>G

3943589|F|0-22:G>C-22:G>C	TGCAGAGCAGCAGCGCCAAGGGGGAACTGAGCCG	22:G>C
3949973|F|0-5:C>T-5:C>T	TGCAGCACGAGGCCATCACATAGTCTCTGCGGCAGTTCGCCG	5:C>T
1025833|F|0-53:G>T-53:G>T	TGCAGCCTCGCCGCGTGAGCGGCCGCGAGGGCGACGCGCAAGGTGTAGGTGCCGCCG	53:G>T
3961378|F|0-45:A>G-45:A>G	TGCAGTTGGCCTGACTGATTTTGTGCTTGGTTATGCTTGATGATGACCG	45:A>G
1065865|F|0-51:C>G-51:C>G	TGCAGGTTAATTTGGATCCTGCTAACATTACAGAAGAAGCTTCCATTTCCTCTCTTAGATCCGTTCGTC	51:C>G
3959679|F|0-32:G>A-32:G>A	TGCAGACCCACCACTTCGCCTACGAGGAGCTGGAGGAGGCCACCG	32:G>A
1221171|F|0-8:G>T-8:G>T	TGCAGCGAGCCCTCAGAGATTTTGACCTCCACGAGCGCAAGCTTCTTGAGGAGGGGAAACTGAAGCGTC	8:G>T
4262689|F|0-10:C>T-10:C>T	TGCAGTATCTCGGACTCGATGGTGGTGACCCGCTCCAGGACCTCCG	10:C>T
1311288|F|0-32:T>A-32:T>A	TGCAGCTTACCCTCTTATATAGCTGAGCTACCTGCGCTCCTTCACGTCGCCAGCCGAGGTTGGACTAAT	32:T>A
1019955|F|0-44:C>G-44:C>G	TGCAGCTTACCCTCTTATATAGCTGAGCTACCTGCGCTCCTTCACGTCGCCAGCCGAGGTGGGACTAAT	44:C>G
1864512|F|0-41:G>A-41:G>A	TGCAGATGGCGAAGCTCGAAGGCGGCAGCAAGGATGTTGACGAAGCCATGGAGAGGTGGCACAACCGTT	41:G>A
1110864|F|0-42:A>T-42:A>T	TGCAGCAGGCGATTGAGCAGAGAAGCGCTGCGAGGAAGAAGAAGGACTTTGCGGAGTCGGACCG	42:A>T
3934851|F|0-34:G>A-34:G>A	TGCAGCTTATGATCATCGTGGACGACGCTGGATCGTTTCTTCCG	34:G>A
1088182|F|0-52:C>G-52:C>G	TGCAGACGCTATGCGCGATGAGCCAAGTGTCGCAGGACATCATCGTGGGGGACGGCGAGTTCTTCGTGG	52:C>G
1218104|F|0-8:A>G-8:A>G	TGCAGCCAACACCAAGCAGAGACCGATCAGGAGGAGCCTGAGCAAGGAGCTAGCCG	8:A>G
1087814|F|0-8:G>A-8:G>A	TGCAGGTCGGGTGGTGTTGCAGCCGTCGTTGCAAACACTGACGACAGCGACGCAGCCG	8:G>A
3021289|F|0-21:T>C-21:T>C	TGCAGGTCGCCATTGAGGTCCTGCCTGGGGCAGATTCCG	21:T>C
3022157|F|0-31:A>T-31:A>T	TGCAGCGCATTAGGCAGCGGAAACGTATTTAATTGCTCCG	31:A>T
1020247|F|0-56:G>C-56:G>C	TGCAGATCCATCGTCGCCAAGCTGCACTACTACCTGCCCCTCTTCGGCGGCTCCTTGGAGAAGCTGCTC	56:G>C
991920|F|0-6:T>C-6:T>C	TGCAGGTTGGTTTCAACAATGGAGTCCCCATCACCGAGGAGTTCTACAGCGCCAACATCAGCGGGTGGC	6:T>C
1863933|F|0-19:C>T-19:C>T	TGCAGGTAGGTGTCGTCGCCGTCGATGAAGTAGGCCGTCCAGCCGCTGTCGTCGGAAGCCACGGCGGTG	19:C>T
1065492|F|0-26:G>A-26:G>A	TGCAGTTGATTAGAGTACTGTTTCCTGCGATTTGTGCTTTAATGGCGAGACCG	26:G>A
1295871|F|0-16:A>G-16:A>G	TGCAGCCGTAGTTGTCAACGCGGAAGGCGACGGCGCCGTCGGGGCCGTACACCGCGCAGCCGTTGCCGT	16:A>G
2241644|F|0-18:T>C-18:T>C	TGCAGGTTCATGGAGACGTAACGACTCCAGAATGTCGACAGATGAGAACGTGAACCG	18:T>C
3934951|F|0-5:C>T-5:C>T	TGCAGCTACGCAGCGCTCACGTCTCCTGCGCATACATGCACGTAGCGGAGCGAGCGATCCACCCGAGAT	5:C>T
3947951|F|0-17:G>A-17:G>A	TGCAGGCTCTGGATGGCGCGGACACGCGGCTCCG	17:G>A
1036788|F|0-5:G>A-5:G>A	TGCAGGGCGAGGAAGCCCTTGTAGTAGAGGAAGCAGTGGACCATCTTGGCGCAGGCCG	5:G>A
1065865|F|0-9:A>G-9:A>G	TGCAGGTTAATTTGGATCCTGCTAACATTACAGAAGAAGCTTCCATTTCCTCACTTAGATCCGTTCGTC	9:A>G
5009032|F|0-28:G>A-28:G>A	TGCAGACGTGGCAGACGAACTGCACCGCGCCGATGAGTATCTGCGCCG	28:G>A
4262813|F|0-25:C>T-25:C>T	TGCAGATGTTGTCGCGCTTCACCGTCAGGATCATGATGGCGTCCG	25:C>T
4439426|F|0-23:G>A-23:G>A	TGCAGCCTGCCGTTCCCGCTGAAGATCCG	23:G>A
994295|F|0-9:A>G-9:A>G	TGCAGTGGAACCACCAAGAACAAGCCTGTCCAAGAGGCCATCGCTTGGAGCTTCTGCAAAGGTCCAGTC	9:A>G
1037126|F|0-25:T>C-25:T>C	TGCAGTTTTCGTGGTAAGTTGTCCGTCTTGAAAGGAAGCCTATTCAATTCCG	25:T>C
4261842|F|0-16:G>C-16:G>C	TGCAGGATCTCAACGTGTCCAACAATCTCCTCTCGGGGAGGATTCCG	16:G>C
5577104|F|0-7:C>T-7:C>T	TGCAGGCCGCTTCGGCTTCTTGGCAGCGGCGGTGGGCTTGGCGACGCAGGCGGGGTTGATCCG	7:C>T
12469808|F|0-24:G>A-24:G>A	TGCAGGTGGTGGACTGGGACAACAGCATCATCCG	24:G>A
1149760|F|0-20:G>A-20:G>A	TGCAGCACGCTCATGTTGTGGTGCTGGTGATCATCCGTCGTCCCCTCCG	20:G>A
3026393|F|0-24:A>T-24:A>T	TGCAGTCGTAATGGAGGGCAGGGCAGTTCGTGAGGTACCG	24:A>T
1022972|F|0-33:G>A-33:G>A	TGCAGTGCGGCGCCGACTCGCTCTACGGCGACAGGCTGGGCGAGTTCAGACTGTCCGTGAAAGGCCACG	33:G>A
6048900|F|0-37:G>A-37:G>A	TGCAGCCTTCCTGGAACATGGTGACCGCCTTTTCTCCGTCAGATTCCCCGCCG	37:G>A
2242104|F|0-34:C>T-34:C>T	TGCAGTTTGGTGCGATGACCTAATAATCCTAGCTCAGCTAGCAGCACTTCACTCCCTCCCTTGCGCCCT	34:C>T
32025840|F|0-52:A>G-52:A>G	TGCAGCTGCTTCCTGAAGCTGCTAGATTCTCCATTGCTTGCTTCAGGGTGTCAGACCTCCG	52:A>G
1688504|F|0-22:T>C-22:T>C	TGCAGCCCAACATGAGGGAGATTGAGTCCGCTCAGGACCTCGTCGACTCGCTGGCCAACGCCG	22:T>C
5364112|F|0-40:A>G-40:A>G	TGCAGTGTCTGAAGAACATGACGAGCATGACCAAACGAGCAGCACGCCG	40:A>G
32025179|F|0-6:A>C-6:A>C	TGCAGTATCTGAGTGGAATCAAGTGCCAAGCTCTGTTCGATCCTAAAATGTTTTTCCGTGGGGGTATAT	6:A>C
5001676|F|0-40:T>A-40:T>A	TGCAGCTACCTAGAGAGTAGTGTGAGTGTGTGACGGAGCTTGGCAGCCG	40:T>A
5005085|F|0-9:T>G-9:T>G	TGCAGGCAGTTGCATTGCAGGGCCGCGTGGCCACCCTGTCTGCGGCGACGGAGGAATGGCGCTTTCCTT	9:T>G
4994208|F|0-13:T>C-13:T>C	TGCAGGACGTGTTTCTCAGGTTCACGTTTGACACCACCACCACGCTGGTCTTCGGCCTCGACCCCGAGA	13:T>C
1096506|F|0-11:T>C-11:T>C	TGCAGACACGATGACGATTCAATCACGCAGAGGCGCAGTTCGCAGGATGACTCACCCTTCGTGGCGCCC	11:T>C
6038481|F|0-10:A>G-10:A>G	TGCAGCTGTGACCGTGCATGGTGAGCTCGTCGTGTTTTCCTGTGAGATGACCG	10:A>G
5001676|F|0-20:T>C-20:T>C	TGCAGCTACCTAGAGAGTAGTGTGAGTGTGTGACGGAGCTTGGCAGCCG	20:T>C
32025635|F|0-33:C>G-33:C>G	TGCAGGGGATTTGGACACCGCCCACAGTCTGCTCGACGAGATGCCCGTCAAGGATGCCGTGGCTTTCAA	33:C>G
3027484|F|0-8:G>C-8:G>C	TGCAGCCCGAGCTTGCGGGCCAGCTCCGTCTTCCTCTCCG	8:G>C
32021872|F|0-16:T>C-16:T>C	TGCAGCGAGACGTCGATGACCTCCACGTCGTAATAAAACGGCCTGCGGCGCCCGCCATTCACATCGGTG	16:T>C
5371405|F|0-19:A>G-19:A>G	TGCAGAGCTCTCTGAACGAACGGTTCAACAGAAGTTCTCGACATGGACAAGAGGAGGGGATTGGAAAGA	19:A>G
5361800|F|0-68:G>A-68:G>A	TGCAGTGCCGCTACATGGCGAGCGCGAGAACCAAGAAACAGAGCTCGTAATGGGGAAGGGGTTAAGGCG	68:G>A
16662456|F|0-44:C>T-44:C>T	TGCAGCGTGCCCCTTCCCTTATTCCCTTCACCAAAACAACCTAACGAGGATCAAGACGGCCGCCGTGGA	44:C>T
5373632|F|0-11:T>G-11:T>G	TGCAGGGCACATGACAGCCACGGAGCGCGGGCGGGCACGGCCAGAGGCTGCACGGCGCGGAGGCCGAGA	11:T>G
32025305|F|0-32:A>C-32:A>C	TGCAGACCAAAGACGAGATCTCCACGTGGCGGAGGTTCTGGCCG	32:A>C

32022457|F|0-41:C>T-41:C>T	TGCAGCGTGGACACGTGTTCCGCGCCGCGGCAGGACAGGTACCGTGCGCCG	41:C>T
1018559|F|0-34:T>A-34:T>A	TGCAGCCACACCAAACACCACGAATCAATCCCCATTGCCGAAATTTTGCTCTTACCCGCTCCATCTCAA	34:T>A
7171286|F|0-26:A>G-26:A>G	TGCAGGGAGAAGGTGAGCATCTCGCCATTGCGCGCGTTCGTGACGGTGGAGGGCGCGGGGGCCGACCGA	26:A>G
32024848|F|0-8:T>C-8:T>C	TGCAGGAATTCGTTGTCGCAGAGGAGGCGGCCCATGGTGTCCG	8:T>C
1392022|F|0-16:A>G-16:A>G	TGCAGATACTGTGGGGACAGACGCAGGTGCTCAACGACGGCAACGGCGACCAGACCATCGCGCTGATGC	16:A>G
1767101|F|0-9:G>A-9:G>A	TGCAGTACTGCGCCTACGCGACATGGGAAATTTGGTCCTCCGATTCTAAAATAGCTCAACTACATGGAA	9:G>A
100222414|F|0-24:C>G-24:C>G	TGCAGTGAGCAGCAAATTTGCTTTCCAAAAGGCCTTCCTTCCACCGCCG	24:C>G
5355371|F|0-11:G>A-11:G>A	TGCAGTAACAAGAACTAGTCGAAGATATTGAGGATGCACACAACCAAAATAATCATGATATTGCAGAAA	11:G>A
7900449|F|0-8:A>G-8:A>G	TGCAGCAGAGCCATTCTTAGGCACGCACATCCCTGACAAAGTGACAATTCCAACTAAACCCCAAACTAC	8:A>G
7920893|F|0-29:T>C-29:T>C	TGCAGGAGCCCGAGCTTCTCTTCCCGTCGTTCCTCTCCAGCAACAGCACCG	29:T>C
5372397|F|0-12:T>C-12:T>C	TGCAGCAGGGCATGGAGGACGACGGCTACCAGCGCAGGTGCTCGAGGAGAGGGAGCTGCTCGGCGGTGC	12:T>C
5005314|F|0-44:A>G-44:A>G	TGCAGCTCGGCTGCTTCCTCCTCATCGCCTTCCCACCGCTTCCTATGAGCCTTGGCCGCGCTTGCCGAC	44:A>G
100402623|F|0-17:C>T-17:C>T	TGCAGGTTTGGAGAAAACTTGGGGATCGTGATTTAGGACATACGCCCCGGTCATCTGTGTTTTGGACTA	17:C>T
5360475|F|0-57:T>C-57:T>C	TGCAGAACAACCGAGAAGATGTTCAAAGGTCACTGCCACAGACCAACTTGTCTCTGCTCAAGATCCTTG	57:T>C
7907694|F|0-53:A>G-53:A>G	TGCAGCCTGATGGGTGCGTGTTCATGTATTTGGTCCTTGTGTTGGAGTTGCATAATGAAACATGGATAG	53:A>G
3574170|F|0-32:A>G-32:A>G	TGCAGCCCCCTCCCACGGCTTTCCTCTTATATACAAAGCGCGCGGCCCG	32:A>G
100221304|F|0-7:T>A-7:T>A	TGCAGCTTTCTACGAATGGAAGAAGTGGCTAGTGTGTGTGGCACTACCG	7:T>A
5360884|F|0-34:T>G-34:T>G	TGCAGCCAAACGAGAGCCTGAACCGCTAGAAACATGGCACAAATAGCTTCCAGATTTTTACCG	34:T>G
5366198|F|0-19:A>G-19:A>G	TGCAGATGGACAGGGAAAGAGCACAAATGGAGCCGCGCATGCCGTGCCCAGCCACTCATTCTGCGTCGC	19:A>G
7921453|F|0-17:C>G-17:C>G	TGCAGGGGAGCATTTTGCAATTTGGACAGCATCGCCTCACTGCCGCTGGCTCGATGTAGAAATGTCAGA	17:C>G
5370373|F|0-17:C>G-17:C>G	TGCAGTAAAATGACTCTCCCTGCTTGCCTTGGGGGCCATGGCCACGGCCGCGGCGGTGGCCG	17:C>G
5360954|F|0-66:T>C-66:T>C	TGCAGCCGCAGAAACATGACCGCGCTGACACCGACCATCCTGCCCGCCGCGCCGTCGCCGCCGTCGTAG	66:T>C
100351384|F|0-28:C>T-28:C>T	TGCAGCGTCAAGCCCCCTCACCAACCCGCTCGCTCACCG	28:C>T
1739881|F|0-18:C>G-18:C>G	TGCAGGGGAATCCGAGGACTGACGGGAAATCCGAGGGAGGAAGAAGCCTTCGGCGTCGCGGATCCGAGA	18:C>G
5362458|F|0-55:T>G-55:T>G	TGCAGGGACACATCGACGTCATCAAGCAGATACAAGATCAGCTCCCCGAGCACGTTAGAGGCTTGAACA	55:T>G
7919615|F|0-13:A>C-13:A>C	TGCAGACAGATCCAACGAGACCAGGCTGGTTTACATGTGAATCGATGGAACGTTCTCCG	13:A>C
100473705|F|0-53:T>G-53:T>G	TGCAGTTAGCACACACCTACACGCACGAAAGGCACGTACGTCGTAGATCGATCTAAGAGGCTTGCTGAG	53:T>G
7928034|F|0-14:T>A-14:T>A	TGCAGCAACCAAACTGGCCATGTCATCAAGCTTCACCTTCCCAATACAAGTCCG	14:T>A
100319734|F|0-27:C>G-27:C>G	TGCAGAGGTGAAGATGGCGGAGTTCTCCCCGTCCGTGGTGGCCGCGTCGGCGCTGCTGGCCGCCGCCGA	27:C>G
13880700|F|0-24:C>G-24:C>G	TGCAGTCATGGGGTGCTCCCTCGTCGTGTACCTCGCGGGGTGGCGGACGTACCG	24:C>G
5368644|F|0-67:C>T-67:C>T	TGCAGGCCGTTCGACGCCACCACCGCATGCTGTCCTCCTCGGCAAAGGTGACCCGTGACCGAATTCACT	67:C>T
4985936|F|0-13:G>C-13:G>C	TGCAGATCGGCGAGCCGTTCTCCTCCTCATCACCACGACCACCACCG	13:G>C
100404031|F|0-9:A>G-9:A>G	TGCAGTTGAATCCAAAAGAACCACCATGGTTAGGTTCAAGAGATCAAAGAAGGTCTCAGGAATCGCAAA	9:A>G
100385138|F|0-36:T>C-36:T>C	TGCAGACCTACATTGTGCAGCTGCACCCACGCGAATTTACCG	36:T>C
5354913|F|0-18:G>C-18:G>C	TGCAGCTCCTTGTTTATGGCCAAAAGTTCTAGTTTTACCATTACTGTGCCGCAGCTGAAGCTTTCAACC	18:G>C
100452307|F|0-36:T>C-36:T>C	TGCAGGGCTGGAGGCCGCCGATGAGCTCAAGCTGGATAACTCCATCGCGGCCTGAGCTCTGAGCACTCG	36:T>C
100507982|F|0-25:C>G-25:C>G	TGCAGATGGGGAGTAGAGAAACACACTCACGCACACACATTTGCATCTCCG	25:C>G
100397430|F|0-68:G>C-68:G>C	TGCAGCACCTTGATGCGCGCCGCGTTCAGCTCCATCGAGTACTCGCTGCATGCATGCATCATGCTAGTG	68:G>C
7918053|F|0-44:T>G-44:T>G	TGCAGGAACTGGGTCGCCGTGGGCACGCTAGGGGCCGCGACGGCTACGCCATTGTTGTTGTTAATGCTG	44:T>G
1741938|F|0-50:T>C-50:T>C	TGCAGGTCCGAGGAGCTGCCGCCGCTTGTGACGCAGCTGGAAGACGACGATGATGGCGATGGTGGCATC	50:T>C
1382563|F|0-6:C>A-6:C>A	TGCAGACAACGGCATTAAAATCATCGGTGACCATCTACACCGCCAGATCCCGTCCGATACTCCGAGCGC	6:C>A
100526392|F|0-26:C>G-26:C>G	TGCAGGAAGGCGTCCTGGACGATGCACCTGCCATATTTGCCGTGCACATCAACACGAGTATGCCGAGAT	26:C>G
7920366|F|0-13:C>T-13:C>T	TGCAGCAAAAACACAAACCCAAACTTAGTAATTAGCCATCCG	13:C>T
100557016|F|0-19:C>T-19:C>T	TGCAGCCTCAACACCACAGCCGTGGAGCCAGCCCGCTTCTTGGCGGCCAAGTACCCGCACTACAACGTC	19:C>T
5372213|F|0-28:T>C-28:T>C	TGCAGCGGTTGGGGCGGCGGCCGAGTCATGGGAACCG	28:T>C
5357432|F|0-46:C>T-46:C>T	TGCAGATACGATTTGGTGTGGAGGTGAGGTGAAGCATCTGGCACATCCGAGGTCCG	46:C>T
22834929|F|0-64:A>T-64:A>T	TGCAGCGTCCATAAAGCCATGTTTCATCGTTTCGGGGATCGGCTCGGACCACACGGCCGCGCCG	64:A>T
100536015|F|0-60:A>G-60:A>G	TGCAGGAAATGGCAGACATGGCAAGTTAGTTAGATTTATGCAAGAAGCGGCGGTGATTATAGTAGTAAT	60:A>G
100545159|F|0-51:T>C-51:T>C	TGCAGGCTGGTAAGATGCGTAAGGTGAACCACGTCTACTTCTTTCTCTTTGTCCTTGAGTGAGTTTTCA	51:T>C
100590175|F|0-41:C>T-41:C>T	TGCAGTCAGCTCTTCTGATCCATATCACACTCACATTTAATCAGCAAAGCAGAGTTTGATCGGGCTTGT	41:C>T
100467685|F|0-19:T>C-19:T>C	TGCAGATTAAGAAGGCCCTTAGGGGTCTGAGGGTTGAGGTCACCCACCGAGGAGAGATGCGCAGGAAAT	19:T>C
7914955|F|0-62:T>C-62:T>C	TGCAGCGGCAAAGTAACCAATCAGTTAGGTTCATCGCAAAACAAAAGAAAATAAGCACAGAATAGGCAC	62:T>C
2246704|F|0-35:A>G-35:A>G	TGCAGGATGCCGACCCAATGAGCTGTGCAATCATCAAGTGCTGCTCCTTGAGCTCTGACTGTGCCAAGA	35:A>G
100488132|F|0-67:G>C-67:G>C	TGCAGGACGCGTTCGACGGCGCCAGCGGGCGACGGCAGGCCCAAGACGACGGGAGCGGCAGTGGGCGGC	67:G>C
100459862|F|0-36:G>C-36:G>C	TGCAGAACCCAAGAAGACTTGCGTCCTACGATGCTTGAGCCG	36:G>C
100485720|F|0-37:A>G-37:A>G	TGCAGCAGGTCGACCACCGTGAGCTCATGGACCACGTATTCAGCGAGAGCTACAGGCCCATGATGCCGA	37:A>G
1388794|F|0-30:C>T-30:C>T	TGCAGCCGACTTGTAAGCATTCTCTGGTCTCTACTCCTCGCCTGCGCTAGAATCTTCAAATGTGACCGA	30:C>T
100556131|F|0-48:C>G-48:C>G	TGCAGAGGCCATTGATGAAGAAGTAGGAGGAGCCTCCAATGGCGCCCACGATGAGGCCAAGGCCTGTCT	48:C>G
100466207|F|0-31:T>G-31:T>G	TGCAGGTGATGGCGTACGTGATAATCGGGGCTGTGGCGGTGGCGATGGAGGCGGCGCTGCTGGGCAAGA	31:T>G

100581195|F|0-19:T>G-19:T>G	TGCAGCTTCTCCAGCTCTTTTTTTGTCCTGTTAGGATTGCCATACAAGGCATGGAGGAACATCTTCATG	19:T>G
100480673|F|0-6:T>C-6:T>C	TGCAGGTACTAAGATAGAAATGGAAAATTACTAGCTTCGGTAGCTGCTACCG	6:T>C
100534206|F|0-45:G>C-45:G>C	TGCAGCAACGGCAACCAGCAGCTTCTTATCCCCCACAAGACGGAGGTCTCTCATGCACGTGCTGCTGCC	45:G>C
100503450|F|0-35:C>T-35:C>T	TGCAGGCGCCACGTGAGACGGGACTCGACGACGGGCGGTAACAGCGGCAACGTGTCGACCCG	35:C>T
100564257|F|0-5:A>C-5:A>C	TGCAGAGACGCTTGCAGGACCCCTCGGTGGAGATCATGACGCATCCGCCGCCCAGGAACCAGTTCCGCT	5:A>C
3953788|F|0-8:G>C-8:G>C	TGCAGTCAGCTCCCCGACAGTCACCGTCTTGAGCTCGCCGTCGGCCG	8:G>C
4734062|F|0-30:C>T-30:C>T	TGCAGAAAGCGCATCCCAGCTGCCATCTCTCTGGTCGAGAGTTGATCTTCGCAGGTAGCTAGCTTCTTA	30:C>T
4734062|F|0-10:G>A-10:G>A	TGCAGAAAGCGCATCCCAGCTGCCATCTCTCTGGTCGAGAGTTGATCTTCGCAGGTAGCTAGCTTCTTA	10:G>A
3022742|F|0-11:G>T-11:G>T	TGCAGCACCGTGCACTCCATGCTCCCTTCCGCCTCCG	11:G>T
7344371|F|0-26:C>T-26:C>T	TGCAGGTCGCGGGTTCGAATCGCGCGCGCGTCCGTTTTTTCGTTCCG	26:C>T
1022281|F|0-63:T>C-63:T>C	TGCAGTCGCAGAGCCTCATCATGCCGCTGCTCCTGTCGTCCGTGGTCACGCTGGCGCTCCACGTCCCCC	63:T>C
1268029|F|0-49:A>C-49:A>C	TGCAGTCGACTAGAGCTGCTCTCCTTGAGCCAGTGTGGCTCTAGCTCCGAAGACATGCTACTACGCATA	49:A>C
1043022|F|0-23:C>T-23:C>T	TGCAGGCGTTTCAATGAACCACACGCAAGGCATCAGTGATCACCAGCTCACTGCCATACTGAAACCGAG	23:C>T
7170533|F|0-28:G>A-28:G>A	TGCAGCACCACCTCCACCACGTCGCTGTGGTTCACCCTCTTCACCGACGTGGACTTCCG	28:G>A
1017810|F|0-41:G>A-41:G>A	TGCAGACCAATTGTCGCGACCAGTCCATCGCATTCCATTCTGCCCCATTTGCATAAGGAGCTGGCTGCC	41:G>A
1242815|F|0-5:A>G-5:A>G	TGCAGACGGGCAGGACCATGACCTACGACATTCATGGCGACGGCGGCCAGCTGACATTCGAGCCGAGAT	5:A>G
1106928|F|0-45:T>A-45:T>A	TGCAGGTCATCTCCACCAACGTCGCCTGGTCCACATCGCTGGATTTAAACGGGCAGTGGACCTCCCACG	45:T>A
3023834|F|0-19:A>G-19:A>G	TGCAGAATTCGTTCAGCACAGCAGAACACATCGGGAGCCG	19:A>G
985496|F|0-46:G>A-46:G>A	TGCAGGTCGGCGCGCCCGTGCACGGCACGGCGTTCCAGCGCGTCGCGTCGTGCTTCGTGCAGGGCCTGG	46:G>A
3023743|F|0-29:T>C-29:T>C	TGCAGGACCTCAACGTGTCCAACAATCTCTTGTCTGGGAGGATTCCG	29:T>C
1221399|F|0-50:C>A-50:C>A	TGCAGCAGCATTGCGCACACCACGAGCGCCACCGCCATGGAACCGAAGGACGCCATTGCCGCAGCTCTG	50:C>A
3022415|F|0-9:T>C-9:T>C	TGCAGCCGTTGATCATAGTGTTGTGTGAATTTCGTCCG	9:T>C
5364119|F|0-5:T>C-5:T>C	TGCAGTTTTGGCCTGGTTGCTGTCGTGATACCGCCG	5:T>C
3064809|F|0-22:A>G-22:A>G	TGCAGGGTGGTGTCGTCGCCCAAGAACGGGACGAAGCTGGACCG	22:A>G
1034182|F|0-65:C>G-65:C>G	TGCAGGCCCCCGACCTGGTCCTGGCAGAGGATGGTGAGGAAGTCGCTGTCCGTGTGCGGCACCAGCCCG	65:C>G
5010988|F|0-22:C>T-22:C>T	TGCAGAACTCCCTCAGCTTCCTCTGCTGCCACACCG	22:C>T
3936902|F|0-17:G>C-17:G>C	TGCAGGAGTTCTTCTACGACCAGGTGCCG	17:G>C
1017487|F|0-43:A>G-43:A>G	TGCAGGATGTATTGAGAAAGGTCTCACCACCCATGGAAACTGAAACTGTGGTGAGAAATTTGCCGAGAT	43:A>G
978268|F|0-30:A>G-30:A>G	TGCAGTTATGCCGAGAAAGATACTGGCAACAGCGCGCATTCATTTCTAGCCG	30:A>G
1120672|F|0-24:G>A-24:G>A	TGCAGCTCTGTGATGCTGGCATCAGAGATCAAGTGGCGCGGCGCTTTGTCTTCTCCG	24:G>A
3028037|F|0-25:C>G-25:C>G	TGCAGCACCACCACGACTACCTGCCCGCCTTCGACGCCGCCACCG	25:C>G
4002821|F|0-24:C>G-24:C>G	TGCAGCTGCAAGTCAAGGTGCTGGCAACGGACGAGTTCTGGCCG	24:C>G
1125663|F|0-50:G>C-50:G>C	TGCAGACATTGTTTCAGTCGTTGCTTGGGTCTTCTAGAGGGTATACTTCTGGCCG	50:G>C
1055737|F|0-36:G>A-36:G>A	TGCAGCTTCTGCCCCTTCTCCTCCGCAGCCTCCTTCGCTTTGCTAAGCTCACTACGTAGTTTGTCCGAG	36:G>A
100495945|F|0-5:C>G-5:C>G	TGCAGCTGGAGGTCAACGTCAAGCGTGGAGAGCTCCG	5:C>G
1052520|F|0-25:G>C-25:G>C	TGCAGGACAAGAAGCACATCGTGGGGATGACGGGCGACGGCGTGAACGACGCGCCG	25:G>C
1022595|F|0-16:C>T-16:C>T	TGCAGGCAACAAATTCCTGTGTGGCAAGCCAACGGACGTGGTCTGCGAATCCGCGTCGTCGCCCGCCGA	16:C>T
3958486|F|0-23:C>A-23:C>A	TGCAGCAGGAGAGAGACGAGGTGCTCCATCGAACGTCACCGTGGGAATGACATGGTGGTAGCCAGATCC	23:C>A
1383267|F|0-24:A>T-24:A>T	TGCAGAGCGCGATGCCGCAGTTGTATGTTAAGGCGGCAAGGATACACTCGCCTGATGTGGGCGGCCCCG	24:A>T
1111995|F|0-12:A>G-12:A>G	TGCAGGTCGGGGAAGAATCGGAGCAAGAGGAATCAGTTCAGAGAGACATCCG	12:A>G
1025257|F|0-29:G>A-29:G>A	TGCAGGAGCCTCGTGATCATGGCGGCGTCGGTGACGCGGTTACGGCTCAGGCGCACGGTCCG	29:G>A
1083636|F|0-38:G>C-38:G>C	TGCAGCGACATGTCCCTGGACGAGATGGACGCCGCGGTGAGGAGGATGGAGTTCGTCGTCTTCGACCGA	38:G>C
1056584|F|0-37:C>G-37:C>G	TGCAGTACGACGTGGACTCCCTGGACCAGAACCTGGCCCAGAACCTGGCGGGCGAGATCATCGGCACCC	37:C>G
1020174|F|0-34:C>T-34:C>T	TGCAGGCCCTGAAGCAGGCCTCGGAACCTCTCCACGTTGCCACCGAACCGCCCGTCCTTCAAAATCCTG	34:C>T
2243154|F|0-19:A>G-19:A>G	TGCAGAACACGTATCCACAAGTAGTCGACCCTGTCTGTCGGGTCGCCATCCATCCTTCCACATTTGAGC	19:A>G
1087814|F|0-11:T>A-11:T>A	TGCAGGTCGGGTGGTGTTGCAGCCGTCGTTGCAAACACTGACGACAGCGACGCAGCCG	11:T>A
3026532|F|0-8:C>G-8:C>G	TGCAGCCACAAGCAAGAACGCACGCACCCTGAGTGAAAATCGCCG	8:C>G
1051236|F|0-12:G>A-12:G>A	TGCAGGCATCGCGCTGCTTCTCCAAGTTAGTATCTGATCCTAATCAGCTCTGTTGTCTTTCCCCGCACC	12:G>A
1022972|F|0-35:C>T-35:C>T	TGCAGTGCGGCGCCGACTCGCTCTACGGCGACAGGCTGGGCGAGTTCAGACTGTCCGTGAAAGGCCACG	35:C>T
3030373|F|0-17:C>T-17:C>T	TGCAGGCCGAGGTCGCGCGCCAGCCGCGCCTTGCGCTCCG	17:C>T
4404145|F|0-7:G>A-7:G>A	TGCAGACGACCTGGTGCGTGGCGTTCCG	7:G>A
1021437|F|0-35:C>G-35:C>G	TGCAGCCAGTTGAGCAGCCTGCCGATCTTCTTGGACACGAAGACCCAGCCG	35:C>G
1021132|F|0-28:C>T-28:C>T	TGCAGGCGTACACCAGGGCACGCTACGGCGAGGATGCGATGATCGTGTACCG	28:C>T
1015860|F|0-36:G>T-36:G>T	TGCAGGCACCATCGCCGACATGCAGCAATATACACAGTTCCAACGCTGTTACACCGTCTACTGGAAAGG	36:G>T
1037126|F|0-10:G>C-10:G>C	TGCAGTTTTCGTGGTAAGTTGTCCGTCTTGAAAGGAAGCCTATTCAATTCCG	10:G>C
1004053|F|0-6:G>A-6:G>A	TGCAGAGAGGGCACATCTCGCTCCCTTTGAGGGTGTCCGCCAGGCATGCGCCATGGAAGACGTGGGGCA	6:G>A
1063727|F|0-49:G>A-49:G>A	TGCAGGGTTGGAGCTACGTTGGTGACAGGCTATTGTCGGCAGTCGTGCCGTATGAAGAAACCG	49:G>A
1022190|F|0-18:C>T-18:C>T	TGCAGGACCTCCTGCTGGCCATCATGGCGGCGCTGGATGTCCCCAGCCTCGTCCGTTCCG	18:C>T
1051914|F|0-46:G>A-46:G>A	TGCAGTTGCTGATACGATTTTTTTGCTTCCAATGCTTTGTTAGATTGATAAGGGCCG	46:G>A

1036788|F|0-53:G>T-53:G>T	TGCAGGGCGAGGAAGCCCTTGTAGTAGAGGAAGCAGTGGACCATCTTGGCGCAGGCCG	53:G>T
1051236|F|0-13:C>T-13:C>T	TGCAGGCATCGCGCTGCTTCTCCAAGTTAGTATCTGATCCTAATCAGCTCTGTTGTCTTTCCCCGCACC	13:C>T
1089281|F|0-11:C>A-11:C>A	TGCAGGATGGACACGACGGGATCAAAACCATCGGCGCCGCCGCTCTTCAAGAATGGACTCCG	11:C>A
4261934|F|0-35:G>C-35:G>C	TGCAGGGGCCTCGAGAAGAAGGAGAAGTTCATGACGCCCTCGCCG	35:G>C
1089877|F|0-54:T>C-54:T>C	TGCAGGCGGAGGAGGACGGCGACGAACATGTCGTTTTTGCATCCAAGCCTGCGTTCTCTCCG	54:T>C
1111995|F|0-28:G>A-28:G>A	TGCAGGTCGGGGAAAAATCGGAGCAAGAGGAATCAGTTCAGAGAGACATCCG	28:G>A
3064870|F|0-8:G>A-8:G>A	TGCAGGAGGGGTGCCGTTTCGGGGAGCAGCGGCTCTTGGTCCG	8:G>A
3020587|F|0-6:T>A-6:T>A	TGCAGCTACGTTTCTTGTAAAGCAGAATAAGTTGCATGCCG	6:T>A
32024217|F|0-9:C>G-9:C>G	TGCAGGTGCCTGCAAAGACTGTCGGTATGTCTAGCCACGTCCGTGGCCG	9:C>G
3020862|F|0-14:G>T-14:G>T	TGCAGAGCAAACCAGAATAAGCCCCCAAAAAGTCAATGTTCCG	14:G>T
1017337|F|0-62:T>C-62:T>C	TGCAGTACTGGAGAGGTGAGGTACGCTCTACGTACCTTTGAGGGGAGCGATGCCCTCTTGCATGAGGAA	62:T>C
3952350|F|0-22:G>C-22:G>C	TGCAGTGGATCATGGCGGAGCTGGTCAAGAACCCG	22:G>C
3023834|F|0-23:G>T-23:G>T	TGCAGAATTCGTTCAGCACAGCAGAACACATCGGGAGCCG	23:G>T
4261652|F|0-15:A>G-15:A>G	TGCAGCCGCGCGTGTAAGGGTTGGCCACCGCAGAAGGCCG	15:A>G
7173621|F|0-26:T>C-26:T>C	TGCAGCAGCAGCCAGTTCACCTTGGCTAGCACCAGCTGGTCGTCGTTGCAGTAGAAGAGCCG	26:T>C
7173059|F|0-11:G>A-11:G>A	TGCAGTTGGGTGAGTAGGCCCCGTTGCAGCGGAAGATTTGCAGTGTGGTTTGCGGCGCCCTTGCCTCCG	11:G>A
1234784|F|0-39:G>A-39:G>A	TGCAGCTTGGCCATTGCCTGTGGGTTCCCGACCAGCTCCGCCATGGCCCATTCCAGCGTGAGGGACGAC	39:G>A
3938585|F|0-14:A>G-14:A>G	TGCAGGACAAGGAGAAGGAGCTTTCGACCCAAAGCCAAATTCCATGGATCCG	14:A>G
3020451|F|0-47:A>G-47:A>G	TGCAGATTCCCAACAGTGCTTAAGCTCCTCGAGGCCACTGGATGCTGACCAATGGGCAACTTCAGATTC	47:A>G
3939103|F|0-36:T>C-36:T>C	TGCAGGAGCGGGTGCTGAGTTGCCATGCAGCGATGATCCAGGCCACCG	36:T>C
3939504|F|0-6:C>G-6:C>G	TGCAGACGGAGGAATCACACAAAATACACCG	6:C>G
1056056|F|0-35:G>C-35:G>C	TGCAGGTGGTGGCTCCTCATCTTGTCGCCGACGCCGAGGGCGTCAAGCGCGCGGAAGGCGTTGGTCCAT	35:G>C
6038593|F|0-14:C>T-14:C>T	TGCAGTTCATACAACGCCAATTATGGATATGGATCCATCTTGCTGCGCCG	14:C>T
22836108|F|0-7:G>A-7:G>A	TGCAGCCGAGGCGGCCACTCCGCCGACGCCCTCGGGCTCGGAGTCGCCG	7:G>A
32023394|F|0-16:C>G-16:C>G	TGCAGGGGCTCCAGGGCTACGTGCAGAACGTCAGGTTCCTGGCCG	16:C>G
7170082|F|0-19:T>C-19:T>C	TGCAGCCCAAGCCGCTCCGTCGCGTGCTTCGCTACCTCGAGGAGGACAAGATAGTGTCGAGGGACCACC	19:T>C
4262843|F|0-10:C>T-10:C>T	TGCAGACTATCGGCGCTCATGGCAGAGACCG	10:C>T
1228670|F|0-6:T>A-6:T>A	TGCAGCTGCTGGTGGTAGCTGCACGACTCGTTGTGGTTGGGCGTCTTGCACGCAACGTGCGGGAAGCGG	6:T>A
3533831|F|0-29:C>G-29:C>G	TGCAGCTCCTTGAAGTAGACGCCGTCGAACGCCGTGCTCGTCCG	29:C>G
4409403|F|0-20:C>T-20:C>T	TGCAGGCCGTTTCTCGTCGGCGAAGCCACCG	20:C>T
4409403|F|0-18:G>A-18:G>A	TGCAGGCCGTTTCTCGTCGGCGAAGCCACCG	18:G>A
2244421|F|0-8:C>T-8:C>T	TGCAGAGGCACCCCATCGAGCGCGTCGTCGTCGAAGTGCTCGGGTAGCTTGTCGAGGAGGAGCCCCAGG	8:C>T
1065865|F|0-13:G>T-13:G>T	TGCAGGTTAATTTGGATCCTGCTAACATTACAGAAGAAGCTTCCATTTCCTCTCTTAGATCCGTTCGTC	13:G>T
1036788|F|0-38:G>C-38:G>C	TGCAGGGCGAGGAAGCCCTTGTAGTAGAGGAAGCAGTGGACCATCTTGGCGCAGGCCG	38:G>C
1394604|F|0-31:G>A-31:G>A	TGCAGGTGATCACCGCGCGGCCGCCCATGGGGCTGACGCATCACGTCGAGAAGGCCATCGAGTCCGAGA	31:G>A
7170262|F|0-6:C>G-6:C>G	TGCAGGCACAGTGTATTCGAAATCATCTGTATACCTGCGCGGCATCGACCGATGCCGCATGTACATTAG	6:C>G
3941110|F|0-6:G>A-6:G>A	TGCAGCGGCACAGCAACGGGCCGATGCGGTCAGACTCTCTGCGGACGCCG	6:G>A
3027456|F|0-27:C>A-27:C>A	TGCAGGCACGCACATGAGCATGCAAGGCATGCCTAAGAACGCCG	27:C>A
977299|F|0-27:A>G-27:A>G	TGCAGGAGTTCACCCGATGGACCGAGGACAGGGTACATGGTACGATATCCG	27:A>G
1052058|F|0-25:A>G-25:A>G	TGCAGGGAGTTTGTGATCATCGTCAAGTCCATTTGGATCCTGTCACCTCAAGAGGTTCTTCAACCGATG	25:A>G
7916077|F|0-63:C>G-63:C>G	TGCAGACCTGGGCACGAACAGAGGGCTGTCGTACATCGACATGAGCGACAACCTTCTTGAAGGCAGGAT	63:C>G
3948138|F|0-9:G>A-9:G>A	TGCAGGGTCGCCAGCGCGAGCACCATGGCCG	9:G>A
3934308|F|0-47:C>T-47:C>T	TGCAGGTAGTCCAGCTTCGCCTTCTCCAGATCCGCCATCAAGCGATCCGCCTTGTATATCCG	47:C>T
1006991|F|0-29:G>C-29:G>C	TGCAGGAGGCCGATCTTGTCCGAGACCTCGGCGTCGGCCGCCGCGTCCTCCG	29:G>C
1004991|F|0-14:C>G-14:C>G	TGCAGGCTTGTCAGCAGTGCGGATTTAAAGTAGCATGGCGACGGCGACATGGCGTGCCACTGTGAACGG	14:C>G
1028890|F|0-20:C>T-20:C>T	TGCAGGTGGAGGTGGTGCTCCTGCTGCTGCCTCCTGCTCCCCGCCGCCAACTCCTCCATGGCCGCCGCG	20:C>T
1056584|F|0-7:C>T-7:C>T	TGCAGTACGACGTGGACTCCCTGGACCAGAACCTGGCCCAGAACCTGGCGGGCGAGATCATCGGCACCC	7:C>T
1062875|F|0-43:G>A-43:G>A	TGCAGCTCTTCCGTGGAGACACCGTCCTGATCAAGGGCAAGAAGCGAAAAGACACGGTCTGCATTATGC	43:G>A
1021636|F|0-34:C>G-34:C>G	TGCAGCCTGCTGCTGTTGGCGATCTTCTTGACGACCTTCTTGGCGTCGGGGTCGCCG	34:C>G
3534358|F|0-10:G>A-10:G>A	TGCAGAACACGGTGACTCGAAGCACCAGCAACCCATCATGTACATTGTCCCCGAGGAGCTTAAAAACTC	10:G>A
3950290|F|0-21:C>T-21:C>T	TGCAGATCAACTAAGAGTTCACGCAGCCG	21:C>T
1058625|F|0-12:A>G-12:A>G	TGCAGCCCGCTCAGCAGCGTGAGGCCCTCTGCTTCTTCCATTGTCGTACATTTGCCCAGTTTGCATCCC	12:A>G
2244421|F|0-7:G>A-7:G>A	TGCAGAGGCACCCCATCGAGCGCGTCGTCGTCGAAGTGCTCGGGTAGCTTGTCGAGGAGGAGCCCCAGG	7:G>A
1020676|F|0-33:C>G-33:C>G	TGCAGATTTCAGGTTTTTGCTTGGCTGGGTTCACCTGTGGGATCAGGCCG	33:C>G
3934308|F|0-34:C>T-34:C>T	TGCAGGTAGTCCAGCTTCGCCTTCTCCAGATCCGCCATCAAGCGATCCGCCTTGTATATCCG	34:C>T
3941675|F|0-16:G>A-16:G>A	TGCAGATGAAGTTAGCGCGGCAGCTCATAAGTTGCCG	16:G>A
32026070|F|0-44:G>A-44:G>A	TGCAGGTAGTCCAGCTTCGCCTTCTCCAGATCCGCCATCACGCGGTCCGCCTTGTATATCCG	44:G>A
4009208|F|0-19:G>C-19:G>C	TGCAGCGCGTCGGCAGCGGGGCCAGCGCGGTCATGGACGCGCCG	19:G>C
1026877|F|0-47:G>T-47:G>T	TGCAGGGAGCACCTCCCCTGACAAAGAGGTTCCTCCTGGTTCTCACTGCGGCATGAATCGTCACAGTTG	47:G>T

988084|F|0-12:G>T-12:G>T	TGCAGCACTGCGGTGATGGAAGAAGTGAACGACAGCCAGCTTCCGATGGAGCCGCTTCACTACCCGAGA	12:G>T
4261131|F|0-16:A>G-16:A>G	TGCAGAGAAACAGAGAATTCAGCATGAGACCG	16:A>G
7170990|F|0-51:T>C-51:T>C	TGCAGAACCGACGACGACAAGGTGAATACTACCGCTGATCCCAAGAAGAAGTTTAGTAGCCCG	51:T>C
7170779|F|0-45:T>C-45:T>C	TGCAGGAGCAGTGGCGCTGCCCACTGTCCATCCTGCTGCTTACTATTTGTGCGCTTACCCG	45:T>C
1022190|F|0-10:C>T-10:C>T	TGCAGGACCTCCTGCTGGCCATCATGGCGGCGCTGGATGTCCCCAGCCTCGTCCGTTCCG	10:C>T
1089877|F|0-19:C>A-19:C>A	TGCAGGCGGAGGAGGACGGCGACGAACATGTCGTTTTTGCATCCAAGCCTGCGTTCTCTCCG	19:C>A
6041850|F|0-5:G>C-5:G>C	TGCAGGTCCACGCCGACGGCCAGATCTCCCGCCCG	5:G>C
1062875|F|0-12:G>A-12:G>A	TGCAGCTCTTCCGTGGAGACACCGTCCTGATCAAGGGCAAGAAGCGAAAAGACACGGTCTGCATTATGC	12:G>A
1053458|F|0-48:T>G-48:T>G	TGCAGGCTTCTGGCACTACAAACAAAACAATCCAAGCATATGTGTCAGTATAAAACCAGTAACCGAGAT	48:T>G
3021203|F|0-25:A>G-25:A>G	TGCAGCAAGTACACACACAAGGAAAAATTCAGACTTCGCCG	25:A>G
4396785|F|0-20:G>A-20:G>A	TGCAGACGACCTGGTGCATGGCGTTCCG	20:G>A
1090065|F|0-41:G>C-41:G>C	TGCAGGCCCCGCACGAACGCCACGGCGTACTTGCTCGCCGTGGTGGGGTCCTCGCCG	41:G>C
1696698|F|0-42:G>A-42:G>A	TGCAGGTCCTGCTCGCTGTTCTTGTCGATGTTGATGGACAGCGCCAGGTTCCG	42:G>A
3026558|F|0-30:G>C-30:G>C	TGCAGAAATTCCAGCTGCACGCGTAGCGTAGACGATCCG	30:G>C
3936766|F|0-13:A>G-13:A>G	TGCAGAACCCAACAAGACTTGCGTCCTACGATGCTTCAGCCG	13:A>G
1018140|F|0-56:C>T-56:C>T	TGCAGGATTGGAGGAATGCTCAGCGAGAGCCCTTTGGCGATGCGTCAGGAGATGGGCGGCGTGAGAGGG	56:C>T
1020676|F|0-12:G>C-12:G>C	TGCAGATTTCAGGTTTTTGCTTGGCTGGGTTCACCTGTGGGATCAGGCCG	12:G>C
32023349|F|0-32:C>G-32:C>G	TGCAGCTGGGCCGCGTGGCCGCGGCGTCTCGACCGTCTCCG	32:C>G
7478638|F|0-23:G>A-23:G>A	TGCAGGCTGGCCCTCGCGAGCACGCACCCG	23:G>A
1051733|F|0-41:G>A-41:G>A	TGCAGGAGGGAGAAGCTTACTCCTGGGAGTTTGATGAAGGCGGTTATCCG	41:G>A
3571299|F|0-35:G>T-35:G>T	TGCAGATGCCGCCGATGGCCATGCACGGTCACGGTGACGTGTGCCCG	35:G>T
32024857|F|0-7:T>G-7:T>G	TGCAGGATGGTGGGCCGCCACGTGGAGGAGAATGATATGGGAGGCGCCG	7:T>G
4005207|F|0-5:G>T-5:G>T	TGCAGGGTCTGTGTCGAGGTTGCTTCGGTATGCTCCG	5:G>T
3942396|F|0-19:G>A-19:G>A	TGCAGAACATCCAGAACGCGCTCAACGACGCCG	19:G>A
4261194|F|0-26:C>T-26:C>T	TGCAGCTTCTTGGATCTCATGGAGTCCATGTGCACGACCG	26:C>T
3021289|F|0-10:C>T-10:C>T	TGCAGGTCGCCATTGAGGTCCTGCCTGGGGCAGATTCCG	10:C>T
3021289|F|0-30:C>A-30:C>A	TGCAGGTCGCCATTGAGGTCCTGCCTGGGGCAGATTCCG	30:C>A
1107007|F|0-20:C>G-20:C>G	TGCAGCATGGGGCTCCTGAACCGCCTCCCTCCTCGGAACCACGCGCGCTCCGACCCCTGCGCCG	20:C>G
3950610|F|0-19:G>A-19:G>A	TGCAGCTGCATCTGCATCAGGAGAGGTCCATCCG	19:G>A
1864913|F|0-62:G>A-62:G>A	TGCAGGGTGCTCGAGAGGCAGAAAGTGGACAGCAAGCAAGACACCGTCCGCTCCTCCTCGCTGACCTTC	62:G>A
7173582|F|0-10:C>A-10:C>A	TGCAGATCGCCAGGACCCTGGGCAGCCTCGTGGCGCCG	10:C>A
2253153|F|0-10:C>A-10:C>A	TGCAGCGCCGCCGACGACGCCCAGGCGGAGCGCATCTGCGGGATTCTCGACGACATCATGGCGGAGTCT	10:C>A
1103411|F|0-8:T>A-8:T>A	TGCAGCACTTTGCCAGCGCCTCCTCCCTTCCTCCAGCAACCCGATCCCCG	8:T>A
5581268|F|0-10:C>G-10:C>G	TGCAGAACCTCACGACGGGCGGCGACAGCTTCCG	10:C>G
3030373|F|0-25:G>T-25:G>T	TGCAGGCCGAGGTCGCGCGCCAGCCGCGCCTTGCGCTCCG	25:G>T
3938720|F|0-9:C>T-9:C>T	TGCAGATCACGTTATTCCGTTTTGTCCG	9:C>T
3939504|F|0-9:A>G-9:A>G	TGCAGACGGAGGAATCACACAAAATACACCG	9:A>G
3020372|F|0-5:G>A-5:G>A	TGCAGGTGTTTTCACACATGATACATGACATTTACCCTACCCG	5:G>A
1021070|F|0-22:G>C-22:G>C	TGCAGCGCGAGCTCGGTGAGGCGGAAGCCCTTGCGGTGCAGGTACTGCTGCACCTTCCTCTCCATCTCC	22:G>C
7161352|F|0-34:G>C-34:G>C	TGCAGCGGCTCTAAGAAGAGTGAGTGCCGTCGTGGCTTATTTTCGGGTCAGTCCG	34:G>C
6036196|F|0-29:A>G-29:A>G	TGCAGGCGGAAGTAGAGCAACCCCTCGACAGCGTCGGTGCGGAGCGTGGCGATGCCGCGCGCCATGAAG	29:A>G
100497038|F|0-40:G>C-40:G>C	TGCAGGTGAGCATCACCAGCCAGGCCCTGATATTCGTGACGCGGAGCCG	40:G>C
1052058|F|0-15:A>G-15:A>G	TGCAGGGAGTTTGTGATCATCGTCAAGTCCATTTGGATCCTGTCACCTCAAGAGGTTCTTCAACCGATG	15:A>G
1149760|F|0-38:C>A-38:C>A	TGCAGCACGCTCATGTTGTGGTGCTGGTGATCATCCGTCGTCCCCTCCG	38:C>A
1020174|F|0-5:G>T-5:G>T	TGCAGGCCCTGAAGCAGGCCTCGGAACCTCTCCACGTTGCCACCGAACCGCCCGTCCTTCAAAATCCTG	5:G>T
4992635|F|0-24:G>T-24:G>T	TGCAGGTCCCGAGTTCGAACTCTGGTAACCTGCACGTTTTTTACCTCGTTTCCTTTGTATTACAACCGA	24:G>T
1018357|F|0-18:T>C-18:T>C	TGCAGCCTCAAAGGATCCTGCGTCATGCAGGGCCTGCCCTACTATAACGCGTCCAAGTCGGACAGCTTC	18:T>C
3575160|F|0-15:G>A-15:G>A	TGCAGGAAGCAGCAGGGCGAGAGGGTGGACTTCTCCG	15:G>A
1017337|F|0-23:C>T-23:C>T	TGCAGTACTGGAGAGGTGAGGTACGCTCTACGTACCTTTGAGGGGAGCGATGCCCTCTTGCATGAGGAA	23:C>T
1001583|F|0-36:T>G-36:T>G	TGCAGCCACCAGGTCGCTCACTTCCCTGCCACCGCCTCCCCCATTGGCTTGTAGCGCTTTGCAATTGCA	36:T>G
4005727|F|0-23:G>A-23:G>A	TGCAGGCTCAGCTCCGTGACGCCGCCGTCGGCATTGCACGACACGCCG	23:G>A
1150315|F|0-22:G>T-22:G>T	TGCAGCCTCACCAGCGGCTCTCGCACGGCGGCGGCGAACCCAAGCTCCG	22:G>T
1090620|F|0-33:A>G-33:A>G	TGCAGCACCAAGACGGCGAGGCGTCGTCTGCGCAGCCTCCTCGTCCTCGCCGCAGACTACCTCAAGTAC	33:A>G
2254157|F|0-14:A>G-14:A>G	TGCAGTCGCTCGGGACGAACACCGACGTCTGCCATGTCCTGCCTCCTAGACGCCGACCTCGATCAGGCT	14:A>G
3957524|F|0-6:C>G-6:C>G	TGCAGGCAGCGACCTAACTCTCGCAGCGCCTGACAAGTTAACCG	6:C>G
7902441|F|0-39:G>C-39:G>C	TGCAGTCGGCGGCGCTGTCCTTCTTCAGCTTCACCAACAGCAAGCCG	39:G>C
32024495|F|0-67:C>T-67:C>T	TGCAGCTGGACGAATGCCAACCTGATGATGATCGAATGGGAGGGGAGGATACCATTACCG	67:C>T
1075105|F|0-18:C>T-18:C>T	TGCAGGTCAGAGAAAAATCGGAGAAAGAGGAATCAGTTCAGAGAGACATCCG	18:C>T
3957680|F|0-9:T>C-9:T>C	TGCAGCCGCTTCACGATCTCGTACTTGTGCTCCG	9:T>C

1161028|F|0-23:T>C-23:T>C	TGCAGTTGGCCTGACTGATTTTGTGCTTCGTTTTGCTTGATGATGACCG	23:T>C
1285750|F|0-27:T>G-27:T>G	TGCAGCTGTTAGTTAACGAACCTTTTTTGGCCGATGAGTACACAGTTTCAACGAGTTCATCATGCTCAT	27:T>G
3029476|F|0-11:G>A-11:G>A	TGCAGCCTCTCGCCGCTGGTGAGGTTCAAGGCGTTGAAGGAGCCG	11:G>A
1219140|F|0-41:G>A-41:G>A	TGCAGATCCAGGGCGCGATGAGGGTGGTCGAGGCCACCAACGACGACAGGGAGCTGAGGATATACATGT	41:G>A
4261931|F|0-22:C>T-22:C>T	TGCAGCTGCACCGCCGCGGCCACAACCTGCCGTGGAAGCTCCG	22:C>T
2243154|F|0-30:C>G-30:C>G	TGCAGAACACGTATCCACAAGTAGTCGACCCTGTCTGTCGGGTCGCCATCCATCCTTCCACATTTGAGC	30:C>G
3023834|F|0-26:C>G-26:C>G	TGCAGAATTCGTTCAGCACAGCAGAACACATCGGGAGCCG	26:C>G
1019452|F|0-61:C>T-61:C>T	TGCAGGTGAGCATCATCAGCCAGGCGCTCATCTTCGTGACGCGGTCGCGGAGCTGGTCGTTCGTGGAGC	61:C>T
1002614|F|0-18:T>C-18:T>C	TGCAGCGCGGTGGACACGTCGCGCATGCGTTCCTGCGTGTAGTCCTCACGCCG	18:T>C
4664225|F|0-12:T>C-12:T>C	TGCAGAACAGAATCACAAACCAATCCACCG	12:T>C
1862723|F|0-11:G>C-11:G>C	TGCAGGGGCTGGCTTTGGGTCGGGCACTTCGAACGACCTTGTCAGCACGGAGCCTGACCGCTCCAGGCT	11:G>C
1862723|F|0-25:A>G-25:A>G	TGCAGGGGCTGGCTTTGGGTCGGGCACTTCGAACGACCTTGTCAGCACGGAGCCTGACCGCTCCAGGCT	25:A>G
1695427|F|0-12:A>G-12:A>G	TGCAGGAGAAGAACCAGGACGCCCTCGAGGCCAACCTACGCTTCGTGGAGGTGCCG	12:A>G
1020753|F|0-11:C>G-11:C>G	TGCAGATTGCCCAATGGATAAAGCAATATCTTGAAGGTTTTTGCCGCTGCCG	11:C>G
5578229|F|0-10:G>C-10:G>C	TGCAGGAGGTGAAGGAGAACTCGGTCCG	10:G>C
32024193|F|0-14:A>G-14:A>G	TGCAGGGATGGCCAACGGGAGGCGGGTGGGAAGAGGAAAGAGAAGCCG	14:A>G
6046183|F|0-9:G>A-9:G>A	TGCAGAGACGCTCTGCGAAATTTGTCAGTTTGACCGAAGCTGAAGATATCCAATGGAGTCATGTAGCGT	9:G>A
2247474|F|0-49:G>A-49:G>A	TGCAGCTCCTCCATCGTGCTCTCCTCCGACGACGTCGACACCTTCACCAGCGACCCGCCCACCGACAGG	49:G>A
2244360|F|0-17:C>G-17:C>G	TGCAGTAACCCGACGTTCCGCTTGACCACCGTCTCGAGGTTGTGCGAGAGGAGATTGTGGTTATGCTTC	17:C>G
6046910|F|0-23:G>A-23:G>A	TGCAGTCCCACGCCGACGAGACGGTCCG	23:G>A
1114850|F|0-15:G>T-15:G>T	TGCAGAGCAGAGCACGGAGACCATGGTGGCCGTCACGGTCGCCGTGACCGCTGGCGCCG	15:G>T
1079000|F|0-53:G>T-53:G>T	TGCAGGGCCAGAGTCACCTGGGAGACAACGCCGAGGACCCCAAGGGAGACCTTGGCGGCGTCCAGGTCC	53:G>T
3946756|F|0-10:G>C-10:G>C	TGCAGCGGCTGCTCCTTGCAAACAATGGGTTCTCCG	10:G>C
1018140|F|0-13:G>A-13:G>A	TGCAGGATTGGAGGAATGCTCAGCGAGAGCCCTTTGGCGATGCGTCAGGAGATGGGTGGCGTGAGAGGG	13:G>A
3021289|F|0-9:C>T-9:C>T	TGCAGGTCGCCATTGAGGTCCTGCCTGGGGCAGATTCCG	9:C>T
3940072|F|0-10:C>T-10:C>T	TGCAGTGGCTCCCCGTCAAATCCGTCCTCCGCTTCCG	10:C>T
1027099|F|0-14:T>C-14:T>C	TGCAGGCGGTGGTGTCCGAAGCTGATCTGCGCAGCGGTGCTGCCGCTCGCGGCCG	14:T>C
979340|F|0-28:T>C-28:T>C	TGCAGATTCCATCCTGGACTTTGAGGTGTAGTGCTGGGATGATGGCCGCACCTTTCTCGGAGCCGAGAT	28:T>C
1036567|F|0-34:G>C-34:G>C	TGCAGTAGAACCAGAGGGCGAGCTTGACGGCGGTGGCCGAGAGCATGATGGAGTAGAGCCATATCAGCT	34:G>C
6042716|F|0-58:G>C-58:G>C	TGCAGCCCATATTTGGCCTGATAAGCGTTGCCACGGACATCCTCGCCTTGGCCGAGTTGGTCACCACCC	58:G>C
3027653|F|0-25:G>T-25:G>T	TGCAGCTCTCCAACCTCTCCTCGGGGCCCCTGTTCCTCTCCG	25:G>T
1020985|F|0-20:T>C-20:T>C	TGCAGCCTATGCAGGCTGGCTAGCGAGCAGTGACAGCGGGTCAGCGAGTGCCACAGAGACCG	20:T>C
1125663|F|0-45:C>T-45:C>T	TGCAGACATTGTTTCAGTCGTTGCTTGGGTCTTCTAGAGGGTATACTTCTGGCCG	45:C>T
2243154|F|0-10:G>T-10:G>T	TGCAGAACACGTATCCACAAGTAGTCGACCGTGTCTGTCGGGTCGCCATCCATCCTTCCACATTTGAGC	10:G>T
1024352|F|0-53:G>C-53:G>C	TGCAGGCCCCTCACGAAGGCGACGGCGTACTTGCTCGCCGTGATCGGGTCCTCGCCG	53:G>C
1208994|F|0-16:G>A-16:G>A	TGCAGGAGATGTCGCAGGACGGCGTCATGGGCAAGCTTGGCCGCGGCACGACGAACCG	16:G>A
4261842|F|0-8:C>G-8:C>G	TGCAGGATCTCAACGTGTCCAACAATCTCCTCTCGGGGAGGATTCCG	8:C>G
991920|F|0-46:C>T-46:C>T	TGCAGGTTGGTTTCAACAATGGAGTCCCCATCACCGAGGAGTTCTACAGCGCCAACATCAGCGGGTGGC	46:C>T
1022186|F|0-7:G>T-7:G>T	TGCAGGAGGCGGCGATGCCGTCGGCAGTGAGGGAGGATCGTCGGATCTGATCACCGATGATCAGCCGAG	7:G>T
1066920|F|0-49:C>T-49:C>T	TGCAGGAAGGAAAGAGGATCTTTGAGGTGTTGGATTGATCGGAGATGAGCGCCCG	49:C>T
4261605|F|0-31:C>G-31:C>G	TGCAGCTGGAGGTCAACGTCAAGCGTGGAGACCTCCG	31:C>G
3949806|F|0-12:T>C-12:T>C	TGCAGGCACCCATATCGATCCGTCTCACCTCCG	12:T>C
1151894|F|0-38:C>A-38:C>A	TGCAGTGTGCTCATGCATAATTGATTCAACAACACTGGCAGGCATGCAACAGCCATGCATGCATGGATG	38:C>A
3942162|F|0-28:G>A-28:G>A	TGCAGCTGACGTGGAACTACAACTACGGGGCGGCGGGGAAGAGCGTGGGATTCGACGGGCTGAACAACC	28:G>A
4539309|F|0-5:C>T-5:C>T	TGCAGCAATCCCGCGCCGTGGTCGACGAGCCG	5:C>T
5370977|F|0-11:C>T-11:C>T	TGCAGCGCACTCCCTCTGCCGCGAGATGGGCCG	11:C>T
3953058|F|0-6:T>G-6:T>G	TGCAGTTGAGGACGAGCACGCCGATCGCGTCTGGGGCGACGCCG	6:T>G
5004301|F|0-13:C>T-13:C>T	TGCAGGTGCTCAACCTGTCGTCGAACCG	13:C>T
3941675|F|0-14:G>C-14:G>C	TGCAGATGAAGTTAGCGCGGCAGCTCATAAGTTGCCG	14:G>C
4261612|F|0-7:G>C-7:G>C	TGCAGCTGGGCACGGAGTGCACCGAGCGGCGGCCCGACCG	7:G>C
1049073|F|0-23:G>A-23:G>A	TGCAGCTCATAGCGGTTCCAACTGAAATTCGTAATCTTTACCACCTTGAGGTTTTCCAAAACATCCGAG	23:G>A
2241644|F|0-21:C>G-21:C>G	TGCAGGTTCATGGAGACGTAACGACTCCAGAATGTCGACAGATGAGAACGTGAACCG	21:C>G
3021177|F|0-41:C>G-41:C>G	TGCAGCTGTACTGTACGCCATATGATGAGCCCAGCTAGGTTCATCCG	41:C>G
984930|F|0-28:G>T-28:G>T	TGCAGCACCTGACGGACAAGAGCGACGTGTACAGCTTCGGCGTGGTGCTGCTGGAGCTCATGTCCGAGA	28:G>T
5580512|F|0-26:G>A-26:G>A	TGCAGGTCGATCTTGCGCGCCGACTCGGCCCGCCG	26:G>A
1032411|F|0-22:G>A-22:G>A	TGCAGAAAGAGAAGAAATAAGAGACCAAGGCGAGAGGAGAAAGATTTTCCCTTAAATAGAGGAAAGAGG	22:G>A
1263626|F|0-44:G>C-44:G>C	TGCAGCAAGCTAAGCATGCACGAGCACGAGTCGTCCGTCCTCGCGACCAAGGTCATCACCATCTCCGAG	44:G>C
5582389|F|0-8:C>T-8:C>T	TGCAGGTACGGCATCTGGGCCACGTCCG	8:C>T
978884|F|0-16:C>T-16:C>T	TGCAGCTTCCCCTCCTCGTCCTCCACCTCCTCGTCGTTCCGTTCCGCCTCGCTCTCTTGCAGCCCGAGA	16:C>T

<table>
<tr><td>3945741|F|0-27:C>T-27:C>T</td><td>TGCAGATGGGCGTGATGCTCACGACGGCGACCGTGGTGGTCACCGCTGGCATCGTCGTAGTGAGCTTGT</td><td>27:C>T</td></tr>
<tr><td>1019021|F|0-7:G>C-7:G>C</td><td>TGCAGAAGCTGTGCTTGTAGGAGCTCCTGGCGTGCAGCGTGACGTTGAAAGCCG</td><td>7:G>C</td></tr>
<tr><td>1056584|F|0-52:C>T-52:C>T</td><td>TGCAGTACGACGTGGACTCCCTGGACCAGAACCTGGCCCAGAACCTGGCGGGCGAGATCATCGGCACCC</td><td>52:C>T</td></tr>
<tr><td>1099470|F|0-33:C>G-33:C>G</td><td>TGCAGCACTTTGCCAACGCCTCCTCCCTTCCTCCAGCAACCCAATCCCCG</td><td>33:C>G</td></tr>
<tr><td>2243225|F|0-10:C>G-10:C>G</td><td>TGCAGGACTCCGATCGATGTCAACGTCCATACGGAGGAGACGACGAGTGGATGGACTCTAGTAACGACC</td><td>10:C>G</td></tr>
<tr><td>1039417|F|0-10:A>G-10:A>G</td><td>TGCAGTTCTCATCGGCGATTGACACGCTTCGTGCTTGTTTCAGGTGTATCAGGAGGAGTCAGACAAGCC</td><td>10:A>G</td></tr>
<tr><td>3025328|F|0-16:C>G-16:C>G</td><td>TGCAGGGAGCCCTGCCCCACCGTGCTCGTCACCAGGTCCG</td><td>16:C>G</td></tr>
<tr><td>6041850|F|0-30:G>T-30:G>T</td><td>TGCAGGTCCACGCCGACGGCCAGATCTCCCGCCCG</td><td>30:G>T</td></tr>
<tr><td>1037839|F|0-9:T>C-9:T>C</td><td>TGCAGTGCGTGAAGGCTCCTCGGACGGGGAAGGCAAGAAGGTGGATCCCCG</td><td>9:T>C</td></tr>
<tr><td>1071878|F|0-67:A>G-67:A>G</td><td>TGCAGCCCAGGCGGTGTACGGCGACTGCGAGTGGTACTTCTTCAGCCCGCGGGACCGCAAGTACCCCAA</td><td>67:A>G</td></tr>
<tr><td>3533035|F|0-10:T>C-10:T>C</td><td>TGCAGGCACGTAACTTGGTTCCGTCGAGACGAGCTAGCTGATTTCGCCG</td><td>10:T>C</td></tr>
<tr><td>32021886|F|0-29:A>G-29:A>G</td><td>TGCAGCGCGGCGATGGAGTCCCTCCACCCATGGAGGACGCAGGACTCGAAGAAGGTGTCGAGCTTGGAG</td><td>29:A>G</td></tr>
<tr><td>1719681|F|0-49:C>T-49:C>T</td><td>TGCAGCATGTGCGGTATTTGCTTTCAATTTGTGCTCTGCCTCTCTGCTGCTGGCTATAAATACCGAGAT</td><td>49:C>T</td></tr>
<tr><td>32018960|F|0-43:G>C-43:G>C</td><td>TGCAGCCTGGCACTAGCAGGGCTGGATTTGGCTGGTTTCCAATGAAAGCTTGCTACTCCG</td><td>43:G>C</td></tr>
<tr><td>5009201|F|0-59:C>G-59:C>G</td><td>TGCAGTCCATCGGCCGCCGCCATCAGATCAGAGCTCAGCACGACGCAGGAAAATGATTGCTTTCGGTGA</td><td>59:C>G</td></tr>
<tr><td>1388522|F|0-28:C>G-28:C>G</td><td>TGCAGACCGAGATGATTTTTCTCATCATCCGTGAGGTGACCAGCCAACCATTGGTGGATGGGGAACGTT</td><td>28:C>G</td></tr>
<tr><td>4999169|F|0-12:A>C-12:A>C</td><td>TGCAGGTTGTTGAAATGATCAGGTCTATGGAAATGTGATTCTCCAACTAGAACAGTTCGAGTTCAGAAT</td><td>12:A>C</td></tr>
<tr><td>7171759|F|0-15:C>T-15:C>T</td><td>TGCAGAGATATGTGGCAGTTACGACGAAGCGCTGGTAGAACTTGCGCCG</td><td>15:C>T</td></tr>
<tr><td>3029476|F|0-6:C>A-6:C>A</td><td>TGCAGCCTCTCGCCGCTAGTGAGGTTCAAGGCGTTGAAGGAGCCG</td><td>6:C>A</td></tr>
<tr><td>5362378|F|0-32:C>T-32:C>T</td><td>TGCAGGAGCTCGTTGTGTGCCGCGAGCTCAGCCTGACACAGCGTGTCGACATCGTCGCGCCG</td><td>32:C>T</td></tr>
<tr><td>5341544|F|0-34:A>G-34:A>G</td><td>TGCAGCCGTTCTTCGGCGGCGAGGCGCAGACCGCATCCGAGCAGCGGCTCTGCCACGCGCCGTTCGGGG</td><td>34:A>G</td></tr>
<tr><td>100526587|F|0-44:T>C-44:T>C</td><td>TGCAGGAGTGTCGGCTGGCCGTTTCATGAATAACCTTTGCTTGTTGGATAGGGAGGGAATAAAGAGAGA</td><td>44:T>C</td></tr>
<tr><td>4999155|F|0-28:C>T-28:C>T</td><td>TGCAGCTCCCTCGTTAACAGGTCGCTCGCCTTCTCCACGGGCCCCG</td><td>28:C>T</td></tr>
<tr><td>100557696|F|0-14:C>T-14:C>T</td><td>TGCAGCTTATCCAACCCAATACCAGAATCTGCCATCGCCCTCTTGCACGGCTCAACGACTCTCTCCAGC</td><td>14:C>T</td></tr>
<tr><td>5357870|F|0-29:T>C-29:T>C</td><td>TGCAGCGGAGTCGGCCCAAACATGTTGCGTGGCCCG</td><td>29:T>C</td></tr>
<tr><td>5007719|F|0-10:G>T-10:G>T</td><td>TGCAGACGGCGAGCAGCTTCTGCATGAGGTAGTTCCCGAATGGGTTCACCATGAGGTCGACGGCGTGAC</td><td>10:G>T</td></tr>
<tr><td>5361115|F|0-55:T>G-55:T>G</td><td>TGCAGCGGTTGGCCGCCGCCTTCAGGCCGCGAATCGACGCGGCGCTGAAGTGGATTACTCGCTCACGGA</td><td>55:T>G</td></tr>
<tr><td>1083308|F|0-8:A>G-8:A>G</td><td>TGCAGTTCAAACCATCAATGATCATCTACAAACTCAACTTAGTATATCATAACAGAACTAGAATGAAAT</td><td>8:A>G</td></tr>
<tr><td>100323844|F|0-58:C>T-58:C>T</td><td>TGCAGCTTGGGGTGGTTTCAAGTTTGAGTCGCCCCGTTGTGCGTAGGTTGGTGACAACCGACAGTGATG</td><td>58:C>T</td></tr>
<tr><td>100388337|F|0-6:A>G-6:A>G</td><td>TGCAGGAAAGTTTAGGGTCGACGAGACCCCTGGACTTTGGCGGATCCG</td><td>6:A>G</td></tr>
<tr><td>100303917|F|0-7:T>A-7:T>A</td><td>TGCAGTGTACTGATGGATGTTCGTTGTGTTCACCGTGGCCG</td><td>7:T>A</td></tr>
<tr><td>7166645|F|0-60:A>G-60:A>G</td><td>TGCAGCAACATGAGCTCGGCGTCCAAGAAGACGTCGTCGTCGCGAGGTGACCACGGCCACAGCGGCGGC</td><td>60:A>G</td></tr>
<tr><td>100417717|F|0-9:G>T-9:G>T</td><td>TGCAGGGATGCTTTTGTGTCGTCGTCCGCTAATCTGAGGGCGCACGCCG</td><td>9:G>T</td></tr>
<tr><td>100469453|F|0-9:T>C-9:T>C</td><td>TGCAGCGCTTGTGTTCTTTAAATGATCAATCTCCATGAAAGGCCAGCCAGTGTTTCATGTCGAGCCGAG</td><td>9:T>C</td></tr>
<tr><td>3934340|F|0-6:C>A-6:C>A</td><td>TGCAGTCAGCTCCCCGACCGTCACCGTCTTGAGCTCGCCGTCGGCCG</td><td>6:C>A</td></tr>
<tr><td>3947066|F|0-26:G>A-26:G>A</td><td>TGCAGGTTCCTCTCGAACTCCCTCAGGTAGTCCAGGAGCAAAACGATCACGAAGAGGATGAACCCACCG</td><td>26:G>A</td></tr>
<tr><td>3947066|F|0-65:A>G-65:A>G</td><td>TGCAGGTTCCTCTCGAACTCCCTCAGGTAGTCCAGGAGCAAAACGATCACGAAGAGGATGAACCCACCG</td><td>65:A>G</td></tr>
<tr><td>4261098|F|0-34:C>T-34:C>T</td><td>TGCAGCCGTCGTCCTCTGCGCCCGCAGACGTCGCCCGTCGCCG</td><td>34:C>T</td></tr>
<tr><td>3064870|F|0-29:C>T-29:C>T</td><td>TGCAGGAGGGGTGCCGTTTCGGGGAGCAGCGGCTCTTGGTCCG</td><td>29:C>T</td></tr>
<tr><td>6045768|F|0-21:A>G-21:A>G</td><td>TGCAGGGTCATTGTTGCAGGGAAAGAAACGAACGCCG</td><td>21:A>G</td></tr>
<tr><td>1018140|F|0-41:G>A-41:G>A</td><td>TGCAGGATTGGAGGAATGCTCAGCGAGAGCCCTTTGGCGATGCGTCAGGAGATGGGCGGCGTGAGAGGG</td><td>41:G>A</td></tr>
<tr><td>2243154|F|0-28:C>G-28:C>G</td><td>TGCAGAACACGTATCCACAAGTAGTCGACCCTGTCTGTCGGGTCGCCATCCATCCTTCCACATTTGAGC</td><td>28:C>G</td></tr>
<tr><td>3028878|F|0-20:G>C-20:G>C</td><td>TGCAGCGCCTTGTTGTACATGAGCGCGGCGTAGTTCCG</td><td>20:G>C</td></tr>
<tr><td>2244196|F|0-32:C>T-32:C>T</td><td>TGCAGAAATGGGTTCCAGAAGCTCGACAAGATCAAGGATGCCAACCGCCG</td><td>32:C>T</td></tr>
<tr><td>1022972|F|0-27:G>A-27:G>A</td><td>TGCAGTGCGGCGCCGACTCGCTCTACGGCGACAGGCTGGGCGAGTTCAGACTGTCCGTGAAAGGCCACG</td><td>27:G>A</td></tr>
<tr><td>3222222|F|0-29:C>G-29:C>G</td><td>TGCAGCTAGTACAGTCTATAACGCGCACACACAAGCACCG</td><td>29:C>G</td></tr>
<tr><td>3934144|F|0-7:G>A-7:G>A</td><td>TGCAGCAGTGCGTGCAGCGGTGCCAGCAGGACCG</td><td>7:G>A</td></tr>
<tr><td>3028678|F|0-31:C>T-31:C>T</td><td>TGCAGCGTCTTCATGTTGAGCGACATCTCGTCCGCCG</td><td>31:C>T</td></tr>
<tr><td>984689|F|0-44:G>A-44:G>A</td><td>TGCAGAATCGGAGCAGAGGGGAGGGGAATCGGAATGGGGCTCTCGCCGTCGCCGTCCTCGCCG</td><td>44:G>A</td></tr>
<tr><td>6045768|F|0-6:G>A-6:G>A</td><td>TGCAGGGTCATTGTTGCAGGGAAAGAAACGAACGCCG</td><td>6:G>A</td></tr>
<tr><td>987127|F|0-50:A>G-50:A>G</td><td>TGCAGCTAATGACCACCCACACACCAGAGTTCCTTGGGCTAAGGAATGGCACCG</td><td>50:A>G</td></tr>
<tr><td>7169769|F|0-17:G>A-17:G>A</td><td>TGCAGGTCGCTGCACCCGTCGAAGAAGTGGCAGCGGCATGACGCAACCG</td><td>17:G>A</td></tr>
<tr><td>5970430|F|0-64:G>A-64:G>A</td><td>TGCAGGGGTGGCCCTCTTGTCGTGCATTTAGTTGGCTAGTGAGTTGTTGGTGCATGCCTTGGTTGTTAG</td><td>64:G>A</td></tr>
<tr><td>983897|F|0-43:T>G-43:T>G</td><td>TGCAGCTAAACACGCCTCGTGCGGGCAGGGCCTCGCGTAGAACTTCGCGATGTTATCCCCG</td><td>43:T>G</td></tr>
<tr><td>4022577|F|0-24:G>A-24:G>A</td><td>TGCAGGCATCGACGACGAAGCGGCGTGGGCGGAAGCCCG</td><td>24:G>A</td></tr>
<tr><td>4006069|F|0-19:G>A-19:G>A</td><td>TGCAGGATCCCAAGGGGCTGTCCGCCGAGATGGGCGCGTCCG</td><td>19:G>A</td></tr>
<tr><td>7165982|F|0-31:T>C-31:T>C</td><td>TGCAGGTGCACCAGTTCTAATGGCTTTCTTGTTATTTTCTGTAGGAGCTCGATGGACCG</td><td>31:T>C</td></tr>
<tr><td>1163422|F|0-9:C>G-9:C>G</td><td>TGCAGCAGCCGCTCAACTTCAGCAGCAGCCACGTCGCGCGCTACCTCACCG</td><td>9:C>G</td></tr>
<tr><td>2248562|F|0-10:A>G-10:A>G</td><td>TGCAGCATGAATGGCAATGGCACCCAGTCCCAGTGCGGGTATCGAGTCGAGTATGGAGGAGGATTAAAG</td><td>10:A>G</td></tr>
</table>

1069533|F|0-10:G>A-10:G>A	TGCAGCAGTAGCGCGCGCTGCCGTAGGCGTTGCAGGCGCTCTTGCACGCCACCACCTCACCGCTTGAGC	10:G>A
1066920|F|0-26:G>T-26:G>T	TGCAGGAAGGAAAGAGGATCTTTGAGGTGTTGGATTGATCGGAGATGAGCGCCCG	26:G>T
1242815|F|0-30:T>C-30:T>C	TGCAGACGGGCAGGACCATGACCTACGACATTCATGGCGACGGCGGCCAGCTGACATTCGAGCCGAGAT	30:T>C
5004301|F|0-7:G>C-7:G>C	TGCAGGTGCTCAACCTGTCGTCGAACCG	7:G>C
3942062|F|0-13:C>A-13:C>A	TGCAGAGCATTGACGTGTCGTCGAACGAGCTCTCCG	13:C>A
4261100|F|0-11:G>C-11:G>C	TGCAGGGACGTGTCCGCCGCGGTGCCGTGGCCCG	11:G>C
1020207|F|0-7:C>T-7:C>T	TGCAGAACTGTGAGAATTGTCCAAAGTCATGACTTACAGGCCACCGACGATGGCGGAGAAGATGGCCGT	7:C>T
4262346|F|0-22:G>C-22:G>C	TGCAGGCAGAAGGTGGCCCTCTGGAACAGCCGCATGATGTTGCCG	22:G>C
3949579|F|0-5:A>G-5:A>G	TGCAGAAAGCGGCAGCACATGCGTGGTCACCG	5:A>G
4404265|F|0-11:G>A-11:G>A	TGCAGTCCGAAGTAGACGATTCCCACGATGATCAGCATGAACCCAACCAGGAACAGAAGCCCG	11:G>A
1217813|F|0-45:C>T-45:C>T	TGCAGTTCTCTTGGGGCGTCAGTGCGGTGCAGCTGGATGTCCAGTCCACCG	45:C>T
5578229|F|0-22:G>C-22:G>C	TGCAGGAGGTGAAGGAGAACTCGGTCCG	22:G>C
6043774|F|0-14:G>C-14:G>C	TGCAGCATCTCCTGGACCTGCGTGTCCG	14:G>C
1018265|F|0-23:G>C-23:G>C	TGCAGGTTCTGGTCCCGCTGGAAGCCCTTGTGGCAGATCTCGCACACGAACCG	23:G>C
1093570|F|0-46:T>G-46:T>G	TGCAGGTCACAAAGTTTAAGTGCGGCGGCGCCGTCGTCGGCTGCACTTTCGACCACCG	46:T>G
32021417|F|0-31:G>C-31:G>C	TGCAGCTCGCCGTGCTCTACGTCTCGCTGCTGTGCACGTCCCTCGGCTCCG	31:G>C
985496|F|0-13:G>T-13:G>T	TGCAGGTCGGCGCGCCCGTGCACGGCACGGCGTTCCAGCGCGTCGCGTCGTGCTTCGTGCAGGGCCTGG	13:G>T
1018957|F|0-11:T>C-11:T>C	TGCAGGTATTCTTGCACGCCGTGCTGGCGAGGAAGCGGAAGATGCAGCTGCGGTTCCG	11:T>C
1022726|F|0-23:C>T-23:C>T	TGCAGCTTTCATTCATGATGTTGCATTTTCTTTCCCGATTCTAGGCCTCTTCGTCCG	23:C>T
4261250|F|0-28:G>C-28:G>C	TGCAGTGCGAGCCGCCGATGGTGTGCGCGCCG	28:G>C
3945302|F|0-30:C>T-30:C>T	TGCAGAAAAGGGGGTCGCAGTTTCTTTGCACTGGAGAAAGCAACCG	30:C>T
100580264|F|0-6:A>G-6:A>G	TGCAGGAGAAGAACCAGGACGCTCTCGAGGCCAACCTACGCTTCGTGGAGGTGCCG	6:A>G
4262430|F|0-37:C>T-37:C>T	TGCAGCACCAGCTGCGGGGCACGGTGCGCCTCGACCCCTCCG	37:C>T
1055097|F|0-28:G>C-28:G>C	TGCAGTATCAGTACGCCAACTACTCGGCGAACTACCTCAAAGGTGGCAAGGGCAGCATCCG	28:G>C
1022186|F|0-27:T>A-27:T>A	TGCAGGAGGCGGCGATGCCGTCGGCAGTGAGGGAGGATCGTCGGATCTGATCACCGATGATCAGCCGAG	27:T>A
1079008|F|0-17:G>A-17:G>A	TGCAGGGAGTTGAACGCGAACGTCAGAAGTGGGTGTGCGGCGAGGGCGGACGCCG	17:G>A
1260788|F|0-27:A>T-27:A>T	TGCAGGTCCAGGGCCCTGGTTCTACTTATCGCCGTCGCCACCAACACCGCCATCTTCCTCCTCTTCTCC	27:A>T
1079655|F|0-28:C>T-28:C>T	TGCAGAGGATGCACGGAGAGATGTAGTGCCGTGACACGCAGCGTACACGTGAGATGCCG	28:C>T
3025398|F|0-7:C>G-7:C>G	TGCAGGTCGGCGATGCGTGTGTGTTCGAGCTGGTGTCCG	7:C>G
1107007|F|0-26:C>T-26:C>T	TGCAGCATGGGGCTCCTGAACCGCCTCCCTCCTCGGAACCACGCGCGCTCCGACCCCTGCGCCG	26:C>T
3937231|F|0-40:G>A-40:G>A	TGCAGGAACTTGGCGGTGGGGAAGTTGACGGCGAAGGAGAGCGCGCCG	40:G>A
1136196|F|0-31:C>A-31:C>A	TGCAGACGCGCCGTTCAGGGACAGCCTGCCGCGGCACGTGCGCGCGCCG	31:C>A
3934648|F|0-15:C>G-15:C>G	TGCAGCTCTCCCCTCCTCGCCCATGCACAGACGCCG	15:C>G
980037|F|0-8:T>C-8:T>C	TGCAGGTTTTGGGCCTATGGCTGCTGGTTCAAAGCTTATCCTTCATCACTGCTGGGGTTTCGGTAACCG	8:T>C
1099950|F|0-17:C>G-17:C>G	TGCAGAATTCTCGCCCTCCACGCCATGGATCATATTCGTCTCGCCACATGGCGCAATCCG	17:C>G
6048444|F|0-35:G>T-35:G>T	TGCAGCTTGGAGACGATGGATGTGAGATGTAAGTTGCCGTGGCAGTAGGAGACGAGGCGCGCGATCTGA	35:G>T
3934279|F|0-25:T>C-25:T>C	TGCAGGTGCTCGACCTCAGCTACAATAACCTCTCCG	25:T>C
1020984|F|0-50:G>A-50:G>A	TGCAGCCTCGGCGGCGCAGAAGGAGCCTCCGCGCAGAGCATCTCGGACTGGAGCACGGCGCAGTAGTGG	50:G>A
3940964|F|0-17:C>A-17:C>A	TGCAGCTTGGCGAGCCGCGAGATCAGGCCCG	17:C>A
1069178|F|0-34:C>T-34:C>T	TGCAGAGGCAGTTTCTCAAGGAGGTGGTGGGGTTCCAGGTGTTCGGGTCAAGCCCCAAGGTGAGCAGGC	34:C>T
1323764|F|0-6:A>G-6:A>G	TGCAGAATTGCAGGTGTCACCTCCAAAGTCCAACGGCTCCACGTCCAGCCTCTACCACGTCACGCTCAT	6:A>G
1862723|F|0-21:G>T-21:G>T	TGCAGGGGCTGGCTTTGGGTCGGGCACTTCGAACGACCTTGTCAGCACGGAGCCTGACCGCTCCAGGCT	21:G>T
1016719|F|0-60:G>T-60:G>T	TGCAGCTCAGGGTCTCTTATTACATGTGGCTTGACATGACAAGAGCACTCCCATCTTCGGGGAGTATGT	60:G>T
1018967|F|0-47:A>G-47:A>G	TGCAGCTTCGTCGTTGCCGCACAAGCTCATATCTTTTTAGGTGTACCATCTCCTCAGCTCTGCATCTAT	47:A>G
4006298|F|0-11:C>T-11:C>T	TGCAGCACCGACAGCGCCATCAACACCTTAACCTCCG	11:C>T
3575160|F|0-23:G>A-23:G>A	TGCAGGAAGCAGCAGGGCGAGAGGGTGGACTTCTCCG	23:G>A
3958811|F|0-33:T>C-33:T>C	TGCAGATAAAGGAGCAATTACTTGATACGTACCTAGCTGCGTGGCTGCACGCGGGCCTGGAGTGCTTCA	33:T>C
991758|F|0-16:A>G-16:A>G	TGCAGCCAGGTGAGTCACTCACCGTGTGAGTACGACTGGCCTCCGAGGCTCCGACGCCG	16:A>G
1696481|F|0-50:G>A-50:G>A	TGCAGGTACTCTGGCTCGAGCGCGAACAGCACGGACACGCCGTCGTCCTTGTCGGGCGCCTGCGTCACG	50:G>A
990064|F|0-29:T>G-29:T>G	TGCAGCACTCGTTCGGCTGGCAGCCGCAGTTCTGCGCGGCCACCAGCCCG	29:T>G
4395881|F|0-5:G>A-5:G>A	TGCAGGTGGAAGAATGCAATGCCGAAGCCG	5:G>A
1070948|F|0-43:C>T-43:C>T	TGCAGGTCCAGGTGAACGGGGCGACGAGGCGTGTGGGCGGGGTCTTCGGGACGCCG	43:C>T
5008085|F|0-14:G>C-14:G>C	TGCAGGTGCAGCCAGGTGGAGTACTGGTCCG	14:G>C
5582642|F|0-26:G>A-26:G>A	TGCAGCAACAGGAGGTCGATGGCGGAGCAGTAGCAAGACGAAGTGGAGCCG	26:G>A
5355150|F|0-33:A>G-33:A>G	TGCAGGCCAGCAAGGCACCAAAATTGAGTCAGAACGCTACAAGTGTCTACCGCAAACATATGCATGCAT	33:A>G
32016632|F|0-40:C>G-40:C>G	TGCAGTTGAGTATTTTTGACCATTTTGAGTTCAGGCTGGCCTAGCATCCG	40:C>G
32016038|F|0-21:T>C-21:T>C	TGCAGTTCTCTCGCTCGAAGATGACATAGGGAATGGAGAATTGGCTGCTGTGGTGAGGCCTGCCGAGAT	21:T>C
7913482|F|0-28:C>T-28:C>T	TGCAGAGCCCGAGAGCTGATTGGAAGCGCGGACCTCAGTGGACCGCCG	28:C>T
7170823|F|0-53:G>C-53:G>C	TGCAGGCGCTTCTTCAGGCGCTCGGTGGAGACCGCCGCGGCGGCCGCGGTGTCGCCCG	53:G>C

7161156|F|0-10:T>C-10:T>C	TGCAGCCTTCTTGGAACATCGTGACCGCCCTTTCCCCGTCGGATTCCCCGCCG	10:T>C
32023578|F|0-12:A>G-12:A>G	TGCAGCAAAACCATCAACGAAGTGGCCGAAATGAGATTTGGAGCTATCGCCGTCGTCGATCTTTGATCA	12:A>G
1863652|F|0-46:G>C-46:G>C	TGCAGTCGGCCCTGCTCGACGACGTGTCCTCCGTGCCCGTCGCCGTGGCCGTCGCCGCGGCCCGCCTCG	46:G>C
4538596|F|0-10:G>A-10:G>A	TGCAGTGGGTGGACGTCTCGTCGAACGCCTTGACCG	10:G>A
6038044|F|0-8:A>C-8:A>C	TGCAGGCCATATGGCAGGACACGCAGACCCG	8:A>C
1019241|F|0-9:T>C-9:T>C	TGCAGCTTCTTGGTGTCCGTGGTGCTCCAGGAGATGAGGTGGAACAGCCG	9:T>C
3939647|F|0-23:G>T-23:G>T	TGCAGATCGGTCCTGATTTGCATGGCCCG	23:G>T
1047582|F|0-27:C>A-27:C>A	TGCAGCCAGTCTACGGTTGACTTCTGCCCTCTATCCTTAGGATCACGCGGCGCAGGAGCGTACTGCATA	27:C>A
1111995|F|0-6:T>A-6:T>A	TGCAGGTCGGGGAAAAATCGGAGCAAGAGGAATCAGTTCAGAGAGACATCCG	6:T>A
3948297|F|0-17:A>C-17:A>C	TGCAGCTCAAGCATTCCAAGAGCCCTGACCCG	17:A>C
3570851|F|0-13:G>C-13:G>C	TGCAGGACAATGAGAAGGAGCTTTCGACCCAAAGCCAAATTCGATGGATCCG	13:G>C
12854624|F|0-27:G>A-27:G>A	TGCAGCTCATCAGGAGGCGCTCAAGGAGGAGATCGAGAGGCTGCGGCAGGTGTACCAGCAGCAGAACCT	27:G>A
1039566|F|0-33:G>A-33:G>A	TGCAGGCCATGCATGCATGCAGTACAAGGAAATGACACCAAAATCGGTGCAAGAGATGAATCATTAGGT	33:G>A
6043621|F|0-28:G>T-28:G>T	TGCAGACCAAGCTGGCCGCTTACCAGAAGCAACCG	28:G>T
5363107|F|0-10:T>C-10:T>C	TGCAGACCGCTGAAGGGTGGTCGCAGTTCACCG	10:T>C
5365038|F|0-27:C>T-27:C>T	TGCAGACGAGGCGGGCGCGGCGCCTCGCGCTCGGCGGGAGCCGCAACAGGATCTCCACCAGCACGTCCG	27:C>T
1686858|F|0-21:A>G-21:A>G	TGCAGCAACCGTACGTGAACAATCTAAATGGCTGAGTTATCTTCTGCCG	21:A>G
1862920|F|0-24:G>T-24:G>T	TGCAGCAATGCATGCATAATAATGGAGAGTTGTTGAAGCTCGATAGCTAGCTAGCTGCAAGCTGGGGGT	24:G>T
3947302|F|0-40:G>T-40:G>T	TGCAGGAGCGGTTGCTGAGTTGCCATGAAGCGATGCTCCAGGCCACCG	40:G>T
1077816|F|0-11:A>G-11:A>G	TGCAGAAAAGGAGGTAGGGTGGAGGGGAAGCAGGATGTGATGTGAGAGGGATGATGGGCTAACCGAGAT	11:A>G
1147583|F|0-38:G>A-38:G>A	TGCAGGTCCTTGGGGTCCTTCCCGTCGACGGTGCAGCCGACGCTGACGCAGGTGCCGAGGATCTCCTTG	38:G>A
1077970|F|0-33:A>C-33:A>C	TGCAGTTCTACGGGGGCGACAGCAACAGCCCGCACCCATCGGCACCATCTCCTATGTGAGCCAGCCGAG	33:A>C
6036587|F|0-9:T>A-9:T>A	TGCAGAGAATGGCTCCCTCCTGACCTGCATCAAGTATGTAATATGCTCAGCTAAAGGATCCG	9:T>A
1044504|F|0-34:C>A-34:C>A	TGCAGGTCCGTCCTCTCCTCAACACCATCACCAACGCTGCACTAGGCAGCACTACGATCCG	34:C>A
3943527|F|0-16:C>T-16:C>T	TGCAGCTCCACGTCGCCCTGCACCACGCCGTCGTCCG	16:C>T
5324488|F|0-5:G>C-5:G>C	TGCAGGTGCCTCCGCGAGAATGGAACCG	5:G>C
1093570|F|0-40:C>A-40:C>A	TGCAGGTCACAAAGTTTAAGTGCGGCGGCGCCGTCGTCGGCTGCACTTTCGACCACCG	40:C>A
3954188|F|0-11:G>C-11:G>C	TGCAGCACAAAGTGGACACCTAATTACCG	11:G>C
1122234|F|0-8:T>C-8:T>C	TGCAGCTCTGTGATGCTGGCATCAGAGATCAAATGGCGCGGCCCTTTGCCTTCTCCG	8:T>C
1079655|F|0-40:G>A-40:G>A	TGCAGAGGATGCACGGAGAGATGTAGTGCCGTGACACGCAGCGTACACGTGAGATGCCG	40:G>A
1044900|F|0-18:T>C-18:T>C	TGCAGCATGTGCGGTATTTGCTTTCAATTTGTGCTTTGCCTCTCTGCTGCTTGCTATAAATACCGAGAT	18:T>C
4394514|F|0-42:G>A-42:G>A	TGCAGGGTCAGAGAAGTTGAGGTGCTCGGCGAGCGTCCTCTCGATGCAGATGCCACCGTCCGCGCCGAG	42:G>A
5581397|F|0-7:C>A-7:C>A	TGCAGCGCGTCGTCCTCCTCGGGGCTCCACGGGCCCTTGATCCG	7:C>A
1151575|F|0-20:G>T-20:G>T	TGCAGGGGCCTGCGCGGCGCGCCAGGCGCGCCATGAGAGGGCGGAGCGCGAAGAACGTGAACGCCACGA	20:G>T
3936792|F|0-37:C>T-37:C>T	TGCAGGTACGTAACTTGGTTCTGTCGAGACGCGCTAGCTGATTGCACCG	37:C>T
4395665|F|0-7:C>T-7:C>T	TGCAGTACTCGAGCGAGCAGACCTTCAAGGAGGCCG	7:C>T
4398235|F|0-30:C>G-30:C>G	TGCAGGCTGGTTAGTTGATCGGGCGATCGGCCGACGTACGTGGTCCG	30:C>G
3953058|F|0-12:C>T-12:C>T	TGCAGTTGAGGACGAGCACGCCGATCGCGTCTGGGGCGACGCCG	12:C>T
1055207|F|0-9:T>C-9:T>C	TGCAGGTTATTGACTCTGGTCAGAGTTATAGTGGTTATTCTATTCTTCACATGGCGCATGAGGCACCGA	9:T>C
3947359|F|0-26:T>C-26:T>C	TGCAGAGTTAGGTGCAGGGCATGGGGTTGCTCACCG	26:T>C
4262789|F|0-7:T>C-7:T>C	TGCAGTTTGGTAAGGTTACCAAGGACATGTTCACCATGGACTACCG	7:T>C
3944314|F|0-11:C>T-11:C>T	TGCAGCAAGCTCACCAGGCTCTCCACGTCCG	11:C>T
6039792|F|0-17:G>C-17:G>C	TGCAGAACAAGTAAACAGGAGACTGTCAACTAAATCGACACTTCGTTTCAGAAAATAAAACAACCGAGA	17:G>C
3574874|F|0-14:G>A-14:G>A	TGCAGCCACTTGACGTCCTTCCAGCTCAGGGTGCGGTCGATTTGGCCG	14:G>A
1036567|F|0-16:G>A-16:G>A	TGCAGTAGAACCAGAGGGCGAGCTTGACGGCGGTGGCCGAGAGCATGATGGAGTAGAGCCATGTCAGCT	16:G>A
1707968|F|0-40:G>C-40:G>C	TGCAGCTAGCGTCCGCCACGGCCTGCGTCACGCTGTCGGCGGTGCGGCTCGTCAGGCAGGACTTCGGCG	40:G>C
4002282|F|0-28:C>G-28:C>G	TGCAGTGGTCCGACGAGTGCTTGGTGAGCGCCGCGATCTGCGCCG	28:C>G
1061928|F|0-25:G>A-25:G>A	TGCAGGACGTGGAGGAACTCCTACAGTGCTGGGAGCGGCTCAAGTCGCCG	25:G>A
1088486|F|0-14:C>T-14:C>T	TGCAGGGCTGGTAGCGGTCTTATTGCACTTCACTGTGCAAGGCAATGGATGGGTACTAAATTACAGAGG	14:C>T
5581100|F|0-52:C>T-52:C>T	TGCAGTGCACGACGCACATGTGCCCCGTGCGCGTCCACTGGCACGTCAAGCTCAACTACCACGACTACT	52:C>T
3026657|F|0-8:G>A-8:G>A	TGCAGCAGGATGAGGAATGGCTCCATCAGGAACACCGCCG	8:G>A
32027272|F|0-5:A>C-5:A>C	TGCAGAACGTGCTGCTCTATGAGGGCGCGGACATGCCGCCCTTTGACGCCATTCTCCAGTCCCTCCGAG	5:A>C
7350814|F|0-13:G>A-13:G>A	TGCAGGCCGCAGAGCAGCGATCTCGGATTAACAGATAGGTAAGCTCCATGCCGCCGCCG	13:G>A
5010346|F|0-13:G>C-13:G>C	TGCAGGTGCTGCCGCCTCCTCTCCCTGTGTGATCCAGCTTGGACGAGGAGGAGTCCTTGTTGCCGTTGT	13:G>C
32016439|F|0-25:G>A-25:G>A	TGCAGCCATTGTTCAAGTGGTGGTCGGACCG	25:G>A
7170474|F|0-18:C>T-18:C>T	TGCAGAGGAAGAAGCCTGCCGCGGCGGCTTCTGCTCCTTCTGCCAAGTTACCG	18:C>T
7168011|F|0-30:A>G-30:A>G	TGCAGGAACCCACAATAGTTCTCCGCAGACAAGCGAAGACCGCCG	30:A>G
3023010|F|0-8:T>G-8:T>G	TGCAGCGTTAACTTCAAAGTAGTACTTAGTACAGAGGCAGATCCG	8:T>G
3027951|F|0-16:C>G-16:C>G	TGCAGTCTATCGGGACCTGGAACGGACCGTCCAAGCCG	16:C>G

1695299|F|0-14:G>A-14:G>A	TGCAGCCACTGCTCGATGGACGCGCGCTCCAGCGCCCCCGTGCCGATCAGGTCCG	14:G>A
3026128|F|0-14:C>A-14:C>A	TGCAGCCAGCACGACGGTCTGGACACTGATCCGTTGGCCTCTCCG	14:C>A
1111995|F|0-44:G>A-44:G>A	TGCAGGTCGGGGAAAAATCGGAGCAAGAGGAATCAGTTCAGAGAGACATCCG	44:G>A
1002305|F|0-44:C>T-44:C>T	TGCAGGGCAGCGTCAGGCTCCAGCTCGTCTGCCTCGCCGCCGTCCTCCTCGGCGCCG	44:C>T
1030960|F|0-12:T>C-12:T>C	TGCAGATGCTAATTAACTTGCTTGTATTCGTGATTGCAGAAACCAAGCGATGATTTGGTCAAGGGTTCT	12:T>C
1060934|F|0-66:C>T-66:C>T	TGCAGTTGGTCTCATCGGAAAGCAGCCAGGGAAGGTTTAACACAAGAGGGAAGGTCAAAAGAATGGCCA	66:C>T
1032322|F|0-29:C>G-29:C>G	TGCAGACATGCATGGCCCATGCATGCATGCAATGCGTTGACGGAAGGCGGATAATATTCGGCCG	29:C>G
3027406|F|0-19:T>C-19:T>C	TGCAGAGGAGAACCAGCAGTGCTCGGAGTTCTTTGGGCAACCG	19:T>C
1000309|F|0-27:A>G-27:A>G	TGCAGGGTTGGTGATATTGCCCATCTTAACCATGGAGTCCGAGAACTGCTTGAAGAACAGCTCCGAGAT	27:A>G
6046049|F|0-21:T>A-21:T>A	TGCAGACAACAAACGGGGGATTAACTGGGGTGATGGGCGTTGGTTTACCG	21:T>A
1150208|F|0-12:C>T-12:C>T	TGCAGGCTGACCCTGGAGTCGCCTCTGGTGTGCAGCAGTTCCCTGTCCG	12:C>T
6045359|F|0-6:A>T-6:A>T	TGCAGAAATCAACTTCCTCAAGGAACCAAAACTTTCCCAATTAAATCAAGTCCG	6:A>T
4002018|F|0-30:C>T-30:C>T	TGCAGGCGCTGCACCTCCGCGAGCATGCGGCTCTCGTCGACCAGGGGCGGGCCG	30:C>T
1230357|F|0-13:C>T-13:C>T	TGCAGCGTCTCGGCGGCCAGCCGCAGCTGCTGCCCGACGGCGCCCATGCGGAACACGCCG	13:C>T
4262839|F|0-19:C>A-19:C>A	TGCAGAGGCGGCGGCTGGGCAGCGCGCCATTTCCTCCG	19:C>A
1022726|F|0-28:T>C-28:T>C	TGCAGCTTTCATTCATGATGTTGCATTTTCTTTCCCGATTCTAGGCCTCTTCGTCCG	28:T>C
1022726|F|0-34:C>T-34:C>T	TGCAGCTTTCATTCATGATGTTGCATTTTCTTTCCCGATTCTAGGCCTCTTCGTCCG	34:C>T
1056685|F|0-54:A>G-54:A>G	TGCAGGATGACATTTTCTTCAAGAAAAGCTCCCTGTCTCATCGCAAGGGGAAGCATCCG	54:A>G
3937564|F|0-24:C>T-24:C>T	TGCAGGCCCTCCAGATAAGCTTGACCCG	24:C>T
6043621|F|0-19:T>C-19:T>C	TGCAGACCAAGCTGGCCGCTTACCAGAAGCAACCG	19:T>C
1696624|F|0-59:G>C-59:G>C	TGCAGGCACGACGCCGCCGAGTGCGCCAGGCTCCTCCTCGACGGGGAGTACGGGATCACGCCG	59:G>C
1019021|F|0-21:A>T-21:A>T	TGCAGAAGCTGTGCTTGTAGGAGCTCCTGGCGTGCAGCGTGACGTTGAAAGCCG	21:A>T
1018140|F|0-29:C>A-29:C>A	TGCAGGATTGGAGGAATGCTCAGCGAGAGCCCTTTGGCGATGCGTCAGGAGATGGGCGGCGTGAGAGGG	29:C>A
2373883|F|0-9:T>C-9:T>C	TGCAGCTCATCAAGAGGCGCTGAAGGAGGAGATCGAGAGGCTGCGGCAGGTGTACCAGCAGCAGAACCT	9:T>C
2255786|F|0-61:G>T-61:G>T	TGCAGGTGGACGAGTCGAGCATGACGGGCGAGCCCCACCCGATCGAGATAGATGCCGAGAAGAGCCCCT	61:G>T
3939367|F|0-18:A>T-18:A>T	TGCAGAGCAGCAGCGCCAAGGGCGAACTGAGCCG	18:A>T
1099560|F|0-8:C>G-8:C>G	TGCAGGAGCGATGTCTTGGGCATCACGAACGACACGTTGTTGATGGACGCCGCGAACCG	8:C>G
3939647|F|0-8:G>A-8:G>A	TGCAGATCGGTCCTGATTTGCATGGCCCG	8:G>A
4022577|F|0-11:A>G-11:A>G	TGCAGGCATCGACGACGAAGCGGCGTGGGCGGAAGCCCG	11:A>G
32025288|F|0-64:G>A-64:G>A	TGCAGTACACGTACAGGAAATAACATGCAAGGGAAATATCCTGCAATAGAGAGTTCTTAAATAAGATCG	64:G>A
1093570|F|0-16:T>C-16:T>C	TGCAGGTCACAAAGTTTAAGTGCGGCGGCGCCGTCGTCGGCTGCACTTTCGACCACCG	16:T>C
4262839|F|0-13:G>A-13:G>A	TGCAGAGGCGGCGGCTGGGCAGCGCGCCATTTCCTCCG	13:G>A
3023404|F|0-8:C>A-8:C>A	TGCAGAGTCTCCTGACCAAGAAACAAGCAAAGGCGTTAGTATCGCCG	8:C>A
3956768|F|0-13:G>A-13:G>A	TGCAGCGGCTGACGTTGTGGATCTTGGTGGTGTTGAAGCCG	13:G>A
6042705|F|0-57:A>G-57:A>G	TGCAGCAAGCTAGCCATGCACGAGCACGAGTCGTCCGTCCTCGCGACCAAGGTCGTCACCATCTCCGAG	57:A>G
4993845|F|0-30:G>A-30:G>A	TGCAGGCTCAGGATCTCCATCGAGATCCTAGACGAGAGCGGCGAGCGCACCGTTTTCGACAACTACATG	30:G>A
3940173|F|0-37:G>A-37:G>A	TGCAGCAATAACCTTGGACTTGGTAATTGGCACTACAGATGCAGCTCGAGCCCTGACTACAGCACCAAC	37:G>A
3023404|F|0-41:T>C-41:T>C	TGCAGAGTCTCCTGACCAAGAAACAAGCAAAGGCGTCAGTATCGCCG	41:T>C
2275225|F|0-21:G>A-21:G>A	TGCAGCTTCGACGCCTAGCTCGACTAAACGCACGAGGTGGACGTGGCTAATTAACCG	21:G>A
3939504|F|0-19:C>A-19:C>A	TGCAGACGGAGGAATCACACAAAATACACCG	19:C>A
3936766|F|0-11:A>G-11:A>G	TGCAGAACCCAACAAGACTTGCGTCCTACGATGCTTCAGCCG	11:A>G
1018140|F|0-63:A>G-63:A>G	TGCAGGATTGGAGGAATGCTCAGCGAGAGCCCTTTGGCGATGCGTCAGGAGATGGGCGGCGTGAGAGGG	63:A>G
3946714|F|0-18:C>T-18:C>T	TGCAGGTAAAGAAGGCGACGACGACGGACCCG	18:C>T
32025958|F|0-15:C>G-15:C>G	TGCAGATCCATCGTCCCCAAGCTGCACTACTACCTGCCCCTCTTCGGCGGCTCCTCCGAGAAGCTGCTC	15:C>G
32024587|F|0-30:A>G-30:A>G	TGCAGGTCACGGCCACCGTTACGGCGTCGCATGGAGCTCAGAGGTGCTGTTAGCGATGCGTGGCTGTGC	30:A>G
32025755|F|0-46:A>C-46:A>C	TGCAGAAGGACATGCATATGCATTGAGAACTCGCGCGAAATGGCGAAGGGGGATTCAAGAATTGATCGA	46:A>C
1019596|F|0-9:T>C-9:T>C	TGCAGGCGATGTGATAAAGACGCTCAAGGACAACGGGAACGAGTACACCTGGGGGCCG	9:T>C
984689|F|0-55:C>A-55:C>A	TGCAGAATCGGAGCAGAGGGGAGGGGAATCGGAATGGGGCTCTCGCCGTCGCCGTCCTCGCCG	55:C>A
3026888|F|0-18:G>T-18:G>T	TGCAGTAACCGCATTAATGGGGAAAATTATTGCAGTCGACTGCGCCG	18:G>T
2262899|F|0-35:T>A-35:T>A	TGCAGGTGGTGGGGTCGGGCCTCGAGCGCGAGGCCTTGCATTACTACGGCAATCAGGCCAAGGCGGTGA	35:T>A
7171580|F|0-21:C>T-21:C>T	TGCAGCTCGCTGCGGATGAAGCCCACCTCCCCCCG	21:C>T
1047582|F|0-28:C>A-28:C>A	TGCAGCCAGTCTACGGTTGACTTCTGCCCTCTATCCTTAGGATCACGCGGCGCAGGAGCGTACTGCATA	28:C>A
1023518|F|0-44:C>A-44:C>A	TGCAGCTGGTGTCCGTGATGTTCGTGGGCCACCTCGGCGAGCTTCCCCTCGCCG	44:C>A
5363824|F|0-21:G>C-21:G>C	TGCAGGCTACGGCTCGTGTGTGTTCACCG	21:G>C
1035295|F|0-19:C>G-19:C>G	TGCAGCTCAAAGCGGAGCACTGCTACATCGTTGCCGACCTCAATGTCCACCTCAAGAAAGAGGGCGCTA	19:C>G
1019729|F|0-30:A>G-30:A>G	TGCAGATCCGACCACGAGCCTCGTGCCACCAGACTGCAAGCACAATTGCCG	30:A>G
983392|F|0-8:T>C-8:T>C	TGCAGCTTTTGCTTTCGGTACATAAATGATTTTGTCTTCAAAATGAAAGCCTACAGCCATGTGGACCGA	8:T>C
3385268|F|0-12:C>T-12:C>T	TGCAGACTCACACACAAGCTGCTACAACTAAGCGCTGGGCAGATACATCCACCCG	12:C>T
3574722|F|0-6:A>G-6:A>G	TGCAGCATGCTCTCGCGCTGCTCGAGGAACTGCTCCG	6:A>G

1020063|F|0-19:T>C-19:T>C	TGCAGGAGCTCCCTGAGGATCTCCAGGTCGCCGCCCCTCGCCGCGGCGTGCATTGCCCG	19:T>C
4262543|F|0-23:T>C-23:T>C	TGCAGTACCTGTACCAGCAGCAGTATGCCATCTCCAACCAATCCG	23:T>C
1219881|F|0-35:G>A-35:G>A	TGCAGGCCGTGGGCGAGCTCACCCGCGCTGAAGCTGATGCATCGTCGGTGCCGTCCGTCACGCCGAGAT	35:G>A
1070953|F|0-38:G>C-38:G>C	TGCAGCGTGAGGTACTTGTTCTTGGGGTACACGTGCGTGCCCAGCAGCTTCATCCTCCCCTGCGACCGA	38:G>C
4008996|F|0-9:C>T-9:C>T	TGCAGGACTCCGATCGATATCAACATCCATACGGAGGAGACGACGAGTGGATGGACTCTAGTAACGACC	9:C>T
4002821|F|0-16:G>A-16:G>A	TGCAGCTGCAAGTCAAGGTGCTGGCGACGGACGAGTTCTGGCCG	16:G>A
5363824|F|0-18:T>C-18:T>C	TGCAGGCTACGGCTCGTGTGTGTTCACCG	18:T>C
4262614|F|0-37:C>G-37:C>G	TGCAGTCGCCGTTCACGTTCCGCAAGGACGGGCAGGCCAGCCG	37:C>G
12855025|F|0-41:C>G-41:C>G	TGCAGCTCGCCGCCGCGCTCGTACATGCCGTGGCAGCGCACCACGGCCG	41:C>G
13589798|F|0-22:G>A-22:G>A	TGCAGCATGCGCGGGAGGCGGGGCCAACCGACCG	22:G>A
1003407|F|0-63:G>A-63:G>A	TGCAGGGCAGCAGTTACGTGGAGTTGCGTTGACGCGACCCGTCAATTGGCGATGGGGCTTGGCGTCCGA	63:G>A
1069558|F|0-25:A>C-25:A>C	TGCAGTACTGCACGATGGCAATATGAATATCACGCGGATGAGCTCTACTGAACCG	25:A>C
992228|F|0-52:T>C-52:T>C	TGCAGCTTGGAGACGATGGATGTGAGATGTAAGTTGCCGTGGCAGCAGGAGATGAGGCGCGCGATCTGA	52:T>C
1075656|F|0-40:G>T-40:G>T	TGCAGATTCCATCCTGGACTTTGAGGTGTAATGCTGGGATGATGGCCGCACCTTTCTCAGAGCCGAGAT	40:G>T
4261367|F|0-21:A>G-21:A>G	TGCAGTCTACAAGAGACAAAAACTCTACACATATACGTACCG	21:A>G
6026286|F|0-6:T>C-6:T>C	TGCAGGTTCTGAGTTGGGTGCAGAGCAGGCTTCGTGGGGCGCAGCAGCACAGCGCCAAGAAACCGAGAT	6:T>C
4542665|F|0-8:T>C-8:T>C	TGCAGCCATGGGTTCTCATGGTACATCCG	8:T>C
5358113|F|0-14:C>G-14:C>G	TGCAGGAAGTTGCGCGCCAGCCCCACGGAGACGAGCCCG	14:C>G
1231164|F|0-28:G>T-28:G>T	TGCAGCGCGAGGGAGCAGCGGCCGCTGCGGGAGCTGGAGGAGCAGGTGGTGGTGAGGAGCTACGGCCCG	28:G>T
1281079|F|0-66:C>G-66:C>G	TGCAGCTGTTAGTTAACGAACCTTTTTTGGCCAACGAGTACACAGTTTCAACGAGTTCATCATGCTCAT	66:C>G
1219881|F|0-30:A>T-30:A>T	TGCAGGCCGTGGGCGAGCTCACCCGCGCTGAAGCTGATGCATCGTCGGTGCCGTCCGTCACGCCGAGAT	30:A>T
6030746|F|0-31:C>G-31:C>G	TGCAGGCAGGAGACCGCATGCTCAAACTTTCCGCCG	31:C>G
3959524|F|0-11:G>A-11:G>A	TGCAGGTTCGAGGGGTACCAGGACACCCTGTACGTGCACGCCAACCG	11:G>A
1022222|F|0-16:T>C-16:T>C	TGCAGCAGCAGCCAGTTCACCTTGGCCAGCAGGAGCTGGTCGTCGTTGCAGTAGAAGAGCCG	16:T>C
2243225|F|0-56:T>C-56:T>C	TGCAGGACTCCGATCGATATCAACGTCCATACGGAGGAGACGACGAGTGGATGGACTCTAGTAACGACC	56:T>C
3950911|F|0-60:A>C-60:A>C	TGCAGTCGAATCGCTCGCTCATGATCCGTTTCCACTTTCCATAAAACAAGCCAACCTTCCACCG	60:A>C
3944876|F|0-22:A>C-22:A>C	TGCAGTTTTTTGACAGAAAGAAAAGGAAGGAAATGGGCCG	22:A>C
1075656|F|0-19:T>A-19:T>A	TGCAGATTCCATCCTGGACTTTGAGGTGTAATGCTGGGATGATGGCCGCACCTTTCTCAGAGCCGAGAT	19:T>A
1055369|F|0-18:G>A-18:G>A	TGCAGTGGTTCACGTGCAGGTGGTTCTCCGTGCACGCGAACTTGGTGAGCGCCACGATGGCCTCCTTGG	18:G>A
1143285|F|0-56:G>C-56:G>C	TGCAGGACGGTGATGCCACGAGGGTTCCCTGTGCGTTTTCTAGCGAGCGGGACGAGGATCCG	56:G>C
3947125|F|0-10:G>A-10:G>A	TGCAGAAGGCGGACCTGAGCTACAACCG	10:G>A
1120672|F|0-31:A>G-31:A>G	TGCAGCTCTGTGATGCTGGCATCAGAGATCAAGTGGCGCGGCGCTTTGACTTCTCCG	31:A>G
1694967|F|0-7:C>T-7:C>T	TGCAGCGCGGGTGCCTCGACGAGCAGCTGGCTTCTTTCAAGAAAGTGCTCGCACGGATTGCTCCGAGAT	7:C>T
1214870|F|0-51:C>A-51:C>A	TGCAGAGTGGAACTGAATGACCATGACAGTATCTGGTGCAGCTCGGCGGCCCAGCCAGTGGCCGCCGAG	51:C>A
3950969|F|0-14:G>A-14:G>A	TGCAGGGTGCAGCCGCAGCGAGGGGCGGCCTCCG	14:G>A
4261690|F|0-22:C>T-22:C>T	TGCAGGTGGCAAATATCCTTTTCGTTGATACACCG	22:C>T
4262720|F|0-11:G>C-11:G>C	TGCAGTGCAGCGAAGGCAGACGGCCATGGACGGCGACAGCCCG	11:G>C
4262543|F|0-33:C>T-33:C>T	TGCAGTACCTGTACCAGCAGCAGTATGCCATGTCCAACCAATCCG	33:C>T
1695792|F|0-10:G>A-10:G>A	TGCAGCTTCGGCCTTTGTCCAACTCGTGGACAATTTCCTTCGATCAGCAGACCG	10:G>A
2242338|F|0-39:G>C-39:G>C	TGCAGCAGTCGTGGTGTCTGCATTAGCACCATTCTCGTCGATCCTCATCTGGGTCTTGGCTGGCACGAA	39:G>C
1041280|F|0-40:A>C-40:A>C	TGCAGGCCCAGCACCGCCAACTGCTCCGCCACCGCCAAGAACGTCTGCCCGCCCTACCTCGTCGTCTAC	40:A>C
3937855|F|0-26:G>T-26:G>T	TGCAGGCTGACCCTGGAGTCGCCTCTGGTGTGCAGCAGCTCCCTGTCCG	26:G>T
1026148|F|0-49:C>G-49:C>G	TGCAGAACAAGGTGACCCTGGAGTGGCTTCTGGCCCTGGGTGACAAGCTCGGCATGGTCCGTGTGCCGA	49:C>G
3959027|F|0-5:G>A-5:G>A	TGCAGGCCAGATACCCTGTGGAAGGGGAGCTCGCCG	5:G>A
3027456|F|0-32:C>G-32:C>G	TGCAGGCACGCACATGAGCATGCAAGGCATGCCTAGGAACGCCG	32:C>G
3027456|F|0-20:T>C-20:T>C	TGCAGGCACGCACATGAGCATGCAAGGCATGCCTAAGAACGCCG	20:T>C
3960371|F|0-10:A>T-10:A>T	TGCAGATCCAAGGTGAGGAAGCCAGGTTCGTGAACGTCCTCACCG	10:A>T
3022918|F|0-18:C>A-18:C>A	TGCAGCTCCTTCTCTAGCCTCGTCAAATCCCAGCGCAGCCG	18:C>A
1667216|F|0-40:T>G-40:T>G	TGCAGAAAATCTTAAAAATATTCCAGGCACACAGTAATCTTCGATGTAAATTGTGAGAAACATTCCCGA	40:T>G
2243154|F|0-13:T>A-13:T>A	TGCAGAACACGTATCCACAGGTAGTCGACCCTGTCTGTCGGGTCGCCATCCATCCTTCCACATTTGAGC	13:T>A
5369353|F|0-31:G>A-31:G>A	TGCAGCAGCCGCTTCTCCTGGAGCTCCTGGGGCAGGACGGCCG	31:G>A
1066920|F|0-33:A>G-33:A>G	TGCAGGAAGGAAAGAGGATCTTTGAGGTGTTGGATTGATCGGAGATGAGCGCCCG	33:A>G
2256296|F|0-19:G>A-19:G>A	TGCAGGCCACCTCGGCGGCGTCCAGGGCGCTCGACCGCTTCGACGTCCTCGACTGCCG	19:G>A
7173372|F|0-5:G>A-5:G>A	TGCAGGCAGACGTGTGATGCTCGCGACACCGCACAGGAGGCGGAGGCATGCGGGTGAGAACCG	5:G>A
1049073|F|0-9:T>A-9:T>A	TGCAGCTCATAGCGGTTCCAACTGAAATTCGTAATCTTTACCACCTTGAGGTTTTCCAAAACATCCGAG	9:T>A
1026547|F|0-22:A>G-22:A>G	TGCAGGCTTGCGTGATGAATGAATGGAGTGCTGCGTGGTGGATGAACGGGTGCTTGAGGTGTTGTGCGA	22:A>G
1039214|F|0-17:C>G-17:C>G	TGCAGCTTCGGGCACCTCAAGGGCCTGGACGCCAACAAAACTGGGCATTTGGATCCCCTCCTCCCCGTC	17:C>G
983488|F|0-46:G>A-46:G>A	TGCAGGGGGTGTCATGTGACCTCTTCAATGGCCTGTGGTACCCAACGGTGGTCAGCATCGGTCCGAGAT	46:G>A
1264300|F|0-33:T>A-33:T>A	TGCAGCCACGGCGGGCGCTGGGCGTTTGGAGCCTAATGGCGGATGGCGTCGACGAGTCCG	33:T>A

<table>
<tr><td>3944308|F|0-18:C>G-18:C>G</td><td>TGCAGACCGCTCCGTCATCGAACAGCACATCGACGTCGACTGACACCTCCACGACCCGACCGAGCCACG</td><td>18:C>G</td></tr>
<tr><td>1090014|F|0-13:C>T-13:C>T</td><td>TGCAGGCGCGCAGCCACAACTCTCCTCAATTCCCAGACTCATATGGATACGGTGGCCTTATACTAGCGT</td><td>13:C>T</td></tr>
<tr><td>1151575|F|0-27:G>T-27:G>T</td><td>TGCAGGGGCCTGCGCGGCGCGCCAGGCGCGCCATGAGAGGGCGGAGCGCGAAGAACGTGAACGCCACGA</td><td>27:G>T</td></tr>
<tr><td>3939984|F|0-42:A>G-42:A>G</td><td>TGCAGGAGATCATCGACGAGAGGAAGGCCGCCCGCGTCGAGAAGATCAAGGCCG</td><td>42:A>G</td></tr>
<tr><td>1037212|F|0-65:T>C-65:T>C</td><td>TGCAGATGCTAATTAACTTGCTTGTCTTCATGATTGCAGAAACCAAGCGATAGTTTGGTCAAGGGTTCT</td><td>65:T>C</td></tr>
<tr><td>1215407|F|0-47:G>A-47:G>A</td><td>TGCAGGCGGACTCGCTGGAGCGGCAGGCCTACGGCTTCAAGAACGACGGCCTCAACTTCACCG</td><td>47:G>A</td></tr>
<tr><td>3946825|F|0-7:G>A-7:G>A</td><td>TGCAGGAGGCGTACCTCGTGCCGCGGGAGCCG</td><td>7:G>A</td></tr>
<tr><td>4262935|F|0-16:T>C-16:T>C</td><td>TGCAGTGCATGCAGGGTAGGGATCGGAGATCCGCGCGAGCCG</td><td>16:T>C</td></tr>
<tr><td>1071878|F|0-30:G>A-30:G>A</td><td>TGCAGCCCAGGCGGTGTACGGCGACTGCGAGTGGTACTTCTTCAGCCCGCGGGACCGCAAGTACCCCAA</td><td>30:G>A</td></tr>
<tr><td>2250648|F|0-66:T>G-66:T>G</td><td>TGCAGCGCCGCCGACGACGACCAGGCGGAGCGTATCTGCGGGATTCTCGACGACATGATGGCAGAGTCT</td><td>66:T>G</td></tr>
<tr><td>3936378|F|0-14:T>C-14:T>C</td><td>TGCAGCCTTCATCATAGACCTTGCTTCATCCG</td><td>14:T>C</td></tr>
<tr><td>1397609|F|0-8:G>C-8:G>C</td><td>TGCAGGAAGGACTCCTTGGTGCCTTCGTCCTCGCCCAGAATGCCCACGGGCACGGCGCCCACGGACTTG</td><td>8:G>C</td></tr>
<tr><td>4262200|F|0-13:C>T-13:C>T</td><td>TGCAGAAGATGTCCTACTTCAACTGGTGGAACTGCGCGCTCTGCTCCG</td><td>13:C>T</td></tr>
<tr><td>1027228|F|0-26:C>T-26:C>T</td><td>TGCAGCAAGAAGCGACAGCAGCTGGGCGCTTCGTGGGCGTTGGCGCGCGCGGAGGCGCGGCCATGGGTG</td><td>26:C>T</td></tr>
<tr><td>1079952|F|0-10:C>A-10:C>A</td><td>TGCAGTTCATCCCCTAGGGCAAGCAAATATCGGGCGTATGTTACGGTACAGGTACAGGTTTCACCTATC</td><td>10:C>A</td></tr>
<tr><td>1000216|F|0-19:C>G-19:C>G</td><td>TGCAGGCCGTGGGCGAGCTCACCCGCTCTGAAGCTGATGCGTCGGCGGTGCCGTCCATCACGCCGAGAT</td><td>19:C>G</td></tr>
<tr><td>1068170|F|0-11:G>A-11:G>A</td><td>TGCAGCAGGAGGAGGATTAGATAGATTACTCTGTTATTACTATTAGGTCTTCTGTCAAATCGCAGTGGG</td><td>11:G>A</td></tr>
<tr><td>1080252|F|0-36:C>T-36:C>T</td><td>TGCAGGTGCGTGCAGCCAAGCCCCACCGCACGCCTTCTATTCCGCGTCCCCTAGCTTGGCCCG</td><td>36:C>T</td></tr>
<tr><td>2241644|F|0-31:A>T-31:A>T</td><td>TGCAGGTTCATGGAGACGTAACGACTCCAGAATGTCGACAGATGAGAACGTGAACCG</td><td>31:A>T</td></tr>
<tr><td>7171570|F|0-8:C>G-8:C>G</td><td>TGCAGCTCCCCGCCGCGCTCGTACATGCCGTGGCAGCGGACGACGGCCG</td><td>8:C>G</td></tr>
<tr><td>32022605|F|0-8:T>C-8:T>C</td><td>TGCAGAAGTTAACGGATGTATAACAGTCAGTGCCG</td><td>8:T>C</td></tr>
<tr><td>32026161|F|0-35:G>A-35:G>A</td><td>TGCAGTTTGGTGCGATGACCTAATAATCCTAGCTTGGCTAGCAGCACTTCACTCCCTCCCTTGCGCCCT</td><td>35:G>A</td></tr>
<tr><td>32017858|F|0-28:T>C-28:T>C</td><td>TGCAGTTGGAAGCGGGATAGTTCTTGGGTCACCG</td><td>28:T>C</td></tr>
<tr><td>1099538|F|0-38:G>A-38:G>A</td><td>TGCAGGTTTGAAGTCGGCCATAAGAAGTATGCCGCGCTGTTTTCTGTGTGCACACACCCAGAAGCAACC</td><td>38:G>A</td></tr>
<tr><td>1742918|F|0-8:T>C-8:T>C</td><td>TGCAGGCGTTGCCGTATCTGAACGCCGTGGTGCTGGAGGCGCTGCGGCTGCATCCGCCG</td><td>8:T>C</td></tr>
<tr><td>4539686|F|0-6:C>T-6:C>T</td><td>TGCAGGCGCAGGGGGGAATGGAAGGGAAGATTCCG</td><td>6:C>T</td></tr>
<tr><td>5360253|F|0-32:C>G-32:C>G</td><td>TGCAGTTCTCAACATGTTGGAAATCTCACATCCAACATCTGCCG</td><td>32:C>G</td></tr>
<tr><td>5373521|F|0-14:G>A-14:G>A</td><td>TGCAGGTGCTAGTCGTCGTCGGCGACCG</td><td>14:G>A</td></tr>
<tr><td>5361205|F|0-28:C>A-28:C>A</td><td>TGCAGCTCGGCTGCTTCCTCCTCATCGCCTTCCCACCGCTTCCTGTGAGCCTTGGCCGCGCCTGCCGAC</td><td>28:C>A</td></tr>
<tr><td>32024474|F|0-29:T>C-29:T>C</td><td>TGCAGCTCCCCGTAGGGGTCCATGATCCTTGCGAGGCAGAGCGTGTCCAGCAACGCCATGTCCACGCTT</td><td>29:T>C</td></tr>
<tr><td>32027429|F|0-16:T>C-16:T>C</td><td>TGCAGCAGCAGTTGGGTGGAAGCAGTGCCGCTGCTGCCTCGGCGAGCGGCGCGGCCCAGCAGGATCAAG</td><td>16:T>C</td></tr>
<tr><td>5008846|F|0-60:G>C-60:G>C</td><td>TGCAGTGAAGCCCGCTCCCTCCCTTATCGCAAAAGGGAAGGAAATGCAAGCGCTCGTGCAGTGTTTTTT</td><td>60:G>C</td></tr>
<tr><td>5005121|F|0-27:T>A-27:T>A</td><td>TGCAGGTGGAGGTGGAGCCTCGAGACCTGCTTCTGGTGGAGGCGGCGGCAGCTCGCCG</td><td>27:T>A</td></tr>
<tr><td>5010677|F|0-24:A>T-24:A>T</td><td>TGCAGGGGGAATGGGGGATGGCCGAGCACAGGAGCAAGGCGGAAACGGCCTGCGCGGACCCAGCAACAC</td><td>24:A>T</td></tr>
<tr><td>1314787|F|0-7:T>C-7:T>C</td><td>TGCAGAATACTCATCTCTGTCATAGGAACAGTGCGAAAATCCATAGTGGGGTAGTAAGTCGCAGTCACG</td><td>7:T>C</td></tr>
<tr><td>5360765|F|0-24:G>A-24:G>A</td><td>TGCAGCACCGTCGTCGACCAGGCAGGTAGCAAGACATGAGTTGGCTGCCATCAAGAAGGAGGGCGTGAC</td><td>24:G>A</td></tr>
<tr><td>4995480|F|0-29:C>G-29:C>G</td><td>TGCAGTCTCGCCTCCCGCGGGAACCCCTGCTCGCAGACCTCGCACGCGTACGTCTTGGGCTCCAGCAGC</td><td>29:C>G</td></tr>
<tr><td>5004242|F|0-38:G>T-38:G>T</td><td>TGCAGGCCGTAGAGTTGGAGTTTTGCTCTTGTTGTTTGGTGGTATTAGACTCTTGGAAATGAACTTGCC</td><td>38:G>T</td></tr>
<tr><td>7915220|F|0-8:A>T-8:A>T</td><td>TGCAGTTAAGTATGGAGAAGAGATCAAGAGAATGCGTTTTGATCCTGCCTGCAAGGGGAATTTTATGTA</td><td>8:A>T</td></tr>
<tr><td>32018905|F|0-28:G>C-28:G>C</td><td>TGCAGCAATGGTGGTACTGGTATGTGGAGTGTCCG</td><td>28:G>C</td></tr>
<tr><td>5363965|F|0-10:T>C-10:T>C</td><td>TGCAGGTGGATGGCGTCGTGGCGGCCGAGGTGGGGGTGCCG</td><td>10:T>C</td></tr>
<tr><td>32018300|F|0-5:G>C-5:G>C</td><td>TGCAGGTGGGTAGGGTTTAGCGGTGGCTGAGGGAGGTCTCGGCGGCATGGCGCCGTCCAAGGGGGAGCG</td><td>5:G>C</td></tr>
<tr><td>7171420|F|0-14:A>G-14:A>G</td><td>TGCAGATACGATTTAGTGTGGAGGTGAGGTGAAGCATCTGGCTCATTCGAGGTCCG</td><td>14:A>G</td></tr>
<tr><td>4998147|F|0-36:C>T-36:C>T</td><td>TGCAGGCTTGTTCCTCCTTTATTGGGATCTATTGAACACCATCGTATTTGAATACTAACAGAGCCGAGA</td><td>36:C>T</td></tr>
<tr><td>7168640|F|0-59:A>G-59:A>G</td><td>TGCAGCAGGGCGCAATGTCAGGCAGCACCACCGCCAGGCAGGCGCGGAGGCGGCCGCCAACCGCCCTCT</td><td>59:A>G</td></tr>
<tr><td>100527786|F|0-41:T>C-41:T>C</td><td>TGCAGGTGGCGGCGCAGCGGACGACGGTTATGGCGTCGGTGTACACGACTATCTCGAGCAGGAGATCGA</td><td>41:T>C</td></tr>
<tr><td>5008802|F|0-43:A>T-43:A>T</td><td>TGCAGTGCCGCTGCCGCACCATCACCTTTCTCTCTTGTACTGTATCATCAAAACTGACAAGTGACAGGG</td><td>43:A>T</td></tr>
<tr><td>5358460|F|0-7:C>G-7:C>G</td><td>TGCAGGTCGACGAGCTGGAGGCGGAGGCGGCAGCGCTGCGCGCCCG</td><td>7:C>G</td></tr>
<tr><td>5009226|F|0-63:A>G-63:A>G</td><td>TGCAGCTCGGCTCTAGTCTACTCCACAGGCTCTGCTCATCTTGAGGAGCGCCGAGAGCACGCCATTCAT</td><td>63:A>G</td></tr>
<tr><td>1021879|F|0-35:C>T-35:C>T</td><td>TGCAGCAGCCGCTCCACCACGTGGCTGAGGCAGCACCCGCGCCTCCCCACCACCAGCACCG</td><td>35:C>T</td></tr>
<tr><td>1865011|F|0-28:C>G-28:C>G</td><td>TGCAGGAAGATCGGCTCGACGTCGGTGGCGACGAACTCGTCCAACGTTGGCGCAGGAGCCG</td><td>28:C>G</td></tr>
<tr><td>100352384|F|0-34:A>G-34:A>G</td><td>TGCAGGCGGCCAAGAAGCTGCTCGAGGGCAACCTATCGAGAGCTCCG</td><td>34:A>G</td></tr>
<tr><td>982993|F|0-7:A>C-7:A>C</td><td>TGCAGTTATGTTGGGGCGTCAATGCGGTGCGGATGGATGTCCAATCCACCG</td><td>7:A>C</td></tr>
<tr><td>4910866|F|0-29:G>T-29:G>T</td><td>TGCAGTATAGCGTTCAGGACAGAAACATAGTGTTGAGAAGTTCACAAGAGATGGTACCTTTTGGTCGAT</td><td>29:G>T</td></tr>
<tr><td>7921621|F|0-41:T>C-41:T>C</td><td>TGCAGCTCGTGCGGACGGCGGCCGCTCAATCGTGGCCAGAGTAACTGGATCTGCCCGCAACGTGGTAGC</td><td>41:T>C</td></tr>
<tr><td>7915668|F|0-56:T>A-56:T>A</td><td>TGCAGTGGCACCGTGCGTGAGACGAGTCTAAGGTAGGGGTCGGTCTCATGTCATTTTTTTTCGGAGATG</td><td>56:T>A</td></tr>
<tr><td>5373894|F|0-29:T>C-29:T>C</td><td>TGCAGCTTCGCGCTTGCTGCCGTGCCTTGTTGCTGCTTCGTCCGACCGCGGACGACCTCCGTCGAGCTC</td><td>29:T>C</td></tr>
<tr><td>5371588|F|0-7:G>A-7:G>A</td><td>TGCAGCAGGGCGATGACGCTCGGTGAAGTAGGGGAGGCAGAGAAGCGGGACTCCG</td><td>7:G>A</td></tr>
<tr><td>100255668|F|0-17:G>C-17:G>C</td><td>TGCAGCAGCTTTTAATCGTGCACTGCTCCGTATGCACACACACAATATCAATCTGATGTTCACCGAGAT</td><td>17:G>C</td></tr>
</table>

7900186|F|0-16:A>C-16:A>C	TGCAGCTGCATCGGCTAGCAAAAAGCGGCGGCTGACGTGGATCTGCCACCAGCGATGGTTGGCGCGCAA	16:A>C
7919629|F|0-36:T>C-36:T>C	TGCAGAGAGGGATCGCAATCCAGCAAGTTAATCGATTACATAACTCATCTTTCAGAGAAAAAGAACTCA	36:T>C
5360253|F|0-40:A>G-40:A>G	TGCAGTTCTCAACATGTTGGAAATCTCACATCCAACATCTACCG	40:A>G
1390482|F|0-35:G>C-35:G>C	TGCAGCACTCGTCATCGGCGTCAACACATCATCATGGCGCGCGTCCATCTACTCCATGCATCCAGCACA	35:G>C
32015839|F|0-20:C>G-20:C>G	TGCAGCGTGGGCGTTGCAGACACGCAGAGCCCTCTTAACTGGAGTGGCTGGTCGTCCTGTCCG	20:C>G
6027339|F|0-8:T>C-8:T>C	TGCAGTGGTGAACCCATGGCCGTCCTGCGACAACTGCGACTCGTGCACCAAGTCCAACCAACCG	8:T>C
3031627|F|0-33:T>C-33:T>C	TGCAGCTCCACCGCTCCCCTCCCCGCGCGCCCGTAGGCCG	33:T>C
5371681|F|0-40:G>C-40:G>C	TGCAGGAGCTCCGCATCTGTCTTCTTCCTCCTAGGCCGCAGCATTGTCCATGAATCCCCG	40:G>C
7907370|F|0-18:A>G-18:A>G	TGCAGCCACGTGCACGCGAATGCATCATGCGTAGCACGTCATGCATGTGTTATGTGTATGCTACTGTCA	18:A>G
6035661|F|0-27:G>A-27:G>A	TGCAGAGTGCATACAACAAGCGAGGACGGAGCGGAGGAGAGAGACGGGTGGACGGACAGGGACACGCTC	27:G>A
100449286|F|0-8:T>C-8:T>C	TGCAGCCGTAGCCTTGGCGAGGCGGGTCATGGAGGGGCGCATGCCTTGGAAGACTACCTGGTGCCGAGA	8:T>C
1113451|F|0-7:A>C-7:A>C	TGCAGGTAATCTAGATGGTTTTTGGTCCACGTTGTGTCGCTGCGGGTAGATGGCACGTCCG	7:A>C
100575657|F|0-16:T>C-16:T>C	TGCAGGCACAGGCTGATCTGTGGCGCCACAGCCTCTACTACCTCTCCTCCATGGGCCTCCG	16:T>C
5356258|F|0-33:A>G-33:A>G	TGCAGGTCAAGTTCGCGTATTCAGTGACCTATGAATCTCGTAGCCTAGCTATGACAGGATCTGAGCTTT	33:A>G
100496681|F|0-5:A>G-5:A>G	TGCAGAGAGCCTTTCACGCGTCCGACGAGCTTCGGCAGGTCCAGGCCG	5:A>G
100548055|F|0-10:G>A-10:G>A	TGCAGCTAGCGTGCTCGGCCTCGATGCGCAGCTCCCTCCGCCG	10:G>A
1022366|F|0-12:T>C-12:T>C	TGCAGATCTCGGTGGGCTCTGCCATGGCGCCGTTGCCGTAGTCCCCCCAGGCGAGCCGCTTGGTCACCG	12:T>C
100573065|F|0-55:G>A-55:G>A	TGCAGAGGTAGCCGATGTAATCCTCGGGGGTGATGTCGTAGACTAGGCCAGGGTCGGCGGCCTTCATTG	55:G>A
100388568|F|0-28:C>A-28:C>A	TGCAGGGTGAGCAAATCAAGCACGCAGCCTCCGTCCG	28:C>A
1390831|F|0-31:C>T-31:C>T	TGCAGGACGCGGTGCATGACCAGACCAGTACCGCTTCGGTGTTCAACGTAGCTAGACAGCCATGGCCGA	31:C>T
100508866|F|0-7:A>G-7:A>G	TGCAGCGAGGGCACCTGTTGATGCAGGAGAAGAACGGAGCACTGTACGCTCACCG	7:A>G
100452289|F|0-43:A>G-43:A>G	TGCAGGGCGGCGGGCTTGTTGTGGTTGGCCGCCTTGCCGTGGAAAATCTTGGAGAGCAGCAGCCGAGAT	43:A>G
7929223|F|0-58:A>G-58:A>G	TGCAGGAGCTGGTGATGAAGGAGGCGCAGGCGATGGCAGCGGTGGACGACGACGTGCGAAACCTCCGAG	58:A>G
100453489|F|0-26:T>C-26:T>C	TGCAGTCCGCGAGGCAGCAGCAGCAGTGGTCGAAGTCGCCACGCCCAATCTTCCCCATGCGTACCGAGA	26:T>C
5361129|F|0-33:C>G-33:C>G	TGCAGCGTGCTCAGGTCCTTGACGCCGCACCGCCCGAGCAGGGTGATGCCCGACACCACGTTGGACGCC	33:C>G
1106874|F|0-47:C>T-47:C>T	TGCAGTAGTCGCATGGCGCAGTCGTCATCACGTTGCCGTTGTCGTCCCCCTCCTGGTAGACGCAGCGGT	47:C>T
1754318|F|0-25:G>A-25:G>A	TGCAGTTGCCTTTAGGTATTTGTGCGGAAGCCCTTTTTGTCTGGTCAAAGTCTATGGGTGGAAGCGTCG	25:G>A
1862642|F|0-20:C>G-20:C>G	TGCAGGCCATGCATGCATGCCTCAAGAAGGTCTCGAATTGATCGATGCATCTGGTGGAATACATCGTAC	20:C>G
100546151|F|0-27:G>C-27:G>C	TGCAGTTCCTGTCGGAGGGGTCATGGGGGAACCACGACGAGATCGACCTGGAGTTCCTGGGCAACTCCA	27:G>C
100542593|F|0-51:A>C-51:A>C	TGCAGAGTGCATAGGAGTCGACCTTCACTCCAACGCTCAGGAGGTGTCGAGAGTGACGAAGGATGCCGA	51:A>C
100449298|F|0-67:T>C-67:T>C	TGCAGCCGCCGCCCCGTCCATGTTCTGATCGGCTTCGCTACCCACGTCGCGCCGCGCGCCGCCGCCGTC	67:T>C
100516469|F|0-52:C>T-52:C>T	TGCAGGTGTTTGGTATCGTTGCTGCACGTGATTACTTGGATCGTAAACGCAACATTGTTTTCCACCGAG	52:C>T
100516568|F|0-61:C>G-61:C>G	TGCAGGTTTGCGACATGCAGTATAACATGTTTTGCTCCCACGTATCGTCAAAATCGTACATCTAAGCCG	61:C>G
100424812|F|0-53:A>C-53:A>C	TGCAGCAACATGAGCTCGGCGTCCAAGAAGACGTCGTCGTCGCGAGGTGACCAAGGCCACGGCGGCGGC	53:A>C
100504511|F|0-19:A>G-19:A>G	TGCAGTACAACGTGGTGTCAACGGGACGGATGCTGGACCCAGCTGGGATGCGGTCCATGGACGAGTCAC	19:A>G
100557987|F|0-53:T>G-53:T>G	TGCAGGAGTTGAGCAGTGCTCCCCACACGCACAGGTCGGCTCCCATCGGCATCTTCTCGATGAGCTCCG	53:T>G
1753488|F|0-8:G>T-8:G>T	TGCAGGAAGACAAACAAAACTTAGACTGGGCCAAGTTAGGTTGATTTGCTCACGTCCGTTTCGTAGGCA	8:G>T
5360696|F|0-12:T>C-12:T>C	TGCAGATGGGAGTAAAGGGATGGTGAAGGGGTCGTGCCCGAACCGCGCCGAGTCGGCGGCCATGAACGA	12:T>C
1039754|F|0-51:C>T-51:C>T	TGCAGGTGGTCCAAATTTCAAGTTCAAGTTCTGGACTCATTCAGTCATTCTCCAACGAATATTCTCATG	51:C>T
6041199|F|0-8:A>C-8:A>C	TGCAGAGCAACGCATGAATAAAGCAAGGTTAGTTAATTAATTCCG	8:A>C
100504702|F|0-37:G>A-37:G>A	TGCAGTCCTTTCAGGTGGCAAGGGTGTGTCGCCATATGTTCTGCCTGTTCACGCTGCCGCGTGCAATCA	37:G>A
100548291|F|0-8:C>T-8:C>T	TGCAGGGTCCTCACCCTCTCGGGCGACCATCGGTGGAGAGAGACGGGGTGCCCGCCG	8:C>T
22841394|F|0-34:C>A-34:C>A	TGCAGCGACAGCATGCTGCGCCACAACCCGCTGGCGAACGCCCCCGACGACCCCGACGCCCTCGTTTTG	34:C>A
6035810|F|0-51:G>T-51:G>T	TGCAGCATTGCGCTAGTGTCATAGCTCGATCGGCGCAGGCTAGCTAGCTAGGAGCGTCTACCCCCGCCC	51:G>T
100550313|F|0-42:T>C-42:T>C	TGCAGCAGATCGTTTTGCGACGAGAACATCATCGTCGGCATCTGGAATTTGGACGACAATGAAGAAGAA	42:T>C
6045783|F|0-29:A>C-29:A>C	TGCAGGTCGTAGATAGGAGCCTGATGGATACATTCAAACGTTTCCG	29:A>C
100480600|F|0-28:A>C-28:A>C	TGCAGGAGTCAGCTAAGGGACAGCGACAACGACGCCG	28:A>C
32021441|F|0-19:A>G-19:A>G	TGCAGGAAGAGGACTTGAAACAGTTCAGACAGTGGGGAAGCTCCACCCCG	19:A>G
100512377|F|0-11:C>T-11:C>T	TGCAGATGAATCGATTGCTTCAGTGCCAGATAAAAAGGACTCACTCTGCCAACAAGATGATCAGTCACA	11:C>T
1386187|F|0-56:A>T-56:A>T	TGCAGGAGGTTTACCTATGGCCATCTCGCGCAGCTCAAGTACATAATGCCTGAGGCAATTGTCATCAAC	56:A>T
100561597|F|0-45:T>C-45:T>C	TGCAGAGATCACTAGACCCTCCGCCGTTGACAAATGGGAGTTCTCTGGCTCGACCTCGGAACGCCAGTG	45:T>C
100571067|F|0-32:C>G-32:C>G	TGCAGGTGGGTCAATCTATTTGCCAAACGAGTCACTTTTGGCCGTCTCGGAGGTTCATCAACTGGATAC	32:C>G
100417718|F|0-15:C>G-15:C>G	TGCAGGGATTCTTTCCTGTCGTCGTCCGCTAATCTGAGGGCGCACGCCG	15:C>G
100534027|F|0-34:C>G-34:C>G	TGCAGATCTCGGCGGGCTCTGCCATGGCGCCGTTCCCGTAGTCACCGCAGGCGAGCCGCTTGGTCACTG	34:C>G
100566651|F|0-14:G>A-14:G>A	TGCAGCCCCACGTCGCTAAACGCCACCGTGAGGAAGACCACCGCCGTGAAGATGGCCTGGACGATGTCC	14:G>A
100447566|F|0-5:T>A-5:T>A	TGCAGTTCAGGACCAAATAAATTGCAGTTGCTGTATATGTATGCCTTCTGCTGCCTGATCCATGCAATT	5:T>A
1094574|F|0-10:A>C-10:A>C	TGCAGGAGCCAGCCAAGGAGGCGATCGGGCCGTGCACGGCGGTCCCGTCCCTAGACTTGGCCGCCGAGA	10:A>C
3574425|F|0-20:T>C-20:T>C	TGCAGCATCCGCTGACGCGATGGAGACGGTCATCTTGGCCTTCTTCTCCG	20:T>C
5368485|F|0-7:T>G-7:T>G	TGCAGGATCCCGCCGTCGCCGCCCTCTCCCGCGCCCGCGCCGTCGACGTCGAACGCGAAGACGGCGTCG	7:T>G

4982034|F|0-29:T>G-29:T>G	TGCAGCGCCTGGCTCTGCTGATTGGCGCCTTGGCTGCTGCATCACTGCTCCTCCTGTTGCTGTGCTGCT	29:T>G
3027455|F|0-11:C>A-11:C>A	TGCAGGCACGGCGCCAGCCCCACCAGCTGCTCCGCGCACTCGCTCCG	11:C>A
1060497|F|0-26:A>G-26:A>G	TGCAGGGATCTTGCAGTTTGTCCAACAGCAGCGCTCAATTATCATGGTGCCGTTGGACAAATAATCGCC	26:A>G
3952379|F|0-23:G>C-23:G>C	TGCAGCACCTCCGTGATCATCTCGTCCG	23:G>C
3959564|F|0-28:T>G-28:T>G	TGCAGTCATCATCCTTGGTCTTGAGGGCTTCCCG	28:T>G
2255786|F|0-18:G>A-18:G>A	TGCAGGTGGACGAGTCGAGCATGACGGGCGAGCCCCACCCGATCGAGATAGATGCCGAGAAGAGCCCCT	18:G>A
1051236|F|0-9:C>G-9:C>G	TGCAGGCATCGCGCTGCTTCTCCAAGTTAGTATCTGATCCTAATCAGCTCTGTTGTCTTTCCCCGCACC	9:C>G
2275332|F|0-42:G>T-42:G>T	TGCAGAAACTATCCAAAGATTGAGATCGACGACGAATGAAAAGATCATCAGAACCACTACTTACATTGT	42:G>T
5412005|F|0-5:G>C-5:G>C	TGCAGGTTCGCACAGGAGACGGGTCACCTGGGCCG	5:G>C
1049802|F|0-23:G>A-23:G>A	TGCAGAGTAAAAACGACAAGGGAGACAAGCTTTCCTGTGCTGTATACACTTGGAAAATGTCCG	23:G>A
1049959|F|0-60:G>A-60:G>A	TGCAGCCTACAGCAGAAACAACAAAATAGGGCAGGTGGCCACAGGCCACAACCACCCCTCGTCAAGAAC	60:G>A
4993350|F|0-7:G>C-7:G>C	TGCAGGGGGTGCAGGCCAGGCTGCGCCG	7:G>C
1021567|F|0-43:C>A-43:C>A	TGCAGACCTTCTGCACCGTCTACGACTGCAAGCGCTGGTTCGGCGGCATACTGGAACTGCCGCAGATAA	43:C>A
1228279|F|0-24:T>C-24:T>C	TGCAGGAGATGCTGGCCGTCGGGGTGGTGGCGAAGCTGCTGCTGGTTCTACAGGTGGACGCCG	24:T>C
1217579|F|0-12:C>T-12:C>T	TGCAGGAGATGTCGCAGGACGGCGTCGTGGGCAAGCTTGGCCGCAGCACGATGAACCG	12:C>T
5357415|F|0-23:G>A-23:G>A	TGCAGATCGGCAGGCACCGAGTCGAAGCCGAGCCG	23:G>A
2241644|F|0-9:A>C-9:A>C	TGCAGGTTCATGGAGACGTAACGACTCCAGAATGTCGACAGATGAGAACGTGAACCG	9:A>C
3020688|F|0-9:G>A-9:G>A	TGCAGCGATGATTAATGCTGCTGTCCCTCGTATGGACCCG	9:G>A
1129159|F|0-16:A>G-16:A>G	TGCAGAATTCTCTAGCACGAATCTTGAACACTGCCAACGGACGGGATAACCG	16:A>G
991758|F|0-20:A>G-20:A>G	TGCAGCCAGGTGAGTCACTCACCGTGTGAGTACGACTGGCCTCCAAGGCTCCGACGCCG	20:A>G
2244360|F|0-64:G>A-64:G>A	TGCAGTAACCCGACGTTCCGCTTGACCACCGTCTCGAGGTTGTGCGAGAGGAGATTGTGGTTATGCTTC	64:G>A
4002266|F|0-14:G>A-14:G>A	TGCAGTATCTGGTAGTGTTACTCGTGAAGATGTCTCGGTCGCCGTCCG	14:G>A
4406051|F|0-25:T>A-25:T>A	TGCAGAGCTGCTGTCTTCAAGGAATTTTCACACCCG	25:T>A
5367336|F|0-7:G>T-7:G>T	TGCAGGCGCGGCGGAGCTGTGGAACCCG	7:G>T
1096523|F|0-52:G>T-52:G>T	TGCAGGTGATGGCGGACGTGATCCACTGGTGCACCCACAAGGACGGCGGGGCGCGGCCGTCGATGAAGC	52:G>T
1035398|F|0-7:A>G-7:A>G	TGCAGTGAGTTGGGCACTTGAGTTATTCTGCTGAAAGTTTCAGTCCATCAGCAGAAGATGAATTGTGTG	7:A>G
3024176|F|0-25:G>A-25:G>A	TGCAGTGGGAGTACTAAATCCAGACGCCAACTCGCTCAGTCACCG	25:G>A
1037126|F|0-41:A>C-41:A>C	TGCAGTTTTCGTGGTAAGTTGTCCGTCTTGAAAGGAAGCCTATTCAATTCCG	41:A>C
991502|F|0-54:T>A-54:T>A	TGCAGTACGAAGGTGATGCAGTTGGCGTCCTCGTGCGCGCTGCTGCCGTTGTTCTCGTCGGCTGTCGCC	54:T>A
1072851|F|0-22:G>T-22:G>T	TGCAGGCCCTGAAACAGGCCTCGGAACCTCTCCACATTGCCGCCGAACTGCCCGTCCTTCAAAATCCTG	22:G>T
1233721|F|0-11:G>T-11:G>T	TGCAGCTACGAGCTTGTTTTGCCCAAGGTTCTGTCGTCCTCGCGGGCCAGCGTCACCGTCACCATCACC	11:G>T
1021220|F|0-29:A>G-29:A>G	TGCAGGTTGGCGAACACGATGCTCACAGCAGCCAGCACGAGTATCCTCGTCCG	29:A>G
3022593|F|0-34:T>G-34:T>G	TGCAGCACGCCTCCTTGTTTGAGCAAGCTTGCAGTTCCGTCCG	34:T>G
992105|F|0-66:G>T-66:G>T	TGCAGGTGCAGGCTGGACCTAGTATGCACGATGATCCCAAACACCTCGCCCATGTCCAGCGTCTCCGAG	66:G>T
4261818|F|0-9:G>A-9:G>A	TGCAGCGGGGACCCGACCATGTTTGAGGCCTTCTGGAGGGAGACCG	9:G>A
1044504|F|0-24:C>A-24:C>A	TGCAGGTCCGTCCTCTCCTCAACACCATCACCAACGCTGCACTAGGCAGCACTACGATCCG	24:C>A
1060934|F|0-20:A>G-20:A>G	TGCAGTTGGTCTCATCGGAAAGCAGCCAGGGAAGGTTTAACACAAGAGGGAAGGTCAAAAGAATGGCCA	20:A>G
6037762|F|0-22:C>T-22:C>T	TGCAGTGACATCGATCAATCCTCTGTACCCG	22:C>T
3939647|F|0-24:G>T-24:G>T	TGCAGATCGGTCCTGATTTGCATGGCCCG	24:G>T
1015860|F|0-18:C>T-18:C>T	TGCAGGCACCATCGCCGACATGCAGCAATATACACAGTTCCAACGCTGTTACACCGTCTACTGGAAAGG	18:C>T
1008666|F|0-10:G>T-10:G>T	TGCAGCCCACGGCGAAGTTGCGGCTGGCGGCGCCCCGTGGCGACATGCGCAGCGTGTCGGAGACGAGGA	10:G>T
1122234|F|0-23:A>C-23:A>C	TGCAGCTCTGTGATGCTGGCATCAGAGATCAAATGGCGCGGCCCTTTGCCTTCTCCG	23:A>C
991492|F|0-9:T>C-9:T>C	TGCAGGTGCTGAGCAACAAGAAGTTGAAGAGCGCCACGCACAGGGTGGTGAGGAGGCCG	9:T>C
991415|F|0-14:T>C-14:T>C	TGCAGTTGCTCCTCTATGGCTCTTCCGCCCACGAGCAGGGCGGTGGGGACGGAGCGGAAAAGGAACAAC	14:T>C
3952379|F|0-10:C>T-10:C>T	TGCAGCACCTCCGTGATCATCTCGTCCG	10:C>T
1030325|F|0-27:G>A-27:G>A	TGCAGGCGAGCAGGTACTCCAGGTCGCGCTCCTGCATTATGGTGTGGCAGATGCCGAGCTCCTGGAGGC	27:G>A
32022069|F|0-58:A>C-58:A>C	TGCAGGTGGATTTGGTGACGGAGACGGACAAGGCCTGCGAGGATCTCATCTTCAACCAACTCCG	58:A>C
2255786|F|0-35:C>T-35:C>T	TGCAGGTGGACGAGTCGAGCATGACGGGCGAGCCCCACCCGATCGAGATAGATGCCGAGAAGAGCCCCT	35:C>T
3952379|F|0-7:C>T-7:C>T	TGCAGCACCTCCGTGATCATCTCGTCCG	7:C>T
4022577|F|0-29:C>T-29:C>T	TGCAGGCATCGACGACGAAGCGGCGTGGGCGGAAGCCCG	29:C>T
3947178|F|0-43:C>T-43:C>T	TGCAGTACATGTCTAACCTCTTGCTTGCCCATCAACACTTGATCGATTCTGCCCCATGCTACCCCAGGC	43:C>T
1057437|F|0-13:G>T-13:G>T	TGCAGCAAAGTGTGGCGTGCCATATAAAAGTATCGTTAAGAAATGACCCG	13:G>T
1128622|F|0-34:G>A-34:G>A	TGCAGCTGCCGCCGCCGCGTCCTATGCTTGCACCGCCACGAGCAAACCGCCG	34:G>A
32021337|F|0-19:G>T-19:G>T	TGCAGCAGCAGTATTTTAGGCATAGAAAAGTTTCCG	19:G>T
3028099|F|0-18:G>C-18:G>C	TGCAGTACATCCGTGACCGAGTGGCACGATCATCGCCGACCG	18:G>C
1093237|F|0-38:T>A-38:T>A	TGCAGGCAGCAGCGCGCACGCCATGGCTGGACTCTGCCTCCCAAGGGTTTGCGCCAAGAGCGCCGAGAT	38:T>A
4022577|F|0-25:T>C-25:T>C	TGCAGGCATCGACGACGAAGCGGCGTGGGCGGAAGCCCG	25:T>C
1056685|F|0-13:T>C-13:T>C	TGCAGGATGACATTTTCTTCAAGAAAAGCTCCCTGTCTCATCGCAAGGGGAAGCATCCG	13:T>C
3947066|F|0-35:G>A-35:G>A	TGCAGGTTCCTCTCGAACTCCCTCAGGTAGTCCAGGAGCAAAACGATCACGAAGAGGATGAACCCACCG	35:G>A

5367164|F|0-17:G>C-17:G>C	TGCAGCTCCTTCACGTCGGTCACGGTTGGCCG	17:G>C
3026658|F|0-9:G>A-9:G>A	TGCAGTAAAGCCTCACGTCACAGCCGTACACGTCGGCCTTAACCG	9:G>A
1023497|F|0-10:G>A-10:G>A	TGCAGGCCGCGGGCAACGGGTCCTCGCAGAGCGCCGCGGCGAGGGAGCGCCG	10:G>A
1038581|F|0-22:C>G-22:C>G	TGCAGCTGGGCGCCTGGCCGCTCTACTGGGTCGTGCAGGGCTGCGTCATGACCG	22:C>G
1020267|F|0-32:T>A-32:T>A	TGCAGGTGGTGATGGCCCGCTCCGCCTTGTCGTTGTTCTGGGCGCGGGTGTAGATGTACCG	32:T>A
987456|F|0-15:A>G-15:A>G	TGCAGGGATAGAGTCATTTTCCAAGCTTGGGGACTCCATCTACTTTGAGGAGAAAGGCGGCAAACCGAG	15:A>G
1010844|F|0-6:C>T-6:C>T	TGCAGCCTTCGGGAGGCAGGGTTGGAGAGAAGCGATTGCACTTTGGTAAAACCG	6:C>T
1093222|F|0-19:T>C-19:T>C	TGCAGTTCCGTCCGCCTCCTGCCGCACCAAATCCGCCCGCACTGGTTGCTACATCGGAGATTCAAGAAG	19:T>C
985339|F|0-14:G>T-14:G>T	TGCAGAGGTCTCCTGGACGCGAAGCTCGCGCCACACGGCATCGCCGACCCTTAGAATCCG	14:G>T
4022587|F|0-32:G>A-32:G>A	TGCAGGGAGCAGGTGATCCTGGCGCCAGCGTCGACGCCGAACGCCCG	32:G>A
1023767|F|0-16:G>A-16:G>A	TGCAGTGGGTAATGGCGGAGCTCATGAGGAACCCGAGGGTTCGGCATAAGGCGCAGGAGGAGGTCCGAG	16:G>A
4262935|F|0-25:G>A-25:G>A	TGCAGTGCATGCAGGGTAGGGATCGGAGATCCGCGCGAGCCG	25:G>A
2276203|F|0-43:C>T-43:C>T	TGCAGCTGGTTAGCACGTCGCCTGCTAGTGTTTCGAACGGCCGCAGTAGTCTCCATGGTACAGCTGAAC	43:C>T
3025416|F|0-10:G>T-10:G>T	TGCAGCAGCCGCTTCTCCTGGAGCTCCTGGGACAGGACCACCG	10:G>T
1043022|F|0-9:T>C-9:T>C	TGCAGGCGTTTCAATGAACCACACGCAAGGCATCAGTGATCACCAGCTCACTGCCATACTGAAACCGAG	9:T>C
1021400|F|0-24:T>C-24:T>C	TGCAGGTACGGCAGCTTGCTGATGTTGGACTCGGTGATGGGCTCGTCGTCGCCGAGCACGTCCCTGATC	24:T>C
1409211|F|0-41:A>T-41:A>T	TGCAGACGGTCATCAGCTTTCTGTCCTCGGCAAGGGCGTCCATGAAAGAAGATGACAGGAGGAATGTCC	41:A>T
3936395|F|0-21:A>G-21:A>G	TGCAGTCCTGCTTCCTCTTGGACACGTTCCTCCTCTTGTCCG	21:A>G
3960559|F|0-29:C>T-29:C>T	TGCAGAAACTCTAGTCGGATTCGACGGATCGGCCG	29:C>T
3022215|F|0-9:G>C-9:G>C	TGCAGAAATGAAATCACCAGGAATGGAAAAACAACCG	9:G>C
1150987|F|0-26:G>C-26:G>C	TGCAGTTGTGTAAGGGTGCCCTCTGTGAGGTTCACCTGGAACACTGCATTGCACACGGCTAGGTTGCCG	26:G>C
1032900|F|0-9:A>T-9:A>T	TGCAGGATTAGCCGAGAGTAAGAGTGGTCGTGCCTGGACTTGGCTGAGGACGGGAAATGTCTCCATGGA	9:A>T
3960371|F|0-6:T>C-6:T>C	TGCAGATCCAAGGTGAGCAAGCCAGGTTCGTGAACGTCATCACCG	6:T>C
6045125|F|0-8:G>C-8:G>C	TGCAGTAAGACTCAAGAAGGTTTGTAGTGCTATATCGCAAGGCGATCAGTAACTAGCAACAAATGAAAG	8:G>C
3947084|F|0-8:G>T-8:G>T	TGCAGTTGGGATCCCCGTAAACCCTTAACCG	8:G>T
1018433|F|0-6:C>G-6:C>G	TGCAGTCAGCATACCAGGGGCATCGGCGTCAGGGTTGCGGCGGGCTACAACATACTCGCCG	6:C>G
1082372|F|0-15:C>T-15:C>T	TGCAGCTCATGTCCGCCGAATTTCAGATGCCATCGCCGCTGGTGCCGACGCGGGACACCCAGTTCCTGC	15:C>T
1063013|F|0-18:G>C-18:G>C	TGCAGCAGAAGAAGAAGAGGGAGGCGCGACAAAGGCGATGGCACGCGGGTCCG	18:G>C
4262782|F|0-18:C>T-18:C>T	TGCAGGGCAAGGATAGGTCAATACCACCG	18:C>T
1023205|F|0-61:C>T-61:C>T	TGCAGCTGCAACTTCTGGGTTGCCTCAGCGGGGTGGAGATGGTGCTCAAGGACATCGGGTACCCGAGAT	61:C>T
1058625|F|0-30:G>A-30:G>A	TGCAGCCCGCTCAGCAGCGTGAGGCCCTCTGCTTCTTCCATTGTCGTACATTTGCCCAGTTTGCATCCC	30:G>A
7169814|F|0-20:G>A-20:G>A	TGCAGTCGAAGAAACGGCGAGTTCAATTCTCCGCCG	20:G>A
1397609|F|0-59:C>G-59:C>G	TGCAGGAAGGACTCCTTGGTGCCTTCGTCCTCGCCCAGAATGCCCACGGGCACGGCGCCCACGGACTTG	59:C>G
1018433|F|0-24:G>A-24:G>A	TGCAGTCAGCATACCAGGGGCATCGGCGTCAGGGTTGCGGCGGGCTACAACATACTCGCCG	24:G>A
1036388|F|0-40:T>C-40:T>C	TGCAGATCATACATACACGTAATATAATGGCGTAAAGTTGTTGCAAACCCAACGAGCTCCCCTGGAATG	40:T>C
3020356|F|0-10:T>C-10:T>C	TGCAGTGCACTGCTTCCTCCTCCTAATAATGATGTTCCG	10:T>C
3934520|F|0-10:G>T-10:G>T	TGCAGAAGGAGAGGGGGTGGCGAATTGGAGCAGACAGCCG	10:G>T
1069799|F|0-46:A>G-46:A>G	TGCAGGAAACAATTGACCACCTCTTAGTAGCCTGTGTCTTCTCCAGATCGGTTTGGCACATGGTGCTGA	46:A>G
1095881|F|0-32:T>C-32:T>C	TGCAGCAAGCGTACGTGAACAATCTAAATGGCTGAGTTATCTTCTGCCG	32:T>C
3020418|F|0-38:T>C-38:T>C	TGCAGCTGGGGCTATGCTTTCTCTGAAGTTCTCTGCAATGCCCG	38:T>C
3570851|F|0-9:A>G-9:A>G	TGCAGGACAATGAGAAGGAGCTTTCGACCCAAAGCCAAATTCGATGGATCCG	9:A>G
1087945|F|0-61:C>G-61:C>G	TGCAGGCCGCTTGCAAGAACGCATGCATGTGCAGTGTTGAGGACACGGCATTTAATCCAGCCACACGAC	61:C>G
987470|F|0-30:A>T-30:A>T	TGCAGGCCTTCTGGACGGAGGTGCGCACAGAGGGACACGGAGACAAGAAGGACGCGCCGTGGTGGCCTA	30:A>T
981445|F|0-5:C>T-5:C>T	TGCAGCACGCAGCAGAGCTTCCCTACTGCGCCCACGCGGGCCATCTCCCGCACCACCTCCG	5:C>T
2244421|F|0-20:C>T-20:C>T	TGCAGAGGCACCCCATCGAGCGCGTCGTCGTCGAAGTGCTCGGGCAGCTTGTCGAGGAGGAGCCCCAGG	20:C>T
5582002|F|0-7:G>T-7:G>T	TGCAGCTGCATCTCCATCAGGAGAGGTCCATCCG	7:G>T
992947|F|0-42:G>C-42:G>C	TGCAGCGGTTCATGGAGAAGCGCAAGGGAAGGGTCGCCGCACGCGCCGTCCCCTACAGCCG	42:G>C
984822|F|0-8:T>C-8:T>C	TGCAGGCCTAGACCATAAAGGTCCAATGTTTTCAGGTACCGAGAGGCAAGAGGCAGGTCGTCCG	8:T>C
[truncated: 581,479 more chars]
